# Understanding nonlinear vibration behaviours in high-power ultrasonic surgical devices

**DOI:** 10.1098/rspa.2014.0906

**Published:** 2015-04-08

**Authors:** Andrew Mathieson, Andrea Cardoni, Niccolò Cerisola, Margaret Lucas

**Affiliations:** 1 School of Engineering, University of Glasgow, Glasgow, UK; 2 Pusonics SL, Arganda del Rey, Spain; 3 Mectron S.p.A, Carasco, GE, Italy

**Keywords:** power ultrasonics, ultrasonic surgery, experimental modal analysis, nonlinear behaviour

## Abstract

Ultrasonic surgical devices are increasingly used in oral, craniofacial and maxillofacial surgery to cut mineralized tissue, offering the surgeon high accuracy with minimal risk to nerve and vessel tissue. Power ultrasonic devices operate in resonance, requiring their length to be a half-wavelength or multiple-half-wavelength. For bone surgery, devices based on a half-wavelength have seen considerable success, but longer multiple-half-wavelength endoscopic devices have recently been proposed to widen the range of surgeries. To provide context for these developments, some examples of surgical procedures and the associated designs of ultrasonic cutting tips are presented. However, multiple-half-wavelength components, typical of endoscopic devices, have greater potential to exhibit nonlinear dynamic behaviours that have a highly detrimental effect on device performance. Through experimental characterization of the dynamic behaviour of endoscopic devices, it is demonstrated how geometrical features influence nonlinear dynamic responses. Period doubling, a known route to chaotic behaviour, is shown to be significantly influenced by the cutting tip shape, whereas the cutting tip has only a limited effect on Duffing-like responses, particularly the shape of the hysteresis curve, which is important for device stability. These findings underpin design, aiming to pave the way for a new generation of ultrasonic endoscopic surgical devices.

## 1. Introduction

### (a) Power ultrasonic surgical devices

The term high-power ultrasonics (HPU) has been used historically to differentiate applications of ultrasound above a certain power or intensity threshold from those below the threshold. HPU applications include many ultrasonically assisted manufacturing processes, such as cleaning and welding, whereas low-power ultrasonics has defined applications in imaging and non-destructive testing. More recently, the term power ultrasonics has been adopted, which particularly defines applications where there is an irreversible change in the load medium, even though the power level may be comparatively low. Power ultrasonic surgical devices generally operate at a tuned frequency in the low ultrasonic range, approximately 20–100 kHz, and at a power level of tens of watts [1,2]. A typical power ultrasonic surgical device consists of a generator, which transforms mains power to a signal whose frequency corresponds with the resonant frequency of the device, and a transducer which uses a transduction material to convert an electrical signal into micrometric vibration. Transduction materials can exploit the magnetostrictive effect but, more commonly in ultrasonic surgical devices, use the inverse piezoelectric effect to generate the vibrational motion. Ultrasonic surgical devices designed to cut mineralized tissue usually operate at a frequency between 20 and 40 kHz, while ultrasonic scalpels or shears used in soft tissue dissection typically operate in the 50–60 kHz range [3,4].

This study focuses on both commercially available and prototype surgical devices designed for osteotomies during maxillofacial, craniofacial, dental and orthopaedic surgeries. The paper aims to present the background to their design, development and application, followed by experimental characterizations of the dynamic responses, particularly aimed at providing insight into the nonlinear behaviours and the impact these have on the design of reliable novel surgical tools. The devices are tuned to operate at frequencies between 25 and 29 kHz and, although they use a common transducer and similar insert base, they possess different geometric features and differ in wavelength. Identifying the extent to which these features determine the vibrational behaviour of the devices enhances opportunities to design stable and reliable novel devices and facilitates the adoption of ultrasonic devices in a wider range of surgical procedures.

### (b) Power ultrasonics in dental and surgical procedures

Power ultrasonic devices capable of cutting mineralized tissue have only been routinely employed in surgical procedures since the start of this century [3], but the introduction of ultrasonic devices for clinical procedures was initiated in the middle of the last century with the development and trials of an industrial ultrasonic impact grinder to cut cavities in extracted teeth [5]. The success of this trial led to the commercial development of a miniaturized version of the industrial device suitable for use in clinical practice. This early ultrasonic surgical device relied on the vibrational motion of the device impacting against an abrasive paste, positioned on the tooth surface, to remove tissue and form a cavity. *In vitro* and *in vivo* studies using the clinical version of the impact grinder reported a reduction in force required to generate a cavity when compared with the low-speed rotatory devices available at the time, while the device also offered high accuracy and minimal damage to tooth pulp [6]. Even though it was claimed that the device was enthusiastically received by patients, the requirement for an abrasive paste during the cutting process, which restricted the view of the operator, its high cost and the success of the competitor high-speed miniature pneumatic turbine, hampered its commercial development and restricted its use by clinicians.

During the 1950s, power ultrasonic devices were also developed for periodontic and endodontic hygiene procedures, particularly oral prophylaxis [7] and root canal therapy [8]. Early trials reported favourable performance over manual scaling and curette instruments, reducing procedure time, patient discomfort and damage to soft tissue surrounding the tooth. Technological and scientific advances enhanced the capability of ultrasonic devices during the 1970s and 1980s. The introduction of thinner tips in interproximal scaling devices improved tip access to sites between teeth [9], whereas specialized endodontic tips coupled with biocidal agents increased the effectiveness of root canal therapy [10]. This ensured the routine adoption of ultrasonic devices in periodontal and endodontic procedures [11].

Despite Vang patenting a design in 1955 with the claim that a vibrating device could cut both soft and hard tissue without the requirement of an abrasive paste [12], the first ultrasonic surgical devices which incorporated scalpel-like blades and possessed this capability did not become available until the late 1950s and early 1960s. Studies comparing the performance of these devices with conventional surgical mechanical and pneumatic-powered burs and saws, concluded that the ultrasonic devices provided the surgeon with more precision and reduced soft tissue damage, however, their material removal rate and post-operative tissue healing rates were found to be slower [13,14]. Nevertheless, the introduction of saline solution, as a coolant to reduce cutting temperature, during ultrasonic cutting improved recovery rates leading to a satisfactory level of healing by the end of the trial period [14]. With satisfactory healing, it was concluded that the ultrasonic device may be preferable during procedures requiring particularly high precision and accuracy. Volkov reported using an ultrasonic device in human osteotomies in 1974, after first using a device clinically in 1969 [15]. After 311 procedures, which included removing deformations such as juvenile exostoses (bone formations), Volkov commented that osteotomies could be completed through smaller incisions than possible with conventional devices, minimizing soft tissue damage and simplifying the surgical procedure. He also reported that sites cut with an ultrasonic device exhibited normal bone regeneration.

Although studies during the 1970s and early 1980s [16–18] agreed with previous observations that ultrasonic surgical devices provided high accuracy during cutting and that surgical sites exhibited normal healing rates after ultrasonic cutting, the limitations associated with slow cutting rates were again highlighted. Ultrasonic devices available at this time were also awkward to handle, owing to their size and weight, and had a tendency to overheat. Furthermore, they required manual control of the excitation signal and therefore were not always driven at the optimal frequency, in resonance. Technological advancements throughout the 1980s and 1990s, especially in the driving electronics, permitted many of the limitations to be addressed, such as automatic tracking of the resonant frequency. Other generator functions were introduced, such as the capability to incorporate modulated pulses in the excitation signal. This momentarily increases the power delivered to the transducer and increases the vibrational amplitude of the cutting tip, consequently enhancing cutting efficiency and preventing stalling of the cutting tip [19,20]. The integration of an internal cooling system also permits a coolant, such as saline solution, to be channelled through the device directly to the cutting site. As well as preventing heating of the surgical site, it also reduces the likelihood of the transducer overheating and exhibiting unstable behaviour.

Technological advancements have underpinned the design and adoption of reliable and efficient ultrasonic cutting devices which often offer a broad range of interchangeable cutting inserts tailored for specific procedures in oral and maxillofacial surgery, neurosurgery and orthopaedics [2–4,20–23].

### (c) Cutting mineralized tissue

#### (i) Biological response of bone tissue to heat shock

Bone tissue damage is induced during cutting when the tissue is exposed to elevated temperatures, however, the extent of this damage is dependent on both the maximum temperature and the duration of exposure. This is often referred to as heat shock and it has been reported that it can have a significant biological effect on bone cells. The typical response of bone tissue to heat shock is an immediate necrotic response (death induced by cell injury) followed by later stage apoptosis (programmed cell death) [24]. However, the heat shock that bone tissue can be exposed to before necrotic and apoptotic responses occur is also dependent on cell type. Osteoblast-like cells (cells responsible for bone formation) have been found to be more susceptible to heat-induced necrotic response than osteocyte-like cells (cells found in mature bone). Nevertheless, it has been reported that when heat shock is minimal, 45°C for 60 s for osteocyte-like cells and 45°C for 30 s for osteoblast-like cells, cells show signs of recovery a few days post-exposure. Cell responses to higher temperatures (60°C) are characterized by an immediate necrotic response and recovery duration of several weeks. Interestingly, it was also reported that mild heat shock (47°C for 60 s or less) could enhance bone healing through mineralization of osteoblast-like cells as well as differentiation and mineralization of mesenchymal stem cells, stem cells that are capable of differentiating to a variety of cells such as osteoblasts [24].

#### (ii) Controlling thermal tissue damage

It is beneficial for post-operative recovery if the maximum temperature and duration of exposure at the surgical site is controlled. Studies have reported that the temperature of bone tissue and bone mimic materials during ultrasonic cutting can range from as low as 35°C (under *in vitro* conditions) to 160°C [15–18,25,26]. The results of these studies are difficult to compare due to differences in the ultrasonic device used, procedure duration, presence of coolant, or *in vitro* or *in vivo* conditions. Nevertheless, they all conclude that ultrasonic cutting of bone, as with cutting with conventional bone saws and burs, causes thermal shock in tissue sufficient to induce necrotic or apoptotic responses.

Heating during ultrasonic cutting is known to stem from absorption of ultrasonic energy, frictional heating between the blade and the tissue, and the combustion of debris at the cutting interface. Although a solution to maintain temperature below a critical value is the delivery of a coolant to the cutting site, the atomization of the coolant by the vibrating ultrasonic blade has led to concerns related to the potential for cross-contamination. To minimize heating without incorporating a coolant system, it is possible to configure the blade geometry with the aim of reducing the contact area of the bone and blade interaction site while also permitting debris to be directed from the cut site [25,26].

#### (iii) Clinician use of ultrasonic devices

The way a clinician uses an ultrasonic device has the potential not only to significantly affect the heat shock, but also to influence the cutting performance. A study that compared five clinicians in their application of a conventional rotary bur and an ultrasonic device under *in vitro* cutting conditions reported that clinicians cut more efficiently with the bone bur, although it was also observed that they applied similar force when operating both devices [27]. Ultrasonic devices require considerably less force to be applied than conventional devices to perform effectively and too high a load hinders cutting progress. This indicates that the ultrasonic devices were sub-optimally used during this study. Other studies investigating *in vivo* use of ultrasonic devices to complete or partially complete clinical procedures have also recognized that it can take several years for a clinician to gain sufficient experience to use an ultrasonic cutting device optimally [19,21].

#### (iv) Tissue damage and recovery

Recent studies indicate that surgical sites prepared with ultrasonic devices often exhibit less tissue damage, such as inflammation, and exhibit higher levels of morphogenetic proteins (proteins with the ability to induce bone formation) and osteoblasts, which lead to enhanced neo-osteogenesis (formation and development of new bone tissue), than sites prepared with conventional devices such as burs or saws [22,23]. Although trials have shown that conventional cutting devices can exhibit superior debris removal, the benefits of ultrasonic cutting of greater precision [12,14–23,27] and minimal soft tissue damage [19–23], can lead to shorter cumulative surgical durations as well as a reduction in procedures postponed due to damage to delicate structures such as nerves and membranes [19–21]. An *in vivo* study on Wistar rats investigated the consequence of exposing nerve tissue directly to the vibrating ultrasonic blade. It was reported that although some structural and functional damage occurred, the tissue was not dissected and the majority of the animals fully recovered by the end of the trial period [20]. A reduction in soft tissue damage was also reported after bony window osteotomy and sinus membrane elevation procedures where perforation of the Schneiderian membrane (the membranous lining of the maxillary sinus cavity) was absent in 20 of the 21 cases [21]. Similar success was reported in another study which exhibited a complete absence of palatal mucosal injury after 140 palatal expansions using an ultrasonic cutting device [19].

## 2. Linear and nonlinear vibrational responses

The design of an ultrasonic surgical device, which ensures stable and reliable performance, relies critically on delivering the most effective vibration, in terms of frequency, amplitude and operational mode shape, to the cutting tip. Power ultrasonic surgical devices are electromechanical systems which are driven at resonance and, as with all dynamic systems, they can be modelled in terms of their mass, *m*, stiffness, *k* and damping, *c*, and a time-varying excitation force, *F*(*t*) [28]. If these properties do not vary with time or level of excitation force, the system can be described as linear, however all real systems, including ultrasonic devices, exhibit nonlinearity above some threshold excitation level. Nonlinearities that influence the vibrational behaviour of power ultrasonic devices can manifest as changes of the resonant frequency, reduced performance of the device, and premature failure.

### (a) Nonlinear vibrational responses

Nonlinear behaviour can stem from various sources in dynamic systems such as changes in material properties, behaviours associated with the physical geometry of the structure or from nonlinear forces applied to a structure. Systems that exhibit nonlinear behaviour often display multiple states of equilibrium, compared to linear systems that possess a single state of equilibrium. This gives rise to the possibility that the structure could alternate between states, resulting in unstable behaviour [28].

**Figure 1.**
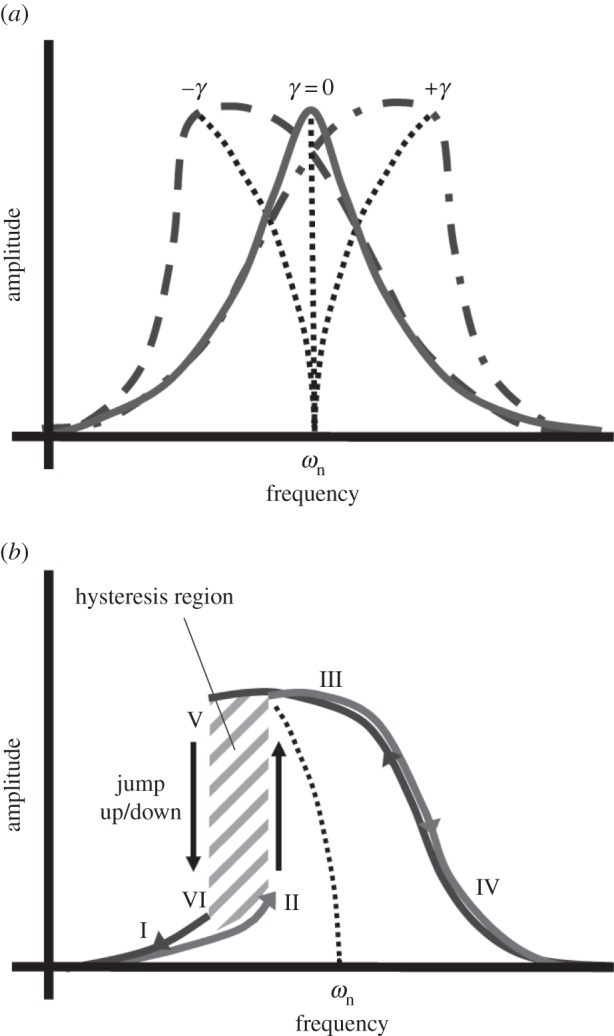
Duffing-like behaviour; (*a*) frequency response curves for varying *γ*: cubic softening: −*γ*, linear: *γ*=0, cubic hardening: +*γ*, (*b*) jump phenomenon and hysteretic region.

Power ultrasonic devices often exhibit Duffing-like behaviour, depicted in figure 1. For a single degree-of-freedom system, the Duffing equation of motion can be represented by equation (2.1). The generalized acceleration, velocity and displacement terms are $\ddot{x},\dot{x}$ and *x*, respectively, however, it is the cubic term, *γx*^3^, which determines whether the spine of the resonance response curve bends to the left or to the right. Under the conditions where *γ*=0, the system is driven as a simple (linear) damped harmonic oscillator. However, when *γ*>0 the stiffness term will increase or ‘harden’ and the resonant frequency will increase, while when *γ*<0 the stiffness term will decrease or ‘soften’, resulting in a lowering of resonant frequency. This behaviour in turn leads to a nonlinear relationship between the input excitation and vibrational amplitude which can lead to saturation of the vibrational amplitude.

2.1 $$m\ddot{x}+c\dot{x}+kx\pm \gamma x^{3}=F(t).$$

Systems exhibiting Duffing-like behaviour will also generally display the jump phenomenon and hysteresis effects, as illustrated in figure 1*b*. The jump phenomenon is characterized by discontinuities in the response of the system (between locations II and III, and VI and V in the figure), while the area between the jumps encloses a hysteretic region where multiple solutions (amplitudes of vibration) are possible. If the system is driven at a frequency in this region, three possible solutions exist; two stable solutions (a vibrational response which lies between III and V or II and VI) and an unstable solution which occurs within the hatched area of figure 1*b*. The unstable solution will never be observed experimentally, however, either stable solution can exist thus providing the possibility that the system becomes unstable if it alternates between them. Away from this region, in regions I and IV, the response of the system is stable and hence only one solution exists [28].

### (b) Sources of nonlinearities in ultrasonic devices

Nonlinear behaviour is known to stem from a range of sources and therefore can be difficult to predict from simulations or theoretical models which do not tend to account for all possible sources. Research has indicated that device geometry can influence nonlinear behaviour, often modifying the threshold excitation level above which nonlinear responses occur [29,30]. Other studies have reported that material properties, such as the mechanical quality factor, *Q*_m_, of alloys used in the manufacture of power ultrasonic devices are strain dependent above a strain threshold consistent with the excitation levels used to drive these devices [31,32]. Furthermore, it is known that transduction materials, such as lead zirconate titanate (PZT), strongly exhibit Duffing-like responses at elevated excitation levels, leading to a loss of performance and efficiency [33–36]. Properties such as the electromechanical loss factor, *k*_eff_, quality factor, *Q*_m_, and dielectric loss factor, tan *δ*, (all indicators of piezoceramic or transducer performance and efficiency) are electric field strength, vibrational amplitude and temperature dependent. The elastic compliance, *s*^E^, dielectric constant, *ε*^T^ and piezoelectric constant, *d*, have also been shown to be temperature and vibrational amplitude dependent [33–36]. The implications of all of these potential sources of nonlinear vibrational responses on device design and performance are not well understood, and there is a need to establish a characterization methodology in order to develop design guidelines for optimal operation.

## 3. Surgical devices

**Figure 2.**
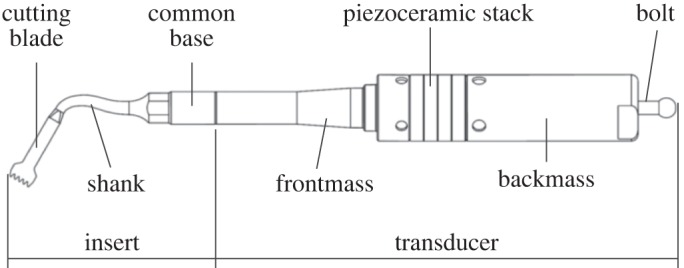
Schematic of transducer and cutting insert.

This study investigates how device design influences the vibrational behaviour, by characterizing and distinguishing the vibrational responses resulting from key geometric features in ultrasonic surgical devices for bone cutting. The investigated devices are based on a device developed by Mectron S.p.A, GE, Italy, and incorporate a Langevin transducer and a cutting insert (figure 2). The transducer contains four piezoceramic rings, with properties similar to PZT-4, arranged to form a stack which is held under a compressive preload by an internal bolt. The front mass and back mass encourage the generated longitudinal wave towards the working face, while the profile of the front mass is designed to amplify the vibrational amplitude.

**Figure 3.**
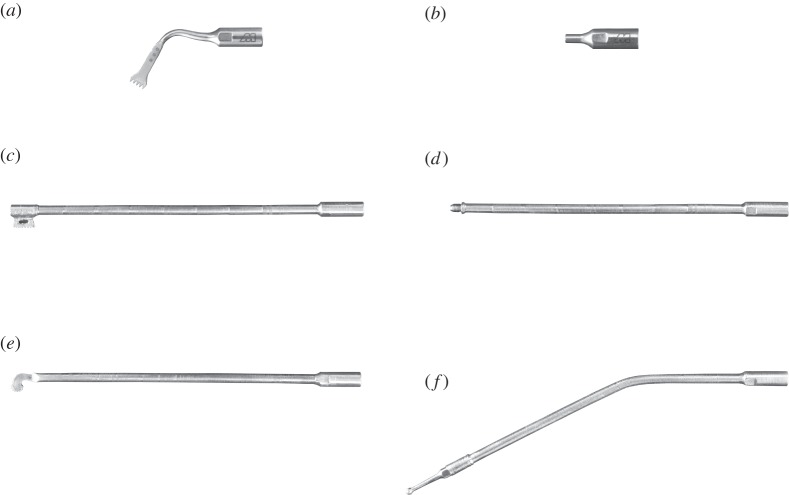
Investigated inserts (*a*) OT7, (*b*) BI, (*c*) I1, (*d*) I2, (*e*) I3 and (*f*) I4.

The investigated commercial (OT7) and novel telescopic (I1, I3 and I4) inserts, figure 3, all have a similar threaded joint section (the common base in figure 2) which allows the insert to be connected to the transducer. This is referred to as BI (the base insert) and is one of the key geometric features investigated in this study. An insert, I2, has been created by the removal of the cutting tip from I1. OT7 and BI assembled with the transducer form a half-wavelength device, while devices assembled incorporating I1–I4 with BI and the transducer, are full-wavelength.

### (a) Vibratory motion of ultrasonic devices

**Figure 4.**
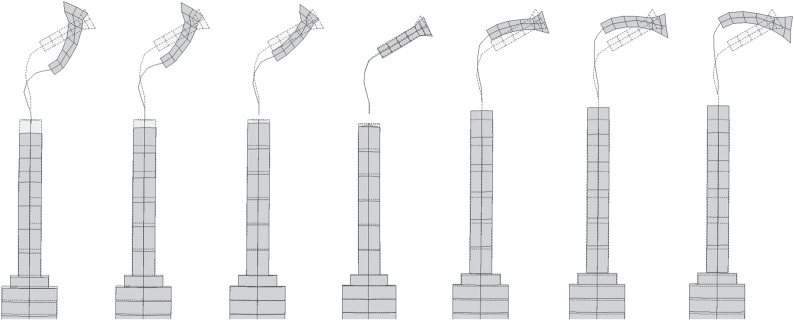
Images of deformation measurements (shaded and superimposed on dashed stationary image) through a half-cycle of oscillation of the OT7 insert.

Although the Langevin transducer generates a longitudinal motion, the OT7 is shaped to exhibit a flexural motion in the plane of the cutting blade. This enhances cutting where the cut line is perpendicular to the cutting tip axis, compared to blades cutting with only longitudinal vibratory motion. Figure 4 illustrates the motion of the front mass of the transducer and OT7 insert through a half-cycle, measured experimentally as described in §4. It can be observed that the front mass and common base vibrate longitudinally, while the shank induces an additional flexural motion.

### (b) Surgical procedures

#### (i) OT7 insert

**Figure 5.**
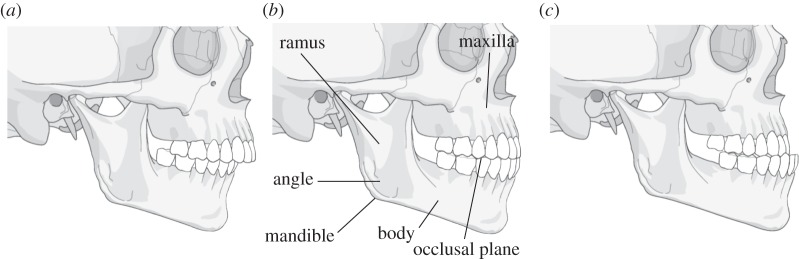
Diagrams of the skull showing (*a*) mandible exhibiting prognathism (*b*) normal mandible position (*c*) mandible exhibiting retrognathism.

The surgical device incorporating OT7 is currently used in craniofacial, maxillofacial, reconstructive and orthopaedic surgeries [3,4,19–23]. An example of a procedure that can be performed using the OT7 is the bilateral sagittal split osteotomy (BSSO). BSSO corrects malocclusion (the misalignment of teeth or the incorrect relationship between teeth and the dental arch) which can arise due to congenital abnormal skeletal development or through trauma sequelea. Malocclusion can be identified where the mandible or maxilla exhibits two conditions; prognathism (skeletal protrusion) or retrognathism (skeletal retrusion) (figure 5). Prognathism or retrognathism of the mandibular is corrected through its mobilization to achieve facial balance before it is fixed in its corrected position.

**Figure 6.**
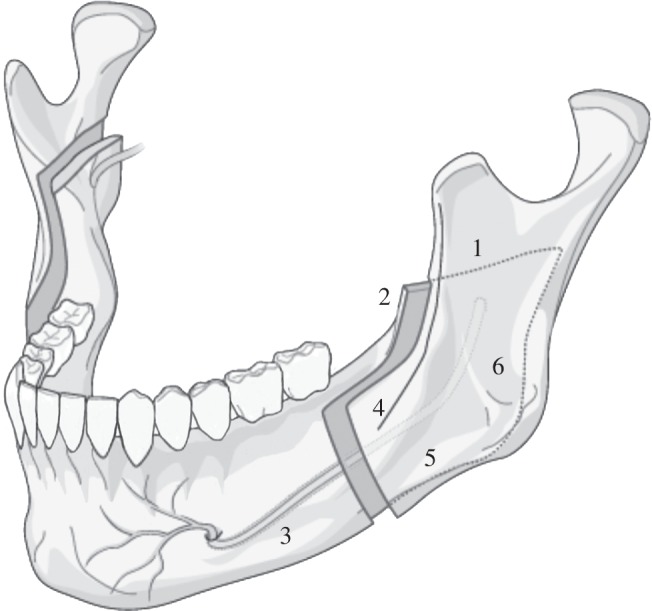
The mandibular and osteotomies during the BSSO procedure.

To mobilize the mandibular through BSSO, the surgeon uses the OT7 during the following steps shown in figure 6:

1. Osteotomy of the mandibular ramus: is made via a cut which is parallel to the occlusal plane and that cuts through the lingual cortex to the cancellous bone terminating just posterior to the lingual.
2. Vertical osteotomy: this cut starts from the osteotomy of step 1, cutting vertically through the centre of the ramus, passing through the cortex and into the cancellous bone. The cut is terminated at the second molar.
3. Mandibular body buccal osteotomy: commences at the lower border of the mandibular body and terminates at the endpoint of the vertical osteotomy.
4. Sagittal split commencement: cancellous bone is cut up to the neurovascular bundle.
5. Nerve protection: before a deeper cut to the cancellous bone of the lower border of the mandibular is performed, the neurovascular nerve is located and protected by a separator.
6. Completion of the Sagittal split: an osteotome is made to the mandibular body that allows its mobilization and for its position to be corrected.

#### (ii) Telescopic inserts

**Figure 7.**
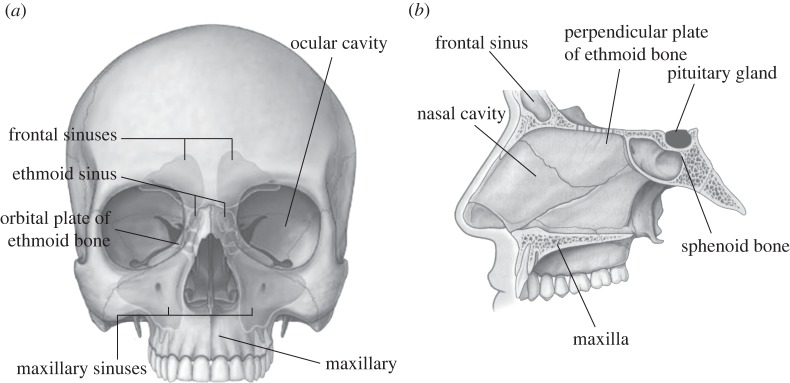
The anatomy of the skull.

I1, I3 and I4 have been developed for endoscopic procedures, one of which is functional endoscopic sinus surgery (FESS). FESS is possibly the most regularly performed otolaryngology procedure (procedures treating disorders of the ear, nose and throat), and it is estimated that around 300 000 are performed each year [37]. FESS is a minimally invasive procedure and surgical sites are accessed through the nasal cavity (figure 7). FESS treats paranasal disease and restores normal drainage of the sinuses through clearing blockages or enlarging the nasal ostia (small orifices that join the paranasal sinuses with the nasal cavity).

Trans-sphenoidal surgery and optic nerve decompression are procedures that could also use ultrasonic devices with telescopic capability. Trans-sphenoidal surgery, a procedure that removes tumours of the pituitary gland, requires access to the base of the skull through the sphenoid bone via the nasal cavity in order to reach the pituitary gland. Decompression of the optic nerve could also be completed through accessing the nerve through the orbital floor from the nasal cavity.

For power ultrasonic devices to access anatomical structures in the nasal cavity region, inserts require to be approximately 10–15 cm in length. Straight inserts can be used in FESS procedures that require access to the ethmoid bone, while to access the frontal sinuses (figure 7) the cutting tip is required to make an angle with the shaft of 50°–70°, as in I4. To prevent tissue damage via frictional heating resulting from the vibrating insert, the shaft of the insert must be contained within a sheath.

## 4. Characterization of devices through experimental modal analysis

The distinctive nature of these surgical procedures results in ultrasonic device designs requiring the incorporation of distinctive tailored features. It is therefore important to understand how vibration behaviours of both the half-wavelength and full-wavelength surgical devices are influenced both by their distinct and common geometric features. The basis of all the vibration characterizations, for linear and nonlinear responses, is an identification of the modal frequencies and mode shapes of each measured device configuration (as presented in figure 4). This allows behaviours to be associated with the tuned operational mode and/or other modal responses of each device. Identification of modal frequencies and mode shapes is carried out through an experimental modal analysis (EMA) [38].

For the EMA, the devices were excited with a random excitation signal, in a frequency range of 0–80 kHz, by a function generator built into the data acquisition hardware (Data Physics Quattro), and amplified through a power amplifier (QSC RMX). Vibration measurements were acquired in the form of frequency response functions (FRFs), which are a measure of the relationship between the input excitation force and output vibration velocity in the Fourier domain, providing complex functions from which magnitude and phase data can be extracted over a grid of measurement points. FRFs were measured from grid points located on the surface of the transducer and surgical inserts using a three-dimensional laser Doppler vibrometer (Polytec CLV-3D) and were acquired with a resolution of 1.6 Hz using Signal Calc ACE data acquisition software (Data Physics). Once the FRF traces had been collected from every measurement point, they were imported to modal analysis software, ME'ScopeVES (Vibrant Technology), where a curve-fitting process generated a single mathematical expression which represents the vibrational responses for the whole device from the experimental data. To visualize modes of vibration, the curve-fitted modal data were assigned to a three-dimensional model, representing the geometry of the device, which subsequently could be animated through the cycles of vibration in each of the measured modes.

**Figure 8.**
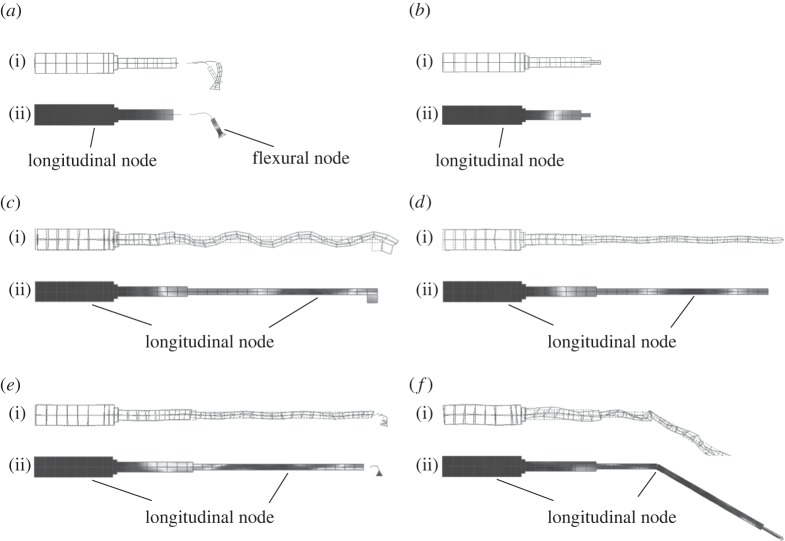
Modes of vibration of operational frequencies showing (i) deformed mode shape superimposed on undeformed shape and (ii) normalized contours of deformation superimposed on undeformed shape, for (*a*) OT7, (*b*) BI, (*c*) I1, (*d*) I2, (*e*) I3 and (*f*) I4.

The mode shapes of the tuned modes of vibration measured through EMA are shown in figure 8. The contour plots, where lighter shading represents higher vibrational amplitude and darker shading represents lower vibrational amplitudes, identify the nodal and antinodal planes. It can be observed that half-wavelength devices, containing OT7 and BI, exhibit one longitudinal nodal plane, while the full-wavelength devices, incorporating I1–I4, exhibit two. Comparison of the mode shapes of the half-wavelength and full-wavelength devices with and without cutting blades illustrates how the cutting blade introduces a flexural motion to the longitudinal mode of vibration. These modes of vibration exhibit a nodal plane associated with the flexural motion in addition to the longitudinal mode nodal plane. The location of this node is an important consideration, as location in a high stress region can result in failure of the insert and therefore poor reliability of the device.

**Figure 9.**
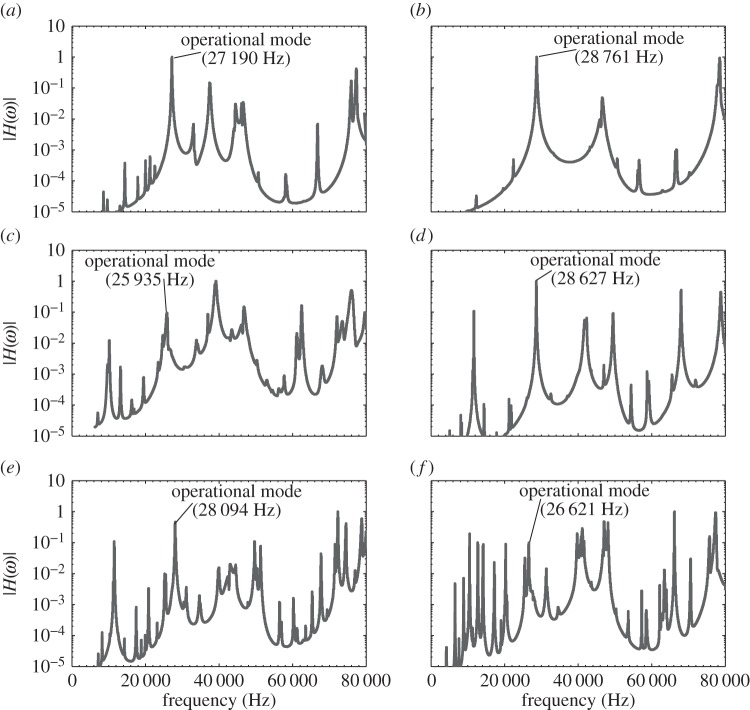
The FRF's after curving fitting for (*a*) OT7, (*b*) BI, (*c*) I1, (*d*) I2, (*e*) I3 and (*f*) I4.

The curve-fitted FRFs, identifying the frequencies of the tuned mode of vibration of each device, are shown in figure 9. The FRFs of the full-wavelength devices contain a much higher spectral density than the half-wavelength device FRFs, and this is consistent with more lower order flexural and torsional modes being excited at lower resonant frequencies for the full-wavelength devices. The addition of a cutting blade, or other device component, to the transducer and base insert also increases the spectral density. These additional modes, especially those neighbouring the tuned mode of vibration, must be considered during the design phase in order to implement strategies to avoid the device exhibiting modal coupling. Modal coupling exists where two or more modes of vibration are excited simultaneously due to the close proximity of their resonant frequencies. The operating vibrational motion is then a combination of the tuned response modified by undesired modal responses from the neighbouring modes. These responses, which may be flexural or torsional, can result in high stress locations in the device and cause premature failure.

## 5. Harmonic characterization

To investigate the influence that device geometry has on the vibrational response of the surgical devices, such as Duffing-like responses, bi-directional frequency sweeps were performed through the tuned resonant frequency of each device, for a series of incremented excitation voltage levels. The devices were excited by a burst sine signal which was applied for a fixed number of 4000 cycles at each frequency increment. The aim of using a burst sine signal was to eliminate, as far as possible, heating within the piezoceramic elements, and thus prevent misinterpretation of responses largely associated with heating as responses due to high strain and electric field.

The excitation signal was generated by a function generator (Agilent 3322A) and amplified through a power amplifier (QSC RMX). The vibration velocity response of each device was measured from the end of the back mass of the transducer (figure 1) using a one-dimensional laser Doppler vibrometer (Polytec CFV 055), while the temperature of the piezoceramic elements was monitored using an infrared thermometer. Data acquisition hardware and interface in conjunction with Labview software (National Instruments) were used to coordinate the experimental protocol and data collection, while the time domain signals of current and voltage as well as the frequency spectra of the velocity response were viewed on an oscilloscope (Tektronic DPO 7054).

**Figure 10.**
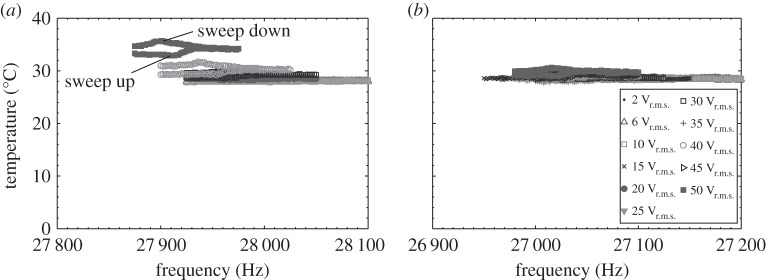
Surface temperature of piezoceramic stack of I3 with (*a*) 1 s and (*b*) 10 s between successive excitation bursts.

For the devices under investigation, it was possible to capture small changes in the vibrational response and exercise sufficient control of the excitation by adopting a frequency step of 1 Hz between successive bursts for excitation levels in the 2–10 V_r.m.s._ range, and 2 Hz for excitation levels in the 10–50 V_r.m.s._ range. It was also essential that any heat generated during an excitation burst was allowed to dissipate by incorporating a time interval between successive bursts. In order to determine an acceptable time interval, I3 was excited through burst excitation in the 2–10 V_r.m.s._ range incorporating a 1 s time delay. From figure 10, it is evident that no significant temperature increase occurred in the piezoceramic stack for this excitation range, indicating that this 1 s interval was an adequate duration for heat dissipation. However, for the 10–50 V_r.m.s._ range, it can be observed that the stack temperature increased as the frequency of excitation signal increased, reaching a maximum value (7.6°C above ambient) at resonance. Since a 1 s time delay is not a sufficient interval for heat dissipation at higher excitation levels, for the 10–50 V_r.m.s._ range this was increased to 10 s, ensuring that the increase in stack temperature across the excitation range was small (the highest temperature recorded at 1.9°C above ambient; figure 10*b*).

**Figure 11.**
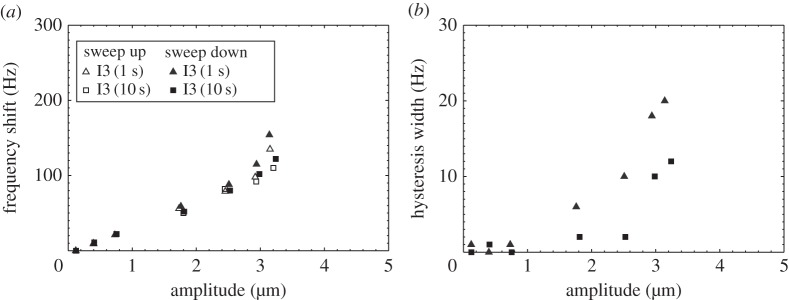
Nonlinear responses of I3 measured at resonance with respect to vibrational amplitude (*a*) resonant frequency shift and (*b*) hysteresis width.

Figure 11 presents the amplitude of vibration measured at resonance during a bi-directional frequency sweep of I3, recording the frequency shifts and hysteresis widths associated with Duffing responses. Although these characteristics are exhibited when both 1 and 10 s intervals are incorporated into the experimental protocol, there are larger frequency shifts and greater hysteresis measured when the vibration amplitude of the device is high for a 1 s interval, because the piezoceramic stack is at a higher temperature. This demonstrates that the selection of appropriate time delays between successive bursts is essential to prevent misinterpretation of responses due to heating effects as Duffing-like responses.

### (a) Insert effect on vibrational response

**Figure 12.**
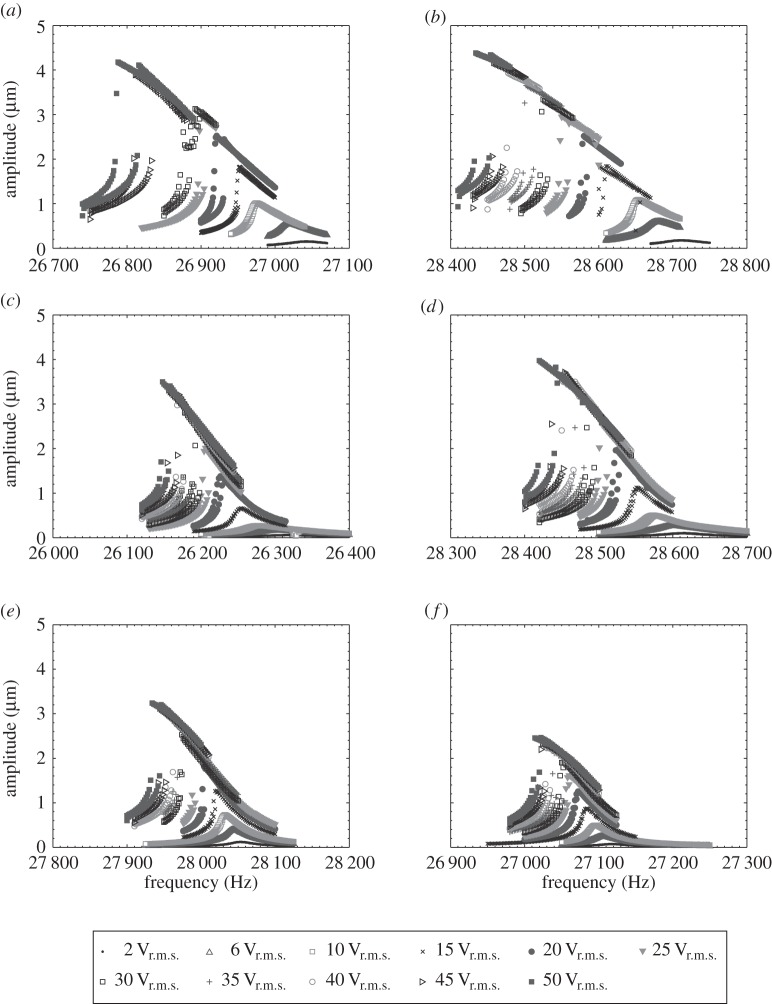
Response of devices excited through resonance for (*a*) OT7, (*b*) BI, (*c*) I1, (*d*) I2, (*e*) I3 and (*f*) I4.

The vibrational responses of the devices excited through resonance are presented in figure 12, and it can be observed that all the devices exhibit Duffing-like behaviour. Resonant frequency shifts and amplitude jumps can be seen in all measurements, the curvature of the spine of all the response curves being consistent with stiffness softening. In order to compare these behaviours for the different devices, the frequency shifts and widths of hysteresis regions were extracted from the data in figure 12.

**Figure 13.**
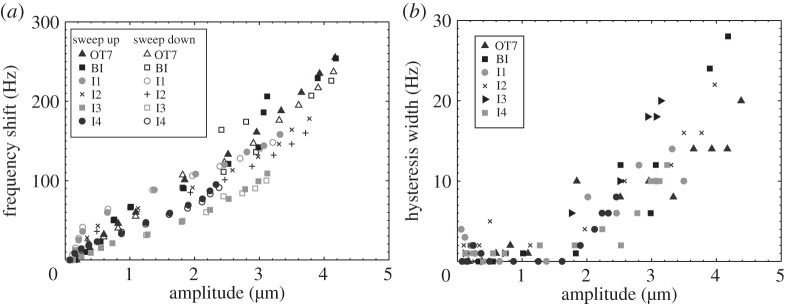
Nonlinear responses of devices with respect to vibrational amplitude for (*a*) resonant frequency shift and (*b*) width of hysteresis region.

 Figure 13*a* illustrates that for all devices, the larger the amplitude of vibration the larger the resonant frequency shift. However, it can also be seen from figure 13*a* and table 1 that at elevated amplitudes of vibration the half-wavelength devices (OT7and BI) exhibit larger resonant frequency shifts than the full-wavelength devices (I1–I4). This is consistent with a previous study, of ultrasonic food-cutting devices, that reported larger frequency shifts with increasing excitation level for longer devices [39]. It is known that the *Q*_m_ of alloys commonly used to manufacture ultrasonic devices, particularly TiV6Al4 and 300 series stainless steel, drops off rapidly once a vibrational amplitude threshold has been reached. *Q*_m_ is a measure of damping and hence it can be expected that a lowering of *Q*_m_ will result in a lowering of resonant frequency [31,32].

**Table 1.** Selected data from figure 12, illustrative of the comparative Duffing-like behaviour exhibited by the devices.

	measured at 2.5 μm		jump first observed at:	
	resonant frequency shift (Hz)	width of hysteresis region (Hz)	amplitude (μm)	voltage (V_r.m.s._)
OT7	121	12	1.81	15
BI	133	8	1.81	15
I1	120	6	1.97	25
I2	113	10	2.47	25
I3	80	2	1.26	15
I4	105	8	1.60	25

Although all the devices displayed the jump phenomenon and hysteresis, as observed in figure 12, a definite relationship does not appear to exist with the device geometry, including the presence of a flexural as well as axial vibrational motion. Figure 13*a* illustrates that the widths of the hysteresis regions are small, if not insignificant, below a vibrational threshold (around 1.8 μm for these devices). Above this threshold, the hysteresis widths all increase with excitation level. Meanwhile, the excitation level at which the jump phenomenon is first observed is presented in table 1, where it can be seen that, again, no relationship is emerging relating the geometry of the device to the excitation threshold.

Duffing-like behaviour is known to stem from several sources in power ultrasonic devices and, although the devices exhibited differing levels of cubic softening, these have been accredited to nonlinear material properties of the inserts induced by elevated strain levels. However, Duffing-like behaviour can also be induced in power ultrasonic devices through sub-optimal tightening of threaded joints and from non-constant piezoceramic properties [33–35,40–42]. It is known that piezoceramic materials exhibit an increase in their elastic compliance, $s_{11}^{E}$, under elevated stress, which has the effect of lowering the resonant frequency and resulting in the jump phenomenon [35]. This is exacerbated when the piezoceramic material is exposed to higher temperatures. High stress and elevated temperatures have also shown to increase both the mechanical and dielectric losses [34,41] and these losses, specifically the dielectric losses, have also been suggested as the cause of Duffing-like behaviour in piezoceramic-based devices [41].

### (b) Harmonic responses

**Figure 14.**
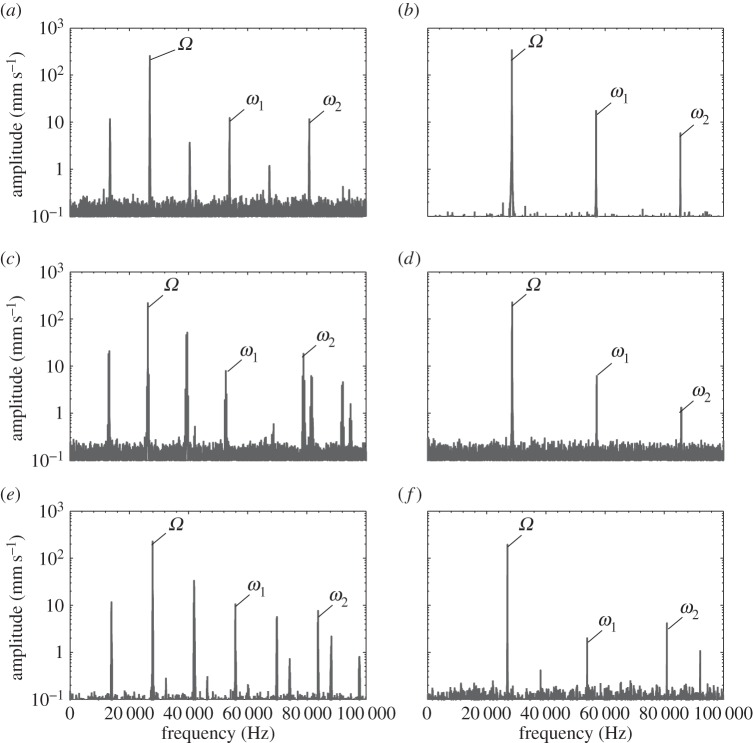
Power spectra of devices excited close to resonance at 50 V_r.m.s._ (*a*) OT7, (*b*) BI, (*c*) I1, (*d*) I2, (*e*) I3 and (*f*) I4.

The power spectra of the devices when excited at 50 V_r.m.s._ close to their resonant frequency are presented in figure 14. It can be observed that the spectral responses of BI and I2 are similar, while OT7, I1 and I3 show similarities. The excitation frequency, *Ω*, as well as the first and second harmonics, *ω*_1_, and *ω*_2_, are visible in the power spectra of BI and I2. However, responses in the spectra of OT7, I1 and I3 other than *ω*_1_ and *ω*_2_, and with a frequency relationship of *ω*_*k*_=*k*0.5*Ω* (where *k* is the harmonic number) can also be observed.

These responses can be accredited to the presence of the flexural motion induced by the cutting blade in OT7, I1 and I3, but also indicate a system exhibiting period-2 motion. Period-2 motion is the first step of the period doubling route to chaotic behaviour, which if reached would result in unpredictable and uncontrollable behaviour in the devices [28]. Period-2 motion will only occur above an excitation threshold and from the power spectra it is clear that this threshold has been exceeded for OT7, I1 and I3. The spectral response of I4 appears to indicate that the device is close to the threshold of period-2 motion, and if the excitation level was higher, then I4 would also exhibit this route to chaos.

## 6. Conclusion

Although power ultrasonic surgical devices have been routinely adopted to cut hard tissue for over a decade, their use in surgery is still limited when compared with the more conventional mechanical or pneumatic-powered devices. To enable the wider adoption of power ultrasonic devices, a better understanding of the design of stable, effective and efficient devices is required.

This investigation illustrates the influence that device geometry has on some nonlinear behaviours, while having a limited effect on others. Certain behaviours were observed to be influenced by device length; at higher vibrational amplitudes longer devices (full-wavelength) were found to exhibit smaller shifts in resonant frequency than shorter devices (half-wavelength). However, the device length or the presence of geometric features, such as a cutting blade, had limited influence on the formation of hysteretic regions and the jump phenomenon. Nevertheless, all the Duffing-like behaviours were influenced by piezoceramic stack temperature and therefore one effective method of reducing the adverse effects of nonlinear responses in power ultrasonic devices is through the careful control of the stack temperature.

As expected, longer devices exhibited higher modal density than the shorter devices within the frequency range of the investigation. However, incorporating an off-axis geometric feature, such as a curved insert, further increased the number of modes of vibration identified through EMA. This increases the possibility of modal coupling occurring between the tuned mode and a neighbouring mode of vibration. Modal coupling can be minimized by ensuring there is a sufficient frequency separation between the operational mode and its neighbouring modes of vibration. However, incorporating an off-axis geometrical feature also resulted in a spectral response exhibiting the period doubling route to chaos. While chaotic behaviour was not observed in this study, the presence of period doubling implies that these devices, especially those with features that lie outside their axis, should be monitored to ensure that chaotic behaviour does not manifest in the device during operation. The future is likely to see a significant increase in power ultrasonic devices used in surgical procedures, especially longer more slender devices for endoscopic procedures and higher power devices capable of cutting through large bones. For both, it becomes more likely that the vibrational threshold at which chaotic behaviour manifests is reached under operating conditions. The nonlinear response characterizations reported here will need to become a routine part of the design process if reliable novel power ultrasonic devices exhibiting stable behaviour are to be realized.

## Supplementary Material

FRF Data

## Supplementary Material

Power Spectrum Data

## Supplementary Material

Bidirectional Sweep Data

## Data accessibility

The datasets supporting this article have been uploaded as part of the electronic supplementary material.
