# Supplementary material for: Understanding nonlinear vibration behaviours in high-power ultrasonic surgical devices
Source: Proc Math Phys Eng Sci. 2015 Apr 8;471(2176):20140906. doi: 10.1098/rspa.2014.0906 (PMC4991263; doi:10.1098/rspa.2014.0906)
Supplement: FRF Data [file rspa20140906supp1.pdf]

Curve Fitted FRF traces

I1		I2		I3		I4		OT7		BI	
Frequency	|H(w)|	Frequency	|H(w)|	Frequency	|H(w)|	Frequency	|H(w)|	Frequency	|H(w)|	Frequency	|H(w)|
6080	1.88E-05	4060	1.13E-06	3850	4.55E-06	3450	2.95E-06	5730	5.68E-06	5170	7.22E-06
6090	1.88E-05	4060	1.14E-06	3850	4.56E-06	3450	2.98E-06	5730	5.69E-06	5170	7.22E-06
6090	1.88E-05	4060	1.14E-06	3860	4.56E-06	3450	2.99E-06	5730	5.69E-06	5170	7.21E-06
6090	1.88E-05	4060	1.15E-06	3860	4.57E-06	3450	3.02E-06	5730	5.70E-06	5180	7.22E-06
6090	1.88E-05	4060	1.15E-06	3860	4.57E-06	3450	3.02E-06	5740	5.71E-06	5180	7.22E-06
6090	1.88E-05	4060	1.16E-06	3860	4.57E-06	3450	3.03E-06	5740	5.72E-06	5180	7.22E-06
6090	1.89E-05	4070	1.16E-06	3860	4.57E-06	3460	3.04E-06	5740	5.72E-06	5180	7.22E-06
6100	1.89E-05	4070	1.17E-06	3860	4.57E-06	3460	3.05E-06	5740	5.73E-06	5180	7.24E-06
6100	1.89E-05	4070	1.17E-06	3870	4.56E-06	3460	3.07E-06	5740	5.74E-06	5180	7.22E-06
6100	1.89E-05	4070	1.17E-06	3870	4.56E-06	3460	3.07E-06	5740	5.74E-06	5180	7.23E-06
6100	1.89E-05	4070	1.17E-06	3870	4.56E-06	3460	3.08E-06	5750	5.74E-06	5190	7.22E-06
6100	1.89E-05	4070	1.18E-06	3870	4.57E-06	3460	3.10E-06	5750	5.75E-06	5190	7.22E-06
6100	1.89E-05	4080	1.17E-06	3870	4.57E-06	3470	3.08E-06	5750	5.75E-06	5190	7.23E-06
6100	1.90E-05	4080	1.18E-06	3870	4.58E-06	3470	3.10E-06	5750	5.76E-06	5190	7.21E-06
6110	1.89E-05	4080	1.18E-06	3880	4.58E-06	3470	3.10E-06	5750	5.76E-06	5190	7.22E-06
6110	1.90E-05	4080	1.18E-06	3880	4.58E-06	3470	3.11E-06	5750	5.77E-06	5190	7.23E-06
6110	1.90E-05	4080	1.19E-06	3880	4.58E-06	3470	3.10E-06	5750	5.77E-06	5200	7.22E-06
6110	1.90E-05	4080	1.19E-06	3880	4.57E-06	3470	3.10E-06	5760	5.77E-06	5200	7.22E-06
6110	1.90E-05	4080	1.19E-06	3880	4.57E-06	3480	3.11E-06	5760	5.78E-06	5200	7.22E-06
6110	1.90E-05	4090	1.19E-06	3880	4.59E-06	3480	3.12E-06	5760	5.77E-06	5200	7.21E-06
6120	1.90E-05	4090	1.18E-06	3880	4.60E-06	3480	3.11E-06	5760	5.77E-06	5200	7.22E-06
6120	1.90E-05	4090	1.18E-06	3890	4.60E-06	3480	3.11E-06	5760	5.76E-06	5200	7.20E-06
6120	1.91E-05	4090	1.19E-06	3890	4.61E-06	3480	3.11E-06	5760	5.77E-06	5200	7.22E-06
6120	1.91E-05	4090	1.19E-06	3890	4.61E-06	3480	3.11E-06	5770	5.76E-06	5210	7.22E-06
6120	1.91E-05	4090	1.18E-06	3890	4.61E-06	3480	3.13E-06	5770	5.76E-06	5210	7.21E-06
6120	1.91E-05	4100	1.18E-06	3890	4.60E-06	3490	3.12E-06	5770	5.76E-06	5210	7.21E-06
6130	1.91E-05	4100	1.19E-06	3890	4.61E-06	3490	3.13E-06	5770	5.76E-06	5210	7.19E-06
6130	1.91E-05	4100	1.19E-06	3900	4.60E-06	3490	3.13E-06	5770	5.75E-06	5210	7.20E-06
6130	1.91E-05	4100	1.19E-06	3900	4.59E-06	3490	3.12E-06	5770	5.74E-06	5210	7.19E-06
6130	1.91E-05	4100	1.19E-06	3900	4.57E-06	3490	3.13E-06	5780	5.74E-06	5220	7.19E-06
6130	1.91E-05	4100	1.19E-06	3900	4.58E-06	3490	3.16E-06	5780	5.74E-06	5220	7.19E-06

6130 1.91E-05 4100 1.19E-06 3900 4.58E-06 3500
3.14E-06 5780 5.74E-06 5220 7.20E-06
6130 1.91E-05 4110 1.18E-06 3900 4.59E-06 3500
3.16E-06 5780 5.73E-06 5220 7.19E-06
6140 1.91E-05 4110 1.20E-06 3900 4.57E-06 3500
3.15E-06 5780 5.74E-06 5220 7.20E-06
6140 1.91E-05 4110 1.19E-06 3910 4.56E-06 3500
3.15E-06 5780 5.74E-06 5220 7.21E-06
6140 1.91E-05 4110 1.19E-06 3910 4.55E-06 3500
3.15E-06 5780 5.74E-06 5230 7.21E-06
6140 1.92E-05 4110 1.19E-06 3910 4.55E-06 3500
3.14E-06 5790 5.74E-06 5230 7.21E-06
6140 1.92E-05 4110 1.20E-06 3910 4.57E-06 3500
3.15E-06 5790 5.74E-06 5230 7.20E-06
6140 1.92E-05 4120 1.19E-06 3910 4.56E-06 3510
3.15E-06 5790 5.74E-06 5230 7.21E-06
6150 1.92E-05 4120 1.19E-06 3910 4.57E-06 3510
3.15E-06 5790 5.74E-06 5230 7.21E-06
6150 1.92E-05 4120 1.19E-06 3920 4.58E-06 3510
3.15E-06 5790 5.73E-06 5230 7.20E-06
6150 1.92E-05 4120 1.20E-06 3920 4.57E-06 3510
3.15E-06 5790 5.73E-06 5230 7.21E-06
6150 1.92E-05 4120 1.19E-06 3920 4.58E-06 3510
3.15E-06 5800 5.73E-06 5240 7.20E-06
6150 1.92E-05 4120 1.19E-06 3920 4.59E-06 3510
3.14E-06 5800 5.73E-06 5240 7.21E-06
6150 1.92E-05 4130 1.19E-06 3920 4.56E-06 3520
3.13E-06 5800 5.72E-06 5240 7.20E-06
6150 1.92E-05 4130 1.20E-06 3920 4.57E-06 3520
3.14E-06 5800 5.72E-06 5240 7.21E-06
6160 1.92E-05 4130 1.20E-06 3930 4.56E-06 3520
3.14E-06 5800 5.72E-06 5240 7.21E-06
6160 1.92E-05 4130 1.19E-06 3930 4.56E-06 3520
3.13E-06 5800 5.72E-06 5240 7.22E-06
6160 1.92E-05 4130 1.19E-06 3930 4.54E-06 3520
3.14E-06 5800 5.71E-06 5250 7.24E-06
6160 1.92E-05 4130 1.19E-06 3930 4.55E-06 3520
3.15E-06 5810 5.72E-06 5250 7.23E-06
6160 1.92E-05 4130 1.20E-06 3930 4.55E-06 3530
3.15E-06 5810 5.71E-06 5250 7.24E-06
6160 1.92E-05 4140 1.19E-06 3930 4.55E-06 3530
3.14E-06 5810 5.72E-06 5250 7.23E-06
6170 1.92E-05 4140 1.20E-06 3930 4.59E-06 3530
3.15E-06 5810 5.72E-06 5250 7.24E-06
6170 1.93E-05 4140 1.20E-06 3940 4.58E-06 3530
3.16E-06 5810 5.72E-06 5250 7.24E-06
6170 1.93E-05 4140 1.20E-06 3940 4.60E-06 3530
3.15E-06 5810 5.73E-06 5250 7.24E-06
6170 1.93E-05 4140 1.20E-06 3940 4.59E-06 3530
3.15E-06 5820 5.73E-06 5260 7.24E-06
6170 1.93E-05 4140 1.20E-06 3940 4.61E-06 3530
3.14E-06 5820 5.73E-06 5260 7.24E-06
6170 1.93E-05 4150 1.20E-06 3940 4.61E-06 3540
3.15E-06 5820 5.73E-06 5260 7.24E-06
6180 1.93E-05 4150 1.20E-06 3940 4.60E-06 3540
3.15E-06 5820 5.73E-06 5260 7.23E-06
6180 1.93E-05 4150 1.20E-06 3950 4.63E-06 3540
3.14E-06 5820 5.72E-06 5260 7.24E-06
6180 1.93E-05 4150 1.20E-06 3950 4.63E-06 3540
3.15E-06 5820 5.73E-06 5260 7.23E-06
6180 1.93E-05 4150 1.20E-06 3950 4.63E-06 3540
3.15E-06 5830 5.72E-06 5270 7.21E-06
6180 1.93E-05 4150 1.21E-06 3950 4.62E-06 3540
3.15E-06 5830 5.72E-06 5270 7.23E-06
6180 1.93E-05 4150 1.20E-06 3950 4.63E-06 3550
3.14E-06 5830 5.72E-06 5270 7.22E-06
6180 1.93E-05 4160 1.21E-06 3950 4.64E-06 3550
3.15E-06 5830 5.72E-06 5270 7.21E-06

6190 1.93E-05 4160 1.21E-06 3950 4.62E-06 3550
3.15E-06 5830 5.71E-06 5270 7.22E-06
6190 1.93E-05 4160 1.21E-06 3960 4.63E-06 3550
3.15E-06 5830 5.72E-06 5270 7.21E-06
6190 1.93E-05 4160 1.20E-06 3960 4.61E-06 3550
3.15E-06 5830 5.72E-06 5280 7.22E-06
6190 1.93E-05 4160 1.21E-06 3960 4.62E-06 3550
3.16E-06 5840 5.71E-06 5280 7.21E-06
6190 1.93E-05 4160 1.20E-06 3960 4.64E-06 3550
3.17E-06 5840 5.71E-06 5280 7.21E-06
6190 1.93E-05 4170 1.20E-06 3960 4.63E-06 3560
3.16E-06 5840 5.71E-06 5280 7.21E-06
6200 1.93E-05 4170 1.21E-06 3960 4.62E-06 3560
3.17E-06 5840 5.72E-06 5280 7.22E-06
6200 1.93E-05 4170 1.20E-06 3970 4.63E-06 3560
3.18E-06 5840 5.72E-06 5280 7.24E-06
6200 1.93E-05 4170 1.21E-06 3970 4.64E-06 3560
3.17E-06 5840 5.72E-06 5280 7.24E-06
6200 1.93E-05 4170 1.21E-06 3970 4.63E-06 3560
3.17E-06 5850 5.72E-06 5290 7.23E-06
6200 1.93E-05 4170 1.20E-06 3970 4.65E-06 3560
3.17E-06 5850 5.72E-06 5290 7.25E-06
6200 1.93E-05 4180 1.21E-06 3970 4.66E-06 3570
3.19E-06 5850 5.72E-06 5290 7.22E-06
6200 1.94E-05 4180 1.21E-06 3970 4.65E-06 3570
3.18E-06 5850 5.72E-06 5290 7.21E-06
6210 1.94E-05 4180 1.21E-06 3980 4.67E-06 3570
3.18E-06 5850 5.71E-06 5290 7.22E-06
6210 1.94E-05 4180 1.21E-06 3980 4.65E-06 3570
3.19E-06 5850 5.72E-06 5290 7.23E-06
6210 1.94E-05 4180 1.21E-06 3980 4.65E-06 3570
3.18E-06 5850 5.71E-06 5300 7.21E-06
6210 1.94E-05 4180 1.21E-06 3980 4.67E-06 3570
3.18E-06 5860 5.71E-06 5300 7.22E-06
6210 1.94E-05 4180 1.21E-06 3980 4.66E-06 3580
3.18E-06 5860 5.70E-06 5300 7.22E-06
6210 1.94E-05 4190 1.21E-06 3980 4.66E-06 3580
3.18E-06 5860 5.71E-06 5300 7.22E-06
6220 1.94E-05 4190 1.21E-06 3980 4.66E-06 3580
3.18E-06 5860 5.70E-06 5300 7.22E-06
6220 1.94E-05 4190 1.21E-06 3990 4.67E-06 3580
3.17E-06 5860 5.70E-06 5300 7.23E-06
6220 1.94E-05 4190 1.21E-06 3990 4.65E-06 3580
3.18E-06 5860 5.69E-06 5300 7.23E-06
6220 1.94E-05 4190 1.21E-06 3990 4.66E-06 3580
3.17E-06 5870 5.69E-06 5310 7.22E-06
6220 1.94E-05 4190 1.21E-06 3990 4.68E-06 3580
3.16E-06 5870 5.69E-06 5310 7.24E-06
6220 1.94E-05 4200 1.21E-06 3990 4.68E-06 3590
3.16E-06 5870 5.69E-06 5310 7.24E-06
6230 1.94E-05 4200 1.21E-06 3990 4.69E-06 3590
3.17E-06 5870 5.69E-06 5310 7.23E-06
6230 1.95E-05 4200 1.21E-06 4000 4.68E-06 3590
3.17E-06 5870 5.70E-06 5310 7.23E-06
6230 1.95E-05 4200 1.21E-06 4000 4.69E-06 3590
3.16E-06 5870 5.69E-06 5310 7.25E-06
6230 1.95E-05 4200 1.21E-06 4000 4.69E-06 3590
3.18E-06 5880 5.70E-06 5320 7.24E-06
6230 1.95E-05 4200 1.21E-06 4000 4.70E-06 3590
3.17E-06 5880 5.70E-06 5320 7.24E-06
6230 1.95E-05 4200 1.22E-06 4000 4.70E-06 3600
3.18E-06 5880 5.70E-06 5320 7.24E-06
6230 1.95E-05 4210 1.21E-06 4000 4.71E-06 3600
3.18E-06 5880 5.70E-06 5320 7.22E-06
6240 1.95E-05 4210 1.22E-06 4000 4.71E-06 3600
3.19E-06 5880 5.69E-06 5320 7.22E-06
6240 1.95E-05 4210 1.22E-06 4010 4.71E-06 3600
3.19E-06 5880 5.70E-06 5320 7.21E-06

6240 1.95E-05 4210 1.22E-06 4010 4.70E-06 3600
3.19E-06 5880 5.70E-06 5330 7.22E-06
6240 1.95E-05 4210 1.21E-06 4010 4.71E-06 3600
3.20E-06 5890 5.70E-06 5330 7.22E-06
6240 1.95E-05 4210 1.22E-06 4010 4.72E-06 3600
3.20E-06 5890 5.70E-06 5330 7.22E-06
6240 1.95E-05 4220 1.22E-06 4010 4.73E-06 3610
3.21E-06 5890 5.70E-06 5330 7.21E-06
6250 1.95E-05 4220 1.22E-06 4010 4.70E-06 3610
3.21E-06 5890 5.70E-06 5330 7.21E-06
6250 1.95E-05 4220 1.22E-06 4020 4.71E-06 3610
3.20E-06 5890 5.70E-06 5330 7.21E-06
6250 1.95E-05 4220 1.22E-06 4020 4.70E-06 3610
3.21E-06 5890 5.70E-06 5330 7.19E-06
6250 1.95E-05 4220 1.21E-06 4020 4.72E-06 3610
3.21E-06 5900 5.71E-06 5340 7.21E-06
6250 1.95E-05 4220 1.22E-06 4020 4.71E-06 3610
3.21E-06 5900 5.71E-06 5340 7.22E-06
6250 1.95E-05 4230 1.21E-06 4020 4.71E-06 3620
3.20E-06 5900 5.71E-06 5340 7.21E-06
6250 1.95E-05 4230 1.22E-06 4020 4.70E-06 3620
3.21E-06 5900 5.72E-06 5340 7.21E-06
6260 1.95E-05 4230 1.21E-06 4030 4.71E-06 3620
3.21E-06 5900 5.72E-06 5340 7.21E-06
6260 1.95E-05 4230 1.21E-06 4030 4.71E-06 3620
3.20E-06 5900 5.72E-06 5340 7.21E-06
6260 1.96E-05 4230 1.22E-06 4030 4.71E-06 3620
3.20E-06 5900 5.72E-06 5350 7.21E-06
6260 1.95E-05 4230 1.22E-06 4030 4.72E-06 3620
3.20E-06 5910 5.73E-06 5350 7.21E-06
6260 1.95E-05 4230 1.22E-06 4030 4.72E-06 3630
3.20E-06 5910 5.73E-06 5350 7.21E-06
6260 1.96E-05 4240 1.21E-06 4030 4.73E-06 3630
3.19E-06 5910 5.73E-06 5350 7.22E-06
6270 1.95E-05 4240 1.22E-06 4030 4.75E-06 3630
3.20E-06 5910 5.72E-06 5350 7.22E-06
6270 1.95E-05 4240 1.22E-06 4040 4.73E-06 3630
3.20E-06 5910 5.72E-06 5350 7.20E-06
6270 1.95E-05 4240 1.22E-06 4040 4.74E-06 3630
3.19E-06 5910 5.73E-06 5350 7.22E-06
6270 1.96E-05 4240 1.22E-06 4040 4.73E-06 3630
3.19E-06 5920 5.73E-06 5360 7.21E-06
6270 1.96E-05 4240 1.22E-06 4040 4.73E-06 3630
3.20E-06 5920 5.73E-06 5360 7.23E-06
6270 1.95E-05 4250 1.22E-06 4040 4.74E-06 3640
3.20E-06 5920 5.73E-06 5360 7.22E-06
6280 1.96E-05 4250 1.22E-06 4040 4.73E-06 3640
3.20E-06 5920 5.73E-06 5360 7.23E-06
6280 1.96E-05 4250 1.22E-06 4050 4.72E-06 3640
3.20E-06 5920 5.73E-06 5360 7.22E-06
6280 1.96E-05 4250 1.22E-06 4050 4.71E-06 3640
3.19E-06 5920 5.72E-06 5360 7.23E-06
6280 1.96E-05 4250 1.22E-06 4050 4.72E-06 3640
3.19E-06 5930 5.72E-06 5370 7.23E-06
6280 1.96E-05 4250 1.22E-06 4050 4.73E-06 3640
3.19E-06 5930 5.72E-06 5370 7.25E-06
6280 1.96E-05 4250 1.21E-06 4050 4.72E-06 3650
3.19E-06 5930 5.72E-06 5370 7.24E-06
6280 1.96E-05 4260 1.22E-06 4050 4.73E-06 3650
3.18E-06 5930 5.72E-06 5370 7.24E-06
6290 1.96E-05 4260 1.22E-06 4050 4.73E-06 3650
3.19E-06 5930 5.73E-06 5370 7.25E-06
6290 1.97E-05 4260 1.22E-06 4060 4.74E-06 3650
3.18E-06 5930 5.73E-06 5370 7.27E-06
6290 1.97E-05 4260 1.22E-06 4060 4.73E-06 3650
3.19E-06 5930 5.74E-06 5380 7.25E-06
6290 1.97E-05 4260 1.22E-06 4060 4.74E-06 3650
3.19E-06 5940 5.74E-06 5380 7.26E-06

6290 1.97E-05 4260 1.23E-06 4060 4.74E-06 3650
3.19E-06 5940 5.75E-06 5380 7.25E-06
6290 1.97E-05 4270 1.22E-06 4060 4.75E-06 3660
3.19E-06 5940 5.74E-06 5380 7.27E-06
6300 1.96E-05 4270 1.23E-06 4060 4.76E-06 3660
3.18E-06 5940 5.73E-06 5380 7.25E-06
6300 1.97E-05 4270 1.23E-06 4070 4.75E-06 3660
3.18E-06 5940 5.73E-06 5380 7.25E-06
6300 1.97E-05 4270 1.23E-06 4070 4.76E-06 3660
3.17E-06 5940 5.73E-06 5380 7.25E-06
6300 1.97E-05 4270 1.23E-06 4070 4.76E-06 3660
3.17E-06 5950 5.73E-06 5390 7.27E-06
6300 1.97E-05 4270 1.23E-06 4070 4.77E-06 3660
3.17E-06 5950 5.73E-06 5390 7.26E-06
6300 1.97E-05 4280 1.23E-06 4070 4.77E-06 3670
3.17E-06 5950 5.73E-06 5390 7.25E-06
6300 1.97E-05 4280 1.23E-06 4070 4.76E-06 3670
3.17E-06 5950 5.74E-06 5390 7.26E-06
6310 1.97E-05 4280 1.23E-06 4080 4.76E-06 3670
3.16E-06 5950 5.73E-06 5390 7.25E-06
6310 1.97E-05 4280 1.23E-06 4080 4.75E-06 3670
3.17E-06 5950 5.73E-06 5390 7.26E-06
6310 1.96E-05 4280 1.22E-06 4080 4.77E-06 3670
3.17E-06 5950 5.73E-06 5400 7.25E-06
6310 1.97E-05 4280 1.23E-06 4080 4.77E-06 3670
3.18E-06 5960 5.73E-06 5400 7.27E-06
6310 1.97E-05 4280 1.22E-06 4080 4.78E-06 3680
3.17E-06 5960 5.73E-06 5400 7.27E-06
6310 1.97E-05 4290 1.23E-06 4080 4.78E-06 3680
3.18E-06 5960 5.74E-06 5400 7.26E-06
6320 1.97E-05 4290 1.23E-06 4080 4.77E-06 3680
3.19E-06 5960 5.74E-06 5400 7.27E-06
6320 1.97E-05 4290 1.23E-06 4090 4.77E-06 3680
3.18E-06 5960 5.74E-06 5400 7.29E-06
6320 1.97E-05 4290 1.23E-06 4090 4.77E-06 3680
3.19E-06 5960 5.74E-06 5400 7.28E-06
6320 1.97E-05 4290 1.23E-06 4090 4.77E-06 3680
3.18E-06 5970 5.74E-06 5410 7.28E-06
6320 1.97E-05 4290 1.24E-06 4090 4.78E-06 3680
3.19E-06 5970 5.74E-06 5410 7.27E-06
6320 1.97E-05 4300 1.23E-06 4090 4.79E-06 3690
3.20E-06 5970 5.73E-06 5410 7.28E-06
6330 1.97E-05 4300 1.23E-06 4090 4.80E-06 3690
3.20E-06 5970 5.72E-06 5410 7.28E-06
6330 1.97E-05 4300 1.23E-06 4100 4.79E-06 3690
3.19E-06 5970 5.73E-06 5410 7.28E-06
6330 1.97E-05 4300 1.23E-06 4100 4.80E-06 3690
3.19E-06 5970 5.72E-06 5410 7.29E-06
6330 1.97E-05 4300 1.24E-06 4100 4.79E-06 3690
3.18E-06 5980 5.73E-06 5420 7.31E-06
6330 1.97E-05 4300 1.23E-06 4100 4.80E-06 3690
3.18E-06 5980 5.72E-06 5420 7.29E-06
6330 1.97E-05 4300 1.23E-06 4100 4.80E-06 3700
3.18E-06 5980 5.73E-06 5420 7.30E-06
6330 1.97E-05 4310 1.23E-06 4100 4.78E-06 3700
3.19E-06 5980 5.73E-06 5420 7.30E-06
6340 1.97E-05 4310 1.23E-06 4100 4.79E-06 3700
3.17E-06 5980 5.72E-06 5420 7.29E-06
6340 1.97E-05 4310 1.23E-06 4110 4.79E-06 3700
3.18E-06 5980 5.72E-06 5420 7.30E-06
6340 1.97E-05 4310 1.23E-06 4110 4.80E-06 3700
3.17E-06 5980 5.72E-06 5430 7.30E-06
6340 1.97E-05 4310 1.23E-06 4110 4.81E-06 3700
3.18E-06 5990 5.72E-06 5430 7.31E-06
6340 1.97E-05 4310 1.23E-06 4110 4.81E-06 3700
3.17E-06 5990 5.71E-06 5430 7.30E-06
6340 1.97E-05 4320 1.23E-06 4110 4.81E-06 3710
3.17E-06 5990 5.72E-06 5430 7.30E-06

6350	1.98E-05	4320	1.23E-06	4110	4.81E-06	3710
3.18E-06	5990	5.72E-06	5430	7.31E-06		
6350	1.98E-05	4320	1.23E-06	4120	4.80E-06	3710
3.17E-06	5990	5.72E-06	5430	7.33E-06		
6350	1.98E-05	4320	1.23E-06	4120	4.81E-06	3710
3.17E-06	5990	5.72E-06	5430	7.31E-06		
6350	1.98E-05	4320	1.24E-06	4120	4.82E-06	3710
3.17E-06	6000	5.73E-06	5440	7.33E-06		
6350	1.98E-05	4320	1.24E-06	4120	4.82E-06	3710
3.17E-06	6000	5.73E-06	5440	7.32E-06		
6350	1.98E-05	4330	1.24E-06	4120	4.83E-06	3720
3.18E-06	6000	5.73E-06	5440	7.33E-06		
6350	1.98E-05	4330	1.23E-06	4120	4.84E-06	3720
3.17E-06	6000	5.72E-06	5440	7.32E-06		
6360	1.98E-05	4330	1.24E-06	4130	4.84E-06	3720
3.18E-06	6000	5.72E-06	5440	7.32E-06		
6360	1.98E-05	4330	1.24E-06	4130	4.85E-06	3720
3.18E-06	6000	5.72E-06	5440	7.32E-06		
6360	1.98E-05	4330	1.25E-06	4130	4.84E-06	3720
3.19E-06	6000	5.71E-06	5450	7.33E-06		
6360	1.98E-05	4330	1.25E-06	4130	4.86E-06	3720
3.19E-06	6010	5.71E-06	5450	7.34E-06		
6360	1.98E-05	4330	1.25E-06	4130	4.85E-06	3730
3.18E-06	6010	5.72E-06	5450	7.33E-06		
6360	1.98E-05	4340	1.25E-06	4130	4.83E-06	3730
3.19E-06	6010	5.72E-06	5450	7.33E-06		
6370	1.98E-05	4340	1.25E-06	4130	4.84E-06	3730
3.17E-06	6010	5.72E-06	5450	7.34E-06		
6370	1.98E-05	4340	1.24E-06	4140	4.84E-06	3730
3.19E-06	6010	5.72E-06	5450	7.34E-06		
6370	1.98E-05	4340	1.25E-06	4140	4.84E-06	3730
3.19E-06	6010	5.72E-06	5450	7.35E-06		
6370	1.98E-05	4340	1.24E-06	4140	4.85E-06	3730
3.19E-06	6020	5.71E-06	5460	7.36E-06		
6370	1.98E-05	4340	1.25E-06	4140	4.85E-06	3730
3.19E-06	6020	5.71E-06	5460	7.36E-06		
6370	1.98E-05	4350	1.25E-06	4140	4.85E-06	3740
3.18E-06	6020	5.72E-06	5460	7.36E-06		
6380	1.99E-05	4350	1.25E-06	4140	4.85E-06	3740
3.18E-06	6020	5.71E-06	5460	7.37E-06		
6380	1.99E-05	4350	1.24E-06	4150	4.85E-06	3740
3.17E-06	6020	5.72E-06	5460	7.39E-06		
6380	1.99E-05	4350	1.24E-06	4150	4.86E-06	3740
3.17E-06	6020	5.71E-06	5460	7.38E-06		
6380	1.99E-05	4350	1.24E-06	4150	4.85E-06	3740
3.17E-06	6030	5.72E-06	5470	7.38E-06		
6380	1.99E-05	4350	1.24E-06	4150	4.85E-06	3740
3.19E-06	6030	5.72E-06	5470	7.38E-06		
6380	1.99E-05	4350	1.23E-06	4150	4.88E-06	3750
3.19E-06	6030	5.72E-06	5470	7.37E-06		
6380	1.99E-05	4360	1.23E-06	4150	4.87E-06	3750
3.19E-06	6030	5.71E-06	5470	7.36E-06		
6390	1.99E-05	4360	1.24E-06	4150	4.87E-06	3750
3.20E-06	6030	5.72E-06	5470	7.38E-06		
6390	1.99E-05	4360	1.24E-06	4160	4.87E-06	3750
3.19E-06	6030	5.71E-06	5470	7.38E-06		
6390	1.99E-05	4360	1.25E-06	4160	4.87E-06	3750
3.19E-06	6030	5.71E-06	5480	7.39E-06		
6390	1.99E-05	4360	1.24E-06	4160	4.87E-06	3750
3.18E-06	6040	5.71E-06	5480	7.39E-06		
6390	1.99E-05	4360	1.25E-06	4160	4.87E-06	3750
3.19E-06	6040	5.71E-06	5480	7.39E-06		
6390	1.99E-05	4370	1.24E-06	4160	4.85E-06	3760
3.19E-06	6040	5.72E-06	5480	7.40E-06		
6400	1.99E-05	4370	1.25E-06	4160	4.85E-06	3760
3.18E-06	6040	5.71E-06	5480	7.39E-06		
6400	2.00E-05	4370	1.24E-06	4170	4.86E-06	3760
3.19E-06	6040	5.71E-06	5480	7.40E-06		

6400	1.99E-05	4370	1.24E-06	4170	4.85E-06	3760
3.19E-06	6040	5.71E-06	5480	7.40E-06		
6400	2.00E-05	4370	1.24E-06	4170	4.86E-06	3760
3.21E-06	6050	5.71E-06	5490	7.41E-06		
6400	2.00E-05	4370	1.23E-06	4170	4.86E-06	3760
3.19E-06	6050	5.71E-06	5490	7.41E-06		
6400	2.00E-05	4380	1.24E-06	4170	4.86E-06	3770
3.20E-06	6050	5.71E-06	5490	7.42E-06		
6400	2.00E-05	4380	1.23E-06	4170	4.85E-06	3770
3.21E-06	6050	5.71E-06	5490	7.41E-06		
6410	2.00E-05	4380	1.23E-06	4180	4.86E-06	3770
3.20E-06	6050	5.71E-06	5490	7.42E-06		
6410	2.00E-05	4380	1.24E-06	4180	4.86E-06	3770
3.19E-06	6050	5.71E-06	5490	7.42E-06		
6410	2.00E-05	4380	1.24E-06	4180	4.85E-06	3770
3.20E-06	6050	5.71E-06	5500	7.42E-06		
6410	2.00E-05	4380	1.25E-06	4180	4.85E-06	3770
3.21E-06	6060	5.72E-06	5500	7.42E-06		
6410	2.01E-05	4380	1.24E-06	4180	4.87E-06	3780
3.21E-06	6060	5.71E-06	5500	7.42E-06		
6410	2.00E-05	4390	1.24E-06	4180	4.86E-06	3780
3.21E-06	6060	5.71E-06	5500	7.41E-06		
6420	2.00E-05	4390	1.24E-06	4180	4.87E-06	3780
3.21E-06	6060	5.71E-06	5500	7.42E-06		
6420	2.00E-05	4390	1.25E-06	4190	4.87E-06	3780
3.21E-06	6060	5.71E-06	5500	7.43E-06		
6420	2.01E-05	4390	1.25E-06	4190	4.87E-06	3780
3.20E-06	6060	5.71E-06	5500	7.44E-06		
6420	2.01E-05	4390	1.25E-06	4190	4.87E-06	3780
3.19E-06	6070	5.71E-06	5510	7.44E-06		
6420	2.00E-05	4390	1.25E-06	4190	4.87E-06	3780
3.20E-06	6070	5.71E-06	5510	7.45E-06		
6420	2.01E-05	4400	1.25E-06	4190	4.85E-06	3790
3.20E-06	6070	5.71E-06	5510	7.44E-06		
6430	2.01E-05	4400	1.25E-06	4190	4.86E-06	3790
3.19E-06	6070	5.71E-06	5510	7.45E-06		
6430	2.01E-05	4400	1.25E-06	4200	4.86E-06	3790
3.20E-06	6070	5.70E-06	5510	7.44E-06		
6430	2.01E-05	4400	1.24E-06	4200	4.86E-06	3790
3.21E-06	6070	5.71E-06	5510	7.46E-06		
6430	2.01E-05	4400	1.24E-06	4200	4.86E-06	3790
3.22E-06	6080	5.71E-06	5520	7.46E-06		
6430	2.01E-05	4400	1.24E-06	4200	4.87E-06	3790
3.22E-06	6080	5.71E-06	5520	7.46E-06		
6430	2.01E-05	4400	1.24E-06	4200	4.87E-06	3800
3.21E-06	6080	5.72E-06	5520	7.47E-06		
6430	2.01E-05	4410	1.23E-06	4200	4.86E-06	3800
3.21E-06	6080	5.72E-06	5520	7.46E-06		
6440	2.01E-05	4410	1.24E-06	4200	4.86E-06	3800
3.21E-06	6080	5.72E-06	5520	7.48E-06		
6440	2.02E-05	4410	1.24E-06	4210	4.87E-06	3800
3.21E-06	6080	5.72E-06	5520	7.46E-06		
6440	2.02E-05	4410	1.24E-06	4210	4.87E-06	3800
3.21E-06	6080	5.72E-06	5530	7.48E-06		
6440	2.02E-05	4410	1.24E-06	4210	4.88E-06	3800
3.22E-06	6090	5.72E-06	5530	7.47E-06		
6440	2.02E-05	4410	1.24E-06	4210	4.88E-06	3800
3.23E-06	6090	5.71E-06	5530	7.46E-06		
6440	2.02E-05	4420	1.24E-06	4210	4.89E-06	3810
3.22E-06	6090	5.72E-06	5530	7.47E-06		
6450	2.02E-05	4420	1.24E-06	4210	4.90E-06	3810
3.23E-06	6090	5.72E-06	5530	7.45E-06		
6450	2.02E-05	4420	1.24E-06	4220	4.90E-06	3810
3.22E-06	6090	5.72E-06	5530	7.47E-06		
6450	2.02E-05	4420	1.25E-06	4220	4.90E-06	3810
3.22E-06	6090	5.72E-06	5530	7.47E-06		
6450	2.02E-05	4420	1.25E-06	4220	4.89E-06	3810
3.22E-06	6100	5.72E-06	5540	7.46E-06		

6450 2.02E-05 4420 1.25E-06 4220 4.89E-06 3810
3.22E-06 6100 5.71E-06 5540 7.46E-06
6450 2.02E-05 4430 1.25E-06 4220 4.88E-06 3820
3.21E-06 6100 5.72E-06 5540 7.45E-06
6450 2.02E-05 4430 1.25E-06 4220 4.88E-06 3820
3.22E-06 6100 5.71E-06 5540 7.45E-06
6460 2.02E-05 4430 1.24E-06 4230 4.88E-06 3820
3.22E-06 6100 5.71E-06 5540 7.45E-06
6460 2.02E-05 4430 1.24E-06 4230 4.88E-06 3820
3.22E-06 6100 5.71E-06 5540 7.43E-06
6460 2.02E-05 4430 1.23E-06 4230 4.88E-06 3820
3.24E-06 6100 5.71E-06 5550 7.47E-06
6460 2.02E-05 4430 1.23E-06 4230 4.88E-06 3820
3.24E-06 6110 5.71E-06 5550 7.44E-06
6460 2.02E-05 4430 1.23E-06 4230 4.87E-06 3830
3.24E-06 6110 5.71E-06 5550 7.44E-06
6460 2.02E-05 4440 1.23E-06 4230 4.87E-06 3830
3.24E-06 6110 5.72E-06 5550 7.44E-06
6470 2.03E-05 4440 1.23E-06 4230 4.88E-06 3830
3.23E-06 6110 5.72E-06 5550 7.47E-06
6470 2.03E-05 4440 1.23E-06 4240 4.87E-06 3830
3.24E-06 6110 5.72E-06 5550 7.45E-06
6470 2.03E-05 4440 1.24E-06 4240 4.87E-06 3830
3.24E-06 6110 5.72E-06 5550 7.45E-06
6470 2.03E-05 4440 1.24E-06 4240 4.87E-06 3830
3.24E-06 6120 5.73E-06 5560 7.46E-06
6470 2.03E-05 4440 1.24E-06 4240 4.88E-06 3830
3.25E-06 6120 5.73E-06 5560 7.46E-06
6470 2.03E-05 4450 1.24E-06 4240 4.89E-06 3840
3.25E-06 6120 5.71E-06 5560 7.43E-06
6480 2.03E-05 4450 1.25E-06 4240 4.89E-06 3840
3.24E-06 6120 5.72E-06 5560 7.44E-06
6480 2.03E-05 4450 1.24E-06 4250 4.89E-06 3840
3.23E-06 6120 5.71E-06 5560 7.46E-06
6480 2.03E-05 4450 1.25E-06 4250 4.89E-06 3840
3.23E-06 6120 5.72E-06 5560 7.46E-06
6480 2.03E-05 4450 1.25E-06 4250 4.90E-06 3840
3.23E-06 6130 5.71E-06 5570 7.47E-06
6480 2.03E-05 4450 1.25E-06 4250 4.89E-06 3840
3.23E-06 6130 5.71E-06 5570 7.47E-06
6480 2.03E-05 4450 1.24E-06 4250 4.88E-06 3850
3.23E-06 6130 5.72E-06 5570 7.48E-06
6480 2.03E-05 4460 1.24E-06 4250 4.88E-06 3850
3.23E-06 6130 5.72E-06 5570 7.47E-06
6490 2.03E-05 4460 1.24E-06 4250 4.88E-06 3850
3.24E-06 6130 5.71E-06 5570 7.47E-06
6490 2.04E-05 4460 1.25E-06 4260 4.90E-06 3850
3.24E-06 6130 5.71E-06 5570 7.48E-06
6490 2.04E-05 4460 1.24E-06 4260 4.89E-06 3850
3.26E-06 6130 5.71E-06 5580 7.47E-06
6490 2.04E-05 4460 1.24E-06 4260 4.89E-06 3850
3.26E-06 6140 5.71E-06 5580 7.49E-06
6490 2.04E-05 4460 1.25E-06 4260 4.90E-06 3850
3.26E-06 6140 5.71E-06 5580 7.49E-06
6490 2.04E-05 4470 1.25E-06 4260 4.89E-06 3860
3.27E-06 6140 5.72E-06 5580 7.49E-06
6500 2.04E-05 4470 1.24E-06 4260 4.89E-06 3860
3.27E-06 6140 5.72E-06 5580 7.49E-06
6500 2.04E-05 4470 1.23E-06 4270 4.90E-06 3860
3.27E-06 6140 5.73E-06 5580 7.50E-06
6500 2.04E-05 4470 1.25E-06 4270 4.90E-06 3860
3.26E-06 6140 5.73E-06 5580 7.49E-06
6500 2.04E-05 4470 1.25E-06 4270 4.90E-06 3860
3.27E-06 6150 5.74E-06 5590 7.49E-06
6500 2.04E-05 4470 1.24E-06 4270 4.90E-06 3860
3.27E-06 6150 5.73E-06 5590 7.48E-06
6500 2.05E-05 4480 1.24E-06 4270 4.91E-06 3870
3.27E-06 6150 5.74E-06 5590 7.48E-06

6500 2.04E-05 4480 1.25E-06 4270 4.92E-06 3870
3.26E-06 6150 5.74E-06 5590 7.48E-06
6510 2.05E-05 4480 1.25E-06 4280 4.92E-06 3870
3.26E-06 6150 5.74E-06 5590 7.48E-06
6510 2.04E-05 4480 1.26E-06 4280 4.92E-06 3870
3.26E-06 6150 5.73E-06 5590 7.48E-06
6510 2.04E-05 4480 1.25E-06 4280 4.93E-06 3870
3.25E-06 6150 5.74E-06 5600 7.49E-06
6510 2.04E-05 4480 1.25E-06 4280 4.92E-06 3870
3.26E-06 6160 5.74E-06 5600 7.47E-06
6510 2.04E-05 4480 1.25E-06 4280 4.91E-06 3880
3.26E-06 6160 5.76E-06 5600 7.49E-06
6510 2.05E-05 4490 1.26E-06 4280 4.91E-06 3880
3.28E-06 6160 5.75E-06 5600 7.48E-06
6520 2.05E-05 4490 1.25E-06 4280 4.91E-06 3880
3.27E-06 6160 5.75E-06 5600 7.48E-06
6520 2.04E-05 4490 1.25E-06 4290 4.91E-06 3880
3.28E-06 6160 5.75E-06 5600 7.47E-06
6520 2.04E-05 4490 1.25E-06 4290 4.91E-06 3880
3.28E-06 6160 5.75E-06 5600 7.49E-06
6520 2.05E-05 4490 1.24E-06 4290 4.91E-06 3880
3.29E-06 6170 5.75E-06 5610 7.47E-06
6520 2.04E-05 4490 1.25E-06 4290 4.91E-06 3880
3.29E-06 6170 5.75E-06 5610 7.48E-06
6520 2.04E-05 4500 1.25E-06 4290 4.90E-06 3890
3.30E-06 6170 5.76E-06 5610 7.47E-06
6530 2.05E-05 4500 1.25E-06 4290 4.91E-06 3890
3.29E-06 6170 5.75E-06 5610 7.48E-06
6530 2.05E-05 4500 1.25E-06 4300 4.90E-06 3890
3.29E-06 6170 5.76E-06 5610 7.49E-06
6530 2.05E-05 4500 1.25E-06 4300 4.90E-06 3890
3.29E-06 6170 5.75E-06 5610 7.50E-06
6530 2.05E-05 4500 1.26E-06 4300 4.90E-06 3890
3.30E-06 6180 5.76E-06 5620 7.48E-06
6530 2.05E-05 4500 1.25E-06 4300 4.90E-06 3890
3.30E-06 6180 5.76E-06 5620 7.49E-06
6530 2.05E-05 4500 1.25E-06 4300 4.91E-06 3900
3.29E-06 6180 5.75E-06 5620 7.49E-06
6530 2.05E-05 4510 1.25E-06 4300 4.90E-06 3900
3.30E-06 6180 5.75E-06 5620 7.48E-06
6540 2.05E-05 4510 1.25E-06 4300 4.91E-06 3900
3.29E-06 6180 5.75E-06 5620 7.50E-06
6540 2.05E-05 4510 1.26E-06 4310 4.91E-06 3900
3.28E-06 6180 5.75E-06 5620 7.51E-06
6540 2.05E-05 4510 1.26E-06 4310 4.91E-06 3900
3.30E-06 6180 5.75E-06 5630 7.51E-06
6540 2.05E-05 4510 1.26E-06 4310 4.91E-06 3900
3.29E-06 6190 5.75E-06 5630 7.50E-06
6540 2.06E-05 4510 1.26E-06 4310 4.89E-06 3900
3.30E-06 6190 5.76E-06 5630 7.52E-06
6540 2.06E-05 4520 1.27E-06 4310 4.90E-06 3910
3.30E-06 6190 5.75E-06 5630 7.52E-06
6550 2.06E-05 4520 1.26E-06 4310 4.89E-06 3910
3.31E-06 6190 5.74E-06 5630 7.51E-06
6550 2.06E-05 4520 1.26E-06 4320 4.91E-06 3910
3.31E-06 6190 5.75E-06 5630 7.52E-06
6550 2.06E-05 4520 1.26E-06 4320 4.91E-06 3910
3.31E-06 6190 5.74E-06 5630 7.53E-06
6550 2.07E-05 4520 1.25E-06 4320 4.92E-06 3910
3.32E-06 6200 5.74E-06 5640 7.52E-06
6550 2.06E-05 4520 1.26E-06 4320 4.91E-06 3910
3.33E-06 6200 5.74E-06 5640 7.52E-06
6550 2.07E-05 4530 1.25E-06 4320 4.92E-06 3920
3.32E-06 6200 5.75E-06 5640 7.53E-06
6550 2.07E-05 4530 1.26E-06 4320 4.92E-06 3920
3.32E-06 6200 5.75E-06 5640 7.54E-06
6560 2.08E-05 4530 1.25E-06 4330 4.93E-06 3920
3.33E-06 6200 5.75E-06 5640 7.53E-06

6560	2.07E-05	4530	1.25E-06	4330	4.92E-06	3920	3.32E-06	6200	5.75E-06	5640	7.53E-06
6560	2.07E-05	4530	1.26E-06	4330	4.92E-06	3920	3.32E-06	6200	5.75E-06	5650	7.54E-06
6560	2.08E-05	4530	1.26E-06	4330	4.92E-06	3920	3.33E-06	6210	5.75E-06	5650	7.53E-06
6560	2.07E-05	4530	1.26E-06	4330	4.94E-06	3930	3.32E-06	6210	5.74E-06	5650	7.54E-06
6560	2.07E-05	4540	1.26E-06	4330	4.92E-06	3930	3.31E-06	6210	5.75E-06	5650	7.53E-06
6570	2.07E-05	4540	1.26E-06	4330	4.93E-06	3930	3.31E-06	6210	5.74E-06	5650	7.54E-06
6570	2.07E-05	4540	1.27E-06	4340	4.93E-06	3930	3.31E-06	6210	5.74E-06	5650	7.54E-06
6570	2.08E-05	4540	1.27E-06	4340	4.94E-06	3930	3.33E-06	6210	5.74E-06	5650	7.54E-06
6570	2.07E-05	4540	1.27E-06	4340	4.94E-06	3930	3.32E-06	6220	5.74E-06	5660	7.54E-06
6570	2.08E-05	4540	1.27E-06	4340	4.93E-06	3930	3.33E-06	6220	5.75E-06	5660	7.53E-06
6570	2.07E-05	4550	1.27E-06	4340	4.93E-06	3940	3.35E-06	6220	5.75E-06	5660	7.53E-06
6580	2.08E-05	4550	1.26E-06	4340	4.93E-06	3940	3.34E-06	6220	5.76E-06	5660	7.54E-06
6580	2.07E-05	4550	1.26E-06	4350	4.93E-06	3940	3.35E-06	6220	5.76E-06	5660	7.54E-06
6580	2.08E-05	4550	1.26E-06	4350	4.93E-06	3940	3.36E-06	6220	5.76E-06	5660	7.55E-06
6580	2.08E-05	4550	1.26E-06	4350	4.93E-06	3940	3.36E-06	6230	5.75E-06	5670	7.55E-06
6580	2.08E-05	4550	1.25E-06	4350	4.93E-06	3940	3.38E-06	6230	5.76E-06	5670	7.54E-06
6580	2.08E-05	4550	1.25E-06	4350	4.93E-06	3950	3.39E-06	6230	5.76E-06	5670	7.56E-06
6580	2.08E-05	4560	1.25E-06	4350	4.93E-06	3950	3.38E-06	6230	5.76E-06	5670	7.56E-06
6590	2.08E-05	4560	1.26E-06	4350	4.94E-06	3950	3.40E-06	6230	5.77E-06	5670	7.56E-06
6590	2.08E-05	4560	1.25E-06	4360	4.93E-06	3950	3.39E-06	6230	5.77E-06	5670	7.57E-06
6590	2.08E-05	4560	1.26E-06	4360	4.92E-06	3950	3.41E-06	6230	5.77E-06	5680	7.55E-06
6590	2.08E-05	4560	1.26E-06	4360	4.93E-06	3950	3.42E-06	6240	5.76E-06	5680	7.56E-06
6590	2.08E-05	4560	1.26E-06	4360	4.94E-06	3950	3.41E-06	6240	5.77E-06	5680	7.57E-06
6590	2.08E-05	4570	1.26E-06	4360	4.93E-06	3960	3.42E-06	6240	5.76E-06	5680	7.57E-06
6600	2.08E-05	4570	1.27E-06	4360	4.94E-06	3960	3.42E-06	6240	5.76E-06	5680	7.57E-06
6600	2.09E-05	4570	1.26E-06	4370	4.94E-06	3960	3.42E-06	6240	5.76E-06	5680	7.58E-06
6600	2.09E-05	4570	1.26E-06	4370	4.94E-06	3960	3.43E-06	6240	5.75E-06	5680	7.58E-06
6600	2.09E-05	4570	1.26E-06	4370	4.94E-06	3960	3.43E-06	6250	5.77E-06	5690	7.58E-06
6600	2.09E-05	4570	1.26E-06	4370	4.92E-06	3960	3.44E-06	6250	5.77E-06	5690	7.58E-06
6600	2.09E-05	4580	1.26E-06	4370	4.93E-06	3970	3.44E-06	6250	5.77E-06	5690	7.58E-06
6600	2.09E-05	4580	1.27E-06	4370	4.93E-06	3970	3.44E-06	6250	5.76E-06	5690	7.58E-06
6610	2.09E-05	4580	1.26E-06	4380	4.94E-06	3970	3.46E-06	6250	5.78E-06	5690	7.58E-06
6610	2.10E-05	4580	1.26E-06	4380	4.93E-06	3970	3.46E-06	6250	5.77E-06	5690	7.59E-06
6610	2.10E-05	4580	1.26E-06	4380	4.93E-06	3970	3.48E-06	6250	5.76E-06	5700	7.59E-06

6610 2.10E-05 4580 1.26E-06 4380 4.93E-06 3970
3.50E-06 6260 5.77E-06 5700 7.59E-06
6610 2.10E-05 4580 1.26E-06 4380 4.93E-06 3980
3.49E-06 6260 5.77E-06 5700 7.59E-06
6610 2.10E-05 4590 1.26E-06 4380 4.95E-06 3980
3.50E-06 6260 5.77E-06 5700 7.60E-06
6620 2.11E-05 4590 1.26E-06 4380 4.94E-06 3980
3.51E-06 6260 5.77E-06 5700 7.60E-06
6620 2.11E-05 4590 1.26E-06 4390 4.95E-06 3980
3.51E-06 6260 5.77E-06 5700 7.60E-06
6620 2.11E-05 4590 1.27E-06 4390 4.94E-06 3980
3.51E-06 6260 5.78E-06 5700 7.61E-06
6620 2.11E-05 4590 1.26E-06 4390 4.94E-06 3980
3.53E-06 6270 5.77E-06 5710 7.60E-06
6620 2.11E-05 4590 1.27E-06 4390 4.95E-06 3980
3.53E-06 6270 5.77E-06 5710 7.60E-06
6620 2.11E-05 4600 1.27E-06 4390 4.95E-06 3990
3.54E-06 6270 5.78E-06 5710 7.59E-06
6630 2.11E-05 4600 1.27E-06 4390 4.95E-06 3990
3.53E-06 6270 5.77E-06 5710 7.58E-06
6630 2.11E-05 4600 1.27E-06 4400 4.96E-06 3990
3.54E-06 6270 5.78E-06 5710 7.60E-06
6630 2.11E-05 4600 1.28E-06 4400 4.97E-06 3990
3.55E-06 6270 5.78E-06 5710 7.59E-06
6630 2.11E-05 4600 1.28E-06 4400 4.97E-06 3990
3.56E-06 6280 5.78E-06 5720 7.58E-06
6630 2.11E-05 4600 1.28E-06 4400 4.95E-06 3990
3.56E-06 6280 5.79E-06 5720 7.59E-06
6630 2.11E-05 4600 1.28E-06 4400 4.96E-06 4000
3.58E-06 6280 5.79E-06 5720 7.57E-06
6630 2.11E-05 4610 1.27E-06 4400 4.95E-06 4000
3.61E-06 6280 5.79E-06 5720 7.59E-06
6640 2.11E-05 4610 1.27E-06 4400 4.97E-06 4000
3.60E-06 6280 5.79E-06 5720 7.59E-06
6640 2.11E-05 4610 1.27E-06 4410 4.96E-06 4000
3.63E-06 6280 5.79E-06 5720 7.59E-06
6640 2.11E-05 4610 1.26E-06 4410 4.96E-06 4000
3.65E-06 6280 5.79E-06 5730 7.58E-06
6640 2.11E-05 4610 1.27E-06 4410 4.96E-06 4000
3.67E-06 6290 5.80E-06 5730 7.58E-06
6640 2.12E-05 4610 1.27E-06 4410 4.96E-06 4000
3.69E-06 6290 5.80E-06 5730 7.59E-06
6640 2.12E-05 4620 1.27E-06 4410 4.98E-06 4010
3.70E-06 6290 5.80E-06 5730 7.59E-06
6650 2.12E-05 4620 1.27E-06 4410 4.97E-06 4010
3.71E-06 6290 5.81E-06 5730 7.59E-06
6650 2.12E-05 4620 1.26E-06 4420 4.97E-06 4010
3.71E-06 6290 5.81E-06 5730 7.58E-06
6650 2.12E-05 4620 1.27E-06 4420 4.96E-06 4010
3.72E-06 6290 5.81E-06 5730 7.59E-06
6650 2.12E-05 4620 1.27E-06 4420 4.97E-06 4010
3.74E-06 6300 5.81E-06 5740 7.59E-06
6650 2.12E-05 4620 1.27E-06 4420 4.98E-06 4010
3.74E-06 6300 5.80E-06 5740 7.59E-06
6650 2.12E-05 4630 1.28E-06 4420 4.97E-06 4020
3.76E-06 6300 5.81E-06 5740 7.59E-06
6650 2.12E-05 4630 1.28E-06 4420 4.97E-06 4020
3.76E-06 6300 5.80E-06 5740 7.59E-06
6660 2.12E-05 4630 1.28E-06 4430 4.98E-06 4020
3.78E-06 6300 5.81E-06 5740 7.59E-06
6660 2.12E-05 4630 1.28E-06 4430 4.97E-06 4020
3.79E-06 6300 5.81E-06 5740 7.60E-06
6660 2.12E-05 4630 1.27E-06 4430 4.97E-06 4020
3.79E-06 6300 5.81E-06 5750 7.60E-06
6660 2.13E-05 4630 1.27E-06 4430 4.97E-06 4020
3.82E-06 6310 5.82E-06 5750 7.61E-06
6660 2.13E-05 4630 1.27E-06 4430 4.96E-06 4030
3.83E-06 6310 5.81E-06 5750 7.60E-06

6660 2.13E-05 4640 1.27E-06 4430 4.97E-06 4030
3.86E-06 6310 5.81E-06 5750 7.63E-06
6670 2.13E-05 4640 1.27E-06 4430 4.98E-06 4030
3.87E-06 6310 5.82E-06 5750 7.62E-06
6670 2.13E-05 4640 1.27E-06 4440 4.97E-06 4030
3.91E-06 6310 5.82E-06 5750 7.62E-06
6670 2.14E-05 4640 1.27E-06 4440 4.98E-06 4030
3.93E-06 6310 5.82E-06 5750 7.62E-06
6670 2.14E-05 4640 1.27E-06 4440 4.98E-06 4030
3.96E-06 6320 5.82E-06 5760 7.63E-06
6670 2.14E-05 4640 1.27E-06 4440 4.99E-06 4030
3.99E-06 6320 5.83E-06 5760 7.63E-06
6670 2.14E-05 4650 1.27E-06 4440 5.00E-06 4040
4.02E-06 6320 5.83E-06 5760 7.64E-06
6680 2.14E-05 4650 1.27E-06 4440 5.00E-06 4040
4.03E-06 6320 5.83E-06 5760 7.63E-06
6680 2.14E-05 4650 1.28E-06 4450 4.99E-06 4040
4.06E-06 6320 5.83E-06 5760 7.65E-06
6680 2.14E-05 4650 1.28E-06 4450 5.00E-06 4040
4.08E-06 6320 5.84E-06 5760 7.64E-06
6680 2.14E-05 4650 1.27E-06 4450 5.01E-06 4040
4.09E-06 6330 5.84E-06 5770 7.65E-06
6680 2.14E-05 4650 1.28E-06 4450 5.00E-06 4040
4.12E-06 6330 5.83E-06 5770 7.65E-06
6680 2.14E-05 4650 1.28E-06 4450 5.01E-06 4050
4.15E-06 6330 5.84E-06 5770 7.67E-06
6680 2.14E-05 4660 1.28E-06 4450 5.02E-06 4050
4.17E-06 6330 5.83E-06 5770 7.66E-06
6690 2.15E-05 4660 1.29E-06 4450 5.02E-06 4050
4.21E-06 6330 5.83E-06 5770 7.68E-06
6690 2.15E-05 4660 1.28E-06 4460 5.02E-06 4050
4.25E-06 6330 5.83E-06 5770 7.67E-06
6690 2.15E-05 4660 1.28E-06 4460 5.02E-06 4050
4.29E-06 6330 5.84E-06 5780 7.68E-06
6690 2.15E-05 4660 1.28E-06 4460 5.01E-06 4050
4.33E-06 6340 5.83E-06 5780 7.69E-06
6690 2.15E-05 4660 1.29E-06 4460 5.02E-06 4050
4.38E-06 6340 5.83E-06 5780 7.68E-06
6690 2.15E-05 4670 1.28E-06 4460 5.02E-06 4060
4.43E-06 6340 5.82E-06 5780 7.69E-06
6700 2.15E-05 4670 1.28E-06 4460 5.03E-06 4060
4.47E-06 6340 5.84E-06 5780 7.70E-06
6700 2.15E-05 4670 1.28E-06 4470 5.03E-06 4060
4.53E-06 6340 5.83E-06 5780 7.71E-06
6700 2.15E-05 4670 1.28E-06 4470 5.03E-06 4060
4.58E-06 6340 5.83E-06 5780 7.70E-06
6700 2.15E-05 4670 1.29E-06 4470 5.03E-06 4060
4.66E-06 6350 5.84E-06 5790 7.70E-06
6700 2.15E-05 4670 1.28E-06 4470 5.04E-06 4060
4.72E-06 6350 5.84E-06 5790 7.71E-06
6700 2.16E-05 4680 1.28E-06 4470 5.05E-06 4070
4.78E-06 6350 5.84E-06 5790 7.72E-06
6700 2.16E-05 4680 1.28E-06 4470 5.04E-06 4070
4.85E-06 6350 5.85E-06 5790 7.72E-06
6710 2.16E-05 4680 1.27E-06 4480 5.03E-06 4070
4.93E-06 6350 5.85E-06 5790 7.73E-06
6710 2.16E-05 4680 1.28E-06 4480 5.03E-06 4070
5.01E-06 6350 5.85E-06 5790 7.73E-06
6710 2.17E-05 4680 1.27E-06 4480 5.04E-06 4070
5.10E-06 6350 5.85E-06 5800 7.74E-06
6710 2.17E-05 4680 1.28E-06 4480 5.05E-06 4070
5.17E-06 6360 5.85E-06 5800 7.74E-06
6710 2.17E-05 4680 1.28E-06 4480 5.05E-06 4080
5.26E-06 6360 5.85E-06 5800 7.73E-06
6710 2.17E-05 4690 1.29E-06 4480 5.05E-06 4080
5.37E-06 6360 5.85E-06 5800 7.74E-06
6720 2.17E-05 4690 1.29E-06 4480 5.05E-06 4080
5.48E-06 6360 5.84E-06 5800 7.76E-06

6720 2.17E-05 4690 1.29E-06 4490 5.05E-06 4080
5.61E-06 6360 5.84E-06 5800 7.75E-06
6720 2.17E-05 4690 1.30E-06 4490 5.04E-06 4080
5.73E-06 6360 5.84E-06 5800 7.75E-06
6720 2.18E-05 4690 1.29E-06 4490 5.03E-06 4080
5.87E-06 6370 5.85E-06 5810 7.76E-06
6720 2.18E-05 4690 1.29E-06 4490 5.03E-06 4080
6.03E-06 6370 5.85E-06 5810 7.77E-06
6720 2.18E-05 4700 1.28E-06 4490 5.03E-06 4090
6.19E-06 6370 5.85E-06 5810 7.78E-06
6730 2.18E-05 4700 1.29E-06 4490 5.04E-06 4090
6.37E-06 6370 5.85E-06 5810 7.78E-06
6730 2.19E-05 4700 1.29E-06 4500 5.04E-06 4090
6.56E-06 6370 5.84E-06 5810 7.79E-06
6730 2.19E-05 4700 1.28E-06 4500 5.04E-06 4090
6.77E-06 6370 5.85E-06 5810 7.78E-06
6730 2.19E-05 4700 1.30E-06 4500 5.04E-06 4090
7.00E-06 6380 5.85E-06 5820 7.78E-06
6730 2.19E-05 4700 1.29E-06 4500 5.03E-06 4090
7.26E-06 6380 5.86E-06 5820 7.79E-06
6730 2.20E-05 4700 1.30E-06 4500 5.03E-06 4100
7.53E-06 6380 5.85E-06 5820 7.80E-06
6730 2.19E-05 4710 1.30E-06 4500 5.03E-06 4100
7.82E-06 6380 5.86E-06 5820 7.81E-06
6740 2.20E-05 4710 1.29E-06 4500 5.03E-06 4100
8.15E-06 6380 5.87E-06 5820 7.81E-06
6740 2.20E-05 4710 1.30E-06 4510 5.03E-06 4100
8.50E-06 6380 5.87E-06 5820 7.80E-06
6740 2.20E-05 4710 1.29E-06 4510 5.04E-06 4100
8.90E-06 6380 5.87E-06 5830 7.82E-06
6740 2.21E-05 4710 1.29E-06 4510 5.05E-06 4100
9.32E-06 6390 5.86E-06 5830 7.81E-06
6740 2.21E-05 4710 1.29E-06 4510 5.05E-06 4100
9.80E-06 6390 5.85E-06 5830 7.81E-06
6740 2.21E-05 4720 1.29E-06 4510 5.05E-06 4110
1.03E-05 6390 5.86E-06 5830 7.83E-06
6750 2.21E-05 4720 1.30E-06 4510 5.05E-06 4110
1.09E-05 6390 5.86E-06 5830 7.83E-06
6750 2.22E-05 4720 1.30E-06 4520 5.05E-06 4110
1.15E-05 6390 5.86E-06 5830 7.84E-06
6750 2.21E-05 4720 1.30E-06 4520 5.05E-06 4110
1.22E-05 6390 5.86E-06 5830 7.84E-06
6750 2.22E-05 4720 1.30E-06 4520 5.04E-06 4110
1.29E-05 6400 5.87E-06 5840 7.86E-06
6750 2.22E-05 4720 1.31E-06 4520 5.04E-06 4110
1.37E-05 6400 5.87E-06 5840 7.85E-06
6750 2.22E-05 4730 1.30E-06 4520 5.04E-06 4120
1.46E-05 6400 5.87E-06 5840 7.86E-06
6750 2.22E-05 4730 1.30E-06 4520 5.04E-06 4120
1.55E-05 6400 5.87E-06 5840 7.88E-06
6760 2.23E-05 4730 1.30E-06 4530 5.06E-06 4120
1.66E-05 6400 5.88E-06 5840 7.87E-06
6760 2.23E-05 4730 1.30E-06 4530 5.05E-06 4120
1.77E-05 6400 5.87E-06 5840 7.87E-06
6760 2.23E-05 4730 1.31E-06 4530 5.05E-06 4120
1.88E-05 6400 5.89E-06 5850 7.90E-06
6760 2.24E-05 4730 1.30E-06 4530 5.06E-06 4120
2.01E-05 6410 5.90E-06 5850 7.91E-06
6760 2.24E-05 4730 1.31E-06 4530 5.06E-06 4130
2.14E-05 6410 5.90E-06 5850 7.92E-06
6760 2.25E-05 4740 1.31E-06 4530 5.05E-06 4130
2.28E-05 6410 5.90E-06 5850 7.92E-06
6770 2.25E-05 4740 1.31E-06 4530 5.06E-06 4130
2.42E-05 6410 5.91E-06 5850 7.93E-06
6770 2.25E-05 4740 1.31E-06 4540 5.05E-06 4130
2.58E-05 6410 5.91E-06 5850 7.92E-06
6770 2.26E-05 4740 1.30E-06 4540 5.07E-06 4130
2.73E-05 6410 5.91E-06 5850 7.95E-06

6770	2.26E-05	4740	1.30E-06	4540	5.07E-06	4130	2.89E-05	6420	5.90E-06	5860	7.94E-06
6770	2.26E-05	4740	1.31E-06	4540	5.07E-06	4130	3.05E-05	6420	5.91E-06	5860	7.95E-06
6770	2.26E-05	4750	1.31E-06	4540	5.08E-06	4140	3.21E-05	6420	5.91E-06	5860	7.94E-06
6780	2.27E-05	4750	1.31E-06	4540	5.07E-06	4140	3.36E-05	6420	5.90E-06	5860	7.96E-06
6780	2.27E-05	4750	1.31E-06	4550	5.08E-06	4140	3.51E-05	6420	5.89E-06	5860	7.96E-06
6780	2.27E-05	4750	1.31E-06	4550	5.07E-06	4140	3.65E-05	6420	5.90E-06	5860	7.94E-06
6780	2.28E-05	4750	1.31E-06	4550	5.07E-06	4140	3.78E-05	6430	5.90E-06	5870	7.97E-06
6780	2.28E-05	4750	1.32E-06	4550	5.07E-06	4140	3.89E-05	6430	5.90E-06	5870	7.96E-06
6780	2.28E-05	4750	1.31E-06	4550	5.06E-06	4150	3.98E-05	6430	5.90E-06	5870	7.97E-06
6780	2.28E-05	4760	1.31E-06	4550	5.06E-06	4150	4.06E-05	6430	5.89E-06	5870	7.96E-06
6790	2.29E-05	4760	1.31E-06	4550	5.06E-06	4150	4.11E-05	6430	5.88E-06	5870	7.95E-06
6790	2.29E-05	4760	1.31E-06	4560	5.06E-06	4150	4.15E-05	6430	5.88E-06	5870	7.96E-06
6790	2.29E-05	4760	1.32E-06	4560	5.06E-06	4150	4.16E-05	6430	5.89E-06	5880	7.95E-06
6790	2.30E-05	4760	1.32E-06	4560	5.06E-06	4150	4.16E-05	6440	5.90E-06	5880	7.95E-06
6790	2.30E-05	4760	1.32E-06	4560	5.06E-06	4150	4.14E-05	6440	5.90E-06	5880	7.97E-06
6790	2.30E-05	4770	1.32E-06	4560	5.06E-06	4160	4.11E-05	6440	5.90E-06	5880	7.96E-06
6800	2.31E-05	4770	1.32E-06	4560	5.06E-06	4160	4.06E-05	6440	5.90E-06	5880	7.96E-06
6800	2.31E-05	4770	1.33E-06	4570	5.06E-06	4160	4.00E-05	6440	5.90E-06	5880	7.97E-06
6800	2.32E-05	4770	1.32E-06	4570	5.07E-06	4160	3.93E-05	6440	5.90E-06	5880	7.97E-06
6800	2.32E-05	4770	1.32E-06	4570	5.07E-06	4160	3.85E-05	6450	5.89E-06	5890	7.95E-06
6800	2.33E-05	4770	1.33E-06	4570	5.09E-06	4160	3.76E-05	6450	5.89E-06	5890	7.96E-06
6800	2.33E-05	4780	1.32E-06	4570	5.08E-06	4170	3.67E-05	6450	5.88E-06	5890	7.97E-06
6800	2.34E-05	4780	1.34E-06	4570	5.09E-06	4170	3.57E-05	6450	5.89E-06	5890	7.98E-06
6810	2.35E-05	4780	1.33E-06	4580	5.08E-06	4170	3.46E-05	6450	5.88E-06	5890	7.96E-06
6810	2.35E-05	4780	1.34E-06	4580	5.07E-06	4170	3.35E-05	6450	5.90E-06	5890	7.97E-06
6810	2.36E-05	4780	1.33E-06	4580	5.07E-06	4170	3.23E-05	6450	5.89E-06	5900	7.97E-06
6810	2.36E-05	4780	1.34E-06	4580	5.07E-06	4170	3.12E-05	6460	5.90E-06	5900	7.96E-06
6810	2.37E-05	4780	1.33E-06	4580	5.07E-06	4180	3.00E-05	6460	5.90E-06	5900	7.95E-06
6810	2.37E-05	4790	1.34E-06	4580	5.07E-06	4180	2.89E-05	6460	5.89E-06	5900	7.94E-06
6820	2.37E-05	4790	1.34E-06	4580	5.07E-06	4180	2.77E-05	6460	5.89E-06	5900	7.94E-06
6820	2.38E-05	4790	1.34E-06	4590	5.07E-06	4180	2.67E-05	6460	5.88E-06	5900	7.92E-06
6820	2.39E-05	4790	1.34E-06	4590	5.08E-06	4180	2.57E-05	6460	5.88E-06	5900	7.91E-06
6820	2.40E-05	4790	1.34E-06	4590	5.08E-06	4180	2.47E-05	6470	5.89E-06	5910	7.92E-06
6820	2.40E-05	4790	1.34E-06	4590	5.08E-06	4180	2.38E-05	6470	5.89E-06	5910	7.93E-06

6820 2.41E-05 4800 1.34E-06 4590 5.09E-06 4190
2.30E-05 6470 5.89E-06 5910 7.92E-06
6830 2.41E-05 4800 1.34E-06 4590 5.09E-06 4190
2.23E-05 6470 5.91E-06 5910 7.90E-06
6830 2.42E-05 4800 1.35E-06 4600 5.10E-06 4190
2.17E-05 6470 5.90E-06 5910 7.90E-06
6830 2.43E-05 4800 1.35E-06 4600 5.11E-06 4190
2.12E-05 6470 5.90E-06 5910 7.89E-06
6830 2.43E-05 4800 1.33E-06 4600 5.11E-06 4190
2.07E-05 6480 5.88E-06 5920 7.88E-06
6830 2.44E-05 4800 1.34E-06 4600 5.11E-06 4190
2.03E-05 6480 5.88E-06 5920 7.88E-06
6830 2.44E-05 4800 1.33E-06 4600 5.12E-06 4200
2.00E-05 6480 5.89E-06 5920 7.88E-06
6830 2.45E-05 4810 1.34E-06 4600 5.12E-06 4200
1.98E-05 6480 5.88E-06 5920 7.89E-06
6840 2.45E-05 4810 1.34E-06 4600 5.12E-06 4200
1.97E-05 6480 5.87E-06 5920 7.88E-06
6840 2.46E-05 4810 1.34E-06 4610 5.11E-06 4200
1.96E-05 6480 5.87E-06 5920 7.87E-06
6840 2.47E-05 4810 1.34E-06 4610 5.10E-06 4200
1.97E-05 6480 5.87E-06 5930 7.87E-06
6840 2.47E-05 4810 1.35E-06 4610 5.11E-06 4200
1.99E-05 6490 5.86E-06 5930 7.85E-06
6840 2.48E-05 4810 1.34E-06 4610 5.11E-06 4200
2.02E-05 6490 5.85E-06 5930 7.85E-06
6840 2.48E-05 4820 1.34E-06 4610 5.10E-06 4210
2.06E-05 6490 5.85E-06 5930 7.84E-06
6850 2.49E-05 4820 1.34E-06 4610 5.11E-06 4210
2.11E-05 6490 5.85E-06 5930 7.84E-06
6850 2.49E-05 4820 1.34E-06 4620 5.12E-06 4210
2.17E-05 6490 5.85E-06 5930 7.83E-06
6850 2.50E-05 4820 1.35E-06 4620 5.11E-06 4210
2.24E-05 6490 5.85E-06 5930 7.82E-06
6850 2.51E-05 4820 1.34E-06 4620 5.10E-06 4210
2.32E-05 6500 5.85E-06 5940 7.83E-06
6850 2.51E-05 4820 1.34E-06 4620 5.10E-06 4210
2.41E-05 6500 5.84E-06 5940 7.84E-06
6850 2.52E-05 4830 1.34E-06 4620 5.11E-06 4220
2.52E-05 6500 5.85E-06 5940 7.83E-06
6850 2.53E-05 4830 1.35E-06 4620 5.11E-06 4220
2.63E-05 6500 5.85E-06 5940 7.83E-06
6860 2.54E-05 4830 1.35E-06 4630 5.11E-06 4220
2.76E-05 6500 5.85E-06 5940 7.83E-06
6860 2.55E-05 4830 1.35E-06 4630 5.12E-06 4220
2.90E-05 6500 5.84E-06 5940 7.82E-06
6860 2.56E-05 4830 1.34E-06 4630 5.13E-06 4220
3.04E-05 6500 5.83E-06 5950 7.81E-06
6860 2.57E-05 4830 1.35E-06 4630 5.12E-06 4220
3.20E-05 6510 5.82E-06 5950 7.82E-06
6860 2.58E-05 4830 1.34E-06 4630 5.13E-06 4230
3.36E-05 6510 5.82E-06 5950 7.82E-06
6860 2.59E-05 4840 1.35E-06 4630 5.13E-06 4230
3.52E-05 6510 5.81E-06 5950 7.83E-06
6870 2.60E-05 4840 1.35E-06 4630 5.13E-06 4230
3.67E-05 6510 5.81E-06 5950 7.83E-06
6870 2.61E-05 4840 1.35E-06 4640 5.13E-06 4230
3.82E-05 6510 5.80E-06 5950 7.83E-06
6870 2.62E-05 4840 1.35E-06 4640 5.12E-06 4230
3.95E-05 6510 5.79E-06 5950 7.83E-06
6870 2.63E-05 4840 1.36E-06 4640 5.12E-06 4230
4.05E-05 6520 5.78E-06 5960 7.83E-06
6870 2.64E-05 4840 1.36E-06 4640 5.12E-06 4230
4.13E-05 6520 5.78E-06 5960 7.83E-06
6870 2.65E-05 4850 1.35E-06 4640 5.12E-06 4240
4.16E-05 6520 5.77E-06 5960 7.84E-06
6880 2.67E-05 4850 1.35E-06 4640 5.13E-06 4240
4.16E-05 6520 5.75E-06 5960 7.83E-06

6880 2.68E-05 4850 1.35E-06 4650 5.14E-06 4240
4.12E-05 6520 5.76E-06 5960 7.84E-06
6880 2.70E-05 4850 1.36E-06 4650 5.15E-06 4240
4.03E-05 6520 5.75E-06 5960 7.84E-06
6880 2.71E-05 4850 1.35E-06 4650 5.14E-06 4240
3.91E-05 6530 5.75E-06 5970 7.84E-06
6880 2.72E-05 4850 1.36E-06 4650 5.15E-06 4240
3.76E-05 6530 5.75E-06 5970 7.85E-06
6880 2.73E-05 4850 1.36E-06 4650 5.16E-06 4250
3.58E-05 6530 5.75E-06 5970 7.85E-06
6880 2.74E-05 4860 1.36E-06 4650 5.16E-06 4250
3.38E-05 6530 5.74E-06 5970 7.84E-06
6890 2.76E-05 4860 1.37E-06 4650 5.16E-06 4250
3.17E-05 6530 5.74E-06 5970 7.85E-06
6890 2.77E-05 4860 1.37E-06 4660 5.18E-06 4250
2.96E-05 6530 5.74E-06 5970 7.84E-06
6890 2.79E-05 4860 1.37E-06 4660 5.18E-06 4250
2.75E-05 6530 5.72E-06 5980 7.85E-06
6890 2.81E-05 4860 1.37E-06 4660 5.19E-06 4250
2.55E-05 6540 5.71E-06 5980 7.86E-06
6890 2.82E-05 4860 1.38E-06 4660 5.19E-06 4250
2.35E-05 6540 5.70E-06 5980 7.85E-06
6890 2.84E-05 4870 1.38E-06 4660 5.19E-06 4260
2.17E-05 6540 5.70E-06 5980 7.86E-06
6900 2.86E-05 4870 1.39E-06 4660 5.19E-06 4260
2.00E-05 6540 5.68E-06 5980 7.86E-06
6900 2.87E-05 4870 1.38E-06 4670 5.19E-06 4260
1.85E-05 6540 5.68E-06 5980 7.85E-06
6900 2.89E-05 4870 1.39E-06 4670 5.18E-06 4260
1.71E-05 6540 5.67E-06 5980 7.86E-06
6900 2.91E-05 4870 1.39E-06 4670 5.19E-06 4260
1.59E-05 6550 5.66E-06 5990 7.85E-06
6900 2.92E-05 4870 1.39E-06 4670 5.19E-06 4260
1.47E-05 6550 5.66E-06 5990 7.85E-06
6900 2.94E-05 4880 1.39E-06 4670 5.19E-06 4270
1.37E-05 6550 5.65E-06 5990 7.86E-06
6900 2.96E-05 4880 1.38E-06 4670 5.19E-06 4270
1.28E-05 6550 5.64E-06 5990 7.86E-06
6910 2.98E-05 4880 1.38E-06 4680 5.20E-06 4270
1.20E-05 6550 5.64E-06 5990 7.86E-06
6910 3.00E-05 4880 1.39E-06 4680 5.19E-06 4270
1.13E-05 6550 5.63E-06 5990 7.87E-06
6910 3.02E-05 4880 1.39E-06 4680 5.19E-06 4270
1.07E-05 6550 5.61E-06 6000 7.85E-06
6910 3.04E-05 4880 1.39E-06 4680 5.19E-06 4270
1.01E-05 6560 5.60E-06 6000 7.87E-06
6910 3.06E-05 4880 1.40E-06 4680 5.20E-06 4280
9.60E-06 6560 5.60E-06 6000 7.86E-06
6910 3.08E-05 4890 1.40E-06 4680 5.19E-06 4280
9.11E-06 6560 5.58E-06 6000 7.87E-06
6920 3.10E-05 4890 1.40E-06 4680 5.19E-06 4280
8.68E-06 6560 5.59E-06 6000 7.86E-06
6920 3.12E-05 4890 1.40E-06 4690 5.20E-06 4280
8.32E-06 6560 5.59E-06 6000 7.87E-06
6920 3.15E-05 4890 1.40E-06 4690 5.21E-06 4280
7.98E-06 6560 5.59E-06 6000 7.86E-06
6920 3.17E-05 4890 1.40E-06 4690 5.20E-06 4280
7.68E-06 6570 5.55E-06 6010 7.86E-06
6920 3.19E-05 4890 1.41E-06 4690 5.20E-06 4280
7.40E-06 6570 5.54E-06 6010 7.88E-06
6920 3.22E-05 4900 1.41E-06 4690 5.20E-06 4290
7.12E-06 6570 5.54E-06 6010 7.87E-06
6930 3.25E-05 4900 1.42E-06 4690 5.21E-06 4290
6.90E-06 6570 5.52E-06 6010 7.87E-06
6930 3.28E-05 4900 1.41E-06 4700 5.20E-06 4290
6.70E-06 6570 5.51E-06 6010 7.87E-06
6930 3.30E-05 4900 1.42E-06 4700 5.19E-06 4290
6.50E-06 6570 5.49E-06 6010 7.87E-06

6930	3.33E-05	4900	1.42E-06	4700	5.19E-06	4290
6.29E-06	6580	5.47E-06	6020	7.87E-06		
6930	3.36E-05	4900	1.43E-06	4700	5.20E-06	4290
6.15E-06	6580	5.45E-06	6020	7.87E-06		
6930	3.39E-05	4900	1.42E-06	4700	5.19E-06	4300
5.99E-06	6580	5.45E-06	6020	7.87E-06		
6930	3.42E-05	4910	1.42E-06	4700	5.19E-06	4300
5.87E-06	6580	5.43E-06	6020	7.88E-06		
6940	3.46E-05	4910	1.43E-06	4700	5.19E-06	4300
5.74E-06	6580	5.44E-06	6020	7.88E-06		
6940	3.49E-05	4910	1.43E-06	4710	5.19E-06	4300
5.62E-06	6580	5.43E-06	6020	7.88E-06		
6940	3.52E-05	4910	1.43E-06	4710	5.20E-06	4300
5.55E-06	6580	5.42E-06	6030	7.88E-06		
6940	3.56E-05	4910	1.44E-06	4710	5.20E-06	4300
5.44E-06	6590	5.40E-06	6030	7.89E-06		
6940	3.59E-05	4910	1.44E-06	4710	5.20E-06	4300
5.35E-06	6590	5.41E-06	6030	7.88E-06		
6940	3.63E-05	4920	1.44E-06	4710	5.21E-06	4310
5.25E-06	6590	5.40E-06	6030	7.89E-06		
6950	3.66E-05	4920	1.45E-06	4710	5.20E-06	4310
5.14E-06	6590	5.39E-06	6030	7.89E-06		
6950	3.70E-05	4920	1.45E-06	4720	5.21E-06	4310
5.07E-06	6590	5.41E-06	6030	7.89E-06		
6950	3.73E-05	4920	1.45E-06	4720	5.23E-06	4310
4.99E-06	6590	5.39E-06	6030	7.89E-06		
6950	3.77E-05	4920	1.47E-06	4720	5.22E-06	4310
4.92E-06	6600	5.40E-06	6040	7.89E-06		
6950	3.81E-05	4920	1.46E-06	4720	5.22E-06	4310
4.85E-06	6600	5.38E-06	6040	7.89E-06		
6950	3.85E-05	4930	1.47E-06	4720	5.22E-06	4320
4.80E-06	6600	5.40E-06	6040	7.91E-06		
6950	3.88E-05	4930	1.48E-06	4720	5.23E-06	4320
4.73E-06	6600	5.40E-06	6040	7.91E-06		
6960	3.92E-05	4930	1.48E-06	4730	5.22E-06	4320
4.67E-06	6600	5.41E-06	6040	7.90E-06		
6960	3.97E-05	4930	1.49E-06	4730	5.22E-06	4320
4.62E-06	6600	5.42E-06	6040	7.90E-06		
6960	4.00E-05	4930	1.49E-06	4730	5.22E-06	4320
4.56E-06	6600	5.42E-06	6050	7.89E-06		
6960	4.05E-05	4930	1.49E-06	4730	5.21E-06	4320
4.53E-06	6610	5.42E-06	6050	7.88E-06		
6960	4.09E-05	4930	1.49E-06	4730	5.22E-06	4330
4.49E-06	6610	5.42E-06	6050	7.88E-06		
6960	4.13E-05	4940	1.49E-06	4730	5.22E-06	4330
4.44E-06	6610	5.43E-06	6050	7.89E-06		
6970	4.18E-05	4940	1.49E-06	4730	5.22E-06	4330
4.41E-06	6610	5.44E-06	6050	7.89E-06		
6970	4.22E-05	4940	1.51E-06	4740	5.22E-06	4330
4.37E-06	6610	5.46E-06	6050	7.90E-06		
6970	4.26E-05	4940	1.50E-06	4740	5.23E-06	4330
4.36E-06	6610	5.47E-06	6050	7.90E-06		
6970	4.31E-05	4940	1.51E-06	4740	5.24E-06	4330
4.32E-06	6620	5.47E-06	6060	7.91E-06		
6970	4.35E-05	4940	1.51E-06	4740	5.24E-06	4330
4.30E-06	6620	5.49E-06	6060	7.91E-06		
6970	4.40E-05	4950	1.51E-06	4740	5.24E-06	4340
4.26E-06	6620	5.49E-06	6060	7.90E-06		
6980	4.44E-05	4950	1.53E-06	4740	5.25E-06	4340
4.25E-06	6620	5.52E-06	6060	7.88E-06		
6980	4.49E-05	4950	1.53E-06	4750	5.25E-06	4340
4.23E-06	6620	5.53E-06	6060	7.88E-06		
6980	4.53E-05	4950	1.53E-06	4750	5.27E-06	4340
4.19E-06	6620	5.54E-06	6060	7.89E-06		
6980	4.58E-05	4950	1.55E-06	4750	5.26E-06	4340
4.17E-06	6630	5.57E-06	6070	7.89E-06		
6980	4.63E-05	4950	1.55E-06	4750	5.26E-06	4340
4.15E-06	6630	5.58E-06	6070	7.87E-06		

6980 4.67E-05 4950 1.56E-06 4750 5.26E-06 4350
4.12E-06 6630 5.59E-06 6070 7.90E-06
6980 4.71E-05 4960 1.57E-06 4750 5.27E-06 4350
4.10E-06 6630 5.61E-06 6070 7.89E-06
6990 4.76E-05 4960 1.57E-06 4750 5.26E-06 4350
4.08E-06 6630 5.62E-06 6070 7.87E-06
6990 4.80E-05 4960 1.59E-06 4760 5.25E-06 4350
4.07E-06 6630 5.64E-06 6070 7.89E-06
6990 4.85E-05 4960 1.59E-06 4760 5.26E-06 4350
4.05E-06 6630 5.65E-06 6080 7.90E-06
6990 4.90E-05 4960 1.59E-06 4760 5.25E-06 4350
4.05E-06 6640 5.66E-06 6080 7.90E-06
6990 4.94E-05 4960 1.60E-06 4760 5.25E-06 4350
4.02E-06 6640 5.66E-06 6080 7.89E-06
6990 4.98E-05 4970 1.60E-06 4760 5.25E-06 4360
4.01E-06 6640 5.68E-06 6080 7.90E-06
7000 5.02E-05 4970 1.61E-06 4760 5.25E-06 4360
3.98E-06 6640 5.69E-06 6080 7.90E-06
7000 5.06E-05 4970 1.62E-06 4770 5.25E-06 4360
3.97E-06 6640 5.71E-06 6080 7.92E-06
7000 5.10E-05 4970 1.62E-06 4770 5.26E-06 4360
3.95E-06 6640 5.72E-06 6080 7.92E-06
7000 5.14E-05 4970 1.64E-06 4770 5.27E-06 4360
3.96E-06 6650 5.73E-06 6090 7.93E-06
7000 5.18E-05 4970 1.65E-06 4770 5.28E-06 4360
3.94E-06 6650 5.74E-06 6090 7.92E-06
7000 5.22E-05 4980 1.65E-06 4770 5.28E-06 4370
3.92E-06 6650 5.74E-06 6090 7.92E-06
7000 5.26E-05 4980 1.67E-06 4770 5.28E-06 4370
3.89E-06 6650 5.75E-06 6090 7.92E-06
7010 5.29E-05 4980 1.67E-06 4780 5.29E-06 4370
3.90E-06 6650 5.75E-06 6090 7.93E-06
7010 5.32E-05 4980 1.68E-06 4780 5.31E-06 4370
3.86E-06 6650 5.76E-06 6090 7.91E-06
7010 5.35E-05 4980 1.70E-06 4780 5.31E-06 4370
3.87E-06 6650 5.77E-06 6100 7.92E-06
7010 5.38E-05 4980 1.71E-06 4780 5.31E-06 4370
3.86E-06 6660 5.77E-06 6100 7.93E-06
7010 5.41E-05 4980 1.73E-06 4780 5.31E-06 4380
3.86E-06 6660 5.78E-06 6100 7.94E-06
7010 5.43E-05 4990 1.74E-06 4780 5.31E-06 4380
3.85E-06 6660 5.78E-06 6100 7.92E-06
7020 5.46E-05 4990 1.76E-06 4780 5.30E-06 4380
3.84E-06 6660 5.79E-06 6100 7.92E-06
7020 5.48E-05 4990 1.77E-06 4790 5.29E-06 4380
3.82E-06 6660 5.80E-06 6100 7.93E-06
7020 5.50E-05 4990 1.80E-06 4790 5.30E-06 4380
3.80E-06 6660 5.79E-06 6100 7.92E-06
7020 5.52E-05 4990 1.81E-06 4790 5.29E-06 4380
3.82E-06 6670 5.79E-06 6110 7.94E-06
7020 5.54E-05 4990 1.81E-06 4790 5.29E-06 4380
3.81E-06 6670 5.80E-06 6110 7.94E-06
7020 5.55E-05 5000 1.83E-06 4790 5.29E-06 4390
3.79E-06 6670 5.80E-06 6110 7.93E-06
7030 5.57E-05 5000 1.85E-06 4790 5.29E-06 4390
3.78E-06 6670 5.81E-06 6110 7.94E-06
7030 5.58E-05 5000 1.88E-06 4800 5.30E-06 4390
3.79E-06 6670 5.81E-06 6110 7.92E-06
7030 5.58E-05 5000 1.90E-06 4800 5.31E-06 4390
3.79E-06 6670 5.82E-06 6110 7.93E-06
7030 5.59E-05 5000 1.93E-06 4800 5.32E-06 4390
3.77E-06 6680 5.83E-06 6120 7.94E-06
7030 5.60E-05 5000 1.95E-06 4800 5.32E-06 4390
3.79E-06 6680 5.83E-06 6120 7.93E-06
7030 5.60E-05 5000 1.97E-06 4800 5.32E-06 4400
3.76E-06 6680 5.84E-06 6120 7.93E-06
7030 5.60E-05 5010 2.01E-06 4800 5.32E-06 4400
3.75E-06 6680 5.85E-06 6120 7.94E-06

7040 5.60E-05 5010 2.04E-06 4800 5.34E-06 4400
3.74E-06 6680 5.84E-06 6120 7.93E-06
7040 5.59E-05 5010 2.07E-06 4810 5.35E-06 4400
3.74E-06 6680 5.84E-06 6120 7.92E-06
7040 5.58E-05 5010 2.09E-06 4810 5.34E-06 4400
3.74E-06 6680 5.84E-06 6130 7.93E-06
7040 5.58E-05 5010 2.13E-06 4810 5.34E-06 4400
3.72E-06 6690 5.85E-06 6130 7.93E-06
7040 5.57E-05 5010 2.18E-06 4810 5.34E-06 4400
3.74E-06 6690 5.85E-06 6130 7.93E-06
7040 5.55E-05 5020 2.21E-06 4810 5.35E-06 4410
3.73E-06 6690 5.85E-06 6130 7.92E-06
7050 5.54E-05 5020 2.27E-06 4810 5.34E-06 4410
3.73E-06 6690 5.85E-06 6130 7.90E-06
7050 5.52E-05 5020 2.31E-06 4820 5.32E-06 4410
3.71E-06 6690 5.86E-06 6130 7.92E-06
7050 5.51E-05 5020 2.36E-06 4820 5.32E-06 4410
3.70E-06 6690 5.86E-06 6130 7.92E-06
7050 5.48E-05 5020 2.41E-06 4820 5.32E-06 4410
3.71E-06 6700 5.86E-06 6140 7.91E-06
7050 5.47E-05 5020 2.46E-06 4820 5.33E-06 4410
3.69E-06 6700 5.86E-06 6140 7.92E-06
7050 5.45E-05 5030 2.52E-06 4820 5.33E-06 4420
3.69E-06 6700 5.85E-06 6140 7.91E-06
7050 5.42E-05 5030 2.59E-06 4820 5.32E-06 4420
3.68E-06 6700 5.87E-06 6140 7.91E-06
7060 5.39E-05 5030 2.66E-06 4830 5.32E-06 4420
3.68E-06 6700 5.86E-06 6140 7.92E-06
7060 5.37E-05 5030 2.74E-06 4830 5.33E-06 4420
3.66E-06 6700 5.87E-06 6140 7.91E-06
7060 5.34E-05 5030 2.83E-06 4830 5.33E-06 4420
3.66E-06 6700 5.87E-06 6150 7.93E-06
7060 5.31E-05 5030 2.92E-06 4830 5.33E-06 4420
3.67E-06 6710 5.87E-06 6150 7.92E-06
7060 5.28E-05 5030 3.02E-06 4830 5.33E-06 4430
3.67E-06 6710 5.88E-06 6150 7.90E-06
7060 5.25E-05 5040 3.13E-06 4830 5.33E-06 4430
3.64E-06 6710 5.88E-06 6150 7.90E-06
7070 5.22E-05 5040 3.25E-06 4830 5.35E-06 4430
3.64E-06 6710 5.88E-06 6150 7.90E-06
7070 5.19E-05 5040 3.38E-06 4840 5.36E-06 4430
3.64E-06 6710 5.88E-06 6150 7.90E-06
7070 5.16E-05 5040 3.52E-06 4840 5.36E-06 4430
3.64E-06 6710 5.88E-06 6150 7.90E-06
7070 5.12E-05 5040 3.69E-06 4840 5.37E-06 4430
3.61E-06 6720 5.89E-06 6160 7.91E-06
7070 5.09E-05 5040 3.87E-06 4840 5.36E-06 4430
3.62E-06 6720 5.89E-06 6160 7.90E-06
7070 5.05E-05 5050 4.06E-06 4840 5.36E-06 4440
3.61E-06 6720 5.89E-06 6160 7.91E-06
7080 5.01E-05 5050 4.28E-06 4840 5.36E-06 4440
3.61E-06 6720 5.89E-06 6160 7.90E-06
7080 4.97E-05 5050 4.52E-06 4850 5.35E-06 4440
3.60E-06 6720 5.90E-06 6160 7.90E-06
7080 4.93E-05 5050 4.80E-06 4850 5.34E-06 4440
3.61E-06 6720 5.90E-06 6160 7.89E-06
7080 4.89E-05 5050 5.10E-06 4850 5.35E-06 4440
3.61E-06 6730 5.90E-06 6170 7.90E-06
7080 4.86E-05 5050 5.43E-06 4850 5.36E-06 4440
3.61E-06 6730 5.90E-06 6170 7.89E-06
7080 4.81E-05 5050 5.81E-06 4850 5.36E-06 4450
3.62E-06 6730 5.90E-06 6170 7.88E-06
7080 4.78E-05 5060 6.23E-06 4850 5.36E-06 4450
3.63E-06 6730 5.91E-06 6170 7.87E-06
7090 4.74E-05 5060 6.70E-06 4850 5.36E-06 4450
3.62E-06 6730 5.91E-06 6170 7.88E-06
7090 4.70E-05 5060 7.22E-06 4860 5.37E-06 4450
3.65E-06 6730 5.91E-06 6170 7.88E-06

7090 4.66E-05 5060 7.80E-06 4860 5.37E-06 4450
3.64E-06 6730 5.92E-06 6180 7.88E-06
7090 4.62E-05 5060 8.43E-06 4860 5.37E-06 4450
3.64E-06 6740 5.92E-06 6180 7.87E-06
7090 4.58E-05 5060 9.12E-06 4860 5.38E-06 4450
3.62E-06 6740 5.92E-06 6180 7.87E-06
7090 4.53E-05 5070 9.88E-06 4860 5.38E-06 4460
3.62E-06 6740 5.93E-06 6180 7.85E-06
7100 4.50E-05 5070 1.07E-05 4860 5.40E-06 4460
3.62E-06 6740 5.93E-06 6180 7.85E-06
7100 4.46E-05 5070 1.15E-05 4870 5.40E-06 4460
3.61E-06 6740 5.93E-06 6180 7.86E-06
7100 4.42E-05 5070 1.23E-05 4870 5.40E-06 4460
3.61E-06 6740 5.93E-06 6180 7.85E-06
7100 4.37E-05 5070 1.30E-05 4870 5.42E-06 4460
3.59E-06 6750 5.93E-06 6190 7.85E-06
7100 4.33E-05 5070 1.37E-05 4870 5.40E-06 4460
3.59E-06 6750 5.93E-06 6190 7.85E-06
7100 4.28E-05 5080 1.42E-05 4870 5.40E-06 4470
3.60E-06 6750 5.94E-06 6190 7.84E-06
7100 4.24E-05 5080 1.46E-05 4870 5.40E-06 4470
3.58E-06 6750 5.94E-06 6190 7.83E-06
7110 4.20E-05 5080 1.49E-05 4880 5.39E-06 4470
3.58E-06 6750 5.95E-06 6190 7.85E-06
7110 4.15E-05 5080 1.50E-05 4880 5.39E-06 4470
3.57E-06 6750 5.94E-06 6190 7.84E-06
7110 4.11E-05 5080 1.49E-05 4880 5.39E-06 4470
3.59E-06 6750 5.94E-06 6200 7.81E-06
7110 4.07E-05 5080 1.48E-05 4880 5.39E-06 4470
3.59E-06 6760 5.94E-06 6200 7.82E-06
7110 4.03E-05 5080 1.46E-05 4880 5.39E-06 4480
3.61E-06 6760 5.94E-06 6200 7.80E-06
7110 3.99E-05 5090 1.43E-05 4880 5.39E-06 4480
3.60E-06 6760 5.95E-06 6200 7.81E-06
7120 3.95E-05 5090 1.39E-05 4880 5.38E-06 4480
3.59E-06 6760 5.95E-06 6200 7.82E-06
7120 3.91E-05 5090 1.35E-05 4890 5.39E-06 4480
3.60E-06 6760 5.96E-06 6200 7.81E-06
7120 3.87E-05 5090 1.30E-05 4890 5.39E-06 4480
3.57E-06 6760 5.96E-06 6200 7.82E-06
7120 3.84E-05 5090 1.24E-05 4890 5.39E-06 4480
3.58E-06 6770 5.97E-06 6210 7.81E-06
7120 3.80E-05 5090 1.17E-05 4890 5.40E-06 4480
3.55E-06 6770 5.96E-06 6210 7.82E-06
7120 3.76E-05 5100 1.10E-05 4890 5.40E-06 4490
3.55E-06 6770 5.97E-06 6210 7.82E-06
7130 3.73E-05 5100 1.03E-05 4890 5.41E-06 4490
3.56E-06 6770 5.97E-06 6210 7.81E-06
7130 3.68E-05 5100 9.56E-06 4900 5.41E-06 4490
3.57E-06 6770 5.97E-06 6210 7.81E-06
7130 3.65E-05 5100 8.85E-06 4900 5.42E-06 4490
3.57E-06 6770 5.97E-06 6210 7.81E-06
7130 3.61E-05 5100 8.19E-06 4900 5.42E-06 4490
3.55E-06 6780 5.97E-06 6220 7.82E-06
7130 3.57E-05 5100 7.57E-06 4900 5.42E-06 4490
3.55E-06 6780 5.98E-06 6220 7.82E-06
7130 3.54E-05 5100 7.01E-06 4900 5.41E-06 4500
3.55E-06 6780 5.98E-06 6220 7.82E-06
7130 3.50E-05 5110 6.52E-06 4900 5.41E-06 4500
3.54E-06 6780 5.98E-06 6220 7.83E-06
7140 3.47E-05 5110 6.07E-06 4900 5.41E-06 4500
3.54E-06 6780 5.99E-06 6220 7.84E-06
7140 3.44E-05 5110 5.68E-06 4910 5.42E-06 4500
3.53E-06 6780 5.99E-06 6220 7.84E-06
7140 3.41E-05 5110 5.33E-06 4910 5.41E-06 4500
3.55E-06 6780 5.98E-06 6230 7.83E-06
7140 3.38E-05 5110 5.01E-06 4910 5.41E-06 4500
3.54E-06 6790 5.99E-06 6230 7.83E-06

7140 3.35E-05 5110 4.74E-06 4910 5.42E-06 4500
3.55E-06 6790 5.99E-06 6230 7.84E-06
7140 3.32E-05 5120 4.51E-06 4910 5.41E-06 4510
3.53E-06 6790 6.00E-06 6230 7.84E-06
7150 3.29E-05 5120 4.29E-06 4910 5.42E-06 4510
3.53E-06 6790 6.00E-06 6230 7.82E-06
7150 3.26E-05 5120 4.09E-06 4920 5.43E-06 4510
3.54E-06 6790 6.00E-06 6230 7.85E-06
7150 3.24E-05 5120 3.90E-06 4920 5.43E-06 4510
3.53E-06 6790 6.01E-06 6230 7.85E-06
7150 3.20E-05 5120 3.74E-06 4920 5.43E-06 4510
3.53E-06 6800 6.01E-06 6240 7.87E-06
7150 3.18E-05 5120 3.59E-06 4920 5.44E-06 4510
3.53E-06 6800 6.02E-06 6240 7.85E-06
7150 3.16E-05 5130 3.46E-06 4920 5.44E-06 4520
3.54E-06 6800 6.01E-06 6240 7.85E-06
7150 3.13E-05 5130 3.33E-06 4920 5.45E-06 4520
3.53E-06 6800 6.02E-06 6240 7.85E-06
7160 3.10E-05 5130 3.20E-06 4930 5.46E-06 4520
3.53E-06 6800 6.02E-06 6240 7.85E-06
7160 3.08E-05 5130 3.10E-06 4930 5.47E-06 4520
3.54E-06 6800 6.02E-06 6240 7.85E-06
7160 3.06E-05 5130 2.99E-06 4930 5.47E-06 4520
3.52E-06 6800 6.02E-06 6250 7.86E-06
7160 3.03E-05 5130 2.90E-06 4930 5.47E-06 4520
3.53E-06 6810 6.03E-06 6250 7.87E-06
7160 3.01E-05 5130 2.82E-06 4930 5.47E-06 4530
3.54E-06 6810 6.02E-06 6250 7.86E-06
7160 2.99E-05 5140 2.74E-06 4930 5.46E-06 4530
3.53E-06 6810 6.03E-06 6250 7.86E-06
7170 2.97E-05 5140 2.67E-06 4930 5.46E-06 4530
3.53E-06 6810 6.04E-06 6250 7.87E-06
7170 2.95E-05 5140 2.61E-06 4940 5.46E-06 4530
3.54E-06 6810 6.04E-06 6250 7.87E-06
7170 2.93E-05 5140 2.55E-06 4940 5.46E-06 4530
3.54E-06 6810 6.04E-06 6250 7.86E-06
7170 2.90E-05 5140 2.48E-06 4940 5.46E-06 4530
3.54E-06 6820 6.04E-06 6260 7.87E-06
7170 2.88E-05 5140 2.44E-06 4940 5.46E-06 4530
3.54E-06 6820 6.04E-06 6260 7.86E-06
7170 2.87E-05 5150 2.40E-06 4940 5.47E-06 4540
3.53E-06 6820 6.04E-06 6260 7.87E-06
7180 2.85E-05 5150 2.35E-06 4940 5.46E-06 4540
3.53E-06 6820 6.04E-06 6260 7.87E-06
7180 2.83E-05 5150 2.30E-06 4950 5.47E-06 4540
3.54E-06 6820 6.04E-06 6260 7.87E-06
7180 2.82E-05 5150 2.28E-06 4950 5.47E-06 4540
3.53E-06 6820 6.05E-06 6260 7.87E-06
7180 2.81E-05 5150 2.23E-06 4950 5.46E-06 4540
3.52E-06 6830 6.05E-06 6270 7.87E-06
7180 2.79E-05 5150 2.18E-06 4950 5.46E-06 4540
3.52E-06 6830 6.05E-06 6270 7.87E-06
7180 2.78E-05 5150 2.16E-06 4950 5.48E-06 4550
3.51E-06 6830 6.06E-06 6270 7.88E-06
7180 2.77E-05 5160 2.14E-06 4950 5.49E-06 4550
3.51E-06 6830 6.06E-06 6270 7.86E-06
7190 2.75E-05 5160 2.10E-06 4950 5.49E-06 4550
3.51E-06 6830 6.06E-06 6270 7.88E-06
7190 2.74E-05 5160 2.08E-06 4960 5.49E-06 4550
3.52E-06 6830 6.06E-06 6270 7.87E-06
7190 2.73E-05 5160 2.07E-06 4960 5.49E-06 4550
3.52E-06 6830 6.06E-06 6280 7.89E-06
7190 2.72E-05 5160 2.03E-06 4960 5.50E-06 4550
3.52E-06 6840 6.06E-06 6280 7.89E-06
7190 2.70E-05 5160 2.01E-06 4960 5.50E-06 4550
3.52E-06 6840 6.06E-06 6280 7.89E-06
7190 2.69E-05 5170 1.99E-06 4960 5.50E-06 4560
3.50E-06 6840 6.07E-06 6280 7.90E-06

7200 2.68E-05 5170 1.97E-06 4960 5.50E-06 4560
3.51E-06 6840 6.07E-06 6280 7.91E-06
7200 2.67E-05 5170 1.95E-06 4970 5.49E-06 4560
3.49E-06 6840 6.07E-06 6280 7.89E-06
7200 2.66E-05 5170 1.93E-06 4970 5.49E-06 4560
3.49E-06 6840 6.07E-06 6280 7.89E-06
7200 2.65E-05 5170 1.91E-06 4970 5.51E-06 4560
3.49E-06 6850 6.06E-06 6290 7.89E-06
7200 2.64E-05 5170 1.89E-06 4970 5.51E-06 4560
3.49E-06 6850 6.06E-06 6290 7.89E-06
7200 2.64E-05 5180 1.89E-06 4970 5.52E-06 4570
3.50E-06 6850 6.06E-06 6290 7.89E-06
7200 2.63E-05 5180 1.87E-06 4970 5.52E-06 4570
3.50E-06 6850 6.06E-06 6290 7.89E-06
7210 2.62E-05 5180 1.84E-06 4980 5.52E-06 4570
3.49E-06 6850 6.06E-06 6290 7.89E-06
7210 2.62E-05 5180 1.83E-06 4980 5.53E-06 4570
3.49E-06 6850 6.06E-06 6290 7.90E-06
7210 2.61E-05 5180 1.82E-06 4980 5.53E-06 4570
3.49E-06 6850 6.06E-06 6300 7.89E-06
7210 2.60E-05 5180 1.81E-06 4980 5.53E-06 4570
3.48E-06 6860 6.06E-06 6300 7.89E-06
7210 2.60E-05 5180 1.79E-06 4980 5.54E-06 4580
3.47E-06 6860 6.07E-06 6300 7.91E-06
7210 2.59E-05 5190 1.79E-06 4980 5.56E-06 4580
3.48E-06 6860 6.06E-06 6300 7.90E-06
7220 2.59E-05 5190 1.76E-06 4980 5.56E-06 4580
3.48E-06 6860 6.06E-06 6300 7.90E-06
7220 2.58E-05 5190 1.76E-06 4990 5.56E-06 4580
3.48E-06 6860 6.06E-06 6300 7.91E-06
7220 2.57E-05 5190 1.77E-06 4990 5.57E-06 4580
3.48E-06 6860 6.06E-06 6300 7.92E-06
7220 2.57E-05 5190 1.74E-06 4990 5.57E-06 4580
3.48E-06 6870 6.06E-06 6310 7.91E-06
7220 2.57E-05 5190 1.73E-06 4990 5.58E-06 4580
3.49E-06 6870 6.06E-06 6310 7.91E-06
7220 2.55E-05 5200 1.74E-06 4990 5.58E-06 4590
3.49E-06 6870 6.07E-06 6310 7.91E-06
7230 2.55E-05 5200 1.72E-06 4990 5.57E-06 4590
3.48E-06 6870 6.07E-06 6310 7.91E-06
7230 2.54E-05 5200 1.72E-06 5000 5.57E-06 4590
3.49E-06 6870 6.07E-06 6310 7.90E-06
7230 2.54E-05 5200 1.72E-06 5000 5.58E-06 4590
3.50E-06 6870 6.07E-06 6310 7.91E-06
7230 2.54E-05 5200 1.69E-06 5000 5.59E-06 4590
3.51E-06 6880 6.07E-06 6320 7.91E-06
7230 2.53E-05 5200 1.68E-06 5000 5.59E-06 4590
3.50E-06 6880 6.07E-06 6320 7.90E-06
7230 2.53E-05 5200 1.67E-06 5000 5.59E-06 4600
3.49E-06 6880 6.07E-06 6320 7.90E-06
7230 2.52E-05 5210 1.68E-06 5000 5.59E-06 4600
3.49E-06 6880 6.06E-06 6320 7.90E-06
7240 2.52E-05 5210 1.67E-06 5000 5.59E-06 4600
3.49E-06 6880 6.07E-06 6320 7.90E-06
7240 2.52E-05 5210 1.66E-06 5010 5.59E-06 4600
3.49E-06 6880 6.06E-06 6320 7.91E-06
7240 2.51E-05 5210 1.66E-06 5010 5.59E-06 4600
3.49E-06 6880 6.07E-06 6330 7.90E-06
7240 2.51E-05 5210 1.65E-06 5010 5.59E-06 4600
3.49E-06 6890 6.07E-06 6330 7.92E-06
7240 2.51E-05 5210 1.64E-06 5010 5.59E-06 4600
3.49E-06 6890 6.07E-06 6330 7.92E-06
7240 2.50E-05 5220 1.64E-06 5010 5.62E-06 4610
3.49E-06 6890 6.07E-06 6330 7.91E-06
7250 2.49E-05 5220 1.63E-06 5010 5.63E-06 4610
3.49E-06 6890 6.07E-06 6330 7.92E-06
7250 2.49E-05 5220 1.62E-06 5020 5.64E-06 4610
3.50E-06 6890 6.06E-06 6330 7.91E-06

7250	2.49E-05	5220	1.62E-06	5020	5.64E-06	4610
3.49E-06	6890	6.07E-06	6330	7.92E-06		
7250	2.49E-05	5220	1.61E-06	5020	5.65E-06	4610
3.50E-06	6900	6.06E-06	6340	7.92E-06		
7250	2.48E-05	5220	1.60E-06	5020	5.65E-06	4610
3.50E-06	6900	6.06E-06	6340	7.92E-06		
7250	2.48E-05	5230	1.60E-06	5020	5.64E-06	4620
3.49E-06	6900	6.06E-06	6340	7.92E-06		
7250	2.48E-05	5230	1.60E-06	5020	5.64E-06	4620
3.48E-06	6900	6.06E-06	6340	7.92E-06		
7260	2.47E-05	5230	1.60E-06	5030	5.64E-06	4620
3.47E-06	6900	6.06E-06	6340	7.91E-06		
7260	2.47E-05	5230	1.60E-06	5030	5.66E-06	4620
3.48E-06	6900	6.05E-06	6340	7.92E-06		
7260	2.47E-05	5230	1.60E-06	5030	5.67E-06	4620
3.46E-06	6900	6.06E-06	6350	7.92E-06		
7260	2.47E-05	5230	1.59E-06	5030	5.68E-06	4620
3.48E-06	6910	6.05E-06	6350	7.91E-06		
7260	2.47E-05	5230	1.59E-06	5030	5.69E-06	4630
3.47E-06	6910	6.05E-06	6350	7.92E-06		
7260	2.47E-05	5240	1.58E-06	5030	5.69E-06	4630
3.47E-06	6910	6.05E-06	6350	7.93E-06		
7270	2.47E-05	5240	1.58E-06	5030	5.71E-06	4630
3.48E-06	6910	6.05E-06	6350	7.93E-06		
7270	2.47E-05	5240	1.59E-06	5040	5.72E-06	4630
3.47E-06	6910	6.05E-06	6350	7.93E-06		
7270	2.46E-05	5240	1.57E-06	5040	5.72E-06	4630
3.47E-06	6910	6.05E-06	6350	7.94E-06		
7270	2.47E-05	5240	1.57E-06	5040	5.74E-06	4630
3.46E-06	6920	6.05E-06	6360	7.94E-06		
7270	2.46E-05	5240	1.57E-06	5040	5.75E-06	4630
3.46E-06	6920	6.05E-06	6360	7.94E-06		
7270	2.46E-05	5250	1.57E-06	5040	5.76E-06	4640
3.45E-06	6920	6.05E-06	6360	7.93E-06		
7280	2.46E-05	5250	1.57E-06	5040	5.78E-06	4640
3.45E-06	6920	6.04E-06	6360	7.94E-06		
7280	2.46E-05	5250	1.56E-06	5050	5.79E-06	4640
3.47E-06	6920	6.05E-06	6360	7.95E-06		
7280	2.46E-05	5250	1.56E-06	5050	5.82E-06	4640
3.46E-06	6920	6.05E-06	6360	7.94E-06		
7280	2.46E-05	5250	1.55E-06	5050	5.82E-06	4640
3.47E-06	6930	6.04E-06	6370	7.95E-06		
7280	2.45E-05	5250	1.55E-06	5050	5.83E-06	4640
3.47E-06	6930	6.05E-06	6370	7.96E-06		
7280	2.45E-05	5250	1.55E-06	5050	5.83E-06	4650
3.47E-06	6930	6.05E-06	6370	7.95E-06		
7280	2.45E-05	5260	1.55E-06	5050	5.85E-06	4650
3.47E-06	6930	6.05E-06	6370	7.95E-06		
7290	2.45E-05	5260	1.54E-06	5050	5.86E-06	4650
3.47E-06	6930	6.04E-06	6370	7.94E-06		
7290	2.44E-05	5260	1.54E-06	5060	5.86E-06	4650
3.47E-06	6930	6.05E-06	6370	7.94E-06		
7290	2.44E-05	5260	1.54E-06	5060	5.88E-06	4650
3.47E-06	6930	6.05E-06	6380	7.93E-06		
7290	2.44E-05	5260	1.53E-06	5060	5.90E-06	4650
3.48E-06	6940	6.04E-06	6380	7.92E-06		
7290	2.45E-05	5260	1.53E-06	5060	5.91E-06	4650
3.46E-06	6940	6.04E-06	6380	7.93E-06		
7290	2.44E-05	5270	1.53E-06	5060	5.92E-06	4660
3.47E-06	6940	6.04E-06	6380	7.94E-06		
7300	2.44E-05	5270	1.52E-06	5060	5.94E-06	4660
3.47E-06	6940	6.04E-06	6380	7.95E-06		
7300	2.45E-05	5270	1.52E-06	5070	5.95E-06	4660
3.47E-06	6940	6.04E-06	6380	7.94E-06		
7300	2.44E-05	5270	1.52E-06	5070	5.96E-06	4660
3.48E-06	6940	6.04E-06	6380	7.95E-06		
7300	2.44E-05	5270	1.51E-06	5070	5.97E-06	4660
3.47E-06	6950	6.05E-06	6390	7.94E-06		

7300 2.44E-05 5270 1.52E-06 5070 5.99E-06 4660
3.48E-06 6950 6.04E-06 6390 7.94E-06
7300 2.44E-05 5280 1.52E-06 5070 5.99E-06 4670
3.46E-06 6950 6.04E-06 6390 7.93E-06
7300 2.43E-05 5280 1.51E-06 5070 6.01E-06 4670
3.47E-06 6950 6.05E-06 6390 7.93E-06
7310 2.44E-05 5280 1.51E-06 5080 6.02E-06 4670
3.49E-06 6950 6.04E-06 6390 7.94E-06
7310 2.43E-05 5280 1.51E-06 5080 6.02E-06 4670
3.47E-06 6950 6.04E-06 6390 7.95E-06
7310 2.43E-05 5280 1.51E-06 5080 6.02E-06 4670
3.47E-06 6950 6.05E-06 6400 7.96E-06
7310 2.43E-05 5280 1.50E-06 5080 6.02E-06 4670
3.46E-06 6960 6.04E-06 6400 7.96E-06
7310 2.43E-05 5280 1.51E-06 5080 6.01E-06 4680
3.46E-06 6960 6.04E-06 6400 7.96E-06
7310 2.43E-05 5290 1.50E-06 5080 6.02E-06 4680
3.46E-06 6960 6.05E-06 6400 7.96E-06
7320 2.43E-05 5290 1.50E-06 5080 6.01E-06 4680
3.45E-06 6960 6.05E-06 6400 7.95E-06
7320 2.42E-05 5290 1.50E-06 5090 6.01E-06 4680
3.45E-06 6960 6.04E-06 6400 7.96E-06
7320 2.42E-05 5290 1.50E-06 5090 6.02E-06 4680
3.45E-06 6960 6.04E-06 6400 7.96E-06
7320 2.42E-05 5290 1.49E-06 5090 6.02E-06 4680
3.46E-06 6970 6.04E-06 6410 7.96E-06
7320 2.42E-05 5290 1.49E-06 5090 6.02E-06 4680
3.45E-06 6970 6.04E-06 6410 7.97E-06
7320 2.42E-05 5300 1.50E-06 5090 6.02E-06 4690
3.46E-06 6970 6.04E-06 6410 7.96E-06
7330 2.42E-05 5300 1.49E-06 5090 6.03E-06 4690
3.46E-06 6970 6.03E-06 6410 7.96E-06
7330 2.42E-05 5300 1.49E-06 5100 6.04E-06 4690
3.45E-06 6970 6.03E-06 6410 7.96E-06
7330 2.42E-05 5300 1.49E-06 5100 6.04E-06 4690
3.45E-06 6970 6.03E-06 6410 7.96E-06
7330 2.42E-05 5300 1.49E-06 5100 6.04E-06 4690
3.45E-06 6980 6.03E-06 6420 7.96E-06
7330 2.42E-05 5300 1.49E-06 5100 6.05E-06 4690
3.44E-06 6980 6.03E-06 6420 7.96E-06
7330 2.42E-05 5300 1.49E-06 5100 6.06E-06 4700
3.43E-06 6980 6.03E-06 6420 7.96E-06
7330 2.42E-05 5310 1.48E-06 5100 6.06E-06 4700
3.42E-06 6980 6.03E-06 6420 7.97E-06
7340 2.42E-05 5310 1.48E-06 5100 6.08E-06 4700
3.45E-06 6980 6.03E-06 6420 7.97E-06
7340 2.42E-05 5310 1.49E-06 5110 6.10E-06 4700
3.43E-06 6980 6.03E-06 6420 7.96E-06
7340 2.42E-05 5310 1.48E-06 5110 6.12E-06 4700
3.45E-06 6980 6.03E-06 6430 7.97E-06
7340 2.42E-05 5310 1.48E-06 5110 6.12E-06 4700
3.47E-06 6990 6.02E-06 6430 7.98E-06
7340 2.42E-05 5310 1.48E-06 5110 6.13E-06 4700
3.46E-06 6990 6.02E-06 6430 7.98E-06
7340 2.42E-05 5320 1.48E-06 5110 6.14E-06 4710
3.47E-06 6990 6.03E-06 6430 7.99E-06
7350 2.42E-05 5320 1.48E-06 5110 6.15E-06 4710
3.45E-06 6990 6.02E-06 6430 7.97E-06
7350 2.42E-05 5320 1.48E-06 5120 6.17E-06 4710
3.46E-06 6990 6.02E-06 6430 7.97E-06
7350 2.42E-05 5320 1.48E-06 5120 6.19E-06 4710
3.45E-06 6990 6.03E-06 6430 7.98E-06
7350 2.42E-05 5320 1.47E-06 5120 6.21E-06 4710
3.46E-06 7000 6.02E-06 6440 7.98E-06
7350 2.42E-05 5320 1.48E-06 5120 6.23E-06 4710
3.46E-06 7000 6.03E-06 6440 7.98E-06
7350 2.42E-05 5330 1.48E-06 5120 6.26E-06 4720
3.46E-06 7000 6.03E-06 6440 7.98E-06

7350 2.42E-05 5330 1.47E-06 5120 6.29E-06 4720
3.48E-06 7000 6.03E-06 6440 7.98E-06
7360 2.42E-05 5330 1.47E-06 5130 6.32E-06 4720
3.47E-06 7000 6.03E-06 6440 7.98E-06
7360 2.42E-05 5330 1.47E-06 5130 6.34E-06 4720
3.49E-06 7000 6.03E-06 6440 7.98E-06
7360 2.42E-05 5330 1.46E-06 5130 6.37E-06 4720
3.48E-06 7000 6.03E-06 6450 7.99E-06
7360 2.42E-05 5330 1.47E-06 5130 6.41E-06 4720
3.49E-06 7010 6.03E-06 6450 7.99E-06
7360 2.42E-05 5330 1.47E-06 5130 6.47E-06 4730
3.50E-06 7010 6.03E-06 6450 7.98E-06
7360 2.42E-05 5340 1.47E-06 5130 6.50E-06 4730
3.48E-06 7010 6.02E-06 6450 7.98E-06
7370 2.42E-05 5340 1.46E-06 5130 6.55E-06 4730
3.51E-06 7010 6.02E-06 6450 7.97E-06
7370 2.42E-05 5340 1.47E-06 5140 6.59E-06 4730
3.49E-06 7010 6.02E-06 6450 7.97E-06
7370 2.42E-05 5340 1.46E-06 5140 6.64E-06 4730
3.50E-06 7010 6.02E-06 6450 7.98E-06
7370 2.42E-05 5340 1.46E-06 5140 6.69E-06 4730
3.49E-06 7020 6.02E-06 6460 7.98E-06
7370 2.42E-05 5340 1.47E-06 5140 6.74E-06 4730
3.50E-06 7020 6.02E-06 6460 7.99E-06
7370 2.42E-05 5350 1.46E-06 5140 6.78E-06 4740
3.49E-06 7020 6.02E-06 6460 7.99E-06
7380 2.42E-05 5350 1.46E-06 5140 6.83E-06 4740
3.49E-06 7020 6.02E-06 6460 7.98E-06
7380 2.42E-05 5350 1.46E-06 5150 6.90E-06 4740
3.51E-06 7020 6.01E-06 6460 7.98E-06
7380 2.42E-05 5350 1.46E-06 5150 6.96E-06 4740
3.49E-06 7020 6.02E-06 6460 7.98E-06
7380 2.42E-05 5350 1.46E-06 5150 7.03E-06 4740
3.51E-06 7030 6.01E-06 6470 7.97E-06
7380 2.42E-05 5350 1.46E-06 5150 7.10E-06 4740
3.51E-06 7030 6.02E-06 6470 7.98E-06
7380 2.42E-05 5350 1.46E-06 5150 7.18E-06 4750
3.52E-06 7030 6.02E-06 6470 7.98E-06
7380 2.42E-05 5360 1.46E-06 5150 7.25E-06 4750
3.52E-06 7030 6.02E-06 6470 7.99E-06
7390 2.42E-05 5360 1.46E-06 5150 7.33E-06 4750
3.51E-06 7030 6.02E-06 6470 7.99E-06
7390 2.42E-05 5360 1.45E-06 5160 7.40E-06 4750
3.51E-06 7030 6.02E-06 6470 7.99E-06
7390 2.42E-05 5360 1.47E-06 5160 7.49E-06 4750
3.51E-06 7030 6.02E-06 6480 7.99E-06
7390 2.42E-05 5360 1.47E-06 5160 7.57E-06 4750
3.52E-06 7040 6.01E-06 6480 8.00E-06
7390 2.43E-05 5360 1.46E-06 5160 7.67E-06 4750
3.52E-06 7040 6.01E-06 6480 7.99E-06
7390 2.42E-05 5370 1.46E-06 5160 7.75E-06 4760
3.52E-06 7040 6.01E-06 6480 8.00E-06
7400 2.42E-05 5370 1.46E-06 5160 7.84E-06 4760
3.52E-06 7040 6.01E-06 6480 8.00E-06
7400 2.42E-05 5370 1.47E-06 5170 7.91E-06 4760
3.52E-06 7040 6.01E-06 6480 8.00E-06
7400 2.43E-05 5370 1.46E-06 5170 7.99E-06 4760
3.53E-06 7040 6.01E-06 6480 8.01E-06
7400 2.42E-05 5370 1.46E-06 5170 8.07E-06 4760
3.51E-06 7050 6.01E-06 6490 8.01E-06
7400 2.42E-05 5370 1.47E-06 5170 8.13E-06 4760
3.52E-06 7050 6.01E-06 6490 8.02E-06
7400 2.42E-05 5380 1.47E-06 5170 8.18E-06 4770
3.53E-06 7050 6.01E-06 6490 8.03E-06
7400 2.42E-05 5380 1.46E-06 5170 8.23E-06 4770
3.52E-06 7050 6.01E-06 6490 8.01E-06
7410 2.43E-05 5380 1.46E-06 5180 8.27E-06 4770
3.53E-06 7050 6.01E-06 6490 8.02E-06

7410	2.42E-05	5380	1.46E-06	5180	8.33E-06	4770
3.52E-06	7050	6.01E-06	6490	8.02E-06		
7410	2.43E-05	5380	1.46E-06	5180	8.36E-06	4770
3.53E-06	7050	6.01E-06	6500	8.02E-06		
7410	2.43E-05	5380	1.46E-06	5180	8.38E-06	4770
3.53E-06	7060	6.01E-06	6500	8.02E-06		
7410	2.43E-05	5380	1.47E-06	5180	8.40E-06	4780
3.54E-06	7060	6.00E-06	6500	8.03E-06		
7410	2.43E-05	5390	1.46E-06	5180	8.41E-06	4780
3.56E-06	7060	6.00E-06	6500	8.03E-06		
7420	2.43E-05	5390	1.46E-06	5180	8.41E-06	4780
3.54E-06	7060	6.00E-06	6500	8.03E-06		
7420	2.43E-05	5390	1.46E-06	5190	8.38E-06	4780
3.53E-06	7060	6.00E-06	6500	8.04E-06		
7420	2.43E-05	5390	1.47E-06	5190	8.37E-06	4780
3.53E-06	7060	6.00E-06	6500	8.04E-06		
7420	2.43E-05	5390	1.47E-06	5190	8.35E-06	4780
3.54E-06	7070	5.99E-06	6510	8.03E-06		
7420	2.43E-05	5390	1.47E-06	5190	8.33E-06	4780
3.54E-06	7070	6.00E-06	6510	8.03E-06		
7420	2.43E-05	5400	1.46E-06	5190	8.29E-06	4790
3.53E-06	7070	5.99E-06	6510	8.03E-06		
7430	2.43E-05	5400	1.46E-06	5190	8.24E-06	4790
3.54E-06	7070	5.98E-06	6510	8.02E-06		
7430	2.43E-05	5400	1.47E-06	5200	8.19E-06	4790
3.53E-06	7070	5.99E-06	6510	8.02E-06		
7430	2.43E-05	5400	1.46E-06	5200	8.13E-06	4790
3.54E-06	7070	5.98E-06	6510	8.04E-06		
7430	2.43E-05	5400	1.47E-06	5200	8.06E-06	4790
3.56E-06	7080	5.98E-06	6520	8.04E-06		
7430	2.43E-05	5400	1.47E-06	5200	7.98E-06	4790
3.56E-06	7080	5.97E-06	6520	8.02E-06		
7430	2.43E-05	5400	1.47E-06	5200	7.89E-06	4800
3.55E-06	7080	5.97E-06	6520	8.02E-06		
7430	2.43E-05	5410	1.46E-06	5200	7.81E-06	4800
3.55E-06	7080	5.96E-06	6520	8.02E-06		
7440	2.43E-05	5410	1.47E-06	5200	7.73E-06	4800
3.55E-06	7080	5.96E-06	6520	8.02E-06		
7440	2.43E-05	5410	1.46E-06	5210	7.66E-06	4800
3.56E-06	7080	5.94E-06	6520	8.02E-06		
7440	2.43E-05	5410	1.46E-06	5210	7.58E-06	4800
3.56E-06	7080	5.95E-06	6530	8.03E-06		
7440	2.44E-05	5410	1.46E-06	5210	7.49E-06	4800
3.55E-06	7090	5.95E-06	6530	8.04E-06		
7440	2.44E-05	5410	1.48E-06	5210	7.41E-06	4800
3.56E-06	7090	5.95E-06	6530	8.05E-06		
7440	2.44E-05	5420	1.47E-06	5210	7.33E-06	4810
3.58E-06	7090	5.94E-06	6530	8.05E-06		
7450	2.44E-05	5420	1.46E-06	5210	7.26E-06	4810
3.56E-06	7090	5.93E-06	6530	8.05E-06		
7450	2.44E-05	5420	1.46E-06	5220	7.18E-06	4810
3.56E-06	7090	5.94E-06	6530	8.05E-06		
7450	2.44E-05	5420	1.47E-06	5220	7.11E-06	4810
3.55E-06	7090	5.94E-06	6530	8.05E-06		
7450	2.44E-05	5420	1.46E-06	5220	7.05E-06	4810
3.56E-06	7100	5.94E-06	6540	8.04E-06		
7450	2.44E-05	5420	1.47E-06	5220	6.99E-06	4810
3.55E-06	7100	5.94E-06	6540	8.04E-06		
7450	2.44E-05	5430	1.47E-06	5220	6.93E-06	4820
3.55E-06	7100	5.92E-06	6540	8.02E-06		
7450	2.44E-05	5430	1.47E-06	5220	6.87E-06	4820
3.56E-06	7100	5.92E-06	6540	8.03E-06		
7460	2.44E-05	5430	1.48E-06	5230	6.82E-06	4820
3.56E-06	7100	5.91E-06	6540	8.02E-06		
7460	2.44E-05	5430	1.48E-06	5230	6.78E-06	4820
3.58E-06	7100	5.91E-06	6540	8.03E-06		
7460	2.44E-05	5430	1.48E-06	5230	6.72E-06	4820
3.56E-06	7100	5.90E-06	6550	8.03E-06		

7460 2.44E-05 5430 1.48E-06 5230 6.67E-06 4820
3.57E-06 7110 5.89E-06 6550 8.04E-06
7460 2.44E-05 5430 1.47E-06 5230 6.61E-06 4830
3.57E-06 7110 5.90E-06 6550 8.05E-06
7460 2.44E-05 5440 1.49E-06 5230 6.57E-06 4830
3.57E-06 7110 5.89E-06 6550 8.04E-06
7470 2.44E-05 5440 1.48E-06 5230 6.52E-06 4830
3.58E-06 7110 5.88E-06 6550 8.05E-06
7470 2.44E-05 5440 1.49E-06 5240 6.50E-06 4830
3.58E-06 7110 5.87E-06 6550 8.05E-06
7470 2.44E-05 5440 1.48E-06 5240 6.46E-06 4830
3.59E-06 7110 5.88E-06 6550 8.06E-06
7470 2.45E-05 5440 1.48E-06 5240 6.42E-06 4830
3.59E-06 7120 5.88E-06 6560 8.06E-06
7470 2.45E-05 5440 1.49E-06 5240 6.39E-06 4830
3.59E-06 7120 5.88E-06 6560 8.07E-06
7470 2.45E-05 5450 1.48E-06 5240 6.35E-06 4840
3.61E-06 7120 5.89E-06 6560 8.07E-06
7480 2.45E-05 5450 1.48E-06 5240 6.33E-06 4840
3.60E-06 7120 5.89E-06 6560 8.06E-06
7480 2.45E-05 5450 1.47E-06 5250 6.30E-06 4840
3.60E-06 7120 5.88E-06 6560 8.06E-06
7480 2.45E-05 5450 1.48E-06 5250 6.28E-06 4840
3.60E-06 7120 5.89E-06 6560 8.06E-06
7480 2.45E-05 5450 1.49E-06 5250 6.26E-06 4840
3.60E-06 7130 5.88E-06 6570 8.05E-06
7480 2.45E-05 5450 1.47E-06 5250 6.25E-06 4840
3.60E-06 7130 5.89E-06 6570 8.05E-06
7480 2.46E-05 5450 1.48E-06 5250 6.23E-06 4850
3.59E-06 7130 5.88E-06 6570 8.05E-06
7480 2.45E-05 5460 1.48E-06 5250 6.22E-06 4850
3.59E-06 7130 5.88E-06 6570 8.05E-06
7490 2.45E-05 5460 1.48E-06 5250 6.20E-06 4850
3.60E-06 7130 5.87E-06 6570 8.04E-06
7490 2.46E-05 5460 1.48E-06 5260 6.17E-06 4850
3.60E-06 7130 5.88E-06 6570 8.05E-06
7490 2.45E-05 5460 1.47E-06 5260 6.16E-06 4850
3.60E-06 7130 5.88E-06 6580 8.04E-06
7490 2.45E-05 5460 1.46E-06 5260 6.13E-06 4850
3.61E-06 7140 5.87E-06 6580 8.04E-06
7490 2.46E-05 5460 1.47E-06 5260 6.11E-06 4850
3.60E-06 7140 5.89E-06 6580 8.05E-06
7490 2.45E-05 5470 1.48E-06 5260 6.09E-06 4860
3.61E-06 7140 5.89E-06 6580 8.03E-06
7500 2.45E-05 5470 1.48E-06 5260 6.07E-06 4860
3.62E-06 7140 5.89E-06 6580 8.04E-06
7500 2.45E-05 5470 1.47E-06 5270 6.06E-06 4860
3.63E-06 7140 5.89E-06 6580 8.03E-06
7500 2.45E-05 5470 1.47E-06 5270 6.04E-06 4860
3.62E-06 7140 5.89E-06 6580 8.05E-06
7500 2.45E-05 5470 1.47E-06 5270 6.04E-06 4860
3.62E-06 7150 5.91E-06 6590 8.05E-06
7500 2.45E-05 5470 1.47E-06 5270 6.03E-06 4860
3.64E-06 7150 5.92E-06 6590 8.05E-06
7500 2.45E-05 5480 1.46E-06 5270 6.01E-06 4870
3.65E-06 7150 5.92E-06 6590 8.05E-06
7500 2.46E-05 5480 1.46E-06 5270 6.00E-06 4870
3.63E-06 7150 5.92E-06 6590 8.04E-06
7510 2.46E-05 5480 1.46E-06 5280 6.00E-06 4870
3.64E-06 7150 5.93E-06 6590 8.05E-06
7510 2.46E-05 5480 1.46E-06 5280 5.99E-06 4870
3.62E-06 7150 5.95E-06 6590 8.04E-06
7510 2.46E-05 5480 1.47E-06 5280 5.98E-06 4870
3.64E-06 7150 5.96E-06 6600 8.04E-06
7510 2.46E-05 5480 1.46E-06 5280 5.98E-06 4870
3.63E-06 7160 5.97E-06 6600 8.04E-06
7510 2.46E-05 5480 1.46E-06 5280 5.98E-06 4880
3.62E-06 7160 5.98E-06 6600 8.02E-06

7510 2.46E-05 5490 1.46E-06 5280 5.97E-06 4880
3.63E-06 7160 5.98E-06 6600 8.03E-06
7520 2.46E-05 5490 1.46E-06 5280 5.96E-06 4880
3.63E-06 7160 5.98E-06 6600 8.03E-06
7520 2.46E-05 5490 1.46E-06 5290 5.95E-06 4880
3.62E-06 7160 5.99E-06 6600 8.04E-06
7520 2.46E-05 5490 1.45E-06 5290 5.94E-06 4880
3.61E-06 7160 5.99E-06 6600 8.04E-06
7520 2.46E-05 5490 1.46E-06 5290 5.93E-06 4880
3.61E-06 7170 6.00E-06 6610 8.04E-06
7520 2.47E-05 5490 1.46E-06 5290 5.92E-06 4880
3.63E-06 7170 6.01E-06 6610 8.04E-06
7520 2.46E-05 5500 1.46E-06 5290 5.91E-06 4890
3.62E-06 7170 6.00E-06 6610 8.03E-06
7530 2.47E-05 5500 1.46E-06 5290 5.90E-06 4890
3.63E-06 7170 6.02E-06 6610 8.04E-06
7530 2.47E-05 5500 1.46E-06 5300 5.90E-06 4890
3.62E-06 7170 6.00E-06 6610 8.04E-06
7530 2.47E-05 5500 1.47E-06 5300 5.89E-06 4890
3.63E-06 7170 6.02E-06 6610 8.05E-06
7530 2.47E-05 5500 1.47E-06 5300 5.89E-06 4890
3.62E-06 7180 6.02E-06 6620 8.05E-06
7530 2.47E-05 5500 1.47E-06 5300 5.88E-06 4890
3.64E-06 7180 6.02E-06 6620 8.05E-06
7530 2.48E-05 5500 1.45E-06 5300 5.88E-06 4900
3.64E-06 7180 6.03E-06 6620 8.05E-06
7530 2.48E-05 5510 1.46E-06 5300 5.88E-06 4900
3.62E-06 7180 6.03E-06 6620 8.05E-06
7540 2.48E-05 5510 1.46E-06 5300 5.88E-06 4900
3.64E-06 7180 6.03E-06 6620 8.05E-06
7540 2.48E-05 5510 1.46E-06 5310 5.88E-06 4900
3.62E-06 7180 6.03E-06 6620 8.05E-06
7540 2.48E-05 5510 1.46E-06 5310 5.87E-06 4900
3.62E-06 7180 6.04E-06 6630 8.05E-06
7540 2.48E-05 5510 1.47E-06 5310 5.88E-06 4900
3.65E-06 7190 6.04E-06 6630 8.04E-06
7540 2.48E-05 5510 1.46E-06 5310 5.87E-06 4900
3.63E-06 7190 6.05E-06 6630 8.05E-06
7540 2.48E-05 5520 1.47E-06 5310 5.87E-06 4910
3.64E-06 7190 6.05E-06 6630 8.04E-06
7550 2.48E-05 5520 1.48E-06 5310 5.88E-06 4910
3.64E-06 7190 6.06E-06 6630 8.04E-06
7550 2.48E-05 5520 1.47E-06 5320 5.88E-06 4910
3.64E-06 7190 6.06E-06 6630 8.05E-06
7550 2.48E-05 5520 1.47E-06 5320 5.87E-06 4910
3.63E-06 7190 6.07E-06 6630 8.05E-06
7550 2.48E-05 5520 1.47E-06 5320 5.87E-06 4910
3.63E-06 7200 6.07E-06 6640 8.04E-06
7550 2.48E-05 5520 1.47E-06 5320 5.85E-06 4910
3.64E-06 7200 6.07E-06 6640 8.04E-06
7550 2.48E-05 5530 1.47E-06 5320 5.85E-06 4920
3.65E-06 7200 6.08E-06 6640 8.03E-06
7550 2.48E-05 5530 1.46E-06 5320 5.85E-06 4920
3.67E-06 7200 6.08E-06 6640 8.04E-06
7560 2.48E-05 5530 1.46E-06 5330 5.84E-06 4920
3.66E-06 7200 6.08E-06 6640 8.03E-06
7560 2.48E-05 5530 1.46E-06 5330 5.83E-06 4920
3.68E-06 7200 6.08E-06 6640 8.04E-06
7560 2.48E-05 5530 1.46E-06 5330 5.83E-06 4920
3.68E-06 7200 6.10E-06 6650 8.04E-06
7560 2.49E-05 5530 1.47E-06 5330 5.82E-06 4920
3.68E-06 7210 6.09E-06 6650 8.04E-06
7560 2.49E-05 5530 1.46E-06 5330 5.81E-06 4930
3.70E-06 7210 6.10E-06 6650 8.04E-06
7560 2.49E-05 5540 1.47E-06 5330 5.82E-06 4930
3.69E-06 7210 6.11E-06 6650 8.05E-06
7570 2.49E-05 5540 1.46E-06 5330 5.84E-06 4930
3.68E-06 7210 6.11E-06 6650 8.04E-06

7570 2.49E-05 5540 1.46E-06 5340 5.82E-06 4930
3.67E-06 7210 6.11E-06 6650 8.05E-06
7570 2.49E-05 5540 1.46E-06 5340 5.81E-06 4930
3.69E-06 7210 6.11E-06 6650 8.04E-06
7570 2.49E-05 5540 1.47E-06 5340 5.82E-06 4930
3.68E-06 7220 6.11E-06 6660 8.05E-06
7570 2.49E-05 5540 1.46E-06 5340 5.82E-06 4930
3.68E-06 7220 6.11E-06 6660 8.04E-06
7570 2.49E-05 5550 1.47E-06 5340 5.83E-06 4940
3.67E-06 7220 6.11E-06 6660 8.05E-06
7580 2.49E-05 5550 1.47E-06 5340 5.83E-06 4940
3.68E-06 7220 6.12E-06 6660 8.04E-06
7580 2.50E-05 5550 1.47E-06 5350 5.82E-06 4940
3.68E-06 7220 6.11E-06 6660 8.05E-06
7580 2.50E-05 5550 1.45E-06 5350 5.82E-06 4940
3.67E-06 7220 6.12E-06 6660 8.06E-06
7580 2.50E-05 5550 1.47E-06 5350 5.82E-06 4940
3.69E-06 7230 6.12E-06 6670 8.05E-06
7580 2.50E-05 5550 1.46E-06 5350 5.80E-06 4940
3.67E-06 7230 6.12E-06 6670 8.06E-06
7580 2.50E-05 5550 1.46E-06 5350 5.82E-06 4950
3.68E-06 7230 6.13E-06 6670 8.05E-06
7580 2.50E-05 5560 1.46E-06 5350 5.82E-06 4950
3.69E-06 7230 6.12E-06 6670 8.07E-06
7590 2.50E-05 5560 1.45E-06 5350 5.82E-06 4950
3.69E-06 7230 6.12E-06 6670 8.05E-06
7590 2.50E-05 5560 1.46E-06 5360 5.82E-06 4950
3.69E-06 7230 6.12E-06 6670 8.07E-06
7590 2.50E-05 5560 1.47E-06 5360 5.82E-06 4950
3.69E-06 7230 6.12E-06 6680 8.07E-06
7590 2.51E-05 5560 1.47E-06 5360 5.82E-06 4950
3.70E-06 7240 6.12E-06 6680 8.07E-06
7590 2.51E-05 5560 1.46E-06 5360 5.83E-06 4950
3.71E-06 7240 6.13E-06 6680 8.07E-06
7590 2.51E-05 5570 1.47E-06 5360 5.82E-06 4960
3.71E-06 7240 6.14E-06 6680 8.07E-06
7600 2.51E-05 5570 1.47E-06 5360 5.83E-06 4960
3.71E-06 7240 6.14E-06 6680 8.08E-06
7600 2.51E-05 5570 1.46E-06 5370 5.83E-06 4960
3.70E-06 7240 6.14E-06 6680 8.08E-06
7600 2.51E-05 5570 1.47E-06 5370 5.83E-06 4960
3.71E-06 7240 6.13E-06 6680 8.07E-06
7600 2.51E-05 5570 1.47E-06 5370 5.84E-06 4960
3.69E-06 7250 6.15E-06 6690 8.07E-06
7600 2.51E-05 5570 1.47E-06 5370 5.84E-06 4960
3.70E-06 7250 6.14E-06 6690 8.06E-06
7600 2.51E-05 5580 1.47E-06 5370 5.84E-06 4970
3.70E-06 7250 6.13E-06 6690 8.08E-06
7600 2.51E-05 5580 1.47E-06 5370 5.85E-06 4970
3.71E-06 7250 6.15E-06 6690 8.07E-06
7610 2.51E-05 5580 1.47E-06 5380 5.85E-06 4970
3.70E-06 7250 6.13E-06 6690 8.07E-06
7610 2.51E-05 5580 1.47E-06 5380 5.85E-06 4970
3.70E-06 7250 6.14E-06 6690 8.07E-06
7610 2.52E-05 5580 1.47E-06 5380 5.85E-06 4970
3.71E-06 7250 6.14E-06 6700 8.07E-06
7610 2.52E-05 5580 1.47E-06 5380 5.85E-06 4970
3.70E-06 7260 6.14E-06 6700 8.07E-06
7610 2.52E-05 5580 1.47E-06 5380 5.84E-06 4980
3.71E-06 7260 6.15E-06 6700 8.06E-06
7610 2.52E-05 5590 1.48E-06 5380 5.84E-06 4980
3.71E-06 7260 6.15E-06 6700 8.08E-06
7620 2.52E-05 5590 1.47E-06 5380 5.85E-06 4980
3.72E-06 7260 6.15E-06 6700 8.06E-06
7620 2.52E-05 5590 1.48E-06 5390 5.85E-06 4980
3.71E-06 7260 6.16E-06 6700 8.07E-06
7620 2.52E-05 5590 1.47E-06 5390 5.84E-06 4980
3.70E-06 7260 6.16E-06 6700 8.07E-06

7620 2.52E-05 5590 1.46E-06 5390 5.85E-06 4980
3.71E-06 7270 6.16E-06 6710 8.08E-06
7620 2.52E-05 5590 1.46E-06 5390 5.87E-06 4980
3.75E-06 7270 6.17E-06 6710 8.08E-06
7620 2.52E-05 5600 1.46E-06 5390 5.85E-06 4990
3.73E-06 7270 6.18E-06 6710 8.08E-06
7630 2.52E-05 5600 1.47E-06 5390 5.85E-06 4990
3.75E-06 7270 6.18E-06 6710 8.09E-06
7630 2.53E-05 5600 1.46E-06 5400 5.86E-06 4990
3.74E-06 7270 6.19E-06 6710 8.08E-06
7630 2.53E-05 5600 1.47E-06 5400 5.86E-06 4990
3.76E-06 7270 6.18E-06 6710 8.08E-06
7630 2.53E-05 5600 1.47E-06 5400 5.87E-06 4990
3.75E-06 7280 6.18E-06 6720 8.08E-06
7630 2.53E-05 5600 1.47E-06 5400 5.88E-06 4990
3.75E-06 7280 6.19E-06 6720 8.07E-06
7630 2.53E-05 5600 1.47E-06 5400 5.87E-06 5000
3.75E-06 7280 6.19E-06 6720 8.08E-06
7630 2.53E-05 5610 1.47E-06 5400 5.88E-06 5000
3.74E-06 7280 6.18E-06 6720 8.06E-06
7640 2.53E-05 5610 1.47E-06 5400 5.88E-06 5000
3.75E-06 7280 6.18E-06 6720 8.07E-06
7640 2.53E-05 5610 1.47E-06 5410 5.89E-06 5000
3.73E-06 7280 6.18E-06 6720 8.07E-06
7640 2.53E-05 5610 1.46E-06 5410 5.86E-06 5000
3.74E-06 7280 6.18E-06 6730 8.07E-06
7640 2.53E-05 5610 1.45E-06 5410 5.86E-06 5000
3.74E-06 7290 6.18E-06 6730 8.08E-06
7640 2.53E-05 5610 1.46E-06 5410 5.86E-06 5000
3.74E-06 7290 6.19E-06 6730 8.08E-06
7640 2.53E-05 5620 1.46E-06 5410 5.85E-06 5010
3.75E-06 7290 6.17E-06 6730 8.09E-06
7650 2.53E-05 5620 1.46E-06 5410 5.85E-06 5010
3.75E-06 7290 6.17E-06 6730 8.08E-06
7650 2.53E-05 5620 1.47E-06 5420 5.86E-06 5010
3.74E-06 7290 6.17E-06 6730 8.10E-06
7650 2.54E-05 5620 1.47E-06 5420 5.84E-06 5010
3.74E-06 7290 6.17E-06 6730 8.10E-06
7650 2.54E-05 5620 1.46E-06 5420 5.85E-06 5010
3.73E-06 7300 6.17E-06 6740 8.10E-06
7650 2.54E-05 5620 1.46E-06 5420 5.86E-06 5010
3.76E-06 7300 6.17E-06 6740 8.11E-06
7650 2.54E-05 5630 1.47E-06 5420 5.86E-06 5020
3.73E-06 7300 6.17E-06 6740 8.11E-06
7650 2.54E-05 5630 1.46E-06 5420 5.87E-06 5020
3.73E-06 7300 6.16E-06 6740 8.10E-06
7660 2.54E-05 5630 1.46E-06 5430 5.87E-06 5020
3.73E-06 7300 6.17E-06 6740 8.10E-06
7660 2.54E-05 5630 1.46E-06 5430 5.86E-06 5020
3.74E-06 7300 6.17E-06 6740 8.10E-06
7660 2.55E-05 5630 1.46E-06 5430 5.85E-06 5020
3.72E-06 7300 6.17E-06 6750 8.11E-06
7660 2.55E-05 5630 1.46E-06 5430 5.89E-06 5020
3.73E-06 7310 6.18E-06 6750 8.10E-06
7660 2.55E-05 5630 1.46E-06 5430 5.88E-06 5030
3.74E-06 7310 6.18E-06 6750 8.11E-06
7660 2.55E-05 5640 1.47E-06 5430 5.85E-06 5030
3.74E-06 7310 6.17E-06 6750 8.10E-06
7670 2.55E-05 5640 1.47E-06 5430 5.87E-06 5030
3.73E-06 7310 6.16E-06 6750 8.10E-06
7670 2.55E-05 5640 1.47E-06 5440 5.89E-06 5030
3.72E-06 7310 6.17E-06 6750 8.09E-06
7670 2.55E-05 5640 1.46E-06 5440 5.87E-06 5030
3.73E-06 7310 6.18E-06 6750 8.10E-06
7670 2.55E-05 5640 1.46E-06 5440 5.87E-06 5030
3.73E-06 7320 6.18E-06 6760 8.10E-06
7670 2.55E-05 5640 1.46E-06 5440 5.85E-06 5030
3.73E-06 7320 6.18E-06 6760 8.11E-06

7670 2.55E-05 5650 1.46E-06 5440 5.86E-06 5040
3.74E-06 7320 6.18E-06 6760 8.11E-06
7680 2.55E-05 5650 1.46E-06 5440 5.85E-06 5040
3.74E-06 7320 6.18E-06 6760 8.11E-06
7680 2.55E-05 5650 1.46E-06 5450 5.84E-06 5040
3.73E-06 7320 6.18E-06 6760 8.11E-06
7680 2.55E-05 5650 1.46E-06 5450 5.81E-06 5040
3.73E-06 7320 6.18E-06 6760 8.11E-06
7680 2.56E-05 5650 1.46E-06 5450 5.85E-06 5040
3.74E-06 7330 6.17E-06 6770 8.11E-06
7680 2.56E-05 5650 1.45E-06 5450 5.86E-06 5040
3.76E-06 7330 6.18E-06 6770 8.11E-06
7680 2.56E-05 5650 1.45E-06 5450 5.83E-06 5050
3.74E-06 7330 6.19E-06 6770 8.12E-06
7680 2.56E-05 5660 1.45E-06 5450 5.82E-06 5050
3.76E-06 7330 6.19E-06 6770 8.11E-06
7690 2.56E-05 5660 1.44E-06 5450 5.79E-06 5050
3.75E-06 7330 6.20E-06 6770 8.11E-06
7690 2.56E-05 5660 1.44E-06 5460 5.78E-06 5050
3.76E-06 7330 6.19E-06 6770 8.12E-06
7690 2.57E-05 5660 1.45E-06 5460 5.83E-06 5050
3.77E-06 7330 6.19E-06 6780 8.11E-06
7690 2.56E-05 5660 1.46E-06 5460 5.80E-06 5050
3.75E-06 7340 6.19E-06 6780 8.11E-06
7690 2.56E-05 5660 1.47E-06 5460 5.80E-06 5050
3.75E-06 7340 6.19E-06 6780 8.10E-06
7690 2.57E-05 5670 1.48E-06 5460 5.82E-06 5060
3.76E-06 7340 6.19E-06 6780 8.10E-06
7700 2.57E-05 5670 1.48E-06 5460 5.81E-06 5060
3.76E-06 7340 6.19E-06 6780 8.10E-06
7700 2.57E-05 5670 1.47E-06 5470 5.80E-06 5060
3.75E-06 7340 6.20E-06 6780 8.09E-06
7700 2.57E-05 5670 1.47E-06 5470 5.78E-06 5060
3.76E-06 7340 6.20E-06 6780 8.10E-06
7700 2.57E-05 5670 1.47E-06 5470 5.75E-06 5060
3.76E-06 7350 6.20E-06 6790 8.10E-06
7700 2.57E-05 5670 1.48E-06 5470 5.75E-06 5060
3.76E-06 7350 6.20E-06 6790 8.10E-06
7700 2.57E-05 5680 1.49E-06 5470 5.75E-06 5070
3.77E-06 7350 6.20E-06 6790 8.11E-06
7700 2.57E-05 5680 1.48E-06 5470 5.75E-06 5070
3.76E-06 7350 6.20E-06 6790 8.10E-06
7710 2.57E-05 5680 1.48E-06 5480 5.73E-06 5070
3.77E-06 7350 6.20E-06 6790 8.11E-06
7710 2.58E-05 5680 1.49E-06 5480 5.75E-06 5070
3.77E-06 7350 6.20E-06 6790 8.11E-06
7710 2.58E-05 5680 1.48E-06 5480 5.74E-06 5070
3.77E-06 7350 6.20E-06 6800 8.12E-06
7710 2.58E-05 5680 1.48E-06 5480 5.76E-06 5070
3.78E-06 7360 6.21E-06 6800 8.12E-06
7710 2.58E-05 5680 1.48E-06 5480 5.77E-06 5080
3.78E-06 7360 6.21E-06 6800 8.12E-06
7710 2.58E-05 5690 1.49E-06 5480 5.75E-06 5080
3.78E-06 7360 6.21E-06 6800 8.12E-06
7720 2.58E-05 5690 1.48E-06 5480 5.75E-06 5080
3.78E-06 7360 6.22E-06 6800 8.12E-06
7720 2.59E-05 5690 1.49E-06 5490 5.73E-06 5080
3.78E-06 7360 6.21E-06 6800 8.12E-06
7720 2.59E-05 5690 1.49E-06 5490 5.71E-06 5080
3.79E-06 7360 6.22E-06 6800 8.12E-06
7720 2.59E-05 5690 1.49E-06 5490 5.73E-06 5080
3.79E-06 7370 6.21E-06 6810 8.12E-06
7720 2.59E-05 5690 1.50E-06 5490 5.73E-06 5080
3.79E-06 7370 6.21E-06 6810 8.12E-06
7720 2.59E-05 5700 1.52E-06 5490 5.75E-06 5090
3.79E-06 7370 6.21E-06 6810 8.11E-06
7730 2.59E-05 5700 1.51E-06 5490 5.75E-06 5090
3.80E-06 7370 6.21E-06 6810 8.12E-06

7730 2.59E-05 5700 1.51E-06 5500 5.73E-06 5090
3.79E-06 7370 6.21E-06 6810 8.12E-06
7730 2.59E-05 5700 1.51E-06 5500 5.71E-06 5090
3.79E-06 7370 6.21E-06 6810 8.12E-06
7730 2.59E-05 5700 1.51E-06 5500 5.69E-06 5090
3.80E-06 7380 6.22E-06 6820 8.12E-06
7730 2.59E-05 5700 1.52E-06 5500 5.73E-06 5090
3.80E-06 7380 6.22E-06 6820 8.13E-06
7730 2.59E-05 5700 1.52E-06 5500 5.69E-06 5100
3.81E-06 7380 6.22E-06 6820 8.14E-06
7730 2.59E-05 5710 1.51E-06 5500 5.69E-06 5100
3.81E-06 7380 6.22E-06 6820 8.14E-06
7740 2.60E-05 5710 1.51E-06 5500 5.73E-06 5100
3.80E-06 7380 6.22E-06 6820 8.14E-06
7740 2.60E-05 5710 1.51E-06 5510 5.73E-06 5100
3.82E-06 7380 6.23E-06 6820 8.14E-06
7740 2.60E-05 5710 1.50E-06 5510 5.76E-06 5100
3.81E-06 7380 6.22E-06 6830 8.15E-06
7740 2.60E-05 5710 1.51E-06 5510 5.79E-06 5100
3.83E-06 7390 6.23E-06 6830 8.16E-06
7740 2.60E-05 5710 1.51E-06 5510 5.78E-06 5100
3.82E-06 7390 6.23E-06 6830 8.15E-06
7740 2.60E-05 5720 1.50E-06 5510 5.78E-06 5110
3.83E-06 7390 6.23E-06 6830 8.15E-06
7750 2.60E-05 5720 1.51E-06 5510 5.78E-06 5110
3.82E-06 7390 6.24E-06 6830 8.15E-06
7750 2.60E-05 5720 1.51E-06 5520 5.76E-06 5110
3.84E-06 7390 6.23E-06 6830 8.14E-06
7750 2.61E-05 5720 1.52E-06 5520 5.80E-06 5110
3.83E-06 7390 6.24E-06 6830 8.14E-06
7750 2.61E-05 5720 1.51E-06 5520 5.80E-06 5110
3.82E-06 7400 6.23E-06 6840 8.14E-06
7750 2.61E-05 5720 1.52E-06 5520 5.82E-06 5110
3.82E-06 7400 6.22E-06 6840 8.15E-06
7750 2.61E-05 5730 1.54E-06 5520 5.84E-06 5120
3.83E-06 7400 6.23E-06 6840 8.13E-06
7750 2.61E-05 5730 1.53E-06 5520 5.88E-06 5120
3.84E-06 7400 6.22E-06 6840 8.13E-06
7760 2.61E-05 5730 1.52E-06 5530 5.90E-06 5120
3.84E-06 7400 6.23E-06 6840 8.13E-06
7760 2.61E-05 5730 1.53E-06 5530 5.86E-06 5120
3.83E-06 7400 6.23E-06 6840 8.13E-06
7760 2.61E-05 5730 1.53E-06 5530 5.86E-06 5120
3.84E-06 7400 6.23E-06 6850 8.12E-06
7760 2.61E-05 5730 1.53E-06 5530 5.86E-06 5120
3.84E-06 7410 6.23E-06 6850 8.12E-06
7760 2.61E-05 5730 1.53E-06 5530 5.82E-06 5130
3.85E-06 7410 6.23E-06 6850 8.13E-06
7760 2.61E-05 5740 1.53E-06 5530 5.82E-06 5130
3.86E-06 7410 6.23E-06 6850 8.13E-06
7770 2.62E-05 5740 1.53E-06 5530 5.78E-06 5130
3.86E-06 7410 6.23E-06 6850 8.14E-06
7770 2.62E-05 5740 1.54E-06 5540 5.78E-06 5130
3.86E-06 7410 6.23E-06 6850 8.14E-06
7770 2.62E-05 5740 1.53E-06 5540 5.80E-06 5130
3.87E-06 7410 6.22E-06 6850 8.14E-06
7770 2.62E-05 5740 1.53E-06 5540 5.78E-06 5130
3.88E-06 7420 6.24E-06 6860 8.14E-06
7770 2.62E-05 5740 1.54E-06 5540 5.78E-06 5130
3.87E-06 7420 6.23E-06 6860 8.14E-06
7770 2.62E-05 5750 1.54E-06 5540 5.80E-06 5140
3.87E-06 7420 6.23E-06 6860 8.15E-06
7780 2.62E-05 5750 1.54E-06 5540 5.74E-06 5140
3.87E-06 7420 6.24E-06 6860 8.15E-06
7780 2.63E-05 5750 1.56E-06 5550 5.82E-06 5140
3.88E-06 7420 6.25E-06 6860 8.15E-06
7780 2.63E-05 5750 1.56E-06 5550 5.80E-06 5140
3.88E-06 7420 6.25E-06 6860 8.15E-06

7780 2.63E-05 5750 1.57E-06 5550 5.82E-06 5140
3.88E-06 7430 6.23E-06 6870 8.13E-06
7780 2.63E-05 5750 1.57E-06 5550 5.86E-06 5140
3.88E-06 7430 6.23E-06 6870 8.15E-06
7780 2.63E-05 5750 1.58E-06 5550 5.86E-06 5150
3.87E-06 7430 6.23E-06 6870 8.13E-06
7780 2.63E-05 5760 1.58E-06 5550 5.84E-06 5150
3.88E-06 7430 6.23E-06 6870 8.14E-06
7790 2.63E-05 5760 1.58E-06 5550 5.84E-06 5150
3.87E-06 7430 6.23E-06 6870 8.15E-06
7790 2.63E-05 5760 1.58E-06 5560 5.80E-06 5150
3.87E-06 7430 6.24E-06 6870 8.15E-06
7790 2.64E-05 5760 1.58E-06 5560 5.78E-06 5150
3.88E-06 7430 6.23E-06 6880 8.15E-06
7790 2.64E-05 5760 1.58E-06 5560 5.76E-06 5150
3.88E-06 7440 6.23E-06 6880 8.16E-06
7790 2.64E-05 5760 1.59E-06 5560 5.76E-06 5150
3.89E-06 7440 6.24E-06 6880 8.18E-06
7790 2.64E-05 5770 1.60E-06 5560 5.78E-06 5160
3.89E-06 7440 6.22E-06 6880 8.17E-06
7800 2.64E-05 5770 1.59E-06 5560 5.76E-06 5160
3.90E-06 7440 6.23E-06 6880 8.18E-06
7800 2.64E-05 5770 1.61E-06 5570 5.78E-06 5160
3.90E-06 7440 6.23E-06 6880 8.18E-06
7800 2.64E-05 5770 1.61E-06 5570 5.75E-06 5160
3.89E-06 7440 6.23E-06 6880 8.19E-06
7800 2.65E-05 5770 1.61E-06 5570 5.75E-06 5160
3.91E-06 7450 6.24E-06 6890 8.18E-06
7800 2.65E-05 5770 1.58E-06 5570 5.76E-06 5160
3.90E-06 7450 6.24E-06 6890 8.18E-06
7800 2.65E-05 5780 1.59E-06 5570 5.74E-06 5170
3.90E-06 7450 6.24E-06 6890 8.19E-06
7800 2.65E-05 5780 1.60E-06 5570 5.75E-06 5170
3.89E-06 7450 6.24E-06 6890 8.18E-06
7810 2.65E-05 5780 1.59E-06 5580 5.77E-06 5170
3.91E-06 7450 6.25E-06 6890 8.18E-06
7810 2.66E-05 5780 1.60E-06 5580 5.78E-06 5170
3.91E-06 7450 6.25E-06 6890 8.19E-06
7810 2.66E-05 5780 1.59E-06 5580 5.79E-06 5170
3.91E-06 7450 6.25E-06 6900 8.18E-06
7810 2.66E-05 5780 1.59E-06 5580 5.83E-06 5170
3.92E-06 7460 6.25E-06 6900 8.19E-06
7810 2.66E-05 5780 1.58E-06 5580 5.85E-06 5180
3.92E-06 7460 6.26E-06 6900 8.18E-06
7810 2.66E-05 5790 1.59E-06 5580 5.88E-06 5180
3.92E-06 7460 6.24E-06 6900 8.18E-06
7820 2.66E-05 5790 1.59E-06 5580 5.86E-06 5180
3.92E-06 7460 6.25E-06 6900 8.18E-06
7820 2.66E-05 5790 1.59E-06 5590 5.85E-06 5180
3.92E-06 7460 6.25E-06 6900 8.18E-06
7820 2.66E-05 5790 1.58E-06 5590 5.84E-06 5180
3.91E-06 7460 6.25E-06 6900 8.18E-06
7820 2.66E-05 5790 1.58E-06 5590 5.85E-06 5180
3.93E-06 7470 6.26E-06 6910 8.17E-06
7820 2.66E-05 5790 1.57E-06 5590 5.83E-06 5180
3.94E-06 7470 6.26E-06 6910 8.19E-06
7820 2.66E-05 5800 1.58E-06 5590 5.82E-06 5190
3.94E-06 7470 6.26E-06 6910 8.18E-06
7830 2.66E-05 5800 1.58E-06 5590 5.82E-06 5190
3.94E-06 7470 6.26E-06 6910 8.19E-06
7830 2.67E-05 5800 1.58E-06 5600 5.80E-06 5190
3.93E-06 7470 6.26E-06 6910 8.18E-06
7830 2.67E-05 5800 1.58E-06 5600 5.81E-06 5190
3.92E-06 7470 6.26E-06 6910 8.19E-06
7830 2.67E-05 5800 1.58E-06 5600 5.82E-06 5190
3.94E-06 7480 6.26E-06 6920 8.19E-06
7830 2.67E-05 5800 1.57E-06 5600 5.81E-06 5190
3.93E-06 7480 6.27E-06 6920 8.18E-06

7830 2.67E-05 5800 1.57E-06 5600 5.81E-06 5200
3.93E-06 7480 6.27E-06 6920 8.19E-06
7830 2.67E-05 5810 1.56E-06 5600 5.82E-06 5200
3.93E-06 7480 6.28E-06 6920 8.18E-06
7840 2.67E-05 5810 1.56E-06 5600 5.81E-06 5200
3.94E-06 7480 6.28E-06 6920 8.17E-06
7840 2.68E-05 5810 1.57E-06 5610 5.81E-06 5200
3.93E-06 7480 6.29E-06 6920 8.18E-06
7840 2.68E-05 5810 1.57E-06 5610 5.83E-06 5200
3.93E-06 7480 6.28E-06 6930 8.18E-06
7840 2.68E-05 5810 1.56E-06 5610 5.85E-06 5200
3.94E-06 7490 6.28E-06 6930 8.18E-06
7840 2.68E-05 5810 1.57E-06 5610 5.84E-06 5200
3.94E-06 7490 6.28E-06 6930 8.17E-06
7840 2.68E-05 5820 1.57E-06 5610 5.84E-06 5210
3.95E-06 7490 6.28E-06 6930 8.18E-06
7850 2.68E-05 5820 1.56E-06 5610 5.84E-06 5210
3.94E-06 7490 6.28E-06 6930 8.17E-06
7850 2.68E-05 5820 1.57E-06 5620 5.84E-06 5210
3.92E-06 7490 6.29E-06 6930 8.18E-06
7850 2.69E-05 5820 1.56E-06 5620 5.86E-06 5210
3.93E-06 7490 6.29E-06 6930 8.18E-06
7850 2.69E-05 5820 1.56E-06 5620 5.85E-06 5210
3.94E-06 7500 6.29E-06 6940 8.18E-06
7850 2.69E-05 5820 1.56E-06 5620 5.87E-06 5210
3.94E-06 7500 6.29E-06 6940 8.18E-06
7850 2.69E-05 5830 1.55E-06 5620 5.89E-06 5220
3.92E-06 7500 6.29E-06 6940 8.19E-06
7850 2.69E-05 5830 1.55E-06 5620 5.90E-06 5220
3.92E-06 7500 6.29E-06 6940 8.20E-06
7860 2.69E-05 5830 1.57E-06 5630 5.90E-06 5220
3.92E-06 7500 6.29E-06 6940 8.20E-06
7860 2.70E-05 5830 1.56E-06 5630 5.89E-06 5220
3.92E-06 7500 6.29E-06 6940 8.20E-06
7860 2.70E-05 5830 1.55E-06 5630 5.92E-06 5220
3.93E-06 7500 6.30E-06 6950 8.19E-06
7860 2.70E-05 5830 1.55E-06 5630 5.93E-06 5220
3.93E-06 7510 6.30E-06 6950 8.20E-06
7860 2.70E-05 5830 1.54E-06 5630 5.95E-06 5230
3.93E-06 7510 6.30E-06 6950 8.20E-06
7860 2.70E-05 5840 1.54E-06 5630 5.93E-06 5230
3.92E-06 7510 6.31E-06 6950 8.20E-06
7870 2.71E-05 5840 1.55E-06 5630 5.94E-06 5230
3.94E-06 7510 6.31E-06 6950 8.20E-06
7870 2.71E-05 5840 1.56E-06 5640 5.94E-06 5230
3.94E-06 7510 6.31E-06 6950 8.20E-06
7870 2.71E-05 5840 1.57E-06 5640 5.94E-06 5230
3.93E-06 7510 6.31E-06 6950 8.20E-06
7870 2.71E-05 5840 1.56E-06 5640 5.95E-06 5230
3.94E-06 7520 6.31E-06 6960 8.20E-06
7870 2.71E-05 5840 1.57E-06 5640 5.94E-06 5230
3.95E-06 7520 6.31E-06 6960 8.20E-06
7870 2.71E-05 5850 1.57E-06 5640 5.97E-06 5240
3.96E-06 7520 6.31E-06 6960 8.20E-06
7880 2.71E-05 5850 1.56E-06 5640 5.98E-06 5240
3.97E-06 7520 6.31E-06 6960 8.20E-06
7880 2.71E-05 5850 1.57E-06 5650 5.96E-06 5240
3.97E-06 7520 6.32E-06 6960 8.19E-06
7880 2.71E-05 5850 1.56E-06 5650 5.96E-06 5240
3.96E-06 7520 6.33E-06 6960 8.19E-06
7880 2.72E-05 5850 1.57E-06 5650 5.95E-06 5240
3.97E-06 7530 6.33E-06 6970 8.19E-06
7880 2.72E-05 5850 1.56E-06 5650 5.97E-06 5240
3.98E-06 7530 6.34E-06 6970 8.20E-06
7880 2.72E-05 5850 1.56E-06 5650 5.97E-06 5250
3.98E-06 7530 6.33E-06 6970 8.19E-06
7880 2.72E-05 5860 1.56E-06 5650 5.97E-06 5250
3.98E-06 7530 6.34E-06 6970 8.19E-06

7890 2.72E-05 5860 1.57E-06 5650 5.98E-06 5250
3.97E-06 7530 6.34E-06 6970 8.19E-06
7890 2.72E-05 5860 1.56E-06 5660 5.95E-06 5250
3.99E-06 7530 6.34E-06 6970 8.19E-06
7890 2.72E-05 5860 1.56E-06 5660 5.98E-06 5250
4.00E-06 7530 6.34E-06 6980 8.20E-06
7890 2.73E-05 5860 1.56E-06 5660 5.97E-06 5250
3.99E-06 7540 6.35E-06 6980 8.20E-06
7890 2.73E-05 5860 1.56E-06 5660 6.02E-06 5250
3.99E-06 7540 6.35E-06 6980 8.20E-06
7890 2.73E-05 5870 1.56E-06 5660 5.99E-06 5260
3.99E-06 7540 6.36E-06 6980 8.19E-06
7900 2.73E-05 5870 1.56E-06 5660 6.00E-06 5260
4.00E-06 7540 6.36E-06 6980 8.19E-06
7900 2.73E-05 5870 1.57E-06 5670 6.03E-06 5260
4.01E-06 7540 6.36E-06 6980 8.19E-06
7900 2.73E-05 5870 1.57E-06 5670 6.04E-06 5260
4.00E-06 7540 6.36E-06 6980 8.20E-06
7900 2.73E-05 5870 1.56E-06 5670 6.04E-06 5260
4.03E-06 7550 6.36E-06 6990 8.20E-06
7900 2.73E-05 5870 1.57E-06 5670 6.04E-06 5260
4.03E-06 7550 6.35E-06 6990 8.19E-06
7900 2.74E-05 5880 1.57E-06 5670 6.06E-06 5270
4.02E-06 7550 6.36E-06 6990 8.20E-06
7900 2.74E-05 5880 1.56E-06 5670 6.07E-06 5270
4.02E-06 7550 6.35E-06 6990 8.20E-06
7910 2.74E-05 5880 1.57E-06 5680 6.07E-06 5270
4.02E-06 7550 6.36E-06 6990 8.20E-06
7910 2.74E-05 5880 1.56E-06 5680 6.05E-06 5270
4.03E-06 7550 6.36E-06 6990 8.21E-06
7910 2.74E-05 5880 1.57E-06 5680 6.05E-06 5270
4.03E-06 7550 6.37E-06 7000 8.20E-06
7910 2.74E-05 5880 1.57E-06 5680 6.07E-06 5270
4.02E-06 7560 6.37E-06 7000 8.22E-06
7910 2.74E-05 5880 1.56E-06 5680 6.07E-06 5280
4.03E-06 7560 6.36E-06 7000 8.21E-06
7910 2.75E-05 5890 1.57E-06 5680 6.07E-06 5280
4.03E-06 7560 6.36E-06 7000 8.22E-06
7920 2.75E-05 5890 1.57E-06 5680 6.07E-06 5280
4.03E-06 7560 6.37E-06 7000 8.22E-06
7920 2.75E-05 5890 1.57E-06 5690 6.08E-06 5280
4.03E-06 7560 6.37E-06 7000 8.22E-06
7920 2.75E-05 5890 1.57E-06 5690 6.09E-06 5280
4.04E-06 7560 6.36E-06 7000 8.22E-06
7920 2.75E-05 5890 1.58E-06 5690 6.10E-06 5280
4.02E-06 7570 6.36E-06 7010 8.21E-06
7920 2.76E-05 5890 1.57E-06 5690 6.10E-06 5280
4.03E-06 7570 6.36E-06 7010 8.23E-06
7920 2.76E-05 5900 1.57E-06 5690 6.11E-06 5290
4.02E-06 7570 6.37E-06 7010 8.22E-06
7930 2.76E-05 5900 1.57E-06 5690 6.11E-06 5290
4.03E-06 7570 6.37E-06 7010 8.22E-06
7930 2.76E-05 5900 1.57E-06 5700 6.10E-06 5290
4.03E-06 7570 6.37E-06 7010 8.22E-06
7930 2.76E-05 5900 1.57E-06 5700 6.11E-06 5290
4.04E-06 7570 6.37E-06 7010 8.22E-06
7930 2.76E-05 5900 1.57E-06 5700 6.12E-06 5290
4.05E-06 7580 6.37E-06 7020 8.23E-06
7930 2.76E-05 5900 1.58E-06 5700 6.11E-06 5290
4.05E-06 7580 6.36E-06 7020 8.22E-06
7930 2.77E-05 5900 1.58E-06 5700 6.13E-06 5300
4.04E-06 7580 6.37E-06 7020 8.23E-06
7930 2.77E-05 5910 1.59E-06 5700 6.12E-06 5300
4.06E-06 7580 6.37E-06 7020 8.23E-06
7940 2.77E-05 5910 1.58E-06 5700 6.11E-06 5300
4.07E-06 7580 6.36E-06 7020 8.22E-06
7940 2.77E-05 5910 1.58E-06 5710 6.12E-06 5300
4.06E-06 7580 6.36E-06 7020 8.23E-06

7940 2.77E-05 5910 1.58E-06 5710 6.10E-06 5300
4.06E-06 7580 6.37E-06 7030 8.22E-06
7940 2.77E-05 5910 1.58E-06 5710 6.11E-06 5300
4.06E-06 7590 6.36E-06 7030 8.24E-06
7940 2.77E-05 5910 1.58E-06 5710 6.11E-06 5300
4.06E-06 7590 6.36E-06 7030 8.22E-06
7940 2.77E-05 5920 1.58E-06 5710 6.11E-06 5310
4.07E-06 7590 6.37E-06 7030 8.22E-06
7950 2.78E-05 5920 1.58E-06 5710 6.11E-06 5310
4.06E-06 7590 6.37E-06 7030 8.23E-06
7950 2.78E-05 5920 1.58E-06 5720 6.11E-06 5310
4.07E-06 7590 6.37E-06 7030 8.22E-06
7950 2.78E-05 5920 1.57E-06 5720 6.11E-06 5310
4.07E-06 7590 6.37E-06 7030 8.23E-06
7950 2.78E-05 5920 1.57E-06 5720 6.12E-06 5310
4.07E-06 7600 6.37E-06 7040 8.23E-06
7950 2.78E-05 5920 1.57E-06 5720 6.12E-06 5310
4.06E-06 7600 6.37E-06 7040 8.23E-06
7950 2.78E-05 5930 1.57E-06 5720 6.12E-06 5320
4.07E-06 7600 6.38E-06 7040 8.24E-06
7950 2.78E-05 5930 1.59E-06 5720 6.13E-06 5320
4.08E-06 7600 6.38E-06 7040 8.24E-06
7960 2.79E-05 5930 1.58E-06 5730 6.13E-06 5320
4.09E-06 7600 6.38E-06 7040 8.23E-06
7960 2.79E-05 5930 1.58E-06 5730 6.14E-06 5320
4.08E-06 7600 6.37E-06 7040 8.23E-06
7960 2.79E-05 5930 1.57E-06 5730 6.14E-06 5320
4.10E-06 7600 6.37E-06 7050 8.25E-06
7960 2.79E-05 5930 1.58E-06 5730 6.14E-06 5320
4.09E-06 7610 6.37E-06 7050 8.24E-06
7960 2.79E-05 5930 1.58E-06 5730 6.16E-06 5330
4.10E-06 7610 6.37E-06 7050 8.24E-06
7960 2.79E-05 5940 1.59E-06 5730 6.15E-06 5330
4.10E-06 7610 6.38E-06 7050 8.23E-06
7970 2.79E-05 5940 1.58E-06 5730 6.15E-06 5330
4.11E-06 7610 6.38E-06 7050 8.23E-06
7970 2.80E-05 5940 1.58E-06 5740 6.14E-06 5330
4.09E-06 7610 6.38E-06 7050 8.24E-06
7970 2.80E-05 5940 1.58E-06 5740 6.12E-06 5330
4.09E-06 7610 6.38E-06 7050 8.24E-06
7970 2.80E-05 5940 1.58E-06 5740 6.13E-06 5330
4.09E-06 7620 6.38E-06 7060 8.25E-06
7970 2.80E-05 5940 1.57E-06 5740 6.14E-06 5330
4.10E-06 7620 6.37E-06 7060 8.25E-06
7970 2.80E-05 5950 1.58E-06 5740 6.14E-06 5340
4.10E-06 7620 6.38E-06 7060 8.25E-06
7980 2.80E-05 5950 1.58E-06 5740 6.14E-06 5340
4.10E-06 7620 6.38E-06 7060 8.26E-06
7980 2.81E-05 5950 1.59E-06 5750 6.15E-06 5340
4.09E-06 7620 6.38E-06 7060 8.26E-06
7980 2.81E-05 5950 1.59E-06 5750 6.17E-06 5340
4.09E-06 7620 6.38E-06 7060 8.27E-06
7980 2.81E-05 5950 1.59E-06 5750 6.16E-06 5340
4.09E-06 7630 6.38E-06 7070 8.26E-06
7980 2.81E-05 5950 1.59E-06 5750 6.17E-06 5340
4.09E-06 7630 6.39E-06 7070 8.27E-06
7980 2.81E-05 5950 1.59E-06 5750 6.16E-06 5350
4.09E-06 7630 6.39E-06 7070 8.27E-06
7980 2.82E-05 5960 1.59E-06 5750 6.17E-06 5350
4.09E-06 7630 6.40E-06 7070 8.27E-06
7990 2.82E-05 5960 1.59E-06 5750 6.17E-06 5350
4.11E-06 7630 6.40E-06 7070 8.26E-06
7990 2.82E-05 5960 1.59E-06 5760 6.17E-06 5350
4.10E-06 7630 6.40E-06 7070 8.26E-06
7990 2.82E-05 5960 1.60E-06 5760 6.19E-06 5350
4.12E-06 7630 6.39E-06 7080 8.27E-06
7990 2.82E-05 5960 1.60E-06 5760 6.19E-06 5350
4.12E-06 7640 6.38E-06 7080 8.27E-06

7990 2.82E-05 5960 1.60E-06 5760 6.19E-06 5350
4.12E-06 7640 6.39E-06 7080 8.26E-06
7990 2.82E-05 5970 1.61E-06 5760 6.20E-06 5360
4.13E-06 7640 6.39E-06 7080 8.26E-06
8000 2.83E-05 5970 1.61E-06 5760 6.20E-06 5360
4.13E-06 7640 6.40E-06 7080 8.26E-06
8000 2.83E-05 5970 1.60E-06 5770 6.19E-06 5360
4.13E-06 7640 6.40E-06 7080 8.27E-06
8000 2.83E-05 5970 1.61E-06 5770 6.19E-06 5360
4.12E-06 7640 6.40E-06 7080 8.26E-06
8000 2.83E-05 5970 1.61E-06 5770 6.19E-06 5360
4.13E-06 7650 6.40E-06 7090 8.27E-06
8000 2.83E-05 5970 1.61E-06 5770 6.19E-06 5360
4.14E-06 7650 6.40E-06 7090 8.27E-06
8000 2.83E-05 5980 1.61E-06 5770 6.21E-06 5370
4.13E-06 7650 6.40E-06 7090 8.28E-06
8000 2.83E-05 5980 1.61E-06 5770 6.21E-06 5370
4.13E-06 7650 6.40E-06 7090 8.27E-06
8010 2.83E-05 5980 1.61E-06 5780 6.22E-06 5370
4.13E-06 7650 6.40E-06 7090 8.27E-06
8010 2.84E-05 5980 1.61E-06 5780 6.22E-06 5370
4.13E-06 7650 6.40E-06 7090 8.27E-06
8010 2.84E-05 5980 1.61E-06 5780 6.25E-06 5370
4.12E-06 7650 6.40E-06 7100 8.27E-06
8010 2.84E-05 5980 1.62E-06 5780 6.24E-06 5370
4.12E-06 7660 6.41E-06 7100 8.28E-06
8010 2.84E-05 5980 1.61E-06 5780 6.23E-06 5380
4.12E-06 7660 6.42E-06 7100 8.28E-06
8010 2.84E-05 5990 1.61E-06 5780 6.23E-06 5380
4.13E-06 7660 6.42E-06 7100 8.28E-06
8020 2.85E-05 5990 1.62E-06 5780 6.23E-06 5380
4.14E-06 7660 6.41E-06 7100 8.28E-06
8020 2.85E-05 5990 1.62E-06 5790 6.23E-06 5380
4.15E-06 7660 6.41E-06 7100 8.29E-06
8020 2.85E-05 5990 1.62E-06 5790 6.24E-06 5380
4.15E-06 7660 6.42E-06 7100 8.28E-06
8020 2.85E-05 5990 1.62E-06 5790 6.24E-06 5380
4.15E-06 7670 6.42E-06 7110 8.28E-06
8020 2.85E-05 5990 1.62E-06 5790 6.25E-06 5380
4.16E-06 7670 6.42E-06 7110 8.28E-06
8020 2.85E-05 6000 1.62E-06 5790 6.24E-06 5390
4.16E-06 7670 6.42E-06 7110 8.27E-06
8030 2.85E-05 6000 1.61E-06 5790 6.24E-06 5390
4.18E-06 7670 6.42E-06 7110 8.26E-06
8030 2.86E-05 6000 1.63E-06 5800 6.24E-06 5390
4.17E-06 7670 6.43E-06 7110 8.29E-06
8030 2.86E-05 6000 1.62E-06 5800 6.23E-06 5390
4.15E-06 7670 6.42E-06 7110 8.27E-06
8030 2.86E-05 6000 1.62E-06 5800 6.24E-06 5390
4.16E-06 7680 6.42E-06 7120 8.29E-06
8030 2.86E-05 6000 1.62E-06 5800 6.25E-06 5390
4.16E-06 7680 6.42E-06 7120 8.30E-06
8030 2.87E-05 6000 1.62E-06 5800 6.25E-06 5400
4.16E-06 7680 6.42E-06 7120 8.30E-06
8030 2.87E-05 6010 1.62E-06 5800 6.26E-06 5400
4.15E-06 7680 6.43E-06 7120 8.29E-06
8040 2.87E-05 6010 1.62E-06 5800 6.26E-06 5400
4.15E-06 7680 6.43E-06 7120 8.30E-06
8040 2.88E-05 6010 1.61E-06 5810 6.26E-06 5400
4.16E-06 7680 6.43E-06 7120 8.30E-06
8040 2.88E-05 6010 1.62E-06 5810 6.27E-06 5400
4.15E-06 7680 6.43E-06 7130 8.30E-06
8040 2.88E-05 6010 1.61E-06 5810 6.26E-06 5400
4.16E-06 7690 6.44E-06 7130 8.31E-06
8040 2.88E-05 6010 1.61E-06 5810 6.27E-06 5400
4.16E-06 7690 6.44E-06 7130 8.31E-06
8040 2.88E-05 6020 1.62E-06 5810 6.26E-06 5410
4.16E-06 7690 6.44E-06 7130 8.30E-06

8050 2.89E-05 6020 1.61E-06 5810 6.28E-06 5410
4.17E-06 7690 6.44E-06 7130 8.31E-06
8050 2.89E-05 6020 1.61E-06 5820 6.28E-06 5410
4.18E-06 7690 6.44E-06 7130 8.32E-06
8050 2.89E-05 6020 1.61E-06 5820 6.28E-06 5410
4.19E-06 7690 6.44E-06 7130 8.31E-06
8050 2.89E-05 6020 1.62E-06 5820 6.29E-06 5410
4.19E-06 7700 6.44E-06 7140 8.31E-06
8050 2.89E-05 6020 1.61E-06 5820 6.29E-06 5410
4.20E-06 7700 6.44E-06 7140 8.32E-06
8050 2.90E-05 6030 1.62E-06 5820 6.28E-06 5420
4.20E-06 7700 6.44E-06 7140 8.30E-06
8050 2.90E-05 6030 1.62E-06 5820 6.29E-06 5420
4.22E-06 7700 6.45E-06 7140 8.31E-06
8060 2.90E-05 6030 1.62E-06 5830 6.30E-06 5420
4.22E-06 7700 6.45E-06 7140 8.31E-06
8060 2.90E-05 6030 1.62E-06 5830 6.29E-06 5420
4.22E-06 7700 6.45E-06 7140 8.32E-06
8060 2.90E-05 6030 1.62E-06 5830 6.29E-06 5420
4.23E-06 7700 6.45E-06 7150 8.32E-06
8060 2.90E-05 6030 1.62E-06 5830 6.28E-06 5420
4.23E-06 7710 6.46E-06 7150 8.33E-06
8060 2.91E-05 6030 1.61E-06 5830 6.30E-06 5430
4.24E-06 7710 6.46E-06 7150 8.33E-06
8060 2.91E-05 6040 1.62E-06 5830 6.30E-06 5430
4.23E-06 7710 6.46E-06 7150 8.33E-06
8070 2.91E-05 6040 1.61E-06 5830 6.30E-06 5430
4.24E-06 7710 6.46E-06 7150 8.34E-06
8070 2.91E-05 6040 1.61E-06 5840 6.31E-06 5430
4.24E-06 7710 6.47E-06 7150 8.34E-06
8070 2.92E-05 6040 1.62E-06 5840 6.32E-06 5430
4.24E-06 7710 6.46E-06 7150 8.34E-06
8070 2.92E-05 6040 1.62E-06 5840 6.31E-06 5430
4.25E-06 7720 6.47E-06 7160 8.35E-06
8070 2.92E-05 6040 1.61E-06 5840 6.32E-06 5430
4.24E-06 7720 6.47E-06 7160 8.36E-06
8070 2.92E-05 6050 1.62E-06 5840 6.31E-06 5440
4.25E-06 7720 6.47E-06 7160 8.34E-06
8080 2.93E-05 6050 1.62E-06 5840 6.32E-06 5440
4.27E-06 7720 6.48E-06 7160 8.34E-06
8080 2.93E-05 6050 1.62E-06 5850 6.33E-06 5440
4.27E-06 7720 6.47E-06 7160 8.34E-06
8080 2.93E-05 6050 1.62E-06 5850 6.35E-06 5440
4.27E-06 7720 6.47E-06 7160 8.34E-06
8080 2.93E-05 6050 1.62E-06 5850 6.33E-06 5440
4.27E-06 7730 6.48E-06 7170 8.34E-06
8080 2.93E-05 6050 1.62E-06 5850 6.36E-06 5440
4.27E-06 7730 6.47E-06 7170 8.34E-06
8080 2.94E-05 6050 1.62E-06 5850 6.35E-06 5450
4.29E-06 7730 6.48E-06 7170 8.34E-06
8080 2.94E-05 6060 1.62E-06 5850 6.35E-06 5450
4.30E-06 7730 6.48E-06 7170 8.35E-06
8090 2.94E-05 6060 1.62E-06 5850 6.34E-06 5450
4.31E-06 7730 6.48E-06 7170 8.35E-06
8090 2.94E-05 6060 1.61E-06 5860 6.34E-06 5450
4.31E-06 7730 6.48E-06 7170 8.33E-06
8090 2.94E-05 6060 1.61E-06 5860 6.33E-06 5450
4.33E-06 7730 6.48E-06 7180 8.34E-06
8090 2.94E-05 6060 1.61E-06 5860 6.35E-06 5450
4.32E-06 7740 6.48E-06 7180 8.34E-06
8090 2.95E-05 6060 1.61E-06 5860 6.35E-06 5450
4.33E-06 7740 6.49E-06 7180 8.34E-06
8090 2.95E-05 6070 1.62E-06 5860 6.34E-06 5460
4.32E-06 7740 6.49E-06 7180 8.34E-06
8100 2.95E-05 6070 1.62E-06 5860 6.35E-06 5460
4.32E-06 7740 6.49E-06 7180 8.35E-06
8100 2.95E-05 6070 1.62E-06 5870 6.34E-06 5460
4.32E-06 7740 6.50E-06 7180 8.35E-06

8100 2.96E-05 6070 1.62E-06 5870 6.34E-06 5460
4.32E-06 7740 6.50E-06 7180 8.35E-06
8100 2.96E-05 6070 1.62E-06 5870 6.34E-06 5460
4.32E-06 7750 6.51E-06 7190 8.35E-06
8100 2.96E-05 6070 1.62E-06 5870 6.34E-06 5460
4.32E-06 7750 6.51E-06 7190 8.36E-06
8100 2.96E-05 6080 1.61E-06 5870 6.33E-06 5470
4.32E-06 7750 6.51E-06 7190 8.35E-06
8100 2.97E-05 6080 1.61E-06 5870 6.35E-06 5470
4.34E-06 7750 6.52E-06 7190 8.35E-06
8110 2.97E-05 6080 1.61E-06 5880 6.36E-06 5470
4.34E-06 7750 6.52E-06 7190 8.36E-06
8110 2.97E-05 6080 1.62E-06 5880 6.36E-06 5470
4.35E-06 7750 6.51E-06 7190 8.36E-06
8110 2.97E-05 6080 1.62E-06 5880 6.37E-06 5470
4.34E-06 7750 6.52E-06 7200 8.36E-06
8110 2.98E-05 6080 1.62E-06 5880 6.38E-06 5470
4.35E-06 7760 6.51E-06 7200 8.37E-06
8110 2.98E-05 6080 1.63E-06 5880 6.37E-06 5480
4.36E-06 7760 6.51E-06 7200 8.37E-06
8110 2.98E-05 6090 1.63E-06 5880 6.37E-06 5480
4.36E-06 7760 6.53E-06 7200 8.37E-06
8120 2.98E-05 6090 1.63E-06 5880 6.36E-06 5480
4.37E-06 7760 6.52E-06 7200 8.37E-06
8120 2.98E-05 6090 1.63E-06 5890 6.36E-06 5480
4.37E-06 7760 6.52E-06 7200 8.37E-06
8120 2.99E-05 6090 1.63E-06 5890 6.35E-06 5480
4.37E-06 7760 6.52E-06 7200 8.37E-06
8120 2.99E-05 6090 1.63E-06 5890 6.35E-06 5480
4.37E-06 7770 6.52E-06 7210 8.37E-06
8120 2.99E-05 6090 1.61E-06 5890 6.35E-06 5480
4.38E-06 7770 6.52E-06 7210 8.38E-06
8120 2.99E-05 6100 1.62E-06 5890 6.36E-06 5490
4.37E-06 7770 6.53E-06 7210 8.38E-06
8130 3.00E-05 6100 1.62E-06 5890 6.36E-06 5490
4.37E-06 7770 6.53E-06 7210 8.40E-06
8130 3.00E-05 6100 1.63E-06 5900 6.38E-06 5490
4.37E-06 7770 6.53E-06 7210 8.40E-06
8130 3.00E-05 6100 1.63E-06 5900 6.37E-06 5490
4.37E-06 7770 6.53E-06 7210 8.39E-06
8130 3.00E-05 6100 1.61E-06 5900 6.38E-06 5490
4.38E-06 7780 6.54E-06 7220 8.39E-06
8130 3.00E-05 6100 1.62E-06 5900 6.38E-06 5490
4.36E-06 7780 6.54E-06 7220 8.40E-06
8130 3.01E-05 6100 1.62E-06 5900 6.37E-06 5500
4.38E-06 7780 6.54E-06 7220 8.39E-06
8130 3.01E-05 6110 1.62E-06 5900 6.39E-06 5500
4.38E-06 7780 6.53E-06 7220 8.39E-06
8140 3.01E-05 6110 1.61E-06 5900 6.40E-06 5500
4.39E-06 7780 6.53E-06 7220 8.39E-06
8140 3.02E-05 6110 1.63E-06 5910 6.41E-06 5500
4.41E-06 7780 6.53E-06 7220 8.39E-06
8140 3.02E-05 6110 1.63E-06 5910 6.42E-06 5500
4.40E-06 7780 6.53E-06 7230 8.39E-06
8140 3.02E-05 6110 1.63E-06 5910 6.43E-06 5500
4.41E-06 7790 6.53E-06 7230 8.39E-06
8140 3.02E-05 6110 1.63E-06 5910 6.43E-06 5500
4.42E-06 7790 6.53E-06 7230 8.38E-06
8140 3.02E-05 6120 1.63E-06 5910 6.41E-06 5510
4.43E-06 7790 6.53E-06 7230 8.39E-06
8150 3.03E-05 6120 1.63E-06 5910 6.40E-06 5510
4.43E-06 7790 6.54E-06 7230 8.40E-06
8150 3.03E-05 6120 1.63E-06 5920 6.41E-06 5510
4.43E-06 7790 6.53E-06 7230 8.38E-06
8150 3.03E-05 6120 1.64E-06 5920 6.41E-06 5510
4.44E-06 7790 6.54E-06 7230 8.40E-06
8150 3.03E-05 6120 1.64E-06 5920 6.40E-06 5510
4.44E-06 7800 6.54E-06 7240 8.39E-06

8150 3.03E-05 6120 1.63E-06 5920 6.41E-06 5510
4.45E-06 7800 6.55E-06 7240 8.40E-06
8150 3.03E-05 6130 1.63E-06 5920 6.43E-06 5520
4.44E-06 7800 6.54E-06 7240 8.40E-06
8150 3.04E-05 6130 1.64E-06 5920 6.42E-06 5520
4.44E-06 7800 6.55E-06 7240 8.40E-06
8160 3.04E-05 6130 1.63E-06 5930 6.43E-06 5520
4.45E-06 7800 6.55E-06 7240 8.41E-06
8160 3.04E-05 6130 1.64E-06 5930 6.44E-06 5520
4.46E-06 7800 6.55E-06 7240 8.40E-06
8160 3.04E-05 6130 1.64E-06 5930 6.43E-06 5520
4.46E-06 7800 6.55E-06 7250 8.40E-06
8160 3.04E-05 6130 1.63E-06 5930 6.44E-06 5520
4.46E-06 7810 6.55E-06 7250 8.41E-06
8160 3.05E-05 6130 1.64E-06 5930 6.43E-06 5530
4.47E-06 7810 6.56E-06 7250 8.40E-06
8160 3.05E-05 6140 1.64E-06 5930 6.44E-06 5530
4.47E-06 7810 6.55E-06 7250 8.41E-06
8170 3.05E-05 6140 1.63E-06 5930 6.45E-06 5530
4.47E-06 7810 6.55E-06 7250 8.41E-06
8170 3.05E-05 6140 1.64E-06 5940 6.46E-06 5530
4.49E-06 7810 6.55E-06 7250 8.41E-06
8170 3.06E-05 6140 1.64E-06 5940 6.47E-06 5530
4.48E-06 7810 6.55E-06 7250 8.40E-06
8170 3.06E-05 6140 1.65E-06 5940 6.48E-06 5530
4.47E-06 7820 6.54E-06 7260 8.41E-06
8170 3.06E-05 6140 1.64E-06 5940 6.49E-06 5530
4.48E-06 7820 6.54E-06 7260 8.40E-06
8170 3.06E-05 6150 1.64E-06 5940 6.48E-06 5540
4.48E-06 7820 6.54E-06 7260 8.40E-06
8180 3.07E-05 6150 1.65E-06 5940 6.48E-06 5540
4.48E-06 7820 6.54E-06 7260 8.41E-06
8180 3.07E-05 6150 1.65E-06 5950 6.48E-06 5540
4.48E-06 7820 6.54E-06 7260 8.40E-06
8180 3.07E-05 6150 1.65E-06 5950 6.47E-06 5540
4.50E-06 7820 6.54E-06 7260 8.41E-06
8180 3.07E-05 6150 1.65E-06 5950 6.48E-06 5540
4.48E-06 7830 6.54E-06 7270 8.42E-06
8180 3.08E-05 6150 1.64E-06 5950 6.49E-06 5540
4.49E-06 7830 6.54E-06 7270 8.42E-06
8180 3.08E-05 6150 1.65E-06 5950 6.52E-06 5550
4.49E-06 7830 6.54E-06 7270 8.43E-06
8180 3.08E-05 6160 1.65E-06 5950 6.54E-06 5550
4.50E-06 7830 6.53E-06 7270 8.43E-06
8190 3.09E-05 6160 1.65E-06 5950 6.52E-06 5550
4.49E-06 7830 6.54E-06 7270 8.43E-06
8190 3.09E-05 6160 1.65E-06 5960 6.54E-06 5550
4.50E-06 7830 6.54E-06 7270 8.43E-06
8190 3.09E-05 6160 1.65E-06 5960 6.53E-06 5550
4.50E-06 7830 6.55E-06 7280 8.43E-06
8190 3.09E-05 6160 1.65E-06 5960 6.54E-06 5550
4.50E-06 7840 6.55E-06 7280 8.44E-06
8190 3.10E-05 6160 1.66E-06 5960 6.55E-06 5550
4.50E-06 7840 6.55E-06 7280 8.43E-06
8190 3.10E-05 6170 1.66E-06 5960 6.56E-06 5560
4.53E-06 7840 6.56E-06 7280 8.44E-06
8200 3.10E-05 6170 1.66E-06 5960 6.57E-06 5560
4.53E-06 7840 6.55E-06 7280 8.43E-06
8200 3.10E-05 6170 1.66E-06 5970 6.59E-06 5560
4.54E-06 7840 6.55E-06 7280 8.43E-06
8200 3.10E-05 6170 1.66E-06 5970 6.59E-06 5560
4.54E-06 7840 6.55E-06 7280 8.44E-06
8200 3.11E-05 6170 1.66E-06 5970 6.62E-06 5560
4.55E-06 7850 6.54E-06 7290 8.44E-06
8200 3.11E-05 6170 1.66E-06 5970 6.61E-06 5560
4.56E-06 7850 6.55E-06 7290 8.43E-06
8200 3.11E-05 6180 1.66E-06 5970 6.61E-06 5570
4.57E-06 7850 6.55E-06 7290 8.43E-06

8200 3.11E-05 6180 1.66E-06 5970 6.61E-06 5570
4.57E-06 7850 6.56E-06 7290 8.44E-06
8210 3.12E-05 6180 1.66E-06 5980 6.61E-06 5570
4.58E-06 7850 6.56E-06 7290 8.44E-06
8210 3.12E-05 6180 1.67E-06 5980 6.62E-06 5570
4.59E-06 7850 6.56E-06 7290 8.45E-06
8210 3.12E-05 6180 1.67E-06 5980 6.64E-06 5570
4.60E-06 7850 6.56E-06 7300 8.46E-06
8210 3.12E-05 6180 1.67E-06 5980 6.65E-06 5570
4.61E-06 7860 6.57E-06 7300 8.44E-06
8210 3.12E-05 6180 1.68E-06 5980 6.65E-06 5580
4.59E-06 7860 6.57E-06 7300 8.44E-06
8210 3.13E-05 6190 1.68E-06 5980 6.65E-06 5580
4.60E-06 7860 6.57E-06 7300 8.45E-06
8220 3.13E-05 6190 1.68E-06 5980 6.65E-06 5580
4.60E-06 7860 6.58E-06 7300 8.46E-06
8220 3.13E-05 6190 1.68E-06 5990 6.67E-06 5580
4.60E-06 7860 6.58E-06 7300 8.46E-06
8220 3.13E-05 6190 1.68E-06 5990 6.68E-06 5580
4.59E-06 7860 6.59E-06 7300 8.46E-06
8220 3.14E-05 6190 1.68E-06 5990 6.67E-06 5580
4.60E-06 7870 6.58E-06 7310 8.45E-06
8220 3.14E-05 6190 1.69E-06 5990 6.68E-06 5580
4.60E-06 7870 6.60E-06 7310 8.46E-06
8220 3.14E-05 6200 1.68E-06 5990 6.68E-06 5590
4.61E-06 7870 6.59E-06 7310 8.46E-06
8230 3.14E-05 6200 1.68E-06 5990 6.71E-06 5590
4.60E-06 7870 6.59E-06 7310 8.47E-06
8230 3.15E-05 6200 1.68E-06 6000 6.73E-06 5590
4.61E-06 7870 6.59E-06 7310 8.46E-06
8230 3.15E-05 6200 1.69E-06 6000 6.73E-06 5590
4.61E-06 7870 6.59E-06 7310 8.46E-06
8230 3.15E-05 6200 1.68E-06 6000 6.73E-06 5590
4.60E-06 7880 6.59E-06 7320 8.46E-06
8230 3.15E-05 6200 1.68E-06 6000 6.73E-06 5590
4.60E-06 7880 6.59E-06 7320 8.45E-06
8230 3.16E-05 6200 1.68E-06 6000 6.74E-06 5600
4.61E-06 7880 6.59E-06 7320 8.46E-06
8230 3.16E-05 6210 1.68E-06 6000 6.71E-06 5600
4.61E-06 7880 6.59E-06 7320 8.47E-06
8240 3.16E-05 6210 1.68E-06 6000 6.74E-06 5600
4.61E-06 7880 6.60E-06 7320 8.47E-06
8240 3.17E-05 6210 1.68E-06 6010 6.76E-06 5600
4.61E-06 7880 6.59E-06 7320 8.48E-06
8240 3.17E-05 6210 1.68E-06 6010 6.73E-06 5600
4.60E-06 7880 6.59E-06 7330 8.49E-06
8240 3.17E-05 6210 1.68E-06 6010 6.74E-06 5600
4.61E-06 7890 6.59E-06 7330 8.48E-06
8240 3.18E-05 6210 1.68E-06 6010 6.76E-06 5600
4.61E-06 7890 6.59E-06 7330 8.48E-06
8240 3.18E-05 6220 1.70E-06 6010 6.75E-06 5610
4.61E-06 7890 6.60E-06 7330 8.48E-06
8250 3.18E-05 6220 1.68E-06 6010 6.74E-06 5610
4.61E-06 7890 6.60E-06 7330 8.49E-06
8250 3.19E-05 6220 1.69E-06 6020 6.72E-06 5610
4.62E-06 7890 6.59E-06 7330 8.49E-06
8250 3.19E-05 6220 1.68E-06 6020 6.77E-06 5610
4.61E-06 7890 6.61E-06 7330 8.49E-06
8250 3.19E-05 6220 1.68E-06 6020 6.74E-06 5610
4.62E-06 7900 6.60E-06 7340 8.49E-06
8250 3.20E-05 6220 1.68E-06 6020 6.73E-06 5610
4.62E-06 7900 6.60E-06 7340 8.49E-06
8250 3.20E-05 6230 1.68E-06 6020 6.74E-06 5620
4.62E-06 7900 6.61E-06 7340 8.49E-06
8250 3.20E-05 6230 1.68E-06 6020 6.75E-06 5620
4.62E-06 7900 6.61E-06 7340 8.50E-06
8260 3.20E-05 6230 1.69E-06 6030 6.75E-06 5620
4.63E-06 7900 6.60E-06 7340 8.50E-06

8260 3.21E-05 6230 1.68E-06 6030 6.76E-06 5620
4.63E-06 7900 6.61E-06 7340 8.50E-06
8260 3.21E-05 6230 1.68E-06 6030 6.77E-06 5620
4.63E-06 7900 6.60E-06 7350 8.49E-06
8260 3.21E-05 6230 1.69E-06 6030 6.76E-06 5620
4.65E-06 7910 6.61E-06 7350 8.49E-06
8260 3.22E-05 6230 1.69E-06 6030 6.76E-06 5630
4.65E-06 7910 6.60E-06 7350 8.49E-06
8260 3.22E-05 6240 1.69E-06 6030 6.75E-06 5630
4.65E-06 7910 6.61E-06 7350 8.51E-06
8270 3.22E-05 6240 1.68E-06 6030 6.74E-06 5630
4.65E-06 7910 6.62E-06 7350 8.50E-06
8270 3.22E-05 6240 1.69E-06 6040 6.74E-06 5630
4.66E-06 7910 6.61E-06 7350 8.51E-06
8270 3.23E-05 6240 1.68E-06 6040 6.75E-06 5630
4.66E-06 7910 6.61E-06 7350 8.51E-06
8270 3.23E-05 6240 1.68E-06 6040 6.73E-06 5630
4.67E-06 7920 6.61E-06 7360 8.50E-06
8270 3.24E-05 6240 1.68E-06 6040 6.73E-06 5630
4.66E-06 7920 6.61E-06 7360 8.50E-06
8270 3.24E-05 6250 1.69E-06 6040 6.71E-06 5640
4.67E-06 7920 6.61E-06 7360 8.50E-06
8280 3.24E-05 6250 1.69E-06 6040 6.71E-06 5640
4.67E-06 7920 6.61E-06 7360 8.51E-06
8280 3.25E-05 6250 1.68E-06 6050 6.74E-06 5640
4.66E-06 7920 6.61E-06 7360 8.51E-06
8280 3.25E-05 6250 1.68E-06 6050 6.74E-06 5640
4.67E-06 7920 6.61E-06 7360 8.52E-06
8280 3.25E-05 6250 1.68E-06 6050 6.73E-06 5640
4.68E-06 7930 6.61E-06 7370 8.51E-06
8280 3.25E-05 6250 1.69E-06 6050 6.72E-06 5640
4.68E-06 7930 6.61E-06 7370 8.50E-06
8280 3.26E-05 6250 1.67E-06 6050 6.71E-06 5650
4.69E-06 7930 6.61E-06 7370 8.51E-06
8280 3.26E-05 6260 1.68E-06 6050 6.71E-06 5650
4.70E-06 7930 6.60E-06 7370 8.52E-06
8290 3.26E-05 6260 1.68E-06 6050 6.71E-06 5650
4.71E-06 7930 6.60E-06 7370 8.53E-06
8290 3.27E-05 6260 1.68E-06 6060 6.72E-06 5650
4.70E-06 7930 6.60E-06 7370 8.53E-06
8290 3.27E-05 6260 1.69E-06 6060 6.71E-06 5650
4.70E-06 7930 6.60E-06 7380 8.52E-06
8290 3.27E-05 6260 1.69E-06 6060 6.71E-06 5650
4.71E-06 7940 6.61E-06 7380 8.52E-06
8290 3.28E-05 6260 1.69E-06 6060 6.72E-06 5650
4.71E-06 7940 6.60E-06 7380 8.52E-06
8290 3.28E-05 6270 1.68E-06 6060 6.71E-06 5660
4.71E-06 7940 6.61E-06 7380 8.53E-06
8300 3.28E-05 6270 1.69E-06 6060 6.71E-06 5660
4.71E-06 7940 6.60E-06 7380 8.53E-06
8300 3.29E-05 6270 1.68E-06 6070 6.70E-06 5660
4.71E-06 7940 6.60E-06 7380 8.55E-06
8300 3.29E-05 6270 1.68E-06 6070 6.69E-06 5660
4.72E-06 7940 6.59E-06 7380 8.54E-06
8300 3.29E-05 6270 1.68E-06 6070 6.70E-06 5660
4.73E-06 7950 6.59E-06 7390 8.55E-06
8300 3.30E-05 6270 1.68E-06 6070 6.70E-06 5660
4.73E-06 7950 6.59E-06 7390 8.55E-06
8300 3.30E-05 6280 1.69E-06 6070 6.70E-06 5670
4.73E-06 7950 6.59E-06 7390 8.56E-06
8300 3.30E-05 6280 1.69E-06 6070 6.68E-06 5670
4.72E-06 7950 6.59E-06 7390 8.56E-06
8310 3.31E-05 6280 1.70E-06 6080 6.70E-06 5670
4.73E-06 7950 6.59E-06 7390 8.55E-06
8310 3.31E-05 6280 1.69E-06 6080 6.69E-06 5670
4.73E-06 7950 6.59E-06 7390 8.56E-06
8310 3.31E-05 6280 1.70E-06 6080 6.69E-06 5670
4.74E-06 7950 6.59E-06 7400 8.56E-06

8310 3.32E-05 6280 1.70E-06 6080 6.68E-06 5670
4.74E-06 7960 6.59E-06 7400 8.56E-06
8310 3.32E-05 6280 1.69E-06 6080 6.70E-06 5680
4.75E-06 7960 6.59E-06 7400 8.56E-06
8310 3.33E-05 6290 1.69E-06 6080 6.69E-06 5680
4.76E-06 7960 6.59E-06 7400 8.57E-06
8320 3.33E-05 6290 1.69E-06 6080 6.69E-06 5680
4.77E-06 7960 6.59E-06 7400 8.56E-06
8320 3.33E-05 6290 1.70E-06 6090 6.70E-06 5680
4.76E-06 7960 6.58E-06 7400 8.56E-06
8320 3.34E-05 6290 1.69E-06 6090 6.70E-06 5680
4.77E-06 7960 6.58E-06 7400 8.56E-06
8320 3.34E-05 6290 1.70E-06 6090 6.69E-06 5680
4.78E-06 7970 6.59E-06 7410 8.55E-06
8320 3.34E-05 6290 1.70E-06 6090 6.70E-06 5680
4.79E-06 7970 6.58E-06 7410 8.56E-06
8320 3.34E-05 6300 1.70E-06 6090 6.67E-06 5690
4.78E-06 7970 6.58E-06 7410 8.56E-06
8330 3.35E-05 6300 1.71E-06 6090 6.68E-06 5690
4.78E-06 7970 6.58E-06 7410 8.56E-06
8330 3.35E-05 6300 1.70E-06 6100 6.68E-06 5690
4.80E-06 7970 6.58E-06 7410 8.57E-06
8330 3.35E-05 6300 1.70E-06 6100 6.67E-06 5690
4.80E-06 7970 6.58E-06 7410 8.58E-06
8330 3.36E-05 6300 1.70E-06 6100 6.67E-06 5690
4.81E-06 7980 6.58E-06 7420 8.58E-06
8330 3.36E-05 6300 1.69E-06 6100 6.67E-06 5690
4.82E-06 7980 6.58E-06 7420 8.58E-06
8330 3.36E-05 6300 1.71E-06 6100 6.68E-06 5700
4.82E-06 7980 6.58E-06 7420 8.58E-06
8330 3.37E-05 6310 1.70E-06 6100 6.68E-06 5700
4.82E-06 7980 6.59E-06 7420 8.59E-06
8340 3.37E-05 6310 1.70E-06 6100 6.68E-06 5700
4.83E-06 7980 6.59E-06 7420 8.58E-06
8340 3.37E-05 6310 1.70E-06 6110 6.68E-06 5700
4.83E-06 7980 6.60E-06 7420 8.59E-06
8340 3.38E-05 6310 1.69E-06 6110 6.67E-06 5700
4.84E-06 7980 6.59E-06 7430 8.59E-06
8340 3.38E-05 6310 1.70E-06 6110 6.68E-06 5700
4.85E-06 7990 6.59E-06 7430 8.60E-06
8340 3.38E-05 6310 1.69E-06 6110 6.69E-06 5700
4.85E-06 7990 6.59E-06 7430 8.60E-06
8340 3.39E-05 6320 1.70E-06 6110 6.68E-06 5710
4.86E-06 7990 6.60E-06 7430 8.59E-06
8350 3.39E-05 6320 1.70E-06 6110 6.69E-06 5710
4.87E-06 7990 6.60E-06 7430 8.59E-06
8350 3.39E-05 6320 1.70E-06 6120 6.69E-06 5710
4.88E-06 7990 6.59E-06 7430 8.59E-06
8350 3.40E-05 6320 1.70E-06 6120 6.69E-06 5710
4.89E-06 7990 6.59E-06 7430 8.59E-06
8350 3.40E-05 6320 1.70E-06 6120 6.70E-06 5710
4.89E-06 8000 6.59E-06 7440 8.59E-06
8350 3.40E-05 6320 1.71E-06 6120 6.70E-06 5710
4.92E-06 8000 6.59E-06 7440 8.58E-06
8350 3.41E-05 6330 1.70E-06 6120 6.68E-06 5720
4.91E-06 8000 6.59E-06 7440 8.59E-06
8350 3.41E-05 6330 1.70E-06 6120 6.68E-06 5720
4.90E-06 8000 6.59E-06 7440 8.58E-06
8360 3.41E-05 6330 1.70E-06 6130 6.68E-06 5720
4.92E-06 8000 6.58E-06 7440 8.60E-06
8360 3.42E-05 6330 1.70E-06 6130 6.67E-06 5720
4.92E-06 8000 6.58E-06 7440 8.59E-06
8360 3.42E-05 6330 1.70E-06 6130 6.67E-06 5720
4.93E-06 8000 6.58E-06 7450 8.61E-06
8360 3.42E-05 6330 1.70E-06 6130 6.68E-06 5720
4.93E-06 8010 6.58E-06 7450 8.60E-06
8360 3.43E-05 6330 1.72E-06 6130 6.67E-06 5730
4.93E-06 8010 6.59E-06 7450 8.61E-06

8360 3.43E-05 6340 1.71E-06 6130 6.67E-06 5730
4.94E-06 8010 6.60E-06 7450 8.61E-06
8370 3.44E-05 6340 1.72E-06 6130 6.68E-06 5730
4.94E-06 8010 6.60E-06 7450 8.60E-06
8370 3.44E-05 6340 1.72E-06 6140 6.68E-06 5730
4.94E-06 8010 6.60E-06 7450 8.61E-06
8370 3.44E-05 6340 1.71E-06 6140 6.68E-06 5730
4.95E-06 8010 6.60E-06 7450 8.61E-06
8370 3.45E-05 6340 1.72E-06 6140 6.68E-06 5730
4.95E-06 8020 6.60E-06 7460 8.60E-06
8370 3.45E-05 6340 1.71E-06 6140 6.70E-06 5730
4.96E-06 8020 6.60E-06 7460 8.61E-06
8370 3.46E-05 6350 1.72E-06 6140 6.70E-06 5740
4.96E-06 8020 6.60E-06 7460 8.60E-06
8380 3.46E-05 6350 1.72E-06 6140 6.70E-06 5740
4.96E-06 8020 6.60E-06 7460 8.59E-06
8380 3.46E-05 6350 1.72E-06 6150 6.70E-06 5740
4.97E-06 8020 6.60E-06 7460 8.60E-06
8380 3.47E-05 6350 1.73E-06 6150 6.71E-06 5740
4.97E-06 8020 6.60E-06 7460 8.61E-06
8380 3.47E-05 6350 1.73E-06 6150 6.71E-06 5740
4.97E-06 8030 6.60E-06 7470 8.61E-06
8380 3.47E-05 6350 1.73E-06 6150 6.72E-06 5740
4.99E-06 8030 6.61E-06 7470 8.61E-06
8380 3.48E-05 6350 1.72E-06 6150 6.70E-06 5750
5.00E-06 8030 6.61E-06 7470 8.62E-06
8380 3.48E-05 6360 1.72E-06 6150 6.70E-06 5750
4.99E-06 8030 6.61E-06 7470 8.60E-06
8390 3.48E-05 6360 1.72E-06 6150 6.70E-06 5750
5.01E-06 8030 6.61E-06 7470 8.62E-06
8390 3.49E-05 6360 1.72E-06 6160 6.71E-06 5750
5.01E-06 8030 6.61E-06 7470 8.63E-06
8390 3.49E-05 6360 1.72E-06 6160 6.71E-06 5750
5.02E-06 8030 6.62E-06 7480 8.64E-06
8390 3.49E-05 6360 1.72E-06 6160 6.72E-06 5750
5.04E-06 8040 6.62E-06 7480 8.65E-06
8390 3.50E-05 6360 1.73E-06 6160 6.71E-06 5750
5.05E-06 8040 6.62E-06 7480 8.66E-06
8390 3.50E-05 6370 1.73E-06 6160 6.72E-06 5760
5.04E-06 8040 6.63E-06 7480 8.65E-06
8400 3.51E-05 6370 1.74E-06 6160 6.72E-06 5760
5.05E-06 8040 6.63E-06 7480 8.65E-06
8400 3.51E-05 6370 1.74E-06 6170 6.73E-06 5760
5.06E-06 8040 6.63E-06 7480 8.67E-06
8400 3.51E-05 6370 1.74E-06 6170 6.73E-06 5760
5.06E-06 8040 6.63E-06 7480 8.67E-06
8400 3.52E-05 6370 1.74E-06 6170 6.73E-06 5760
5.06E-06 8050 6.64E-06 7490 8.67E-06
8400 3.53E-05 6370 1.74E-06 6170 6.74E-06 5760
5.08E-06 8050 6.64E-06 7490 8.67E-06
8400 3.53E-05 6380 1.74E-06 6170 6.74E-06 5770
5.08E-06 8050 6.65E-06 7490 8.68E-06
8400 3.53E-05 6380 1.74E-06 6170 6.75E-06 5770
5.08E-06 8050 6.65E-06 7490 8.66E-06
8410 3.54E-05 6380 1.74E-06 6180 6.74E-06 5770
5.08E-06 8050 6.64E-06 7490 8.67E-06
8410 3.54E-05 6380 1.75E-06 6180 6.75E-06 5770
5.09E-06 8050 6.65E-06 7490 8.65E-06
8410 3.54E-05 6380 1.75E-06 6180 6.74E-06 5770
5.11E-06 8050 6.65E-06 7500 8.65E-06
8410 3.55E-05 6380 1.75E-06 6180 6.75E-06 5770
5.11E-06 8060 6.66E-06 7500 8.65E-06
8410 3.55E-05 6380 1.75E-06 6180 6.74E-06 5780
5.12E-06 8060 6.66E-06 7500 8.66E-06
8410 3.56E-05 6390 1.75E-06 6180 6.73E-06 5780
5.12E-06 8060 6.66E-06 7500 8.65E-06
8420 3.56E-05 6390 1.75E-06 6180 6.73E-06 5780
5.13E-06 8060 6.66E-06 7500 8.66E-06

8420 3.56E-05 6390 1.75E-06 6190 6.73E-06 5780
5.14E-06 8060 6.66E-06 7500 8.66E-06
8420 3.56E-05 6390 1.74E-06 6190 6.74E-06 5780
5.15E-06 8060 6.67E-06 7500 8.67E-06
8420 3.57E-05 6390 1.74E-06 6190 6.73E-06 5780
5.16E-06 8070 6.67E-06 7510 8.67E-06
8420 3.57E-05 6390 1.76E-06 6190 6.73E-06 5780
5.16E-06 8070 6.67E-06 7510 8.68E-06
8420 3.58E-05 6400 1.75E-06 6190 6.72E-06 5790
5.16E-06 8070 6.68E-06 7510 8.69E-06
8430 3.58E-05 6400 1.76E-06 6190 6.72E-06 5790
5.17E-06 8070 6.69E-06 7510 8.69E-06
8430 3.59E-05 6400 1.76E-06 6200 6.71E-06 5790
5.18E-06 8070 6.69E-06 7510 8.69E-06
8430 3.59E-05 6400 1.76E-06 6200 6.72E-06 5790
5.18E-06 8070 6.69E-06 7510 8.70E-06
8430 3.60E-05 6400 1.76E-06 6200 6.71E-06 5790
5.19E-06 8080 6.69E-06 7520 8.69E-06
8430 3.60E-05 6400 1.75E-06 6200 6.71E-06 5790
5.20E-06 8080 6.69E-06 7520 8.70E-06
8430 3.60E-05 6400 1.76E-06 6200 6.72E-06 5800
5.21E-06 8080 6.70E-06 7520 8.70E-06
8430 3.61E-05 6410 1.76E-06 6200 6.72E-06 5800
5.20E-06 8080 6.70E-06 7520 8.71E-06
8440 3.61E-05 6410 1.76E-06 6200 6.72E-06 5800
5.21E-06 8080 6.70E-06 7520 8.71E-06
8440 3.62E-05 6410 1.76E-06 6210 6.73E-06 5800
5.22E-06 8080 6.70E-06 7520 8.71E-06
8440 3.62E-05 6410 1.76E-06 6210 6.72E-06 5800
5.22E-06 8080 6.71E-06 7530 8.72E-06
8440 3.63E-05 6410 1.77E-06 6210 6.72E-06 5800
5.25E-06 8090 6.71E-06 7530 8.72E-06
8440 3.63E-05 6410 1.76E-06 6210 6.71E-06 5800
5.25E-06 8090 6.72E-06 7530 8.71E-06
8440 3.63E-05 6420 1.77E-06 6210 6.72E-06 5810
5.24E-06 8090 6.72E-06 7530 8.73E-06
8450 3.64E-05 6420 1.76E-06 6210 6.72E-06 5810
5.25E-06 8090 6.72E-06 7530 8.75E-06
8450 3.64E-05 6420 1.76E-06 6220 6.72E-06 5810
5.26E-06 8090 6.72E-06 7530 8.77E-06
8450 3.65E-05 6420 1.76E-06 6220 6.73E-06 5810
5.26E-06 8090 6.73E-06 7530 8.77E-06
8450 3.65E-05 6420 1.76E-06 6220 6.73E-06 5810
5.27E-06 8100 6.73E-06 7540 8.79E-06
8450 3.66E-05 6420 1.76E-06 6220 6.74E-06 5810
5.27E-06 8100 6.72E-06 7540 8.78E-06
8450 3.66E-05 6430 1.76E-06 6220 6.74E-06 5820
5.28E-06 8100 6.73E-06 7540 8.78E-06
8450 3.67E-05 6430 1.77E-06 6220 6.75E-06 5820
5.29E-06 8100 6.74E-06 7540 8.78E-06
8460 3.67E-05 6430 1.76E-06 6230 6.75E-06 5820
5.29E-06 8100 6.75E-06 7540 8.79E-06
8460 3.67E-05 6430 1.76E-06 6230 6.75E-06 5820
5.29E-06 8100 6.75E-06 7540 8.79E-06
8460 3.68E-05 6430 1.76E-06 6230 6.77E-06 5820
5.31E-06 8100 6.75E-06 7550 8.79E-06
8460 3.69E-05 6430 1.75E-06 6230 6.78E-06 5820
5.31E-06 8110 6.75E-06 7550 8.80E-06
8460 3.69E-05 6430 1.76E-06 6230 6.79E-06 5830
5.32E-06 8110 6.75E-06 7550 8.79E-06
8460 3.69E-05 6440 1.76E-06 6230 6.79E-06 5830
5.32E-06 8110 6.75E-06 7550 8.79E-06
8470 3.70E-05 6440 1.77E-06 6230 6.79E-06 5830
5.33E-06 8110 6.75E-06 7550 8.78E-06
8470 3.70E-05 6440 1.78E-06 6240 6.79E-06 5830
5.33E-06 8110 6.75E-06 7550 8.78E-06
8470 3.71E-05 6440 1.78E-06 6240 6.80E-06 5830
5.35E-06 8110 6.76E-06 7550 8.79E-06

8470 3.71E-05 6440 1.78E-06 6240 6.80E-06 5830
5.36E-06 8120 6.76E-06 7560 8.80E-06
8470 3.71E-05 6440 1.78E-06 6240 6.79E-06 5830
5.37E-06 8120 6.76E-06 7560 8.81E-06
8470 3.72E-05 6450 1.78E-06 6240 6.79E-06 5840
5.37E-06 8120 6.76E-06 7560 8.80E-06
8480 3.72E-05 6450 1.77E-06 6240 6.80E-06 5840
5.39E-06 8120 6.76E-06 7560 8.79E-06
8480 3.73E-05 6450 1.77E-06 6250 6.80E-06 5840
5.39E-06 8120 6.76E-06 7560 8.80E-06
8480 3.73E-05 6450 1.77E-06 6250 6.80E-06 5840
5.40E-06 8120 6.77E-06 7560 8.81E-06
8480 3.73E-05 6450 1.76E-06 6250 6.81E-06 5840
5.41E-06 8130 6.77E-06 7570 8.83E-06
8480 3.74E-05 6450 1.78E-06 6250 6.80E-06 5840
5.42E-06 8130 6.77E-06 7570 8.82E-06
8480 3.74E-05 6450 1.78E-06 6250 6.80E-06 5850
5.42E-06 8130 6.77E-06 7570 8.82E-06
8480 3.75E-05 6460 1.78E-06 6250 6.81E-06 5850
5.43E-06 8130 6.77E-06 7570 8.82E-06
8490 3.75E-05 6460 1.78E-06 6250 6.81E-06 5850
5.44E-06 8130 6.78E-06 7570 8.83E-06
8490 3.76E-05 6460 1.77E-06 6260 6.81E-06 5850
5.45E-06 8130 6.78E-06 7570 8.84E-06
8490 3.76E-05 6460 1.77E-06 6260 6.82E-06 5850
5.46E-06 8130 6.78E-06 7580 8.85E-06
8490 3.77E-05 6460 1.78E-06 6260 6.83E-06 5850
5.46E-06 8140 6.78E-06 7580 8.84E-06
8490 3.77E-05 6460 1.78E-06 6260 6.84E-06 5850
5.47E-06 8140 6.78E-06 7580 8.82E-06
8490 3.77E-05 6470 1.78E-06 6260 6.85E-06 5860
5.47E-06 8140 6.78E-06 7580 8.84E-06
8500 3.78E-05 6470 1.78E-06 6260 6.85E-06 5860
5.49E-06 8140 6.78E-06 7580 8.84E-06
8500 3.78E-05 6470 1.79E-06 6270 6.85E-06 5860
5.49E-06 8140 6.78E-06 7580 8.83E-06
8500 3.79E-05 6470 1.79E-06 6270 6.85E-06 5860
5.50E-06 8140 6.79E-06 7580 8.85E-06
8500 3.79E-05 6470 1.79E-06 6270 6.86E-06 5860
5.49E-06 8150 6.79E-06 7590 8.86E-06
8500 3.80E-05 6470 1.79E-06 6270 6.85E-06 5860
5.51E-06 8150 6.79E-06 7590 8.85E-06
8500 3.80E-05 6480 1.79E-06 6270 6.85E-06 5870
5.48E-06 8150 6.78E-06 7590 8.85E-06
8500 3.81E-05 6480 1.78E-06 6270 6.86E-06 5870
5.50E-06 8150 6.78E-06 7590 8.87E-06
8510 3.81E-05 6480 1.79E-06 6280 6.86E-06 5870
5.50E-06 8150 6.78E-06 7590 8.87E-06
8510 3.82E-05 6480 1.78E-06 6280 6.87E-06 5870
5.50E-06 8150 6.79E-06 7590 8.89E-06
8510 3.82E-05 6480 1.79E-06 6280 6.88E-06 5870
5.51E-06 8150 6.79E-06 7600 8.90E-06
8510 3.83E-05 6480 1.78E-06 6280 6.87E-06 5870
5.51E-06 8160 6.79E-06 7600 8.88E-06
8510 3.84E-05 6480 1.78E-06 6280 6.88E-06 5880
5.51E-06 8160 6.79E-06 7600 8.88E-06
8510 3.84E-05 6490 1.79E-06 6280 6.88E-06 5880
5.52E-06 8160 6.79E-06 7600 8.89E-06
8520 3.85E-05 6490 1.79E-06 6280 6.89E-06 5880
5.53E-06 8160 6.80E-06 7600 8.89E-06
8520 3.85E-05 6490 1.78E-06 6290 6.89E-06 5880
5.51E-06 8160 6.81E-06 7600 8.90E-06
8520 3.86E-05 6490 1.79E-06 6290 6.90E-06 5880
5.53E-06 8160 6.81E-06 7600 8.90E-06
8520 3.87E-05 6490 1.79E-06 6290 6.91E-06 5880
5.53E-06 8170 6.82E-06 7610 8.88E-06
8520 3.87E-05 6490 1.79E-06 6290 6.92E-06 5880
5.53E-06 8170 6.81E-06 7610 8.89E-06

```
8520    3.87E-05        6500    1.80E-06        6290    6.92E-06        5890
5.53E-06        8170    6.82E-06        7610    8.89E-06
8530    3.88E-05        6500    1.80E-06        6290    6.93E-06        5890
5.53E-06        8170    6.82E-06        7610    8.88E-06
8530    3.88E-05        6500    1.80E-06        6300    6.93E-06        5890
5.54E-06        8170    6.82E-06        7610    8.88E-06
8530    3.89E-05        6500    1.81E-06        6300    6.94E-06        5890
5.53E-06        8170    6.83E-06        7610    8.89E-06
8530    3.89E-05        6500    1.81E-06        6300    6.93E-06        5890
5.55E-06        8180    6.83E-06        7620    8.88E-06
8530    3.90E-05        6500    1.81E-06        6300    6.93E-06        5890
5.56E-06        8180    6.84E-06        7620    8.88E-06
8530    3.90E-05        6500    1.80E-06        6300    6.93E-06        5900
5.57E-06        8180    6.84E-06        7620    8.89E-06
8530    3.91E-05        6510    1.81E-06        6300    6.94E-06        5900
5.59E-06        8180    6.84E-06        7620    8.90E-06
8540    3.91E-05        6510    1.81E-06        6300    6.93E-06        5900
5.60E-06        8180    6.84E-06        7620    8.89E-06
8540    3.92E-05        6510    1.80E-06        6310    6.93E-06        5900
5.61E-06        8180    6.85E-06        7620    8.90E-06
8540    3.92E-05        6510    1.80E-06        6310    6.94E-06        5900
5.63E-06        8180    6.85E-06        7630    8.90E-06
8540    3.93E-05        6510    1.80E-06        6310    6.93E-06        5900
5.63E-06        8190    6.85E-06        7630    8.91E-06
8540    3.93E-05        6510    1.80E-06        6310    6.93E-06        5900
5.65E-06        8190    6.86E-06        7630    8.92E-06
8540    3.94E-05        6520    1.80E-06        6310    6.94E-06        5910
5.67E-06        8190    6.86E-06        7630    8.92E-06
8550    3.95E-05        6520    1.80E-06        6310    6.93E-06        5910
5.69E-06        8190    6.87E-06        7630    8.92E-06
8550    3.95E-05        6520    1.80E-06        6320    6.94E-06        5910
5.70E-06        8190    6.88E-06        7630    8.93E-06
8550    3.96E-05        6520    1.80E-06        6320    6.95E-06        5910
5.72E-06        8190    6.88E-06        7630    8.94E-06
8550    3.96E-05        6520    1.80E-06        6320    6.95E-06        5910
5.74E-06        8200    6.89E-06        7640    8.93E-06
8550    3.97E-05        6520    1.80E-06        6320    6.97E-06        5910
5.75E-06        8200    6.89E-06        7640    8.94E-06
8550    3.97E-05        6530    1.80E-06        6320    6.97E-06        5920
5.77E-06        8200    6.89E-06        7640    8.95E-06
8550    3.98E-05        6530    1.80E-06        6320    6.98E-06        5920
5.78E-06        8200    6.90E-06        7640    8.94E-06
8560    3.98E-05        6530    1.81E-06        6330    6.97E-06        5920
5.79E-06        8200    6.90E-06        7640    8.92E-06
8560    3.99E-05        6530    1.81E-06        6330    6.98E-06        5920
5.80E-06        8200    6.90E-06        7640    8.93E-06
8560    3.99E-05        6530    1.81E-06        6330    6.97E-06        5920
5.81E-06        8200    6.91E-06        7650    8.94E-06
8560    4.00E-05        6530    1.81E-06        6330    6.97E-06        5920
5.82E-06        8210    6.91E-06        7650    8.93E-06
8560    4.01E-05        6530    1.81E-06        6330    6.97E-06        5930
5.84E-06        8210    6.91E-06        7650    8.94E-06
8560    4.01E-05        6540    1.81E-06        6330    6.97E-06        5930
5.85E-06        8210    6.91E-06        7650    8.94E-06
8570    4.02E-05        6540    1.81E-06        6330    6.98E-06        5930
5.86E-06        8210    6.90E-06        7650    8.94E-06
8570    4.03E-05        6540    1.81E-06        6340    6.98E-06        5930
5.86E-06        8210    6.91E-06        7650    8.95E-06
8570    4.03E-05        6540    1.81E-06        6340    6.98E-06        5930
5.88E-06        8210    6.91E-06        7650    8.95E-06
8570    4.04E-05        6540    1.81E-06        6340    6.98E-06        5930
5.89E-06        8220    6.91E-06        7660    8.95E-06
8570    4.04E-05        6540    1.81E-06        6340    6.99E-06        5930
5.91E-06        8220    6.93E-06        7660    8.95E-06
8570    4.05E-05        6550    1.81E-06        6340    7.01E-06        5940
5.92E-06        8220    6.93E-06        7660    8.96E-06
8580    4.06E-05        6550    1.82E-06        6340    7.01E-06        5940
5.94E-06        8220    6.94E-06        7660    8.95E-06
```

8580 4.06E-05 6550 1.81E-06 6350 7.01E-06 5940
5.93E-06 8220 6.94E-06 7660 8.95E-06
8580 4.07E-05 6550 1.81E-06 6350 7.02E-06 5940
5.94E-06 8220 6.95E-06 7660 8.94E-06
8580 4.08E-05 6550 1.82E-06 6350 7.03E-06 5940
5.96E-06 8230 6.95E-06 7670 8.93E-06
8580 4.08E-05 6550 1.82E-06 6350 7.03E-06 5940
5.97E-06 8230 6.95E-06 7670 8.95E-06
8580 4.08E-05 6550 1.83E-06 6350 7.04E-06 5950
5.98E-06 8230 6.95E-06 7670 8.95E-06
8580 4.09E-05 6560 1.83E-06 6350 7.05E-06 5950
5.99E-06 8230 6.95E-06 7670 8.94E-06
8590 4.10E-05 6560 1.83E-06 6350 7.04E-06 5950
6.00E-06 8230 6.95E-06 7670 8.94E-06
8590 4.10E-05 6560 1.84E-06 6360 7.05E-06 5950
6.01E-06 8230 6.96E-06 7670 8.94E-06
8590 4.11E-05 6560 1.84E-06 6360 7.05E-06 5950
6.03E-06 8230 6.97E-06 7680 8.94E-06
8590 4.12E-05 6560 1.84E-06 6360 7.04E-06 5950
6.04E-06 8240 6.97E-06 7680 8.92E-06
8590 4.12E-05 6560 1.83E-06 6360 7.05E-06 5950
6.04E-06 8240 6.97E-06 7680 8.94E-06
8590 4.13E-05 6570 1.83E-06 6360 7.05E-06 5960
6.06E-06 8240 6.97E-06 7680 8.93E-06
8600 4.13E-05 6570 1.83E-06 6360 7.05E-06 5960
6.07E-06 8240 6.97E-06 7680 8.94E-06
8600 4.14E-05 6570 1.83E-06 6370 7.05E-06 5960
6.09E-06 8240 6.98E-06 7680 8.93E-06
8600 4.14E-05 6570 1.83E-06 6370 7.05E-06 5960
6.11E-06 8240 6.97E-06 7680 8.93E-06
8600 4.15E-05 6570 1.84E-06 6370 7.05E-06 5960
6.11E-06 8250 6.98E-06 7690 8.93E-06
8600 4.16E-05 6570 1.84E-06 6370 7.05E-06 5960
6.13E-06 8250 6.98E-06 7690 8.94E-06
8600 4.17E-05 6580 1.84E-06 6370 7.07E-06 5970
6.15E-06 8250 6.99E-06 7690 8.95E-06
8600 4.17E-05 6580 1.84E-06 6370 7.07E-06 5970
6.17E-06 8250 7.00E-06 7690 8.95E-06
8610 4.18E-05 6580 1.84E-06 6380 7.08E-06 5970
6.16E-06 8250 7.00E-06 7690 8.94E-06
8610 4.19E-05 6580 1.84E-06 6380 7.08E-06 5970
6.17E-06 8250 7.01E-06 7690 8.93E-06
8610 4.19E-05 6580 1.84E-06 6380 7.09E-06 5970
6.18E-06 8250 7.01E-06 7700 8.94E-06
8610 4.20E-05 6580 1.84E-06 6380 7.10E-06 5970
6.20E-06 8260 7.01E-06 7700 8.94E-06
8610 4.20E-05 6580 1.85E-06 6380 7.10E-06 5980
6.21E-06 8260 7.01E-06 7700 8.96E-06
8610 4.21E-05 6590 1.85E-06 6380 7.10E-06 5980
6.23E-06 8260 7.01E-06 7700 8.95E-06
8620 4.21E-05 6590 1.86E-06 6380 7.11E-06 5980
6.22E-06 8260 7.01E-06 7700 8.94E-06
8620 4.22E-05 6590 1.86E-06 6390 7.11E-06 5980
6.24E-06 8260 7.01E-06 7700 8.95E-06
8620 4.23E-05 6590 1.86E-06 6390 7.10E-06 5980
6.26E-06 8260 7.01E-06 7700 8.93E-06
8620 4.24E-05 6590 1.86E-06 6390 7.10E-06 5980
6.27E-06 8270 7.01E-06 7710 8.93E-06
8620 4.24E-05 6590 1.85E-06 6390 7.10E-06 5980
6.27E-06 8270 7.01E-06 7710 8.94E-06
8620 4.25E-05 6600 1.85E-06 6390 7.10E-06 5990
6.29E-06 8270 7.01E-06 7710 8.94E-06
8630 4.26E-05 6600 1.85E-06 6390 7.12E-06 5990
6.30E-06 8270 7.01E-06 7710 8.94E-06
8630 4.26E-05 6600 1.85E-06 6400 7.11E-06 5990
6.33E-06 8270 7.02E-06 7710 8.93E-06
8630 4.27E-05 6600 1.85E-06 6400 7.11E-06 5990
6.35E-06 8270 7.02E-06 7710 8.95E-06

8630 4.28E-05 6600 1.86E-06 6400 7.11E-06 5990
6.36E-06 8280 7.02E-06 7720 8.95E-06
8630 4.29E-05 6600 1.85E-06 6400 7.12E-06 5990
6.36E-06 8280 7.02E-06 7720 8.94E-06
8630 4.29E-05 6600 1.86E-06 6400 7.14E-06 6000
6.38E-06 8280 7.04E-06 7720 8.94E-06
8630 4.30E-05 6610 1.87E-06 6400 7.14E-06 6000
6.41E-06 8280 7.03E-06 7720 8.94E-06
8640 4.31E-05 6610 1.86E-06 6400 7.14E-06 6000
6.41E-06 8280 7.04E-06 7720 8.94E-06
8640 4.32E-05 6610 1.86E-06 6410 7.15E-06 6000
6.42E-06 8280 7.05E-06 7720 8.95E-06
8640 4.32E-05 6610 1.85E-06 6410 7.15E-06 6000
6.44E-06 8280 7.05E-06 7730 8.93E-06
8640 4.33E-05 6610 1.86E-06 6410 7.16E-06 6000
6.46E-06 8290 7.05E-06 7730 8.95E-06
8640 4.33E-05 6610 1.86E-06 6410 7.17E-06 6000
6.46E-06 8290 7.06E-06 7730 8.95E-06
8640 4.34E-05 6620 1.86E-06 6410 7.18E-06 6010
6.48E-06 8290 7.05E-06 7730 8.95E-06
8650 4.35E-05 6620 1.87E-06 6410 7.17E-06 6010
6.50E-06 8290 7.06E-06 7730 8.94E-06
8650 4.36E-05 6620 1.87E-06 6420 7.18E-06 6010
6.51E-06 8290 7.06E-06 7730 8.94E-06
8650 4.36E-05 6620 1.87E-06 6420 7.17E-06 6010
6.54E-06 8290 7.08E-06 7730 8.94E-06
8650 4.37E-05 6620 1.86E-06 6420 7.18E-06 6010
6.55E-06 8300 7.08E-06 7740 8.94E-06
8650 4.38E-05 6620 1.87E-06 6420 7.17E-06 6010
6.55E-06 8300 7.08E-06 7740 8.95E-06
8650 4.38E-05 6630 1.86E-06 6420 7.17E-06 6020
6.58E-06 8300 7.08E-06 7740 8.95E-06
8650 4.39E-05 6630 1.86E-06 6420 7.18E-06 6020
6.59E-06 8300 7.09E-06 7740 8.95E-06
8660 4.40E-05 6630 1.86E-06 6430 7.18E-06 6020
6.61E-06 8300 7.10E-06 7740 8.95E-06
8660 4.40E-05 6630 1.86E-06 6430 7.18E-06 6020
6.64E-06 8300 7.11E-06 7740 8.96E-06
8660 4.41E-05 6630 1.86E-06 6430 7.18E-06 6020
6.65E-06 8300 7.11E-06 7750 8.95E-06
8660 4.42E-05 6630 1.86E-06 6430 7.18E-06 6020
6.66E-06 8310 7.11E-06 7750 8.96E-06
8660 4.43E-05 6630 1.87E-06 6430 7.19E-06 6030
6.68E-06 8310 7.13E-06 7750 8.95E-06
8660 4.43E-05 6640 1.86E-06 6430 7.21E-06 6030
6.71E-06 8310 7.13E-06 7750 8.97E-06
8670 4.44E-05 6640 1.86E-06 6430 7.21E-06 6030
6.73E-06 8310 7.14E-06 7750 8.96E-06
8670 4.45E-05 6640 1.86E-06 6440 7.21E-06 6030
6.75E-06 8310 7.14E-06 7750 8.97E-06
8670 4.46E-05 6640 1.86E-06 6440 7.21E-06 6030
6.77E-06 8310 7.15E-06 7750 8.98E-06
8670 4.46E-05 6640 1.86E-06 6440 7.22E-06 6030
6.79E-06 8320 7.16E-06 7760 8.97E-06
8670 4.47E-05 6640 1.86E-06 6440 7.23E-06 6030
6.80E-06 8320 7.17E-06 7760 8.96E-06
8670 4.48E-05 6650 1.86E-06 6440 7.23E-06 6040
6.82E-06 8320 7.16E-06 7760 8.97E-06
8680 4.48E-05 6650 1.87E-06 6440 7.23E-06 6040
6.86E-06 8320 7.17E-06 7760 8.97E-06
8680 4.49E-05 6650 1.87E-06 6450 7.23E-06 6040
6.86E-06 8320 7.18E-06 7760 8.97E-06
8680 4.50E-05 6650 1.89E-06 6450 7.24E-06 6040
6.88E-06 8320 7.19E-06 7760 8.98E-06
8680 4.51E-05 6650 1.88E-06 6450 7.23E-06 6040
6.90E-06 8330 7.20E-06 7770 8.98E-06
8680 4.51E-05 6650 1.88E-06 6450 7.22E-06 6040
6.92E-06 8330 7.20E-06 7770 8.99E-06

8680 4.52E-05 6650 1.87E-06 6450 7.23E-06 6050
6.96E-06 8330 7.20E-06 7770 9.00E-06
8680 4.53E-05 6660 1.87E-06 6450 7.23E-06 6050
6.96E-06 8330 7.22E-06 7770 9.00E-06
8690 4.54E-05 6660 1.87E-06 6450 7.23E-06 6050
6.97E-06 8330 7.23E-06 7770 9.00E-06
8690 4.54E-05 6660 1.88E-06 6460 7.23E-06 6050
6.99E-06 8330 7.23E-06 7770 9.02E-06
8690 4.55E-05 6660 1.88E-06 6460 7.23E-06 6050
7.01E-06 8330 7.24E-06 7780 9.02E-06
8690 4.56E-05 6660 1.88E-06 6460 7.23E-06 6050
7.03E-06 8340 7.24E-06 7780 9.02E-06
8690 4.57E-05 6660 1.88E-06 6460 7.23E-06 6050
7.05E-06 8340 7.26E-06 7780 9.03E-06
8690 4.58E-05 6670 1.88E-06 6460 7.24E-06 6060
7.07E-06 8340 7.27E-06 7780 9.02E-06
8700 4.59E-05 6670 1.88E-06 6460 7.24E-06 6060
7.09E-06 8340 7.28E-06 7780 9.03E-06
8700 4.60E-05 6670 1.88E-06 6470 7.25E-06 6060
7.10E-06 8340 7.28E-06 7780 9.03E-06
8700 4.61E-05 6670 1.88E-06 6470 7.26E-06 6060
7.12E-06 8340 7.29E-06 7780 9.03E-06
8700 4.61E-05 6670 1.88E-06 6470 7.26E-06 6060
7.14E-06 8350 7.30E-06 7790 9.04E-06
8700 4.62E-05 6670 1.89E-06 6470 7.27E-06 6060
7.16E-06 8350 7.31E-06 7790 9.05E-06
8700 4.63E-05 6680 1.89E-06 6470 7.27E-06 6070
7.18E-06 8350 7.31E-06 7790 9.06E-06
8700 4.64E-05 6680 1.89E-06 6470 7.27E-06 6070
7.21E-06 8350 7.32E-06 7790 9.07E-06
8710 4.65E-05 6680 1.90E-06 6480 7.27E-06 6070
7.23E-06 8350 7.33E-06 7790 9.07E-06
8710 4.66E-05 6680 1.90E-06 6480 7.28E-06 6070
7.25E-06 8350 7.35E-06 7790 9.08E-06
8710 4.67E-05 6680 1.90E-06 6480 7.27E-06 6070
7.28E-06 8350 7.36E-06 7800 9.09E-06
8710 4.68E-05 6680 1.90E-06 6480 7.27E-06 6070
7.29E-06 8360 7.37E-06 7800 9.12E-06
8710 4.68E-05 6680 1.89E-06 6480 7.27E-06 6080
7.32E-06 8360 7.39E-06 7800 9.13E-06
8710 4.69E-05 6690 1.89E-06 6480 7.27E-06 6080
7.35E-06 8360 7.40E-06 7800 9.13E-06
8720 4.70E-05 6690 1.89E-06 6480 7.28E-06 6080
7.38E-06 8360 7.42E-06 7800 9.14E-06
8720 4.71E-05 6690 1.90E-06 6490 7.28E-06 6080
7.40E-06 8360 7.43E-06 7800 9.15E-06
8720 4.72E-05 6690 1.90E-06 6490 7.29E-06 6080
7.43E-06 8360 7.44E-06 7800 9.18E-06
8720 4.73E-05 6690 1.90E-06 6490 7.29E-06 6080
7.45E-06 8370 7.45E-06 7810 9.18E-06
8720 4.74E-05 6690 1.90E-06 6490 7.29E-06 6080
7.48E-06 8370 7.46E-06 7810 9.19E-06
8720 4.75E-05 6700 1.90E-06 6490 7.30E-06 6090
7.51E-06 8370 7.48E-06 7810 9.20E-06
8730 4.76E-05 6700 1.89E-06 6490 7.30E-06 6090
7.52E-06 8370 7.49E-06 7810 9.22E-06
8730 4.77E-05 6700 1.90E-06 6500 7.31E-06 6090
7.55E-06 8370 7.51E-06 7810 9.20E-06
8730 4.78E-05 6700 1.89E-06 6500 7.32E-06 6090
7.57E-06 8370 7.52E-06 7810 9.20E-06
8730 4.79E-05 6700 1.89E-06 6500 7.32E-06 6090
7.59E-06 8380 7.53E-06 7820 9.21E-06
8730 4.80E-05 6700 1.89E-06 6500 7.32E-06 6090
7.62E-06 8380 7.55E-06 7820 9.22E-06
8730 4.81E-05 6700 1.90E-06 6500 7.33E-06 6100
7.64E-06 8380 7.56E-06 7820 9.25E-06
8730 4.82E-05 6710 1.90E-06 6500 7.33E-06 6100
7.67E-06 8380 7.57E-06 7820 9.22E-06

8740 4.83E-05 6710 1.90E-06 6500 7.34E-06 6100
7.70E-06 8380 7.59E-06 7820 9.23E-06
8740 4.84E-05 6710 1.90E-06 6510 7.34E-06 6100
7.73E-06 8380 7.60E-06 7820 9.25E-06
8740 4.84E-05 6710 1.90E-06 6510 7.33E-06 6100
7.76E-06 8380 7.62E-06 7830 9.23E-06
8740 4.86E-05 6710 1.89E-06 6510 7.33E-06 6100
7.79E-06 8390 7.63E-06 7830 9.25E-06
8740 4.87E-05 6710 1.90E-06 6510 7.32E-06 6100
7.82E-06 8390 7.64E-06 7830 9.27E-06
8740 4.87E-05 6720 1.89E-06 6510 7.33E-06 6110
7.85E-06 8390 7.66E-06 7830 9.27E-06
8750 4.88E-05 6720 1.89E-06 6510 7.33E-06 6110
7.87E-06 8390 7.69E-06 7830 9.28E-06
8750 4.89E-05 6720 1.89E-06 6520 7.34E-06 6110
7.91E-06 8390 7.71E-06 7830 9.29E-06
8750 4.90E-05 6720 1.89E-06 6520 7.34E-06 6110
7.93E-06 8390 7.73E-06 7830 9.29E-06
8750 4.91E-05 6720 1.90E-06 6520 7.34E-06 6110
7.97E-06 8400 7.74E-06 7840 9.29E-06
8750 4.92E-05 6720 1.89E-06 6520 7.34E-06 6110
7.99E-06 8400 7.76E-06 7840 9.28E-06
8750 4.93E-05 6730 1.90E-06 6520 7.34E-06 6120
8.04E-06 8400 7.78E-06 7840 9.29E-06
8750 4.95E-05 6730 1.90E-06 6520 7.34E-06 6120
8.05E-06 8400 7.81E-06 7840 9.31E-06
8760 4.95E-05 6730 1.90E-06 6530 7.35E-06 6120
8.09E-06 8400 7.83E-06 7840 9.31E-06
8760 4.97E-05 6730 1.89E-06 6530 7.36E-06 6120
8.11E-06 8400 7.85E-06 7840 9.29E-06
8760 4.97E-05 6730 1.89E-06 6530 7.36E-06 6120
8.14E-06 8400 7.87E-06 7850 9.31E-06
8760 4.98E-05 6730 1.90E-06 6530 7.36E-06 6120
8.17E-06 8410 7.90E-06 7850 9.31E-06
8760 4.99E-05 6730 1.91E-06 6530 7.37E-06 6130
8.20E-06 8410 7.91E-06 7850 9.29E-06
8760 5.00E-05 6740 1.91E-06 6530 7.37E-06 6130
8.24E-06 8410 7.93E-06 7850 9.29E-06
8770 5.02E-05 6740 1.91E-06 6530 7.38E-06 6130
8.27E-06 8410 7.95E-06 7850 9.31E-06
8770 5.03E-05 6740 1.92E-06 6540 7.38E-06 6130
8.28E-06 8410 7.98E-06 7850 9.28E-06
8770 5.03E-05 6740 1.91E-06 6540 7.38E-06 6130
8.32E-06 8410 8.01E-06 7850 9.29E-06
8770 5.05E-05 6740 1.91E-06 6540 7.37E-06 6130
8.35E-06 8420 8.03E-06 7860 9.29E-06
8770 5.06E-05 6740 1.90E-06 6540 7.36E-06 6130
8.39E-06 8420 8.06E-06 7860 9.28E-06
8770 5.07E-05 6750 1.90E-06 6540 7.38E-06 6140
8.43E-06 8420 8.09E-06 7860 9.28E-06
8780 5.08E-05 6750 1.91E-06 6540 7.39E-06 6140
8.47E-06 8420 8.11E-06 7860 9.28E-06
8780 5.09E-05 6750 1.91E-06 6550 7.40E-06 6140
8.50E-06 8420 8.14E-06 7860 9.28E-06
8780 5.10E-05 6750 1.90E-06 6550 7.40E-06 6140
8.53E-06 8420 8.17E-06 7860 9.28E-06
8780 5.11E-05 6750 1.91E-06 6550 7.39E-06 6140
8.58E-06 8430 8.21E-06 7870 9.28E-06
8780 5.13E-05 6750 1.92E-06 6550 7.41E-06 6140
8.61E-06 8430 8.25E-06 7870 9.26E-06
8780 5.14E-05 6750 1.91E-06 6550 7.41E-06 6150
8.65E-06 8430 8.29E-06 7870 9.26E-06
8780 5.15E-05 6760 1.91E-06 6550 7.42E-06 6150
8.67E-06 8430 8.32E-06 7870 9.26E-06
8790 5.16E-05 6760 1.92E-06 6550 7.42E-06 6150
8.72E-06 8430 8.36E-06 7870 9.24E-06
8790 5.18E-05 6760 1.91E-06 6560 7.43E-06 6150
8.74E-06 8430 8.39E-06 7870 9.24E-06

8790 5.19E-05 6760 1.91E-06 6560 7.43E-06 6150
8.78E-06 8430 8.43E-06 7880 9.22E-06
8790 5.20E-05 6760 1.91E-06 6560 7.44E-06 6150
8.83E-06 8440 8.48E-06 7880 9.23E-06
8790 5.21E-05 6760 1.92E-06 6560 7.45E-06 6150
8.88E-06 8440 8.50E-06 7880 9.22E-06
8790 5.22E-05 6770 1.93E-06 6560 7.45E-06 6160
8.92E-06 8440 8.54E-06 7880 9.20E-06
8800 5.24E-05 6770 1.93E-06 6560 7.45E-06 6160
8.96E-06 8440 8.58E-06 7880 9.20E-06
8800 5.25E-05 6770 1.92E-06 6570 7.46E-06 6160
9.00E-06 8440 8.62E-06 7880 9.17E-06
8800 5.26E-05 6770 1.93E-06 6570 7.46E-06 6160
9.04E-06 8440 8.68E-06 7880 9.18E-06
8800 5.27E-05 6770 1.93E-06 6570 7.45E-06 6160
9.09E-06 8450 8.72E-06 7890 9.15E-06
8800 5.28E-05 6770 1.92E-06 6570 7.45E-06 6160
9.13E-06 8450 8.77E-06 7890 9.15E-06
8800 5.29E-05 6780 1.92E-06 6570 7.45E-06 6170
9.18E-06 8450 8.82E-06 7890 9.14E-06
8800 5.30E-05 6780 1.92E-06 6570 7.46E-06 6170
9.22E-06 8450 8.88E-06 7890 9.14E-06
8810 5.31E-05 6780 1.92E-06 6580 7.46E-06 6170
9.26E-06 8450 8.93E-06 7890 9.15E-06
8810 5.32E-05 6780 1.93E-06 6580 7.45E-06 6170
9.31E-06 8450 8.98E-06 7890 9.15E-06
8810 5.34E-05 6780 1.93E-06 6580 7.46E-06 6170
9.36E-06 8450 9.05E-06 7900 9.13E-06
8810 5.35E-05 6780 1.94E-06 6580 7.46E-06 6170
9.39E-06 8460 9.11E-06 7900 9.13E-06
8810 5.36E-05 6780 1.94E-06 6580 7.47E-06 6180
9.44E-06 8460 9.17E-06 7900 9.12E-06
8810 5.38E-05 6790 1.93E-06 6580 7.48E-06 6180
9.49E-06 8460 9.23E-06 7900 9.12E-06
8820 5.39E-05 6790 1.93E-06 6580 7.49E-06 6180
9.54E-06 8460 9.31E-06 7900 9.11E-06
8820 5.40E-05 6790 1.93E-06 6590 7.49E-06 6180
9.57E-06 8460 9.37E-06 7900 9.09E-06
8820 5.42E-05 6790 1.93E-06 6590 7.50E-06 6180
9.62E-06 8460 9.44E-06 7900 9.10E-06
8820 5.43E-05 6790 1.93E-06 6590 7.50E-06 6180
9.68E-06 8470 9.53E-06 7910 9.09E-06
8820 5.44E-05 6790 1.93E-06 6590 7.51E-06 6180
9.72E-06 8470 9.59E-06 7910 9.08E-06
8820 5.45E-05 6800 1.95E-06 6590 7.51E-06 6190
9.76E-06 8470 9.67E-06 7910 9.08E-06
8830 5.47E-05 6800 1.95E-06 6590 7.51E-06 6190
9.82E-06 8470 9.76E-06 7910 9.08E-06
8830 5.48E-05 6800 1.95E-06 6600 7.52E-06 6190
9.86E-06 8470 9.85E-06 7910 9.06E-06
8830 5.50E-05 6800 1.95E-06 6600 7.51E-06 6190
9.91E-06 8470 9.94E-06 7910 9.06E-06
8830 5.52E-05 6800 1.95E-06 6600 7.51E-06 6190
9.96E-06 8480 1.00E-05 7920 9.06E-06
8830 5.52E-05 6800 1.94E-06 6600 7.51E-06 6190
1.00E-05 8480 1.01E-05 7920 9.06E-06
8830 5.54E-05 6800 1.94E-06 6600 7.50E-06 6200
1.01E-05 8480 1.02E-05 7920 9.06E-06
8830 5.55E-05 6810 1.94E-06 6600 7.51E-06 6200
1.01E-05 8480 1.03E-05 7920 9.06E-06
8840 5.56E-05 6810 1.95E-06 6600 7.51E-06 6200
1.02E-05 8480 1.05E-05 7920 9.06E-06
8840 5.58E-05 6810 1.95E-06 6610 7.52E-06 6200
1.02E-05 8480 1.06E-05 7920 9.05E-06
8840 5.59E-05 6810 1.94E-06 6610 7.53E-06 6200
1.03E-05 8480 1.07E-05 7930 9.05E-06
8840 5.61E-05 6810 1.95E-06 6610 7.54E-06 6200
1.04E-05 8490 1.08E-05 7930 9.04E-06

8840 5.62E-05 6810 1.95E-06 6610 7.54E-06 6200
1.04E-05 8490 1.10E-05 7930 9.05E-06
8840 5.64E-05 6820 1.95E-06 6610 7.54E-06 6210
1.05E-05 8490 1.11E-05 7930 9.03E-06
8850 5.66E-05 6820 1.95E-06 6610 7.55E-06 6210
1.05E-05 8490 1.13E-05 7930 9.03E-06
8850 5.67E-05 6820 1.95E-06 6620 7.56E-06 6210
1.06E-05 8490 1.14E-05 7930 9.02E-06
8850 5.68E-05 6820 1.95E-06 6620 7.57E-06 6210
1.06E-05 8490 1.16E-05 7930 9.01E-06
8850 5.69E-05 6820 1.95E-06 6620 7.56E-06 6210
1.07E-05 8500 1.18E-05 7940 9.02E-06
8850 5.71E-05 6820 1.96E-06 6620 7.57E-06 6210
1.08E-05 8500 1.19E-05 7940 9.01E-06
8850 5.73E-05 6830 1.96E-06 6620 7.58E-06 6220
1.08E-05 8500 1.21E-05 7940 9.02E-06
8850 5.74E-05 6830 1.97E-06 6620 7.58E-06 6220
1.09E-05 8500 1.23E-05 7940 9.00E-06
8860 5.75E-05 6830 1.97E-06 6630 7.58E-06 6220
1.10E-05 8500 1.26E-05 7940 8.99E-06
8860 5.76E-05 6830 1.97E-06 6630 7.58E-06 6220
1.10E-05 8500 1.28E-05 7940 9.00E-06
8860 5.78E-05 6830 1.96E-06 6630 7.58E-06 6220
1.11E-05 8500 1.30E-05 7950 8.99E-06
8860 5.80E-05 6830 1.96E-06 6630 7.58E-06 6220
1.12E-05 8510 1.33E-05 7950 8.99E-06
8860 5.81E-05 6830 1.96E-06 6630 7.59E-06 6230
1.13E-05 8510 1.35E-05 7950 8.98E-06
8860 5.82E-05 6840 1.96E-06 6630 7.58E-06 6230
1.14E-05 8510 1.38E-05 7950 8.97E-06
8870 5.84E-05 6840 1.96E-06 6630 7.58E-06 6230
1.14E-05 8510 1.41E-05 7950 8.98E-06
8870 5.85E-05 6840 1.96E-06 6640 7.58E-06 6230
1.15E-05 8510 1.44E-05 7950 8.98E-06
8870 5.87E-05 6840 1.96E-06 6640 7.58E-06 6230
1.16E-05 8510 1.47E-05 7950 8.97E-06
8870 5.88E-05 6840 1.96E-06 6640 7.60E-06 6230
1.17E-05 8520 1.51E-05 7960 8.97E-06
8870 5.90E-05 6840 1.97E-06 6640 7.60E-06 6230
1.17E-05 8520 1.55E-05 7960 8.96E-06
8870 5.91E-05 6850 1.97E-06 6640 7.59E-06 6240
1.18E-05 8520 1.58E-05 7960 8.97E-06
8880 5.93E-05 6850 1.97E-06 6640 7.59E-06 6240
1.19E-05 8520 1.62E-05 7960 8.95E-06
8880 5.95E-05 6850 1.97E-06 6650 7.61E-06 6240
1.20E-05 8520 1.67E-05 7960 8.96E-06
8880 5.96E-05 6850 1.97E-06 6650 7.61E-06 6240
1.21E-05 8520 1.71E-05 7960 8.95E-06
8880 5.98E-05 6850 1.97E-06 6650 7.61E-06 6240
1.22E-05 8530 1.76E-05 7970 8.96E-06
8880 5.99E-05 6850 1.97E-06 6650 7.62E-06 6240
1.22E-05 8530 1.81E-05 7970 8.95E-06
8880 6.01E-05 6850 1.97E-06 6650 7.62E-06 6250
1.23E-05 8530 1.87E-05 7970 8.95E-06
8880 6.02E-05 6860 1.98E-06 6650 7.63E-06 6250
1.24E-05 8530 1.92E-05 7970 8.94E-06
8890 6.04E-05 6860 1.98E-06 6650 7.63E-06 6250
1.25E-05 8530 1.99E-05 7970 8.94E-06
8890 6.06E-05 6860 1.98E-06 6660 7.62E-06 6250
1.26E-05 8530 2.05E-05 7970 8.94E-06
8890 6.07E-05 6860 1.97E-06 6660 7.62E-06 6250
1.27E-05 8530 2.12E-05 7980 8.94E-06
8890 6.09E-05 6860 1.97E-06 6660 7.63E-06 6250
1.28E-05 8540 2.19E-05 7980 8.94E-06
8890 6.11E-05 6860 1.97E-06 6660 7.64E-06 6250
1.29E-05 8540 2.27E-05 7980 8.93E-06
8890 6.13E-05 6870 1.98E-06 6660 7.63E-06 6260
1.30E-05 8540 2.34E-05 7980 8.95E-06

8900 6.14E-05 6870 1.97E-06 6660 7.64E-06 6260
1.31E-05 8540 2.43E-05 7980 8.95E-06
8900 6.16E-05 6870 1.98E-06 6670 7.64E-06 6260
1.32E-05 8540 2.51E-05 7980 8.95E-06
8900 6.18E-05 6870 1.98E-06 6670 7.64E-06 6260
1.33E-05 8540 2.61E-05 7980 8.95E-06
8900 6.20E-05 6870 1.98E-06 6670 7.65E-06 6260
1.34E-05 8550 2.70E-05 7990 8.94E-06
8900 6.22E-05 6870 1.98E-06 6670 7.65E-06 6260
1.35E-05 8550 2.80E-05 7990 8.94E-06
8900 6.24E-05 6880 1.98E-06 6670 7.66E-06 6270
1.36E-05 8550 2.90E-05 7990 8.93E-06
8900 6.26E-05 6880 1.98E-06 6670 7.67E-06 6270
1.37E-05 8550 3.01E-05 7990 8.93E-06
8910 6.28E-05 6880 1.98E-06 6680 7.67E-06 6270
1.38E-05 8550 3.11E-05 7990 8.92E-06
8910 6.29E-05 6880 1.99E-06 6680 7.67E-06 6270
1.40E-05 8550 3.22E-05 7990 8.93E-06
8910 6.31E-05 6880 1.99E-06 6680 7.68E-06 6270
1.41E-05 8550 3.34E-05 8000 8.93E-06
8910 6.33E-05 6880 1.99E-06 6680 7.68E-06 6270
1.42E-05 8560 3.45E-05 8000 8.94E-06
8910 6.35E-05 6880 1.99E-06 6680 7.69E-06 6280
1.43E-05 8560 3.56E-05 8000 8.93E-06
8910 6.37E-05 6890 1.99E-06 6680 7.68E-06 6280
1.44E-05 8560 3.67E-05 8000 8.93E-06
8920 6.39E-05 6890 2.00E-06 6680 7.69E-06 6280
1.46E-05 8560 3.78E-05 8000 8.92E-06
8920 6.41E-05 6890 2.00E-06 6690 7.68E-06 6280
1.47E-05 8560 3.88E-05 8000 8.92E-06
8920 6.43E-05 6890 1.99E-06 6690 7.69E-06 6280
1.48E-05 8560 3.98E-05 8000 8.93E-06
8920 6.45E-05 6890 1.99E-06 6690 7.68E-06 6280
1.50E-05 8570 4.07E-05 8010 8.93E-06
8920 6.46E-05 6890 1.99E-06 6690 7.68E-06 6280
1.51E-05 8570 4.16E-05 8010 8.93E-06
8920 6.49E-05 6900 1.99E-06 6690 7.69E-06 6290
1.52E-05 8570 4.23E-05 8010 8.93E-06
8930 6.51E-05 6900 1.99E-06 6690 7.69E-06 6290
1.54E-05 8570 4.29E-05 8010 8.93E-06
8930 6.53E-05 6900 1.99E-06 6700 7.69E-06 6290
1.55E-05 8570 4.35E-05 8010 8.95E-06
8930 6.55E-05 6900 1.99E-06 6700 7.69E-06 6290
1.57E-05 8570 4.39E-05 8010 8.95E-06
8930 6.57E-05 6900 1.99E-06 6700 7.70E-06 6290
1.58E-05 8580 4.41E-05 8020 8.95E-06
8930 6.59E-05 6900 1.99E-06 6700 7.70E-06 6290
1.60E-05 8580 4.42E-05 8020 8.95E-06
8930 6.61E-05 6900 1.99E-06 6700 7.70E-06 6300
1.61E-05 8580 4.42E-05 8020 8.95E-06
8930 6.63E-05 6910 1.99E-06 6700 7.71E-06 6300
1.63E-05 8580 4.41E-05 8020 8.94E-06
8940 6.66E-05 6910 1.99E-06 6700 7.72E-06 6300
1.64E-05 8580 4.38E-05 8020 8.95E-06
8940 6.67E-05 6910 1.99E-06 6710 7.72E-06 6300
1.66E-05 8580 4.33E-05 8020 8.95E-06
8940 6.69E-05 6910 2.00E-06 6710 7.73E-06 6300
1.68E-05 8580 4.28E-05 8030 8.96E-06
8940 6.71E-05 6910 2.00E-06 6710 7.72E-06 6300
1.70E-05 8590 4.21E-05 8030 8.97E-06
8940 6.74E-05 6910 2.01E-06 6710 7.73E-06 6300
1.71E-05 8590 4.14E-05 8030 8.97E-06
8940 6.76E-05 6920 2.01E-06 6710 7.72E-06 6310
1.73E-05 8590 4.05E-05 8030 8.97E-06
8950 6.78E-05 6920 2.01E-06 6710 7.73E-06 6310
1.75E-05 8590 3.96E-05 8030 8.97E-06
8950 6.80E-05 6920 2.01E-06 6720 7.73E-06 6310
1.77E-05 8590 3.86E-05 8030 8.98E-06

8950 6.82E-05 6920 2.01E-06 6720 7.73E-06 6310
1.78E-05 8590 3.76E-05 8030 8.99E-06
8950 6.84E-05 6920 2.00E-06 6720 7.73E-06 6310
1.80E-05 8600 3.65E-05 8040 9.00E-06
8950 6.87E-05 6920 2.00E-06 6720 7.73E-06 6310
1.82E-05 8600 3.54E-05 8040 9.00E-06
8950 6.89E-05 6930 2.00E-06 6720 7.73E-06 6320
1.85E-05 8600 3.43E-05 8040 9.01E-06
8950 6.91E-05 6930 2.00E-06 6720 7.74E-06 6320
1.87E-05 8600 3.32E-05 8040 9.01E-06
8960 6.93E-05 6930 2.01E-06 6730 7.74E-06 6320
1.89E-05 8600 3.21E-05 8040 9.04E-06
8960 6.96E-05 6930 2.00E-06 6730 7.75E-06 6320
1.91E-05 8600 3.10E-05 8040 9.02E-06
8960 6.98E-05 6930 2.01E-06 6730 7.75E-06 6320
1.93E-05 8600 2.99E-05 8050 9.03E-06
8960 7.01E-05 6930 2.01E-06 6730 7.76E-06 6320
1.95E-05 8610 2.89E-05 8050 9.03E-06
8960 7.03E-05 6930 2.02E-06 6730 7.76E-06 6330
1.98E-05 8610 2.79E-05 8050 9.05E-06
8960 7.06E-05 6940 2.02E-06 6730 7.76E-06 6330
2.00E-05 8610 2.70E-05 8050 9.03E-06
8970 7.08E-05 6940 2.01E-06 6730 7.77E-06 6330
2.02E-05 8610 2.60E-05 8050 9.04E-06
8970 7.10E-05 6940 2.01E-06 6740 7.77E-06 6330
2.05E-05 8610 2.51E-05 8050 9.05E-06
8970 7.13E-05 6940 2.01E-06 6740 7.78E-06 6330
2.08E-05 8610 2.43E-05 8050 9.06E-06
8970 7.15E-05 6940 2.02E-06 6740 7.78E-06 6330
2.10E-05 8620 2.34E-05 8060 9.07E-06
8970 7.17E-05 6940 2.03E-06 6740 7.79E-06 6330
2.13E-05 8620 2.27E-05 8060 9.07E-06
8970 7.20E-05 6950 2.02E-06 6740 7.78E-06 6340
2.16E-05 8620 2.19E-05 8060 9.08E-06
8980 7.22E-05 6950 2.03E-06 6740 7.79E-06 6340
2.18E-05 8620 2.12E-05 8060 9.08E-06
8980 7.24E-05 6950 2.02E-06 6750 7.77E-06 6340
2.21E-05 8620 2.06E-05 8060 9.09E-06
8980 7.27E-05 6950 2.02E-06 6750 7.78E-06 6340
2.24E-05 8620 1.99E-05 8060 9.10E-06
8980 7.29E-05 6950 2.02E-06 6750 7.78E-06 6340
2.27E-05 8630 1.93E-05 8070 9.11E-06
8980 7.32E-05 6950 2.02E-06 6750 7.78E-06 6340
2.30E-05 8630 1.88E-05 8070 9.11E-06
8980 7.34E-05 6950 2.02E-06 6750 7.78E-06 6350
2.33E-05 8630 1.82E-05 8070 9.13E-06
8980 7.37E-05 6960 2.03E-06 6750 7.79E-06 6350
2.37E-05 8630 1.77E-05 8070 9.14E-06
8990 7.40E-05 6960 2.03E-06 6750 7.79E-06 6350
2.40E-05 8630 1.72E-05 8070 9.16E-06
8990 7.42E-05 6960 2.02E-06 6760 7.80E-06 6350
2.44E-05 8630 1.68E-05 8070 9.16E-06
8990 7.45E-05 6960 2.03E-06 6760 7.80E-06 6350
2.47E-05 8630 1.64E-05 8080 9.16E-06
8990 7.48E-05 6960 2.03E-06 6760 7.81E-06 6350
2.51E-05 8640 1.60E-05 8080 9.18E-06
8990 7.50E-05 6960 2.03E-06 6760 7.81E-06 6350
2.54E-05 8640 1.56E-05 8080 9.19E-06
8990 7.53E-05 6970 2.03E-06 6760 7.81E-06 6360
2.58E-05 8640 1.52E-05 8080 9.18E-06
9000 7.56E-05 6970 2.03E-06 6760 7.82E-06 6360
2.62E-05 8640 1.49E-05 8080 9.18E-06
9000 7.59E-05 6970 2.03E-06 6770 7.83E-06 6360
2.66E-05 8640 1.46E-05 8080 9.18E-06
9000 7.61E-05 6970 2.03E-06 6770 7.84E-06 6360
2.70E-05 8640 1.43E-05 8080 9.20E-06
9000 7.64E-05 6970 2.03E-06 6770 7.84E-06 6360
2.74E-05 8650 1.40E-05 8090 9.22E-06

9000	7.67E-05	6970	2.04E-06	6770	7.84E-06	6360
2.79E-05	8650	1.37E-05	8090	9.22E-06		
9000	7.69E-05	6980	2.04E-06	6770	7.85E-06	6370
2.83E-05	8650	1.34E-05	8090	9.23E-06		
9000	7.72E-05	6980	2.05E-06	6770	7.85E-06	6370
2.88E-05	8650	1.32E-05	8090	9.24E-06		
9010	7.75E-05	6980	2.05E-06	6780	7.84E-06	6370
2.93E-05	8650	1.29E-05	8090	9.24E-06		
9010	7.78E-05	6980	2.04E-06	6780	7.85E-06	6370
2.97E-05	8650	1.27E-05	8090	9.26E-06		
9010	7.81E-05	6980	2.04E-06	6780	7.85E-06	6370
3.03E-05	8650	1.25E-05	8100	9.27E-06		
9010	7.84E-05	6980	2.04E-06	6780	7.85E-06	6370
3.08E-05	8660	1.23E-05	8100	9.28E-06		
9010	7.87E-05	6980	2.04E-06	6780	7.86E-06	6380
3.13E-05	8660	1.21E-05	8100	9.30E-06		
9010	7.90E-05	6990	2.05E-06	6780	7.86E-06	6380
3.18E-05	8660	1.19E-05	8100	9.30E-06		
9020	7.93E-05	6990	2.04E-06	6780	7.86E-06	6380
3.24E-05	8660	1.18E-05	8100	9.32E-06		
9020	7.96E-05	6990	2.05E-06	6790	7.87E-06	6380
3.30E-05	8660	1.16E-05	8100	9.33E-06		
9020	7.99E-05	6990	2.05E-06	6790	7.87E-06	6380
3.36E-05	8660	1.14E-05	8100	9.33E-06		
9020	8.02E-05	6990	2.05E-06	6790	7.88E-06	6380
3.42E-05	8670	1.13E-05	8110	9.34E-06		
9020	8.05E-05	6990	2.05E-06	6790	7.89E-06	6380
3.48E-05	8670	1.11E-05	8110	9.36E-06		
9020	8.09E-05	7000	2.05E-06	6790	7.89E-06	6390
3.55E-05	8670	1.10E-05	8110	9.36E-06		
9030	8.12E-05	7000	2.05E-06	6790	7.90E-06	6390
3.62E-05	8670	1.09E-05	8110	9.37E-06		
9030	8.14E-05	7000	2.06E-06	6800	7.91E-06	6390
3.69E-05	8670	1.08E-05	8110	9.37E-06		
9030	8.18E-05	7000	2.06E-06	6800	7.92E-06	6390
3.76E-05	8670	1.06E-05	8110	9.39E-06		
9030	8.21E-05	7000	2.06E-06	6800	7.93E-06	6390
3.84E-05	8680	1.05E-05	8120	9.40E-06		
9030	8.24E-05	7000	2.06E-06	6800	7.94E-06	6390
3.92E-05	8680	1.04E-05	8120	9.42E-06		
9030	8.28E-05	7000	2.07E-06	6800	7.94E-06	6400
4.00E-05	8680	1.03E-05	8120	9.43E-06		
9030	8.31E-05	7010	2.06E-06	6800	7.93E-06	6400
4.09E-05	8680	1.02E-05	8120	9.44E-06		
9040	8.34E-05	7010	2.07E-06	6800	7.92E-06	6400
4.17E-05	8680	1.01E-05	8120	9.46E-06		
9040	8.37E-05	7010	2.06E-06	6810	7.94E-06	6400
4.26E-05	8680	1.00E-05	8120	9.48E-06		
9040	8.40E-05	7010	2.06E-06	6810	7.94E-06	6400
4.36E-05	8680	9.95E-06	8130	9.48E-06		
9040	8.44E-05	7010	2.07E-06	6810	7.93E-06	6400
4.45E-05	8690	9.86E-06	8130	9.50E-06		
9040	8.47E-05	7010	2.07E-06	6810	7.94E-06	6400
4.56E-05	8690	9.79E-06	8130	9.51E-06		
9040	8.51E-05	7020	2.07E-06	6810	7.95E-06	6410
4.66E-05	8690	9.71E-06	8130	9.50E-06		
9050	8.54E-05	7020	2.07E-06	6810	7.96E-06	6410
4.77E-05	8690	9.63E-06	8130	9.53E-06		
9050	8.58E-05	7020	2.07E-06	6820	7.97E-06	6410
4.88E-05	8690	9.56E-06	8130	9.52E-06		
9050	8.61E-05	7020	2.08E-06	6820	7.98E-06	6410
5.00E-05	8690	9.50E-06	8130	9.54E-06		
9050	8.64E-05	7020	2.08E-06	6820	7.98E-06	6410
5.13E-05	8700	9.45E-06	8140	9.55E-06		
9050	8.68E-05	7020	2.08E-06	6820	7.99E-06	6410
5.25E-05	8700	9.38E-06	8140	9.56E-06		
9050	8.72E-05	7030	2.08E-06	6820	7.99E-06	6420
5.39E-05	8700	9.32E-06	8140	9.54E-06		

9050 8.76E-05 7030 2.08E-06 6820 8.00E-06 6420
5.53E-05 8700 9.25E-06 8140 9.56E-06
9060 8.79E-05 7030 2.09E-06 6830 8.01E-06 6420
5.67E-05 8700 9.19E-06 8140 9.56E-06
9060 8.82E-05 7030 2.08E-06 6830 8.02E-06 6420
5.82E-05 8700 9.14E-06 8140 9.56E-06
9060 8.85E-05 7030 2.08E-06 6830 8.02E-06 6420
5.98E-05 8700 9.08E-06 8150 9.58E-06
9060 8.89E-05 7030 2.09E-06 6830 8.02E-06 6420
6.15E-05 8710 9.03E-06 8150 9.58E-06
9060 8.93E-05 7030 2.09E-06 6830 8.03E-06 6430
6.32E-05 8710 8.99E-06 8150 9.56E-06
9060 8.97E-05 7040 2.09E-06 6830 8.02E-06 6430
6.50E-05 8710 8.94E-06 8150 9.56E-06
9070 9.00E-05 7040 2.09E-06 6830 8.02E-06 6430
6.69E-05 8710 8.90E-06 8150 9.55E-06
9070 9.05E-05 7040 2.08E-06 6840 8.04E-06 6430
6.89E-05 8710 8.86E-06 8150 9.55E-06
9070 9.08E-05 7040 2.08E-06 6840 8.04E-06 6430
7.10E-05 8710 8.81E-06 8150 9.59E-06
9070 9.12E-05 7040 2.08E-06 6840 8.04E-06 6430
7.32E-05 8720 8.77E-06 8160 9.56E-06
9070 9.16E-05 7040 2.09E-06 6840 8.04E-06 6430
7.55E-05 8720 8.73E-06 8160 9.58E-06
9070 9.20E-05 7050 2.09E-06 6840 8.05E-06 6440
7.79E-05 8720 8.69E-06 8160 9.58E-06
9080 9.24E-05 7050 2.09E-06 6840 8.05E-06 6440
8.05E-05 8720 8.66E-06 8160 9.58E-06
9080 9.28E-05 7050 2.08E-06 6850 8.05E-06 6440
8.32E-05 8720 8.62E-06 8160 9.59E-06
9080 9.32E-05 7050 2.09E-06 6850 8.05E-06 6440
8.60E-05 8720 8.59E-06 8160 9.57E-06
9080 9.36E-05 7050 2.08E-06 6850 8.06E-06 6440
8.90E-05 8730 8.55E-06 8170 9.57E-06
9080 9.40E-05 7050 2.08E-06 6850 8.07E-06 6440
9.22E-05 8730 8.52E-06 8170 9.57E-06
9080 9.44E-05 7050 2.08E-06 6850 8.07E-06 6450
9.56E-05 8730 8.49E-06 8170 9.54E-06
9080 9.48E-05 7060 2.08E-06 6850 8.08E-06 6450
9.92E-05 8730 8.45E-06 8170 9.54E-06
9090 9.52E-05 7060 2.08E-06 6850 8.08E-06 6450
0.000102949 8730 8.42E-06 8170 9.53E-06
9090 9.56E-05 7060 2.08E-06 6860 8.10E-06 6450
0.000107021 8730 8.38E-06 8170 9.55E-06
9090 9.61E-05 7060 2.09E-06 6860 8.10E-06 6450
0.00011132 8730 8.35E-06 8180 9.55E-06
9090 9.65E-05 7060 2.09E-06 6860 8.11E-06 6450
0.000115914 8740 8.33E-06 8180 9.53E-06
9090 9.69E-05 7060 2.09E-06 6860 8.10E-06 6450
0.000120832 8740 8.30E-06 8180 9.53E-06
9090 9.74E-05 7070 2.09E-06 6860 8.10E-06 6460
0.000126077 8740 8.28E-06 8180 9.52E-06
9100 9.78E-05 7070 2.08E-06 6860 8.10E-06 6460
0.000131708 8740 8.26E-06 8180 9.50E-06
9100 9.83E-05 7070 2.08E-06 6870 8.11E-06 6460
0.000137752 8740 8.24E-06 8180 9.49E-06
9100 9.87E-05 7070 2.08E-06 6870 8.11E-06 6460
0.000144257 8740 8.20E-06 8180 9.48E-06
9100 9.92E-05 7070 2.06E-06 6870 8.12E-06 6460
0.000151266 8750 8.17E-06 8190 9.50E-06
9100 9.96E-05 7070 2.08E-06 6870 8.12E-06 6460
0.000158848 8750 8.15E-06 8190 9.48E-06
9100 0.0001 7080 2.08E-06 6870 8.13E-06 6470
0.000167069 8750 8.14E-06 8190 9.48E-06
9100 0.000101 7080 2.07E-06 6870 8.14E-06 6470
0.000175997 8750 8.11E-06 8190 9.48E-06
9110 0.000101 7080 2.08E-06 6880 8.14E-06 6470
0.0001857 8750 8.08E-06 8190 9.45E-06

9110 0.000102 7080 2.07E-06 6880 8.16E-06 6470
0.00019628 8750 8.07E-06 8190 9.44E-06
9110 0.000102 7080 2.08E-06 6880 8.16E-06 6470
0.000207863 8750 8.06E-06 8200 9.44E-06
9110 0.000103 7080 2.08E-06 6880 8.17E-06 6470
0.000220549 8760 8.04E-06 8200 9.43E-06
9110 0.000103 7080 2.09E-06 6880 8.17E-06 6480
0.000234473 8760 8.01E-06 8200 9.40E-06
9110 0.000104 7090 2.09E-06 6880 8.17E-06 6480
0.000249777 8760 8.00E-06 8200 9.41E-06
9120 0.000104 7090 2.09E-06 6880 8.19E-06 6480
0.000266647 8760 7.97E-06 8200 9.41E-06
9120 0.000105 7090 2.09E-06 6890 8.20E-06 6480
0.000285216 8760 7.95E-06 8200 9.39E-06
9120 0.000105 7090 2.09E-06 6890 8.21E-06 6480
0.000305684 8760 7.93E-06 8200 9.39E-06
9120 0.000105 7090 2.10E-06 6890 8.21E-06 6480
0.000328174 8770 7.90E-06 8210 9.37E-06
9120 0.000106 7090 2.10E-06 6890 8.21E-06 6480
0.000352846 8770 7.90E-06 8210 9.36E-06
9120 0.000107 7100 2.11E-06 6890 8.20E-06 6490
0.000379818 8770 7.88E-06 8210 9.35E-06
9130 0.000107 7100 2.11E-06 6890 8.20E-06 6490
0.000409195 8770 7.87E-06 8210 9.33E-06
9130 0.000108 7100 2.11E-06 6900 8.21E-06 6490
0.000441112 8770 7.85E-06 8210 9.32E-06
9130 0.000108 7100 2.12E-06 6900 8.21E-06 6490
0.000475687 8770 7.83E-06 8210 9.32E-06
9130 0.000109 7100 2.11E-06 6900 8.21E-06 6490
0.000513155 8780 7.82E-06 8220 9.30E-06
9130 0.000109 7100 2.12E-06 6900 8.21E-06 6490
0.000553895 8780 7.80E-06 8220 9.31E-06
9130 0.00011 7100 2.12E-06 6900 8.22E-06 6500
0.000598462 8780 7.78E-06 8220 9.31E-06
9130 0.00011 7110 2.12E-06 6900 8.21E-06 6500
0.000647581 8780 7.76E-06 8220 9.29E-06
9140 0.000111 7110 2.12E-06 6900 8.22E-06 6500
0.000702257 8780 7.74E-06 8220 9.28E-06
9140 0.000111 7110 2.12E-06 6910 8.24E-06 6500
0.00076361 8780 7.74E-06 8220 9.28E-06
9140 0.000112 7110 2.14E-06 6910 8.24E-06 6500
0.000832991 8780 7.71E-06 8230 9.26E-06
9140 0.000113 7110 2.13E-06 6910 8.24E-06 6500
0.00091187 8790 7.71E-06 8230 9.27E-06
9140 0.000113 7110 2.13E-06 6910 8.24E-06 6500
0.001001934 8790 7.69E-06 8230 9.26E-06
9140 0.000114 7120 2.13E-06 6910 8.24E-06 6510
0.001104962 8790 7.67E-06 8230 9.26E-06
9150 0.000114 7120 2.13E-06 6910 8.26E-06 6510
0.001223078 8790 7.65E-06 8230 9.25E-06
9150 0.000115 7120 2.13E-06 6920 8.27E-06 6510
0.001358773 8790 7.65E-06 8230 9.26E-06
9150 0.000116 7120 2.14E-06 6920 8.27E-06 6510
0.001514913 8790 7.63E-06 8230 9.26E-06
9150 0.000116 7120 2.15E-06 6920 8.27E-06 6510
0.001694794 8800 7.61E-06 8240 9.25E-06
9150 0.000117 7120 2.14E-06 6920 8.27E-06 6510
0.00190178 8800 7.61E-06 8240 9.24E-06
9150 0.000117 7130 2.14E-06 6920 8.26E-06 6520
0.002138954 8800 7.58E-06 8240 9.25E-06
9150 0.000118 7130 2.15E-06 6920 8.26E-06 6520
0.00240835 8800 7.57E-06 8240 9.24E-06
9160 0.000119 7130 2.15E-06 6930 8.27E-06 6520
0.002709734 8800 7.56E-06 8240 9.25E-06
9160 0.000119 7130 2.15E-06 6930 8.26E-06 6520
0.003039393 8800 7.54E-06 8240 9.24E-06
9160 0.00012 7130 2.14E-06 6930 8.26E-06 6520
0.003388614 8800 7.53E-06 8250 9.24E-06

9160 0.000121 7130 2.14E-06 6930 8.27E-06 6520
0.003742782 8810 7.51E-06 8250 9.23E-06
9160 0.000121 7130 2.14E-06 6930 8.27E-06 6530
0.004080981 8810 7.49E-06 8250 9.23E-06
9160 0.000122 7140 2.15E-06 6930 8.27E-06 6530
0.004377683 8810 7.47E-06 8250 9.25E-06
9170 0.000123 7140 2.15E-06 6930 8.28E-06 6530
0.004605766 8810 7.46E-06 8250 9.25E-06
9170 0.000123 7140 2.15E-06 6940 8.30E-06 6530
0.004741275 8810 7.46E-06 8250 9.23E-06
9170 0.000124 7140 2.15E-06 6940 8.31E-06 6530
0.004768579 8810 7.43E-06 8250 9.23E-06
9170 0.000125 7140 2.15E-06 6940 8.31E-06 6530
0.004683989 8820 7.42E-06 8260 9.25E-06
9170 0.000126 7140 2.16E-06 6940 8.30E-06 6530
0.00449696 8820 7.41E-06 8260 9.22E-06
9170 0.000126 7150 2.16E-06 6940 8.31E-06 6540
0.004227654 8820 7.39E-06 8260 9.22E-06
9180 0.000127 7150 2.16E-06 6940 8.31E-06 6540
0.003902421 8820 7.38E-06 8260 9.22E-06
9180 0.000128 7150 2.17E-06 6950 8.33E-06 6540
0.003548563 8820 7.36E-06 8260 9.23E-06
9180 0.000128 7150 2.17E-06 6950 8.33E-06 6540
0.003189983 8820 7.34E-06 8260 9.23E-06
9180 0.000129 7150 2.18E-06 6950 8.34E-06 6540
0.002845004 8830 7.33E-06 8270 9.23E-06
9180 0.00013 7150 2.17E-06 6950 8.33E-06 6540
0.002525577 8830 7.32E-06 8270 9.24E-06
9180 0.000131 7150 2.18E-06 6950 8.33E-06 6550
0.002238134 8830 7.31E-06 8270 9.24E-06
9180 0.000131 7160 2.17E-06 6950 8.34E-06 6550
0.001984864 8830 7.30E-06 8270 9.23E-06
9190 0.000132 7160 2.18E-06 6950 8.36E-06 6550
0.001765108 8830 7.28E-06 8270 9.23E-06
9190 0.000133 7160 2.17E-06 6960 8.36E-06 6550
0.001576298 8830 7.27E-06 8270 9.23E-06
9190 0.000134 7160 2.17E-06 6960 8.37E-06 6550
0.001414928 8830 7.24E-06 8280 9.23E-06
9190 0.000135 7160 2.17E-06 6960 8.37E-06 6550
0.001277025 8840 7.23E-06 8280 9.24E-06
9190 0.000135 7160 2.17E-06 6960 8.38E-06 6550
0.001158461 8840 7.23E-06 8280 9.24E-06
9190 0.000136 7170 2.17E-06 6960 8.39E-06 6560
0.001055348 8840 7.22E-06 8280 9.25E-06
9200 0.000137 7170 2.17E-06 6960 8.40E-06 6560
0.000964252 8840 7.21E-06 8280 9.24E-06
9200 0.000138 7170 2.18E-06 6970 8.42E-06 6560
0.000882418 8840 7.20E-06 8280 9.24E-06
9200 0.000139 7170 2.18E-06 6970 8.45E-06 6560
0.0008079 8840 7.19E-06 8280 9.24E-06
9200 0.000139 7170 2.17E-06 6970 8.47E-06 6560
0.000739481 8850 7.18E-06 8290 9.24E-06
9200 0.00014 7170 2.18E-06 6970 8.47E-06 6560
0.000676563 8850 7.14E-06 8290 9.24E-06
9200 0.000141 7180 2.17E-06 6970 8.49E-06 6570
0.000618867 8850 7.15E-06 8290 9.24E-06
9200 0.000142 7180 2.18E-06 6970 8.50E-06 6570
0.000566317 8850 7.12E-06 8290 9.24E-06
9210 0.000143 7180 2.17E-06 6980 8.53E-06 6570
0.000518783 8850 7.11E-06 8290 9.24E-06
9210 0.000144 7180 2.18E-06 6980 8.55E-06 6570
0.000476057 8850 7.08E-06 8290 9.25E-06
9210 0.000145 7180 2.18E-06 6980 8.57E-06 6570
0.000437839 8850 7.06E-06 8300 9.25E-06
9210 0.000146 7180 2.18E-06 6980 8.59E-06 6570
0.000403737 8860 7.04E-06 8300 9.26E-06
9210 0.000146 7180 2.18E-06 6980 8.59E-06 6580
0.000373314 8860 7.03E-06 8300 9.25E-06

9210 0.000147 7190 2.18E-06 6980 8.61E-06 6580
0.000346187 8860 7.04E-06 8300 9.26E-06
9220 0.000148 7190 2.18E-06 6980 8.64E-06 6580
0.000321924 8860 7.02E-06 8300 9.25E-06
9220 0.000149 7190 2.19E-06 6990 8.66E-06 6580
0.000300189 8860 7.02E-06 8300 9.25E-06
9220 0.00015 7190 2.18E-06 6990 8.68E-06 6580
0.00028064 8860 6.99E-06 8300 9.25E-06
9220 0.000151 7190 2.18E-06 6990 8.68E-06 6580
0.000262997 8870 6.98E-06 8310 9.26E-06
9220 0.000152 7190 2.18E-06 6990 8.70E-06 6580
0.000247047 8870 6.97E-06 8310 9.26E-06
9220 0.000153 7200 2.18E-06 6990 8.72E-06 6590
0.000232554 8870 6.96E-06 8310 9.27E-06
9230 0.000154 7200 2.18E-06 6990 8.75E-06 6590
0.000219389 8870 6.95E-06 8310 9.27E-06
9230 0.000155 7200 2.19E-06 7000 8.77E-06 6590
0.000207329 8870 6.96E-06 8310 9.28E-06
9230 0.000156 7200 2.19E-06 7000 8.79E-06 6590
0.000196282 8870 6.96E-06 8310 9.27E-06
9230 0.000157 7200 2.19E-06 7000 8.82E-06 6590
0.000186132 8880 6.96E-06 8320 9.28E-06
9230 0.000158 7200 2.19E-06 7000 8.83E-06 6590
0.000176795 8880 6.94E-06 8320 9.29E-06
9230 0.000159 7200 2.19E-06 7000 8.84E-06 6600
0.000168177 8880 6.93E-06 8320 9.28E-06
9230 0.00016 7210 2.20E-06 7000 8.87E-06 6600
0.000160191 8880 6.93E-06 8320 9.29E-06
9240 0.000161 7210 2.20E-06 7000 8.90E-06 6600
0.000152796 8880 6.91E-06 8320 9.29E-06
9240 0.000162 7210 2.20E-06 7010 8.92E-06 6600
0.000145911 8880 6.92E-06 8320 9.30E-06
9240 0.000163 7210 2.20E-06 7010 8.94E-06 6600
0.000139524 8880 6.90E-06 8330 9.31E-06
9240 0.000165 7210 2.20E-06 7010 8.96E-06 6600
0.000133581 8890 6.90E-06 8330 9.29E-06
9240 0.000166 7210 2.21E-06 7010 8.97E-06 6600
0.000128014 8890 6.90E-06 8330 9.30E-06
9240 0.000167 7220 2.21E-06 7010 9.00E-06 6610
0.000122806 8890 6.90E-06 8330 9.29E-06
9250 0.000168 7220 2.21E-06 7010 9.02E-06 6610
0.000117924 8890 6.91E-06 8330 9.28E-06
9250 0.000169 7220 2.21E-06 7020 9.04E-06 6610
0.000113346 8890 6.91E-06 8330 9.29E-06
9250 0.00017 7220 2.21E-06 7020 9.06E-06 6610
0.000109027 8890 6.92E-06 8330 9.32E-06
9250 0.000171 7220 2.21E-06 7020 9.09E-06 6610
0.000104997 8900 6.91E-06 8340 9.31E-06
9250 0.000173 7220 2.21E-06 7020 9.11E-06 6610
0.000101167 8900 6.92E-06 8340 9.31E-06
9250 0.000174 7230 2.21E-06 7020 9.14E-06 6620
9.76E-05 8900 6.93E-06 8340 9.31E-06
9250 0.000175 7230 2.21E-06 7020 9.17E-06 6620
9.42E-05 8900 6.94E-06 8340 9.31E-06
9260 0.000176 7230 2.22E-06 7030 9.20E-06 6620
9.09E-05 8900 6.96E-06 8340 9.30E-06
9260 0.000178 7230 2.22E-06 7030 9.22E-06 6620
8.79E-05 8900 6.97E-06 8340 9.32E-06
9260 0.000179 7230 2.21E-06 7030 9.26E-06 6620
8.50E-05 8900 6.99E-06 8350 9.32E-06
9260 0.00018 7230 2.22E-06 7030 9.28E-06 6620 8.23E-05
8910 6.98E-06 8350 9.31E-06
9260 0.000181 7230 2.21E-06 7030 9.30E-06 6630
7.97E-05 8910 7.00E-06 8350 9.32E-06
9260 0.000183 7240 2.22E-06 7030 9.34E-06 6630
7.72E-05 8910 7.01E-06 8350 9.32E-06
9270 0.000184 7240 2.22E-06 7030 9.38E-06 6630
7.49E-05 8910 7.02E-06 8350 9.31E-06

9270 0.000185 7240 2.22E-06 7040 9.41E-06 6630
7.26E-05 8910 7.02E-06 8350 9.32E-06
9270 0.000187 7240 2.23E-06 7040 9.44E-06 6630
7.05E-05 8910 7.03E-06 8350 9.31E-06
9270 0.000188 7240 2.23E-06 7040 9.47E-06 6630
6.85E-05 8920 7.03E-06 8360 9.31E-06
9270 0.000189 7240 2.22E-06 7040 9.49E-06 6630
6.65E-05 8920 7.05E-06 8360 9.31E-06
9270 0.000191 7250 2.22E-06 7040 9.54E-06 6640
6.47E-05 8920 7.06E-06 8360 9.30E-06
9280 0.000192 7250 2.23E-06 7040 9.57E-06 6640
6.29E-05 8920 7.08E-06 8360 9.31E-06
9280 0.000193 7250 2.22E-06 7050 9.60E-06 6640
6.12E-05 8920 7.08E-06 8360 9.31E-06
9280 0.000195 7250 2.23E-06 7050 9.65E-06 6640
5.96E-05 8920 7.08E-06 8360 9.31E-06
9280 0.000196 7250 2.23E-06 7050 9.69E-06 6640
5.81E-05 8930 7.09E-06 8370 9.33E-06
9280 0.000198 7250 2.22E-06 7050 9.73E-06 6640
5.66E-05 8930 7.09E-06 8370 9.34E-06
9280 0.000199 7250 2.23E-06 7050 9.78E-06 6650
5.52E-05 8930 7.10E-06 8370 9.31E-06
9280 0.000201 7260 2.23E-06 7050 9.83E-06 6650
5.38E-05 8930 7.10E-06 8370 9.33E-06
9290 0.000202 7260 2.24E-06 7050 9.88E-06 6650
5.25E-05 8930 7.10E-06 8370 9.31E-06
9290 0.000204 7260 2.24E-06 7060 9.94E-06 6650
5.13E-05 8930 7.11E-06 8370 9.31E-06
9290 0.000205 7260 2.23E-06 7060 9.99E-06 6650
5.01E-05 8930 7.12E-06 8380 9.31E-06
9290 0.000207 7260 2.24E-06 7060 1.00E-05 6650
4.89E-05 8940 7.12E-06 8380 9.32E-06
9290 0.000209 7260 2.23E-06 7060 1.01E-05 6650
4.78E-05 8940 7.14E-06 8380 9.32E-06
9290 0.00021 7270 2.24E-06 7060 1.02E-05 6660 4.67E-05
8940 7.13E-06 8380 9.31E-06
9300 0.000212 7270 2.24E-06 7060 1.02E-05 6660
4.57E-05 8940 7.13E-06 8380 9.31E-06
9300 0.000213 7270 2.24E-06 7070 1.03E-05 6660
4.47E-05 8940 7.13E-06 8380 9.31E-06
9300 0.000215 7270 2.24E-06 7070 1.04E-05 6660
4.38E-05 8940 7.13E-06 8380 9.31E-06
9300 0.000217 7270 2.24E-06 7070 1.04E-05 6660
4.29E-05 8950 7.13E-06 8390 9.31E-06
9300 0.000218 7270 2.24E-06 7070 1.05E-05 6660
4.20E-05 8950 7.15E-06 8390 9.31E-06
9300 0.00022 7280 2.24E-06 7070 1.06E-05 6670 4.11E-05
8950 7.14E-06 8390 9.31E-06
9300 0.000222 7280 2.25E-06 7070 1.07E-05 6670
4.03E-05 8950 7.15E-06 8390 9.30E-06
9310 0.000224 7280 2.25E-06 7080 1.07E-05 6670
3.95E-05 8950 7.14E-06 8390 9.31E-06
9310 0.000225 7280 2.24E-06 7080 1.08E-05 6670
3.88E-05 8950 7.14E-06 8390 9.31E-06
9310 0.000227 7280 2.24E-06 7080 1.09E-05 6670
3.80E-05 8950 7.14E-06 8400 9.33E-06
9310 0.000229 7280 2.25E-06 7080 1.10E-05 6670
3.73E-05 8960 7.14E-06 8400 9.34E-06
9310 0.000231 7280 2.25E-06 7080 1.11E-05 6680
3.66E-05 8960 7.14E-06 8400 9.34E-06
9310 0.000233 7290 2.25E-06 7080 1.12E-05 6680
3.60E-05 8960 7.15E-06 8400 9.34E-06
9320 0.000234 7290 2.26E-06 7080 1.13E-05 6680
3.53E-05 8960 7.16E-06 8400 9.33E-06
9320 0.000236 7290 2.26E-06 7090 1.15E-05 6680
3.47E-05 8960 7.17E-06 8400 9.34E-06
9320 0.000238 7290 2.26E-06 7090 1.16E-05 6680
3.41E-05 8960 7.17E-06 8400 9.34E-06

9320 0.00024 7290 2.27E-06 7090 1.17E-05 6680 3.35E-05
8970 7.17E-06 8410 9.33E-06
9320 0.000242 7290 2.27E-06 7090 1.19E-05 6680
3.30E-05 8970 7.17E-06 8410 9.34E-06
9320 0.000244 7300 2.27E-06 7090 1.20E-05 6690
3.24E-05 8970 7.17E-06 8410 9.33E-06
9330 0.000246 7300 2.27E-06 7090 1.22E-05 6690
3.19E-05 8970 7.17E-06 8410 9.31E-06
9330 0.000248 7300 2.26E-06 7100 1.24E-05 6690
3.13E-05 8970 7.17E-06 8410 9.34E-06
9330 0.00025 7300 2.27E-06 7100 1.26E-05 6690 3.09E-05
8970 7.16E-06 8410 9.33E-06
9330 0.000252 7300 2.27E-06 7100 1.28E-05 6690
3.04E-05 8980 7.16E-06 8420 9.33E-06
9330 0.000254 7300 2.28E-06 7100 1.29E-05 6690
2.99E-05 8980 7.17E-06 8420 9.33E-06
9330 0.000257 7300 2.28E-06 7100 1.32E-05 6700
2.94E-05 8980 7.17E-06 8420 9.31E-06
9330 0.000259 7310 2.28E-06 7100 1.34E-05 6700
2.90E-05 8980 7.18E-06 8420 9.31E-06
9340 0.000261 7310 2.28E-06 7100 1.36E-05 6700
2.85E-05 8980 7.18E-06 8420 9.31E-06
9340 0.000263 7310 2.28E-06 7110 1.39E-05 6700
2.81E-05 8980 7.17E-06 8420 9.32E-06
9340 0.000265 7310 2.28E-06 7110 1.42E-05 6700
2.77E-05 8980 7.16E-06 8430 9.32E-06
9340 0.000268 7310 2.28E-06 7110 1.45E-05 6700
2.73E-05 8990 7.16E-06 8430 9.34E-06
9340 0.00027 7310 2.29E-06 7110 1.48E-05 6700 2.69E-05
8990 7.17E-06 8430 9.34E-06
9340 0.000272 7320 2.29E-06 7110 1.51E-05 6710
2.65E-05 8990 7.17E-06 8430 9.33E-06
9350 0.000275 7320 2.29E-06 7110 1.55E-05 6710
2.62E-05 8990 7.18E-06 8430 9.33E-06
9350 0.000277 7320 2.29E-06 7120 1.58E-05 6710
2.58E-05 8990 7.18E-06 8430 9.32E-06
9350 0.000279 7320 2.29E-06 7120 1.62E-05 6710
2.54E-05 8990 7.18E-06 8430 9.31E-06
9350 0.000282 7320 2.29E-06 7120 1.67E-05 6710
2.51E-05 9000 7.18E-06 8440 9.30E-06
9350 0.000284 7320 2.30E-06 7120 1.71E-05 6710
2.47E-05 9000 7.18E-06 8440 9.29E-06
9350 0.000287 7330 2.30E-06 7120 1.76E-05 6720
2.44E-05 9000 7.18E-06 8440 9.29E-06
9350 0.000289 7330 2.31E-06 7120 1.81E-05 6720
2.40E-05 9000 7.18E-06 8440 9.29E-06
9360 0.000292 7330 2.31E-06 7130 1.86E-05 6720
2.37E-05 9000 7.18E-06 8440 9.30E-06
9360 0.000294 7330 2.30E-06 7130 1.91E-05 6720
2.34E-05 9000 7.17E-06 8440 9.29E-06
9360 0.000297 7330 2.31E-06 7130 1.97E-05 6720
2.31E-05 9000 7.18E-06 8450 9.30E-06
9360 0.0003 7330 2.30E-06 7130 2.03E-05 6720 2.28E-05
9010 7.18E-06 8450 9.29E-06
9360 0.000302 7330 2.30E-06 7130 2.08E-05 6730
2.25E-05 9010 7.18E-06 8450 9.29E-06
9360 0.000305 7340 2.30E-06 7130 2.14E-05 6730
2.22E-05 9010 7.18E-06 8450 9.29E-06
9370 0.000308 7340 2.31E-06 7130 2.20E-05 6730
2.20E-05 9010 7.19E-06 8450 9.28E-06
9370 0.000311 7340 2.31E-06 7140 2.26E-05 6730
2.17E-05 9010 7.19E-06 8450 9.29E-06
9370 0.000313 7340 2.31E-06 7140 2.31E-05 6730
2.14E-05 9010 7.19E-06 8450 9.29E-06
9370 0.000316 7340 2.30E-06 7140 2.37E-05 6730
2.12E-05 9020 7.19E-06 8460 9.30E-06
9370 0.000319 7340 2.31E-06 7140 2.41E-05 6730
2.09E-05 9020 7.20E-06 8460 9.31E-06

9370 0.000322 7350 2.30E-06 7140 2.46E-05 6740
2.07E-05 9020 7.20E-06 8460 9.31E-06
9380 0.000325 7350 2.31E-06 7140 2.50E-05 6740
2.05E-05 9020 7.21E-06 8460 9.29E-06
9380 0.000328 7350 2.30E-06 7150 2.53E-05 6740
2.02E-05 9020 7.21E-06 8460 9.30E-06
9380 0.000331 7350 2.30E-06 7150 2.55E-05 6740
2.00E-05 9020 7.21E-06 8460 9.30E-06
9380 0.000334 7350 2.31E-06 7150 2.56E-05 6740
1.98E-05 9030 7.21E-06 8470 9.28E-06
9380 0.000337 7350 2.31E-06 7150 2.57E-05 6740
1.95E-05 9030 7.22E-06 8470 9.28E-06
9380 0.00034 7350 2.32E-06 7150 2.56E-05 6750 1.93E-05
9030 7.21E-06 8470 9.28E-06
9380 0.000344 7360 2.32E-06 7150 2.55E-05 6750
1.91E-05 9030 7.21E-06 8470 9.29E-06
9390 0.000347 7360 2.31E-06 7150 2.52E-05 6750
1.89E-05 9030 7.21E-06 8470 9.28E-06
9390 0.00035 7360 2.31E-06 7160 2.49E-05 6750 1.87E-05
9030 7.21E-06 8470 9.26E-06
9390 0.000353 7360 2.31E-06 7160 2.46E-05 6750
1.85E-05 9030 7.21E-06 8480 9.27E-06
9390 0.000357 7360 2.31E-06 7160 2.41E-05 6750
1.83E-05 9040 7.21E-06 8480 9.25E-06
9390 0.00036 7360 2.32E-06 7160 2.36E-05 6750 1.81E-05
9040 7.21E-06 8480 9.26E-06
9390 0.000364 7370 2.31E-06 7160 2.31E-05 6760
1.79E-05 9040 7.21E-06 8480 9.25E-06
9400 0.000367 7370 2.32E-06 7160 2.25E-05 6760
1.78E-05 9040 7.21E-06 8480 9.27E-06
9400 0.000371 7370 2.32E-06 7170 2.20E-05 6760
1.76E-05 9040 7.22E-06 8480 9.25E-06
9400 0.000374 7370 2.32E-06 7170 2.14E-05 6760
1.74E-05 9040 7.22E-06 8480 9.27E-06
9400 0.000378 7370 2.31E-06 7170 2.08E-05 6760
1.73E-05 9050 7.22E-06 8490 9.27E-06
9400 0.000381 7370 2.32E-06 7170 2.03E-05 6760
1.71E-05 9050 7.21E-06 8490 9.27E-06
9400 0.000385 7380 2.31E-06 7170 1.97E-05 6770
1.70E-05 9050 7.22E-06 8490 9.26E-06
9400 0.000389 7380 2.31E-06 7170 1.92E-05 6770
1.68E-05 9050 7.22E-06 8490 9.26E-06
9410 0.000393 7380 2.32E-06 7180 1.86E-05 6770
1.67E-05 9050 7.22E-06 8490 9.27E-06
9410 0.000396 7380 2.31E-06 7180 1.81E-05 6770
1.65E-05 9050 7.23E-06 8490 9.26E-06
9410 0.0004 7380 2.31E-06 7180 1.77E-05 6770 1.64E-05
9050 7.22E-06 8500 9.25E-06
9410 0.000404 7380 2.31E-06 7180 1.72E-05 6770
1.62E-05 9060 7.23E-06 8500 9.25E-06
9410 0.000408 7380 2.31E-06 7180 1.68E-05 6780
1.61E-05 9060 7.22E-06 8500 9.23E-06
9410 0.000412 7390 2.31E-06 7180 1.64E-05 6780
1.59E-05 9060 7.22E-06 8500 9.24E-06
9420 0.000416 7390 2.31E-06 7180 1.60E-05 6780
1.58E-05 9060 7.22E-06 8500 9.25E-06
9420 0.00042 7390 2.32E-06 7190 1.56E-05 6780 1.56E-05
9060 7.21E-06 8500 9.25E-06
9420 0.000425 7390 2.32E-06 7190 1.53E-05 6780
1.55E-05 9060 7.22E-06 8500 9.23E-06
9420 0.000429 7390 2.32E-06 7190 1.50E-05 6780
1.54E-05 9070 7.22E-06 8510 9.25E-06
9420 0.000433 7390 2.32E-06 7190 1.46E-05 6780
1.53E-05 9070 7.22E-06 8510 9.25E-06
9420 0.000437 7400 2.32E-06 7190 1.44E-05 6790
1.52E-05 9070 7.23E-06 8510 9.26E-06
9430 0.000442 7400 2.32E-06 7190 1.41E-05 6790
1.50E-05 9070 7.23E-06 8510 9.27E-06

9430 0.000446 7400 2.32E-06 7200 1.39E-05 6790
1.49E-05 9070 7.23E-06 8510 9.28E-06
9430 0.000451 7400 2.32E-06 7200 1.36E-05 6790
1.48E-05 9070 7.24E-06 8510 9.27E-06
9430 0.000455 7400 2.32E-06 7200 1.34E-05 6790
1.47E-05 9080 7.23E-06 8520 9.27E-06
9430 0.00046 7400 2.33E-06 7200 1.32E-05 6790 1.46E-05
9080 7.23E-06 8520 9.28E-06
9430 0.000465 7400 2.33E-06 7200 1.30E-05 6800
1.45E-05 9080 7.24E-06 8520 9.29E-06
9430 0.000469 7410 2.33E-06 7200 1.28E-05 6800
1.44E-05 9080 7.24E-06 8520 9.28E-06
9440 0.000474 7410 2.32E-06 7200 1.27E-05 6800
1.43E-05 9080 7.24E-06 8520 9.29E-06
9440 0.000479 7410 2.32E-06 7210 1.25E-05 6800
1.42E-05 9080 7.25E-06 8520 9.28E-06
9440 0.000484 7410 2.33E-06 7210 1.24E-05 6800
1.41E-05 9080 7.24E-06 8530 9.27E-06
9440 0.000489 7410 2.33E-06 7210 1.22E-05 6800
1.40E-05 9090 7.25E-06 8530 9.28E-06
9440 0.000494 7410 2.33E-06 7210 1.21E-05 6800
1.39E-05 9090 7.24E-06 8530 9.28E-06
9440 0.000499 7420 2.33E-06 7210 1.20E-05 6810
1.38E-05 9090 7.25E-06 8530 9.28E-06
9450 0.000504 7420 2.33E-06 7210 1.19E-05 6810
1.37E-05 9090 7.24E-06 8530 9.29E-06
9450 0.00051 7420 2.34E-06 7220 1.17E-05 6810 1.36E-05
9090 7.24E-06 8530 9.28E-06
9450 0.000515 7420 2.34E-06 7220 1.16E-05 6810
1.35E-05 9090 7.24E-06 8530 9.28E-06
9450 0.00052 7420 2.34E-06 7220 1.15E-05 6810 1.34E-05
9100 7.24E-06 8540 9.29E-06
9450 0.000526 7420 2.35E-06 7220 1.14E-05 6810
1.33E-05 9100 7.25E-06 8540 9.28E-06
9450 0.000531 7430 2.36E-06 7220 1.14E-05 6820
1.32E-05 9100 7.25E-06 8540 9.28E-06
9450 0.000537 7430 2.35E-06 7220 1.13E-05 6820
1.32E-05 9100 7.24E-06 8540 9.28E-06
9460 0.000542 7430 2.35E-06 7230 1.12E-05 6820
1.31E-05 9100 7.25E-06 8540 9.28E-06
9460 0.000548 7430 2.36E-06 7230 1.11E-05 6820
1.30E-05 9100 7.25E-06 8540 9.28E-06
9460 0.000554 7430 2.36E-06 7230 1.10E-05 6820
1.29E-05 9100 7.25E-06 8550 9.29E-06
9460 0.00056 7430 2.37E-06 7230 1.10E-05 6820 1.29E-05
9110 7.25E-06 8550 9.30E-06
9460 0.000566 7430 2.37E-06 7230 1.09E-05 6830
1.28E-05 9110 7.25E-06 8550 9.30E-06
9460 0.000572 7440 2.36E-06 7230 1.08E-05 6830
1.27E-05 9110 7.26E-06 8550 9.29E-06
9470 0.000578 7440 2.36E-06 7230 1.08E-05 6830
1.26E-05 9110 7.26E-06 8550 9.29E-06
9470 0.000584 7440 2.37E-06 7240 1.07E-05 6830
1.26E-05 9110 7.26E-06 8550 9.30E-06
9470 0.00059 7440 2.37E-06 7240 1.07E-05 6830 1.25E-05
9110 7.26E-06 8550 9.30E-06
9470 0.000596 7440 2.37E-06 7240 1.06E-05 6830
1.24E-05 9120 7.26E-06 8560 9.29E-06
9470 0.000603 7440 2.37E-06 7240 1.06E-05 6830
1.24E-05 9120 7.25E-06 8560 9.29E-06
9470 0.000609 7450 2.37E-06 7240 1.05E-05 6840
1.23E-05 9120 7.25E-06 8560 9.28E-06
9480 0.000616 7450 2.36E-06 7240 1.05E-05 6840
1.22E-05 9120 7.25E-06 8560 9.30E-06
9480 0.000622 7450 2.37E-06 7250 1.05E-05 6840
1.21E-05 9120 7.25E-06 8560 9.28E-06
9480 0.000629 7450 2.37E-06 7250 1.04E-05 6840
1.21E-05 9120 7.24E-06 8560 9.29E-06

9480 0.000636 7450 2.38E-06 7250 1.04E-05 6840
1.20E-05 9130 7.26E-06 8570 9.29E-06
9480 0.000643 7450 2.38E-06 7250 1.03E-05 6840
1.20E-05 9130 7.25E-06 8570 9.30E-06
9480 0.00065 7450 2.38E-06 7250 1.03E-05 6850 1.19E-05
9130 7.25E-06 8570 9.30E-06
9480 0.000657 7460 2.37E-06 7250 1.03E-05 6850
1.18E-05 9130 7.25E-06 8570 9.29E-06
9490 0.000664 7460 2.38E-06 7250 1.03E-05 6850
1.18E-05 9130 7.24E-06 8570 9.30E-06
9490 0.000671 7460 2.38E-06 7260 1.02E-05 6850
1.17E-05 9130 7.25E-06 8570 9.31E-06
9490 0.000678 7460 2.38E-06 7260 1.02E-05 6850
1.16E-05 9130 7.25E-06 8580 9.31E-06
9490 0.000685 7460 2.38E-06 7260 1.02E-05 6850
1.16E-05 9140 7.26E-06 8580 9.30E-06
9490 0.000693 7460 2.38E-06 7260 1.02E-05 6850
1.15E-05 9140 7.25E-06 8580 9.31E-06
9490 0.0007 7470 2.38E-06 7260 1.01E-05 6860 1.15E-05
9140 7.26E-06 8580 9.30E-06
9500 0.000708 7470 2.38E-06 7260 1.01E-05 6860
1.14E-05 9140 7.26E-06 8580 9.31E-06
9500 0.000716 7470 2.39E-06 7270 1.01E-05 6860
1.14E-05 9140 7.26E-06 8580 9.30E-06
9500 0.000723 7470 2.40E-06 7270 1.01E-05 6860
1.13E-05 9140 7.27E-06 8580 9.30E-06
9500 0.000731 7470 2.40E-06 7270 1.01E-05 6860
1.13E-05 9150 7.27E-06 8590 9.31E-06
9500 0.000739 7470 2.40E-06 7270 1.01E-05 6860
1.12E-05 9150 7.26E-06 8590 9.30E-06
9500 0.000747 7480 2.40E-06 7270 1.00E-05 6870
1.12E-05 9150 7.26E-06 8590 9.28E-06
9500 0.000755 7480 2.39E-06 7270 1.00E-05 6870
1.11E-05 9150 7.26E-06 8590 9.30E-06
9510 0.000763 7480 2.41E-06 7280 1.00E-05 6870
1.10E-05 9150 7.26E-06 8590 9.30E-06
9510 0.000772 7480 2.41E-06 7280 9.99E-06 6870
1.10E-05 9150 7.26E-06 8590 9.30E-06
9510 0.00078 7480 2.42E-06 7280 9.97E-06 6870 1.10E-05
9150 7.27E-06 8600 9.30E-06
9510 0.000789 7480 2.42E-06 7280 9.95E-06 6870
1.09E-05 9160 7.26E-06 8600 9.29E-06
9510 0.000797 7480 2.42E-06 7280 9.94E-06 6880
1.09E-05 9160 7.27E-06 8600 9.30E-06
9510 0.000806 7490 2.42E-06 7280 9.93E-06 6880
1.08E-05 9160 7.27E-06 8600 9.29E-06
9520 0.000815 7490 2.41E-06 7280 9.92E-06 6880
1.08E-05 9160 7.28E-06 8600 9.30E-06
9520 0.000823 7490 2.42E-06 7290 9.90E-06 6880
1.07E-05 9160 7.29E-06 8600 9.30E-06
9520 0.000832 7490 2.42E-06 7290 9.89E-06 6880
1.07E-05 9160 7.28E-06 8600 9.30E-06
9520 0.000841 7490 2.42E-06 7290 9.88E-06 6880
1.06E-05 9170 7.29E-06 8610 9.29E-06
9520 0.00085 7490 2.43E-06 7290 9.86E-06 6880 1.06E-05
9170 7.28E-06 8610 9.30E-06
9520 0.00086 7500 2.42E-06 7290 9.85E-06 6890 1.05E-05
9170 7.30E-06 8610 9.30E-06
9530 0.000869 7500 2.42E-06 7290 9.84E-06 6890
1.05E-05 9170 7.30E-06 8610 9.30E-06
9530 0.000878 7500 2.43E-06 7300 9.83E-06 6890
1.05E-05 9170 7.30E-06 8610 9.29E-06
9530 0.000888 7500 2.43E-06 7300 9.81E-06 6890
1.04E-05 9170 7.31E-06 8610 9.29E-06
9530 0.000897 7500 2.44E-06 7300 9.82E-06 6890
1.04E-05 9180 7.31E-06 8620 9.30E-06
9530 0.000907 7500 2.44E-06 7300 9.81E-06 6890
1.03E-05 9180 7.30E-06 8620 9.30E-06

9530 0.000917 7500 2.44E-06 7300 9.81E-06 6900
1.03E-05 9180 7.30E-06 8620 9.30E-06
9530 0.000927 7510 2.45E-06 7300 9.80E-06 6900
1.02E-05 9180 7.30E-06 8620 9.31E-06
9540 0.000936 7510 2.45E-06 7300 9.80E-06 6900
1.02E-05 9180 7.31E-06 8620 9.30E-06
9540 0.000946 7510 2.46E-06 7310 9.79E-06 6900
1.01E-05 9180 7.30E-06 8620 9.32E-06
9540 0.000957 7510 2.46E-06 7310 9.77E-06 6900
1.01E-05 9180 7.31E-06 8630 9.32E-06
9540 0.000967 7510 2.47E-06 7310 9.76E-06 6900
1.01E-05 9190 7.31E-06 8630 9.33E-06
9540 0.000977 7510 2.46E-06 7310 9.76E-06 6900
1.00E-05 9190 7.32E-06 8630 9.33E-06
9540 0.000987 7520 2.47E-06 7310 9.75E-06 6910
1.00E-05 9190 7.32E-06 8630 9.33E-06
9550 0.000998 7520 2.46E-06 7310 9.76E-06 6910
9.96E-06 9190 7.32E-06 8630 9.34E-06
9550 0.00101 7520 2.46E-06 7320 9.76E-06 6910 9.93E-06
9190 7.32E-06 8630 9.34E-06
9550 0.00102 7520 2.46E-06 7320 9.75E-06 6910 9.92E-06
9190 7.32E-06 8630 9.34E-06
9550 0.00103 7520 2.46E-06 7320 9.74E-06 6910 9.86E-06
9200 7.33E-06 8640 9.35E-06
9550 0.00104 7520 2.47E-06 7320 9.74E-06 6910 9.84E-06
9200 7.32E-06 8640 9.34E-06
9550 0.00105 7530 2.47E-06 7320 9.73E-06 6920 9.81E-06
9200 7.33E-06 8640 9.34E-06
9550 0.00106 7530 2.47E-06 7320 9.73E-06 6920 9.78E-06
9200 7.34E-06 8640 9.34E-06
9560 0.00107 7530 2.48E-06 7330 9.73E-06 6920 9.75E-06
9200 7.33E-06 8640 9.36E-06
9560 0.00108 7530 2.48E-06 7330 9.72E-06 6920 9.73E-06
9200 7.33E-06 8640 9.35E-06
9560 0.0011 7530 2.49E-06 7330 9.72E-06 6920 9.69E-06
9200 7.33E-06 8650 9.35E-06
9560 0.00111 7530 2.49E-06 7330 9.72E-06 6920 9.66E-06
9210 7.33E-06 8650 9.36E-06
9560 0.00112 7530 2.49E-06 7330 9.73E-06 6930 9.60E-06
9210 7.32E-06 8650 9.35E-06
9560 0.00113 7540 2.50E-06 7330 9.73E-06 6930 9.56E-06
9210 7.32E-06 8650 9.35E-06
9570 0.00114 7540 2.50E-06 7330 9.73E-06 6930 9.54E-06
9210 7.33E-06 8650 9.35E-06
9570 0.00115 7540 2.50E-06 7340 9.72E-06 6930 9.54E-06
9210 7.33E-06 8650 9.35E-06
9570 0.00116 7540 2.51E-06 7340 9.70E-06 6930 9.50E-06
9210 7.34E-06 8650 9.34E-06
9570 0.00118 7540 2.51E-06 7340 9.70E-06 6930 9.47E-06
9220 7.34E-06 8660 9.36E-06
9570 0.00119 7540 2.51E-06 7340 9.70E-06 6930 9.45E-06
9220 7.34E-06 8660 9.35E-06
9570 0.0012 7550 2.51E-06 7340 9.69E-06 6940 9.41E-06
9220 7.34E-06 8660 9.36E-06
9580 0.00121 7550 2.51E-06 7340 9.69E-06 6940 9.41E-06
9220 7.34E-06 8660 9.36E-06
9580 0.00122 7550 2.51E-06 7350 9.69E-06 6940 9.39E-06
9220 7.34E-06 8660 9.36E-06
9580 0.00124 7550 2.52E-06 7350 9.69E-06 6940 9.35E-06
9220 7.34E-06 8660 9.36E-06
9580 0.00125 7550 2.51E-06 7350 9.68E-06 6940 9.34E-06
9230 7.34E-06 8670 9.36E-06
9580 0.00126 7550 2.51E-06 7350 9.68E-06 6940 9.32E-06
9230 7.33E-06 8670 9.34E-06
9580 0.00127 7550 2.51E-06 7350 9.67E-06 6950 9.28E-06
9230 7.34E-06 8670 9.35E-06
9580 0.00128 7560 2.51E-06 7350 9.69E-06 6950 9.27E-06
9230 7.34E-06 8670 9.34E-06

9590 0.0013 7560 2.51E-06 7350 9.68E-06 6950 9.23E-06
9230 7.34E-06 8670 9.36E-06
9590 0.00131 7560 2.52E-06 7360 9.68E-06 6950 9.21E-06
9230 7.35E-06 8670 9.34E-06
9590 0.00132 7560 2.51E-06 7360 9.68E-06 6950 9.20E-06
9230 7.34E-06 8680 9.35E-06
9590 0.00133 7560 2.51E-06 7360 9.70E-06 6950 9.17E-06
9240 7.33E-06 8680 9.36E-06
9590 0.00135 7560 2.52E-06 7360 9.69E-06 6950 9.15E-06
9240 7.34E-06 8680 9.35E-06
9590 0.00136 7570 2.52E-06 7360 9.68E-06 6960 9.12E-06
9240 7.33E-06 8680 9.35E-06
9600 0.00137 7570 2.52E-06 7360 9.68E-06 6960 9.09E-06
9240 7.33E-06 8680 9.36E-06
9600 0.00138 7570 2.52E-06 7370 9.67E-06 6960 9.08E-06
9240 7.34E-06 8680 9.36E-06
9600 0.0014 7570 2.53E-06 7370 9.66E-06 6960 9.06E-06
9240 7.34E-06 8680 9.36E-06
9600 0.00141 7570 2.53E-06 7370 9.67E-06 6960 9.05E-06
9250 7.34E-06 8690 9.36E-06
9600 0.00142 7570 2.53E-06 7370 9.66E-06 6960 9.03E-06
9250 7.35E-06 8690 9.36E-06
9600 0.00143 7580 2.53E-06 7370 9.67E-06 6970 9.01E-06
9250 7.34E-06 8690 9.38E-06
9600 0.00145 7580 2.53E-06 7370 9.67E-06 6970 9.01E-06
9250 7.33E-06 8690 9.38E-06
9610 0.00146 7580 2.54E-06 7380 9.67E-06 6970 8.97E-06
9250 7.34E-06 8690 9.38E-06
9610 0.00147 7580 2.54E-06 7380 9.68E-06 6970 8.95E-06
9250 7.34E-06 8690 9.38E-06
9610 0.00148 7580 2.55E-06 7380 9.67E-06 6970 8.92E-06
9250 7.34E-06 8700 9.37E-06
9610 0.0015 7580 2.55E-06 7380 9.68E-06 6970 8.90E-06
9260 7.34E-06 8700 9.37E-06
9610 0.00151 7580 2.55E-06 7380 9.69E-06 6980 8.90E-06
9260 7.34E-06 8700 9.38E-06
9610 0.00152 7590 2.55E-06 7380 9.69E-06 6980 8.89E-06
9260 7.34E-06 8700 9.37E-06
9620 0.00154 7590 2.55E-06 7380 9.69E-06 6980 8.88E-06
9260 7.34E-06 8700 9.38E-06
9620 0.00155 7590 2.56E-06 7390 9.68E-06 6980 8.85E-06
9260 7.34E-06 8700 9.38E-06
9620 0.00156 7590 2.56E-06 7390 9.69E-06 6980 8.83E-06
9260 7.34E-06 8700 9.38E-06
9620 0.00157 7590 2.55E-06 7390 9.70E-06 6980 8.81E-06
9270 7.34E-06 8710 9.38E-06
9620 0.00159 7590 2.56E-06 7390 9.69E-06 6980 8.81E-06
9270 7.33E-06 8710 9.39E-06
9620 0.0016 7600 2.56E-06 7390 9.70E-06 6990 8.79E-06
9270 7.33E-06 8710 9.38E-06
9630 0.00161 7600 2.56E-06 7390 9.70E-06 6990 8.75E-06
9270 7.34E-06 8710 9.38E-06
9630 0.00162 7600 2.56E-06 7400 9.71E-06 6990 8.73E-06
9270 7.34E-06 8710 9.38E-06
9630 0.00163 7600 2.56E-06 7400 9.69E-06 6990 8.72E-06
9270 7.34E-06 8710 9.38E-06
9630 0.00165 7600 2.56E-06 7400 9.69E-06 6990 8.71E-06
9280 7.34E-06 8720 9.40E-06
9630 0.00166 7600 2.57E-06 7400 9.68E-06 6990 8.69E-06
9280 7.35E-06 8720 9.38E-06
9630 0.00167 7600 2.57E-06 7400 9.70E-06 7000 8.67E-06
9280 7.34E-06 8720 9.39E-06
9630 0.00168 7610 2.57E-06 7400 9.70E-06 7000 8.67E-06
9280 7.35E-06 8720 9.40E-06
9640 0.00169 7610 2.57E-06 7400 9.70E-06 7000 8.64E-06
9280 7.35E-06 8720 9.40E-06
9640 0.00171 7610 2.58E-06 7410 9.71E-06 7000 8.62E-06
9280 7.35E-06 8720 9.41E-06

9640	0.00172	7610	2.57E-06	7410	9.71E-06	7000	8.60E-06
9280	7.35E-06		8730	9.40E-06			
9640	0.00173	7610	2.58E-06	7410	9.70E-06	7000	8.59E-06
9290	7.34E-06		8730	9.41E-06			
9640	0.00174	7610	2.57E-06	7410	9.71E-06	7000	8.57E-06
9290	7.34E-06		8730	9.42E-06			
9640	0.00175	7620	2.58E-06	7410	9.72E-06	7010	8.56E-06
9290	7.35E-06		8730	9.41E-06			
9650	0.00176	7620	2.58E-06	7410	9.71E-06	7010	8.54E-06
9290	7.35E-06		8730	9.40E-06			
9650	0.00177	7620	2.59E-06	7420	9.72E-06	7010	8.53E-06
9290	7.35E-06		8730	9.40E-06			
9650	0.00179	7620	2.58E-06	7420	9.73E-06	7010	8.52E-06
9290	7.35E-06		8730	9.41E-06			
9650	0.0018	7620	2.59E-06	7420	9.72E-06	7010	8.49E-06
9300	7.34E-06		8740	9.41E-06			
9650	0.00181	7620	2.59E-06	7420	9.73E-06	7010	8.48E-06
9300	7.34E-06		8740	9.40E-06			
9650	0.00182	7630	2.60E-06	7420	9.72E-06	7020	8.45E-06
9300	7.34E-06		8740	9.41E-06			
9650	0.00183	7630	2.60E-06	7420	9.72E-06	7020	8.43E-06
9300	7.35E-06		8740	9.41E-06			
9660	0.00184	7630	2.60E-06	7430	9.73E-06	7020	8.42E-06
9300	7.34E-06		8740	9.41E-06			
9660	0.00185	7630	2.60E-06	7430	9.74E-06	7020	8.42E-06
9300	7.35E-06		8740	9.41E-06			
9660	0.00186	7630	2.60E-06	7430	9.72E-06	7020	8.40E-06
9300	7.35E-06		8750	9.41E-06			
9660	0.00187	7630	2.60E-06	7430	9.71E-06	7020	8.37E-06
9310	7.35E-06		8750	9.40E-06			
9660	0.00188	7630	2.60E-06	7430	9.72E-06	7030	8.36E-06
9310	7.34E-06		8750	9.41E-06			
9660	0.00189	7640	2.60E-06	7430	9.72E-06	7030	8.36E-06
9310	7.35E-06		8750	9.42E-06			
9670	0.0019	7640	2.61E-06	7430	9.72E-06	7030	8.34E-06
9310	7.35E-06		8750	9.42E-06			
9670	0.0019	7640	2.60E-06	7440	9.72E-06	7030	8.32E-06
9310	7.35E-06		8750	9.43E-06			
9670	0.00191	7640	2.61E-06	7440	9.72E-06	7030	8.30E-06
9310	7.35E-06		8750	9.43E-06			
9670	0.00192	7640	2.62E-06	7440	9.72E-06	7030	8.30E-06
9320	7.35E-06		8760	9.43E-06			
9670	0.00193	7640	2.62E-06	7440	9.72E-06	7030	8.28E-06
9320	7.35E-06		8760	9.44E-06			
9670	0.00194	7650	2.62E-06	7440	9.73E-06	7040	8.27E-06
9320	7.35E-06		8760	9.43E-06			
9680	0.00195	7650	2.62E-06	7440	9.72E-06	7040	8.25E-06
9320	7.35E-06		8760	9.44E-06			
9680	0.00195	7650	2.62E-06	7450	9.72E-06	7040	8.25E-06
9320	7.36E-06		8760	9.42E-06			
9680	0.00196	7650	2.63E-06	7450	9.74E-06	7040	8.22E-06
9320	7.35E-06		8760	9.43E-06			
9680	0.00197	7650	2.63E-06	7450	9.73E-06	7040	8.21E-06
9330	7.34E-06		8770	9.43E-06			
9680	0.00197	7650	2.63E-06	7450	9.73E-06	7040	8.19E-06
9330	7.34E-06		8770	9.43E-06			
9680	0.00198	7650	2.63E-06	7450	9.73E-06	7050	8.17E-06
9330	7.34E-06		8770	9.43E-06			
9680	0.00199	7660	2.64E-06	7450	9.74E-06	7050	8.17E-06
9330	7.34E-06		8770	9.44E-06			
9690	0.00199	7660	2.65E-06	7450	9.74E-06	7050	8.16E-06
9330	7.34E-06		8770	9.44E-06			
9690	0.002	7660	2.65E-06	7460	9.74E-06	7050	8.16E-06
9330	7.35E-06		8770	9.45E-06			
9690	0.00201	7660	2.65E-06	7460	9.73E-06	7050	8.13E-06
9330	7.35E-06		8780	9.45E-06			
9690	0.00201	7660	2.65E-06	7460	9.73E-06	7050	8.13E-06
9340	7.35E-06		8780	9.45E-06			

9690	0.00202	7660	2.66E-06	7460	9.73E-06	7050	8.12E-06
9340	7.35E-06	8780	9.46E-06				
9690	0.00202	7670	2.66E-06	7460	9.74E-06	7060	8.12E-06
9340	7.34E-06	8780	9.47E-06				
9700	0.00203	7670	2.66E-06	7460	9.74E-06	7060	8.12E-06
9340	7.35E-06	8780	9.46E-06				
9700	0.00203	7670	2.66E-06	7470	9.75E-06	7060	8.10E-06
9340	7.35E-06	8780	9.47E-06				
9700	0.00203	7670	2.67E-06	7470	9.74E-06	7060	8.09E-06
9340	7.35E-06	8780	9.47E-06				
9700	0.00204	7670	2.67E-06	7470	9.74E-06	7060	8.09E-06
9350	7.35E-06	8790	9.46E-06				
9700	0.00204	7670	2.67E-06	7470	9.74E-06	7060	8.08E-06
9350	7.35E-06	8790	9.48E-06				
9700	0.00204	7680	2.67E-06	7470	9.74E-06	7070	8.07E-06
9350	7.34E-06	8790	9.47E-06				
9700	0.00205	7680	2.67E-06	7470	9.74E-06	7070	8.05E-06
9350	7.34E-06	8790	9.48E-06				
9710	0.00205	7680	2.67E-06	7480	9.75E-06	7070	8.05E-06
9350	7.34E-06	8790	9.47E-06				
9710	0.00205	7680	2.67E-06	7480	9.76E-06	7070	8.04E-06
9350	7.35E-06	8790	9.47E-06				
9710	0.00205	7680	2.68E-06	7480	9.75E-06	7070	8.03E-06
9350	7.34E-06	8800	9.48E-06				
9710	0.00205	7680	2.68E-06	7480	9.75E-06	7070	8.02E-06
9360	7.33E-06	8800	9.49E-06				
9710	0.00206	7680	2.69E-06	7480	9.74E-06	7080	8.01E-06
9360	7.32E-06	8800	9.49E-06				
9710	0.00206	7690	2.68E-06	7480	9.74E-06	7080	7.99E-06
9360	7.32E-06	8800	9.48E-06				
9720	0.00206	7690	2.69E-06	7480	9.76E-06	7080	7.97E-06
9360	7.32E-06	8800	9.48E-06				
9720	0.00206	7690	2.70E-06	7490	9.76E-06	7080	7.97E-06
9360	7.31E-06	8800	9.47E-06				
9720	0.00206	7690	2.70E-06	7490	9.75E-06	7080	7.95E-06
9360	7.30E-06	8800	9.47E-06				
9720	0.00206	7690	2.69E-06	7490	9.74E-06	7080	7.94E-06
9370	7.31E-06	8810	9.47E-06				
9720	0.00206	7690	2.71E-06	7490	9.76E-06	7080	7.94E-06
9370	7.30E-06	8810	9.47E-06				
9720	0.00206	7700	2.70E-06	7490	9.77E-06	7090	7.94E-06
9370	7.30E-06	8810	9.48E-06				
9730	0.00205	7700	2.71E-06	7490	9.77E-06	7090	7.94E-06
9370	7.29E-06	8810	9.47E-06				
9730	0.00205	7700	2.71E-06	7500	9.77E-06	7090	7.93E-06
9370	7.29E-06	8810	9.48E-06				
9730	0.00205	7700	2.72E-06	7500	9.77E-06	7090	7.92E-06
9370	7.28E-06	8810	9.49E-06				
9730	0.00205	7700	2.72E-06	7500	9.78E-06	7090	7.92E-06
9380	7.27E-06	8820	9.48E-06				
9730	0.00205	7700	2.73E-06	7500	9.78E-06	7090	7.91E-06
9380	7.27E-06	8820	9.49E-06				
9730	0.00204	7700	2.73E-06	7500	9.78E-06	7100	7.91E-06
9380	7.25E-06	8820	9.48E-06				
9730	0.00204	7710	2.72E-06	7500	9.79E-06	7100	7.90E-06
9380	7.24E-06	8820	9.49E-06				
9740	0.00204	7710	2.73E-06	7500	9.79E-06	7100	7.90E-06
9380	7.24E-06	8820	9.48E-06				
9740	0.00203	7710	2.73E-06	7510	9.81E-06	7100	7.90E-06
9380	7.24E-06	8820	9.48E-06				
9740	0.00203	7710	2.72E-06	7510	9.81E-06	7100	7.87E-06
9380	7.23E-06	8830	9.48E-06				
9740	0.00203	7710	2.73E-06	7510	9.83E-06	7100	7.87E-06
9390	7.22E-06	8830	9.49E-06				
9740	0.00202	7710	2.73E-06	7510	9.83E-06	7100	7.86E-06
9390	7.21E-06	8830	9.50E-06				
9740	0.00202	7720	2.73E-06	7510	9.84E-06	7110	7.84E-06
9390	7.22E-06	8830	9.50E-06				

9750 0.00201 7720 2.74E-06 7510 9.85E-06 7110 7.83E-06
9390 7.22E-06 8830 9.50E-06
9750 0.00201 7720 2.75E-06 7520 9.87E-06 7110 7.83E-06
9390 7.21E-06 8830 9.50E-06
9750 0.002 7720 2.75E-06 7520 9.86E-06 7110 7.83E-06
9390 7.22E-06 8830 9.51E-06
9750 0.002 7720 2.74E-06 7520 9.88E-06 7110 7.84E-06
9400 7.21E-06 8840 9.51E-06
9750 0.00199 7720 2.76E-06 7520 9.88E-06 7110 7.84E-06
9400 7.20E-06 8840 9.52E-06
9750 0.00198 7730 2.76E-06 7520 9.90E-06 7120 7.83E-06
9400 7.22E-06 8840 9.52E-06
9750 0.00198 7730 2.76E-06 7520 9.91E-06 7120 7.83E-06
9400 7.22E-06 8840 9.52E-06
9760 0.00197 7730 2.75E-06 7530 9.93E-06 7120 7.84E-06
9400 7.21E-06 8840 9.51E-06
9760 0.00196 7730 2.77E-06 7530 9.95E-06 7120 7.81E-06
9400 7.20E-06 8840 9.53E-06
9760 0.00196 7730 2.76E-06 7530 9.96E-06 7120 7.82E-06
9400 7.20E-06 8850 9.52E-06
9760 0.00195 7730 2.76E-06 7530 9.98E-06 7120 7.82E-06
9410 7.22E-06 8850 9.53E-06
9760 0.00194 7730 2.75E-06 7530 9.97E-06 7130 7.79E-06
9410 7.23E-06 8850 9.52E-06
9760 0.00193 7740 2.75E-06 7530 9.98E-06 7130 7.78E-06
9410 7.24E-06 8850 9.53E-06
9770 0.00193 7740 2.76E-06 7530 1.00E-05 7130 7.80E-06
9410 7.24E-06 8850 9.52E-06
9770 0.00192 7740 2.76E-06 7540 1.00E-05 7130 7.78E-06
9410 7.26E-06 8850 9.52E-06
9770 0.00191 7740 2.77E-06 7540 1.00E-05 7130 7.78E-06
9410 7.25E-06 8850 9.53E-06
9770 0.0019 7740 2.76E-06 7540 1.00E-05 7130 7.78E-06
9420 7.27E-06 8860 9.52E-06
9770 0.00189 7740 2.78E-06 7540 1.00E-05 7130 7.77E-06
9420 7.27E-06 8860 9.52E-06
9770 0.00188 7750 2.77E-06 7540 1.01E-05 7140 7.78E-06
9420 7.30E-06 8860 9.53E-06
9780 0.00188 7750 2.78E-06 7540 1.01E-05 7140 7.77E-06
9420 7.32E-06 8860 9.52E-06
9780 0.00187 7750 2.79E-06 7550 1.01E-05 7140 7.77E-06
9420 7.33E-06 8860 9.52E-06
9780 0.00186 7750 2.79E-06 7550 1.01E-05 7140 7.77E-06
9420 7.34E-06 8860 9.52E-06
9780 0.00185 7750 2.79E-06 7550 1.01E-05 7140 7.77E-06
9430 7.35E-06 8870 9.50E-06
9780 0.00184 7750 2.79E-06 7550 1.01E-05 7140 7.76E-06
9430 7.38E-06 8870 9.51E-06
9780 0.00183 7750 2.80E-06 7550 1.01E-05 7150 7.76E-06
9430 7.38E-06 8870 9.51E-06
9780 0.00182 7760 2.79E-06 7550 1.01E-05 7150 7.77E-06
9430 7.41E-06 8870 9.52E-06
9790 0.00181 7760 2.80E-06 7550 1.01E-05 7150 7.76E-06
9430 7.43E-06 8870 9.51E-06
9790 0.0018 7760 2.81E-06 7560 1.01E-05 7150 7.74E-06
9430 7.45E-06 8870 9.52E-06
9790 0.00179 7760 2.81E-06 7560 1.01E-05 7150 7.75E-06
9430 7.46E-06 8880 9.52E-06
9790 0.00178 7760 2.80E-06 7560 1.01E-05 7150 7.74E-06
9440 7.48E-06 8880 9.53E-06
9790 0.00177 7760 2.81E-06 7560 1.01E-05 7150 7.73E-06
9440 7.49E-06 8880 9.53E-06
9790 0.00176 7770 2.80E-06 7560 1.01E-05 7160 7.72E-06
9440 7.51E-06 8880 9.53E-06
9800 0.00175 7770 2.81E-06 7560 1.01E-05 7160 7.72E-06
9440 7.53E-06 8880 9.51E-06
9800 0.00174 7770 2.81E-06 7570 1.01E-05 7160 7.72E-06
9440 7.54E-06 8880 9.52E-06

9800 0.00173 7770 2.81E-06 7570 1.01E-05 7160 7.71E-06
9440 7.56E-06 8880 9.51E-06
9800 0.00172 7770 2.82E-06 7570 1.02E-05 7160 7.71E-06
9450 7.57E-06 8890 9.52E-06
9800 0.00171 7770 2.81E-06 7570 1.02E-05 7160 7.69E-06
9450 7.58E-06 8890 9.51E-06
9800 0.00169 7780 2.81E-06 7570 1.02E-05 7170 7.69E-06
9450 7.60E-06 8890 9.52E-06
9800 0.00168 7780 2.82E-06 7570 1.02E-05 7170 7.69E-06
9450 7.61E-06 8890 9.52E-06
9810 0.00167 7780 2.83E-06 7580 1.02E-05 7170 7.68E-06
9450 7.64E-06 8890 9.52E-06
9810 0.00166 7780 2.83E-06 7580 1.02E-05 7170 7.67E-06
9450 7.65E-06 8890 9.53E-06
9810 0.00165 7780 2.84E-06 7580 1.02E-05 7170 7.66E-06
9450 7.67E-06 8900 9.52E-06
9810 0.00164 7780 2.84E-06 7580 1.02E-05 7170 7.68E-06
9460 7.69E-06 8900 9.53E-06
9810 0.00163 7780 2.84E-06 7580 1.02E-05 7180 7.68E-06
9460 7.70E-06 8900 9.53E-06
9810 0.00162 7790 2.84E-06 7580 1.02E-05 7180 7.68E-06
9460 7.72E-06 8900 9.54E-06
9820 0.00161 7790 2.85E-06 7580 1.02E-05 7180 7.68E-06
9460 7.74E-06 8900 9.54E-06
9820 0.0016 7790 2.86E-06 7590 1.02E-05 7180 7.67E-06
9460 7.76E-06 8900 9.53E-06
9820 0.00159 7790 2.86E-06 7590 1.02E-05 7180 7.67E-06
9460 7.77E-06 8900 9.54E-06
9820 0.00158 7790 2.87E-06 7590 1.02E-05 7180 7.67E-06
9470 7.79E-06 8910 9.55E-06
9820 0.00157 7790 2.86E-06 7590 1.02E-05 7180 7.66E-06
9470 7.82E-06 8910 9.55E-06
9820 0.00156 7800 2.87E-06 7590 1.02E-05 7190 7.67E-06
9470 7.84E-06 8910 9.55E-06
9830 0.00154 7800 2.88E-06 7590 1.02E-05 7190 7.67E-06
9470 7.85E-06 8910 9.53E-06
9830 0.00153 7800 2.87E-06 7600 1.03E-05 7190 7.67E-06
9470 7.88E-06 8910 9.54E-06
9830 0.00152 7800 2.89E-06 7600 1.02E-05 7190 7.66E-06
9470 7.88E-06 8910 9.53E-06
9830 0.00151 7800 2.89E-06 7600 1.02E-05 7190 7.68E-06
9480 7.90E-06 8920 9.54E-06
9830 0.0015 7800 2.89E-06 7600 1.03E-05 7190 7.67E-06
9480 7.91E-06 8920 9.53E-06
9830 0.00149 7800 2.89E-06 7600 1.03E-05 7200 7.65E-06
9480 7.94E-06 8920 9.54E-06
9830 0.00148 7810 2.89E-06 7600 1.03E-05 7200 7.66E-06
9480 7.96E-06 8920 9.54E-06
9840 0.00147 7810 2.90E-06 7600 1.03E-05 7200 7.66E-06
9480 7.99E-06 8920 9.53E-06
9840 0.00146 7810 2.90E-06 7610 1.03E-05 7200 7.63E-06
9480 8.00E-06 8920 9.53E-06
9840 0.00145 7810 2.92E-06 7610 1.03E-05 7200 7.65E-06
9480 8.02E-06 8930 9.52E-06
9840 0.00144 7810 2.91E-06 7610 1.03E-05 7200 7.65E-06
9490 8.04E-06 8930 9.53E-06
9840 0.00143 7810 2.91E-06 7610 1.03E-05 7200 7.66E-06
9490 8.06E-06 8930 9.53E-06
9840 0.00142 7820 2.92E-06 7610 1.03E-05 7210 7.66E-06
9490 8.09E-06 8930 9.53E-06
9850 0.00141 7820 2.92E-06 7610 1.03E-05 7210 7.66E-06
9490 8.12E-06 8930 9.53E-06
9850 0.0014 7820 2.93E-06 7620 1.03E-05 7210 7.65E-06
9490 8.14E-06 8930 9.53E-06
9850 0.0014 7820 2.93E-06 7620 1.03E-05 7210 7.66E-06
9490 8.17E-06 8930 9.54E-06
9850 0.00139 7820 2.92E-06 7620 1.03E-05 7210 7.67E-06
9500 8.20E-06 8940 9.55E-06

9850	0.00138	7820	2.92E-06	7620	1.03E-05	7210	7.66E-06
9500	8.23E-06		8940	9.54E-06			
9850	0.00137	7830	2.93E-06	7620	1.04E-05	7220	7.66E-06
9500	8.23E-06		8940	9.54E-06			
9850	0.00136	7830	2.93E-06	7620	1.04E-05	7220	7.66E-06
9500	8.27E-06		8940	9.53E-06			
9860	0.00135	7830	2.93E-06	7630	1.03E-05	7220	7.64E-06
9500	8.29E-06		8940	9.54E-06			
9860	0.00134	7830	2.93E-06	7630	1.03E-05	7220	7.63E-06
9500	8.32E-06		8940	9.53E-06			
9860	0.00133	7830	2.93E-06	7630	1.04E-05	7220	7.63E-06
9500	8.34E-06		8950	9.53E-06			
9860	0.00133	7830	2.94E-06	7630	1.04E-05	7220	7.63E-06
9510	8.36E-06		8950	9.54E-06			
9860	0.00132	7830	2.94E-06	7630	1.04E-05	7230	7.63E-06
9510	8.39E-06		8950	9.55E-06			
9860	0.00131	7840	2.94E-06	7630	1.04E-05	7230	7.64E-06
9510	8.43E-06		8950	9.56E-06			
9870	0.0013	7840	2.95E-06	7630	1.04E-05	7230	7.63E-06
9510	8.46E-06		8950	9.55E-06			
9870	0.00129	7840	2.95E-06	7640	1.04E-05	7230	7.60E-06
9510	8.49E-06		8950	9.55E-06			
9870	0.00129	7840	2.96E-06	7640	1.04E-05	7230	7.61E-06
9510	8.51E-06		8950	9.54E-06			
9870	0.00128	7840	2.97E-06	7640	1.04E-05	7230	7.59E-06
9520	8.54E-06		8960	9.56E-06			
9870	0.00127	7840	2.95E-06	7640	1.04E-05	7230	7.60E-06
9520	8.59E-06		8960	9.56E-06			
9870	0.00127	7850	2.96E-06	7640	1.04E-05	7240	7.60E-06
9520	8.62E-06		8960	9.56E-06			
9880	0.00126	7850	2.97E-06	7640	1.04E-05	7240	7.60E-06
9520	8.66E-06		8960	9.56E-06			
9880	0.00125	7850	2.98E-06	7650	1.04E-05	7240	7.60E-06
9520	8.70E-06		8960	9.56E-06			
9880	0.00125	7850	2.99E-06	7650	1.04E-05	7240	7.60E-06
9520	8.73E-06		8960	9.57E-06			
9880	0.00124	7850	2.99E-06	7650	1.04E-05	7240	7.61E-06
9530	8.76E-06		8970	9.59E-06			
9880	0.00123	7850	2.99E-06	7650	1.03E-05	7240	7.61E-06
9530	8.81E-06		8970	9.58E-06			
9880	0.00123	7850	3.00E-06	7650	1.04E-05	7250	7.63E-06
9530	8.85E-06		8970	9.58E-06			
9880	0.00122	7860	3.00E-06	7650	1.04E-05	7250	7.62E-06
9530	8.90E-06		8970	9.57E-06			
9890	0.00122	7860	3.01E-06	7650	1.03E-05	7250	7.60E-06
9530	8.94E-06		8970	9.57E-06			
9890	0.00121	7860	3.02E-06	7660	1.03E-05	7250	7.61E-06
9530	9.00E-06		8970	9.57E-06			
9890	0.00121	7860	3.03E-06	7660	1.04E-05	7250	7.59E-06
9530	9.03E-06		8980	9.57E-06			
9890	0.0012	7860	3.04E-06	7660	1.04E-05	7250	7.59E-06
9540	9.08E-06		8980	9.57E-06			
9890	0.0012	7860	3.05E-06	7660	1.04E-05	7250	7.59E-06
9540	9.14E-06		8980	9.58E-06			
9890	0.00119	7870	3.05E-06	7660	1.04E-05	7260	7.61E-06
9540	9.21E-06		8980	9.58E-06			
9900	0.00119	7870	3.06E-06	7660	1.04E-05	7260	7.59E-06
9540	9.26E-06		8980	9.58E-06			
9900	0.00119	7870	3.06E-06	7670	1.04E-05	7260	7.59E-06
9540	9.32E-06		8980	9.58E-06			
9900	0.00118	7870	3.06E-06	7670	1.04E-05	7260	7.60E-06
9540	9.40E-06		8980	9.57E-06			
9900	0.00118	7870	3.09E-06	7670	1.04E-05	7260	7.60E-06
9550	9.47E-06		8990	9.58E-06			
9900	0.00118	7870	3.08E-06	7670	1.04E-05	7260	7.61E-06
9550	9.53E-06		8990	9.58E-06			
9900	0.00117	7880	3.10E-06	7670	1.04E-05	7270	7.61E-06
9550	9.60E-06		8990	9.58E-06			

9900 0.00117 7880 3.10E-06 7670 1.04E-05 7270 7.62E-06
9550 9.70E-06 8990 9.58E-06
9910 0.00117 7880 3.10E-06 7680 1.04E-05 7270 7.60E-06
9550 9.77E-06 8990 9.58E-06
9910 0.00117 7880 3.11E-06 7680 1.04E-05 7270 7.62E-06
9550 9.85E-06 8990 9.58E-06
9910 0.00116 7880 3.11E-06 7680 1.04E-05 7270 7.64E-06
9550 9.95E-06 9000 9.60E-06
9910 0.00116 7880 3.11E-06 7680 1.04E-05 7270 7.64E-06
9560 1.00E-05 9000 9.59E-06
9910 0.00116 7880 3.12E-06 7680 1.04E-05 7280 7.64E-06
9560 1.01E-05 9000 9.60E-06
9910 0.00116 7890 3.13E-06 7680 1.04E-05 7280 7.64E-06
9560 1.02E-05 9000 9.59E-06
9920 0.00116 7890 3.14E-06 7680 1.05E-05 7280 7.64E-06
9560 1.03E-05 9000 9.60E-06
9920 0.00116 7890 3.15E-06 7690 1.05E-05 7280 7.64E-06
9560 1.05E-05 9000 9.59E-06
9920 0.00116 7890 3.14E-06 7690 1.04E-05 7280 7.64E-06
9560 1.06E-05 9000 9.59E-06
9920 0.00116 7890 3.16E-06 7690 1.05E-05 7280 7.63E-06
9570 1.07E-05 9010 9.59E-06
9920 0.00116 7890 3.17E-06 7690 1.05E-05 7280 7.63E-06
9570 1.08E-05 9010 9.59E-06
9920 0.00116 7900 3.18E-06 7690 1.05E-05 7290 7.64E-06
9570 1.10E-05 9010 9.60E-06
9930 0.00116 7900 3.18E-06 7690 1.05E-05 7290 7.62E-06
9570 1.11E-05 9010 9.60E-06
9930 0.00116 7900 3.18E-06 7700 1.05E-05 7290 7.64E-06
9570 1.12E-05 9010 9.61E-06
9930 0.00116 7900 3.19E-06 7700 1.05E-05 7290 7.65E-06
9570 1.14E-05 9010 9.60E-06
9930 0.00116 7900 3.21E-06 7700 1.06E-05 7290 7.63E-06
9580 1.16E-05 9020 9.61E-06
9930 0.00117 7900 3.21E-06 7700 1.06E-05 7290 7.64E-06
9580 1.17E-05 9020 9.61E-06
9930 0.00117 7900 3.22E-06 7700 1.07E-05 7300 7.65E-06
9580 1.19E-05 9020 9.61E-06
9930 0.00117 7910 3.23E-06 7700 1.08E-05 7300 7.64E-06
9580 1.21E-05 9020 9.61E-06
9940 0.00117 7910 3.24E-06 7700 1.08E-05 7300 7.65E-06
9580 1.23E-05 9020 9.61E-06
9940 0.00118 7910 3.23E-06 7710 1.08E-05 7300 7.65E-06
9580 1.25E-05 9020 9.62E-06
9940 0.00118 7910 3.26E-06 7710 1.08E-05 7300 7.65E-06
9580 1.28E-05 9030 9.63E-06
9940 0.00118 7910 3.26E-06 7710 1.08E-05 7300 7.64E-06
9590 1.30E-05 9030 9.62E-06
9940 0.00119 7910 3.28E-06 7710 1.08E-05 7300 7.65E-06
9590 1.33E-05 9030 9.63E-06
9940 0.00119 7920 3.29E-06 7710 1.08E-05 7310 7.65E-06
9590 1.35E-05 9030 9.62E-06
9950 0.0012 7920 3.31E-06 7710 1.09E-05 7310 7.64E-06
9590 1.38E-05 9030 9.63E-06
9950 0.0012 7920 3.33E-06 7720 1.09E-05 7310 7.65E-06
9590 1.41E-05 9030 9.61E-06
9950 0.00121 7920 3.34E-06 7720 1.08E-05 7310 7.66E-06
9590 1.44E-05 9030 9.62E-06
9950 0.00122 7920 3.35E-06 7720 1.09E-05 7310 7.65E-06
9600 1.47E-05 9040 9.62E-06
9950 0.00122 7920 3.36E-06 7720 1.08E-05 7310 7.65E-06
9600 1.50E-05 9040 9.62E-06
9950 0.00123 7930 3.37E-06 7720 1.08E-05 7320 7.66E-06
9600 1.54E-05 9040 9.62E-06
9950 0.00124 7930 3.37E-06 7720 1.08E-05 7320 7.64E-06
9600 1.57E-05 9040 9.63E-06
9960 0.00124 7930 3.39E-06 7730 1.08E-05 7320 7.65E-06
9600 1.61E-05 9040 9.63E-06

9960	0.00125	7930	3.40E-06	7730	1.09E-05	7320	7.66E-06
9600	1.65E-05	9040	9.63E-06				
9960	0.00126	7930	3.42E-06	7730	1.09E-05	7320	7.65E-06
9600	1.69E-05	9050	9.64E-06				
9960	0.00127	7930	3.43E-06	7730	1.08E-05	7320	7.66E-06
9610	1.73E-05	9050	9.64E-06				
9960	0.00128	7930	3.45E-06	7730	1.08E-05	7330	7.66E-06
9610	1.78E-05	9050	9.65E-06				
9960	0.00129	7940	3.45E-06	7730	1.08E-05	7330	7.67E-06
9610	1.82E-05	9050	9.65E-06				
9970	0.0013	7940	3.48E-06	7730	1.08E-05	7330	7.66E-06
9610	1.86E-05	9050	9.65E-06				
9970	0.00131	7940	3.47E-06	7740	1.08E-05	7330	7.67E-06
9610	1.91E-05	9050	9.65E-06				
9970	0.00132	7940	3.48E-06	7740	1.08E-05	7330	7.67E-06
9610	1.96E-05	9050	9.66E-06				
9970	0.00133	7940	3.49E-06	7740	1.08E-05	7330	7.67E-06
9620	2.00E-05	9060	9.65E-06				
9970	0.00134	7940	3.51E-06	7740	1.09E-05	7330	7.67E-06
9620	2.05E-05	9060	9.65E-06				
9970	0.00136	7950	3.53E-06	7740	1.08E-05	7340	7.67E-06
9620	2.09E-05	9060	9.65E-06				
9980	0.00137	7950	3.54E-06	7740	1.08E-05	7340	7.67E-06
9620	2.14E-05	9060	9.65E-06				
9980	0.00139	7950	3.56E-06	7750	1.08E-05	7340	7.67E-06
9620	2.18E-05	9060	9.64E-06				
9980	0.0014	7950	3.57E-06	7750	1.08E-05	7340	7.67E-06
9620	2.22E-05	9060	9.64E-06				
9980	0.00141	7950	3.60E-06	7750	1.08E-05	7340	7.67E-06
9630	2.26E-05	9070	9.64E-06				
9980	0.00143	7950	3.60E-06	7750	1.08E-05	7340	7.68E-06
9630	2.30E-05	9070	9.63E-06				
9980	0.00145	7950	3.60E-06	7750	1.09E-05	7350	7.69E-06
9630	2.33E-05	9070	9.64E-06				
9980	0.00146	7960	3.62E-06	7750	1.09E-05	7350	7.69E-06
9630	2.36E-05	9070	9.64E-06				
9990	0.00148	7960	3.63E-06	7750	1.09E-05	7350	7.68E-06
9630	2.39E-05	9070	9.64E-06				
9990	0.0015	7960	3.65E-06	7760	1.09E-05	7350	7.68E-06
9630	2.41E-05	9070	9.64E-06				
9990	0.00152	7960	3.66E-06	7760	1.09E-05	7350	7.68E-06
9630	2.43E-05	9080	9.65E-06				
9990	0.00154	7960	3.69E-06	7760	1.10E-05	7350	7.69E-06
9640	2.44E-05	9080	9.65E-06				
9990	0.00156	7960	3.71E-06	7760	1.10E-05	7350	7.70E-06
9640	2.45E-05	9080	9.68E-06				
9990	0.00158	7970	3.72E-06	7760	1.09E-05	7360	7.70E-06
9640	2.45E-05	9080	9.66E-06				
10000	0.0016	7970	3.72E-06	7760	1.10E-05	7360	7.70E-06
9640	2.45E-05	9080	9.67E-06				
10000	0.00163	7970	3.74E-06	7770	1.10E-05	7360	7.70E-06
9640	2.44E-05	9080	9.68E-06				
10000	0.00165	7970	3.75E-06	7770	1.11E-05	7360	7.70E-06
9640	2.42E-05	9080	9.68E-06				
10000	0.00167	7970	3.77E-06	7770	1.11E-05	7360	7.70E-06
9650	2.41E-05	9090	9.68E-06				
10000	0.0017	7970	3.79E-06	7770	1.11E-05	7360	7.71E-06
9650	2.38E-05	9090	9.68E-06				
10000	0.00173	7980	3.81E-06	7770	1.11E-05	7370	7.70E-06
9650	2.35E-05	9090	9.67E-06				
10000	0.00175	7980	3.84E-06	7770	1.12E-05	7370	7.72E-06
9650	2.32E-05	9090	9.69E-06				
10000	0.00178	7980	3.86E-06	7780	1.12E-05	7370	7.72E-06
9650	2.29E-05	9090	9.67E-06				
10000	0.00181	7980	3.89E-06	7780	1.12E-05	7370	7.72E-06
9650	2.25E-05	9090	9.67E-06				
10000	0.00184	7980	3.91E-06	7780	1.12E-05	7370	7.72E-06
9650	2.21E-05	9100	9.67E-06				

10000	0.00187	7980	3.93E-06	7780	1.13E-05	7370	7.72E-06
9660	2.16E-05	9100	9.67E-06				
10000	0.00191	7980	3.97E-06	7780	1.13E-05	7380	7.72E-06
9660	2.12E-05	9100	9.67E-06				
10000	0.00194	7990	3.99E-06	7780	1.13E-05	7380	7.72E-06
9660	2.07E-05	9100	9.67E-06				
10000	0.00197	7990	4.02E-06	7780	1.13E-05	7380	7.71E-06
9660	2.03E-05	9100	9.69E-06				
10000	0.00201	7990	4.05E-06	7790	1.13E-05	7380	7.73E-06
9660	1.98E-05	9100	9.69E-06				
10000	0.00205	7990	4.09E-06	7790	1.13E-05	7380	7.73E-06
9660	1.94E-05	9100	9.69E-06				
10000	0.00209	7990	4.12E-06	7790	1.13E-05	7380	7.74E-06
9670	1.89E-05	9110	9.69E-06				
10000	0.00213	7990	4.15E-06	7790	1.13E-05	7380	7.75E-06
9670	1.84E-05	9110	9.70E-06				
10000	0.00217	8000	4.18E-06	7790	1.14E-05	7390	7.74E-06
9670	1.80E-05	9110	9.70E-06				
10000	0.00221	8000	4.23E-06	7790	1.14E-05	7390	7.75E-06
9670	1.76E-05	9110	9.70E-06				
10000	0.00225	8000	4.27E-06	7800	1.14E-05	7390	7.75E-06
9670	1.71E-05	9110	9.71E-06				
10000	0.0023	8000	4.31E-06	7800	1.14E-05	7390	7.76E-06
9670	1.67E-05	9110	9.72E-06				
10000	0.00235	8000	4.35E-06	7800	1.14E-05	7390	7.76E-06
9680	1.63E-05	9120	9.72E-06				
10000	0.0024	8000	4.41E-06	7800	1.14E-05	7390	7.77E-06
9680	1.60E-05	9120	9.71E-06				
10000	0.00245	8000	4.46E-06	7800	1.15E-05	7400	7.77E-06
9680	1.56E-05	9120	9.71E-06				
10000	0.0025	8010	4.51E-06	7800	1.15E-05	7400	7.78E-06
9680	1.52E-05	9120	9.72E-06				
10000	0.00255	8010	4.57E-06	7800	1.15E-05	7400	7.78E-06
9680	1.49E-05	9120	9.71E-06				
10000	0.00261	8010	4.63E-06	7810	1.15E-05	7400	7.80E-06
9680	1.46E-05	9120	9.71E-06				
10000	0.00267	8010	4.69E-06	7810	1.15E-05	7400	7.79E-06
9680	1.43E-05	9130	9.71E-06				
10000	0.00273	8010	4.75E-06	7810	1.15E-05	7400	7.79E-06
9690	1.40E-05	9130	9.70E-06				
10000	0.00279	8010	4.82E-06	7810	1.15E-05	7400	7.82E-06
9690	1.37E-05	9130	9.70E-06				
10000	0.00285	8020	4.90E-06	7810	1.15E-05	7410	7.82E-06
9690	1.34E-05	9130	9.70E-06				
10000	0.00292	8020	4.98E-06	7810	1.15E-05	7410	7.81E-06
9690	1.32E-05	9130	9.70E-06				
10000	0.00299	8020	5.04E-06	7820	1.15E-05	7410	7.82E-06
9690	1.29E-05	9130	9.71E-06				
10000	0.00306	8020	5.14E-06	7820	1.16E-05	7410	7.83E-06
9690	1.27E-05	9130	9.71E-06				
10100	0.00313	8020	5.22E-06	7820	1.16E-05	7410	7.83E-06
9700	1.25E-05	9140	9.70E-06				
10100	0.0032	8020	5.33E-06	7820	1.16E-05	7410	7.85E-06
9700	1.23E-05	9140	9.71E-06				
10100	0.00328	8030	5.42E-06	7820	1.16E-05	7420	7.86E-06
9700	1.21E-05	9140	9.71E-06				
10100	0.00336	8030	5.54E-06	7820	1.16E-05	7420	7.86E-06
9700	1.19E-05	9140	9.70E-06				
10100	0.00345	8030	5.65E-06	7830	1.16E-05	7420	7.87E-06
9700	1.17E-05	9140	9.71E-06				
10100	0.00353	8030	5.78E-06	7830	1.16E-05	7420	7.87E-06
9700	1.15E-05	9140	9.72E-06				
10100	0.00362	8030	5.91E-06	7830	1.17E-05	7420	7.89E-06
9700	1.14E-05	9150	9.71E-06				
10100	0.00371	8030	6.05E-06	7830	1.17E-05	7420	7.88E-06
9710	1.12E-05	9150	9.71E-06				
10100	0.00381	8030	6.19E-06	7830	1.17E-05	7430	7.89E-06
9710	1.11E-05	9150	9.71E-06				

10100	0.0039	8040	6.37E-06	7830	1.17E-05	7430	7.90E-06
9710	1.10E-05		9150	9.72E-06			
10100	0.004	8040	6.55E-06	7830	1.17E-05	7430	7.90E-06
9710	1.08E-05		9150	9.71E-06			
10100	0.00411	8040	6.74E-06	7840	1.17E-05	7430	7.90E-06
9710	1.07E-05		9150	9.72E-06			
10100	0.00421	8040	6.93E-06	7840	1.18E-05	7430	7.90E-06
9710	1.06E-05		9150	9.73E-06			
10100	0.00432	8040	7.14E-06	7840	1.18E-05	7430	7.91E-06
9720	1.05E-05		9160	9.72E-06			
10100	0.00444	8040	7.38E-06	7840	1.18E-05	7430	7.92E-06
9720	1.03E-05		9160	9.72E-06			
10100	0.00455	8050	7.61E-06	7840	1.18E-05	7440	7.93E-06
9720	1.02E-05		9160	9.71E-06			
10100	0.00467	8050	7.87E-06	7840	1.18E-05	7440	7.94E-06
9720	1.01E-05		9160	9.73E-06			
10100	0.00479	8050	8.17E-06	7850	1.18E-05	7440	7.95E-06
9720	1.01E-05		9160	9.74E-06			
10100	0.00492	8050	8.48E-06	7850	1.18E-05	7440	7.96E-06
9720	9.97E-06		9160	9.75E-06			
10100	0.00505	8050	8.83E-06	7850	1.18E-05	7440	7.97E-06
9730	9.89E-06		9170	9.75E-06			
10100	0.00519	8050	9.18E-06	7850	1.19E-05	7440	7.97E-06
9730	9.80E-06		9170	9.75E-06			
10100	0.00532	8050	9.61E-06	7850	1.19E-05	7450	8.00E-06
9730	9.73E-06		9170	9.75E-06			
10100	0.00546	8060	1.01E-05	7850	1.19E-05	7450	8.00E-06
9730	9.66E-06		9170	9.74E-06			
10100	0.00561	8060	1.05E-05	7850	1.19E-05	7450	8.01E-06
9730	9.58E-06		9170	9.76E-06			
10100	0.00576	8060	1.11E-05	7860	1.19E-05	7450	8.03E-06
9730	9.52E-06		9170	9.76E-06			
10100	0.00591	8060	1.17E-05	7860	1.19E-05	7450	8.04E-06
9730	9.46E-06		9180	9.76E-06			
10100	0.00606	8060	1.23E-05	7860	1.20E-05	7450	8.04E-06
9740	9.40E-06		9180	9.75E-06			
10100	0.00622	8060	1.30E-05	7860	1.20E-05	7450	8.06E-06
9740	9.35E-06		9180	9.75E-06			
10100	0.00638	8070	1.38E-05	7860	1.20E-05	7460	8.07E-06
9740	9.29E-06		9180	9.76E-06			
10100	0.00655	8070	1.48E-05	7860	1.20E-05	7460	8.08E-06
9740	9.23E-06		9180	9.75E-06			
10100	0.00671	8070	1.58E-05	7870	1.20E-05	7460	8.10E-06
9740	9.18E-06		9180	9.75E-06			
10100	0.00688	8070	1.69E-05	7870	1.21E-05	7460	8.11E-06
9740	9.13E-06		9180	9.74E-06			
10100	0.00706	8070	1.81E-05	7870	1.21E-05	7460	8.14E-06
9750	9.08E-06		9190	9.74E-06			
10100	0.00723	8070	1.95E-05	7870	1.21E-05	7460	8.15E-06
9750	9.05E-06		9190	9.73E-06			
10100	0.00741	8080	2.10E-05	7870	1.21E-05	7470	8.16E-06
9750	9.00E-06		9190	9.73E-06			
10100	0.00759	8080	2.28E-05	7870	1.21E-05	7470	8.17E-06
9750	8.97E-06		9190	9.74E-06			
10100	0.00778	8080	2.46E-05	7880	1.21E-05	7470	8.19E-06
9750	8.93E-06		9190	9.73E-06			
10100	0.00796	8080	2.67E-05	7880	1.21E-05	7470	8.21E-06
9750	8.89E-06		9190	9.75E-06			
10100	0.00815	8080	2.90E-05	7880	1.22E-05	7470	8.22E-06
9750	8.85E-06		9200	9.75E-06			
10100	0.00833	8080	3.14E-05	7880	1.22E-05	7470	8.24E-06
9760	8.81E-06		9200	9.76E-06			
10100	0.00852	8080	3.40E-05	7880	1.22E-05	7480	8.26E-06
9760	8.79E-06		9200	9.75E-06			
10100	0.00871	8090	3.66E-05	7880	1.22E-05	7480	8.28E-06
9760	8.76E-06		9200	9.75E-06			
10100	0.0089	8090	3.92E-05	7880	1.22E-05	7480	8.31E-06
9760	8.73E-06		9200	9.76E-06			

10100	0.00909	8090	4.16E-05	7890	1.22E-05	7480	8.31E-06
9760	8.70E-06		9200	9.76E-06			
10100	0.00927	8090	4.37E-05	7890	1.23E-05	7480	8.32E-06
9760	8.67E-06		9200	9.76E-06			
10100	0.00946	8090	4.55E-05	7890	1.23E-05	7480	8.35E-06
9770	8.65E-06		9210	9.76E-06			
10100	0.00964	8090	4.66E-05	7890	1.23E-05	7480	8.38E-06
9770	8.62E-06		9210	9.76E-06			
10100	0.00983	8100	4.72E-05	7890	1.23E-05	7490	8.39E-06
9770	8.60E-06		9210	9.77E-06			
10100	0.01	8100	4.70E-05	7890	1.23E-05	7490	8.42E-06
9770	8.57E-06		9210	9.76E-06			
10100	0.0102	8100	4.63E-05	7900	1.24E-05	7490	8.44E-06
9770	8.54E-06		9210	9.76E-06			
10100	0.0104	8100	4.49E-05	7900	1.24E-05	7490	8.45E-06
9770	8.52E-06		9210	9.75E-06			
10100	0.0105	8100	4.31E-05	7900	1.24E-05	7490	8.49E-06
9780	8.49E-06		9220	9.76E-06			
10100	0.0107	8100	4.09E-05	7900	1.24E-05	7490	8.51E-06
9780	8.47E-06		9220	9.75E-06			
10100	0.0108	8100	3.86E-05	7900	1.24E-05	7500	8.52E-06
9780	8.45E-06		9220	9.76E-06			
10100	0.011	8110	3.62E-05	7900	1.24E-05	7500	8.54E-06
9780	8.43E-06		9220	9.76E-06			
10100	0.0111	8110	3.37E-05	7900	1.25E-05	7500	8.56E-06
9780	8.42E-06		9220	9.77E-06			
10100	0.0113	8110	3.14E-05	7910	1.25E-05	7500	8.61E-06
9780	8.40E-06		9220	9.78E-06			
10100	0.0114	8110	2.92E-05	7910	1.25E-05	7500	8.64E-06
9780	8.37E-06		9230	9.78E-06			
10100	0.0115	8110	2.71E-05	7910	1.25E-05	7500	8.68E-06
9790	8.36E-06		9230	9.78E-06			
10100	0.0116	8110	2.52E-05	7910	1.26E-05	7500	8.72E-06
9790	8.34E-06		9230	9.78E-06			
10100	0.0117	8120	2.35E-05	7910	1.26E-05	7510	8.76E-06
9790	8.32E-06		9230	9.77E-06			
10100	0.0118	8120	2.19E-05	7910	1.26E-05	7510	8.78E-06
9790	8.32E-06		9230	9.78E-06			
10100	0.0119	8120	2.04E-05	7920	1.26E-05	7510	8.81E-06
9790	8.30E-06		9230	9.79E-06			
10100	0.012	8120	1.91E-05	7920	1.26E-05	7510	8.86E-06
9790	8.29E-06		9230	9.79E-06			
10200	0.0121	8120	1.78E-05	7920	1.27E-05	7510	8.90E-06
9800	8.28E-06		9240	9.79E-06			
10200	0.0121	8120	1.67E-05	7920	1.27E-05	7510	8.94E-06
9800	8.27E-06		9240	9.79E-06			
10200	0.0122	8130	1.57E-05	7920	1.27E-05	7520	9.01E-06
9800	8.25E-06		9240	9.79E-06			
10200	0.0122	8130	1.48E-05	7920	1.27E-05	7520	9.04E-06
9800	8.24E-06		9240	9.79E-06			
10200	0.0122	8130	1.39E-05	7930	1.28E-05	7520	9.09E-06
9800	8.23E-06		9240	9.80E-06			
10200	0.0122	8130	1.31E-05	7930	1.28E-05	7520	9.15E-06
9800	8.21E-06		9240	9.79E-06			
10200	0.0122	8130	1.24E-05	7930	1.28E-05	7520	9.21E-06
9800	8.19E-06		9250	9.79E-06			
10200	0.0122	8130	1.18E-05	7930	1.28E-05	7520	9.27E-06
9810	8.18E-06		9250	9.78E-06			
10200	0.0122	8130	1.12E-05	7930	1.28E-05	7530	9.33E-06
9810	8.19E-06		9250	9.77E-06			
10200	0.0121	8140	1.07E-05	7930	1.28E-05	7530	9.40E-06
9810	8.17E-06		9250	9.78E-06			
10200	0.0121	8140	1.02E-05	7930	1.28E-05	7530	9.49E-06
9810	8.17E-06		9250	9.77E-06			
10200	0.012	8140	9.73E-06	7940	1.29E-05	7530	9.58E-06
9810	8.16E-06		9250	9.79E-06			
10200	0.012	8140	9.32E-06	7940	1.29E-05	7530	9.66E-06
9810	8.15E-06		9250	9.79E-06			

10200	0.0119	8140	8.95E-06	7940	1.29E-05	7530	9.77E-06	9820	8.14E-06	9260	9.80E-06
10200	0.0118	8140	8.61E-06	7940	1.29E-05	7530	9.87E-06	9820	8.13E-06	9260	9.79E-06
10200	0.0117	8150	8.32E-06	7940	1.29E-05	7540	9.98E-06	9820	8.12E-06	9260	9.78E-06
10200	0.0116	8150	8.03E-06	7940	1.30E-05	7540	1.01E-05	9820	8.12E-06	9260	9.79E-06
10200	0.0115	8150	7.77E-06	7950	1.30E-05	7540	1.02E-05	9820	8.11E-06	9260	9.79E-06
10200	0.0114	8150	7.54E-06	7950	1.30E-05	7540	1.04E-05	9820	8.10E-06	9260	9.80E-06
10200	0.0112	8150	7.32E-06	7950	1.30E-05	7540	1.05E-05	9830	8.10E-06	9270	9.80E-06
10200	0.0111	8150	7.11E-06	7950	1.31E-05	7540	1.07E-05	9830	8.10E-06	9270	9.79E-06
10200	0.011	8150	6.93E-06	7950	1.31E-05	7550	1.08E-05	9830	8.09E-06	9270	9.81E-06
10200	0.0108	8160	6.76E-06	7950	1.31E-05	7550	1.10E-05	9830	8.08E-06	9270	9.80E-06
10200	0.0107	8160	6.59E-06	7950	1.31E-05	7550	1.12E-05	9830	8.07E-06	9270	9.80E-06
10200	0.0105	8160	6.44E-06	7960	1.32E-05	7550	1.14E-05	9830	8.07E-06	9270	9.80E-06
10200	0.0103	8160	6.30E-06	7960	1.32E-05	7550	1.16E-05	9830	8.06E-06	9280	9.80E-06
10200	0.0102	8160	6.16E-06	7960	1.32E-05	7550	1.19E-05	9840	8.05E-06	9280	9.80E-06
10200	0.00999	8160	6.05E-06	7960	1.32E-05	7550	1.22E-05	9840	8.04E-06	9280	9.79E-06
10200	0.00982	8170	5.92E-06	7960	1.32E-05	7560	1.25E-05	9840	8.04E-06	9280	9.80E-06
10200	0.00964	8170	5.82E-06	7960	1.33E-05	7560	1.28E-05	9840	8.04E-06	9280	9.80E-06
10200	0.00946	8170	5.72E-06	7970	1.33E-05	7560	1.32E-05	9840	8.03E-06	9280	9.81E-06
10200	0.00928	8170	5.63E-06	7970	1.33E-05	7560	1.37E-05	9840	8.02E-06	9280	9.81E-06
10200	0.0091	8170	5.54E-06	7970	1.33E-05	7560	1.41E-05	9850	8.02E-06	9290	9.82E-06
10200	0.00892	8170	5.46E-06	7970	1.34E-05	7560	1.47E-05	9850	8.01E-06	9290	9.82E-06
10200	0.00874	8180	5.39E-06	7970	1.34E-05	7570	1.53E-05	9850	8.01E-06	9290	9.82E-06
10200	0.00856	8180	5.32E-06	7970	1.34E-05	7570	1.60E-05	9850	8.01E-06	9290	9.84E-06
10200	0.00838	8180	5.25E-06	7980	1.35E-05	7570	1.68E-05	9850	8.01E-06	9290	9.83E-06
10200	0.0082	8180	5.18E-06	7980	1.35E-05	7570	1.77E-05	9850	8.00E-06	9290	9.84E-06
10200	0.00802	8180	5.13E-06	7980	1.35E-05	7570	1.87E-05	9850	7.99E-06	9300	9.83E-06
10200	0.00784	8180	5.07E-06	7980	1.35E-05	7570	1.99E-05	9860	7.99E-06	9300	9.84E-06
10200	0.00767	8180	5.02E-06	7980	1.36E-05	7580	2.12E-05	9860	7.98E-06	9300	9.84E-06
10200	0.00749	8190	4.97E-06	7980	1.36E-05	7580	2.28E-05	9860	7.98E-06	9300	9.84E-06
10200	0.00732	8190	4.93E-06	7980	1.36E-05	7580	2.46E-05	9860	7.98E-06	9300	9.85E-06
10200	0.00715	8190	4.89E-06	7990	1.37E-05	7580	2.68E-05	9860	7.98E-06	9300	9.85E-06
10200	0.00698	8190	4.85E-06	7990	1.37E-05	7580	2.92E-05	9860	7.96E-06	9300	9.85E-06
10200	0.00682	8190	4.81E-06	7990	1.37E-05	7580	3.21E-05	9870	7.96E-06	9310	9.85E-06
10200	0.00666	8190	4.78E-06	7990	1.38E-05	7580	3.55E-05	9870	7.96E-06	9310	9.84E-06

10200	0.0065	8200	4.74E-06	7990	1.38E-05	7590	3.94E-05
9870	7.96E-06		9310	9.84E-06			
10200	0.00634	8200	4.70E-06	7990	1.38E-05	7590	4.38E-05
9870	7.96E-06		9310	9.84E-06			
10200	0.00619	8200	4.67E-06	8000	1.38E-05	7590	4.87E-05
9870	7.95E-06		9310	9.85E-06			
10200	0.00604	8200	4.65E-06	8000	1.39E-05	7590	5.40E-05
9870	7.95E-06		9310	9.85E-06			
10200	0.00589	8200	4.61E-06	8000	1.39E-05	7590	5.96E-05
9880	7.95E-06		9320	9.85E-06			
10200	0.00574	8200	4.59E-06	8000	1.39E-05	7590	6.53E-05
9880	7.95E-06		9320	9.85E-06			
10200	0.0056	8200	4.56E-06	8000	1.40E-05	7600	7.06E-05
9880	7.94E-06		9320	9.84E-06			
10200	0.00546	8210	4.54E-06	8000	1.40E-05	7600	7.51E-05
9880	7.94E-06		9320	9.86E-06			
10200	0.00533	8210	4.51E-06	8000	1.40E-05	7600	7.84E-05
9880	7.94E-06		9320	9.86E-06			
10200	0.0052	8210	4.49E-06	8010	1.41E-05	7600	8.00E-05
9880	7.94E-06		9320	9.87E-06			
10200	0.00507	8210	4.46E-06	8010	1.41E-05	7600	7.99E-05
9880	7.94E-06		9330	9.85E-06			
10200	0.00494	8210	4.46E-06	8010	1.42E-05	7600	7.79E-05
9890	7.94E-06		9330	9.85E-06			
10200	0.00482	8210	4.44E-06	8010	1.42E-05	7600	7.43E-05
9890	7.93E-06		9330	9.87E-06			
10200	0.0047	8220	4.42E-06	8010	1.43E-05	7610	6.96E-05
9890	7.94E-06		9330	9.86E-06			
10200	0.00459	8220	4.42E-06	8010	1.43E-05	7610	6.41E-05
9890	7.93E-06		9330	9.87E-06			
10200	0.00447	8220	4.41E-06	8020	1.44E-05	7610	5.83E-05
9890	7.92E-06		9330	9.86E-06			
10200	0.00436	8220	4.40E-06	8020	1.44E-05	7610	5.27E-05
9890	7.91E-06		9330	9.87E-06			
10300	0.00426	8220	4.40E-06	8020	1.44E-05	7610	4.73E-05
9900	7.92E-06		9340	9.87E-06			
10300	0.00415	8220	4.39E-06	8020	1.45E-05	7610	4.25E-05
9900	7.92E-06		9340	9.86E-06			
10300	0.00405	8230	4.37E-06	8020	1.46E-05	7620	3.82E-05
9900	7.92E-06		9340	9.87E-06			
10300	0.00395	8230	4.36E-06	8020	1.46E-05	7620	3.45E-05
9900	7.91E-06		9340	9.87E-06			
10300	0.00385	8230	4.37E-06	8030	1.46E-05	7620	3.12E-05
9900	7.91E-06		9340	9.88E-06			
10300	0.00376	8230	4.37E-06	8030	1.47E-05	7620	2.85E-05
9900	7.90E-06		9340	9.88E-06			
10300	0.00367	8230	4.36E-06	8030	1.48E-05	7620	2.61E-05
9900	7.89E-06		9350	9.88E-06			
10300	0.00358	8230	4.37E-06	8030	1.48E-05	7620	2.41E-05
9910	7.89E-06		9350	9.88E-06			
10300	0.0035	8230	4.38E-06	8030	1.49E-05	7630	2.23E-05
9910	7.89E-06		9350	9.87E-06			
10300	0.00341	8240	4.37E-06	8030	1.49E-05	7630	2.09E-05
9910	7.90E-06		9350	9.88E-06			
10300	0.00333	8240	4.38E-06	8030	1.50E-05	7630	1.96E-05
9910	7.90E-06		9350	9.88E-06			
10300	0.00325	8240	4.39E-06	8040	1.51E-05	7630	1.85E-05
9910	7.90E-06		9350	9.89E-06			
10300	0.00318	8240	4.38E-06	8040	1.51E-05	7630	1.75E-05
9910	7.90E-06		9350	9.88E-06			
10300	0.0031	8240	4.38E-06	8040	1.52E-05	7630	1.67E-05
9920	7.90E-06		9360	9.88E-06			
10300	0.00303	8240	4.40E-06	8040	1.52E-05	7630	1.59E-05
9920	7.90E-06		9360	9.90E-06			
10300	0.00296	8250	4.41E-06	8040	1.53E-05	7640	1.53E-05
9920	7.90E-06		9360	9.90E-06			
10300	0.00289	8250	4.44E-06	8040	1.54E-05	7640	1.47E-05
9920	7.89E-06		9360	9.90E-06			

10300	0.00282	8250	4.45E-06	8050	1.55E-05	7640	1.42E-05
9920	7.89E-06		9360	9.90E-06			
10300	0.00276	8250	4.47E-06	8050	1.55E-05	7640	1.38E-05
9920	7.88E-06		9360	9.90E-06			
10300	0.0027	8250	4.49E-06	8050	1.56E-05	7640	1.33E-05
9930	7.89E-06		9370	9.91E-06			
10300	0.00264	8250	4.51E-06	8050	1.57E-05	7640	1.30E-05
9930	7.89E-06		9370	9.89E-06			
10300	0.00258	8250	4.53E-06	8050	1.57E-05	7650	1.27E-05
9930	7.89E-06		9370	9.90E-06			
10300	0.00252	8260	4.55E-06	8050	1.58E-05	7650	1.24E-05
9930	7.89E-06		9370	9.91E-06			
10300	0.00247	8260	4.57E-06	8050	1.59E-05	7650	1.21E-05
9930	7.89E-06		9370	9.91E-06			
10300	0.00241	8260	4.61E-06	8060	1.60E-05	7650	1.18E-05
9930	7.88E-06		9370	9.92E-06			
10300	0.00236	8260	4.64E-06	8060	1.61E-05	7650	1.16E-05
9930	7.88E-06		9380	9.91E-06			
10300	0.00231	8260	4.67E-06	8060	1.62E-05	7650	1.14E-05
9940	7.88E-06		9380	9.91E-06			
10300	0.00226	8260	4.71E-06	8060	1.63E-05	7650	1.13E-05
9940	7.88E-06		9380	9.91E-06			
10300	0.00221	8270	4.77E-06	8060	1.63E-05	7660	1.11E-05
9940	7.89E-06		9380	9.92E-06			
10300	0.00216	8270	4.81E-06	8060	1.65E-05	7660	1.09E-05
9940	7.88E-06		9380	9.92E-06			
10300	0.00212	8270	4.86E-06	8070	1.65E-05	7660	1.08E-05
9940	7.89E-06		9380	9.93E-06			
10300	0.00207	8270	4.92E-06	8070	1.66E-05	7660	1.07E-05
9940	7.89E-06		9380	9.92E-06			
10300	0.00203	8270	4.98E-06	8070	1.67E-05	7660	1.06E-05
9950	7.89E-06		9390	9.92E-06			
10300	0.00199	8270	5.06E-06	8070	1.69E-05	7660	1.04E-05
9950	7.89E-06		9390	9.93E-06			
10300	0.00195	8280	5.14E-06	8070	1.70E-05	7670	1.03E-05
9950	7.89E-06		9390	9.94E-06			
10300	0.00191	8280	5.25E-06	8070	1.71E-05	7670	1.02E-05
9950	7.88E-06		9390	9.94E-06			
10300	0.00187	8280	5.32E-06	8080	1.72E-05	7670	1.02E-05
9950	7.89E-06		9390	9.94E-06			
10300	0.00183	8280	5.45E-06	8080	1.73E-05	7670	1.01E-05
9950	7.88E-06		9390	9.94E-06			
10300	0.00179	8280	5.57E-06	8080	1.75E-05	7670	1.00E-05
9950	7.88E-06		9400	9.94E-06			
10300	0.00176	8280	5.70E-06	8080	1.76E-05	7670	9.93E-06
9960	7.88E-06		9400	9.93E-06			
10300	0.00172	8280	5.85E-06	8080	1.77E-05	7680	9.86E-06
9960	7.88E-06		9400	9.95E-06			
10300	0.00169	8290	6.02E-06	8080	1.78E-05	7680	9.79E-06
9960	7.89E-06		9400	9.94E-06			
10300	0.00166	8290	6.21E-06	8080	1.80E-05	7680	9.74E-06
9960	7.89E-06		9400	9.95E-06			
10300	0.00163	8290	6.45E-06	8090	1.81E-05	7680	9.69E-06
9960	7.89E-06		9400	9.95E-06			
10300	0.00159	8290	6.68E-06	8090	1.83E-05	7680	9.65E-06
9960	7.88E-06		9400	9.96E-06			
10300	0.00156	8290	6.98E-06	8090	1.84E-05	7680	9.59E-06
9970	7.89E-06		9410	9.96E-06			
10300	0.00153	8290	7.29E-06	8090	1.86E-05	7680	9.55E-06
9970	7.89E-06		9410	9.96E-06			
10300	0.00151	8300	7.67E-06	8090	1.88E-05	7690	9.51E-06
9970	7.89E-06		9410	9.97E-06			
10300	0.00148	8300	8.10E-06	8090	1.89E-05	7690	9.47E-06
9970	7.89E-06		9410	9.97E-06			
10300	0.00145	8300	8.60E-06	8100	1.91E-05	7690	9.43E-06
9970	7.90E-06		9410	9.98E-06			
10300	0.00142	8300	9.18E-06	8100	1.93E-05	7690	9.39E-06
9970	7.89E-06		9410	9.97E-06			

10300 0.0014 8300 9.86E-06 8100 1.95E-05 7690 9.36E-06
9980 7.89E-06 9420 9.97E-06
10300 0.00137 8300 1.07E-05 8100 1.97E-05 7690 9.32E-06
9980 7.89E-06 9420 9.97E-06
10300 0.00135 8300 1.16E-05 8100 1.99E-05 7700 9.30E-06
9980 7.89E-06 9420 9.98E-06
10300 0.00132 8310 1.27E-05 8100 2.01E-05 7700 9.27E-06
9980 7.88E-06 9420 9.98E-06
10300 0.0013 8310 1.40E-05 8100 2.03E-05 7700 9.25E-06
9980 7.89E-06 9420 9.99E-06
10300 0.00128 8310 1.56E-05 8110 2.06E-05 7700 9.22E-06
9980 7.89E-06 9420 9.98E-06
10300 0.00126 8310 1.73E-05 8110 2.08E-05 7700 9.18E-06
9980 7.89E-06 9430 9.98E-06
10300 0.00123 8310 1.93E-05 8110 2.11E-05 7700 9.17E-06
9990 7.89E-06 9430 9.98E-06
10300 0.00121 8310 2.15E-05 8110 2.13E-05 7700 9.15E-06
9990 7.90E-06 9430 9.99E-06
10300 0.00119 8320 2.37E-05 8110 2.16E-05 7710 9.13E-06
9990 7.91E-06 9430 9.98E-06
10300 0.00117 8320 2.57E-05 8110 2.18E-05 7710 9.12E-06
9990 7.91E-06 9430 9.99E-06
10300 0.00115 8320 2.73E-05 8120 2.22E-05 7710 9.11E-06
9990 7.91E-06 9430 1.00E-05
10300 0.00113 8320 2.83E-05 8120 2.24E-05 7710 9.09E-06
9990 7.90E-06 9430 1.00E-05
10400 0.00112 8320 2.84E-05 8120 2.28E-05 7710 9.09E-06
10000 7.90E-06 9440 1.00E-05
10400 0.0011 8320 2.77E-05 8120 2.31E-05 7710 9.08E-06
10000 7.91E-06 9440 1.00E-05
10400 0.00108 8330 2.63E-05 8120 2.34E-05 7720 9.07E-06
10000 7.91E-06 9440 1.00E-05
10400 0.00106 8330 2.43E-05 8120 2.38E-05 7720 9.06E-06
10000 7.91E-06 9440 1.00E-05
10400 0.00104 8330 2.21E-05 8130 2.42E-05 7720 9.06E-06
10000 7.92E-06 9440 1.00E-05
10400 0.00103 8330 1.99E-05 8130 2.46E-05 7720 9.04E-06
10000 7.91E-06 9440 1.00E-05
10400 0.00101 8330 1.78E-05 8130 2.50E-05 7720 9.04E-06
10000 7.92E-06 9450 1.00E-05
10400 0.000996 8330 1.60E-05 8130 2.55E-05 7720
9.02E-06 10000 7.92E-06 9450 1.00E-05
10400 0.00098 8330 1.44E-05 8130 2.59E-05 7730 9.00E-06
10000 7.92E-06 9450 1.00E-05
10400 0.000965 8340 1.30E-05 8130 2.64E-05 7730
9.01E-06 10000 7.92E-06 9450 1.00E-05
10400 0.00095 8340 1.18E-05 8130 2.69E-05 7730 8.99E-06
10000 7.91E-06 9450 1.00E-05
10400 0.000936 8340 1.08E-05 8140 2.74E-05 7730
8.99E-06 10000 7.92E-06 9450 1.00E-05
10400 0.000922 8340 9.96E-06 8140 2.80E-05 7730
8.99E-06 10000 7.91E-06 9450 1.00E-05
10400 0.000908 8340 9.23E-06 8140 2.86E-05 7730
8.99E-06 10000 7.92E-06 9460 1.00E-05
10400 0.000894 8340 8.62E-06 8140 2.92E-05 7730
8.97E-06 10000 7.92E-06 9460 1.00E-05
10400 0.000881 8350 8.08E-06 8140 2.98E-05 7740
8.97E-06 10000 7.93E-06 9460 1.00E-05
10400 0.000868 8350 7.63E-06 8140 3.05E-05 7740
8.96E-06 10000 7.92E-06 9460 1.00E-05
10400 0.000855 8350 7.25E-06 8150 3.13E-05 7740
8.96E-06 10000 7.92E-06 9460 1.00E-05
10400 0.000843 8350 6.91E-06 8150 3.20E-05 7740
8.94E-06 10000 7.92E-06 9460 1.00E-05
10400 0.000831 8350 6.61E-06 8150 3.28E-05 7740
8.96E-06 10000 7.91E-06 9470 1.00E-05
10400 0.000819 8350 6.35E-06 8150 3.37E-05 7740
8.96E-06 10000 7.92E-06 9470 1.00E-05

10400 0.000807 8350 6.13E-06 8150 3.46E-05 7750
8.95E-06 10000 7.92E-06 9470 1.00E-05
10400 0.000796 8360 5.92E-06 8150 3.55E-05 7750
8.96E-06 10000 7.92E-06 9470 1.00E-05
10400 0.000785 8360 5.75E-06 8150 3.65E-05 7750
8.95E-06 10000 7.93E-06 9470 1.00E-05
10400 0.000774 8360 5.59E-06 8160 3.76E-05 7750
8.94E-06 10000 7.93E-06 9470 1.00E-05
10400 0.000763 8360 5.43E-06 8160 3.88E-05 7750
8.95E-06 10000 7.93E-06 9480 1.00E-05
10400 0.000753 8360 5.31E-06 8160 4.00E-05 7750
8.94E-06 10000 7.93E-06 9480 1.00E-05
10400 0.000743 8360 5.19E-06 8160 4.12E-05 7750
8.92E-06 10000 7.93E-06 9480 1.00E-05
10400 0.000732 8370 5.09E-06 8160 4.26E-05 7760
8.93E-06 10000 7.93E-06 9480 1.00E-05
10400 0.000723 8370 4.99E-06 8160 4.40E-05 7760
8.93E-06 10000 7.92E-06 9480 1.00E-05
10400 0.000713 8370 4.91E-06 8170 4.56E-05 7760
8.94E-06 10000 7.93E-06 9480 1.00E-05
10400 0.000704 8370 4.82E-06 8170 4.72E-05 7760
8.93E-06 10000 7.92E-06 9480 1.00E-05
10400 0.000694 8370 4.75E-06 8170 4.89E-05 7760
8.93E-06 10000 7.93E-06 9490 1.00E-05
10400 0.000685 8370 4.68E-06 8170 5.07E-05 7760
8.93E-06 10000 7.93E-06 9490 1.01E-05
10400 0.000676 8380 4.62E-06 8170 5.27E-05 7770
8.93E-06 10000 7.94E-06 9490 1.01E-05
10400 0.000668 8380 4.56E-06 8170 5.47E-05 7770
8.93E-06 10100 7.94E-06 9490 1.01E-05
10400 0.000659 8380 4.52E-06 8180 5.69E-05 7770
8.93E-06 10100 7.94E-06 9490 1.01E-05
10400 0.000651 8380 4.47E-06 8180 5.92E-05 7770
8.93E-06 10100 7.93E-06 9490 1.01E-05
10400 0.000642 8380 4.44E-06 8180 6.17E-05 7770
8.93E-06 10100 7.93E-06 9500 1.01E-05
10400 0.000634 8380 4.40E-06 8180 6.43E-05 7770
8.94E-06 10100 7.94E-06 9500 1.01E-05
10400 0.000626 8380 4.37E-06 8180 6.70E-05 7780
8.95E-06 10100 7.93E-06 9500 1.01E-05
10400 0.000619 8390 4.33E-06 8180 6.98E-05 7780
8.95E-06 10100 7.94E-06 9500 1.01E-05
10400 0.000611 8390 4.30E-06 8180 7.28E-05 7780
8.95E-06 10100 7.95E-06 9500 1.01E-05
10400 0.000603 8390 4.27E-06 8190 7.60E-05 7780
8.96E-06 10100 7.94E-06 9500 1.01E-05
10400 0.000596 8390 4.24E-06 8190 7.92E-05 7780
8.96E-06 10100 7.94E-06 9500 1.01E-05
10400 0.000589 8390 4.21E-06 8190 8.26E-05 7780
8.95E-06 10100 7.94E-06 9510 1.01E-05
10400 0.000582 8390 4.20E-06 8190 8.61E-05 7780
8.96E-06 10100 7.94E-06 9510 1.01E-05
10400 0.000575 8400 4.18E-06 8190 8.96E-05 7790
8.95E-06 10100 7.94E-06 9510 1.01E-05
10400 0.000568 8400 4.15E-06 8190 9.32E-05 7790
8.95E-06 10100 7.93E-06 9510 1.01E-05
10400 0.000561 8400 4.14E-06 8200 9.68E-05 7790
8.96E-06 10100 7.94E-06 9510 1.01E-05
10400 0.000555 8400 4.12E-06 8200 1.00E-04 7790
8.96E-06 10100 7.94E-06 9510 1.01E-05
10400 0.000548 8400 4.11E-06 8200 1.04E-04 7790
8.97E-06 10100 7.94E-06 9520 1.01E-05
10400 0.000542 8400 4.10E-06 8200 1.07E-04 7790
8.97E-06 10100 7.94E-06 9520 1.01E-05
10400 0.000536 8400 4.08E-06 8200 1.11E-04 7800
8.98E-06 10100 7.95E-06 9520 1.01E-05
10400 0.00053 8410 4.06E-06 8200 1.14E-04 7800 8.99E-06
10100 7.94E-06 9520 1.01E-05

10400 0.000524 8410 4.05E-06 8200 1.16E-04 7800
8.98E-06 10100 7.94E-06 9520 1.01E-05
10400 0.000518 8410 4.03E-06 8210 1.19E-04 7800
8.99E-06 10100 7.93E-06 9520 1.01E-05
10400 0.000512 8410 4.02E-06 8210 1.21E-04 7800
8.99E-06 10100 7.94E-06 9530 1.01E-05
10400 0.000506 8410 4.01E-06 8210 1.23E-04 7800
9.00E-06 10100 7.93E-06 9530 1.01E-05
10400 0.000501 8410 4.01E-06 8210 1.24E-04 7800
9.00E-06 10100 7.94E-06 9530 1.01E-05
10400 0.000495 8420 4.00E-06 8210 1.24E-04 7810
9.00E-06 10100 7.94E-06 9530 1.01E-05
10400 0.00049 8420 3.98E-06 8210 1.25E-04 7810 9.01E-06
10100 7.95E-06 9530 1.01E-05
10400 0.000484 8420 3.98E-06 8220 1.24E-04 7810
9.01E-06 10100 7.95E-06 9530 1.01E-05
10400 0.000479 8420 3.97E-06 8220 1.23E-04 7810
9.04E-06 10100 7.95E-06 9530 1.01E-05
10500 0.000474 8420 3.96E-06 8220 1.22E-04 7810
9.03E-06 10100 7.95E-06 9540 1.01E-05
10500 0.000469 8420 3.96E-06 8220 1.20E-04 7810
9.03E-06 10100 7.95E-06 9540 1.01E-05
10500 0.000464 8430 3.95E-06 8220 1.18E-04 7820
9.03E-06 10100 7.94E-06 9540 1.01E-05
10500 0.000459 8430 3.94E-06 8220 1.15E-04 7820
9.03E-06 10100 7.94E-06 9540 1.01E-05
10500 0.000454 8430 3.94E-06 8230 1.12E-04 7820
9.05E-06 10100 7.94E-06 9540 1.01E-05
10500 0.000449 8430 3.94E-06 8230 1.09E-04 7820
9.06E-06 10100 7.94E-06 9540 1.01E-05
10500 0.000445 8430 3.93E-06 8230 1.06E-04 7820
9.07E-06 10100 7.94E-06 9550 1.01E-05
10500 0.00044 8430 3.93E-06 8230 1.02E-04 7820 9.08E-06
10100 7.94E-06 9550 1.01E-05
10500 0.000436 8430 3.92E-06 8230 9.88E-05 7830
9.07E-06 10100 7.94E-06 9550 1.01E-05
10500 0.000431 8440 3.91E-06 8230 9.52E-05 7830
9.10E-06 10100 7.94E-06 9550 1.01E-05
10500 0.000427 8440 3.91E-06 8230 9.16E-05 7830
9.11E-06 10100 7.95E-06 9550 1.01E-05
10500 0.000422 8440 3.91E-06 8240 8.80E-05 7830
9.12E-06 10100 7.94E-06 9550 1.01E-05
10500 0.000418 8440 3.90E-06 8240 8.45E-05 7830
9.14E-06 10100 7.94E-06 9550 1.01E-05
10500 0.000414 8440 3.90E-06 8240 8.12E-05 7830
9.16E-06 10100 7.94E-06 9560 1.01E-05
10500 0.00041 8440 3.90E-06 8240 7.79E-05 7830 9.16E-06
10100 7.94E-06 9560 1.01E-05
10500 0.000406 8450 3.89E-06 8240 7.47E-05 7840
9.17E-06 10100 7.94E-06 9560 1.01E-05
10500 0.000402 8450 3.89E-06 8240 7.16E-05 7840
9.18E-06 10100 7.94E-06 9560 1.01E-05
10500 0.000398 8450 3.89E-06 8250 6.87E-05 7840
9.19E-06 10100 7.94E-06 9560 1.01E-05
10500 0.000394 8450 3.89E-06 8250 6.59E-05 7840
9.20E-06 10100 7.95E-06 9560 1.01E-05
10500 0.00039 8450 3.88E-06 8250 6.33E-05 7840 9.20E-06
10100 7.95E-06 9570 1.01E-05
10500 0.000387 8450 3.88E-06 8250 6.08E-05 7840
9.21E-06 10100 7.95E-06 9570 1.01E-05
10500 0.000383 8450 3.88E-06 8250 5.84E-05 7850
9.21E-06 10100 7.95E-06 9570 1.01E-05
10500 0.000379 8460 3.87E-06 8250 5.62E-05 7850
9.23E-06 10100 7.95E-06 9570 1.01E-05
10500 0.000376 8460 3.88E-06 8250 5.40E-05 7850
9.25E-06 10100 7.95E-06 9570 1.01E-05
10500 0.000372 8460 3.88E-06 8260 5.21E-05 7850
9.26E-06 10100 7.95E-06 9570 1.01E-05

10500 0.000369 8460 3.88E-06 8260 5.02E-05 7850
9.27E-06 10100 7.96E-06 9580 1.01E-05
10500 0.000365 8460 3.87E-06 8260 4.84E-05 7850
9.29E-06 10100 7.97E-06 9580 1.01E-05
10500 0.000362 8460 3.87E-06 8260 4.67E-05 7850
9.30E-06 10100 7.97E-06 9580 1.01E-05
10500 0.000359 8470 3.87E-06 8260 4.51E-05 7860
9.30E-06 10100 7.97E-06 9580 1.01E-05
10500 0.000355 8470 3.87E-06 8260 4.37E-05 7860
9.30E-06 10100 7.97E-06 9580 1.01E-05
10500 0.000352 8470 3.86E-06 8270 4.23E-05 7860
9.32E-06 10100 7.96E-06 9580 1.02E-05
10500 0.000349 8470 3.86E-06 8270 4.10E-05 7860
9.33E-06 10100 7.97E-06 9580 1.01E-05
10500 0.000346 8470 3.86E-06 8270 3.97E-05 7860
9.34E-06 10100 7.97E-06 9590 1.02E-05
10500 0.000343 8470 3.86E-06 8270 3.86E-05 7860
9.34E-06 10100 7.97E-06 9590 1.02E-05
10500 0.00034 8480 3.86E-06 8270 3.75E-05 7870 9.34E-06
10100 7.98E-06 9590 1.02E-05
10500 0.000337 8480 3.85E-06 8270 3.65E-05 7870
9.34E-06 10200 7.99E-06 9590 1.02E-05
10500 0.000334 8480 3.86E-06 8280 3.55E-05 7870
9.35E-06 10200 7.98E-06 9590 1.02E-05
10500 0.000331 8480 3.86E-06 8280 3.47E-05 7870
9.35E-06 10200 7.99E-06 9590 1.02E-05
10500 0.000328 8480 3.85E-06 8280 3.38E-05 7870
9.35E-06 10200 7.99E-06 9600 1.02E-05
10500 0.000325 8480 3.86E-06 8280 3.29E-05 7870
9.35E-06 10200 7.99E-06 9600 1.02E-05
10500 0.000322 8480 3.86E-06 8280 3.22E-05 7880
9.35E-06 10200 7.99E-06 9600 1.02E-05
10500 0.000319 8490 3.85E-06 8280 3.14E-05 7880
9.36E-06 10200 7.99E-06 9600 1.02E-05
10500 0.000317 8490 3.85E-06 8280 3.08E-05 7880
9.37E-06 10200 7.98E-06 9600 1.02E-05
10500 0.000314 8490 3.86E-06 8290 3.02E-05 7880
9.38E-06 10200 7.99E-06 9600 1.02E-05
10500 0.000311 8490 3.86E-06 8290 2.95E-05 7880
9.37E-06 10200 7.99E-06 9600 1.02E-05
10500 0.000309 8490 3.86E-06 8290 2.90E-05 7880
9.38E-06 10200 8.00E-06 9610 1.02E-05
10500 0.000306 8490 3.85E-06 8290 2.84E-05 7880
9.38E-06 10200 8.01E-06 9610 1.02E-05
10500 0.000304 8500 3.86E-06 8290 2.79E-05 7890
9.39E-06 10200 8.01E-06 9610 1.02E-05
10500 0.000301 8500 3.86E-06 8290 2.74E-05 7890
9.39E-06 10200 7.99E-06 9610 1.02E-05
10500 0.000299 8500 3.86E-06 8300 2.69E-05 7890
9.40E-06 10200 7.99E-06 9610 1.02E-05
10500 0.000296 8500 3.86E-06 8300 2.65E-05 7890
9.42E-06 10200 8.00E-06 9610 1.02E-05
10500 0.000294 8500 3.86E-06 8300 2.61E-05 7890
9.43E-06 10200 8.00E-06 9620 1.02E-05
10500 0.000292 8500 3.87E-06 8300 2.57E-05 7890
9.44E-06 10200 8.00E-06 9620 1.02E-05
10500 0.000289 8500 3.86E-06 8300 2.52E-05 7900
9.45E-06 10200 8.00E-06 9620 1.02E-05
10500 0.000287 8510 3.86E-06 8300 2.49E-05 7900
9.46E-06 10200 8.01E-06 9620 1.02E-05
10500 0.000285 8510 3.87E-06 8300 2.45E-05 7900
9.47E-06 10200 8.00E-06 9620 1.02E-05
10500 0.000282 8510 3.87E-06 8310 2.42E-05 7900
9.48E-06 10200 8.00E-06 9620 1.02E-05
10500 0.00028 8510 3.87E-06 8310 2.38E-05 7900 9.48E-06
10200 8.00E-06 9630 1.02E-05
10500 0.000278 8510 3.87E-06 8310 2.35E-05 7900
9.48E-06 10200 8.00E-06 9630 1.02E-05

10500 0.000276 8510 3.87E-06 8310 2.32E-05 7900
9.49E-06 10200 8.00E-06 9630 1.02E-05
10500 0.000274 8520 3.88E-06 8310 2.29E-05 7910
9.50E-06 10200 8.00E-06 9630 1.02E-05
10500 0.000271 8520 3.88E-06 8310 2.27E-05 7910
9.51E-06 10200 8.00E-06 9630 1.02E-05
10500 0.000269 8520 3.88E-06 8320 2.24E-05 7910
9.52E-06 10200 8.00E-06 9630 1.02E-05
10500 0.000267 8520 3.87E-06 8320 2.22E-05 7910
9.53E-06 10200 8.00E-06 9630 1.02E-05
10600 0.000265 8520 3.88E-06 8320 2.20E-05 7910
9.53E-06 10200 7.99E-06 9640 1.02E-05
10600 0.000263 8520 3.88E-06 8320 2.17E-05 7910
9.54E-06 10200 8.01E-06 9640 1.02E-05
10600 0.000261 8530 3.88E-06 8320 2.15E-05 7920
9.56E-06 10200 8.01E-06 9640 1.02E-05
10600 0.000259 8530 3.87E-06 8320 2.13E-05 7920
9.56E-06 10200 8.00E-06 9640 1.02E-05
10600 0.000257 8530 3.88E-06 8330 2.11E-05 7920
9.58E-06 10200 8.00E-06 9640 1.02E-05
10600 0.000255 8530 3.88E-06 8330 2.09E-05 7920
9.58E-06 10200 8.00E-06 9640 1.02E-05
10600 0.000254 8530 3.88E-06 8330 2.07E-05 7920
9.62E-06 10200 8.00E-06 9650 1.02E-05
10600 0.000252 8530 3.89E-06 8330 2.06E-05 7920
9.62E-06 10200 8.01E-06 9650 1.02E-05
10600 0.00025 8530 3.89E-06 8330 2.04E-05 7930 9.63E-06
10200 8.00E-06 9650 1.02E-05
10600 0.000248 8540 3.89E-06 8330 2.02E-05 7930
9.65E-06 10200 8.00E-06 9650 1.02E-05
10600 0.000246 8540 3.90E-06 8330 2.01E-05 7930
9.66E-06 10200 7.99E-06 9650 1.02E-05
10600 0.000244 8540 3.89E-06 8340 1.99E-05 7930
9.67E-06 10200 8.00E-06 9650 1.02E-05
10600 0.000243 8540 3.90E-06 8340 1.98E-05 7930
9.68E-06 10200 8.00E-06 9650 1.02E-05
10600 0.000241 8540 3.90E-06 8340 1.96E-05 7930
9.67E-06 10200 8.00E-06 9660 1.02E-05
10600 0.000239 8540 3.89E-06 8340 1.95E-05 7930
9.68E-06 10200 7.99E-06 9660 1.02E-05
10600 0.000237 8550 3.90E-06 8340 1.94E-05 7940
9.68E-06 10200 7.99E-06 9660 1.02E-05
10600 0.000236 8550 3.90E-06 8340 1.93E-05 7940
9.70E-06 10200 7.99E-06 9660 1.02E-05
10600 0.000234 8550 3.91E-06 8350 1.92E-05 7940
9.71E-06 10200 7.99E-06 9660 1.02E-05
10600 0.000232 8550 3.91E-06 8350 1.90E-05 7940
9.72E-06 10200 7.99E-06 9660 1.02E-05
10600 0.000231 8550 3.91E-06 8350 1.89E-05 7940
9.74E-06 10200 7.99E-06 9670 1.02E-05
10600 0.000229 8550 3.91E-06 8350 1.88E-05 7940
9.74E-06 10200 8.00E-06 9670 1.03E-05
10600 0.000228 8550 3.91E-06 8350 1.87E-05 7950
9.76E-06 10200 7.99E-06 9670 1.03E-05
10600 0.000226 8560 3.91E-06 8350 1.86E-05 7950
9.77E-06 10200 7.98E-06 9670 1.03E-05
10600 0.000225 8560 3.92E-06 8350 1.86E-05 7950
9.78E-06 10200 7.98E-06 9670 1.03E-05
10600 0.000223 8560 3.92E-06 8360 1.85E-05 7950
9.79E-06 10200 7.98E-06 9670 1.03E-05
10600 0.000222 8560 3.92E-06 8360 1.84E-05 7950
9.81E-06 10200 7.98E-06 9680 1.03E-05
10600 0.00022 8560 3.93E-06 8360 1.83E-05 7950 9.81E-06
10200 7.98E-06 9680 1.03E-05
10600 0.000219 8560 3.92E-06 8360 1.82E-05 7950
9.82E-06 10200 7.98E-06 9680 1.03E-05
10600 0.000217 8570 3.93E-06 8360 1.81E-05 7960
9.83E-06 10200 7.98E-06 9680 1.03E-05

10600 0.000216 8570 3.93E-06 8360 1.81E-05 7960
9.84E-06 10200 7.98E-06 9680 1.03E-05
10600 0.000214 8570 3.93E-06 8370 1.80E-05 7960
9.86E-06 10200 7.98E-06 9680 1.03E-05
10600 0.000213 8570 3.93E-06 8370 1.79E-05 7960
9.86E-06 10200 7.98E-06 9680 1.03E-05
10600 0.000211 8570 3.94E-06 8370 1.78E-05 7960
9.87E-06 10200 7.98E-06 9690 1.03E-05
10600 0.00021 8570 3.94E-06 8370 1.78E-05 7960 9.87E-06
10200 7.97E-06 9690 1.03E-05
10600 0.000209 8580 3.94E-06 8370 1.77E-05 7970
9.88E-06 10200 7.97E-06 9690 1.03E-05
10600 0.000207 8580 3.94E-06 8370 1.76E-05 7970
9.89E-06 10300 7.97E-06 9690 1.03E-05
10600 0.000206 8580 3.94E-06 8380 1.76E-05 7970
9.91E-06 10300 7.97E-06 9690 1.03E-05
10600 0.000205 8580 3.94E-06 8380 1.75E-05 7970
9.92E-06 10300 7.97E-06 9690 1.03E-05
10600 0.000203 8580 3.94E-06 8380 1.75E-05 7970
9.93E-06 10300 7.97E-06 9700 1.03E-05
10600 0.000202 8580 3.94E-06 8380 1.74E-05 7970
9.93E-06 10300 7.98E-06 9700 1.03E-05
10600 0.000201 8580 3.95E-06 8380 1.74E-05 7980
9.96E-06 10300 7.97E-06 9700 1.03E-05
10600 0.0002 8590 3.96E-06 8380 1.73E-05 7980 9.97E-06
10300 7.97E-06 9700 1.03E-05
10600 0.000198 8590 3.96E-06 8380 1.73E-05 7980
9.97E-06 10300 7.97E-06 9700 1.03E-05
10600 0.000197 8590 3.96E-06 8390 1.72E-05 7980
9.99E-06 10300 7.97E-06 9700 1.03E-05
10600 0.000196 8590 3.96E-06 8390 1.72E-05 7980
1.00E-05 10300 7.97E-06 9700 1.03E-05
10600 0.000195 8590 3.96E-06 8390 1.71E-05 7980
1.00E-05 10300 7.97E-06 9710 1.03E-05
10600 0.000193 8590 3.96E-06 8390 1.71E-05 7980
1.00E-05 10300 7.96E-06 9710 1.03E-05
10600 0.000192 8600 3.97E-06 8390 1.70E-05 7990
1.00E-05 10300 7.97E-06 9710 1.03E-05
10600 0.000191 8600 3.97E-06 8390 1.70E-05 7990
1.01E-05 10300 7.97E-06 9710 1.03E-05
10600 0.00019 8600 3.97E-06 8400 1.70E-05 7990 1.01E-05
10300 7.98E-06 9710 1.03E-05
10600 0.000189 8600 3.98E-06 8400 1.69E-05 7990
1.01E-05 10300 7.97E-06 9710 1.03E-05
10600 0.000188 8600 3.98E-06 8400 1.69E-05 7990
1.01E-05 10300 7.97E-06 9720 1.03E-05
10600 0.000187 8600 3.98E-06 8400 1.69E-05 7990
1.01E-05 10300 7.96E-06 9720 1.03E-05
10600 0.000185 8600 3.98E-06 8400 1.68E-05 8000
1.01E-05 10300 7.97E-06 9720 1.03E-05
10600 0.000184 8610 3.99E-06 8400 1.68E-05 8000
1.01E-05 10300 7.97E-06 9720 1.03E-05
10600 0.000183 8610 3.99E-06 8400 1.68E-05 8000
1.01E-05 10300 7.96E-06 9720 1.03E-05
10600 0.000182 8610 3.99E-06 8410 1.67E-05 8000
1.01E-05 10300 7.97E-06 9720 1.03E-05
10600 0.000181 8610 3.99E-06 8410 1.67E-05 8000
1.01E-05 10300 7.96E-06 9730 1.03E-05
10600 0.00018 8610 4.01E-06 8410 1.67E-05 8000 1.02E-05
10300 7.97E-06 9730 1.03E-05
10600 0.000179 8610 4.01E-06 8410 1.66E-05 8000
1.02E-05 10300 7.97E-06 9730 1.03E-05
10600 0.000178 8620 4.01E-06 8410 1.66E-05 8010
1.02E-05 10300 7.96E-06 9730 1.03E-05
10600 0.000177 8620 4.02E-06 8410 1.66E-05 8010
1.02E-05 10300 7.96E-06 9730 1.03E-05
10600 0.000176 8620 4.01E-06 8420 1.65E-05 8010
1.02E-05 10300 7.95E-06 9730 1.03E-05

10600 0.000175 8620 4.02E-06 8420 1.65E-05 8010
1.02E-05 10300 7.96E-06 9730 1.03E-05
10700 0.000174 8620 4.02E-06 8420 1.65E-05 8010
1.02E-05 10300 7.96E-06 9740 1.03E-05
10700 0.000173 8620 4.03E-06 8420 1.65E-05 8010
1.02E-05 10300 7.95E-06 9740 1.03E-05
10700 0.000172 8630 4.03E-06 8420 1.64E-05 8020
1.03E-05 10300 7.96E-06 9740 1.03E-05
10700 0.000171 8630 4.03E-06 8420 1.64E-05 8020
1.03E-05 10300 7.96E-06 9740 1.04E-05
10700 0.00017 8630 4.03E-06 8430 1.64E-05 8020 1.03E-05
10300 7.97E-06 9740 1.04E-05
10700 0.000169 8630 4.03E-06 8430 1.64E-05 8020
1.03E-05 10300 7.97E-06 9740 1.04E-05
10700 0.000168 8630 4.04E-06 8430 1.64E-05 8020
1.03E-05 10300 7.97E-06 9750 1.04E-05
10700 0.000167 8630 4.04E-06 8430 1.63E-05 8020
1.03E-05 10300 7.96E-06 9750 1.04E-05
10700 0.000166 8630 4.04E-06 8430 1.63E-05 8030
1.03E-05 10300 7.97E-06 9750 1.04E-05
10700 0.000165 8640 4.04E-06 8430 1.63E-05 8030
1.03E-05 10300 7.97E-06 9750 1.04E-05
10700 0.000164 8640 4.05E-06 8430 1.63E-05 8030
1.04E-05 10300 7.97E-06 9750 1.04E-05
10700 0.000164 8640 4.05E-06 8440 1.63E-05 8030
1.04E-05 10300 7.97E-06 9750 1.04E-05
10700 0.000163 8640 4.05E-06 8440 1.62E-05 8030
1.04E-05 10300 7.97E-06 9750 1.04E-05
10700 0.000162 8640 4.05E-06 8440 1.62E-05 8030
1.04E-05 10300 7.98E-06 9760 1.04E-05
10700 0.000161 8640 4.06E-06 8440 1.62E-05 8030
1.04E-05 10300 7.98E-06 9760 1.04E-05
10700 0.00016 8650 4.06E-06 8440 1.62E-05 8040 1.04E-05
10300 7.98E-06 9760 1.04E-05
10700 0.000159 8650 4.06E-06 8440 1.62E-05 8040
1.04E-05 10300 7.97E-06 9760 1.04E-05
10700 0.000158 8650 4.07E-06 8450 1.62E-05 8040
1.05E-05 10300 7.97E-06 9760 1.04E-05
10700 0.000158 8650 4.07E-06 8450 1.62E-05 8040
1.05E-05 10300 7.98E-06 9760 1.04E-05
10700 0.000157 8650 4.08E-06 8450 1.61E-05 8040
1.05E-05 10300 7.98E-06 9770 1.04E-05
10700 0.000156 8650 4.08E-06 8450 1.61E-05 8040
1.05E-05 10300 7.98E-06 9770 1.04E-05
10700 0.000155 8650 4.09E-06 8450 1.61E-05 8050
1.05E-05 10300 7.98E-06 9770 1.04E-05
10700 0.000154 8660 4.09E-06 8450 1.61E-05 8050
1.05E-05 10300 7.99E-06 9770 1.04E-05
10700 0.000153 8660 4.09E-06 8450 1.61E-05 8050
1.05E-05 10300 7.99E-06 9770 1.04E-05
10700 0.000153 8660 4.10E-06 8460 1.61E-05 8050
1.05E-05 10300 8.00E-06 9770 1.04E-05
10700 0.000152 8660 4.10E-06 8460 1.61E-05 8050
1.06E-05 10300 8.00E-06 9780 1.04E-05
10700 0.000151 8660 4.11E-06 8460 1.61E-05 8050
1.06E-05 10300 7.98E-06 9780 1.04E-05
10700 0.00015 8660 4.11E-06 8460 1.61E-05 8050 1.06E-05
10300 7.99E-06 9780 1.04E-05
10700 0.00015 8670 4.11E-06 8460 1.61E-05 8060 1.06E-05
10300 7.99E-06 9780 1.04E-05
10700 0.000149 8670 4.12E-06 8460 1.60E-05 8060
1.06E-05 10300 7.98E-06 9780 1.04E-05
10700 0.000148 8670 4.12E-06 8470 1.60E-05 8060
1.06E-05 10300 7.99E-06 9780 1.04E-05
10700 0.000147 8670 4.12E-06 8470 1.60E-05 8060
1.06E-05 10300 7.99E-06 9780 1.04E-05
10700 0.000147 8670 4.13E-06 8470 1.60E-05 8060
1.07E-05 10300 8.00E-06 9790 1.04E-05

10700 0.000146 8670 4.13E-06 8470 1.60E-05 8060
1.07E-05 10300 8.00E-06 9790 1.04E-05
10700 0.000145 8680 4.13E-06 8470 1.60E-05 8070
1.07E-05 10300 8.01E-06 9790 1.04E-05
10700 0.000144 8680 4.14E-06 8470 1.59E-05 8070
1.07E-05 10400 8.00E-06 9790 1.04E-05
10700 0.000144 8680 4.14E-06 8480 1.59E-05 8070
1.07E-05 10400 8.00E-06 9790 1.04E-05
10700 0.000143 8680 4.15E-06 8480 1.59E-05 8070
1.08E-05 10400 8.01E-06 9790 1.04E-05
10700 0.000142 8680 4.15E-06 8480 1.59E-05 8070
1.08E-05 10400 8.01E-06 9800 1.04E-05
10700 0.000142 8680 4.15E-06 8480 1.59E-05 8070
1.08E-05 10400 8.00E-06 9800 1.04E-05
10700 0.000141 8680 4.16E-06 8480 1.59E-05 8080
1.08E-05 10400 8.00E-06 9800 1.04E-05
10700 0.00014 8690 4.17E-06 8480 1.59E-05 8080 1.08E-05
10400 8.01E-06 9800 1.04E-05
10700 0.00014 8690 4.17E-06 8480 1.59E-05 8080 1.08E-05
10400 8.02E-06 9800 1.04E-05
10700 0.000139 8690 4.17E-06 8490 1.59E-05 8080
1.08E-05 10400 8.02E-06 9800 1.04E-05
10700 0.000138 8690 4.18E-06 8490 1.59E-05 8080
1.08E-05 10400 8.02E-06 9800 1.04E-05
10700 0.000138 8690 4.18E-06 8490 1.59E-05 8080
1.09E-05 10400 8.02E-06 9810 1.05E-05
10700 0.000137 8690 4.19E-06 8490 1.59E-05 8080
1.09E-05 10400 8.03E-06 9810 1.04E-05
10700 0.000136 8700 4.19E-06 8490 1.59E-05 8090
1.09E-05 10400 8.03E-06 9810 1.05E-05
10700 0.000136 8700 4.19E-06 8490 1.59E-05 8090
1.09E-05 10400 8.02E-06 9810 1.05E-05
10700 0.000135 8700 4.20E-06 8500 1.59E-05 8090
1.09E-05 10400 8.03E-06 9810 1.05E-05
10700 0.000134 8700 4.20E-06 8500 1.59E-05 8090
1.09E-05 10400 8.03E-06 9810 1.05E-05
10700 0.000134 8700 4.20E-06 8500 1.59E-05 8090
1.09E-05 10400 8.04E-06 9820 1.05E-05
10700 0.000133 8700 4.20E-06 8500 1.59E-05 8090
1.10E-05 10400 8.04E-06 9820 1.05E-05
10700 0.000133 8700 4.21E-06 8500 1.59E-05 8100
1.10E-05 10400 8.03E-06 9820 1.05E-05
10700 0.000132 8710 4.21E-06 8500 1.59E-05 8100
1.10E-05 10400 8.03E-06 9820 1.05E-05
10700 0.000131 8710 4.21E-06 8500 1.59E-05 8100
1.10E-05 10400 8.03E-06 9820 1.05E-05
10700 0.000131 8710 4.22E-06 8510 1.59E-05 8100
1.10E-05 10400 8.04E-06 9820 1.05E-05
10700 0.00013 8710 4.22E-06 8510 1.59E-05 8100 1.10E-05
10400 8.03E-06 9830 1.05E-05
10700 0.00013 8710 4.22E-06 8510 1.59E-05 8100 1.10E-05
10400 8.03E-06 9830 1.05E-05
10700 0.000129 8710 4.23E-06 8510 1.59E-05 8100
1.11E-05 10400 8.04E-06 9830 1.05E-05
10700 0.000128 8720 4.24E-06 8510 1.59E-05 8110
1.11E-05 10400 8.04E-06 9830 1.05E-05
10700 0.000128 8720 4.24E-06 8510 1.59E-05 8110
1.11E-05 10400 8.05E-06 9830 1.05E-05
10700 0.000127 8720 4.23E-06 8520 1.59E-05 8110
1.11E-05 10400 8.05E-06 9830 1.05E-05
10700 0.000127 8720 4.24E-06 8520 1.60E-05 8110
1.11E-05 10400 8.06E-06 9830 1.05E-05
10800 0.000126 8720 4.24E-06 8520 1.60E-05 8110
1.11E-05 10400 8.05E-06 9840 1.06E-05
10800 0.000126 8720 4.24E-06 8520 1.60E-05 8110
1.11E-05 10400 8.06E-06 9840 1.05E-05
10800 0.000125 8730 4.25E-06 8520 1.60E-05 8120
1.12E-05 10400 8.06E-06 9840 1.05E-05

10800 0.000125 8730 4.26E-06 8520 1.60E-05 8120
1.12E-05 10400 8.06E-06 9840 1.05E-05
10800 0.000124 8730 4.25E-06 8530 1.60E-05 8120
1.12E-05 10400 8.06E-06 9840 1.05E-05
10800 0.000123 8730 4.26E-06 8530 1.60E-05 8120
1.12E-05 10400 8.06E-06 9840 1.05E-05
10800 0.000123 8730 4.26E-06 8530 1.60E-05 8120
1.13E-05 10400 8.06E-06 9850 1.05E-05
10800 0.000122 8730 4.26E-06 8530 1.60E-05 8120
1.13E-05 10400 8.06E-06 9850 1.06E-05
10800 0.000122 8730 4.26E-06 8530 1.61E-05 8130
1.13E-05 10400 8.07E-06 9850 1.06E-05
10800 0.000121 8740 4.26E-06 8530 1.61E-05 8130
1.13E-05 10400 8.07E-06 9850 1.06E-05
10800 0.000121 8740 4.26E-06 8530 1.61E-05 8130
1.13E-05 10400 8.06E-06 9850 1.06E-05
10800 0.00012 8740 4.27E-06 8540 1.61E-05 8130 1.14E-05
10400 8.07E-06 9850 1.06E-05
10800 0.00012 8740 4.27E-06 8540 1.61E-05 8130 1.14E-05
10400 8.06E-06 9850 1.06E-05
10800 0.000119 8740 4.28E-06 8540 1.61E-05 8130
1.14E-05 10400 8.05E-06 9860 1.06E-05
10800 0.000119 8740 4.28E-06 8540 1.61E-05 8130
1.14E-05 10400 8.06E-06 9860 1.06E-05
10800 0.000118 8750 4.28E-06 8540 1.61E-05 8140
1.14E-05 10400 8.06E-06 9860 1.06E-05
10800 0.000118 8750 4.29E-06 8540 1.61E-05 8140
1.15E-05 10400 8.07E-06 9860 1.06E-05
10800 0.000117 8750 4.29E-06 8550 1.62E-05 8140
1.15E-05 10400 8.06E-06 9860 1.06E-05
10800 0.000117 8750 4.30E-06 8550 1.62E-05 8140
1.15E-05 10400 8.06E-06 9860 1.06E-05
10800 0.000116 8750 4.31E-06 8550 1.62E-05 8140
1.15E-05 10400 8.06E-06 9870 1.06E-05
10800 0.000116 8750 4.32E-06 8550 1.62E-05 8140
1.15E-05 10400 8.06E-06 9870 1.06E-05
10800 0.000115 8750 4.32E-06 8550 1.62E-05 8150
1.15E-05 10400 8.07E-06 9870 1.06E-05
10800 0.000115 8760 4.33E-06 8550 1.62E-05 8150
1.16E-05 10400 8.06E-06 9870 1.06E-05
10800 0.000114 8760 4.33E-06 8550 1.62E-05 8150
1.16E-05 10400 8.07E-06 9870 1.06E-05
10800 0.000114 8760 4.33E-06 8560 1.62E-05 8150
1.16E-05 10400 8.07E-06 9870 1.06E-05
10800 0.000114 8760 4.34E-06 8560 1.62E-05 8150
1.16E-05 10400 8.06E-06 9880 1.06E-05
10800 0.000113 8760 4.34E-06 8560 1.62E-05 8150
1.16E-05 10400 8.07E-06 9880 1.06E-05
10800 0.000113 8760 4.34E-06 8560 1.62E-05 8150
1.16E-05 10400 8.07E-06 9880 1.06E-05
10800 0.000112 8770 4.35E-06 8560 1.62E-05 8160
1.17E-05 10400 8.07E-06 9880 1.06E-05
10800 0.000112 8770 4.35E-06 8560 1.62E-05 8160
1.17E-05 10400 8.06E-06 9880 1.06E-05
10800 0.000111 8770 4.35E-06 8570 1.62E-05 8160
1.17E-05 10400 8.06E-06 9880 1.06E-05
10800 0.000111 8770 4.35E-06 8570 1.62E-05 8160
1.17E-05 10400 8.06E-06 9880 1.06E-05
10800 0.00011 8770 4.36E-06 8570 1.62E-05 8160 1.17E-05
10400 8.05E-06 9890 1.06E-05
10800 0.00011 8770 4.37E-06 8570 1.62E-05 8160 1.17E-05
10400 8.06E-06 9890 1.06E-05
10800 0.00011 8780 4.37E-06 8570 1.62E-05 8170 1.17E-05
10400 8.06E-06 9890 1.06E-05
10800 0.000109 8780 4.37E-06 8570 1.62E-05 8170
1.17E-05 10500 8.07E-06 9890 1.07E-05
10800 0.000109 8780 4.37E-06 8580 1.62E-05 8170
1.17E-05 10500 8.06E-06 9890 1.06E-05

10800 0.000108 8780 4.38E-06 8580 1.63E-05 8170
1.17E-05 10500 8.07E-06 9890 1.07E-05
10800 0.000108 8780 4.38E-06 8580 1.63E-05 8170
1.17E-05 10500 8.07E-06 9900 1.07E-05
10800 0.000107 8780 4.39E-06 8580 1.63E-05 8170
1.18E-05 10500 8.07E-06 9900 1.06E-05
10800 0.000107 8780 4.39E-06 8580 1.63E-05 8180
1.18E-05 10500 8.07E-06 9900 1.07E-05
10800 0.000107 8790 4.40E-06 8580 1.63E-05 8180
1.18E-05 10500 8.08E-06 9900 1.07E-05
10800 0.000106 8790 4.40E-06 8580 1.63E-05 8180
1.18E-05 10500 8.08E-06 9900 1.07E-05
10800 0.000106 8790 4.41E-06 8590 1.63E-05 8180
1.18E-05 10500 8.08E-06 9900 1.06E-05
10800 0.000105 8790 4.41E-06 8590 1.63E-05 8180
1.18E-05 10500 8.08E-06 9900 1.07E-05
10800 0.000105 8790 4.41E-06 8590 1.63E-05 8180
1.18E-05 10500 8.09E-06 9910 1.07E-05
10800 0.000105 8790 4.41E-06 8590 1.63E-05 8180
1.19E-05 10500 8.09E-06 9910 1.07E-05
10800 0.000104 8800 4.41E-06 8590 1.63E-05 8190
1.19E-05 10500 8.10E-06 9910 1.07E-05
10800 0.000104 8800 4.41E-06 8590 1.63E-05 8190
1.19E-05 10500 8.10E-06 9910 1.07E-05
10800 0.000103 8800 4.41E-06 8600 1.63E-05 8190
1.19E-05 10500 8.11E-06 9910 1.07E-05
10800 0.000103 8800 4.42E-06 8600 1.63E-05 8190
1.20E-05 10500 8.10E-06 9910 1.07E-05
10800 0.000103 8800 4.42E-06 8600 1.63E-05 8190
1.20E-05 10500 8.10E-06 9920 1.07E-05
10800 0.000102 8800 4.43E-06 8600 1.63E-05 8190
1.20E-05 10500 8.12E-06 9920 1.07E-05
10800 0.000102 8800 4.43E-06 8600 1.63E-05 8200
1.20E-05 10500 8.12E-06 9920 1.07E-05
10800 0.000102 8810 4.44E-06 8600 1.63E-05 8200
1.21E-05 10500 8.13E-06 9920 1.07E-05
10800 0.000101 8810 4.44E-06 8600 1.63E-05 8200
1.21E-05 10500 8.12E-06 9920 1.07E-05
10800 0.000101 8810 4.44E-06 8610 1.63E-05 8200
1.22E-05 10500 8.13E-06 9920 1.07E-05
10800 1.00E-04 8810 4.44E-06 8610 1.63E-05 8200
1.22E-05 10500 8.12E-06 9930 1.07E-05
10800 1.00E-04 8810 4.46E-06 8610 1.64E-05 8200
1.22E-05 10500 8.13E-06 9930 1.07E-05
10800 9.97E-05 8810 4.46E-06 8610 1.64E-05 8200
1.22E-05 10500 8.13E-06 9930 1.07E-05
10800 9.94E-05 8820 4.46E-06 8610 1.64E-05 8210
1.23E-05 10500 8.13E-06 9930 1.07E-05
10800 9.90E-05 8820 4.47E-06 8610 1.64E-05 8210
1.23E-05 10500 8.13E-06 9930 1.07E-05
10800 9.87E-05 8820 4.47E-06 8620 1.64E-05 8210
1.23E-05 10500 8.13E-06 9930 1.07E-05
10800 9.83E-05 8820 4.48E-06 8620 1.64E-05 8210
1.23E-05 10500 8.13E-06 9930 1.07E-05
10900 9.80E-05 8820 4.49E-06 8620 1.64E-05 8210
1.24E-05 10500 8.13E-06 9940 1.07E-05
10900 9.77E-05 8820 4.48E-06 8620 1.64E-05 8210
1.24E-05 10500 8.13E-06 9940 1.07E-05
10900 9.73E-05 8830 4.49E-06 8620 1.64E-05 8220
1.24E-05 10500 8.12E-06 9940 1.07E-05
10900 9.70E-05 8830 4.49E-06 8620 1.64E-05 8220
1.24E-05 10500 8.14E-06 9940 1.07E-05
10900 9.67E-05 8830 4.50E-06 8630 1.64E-05 8220
1.25E-05 10500 8.14E-06 9940 1.07E-05
10900 9.63E-05 8830 4.50E-06 8630 1.64E-05 8220
1.25E-05 10500 8.15E-06 9940 1.07E-05
10900 9.60E-05 8830 4.51E-06 8630 1.65E-05 8220
1.25E-05 10500 8.14E-06 9950 1.07E-05

10900 9.57E-05 8830 4.51E-06 8630 1.65E-05 8220
1.25E-05 10500 8.15E-06 9950 1.07E-05
10900 9.54E-05 8830 4.51E-06 8630 1.65E-05 8230
1.25E-05 10500 8.15E-06 9950 1.07E-05
10900 9.51E-05 8840 4.52E-06 8630 1.65E-05 8230
1.26E-05 10500 8.16E-06 9950 1.07E-05
10900 9.47E-05 8840 4.51E-06 8630 1.65E-05 8230
1.26E-05 10500 8.15E-06 9950 1.07E-05
10900 9.44E-05 8840 4.53E-06 8640 1.65E-05 8230
1.26E-05 10500 8.15E-06 9950 1.07E-05
10900 9.41E-05 8840 4.53E-06 8640 1.65E-05 8230
1.27E-05 10500 8.14E-06 9950 1.07E-05
10900 9.38E-05 8840 4.54E-06 8640 1.65E-05 8230
1.27E-05 10500 8.15E-06 9960 1.07E-05
10900 9.34E-05 8840 4.55E-06 8640 1.66E-05 8230
1.27E-05 10500 8.15E-06 9960 1.07E-05
10900 9.32E-05 8850 4.55E-06 8640 1.66E-05 8240
1.27E-05 10500 8.15E-06 9960 1.07E-05
10900 9.28E-05 8850 4.55E-06 8640 1.66E-05 8240
1.27E-05 10500 8.16E-06 9960 1.07E-05
10900 9.25E-05 8850 4.55E-06 8650 1.66E-05 8240
1.28E-05 10500 8.15E-06 9960 1.07E-05
10900 9.22E-05 8850 4.56E-06 8650 1.66E-05 8240
1.28E-05 10500 8.16E-06 9960 1.07E-05
10900 9.19E-05 8850 4.56E-06 8650 1.66E-05 8240
1.28E-05 10500 8.16E-06 9970 1.07E-05
10900 9.16E-05 8850 4.56E-06 8650 1.66E-05 8240
1.29E-05 10500 8.16E-06 9970 1.07E-05
10900 9.13E-05 8850 4.57E-06 8650 1.66E-05 8250
1.29E-05 10500 8.16E-06 9970 1.07E-05
10900 9.10E-05 8860 4.56E-06 8650 1.66E-05 8250
1.29E-05 10500 8.17E-06 9970 1.07E-05
10900 9.07E-05 8860 4.57E-06 8650 1.66E-05 8250
1.29E-05 10500 8.17E-06 9970 1.07E-05
10900 9.04E-05 8860 4.58E-06 8660 1.66E-05 8250
1.30E-05 10500 8.16E-06 9970 1.07E-05
10900 9.02E-05 8860 4.58E-06 8660 1.67E-05 8250
1.30E-05 10500 8.16E-06 9980 1.07E-05
10900 8.99E-05 8860 4.58E-06 8660 1.67E-05 8250
1.30E-05 10500 8.17E-06 9980 1.07E-05
10900 8.96E-05 8860 4.58E-06 8660 1.67E-05 8250
1.31E-05 10500 8.16E-06 9980 1.07E-05
10900 8.93E-05 8870 4.59E-06 8660 1.67E-05 8260
1.31E-05 10500 8.17E-06 9980 1.07E-05
10900 8.90E-05 8870 4.60E-06 8660 1.67E-05 8260
1.31E-05 10500 8.16E-06 9980 1.07E-05
10900 8.87E-05 8870 4.60E-06 8670 1.67E-05 8260
1.31E-05 10500 8.15E-06 9980 1.07E-05
10900 8.85E-05 8870 4.60E-06 8670 1.67E-05 8260
1.32E-05 10500 8.15E-06 9980 1.07E-05
10900 8.82E-05 8870 4.62E-06 8670 1.67E-05 8260
1.32E-05 10500 8.15E-06 9990 1.07E-05
10900 8.79E-05 8870 4.62E-06 8670 1.67E-05 8260
1.32E-05 10500 8.15E-06 9990 1.07E-05
10900 8.76E-05 8880 4.63E-06 8670 1.68E-05 8270
1.32E-05 10500 8.15E-06 9990 1.07E-05
10900 8.73E-05 8880 4.63E-06 8670 1.68E-05 8270
1.32E-05 10600 8.15E-06 9990 1.07E-05
10900 8.71E-05 8880 4.64E-06 8680 1.68E-05 8270
1.33E-05 10600 8.15E-06 9990 1.07E-05
10900 8.68E-05 8880 4.64E-06 8680 1.68E-05 8270
1.33E-05 10600 8.15E-06 9990 1.07E-05
10900 8.66E-05 8880 4.64E-06 8680 1.68E-05 8270
1.33E-05 10600 8.14E-06 10000 1.07E-05
10900 8.63E-05 8880 4.64E-06 8680 1.68E-05 8270
1.34E-05 10600 8.14E-06 10000 1.07E-05
10900 8.60E-05 8880 4.65E-06 8680 1.68E-05 8280
1.34E-05 10600 8.14E-06 10000 1.07E-05

10900 8.58E-05 8890 4.65E-06 8680 1.68E-05 8280
1.34E-05 10600 8.13E-06 10000 1.07E-05
10900 8.55E-05 8890 4.66E-06 8680 1.68E-05 8280
1.35E-05 10600 8.14E-06 10000 1.07E-05
10900 8.53E-05 8890 4.66E-06 8690 1.69E-05 8280
1.35E-05 10600 8.14E-06 10000 1.07E-05
10900 8.50E-05 8890 4.66E-06 8690 1.69E-05 8280
1.35E-05 10600 8.14E-06 10000 1.07E-05
10900 8.48E-05 8890 4.67E-06 8690 1.69E-05 8280
1.36E-05 10600 8.13E-06 10000 1.07E-05
10900 8.45E-05 8890 4.68E-06 8690 1.69E-05 8280
1.36E-05 10600 8.14E-06 10000 1.07E-05
10900 8.43E-05 8900 4.69E-06 8690 1.69E-05 8290
1.36E-05 10600 8.13E-06 10000 1.07E-05
10900 8.40E-05 8900 4.69E-06 8690 1.69E-05 8290
1.36E-05 10600 8.14E-06 10000 1.07E-05
10900 8.38E-05 8900 4.70E-06 8700 1.69E-05 8290
1.37E-05 10600 8.13E-06 10000 1.07E-05
10900 8.35E-05 8900 4.70E-06 8700 1.70E-05 8290
1.37E-05 10600 8.12E-06 10000 1.07E-05
10900 8.33E-05 8900 4.71E-06 8700 1.70E-05 8290
1.38E-05 10600 8.13E-06 10000 1.07E-05
10900 8.30E-05 8900 4.72E-06 8700 1.70E-05 8290
1.38E-05 10600 8.14E-06 10000 1.07E-05
10900 8.28E-05 8900 4.72E-06 8700 1.70E-05 8300
1.38E-05 10600 8.14E-06 10000 1.07E-05
10900 8.25E-05 8910 4.72E-06 8700 1.70E-05 8300
1.38E-05 10600 8.14E-06 10000 1.07E-05
10900 8.23E-05 8910 4.73E-06 8700 1.70E-05 8300
1.39E-05 10600 8.14E-06 10000 1.07E-05
10900 8.21E-05 8910 4.73E-06 8710 1.70E-05 8300
1.39E-05 10600 8.14E-06 10000 1.07E-05
10900 8.18E-05 8910 4.72E-06 8710 1.70E-05 8300
1.39E-05 10600 8.13E-06 10000 1.07E-05
10900 8.16E-05 8910 4.73E-06 8710 1.71E-05 8300
1.40E-05 10600 8.13E-06 10000 1.07E-05
10900 8.14E-05 8910 4.73E-06 8710 1.71E-05 8300
1.40E-05 10600 8.13E-06 10000 1.07E-05
10900 8.11E-05 8920 4.73E-06 8710 1.71E-05 8310
1.41E-05 10600 8.13E-06 10000 1.07E-05
10900 8.09E-05 8920 4.74E-06 8710 1.71E-05 8310
1.41E-05 10600 8.14E-06 10000 1.07E-05
10900 8.07E-05 8920 4.74E-06 8720 1.71E-05 8310
1.41E-05 10600 8.14E-06 10000 1.07E-05
10900 8.04E-05 8920 4.74E-06 8720 1.71E-05 8310
1.42E-05 10600 8.14E-06 10000 1.07E-05
11000 8.02E-05 8920 4.74E-06 8720 1.71E-05 8310
1.42E-05 10600 8.14E-06 10000 1.07E-05
11000 8.00E-05 8920 4.75E-06 8720 1.71E-05 8310
1.42E-05 10600 8.15E-06 10000 1.07E-05
11000 7.98E-05 8930 4.75E-06 8720 1.72E-05 8320
1.43E-05 10600 8.14E-06 10000 1.07E-05
11000 7.96E-05 8930 4.76E-06 8720 1.72E-05 8320
1.43E-05 10600 8.15E-06 10000 1.07E-05
11000 7.93E-05 8930 4.77E-06 8730 1.72E-05 8320
1.43E-05 10600 8.14E-06 10000 1.07E-05
11000 7.91E-05 8930 4.77E-06 8730 1.72E-05 8320
1.44E-05 10600 8.16E-06 10000 1.07E-05
11000 7.89E-05 8930 4.78E-06 8730 1.72E-05 8320
1.44E-05 10600 8.15E-06 10000 1.07E-05
11000 7.87E-05 8930 4.79E-06 8730 1.72E-05 8320
1.45E-05 10600 8.15E-06 10000 1.07E-05
11000 7.84E-05 8930 4.79E-06 8730 1.72E-05 8330
1.45E-05 10600 8.15E-06 10000 1.07E-05
11000 7.82E-05 8940 4.80E-06 8730 1.73E-05 8330
1.45E-05 10600 8.16E-06 10100 1.07E-05
11000 7.80E-05 8940 4.80E-06 8730 1.73E-05 8330
1.46E-05 10600 8.16E-06 10100 1.07E-05

11000 7.78E-05 8940 4.81E-06 8740 1.73E-05 8330
1.46E-05 10600 8.16E-06 10100 1.07E-05
11000 7.76E-05 8940 4.81E-06 8740 1.73E-05 8330
1.46E-05 10600 8.15E-06 10100 1.07E-05
11000 7.74E-05 8940 4.81E-06 8740 1.73E-05 8330
1.47E-05 10600 8.15E-06 10100 1.07E-05
11000 7.72E-05 8940 4.82E-06 8740 1.73E-05 8330
1.47E-05 10600 8.16E-06 10100 1.07E-05
11000 7.70E-05 8950 4.82E-06 8740 1.73E-05 8340
1.48E-05 10600 8.15E-06 10100 1.07E-05
11000 7.68E-05 8950 4.83E-06 8740 1.73E-05 8340
1.48E-05 10600 8.15E-06 10100 1.07E-05
11000 7.66E-05 8950 4.84E-06 8750 1.73E-05 8340
1.48E-05 10600 8.14E-06 10100 1.07E-05
11000 7.64E-05 8950 4.83E-06 8750 1.74E-05 8340
1.49E-05 10600 8.14E-06 10100 1.08E-05
11000 7.62E-05 8950 4.83E-06 8750 1.74E-05 8340
1.49E-05 10600 8.15E-06 10100 1.07E-05
11000 7.60E-05 8950 4.84E-06 8750 1.74E-05 8340
1.50E-05 10600 8.14E-06 10100 1.08E-05
11000 7.58E-05 8950 4.85E-06 8750 1.74E-05 8350
1.50E-05 10600 8.15E-06 10100 1.08E-05
11000 7.56E-05 8960 4.85E-06 8750 1.74E-05 8350
1.50E-05 10600 8.15E-06 10100 1.08E-05
11000 7.54E-05 8960 4.86E-06 8750 1.74E-05 8350
1.51E-05 10600 8.15E-06 10100 1.08E-05
11000 7.52E-05 8960 4.86E-06 8760 1.75E-05 8350
1.51E-05 10600 8.16E-06 10100 1.08E-05
11000 7.50E-05 8960 4.87E-06 8760 1.75E-05 8350
1.52E-05 10600 8.16E-06 10100 1.08E-05
11000 7.48E-05 8960 4.88E-06 8760 1.75E-05 8350
1.52E-05 10600 8.16E-06 10100 1.08E-05
11000 7.46E-05 8960 4.88E-06 8760 1.75E-05 8350
1.53E-05 10600 8.16E-06 10100 1.08E-05
11000 7.44E-05 8970 4.89E-06 8760 1.75E-05 8360
1.53E-05 10600 8.16E-06 10100 1.08E-05
11000 7.43E-05 8970 4.90E-06 8760 1.75E-05 8360
1.54E-05 10600 8.16E-06 10100 1.08E-05
11000 7.41E-05 8970 4.90E-06 8770 1.75E-05 8360
1.54E-05 10600 8.17E-06 10100 1.08E-05
11000 7.39E-05 8970 4.90E-06 8770 1.75E-05 8360
1.54E-05 10600 8.16E-06 10100 1.08E-05
11000 7.37E-05 8970 4.91E-06 8770 1.76E-05 8360
1.55E-05 10600 8.16E-06 10100 1.09E-05
11000 7.35E-05 8970 4.91E-06 8770 1.76E-05 8360
1.55E-05 10600 8.17E-06 10100 1.09E-05
11000 7.34E-05 8980 4.92E-06 8770 1.76E-05 8370
1.56E-05 10600 8.17E-06 10100 1.09E-05
11000 7.32E-05 8980 4.93E-06 8770 1.76E-05 8370
1.56E-05 10700 8.17E-06 10100 1.09E-05
11000 7.30E-05 8980 4.93E-06 8780 1.76E-05 8370
1.57E-05 10700 8.17E-06 10100 1.09E-05
11000 7.28E-05 8980 4.93E-06 8780 1.76E-05 8370
1.57E-05 10700 8.17E-06 10100 1.09E-05
11000 7.27E-05 8980 4.93E-06 8780 1.76E-05 8370
1.58E-05 10700 8.17E-06 10100 1.09E-05
11000 7.25E-05 8980 4.94E-06 8780 1.77E-05 8370
1.58E-05 10700 8.17E-06 10100 1.09E-05
11000 7.23E-05 8980 4.95E-06 8780 1.77E-05 8380
1.58E-05 10700 8.17E-06 10100 1.09E-05
11000 7.21E-05 8990 4.95E-06 8780 1.77E-05 8380
1.59E-05 10700 8.18E-06 10100 1.09E-05
11000 7.20E-05 8990 4.96E-06 8780 1.77E-05 8380
1.59E-05 10700 8.18E-06 10100 1.09E-05
11000 7.18E-05 8990 4.97E-06 8790 1.77E-05 8380
1.60E-05 10700 8.17E-06 10100 1.09E-05
11000 7.16E-05 8990 4.98E-06 8790 1.77E-05 8380
1.60E-05 10700 8.17E-06 10100 1.10E-05

11000 7.14E-05 8990 4.98E-06 8790 1.78E-05 8380
1.61E-05 10700 8.17E-06 10100 1.10E-05
11000 7.13E-05 8990 4.99E-06 8790 1.78E-05 8380
1.61E-05 10700 8.17E-06 10100 1.10E-05
11000 7.11E-05 9000 4.99E-06 8790 1.78E-05 8390
1.62E-05 10700 8.17E-06 10100 1.09E-05
11000 7.09E-05 9000 4.99E-06 8790 1.78E-05 8390
1.62E-05 10700 8.18E-06 10100 1.10E-05
11000 7.08E-05 9000 5.00E-06 8800 1.78E-05 8390
1.63E-05 10700 8.18E-06 10100 1.10E-05
11000 7.06E-05 9000 4.99E-06 8800 1.78E-05 8390
1.63E-05 10700 8.18E-06 10100 1.10E-05
11000 7.04E-05 9000 5.00E-06 8800 1.78E-05 8390
1.64E-05 10700 8.18E-06 10100 1.10E-05
11000 7.02E-05 9000 5.00E-06 8800 1.78E-05 8390
1.64E-05 10700 8.18E-06 10100 1.10E-05
11000 7.01E-05 9000 5.01E-06 8800 1.78E-05 8400
1.65E-05 10700 8.19E-06 10100 1.10E-05
11000 6.99E-05 9010 5.02E-06 8800 1.78E-05 8400
1.66E-05 10700 8.20E-06 10100 1.10E-05
11000 6.98E-05 9010 5.02E-06 8800 1.79E-05 8400
1.66E-05 10700 8.19E-06 10100 1.10E-05
11000 6.96E-05 9010 5.02E-06 8810 1.79E-05 8400
1.67E-05 10700 8.19E-06 10100 1.10E-05
11000 6.94E-05 9010 5.03E-06 8810 1.79E-05 8400
1.67E-05 10700 8.19E-06 10100 1.10E-05
11000 6.93E-05 9010 5.04E-06 8810 1.79E-05 8400
1.68E-05 10700 8.19E-06 10100 1.10E-05
11000 6.91E-05 9010 5.05E-06 8810 1.79E-05 8400
1.68E-05 10700 8.20E-06 10100 1.10E-05
11000 6.90E-05 9020 5.05E-06 8810 1.80E-05 8410
1.69E-05 10700 8.19E-06 10100 1.10E-05
11000 6.88E-05 9020 5.05E-06 8810 1.80E-05 8410
1.69E-05 10700 8.19E-06 10100 1.10E-05
11000 6.87E-05 9020 5.07E-06 8820 1.80E-05 8410
1.70E-05 10700 8.20E-06 10100 1.10E-05
11000 6.85E-05 9020 5.08E-06 8820 1.80E-05 8410
1.70E-05 10700 8.19E-06 10100 1.10E-05
11100 6.83E-05 9020 5.08E-06 8820 1.80E-05 8410
1.71E-05 10700 8.20E-06 10100 1.10E-05
11100 6.82E-05 9020 5.09E-06 8820 1.80E-05 8410
1.71E-05 10700 8.19E-06 10100 1.10E-05
11100 6.80E-05 9030 5.10E-06 8820 1.80E-05 8420
1.72E-05 10700 8.19E-06 10100 1.10E-05
11100 6.79E-05 9030 5.10E-06 8820 1.81E-05 8420
1.72E-05 10700 8.19E-06 10100 1.10E-05
11100 6.77E-05 9030 5.10E-06 8830 1.81E-05 8420
1.73E-05 10700 8.19E-06 10100 1.10E-05
11100 6.76E-05 9030 5.10E-06 8830 1.81E-05 8420
1.73E-05 10700 8.19E-06 10100 1.10E-05
11100 6.75E-05 9030 5.11E-06 8830 1.81E-05 8420
1.74E-05 10700 8.19E-06 10100 1.10E-05
11100 6.73E-05 9030 5.11E-06 8830 1.81E-05 8420
1.75E-05 10700 8.19E-06 10100 1.10E-05
11100 6.72E-05 9030 5.12E-06 8830 1.81E-05 8430
1.75E-05 10700 8.19E-06 10100 1.10E-05
11100 6.70E-05 9040 5.12E-06 8830 1.81E-05 8430
1.76E-05 10700 8.20E-06 10200 1.10E-05
11100 6.69E-05 9040 5.13E-06 8830 1.82E-05 8430
1.77E-05 10700 8.19E-06 10200 1.10E-05
11100 6.67E-05 9040 5.13E-06 8840 1.82E-05 8430
1.77E-05 10700 8.20E-06 10200 1.10E-05
11100 6.66E-05 9040 5.15E-06 8840 1.82E-05 8430
1.78E-05 10700 8.21E-06 10200 1.10E-05
11100 6.65E-05 9040 5.15E-06 8840 1.82E-05 8430
1.78E-05 10700 8.21E-06 10200 1.09E-05
11100 6.63E-05 9040 5.17E-06 8840 1.82E-05 8430
1.79E-05 10700 8.20E-06 10200 1.09E-05

11100 6.62E-05 9050 5.16E-06 8840 1.83E-05 8440
1.80E-05 10700 8.20E-06 10200 1.09E-05
11100 6.60E-05 9050 5.17E-06 8840 1.83E-05 8440
1.80E-05 10700 8.21E-06 10200 1.09E-05
11100 6.59E-05 9050 5.17E-06 8850 1.83E-05 8440
1.81E-05 10700 8.21E-06 10200 1.09E-05
11100 6.57E-05 9050 5.19E-06 8850 1.83E-05 8440
1.82E-05 10700 8.21E-06 10200 1.09E-05
11100 6.56E-05 9050 5.18E-06 8850 1.83E-05 8440
1.82E-05 10700 8.21E-06 10200 1.09E-05
11100 6.55E-05 9050 5.20E-06 8850 1.83E-05 8440
1.83E-05 10700 8.21E-06 10200 1.09E-05
11100 6.53E-05 9050 5.20E-06 8850 1.84E-05 8450
1.84E-05 10700 8.21E-06 10200 1.09E-05
11100 6.52E-05 9060 5.21E-06 8850 1.84E-05 8450
1.85E-05 10700 8.21E-06 10200 1.09E-05
11100 6.50E-05 9060 5.20E-06 8850 1.84E-05 8450
1.85E-05 10700 8.22E-06 10200 1.09E-05
11100 6.49E-05 9060 5.21E-06 8860 1.84E-05 8450
1.86E-05 10700 8.22E-06 10200 1.09E-05
11100 6.48E-05 9060 5.21E-06 8860 1.84E-05 8450
1.87E-05 10700 8.22E-06 10200 1.09E-05
11100 6.46E-05 9060 5.21E-06 8860 1.84E-05 8450
1.88E-05 10700 8.22E-06 10200 1.09E-05
11100 6.45E-05 9060 5.22E-06 8860 1.84E-05 8450
1.88E-05 10700 8.22E-06 10200 1.09E-05
11100 6.43E-05 9070 5.22E-06 8860 1.84E-05 8460
1.89E-05 10700 8.23E-06 10200 1.09E-05
11100 6.42E-05 9070 5.23E-06 8860 1.85E-05 8460
1.90E-05 10700 8.23E-06 10200 1.09E-05
11100 6.41E-05 9070 5.23E-06 8870 1.85E-05 8460
1.91E-05 10700 8.23E-06 10200 1.09E-05
11100 6.40E-05 9070 5.24E-06 8870 1.85E-05 8460
1.91E-05 10700 8.23E-06 10200 1.08E-05
11100 6.38E-05 9070 5.25E-06 8870 1.85E-05 8460
1.92E-05 10700 8.24E-06 10200 1.08E-05
11100 6.37E-05 9070 5.25E-06 8870 1.85E-05 8460
1.93E-05 10700 8.24E-06 10200 1.08E-05
11100 6.36E-05 9080 5.27E-06 8870 1.86E-05 8470
1.94E-05 10700 8.24E-06 10200 1.08E-05
11100 6.34E-05 9080 5.27E-06 8870 1.86E-05 8470
1.94E-05 10800 8.24E-06 10200 1.08E-05
11100 6.33E-05 9080 5.27E-06 8880 1.86E-05 8470
1.95E-05 10800 8.24E-06 10200 1.08E-05
11100 6.32E-05 9080 5.28E-06 8880 1.86E-05 8470
1.96E-05 10800 8.24E-06 10200 1.08E-05
11100 6.30E-05 9080 5.29E-06 8880 1.86E-05 8470
1.97E-05 10800 8.24E-06 10200 1.08E-05
11100 6.29E-05 9080 5.31E-06 8880 1.86E-05 8470
1.98E-05 10800 8.24E-06 10200 1.08E-05
11100 6.28E-05 9080 5.31E-06 8880 1.86E-05 8480
1.98E-05 10800 8.24E-06 10200 1.08E-05
11100 6.27E-05 9090 5.31E-06 8880 1.87E-05 8480
1.99E-05 10800 8.24E-06 10200 1.08E-05
11100 6.25E-05 9090 5.31E-06 8880 1.87E-05 8480
2.00E-05 10800 8.25E-06 10200 1.08E-05
11100 6.24E-05 9090 5.31E-06 8890 1.87E-05 8480
2.01E-05 10800 8.24E-06 10200 1.08E-05
11100 6.23E-05 9090 5.32E-06 8890 1.87E-05 8480
2.02E-05 10800 8.24E-06 10200 1.08E-05
11100 6.22E-05 9090 5.33E-06 8890 1.87E-05 8480
2.03E-05 10800 8.24E-06 10200 1.08E-05
11100 6.21E-05 9090 5.33E-06 8890 1.87E-05 8480
2.03E-05 10800 8.24E-06 10200 1.08E-05
11100 6.20E-05 9100 5.33E-06 8890 1.87E-05 8490
2.04E-05 10800 8.24E-06 10200 1.08E-05
11100 6.18E-05 9100 5.34E-06 8890 1.88E-05 8490
2.05E-05 10800 8.24E-06 10200 1.08E-05

11100 6.17E-05 9100 5.34E-06 8900 1.88E-05 8490
2.06E-05 10800 8.25E-06 10200 1.08E-05
11100 6.16E-05 9100 5.35E-06 8900 1.88E-05 8490
2.07E-05 10800 8.24E-06 10200 1.08E-05
11100 6.15E-05 9100 5.35E-06 8900 1.88E-05 8490
2.08E-05 10800 8.24E-06 10200 1.08E-05
11100 6.14E-05 9100 5.36E-06 8900 1.88E-05 8490
2.09E-05 10800 8.24E-06 10200 1.08E-05
11100 6.13E-05 9100 5.37E-06 8900 1.89E-05 8500
2.10E-05 10800 8.24E-06 10200 1.08E-05
11100 6.11E-05 9110 5.38E-06 8900 1.89E-05 8500
2.11E-05 10800 8.24E-06 10200 1.08E-05
11100 6.10E-05 9110 5.38E-06 8900 1.89E-05 8500
2.12E-05 10800 8.25E-06 10200 1.08E-05
11100 6.09E-05 9110 5.39E-06 8910 1.89E-05 8500
2.13E-05 10800 8.24E-06 10200 1.08E-05
11100 6.08E-05 9110 5.39E-06 8910 1.89E-05 8500
2.14E-05 10800 8.25E-06 10200 1.07E-05
11100 6.07E-05 9110 5.41E-06 8910 1.89E-05 8500
2.15E-05 10800 8.25E-06 10200 1.07E-05
11100 6.06E-05 9110 5.41E-06 8910 1.89E-05 8500
2.16E-05 10800 8.25E-06 10200 1.07E-05
11100 6.04E-05 9120 5.42E-06 8910 1.90E-05 8510
2.17E-05 10800 8.25E-06 10200 1.07E-05
11100 6.03E-05 9120 5.41E-06 8910 1.90E-05 8510
2.18E-05 10800 8.26E-06 10200 1.07E-05
11100 6.02E-05 9120 5.42E-06 8920 1.90E-05 8510
2.19E-05 10800 8.26E-06 10200 1.07E-05
11100 6.01E-05 9120 5.43E-06 8920 1.90E-05 8510
2.20E-05 10800 8.27E-06 10200 1.07E-05
11200 6.00E-05 9120 5.43E-06 8920 1.90E-05 8510
2.22E-05 10800 8.27E-06 10200 1.07E-05
11200 5.99E-05 9120 5.44E-06 8920 1.90E-05 8510
2.23E-05 10800 8.27E-06 10200 1.07E-05
11200 5.97E-05 9130 5.43E-06 8920 1.91E-05 8520
2.24E-05 10800 8.27E-06 10200 1.07E-05
11200 5.96E-05 9130 5.44E-06 8920 1.91E-05 8520
2.25E-05 10800 8.27E-06 10200 1.07E-05
11200 5.95E-05 9130 5.44E-06 8930 1.91E-05 8520
2.27E-05 10800 8.27E-06 10200 1.07E-05
11200 5.94E-05 9130 5.45E-06 8930 1.91E-05 8520
2.28E-05 10800 8.27E-06 10200 1.07E-05
11200 5.93E-05 9130 5.46E-06 8930 1.91E-05 8520
2.29E-05 10800 8.27E-06 10200 1.07E-05
11200 5.92E-05 9130 5.46E-06 8930 1.91E-05 8520
2.30E-05 10800 8.28E-06 10200 1.07E-05
11200 5.91E-05 9130 5.47E-06 8930 1.92E-05 8530
2.31E-05 10800 8.27E-06 10200 1.07E-05
11200 5.90E-05 9140 5.48E-06 8930 1.92E-05 8530
2.33E-05 10800 8.28E-06 10300 1.07E-05
11200 5.89E-05 9140 5.48E-06 8930 1.92E-05 8530
2.34E-05 10800 8.28E-06 10300 1.07E-05
11200 5.88E-05 9140 5.49E-06 8940 1.92E-05 8530
2.35E-05 10800 8.27E-06 10300 1.07E-05
11200 5.86E-05 9140 5.49E-06 8940 1.92E-05 8530
2.37E-05 10800 8.28E-06 10300 1.07E-05
11200 5.85E-05 9140 5.50E-06 8940 1.93E-05 8530
2.38E-05 10800 8.28E-06 10300 1.07E-05
11200 5.84E-05 9140 5.51E-06 8940 1.93E-05 8530
2.39E-05 10800 8.28E-06 10300 1.07E-05
11200 5.83E-05 9150 5.51E-06 8940 1.93E-05 8540
2.41E-05 10800 8.28E-06 10300 1.07E-05
11200 5.82E-05 9150 5.51E-06 8940 1.93E-05 8540
2.42E-05 10800 8.28E-06 10300 1.07E-05
11200 5.81E-05 9150 5.52E-06 8950 1.93E-05 8540
2.44E-05 10800 8.28E-06 10300 1.07E-05
11200 5.80E-05 9150 5.52E-06 8950 1.93E-05 8540
2.45E-05 10800 8.29E-06 10300 1.07E-05

11200 5.79E-05 9150 5.53E-06 8950 1.93E-05 8540
2.46E-05 10800 8.29E-06 10300 1.07E-05
11200 5.78E-05 9150 5.54E-06 8950 1.94E-05 8540
2.48E-05 10800 8.29E-06 10300 1.07E-05
11200 5.78E-05 9150 5.54E-06 8950 1.94E-05 8550
2.50E-05 10800 8.29E-06 10300 1.07E-05
11200 5.77E-05 9160 5.55E-06 8950 1.94E-05 8550
2.51E-05 10800 8.29E-06 10300 1.07E-05
11200 5.75E-05 9160 5.54E-06 8950 1.94E-05 8550
2.53E-05 10800 8.29E-06 10300 1.07E-05
11200 5.74E-05 9160 5.55E-06 8960 1.94E-05 8550
2.54E-05 10800 8.29E-06 10300 1.07E-05
11200 5.74E-05 9160 5.56E-06 8960 1.95E-05 8550
2.56E-05 10800 8.29E-06 10300 1.07E-05
11200 5.73E-05 9160 5.56E-06 8960 1.95E-05 8550
2.57E-05 10800 8.30E-06 10300 1.07E-05
11200 5.71E-05 9160 5.58E-06 8960 1.95E-05 8550
2.59E-05 10800 8.29E-06 10300 1.07E-05
11200 5.70E-05 9170 5.59E-06 8960 1.95E-05 8560
2.60E-05 10800 8.30E-06 10300 1.07E-05
11200 5.69E-05 9170 5.60E-06 8960 1.95E-05 8560
2.62E-05 10800 8.30E-06 10300 1.07E-05
11200 5.68E-05 9170 5.61E-06 8970 1.96E-05 8560
2.64E-05 10800 8.29E-06 10300 1.07E-05
11200 5.67E-05 9170 5.62E-06 8970 1.96E-05 8560
2.66E-05 10800 8.30E-06 10300 1.07E-05
11200 5.66E-05 9170 5.63E-06 8970 1.96E-05 8560
2.67E-05 10800 8.30E-06 10300 1.07E-05
11200 5.65E-05 9170 5.64E-06 8970 1.96E-05 8560
2.69E-05 10800 8.30E-06 10300 1.07E-05
11200 5.64E-05 9180 5.64E-06 8970 1.96E-05 8570
2.71E-05 10800 8.30E-06 10300 1.07E-05
11200 5.63E-05 9180 5.64E-06 8970 1.96E-05 8570
2.73E-05 10900 8.31E-06 10300 1.07E-05
11200 5.62E-05 9180 5.64E-06 8980 1.97E-05 8570
2.75E-05 10900 8.31E-06 10300 1.07E-05
11200 5.61E-05 9180 5.65E-06 8980 1.97E-05 8570
2.76E-05 10900 8.32E-06 10300 1.07E-05
11200 5.60E-05 9180 5.66E-06 8980 1.97E-05 8570
2.78E-05 10900 8.32E-06 10300 1.07E-05
11200 5.59E-05 9180 5.66E-06 8980 1.97E-05 8570
2.80E-05 10900 8.33E-06 10300 1.07E-05
11200 5.58E-05 9180 5.66E-06 8980 1.97E-05 8580
2.82E-05 10900 8.33E-06 10300 1.07E-05
11200 5.57E-05 9190 5.67E-06 8980 1.97E-05 8580
2.85E-05 10900 8.33E-06 10300 1.07E-05
11200 5.56E-05 9190 5.67E-06 8980 1.98E-05 8580
2.87E-05 10900 8.34E-06 10300 1.07E-05
11200 5.55E-05 9190 5.67E-06 8990 1.98E-05 8580
2.88E-05 10900 8.34E-06 10300 1.07E-05
11200 5.54E-05 9190 5.69E-06 8990 1.98E-05 8580
2.91E-05 10900 8.34E-06 10300 1.07E-05
11200 5.54E-05 9190 5.69E-06 8990 1.98E-05 8580
2.93E-05 10900 8.35E-06 10300 1.07E-05
11200 5.53E-05 9190 5.70E-06 8990 1.98E-05 8580
2.95E-05 10900 8.35E-06 10300 1.07E-05
11200 5.52E-05 9200 5.71E-06 8990 1.99E-05 8590
2.97E-05 10900 8.35E-06 10300 1.07E-05
11200 5.51E-05 9200 5.72E-06 8990 1.99E-05 8590
2.99E-05 10900 8.36E-06 10300 1.07E-05
11200 5.50E-05 9200 5.73E-06 9000 1.99E-05 8590
3.01E-05 10900 8.34E-06 10300 1.07E-05
11200 5.49E-05 9200 5.73E-06 9000 1.99E-05 8590
3.04E-05 10900 8.35E-06 10300 1.07E-05
11200 5.48E-05 9200 5.75E-06 9000 1.99E-05 8590
3.06E-05 10900 8.35E-06 10300 1.07E-05
11200 5.47E-05 9200 5.76E-06 9000 1.99E-05 8590
3.08E-05 10900 8.35E-06 10300 1.07E-05

11200	5.47E-05	9200	5.76E-06	9000	2.00E-05	8600	3.11E-05	10900	8.35E-06	10300	1.07E-05
11200	5.46E-05	9210	5.76E-06	9000	2.00E-05	8600	3.13E-05	10900	8.35E-06	10300	1.07E-05
11200	5.45E-05	9210	5.76E-06	9000	2.00E-05	8600	3.16E-05	10900	8.35E-06	10300	1.07E-05
11200	5.44E-05	9210	5.77E-06	9010	2.00E-05	8600	3.18E-05	10900	8.35E-06	10300	1.07E-05
11200	5.43E-05	9210	5.78E-06	9010	2.00E-05	8600	3.21E-05	10900	8.35E-06	10300	1.07E-05
11200	5.42E-05	9210	5.79E-06	9010	2.01E-05	8600	3.24E-05	10900	8.36E-06	10300	1.07E-05
11200	5.41E-05	9210	5.79E-06	9010	2.01E-05	8600	3.27E-05	10900	8.36E-06	10300	1.07E-05
11200	5.41E-05	9220	5.80E-06	9010	2.01E-05	8610	3.29E-05	10900	8.36E-06	10300	1.07E-05
11200	5.40E-05	9220	5.80E-06	9010	2.01E-05	8610	3.32E-05	10900	8.37E-06	10300	1.07E-05
11200	5.39E-05	9220	5.80E-06	9020	2.01E-05	8610	3.35E-05	10900	8.37E-06	10300	1.07E-05
11200	5.38E-05	9220	5.82E-06	9020	2.02E-05	8610	3.38E-05	10900	8.37E-06	10300	1.07E-05
11300	5.37E-05	9220	5.82E-06	9020	2.02E-05	8610	3.41E-05	10900	8.37E-06	10300	1.07E-05
11300	5.37E-05	9220	5.83E-06	9020	2.02E-05	8610	3.44E-05	10900	8.37E-06	10300	1.07E-05
11300	5.36E-05	9230	5.84E-06	9020	2.02E-05	8620	3.47E-05	10900	8.36E-06	10300	1.07E-05
11300	5.35E-05	9230	5.84E-06	9020	2.02E-05	8620	3.50E-05	10900	8.37E-06	10300	1.07E-05
11300	5.34E-05	9230	5.85E-06	9030	2.03E-05	8620	3.54E-05	10900	8.36E-06	10300	1.07E-05
11300	5.33E-05	9230	5.85E-06	9030	2.03E-05	8620	3.57E-05	10900	8.36E-06	10300	1.07E-05
11300	5.32E-05	9230	5.87E-06	9030	2.03E-05	8620	3.60E-05	10900	8.37E-06	10300	1.07E-05
11300	5.31E-05	9230	5.87E-06	9030	2.03E-05	8620	3.63E-05	10900	8.37E-06	10300	1.07E-05
11300	5.31E-05	9230	5.87E-06	9030	2.03E-05	8630	3.67E-05	10900	8.38E-06	10300	1.07E-05
11300	5.30E-05	9240	5.88E-06	9030	2.03E-05	8630	3.70E-05	10900	8.38E-06	10400	1.07E-05
11300	5.29E-05	9240	5.88E-06	9030	2.04E-05	8630	3.74E-05	10900	8.38E-06	10400	1.07E-05
11300	5.28E-05	9240	5.89E-06	9040	2.04E-05	8630	3.78E-05	10900	8.38E-06	10400	1.07E-05
11300	5.27E-05	9240	5.90E-06	9040	2.04E-05	8630	3.81E-05	10900	8.39E-06	10400	1.07E-05
11300	5.27E-05	9240	5.91E-06	9040	2.04E-05	8630	3.85E-05	10900	8.39E-06	10400	1.07E-05
11300	5.26E-05	9240	5.91E-06	9040	2.04E-05	8630	3.89E-05	10900	8.39E-06	10400	1.07E-05
11300	5.25E-05	9250	5.92E-06	9040	2.05E-05	8640	3.93E-05	10900	8.39E-06	10400	1.07E-05
11300	5.25E-05	9250	5.92E-06	9040	2.05E-05	8640	3.97E-05	10900	8.39E-06	10400	1.07E-05
11300	5.24E-05	9250	5.92E-06	9050	2.05E-05	8640	4.02E-05	10900	8.40E-06	10400	1.07E-05
11300	5.23E-05	9250	5.94E-06	9050	2.05E-05	8640	4.06E-05	10900	8.39E-06	10400	1.07E-05
11300	5.23E-05	9250	5.94E-06	9050	2.06E-05	8640	4.10E-05	10900	8.40E-06	10400	1.07E-05
11300	5.22E-05	9250	5.95E-06	9050	2.06E-05	8640	4.15E-05	10900	8.40E-06	10400	1.07E-05
11300	5.21E-05	9250	5.96E-06	9050	2.06E-05	8650	4.19E-05	10900	8.40E-06	10400	1.07E-05
11300	5.20E-05	9260	5.97E-06	9050	2.06E-05	8650	4.24E-05	10900	8.40E-06	10400	1.07E-05

11300 5.20E-05 9260 5.98E-06 9050 2.07E-05 8650
4.29E-05 10900 8.39E-06 10400 1.07E-05
11300 5.19E-05 9260 5.98E-06 9060 2.07E-05 8650
4.33E-05 10900 8.39E-06 10400 1.07E-05
11300 5.18E-05 9260 6.00E-06 9060 2.07E-05 8650
4.38E-05 10900 8.40E-06 10400 1.07E-05
11300 5.17E-05 9260 6.01E-06 9060 2.07E-05 8650
4.44E-05 10900 8.39E-06 10400 1.07E-05
11300 5.17E-05 9260 6.01E-06 9060 2.07E-05 8650
4.49E-05 10900 8.40E-06 10400 1.07E-05
11300 5.16E-05 9270 6.02E-06 9060 2.07E-05 8660
4.54E-05 10900 8.40E-06 10400 1.07E-05
11300 5.15E-05 9270 6.02E-06 9060 2.08E-05 8660
4.60E-05 10900 8.40E-06 10400 1.07E-05
11300 5.14E-05 9270 6.03E-06 9070 2.08E-05 8660
4.65E-05 10900 8.40E-06 10400 1.07E-05
11300 5.14E-05 9270 6.04E-06 9070 2.08E-05 8660
4.71E-05 10900 8.40E-06 10400 1.07E-05
11300 5.13E-05 9270 6.05E-06 9070 2.08E-05 8660
4.77E-05 10900 8.40E-06 10400 1.07E-05
11300 5.12E-05 9270 6.06E-06 9070 2.09E-05 8660
4.82E-05 10900 8.40E-06 10400 1.07E-05
11300 5.12E-05 9280 6.06E-06 9070 2.09E-05 8670
4.89E-05 10900 8.40E-06 10400 1.07E-05
11300 5.11E-05 9280 6.07E-06 9070 2.09E-05 8670
4.95E-05 11000 8.41E-06 10400 1.07E-05
11300 5.11E-05 9280 6.07E-06 9080 2.09E-05 8670
5.01E-05 11000 8.42E-06 10400 1.07E-05
11300 5.10E-05 9280 6.08E-06 9080 2.10E-05 8670
5.08E-05 11000 8.41E-06 10400 1.07E-05
11300 5.10E-05 9280 6.09E-06 9080 2.10E-05 8670
5.15E-05 11000 8.41E-06 10400 1.07E-05
11300 5.09E-05 9280 6.10E-06 9080 2.10E-05 8670
5.22E-05 11000 8.41E-06 10400 1.07E-05
11300 5.08E-05 9280 6.11E-06 9080 2.10E-05 8680
5.29E-05 11000 8.41E-06 10400 1.07E-05
11300 5.08E-05 9290 6.11E-06 9080 2.11E-05 8680
5.36E-05 11000 8.42E-06 10400 1.07E-05
11300 5.07E-05 9290 6.11E-06 9080 2.11E-05 8680
5.44E-05 11000 8.41E-06 10400 1.07E-05
11300 5.06E-05 9290 6.13E-06 9090 2.11E-05 8680
5.52E-05 11000 8.41E-06 10400 1.07E-05
11300 5.06E-05 9290 6.14E-06 9090 2.11E-05 8680
5.60E-05 11000 8.41E-06 10400 1.07E-05
11300 5.05E-05 9290 6.15E-06 9090 2.12E-05 8680
5.68E-05 11000 8.42E-06 10400 1.07E-05
11300 5.04E-05 9290 6.16E-06 9090 2.12E-05 8680
5.76E-05 11000 8.42E-06 10400 1.07E-05
11300 5.04E-05 9300 6.16E-06 9090 2.12E-05 8690
5.85E-05 11000 8.42E-06 10400 1.07E-05
11300 5.03E-05 9300 6.17E-06 9090 2.12E-05 8690
5.94E-05 11000 8.42E-06 10400 1.07E-05
11300 5.02E-05 9300 6.18E-06 9100 2.12E-05 8690
6.04E-05 11000 8.43E-06 10400 1.07E-05
11300 5.02E-05 9300 6.18E-06 9100 2.13E-05 8690
6.13E-05 11000 8.43E-06 10400 1.07E-05
11300 5.01E-05 9300 6.19E-06 9100 2.13E-05 8690
6.23E-05 11000 8.43E-06 10400 1.07E-05
11300 5.00E-05 9300 6.20E-06 9100 2.13E-05 8690
6.33E-05 11000 8.44E-06 10400 1.07E-05
11300 5.00E-05 9300 6.20E-06 9100 2.13E-05 8700
6.44E-05 11000 8.43E-06 10400 1.07E-05
11300 4.99E-05 9310 6.22E-06 9100 2.14E-05 8700
6.55E-05 11000 8.44E-06 10400 1.07E-05
11300 4.99E-05 9310 6.22E-06 9100 2.14E-05 8700
6.66E-05 11000 8.44E-06 10400 1.07E-05
11300 4.98E-05 9310 6.23E-06 9110 2.14E-05 8700
6.77E-05 11000 8.44E-06 10400 1.07E-05

11300 4.97E-05 9310 6.24E-06 9110 2.14E-05 8700
6.89E-05 11000 8.45E-06 10400 1.07E-05
11300 4.97E-05 9310 6.25E-06 9110 2.15E-05 8700
7.01E-05 11000 8.44E-06 10400 1.07E-05
11300 4.96E-05 9310 6.25E-06 9110 2.15E-05 8700
7.14E-05 11000 8.45E-06 10400 1.07E-05
11300 4.96E-05 9320 6.26E-06 9110 2.15E-05 8710
7.27E-05 11000 8.45E-06 10400 1.07E-05
11300 4.95E-05 9320 6.27E-06 9110 2.15E-05 8710
7.40E-05 11000 8.44E-06 10400 1.07E-05
11300 4.94E-05 9320 6.28E-06 9120 2.16E-05 8710
7.54E-05 11000 8.45E-06 10400 1.07E-05
11300 4.94E-05 9320 6.28E-06 9120 2.16E-05 8710
7.68E-05 11000 8.44E-06 10400 1.07E-05
11400 4.93E-05 9320 6.29E-06 9120 2.16E-05 8710
7.83E-05 11000 8.45E-06 10400 1.07E-05
11400 4.92E-05 9320 6.30E-06 9120 2.16E-05 8710
7.99E-05 11000 8.45E-06 10400 1.07E-05
11400 4.92E-05 9330 6.30E-06 9120 2.16E-05 8720
8.15E-05 11000 8.46E-06 10400 1.07E-05
11400 4.91E-05 9330 6.30E-06 9120 2.17E-05 8720
8.32E-05 11000 8.47E-06 10400 1.07E-05
11400 4.90E-05 9330 6.30E-06 9130 2.17E-05 8720
8.49E-05 11000 8.47E-06 10400 1.07E-05
11400 4.90E-05 9330 6.31E-06 9130 2.17E-05 8720
8.66E-05 11000 8.46E-06 10400 1.07E-05
11400 4.89E-05 9330 6.31E-06 9130 2.17E-05 8720
8.85E-05 11000 8.46E-06 10400 1.07E-05
11400 4.89E-05 9330 6.32E-06 9130 2.17E-05 8720
9.04E-05 11000 8.46E-06 10400 1.07E-05
11400 4.88E-05 9330 6.33E-06 9130 2.18E-05 8730
9.24E-05 11000 8.47E-06 10400 1.07E-05
11400 4.88E-05 9340 6.33E-06 9130 2.18E-05 8730
9.44E-05 11000 8.47E-06 10500 1.07E-05
11400 4.87E-05 9340 6.34E-06 9130 2.18E-05 8730
9.65E-05 11000 8.47E-06 10500 1.07E-05
11400 4.87E-05 9340 6.34E-06 9140 2.18E-05 8730
9.87E-05 11000 8.47E-06 10500 1.07E-05
11400 4.86E-05 9340 6.35E-06 9140 2.19E-05 8730
0.000101021 11000 8.47E-06 10500 1.07E-05
11400 4.85E-05 9340 6.35E-06 9140 2.19E-05 8730
0.000103402 11000 8.46E-06 10500 1.07E-05
11400 4.85E-05 9340 6.36E-06 9140 2.19E-05 8730
0.00010591 11000 8.46E-06 10500 1.07E-05
11400 4.84E-05 9350 6.37E-06 9140 2.19E-05 8740
0.000108513 11000 8.46E-06 10500 1.07E-05
11400 4.84E-05 9350 6.38E-06 9140 2.20E-05 8740
0.000111223 11000 8.44E-06 10500 1.07E-05
11400 4.83E-05 9350 6.38E-06 9150 2.20E-05 8740
0.000114092 11000 8.46E-06 10500 1.07E-05
11400 4.83E-05 9350 6.38E-06 9150 2.20E-05 8740
0.000117071 11000 8.46E-06 10500 1.07E-05
11400 4.82E-05 9350 6.39E-06 9150 2.20E-05 8740
0.000120189 11000 8.46E-06 10500 1.07E-05
11400 4.82E-05 9350 6.39E-06 9150 2.20E-05 8740
0.000123456 11000 8.47E-06 10500 1.07E-05
11400 4.81E-05 9350 6.39E-06 9150 2.21E-05 8750
0.000126858 11000 8.47E-06 10500 1.07E-05
11400 4.81E-05 9360 6.40E-06 9150 2.21E-05 8750
0.000130436 11000 8.47E-06 10500 1.07E-05
11400 4.80E-05 9360 6.38E-06 9150 2.21E-05 8750
0.000134154 11000 8.47E-06 10500 1.07E-05
11400 4.80E-05 9360 6.39E-06 9160 2.21E-05 8750
0.00013805 11000 8.48E-06 10500 1.07E-05
11400 4.79E-05 9360 6.38E-06 9160 2.22E-05 8750
0.000142131 11000 8.47E-06 10500 1.07E-05
11400 4.79E-05 9360 6.40E-06 9160 2.22E-05 8750
0.000146395 11000 8.48E-06 10500 1.08E-05

11400 4.78E-05 9360 6.41E-06 9160 2.22E-05 8750
0.000150886 11000 8.47E-06 10500 1.08E-05
11400 4.78E-05 9370 6.42E-06 9160 2.22E-05 8760
0.000155593 11000 8.48E-06 10500 1.08E-05
11400 4.77E-05 9370 6.43E-06 9160 2.23E-05 8760
0.000160541 11000 8.49E-06 10500 1.08E-05
11400 4.77E-05 9370 6.45E-06 9170 2.23E-05 8760
0.000165765 11000 8.50E-06 10500 1.08E-05
11400 4.76E-05 9370 6.46E-06 9170 2.23E-05 8760
0.000171292 11000 8.51E-06 10500 1.08E-05
11400 4.76E-05 9370 6.47E-06 9170 2.23E-05 8760
0.000177141 11000 8.50E-06 10500 1.08E-05
11400 4.75E-05 9370 6.49E-06 9170 2.23E-05 8760
0.000183303 11000 8.50E-06 10500 1.08E-05
11400 4.75E-05 9380 6.49E-06 9170 2.24E-05 8770
0.000189831 11000 8.50E-06 10500 1.08E-05
11400 4.74E-05 9380 6.51E-06 9170 2.24E-05 8770
0.000196731 11100 8.50E-06 10500 1.08E-05
11400 4.74E-05 9380 6.52E-06 9180 2.24E-05 8770
0.000204041 11100 8.49E-06 10500 1.08E-05
11400 4.73E-05 9380 6.52E-06 9180 2.24E-05 8770
0.000211805 11100 8.50E-06 10500 1.08E-05
11400 4.72E-05 9380 6.55E-06 9180 2.25E-05 8770
0.000220019 11100 8.49E-06 10500 1.08E-05
11400 4.72E-05 9380 6.56E-06 9180 2.25E-05 8770
0.000228717 11100 8.50E-06 10500 1.08E-05
11400 4.71E-05 9380 6.57E-06 9180 2.25E-05 8780
0.000237963 11100 8.50E-06 10500 1.08E-05
11400 4.70E-05 9390 6.58E-06 9180 2.25E-05 8780
0.000247781 11100 8.50E-06 10500 1.08E-05
11400 4.70E-05 9390 6.59E-06 9180 2.25E-05 8780
0.000258241 11100 8.50E-06 10500 1.08E-05
11400 4.69E-05 9390 6.60E-06 9190 2.26E-05 8780
0.000269347 11100 8.50E-06 10500 1.08E-05
11400 4.69E-05 9390 6.61E-06 9190 2.26E-05 8780
0.000281139 11100 8.49E-06 10500 1.08E-05
11400 4.68E-05 9390 6.63E-06 9190 2.26E-05 8780
0.000293765 11100 8.50E-06 10500 1.08E-05
11400 4.68E-05 9390 6.64E-06 9190 2.26E-05 8780
0.000307273 11100 8.49E-06 10500 1.08E-05
11400 4.67E-05 9400 6.65E-06 9190 2.26E-05 8790
0.000321725 11100 8.51E-06 10500 1.08E-05
11400 4.67E-05 9400 6.65E-06 9190 2.27E-05 8790
0.000337299 11100 8.51E-06 10500 1.08E-05
11400 4.66E-05 9400 6.67E-06 9200 2.27E-05 8790
0.000354092 11100 8.51E-06 10500 1.08E-05
11400 4.66E-05 9400 6.67E-06 9200 2.27E-05 8790
0.000372248 11100 8.53E-06 10500 1.08E-05
11400 4.65E-05 9400 6.69E-06 9200 2.27E-05 8790
0.000391863 11100 8.52E-06 10500 1.08E-05
11400 4.65E-05 9400 6.70E-06 9200 2.27E-05 8790
0.000413115 11100 8.51E-06 10500 1.08E-05
11400 4.64E-05 9400 6.71E-06 9200 2.28E-05 8800
0.000436168 11100 8.51E-06 10500 1.08E-05
11400 4.64E-05 9410 6.72E-06 9200 2.28E-05 8800
0.000461159 11100 8.48E-06 10500 1.08E-05
11400 4.63E-05 9410 6.73E-06 9200 2.28E-05 8800
0.00048834 11100 8.50E-06 10500 1.08E-05
11400 4.63E-05 9410 6.74E-06 9210 2.28E-05 8800
0.000517966 11100 8.49E-06 10500 1.08E-05
11400 4.63E-05 9410 6.75E-06 9210 2.28E-05 8800
0.000550326 11100 8.50E-06 10500 1.08E-05
11400 4.62E-05 9410 6.76E-06 9210 2.29E-05 8800
0.000585751 11100 8.49E-06 10500 1.08E-05
11400 4.62E-05 9410 6.77E-06 9210 2.29E-05 8800
0.000624583 11100 8.48E-06 10500 1.08E-05
11400 4.61E-05 9420 6.78E-06 9210 2.29E-05 8810
0.000667241 11100 8.46E-06 10500 1.08E-05

11400 4.61E-05 9420 6.79E-06 9210 2.29E-05 8810
0.000714138 11100 8.46E-06 10500 1.08E-05
11400 4.60E-05 9420 6.80E-06 9220 2.29E-05 8810
0.000765821 11100 8.46E-06 10500 1.08E-05
11400 4.60E-05 9420 6.81E-06 9220 2.29E-05 8810
0.000822838 11100 8.45E-06 10500 1.08E-05
11500 4.60E-05 9420 6.81E-06 9220 2.30E-05 8810
0.000885865 11100 8.45E-06 10500 1.08E-05
11500 4.59E-05 9420 6.82E-06 9220 2.30E-05 8810
0.000955662 11100 8.45E-06 10500 1.08E-05
11500 4.59E-05 9430 6.82E-06 9220 2.30E-05 8820
0.001033 11100 8.45E-06 10500 1.08E-05
11500 4.59E-05 9430 6.83E-06 9220 2.30E-05 8820
0.001118869 11100 8.46E-06 10500 1.08E-05
11500 4.58E-05 9430 6.84E-06 9230 2.30E-05 8820
0.001214325 11100 8.46E-06 10500 1.08E-05
11500 4.58E-05 9430 6.85E-06 9230 2.30E-05 8820
0.001320638 11100 8.48E-06 10500 1.08E-05
11500 4.58E-05 9430 6.86E-06 9230 2.31E-05 8820
0.001439121 11100 8.46E-06 10500 1.08E-05
11500 4.57E-05 9430 6.88E-06 9230 2.31E-05 8820
0.001571284 11100 8.48E-06 10500 1.08E-05
11500 4.56E-05 9430 6.88E-06 9230 2.31E-05 8830
0.001718666 11100 8.47E-06 10500 1.08E-05
11500 4.56E-05 9440 6.90E-06 9230 2.31E-05 8830
0.001882787 11100 8.47E-06 10600 1.08E-05
11500 4.55E-05 9440 6.90E-06 9230 2.32E-05 8830
0.002065248 11100 8.47E-06 10600 1.08E-05
11500 4.55E-05 9440 6.91E-06 9240 2.32E-05 8830
0.00226759 11100 8.49E-06 10600 1.08E-05
11500 4.54E-05 9440 6.92E-06 9240 2.32E-05 8830
0.002491526 11100 8.51E-06 10600 1.08E-05
11500 4.54E-05 9440 6.93E-06 9240 2.32E-05 8830
0.002738681 11100 8.49E-06 10600 1.08E-05
11500 4.53E-05 9440 6.93E-06 9240 2.32E-05 8830
0.003010468 11100 8.48E-06 10600 1.08E-05
11500 4.53E-05 9450 6.95E-06 9240 2.33E-05 8840
0.0033076 11100 8.48E-06 10600 1.08E-05
11500 4.52E-05 9450 6.96E-06 9240 2.33E-05 8840
0.00362968 11100 8.49E-06 10600 1.08E-05
11500 4.52E-05 9450 6.97E-06 9250 2.33E-05 8840
0.003974969 11100 8.49E-06 10600 1.08E-05
11500 4.51E-05 9450 6.98E-06 9250 2.34E-05 8840
0.004340042 11100 8.50E-06 10600 1.08E-05
11500 4.51E-05 9450 6.98E-06 9250 2.34E-05 8840
0.004719755 11100 8.45E-06 10600 1.08E-05
11500 4.50E-05 9450 6.99E-06 9250 2.34E-05 8840
0.005106976 11100 8.45E-06 10600 1.08E-05
11500 4.50E-05 9450 7.00E-06 9250 2.35E-05 8850
0.005492202 11100 8.46E-06 10600 1.08E-05
11500 4.50E-05 9460 7.01E-06 9250 2.35E-05 8850
0.005863896 11100 8.44E-06 10600 1.08E-05
11500 4.49E-05 9460 7.01E-06 9250 2.35E-05 8850
0.006208804 11100 8.44E-06 10600 1.08E-05
11500 4.49E-05 9460 7.02E-06 9260 2.36E-05 8850
0.006513019 11100 8.45E-06 10600 1.08E-05
11500 4.48E-05 9460 7.03E-06 9260 2.36E-05 8850
0.006763181 11100 8.45E-06 10600 1.08E-05
11500 4.48E-05 9460 7.04E-06 9260 2.37E-05 8850
0.006947901 11100 8.43E-06 10600 1.08E-05
11500 4.47E-05 9460 7.05E-06 9260 2.37E-05 8850
0.007058778 11100 8.43E-06 10600 1.08E-05
11500 4.47E-05 9470 7.06E-06 9260 2.38E-05 8860
0.007091312 11100 8.42E-06 10600 1.08E-05
11500 4.47E-05 9470 7.08E-06 9260 2.38E-05 8860
0.007045389 11100 8.43E-06 10600 1.08E-05
11500 4.46E-05 9470 7.08E-06 9270 2.38E-05 8860
0.006924838 11100 8.44E-06 10600 1.08E-05

11500 4.46E-05 9470 7.10E-06 9270 2.39E-05 8860
0.006736986 11100 8.44E-06 10600 1.08E-05
11500 4.45E-05 9470 7.10E-06 9270 2.39E-05 8860
0.006491674 11100 8.42E-06 10600 1.08E-05
11500 4.45E-05 9470 7.11E-06 9270 2.39E-05 8860
0.006200418 11100 8.40E-06 10600 1.08E-05
11500 4.45E-05 9480 7.12E-06 9270 2.40E-05 8870
0.005875443 11100 8.39E-06 10600 1.08E-05
11500 4.44E-05 9480 7.12E-06 9270 2.40E-05 8870
0.005528983 11200 8.39E-06 10600 1.08E-05
11500 4.44E-05 9480 7.13E-06 9280 2.40E-05 8870
0.005172439 11200 8.39E-06 10600 1.08E-05
11500 4.43E-05 9480 7.14E-06 9280 2.41E-05 8870
0.004815812 11200 8.37E-06 10600 1.08E-05
11500 4.43E-05 9480 7.15E-06 9280 2.41E-05 8870
0.004467416 11200 8.36E-06 10600 1.08E-05
11500 4.43E-05 9480 7.17E-06 9280 2.42E-05 8870
0.004133543 11200 8.40E-06 10600 1.08E-05
11500 4.42E-05 9480 7.18E-06 9280 2.42E-05 8880
0.003818441 11200 8.38E-06 10600 1.08E-05
11500 4.42E-05 9490 7.19E-06 9280 2.42E-05 8880
0.003524596 11200 8.36E-06 10600 1.08E-05
11500 4.42E-05 9490 7.19E-06 9280 2.43E-05 8880
0.003252795 11200 8.40E-06 10600 1.08E-05
11500 4.41E-05 9490 7.20E-06 9290 2.43E-05 8880
0.003002585 11200 8.41E-06 10600 1.08E-05
11500 4.41E-05 9490 7.21E-06 9290 2.43E-05 8880
0.002772584 11200 8.37E-06 10600 1.08E-05
11500 4.40E-05 9490 7.23E-06 9290 2.44E-05 8880
0.002560973 11200 8.35E-06 10600 1.08E-05
11500 4.40E-05 9490 7.24E-06 9290 2.44E-05 8880
0.002365733 11200 8.34E-06 10600 1.08E-05
11500 4.39E-05 9500 7.25E-06 9290 2.45E-05 8890
0.002185127 11200 8.35E-06 10600 1.08E-05
11500 4.39E-05 9500 7.26E-06 9290 2.45E-05 8890
0.002017703 11200 8.33E-06 10600 1.08E-05
11500 4.39E-05 9500 7.26E-06 9300 2.45E-05 8890
0.001862316 11200 8.41E-06 10600 1.08E-05
11500 4.38E-05 9500 7.28E-06 9300 2.46E-05 8890
0.001718189 11200 8.38E-06 10600 1.08E-05
11500 4.38E-05 9500 7.29E-06 9300 2.46E-05 8890
0.00158472 11200 8.41E-06 10600 1.08E-05
11500 4.37E-05 9500 7.30E-06 9300 2.46E-05 8890
0.001461423 11200 8.40E-06 10600 1.08E-05
11500 4.37E-05 9500 7.31E-06 9300 2.47E-05 8900
0.001347859 11200 8.36E-06 10600 1.08E-05
11500 4.36E-05 9510 7.32E-06 9300 2.47E-05 8900
0.001243573 11200 8.38E-06 10600 1.08E-05
11500 4.36E-05 9510 7.33E-06 9300 2.47E-05 8900
0.001148067 11200 8.36E-06 10600 1.08E-05
11500 4.36E-05 9510 7.34E-06 9310 2.48E-05 8900
0.001060862 11200 8.36E-06 10600 1.08E-05
11500 4.35E-05 9510 7.35E-06 9310 2.48E-05 8900
0.000981454 11200 8.37E-06 10600 1.08E-05
11500 4.35E-05 9510 7.36E-06 9310 2.48E-05 8900
0.000909292 11200 8.35E-06 10600 1.08E-05
11500 4.34E-05 9510 7.38E-06 9310 2.49E-05 8900
0.000843806 11200 8.38E-06 10600 1.08E-05
11500 4.34E-05 9520 7.39E-06 9310 2.49E-05 8910
0.000784431 11200 8.39E-06 10600 1.08E-05
11500 4.33E-05 9520 7.40E-06 9310 2.49E-05 8910
0.000730569 11200 8.43E-06 10600 1.08E-05
11500 4.33E-05 9520 7.40E-06 9320 2.50E-05 8910
0.000681637 11200 8.44E-06 10600 1.08E-05
11500 4.33E-05 9520 7.41E-06 9320 2.50E-05 8910
0.000637114 11200 8.45E-06 10600 1.08E-05
11600 4.32E-05 9520 7.43E-06 9320 2.50E-05 8910
0.00059652 11200 8.46E-06 10600 1.08E-05

11600 4.32E-05 9520 7.44E-06 9320 2.51E-05 8910
0.00055944 11200 8.45E-06 10600 1.08E-05
11600 4.32E-05 9530 7.45E-06 9320 2.51E-05 8920
0.000525533 11200 8.43E-06 10600 1.08E-05
11600 4.31E-05 9530 7.46E-06 9320 2.51E-05 8920
0.000494535 11200 8.44E-06 10600 1.08E-05
11600 4.31E-05 9530 7.46E-06 9330 2.52E-05 8920
0.000466152 11200 8.45E-06 10600 1.08E-05
11600 4.30E-05 9530 7.47E-06 9330 2.52E-05 8920
0.000440137 11200 8.46E-06 10600 1.08E-05
11600 4.30E-05 9530 7.48E-06 9330 2.52E-05 8920
0.000416265 11200 8.47E-06 10600 1.08E-05
11600 4.30E-05 9530 7.49E-06 9330 2.53E-05 8920
0.000394328 11200 8.48E-06 10600 1.08E-05
11600 4.29E-05 9530 7.50E-06 9330 2.53E-05 8930
0.000374116 11200 8.49E-06 10600 1.08E-05
11600 4.29E-05 9540 7.50E-06 9330 2.53E-05 8930
0.000355463 11200 8.52E-06 10700 1.08E-05
11600 4.29E-05 9540 7.51E-06 9330 2.54E-05 8930
0.000338216 11200 8.54E-06 10700 1.08E-05
11600 4.28E-05 9540 7.52E-06 9340 2.54E-05 8930
0.000322248 11200 8.54E-06 10700 1.08E-05
11600 4.28E-05 9540 7.54E-06 9340 2.54E-05 8930
0.000307451 11200 8.55E-06 10700 1.08E-05
11600 4.28E-05 9540 7.55E-06 9340 2.55E-05 8930
0.000293758 11200 8.55E-06 10700 1.08E-05
11600 4.27E-05 9540 7.56E-06 9340 2.55E-05 8930
0.000280994 11200 8.58E-06 10700 1.09E-05
11600 4.27E-05 9550 7.58E-06 9340 2.55E-05 8940
0.000269143 11200 8.55E-06 10700 1.08E-05
11600 4.27E-05 9550 7.59E-06 9340 2.56E-05 8940
0.000258077 11200 8.56E-06 10700 1.08E-05
11600 4.27E-05 9550 7.59E-06 9350 2.56E-05 8940
0.000247709 11200 8.56E-06 10700 1.09E-05
11600 4.27E-05 9550 7.60E-06 9350 2.56E-05 8940
0.000237968 11200 8.58E-06 10700 1.08E-05
11600 4.26E-05 9550 7.61E-06 9350 2.57E-05 8940
0.000228814 11200 8.56E-06 10700 1.09E-05
11600 4.26E-05 9550 7.63E-06 9350 2.57E-05 8940
0.000220198 11200 8.56E-06 10700 1.08E-05
11600 4.25E-05 9550 7.63E-06 9350 2.58E-05 8950
0.000212087 11200 8.57E-06 10700 1.08E-05
11600 4.25E-05 9560 7.65E-06 9350 2.58E-05 8950
0.000204482 11200 8.56E-06 10700 1.09E-05
11600 4.24E-05 9560 7.66E-06 9350 2.58E-05 8950
0.000197343 11200 8.57E-06 10700 1.08E-05
11600 4.24E-05 9560 7.66E-06 9360 2.59E-05 8950
0.000190647 11200 8.57E-06 10700 1.08E-05
11600 4.24E-05 9560 7.67E-06 9360 2.59E-05 8950
0.000184374 11200 8.58E-06 10700 1.08E-05
11600 4.23E-05 9560 7.68E-06 9360 2.59E-05 8950
0.000178457 11200 8.60E-06 10700 1.08E-05
11600 4.23E-05 9560 7.69E-06 9360 2.59E-05 8950
0.000172875 11200 8.60E-06 10700 1.08E-05
11600 4.23E-05 9570 7.70E-06 9360 2.60E-05 8960
0.000167585 11200 8.61E-06 10700 1.08E-05
11600 4.23E-05 9570 7.72E-06 9360 2.60E-05 8960
0.000162522 11200 8.61E-06 10700 1.09E-05
11600 4.22E-05 9570 7.73E-06 9370 2.60E-05 8960
0.00015771 11200 8.61E-06 10700 1.09E-05
11600 4.22E-05 9570 7.74E-06 9370 2.61E-05 8960
0.000153115 11200 8.61E-06 10700 1.09E-05
11600 4.22E-05 9570 7.76E-06 9370 2.61E-05 8960
0.000148764 11200 8.62E-06 10700 1.08E-05
11600 4.22E-05 9570 7.77E-06 9370 2.62E-05 8960
0.000144608 11200 8.61E-06 10700 1.09E-05
11600 4.21E-05 9580 7.78E-06 9370 2.62E-05 8970
0.000140664 11200 8.62E-06 10700 1.09E-05

11600	4.21E-05	9580	7.80E-06	9370	2.62E-05	8970
0.000136915	11300	8.62E-06	10700	1.09E-05		
11600	4.21E-05	9580	7.80E-06	9380	2.63E-05	8970
0.000133351	11300	8.61E-06	10700	1.09E-05		
11600	4.20E-05	9580	7.82E-06	9380	2.63E-05	8970
0.000129952	11300	8.62E-06	10700	1.09E-05		
11600	4.20E-05	9580	7.83E-06	9380	2.63E-05	8970
0.000126712	11300	8.62E-06	10700	1.08E-05		
11600	4.20E-05	9580	7.84E-06	9380	2.64E-05	8970
0.000123629	11300	8.62E-06	10700	1.08E-05		
11600	4.20E-05	9580	7.85E-06	9380	2.64E-05	8980
0.000120662	11300	8.62E-06	10700	1.09E-05		
11600	4.19E-05	9590	7.87E-06	9380	2.65E-05	8980
0.000117819	11300	8.62E-06	10700	1.09E-05		
11600	4.19E-05	9590	7.88E-06	9380	2.65E-05	8980
0.000115095	11300	8.63E-06	10700	1.09E-05		
11600	4.19E-05	9590	7.89E-06	9390	2.65E-05	8980
0.000112481	11300	8.64E-06	10700	1.08E-05		
11600	4.18E-05	9590	7.90E-06	9390	2.65E-05	8980
0.000109965	11300	8.63E-06	10700	1.09E-05		
11600	4.18E-05	9590	7.90E-06	9390	2.66E-05	8980
0.000107558	11300	8.63E-06	10700	1.09E-05		
11600	4.18E-05	9590	7.91E-06	9390	2.66E-05	8980
0.00010524	11300	8.64E-06	10700	1.09E-05		
11600	4.17E-05	9600	7.92E-06	9390	2.66E-05	8990
0.000103035	11300	8.63E-06	10700	1.09E-05		
11600	4.17E-05	9600	7.93E-06	9390	2.67E-05	8990
0.000100903	11300	8.63E-06	10700	1.09E-05		
11600	4.17E-05	9600	7.93E-06	9400	2.67E-05	8990
9.89E-05	11300	8.63E-06	10700	1.09E-05		
11600	4.17E-05	9600	7.95E-06	9400	2.67E-05	8990
9.69E-05	11300	8.65E-06	10700	1.09E-05		
11600	4.16E-05	9600	7.97E-06	9400	2.68E-05	8990
9.50E-05	11300	8.64E-06	10700	1.09E-05		
11600	4.16E-05	9600	7.97E-06	9400	2.68E-05	8990
9.32E-05	11300	8.65E-06	10700	1.09E-05		
11600	4.16E-05	9600	7.99E-06	9400	2.68E-05	9000
9.14E-05	11300	8.66E-06	10700	1.09E-05		
11600	4.16E-05	9610	8.00E-06	9400	2.69E-05	9000
8.97E-05	11300	8.66E-06	10700	1.09E-05		
11600	4.16E-05	9610	8.00E-06	9400	2.69E-05	9000
8.81E-05	11300	8.66E-06	10700	1.09E-05		
11600	4.15E-05	9610	8.02E-06	9410	2.69E-05	9000
8.65E-05	11300	8.67E-06	10700	1.09E-05		
11600	4.15E-05	9610	8.04E-06	9410	2.70E-05	9000
8.50E-05	11300	8.66E-06	10700	1.09E-05		
11600	4.15E-05	9610	8.05E-06	9410	2.70E-05	9000
8.35E-05	11300	8.66E-06	10700	1.09E-05		
11600	4.14E-05	9610	8.06E-06	9410	2.71E-05	9000
8.21E-05	11300	8.66E-06	10700	1.09E-05		
11600	4.14E-05	9620	8.08E-06	9410	2.71E-05	9010
8.07E-05	11300	8.66E-06	10700	1.09E-05		
11600	4.14E-05	9620	8.08E-06	9410	2.71E-05	9010
7.94E-05	11300	8.67E-06	10700	1.09E-05		
11600	4.13E-05	9620	8.09E-06	9420	2.72E-05	9010
7.82E-05	11300	8.67E-06	10700	1.09E-05		
11600	4.13E-05	9620	8.11E-06	9420	2.72E-05	9010
7.70E-05	11300	8.67E-06	10700	1.09E-05		
11700	4.13E-05	9620	8.12E-06	9420	2.72E-05	9010
7.58E-05	11300	8.68E-06	10700	1.09E-05		
11700	4.13E-05	9620	8.13E-06	9420	2.73E-05	9010
7.47E-05	11300	8.68E-06	10700	1.09E-05		
11700	4.12E-05	9630	8.15E-06	9420	2.73E-05	9020
7.36E-05	11300	8.69E-06	10700	1.09E-05		
11700	4.12E-05	9630	8.15E-06	9420	2.73E-05	9020
7.26E-05	11300	8.68E-06	10700	1.09E-05		
11700	4.12E-05	9630	8.17E-06	9430	2.74E-05	9020
7.16E-05	11300	8.69E-06	10700	1.09E-05		

11700	4.12E-05	9630	8.17E-06	9430	2.74E-05	9020	7.06E-05	11300	8.69E-06	10700	1.09E-05
11700	4.12E-05	9630	8.18E-06	9430	2.74E-05	9020	6.97E-05	11300	8.69E-06	10700	1.09E-05
11700	4.11E-05	9630	8.20E-06	9430	2.75E-05	9020	6.88E-05	11300	8.69E-06	10700	1.09E-05
11700	4.11E-05	9630	8.22E-06	9430	2.75E-05	9030	6.79E-05	11300	8.69E-06	10700	1.09E-05
11700	4.11E-05	9640	8.22E-06	9430	2.76E-05	9030	6.71E-05	11300	8.70E-06	10800	1.10E-05
11700	4.11E-05	9640	8.23E-06	9430	2.76E-05	9030	6.63E-05	11300	8.70E-06	10800	1.10E-05
11700	4.11E-05	9640	8.25E-06	9440	2.76E-05	9030	6.56E-05	11300	8.71E-06	10800	1.10E-05
11700	4.10E-05	9640	8.26E-06	9440	2.77E-05	9030	6.48E-05	11300	8.70E-06	10800	1.10E-05
11700	4.10E-05	9640	8.28E-06	9440	2.77E-05	9030	6.41E-05	11300	8.71E-06	10800	1.10E-05
11700	4.10E-05	9640	8.30E-06	9440	2.78E-05	9030	6.34E-05	11300	8.71E-06	10800	1.10E-05
11700	4.09E-05	9650	8.31E-06	9440	2.78E-05	9040	6.28E-05	11300	8.72E-06	10800	1.10E-05
11700	4.09E-05	9650	8.32E-06	9440	2.78E-05	9040	6.21E-05	11300	8.73E-06	10800	1.10E-05
11700	4.09E-05	9650	8.34E-06	9450	2.79E-05	9040	6.15E-05	11300	8.73E-06	10800	1.10E-05
11700	4.09E-05	9650	8.35E-06	9450	2.79E-05	9040	6.09E-05	11300	8.73E-06	10800	1.10E-05
11700	4.08E-05	9650	8.36E-06	9450	2.79E-05	9040	6.03E-05	11300	8.73E-06	10800	1.10E-05
11700	4.08E-05	9650	8.38E-06	9450	2.80E-05	9040	5.98E-05	11300	8.73E-06	10800	1.10E-05
11700	4.08E-05	9650	8.38E-06	9450	2.80E-05	9050	5.93E-05	11300	8.74E-06	10800	1.10E-05
11700	4.08E-05	9660	8.39E-06	9450	2.80E-05	9050	5.88E-05	11300	8.73E-06	10800	1.10E-05
11700	4.07E-05	9660	8.41E-06	9450	2.81E-05	9050	5.84E-05	11300	8.74E-06	10800	1.10E-05
11700	4.07E-05	9660	8.42E-06	9460	2.81E-05	9050	5.80E-05	11300	8.74E-06	10800	1.10E-05
11700	4.07E-05	9660	8.43E-06	9460	2.81E-05	9050	5.75E-05	11300	8.75E-06	10800	1.10E-05
11700	4.07E-05	9660	8.45E-06	9460	2.82E-05	9050	5.72E-05	11300	8.74E-06	10800	1.10E-05
11700	4.07E-05	9660	8.46E-06	9460	2.82E-05	9050	5.68E-05	11300	8.74E-06	10800	1.10E-05
11700	4.06E-05	9670	8.47E-06	9460	2.83E-05	9060	5.65E-05	11300	8.75E-06	10800	1.10E-05
11700	4.06E-05	9670	8.47E-06	9460	2.83E-05	9060	5.61E-05	11300	8.75E-06	10800	1.10E-05
11700	4.06E-05	9670	8.48E-06	9470	2.83E-05	9060	5.58E-05	11300	8.75E-06	10800	1.10E-05
11700	4.06E-05	9670	8.50E-06	9470	2.84E-05	9060	5.55E-05	11300	8.75E-06	10800	1.10E-05
11700	4.06E-05	9670	8.51E-06	9470	2.84E-05	9060	5.52E-05	11300	8.75E-06	10800	1.10E-05
11700	4.05E-05	9670	8.52E-06	9470	2.85E-05	9060	5.50E-05	11300	8.76E-06	10800	1.10E-05
11700	4.05E-05	9680	8.53E-06	9470	2.85E-05	9070	5.47E-05	11300	8.75E-06	10800	1.10E-05
11700	4.05E-05	9680	8.54E-06	9470	2.85E-05	9070	5.45E-05	11400	8.76E-06	10800	1.10E-05
11700	4.05E-05	9680	8.56E-06	9480	2.86E-05	9070	5.43E-05	11400	8.76E-06	10800	1.10E-05
11700	4.05E-05	9680	8.56E-06	9480	2.86E-05	9070	5.41E-05	11400	8.76E-06	10800	1.10E-05
11700	4.04E-05	9680	8.58E-06	9480	2.86E-05	9070	5.40E-05	11400	8.76E-06	10800	1.10E-05

11700 4.04E-05 9680 8.59E-06 9480 2.87E-05 9070
5.39E-05 11400 8.76E-06 10800 1.11E-05
11700 4.04E-05 9680 8.61E-06 9480 2.87E-05 9080
5.38E-05 11400 8.76E-06 10800 1.11E-05
11700 4.04E-05 9690 8.62E-06 9480 2.88E-05 9080
5.38E-05 11400 8.76E-06 10800 1.11E-05
11700 4.03E-05 9690 8.63E-06 9480 2.88E-05 9080
5.37E-05 11400 8.78E-06 10800 1.11E-05
11700 4.03E-05 9690 8.65E-06 9490 2.88E-05 9080
5.37E-05 11400 8.78E-06 10800 1.11E-05
11700 4.03E-05 9690 8.66E-06 9490 2.89E-05 9080
5.38E-05 11400 8.78E-06 10800 1.11E-05
11700 4.02E-05 9690 8.67E-06 9490 2.89E-05 9080
5.39E-05 11400 8.79E-06 10800 1.11E-05
11700 4.03E-05 9690 8.68E-06 9490 2.90E-05 9080
5.40E-05 11400 8.79E-06 10800 1.11E-05
11700 4.02E-05 9700 8.69E-06 9490 2.90E-05 9090
5.42E-05 11400 8.79E-06 10800 1.11E-05
11700 4.02E-05 9700 8.71E-06 9490 2.90E-05 9090
5.44E-05 11400 8.79E-06 10800 1.11E-05
11700 4.02E-05 9700 8.72E-06 9500 2.91E-05 9090
5.47E-05 11400 8.80E-06 10800 1.11E-05
11700 4.01E-05 9700 8.73E-06 9500 2.91E-05 9090
5.50E-05 11400 8.79E-06 10800 1.11E-05
11700 4.01E-05 9700 8.75E-06 9500 2.92E-05 9090
5.54E-05 11400 8.80E-06 10800 1.11E-05
11700 4.01E-05 9700 8.76E-06 9500 2.92E-05 9090
5.58E-05 11400 8.80E-06 10800 1.11E-05
11700 4.01E-05 9700 8.79E-06 9500 2.93E-05 9100
5.63E-05 11400 8.80E-06 10800 1.11E-05
11700 4.01E-05 9710 8.79E-06 9500 2.93E-05 9100
5.68E-05 11400 8.80E-06 10800 1.11E-05
11700 4.00E-05 9710 8.80E-06 9500 2.93E-05 9100
5.75E-05 11400 8.80E-06 10800 1.11E-05
11700 4.00E-05 9710 8.82E-06 9510 2.94E-05 9100
5.82E-05 11400 8.80E-06 10800 1.11E-05
11700 4.00E-05 9710 8.83E-06 9510 2.94E-05 9100
5.90E-05 11400 8.82E-06 10800 1.11E-05
11700 4.00E-05 9710 8.85E-06 9510 2.94E-05 9100
5.99E-05 11400 8.81E-06 10800 1.11E-05
11700 3.99E-05 9710 8.86E-06 9510 2.95E-05 9100
6.09E-05 11400 8.82E-06 10800 1.11E-05
11700 3.99E-05 9720 8.88E-06 9510 2.95E-05 9110
6.19E-05 11400 8.81E-06 10800 1.11E-05
11700 3.99E-05 9720 8.88E-06 9510 2.96E-05 9110
6.31E-05 11400 8.81E-06 10800 1.11E-05
11700 3.99E-05 9720 8.89E-06 9520 2.96E-05 9110
6.43E-05 11400 8.82E-06 10800 1.11E-05
11700 3.99E-05 9720 8.90E-06 9520 2.96E-05 9110
6.57E-05 11400 8.82E-06 10800 1.11E-05
11800 3.98E-05 9720 8.92E-06 9520 2.97E-05 9110
6.71E-05 11400 8.83E-06 10800 1.11E-05
11800 3.98E-05 9720 8.93E-06 9520 2.97E-05 9110
6.87E-05 11400 8.83E-06 10800 1.11E-05
11800 3.98E-05 9730 8.94E-06 9520 2.97E-05 9120
7.03E-05 11400 8.83E-06 10800 1.12E-05
11800 3.97E-05 9730 8.96E-06 9520 2.98E-05 9120
7.20E-05 11400 8.83E-06 10800 1.12E-05
11800 3.97E-05 9730 8.97E-06 9530 2.98E-05 9120
7.38E-05 11400 8.84E-06 10800 1.12E-05
11800 3.97E-05 9730 8.98E-06 9530 2.99E-05 9120
7.56E-05 11400 8.83E-06 10800 1.12E-05
11800 3.97E-05 9730 9.00E-06 9530 2.99E-05 9120
7.75E-05 11400 8.84E-06 10800 1.12E-05
11800 3.97E-05 9730 9.01E-06 9530 3.00E-05 9120
7.95E-05 11400 8.84E-06 10800 1.12E-05
11800 3.97E-05 9730 9.03E-06 9530 3.00E-05 9130
8.15E-05 11400 8.84E-06 10800 1.12E-05

11800	3.96E-05	9740	9.05E-06	9530	3.00E-05	9130	8.36E-05	11400	8.84E-06	10900	1.12E-05
11800	3.96E-05	9740	9.07E-06	9530	3.01E-05	9130	8.57E-05	11400	8.84E-06	10900	1.12E-05
11800	3.96E-05	9740	9.09E-06	9540	3.01E-05	9130	8.78E-05	11400	8.84E-06	10900	1.12E-05
11800	3.96E-05	9740	9.10E-06	9540	3.02E-05	9130	8.99E-05	11400	8.83E-06	10900	1.12E-05
11800	3.96E-05	9740	9.12E-06	9540	3.02E-05	9130	9.20E-05	11400	8.84E-06	10900	1.12E-05
11800	3.95E-05	9740	9.13E-06	9540	3.02E-05	9130	9.41E-05	11400	8.85E-06	10900	1.12E-05
11800	3.96E-05	9750	9.15E-06	9540	3.03E-05	9140	9.61E-05	11400	8.84E-06	10900	1.12E-05
11800	3.95E-05	9750	9.15E-06	9540	3.03E-05	9140	9.81E-05	11400	8.84E-06	10900	1.12E-05
11800	3.95E-05	9750	9.17E-06	9550	3.04E-05	9140	9.99E-05	11400	8.85E-06	10900	1.12E-05
11800	3.95E-05	9750	9.17E-06	9550	3.04E-05	9140	0.000101647	11400	8.85E-06	10900	1.12E-05
11800	3.95E-05	9750	9.19E-06	9550	3.05E-05	9140	0.000103293	11400	8.85E-06	10900	1.12E-05
11800	3.95E-05	9750	9.20E-06	9550	3.05E-05	9140	0.000104873	11400	8.85E-06	10900	1.12E-05
11800	3.95E-05	9750	9.20E-06	9550	3.06E-05	9150	0.000106375	11400	8.86E-06	10900	1.12E-05
11800	3.95E-05	9760	9.22E-06	9550	3.06E-05	9150	0.000107784	11400	8.86E-06	10900	1.12E-05
11800	3.94E-05	9760	9.23E-06	9550	3.07E-05	9150	0.000109042	11400	8.86E-06	10900	1.12E-05
11800	3.94E-05	9760	9.26E-06	9560	3.07E-05	9150	0.000110164	11400	8.85E-06	10900	1.12E-05
11800	3.94E-05	9760	9.27E-06	9560	3.08E-05	9150	0.000111102	11400	8.86E-06	10900	1.12E-05
11800	3.94E-05	9760	9.29E-06	9560	3.08E-05	9150	0.000111886	11400	8.86E-06	10900	1.12E-05
11800	3.93E-05	9760	9.32E-06	9560	3.08E-05	9150	0.000112505	11400	8.87E-06	10900	1.12E-05
11800	3.93E-05	9770	9.34E-06	9560	3.09E-05	9160	0.00011301	11400	8.87E-06	10900	1.12E-05
11800	3.93E-05	9770	9.35E-06	9560	3.09E-05	9160	0.000113402	11400	8.87E-06	10900	1.12E-05
11800	3.93E-05	9770	9.37E-06	9570	3.10E-05	9160	0.000113739	11400	8.88E-06	10900	1.12E-05
11800	3.93E-05	9770	9.39E-06	9570	3.10E-05	9160	0.000114035	11400	8.87E-06	10900	1.12E-05
11800	3.93E-05	9770	9.41E-06	9570	3.11E-05	9160	0.00011427	11400	8.87E-06	10900	1.12E-05
11800	3.92E-05	9770	9.42E-06	9570	3.11E-05	9160	0.000114516	11400	8.88E-06	10900	1.12E-05
11800	3.92E-05	9780	9.44E-06	9570	3.11E-05	9170	0.000114758	11400	8.88E-06	10900	1.12E-05
11800	3.92E-05	9780	9.45E-06	9570	3.12E-05	9170	0.000114989	11500	8.88E-06	10900	1.13E-05
11800	3.92E-05	9780	9.46E-06	9580	3.12E-05	9170	0.000115184	11500	8.89E-06	10900	1.13E-05
11800	3.92E-05	9780	9.48E-06	9580	3.12E-05	9170	0.000115357	11500	8.90E-06	10900	1.13E-05
11800	3.92E-05	9780	9.50E-06	9580	3.13E-05	9170	0.000115425	11500	8.90E-06	10900	1.13E-05
11800	3.91E-05	9780	9.51E-06	9580	3.14E-05	9170	0.000115383	11500	8.90E-06	10900	1.13E-05
11800	3.91E-05	9780	9.51E-06	9580	3.14E-05	9180	0.000115215	11500	8.91E-06	10900	1.13E-05
11800	3.91E-05	9790	9.52E-06	9580	3.14E-05	9180	0.000114871	11500	8.91E-06	10900	1.13E-05
11800	3.90E-05	9790	9.53E-06	9580	3.15E-05	9180	0.00011435	11500	8.91E-06	10900	1.13E-05

11800 3.90E-05 9790 9.55E-06 9590 3.15E-05 9180
0.000113683 11500 8.91E-06 10900 1.13E-05
11800 3.90E-05 9790 9.57E-06 9590 3.16E-05 9180
0.000112843 11500 8.91E-06 10900 1.13E-05
11800 3.90E-05 9790 9.58E-06 9590 3.16E-05 9180
0.000111801 11500 8.92E-06 10900 1.13E-05
11800 3.90E-05 9790 9.59E-06 9590 3.17E-05 9180
0.000110627 11500 8.91E-06 10900 1.13E-05
11800 3.90E-05 9800 9.61E-06 9590 3.17E-05 9190
0.000109262 11500 8.92E-06 10900 1.13E-05
11800 3.90E-05 9800 9.63E-06 9590 3.18E-05 9190
0.000107737 11500 8.92E-06 10900 1.13E-05
11800 3.90E-05 9800 9.64E-06 9600 3.18E-05 9190
0.000106078 11500 8.92E-06 10900 1.13E-05
11800 3.89E-05 9800 9.66E-06 9600 3.18E-05 9190
0.000104261 11500 8.92E-06 10900 1.13E-05
11800 3.89E-05 9800 9.67E-06 9600 3.19E-05 9190
0.000102298 11500 8.92E-06 10900 1.13E-05
11800 3.89E-05 9800 9.69E-06 9600 3.19E-05 9190
0.00010022 11500 8.92E-06 10900 1.13E-05
11800 3.89E-05 9800 9.71E-06 9600 3.20E-05 9200
9.80E-05 11500 8.92E-06 10900 1.13E-05
11800 3.89E-05 9810 9.72E-06 9600 3.20E-05 9200
9.58E-05 11500 8.92E-06 10900 1.13E-05
11800 3.89E-05 9810 9.74E-06 9600 3.21E-05 9200
9.34E-05 11500 8.93E-06 10900 1.13E-05
11800 3.88E-05 9810 9.76E-06 9610 3.21E-05 9200
9.11E-05 11500 8.94E-06 10900 1.13E-05
11800 3.88E-05 9810 9.78E-06 9610 3.21E-05 9200
8.87E-05 11500 8.94E-06 10900 1.13E-05
11800 3.88E-05 9810 9.80E-06 9610 3.22E-05 9200
8.63E-05 11500 8.94E-06 10900 1.13E-05
11800 3.88E-05 9810 9.82E-06 9610 3.22E-05 9200
8.39E-05 11500 8.94E-06 10900 1.13E-05
11800 3.88E-05 9820 9.83E-06 9610 3.23E-05 9210
8.15E-05 11500 8.95E-06 10900 1.13E-05
11800 3.88E-05 9820 9.86E-06 9610 3.23E-05 9210
7.91E-05 11500 8.94E-06 10900 1.13E-05
11800 3.88E-05 9820 9.88E-06 9620 3.24E-05 9210
7.67E-05 11500 8.94E-06 10900 1.14E-05
11800 3.87E-05 9820 9.90E-06 9620 3.24E-05 9210
7.43E-05 11500 8.94E-06 10900 1.14E-05
11900 3.87E-05 9820 9.92E-06 9620 3.25E-05 9210
7.21E-05 11500 8.94E-06 10900 1.14E-05
11900 3.87E-05 9820 9.93E-06 9620 3.25E-05 9210
6.98E-05 11500 8.93E-06 10900 1.14E-05
11900 3.87E-05 9830 9.95E-06 9620 3.26E-05 9220
6.77E-05 11500 8.94E-06 10900 1.14E-05
11900 3.87E-05 9830 9.97E-06 9620 3.26E-05 9220
6.56E-05 11500 8.94E-06 10900 1.14E-05
11900 3.87E-05 9830 9.98E-06 9630 3.27E-05 9220
6.37E-05 11500 8.95E-06 10900 1.14E-05
11900 3.86E-05 9830 9.99E-06 9630 3.27E-05 9220
6.19E-05 11500 8.94E-06 10900 1.14E-05
11900 3.86E-05 9830 1.00E-05 9630 3.28E-05 9220
6.03E-05 11500 8.94E-06 10900 1.14E-05
11900 3.86E-05 9830 1.00E-05 9630 3.28E-05 9220
5.89E-05 11500 8.95E-06 10900 1.14E-05
11900 3.86E-05 9830 1.00E-05 9630 3.29E-05 9230
5.77E-05 11500 8.95E-06 10900 1.14E-05
11900 3.85E-05 9840 1.00E-05 9630 3.29E-05 9230
5.66E-05 11500 8.95E-06 11000 1.14E-05
11900 3.85E-05 9840 1.01E-05 9630 3.30E-05 9230
5.58E-05 11500 8.96E-06 11000 1.14E-05
11900 3.85E-05 9840 1.01E-05 9640 3.30E-05 9230
5.52E-05 11500 8.96E-06 11000 1.14E-05
11900 3.85E-05 9840 1.01E-05 9640 3.30E-05 9230
5.48E-05 11500 8.97E-06 11000 1.14E-05

11900 3.85E-05 9840 1.01E-05 9640 3.31E-05 9230
5.46E-05 11500 8.97E-06 11000 1.14E-05
11900 3.85E-05 9840 1.01E-05 9640 3.32E-05 9230
5.46E-05 11500 8.97E-06 11000 1.14E-05
11900 3.85E-05 9850 1.01E-05 9640 3.32E-05 9240
5.48E-05 11500 8.98E-06 11000 1.14E-05
11900 3.84E-05 9850 1.02E-05 9640 3.33E-05 9240
5.52E-05 11500 8.98E-06 11000 1.14E-05
11900 3.84E-05 9850 1.02E-05 9650 3.33E-05 9240
5.58E-05 11500 8.97E-06 11000 1.14E-05
11900 3.84E-05 9850 1.02E-05 9650 3.34E-05 9240
5.64E-05 11500 8.97E-06 11000 1.14E-05
11900 3.84E-05 9850 1.02E-05 9650 3.34E-05 9240
5.72E-05 11500 8.98E-06 11000 1.14E-05
11900 3.84E-05 9850 1.02E-05 9650 3.35E-05 9240
5.79E-05 11500 8.98E-06 11000 1.14E-05
11900 3.84E-05 9850 1.03E-05 9650 3.35E-05 9250
5.85E-05 11500 8.98E-06 11000 1.14E-05
11900 3.83E-05 9860 1.03E-05 9650 3.36E-05 9250
5.89E-05 11500 8.98E-06 11000 1.14E-05
11900 3.84E-05 9860 1.03E-05 9650 3.36E-05 9250
5.92E-05 11500 8.98E-06 11000 1.14E-05
11900 3.83E-05 9860 1.03E-05 9660 3.36E-05 9250
5.93E-05 11500 8.97E-06 11000 1.14E-05
11900 3.83E-05 9860 1.03E-05 9660 3.37E-05 9250
5.92E-05 11500 8.97E-06 11000 1.14E-05
11900 3.83E-05 9860 1.03E-05 9660 3.37E-05 9250
5.90E-05 11500 8.98E-06 11000 1.14E-05
11900 3.83E-05 9860 1.03E-05 9660 3.38E-05 9250
5.85E-05 11500 8.98E-06 11000 1.14E-05
11900 3.83E-05 9870 1.04E-05 9660 3.39E-05 9260
5.80E-05 11500 8.98E-06 11000 1.14E-05
11900 3.83E-05 9870 1.04E-05 9660 3.39E-05 9260
5.73E-05 11500 8.99E-06 11000 1.14E-05
11900 3.82E-05 9870 1.04E-05 9670 3.39E-05 9260
5.65E-05 11500 8.98E-06 11000 1.14E-05
11900 3.82E-05 9870 1.04E-05 9670 3.40E-05 9260
5.56E-05 11500 8.99E-06 11000 1.14E-05
11900 3.82E-05 9870 1.04E-05 9670 3.40E-05 9260
5.47E-05 11500 8.99E-06 11000 1.14E-05
11900 3.82E-05 9870 1.04E-05 9670 3.41E-05 9260
5.37E-05 11500 9.00E-06 11000 1.14E-05
11900 3.82E-05 9880 1.05E-05 9670 3.42E-05 9270
5.27E-05 11500 8.99E-06 11000 1.14E-05
11900 3.82E-05 9880 1.05E-05 9670 3.42E-05 9270
5.17E-05 11600 9.00E-06 11000 1.15E-05
11900 3.82E-05 9880 1.05E-05 9680 3.43E-05 9270
5.06E-05 11600 9.00E-06 11000 1.15E-05
11900 3.82E-05 9880 1.05E-05 9680 3.43E-05 9270
4.96E-05 11600 9.00E-06 11000 1.15E-05
11900 3.82E-05 9880 1.05E-05 9680 3.44E-05 9270
4.86E-05 11600 9.00E-06 11000 1.15E-05
11900 3.81E-05 9880 1.06E-05 9680 3.44E-05 9270
4.77E-05 11600 9.00E-06 11000 1.15E-05
11900 3.81E-05 9880 1.06E-05 9680 3.45E-05 9280
4.67E-05 11600 9.00E-06 11000 1.15E-05
11900 3.81E-05 9890 1.06E-05 9680 3.45E-05 9280
4.58E-05 11600 9.00E-06 11000 1.15E-05
11900 3.81E-05 9890 1.06E-05 9680 3.46E-05 9280
4.50E-05 11600 9.00E-06 11000 1.15E-05
11900 3.81E-05 9890 1.06E-05 9690 3.46E-05 9280
4.42E-05 11600 9.00E-06 11000 1.15E-05
11900 3.81E-05 9890 1.07E-05 9690 3.47E-05 9280
4.34E-05 11600 9.00E-06 11000 1.15E-05
11900 3.80E-05 9890 1.07E-05 9690 3.47E-05 9280
4.27E-05 11600 9.01E-06 11000 1.15E-05
11900 3.80E-05 9890 1.07E-05 9690 3.48E-05 9280
4.20E-05 11600 9.02E-06 11000 1.15E-05

11900 3.80E-05 9900 1.07E-05 9690 3.48E-05 9290
4.14E-05 11600 9.02E-06 11000 1.15E-05
11900 3.80E-05 9900 1.07E-05 9690 3.49E-05 9290
4.09E-05 11600 9.02E-06 11000 1.15E-05
11900 3.80E-05 9900 1.07E-05 9700 3.49E-05 9290
4.04E-05 11600 9.03E-06 11000 1.15E-05
11900 3.80E-05 9900 1.08E-05 9700 3.50E-05 9290
3.99E-05 11600 9.04E-06 11000 1.15E-05
11900 3.80E-05 9900 1.08E-05 9700 3.51E-05 9290
3.94E-05 11600 9.04E-06 11000 1.15E-05
11900 3.80E-05 9900 1.08E-05 9700 3.51E-05 9290
3.90E-05 11600 9.04E-06 11000 1.15E-05
11900 3.80E-05 9900 1.08E-05 9700 3.52E-05 9300
3.86E-05 11600 9.04E-06 11000 1.15E-05
11900 3.80E-05 9910 1.08E-05 9700 3.52E-05 9300
3.82E-05 11600 9.04E-06 11000 1.15E-05
11900 3.79E-05 9910 1.09E-05 9700 3.53E-05 9300
3.79E-05 11600 9.04E-06 11000 1.15E-05
11900 3.80E-05 9910 1.09E-05 9710 3.53E-05 9300
3.76E-05 11600 9.05E-06 11000 1.15E-05
11900 3.79E-05 9910 1.09E-05 9710 3.54E-05 9300
3.73E-05 11600 9.06E-06 11000 1.15E-05
11900 3.79E-05 9910 1.09E-05 9710 3.54E-05 9300
3.70E-05 11600 9.05E-06 11000 1.15E-05
11900 3.79E-05 9910 1.10E-05 9710 3.55E-05 9300
3.68E-05 11600 9.05E-06 11000 1.15E-05
11900 3.79E-05 9920 1.10E-05 9710 3.55E-05 9310
3.66E-05 11600 9.06E-06 11000 1.15E-05
11900 3.79E-05 9920 1.10E-05 9710 3.56E-05 9310
3.63E-05 11600 9.06E-06 11000 1.15E-05
11900 3.79E-05 9920 1.10E-05 9720 3.56E-05 9310
3.61E-05 11600 9.05E-06 11000 1.15E-05
11900 3.79E-05 9920 1.10E-05 9720 3.57E-05 9310
3.60E-05 11600 9.06E-06 11000 1.15E-05
12000 3.78E-05 9920 1.10E-05 9720 3.57E-05 9310
3.58E-05 11600 9.06E-06 11000 1.15E-05
12000 3.78E-05 9920 1.11E-05 9720 3.58E-05 9310
3.57E-05 11600 9.07E-06 11000 1.15E-05
12000 3.78E-05 9930 1.11E-05 9720 3.58E-05 9320
3.55E-05 11600 9.07E-06 11000 1.15E-05
12000 3.78E-05 9930 1.11E-05 9720 3.59E-05 9320
3.54E-05 11600 9.08E-06 11000 1.15E-05
12000 3.78E-05 9930 1.11E-05 9730 3.60E-05 9320
3.53E-05 11600 9.08E-06 11000 1.15E-05
12000 3.78E-05 9930 1.11E-05 9730 3.60E-05 9320
3.52E-05 11600 9.08E-06 11000 1.15E-05
12000 3.78E-05 9930 1.12E-05 9730 3.61E-05 9320
3.51E-05 11600 9.09E-06 11000 1.15E-05
12000 3.78E-05 9930 1.12E-05 9730 3.61E-05 9320
3.50E-05 11600 9.09E-06 11000 1.15E-05
12000 3.78E-05 9930 1.12E-05 9730 3.62E-05 9330
3.49E-05 11600 9.10E-06 11000 1.15E-05
12000 3.78E-05 9940 1.12E-05 9730 3.62E-05 9330
3.49E-05 11600 9.10E-06 11100 1.15E-05
12000 3.78E-05 9940 1.13E-05 9730 3.63E-05 9330
3.48E-05 11600 9.10E-06 11100 1.15E-05
12000 3.78E-05 9940 1.13E-05 9740 3.64E-05 9330
3.48E-05 11600 9.10E-06 11100 1.15E-05
12000 3.78E-05 9940 1.13E-05 9740 3.64E-05 9330
3.47E-05 11600 9.11E-06 11100 1.15E-05
12000 3.77E-05 9940 1.13E-05 9740 3.65E-05 9330
3.46E-05 11600 9.11E-06 11100 1.15E-05
12000 3.77E-05 9940 1.13E-05 9740 3.65E-05 9330
3.46E-05 11600 9.11E-06 11100 1.15E-05
12000 3.77E-05 9950 1.14E-05 9740 3.66E-05 9340
3.45E-05 11600 9.12E-06 11100 1.15E-05
12000 3.77E-05 9950 1.14E-05 9740 3.66E-05 9340
3.45E-05 11600 9.12E-06 11100 1.15E-05

12000 3.77E-05 9950 1.14E-05 9750 3.67E-05 9340
3.44E-05 11600 9.12E-06 11100 1.15E-05
12000 3.77E-05 9950 1.14E-05 9750 3.67E-05 9340
3.44E-05 11600 9.13E-06 11100 1.15E-05
12000 3.77E-05 9950 1.14E-05 9750 3.68E-05 9340
3.44E-05 11600 9.14E-06 11100 1.15E-05
12000 3.76E-05 9950 1.14E-05 9750 3.68E-05 9340
3.44E-05 11600 9.13E-06 11100 1.15E-05
12000 3.76E-05 9950 1.15E-05 9750 3.69E-05 9350
3.43E-05 11600 9.14E-06 11100 1.15E-05
12000 3.77E-05 9960 1.15E-05 9750 3.70E-05 9350
3.43E-05 11600 9.15E-06 11100 1.15E-05
12000 3.76E-05 9960 1.15E-05 9750 3.70E-05 9350
3.43E-05 11600 9.15E-06 11100 1.15E-05
12000 3.76E-05 9960 1.15E-05 9760 3.71E-05 9350
3.43E-05 11600 9.16E-06 11100 1.15E-05
12000 3.76E-05 9960 1.15E-05 9760 3.72E-05 9350
3.43E-05 11600 9.16E-06 11100 1.15E-05
12000 3.76E-05 9960 1.16E-05 9760 3.72E-05 9350
3.43E-05 11600 9.17E-06 11100 1.16E-05
12000 3.76E-05 9960 1.16E-05 9760 3.73E-05 9350
3.43E-05 11600 9.17E-06 11100 1.15E-05
12000 3.76E-05 9970 1.16E-05 9760 3.73E-05 9360
3.43E-05 11600 9.17E-06 11100 1.15E-05
12000 3.76E-05 9970 1.16E-05 9760 3.74E-05 9360
3.43E-05 11600 9.17E-06 11100 1.15E-05
12000 3.76E-05 9970 1.17E-05 9770 3.74E-05 9360
3.43E-05 11600 9.18E-06 11100 1.16E-05
12000 3.76E-05 9970 1.17E-05 9770 3.75E-05 9360
3.43E-05 11600 9.18E-06 11100 1.16E-05
12000 3.76E-05 9970 1.17E-05 9770 3.76E-05 9360
3.43E-05 11600 9.19E-06 11100 1.16E-05
12000 3.76E-05 9970 1.17E-05 9770 3.76E-05 9360
3.43E-05 11600 9.19E-06 11100 1.16E-05
12000 3.75E-05 9980 1.18E-05 9770 3.77E-05 9370
3.43E-05 11600 9.20E-06 11100 1.16E-05
12000 3.75E-05 9980 1.18E-05 9770 3.77E-05 9370
3.43E-05 11700 9.20E-06 11100 1.16E-05
12000 3.75E-05 9980 1.18E-05 9780 3.78E-05 9370
3.43E-05 11700 9.21E-06 11100 1.16E-05
12000 3.75E-05 9980 1.18E-05 9780 3.78E-05 9370
3.43E-05 11700 9.21E-06 11100 1.16E-05
12000 3.75E-05 9980 1.18E-05 9780 3.79E-05 9370
3.44E-05 11700 9.22E-06 11100 1.16E-05
12000 3.75E-05 9980 1.19E-05 9780 3.80E-05 9370
3.44E-05 11700 9.23E-06 11100 1.16E-05
12000 3.75E-05 9980 1.19E-05 9780 3.80E-05 9380
3.44E-05 11700 9.24E-06 11100 1.16E-05
12000 3.75E-05 9990 1.19E-05 9780 3.81E-05 9380
3.44E-05 11700 9.25E-06 11100 1.16E-05
12000 3.75E-05 9990 1.19E-05 9780 3.81E-05 9380
3.44E-05 11700 9.28E-06 11100 1.16E-05
12000 3.75E-05 9990 1.19E-05 9790 3.82E-05 9380
3.44E-05 11700 9.26E-06 11100 1.16E-05
12000 3.75E-05 9990 1.19E-05 9790 3.83E-05 9380
3.45E-05 11700 9.28E-06 11100 1.16E-05
12000 3.75E-05 9990 1.20E-05 9790 3.83E-05 9380
3.45E-05 11700 9.29E-06 11100 1.16E-05
12000 3.75E-05 9990 1.20E-05 9790 3.84E-05 9380
3.45E-05 11700 9.28E-06 11100 1.16E-05
12000 3.75E-05 10000 1.20E-05 9790 3.85E-05 9390
3.45E-05 11700 9.30E-06 11100 1.16E-05
12000 3.74E-05 10000 1.21E-05 9790 3.85E-05 9390
3.45E-05 11700 9.30E-06 11100 1.16E-05
12000 3.74E-05 10000 1.21E-05 9800 3.86E-05 9390
3.46E-05 11700 9.32E-06 11100 1.16E-05
12000 3.74E-05 10000 1.21E-05 9800 3.87E-05 9390
3.46E-05 11700 9.32E-06 11100 1.16E-05

12000 3.74E-05 10000 1.21E-05 9800 3.88E-05 9390
3.46E-05 11700 9.34E-06 11100 1.16E-05
12000 3.74E-05 10000 1.22E-05 9800 3.88E-05 9390
3.47E-05 11700 9.34E-06 11100 1.16E-05
12000 3.74E-05 10000 1.22E-05 9800 3.89E-05 9400
3.47E-05 11700 9.35E-06 11100 1.16E-05
12000 3.74E-05 10000 1.22E-05 9800 3.89E-05 9400
3.47E-05 11700 9.35E-06 11100 1.16E-05
12000 3.74E-05 10000 1.22E-05 9800 3.90E-05 9400
3.47E-05 11700 9.35E-06 11100 1.16E-05
12000 3.74E-05 10000 1.23E-05 9810 3.91E-05 9400
3.48E-05 11700 9.34E-06 11100 1.16E-05
12000 3.73E-05 10000 1.23E-05 9810 3.92E-05 9400
3.48E-05 11700 9.35E-06 11100 1.16E-05
12000 3.74E-05 10000 1.23E-05 9810 3.92E-05 9400
3.49E-05 11700 9.35E-06 11100 1.16E-05
12000 3.74E-05 10000 1.23E-05 9810 3.93E-05 9400
3.49E-05 11700 9.36E-06 11100 1.16E-05
12000 3.74E-05 10000 1.23E-05 9810 3.94E-05 9410
3.50E-05 11700 9.36E-06 11100 1.16E-05
12000 3.73E-05 10000 1.24E-05 9810 3.94E-05 9410
3.50E-05 11700 9.36E-06 11100 1.16E-05
12000 3.73E-05 10000 1.24E-05 9820 3.95E-05 9410
3.50E-05 11700 9.37E-06 11100 1.16E-05
12000 3.74E-05 10000 1.24E-05 9820 3.96E-05 9410
3.51E-05 11700 9.38E-06 11100 1.16E-05
12100 3.73E-05 10000 1.24E-05 9820 3.97E-05 9410
3.51E-05 11700 9.39E-06 11100 1.16E-05
12100 3.74E-05 10000 1.25E-05 9820 3.98E-05 9410
3.52E-05 11700 9.38E-06 11100 1.16E-05
12100 3.74E-05 10000 1.25E-05 9820 3.98E-05 9420
3.52E-05 11700 9.37E-06 11100 1.16E-05
12100 3.73E-05 10000 1.25E-05 9820 3.99E-05 9420
3.53E-05 11700 9.37E-06 11100 1.16E-05
12100 3.73E-05 10000 1.25E-05 9830 4.00E-05 9420
3.53E-05 11700 9.37E-06 11100 1.16E-05
12100 3.73E-05 10000 1.25E-05 9830 4.00E-05 9420
3.54E-05 11700 9.37E-06 11100 1.16E-05
12100 3.73E-05 10000 1.26E-05 9830 4.01E-05 9420
3.54E-05 11700 9.38E-06 11100 1.16E-05
12100 3.73E-05 10000 1.26E-05 9830 4.02E-05 9420
3.55E-05 11700 9.36E-06 11100 1.16E-05
12100 3.73E-05 10000 1.26E-05 9830 4.02E-05 9430
3.55E-05 11700 9.36E-06 11100 1.16E-05
12100 3.73E-05 10000 1.26E-05 9830 4.03E-05 9430
3.55E-05 11700 9.35E-06 11200 1.16E-05
12100 3.73E-05 10000 1.27E-05 9830 4.04E-05 9430
3.56E-05 11700 9.36E-06 11200 1.16E-05
12100 3.72E-05 10000 1.27E-05 9840 4.05E-05 9430
3.57E-05 11700 9.34E-06 11200 1.16E-05
12100 3.73E-05 10000 1.27E-05 9840 4.05E-05 9430
3.57E-05 11700 9.33E-06 11200 1.16E-05
12100 3.72E-05 10000 1.27E-05 9840 4.06E-05 9430
3.58E-05 11700 9.35E-06 11200 1.16E-05
12100 3.72E-05 10000 1.27E-05 9840 4.07E-05 9430
3.58E-05 11700 9.34E-06 11200 1.16E-05
12100 3.72E-05 10000 1.28E-05 9840 4.07E-05 9440
3.59E-05 11700 9.35E-06 11200 1.16E-05
12100 3.72E-05 10000 1.28E-05 9840 4.08E-05 9440
3.59E-05 11700 9.35E-06 11200 1.16E-05
12100 3.72E-05 10000 1.28E-05 9850 4.09E-05 9440
3.60E-05 11700 9.35E-06 11200 1.16E-05
12100 3.72E-05 10100 1.28E-05 9850 4.10E-05 9440
3.61E-05 11700 9.35E-06 11200 1.16E-05
12100 3.72E-05 10100 1.29E-05 9850 4.10E-05 9440
3.61E-05 11700 9.36E-06 11200 1.16E-05
12100 3.72E-05 10100 1.29E-05 9850 4.11E-05 9440
3.62E-05 11700 9.35E-06 11200 1.16E-05

12100 3.72E-05 10100 1.29E-05 9850 4.12E-05 9450
3.62E-05 11700 9.35E-06 11200 1.16E-05
12100 3.71E-05 10100 1.29E-05 9850 4.12E-05 9450
3.63E-05 11700 9.33E-06 11200 1.16E-05
12100 3.72E-05 10100 1.30E-05 9850 4.13E-05 9450
3.63E-05 11700 9.33E-06 11200 1.16E-05
12100 3.72E-05 10100 1.30E-05 9860 4.14E-05 9450
3.64E-05 11700 9.32E-06 11200 1.16E-05
12100 3.72E-05 10100 1.30E-05 9860 4.15E-05 9450
3.65E-05 11700 9.33E-06 11200 1.16E-05
12100 3.72E-05 10100 1.31E-05 9860 4.15E-05 9450
3.65E-05 11700 9.31E-06 11200 1.16E-05
12100 3.72E-05 10100 1.31E-05 9860 4.16E-05 9450
3.66E-05 11700 9.29E-06 11200 1.16E-05
12100 3.72E-05 10100 1.31E-05 9860 4.17E-05 9460
3.66E-05 11700 9.30E-06 11200 1.16E-05
12100 3.71E-05 10100 1.31E-05 9860 4.17E-05 9460
3.67E-05 11700 9.29E-06 11200 1.16E-05
12100 3.71E-05 10100 1.31E-05 9870 4.18E-05 9460
3.68E-05 11700 9.29E-06 11200 1.16E-05
12100 3.71E-05 10100 1.32E-05 9870 4.19E-05 9460
3.69E-05 11700 9.29E-06 11200 1.16E-05
12100 3.71E-05 10100 1.32E-05 9870 4.20E-05 9460
3.69E-05 11700 9.28E-06 11200 1.17E-05
12100 3.72E-05 10100 1.32E-05 9870 4.20E-05 9460
3.70E-05 11700 9.29E-06 11200 1.17E-05
12100 3.71E-05 10100 1.32E-05 9870 4.21E-05 9470
3.71E-05 11700 9.29E-06 11200 1.17E-05
12100 3.72E-05 10100 1.33E-05 9870 4.22E-05 9470
3.71E-05 11800 9.27E-06 11200 1.17E-05
12100 3.71E-05 10100 1.33E-05 9880 4.23E-05 9470
3.72E-05 11800 9.28E-06 11200 1.17E-05
12100 3.71E-05 10100 1.33E-05 9880 4.24E-05 9470
3.73E-05 11800 9.27E-06 11200 1.17E-05
12100 3.71E-05 10100 1.33E-05 9880 4.24E-05 9470
3.73E-05 11800 9.28E-06 11200 1.16E-05
12100 3.71E-05 10100 1.34E-05 9880 4.25E-05 9470
3.74E-05 11800 9.27E-06 11200 1.17E-05
12100 3.71E-05 10100 1.34E-05 9880 4.26E-05 9480
3.75E-05 11800 9.27E-06 11200 1.17E-05
12100 3.71E-05 10100 1.34E-05 9880 4.26E-05 9480
3.75E-05 11800 9.27E-06 11200 1.17E-05
12100 3.71E-05 10100 1.34E-05 9880 4.27E-05 9480
3.76E-05 11800 9.27E-06 11200 1.17E-05
12100 3.71E-05 10100 1.35E-05 9890 4.28E-05 9480
3.77E-05 11800 9.26E-06 11200 1.17E-05
12100 3.71E-05 10100 1.35E-05 9890 4.29E-05 9480
3.78E-05 11800 9.26E-06 11200 1.17E-05
12100 3.71E-05 10100 1.35E-05 9890 4.30E-05 9480
3.78E-05 11800 9.25E-06 11200 1.17E-05
12100 3.71E-05 10100 1.36E-05 9890 4.30E-05 9480
3.79E-05 11800 9.24E-06 11200 1.17E-05
12100 3.71E-05 10100 1.36E-05 9890 4.31E-05 9490
3.80E-05 11800 9.24E-06 11200 1.17E-05
12100 3.71E-05 10100 1.36E-05 9890 4.32E-05 9490
3.81E-05 11800 9.25E-06 11200 1.17E-05
12100 3.71E-05 10100 1.36E-05 9900 4.33E-05 9490
3.81E-05 11800 9.24E-06 11200 1.17E-05
12100 3.71E-05 10100 1.37E-05 9900 4.33E-05 9490
3.82E-05 11800 9.24E-06 11200 1.17E-05
12100 3.70E-05 10100 1.37E-05 9900 4.34E-05 9490
3.83E-05 11800 9.24E-06 11200 1.17E-05
12100 3.70E-05 10100 1.37E-05 9900 4.35E-05 9490
3.84E-05 11800 9.24E-06 11200 1.17E-05
12100 3.70E-05 10100 1.37E-05 9900 4.36E-05 9500
3.85E-05 11800 9.24E-06 11200 1.17E-05
12100 3.70E-05 10100 1.38E-05 9900 4.37E-05 9500
3.86E-05 11800 9.25E-06 11200 1.17E-05

12100 3.70E-05 10100 1.38E-05 9900 4.37E-05 9500
3.86E-05 11800 9.25E-06 11200 1.17E-05
12100 3.71E-05 10100 1.38E-05 9910 4.38E-05 9500
3.87E-05 11800 9.24E-06 11200 1.17E-05
12100 3.70E-05 10100 1.38E-05 9910 4.39E-05 9500
3.88E-05 11800 9.25E-06 11200 1.17E-05
12100 3.70E-05 10100 1.39E-05 9910 4.40E-05 9500
3.89E-05 11800 9.25E-06 11200 1.17E-05
12100 3.70E-05 10100 1.39E-05 9910 4.41E-05 9500
3.89E-05 11800 9.24E-06 11200 1.17E-05
12100 3.70E-05 10100 1.39E-05 9910 4.42E-05 9510
3.90E-05 11800 9.25E-06 11200 1.17E-05
12100 3.70E-05 10100 1.40E-05 9910 4.42E-05 9510
3.91E-05 11800 9.24E-06 11200 1.17E-05
12100 3.70E-05 10100 1.40E-05 9920 4.43E-05 9510
3.92E-05 11800 9.23E-06 11200 1.17E-05
12100 3.70E-05 10100 1.40E-05 9920 4.44E-05 9510
3.93E-05 11800 9.23E-06 11200 1.17E-05
12200 3.70E-05 10100 1.41E-05 9920 4.45E-05 9510
3.94E-05 11800 9.22E-06 11200 1.17E-05
12200 3.70E-05 10100 1.41E-05 9920 4.46E-05 9510
3.95E-05 11800 9.23E-06 11200 1.17E-05
12200 3.70E-05 10100 1.41E-05 9920 4.47E-05 9520
3.95E-05 11800 9.23E-06 11200 1.17E-05
12200 3.70E-05 10100 1.41E-05 9920 4.47E-05 9520
3.96E-05 11800 9.22E-06 11200 1.17E-05
12200 3.70E-05 10100 1.42E-05 9930 4.48E-05 9520
3.97E-05 11800 9.23E-06 11200 1.17E-05
12200 3.70E-05 10100 1.42E-05 9930 4.49E-05 9520
3.98E-05 11800 9.21E-06 11200 1.17E-05
12200 3.70E-05 10100 1.42E-05 9930 4.50E-05 9520
3.99E-05 11800 9.21E-06 11200 1.17E-05
12200 3.70E-05 10100 1.42E-05 9930 4.51E-05 9520
4.00E-05 11800 9.22E-06 11200 1.17E-05
12200 3.70E-05 10100 1.43E-05 9930 4.52E-05 9530
4.01E-05 11800 9.23E-06 11200 1.17E-05
12200 3.70E-05 10100 1.43E-05 9930 4.53E-05 9530
4.02E-05 11800 9.22E-06 11300 1.17E-05
12200 3.70E-05 10100 1.43E-05 9930 4.53E-05 9530
4.03E-05 11800 9.22E-06 11300 1.17E-05
12200 3.71E-05 10100 1.44E-05 9940 4.54E-05 9530
4.04E-05 11800 9.22E-06 11300 1.17E-05
12200 3.71E-05 10100 1.44E-05 9940 4.55E-05 9530
4.05E-05 11800 9.22E-06 11300 1.17E-05
12200 3.71E-05 10100 1.44E-05 9940 4.56E-05 9530
4.06E-05 11800 9.21E-06 11300 1.17E-05
12200 3.71E-05 10100 1.45E-05 9940 4.57E-05 9530
4.07E-05 11800 9.22E-06 11300 1.17E-05
12200 3.71E-05 10100 1.45E-05 9940 4.58E-05 9540
4.08E-05 11800 9.23E-06 11300 1.17E-05
12200 3.71E-05 10100 1.45E-05 9940 4.59E-05 9540
4.09E-05 11800 9.22E-06 11300 1.17E-05
12200 3.71E-05 10100 1.46E-05 9950 4.60E-05 9540
4.10E-05 11800 9.22E-06 11300 1.17E-05
12200 3.71E-05 10200 1.46E-05 9950 4.61E-05 9540
4.11E-05 11800 9.22E-06 11300 1.17E-05
12200 3.71E-05 10200 1.46E-05 9950 4.61E-05 9540
4.12E-05 11800 9.22E-06 11300 1.17E-05
12200 3.71E-05 10200 1.47E-05 9950 4.62E-05 9540
4.13E-05 11800 9.21E-06 11300 1.17E-05
12200 3.71E-05 10200 1.47E-05 9950 4.63E-05 9550
4.14E-05 11800 9.21E-06 11300 1.17E-05
12200 3.71E-05 10200 1.47E-05 9950 4.64E-05 9550
4.15E-05 11800 9.22E-06 11300 1.17E-05
12200 3.71E-05 10200 1.47E-05 9950 4.65E-05 9550
4.16E-05 11800 9.22E-06 11300 1.17E-05
12200 3.71E-05 10200 1.48E-05 9960 4.66E-05 9550
4.17E-05 11800 9.21E-06 11300 1.18E-05

12200 3.71E-05 10200 1.48E-05 9960 4.67E-05 9550
4.18E-05 11800 9.22E-06 11300 1.18E-05
12200 3.71E-05 10200 1.48E-05 9960 4.68E-05 9550
4.19E-05 11800 9.22E-06 11300 1.18E-05
12200 3.71E-05 10200 1.49E-05 9960 4.69E-05 9550
4.20E-05 11800 9.22E-06 11300 1.18E-05
12200 3.71E-05 10200 1.49E-05 9960 4.70E-05 9560
4.21E-05 11800 9.23E-06 11300 1.18E-05
12200 3.71E-05 10200 1.50E-05 9960 4.71E-05 9560
4.22E-05 11800 9.23E-06 11300 1.18E-05
12200 3.71E-05 10200 1.50E-05 9970 4.72E-05 9560
4.23E-05 11800 9.23E-06 11300 1.18E-05
12200 3.71E-05 10200 1.50E-05 9970 4.72E-05 9560
4.24E-05 11800 9.23E-06 11300 1.18E-05
12200 3.71E-05 10200 1.50E-05 9970 4.73E-05 9560
4.25E-05 11800 9.25E-06 11300 1.18E-05
12200 3.71E-05 10200 1.51E-05 9970 4.74E-05 9560
4.27E-05 11800 9.25E-06 11300 1.18E-05
12200 3.71E-05 10200 1.51E-05 9970 4.75E-05 9570
4.28E-05 11800 9.25E-06 11300 1.18E-05
12200 3.71E-05 10200 1.51E-05 9970 4.76E-05 9570
4.29E-05 11900 9.25E-06 11300 1.18E-05
12200 3.71E-05 10200 1.52E-05 9980 4.77E-05 9570
4.30E-05 11900 9.25E-06 11300 1.18E-05
12200 3.71E-05 10200 1.52E-05 9980 4.78E-05 9570
4.31E-05 11900 9.24E-06 11300 1.18E-05
12200 3.71E-05 10200 1.52E-05 9980 4.79E-05 9570
4.32E-05 11900 9.24E-06 11300 1.18E-05
12200 3.71E-05 10200 1.53E-05 9980 4.80E-05 9570
4.33E-05 11900 9.25E-06 11300 1.18E-05
12200 3.71E-05 10200 1.53E-05 9980 4.81E-05 9580
4.34E-05 11900 9.25E-06 11300 1.18E-05
12200 3.71E-05 10200 1.53E-05 9980 4.82E-05 9580
4.35E-05 11900 9.24E-06 11300 1.18E-05
12200 3.71E-05 10200 1.53E-05 9980 4.83E-05 9580
4.36E-05 11900 9.25E-06 11300 1.18E-05
12200 3.71E-05 10200 1.54E-05 9990 4.84E-05 9580
4.38E-05 11900 9.23E-06 11300 1.18E-05
12200 3.71E-05 10200 1.54E-05 9990 4.85E-05 9580
4.39E-05 11900 9.24E-06 11300 1.18E-05
12200 3.71E-05 10200 1.55E-05 9990 4.86E-05 9580
4.40E-05 11900 9.25E-06 11300 1.18E-05
12200 3.71E-05 10200 1.55E-05 9990 4.87E-05 9580
4.41E-05 11900 9.25E-06 11300 1.18E-05
12200 3.72E-05 10200 1.55E-05 9990 4.87E-05 9590
4.42E-05 11900 9.25E-06 11300 1.18E-05
12200 3.72E-05 10200 1.56E-05 9990 4.89E-05 9590
4.43E-05 11900 9.24E-06 11300 1.18E-05
12200 3.72E-05 10200 1.56E-05 10000 4.90E-05 9590
4.45E-05 11900 9.25E-06 11300 1.18E-05
12200 3.72E-05 10200 1.56E-05 10000 4.90E-05 9590
4.46E-05 11900 9.25E-06 11300 1.18E-05
12200 3.72E-05 10200 1.57E-05 10000 4.91E-05 9590
4.47E-05 11900 9.27E-06 11300 1.18E-05
12200 3.72E-05 10200 1.57E-05 10000 4.93E-05 9590
4.48E-05 11900 9.27E-06 11300 1.18E-05
12200 3.72E-05 10200 1.57E-05 10000 4.94E-05 9600
4.49E-05 11900 9.26E-06 11300 1.18E-05
12200 3.72E-05 10200 1.58E-05 10000 4.94E-05 9600
4.51E-05 11900 9.26E-06 11300 1.18E-05
12200 3.72E-05 10200 1.58E-05 10000 4.96E-05 9600
4.52E-05 11900 9.25E-06 11300 1.19E-05
12200 3.72E-05 10200 1.59E-05 10000 4.96E-05 9600
4.53E-05 11900 9.26E-06 11300 1.19E-05
12200 3.72E-05 10200 1.59E-05 10000 4.98E-05 9600
4.54E-05 11900 9.25E-06 11300 1.19E-05
12200 3.72E-05 10200 1.59E-05 10000 4.98E-05 9600
4.55E-05 11900 9.26E-06 11300 1.19E-05

12200 3.72E-05 10200 1.60E-05 10000 4.99E-05 9600
4.57E-05 11900 9.25E-06 11300 1.19E-05
12200 3.72E-05 10200 1.60E-05 10000 5.00E-05 9610
4.58E-05 11900 9.27E-06 11300 1.19E-05
12200 3.72E-05 10200 1.60E-05 10000 5.01E-05 9610
4.59E-05 11900 9.26E-06 11300 1.19E-05
12200 3.72E-05 10200 1.60E-05 10000 5.02E-05 9610
4.60E-05 11900 9.27E-06 11300 1.19E-05
12200 3.72E-05 10200 1.61E-05 10000 5.03E-05 9610
4.62E-05 11900 9.26E-06 11300 1.19E-05
12300 3.72E-05 10200 1.61E-05 10000 5.04E-05 9610
4.63E-05 11900 9.27E-06 11300 1.19E-05
12300 3.72E-05 10200 1.61E-05 10000 5.05E-05 9610
4.65E-05 11900 9.29E-06 11300 1.19E-05
12300 3.73E-05 10200 1.62E-05 10000 5.06E-05 9620
4.66E-05 11900 9.29E-06 11300 1.19E-05
12300 3.73E-05 10200 1.62E-05 10000 5.07E-05 9620
4.67E-05 11900 9.29E-06 11300 1.19E-05
12300 3.73E-05 10200 1.62E-05 10000 5.08E-05 9620
4.69E-05 11900 9.29E-06 11300 1.19E-05
12300 3.73E-05 10200 1.63E-05 10000 5.09E-05 9620
4.70E-05 11900 9.30E-06 11300 1.19E-05
12300 3.73E-05 10200 1.63E-05 10000 5.10E-05 9620
4.72E-05 11900 9.32E-06 11300 1.19E-05
12300 3.73E-05 10200 1.64E-05 10000 5.11E-05 9620
4.73E-05 11900 9.32E-06 11300 1.19E-05
12300 3.73E-05 10200 1.64E-05 10000 5.12E-05 9630
4.74E-05 11900 9.33E-06 11300 1.19E-05
12300 3.73E-05 10200 1.64E-05 10000 5.13E-05 9630
4.76E-05 11900 9.32E-06 11400 1.19E-05
12300 3.74E-05 10200 1.65E-05 10000 5.14E-05 9630
4.77E-05 11900 9.32E-06 11400 1.19E-05
12300 3.73E-05 10200 1.65E-05 10000 5.15E-05 9630
4.79E-05 11900 9.32E-06 11400 1.19E-05
12300 3.74E-05 10200 1.66E-05 10000 5.17E-05 9630
4.80E-05 11900 9.31E-06 11400 1.19E-05
12300 3.73E-05 10200 1.66E-05 10000 5.17E-05 9630
4.81E-05 11900 9.31E-06 11400 1.19E-05
12300 3.74E-05 10200 1.66E-05 10000 5.18E-05 9630
4.83E-05 11900 9.32E-06 11400 1.19E-05
12300 3.74E-05 10200 1.67E-05 10000 5.19E-05 9640
4.84E-05 11900 9.33E-06 11400 1.19E-05
12300 3.74E-05 10200 1.67E-05 10000 5.20E-05 9640
4.86E-05 11900 9.31E-06 11400 1.19E-05
12300 3.74E-05 10200 1.67E-05 10000 5.21E-05 9640
4.87E-05 11900 9.31E-06 11400 1.19E-05
12300 3.74E-05 10300 1.68E-05 10000 5.23E-05 9640
4.88E-05 11900 9.32E-06 11400 1.19E-05
12300 3.74E-05 10300 1.68E-05 10000 5.24E-05 9640
4.90E-05 11900 9.32E-06 11400 1.19E-05
12300 3.74E-05 10300 1.68E-05 10100 5.25E-05 9640
4.91E-05 11900 9.33E-06 11400 1.19E-05
12300 3.75E-05 10300 1.69E-05 10100 5.26E-05 9650
4.93E-05 11900 9.33E-06 11400 1.19E-05
12300 3.75E-05 10300 1.69E-05 10100 5.27E-05 9650
4.94E-05 11900 9.33E-06 11400 1.19E-05
12300 3.75E-05 10300 1.69E-05 10100 5.28E-05 9650
4.96E-05 11900 9.33E-06 11400 1.19E-05
12300 3.75E-05 10300 1.69E-05 10100 5.29E-05 9650
4.97E-05 11900 9.34E-06 11400 1.19E-05
12300 3.75E-05 10300 1.70E-05 10100 5.30E-05 9650
4.99E-05 11900 9.35E-06 11400 1.19E-05
12300 3.75E-05 10300 1.70E-05 10100 5.31E-05 9650
5.01E-05 11900 9.34E-06 11400 1.19E-05
12300 3.75E-05 10300 1.71E-05 10100 5.32E-05 9650
5.02E-05 11900 9.34E-06 11400 1.19E-05
12300 3.75E-05 10300 1.71E-05 10100 5.33E-05 9660
5.04E-05 11900 9.34E-06 11400 1.19E-05

12300 3.75E-05 10300 1.71E-05 10100 5.35E-05 9660
5.05E-05 11900 9.34E-06 11400 1.19E-05
12300 3.75E-05 10300 1.72E-05 10100 5.36E-05 9660
5.07E-05 11900 9.34E-06 11400 1.20E-05
12300 3.75E-05 10300 1.72E-05 10100 5.37E-05 9660
5.09E-05 11900 9.34E-06 11400 1.20E-05
12300 3.75E-05 10300 1.72E-05 10100 5.38E-05 9660
5.10E-05 11900 9.35E-06 11400 1.20E-05
12300 3.75E-05 10300 1.73E-05 10100 5.39E-05 9660
5.12E-05 11900 9.35E-06 11400 1.20E-05
12300 3.75E-05 10300 1.73E-05 10100 5.40E-05 9670
5.13E-05 11900 9.35E-06 11400 1.20E-05
12300 3.76E-05 10300 1.74E-05 10100 5.41E-05 9670
5.15E-05 12000 9.35E-06 11400 1.20E-05
12300 3.76E-05 10300 1.74E-05 10100 5.42E-05 9670
5.17E-05 12000 9.36E-06 11400 1.20E-05
12300 3.76E-05 10300 1.74E-05 10100 5.44E-05 9670
5.19E-05 12000 9.35E-06 11400 1.20E-05
12300 3.76E-05 10300 1.75E-05 10100 5.45E-05 9670
5.20E-05 12000 9.37E-06 11400 1.20E-05
12300 3.76E-05 10300 1.75E-05 10100 5.46E-05 9670
5.22E-05 12000 9.38E-06 11400 1.20E-05
12300 3.76E-05 10300 1.75E-05 10100 5.47E-05 9680
5.24E-05 12000 9.38E-06 11400 1.20E-05
12300 3.76E-05 10300 1.76E-05 10100 5.48E-05 9680
5.25E-05 12000 9.38E-06 11400 1.20E-05
12300 3.76E-05 10300 1.76E-05 10100 5.50E-05 9680
5.27E-05 12000 9.37E-06 11400 1.20E-05
12300 3.76E-05 10300 1.77E-05 10100 5.51E-05 9680
5.29E-05 12000 9.38E-06 11400 1.20E-05
12300 3.77E-05 10300 1.77E-05 10100 5.52E-05 9680
5.31E-05 12000 9.38E-06 11400 1.20E-05
12300 3.77E-05 10300 1.77E-05 10100 5.53E-05 9680
5.33E-05 12000 9.38E-06 11400 1.20E-05
12300 3.77E-05 10300 1.78E-05 10100 5.55E-05 9680
5.34E-05 12000 9.39E-06 11400 1.20E-05
12300 3.77E-05 10300 1.78E-05 10100 5.56E-05 9690
5.36E-05 12000 9.39E-06 11400 1.20E-05
12300 3.77E-05 10300 1.79E-05 10100 5.57E-05 9690
5.38E-05 12000 9.39E-06 11400 1.20E-05
12300 3.77E-05 10300 1.79E-05 10100 5.58E-05 9690
5.40E-05 12000 9.41E-06 11400 1.20E-05
12300 3.77E-05 10300 1.79E-05 10100 5.60E-05 9690
5.42E-05 12000 9.39E-06 11400 1.20E-05
12300 3.77E-05 10300 1.80E-05 10100 5.61E-05 9690
5.44E-05 12000 9.39E-06 11400 1.20E-05
12300 3.77E-05 10300 1.80E-05 10100 5.62E-05 9690
5.45E-05 12000 9.39E-06 11400 1.20E-05
12300 3.77E-05 10300 1.81E-05 10100 5.63E-05 9700
5.47E-05 12000 9.39E-06 11400 1.20E-05
12300 3.77E-05 10300 1.81E-05 10100 5.64E-05 9700
5.49E-05 12000 9.39E-06 11400 1.20E-05
12300 3.77E-05 10300 1.81E-05 10100 5.65E-05 9700
5.51E-05 12000 9.39E-06 11400 1.20E-05
12300 3.78E-05 10300 1.82E-05 10100 5.67E-05 9700
5.53E-05 12000 9.40E-06 11400 1.20E-05
12300 3.78E-05 10300 1.82E-05 10100 5.68E-05 9700
5.55E-05 12000 9.41E-06 11400 1.20E-05
12300 3.78E-05 10300 1.83E-05 10100 5.69E-05 9700
5.57E-05 12000 9.41E-06 11400 1.20E-05
12300 3.78E-05 10300 1.83E-05 10100 5.70E-05 9700
5.59E-05 12000 9.41E-06 11400 1.20E-05
12300 3.78E-05 10300 1.83E-05 10100 5.72E-05 9710
5.61E-05 12000 9.41E-06 11400 1.20E-05
12300 3.79E-05 10300 1.84E-05 10100 5.73E-05 9710
5.63E-05 12000 9.41E-06 11400 1.20E-05
12300 3.79E-05 10300 1.84E-05 10100 5.74E-05 9710
5.65E-05 12000 9.41E-06 11400 1.20E-05

12300 3.79E-05 10300 1.85E-05 10100 5.76E-05 9710
5.67E-05 12000 9.42E-06 11400 1.20E-05
12400 3.79E-05 10300 1.85E-05 10100 5.77E-05 9710
5.69E-05 12000 9.42E-06 11400 1.20E-05
12400 3.79E-05 10300 1.86E-05 10100 5.78E-05 9710
5.71E-05 12000 9.43E-06 11400 1.20E-05
12400 3.79E-05 10300 1.86E-05 10100 5.80E-05 9720
5.73E-05 12000 9.42E-06 11400 1.20E-05
12400 3.79E-05 10300 1.86E-05 10100 5.81E-05 9720
5.75E-05 12000 9.44E-06 11400 1.20E-05
12400 3.79E-05 10300 1.87E-05 10100 5.82E-05 9720
5.77E-05 12000 9.43E-06 11400 1.20E-05
12400 3.79E-05 10300 1.87E-05 10100 5.83E-05 9720
5.79E-05 12000 9.43E-06 11400 1.20E-05
12400 3.80E-05 10300 1.88E-05 10100 5.85E-05 9720
5.81E-05 12000 9.43E-06 11400 1.20E-05
12400 3.80E-05 10300 1.88E-05 10100 5.86E-05 9720
5.83E-05 12000 9.43E-06 11400 1.21E-05
12400 3.80E-05 10300 1.89E-05 10100 5.87E-05 9730
5.85E-05 12000 9.43E-06 11400 1.20E-05
12400 3.80E-05 10300 1.89E-05 10100 5.89E-05 9730
5.88E-05 12000 9.44E-06 11500 1.21E-05
12400 3.80E-05 10300 1.90E-05 10100 5.90E-05 9730
5.90E-05 12000 9.43E-06 11500 1.20E-05
12400 3.80E-05 10300 1.90E-05 10100 5.92E-05 9730
5.92E-05 12000 9.43E-06 11500 1.20E-05
12400 3.81E-05 10300 1.90E-05 10100 5.93E-05 9730
5.94E-05 12000 9.45E-06 11500 1.20E-05
12400 3.81E-05 10300 1.91E-05 10100 5.95E-05 9730
5.96E-05 12000 9.45E-06 11500 1.21E-05
12400 3.81E-05 10300 1.91E-05 10100 5.96E-05 9730
5.98E-05 12000 9.45E-06 11500 1.21E-05
12400 3.81E-05 10300 1.92E-05 10100 5.97E-05 9740
6.00E-05 12000 9.45E-06 11500 1.21E-05
12400 3.81E-05 10300 1.92E-05 10100 5.99E-05 9740
6.03E-05 12000 9.46E-06 11500 1.21E-05
12400 3.81E-05 10300 1.92E-05 10100 6.00E-05 9740
6.05E-05 12000 9.47E-06 11500 1.21E-05
12400 3.82E-05 10400 1.93E-05 10100 6.02E-05 9740
6.07E-05 12000 9.47E-06 11500 1.21E-05
12400 3.82E-05 10400 1.93E-05 10100 6.03E-05 9740
6.09E-05 12000 9.47E-06 11500 1.21E-05
12400 3.82E-05 10400 1.94E-05 10200 6.05E-05 9740
6.12E-05 12000 9.47E-06 11500 1.21E-05
12400 3.82E-05 10400 1.95E-05 10200 6.06E-05 9750
6.14E-05 12000 9.47E-06 11500 1.21E-05
12400 3.82E-05 10400 1.95E-05 10200 6.08E-05 9750
6.16E-05 12000 9.48E-06 11500 1.21E-05
12400 3.82E-05 10400 1.96E-05 10200 6.09E-05 9750
6.19E-05 12000 9.48E-06 11500 1.21E-05
12400 3.82E-05 10400 1.96E-05 10200 6.11E-05 9750
6.21E-05 12000 9.48E-06 11500 1.21E-05
12400 3.82E-05 10400 1.97E-05 10200 6.12E-05 9750
6.24E-05 12000 9.47E-06 11500 1.21E-05
12400 3.82E-05 10400 1.97E-05 10200 6.14E-05 9750
6.26E-05 12000 9.48E-06 11500 1.21E-05
12400 3.83E-05 10400 1.98E-05 10200 6.15E-05 9750
6.28E-05 12000 9.49E-06 11500 1.21E-05
12400 3.83E-05 10400 1.98E-05 10200 6.16E-05 9760
6.31E-05 12000 9.49E-06 11500 1.21E-05
12400 3.83E-05 10400 1.99E-05 10200 6.18E-05 9760
6.33E-05 12000 9.49E-06 11500 1.21E-05
12400 3.83E-05 10400 1.99E-05 10200 6.20E-05 9760
6.36E-05 12000 9.48E-06 11500 1.21E-05
12400 3.83E-05 10400 2.00E-05 10200 6.21E-05 9760
6.38E-05 12000 9.49E-06 11500 1.21E-05
12400 3.83E-05 10400 2.00E-05 10200 6.23E-05 9760
6.41E-05 12000 9.50E-06 11500 1.21E-05

12400 3.84E-05 10400 2.01E-05 10200 6.24E-05 9760
6.43E-05 12000 9.50E-06 11500 1.21E-05
12400 3.84E-05 10400 2.02E-05 10200 6.26E-05 9770
6.46E-05 12000 9.51E-06 11500 1.21E-05
12400 3.84E-05 10400 2.02E-05 10200 6.27E-05 9770
6.48E-05 12100 9.51E-06 11500 1.21E-05
12400 3.84E-05 10400 2.02E-05 10200 6.29E-05 9770
6.51E-05 12100 9.51E-06 11500 1.21E-05
12400 3.84E-05 10400 2.03E-05 10200 6.31E-05 9770
6.53E-05 12100 9.52E-06 11500 1.21E-05
12400 3.84E-05 10400 2.03E-05 10200 6.32E-05 9770
6.56E-05 12100 9.52E-06 11500 1.21E-05
12400 3.84E-05 10400 2.04E-05 10200 6.34E-05 9770
6.58E-05 12100 9.52E-06 11500 1.21E-05
12400 3.85E-05 10400 2.05E-05 10200 6.36E-05 9780
6.61E-05 12100 9.52E-06 11500 1.21E-05
12400 3.85E-05 10400 2.05E-05 10200 6.37E-05 9780
6.64E-05 12100 9.52E-06 11500 1.21E-05
12400 3.85E-05 10400 2.06E-05 10200 6.39E-05 9780
6.66E-05 12100 9.52E-06 11500 1.21E-05
12400 3.85E-05 10400 2.06E-05 10200 6.41E-05 9780
6.69E-05 12100 9.53E-06 11500 1.21E-05
12400 3.85E-05 10400 2.07E-05 10200 6.42E-05 9780
6.72E-05 12100 9.52E-06 11500 1.21E-05
12400 3.86E-05 10400 2.07E-05 10200 6.44E-05 9780
6.74E-05 12100 9.53E-06 11500 1.21E-05
12400 3.86E-05 10400 2.08E-05 10200 6.46E-05 9780
6.77E-05 12100 9.53E-06 11500 1.21E-05
12400 3.86E-05 10400 2.08E-05 10200 6.47E-05 9790
6.80E-05 12100 9.53E-06 11500 1.21E-05
12400 3.86E-05 10400 2.09E-05 10200 6.49E-05 9790
6.82E-05 12100 9.53E-06 11500 1.21E-05
12400 3.87E-05 10400 2.09E-05 10200 6.51E-05 9790
6.85E-05 12100 9.53E-06 11500 1.21E-05
12400 3.87E-05 10400 2.10E-05 10200 6.53E-05 9790
6.88E-05 12100 9.54E-06 11500 1.21E-05
12400 3.87E-05 10400 2.10E-05 10200 6.55E-05 9790
6.91E-05 12100 9.54E-06 11500 1.21E-05
12400 3.87E-05 10400 2.11E-05 10200 6.57E-05 9790
6.94E-05 12100 9.55E-06 11500 1.21E-05
12400 3.87E-05 10400 2.11E-05 10200 6.58E-05 9800
6.96E-05 12100 9.55E-06 11500 1.21E-05
12400 3.88E-05 10400 2.12E-05 10200 6.60E-05 9800
6.99E-05 12100 9.55E-06 11500 1.21E-05
12400 3.88E-05 10400 2.12E-05 10200 6.62E-05 9800
7.02E-05 12100 9.56E-06 11500 1.21E-05
12400 3.88E-05 10400 2.13E-05 10200 6.64E-05 9800
7.05E-05 12100 9.56E-06 11500 1.21E-05
12400 3.88E-05 10400 2.13E-05 10200 6.66E-05 9800
7.08E-05 12100 9.56E-06 11500 1.22E-05
12400 3.89E-05 10400 2.14E-05 10200 6.68E-05 9800
7.11E-05 12100 9.56E-06 11500 1.21E-05
12400 3.89E-05 10400 2.14E-05 10200 6.70E-05 9800
7.14E-05 12100 9.57E-06 11500 1.21E-05
12400 3.89E-05 10400 2.15E-05 10200 6.72E-05 9810
7.17E-05 12100 9.57E-06 11500 1.22E-05
12400 3.89E-05 10400 2.15E-05 10200 6.74E-05 9810
7.20E-05 12100 9.57E-06 11500 1.22E-05
12400 3.89E-05 10400 2.16E-05 10200 6.76E-05 9810
7.23E-05 12100 9.56E-06 11500 1.22E-05
12400 3.89E-05 10400 2.17E-05 10200 6.78E-05 9810
7.26E-05 12100 9.57E-06 11500 1.22E-05
12500 3.90E-05 10400 2.17E-05 10200 6.80E-05 9810
7.29E-05 12100 9.57E-06 11500 1.22E-05
12500 3.90E-05 10400 2.18E-05 10200 6.82E-05 9810
7.33E-05 12100 9.57E-06 11500 1.22E-05
12500 3.90E-05 10400 2.18E-05 10200 6.84E-05 9820
7.36E-05 12100 9.58E-06 11500 1.22E-05

12500 3.90E-05 10400 2.19E-05 10200 6.86E-05 9820
7.39E-05 12100 9.59E-06 11500 1.22E-05
12500 3.90E-05 10400 2.19E-05 10200 6.89E-05 9820
7.42E-05 12100 9.59E-06 11500 1.22E-05
12500 3.90E-05 10400 2.20E-05 10200 6.91E-05 9820
7.46E-05 12100 9.60E-06 11500 1.22E-05
12500 3.91E-05 10400 2.20E-05 10200 6.93E-05 9820
7.49E-05 12100 9.60E-06 11500 1.22E-05
12500 3.91E-05 10400 2.21E-05 10200 6.95E-05 9820
7.52E-05 12100 9.61E-06 11500 1.22E-05
12500 3.91E-05 10400 2.22E-05 10200 6.98E-05 9830
7.55E-05 12100 9.61E-06 11500 1.22E-05
12500 3.91E-05 10400 2.22E-05 10200 7.00E-05 9830
7.59E-05 12100 9.61E-06 11600 1.22E-05
12500 3.91E-05 10400 2.23E-05 10200 7.02E-05 9830
7.62E-05 12100 9.62E-06 11600 1.22E-05
12500 3.92E-05 10400 2.23E-05 10200 7.05E-05 9830
7.66E-05 12100 9.61E-06 11600 1.22E-05
12500 3.92E-05 10400 2.24E-05 10200 7.08E-05 9830
7.69E-05 12100 9.62E-06 11600 1.22E-05
12500 3.92E-05 10400 2.25E-05 10200 7.10E-05 9830
7.73E-05 12100 9.62E-06 11600 1.22E-05
12500 3.92E-05 10400 2.25E-05 10200 7.13E-05 9830
7.76E-05 12100 9.63E-06 11600 1.22E-05
12500 3.92E-05 10400 2.26E-05 10200 7.16E-05 9840
7.80E-05 12100 9.63E-06 11600 1.22E-05
12500 3.92E-05 10400 2.26E-05 10200 7.18E-05 9840
7.83E-05 12100 9.63E-06 11600 1.22E-05
12500 3.93E-05 10400 2.27E-05 10200 7.21E-05 9840
7.87E-05 12100 9.63E-06 11600 1.22E-05
12500 3.93E-05 10500 2.28E-05 10200 7.24E-05 9840
7.91E-05 12100 9.63E-06 11600 1.22E-05
12500 3.93E-05 10500 2.28E-05 10200 7.27E-05 9840
7.94E-05 12100 9.63E-06 11600 1.22E-05
12500 3.93E-05 10500 2.29E-05 10300 7.30E-05 9840
7.98E-05 12100 9.63E-06 11600 1.22E-05
12500 3.94E-05 10500 2.29E-05 10300 7.33E-05 9850
8.02E-05 12100 9.62E-06 11600 1.22E-05
12500 3.94E-05 10500 2.30E-05 10300 7.36E-05 9850
8.05E-05 12100 9.63E-06 11600 1.22E-05
12500 3.94E-05 10500 2.31E-05 10300 7.39E-05 9850
8.09E-05 12100 9.62E-06 11600 1.22E-05
12500 3.94E-05 10500 2.31E-05 10300 7.42E-05 9850
8.13E-05 12100 9.63E-06 11600 1.22E-05
12500 3.95E-05 10500 2.32E-05 10300 7.46E-05 9850
8.17E-05 12100 9.64E-06 11600 1.22E-05
12500 3.95E-05 10500 2.33E-05 10300 7.49E-05 9850
8.20E-05 12100 9.64E-06 11600 1.22E-05
12500 3.95E-05 10500 2.33E-05 10300 7.53E-05 9850
8.24E-05 12100 9.65E-06 11600 1.22E-05
12500 3.96E-05 10500 2.34E-05 10300 7.57E-05 9860
8.28E-05 12100 9.65E-06 11600 1.23E-05
12500 3.96E-05 10500 2.35E-05 10300 7.60E-05 9860
8.32E-05 12100 9.65E-06 11600 1.23E-05
12500 3.96E-05 10500 2.35E-05 10300 7.65E-05 9860
8.36E-05 12100 9.65E-06 11600 1.23E-05
12500 3.96E-05 10500 2.36E-05 10300 7.69E-05 9860
8.40E-05 12100 9.65E-06 11600 1.23E-05
12500 3.97E-05 10500 2.37E-05 10300 7.73E-05 9860
8.44E-05 12100 9.66E-06 11600 1.23E-05
12500 3.97E-05 10500 2.37E-05 10300 7.78E-05 9860
8.48E-05 12100 9.66E-06 11600 1.23E-05
12500 3.97E-05 10500 2.38E-05 10300 7.82E-05 9870
8.52E-05 12100 9.66E-06 11600 1.23E-05
12500 3.97E-05 10500 2.39E-05 10300 7.87E-05 9870
8.56E-05 12200 9.66E-06 11600 1.23E-05
12500 3.97E-05 10500 2.39E-05 10300 7.92E-05 9870
8.60E-05 12200 9.66E-06 11600 1.23E-05

12500	3.98E-05	10500	2.40E-05	10300	7.97E-05	9870	8.64E-05	12200	9.64E-06	11600	1.23E-05
12500	3.98E-05	10500	2.40E-05	10300	8.03E-05	9870	8.68E-05	12200	9.65E-06	11600	1.23E-05
12500	3.98E-05	10500	2.41E-05	10300	8.08E-05	9870	8.73E-05	12200	9.65E-06	11600	1.23E-05
12500	3.98E-05	10500	2.42E-05	10300	8.14E-05	9880	8.77E-05	12200	9.65E-06	11600	1.23E-05
12500	3.99E-05	10500	2.42E-05	10300	8.20E-05	9880	8.81E-05	12200	9.66E-06	11600	1.23E-05
12500	3.99E-05	10500	2.43E-05	10300	8.26E-05	9880	8.86E-05	12200	9.66E-06	11600	1.23E-05
12500	3.99E-05	10500	2.44E-05	10300	8.33E-05	9880	8.90E-05	12200	9.67E-06	11600	1.23E-05
12500	3.99E-05	10500	2.44E-05	10300	8.40E-05	9880	8.94E-05	12200	9.67E-06	11600	1.23E-05
12500	4.00E-05	10500	2.45E-05	10300	8.47E-05	9880	8.99E-05	12200	9.67E-06	11600	1.23E-05
12500	4.00E-05	10500	2.46E-05	10300	8.55E-05	9880	9.03E-05	12200	9.67E-06	11600	1.23E-05
12500	4.01E-05	10500	2.46E-05	10300	8.64E-05	9890	9.08E-05	12200	9.67E-06	11600	1.23E-05
12500	4.01E-05	10500	2.47E-05	10300	8.72E-05	9890	9.12E-05	12200	9.68E-06	11600	1.23E-05
12500	4.01E-05	10500	2.48E-05	10300	8.81E-05	9890	9.17E-05	12200	9.68E-06	11600	1.23E-05
12500	4.02E-05	10500	2.48E-05	10300	8.90E-05	9890	9.22E-05	12200	9.68E-06	11600	1.23E-05
12500	4.02E-05	10500	2.49E-05	10300	9.00E-05	9890	9.26E-05	12200	9.68E-06	11600	1.23E-05
12500	4.02E-05	10500	2.50E-05	10300	9.10E-05	9890	9.31E-05	12200	9.69E-06	11600	1.23E-05
12500	4.02E-05	10500	2.50E-05	10300	9.21E-05	9900	9.36E-05	12200	9.68E-06	11600	1.23E-05
12500	4.02E-05	10500	2.51E-05	10300	9.32E-05	9900	9.41E-05	12200	9.69E-06	11600	1.23E-05
12500	4.03E-05	10500	2.52E-05	10300	9.44E-05	9900	9.46E-05	12200	9.69E-06	11600	1.23E-05
12500	4.03E-05	10500	2.53E-05	10300	9.56E-05	9900	9.50E-05	12200	9.69E-06	11600	1.23E-05
12500	4.04E-05	10500	2.53E-05	10300	9.68E-05	9900	9.55E-05	12200	9.69E-06	11600	1.23E-05
12500	4.04E-05	10500	2.54E-05	10300	9.80E-05	9900	9.60E-05	12200	9.68E-06	11600	1.23E-05
12500	4.04E-05	10500	2.55E-05	10300	9.93E-05	9900	9.65E-05	12200	9.68E-06	11600	1.23E-05
12500	4.04E-05	10500	2.56E-05	10300	1.01E-04	9910	9.70E-05	12200	9.68E-06	11600	1.23E-05
12500	4.05E-05	10500	2.56E-05	10300	1.02E-04	9910	9.75E-05	12200	9.68E-06	11600	1.23E-05
12500	4.05E-05	10500	2.57E-05	10300	1.03E-04	9910	9.80E-05	12200	9.69E-06	11600	1.23E-05
12500	4.06E-05	10500	2.58E-05	10300	1.05E-04	9910	9.85E-05	12200	9.70E-06	11600	1.23E-05
12600	4.06E-05	10500	2.58E-05	10300	1.06E-04	9910	9.90E-05	12200	9.69E-06	11600	1.23E-05
12600	4.06E-05	10500	2.59E-05	10300	1.07E-04	9910	9.95E-05	12200	9.69E-06	11600	1.23E-05
12600	4.07E-05	10500	2.60E-05	10300	1.08E-04	9920	0.000100058	12200	9.69E-06	11600	1.23E-05
12600	4.07E-05	10500	2.61E-05	10300	1.09E-04	9920	0.000100579	12200	9.69E-06	11600	1.23E-05
12600	4.08E-05	10500	2.61E-05	10300	1.10E-04	9920	0.000101111	12200	9.69E-06	11600	1.24E-05
12600	4.08E-05	10500	2.62E-05	10300	1.11E-04	9920	0.000101645	12200	9.69E-06	11600	1.24E-05
12600	4.08E-05	10500	2.63E-05	10300	1.12E-04	9920	0.000102181	12200	9.70E-06	11600	1.24E-05

12600 4.09E-05 10500 2.63E-05 10300 1.12E-04 9920
0.000102729 12200 9.70E-06 11600 1.24E-05
12600 4.09E-05 10500 2.64E-05 10300 1.13E-04 9930
0.000103293 12200 9.69E-06 11600 1.24E-05
12600 4.09E-05 10500 2.65E-05 10300 1.13E-04 9930
0.000103851 12200 9.70E-06 11700 1.24E-05
12600 4.10E-05 10500 2.66E-05 10300 1.13E-04 9930
0.00010442 12200 9.70E-06 11700 1.24E-05
12600 4.10E-05 10500 2.67E-05 10300 1.13E-04 9930
0.000104988 12200 9.71E-06 11700 1.24E-05
12600 4.10E-05 10500 2.67E-05 10300 1.13E-04 9930
0.000105549 12200 9.70E-06 11700 1.24E-05
12600 4.11E-05 10500 2.68E-05 10300 1.12E-04 9930
0.000106145 12200 9.70E-06 11700 1.24E-05
12600 4.11E-05 10500 2.69E-05 10300 1.12E-04 9930
0.000106728 12200 9.70E-06 11700 1.24E-05
12600 4.11E-05 10500 2.70E-05 10300 1.11E-04 9940
0.000107323 12200 9.71E-06 11700 1.24E-05
12600 4.12E-05 10500 2.71E-05 10300 1.10E-04 9940
0.000107911 12200 9.71E-06 11700 1.24E-05
12600 4.12E-05 10500 2.71E-05 10300 1.10E-04 9940
0.000108513 12200 9.71E-06 11700 1.24E-05
12600 4.12E-05 10600 2.72E-05 10300 1.09E-04 9940
0.000109135 12200 9.72E-06 11700 1.24E-05
12600 4.13E-05 10600 2.73E-05 10300 1.08E-04 9940
0.000109734 12200 9.73E-06 11700 1.24E-05
12600 4.13E-05 10600 2.74E-05 10400 1.07E-04 9940
0.000110356 12200 9.73E-06 11700 1.24E-05
12600 4.14E-05 10600 2.75E-05 10400 1.06E-04 9950
0.000110968 12200 9.73E-06 11700 1.24E-05
12600 4.14E-05 10600 2.75E-05 10400 1.05E-04 9950
0.000111598 12200 9.73E-06 11700 1.24E-05
12600 4.15E-05 10600 2.76E-05 10400 1.04E-04 9950
0.000112229 12200 9.73E-06 11700 1.24E-05
12600 4.15E-05 10600 2.77E-05 10400 1.03E-04 9950
0.000112871 12200 9.73E-06 11700 1.24E-05
12600 4.16E-05 10600 2.78E-05 10400 1.03E-04 9950
0.000113509 12200 9.73E-06 11700 1.24E-05
12600 4.16E-05 10600 2.78E-05 10400 1.02E-04 9950
0.000114159 12200 9.74E-06 11700 1.24E-05
12600 4.17E-05 10600 2.79E-05 10400 1.01E-04 9950
0.000114833 12200 9.74E-06 11700 1.24E-05
12600 4.17E-05 10600 2.80E-05 10400 1.00E-04 9960
0.000115493 12200 9.74E-06 11700 1.24E-05
12600 4.17E-05 10600 2.81E-05 10400 9.95E-05 9960
0.000116171 12200 9.75E-06 11700 1.24E-05
12600 4.18E-05 10600 2.82E-05 10400 9.88E-05 9960
0.000116847 12200 9.75E-06 11700 1.24E-05
12600 4.18E-05 10600 2.83E-05 10400 9.82E-05 9960
0.00011751 12200 9.75E-06 11700 1.24E-05
12600 4.19E-05 10600 2.84E-05 10400 9.77E-05 9960
0.000118197 12200 9.74E-06 11700 1.24E-05
12600 4.19E-05 10600 2.85E-05 10400 9.71E-05 9960
0.000118897 12200 9.75E-06 11700 1.24E-05
12600 4.20E-05 10600 2.85E-05 10400 9.67E-05 9970
0.000119602 12200 9.75E-06 11700 1.24E-05
12600 4.20E-05 10600 2.86E-05 10400 9.62E-05 9970
0.000120313 12300 9.75E-06 11700 1.24E-05
12600 4.21E-05 10600 2.87E-05 10400 9.58E-05 9970
0.000121023 12300 9.75E-06 11700 1.25E-05
12600 4.21E-05 10600 2.88E-05 10400 9.55E-05 9970
0.000121742 12300 9.75E-06 11700 1.24E-05
12600 4.22E-05 10600 2.89E-05 10400 9.51E-05 9970
0.00012247 12300 9.76E-06 11700 1.25E-05
12600 4.22E-05 10600 2.90E-05 10400 9.49E-05 9970
0.000123208 12300 9.77E-06 11700 1.25E-05
12600 4.23E-05 10600 2.90E-05 10400 9.46E-05 9980
0.000123944 12300 9.76E-06 11700 1.25E-05

12600	4.23E-05	10600	2.91E-05	10400	9.44E-05	9980	0.00012469	12300	9.77E-06	11700	1.25E-05
12600	4.24E-05	10600	2.92E-05	10400	9.42E-05	9980	0.000125458	12300	9.77E-06	11700	1.25E-05
12600	4.25E-05	10600	2.93E-05	10400	9.40E-05	9980	0.000126234	12300	9.77E-06	11700	1.25E-05
12600	4.25E-05	10600	2.94E-05	10400	9.39E-05	9980	0.000126997	12300	9.78E-06	11700	1.25E-05
12600	4.25E-05	10600	2.95E-05	10400	9.37E-05	9980	0.000127783	12300	9.78E-06	11700	1.25E-05
12600	4.26E-05	10600	2.96E-05	10400	9.36E-05	9980	0.000128583	12300	9.78E-06	11700	1.25E-05
12600	4.26E-05	10600	2.97E-05	10400	9.35E-05	9990	0.000129382	12300	9.78E-06	11700	1.25E-05
12600	4.27E-05	10600	2.97E-05	10400	9.35E-05	9990	0.000130182	12300	9.78E-06	11700	1.25E-05
12600	4.27E-05	10600	2.98E-05	10400	9.34E-05	9990	0.000130992	12300	9.79E-06	11700	1.25E-05
12600	4.28E-05	10600	2.99E-05	10400	9.34E-05	9990	0.000131802	12300	9.79E-06	11700	1.25E-05
12600	4.28E-05	10600	3.00E-05	10400	9.34E-05	9990	0.000132621	12300	9.79E-06	11700	1.25E-05
12600	4.29E-05	10600	3.01E-05	10400	9.34E-05	9990	0.00013346	12300	9.79E-06	11700	1.25E-05
12600	4.30E-05	10600	3.02E-05	10400	9.34E-05	10000	0.0001343	12300	9.80E-06	11700	1.25E-05
12600	4.30E-05	10600	3.03E-05	10400	9.34E-05	10000	0.000135149	12300	9.81E-06	11700	1.25E-05
12600	4.31E-05	10600	3.04E-05	10400	9.34E-05	10000	0.000136014	12300	9.81E-06	11700	1.25E-05
12600	4.31E-05	10600	3.05E-05	10400	9.35E-05	10000	0.00013688	12300	9.81E-06	11700	1.25E-05
12600	4.32E-05	10600	3.06E-05	10400	9.35E-05	10000	0.000137761	12300	9.81E-06	11700	1.25E-05
12600	4.33E-05	10600	3.07E-05	10400	9.36E-05	10000	0.000138626	12300	9.82E-06	11700	1.25E-05
12600	4.33E-05	10600	3.07E-05	10400	9.37E-05	10000	0.000139526	12300	9.82E-06	11700	1.26E-05
12600	4.34E-05	10600	3.08E-05	10400	9.38E-05	10000	0.000140432	12300	9.83E-06	11700	1.26E-05
12600	4.35E-05	10600	3.09E-05	10400	9.39E-05	10000	0.000141346	12300	9.83E-06	11700	1.26E-05
12600	4.35E-05	10600	3.10E-05	10400	9.40E-05	10000	0.000142284	12300	9.83E-06	11700	1.26E-05
12600	4.36E-05	10600	3.11E-05	10400	9.41E-05	10000	0.000143216	12300	9.84E-06	11700	1.26E-05
12700	4.36E-05	10600	3.12E-05	10400	9.42E-05	10000	0.000144165	12300	9.84E-06	11700	1.26E-05
12700	4.37E-05	10600	3.13E-05	10400	9.44E-05	10000	0.000145123	12300	9.84E-06	11700	1.26E-05
12700	4.37E-05	10600	3.14E-05	10400	9.45E-05	10000	0.000146092	12300	9.84E-06	11700	1.26E-05
12700	4.38E-05	10600	3.15E-05	10400	9.47E-05	10000	0.000147081	12300	9.84E-06	11700	1.26E-05
12700	4.39E-05	10600	3.16E-05	10400	9.48E-05	10000	0.000148068	12300	9.84E-06	11700	1.26E-05
12700	4.39E-05	10600	3.17E-05	10400	9.50E-05	10000	0.000149054	12300	9.84E-06	11700	1.26E-05
12700	4.40E-05	10600	3.19E-05	10400	9.51E-05	10000	0.000150066	12300	9.84E-06	11700	1.26E-05
12700	4.41E-05	10600	3.19E-05	10400	9.53E-05	10000	0.000151101	12300	9.84E-06	11700	1.26E-05
12700	4.41E-05	10600	3.21E-05	10400	9.55E-05	10000	0.000152138	12300	9.84E-06	11700	1.26E-05
12700	4.42E-05	10600	3.21E-05	10400	9.56E-05	10000	0.000153172	12300	9.85E-06	11800	1.26E-05
12700	4.43E-05	10600	3.22E-05	10400	9.58E-05	10000	0.000154211	12300	9.86E-06	11800	1.26E-05

12700	4.43E-05	10600	3.23E-05	10400	9.60E-05	10000	0.000155257	12300	9.86E-06	11800	1.26E-05
12700	4.44E-05	10600	3.24E-05	10400	9.62E-05	10000	0.000156321	12300	9.86E-06	11800	1.26E-05
12700	4.45E-05	10600	3.25E-05	10400	9.64E-05	10000	0.000157406	12300	9.87E-06	11800	1.26E-05
12700	4.46E-05	10600	3.26E-05	10400	9.65E-05	10000	0.000158483	12300	9.87E-06	11800	1.26E-05
12700	4.47E-05	10600	3.27E-05	10400	9.67E-05	10000	0.000159587	12300	9.88E-06	11800	1.26E-05
12700	4.47E-05	10600	3.28E-05	10400	9.70E-05	10000	0.000160697	12300	9.89E-06	11800	1.26E-05
12700	4.48E-05	10600	3.29E-05	10400	9.72E-05	10000	0.000161833	12300	9.88E-06	11800	1.26E-05
12700	4.49E-05	10700	3.31E-05	10400	9.74E-05	10000	0.000162955	12300	9.89E-06	11800	1.26E-05
12700	4.50E-05	10700	3.32E-05	10400	9.76E-05	10000	0.000164118	12300	9.88E-06	11800	1.26E-05
12700	4.51E-05	10700	3.33E-05	10500	9.78E-05	10000	0.000165274	12300	9.89E-06	11800	1.26E-05
12700	4.51E-05	10700	3.34E-05	10500	9.81E-05	10000	0.000166445	12300	9.89E-06	11800	1.26E-05
12700	4.52E-05	10700	3.35E-05	10500	9.83E-05	10000	0.000167636	12300	9.89E-06	11800	1.26E-05
12700	4.53E-05	10700	3.36E-05	10500	9.85E-05	10000	0.000168829	12300	9.89E-06	11800	1.26E-05
12700	4.54E-05	10700	3.37E-05	10500	9.87E-05	10100	0.000170059	12300	9.90E-06	11800	1.27E-05
12700	4.55E-05	10700	3.38E-05	10500	9.90E-05	10100	0.0001713	12300	9.90E-06	11800	1.27E-05
12700	4.55E-05	10700	3.39E-05	10500	9.92E-05	10100	0.00017256	12300	9.90E-06	11800	1.27E-05
12700	4.56E-05	10700	3.40E-05	10500	9.94E-05	10100	0.000173851	12300	9.91E-06	11800	1.27E-05
12700	4.57E-05	10700	3.41E-05	10500	9.97E-05	10100	0.000175148	12300	9.91E-06	11800	1.27E-05
12700	4.58E-05	10700	3.43E-05	10500	9.99E-05	10100	0.000176452	12300	9.92E-06	11800	1.27E-05
12700	4.59E-05	10700	3.44E-05	10500	1.00E-04	10100	0.000177786	12300	9.92E-06	11800	1.27E-05
12700	4.60E-05	10700	3.45E-05	10500	1.00E-04	10100	0.000179128	12300	9.92E-06	11800	1.27E-05
12700	4.61E-05	10700	3.46E-05	10500	1.01E-04	10100	0.000180481	12300	9.92E-06	11800	1.27E-05
12700	4.62E-05	10700	3.47E-05	10500	1.01E-04	10100	0.000181849	12300	9.93E-06	11800	1.27E-05
12700	4.62E-05	10700	3.49E-05	10500	1.01E-04	10100	0.000183201	12300	9.93E-06	11800	1.27E-05
12700	4.63E-05	10700	3.50E-05	10500	1.01E-04	10100	0.000184597	12400	9.94E-06	11800	1.27E-05
12700	4.64E-05	10700	3.51E-05	10500	1.02E-04	10100	0.000186006	12400	9.94E-06	11800	1.27E-05
12700	4.65E-05	10700	3.52E-05	10500	1.02E-04	10100	0.000187425	12400	9.94E-06	11800	1.27E-05
12700	4.66E-05	10700	3.53E-05	10500	1.02E-04	10100	0.000188855	12400	9.94E-06	11800	1.27E-05
12700	4.67E-05	10700	3.55E-05	10500	1.03E-04	10100	0.000190312	12400	9.94E-06	11800	1.27E-05
12700	4.68E-05	10700	3.56E-05	10500	1.03E-04	10100	0.000191787	12400	9.94E-06	11800	1.27E-05
12700	4.69E-05	10700	3.57E-05	10500	1.03E-04	10100	0.000193278	12400	9.94E-06	11800	1.27E-05
12700	4.69E-05	10700	3.58E-05	10500	1.03E-04	10100	0.000194785	12400	9.94E-06	11800	1.27E-05
12700	4.70E-05	10700	3.59E-05	10500	1.04E-04	10100	0.000196319	12400	9.95E-06	11800	1.27E-05
12700	4.71E-05	10700	3.61E-05	10500	1.04E-04	10100	0.000197876	12400	9.94E-06	11800	1.27E-05

12700 4.72E-05 10700 3.62E-05 10500 1.04E-04 10100
0.000199455 12400 9.95E-06 11800 1.27E-05
12700 4.73E-05 10700 3.63E-05 10500 1.04E-04 10100
0.000201051 12400 9.94E-06 11800 1.27E-05
12700 4.74E-05 10700 3.64E-05 10500 1.05E-04 10100
0.000202672 12400 9.94E-06 11800 1.27E-05
12700 4.75E-05 10700 3.65E-05 10500 1.05E-04 10100
0.000204294 12400 9.95E-06 11800 1.27E-05
12700 4.76E-05 10700 3.67E-05 10500 1.05E-04 10100
0.000205938 12400 9.95E-06 11800 1.27E-05
12700 4.77E-05 10700 3.68E-05 10500 1.06E-04 10100
0.000207595 12400 9.95E-06 11800 1.27E-05
12700 4.79E-05 10700 3.69E-05 10500 1.06E-04 10100
0.000209261 12400 9.96E-06 11800 1.27E-05
12700 4.80E-05 10700 3.70E-05 10500 1.06E-04 10100
0.000210951 12400 9.96E-06 11800 1.27E-05
12700 4.81E-05 10700 3.72E-05 10500 1.07E-04 10100
0.000212659 12400 9.97E-06 11800 1.27E-05
12700 4.82E-05 10700 3.73E-05 10500 1.07E-04 10100
0.000214392 12400 9.97E-06 11800 1.27E-05
12700 4.83E-05 10700 3.74E-05 10500 1.07E-04 10100
0.000216143 12400 9.96E-06 11800 1.27E-05
12700 4.84E-05 10700 3.76E-05 10500 1.07E-04 10100
0.000217919 12400 9.97E-06 11800 1.27E-05
12700 4.85E-05 10700 3.77E-05 10500 1.08E-04 10100
0.000219737 12400 9.97E-06 11800 1.27E-05
12700 4.86E-05 10700 3.78E-05 10500 1.08E-04 10100
0.00022156 12400 9.96E-06 11800 1.28E-05
12700 4.87E-05 10700 3.80E-05 10500 1.08E-04 10100
0.000223423 12400 9.97E-06 11800 1.28E-05
12700 4.88E-05 10700 3.81E-05 10500 1.09E-04 10100
0.000225297 12400 9.96E-06 11800 1.28E-05
12700 4.89E-05 10700 3.82E-05 10500 1.09E-04 10100
0.000227215 12400 9.96E-06 11800 1.28E-05
12700 4.91E-05 10700 3.84E-05 10500 1.09E-04 10100
0.000229143 12400 9.96E-06 11800 1.28E-05
12700 4.92E-05 10700 3.85E-05 10500 1.10E-04 10100
0.000231118 12400 9.96E-06 11800 1.28E-05
12800 4.93E-05 10700 3.87E-05 10500 1.10E-04 10100
0.000233125 12400 9.95E-06 11800 1.28E-05
12800 4.94E-05 10700 3.88E-05 10500 1.10E-04 10100
0.000235159 12400 9.95E-06 11800 1.28E-05
12800 4.95E-05 10700 3.89E-05 10500 1.11E-04 10100
0.000237217 12400 9.96E-06 11800 1.28E-05
12800 4.96E-05 10700 3.91E-05 10500 1.11E-04 10100
0.000239302 12400 9.96E-06 11800 1.28E-05
12800 4.98E-05 10700 3.92E-05 10500 1.11E-04 10100
0.000241421 12400 9.97E-06 11800 1.28E-05
12800 4.99E-05 10700 3.94E-05 10500 1.12E-04 10100
0.000243554 12400 9.97E-06 11800 1.28E-05
12800 5.00E-05 10700 3.95E-05 10500 1.12E-04 10100
0.000245726 12400 9.98E-06 11800 1.28E-05
12800 5.02E-05 10700 3.96E-05 10500 1.12E-04 10100
0.000247944 12400 9.97E-06 11800 1.28E-05
12800 5.03E-05 10700 3.98E-05 10500 1.13E-04 10100
0.000250166 12400 9.98E-06 11800 1.28E-05
12800 5.04E-05 10700 3.99E-05 10500 1.13E-04 10100
0.000252433 12400 9.98E-06 11900 1.28E-05
12800 5.06E-05 10700 4.01E-05 10500 1.13E-04 10100
0.00025474 12400 9.98E-06 11900 1.28E-05
12800 5.07E-05 10700 4.02E-05 10500 1.14E-04 10100
0.000257058 12400 9.98E-06 11900 1.28E-05
12800 5.08E-05 10700 4.03E-05 10500 1.14E-04 10100
0.000259423 12400 9.98E-06 11900 1.28E-05
12800 5.10E-05 10700 4.05E-05 10500 1.14E-04 10100
0.000261828 12400 9.98E-06 11900 1.28E-05
12800 5.11E-05 10700 4.06E-05 10500 1.15E-04 10100
0.000264244 12400 9.98E-06 11900 1.28E-05

12800 5.12E-05 10700 4.08E-05 10500 1.15E-04 10100
0.000266707 12400 9.97E-06 11900 1.28E-05
12800 5.14E-05 10700 4.09E-05 10500 1.15E-04 10100
0.000269204 12400 9.98E-06 11900 1.28E-05
12800 5.15E-05 10700 4.11E-05 10500 1.16E-04 10100
0.000271728 12400 9.98E-06 11900 1.28E-05
12800 5.17E-05 10800 4.12E-05 10500 1.16E-04 10100
0.000274298 12400 9.98E-06 11900 1.28E-05
12800 5.18E-05 10800 4.14E-05 10500 1.16E-04 10100
0.000276903 12400 9.98E-06 11900 1.28E-05
12800 5.19E-05 10800 4.15E-05 10600 1.17E-04 10100
0.000279533 12400 9.99E-06 11900 1.28E-05
12800 5.20E-05 10800 4.16E-05 10600 1.17E-04 10100
0.000282203 12400 9.99E-06 11900 1.29E-05
12800 5.22E-05 10800 4.18E-05 10600 1.18E-04 10100
0.000284891 12400 9.99E-06 11900 1.28E-05
12800 5.23E-05 10800 4.20E-05 10600 1.18E-04 10100
0.000287623 12400 1.00E-05 11900 1.29E-05
12800 5.25E-05 10800 4.21E-05 10600 1.18E-04 10200
0.000290419 12400 1.00E-05 11900 1.29E-05
12800 5.27E-05 10800 4.23E-05 10600 1.19E-04 10200
0.00029323 12400 1.00E-05 11900 1.29E-05
12800 5.28E-05 10800 4.24E-05 10600 1.19E-04 10200
0.000296106 12400 1.00E-05 11900 1.29E-05
12800 5.30E-05 10800 4.26E-05 10600 1.19E-04 10200
0.000299012 12400 1.00E-05 11900 1.29E-05
12800 5.31E-05 10800 4.27E-05 10600 1.20E-04 10200
0.000301969 12400 1.00E-05 11900 1.29E-05
12800 5.33E-05 10800 4.29E-05 10600 1.20E-04 10200
0.000304987 12400 1.00E-05 11900 1.29E-05
12800 5.34E-05 10800 4.30E-05 10600 1.21E-04 10200
0.00030804 12400 1.00E-05 11900 1.29E-05
12800 5.36E-05 10800 4.32E-05 10600 1.21E-04 10200
0.000311152 12400 1.00E-05 11900 1.29E-05
12800 5.38E-05 10800 4.34E-05 10600 1.21E-04 10200
0.000314293 12400 1.00E-05 11900 1.29E-05
12800 5.39E-05 10800 4.35E-05 10600 1.22E-04 10200
0.000317484 12400 1.00E-05 11900 1.29E-05
12800 5.41E-05 10800 4.37E-05 10600 1.22E-04 10200
0.000320724 12400 1.00E-05 11900 1.29E-05
12800 5.43E-05 10800 4.39E-05 10600 1.23E-04 10200
0.000323984 12500 1.00E-05 11900 1.29E-05
12800 5.45E-05 10800 4.40E-05 10600 1.23E-04 10200
0.000327296 12500 1.00E-05 11900 1.29E-05
12800 5.46E-05 10800 4.42E-05 10600 1.23E-04 10200
0.000330668 12500 1.00E-05 11900 1.29E-05
12800 5.48E-05 10800 4.44E-05 10600 1.24E-04 10200
0.000334097 12500 1.00E-05 11900 1.29E-05
12800 5.50E-05 10800 4.45E-05 10600 1.24E-04 10200
0.000337581 12500 1.00E-05 11900 1.29E-05
12800 5.52E-05 10800 4.47E-05 10600 1.25E-04 10200
0.000341131 12500 1.00E-05 11900 1.29E-05
12800 5.53E-05 10800 4.49E-05 10600 1.25E-04 10200
0.000344729 12500 1.00E-05 11900 1.29E-05
12800 5.55E-05 10800 4.50E-05 10600 1.25E-04 10200
0.000348391 12500 1.00E-05 11900 1.29E-05
12800 5.57E-05 10800 4.52E-05 10600 1.26E-04 10200
0.000352111 12500 1.00E-05 11900 1.30E-05
12800 5.59E-05 10800 4.54E-05 10600 1.26E-04 10200
0.000355875 12500 1.00E-05 11900 1.30E-05
12800 5.61E-05 10800 4.56E-05 10600 1.27E-04 10200
0.000359713 12500 1.00E-05 11900 1.30E-05
12800 5.63E-05 10800 4.57E-05 10600 1.27E-04 10200
0.000363614 12500 1.00E-05 11900 1.30E-05
12800 5.65E-05 10800 4.59E-05 10600 1.27E-04 10200
0.000367575 12500 1.00E-05 11900 1.30E-05
12800 5.67E-05 10800 4.61E-05 10600 1.28E-04 10200
0.000371617 12500 1.00E-05 11900 1.30E-05

12800 5.69E-05 10800 4.63E-05 10600 1.28E-04 10200
0.000375739 12500 1.00E-05 11900 1.30E-05
12800 5.71E-05 10800 4.65E-05 10600 1.29E-04 10200
0.000379957 12500 1.00E-05 11900 1.30E-05
12800 5.73E-05 10800 4.66E-05 10600 1.29E-04 10200
0.000384252 12500 1.00E-05 11900 1.30E-05
12800 5.75E-05 10800 4.68E-05 10600 1.30E-04 10200
0.000388626 12500 1.01E-05 11900 1.30E-05
12800 5.77E-05 10800 4.70E-05 10600 1.30E-04 10200
0.000393089 12500 1.01E-05 11900 1.30E-05
12800 5.80E-05 10800 4.72E-05 10600 1.30E-04 10200
0.000397634 12500 1.01E-05 11900 1.30E-05
12800 5.82E-05 10800 4.73E-05 10600 1.31E-04 10200
0.000402248 12500 1.01E-05 11900 1.30E-05
12800 5.84E-05 10800 4.75E-05 10600 1.31E-04 10200
0.000406949 12500 1.01E-05 11900 1.30E-05
12800 5.86E-05 10800 4.77E-05 10600 1.32E-04 10200
0.000411727 12500 1.01E-05 11900 1.30E-05
12800 5.88E-05 10800 4.79E-05 10600 1.32E-04 10200
0.000416575 12500 1.01E-05 11900 1.30E-05
12800 5.90E-05 10800 4.81E-05 10600 1.33E-04 10200
0.000421518 12500 1.01E-05 11900 1.30E-05
12800 5.93E-05 10800 4.83E-05 10600 1.33E-04 10200
0.000426526 12500 1.01E-05 11900 1.30E-05
12800 5.95E-05 10800 4.85E-05 10600 1.34E-04 10200
0.000431639 12500 1.01E-05 11900 1.30E-05
12800 5.98E-05 10800 4.87E-05 10600 1.34E-04 10200
0.000436846 12500 1.01E-05 11900 1.30E-05
12800 6.00E-05 10800 4.89E-05 10600 1.35E-04 10200
0.000442136 12500 1.01E-05 11900 1.31E-05
12900 6.03E-05 10800 4.91E-05 10600 1.35E-04 10200
0.000447525 12500 1.01E-05 11900 1.31E-05
12900 6.05E-05 10800 4.93E-05 10600 1.35E-04 10200
0.000453031 12500 1.01E-05 11900 1.31E-05
12900 6.08E-05 10800 4.95E-05 10600 1.36E-04 10200
0.000458631 12500 1.01E-05 11900 1.31E-05
12900 6.11E-05 10800 4.97E-05 10600 1.36E-04 10200
0.000464337 12500 1.01E-05 11900 1.31E-05
12900 6.13E-05 10800 4.98E-05 10600 1.37E-04 10200
0.000470155 12500 1.01E-05 11900 1.31E-05
12900 6.15E-05 10800 5.01E-05 10600 1.37E-04 10200
0.00047608 12500 1.01E-05 11900 1.31E-05
12900 6.18E-05 10800 5.02E-05 10600 1.38E-04 10200
0.000482119 12500 1.01E-05 11900 1.31E-05
12900 6.21E-05 10800 5.04E-05 10600 1.38E-04 10200
0.000488278 12500 1.01E-05 11900 1.31E-05
12900 6.23E-05 10800 5.07E-05 10600 1.39E-04 10200
0.000494551 12500 1.01E-05 11900 1.31E-05
12900 6.26E-05 10800 5.09E-05 10600 1.39E-04 10200
0.000500963 12500 1.01E-05 12000 1.31E-05
12900 6.29E-05 10800 5.11E-05 10600 1.40E-04 10200
0.000507498 12500 1.01E-05 12000 1.31E-05
12900 6.32E-05 10800 5.13E-05 10600 1.40E-04 10200
0.00051417 12500 1.01E-05 12000 1.31E-05
12900 6.35E-05 10800 5.15E-05 10600 1.41E-04 10200
0.000521002 12500 1.01E-05 12000 1.31E-05
12900 6.38E-05 10800 5.17E-05 10600 1.41E-04 10200
0.000527974 12500 1.01E-05 12000 1.31E-05
12900 6.41E-05 10800 5.19E-05 10600 1.42E-04 10200
0.0005351 12500 1.01E-05 12000 1.32E-05
12900 6.44E-05 10800 5.21E-05 10600 1.42E-04 10200
0.000542357 12500 1.01E-05 12000 1.32E-05
12900 6.47E-05 10800 5.23E-05 10600 1.43E-04 10200
0.000549781 12500 1.01E-05 12000 1.32E-05
12900 6.50E-05 10800 5.25E-05 10600 1.43E-04 10200
0.000557362 12500 1.01E-05 12000 1.32E-05
12900 6.54E-05 10900 5.27E-05 10600 1.44E-04 10200
0.0005651 12500 1.01E-05 12000 1.32E-05

12900 6.57E-05 10900 5.30E-05 10600 1.44E-04 10200
0.000573 12500 1.01E-05 12000 1.32E-05
12900 6.60E-05 10900 5.32E-05 10700 1.45E-04 10200
0.00058108 12500 1.01E-05 12000 1.32E-05
12900 6.64E-05 10900 5.34E-05 10700 1.45E-04 10200
0.00058933 12500 1.01E-05 12000 1.32E-05
12900 6.67E-05 10900 5.36E-05 10700 1.46E-04 10200
0.000597754 12500 1.01E-05 12000 1.32E-05
12900 6.70E-05 10900 5.38E-05 10700 1.46E-04 10200
0.000606349 12500 1.01E-05 12000 1.32E-05
12900 6.74E-05 10900 5.41E-05 10700 1.47E-04 10300
0.00061513 12500 1.01E-05 12000 1.32E-05
12900 6.77E-05 10900 5.43E-05 10700 1.47E-04 10300
0.000624091 12500 1.01E-05 12000 1.32E-05
12900 6.81E-05 10900 5.45E-05 10700 1.48E-04 10300
0.000633246 12500 1.01E-05 12000 1.32E-05
12900 6.84E-05 10900 5.48E-05 10700 1.49E-04 10300
0.000642589 12500 1.01E-05 12000 1.33E-05
12900 6.88E-05 10900 5.50E-05 10700 1.49E-04 10300
0.000652157 12500 1.01E-05 12000 1.33E-05
12900 6.92E-05 10900 5.52E-05 10700 1.50E-04 10300
0.000661918 12500 1.01E-05 12000 1.33E-05
12900 6.96E-05 10900 5.55E-05 10700 1.50E-04 10300
0.000671921 12500 1.01E-05 12000 1.33E-05
12900 6.99E-05 10900 5.57E-05 10700 1.51E-04 10300
0.000682161 12500 1.01E-05 12000 1.33E-05
12900 7.03E-05 10900 5.59E-05 10700 1.51E-04 10300
0.000692627 12500 1.01E-05 12000 1.33E-05
12900 7.07E-05 10900 5.62E-05 10700 1.52E-04 10300
0.000703339 12500 1.01E-05 12000 1.33E-05
12900 7.11E-05 10900 5.64E-05 10700 1.52E-04 10300
0.00071431 12500 1.01E-05 12000 1.33E-05
12900 7.15E-05 10900 5.67E-05 10700 1.53E-04 10300
0.000725542 12600 1.01E-05 12000 1.33E-05
12900 7.20E-05 10900 5.69E-05 10700 1.54E-04 10300
0.000737059 12600 1.01E-05 12000 1.33E-05
12900 7.24E-05 10900 5.72E-05 10700 1.54E-04 10300
0.000748859 12600 1.01E-05 12000 1.33E-05
12900 7.28E-05 10900 5.74E-05 10700 1.55E-04 10300
0.000760982 12600 1.01E-05 12000 1.34E-05
12900 7.33E-05 10900 5.77E-05 10700 1.55E-04 10300
0.000773416 12600 1.01E-05 12000 1.34E-05
12900 7.37E-05 10900 5.79E-05 10700 1.56E-04 10300
0.000786196 12600 1.02E-05 12000 1.34E-05
12900 7.42E-05 10900 5.82E-05 10700 1.56E-04 10300
0.000799312 12600 1.02E-05 12000 1.34E-05
12900 7.46E-05 10900 5.84E-05 10700 1.57E-04 10300
0.000812778 12600 1.01E-05 12000 1.34E-05
12900 7.51E-05 10900 5.87E-05 10700 1.58E-04 10300
0.000826597 12600 1.02E-05 12000 1.34E-05
12900 7.55E-05 10900 5.89E-05 10700 1.58E-04 10300
0.000840792 12600 1.02E-05 12000 1.34E-05
12900 7.60E-05 10900 5.92E-05 10700 1.59E-04 10300
0.000855326 12600 1.02E-05 12000 1.34E-05
12900 7.65E-05 10900 5.95E-05 10700 1.59E-04 10300
0.000870237 12600 1.01E-05 12000 1.34E-05
12900 7.70E-05 10900 5.97E-05 10700 1.60E-04 10300
0.000885538 12600 1.01E-05 12000 1.34E-05
12900 7.75E-05 10900 6.00E-05 10700 1.61E-04 10300
0.000901222 12600 1.02E-05 12000 1.34E-05
12900 7.80E-05 10900 6.03E-05 10700 1.61E-04 10300
0.00091734 12600 1.02E-05 12000 1.35E-05
12900 7.85E-05 10900 6.05E-05 10700 1.62E-04 10300
0.000933889 12600 1.02E-05 12000 1.35E-05
12900 7.91E-05 10900 6.08E-05 10700 1.63E-04 10300
0.000950886 12600 1.02E-05 12000 1.35E-05
12900 7.96E-05 10900 6.11E-05 10700 1.63E-04 10300
0.000968344 12600 1.02E-05 12000 1.35E-05

12900 8.02E-05 10900 6.14E-05 10700 1.64E-04 10300
0.000986295 12600 1.02E-05 12000 1.35E-05
12900 8.07E-05 10900 6.16E-05 10700 1.64E-04 10300
0.001004745 12600 1.02E-05 12000 1.35E-05
12900 8.13E-05 10900 6.19E-05 10700 1.65E-04 10300
0.001023746 12600 1.02E-05 12000 1.35E-05
12900 8.19E-05 10900 6.22E-05 10700 1.66E-04 10300
0.001043269 12600 1.02E-05 12000 1.35E-05
12900 8.25E-05 10900 6.25E-05 10700 1.66E-04 10300
0.001063375 12600 1.02E-05 12000 1.36E-05
12900 8.31E-05 10900 6.28E-05 10700 1.67E-04 10300
0.001084062 12600 1.02E-05 12000 1.36E-05
12900 8.37E-05 10900 6.30E-05 10700 1.68E-04 10300
0.001105397 12600 1.02E-05 12000 1.36E-05
12900 8.43E-05 10900 6.33E-05 10700 1.68E-04 10300
0.00112737 12600 1.02E-05 12000 1.36E-05
12900 8.49E-05 10900 6.36E-05 10700 1.69E-04 10300
0.001150037 12600 1.02E-05 12000 1.36E-05
12900 8.56E-05 10900 6.39E-05 10700 1.70E-04 10300
0.001173411 12600 1.02E-05 12000 1.36E-05
12900 8.62E-05 10900 6.42E-05 10700 1.70E-04 10300
0.001197567 12600 1.02E-05 12000 1.36E-05
13000 8.69E-05 10900 6.45E-05 10700 1.71E-04 10300
0.00122252 12600 1.02E-05 12000 1.36E-05
13000 8.75E-05 10900 6.48E-05 10700 1.72E-04 10300
0.001248301 12600 1.02E-05 12000 1.37E-05
13000 8.82E-05 10900 6.51E-05 10700 1.72E-04 10300
0.001274976 12600 1.02E-05 12000 1.37E-05
13000 8.89E-05 10900 6.54E-05 10700 1.73E-04 10300
0.001302568 12600 1.02E-05 12000 1.37E-05
13000 8.96E-05 10900 6.57E-05 10700 1.74E-04 10300
0.001331138 12600 1.02E-05 12000 1.37E-05
13000 9.04E-05 10900 6.60E-05 10700 1.74E-04 10300
0.001360733 12600 1.02E-05 12000 1.37E-05
13000 9.11E-05 10900 6.63E-05 10700 1.75E-04 10300
0.001391343 12600 1.02E-05 12000 1.37E-05
13000 9.19E-05 10900 6.66E-05 10700 1.76E-04 10300
0.001423026 12600 1.02E-05 12000 1.38E-05
13000 9.26E-05 10900 6.69E-05 10700 1.77E-04 10300
0.001455822 12600 1.02E-05 12000 1.38E-05
13000 9.34E-05 10900 6.73E-05 10700 1.77E-04 10300
0.001489781 12600 1.02E-05 12100 1.38E-05
13000 9.42E-05 10900 6.76E-05 10700 1.78E-04 10300
0.001524986 12600 1.02E-05 12100 1.38E-05
13000 9.51E-05 10900 6.79E-05 10700 1.79E-04 10300
0.001561456 12600 1.02E-05 12100 1.38E-05
13000 9.59E-05 10900 6.83E-05 10700 1.79E-04 10300
0.001599311 12600 1.02E-05 12100 1.38E-05
13000 9.68E-05 10900 6.86E-05 10700 1.80E-04 10300
0.00163858 12600 1.02E-05 12100 1.39E-05
13000 9.76E-05 10900 6.89E-05 10700 1.81E-04 10300
0.001679346 12600 1.02E-05 12100 1.39E-05
13000 9.85E-05 10900 6.93E-05 10700 1.82E-04 10300
0.001721638 12600 1.02E-05 12100 1.39E-05
13000 9.94E-05 10900 6.96E-05 10700 1.82E-04 10300
0.001765545 12600 1.02E-05 12100 1.39E-05
13000 0.0001 10900 6.99E-05 10700 1.83E-04 10300
0.001811135 12600 1.02E-05 12100 1.39E-05
13000 0.000101 11000 7.03E-05 10700 1.84E-04 10300
0.00185847 12600 1.02E-05 12100 1.40E-05
13000 0.000102 11000 7.06E-05 10700 1.85E-04 10300
0.001907662 12600 1.02E-05 12100 1.40E-05
13000 0.000103 11000 7.10E-05 10800 1.86E-04 10300
0.001958785 12600 1.02E-05 12100 1.40E-05
13000 0.000104 11000 7.13E-05 10800 1.86E-04 10300
0.002011944 12600 1.02E-05 12100 1.40E-05
13000 0.000105 11000 7.16E-05 10800 1.87E-04 10300
0.002067253 12600 1.03E-05 12100 1.40E-05

13000 0.000106 11000 7.20E-05 10800 1.88E-04 10300
0.002124871 12600 1.03E-05 12100 1.40E-05
13000 0.000107 11000 7.23E-05 10800 1.89E-04 10400
0.002184914 12600 1.03E-05 12100 1.41E-05
13000 0.000108 11000 7.27E-05 10800 1.89E-04 10400
0.002247508 12600 1.03E-05 12100 1.41E-05
13000 0.000109 11000 7.31E-05 10800 1.90E-04 10400
0.002312802 12600 1.03E-05 12100 1.41E-05
13000 0.000111 11000 7.34E-05 10800 1.91E-04 10400
0.002380936 12600 1.03E-05 12100 1.41E-05
13000 0.000112 11000 7.38E-05 10800 1.92E-04 10400
0.002452024 12600 1.03E-05 12100 1.42E-05
13000 0.000113 11000 7.42E-05 10800 1.93E-04 10400
0.002526215 12600 1.03E-05 12100 1.42E-05
13000 0.000114 11000 7.45E-05 10800 1.94E-04 10400
0.002603623 12600 1.03E-05 12100 1.42E-05
13000 0.000115 11000 7.49E-05 10800 1.94E-04 10400
0.002684411 12600 1.03E-05 12100 1.42E-05
13000 0.000117 11000 7.53E-05 10800 1.95E-04 10400
0.002768768 12600 1.03E-05 12100 1.43E-05
13000 0.000118 11000 7.57E-05 10800 1.96E-04 10400
0.002856898 12600 1.03E-05 12100 1.43E-05
13000 0.000119 11000 7.61E-05 10800 1.97E-04 10400
0.002948997 12600 1.03E-05 12100 1.43E-05
13000 0.00012 11000 7.65E-05 10800 1.98E-04 10400
0.003045362 12700 1.03E-05 12100 1.44E-05
13000 0.000122 11000 7.69E-05 10800 1.99E-04 10400
0.003146253 12700 1.03E-05 12100 1.44E-05
13000 0.000123 11000 7.73E-05 10800 1.99E-04 10400
0.003251982 12700 1.03E-05 12100 1.44E-05
13000 0.000125 11000 7.77E-05 10800 2.00E-04 10400
0.003362882 12700 1.03E-05 12100 1.45E-05
13000 0.000126 11000 7.81E-05 10800 2.01E-04 10400
0.003479296 12700 1.03E-05 12100 1.45E-05
13000 0.000128 11000 7.85E-05 10800 2.02E-04 10400
0.003601631 12700 1.03E-05 12100 1.46E-05
13000 0.000129 11000 7.89E-05 10800 2.03E-04 10400
0.003730342 12700 1.03E-05 12100 1.46E-05
13000 0.000131 11000 7.94E-05 10800 2.04E-04 10400
0.0038659 12700 1.03E-05 12100 1.46E-05
13000 0.000132 11000 7.98E-05 10800 2.05E-04 10400
0.004008818 12700 1.03E-05 12100 1.47E-05
13000 0.000134 11000 8.02E-05 10800 2.06E-04 10400
0.004159558 12700 1.03E-05 12100 1.47E-05
13000 0.000136 11000 8.06E-05 10800 2.07E-04 10400
0.004318718 12700 1.03E-05 12100 1.47E-05
13000 0.000137 11000 8.10E-05 10800 2.07E-04 10400
0.004486877 12700 1.03E-05 12100 1.48E-05
13000 0.000139 11000 8.15E-05 10800 2.08E-04 10400
0.00466468 12700 1.03E-05 12100 1.48E-05
13000 0.000141 11000 8.19E-05 10800 2.09E-04 10400
0.004852871 12700 1.03E-05 12100 1.49E-05
13000 0.000143 11000 8.24E-05 10800 2.10E-04 10400
0.005052264 12700 1.03E-05 12100 1.49E-05
13000 0.000145 11000 8.28E-05 10800 2.11E-04 10400
0.005263803 12700 1.03E-05 12100 1.49E-05
13000 0.000147 11000 8.32E-05 10800 2.12E-04 10400
0.005488528 12700 1.03E-05 12100 1.50E-05
13000 0.000149 11000 8.37E-05 10800 2.13E-04 10400
0.00572762 12700 1.04E-05 12100 1.50E-05
13000 0.000151 11000 8.42E-05 10800 2.14E-04 10400
0.0059823 12700 1.04E-05 12100 1.51E-05
13000 0.000153 11000 8.46E-05 10800 2.15E-04 10400
0.006253972 12700 1.04E-05 12100 1.51E-05
13000 0.000155 11000 8.51E-05 10800 2.16E-04 10400
0.006544186 12700 1.04E-05 12100 1.52E-05
13000 0.000157 11000 8.55E-05 10800 2.17E-04 10400
0.00685479 12700 1.04E-05 12100 1.52E-05

13000 0.000159 11000 8.60E-05 10800 2.18E-04 10400
0.007187814 12700 1.04E-05 12100 1.53E-05
13000 0.000162 11000 8.65E-05 10800 2.19E-04 10400
0.007545576 12700 1.04E-05 12100 1.53E-05
13000 0.000164 11000 8.70E-05 10800 2.20E-04 10400
0.007930688 12700 1.04E-05 12100 1.54E-05
13000 0.000167 11000 8.75E-05 10800 2.21E-04 10400
0.00834601 12700 1.04E-05 12100 1.54E-05
13000 0.000169 11000 8.80E-05 10800 2.22E-04 10400
0.008794754 12700 1.04E-05 12100 1.55E-05
13000 0.000172 11000 8.85E-05 10800 2.23E-04 10400
0.009280412 12700 1.04E-05 12100 1.55E-05
13000 0.000174 11000 8.90E-05 10800 2.24E-04 10400
0.009806912 12700 1.04E-05 12100 1.56E-05
13100 0.000177 11000 8.95E-05 10800 2.25E-04 10400
0.01037881 12700 1.04E-05 12100 1.57E-05
13100 0.00018 11000 9.01E-05 10800 2.26E-04 10400
0.01100133 12700 1.04E-05 12100 1.57E-05
13100 0.000183 11000 9.06E-05 10800 2.27E-04 10400
0.01168059 12700 1.04E-05 12100 1.58E-05
13100 0.000186 11000 9.11E-05 10800 2.28E-04 10400
0.01242354 12700 1.04E-05 12100 1.59E-05
13100 0.000189 11000 9.17E-05 10800 2.29E-04 10400
0.01323827 12700 1.04E-05 12100 1.59E-05
13100 0.000192 11000 9.22E-05 10800 2.31E-04 10400
0.01413394 12700 1.04E-05 12100 1.60E-05
13100 0.000195 11000 9.27E-05 10800 2.32E-04 10400
0.01512101 12700 1.04E-05 12100 1.61E-05
13100 0.000198 11000 9.33E-05 10800 2.33E-04 10400
0.01621149 12700 1.04E-05 12100 1.62E-05
13100 0.000202 11000 9.38E-05 10800 2.34E-04 10400
0.01741923 12700 1.04E-05 12100 1.62E-05
13100 0.000205 11000 9.43E-05 10800 2.35E-04 10400
0.01876025 12700 1.04E-05 12200 1.63E-05
13100 0.000209 11000 9.49E-05 10800 2.36E-04 10400
0.0202532 12700 1.04E-05 12200 1.64E-05
13100 0.000213 11000 9.55E-05 10800 2.37E-04 10400
0.02191969 12700 1.04E-05 12200 1.64E-05
13100 0.000216 11000 9.60E-05 10800 2.38E-04 10400
0.02378481 12700 1.04E-05 12200 1.65E-05
13100 0.00022 11000 9.66E-05 10800 2.40E-04 10400
0.0258775 12700 1.04E-05 12200 1.66E-05
13100 0.000224 11000 9.72E-05 10800 2.41E-04 10400
0.02823144 12700 1.04E-05 12200 1.67E-05
13100 0.000229 11000 9.78E-05 10800 2.42E-04 10400
0.03088568 12700 1.04E-05 12200 1.68E-05
13100 0.000233 11000 9.83E-05 10800 2.43E-04 10400
0.03388572 12700 1.04E-05 12200 1.69E-05
13100 0.000237 11000 9.89E-05 10800 2.44E-04 10400
0.03728418 12700 1.04E-05 12200 1.70E-05
13100 0.000242 11100 9.95E-05 10800 2.45E-04 10400
0.04114157 12700 1.04E-05 12200 1.71E-05
13100 0.000247 11100 0.000100127 10800 2.47E-04 10400
0.04552646 12700 1.04E-05 12200 1.72E-05
13100 0.000252 11100 0.000100739 10900 2.48E-04 10400
0.05051494 12700 1.04E-05 12200 1.73E-05
13100 0.000257 11100 0.000101351 10900 2.49E-04 10400
0.0561889 12700 1.04E-05 12200 1.74E-05
13100 0.000262 11100 0.000101979 10900 2.50E-04 10400
0.06263316 12700 1.04E-05 12200 1.75E-05
13100 0.000267 11100 0.000102602 10900 2.52E-04 10400
0.06993031 12700 1.04E-05 12200 1.76E-05
13100 0.000273 11100 0.000103237 10900 2.53E-04 10500
0.07815271 12700 1.05E-05 12200 1.77E-05
13100 0.000278 11100 0.000103881 10900 2.54E-04 10500
0.08735064 12700 1.05E-05 12200 1.78E-05
13100 0.000284 11100 0.000104542 10900 2.55E-04 10500
0.09753555 12700 1.05E-05 12200 1.79E-05

13100 0.00029 11100 0.000105208 10900 2.57E-04 10500
0.1086581 12700 1.05E-05 12200 1.80E-05
13100 0.000297 11100 0.000105887 10900 2.58E-04 10500
0.1205826 12700 1.05E-05 12200 1.81E-05
13100 0.000303 11100 0.000106567 10900 0.000259301 10500
0.1330611 12700 1.05E-05 12200 1.83E-05
13100 0.00031 11100 0.000107272 10900 0.000260633 10500
0.1457142 12700 1.05E-05 12200 1.84E-05
13100 0.000317 11100 0.000107975 10900 0.000261957 10500
0.1580286 12700 1.05E-05 12200 1.85E-05
13100 0.000324 11100 0.000108688 10900 0.000263302 10500
0.1693793 12700 1.05E-05 12200 1.86E-05
13100 0.000331 11100 0.000109414 10900 0.000264665 10500
0.1790835 12700 1.05E-05 12200 1.88E-05
13100 0.000339 11100 0.00011014 10900 0.000266003 10500
0.1864817 12700 1.05E-05 12200 1.89E-05
13100 0.000347 11100 0.000110879 10900 0.000267385 10500
0.1910308 12800 1.05E-05 12200 1.90E-05
13100 0.000355 11100 0.000111634 10900 0.000268771 10500
0.1923897 12800 1.05E-05 12200 1.92E-05
13100 0.000364 11100 0.000112383 10900 0.000270165 10500
0.1904723 12800 1.05E-05 12200 1.93E-05
13100 0.000373 11100 0.000113157 10900 0.000271579 10500
0.1854545 12800 1.05E-05 12200 1.94E-05
13100 0.000382 11100 0.000113925 10900 0.000273002 10500
0.1777364 12800 1.05E-05 12200 1.96E-05
13100 0.000391 11100 0.00011471 10900 0.000274432 10500
0.1678733 12800 1.05E-05 12200 1.97E-05
13100 0.000401 11100 0.000115502 10900 0.000275875 10500
0.1564947 12800 1.05E-05 12200 1.99E-05
13100 0.000411 11100 0.000116299 10900 0.000277338 10500
0.1442283 12800 1.05E-05 12200 2.00E-05
13100 0.000421 11100 0.000117102 10900 0.000278809 10500
0.131642 12800 1.05E-05 12200 2.01E-05
13100 0.000432 11100 0.000117912 10900 0.000280291 10500
0.1192071 12800 1.05E-05 12200 2.03E-05
13100 0.000443 11100 0.000118738 10900 0.000281777 10500
0.1072826 12800 1.06E-05 12200 2.04E-05
13100 0.000455 11100 0.000119566 10900 0.000283299 10500
0.09611622 12800 1.06E-05 12200 2.06E-05
13100 0.000467 11100 0.000120414 10900 0.000284816 10500
0.08585579 12800 1.06E-05 12200 2.07E-05
13100 0.000479 11100 0.000121271 10900 0.000286353 10500
0.07656708 12800 1.06E-05 12200 2.09E-05
13100 0.000492 11100 0.000122144 10900 0.000287902 10500
0.06825369 12800 1.06E-05 12200 2.10E-05
13100 0.000505 11100 0.000123025 10900 0.000289463 10500
0.06087611 12800 1.06E-05 12200 2.12E-05
13100 0.000519 11100 0.000123924 10900 0.000291044 10500
0.05436827 12800 1.06E-05 12200 2.13E-05
13100 0.000533 11100 0.00012483 10900 0.000292633 10500
0.04865022 12800 1.06E-05 12200 2.15E-05
13100 0.000548 11100 0.000125746 10900 0.000294215 10500
0.04363746 12800 1.06E-05 12200 2.16E-05
13100 0.000563 11100 0.00012667 10900 0.000295844 10500
0.03924687 12800 1.06E-05 12200 2.18E-05
13100 0.000578 11100 0.000127611 10900 0.000297471 10500
0.03540064 12800 1.06E-05 12200 2.19E-05
13100 0.000594 11100 0.000128565 10900 0.000299133 10500
0.03202826 12800 1.06E-05 12200 2.21E-05
13100 0.000611 11100 0.000129533 10900 0.000300788 10500
0.02906704 12800 1.06E-05 12200 2.23E-05
13100 0.000629 11100 0.000130512 10900 0.000302466 10500
0.02646202 12800 1.06E-05 12200 2.24E-05
13100 0.000646 11100 0.000131515 10900 0.000304159 10500
0.0241652 12800 1.06E-05 12200 2.26E-05
13100 0.000665 11100 0.00013253 10900 0.000305867 10500
0.022135 12800 1.06E-05 12200 2.28E-05

13100 0.000684 11100 0.000133563 10900 0.00030761 10500
0.02033549 12800 1.06E-05 12200 2.29E-05
13100 0.000703 11100 0.000134605 10900 0.000309351 10500
0.01873583 12800 1.06E-05 12200 2.31E-05
13100 0.000723 11100 0.000135669 10900 0.000311117 10500
0.01730959 12800 1.06E-05 12200 2.33E-05
13200 0.000744 11100 0.00013676 10900 0.000312878 10500
0.01603419 12800 1.06E-05 12200 2.34E-05
13200 0.000766 11100 0.000137864 10900 0.000314677 10500
0.01489034 12800 1.06E-05 12200 2.36E-05
13200 0.000787 11100 0.000138991 10900 0.00031648 10500
0.01386153 12800 1.06E-05 12200 2.38E-05
13200 0.00081 11100 0.000140128 10900 0.000318314 10500
0.01293354 12800 1.06E-05 12200 2.39E-05
13200 0.000833 11100 0.000141296 10900 0.000320157 10500
0.01209413 12800 1.06E-05 12200 2.41E-05
13200 0.000857 11100 0.000142472 10900 0.000322014 10500
0.01133271 12800 1.07E-05 12200 2.43E-05
13200 0.000881 11100 0.000143678 10900 0.000323894 10500
0.01064019 12800 1.07E-05 12200 2.45E-05
13200 0.000906 11100 0.000144899 10900 0.000325794 10500
0.01000869 12800 1.07E-05 12200 2.47E-05
13200 0.000931 11100 0.000146146 10900 0.000327699 10500
0.009431477 12800 1.07E-05 12200 2.49E-05
13200 0.000957 11100 0.000147413 10900 0.000329636 10500
0.008902603 12800 1.07E-05 12300 2.51E-05
13200 0.000983 11100 0.000148712 10900 0.000331582 10500
0.008416937 12800 1.07E-05 12300 2.53E-05
13200 0.00101 11100 0.000150026 10900 0.000333563 10500
0.007969988 12800 1.07E-05 12300 2.55E-05
13200 0.00104 11100 0.00015137 10900 0.000335541 10500
0.007557787 12800 1.07E-05 12300 2.57E-05
13200 0.00107 11100 0.000152739 10900 0.000337561 10500
0.007176865 12800 1.07E-05 12300 2.59E-05
13200 0.00109 11100 0.000154128 10900 0.000339578 10500
0.006824142 12800 1.07E-05 12300 2.61E-05
13200 0.00112 11100 0.00015554 10900 0.000341633 10500
0.006496914 12800 1.07E-05 12300 2.63E-05
13200 0.00115 11100 0.000156983 10900 0.000343715 10500
0.006192747 12800 1.07E-05 12300 2.66E-05
13200 0.00118 11100 0.000158449 10900 0.000345801 10500
0.005909583 12800 1.07E-05 12300 2.68E-05
13200 0.00121 11200 0.000159939 10900 0.000347923 10500
0.005645528 12800 1.07E-05 12300 2.70E-05
13200 0.00124 11200 0.000161463 10900 0.00035005 10500
0.005398894 12800 1.07E-05 12300 2.73E-05
13200 0.00126 11200 0.000163025 11000 0.00035221 10500
0.005168207 12800 1.07E-05 12300 2.75E-05
13200 0.00129 11200 0.000164617 11000 0.000354382 10500
0.004952107 12800 1.07E-05 12300 2.77E-05
13200 0.00132 11200 0.000166242 11000 0.000356589 10500
0.004749381 12800 1.07E-05 12300 2.80E-05
13200 0.00135 11200 0.000167906 11000 0.000358819 10500
0.004558985 12800 1.07E-05 12300 2.82E-05
13200 0.00138 11200 0.000169608 11000 0.000361065 10600
0.004379889 12800 1.07E-05 12300 2.85E-05
13200 0.0014 11200 0.000171364 11000 0.00036333 10600
0.004211256 12800 1.07E-05 12300 2.87E-05
13200 0.00143 11200 0.000173142 11000 0.000365628 10600
0.004052281 12800 1.07E-05 12300 2.90E-05
13200 0.00146 11200 0.000174965 11000 0.000367942 10600
0.003902241 12800 1.07E-05 12300 2.92E-05
13200 0.00148 11200 0.000176828 11000 0.000370292 10600
0.003760496 12800 1.07E-05 12300 2.95E-05
13200 0.00151 11200 0.000178744 11000 0.000372658 10600
0.003626432 12800 1.07E-05 12300 2.97E-05
13200 0.00153 11200 0.000180706 11000 0.000375074 10600
0.003499498 12800 1.07E-05 12300 3.00E-05

13200	0.00155	11200	0.000182723	11000	0.000377488	10600
0.00337919		12800	1.07E-05	12300	3.02E-05	
13200	0.00157	11200	0.000184788	11000	0.000379942	10600
0.003265081		12800	1.07E-05	12300	3.05E-05	
13200	0.00159	11200	0.000186908	11000	0.000382418	10600
0.00315675		12800	1.07E-05	12300	3.07E-05	
13200	0.00161	11200	0.000189074	11000	0.000384927	10600
0.00305378		12800	1.07E-05	12300	3.09E-05	
13200	0.00162	11200	0.00019131	11000	0.000387476	10600
0.002955841		12900	1.07E-05	12300	3.12E-05	
13200	0.00164	11200	0.000193589	11000	0.000390038	10600
0.002862607		12900	1.07E-05	12300	3.14E-05	
13200	0.00165	11200	0.000195931	11000	0.000392632	10600
0.002773745		12900	1.07E-05	12300	3.16E-05	
13200	0.00166	11200	0.000198342	11000	0.000395241	10600
0.002689013		12900	1.07E-05	12300	3.18E-05	
13200	0.00167	11200	0.000200818	11000	0.000397904	10600
0.002608143		12900	1.07E-05	12300	3.20E-05	
13200	0.00168	11200	0.000203373	11000	0.000400566	10600
0.002530911		12900	1.08E-05	12300	3.22E-05	
13200	0.00169	11200	0.000206003	11000	0.000403293	10600
0.002457083		12900	1.08E-05	12300	3.23E-05	
13200	0.00169	11200	0.000208726	11000	0.000406043	10600
0.002386494		12900	1.08E-05	12300	3.25E-05	
13200	0.0017	11200	0.000211542	11000	0.000408808	10600
0.002318936		12900	1.08E-05	12300	3.26E-05	
13200	0.0017	11200	0.000214471	11000	0.000411609	10600
0.002254258		12900	1.08E-05	12300	3.28E-05	
13200	0.00169	11200	0.000217498	11000	0.00041445	10600
0.002192296		12900	1.08E-05	12300	3.29E-05	
13200	0.00169	11200	0.000220637	11000	0.000417317	10600
0.002132909		12900	1.08E-05	12300	3.30E-05	
13200	0.00169	11200	0.00022389	11000	0.000420234	10600
0.002075927		12900	1.08E-05	12300	3.31E-05	
13200	0.00168	11200	0.000227276	11000	0.000423177	10600
0.002021225		12900	1.08E-05	12300	3.32E-05	
13200	0.00167	11200	0.00023078	11000	0.000426169	10600
0.001968666		12900	1.08E-05	12300	3.32E-05	
13200	0.00166	11200	0.000234424	11000	0.000429183	10600
0.001918161		12900	1.08E-05	12300	3.32E-05	
13200	0.00165	11200	0.000238192	11000	0.000432233	10600
0.00186961		12900	1.08E-05	12300	3.32E-05	
13200	0.00163	11200	0.000242111	11000	0.000435313	10600
0.001822866		12900	1.08E-05	12300	3.32E-05	
13200	0.00161	11200	0.000246183	11000	0.000438447	10600
0.00177788		12900	1.08E-05	12300	3.32E-05	
13200	0.0016	11200	0.000250432	11000	0.000441621	10600
0.001734543		12900	1.08E-05	12300	3.32E-05	
13200	0.00158	11200	0.000254849	11000	0.000444826	10600
0.001692808		12900	1.08E-05	12300	3.31E-05	
13200	0.00155	11200	0.000259465	11000	0.00044808	10600
0.00165256		12900	1.09E-05	12300	3.30E-05	
13200	0.00153	11200	0.00026428	11000	0.000451359	10600
0.001613757		12900	1.09E-05	12300	3.29E-05	
13200	0.00151	11200	0.000269322	11000	0.000454704	10600
0.001576319		12900	1.09E-05	12300	3.28E-05	
13200	0.00148	11200	0.000274606	11000	0.000458078	10600
0.001540184		12900	1.09E-05	12300	3.26E-05	
13200	0.00146	11200	0.000280155	11000	0.000461503	10600
0.001505265		12900	1.09E-05	12300	3.25E-05	
13200	0.00143	11200	0.00028598	11000	0.000464968	10600
0.001471539		12900	1.09E-05	12300	3.23E-05	
13200	0.0014	11200	0.000292106	11000	0.000468478	10600
0.001438942		12900	1.09E-05	12300	3.21E-05	
13200	0.00137	11200	0.000298556	11000	0.000472039	10600
0.001407432		12900	1.09E-05	12300	3.19E-05	
13300	0.00134	11200	0.000305349	11000	0.000475637	10600
0.001376932		12900	1.09E-05	12300	3.16E-05	

13300 0.00131 11200 0.000312504 11000 0.000479288 10600
0.00134744 12900 1.09E-05 12300 3.13E-05
13300 0.00128 11200 0.000320076 11000 0.000483002 10600
0.001318881 12900 1.09E-05 12300 3.11E-05
13300 0.00125 11200 0.000328074 11000 0.00048675 10600
0.001291215 12900 1.09E-05 12300 3.08E-05
13300 0.00122 11200 0.000336557 11000 0.00049058 10600
0.0012644 12900 1.09E-05 12300 3.05E-05
13300 0.00118 11200 0.000345561 11000 0.000494446 10600
0.001238429 12900 1.09E-05 12300 3.03E-05
13300 0.00115 11200 0.000355127 11000 0.000498361 10600
0.001213246 12900 1.09E-05 12300 2.99E-05
13300 0.00112 11200 0.000365303 11000 0.000502327 10600
0.001188822 12900 1.10E-05 12300 2.96E-05
13300 0.00109 11200 0.000376138 11000 0.000506361 10600
0.001165122 12900 1.10E-05 12300 2.93E-05
13300 0.00106 11200 0.00038767 11000 0.000510462 10600
0.001142123 12900 1.10E-05 12400 2.90E-05
13300 0.00103 11200 0.000399967 11000 0.000514613 10600
0.001119814 12900 1.10E-05 12400 2.87E-05
13300 0.000994 11200 0.000413098 11000 0.000518831 10600
0.001098142 12900 1.10E-05 12400 2.83E-05
13300 0.000964 11200 0.000427131 11000 0.000523097 10600
0.001077119 12900 1.10E-05 12400 2.80E-05
13300 0.000935 11200 0.000442138 11000 0.000527454 10600
0.001056714 12900 1.10E-05 12400 2.76E-05
13300 0.000906 11200 0.000458202 11000 0.000531857 10600
0.001036889 12900 1.10E-05 12400 2.73E-05
13300 0.000877 11200 0.000475395 11000 0.000536346 10600
0.001017609 12900 1.10E-05 12400 2.69E-05
13300 0.00085 11200 0.000493807 11000 0.000540888 10600
0.000998881 12900 1.11E-05 12400 2.66E-05
13300 0.000823 11200 0.000513505 11000 0.000545508 10600
0.000980671 12900 1.11E-05 12400 2.62E-05
13300 0.000797 11300 0.00053457 11000 0.000550192 10600
0.000962963 12900 1.11E-05 12400 2.59E-05
13300 0.000771 11300 0.000557068 11000 0.000554955 10600
0.000945743 12900 1.11E-05 12400 2.56E-05
13300 0.000747 11300 0.000581057 11100 0.000559787 10600
0.000929002 12900 1.11E-05 12400 2.52E-05
13300 0.000723 11300 0.000606581 11100 0.000564729 10600
0.000912696 12900 1.11E-05 12400 2.49E-05
13300 0.0007 11300 0.000633637 11100 0.00056972 10600
0.000896825 12900 1.11E-05 12400 2.46E-05
13300 0.000678 11300 0.000662211 11100 0.000574816 10600
0.000881386 12900 1.12E-05 12400 2.43E-05
13300 0.000656 11300 0.00069224 11100 0.00057999 10700
0.000866342 12900 1.12E-05 12400 2.40E-05
13300 0.000636 11300 0.000723574 11100 0.00058525 10700
0.00085169 12900 1.12E-05 12400 2.37E-05
13300 0.000616 11300 0.000756037 11100 0.00059059 10700
0.000837415 12900 1.12E-05 12400 2.34E-05
13300 0.000596 11300 0.00078936 11100 0.000596033 10700
0.000823508 12900 1.12E-05 12400 2.31E-05
13300 0.000578 11300 0.000823258 11100 0.00060158 10700
0.000809936 12900 1.12E-05 12400 2.28E-05
13300 0.00056 11300 0.000857403 11100 0.000607212 10700
0.000796725 12900 1.13E-05 12400 2.25E-05
13300 0.000543 11300 0.000891461 11100 0.000612959 10700
0.000783821 12900 1.13E-05 12400 2.22E-05
13300 0.000527 11300 0.000925139 11100 0.000618785 10700
0.000771239 12900 1.13E-05 12400 2.19E-05
13300 0.000511 11300 0.000958161 11100 0.000624743 10700
0.000758987 12900 1.13E-05 12400 2.17E-05
13300 0.000496 11300 0.000990306 11100 0.000630795 10700
0.000747017 12900 1.13E-05 12400 2.14E-05
13300 0.000482 11300 0.001021412 11100 0.000636979 10700
0.000735331 12900 1.14E-05 12400 2.12E-05

13300 0.000468 11300 0.001051422 11100 0.000643261 10700
0.000723932 13000 1.14E-05 12400 2.09E-05
13300 0.000454 11300 0.001080292 11100 0.000649666 10700
0.000712799 13000 1.14E-05 12400 2.07E-05
13300 0.000441 11300 0.001108057 11100 0.00065619 10700
0.000701921 13000 1.14E-05 12400 2.05E-05
13300 0.000429 11300 0.001134739 11100 0.000662853 10700
0.000691304 13000 1.14E-05 12400 2.03E-05
13300 0.000417 11300 0.001160312 11100 0.000669636 10700
0.000680938 13000 1.15E-05 12400 2.00E-05
13300 0.000406 11300 0.001184669 11100 0.000676573 10700
0.000670799 13000 1.15E-05 12400 1.98E-05
13300 0.000395 11300 0.001207566 11100 0.000683634 10700
0.000660906 13000 1.15E-05 12400 1.96E-05
13300 0.000384 11300 0.001228672 11100 0.000690857 10700
0.000651228 13000 1.16E-05 12400 1.95E-05
13300 0.000374 11300 0.001247542 11100 0.000698219 10700
0.000641777 13000 1.16E-05 12400 1.93E-05
13300 0.000365 11300 0.001263606 11100 0.000705742 10700
0.000632523 13000 1.16E-05 12400 1.91E-05
13300 0.000355 11300 0.001276245 11100 0.000713414 10700
0.000623483 13000 1.17E-05 12400 1.89E-05
13300 0.000346 11300 0.00128477 11100 0.00072126 10700
0.000614633 13000 1.17E-05 12400 1.87E-05
13300 0.000338 11300 0.001288535 11100 0.000729297 10700
0.00060596 13000 1.17E-05 12400 1.86E-05
13300 0.00033 11300 0.00128697 11100 0.000737495 10700
0.00059747 13000 1.18E-05 12400 1.84E-05
13300 0.000322 11300 0.001279729 11100 0.000745879 10700
0.000589164 13000 1.18E-05 12400 1.83E-05
13300 0.000314 11300 0.00126674 11100 0.000754465 10700
0.000581049 13000 1.18E-05 12400 1.81E-05
13300 0.000306 11300 0.001248217 11100 0.000763254 10700
0.000573091 13000 1.19E-05 12400 1.80E-05
13300 0.000299 11300 0.001224644 11100 0.000772236 10700
0.000565292 13000 1.19E-05 12400 1.79E-05
13300 0.000292 11300 0.00119674 11100 0.000781452 10700
0.000557654 13000 1.20E-05 12400 1.77E-05
13300 0.000286 11300 0.001165362 11100 0.000790877 10700
0.000550161 13000 1.20E-05 12400 1.76E-05
13300 0.000279 11300 0.001131398 11100 0.000800535 10700
0.000542837 13000 1.21E-05 12400 1.75E-05
13300 0.000273 11300 0.001095721 11100 0.000810427 10700
0.000535641 13000 1.21E-05 12400 1.74E-05
13300 0.000267 11300 0.00105911 11100 0.00082058 10700
0.000528611 13000 1.22E-05 12400 1.73E-05
13300 0.000262 11300 0.001022228 11100 0.000830987 10700
0.000521709 13000 1.23E-05 12400 1.72E-05
13300 0.000256 11300 0.000985657 11100 0.00084167 10700
0.000514941 13000 1.23E-05 12400 1.70E-05
13300 0.000251 11300 0.00094984 11100 0.000852643 10700
0.00050832 13000 1.24E-05 12400 1.69E-05
13300 0.000246 11300 0.000915174 11100 0.000863912 10700
0.000501818 13000 1.24E-05 12400 1.69E-05
13300 0.000241 11300 0.000881956 11100 0.000875481 10700
0.000495457 13000 1.25E-05 12400 1.68E-05
13300 0.000236 11300 0.00085041 11100 0.000887381 10700
0.000489205 13000 1.26E-05 12400 1.67E-05
13400 0.000231 11300 0.000820675 11100 0.000899605 10700
0.000483082 13000 1.27E-05 12400 1.66E-05
13400 0.000227 11300 0.000792831 11100 0.000912184 10700
0.00047708 13000 1.27E-05 12400 1.65E-05
13400 0.000222 11300 0.000766881 11100 0.000925146 10700
0.000471188 13000 1.28E-05 12400 1.64E-05
13400 0.000218 11300 0.000742796 11100 0.000938472 10700
0.000465417 13000 1.29E-05 12400 1.64E-05
13400 0.000214 11300 0.000720516 11100 0.000952202 10700
0.00045975 13000 1.30E-05 12400 1.63E-05

13400 0.00021 11300 0.000699999 11100 0.000966346 10700
0.000454194 13000 1.31E-05 12400 1.62E-05
13400 0.000206 11300 0.000681164 11100 0.000980937 10700
0.000448754 13000 1.32E-05 12400 1.61E-05
13400 0.000203 11300 0.000663918 11100 0.000995981 10700
0.000443413 13000 1.33E-05 12400 1.61E-05
13400 0.000199 11300 0.000648166 11100 0.001011519 10700
0.00043817 13000 1.34E-05 12400 1.60E-05
13400 0.000195 11300 0.000633843 11100 0.001027546 10700
0.000433018 13000 1.35E-05 12500 1.59E-05
13400 0.000192 11300 0.000620829 11100 0.001044105 10700
0.000427951 13000 1.36E-05 12500 1.59E-05
13400 0.000189 11300 0.000609063 11100 0.001061212 10700
0.000422965 13000 1.37E-05 12500 1.58E-05
13400 0.000186 11300 0.000598424 11100 0.001078902 10700
0.000418079 13000 1.38E-05 12500 1.58E-05
13400 0.000182 11300 0.000588845 11100 0.001097203 10700
0.000413279 13000 1.40E-05 12500 1.57E-05
13400 0.000179 11300 0.000580228 11100 0.001116167 10700
0.000408552 13000 1.41E-05 12500 1.57E-05
13400 0.000177 11300 0.000572508 11100 0.001135797 10700
0.000403931 13000 1.42E-05 12500 1.56E-05
13400 0.000174 11300 0.000565603 11100 0.001156147 10700
0.000399386 13000 1.43E-05 12500 1.56E-05
13400 0.000171 11300 0.000559472 11100 0.001177245 10700
0.000394923 13000 1.44E-05 12500 1.55E-05
13400 0.000168 11400 0.000554039 11100 0.001199137 10700
0.000390534 13000 1.45E-05 12500 1.55E-05
13400 0.000166 11400 0.000549264 11100 0.001221856 10700
0.000386216 13000 1.47E-05 12500 1.54E-05
13400 0.000163 11400 0.000545105 11200 0.001245468 10700
0.000381983 13000 1.47E-05 12500 1.54E-05
13400 0.000161 11400 0.000541534 11200 0.001270022 10700
0.000377808 13000 1.49E-05 12500 1.53E-05
13400 0.000158 11400 0.000538501 11200 0.001295538 10700
0.000373721 13000 1.50E-05 12500 1.53E-05
13400 0.000156 11400 0.000535968 11200 0.001322106 10700
0.000369684 13000 1.51E-05 12500 1.53E-05
13400 0.000154 11400 0.000533929 11200 0.001349745 10800
0.000365734 13000 1.51E-05 12500 1.52E-05
13400 0.000152 11400 0.00053234 11200 0.001378559 10800
0.000361838 13000 1.52E-05 12500 1.52E-05
13400 0.000149 11400 0.000531171 11200 0.001408592 10800
0.000358013 13000 1.53E-05 12500 1.52E-05
13400 0.000147 11400 0.000530378 11200 0.001439926 10800
0.000354259 13000 1.54E-05 12500 1.51E-05
13400 0.000145 11400 0.000529969 11200 0.00147261 10800
0.000350555 13000 1.54E-05 12500 1.51E-05
13400 0.000143 11400 0.000529912 11200 0.001506748 10800
0.0003469 13000 1.55E-05 12500 1.51E-05
13400 0.000141 11400 0.000530179 11200 0.001542404 10800
0.000343294 13000 1.55E-05 12500 1.50E-05
13400 0.00014 11400 0.000530761 11200 0.001579682 10800
0.000339757 13000 1.55E-05 12500 1.50E-05
13400 0.000138 11400 0.000531634 11200 0.001618658 10800
0.000336282 13000 1.55E-05 12500 1.50E-05
13400 0.000136 11400 0.000532793 11200 0.001659458 10800
0.000332873 13000 1.55E-05 12500 1.49E-05
13400 0.000134 11400 0.000534214 11200 0.001702154 10800
0.000329499 13000 1.55E-05 12500 1.49E-05
13400 0.000133 11400 0.000535906 11200 0.001746879 10800
0.000326192 13100 1.54E-05 12500 1.49E-05
13400 0.000131 11400 0.000537858 11200 0.001793722 10800
0.000322934 13100 1.54E-05 12500 1.49E-05
13400 0.000129 11400 0.000540036 11200 0.00184282 10800
0.000319727 13100 1.53E-05 12500 1.48E-05
13400 0.000128 11400 0.000542458 11200 0.001894275 10800
0.000316588 13100 1.53E-05 12500 1.48E-05

13400 0.000126 11400 0.000545114 11200 0.001948234 10800
0.000313478 13100 1.52E-05 12500 1.48E-05
13400 0.000125 11400 0.000547999 11200 0.002004823 10800
0.000310421 13100 1.51E-05 12500 1.48E-05
13400 0.000123 11400 0.000551105 11200 0.002064149 10800
0.000307426 13100 1.50E-05 12500 1.48E-05
13400 0.000122 11400 0.000554424 11200 0.002126366 10800
0.000304461 13100 1.49E-05 12500 1.47E-05
13400 0.00012 11400 0.000557974 11200 0.002191588 10800
0.000301555 13100 1.48E-05 12500 1.47E-05
13400 0.000119 11400 0.000561728 11200 0.00225997 10800
0.000298703 13100 1.47E-05 12500 1.47E-05
13400 0.000118 11400 0.000565696 11200 0.002331632 10800
0.00029587 13100 1.46E-05 12500 1.47E-05
13400 0.000116 11400 0.000569849 11200 0.002406689 10800
0.0002931 13100 1.44E-05 12500 1.47E-05
13400 0.000115 11400 0.000574214 11200 0.002485246 10800
0.00029036 13100 1.43E-05 12500 1.47E-05
13400 0.000114 11400 0.000578771 11200 0.002567417 10800
0.000287658 13100 1.42E-05 12500 1.46E-05
13400 0.000113 11400 0.000583521 11200 0.002653271 10800
0.000285003 13100 1.40E-05 12500 1.46E-05
13400 0.000112 11400 0.000588465 11200 0.002742874 10800
0.000282358 13100 1.39E-05 12500 1.46E-05
13400 0.00011 11400 0.000593591 11200 0.002836263 10800
0.00027977 13100 1.38E-05 12500 1.46E-05
13400 0.000109 11400 0.000598906 11200 0.002933455 10800
0.000277214 13100 1.37E-05 12500 1.46E-05
13400 0.000108 11400 0.000604405 11200 0.003034388 10800
0.000274689 13100 1.36E-05 12500 1.46E-05
13400 0.000107 11400 0.000610088 11200 0.003138978 10800
0.000272197 13100 1.35E-05 12500 1.45E-05
13400 0.000106 11400 0.000615973 11200 0.003247083 10800
0.000269719 13100 1.33E-05 12500 1.45E-05
13400 0.000105 11400 0.000622036 11200 0.003358491 10800
0.000267282 13100 1.32E-05 12500 1.45E-05
13400 0.000104 11400 0.000628294 11200 0.003472938 10800
0.00026488 13100 1.31E-05 12500 1.45E-05
13400 0.000103 11400 0.000634748 11200 0.003590081 10800
0.000262516 13100 1.30E-05 12500 1.45E-05
13400 0.000102 11400 0.000641413 11200 0.003709463 10800
0.000260184 13100 1.29E-05 12500 1.45E-05
13400 1.01E-04 11400 0.000648266 11200 0.003830545 10800
0.000257888 13100 1.29E-05 12500 1.45E-05
13400 1.00E-04 11400 0.000655327 11200 0.003952693 10800
0.000255611 13100 1.28E-05 12500 1.45E-05
13400 9.90E-05 11400 0.000662603 11200 0.00407519 10800
0.000253381 13100 1.27E-05 12500 1.45E-05
13400 9.81E-05 11400 0.000670082 11200 0.004197216 10800
0.000251172 13100 1.26E-05 12500 1.44E-05
13500 9.72E-05 11400 0.000677768 11200 0.004317824 10800
0.000249003 13100 1.25E-05 12500 1.44E-05
13500 9.64E-05 11400 0.000685655 11200 0.004436024 10800
0.000246874 13100 1.25E-05 12500 1.44E-05
13500 9.55E-05 11400 0.00069376 11200 0.004550714 10800
0.000244764 13100 1.24E-05 12500 1.44E-05
13500 9.47E-05 11400 0.000702085 11200 0.004660768 10800
0.000242685 13100 1.23E-05 12500 1.44E-05
13500 9.38E-05 11400 0.000710631 11200 0.004764994 10800
0.000240609 13100 1.23E-05 12500 1.44E-05
13500 9.30E-05 11400 0.000719411 11200 0.004862213 10800
0.000238573 13100 1.22E-05 12500 1.44E-05
13500 9.22E-05 11400 0.000728414 11200 0.004951256 10800
0.000236568 13100 1.22E-05 12500 1.44E-05
13500 9.15E-05 11400 0.000737663 11200 0.005031029 10800
0.000234594 13100 1.21E-05 12500 1.44E-05
13500 9.07E-05 11400 0.000747155 11200 0.005100487 10800
0.000232646 13100 1.21E-05 12500 1.43E-05

13500 9.00E-05 11400 0.000756906 11200 0.005158731 10800
0.000230723 13100 1.20E-05 12600 1.43E-05
13500 8.93E-05 11400 0.000766924 11200 0.005205008 10800
0.000228827 13100 1.20E-05 12600 1.43E-05
13500 8.86E-05 11400 0.000777194 11200 0.005238719 10800
0.000226943 13100 1.19E-05 12600 1.43E-05
13500 8.78E-05 11400 0.00078773 11200 0.005259522 10800
0.000225108 13100 1.19E-05 12600 1.43E-05
13500 8.72E-05 11400 0.00079853 11200 0.005267248 10800
0.000223282 13100 1.19E-05 12600 1.43E-05
13500 8.65E-05 11400 0.000809607 11200 0.005261946 10800
0.000221489 13100 1.18E-05 12600 1.43E-05
13500 8.58E-05 11400 0.000820949 11200 0.005243912 10800
0.000219726 13100 1.18E-05 12600 1.43E-05
13500 8.52E-05 11400 0.000832568 11200 0.005213635 10800
0.00021796 13100 1.18E-05 12600 1.43E-05
13500 8.46E-05 11400 0.000844498 11200 0.005171813 10800
0.000216243 13100 1.17E-05 12600 1.43E-05
13500 8.39E-05 11500 0.000856729 11200 0.005119321 10800
0.000214533 13100 1.17E-05 12600 1.43E-05
13500 8.33E-05 11500 0.000869261 11200 0.005057136 10800
0.000212849 13100 1.17E-05 12600 1.43E-05
13500 8.27E-05 11500 0.000882103 11300 0.004986367 10800
0.000211194 13100 1.17E-05 12600 1.42E-05
13500 8.21E-05 11500 0.000895288 11300 0.004908181 10800
0.000209553 13100 1.16E-05 12600 1.42E-05
13500 8.15E-05 11500 0.000908814 11300 0.004823809 10800
0.000207933 13100 1.16E-05 12600 1.42E-05
13500 8.09E-05 11500 0.000922695 11300 0.004734456 10800
0.000206319 13100 1.16E-05 12600 1.42E-05
13500 8.04E-05 11500 0.000936956 11300 0.004641313 10900
0.000204731 13100 1.16E-05 12600 1.42E-05
13500 7.98E-05 11500 0.000951586 11300 0.004545545 10900
0.000203163 13100 1.16E-05 12600 1.42E-05
13500 7.93E-05 11500 0.000966619 11300 0.004448223 10900
0.000201621 13100 1.16E-05 12600 1.42E-05
13500 7.87E-05 11500 0.000982049 11300 0.004350339 10900
0.000200088 13100 1.15E-05 12600 1.42E-05
13500 7.82E-05 11500 0.000997901 11300 0.004252796 10900
0.000198579 13100 1.15E-05 12600 1.42E-05
13500 7.77E-05 11500 0.001014187 11300 0.004156373 10900
0.000197091 13100 1.15E-05 12600 1.42E-05
13500 7.72E-05 11500 0.001030913 11300 0.004061758 10900
0.000195619 13100 1.15E-05 12600 1.42E-05
13500 7.67E-05 11500 0.001048092 11300 0.003969553 10900
0.000194166 13100 1.15E-05 12600 1.42E-05
13500 7.62E-05 11500 0.001065745 11300 0.003880253 10900
0.000192733 13100 1.15E-05 12600 1.42E-05
13500 7.57E-05 11500 0.001083889 11300 0.003794244 10900
0.000191301 13100 1.15E-05 12600 1.41E-05
13500 7.52E-05 11500 0.001102512 11300 0.003711835 10900
0.000189903 13100 1.15E-05 12600 1.41E-05
13500 7.48E-05 11500 0.00112167 11300 0.00363326 10900
0.000188519 13200 1.14E-05 12600 1.41E-05
13500 7.43E-05 11500 0.001141374 11300 0.0035587 10900
0.000187154 13200 1.14E-05 12600 1.41E-05
13500 7.39E-05 11500 0.001161642 11300 0.003488256 10900
0.0001858 13200 1.14E-05 12600 1.41E-05
13500 7.34E-05 11500 0.001182481 11300 0.00342199 10900
0.000184462 13200 1.14E-05 12600 1.41E-05
13500 7.30E-05 11500 0.001203909 11300 0.003359891 10900
0.00018314 13200 1.14E-05 12600 1.41E-05
13500 7.25E-05 11500 0.001225949 11300 0.00330196 10900
0.000181827 13200 1.14E-05 12600 1.41E-05
13500 7.21E-05 11500 0.001248611 11300 0.003248134 10900
0.000180535 13200 1.14E-05 12600 1.41E-05
13500 7.17E-05 11500 0.001271929 11300 0.003198339 10900
0.000179245 13200 1.14E-05 12600 1.40E-05

13500 7.12E-05 11500 0.001295935 11300 0.003152467 10900
0.000177978 13200 1.14E-05 12600 1.40E-05
13500 7.08E-05 11500 0.001320638 11300 0.003110429 10900
0.000176721 13200 1.14E-05 12600 1.40E-05
13500 7.05E-05 11500 0.001346097 11300 0.003072111 10900
0.000175491 13200 1.14E-05 12600 1.40E-05
13500 7.00E-05 11500 0.001372323 11300 0.003037393 10900
0.000174278 13200 1.14E-05 12600 1.40E-05
13500 6.97E-05 11500 0.001399358 11300 0.00300613 10900
0.000173075 13200 1.13E-05 12600 1.40E-05
13500 6.93E-05 11500 0.001427245 11300 0.002978204 10900
0.000171889 13200 1.14E-05 12600 1.40E-05
13500 6.89E-05 11500 0.001456003 11300 0.002953479 10900
0.000170714 13200 1.13E-05 12600 1.40E-05
13500 6.86E-05 11500 0.001485671 11300 0.002931825 10900
0.000169552 13200 1.13E-05 12600 1.40E-05
13500 6.82E-05 11500 0.001516287 11300 0.002913159 10900
0.000168396 13200 1.13E-05 12600 1.40E-05
13500 6.79E-05 11500 0.001547906 11300 0.00289734 10900
0.00016728 13200 1.13E-05 12600 1.40E-05
13500 6.75E-05 11500 0.001580533 11300 0.002884248 10900
0.00016614 13200 1.13E-05 12600 1.40E-05
13500 6.72E-05 11500 0.001614236 11300 0.002873772 10900
0.000165047 13200 1.13E-05 12600 1.40E-05
13500 6.69E-05 11500 0.001649052 11300 0.002865826 10900
0.000163949 13200 1.13E-05 12600 1.40E-05
13500 6.65E-05 11500 0.001685028 11300 0.00286031 10900
0.00016286 13200 1.13E-05 12600 1.39E-05
13500 6.62E-05 11500 0.001722204 11300 0.002857142 10900
0.000161786 13200 1.13E-05 12600 1.39E-05
13500 6.58E-05 11500 0.001760642 11300 0.002856235 10900
0.000160737 13200 1.13E-05 12600 1.39E-05
13500 6.55E-05 11500 0.0018004 11300 0.002857499 10900
0.000159674 13200 1.13E-05 12600 1.39E-05
13500 6.52E-05 11500 0.001841535 11300 0.002860865 10900
0.000158629 13200 1.13E-05 12600 1.39E-05
13500 6.49E-05 11500 0.00188412 11300 0.002866275 10900
0.000157593 13200 1.13E-05 12600 1.39E-05
13500 6.45E-05 11500 0.001928211 11300 0.002873666 10900
0.000156573 13200 1.13E-05 12600 1.39E-05
13500 6.42E-05 11500 0.001973878 11300 0.002882987 10900
0.000155554 13200 1.13E-05 12600 1.39E-05
13600 6.40E-05 11500 0.0020212 11300 0.002894196 10900
0.00015457 13200 1.13E-05 12600 1.39E-05
13600 6.37E-05 11500 0.002070261 11300 0.002907242 10900
0.000153577 13200 1.13E-05 12600 1.39E-05
13600 6.34E-05 11500 0.00212115 11300 0.002922069 10900
0.000152604 13200 1.13E-05 12600 1.39E-05
13600 6.31E-05 11500 0.002173962 11300 0.002938668 10900
0.000151653 13200 1.13E-05 12600 1.39E-05
13600 6.28E-05 11500 0.002228781 11300 0.002956979 10900
0.000150682 13200 1.13E-05 12600 1.39E-05
13600 6.25E-05 11500 0.0022857 11300 0.002976997 10900
0.000149746 13200 1.13E-05 12600 1.39E-05
13600 6.22E-05 11500 0.002344844 11300 0.002998717 10900
0.000148801 13200 1.13E-05 12600 1.39E-05
13600 6.20E-05 11500 0.002406328 11300 0.003022086 10900
0.000147888 13200 1.13E-05 12600 1.39E-05
13600 6.17E-05 11500 0.00247026 11300 0.003047105 10900
0.000146969 13200 1.13E-05 12600 1.39E-05
13600 6.14E-05 11500 0.002536786 11300 0.003073749 10900
0.000146064 13200 1.13E-05 12700 1.39E-05
13600 6.12E-05 11500 0.00260603 11300 0.003102034 10900
0.000145167 13200 1.13E-05 12700 1.39E-05
13600 6.09E-05 11500 0.002678154 11300 0.003131946 10900
0.000144291 13200 1.13E-05 12700 1.39E-05
13600 6.07E-05 11500 0.002753293 11300 0.003163487 10900
0.000143408 13200 1.13E-05 12700 1.39E-05

13600 6.04E-05 11500 0.002831612 11300 0.003196656 10900
0.000142563 13200 1.13E-05 12700 1.39E-05
13600 6.01E-05 11500 0.002913302 11300 0.003231462 10900
0.000141698 13200 1.13E-05 12700 1.39E-05
13600 5.99E-05 11500 0.002998562 11300 0.00326792 10900
0.000140853 13200 1.13E-05 12700 1.38E-05
13600 5.97E-05 11500 0.003087601 11300 0.003306032 10900
0.000140025 13200 1.13E-05 12700 1.38E-05
13600 5.94E-05 11500 0.003180652 11300 0.003345825 10900
0.000139185 13200 1.13E-05 12700 1.38E-05
13600 5.92E-05 11600 0.003277952 11300 0.003387311 10900
0.000138356 13200 1.13E-05 12700 1.38E-05
13600 5.90E-05 11600 0.003379785 11300 0.003430522 10900
0.00013754 13200 1.13E-05 12700 1.38E-05
13600 5.88E-05 11600 0.003486402 11400 0.003475491 10900
0.000136733 13200 1.13E-05 12700 1.38E-05
13600 5.85E-05 11600 0.003598123 11400 0.003522223 10900
0.000135923 13200 1.13E-05 12700 1.38E-05
13600 5.83E-05 11600 0.003715242 11400 0.003570764 10900
0.000135139 13200 1.13E-05 12700 1.38E-05
13600 5.81E-05 11600 0.00383812 11400 0.003621138 10900
0.000134339 13200 1.13E-05 12700 1.38E-05
13600 5.79E-05 11600 0.003967104 11400 0.003673381 11000
0.000133566 13200 1.13E-05 12700 1.38E-05
13600 5.77E-05 11600 0.004102641 11400 0.003727573 11000
0.000132772 13200 1.13E-05 12700 1.38E-05
13600 5.75E-05 11600 0.004245142 11400 0.003783728 11000
0.000132038 13200 1.13E-05 12700 1.38E-05
13600 5.73E-05 11600 0.00439512 11400 0.003841891 11000
0.000131263 13200 1.13E-05 12700 1.38E-05
13600 5.71E-05 11600 0.004553108 11400 0.003902122 11000
0.000130514 13200 1.13E-05 12700 1.38E-05
13600 5.69E-05 11600 0.00471964 11400 0.003964469 11000
0.000129762 13200 1.13E-05 12700 1.37E-05
13600 5.66E-05 11600 0.004895377 11400 0.004029001 11000
0.000129039 13200 1.13E-05 12700 1.37E-05
13600 5.65E-05 11600 0.005080967 11400 0.004095786 11000
0.000128311 13200 1.13E-05 12700 1.37E-05
13600 5.63E-05 11600 0.005277154 11400 0.004164869 11000
0.000127614 13200 1.13E-05 12700 1.37E-05
13600 5.61E-05 11600 0.005484737 11400 0.004236346 11000
0.000126896 13200 1.13E-05 12700 1.37E-05
13600 5.59E-05 11600 0.005704582 11400 0.004310269 11000
0.000126195 13200 1.13E-05 12700 1.37E-05
13600 5.57E-05 11600 0.005937646 11400 0.004386735 11000
0.000125497 13300 1.13E-05 12700 1.37E-05
13600 5.55E-05 11600 0.006184989 11400 0.004465824 11000
0.000124804 13300 1.13E-05 12700 1.37E-05
13600 5.53E-05 11600 0.006447776 11400 0.004547634 11000
0.000124131 13300 1.13E-05 12700 1.37E-05
13600 5.51E-05 11600 0.006727318 11400 0.004632249 11000
0.000123435 13300 1.13E-05 12700 1.37E-05
13600 5.49E-05 11600 0.007025031 11400 0.004719779 11000
0.000122767 13300 1.13E-05 12700 1.37E-05
13600 5.48E-05 11600 0.007342499 11400 0.004810329 11000
0.000122106 13300 1.13E-05 12700 1.37E-05
13600 5.46E-05 11600 0.007681457 11400 0.00490401 11000
0.000121468 13300 1.13E-05 12700 1.36E-05
13600 5.44E-05 11600 0.00804382 11400 0.005000929 11000
0.000120802 13300 1.13E-05 12700 1.37E-05
13600 5.42E-05 11600 0.008431707 11400 0.005101211 11000
0.00012015 13300 1.13E-05 12700 1.36E-05
13600 5.41E-05 11600 0.00884751 11400 0.005205023 11000
0.000119511 13300 1.13E-05 12700 1.36E-05
13600 5.39E-05 11600 0.009293836 11400 0.00531248 11000
0.00011887 13300 1.13E-05 12700 1.37E-05
13600 5.38E-05 11600 0.009773673 11400 0.005423727 11000
0.000118232 13300 1.13E-05 12700 1.37E-05

13600	5.36E-05	11600	0.01029034	11400	0.005538931	11000	0.000117603	13300	1.14E-05	12700	1.36E-05
13600	5.34E-05	11600	0.0108475	11400	0.005658234	11000	0.000116989	13300	1.14E-05	12700	1.37E-05
13600	5.33E-05	11600	0.01144936	11400	0.00578185	11000	0.000116376	13300	1.13E-05	12700	1.36E-05
13600	5.31E-05	11600	0.01210055	11400	0.005909933	11000	0.000115764	13300	1.13E-05	12700	1.36E-05
13600	5.30E-05	11600	0.01280636	11400	0.006042683	11000	0.000115172	13300	1.14E-05	12700	1.37E-05
13600	5.28E-05	11600	0.01357273	11400	0.006180322	11000	0.000114576	13300	1.13E-05	12700	1.36E-05
13600	5.26E-05	11600	0.01440637	11400	0.006323053	11000	0.000113987	13300	1.14E-05	12700	1.36E-05
13600	5.25E-05	11600	0.01531494	11400	0.006471122	11000	0.000113407	13300	1.13E-05	12700	1.36E-05
13600	5.23E-05	11600	0.01630709	11400	0.006624765	11000	0.000112831	13300	1.14E-05	12700	1.36E-05
13600	5.22E-05	11600	0.01739261	11400	0.006784232	11000	0.000112257	13300	1.14E-05	12700	1.36E-05
13600	5.21E-05	11600	0.01858258	11400	0.006949839	11000	0.000111691	13300	1.14E-05	12700	1.36E-05
13600	5.19E-05	11600	0.01988953	11400	0.007121854	11000	0.000111132	13300	1.14E-05	12700	1.36E-05
13600	5.18E-05	11600	0.02132767	11400	0.007300574	11000	0.000110572	13300	1.14E-05	12700	1.36E-05
13600	5.16E-05	11600	0.02291306	11400	0.007486358	11000	0.000110025	13300	1.13E-05	12700	1.36E-05
13600	5.15E-05	11600	0.02466387	11400	0.007679524	11000	0.000109478	13300	1.13E-05	12700	1.36E-05
13600	5.13E-05	11600	0.02660053	11400	0.00788045	11000	0.000108939	13300	1.14E-05	12700	1.36E-05
13600	5.12E-05	11600	0.02874593	11400	0.008089582	11000	0.000108389	13300	1.14E-05	12700	1.36E-05
13700	5.11E-05	11600	0.03112537	11400	0.008307286	11000	0.000107858	13300	1.14E-05	12700	1.36E-05
13700	5.10E-05	11600	0.03376658	11400	0.008534036	11000	0.000107325	13300	1.14E-05	12700	1.36E-05
13700	5.08E-05	11600	0.03669911	11400	0.008770295	11000	0.000106808	13300	1.14E-05	12700	1.36E-05
13700	5.07E-05	11600	0.03995346	11400	0.009016575	11000	0.000106298	13300	1.14E-05	12700	1.36E-05
13700	5.06E-05	11600	0.0435597	11400	0.009273429	11000	0.000105782	13300	1.13E-05	12700	1.37E-05
13700	5.04E-05	11600	0.04754508	11400	0.009541415	11000	0.000105281	13300	1.14E-05	12700	1.37E-05
13700	5.03E-05	11600	0.05193074	11400	0.00982117	11000	0.000104797	13300	1.14E-05	12700	1.37E-05
13700	5.02E-05	11600	0.05672675	11400	0.01011333	11000	0.000104296	13300	1.14E-05	12700	1.37E-05
13700	5.01E-05	11600	0.06192536	11400	0.01041862	11000	0.000103803	13300	1.14E-05	12700	1.37E-05
13700	4.99E-05	11600	0.06749181	11400	0.01073776	11000	0.000103313	13300	1.14E-05	12800	1.37E-05
13700	4.98E-05	11600	0.07335357	11400	0.01107159	11000	0.000102815	13300	1.14E-05	12800	1.37E-05
13700	4.97E-05	11600	0.07938925	11400	0.01142094	11000	0.000102328	13300	1.14E-05	12800	1.37E-05
13700	4.96E-05	11600	0.08542048	11400	0.01178674	11000	0.000101859	13300	1.14E-05	12800	1.38E-05
13700	4.95E-05	11600	0.09121093	11400	0.01216998	11000	0.000101381	13300	1.14E-05	12800	1.38E-05
13700	4.94E-05	11600	0.09647737	11400	0.01257169	11000	0.000100919	13300	1.14E-05	12800	1.38E-05
13700	4.93E-05	11600	0.1009143	11400	0.01299301	11000	0.000100447	13300	1.14E-05	12800	1.38E-05
13700	4.92E-05	11600	0.1042309	11400	0.01343513	11000	0.0001	13300	1.14E-05	12800	1.38E-05

13700 4.90E-05 11600 0.1061933 11400 0.01389936 11000
9.95E-05 13300 1.14E-05 12800 1.38E-05
13700 4.89E-05 11700 0.1066619 11400 0.01438711 11000
9.91E-05 13300 1.14E-05 12800 1.38E-05
13700 4.88E-05 11700 0.1056146 11400 0.01489983 11000
9.86E-05 13300 1.14E-05 12800 1.38E-05
13700 4.87E-05 11700 0.1031473 11500 0.01543916 11000
9.82E-05 13300 1.14E-05 12800 1.39E-05
13700 4.86E-05 11700 0.09945457 11500 0.01600677 11000
9.77E-05 13300 1.14E-05 12800 1.39E-05
13700 4.85E-05 11700 0.09479455 11500 0.01660452 11000
9.73E-05 13300 1.14E-05 12800 1.39E-05
13700 4.84E-05 11700 0.08945203 11500 0.01723441 11000
9.69E-05 13300 1.14E-05 12800 1.39E-05
13700 4.83E-05 11700 0.08370444 11500 0.0178985 11100
9.64E-05 13300 1.14E-05 12800 1.39E-05
13700 4.82E-05 11700 0.07779777 11500 0.01859911 11100
9.60E-05 13300 1.14E-05 12800 1.39E-05
13700 4.81E-05 11700 0.07193237 11500 0.01933866 11100
9.56E-05 13300 1.14E-05 12800 1.39E-05
13700 4.80E-05 11700 0.06625839 11500 0.0201198 11100
9.52E-05 13300 1.14E-05 12800 1.39E-05
13700 4.79E-05 11700 0.06087822 11500 0.02094528 11100
9.47E-05 13300 1.14E-05 12800 1.39E-05
13700 4.78E-05 11700 0.05585321 11500 0.02181811 11100
9.43E-05 13300 1.14E-05 12800 1.39E-05
13700 4.77E-05 11700 0.05121257 11500 0.02274151 11100
9.39E-05 13300 1.14E-05 12800 1.39E-05
13700 4.76E-05 11700 0.04696213 11500 0.02371888 11100
9.35E-05 13300 1.14E-05 12800 1.39E-05
13700 4.75E-05 11700 0.04309206 11500 0.02475388 11100
9.31E-05 13300 1.14E-05 12800 1.39E-05
13700 4.74E-05 11700 0.03958271 11500 0.02585034 11100
9.27E-05 13300 1.14E-05 12800 1.39E-05
13700 4.73E-05 11700 0.03640889 11500 0.02701239 11100
9.23E-05 13300 1.14E-05 12800 1.39E-05
13700 4.72E-05 11700 0.03354277 11500 0.02824433 11100
9.19E-05 13400 1.14E-05 12800 1.40E-05
13700 4.71E-05 11700 0.03095595 11500 0.02955072 11100
9.15E-05 13400 1.14E-05 12800 1.40E-05
13700 4.70E-05 11700 0.02862092 11500 0.03093638 11100
9.11E-05 13400 1.14E-05 12800 1.40E-05
13700 4.69E-05 11700 0.02651178 11500 0.03240624 11100
9.08E-05 13400 1.14E-05 12800 1.40E-05
13700 4.69E-05 11700 0.02460474 11500 0.03396543 11100
9.04E-05 13400 1.14E-05 12800 1.40E-05
13700 4.67E-05 11700 0.02287817 11500 0.03561919 11100
9.00E-05 13400 1.14E-05 12800 1.40E-05
13700 4.67E-05 11700 0.02131257 11500 0.03737285 11100
8.96E-05 13400 1.14E-05 12800 1.40E-05
13700 4.66E-05 11700 0.01989063 11500 0.03923168 11100
8.93E-05 13400 1.14E-05 12800 1.40E-05
13700 4.65E-05 11700 0.01859688 11500 0.04120085 11100
8.89E-05 13400 1.14E-05 12800 1.40E-05
13700 4.64E-05 11700 0.01741766 11500 0.0432853 11100
8.86E-05 13400 1.14E-05 12800 1.40E-05
13700 4.63E-05 11700 0.01634087 11500 0.0454895 11100
8.82E-05 13400 1.14E-05 12800 1.40E-05
13700 4.62E-05 11700 0.01535579 11500 0.04781737 11100
8.78E-05 13400 1.14E-05 12800 1.40E-05
13700 4.61E-05 11700 0.01445293 11500 0.05027189 11100
8.75E-05 13400 1.14E-05 12800 1.40E-05
13700 4.61E-05 11700 0.01362389 11500 0.05285495 11100
8.71E-05 13400 1.14E-05 12800 1.40E-05
13700 4.60E-05 11700 0.01286121 11500 0.05556691 11100
8.68E-05 13400 1.14E-05 12800 1.40E-05
13700 4.59E-05 11700 0.01215835 11500 0.05840621 11100
8.64E-05 13400 1.14E-05 12800 1.40E-05

13700 4.58E-05 11700 0.01150945 11500 0.06136903 11100
8.61E-05 13400 1.14E-05 12800 1.40E-05
13700 4.57E-05 11700 0.01090939 11500 0.06444865 11100
8.58E-05 13400 1.14E-05 12800 1.40E-05
13700 4.57E-05 11700 0.01035358 11500 0.06763504 11100
8.54E-05 13400 1.14E-05 12800 1.40E-05
13700 4.56E-05 11700 0.009837939 11500 0.07091429 11100
8.51E-05 13400 1.14E-05 12800 1.40E-05
13700 4.55E-05 11700 0.009358841 11500 0.07426822 11100
8.48E-05 13400 1.14E-05 12800 1.40E-05
13700 4.55E-05 11700 0.008913019 11500 0.07767384 11100
8.44E-05 13400 1.14E-05 12800 1.40E-05
13700 4.54E-05 11700 0.00849756 11500 0.08110287 11100
8.41E-05 13400 1.15E-05 12800 1.41E-05
13700 4.54E-05 11700 0.008109853 11500 0.08452189 11100
8.38E-05 13400 1.14E-05 12800 1.41E-05
13700 4.53E-05 11700 0.00774749 11500 0.08789212 11100
8.35E-05 13400 1.14E-05 12800 1.41E-05
13700 4.52E-05 11700 0.007408348 11500 0.09116995 11100
8.31E-05 13400 1.14E-05 12800 1.41E-05
13700 4.52E-05 11700 0.007090544 11500 0.09430753 11100
8.28E-05 13400 1.14E-05 12800 1.41E-05
13700 4.51E-05 11700 0.00679238 11500 0.09725383 11100
8.25E-05 13400 1.14E-05 12800 1.41E-05
13700 4.50E-05 11700 0.006512312 11500 0.09995609 11100
8.22E-05 13400 1.14E-05 12800 1.41E-05
13800 4.49E-05 11700 0.006248947 11500 0.1023616 11100
8.19E-05 13400 1.14E-05 12800 1.41E-05
13800 4.48E-05 11700 0.006001019 11500 0.10442 11100 8.16E-05
13400 1.14E-05 12800 1.41E-05
13800 4.48E-05 11700 0.00576737 11500 0.106085 11100
8.13E-05 13400 1.14E-05 12800 1.41E-05
13800 4.47E-05 11700 0.005546933 11500 0.1073171 11100
8.10E-05 13400 1.14E-05 12800 1.41E-05
13800 4.46E-05 11700 0.005338735 11500 0.1080856 11100
8.07E-05 13400 1.14E-05 12800 1.40E-05
13800 4.46E-05 11700 0.00514191 11500 0.1083701 11100
8.04E-05 13400 1.14E-05 12800 1.41E-05
13800 4.45E-05 11700 0.00495565 11500 0.108162 11100
8.01E-05 13400 1.14E-05 12800 1.41E-05
13800 4.44E-05 11700 0.004779218 11500 0.1074646 11100
7.99E-05 13400 1.14E-05 12800 1.41E-05
13800 4.43E-05 11700 0.004611947 11500 0.1062937 11100
7.96E-05 13400 1.14E-05 12800 1.41E-05
13800 4.43E-05 11700 0.004453214 11500 0.1046759 11100
7.93E-05 13400 1.14E-05 12900 1.41E-05
13800 4.42E-05 11700 0.004302439 11500 0.1026478 11100
7.90E-05 13400 1.14E-05 12900 1.41E-05
13800 4.41E-05 11700 0.004159115 11500 0.100254 11100
7.88E-05 13400 1.14E-05 12900 1.41E-05
13800 4.41E-05 11700 0.004022753 11500 0.09754423 11100
7.85E-05 13400 1.14E-05 12900 1.41E-05
13800 4.40E-05 11700 0.003892924 11500 0.09457197 11100
7.82E-05 13400 1.14E-05 12900 1.41E-05
13800 4.39E-05 11700 0.003769219 11500 0.09139161 11100
7.79E-05 13400 1.14E-05 12900 1.42E-05
13800 4.39E-05 11700 0.003651267 11500 0.08805661 11100
7.77E-05 13400 1.14E-05 12900 1.42E-05
13800 4.38E-05 11700 0.003538738 11500 0.0846177 11100
7.74E-05 13400 1.14E-05 12900 1.42E-05
13800 4.38E-05 11700 0.003431289 11500 0.08112164 11100
7.71E-05 13400 1.14E-05 12900 1.42E-05
13800 4.37E-05 11800 0.003328641 11500 0.07761028 11100
7.69E-05 13400 1.14E-05 12900 1.41E-05
13800 4.36E-05 11800 0.003230506 11500 0.07412002 11100
7.66E-05 13400 1.14E-05 12900 1.42E-05
13800 4.36E-05 11800 0.003136622 11600 0.07068171 11100
7.64E-05 13400 1.14E-05 12900 1.42E-05

```
13800   4.35E-05        11800   0.00304677      11600   0.06732055      11100
7.61E-05        13400   1.14E-05        12900   1.42E-05
13800   4.35E-05        11800   0.00296071      11600   0.06405661      11100
7.59E-05        13400   1.14E-05        12900   1.42E-05
13800   4.34E-05        11800   0.002878234     11600   0.06090514      11100
7.56E-05        13400   1.14E-05        12900   1.42E-05
13800   4.34E-05        11800   0.002799154     11600   0.05787704      11200
7.54E-05        13400   1.14E-05        12900   1.42E-05
13800   4.33E-05        11800   0.002723284     11600   0.05497965      11200
7.51E-05        13400   1.14E-05        12900   1.42E-05
13800   4.33E-05        11800   0.002650451     11600   0.05221705      11200
7.49E-05        13400   1.14E-05        12900   1.42E-05
13800   4.32E-05        11800   0.002580508     11600   0.04959082      11200
7.47E-05        13400   1.14E-05        12900   1.42E-05
13800   4.32E-05        11800   0.002513303     11600   0.04710035      11200
7.44E-05        13400   1.14E-05        12900   1.42E-05
13800   4.31E-05        11800   0.002448691     11600   0.0447435       11200
7.42E-05        13400   1.14E-05        12900   1.42E-05
13800   4.31E-05        11800   0.002386552     11600   0.04251678      11200
7.40E-05        13400   1.14E-05        12900   1.42E-05
13800   4.30E-05        11800   0.002326746     11600   0.0404157       11200
7.37E-05        13400   1.14E-05        12900   1.42E-05
13800   4.30E-05        11800   0.002269163     11600   0.03843527      11200
7.35E-05        13400   1.14E-05        12900   1.42E-05
13800   4.29E-05        11800   0.002213662     11600   0.03656997      11200
7.33E-05        13400   1.14E-05        12900   1.42E-05
13800   4.29E-05        11800   0.002160161     11600   0.03481413      11200
7.30E-05        13400   1.14E-05        12900   1.42E-05
13800   4.28E-05        11800   0.002108574     11600   0.03316189      11200
7.28E-05        13500   1.14E-05        12900   1.42E-05
13800   4.28E-05        11800   0.002058806     11600   0.03160738      11200
7.26E-05        13500   1.14E-05        12900   1.42E-05
13800   4.27E-05        11800   0.002010775     11600   0.03014494      11200
7.24E-05        13500   1.14E-05        12900   1.42E-05
13800   4.27E-05        11800   0.001964403     11600   0.02876897      11200
7.22E-05        13500   1.14E-05        12900   1.42E-05
13800   4.26E-05        11800   0.001919608     11600   0.02747412      11200
7.20E-05        13500   1.14E-05        12900   1.42E-05
13800   4.25E-05        11800   0.001876324     11600   0.02625526      11200
7.18E-05        13500   1.14E-05        12900   1.42E-05
13800   4.25E-05        11800   0.001834483     11600   0.02510752      11200
7.16E-05        13500   1.15E-05        12900   1.42E-05
13800   4.25E-05        11800   0.001794017     11600   0.02402629      11200
7.14E-05        13500   1.15E-05        12900   1.42E-05
13800   4.24E-05        11800   0.001754883     11600   0.02300723      11200
7.12E-05        13500   1.15E-05        12900   1.42E-05
13800   4.24E-05        11800   0.001717028     11600   0.02204628      11200
7.10E-05        13500   1.15E-05        12900   1.42E-05
13800   4.23E-05        11800   0.001680392     11600   0.0211396       11200
7.08E-05        13500   1.15E-05        12900   1.42E-05
13800   4.22E-05        11800   0.001644937     11600   0.02028364      11200
7.07E-05        13500   1.15E-05        12900   1.42E-05
13800   4.22E-05        11800   0.001610578     11600   0.01947504      11200
7.05E-05        13500   1.15E-05        12900   1.42E-05
13800   4.22E-05        11800   0.001577306     11600   0.01871075      11200
7.03E-05        13500   1.15E-05        12900   1.42E-05
13800   4.21E-05        11800   0.001545054     11600   0.01798783      11200
7.01E-05        13500   1.15E-05        12900   1.42E-05
13800   4.21E-05        11800   0.001513781     11600   0.01730358      11200
6.99E-05        13500   1.15E-05        12900   1.42E-05
13800   4.20E-05        11800   0.001483454     11600   0.01665553      11200
6.98E-05        13500   1.15E-05        12900   1.42E-05
13800   4.20E-05        11800   0.001454027     11600   0.01604132      11200
6.96E-05        13500   1.16E-05        12900   1.43E-05
13800   4.20E-05        11800   0.001425464     11600   0.01545886      11200
6.94E-05        13500   1.16E-05        12900   1.43E-05
13800   4.19E-05        11800   0.001397737     11600   0.01490608      11200
6.93E-05        13500   1.16E-05        12900   1.43E-05
```

13800 4.19E-05 11800 0.001370809 11600 0.01438114 11200
6.91E-05 13500 1.16E-05 12900 1.43E-05
13800 4.18E-05 11800 0.001344655 11600 0.01388231 11200
6.90E-05 13500 1.16E-05 12900 1.43E-05
13800 4.18E-05 11800 0.001319253 11600 0.01340797 11200
6.89E-05 13500 1.16E-05 12900 1.43E-05
13800 4.17E-05 11800 0.001294561 11600 0.01295662 11200
6.87E-05 13500 1.16E-05 12900 1.43E-05
13800 4.17E-05 11800 0.001270567 11600 0.01252689 11200
6.86E-05 13500 1.16E-05 12900 1.43E-05
13800 4.16E-05 11800 0.001247237 11600 0.01211745 11200
6.84E-05 13500 1.16E-05 12900 1.43E-05
13800 4.16E-05 11800 0.001224545 11600 0.01172712 11200
6.83E-05 13500 1.16E-05 12900 1.43E-05
13800 4.16E-05 11800 0.001202465 11600 0.01135476 11200
6.81E-05 13500 1.16E-05 12900 1.43E-05
13800 4.15E-05 11800 0.001180975 11600 0.01099936 11200
6.80E-05 13500 1.16E-05 12900 1.43E-05
13900 4.15E-05 11800 0.001160058 11600 0.01065991 11200
6.79E-05 13500 1.17E-05 12900 1.43E-05
13900 4.14E-05 11800 0.001139695 11600 0.01033552 11200
6.78E-05 13500 1.17E-05 12900 1.43E-05
13900 4.14E-05 11800 0.001119867 11600 0.01002533 11200
6.77E-05 13500 1.17E-05 12900 1.43E-05
13900 4.14E-05 11800 0.001100562 11600 0.009728568 11200
6.76E-05 13500 1.17E-05 12900 1.43E-05
13900 4.14E-05 11800 0.001081768 11600 0.009444492 11200
6.75E-05 13500 1.17E-05 12900 1.43E-05
13900 4.13E-05 11800 0.001063437 11600 0.009172382 11200
6.74E-05 13500 1.17E-05 12900 1.44E-05
13900 4.13E-05 11800 0.001045593 11600 0.008911642 11200
6.73E-05 13500 1.17E-05 12900 1.44E-05
13900 4.13E-05 11800 0.001028195 11600 0.008661615 11200
6.73E-05 13500 1.17E-05 12900 1.43E-05
13900 4.12E-05 11800 0.001011224 11600 0.008421782 11200
6.72E-05 13500 1.17E-05 12900 1.44E-05
13900 4.12E-05 11800 0.000994677 11600 0.008191589 11200
6.71E-05 13500 1.17E-05 13000 1.43E-05
13900 4.12E-05 11800 0.000978542 11600 0.007970524 11200
6.71E-05 13500 1.17E-05 13000 1.43E-05
13900 4.11E-05 11800 0.0009628 11600 0.007758166 11200
6.71E-05 13500 1.18E-05 13000 1.43E-05
13900 4.11E-05 11800 0.000947451 11600 0.007554023 11200
6.70E-05 13500 1.18E-05 13000 1.43E-05
13900 4.10E-05 11800 0.000932474 11600 0.007357717 11200
6.70E-05 13500 1.18E-05 13000 1.43E-05
13900 4.10E-05 11800 0.000917852 11600 0.007168843 11200
6.70E-05 13500 1.18E-05 13000 1.43E-05
13900 4.09E-05 11800 0.000903582 11600 0.006987063 11200
6.70E-05 13500 1.18E-05 13000 1.43E-05
13900 4.09E-05 11800 0.000889639 11600 0.00681199 11200
6.70E-05 13500 1.18E-05 13000 1.43E-05
13900 4.09E-05 11800 0.000876013 11600 0.006643331 11200
6.71E-05 13500 1.18E-05 13000 1.43E-05
13900 4.09E-05 11900 0.000862698 11600 0.006480793 11200
6.72E-05 13500 1.18E-05 13000 1.44E-05
13900 4.08E-05 11900 0.000849666 11600 0.006324062 11200
6.72E-05 13500 1.18E-05 13000 1.44E-05
13900 4.08E-05 11900 0.000836922 11700 0.006172893 11200
6.73E-05 13500 1.18E-05 13000 1.44E-05
13900 4.07E-05 11900 0.000824443 11700 0.006027015 11200
6.75E-05 13500 1.18E-05 13000 1.43E-05
13900 4.07E-05 11900 0.000812243 11700 0.005886216 11200
6.76E-05 13500 1.18E-05 13000 1.43E-05
13900 4.07E-05 11900 0.000800302 11700 0.00575023 11200
6.77E-05 13500 1.18E-05 13000 1.43E-05
13900 4.07E-05 11900 0.000788621 11700 0.005618844 11300
6.79E-05 13500 1.18E-05 13000 1.43E-05

13900 4.06E-05 11900 0.00077721 11700 0.00549188 11300
6.81E-05 13500 1.19E-05 13000 1.43E-05
13900 4.06E-05 11900 0.000766042 11700 0.005369118 11300
6.83E-05 13500 1.19E-05 13000 1.43E-05
13900 4.06E-05 11900 0.000755117 11700 0.005250405 11300
6.86E-05 13500 1.19E-05 13000 1.43E-05
13900 4.05E-05 11900 0.000744458 11700 0.005135565 11300
6.89E-05 13500 1.19E-05 13000 1.43E-05
13900 4.05E-05 11900 0.000734033 11700 0.005024401 11300
6.92E-05 13500 1.19E-05 13000 1.43E-05
13900 4.05E-05 11900 0.000723835 11700 0.004916789 11300
6.96E-05 13500 1.19E-05 13000 1.44E-05
13900 4.05E-05 11900 0.000713865 11700 0.004812571 11300
7.00E-05 13500 1.19E-05 13000 1.44E-05
13900 4.04E-05 11900 0.000704112 11700 0.004711617 11300
7.05E-05 13500 1.19E-05 13000 1.44E-05
13900 4.04E-05 11900 0.00069455 11700 0.004613772 11300
7.10E-05 13500 1.19E-05 13000 1.44E-05
13900 4.03E-05 11900 0.000685195 11700 0.004518936 11300
7.16E-05 13500 1.19E-05 13000 1.44E-05
13900 4.03E-05 11900 0.000676018 11700 0.004426955 11300
7.22E-05 13600 1.19E-05 13000 1.44E-05
13900 4.03E-05 11900 0.00066702 11700 0.004337742 11300
7.30E-05 13600 1.19E-05 13000 1.44E-05
13900 4.03E-05 11900 0.00065819 11700 0.004251191 11300
7.38E-05 13600 1.19E-05 13000 1.44E-05
13900 4.02E-05 11900 0.000649531 11700 0.004167184 11300
7.48E-05 13600 1.19E-05 13000 1.44E-05
13900 4.02E-05 11900 0.000641039 11700 0.004085628 11300
7.59E-05 13600 1.19E-05 13000 1.44E-05
13900 4.02E-05 11900 0.000632708 11700 0.004006442 11300
7.71E-05 13600 1.19E-05 13000 1.44E-05
13900 4.01E-05 11900 0.000624542 11700 0.003929531 11300
7.84E-05 13600 1.19E-05 13000 1.44E-05
13900 4.01E-05 11900 0.000616541 11700 0.003854789 11300
7.98E-05 13600 1.19E-05 13000 1.44E-05
13900 4.01E-05 11900 0.000608682 11700 0.00378215 11300
8.14E-05 13600 1.20E-05 13000 1.44E-05
13900 4.01E-05 11900 0.000600993 11700 0.003711541 11300
8.32E-05 13600 1.20E-05 13000 1.44E-05
13900 4.01E-05 11900 0.000593459 11700 0.003642884 11300
8.52E-05 13600 1.20E-05 13000 1.44E-05
13900 4.01E-05 11900 0.000586061 11700 0.003576104 11300
8.74E-05 13600 1.20E-05 13000 1.44E-05
13900 4.00E-05 11900 0.000578819 11700 0.003511146 11300
8.99E-05 13600 1.20E-05 13000 1.44E-05
13900 4.00E-05 11900 0.000571713 11700 0.003447929 11300
9.26E-05 13600 1.20E-05 13000 1.44E-05
13900 4.00E-05 11900 0.000564728 11700 0.003386408 11300
9.57E-05 13600 1.20E-05 13000 1.44E-05
13900 3.99E-05 11900 0.000557871 11700 0.003326515 11300
9.91E-05 13600 1.20E-05 13000 1.44E-05
13900 3.99E-05 11900 0.000551145 11700 0.003268187 11300
0.000102976 13600 1.20E-05 13000 1.44E-05
13900 3.99E-05 11900 0.00054452 11700 0.003211376 11300
0.000107185 13600 1.20E-05 13000 1.44E-05
13900 3.98E-05 11900 0.000538018 11700 0.00315603 11300
0.000111856 13600 1.20E-05 13000 1.44E-05
13900 3.98E-05 11900 0.000531626 11700 0.003102093 11300
0.000116936 13600 1.20E-05 13000 1.44E-05
13900 3.98E-05 11900 0.000525343 11700 0.003049518 11300
0.000122406 13600 1.20E-05 13000 1.44E-05
13900 3.98E-05 11900 0.000519163 11700 0.002998276 11300
0.000128245 13600 1.20E-05 13000 1.44E-05
13900 3.98E-05 11900 0.000513102 11700 0.002948317 11300
0.000134317 13600 1.20E-05 13000 1.44E-05
13900 3.97E-05 11900 0.000507151 11700 0.002899582 11300
0.00014053 13600 1.20E-05 13000 1.44E-05

```
13900	3.97E-05		11900	0.000501293	11700	0.002852058	11300
0.000146713	13600	1.20E-05	13000	1.44E-05
13900	3.97E-05		11900	0.00049554	11700	0.002805689	11300
0.000152743	13600	1.21E-05	13000	1.44E-05
13900	3.97E-05		11900	0.000489884	11700	0.002760442	11300
0.000158431	13600	1.21E-05	13000	1.44E-05
13900	3.96E-05		11900	0.000484328	11700	0.002716281	11300
0.00016354	13600	1.21E-05	13000	1.44E-05
13900	3.96E-05		11900	0.000478858	11700	0.002673155	11300
0.000167903	13600	1.21E-05	13000	1.44E-05
14000	3.96E-05		11900	0.000473508	11700	0.002631058	11300
0.000171322	13600	1.21E-05	13000	1.44E-05
14000	3.96E-05		11900	0.000468219	11700	0.002589936	11300
0.000173679	13600	1.21E-05	13000	1.44E-05
14000	3.95E-05		11900	0.000463045	11700	0.002549792	11300
0.000174863	13600	1.21E-05	13000	1.44E-05
14000	3.95E-05		11900	0.000457962	11700	0.00251055	11300
0.000174814	13600	1.21E-05	13000	1.44E-05
14000	3.95E-05		11900	0.00045297	11700	0.002472219	11300
0.000173522	13600	1.21E-05	13000	1.44E-05
14000	3.95E-05		11900	0.000448053	11700	0.002434766	11300
0.000171036	13600	1.21E-05	13000	1.44E-05
14000	3.94E-05		11900	0.000443232	11700	0.002398144	11300
0.000167445	13600	1.21E-05	13000	1.44E-05
14000	3.94E-05		11900	0.000438491	11700	0.002362356	11300
0.00016291	13600	1.21E-05	13000	1.44E-05
14000	3.94E-05		11900	0.000433811	11700	0.002327356	11300
0.000157612	13600	1.21E-05	13000	1.44E-05
14000	3.94E-05		11900	0.000429211	11700	0.00229312	11300
0.000151753	13600	1.21E-05	13100	1.44E-05
14000	3.94E-05		11900	0.000424692	11700	0.002259644	11300
0.000145531	13600	1.21E-05	13100	1.44E-05
14000	3.94E-05		11900	0.000420223	11700	0.002226882	11300
0.000139177	13600	1.21E-05	13100	1.44E-05
14000	3.93E-05		11900	0.00041584	11700	0.00219484	11300
0.000132843	13600	1.21E-05	13100	1.44E-05
14000	3.93E-05		11900	0.000411512	11700	0.002163479	11300
0.000126681	13600	1.21E-05	13100	1.44E-05
14000	3.93E-05		11900	0.000407259	11700	0.00213278	11300
0.000120771	13600	1.22E-05	13100	1.44E-05
14000	3.93E-05		11900	0.000403057	11700	0.002102748	11300
0.000115207	13600	1.22E-05	13100	1.44E-05
14000	3.92E-05		11900	0.00039892	11700	0.002073344	11300
0.000109998	13600	1.22E-05	13100	1.44E-05
14000	3.92E-05		11900	0.000394849	11700	0.002044551	11300
0.000105154	13600	1.22E-05	13100	1.44E-05
14000	3.92E-05		12000	0.000390828	11700	0.00201635	11300
0.000100679	13600	1.22E-05	13100	1.44E-05
14000	3.92E-05		12000	0.000386908	11700	0.001988732	11300
9.65E-05	13600	1.22E-05	13100	1.44E-05
14000	3.92E-05		12000	0.000383028	11800	0.001961674	11300
9.27E-05	13600	1.22E-05	13100	1.44E-05
14000	3.92E-05		12000	0.000379218	11800	0.001935183	11300
8.92E-05	13600	1.22E-05	13100	1.44E-05
14000	3.91E-05		12000	0.000375459	11800	0.001909208	11300
8.60E-05	13600	1.22E-05	13100	1.44E-05
14000	3.91E-05		12000	0.00037177	11800	0.001883773	11300
8.30E-05	13600	1.22E-05	13100	1.44E-05
14000	3.91E-05		12000	0.000368121	11800	0.001858828	11400
8.03E-05	13600	1.22E-05	13100	1.44E-05
14000	3.90E-05		12000	0.000364548	11800	0.001834384	11400
7.79E-05	13600	1.22E-05	13100	1.44E-05
14000	3.90E-05		12000	0.000361035	11800	0.00181041	11400
7.57E-05	13600	1.22E-05	13100	1.44E-05
14000	3.90E-05		12000	0.000357553	11800	0.001786914	11400
7.37E-05	13600	1.22E-05	13100	1.44E-05
14000	3.90E-05		12000	0.000354151	11800	0.001763857	11400
7.20E-05	13600	1.23E-05	13100	1.44E-05
```

14000	3.90E-05	12000	0.000350779	11800	0.001741257	11400
7.03E-05	13600	1.23E-05	13100	1.44E-05		
14000	3.89E-05	12000	0.000347448	11800	0.001719088	11400
6.89E-05	13600	1.23E-05	13100	1.45E-05		
14000	3.89E-05	12000	0.000344156	11800	0.001697339	11400
6.76E-05	13600	1.23E-05	13100	1.45E-05		
14000	3.89E-05	12000	0.000340905	11800	0.001675999	11400
6.64E-05	13600	1.23E-05	13100	1.44E-05		
14000	3.89E-05	12000	0.000337715	11800	0.001655056	11400
6.53E-05	13600	1.23E-05	13100	1.45E-05		
14000	3.89E-05	12000	0.000334564	11800	0.001634518	11400
6.43E-05	13600	1.23E-05	13100	1.45E-05		
14000	3.89E-05	12000	0.000331444	11800	0.001614364	11400
6.33E-05	13700	1.23E-05	13100	1.45E-05		
14000	3.89E-05	12000	0.000328354	11800	0.001594569	11400
6.25E-05	13700	1.23E-05	13100	1.45E-05		
14000	3.89E-05	12000	0.000325335	11800	0.001575135	11400
6.17E-05	13700	1.23E-05	13100	1.45E-05		
14000	3.88E-05	12000	0.000322365	11800	0.001556071	11400
6.10E-05	13700	1.23E-05	13100	1.45E-05		
14000	3.88E-05	12000	0.000319386	11800	0.001537336	11400
6.03E-05	13700	1.23E-05	13100	1.45E-05		
14000	3.88E-05	12000	0.000316507	11800	0.00151895	11400
5.97E-05	13700	1.24E-05	13100	1.45E-05		
14000	3.87E-05	12000	0.000313649	11800	0.001500878	11400
5.92E-05	13700	1.24E-05	13100	1.45E-05		
14000	3.87E-05	12000	0.000310821	11800	0.00148314	11400
5.86E-05	13700	1.24E-05	13100	1.45E-05		
14000	3.87E-05	12000	0.000308063	11800	0.001465715	11400
5.81E-05	13700	1.24E-05	13100	1.45E-05		
14000	3.87E-05	12000	0.000305315	11800	0.001448591	11400
5.77E-05	13700	1.24E-05	13100	1.45E-05		
14000	3.86E-05	12000	0.000302628	11800	0.001431764	11400
5.72E-05	13700	1.24E-05	13100	1.45E-05		
14000	3.86E-05	12000	0.000299981	11800	0.001415241	11400
5.68E-05	13700	1.24E-05	13100	1.45E-05		
14000	3.86E-05	12000	0.000297343	11800	0.001398982	11400
5.64E-05	13700	1.24E-05	13100	1.45E-05		
14000	3.86E-05	12000	0.000294747	11800	0.001383014	11400
5.61E-05	13700	1.24E-05	13100	1.45E-05		
14000	3.86E-05	12000	0.00029219	11800	0.001367319	11400
5.58E-05	13700	1.24E-05	13100	1.45E-05		
14000	3.85E-05	12000	0.000289674	11800	0.001351892	11400
5.55E-05	13700	1.24E-05	13100	1.45E-05		
14000	3.85E-05	12000	0.000287198	11800	0.001336718	11400
5.52E-05	13700	1.24E-05	13100	1.45E-05		
14000	3.85E-05	12000	0.000284742	11800	0.001321807	11400
5.49E-05	13700	1.24E-05	13100	1.45E-05		
14000	3.85E-05	12000	0.000282286	11800	0.001307151	11400
5.46E-05	13700	1.24E-05	13100	1.45E-05		
14000	3.85E-05	12000	0.000279891	11800	0.001292728	11400
5.44E-05	13700	1.24E-05	13100	1.45E-05		
14000	3.85E-05	12000	0.000277495	11800	0.001278554	11400
5.41E-05	13700	1.25E-05	13100	1.45E-05		
14000	3.85E-05	12000	0.00027516	11800	0.001264602	11400
5.39E-05	13700	1.25E-05	13100	1.45E-05		
14000	3.84E-05	12000	0.000272865	11800	0.001250877	11400
5.37E-05	13700	1.25E-05	13100	1.46E-05		
14000	3.85E-05	12000	0.000270611	11800	0.001237388	11400
5.35E-05	13700	1.25E-05	13100	1.45E-05		
14000	3.84E-05	12000	0.000268376	11800	0.001224116	11400
5.33E-05	13700	1.25E-05	13100	1.45E-05		
14000	3.84E-05	12000	0.000266162	11800	0.001211051	11400
5.31E-05	13700	1.25E-05	13100	1.45E-05		
14000	3.84E-05	12000	0.000264028	11800	0.001198206	11400
5.29E-05	13700	1.25E-05	13100	1.45E-05		
14000	3.84E-05	12000	0.000261894	11800	0.001185557	11400
5.27E-05	13700	1.25E-05	13100	1.46E-05		

14000 3.83E-05 12000 0.00025972 11800 0.001173101 11400
5.25E-05 13700 1.25E-05 13100 1.46E-05
14100 3.83E-05 12000 0.000257667 11800 0.001160847 11400
5.23E-05 13700 1.25E-05 13100 1.45E-05
14100 3.83E-05 12000 0.000255573 11800 0.001148777 11400
5.22E-05 13700 1.25E-05 13100 1.46E-05
14100 3.83E-05 12000 0.00025354 11800 0.001136897 11400
5.20E-05 13700 1.25E-05 13100 1.46E-05
14100 3.83E-05 12000 0.000251527 11800 0.001125203 11400
5.19E-05 13700 1.25E-05 13100 1.46E-05
14100 3.83E-05 12000 0.000249514 11800 0.001113689 11400
5.17E-05 13700 1.25E-05 13100 1.46E-05
14100 3.82E-05 12000 0.000247521 11800 0.001102349 11400
5.16E-05 13700 1.25E-05 13100 1.46E-05
14100 3.82E-05 12000 0.000245569 11800 0.001091188 11400
5.14E-05 13700 1.25E-05 13100 1.46E-05
14100 3.82E-05 12000 0.000243656 11800 0.001080201 11400
5.13E-05 13700 1.25E-05 13100 1.46E-05
14100 3.82E-05 12000 0.000241724 11800 0.001069371 11400
5.12E-05 13700 1.26E-05 13100 1.46E-05
14100 3.82E-05 12000 0.000239852 11800 0.001058705 11400
5.10E-05 13700 1.26E-05 13200 1.46E-05
14100 3.82E-05 12000 0.000237939 11800 0.001048209 11400
5.09E-05 13700 1.26E-05 13200 1.46E-05
14100 3.82E-05 12000 0.000236087 11800 0.001037856 11400
5.08E-05 13700 1.26E-05 13200 1.46E-05
14100 3.82E-05 12000 0.000234235 11800 0.001027657 11400
5.07E-05 13700 1.26E-05 13200 1.46E-05
14100 3.82E-05 12000 0.000232504 11800 0.001017614 11400
5.06E-05 13700 1.26E-05 13200 1.46E-05
14100 3.82E-05 12000 0.000230733 11800 0.001007732 11400
5.04E-05 13700 1.26E-05 13200 1.46E-05
14100 3.82E-05 12000 0.000228961 11800 0.000997979 11400
5.03E-05 13700 1.26E-05 13200 1.46E-05
14100 3.82E-05 12000 0.00022719 11800 0.00098838 11400
5.02E-05 13700 1.26E-05 13200 1.46E-05
14100 3.81E-05 12000 0.000225479 11800 0.000978904 11400
5.01E-05 13700 1.26E-05 13200 1.46E-05
14100 3.81E-05 12100 0.000223767 11800 0.000969575 11400
5.00E-05 13700 1.26E-05 13200 1.46E-05
14100 3.81E-05 12100 0.000222077 11800 0.000960383 11400
4.99E-05 13700 1.26E-05 13200 1.46E-05
14100 3.81E-05 12100 0.000220486 11900 0.000951304 11400
4.98E-05 13700 1.26E-05 13200 1.46E-05
14100 3.81E-05 12100 0.000218856 11900 0.000942347 11400
4.97E-05 13700 1.27E-05 13200 1.46E-05
14100 3.81E-05 12100 0.000217245 11900 0.000933526 11400
4.96E-05 13700 1.27E-05 13200 1.46E-05
14100 3.80E-05 12100 0.000215635 11900 0.000924845 11400
4.95E-05 13700 1.27E-05 13200 1.46E-05
14100 3.80E-05 12100 0.000214025 11900 0.000916275 11500
4.94E-05 13700 1.27E-05 13200 1.47E-05
14100 3.80E-05 12100 0.000212495 11900 0.000907829 11500
4.93E-05 13700 1.27E-05 13200 1.47E-05
14100 3.80E-05 12100 0.000210924 11900 0.000899504 11500
4.93E-05 13700 1.27E-05 13200 1.47E-05
14100 3.80E-05 12100 0.000209415 11900 0.000891293 11500
4.92E-05 13700 1.27E-05 13200 1.47E-05
14100 3.79E-05 12100 0.000207885 11900 0.000883198 11500
4.91E-05 13700 1.27E-05 13200 1.47E-05
14100 3.79E-05 12100 0.000206355 11900 0.00087521 11500
4.90E-05 13700 1.27E-05 13200 1.47E-05
14100 3.79E-05 12100 0.000204885 11900 0.000867335 11500
4.89E-05 13700 1.27E-05 13200 1.47E-05
14100 3.79E-05 12100 0.000203416 11900 0.000859546 11500
4.88E-05 13700 1.27E-05 13200 1.47E-05
14100 3.79E-05 12100 0.000201967 11900 0.000851885 11500
4.88E-05 13700 1.28E-05 13200 1.47E-05

14100 3.79E-05 12100 0.000200537 11900 0.000844334 11500
4.87E-05 13700 1.28E-05 13200 1.47E-05
14100 3.79E-05 12100 0.000199128 11900 0.00083688 11500
4.86E-05 13700 1.28E-05 13200 1.47E-05
14100 3.79E-05 12100 0.000197699 11900 0.000829522 11500
4.85E-05 13800 1.28E-05 13200 1.47E-05
14100 3.79E-05 12100 0.00019635 11900 0.000822259 11500
4.84E-05 13800 1.28E-05 13200 1.47E-05
14100 3.78E-05 12100 0.000194961 11900 0.000815093 11500
4.84E-05 13800 1.28E-05 13200 1.47E-05
14100 3.78E-05 12100 0.000193592 11900 0.000808016 11500
4.83E-05 13800 1.28E-05 13200 1.47E-05
14100 3.78E-05 12100 0.000192264 11900 0.000801027 11500
4.82E-05 13800 1.28E-05 13200 1.47E-05
14100 3.78E-05 12100 0.000190945 11900 0.000794126 11500
4.81E-05 13800 1.28E-05 13200 1.47E-05
14100 3.78E-05 12100 0.000189617 11900 0.000787319 11500
4.81E-05 13800 1.28E-05 13200 1.47E-05
14100 3.78E-05 12100 0.000188318 11900 0.00078062 11500
4.80E-05 13800 1.28E-05 13200 1.47E-05
14100 3.78E-05 12100 0.00018704 11900 0.000773982 11500
4.79E-05 13800 1.28E-05 13200 1.47E-05
14100 3.78E-05 12100 0.000185762 11900 0.000767437 11500
4.79E-05 13800 1.28E-05 13200 1.47E-05
14100 3.78E-05 12100 0.000184493 11900 0.000760978 11500
4.78E-05 13800 1.28E-05 13200 1.47E-05
14100 3.78E-05 12100 0.000183255 11900 0.000754601 11500
4.77E-05 13800 1.29E-05 13200 1.47E-05
14100 3.78E-05 12100 0.000182017 11900 0.000748296 11500
4.77E-05 13800 1.29E-05 13200 1.47E-05
14100 3.78E-05 12100 0.000180779 11900 0.000742089 11500
4.76E-05 13800 1.29E-05 13200 1.47E-05
14100 3.78E-05 12100 0.000179582 11900 0.00073593 11500
4.76E-05 13800 1.29E-05 13200 1.47E-05
14100 3.77E-05 12100 0.000178374 11900 0.000729867 11500
4.75E-05 13800 1.29E-05 13200 1.47E-05
14100 3.77E-05 12100 0.000177206 11900 0.000723883 11500
4.74E-05 13800 1.29E-05 13200 1.48E-05
14100 3.78E-05 12100 0.000176039 11900 0.000717979 11500
4.74E-05 13800 1.29E-05 13200 1.48E-05
14100 3.77E-05 12100 0.000174891 11900 0.000712137 11500
4.73E-05 13800 1.29E-05 13200 1.48E-05
14100 3.77E-05 12100 0.000173734 11900 0.000706374 11500
4.73E-05 13800 1.29E-05 13200 1.48E-05
14100 3.77E-05 12100 0.000172596 11900 0.000700678 11500
4.73E-05 13800 1.29E-05 13200 1.48E-05
14100 3.77E-05 12100 0.000171469 11900 0.000695047 11500
4.72E-05 13800 1.30E-05 13200 1.48E-05
14100 3.77E-05 12100 0.000170372 11900 0.000689483 11500
4.72E-05 13800 1.30E-05 13200 1.48E-05
14100 3.77E-05 12100 0.000169275 11900 0.00068398 11500
4.71E-05 13800 1.30E-05 13200 1.48E-05
14100 3.77E-05 12100 0.000168168 11900 0.000678546 11500
4.71E-05 13800 1.30E-05 13200 1.48E-05
14100 3.77E-05 12100 0.000167101 11900 0.000673173 11500
4.70E-05 13800 1.30E-05 13200 1.48E-05
14100 3.76E-05 12100 0.000166054 11900 0.000667874 11500
4.70E-05 13800 1.30E-05 13200 1.48E-05
14100 3.76E-05 12100 0.000164987 11900 0.000662637 11500
4.70E-05 13800 1.30E-05 13200 1.48E-05
14100 3.76E-05 12100 0.000163941 11900 0.000657461 11500
4.69E-05 13800 1.30E-05 13200 1.48E-05
14200 3.76E-05 12100 0.000162914 11900 0.000652349 11500
4.69E-05 13800 1.30E-05 13200 1.48E-05
14200 3.76E-05 12100 0.000161902 11900 0.000647296 11500
4.68E-05 13800 1.30E-05 13200 1.48E-05
14200 3.76E-05 12100 0.000160886 11900 0.000642301 11500
4.68E-05 13800 1.31E-05 13200 1.48E-05

14200 3.76E-05 12100 0.000159874 11900 0.000637361 11500
4.67E-05 13800 1.31E-05 13200 1.48E-05
14200 3.76E-05 12100 0.000158873 11900 0.000632473 11500
4.67E-05 13800 1.31E-05 13200 1.48E-05
14200 3.75E-05 12100 0.000157886 11900 0.000627642 11500
4.66E-05 13800 1.31E-05 13200 1.48E-05
14200 3.76E-05 12100 0.000156915 11900 0.000622868 11500
4.66E-05 13800 1.31E-05 13200 1.48E-05
14200 3.75E-05 12100 0.000155924 11900 0.000618158 11500
4.65E-05 13800 1.31E-05 13200 1.48E-05
14200 3.75E-05 12100 0.000154967 11900 0.000613496 11500
4.65E-05 13800 1.31E-05 13200 1.48E-05
14200 3.75E-05 12100 0.000154006 11900 0.000608891 11500
4.65E-05 13800 1.31E-05 13300 1.48E-05
14200 3.75E-05 12100 0.000153055 11900 0.000604351 11500
4.64E-05 13800 1.31E-05 13300 1.48E-05
14200 3.75E-05 12100 0.000152129 11900 0.000599844 11500
4.64E-05 13800 1.32E-05 13300 1.48E-05
14200 3.75E-05 12100 0.000151198 11900 0.000595379 11500
4.63E-05 13800 1.32E-05 13300 1.48E-05
14200 3.74E-05 12100 0.000150272 11900 0.000590972 11500
4.63E-05 13800 1.32E-05 13300 1.48E-05
14200 3.75E-05 12100 0.000149371 11900 0.000586615 11500
4.62E-05 13800 1.32E-05 13300 1.48E-05
14200 3.75E-05 12100 0.000148465 11900 0.0005823 11500
4.62E-05 13800 1.32E-05 13300 1.48E-05
14200 3.74E-05 12100 0.000147585 11900 0.000578044 11500
4.61E-05 13800 1.32E-05 13300 1.48E-05
14200 3.74E-05 12100 0.000146684 11900 0.000573831 11500
4.61E-05 13800 1.32E-05 13300 1.48E-05
14200 3.74E-05 12200 0.000145808 11900 0.000569665 11500
4.61E-05 13800 1.32E-05 13300 1.48E-05
14200 3.74E-05 12200 0.000144938 11900 0.000565542 11500
4.60E-05 13800 1.32E-05 13300 1.49E-05
14200 3.74E-05 12200 0.000144077 12000 0.000561471 11500
4.60E-05 13800 1.33E-05 13300 1.49E-05
14200 3.74E-05 12200 0.000143232 12000 0.000557447 11500
4.60E-05 13800 1.33E-05 13300 1.49E-05
14200 3.74E-05 12200 0.000142381 12000 0.000553455 11500
4.60E-05 13800 1.33E-05 13300 1.49E-05
14200 3.75E-05 12200 0.000141531 12000 0.000549509 11500
4.59E-05 13800 1.33E-05 13300 1.49E-05
14200 3.74E-05 12200 0.000140715 12000 0.000545596 11600
4.59E-05 13800 1.33E-05 13300 1.49E-05
14200 3.74E-05 12200 0.000139875 12000 0.000541742 11600
4.59E-05 13800 1.33E-05 13300 1.49E-05
14200 3.74E-05 12200 0.00013907 12000 0.000537927 11600
4.58E-05 13800 1.33E-05 13300 1.49E-05
14200 3.74E-05 12200 0.000138249 12000 0.000534141 11600
4.58E-05 13800 1.33E-05 13300 1.49E-05
14200 3.74E-05 12200 0.000137459 12000 0.000530411 11600
4.58E-05 13800 1.34E-05 13300 1.49E-05
14200 3.74E-05 12200 0.000136669 12000 0.000526724 11600
4.58E-05 13800 1.34E-05 13300 1.49E-05
14200 3.74E-05 12200 0.000135889 12000 0.000523066 11600
4.57E-05 13800 1.34E-05 13300 1.49E-05
14200 3.74E-05 12200 0.000135124 12000 0.000519432 11600
4.57E-05 13800 1.34E-05 13300 1.49E-05
14200 3.74E-05 12200 0.000134369 12000 0.000515848 11600
4.57E-05 13800 1.34E-05 13300 1.49E-05
14200 3.73E-05 12200 0.000133612 12000 0.000512303 11600
4.56E-05 13800 1.34E-05 13300 1.49E-05
14200 3.74E-05 12200 0.000132877 12000 0.000508793 11600
4.56E-05 13800 1.34E-05 13300 1.49E-05
14200 3.73E-05 12200 0.000132137 12000 0.000505327 11600
4.56E-05 13900 1.35E-05 13300 1.49E-05
14200 3.73E-05 12200 0.000131423 12000 0.00050188 11600
4.56E-05 13900 1.35E-05 13300 1.49E-05

14200 3.73E-05 12200 0.000130693 12000 0.00049849 11600
4.56E-05 13900 1.35E-05 13300 1.49E-05
14200 3.73E-05 12200 0.000129968 12000 0.000495118 11600
4.55E-05 13900 1.35E-05 13300 1.49E-05
14200 3.73E-05 12200 0.000129266 12000 0.000491786 11600
4.55E-05 13900 1.35E-05 13300 1.49E-05
14200 3.73E-05 12200 0.000128541 12000 0.000488498 11600
4.55E-05 13900 1.35E-05 13300 1.49E-05
14200 3.73E-05 12200 0.000127827 12000 0.000485231 11600
4.55E-05 13900 1.35E-05 13300 1.49E-05
14200 3.73E-05 12200 0.000127122 12000 0.000482013 11600
4.55E-05 13900 1.36E-05 13300 1.49E-05
14200 3.73E-05 12200 0.00012641 12000 0.00047881 11600
4.54E-05 13900 1.36E-05 13300 1.49E-05
14200 3.73E-05 12200 0.000125711 12000 0.000475647 11600
4.54E-05 13900 1.36E-05 13300 1.49E-05
14200 3.73E-05 12200 0.000125021 12000 0.000472514 11600
4.54E-05 13900 1.36E-05 13300 1.49E-05
14200 3.73E-05 12200 0.000124342 12000 0.000469424 11600
4.54E-05 13900 1.36E-05 13300 1.50E-05
14200 3.72E-05 12200 0.00012367 12000 0.000466353 11600
4.54E-05 13900 1.36E-05 13300 1.49E-05
14200 3.72E-05 12200 0.000122986 12000 0.000463314 11600
4.54E-05 13900 1.36E-05 13300 1.50E-05
14200 3.72E-05 12200 0.000122336 12000 0.000460304 11600
4.54E-05 13900 1.37E-05 13300 1.50E-05
14200 3.72E-05 12200 0.00012167 12000 0.00045732 11600
4.53E-05 13900 1.37E-05 13300 1.50E-05
14200 3.72E-05 12200 0.000121023 12000 0.000454367 11600
4.53E-05 13900 1.37E-05 13300 1.49E-05
14200 3.72E-05 12200 0.000120376 12000 0.000451439 11600
4.53E-05 13900 1.37E-05 13300 1.50E-05
14200 3.72E-05 12200 0.000119737 12000 0.000448533 11600
4.53E-05 13900 1.37E-05 13300 1.50E-05
14200 3.72E-05 12200 0.000119098 12000 0.000445668 11600
4.53E-05 13900 1.37E-05 13300 1.50E-05
14200 3.72E-05 12200 0.000118481 12000 0.000442826 11600
4.53E-05 13900 1.37E-05 13300 1.50E-05
14200 3.72E-05 12200 0.00011786 12000 0.000440024 11600
4.52E-05 13900 1.38E-05 13300 1.50E-05
14200 3.72E-05 12200 0.000117241 12000 0.000437222 11600
4.52E-05 13900 1.38E-05 13300 1.50E-05
14200 3.72E-05 12200 0.000116624 12000 0.000434461 11600
4.52E-05 13900 1.38E-05 13300 1.50E-05
14200 3.72E-05 12200 0.000116013 12000 0.000431717 11600
4.52E-05 13900 1.38E-05 13300 1.50E-05
14200 3.72E-05 12200 0.000115406 12000 0.000429029 11600
4.52E-05 13900 1.38E-05 13300 1.50E-05
14200 3.72E-05 12200 0.000114803 12000 0.000426339 11600
4.52E-05 13900 1.38E-05 13300 1.50E-05
14200 3.72E-05 12200 0.000114199 12000 0.000423679 11600
4.52E-05 13900 1.39E-05 13300 1.50E-05
14200 3.72E-05 12200 0.0001136 12000 0.000421055 11600
4.51E-05 13900 1.39E-05 13300 1.50E-05
14300 3.72E-05 12200 0.000112993 12000 0.000418463 11600
4.51E-05 13900 1.39E-05 13300 1.50E-05
14300 3.72E-05 12200 0.000112415 12000 0.000415887 11600
4.51E-05 13900 1.39E-05 13300 1.50E-05
14300 3.72E-05 12200 0.00011183 12000 0.000413329 11600
4.51E-05 13900 1.39E-05 13300 1.50E-05
14300 3.72E-05 12200 0.000111257 12000 0.000410808 11600
4.51E-05 13900 1.39E-05 13300 1.50E-05
14300 3.71E-05 12200 0.000110686 12000 0.000408303 11600
4.51E-05 13900 1.40E-05 13300 1.50E-05
14300 3.71E-05 12200 0.000110116 12000 0.000405813 11600
4.51E-05 13900 1.40E-05 13300 1.50E-05
14300 3.71E-05 12200 0.00010955 12000 0.000403349 11600
4.51E-05 13900 1.40E-05 13300 1.50E-05

14300 3.71E-05 12200 0.000108992 12000 0.000400913 11600
4.51E-05 13900 1.40E-05 13300 1.50E-05
14300 3.72E-05 12200 0.000108438 12000 0.00039848 11600
4.51E-05 13900 1.40E-05 13300 1.50E-05
14300 3.71E-05 12200 0.000107888 12000 0.000396086 11600
4.51E-05 13900 1.40E-05 13400 1.50E-05
14300 3.71E-05 12200 0.000107336 12000 0.000393706 11600
4.51E-05 13900 1.41E-05 13400 1.50E-05
14300 3.71E-05 12200 0.000106792 12000 0.000391375 11600
4.51E-05 13900 1.41E-05 13400 1.50E-05
14300 3.71E-05 12200 0.000106251 12000 0.000389041 11600
4.51E-05 13900 1.41E-05 13400 1.51E-05
14300 3.71E-05 12200 0.000105726 12000 0.00038674 11600
4.51E-05 13900 1.41E-05 13400 1.51E-05
14300 3.71E-05 12200 0.000105195 12000 0.000384 11600
4.51E-05 13900 1.41E-05 13400 1.51E-05
14300 3.71E-05 12200 0.00010468 12000 0.000382 11600
4.51E-05 13900 1.41E-05 13400 1.51E-05
14300 3.71E-05 12200 0.000104164 12000 0.00038 11600 4.51E-05
13900 1.42E-05 13400 1.51E-05
14300 3.71E-05 12200 0.000103653 12000 0.000378 11600
4.51E-05 13900 1.42E-05 13400 1.51E-05
14300 3.71E-05 12300 0.000103152 12000 0.000375 11600
4.50E-05 13900 1.42E-05 13400 1.51E-05
14300 3.71E-05 12300 0.00010264 12000 0.000373 11600
4.50E-05 13900 1.42E-05 13400 1.51E-05
14300 3.71E-05 12300 0.000102154 12100 0.000371 11600
4.50E-05 13900 1.42E-05 13400 1.51E-05
14300 3.71E-05 12300 0.00010166 12100 0.000369 11600
4.51E-05 13900 1.42E-05 13400 1.51E-05
14300 3.71E-05 12300 0.000101176 12100 0.000367 11600
4.51E-05 13900 1.43E-05 13400 1.51E-05
14300 3.71E-05 12300 0.000100681 12100 0.000365 11600
4.51E-05 13900 1.43E-05 13400 1.51E-05
14300 3.71E-05 12300 0.000100191 12100 0.000363 11700
4.51E-05 13900 1.43E-05 13400 1.51E-05
14300 3.71E-05 12300 9.97E-05 12100 0.000361 11700
4.51E-05 13900 1.43E-05 13400 1.51E-05
14300 3.71E-05 12300 9.92E-05 12100 0.000359 11700
4.51E-05 13900 1.43E-05 13400 1.51E-05
14300 3.71E-05 12300 9.88E-05 12100 0.000356 11700
4.51E-05 13900 1.43E-05 13400 1.51E-05
14300 3.71E-05 12300 9.83E-05 12100 0.000354 11700
4.51E-05 13900 1.44E-05 13400 1.51E-05
14300 3.70E-05 12300 9.78E-05 12100 0.000352 11700
4.51E-05 13900 1.44E-05 13400 1.51E-05
14300 3.70E-05 12300 9.73E-05 12100 0.00035 11700 4.51E-05
13900 1.44E-05 13400 1.51E-05
14300 3.70E-05 12300 9.69E-05 12100 0.000349 11700
4.51E-05 13900 1.44E-05 13400 1.51E-05
14300 3.70E-05 12300 9.64E-05 12100 0.000347 11700
4.51E-05 13900 1.44E-05 13400 1.51E-05
14300 3.70E-05 12300 9.60E-05 12100 0.000345 11700
4.51E-05 13900 1.45E-05 13400 1.51E-05
14300 3.70E-05 12300 9.55E-05 12100 0.000343 11700
4.51E-05 13900 1.45E-05 13400 1.51E-05
14300 3.70E-05 12300 9.51E-05 12100 3.41E-04 11700
4.51E-05 14000 1.45E-05 13400 1.51E-05
14300 3.70E-05 12300 9.46E-05 12100 3.39E-04 11700
4.51E-05 14000 1.45E-05 13400 1.51E-05
14300 3.70E-05 12300 9.42E-05 12100 3.37E-04 11700
4.51E-05 14000 1.45E-05 13400 1.51E-05
14300 3.70E-05 12300 9.38E-05 12100 3.35E-04 11700
4.52E-05 14000 1.46E-05 13400 1.51E-05
14300 3.70E-05 12300 9.33E-05 12100 3.33E-04 11700
4.52E-05 14000 1.46E-05 13400 1.51E-05
14300 3.70E-05 12300 9.29E-05 12100 3.32E-04 11700
4.52E-05 14000 1.46E-05 13400 1.51E-05

```
14300   3.70E-05          12300   9.25E-05          12100   3.30E-04          11700
4.52E-05          14000   1.46E-05          13400   1.51E-05
14300   3.70E-05          12300   9.21E-05          12100   3.28E-04          11700
4.52E-05          14000   1.46E-05          13400   1.51E-05
14300   3.70E-05          12300   9.17E-05          12100   3.26E-04          11700
4.52E-05          14000   1.47E-05          13400   1.51E-05
14300   3.70E-05          12300   9.13E-05          12100   3.24E-04          11700
4.52E-05          14000   1.47E-05          13400   1.51E-05
14300   3.70E-05          12300   9.09E-05          12100   3.23E-04          11700
4.52E-05          14000   1.47E-05          13400   1.51E-05
14300   3.70E-05          12300   9.05E-05          12100   3.21E-04          11700
4.53E-05          14000   1.47E-05          13400   1.51E-05
14300   3.70E-05          12300   9.01E-05          12100   3.19E-04          11700
4.53E-05          14000   1.48E-05          13400   1.51E-05
14300   3.70E-05          12300   8.96E-05          12100   3.18E-04          11700
4.53E-05          14000   1.48E-05          13400   1.52E-05
14300   3.70E-05          12300   8.92E-05          12100   3.16E-04          11700
4.53E-05          14000   1.48E-05          13400   1.52E-05
14300   3.70E-05          12300   8.88E-05          12100   3.14E-04          11700
4.53E-05          14000   1.48E-05          13400   1.52E-05
14300   3.70E-05          12300   8.85E-05          12100   3.12E-04          11700
4.53E-05          14000   1.49E-05          13400   1.52E-05
14300   3.70E-05          12300   8.81E-05          12100   3.11E-04          11700
4.53E-05          14000   1.49E-05          13400   1.52E-05
14300   3.70E-05          12300   8.77E-05          12100   3.09E-04          11700
4.54E-05          14000   1.49E-05          13400   1.52E-05
14300   3.70E-05          12300   8.73E-05          12100   3.08E-04          11700
4.54E-05          14000   1.50E-05          13400   1.52E-05
14300   3.70E-05          12300   8.69E-05          12100   3.06E-04          11700
4.54E-05          14000   1.50E-05          13400   1.52E-05
14300   3.70E-05          12300   8.65E-05          12100   3.04E-04          11700
4.54E-05          14000   1.50E-05          13400   1.52E-05
14300   3.70E-05          12300   8.61E-05          12100   3.03E-04          11700
4.54E-05          14000   1.51E-05          13400   1.52E-05
14300   3.70E-05          12300   8.58E-05          12100   3.01E-04          11700
4.54E-05          14000   1.51E-05          13400   1.52E-05
14300   3.70E-05          12300   8.54E-05          12100   3.00E-04          11700
4.55E-05          14000   1.51E-05          13400   1.52E-05
14300   3.70E-05          12300   8.50E-05          12100   2.98E-04          11700
4.55E-05          14000   1.51E-05          13400   1.52E-05
14300   3.70E-05          12300   8.46E-05          12100   2.97E-04          11700
4.55E-05          14000   1.51E-05          13400   1.52E-05
14300   3.70E-05          12300   8.43E-05          12100   2.95E-04          11700
4.56E-05          14000   1.52E-05          13400   1.52E-05
14300   3.70E-05          12300   8.39E-05          12100   2.94E-04          11700
4.56E-05          14000   1.52E-05          13400   1.52E-05
14400   3.69E-05          12300   8.35E-05          12100   2.92E-04          11700
4.56E-05          14000   1.53E-05          13400   1.52E-05
14400   3.69E-05          12300   8.32E-05          12100   2.91E-04          11700
4.56E-05          14000   1.53E-05          13400   1.52E-05
14400   3.69E-05          12300   8.28E-05          12100   2.89E-04          11700
4.56E-05          14000   1.53E-05          13400   1.52E-05
14400   3.69E-05          12300   8.24E-05          12100   2.88E-04          11700
4.56E-05          14000   1.53E-05          13400   1.52E-05
14400   3.69E-05          12300   8.21E-05          12100   2.86E-04          11700
4.57E-05          14000   1.54E-05          13400   1.52E-05
14400   3.69E-05          12300   8.17E-05          12100   2.85E-04          11700
4.57E-05          14000   1.54E-05          13400   1.52E-05
14400   3.69E-05          12300   8.14E-05          12100   2.83E-04          11700
4.57E-05          14000   1.55E-05          13400   1.52E-05
14400   3.69E-05          12300   8.10E-05          12100   2.82E-04          11700
4.58E-05          14000   1.55E-05          13400   1.52E-05
14400   3.69E-05          12300   8.07E-05          12100   2.80E-04          11700
4.58E-05          14000   1.55E-05          13400   1.52E-05
14400   3.69E-05          12300   8.03E-05          12100   2.79E-04          11700
4.58E-05          14000   1.56E-05          13500   1.52E-05
14400   3.69E-05          12300   8.00E-05          12100   2.78E-04          11700
4.58E-05          14000   1.56E-05          13500   1.52E-05
```

14400 3.69E-05 12300 7.96E-05 12100 2.76E-04 11700
4.59E-05 14000 1.56E-05 13500 1.52E-05
14400 3.70E-05 12300 7.93E-05 12100 2.75E-04 11700
4.59E-05 14000 1.57E-05 13500 1.52E-05
14400 3.69E-05 12300 7.90E-05 12100 2.74E-04 11700
4.59E-05 14000 1.57E-05 13500 1.52E-05
14400 3.69E-05 12300 7.86E-05 12100 2.72E-04 11700
4.59E-05 14000 1.58E-05 13500 1.52E-05
14400 3.69E-05 12300 7.83E-05 12100 2.71E-04 11700
4.60E-05 14000 1.58E-05 13500 1.52E-05
14400 3.69E-05 12300 7.80E-05 12100 2.70E-04 11700
4.60E-05 14000 1.58E-05 13500 1.52E-05
14400 3.69E-05 12300 7.77E-05 12100 2.68E-04 11700
4.61E-05 14000 1.59E-05 13500 1.52E-05
14400 3.69E-05 12400 7.73E-05 12100 2.67E-04 11700
4.61E-05 14000 1.59E-05 13500 1.52E-05
14400 3.69E-05 12400 7.70E-05 12100 2.66E-04 11700
4.61E-05 14000 1.60E-05 13500 1.52E-05
14400 3.69E-05 12400 7.67E-05 12200 2.64E-04 11700
4.61E-05 14000 1.60E-05 13500 1.52E-05
14400 3.69E-05 12400 7.63E-05 12200 2.63E-04 11700
4.62E-05 14000 1.60E-05 13500 1.52E-05
14400 3.69E-05 12400 7.60E-05 12200 2.62E-04 11700
4.62E-05 14000 1.61E-05 13500 1.52E-05
14400 3.69E-05 12400 7.57E-05 12200 2.61E-04 11700
4.62E-05 14000 1.61E-05 13500 1.52E-05
14400 3.69E-05 12400 7.54E-05 12200 2.59E-04 11800
4.63E-05 14000 1.62E-05 13500 1.52E-05
14400 3.69E-05 12400 7.51E-05 12200 2.58E-04 11800
4.63E-05 14000 1.62E-05 13500 1.52E-05
14400 3.70E-05 12400 7.48E-05 12200 2.57E-04 11800
4.63E-05 14000 1.63E-05 13500 1.52E-05
14400 3.69E-05 12400 7.44E-05 12200 2.56E-04 11800
4.64E-05 14000 1.63E-05 13500 1.52E-05
14400 3.70E-05 12400 7.41E-05 12200 2.54E-04 11800
4.64E-05 14000 1.63E-05 13500 1.52E-05
14400 3.69E-05 12400 7.38E-05 12200 2.53E-04 11800
4.64E-05 14000 1.64E-05 13500 1.52E-05
14400 3.70E-05 12400 7.35E-05 12200 2.52E-04 11800
4.65E-05 14000 1.64E-05 13500 1.52E-05
14400 3.70E-05 12400 7.32E-05 12200 2.51E-04 11800
4.65E-05 14000 1.65E-05 13500 1.52E-05
14400 3.69E-05 12400 7.29E-05 12200 2.50E-04 11800
4.65E-05 14000 1.65E-05 13500 1.52E-05
14400 3.69E-05 12400 7.26E-05 12200 2.48E-04 11800
4.66E-05 14000 1.66E-05 13500 1.53E-05
14400 3.69E-05 12400 7.24E-05 12200 2.47E-04 11800
4.66E-05 14000 1.66E-05 13500 1.53E-05
14400 3.69E-05 12400 7.21E-05 12200 2.46E-04 11800
4.67E-05 14100 1.67E-05 13500 1.53E-05
14400 3.69E-05 12400 7.18E-05 12200 2.45E-04 11800
4.67E-05 14100 1.67E-05 13500 1.53E-05
14400 3.69E-05 12400 7.15E-05 12200 2.44E-04 11800
4.67E-05 14100 1.68E-05 13500 1.52E-05
14400 3.69E-05 12400 7.12E-05 12200 2.43E-04 11800
4.68E-05 14100 1.68E-05 13500 1.52E-05
14400 3.69E-05 12400 7.09E-05 12200 2.42E-04 11800
4.68E-05 14100 1.69E-05 13500 1.52E-05
14400 3.69E-05 12400 7.06E-05 12200 2.41E-04 11800
4.69E-05 14100 1.69E-05 13500 1.53E-05
14400 3.69E-05 12400 7.04E-05 12200 2.39E-04 11800
4.69E-05 14100 1.70E-05 13500 1.53E-05
14400 3.69E-05 12400 7.01E-05 12200 2.38E-04 11800
4.70E-05 14100 1.70E-05 13500 1.53E-05
14400 3.69E-05 12400 6.98E-05 12200 2.37E-04 11800
4.70E-05 14100 1.71E-05 13500 1.53E-05
14400 3.69E-05 12400 6.95E-05 12200 2.36E-04 11800
4.71E-05 14100 1.71E-05 13500 1.53E-05

14400 3.69E-05 12400 6.93E-05 12200 2.35E-04 11800
4.71E-05 14100 1.72E-05 13500 1.53E-05
14400 3.68E-05 12400 6.90E-05 12200 2.34E-04 11800
4.72E-05 14100 1.73E-05 13500 1.53E-05
14400 3.68E-05 12400 6.87E-05 12200 2.33E-04 11800
4.72E-05 14100 1.73E-05 13500 1.53E-05
14400 3.68E-05 12400 6.85E-05 12200 2.32E-04 11800
4.73E-05 14100 1.74E-05 13500 1.53E-05
14400 3.68E-05 12400 6.82E-05 12200 2.31E-04 11800
4.73E-05 14100 1.74E-05 13500 1.53E-05
14400 3.69E-05 12400 6.80E-05 12200 2.30E-04 11800
4.74E-05 14100 1.75E-05 13500 1.53E-05
14400 3.68E-05 12400 6.77E-05 12200 2.29E-04 11800
4.74E-05 14100 1.75E-05 13500 1.53E-05
14400 3.68E-05 12400 6.74E-05 12200 2.28E-04 11800
4.75E-05 14100 1.76E-05 13500 1.53E-05
14400 3.68E-05 12400 6.72E-05 12200 2.27E-04 11800
4.75E-05 14100 1.77E-05 13500 1.53E-05
14400 3.68E-05 12400 6.69E-05 12200 2.26E-04 11800
4.76E-05 14100 1.77E-05 13500 1.53E-05
14400 3.68E-05 12400 6.67E-05 12200 2.25E-04 11800
4.77E-05 14100 1.78E-05 13500 1.53E-05
14400 3.68E-05 12400 6.64E-05 12200 2.24E-04 11800
4.77E-05 14100 1.78E-05 13500 1.53E-05
14400 3.68E-05 12400 6.62E-05 12200 2.23E-04 11800
4.78E-05 14100 1.79E-05 13500 1.53E-05
14400 3.68E-05 12400 6.59E-05 12200 2.22E-04 11800
4.78E-05 14100 1.80E-05 13500 1.53E-05
14400 3.68E-05 12400 6.57E-05 12200 2.21E-04 11800
4.79E-05 14100 1.81E-05 13500 1.53E-05
14400 3.68E-05 12400 6.54E-05 12200 2.20E-04 11800
4.79E-05 14100 1.81E-05 13500 1.53E-05
14400 3.68E-05 12400 6.52E-05 12200 2.19E-04 11800
4.80E-05 14100 1.82E-05 13500 1.53E-05
14400 3.68E-05 12400 6.50E-05 12200 2.18E-04 11800
4.80E-05 14100 1.83E-05 13500 1.53E-05
14400 3.68E-05 12400 6.47E-05 12200 2.17E-04 11800
4.81E-05 14100 1.83E-05 13500 1.53E-05
14500 3.68E-05 12400 6.45E-05 12200 2.16E-04 11800
4.81E-05 14100 1.84E-05 13500 1.53E-05
14500 3.68E-05 12400 6.42E-05 12200 2.15E-04 11800
4.82E-05 14100 1.85E-05 13500 1.53E-05
14500 3.68E-05 12400 6.40E-05 12200 2.14E-04 11800
4.82E-05 14100 1.86E-05 13500 1.53E-05
14500 3.68E-05 12400 6.37E-05 12200 2.13E-04 11800
4.83E-05 14100 1.86E-05 13500 1.53E-05
14500 3.68E-05 12400 6.35E-05 12200 2.12E-04 11800
4.84E-05 14100 1.87E-05 13500 1.53E-05
14500 3.68E-05 12400 6.33E-05 12200 2.11E-04 11800
4.84E-05 14100 1.88E-05 13500 1.53E-05
14500 3.68E-05 12400 6.30E-05 12200 2.10E-04 11800
4.85E-05 14100 1.89E-05 13500 1.53E-05
14500 3.68E-05 12400 6.28E-05 12200 2.10E-04 11800
4.85E-05 14100 1.90E-05 13500 1.53E-05
14500 3.68E-05 12400 6.26E-05 12200 2.09E-04 11800
4.86E-05 14100 1.91E-05 13500 1.54E-05
14500 3.68E-05 12400 6.24E-05 12200 2.08E-04 11800
4.87E-05 14100 1.91E-05 13600 1.53E-05
14500 3.68E-05 12400 6.21E-05 12200 2.07E-04 11800
4.87E-05 14100 1.92E-05 13600 1.53E-05
14500 3.68E-05 12400 6.19E-05 12200 2.06E-04 11800
4.88E-05 14100 1.93E-05 13600 1.53E-05
14500 3.68E-05 12400 6.17E-05 12200 2.05E-04 11800
4.89E-05 14100 1.94E-05 13600 1.53E-05
14500 3.68E-05 12400 6.15E-05 12200 2.04E-04 11800
4.89E-05 14100 1.95E-05 13600 1.53E-05
14500 3.68E-05 12400 6.12E-05 12200 2.03E-04 11800
4.90E-05 14100 1.96E-05 13600 1.53E-05

14500 3.68E-05 12400 6.10E-05 12200 2.03E-04 11800
4.90E-05 14100 1.97E-05 13600 1.54E-05
14500 3.68E-05 12400 6.08E-05 12200 2.02E-04 11800
4.91E-05 14100 1.98E-05 13600 1.54E-05
14500 3.68E-05 12400 6.06E-05 12200 2.01E-04 11800
4.92E-05 14100 1.99E-05 13600 1.54E-05
14500 3.68E-05 12500 6.04E-05 12200 2.00E-04 11800
4.93E-05 14100 2.00E-05 13600 1.54E-05
14500 3.68E-05 12500 6.01E-05 12200 1.99E-04 11800
4.93E-05 14100 2.01E-05 13600 1.54E-05
14500 3.68E-05 12500 5.99E-05 12300 1.98E-04 11800
4.94E-05 14100 2.02E-05 13600 1.54E-05
14500 3.68E-05 12500 5.97E-05 12300 1.97E-04 11800
4.95E-05 14100 2.03E-05 13600 1.54E-05
14500 3.68E-05 12500 5.95E-05 12300 1.97E-04 11800
4.95E-05 14100 2.04E-05 13600 1.54E-05
14500 3.68E-05 12500 5.93E-05 12300 1.96E-04 11800
4.96E-05 14100 2.05E-05 13600 1.54E-05
14500 3.68E-05 12500 5.90E-05 12300 1.95E-04 11900
4.97E-05 14100 2.06E-05 13600 1.54E-05
14500 3.68E-05 12500 5.88E-05 12300 1.94E-04 11900
4.98E-05 14100 2.08E-05 13600 1.54E-05
14500 3.68E-05 12500 5.86E-05 12300 1.93E-04 11900
4.98E-05 14100 2.09E-05 13600 1.54E-05
14500 3.68E-05 12500 5.84E-05 12300 1.93E-04 11900
4.99E-05 14100 2.10E-05 13600 1.54E-05
14500 3.68E-05 12500 5.82E-05 12300 1.92E-04 11900
5.00E-05 14100 2.11E-05 13600 1.54E-05
14500 3.68E-05 12500 5.80E-05 12300 1.91E-04 11900
5.01E-05 14100 2.13E-05 13600 1.54E-05
14500 3.68E-05 12500 5.78E-05 12300 1.90E-04 11900
5.01E-05 14100 2.14E-05 13600 1.54E-05
14500 3.68E-05 12500 5.77E-05 12300 1.90E-04 11900
5.02E-05 14100 2.15E-05 13600 1.54E-05
14500 3.68E-05 12500 5.74E-05 12300 1.89E-04 11900
5.03E-05 14100 2.16E-05 13600 1.54E-05
14500 3.68E-05 12500 5.73E-05 12300 1.88E-04 11900
5.03E-05 14100 2.18E-05 13600 1.54E-05
14500 3.68E-05 12500 5.71E-05 12300 1.87E-04 11900
5.04E-05 14100 2.19E-05 13600 1.54E-05
14500 3.68E-05 12500 5.69E-05 12300 1.87E-04 11900
5.05E-05 14200 2.21E-05 13600 1.54E-05
14500 3.68E-05 12500 5.67E-05 12300 1.86E-04 11900
5.06E-05 14200 2.22E-05 13600 1.54E-05
14500 3.68E-05 12500 5.65E-05 12300 1.85E-04 11900
5.06E-05 14200 2.23E-05 13600 1.54E-05
14500 3.68E-05 12500 5.63E-05 12300 1.84E-04 11900
5.07E-05 14200 2.25E-05 13600 1.54E-05
14500 3.68E-05 12500 5.61E-05 12300 1.84E-04 11900
5.08E-05 14200 2.27E-05 13600 1.54E-05
14500 3.68E-05 12500 5.59E-05 12300 1.83E-04 11900
5.09E-05 14200 2.28E-05 13600 1.54E-05
14500 3.68E-05 12500 5.57E-05 12300 1.82E-04 11900
5.10E-05 14200 2.30E-05 13600 1.54E-05
14500 3.69E-05 12500 5.55E-05 12300 1.81E-04 11900
5.11E-05 14200 2.31E-05 13600 1.54E-05
14500 3.68E-05 12500 5.53E-05 12300 1.81E-04 11900
5.11E-05 14200 2.33E-05 13600 1.54E-05
14500 3.69E-05 12500 5.51E-05 12300 1.80E-04 11900
5.12E-05 14200 2.35E-05 13600 1.54E-05
14500 3.68E-05 12500 5.50E-05 12300 1.79E-04 11900
5.13E-05 14200 2.36E-05 13600 1.55E-05
14500 3.68E-05 12500 5.48E-05 12300 1.79E-04 11900
5.14E-05 14200 2.38E-05 13600 1.55E-05
14500 3.68E-05 12500 5.46E-05 12300 1.78E-04 11900
5.15E-05 14200 2.40E-05 13600 1.55E-05
14500 3.68E-05 12500 5.44E-05 12300 1.77E-04 11900
5.16E-05 14200 2.42E-05 13600 1.55E-05

14500 3.68E-05 12500 5.42E-05 12300 1.77E-04 11900
5.17E-05 14200 2.44E-05 13600 1.55E-05
14500 3.68E-05 12500 5.41E-05 12300 1.76E-04 11900
5.18E-05 14200 2.46E-05 13600 1.55E-05
14500 3.68E-05 12500 5.39E-05 12300 1.75E-04 11900
5.19E-05 14200 2.48E-05 13600 1.55E-05
14500 3.68E-05 12500 5.37E-05 12300 1.75E-04 11900
5.20E-05 14200 2.50E-05 13600 1.55E-05
14500 3.68E-05 12500 5.35E-05 12300 1.74E-04 11900
5.21E-05 14200 2.52E-05 13600 1.55E-05
14500 3.68E-05 12500 5.33E-05 12300 1.73E-04 11900
5.22E-05 14200 2.54E-05 13600 1.55E-05
14500 3.68E-05 12500 5.31E-05 12300 1.73E-04 11900
5.23E-05 14200 2.56E-05 13600 1.55E-05
14500 3.68E-05 12500 5.30E-05 12300 1.72E-04 11900
5.24E-05 14200 2.58E-05 13600 1.55E-05
14500 3.68E-05 12500 5.28E-05 12300 1.71E-04 11900
5.25E-05 14200 2.61E-05 13600 1.55E-05
14500 3.68E-05 12500 5.26E-05 12300 1.71E-04 11900
5.26E-05 14200 2.63E-05 13600 1.55E-05
14500 3.69E-05 12500 5.24E-05 12300 1.70E-04 11900
5.27E-05 14200 2.65E-05 13600 1.55E-05
14500 3.68E-05 12500 5.23E-05 12300 1.69E-04 11900
5.28E-05 14200 2.68E-05 13600 1.55E-05
14500 3.69E-05 12500 5.21E-05 12300 1.69E-04 11900
5.29E-05 14200 2.70E-05 13600 1.55E-05
14500 3.69E-05 12500 5.19E-05 12300 1.68E-04 11900
5.30E-05 14200 2.73E-05 13600 1.55E-05
14500 3.69E-05 12500 5.18E-05 12300 1.67E-04 11900
5.31E-05 14200 2.76E-05 13600 1.55E-05
14600 3.69E-05 12500 5.16E-05 12300 1.67E-04 11900
5.33E-05 14200 2.78E-05 13600 1.55E-05
14600 3.68E-05 12500 5.14E-05 12300 1.66E-04 11900
5.33E-05 14200 2.81E-05 13600 1.55E-05
14600 3.68E-05 12500 5.13E-05 12300 1.66E-04 11900
5.35E-05 14200 2.84E-05 13600 1.55E-05
14600 3.68E-05 12500 5.11E-05 12300 1.65E-04 11900
5.36E-05 14200 2.87E-05 13600 1.55E-05
14600 3.69E-05 12500 5.10E-05 12300 1.64E-04 11900
5.37E-05 14200 2.90E-05 13600 1.55E-05
14600 3.69E-05 12500 5.08E-05 12300 1.64E-04 11900
5.38E-05 14200 2.93E-05 13600 1.55E-05
14600 3.69E-05 12500 5.07E-05 12300 1.63E-04 11900
5.39E-05 14200 2.96E-05 13600 1.55E-05
14600 3.69E-05 12500 5.05E-05 12300 1.62E-04 11900
5.40E-05 14200 2.99E-05 13600 1.56E-05
14600 3.69E-05 12500 5.03E-05 12300 1.62E-04 11900
5.41E-05 14200 3.03E-05 13600 1.56E-05
14600 3.69E-05 12500 5.02E-05 12300 1.61E-04 11900
5.42E-05 14200 3.06E-05 13700 1.56E-05
14600 3.69E-05 12500 5.00E-05 12300 1.61E-04 11900
5.44E-05 14200 3.10E-05 13700 1.56E-05
14600 3.69E-05 12500 4.99E-05 12300 1.60E-04 11900
5.45E-05 14200 3.13E-05 13700 1.56E-05
14600 3.69E-05 12500 4.97E-05 12300 1.60E-04 11900
5.46E-05 14200 3.17E-05 13700 1.56E-05
14600 3.69E-05 12500 4.95E-05 12300 1.59E-04 11900
5.47E-05 14200 3.21E-05 13700 1.56E-05
14600 3.69E-05 12500 4.94E-05 12300 1.58E-04 11900
5.48E-05 14200 3.25E-05 13700 1.56E-05
14600 3.69E-05 12500 4.92E-05 12300 1.58E-04 11900
5.49E-05 14200 3.29E-05 13700 1.56E-05
14600 3.70E-05 12500 4.91E-05 12300 1.57E-04 11900
5.51E-05 14200 3.33E-05 13700 1.56E-05
14600 3.70E-05 12500 4.89E-05 12300 1.57E-04 11900
5.52E-05 14200 3.37E-05 13700 1.56E-05
14600 3.70E-05 12600 4.88E-05 12300 1.56E-04 11900
5.53E-05 14200 3.42E-05 13700 1.56E-05

14600 3.69E-05 12600 4.86E-05 12300 1.56E-04 11900
5.54E-05 14200 3.46E-05 13700 1.56E-05
14600 3.69E-05 12600 4.85E-05 12400 1.55E-04 11900
5.55E-05 14200 3.51E-05 13700 1.56E-05
14600 3.69E-05 12600 4.83E-05 12400 1.54E-04 11900
5.56E-05 14200 3.55E-05 13700 1.56E-05
14600 3.69E-05 12600 4.81E-05 12400 1.54E-04 11900
5.58E-05 14200 3.60E-05 13700 1.56E-05
14600 3.69E-05 12600 4.80E-05 12400 1.53E-04 11900
5.59E-05 14200 3.65E-05 13700 1.56E-05
14600 3.69E-05 12600 4.78E-05 12400 1.53E-04 12000
5.60E-05 14200 3.71E-05 13700 1.56E-05
14600 3.70E-05 12600 4.77E-05 12400 1.52E-04 12000
5.61E-05 14200 3.76E-05 13700 1.56E-05
14600 3.70E-05 12600 4.76E-05 12400 1.52E-04 12000
5.62E-05 14200 3.82E-05 13700 1.56E-05
14600 3.70E-05 12600 4.74E-05 12400 1.51E-04 12000
5.64E-05 14200 3.87E-05 13700 1.57E-05
14600 3.70E-05 12600 4.72E-05 12400 1.51E-04 12000
5.65E-05 14200 3.93E-05 13700 1.57E-05
14600 3.70E-05 12600 4.71E-05 12400 1.50E-04 12000
5.66E-05 14200 3.99E-05 13700 1.57E-05
14600 3.70E-05 12600 4.70E-05 12400 1.50E-04 12000
5.68E-05 14200 4.05E-05 13700 1.57E-05
14600 3.70E-05 12600 4.68E-05 12400 1.49E-04 12000
5.69E-05 14200 4.12E-05 13700 1.57E-05
14600 3.70E-05 12600 4.67E-05 12400 1.49E-04 12000
5.71E-05 14200 4.19E-05 13700 1.57E-05
14600 3.70E-05 12600 4.65E-05 12400 1.48E-04 12000
5.72E-05 14200 4.25E-05 13700 1.57E-05
14600 3.70E-05 12600 4.64E-05 12400 1.48E-04 12000
5.73E-05 14200 4.33E-05 13700 1.57E-05
14600 3.70E-05 12600 4.63E-05 12400 1.47E-04 12000
5.75E-05 14300 4.40E-05 13700 1.57E-05
14600 3.70E-05 12600 4.61E-05 12400 1.47E-04 12000
5.76E-05 14300 4.47E-05 13700 1.57E-05
14600 3.70E-05 12600 4.60E-05 12400 1.46E-04 12000
5.77E-05 14300 4.55E-05 13700 1.57E-05
14600 3.70E-05 12600 4.58E-05 12400 1.46E-04 12000
5.79E-05 14300 4.63E-05 13700 1.57E-05
14600 3.70E-05 12600 4.57E-05 12400 1.45E-04 12000
5.80E-05 14300 4.72E-05 13700 1.57E-05
14600 3.70E-05 12600 4.56E-05 12400 1.45E-04 12000
5.82E-05 14300 4.80E-05 13700 1.57E-05
14600 3.70E-05 12600 4.54E-05 12400 1.44E-04 12000
5.83E-05 14300 4.89E-05 13700 1.57E-05
14600 3.69E-05 12600 4.53E-05 12400 1.44E-04 12000
5.85E-05 14300 4.98E-05 13700 1.57E-05
14600 3.70E-05 12600 4.51E-05 12400 1.43E-04 12000
5.86E-05 14300 5.08E-05 13700 1.57E-05
14600 3.70E-05 12600 4.50E-05 12400 1.43E-04 12000
5.88E-05 14300 5.18E-05 13700 1.57E-05
14600 3.70E-05 12600 4.49E-05 12400 1.42E-04 12000
5.89E-05 14300 5.28E-05 13700 1.57E-05
14600 3.70E-05 12600 4.47E-05 12400 1.42E-04 12000
5.91E-05 14300 5.38E-05 13700 1.57E-05
14600 3.70E-05 12600 4.46E-05 12400 1.41E-04 12000
5.93E-05 14300 5.49E-05 13700 1.57E-05
14600 3.70E-05 12600 4.45E-05 12400 1.41E-04 12000
5.94E-05 14300 5.61E-05 13700 1.57E-05
14600 3.70E-05 12600 4.43E-05 12400 1.40E-04 12000
5.96E-05 14300 5.72E-05 13700 1.57E-05
14600 3.70E-05 12600 4.42E-05 12400 1.40E-04 12000
5.97E-05 14300 5.85E-05 13700 1.57E-05
14600 3.70E-05 12600 4.40E-05 12400 1.39E-04 12000
5.99E-05 14300 5.97E-05 13700 1.57E-05
14600 3.70E-05 12600 4.39E-05 12400 1.39E-04 12000
6.00E-05 14300 6.10E-05 13700 1.57E-05

14600 3.70E-05 12600 4.37E-05 12400 1.38E-04 12000
6.02E-05 14300 6.24E-05 13700 1.57E-05
14600 3.70E-05 12600 4.36E-05 12400 1.38E-04 12000
6.03E-05 14300 6.38E-05 13700 1.57E-05
14600 3.70E-05 12600 4.35E-05 12400 1.37E-04 12000
6.05E-05 14300 6.53E-05 13700 1.57E-05
14600 3.70E-05 12600 4.33E-05 12400 1.37E-04 12000
6.07E-05 14300 6.68E-05 13700 1.57E-05
14600 3.70E-05 12600 4.32E-05 12400 1.37E-04 12000
6.08E-05 14300 6.83E-05 13700 1.57E-05
14600 3.70E-05 12600 4.30E-05 12400 1.36E-04 12000
6.10E-05 14300 7.00E-05 13700 1.57E-05
14600 3.70E-05 12600 4.29E-05 12400 1.36E-04 12000
6.12E-05 14300 7.17E-05 13700 1.57E-05
14600 3.70E-05 12600 4.28E-05 12400 1.35E-04 12000
6.14E-05 14300 7.34E-05 13700 1.57E-05
14600 3.70E-05 12600 4.27E-05 12400 1.35E-04 12000
6.15E-05 14300 7.53E-05 13700 1.58E-05
14600 3.70E-05 12600 4.25E-05 12400 1.34E-04 12000
6.17E-05 14300 7.72E-05 13700 1.58E-05
14600 3.70E-05 12600 4.24E-05 12400 1.34E-04 12000
6.19E-05 14300 7.91E-05 13700 1.58E-05
14700 3.70E-05 12600 4.23E-05 12400 1.33E-04 12000
6.21E-05 14300 8.12E-05 13700 1.58E-05
14700 3.70E-05 12600 4.21E-05 12400 1.33E-04 12000
6.22E-05 14300 8.33E-05 13700 1.58E-05
14700 3.71E-05 12600 4.21E-05 12400 1.33E-04 12000
6.24E-05 14300 8.55E-05 13700 1.58E-05
14700 3.71E-05 12600 4.19E-05 12400 1.32E-04 12000
6.26E-05 14300 8.79E-05 13700 1.58E-05
14700 3.71E-05 12600 4.18E-05 12400 1.32E-04 12000
6.28E-05 14300 9.03E-05 13700 1.58E-05
14700 3.71E-05 12600 4.17E-05 12400 1.31E-04 12000
6.29E-05 14300 9.28E-05 13700 1.58E-05
14700 3.71E-05 12600 4.16E-05 12400 1.31E-04 12000
6.31E-05 14300 9.54E-05 13700 1.58E-05
14700 3.71E-05 12600 4.15E-05 12400 1.31E-04 12000
6.33E-05 14300 9.81E-05 13700 1.58E-05
14700 3.71E-05 12600 4.13E-05 12400 1.30E-04 12000
6.35E-05 14300 0.000101 13700 1.58E-05
14700 3.71E-05 12600 4.12E-05 12400 1.30E-04 12000
6.36E-05 14300 0.000104 13800 1.58E-05
14700 3.71E-05 12600 4.11E-05 12400 1.29E-04 12000
6.38E-05 14300 0.000107 13800 1.58E-05
14700 3.71E-05 12600 4.11E-05 12400 1.29E-04 12000
6.40E-05 14300 0.00011 13800 1.58E-05
14700 3.71E-05 12600 4.09E-05 12400 1.28E-04 12000
6.42E-05 14300 0.000113 13800 1.58E-05
14700 3.71E-05 12600 4.08E-05 12400 1.28E-04 12000
6.44E-05 14300 0.000117 13800 1.58E-05
14700 3.71E-05 12600 4.07E-05 12400 1.28E-04 12000
6.46E-05 14300 0.00012 13800 1.58E-05
14700 3.71E-05 12600 4.06E-05 12400 1.27E-04 12000
6.48E-05 14300 0.000124 13800 1.58E-05
14700 3.71E-05 12600 4.05E-05 12400 1.27E-04 12000
6.50E-05 14300 0.000128 13800 1.58E-05
14700 3.71E-05 12600 4.04E-05 12400 1.27E-04 12000
6.51E-05 14300 0.000132 13800 1.58E-05
14700 3.71E-05 12700 4.03E-05 12400 1.26E-04 12000
6.54E-05 14300 0.000136 13800 1.58E-05
14700 3.71E-05 12700 4.02E-05 12400 1.26E-04 12000
6.56E-05 14300 0.000141 13800 1.58E-05
14700 3.71E-05 12700 4.01E-05 12500 1.25E-04 12000
6.58E-05 14300 0.000145 13800 1.58E-05
14700 3.71E-05 12700 3.99E-05 12500 1.25E-04 12000
6.60E-05 14300 0.00015 13800 1.58E-05
14700 3.71E-05 12700 3.98E-05 12500 1.25E-04 12000
6.62E-05 14300 0.000155 13800 1.58E-05

14700 3.71E-05 12700 3.97E-05 12500 1.24E-04 12000
6.64E-05 14300 0.00016 13800 1.58E-05
14700 3.71E-05 12700 3.96E-05 12500 1.24E-04 12100
6.66E-05 14300 0.000165 13800 1.58E-05
14700 3.71E-05 12700 3.95E-05 12500 1.23E-04 12100
6.68E-05 14300 0.000171 13800 1.58E-05
14700 3.72E-05 12700 3.94E-05 12500 1.23E-04 12100
6.70E-05 14300 0.000176 13800 1.58E-05
14700 3.72E-05 12700 3.93E-05 12500 1.23E-04 12100
6.72E-05 14300 0.000182 13800 1.58E-05
14700 3.72E-05 12700 3.92E-05 12500 1.22E-04 12100
6.74E-05 14300 0.000188 13800 1.58E-05
14700 3.72E-05 12700 3.91E-05 12500 1.22E-04 12100
6.76E-05 14300 0.000195 13800 1.58E-05
14700 3.72E-05 12700 3.90E-05 12500 1.22E-04 12100
6.78E-05 14300 0.000201 13800 1.58E-05
14700 3.72E-05 12700 3.89E-05 12500 1.21E-04 12100
6.81E-05 14300 0.000208 13800 1.58E-05
14700 3.72E-05 12700 3.88E-05 12500 1.21E-04 12100
6.83E-05 14300 0.000215 13800 1.58E-05
14700 3.72E-05 12700 3.87E-05 12500 1.21E-04 12100
6.85E-05 14300 0.000222 13800 1.58E-05
14700 3.72E-05 12700 3.86E-05 12500 1.20E-04 12100
6.88E-05 14300 0.000229 13800 1.59E-05
14700 3.72E-05 12700 3.85E-05 12500 1.20E-04 12100
6.90E-05 14400 0.000236 13800 1.58E-05
14700 3.72E-05 12700 3.84E-05 12500 1.20E-04 12100
6.92E-05 14400 0.000243 13800 1.58E-05
14700 3.72E-05 12700 3.83E-05 12500 1.19E-04 12100
6.94E-05 14400 0.000251 13800 1.59E-05
14700 3.72E-05 12700 3.82E-05 12500 1.19E-04 12100
6.97E-05 14400 0.000259 13800 1.59E-05
14700 3.72E-05 12700 3.81E-05 12500 1.19E-04 12100
6.99E-05 14400 0.000266 13800 1.59E-05
14700 3.72E-05 12700 3.80E-05 12500 1.18E-04 12100
7.02E-05 14400 0.000274 13800 1.59E-05
14700 3.73E-05 12700 3.79E-05 12500 1.18E-04 12100
7.04E-05 14400 0.000282 13800 1.59E-05
14700 3.73E-05 12700 3.78E-05 12500 1.18E-04 12100
7.06E-05 14400 0.00029 13800 1.59E-05
14700 3.73E-05 12700 3.77E-05 12500 1.17E-04 12100
7.09E-05 14400 0.000298 13800 1.59E-05
14700 3.73E-05 12700 3.76E-05 12500 1.17E-04 12100
7.11E-05 14400 0.000305 13800 1.59E-05
14700 3.73E-05 12700 3.75E-05 12500 1.17E-04 12100
7.14E-05 14400 0.000313 13800 1.59E-05
14700 3.73E-05 12700 3.74E-05 12500 1.16E-04 12100
7.16E-05 14400 0.00032 13800 1.59E-05
14700 3.73E-05 12700 3.73E-05 12500 1.16E-04 12100
7.19E-05 14400 0.000327 13800 1.59E-05
14700 3.73E-05 12700 3.72E-05 12500 1.16E-04 12100
7.21E-05 14400 0.000334 13800 1.59E-05
14700 3.73E-05 12700 3.71E-05 12500 1.15E-04 12100
7.24E-05 14400 0.000341 13800 1.59E-05
14700 3.73E-05 12700 3.70E-05 12500 1.15E-04 12100
7.26E-05 14400 0.000347 13800 1.59E-05
14700 3.73E-05 12700 3.69E-05 12500 1.15E-04 12100
7.29E-05 14400 0.000353 13800 1.59E-05
14700 3.73E-05 12700 3.68E-05 12500 1.15E-04 12100
7.32E-05 14400 0.000358 13800 1.59E-05
14700 3.73E-05 12700 3.67E-05 12500 1.14E-04 12100
7.34E-05 14400 0.000363 13800 1.59E-05
14700 3.73E-05 12700 3.67E-05 12500 1.14E-04 12100
7.37E-05 14400 0.000367 13800 1.59E-05
14700 3.73E-05 12700 3.66E-05 12500 1.14E-04 12100
7.40E-05 14400 0.000371 13800 1.59E-05
14700 3.73E-05 12700 3.65E-05 12500 1.14E-04 12100
7.43E-05 14400 0.000374 13800 1.59E-05

14700 3.73E-05 12700 3.64E-05 12500 1.13E-04 12100
7.45E-05 14400 0.000376 13800 1.59E-05
14700 3.73E-05 12700 3.63E-05 12500 1.13E-04 12100
7.48E-05 14400 0.000378 13800 1.59E-05
14700 3.73E-05 12700 3.62E-05 12500 1.13E-04 12100
7.51E-05 14400 0.000379 13800 1.59E-05
14700 3.73E-05 12700 3.61E-05 12500 1.12E-04 12100
7.54E-05 14400 0.000379 13800 1.59E-05
14700 3.73E-05 12700 3.60E-05 12500 1.12E-04 12100
7.57E-05 14400 0.000378 13800 1.59E-05
14700 3.73E-05 12700 3.59E-05 12500 1.12E-04 12100
7.60E-05 14400 0.000376 13800 1.59E-05
14700 3.73E-05 12700 3.58E-05 12500 1.12E-04 12100
7.62E-05 14400 0.000374 13800 1.59E-05
14800 3.73E-05 12700 3.57E-05 12500 1.12E-04 12100
7.65E-05 14400 0.000371 13800 1.59E-05
14800 3.73E-05 12700 3.56E-05 12500 1.11E-04 12100
7.68E-05 14400 0.000367 13800 1.59E-05
14800 3.73E-05 12700 3.55E-05 12500 1.11E-04 12100
7.71E-05 14400 0.000363 13800 1.59E-05
14800 3.73E-05 12700 3.54E-05 12500 1.11E-04 12100
7.74E-05 14400 0.000358 13800 1.60E-05
14800 3.73E-05 12700 3.54E-05 12500 1.11E-04 12100
7.77E-05 14400 0.000352 13800 1.60E-05
14800 3.73E-05 12700 3.53E-05 12500 1.10E-04 12100
7.80E-05 14400 0.000346 13800 1.60E-05
14800 3.73E-05 12700 3.52E-05 12500 1.10E-04 12100
7.83E-05 14400 0.000339 13800 1.60E-05
14800 3.73E-05 12700 3.51E-05 12500 1.10E-04 12100
7.86E-05 14400 0.000332 13800 1.60E-05
14800 3.73E-05 12700 3.50E-05 12500 1.10E-04 12100
7.89E-05 14400 0.000324 13800 1.60E-05
14800 3.73E-05 12700 3.49E-05 12500 1.10E-04 12100
7.92E-05 14400 0.000316 13900 1.60E-05
14800 3.73E-05 12700 3.48E-05 12500 1.10E-04 12100
7.96E-05 14400 0.000307 13900 1.60E-05
14800 3.73E-05 12700 3.47E-05 12500 1.09E-04 12100
7.99E-05 14400 0.000299 13900 1.60E-05
14800 3.73E-05 12700 3.46E-05 12500 1.09E-04 12100
8.02E-05 14400 0.00029 13900 1.60E-05
14800 3.73E-05 12700 3.46E-05 12500 1.09E-04 12100
8.06E-05 14400 0.000281 13900 1.60E-05
14800 3.73E-05 12700 3.45E-05 12500 1.09E-04 12100
8.09E-05 14400 0.000272 13900 1.60E-05
14800 3.74E-05 12700 3.44E-05 12500 1.09E-04 12100
8.12E-05 14400 0.000263 13900 1.60E-05
14800 3.74E-05 12700 3.43E-05 12500 1.09E-04 12100
8.15E-05 14400 0.000254 13900 1.60E-05
14800 3.74E-05 12700 3.42E-05 12500 1.09E-04 12100
8.19E-05 14400 0.000246 13900 1.60E-05
14800 3.74E-05 12800 3.41E-05 12500 1.09E-04 12100
8.22E-05 14400 0.000237 13900 1.60E-05
14800 3.74E-05 12800 3.41E-05 12500 1.08E-04 12100
8.26E-05 14400 0.000228 13900 1.60E-05
14800 3.74E-05 12800 3.40E-05 12600 1.08E-04 12100
8.29E-05 14400 0.00022 13900 1.60E-05
14800 3.74E-05 12800 3.39E-05 12600 1.08E-04 12100
8.32E-05 14400 0.000212 13900 1.60E-05
14800 3.74E-05 12800 3.38E-05 12600 1.08E-04 12100
8.36E-05 14400 0.000204 13900 1.60E-05
14800 3.74E-05 12800 3.38E-05 12600 1.08E-04 12100
8.39E-05 14400 0.000196 13900 1.60E-05
14800 3.74E-05 12800 3.37E-05 12600 1.08E-04 12200
8.43E-05 14400 0.000188 13900 1.60E-05
14800 3.74E-05 12800 3.36E-05 12600 1.08E-04 12200
8.46E-05 14400 0.000181 13900 1.60E-05
14800 3.74E-05 12800 3.35E-05 12600 1.08E-04 12200
8.50E-05 14400 0.000174 13900 1.60E-05

14800 3.74E-05 12800 3.34E-05 12600 1.08E-04 12200
8.53E-05 14400 0.000167 13900 1.60E-05
14800 3.74E-05 12800 3.33E-05 12600 1.08E-04 12200
8.57E-05 14400 0.00016 13900 1.60E-05
14800 3.75E-05 12800 3.33E-05 12600 1.09E-04 12200
8.61E-05 14400 0.000154 13900 1.60E-05
14800 3.74E-05 12800 3.32E-05 12600 1.09E-04 12200
8.65E-05 14400 0.000148 13900 1.61E-05
14800 3.74E-05 12800 3.31E-05 12600 1.09E-04 12200
8.69E-05 14400 0.000142 13900 1.61E-05
14800 3.74E-05 12800 3.30E-05 12600 1.09E-04 12200
8.73E-05 14400 0.000137 13900 1.61E-05
14800 3.74E-05 12800 3.29E-05 12600 1.09E-04 12200
8.77E-05 14400 0.000132 13900 1.61E-05
14800 3.75E-05 12800 3.29E-05 12600 1.09E-04 12200
8.81E-05 14400 0.000127 13900 1.61E-05
14800 3.74E-05 12800 3.28E-05 12600 1.10E-04 12200
8.85E-05 14500 0.000122 13900 1.61E-05
14800 3.74E-05 12800 3.27E-05 12600 1.10E-04 12200
8.89E-05 14500 0.000117 13900 1.61E-05
14800 3.75E-05 12800 3.26E-05 12600 1.10E-04 12200
8.93E-05 14500 0.000113 13900 1.61E-05
14800 3.75E-05 12800 3.25E-05 12600 1.10E-04 12200
8.97E-05 14500 0.000109 13900 1.61E-05
14800 3.75E-05 12800 3.24E-05 12600 1.11E-04 12200
9.01E-05 14500 0.000105 13900 1.61E-05
14800 3.75E-05 12800 3.24E-05 12600 1.11E-04 12200
9.05E-05 14500 0.000101 13900 1.61E-05
14800 3.75E-05 12800 3.23E-05 12600 1.11E-04 12200
9.09E-05 14500 9.77E-05 13900 1.61E-05
14800 3.75E-05 12800 3.22E-05 12600 1.12E-04 12200
9.14E-05 14500 9.43E-05 13900 1.61E-05
14800 3.75E-05 12800 3.21E-05 12600 1.12E-04 12200
9.18E-05 14500 9.11E-05 13900 1.61E-05
14800 3.75E-05 12800 3.20E-05 12600 1.12E-04 12200
9.22E-05 14500 8.81E-05 13900 1.61E-05
14800 3.75E-05 12800 3.20E-05 12600 1.13E-04 12200
9.27E-05 14500 8.52E-05 13900 1.61E-05
14800 3.75E-05 12800 3.19E-05 12600 1.13E-04 12200
9.31E-05 14500 8.25E-05 13900 1.61E-05
14800 3.75E-05 12800 3.18E-05 12600 1.13E-04 12200
9.35E-05 14500 7.98E-05 13900 1.61E-05
14800 3.75E-05 12800 3.17E-05 12600 1.14E-04 12200
9.40E-05 14500 7.74E-05 13900 1.61E-05
14800 3.75E-05 12800 3.17E-05 12600 1.14E-04 12200
9.44E-05 14500 7.50E-05 13900 1.61E-05
14800 3.75E-05 12800 3.16E-05 12600 1.14E-04 12200
9.49E-05 14500 7.28E-05 13900 1.61E-05
14800 3.75E-05 12800 3.15E-05 12600 1.15E-04 12200
9.53E-05 14500 7.06E-05 13900 1.61E-05
14800 3.76E-05 12800 3.14E-05 12600 1.15E-04 12200
9.57E-05 14500 6.86E-05 13900 1.61E-05
14800 3.76E-05 12800 3.14E-05 12600 1.15E-04 12200
9.62E-05 14500 6.67E-05 13900 1.61E-05
14800 3.76E-05 12800 3.13E-05 12600 1.15E-04 12200
9.67E-05 14500 6.48E-05 13900 1.61E-05
14800 3.76E-05 12800 3.12E-05 12600 1.15E-04 12200
9.71E-05 14500 6.31E-05 13900 1.61E-05
14800 3.76E-05 12800 3.12E-05 12600 1.15E-04 12200
9.76E-05 14500 6.14E-05 13900 1.61E-05
14800 3.76E-05 12800 3.11E-05 12600 1.15E-04 12200
9.81E-05 14500 5.98E-05 13900 1.61E-05
14800 3.76E-05 12800 3.11E-05 12600 1.15E-04 12200
9.86E-05 14500 5.83E-05 13900 1.62E-05
14800 3.76E-05 12800 3.10E-05 12600 1.15E-04 12200
9.91E-05 14500 5.68E-05 13900 1.62E-05
14800 3.77E-05 12800 3.09E-05 12600 1.14E-04 12200
9.96E-05 14500 5.55E-05 13900 1.62E-05

14800 3.77E-05 12800 3.09E-05 12600 1.14E-04 12200
0.000100054 14500 5.41E-05 13900 1.62E-05
14800 3.77E-05 12800 3.08E-05 12600 1.14E-04 12200
0.000100547 14500 5.29E-05 13900 1.62E-05
14800 3.77E-05 12800 3.07E-05 12600 1.13E-04 12200
0.000101054 14500 5.17E-05 13900 1.62E-05
14900 3.77E-05 12800 3.07E-05 12600 1.12E-04 12200
0.00010157 14500 5.05E-05 13900 1.62E-05
14900 3.76E-05 12800 3.06E-05 12600 1.12E-04 12200
0.000102075 14500 4.94E-05 13900 1.62E-05
14900 3.76E-05 12800 3.05E-05 12600 1.11E-04 12200
0.000102616 14500 4.84E-05 13900 1.62E-05
14900 3.77E-05 12800 3.05E-05 12600 1.10E-04 12200
0.000103128 14500 4.73E-05 13900 1.62E-05
14900 3.77E-05 12800 3.04E-05 12600 1.09E-04 12200
0.000103654 14500 4.64E-05 13900 1.62E-05
14900 3.77E-05 12800 3.03E-05 12600 1.08E-04 12200
0.000104202 14500 4.55E-05 13900 1.62E-05
14900 3.76E-05 12800 3.03E-05 12600 1.08E-04 12200
0.000104755 14500 4.46E-05 13900 1.62E-05
14900 3.76E-05 12800 3.02E-05 12600 1.07E-04 12200
0.000105303 14500 4.37E-05 13900 1.62E-05
14900 3.77E-05 12800 3.01E-05 12600 1.06E-04 12200
0.000105847 14500 4.29E-05 13900 1.62E-05
14900 3.76E-05 12800 3.00E-05 12600 1.05E-04 12200
0.000106421 14500 4.21E-05 14000 1.62E-05
14900 3.76E-05 12800 3.00E-05 12600 1.04E-04 12200
0.000106997 14500 4.14E-05 14000 1.62E-05
14900 3.76E-05 12800 2.99E-05 12600 1.03E-04 12200
0.000107569 14500 4.07E-05 14000 1.62E-05
14900 3.77E-05 12800 2.98E-05 12600 1.02E-04 12200
0.000108161 14500 4.00E-05 14000 1.62E-05
14900 3.77E-05 12800 2.98E-05 12600 1.01E-04 12200
0.000108742 14500 3.93E-05 14000 1.62E-05
14900 3.77E-05 12800 2.97E-05 12600 1.00E-04 12200
0.000109325 14500 3.86E-05 14000 1.62E-05
14900 3.77E-05 12800 2.96E-05 12600 9.95E-05 12200
0.000109914 14500 3.80E-05 14000 1.62E-05
14900 3.77E-05 12800 2.95E-05 12600 9.87E-05 12200
0.000110512 14500 3.74E-05 14000 1.62E-05
14900 3.77E-05 12800 2.95E-05 12600 9.79E-05 12200
0.000111121 14500 3.68E-05 14000 1.62E-05
14900 3.77E-05 12900 2.94E-05 12600 9.72E-05 12200
0.000111722 14500 3.63E-05 14000 1.62E-05
14900 3.77E-05 12900 2.93E-05 12600 9.65E-05 12200
0.000112338 14500 3.58E-05 14000 1.62E-05
14900 3.76E-05 12900 2.93E-05 12700 9.58E-05 12200
0.000112958 14500 3.52E-05 14000 1.62E-05
14900 3.77E-05 12900 2.92E-05 12700 9.51E-05 12200
0.0001136 14500 3.47E-05 14000 1.62E-05
14900 3.77E-05 12900 2.91E-05 12700 9.44E-05 12200
0.000114233 14500 3.43E-05 14000 1.63E-05
14900 3.77E-05 12900 2.91E-05 12700 9.38E-05 12200
0.000114872 14500 3.38E-05 14000 1.63E-05
14900 3.77E-05 12900 2.90E-05 12700 9.32E-05 12300
0.000115534 14500 3.33E-05 14000 1.63E-05
14900 3.77E-05 12900 2.90E-05 12700 9.26E-05 12300
0.000116201 14500 3.29E-05 14000 1.63E-05
14900 3.77E-05 12900 2.89E-05 12700 9.20E-05 12300
0.000116877 14500 3.25E-05 14000 1.63E-05
14900 3.77E-05 12900 2.88E-05 12700 9.15E-05 12300
0.000117546 14500 3.21E-05 14000 1.63E-05
14900 3.77E-05 12900 2.88E-05 12700 9.10E-05 12300
0.000118246 14500 3.17E-05 14000 1.63E-05
14900 3.77E-05 12900 2.87E-05 12700 9.05E-05 12300
0.000118946 14500 3.13E-05 14000 1.63E-05
14900 3.77E-05 12900 2.86E-05 12700 9.00E-05 12300
0.000119649 14500 3.09E-05 14000 1.63E-05

14900 3.77E-05 12900 2.86E-05 12700 8.95E-05 12300
0.000120345 14500 3.06E-05 14000 1.63E-05
14900 3.77E-05 12900 2.85E-05 12700 8.90E-05 12300
0.000121049 14500 3.02E-05 14000 1.63E-05
14900 3.78E-05 12900 2.85E-05 12700 8.86E-05 12300
0.000121756 14500 2.99E-05 14000 1.63E-05
14900 3.78E-05 12900 2.84E-05 12700 8.82E-05 12300
0.000122466 14500 2.96E-05 14000 1.63E-05
14900 3.78E-05 12900 2.83E-05 12700 8.78E-05 12300
0.000123187 14600 2.93E-05 14000 1.63E-05
14900 3.78E-05 12900 2.83E-05 12700 8.74E-05 12300
0.000123929 14600 2.90E-05 14000 1.63E-05
14900 3.78E-05 12900 2.82E-05 12700 8.70E-05 12300
0.000124663 14600 2.87E-05 14000 1.63E-05
14900 3.78E-05 12900 2.82E-05 12700 8.66E-05 12300
0.000125412 14600 2.84E-05 14000 1.63E-05
14900 3.78E-05 12900 2.81E-05 12700 8.63E-05 12300
0.000126179 14600 2.81E-05 14000 1.63E-05
14900 3.78E-05 12900 2.80E-05 12700 8.59E-05 12300
0.000126957 14600 2.78E-05 14000 1.63E-05
14900 3.78E-05 12900 2.80E-05 12700 8.55E-05 12300
0.000127739 14600 2.76E-05 14000 1.63E-05
14900 3.78E-05 12900 2.79E-05 12700 8.52E-05 12300
0.000128522 14600 2.73E-05 14000 1.63E-05
14900 3.78E-05 12900 2.79E-05 12700 8.49E-05 12300
0.000129341 14600 2.71E-05 14000 1.63E-05
14900 3.78E-05 12900 2.78E-05 12700 8.45E-05 12300
0.000130147 14600 2.68E-05 14000 1.64E-05
14900 3.78E-05 12900 2.77E-05 12700 8.42E-05 12300
0.00013097 14600 2.66E-05 14000 1.64E-05
14900 3.78E-05 12900 2.77E-05 12700 8.39E-05 12300
0.000131793 14600 2.64E-05 14000 1.64E-05
14900 3.78E-05 12900 2.76E-05 12700 8.36E-05 12300
0.000132626 14600 2.61E-05 14000 1.64E-05
14900 3.78E-05 12900 2.76E-05 12700 8.33E-05 12300
0.000133477 14600 2.59E-05 14000 1.64E-05
14900 3.78E-05 12900 2.75E-05 12700 8.30E-05 12300
0.000134345 14600 2.57E-05 14000 1.64E-05
14900 3.78E-05 12900 2.74E-05 12700 8.27E-05 12300
0.0001352 14600 2.55E-05 14000 1.64E-05
14900 3.78E-05 12900 2.74E-05 12700 8.24E-05 12300
0.000136073 14600 2.53E-05 14000 1.64E-05
14900 3.78E-05 12900 2.73E-05 12700 8.22E-05 12300
0.000136941 14600 2.51E-05 14000 1.64E-05
14900 3.79E-05 12900 2.73E-05 12700 8.19E-05 12300
0.000137834 14600 2.49E-05 14000 1.64E-05
14900 3.79E-05 12900 2.72E-05 12700 8.16E-05 12300
0.000138736 14600 2.47E-05 14000 1.64E-05
14900 3.79E-05 12900 2.71E-05 12700 8.13E-05 12300
0.000139659 14600 2.46E-05 14000 1.64E-05
14900 3.79E-05 12900 2.71E-05 12700 8.11E-05 12300
0.000140577 14600 2.44E-05 14000 1.64E-05
14900 3.79E-05 12900 2.70E-05 12700 8.08E-05 12300
0.000141506 14600 2.42E-05 14000 1.64E-05
14900 3.79E-05 12900 2.70E-05 12700 8.06E-05 12300
0.000142453 14600 2.40E-05 14000 1.64E-05
14900 3.79E-05 12900 2.69E-05 12700 8.03E-05 12300
0.000143408 14600 2.39E-05 14000 1.64E-05
14900 3.79E-05 12900 2.69E-05 12700 8.01E-05 12300
0.000144366 14600 2.37E-05 14000 1.64E-05
14900 3.79E-05 12900 2.68E-05 12700 7.98E-05 12300
0.000145333 14600 2.36E-05 14000 1.64E-05
14900 3.79E-05 12900 2.67E-05 12700 7.96E-05 12300
0.000146325 14600 2.34E-05 14000 1.64E-05
14900 3.79E-05 12900 2.67E-05 12700 7.94E-05 12300
0.00014732 14600 2.33E-05 14000 1.64E-05
15000 3.79E-05 12900 2.66E-05 12700 7.91E-05 12300
0.000148335 14600 2.31E-05 14000 1.64E-05

15000 3.79E-05 12900 2.66E-05 12700 7.89E-05 12300
0.000149339 14600 2.30E-05 14000 1.64E-05
15000 3.79E-05 12900 2.65E-05 12700 7.87E-05 12300
0.000150366 14600 2.28E-05 14000 1.64E-05
15000 3.79E-05 12900 2.65E-05 12700 7.84E-05 12300
0.000151413 14600 2.27E-05 14000 1.64E-05
15000 3.79E-05 12900 2.64E-05 12700 7.82E-05 12300
0.000152473 14600 2.26E-05 14000 1.64E-05
15000 3.79E-05 12900 2.64E-05 12700 7.80E-05 12300
0.00015354 14600 2.25E-05 14000 1.64E-05
15000 3.79E-05 12900 2.63E-05 12700 7.77E-05 12300
0.000154618 14600 2.23E-05 14000 1.64E-05
15000 3.79E-05 12900 2.62E-05 12700 7.75E-05 12300
0.000155697 14600 2.22E-05 14000 1.64E-05
15000 3.80E-05 12900 2.62E-05 12700 7.73E-05 12300
0.000156816 14600 2.21E-05 14000 1.64E-05
15000 3.80E-05 12900 2.61E-05 12700 7.71E-05 12300
0.000157922 14600 2.20E-05 14100 1.65E-05
15000 3.79E-05 12900 2.61E-05 12700 7.69E-05 12300
0.000159063 14600 2.19E-05 14100 1.65E-05
15000 3.80E-05 12900 2.60E-05 12700 7.67E-05 12300
0.000160202 14600 2.17E-05 14100 1.65E-05
15000 3.80E-05 12900 2.60E-05 12700 7.65E-05 12300
0.000161362 14600 2.16E-05 14100 1.65E-05
15000 3.80E-05 12900 2.59E-05 12700 7.63E-05 12300
0.00016255 14600 2.15E-05 14100 1.65E-05
15000 3.80E-05 12900 2.59E-05 12700 7.61E-05 12300
0.000163732 14600 2.14E-05 14100 1.65E-05
15000 3.80E-05 12900 2.58E-05 12700 7.59E-05 12300
0.000164928 14600 2.13E-05 14100 1.65E-05
15000 3.80E-05 12900 2.58E-05 12700 7.56E-05 12300
0.000166148 14600 2.12E-05 14100 1.65E-05
15000 3.80E-05 12900 2.57E-05 12700 7.54E-05 12300
0.000167387 14600 2.11E-05 14100 1.65E-05
15000 3.80E-05 13000 2.57E-05 12700 7.52E-05 12300
0.000168643 14600 2.10E-05 14100 1.65E-05
15000 3.80E-05 13000 2.56E-05 12700 7.50E-05 12300
0.00016991 14600 2.09E-05 14100 1.65E-05
15000 3.81E-05 13000 2.56E-05 12800 7.49E-05 12300
0.000171209 14600 2.08E-05 14100 1.65E-05
15000 3.81E-05 13000 2.55E-05 12800 7.47E-05 12300
0.000172541 14600 2.07E-05 14100 1.65E-05
15000 3.81E-05 13000 2.55E-05 12800 7.45E-05 12300
0.000173851 14600 2.06E-05 14100 1.65E-05
15000 3.81E-05 13000 2.54E-05 12800 7.43E-05 12300
0.000175208 14600 2.05E-05 14100 1.65E-05
15000 3.81E-05 13000 2.54E-05 12800 7.41E-05 12400
0.000176568 14600 2.05E-05 14100 1.65E-05
15000 3.81E-05 13000 2.53E-05 12800 7.39E-05 12400
0.000177946 14600 2.04E-05 14100 1.65E-05
15000 3.81E-05 13000 2.53E-05 12800 7.37E-05 12400
0.000179337 14600 2.03E-05 14100 1.65E-05
15000 3.81E-05 13000 2.52E-05 12800 7.35E-05 12400
0.000180759 14600 2.02E-05 14100 1.65E-05
15000 3.81E-05 13000 2.52E-05 12800 7.33E-05 12400
0.000182197 14600 2.01E-05 14100 1.65E-05
15000 3.81E-05 13000 2.51E-05 12800 7.31E-05 12400
0.000183648 14600 2.00E-05 14100 1.65E-05
15000 3.81E-05 13000 2.51E-05 12800 7.30E-05 12400
0.000185126 14600 2.00E-05 14100 1.65E-05
15000 3.81E-05 13000 2.50E-05 12800 7.28E-05 12400
0.00018661 14600 1.99E-05 14100 1.65E-05
15000 3.81E-05 13000 2.50E-05 12800 7.26E-05 12400
0.000188139 14600 1.98E-05 14100 1.65E-05
15000 3.81E-05 13000 2.49E-05 12800 7.24E-05 12400
0.000189668 14600 1.97E-05 14100 1.65E-05
15000 3.81E-05 13000 2.49E-05 12800 7.22E-05 12400
0.000191214 14600 1.97E-05 14100 1.65E-05

15000 3.81E-05 13000 2.48E-05 12800 7.21E-05 12400
0.000192795 14700 1.96E-05 14100 1.65E-05
15000 3.81E-05 13000 2.48E-05 12800 7.19E-05 12400
0.000194376 14700 1.95E-05 14100 1.65E-05
15000 3.81E-05 13000 2.47E-05 12800 7.17E-05 12400
0.000196002 14700 1.94E-05 14100 1.65E-05
15000 3.81E-05 13000 2.47E-05 12800 7.15E-05 12400
0.000197645 14700 1.94E-05 14100 1.65E-05
15000 3.81E-05 13000 2.47E-05 12800 7.14E-05 12400
0.00019931 14700 1.93E-05 14100 1.66E-05
15000 3.81E-05 13000 2.46E-05 12800 7.12E-05 12400
0.000201025 14700 1.93E-05 14100 1.66E-05
15000 3.81E-05 13000 2.46E-05 12800 7.10E-05 12400
0.000202744 14700 1.92E-05 14100 1.66E-05
15000 3.81E-05 13000 2.45E-05 12800 7.08E-05 12400
0.000204503 14700 1.91E-05 14100 1.66E-05
15000 3.81E-05 13000 2.45E-05 12800 7.07E-05 12400
0.000206285 14700 1.91E-05 14100 1.66E-05
15000 3.81E-05 13000 2.44E-05 12800 7.05E-05 12400
0.000208104 14700 1.90E-05 14100 1.66E-05
15000 3.82E-05 13000 2.44E-05 12800 7.03E-05 12400
0.000209928 14700 1.89E-05 14100 1.66E-05
15000 3.82E-05 13000 2.43E-05 12800 7.02E-05 12400
0.000211788 14700 1.89E-05 14100 1.66E-05
15000 3.82E-05 13000 2.43E-05 12800 7.00E-05 12400
0.000213662 14700 1.88E-05 14100 1.66E-05
15000 3.82E-05 13000 2.42E-05 12800 6.98E-05 12400
0.000215571 14700 1.87E-05 14100 1.66E-05
15000 3.82E-05 13000 2.42E-05 12800 6.97E-05 12400
0.000217482 14700 1.87E-05 14100 1.66E-05
15000 3.82E-05 13000 2.41E-05 12800 6.95E-05 12400
0.000219422 14700 1.86E-05 14100 1.66E-05
15000 3.82E-05 13000 2.41E-05 12800 6.93E-05 12400
0.000221417 14700 1.86E-05 14100 1.66E-05
15000 3.82E-05 13000 2.40E-05 12800 6.92E-05 12400
0.000223394 14700 1.85E-05 14100 1.66E-05
15000 3.82E-05 13000 2.40E-05 12800 6.90E-05 12400
0.00022542 14700 1.85E-05 14100 1.66E-05
15000 3.82E-05 13000 2.40E-05 12800 6.88E-05 12400
0.000227478 14700 1.84E-05 14100 1.66E-05
15000 3.82E-05 13000 2.39E-05 12800 6.87E-05 12400
0.000229564 14700 1.84E-05 14100 1.66E-05
15000 3.82E-05 13000 2.39E-05 12800 6.86E-05 12400
0.000231701 14700 1.83E-05 14100 1.66E-05
15000 3.82E-05 13000 2.38E-05 12800 6.84E-05 12400
0.000233855 14700 1.83E-05 14100 1.66E-05
15000 3.82E-05 13000 2.38E-05 12800 6.82E-05 12400
0.000236035 14700 1.82E-05 14100 1.66E-05
15000 3.82E-05 13000 2.37E-05 12800 6.81E-05 12400
0.000238258 14700 1.82E-05 14100 1.66E-05
15000 3.83E-05 13000 2.37E-05 12800 6.79E-05 12400
0.000240491 14700 1.81E-05 14100 1.66E-05
15000 3.83E-05 13000 2.36E-05 12800 6.78E-05 12400
0.000242767 14700 1.81E-05 14100 1.66E-05
15000 3.83E-05 13000 2.36E-05 12800 6.76E-05 12400
0.000245069 14700 1.80E-05 14100 1.66E-05
15000 3.83E-05 13000 2.35E-05 12800 6.74E-05 12400
0.000247388 14700 1.80E-05 14100 1.66E-05
15100 3.83E-05 13000 2.35E-05 12800 6.73E-05 12400
0.000249763 14700 1.80E-05 14100 1.67E-05
15100 3.83E-05 13000 2.35E-05 12800 6.72E-05 12400
0.000252162 14700 1.79E-05 14100 1.67E-05
15100 3.83E-05 13000 2.34E-05 12800 6.70E-05 12400
0.000254586 14700 1.79E-05 14100 1.67E-05
15100 3.83E-05 13000 2.34E-05 12800 6.69E-05 12400
0.000257063 14700 1.78E-05 14100 1.67E-05
15100 3.83E-05 13000 2.33E-05 12800 6.67E-05 12400
0.000259562 14700 1.78E-05 14100 1.67E-05

15100 3.83E-05 13000 2.33E-05 12800 6.66E-05 12400
0.000262109 14700 1.77E-05 14100 1.67E-05
15100 3.83E-05 13000 2.32E-05 12800 6.64E-05 12400
0.000264718 14700 1.77E-05 14100 1.67E-05
15100 3.83E-05 13000 2.32E-05 12800 6.63E-05 12400
0.000267358 14700 1.77E-05 14100 1.67E-05
15100 3.83E-05 13000 2.32E-05 12800 6.61E-05 12400
0.000270046 14700 1.76E-05 14100 1.67E-05
15100 3.83E-05 13000 2.31E-05 12800 6.59E-05 12400
0.000272774 14700 1.76E-05 14200 1.67E-05
15100 3.83E-05 13000 2.31E-05 12800 6.58E-05 12400
0.000275531 14700 1.76E-05 14200 1.67E-05
15100 3.83E-05 13000 2.30E-05 12800 6.57E-05 12400
0.000278349 14700 1.75E-05 14200 1.67E-05
15100 3.83E-05 13000 2.30E-05 12800 6.55E-05 12400
0.000281208 14700 1.75E-05 14200 1.67E-05
15100 3.84E-05 13000 2.29E-05 12800 6.54E-05 12400
0.000284093 14700 1.75E-05 14200 1.67E-05
15100 3.84E-05 13000 2.29E-05 12800 6.52E-05 12400
0.000287037 14700 1.74E-05 14200 1.67E-05
15100 3.84E-05 13000 2.28E-05 12800 6.51E-05 12400
0.000290023 14700 1.74E-05 14200 1.67E-05
15100 3.84E-05 13000 2.28E-05 12800 6.49E-05 12400
0.000293074 14700 1.73E-05 14200 1.67E-05
15100 3.84E-05 13000 2.28E-05 12800 6.48E-05 12400
0.000296162 14700 1.73E-05 14200 1.67E-05
15100 3.84E-05 13100 2.27E-05 12800 6.46E-05 12400
0.000299311 14700 1.73E-05 14200 1.67E-05
15100 3.84E-05 13100 2.27E-05 12800 6.45E-05 12400
0.00030251 14700 1.73E-05 14200 1.67E-05
15100 3.84E-05 13100 2.26E-05 12900 6.44E-05 12400
0.000305786 14700 1.72E-05 14200 1.67E-05
15100 3.84E-05 13100 2.26E-05 12900 6.42E-05 12400
0.000309102 14700 1.72E-05 14200 1.67E-05
15100 3.84E-05 13100 2.26E-05 12900 6.41E-05 12400
0.000312491 14700 1.72E-05 14200 1.67E-05
15100 3.84E-05 13100 2.25E-05 12900 6.40E-05 12400
0.000315931 14700 1.71E-05 14200 1.67E-05
15100 3.84E-05 13100 2.25E-05 12900 6.38E-05 12500
0.000319442 14700 1.71E-05 14200 1.67E-05
15100 3.84E-05 13100 2.24E-05 12900 6.37E-05 12500
0.000323016 14700 1.71E-05 14200 1.67E-05
15100 3.84E-05 13100 2.24E-05 12900 6.35E-05 12500
0.000326642 14700 1.70E-05 14200 1.67E-05
15100 3.84E-05 13100 2.24E-05 12900 6.34E-05 12500
0.000330326 14700 1.70E-05 14200 1.67E-05
15100 3.84E-05 13100 2.23E-05 12900 6.32E-05 12500
0.000334092 14700 1.70E-05 14200 1.67E-05
15100 3.84E-05 13100 2.23E-05 12900 6.31E-05 12500
0.000337921 14700 1.70E-05 14200 1.67E-05
15100 3.85E-05 13100 2.22E-05 12900 6.30E-05 12500
0.000341815 14700 1.69E-05 14200 1.67E-05
15100 3.85E-05 13100 2.22E-05 12900 6.28E-05 12500
0.000345791 14700 1.69E-05 14200 1.68E-05
15100 3.85E-05 13100 2.21E-05 12900 6.27E-05 12500
0.000349854 14700 1.69E-05 14200 1.68E-05
15100 3.85E-05 13100 2.21E-05 12900 6.26E-05 12500
0.00035399 14700 1.69E-05 14200 1.68E-05
15100 3.85E-05 13100 2.21E-05 12900 6.24E-05 12500
0.000358191 14700 1.68E-05 14200 1.68E-05
15100 3.85E-05 13100 2.20E-05 12900 6.23E-05 12500
0.000362497 14800 1.68E-05 14200 1.68E-05
15100 3.85E-05 13100 2.20E-05 12900 6.22E-05 12500
0.000366875 14800 1.68E-05 14200 1.68E-05
15100 3.85E-05 13100 2.19E-05 12900 6.20E-05 12500
0.000371339 14800 1.68E-05 14200 1.68E-05
15100 3.85E-05 13100 2.19E-05 12900 6.19E-05 12500
0.00037587 14800 1.67E-05 14200 1.68E-05

15100	3.86E-05	13100	2.19E-05	12900	6.18E-05	12500	0.000380506	14800	1.67E-05	14200	1.68E-05
15100	3.86E-05	13100	2.18E-05	12900	6.16E-05	12500	0.000385196	14800	1.67E-05	14200	1.68E-05
15100	3.86E-05	13100	2.18E-05	12900	6.15E-05	12500	0.000389975	14800	1.67E-05	14200	1.68E-05
15100	3.86E-05	13100	2.18E-05	12900	6.14E-05	12500	0.000394843	14800	1.66E-05	14200	1.68E-05
15100	3.86E-05	13100	2.17E-05	12900	6.13E-05	12500	0.000399809	14800	1.66E-05	14200	1.68E-05
15100	3.86E-05	13100	2.17E-05	12900	6.12E-05	12500	0.000404869	14800	1.66E-05	14200	1.68E-05
15100	3.86E-05	13100	2.17E-05	12900	6.10E-05	12500	0.000410008	14800	1.66E-05	14200	1.68E-05
15100	3.86E-05	13100	2.16E-05	12900	6.09E-05	12500	0.00041526	14800	1.65E-05	14200	1.68E-05
15100	3.86E-05	13100	2.16E-05	12900	6.08E-05	12500	0.000420628	14800	1.65E-05	14200	1.68E-05
15100	3.86E-05	13100	2.16E-05	12900	6.06E-05	12500	0.000426108	14800	1.65E-05	14200	1.68E-05
15100	3.86E-05	13100	2.15E-05	12900	6.05E-05	12500	0.0004317	14800	1.65E-05	14200	1.68E-05
15100	3.86E-05	13100	2.15E-05	12900	6.04E-05	12500	0.000437413	14800	1.65E-05	14200	1.68E-05
15100	3.86E-05	13100	2.14E-05	12900	6.02E-05	12500	0.000443229	14800	1.64E-05	14200	1.68E-05
15100	3.86E-05	13100	2.14E-05	12900	6.01E-05	12500	0.00044919	14800	1.64E-05	14200	1.68E-05
15100	3.86E-05	13100	2.14E-05	12900	6.00E-05	12500	0.000455241	14800	1.64E-05	14200	1.68E-05
15100	3.86E-05	13100	2.13E-05	12900	5.99E-05	12500	0.00046146	14800	1.64E-05	14200	1.68E-05
15100	3.86E-05	13100	2.13E-05	12900	5.98E-05	12500	0.00046781	14800	1.64E-05	14200	1.68E-05
15100	3.86E-05	13100	2.12E-05	12900	5.96E-05	12500	0.000474296	14800	1.64E-05	14200	1.68E-05
15100	3.87E-05	13100	2.12E-05	12900	5.95E-05	12500	0.000480926	14800	1.63E-05	14200	1.68E-05
15100	3.87E-05	13100	2.11E-05	12900	5.94E-05	12500	0.000487718	14800	1.63E-05	14200	1.68E-05
15100	3.87E-05	13100	2.11E-05	12900	5.93E-05	12500	0.000494638	14800	1.63E-05	14200	1.68E-05
15100	3.87E-05	13100	2.11E-05	12900	5.91E-05	12500	0.000501721	14800	1.63E-05	14200	1.68E-05
15100	3.87E-05	13100	2.10E-05	12900	5.90E-05	12500	0.000508935	14800	1.62E-05	14200	1.68E-05
15100	3.87E-05	13100	2.10E-05	12900	5.89E-05	12500	0.000516327	14800	1.62E-05	14200	1.68E-05
15100	3.87E-05	13100	2.10E-05	12900	5.88E-05	12500	0.00052388	14800	1.62E-05	14200	1.68E-05
15200	3.87E-05	13100	2.09E-05	12900	5.87E-05	12500	0.000531597	14800	1.62E-05	14200	1.68E-05
15200	3.87E-05	13100	2.09E-05	12900	5.85E-05	12500	0.000539499	14800	1.62E-05	14200	1.68E-05
15200	3.87E-05	13100	2.09E-05	12900	5.84E-05	12500	0.000547581	14800	1.61E-05	14200	1.68E-05
15200	3.87E-05	13100	2.08E-05	12900	5.83E-05	12500	0.000555852	14800	1.61E-05	14200	1.68E-05
15200	3.87E-05	13100	2.08E-05	12900	5.82E-05	12500	0.000564324	14800	1.61E-05	14200	1.69E-05
15200	3.87E-05	13100	2.08E-05	12900	5.81E-05	12500	0.000572984	14800	1.61E-05	14200	1.68E-05
15200	3.88E-05	13100	2.07E-05	12900	5.80E-05	12500	0.000581855	14800	1.61E-05	14200	1.69E-05
15200	3.88E-05	13100	2.07E-05	12900	5.78E-05	12500	0.000590937	14800	1.61E-05	14200	1.69E-05
15200	3.88E-05	13100	2.07E-05	12900	5.77E-05	12500	0.000600219	14800	1.60E-05	14200	1.69E-05

15200 3.88E-05 13100 2.06E-05 12900 5.76E-05 12500
0.00060975 14800 1.60E-05 14300 1.69E-05
15200 3.88E-05 13100 2.06E-05 12900 5.75E-05 12500
0.000619507 14800 1.60E-05 14300 1.69E-05
15200 3.88E-05 13100 2.06E-05 12900 5.74E-05 12500
0.000629508 14800 1.60E-05 14300 1.69E-05
15200 3.88E-05 13100 2.05E-05 12900 5.73E-05 12500
0.000639756 14800 1.60E-05 14300 1.69E-05
15200 3.88E-05 13100 2.05E-05 12900 5.72E-05 12500
0.000650261 14800 1.60E-05 14300 1.69E-05
15200 3.88E-05 13100 2.04E-05 12900 5.70E-05 12500
0.000661045 14800 1.59E-05 14300 1.69E-05
15200 3.88E-05 13100 2.04E-05 12900 5.69E-05 12500
0.000672097 14800 1.59E-05 14300 1.69E-05
15200 3.89E-05 13100 2.04E-05 12900 5.68E-05 12500
0.000683444 14800 1.59E-05 14300 1.69E-05
15200 3.89E-05 13100 2.03E-05 12900 5.67E-05 12500
0.000695087 14800 1.59E-05 14300 1.69E-05
15200 3.89E-05 13200 2.03E-05 12900 5.66E-05 12500
0.000707054 14800 1.59E-05 14300 1.69E-05
15200 3.89E-05 13200 2.03E-05 12900 5.65E-05 12500
0.000719328 14800 1.59E-05 14300 1.69E-05
15200 3.89E-05 13200 2.03E-05 13000 5.64E-05 12500
0.00073192 14800 1.59E-05 14300 1.69E-05
15200 3.89E-05 13200 2.02E-05 13000 5.63E-05 12500
0.000744859 14800 1.59E-05 14300 1.69E-05
15200 3.89E-05 13200 2.02E-05 13000 5.62E-05 12500
0.000758145 14800 1.58E-05 14300 1.69E-05
15200 3.89E-05 13200 2.02E-05 13000 5.60E-05 12500
0.0007718 14800 1.58E-05 14300 1.69E-05
15200 3.89E-05 13200 2.01E-05 13000 5.59E-05 12600
0.000785821 14800 1.58E-05 14300 1.69E-05
15200 3.89E-05 13200 2.01E-05 13000 5.58E-05 12600
0.000800219 14800 1.58E-05 14300 1.69E-05
15200 3.89E-05 13200 2.01E-05 13000 5.57E-05 12600
0.000815034 14800 1.58E-05 14300 1.69E-05
15200 3.89E-05 13200 2.01E-05 13000 5.56E-05 12600
0.000830238 14800 1.58E-05 14300 1.69E-05
15200 3.89E-05 13200 2.00E-05 13000 5.55E-05 12600
0.000845918 14800 1.58E-05 14300 1.69E-05
15200 3.89E-05 13200 2.00E-05 13000 5.54E-05 12600
0.00086206 14800 1.58E-05 14300 1.69E-05
15200 3.89E-05 13200 2.00E-05 13000 5.53E-05 12600
0.000878668 14800 1.58E-05 14300 1.69E-05
15200 3.89E-05 13200 1.99E-05 13000 5.52E-05 12600
0.000895775 14800 1.57E-05 14300 1.70E-05
15200 3.90E-05 13200 1.99E-05 13000 5.51E-05 12600
0.00091339 14800 1.57E-05 14300 1.70E-05
15200 3.90E-05 13200 1.98E-05 13000 5.50E-05 12600
0.000931535 14800 1.57E-05 14300 1.69E-05
15200 3.90E-05 13200 1.98E-05 13000 5.49E-05 12600
0.000950237 14800 1.57E-05 14300 1.69E-05
15200 3.90E-05 13200 1.98E-05 13000 5.48E-05 12600
0.000969499 14900 1.57E-05 14300 1.70E-05
15200 3.90E-05 13200 1.98E-05 13000 5.47E-05 12600
0.00098936 14900 1.57E-05 14300 1.70E-05
15200 3.90E-05 13200 1.97E-05 13000 5.46E-05 12600
0.001009859 14900 1.57E-05 14300 1.70E-05
15200 3.90E-05 13200 1.97E-05 13000 5.45E-05 12600
0.001030996 14900 1.57E-05 14300 1.70E-05
15200 3.90E-05 13200 1.97E-05 13000 5.43E-05 12600
0.001052789 14900 1.57E-05 14300 1.70E-05
15200 3.90E-05 13200 1.96E-05 13000 5.42E-05 12600
0.001075298 14900 1.56E-05 14300 1.70E-05
15200 3.90E-05 13200 1.96E-05 13000 5.41E-05 12600
0.001098531 14900 1.56E-05 14300 1.70E-05
15200 3.90E-05 13200 1.96E-05 13000 5.40E-05 12600
0.001122563 14900 1.56E-05 14300 1.70E-05

15200 3.90E-05 13200 1.95E-05 13000 5.39E-05 12600
0.001147386 14900 1.56E-05 14300 1.70E-05
15200 3.90E-05 13200 1.95E-05 13000 5.38E-05 12600
0.001173024 14900 1.56E-05 14300 1.70E-05
15200 3.91E-05 13200 1.95E-05 13000 5.37E-05 12600
0.001199549 14900 1.56E-05 14300 1.70E-05
15200 3.91E-05 13200 1.94E-05 13000 5.36E-05 12600
0.001226956 14900 1.56E-05 14300 1.70E-05
15200 3.91E-05 13200 1.94E-05 13000 5.35E-05 12600
0.001255327 14900 1.56E-05 14300 1.70E-05
15200 3.91E-05 13200 1.94E-05 13000 5.34E-05 12600
0.00128469 14900 1.56E-05 14300 1.70E-05
15200 3.91E-05 13200 1.93E-05 13000 5.33E-05 12600
0.001315076 14900 1.56E-05 14300 1.70E-05
15200 3.91E-05 13200 1.93E-05 13000 5.32E-05 12600
0.001346563 14900 1.56E-05 14300 1.70E-05
15200 3.92E-05 13200 1.93E-05 13000 5.31E-05 12600
0.001379185 14900 1.55E-05 14300 1.70E-05
15200 3.91E-05 13200 1.92E-05 13000 5.30E-05 12600
0.001412995 14900 1.55E-05 14300 1.70E-05
15200 3.92E-05 13200 1.92E-05 13000 5.29E-05 12600
0.001448058 14900 1.55E-05 14300 1.70E-05
15200 3.92E-05 13200 1.92E-05 13000 5.28E-05 12600
0.001484411 14900 1.55E-05 14300 1.70E-05
15200 3.92E-05 13200 1.92E-05 13000 5.27E-05 12600
0.001522144 14900 1.55E-05 14300 1.71E-05
15200 3.92E-05 13200 1.91E-05 13000 5.26E-05 12600
0.001561366 14900 1.55E-05 14300 1.71E-05
15200 3.92E-05 13200 1.91E-05 13000 5.25E-05 12600
0.001602076 14900 1.55E-05 14300 1.71E-05
15200 3.92E-05 13200 1.91E-05 13000 5.24E-05 12600
0.001644378 14900 1.55E-05 14300 1.71E-05
15200 3.92E-05 13200 1.91E-05 13000 5.23E-05 12600
0.001688381 14900 1.55E-05 14300 1.70E-05
15200 3.92E-05 13200 1.90E-05 13000 5.22E-05 12600
0.001734147 14900 1.55E-05 14300 1.71E-05
15200 3.92E-05 13200 1.90E-05 13000 5.21E-05 12600
0.001781773 14900 1.55E-05 14300 1.70E-05
15200 3.92E-05 13200 1.90E-05 13000 5.20E-05 12600
0.001831337 14900 1.55E-05 14300 1.70E-05
15200 3.93E-05 13200 1.90E-05 13000 5.20E-05 12600
0.001882929 14900 1.54E-05 14300 1.71E-05
15300 3.93E-05 13200 1.89E-05 13000 5.19E-05 12600
0.001936704 14900 1.54E-05 14300 1.71E-05
15300 3.93E-05 13200 1.89E-05 13000 5.18E-05 12600
0.001992724 14900 1.54E-05 14300 1.71E-05
15300 3.93E-05 13200 1.89E-05 13000 5.17E-05 12600
0.002051159 14900 1.54E-05 14300 1.71E-05
15300 3.93E-05 13200 1.88E-05 13000 5.16E-05 12600
0.002112136 14900 1.54E-05 14300 1.71E-05
15300 3.93E-05 13200 1.88E-05 13000 5.15E-05 12600
0.002175772 14900 1.54E-05 14300 1.71E-05
15300 3.93E-05 13200 1.88E-05 13000 5.14E-05 12600
0.002242265 14900 1.54E-05 14300 1.71E-05
15300 3.94E-05 13200 1.87E-05 13000 5.13E-05 12600
0.002311765 14900 1.54E-05 14300 1.71E-05
15300 3.94E-05 13200 1.87E-05 13000 5.12E-05 12600
0.002384435 14900 1.54E-05 14300 1.71E-05
15300 3.94E-05 13200 1.87E-05 13000 5.11E-05 12600
0.002460514 14900 1.54E-05 14300 1.71E-05
15300 3.94E-05 13200 1.87E-05 13000 5.10E-05 12600
0.002540154 14900 1.54E-05 14400 1.71E-05
15300 3.94E-05 13200 1.86E-05 13000 5.09E-05 12600
0.002623622 14900 1.54E-05 14400 1.71E-05
15300 3.94E-05 13200 1.86E-05 13000 5.08E-05 12600
0.002711143 14900 1.54E-05 14400 1.71E-05
15300 3.94E-05 13200 1.86E-05 13000 5.08E-05 12600
0.002802933 14900 1.54E-05 14400 1.71E-05

15300 3.94E-05 13200 1.85E-05 13000 5.07E-05 12600
0.002899265 14900 1.54E-05 14400 1.71E-05
15300 3.94E-05 13200 1.85E-05 13000 5.06E-05 12600
0.003000447 14900 1.53E-05 14400 1.71E-05
15300 3.94E-05 13200 1.85E-05 13000 5.05E-05 12600
0.003106775 14900 1.53E-05 14400 1.71E-05
15300 3.94E-05 13200 1.84E-05 13000 5.04E-05 12600
0.003218582 14900 1.53E-05 14400 1.71E-05
15300 3.94E-05 13200 1.84E-05 13000 5.03E-05 12600
0.003336213 14900 1.53E-05 14400 1.71E-05
15300 3.95E-05 13300 1.84E-05 13000 5.02E-05 12600
0.00346004 14900 1.53E-05 14400 1.71E-05
15300 3.95E-05 13300 1.84E-05 13000 5.01E-05 12600
0.003590534 14900 1.53E-05 14400 1.71E-05
15300 3.95E-05 13300 1.83E-05 13100 5.00E-05 12600
0.003728106 14900 1.53E-05 14400 1.71E-05
15300 3.95E-05 13300 1.83E-05 13100 4.99E-05 12600
0.003873304 14900 1.53E-05 14400 1.71E-05
15300 3.95E-05 13300 1.83E-05 13100 4.98E-05 12600
0.004026667 14900 1.53E-05 14400 1.72E-05
15300 3.95E-05 13300 1.83E-05 13100 4.97E-05 12600
0.004188775 14900 1.53E-05 14400 1.72E-05
15300 3.96E-05 13300 1.82E-05 13100 4.96E-05 12700
0.00436026 14900 1.53E-05 14400 1.72E-05
15300 3.96E-05 13300 1.82E-05 13100 4.96E-05 12700
0.004541822 14900 1.53E-05 14400 1.72E-05
15300 3.96E-05 13300 1.82E-05 13100 4.95E-05 12700
0.00473413 14900 1.53E-05 14400 1.72E-05
15300 3.96E-05 13300 1.82E-05 13100 4.94E-05 12700
0.004938019 14900 1.53E-05 14400 1.72E-05
15300 3.96E-05 13300 1.82E-05 13100 4.93E-05 12700
0.005154305 14900 1.53E-05 14400 1.72E-05
15300 3.96E-05 13300 1.81E-05 13100 4.92E-05 12700
0.005383968 14900 1.53E-05 14400 1.72E-05
15300 3.96E-05 13300 1.81E-05 13100 4.91E-05 12700
0.005628088 14900 1.53E-05 14400 1.72E-05
15300 3.96E-05 13300 1.81E-05 13100 4.90E-05 12700
0.005887788 14900 1.53E-05 14400 1.72E-05
15300 3.96E-05 13300 1.80E-05 13100 4.89E-05 12700
0.006164368 14900 1.53E-05 14400 1.71E-05
15300 3.97E-05 13300 1.80E-05 13100 4.89E-05 12700
0.006459266 14900 1.52E-05 14400 1.72E-05
15300 3.96E-05 13300 1.80E-05 13100 4.88E-05 12700
0.006773993 14900 1.52E-05 14400 1.71E-05
15300 3.97E-05 13300 1.80E-05 13100 4.87E-05 12700
0.007110235 15000 1.52E-05 14400 1.72E-05
15300 3.97E-05 13300 1.79E-05 13100 4.86E-05 12700
0.007469812 15000 1.52E-05 14400 1.72E-05
15300 3.97E-05 13300 1.79E-05 13100 4.85E-05 12700
0.007854765 15000 1.52E-05 14400 1.72E-05
15300 3.97E-05 13300 1.79E-05 13100 4.84E-05 12700
0.008267301 15000 1.52E-05 14400 1.72E-05
15300 3.97E-05 13300 1.79E-05 13100 4.83E-05 12700
0.008709778 15000 1.52E-05 14400 1.72E-05
15300 3.98E-05 13300 1.78E-05 13100 4.83E-05 12700
0.009184869 15000 1.52E-05 14400 1.72E-05
15300 3.98E-05 13300 1.78E-05 13100 4.82E-05 12700
0.009695383 15000 1.52E-05 14400 1.72E-05
15300 3.98E-05 13300 1.78E-05 13100 4.81E-05 12700
0.01024446 15000 1.52E-05 14400 1.72E-05
15300 3.98E-05 13300 1.77E-05 13100 4.80E-05 12700
0.01083549 15000 1.52E-05 14400 1.72E-05
15300 3.98E-05 13300 1.77E-05 13100 4.80E-05 12700
0.01147222 15000 1.52E-05 14400 1.72E-05
15300 3.98E-05 13300 1.77E-05 13100 4.79E-05 12700
0.01215871 15000 1.52E-05 14400 1.72E-05
15300 3.98E-05 13300 1.77E-05 13100 4.78E-05 12700
0.01289949 15000 1.52E-05 14400 1.72E-05

15300 3.98E-05 13300 1.76E-05 13100 4.77E-05 12700
0.01369954 15000 1.52E-05 14400 1.72E-05
15300 3.99E-05 13300 1.76E-05 13100 4.76E-05 12700
0.01456427 15000 1.52E-05 14400 1.72E-05
15300 3.99E-05 13300 1.76E-05 13100 4.75E-05 12700
0.01549961 15000 1.52E-05 14400 1.72E-05
15300 3.98E-05 13300 1.76E-05 13100 4.75E-05 12700
0.01651194 15000 1.52E-05 14400 1.72E-05
15300 3.99E-05 13300 1.75E-05 13100 4.74E-05 12700
0.01760825 15000 1.52E-05 14400 1.72E-05
15300 3.99E-05 13300 1.75E-05 13100 4.73E-05 12700
0.01879608 15000 1.52E-05 14400 1.72E-05
15300 3.98E-05 13300 1.75E-05 13100 4.72E-05 12700
0.02008344 15000 1.52E-05 14400 1.72E-05
15300 3.98E-05 13300 1.75E-05 13100 4.71E-05 12700
0.02147898 15000 1.52E-05 14400 1.72E-05
15300 3.98E-05 13300 1.74E-05 13100 4.70E-05 12700
0.02299183 15000 1.52E-05 14400 1.72E-05
15300 3.99E-05 13300 1.74E-05 13100 4.70E-05 12700
0.02463161 15000 1.52E-05 14400 1.73E-05
15300 3.99E-05 13300 1.74E-05 13100 4.69E-05 12700
0.02640831 15000 1.52E-05 14400 1.73E-05
15300 3.99E-05 13300 1.73E-05 13100 4.68E-05 12700
0.02833205 15000 1.52E-05 14400 1.73E-05
15300 3.99E-05 13300 1.73E-05 13100 4.67E-05 12700
0.03041302 15000 1.52E-05 14400 1.73E-05
15300 3.99E-05 13300 1.73E-05 13100 4.66E-05 12700
0.03266108 15000 1.51E-05 14400 1.73E-05
15300 3.99E-05 13300 1.73E-05 13100 4.66E-05 12700
0.03508547 15000 1.51E-05 14400 1.73E-05
15300 3.99E-05 13300 1.73E-05 13100 4.65E-05 12700
0.03769436 15000 1.51E-05 14400 1.73E-05
15300 4.00E-05 13300 1.72E-05 13100 4.64E-05 12700
0.04049427 15000 1.51E-05 14400 1.73E-05
15400 4.00E-05 13300 1.72E-05 13100 4.63E-05 12700
0.04348955 15000 1.51E-05 14400 1.73E-05
15400 4.00E-05 13300 1.72E-05 13100 4.63E-05 12700
0.04668159 15000 1.51E-05 14400 1.73E-05
15400 4.00E-05 13300 1.71E-05 13100 4.62E-05 12700
0.05006796 15000 1.51E-05 14400 1.73E-05
15400 4.00E-05 13300 1.71E-05 13100 4.61E-05 12700
0.05364125 15000 1.51E-05 14400 1.73E-05
15400 4.00E-05 13300 1.71E-05 13100 4.60E-05 12700
0.0573879 15000 1.51E-05 14400 1.73E-05
15400 4.00E-05 13300 1.71E-05 13100 4.59E-05 12700
0.06128679 15000 1.51E-05 14400 1.74E-05
15400 4.00E-05 13300 1.71E-05 13100 4.59E-05 12700
0.06530844 15000 1.51E-05 14400 1.74E-05
15400 4.00E-05 13300 1.70E-05 13100 4.58E-05 12700
0.06941357 15000 1.51E-05 14400 1.74E-05
15400 4.00E-05 13300 1.70E-05 13100 4.57E-05 12700
0.07355259 15000 1.51E-05 14400 1.73E-05
15400 4.00E-05 13300 1.70E-05 13100 4.56E-05 12700
0.07766504 15000 1.51E-05 14500 1.73E-05
15400 4.00E-05 13300 1.70E-05 13100 4.55E-05 12700
0.08167977 15000 1.51E-05 14500 1.73E-05
15400 4.01E-05 13300 1.69E-05 13100 4.55E-05 12700
0.08551568 15000 1.51E-05 14500 1.74E-05
15400 4.01E-05 13300 1.69E-05 13100 4.54E-05 12700
0.08908359 15000 1.51E-05 14500 1.74E-05
15400 4.01E-05 13300 1.69E-05 13100 4.53E-05 12700
0.09228913 15000 1.51E-05 14500 1.74E-05
15400 4.01E-05 13300 1.69E-05 13100 4.52E-05 12700
0.09503663 15000 1.51E-05 14500 1.74E-05
15400 4.01E-05 13300 1.69E-05 13100 4.52E-05 12700
0.09723376 15000 1.51E-05 14500 1.74E-05
15400 4.02E-05 13300 1.68E-05 13100 4.51E-05 12700
0.09879706 15000 1.51E-05 14500 1.74E-05

15400 4.02E-05 13300 1.68E-05 13100 4.50E-05 12700
0.09965727 15000 1.51E-05 14500 1.74E-05
15400 4.02E-05 13400 1.68E-05 13100 4.49E-05 12700
0.09976492 15000 1.51E-05 14500 1.74E-05
15400 4.02E-05 13400 1.68E-05 13100 4.49E-05 12700
0.09909467 15000 1.51E-05 14500 1.74E-05
15400 4.02E-05 13400 1.68E-05 13200 4.48E-05 12700
0.09764874 15000 1.51E-05 14500 1.74E-05
15400 4.02E-05 13400 1.67E-05 13200 4.47E-05 12700
0.09545814 15000 1.51E-05 14500 1.74E-05
15400 4.02E-05 13400 1.67E-05 13200 4.47E-05 12700
0.09258178 15000 1.51E-05 14500 1.75E-05
15400 4.02E-05 13400 1.67E-05 13200 4.46E-05 12700
0.08910303 15000 1.51E-05 14500 1.74E-05
15400 4.02E-05 13400 1.67E-05 13200 4.45E-05 12800
0.08512422 15000 1.51E-05 14500 1.74E-05
15400 4.02E-05 13400 1.67E-05 13200 4.44E-05 12800
0.08075979 15000 1.51E-05 14500 1.74E-05
15400 4.02E-05 13400 1.66E-05 13200 4.44E-05 12800
0.07612829 15000 1.51E-05 14500 1.74E-05
15400 4.02E-05 13400 1.66E-05 13200 4.43E-05 12800
0.07134509 15000 1.51E-05 14500 1.75E-05
15400 4.02E-05 13400 1.66E-05 13200 4.42E-05 12800
0.06651638 15000 1.51E-05 14500 1.75E-05
15400 4.02E-05 13400 1.66E-05 13200 4.41E-05 12800
0.06173472 15000 1.51E-05 14500 1.75E-05
15400 4.03E-05 13400 1.65E-05 13200 4.41E-05 12800
0.05707676 15000 1.51E-05 14500 1.75E-05
15400 4.02E-05 13400 1.65E-05 13200 4.40E-05 12800
0.05260246 15000 1.51E-05 14500 1.75E-05
15400 4.03E-05 13400 1.65E-05 13200 4.39E-05 12800
0.04835565 15000 1.51E-05 14500 1.75E-05
15400 4.03E-05 13400 1.65E-05 13200 4.38E-05 12800
0.04436541 15000 1.51E-05 14500 1.75E-05
15400 4.03E-05 13400 1.64E-05 13200 4.38E-05 12800
0.04064807 15000 1.51E-05 14500 1.75E-05
15400 4.03E-05 13400 1.64E-05 13200 4.37E-05 12800
0.03720944 15100 1.51E-05 14500 1.75E-05
15400 4.03E-05 13400 1.64E-05 13200 4.36E-05 12800
0.03404707 15100 1.51E-05 14500 1.75E-05
15400 4.03E-05 13400 1.64E-05 13200 4.36E-05 12800
0.03115237 15100 1.51E-05 14500 1.75E-05
15400 4.03E-05 13400 1.63E-05 13200 4.35E-05 12800
0.02851248 15100 1.51E-05 14500 1.75E-05
15400 4.03E-05 13400 1.63E-05 13200 4.34E-05 12800
0.02611188 15100 1.51E-05 14500 1.75E-05
15400 4.03E-05 13400 1.63E-05 13200 4.34E-05 12800
0.02393353 15100 1.51E-05 14500 1.75E-05
15400 4.03E-05 13400 1.63E-05 13200 4.33E-05 12800
0.02195989 15100 1.51E-05 14500 1.75E-05
15400 4.04E-05 13400 1.63E-05 13200 4.32E-05 12800
0.02017358 15100 1.51E-05 14500 1.76E-05
15400 4.04E-05 13400 1.62E-05 13200 4.32E-05 12800
0.01855771 15100 1.51E-05 14500 1.75E-05
15400 4.04E-05 13400 1.62E-05 13200 4.31E-05 12800
0.01709638 15100 1.51E-05 14500 1.75E-05
15400 4.04E-05 13400 1.62E-05 13200 4.30E-05 12800
0.0157746 15100 1.51E-05 14500 1.75E-05
15400 4.04E-05 13400 1.62E-05 13200 4.30E-05 12800
0.01457842 15100 1.51E-05 14500 1.76E-05
15400 4.04E-05 13400 1.61E-05 13200 4.29E-05 12800
0.01349517 15100 1.51E-05 14500 1.76E-05
15400 4.04E-05 13400 1.61E-05 13200 4.28E-05 12800
0.0125133 15100 1.51E-05 14500 1.75E-05
15400 4.05E-05 13400 1.61E-05 13200 4.28E-05 12800
0.01162235 15100 1.50E-05 14500 1.76E-05
15400 4.05E-05 13400 1.61E-05 13200 4.27E-05 12800
0.01081301 15100 1.51E-05 14500 1.76E-05

15400 4.05E-05 13400 1.61E-05 13200 4.26E-05 12800
0.01007688 15100 1.51E-05 14500 1.76E-05
15400 4.05E-05 13400 1.60E-05 13200 4.26E-05 12800
0.009406466 15100 1.51E-05 14500 1.76E-05
15400 4.05E-05 13400 1.60E-05 13200 4.25E-05 12800
0.008795097 15100 1.51E-05 14500 1.76E-05
15400 4.05E-05 13400 1.60E-05 13200 4.24E-05 12800
0.008236825 15100 1.51E-05 14500 1.76E-05
15400 4.05E-05 13400 1.60E-05 13200 4.24E-05 12800
0.007726311 15100 1.51E-05 14500 1.76E-05
15400 4.06E-05 13400 1.59E-05 13200 4.23E-05 12800
0.007258826 15100 1.51E-05 14500 1.76E-05
15400 4.06E-05 13400 1.59E-05 13200 4.22E-05 12800
0.006830064 15100 1.51E-05 14500 1.76E-05
15400 4.06E-05 13400 1.59E-05 13200 4.22E-05 12800
0.006436267 15100 1.51E-05 14500 1.76E-05
15400 4.06E-05 13400 1.59E-05 13200 4.21E-05 12800
0.006073998 15100 1.51E-05 14500 1.76E-05
15400 4.06E-05 13400 1.59E-05 13200 4.21E-05 12800
0.005740222 15100 1.51E-05 14500 1.76E-05
15400 4.06E-05 13400 1.58E-05 13200 4.20E-05 12800
0.005432209 15100 1.51E-05 14500 1.76E-05
15400 4.06E-05 13400 1.58E-05 13200 4.19E-05 12800
0.005147539 15100 1.51E-05 14500 1.76E-05
15400 4.06E-05 13400 1.58E-05 13200 4.19E-05 12800
0.00488406 15100 1.51E-05 14500 1.76E-05
15500 4.06E-05 13400 1.58E-05 13200 4.18E-05 12800
0.004639813 15100 1.51E-05 14500 1.76E-05
15500 4.06E-05 13400 1.57E-05 13200 4.17E-05 12800
0.004413055 15100 1.51E-05 14500 1.76E-05
15500 4.06E-05 13400 1.57E-05 13200 4.17E-05 12800
0.004202266 15100 1.51E-05 14500 1.76E-05
15500 4.06E-05 13400 1.57E-05 13200 4.16E-05 12800
0.00400605 15100 1.51E-05 14500 1.76E-05
15500 4.07E-05 13400 1.57E-05 13200 4.15E-05 12800
0.003823127 15100 1.50E-05 14500 1.76E-05
15500 4.07E-05 13400 1.57E-05 13200 4.15E-05 12800
0.003652406 15100 1.50E-05 14500 1.76E-05
15500 4.07E-05 13400 1.57E-05 13200 4.14E-05 12800
0.003492843 15100 1.50E-05 14500 1.76E-05
15500 4.07E-05 13400 1.56E-05 13200 4.13E-05 12800
0.003343533 15100 1.50E-05 14500 1.76E-05
15500 4.07E-05 13400 1.56E-05 13200 4.13E-05 12800
0.003203634 15100 1.50E-05 14500 1.76E-05
15500 4.07E-05 13400 1.56E-05 13200 4.12E-05 12800
0.003072387 15100 1.50E-05 14600 1.76E-05
15500 4.08E-05 13400 1.56E-05 13200 4.12E-05 12800
0.002949144 15100 1.50E-05 14600 1.76E-05
15500 4.08E-05 13400 1.56E-05 13200 4.11E-05 12800
0.002833273 15100 1.50E-05 14600 1.76E-05
15500 4.08E-05 13400 1.55E-05 13200 4.10E-05 12800
0.002724231 15100 1.50E-05 14600 1.76E-05
15500 4.08E-05 13400 1.55E-05 13200 4.10E-05 12800
0.002621521 15100 1.50E-05 14600 1.76E-05
15500 4.08E-05 13400 1.55E-05 13200 4.09E-05 12800
0.002524662 15100 1.50E-05 14600 1.76E-05
15500 4.08E-05 13400 1.55E-05 13200 4.09E-05 12800
0.002433221 15100 1.50E-05 14600 1.76E-05
15500 4.08E-05 13400 1.55E-05 13200 4.08E-05 12800
0.002346847 15100 1.50E-05 14600 1.76E-05
15500 4.08E-05 13400 1.54E-05 13200 4.07E-05 12800
0.00226514 15100 1.50E-05 14600 1.76E-05
15500 4.09E-05 13500 1.54E-05 13200 4.07E-05 12800
0.002187792 15100 1.50E-05 14600 1.76E-05
15500 4.09E-05 13500 1.54E-05 13200 4.06E-05 12800
0.002114475 15100 1.50E-05 14600 1.76E-05
15500 4.09E-05 13500 1.54E-05 13300 4.05E-05 12800
0.002044919 15100 1.50E-05 14600 1.77E-05

15500 4.09E-05 13500 1.54E-05 13300 4.05E-05 12800
0.001978856 15100 1.50E-05 14600 1.77E-05
15500 4.09E-05 13500 1.53E-05 13300 4.04E-05 12800
0.001916092 15100 1.50E-05 14600 1.76E-05
15500 4.10E-05 13500 1.53E-05 13300 4.04E-05 12800
0.001856341 15100 1.50E-05 14600 1.77E-05
15500 4.10E-05 13500 1.53E-05 13300 4.03E-05 12900
0.001799475 15100 1.50E-05 14600 1.77E-05
15500 4.10E-05 13500 1.53E-05 13300 4.03E-05 12900
0.001745284 15100 1.50E-05 14600 1.76E-05
15500 4.10E-05 13500 1.53E-05 13300 4.02E-05 12900
0.001693607 15100 1.50E-05 14600 1.77E-05
15500 4.11E-05 13500 1.53E-05 13300 4.01E-05 12900
0.001644303 15100 1.50E-05 14600 1.77E-05
15500 4.11E-05 13500 1.52E-05 13300 4.01E-05 12900
0.001597216 15100 1.50E-05 14600 1.77E-05
15500 4.11E-05 13500 1.52E-05 13300 4.00E-05 12900
0.001552228 15100 1.50E-05 14600 1.77E-05
15500 4.11E-05 13500 1.52E-05 13300 4.00E-05 12900
0.001509209 15100 1.50E-05 14600 1.77E-05
15500 4.11E-05 13500 1.52E-05 13300 3.99E-05 12900
0.001468042 15100 1.50E-05 14600 1.77E-05
15500 4.11E-05 13500 1.52E-05 13300 3.98E-05 12900
0.001428632 15100 1.50E-05 14600 1.77E-05
15500 4.12E-05 13500 1.51E-05 13300 3.98E-05 12900
0.001390842 15100 1.50E-05 14600 1.78E-05
15500 4.12E-05 13500 1.51E-05 13300 3.97E-05 12900
0.001354629 15100 1.50E-05 14600 1.77E-05
15500 4.12E-05 13500 1.51E-05 13300 3.97E-05 12900
0.001319879 15200 1.50E-05 14600 1.78E-05
15500 4.12E-05 13500 1.51E-05 13300 3.96E-05 12900
0.001286528 15200 1.50E-05 14600 1.77E-05
15500 4.12E-05 13500 1.51E-05 13300 3.95E-05 12900
0.001254509 15200 1.50E-05 14600 1.78E-05
15500 4.12E-05 13500 1.51E-05 13300 3.95E-05 12900
0.001223719 15200 1.50E-05 14600 1.78E-05
15500 4.12E-05 13500 1.50E-05 13300 3.94E-05 12900
0.00119413 15200 1.50E-05 14600 1.78E-05
15500 4.13E-05 13500 1.50E-05 13300 3.94E-05 12900
0.001165677 15200 1.50E-05 14600 1.78E-05
15500 4.13E-05 13500 1.50E-05 13300 3.93E-05 12900
0.001138289 15200 1.50E-05 14600 1.78E-05
15500 4.13E-05 13500 1.50E-05 13300 3.93E-05 12900
0.001111903 15200 1.50E-05 14600 1.78E-05
15500 4.13E-05 13500 1.50E-05 13300 3.92E-05 12900
0.00108648 15200 1.50E-05 14600 1.78E-05
15500 4.13E-05 13500 1.50E-05 13300 3.92E-05 12900
0.001061979 15200 1.50E-05 14600 1.78E-05
15500 4.14E-05 13500 1.50E-05 13300 3.91E-05 12900
0.001038343 15200 1.50E-05 14600 1.78E-05
15500 4.14E-05 13500 1.49E-05 13300 3.90E-05 12900
0.001015538 15200 1.50E-05 14600 1.78E-05
15500 4.14E-05 13500 1.49E-05 13300 3.90E-05 12900
0.000993528 15200 1.50E-05 14600 1.78E-05
15500 4.14E-05 13500 1.49E-05 13300 3.89E-05 12900
0.00097227 15200 1.50E-05 14600 1.78E-05
15500 4.14E-05 13500 1.49E-05 13300 3.89E-05 12900
0.000951709 15200 1.50E-05 14600 1.78E-05
15500 4.14E-05 13500 1.49E-05 13300 3.88E-05 12900
0.000931846 15200 1.50E-05 14600 1.78E-05
15500 4.15E-05 13500 1.49E-05 13300 3.88E-05 12900
0.000912636 15200 1.50E-05 14600 1.78E-05
15500 4.15E-05 13500 1.48E-05 13300 3.87E-05 12900
0.000894041 15200 1.50E-05 14600 1.78E-05
15500 4.15E-05 13500 1.48E-05 13300 3.87E-05 12900
0.000876044 15200 1.50E-05 14600 1.79E-05
15500 4.15E-05 13500 1.48E-05 13300 3.86E-05 12900
0.00085862 15200 1.50E-05 14600 1.79E-05

15500 4.15E-05 13500 1.48E-05 13300 3.85E-05 12900
0.000841758 15200 1.50E-05 14600 1.78E-05
15500 4.15E-05 13500 1.48E-05 13300 3.85E-05 12900
0.000825425 15200 1.50E-05 14600 1.78E-05
15500 4.15E-05 13500 1.47E-05 13300 3.84E-05 12900
0.000809576 15200 1.50E-05 14600 1.79E-05
15500 4.16E-05 13500 1.47E-05 13300 3.84E-05 12900
0.000794221 15200 1.50E-05 14600 1.79E-05
15500 4.16E-05 13500 1.47E-05 13300 3.83E-05 12900
0.000779337 15200 1.50E-05 14600 1.79E-05
15500 4.16E-05 13500 1.47E-05 13300 3.83E-05 12900
0.000764893 15200 1.50E-05 14600 1.79E-05
15500 4.17E-05 13500 1.47E-05 13300 3.82E-05 12900
0.00075086 15200 1.50E-05 14600 1.79E-05
15500 4.17E-05 13500 1.46E-05 13300 3.81E-05 12900
0.000737245 15200 1.50E-05 14600 1.79E-05
15500 4.17E-05 13500 1.46E-05 13300 3.81E-05 12900
0.000724023 15200 1.50E-05 14600 1.79E-05
15600 4.17E-05 13500 1.46E-05 13300 3.80E-05 12900
0.000711177 15200 1.50E-05 14600 1.79E-05
15600 4.17E-05 13500 1.46E-05 13300 3.80E-05 12900
0.000698683 15200 1.50E-05 14600 1.79E-05
15600 4.17E-05 13500 1.46E-05 13300 3.79E-05 12900
0.000686556 15200 1.50E-05 14600 1.79E-05
15600 4.18E-05 13500 1.46E-05 13300 3.79E-05 12900
0.000674751 15200 1.50E-05 14600 1.79E-05
15600 4.18E-05 13500 1.46E-05 13300 3.78E-05 12900
0.000663272 15200 1.50E-05 14600 1.79E-05
15600 4.18E-05 13500 1.45E-05 13300 3.78E-05 12900
0.000652115 15200 1.50E-05 14600 1.80E-05
15600 4.18E-05 13500 1.45E-05 13300 3.77E-05 12900
0.000641241 15200 1.50E-05 14600 1.80E-05
15600 4.19E-05 13500 1.45E-05 13300 3.77E-05 12900
0.000630672 15200 1.50E-05 14600 1.80E-05
15600 4.19E-05 13500 1.45E-05 13300 3.76E-05 12900
0.000620352 15200 1.50E-05 14600 1.80E-05
15600 4.19E-05 13500 1.45E-05 13300 3.76E-05 12900
0.000610317 15200 1.50E-05 14700 1.80E-05
15600 4.19E-05 13500 1.45E-05 13300 3.75E-05 12900
0.000600552 15200 1.50E-05 14700 1.80E-05
15600 4.19E-05 13500 1.44E-05 13300 3.74E-05 12900
0.000591024 15200 1.50E-05 14700 1.80E-05
15600 4.20E-05 13500 1.44E-05 13300 3.74E-05 12900
0.000581739 15200 1.50E-05 14700 1.80E-05
15600 4.20E-05 13500 1.44E-05 13300 3.73E-05 12900
0.000572691 15200 1.50E-05 14700 1.81E-05
15600 4.20E-05 13500 1.44E-05 13300 3.73E-05 12900
0.000563879 15200 1.50E-05 14700 1.81E-05
15600 4.20E-05 13500 1.43E-05 13300 3.72E-05 12900
0.000555261 15200 1.50E-05 14700 1.81E-05
15600 4.20E-05 13500 1.43E-05 13300 3.72E-05 12900
0.00054685 15200 1.50E-05 14700 1.81E-05
15600 4.20E-05 13500 1.43E-05 13300 3.71E-05 12900
0.00053866 15200 1.50E-05 14700 1.81E-05
15600 4.21E-05 13600 1.43E-05 13300 3.71E-05 12900
0.000530667 15200 1.50E-05 14700 1.81E-05
15600 4.21E-05 13600 1.43E-05 13300 3.70E-05 12900
0.00052287 15200 1.50E-05 14700 1.81E-05
15600 4.21E-05 13600 1.43E-05 13400 3.70E-05 12900
0.000515249 15200 1.50E-05 14700 1.81E-05
15600 4.21E-05 13600 1.43E-05 13400 3.69E-05 12900
0.0005078 15200 1.50E-05 14700 1.81E-05
15600 4.22E-05 13600 1.42E-05 13400 3.69E-05 12900
0.000500531 15200 1.50E-05 14700 1.81E-05
15600 4.22E-05 13600 1.42E-05 13400 3.68E-05 12900
0.000493419 15200 1.50E-05 14700 1.81E-05
15600 4.22E-05 13600 1.42E-05 13400 3.68E-05 13000
0.000486461 15200 1.50E-05 14700 1.81E-05

15600 4.22E-05 13600 1.42E-05 13400 3.67E-05 13000
0.000479687 15200 1.50E-05 14700 1.81E-05
15600 4.22E-05 13600 1.42E-05 13400 3.67E-05 13000
0.000473053 15200 1.50E-05 14700 1.82E-05
15600 4.22E-05 13600 1.41E-05 13400 3.67E-05 13000
0.000466566 15200 1.50E-05 14700 1.81E-05
15600 4.23E-05 13600 1.41E-05 13400 3.66E-05 13000
0.000460222 15200 1.50E-05 14700 1.81E-05
15600 4.23E-05 13600 1.41E-05 13400 3.66E-05 13000
0.000454032 15200 1.50E-05 14700 1.81E-05
15600 4.23E-05 13600 1.41E-05 13400 3.65E-05 13000
0.000447965 15200 1.50E-05 14700 1.81E-05
15600 4.23E-05 13600 1.41E-05 13400 3.64E-05 13000
0.000442035 15200 1.50E-05 14700 1.81E-05
15600 4.23E-05 13600 1.41E-05 13400 3.64E-05 13000
0.000436236 15200 1.50E-05 14700 1.81E-05
15600 4.23E-05 13600 1.40E-05 13400 3.63E-05 13000
0.000430575 15200 1.50E-05 14700 1.82E-05
15600 4.24E-05 13600 1.40E-05 13400 3.63E-05 13000
0.000425023 15200 1.50E-05 14700 1.82E-05
15600 4.24E-05 13600 1.40E-05 13400 3.62E-05 13000
0.000419588 15300 1.50E-05 14700 1.82E-05
15600 4.24E-05 13600 1.40E-05 13400 3.62E-05 13000
0.000414278 15300 1.50E-05 14700 1.82E-05
15600 4.24E-05 13600 1.40E-05 13400 3.61E-05 13000
0.000409064 15300 1.50E-05 14700 1.82E-05
15600 4.24E-05 13600 1.40E-05 13400 3.61E-05 13000
0.000403952 15300 1.50E-05 14700 1.82E-05
15600 4.25E-05 13600 1.40E-05 13400 3.60E-05 13000
0.000398944 15300 1.50E-05 14700 1.82E-05
15600 4.25E-05 13600 1.39E-05 13400 3.60E-05 13000
0.000394038 15300 1.50E-05 14700 1.82E-05
15600 4.25E-05 13600 1.39E-05 13400 3.59E-05 13000
0.000389231 15300 1.50E-05 14700 1.82E-05
15600 4.25E-05 13600 1.39E-05 13400 3.59E-05 13000
0.000384505 15300 1.50E-05 14700 1.82E-05
15600 4.25E-05 13600 1.39E-05 13400 3.58E-05 13000
0.000379888 15300 1.50E-05 14700 1.82E-05
15600 4.25E-05 13600 1.39E-05 13400 3.58E-05 13000
0.000375354 15300 1.50E-05 14700 1.82E-05
15600 4.26E-05 13600 1.39E-05 13400 3.58E-05 13000
0.000370905 15300 1.50E-05 14700 1.82E-05
15600 4.26E-05 13600 1.39E-05 13400 3.57E-05 13000
0.000366542 15300 1.50E-05 14700 1.82E-05
15600 4.26E-05 13600 1.38E-05 13400 3.57E-05 13000
0.000362265 15300 1.50E-05 14700 1.82E-05
15600 4.26E-05 13600 1.38E-05 13400 3.56E-05 13000
0.000358071 15300 1.50E-05 14700 1.82E-05
15600 4.26E-05 13600 1.38E-05 13400 3.56E-05 13000
0.000353955 15300 1.50E-05 14700 1.82E-05
15600 4.27E-05 13600 1.38E-05 13400 3.55E-05 13000
0.000349918 15300 1.50E-05 14700 1.82E-05
15600 4.27E-05 13600 1.38E-05 13400 3.55E-05 13000
0.00034595 15300 1.50E-05 14700 1.82E-05
15600 4.27E-05 13600 1.38E-05 13400 3.54E-05 13000
0.000342071 15300 1.50E-05 14700 1.82E-05
15600 4.27E-05 13600 1.38E-05 13400 3.54E-05 13000
0.000338255 15300 1.50E-05 14700 1.82E-05
15600 4.28E-05 13600 1.37E-05 13400 3.53E-05 13000
0.000334514 15300 1.50E-05 14700 1.82E-05
15600 4.28E-05 13600 1.37E-05 13400 3.53E-05 13000
0.000330854 15300 1.50E-05 14700 1.82E-05
15600 4.28E-05 13600 1.37E-05 13400 3.52E-05 13000
0.000327254 15300 1.50E-05 14700 1.82E-05
15600 4.28E-05 13600 1.37E-05 13400 3.52E-05 13000
0.000323721 15300 1.50E-05 14700 1.82E-05
15600 4.28E-05 13600 1.37E-05 13400 3.51E-05 13000
0.000320239 15300 1.50E-05 14700 1.82E-05

15600 4.29E-05 13600 1.37E-05 13400 3.51E-05 13000
0.00031682 15300 1.50E-05 14700 1.82E-05
15600 4.29E-05 13600 1.36E-05 13400 3.51E-05 13000
0.000313466 15300 1.50E-05 14700 1.82E-05
15600 4.29E-05 13600 1.36E-05 13400 3.50E-05 13000
0.000310166 15300 1.50E-05 14700 1.82E-05
15600 4.30E-05 13600 1.36E-05 13400 3.50E-05 13000
0.000306925 15300 1.50E-05 14700 1.82E-05
15600 4.30E-05 13600 1.36E-05 13400 3.49E-05 13000
0.000303753 15300 1.50E-05 14700 1.82E-05
15700 4.30E-05 13600 1.36E-05 13400 3.49E-05 13000
0.000300626 15300 1.50E-05 14700 1.82E-05
15700 4.30E-05 13600 1.36E-05 13400 3.48E-05 13000
0.000297563 15300 1.50E-05 14700 1.82E-05
15700 4.30E-05 13600 1.36E-05 13400 3.48E-05 13000
0.000294535 15300 1.50E-05 14700 1.82E-05
15700 4.30E-05 13600 1.36E-05 13400 3.48E-05 13000
0.000291571 15300 1.50E-05 14700 1.82E-05
15700 4.30E-05 13600 1.35E-05 13400 3.47E-05 13000
0.000288668 15300 1.50E-05 14700 1.82E-05
15700 4.31E-05 13600 1.35E-05 13400 3.46E-05 13000
0.000285815 15300 1.50E-05 14700 1.82E-05
15700 4.31E-05 13600 1.35E-05 13400 3.46E-05 13000
0.000283018 15300 1.50E-05 14700 1.82E-05
15700 4.31E-05 13600 1.35E-05 13400 3.46E-05 13000
0.000280265 15300 1.50E-05 14700 1.82E-05
15700 4.31E-05 13600 1.35E-05 13400 3.45E-05 13000
0.000277561 15300 1.50E-05 14700 1.82E-05
15700 4.31E-05 13600 1.35E-05 13400 3.45E-05 13000
0.000274887 15300 1.51E-05 14800 1.82E-05
15700 4.32E-05 13600 1.34E-05 13400 3.44E-05 13000
0.000272275 15300 1.51E-05 14800 1.82E-05
15700 4.32E-05 13600 1.34E-05 13400 3.44E-05 13000
0.000269693 15300 1.51E-05 14800 1.82E-05
15700 4.32E-05 13600 1.34E-05 13400 3.43E-05 13000
0.000267151 15300 1.51E-05 14800 1.82E-05
15700 4.32E-05 13600 1.34E-05 13400 3.43E-05 13000
0.000264653 15300 1.51E-05 14800 1.82E-05
15700 4.33E-05 13600 1.34E-05 13400 3.43E-05 13000
0.000262193 15300 1.51E-05 14800 1.82E-05
15700 4.33E-05 13600 1.34E-05 13400 3.42E-05 13000
0.000259771 15300 1.51E-05 14800 1.82E-05
15700 4.33E-05 13600 1.34E-05 13400 3.42E-05 13000
0.000257375 15300 1.51E-05 14800 1.82E-05
15700 4.33E-05 13600 1.33E-05 13400 3.41E-05 13000
0.000255029 15300 1.51E-05 14800 1.82E-05
15700 4.34E-05 13700 1.33E-05 13400 3.41E-05 13000
0.000252725 15300 1.51E-05 14800 1.82E-05
15700 4.34E-05 13700 1.33E-05 13400 3.40E-05 13000
0.000250449 15300 1.51E-05 14800 1.82E-05
15700 4.34E-05 13700 1.33E-05 13500 3.40E-05 13000
0.000248199 15300 1.51E-05 14800 1.82E-05
15700 4.34E-05 13700 1.33E-05 13500 3.40E-05 13000
0.000246005 15300 1.51E-05 14800 1.82E-05
15700 4.34E-05 13700 1.33E-05 13500 3.39E-05 13000
0.000243821 15300 1.51E-05 14800 1.82E-05
15700 4.35E-05 13700 1.33E-05 13500 3.39E-05 13000
0.000241687 15300 1.51E-05 14800 1.82E-05
15700 4.35E-05 13700 1.33E-05 13500 3.38E-05 13100
0.000239591 15300 1.51E-05 14800 1.82E-05
15700 4.35E-05 13700 1.32E-05 13500 3.38E-05 13100
0.00023751 15300 1.51E-05 14800 1.82E-05
15700 4.36E-05 13700 1.32E-05 13500 3.37E-05 13100
0.000235475 15300 1.51E-05 14800 1.83E-05
15700 4.36E-05 13700 1.32E-05 13500 3.37E-05 13100
0.000233464 15300 1.51E-05 14800 1.82E-05
15700 4.37E-05 13700 1.32E-05 13500 3.37E-05 13100
0.000231487 15300 1.51E-05 14800 1.82E-05

15700 4.37E-05 13700 1.32E-05 13500 3.36E-05 13100
0.000229542 15300 1.51E-05 14800 1.82E-05
15700 4.37E-05 13700 1.32E-05 13500 3.36E-05 13100
0.000227615 15300 1.51E-05 14800 1.82E-05
15700 4.38E-05 13700 1.32E-05 13500 3.36E-05 13100
0.000225715 15300 1.51E-05 14800 1.83E-05
15700 4.38E-05 13700 1.32E-05 13500 3.35E-05 13100
0.000223842 15300 1.51E-05 14800 1.82E-05
15700 4.38E-05 13700 1.32E-05 13500 3.35E-05 13100
0.000221988 15300 1.51E-05 14800 1.83E-05
15700 4.39E-05 13700 1.32E-05 13500 3.34E-05 13100
0.000220175 15300 1.51E-05 14800 1.82E-05
15700 4.39E-05 13700 1.31E-05 13500 3.34E-05 13100
0.000218371 15400 1.51E-05 14800 1.83E-05
15700 4.39E-05 13700 1.31E-05 13500 3.33E-05 13100
0.000216589 15400 1.51E-05 14800 1.83E-05
15700 4.39E-05 13700 1.31E-05 13500 3.33E-05 13100
0.000214847 15400 1.51E-05 14800 1.83E-05
15700 4.40E-05 13700 1.31E-05 13500 3.33E-05 13100
0.000213119 15400 1.51E-05 14800 1.83E-05
15700 4.40E-05 13700 1.31E-05 13500 3.32E-05 13100
0.00021141 15400 1.51E-05 14800 1.83E-05
15700 4.40E-05 13700 1.31E-05 13500 3.32E-05 13100
0.000209741 15400 1.51E-05 14800 1.83E-05
15700 4.40E-05 13700 1.31E-05 13500 3.31E-05 13100
0.000208068 15400 1.51E-05 14800 1.83E-05
15700 4.41E-05 13700 1.30E-05 13500 3.31E-05 13100
0.000206433 15400 1.51E-05 14800 1.83E-05
15700 4.41E-05 13700 1.30E-05 13500 3.30E-05 13100
0.000204841 15400 1.51E-05 14800 1.83E-05
15700 4.41E-05 13700 1.30E-05 13500 3.30E-05 13100
0.000203259 15400 1.51E-05 14800 1.83E-05
15700 4.41E-05 13700 1.30E-05 13500 3.30E-05 13100
0.000201689 15400 1.51E-05 14800 1.83E-05
15700 4.42E-05 13700 1.30E-05 13500 3.29E-05 13100
0.000200165 15400 1.51E-05 14800 1.83E-05
15700 4.42E-05 13700 1.30E-05 13500 3.29E-05 13100
0.000198659 15400 1.51E-05 14800 1.83E-05
15700 4.42E-05 13700 1.30E-05 13500 3.28E-05 13100
0.000197166 15400 1.51E-05 14800 1.83E-05
15700 4.43E-05 13700 1.30E-05 13500 3.28E-05 13100
0.000195683 15400 1.51E-05 14800 1.83E-05
15700 4.43E-05 13700 1.29E-05 13500 3.28E-05 13100
0.000194233 15400 1.51E-05 14800 1.83E-05
15700 4.43E-05 13700 1.29E-05 13500 3.27E-05 13100
0.000192812 15400 1.51E-05 14800 1.83E-05
15700 4.44E-05 13700 1.29E-05 13500 3.27E-05 13100
0.000191388 15400 1.51E-05 14800 1.83E-05
15700 4.44E-05 13700 1.29E-05 13500 3.26E-05 13100
0.00019001 15400 1.51E-05 14800 1.83E-05
15700 4.44E-05 13700 1.29E-05 13500 3.26E-05 13100
0.000188611 15400 1.51E-05 14800 1.83E-05
15700 4.45E-05 13700 1.29E-05 13500 3.26E-05 13100
0.000187265 15400 1.51E-05 14800 1.83E-05
15700 4.45E-05 13700 1.29E-05 13500 3.25E-05 13100
0.000185932 15400 1.51E-05 14800 1.83E-05
15700 4.45E-05 13700 1.28E-05 13500 3.25E-05 13100
0.000184614 15400 1.51E-05 14800 1.84E-05
15700 4.46E-05 13700 1.28E-05 13500 3.24E-05 13100
0.000183292 15400 1.51E-05 14800 1.83E-05
15700 4.46E-05 13700 1.28E-05 13500 3.24E-05 13100
0.000181989 15400 1.51E-05 14800 1.83E-05
15700 4.46E-05 13700 1.28E-05 13500 3.23E-05 13100
0.000180723 15400 1.51E-05 14800 1.83E-05
15700 4.46E-05 13700 1.28E-05 13500 3.23E-05 13100
0.000179477 15400 1.52E-05 14800 1.83E-05
15700 4.47E-05 13700 1.28E-05 13500 3.23E-05 13100
0.000178259 15400 1.51E-05 14800 1.84E-05

15700 4.47E-05 13700 1.28E-05 13500 3.22E-05 13100
0.000177053 15400 1.51E-05 14800 1.84E-05
15800 4.48E-05 13700 1.27E-05 13500 3.22E-05 13100
0.000175847 15400 1.51E-05 14800 1.84E-05
15800 4.48E-05 13700 1.27E-05 13500 3.21E-05 13100
0.000174669 15400 1.51E-05 14800 1.84E-05
15800 4.48E-05 13700 1.27E-05 13500 3.21E-05 13100
0.000173514 15400 1.51E-05 14800 1.84E-05
15800 4.49E-05 13700 1.27E-05 13500 3.21E-05 13100
0.00017237 15400 1.52E-05 14800 1.84E-05
15800 4.49E-05 13700 1.27E-05 13500 3.20E-05 13100
0.000171226 15400 1.52E-05 14800 1.84E-05
15800 4.50E-05 13700 1.27E-05 13500 3.20E-05 13100
0.00017011 15400 1.52E-05 14800 1.84E-05
15800 4.50E-05 13700 1.26E-05 13500 3.20E-05 13100
0.000168994 15400 1.52E-05 14800 1.84E-05
15800 4.50E-05 13700 1.26E-05 13500 3.19E-05 13100
0.000167883 15400 1.52E-05 14800 1.84E-05
15800 4.51E-05 13700 1.26E-05 13500 3.19E-05 13100
0.000166796 15400 1.52E-05 14800 1.84E-05
15800 4.51E-05 13700 1.26E-05 13500 3.18E-05 13100
0.000165708 15400 1.52E-05 14900 1.84E-05
15800 4.52E-05 13700 1.26E-05 13500 3.18E-05 13100
0.000164644 15400 1.52E-05 14900 1.84E-05
15800 4.52E-05 13700 1.26E-05 13500 3.18E-05 13100
0.000163609 15400 1.52E-05 14900 1.84E-05
15800 4.52E-05 13700 1.26E-05 13500 3.17E-05 13100
0.000162582 15400 1.52E-05 14900 1.84E-05
15800 4.53E-05 13700 1.26E-05 13500 3.17E-05 13100
0.000161555 15400 1.52E-05 14900 1.84E-05
15800 4.53E-05 13700 1.26E-05 13500 3.16E-05 13100
0.000160533 15400 1.52E-05 14900 1.84E-05
15800 4.54E-05 13700 1.26E-05 13500 3.16E-05 13100
0.000159546 15400 1.52E-05 14900 1.84E-05
15800 4.54E-05 13700 1.25E-05 13500 3.16E-05 13100
0.000158573 15400 1.52E-05 14900 1.84E-05
15800 4.54E-05 13700 1.25E-05 13500 3.15E-05 13100
0.000157595 15400 1.52E-05 14900 1.84E-05
15800 4.55E-05 13800 1.25E-05 13500 3.15E-05 13100
0.000156642 15400 1.52E-05 14900 1.84E-05
15800 4.55E-05 13800 1.25E-05 13500 3.14E-05 13100
0.0001557 15400 1.52E-05 14900 1.84E-05
15800 4.55E-05 13800 1.25E-05 13600 3.14E-05 13100
0.000154783 15400 1.52E-05 14900 1.84E-05
15800 4.56E-05 13800 1.25E-05 13600 3.14E-05 13100
0.000153864 15400 1.52E-05 14900 1.84E-05
15800 4.56E-05 13800 1.25E-05 13600 3.13E-05 13100
0.000152967 15400 1.52E-05 14900 1.84E-05
15800 4.57E-05 13800 1.25E-05 13600 3.13E-05 13100
0.000152079 15400 1.52E-05 14900 1.84E-05
15800 4.57E-05 13800 1.24E-05 13600 3.12E-05 13200
0.000151191 15400 1.52E-05 14900 1.84E-05
15800 4.58E-05 13800 1.24E-05 13600 3.12E-05 13200
0.000150305 15400 1.52E-05 14900 1.84E-05
15800 4.58E-05 13800 1.24E-05 13600 3.12E-05 13200
0.000149437 15400 1.52E-05 14900 1.84E-05
15800 4.59E-05 13800 1.24E-05 13600 3.11E-05 13200
0.000148576 15400 1.52E-05 14900 1.84E-05
15800 4.59E-05 13800 1.24E-05 13600 3.11E-05 13200
0.000147717 15400 1.52E-05 14900 1.84E-05
15800 4.60E-05 13800 1.24E-05 13600 3.11E-05 13200
0.000146888 15400 1.52E-05 14900 1.84E-05
15800 4.60E-05 13800 1.24E-05 13600 3.10E-05 13200
0.000146061 15400 1.52E-05 14900 1.84E-05
15800 4.61E-05 13800 1.24E-05 13600 3.10E-05 13200
0.00014524 15400 1.52E-05 14900 1.85E-05
15800 4.61E-05 13800 1.24E-05 13600 3.10E-05 13200
0.000144428 15400 1.52E-05 14900 1.85E-05

15800 4.62E-05 13800 1.24E-05 13600 3.09E-05 13200
0.000143623 15400 1.52E-05 14900 1.85E-05
15800 4.62E-05 13800 1.24E-05 13600 3.09E-05 13200
0.000142834 15400 1.52E-05 14900 1.85E-05
15800 4.63E-05 13800 1.24E-05 13600 3.08E-05 13200
0.000142043 15500 1.52E-05 14900 1.85E-05
15800 4.63E-05 13800 1.23E-05 13600 3.08E-05 13200
0.000141264 15500 1.52E-05 14900 1.85E-05
15800 4.64E-05 13800 1.23E-05 13600 3.08E-05 13200
0.00014047 15500 1.52E-05 14900 1.85E-05
15800 4.64E-05 13800 1.23E-05 13600 3.07E-05 13200
0.000139746 15500 1.52E-05 14900 1.85E-05
15800 4.65E-05 13800 1.23E-05 13600 3.07E-05 13200
0.000139011 15500 1.52E-05 14900 1.85E-05
15800 4.65E-05 13800 1.23E-05 13600 3.07E-05 13200
0.000138272 15500 1.52E-05 14900 1.85E-05
15800 4.66E-05 13800 1.23E-05 13600 3.06E-05 13200
0.000137524 15500 1.53E-05 14900 1.85E-05
15800 4.67E-05 13800 1.23E-05 13600 3.06E-05 13200
0.000136818 15500 1.52E-05 14900 1.85E-05
15800 4.67E-05 13800 1.23E-05 13600 3.06E-05 13200
0.000136088 15500 1.52E-05 14900 1.85E-05
15800 4.68E-05 13800 1.23E-05 13600 3.05E-05 13200
0.000135396 15500 1.53E-05 14900 1.85E-05
15800 4.68E-05 13800 1.23E-05 13600 3.05E-05 13200
0.000134686 15500 1.53E-05 14900 1.85E-05
15800 4.69E-05 13800 1.22E-05 13600 3.04E-05 13200
0.000133997 15500 1.53E-05 14900 1.85E-05
15800 4.70E-05 13800 1.22E-05 13600 3.04E-05 13200
0.000133311 15500 1.53E-05 14900 1.85E-05
15800 4.70E-05 13800 1.22E-05 13600 3.04E-05 13200
0.00013265 15500 1.53E-05 14900 1.85E-05
15800 4.71E-05 13800 1.22E-05 13600 3.03E-05 13200
0.000131973 15500 1.53E-05 14900 1.85E-05
15800 4.71E-05 13800 1.22E-05 13600 3.03E-05 13200
0.000131333 15500 1.53E-05 14900 1.86E-05
15800 4.72E-05 13800 1.22E-05 13600 3.03E-05 13200
0.000130699 15500 1.53E-05 14900 1.86E-05
15800 4.72E-05 13800 1.22E-05 13600 3.03E-05 13200
0.000130063 15500 1.53E-05 14900 1.86E-05
15800 4.73E-05 13800 1.22E-05 13600 3.02E-05 13200
0.000129441 15500 1.53E-05 14900 1.86E-05
15800 4.73E-05 13800 1.22E-05 13600 3.02E-05 13200
0.000128837 15500 1.53E-05 14900 1.86E-05
15800 4.74E-05 13800 1.21E-05 13600 3.01E-05 13200
0.000128219 15500 1.53E-05 14900 1.86E-05
15800 4.74E-05 13800 1.21E-05 13600 3.01E-05 13200
0.00012761 15500 1.53E-05 14900 1.86E-05
15800 4.75E-05 13800 1.21E-05 13600 3.01E-05 13200
0.000127023 15500 1.53E-05 14900 1.86E-05
15800 4.75E-05 13800 1.21E-05 13600 3.00E-05 13200
0.000126451 15500 1.53E-05 14900 1.86E-05
15800 4.76E-05 13800 1.21E-05 13600 3.00E-05 13200
0.000125872 15500 1.53E-05 14900 1.86E-05
15800 4.77E-05 13800 1.21E-05 13600 3.00E-05 13200
0.000125294 15500 1.53E-05 14900 1.86E-05
15800 4.77E-05 13800 1.21E-05 13600 2.99E-05 13200
0.000124749 15500 1.53E-05 14900 1.86E-05
15800 4.78E-05 13800 1.21E-05 13600 2.99E-05 13200
0.00012419 15500 1.53E-05 14900 1.86E-05
15800 4.78E-05 13800 1.21E-05 13600 2.99E-05 13200
0.000123636 15500 1.53E-05 14900 1.86E-05
15900 4.79E-05 13800 1.20E-05 13600 2.99E-05 13200
0.000123093 15500 1.53E-05 14900 1.86E-05
15900 4.80E-05 13800 1.20E-05 13600 2.98E-05 13200
0.000122549 15500 1.53E-05 14900 1.86E-05
15900 4.81E-05 13800 1.20E-05 13600 2.98E-05 13200
0.000121967 15500 1.53E-05 14900 1.86E-05

15900	4.81E-05	13800	1.20E-05	13600	2.98E-05	13200	0.000121408	15500	1.53E-05	14900	1.86E-05		
15900	4.82E-05	13800	1.20E-05	13600	2.97E-05	13200	0.000120898	15500	1.53E-05	14900	1.86E-05		
15900	4.83E-05	13800	1.20E-05	13600	2.97E-05	13200	0.000120373	15500	1.53E-05	14900	1.86E-05		
15900	4.83E-05	13800	1.20E-05	13600	2.97E-05	13200	0.000119833	15500	1.53E-05	14900	1.86E-05		
15900	4.84E-05	13800	1.20E-05	13600	2.96E-05	13200	0.000119325	15500	1.53E-05	14900	1.86E-05		
15900	4.84E-05	13800	1.20E-05	13600	2.96E-05	13200	0.000118787	15500	1.54E-05	14900	1.86E-05		
15900	4.85E-05	13800	1.19E-05	13600	2.96E-05	13200	0.000118289	15500	1.54E-05	15000	1.86E-05		
15900	4.86E-05	13800	1.19E-05	13600	2.95E-05	13200	0.000117796	15500	1.54E-05	15000	1.86E-05		
15900	4.86E-05	13800	1.19E-05	13600	2.95E-05	13200	0.000117302	15500	1.54E-05	15000	1.86E-05		
15900	4.87E-05	13800	1.19E-05	13600	2.95E-05	13200	0.000116822	15500	1.54E-05	15000	1.86E-05		
15900	4.88E-05	13800	1.19E-05	13600	2.94E-05	13200	0.000116364	15500	1.54E-05	15000	1.87E-05		
15900	4.89E-05	13800	1.19E-05	13600	2.94E-05	13200	0.000115872	15500	1.54E-05	15000	1.86E-05		
15900	4.90E-05	13800	1.19E-05	13600	2.94E-05	13200	0.000115447	15500	1.54E-05	15000	1.87E-05		
15900	4.91E-05	13800	1.19E-05	13600	2.93E-05	13200	0.00011497	15500	1.54E-05	15000	1.87E-05		
15900	4.91E-05	13800	1.19E-05	13600	2.93E-05	13200	0.000114527	15500	1.54E-05	15000	1.87E-05		
15900	4.92E-05	13900	1.18E-05	13600	2.93E-05	13200	0.000114088	15500	1.54E-05	15000	1.87E-05		
15900	4.93E-05	13900	1.18E-05	13600	2.93E-05	13200	0.000113645	15500	1.54E-05	15000	1.87E-05		
15900	4.94E-05	13900	1.18E-05	13700	2.92E-05	13200	0.000113226	15500	1.54E-05	15000	1.87E-05		
15900	4.95E-05	13900	1.18E-05	13700	2.92E-05	13200	0.000112775	15500	1.54E-05	15000	1.87E-05		
15900	4.95E-05	13900	1.18E-05	13700	2.92E-05	13200	0.000112345	15500	1.54E-05	15000	1.87E-05		
15900	4.96E-05	13900	1.18E-05	13700	2.91E-05	13200	0.000111892	15500	1.54E-05	15000	1.87E-05		
15900	4.97E-05	13900	1.18E-05	13700	2.91E-05	13300	0.00011147	15500	1.54E-05	15000	1.87E-05		
15900	4.98E-05	13900	1.18E-05	13700	2.91E-05	13300	0.00011104	15500	1.54E-05	15000	1.87E-05		
15900	4.99E-05	13900	1.18E-05	13700	2.90E-05	13300	0.00011064	15500	1.54E-05	15000	1.87E-05		
15900	4.99E-05	13900	1.18E-05	13700	2.90E-05	13300	0.000110249	15500	1.54E-05	15000	1.87E-05		
15900	5.00E-05	13900	1.18E-05	13700	2.90E-05	13300	0.000109853	15500	1.54E-05	15000	1.88E-05		
15900	5.01E-05	13900	1.18E-05	13700	2.90E-05	13300	0.000109455	15500	1.54E-05	15000	1.88E-05		
15900	5.02E-05	13900	1.18E-05	13700	2.89E-05	13300	0.000109062	15500	1.54E-05	15000	1.88E-05		
15900	5.03E-05	13900	1.18E-05	13700	2.89E-05	13300	0.000108692	15500	1.54E-05	15000	1.88E-05		
15900	5.04E-05	13900	1.18E-05	13700	2.89E-05	13300	0.000108327	15500	1.54E-05	15000	1.88E-05		
15900	5.05E-05	13900	1.17E-05	13700	2.88E-05	13300	0.000107939	15500	1.54E-05	15000	1.88E-05		
15900	5.06E-05	13900	1.17E-05	13700	2.88E-05	13300	0.00010758	15500	1.54E-05	15000	1.88E-05		
15900	5.07E-05	13900	1.17E-05	13700	2.88E-05	13300	0.000107215	15600	1.54E-05	15000	1.88E-05		
15900	5.08E-05	13900	1.17E-05	13700	2.88E-05	13300	0.000106827	15600	1.54E-05	15000	1.88E-05		

15900	5.09E-05	13900	1.17E-05	13700	2.87E-05	13300	0.000106477	15600	1.54E-05	15000	1.88E-05
15900	5.09E-05	13900	1.17E-05	13700	2.87E-05	13300	0.000106086	15600	1.54E-05	15000	1.88E-05
15900	5.10E-05	13900	1.17E-05	13700	2.87E-05	13300	0.000105728	15600	1.55E-05	15000	1.88E-05
15900	5.12E-05	13900	1.17E-05	13700	2.87E-05	13300	0.000105375	15600	1.54E-05	15000	1.88E-05
15900	5.12E-05	13900	1.17E-05	13700	2.86E-05	13300	0.000105041	15600	1.54E-05	15000	1.88E-05
15900	5.13E-05	13900	1.17E-05	13700	2.86E-05	13300	0.000104712	15600	1.54E-05	15000	1.88E-05
15900	5.14E-05	13900	1.16E-05	13700	2.86E-05	13300	0.000104359	15600	1.54E-05	15000	1.88E-05
15900	5.15E-05	13900	1.16E-05	13700	2.85E-05	13300	0.000104044	15600	1.55E-05	15000	1.88E-05
15900	5.16E-05	13900	1.16E-05	13700	2.85E-05	13300	0.000103707	15600	1.54E-05	15000	1.88E-05
15900	5.17E-05	13900	1.16E-05	13700	2.85E-05	13300	0.000103419	15600	1.55E-05	15000	1.88E-05
15900	5.18E-05	13900	1.16E-05	13700	2.84E-05	13300	0.000103111	15600	1.55E-05	15000	1.88E-05
15900	5.19E-05	13900	1.16E-05	13700	2.84E-05	13300	0.00010277	15600	1.55E-05	15000	1.88E-05
15900	5.20E-05	13900	1.16E-05	13700	2.84E-05	13300	0.000102487	15600	1.55E-05	15000	1.89E-05
15900	5.21E-05	13900	1.16E-05	13700	2.84E-05	13300	0.000102187	15600	1.55E-05	15000	1.88E-05
15900	5.23E-05	13900	1.16E-05	13700	2.83E-05	13300	0.000101885	15600	1.55E-05	15000	1.89E-05
15900	5.24E-05	13900	1.16E-05	13700	2.83E-05	13300	0.000101587	15600	1.55E-05	15000	1.89E-05
15900	5.25E-05	13900	1.16E-05	13700	2.83E-05	13300	0.000101305	15600	1.55E-05	15000	1.89E-05
15900	5.26E-05	13900	1.16E-05	13700	2.82E-05	13300	0.000101015	15600	1.55E-05	15000	1.89E-05
15900	5.27E-05	13900	1.16E-05	13700	2.82E-05	13300	0.000100708	15600	1.55E-05	15000	1.89E-05
15900	5.28E-05	13900	1.16E-05	13700	2.82E-05	13300	0.000100442	15600	1.55E-05	15000	1.89E-05
15900	5.30E-05	13900	1.16E-05	13700	2.82E-05	13300	0.000100143	15600	1.55E-05	15000	1.89E-05
15900	5.31E-05	13900	1.16E-05	13700	2.81E-05	13300	9.98E-05	15600	1.55E-05	15000	1.89E-05
15900	5.32E-05	13900	1.16E-05	13700	2.81E-05	13300	9.96E-05	15600	1.55E-05	15000	1.89E-05
15900	5.33E-05	13900	1.16E-05	13700	2.81E-05	13300	9.93E-05	15600	1.55E-05	15000	1.89E-05
15900	5.35E-05	13900	1.16E-05	13700	2.81E-05	13300	9.90E-05	15600	1.55E-05	15000	1.89E-05
15900	5.36E-05	13900	1.15E-05	13700	2.80E-05	13300	9.88E-05	15600	1.55E-05	15000	1.89E-05
15900	5.37E-05	13900	1.15E-05	13700	2.80E-05	13300	9.85E-05	15600	1.55E-05	15000	1.89E-05
16000	5.39E-05	13900	1.15E-05	13700	2.80E-05	13300	9.82E-05	15600	1.55E-05	15000	1.89E-05
16000	5.40E-05	13900	1.15E-05	13700	2.79E-05	13300	9.80E-05	15600	1.55E-05	15000	1.89E-05
16000	5.41E-05	13900	1.15E-05	13700	2.79E-05	13300	9.78E-05	15600	1.55E-05	15000	1.89E-05
16000	5.42E-05	13900	1.15E-05	13700	2.79E-05	13300	9.75E-05	15600	1.55E-05	15000	1.89E-05
16000	5.44E-05	13900	1.15E-05	13700	2.79E-05	13300	9.72E-05	15600	1.55E-05	15000	1.90E-05
16000	5.45E-05	13900	1.15E-05	13700	2.78E-05	13300	9.70E-05	15600	1.55E-05	15000	1.90E-05
16000	5.47E-05	13900	1.15E-05	13700	2.78E-05	13300	9.67E-05	15600	1.55E-05	15000	1.90E-05

16000 5.48E-05 13900 1.15E-05 13700 2.78E-05 13300
9.65E-05 15600 1.55E-05 15000 1.90E-05
16000 5.50E-05 13900 1.15E-05 13700 2.78E-05 13300
9.62E-05 15600 1.55E-05 15000 1.90E-05
16000 5.51E-05 13900 1.15E-05 13700 2.77E-05 13300
9.60E-05 15600 1.55E-05 15100 1.90E-05
16000 5.53E-05 13900 1.14E-05 13700 2.77E-05 13300
9.58E-05 15600 1.55E-05 15100 1.90E-05
16000 5.54E-05 13900 1.14E-05 13700 2.77E-05 13300
9.56E-05 15600 1.55E-05 15100 1.90E-05
16000 5.56E-05 13900 1.14E-05 13700 2.77E-05 13300
9.54E-05 15600 1.56E-05 15100 1.90E-05
16000 5.58E-05 13900 1.14E-05 13700 2.76E-05 13300
9.51E-05 15600 1.56E-05 15100 1.90E-05
16000 5.59E-05 13900 1.14E-05 13700 2.76E-05 13300
9.49E-05 15600 1.56E-05 15100 1.90E-05
16000 5.61E-05 13900 1.14E-05 13700 2.76E-05 13300
9.47E-05 15600 1.56E-05 15100 1.90E-05
16000 5.63E-05 13900 1.14E-05 13700 2.76E-05 13300
9.45E-05 15600 1.56E-05 15100 1.90E-05
16000 5.64E-05 13900 1.14E-05 13700 2.75E-05 13300
9.43E-05 15600 1.56E-05 15100 1.90E-05
16000 5.66E-05 14000 1.14E-05 13700 2.75E-05 13300
9.41E-05 15600 1.56E-05 15100 1.90E-05
16000 5.67E-05 14000 1.14E-05 13700 2.75E-05 13300
9.39E-05 15600 1.55E-05 15100 1.90E-05
16000 5.69E-05 14000 1.14E-05 13800 2.75E-05 13300
9.37E-05 15600 1.56E-05 15100 1.90E-05
16000 5.70E-05 14000 1.13E-05 13800 2.74E-05 13300
9.35E-05 15600 1.56E-05 15100 1.90E-05
16000 5.72E-05 14000 1.13E-05 13800 2.74E-05 13300
9.33E-05 15600 1.56E-05 15100 1.90E-05
16000 5.74E-05 14000 1.13E-05 13800 2.74E-05 13300
9.31E-05 15600 1.56E-05 15100 1.90E-05
16000 5.75E-05 14000 1.13E-05 13800 2.74E-05 13400
9.29E-05 15600 1.56E-05 15100 1.90E-05
16000 5.77E-05 14000 1.13E-05 13800 2.73E-05 13400
9.28E-05 15600 1.56E-05 15100 1.90E-05
16000 5.79E-05 14000 1.13E-05 13800 2.73E-05 13400
9.26E-05 15600 1.56E-05 15100 1.90E-05
16000 5.81E-05 14000 1.13E-05 13800 2.73E-05 13400
9.24E-05 15600 1.56E-05 15100 1.90E-05
16000 5.83E-05 14000 1.13E-05 13800 2.73E-05 13400
9.22E-05 15600 1.56E-05 15100 1.90E-05
16000 5.85E-05 14000 1.13E-05 13800 2.73E-05 13400
9.20E-05 15600 1.56E-05 15100 1.90E-05
16000 5.87E-05 14000 1.13E-05 13800 2.72E-05 13400
9.19E-05 15600 1.56E-05 15100 1.90E-05
16000 5.89E-05 14000 1.13E-05 13800 2.72E-05 13400
9.18E-05 15600 1.56E-05 15100 1.90E-05
16000 5.91E-05 14000 1.13E-05 13800 2.72E-05 13400
9.16E-05 15600 1.56E-05 15100 1.90E-05
16000 5.93E-05 14000 1.12E-05 13800 2.72E-05 13400
9.14E-05 15600 1.56E-05 15100 1.90E-05
16000 5.95E-05 14000 1.12E-05 13800 2.71E-05 13400
9.12E-05 15600 1.56E-05 15100 1.90E-05
16000 5.97E-05 14000 1.12E-05 13800 2.71E-05 13400
9.11E-05 15700 1.56E-05 15100 1.91E-05
16000 5.99E-05 14000 1.12E-05 13800 2.71E-05 13400
9.09E-05 15700 1.56E-05 15100 1.91E-05
16000 6.00E-05 14000 1.12E-05 13800 2.71E-05 13400
9.08E-05 15700 1.56E-05 15100 1.91E-05
16000 6.03E-05 14000 1.12E-05 13800 2.71E-05 13400
9.06E-05 15700 1.56E-05 15100 1.91E-05
16000 6.05E-05 14000 1.12E-05 13800 2.70E-05 13400
9.05E-05 15700 1.56E-05 15100 1.91E-05
16000 6.07E-05 14000 1.12E-05 13800 2.70E-05 13400
9.03E-05 15700 1.56E-05 15100 1.91E-05

16000 6.09E-05 14000 1.12E-05 13800 2.70E-05 13400
9.01E-05 15700 1.56E-05 15100 1.91E-05
16000 6.11E-05 14000 1.12E-05 13800 2.70E-05 13400
9.00E-05 15700 1.56E-05 15100 1.91E-05
16000 6.14E-05 14000 1.12E-05 13800 2.69E-05 13400
8.99E-05 15700 1.56E-05 15100 1.91E-05
16000 6.16E-05 14000 1.12E-05 13800 2.69E-05 13400
8.98E-05 15700 1.56E-05 15100 1.91E-05
16000 6.18E-05 14000 1.12E-05 13800 2.69E-05 13400
8.96E-05 15700 1.56E-05 15100 1.91E-05
16000 6.21E-05 14000 1.12E-05 13800 2.69E-05 13400
8.95E-05 15700 1.56E-05 15100 1.91E-05
16000 6.23E-05 14000 1.11E-05 13800 2.69E-05 13400
8.94E-05 15700 1.56E-05 15100 1.91E-05
16000 6.26E-05 14000 1.11E-05 13800 2.69E-05 13400
8.93E-05 15700 1.56E-05 15100 1.91E-05
16000 6.28E-05 14000 1.11E-05 13800 2.69E-05 13400
8.91E-05 15700 1.56E-05 15100 1.91E-05
16000 6.31E-05 14000 1.11E-05 13800 2.68E-05 13400
8.90E-05 15700 1.56E-05 15100 1.91E-05
16000 6.33E-05 14000 1.11E-05 13800 2.68E-05 13400
8.89E-05 15700 1.56E-05 15100 1.91E-05
16000 6.35E-05 14000 1.11E-05 13800 2.68E-05 13400
8.88E-05 15700 1.56E-05 15100 1.91E-05
16000 6.38E-05 14000 1.11E-05 13800 2.68E-05 13400
8.87E-05 15700 1.57E-05 15100 1.91E-05
16000 6.40E-05 14000 1.11E-05 13800 2.68E-05 13400
8.86E-05 15700 1.56E-05 15100 1.91E-05
16000 6.43E-05 14000 1.11E-05 13800 2.67E-05 13400
8.85E-05 15700 1.57E-05 15100 1.91E-05
16000 6.46E-05 14000 1.11E-05 13800 2.67E-05 13400
8.85E-05 15700 1.57E-05 15100 1.92E-05
16000 6.48E-05 14000 1.11E-05 13800 2.67E-05 13400
8.83E-05 15700 1.57E-05 15100 1.92E-05
16000 6.51E-05 14000 1.11E-05 13800 2.67E-05 13400
8.82E-05 15700 1.57E-05 15100 1.91E-05
16000 6.53E-05 14000 1.11E-05 13800 2.67E-05 13400
8.81E-05 15700 1.57E-05 15100 1.91E-05
16000 6.56E-05 14000 1.11E-05 13800 2.66E-05 13400
8.81E-05 15700 1.57E-05 15100 1.92E-05
16000 6.58E-05 14000 1.11E-05 13800 2.66E-05 13400
8.79E-05 15700 1.57E-05 15100 1.92E-05
16000 6.61E-05 14000 1.10E-05 13800 2.66E-05 13400
8.79E-05 15700 1.57E-05 15100 1.92E-05
16000 6.64E-05 14000 1.10E-05 13800 2.66E-05 13400
8.78E-05 15700 1.57E-05 15100 1.92E-05
16100 6.67E-05 14000 1.10E-05 13800 2.65E-05 13400
8.77E-05 15700 1.57E-05 15100 1.92E-05
16100 6.70E-05 14000 1.10E-05 13800 2.65E-05 13400
8.76E-05 15700 1.57E-05 15100 1.92E-05
16100 6.73E-05 14000 1.10E-05 13800 2.65E-05 13400
8.76E-05 15700 1.57E-05 15100 1.92E-05
16100 6.76E-05 14000 1.10E-05 13800 2.65E-05 13400
8.75E-05 15700 1.57E-05 15100 1.92E-05
16100 6.79E-05 14000 1.10E-05 13800 2.64E-05 13400
8.74E-05 15700 1.57E-05 15100 1.92E-05
16100 6.82E-05 14000 1.10E-05 13800 2.64E-05 13400
8.73E-05 15700 1.57E-05 15100 1.92E-05
16100 6.85E-05 14000 1.10E-05 13800 2.64E-05 13400
8.73E-05 15700 1.57E-05 15100 1.92E-05
16100 6.88E-05 14000 1.10E-05 13800 2.63E-05 13400
8.72E-05 15700 1.57E-05 15100 1.92E-05
16100 6.91E-05 14000 1.10E-05 13800 2.63E-05 13400
8.72E-05 15700 1.57E-05 15100 1.92E-05
16100 6.95E-05 14000 1.10E-05 13800 2.63E-05 13400
8.71E-05 15700 1.57E-05 15200 1.92E-05
16100 6.98E-05 14000 1.10E-05 13800 2.62E-05 13400
8.71E-05 15700 1.57E-05 15200 1.92E-05

16100 7.01E-05 14000 1.10E-05 13800 2.62E-05 13400
8.70E-05 15700 1.58E-05 15200 1.92E-05
16100 7.05E-05 14000 1.10E-05 13800 2.62E-05 13400
8.69E-05 15700 1.57E-05 15200 1.92E-05
16100 7.08E-05 14000 1.10E-05 13800 2.62E-05 13400
8.69E-05 15700 1.57E-05 15200 1.92E-05
16100 7.11E-05 14000 1.09E-05 13800 2.61E-05 13400
8.68E-05 15700 1.58E-05 15200 1.92E-05
16100 7.15E-05 14000 1.09E-05 13800 2.61E-05 13400
8.68E-05 15700 1.58E-05 15200 1.92E-05
16100 7.18E-05 14000 1.09E-05 13800 2.61E-05 13400
8.68E-05 15700 1.58E-05 15200 1.92E-05
16100 7.22E-05 14000 1.09E-05 13800 2.60E-05 13400
8.68E-05 15700 1.58E-05 15200 1.92E-05
16100 7.26E-05 14100 1.09E-05 13800 2.60E-05 13400
8.67E-05 15700 1.58E-05 15200 1.92E-05
16100 7.29E-05 14100 1.09E-05 13800 2.60E-05 13400
8.67E-05 15700 1.58E-05 15200 1.92E-05
16100 7.33E-05 14100 1.09E-05 13900 2.60E-05 13400
8.66E-05 15700 1.58E-05 15200 1.93E-05
16100 7.37E-05 14100 1.09E-05 13900 2.59E-05 13400
8.66E-05 15700 1.58E-05 15200 1.93E-05
16100 7.41E-05 14100 1.09E-05 13900 2.59E-05 13400
8.66E-05 15700 1.58E-05 15200 1.93E-05
16100 7.45E-05 14100 1.09E-05 13900 2.59E-05 13400
8.65E-05 15700 1.58E-05 15200 1.93E-05
16100 7.49E-05 14100 1.09E-05 13900 2.59E-05 13500
8.65E-05 15700 1.58E-05 15200 1.93E-05
16100 7.53E-05 14100 1.09E-05 13900 2.58E-05 13500
8.65E-05 15700 1.58E-05 15200 1.93E-05
16100 7.57E-05 14100 1.09E-05 13900 2.58E-05 13500
8.65E-05 15700 1.58E-05 15200 1.93E-05
16100 7.61E-05 14100 1.09E-05 13900 2.58E-05 13500
8.65E-05 15700 1.58E-05 15200 1.93E-05
16100 7.65E-05 14100 1.09E-05 13900 2.58E-05 13500
8.64E-05 15700 1.58E-05 15200 1.93E-05
16100 7.69E-05 14100 1.09E-05 13900 2.58E-05 13500
8.64E-05 15700 1.58E-05 15200 1.93E-05
16100 7.73E-05 14100 1.09E-05 13900 2.57E-05 13500
8.64E-05 15700 1.58E-05 15200 1.93E-05
16100 7.78E-05 14100 1.09E-05 13900 2.57E-05 13500
8.64E-05 15700 1.58E-05 15200 1.93E-05
16100 7.82E-05 14100 1.09E-05 13900 2.57E-05 13500
8.64E-05 15700 1.58E-05 15200 1.93E-05
16100 7.86E-05 14100 1.09E-05 13900 2.57E-05 13500
8.64E-05 15700 1.58E-05 15200 1.93E-05
16100 7.91E-05 14100 1.09E-05 13900 2.56E-05 13500
8.64E-05 15700 1.58E-05 15200 1.93E-05
16100 7.95E-05 14100 1.09E-05 13900 2.56E-05 13500
8.64E-05 15800 1.58E-05 15200 1.93E-05
16100 8.00E-05 14100 1.09E-05 13900 2.56E-05 13500
8.64E-05 15800 1.58E-05 15200 1.93E-05
16100 8.05E-05 14100 1.09E-05 13900 2.56E-05 13500
8.64E-05 15800 1.58E-05 15200 1.93E-05
16100 8.09E-05 14100 1.09E-05 13900 2.56E-05 13500
8.64E-05 15800 1.58E-05 15200 1.93E-05
16100 8.14E-05 14100 1.09E-05 13900 2.55E-05 13500
8.65E-05 15800 1.58E-05 15200 1.93E-05
16100 8.19E-05 14100 1.09E-05 13900 2.55E-05 13500
8.65E-05 15800 1.59E-05 15200 1.94E-05
16100 8.24E-05 14100 1.08E-05 13900 2.55E-05 13500
8.65E-05 15800 1.59E-05 15200 1.93E-05
16100 8.29E-05 14100 1.08E-05 13900 2.55E-05 13500
8.65E-05 15800 1.59E-05 15200 1.93E-05
16100 8.34E-05 14100 1.08E-05 13900 2.54E-05 13500
8.66E-05 15800 1.59E-05 15200 1.93E-05
16100 8.39E-05 14100 1.08E-05 13900 2.54E-05 13500
8.66E-05 15800 1.59E-05 15200 1.93E-05

16100 8.44E-05 14100 1.08E-05 13900 2.54E-05 13500
8.66E-05 15800 1.59E-05 15200 1.94E-05
16100 8.49E-05 14100 1.08E-05 13900 2.54E-05 13500
8.66E-05 15800 1.59E-05 15200 1.94E-05
16100 8.54E-05 14100 1.08E-05 13900 2.54E-05 13500
8.67E-05 15800 1.59E-05 15200 1.94E-05
16100 8.60E-05 14100 1.08E-05 13900 2.54E-05 13500
8.67E-05 15800 1.59E-05 15200 1.94E-05
16100 8.65E-05 14100 1.08E-05 13900 2.54E-05 13500
8.67E-05 15800 1.59E-05 15200 1.94E-05
16100 8.71E-05 14100 1.08E-05 13900 2.53E-05 13500
8.68E-05 15800 1.59E-05 15200 1.94E-05
16100 8.76E-05 14100 1.08E-05 13900 2.53E-05 13500
8.68E-05 15800 1.59E-05 15200 1.94E-05
16100 8.82E-05 14100 1.08E-05 13900 2.53E-05 13500
8.69E-05 15800 1.59E-05 15200 1.94E-05
16100 8.87E-05 14100 1.08E-05 13900 2.53E-05 13500
8.69E-05 15800 1.59E-05 15200 1.94E-05
16100 8.93E-05 14100 1.08E-05 13900 2.53E-05 13500
8.70E-05 15800 1.59E-05 15200 1.94E-05
16100 8.99E-05 14100 1.08E-05 13900 2.52E-05 13500
8.70E-05 15800 1.59E-05 15200 1.94E-05
16100 9.05E-05 14100 1.08E-05 13900 2.52E-05 13500
8.71E-05 15800 1.59E-05 15200 1.94E-05
16100 9.11E-05 14100 1.08E-05 13900 2.52E-05 13500
8.72E-05 15800 1.59E-05 15200 1.94E-05
16100 9.17E-05 14100 1.08E-05 13900 2.52E-05 13500
8.73E-05 15800 1.59E-05 15200 1.94E-05
16100 9.23E-05 14100 1.08E-05 13900 2.52E-05 13500
8.73E-05 15800 1.59E-05 15200 1.94E-05
16100 9.29E-05 14100 1.08E-05 13900 2.52E-05 13500
8.74E-05 15800 1.59E-05 15200 1.95E-05
16100 9.36E-05 14100 1.08E-05 13900 2.51E-05 13500
8.75E-05 15800 1.59E-05 15200 1.95E-05
16100 9.42E-05 14100 1.08E-05 13900 2.51E-05 13500
8.75E-05 15800 1.59E-05 15200 1.95E-05
16100 9.49E-05 14100 1.08E-05 13900 2.51E-05 13500
8.76E-05 15800 1.59E-05 15200 1.94E-05
16200 9.55E-05 14100 1.08E-05 13900 2.51E-05 13500
8.77E-05 15800 1.59E-05 15200 1.94E-05
16200 9.62E-05 14100 1.08E-05 13900 2.51E-05 13500
8.78E-05 15800 1.59E-05 15200 1.95E-05
16200 9.68E-05 14100 1.08E-05 13900 2.51E-05 13500
8.78E-05 15800 1.59E-05 15200 1.95E-05
16200 9.75E-05 14100 1.08E-05 13900 2.50E-05 13500
8.79E-05 15800 1.59E-05 15200 1.95E-05
16200 9.82E-05 14100 1.08E-05 13900 2.50E-05 13500
8.80E-05 15800 1.59E-05 15200 1.95E-05
16200 9.88E-05 14100 1.08E-05 13900 2.50E-05 13500
8.81E-05 15800 1.59E-05 15200 1.95E-05
16200 9.95E-05 14100 1.08E-05 13900 2.50E-05 13500
8.82E-05 15800 1.59E-05 15200 1.95E-05
16200 0.0001 14100 1.08E-05 13900 2.50E-05 13500 8.83E-05
15800 1.59E-05 15200 1.95E-05
16200 0.000101 14100 1.08E-05 13900 2.49E-05 13500
8.84E-05 15800 1.59E-05 15200 1.95E-05
16200 0.000102 14100 1.08E-05 13900 2.49E-05 13500
8.84E-05 15800 1.59E-05 15300 1.95E-05
16200 0.000102 14100 1.08E-05 13900 2.49E-05 13500
8.85E-05 15800 1.59E-05 15300 1.95E-05
16200 0.000103 14100 1.08E-05 13900 2.49E-05 13500
8.87E-05 15800 1.60E-05 15300 1.95E-05
16200 0.000104 14100 1.08E-05 13900 2.49E-05 13500
8.87E-05 15800 1.60E-05 15300 1.95E-05
16200 0.000105 14100 1.08E-05 13900 2.49E-05 13500
8.89E-05 15800 1.60E-05 15300 1.95E-05
16200 0.000105 14100 1.08E-05 13900 2.49E-05 13500
8.90E-05 15800 1.60E-05 15300 1.95E-05

16200 0.000106 14100 1.08E-05 13900 2.49E-05 13500
8.91E-05 15800 1.60E-05 15300 1.95E-05
16200 0.000107 14100 1.08E-05 13900 2.48E-05 13500
8.92E-05 15800 1.60E-05 15300 1.95E-05
16200 0.000108 14100 1.08E-05 13900 2.48E-05 13500
8.93E-05 15800 1.60E-05 15300 1.95E-05
16200 0.000108 14200 1.08E-05 13900 2.48E-05 13500
8.94E-05 15800 1.60E-05 15300 1.95E-05
16200 0.000109 14200 1.08E-05 13900 2.48E-05 13500
8.96E-05 15800 1.60E-05 15300 1.95E-05
16200 0.00011 14200 1.08E-05 14000 2.48E-05 13500 8.97E-05
15800 1.60E-05 15300 1.95E-05
16200 0.000111 14200 1.08E-05 14000 2.48E-05 13500
8.98E-05 15800 1.60E-05 15300 1.95E-05
16200 0.000111 14200 1.08E-05 14000 2.48E-05 13500
9.00E-05 15800 1.60E-05 15300 1.96E-05
16200 0.000112 14200 1.08E-05 14000 2.48E-05 13500
9.01E-05 15800 1.60E-05 15300 1.95E-05
16200 0.000113 14200 1.09E-05 14000 2.47E-05 13600
9.03E-05 15800 1.60E-05 15300 1.95E-05
16200 0.000114 14200 1.09E-05 14000 2.47E-05 13600
9.04E-05 15800 1.60E-05 15300 1.96E-05
16200 0.000115 14200 1.09E-05 14000 2.47E-05 13600
9.06E-05 15800 1.60E-05 15300 1.95E-05
16200 0.000115 14200 1.09E-05 14000 2.47E-05 13600
9.07E-05 15800 1.60E-05 15300 1.96E-05
16200 0.000116 14200 1.09E-05 14000 2.47E-05 13600
9.09E-05 15800 1.60E-05 15300 1.96E-05
16200 0.000117 14200 1.09E-05 14000 2.47E-05 13600
9.10E-05 15800 1.60E-05 15300 1.96E-05
16200 0.000118 14200 1.09E-05 14000 2.47E-05 13600
9.12E-05 15800 1.60E-05 15300 1.96E-05
16200 0.000119 14200 1.09E-05 14000 2.46E-05 13600
9.14E-05 15800 1.60E-05 15300 1.96E-05
16200 0.00012 14200 1.09E-05 14000 2.46E-05 13600 9.15E-05
15800 1.60E-05 15300 1.96E-05
16200 0.00012 14200 1.09E-05 14000 2.46E-05 13600 9.17E-05
15800 1.60E-05 15300 1.96E-05
16200 0.000121 14200 1.09E-05 14000 2.46E-05 13600
9.19E-05 15800 1.60E-05 15300 1.96E-05
16200 0.000122 14200 1.09E-05 14000 2.46E-05 13600
9.21E-05 15900 1.60E-05 15300 1.96E-05
16200 0.000123 14200 1.09E-05 14000 2.46E-05 13600
9.22E-05 15900 1.60E-05 15300 1.96E-05
16200 0.000124 14200 1.09E-05 14000 2.46E-05 13600
9.24E-05 15900 1.61E-05 15300 1.96E-05
16200 0.000125 14200 1.09E-05 14000 2.46E-05 13600
9.26E-05 15900 1.61E-05 15300 1.96E-05
16200 0.000125 14200 1.09E-05 14000 2.46E-05 13600
9.28E-05 15900 1.61E-05 15300 1.96E-05
16200 0.000126 14200 1.10E-05 14000 2.46E-05 13600
9.30E-05 15900 1.61E-05 15300 1.96E-05
16200 0.000127 14200 1.10E-05 14000 2.46E-05 13600
9.32E-05 15900 1.61E-05 15300 1.96E-05
16200 0.000128 14200 1.10E-05 14000 2.46E-05 13600
9.34E-05 15900 1.61E-05 15300 1.96E-05
16200 0.000129 14200 1.10E-05 14000 2.45E-05 13600
9.37E-05 15900 1.61E-05 15300 1.96E-05
16200 0.000129 14200 1.10E-05 14000 2.45E-05 13600
9.39E-05 15900 1.61E-05 15300 1.96E-05
16200 0.00013 14200 1.10E-05 14000 2.45E-05 13600 9.41E-05
15900 1.61E-05 15300 1.96E-05
16200 0.000131 14200 1.10E-05 14000 2.45E-05 13600
9.43E-05 15900 1.61E-05 15300 1.96E-05
16200 0.000132 14200 1.10E-05 14000 2.45E-05 13600
9.45E-05 15900 1.61E-05 15300 1.96E-05
16200 0.000133 14200 1.10E-05 14000 2.45E-05 13600
9.48E-05 15900 1.61E-05 15300 1.96E-05

16200 0.000133 14200 1.10E-05 14000 2.45E-05 13600
9.50E-05 15900 1.61E-05 15300 1.96E-05
16200 0.000134 14200 1.10E-05 14000 2.45E-05 13600
9.52E-05 15900 1.61E-05 15300 1.96E-05
16200 0.000135 14200 1.11E-05 14000 2.45E-05 13600
9.55E-05 15900 1.61E-05 15300 1.96E-05
16200 0.000136 14200 1.11E-05 14000 2.45E-05 13600
9.57E-05 15900 1.61E-05 15300 1.96E-05
16200 0.000136 14200 1.11E-05 14000 2.45E-05 13600
9.59E-05 15900 1.61E-05 15300 1.96E-05
16200 0.000137 14200 1.11E-05 14000 2.45E-05 13600
9.62E-05 15900 1.61E-05 15300 1.96E-05
16200 0.000138 14200 1.11E-05 14000 2.44E-05 13600
9.64E-05 15900 1.61E-05 15300 1.97E-05
16200 0.000138 14200 1.11E-05 14000 2.44E-05 13600
9.67E-05 15900 1.61E-05 15300 1.96E-05
16200 0.000139 14200 1.11E-05 14000 2.44E-05 13600
9.69E-05 15900 1.61E-05 15300 1.96E-05
16200 0.00014 14200 1.11E-05 14000 2.44E-05 13600 9.72E-05
15900 1.61E-05 15300 1.97E-05
16200 0.00014 14200 1.11E-05 14000 2.44E-05 13600 9.74E-05
15900 1.61E-05 15300 1.97E-05
16200 0.000141 14200 1.11E-05 14000 2.44E-05 13600
9.77E-05 15900 1.61E-05 15300 1.97E-05
16200 0.000142 14200 1.12E-05 14000 2.44E-05 13600
9.79E-05 15900 1.62E-05 15300 1.96E-05
16200 0.000142 14200 1.12E-05 14000 2.44E-05 13600
9.82E-05 15900 1.62E-05 15300 1.96E-05
16200 0.000143 14200 1.12E-05 14000 2.44E-05 13600
9.85E-05 15900 1.62E-05 15300 1.97E-05
16300 0.000143 14200 1.12E-05 14000 2.44E-05 13600
9.88E-05 15900 1.62E-05 15300 1.96E-05
16300 0.000144 14200 1.12E-05 14000 2.44E-05 13600
9.91E-05 15900 1.62E-05 15300 1.97E-05
16300 0.000144 14200 1.13E-05 14000 2.44E-05 13600
9.93E-05 15900 1.62E-05 15300 1.97E-05
16300 0.000145 14200 1.13E-05 14000 2.44E-05 13600
9.96E-05 15900 1.62E-05 15300 1.97E-05
16300 0.000145 14200 1.13E-05 14000 2.44E-05 13600
9.99E-05 15900 1.62E-05 15300 1.97E-05
16300 0.000145 14200 1.13E-05 14000 2.44E-05 13600
0.000100257 15900 1.62E-05 15300 1.97E-05
16300 0.000146 14200 1.13E-05 14000 2.44E-05 13600
0.000100569 15900 1.62E-05 15300 1.97E-05
16300 0.000146 14200 1.14E-05 14000 2.44E-05 13600
0.000100897 15900 1.62E-05 15300 1.97E-05
16300 0.000146 14200 1.14E-05 14000 2.43E-05 13600
0.000101217 15900 1.62E-05 15300 1.97E-05
16300 0.000147 14200 1.14E-05 14000 2.43E-05 13600
0.000101532 15900 1.62E-05 15400 1.97E-05
16300 0.000147 14200 1.14E-05 14000 2.43E-05 13600
0.000101867 15900 1.62E-05 15400 1.97E-05
16300 0.000147 14200 1.14E-05 14000 2.43E-05 13600
0.0001022 15900 1.62E-05 15400 1.97E-05
16300 0.000147 14200 1.14E-05 14000 2.44E-05 13600
0.00010253 15900 1.62E-05 15400 1.97E-05
16300 0.000147 14200 1.15E-05 14000 2.44E-05 13600
0.000102857 15900 1.62E-05 15400 1.97E-05
16300 0.000147 14200 1.15E-05 14000 2.44E-05 13600
0.000103228 15900 1.62E-05 15400 1.97E-05
16300 0.000147 14200 1.15E-05 14000 2.44E-05 13600
0.000103578 15900 1.62E-05 15400 1.97E-05
16300 0.000147 14200 1.15E-05 14000 2.44E-05 13600
0.000103935 15900 1.62E-05 15400 1.98E-05
16300 0.000147 14200 1.16E-05 14000 2.44E-05 13600
0.000104304 15900 1.62E-05 15400 1.98E-05
16300 0.000147 14300 1.16E-05 14000 2.44E-05 13600
0.000104669 15900 1.62E-05 15400 1.98E-05

16300 0.000147 14300 1.16E-05 14000 2.44E-05 13600
0.000105038 15900 1.62E-05 15400 1.98E-05
16300 0.000147 14300 1.17E-05 14100 2.44E-05 13600
0.00010542 15900 1.62E-05 15400 1.98E-05
16300 0.000147 14300 1.17E-05 14100 2.44E-05 13600
0.000105796 15900 1.62E-05 15400 1.98E-05
16300 0.000146 14300 1.17E-05 14100 2.44E-05 13600
0.000106181 15900 1.63E-05 15400 1.98E-05
16300 0.000146 14300 1.18E-05 14100 2.44E-05 13600
0.000106572 15900 1.63E-05 15400 1.98E-05
16300 0.000146 14300 1.18E-05 14100 2.44E-05 13700
0.000106947 15900 1.63E-05 15400 1.98E-05
16300 0.000145 14300 1.19E-05 14100 2.44E-05 13700
0.000107358 15900 1.63E-05 15400 1.99E-05
16300 0.000145 14300 1.19E-05 14100 2.44E-05 13700
0.000107771 15900 1.63E-05 15400 1.99E-05
16300 0.000144 14300 1.19E-05 14100 2.44E-05 13700
0.000108186 15900 1.63E-05 15400 1.99E-05
16300 0.000144 14300 1.20E-05 14100 2.44E-05 13700
0.000108611 15900 1.63E-05 15400 1.99E-05
16300 0.000143 14300 1.20E-05 14100 2.44E-05 13700
0.000109027 15900 1.63E-05 15400 1.99E-05
16300 0.000143 14300 1.21E-05 14100 2.44E-05 13700
0.000109453 15900 1.63E-05 15400 1.99E-05
16300 0.000142 14300 1.21E-05 14100 2.44E-05 13700
0.0001099 15900 1.63E-05 15400 1.99E-05
16300 0.000141 14300 1.21E-05 14100 2.45E-05 13700
0.000110343 15900 1.63E-05 15400 1.99E-05
16300 0.00014 14300 1.22E-05 14100 2.45E-05 13700
0.000110804 15900 1.63E-05 15400 1.99E-05
16300 0.00014 14300 1.22E-05 14100 2.45E-05 13700
0.000111265 15900 1.63E-05 15400 1.99E-05
16300 0.000139 14300 1.23E-05 14100 2.45E-05 13700
0.000111743 16000 1.63E-05 15400 1.99E-05
16300 0.000138 14300 1.24E-05 14100 2.45E-05 13700
0.000112231 16000 1.63E-05 15400 1.99E-05
16300 0.000137 14300 1.24E-05 14100 2.45E-05 13700
0.000112726 16000 1.63E-05 15400 1.99E-05
16300 0.000136 14300 1.25E-05 14100 2.45E-05 13700
0.000113212 16000 1.63E-05 15400 1.99E-05
16300 0.000135 14300 1.25E-05 14100 2.45E-05 13700
0.000113719 16000 1.63E-05 15400 1.99E-05
16300 0.000134 14300 1.26E-05 14100 2.45E-05 13700
0.000114218 16000 1.63E-05 15400 1.99E-05
16300 0.000133 14300 1.26E-05 14100 2.46E-05 13700
0.000114725 16000 1.63E-05 15400 1.99E-05
16300 0.000132 14300 1.27E-05 14100 2.46E-05 13700
0.000115241 16000 1.63E-05 15400 2.00E-05
16300 0.000131 14300 1.28E-05 14100 2.46E-05 13700
0.000115737 16000 1.63E-05 15400 2.00E-05
16300 0.00013 14300 1.28E-05 14100 2.46E-05 13700
0.000116273 16000 1.63E-05 15400 2.00E-05
16300 0.000129 14300 1.29E-05 14100 2.46E-05 13700
0.000116796 16000 1.63E-05 15400 2.00E-05
16300 0.000128 14300 1.30E-05 14100 2.47E-05 13700
0.00011733 16000 1.63E-05 15400 2.00E-05
16300 0.000126 14300 1.31E-05 14100 2.47E-05 13700
0.000117873 16000 1.63E-05 15400 2.00E-05
16300 0.000125 14300 1.31E-05 14100 2.47E-05 13700
0.000118421 16000 1.63E-05 15400 2.00E-05
16300 0.000124 14300 1.32E-05 14100 2.47E-05 13700
0.000118982 16000 1.63E-05 15400 2.00E-05
16300 0.000123 14300 1.33E-05 14100 2.47E-05 13700
0.000119535 16000 1.63E-05 15400 2.00E-05
16300 0.000122 14300 1.34E-05 14100 2.48E-05 13700
0.000120098 16000 1.64E-05 15400 2.00E-05
16300 0.00012 14300 1.35E-05 14100 2.48E-05 13700
0.000120706 16000 1.64E-05 15400 2.00E-05

16300 0.000119 14300 1.36E-05 14100 2.48E-05 13700
0.000121296 16000 1.64E-05 15400 2.00E-05
16300 0.000118 14300 1.37E-05 14100 2.49E-05 13700
0.000121886 16000 1.64E-05 15400 2.00E-05
16300 0.000116 14300 1.38E-05 14100 2.49E-05 13700
0.000122499 16000 1.64E-05 15400 2.01E-05
16300 0.000115 14300 1.39E-05 14100 2.49E-05 13700
0.000123119 16000 1.64E-05 15400 2.01E-05
16300 0.000114 14300 1.40E-05 14100 2.50E-05 13700
0.000123741 16000 1.64E-05 15400 2.01E-05
16300 0.000113 14300 1.41E-05 14100 2.50E-05 13700
0.000124363 16000 1.64E-05 15400 2.01E-05
16300 0.000111 14300 1.43E-05 14100 2.50E-05 13700
0.000124998 16000 1.64E-05 15400 2.01E-05
16300 0.00011 14300 1.44E-05 14100 2.51E-05 13700
0.000125659 16000 1.64E-05 15400 2.01E-05
16300 0.000109 14300 1.45E-05 14100 2.51E-05 13700
0.000126314 16000 1.64E-05 15400 2.01E-05
16300 0.000107 14300 1.46E-05 14100 2.52E-05 13700
0.000126977 16000 1.64E-05 15400 2.01E-05
16300 0.000106 14300 1.48E-05 14100 2.52E-05 13700
0.000127669 16000 1.64E-05 15400 2.01E-05
16400 0.000105 14300 1.49E-05 14100 2.52E-05 13700
0.000128371 16000 1.64E-05 15400 2.01E-05
16400 0.000104 14300 1.51E-05 14100 2.53E-05 13700
0.000129059 16000 1.64E-05 15400 2.01E-05
16400 0.000102 14300 1.53E-05 14100 2.53E-05 13700
0.000129758 16000 1.64E-05 15400 2.01E-05
16400 1.01E-04 14300 1.54E-05 14100 2.54E-05 13700
0.000130482 16000 1.64E-05 15400 2.01E-05
16400 1.00E-04 14300 1.56E-05 14100 2.54E-05 13700
0.000131212 16000 1.64E-05 15400 2.01E-05
16400 9.87E-05 14300 1.58E-05 14100 2.55E-05 13700
0.000131933 16000 1.64E-05 15400 2.01E-05
16400 9.76E-05 14300 1.60E-05 14100 2.56E-05 13700
0.000132661 16000 1.64E-05 15400 2.02E-05
16400 9.64E-05 14300 1.62E-05 14100 2.56E-05 13700
0.000133424 16000 1.64E-05 15400 2.02E-05
16400 9.52E-05 14300 1.64E-05 14100 2.57E-05 13700
0.000134195 16000 1.64E-05 15400 2.02E-05
16400 9.40E-05 14300 1.66E-05 14100 2.58E-05 13700
0.000134958 16000 1.64E-05 15500 2.02E-05
16400 9.29E-05 14300 1.68E-05 14100 2.58E-05 13700
0.000135761 16000 1.64E-05 15500 2.02E-05
16400 9.18E-05 14300 1.70E-05 14100 2.59E-05 13700
0.000136571 16000 1.65E-05 15500 2.02E-05
16400 9.07E-05 14300 1.73E-05 14100 2.60E-05 13700
0.000137378 16000 1.64E-05 15500 2.02E-05
16400 8.96E-05 14300 1.75E-05 14100 2.61E-05 13700
0.00013821 16000 1.65E-05 15500 2.02E-05
16400 8.85E-05 14300 1.78E-05 14100 2.61E-05 13700
0.000139053 16000 1.65E-05 15500 2.02E-05
16400 8.74E-05 14300 1.81E-05 14100 2.62E-05 13700
0.000139911 16000 1.65E-05 15500 2.02E-05
16400 8.64E-05 14300 1.84E-05 14100 2.63E-05 13700
0.000140784 16000 1.65E-05 15500 2.02E-05
16400 8.54E-05 14300 1.87E-05 14100 2.64E-05 13700
0.00014164 16000 1.65E-05 15500 2.02E-05
16400 8.44E-05 14400 1.91E-05 14100 2.65E-05 13700
0.000142544 16000 1.65E-05 15500 2.02E-05
16400 8.35E-05 14400 1.94E-05 14100 2.66E-05 13700
0.000143458 16000 1.65E-05 15500 2.02E-05
16400 8.26E-05 14400 1.98E-05 14200 2.67E-05 13700
0.000144356 16000 1.65E-05 15500 2.02E-05
16400 8.16E-05 14400 2.01E-05 14200 2.68E-05 13700
0.000145295 16000 1.65E-05 15500 2.02E-05
16400 8.07E-05 14400 2.06E-05 14200 2.69E-05 13700
0.000146231 16000 1.65E-05 15500 2.02E-05

16400	7.98E-05	14400	2.10E-05	14200	2.71E-05	13700	0.000147188	16000	1.65E-05	15500	2.02E-05
16400	7.90E-05	14400	2.14E-05	14200	2.72E-05	13800	0.000148162	16000	1.65E-05	15500	2.02E-05
16400	7.82E-05	14400	2.19E-05	14200	2.73E-05	13800	0.00014913	16000	1.65E-05	15500	2.02E-05
16400	7.73E-05	14400	2.24E-05	14200	2.75E-05	13800	0.000150142	16000	1.65E-05	15500	2.02E-05
16400	7.66E-05	14400	2.30E-05	14200	2.76E-05	13800	0.000151158	16000	1.65E-05	15500	2.03E-05
16400	7.58E-05	14400	2.36E-05	14200	2.78E-05	13800	0.000152185	16000	1.65E-05	15500	2.03E-05
16400	7.50E-05	14400	2.42E-05	14200	2.79E-05	13800	0.000153224	16000	1.65E-05	15500	2.03E-05
16400	7.43E-05	14400	2.48E-05	14200	2.81E-05	13800	0.00015428	16000	1.65E-05	15500	2.03E-05
16400	7.36E-05	14400	2.55E-05	14200	2.83E-05	13800	0.000155359	16000	1.65E-05	15500	2.03E-05
16400	7.29E-05	14400	2.62E-05	14200	2.84E-05	13800	0.000156456	16000	1.65E-05	15500	2.03E-05
16400	7.22E-05	14400	2.70E-05	14200	2.86E-05	13800	0.000157557	16000	1.65E-05	15500	2.03E-05
16400	7.15E-05	14400	2.78E-05	14200	2.88E-05	13800	0.000158699	16000	1.66E-05	15500	2.03E-05
16400	7.09E-05	14400	2.87E-05	14200	2.91E-05	13800	0.000159836	16100	1.66E-05	15500	2.03E-05
16400	7.03E-05	14400	2.96E-05	14200	2.93E-05	13800	0.000161007	16100	1.66E-05	15500	2.03E-05
16400	6.97E-05	14400	3.06E-05	14200	2.95E-05	13800	0.000162194	16100	1.66E-05	15500	2.03E-05
16400	6.91E-05	14400	3.17E-05	14200	2.97E-05	13800	0.0001634	16100	1.66E-05	15500	2.03E-05
16400	6.86E-05	14400	3.28E-05	14200	3.00E-05	13800	0.000164607	16100	1.66E-05	15500	2.03E-05
16400	6.80E-05	14400	3.41E-05	14200	3.02E-05	13800	0.000165839	16100	1.66E-05	15500	2.03E-05
16400	6.75E-05	14400	3.54E-05	14200	3.05E-05	13800	0.00016708	16100	1.66E-05	15500	2.04E-05
16400	6.70E-05	14400	3.68E-05	14200	3.08E-05	13800	0.000168337	16100	1.66E-05	15500	2.03E-05
16400	6.65E-05	14400	3.83E-05	14200	3.11E-05	13800	0.000169604	16100	1.66E-05	15500	2.04E-05
16400	6.60E-05	14400	3.99E-05	14200	3.15E-05	13800	0.000170876	16100	1.66E-05	15500	2.03E-05
16400	6.55E-05	14400	4.16E-05	14200	3.18E-05	13800	0.000172218	16100	1.66E-05	15500	2.04E-05
16400	6.51E-05	14400	4.35E-05	14200	3.21E-05	13800	0.000173569	16100	1.66E-05	15500	2.04E-05
16400	6.46E-05	14400	4.54E-05	14200	3.25E-05	13800	0.000174922	16100	1.66E-05	15500	2.04E-05
16400	6.42E-05	14400	4.75E-05	14200	3.29E-05	13800	0.000176333	16100	1.66E-05	15500	2.04E-05
16400	6.38E-05	14400	4.98E-05	14200	3.33E-05	13800	0.000177755	16100	1.66E-05	15500	2.04E-05
16400	6.34E-05	14400	5.22E-05	14200	3.37E-05	13800	0.000179217	16100	1.66E-05	15500	2.04E-05
16400	6.30E-05	14400	5.47E-05	14200	3.41E-05	13800	0.000180699	16100	1.66E-05	15500	2.04E-05
16400	6.26E-05	14400	5.74E-05	14200	3.46E-05	13800	0.000182202	16100	1.66E-05	15500	2.04E-05
16400	6.22E-05	14400	6.02E-05	14200	3.51E-05	13800	0.000183704	16100	1.66E-05	15500	2.04E-05
16400	6.19E-05	14400	6.32E-05	14200	3.56E-05	13800	0.000185246	16100	1.66E-05	15500	2.04E-05
16400	6.15E-05	14400	6.64E-05	14200	3.62E-05	13800	0.000186788	16100	1.66E-05	15500	2.04E-05
16400	6.12E-05	14400	6.97E-05	14200	3.67E-05	13800	0.00018842	16100	1.67E-05	15500	2.04E-05

16400 6.09E-05 14400 7.31E-05 14200 3.73E-05 13800
0.000190022 16100 1.67E-05 15500 2.04E-05
16400 6.06E-05 14400 7.66E-05 14200 3.80E-05 13800
0.000191655 16100 1.67E-05 15500 2.04E-05
16400 6.03E-05 14400 8.01E-05 14200 3.86E-05 13800
0.000193297 16100 1.67E-05 15500 2.04E-05
16400 6.00E-05 14400 8.37E-05 14200 3.93E-05 13800
0.000194999 16100 1.67E-05 15500 2.04E-05
16400 5.97E-05 14400 8.72E-05 14200 4.01E-05 13800
0.000196762 16100 1.67E-05 15500 2.04E-05
16400 5.95E-05 14400 9.06E-05 14200 4.08E-05 13800
0.000198524 16100 1.67E-05 15500 2.04E-05
16400 5.92E-05 14400 9.39E-05 14200 4.17E-05 13800
0.000200287 16100 1.67E-05 15500 2.04E-05
16500 5.90E-05 14400 9.69E-05 14200 4.25E-05 13800
0.000202109 16100 1.67E-05 15500 2.05E-05
16500 5.87E-05 14400 9.96E-05 14200 4.34E-05 13800
0.000203972 16100 1.67E-05 15500 2.05E-05
16500 5.85E-05 14400 0.000101872 14200 4.43E-05 13800
0.000205835 16100 1.67E-05 15500 2.05E-05
16500 5.83E-05 14400 0.000103724 14200 4.53E-05 13800
0.000207777 16100 1.67E-05 15500 2.05E-05
16500 5.81E-05 14400 0.000105077 14200 4.63E-05 13800
0.00020976 16100 1.67E-05 15500 2.05E-05
16500 5.79E-05 14400 0.00010589 14200 4.74E-05 13800
0.000211723 16100 1.67E-05 15500 2.05E-05
16500 5.76E-05 14400 0.000106135 14200 4.85E-05 13800
0.000213786 16100 1.67E-05 15500 2.05E-05
16500 5.74E-05 14400 0.000105807 14200 4.96E-05 13800
0.000215828 16100 1.67E-05 15500 2.05E-05
16500 5.72E-05 14400 0.000104919 14200 5.08E-05 13800
0.000217911 16100 1.67E-05 15500 2.05E-05
16500 5.71E-05 14400 0.000103496 14200 5.21E-05 13800
0.000220154 16100 1.67E-05 15600 2.05E-05
16500 5.69E-05 14400 0.000101602 14200 5.34E-05 13800
0.000222317 16100 1.67E-05 15600 2.05E-05
16500 5.67E-05 14400 9.93E-05 14200 5.47E-05 13800
0.00022448 16100 1.67E-05 15600 2.05E-05
16500 5.66E-05 14400 9.66E-05 14200 5.61E-05 13800
0.000226804 16100 1.67E-05 15600 2.06E-05
16500 5.64E-05 14400 9.37E-05 14200 5.75E-05 13800
0.000229047 16100 1.67E-05 15600 2.06E-05
16500 5.63E-05 14400 9.05E-05 14200 5.89E-05 13800
0.00023145 16100 1.67E-05 15600 2.06E-05
16500 5.61E-05 14400 8.72E-05 14200 6.04E-05 13800
0.000233893 16100 1.68E-05 15600 2.06E-05
16500 5.60E-05 14400 8.38E-05 14200 6.18E-05 13800
0.000236257 16100 1.68E-05 15600 2.06E-05
16500 5.59E-05 14400 8.04E-05 14200 6.33E-05 13800
0.00023874 16100 1.68E-05 15600 2.06E-05
16500 5.57E-05 14500 7.70E-05 14200 6.48E-05 13800
0.000241384 16100 1.68E-05 15600 2.06E-05
16500 5.56E-05 14500 7.36E-05 14200 6.63E-05 13800
0.000243908 16100 1.68E-05 15600 2.06E-05
16500 5.55E-05 14500 7.04E-05 14300 6.77E-05 13800
0.000246591 16100 1.68E-05 15600 2.06E-05
16500 5.53E-05 14500 6.73E-05 14300 6.91E-05 13800
0.000249275 16100 1.68E-05 15600 2.06E-05
16500 5.52E-05 14500 6.43E-05 14300 7.05E-05 13800
0.000252039 16100 1.68E-05 15600 2.06E-05
16500 5.51E-05 14500 6.14E-05 14300 7.18E-05 13800
0.000254883 16100 1.68E-05 15600 2.06E-05
16500 5.50E-05 14500 5.87E-05 14300 7.31E-05 13900
0.000257807 16100 1.68E-05 15600 2.06E-05
16500 5.49E-05 14500 5.61E-05 14300 7.42E-05 13900
0.000260731 16100 1.68E-05 15600 2.06E-05
16500 5.48E-05 14500 5.36E-05 14300 7.53E-05 13900
0.000263735 16100 1.68E-05 15600 2.06E-05

16500 5.46E-05 14500 5.13E-05 14300 7.62E-05 13900
0.000266779 16100 1.68E-05 15600 2.06E-05
16500 5.46E-05 14500 4.91E-05 14300 7.70E-05 13900
0.000269783 16100 1.68E-05 15600 2.06E-05
16500 5.45E-05 14500 4.71E-05 14300 7.77E-05 13900
0.000273028 16100 1.68E-05 15600 2.06E-05
16500 5.44E-05 14500 4.52E-05 14300 7.82E-05 13900
0.000276272 16100 1.68E-05 15600 2.07E-05
16500 5.43E-05 14500 4.33E-05 14300 7.85E-05 13900
0.000279597 16100 1.68E-05 15600 2.07E-05
16500 5.42E-05 14500 4.16E-05 14300 7.87E-05 13900
0.000282962 16100 1.68E-05 15600 2.07E-05
16500 5.41E-05 14500 4.00E-05 14300 7.87E-05 13900
0.000286367 16100 1.68E-05 15600 2.07E-05
16500 5.40E-05 14500 3.84E-05 14300 7.85E-05 13900
0.000289932 16100 1.68E-05 15600 2.07E-05
16500 5.40E-05 14500 3.70E-05 14300 7.82E-05 13900
0.000293497 16200 1.68E-05 15600 2.07E-05
16500 5.39E-05 14500 3.56E-05 14300 7.77E-05 13900
0.000297182 16200 1.69E-05 15600 2.07E-05
16500 5.38E-05 14500 3.43E-05 14300 7.70E-05 13900
0.000300987 16200 1.68E-05 15600 2.07E-05
16500 5.37E-05 14500 3.31E-05 14300 7.62E-05 13900
0.000304873 16200 1.69E-05 15600 2.07E-05
16500 5.37E-05 14500 3.20E-05 14300 7.52E-05 13900
0.000308878 16200 1.69E-05 15600 2.07E-05
16500 5.36E-05 14500 3.09E-05 14300 7.41E-05 13900
0.000313004 16200 1.69E-05 15600 2.07E-05
16500 5.35E-05 14500 2.98E-05 14300 7.29E-05 13900
0.00031717 16200 1.69E-05 15600 2.07E-05
16500 5.35E-05 14500 2.89E-05 14300 7.16E-05 13900
0.000321496 16200 1.69E-05 15600 2.07E-05
16500 5.34E-05 14500 2.80E-05 14300 7.01E-05 13900
0.000325942 16200 1.69E-05 15600 2.07E-05
16500 5.34E-05 14500 2.71E-05 14300 6.87E-05 13900
0.000330468 16200 1.69E-05 15600 2.07E-05
16500 5.33E-05 14500 2.63E-05 14300 6.71E-05 13900
0.000335135 16200 1.69E-05 15600 2.07E-05
16500 5.33E-05 14500 2.55E-05 14300 6.55E-05 13900
0.000339881 16200 1.69E-05 15600 2.07E-05
16500 5.32E-05 14500 2.48E-05 14300 6.39E-05 13900
0.000344788 16200 1.69E-05 15600 2.08E-05
16500 5.32E-05 14500 2.41E-05 14300 6.23E-05 13900
0.000349815 16200 1.69E-05 15600 2.08E-05
16500 5.32E-05 14500 2.35E-05 14300 6.06E-05 13900
0.000354962 16200 1.69E-05 15600 2.08E-05
16500 5.31E-05 14500 2.29E-05 14300 5.90E-05 13900
0.00036019 16200 1.69E-05 15600 2.08E-05
16500 5.31E-05 14500 2.23E-05 14300 5.74E-05 13900
0.000365637 16200 1.69E-05 15600 2.08E-05
16500 5.31E-05 14500 2.18E-05 14300 5.58E-05 13900
0.000371205 16200 1.69E-05 15600 2.08E-05
16500 5.30E-05 14500 2.12E-05 14300 5.43E-05 13900
0.000376873 16200 1.69E-05 15600 2.08E-05
16500 5.30E-05 14500 2.08E-05 14300 5.28E-05 13900
0.000382761 16200 1.69E-05 15600 2.08E-05
16500 5.30E-05 14500 2.03E-05 14300 5.13E-05 13900
0.00038881 16200 1.69E-05 15600 2.08E-05
16500 5.29E-05 14500 1.99E-05 14300 4.99E-05 13900
0.000394978 16200 1.69E-05 15600 2.08E-05
16500 5.29E-05 14500 1.95E-05 14300 4.85E-05 13900
0.000401347 16200 1.69E-05 15600 2.08E-05
16500 5.29E-05 14500 1.91E-05 14300 4.72E-05 13900
0.000407906 16200 1.69E-05 15600 2.08E-05
16500 5.28E-05 14500 1.87E-05 14300 4.59E-05 13900
0.000414626 16200 1.69E-05 15600 2.08E-05
16500 5.28E-05 14500 1.84E-05 14300 4.47E-05 13900
0.000421575 16200 1.69E-05 15600 2.08E-05

16500 5.28E-05 14500 1.80E-05 14300 4.36E-05 13900
0.000428735 16200 1.69E-05 15600 2.08E-05
16500 5.27E-05 14500 1.77E-05 14300 4.25E-05 13900
0.000436106 16200 1.69E-05 15600 2.08E-05
16500 5.27E-05 14500 1.74E-05 14300 4.15E-05 13900
0.000443706 16200 1.70E-05 15600 2.08E-05
16600 5.27E-05 14500 1.71E-05 14300 4.05E-05 13900
0.000451577 16200 1.70E-05 15600 2.08E-05
16600 5.27E-05 14500 1.69E-05 14300 3.95E-05 13900
0.000459689 16200 1.70E-05 15600 2.09E-05
16600 5.26E-05 14500 1.66E-05 14300 3.87E-05 13900
0.000468055 16200 1.70E-05 15600 2.09E-05
16600 5.26E-05 14500 1.64E-05 14300 3.78E-05 13900
0.000476712 16200 1.70E-05 15600 2.09E-05
16600 5.26E-05 14500 1.61E-05 14300 3.70E-05 13900
0.000485665 16200 1.70E-05 15600 2.09E-05
16600 5.26E-05 14500 1.59E-05 14300 3.62E-05 13900
0.000494938 16200 1.70E-05 15600 2.09E-05
16600 5.26E-05 14500 1.57E-05 14300 3.55E-05 13900
0.000504526 16200 1.70E-05 15600 2.09E-05
16600 5.26E-05 14500 1.55E-05 14300 3.49E-05 13900
0.000514435 16200 1.70E-05 15600 2.09E-05
16600 5.25E-05 14500 1.53E-05 14300 3.42E-05 13900
0.00052473 16200 1.70E-05 15600 2.09E-05
16600 5.25E-05 14500 1.51E-05 14300 3.36E-05 13900
0.000535374 16200 1.70E-05 15700 2.09E-05
16600 5.25E-05 14500 1.49E-05 14300 3.30E-05 13900
0.000546415 16200 1.70E-05 15700 2.09E-05
16600 5.25E-05 14500 1.47E-05 14300 3.25E-05 13900
0.000557876 16200 1.70E-05 15700 2.09E-05
16600 5.25E-05 14500 1.46E-05 14300 3.19E-05 13900
0.000569798 16200 1.70E-05 15700 2.09E-05
16600 5.25E-05 14500 1.44E-05 14300 3.15E-05 13900
0.00058219 16200 1.70E-05 15700 2.09E-05
16600 5.24E-05 14500 1.42E-05 14300 3.10E-05 13900
0.000595053 16200 1.70E-05 15700 2.09E-05
16600 5.24E-05 14500 1.41E-05 14300 3.05E-05 13900
0.000608447 16200 1.70E-05 15700 2.09E-05
16600 5.24E-05 14500 1.40E-05 14300 3.01E-05 13900
0.000622386 16200 1.70E-05 15700 2.09E-05
16600 5.24E-05 14500 1.38E-05 14300 2.97E-05 13900
0.000636874 16200 1.70E-05 15700 2.09E-05
16600 5.24E-05 14600 1.37E-05 14300 2.93E-05 13900
0.000651993 16200 1.71E-05 15700 2.09E-05
16600 5.24E-05 14600 1.36E-05 14300 2.90E-05 13900
0.000667767 16200 1.71E-05 15700 2.09E-05
16600 5.24E-05 14600 1.35E-05 14400 2.86E-05 13900
0.00068423 16200 1.71E-05 15700 2.10E-05
16600 5.24E-05 14600 1.33E-05 14400 2.83E-05 13900
0.000701457 16200 1.71E-05 15700 2.10E-05
16600 5.24E-05 14600 1.32E-05 14400 2.80E-05 13900
0.000719442 16200 1.71E-05 15700 2.10E-05
16600 5.24E-05 14600 1.31E-05 14400 2.77E-05 13900
0.000738311 16200 1.71E-05 15700 2.10E-05
16600 5.24E-05 14600 1.30E-05 14400 2.74E-05 14000
0.000758073 16200 1.71E-05 15700 2.10E-05
16600 5.24E-05 14600 1.29E-05 14400 2.71E-05 14000
0.000778795 16200 1.71E-05 15700 2.10E-05
16600 5.24E-05 14600 1.28E-05 14400 2.69E-05 14000
0.00080054 16200 1.71E-05 15700 2.10E-05
16600 5.24E-05 14600 1.27E-05 14400 2.66E-05 14000
0.000823402 16200 1.71E-05 15700 2.10E-05
16600 5.24E-05 14600 1.26E-05 14400 2.64E-05 14000
0.000847441 16200 1.71E-05 15700 2.10E-05
16600 5.24E-05 14600 1.25E-05 14400 2.62E-05 14000
0.000872729 16200 1.71E-05 15700 2.10E-05
16600 5.24E-05 14600 1.24E-05 14400 2.59E-05 14000
0.000899356 16200 1.71E-05 15700 2.10E-05

16600 5.24E-05 14600 1.24E-05 14400 2.57E-05 14000
0.000927493 16200 1.71E-05 15700 2.10E-05
16600 5.24E-05 14600 1.23E-05 14400 2.56E-05 14000
0.000957172 16200 1.71E-05 15700 2.10E-05
16600 5.24E-05 14600 1.22E-05 14400 2.54E-05 14000
0.000988558 16200 1.71E-05 15700 2.10E-05
16600 5.24E-05 14600 1.21E-05 14400 2.52E-05 14000
0.001021766 16200 1.71E-05 15700 2.10E-05
16600 5.24E-05 14600 1.20E-05 14400 2.50E-05 14000
0.001056931 16300 1.71E-05 15700 2.10E-05
16600 5.24E-05 14600 1.20E-05 14400 2.48E-05 14000
0.001094218 16300 1.71E-05 15700 2.10E-05
16600 5.24E-05 14600 1.19E-05 14400 2.47E-05 14000
0.001133814 16300 1.71E-05 15700 2.10E-05
16600 5.24E-05 14600 1.18E-05 14400 2.45E-05 14000
0.001175905 16300 1.71E-05 15700 2.11E-05
16600 5.24E-05 14600 1.18E-05 14400 2.44E-05 14000
0.001220683 16300 1.71E-05 15700 2.11E-05
16600 5.24E-05 14600 1.17E-05 14400 2.43E-05 14000
0.001268423 16300 1.72E-05 15700 2.11E-05
16600 5.25E-05 14600 1.16E-05 14400 2.41E-05 14000
0.001319358 16300 1.72E-05 15700 2.11E-05
16600 5.25E-05 14600 1.16E-05 14400 2.40E-05 14000
0.001373795 16300 1.72E-05 15700 2.11E-05
16600 5.25E-05 14600 1.15E-05 14400 2.39E-05 14000
0.001432021 16300 1.72E-05 15700 2.11E-05
16600 5.25E-05 14600 1.14E-05 14400 2.37E-05 14000
0.001494353 16300 1.72E-05 15700 2.11E-05
16600 5.25E-05 14600 1.14E-05 14400 2.36E-05 14000
0.001561222 16300 1.72E-05 15700 2.11E-05
16600 5.25E-05 14600 1.13E-05 14400 2.35E-05 14000
0.001632976 16300 1.72E-05 15700 2.11E-05
16600 5.25E-05 14600 1.13E-05 14400 2.34E-05 14000
0.001710078 16300 1.72E-05 15700 2.11E-05
16600 5.26E-05 14600 1.12E-05 14400 2.33E-05 14000
0.001793018 16300 1.72E-05 15700 2.11E-05
16600 5.26E-05 14600 1.11E-05 14400 2.32E-05 14000
0.001882279 16300 1.72E-05 15700 2.11E-05
16600 5.26E-05 14600 1.11E-05 14400 2.31E-05 14000
0.00197845 16300 1.72E-05 15700 2.11E-05
16600 5.26E-05 14600 1.11E-05 14400 2.30E-05 14000
0.002082095 16300 1.72E-05 15700 2.11E-05
16600 5.26E-05 14600 1.10E-05 14400 2.29E-05 14000
0.002193854 16300 1.72E-05 15700 2.11E-05
16600 5.26E-05 14600 1.09E-05 14400 2.28E-05 14000
0.00231436 16300 1.72E-05 15700 2.11E-05
16600 5.26E-05 14600 1.09E-05 14400 2.27E-05 14000
0.002444365 16300 1.72E-05 15700 2.11E-05
16600 5.27E-05 14600 1.09E-05 14400 2.26E-05 14000
0.002584483 16300 1.72E-05 15700 2.11E-05
16600 5.27E-05 14600 1.08E-05 14400 2.25E-05 14000
0.002735495 16300 1.72E-05 15700 2.11E-05
16600 5.27E-05 14600 1.08E-05 14400 2.25E-05 14000
0.002898063 16300 1.72E-05 15700 2.11E-05
16600 5.27E-05 14600 1.07E-05 14400 2.24E-05 14000
0.003072897 16300 1.72E-05 15700 2.11E-05
16600 5.27E-05 14600 1.07E-05 14400 2.23E-05 14000
0.003260595 16300 1.72E-05 15700 2.11E-05
16600 5.28E-05 14600 1.06E-05 14400 2.22E-05 14000
0.003461663 16300 1.72E-05 15700 2.12E-05
16600 5.28E-05 14600 1.06E-05 14400 2.22E-05 14000
0.003676484 16300 1.73E-05 15700 2.12E-05
16600 5.28E-05 14600 1.06E-05 14400 2.21E-05 14000
0.003905073 16300 1.73E-05 15700 2.12E-05
16600 5.28E-05 14600 1.05E-05 14400 2.20E-05 14000
0.004147196 16300 1.73E-05 15700 2.12E-05
16700 5.29E-05 14600 1.05E-05 14400 2.20E-05 14000
0.004402183 16300 1.73E-05 15700 2.12E-05

16700	5.29E-05	14600	1.05E-05	14400	2.19E-05	14000	0.004668907	16300	1.73E-05	15700	2.12E-05
16700	5.29E-05	14600	1.04E-05	14400	2.18E-05	14000	0.004945694	16300	1.73E-05	15700	2.12E-05
16700	5.29E-05	14600	1.04E-05	14400	2.18E-05	14000	0.005230177	16300	1.73E-05	15700	2.12E-05
16700	5.30E-05	14600	1.03E-05	14400	2.17E-05	14000	0.005519319	16300	1.73E-05	15700	2.12E-05
16700	5.30E-05	14600	1.03E-05	14400	2.17E-05	14000	0.00580933	16300	1.73E-05	15700	2.12E-05
16700	5.30E-05	14600	1.03E-05	14400	2.16E-05	14000	0.006095538	16300	1.73E-05	15700	2.12E-05
16700	5.30E-05	14600	1.02E-05	14400	2.15E-05	14000	0.006372825	16300	1.73E-05	15700	2.12E-05
16700	5.31E-05	14600	1.02E-05	14400	2.15E-05	14000	0.006635414	16300	1.73E-05	15700	2.12E-05
16700	5.31E-05	14600	1.02E-05	14400	2.14E-05	14000	0.006877339	16300	1.73E-05	15800	2.12E-05
16700	5.31E-05	14600	1.01E-05	14400	2.14E-05	14000	0.007092752	16300	1.73E-05	15800	2.12E-05
16700	5.32E-05	14600	1.01E-05	14400	2.13E-05	14000	0.00727602	16300	1.73E-05	15800	2.12E-05
16700	5.32E-05	14600	1.01E-05	14400	2.13E-05	14000	0.007422105	16300	1.73E-05	15800	2.12E-05
16700	5.32E-05	14600	1.01E-05	14400	2.12E-05	14000	0.007526902	16300	1.73E-05	15800	2.12E-05
16700	5.33E-05	14600	1.00E-05	14400	2.12E-05	14000	0.007587477	16300	1.73E-05	15800	2.12E-05
16700	5.33E-05	14600	1.00E-05	14400	2.11E-05	14000	0.007602261	16300	1.73E-05	15800	2.12E-05
16700	5.34E-05	14600	9.99E-06	14400	2.11E-05	14000	0.00757122	16300	1.73E-05	15800	2.13E-05
16700	5.34E-05	14600	9.95E-06	14400	2.11E-05	14000	0.007495857	16300	1.73E-05	15800	2.13E-05
16700	5.35E-05	14700	9.94E-06	14400	2.10E-05	14000	0.007379106	16300	1.73E-05	15800	2.13E-05
16700	5.35E-05	14700	9.91E-06	14400	2.10E-05	14000	0.007225168	16300	1.74E-05	15800	2.13E-05
16700	5.36E-05	14700	9.88E-06	14500	2.09E-05	14000	0.007039201	16300	1.73E-05	15800	2.13E-05
16700	5.36E-05	14700	9.84E-06	14500	2.09E-05	14000	0.006826974	16300	1.74E-05	15800	2.13E-05
16700	5.37E-05	14700	9.82E-06	14500	2.09E-05	14000	0.006594695	16300	1.74E-05	15800	2.13E-05
16700	5.37E-05	14700	9.80E-06	14500	2.08E-05	14000	0.006348638	16300	1.74E-05	15800	2.13E-05
16700	5.38E-05	14700	9.77E-06	14500	2.08E-05	14100	0.006095103	16300	1.74E-05	15800	2.13E-05
16700	5.38E-05	14700	9.74E-06	14500	2.07E-05	14100	0.005839965	16300	1.74E-05	15800	2.13E-05
16700	5.39E-05	14700	9.72E-06	14500	2.07E-05	14100	0.00558871	16300	1.74E-05	15800	2.13E-05
16700	5.39E-05	14700	9.70E-06	14500	2.07E-05	14100	0.005346272	16300	1.74E-05	15800	2.13E-05
16700	5.40E-05	14700	9.68E-06	14500	2.06E-05	14100	0.005116867	16300	1.74E-05	15800	2.13E-05
16700	5.40E-05	14700	9.65E-06	14500	2.06E-05	14100	0.004903946	16300	1.74E-05	15800	2.13E-05
16700	5.41E-05	14700	9.62E-06	14500	2.05E-05	14100	0.004710167	16300	1.74E-05	15800	2.13E-05
16700	5.42E-05	14700	9.61E-06	14500	2.05E-05	14100	0.004537408	16300	1.74E-05	15800	2.13E-05
16700	5.42E-05	14700	9.58E-06	14500	2.05E-05	14100	0.004386808	16300	1.74E-05	15800	2.13E-05
16700	5.43E-05	14700	9.56E-06	14500	2.05E-05	14100	0.004258938	16300	1.74E-05	15800	2.13E-05
16700	5.43E-05	14700	9.54E-06	14500	2.04E-05	14100	0.004153805	16300	1.74E-05	15800	2.13E-05

16700	5.44E-05	14700	9.53E-06	14500	2.04E-05	14100	0.004071101	16400	1.74E-05	15800	2.13E-05
16700	5.45E-05	14700	9.50E-06	14500	2.04E-05	14100	0.004010237	16400	1.74E-05	15800	2.14E-05
16700	5.45E-05	14700	9.50E-06	14500	2.03E-05	14100	0.003970476	16400	1.74E-05	15800	2.14E-05
16700	5.46E-05	14700	9.50E-06	14500	2.03E-05	14100	0.003950988	16400	1.74E-05	15800	2.14E-05
16700	5.47E-05	14700	9.47E-06	14500	2.03E-05	14100	0.00395095	16400	1.74E-05	15800	2.14E-05
16700	5.48E-05	14700	9.46E-06	14500	2.02E-05	14100	0.003969662	16400	1.74E-05	15800	2.14E-05
16700	5.48E-05	14700	9.44E-06	14500	2.02E-05	14100	0.004006478	16400	1.74E-05	15800	2.14E-05
16700	5.49E-05	14700	9.43E-06	14500	2.02E-05	14100	0.004060765	16400	1.74E-05	15800	2.14E-05
16700	5.50E-05	14700	9.40E-06	14500	2.01E-05	14100	0.004132266	16400	1.74E-05	15800	2.14E-05
16700	5.51E-05	14700	9.38E-06	14500	2.01E-05	14100	0.004220721	16400	1.74E-05	15800	2.14E-05
16700	5.52E-05	14700	9.37E-06	14500	2.01E-05	14100	0.004326085	16400	1.74E-05	15800	2.14E-05
16700	5.53E-05	14700	9.34E-06	14500	2.01E-05	14100	0.004448501	16400	1.74E-05	15800	2.14E-05
16700	5.54E-05	14700	9.33E-06	14500	2.00E-05	14100	0.004588344	16400	1.75E-05	15800	2.14E-05
16700	5.55E-05	14700	9.29E-06	14500	2.00E-05	14100	0.004746154	16400	1.75E-05	15800	2.14E-05
16700	5.56E-05	14700	9.28E-06	14500	2.00E-05	14100	0.004922684	16400	1.75E-05	15800	2.14E-05
16700	5.57E-05	14700	9.25E-06	14500	2.00E-05	14100	0.005118821	16400	1.75E-05	15800	2.14E-05
16700	5.57E-05	14700	9.25E-06	14500	1.99E-05	14100	0.005335745	16400	1.75E-05	15800	2.14E-05
16700	5.59E-05	14700	9.22E-06	14500	1.99E-05	14100	0.005574823	16400	1.75E-05	15800	2.14E-05
16700	5.60E-05	14700	9.20E-06	14500	1.99E-05	14100	0.005837723	16400	1.75E-05	15800	2.14E-05
16700	5.61E-05	14700	9.19E-06	14500	1.99E-05	14100	0.006126408	16400	1.75E-05	15800	2.14E-05
16700	5.62E-05	14700	9.18E-06	14500	1.99E-05	14100	0.006442998	16400	1.75E-05	15800	2.14E-05
16700	5.63E-05	14700	9.17E-06	14500	1.98E-05	14100	0.006790114	16400	1.75E-05	15800	2.14E-05
16700	5.65E-05	14700	9.15E-06	14500	1.98E-05	14100	0.007170698	16400	1.75E-05	15800	2.15E-05
16700	5.66E-05	14700	9.14E-06	14500	1.98E-05	14100	0.007588051	16400	1.75E-05	15800	2.15E-05
16700	5.67E-05	14700	9.11E-06	14500	1.98E-05	14100	0.008046	16400	1.75E-05	15800	2.15E-05
16700	5.68E-05	14700	9.09E-06	14500	1.98E-05	14100	0.008548873	16400	1.75E-05	15800	2.15E-05
16700	5.69E-05	14700	9.08E-06	14500	1.97E-05	14100	0.009101699	16400	1.75E-05	15800	2.14E-05
16700	5.71E-05	14700	9.06E-06	14500	1.97E-05	14100	0.009710163	16400	1.75E-05	15800	2.15E-05
16700	5.72E-05	14700	9.04E-06	14500	1.97E-05	14100	0.01038073	16400	1.75E-05	15800	2.15E-05
16800	5.73E-05	14700	9.04E-06	14500	1.97E-05	14100	0.0111208	16400	1.75E-05	15800	2.15E-05
16800	5.75E-05	14700	9.05E-06	14500	1.97E-05	14100	0.01193869	16400	1.75E-05	15800	2.15E-05
16800	5.76E-05	14700	9.03E-06	14500	1.97E-05	14100	0.01284396	16400	1.75E-05	15800	2.15E-05
16800	5.78E-05	14700	9.01E-06	14500	1.96E-05	14100	0.01384731	16400	1.75E-05	15800	2.15E-05
16800	5.79E-05	14700	8.99E-06	14500	1.96E-05	14100	0.01496098	16400	1.75E-05	15800	2.15E-05

16800	5.81E-05	14700	8.99E-06	14500	1.96E-05	14100	0.01619871	16400	1.75E-05	15800	2.15E-05
16800	5.82E-05	14700	8.98E-06	14500	1.95E-05	14100	0.0175761	16400	1.76E-05	15800	2.15E-05
16800	5.84E-05	14700	8.97E-06	14500	1.95E-05	14100	0.01911071	16400	1.76E-05	15800	2.15E-05
16800	5.86E-05	14700	8.97E-06	14500	1.95E-05	14100	0.02082187	16400	1.76E-05	15800	2.15E-05
16800	5.87E-05	14700	8.96E-06	14500	1.95E-05	14100	0.0227309	16400	1.76E-05	15900	2.15E-05
16800	5.89E-05	14700	8.93E-06	14500	1.95E-05	14100	0.02486058	16400	1.76E-05	15900	2.15E-05
16800	5.91E-05	14700	8.92E-06	14500	1.95E-05	14100	0.02723468	16400	1.76E-05	15900	2.15E-05
16800	5.93E-05	14700	8.90E-06	14500	1.94E-05	14100	0.0298772	16400	1.76E-05	15900	2.15E-05
16800	5.95E-05	14700	8.88E-06	14500	1.94E-05	14100	0.03281111	16400	1.76E-05	15900	2.15E-05
16800	5.96E-05	14700	8.90E-06	14500	1.94E-05	14100	0.03605649	16400	1.76E-05	15900	2.15E-05
16800	5.98E-05	14700	8.87E-06	14500	1.94E-05	14100	0.03962792	16400	1.76E-05	15900	2.15E-05
16800	6.00E-05	14700	8.86E-06	14500	1.94E-05	14100	0.04353115	16400	1.76E-05	15900	2.15E-05
16800	6.02E-05	14700	8.84E-06	14500	1.94E-05	14100	0.04775865	16400	1.76E-05	15900	2.15E-05
16800	6.04E-05	14800	8.83E-06	14500	1.94E-05	14100	0.05228411	16400	1.76E-05	15900	2.16E-05
16800	6.06E-05	14800	8.84E-06	14500	1.93E-05	14100	0.05705651	16400	1.76E-05	15900	2.16E-05
16800	6.08E-05	14800	8.83E-06	14600	1.93E-05	14100	0.06199385	16400	1.76E-05	15900	2.16E-05
16800	6.10E-05	14800	8.82E-06	14600	1.93E-05	14100	0.06697827	16400	1.76E-05	15900	2.16E-05
16800	6.13E-05	14800	8.81E-06	14600	1.93E-05	14100	0.07185386	16400	1.77E-05	15900	2.16E-05
16800	6.15E-05	14800	8.81E-06	14600	1.93E-05	14100	0.0764292	16400	1.77E-05	15900	2.16E-05
16800	6.17E-05	14800	8.80E-06	14600	1.92E-05	14200	0.08048659	16400	1.77E-05	15900	2.16E-05
16800	6.19E-05	14800	8.78E-06	14600	1.92E-05	14200	0.08379889	16400	1.76E-05	15900	2.16E-05
16800	6.21E-05	14800	8.78E-06	14600	1.92E-05	14200	0.0861529	16400	1.77E-05	15900	2.16E-05
16800	6.24E-05	14800	8.77E-06	14600	1.92E-05	14200	0.0873761	16400	1.77E-05	15900	2.16E-05
16800	6.26E-05	14800	8.78E-06	14600	1.92E-05	14200	0.08736174	16400	1.77E-05	15900	2.17E-05
16800	6.29E-05	14800	8.77E-06	14600	1.92E-05	14200	0.08608625	16400	1.77E-05	15900	2.17E-05
16800	6.32E-05	14800	8.76E-06	14600	1.91E-05	14200	0.08361389	16400	1.77E-05	15900	2.17E-05
16800	6.34E-05	14800	8.75E-06	14600	1.91E-05	14200	0.08008765	16400	1.77E-05	15900	2.17E-05
16800	6.37E-05	14800	8.76E-06	14600	1.91E-05	14200	0.0757091	16400	1.77E-05	15900	2.17E-05
16800	6.39E-05	14800	8.74E-06	14600	1.91E-05	14200	0.07071196	16400	1.77E-05	15900	2.17E-05
16800	6.42E-05	14800	8.72E-06	14600	1.91E-05	14200	0.06533592	16400	1.77E-05	15900	2.17E-05
16800	6.45E-05	14800	8.71E-06	14600	1.90E-05	14200	0.0598045	16500	1.77E-05	15900	2.17E-05
16800	6.47E-05	14800	8.71E-06	14600	1.90E-05	14200	0.05430968	16500	1.77E-05	15900	2.17E-05
16800	6.50E-05	14800	8.70E-06	14600	1.90E-05	14200	0.04900368	16500	1.77E-05	15900	2.17E-05
16800	6.53E-05	14800	8.69E-06	14600	1.90E-05	14200	0.04399697	16500	1.77E-05	15900	2.17E-05

16800 6.56E-05 14800 8.67E-06 14600 1.90E-05 14200
0.0393608 16500 1.77E-05 15900 2.17E-05
16800 6.59E-05 14800 8.68E-06 14600 1.90E-05 14200
0.03513289 16500 1.77E-05 15900 2.17E-05
16800 6.62E-05 14800 8.66E-06 14600 1.90E-05 14200
0.03132424 16500 1.78E-05 15900 2.17E-05
16800 6.64E-05 14800 8.64E-06 14600 1.89E-05 14200
0.0279263 16500 1.78E-05 15900 2.17E-05
16800 6.67E-05 14800 8.66E-06 14600 1.89E-05 14200
0.0249171 16500 1.78E-05 15900 2.17E-05
16800 6.70E-05 14800 8.64E-06 14600 1.89E-05 14200
0.02226671 16500 1.78E-05 15900 2.17E-05
16800 6.73E-05 14800 8.60E-06 14600 1.89E-05 14200
0.01994112 16500 1.78E-05 15900 2.17E-05
16800 6.76E-05 14800 8.59E-06 14600 1.89E-05 14200
0.01790513 16500 1.78E-05 15900 2.18E-05
16800 6.79E-05 14800 8.61E-06 14600 1.89E-05 14200
0.01612456 16500 1.78E-05 15900 2.18E-05
16800 6.82E-05 14800 8.55E-06 14600 1.88E-05 14200
0.01456729 16500 1.78E-05 15900 2.18E-05
16800 6.86E-05 14800 8.55E-06 14600 1.88E-05 14200
0.01320413 16500 1.78E-05 15900 2.18E-05
16800 6.89E-05 14800 8.56E-06 14600 1.88E-05 14200
0.01200906 16500 1.78E-05 15900 2.18E-05
16800 6.92E-05 14800 8.58E-06 14600 1.88E-05 14200
0.0109592 16500 1.78E-05 15900 2.18E-05
16800 6.95E-05 14800 8.57E-06 14600 1.88E-05 14200
0.0100346 16500 1.78E-05 15900 2.18E-05
16800 6.98E-05 14800 8.58E-06 14600 1.88E-05 14200
0.009218136 16500 1.78E-05 15900 2.18E-05
16800 7.01E-05 14800 8.57E-06 14600 1.87E-05 14200
0.008494988 16500 1.78E-05 15900 2.18E-05
16800 7.04E-05 14800 8.54E-06 14600 1.87E-05 14200
0.007852586 16500 1.78E-05 15900 2.18E-05
16800 7.07E-05 14800 8.53E-06 14600 1.87E-05 14200
0.007280075 16500 1.78E-05 15900 2.18E-05
16800 7.09E-05 14800 8.55E-06 14600 1.87E-05 14200
0.006768244 16500 1.78E-05 15900 2.18E-05
16800 7.12E-05 14800 8.55E-06 14600 1.87E-05 14200
0.006309163 16500 1.78E-05 15900 2.18E-05
16800 7.15E-05 14800 8.56E-06 14600 1.87E-05 14200
0.005896134 16500 1.78E-05 15900 2.18E-05
16800 7.18E-05 14800 8.53E-06 14600 1.86E-05 14200
0.005523352 16500 1.79E-05 15900 2.18E-05
16800 7.21E-05 14800 8.52E-06 14600 1.86E-05 14200
0.005185841 16500 1.79E-05 15900 2.19E-05
16800 7.23E-05 14800 8.50E-06 14600 1.86E-05 14200
0.004879422 16500 1.79E-05 15900 2.19E-05
16800 7.26E-05 14800 8.49E-06 14600 1.86E-05 14200
0.004600374 16500 1.79E-05 15900 2.19E-05
16900 7.28E-05 14800 8.48E-06 14600 1.86E-05 14200
0.004345572 16500 1.79E-05 15900 2.19E-05
16900 7.31E-05 14800 8.47E-06 14600 1.86E-05 14200
0.0041123 16500 1.79E-05 15900 2.19E-05
16900 7.33E-05 14800 8.46E-06 14600 1.85E-05 14200
0.003898195 16500 1.79E-05 15900 2.19E-05
16900 7.35E-05 14800 8.47E-06 14600 1.85E-05 14200
0.003701171 16500 1.79E-05 15900 2.19E-05
16900 7.38E-05 14800 8.46E-06 14600 1.85E-05 14200
0.003519446 16500 1.79E-05 15900 2.19E-05
16900 7.40E-05 14800 8.44E-06 14600 1.85E-05 14200
0.003351457 16500 1.79E-05 15900 2.19E-05
16900 7.41E-05 14800 8.43E-06 14600 1.85E-05 14200
0.003195824 16500 1.79E-05 15900 2.20E-05
16900 7.43E-05 14800 8.43E-06 14600 1.85E-05 14200
0.003051373 16500 1.79E-05 15900 2.19E-05
16900 7.45E-05 14800 8.41E-06 14600 1.85E-05 14200
0.002917019 16500 1.79E-05 15900 2.19E-05

16900 7.46E-05 14800 8.40E-06 14600 1.84E-05 14200
0.002791834 16500 1.79E-05 16000 2.19E-05
16900 7.47E-05 14800 8.39E-06 14600 1.84E-05 14200
0.002674989 16500 1.79E-05 16000 2.20E-05
16900 7.48E-05 14800 8.40E-06 14600 1.84E-05 14200
0.002565751 16500 1.79E-05 16000 2.20E-05
16900 7.49E-05 14800 8.38E-06 14600 1.84E-05 14200
0.002463442 16500 1.79E-05 16000 2.20E-05
16900 7.50E-05 14800 8.40E-06 14600 1.84E-05 14200
0.002367466 16500 1.79E-05 16000 2.20E-05
16900 7.50E-05 14800 8.40E-06 14600 1.84E-05 14200
0.002277301 16500 1.79E-05 16000 2.20E-05
16900 7.51E-05 14800 8.39E-06 14600 1.84E-05 14200
0.002192449 16500 1.80E-05 16000 2.20E-05
16900 7.51E-05 14800 8.38E-06 14600 1.84E-05 14200
0.002112506 16500 1.80E-05 16000 2.20E-05
16900 7.51E-05 14800 8.37E-06 14600 1.84E-05 14200
0.002037088 16500 1.80E-05 16000 2.20E-05
16900 7.51E-05 14900 8.37E-06 14600 1.83E-05 14200
0.00196583 16500 1.80E-05 16000 2.20E-05
16900 7.51E-05 14900 8.35E-06 14600 1.83E-05 14200
0.001898451 16500 1.80E-05 16000 2.20E-05
16900 7.51E-05 14900 8.34E-06 14700 1.83E-05 14200
0.001834657 16500 1.80E-05 16000 2.20E-05
16900 7.50E-05 14900 8.32E-06 14700 1.83E-05 14200
0.001774185 16500 1.80E-05 16000 2.20E-05
16900 7.49E-05 14900 8.32E-06 14700 1.83E-05 14200
0.001716827 16500 1.80E-05 16000 2.21E-05
16900 7.48E-05 14900 8.32E-06 14700 1.83E-05 14200
0.001662339 16500 1.80E-05 16000 2.21E-05
16900 7.47E-05 14900 8.31E-06 14700 1.83E-05 14300
0.001610526 16500 1.80E-05 16000 2.21E-05
16900 7.46E-05 14900 8.30E-06 14700 1.82E-05 14300
0.001561244 16500 1.80E-05 16000 2.21E-05
16900 7.44E-05 14900 8.28E-06 14700 1.82E-05 14300
0.001514302 16500 1.80E-05 16000 2.21E-05
16900 7.42E-05 14900 8.28E-06 14700 1.82E-05 14300
0.00146955 16500 1.80E-05 16000 2.21E-05
16900 7.41E-05 14900 8.28E-06 14700 1.82E-05 14300
0.001426828 16500 1.80E-05 16000 2.21E-05
16900 7.38E-05 14900 8.26E-06 14700 1.82E-05 14300
0.00138607 16500 1.80E-05 16000 2.21E-05
16900 7.36E-05 14900 8.26E-06 14700 1.82E-05 14300
0.001347101 16500 1.80E-05 16000 2.21E-05
16900 7.34E-05 14900 8.25E-06 14700 1.82E-05 14300
0.001309838 16500 1.80E-05 16000 2.21E-05
16900 7.32E-05 14900 8.24E-06 14700 1.82E-05 14300
0.001274173 16500 1.80E-05 16000 2.21E-05
16900 7.29E-05 14900 8.23E-06 14700 1.81E-05 14300
0.001240009 16500 1.81E-05 16000 2.21E-05
16900 7.27E-05 14900 8.25E-06 14700 1.81E-05 14300
0.001207278 16500 1.81E-05 16000 2.21E-05
16900 7.24E-05 14900 8.22E-06 14700 1.81E-05 14300
0.001175906 16600 1.81E-05 16000 2.21E-05
16900 7.21E-05 14900 8.22E-06 14700 1.81E-05 14300
0.001145794 16600 1.81E-05 16000 2.21E-05
16900 7.17E-05 14900 8.21E-06 14700 1.81E-05 14300
0.001116863 16600 1.81E-05 16000 2.21E-05
16900 7.15E-05 14900 8.20E-06 14700 1.81E-05 14300
0.001089079 16600 1.81E-05 16000 2.21E-05
16900 7.11E-05 14900 8.20E-06 14700 1.81E-05 14300
0.001062346 16600 1.81E-05 16000 2.21E-05
16900 7.08E-05 14900 8.21E-06 14700 1.80E-05 14300
0.001036635 16600 1.81E-05 16000 2.21E-05
16900 7.05E-05 14900 8.19E-06 14700 1.80E-05 14300
0.001011878 16600 1.81E-05 16000 2.21E-05
16900 7.01E-05 14900 8.20E-06 14700 1.80E-05 14300
0.000988022 16600 1.81E-05 16000 2.21E-05

16900	6.98E-05	14900	8.20E-06	14700	1.80E-05	14300	0.000965041	16600	1.81E-05	16000	2.21E-05
16900	6.94E-05	14900	8.20E-06	14700	1.80E-05	14300	0.000942887	16600	1.81E-05	16000	2.21E-05
16900	6.91E-05	14900	8.18E-06	14700	1.80E-05	14300	0.000921525	16600	1.81E-05	16000	2.21E-05
16900	6.87E-05	14900	8.18E-06	14700	1.80E-05	14300	0.000900913	16600	1.81E-05	16000	2.21E-05
16900	6.84E-05	14900	8.18E-06	14700	1.79E-05	14300	0.000881011	16600	1.81E-05	16000	2.22E-05
16900	6.80E-05	14900	8.18E-06	14700	1.79E-05	14300	0.000861821	16600	1.81E-05	16000	2.22E-05
16900	6.77E-05	14900	8.18E-06	14700	1.79E-05	14300	0.00084328	16600	1.81E-05	16000	2.22E-05
16900	6.73E-05	14900	8.18E-06	14700	1.79E-05	14300	0.000825364	16600	1.81E-05	16000	2.22E-05
16900	6.70E-05	14900	8.17E-06	14700	1.79E-05	14300	0.000808035	16600	1.81E-05	16000	2.22E-05
16900	6.66E-05	14900	8.17E-06	14700	1.79E-05	14300	0.000791276	16600	1.81E-05	16000	2.22E-05
16900	6.63E-05	14900	8.16E-06	14700	1.79E-05	14300	0.000775032	16600	1.82E-05	16000	2.22E-05
16900	6.59E-05	14900	8.16E-06	14700	1.79E-05	14300	0.000759331	16600	1.82E-05	16000	2.22E-05
16900	6.55E-05	14900	8.14E-06	14700	1.79E-05	14300	0.00074408	16600	1.81E-05	16000	2.22E-05
16900	6.52E-05	14900	8.13E-06	14700	1.78E-05	14300	0.000729322	16600	1.82E-05	16000	2.22E-05
16900	6.48E-05	14900	8.14E-06	14700	1.78E-05	14300	0.000715002	16600	1.82E-05	16000	2.22E-05
16900	6.45E-05	14900	8.13E-06	14700	1.78E-05	14300	0.000701119	16600	1.82E-05	16000	2.23E-05
16900	6.42E-05	14900	8.12E-06	14700	1.78E-05	14300	0.000687663	16600	1.82E-05	16000	2.23E-05
16900	6.39E-05	14900	8.12E-06	14700	1.78E-05	14300	0.00067461	16600	1.82E-05	16000	2.23E-05
16900	6.36E-05	14900	8.12E-06	14700	1.78E-05	14300	0.000661942	16600	1.82E-05	16000	2.23E-05
16900	6.32E-05	14900	8.11E-06	14700	1.78E-05	14300	0.000649658	16600	1.82E-05	16000	2.23E-05
16900	6.29E-05	14900	8.09E-06	14700	1.78E-05	14300	0.000637715	16600	1.82E-05	16000	2.23E-05
17000	6.26E-05	14900	8.10E-06	14700	1.78E-05	14300	0.000626118	16600	1.82E-05	16000	2.23E-05
17000	6.23E-05	14900	8.10E-06	14700	1.77E-05	14300	0.000614855	16600	1.82E-05	16000	2.23E-05
17000	6.20E-05	14900	8.07E-06	14700	1.77E-05	14300	0.000603871	16600	1.82E-05	16000	2.23E-05
17000	6.17E-05	14900	8.10E-06	14700	1.77E-05	14300	0.000593217	16600	1.82E-05	16000	2.23E-05
17000	6.15E-05	14900	8.08E-06	14700	1.77E-05	14300	0.000582871	16600	1.82E-05	16000	2.23E-05
17000	6.12E-05	14900	8.06E-06	14700	1.77E-05	14300	0.000572798	16600	1.82E-05	16000	2.23E-05
17000	6.09E-05	14900	8.08E-06	14700	1.77E-05	14300	0.000563016	16600	1.82E-05	16000	2.23E-05
17000	6.07E-05	14900	8.08E-06	14700	1.77E-05	14300	0.0005535	16600	1.82E-05	16000	2.23E-05
17000	6.04E-05	14900	8.07E-06	14700	1.76E-05	14300	0.000544246	16600	1.82E-05	16000	2.23E-05
17000	6.01E-05	14900	8.06E-06	14700	1.77E-05	14300	0.000535254	16600	1.82E-05	16100	2.23E-05
17000	5.99E-05	14900	8.05E-06	14700	1.77E-05	14300	0.00052648	16600	1.83E-05	16100	2.23E-05
17000	5.97E-05	14900	8.05E-06	14700	1.76E-05	14300	0.000517968	16600	1.83E-05	16100	2.23E-05
17000	5.94E-05	14900	8.04E-06	14700	1.76E-05	14300	0.000509659	16600	1.83E-05	16100	2.23E-05

17000 5.92E-05 14900 8.02E-06 14700 1.76E-05 14300
0.000501578 16600 1.83E-05 16100 2.23E-05
17000 5.90E-05 14900 8.04E-06 14700 1.76E-05 14300
0.000493705 16600 1.83E-05 16100 2.23E-05
17000 5.88E-05 14900 8.02E-06 14700 1.76E-05 14300
0.000486042 16600 1.83E-05 16100 2.23E-05
17000 5.86E-05 14900 8.01E-06 14700 1.76E-05 14300
0.000478532 16600 1.83E-05 16100 2.23E-05
17000 5.84E-05 14900 8.02E-06 14700 1.76E-05 14300
0.000471217 16600 1.83E-05 16100 2.24E-05
17000 5.82E-05 15000 8.00E-06 14700 1.76E-05 14300
0.000464087 16600 1.83E-05 16100 2.24E-05
17000 5.80E-05 15000 8.02E-06 14700 1.76E-05 14300
0.000457119 16600 1.83E-05 16100 2.24E-05
17000 5.79E-05 15000 8.02E-06 14800 1.76E-05 14300
0.000450313 16600 1.83E-05 16100 2.24E-05
17000 5.77E-05 15000 7.99E-06 14800 1.76E-05 14300
0.000443622 16600 1.83E-05 16100 2.24E-05
17000 5.75E-05 15000 8.00E-06 14800 1.75E-05 14300
0.000437109 16600 1.83E-05 16100 2.24E-05
17000 5.74E-05 15000 8.00E-06 14800 1.75E-05 14300
0.000430729 16600 1.83E-05 16100 2.24E-05
17000 5.72E-05 15000 7.99E-06 14800 1.75E-05 14400
0.000424467 16600 1.83E-05 16100 2.24E-05
17000 5.71E-05 15000 8.01E-06 14800 1.75E-05 14400
0.000418364 16600 1.83E-05 16100 2.24E-05
17000 5.69E-05 15000 7.99E-06 14800 1.75E-05 14400
0.000412369 16600 1.83E-05 16100 2.24E-05
17000 5.68E-05 15000 7.99E-06 14800 1.75E-05 14400
0.000406515 16600 1.84E-05 16100 2.24E-05
17000 5.67E-05 15000 7.98E-06 14800 1.74E-05 14400
0.000400777 16600 1.84E-05 16100 2.24E-05
17000 5.65E-05 15000 7.98E-06 14800 1.74E-05 14400
0.000395156 16600 1.84E-05 16100 2.24E-05
17000 5.64E-05 15000 7.96E-06 14800 1.75E-05 14400
0.000389671 16600 1.84E-05 16100 2.24E-05
17000 5.63E-05 15000 7.96E-06 14800 1.74E-05 14400
0.000384296 16600 1.84E-05 16100 2.24E-05
17000 5.62E-05 15000 7.96E-06 14800 1.74E-05 14400
0.000379046 16600 1.84E-05 16100 2.24E-05
17000 5.61E-05 15000 7.96E-06 14800 1.74E-05 14400
0.000373909 16600 1.84E-05 16100 2.24E-05
17000 5.60E-05 15000 7.94E-06 14800 1.74E-05 14400
0.00036888 16600 1.84E-05 16100 2.24E-05
17000 5.59E-05 15000 7.95E-06 14800 1.74E-05 14400
0.000363954 16700 1.84E-05 16100 2.24E-05
17000 5.58E-05 15000 7.95E-06 14800 1.74E-05 14400
0.000359141 16700 1.84E-05 16100 2.24E-05
17000 5.57E-05 15000 7.93E-06 14800 1.74E-05 14400
0.000354428 16700 1.84E-05 16100 2.25E-05
17000 5.56E-05 15000 7.94E-06 14800 1.74E-05 14400
0.000349829 16700 1.84E-05 16100 2.25E-05
17000 5.55E-05 15000 7.92E-06 14800 1.74E-05 14400
0.000345321 16700 1.84E-05 16100 2.24E-05
17000 5.54E-05 15000 7.92E-06 14800 1.74E-05 14400
0.00034089 16700 1.84E-05 16100 2.25E-05
17000 5.53E-05 15000 7.93E-06 14800 1.74E-05 14400
0.000336548 16700 1.84E-05 16100 2.25E-05
17000 5.52E-05 15000 7.93E-06 14800 1.74E-05 14400
0.000332292 16700 1.84E-05 16100 2.25E-05
17000 5.51E-05 15000 7.92E-06 14800 1.74E-05 14400
0.000328114 16700 1.84E-05 16100 2.25E-05
17000 5.51E-05 15000 7.91E-06 14800 1.74E-05 14400
0.000324029 16700 1.84E-05 16100 2.25E-05
17000 5.50E-05 15000 7.91E-06 14800 1.73E-05 14400
0.000320037 16700 1.85E-05 16100 2.25E-05
17000 5.49E-05 15000 7.91E-06 14800 1.73E-05 14400
0.000316107 16700 1.85E-05 16100 2.25E-05

17000 5.48E-05 15000 7.91E-06 14800 1.73E-05 14400
0.000312285 16700 1.85E-05 16100 2.25E-05
17000 5.48E-05 15000 7.89E-06 14800 1.73E-05 14400
0.000308524 16700 1.85E-05 16100 2.25E-05
17000 5.47E-05 15000 7.89E-06 14800 1.73E-05 14400
0.000304871 16700 1.85E-05 16100 2.25E-05
17000 5.46E-05 15000 7.88E-06 14800 1.73E-05 14400
0.000301267 16700 1.85E-05 16100 2.25E-05
17000 5.46E-05 15000 7.88E-06 14800 1.73E-05 14400
0.000297731 16700 1.85E-05 16100 2.25E-05
17000 5.45E-05 15000 7.88E-06 14800 1.73E-05 14400
0.000294276 16700 1.85E-05 16100 2.25E-05
17000 5.45E-05 15000 7.87E-06 14800 1.73E-05 14400
0.000290864 16700 1.85E-05 16100 2.25E-05
17000 5.44E-05 15000 7.87E-06 14800 1.73E-05 14400
0.000287506 16700 1.85E-05 16100 2.25E-05
17000 5.44E-05 15000 7.87E-06 14800 1.72E-05 14400
0.000284193 16700 1.85E-05 16100 2.25E-05
17000 5.43E-05 15000 7.85E-06 14800 1.73E-05 14400
0.000280935 16700 1.85E-05 16100 2.26E-05
17000 5.42E-05 15000 7.85E-06 14800 1.72E-05 14400
0.000277748 16700 1.85E-05 16100 2.26E-05
17000 5.42E-05 15000 7.84E-06 14800 1.73E-05 14400
0.000274616 16700 1.85E-05 16100 2.26E-05
17000 5.41E-05 15000 7.84E-06 14800 1.73E-05 14400
0.000271521 16700 1.86E-05 16100 2.26E-05
17000 5.41E-05 15000 7.83E-06 14800 1.72E-05 14400
0.000268515 16700 1.86E-05 16100 2.26E-05
17000 5.41E-05 15000 7.82E-06 14800 1.73E-05 14400
0.000265561 16700 1.86E-05 16100 2.26E-05
17000 5.40E-05 15000 7.82E-06 14800 1.72E-05 14400
0.00026264 16700 1.86E-05 16100 2.26E-05
17000 5.40E-05 15000 7.82E-06 14800 1.72E-05 14400
0.000259792 16700 1.86E-05 16100 2.26E-05
17100 5.40E-05 15000 7.81E-06 14800 1.72E-05 14400
0.000256993 16700 1.86E-05 16100 2.26E-05
17100 5.39E-05 15000 7.81E-06 14800 1.72E-05 14400
0.000254246 16700 1.86E-05 16100 2.26E-05
17100 5.39E-05 15000 7.81E-06 14800 1.72E-05 14400
0.000251562 16700 1.86E-05 16100 2.26E-05
17100 5.39E-05 15000 7.80E-06 14800 1.72E-05 14400
0.000248936 16700 1.86E-05 16100 2.26E-05
17100 5.38E-05 15000 7.79E-06 14800 1.72E-05 14400
0.00024636 16700 1.86E-05 16100 2.26E-05
17100 5.38E-05 15000 7.79E-06 14800 1.72E-05 14400
0.000243802 16700 1.86E-05 16100 2.26E-05
17100 5.38E-05 15000 7.78E-06 14800 1.72E-05 14400
0.000241302 16700 1.86E-05 16100 2.26E-05
17100 5.38E-05 15000 7.77E-06 14800 1.72E-05 14400
0.000238855 16700 1.86E-05 16100 2.26E-05
17100 5.38E-05 15000 7.77E-06 14800 1.72E-05 14400
0.000236451 16700 1.86E-05 16100 2.27E-05
17100 5.38E-05 15000 7.78E-06 14800 1.72E-05 14400
0.000234058 16700 1.86E-05 16200 2.27E-05
17100 5.37E-05 15000 7.78E-06 14800 1.72E-05 14400
0.000231725 16700 1.86E-05 16200 2.27E-05
17100 5.37E-05 15000 7.78E-06 14800 1.71E-05 14400
0.000229412 16700 1.86E-05 16200 2.27E-05
17100 5.37E-05 15000 7.78E-06 14800 1.71E-05 14400
0.000227159 16700 1.87E-05 16200 2.27E-05
17100 5.37E-05 15000 7.77E-06 14800 1.71E-05 14400
0.000224939 16700 1.87E-05 16200 2.27E-05
17100 5.36E-05 15000 7.76E-06 14800 1.71E-05 14400
0.000222734 16700 1.87E-05 16200 2.27E-05
17100 5.36E-05 15000 7.76E-06 14800 1.71E-05 14400
0.000220576 16700 1.87E-05 16200 2.27E-05
17100 5.36E-05 15000 7.76E-06 14800 1.71E-05 14400
0.000218464 16700 1.87E-05 16200 2.27E-05

17100 5.36E-05 15000 7.77E-06 14800 1.71E-05 14400
0.000216339 16700 1.87E-05 16200 2.27E-05
17100 5.36E-05 15100 7.77E-06 14800 1.71E-05 14400
0.000214248 16700 1.87E-05 16200 2.27E-05
17100 5.36E-05 15100 7.77E-06 14800 1.71E-05 14400
0.000212191 16700 1.87E-05 16200 2.27E-05
17100 5.36E-05 15100 7.75E-06 14900 1.71E-05 14400
0.000210151 16700 1.87E-05 16200 2.27E-05
17100 5.35E-05 15100 7.75E-06 14900 1.70E-05 14400
0.000208154 16700 1.87E-05 16200 2.27E-05
17100 5.35E-05 15100 7.75E-06 14900 1.70E-05 14400
0.000206195 16700 1.87E-05 16200 2.27E-05
17100 5.35E-05 15100 7.74E-06 14900 1.70E-05 14400
0.000204266 16700 1.87E-05 16200 2.27E-05
17100 5.35E-05 15100 7.73E-06 14900 1.70E-05 14500
0.000202366 16700 1.87E-05 16200 2.27E-05
17100 5.35E-05 15100 7.74E-06 14900 1.70E-05 14500
0.000200497 16700 1.87E-05 16200 2.28E-05
17100 5.35E-05 15100 7.74E-06 14900 1.70E-05 14500
0.000198691 16700 1.87E-05 16200 2.28E-05
17100 5.35E-05 15100 7.73E-06 14900 1.70E-05 14500
0.000196897 16700 1.87E-05 16200 2.28E-05
17100 5.35E-05 15100 7.73E-06 14900 1.70E-05 14500
0.00019513 16700 1.87E-05 16200 2.28E-05
17100 5.35E-05 15100 7.74E-06 14900 1.69E-05 14500
0.000193379 16700 1.88E-05 16200 2.28E-05
17100 5.35E-05 15100 7.72E-06 14900 1.70E-05 14500
0.000191654 16700 1.88E-05 16200 2.28E-05
17100 5.34E-05 15100 7.73E-06 14900 1.69E-05 14500
0.000189951 16700 1.88E-05 16200 2.28E-05
17100 5.35E-05 15100 7.72E-06 14900 1.69E-05 14500
0.000188277 16700 1.88E-05 16200 2.28E-05
17100 5.34E-05 15100 7.72E-06 14900 1.69E-05 14500
0.000186602 16700 1.88E-05 16200 2.28E-05
17100 5.34E-05 15100 7.71E-06 14900 1.69E-05 14500
0.000184963 16700 1.88E-05 16200 2.28E-05
17100 5.34E-05 15100 7.71E-06 14900 1.69E-05 14500
0.00018334 16800 1.88E-05 16200 2.28E-05
17100 5.34E-05 15100 7.71E-06 14900 1.69E-05 14500
0.000181727 16800 1.88E-05 16200 2.28E-05
17100 5.34E-05 15100 7.70E-06 14900 1.69E-05 14500
0.000180142 16800 1.88E-05 16200 2.28E-05
17100 5.34E-05 15100 7.70E-06 14900 1.69E-05 14500
0.000178584 16800 1.88E-05 16200 2.28E-05
17100 5.35E-05 15100 7.69E-06 14900 1.69E-05 14500
0.000177051 16800 1.88E-05 16200 2.28E-05
17100 5.34E-05 15100 7.69E-06 14900 1.69E-05 14500
0.000175561 16800 1.88E-05 16200 2.29E-05
17100 5.34E-05 15100 7.69E-06 14900 1.69E-05 14500
0.000174087 16800 1.88E-05 16200 2.29E-05
17100 5.34E-05 15100 7.67E-06 14900 1.69E-05 14500
0.000172631 16800 1.88E-05 16200 2.29E-05
17100 5.34E-05 15100 7.67E-06 14900 1.69E-05 14500
0.000171208 16800 1.88E-05 16200 2.29E-05
17100 5.35E-05 15100 7.67E-06 14900 1.68E-05 14500
0.000169803 16800 1.88E-05 16200 2.29E-05
17100 5.35E-05 15100 7.68E-06 14900 1.68E-05 14500
0.00016843 16800 1.88E-05 16200 2.29E-05
17100 5.35E-05 15100 7.67E-06 14900 1.68E-05 14500
0.000167073 16800 1.88E-05 16200 2.29E-05
17100 5.35E-05 15100 7.68E-06 14900 1.68E-05 14500
0.000165713 16800 1.88E-05 16200 2.29E-05
17100 5.35E-05 15100 7.67E-06 14900 1.68E-05 14500
0.000164377 16800 1.88E-05 16200 2.29E-05
17100 5.35E-05 15100 7.67E-06 14900 1.68E-05 14500
0.000163069 16800 1.88E-05 16200 2.29E-05
17100 5.35E-05 15100 7.68E-06 14900 1.68E-05 14500
0.000161781 16800 1.88E-05 16200 2.29E-05

17100	5.35E-05	15100	7.67E-06	14900	1.68E-05	14500
0.000160493	16800	1.88E-05	16200	2.29E-05		
17100	5.35E-05	15100	7.65E-06	14900	1.68E-05	14500
0.000159231	16800	1.89E-05	16200	2.29E-05		
17100	5.35E-05	15100	7.66E-06	14900	1.68E-05	14500
0.000157982	16800	1.89E-05	16200	2.30E-05		
17100	5.35E-05	15100	7.66E-06	14900	1.68E-05	14500
0.000156749	16800	1.89E-05	16200	2.30E-05		
17100	5.35E-05	15100	7.65E-06	14900	1.68E-05	14500
0.000155517	16800	1.89E-05	16200	2.30E-05		
17100	5.35E-05	15100	7.65E-06	14900	1.68E-05	14500
0.000154306	16800	1.89E-05	16200	2.30E-05		
17100	5.35E-05	15100	7.65E-06	14900	1.68E-05	14500
0.000153116	16800	1.89E-05	16200	2.30E-05		
17100	5.35E-05	15100	7.65E-06	14900	1.67E-05	14500
0.000151944	16800	1.89E-05	16200	2.30E-05		
17100	5.35E-05	15100	7.64E-06	14900	1.67E-05	14500
0.000150787	16800	1.89E-05	16200	2.30E-05		
17100	5.35E-05	15100	7.65E-06	14900	1.67E-05	14500
0.000149655	16800	1.89E-05	16200	2.30E-05		
17100	5.35E-05	15100	7.65E-06	14900	1.67E-05	14500
0.000148534	16800	1.89E-05	16200	2.30E-05		
17100	5.35E-05	15100	7.62E-06	14900	1.67E-05	14500
0.000147418	16800	1.89E-05	16200	2.30E-05		
17100	5.35E-05	15100	7.64E-06	14900	1.67E-05	14500
0.000146329	16800	1.89E-05	16200	2.30E-05		
17200	5.35E-05	15100	7.64E-06	14900	1.67E-05	14500
0.000145244	16800	1.89E-05	16200	2.30E-05		
17200	5.35E-05	15100	7.63E-06	14900	1.67E-05	14500
0.00014417	16800	1.89E-05	16200	2.30E-05		
17200	5.35E-05	15100	7.63E-06	14900	1.67E-05	14500
0.000143117	16800	1.89E-05	16200	2.30E-05		
17200	5.36E-05	15100	7.62E-06	14900	1.67E-05	14500
0.000142072	16800	1.89E-05	16200	2.30E-05		
17200	5.35E-05	15100	7.62E-06	14900	1.67E-05	14500
0.000141029	16800	1.89E-05	16200	2.30E-05		
17200	5.35E-05	15100	7.63E-06	14900	1.67E-05	14500
0.000140013	16800	1.89E-05	16200	2.30E-05		
17200	5.36E-05	15100	7.62E-06	14900	1.67E-05	14500
0.000139003	16800	1.90E-05	16200	2.31E-05		
17200	5.36E-05	15100	7.61E-06	14900	1.67E-05	14500
0.000137994	16800	1.90E-05	16200	2.31E-05		
17200	5.36E-05	15100	7.61E-06	14900	1.67E-05	14500
0.000137024	16800	1.90E-05	16200	2.31E-05		
17200	5.36E-05	15100	7.61E-06	14900	1.67E-05	14500
0.000136054	16800	1.90E-05	16300	2.31E-05		
17200	5.36E-05	15100	7.62E-06	14900	1.67E-05	14500
0.000135079	16800	1.90E-05	16300	2.31E-05		
17200	5.36E-05	15100	7.61E-06	14900	1.66E-05	14500
0.000134117	16800	1.90E-05	16300	2.31E-05		
17200	5.36E-05	15100	7.61E-06	14900	1.66E-05	14500
0.000133177	16800	1.90E-05	16300	2.31E-05		
17200	5.36E-05	15100	7.61E-06	14900	1.66E-05	14500
0.000132249	16800	1.90E-05	16300	2.31E-05		
17200	5.36E-05	15100	7.60E-06	14900	1.66E-05	14500
0.000131338	16800	1.90E-05	16300	2.31E-05		
17200	5.36E-05	15100	7.59E-06	14900	1.66E-05	14500
0.000130435	16800	1.90E-05	16300	2.31E-05		
17200	5.36E-05	15100	7.58E-06	14900	1.66E-05	14500
0.000129559	16800	1.90E-05	16300	2.31E-05		
17200	5.36E-05	15100	7.57E-06	14900	1.66E-05	14500
0.000128672	16800	1.90E-05	16300	2.31E-05		
17200	5.37E-05	15200	7.57E-06	14900	1.66E-05	14500
0.000127792	16800	1.90E-05	16300	2.31E-05		
17200	5.37E-05	15200	7.56E-06	14900	1.66E-05	14500
0.000126923	16800	1.90E-05	16300	2.31E-05		
17200	5.37E-05	15200	7.55E-06	15000	1.66E-05	14500
0.000126064	16800	1.90E-05	16300	2.32E-05		

17200 5.37E-05 15200 7.55E-06 15000 1.66E-05 14500
0.000125208 16800 1.90E-05 16300 2.32E-05
17200 5.37E-05 15200 7.55E-06 15000 1.66E-05 14500
0.000124367 16800 1.91E-05 16300 2.32E-05
17200 5.37E-05 15200 7.56E-06 15000 1.66E-05 14500
0.000123536 16800 1.91E-05 16300 2.32E-05
17200 5.37E-05 15200 7.56E-06 15000 1.65E-05 14600
0.000122708 16800 1.91E-05 16300 2.32E-05
17200 5.37E-05 15200 7.56E-06 15000 1.66E-05 14600
0.000121915 16800 1.91E-05 16300 2.32E-05
17200 5.37E-05 15200 7.55E-06 15000 1.65E-05 14600
0.000121117 16800 1.91E-05 16300 2.32E-05
17200 5.37E-05 15200 7.55E-06 15000 1.65E-05 14600
0.000120329 16800 1.91E-05 16300 2.32E-05
17200 5.38E-05 15200 7.54E-06 15000 1.65E-05 14600
0.000119551 16800 1.91E-05 16300 2.32E-05
17200 5.38E-05 15200 7.54E-06 15000 1.65E-05 14600
0.000118777 16800 1.91E-05 16300 2.32E-05
17200 5.38E-05 15200 7.54E-06 15000 1.65E-05 14600
0.000118001 16800 1.91E-05 16300 2.32E-05
17200 5.38E-05 15200 7.54E-06 15000 1.65E-05 14600
0.000117254 16800 1.91E-05 16300 2.32E-05
17200 5.38E-05 15200 7.54E-06 15000 1.65E-05 14600
0.000116509 16800 1.91E-05 16300 2.32E-05
17200 5.38E-05 15200 7.54E-06 15000 1.65E-05 14600
0.000115776 16800 1.91E-05 16300 2.33E-05
17200 5.38E-05 15200 7.55E-06 15000 1.65E-05 14600
0.000115066 16800 1.91E-05 16300 2.33E-05
17200 5.38E-05 15200 7.53E-06 15000 1.65E-05 14600
0.000114355 16900 1.91E-05 16300 2.33E-05
17200 5.38E-05 15200 7.52E-06 15000 1.65E-05 14600
0.000113643 16900 1.91E-05 16300 2.33E-05
17200 5.38E-05 15200 7.53E-06 15000 1.65E-05 14600
0.000112938 16900 1.91E-05 16300 2.33E-05
17200 5.38E-05 15200 7.54E-06 15000 1.65E-05 14600
0.000112234 16900 1.91E-05 16300 2.33E-05
17200 5.38E-05 15200 7.52E-06 15000 1.65E-05 14600
0.000111546 16900 1.92E-05 16300 2.33E-05
17200 5.39E-05 15200 7.52E-06 15000 1.65E-05 14600
0.000110856 16900 1.92E-05 16300 2.33E-05
17200 5.39E-05 15200 7.52E-06 15000 1.65E-05 14600
0.00011017 16900 1.92E-05 16300 2.33E-05
17200 5.39E-05 15200 7.52E-06 15000 1.65E-05 14600
0.000109497 16900 1.92E-05 16300 2.33E-05
17200 5.39E-05 15200 7.52E-06 15000 1.64E-05 14600
0.00010882 16900 1.92E-05 16300 2.33E-05
17200 5.39E-05 15200 7.52E-06 15000 1.64E-05 14600
0.000108174 16900 1.92E-05 16300 2.33E-05
17200 5.39E-05 15200 7.50E-06 15000 1.64E-05 14600
0.00010752 16900 1.92E-05 16300 2.33E-05
17200 5.39E-05 15200 7.50E-06 15000 1.64E-05 14600
0.000106901 16900 1.92E-05 16300 2.34E-05
17200 5.39E-05 15200 7.50E-06 15000 1.64E-05 14600
0.000106275 16900 1.92E-05 16300 2.34E-05
17200 5.39E-05 15200 7.50E-06 15000 1.64E-05 14600
0.000105647 16900 1.92E-05 16300 2.34E-05
17200 5.39E-05 15200 7.49E-06 15000 1.64E-05 14600
0.000105017 16900 1.92E-05 16300 2.34E-05
17200 5.39E-05 15200 7.49E-06 15000 1.64E-05 14600
0.000104406 16900 1.92E-05 16300 2.33E-05
17200 5.39E-05 15200 7.48E-06 15000 1.64E-05 14600
0.000103783 16900 1.92E-05 16300 2.34E-05
17200 5.39E-05 15200 7.48E-06 15000 1.64E-05 14600
0.000103196 16900 1.92E-05 16300 2.34E-05
17200 5.39E-05 15200 7.47E-06 15000 1.64E-05 14600
0.000102588 16900 1.93E-05 16300 2.34E-05
17200 5.39E-05 15200 7.47E-06 15000 1.64E-05 14600
0.00010202 16900 1.93E-05 16300 2.34E-05

17200 5.39E-05 15200 7.46E-06 15000 1.64E-05 14600
0.00010143 16900 1.93E-05 16300 2.34E-05
17200 5.40E-05 15200 7.46E-06 15000 1.64E-05 14600
0.000100852 16900 1.93E-05 16300 2.34E-05
17200 5.40E-05 15200 7.45E-06 15000 1.64E-05 14600
0.000100273 16900 1.93E-05 16300 2.34E-05
17200 5.40E-05 15200 7.44E-06 15000 1.63E-05 14600
9.97E-05 16900 1.93E-05 16300 2.34E-05
17200 5.40E-05 15200 7.44E-06 15000 1.63E-05 14600
9.91E-05 16900 1.93E-05 16300 2.34E-05
17200 5.40E-05 15200 7.44E-06 15000 1.63E-05 14600
9.86E-05 16900 1.93E-05 16300 2.34E-05
17200 5.40E-05 15200 7.44E-06 15000 1.63E-05 14600
9.80E-05 16900 1.93E-05 16300 2.34E-05
17200 5.40E-05 15200 7.44E-06 15000 1.63E-05 14600
9.75E-05 16900 1.93E-05 16300 2.34E-05
17200 5.40E-05 15200 7.43E-06 15000 1.63E-05 14600
9.69E-05 16900 1.93E-05 16300 2.34E-05
17300 5.40E-05 15200 7.43E-06 15000 1.63E-05 14600
9.64E-05 16900 1.93E-05 16300 2.34E-05
17300 5.40E-05 15200 7.44E-06 15000 1.63E-05 14600
9.59E-05 16900 1.93E-05 16300 2.34E-05
17300 5.41E-05 15200 7.44E-06 15000 1.63E-05 14600
9.53E-05 16900 1.93E-05 16300 2.34E-05
17300 5.41E-05 15200 7.44E-06 15000 1.63E-05 14600
9.48E-05 16900 1.93E-05 16300 2.35E-05
17300 5.41E-05 15200 7.42E-06 15000 1.63E-05 14600
9.43E-05 16900 1.93E-05 16300 2.35E-05
17300 5.41E-05 15200 7.42E-06 15000 1.63E-05 14600
9.38E-05 16900 1.93E-05 16300 2.35E-05
17300 5.41E-05 15200 7.41E-06 15000 1.63E-05 14600
9.33E-05 16900 1.94E-05 16300 2.35E-05
17300 5.41E-05 15200 7.43E-06 15000 1.63E-05 14600
9.28E-05 16900 1.94E-05 16300 2.35E-05
17300 5.41E-05 15200 7.41E-06 15000 1.63E-05 14600
9.23E-05 16900 1.94E-05 16300 2.35E-05
17300 5.41E-05 15200 7.42E-06 15000 1.63E-05 14600
9.18E-05 16900 1.94E-05 16400 2.35E-05
17300 5.41E-05 15200 7.40E-06 15000 1.63E-05 14600
9.13E-05 16900 1.94E-05 16400 2.35E-05
17300 5.42E-05 15200 7.41E-06 15000 1.63E-05 14600
9.08E-05 16900 1.94E-05 16400 2.35E-05
17300 5.42E-05 15200 7.40E-06 15000 1.63E-05 14600
9.04E-05 16900 1.94E-05 16400 2.35E-05
17300 5.42E-05 15200 7.40E-06 15000 1.63E-05 14600
8.99E-05 16900 1.94E-05 16400 2.35E-05
17300 5.42E-05 15200 7.39E-06 15000 1.62E-05 14600
8.94E-05 16900 1.94E-05 16400 2.35E-05
17300 5.42E-05 15200 7.39E-06 15000 1.62E-05 14600
8.90E-05 16900 1.94E-05 16400 2.36E-05
17300 5.42E-05 15200 7.39E-06 15000 1.62E-05 14600
8.85E-05 16900 1.94E-05 16400 2.36E-05
17300 5.42E-05 15200 7.39E-06 15000 1.62E-05 14600
8.80E-05 16900 1.94E-05 16400 2.36E-05
17300 5.43E-05 15300 7.39E-06 15000 1.62E-05 14600
8.76E-05 16900 1.94E-05 16400 2.36E-05
17300 5.43E-05 15300 7.37E-06 15000 1.62E-05 14600
8.71E-05 16900 1.94E-05 16400 2.36E-05
17300 5.43E-05 15300 7.39E-06 15100 1.62E-05 14600
8.67E-05 16900 1.94E-05 16400 2.36E-05
17300 5.43E-05 15300 7.38E-06 15100 1.62E-05 14600
8.63E-05 16900 1.94E-05 16400 2.36E-05
17300 5.43E-05 15300 7.37E-06 15100 1.62E-05 14600
8.58E-05 16900 1.95E-05 16400 2.36E-05
17300 5.43E-05 15300 7.38E-06 15100 1.62E-05 14600
8.54E-05 16900 1.95E-05 16400 2.36E-05
17300 5.43E-05 15300 7.37E-06 15100 1.62E-05 14700
8.50E-05 16900 1.95E-05 16400 2.36E-05

17300 5.43E-05 15300 7.37E-06 15100 1.62E-05 14700
8.46E-05 16900 1.95E-05 16400 2.36E-05
17300 5.43E-05 15300 7.37E-06 15100 1.62E-05 14700
8.42E-05 16900 1.95E-05 16400 2.36E-05
17300 5.43E-05 15300 7.37E-06 15100 1.62E-05 14700
8.38E-05 16900 1.95E-05 16400 2.36E-05
17300 5.44E-05 15300 7.37E-06 15100 1.61E-05 14700
8.34E-05 16900 1.95E-05 16400 2.36E-05
17300 5.44E-05 15300 7.36E-06 15100 1.61E-05 14700
8.30E-05 16900 1.95E-05 16400 2.36E-05
17300 5.44E-05 15300 7.36E-06 15100 1.61E-05 14700
8.26E-05 16900 1.95E-05 16400 2.36E-05
17300 5.44E-05 15300 7.36E-06 15100 1.61E-05 14700
8.22E-05 16900 1.95E-05 16400 2.36E-05
17300 5.44E-05 15300 7.36E-06 15100 1.61E-05 14700
8.18E-05 16900 1.95E-05 16400 2.36E-05
17300 5.44E-05 15300 7.35E-06 15100 1.61E-05 14700
8.14E-05 16900 1.95E-05 16400 2.36E-05
17300 5.45E-05 15300 7.35E-06 15100 1.61E-05 14700
8.10E-05 16900 1.95E-05 16400 2.36E-05
17300 5.44E-05 15300 7.35E-06 15100 1.61E-05 14700
8.06E-05 17000 1.95E-05 16400 2.36E-05
17300 5.45E-05 15300 7.35E-06 15100 1.61E-05 14700
8.02E-05 17000 1.96E-05 16400 2.37E-05
17300 5.45E-05 15300 7.35E-06 15100 1.61E-05 14700
7.98E-05 17000 1.96E-05 16400 2.37E-05
17300 5.45E-05 15300 7.35E-06 15100 1.61E-05 14700
7.94E-05 17000 1.95E-05 16400 2.37E-05
17300 5.45E-05 15300 7.34E-06 15100 1.61E-05 14700
7.91E-05 17000 1.96E-05 16400 2.37E-05
17300 5.45E-05 15300 7.35E-06 15100 1.61E-05 14700
7.87E-05 17000 1.96E-05 16400 2.37E-05
17300 5.45E-05 15300 7.35E-06 15100 1.61E-05 14700
7.83E-05 17000 1.96E-05 16400 2.37E-05
17300 5.46E-05 15300 7.35E-06 15100 1.61E-05 14700
7.79E-05 17000 1.96E-05 16400 2.37E-05
17300 5.46E-05 15300 7.34E-06 15100 1.61E-05 14700
7.76E-05 17000 1.96E-05 16400 2.37E-05
17300 5.46E-05 15300 7.34E-06 15100 1.60E-05 14700
7.72E-05 17000 1.96E-05 16400 2.37E-05
17300 5.46E-05 15300 7.35E-06 15100 1.60E-05 14700
7.69E-05 17000 1.96E-05 16400 2.37E-05
17300 5.46E-05 15300 7.36E-06 15100 1.60E-05 14700
7.65E-05 17000 1.96E-05 16400 2.37E-05
17300 5.46E-05 15300 7.34E-06 15100 1.60E-05 14700
7.62E-05 17000 1.96E-05 16400 2.37E-05
17300 5.46E-05 15300 7.34E-06 15100 1.60E-05 14700
7.58E-05 17000 1.96E-05 16400 2.37E-05
17300 5.47E-05 15300 7.34E-06 15100 1.60E-05 14700
7.55E-05 17000 1.96E-05 16400 2.38E-05
17300 5.47E-05 15300 7.35E-06 15100 1.60E-05 14700
7.52E-05 17000 1.96E-05 16400 2.38E-05
17300 5.47E-05 15300 7.34E-06 15100 1.60E-05 14700
7.48E-05 17000 1.96E-05 16400 2.38E-05
17300 5.47E-05 15300 7.33E-06 15100 1.60E-05 14700
7.45E-05 17000 1.96E-05 16400 2.38E-05
17300 5.48E-05 15300 7.33E-06 15100 1.60E-05 14700
7.42E-05 17000 1.96E-05 16400 2.38E-05
17300 5.48E-05 15300 7.34E-06 15100 1.60E-05 14700
7.38E-05 17000 1.97E-05 16400 2.38E-05
17300 5.48E-05 15300 7.35E-06 15100 1.60E-05 14700
7.35E-05 17000 1.97E-05 16400 2.38E-05
17300 5.48E-05 15300 7.34E-06 15100 1.60E-05 14700
7.32E-05 17000 1.97E-05 16400 2.38E-05
17300 5.48E-05 15300 7.33E-06 15100 1.60E-05 14700
7.29E-05 17000 1.97E-05 16400 2.38E-05
17300 5.48E-05 15300 7.34E-06 15100 1.60E-05 14700
7.25E-05 17000 1.97E-05 16400 2.38E-05

17300 5.48E-05 15300 7.35E-06 15100 1.60E-05 14700
7.22E-05 17000 1.97E-05 16400 2.38E-05
17300 5.49E-05 15300 7.35E-06 15100 1.60E-05 14700
7.19E-05 17000 1.97E-05 16400 2.38E-05
17300 5.49E-05 15300 7.34E-06 15100 1.60E-05 14700
7.16E-05 17000 1.97E-05 16400 2.38E-05
17300 5.49E-05 15300 7.35E-06 15100 1.60E-05 14700
7.13E-05 17000 1.97E-05 16400 2.38E-05
17300 5.49E-05 15300 7.34E-06 15100 1.60E-05 14700
7.10E-05 17000 1.97E-05 16400 2.38E-05
17400 5.49E-05 15300 7.34E-06 15100 1.60E-05 14700
7.07E-05 17000 1.97E-05 16400 2.39E-05
17400 5.49E-05 15300 7.35E-06 15100 1.59E-05 14700
7.04E-05 17000 1.97E-05 16400 2.39E-05
17400 5.49E-05 15300 7.34E-06 15100 1.60E-05 14700
7.01E-05 17000 1.97E-05 16400 2.39E-05
17400 5.49E-05 15300 7.33E-06 15100 1.59E-05 14700
6.98E-05 17000 1.97E-05 16400 2.39E-05
17400 5.50E-05 15300 7.33E-06 15100 1.59E-05 14700
6.95E-05 17000 1.98E-05 16400 2.39E-05
17400 5.50E-05 15300 7.33E-06 15100 1.59E-05 14700
6.92E-05 17000 1.98E-05 16400 2.39E-05
17400 5.50E-05 15300 7.33E-06 15100 1.59E-05 14700
6.89E-05 17000 1.98E-05 16400 2.39E-05
17400 5.50E-05 15300 7.32E-06 15100 1.59E-05 14700
6.86E-05 17000 1.98E-05 16400 2.39E-05
17400 5.50E-05 15300 7.31E-06 15100 1.59E-05 14700
6.83E-05 17000 1.98E-05 16400 2.39E-05
17400 5.50E-05 15300 7.31E-06 15100 1.59E-05 14700
6.80E-05 17000 1.98E-05 16500 2.39E-05
17400 5.51E-05 15300 7.31E-06 15100 1.59E-05 14700
6.77E-05 17000 1.98E-05 16500 2.39E-05
17400 5.51E-05 15300 7.30E-06 15100 1.59E-05 14700
6.74E-05 17000 1.98E-05 16500 2.39E-05
17400 5.51E-05 15300 7.30E-06 15100 1.59E-05 14700
6.72E-05 17000 1.98E-05 16500 2.40E-05
17400 5.51E-05 15300 7.31E-06 15100 1.59E-05 14700
6.69E-05 17000 1.98E-05 16500 2.40E-05
17400 5.51E-05 15300 7.31E-06 15100 1.59E-05 14700
6.66E-05 17000 1.98E-05 16500 2.40E-05
17400 5.52E-05 15300 7.31E-06 15100 1.59E-05 14700
6.64E-05 17000 1.98E-05 16500 2.40E-05
17400 5.52E-05 15300 7.30E-06 15100 1.59E-05 14700
6.61E-05 17000 1.98E-05 16500 2.40E-05
17400 5.52E-05 15300 7.30E-06 15100 1.59E-05 14700
6.58E-05 17000 1.98E-05 16500 2.40E-05
17400 5.52E-05 15400 7.30E-06 15100 1.59E-05 14700
6.56E-05 17000 1.99E-05 16500 2.40E-05
17400 5.53E-05 15400 7.30E-06 15100 1.58E-05 14700
6.53E-05 17000 1.99E-05 16500 2.40E-05
17400 5.53E-05 15400 7.30E-06 15200 1.58E-05 14700
6.50E-05 17000 1.99E-05 16500 2.40E-05
17400 5.53E-05 15400 7.29E-06 15200 1.58E-05 14700
6.48E-05 17000 1.99E-05 16500 2.40E-05
17400 5.53E-05 15400 7.29E-06 15200 1.58E-05 14700
6.45E-05 17000 1.99E-05 16500 2.40E-05
17400 5.53E-05 15400 7.28E-06 15200 1.58E-05 14700
6.43E-05 17000 1.99E-05 16500 2.41E-05
17400 5.53E-05 15400 7.28E-06 15200 1.58E-05 14800
6.41E-05 17000 1.99E-05 16500 2.41E-05
17400 5.54E-05 15400 7.28E-06 15200 1.58E-05 14800
6.38E-05 17000 1.99E-05 16500 2.41E-05
17400 5.54E-05 15400 7.28E-06 15200 1.58E-05 14800
6.36E-05 17000 1.99E-05 16500 2.41E-05
17400 5.54E-05 15400 7.28E-06 15200 1.58E-05 14800
6.33E-05 17000 1.99E-05 16500 2.41E-05
17400 5.54E-05 15400 7.28E-06 15200 1.58E-05 14800
6.31E-05 17000 1.99E-05 16500 2.41E-05

17400	5.54E-05	15400	7.28E-06	15200	1.58E-05	14800	6.29E-05	17000	1.99E-05	16500	2.41E-05
17400	5.55E-05	15400	7.27E-06	15200	1.58E-05	14800	6.26E-05	17000	1.99E-05	16500	2.41E-05
17400	5.55E-05	15400	7.28E-06	15200	1.58E-05	14800	6.24E-05	17000	1.99E-05	16500	2.41E-05
17400	5.55E-05	15400	7.27E-06	15200	1.58E-05	14800	6.21E-05	17000	1.99E-05	16500	2.41E-05
17400	5.55E-05	15400	7.27E-06	15200	1.58E-05	14800	6.19E-05	17000	1.99E-05	16500	2.41E-05
17400	5.55E-05	15400	7.27E-06	15200	1.58E-05	14800	6.17E-05	17000	1.99E-05	16500	2.41E-05
17400	5.55E-05	15400	7.27E-06	15200	1.58E-05	14800	6.14E-05	17100	2.00E-05	16500	2.41E-05
17400	5.55E-05	15400	7.27E-06	15200	1.58E-05	14800	6.12E-05	17100	2.00E-05	16500	2.41E-05
17400	5.55E-05	15400	7.26E-06	15200	1.58E-05	14800	6.10E-05	17100	2.00E-05	16500	2.41E-05
17400	5.56E-05	15400	7.26E-06	15200	1.57E-05	14800	6.07E-05	17100	2.00E-05	16500	2.41E-05
17400	5.56E-05	15400	7.25E-06	15200	1.57E-05	14800	6.05E-05	17100	2.00E-05	16500	2.41E-05
17400	5.55E-05	15400	7.24E-06	15200	1.57E-05	14800	6.03E-05	17100	2.00E-05	16500	2.42E-05
17400	5.56E-05	15400	7.25E-06	15200	1.57E-05	14800	6.01E-05	17100	2.00E-05	16500	2.42E-05
17400	5.56E-05	15400	7.25E-06	15200	1.57E-05	14800	5.98E-05	17100	2.00E-05	16500	2.42E-05
17400	5.56E-05	15400	7.24E-06	15200	1.57E-05	14800	5.96E-05	17100	2.00E-05	16500	2.42E-05
17400	5.56E-05	15400	7.24E-06	15200	1.57E-05	14800	5.94E-05	17100	2.00E-05	16500	2.42E-05
17400	5.56E-05	15400	7.25E-06	15200	1.57E-05	14800	5.92E-05	17100	2.00E-05	16500	2.42E-05
17400	5.56E-05	15400	7.24E-06	15200	1.57E-05	14800	5.90E-05	17100	2.00E-05	16500	2.42E-05
17400	5.57E-05	15400	7.24E-06	15200	1.57E-05	14800	5.88E-05	17100	2.00E-05	16500	2.42E-05
17400	5.57E-05	15400	7.24E-06	15200	1.57E-05	14800	5.86E-05	17100	2.00E-05	16500	2.42E-05
17400	5.57E-05	15400	7.24E-06	15200	1.57E-05	14800	5.84E-05	17100	2.00E-05	16500	2.42E-05
17400	5.57E-05	15400	7.24E-06	15200	1.57E-05	14800	5.81E-05	17100	2.00E-05	16500	2.42E-05
17400	5.57E-05	15400	7.24E-06	15200	1.57E-05	14800	5.80E-05	17100	2.00E-05	16500	2.42E-05
17400	5.57E-05	15400	7.25E-06	15200	1.57E-05	14800	5.78E-05	17100	2.01E-05	16500	2.42E-05
17400	5.57E-05	15400	7.25E-06	15200	1.57E-05	14800	5.76E-05	17100	2.01E-05	16500	2.42E-05
17400	5.58E-05	15400	7.24E-06	15200	1.57E-05	14800	5.74E-05	17100	2.01E-05	16500	2.42E-05
17400	5.58E-05	15400	7.23E-06	15200	1.57E-05	14800	5.72E-05	17100	2.01E-05	16500	2.43E-05
17400	5.58E-05	15400	7.23E-06	15200	1.57E-05	14800	5.70E-05	17100	2.01E-05	16500	2.43E-05
17400	5.58E-05	15400	7.23E-06	15200	1.57E-05	14800	5.68E-05	17100	2.01E-05	16500	2.43E-05
17400	5.58E-05	15400	7.23E-06	15200	1.57E-05	14800	5.66E-05	17100	2.01E-05	16500	2.43E-05
17400	5.58E-05	15400	7.22E-06	15200	1.57E-05	14800	5.64E-05	17100	2.01E-05	16500	2.43E-05
17400	5.59E-05	15400	7.23E-06	15200	1.57E-05	14800	5.62E-05	17100	2.01E-05	16500	2.43E-05
17400	5.59E-05	15400	7.23E-06	15200	1.57E-05	14800	5.60E-05	17100	2.01E-05	16500	2.43E-05
17400	5.59E-05	15400	7.23E-06	15200	1.57E-05	14800	5.58E-05	17100	2.01E-05	16500	2.43E-05

17400 5.59E-05 15400 7.23E-06 15200 1.57E-05 14800
5.56E-05 17100 2.01E-05 16500 2.43E-05
17500 5.60E-05 15400 7.22E-06 15200 1.56E-05 14800
5.54E-05 17100 2.01E-05 16500 2.43E-05
17500 5.60E-05 15400 7.21E-06 15200 1.57E-05 14800
5.52E-05 17100 2.01E-05 16500 2.43E-05
17500 5.60E-05 15400 7.22E-06 15200 1.57E-05 14800
5.50E-05 17100 2.01E-05 16500 2.44E-05
17500 5.60E-05 15400 7.22E-06 15200 1.56E-05 14800
5.48E-05 17100 2.01E-05 16500 2.43E-05
17500 5.60E-05 15400 7.22E-06 15200 1.57E-05 14800
5.46E-05 17100 2.01E-05 16500 2.44E-05
17500 5.60E-05 15400 7.22E-06 15200 1.56E-05 14800
5.45E-05 17100 2.01E-05 16500 2.44E-05
17500 5.60E-05 15400 7.22E-06 15200 1.56E-05 14800
5.43E-05 17100 2.01E-05 16500 2.44E-05
17500 5.61E-05 15400 7.22E-06 15200 1.56E-05 14800
5.41E-05 17100 2.02E-05 16500 2.44E-05
17500 5.61E-05 15400 7.22E-06 15200 1.56E-05 14800
5.39E-05 17100 2.02E-05 16500 2.44E-05
17500 5.61E-05 15400 7.21E-06 15200 1.56E-05 14800
5.37E-05 17100 2.02E-05 16600 2.44E-05
17500 5.61E-05 15400 7.21E-06 15200 1.56E-05 14800
5.35E-05 17100 2.02E-05 16600 2.44E-05
17500 5.61E-05 15400 7.20E-06 15200 1.56E-05 14800
5.34E-05 17100 2.02E-05 16600 2.44E-05
17500 5.61E-05 15400 7.19E-06 15200 1.56E-05 14800
5.32E-05 17100 2.02E-05 16600 2.44E-05
17500 5.62E-05 15400 7.19E-06 15200 1.56E-05 14800
5.30E-05 17100 2.02E-05 16600 2.44E-05
17500 5.62E-05 15400 7.18E-06 15200 1.56E-05 14800
5.29E-05 17100 2.02E-05 16600 2.44E-05
17500 5.62E-05 15400 7.18E-06 15200 1.56E-05 14800
5.27E-05 17100 2.02E-05 16600 2.44E-05
17500 5.62E-05 15400 7.17E-06 15200 1.56E-05 14800
5.25E-05 17100 2.02E-05 16600 2.44E-05
17500 5.62E-05 15400 7.18E-06 15200 1.56E-05 14800
5.24E-05 17100 2.02E-05 16600 2.45E-05
17500 5.62E-05 15500 7.17E-06 15200 1.56E-05 14800
5.22E-05 17100 2.02E-05 16600 2.45E-05
17500 5.62E-05 15500 7.17E-06 15200 1.56E-05 14800
5.20E-05 17100 2.02E-05 16600 2.45E-05
17500 5.63E-05 15500 7.16E-06 15300 1.56E-05 14800
5.18E-05 17100 2.03E-05 16600 2.45E-05
17500 5.63E-05 15500 7.17E-06 15300 1.56E-05 14800
5.17E-05 17100 2.03E-05 16600 2.45E-05
17500 5.63E-05 15500 7.17E-06 15300 1.56E-05 14800
5.15E-05 17100 2.03E-05 16600 2.45E-05
17500 5.63E-05 15500 7.16E-06 15300 1.56E-05 14800
5.14E-05 17100 2.03E-05 16600 2.45E-05
17500 5.63E-05 15500 7.16E-06 15300 1.56E-05 14900
5.12E-05 17100 2.03E-05 16600 2.45E-05
17500 5.63E-05 15500 7.16E-06 15300 1.56E-05 14900
5.10E-05 17100 2.03E-05 16600 2.45E-05
17500 5.64E-05 15500 7.16E-06 15300 1.56E-05 14900
5.09E-05 17100 2.03E-05 16600 2.45E-05
17500 5.64E-05 15500 7.16E-06 15300 1.56E-05 14900
5.07E-05 17100 2.03E-05 16600 2.45E-05
17500 5.64E-05 15500 7.15E-06 15300 1.56E-05 14900
5.05E-05 17100 2.03E-05 16600 2.45E-05
17500 5.64E-05 15500 7.15E-06 15300 1.56E-05 14900
5.04E-05 17100 2.03E-05 16600 2.45E-05
17500 5.65E-05 15500 7.15E-06 15300 1.56E-05 14900
5.02E-05 17100 2.03E-05 16600 2.45E-05
17500 5.65E-05 15500 7.16E-06 15300 1.56E-05 14900
5.01E-05 17100 2.03E-05 16600 2.45E-05
17500 5.65E-05 15500 7.16E-06 15300 1.56E-05 14900
4.99E-05 17100 2.03E-05 16600 2.45E-05

17500 5.65E-05 15500 7.16E-06 15300 1.55E-05 14900
4.98E-05 17100 2.03E-05 16600 2.45E-05
17500 5.65E-05 15500 7.15E-06 15300 1.55E-05 14900
4.96E-05 17100 2.03E-05 16600 2.45E-05
17500 5.66E-05 15500 7.14E-06 15300 1.55E-05 14900
4.95E-05 17200 2.03E-05 16600 2.46E-05
17500 5.66E-05 15500 7.16E-06 15300 1.55E-05 14900
4.93E-05 17200 2.03E-05 16600 2.46E-05
17500 5.66E-05 15500 7.15E-06 15300 1.55E-05 14900
4.92E-05 17200 2.04E-05 16600 2.46E-05
17500 5.66E-05 15500 7.16E-06 15300 1.55E-05 14900
4.90E-05 17200 2.04E-05 16600 2.46E-05
17500 5.67E-05 15500 7.16E-06 15300 1.55E-05 14900
4.89E-05 17200 2.04E-05 16600 2.46E-05
17500 5.67E-05 15500 7.16E-06 15300 1.55E-05 14900
4.87E-05 17200 2.04E-05 16600 2.46E-05
17500 5.67E-05 15500 7.16E-06 15300 1.55E-05 14900
4.86E-05 17200 2.04E-05 16600 2.46E-05
17500 5.67E-05 15500 7.17E-06 15300 1.55E-05 14900
4.84E-05 17200 2.04E-05 16600 2.46E-05
17500 5.67E-05 15500 7.17E-06 15300 1.55E-05 14900
4.83E-05 17200 2.04E-05 16600 2.46E-05
17500 5.67E-05 15500 7.17E-06 15300 1.55E-05 14900
4.81E-05 17200 2.04E-05 16600 2.46E-05
17500 5.68E-05 15500 7.17E-06 15300 1.55E-05 14900
4.80E-05 17200 2.04E-05 16600 2.46E-05
17500 5.68E-05 15500 7.17E-06 15300 1.55E-05 14900
4.78E-05 17200 2.04E-05 16600 2.46E-05
17500 5.68E-05 15500 7.17E-06 15300 1.55E-05 14900
4.77E-05 17200 2.04E-05 16600 2.46E-05
17500 5.68E-05 15500 7.16E-06 15300 1.55E-05 14900
4.75E-05 17200 2.04E-05 16600 2.46E-05
17500 5.68E-05 15500 7.16E-06 15300 1.55E-05 14900
4.74E-05 17200 2.04E-05 16600 2.46E-05
17500 5.69E-05 15500 7.17E-06 15300 1.55E-05 14900
4.73E-05 17200 2.05E-05 16600 2.46E-05
17500 5.69E-05 15500 7.16E-06 15300 1.55E-05 14900
4.71E-05 17200 2.05E-05 16600 2.46E-05
17500 5.69E-05 15500 7.15E-06 15300 1.55E-05 14900
4.70E-05 17200 2.05E-05 16600 2.47E-05
17500 5.69E-05 15500 7.15E-06 15300 1.55E-05 14900
4.68E-05 17200 2.05E-05 16600 2.47E-05
17500 5.69E-05 15500 7.15E-06 15300 1.55E-05 14900
4.67E-05 17200 2.05E-05 16600 2.47E-05
17500 5.69E-05 15500 7.15E-06 15300 1.55E-05 14900
4.66E-05 17200 2.05E-05 16600 2.47E-05
17500 5.70E-05 15500 7.15E-06 15300 1.55E-05 14900
4.64E-05 17200 2.05E-05 16600 2.47E-05
17500 5.70E-05 15500 7.15E-06 15300 1.55E-05 14900
4.63E-05 17200 2.05E-05 16600 2.47E-05
17500 5.70E-05 15500 7.14E-06 15300 1.55E-05 14900
4.62E-05 17200 2.05E-05 16600 2.47E-05
17500 5.70E-05 15500 7.14E-06 15300 1.55E-05 14900
4.61E-05 17200 2.05E-05 16600 2.47E-05
17500 5.70E-05 15500 7.13E-06 15300 1.55E-05 14900
4.59E-05 17200 2.05E-05 16600 2.47E-05
17500 5.71E-05 15500 7.13E-06 15300 1.55E-05 14900
4.58E-05 17200 2.05E-05 16600 2.47E-05
17500 5.71E-05 15500 7.13E-06 15300 1.55E-05 14900
4.57E-05 17200 2.05E-05 16600 2.47E-05
17500 5.71E-05 15500 7.12E-06 15300 1.55E-05 14900
4.56E-05 17200 2.05E-05 16600 2.47E-05
17600 5.71E-05 15500 7.13E-06 15300 1.55E-05 14900
4.54E-05 17200 2.05E-05 16600 2.47E-05
17600 5.71E-05 15500 7.13E-06 15300 1.54E-05 14900
4.53E-05 17200 2.05E-05 16600 2.47E-05
17600 5.71E-05 15500 7.12E-06 15300 1.54E-05 14900
4.52E-05 17200 2.06E-05 16600 2.47E-05

17600 5.72E-05 15500 7.11E-06 15300 1.54E-05 14900
4.50E-05 17200 2.06E-05 16600 2.47E-05
17600 5.72E-05 15500 7.12E-06 15300 1.54E-05 14900
4.49E-05 17200 2.06E-05 16600 2.48E-05
17600 5.72E-05 15500 7.12E-06 15300 1.54E-05 14900
4.48E-05 17200 2.06E-05 16600 2.47E-05
17600 5.72E-05 15500 7.11E-06 15300 1.54E-05 14900
4.47E-05 17200 2.06E-05 16600 2.48E-05
17600 5.72E-05 15500 7.10E-06 15300 1.54E-05 14900
4.45E-05 17200 2.06E-05 16600 2.48E-05
17600 5.72E-05 15500 7.10E-06 15300 1.54E-05 14900
4.44E-05 17200 2.06E-05 16600 2.48E-05
17600 5.73E-05 15500 7.11E-06 15300 1.54E-05 14900
4.43E-05 17200 2.06E-05 16700 2.48E-05
17600 5.73E-05 15500 7.10E-06 15300 1.54E-05 14900
4.42E-05 17200 2.06E-05 16700 2.48E-05
17600 5.73E-05 15500 7.10E-06 15300 1.54E-05 14900
4.41E-05 17200 2.06E-05 16700 2.48E-05
17600 5.73E-05 15500 7.10E-06 15300 1.54E-05 14900
4.39E-05 17200 2.06E-05 16700 2.48E-05
17600 5.74E-05 15500 7.10E-06 15300 1.54E-05 14900
4.38E-05 17200 2.06E-05 16700 2.48E-05
17600 5.74E-05 15500 7.10E-06 15300 1.54E-05 14900
4.37E-05 17200 2.06E-05 16700 2.48E-05
17600 5.74E-05 15500 7.10E-06 15300 1.54E-05 14900
4.36E-05 17200 2.06E-05 16700 2.48E-05
17600 5.74E-05 15500 7.09E-06 15300 1.54E-05 14900
4.35E-05 17200 2.07E-05 16700 2.48E-05
17600 5.74E-05 15500 7.09E-06 15300 1.54E-05 14900
4.34E-05 17200 2.07E-05 16700 2.48E-05
17600 5.74E-05 15600 7.10E-06 15300 1.54E-05 14900
4.33E-05 17200 2.07E-05 16700 2.48E-05
17600 5.74E-05 15600 7.10E-06 15300 1.54E-05 14900
4.31E-05 17200 2.07E-05 16700 2.48E-05
17600 5.75E-05 15600 7.10E-06 15400 1.54E-05 14900
4.30E-05 17200 2.07E-05 16700 2.49E-05
17600 5.75E-05 15600 7.10E-06 15400 1.54E-05 14900
4.29E-05 17200 2.07E-05 16700 2.49E-05
17600 5.75E-05 15600 7.10E-06 15400 1.53E-05 14900
4.28E-05 17200 2.07E-05 16700 2.49E-05
17600 5.76E-05 15600 7.10E-06 15400 1.53E-05 14900
4.27E-05 17200 2.07E-05 16700 2.49E-05
17600 5.76E-05 15600 7.10E-06 15400 1.53E-05 15000
4.26E-05 17200 2.07E-05 16700 2.49E-05
17600 5.76E-05 15600 7.09E-06 15400 1.53E-05 15000
4.25E-05 17200 2.07E-05 16700 2.49E-05
17600 5.76E-05 15600 7.09E-06 15400 1.53E-05 15000
4.24E-05 17200 2.07E-05 16700 2.49E-05
17600 5.76E-05 15600 7.08E-06 15400 1.53E-05 15000
4.23E-05 17200 2.07E-05 16700 2.49E-05
17600 5.77E-05 15600 7.08E-06 15400 1.53E-05 15000
4.22E-05 17200 2.08E-05 16700 2.49E-05
17600 5.77E-05 15600 7.08E-06 15400 1.53E-05 15000
4.21E-05 17200 2.08E-05 16700 2.50E-05
17600 5.77E-05 15600 7.08E-06 15400 1.53E-05 15000
4.20E-05 17200 2.08E-05 16700 2.50E-05
17600 5.77E-05 15600 7.08E-06 15400 1.53E-05 15000
4.19E-05 17200 2.08E-05 16700 2.50E-05
17600 5.78E-05 15600 7.08E-06 15400 1.53E-05 15000
4.18E-05 17200 2.08E-05 16700 2.50E-05
17600 5.78E-05 15600 7.09E-06 15400 1.53E-05 15000
4.17E-05 17200 2.08E-05 16700 2.50E-05
17600 5.78E-05 15600 7.09E-06 15400 1.53E-05 15000
4.16E-05 17200 2.08E-05 16700 2.50E-05
17600 5.78E-05 15600 7.08E-06 15400 1.53E-05 15000
4.15E-05 17300 2.08E-05 16700 2.50E-05
17600 5.79E-05 15600 7.08E-06 15400 1.53E-05 15000
4.14E-05 17300 2.08E-05 16700 2.50E-05

17600 5.79E-05 15600 7.08E-06 15400 1.53E-05 15000
4.13E-05 17300 2.08E-05 16700 2.50E-05
17600 5.79E-05 15600 7.08E-06 15400 1.53E-05 15000
4.12E-05 17300 2.08E-05 16700 2.50E-05
17600 5.79E-05 15600 7.08E-06 15400 1.53E-05 15000
4.11E-05 17300 2.08E-05 16700 2.50E-05
17600 5.79E-05 15600 7.08E-06 15400 1.53E-05 15000
4.10E-05 17300 2.09E-05 16700 2.51E-05
17600 5.80E-05 15600 7.07E-06 15400 1.53E-05 15000
4.09E-05 17300 2.09E-05 16700 2.51E-05
17600 5.80E-05 15600 7.07E-06 15400 1.53E-05 15000
4.08E-05 17300 2.09E-05 16700 2.51E-05
17600 5.80E-05 15600 7.06E-06 15400 1.53E-05 15000
4.07E-05 17300 2.09E-05 16700 2.51E-05
17600 5.80E-05 15600 7.06E-06 15400 1.53E-05 15000
4.06E-05 17300 2.09E-05 16700 2.51E-05
17600 5.80E-05 15600 7.05E-06 15400 1.53E-05 15000
4.05E-05 17300 2.09E-05 16700 2.51E-05
17600 5.80E-05 15600 7.05E-06 15400 1.53E-05 15000
4.04E-05 17300 2.09E-05 16700 2.51E-05
17600 5.81E-05 15600 7.05E-06 15400 1.53E-05 15000
4.03E-05 17300 2.09E-05 16700 2.51E-05
17600 5.81E-05 15600 7.05E-06 15400 1.53E-05 15000
4.02E-05 17300 2.09E-05 16700 2.51E-05
17600 5.81E-05 15600 7.05E-06 15400 1.53E-05 15000
4.01E-05 17300 2.09E-05 16700 2.51E-05
17600 5.81E-05 15600 7.04E-06 15400 1.53E-05 15000
4.00E-05 17300 2.09E-05 16700 2.51E-05
17600 5.82E-05 15600 7.03E-06 15400 1.53E-05 15000
3.99E-05 17300 2.09E-05 16700 2.51E-05
17600 5.82E-05 15600 7.03E-06 15400 1.53E-05 15000
3.99E-05 17300 2.09E-05 16700 2.52E-05
17600 5.82E-05 15600 7.03E-06 15400 1.53E-05 15000
3.98E-05 17300 2.10E-05 16700 2.51E-05
17600 5.82E-05 15600 7.03E-06 15400 1.53E-05 15000
3.97E-05 17300 2.10E-05 16700 2.51E-05
17600 5.82E-05 15600 7.03E-06 15400 1.53E-05 15000
3.96E-05 17300 2.10E-05 16700 2.52E-05
17600 5.82E-05 15600 7.03E-06 15400 1.53E-05 15000
3.95E-05 17300 2.10E-05 16700 2.52E-05
17600 5.83E-05 15600 7.03E-06 15400 1.53E-05 15000
3.94E-05 17300 2.10E-05 16700 2.52E-05
17600 5.83E-05 15600 7.03E-06 15400 1.53E-05 15000
3.93E-05 17300 2.10E-05 16700 2.52E-05
17600 5.83E-05 15600 7.03E-06 15400 1.53E-05 15000
3.92E-05 17300 2.10E-05 16700 2.52E-05
17600 5.83E-05 15600 7.03E-06 15400 1.53E-05 15000
3.91E-05 17300 2.10E-05 16700 2.52E-05
17600 5.84E-05 15600 7.02E-06 15400 1.52E-05 15000
3.90E-05 17300 2.10E-05 16700 2.52E-05
17600 5.84E-05 15600 7.03E-06 15400 1.52E-05 15000
3.89E-05 17300 2.10E-05 16700 2.52E-05
17600 5.84E-05 15600 7.04E-06 15400 1.52E-05 15000
3.88E-05 17300 2.10E-05 16700 2.52E-05
17700 5.84E-05 15600 7.03E-06 15400 1.52E-05 15000
3.88E-05 17300 2.10E-05 16700 2.52E-05
17700 5.84E-05 15600 7.02E-06 15400 1.52E-05 15000
3.87E-05 17300 2.11E-05 16700 2.52E-05
17700 5.85E-05 15600 7.03E-06 15400 1.52E-05 15000
3.86E-05 17300 2.11E-05 16700 2.53E-05
17700 5.85E-05 15600 7.02E-06 15400 1.52E-05 15000
3.85E-05 17300 2.11E-05 16700 2.53E-05
17700 5.85E-05 15600 7.02E-06 15400 1.52E-05 15000
3.84E-05 17300 2.11E-05 16700 2.53E-05
17700 5.85E-05 15600 7.02E-06 15400 1.52E-05 15000
3.83E-05 17300 2.11E-05 16700 2.53E-05
17700 5.86E-05 15600 7.02E-06 15400 1.52E-05 15000
3.82E-05 17300 2.11E-05 16700 2.53E-05

17700 5.86E-05 15600 7.02E-06 15400 1.52E-05 15000
3.81E-05 17300 2.11E-05 16700 2.53E-05
17700 5.86E-05 15600 7.02E-06 15400 1.52E-05 15000
3.81E-05 17300 2.11E-05 16700 2.53E-05
17700 5.86E-05 15600 7.01E-06 15400 1.52E-05 15000
3.80E-05 17300 2.11E-05 16800 2.53E-05
17700 5.87E-05 15600 7.01E-06 15400 1.52E-05 15000
3.79E-05 17300 2.11E-05 16800 2.53E-05
17700 5.87E-05 15600 7.01E-06 15400 1.52E-05 15000
3.78E-05 17300 2.12E-05 16800 2.53E-05
17700 5.87E-05 15600 7.01E-06 15400 1.52E-05 15000
3.77E-05 17300 2.12E-05 16800 2.53E-05
17700 5.87E-05 15600 7.01E-06 15400 1.52E-05 15000
3.76E-05 17300 2.12E-05 16800 2.53E-05
17700 5.87E-05 15600 7.02E-06 15400 1.52E-05 15000
3.75E-05 17300 2.12E-05 16800 2.53E-05
17700 5.87E-05 15600 7.01E-06 15400 1.52E-05 15000
3.75E-05 17300 2.12E-05 16800 2.53E-05
17700 5.88E-05 15600 7.02E-06 15400 1.52E-05 15000
3.74E-05 17300 2.12E-05 16800 2.53E-05
17700 5.88E-05 15600 7.02E-06 15400 1.52E-05 15000
3.73E-05 17300 2.12E-05 16800 2.53E-05
17700 5.88E-05 15700 7.02E-06 15400 1.52E-05 15000
3.72E-05 17300 2.12E-05 16800 2.53E-05
17700 5.88E-05 15700 7.02E-06 15400 1.52E-05 15000
3.72E-05 17300 2.12E-05 16800 2.53E-05
17700 5.88E-05 15700 7.01E-06 15500 1.52E-05 15000
3.71E-05 17300 2.12E-05 16800 2.54E-05
17700 5.88E-05 15700 7.01E-06 15500 1.52E-05 15000
3.70E-05 17300 2.13E-05 16800 2.54E-05
17700 5.89E-05 15700 7.01E-06 15500 1.52E-05 15000
3.69E-05 17300 2.13E-05 16800 2.54E-05
17700 5.89E-05 15700 7.00E-06 15500 1.51E-05 15000
3.69E-05 17300 2.13E-05 16800 2.54E-05
17700 5.89E-05 15700 7.00E-06 15500 1.52E-05 15100
3.68E-05 17300 2.13E-05 16800 2.54E-05
17700 5.89E-05 15700 7.00E-06 15500 1.51E-05 15100
3.67E-05 17300 2.13E-05 16800 2.54E-05
17700 5.89E-05 15700 6.99E-06 15500 1.51E-05 15100
3.67E-05 17300 2.13E-05 16800 2.54E-05
17700 5.90E-05 15700 6.99E-06 15500 1.51E-05 15100
3.66E-05 17300 2.13E-05 16800 2.54E-05
17700 5.90E-05 15700 6.98E-06 15500 1.51E-05 15100
3.65E-05 17300 2.13E-05 16800 2.54E-05
17700 5.90E-05 15700 6.98E-06 15500 1.52E-05 15100
3.64E-05 17300 2.13E-05 16800 2.54E-05
17700 5.90E-05 15700 6.99E-06 15500 1.51E-05 15100
3.63E-05 17300 2.13E-05 16800 2.54E-05
17700 5.90E-05 15700 6.98E-06 15500 1.51E-05 15100
3.63E-05 17300 2.14E-05 16800 2.54E-05
17700 5.90E-05 15700 6.98E-06 15500 1.51E-05 15100
3.62E-05 17300 2.14E-05 16800 2.54E-05
17700 5.91E-05 15700 6.98E-06 15500 1.51E-05 15100
3.61E-05 17300 2.14E-05 16800 2.54E-05
17700 5.91E-05 15700 6.98E-06 15500 1.51E-05 15100
3.60E-05 17300 2.14E-05 16800 2.54E-05
17700 5.91E-05 15700 6.98E-06 15500 1.51E-05 15100
3.59E-05 17400 2.14E-05 16800 2.55E-05
17700 5.92E-05 15700 6.98E-06 15500 1.51E-05 15100
3.59E-05 17400 2.14E-05 16800 2.55E-05
17700 5.92E-05 15700 6.98E-06 15500 1.51E-05 15100
3.58E-05 17400 2.14E-05 16800 2.55E-05
17700 5.92E-05 15700 6.97E-06 15500 1.51E-05 15100
3.57E-05 17400 2.14E-05 16800 2.55E-05
17700 5.92E-05 15700 6.97E-06 15500 1.51E-05 15100
3.56E-05 17400 2.14E-05 16800 2.55E-05
17700 5.92E-05 15700 6.97E-06 15500 1.51E-05 15100
3.56E-05 17400 2.14E-05 16800 2.55E-05

17700 5.93E-05 15700 6.97E-06 15500 1.51E-05 15100
3.55E-05 17400 2.15E-05 16800 2.55E-05
17700 5.93E-05 15700 6.96E-06 15500 1.51E-05 15100
3.54E-05 17400 2.15E-05 16800 2.55E-05
17700 5.93E-05 15700 6.97E-06 15500 1.51E-05 15100
3.53E-05 17400 2.15E-05 16800 2.55E-05
17700 5.93E-05 15700 6.97E-06 15500 1.51E-05 15100
3.53E-05 17400 2.15E-05 16800 2.55E-05
17700 5.93E-05 15700 6.97E-06 15500 1.51E-05 15100
3.52E-05 17400 2.15E-05 16800 2.55E-05
17700 5.94E-05 15700 6.97E-06 15500 1.51E-05 15100
3.52E-05 17400 2.15E-05 16800 2.55E-05
17700 5.94E-05 15700 6.98E-06 15500 1.51E-05 15100
3.51E-05 17400 2.15E-05 16800 2.56E-05
17700 5.94E-05 15700 6.97E-06 15500 1.51E-05 15100
3.50E-05 17400 2.15E-05 16800 2.56E-05
17700 5.94E-05 15700 6.97E-06 15500 1.51E-05 15100
3.50E-05 17400 2.15E-05 16800 2.56E-05
17700 5.94E-05 15700 6.97E-06 15500 1.51E-05 15100
3.49E-05 17400 2.15E-05 16800 2.56E-05
17700 5.94E-05 15700 6.97E-06 15500 1.51E-05 15100
3.48E-05 17400 2.15E-05 16800 2.56E-05
17700 5.94E-05 15700 6.98E-06 15500 1.51E-05 15100
3.47E-05 17400 2.15E-05 16800 2.56E-05
17700 5.95E-05 15700 6.97E-06 15500 1.51E-05 15100
3.47E-05 17400 2.16E-05 16800 2.56E-05
17700 5.95E-05 15700 6.97E-06 15500 1.51E-05 15100
3.46E-05 17400 2.16E-05 16800 2.56E-05
17700 5.95E-05 15700 6.98E-06 15500 1.51E-05 15100
3.46E-05 17400 2.16E-05 16800 2.56E-05
17700 5.95E-05 15700 6.97E-06 15500 1.51E-05 15100
3.45E-05 17400 2.16E-05 16800 2.56E-05
17700 5.95E-05 15700 6.96E-06 15500 1.51E-05 15100
3.44E-05 17400 2.16E-05 16800 2.56E-05
17700 5.95E-05 15700 6.96E-06 15500 1.51E-05 15100
3.44E-05 17400 2.16E-05 16800 2.56E-05
17700 5.95E-05 15700 6.96E-06 15500 1.51E-05 15100
3.43E-05 17400 2.16E-05 16800 2.57E-05
17700 5.96E-05 15700 6.96E-06 15500 1.51E-05 15100
3.43E-05 17400 2.16E-05 16800 2.57E-05
17700 5.96E-05 15700 6.95E-06 15500 1.51E-05 15100
3.42E-05 17400 2.16E-05 16800 2.57E-05
17700 5.96E-05 15700 6.95E-06 15500 1.51E-05 15100
3.41E-05 17400 2.16E-05 16800 2.57E-05
17700 5.96E-05 15700 6.94E-06 15500 1.51E-05 15100
3.41E-05 17400 2.16E-05 16800 2.57E-05
17800 5.96E-05 15700 6.95E-06 15500 1.51E-05 15100
3.40E-05 17400 2.16E-05 16800 2.57E-05
17800 5.97E-05 15700 6.95E-06 15500 1.51E-05 15100
3.39E-05 17400 2.16E-05 16800 2.57E-05
17800 5.97E-05 15700 6.95E-06 15500 1.51E-05 15100
3.39E-05 17400 2.16E-05 16800 2.57E-05
17800 5.97E-05 15700 6.94E-06 15500 1.51E-05 15100
3.38E-05 17400 2.17E-05 16800 2.57E-05
17800 5.97E-05 15700 6.95E-06 15500 1.51E-05 15100
3.37E-05 17400 2.17E-05 16800 2.57E-05
17800 5.97E-05 15700 6.95E-06 15500 1.51E-05 15100
3.37E-05 17400 2.17E-05 16800 2.58E-05
17800 5.97E-05 15700 6.95E-06 15500 1.51E-05 15100
3.36E-05 17400 2.17E-05 16800 2.57E-05
17800 5.97E-05 15700 6.95E-06 15500 1.51E-05 15100
3.35E-05 17400 2.17E-05 16800 2.58E-05
17800 5.98E-05 15700 6.95E-06 15500 1.51E-05 15100
3.35E-05 17400 2.17E-05 16800 2.58E-05
17800 5.98E-05 15700 6.96E-06 15500 1.51E-05 15100
3.34E-05 17400 2.17E-05 16900 2.58E-05
17800 5.98E-05 15700 6.96E-06 15500 1.51E-05 15100
3.33E-05 17400 2.17E-05 16900 2.58E-05

17800 5.99E-05 15700 6.96E-06 15500 1.51E-05 15100
3.33E-05 17400 2.17E-05 16900 2.58E-05
17800 5.99E-05 15700 6.95E-06 15500 1.51E-05 15100
3.32E-05 17400 2.17E-05 16900 2.58E-05
17800 5.99E-05 15700 6.95E-06 15500 1.51E-05 15100
3.31E-05 17400 2.17E-05 16900 2.58E-05
17800 5.99E-05 15700 6.95E-06 15500 1.51E-05 15100
3.31E-05 17400 2.17E-05 16900 2.58E-05
17800 5.99E-05 15700 6.95E-06 15500 1.51E-05 15100
3.30E-05 17400 2.17E-05 16900 2.58E-05
17800 6.00E-05 15700 6.95E-06 15500 1.50E-05 15100
3.30E-05 17400 2.17E-05 16900 2.59E-05
17800 6.00E-05 15700 6.94E-06 15500 1.51E-05 15100
3.29E-05 17400 2.17E-05 16900 2.59E-05
17800 6.01E-05 15800 6.95E-06 15500 1.51E-05 15100
3.28E-05 17400 2.17E-05 16900 2.59E-05
17800 6.00E-05 15800 6.94E-06 15500 1.50E-05 15100
3.28E-05 17400 2.18E-05 16900 2.59E-05
17800 6.01E-05 15800 6.95E-06 15600 1.51E-05 15100
3.28E-05 17400 2.18E-05 16900 2.59E-05
17800 6.01E-05 15800 6.94E-06 15600 1.51E-05 15100
3.27E-05 17400 2.18E-05 16900 2.59E-05
17800 6.01E-05 15800 6.94E-06 15600 1.51E-05 15100
3.26E-05 17400 2.18E-05 16900 2.59E-05
17800 6.01E-05 15800 6.95E-06 15600 1.50E-05 15100
3.26E-05 17400 2.18E-05 16900 2.59E-05
17800 6.02E-05 15800 6.94E-06 15600 1.51E-05 15200
3.26E-05 17400 2.18E-05 16900 2.59E-05
17800 6.02E-05 15800 6.94E-06 15600 1.50E-05 15200
3.25E-05 17400 2.18E-05 16900 2.59E-05
17800 6.02E-05 15800 6.95E-06 15600 1.50E-05 15200
3.24E-05 17400 2.18E-05 16900 2.59E-05
17800 6.02E-05 15800 6.95E-06 15600 1.50E-05 15200
3.24E-05 17400 2.18E-05 16900 2.59E-05
17800 6.02E-05 15800 6.95E-06 15600 1.50E-05 15200
3.23E-05 17400 2.18E-05 16900 2.59E-05
17800 6.03E-05 15800 6.96E-06 15600 1.50E-05 15200
3.23E-05 17400 2.18E-05 16900 2.59E-05
17800 6.03E-05 15800 6.95E-06 15600 1.50E-05 15200
3.22E-05 17400 2.19E-05 16900 2.60E-05
17800 6.03E-05 15800 6.96E-06 15600 1.50E-05 15200
3.22E-05 17400 2.19E-05 16900 2.60E-05
17800 6.03E-05 15800 6.95E-06 15600 1.50E-05 15200
3.21E-05 17400 2.19E-05 16900 2.60E-05
17800 6.03E-05 15800 6.95E-06 15600 1.50E-05 15200
3.20E-05 17400 2.19E-05 16900 2.60E-05
17800 6.03E-05 15800 6.95E-06 15600 1.50E-05 15200
3.20E-05 17400 2.19E-05 16900 2.60E-05
17800 6.04E-05 15800 6.94E-06 15600 1.50E-05 15200
3.19E-05 17500 2.19E-05 16900 2.60E-05
17800 6.04E-05 15800 6.95E-06 15600 1.50E-05 15200
3.19E-05 17500 2.19E-05 16900 2.60E-05
17800 6.04E-05 15800 6.94E-06 15600 1.50E-05 15200
3.18E-05 17500 2.19E-05 16900 2.61E-05
17800 6.04E-05 15800 6.94E-06 15600 1.50E-05 15200
3.18E-05 17500 2.20E-05 16900 2.61E-05
17800 6.05E-05 15800 6.94E-06 15600 1.50E-05 15200
3.18E-05 17500 2.20E-05 16900 2.61E-05
17800 6.05E-05 15800 6.94E-06 15600 1.50E-05 15200
3.17E-05 17500 2.20E-05 16900 2.61E-05
17800 6.05E-05 15800 6.94E-06 15600 1.50E-05 15200
3.17E-05 17500 2.20E-05 16900 2.61E-05
17800 6.05E-05 15800 6.95E-06 15600 1.50E-05 15200
3.16E-05 17500 2.20E-05 16900 2.61E-05
17800 6.06E-05 15800 6.95E-06 15600 1.50E-05 15200
3.16E-05 17500 2.20E-05 16900 2.61E-05
17800 6.06E-05 15800 6.95E-06 15600 1.50E-05 15200
3.15E-05 17500 2.20E-05 16900 2.61E-05

17800	6.06E-05	15800	6.95E-06	15600	1.50E-05	15200	3.15E-05	17500	2.20E-05	16900	2.61E-05
17800	6.07E-05	15800	6.95E-06	15600	1.50E-05	15200	3.14E-05	17500	2.20E-05	16900	2.61E-05
17800	6.07E-05	15800	6.95E-06	15600	1.50E-05	15200	3.14E-05	17500	2.20E-05	16900	2.61E-05
17800	6.07E-05	15800	6.95E-06	15600	1.50E-05	15200	3.13E-05	17500	2.21E-05	16900	2.61E-05
17800	6.07E-05	15800	6.94E-06	15600	1.50E-05	15200	3.13E-05	17500	2.21E-05	16900	2.62E-05
17800	6.08E-05	15800	6.94E-06	15600	1.50E-05	15200	3.12E-05	17500	2.21E-05	16900	2.62E-05
17800	6.08E-05	15800	6.93E-06	15600	1.50E-05	15200	3.12E-05	17500	2.21E-05	16900	2.62E-05
17800	6.08E-05	15800	6.93E-06	15600	1.50E-05	15200	3.11E-05	17500	2.21E-05	16900	2.62E-05
17800	6.09E-05	15800	6.93E-06	15600	1.50E-05	15200	3.10E-05	17500	2.21E-05	16900	2.62E-05
17800	6.09E-05	15800	6.93E-06	15600	1.50E-05	15200	3.10E-05	17500	2.21E-05	16900	2.62E-05
17800	6.09E-05	15800	6.93E-06	15600	1.50E-05	15200	3.09E-05	17500	2.21E-05	16900	2.62E-05
17800	6.10E-05	15800	6.92E-06	15600	1.50E-05	15200	3.09E-05	17500	2.21E-05	16900	2.62E-05
17800	6.10E-05	15800	6.93E-06	15600	1.50E-05	15200	3.08E-05	17500	2.22E-05	16900	2.62E-05
17800	6.10E-05	15800	6.93E-06	15600	1.50E-05	15200	3.08E-05	17500	2.22E-05	16900	2.62E-05
17800	6.11E-05	15800	6.93E-06	15600	1.50E-05	15200	3.07E-05	17500	2.22E-05	16900	2.63E-05
17800	6.11E-05	15800	6.92E-06	15600	1.50E-05	15200	3.07E-05	17500	2.22E-05	16900	2.63E-05
17800	6.11E-05	15800	6.92E-06	15600	1.50E-05	15200	3.06E-05	17500	2.22E-05	16900	2.63E-05
17800	6.11E-05	15800	6.92E-06	15600	1.50E-05	15200	3.06E-05	17500	2.22E-05	16900	2.63E-05
17800	6.11E-05	15800	6.93E-06	15600	1.50E-05	15200	3.05E-05	17500	2.22E-05	16900	2.63E-05
17900	6.12E-05	15800	6.92E-06	15600	1.50E-05	15200	3.05E-05	17500	2.22E-05	16900	2.63E-05
17900	6.12E-05	15800	6.91E-06	15600	1.50E-05	15200	3.04E-05	17500	2.22E-05	16900	2.63E-05
17900	6.12E-05	15800	6.91E-06	15600	1.50E-05	15200	3.04E-05	17500	2.23E-05	16900	2.63E-05
17900	6.13E-05	15800	6.92E-06	15600	1.50E-05	15200	3.03E-05	17500	2.23E-05	16900	2.63E-05
17900	6.13E-05	15800	6.92E-06	15600	1.50E-05	15200	3.03E-05	17500	2.23E-05	16900	2.64E-05
17900	6.13E-05	15800	6.91E-06	15600	1.50E-05	15200	3.03E-05	17500	2.23E-05	16900	2.64E-05
17900	6.13E-05	15800	6.91E-06	15600	1.50E-05	15200	3.02E-05	17500	2.23E-05	16900	2.64E-05
17900	6.14E-05	15800	6.91E-06	15600	1.50E-05	15200	3.02E-05	17500	2.23E-05	16900	2.64E-05
17900	6.14E-05	15800	6.92E-06	15600	1.50E-05	15200	3.01E-05	17500	2.23E-05	16900	2.64E-05
17900	6.14E-05	15800	6.91E-06	15600	1.50E-05	15200	3.01E-05	17500	2.24E-05	17000	2.64E-05
17900	6.15E-05	15800	6.91E-06	15600	1.50E-05	15200	3.00E-05	17500	2.24E-05	17000	2.64E-05
17900	6.15E-05	15800	6.90E-06	15600	1.50E-05	15200	3.00E-05	17500	2.24E-05	17000	2.64E-05
17900	6.15E-05	15800	6.90E-06	15600	1.50E-05	15200	3.00E-05	17500	2.24E-05	17000	2.64E-05
17900	6.16E-05	15800	6.91E-06	15600	1.50E-05	15200	2.99E-05	17500	2.24E-05	17000	2.64E-05
17900	6.16E-05	15800	6.91E-06	15600	1.50E-05	15200	2.99E-05	17500	2.24E-05	17000	2.65E-05

17900 6.16E-05 15800 6.90E-06 15600 1.50E-05 15200
2.99E-05 17500 2.24E-05 17000 2.65E-05
17900 6.16E-05 15800 6.91E-06 15600 1.50E-05 15200
2.98E-05 17500 2.25E-05 17000 2.65E-05
17900 6.16E-05 15800 6.90E-06 15600 1.50E-05 15200
2.98E-05 17500 2.25E-05 17000 2.65E-05
17900 6.17E-05 15900 6.91E-06 15600 1.50E-05 15200
2.98E-05 17500 2.25E-05 17000 2.65E-05
17900 6.17E-05 15900 6.90E-06 15600 1.50E-05 15200
2.97E-05 17500 2.25E-05 17000 2.65E-05
17900 6.17E-05 15900 6.90E-06 15700 1.50E-05 15200
2.97E-05 17500 2.25E-05 17000 2.65E-05
17900 6.17E-05 15900 6.90E-06 15700 1.50E-05 15200
2.96E-05 17500 2.25E-05 17000 2.65E-05
17900 6.18E-05 15900 6.90E-06 15700 1.50E-05 15200
2.96E-05 17500 2.25E-05 17000 2.65E-05
17900 6.18E-05 15900 6.89E-06 15700 1.50E-05 15200
2.95E-05 17500 2.25E-05 17000 2.65E-05
17900 6.18E-05 15900 6.88E-06 15700 1.50E-05 15300
2.95E-05 17500 2.26E-05 17000 2.65E-05
17900 6.18E-05 15900 6.88E-06 15700 1.50E-05 15300
2.95E-05 17500 2.26E-05 17000 2.66E-05
17900 6.18E-05 15900 6.88E-06 15700 1.50E-05 15300
2.94E-05 17500 2.26E-05 17000 2.66E-05
17900 6.19E-05 15900 6.88E-06 15700 1.50E-05 15300
2.94E-05 17500 2.26E-05 17000 2.66E-05
17900 6.19E-05 15900 6.87E-06 15700 1.50E-05 15300
2.94E-05 17500 2.26E-05 17000 2.66E-05
17900 6.19E-05 15900 6.87E-06 15700 1.50E-05 15300
2.93E-05 17500 2.26E-05 17000 2.66E-05
17900 6.20E-05 15900 6.88E-06 15700 1.50E-05 15300
2.93E-05 17500 2.26E-05 17000 2.66E-05
17900 6.20E-05 15900 6.87E-06 15700 1.50E-05 15300
2.92E-05 17500 2.27E-05 17000 2.66E-05
17900 6.20E-05 15900 6.87E-06 15700 1.50E-05 15300
2.92E-05 17500 2.27E-05 17000 2.66E-05
17900 6.20E-05 15900 6.87E-06 15700 1.50E-05 15300
2.92E-05 17500 2.27E-05 17000 2.66E-05
17900 6.20E-05 15900 6.87E-06 15700 1.50E-05 15300
2.91E-05 17500 2.27E-05 17000 2.66E-05
17900 6.21E-05 15900 6.88E-06 15700 1.50E-05 15300
2.91E-05 17600 2.27E-05 17000 2.66E-05
17900 6.21E-05 15900 6.88E-06 15700 1.50E-05 15300
2.91E-05 17600 2.27E-05 17000 2.66E-05
17900 6.21E-05 15900 6.89E-06 15700 1.50E-05 15300
2.90E-05 17600 2.28E-05 17000 2.67E-05
17900 6.22E-05 15900 6.89E-06 15700 1.50E-05 15300
2.90E-05 17600 2.28E-05 17000 2.67E-05
17900 6.22E-05 15900 6.88E-06 15700 1.50E-05 15300
2.89E-05 17600 2.28E-05 17000 2.66E-05
17900 6.22E-05 15900 6.88E-06 15700 1.50E-05 15300
2.89E-05 17600 2.28E-05 17000 2.67E-05
17900 6.23E-05 15900 6.88E-06 15700 1.50E-05 15300
2.88E-05 17600 2.28E-05 17000 2.67E-05
17900 6.23E-05 15900 6.88E-06 15700 1.50E-05 15300
2.88E-05 17600 2.28E-05 17000 2.67E-05
17900 6.23E-05 15900 6.87E-06 15700 1.50E-05 15300
2.88E-05 17600 2.29E-05 17000 2.67E-05
17900 6.23E-05 15900 6.87E-06 15700 1.50E-05 15300
2.87E-05 17600 2.29E-05 17000 2.67E-05
17900 6.24E-05 15900 6.87E-06 15700 1.50E-05 15300
2.87E-05 17600 2.29E-05 17000 2.67E-05
17900 6.24E-05 15900 6.88E-06 15700 1.50E-05 15300
2.87E-05 17600 2.29E-05 17000 2.67E-05
17900 6.24E-05 15900 6.87E-06 15700 1.50E-05 15300
2.86E-05 17600 2.29E-05 17000 2.67E-05
17900 6.24E-05 15900 6.87E-06 15700 1.50E-05 15300
2.86E-05 17600 2.30E-05 17000 2.67E-05

17900 6.25E-05 15900 6.86E-06 15700 1.50E-05 15300
2.85E-05 17600 2.30E-05 17000 2.67E-05
17900 6.25E-05 15900 6.86E-06 15700 1.51E-05 15300
2.85E-05 17600 2.30E-05 17000 2.68E-05
17900 6.25E-05 15900 6.86E-06 15700 1.51E-05 15300
2.85E-05 17600 2.30E-05 17000 2.67E-05
17900 6.26E-05 15900 6.86E-06 15700 1.51E-05 15300
2.84E-05 17600 2.30E-05 17000 2.67E-05
17900 6.26E-05 15900 6.86E-06 15700 1.51E-05 15300
2.84E-05 17600 2.30E-05 17000 2.67E-05
17900 6.26E-05 15900 6.86E-06 15700 1.51E-05 15300
2.83E-05 17600 2.31E-05 17000 2.68E-05
17900 6.27E-05 15900 6.86E-06 15700 1.51E-05 15300
2.83E-05 17600 2.31E-05 17000 2.68E-05
17900 6.27E-05 15900 6.86E-06 15700 1.51E-05 15300
2.83E-05 17600 2.31E-05 17000 2.68E-05
17900 6.27E-05 15900 6.86E-06 15700 1.51E-05 15300
2.82E-05 17600 2.31E-05 17000 2.68E-05
17900 6.27E-05 15900 6.86E-06 15700 1.51E-05 15300
2.82E-05 17600 2.31E-05 17000 2.68E-05
17900 6.28E-05 15900 6.85E-06 15700 1.51E-05 15300
2.82E-05 17600 2.32E-05 17000 2.68E-05
17900 6.28E-05 15900 6.86E-06 15700 1.51E-05 15300
2.81E-05 17600 2.32E-05 17000 2.68E-05
17900 6.28E-05 15900 6.85E-06 15700 1.51E-05 15300
2.81E-05 17600 2.32E-05 17000 2.68E-05
17900 6.28E-05 15900 6.85E-06 15700 1.52E-05 15300
2.81E-05 17600 2.32E-05 17000 2.68E-05
17900 6.29E-05 15900 6.86E-06 15700 1.52E-05 15300
2.81E-05 17600 2.32E-05 17000 2.68E-05
18000 6.29E-05 15900 6.86E-06 15700 1.52E-05 15300
2.80E-05 17600 2.33E-05 17000 2.68E-05
18000 6.29E-05 15900 6.86E-06 15700 1.52E-05 15300
2.80E-05 17600 2.33E-05 17000 2.68E-05
18000 6.29E-05 15900 6.86E-06 15700 1.52E-05 15300
2.80E-05 17600 2.33E-05 17000 2.68E-05
18000 6.29E-05 15900 6.87E-06 15700 1.52E-05 15300
2.80E-05 17600 2.33E-05 17000 2.68E-05
18000 6.30E-05 15900 6.86E-06 15700 1.52E-05 15300
2.79E-05 17600 2.33E-05 17000 2.68E-05
18000 6.30E-05 15900 6.87E-06 15700 1.52E-05 15300
2.79E-05 17600 2.34E-05 17000 2.68E-05
18000 6.31E-05 15900 6.87E-06 15700 1.52E-05 15300
2.79E-05 17600 2.34E-05 17000 2.68E-05
18000 6.31E-05 15900 6.89E-06 15700 1.52E-05 15300
2.78E-05 17600 2.34E-05 17000 2.68E-05
18000 6.31E-05 15900 6.87E-06 15700 1.52E-05 15300
2.78E-05 17600 2.34E-05 17000 2.69E-05
18000 6.31E-05 15900 6.87E-06 15700 1.52E-05 15300
2.78E-05 17600 2.34E-05 17100 2.69E-05
18000 6.31E-05 15900 6.87E-06 15700 1.52E-05 15300
2.77E-05 17600 2.35E-05 17100 2.69E-05
18000 6.31E-05 15900 6.87E-06 15700 1.53E-05 15300
2.77E-05 17600 2.35E-05 17100 2.69E-05
18000 6.32E-05 15900 6.88E-06 15700 1.53E-05 15300
2.77E-05 17600 2.35E-05 17100 2.69E-05
18000 6.32E-05 15900 6.88E-06 15700 1.52E-05 15300
2.77E-05 17600 2.35E-05 17100 2.69E-05
18000 6.32E-05 15900 6.87E-06 15700 1.52E-05 15300
2.76E-05 17600 2.36E-05 17100 2.69E-05
18000 6.33E-05 15900 6.87E-06 15700 1.52E-05 15300
2.76E-05 17600 2.36E-05 17100 2.69E-05
18000 6.33E-05 15900 6.87E-06 15700 1.52E-05 15300
2.76E-05 17600 2.36E-05 17100 2.69E-05
18000 6.33E-05 15900 6.86E-06 15700 1.52E-05 15300
2.76E-05 17600 2.36E-05 17100 2.69E-05
18000 6.33E-05 16000 6.86E-06 15700 1.52E-05 15300
2.75E-05 17600 2.36E-05 17100 2.69E-05

18000 6.34E-05 16000 6.87E-06 15700 1.52E-05 15300
2.75E-05 17600 2.37E-05 17100 2.69E-05
18000 6.34E-05 16000 6.86E-06 15800 1.52E-05 15300
2.75E-05 17600 2.37E-05 17100 2.69E-05
18000 6.34E-05 16000 6.86E-06 15800 1.52E-05 15300
2.74E-05 17600 2.37E-05 17100 2.70E-05
18000 6.34E-05 16000 6.85E-06 15800 1.52E-05 15300
2.74E-05 17600 2.37E-05 17100 2.70E-05
18000 6.35E-05 16000 6.85E-06 15800 1.52E-05 15300
2.73E-05 17600 2.38E-05 17100 2.70E-05
18000 6.35E-05 16000 6.84E-06 15800 1.52E-05 15400
2.73E-05 17600 2.38E-05 17100 2.70E-05
18000 6.35E-05 16000 6.84E-06 15800 1.51E-05 15400
2.73E-05 17600 2.38E-05 17100 2.70E-05
18000 6.35E-05 16000 6.85E-06 15800 1.51E-05 15400
2.72E-05 17600 2.38E-05 17100 2.70E-05
18000 6.35E-05 16000 6.84E-06 15800 1.51E-05 15400
2.72E-05 17600 2.39E-05 17100 2.70E-05
18000 6.35E-05 16000 6.84E-06 15800 1.51E-05 15400
2.72E-05 17600 2.39E-05 17100 2.70E-05
18000 6.36E-05 16000 6.84E-06 15800 1.51E-05 15400
2.71E-05 17600 2.39E-05 17100 2.70E-05
18000 6.36E-05 16000 6.83E-06 15800 1.51E-05 15400
2.71E-05 17600 2.39E-05 17100 2.70E-05
18000 6.36E-05 16000 6.84E-06 15800 1.51E-05 15400
2.71E-05 17600 2.40E-05 17100 2.70E-05
18000 6.36E-05 16000 6.85E-06 15800 1.51E-05 15400
2.70E-05 17600 2.40E-05 17100 2.70E-05
18000 6.37E-05 16000 6.84E-06 15800 1.50E-05 15400
2.70E-05 17600 2.40E-05 17100 2.70E-05
18000 6.37E-05 16000 6.84E-06 15800 1.50E-05 15400
2.70E-05 17600 2.40E-05 17100 2.70E-05
18000 6.37E-05 16000 6.84E-06 15800 1.50E-05 15400
2.69E-05 17700 2.41E-05 17100 2.70E-05
18000 6.37E-05 16000 6.83E-06 15800 1.50E-05 15400
2.69E-05 17700 2.41E-05 17100 2.70E-05
18000 6.37E-05 16000 6.84E-06 15800 1.50E-05 15400
2.69E-05 17700 2.41E-05 17100 2.71E-05
18000 6.37E-05 16000 6.84E-06 15800 1.50E-05 15400
2.68E-05 17700 2.42E-05 17100 2.71E-05
18000 6.38E-05 16000 6.84E-06 15800 1.50E-05 15400
2.68E-05 17700 2.42E-05 17100 2.71E-05
18000 6.38E-05 16000 6.83E-06 15800 1.49E-05 15400
2.68E-05 17700 2.42E-05 17100 2.71E-05
18000 6.38E-05 16000 6.83E-06 15800 1.49E-05 15400
2.68E-05 17700 2.43E-05 17100 2.71E-05
18000 6.38E-05 16000 6.83E-06 15800 1.49E-05 15400
2.67E-05 17700 2.43E-05 17100 2.71E-05
18000 6.38E-05 16000 6.83E-06 15800 1.49E-05 15400
2.67E-05 17700 2.43E-05 17100 2.71E-05
18000 6.38E-05 16000 6.83E-06 15800 1.49E-05 15400
2.67E-05 17700 2.43E-05 17100 2.71E-05
18000 6.39E-05 16000 6.84E-06 15800 1.49E-05 15400
2.66E-05 17700 2.44E-05 17100 2.71E-05
18000 6.39E-05 16000 6.84E-06 15800 1.49E-05 15400
2.66E-05 17700 2.44E-05 17100 2.71E-05
18000 6.39E-05 16000 6.83E-06 15800 1.49E-05 15400
2.65E-05 17700 2.44E-05 17100 2.71E-05
18000 6.39E-05 16000 6.84E-06 15800 1.49E-05 15400
2.65E-05 17700 2.45E-05 17100 2.71E-05
18000 6.39E-05 16000 6.84E-06 15800 1.49E-05 15400
2.65E-05 17700 2.45E-05 17100 2.71E-05
18000 6.40E-05 16000 6.84E-06 15800 1.49E-05 15400
2.65E-05 17700 2.45E-05 17100 2.71E-05
18000 6.40E-05 16000 6.84E-06 15800 1.49E-05 15400
2.64E-05 17700 2.46E-05 17100 2.71E-05
18000 6.40E-05 16000 6.84E-06 15800 1.49E-05 15400
2.64E-05 17700 2.46E-05 17100 2.71E-05

18000 6.40E-05 16000 6.84E-06 15800 1.48E-05 15400
2.64E-05 17700 2.46E-05 17100 2.72E-05
18000 6.41E-05 16000 6.84E-06 15800 1.48E-05 15400
2.64E-05 17700 2.47E-05 17100 2.72E-05
18000 6.40E-05 16000 6.84E-06 15800 1.48E-05 15400
2.63E-05 17700 2.47E-05 17100 2.72E-05
18000 6.41E-05 16000 6.84E-06 15800 1.48E-05 15400
2.63E-05 17700 2.48E-05 17100 2.72E-05
18000 6.41E-05 16000 6.84E-06 15800 1.48E-05 15400
2.63E-05 17700 2.48E-05 17100 2.72E-05
18000 6.42E-05 16000 6.83E-06 15800 1.48E-05 15400
2.63E-05 17700 2.48E-05 17100 2.72E-05
18000 6.42E-05 16000 6.83E-06 15800 1.48E-05 15400
2.62E-05 17700 2.49E-05 17100 2.72E-05
18000 6.42E-05 16000 6.83E-06 15800 1.48E-05 15400
2.62E-05 17700 2.49E-05 17100 2.72E-05
18000 6.43E-05 16000 6.83E-06 15800 1.48E-05 15400
2.62E-05 17700 2.50E-05 17100 2.72E-05
18000 6.43E-05 16000 6.83E-06 15800 1.48E-05 15400
2.61E-05 17700 2.50E-05 17100 2.72E-05
18000 6.43E-05 16000 6.84E-06 15800 1.48E-05 15400
2.61E-05 17700 2.51E-05 17100 2.72E-05
18100 6.43E-05 16000 6.84E-06 15800 1.48E-05 15400
2.61E-05 17700 2.51E-05 17100 2.72E-05
18100 6.43E-05 16000 6.84E-06 15800 1.48E-05 15400
2.60E-05 17700 2.51E-05 17100 2.72E-05
18100 6.43E-05 16000 6.84E-06 15800 1.48E-05 15400
2.60E-05 17700 2.52E-05 17100 2.72E-05
18100 6.44E-05 16000 6.84E-06 15800 1.48E-05 15400
2.60E-05 17700 2.52E-05 17100 2.73E-05
18100 6.44E-05 16000 6.84E-06 15800 1.48E-05 15400
2.60E-05 17700 2.53E-05 17100 2.73E-05
18100 6.44E-05 16000 6.84E-06 15800 1.48E-05 15400
2.60E-05 17700 2.53E-05 17100 2.73E-05
18100 6.44E-05 16000 6.84E-06 15800 1.48E-05 15400
2.60E-05 17700 2.54E-05 17100 2.72E-05
18100 6.44E-05 16000 6.83E-06 15800 1.48E-05 15400
2.59E-05 17700 2.55E-05 17100 2.72E-05
18100 6.45E-05 16000 6.82E-06 15800 1.48E-05 15400
2.59E-05 17700 2.55E-05 17100 2.72E-05
18100 6.45E-05 16000 6.82E-06 15800 1.48E-05 15400
2.59E-05 17700 2.56E-05 17200 2.73E-05
18100 6.45E-05 16000 6.82E-06 15800 1.48E-05 15400
2.59E-05 17700 2.56E-05 17200 2.73E-05
18100 6.45E-05 16000 6.82E-06 15800 1.48E-05 15400
2.59E-05 17700 2.57E-05 17200 2.73E-05
18100 6.45E-05 16000 6.82E-06 15800 1.48E-05 15400
2.59E-05 17700 2.57E-05 17200 2.73E-05
18100 6.45E-05 16000 6.82E-06 15800 1.48E-05 15400
2.59E-05 17700 2.58E-05 17200 2.73E-05
18100 6.46E-05 16000 6.81E-06 15800 1.47E-05 15400
2.58E-05 17700 2.59E-05 17200 2.73E-05
18100 6.46E-05 16000 6.81E-06 15800 1.47E-05 15400
2.58E-05 17700 2.59E-05 17200 2.73E-05
18100 6.46E-05 16000 6.80E-06 15800 1.47E-05 15400
2.58E-05 17700 2.60E-05 17200 2.73E-05
18100 6.47E-05 16000 6.80E-06 15800 1.48E-05 15400
2.58E-05 17700 2.60E-05 17200 2.73E-05
18100 6.47E-05 16100 6.80E-06 15800 1.47E-05 15400
2.57E-05 17700 2.61E-05 17200 2.73E-05
18100 6.47E-05 16100 6.81E-06 15800 1.47E-05 15400
2.57E-05 17700 2.62E-05 17200 2.73E-05
18100 6.47E-05 16100 6.80E-06 15900 1.47E-05 15400
2.57E-05 17700 2.62E-05 17200 2.73E-05
18100 6.47E-05 16100 6.81E-06 15900 1.47E-05 15400
2.57E-05 17700 2.63E-05 17200 2.73E-05
18100 6.48E-05 16100 6.81E-06 15900 1.47E-05 15400
2.56E-05 17700 2.64E-05 17200 2.73E-05

18100 6.48E-05 16100 6.80E-06 15900 1.47E-05 15400
2.56E-05 17700 2.65E-05 17200 2.73E-05
18100 6.48E-05 16100 6.81E-06 15900 1.47E-05 15500
2.56E-05 17700 2.65E-05 17200 2.73E-05
18100 6.49E-05 16100 6.81E-06 15900 1.47E-05 15500
2.56E-05 17700 2.66E-05 17200 2.73E-05
18100 6.49E-05 16100 6.81E-06 15900 1.47E-05 15500
2.56E-05 17700 2.67E-05 17200 2.73E-05
18100 6.49E-05 16100 6.81E-06 15900 1.47E-05 15500
2.55E-05 17700 2.68E-05 17200 2.74E-05
18100 6.49E-05 16100 6.81E-06 15900 1.47E-05 15500
2.55E-05 17700 2.68E-05 17200 2.73E-05
18100 6.50E-05 16100 6.81E-06 15900 1.47E-05 15500
2.55E-05 17700 2.69E-05 17200 2.74E-05
18100 6.50E-05 16100 6.82E-06 15900 1.47E-05 15500
2.55E-05 17700 2.70E-05 17200 2.74E-05
18100 6.50E-05 16100 6.83E-06 15900 1.47E-05 15500
2.54E-05 17700 2.71E-05 17200 2.74E-05
18100 6.51E-05 16100 6.82E-06 15900 1.47E-05 15500
2.54E-05 17700 2.72E-05 17200 2.74E-05
18100 6.51E-05 16100 6.82E-06 15900 1.47E-05 15500
2.54E-05 17700 2.73E-05 17200 2.74E-05
18100 6.51E-05 16100 6.82E-06 15900 1.47E-05 15500
2.54E-05 17700 2.74E-05 17200 2.74E-05
18100 6.52E-05 16100 6.82E-06 15900 1.47E-05 15500
2.54E-05 17800 2.75E-05 17200 2.74E-05
18100 6.52E-05 16100 6.82E-06 15900 1.47E-05 15500
2.54E-05 17800 2.76E-05 17200 2.74E-05
18100 6.52E-05 16100 6.82E-06 15900 1.47E-05 15500
2.53E-05 17800 2.77E-05 17200 2.74E-05
18100 6.53E-05 16100 6.82E-06 15900 1.47E-05 15500
2.53E-05 17800 2.78E-05 17200 2.74E-05
18100 6.53E-05 16100 6.83E-06 15900 1.47E-05 15500
2.53E-05 17800 2.79E-05 17200 2.74E-05
18100 6.53E-05 16100 6.83E-06 15900 1.47E-05 15500
2.53E-05 17800 2.80E-05 17200 2.74E-05
18100 6.53E-05 16100 6.83E-06 15900 1.47E-05 15500
2.53E-05 17800 2.81E-05 17200 2.74E-05
18100 6.54E-05 16100 6.82E-06 15900 1.47E-05 15500
2.52E-05 17800 2.82E-05 17200 2.74E-05
18100 6.54E-05 16100 6.83E-06 15900 1.47E-05 15500
2.52E-05 17800 2.83E-05 17200 2.74E-05
18100 6.54E-05 16100 6.83E-06 15900 1.47E-05 15500
2.52E-05 17800 2.85E-05 17200 2.74E-05
18100 6.55E-05 16100 6.83E-06 15900 1.47E-05 15500
2.52E-05 17800 2.86E-05 17200 2.74E-05
18100 6.55E-05 16100 6.82E-06 15900 1.47E-05 15500
2.52E-05 17800 2.87E-05 17200 2.74E-05
18100 6.55E-05 16100 6.82E-06 15900 1.47E-05 15500
2.52E-05 17800 2.88E-05 17200 2.74E-05
18100 6.55E-05 16100 6.81E-06 15900 1.47E-05 15500
2.51E-05 17800 2.90E-05 17200 2.74E-05
18100 6.56E-05 16100 6.82E-06 15900 1.47E-05 15500
2.51E-05 17800 2.91E-05 17200 2.75E-05
18100 6.56E-05 16100 6.81E-06 15900 1.47E-05 15500
2.51E-05 17800 2.93E-05 17200 2.75E-05
18100 6.56E-05 16100 6.81E-06 15900 1.47E-05 15500
2.51E-05 17800 2.94E-05 17200 2.75E-05
18100 6.57E-05 16100 6.81E-06 15900 1.47E-05 15500
2.51E-05 17800 2.96E-05 17200 2.75E-05
18100 6.57E-05 16100 6.81E-06 15900 1.47E-05 15500
2.51E-05 17800 2.97E-05 17200 2.75E-05
18100 6.57E-05 16100 6.80E-06 15900 1.47E-05 15500
2.50E-05 17800 2.99E-05 17200 2.75E-05
18100 6.57E-05 16100 6.80E-06 15900 1.47E-05 15500
2.50E-05 17800 3.01E-05 17200 2.75E-05
18100 6.58E-05 16100 6.79E-06 15900 1.47E-05 15500
2.50E-05 17800 3.02E-05 17200 2.75E-05

18100 6.58E-05 16100 6.79E-06 15900 1.47E-05 15500
2.50E-05 17800 3.04E-05 17200 2.75E-05
18100 6.58E-05 16100 6.80E-06 15900 1.47E-05 15500
2.49E-05 17800 3.06E-05 17200 2.75E-05
18100 6.58E-05 16100 6.79E-06 15900 1.47E-05 15500
2.49E-05 17800 3.08E-05 17200 2.75E-05
18100 6.59E-05 16100 6.79E-06 15900 1.47E-05 15500
2.49E-05 17800 3.10E-05 17200 2.75E-05
18100 6.59E-05 16100 6.78E-06 15900 1.47E-05 15500
2.49E-05 17800 3.12E-05 17200 2.75E-05
18100 6.60E-05 16100 6.79E-06 15900 1.47E-05 15500
2.49E-05 17800 3.14E-05 17200 2.75E-05
18100 6.60E-05 16100 6.78E-06 15900 1.47E-05 15500
2.49E-05 17800 3.16E-05 17200 2.75E-05
18200 6.60E-05 16100 6.78E-06 15900 1.47E-05 15500
2.48E-05 17800 3.18E-05 17200 2.75E-05
18200 6.61E-05 16100 6.78E-06 15900 1.47E-05 15500
2.48E-05 17800 3.21E-05 17200 2.75E-05
18200 6.61E-05 16100 6.77E-06 15900 1.47E-05 15500
2.48E-05 17800 3.23E-05 17200 2.75E-05
18200 6.61E-05 16100 6.78E-06 15900 1.47E-05 15500
2.48E-05 17800 3.26E-05 17200 2.75E-05
18200 6.62E-05 16100 6.77E-06 15900 1.47E-05 15500
2.47E-05 17800 3.28E-05 17200 2.75E-05
18200 6.62E-05 16100 6.78E-06 15900 1.47E-05 15500
2.47E-05 17800 3.31E-05 17200 2.75E-05
18200 6.62E-05 16100 6.77E-06 15900 1.47E-05 15500
2.47E-05 17800 3.34E-05 17200 2.75E-05
18200 6.63E-05 16100 6.78E-06 15900 1.47E-05 15500
2.47E-05 17800 3.37E-05 17200 2.75E-05
18200 6.63E-05 16100 6.77E-06 15900 1.47E-05 15500
2.47E-05 17800 3.40E-05 17200 2.75E-05
18200 6.64E-05 16100 6.77E-06 15900 1.47E-05 15500
2.47E-05 17800 3.43E-05 17300 2.75E-05
18200 6.64E-05 16100 6.78E-06 15900 1.47E-05 15500
2.47E-05 17800 3.46E-05 17300 2.75E-05
18200 6.64E-05 16100 6.78E-06 15900 1.47E-05 15500
2.46E-05 17800 3.50E-05 17300 2.75E-05
18200 6.65E-05 16100 6.77E-06 15900 1.47E-05 15500
2.46E-05 17800 3.53E-05 17300 2.76E-05
18200 6.65E-05 16100 6.77E-06 15900 1.47E-05 15500
2.46E-05 17800 3.57E-05 17300 2.76E-05
18200 6.65E-05 16100 6.77E-06 15900 1.47E-05 15500
2.46E-05 17800 3.61E-05 17300 2.76E-05
18200 6.65E-05 16100 6.78E-06 15900 1.47E-05 15500
2.46E-05 17800 3.65E-05 17300 2.76E-05
18200 6.66E-05 16100 6.78E-06 15900 1.47E-05 15500
2.46E-05 17800 3.69E-05 17300 2.76E-05
18200 6.66E-05 16100 6.78E-06 15900 1.47E-05 15500
2.46E-05 17800 3.73E-05 17300 2.76E-05
18200 6.66E-05 16200 6.78E-06 15900 1.47E-05 15500
2.46E-05 17800 3.78E-05 17300 2.76E-05
18200 6.67E-05 16200 6.79E-06 15900 1.47E-05 15500
2.45E-05 17800 3.83E-05 17300 2.76E-05
18200 6.67E-05 16200 6.78E-06 16000 1.47E-05 15500
2.45E-05 17800 3.87E-05 17300 2.76E-05
18200 6.67E-05 16200 6.78E-06 16000 1.47E-05 15500
2.45E-05 17800 3.93E-05 17300 2.76E-05
18200 6.67E-05 16200 6.78E-06 16000 1.47E-05 15500
2.45E-05 17800 3.98E-05 17300 2.76E-05
18200 6.68E-05 16200 6.77E-06 16000 1.47E-05 15500
2.45E-05 17800 4.03E-05 17300 2.76E-05
18200 6.68E-05 16200 6.77E-06 16000 1.47E-05 15600
2.45E-05 17800 4.09E-05 17300 2.76E-05
18200 6.68E-05 16200 6.77E-06 16000 1.47E-05 15600
2.45E-05 17800 4.15E-05 17300 2.76E-05
18200 6.69E-05 16200 6.78E-06 16000 1.47E-05 15600
2.44E-05 17800 4.22E-05 17300 2.76E-05

18200 6.69E-05 16200 6.77E-06 16000 1.47E-05 15600
2.44E-05 17800 4.29E-05 17300 2.76E-05
18200 6.69E-05 16200 6.77E-06 16000 1.47E-05 15600
2.44E-05 17800 4.36E-05 17300 2.76E-05
18200 6.70E-05 16200 6.77E-06 16000 1.47E-05 15600
2.44E-05 17800 4.43E-05 17300 2.76E-05
18200 6.70E-05 16200 6.77E-06 16000 1.47E-05 15600
2.44E-05 17800 4.51E-05 17300 2.76E-05
18200 6.70E-05 16200 6.77E-06 16000 1.47E-05 15600
2.44E-05 17800 4.59E-05 17300 2.76E-05
18200 6.71E-05 16200 6.77E-06 16000 1.47E-05 15600
2.43E-05 17800 4.67E-05 17300 2.76E-05
18200 6.71E-05 16200 6.77E-06 16000 1.47E-05 15600
2.43E-05 17800 4.76E-05 17300 2.77E-05
18200 6.71E-05 16200 6.78E-06 16000 1.47E-05 15600
2.43E-05 17800 4.85E-05 17300 2.77E-05
18200 6.71E-05 16200 6.78E-06 16000 1.47E-05 15600
2.43E-05 17900 4.95E-05 17300 2.76E-05
18200 6.72E-05 16200 6.78E-06 16000 1.47E-05 15600
2.43E-05 17900 5.05E-05 17300 2.76E-05
18200 6.72E-05 16200 6.78E-06 16000 1.47E-05 15600
2.43E-05 17900 5.16E-05 17300 2.76E-05
18200 6.72E-05 16200 6.78E-06 16000 1.47E-05 15600
2.42E-05 17900 5.27E-05 17300 2.76E-05
18200 6.72E-05 16200 6.78E-06 16000 1.47E-05 15600
2.42E-05 17900 5.39E-05 17300 2.77E-05
18200 6.73E-05 16200 6.77E-06 16000 1.47E-05 15600
2.42E-05 17900 5.51E-05 17300 2.77E-05
18200 6.73E-05 16200 6.78E-06 16000 1.47E-05 15600
2.42E-05 17900 5.65E-05 17300 2.77E-05
18200 6.73E-05 16200 6.78E-06 16000 1.47E-05 15600
2.42E-05 17900 5.78E-05 17300 2.77E-05
18200 6.73E-05 16200 6.78E-06 16000 1.47E-05 15600
2.42E-05 17900 5.93E-05 17300 2.77E-05
18200 6.74E-05 16200 6.79E-06 16000 1.47E-05 15600
2.42E-05 17900 6.08E-05 17300 2.77E-05
18200 6.74E-05 16200 6.80E-06 16000 1.46E-05 15600
2.42E-05 17900 6.24E-05 17300 2.78E-05
18200 6.74E-05 16200 6.78E-06 16000 1.47E-05 15600
2.42E-05 17900 6.41E-05 17300 2.78E-05
18200 6.74E-05 16200 6.79E-06 16000 1.47E-05 15600
2.42E-05 17900 6.59E-05 17300 2.78E-05
18200 6.75E-05 16200 6.79E-06 16000 1.47E-05 15600
2.42E-05 17900 6.77E-05 17300 2.78E-05
18200 6.75E-05 16200 6.79E-06 16000 1.47E-05 15600
2.42E-05 17900 6.96E-05 17300 2.78E-05
18200 6.75E-05 16200 6.79E-06 16000 1.47E-05 15600
2.42E-05 17900 7.17E-05 17300 2.78E-05
18200 6.75E-05 16200 6.80E-06 16000 1.46E-05 15600
2.41E-05 17900 7.38E-05 17300 2.78E-05
18200 6.76E-05 16200 6.80E-06 16000 1.46E-05 15600
2.41E-05 17900 7.60E-05 17300 2.79E-05
18200 6.76E-05 16200 6.80E-06 16000 1.46E-05 15600
2.41E-05 17900 7.83E-05 17300 2.79E-05
18200 6.76E-05 16200 6.79E-06 16000 1.46E-05 15600
2.41E-05 17900 8.07E-05 17300 2.79E-05
18200 6.77E-05 16200 6.79E-06 16000 1.46E-05 15600
2.41E-05 17900 8.32E-05 17300 2.79E-05
18200 6.77E-05 16200 6.79E-06 16000 1.46E-05 15600
2.41E-05 17900 8.58E-05 17300 2.79E-05
18200 6.77E-05 16200 6.80E-06 16000 1.47E-05 15600
2.41E-05 17900 8.84E-05 17300 2.79E-05
18200 6.77E-05 16200 6.80E-06 16000 1.47E-05 15600
2.41E-05 17900 9.12E-05 17300 2.80E-05
18200 6.77E-05 16200 6.79E-06 16000 1.47E-05 15600
2.41E-05 17900 9.40E-05 17300 2.80E-05
18200 6.77E-05 16200 6.79E-06 16000 1.47E-05 15600
2.40E-05 17900 9.69E-05 17300 2.80E-05

18200 6.78E-05 16200 6.79E-06 16000 1.47E-05 15600
2.40E-05 17900 9.98E-05 17300 2.80E-05
18200 6.78E-05 16200 6.78E-06 16000 1.47E-05 15600
2.40E-05 17900 0.000103 17300 2.81E-05
18200 6.79E-05 16200 6.77E-06 16000 1.47E-05 15600
2.40E-05 17900 0.000106 17300 2.81E-05
18300 6.79E-05 16200 6.77E-06 16000 1.47E-05 15600
2.40E-05 17900 0.000109 17300 2.81E-05
18300 6.79E-05 16200 6.76E-06 16000 1.47E-05 15600
2.40E-05 17900 0.000112 17300 2.81E-05
18300 6.80E-05 16200 6.77E-06 16000 1.47E-05 15600
2.39E-05 17900 0.000114 17300 2.81E-05
18300 6.80E-05 16200 6.78E-06 16000 1.47E-05 15600
2.39E-05 17900 0.000117 17300 2.81E-05
18300 6.80E-05 16200 6.78E-06 16000 1.47E-05 15600
2.39E-05 17900 0.00012 17300 2.82E-05
18300 6.81E-05 16200 6.79E-06 16000 1.47E-05 15600
2.39E-05 17900 0.000122 17300 2.82E-05
18300 6.81E-05 16200 6.79E-06 16000 1.47E-05 15600
2.38E-05 17900 0.000125 17300 2.82E-05
18300 6.81E-05 16200 6.78E-06 16000 1.47E-05 15600
2.39E-05 17900 0.000127 17300 2.82E-05
18300 6.81E-05 16200 6.78E-06 16000 1.47E-05 15600
2.39E-05 17900 0.000129 17300 2.82E-05
18300 6.81E-05 16200 6.77E-06 16000 1.47E-05 15600
2.39E-05 17900 0.00013 17400 2.82E-05
18300 6.82E-05 16200 6.78E-06 16000 1.47E-05 15600
2.39E-05 17900 0.000131 17400 2.82E-05
18300 6.82E-05 16200 6.77E-06 16000 1.47E-05 15600
2.38E-05 17900 0.000132 17400 2.82E-05
18300 6.83E-05 16200 6.78E-06 16000 1.47E-05 15600
2.38E-05 17900 0.000133 17400 2.82E-05
18300 6.83E-05 16200 6.77E-06 16000 1.47E-05 15600
2.38E-05 17900 0.000133 17400 2.83E-05
18300 6.83E-05 16200 6.77E-06 16000 1.47E-05 15600
2.38E-05 17900 0.000132 17400 2.83E-05
18300 6.84E-05 16200 6.77E-06 16000 1.47E-05 15600
2.38E-05 17900 0.000132 17400 2.83E-05
18300 6.84E-05 16200 6.77E-06 16000 1.47E-05 15600
2.38E-05 17900 0.000131 17400 2.83E-05
18300 6.84E-05 16200 6.77E-06 16000 1.46E-05 15600
2.38E-05 17900 0.000129 17400 2.83E-05
18300 6.85E-05 16300 6.77E-06 16000 1.46E-05 15600
2.38E-05 17900 0.000127 17400 2.83E-05
18300 6.85E-05 16300 6.77E-06 16000 1.46E-05 15600
2.38E-05 17900 0.000125 17400 2.83E-05
18300 6.85E-05 16300 6.76E-06 16100 1.46E-05 15600
2.38E-05 17900 0.000123 17400 2.83E-05
18300 6.86E-05 16300 6.77E-06 16100 1.46E-05 15600
2.38E-05 17900 0.00012 17400 2.84E-05
18300 6.86E-05 16300 6.77E-06 16100 1.46E-05 15600
2.38E-05 17900 0.000117 17400 2.84E-05
18300 6.87E-05 16300 6.78E-06 16100 1.46E-05 15600
2.38E-05 17900 0.000114 17400 2.84E-05
18300 6.87E-05 16300 6.78E-06 16100 1.46E-05 15700
2.38E-05 17900 0.000111 17400 2.84E-05
18300 6.87E-05 16300 6.77E-06 16100 1.47E-05 15700
2.37E-05 17900 0.000108 17400 2.84E-05
18300 6.88E-05 16300 6.77E-06 16100 1.46E-05 15700
2.37E-05 17900 0.000104 17400 2.84E-05
18300 6.88E-05 16300 6.77E-06 16100 1.46E-05 15700
2.37E-05 17900 0.000101 17400 2.84E-05
18300 6.88E-05 16300 6.76E-06 16100 1.47E-05 15700
2.37E-05 17900 9.75E-05 17400 2.84E-05
18300 6.89E-05 16300 6.76E-06 16100 1.46E-05 15700
2.37E-05 17900 9.42E-05 17400 2.85E-05
18300 6.89E-05 16300 6.76E-06 16100 1.46E-05 15700
2.37E-05 17900 9.10E-05 17400 2.85E-05

18300 6.90E-05	16300 6.76E-06	16100 1.47E-05	15700
2.37E-05 17900	8.78E-05 17400	2.85E-05	
18300 6.90E-05	16300 6.76E-06	16100 1.46E-05	15700
2.37E-05 17900	8.48E-05 17400	2.85E-05	
18300 6.91E-05	16300 6.75E-06	16100 1.47E-05	15700
2.37E-05 17900	8.18E-05 17400	2.85E-05	
18300 6.91E-05	16300 6.76E-06	16100 1.46E-05	15700
2.37E-05 17900	7.90E-05 17400	2.85E-05	
18300 6.92E-05	16300 6.76E-06	16100 1.46E-05	15700
2.37E-05 18000	7.63E-05 17400	2.85E-05	
18300 6.92E-05	16300 6.77E-06	16100 1.47E-05	15700
2.36E-05 18000	7.37E-05 17400	2.86E-05	
18300 6.92E-05	16300 6.77E-06	16100 1.47E-05	15700
2.36E-05 18000	7.13E-05 17400	2.86E-05	
18300 6.93E-05	16300 6.77E-06	16100 1.47E-05	15700
2.36E-05 18000	6.90E-05 17400	2.86E-05	
18300 6.93E-05	16300 6.76E-06	16100 1.47E-05	15700
2.36E-05 18000	6.68E-05 17400	2.86E-05	
18300 6.94E-05	16300 6.76E-06	16100 1.47E-05	15700
2.36E-05 18000	6.47E-05 17400	2.86E-05	
18300 6.94E-05	16300 6.77E-06	16100 1.47E-05	15700
2.36E-05 18000	6.27E-05 17400	2.86E-05	
18300 6.94E-05	16300 6.77E-06	16100 1.47E-05	15700
2.36E-05 18000	6.09E-05 17400	2.86E-05	
18300 6.95E-05	16300 6.77E-06	16100 1.47E-05	15700
2.36E-05 18000	5.92E-05 17400	2.86E-05	
18300 6.95E-05	16300 6.77E-06	16100 1.47E-05	15700
2.35E-05 18000	5.75E-05 17400	2.86E-05	
18300 6.95E-05	16300 6.77E-06	16100 1.47E-05	15700
2.35E-05 18000	5.60E-05 17400	2.86E-05	
18300 6.96E-05	16300 6.77E-06	16100 1.47E-05	15700
2.35E-05 18000	5.45E-05 17400	2.86E-05	
18300 6.96E-05	16300 6.76E-06	16100 1.47E-05	15700
2.35E-05 18000	5.32E-05 17400	2.87E-05	
18300 6.96E-05	16300 6.77E-06	16100 1.47E-05	15700
2.35E-05 18000	5.19E-05 17400	2.87E-05	
18300 6.97E-05	16300 6.77E-06	16100 1.47E-05	15700
2.35E-05 18000	5.07E-05 17400	2.87E-05	
18300 6.97E-05	16300 6.77E-06	16100 1.47E-05	15700
2.35E-05 18000	4.96E-05 17400	2.87E-05	
18300 6.97E-05	16300 6.77E-06	16100 1.47E-05	15700
2.35E-05 18000	4.85E-05 17400	2.87E-05	
18300 6.97E-05	16300 6.77E-06	16100 1.47E-05	15700
2.35E-05 18000	4.75E-05 17400	2.87E-05	
18300 6.98E-05	16300 6.77E-06	16100 1.47E-05	15700
2.35E-05 18000	4.66E-05 17400	2.87E-05	
18300 6.98E-05	16300 6.77E-06	16100 1.47E-05	15700
2.35E-05 18000	4.57E-05 17400	2.87E-05	
18300 6.98E-05	16300 6.78E-06	16100 1.47E-05	15700
2.35E-05 18000	4.48E-05 17400	2.87E-05	
18300 6.99E-05	16300 6.77E-06	16100 1.47E-05	15700
2.35E-05 18000	4.40E-05 17400	2.87E-05	
18300 6.99E-05	16300 6.77E-06	16100 1.47E-05	15700
2.35E-05 18000	4.33E-05 17400	2.87E-05	
18300 6.99E-05	16300 6.76E-06	16100 1.47E-05	15700
2.35E-05 18000	4.26E-05 17400	2.87E-05	
18300 7.00E-05	16300 6.76E-06	16100 1.47E-05	15700
2.35E-05 18000	4.19E-05 17400	2.87E-05	
18300 7.00E-05	16300 6.76E-06	16100 1.47E-05	15700
2.35E-05 18000	4.13E-05 17400	2.87E-05	
18300 7.00E-05	16300 6.76E-06	16100 1.47E-05	15700
2.35E-05 18000	4.07E-05 17400	2.87E-05	
18300 7.01E-05	16300 6.77E-06	16100 1.47E-05	15700
2.35E-05 18000	4.01E-05 17400	2.88E-05	
18300 7.01E-05	16300 6.76E-06	16100 1.47E-05	15700
2.34E-05 18000	3.96E-05 17400	2.88E-05	
18400 7.01E-05	16300 6.76E-06	16100 1.47E-05	15700
2.35E-05 18000	3.90E-05 17400	2.88E-05	

18400 7.02E-05 16300 6.76E-06 16100 1.47E-05 15700
2.34E-05 18000 3.86E-05 17400 2.88E-05
18400 7.02E-05 16300 6.74E-06 16100 1.47E-05 15700
2.34E-05 18000 3.81E-05 17400 2.88E-05
18400 7.03E-05 16300 6.74E-06 16100 1.47E-05 15700
2.34E-05 18000 3.77E-05 17400 2.88E-05
18400 7.03E-05 16300 6.75E-06 16100 1.47E-05 15700
2.34E-05 18000 3.72E-05 17400 2.88E-05
18400 7.03E-05 16300 6.74E-06 16100 1.47E-05 15700
2.34E-05 18000 3.69E-05 17400 2.88E-05
18400 7.03E-05 16300 6.75E-06 16100 1.47E-05 15700
2.34E-05 18000 3.65E-05 17400 2.88E-05
18400 7.04E-05 16300 6.75E-06 16100 1.47E-05 15700
2.34E-05 18000 3.61E-05 17400 2.88E-05
18400 7.04E-05 16300 6.76E-06 16100 1.47E-05 15700
2.34E-05 18000 3.57E-05 17400 2.89E-05
18400 7.04E-05 16300 6.76E-06 16100 1.47E-05 15700
2.34E-05 18000 3.54E-05 17500 2.89E-05
18400 7.05E-05 16300 6.77E-06 16100 1.47E-05 15700
2.33E-05 18000 3.51E-05 17500 2.89E-05
18400 7.05E-05 16300 6.76E-06 16100 1.47E-05 15700
2.34E-05 18000 3.48E-05 17500 2.89E-05
18400 7.06E-05 16300 6.76E-06 16100 1.47E-05 15700
2.33E-05 18000 3.45E-05 17500 2.89E-05
18400 7.06E-05 16300 6.76E-06 16100 1.47E-05 15700
2.33E-05 18000 3.42E-05 17500 2.90E-05
18400 7.06E-05 16300 6.76E-06 16100 1.47E-05 15700
2.33E-05 18000 3.40E-05 17500 2.90E-05
18400 7.07E-05 16300 6.76E-06 16100 1.47E-05 15700
2.33E-05 18000 3.37E-05 17500 2.89E-05
18400 7.07E-05 16300 6.76E-06 16100 1.47E-05 15700
2.33E-05 18000 3.35E-05 17500 2.90E-05
18400 7.08E-05 16300 6.76E-06 16100 1.47E-05 15700
2.33E-05 18000 3.33E-05 17500 2.90E-05
18400 7.08E-05 16400 6.77E-06 16100 1.47E-05 15700
2.33E-05 18000 3.30E-05 17500 2.90E-05
18400 7.08E-05 16400 6.76E-06 16100 1.47E-05 15700
2.33E-05 18000 3.28E-05 17500 2.90E-05
18400 7.08E-05 16400 6.76E-06 16200 1.47E-05 15700
2.33E-05 18000 3.26E-05 17500 2.90E-05
18400 7.09E-05 16400 6.76E-06 16200 1.47E-05 15700
2.34E-05 18000 3.24E-05 17500 2.90E-05
18400 7.09E-05 16400 6.76E-06 16200 1.47E-05 15700
2.33E-05 18000 3.22E-05 17500 2.90E-05
18400 7.09E-05 16400 6.75E-06 16200 1.47E-05 15700
2.33E-05 18000 3.20E-05 17500 2.90E-05
18400 7.10E-05 16400 6.75E-06 16200 1.47E-05 15800
2.33E-05 18000 3.18E-05 17500 2.90E-05
18400 7.10E-05 16400 6.75E-06 16200 1.47E-05 15800
2.33E-05 18000 3.16E-05 17500 2.91E-05
18400 7.10E-05 16400 6.75E-06 16200 1.47E-05 15800
2.33E-05 18000 3.15E-05 17500 2.90E-05
18400 7.11E-05 16400 6.75E-06 16200 1.47E-05 15800
2.33E-05 18000 3.13E-05 17500 2.91E-05
18400 7.11E-05 16400 6.75E-06 16200 1.47E-05 15800
2.33E-05 18000 3.11E-05 17500 2.91E-05
18400 7.11E-05 16400 6.75E-06 16200 1.47E-05 15800
2.33E-05 18000 3.10E-05 17500 2.91E-05
18400 7.12E-05 16400 6.75E-06 16200 1.47E-05 15800
2.33E-05 18000 3.08E-05 17500 2.92E-05
18400 7.12E-05 16400 6.75E-06 16200 1.47E-05 15800
2.33E-05 18000 3.07E-05 17500 2.92E-05
18400 7.12E-05 16400 6.74E-06 16200 1.47E-05 15800
2.33E-05 18000 3.06E-05 17500 2.92E-05
18400 7.13E-05 16400 6.74E-06 16200 1.47E-05 15800
2.33E-05 18000 3.04E-05 17500 2.92E-05
18400 7.13E-05 16400 6.74E-06 16200 1.47E-05 15800
2.33E-05 18000 3.03E-05 17500 2.92E-05

18400 7.14E-05 16400 6.74E-06 16200 1.47E-05 15800
2.33E-05 18100 3.02E-05 17500 2.92E-05
18400 7.14E-05 16400 6.75E-06 16200 1.47E-05 15800
2.33E-05 18100 3.01E-05 17500 2.92E-05
18400 7.14E-05 16400 6.75E-06 16200 1.48E-05 15800
2.33E-05 18100 2.99E-05 17500 2.92E-05
18400 7.15E-05 16400 6.75E-06 16200 1.47E-05 15800
2.33E-05 18100 2.98E-05 17500 2.92E-05
18400 7.15E-05 16400 6.75E-06 16200 1.48E-05 15800
2.33E-05 18100 2.97E-05 17500 2.92E-05
18400 7.15E-05 16400 6.75E-06 16200 1.48E-05 15800
2.33E-05 18100 2.96E-05 17500 2.92E-05
18400 7.16E-05 16400 6.76E-06 16200 1.48E-05 15800
2.33E-05 18100 2.95E-05 17500 2.92E-05
18400 7.16E-05 16400 6.75E-06 16200 1.47E-05 15800
2.33E-05 18100 2.94E-05 17500 2.92E-05
18400 7.17E-05 16400 6.76E-06 16200 1.48E-05 15800
2.33E-05 18100 2.93E-05 17500 2.92E-05
18400 7.17E-05 16400 6.76E-06 16200 1.48E-05 15800
2.32E-05 18100 2.92E-05 17500 2.92E-05
18400 7.17E-05 16400 6.75E-06 16200 1.47E-05 15800
2.33E-05 18100 2.91E-05 17500 2.92E-05
18400 7.18E-05 16400 6.75E-06 16200 1.48E-05 15800
2.32E-05 18100 2.90E-05 17500 2.92E-05
18400 7.18E-05 16400 6.75E-06 16200 1.48E-05 15800
2.32E-05 18100 2.89E-05 17500 2.93E-05
18400 7.19E-05 16400 6.76E-06 16200 1.48E-05 15800
2.32E-05 18100 2.89E-05 17500 2.93E-05
18400 7.19E-05 16400 6.75E-06 16200 1.48E-05 15800
2.32E-05 18100 2.88E-05 17500 2.93E-05
18400 7.20E-05 16400 6.76E-06 16200 1.48E-05 15800
2.33E-05 18100 2.87E-05 17500 2.93E-05
18400 7.20E-05 16400 6.76E-06 16200 1.48E-05 15800
2.33E-05 18100 2.86E-05 17500 2.93E-05
18400 7.21E-05 16400 6.75E-06 16200 1.48E-05 15800
2.33E-05 18100 2.86E-05 17500 2.93E-05
18400 7.21E-05 16400 6.76E-06 16200 1.48E-05 15800
2.33E-05 18100 2.85E-05 17500 2.93E-05
18400 7.21E-05 16400 6.75E-06 16200 1.48E-05 15800
2.33E-05 18100 2.84E-05 17500 2.93E-05
18400 7.22E-05 16400 6.76E-06 16200 1.48E-05 15800
2.33E-05 18100 2.84E-05 17500 2.93E-05
18400 7.22E-05 16400 6.75E-06 16200 1.48E-05 15800
2.32E-05 18100 2.83E-05 17500 2.93E-05
18400 7.22E-05 16400 6.76E-06 16200 1.48E-05 15800
2.33E-05 18100 2.82E-05 17500 2.94E-05
18400 7.23E-05 16400 6.75E-06 16200 1.48E-05 15800
2.33E-05 18100 2.82E-05 17500 2.94E-05
18400 7.23E-05 16400 6.75E-06 16200 1.48E-05 15800
2.33E-05 18100 2.81E-05 17500 2.93E-05
18400 7.23E-05 16400 6.76E-06 16200 1.48E-05 15800
2.33E-05 18100 2.81E-05 17500 2.94E-05
18400 7.24E-05 16400 6.75E-06 16200 1.48E-05 15800
2.33E-05 18100 2.80E-05 17500 2.94E-05
18400 7.24E-05 16400 6.75E-06 16200 1.48E-05 15800
2.32E-05 18100 2.80E-05 17500 2.94E-05
18400 7.25E-05 16400 6.74E-06 16200 1.48E-05 15800
2.32E-05 18100 2.79E-05 17500 2.94E-05
18500 7.25E-05 16400 6.75E-06 16200 1.48E-05 15800
2.32E-05 18100 2.79E-05 17500 2.94E-05
18500 7.25E-05 16400 6.75E-06 16200 1.48E-05 15800
2.32E-05 18100 2.78E-05 17500 2.94E-05
18500 7.26E-05 16400 6.74E-06 16200 1.48E-05 15800
2.32E-05 18100 2.78E-05 17500 2.94E-05
18500 7.26E-05 16400 6.75E-06 16200 1.48E-05 15800
2.32E-05 18100 2.77E-05 17500 2.94E-05
18500 7.27E-05 16400 6.75E-06 16200 1.48E-05 15800
2.32E-05 18100 2.77E-05 17500 2.94E-05

18500 7.27E-05 16400 6.76E-06 16200 1.48E-05 15800
2.32E-05 18100 2.76E-05 17500 2.94E-05
18500 7.27E-05 16400 6.75E-06 16200 1.48E-05 15800
2.32E-05 18100 2.76E-05 17500 2.94E-05
18500 7.28E-05 16400 6.75E-06 16200 1.48E-05 15800
2.32E-05 18100 2.76E-05 17500 2.94E-05
18500 7.28E-05 16400 6.77E-06 16200 1.48E-05 15800
2.32E-05 18100 2.75E-05 17500 2.94E-05
18500 7.29E-05 16400 6.76E-06 16200 1.48E-05 15800
2.32E-05 18100 2.75E-05 17600 2.94E-05
18500 7.29E-05 16400 6.76E-06 16200 1.48E-05 15800
2.32E-05 18100 2.74E-05 17600 2.94E-05
18500 7.29E-05 16400 6.76E-06 16200 1.48E-05 15800
2.32E-05 18100 2.74E-05 17600 2.95E-05
18500 7.30E-05 16400 6.78E-06 16200 1.48E-05 15800
2.33E-05 18100 2.74E-05 17600 2.95E-05
18500 7.30E-05 16400 6.78E-06 16200 1.48E-05 15800
2.33E-05 18100 2.73E-05 17600 2.95E-05
18500 7.31E-05 16400 6.77E-06 16200 1.48E-05 15800
2.33E-05 18100 2.73E-05 17600 2.95E-05
18500 7.31E-05 16400 6.78E-06 16200 1.48E-05 15800
2.33E-05 18100 2.73E-05 17600 2.95E-05
18500 7.32E-05 16400 6.78E-06 16200 1.48E-05 15800
2.33E-05 18100 2.72E-05 17600 2.95E-05
18500 7.32E-05 16400 6.78E-06 16200 1.48E-05 15800
2.33E-05 18100 2.72E-05 17600 2.95E-05
18500 7.32E-05 16500 6.77E-06 16200 1.48E-05 15800
2.33E-05 18100 2.72E-05 17600 2.95E-05
18500 7.33E-05 16500 6.77E-06 16200 1.48E-05 15800
2.33E-05 18100 2.71E-05 17600 2.96E-05
18500 7.33E-05 16500 6.77E-06 16300 1.48E-05 15800
2.33E-05 18100 2.71E-05 17600 2.96E-05
18500 7.34E-05 16500 6.77E-06 16300 1.48E-05 15800
2.33E-05 18100 2.71E-05 17600 2.96E-05
18500 7.34E-05 16500 6.77E-06 16300 1.48E-05 15800
2.33E-05 18100 2.70E-05 17600 2.96E-05
18500 7.35E-05 16500 6.77E-06 16300 1.48E-05 15800
2.33E-05 18100 2.70E-05 17600 2.96E-05
18500 7.35E-05 16500 6.78E-06 16300 1.48E-05 15900
2.33E-05 18100 2.70E-05 17600 2.96E-05
18500 7.36E-05 16500 6.78E-06 16300 1.48E-05 15900
2.33E-05 18100 2.70E-05 17600 2.96E-05
18500 7.36E-05 16500 6.78E-06 16300 1.48E-05 15900
2.33E-05 18100 2.70E-05 17600 2.96E-05
18500 7.37E-05 16500 6.79E-06 16300 1.48E-05 15900
2.33E-05 18100 2.69E-05 17600 2.96E-05
18500 7.37E-05 16500 6.78E-06 16300 1.48E-05 15900
2.33E-05 18100 2.69E-05 17600 2.96E-05
18500 7.38E-05 16500 6.79E-06 16300 1.49E-05 15900
2.33E-05 18100 2.69E-05 17600 2.96E-05
18500 7.38E-05 16500 6.78E-06 16300 1.48E-05 15900
2.33E-05 18100 2.69E-05 17600 2.96E-05
18500 7.39E-05 16500 6.79E-06 16300 1.48E-05 15900
2.33E-05 18100 2.69E-05 17600 2.96E-05
18500 7.39E-05 16500 6.79E-06 16300 1.49E-05 15900
2.33E-05 18100 2.68E-05 17600 2.96E-05
18500 7.39E-05 16500 6.78E-06 16300 1.49E-05 15900
2.33E-05 18100 2.68E-05 17600 2.96E-05
18500 7.40E-05 16500 6.78E-06 16300 1.49E-05 15900
2.33E-05 18100 2.68E-05 17600 2.96E-05
18500 7.40E-05 16500 6.77E-06 16300 1.49E-05 15900
2.33E-05 18200 2.68E-05 17600 2.96E-05
18500 7.41E-05 16500 6.78E-06 16300 1.49E-05 15900
2.32E-05 18200 2.68E-05 17600 2.96E-05
18500 7.41E-05 16500 6.77E-06 16300 1.49E-05 15900
2.32E-05 18200 2.68E-05 17600 2.96E-05
18500 7.42E-05 16500 6.77E-06 16300 1.49E-05 15900
2.32E-05 18200 2.67E-05 17600 2.96E-05

18500 7.42E-05 16500 6.76E-06 16300 1.49E-05 15900
2.32E-05 18200 2.67E-05 17600 2.97E-05
18500 7.43E-05 16500 6.76E-06 16300 1.49E-05 15900
2.33E-05 18200 2.67E-05 17600 2.97E-05
18500 7.43E-05 16500 6.76E-06 16300 1.49E-05 15900
2.33E-05 18200 2.67E-05 17600 2.97E-05
18500 7.44E-05 16500 6.76E-06 16300 1.49E-05 15900
2.33E-05 18200 2.67E-05 17600 2.97E-05
18500 7.44E-05 16500 6.77E-06 16300 1.49E-05 15900
2.33E-05 18200 2.67E-05 17600 2.97E-05
18500 7.45E-05 16500 6.76E-06 16300 1.49E-05 15900
2.33E-05 18200 2.67E-05 17600 2.97E-05
18500 7.45E-05 16500 6.76E-06 16300 1.49E-05 15900
2.33E-05 18200 2.67E-05 17600 2.98E-05
18500 7.45E-05 16500 6.76E-06 16300 1.49E-05 15900
2.33E-05 18200 2.66E-05 17600 2.97E-05
18500 7.46E-05 16500 6.75E-06 16300 1.49E-05 15900
2.33E-05 18200 2.66E-05 17600 2.98E-05
18500 7.47E-05 16500 6.76E-06 16300 1.49E-05 15900
2.33E-05 18200 2.66E-05 17600 2.97E-05
18500 7.47E-05 16500 6.76E-06 16300 1.49E-05 15900
2.33E-05 18200 2.66E-05 17600 2.98E-05
18500 7.48E-05 16500 6.76E-06 16300 1.49E-05 15900
2.33E-05 18200 2.66E-05 17600 2.98E-05
18500 7.48E-05 16500 6.76E-06 16300 1.49E-05 15900
2.33E-05 18200 2.66E-05 17600 2.98E-05
18500 7.48E-05 16500 6.76E-06 16300 1.49E-05 15900
2.33E-05 18200 2.66E-05 17600 2.98E-05
18500 7.49E-05 16500 6.76E-06 16300 1.49E-05 15900
2.33E-05 18200 2.66E-05 17600 2.98E-05
18500 7.49E-05 16500 6.75E-06 16300 1.49E-05 15900
2.33E-05 18200 2.66E-05 17600 2.98E-05
18500 7.50E-05 16500 6.76E-06 16300 1.49E-05 15900
2.33E-05 18200 2.65E-05 17600 2.98E-05
18500 7.50E-05 16500 6.75E-06 16300 1.49E-05 15900
2.33E-05 18200 2.65E-05 17600 2.99E-05
18500 7.51E-05 16500 6.75E-06 16300 1.49E-05 15900
2.33E-05 18200 2.65E-05 17600 2.99E-05
18500 7.51E-05 16500 6.75E-06 16300 1.49E-05 15900
2.34E-05 18200 2.65E-05 17600 2.99E-05
18500 7.52E-05 16500 6.74E-06 16300 1.49E-05 15900
2.33E-05 18200 2.65E-05 17600 2.99E-05
18500 7.52E-05 16500 6.75E-06 16300 1.49E-05 15900
2.33E-05 18200 2.65E-05 17600 2.99E-05
18500 7.53E-05 16500 6.75E-06 16300 1.49E-05 15900
2.33E-05 18200 2.65E-05 17600 2.99E-05
18500 7.53E-05 16500 6.75E-06 16300 1.49E-05 15900
2.34E-05 18200 2.65E-05 17600 2.99E-05
18500 7.54E-05 16500 6.75E-06 16300 1.49E-05 15900
2.34E-05 18200 2.65E-05 17600 2.99E-05
18600 7.54E-05 16500 6.75E-06 16300 1.49E-05 15900
2.34E-05 18200 2.65E-05 17600 2.99E-05
18600 7.55E-05 16500 6.75E-06 16300 1.49E-05 15900
2.34E-05 18200 2.65E-05 17600 2.99E-05
18600 7.55E-05 16500 6.75E-06 16300 1.49E-05 15900
2.34E-05 18200 2.65E-05 17600 2.99E-05
18600 7.56E-05 16500 6.75E-06 16300 1.49E-05 15900
2.34E-05 18200 2.64E-05 17600 3.00E-05
18600 7.56E-05 16500 6.76E-06 16300 1.49E-05 15900
2.34E-05 18200 2.64E-05 17600 3.00E-05
18600 7.56E-05 16500 6.75E-06 16300 1.49E-05 15900
2.34E-05 18200 2.64E-05 17600 3.00E-05
18600 7.57E-05 16500 6.76E-06 16300 1.49E-05 15900
2.35E-05 18200 2.64E-05 17600 3.00E-05
18600 7.57E-05 16500 6.75E-06 16300 1.49E-05 15900
2.35E-05 18200 2.64E-05 17600 3.00E-05
18600 7.58E-05 16500 6.76E-06 16300 1.49E-05 15900
2.35E-05 18200 2.64E-05 17600 3.00E-05

```
18600   7.59E-05        16500   6.73E-06        16300   1.49E-05        15900
2.35E-05        18200   2.64E-05        17700   3.00E-05
18600   7.59E-05        16500   6.74E-06        16300   1.50E-05        15900
2.35E-05        18200   2.64E-05        17700   3.00E-05
18600   7.60E-05        16500   6.74E-06        16300   1.49E-05        15900
2.35E-05        18200   2.64E-05        17700   3.01E-05
18600   7.60E-05        16500   6.75E-06        16300   1.50E-05        15900
2.35E-05        18200   2.64E-05        17700   3.01E-05
18600   7.60E-05        16500   6.74E-06        16300   1.50E-05        15900
2.35E-05        18200   2.64E-05        17700   3.01E-05
18600   7.61E-05        16500   6.73E-06        16300   1.50E-05        15900
2.36E-05        18200   2.64E-05        17700   3.01E-05
18600   7.62E-05        16500   6.73E-06        16300   1.50E-05        15900
2.36E-05        18200   2.64E-05        17700   3.01E-05
18600   7.62E-05        16500   6.73E-06        16300   1.50E-05        15900
2.36E-05        18200   2.64E-05        17700   3.02E-05
18600   7.63E-05        16500   6.73E-06        16300   1.50E-05        15900
2.36E-05        18200   2.64E-05        17700   3.02E-05
18600   7.63E-05        16600   6.73E-06        16300   1.50E-05        15900
2.36E-05        18200   2.64E-05        17700   3.02E-05
18600   7.64E-05        16600   6.72E-06        16300   1.50E-05        15900
2.36E-05        18200   2.64E-05        17700   3.02E-05
18600   7.64E-05        16600   6.73E-06        16400   1.50E-05        15900
2.36E-05        18200   2.64E-05        17700   3.02E-05
18600   7.65E-05        16600   6.73E-06        16400   1.50E-05        15900
2.36E-05        18200   2.64E-05        17700   3.02E-05
18600   7.65E-05        16600   6.74E-06        16400   1.50E-05        15900
2.36E-05        18200   2.64E-05        17700   3.02E-05
18600   7.65E-05        16600   6.74E-06        16400   1.50E-05        15900
2.36E-05        18200   2.64E-05        17700   3.02E-05
18600   7.66E-05        16600   6.75E-06        16400   1.50E-05        16000
2.36E-05        18200   2.64E-05        17700   3.03E-05
18600   7.66E-05        16600   6.75E-06        16400   1.50E-05        16000
2.36E-05        18200   2.64E-05        17700   3.03E-05
18600   7.67E-05        16600   6.74E-06        16400   1.50E-05        16000
2.36E-05        18200   2.64E-05        17700   3.03E-05
18600   7.67E-05        16600   6.76E-06        16400   1.50E-05        16000
2.36E-05        18200   2.64E-05        17700   3.03E-05
18600   7.68E-05        16600   6.75E-06        16400   1.50E-05        16000
2.36E-05        18200   2.64E-05        17700   3.03E-05
18600   7.68E-05        16600   6.77E-06        16400   1.50E-05        16000
2.36E-05        18200   2.64E-05        17700   3.03E-05
18600   7.69E-05        16600   6.75E-06        16400   1.50E-05        16000
2.36E-05        18200   2.64E-05        17700   3.03E-05
18600   7.69E-05        16600   6.76E-06        16400   1.50E-05        16000
2.36E-05        18200   2.64E-05        17700   3.04E-05
18600   7.70E-05        16600   6.76E-06        16400   1.50E-05        16000
2.37E-05        18200   2.64E-05        17700   3.04E-05
18600   7.70E-05        16600   6.76E-06        16400   1.50E-05        16000
2.37E-05        18200   2.64E-05        17700   3.04E-05
18600   7.71E-05        16600   6.76E-06        16400   1.50E-05        16000
2.37E-05        18200   2.64E-05        17700   3.04E-05
18600   7.71E-05        16600   6.75E-06        16400   1.50E-05        16000
2.37E-05        18300   2.64E-05        17700   3.04E-05
18600   7.72E-05        16600   6.76E-06        16400   1.50E-05        16000
2.37E-05        18300   2.64E-05        17700   3.04E-05
18600   7.72E-05        16600   6.76E-06        16400   1.50E-05        16000
2.37E-05        18300   2.64E-05        17700   3.04E-05
18600   7.73E-05        16600   6.77E-06        16400   1.50E-05        16000
2.37E-05        18300   2.64E-05        17700   3.04E-05
18600   7.73E-05        16600   6.78E-06        16400   1.50E-05        16000
2.37E-05        18300   2.64E-05        17700   3.05E-05
18600   7.74E-05        16600   6.78E-06        16400   1.50E-05        16000
2.37E-05        18300   2.64E-05        17700   3.05E-05
18600   7.74E-05        16600   6.78E-06        16400   1.50E-05        16000
2.37E-05        18300   2.64E-05        17700   3.05E-05
18600   7.75E-05        16600   6.79E-06        16400   1.50E-05        16000
2.37E-05        18300   2.64E-05        17700   3.05E-05
```

18600 7.75E-05 16600 6.80E-06 16400 1.50E-05 16000
2.37E-05 18300 2.64E-05 17700 3.05E-05
18600 7.76E-05 16600 6.80E-06 16400 1.51E-05 16000
2.37E-05 18300 2.64E-05 17700 3.05E-05
18600 7.76E-05 16600 6.79E-06 16400 1.50E-05 16000
2.37E-05 18300 2.64E-05 17700 3.05E-05
18600 7.77E-05 16600 6.80E-06 16400 1.50E-05 16000
2.37E-05 18300 2.64E-05 17700 3.05E-05
18600 7.78E-05 16600 6.78E-06 16400 1.51E-05 16000
2.37E-05 18300 2.64E-05 17700 3.05E-05
18600 7.78E-05 16600 6.80E-06 16400 1.51E-05 16000
2.37E-05 18300 2.64E-05 17700 3.05E-05
18600 7.79E-05 16600 6.78E-06 16400 1.51E-05 16000
2.37E-05 18300 2.64E-05 17700 3.06E-05
18600 7.80E-05 16600 6.79E-06 16400 1.51E-05 16000
2.37E-05 18300 2.64E-05 17700 3.05E-05
18600 7.80E-05 16600 6.79E-06 16400 1.51E-05 16000
2.38E-05 18300 2.64E-05 17700 3.06E-05
18600 7.81E-05 16600 6.78E-06 16400 1.51E-05 16000
2.38E-05 18300 2.64E-05 17700 3.06E-05
18600 7.82E-05 16600 6.78E-06 16400 1.51E-05 16000
2.38E-05 18300 2.64E-05 17700 3.06E-05
18600 7.82E-05 16600 6.78E-06 16400 1.51E-05 16000
2.38E-05 18300 2.64E-05 17700 3.06E-05
18600 7.83E-05 16600 6.78E-06 16400 1.51E-05 16000
2.38E-05 18300 2.64E-05 17700 3.06E-05
18600 7.83E-05 16600 6.77E-06 16400 1.51E-05 16000
2.39E-05 18300 2.64E-05 17700 3.06E-05
18600 7.84E-05 16600 6.77E-06 16400 1.51E-05 16000
2.39E-05 18300 2.64E-05 17700 3.06E-05
18600 7.85E-05 16600 6.77E-06 16400 1.51E-05 16000
2.39E-05 18300 2.64E-05 17700 3.07E-05
18600 7.85E-05 16600 6.77E-06 16400 1.51E-05 16000
2.39E-05 18300 2.64E-05 17700 3.07E-05
18600 7.86E-05 16600 6.78E-06 16400 1.51E-05 16000
2.39E-05 18300 2.64E-05 17700 3.07E-05
18600 7.87E-05 16600 6.80E-06 16400 1.51E-05 16000
2.39E-05 18300 2.64E-05 17700 3.07E-05
18600 7.87E-05 16600 6.79E-06 16400 1.51E-05 16000
2.40E-05 18300 2.64E-05 17700 3.07E-05
18600 7.88E-05 16600 6.78E-06 16400 1.51E-05 16000
2.40E-05 18300 2.64E-05 17700 3.07E-05
18700 7.88E-05 16600 6.78E-06 16400 1.51E-05 16000
2.40E-05 18300 2.64E-05 17700 3.07E-05
18700 7.89E-05 16600 6.79E-06 16400 1.51E-05 16000
2.40E-05 18300 2.64E-05 17700 3.07E-05
18700 7.89E-05 16600 6.77E-06 16400 1.51E-05 16000
2.40E-05 18300 2.64E-05 17700 3.07E-05
18700 7.90E-05 16600 6.79E-06 16400 1.51E-05 16000
2.40E-05 18300 2.65E-05 17700 3.07E-05
18700 7.91E-05 16600 6.77E-06 16400 1.51E-05 16000
2.40E-05 18300 2.65E-05 17700 3.07E-05
18700 7.91E-05 16600 6.79E-06 16400 1.51E-05 16000
2.40E-05 18300 2.65E-05 17700 3.07E-05
18700 7.92E-05 16600 6.78E-06 16400 1.52E-05 16000
2.40E-05 18300 2.65E-05 17700 3.08E-05
18700 7.92E-05 16600 6.78E-06 16400 1.52E-05 16000
2.41E-05 18300 2.65E-05 17700 3.08E-05
18700 7.93E-05 16600 6.78E-06 16400 1.52E-05 16000
2.41E-05 18300 2.65E-05 17700 3.08E-05
18700 7.94E-05 16600 6.77E-06 16400 1.52E-05 16000
2.41E-05 18300 2.65E-05 17800 3.08E-05
18700 7.95E-05 16600 6.79E-06 16400 1.52E-05 16000
2.41E-05 18300 2.65E-05 17800 3.08E-05
18700 7.95E-05 16600 6.78E-06 16400 1.52E-05 16000
2.41E-05 18300 2.65E-05 17800 3.08E-05
18700 7.96E-05 16600 6.78E-06 16400 1.52E-05 16000
2.41E-05 18300 2.65E-05 17800 3.08E-05

18700 7.96E-05 16600 6.78E-06 16400 1.52E-05 16000
2.41E-05 18300 2.65E-05 17800 3.09E-05
18700 7.97E-05 16600 6.78E-06 16400 1.52E-05 16000
2.41E-05 18300 2.65E-05 17800 3.08E-05
18700 7.98E-05 16600 6.79E-06 16400 1.52E-05 16000
2.41E-05 18300 2.65E-05 17800 3.09E-05
18700 7.98E-05 16600 6.80E-06 16400 1.52E-05 16000
2.42E-05 18300 2.65E-05 17800 3.09E-05
18700 7.99E-05 16600 6.80E-06 16400 1.52E-05 16000
2.42E-05 18300 2.65E-05 17800 3.09E-05
18700 8.00E-05 16700 6.80E-06 16400 1.52E-05 16000
2.42E-05 18300 2.65E-05 17800 3.09E-05
18700 8.00E-05 16700 6.80E-06 16400 1.52E-05 16000
2.42E-05 18300 2.65E-05 17800 3.09E-05
18700 8.01E-05 16700 6.79E-06 16500 1.52E-05 16000
2.42E-05 18300 2.65E-05 17800 3.09E-05
18700 8.02E-05 16700 6.79E-06 16500 1.52E-05 16000
2.43E-05 18300 2.65E-05 17800 3.09E-05
18700 8.02E-05 16700 6.79E-06 16500 1.52E-05 16000
2.42E-05 18300 2.65E-05 17800 3.09E-05
18700 8.03E-05 16700 6.79E-06 16500 1.52E-05 16000
2.43E-05 18300 2.65E-05 17800 3.10E-05
18700 8.03E-05 16700 6.80E-06 16500 1.52E-05 16100
2.43E-05 18300 2.65E-05 17800 3.10E-05
18700 8.04E-05 16700 6.79E-06 16500 1.52E-05 16100
2.43E-05 18300 2.65E-05 17800 3.10E-05
18700 8.05E-05 16700 6.79E-06 16500 1.52E-05 16100
2.43E-05 18300 2.65E-05 17800 3.10E-05
18700 8.05E-05 16700 6.79E-06 16500 1.52E-05 16100
2.43E-05 18300 2.65E-05 17800 3.10E-05
18700 8.06E-05 16700 6.79E-06 16500 1.52E-05 16100
2.43E-05 18300 2.65E-05 17800 3.10E-05
18700 8.07E-05 16700 6.79E-06 16500 1.52E-05 16100
2.43E-05 18300 2.66E-05 17800 3.11E-05
18700 8.08E-05 16700 6.79E-06 16500 1.52E-05 16100
2.44E-05 18300 2.66E-05 17800 3.11E-05
18700 8.09E-05 16700 6.78E-06 16500 1.52E-05 16100
2.44E-05 18300 2.66E-05 17800 3.11E-05
18700 8.09E-05 16700 6.79E-06 16500 1.53E-05 16100
2.44E-05 18300 2.66E-05 17800 3.11E-05
18700 8.10E-05 16700 6.79E-06 16500 1.53E-05 16100
2.44E-05 18300 2.66E-05 17800 3.11E-05
18700 8.11E-05 16700 6.79E-06 16500 1.53E-05 16100
2.44E-05 18300 2.66E-05 17800 3.11E-05
18700 8.11E-05 16700 6.79E-06 16500 1.53E-05 16100
2.44E-05 18400 2.66E-05 17800 3.11E-05
18700 8.12E-05 16700 6.80E-06 16500 1.53E-05 16100
2.44E-05 18400 2.66E-05 17800 3.11E-05
18700 8.13E-05 16700 6.80E-06 16500 1.53E-05 16100
2.45E-05 18400 2.66E-05 17800 3.11E-05
18700 8.13E-05 16700 6.79E-06 16500 1.53E-05 16100
2.45E-05 18400 2.66E-05 17800 3.12E-05
18700 8.14E-05 16700 6.79E-06 16500 1.53E-05 16100
2.45E-05 18400 2.66E-05 17800 3.12E-05
18700 8.14E-05 16700 6.78E-06 16500 1.53E-05 16100
2.46E-05 18400 2.66E-05 17800 3.12E-05
18700 8.15E-05 16700 6.78E-06 16500 1.53E-05 16100
2.46E-05 18400 2.66E-05 17800 3.12E-05
18700 8.16E-05 16700 6.78E-06 16500 1.53E-05 16100
2.46E-05 18400 2.66E-05 17800 3.12E-05
18700 8.16E-05 16700 6.78E-06 16500 1.53E-05 16100
2.46E-05 18400 2.66E-05 17800 3.12E-05
18700 8.17E-05 16700 6.79E-06 16500 1.53E-05 16100
2.46E-05 18400 2.66E-05 17800 3.12E-05
18700 8.17E-05 16700 6.78E-06 16500 1.53E-05 16100
2.46E-05 18400 2.66E-05 17800 3.12E-05
18700 8.18E-05 16700 6.79E-06 16500 1.53E-05 16100
2.46E-05 18400 2.66E-05 17800 3.13E-05

18700 8.19E-05 16700 6.78E-06 16500 1.53E-05 16100
2.47E-05 18400 2.66E-05 17800 3.13E-05
18700 8.20E-05 16700 6.79E-06 16500 1.53E-05 16100
2.47E-05 18400 2.67E-05 17800 3.13E-05
18700 8.20E-05 16700 6.78E-06 16500 1.53E-05 16100
2.47E-05 18400 2.67E-05 17800 3.13E-05
18700 8.21E-05 16700 6.78E-06 16500 1.53E-05 16100
2.47E-05 18400 2.67E-05 17800 3.13E-05
18700 8.22E-05 16700 6.78E-06 16500 1.53E-05 16100
2.48E-05 18400 2.67E-05 17800 3.13E-05
18700 8.23E-05 16700 6.78E-06 16500 1.54E-05 16100
2.48E-05 18400 2.67E-05 17800 3.13E-05
18700 8.23E-05 16700 6.79E-06 16500 1.54E-05 16100
2.48E-05 18400 2.67E-05 17800 3.13E-05
18700 8.24E-05 16700 6.78E-06 16500 1.54E-05 16100
2.48E-05 18400 2.67E-05 17800 3.13E-05
18700 8.25E-05 16700 6.79E-06 16500 1.54E-05 16100
2.48E-05 18400 2.67E-05 17800 3.13E-05
18700 8.25E-05 16700 6.79E-06 16500 1.54E-05 16100
2.49E-05 18400 2.67E-05 17800 3.13E-05
18700 8.26E-05 16700 6.79E-06 16500 1.54E-05 16100
2.49E-05 18400 2.67E-05 17800 3.14E-05
18700 8.27E-05 16700 6.79E-06 16500 1.54E-05 16100
2.49E-05 18400 2.67E-05 17800 3.14E-05
18700 8.27E-05 16700 6.79E-06 16500 1.54E-05 16100
2.49E-05 18400 2.67E-05 17800 3.14E-05
18700 8.28E-05 16700 6.80E-06 16500 1.54E-05 16100
2.49E-05 18400 2.67E-05 17800 3.14E-05
18700 8.29E-05 16700 6.80E-06 16500 1.54E-05 16100
2.50E-05 18400 2.67E-05 17800 3.14E-05
18700 8.30E-05 16700 6.79E-06 16500 1.54E-05 16100
2.50E-05 18400 2.67E-05 17800 3.14E-05
18700 8.30E-05 16700 6.79E-06 16500 1.54E-05 16100
2.50E-05 18400 2.67E-05 17800 3.14E-05
18800 8.31E-05 16700 6.79E-06 16500 1.54E-05 16100
2.50E-05 18400 2.67E-05 17800 3.15E-05
18800 8.32E-05 16700 6.80E-06 16500 1.54E-05 16100
2.50E-05 18400 2.67E-05 17800 3.15E-05
18800 8.33E-05 16700 6.79E-06 16500 1.54E-05 16100
2.50E-05 18400 2.67E-05 17800 3.15E-05
18800 8.34E-05 16700 6.79E-06 16500 1.54E-05 16100
2.51E-05 18400 2.68E-05 17800 3.15E-05
18800 8.34E-05 16700 6.78E-06 16500 1.55E-05 16100
2.51E-05 18400 2.68E-05 17800 3.15E-05
18800 8.35E-05 16700 6.78E-06 16500 1.55E-05 16100
2.51E-05 18400 2.68E-05 17800 3.15E-05
18800 8.36E-05 16700 6.79E-06 16500 1.55E-05 16100
2.52E-05 18400 2.68E-05 17800 3.15E-05
18800 8.36E-05 16700 6.78E-06 16500 1.55E-05 16100
2.52E-05 18400 2.68E-05 17800 3.15E-05
18800 8.37E-05 16700 6.79E-06 16500 1.55E-05 16100
2.52E-05 18400 2.68E-05 17800 3.15E-05
18800 8.38E-05 16700 6.79E-06 16500 1.55E-05 16100
2.52E-05 18400 2.68E-05 17900 3.15E-05
18800 8.39E-05 16700 6.80E-06 16500 1.55E-05 16100
2.52E-05 18400 2.68E-05 17900 3.15E-05
18800 8.40E-05 16700 6.79E-06 16500 1.55E-05 16100
2.53E-05 18400 2.68E-05 17900 3.16E-05
18800 8.40E-05 16700 6.79E-06 16500 1.55E-05 16100
2.53E-05 18400 2.68E-05 17900 3.16E-05
18800 8.41E-05 16700 6.79E-06 16500 1.55E-05 16100
2.53E-05 18400 2.68E-05 17900 3.16E-05
18800 8.42E-05 16700 6.78E-06 16500 1.55E-05 16100
2.53E-05 18400 2.68E-05 17900 3.16E-05
18800 8.43E-05 16700 6.79E-06 16500 1.55E-05 16100
2.53E-05 18400 2.68E-05 17900 3.16E-05
18800 8.43E-05 16700 6.79E-06 16500 1.55E-05 16100
2.54E-05 18400 2.68E-05 17900 3.16E-05

18800 8.44E-05 16700 6.79E-06 16500 1.56E-05 16100
2.54E-05 18400 2.68E-05 17900 3.16E-05
18800 8.45E-05 16800 6.79E-06 16500 1.56E-05 16100
2.54E-05 18400 2.69E-05 17900 3.16E-05
18800 8.45E-05 16800 6.78E-06 16500 1.56E-05 16100
2.54E-05 18400 2.69E-05 17900 3.16E-05
18800 8.46E-05 16800 6.79E-06 16600 1.56E-05 16100
2.55E-05 18400 2.69E-05 17900 3.16E-05
18800 8.47E-05 16800 6.78E-06 16600 1.56E-05 16100
2.55E-05 18400 2.69E-05 17900 3.17E-05
18800 8.48E-05 16800 6.78E-06 16600 1.56E-05 16100
2.55E-05 18400 2.69E-05 17900 3.17E-05
18800 8.49E-05 16800 6.77E-06 16600 1.56E-05 16100
2.55E-05 18400 2.69E-05 17900 3.17E-05
18800 8.50E-05 16800 6.77E-06 16600 1.56E-05 16200
2.56E-05 18400 2.69E-05 17900 3.17E-05
18800 8.50E-05 16800 6.78E-06 16600 1.56E-05 16200
2.56E-05 18400 2.69E-05 17900 3.17E-05
18800 8.51E-05 16800 6.78E-06 16600 1.56E-05 16200
2.56E-05 18400 2.69E-05 17900 3.17E-05
18800 8.52E-05 16800 6.78E-06 16600 1.56E-05 16200
2.56E-05 18400 2.70E-05 17900 3.17E-05
18800 8.53E-05 16800 6.78E-06 16600 1.56E-05 16200
2.56E-05 18400 2.70E-05 17900 3.17E-05
18800 8.53E-05 16800 6.79E-06 16600 1.56E-05 16200
2.57E-05 18400 2.70E-05 17900 3.17E-05
18800 8.54E-05 16800 6.78E-06 16600 1.56E-05 16200
2.57E-05 18400 2.70E-05 17900 3.17E-05
18800 8.55E-05 16800 6.79E-06 16600 1.56E-05 16200
2.57E-05 18400 2.70E-05 17900 3.17E-05
18800 8.56E-05 16800 6.79E-06 16600 1.56E-05 16200
2.57E-05 18400 2.70E-05 17900 3.18E-05
18800 8.57E-05 16800 6.78E-06 16600 1.56E-05 16200
2.57E-05 18400 2.70E-05 17900 3.18E-05
18800 8.58E-05 16800 6.79E-06 16600 1.56E-05 16200
2.57E-05 18400 2.70E-05 17900 3.18E-05
18800 8.59E-05 16800 6.80E-06 16600 1.56E-05 16200
2.58E-05 18500 2.70E-05 17900 3.18E-05
18800 8.59E-05 16800 6.81E-06 16600 1.57E-05 16200
2.58E-05 18500 2.70E-05 17900 3.18E-05
18800 8.60E-05 16800 6.80E-06 16600 1.57E-05 16200
2.58E-05 18500 2.70E-05 17900 3.18E-05
18800 8.61E-05 16800 6.81E-06 16600 1.57E-05 16200
2.59E-05 18500 2.70E-05 17900 3.18E-05
18800 8.62E-05 16800 6.81E-06 16600 1.57E-05 16200
2.59E-05 18500 2.70E-05 17900 3.19E-05
18800 8.63E-05 16800 6.80E-06 16600 1.57E-05 16200
2.59E-05 18500 2.70E-05 17900 3.19E-05
18800 8.64E-05 16800 6.81E-06 16600 1.57E-05 16200
2.60E-05 18500 2.71E-05 17900 3.19E-05
18800 8.65E-05 16800 6.80E-06 16600 1.57E-05 16200
2.60E-05 18500 2.71E-05 17900 3.19E-05
18800 8.66E-05 16800 6.80E-06 16600 1.57E-05 16200
2.60E-05 18500 2.71E-05 17900 3.19E-05
18800 8.67E-05 16800 6.82E-06 16600 1.57E-05 16200
2.61E-05 18500 2.71E-05 17900 3.19E-05
18800 8.68E-05 16800 6.81E-06 16600 1.57E-05 16200
2.61E-05 18500 2.71E-05 17900 3.19E-05
18800 8.69E-05 16800 6.81E-06 16600 1.57E-05 16200
2.61E-05 18500 2.71E-05 17900 3.19E-05
18800 8.70E-05 16800 6.81E-06 16600 1.57E-05 16200
2.61E-05 18500 2.71E-05 17900 3.20E-05
18800 8.71E-05 16800 6.82E-06 16600 1.57E-05 16200
2.62E-05 18500 2.71E-05 17900 3.20E-05
18800 8.72E-05 16800 6.82E-06 16600 1.57E-05 16200
2.62E-05 18500 2.71E-05 17900 3.20E-05
18800 8.72E-05 16800 6.81E-06 16600 1.57E-05 16200
2.62E-05 18500 2.71E-05 17900 3.20E-05

18800	8.73E-05	16800	6.81E-06	16600	1.57E-05	16200
2.63E-05	18500	2.71E-05	17900	3.20E-05		
18800	8.74E-05	16800	6.81E-06	16600	1.57E-05	16200
2.63E-05	18500	2.72E-05	17900	3.20E-05		
18800	8.75E-05	16800	6.81E-06	16600	1.57E-05	16200
2.63E-05	18500	2.72E-05	17900	3.20E-05		
18800	8.76E-05	16800	6.81E-06	16600	1.57E-05	16200
2.63E-05	18500	2.72E-05	17900	3.20E-05		
18800	8.77E-05	16800	6.82E-06	16600	1.57E-05	16200
2.64E-05	18500	2.72E-05	17900	3.20E-05		
18800	8.78E-05	16800	6.81E-06	16600	1.57E-05	16200
2.64E-05	18500	2.72E-05	17900	3.21E-05		
18800	8.79E-05	16800	6.81E-06	16600	1.58E-05	16200
2.65E-05	18500	2.72E-05	17900	3.21E-05		
18800	8.80E-05	16800	6.81E-06	16600	1.58E-05	16200
2.65E-05	18500	2.72E-05	17900	3.21E-05		
18800	8.81E-05	16800	6.81E-06	16600	1.58E-05	16200
2.65E-05	18500	2.72E-05	17900	3.21E-05		
18800	8.82E-05	16800	6.82E-06	16600	1.58E-05	16200
2.65E-05	18500	2.72E-05	17900	3.21E-05		
18800	8.83E-05	16800	6.81E-06	16600	1.58E-05	16200
2.66E-05	18500	2.72E-05	17900	3.21E-05		
18800	8.84E-05	16800	6.81E-06	16600	1.58E-05	16200
2.66E-05	18500	2.72E-05	17900	3.21E-05		
18800	8.85E-05	16800	6.82E-06	16600	1.58E-05	16200
2.66E-05	18500	2.72E-05	17900	3.21E-05		
18900	8.87E-05	16800	6.82E-06	16600	1.58E-05	16200
2.66E-05	18500	2.73E-05	17900	3.21E-05		
18900	8.88E-05	16800	6.83E-06	16600	1.58E-05	16200
2.67E-05	18500	2.73E-05	17900	3.21E-05		
18900	8.88E-05	16800	6.83E-06	16600	1.58E-05	16200
2.67E-05	18500	2.73E-05	17900	3.21E-05		
18900	8.89E-05	16800	6.83E-06	16600	1.58E-05	16200
2.67E-05	18500	2.73E-05	17900	3.22E-05		
18900	8.91E-05	16800	6.84E-06	16600	1.58E-05	16200
2.67E-05	18500	2.73E-05	17900	3.22E-05		
18900	8.92E-05	16800	6.85E-06	16600	1.58E-05	16200
2.68E-05	18500	2.73E-05	17900	3.22E-05		
18900	8.93E-05	16800	6.85E-06	16600	1.58E-05	16200
2.68E-05	18500	2.73E-05	17900	3.22E-05		
18900	8.94E-05	16800	6.85E-06	16600	1.58E-05	16200
2.68E-05	18500	2.73E-05	17900	3.22E-05		
18900	8.95E-05	16800	6.86E-06	16600	1.58E-05	16200
2.68E-05	18500	2.73E-05	17900	3.22E-05		
18900	8.96E-05	16800	6.86E-06	16600	1.58E-05	16200
2.69E-05	18500	2.73E-05	18000	3.22E-05		
18900	8.97E-05	16800	6.86E-06	16600	1.58E-05	16200
2.69E-05	18500	2.73E-05	18000	3.22E-05		
18900	8.98E-05	16800	6.86E-06	16600	1.58E-05	16200
2.70E-05	18500	2.73E-05	18000	3.23E-05		
18900	8.99E-05	16800	6.86E-06	16600	1.58E-05	16200
2.70E-05	18500	2.74E-05	18000	3.23E-05		
18900	9.00E-05	16800	6.86E-06	16600	1.59E-05	16200
2.70E-05	18500	2.74E-05	18000	3.23E-05		
18900	9.01E-05	16800	6.87E-06	16600	1.58E-05	16200
2.71E-05	18500	2.74E-05	18000	3.23E-05		
18900	9.03E-05	16800	6.87E-06	16600	1.59E-05	16200
2.71E-05	18500	2.74E-05	18000	3.23E-05		
18900	9.04E-05	16800	6.87E-06	16600	1.59E-05	16200
2.71E-05	18500	2.74E-05	18000	3.23E-05		
18900	9.05E-05	16800	6.87E-06	16600	1.59E-05	16200
2.72E-05	18500	2.74E-05	18000	3.23E-05		
18900	9.06E-05	16900	6.88E-06	16600	1.59E-05	16200
2.72E-05	18500	2.74E-05	18000	3.23E-05		
18900	9.07E-05	16900	6.88E-06	16600	1.59E-05	16200
2.72E-05	18500	2.75E-05	18000	3.23E-05		
18900	9.08E-05	16900	6.88E-06	16700	1.59E-05	16200
2.73E-05	18500	2.75E-05	18000	3.24E-05		

18900 9.09E-05 16900 6.89E-06 16700 1.59E-05 16200
2.73E-05 18500 2.75E-05 18000 3.24E-05
18900 9.11E-05 16900 6.91E-06 16700 1.59E-05 16200
2.73E-05 18500 2.75E-05 18000 3.24E-05
18900 9.12E-05 16900 6.91E-06 16700 1.59E-05 16200
2.74E-05 18500 2.75E-05 18000 3.24E-05
18900 9.13E-05 16900 6.91E-06 16700 1.59E-05 16300
2.74E-05 18500 2.75E-05 18000 3.24E-05
18900 9.14E-05 16900 6.91E-06 16700 1.59E-05 16300
2.74E-05 18500 2.75E-05 18000 3.24E-05
18900 9.15E-05 16900 6.92E-06 16700 1.60E-05 16300
2.75E-05 18500 2.75E-05 18000 3.24E-05
18900 9.17E-05 16900 6.92E-06 16700 1.60E-05 16300
2.75E-05 18500 2.75E-05 18000 3.24E-05
18900 9.18E-05 16900 6.92E-06 16700 1.60E-05 16300
2.75E-05 18500 2.75E-05 18000 3.24E-05
18900 9.19E-05 16900 6.93E-06 16700 1.60E-05 16300
2.76E-05 18500 2.75E-05 18000 3.25E-05
18900 9.20E-05 16900 6.92E-06 16700 1.60E-05 16300
2.76E-05 18500 2.75E-05 18000 3.25E-05
18900 9.22E-05 16900 6.91E-06 16700 1.60E-05 16300
2.76E-05 18500 2.75E-05 18000 3.25E-05
18900 9.23E-05 16900 6.91E-06 16700 1.60E-05 16300
2.77E-05 18500 2.75E-05 18000 3.25E-05
18900 9.24E-05 16900 6.92E-06 16700 1.60E-05 16300
2.77E-05 18500 2.76E-05 18000 3.25E-05
18900 9.25E-05 16900 6.92E-06 16700 1.60E-05 16300
2.78E-05 18500 2.76E-05 18000 3.25E-05
18900 9.27E-05 16900 6.93E-06 16700 1.60E-05 16300
2.78E-05 18600 2.76E-05 18000 3.25E-05
18900 9.28E-05 16900 6.91E-06 16700 1.60E-05 16300
2.79E-05 18600 2.76E-05 18000 3.25E-05
18900 9.29E-05 16900 6.92E-06 16700 1.61E-05 16300
2.79E-05 18600 2.76E-05 18000 3.26E-05
18900 9.31E-05 16900 6.91E-06 16700 1.61E-05 16300
2.79E-05 18600 2.76E-05 18000 3.26E-05
18900 9.32E-05 16900 6.92E-06 16700 1.61E-05 16300
2.80E-05 18600 2.76E-05 18000 3.26E-05
18900 9.33E-05 16900 6.91E-06 16700 1.61E-05 16300
2.80E-05 18600 2.76E-05 18000 3.26E-05
18900 9.34E-05 16900 6.92E-06 16700 1.61E-05 16300
2.80E-05 18600 2.76E-05 18000 3.26E-05
18900 9.36E-05 16900 6.91E-06 16700 1.61E-05 16300
2.81E-05 18600 2.76E-05 18000 3.26E-05
18900 9.37E-05 16900 6.91E-06 16700 1.61E-05 16300
2.81E-05 18600 2.76E-05 18000 3.26E-05
18900 9.38E-05 16900 6.91E-06 16700 1.61E-05 16300
2.81E-05 18600 2.76E-05 18000 3.26E-05
18900 9.39E-05 16900 6.90E-06 16700 1.61E-05 16300
2.82E-05 18600 2.77E-05 18000 3.26E-05
18900 9.41E-05 16900 6.90E-06 16700 1.61E-05 16300
2.82E-05 18600 2.77E-05 18000 3.26E-05
18900 9.42E-05 16900 6.90E-06 16700 1.61E-05 16300
2.82E-05 18600 2.77E-05 18000 3.26E-05
18900 9.43E-05 16900 6.89E-06 16700 1.62E-05 16300
2.83E-05 18600 2.77E-05 18000 3.27E-05
18900 9.45E-05 16900 6.88E-06 16700 1.62E-05 16300
2.83E-05 18600 2.77E-05 18000 3.27E-05
18900 9.46E-05 16900 6.87E-06 16700 1.62E-05 16300
2.84E-05 18600 2.77E-05 18000 3.27E-05
18900 9.47E-05 16900 6.87E-06 16700 1.62E-05 16300
2.84E-05 18600 2.77E-05 18000 3.27E-05
18900 9.49E-05 16900 6.87E-06 16700 1.62E-05 16300
2.85E-05 18600 2.77E-05 18000 3.27E-05
18900 9.50E-05 16900 6.87E-06 16700 1.62E-05 16300
2.86E-05 18600 2.77E-05 18000 3.27E-05
18900 9.52E-05 16900 6.87E-06 16700 1.62E-05 16300
2.86E-05 18600 2.77E-05 18000 3.27E-05

18900 9.53E-05 16900 6.86E-06 16700 1.62E-05 16300
2.87E-05 18600 2.77E-05 18000 3.27E-05
18900 9.54E-05 16900 6.86E-06 16700 1.62E-05 16300
2.87E-05 18600 2.78E-05 18000 3.27E-05
18900 9.56E-05 16900 6.87E-06 16700 1.62E-05 16300
2.88E-05 18600 2.77E-05 18000 3.27E-05
18900 9.57E-05 16900 6.86E-06 16700 1.62E-05 16300
2.88E-05 18600 2.78E-05 18000 3.28E-05
18900 9.59E-05 16900 6.86E-06 16700 1.62E-05 16300
2.88E-05 18600 2.78E-05 18000 3.28E-05
18900 9.60E-05 16900 6.86E-06 16700 1.62E-05 16300
2.89E-05 18600 2.78E-05 18000 3.28E-05
18900 9.62E-05 16900 6.87E-06 16700 1.63E-05 16300
2.90E-05 18600 2.78E-05 18000 3.28E-05
18900 9.63E-05 16900 6.87E-06 16700 1.63E-05 16300
2.90E-05 18600 2.78E-05 18000 3.28E-05
18900 9.65E-05 16900 6.87E-06 16700 1.63E-05 16300
2.91E-05 18600 2.78E-05 18000 3.28E-05
19000 9.66E-05 16900 6.86E-06 16700 1.63E-05 16300
2.91E-05 18600 2.78E-05 18000 3.28E-05
19000 9.68E-05 16900 6.87E-06 16700 1.63E-05 16300
2.91E-05 18600 2.78E-05 18000 3.28E-05
19000 9.69E-05 16900 6.88E-06 16700 1.63E-05 16300
2.92E-05 18600 2.78E-05 18000 3.28E-05
19000 9.71E-05 16900 6.87E-06 16700 1.63E-05 16300
2.92E-05 18600 2.78E-05 18000 3.28E-05
19000 9.72E-05 16900 6.87E-06 16700 1.63E-05 16300
2.93E-05 18600 2.78E-05 18000 3.29E-05
19000 9.74E-05 16900 6.87E-06 16700 1.63E-05 16300
2.93E-05 18600 2.78E-05 18000 3.29E-05
19000 9.76E-05 16900 6.88E-06 16700 1.63E-05 16300
2.94E-05 18600 2.78E-05 18000 3.29E-05
19000 9.77E-05 16900 6.87E-06 16700 1.63E-05 16300
2.94E-05 18600 2.79E-05 18000 3.29E-05
19000 9.79E-05 16900 6.87E-06 16700 1.63E-05 16300
2.95E-05 18600 2.78E-05 18000 3.29E-05
19000 9.80E-05 16900 6.88E-06 16700 1.64E-05 16300
2.95E-05 18600 2.79E-05 18100 3.29E-05
19000 9.83E-05 16900 6.87E-06 16700 1.64E-05 16300
2.96E-05 18600 2.79E-05 18100 3.29E-05
19000 9.84E-05 16900 6.87E-06 16700 1.64E-05 16300
2.96E-05 18600 2.79E-05 18100 3.29E-05
19000 9.86E-05 16900 6.87E-06 16700 1.64E-05 16300
2.97E-05 18600 2.79E-05 18100 3.29E-05
19000 9.87E-05 16900 6.88E-06 16700 1.64E-05 16300
2.97E-05 18600 2.79E-05 18100 3.30E-05
19000 9.89E-05 16900 6.87E-06 16700 1.64E-05 16300
2.97E-05 18600 2.79E-05 18100 3.30E-05
19000 9.90E-05 16900 6.88E-06 16700 1.64E-05 16300
2.98E-05 18600 2.79E-05 18100 3.30E-05
19000 9.92E-05 16900 6.88E-06 16700 1.65E-05 16300
2.99E-05 18600 2.79E-05 18100 3.30E-05
19000 9.94E-05 16900 6.89E-06 16700 1.65E-05 16300
2.99E-05 18600 2.79E-05 18100 3.30E-05
19000 9.96E-05 17000 6.89E-06 16700 1.65E-05 16300
3.00E-05 18600 2.79E-05 18100 3.30E-05
19000 9.97E-05 17000 6.90E-06 16700 1.65E-05 16300
3.00E-05 18600 2.80E-05 18100 3.30E-05
19000 9.99E-05 17000 6.90E-06 16800 1.65E-05 16300
3.00E-05 18600 2.80E-05 18100 3.30E-05
19000 0.0001 17000 6.89E-06 16800 1.65E-05 16300 3.01E-05
18600 2.80E-05 18100 3.30E-05
19000 0.0001 17000 6.91E-06 16800 1.65E-05 16300 3.02E-05
18600 2.80E-05 18100 3.30E-05
19000 0.0001 17000 6.90E-06 16800 1.65E-05 16300 3.02E-05
18600 2.80E-05 18100 3.31E-05
19000 0.000101 17000 6.89E-06 16800 1.65E-05 16400
3.03E-05 18600 2.80E-05 18100 3.31E-05

19000 0.000101 17000 6.89E-06 16800 1.65E-05 16400
3.04E-05 18600 2.80E-05 18100 3.31E-05
19000 0.000101 17000 6.89E-06 16800 1.65E-05 16400
3.04E-05 18600 2.80E-05 18100 3.31E-05
19000 0.000101 17000 6.90E-06 16800 1.65E-05 16400
3.05E-05 18600 2.80E-05 18100 3.31E-05
19000 0.000101 17000 6.88E-06 16800 1.65E-05 16400
3.05E-05 18600 2.80E-05 18100 3.31E-05
19000 0.000102 17000 6.87E-06 16800 1.65E-05 16400
3.06E-05 18600 2.81E-05 18100 3.31E-05
19000 0.000102 17000 6.88E-06 16800 1.65E-05 16400
3.06E-05 18600 2.81E-05 18100 3.31E-05
19000 0.000102 17000 6.88E-06 16800 1.66E-05 16400
3.07E-05 18600 2.81E-05 18100 3.31E-05
19000 0.000102 17000 6.88E-06 16800 1.66E-05 16400
3.07E-05 18600 2.81E-05 18100 3.32E-05
19000 0.000102 17000 6.88E-06 16800 1.66E-05 16400
3.08E-05 18600 2.81E-05 18100 3.32E-05
19000 0.000103 17000 6.89E-06 16800 1.66E-05 16400
3.09E-05 18600 2.81E-05 18100 3.32E-05
19000 0.000103 17000 6.89E-06 16800 1.66E-05 16400
3.09E-05 18700 2.81E-05 18100 3.32E-05
19000 0.000103 17000 6.89E-06 16800 1.66E-05 16400
3.10E-05 18700 2.81E-05 18100 3.32E-05
19000 0.000103 17000 6.90E-06 16800 1.66E-05 16400
3.10E-05 18700 2.81E-05 18100 3.32E-05
19000 0.000103 17000 6.89E-06 16800 1.66E-05 16400
3.10E-05 18700 2.82E-05 18100 3.32E-05
19000 0.000104 17000 6.90E-06 16800 1.66E-05 16400
3.11E-05 18700 2.82E-05 18100 3.33E-05
19000 0.000104 17000 6.89E-06 16800 1.67E-05 16400
3.11E-05 18700 2.82E-05 18100 3.33E-05
19000 0.000104 17000 6.90E-06 16800 1.67E-05 16400
3.12E-05 18700 2.82E-05 18100 3.33E-05
19000 0.000104 17000 6.89E-06 16800 1.67E-05 16400
3.12E-05 18700 2.82E-05 18100 3.33E-05
19000 0.000104 17000 6.89E-06 16800 1.67E-05 16400
3.13E-05 18700 2.82E-05 18100 3.33E-05
19000 0.000105 17000 6.89E-06 16800 1.67E-05 16400
3.13E-05 18700 2.82E-05 18100 3.33E-05
19000 0.000105 17000 6.89E-06 16800 1.67E-05 16400
3.14E-05 18700 2.82E-05 18100 3.34E-05
19000 0.000105 17000 6.89E-06 16800 1.67E-05 16400
3.15E-05 18700 2.82E-05 18100 3.34E-05
19000 0.000105 17000 6.88E-06 16800 1.67E-05 16400
3.15E-05 18700 2.82E-05 18100 3.34E-05
19000 0.000106 17000 6.89E-06 16800 1.67E-05 16400
3.16E-05 18700 2.83E-05 18100 3.34E-05
19000 0.000106 17000 6.88E-06 16800 1.67E-05 16400
3.16E-05 18700 2.83E-05 18100 3.34E-05
19000 0.000106 17000 6.89E-06 16800 1.68E-05 16400
3.16E-05 18700 2.83E-05 18100 3.34E-05
19000 0.000106 17000 6.88E-06 16800 1.68E-05 16400
3.17E-05 18700 2.83E-05 18100 3.34E-05
19000 0.000106 17000 6.88E-06 16800 1.68E-05 16400
3.17E-05 18700 2.83E-05 18100 3.34E-05
19000 0.000107 17000 6.89E-06 16800 1.68E-05 16400
3.18E-05 18700 2.83E-05 18100 3.35E-05
19000 0.000107 17000 6.89E-06 16800 1.68E-05 16400
3.19E-05 18700 2.83E-05 18100 3.35E-05
19000 0.000107 17000 6.89E-06 16800 1.68E-05 16400
3.19E-05 18700 2.83E-05 18100 3.35E-05
19000 0.000107 17000 6.90E-06 16800 1.68E-05 16400
3.20E-05 18700 2.83E-05 18100 3.35E-05
19000 0.000108 17000 6.90E-06 16800 1.69E-05 16400
3.21E-05 18700 2.83E-05 18100 3.35E-05
19000 0.000108 17000 6.90E-06 16800 1.69E-05 16400
3.21E-05 18700 2.84E-05 18100 3.35E-05

19000 0.000108 17000 6.90E-06 16800 1.69E-05 16400
3.22E-05 18700 2.84E-05 18100 3.35E-05
19000 0.000108 17000 6.90E-06 16800 1.69E-05 16400
3.23E-05 18700 2.84E-05 18100 3.35E-05
19000 0.000109 17000 6.90E-06 16800 1.69E-05 16400
3.23E-05 18700 2.84E-05 18100 3.35E-05
19000 0.000109 17000 6.88E-06 16800 1.69E-05 16400
3.24E-05 18700 2.84E-05 18100 3.36E-05
19000 0.000109 17000 6.88E-06 16800 1.69E-05 16400
3.25E-05 18700 2.84E-05 18100 3.35E-05
19100 0.000109 17000 6.89E-06 16800 1.70E-05 16400
3.26E-05 18700 2.84E-05 18100 3.36E-05
19100 0.00011 17000 6.88E-06 16800 1.70E-05 16400 3.27E-05
18700 2.84E-05 18100 3.36E-05
19100 0.00011 17000 6.89E-06 16800 1.70E-05 16400 3.27E-05
18700 2.85E-05 18100 3.36E-05
19100 0.00011 17000 6.88E-06 16800 1.70E-05 16400 3.28E-05
18700 2.85E-05 18100 3.36E-05
19100 0.000111 17000 6.87E-06 16800 1.70E-05 16400
3.28E-05 18700 2.85E-05 18100 3.36E-05
19100 0.000111 17000 6.88E-06 16800 1.70E-05 16400
3.29E-05 18700 2.85E-05 18100 3.36E-05
19100 0.000111 17000 6.88E-06 16800 1.71E-05 16400
3.30E-05 18700 2.85E-05 18100 3.36E-05
19100 0.000111 17000 6.87E-06 16800 1.71E-05 16400
3.30E-05 18700 2.85E-05 18100 3.36E-05
19100 0.000112 17000 6.87E-06 16800 1.71E-05 16400
3.31E-05 18700 2.85E-05 18100 3.37E-05
19100 0.000112 17000 6.89E-06 16800 1.71E-05 16400
3.32E-05 18700 2.85E-05 18200 3.37E-05
19100 0.000112 17000 6.89E-06 16800 1.71E-05 16400
3.33E-05 18700 2.85E-05 18200 3.37E-05
19100 0.000113 17000 6.89E-06 16800 1.71E-05 16400
3.33E-05 18700 2.86E-05 18200 3.37E-05
19100 0.000113 17000 6.90E-06 16800 1.72E-05 16400
3.34E-05 18700 2.86E-05 18200 3.37E-05
19100 0.000113 17000 6.90E-06 16800 1.72E-05 16400
3.35E-05 18700 2.86E-05 18200 3.37E-05
19100 0.000113 17000 6.89E-06 16800 1.72E-05 16400
3.35E-05 18700 2.86E-05 18200 3.37E-05
19100 0.000114 17000 6.90E-06 16800 1.72E-05 16400
3.36E-05 18700 2.86E-05 18200 3.37E-05
19100 0.000114 17000 6.89E-06 16800 1.72E-05 16400
3.37E-05 18700 2.86E-05 18200 3.38E-05
19100 0.000114 17000 6.88E-06 16800 1.72E-05 16400
3.38E-05 18700 2.86E-05 18200 3.38E-05
19100 0.000115 17100 6.88E-06 16800 1.73E-05 16400
3.38E-05 18700 2.86E-05 18200 3.38E-05
19100 0.000115 17100 6.90E-06 16800 1.73E-05 16400
3.39E-05 18700 2.86E-05 18200 3.38E-05
19100 0.000115 17100 6.89E-06 16900 1.73E-05 16400
3.40E-05 18700 2.86E-05 18200 3.38E-05
19100 0.000116 17100 6.91E-06 16900 1.73E-05 16400
3.41E-05 18700 2.87E-05 18200 3.38E-05
19100 0.000116 17100 6.88E-06 16900 1.73E-05 16400
3.42E-05 18700 2.87E-05 18200 3.38E-05
19100 0.000116 17100 6.89E-06 16900 1.73E-05 16400
3.43E-05 18700 2.87E-05 18200 3.38E-05
19100 0.000117 17100 6.89E-06 16900 1.73E-05 16500
3.44E-05 18700 2.87E-05 18200 3.39E-05
19100 0.000117 17100 6.89E-06 16900 1.74E-05 16500
3.44E-05 18700 2.87E-05 18200 3.39E-05
19100 0.000117 17100 6.90E-06 16900 1.74E-05 16500
3.45E-05 18700 2.87E-05 18200 3.39E-05
19100 0.000118 17100 6.91E-06 16900 1.74E-05 16500
3.46E-05 18700 2.87E-05 18200 3.39E-05
19100 0.000118 17100 6.90E-06 16900 1.74E-05 16500
3.47E-05 18700 2.87E-05 18200 3.39E-05

19100 0.000118 17100 6.90E-06 16900 1.75E-05 16500
3.48E-05 18700 2.88E-05 18200 3.39E-05
19100 0.000119 17100 6.92E-06 16900 1.75E-05 16500
3.49E-05 18700 2.87E-05 18200 3.39E-05
19100 0.000119 17100 6.92E-06 16900 1.75E-05 16500
3.49E-05 18700 2.88E-05 18200 3.39E-05
19100 0.00012 17100 6.92E-06 16900 1.75E-05 16500 3.50E-05
18700 2.88E-05 18200 3.39E-05
19100 0.00012 17100 6.93E-06 16900 1.75E-05 16500 3.51E-05
18700 2.88E-05 18200 3.39E-05
19100 0.00012 17100 6.93E-06 16900 1.75E-05 16500 3.52E-05
18700 2.88E-05 18200 3.39E-05
19100 0.000121 17100 6.94E-06 16900 1.76E-05 16500
3.53E-05 18800 2.88E-05 18200 3.40E-05
19100 0.000121 17100 6.91E-06 16900 1.76E-05 16500
3.54E-05 18800 2.88E-05 18200 3.40E-05
19100 0.000121 17100 6.90E-06 16900 1.76E-05 16500
3.54E-05 18800 2.88E-05 18200 3.40E-05
19100 0.000122 17100 6.89E-06 16900 1.76E-05 16500
3.55E-05 18800 2.88E-05 18200 3.40E-05
19100 0.000122 17100 6.92E-06 16900 1.76E-05 16500
3.56E-05 18800 2.89E-05 18200 3.40E-05
19100 0.000123 17100 6.93E-06 16900 1.77E-05 16500
3.57E-05 18800 2.89E-05 18200 3.40E-05
19100 0.000123 17100 6.90E-06 16900 1.77E-05 16500
3.58E-05 18800 2.89E-05 18200 3.40E-05
19100 0.000124 17100 6.89E-06 16900 1.77E-05 16500
3.59E-05 18800 2.89E-05 18200 3.41E-05
19100 0.000124 17100 6.91E-06 16900 1.78E-05 16500
3.59E-05 18800 2.89E-05 18200 3.41E-05
19100 0.000124 17100 6.90E-06 16900 1.78E-05 16500
3.60E-05 18800 2.89E-05 18200 3.41E-05
19100 0.000125 17100 6.90E-06 16900 1.78E-05 16500
3.61E-05 18800 2.89E-05 18200 3.41E-05
19100 0.000125 17100 6.91E-06 16900 1.78E-05 16500
3.62E-05 18800 2.89E-05 18200 3.41E-05
19100 0.000126 17100 6.91E-06 16900 1.78E-05 16500
3.63E-05 18800 2.89E-05 18200 3.41E-05
19100 0.000126 17100 6.93E-06 16900 1.79E-05 16500
3.64E-05 18800 2.89E-05 18200 3.41E-05
19100 0.000127 17100 6.91E-06 16900 1.79E-05 16500
3.65E-05 18800 2.90E-05 18200 3.41E-05
19100 0.000127 17100 6.91E-06 16900 1.79E-05 16500
3.66E-05 18800 2.90E-05 18200 3.42E-05
19100 0.000128 17100 6.92E-06 16900 1.79E-05 16500
3.67E-05 18800 2.90E-05 18200 3.42E-05
19100 0.000128 17100 6.90E-06 16900 1.79E-05 16500
3.68E-05 18800 2.90E-05 18200 3.42E-05
19100 0.000128 17100 6.90E-06 16900 1.80E-05 16500
3.69E-05 18800 2.90E-05 18200 3.42E-05
19100 0.000129 17100 6.91E-06 16900 1.80E-05 16500
3.70E-05 18800 2.90E-05 18200 3.42E-05
19100 0.000129 17100 6.90E-06 16900 1.80E-05 16500
3.71E-05 18800 2.90E-05 18200 3.42E-05
19100 0.00013 17100 6.91E-06 16900 1.80E-05 16500 3.72E-05
18800 2.90E-05 18200 3.42E-05
19100 0.00013 17100 6.91E-06 16900 1.80E-05 16500 3.73E-05
18800 2.91E-05 18200 3.42E-05
19100 0.000131 17100 6.92E-06 16900 1.81E-05 16500
3.74E-05 18800 2.91E-05 18200 3.43E-05
19100 0.000131 17100 6.93E-06 16900 1.81E-05 16500
3.75E-05 18800 2.91E-05 18200 3.43E-05
19100 0.000132 17100 6.92E-06 16900 1.81E-05 16500
3.76E-05 18800 2.91E-05 18200 3.43E-05
19100 0.000133 17100 6.91E-06 16900 1.81E-05 16500
3.78E-05 18800 2.91E-05 18200 3.43E-05
19100 0.000133 17100 6.94E-06 16900 1.81E-05 16500
3.79E-05 18800 2.91E-05 18200 3.43E-05

19100 0.000134 17100 6.93E-06 16900 1.82E-05 16500
3.80E-05 18800 2.91E-05 18200 3.43E-05
19200 0.000134 17100 6.92E-06 16900 1.82E-05 16500
3.81E-05 18800 2.92E-05 18200 3.43E-05
19200 0.000135 17100 6.94E-06 16900 1.82E-05 16500
3.82E-05 18800 2.92E-05 18200 3.43E-05
19200 0.000135 17100 6.93E-06 16900 1.82E-05 16500
3.83E-05 18800 2.92E-05 18200 3.44E-05
19200 0.000136 17100 6.93E-06 16900 1.83E-05 16500
3.84E-05 18800 2.92E-05 18200 3.44E-05
19200 0.000136 17100 6.94E-06 16900 1.83E-05 16500
3.86E-05 18800 2.92E-05 18200 3.44E-05
19200 0.000137 17100 6.93E-06 16900 1.83E-05 16500
3.87E-05 18800 2.92E-05 18200 3.44E-05
19200 0.000138 17100 6.93E-06 16900 1.84E-05 16500
3.88E-05 18800 2.92E-05 18200 3.44E-05
19200 0.000138 17100 6.93E-06 16900 1.84E-05 16500
3.89E-05 18800 2.92E-05 18200 3.44E-05
19200 0.000139 17100 6.93E-06 16900 1.84E-05 16500
3.90E-05 18800 2.93E-05 18200 3.44E-05
19200 0.000139 17100 6.92E-06 16900 1.84E-05 16500
3.91E-05 18800 2.93E-05 18300 3.44E-05
19200 0.00014 17100 6.93E-06 16900 1.84E-05 16500 3.92E-05
18800 2.93E-05 18300 3.45E-05
19200 0.000141 17100 6.93E-06 16900 1.85E-05 16500
3.93E-05 18800 2.93E-05 18300 3.45E-05
19200 0.000141 17100 6.93E-06 16900 1.85E-05 16500
3.94E-05 18800 2.93E-05 18300 3.45E-05
19200 0.000142 17100 6.94E-06 16900 1.85E-05 16500
3.96E-05 18800 2.93E-05 18300 3.45E-05
19200 0.000142 17100 6.94E-06 16900 1.85E-05 16500
3.97E-05 18800 2.93E-05 18300 3.45E-05
19200 0.000143 17100 6.94E-06 16900 1.86E-05 16500
3.98E-05 18800 2.93E-05 18300 3.45E-05
19200 0.000144 17100 6.92E-06 16900 1.86E-05 16500
3.99E-05 18800 2.93E-05 18300 3.45E-05
19200 0.000144 17100 6.93E-06 16900 1.86E-05 16500
4.00E-05 18800 2.94E-05 18300 3.45E-05
19200 0.000145 17200 6.92E-06 16900 1.87E-05 16500
4.01E-05 18800 2.94E-05 18300 3.45E-05
19200 0.000146 17200 6.92E-06 16900 1.87E-05 16500
4.03E-05 18800 2.94E-05 18300 3.46E-05
19200 0.000147 17200 6.93E-06 17000 1.87E-05 16500
4.04E-05 18800 2.94E-05 18300 3.46E-05
19200 0.000147 17200 6.94E-06 17000 1.88E-05 16500
4.05E-05 18800 2.94E-05 18300 3.46E-05
19200 0.000148 17200 6.94E-06 17000 1.88E-05 16500
4.07E-05 18800 2.94E-05 18300 3.46E-05
19200 0.000149 17200 6.95E-06 17000 1.88E-05 16500
4.08E-05 18800 2.94E-05 18300 3.46E-05
19200 0.000149 17200 6.95E-06 17000 1.89E-05 16600
4.09E-05 18800 2.94E-05 18300 3.46E-05
19200 0.00015 17200 6.94E-06 17000 1.89E-05 16600 4.11E-05
18800 2.94E-05 18300 3.46E-05
19200 0.000151 17200 6.92E-06 17000 1.89E-05 16600
4.12E-05 18800 2.95E-05 18300 3.46E-05
19200 0.000152 17200 6.91E-06 17000 1.89E-05 16600
4.14E-05 18800 2.95E-05 18300 3.46E-05
19200 0.000152 17200 6.91E-06 17000 1.90E-05 16600
4.15E-05 18800 2.95E-05 18300 3.47E-05
19200 0.000153 17200 6.90E-06 17000 1.90E-05 16600
4.16E-05 18800 2.95E-05 18300 3.47E-05
19200 0.000154 17200 6.91E-06 17000 1.90E-05 16600
4.18E-05 18800 2.95E-05 18300 3.47E-05
19200 0.000155 17200 6.91E-06 17000 1.90E-05 16600
4.19E-05 18800 2.95E-05 18300 3.47E-05
19200 0.000156 17200 6.90E-06 17000 1.91E-05 16600
4.21E-05 18800 2.96E-05 18300 3.47E-05

19200 0.000157 17200 6.91E-06 17000 1.91E-05 16600
4.22E-05 18800 2.96E-05 18300 3.47E-05
19200 0.000157 17200 6.91E-06 17000 1.91E-05 16600
4.23E-05 18800 2.96E-05 18300 3.47E-05
19200 0.000158 17200 6.93E-06 17000 1.92E-05 16600
4.25E-05 18900 2.96E-05 18300 3.48E-05
19200 0.000159 17200 6.90E-06 17000 1.92E-05 16600
4.26E-05 18900 2.96E-05 18300 3.48E-05
19200 0.00016 17200 6.90E-06 17000 1.93E-05 16600 4.28E-05
18900 2.96E-05 18300 3.48E-05
19200 0.000161 17200 6.92E-06 17000 1.93E-05 16600
4.29E-05 18900 2.96E-05 18300 3.48E-05
19200 0.000162 17200 6.92E-06 17000 1.93E-05 16600
4.30E-05 18900 2.96E-05 18300 3.48E-05
19200 0.000163 17200 6.90E-06 17000 1.94E-05 16600
4.32E-05 18900 2.96E-05 18300 3.48E-05
19200 0.000164 17200 6.93E-06 17000 1.94E-05 16600
4.33E-05 18900 2.97E-05 18300 3.48E-05
19200 0.000165 17200 6.94E-06 17000 1.94E-05 16600
4.35E-05 18900 2.97E-05 18300 3.49E-05
19200 0.000166 17200 6.96E-06 17000 1.95E-05 16600
4.36E-05 18900 2.97E-05 18300 3.49E-05
19200 0.000167 17200 6.93E-06 17000 1.95E-05 16600
4.37E-05 18900 2.97E-05 18300 3.49E-05
19200 0.000168 17200 6.91E-06 17000 1.95E-05 16600
4.39E-05 18900 2.97E-05 18300 3.49E-05
19200 0.000169 17200 6.87E-06 17000 1.96E-05 16600
4.40E-05 18900 2.97E-05 18300 3.49E-05
19200 0.00017 17200 6.89E-06 17000 1.96E-05 16600 4.42E-05
18900 2.97E-05 18300 3.49E-05
19200 0.000171 17200 6.88E-06 17000 1.96E-05 16600
4.43E-05 18900 2.97E-05 18300 3.49E-05
19200 0.000172 17200 6.90E-06 17000 1.97E-05 16600
4.45E-05 18900 2.97E-05 18300 3.49E-05
19200 0.000173 17200 6.91E-06 17000 1.97E-05 16600
4.46E-05 18900 2.98E-05 18300 3.50E-05
19200 0.000174 17200 6.90E-06 17000 1.97E-05 16600
4.48E-05 18900 2.98E-05 18300 3.50E-05
19200 0.000175 17200 6.91E-06 17000 1.98E-05 16600
4.50E-05 18900 2.98E-05 18300 3.50E-05
19200 0.000177 17200 6.91E-06 17000 1.98E-05 16600
4.51E-05 18900 2.98E-05 18300 3.50E-05
19200 0.000178 17200 6.91E-06 17000 1.99E-05 16600
4.53E-05 18900 2.98E-05 18300 3.50E-05
19200 0.000179 17200 6.92E-06 17000 1.99E-05 16600
4.54E-05 18900 2.98E-05 18300 3.50E-05
19200 0.00018 17200 6.90E-06 17000 2.00E-05 16600 4.56E-05
18900 2.99E-05 18300 3.50E-05
19200 0.000181 17200 6.91E-06 17000 2.00E-05 16600
4.57E-05 18900 2.99E-05 18300 3.50E-05
19200 0.000183 17200 6.91E-06 17000 2.00E-05 16600
4.59E-05 18900 2.99E-05 18300 3.50E-05
19200 0.000184 17200 6.92E-06 17000 2.01E-05 16600
4.61E-05 18900 2.99E-05 18300 3.51E-05
19200 0.000185 17200 6.92E-06 17000 2.01E-05 16600
4.62E-05 18900 2.99E-05 18300 3.51E-05
19200 0.000186 17200 6.92E-06 17000 2.02E-05 16600
4.64E-05 18900 2.99E-05 18300 3.51E-05
19200 0.000188 17200 6.92E-06 17000 2.02E-05 16600
4.65E-05 18900 2.99E-05 18300 3.51E-05
19200 0.000189 17200 6.93E-06 17000 2.02E-05 16600
4.67E-05 18900 2.99E-05 18300 3.51E-05
19300 0.00019 17200 6.91E-06 17000 2.03E-05 16600 4.69E-05
18900 3.00E-05 18300 3.51E-05
19300 0.000192 17200 6.93E-06 17000 2.03E-05 16600
4.70E-05 18900 3.00E-05 18300 3.51E-05
19300 0.000193 17200 6.92E-06 17000 2.04E-05 16600
4.72E-05 18900 3.00E-05 18300 3.51E-05

19300 0.000195 17200 6.92E-06 17000 2.04E-05 16600
4.74E-05 18900 3.00E-05 18300 3.51E-05
19300 0.000196 17200 6.93E-06 17000 2.05E-05 16600
4.75E-05 18900 3.00E-05 18300 3.52E-05
19300 0.000198 17200 6.94E-06 17000 2.05E-05 16600
4.78E-05 18900 3.00E-05 18300 3.52E-05
19300 0.000199 17200 6.95E-06 17000 2.05E-05 16600
4.79E-05 18900 3.00E-05 18300 3.52E-05
19300 0.000201 17200 6.96E-06 17000 2.06E-05 16600
4.81E-05 18900 3.01E-05 18300 3.52E-05
19300 0.000203 17200 6.97E-06 17000 2.07E-05 16600
4.83E-05 18900 3.01E-05 18300 3.52E-05
19300 0.000204 17200 6.94E-06 17000 2.07E-05 16600
4.85E-05 18900 3.01E-05 18400 3.52E-05
19300 0.000206 17200 6.95E-06 17000 2.08E-05 16600
4.87E-05 18900 3.01E-05 18400 3.52E-05
19300 0.000207 17200 6.94E-06 17000 2.08E-05 16600
4.89E-05 18900 3.01E-05 18400 3.52E-05
19300 0.000209 17200 6.96E-06 17000 2.08E-05 16600
4.91E-05 18900 3.01E-05 18400 3.52E-05
19300 0.000211 17200 6.95E-06 17000 2.09E-05 16600
4.93E-05 18900 3.01E-05 18400 3.53E-05
19300 0.000213 17200 6.97E-06 17000 2.09E-05 16600
4.95E-05 18900 3.01E-05 18400 3.53E-05
19300 0.000214 17200 6.98E-06 17000 2.10E-05 16600
4.97E-05 18900 3.01E-05 18400 3.53E-05
19300 0.000216 17200 6.96E-06 17000 2.10E-05 16600
4.99E-05 18900 3.02E-05 18400 3.53E-05
19300 0.000218 17200 6.96E-06 17000 2.11E-05 16600
5.01E-05 18900 3.02E-05 18400 3.53E-05
19300 0.00022 17300 6.96E-06 17000 2.11E-05 16600 5.03E-05
18900 3.02E-05 18400 3.53E-05
19300 0.000222 17300 6.98E-06 17000 2.12E-05 16600
5.05E-05 18900 3.02E-05 18400 3.53E-05
19300 0.000224 17300 6.95E-06 17100 2.12E-05 16600
5.07E-05 18900 3.02E-05 18400 3.54E-05
19300 0.000226 17300 6.96E-06 17100 2.13E-05 16600
5.10E-05 18900 3.02E-05 18400 3.54E-05
19300 0.000228 17300 6.98E-06 17100 2.13E-05 16600
5.12E-05 18900 3.02E-05 18400 3.54E-05
19300 0.00023 17300 6.98E-06 17100 2.14E-05 16600 5.14E-05
18900 3.02E-05 18400 3.54E-05
19300 0.000232 17300 6.99E-06 17100 2.15E-05 16700
5.17E-05 18900 3.02E-05 18400 3.54E-05
19300 0.000234 17300 6.98E-06 17100 2.15E-05 16700
5.19E-05 18900 3.02E-05 18400 3.54E-05
19300 0.000237 17300 6.99E-06 17100 2.16E-05 16700
5.22E-05 18900 3.03E-05 18400 3.54E-05
19300 0.000239 17300 6.99E-06 17100 2.17E-05 16700
5.24E-05 18900 3.03E-05 18400 3.54E-05
19300 0.000241 17300 6.99E-06 17100 2.17E-05 16700
5.26E-05 18900 3.03E-05 18400 3.55E-05
19300 0.000244 17300 7.01E-06 17100 2.18E-05 16700
5.28E-05 18900 3.03E-05 18400 3.55E-05
19300 0.000246 17300 7.00E-06 17100 2.18E-05 16700
5.31E-05 18900 3.03E-05 18400 3.55E-05
19300 0.000248 17300 7.02E-06 17100 2.19E-05 16700
5.33E-05 18900 3.03E-05 18400 3.55E-05
19300 0.000251 17300 7.01E-06 17100 2.20E-05 16700
5.35E-05 18900 3.03E-05 18400 3.55E-05
19300 0.000253 17300 7.00E-06 17100 2.20E-05 16700
5.37E-05 18900 3.03E-05 18400 3.55E-05
19300 0.000256 17300 7.00E-06 17100 2.21E-05 16700
5.40E-05 18900 3.04E-05 18400 3.55E-05
19300 0.000258 17300 7.01E-06 17100 2.21E-05 16700
5.42E-05 19000 3.04E-05 18400 3.55E-05
19300 0.000261 17300 7.01E-06 17100 2.22E-05 16700
5.45E-05 19000 3.04E-05 18400 3.55E-05

19300 0.000264 17300 7.02E-06 17100 2.23E-05 16700
5.47E-05 19000 3.04E-05 18400 3.55E-05
19300 0.000266 17300 7.01E-06 17100 2.23E-05 16700
5.49E-05 19000 3.04E-05 18400 3.56E-05
19300 0.000269 17300 6.99E-06 17100 2.24E-05 16700
5.52E-05 19000 3.04E-05 18400 3.56E-05
19300 0.000272 17300 7.01E-06 17100 2.25E-05 16700
5.54E-05 19000 3.04E-05 18400 3.56E-05
19300 0.000275 17300 7.01E-06 17100 2.25E-05 16700
5.57E-05 19000 3.05E-05 18400 3.56E-05
19300 0.000278 17300 7.01E-06 17100 2.26E-05 16700
5.59E-05 19000 3.05E-05 18400 3.56E-05
19300 0.000281 17300 7.01E-06 17100 2.27E-05 16700
5.62E-05 19000 3.05E-05 18400 3.56E-05
19300 0.000284 17300 7.02E-06 17100 2.27E-05 16700
5.65E-05 19000 3.05E-05 18400 3.57E-05
19300 0.000287 17300 7.02E-06 17100 2.28E-05 16700
5.67E-05 19000 3.05E-05 18400 3.57E-05
19300 0.00029 17300 7.02E-06 17100 2.29E-05 16700 5.70E-05
19000 3.05E-05 18400 3.57E-05
19300 0.000293 17300 7.01E-06 17100 2.30E-05 16700
5.73E-05 19000 3.06E-05 18400 3.57E-05
19300 0.000297 17300 7.01E-06 17100 2.31E-05 16700
5.76E-05 19000 3.06E-05 18400 3.57E-05
19300 0.0003 17300 7.00E-06 17100 2.31E-05 16700 5.79E-05
19000 3.06E-05 18400 3.57E-05
19300 0.000303 17300 7.00E-06 17100 2.32E-05 16700
5.81E-05 19000 3.06E-05 18400 3.57E-05
19300 0.000307 17300 7.01E-06 17100 2.33E-05 16700
5.84E-05 19000 3.06E-05 18400 3.57E-05
19300 0.00031 17300 6.99E-06 17100 2.34E-05 16700 5.87E-05
19000 3.06E-05 18400 3.58E-05
19300 0.000314 17300 7.01E-06 17100 2.34E-05 16700
5.90E-05 19000 3.06E-05 18400 3.58E-05
19300 0.000318 17300 7.00E-06 17100 2.35E-05 16700
5.92E-05 19000 3.07E-05 18400 3.58E-05
19300 0.000321 17300 7.02E-06 17100 2.36E-05 16700
5.95E-05 19000 3.07E-05 18400 3.58E-05
19300 0.000325 17300 7.02E-06 17100 2.37E-05 16700
5.98E-05 19000 3.07E-05 18400 3.58E-05
19300 0.000329 17300 7.02E-06 17100 2.37E-05 16700
6.01E-05 19000 3.07E-05 18400 3.58E-05
19300 0.000333 17300 7.02E-06 17100 2.38E-05 16700
6.04E-05 19000 3.07E-05 18400 3.58E-05
19300 0.000337 17300 7.02E-06 17100 2.39E-05 16700
6.07E-05 19000 3.07E-05 18400 3.59E-05
19300 0.000341 17300 7.04E-06 17100 2.40E-05 16700
6.10E-05 19000 3.07E-05 18400 3.59E-05
19300 0.000345 17300 7.03E-06 17100 2.41E-05 16700
6.13E-05 19000 3.07E-05 18400 3.59E-05
19300 0.000349 17300 7.04E-06 17100 2.42E-05 16700
6.16E-05 19000 3.08E-05 18400 3.59E-05
19300 0.000354 17300 7.05E-06 17100 2.43E-05 16700
6.19E-05 19000 3.08E-05 18400 3.59E-05
19400 0.000358 17300 7.06E-06 17100 2.44E-05 16700
6.22E-05 19000 3.08E-05 18400 3.59E-05
19400 0.000363 17300 7.04E-06 17100 2.45E-05 16700
6.25E-05 19000 3.08E-05 18400 3.59E-05
19400 0.000367 17300 7.03E-06 17100 2.46E-05 16700
6.29E-05 19000 3.08E-05 18400 3.60E-05
19400 0.000372 17300 7.04E-06 17100 2.46E-05 16700
6.32E-05 19000 3.08E-05 18400 3.60E-05
19400 0.000376 17300 7.05E-06 17100 2.48E-05 16700
6.35E-05 19000 3.08E-05 18400 3.60E-05
19400 0.000381 17300 7.05E-06 17100 2.49E-05 16700
6.38E-05 19000 3.08E-05 18400 3.60E-05
19400 0.000386 17300 7.05E-06 17100 2.50E-05 16700
6.42E-05 19000 3.08E-05 18400 3.60E-05

19400 0.000391 17300 7.05E-06 17100 2.51E-05 16700
6.45E-05 19000 3.09E-05 18400 3.60E-05
19400 0.000396 17300 7.04E-06 17100 2.52E-05 16700
6.49E-05 19000 3.09E-05 18400 3.60E-05
19400 0.000401 17300 7.05E-06 17100 2.53E-05 16700
6.52E-05 19000 3.09E-05 18500 3.60E-05
19400 0.000406 17300 7.06E-06 17100 2.54E-05 16700
6.56E-05 19000 3.09E-05 18500 3.60E-05
19400 0.000411 17300 7.06E-06 17100 2.55E-05 16700
6.59E-05 19000 3.09E-05 18500 3.60E-05
19400 0.000417 17300 7.04E-06 17100 2.56E-05 16700
6.62E-05 19000 3.09E-05 18500 3.61E-05
19400 0.000422 17300 7.05E-06 17100 2.57E-05 16700
6.66E-05 19000 3.09E-05 18500 3.61E-05
19400 0.000428 17300 7.06E-06 17100 2.58E-05 16700
6.70E-05 19000 3.10E-05 18500 3.61E-05
19400 0.000433 17300 7.07E-06 17100 2.59E-05 16700
6.73E-05 19000 3.10E-05 18500 3.61E-05
19400 0.000439 17300 7.06E-06 17100 2.60E-05 16700
6.77E-05 19000 3.10E-05 18500 3.61E-05
19400 0.000445 17300 7.06E-06 17100 2.61E-05 16700
6.81E-05 19000 3.10E-05 18500 3.61E-05
19400 0.00045 17400 7.07E-06 17100 2.62E-05 16700 6.85E-05
19000 3.10E-05 18500 3.62E-05
19400 0.000456 17400 7.08E-06 17100 2.64E-05 16700
6.89E-05 19000 3.10E-05 18500 3.62E-05
19400 0.000462 17400 7.07E-06 17200 2.65E-05 16700
6.93E-05 19000 3.10E-05 18500 3.62E-05
19400 0.000468 17400 7.06E-06 17200 2.66E-05 16700
6.97E-05 19000 3.10E-05 18500 3.62E-05
19400 0.000475 17400 7.06E-06 17200 2.67E-05 16700
7.01E-05 19000 3.11E-05 18500 3.62E-05
19400 0.000481 17400 7.06E-06 17200 2.69E-05 16700
7.05E-05 19000 3.11E-05 18500 3.62E-05
19400 0.000487 17400 7.07E-06 17200 2.70E-05 16800
7.10E-05 19000 3.11E-05 18500 3.62E-05
19400 0.000494 17400 7.07E-06 17200 2.71E-05 16800
7.14E-05 19000 3.11E-05 18500 3.62E-05
19400 0.0005 17400 7.08E-06 17200 2.73E-05 16800 7.18E-05
19000 3.11E-05 18500 3.63E-05
19400 0.000507 17400 7.07E-06 17200 2.74E-05 16800
7.23E-05 19000 3.11E-05 18500 3.63E-05
19400 0.000513 17400 7.07E-06 17200 2.75E-05 16800
7.27E-05 19000 3.11E-05 18500 3.63E-05
19400 0.00052 17400 7.07E-06 17200 2.77E-05 16800 7.31E-05
19000 3.12E-05 18500 3.63E-05
19400 0.000527 17400 7.09E-06 17200 2.78E-05 16800
7.35E-05 19000 3.12E-05 18500 3.63E-05
19400 0.000533 17400 7.08E-06 17200 2.79E-05 16800
7.40E-05 19000 3.12E-05 18500 3.63E-05
19400 0.00054 17400 7.08E-06 17200 2.81E-05 16800 7.44E-05
19000 3.12E-05 18500 3.63E-05
19400 0.000547 17400 7.09E-06 17200 2.82E-05 16800
7.49E-05 19000 3.12E-05 18500 3.63E-05
19400 0.000554 17400 7.09E-06 17200 2.84E-05 16800
7.53E-05 19000 3.12E-05 18500 3.64E-05
19400 0.000561 17400 7.08E-06 17200 2.85E-05 16800
7.58E-05 19100 3.12E-05 18500 3.64E-05
19400 0.000568 17400 7.10E-06 17200 2.87E-05 16800
7.63E-05 19100 3.13E-05 18500 3.64E-05
19400 0.000575 17400 7.10E-06 17200 2.89E-05 16800
7.67E-05 19100 3.13E-05 18500 3.64E-05
19400 0.000582 17400 7.11E-06 17200 2.90E-05 16800
7.72E-05 19100 3.13E-05 18500 3.64E-05
19400 0.00059 17400 7.10E-06 17200 2.91E-05 16800 7.77E-05
19100 3.13E-05 18500 3.64E-05
19400 0.000597 17400 7.09E-06 17200 2.93E-05 16800
7.82E-05 19100 3.13E-05 18500 3.65E-05

19400 0.000604 17400 7.10E-06 17200 2.95E-05 16800
7.87E-05 19100 3.13E-05 18500 3.65E-05
19400 0.000611 17400 7.08E-06 17200 2.96E-05 16800
7.92E-05 19100 3.13E-05 18500 3.65E-05
19400 0.000618 17400 7.09E-06 17200 2.98E-05 16800
7.97E-05 19100 3.13E-05 18500 3.65E-05
19400 0.000626 17400 7.10E-06 17200 3.00E-05 16800
8.02E-05 19100 3.14E-05 18500 3.65E-05
19400 0.000633 17400 7.10E-06 17200 3.02E-05 16800
8.07E-05 19100 3.14E-05 18500 3.65E-05
19400 0.00064 17400 7.11E-06 17200 3.03E-05 16800 8.13E-05
19100 3.14E-05 18500 3.65E-05
19400 0.000647 17400 7.11E-06 17200 3.05E-05 16800
8.18E-05 19100 3.14E-05 18500 3.65E-05
19400 0.000654 17400 7.11E-06 17200 3.07E-05 16800
8.23E-05 19100 3.14E-05 18500 3.65E-05
19400 0.000661 17400 7.12E-06 17200 3.09E-05 16800
8.29E-05 19100 3.14E-05 18500 3.66E-05
19400 0.000668 17400 7.12E-06 17200 3.11E-05 16800
8.34E-05 19100 3.14E-05 18500 3.66E-05
19400 0.000675 17400 7.14E-06 17200 3.13E-05 16800
8.40E-05 19100 3.14E-05 18500 3.66E-05
19400 0.000682 17400 7.13E-06 17200 3.15E-05 16800
8.46E-05 19100 3.15E-05 18500 3.66E-05
19400 0.000689 17400 7.12E-06 17200 3.17E-05 16800
8.52E-05 19100 3.15E-05 18500 3.66E-05
19400 0.000695 17400 7.13E-06 17200 3.19E-05 16800
8.58E-05 19100 3.15E-05 18500 3.66E-05
19400 0.000702 17400 7.13E-06 17200 3.21E-05 16800
8.64E-05 19100 3.15E-05 18500 3.66E-05
19400 0.000708 17400 7.14E-06 17200 3.23E-05 16800
8.70E-05 19100 3.15E-05 18500 3.67E-05
19400 0.000714 17400 7.14E-06 17200 3.25E-05 16800
8.77E-05 19100 3.15E-05 18500 3.67E-05
19400 0.00072 17400 7.14E-06 17200 3.27E-05 16800 8.83E-05
19100 3.15E-05 18500 3.67E-05
19400 0.000726 17400 7.14E-06 17200 3.30E-05 16800
8.89E-05 19100 3.16E-05 18500 3.67E-05
19400 0.000732 17400 7.14E-06 17200 3.32E-05 16800
8.95E-05 19100 3.16E-05 18500 3.67E-05
19400 0.000738 17400 7.12E-06 17200 3.34E-05 16800
9.02E-05 19100 3.16E-05 18500 3.67E-05
19400 0.000743 17400 7.14E-06 17200 3.37E-05 16800
9.08E-05 19100 3.16E-05 18500 3.67E-05
19400 0.000748 17400 7.14E-06 17200 3.39E-05 16800
9.15E-05 19100 3.16E-05 18500 3.67E-05
19500 0.000753 17400 7.14E-06 17200 3.42E-05 16800
9.22E-05 19100 3.16E-05 18500 3.68E-05
19500 0.000758 17400 7.13E-06 17200 3.44E-05 16800
9.28E-05 19100 3.17E-05 18500 3.68E-05
19500 0.000762 17400 7.13E-06 17200 3.47E-05 16800
9.35E-05 19100 3.17E-05 18500 3.68E-05
19500 0.000766 17400 7.14E-06 17200 3.49E-05 16800
9.42E-05 19100 3.17E-05 18500 3.68E-05
19500 0.00077 17400 7.13E-06 17200 3.52E-05 16800 9.49E-05
19100 3.17E-05 18500 3.68E-05
19500 0.000773 17400 7.13E-06 17200 3.55E-05 16800
9.57E-05 19100 3.17E-05 18500 3.68E-05
19500 0.000777 17400 7.12E-06 17200 3.58E-05 16800
9.64E-05 19100 3.17E-05 18500 3.68E-05
19500 0.00078 17400 7.13E-06 17200 3.60E-05 16800 9.72E-05
19100 3.18E-05 18500 3.68E-05
19500 0.000782 17400 7.12E-06 17200 3.63E-05 16800
9.79E-05 19100 3.18E-05 18500 3.69E-05
19500 0.000784 17400 7.12E-06 17200 3.66E-05 16800
9.87E-05 19100 3.18E-05 18600 3.69E-05
19500 0.000786 17400 7.12E-06 17200 3.69E-05 16800
9.95E-05 19100 3.18E-05 18600 3.69E-05

19500 0.000788 17400 7.12E-06 17200 3.72E-05 16800
0.000100254 19100 3.18E-05 18600 3.69E-05
19500 0.000789 17400 7.13E-06 17200 3.75E-05 16800
0.000101064 19100 3.18E-05 18600 3.69E-05
19500 0.00079 17400 7.12E-06 17200 3.78E-05 16800
0.000101876 19100 3.19E-05 18600 3.69E-05
19500 0.00079 17400 7.13E-06 17200 3.82E-05 16800
0.000102716 19100 3.19E-05 18600 3.69E-05
19500 0.00079 17400 7.14E-06 17200 3.85E-05 16800
0.000103532 19100 3.19E-05 18600 3.70E-05
19500 0.00079 17400 7.12E-06 17200 3.89E-05 16800
0.000104386 19100 3.19E-05 18600 3.70E-05
19500 0.000789 17400 7.14E-06 17200 3.92E-05 16800
0.000105243 19100 3.19E-05 18600 3.70E-05
19500 0.000788 17500 7.14E-06 17200 3.96E-05 16800
0.000106115 19100 3.19E-05 18600 3.70E-05
19500 0.000787 17500 7.14E-06 17200 3.99E-05 16800
0.00010703 19100 3.19E-05 18600 3.70E-05
19500 0.000785 17500 7.12E-06 17300 4.03E-05 16800
0.000107951 19100 3.20E-05 18600 3.70E-05
19500 0.000782 17500 7.14E-06 17300 4.07E-05 16800
0.00010888 19100 3.20E-05 18600 3.70E-05
19500 0.000779 17500 7.14E-06 17300 4.11E-05 16800
0.000109856 19100 3.20E-05 18600 3.70E-05
19500 0.000776 17500 7.13E-06 17300 4.15E-05 16800
0.000110839 19100 3.20E-05 18600 3.71E-05
19500 0.000773 17500 7.13E-06 17300 4.19E-05 16900
0.000111836 19100 3.20E-05 18600 3.71E-05
19500 0.000769 17500 7.14E-06 17300 4.23E-05 16900
0.000112873 19100 3.20E-05 18600 3.71E-05
19500 0.000764 17500 7.13E-06 17300 4.27E-05 16900
0.000113932 19100 3.20E-05 18600 3.71E-05
19500 0.00076 17500 7.13E-06 17300 4.31E-05 16900
0.000115005 19100 3.21E-05 18600 3.71E-05
19500 0.000754 17500 7.13E-06 17300 4.35E-05 16900
0.000116144 19100 3.21E-05 18600 3.71E-05
19500 0.000749 17500 7.14E-06 17300 4.40E-05 16900
0.000117284 19100 3.21E-05 18600 3.71E-05
19500 0.000743 17500 7.13E-06 17300 4.45E-05 16900
0.000118443 19100 3.21E-05 18600 3.72E-05
19500 0.000737 17500 7.13E-06 17300 4.49E-05 16900
0.000119645 19100 3.21E-05 18600 3.72E-05
19500 0.00073 17500 7.13E-06 17300 4.54E-05 16900
0.000120862 19100 3.21E-05 18600 3.72E-05
19500 0.000723 17500 7.13E-06 17300 4.59E-05 16900
0.000122098 19100 3.22E-05 18600 3.72E-05
19500 0.000716 17500 7.14E-06 17300 4.64E-05 16900
0.000123359 19100 3.22E-05 18600 3.72E-05
19500 0.000709 17500 7.14E-06 17300 4.69E-05 16900
0.000124647 19200 3.22E-05 18600 3.72E-05
19500 0.000701 17500 7.15E-06 17300 4.75E-05 16900
0.000125956 19200 3.22E-05 18600 3.72E-05
19500 0.000693 17500 7.15E-06 17300 4.80E-05 16900
0.000127304 19200 3.22E-05 18600 3.72E-05
19500 0.000685 17500 7.16E-06 17300 4.85E-05 16900
0.000128688 19200 3.22E-05 18600 3.73E-05
19500 0.000676 17500 7.15E-06 17300 4.91E-05 16900
0.000130117 19200 3.22E-05 18600 3.73E-05
19500 0.000668 17500 7.16E-06 17300 4.97E-05 16900
0.000131556 19200 3.23E-05 18600 3.73E-05
19500 0.000659 17500 7.16E-06 17300 5.03E-05 16900
0.000133057 19200 3.23E-05 18600 3.73E-05
19500 0.00065 17500 7.17E-06 17300 5.09E-05 16900
0.000134588 19200 3.23E-05 18600 3.73E-05
19500 0.00064 17500 7.17E-06 17300 5.15E-05 16900
0.000136176 19200 3.23E-05 18600 3.73E-05
19500 0.000631 17500 7.17E-06 17300 5.21E-05 16900
0.000137801 19200 3.23E-05 18600 3.73E-05

19500 0.000621 17500 7.17E-06 17300 5.28E-05 16900
0.000139488 19200 3.23E-05 18600 3.74E-05
19500 0.000612 17500 7.17E-06 17300 5.34E-05 16900
0.000141208 19200 3.23E-05 18600 3.74E-05
19500 0.000602 17500 7.17E-06 17300 5.41E-05 16900
0.000143015 19200 3.24E-05 18600 3.74E-05
19500 0.000592 17500 7.17E-06 17300 5.48E-05 16900
0.000144875 19200 3.24E-05 18600 3.74E-05
19500 0.000583 17500 7.18E-06 17300 5.56E-05 16900
0.000146787 19200 3.24E-05 18600 3.74E-05
19500 0.000573 17500 7.17E-06 17300 5.63E-05 16900
0.000148771 19200 3.24E-05 18600 3.74E-05
19500 0.000563 17500 7.17E-06 17300 5.71E-05 16900
0.000150819 19200 3.24E-05 18600 3.75E-05
19500 0.000553 17500 7.17E-06 17300 5.79E-05 16900
0.000152952 19200 3.24E-05 18600 3.75E-05
19500 0.000543 17500 7.17E-06 17300 5.87E-05 16900
0.000155137 19200 3.24E-05 18600 3.75E-05
19500 0.000533 17500 7.16E-06 17300 5.95E-05 16900
0.000157429 19200 3.25E-05 18600 3.75E-05
19500 0.000524 17500 7.16E-06 17300 6.04E-05 16900
0.000159812 19200 3.25E-05 18600 3.75E-05
19500 0.000514 17500 7.16E-06 17300 6.13E-05 16900
0.000162297 19200 3.25E-05 18600 3.75E-05
19500 0.000504 17500 7.15E-06 17300 6.21E-05 16900
0.000164874 19200 3.25E-05 18600 3.75E-05
19500 0.000495 17500 7.16E-06 17300 6.31E-05 16900
0.000167566 19200 3.25E-05 18600 3.76E-05
19500 0.000486 17500 7.16E-06 17300 6.40E-05 16900
0.000170367 19200 3.25E-05 18600 3.76E-05
19500 0.000476 17500 7.15E-06 17300 6.50E-05 16900
0.000173291 19200 3.25E-05 18600 3.76E-05
19500 0.000467 17500 7.16E-06 17300 6.60E-05 16900
0.000176343 19200 3.26E-05 18600 3.76E-05
19500 0.000458 17500 7.17E-06 17300 6.71E-05 16900
0.000179527 19200 3.26E-05 18600 3.76E-05
19500 0.000449 17500 7.16E-06 17300 6.81E-05 16900
0.00018285 19200 3.26E-05 18600 3.76E-05
19600 0.00044 17500 7.17E-06 17300 6.92E-05 16900
0.000186332 19200 3.26E-05 18600 3.77E-05
19600 0.000432 17500 7.17E-06 17300 7.03E-05 16900
0.00018994 19200 3.26E-05 18600 3.77E-05
19600 0.000423 17500 7.16E-06 17300 7.15E-05 16900
0.000193702 19200 3.26E-05 18600 3.77E-05
19600 0.000415 17500 7.17E-06 17300 7.27E-05 16900
0.000197623 19200 3.27E-05 18600 3.77E-05
19600 0.000407 17500 7.18E-06 17300 7.40E-05 16900
0.000201686 19200 3.27E-05 18600 3.77E-05
19600 0.000399 17500 7.18E-06 17300 7.52E-05 16900
0.000205856 19200 3.27E-05 18600 3.77E-05
19600 0.000391 17500 7.18E-06 17300 7.65E-05 16900
0.000210165 19200 3.27E-05 18600 3.78E-05
19600 0.000384 17500 7.19E-06 17300 7.79E-05 16900
0.000214603 19200 3.27E-05 18600 3.78E-05
19600 0.000376 17500 7.20E-06 17300 7.93E-05 16900
0.000219115 19200 3.27E-05 18600 3.78E-05
19600 0.000369 17500 7.20E-06 17300 8.08E-05 16900
0.000223719 19200 3.28E-05 18700 3.78E-05
19600 0.000362 17500 7.20E-06 17300 8.23E-05 16900
0.000228339 19200 3.28E-05 18700 3.78E-05
19600 0.000355 17500 7.21E-06 17300 8.38E-05 16900
0.000232933 19200 3.28E-05 18700 3.78E-05
19600 0.000349 17500 7.21E-06 17300 8.54E-05 16900
0.000237484 19200 3.28E-05 18700 3.78E-05
19600 0.000342 17500 7.22E-06 17300 8.71E-05 16900
0.000241901 19200 3.28E-05 18700 3.78E-05
19600 0.000336 17500 7.22E-06 17300 8.88E-05 16900
0.000246097 19200 3.28E-05 18700 3.78E-05

19600 0.00033 17500 7.22E-06 17300 9.05E-05 16900
0.000250058 19200 3.29E-05 18700 3.78E-05
19600 0.000324 17500 7.22E-06 17300 9.23E-05 16900
0.000253678 19200 3.29E-05 18700 3.79E-05
19600 0.000318 17500 7.22E-06 17300 9.42E-05 16900
0.000256905 19200 3.29E-05 18700 3.79E-05
19600 0.000313 17600 7.22E-06 17300 9.61E-05 16900
0.000259686 19200 3.29E-05 18700 3.79E-05
19600 0.000307 17600 7.23E-06 17300 9.82E-05 16900
0.000261973 19200 3.29E-05 18700 3.79E-05
19600 0.000302 17600 7.22E-06 17400 1.00E-04 16900
0.000263766 19200 3.29E-05 18700 3.79E-05
19600 0.000297 17600 7.22E-06 17400 1.02E-04 16900
0.000265018 19200 3.30E-05 18700 3.79E-05
19600 0.000292 17600 7.24E-06 17400 1.05E-04 16900
0.000265793 19200 3.30E-05 18700 3.80E-05
19600 0.000287 17600 7.24E-06 17400 1.07E-04 16900
0.000266062 19200 3.30E-05 18700 3.80E-05
19600 0.000283 17600 7.24E-06 17400 1.09E-04 17000
0.000265911 19200 3.30E-05 18700 3.80E-05
19600 0.000279 17600 7.24E-06 17400 1.12E-04 17000
0.000265365 19200 3.30E-05 18700 3.80E-05
19600 0.000274 17600 7.24E-06 17400 1.14E-04 17000
0.000264512 19200 3.30E-05 18700 3.80E-05
19600 0.00027 17600 7.25E-06 17400 1.17E-04 17000
0.000263405 19200 3.31E-05 18700 3.80E-05
19600 0.000266 17600 7.24E-06 17400 1.20E-04 17000
0.000262186 19200 3.31E-05 18700 3.80E-05
19600 0.000263 17600 7.25E-06 17400 1.23E-04 17000
0.000260891 19200 3.31E-05 18700 3.80E-05
19600 0.000259 17600 7.26E-06 17400 1.26E-04 17000
0.000259675 19200 3.31E-05 18700 3.81E-05
19600 0.000255 17600 7.26E-06 17400 1.29E-04 17000
0.000258542 19200 3.31E-05 18700 3.81E-05
19600 0.000252 17600 7.26E-06 17400 1.32E-04 17000
0.000257624 19200 3.31E-05 18700 3.81E-05
19600 0.000249 17600 7.28E-06 17400 1.35E-04 17000
0.000256881 19200 3.32E-05 18700 3.81E-05
19600 0.000246 17600 7.27E-06 17400 1.39E-04 17000
0.000256389 19200 3.32E-05 18700 3.81E-05
19600 0.000243 17600 7.28E-06 17400 1.43E-04 17000
0.000256177 19300 3.32E-05 18700 3.82E-05
19600 0.00024 17600 7.28E-06 17400 1.46E-04 17000
0.000256243 19300 3.32E-05 18700 3.82E-05
19600 0.000237 17600 7.29E-06 17400 1.50E-04 17000
0.000256618 19300 3.32E-05 18700 3.82E-05
19600 0.000234 17600 7.29E-06 17400 1.54E-04 17000
0.000257308 19300 3.33E-05 18700 3.82E-05
19600 0.000232 17600 7.29E-06 17400 1.59E-04 17000
0.000258242 19300 3.33E-05 18700 3.82E-05
19600 0.000229 17600 7.29E-06 17400 1.63E-04 17000
0.000259466 19300 3.33E-05 18700 3.82E-05
19600 0.000227 17600 7.29E-06 17400 1.68E-04 17000
0.00026096 19300 3.33E-05 18700 3.82E-05
19600 0.000225 17600 7.29E-06 17400 1.73E-04 17000
0.000262691 19300 3.33E-05 18700 3.83E-05
19600 0.000223 17600 7.29E-06 17400 1.78E-04 17000
0.000264644 19300 3.33E-05 18700 3.83E-05
19600 0.00022 17600 7.30E-06 17400 1.83E-04 17000
0.000266814 19300 3.34E-05 18700 3.83E-05
19600 0.000218 17600 7.30E-06 17400 1.88E-04 17000
0.000269173 19300 3.34E-05 18700 3.83E-05
19600 0.000216 17600 7.31E-06 17400 1.94E-04 17000
0.000271726 19300 3.34E-05 18700 3.83E-05
19600 0.000215 17600 7.31E-06 17400 2.00E-04 17000
0.000274464 19300 3.34E-05 18700 3.83E-05
19600 0.000213 17600 7.31E-06 17400 2.06E-04 17000
0.000277336 19300 3.34E-05 18700 3.83E-05

19600 0.000211 17600 7.32E-06 17400 2.13E-04 17000
0.000280388 19300 3.34E-05 18700 3.83E-05
19600 0.000209 17600 7.32E-06 17400 2.19E-04 17000
0.000283574 19300 3.35E-05 18700 3.84E-05
19600 0.000208 17600 7.33E-06 17400 2.26E-04 17000
0.000286891 19300 3.35E-05 18700 3.84E-05
19600 0.000206 17600 7.33E-06 17400 2.34E-04 17000
0.000290375 19300 3.35E-05 18700 3.84E-05
19600 0.000205 17600 7.34E-06 17400 2.41E-04 17000
0.000293989 19300 3.35E-05 18700 3.84E-05
19600 0.000203 17600 7.34E-06 17400 2.49E-04 17000
0.000297759 19300 3.35E-05 18700 3.84E-05
19600 0.000202 17600 7.34E-06 17400 2.58E-04 17000
0.000301644 19300 3.36E-05 18700 3.84E-05
19600 0.000201 17600 7.34E-06 17400 2.67E-04 17000
0.000305681 19300 3.36E-05 18700 3.84E-05
19600 0.000199 17600 7.36E-06 17400 2.76E-04 17000
0.000309859 19300 3.36E-05 18700 3.84E-05
19600 0.000198 17600 7.36E-06 17400 2.85E-04 17000
0.000314172 19300 3.36E-05 18700 3.85E-05
19600 0.000197 17600 7.36E-06 17400 2.95E-04 17000
0.000318639 19300 3.36E-05 18700 3.85E-05
19600 0.000196 17600 7.36E-06 17400 3.06E-04 17000
0.000323233 19300 3.37E-05 18700 3.85E-05
19600 0.000195 17600 7.36E-06 17400 3.16E-04 17000
0.000327956 19300 3.37E-05 18700 3.85E-05
19600 0.000194 17600 7.37E-06 17400 3.28E-04 17000
0.000332806 19300 3.37E-05 18700 3.85E-05
19600 0.000193 17600 7.38E-06 17400 3.39E-04 17000
0.000337805 19300 3.37E-05 18700 3.85E-05
19700 0.000192 17600 7.37E-06 17400 3.52E-04 17000
0.000342929 19300 3.37E-05 18700 3.85E-05
19700 0.000191 17600 7.38E-06 17400 3.64E-04 17000
0.000348207 19300 3.37E-05 18700 3.86E-05
19700 0.00019 17600 7.38E-06 17400 3.78E-04 17000
0.000353638 19300 3.38E-05 18700 3.86E-05
19700 0.000189 17600 7.39E-06 17400 3.91E-04 17000
0.000359221 19300 3.38E-05 18700 3.86E-05
19700 0.000188 17600 7.39E-06 17400 4.06E-04 17000
0.00036498 19300 3.38E-05 18700 3.86E-05
19700 0.000187 17600 7.39E-06 17400 4.21E-04 17000
0.000370903 19300 3.38E-05 18700 3.86E-05
19700 0.000186 17600 7.41E-06 17400 4.36E-04 17000
0.000377021 19300 3.38E-05 18700 3.86E-05
19700 0.000185 17600 7.41E-06 17400 4.52E-04 17000
0.000383295 19300 3.39E-05 18700 3.87E-05
19700 0.000184 17600 7.41E-06 17400 4.68E-04 17000
0.000389743 19300 3.39E-05 18700 3.87E-05
19700 0.000184 17600 7.41E-06 17400 4.85E-04 17000
0.000396393 19300 3.39E-05 18800 3.87E-05
19700 0.000183 17600 7.42E-06 17400 5.02E-04 17000
0.0004032 19300 3.39E-05 18800 3.87E-05
19700 0.000182 17600 7.42E-06 17400 5.20E-04 17000
0.000410226 19300 3.39E-05 18800 3.87E-05
19700 0.000181 17600 7.43E-06 17400 5.38E-04 17000
0.000417466 19300 3.39E-05 18800 3.87E-05
19700 0.000181 17600 7.44E-06 17400 5.56E-04 17000
0.000424883 19300 3.40E-05 18800 3.87E-05
19700 0.00018 17600 7.44E-06 17400 5.75E-04 17000
0.000432514 19300 3.40E-05 18800 3.87E-05
19700 0.000179 17600 7.45E-06 17400 5.94E-04 17000
0.000440369 19300 3.40E-05 18800 3.88E-05
19700 0.000179 17600 7.45E-06 17400 6.13E-04 17000
0.00044841 19300 3.40E-05 18800 3.88E-05
19700 0.000178 17600 7.46E-06 17400 6.32E-04 17000
0.00045671 19300 3.40E-05 18800 3.88E-05
19700 0.000177 17700 7.46E-06 17400 6.51E-04 17000
0.000465211 19300 3.41E-05 18800 3.88E-05

19700 0.000177 17700 7.47E-06 17400 6.70E-04 17000
0.000473958 19300 3.41E-05 18800 3.88E-05
19700 0.000176 17700 7.48E-06 17500 6.88E-04 17000
0.000482932 19300 3.41E-05 18800 3.88E-05
19700 0.000175 17700 7.48E-06 17500 7.06E-04 17000
0.000492181 19300 3.41E-05 18800 3.88E-05
19700 0.000175 17700 7.49E-06 17500 7.24E-04 17000
0.000501685 19300 3.41E-05 18800 3.88E-05
19700 0.000174 17700 7.50E-06 17500 7.40E-04 17000
0.000511501 19300 3.41E-05 18800 3.89E-05
19700 0.000173 17700 7.51E-06 17500 7.56E-04 17100
0.000521602 19300 3.41E-05 18800 3.89E-05
19700 0.000173 17700 7.51E-06 17500 7.70E-04 17100
0.000532004 19300 3.42E-05 18800 3.89E-05
19700 0.000172 17700 7.52E-06 17500 7.83E-04 17100
0.000542738 19300 3.42E-05 18800 3.89E-05
19700 0.000172 17700 7.52E-06 17500 7.95E-04 17100
0.000553808 19300 3.42E-05 18800 3.89E-05
19700 0.000171 17700 7.53E-06 17500 8.05E-04 17100
0.000565194 19300 3.42E-05 18800 3.89E-05
19700 0.00017 17700 7.53E-06 17500 8.13E-04 17100
0.000576944 19300 3.43E-05 18800 3.89E-05
19700 0.00017 17700 7.54E-06 17500 8.19E-04 17100
0.000589065 19300 3.43E-05 18800 3.89E-05
19700 0.000169 17700 7.54E-06 17500 8.23E-04 17100
0.000601569 19300 3.43E-05 18800 3.90E-05
19700 0.000168 17700 7.56E-06 17500 8.25E-04 17100
0.000614465 19300 3.43E-05 18800 3.90E-05
19700 0.000168 17700 7.56E-06 17500 8.25E-04 17100
0.000627785 19300 3.43E-05 18800 3.90E-05
19700 0.000167 17700 7.57E-06 17500 8.22E-04 17100
0.000641569 19300 3.44E-05 18800 3.90E-05
19700 0.000167 17700 7.57E-06 17500 8.17E-04 17100
0.000655794 19400 3.44E-05 18800 3.90E-05
19700 0.000166 17700 7.58E-06 17500 8.10E-04 17100
0.000670525 19400 3.44E-05 18800 3.90E-05
19700 0.000166 17700 7.58E-06 17500 8.01E-04 17100
0.000685761 19400 3.44E-05 18800 3.90E-05
19700 0.000165 17700 7.60E-06 17500 7.89E-04 17100
0.000701534 19400 3.44E-05 18800 3.91E-05
19700 0.000164 17700 7.60E-06 17500 7.76E-04 17100
0.000717849 19400 3.44E-05 18800 3.91E-05
19700 0.000164 17700 7.60E-06 17500 7.61E-04 17100
0.000734747 19400 3.45E-05 18800 3.91E-05
19700 0.000163 17700 7.63E-06 17500 7.44E-04 17100
0.000752239 19400 3.45E-05 18800 3.91E-05
19700 0.000163 17700 7.63E-06 17500 7.25E-04 17100
0.000770362 19400 3.45E-05 18800 3.91E-05
19700 0.000162 17700 7.64E-06 17500 7.06E-04 17100
0.000789153 19400 3.45E-05 18800 3.91E-05
19700 0.000161 17700 7.65E-06 17500 6.85E-04 17100
0.000808619 19400 3.45E-05 18800 3.91E-05
19700 0.000161 17700 7.65E-06 17500 6.64E-04 17100
0.000828797 19400 3.45E-05 18800 3.91E-05
19700 0.00016 17700 7.67E-06 17500 6.42E-04 17100
0.000849716 19400 3.46E-05 18800 3.92E-05
19700 0.00016 17700 7.68E-06 17500 6.19E-04 17100
0.000871393 19400 3.46E-05 18800 3.92E-05
19700 0.000159 17700 7.69E-06 17500 5.96E-04 17100
0.000893873 19400 3.46E-05 18800 3.92E-05
19700 0.000159 17700 7.70E-06 17500 5.73E-04 17100
0.000917205 19400 3.46E-05 18800 3.92E-05
19700 0.000158 17700 7.71E-06 17500 5.51E-04 17100
0.000941428 19400 3.46E-05 18800 3.92E-05
19700 0.000157 17700 7.72E-06 17500 5.28E-04 17100
0.00096658 19400 3.47E-05 18800 3.93E-05
19700 0.000157 17700 7.73E-06 17500 5.06E-04 17100
0.000992712 19400 3.47E-05 18800 3.93E-05

19700 0.000156 17700 7.75E-06 17500 4.84E-04 17100
0.001019866 19400 3.47E-05 18800 3.93E-05
19700 0.000156 17700 7.76E-06 17500 4.63E-04 17100
0.001048086 19400 3.47E-05 18800 3.93E-05
19700 0.000155 17700 7.79E-06 17500 4.43E-04 17100
0.001077457 19400 3.48E-05 18800 3.93E-05
19700 0.000155 17700 7.80E-06 17500 4.23E-04 17100
0.001108009 19400 3.48E-05 18800 3.93E-05
19700 0.000154 17700 7.82E-06 17500 4.04E-04 17100
0.001139816 19400 3.48E-05 18800 3.93E-05
19700 0.000154 17700 7.83E-06 17500 3.86E-04 17100
0.00117295 19400 3.48E-05 18800 3.94E-05
19700 0.000153 17700 7.85E-06 17500 3.69E-04 17100
0.001207495 19400 3.48E-05 18800 3.94E-05
19700 0.000153 17700 7.87E-06 17500 3.52E-04 17100
0.001243536 19400 3.48E-05 18800 3.94E-05
19700 0.000152 17700 7.89E-06 17500 3.37E-04 17100
0.001281152 19400 3.49E-05 18800 3.94E-05
19700 0.000151 17700 7.91E-06 17500 3.22E-04 17100
0.001320421 19400 3.49E-05 18800 3.94E-05
19700 0.000151 17700 7.92E-06 17500 3.07E-04 17100
0.001361424 19400 3.49E-05 18800 3.94E-05
19800 0.00015 17700 7.94E-06 17500 2.94E-04 17100
0.001404261 19400 3.49E-05 18800 3.94E-05
19800 0.00015 17700 7.96E-06 17500 2.81E-04 17100
0.00144904 19400 3.50E-05 18800 3.94E-05
19800 0.000149 17700 7.98E-06 17500 2.69E-04 17100
0.001495837 19400 3.50E-05 18800 3.95E-05
19800 0.000149 17700 8.00E-06 17500 2.58E-04 17100
0.001544782 19400 3.50E-05 18800 3.95E-05
19800 0.000148 17700 8.02E-06 17500 2.47E-04 17100
0.001596017 19400 3.50E-05 18800 3.95E-05
19800 0.000148 17700 8.05E-06 17500 2.37E-04 17100
0.001649647 19400 3.50E-05 18800 3.95E-05
19800 0.000147 17700 8.06E-06 17500 2.27E-04 17100
0.001705814 19400 3.51E-05 18800 3.95E-05
19800 0.000147 17700 8.09E-06 17500 2.18E-04 17100
0.001764662 19400 3.51E-05 18800 3.95E-05
19800 0.000146 17700 8.12E-06 17500 2.09E-04 17100
0.001826373 19400 3.51E-05 18800 3.96E-05
19800 0.000146 17700 8.15E-06 17500 2.01E-04 17100
0.001891118 19400 3.51E-05 18900 3.96E-05
19800 0.000145 17700 8.17E-06 17500 1.94E-04 17100
0.001959065 19400 3.52E-05 18900 3.96E-05
19800 0.000145 17700 8.21E-06 17500 1.86E-04 17100
0.002030453 19400 3.52E-05 18900 3.96E-05
19800 0.000145 17700 8.25E-06 17500 1.80E-04 17100
0.002105478 19400 3.52E-05 18900 3.96E-05
19800 0.000144 17700 8.28E-06 17500 1.73E-04 17100
0.002184432 19400 3.52E-05 18900 3.97E-05
19800 0.000144 17700 8.31E-06 17500 1.67E-04 17100
0.002267529 19400 3.53E-05 18900 3.97E-05
19800 0.000143 17700 8.36E-06 17500 1.61E-04 17100
0.002355065 19400 3.53E-05 18900 3.97E-05
19800 0.000143 17700 8.40E-06 17500 1.56E-04 17100
0.002447336 19400 3.53E-05 18900 3.97E-05
19800 0.000142 17700 8.44E-06 17500 1.51E-04 17100
0.002544608 19400 3.53E-05 18900 3.97E-05
19800 0.000142 17800 8.48E-06 17500 1.46E-04 17100
0.002647206 19400 3.53E-05 18900 3.97E-05
19800 0.000141 17800 8.52E-06 17500 1.41E-04 17100
0.002755465 19400 3.54E-05 18900 3.97E-05
19800 0.000141 17800 8.57E-06 17600 1.37E-04 17100
0.00286977 19400 3.54E-05 18900 3.97E-05
19800 0.000141 17800 8.63E-06 17600 1.33E-04 17100
0.002990509 19400 3.54E-05 18900 3.97E-05
19800 0.00014 17800 8.69E-06 17600 1.29E-04 17100
0.003118143 19400 3.54E-05 18900 3.98E-05

19800 0.00014 17800 8.75E-06 17600 1.25E-04 17100
0.003253123 19400 3.54E-05 18900 3.98E-05
19800 0.000139 17800 8.83E-06 17600 1.21E-04 17200
0.003395913 19400 3.55E-05 18900 3.98E-05
19800 0.000139 17800 8.89E-06 17600 1.18E-04 17200
0.003547065 19400 3.55E-05 18900 3.98E-05
19800 0.000139 17800 8.96E-06 17600 1.15E-04 17200
0.003707123 19400 3.55E-05 18900 3.98E-05
19800 0.000138 17800 9.04E-06 17600 1.12E-04 17200
0.003876725 19400 3.56E-05 18900 3.98E-05
19800 0.000138 17800 9.12E-06 17600 1.09E-04 17200
0.004056487 19400 3.56E-05 18900 3.99E-05
19800 0.000138 17800 9.20E-06 17600 1.06E-04 17200
0.004247048 19400 3.56E-05 18900 3.99E-05
19800 0.000137 17800 9.29E-06 17600 1.03E-04 17200
0.004449181 19400 3.56E-05 18900 3.99E-05
19800 0.000137 17800 9.40E-06 17600 1.01E-04 17200
0.004663612 19400 3.56E-05 18900 3.99E-05
19800 0.000136 17800 9.49E-06 17600 9.81E-05 17200
0.004891218 19400 3.57E-05 18900 3.99E-05
19800 0.000136 17800 9.60E-06 17600 9.57E-05 17200
0.005132876 19400 3.57E-05 18900 3.99E-05
19800 0.000136 17800 9.73E-06 17600 9.35E-05 17200
0.005389517 19400 3.57E-05 18900 3.99E-05
19800 0.000135 17800 9.85E-06 17600 9.13E-05 17200
0.005662137 19500 3.57E-05 18900 4.00E-05
19800 0.000135 17800 9.97E-06 17600 8.93E-05 17200
0.005951732 19500 3.58E-05 18900 4.00E-05
19800 0.000135 17800 1.01E-05 17600 8.73E-05 17200
0.006259344 19500 3.58E-05 18900 4.00E-05
19800 0.000135 17800 1.02E-05 17600 8.54E-05 17200
0.006586048 19500 3.58E-05 18900 4.00E-05
19800 0.000134 17800 1.04E-05 17600 8.36E-05 17200
0.006932952 19500 3.58E-05 18900 4.00E-05
19800 0.000134 17800 1.05E-05 17600 8.18E-05 17200
0.007301177 19500 3.59E-05 18900 4.00E-05
19800 0.000134 17800 1.07E-05 17600 8.01E-05 17200
0.007691833 19500 3.59E-05 18900 4.00E-05
19800 0.000133 17800 1.09E-05 17600 7.85E-05 17200
0.008105993 19500 3.59E-05 18900 4.01E-05
19800 0.000133 17800 1.10E-05 17600 7.69E-05 17200
0.008544674 19500 3.59E-05 18900 4.01E-05
19800 0.000133 17800 1.12E-05 17600 7.54E-05 17200
0.009008829 19500 3.60E-05 18900 4.01E-05
19800 0.000133 17800 1.14E-05 17600 7.40E-05 17200
0.009499289 19500 3.60E-05 18900 4.01E-05
19800 0.000132 17800 1.15E-05 17600 7.26E-05 17200
0.01001674 19500 3.60E-05 18900 4.01E-05
19800 0.000132 17800 1.17E-05 17600 7.13E-05 17200
0.01056166 19500 3.61E-05 18900 4.01E-05
19800 0.000132 17800 1.19E-05 17600 7.00E-05 17200
0.01113426 19500 3.61E-05 18900 4.02E-05
19800 0.000131 17800 1.21E-05 17600 6.87E-05 17200
0.01173439 19500 3.61E-05 18900 4.02E-05
19800 0.000131 17800 1.22E-05 17600 6.75E-05 17200
0.01236146 19500 3.61E-05 18900 4.02E-05
19800 0.000131 17800 1.24E-05 17600 6.64E-05 17200
0.01301436 19500 3.62E-05 18900 4.02E-05
19800 0.000131 17800 1.25E-05 17600 6.52E-05 17200
0.01369135 19500 3.62E-05 18900 4.02E-05
19800 0.000131 17800 1.26E-05 17600 6.41E-05 17200
0.01438993 19500 3.62E-05 18900 4.02E-05
19800 0.00013 17800 1.27E-05 17600 6.31E-05 17200
0.01510685 19500 3.62E-05 18900 4.02E-05
19800 0.00013 17800 1.28E-05 17600 6.21E-05 17200
0.01583794 19500 3.63E-05 18900 4.02E-05
19800 0.00013 17800 1.29E-05 17600 6.11E-05 17200
0.01657806 19500 3.63E-05 18900 4.02E-05

19800 0.00013 17800 1.29E-05 17600 6.01E-05 17200
0.01732107 19500 3.63E-05 18900 4.03E-05
19800 0.000129 17800 1.29E-05 17600 5.92E-05 17200
0.01805962 19500 3.63E-05 18900 4.03E-05
19800 0.000129 17800 1.29E-05 17600 5.83E-05 17200
0.01878525 19500 3.64E-05 18900 4.03E-05
19800 0.000129 17800 1.29E-05 17600 5.74E-05 17200
0.01948827 19500 3.64E-05 18900 4.03E-05
19800 0.000129 17800 1.28E-05 17600 5.66E-05 17200
0.02015794 19500 3.64E-05 18900 4.03E-05
19800 0.000129 17800 1.27E-05 17600 5.58E-05 17200
0.02078259 19500 3.65E-05 18900 4.03E-05
19800 0.000128 17800 1.27E-05 17600 5.50E-05 17200
0.02134997 19500 3.65E-05 18900 4.04E-05
19900 0.000128 17800 1.26E-05 17600 5.42E-05 17200
0.02184756 19500 3.65E-05 18900 4.04E-05
19900 0.000128 17800 1.25E-05 17600 5.35E-05 17200
0.02226308 19500 3.65E-05 18900 4.04E-05
19900 0.000128 17800 1.24E-05 17600 5.27E-05 17200
0.02258499 19500 3.66E-05 18900 4.04E-05
19900 0.000128 17800 1.22E-05 17600 5.20E-05 17200
0.02280299 19500 3.66E-05 18900 4.04E-05
19900 0.000128 17800 1.21E-05 17600 5.14E-05 17200
0.02290851 19500 3.66E-05 18900 4.04E-05
19900 0.000127 17800 1.20E-05 17600 5.07E-05 17200
0.02289527 19500 3.67E-05 18900 4.04E-05
19900 0.000127 17800 1.18E-05 17600 5.00E-05 17200
0.02275964 19500 3.67E-05 18900 4.04E-05
19900 0.000127 17800 1.17E-05 17600 4.94E-05 17200
0.02250094 19500 3.67E-05 18900 4.04E-05
19900 0.000127 17800 1.16E-05 17600 4.88E-05 17200
0.02212163 19500 3.68E-05 18900 4.05E-05
19900 0.000127 17800 1.14E-05 17600 4.82E-05 17200
0.02162725 19500 3.68E-05 19000 4.05E-05
19900 0.000127 17800 1.13E-05 17600 4.76E-05 17200
0.02102626 19500 3.68E-05 19000 4.05E-05
19900 0.000127 17800 1.12E-05 17600 4.71E-05 17200
0.0203297 19500 3.68E-05 19000 4.05E-05
19900 0.000126 17800 1.10E-05 17600 4.65E-05 17200
0.01955072 19500 3.69E-05 19000 4.06E-05
19900 0.000126 17800 1.09E-05 17600 4.60E-05 17200
0.01870396 19500 3.69E-05 19000 4.06E-05
19900 0.000126 17800 1.08E-05 17600 4.54E-05 17200
0.01780496 19500 3.69E-05 19000 4.06E-05
19900 0.000126 17800 1.07E-05 17600 4.49E-05 17200
0.01686929 19500 3.70E-05 19000 4.06E-05
19900 0.000126 17800 1.06E-05 17600 4.44E-05 17200
0.01591206 19500 3.70E-05 19000 4.06E-05
19900 0.000126 17800 1.04E-05 17600 4.40E-05 17200
0.01494731 19500 3.70E-05 19000 4.06E-05
19900 0.000126 17900 1.03E-05 17600 4.35E-05 17200
0.01398771 19500 3.71E-05 19000 4.06E-05
19900 0.000126 17900 1.02E-05 17600 4.30E-05 17200
0.01304437 19500 3.71E-05 19000 4.07E-05
19900 0.000125 17900 1.01E-05 17700 4.26E-05 17200
0.0121267 19500 3.71E-05 19000 4.07E-05
19900 0.000125 17900 9.98E-06 17700 4.22E-05 17200
0.01124239 19500 3.72E-05 19000 4.07E-05
19900 0.000125 17900 9.87E-06 17700 4.17E-05 17200
0.01039746 19500 3.72E-05 19000 4.07E-05
19900 0.000125 17900 9.76E-06 17700 4.13E-05 17200
0.009596269 19500 3.72E-05 19000 4.07E-05
19900 0.000125 17900 9.67E-06 17700 4.09E-05 17300
0.008841733 19500 3.72E-05 19000 4.08E-05
19900 0.000125 17900 9.57E-06 17700 4.05E-05 17300
0.008135371 19500 3.73E-05 19000 4.08E-05
19900 0.000125 17900 9.48E-06 17700 4.01E-05 17300
0.007477646 19500 3.73E-05 19000 4.08E-05

19900 0.000125 17900 9.39E-06 17700 3.97E-05 17300
0.006868087 19500 3.73E-05 19000 4.08E-05
19900 0.000125 17900 9.30E-06 17700 3.94E-05 17300
0.006305522 19500 3.74E-05 19000 4.08E-05
19900 0.000124 17900 9.23E-06 17700 3.90E-05 17300
0.005788248 19500 3.74E-05 19000 4.08E-05
19900 0.000124 17900 9.15E-06 17700 3.87E-05 17300
0.005314121 19500 3.74E-05 19000 4.09E-05
19900 0.000124 17900 9.08E-06 17700 3.83E-05 17300
0.004880718 19500 3.75E-05 19000 4.09E-05
19900 0.000124 17900 9.01E-06 17700 3.80E-05 17300
0.004485444 19500 3.75E-05 19000 4.09E-05
19900 0.000124 17900 8.95E-06 17700 3.76E-05 17300
0.004125579 19500 3.75E-05 19000 4.09E-05
19900 0.000124 17900 8.88E-06 17700 3.73E-05 17300
0.003798432 19500 3.76E-05 19000 4.09E-05
19900 0.000124 17900 8.82E-06 17700 3.70E-05 17300
0.003501337 19600 3.76E-05 19000 4.09E-05
19900 0.000124 17900 8.77E-06 17700 3.67E-05 17300
0.003231715 19600 3.76E-05 19000 4.10E-05
19900 0.000124 17900 8.71E-06 17700 3.64E-05 17300
0.002987153 19600 3.77E-05 19000 4.10E-05
19900 0.000124 17900 8.67E-06 17700 3.61E-05 17300
0.002765373 19600 3.77E-05 19000 4.10E-05
19900 0.000124 17900 8.63E-06 17700 3.58E-05 17300
0.002564277 19600 3.77E-05 19000 4.10E-05
19900 0.000123 17900 8.58E-06 17700 3.55E-05 17300
0.002381882 19600 3.78E-05 19000 4.10E-05
19900 0.000123 17900 8.53E-06 17700 3.52E-05 17300
0.002216388 19600 3.78E-05 19000 4.11E-05
19900 0.000123 17900 8.50E-06 17700 3.49E-05 17300
0.00206618 19600 3.78E-05 19000 4.11E-05
19900 0.000123 17900 8.46E-06 17700 3.47E-05 17300
0.001929728 19600 3.79E-05 19000 4.11E-05
19900 0.000123 17900 8.43E-06 17700 3.44E-05 17300
0.001805684 19600 3.79E-05 19000 4.11E-05
19900 0.000123 17900 8.39E-06 17700 3.42E-05 17300
0.001692819 19600 3.79E-05 19000 4.11E-05
19900 0.000123 17900 8.36E-06 17700 3.39E-05 17300
0.001589984 19600 3.80E-05 19000 4.12E-05
19900 0.000123 17900 8.33E-06 17700 3.37E-05 17300
0.001496212 19600 3.80E-05 19000 4.11E-05
19900 0.000123 17900 8.30E-06 17700 3.34E-05 17300
0.001410582 19600 3.81E-05 19000 4.12E-05
19900 0.000123 17900 8.27E-06 17700 3.32E-05 17300
0.001332275 19600 3.81E-05 19000 4.12E-05
19900 0.000123 17900 8.24E-06 17700 3.29E-05 17300
0.001260567 19600 3.81E-05 19000 4.12E-05
19900 0.000123 17900 8.21E-06 17700 3.27E-05 17300
0.001194819 19600 3.82E-05 19000 4.12E-05
19900 0.000123 17900 8.19E-06 17700 3.25E-05 17300
0.001134415 19600 3.82E-05 19000 4.13E-05
19900 0.000123 17900 8.17E-06 17700 3.23E-05 17300
0.001078876 19600 3.82E-05 19000 4.13E-05
19900 0.000123 17900 8.14E-06 17700 3.20E-05 17300
0.001027717 19600 3.83E-05 19000 4.13E-05
19900 0.000123 17900 8.12E-06 17700 3.18E-05 17300
0.000980512 19600 3.83E-05 19000 4.14E-05
19900 0.000123 17900 8.10E-06 17700 3.16E-05 17300
0.00093688 19600 3.83E-05 19000 4.14E-05
19900 0.000123 17900 8.07E-06 17700 3.14E-05 17300
0.00089651 19600 3.84E-05 19000 4.14E-05
19900 0.000122 17900 8.06E-06 17700 3.12E-05 17300
0.000859089 19600 3.84E-05 19000 4.14E-05
19900 0.000122 17900 8.05E-06 17700 3.10E-05 17300
0.00082435 19600 3.85E-05 19000 4.14E-05
19900 0.000122 17900 8.03E-06 17700 3.09E-05 17300
0.000792038 19600 3.85E-05 19000 4.14E-05

19900 0.000122 17900 8.01E-06 17700 3.07E-05 17300
0.000761922 19600 3.85E-05 19000 4.15E-05
19900 0.000122 17900 8.00E-06 17700 3.05E-05 17300
0.000733832 19600 3.86E-05 19000 4.14E-05
19900 0.000122 17900 7.98E-06 17700 3.03E-05 17300
0.000707564 19600 3.86E-05 19000 4.15E-05
20000 0.000122 17900 7.97E-06 17700 3.01E-05 17300
0.000682975 19600 3.87E-05 19000 4.15E-05
20000 0.000122 17900 7.95E-06 17700 3.00E-05 17300
0.000659923 19600 3.87E-05 19000 4.15E-05
20000 0.000122 17900 7.94E-06 17700 2.98E-05 17300
0.000638271 19600 3.88E-05 19000 4.15E-05
20000 0.000122 17900 7.93E-06 17700 2.96E-05 17300
0.000617929 19600 3.88E-05 19000 4.16E-05
20000 0.000122 17900 7.92E-06 17700 2.95E-05 17300
0.00059875 19600 3.88E-05 19000 4.16E-05
20000 0.000122 17900 7.91E-06 17700 2.93E-05 17300
0.000580662 19600 3.89E-05 19000 4.16E-05
20000 0.000122 17900 7.90E-06 17700 2.91E-05 17300
0.000563584 19600 3.89E-05 19000 4.16E-05
20000 0.000122 17900 7.88E-06 17700 2.90E-05 17300
0.000547454 19600 3.90E-05 19000 4.16E-05
20000 0.000122 17900 7.88E-06 17700 2.88E-05 17300
0.000532167 19600 3.90E-05 19000 4.16E-05
20000 0.000122 17900 7.86E-06 17700 2.87E-05 17300
0.000517686 19600 3.91E-05 19100 4.16E-05
20000 0.000122 17900 7.85E-06 17700 2.85E-05 17300
0.000503923 19600 3.91E-05 19100 4.17E-05
20000 0.000122 17900 7.85E-06 17700 2.84E-05 17300
0.000490831 19600 3.92E-05 19100 4.17E-05
20000 0.000122 17900 7.83E-06 17700 2.82E-05 17300
0.000478362 19600 3.92E-05 19100 4.17E-05
20000 0.000122 17900 7.83E-06 17700 2.81E-05 17300
0.000466497 19600 3.93E-05 19100 4.17E-05
20000 0.000122 17900 7.83E-06 17700 2.79E-05 17300
0.000455169 19600 3.93E-05 19100 4.17E-05
20000 0.000122 17900 7.82E-06 17700 2.78E-05 17300
0.000444363 19600 3.93E-05 19100 4.18E-05
20000 0.000122 17900 7.82E-06 17700 2.77E-05 17300
0.000434008 19600 3.94E-05 19100 4.18E-05
20000 0.000122 17900 7.80E-06 17700 2.75E-05 17300
0.00042411 19600 3.94E-05 19100 4.18E-05
20000 0.000122 18000 7.80E-06 17700 2.74E-05 17300
0.000414625 19600 3.95E-05 19100 4.18E-05
20000 0.000122 18000 7.78E-06 17700 2.73E-05 17300
0.000405534 19600 3.95E-05 19100 4.18E-05
20000 0.000122 18000 7.78E-06 17800 2.72E-05 17300
0.000396792 19600 3.96E-05 19100 4.19E-05
20000 0.000122 18000 7.78E-06 17800 2.70E-05 17300
0.000388427 19600 3.96E-05 19100 4.19E-05
20000 0.000122 18000 7.77E-06 17800 2.69E-05 17300
0.00038038 19600 3.97E-05 19100 4.19E-05
20000 0.000122 18000 7.76E-06 17800 2.68E-05 17300
0.000372632 19600 3.97E-05 19100 4.19E-05
20000 0.000122 18000 7.76E-06 17800 2.67E-05 17400
0.000365174 19600 3.98E-05 19100 4.19E-05
20000 0.000122 18000 7.75E-06 17800 2.66E-05 17400
0.000357995 19600 3.98E-05 19100 4.19E-05
20000 0.000122 18000 7.74E-06 17800 2.64E-05 17400
0.000351082 19600 3.99E-05 19100 4.19E-05
20000 0.000122 18000 7.73E-06 17800 2.63E-05 17400
0.000344415 19600 3.99E-05 19100 4.20E-05
20000 0.000122 18000 7.72E-06 17800 2.62E-05 17400
0.000338001 19600 4.00E-05 19100 4.20E-05
20000 0.000122 18000 7.72E-06 17800 2.61E-05 17400
0.000331772 19600 4.00E-05 19100 4.20E-05
20000 0.000122 18000 7.72E-06 17800 2.60E-05 17400
0.000325777 19600 4.01E-05 19100 4.20E-05

20000 0.000122 18000 7.71E-06 17800 2.59E-05 17400
0.000319963 19600 4.01E-05 19100 4.20E-05
20000 0.000122 18000 7.71E-06 17800 2.58E-05 17400
0.000314337 19600 4.02E-05 19100 4.20E-05
20000 0.000122 18000 7.71E-06 17800 2.57E-05 17400
0.000308889 19600 4.02E-05 19100 4.21E-05
20000 0.000122 18000 7.71E-06 17800 2.56E-05 17400
0.000303601 19600 4.03E-05 19100 4.21E-05
20000 0.000122 18000 7.70E-06 17800 2.55E-05 17400
0.000298464 19700 4.04E-05 19100 4.21E-05
20000 0.000122 18000 7.70E-06 17800 2.54E-05 17400
0.000293466 19700 4.04E-05 19100 4.21E-05
20000 0.000122 18000 7.70E-06 17800 2.53E-05 17400
0.000288624 19700 4.05E-05 19100 4.21E-05
20000 0.000122 18000 7.70E-06 17800 2.52E-05 17400
0.000283906 19700 4.05E-05 19100 4.22E-05
20000 0.000122 18000 7.71E-06 17800 2.51E-05 17400
0.000279316 19700 4.06E-05 19100 4.22E-05
20000 0.000122 18000 7.70E-06 17800 2.51E-05 17400
0.000274865 19700 4.06E-05 19100 4.22E-05
20000 0.000122 18000 7.70E-06 17800 2.50E-05 17400
0.000270535 19700 4.07E-05 19100 4.22E-05
20000 0.000122 18000 7.71E-06 17800 2.49E-05 17400
0.000266328 19700 4.07E-05 19100 4.22E-05
20000 0.000122 18000 7.71E-06 17800 2.48E-05 17400
0.000262228 19700 4.08E-05 19100 4.22E-05
20000 0.000122 18000 7.71E-06 17800 2.47E-05 17400
0.000258252 19700 4.09E-05 19100 4.23E-05
20000 0.000122 18000 7.71E-06 17800 2.46E-05 17400
0.000254372 19700 4.09E-05 19100 4.23E-05
20000 0.000122 18000 7.71E-06 17800 2.45E-05 17400
0.000250614 19700 4.10E-05 19100 4.23E-05
20000 0.000122 18000 7.70E-06 17800 2.44E-05 17400
0.000246941 19700 4.11E-05 19100 4.23E-05
20000 0.000122 18000 7.70E-06 17800 2.44E-05 17400
0.000243363 19700 4.11E-05 19100 4.23E-05
20000 0.000122 18000 7.70E-06 17800 2.43E-05 17400
0.000239883 19700 4.12E-05 19100 4.23E-05
20000 0.000122 18000 7.70E-06 17800 2.42E-05 17400
0.000236487 19700 4.12E-05 19100 4.24E-05
20000 0.000122 18000 7.70E-06 17800 2.41E-05 17400
0.000233185 19700 4.13E-05 19100 4.24E-05
20000 0.000122 18000 7.70E-06 17800 2.40E-05 17400
0.000229953 19700 4.14E-05 19100 4.24E-05
20000 0.000122 18000 7.70E-06 17800 2.40E-05 17400
0.000226787 19700 4.14E-05 19100 4.24E-05
20000 0.000122 18000 7.68E-06 17800 2.39E-05 17400
0.000223689 19700 4.15E-05 19100 4.24E-05
20000 0.000122 18000 7.69E-06 17800 2.38E-05 17400
0.000220676 19700 4.16E-05 19100 4.25E-05
20000 0.000122 18000 7.69E-06 17800 2.37E-05 17400
0.000217709 19700 4.16E-05 19100 4.25E-05
20000 0.000122 18000 7.68E-06 17800 2.37E-05 17400
0.000214821 19700 4.17E-05 19100 4.25E-05
20000 0.000122 18000 7.68E-06 17800 2.36E-05 17400
0.000211982 19700 4.17E-05 19100 4.25E-05
20000 0.000122 18000 7.69E-06 17800 2.35E-05 17400
0.000209218 19700 4.18E-05 19100 4.25E-05
20000 0.000122 18000 7.68E-06 17800 2.35E-05 17400
0.000206524 19700 4.19E-05 19100 4.26E-05
20000 0.000122 18000 7.68E-06 17800 2.34E-05 17400
0.000203882 19700 4.19E-05 19100 4.26E-05
20000 0.000122 18000 7.69E-06 17800 2.33E-05 17400
0.000201296 19700 4.20E-05 19100 4.26E-05
20000 0.000122 18000 7.67E-06 17800 2.32E-05 17400
0.000198765 19700 4.21E-05 19100 4.26E-05
20100 0.000122 18000 7.67E-06 17800 2.32E-05 17400
0.000196277 19700 4.21E-05 19100 4.27E-05

20100 0.000122 18000 7.67E-06 17800 2.31E-05 17400
0.000193851 19700 4.22E-05 19100 4.27E-05
20100 0.000122 18000 7.66E-06 17800 2.31E-05 17400
0.000191495 19700 4.23E-05 19100 4.27E-05
20100 0.000122 18000 7.66E-06 17800 2.30E-05 17400
0.000189161 19700 4.23E-05 19100 4.27E-05
20100 0.000122 18000 7.66E-06 17800 2.29E-05 17400
0.000186888 19700 4.24E-05 19100 4.27E-05
20100 0.000122 18000 7.65E-06 17800 2.29E-05 17400
0.000184637 19700 4.25E-05 19100 4.28E-05
20100 0.000122 18000 7.65E-06 17800 2.28E-05 17400
0.000182449 19700 4.26E-05 19100 4.28E-05
20100 0.000122 18000 7.65E-06 17800 2.28E-05 17400
0.000180292 19700 4.26E-05 19100 4.28E-05
20100 0.000122 18000 7.65E-06 17800 2.27E-05 17400
0.000178179 19700 4.27E-05 19100 4.28E-05
20100 0.000122 18000 7.64E-06 17800 2.26E-05 17400
0.000176086 19700 4.28E-05 19200 4.28E-05
20100 0.000122 18000 7.65E-06 17800 2.26E-05 17400
0.000174051 19700 4.29E-05 19200 4.29E-05
20100 0.000122 18000 7.65E-06 17800 2.25E-05 17400
0.000172031 19700 4.30E-05 19200 4.29E-05
20100 0.000122 18000 7.65E-06 17800 2.25E-05 17400
0.000170062 19700 4.30E-05 19200 4.29E-05
20100 0.000122 18000 7.65E-06 17800 2.24E-05 17400
0.000168138 19700 4.31E-05 19200 4.29E-05
20100 0.000122 18000 7.66E-06 17800 2.24E-05 17400
0.00016626 19700 4.32E-05 19200 4.29E-05
20100 0.000122 18000 7.65E-06 17800 2.23E-05 17400
0.000164419 19700 4.33E-05 19200 4.29E-05
20100 0.000122 18000 7.66E-06 17800 2.23E-05 17400
0.000162615 19700 4.34E-05 19200 4.30E-05
20100 0.000122 18000 7.66E-06 17800 2.22E-05 17400
0.000160843 19700 4.35E-05 19200 4.30E-05
20100 0.000122 18100 7.66E-06 17800 2.22E-05 17400
0.000159115 19700 4.36E-05 19200 4.30E-05
20100 0.000122 18100 7.65E-06 17800 2.21E-05 17400
0.000157415 19700 4.37E-05 19200 4.31E-05
20100 0.000122 18100 7.66E-06 17900 2.21E-05 17400
0.000155762 19700 4.37E-05 19200 4.31E-05
20100 0.000122 18100 7.65E-06 17900 2.20E-05 17400
0.000154143 19700 4.38E-05 19200 4.31E-05
20100 0.000122 18100 7.65E-06 17900 2.20E-05 17400
0.000152534 19700 4.39E-05 19200 4.31E-05
20100 0.000122 18100 7.65E-06 17900 2.19E-05 17400
0.000150977 19700 4.40E-05 19200 4.31E-05
20100 0.000122 18100 7.65E-06 17900 2.19E-05 17500
0.000149437 19700 4.41E-05 19200 4.32E-05
20100 0.000122 18100 7.66E-06 17900 2.18E-05 17500
0.000147928 19700 4.42E-05 19200 4.32E-05
20100 0.000122 18100 7.66E-06 17900 2.18E-05 17500
0.000146433 19700 4.43E-05 19200 4.32E-05
20100 0.000122 18100 7.66E-06 17900 2.17E-05 17500
0.00014497 19700 4.44E-05 19200 4.32E-05
20100 0.000122 18100 7.66E-06 17900 2.17E-05 17500
0.000143517 19700 4.46E-05 19200 4.32E-05
20100 0.000123 18100 7.65E-06 17900 2.16E-05 17500
0.0001421 19700 4.47E-05 19200 4.33E-05
20100 0.000123 18100 7.66E-06 17900 2.16E-05 17500
0.000140691 19700 4.48E-05 19200 4.33E-05
20100 0.000123 18100 7.65E-06 17900 2.16E-05 17500
0.000139318 19700 4.49E-05 19200 4.33E-05
20100 0.000123 18100 7.65E-06 17900 2.15E-05 17500
0.000137956 19700 4.50E-05 19200 4.33E-05
20100 0.000123 18100 7.67E-06 17900 2.15E-05 17500
0.000136617 19700 4.51E-05 19200 4.33E-05
20100 0.000123 18100 7.67E-06 17900 2.14E-05 17500
0.000135301 19700 4.52E-05 19200 4.34E-05

20100 0.000123 18100 7.67E-06 17900 2.14E-05 17500
0.000134015 19800 4.53E-05 19200 4.34E-05
20100 0.000123 18100 7.67E-06 17900 2.14E-05 17500
0.000132738 19800 4.54E-05 19200 4.34E-05
20100 0.000123 18100 7.67E-06 17900 2.13E-05 17500
0.000131479 19800 4.56E-05 19200 4.34E-05
20100 0.000123 18100 7.67E-06 17900 2.13E-05 17500
0.000130244 19800 4.57E-05 19200 4.34E-05
20100 0.000123 18100 7.67E-06 17900 2.12E-05 17500
0.000129043 19800 4.58E-05 19200 4.35E-05
20100 0.000123 18100 7.67E-06 17900 2.12E-05 17500
0.000127871 19800 4.59E-05 19200 4.35E-05
20100 0.000123 18100 7.67E-06 17900 2.12E-05 17500
0.000126698 19800 4.61E-05 19200 4.35E-05
20100 0.000123 18100 7.67E-06 17900 2.11E-05 17500
0.000125565 19800 4.62E-05 19200 4.36E-05
20100 0.000123 18100 7.67E-06 17900 2.11E-05 17500
0.000124443 19800 4.63E-05 19200 4.36E-05
20100 0.000123 18100 7.68E-06 17900 2.10E-05 17500
0.000123355 19800 4.65E-05 19200 4.36E-05
20100 0.000123 18100 7.68E-06 17900 2.10E-05 17500
0.000122281 19800 4.66E-05 19200 4.36E-05
20100 0.000123 18100 7.69E-06 17900 2.09E-05 17500
0.000121219 19800 4.68E-05 19200 4.36E-05
20100 0.000123 18100 7.69E-06 17900 2.09E-05 17500
0.000120177 19800 4.69E-05 19200 4.36E-05
20100 0.000123 18100 7.69E-06 17900 2.09E-05 17500
0.000119136 19800 4.71E-05 19200 4.37E-05
20100 0.000123 18100 7.69E-06 17900 2.09E-05 17500
0.000118107 19800 4.72E-05 19200 4.37E-05
20100 0.000123 18100 7.69E-06 17900 2.08E-05 17500
0.000117114 19800 4.74E-05 19200 4.37E-05
20100 0.000123 18100 7.69E-06 17900 2.08E-05 17500
0.000116097 19800 4.75E-05 19200 4.37E-05
20100 0.000123 18100 7.70E-06 17900 2.08E-05 17500
0.000115124 19800 4.77E-05 19200 4.37E-05
20100 0.000123 18100 7.70E-06 17900 2.07E-05 17500
0.000114147 19800 4.78E-05 19200 4.38E-05
20100 0.000123 18100 7.70E-06 17900 2.07E-05 17500
0.000113188 19800 4.80E-05 19200 4.38E-05
20100 0.000123 18100 7.70E-06 17900 2.06E-05 17500
0.000112224 19800 4.82E-05 19200 4.38E-05
20100 0.000123 18100 7.70E-06 17900 2.06E-05 17500
0.000111282 19800 4.83E-05 19200 4.38E-05
20100 0.000123 18100 7.69E-06 17900 2.06E-05 17500
0.000110349 19800 4.85E-05 19200 4.38E-05
20100 0.000123 18100 7.69E-06 17900 2.05E-05 17500
0.00010943 19800 4.87E-05 19200 4.39E-05
20100 0.000123 18100 7.69E-06 17900 2.05E-05 17500
0.00010853 19800 4.89E-05 19200 4.39E-05
20100 0.000123 18100 7.69E-06 17900 2.05E-05 17500
0.000107648 19800 4.91E-05 19200 4.39E-05
20100 0.000123 18100 7.69E-06 17900 2.04E-05 17500
0.000106772 19800 4.93E-05 19200 4.40E-05
20100 0.000124 18100 7.69E-06 17900 2.04E-05 17500
0.000105916 19800 4.94E-05 19200 4.40E-05
20100 0.000124 18100 7.70E-06 17900 2.04E-05 17500
0.000105081 19800 4.96E-05 19200 4.40E-05
20200 0.000124 18100 7.71E-06 17900 2.04E-05 17500
0.000104245 19800 4.98E-05 19200 4.40E-05
20200 0.000124 18100 7.71E-06 17900 2.03E-05 17500
0.000103426 19800 5.00E-05 19200 4.40E-05
20200 0.000124 18100 7.70E-06 17900 2.03E-05 17500
0.000102616 19800 5.03E-05 19200 4.41E-05
20200 0.000124 18100 7.70E-06 17900 2.03E-05 17500
0.00010181 19800 5.05E-05 19200 4.41E-05
20200 0.000124 18100 7.70E-06 17900 2.02E-05 17500
0.000101014 19800 5.07E-05 19200 4.41E-05

20200	0.000124	18100	7.70E-06	17900	2.02E-05	17500
0.000100232	19800	5.09E-05	19200	4.41E-05		
20200	0.000124	18100	7.70E-06	17900	2.02E-05	17500
9.94E-05	19800	5.11E-05	19200	4.41E-05		
20200	0.000124	18100	7.71E-06	17900	2.02E-05	17500
9.87E-05	19800	5.14E-05	19200	4.42E-05		
20200	0.000124	18100	7.71E-06	17900	2.01E-05	17500
9.79E-05	19800	5.16E-05	19200	4.42E-05		
20200	0.000124	18100	7.71E-06	17900	2.01E-05	17500
9.72E-05	19800	5.18E-05	19300	4.42E-05		
20200	0.000124	18100	7.71E-06	17900	2.01E-05	17500
9.65E-05	19800	5.21E-05	19300	4.42E-05		
20200	0.000124	18100	7.71E-06	17900	2.01E-05	17500
9.57E-05	19800	5.23E-05	19300	4.43E-05		
20200	0.000124	18100	7.71E-06	17900	2.00E-05	17500
9.50E-05	19800	5.26E-05	19300	4.43E-05		
20200	0.000124	18100	7.71E-06	17900	2.00E-05	17500
9.43E-05	19800	5.29E-05	19300	4.43E-05		
20200	0.000124	18100	7.71E-06	17900	2.00E-05	17500
9.37E-05	19800	5.31E-05	19300	4.43E-05		
20200	0.000124	18100	7.70E-06	17900	1.99E-05	17500
9.30E-05	19800	5.34E-05	19300	4.43E-05		
20200	0.000124	18100	7.70E-06	17900	1.99E-05	17500
9.23E-05	19800	5.37E-05	19300	4.44E-05		
20200	0.000124	18100	7.70E-06	17900	1.99E-05	17500
9.17E-05	19800	5.40E-05	19300	4.44E-05		
20200	0.000124	18200	7.71E-06	17900	1.99E-05	17500
9.10E-05	19800	5.43E-05	19300	4.45E-05		
20200	0.000124	18200	7.71E-06	17900	1.98E-05	17500
9.04E-05	19800	5.46E-05	19300	4.45E-05		
20200	0.000124	18200	7.70E-06	18000	1.98E-05	17500
8.97E-05	19800	5.49E-05	19300	4.45E-05		
20200	0.000124	18200	7.71E-06	18000	1.98E-05	17500
8.91E-05	19800	5.52E-05	19300	4.45E-05		
20200	0.000124	18200	7.71E-06	18000	1.98E-05	17500
8.84E-05	19800	5.55E-05	19300	4.45E-05		
20200	0.000125	18200	7.71E-06	18000	1.98E-05	17500
8.78E-05	19800	5.59E-05	19300	4.45E-05		
20200	0.000125	18200	7.71E-06	18000	1.97E-05	17600
8.72E-05	19800	5.62E-05	19300	4.45E-05		
20200	0.000125	18200	7.72E-06	18000	1.97E-05	17600
8.66E-05	19800	5.65E-05	19300	4.46E-05		
20200	0.000125	18200	7.72E-06	18000	1.97E-05	17600
8.60E-05	19800	5.69E-05	19300	4.46E-05		
20200	0.000125	18200	7.73E-06	18000	1.97E-05	17600
8.54E-05	19800	5.73E-05	19300	4.46E-05		
20200	0.000125	18200	7.73E-06	18000	1.96E-05	17600
8.48E-05	19800	5.76E-05	19300	4.46E-05		
20200	0.000125	18200	7.73E-06	18000	1.96E-05	17600
8.42E-05	19800	5.80E-05	19300	4.46E-05		
20200	0.000125	18200	7.73E-06	18000	1.96E-05	17600
8.36E-05	19800	5.84E-05	19300	4.46E-05		
20200	0.000125	18200	7.73E-06	18000	1.96E-05	17600
8.31E-05	19800	5.88E-05	19300	4.47E-05		
20200	0.000125	18200	7.73E-06	18000	1.96E-05	17600
8.25E-05	19800	5.92E-05	19300	4.46E-05		
20200	0.000125	18200	7.73E-06	18000	1.95E-05	17600
8.20E-05	19800	5.97E-05	19300	4.47E-05		
20200	0.000125	18200	7.73E-06	18000	1.95E-05	17600
8.14E-05	19800	6.01E-05	19300	4.47E-05		
20200	0.000125	18200	7.72E-06	18000	1.95E-05	17600
8.09E-05	19900	6.06E-05	19300	4.47E-05		
20200	0.000125	18200	7.72E-06	18000	1.95E-05	17600
8.04E-05	19900	6.10E-05	19300	4.47E-05		
20200	0.000125	18200	7.73E-06	18000	1.95E-05	17600
7.99E-05	19900	6.15E-05	19300	4.48E-05		
20200	0.000125	18200	7.73E-06	18000	1.94E-05	17600
7.94E-05	19900	6.20E-05	19300	4.48E-05		

20200 0.000125 18200 7.73E-06 18000 1.94E-05 17600
7.89E-05 19900 6.25E-05 19300 4.48E-05
20200 0.000125 18200 7.73E-06 18000 1.94E-05 17600
7.84E-05 19900 6.30E-05 19300 4.48E-05
20200 0.000125 18200 7.73E-06 18000 1.94E-05 17600
7.80E-05 19900 6.36E-05 19300 4.49E-05
20200 0.000126 18200 7.74E-06 18000 1.94E-05 17600
7.75E-05 19900 6.41E-05 19300 4.49E-05
20200 0.000126 18200 7.74E-06 18000 1.94E-05 17600
7.70E-05 19900 6.47E-05 19300 4.49E-05
20200 0.000126 18200 7.74E-06 18000 1.94E-05 17600
7.65E-05 19900 6.53E-05 19300 4.49E-05
20200 0.000126 18200 7.74E-06 18000 1.94E-05 17600
7.61E-05 19900 6.59E-05 19300 4.49E-05
20200 0.000126 18200 7.74E-06 18000 1.93E-05 17600
7.56E-05 19900 6.65E-05 19300 4.49E-05
20200 0.000126 18200 7.75E-06 18000 1.93E-05 17600
7.52E-05 19900 6.71E-05 19300 4.50E-05
20200 0.000126 18200 7.75E-06 18000 1.93E-05 17600
7.47E-05 19900 6.78E-05 19300 4.50E-05
20200 0.000126 18200 7.75E-06 18000 1.93E-05 17600
7.43E-05 19900 6.85E-05 19300 4.51E-05
20200 0.000126 18200 7.75E-06 18000 1.93E-05 17600
7.38E-05 19900 6.92E-05 19300 4.51E-05
20200 0.000126 18200 7.75E-06 18000 1.92E-05 17600
7.34E-05 19900 6.99E-05 19300 4.51E-05
20200 0.000126 18200 7.75E-06 18000 1.92E-05 17600
7.30E-05 19900 7.07E-05 19300 4.51E-05
20200 0.000126 18200 7.74E-06 18000 1.92E-05 17600
7.25E-05 19900 7.15E-05 19300 4.52E-05
20200 0.000126 18200 7.75E-06 18000 1.92E-05 17600
7.21E-05 19900 7.23E-05 19300 4.52E-05
20200 0.000126 18200 7.75E-06 18000 1.92E-05 17600
7.17E-05 19900 7.32E-05 19300 4.52E-05
20200 0.000126 18200 7.75E-06 18000 1.92E-05 17600
7.13E-05 19900 7.40E-05 19300 4.52E-05
20200 0.000126 18200 7.75E-06 18000 1.91E-05 17600
7.09E-05 19900 7.49E-05 19300 4.52E-05
20200 0.000126 18200 7.74E-06 18000 1.91E-05 17600
7.04E-05 19900 7.59E-05 19300 4.53E-05
20200 0.000126 18200 7.75E-06 18000 1.91E-05 17600
7.00E-05 19900 7.68E-05 19300 4.53E-05
20200 0.000126 18200 7.75E-06 18000 1.91E-05 17600
6.96E-05 19900 7.78E-05 19300 4.54E-05
20200 0.000127 18200 7.75E-06 18000 1.91E-05 17600
6.93E-05 19900 7.89E-05 19300 4.54E-05
20200 0.000127 18200 7.76E-06 18000 1.91E-05 17600
6.89E-05 19900 7.99E-05 19300 4.54E-05
20200 0.000127 18200 7.76E-06 18000 1.91E-05 17600
6.85E-05 19900 8.10E-05 19300 4.55E-05
20300 0.000127 18200 7.76E-06 18000 1.90E-05 17600
6.81E-05 19900 8.22E-05 19300 4.55E-05
20300 0.000127 18200 7.76E-06 18000 1.90E-05 17600
6.77E-05 19900 8.34E-05 19300 4.55E-05
20300 0.000127 18200 7.77E-06 18000 1.90E-05 17600
6.74E-05 19900 8.46E-05 19300 4.55E-05
20300 0.000127 18200 7.77E-06 18000 1.90E-05 17600
6.70E-05 19900 8.59E-05 19300 4.56E-05
20300 0.000127 18200 7.76E-06 18000 1.90E-05 17600
6.66E-05 19900 8.72E-05 19300 4.56E-05
20300 0.000127 18200 7.77E-06 18000 1.90E-05 17600
6.63E-05 19900 8.86E-05 19300 4.56E-05
20300 0.000127 18200 7.77E-06 18000 1.89E-05 17600
6.59E-05 19900 9.01E-05 19300 4.57E-05
20300 0.000127 18200 7.77E-06 18000 1.89E-05 17600
6.56E-05 19900 9.15E-05 19300 4.57E-05
20300 0.000127 18200 7.77E-06 18000 1.89E-05 17600
6.53E-05 19900 9.31E-05 19300 4.57E-05

20300	0.000127	18200	7.77E-06	18000	1.89E-05	17600	6.49E-05	19900	9.47E-05	19400	4.57E-05
20300	0.000127	18200	7.77E-06	18000	1.89E-05	17600	6.46E-05	19900	9.64E-05	19400	4.58E-05
20300	0.000127	18200	7.78E-06	18000	1.89E-05	17600	6.43E-05	19900	9.82E-05	19400	4.58E-05
20300	0.000127	18200	7.78E-06	18000	1.89E-05	17600	6.40E-05	19900	0.0001	19400	4.58E-05
20300	0.000128	18200	7.77E-06	18000	1.89E-05	17600	6.36E-05	19900	0.000102	19400	4.59E-05
20300	0.000128	18200	7.77E-06	18000	1.89E-05	17600	6.33E-05	19900	0.000104	19400	4.59E-05
20300	0.000128	18200	7.77E-06	18000	1.88E-05	17600	6.30E-05	19900	0.000106	19400	4.59E-05
20300	0.000128	18200	7.79E-06	18000	1.88E-05	17600	6.27E-05	19900	0.000108	19400	4.59E-05
20300	0.000128	18200	7.78E-06	18000	1.88E-05	17600	6.24E-05	19900	0.000111	19400	4.60E-05
20300	0.000128	18300	7.79E-06	18000	1.88E-05	17600	6.20E-05	19900	0.000113	19400	4.60E-05
20300	0.000128	18300	7.79E-06	18000	1.88E-05	17600	6.17E-05	19900	0.000115	19400	4.60E-05
20300	0.000128	18300	7.79E-06	18100	1.88E-05	17600	6.14E-05	19900	0.000118	19400	4.61E-05
20300	0.000128	18300	7.78E-06	18100	1.88E-05	17600	6.11E-05	19900	0.000121	19400	4.61E-05
20300	0.000128	18300	7.79E-06	18100	1.88E-05	17600	6.08E-05	19900	0.000124	19400	4.61E-05
20300	0.000128	18300	7.79E-06	18100	1.88E-05	17600	6.05E-05	19900	0.000127	19400	4.62E-05
20300	0.000128	18300	7.79E-06	18100	1.87E-05	17700	6.02E-05	19900	0.00013	19400	4.62E-05
20300	0.000128	18300	7.80E-06	18100	1.87E-05	17700	5.99E-05	19900	0.000133	19400	4.62E-05
20300	0.000128	18300	7.80E-06	18100	1.87E-05	17700	5.97E-05	19900	0.000136	19400	4.63E-05
20300	0.000128	18300	7.80E-06	18100	1.87E-05	17700	5.94E-05	19900	0.00014	19400	4.63E-05
20300	0.000128	18300	7.80E-06	18100	1.87E-05	17700	5.91E-05	19900	0.000144	19400	4.63E-05
20300	0.000128	18300	7.80E-06	18100	1.87E-05	17700	5.88E-05	19900	0.000148	19400	4.64E-05
20300	0.000128	18300	7.81E-06	18100	1.87E-05	17700	5.85E-05	19900	0.000152	19400	4.64E-05
20300	0.000128	18300	7.80E-06	18100	1.86E-05	17700	5.82E-05	19900	0.000156	19400	4.64E-05
20300	0.000128	18300	7.80E-06	18100	1.86E-05	17700	5.79E-05	19900	0.000161	19400	4.65E-05
20300	0.000129	18300	7.80E-06	18100	1.86E-05	17700	5.76E-05	19900	0.000166	19400	4.65E-05
20300	0.000129	18300	7.80E-06	18100	1.86E-05	17700	5.74E-05	19900	0.000171	19400	4.65E-05
20300	0.000129	18300	7.80E-06	18100	1.86E-05	17700	5.71E-05	20000	0.000176	19400	4.66E-05
20300	0.000129	18300	7.80E-06	18100	1.86E-05	17700	5.68E-05	20000	0.000181	19400	4.66E-05
20300	0.000129	18300	7.81E-06	18100	1.86E-05	17700	5.66E-05	20000	0.000187	19400	4.66E-05
20300	0.000129	18300	7.82E-06	18100	1.86E-05	17700	5.63E-05	20000	0.000193	19400	4.67E-05
20300	0.000129	18300	7.82E-06	18100	1.86E-05	17700	5.61E-05	20000	0.0002	19400	4.67E-05
20300	0.000129	18300	7.82E-06	18100	1.86E-05	17700	5.59E-05	20000	0.000206	19400	4.67E-05
20300	0.000129	18300	7.83E-06	18100	1.86E-05	17700	5.56E-05	20000	0.000213	19400	4.68E-05
20300	0.000129	18300	7.82E-06	18100	1.86E-05	17700	5.54E-05	20000	0.000221	19400	4.68E-05

20300 0.000129 18300 7.82E-06 18100 1.85E-05 17700
5.51E-05 20000 0.000228 19400 4.69E-05
20300 0.000129 18300 7.82E-06 18100 1.85E-05 17700
5.49E-05 20000 0.000236 19400 4.69E-05
20300 0.000129 18300 7.82E-06 18100 1.85E-05 17700
5.47E-05 20000 0.000245 19400 4.69E-05
20300 0.000129 18300 7.83E-06 18100 1.85E-05 17700
5.44E-05 20000 0.000253 19400 4.70E-05
20300 0.000129 18300 7.83E-06 18100 1.85E-05 17700
5.42E-05 20000 0.000262 19400 4.70E-05
20300 0.000129 18300 7.83E-06 18100 1.85E-05 17700
5.40E-05 20000 0.000272 19400 4.71E-05
20300 0.000129 18300 7.83E-06 18100 1.85E-05 17700
5.38E-05 20000 0.000281 19400 4.71E-05
20300 0.000129 18300 7.84E-06 18100 1.85E-05 17700
5.35E-05 20000 0.000291 19400 4.71E-05
20300 0.000129 18300 7.84E-06 18100 1.85E-05 17700
5.33E-05 20000 0.000302 19400 4.72E-05
20300 0.00013 18300 7.85E-06 18100 1.84E-05 17700 5.31E-05
20000 0.000312 19400 4.72E-05
20300 0.00013 18300 7.85E-06 18100 1.84E-05 17700 5.29E-05
20000 0.000323 19400 4.73E-05
20300 0.00013 18300 7.86E-06 18100 1.84E-05 17700 5.26E-05
20000 0.000334 19400 4.73E-05
20300 0.00013 18300 7.86E-06 18100 1.84E-05 17700 5.24E-05
20000 0.000345 19400 4.73E-05
20300 0.00013 18300 7.87E-06 18100 1.84E-05 17700 5.22E-05
20000 0.000355 19400 4.74E-05
20300 0.00013 18300 7.87E-06 18100 1.84E-05 17700 5.20E-05
20000 0.000366 19400 4.74E-05
20300 0.00013 18300 7.88E-06 18100 1.84E-05 17700 5.18E-05
20000 0.000377 19400 4.75E-05
20300 0.00013 18300 7.87E-06 18100 1.84E-05 17700 5.16E-05
20000 0.000388 19400 4.75E-05
20300 0.00013 18300 7.88E-06 18100 1.84E-05 17700 5.14E-05
20000 0.000398 19400 4.75E-05
20300 0.00013 18300 7.88E-06 18100 1.84E-05 17700 5.12E-05
20000 0.000407 19400 4.76E-05
20300 0.00013 18300 7.87E-06 18100 1.84E-05 17700 5.10E-05
20000 0.000416 19400 4.76E-05
20300 0.00013 18300 7.87E-06 18100 1.84E-05 17700 5.08E-05
20000 0.000424 19400 4.76E-05
20400 0.00013 18300 7.86E-06 18100 1.84E-05 17700 5.06E-05
20000 0.000432 19400 4.77E-05
20400 0.00013 18300 7.87E-06 18100 1.84E-05 17700 5.04E-05
20000 0.000438 19400 4.77E-05
20400 0.00013 18300 7.86E-06 18100 1.84E-05 17700 5.02E-05
20000 0.000443 19400 4.78E-05
20400 0.00013 18300 7.86E-06 18100 1.83E-05 17700 5.00E-05
20000 0.000446 19400 4.78E-05
20400 0.00013 18300 7.87E-06 18100 1.83E-05 17700 4.98E-05
20000 0.000449 19400 4.79E-05
20400 0.00013 18300 7.86E-06 18100 1.83E-05 17700 4.96E-05
20000 0.00045 19400 4.79E-05
20400 0.000131 18300 7.86E-06 18100 1.83E-05 17700
4.94E-05 20000 0.000449 19400 4.79E-05
20400 0.000131 18300 7.86E-06 18100 1.83E-05 17700
4.92E-05 20000 0.000447 19400 4.80E-05
20400 0.000131 18300 7.86E-06 18100 1.83E-05 17700
4.90E-05 20000 0.000443 19400 4.80E-05
20400 0.000131 18300 7.87E-06 18100 1.83E-05 17700
4.88E-05 20000 0.000438 19500 4.81E-05
20400 0.000131 18300 7.87E-06 18100 1.83E-05 17700
4.87E-05 20000 0.000431 19500 4.81E-05
20400 0.000131 18300 7.88E-06 18100 1.83E-05 17700
4.85E-05 20000 0.000423 19500 4.81E-05
20400 0.000131 18300 7.89E-06 18100 1.83E-05 17700
4.83E-05 20000 0.000414 19500 4.82E-05

20400 0.000131 18300 7.89E-06 18100 1.83E-05 17700
4.81E-05 20000 0.000404 19500 4.82E-05
20400 0.000131 18300 7.90E-06 18100 1.83E-05 17700
4.80E-05 20000 0.000393 19500 4.83E-05
20400 0.000131 18300 7.91E-06 18100 1.83E-05 17700
4.78E-05 20000 0.000382 19500 4.83E-05
20400 0.000131 18300 7.91E-06 18100 1.83E-05 17700
4.76E-05 20000 0.00037 19500 4.84E-05
20400 0.000131 18300 7.91E-06 18100 1.83E-05 17700
4.74E-05 20000 0.000357 19500 4.84E-05
20400 0.000131 18400 7.91E-06 18100 1.83E-05 17700
4.73E-05 20000 0.000345 19500 4.85E-05
20400 0.000131 18400 7.90E-06 18100 1.83E-05 17700
4.71E-05 20000 0.000332 19500 4.85E-05
20400 0.000131 18400 7.90E-06 18200 1.83E-05 17700
4.69E-05 20000 0.000319 19500 4.86E-05
20400 0.000131 18400 7.90E-06 18200 1.82E-05 17700
4.68E-05 20000 0.000307 19500 4.86E-05
20400 0.000132 18400 7.90E-06 18200 1.82E-05 17700
4.66E-05 20000 0.000295 19500 4.87E-05
20400 0.000132 18400 7.90E-06 18200 1.82E-05 17700
4.64E-05 20000 0.000283 19500 4.87E-05
20400 0.000132 18400 7.90E-06 18200 1.82E-05 17800
4.63E-05 20000 0.000271 19500 4.87E-05
20400 0.000132 18400 7.90E-06 18200 1.82E-05 17800
4.61E-05 20000 0.00026 19500 4.88E-05
20400 0.000132 18400 7.89E-06 18200 1.82E-05 17800
4.59E-05 20000 0.00025 19500 4.88E-05
20400 0.000132 18400 7.90E-06 18200 1.82E-05 17800
4.58E-05 20000 0.00024 19500 4.89E-05
20400 0.000132 18400 7.90E-06 18200 1.82E-05 17800
4.56E-05 20000 0.00023 19500 4.89E-05
20400 0.000132 18400 7.90E-06 18200 1.82E-05 17800
4.55E-05 20000 0.000221 19500 4.89E-05
20400 0.000132 18400 7.90E-06 18200 1.82E-05 17800
4.53E-05 20000 0.000213 19500 4.90E-05
20400 0.000132 18400 7.90E-06 18200 1.82E-05 17800
4.51E-05 20000 0.000205 19500 4.90E-05
20400 0.000132 18400 7.91E-06 18200 1.82E-05 17800
4.50E-05 20000 0.000197 19500 4.91E-05
20400 0.000132 18400 7.91E-06 18200 1.82E-05 17800
4.48E-05 20000 0.00019 19500 4.91E-05
20400 0.000132 18400 7.92E-06 18200 1.82E-05 17800
4.47E-05 20000 0.000183 19500 4.92E-05
20400 0.000132 18400 7.92E-06 18200 1.82E-05 17800
4.45E-05 20100 0.000177 19500 4.92E-05
20400 0.000132 18400 7.92E-06 18200 1.82E-05 17800
4.43E-05 20100 0.000171 19500 4.92E-05
20400 0.000132 18400 7.92E-06 18200 1.82E-05 17800
4.42E-05 20100 0.000165 19500 4.93E-05
20400 0.000133 18400 7.91E-06 18200 1.82E-05 17800
4.40E-05 20100 0.00016 19500 4.93E-05
20400 0.000133 18400 7.91E-06 18200 1.82E-05 17800
4.39E-05 20100 0.000155 19500 4.94E-05
20400 0.000133 18400 7.91E-06 18200 1.82E-05 17800
4.37E-05 20100 0.00015 19500 4.94E-05
20400 0.000133 18400 7.92E-06 18200 1.82E-05 17800
4.36E-05 20100 0.000146 19500 4.94E-05
20400 0.000133 18400 7.92E-06 18200 1.81E-05 17800
4.35E-05 20100 0.000142 19500 4.95E-05
20400 0.000133 18400 7.92E-06 18200 1.82E-05 17800
4.33E-05 20100 0.000138 19500 4.95E-05
20400 0.000133 18400 7.92E-06 18200 1.82E-05 17800
4.32E-05 20100 0.000134 19500 4.95E-05
20400 0.000133 18400 7.92E-06 18200 1.82E-05 17800
4.31E-05 20100 0.00013 19500 4.96E-05
20400 0.000133 18400 7.92E-06 18200 1.82E-05 17800
4.29E-05 20100 0.000127 19500 4.96E-05

20400 0.000133 18400 7.93E-06 18200 1.82E-05 17800
4.28E-05 20100 0.000124 19500 4.97E-05
20400 0.000133 18400 7.92E-06 18200 1.82E-05 17800
4.26E-05 20100 0.000121 19500 4.97E-05
20400 0.000133 18400 7.94E-06 18200 1.82E-05 17800
4.25E-05 20100 0.000118 19500 4.97E-05
20400 0.000133 18400 7.95E-06 18200 1.82E-05 17800
4.24E-05 20100 0.000115 19500 4.98E-05
20400 0.000133 18400 7.94E-06 18200 1.82E-05 17800
4.22E-05 20100 0.000113 19500 4.98E-05
20400 0.000133 18400 7.95E-06 18200 1.82E-05 17800
4.21E-05 20100 0.000111 19500 4.99E-05
20400 0.000134 18400 7.95E-06 18200 1.82E-05 17800
4.20E-05 20100 0.000108 19500 4.99E-05
20400 0.000134 18400 7.95E-06 18200 1.81E-05 17800
4.18E-05 20100 0.000106 19500 4.99E-05
20400 0.000134 18400 7.95E-06 18200 1.81E-05 17800
4.17E-05 20100 0.000104 19500 5.00E-05
20400 0.000134 18400 7.95E-06 18200 1.81E-05 17800
4.16E-05 20100 0.000102 19500 5.00E-05
20400 0.000134 18400 7.95E-06 18200 1.81E-05 17800
4.15E-05 20100 0.0001 19500 5.00E-05
20400 0.000134 18400 7.95E-06 18200 1.81E-05 17800
4.14E-05 20100 9.83E-05 19500 5.01E-05
20400 0.000134 18400 7.94E-06 18200 1.81E-05 17800
4.13E-05 20100 9.66E-05 19500 5.01E-05
20400 0.000134 18400 7.94E-06 18200 1.81E-05 17800
4.12E-05 20100 9.50E-05 19500 5.02E-05
20400 0.000134 18400 7.95E-06 18200 1.81E-05 17800
4.11E-05 20100 9.34E-05 19500 5.02E-05
20400 0.000134 18400 7.94E-06 18200 1.81E-05 17800
4.10E-05 20100 9.19E-05 19500 5.03E-05
20400 0.000134 18400 7.94E-06 18200 1.81E-05 17800
4.09E-05 20100 9.05E-05 19500 5.03E-05
20500 0.000134 18400 7.94E-06 18200 1.81E-05 17800
4.08E-05 20100 8.91E-05 19500 5.04E-05
20500 0.000135 18400 7.95E-06 18200 1.81E-05 17800
4.07E-05 20100 8.78E-05 19500 5.04E-05
20500 0.000135 18400 7.95E-06 18200 1.81E-05 17800
4.05E-05 20100 8.66E-05 19500 5.05E-05
20500 0.000135 18400 7.95E-06 18200 1.81E-05 17800
4.04E-05 20100 8.53E-05 19500 5.06E-05
20500 0.000135 18400 7.96E-06 18200 1.81E-05 17800
4.03E-05 20100 8.42E-05 19500 5.06E-05
20500 0.000135 18400 7.96E-06 18200 1.81E-05 17800
4.02E-05 20100 8.31E-05 19500 5.07E-05
20500 0.000135 18400 7.96E-06 18200 1.81E-05 17800
4.01E-05 20100 8.20E-05 19500 5.07E-05
20500 0.000135 18400 7.96E-06 18200 1.81E-05 17800
4.00E-05 20100 8.10E-05 19500 5.08E-05
20500 0.000135 18400 7.97E-06 18200 1.81E-05 17800
3.99E-05 20100 8.00E-05 19500 5.09E-05
20500 0.000135 18400 7.97E-06 18200 1.81E-05 17800
3.98E-05 20100 7.90E-05 19600 5.09E-05
20500 0.000135 18400 7.97E-06 18200 1.81E-05 17800
3.97E-05 20100 7.81E-05 19600 5.10E-05
20500 0.000135 18400 7.97E-06 18200 1.81E-05 17800
3.96E-05 20100 7.73E-05 19600 5.11E-05
20500 0.000135 18400 7.97E-06 18200 1.81E-05 17800
3.95E-05 20100 7.64E-05 19600 5.12E-05
20500 0.000135 18400 7.97E-06 18200 1.81E-05 17800
3.94E-05 20100 7.56E-05 19600 5.12E-05
20500 0.000136 18400 7.97E-06 18200 1.81E-05 17800
3.93E-05 20100 7.48E-05 19600 5.13E-05
20500 0.000136 18400 7.97E-06 18200 1.81E-05 17800
3.92E-05 20100 7.41E-05 19600 5.14E-05
20500 0.000136 18400 7.98E-06 18200 1.81E-05 17800
3.91E-05 20100 7.33E-05 19600 5.15E-05

```
20500   0.000136        18400   7.98E-06        18200   1.81E-05        17800
3.90E-05        20100   7.26E-05        19600   5.15E-05
20500   0.000136        18500   7.99E-06        18200   1.81E-05        17800
3.89E-05        20100   7.19E-05        19600   5.16E-05
20500   0.000136        18500   7.99E-06        18200   1.81E-05        17800
3.88E-05        20100   7.13E-05        19600   5.17E-05
20500   0.000136        18500   8.00E-06        18300   1.81E-05        17800
3.87E-05        20100   7.06E-05        19600   5.18E-05
20500   0.000136        18500   8.00E-06        18300   1.81E-05        17800
3.86E-05        20100   7.00E-05        19600   5.19E-05
20500   0.000136        18500   8.00E-06        18300   1.81E-05        17800
3.85E-05        20100   6.94E-05        19600   5.20E-05
20500   0.000136        18500   8.00E-06        18300   1.81E-05        17800
3.84E-05        20100   6.89E-05        19600   5.21E-05
20500   0.000136        18500   8.01E-06        18300   1.81E-05        17900
3.82E-05        20100   6.83E-05        19600   5.22E-05
20500   0.000136        18500   8.01E-06        18300   1.81E-05        17900
3.82E-05        20100   6.78E-05        19600   5.23E-05
20500   0.000137        18500   8.01E-06        18300   1.81E-05        17900
3.80E-05        20100   6.72E-05        19600   5.24E-05
20500   0.000137        18500   8.02E-06        18300   1.81E-05        17900
3.79E-05        20100   6.67E-05        19600   5.24E-05
20500   0.000137        18500   8.02E-06        18300   1.81E-05        17900
3.79E-05        20100   6.63E-05        19600   5.26E-05
20500   0.000137        18500   8.02E-06        18300   1.81E-05        17900
3.77E-05        20100   6.58E-05        19600   5.27E-05
20500   0.000137        18500   8.01E-06        18300   1.81E-05        17900
3.76E-05        20100   6.53E-05        19600   5.28E-05
20500   0.000137        18500   8.02E-06        18300   1.81E-05        17900
3.76E-05        20100   6.49E-05        19600   5.29E-05
20500   0.000137        18500   8.02E-06        18300   1.81E-05        17900
3.75E-05        20100   6.44E-05        19600   5.30E-05
20500   0.000137        18500   8.02E-06        18300   1.81E-05        17900
3.74E-05        20100   6.40E-05        19600   5.32E-05
20500   0.000137        18500   8.02E-06        18300   1.81E-05        17900
3.73E-05        20100   6.36E-05        19600   5.33E-05
20500   0.000137        18500   8.04E-06        18300   1.81E-05        17900
3.73E-05        20200   6.32E-05        19600   5.34E-05
20500   0.000137        18500   8.03E-06        18300   1.81E-05        17900
3.72E-05        20200   6.28E-05        19600   5.36E-05
20500   0.000137        18500   8.03E-06        18300   1.81E-05        17900
3.71E-05        20200   6.25E-05        19600   5.37E-05
20500   0.000137        18500   8.04E-06        18300   1.81E-05        17900
3.70E-05        20200   6.21E-05        19600   5.39E-05
20500   0.000138        18500   8.04E-06        18300   1.81E-05        17900
3.69E-05        20200   6.18E-05        19600   5.40E-05
20500   0.000138        18500   8.06E-06        18300   1.81E-05        17900
3.69E-05        20200   6.14E-05        19600   5.41E-05
20500   0.000138        18500   8.05E-06        18300   1.81E-05        17900
3.68E-05        20200   6.11E-05        19600   5.43E-05
20500   0.000138        18500   8.06E-06        18300   1.81E-05        17900
3.67E-05        20200   6.08E-05        19600   5.44E-05
20500   0.000138        18500   8.07E-06        18300   1.81E-05        17900
3.66E-05        20200   6.05E-05        19600   5.46E-05
20500   0.000138        18500   8.07E-06        18300   1.81E-05        17900
3.65E-05        20200   6.02E-05        19600   5.47E-05
20500   0.000138        18500   8.06E-06        18300   1.81E-05        17900
3.65E-05        20200   5.99E-05        19600   5.48E-05
20500   0.000138        18500   8.07E-06        18300   1.81E-05        17900
3.64E-05        20200   5.96E-05        19600   5.50E-05
20500   0.000138        18500   8.07E-06        18300   1.81E-05        17900
3.63E-05        20200   5.93E-05        19600   5.51E-05
20500   0.000138        18500   8.07E-06        18300   1.81E-05        17900
3.62E-05        20200   5.91E-05        19600   5.52E-05
20500   0.000138        18500   8.06E-06        18300   1.81E-05        17900
3.61E-05        20200   5.88E-05        19600   5.53E-05
20500   0.000138        18500   8.08E-06        18300   1.81E-05        17900
3.60E-05        20200   5.86E-05        19600   5.55E-05
```

20500 0.000138 18500 8.07E-06 18300 1.81E-05 17900
3.59E-05 20200 5.83E-05 19600 5.56E-05
20500 0.000139 18500 8.07E-06 18300 1.81E-05 17900
3.58E-05 20200 5.81E-05 19600 5.58E-05
20500 0.000139 18500 8.06E-06 18300 1.81E-05 17900
3.57E-05 20200 5.78E-05 19600 5.59E-05
20500 0.000139 18500 8.08E-06 18300 1.81E-05 17900
3.57E-05 20200 5.76E-05 19600 5.61E-05
20500 0.000139 18500 8.07E-06 18300 1.81E-05 17900
3.56E-05 20200 5.74E-05 19600 5.62E-05
20500 0.000139 18500 8.07E-06 18300 1.81E-05 17900
3.55E-05 20200 5.72E-05 19600 5.63E-05
20500 0.000139 18500 8.07E-06 18300 1.81E-05 17900
3.54E-05 20200 5.70E-05 19600 5.64E-05
20500 0.000139 18500 8.08E-06 18300 1.81E-05 17900
3.54E-05 20200 5.68E-05 19600 5.65E-05
20500 0.000139 18500 8.10E-06 18300 1.81E-05 17900
3.53E-05 20200 5.66E-05 19600 5.66E-05
20500 0.000139 18500 8.10E-06 18300 1.81E-05 17900
3.52E-05 20200 5.64E-05 19600 5.67E-05
20500 0.000139 18500 8.10E-06 18300 1.81E-05 17900
3.51E-05 20200 5.62E-05 19600 5.68E-05
20500 0.000139 18500 8.11E-06 18300 1.81E-05 17900
3.50E-05 20200 5.60E-05 19600 5.69E-05
20500 0.000139 18500 8.11E-06 18300 1.82E-05 17900
3.50E-05 20200 5.58E-05 19600 5.70E-05
20600 0.00014 18500 8.11E-06 18300 1.82E-05 17900 3.49E-05
20200 5.56E-05 19600 5.71E-05
20600 0.00014 18500 8.12E-06 18300 1.81E-05 17900 3.49E-05
20200 5.55E-05 19600 5.71E-05
20600 0.00014 18500 8.12E-06 18300 1.81E-05 17900 3.48E-05
20200 5.53E-05 19600 5.72E-05
20600 0.00014 18500 8.13E-06 18300 1.81E-05 17900 3.47E-05
20200 5.51E-05 19600 5.72E-05
20600 0.00014 18500 8.12E-06 18300 1.81E-05 17900 3.47E-05
20200 5.50E-05 19600 5.73E-05
20600 0.00014 18500 8.12E-06 18300 1.81E-05 17900 3.46E-05
20200 5.48E-05 19600 5.73E-05
20600 0.00014 18500 8.12E-06 18300 1.81E-05 17900 3.45E-05
20200 5.47E-05 19600 5.73E-05
20600 0.00014 18500 8.12E-06 18300 1.81E-05 17900 3.45E-05
20200 5.45E-05 19600 5.73E-05
20600 0.00014 18500 8.12E-06 18300 1.81E-05 17900 3.44E-05
20200 5.44E-05 19600 5.73E-05
20600 0.00014 18500 8.12E-06 18300 1.81E-05 17900 3.44E-05
20200 5.43E-05 19700 5.73E-05
20600 0.00014 18500 8.12E-06 18300 1.81E-05 17900 3.43E-05
20200 5.41E-05 19700 5.73E-05
20600 0.000141 18500 8.11E-06 18300 1.81E-05 17900
3.43E-05 20200 5.40E-05 19700 5.73E-05
20600 0.000141 18500 8.10E-06 18300 1.81E-05 17900
3.42E-05 20200 5.39E-05 19700 5.73E-05
20600 0.000141 18500 8.10E-06 18300 1.81E-05 17900
3.42E-05 20200 5.38E-05 19700 5.72E-05
20600 0.000141 18500 8.11E-06 18300 1.81E-05 17900
3.41E-05 20200 5.37E-05 19700 5.72E-05
20600 0.000141 18500 8.11E-06 18300 1.81E-05 17900
3.41E-05 20200 5.35E-05 19700 5.71E-05
20600 0.000141 18500 8.10E-06 18300 1.81E-05 17900
3.40E-05 20200 5.34E-05 19700 5.70E-05
20600 0.000141 18500 8.12E-06 18300 1.82E-05 17900
3.40E-05 20200 5.33E-05 19700 5.70E-05
20600 0.000141 18600 8.12E-06 18300 1.82E-05 17900
3.39E-05 20200 5.32E-05 19700 5.69E-05
20600 0.000141 18600 8.12E-06 18300 1.82E-05 17900
3.39E-05 20200 5.31E-05 19700 5.68E-05
20600 0.000141 18600 8.13E-06 18400 1.81E-05 17900
3.38E-05 20200 5.30E-05 19700 5.67E-05

20600	0.000141	18600	8.13E-06	18400	1.81E-05	17900	3.37E-05	20200	5.29E-05	19700	5.66E-05
20600	0.000142	18600	8.13E-06	18400	1.81E-05	17900	3.37E-05	20200	5.28E-05	19700	5.65E-05
20600	0.000142	18600	8.13E-06	18400	1.81E-05	17900	3.36E-05	20200	5.27E-05	19700	5.64E-05
20600	0.000142	18600	8.13E-06	18400	1.82E-05	18000	3.35E-05	20200	5.26E-05	19700	5.63E-05
20600	0.000142	18600	8.13E-06	18400	1.81E-05	18000	3.35E-05	20200	5.25E-05	19700	5.61E-05
20600	0.000142	18600	8.13E-06	18400	1.81E-05	18000	3.34E-05	20200	5.24E-05	19700	5.60E-05
20600	0.000142	18600	8.14E-06	18400	1.81E-05	18000	3.34E-05	20200	5.23E-05	19700	5.59E-05
20600	0.000142	18600	8.15E-06	18400	1.81E-05	18000	3.33E-05	20200	5.23E-05	19700	5.57E-05
20600	0.000142	18600	8.14E-06	18400	1.81E-05	18000	3.33E-05	20200	5.22E-05	19700	5.56E-05
20600	0.000142	18600	8.15E-06	18400	1.81E-05	18000	3.32E-05	20200	5.21E-05	19700	5.55E-05
20600	0.000142	18600	8.15E-06	18400	1.82E-05	18000	3.31E-05	20200	5.20E-05	19700	5.53E-05
20600	0.000143	18600	8.15E-06	18400	1.82E-05	18000	3.31E-05	20200	5.19E-05	19700	5.52E-05
20600	0.000143	18600	8.16E-06	18400	1.82E-05	18000	3.30E-05	20200	5.19E-05	19700	5.51E-05
20600	0.000143	18600	8.16E-06	18400	1.82E-05	18000	3.30E-05	20200	5.18E-05	19700	5.49E-05
20600	0.000143	18600	8.15E-06	18400	1.82E-05	18000	3.29E-05	20300	5.17E-05	19700	5.48E-05
20600	0.000143	18600	8.17E-06	18400	1.82E-05	18000	3.28E-05	20300	5.16E-05	19700	5.47E-05
20600	0.000143	18600	8.17E-06	18400	1.82E-05	18000	3.28E-05	20300	5.16E-05	19700	5.46E-05
20600	0.000143	18600	8.17E-06	18400	1.82E-05	18000	3.27E-05	20300	5.15E-05	19700	5.44E-05
20600	0.000143	18600	8.17E-06	18400	1.82E-05	18000	3.27E-05	20300	5.15E-05	19700	5.43E-05
20600	0.000143	18600	8.18E-06	18400	1.82E-05	18000	3.27E-05	20300	5.14E-05	19700	5.42E-05
20600	0.000143	18600	8.19E-06	18400	1.82E-05	18000	3.26E-05	20300	5.14E-05	19700	5.40E-05
20600	0.000143	18600	8.19E-06	18400	1.82E-05	18000	3.26E-05	20300	5.13E-05	19700	5.39E-05
20600	0.000144	18600	8.19E-06	18400	1.82E-05	18000	3.25E-05	20300	5.12E-05	19700	5.38E-05
20600	0.000144	18600	8.20E-06	18400	1.82E-05	18000	3.25E-05	20300	5.12E-05	19700	5.37E-05
20600	0.000144	18600	8.20E-06	18400	1.82E-05	18000	3.24E-05	20300	5.11E-05	19700	5.36E-05
20600	0.000144	18600	8.21E-06	18400	1.82E-05	18000	3.24E-05	20300	5.11E-05	19700	5.35E-05
20600	0.000144	18600	8.22E-06	18400	1.82E-05	18000	3.23E-05	20300	5.11E-05	19700	5.34E-05
20600	0.000144	18600	8.21E-06	18400	1.82E-05	18000	3.23E-05	20300	5.10E-05	19700	5.33E-05
20600	0.000144	18600	8.21E-06	18400	1.82E-05	18000	3.22E-05	20300	5.10E-05	19700	5.32E-05
20600	0.000144	18600	8.21E-06	18400	1.82E-05	18000	3.22E-05	20300	5.09E-05	19700	5.31E-05
20600	0.000144	18600	8.20E-06	18400	1.82E-05	18000	3.21E-05	20300	5.09E-05	19700	5.30E-05
20600	0.000144	18600	8.21E-06	18400	1.82E-05	18000	3.21E-05	20300	5.08E-05	19700	5.29E-05
20600	0.000144	18600	8.21E-06	18400	1.82E-05	18000	3.20E-05	20300	5.08E-05	19700	5.28E-05
20600	0.000145	18600	8.20E-06	18400	1.82E-05	18000	3.20E-05	20300	5.07E-05	19700	5.28E-05

20600 0.000145 18600 8.21E-06 18400 1.82E-05 18000
3.19E-05 20300 5.07E-05 19700 5.27E-05
20600 0.000145 18600 8.21E-06 18400 1.82E-05 18000
3.19E-05 20300 5.07E-05 19700 5.26E-05
20600 0.000145 18600 8.21E-06 18400 1.82E-05 18000
3.18E-05 20300 5.06E-05 19700 5.26E-05
20600 0.000145 18600 8.21E-06 18400 1.82E-05 18000
3.18E-05 20300 5.06E-05 19700 5.25E-05
20600 0.000145 18600 8.20E-06 18400 1.83E-05 18000
3.17E-05 20300 5.05E-05 19700 5.24E-05
20600 0.000145 18600 8.21E-06 18400 1.82E-05 18000
3.17E-05 20300 5.05E-05 19700 5.24E-05
20600 0.000145 18600 8.21E-06 18400 1.83E-05 18000
3.16E-05 20300 5.05E-05 19700 5.23E-05
20600 0.000145 18600 8.21E-06 18400 1.83E-05 18000
3.16E-05 20300 5.05E-05 19700 5.22E-05
20600 0.000145 18600 8.21E-06 18400 1.83E-05 18000
3.16E-05 20300 5.04E-05 19700 5.22E-05
20700 0.000145 18600 8.21E-06 18400 1.83E-05 18000
3.16E-05 20300 5.04E-05 19700 5.21E-05
20700 0.000146 18600 8.22E-06 18400 1.83E-05 18000
3.15E-05 20300 5.04E-05 19700 5.21E-05
20700 0.000146 18600 8.22E-06 18400 1.83E-05 18000
3.15E-05 20300 5.03E-05 19700 5.20E-05
20700 0.000146 18600 8.22E-06 18400 1.83E-05 18000
3.14E-05 20300 5.03E-05 19700 5.20E-05
20700 0.000146 18600 8.22E-06 18400 1.83E-05 18000
3.14E-05 20300 5.03E-05 19700 5.20E-05
20700 0.000146 18600 8.22E-06 18400 1.83E-05 18000
3.13E-05 20300 5.03E-05 19700 5.19E-05
20700 0.000146 18600 8.21E-06 18400 1.83E-05 18000
3.13E-05 20300 5.02E-05 19700 5.19E-05
20700 0.000146 18600 8.22E-06 18400 1.83E-05 18000
3.12E-05 20300 5.02E-05 19700 5.18E-05
20700 0.000146 18600 8.22E-06 18400 1.83E-05 18000
3.12E-05 20300 5.02E-05 19700 5.18E-05
20700 0.000146 18600 8.22E-06 18400 1.83E-05 18000
3.11E-05 20300 5.01E-05 19800 5.18E-05
20700 0.000146 18600 8.23E-06 18400 1.83E-05 18000
3.11E-05 20300 5.01E-05 19800 5.18E-05
20700 0.000146 18600 8.23E-06 18400 1.84E-05 18000
3.10E-05 20300 5.01E-05 19800 5.18E-05
20700 0.000146 18600 8.22E-06 18400 1.84E-05 18000
3.10E-05 20300 5.00E-05 19800 5.17E-05
20700 0.000146 18600 8.23E-06 18400 1.84E-05 18000
3.09E-05 20300 5.00E-05 19800 5.17E-05
20700 0.000147 18600 8.23E-06 18400 1.84E-05 18000
3.09E-05 20300 5.00E-05 19800 5.17E-05
20700 0.000147 18600 8.23E-06 18400 1.84E-05 18000
3.08E-05 20300 5.00E-05 19800 5.17E-05
20700 0.000147 18600 8.23E-06 18400 1.84E-05 18000
3.08E-05 20300 4.99E-05 19800 5.16E-05
20700 0.000147 18600 8.24E-06 18400 1.84E-05 18000
3.08E-05 20300 4.99E-05 19800 5.16E-05
20700 0.000147 18700 8.24E-06 18400 1.84E-05 18000
3.07E-05 20300 4.99E-05 19800 5.16E-05
20700 0.000147 18700 8.24E-06 18400 1.84E-05 18000
3.07E-05 20300 4.99E-05 19800 5.16E-05
20700 0.000147 18700 8.24E-06 18500 1.84E-05 18000
3.06E-05 20300 4.98E-05 19800 5.16E-05
20700 0.000147 18700 8.25E-06 18500 1.84E-05 18000
3.06E-05 20300 4.98E-05 19800 5.15E-05
20700 0.000147 18700 8.25E-06 18500 1.84E-05 18000
3.06E-05 20300 4.98E-05 19800 5.15E-05
20700 0.000147 18700 8.25E-06 18500 1.84E-05 18000
3.05E-05 20300 4.98E-05 19800 5.15E-05
20700 0.000147 18700 8.25E-06 18500 1.84E-05 18100
3.05E-05 20300 4.97E-05 19800 5.15E-05

20700 0.000147 18700 8.25E-06 18500 1.84E-05 18100
3.05E-05 20300 4.97E-05 19800 5.15E-05
20700 0.000147 18700 8.26E-06 18500 1.84E-05 18100
3.04E-05 20300 4.97E-05 19800 5.15E-05
20700 0.000148 18700 8.26E-06 18500 1.84E-05 18100
3.04E-05 20300 4.97E-05 19800 5.15E-05
20700 0.000148 18700 8.26E-06 18500 1.84E-05 18100
3.04E-05 20300 4.97E-05 19800 5.15E-05
20700 0.000148 18700 8.26E-06 18500 1.84E-05 18100
3.04E-05 20300 4.96E-05 19800 5.15E-05
20700 0.000148 18700 8.26E-06 18500 1.85E-05 18100
3.03E-05 20300 4.96E-05 19800 5.15E-05
20700 0.000148 18700 8.26E-06 18500 1.85E-05 18100
3.03E-05 20300 4.96E-05 19800 5.15E-05
20700 0.000148 18700 8.26E-06 18500 1.85E-05 18100
3.03E-05 20300 4.96E-05 19800 5.15E-05
20700 0.000148 18700 8.27E-06 18500 1.85E-05 18100
3.02E-05 20300 4.96E-05 19800 5.15E-05
20700 0.000148 18700 8.26E-06 18500 1.85E-05 18100
3.02E-05 20300 4.96E-05 19800 5.15E-05
20700 0.000148 18700 8.27E-06 18500 1.85E-05 18100
3.02E-05 20400 4.95E-05 19800 5.15E-05
20700 0.000148 18700 8.27E-06 18500 1.85E-05 18100
3.01E-05 20400 4.95E-05 19800 5.15E-05
20700 0.000148 18700 8.27E-06 18500 1.85E-05 18100
3.01E-05 20400 4.95E-05 19800 5.15E-05
20700 0.000148 18700 8.27E-06 18500 1.85E-05 18100
3.00E-05 20400 4.95E-05 19800 5.15E-05
20700 0.000149 18700 8.28E-06 18500 1.85E-05 18100
3.00E-05 20400 4.95E-05 19800 5.15E-05
20700 0.000149 18700 8.28E-06 18500 1.85E-05 18100
3.00E-05 20400 4.95E-05 19800 5.15E-05
20700 0.000149 18700 8.28E-06 18500 1.85E-05 18100
2.99E-05 20400 4.95E-05 19800 5.15E-05
20700 0.000149 18700 8.29E-06 18500 1.85E-05 18100
2.99E-05 20400 4.95E-05 19800 5.15E-05
20700 0.000149 18700 8.29E-06 18500 1.86E-05 18100
2.99E-05 20400 4.95E-05 19800 5.15E-05
20700 0.000149 18700 8.29E-06 18500 1.86E-05 18100
2.98E-05 20400 4.94E-05 19800 5.15E-05
20700 0.000149 18700 8.30E-06 18500 1.86E-05 18100
2.98E-05 20400 4.95E-05 19800 5.15E-05
20700 0.000149 18700 8.29E-06 18500 1.86E-05 18100
2.98E-05 20400 4.94E-05 19800 5.15E-05
20700 0.000149 18700 8.29E-06 18500 1.86E-05 18100
2.97E-05 20400 4.94E-05 19800 5.15E-05
20700 0.000149 18700 8.29E-06 18500 1.86E-05 18100
2.97E-05 20400 4.94E-05 19800 5.15E-05
20700 0.000149 18700 8.29E-06 18500 1.86E-05 18100
2.97E-05 20400 4.94E-05 19800 5.15E-05
20700 0.00015 18700 8.28E-06 18500 1.86E-05 18100 2.96E-05
20400 4.94E-05 19800 5.15E-05
20700 0.00015 18700 8.29E-06 18500 1.86E-05 18100 2.96E-05
20400 4.94E-05 19800 5.15E-05
20700 0.00015 18700 8.29E-06 18500 1.86E-05 18100 2.96E-05
20400 4.94E-05 19800 5.15E-05
20700 0.00015 18700 8.29E-06 18500 1.86E-05 18100 2.96E-05
20400 4.94E-05 19800 5.16E-05
20700 0.00015 18700 8.29E-06 18500 1.86E-05 18100 2.95E-05
20400 4.94E-05 19800 5.16E-05
20700 0.00015 18700 8.29E-06 18500 1.87E-05 18100 2.95E-05
20400 4.94E-05 19800 5.16E-05
20700 0.00015 18700 8.31E-06 18500 1.87E-05 18100 2.94E-05
20400 4.94E-05 19800 5.16E-05
20700 0.00015 18700 8.30E-06 18500 1.87E-05 18100 2.94E-05
20400 4.94E-05 19800 5.16E-05
20700 0.00015 18700 8.32E-06 18500 1.87E-05 18100 2.93E-05
20400 4.94E-05 19800 5.16E-05

20700 0.00015 18700 8.32E-06 18500 1.87E-05 18100 2.93E-05
20400 4.94E-05 19800 5.16E-05
20700 0.000151 18700 8.32E-06 18500 1.87E-05 18100
2.93E-05 20400 4.94E-05 19800 5.16E-05
20700 0.000151 18700 8.33E-06 18500 1.87E-05 18100
2.93E-05 20400 4.94E-05 19800 5.16E-05
20700 0.000151 18700 8.33E-06 18500 1.87E-05 18100
2.92E-05 20400 4.94E-05 19800 5.16E-05
20700 0.000151 18700 8.33E-06 18500 1.87E-05 18100
2.92E-05 20400 4.94E-05 19800 5.16E-05
20800 0.000151 18700 8.34E-06 18500 1.88E-05 18100
2.92E-05 20400 4.94E-05 19800 5.16E-05
20800 0.000151 18700 8.35E-06 18500 1.88E-05 18100
2.91E-05 20400 4.94E-05 19800 5.17E-05
20800 0.000151 18700 8.34E-06 18500 1.88E-05 18100
2.91E-05 20400 4.94E-05 19800 5.17E-05
20800 0.000151 18700 8.34E-06 18500 1.88E-05 18100
2.91E-05 20400 4.94E-05 19800 5.17E-05
20800 0.000151 18700 8.35E-06 18500 1.88E-05 18100
2.90E-05 20400 4.94E-05 19800 5.17E-05
20800 0.000151 18700 8.35E-06 18500 1.88E-05 18100
2.90E-05 20400 4.94E-05 19800 5.17E-05
20800 0.000152 18700 8.35E-06 18500 1.88E-05 18100
2.89E-05 20400 4.94E-05 19800 5.17E-05
20800 0.000152 18700 8.36E-06 18500 1.88E-05 18100
2.89E-05 20400 4.94E-05 19800 5.17E-05
20800 0.000152 18700 8.35E-06 18500 1.88E-05 18100
2.89E-05 20400 4.94E-05 19800 5.17E-05
20800 0.000152 18700 8.36E-06 18500 1.88E-05 18100
2.88E-05 20400 4.94E-05 19900 5.18E-05
20800 0.000152 18700 8.36E-06 18500 1.89E-05 18100
2.88E-05 20400 4.94E-05 19900 5.18E-05
20800 0.000152 18700 8.36E-06 18500 1.89E-05 18100
2.88E-05 20400 4.94E-05 19900 5.18E-05
20800 0.000152 18700 8.35E-06 18500 1.89E-05 18100
2.88E-05 20400 4.94E-05 19900 5.18E-05
20800 0.000152 18700 8.36E-06 18500 1.89E-05 18100
2.88E-05 20400 4.94E-05 19900 5.18E-05
20800 0.000152 18700 8.36E-06 18500 1.89E-05 18100
2.88E-05 20400 4.95E-05 19900 5.18E-05
20800 0.000153 18700 8.36E-06 18500 1.89E-05 18100
2.87E-05 20400 4.95E-05 19900 5.19E-05
20800 0.000153 18700 8.36E-06 18500 1.89E-05 18100
2.88E-05 20400 4.95E-05 19900 5.19E-05
20800 0.000153 18700 8.36E-06 18500 1.89E-05 18100
2.88E-05 20400 4.95E-05 19900 5.19E-05
20800 0.000153 18800 8.37E-06 18500 1.89E-05 18100
2.87E-05 20400 4.95E-05 19900 5.19E-05
20800 0.000153 18800 8.37E-06 18500 1.89E-05 18100
2.87E-05 20400 4.95E-05 19900 5.19E-05
20800 0.000153 18800 8.37E-06 18600 1.90E-05 18100
2.86E-05 20400 4.95E-05 19900 5.19E-05
20800 0.000153 18800 8.37E-06 18600 1.90E-05 18100
2.86E-05 20400 4.95E-05 19900 5.19E-05
20800 0.000153 18800 8.38E-06 18600 1.90E-05 18100
2.86E-05 20400 4.95E-05 19900 5.20E-05
20800 0.000153 18800 8.39E-06 18600 1.90E-05 18100
2.86E-05 20400 4.95E-05 19900 5.20E-05
20800 0.000154 18800 8.38E-06 18600 1.90E-05 18200
2.86E-05 20400 4.95E-05 19900 5.20E-05
20800 0.000154 18800 8.38E-06 18600 1.90E-05 18200
2.86E-05 20400 4.96E-05 19900 5.20E-05
20800 0.000154 18800 8.39E-06 18600 1.90E-05 18200
2.86E-05 20400 4.96E-05 19900 5.20E-05
20800 0.000154 18800 8.39E-06 18600 1.90E-05 18200
2.86E-05 20400 4.96E-05 19900 5.20E-05
20800 0.000154 18800 8.38E-06 18600 1.91E-05 18200
2.85E-05 20400 4.96E-05 19900 5.20E-05

20800 0.000154 18800 8.39E-06 18600 1.91E-05 18200
2.85E-05 20400 4.96E-05 19900 5.21E-05
20800 0.000154 18800 8.38E-06 18600 1.91E-05 18200
2.85E-05 20400 4.96E-05 19900 5.21E-05
20800 0.000154 18800 8.38E-06 18600 1.91E-05 18200
2.85E-05 20400 4.96E-05 19900 5.21E-05
20800 0.000154 18800 8.38E-06 18600 1.91E-05 18200
2.85E-05 20400 4.97E-05 19900 5.21E-05
20800 0.000154 18800 8.39E-06 18600 1.91E-05 18200
2.85E-05 20400 4.97E-05 19900 5.21E-05
20800 0.000155 18800 8.39E-06 18600 1.91E-05 18200
2.85E-05 20400 4.97E-05 19900 5.22E-05
20800 0.000155 18800 8.38E-06 18600 1.92E-05 18200
2.86E-05 20500 4.97E-05 19900 5.22E-05
20800 0.000155 18800 8.38E-06 18600 1.92E-05 18200
2.85E-05 20500 4.97E-05 19900 5.22E-05
20800 0.000155 18800 8.38E-06 18600 1.92E-05 18200
2.85E-05 20500 4.97E-05 19900 5.22E-05
20800 0.000155 18800 8.39E-06 18600 1.92E-05 18200
2.85E-05 20500 4.98E-05 19900 5.22E-05
20800 0.000155 18800 8.39E-06 18600 1.92E-05 18200
2.85E-05 20500 4.98E-05 19900 5.22E-05
20800 0.000155 18800 8.40E-06 18600 1.92E-05 18200
2.85E-05 20500 4.98E-05 19900 5.22E-05
20800 0.000155 18800 8.40E-06 18600 1.92E-05 18200
2.84E-05 20500 4.98E-05 19900 5.23E-05
20800 0.000155 18800 8.40E-06 18600 1.93E-05 18200
2.84E-05 20500 4.98E-05 19900 5.23E-05
20800 0.000156 18800 8.41E-06 18600 1.93E-05 18200
2.84E-05 20500 4.98E-05 19900 5.23E-05
20800 0.000156 18800 8.41E-06 18600 1.93E-05 18200
2.83E-05 20500 4.98E-05 19900 5.23E-05
20800 0.000156 18800 8.41E-06 18600 1.93E-05 18200
2.83E-05 20500 4.98E-05 19900 5.23E-05
20800 0.000156 18800 8.41E-06 18600 1.93E-05 18200
2.83E-05 20500 4.99E-05 19900 5.23E-05
20800 0.000156 18800 8.41E-06 18600 1.93E-05 18200
2.83E-05 20500 4.99E-05 19900 5.24E-05
20800 0.000156 18800 8.42E-06 18600 1.93E-05 18200
2.83E-05 20500 4.99E-05 19900 5.24E-05
20800 0.000156 18800 8.41E-06 18600 1.94E-05 18200
2.83E-05 20500 4.99E-05 19900 5.24E-05
20800 0.000156 18800 8.41E-06 18600 1.94E-05 18200
2.83E-05 20500 4.99E-05 19900 5.24E-05
20800 0.000156 18800 8.42E-06 18600 1.94E-05 18200
2.83E-05 20500 4.99E-05 19900 5.24E-05
20800 0.000157 18800 8.43E-06 18600 1.94E-05 18200
2.83E-05 20500 4.99E-05 19900 5.24E-05
20800 0.000157 18800 8.42E-06 18600 1.94E-05 18200
2.83E-05 20500 5.00E-05 19900 5.25E-05
20800 0.000157 18800 8.42E-06 18600 1.94E-05 18200
2.83E-05 20500 5.00E-05 19900 5.25E-05
20800 0.000157 18800 8.42E-06 18600 1.94E-05 18200
2.83E-05 20500 5.00E-05 19900 5.25E-05
20800 0.000157 18800 8.43E-06 18600 1.95E-05 18200
2.83E-05 20500 5.00E-05 19900 5.25E-05
20800 0.000157 18800 8.43E-06 18600 1.95E-05 18200
2.83E-05 20500 5.00E-05 19900 5.25E-05
20800 0.000157 18800 8.44E-06 18600 1.95E-05 18200
2.82E-05 20500 5.00E-05 19900 5.25E-05
20800 0.000157 18800 8.44E-06 18600 1.95E-05 18200
2.82E-05 20500 5.00E-05 19900 5.26E-05
20800 0.000157 18800 8.44E-06 18600 1.95E-05 18200
2.82E-05 20500 5.00E-05 19900 5.26E-05
20800 0.000158 18800 8.43E-06 18600 1.96E-05 18200
2.82E-05 20500 5.01E-05 19900 5.26E-05
20800 0.000158 18800 8.43E-06 18600 1.96E-05 18200
2.82E-05 20500 5.01E-05 19900 5.27E-05

20800 0.000158 18800 8.44E-06 18600 1.96E-05 18200
2.82E-05 20500 5.01E-05 19900 5.27E-05
20900 0.000158 18800 8.43E-06 18600 1.96E-05 18200
2.81E-05 20500 5.01E-05 19900 5.27E-05
20900 0.000158 18800 8.44E-06 18600 1.96E-05 18200
2.81E-05 20500 5.01E-05 19900 5.27E-05
20900 0.000158 18800 8.44E-06 18600 1.96E-05 18200
2.81E-05 20500 5.02E-05 19900 5.28E-05
20900 0.000158 18800 8.44E-06 18600 1.97E-05 18200
2.81E-05 20500 5.02E-05 19900 5.28E-05
20900 0.000158 18800 8.44E-06 18600 1.97E-05 18200
2.81E-05 20500 5.02E-05 19900 5.28E-05
20900 0.000158 18800 8.45E-06 18600 1.97E-05 18200
2.80E-05 20500 5.02E-05 19900 5.28E-05
20900 0.000158 18800 8.46E-06 18600 1.97E-05 18200
2.80E-05 20500 5.02E-05 19900 5.28E-05
20900 0.000159 18800 8.46E-06 18600 1.98E-05 18200
2.80E-05 20500 5.02E-05 19900 5.29E-05
20900 0.000159 18800 8.47E-06 18600 1.98E-05 18200
2.80E-05 20500 5.03E-05 19900 5.29E-05
20900 0.000159 18800 8.46E-06 18600 1.98E-05 18200
2.80E-05 20500 5.03E-05 20000 5.29E-05
20900 0.000159 18800 8.46E-06 18600 1.99E-05 18200
2.80E-05 20500 5.03E-05 20000 5.30E-05
20900 0.000159 18800 8.46E-06 18600 1.99E-05 18200
2.80E-05 20500 5.03E-05 20000 5.30E-05
20900 0.000159 18800 8.46E-06 18600 1.99E-05 18200
2.80E-05 20500 5.04E-05 20000 5.30E-05
20900 0.000159 18800 8.47E-06 18600 1.99E-05 18200
2.80E-05 20500 5.04E-05 20000 5.30E-05
20900 0.000159 18800 8.47E-06 18600 2.00E-05 18200
2.80E-05 20500 5.04E-05 20000 5.30E-05
20900 0.000159 18800 8.47E-06 18600 2.00E-05 18200
2.80E-05 20500 5.04E-05 20000 5.31E-05
20900 0.00016 18800 8.47E-06 18600 2.00E-05 18200 2.80E-05
20500 5.04E-05 20000 5.31E-05
20900 0.00016 18800 8.47E-06 18600 2.01E-05 18200 2.80E-05
20500 5.04E-05 20000 5.31E-05
20900 0.00016 18900 8.48E-06 18600 2.01E-05 18200 2.80E-05
20500 5.04E-05 20000 5.32E-05
20900 0.00016 18900 8.47E-06 18600 2.01E-05 18200 2.79E-05
20500 5.05E-05 20000 5.32E-05
20900 0.00016 18900 8.49E-06 18700 2.01E-05 18200 2.79E-05
20500 5.05E-05 20000 5.32E-05
20900 0.00016 18900 8.49E-06 18700 2.02E-05 18200 2.79E-05
20500 5.05E-05 20000 5.32E-05
20900 0.00016 18900 8.49E-06 18700 2.02E-05 18200 2.79E-05
20500 5.06E-05 20000 5.32E-05
20900 0.00016 18900 8.49E-06 18700 2.02E-05 18200 2.79E-05
20500 5.06E-05 20000 5.33E-05
20900 0.00016 18900 8.50E-06 18700 2.03E-05 18300 2.79E-05
20500 5.06E-05 20000 5.33E-05
20900 0.00016 18900 8.50E-06 18700 2.03E-05 18300 2.79E-05
20500 5.06E-05 20000 5.33E-05
20900 0.000161 18900 8.50E-06 18700 2.03E-05 18300
2.79E-05 20500 5.06E-05 20000 5.33E-05
20900 0.000161 18900 8.51E-06 18700 2.04E-05 18300
2.79E-05 20500 5.07E-05 20000 5.34E-05
20900 0.000161 18900 8.50E-06 18700 2.04E-05 18300
2.79E-05 20500 5.07E-05 20000 5.34E-05
20900 0.000161 18900 8.50E-06 18700 2.04E-05 18300
2.79E-05 20500 5.07E-05 20000 5.34E-05
20900 0.000161 18900 8.50E-06 18700 2.05E-05 18300
2.79E-05 20500 5.07E-05 20000 5.35E-05
20900 0.000161 18900 8.51E-06 18700 2.05E-05 18300
2.78E-05 20500 5.08E-05 20000 5.35E-05
20900 0.000161 18900 8.51E-06 18700 2.06E-05 18300
2.78E-05 20500 5.08E-05 20000 5.35E-05

20900	0.000161	18900	8.52E-06	18700	2.06E-05	18300	2.78E-05	20500	5.08E-05	20000	5.35E-05
20900	0.000161	18900	8.53E-06	18700	2.06E-05	18300	2.78E-05	20500	5.08E-05	20000	5.35E-05
20900	0.000161	18900	8.53E-06	18700	2.07E-05	18300	2.78E-05	20600	5.09E-05	20000	5.36E-05
20900	0.000162	18900	8.54E-06	18700	2.07E-05	18300	2.78E-05	20600	5.09E-05	20000	5.36E-05
20900	0.000162	18900	8.54E-06	18700	2.08E-05	18300	2.78E-05	20600	5.09E-05	20000	5.36E-05
20900	0.000162	18900	8.54E-06	18700	2.08E-05	18300	2.78E-05	20600	5.09E-05	20000	5.36E-05
20900	0.000162	18900	8.54E-06	18700	2.08E-05	18300	2.77E-05	20600	5.10E-05	20000	5.37E-05
20900	0.000162	18900	8.55E-06	18700	2.09E-05	18300	2.77E-05	20600	5.10E-05	20000	5.37E-05
20900	0.000162	18900	8.55E-06	18700	2.10E-05	18300	2.77E-05	20600	5.10E-05	20000	5.37E-05
20900	0.000162	18900	8.54E-06	18700	2.10E-05	18300	2.77E-05	20600	5.10E-05	20000	5.37E-05
20900	0.000162	18900	8.54E-06	18700	2.11E-05	18300	2.77E-05	20600	5.11E-05	20000	5.38E-05
20900	0.000162	18900	8.55E-06	18700	2.11E-05	18300	2.77E-05	20600	5.11E-05	20000	5.38E-05
20900	0.000163	18900	8.56E-06	18700	2.12E-05	18300	2.77E-05	20600	5.11E-05	20000	5.38E-05
20900	0.000163	18900	8.55E-06	18700	2.12E-05	18300	2.77E-05	20600	5.12E-05	20000	5.38E-05
20900	0.000163	18900	8.56E-06	18700	2.13E-05	18300	2.77E-05	20600	5.12E-05	20000	5.38E-05
20900	0.000163	18900	8.56E-06	18700	2.13E-05	18300	2.77E-05	20600	5.12E-05	20000	5.39E-05
20900	0.000163	18900	8.56E-06	18700	2.14E-05	18300	2.77E-05	20600	5.12E-05	20000	5.39E-05
20900	0.000163	18900	8.56E-06	18700	2.15E-05	18300	2.77E-05	20600	5.13E-05	20000	5.39E-05
20900	0.000163	18900	8.57E-06	18700	2.15E-05	18300	2.77E-05	20600	5.13E-05	20000	5.39E-05
20900	0.000163	18900	8.57E-06	18700	2.16E-05	18300	2.77E-05	20600	5.13E-05	20000	5.39E-05
20900	0.000163	18900	8.57E-06	18700	2.17E-05	18300	2.77E-05	20600	5.14E-05	20000	5.40E-05
20900	0.000164	18900	8.58E-06	18700	2.17E-05	18300	2.77E-05	20600	5.14E-05	20000	5.40E-05
20900	0.000164	18900	8.58E-06	18700	2.18E-05	18300	2.77E-05	20600	5.14E-05	20000	5.40E-05
20900	0.000164	18900	8.58E-06	18700	2.19E-05	18300	2.77E-05	20600	5.15E-05	20000	5.40E-05
20900	0.000164	18900	8.58E-06	18700	2.19E-05	18300	2.77E-05	20600	5.15E-05	20000	5.40E-05
20900	0.000164	18900	8.58E-06	18700	2.20E-05	18300	2.77E-05	20600	5.16E-05	20000	5.40E-05
20900	0.000164	18900	8.59E-06	18700	2.21E-05	18300	2.77E-05	20600	5.16E-05	20000	5.41E-05
20900	0.000164	18900	8.59E-06	18700	2.22E-05	18300	2.77E-05	20600	5.16E-05	20000	5.41E-05
20900	0.000164	18900	8.59E-06	18700	2.23E-05	18300	2.77E-05	20600	5.17E-05	20000	5.41E-05
20900	0.000164	18900	8.59E-06	18700	2.23E-05	18300	2.77E-05	20600	5.17E-05	20000	5.41E-05
20900	0.000164	18900	8.58E-06	18700	2.24E-05	18300	2.77E-05	20600	5.17E-05	20000	5.41E-05
21000	0.000165	18900	8.60E-06	18700	2.25E-05	18300	2.77E-05	20600	5.18E-05	20000	5.42E-05
21000	0.000165	18900	8.59E-06	18700	2.26E-05	18300	2.77E-05	20600	5.18E-05	20000	5.42E-05
21000	0.000165	18900	8.59E-06	18700	2.27E-05	18300	2.77E-05	20600	5.19E-05	20000	5.42E-05

21000	0.000165	18900	8.58E-06	18700	2.28E-05	18300	2.77E-05	20600	5.19E-05	20000	5.42E-05
21000	0.000165	18900	8.59E-06	18700	2.29E-05	18300	2.77E-05	20600	5.19E-05	20000	5.42E-05
21000	0.000165	18900	8.59E-06	18700	2.30E-05	18300	2.76E-05	20600	5.20E-05	20000	5.42E-05
21000	0.000165	18900	8.59E-06	18700	2.31E-05	18300	2.76E-05	20600	5.20E-05	20000	5.43E-05
21000	0.000165	18900	8.60E-06	18700	2.32E-05	18300	2.76E-05	20600	5.20E-05	20000	5.43E-05
21000	0.000165	18900	8.61E-06	18700	2.34E-05	18300	2.76E-05	20600	5.21E-05	20000	5.43E-05
21000	0.000165	18900	8.61E-06	18700	2.35E-05	18300	2.76E-05	20600	5.21E-05	20100	5.43E-05
21000	0.000166	18900	8.61E-06	18700	2.36E-05	18300	2.76E-05	20600	5.22E-05	20100	5.44E-05
21000	0.000166	18900	8.61E-06	18700	2.38E-05	18300	2.76E-05	20600	5.22E-05	20100	5.44E-05
21000	0.000166	18900	8.62E-06	18700	2.39E-05	18300	2.76E-05	20600	5.22E-05	20100	5.44E-05
21000	0.000166	18900	8.62E-06	18700	2.40E-05	18300	2.76E-05	20600	5.23E-05	20100	5.44E-05
21000	0.000166	18900	8.63E-06	18700	2.42E-05	18300	2.76E-05	20600	5.23E-05	20100	5.44E-05
21000	0.000166	18900	8.64E-06	18700	2.43E-05	18300	2.76E-05	20600	5.23E-05	20100	5.45E-05
21000	0.000166	18900	8.64E-06	18700	2.45E-05	18300	2.76E-05	20600	5.24E-05	20100	5.45E-05
21000	0.000166	18900	8.64E-06	18700	2.47E-05	18300	2.76E-05	20600	5.24E-05	20100	5.45E-05
21000	0.000166	19000	8.64E-06	18700	2.48E-05	18300	2.76E-05	20600	5.24E-05	20100	5.45E-05
21000	0.000166	19000	8.63E-06	18700	2.50E-05	18300	2.76E-05	20600	5.25E-05	20100	5.46E-05
21000	0.000167	19000	8.64E-06	18800	2.52E-05	18300	2.76E-05	20600	5.25E-05	20100	5.46E-05
21000	0.000167	19000	8.65E-06	18800	2.54E-05	18300	2.77E-05	20600	5.26E-05	20100	5.46E-05
21000	0.000167	19000	8.65E-06	18800	2.56E-05	18300	2.76E-05	20600	5.26E-05	20100	5.46E-05
21000	0.000167	19000	8.66E-06	18800	2.58E-05	18300	2.76E-05	20600	5.26E-05	20100	5.47E-05
21000	0.000167	19000	8.66E-06	18800	2.60E-05	18400	2.76E-05	20600	5.27E-05	20100	5.47E-05
21000	0.000167	19000	8.67E-06	18800	2.62E-05	18400	2.76E-05	20600	5.27E-05	20100	5.47E-05
21000	0.000167	19000	8.68E-06	18800	2.65E-05	18400	2.76E-05	20600	5.27E-05	20100	5.47E-05
21000	0.000167	19000	8.69E-06	18800	2.67E-05	18400	2.76E-05	20600	5.28E-05	20100	5.48E-05
21000	0.000167	19000	8.69E-06	18800	2.70E-05	18400	2.76E-05	20600	5.28E-05	20100	5.48E-05
21000	0.000168	19000	8.69E-06	18800	2.72E-05	18400	2.76E-05	20600	5.28E-05	20100	5.48E-05
21000	0.000168	19000	8.70E-06	18800	2.75E-05	18400	2.76E-05	20600	5.29E-05	20100	5.49E-05
21000	0.000168	19000	8.70E-06	18800	2.78E-05	18400	2.76E-05	20600	5.29E-05	20100	5.49E-05
21000	0.000168	19000	8.71E-06	18800	2.81E-05	18400	2.76E-05	20600	5.30E-05	20100	5.49E-05
21000	0.000168	19000	8.71E-06	18800	2.84E-05	18400	2.76E-05	20600	5.30E-05	20100	5.49E-05
21000	0.000168	19000	8.71E-06	18800	2.87E-05	18400	2.76E-05	20600	5.30E-05	20100	5.49E-05
21000	0.000168	19000	8.72E-06	18800	2.91E-05	18400	2.76E-05	20700	5.31E-05	20100	5.50E-05
21000	0.000168	19000	8.71E-06	18800	2.94E-05	18400	2.76E-05	20700	5.31E-05	20100	5.50E-05

21000 0.000168 19000 8.72E-06 18800 2.98E-05 18400
2.76E-05 20700 5.32E-05 20100 5.50E-05
21000 0.000168 19000 8.72E-06 18800 3.02E-05 18400
2.76E-05 20700 5.32E-05 20100 5.50E-05
21000 0.000169 19000 8.72E-06 18800 3.06E-05 18400
2.76E-05 20700 5.33E-05 20100 5.51E-05
21000 0.000169 19000 8.72E-06 18800 3.10E-05 18400
2.76E-05 20700 5.33E-05 20100 5.51E-05
21000 0.000169 19000 8.73E-06 18800 3.14E-05 18400
2.76E-05 20700 5.34E-05 20100 5.51E-05
21000 0.000169 19000 8.72E-06 18800 3.19E-05 18400
2.76E-05 20700 5.34E-05 20100 5.51E-05
21000 0.000169 19000 8.73E-06 18800 3.24E-05 18400
2.76E-05 20700 5.35E-05 20100 5.51E-05
21000 0.000169 19000 8.73E-06 18800 3.29E-05 18400
2.76E-05 20700 5.35E-05 20100 5.52E-05
21000 0.000169 19000 8.73E-06 18800 3.34E-05 18400
2.76E-05 20700 5.35E-05 20100 5.52E-05
21000 0.000169 19000 8.74E-06 18800 3.40E-05 18400
2.76E-05 20700 5.36E-05 20100 5.52E-05
21000 0.00017 19000 8.74E-06 18800 3.46E-05 18400 2.76E-05
20700 5.36E-05 20100 5.53E-05
21000 0.00017 19000 8.74E-06 18800 3.52E-05 18400 2.76E-05
20700 5.37E-05 20100 5.53E-05
21000 0.00017 19000 8.74E-06 18800 3.58E-05 18400 2.76E-05
20700 5.37E-05 20100 5.53E-05
21000 0.00017 19000 8.74E-06 18800 3.65E-05 18400 2.76E-05
20700 5.38E-05 20100 5.53E-05
21000 0.00017 19000 8.74E-06 18800 3.72E-05 18400 2.76E-05
20700 5.38E-05 20100 5.54E-05
21000 0.00017 19000 8.75E-06 18800 3.79E-05 18400 2.76E-05
20700 5.39E-05 20100 5.54E-05
21000 0.00017 19000 8.75E-06 18800 3.87E-05 18400 2.76E-05
20700 5.39E-05 20100 5.54E-05
21000 0.00017 19000 8.75E-06 18800 3.95E-05 18400 2.77E-05
20700 5.40E-05 20100 5.54E-05
21000 0.000171 19000 8.74E-06 18800 4.03E-05 18400
2.77E-05 20700 5.40E-05 20100 5.55E-05
21000 0.000171 19000 8.75E-06 18800 4.12E-05 18400
2.77E-05 20700 5.41E-05 20100 5.55E-05
21000 0.000171 19000 8.75E-06 18800 4.21E-05 18400
2.77E-05 20700 5.41E-05 20100 5.55E-05
21000 0.000171 19000 8.74E-06 18800 4.30E-05 18400
2.77E-05 20700 5.42E-05 20100 5.55E-05
21000 0.000171 19000 8.74E-06 18800 4.40E-05 18400
2.77E-05 20700 5.42E-05 20100 5.56E-05
21000 0.000171 19000 8.74E-06 18800 4.50E-05 18400
2.77E-05 20700 5.43E-05 20100 5.56E-05
21000 0.000171 19000 8.74E-06 18800 4.61E-05 18400
2.77E-05 20700 5.43E-05 20100 5.56E-05
21000 0.000172 19000 8.74E-06 18800 4.72E-05 18400
2.77E-05 20700 5.44E-05 20100 5.56E-05
21000 0.000172 19000 8.74E-06 18800 4.83E-05 18400
2.77E-05 20700 5.45E-05 20100 5.56E-05
21100 0.000172 19000 8.75E-06 18800 4.95E-05 18400
2.77E-05 20700 5.45E-05 20100 5.57E-05
21100 0.000172 19000 8.74E-06 18800 5.07E-05 18400
2.77E-05 20700 5.46E-05 20100 5.57E-05
21100 0.000172 19000 8.74E-06 18800 5.20E-05 18400
2.77E-05 20700 5.46E-05 20100 5.57E-05
21100 0.000172 19000 8.75E-06 18800 5.32E-05 18400
2.77E-05 20700 5.47E-05 20100 5.58E-05
21100 0.000172 19000 8.75E-06 18800 5.45E-05 18400
2.77E-05 20700 5.47E-05 20100 5.58E-05
21100 0.000172 19000 8.75E-06 18800 5.58E-05 18400
2.77E-05 20700 5.48E-05 20100 5.58E-05
21100 0.000173 19000 8.75E-06 18800 5.72E-05 18400
2.77E-05 20700 5.48E-05 20100 5.58E-05

21100 0.000173 19000 8.76E-06 18800 5.85E-05 18400
2.77E-05 20700 5.49E-05 20100 5.58E-05
21100 0.000173 19000 8.77E-06 18800 5.98E-05 18400
2.77E-05 20700 5.49E-05 20100 5.59E-05
21100 0.000173 19000 8.76E-06 18800 6.11E-05 18400
2.78E-05 20700 5.50E-05 20200 5.59E-05
21100 0.000173 19000 8.76E-06 18800 6.24E-05 18400
2.78E-05 20700 5.51E-05 20200 5.59E-05
21100 0.000173 19000 8.77E-06 18800 6.37E-05 18400
2.78E-05 20700 5.51E-05 20200 5.60E-05
21100 0.000173 19000 8.77E-06 18800 6.49E-05 18400
2.78E-05 20700 5.52E-05 20200 5.60E-05
21100 0.000173 19000 8.77E-06 18800 6.60E-05 18400
2.78E-05 20700 5.52E-05 20200 5.60E-05
21100 0.000174 19000 8.77E-06 18800 6.71E-05 18400
2.78E-05 20700 5.53E-05 20200 5.60E-05
21100 0.000174 19000 8.77E-06 18800 6.81E-05 18400
2.78E-05 20700 5.53E-05 20200 5.60E-05
21100 0.000174 19000 8.78E-06 18800 6.90E-05 18400
2.78E-05 20700 5.54E-05 20200 5.61E-05
21100 0.000174 19000 8.79E-06 18800 6.97E-05 18400
2.79E-05 20700 5.55E-05 20200 5.61E-05
21100 0.000174 19100 8.78E-06 18800 7.03E-05 18400
2.79E-05 20700 5.55E-05 20200 5.61E-05
21100 0.000174 19100 8.79E-06 18800 7.08E-05 18400
2.79E-05 20700 5.56E-05 20200 5.61E-05
21100 0.000174 19100 8.80E-06 18900 7.11E-05 18400
2.79E-05 20700 5.56E-05 20200 5.62E-05
21100 0.000175 19100 8.80E-06 18900 7.13E-05 18400
2.79E-05 20700 5.57E-05 20200 5.62E-05
21100 0.000175 19100 8.81E-06 18900 7.13E-05 18400
2.79E-05 20700 5.58E-05 20200 5.62E-05
21100 0.000175 19100 8.81E-06 18900 7.11E-05 18400
2.79E-05 20700 5.58E-05 20200 5.62E-05
21100 0.000175 19100 8.82E-06 18900 7.07E-05 18500
2.79E-05 20700 5.59E-05 20200 5.63E-05
21100 0.000175 19100 8.82E-06 18900 7.02E-05 18500
2.79E-05 20700 5.59E-05 20200 5.63E-05
21100 0.000175 19100 8.81E-06 18900 6.95E-05 18500
2.79E-05 20700 5.60E-05 20200 5.63E-05
21100 0.000175 19100 8.81E-06 18900 6.86E-05 18500
2.79E-05 20700 5.61E-05 20200 5.63E-05
21100 0.000175 19100 8.81E-06 18900 6.77E-05 18500
2.79E-05 20700 5.61E-05 20200 5.64E-05
21100 0.000176 19100 8.81E-06 18900 6.66E-05 18500
2.80E-05 20700 5.62E-05 20200 5.64E-05
21100 0.000176 19100 8.82E-06 18900 6.53E-05 18500
2.80E-05 20700 5.63E-05 20200 5.64E-05
21100 0.000176 19100 8.82E-06 18900 6.40E-05 18500
2.80E-05 20700 5.63E-05 20200 5.65E-05
21100 0.000176 19100 8.83E-06 18900 6.26E-05 18500
2.80E-05 20700 5.64E-05 20200 5.65E-05
21100 0.000176 19100 8.83E-06 18900 6.11E-05 18500
2.80E-05 20700 5.65E-05 20200 5.65E-05
21100 0.000176 19100 8.83E-06 18900 5.96E-05 18500
2.80E-05 20700 5.65E-05 20200 5.65E-05
21100 0.000176 19100 8.84E-06 18900 5.81E-05 18500
2.80E-05 20800 5.66E-05 20200 5.66E-05
21100 0.000176 19100 8.85E-06 18900 5.65E-05 18500
2.80E-05 20800 5.67E-05 20200 5.66E-05
21100 0.000177 19100 8.86E-06 18900 5.50E-05 18500
2.80E-05 20800 5.67E-05 20200 5.66E-05
21100 0.000177 19100 8.86E-06 18900 5.34E-05 18500
2.80E-05 20800 5.68E-05 20200 5.66E-05
21100 0.000177 19100 8.86E-06 18900 5.19E-05 18500
2.80E-05 20800 5.69E-05 20200 5.67E-05
21100 0.000177 19100 8.87E-06 18900 5.04E-05 18500
2.80E-05 20800 5.69E-05 20200 5.67E-05

21100 0.000177 19100 8.86E-06 18900 4.90E-05 18500
2.80E-05 20800 5.70E-05 20200 5.67E-05
21100 0.000177 19100 8.87E-06 18900 4.76E-05 18500
2.80E-05 20800 5.71E-05 20200 5.67E-05
21100 0.000177 19100 8.87E-06 18900 4.62E-05 18500
2.80E-05 20800 5.72E-05 20200 5.68E-05
21100 0.000177 19100 8.86E-06 18900 4.49E-05 18500
2.80E-05 20800 5.72E-05 20200 5.68E-05
21100 0.000178 19100 8.86E-06 18900 4.37E-05 18500
2.81E-05 20800 5.73E-05 20200 5.68E-05
21100 0.000178 19100 8.87E-06 18900 4.25E-05 18500
2.81E-05 20800 5.74E-05 20200 5.68E-05
21100 0.000178 19100 8.87E-06 18900 4.14E-05 18500
2.81E-05 20800 5.75E-05 20200 5.69E-05
21100 0.000178 19100 8.87E-06 18900 4.04E-05 18500
2.81E-05 20800 5.75E-05 20200 5.69E-05
21100 0.000178 19100 8.87E-06 18900 3.94E-05 18500
2.81E-05 20800 5.76E-05 20200 5.69E-05
21100 0.000178 19100 8.87E-06 18900 3.84E-05 18500
2.82E-05 20800 5.77E-05 20200 5.70E-05
21100 0.000178 19100 8.88E-06 18900 3.75E-05 18500
2.82E-05 20800 5.78E-05 20200 5.70E-05
21100 0.000178 19100 8.88E-06 18900 3.67E-05 18500
2.82E-05 20800 5.79E-05 20200 5.70E-05
21100 0.000179 19100 8.88E-06 18900 3.59E-05 18500
2.82E-05 20800 5.79E-05 20200 5.70E-05
21100 0.000179 19100 8.88E-06 18900 3.51E-05 18500
2.82E-05 20800 5.80E-05 20200 5.71E-05
21100 0.000179 19100 8.89E-06 18900 3.44E-05 18500
2.82E-05 20800 5.81E-05 20200 5.71E-05
21100 0.000179 19100 8.89E-06 18900 3.38E-05 18500
2.82E-05 20800 5.82E-05 20200 5.71E-05
21100 0.000179 19100 8.91E-06 18900 3.32E-05 18500
2.82E-05 20800 5.82E-05 20200 5.71E-05
21100 0.000179 19100 8.91E-06 18900 3.26E-05 18500
2.82E-05 20800 5.83E-05 20200 5.72E-05
21100 0.000179 19100 8.92E-06 18900 3.20E-05 18500
2.83E-05 20800 5.84E-05 20200 5.72E-05
21100 0.000179 19100 8.93E-06 18900 3.15E-05 18500
2.82E-05 20800 5.85E-05 20200 5.72E-05
21100 0.00018 19100 8.93E-06 18900 3.10E-05 18500 2.83E-05
20800 5.86E-05 20200 5.72E-05
21100 0.00018 19100 8.94E-06 18900 3.06E-05 18500 2.82E-05
20800 5.86E-05 20200 5.73E-05
21100 0.00018 19100 8.94E-06 18900 3.01E-05 18500 2.83E-05
20800 5.87E-05 20200 5.73E-05
21200 0.00018 19100 8.93E-06 18900 2.97E-05 18500 2.83E-05
20800 5.88E-05 20200 5.73E-05
21200 0.00018 19100 8.94E-06 18900 2.93E-05 18500 2.83E-05
20800 5.89E-05 20200 5.73E-05
21200 0.00018 19100 8.94E-06 18900 2.90E-05 18500 2.83E-05
20800 5.90E-05 20200 5.74E-05
21200 0.00018 19100 8.95E-06 18900 2.86E-05 18500 2.83E-05
20800 5.91E-05 20200 5.74E-05
21200 0.000181 19100 8.94E-06 18900 2.83E-05 18500
2.84E-05 20800 5.92E-05 20200 5.74E-05
21200 0.000181 19100 8.94E-06 18900 2.80E-05 18500
2.84E-05 20800 5.93E-05 20200 5.74E-05
21200 0.000181 19100 8.95E-06 18900 2.77E-05 18500
2.84E-05 20800 5.94E-05 20200 5.75E-05
21200 0.000181 19100 8.95E-06 18900 2.74E-05 18500
2.85E-05 20800 5.95E-05 20200 5.75E-05
21200 0.000181 19100 8.96E-06 18900 2.72E-05 18500
2.85E-05 20800 5.96E-05 20200 5.75E-05
21200 0.000181 19100 8.96E-06 18900 2.69E-05 18500
2.85E-05 20800 5.96E-05 20300 5.75E-05
21200 0.000181 19100 8.97E-06 18900 2.67E-05 18500
2.85E-05 20800 5.97E-05 20300 5.76E-05

21200 0.000181 19100 8.97E-06 18900 2.65E-05 18500
2.85E-05 20800 5.98E-05 20300 5.76E-05
21200 0.000182 19100 8.98E-06 18900 2.62E-05 18500
2.86E-05 20800 5.99E-05 20300 5.76E-05
21200 0.000182 19100 8.99E-06 18900 2.61E-05 18500
2.85E-05 20800 6.00E-05 20300 5.76E-05
21200 0.000182 19100 9.00E-06 18900 2.59E-05 18500
2.85E-05 20800 6.01E-05 20300 5.77E-05
21200 0.000182 19100 9.00E-06 18900 2.57E-05 18500
2.86E-05 20800 6.02E-05 20300 5.77E-05
21200 0.000182 19100 9.00E-06 18900 2.55E-05 18500
2.86E-05 20800 6.03E-05 20300 5.77E-05
21200 0.000182 19100 9.01E-06 18900 2.53E-05 18500
2.86E-05 20800 6.04E-05 20300 5.78E-05
21200 0.000182 19200 9.02E-06 18900 2.52E-05 18500
2.86E-05 20800 6.05E-05 20300 5.78E-05
21200 0.000182 19200 9.02E-06 18900 2.50E-05 18500
2.86E-05 20800 6.06E-05 20300 5.78E-05
21200 0.000183 19200 9.03E-06 19000 2.49E-05 18500
2.86E-05 20800 6.07E-05 20300 5.78E-05
21200 0.000183 19200 9.03E-06 19000 2.47E-05 18500
2.86E-05 20800 6.08E-05 20300 5.79E-05
21200 0.000183 19200 9.03E-06 19000 2.46E-05 18500
2.86E-05 20800 6.09E-05 20300 5.79E-05
21200 0.000183 19200 9.04E-06 19000 2.45E-05 18500
2.87E-05 20800 6.10E-05 20300 5.79E-05
21200 0.000183 19200 9.04E-06 19000 2.43E-05 18600
2.87E-05 20800 6.11E-05 20300 5.79E-05
21200 0.000183 19200 9.05E-06 19000 2.42E-05 18600
2.87E-05 20800 6.13E-05 20300 5.80E-05
21200 0.000183 19200 9.06E-06 19000 2.41E-05 18600
2.87E-05 20800 6.14E-05 20300 5.80E-05
21200 0.000184 19200 9.05E-06 19000 2.40E-05 18600
2.87E-05 20800 6.15E-05 20300 5.80E-05
21200 0.000184 19200 9.05E-06 19000 2.39E-05 18600
2.87E-05 20800 6.16E-05 20300 5.80E-05
21200 0.000184 19200 9.07E-06 19000 2.38E-05 18600
2.88E-05 20800 6.17E-05 20300 5.80E-05
21200 0.000184 19200 9.06E-06 19000 2.37E-05 18600
2.87E-05 20800 6.19E-05 20300 5.81E-05
21200 0.000184 19200 9.05E-06 19000 2.36E-05 18600
2.88E-05 20800 6.19E-05 20300 5.81E-05
21200 0.000184 19200 9.07E-06 19000 2.35E-05 18600
2.88E-05 20800 6.21E-05 20300 5.81E-05
21200 0.000184 19200 9.06E-06 19000 2.34E-05 18600
2.88E-05 20800 6.22E-05 20300 5.82E-05
21200 0.000185 19200 9.06E-06 19000 2.33E-05 18600
2.89E-05 20800 6.23E-05 20300 5.82E-05
21200 0.000185 19200 9.06E-06 19000 2.33E-05 18600
2.89E-05 20900 6.25E-05 20300 5.82E-05
21200 0.000185 19200 9.07E-06 19000 2.32E-05 18600
2.89E-05 20900 6.25E-05 20300 5.82E-05
21200 0.000185 19200 9.07E-06 19000 2.31E-05 18600
2.89E-05 20900 6.27E-05 20300 5.82E-05
21200 0.000185 19200 9.07E-06 19000 2.30E-05 18600
2.89E-05 20900 6.28E-05 20300 5.83E-05
21200 0.000185 19200 9.07E-06 19000 2.30E-05 18600
2.90E-05 20900 6.29E-05 20300 5.83E-05
21200 0.000185 19200 9.06E-06 19000 2.29E-05 18600
2.90E-05 20900 6.31E-05 20300 5.83E-05
21200 0.000185 19200 9.06E-06 19000 2.28E-05 18600
2.90E-05 20900 6.32E-05 20300 5.83E-05
21200 0.000186 19200 9.06E-06 19000 2.28E-05 18600
2.90E-05 20900 6.33E-05 20300 5.84E-05
21200 0.000186 19200 9.07E-06 19000 2.27E-05 18600
2.90E-05 20900 6.34E-05 20300 5.84E-05
21200 0.000186 19200 9.07E-06 19000 2.26E-05 18600
2.90E-05 20900 6.36E-05 20300 5.84E-05

21200 0.000186 19200 9.06E-06 19000 2.26E-05 18600
2.91E-05 20900 6.37E-05 20300 5.84E-05
21200 0.000186 19200 9.07E-06 19000 2.25E-05 18600
2.91E-05 20900 6.39E-05 20300 5.85E-05
21200 0.000186 19200 9.07E-06 19000 2.25E-05 18600
2.91E-05 20900 6.40E-05 20300 5.85E-05
21200 0.000186 19200 9.08E-06 19000 2.24E-05 18600
2.92E-05 20900 6.41E-05 20300 5.85E-05
21200 0.000187 19200 9.08E-06 19000 2.24E-05 18600
2.92E-05 20900 6.43E-05 20300 5.85E-05
21200 0.000187 19200 9.07E-06 19000 2.23E-05 18600
2.92E-05 20900 6.44E-05 20300 5.86E-05
21200 0.000187 19200 9.08E-06 19000 2.23E-05 18600
2.92E-05 20900 6.46E-05 20300 5.86E-05
21200 0.000187 19200 9.08E-06 19000 2.22E-05 18600
2.92E-05 20900 6.47E-05 20300 5.86E-05
21200 0.000187 19200 9.09E-06 19000 2.22E-05 18600
2.93E-05 20900 6.49E-05 20300 5.86E-05
21200 0.000187 19200 9.08E-06 19000 2.22E-05 18600
2.93E-05 20900 6.50E-05 20300 5.87E-05
21200 0.000187 19200 9.08E-06 19000 2.21E-05 18600
2.93E-05 20900 6.52E-05 20300 5.87E-05
21200 0.000188 19200 9.09E-06 19000 2.21E-05 18600
2.93E-05 20900 6.54E-05 20300 5.87E-05
21200 0.000188 19200 9.08E-06 19000 2.20E-05 18600
2.94E-05 20900 6.55E-05 20300 5.87E-05
21200 0.000188 19200 9.08E-06 19000 2.20E-05 18600
2.94E-05 20900 6.57E-05 20300 5.88E-05
21200 0.000188 19200 9.08E-06 19000 2.20E-05 18600
2.94E-05 20900 6.58E-05 20300 5.88E-05
21200 0.000188 19200 9.08E-06 19000 2.19E-05 18600
2.94E-05 20900 6.60E-05 20300 5.88E-05
21200 0.000188 19200 9.08E-06 19000 2.19E-05 18600
2.95E-05 20900 6.62E-05 20300 5.88E-05
21200 0.000188 19200 9.09E-06 19000 2.19E-05 18600
2.95E-05 20900 6.63E-05 20300 5.89E-05
21200 0.000188 19200 9.09E-06 19000 2.19E-05 18600
2.95E-05 20900 6.65E-05 20300 5.89E-05
21300 0.000189 19200 9.09E-06 19000 2.18E-05 18600
2.95E-05 20900 6.67E-05 20300 5.89E-05
21300 0.000189 19200 9.08E-06 19000 2.18E-05 18600
2.95E-05 20900 6.69E-05 20300 5.89E-05
21300 0.000189 19200 9.08E-06 19000 2.18E-05 18600
2.96E-05 20900 6.70E-05 20300 5.90E-05
21300 0.000189 19200 9.08E-06 19000 2.17E-05 18600
2.96E-05 20900 6.72E-05 20300 5.90E-05
21300 0.000189 19200 9.09E-06 19000 2.17E-05 18600
2.96E-05 20900 6.74E-05 20300 5.90E-05
21300 0.000189 19200 9.09E-06 19000 2.17E-05 18600
2.96E-05 20900 6.76E-05 20300 5.90E-05
21300 0.000189 19200 9.09E-06 19000 2.17E-05 18600
2.96E-05 20900 6.77E-05 20300 5.90E-05
21300 0.00019 19200 9.10E-06 19000 2.16E-05 18600 2.96E-05
20900 6.79E-05 20300 5.91E-05
21300 0.00019 19200 9.10E-06 19000 2.16E-05 18600 2.96E-05
20900 6.81E-05 20300 5.91E-05
21300 0.00019 19200 9.10E-06 19000 2.16E-05 18600 2.97E-05
20900 6.83E-05 20400 5.91E-05
21300 0.00019 19200 9.10E-06 19000 2.16E-05 18600 2.97E-05
20900 6.85E-05 20400 5.92E-05
21300 0.00019 19200 9.10E-06 19000 2.16E-05 18600 2.98E-05
20900 6.87E-05 20400 5.92E-05
21300 0.00019 19200 9.10E-06 19000 2.15E-05 18600 2.98E-05
20900 6.89E-05 20400 5.92E-05
21300 0.00019 19200 9.12E-06 19000 2.15E-05 18600 2.98E-05
20900 6.91E-05 20400 5.92E-05
21300 0.000191 19200 9.12E-06 19000 2.15E-05 18600
2.98E-05 20900 6.93E-05 20400 5.93E-05

21300	0.000191	19200	9.12E-06	19000	2.15E-05	18600	2.99E-05	20900	6.95E-05	20400	5.93E-05
21300	0.000191	19200	9.13E-06	19000	2.15E-05	18600	2.99E-05	20900	6.97E-05	20400	5.93E-05
21300	0.000191	19200	9.13E-06	19000	2.14E-05	18600	2.99E-05	20900	6.99E-05	20400	5.93E-05
21300	0.000191	19300	9.14E-06	19000	2.14E-05	18600	3.00E-05	20900	7.02E-05	20400	5.94E-05
21300	0.000191	19300	9.13E-06	19000	2.14E-05	18600	3.00E-05	20900	7.04E-05	20400	5.94E-05
21300	0.000191	19300	9.13E-06	19100	2.14E-05	18600	3.01E-05	20900	7.06E-05	20400	5.94E-05
21300	0.000192	19300	9.14E-06	19100	2.14E-05	18600	3.01E-05	20900	7.08E-05	20400	5.95E-05
21300	0.000192	19300	9.14E-06	19100	2.13E-05	18600	3.01E-05	20900	7.11E-05	20400	5.95E-05
21300	0.000192	19300	9.15E-06	19100	2.13E-05	18600	3.01E-05	20900	7.13E-05	20400	5.95E-05
21300	0.000192	19300	9.16E-06	19100	2.13E-05	18700	3.01E-05	20900	7.15E-05	20400	5.95E-05
21300	0.000192	19300	9.17E-06	19100	2.13E-05	18700	3.02E-05	20900	7.18E-05	20400	5.96E-05
21300	0.000192	19300	9.17E-06	19100	2.13E-05	18700	3.02E-05	20900	7.20E-05	20400	5.96E-05
21300	0.000192	19300	9.16E-06	19100	2.13E-05	18700	3.03E-05	20900	7.23E-05	20400	5.96E-05
21300	0.000193	19300	9.17E-06	19100	2.13E-05	18700	3.03E-05	20900	7.25E-05	20400	5.97E-05
21300	0.000193	19300	9.17E-06	19100	2.13E-05	18700	3.03E-05	20900	7.27E-05	20400	5.97E-05
21300	0.000193	19300	9.17E-06	19100	2.12E-05	18700	3.04E-05	20900	7.30E-05	20400	5.97E-05
21300	0.000193	19300	9.17E-06	19100	2.12E-05	18700	3.04E-05	20900	7.33E-05	20400	5.98E-05
21300	0.000193	19300	9.19E-06	19100	2.12E-05	18700	3.04E-05	20900	7.35E-05	20400	5.98E-05
21300	0.000193	19300	9.19E-06	19100	2.12E-05	18700	3.04E-05	20900	7.38E-05	20400	5.98E-05
21300	0.000194	19300	9.19E-06	19100	2.12E-05	18700	3.05E-05	20900	7.41E-05	20400	5.99E-05
21300	0.000194	19300	9.18E-06	19100	2.12E-05	18700	3.05E-05	21000	7.44E-05	20400	5.99E-05
21300	0.000194	19300	9.18E-06	19100	2.12E-05	18700	3.06E-05	21000	7.46E-05	20400	5.99E-05
21300	0.000194	19300	9.20E-06	19100	2.12E-05	18700	3.06E-05	21000	7.49E-05	20400	6.00E-05
21300	0.000194	19300	9.20E-06	19100	2.12E-05	18700	3.06E-05	21000	7.52E-05	20400	6.00E-05
21300	0.000194	19300	9.19E-06	19100	2.12E-05	18700	3.07E-05	21000	7.55E-05	20400	6.00E-05
21300	0.000195	19300	9.20E-06	19100	2.12E-05	18700	3.06E-05	21000	7.58E-05	20400	6.01E-05
21300	0.000195	19300	9.18E-06	19100	2.12E-05	18700	3.07E-05	21000	7.61E-05	20400	6.01E-05
21300	0.000195	19300	9.19E-06	19100	2.11E-05	18700	3.07E-05	21000	7.64E-05	20400	6.01E-05
21300	0.000195	19300	9.19E-06	19100	2.11E-05	18700	3.07E-05	21000	7.67E-05	20400	6.01E-05
21300	0.000195	19300	9.19E-06	19100	2.11E-05	18700	3.07E-05	21000	7.71E-05	20400	6.02E-05
21300	0.000195	19300	9.18E-06	19100	2.11E-05	18700	3.08E-05	21000	7.74E-05	20400	6.02E-05
21300	0.000196	19300	9.17E-06	19100	2.11E-05	18700	3.08E-05	21000	7.77E-05	20400	6.02E-05
21300	0.000196	19300	9.18E-06	19100	2.11E-05	18700	3.09E-05	21000	7.81E-05	20400	6.03E-05
21300	0.000196	19300	9.19E-06	19100	2.11E-05	18700	3.09E-05	21000	7.84E-05	20400	6.03E-05

```
21300   0.000196        19300   9.20E-06        19100   2.11E-05        18700
3.09E-05        21000   7.88E-05        20400   6.03E-05
21300   0.000196        19300   9.20E-06        19100   2.11E-05        18700
3.10E-05        21000   7.91E-05        20400   6.03E-05
21300   0.000196        19300   9.20E-06        19100   2.11E-05        18700
3.10E-05        21000   7.95E-05        20400   6.04E-05
21300   0.000196        19300   9.20E-06        19100   2.11E-05        18700
3.10E-05        21000   7.99E-05        20400   6.04E-05
21300   0.000197        19300   9.19E-06        19100   2.11E-05        18700
3.10E-05        21000   8.03E-05        20400   6.04E-05
21300   0.000197        19300   9.19E-06        19100   2.11E-05        18700
3.10E-05        21000   8.07E-05        20400   6.05E-05
21300   0.000197        19300   9.19E-06        19100   2.11E-05        18700
3.11E-05        21000   8.11E-05        20400   6.05E-05
21300   0.000197        19300   9.19E-06        19100   2.11E-05        18700
3.11E-05        21000   8.15E-05        20400   6.05E-05
21300   0.000197        19300   9.21E-06        19100   2.11E-05        18700
3.11E-05        21000   8.19E-05        20400   6.06E-05
21300   0.000197        19300   9.20E-06        19100   2.11E-05        18700
3.12E-05        21000   8.23E-05        20400   6.06E-05
21300   0.000198        19300   9.19E-06        19100   2.11E-05        18700
3.12E-05        21000   8.27E-05        20400   6.06E-05
21300   0.000198        19300   9.20E-06        19100   2.11E-05        18700
3.12E-05        21000   8.32E-05        20400   6.06E-05
21300   0.000198        19300   9.20E-06        19100   2.11E-05        18700
3.12E-05        21000   8.36E-05        20400   6.07E-05
21300   0.000198        19300   9.20E-06        19100   2.11E-05        18700
3.12E-05        21000   8.41E-05        20400   6.07E-05
21300   0.000198        19300   9.19E-06        19100   2.11E-05        18700
3.13E-05        21000   8.45E-05        20400   6.07E-05
21400   0.000198        19300   9.20E-06        19100   2.11E-05        18700
3.13E-05        21000   8.50E-05        20400   6.08E-05
21400   0.000198        19300   9.21E-06        19100   2.11E-05        18700
3.13E-05        21000   8.55E-05        20400   6.08E-05
21400   0.000199        19300   9.21E-06        19100   2.11E-05        18700
3.14E-05        21000   8.59E-05        20400   6.08E-05
21400   0.000199        19300   9.21E-06        19100   2.11E-05        18700
3.15E-05        21000   8.64E-05        20400   6.08E-05
21400   0.000199        19300   9.21E-06        19100   2.11E-05        18700
3.15E-05        21000   8.69E-05        20400   6.09E-05
21400   0.000199        19300   9.22E-06        19100   2.11E-05        18700
3.15E-05        21000   8.74E-05        20400   6.09E-05
21400   0.000199        19300   9.23E-06        19100   2.11E-05        18700
3.16E-05        21000   8.79E-05        20400   6.10E-05
21400   0.000199        19300   9.24E-06        19100   2.11E-05        18700
3.16E-05        21000   8.85E-05        20400   6.10E-05
21400   0.000199        19300   9.25E-06        19100   2.11E-05        18700
3.16E-05        21000   8.90E-05        20400   6.10E-05
21400   0.0002  19300   9.24E-06        19100   2.11E-05        18700   3.17E-05
21000   8.96E-05        20500   6.10E-05
21400   0.0002  19300   9.25E-06        19100   2.11E-05        18700   3.17E-05
21000   9.01E-05        20500   6.11E-05
21400   0.0002  19300   9.26E-06        19100   2.11E-05        18700   3.18E-05
21000   9.07E-05        20500   6.11E-05
21400   0.0002  19300   9.27E-06        19100   2.11E-05        18700   3.18E-05
21000   9.13E-05        20500   6.11E-05
21400   0.0002  19300   9.26E-06        19100   2.11E-05        18700   3.19E-05
21000   9.19E-05        20500   6.11E-05
21400   0.0002  19300   9.28E-06        19100   2.11E-05        18700   3.19E-05
21000   9.25E-05        20500   6.12E-05
21400   0.000201        19300   9.27E-06        19100   2.11E-05        18700
3.20E-05        21000   9.31E-05        20500   6.12E-05
21400   0.000201        19300   9.29E-06        19100   2.11E-05        18700
3.20E-05        21000   9.37E-05        20500   6.12E-05
21400   0.000201        19300   9.30E-06        19100   2.11E-05        18700
3.21E-05        21000   9.44E-05        20500   6.13E-05
21400   0.000201        19400   9.28E-06        19100   2.11E-05        18700
3.21E-05        21000   9.50E-05        20500   6.13E-05
```

21400 0.000201 19400 9.29E-06 19100 2.11E-05 18700
3.21E-05 21000 9.57E-05 20500 6.13E-05
21400 0.000201 19400 9.29E-06 19200 2.12E-05 18700
3.22E-05 21000 9.64E-05 20500 6.13E-05
21400 0.000201 19400 9.29E-06 19200 2.12E-05 18700
3.22E-05 21000 9.71E-05 20500 6.14E-05
21400 0.000202 19400 9.29E-06 19200 2.11E-05 18700
3.23E-05 21000 9.78E-05 20500 6.14E-05
21400 0.000202 19400 9.30E-06 19200 2.11E-05 18700
3.23E-05 21000 9.85E-05 20500 6.14E-05
21400 0.000202 19400 9.29E-06 19200 2.11E-05 18800
3.24E-05 21000 9.92E-05 20500 6.14E-05
21400 0.000202 19400 9.31E-06 19200 2.11E-05 18800
3.24E-05 21000 0.0001 20500 6.15E-05
21400 0.000202 19400 9.33E-06 19200 2.11E-05 18800
3.24E-05 21000 0.000101 20500 6.15E-05
21400 0.000202 19400 9.31E-06 19200 2.12E-05 18800
3.25E-05 21000 0.000102 20500 6.15E-05
21400 0.000203 19400 9.31E-06 19200 2.12E-05 18800
3.26E-05 21000 0.000102 20500 6.15E-05
21400 0.000203 19400 9.34E-06 19200 2.12E-05 18800
3.26E-05 21000 0.000103 20500 6.16E-05
21400 0.000203 19400 9.34E-06 19200 2.12E-05 18800
3.27E-05 21000 0.000104 20500 6.16E-05
21400 0.000203 19400 9.35E-06 19200 2.12E-05 18800
3.27E-05 21000 0.000105 20500 6.16E-05
21400 0.000203 19400 9.32E-06 19200 2.12E-05 18800
3.28E-05 21000 0.000106 20500 6.16E-05
21400 0.000203 19400 9.33E-06 19200 2.12E-05 18800
3.28E-05 21000 0.000107 20500 6.17E-05
21400 0.000203 19400 9.36E-06 19200 2.12E-05 18800
3.28E-05 21000 0.000108 20500 6.17E-05
21400 0.000204 19400 9.36E-06 19200 2.12E-05 18800
3.29E-05 21100 0.000109 20500 6.17E-05
21400 0.000204 19400 9.35E-06 19200 2.12E-05 18800
3.29E-05 21100 0.000109 20500 6.17E-05
21400 0.000204 19400 9.35E-06 19200 2.12E-05 18800
3.30E-05 21100 0.00011 20500 6.18E-05
21400 0.000204 19400 9.34E-06 19200 2.12E-05 18800
3.31E-05 21100 0.000111 20500 6.18E-05
21400 0.000204 19400 9.34E-06 19200 2.13E-05 18800
3.31E-05 21100 0.000112 20500 6.18E-05
21400 0.000204 19400 9.36E-06 19200 2.13E-05 18800
3.32E-05 21100 0.000114 20500 6.19E-05
21400 0.000205 19400 9.35E-06 19200 2.13E-05 18800
3.32E-05 21100 0.000115 20500 6.19E-05
21400 0.000205 19400 9.33E-06 19200 2.13E-05 18800
3.32E-05 21100 0.000116 20500 6.19E-05
21400 0.000205 19400 9.34E-06 19200 2.13E-05 18800
3.33E-05 21100 0.000117 20500 6.19E-05
21400 0.000205 19400 9.33E-06 19200 2.13E-05 18800
3.33E-05 21100 0.000118 20500 6.20E-05
21400 0.000205 19400 9.36E-06 19200 2.13E-05 18800
3.34E-05 21100 0.000119 20500 6.20E-05
21400 0.000205 19400 9.35E-06 19200 2.13E-05 18800
3.34E-05 21100 0.00012 20500 6.20E-05
21400 0.000206 19400 9.38E-06 19200 2.13E-05 18800
3.35E-05 21100 0.000122 20500 6.21E-05
21400 0.000206 19400 9.38E-06 19200 2.13E-05 18800
3.35E-05 21100 0.000123 20500 6.21E-05
21400 0.000206 19400 9.41E-06 19200 2.13E-05 18800
3.36E-05 21100 0.000124 20500 6.21E-05
21400 0.000206 19400 9.41E-06 19200 2.13E-05 18800
3.37E-05 21100 0.000126 20500 6.22E-05
21400 0.000206 19400 9.41E-06 19200 2.13E-05 18800
3.37E-05 21100 0.000127 20500 6.22E-05
21400 0.000207 19400 9.40E-06 19200 2.13E-05 18800
3.38E-05 21100 0.000128 20500 6.22E-05

21400 0.000207 19400 9.39E-06 19200 2.13E-05 18800
3.39E-05 21100 0.00013 20500 6.22E-05
21400 0.000207 19400 9.41E-06 19200 2.13E-05 18800
3.39E-05 21100 0.000131 20500 6.23E-05
21400 0.000207 19400 9.42E-06 19200 2.13E-05 18800
3.40E-05 21100 0.000133 20500 6.23E-05
21400 0.000207 19400 9.41E-06 19200 2.14E-05 18800
3.41E-05 21100 0.000134 20500 6.23E-05
21400 0.000207 19400 9.42E-06 19200 2.13E-05 18800
3.41E-05 21100 0.000136 20500 6.24E-05
21400 0.000208 19400 9.40E-06 19200 2.14E-05 18800
3.42E-05 21100 0.000138 20500 6.24E-05
21400 0.000208 19400 9.40E-06 19200 2.14E-05 18800
3.43E-05 21100 0.000139 20500 6.24E-05
21400 0.000208 19400 9.40E-06 19200 2.14E-05 18800
3.43E-05 21100 0.000141 20500 6.24E-05
21400 0.000208 19400 9.41E-06 19200 2.14E-05 18800
3.44E-05 21100 0.000143 20500 6.25E-05
21400 0.000208 19400 9.42E-06 19200 2.14E-05 18800
3.45E-05 21100 0.000145 20500 6.25E-05
21400 0.000209 19400 9.41E-06 19200 2.14E-05 18800
3.46E-05 21100 0.000146 20500 6.26E-05
21500 0.000209 19400 9.42E-06 19200 2.14E-05 18800
3.46E-05 21100 0.000148 20500 6.26E-05
21500 0.000209 19400 9.43E-06 19200 2.14E-05 18800
3.47E-05 21100 0.00015 20500 6.26E-05
21500 0.000209 19400 9.43E-06 19200 2.14E-05 18800
3.47E-05 21100 0.000152 20500 6.27E-05
21500 0.000209 19400 9.43E-06 19200 2.14E-05 18800
3.48E-05 21100 0.000154 20500 6.27E-05
21500 0.000209 19400 9.45E-06 19200 2.14E-05 18800
3.49E-05 21100 0.000157 20500 6.27E-05
21500 0.00021 19400 9.46E-06 19200 2.14E-05 18800 3.50E-05
21100 0.000159 20500 6.27E-05
21500 0.00021 19400 9.45E-06 19200 2.14E-05 18800 3.51E-05
21100 0.000161 20500 6.28E-05
21500 0.00021 19400 9.44E-06 19200 2.15E-05 18800 3.51E-05
21100 0.000163 20500 6.28E-05
21500 0.00021 19400 9.44E-06 19200 2.14E-05 18800 3.52E-05
21100 0.000166 20500 6.28E-05
21500 0.00021 19400 9.45E-06 19200 2.15E-05 18800 3.53E-05
21100 0.000168 20600 6.28E-05
21500 0.00021 19400 9.46E-06 19200 2.15E-05 18800 3.53E-05
21100 0.000171 20600 6.29E-05
21500 0.000211 19400 9.45E-06 19200 2.15E-05 18800
3.54E-05 21100 0.000173 20600 6.29E-05
21500 0.000211 19400 9.46E-06 19200 2.15E-05 18800
3.55E-05 21100 0.000176 20600 6.29E-05
21500 0.000211 19400 9.47E-06 19200 2.15E-05 18800
3.56E-05 21100 0.000179 20600 6.30E-05
21500 0.000211 19400 9.48E-06 19200 2.15E-05 18800
3.57E-05 21100 0.000181 20600 6.30E-05
21500 0.000211 19400 9.48E-06 19200 2.15E-05 18800
3.58E-05 21100 0.000184 20600 6.30E-05
21500 0.000212 19400 9.49E-06 19200 2.15E-05 18800
3.59E-05 21100 0.000187 20600 6.30E-05
21500 0.000212 19400 9.49E-06 19200 2.15E-05 18800
3.59E-05 21100 0.00019 20600 6.31E-05
21500 0.000212 19500 9.50E-06 19200 2.15E-05 18800
3.60E-05 21100 0.000193 20600 6.31E-05
21500 0.000212 19500 9.50E-06 19200 2.15E-05 18800
3.61E-05 21100 0.000197 20600 6.31E-05
21500 0.000212 19500 9.51E-06 19300 2.15E-05 18800
3.62E-05 21100 0.0002 20600 6.32E-05
21500 0.000212 19500 9.52E-06 19300 2.15E-05 18800
3.63E-05 21100 0.000203 20600 6.32E-05
21500 0.000213 19500 9.52E-06 19300 2.15E-05 18800
3.64E-05 21100 0.000207 20600 6.32E-05

21500	0.000213	19500	9.53E-06	19300	2.15E-05	18800	3.65E-05	21100	0.00021	20600	6.32E-05
21500	0.000213	19500	9.55E-06	19300	2.16E-05	18900	3.67E-05	21100	0.000214	20600	6.33E-05
21500	0.000213	19500	9.55E-06	19300	2.16E-05	18900	3.68E-05	21100	0.000218	20600	6.33E-05
21500	0.000213	19500	9.53E-06	19300	2.16E-05	18900	3.69E-05	21100	0.000222	20600	6.33E-05
21500	0.000214	19500	9.54E-06	19300	2.16E-05	18900	3.70E-05	21100	0.000226	20600	6.33E-05
21500	0.000214	19500	9.56E-06	19300	2.16E-05	18900	3.71E-05	21100	0.00023	20600	6.34E-05
21500	0.000214	19500	9.57E-06	19300	2.16E-05	18900	3.72E-05	21100	0.000234	20600	6.34E-05
21500	0.000214	19500	9.59E-06	19300	2.16E-05	18900	3.73E-05	21100	0.000239	20600	6.34E-05
21500	0.000214	19500	9.58E-06	19300	2.16E-05	18900	3.75E-05	21100	0.000243	20600	6.35E-05
21500	0.000215	19500	9.60E-06	19300	2.16E-05	18900	3.76E-05	21100	0.000248	20600	6.35E-05
21500	0.000215	19500	9.59E-06	19300	2.16E-05	18900	3.77E-05	21100	0.000253	20600	6.35E-05
21500	0.000215	19500	9.61E-06	19300	2.16E-05	18900	3.79E-05	21100	0.000257	20600	6.36E-05
21500	0.000215	19500	9.61E-06	19300	2.16E-05	18900	3.80E-05	21200	0.000262	20600	6.36E-05
21500	0.000215	19500	9.60E-06	19300	2.16E-05	18900	3.81E-05	21200	0.000268	20600	6.36E-05
21500	0.000216	19500	9.58E-06	19300	2.16E-05	18900	3.83E-05	21200	0.000273	20600	6.37E-05
21500	0.000216	19500	9.60E-06	19300	2.16E-05	18900	3.84E-05	21200	0.000278	20600	6.37E-05
21500	0.000216	19500	9.59E-06	19300	2.17E-05	18900	3.86E-05	21200	0.000284	20600	6.37E-05
21500	0.000216	19500	9.58E-06	19300	2.17E-05	18900	3.87E-05	21200	0.00029	20600	6.38E-05
21500	0.000216	19500	9.59E-06	19300	2.17E-05	18900	3.89E-05	21200	0.000296	20600	6.38E-05
21500	0.000217	19500	9.58E-06	19300	2.17E-05	18900	3.91E-05	21200	0.000302	20600	6.38E-05
21500	0.000217	19500	9.59E-06	19300	2.17E-05	18900	3.92E-05	21200	0.000308	20600	6.38E-05
21500	0.000217	19500	9.58E-06	19300	2.17E-05	18900	3.94E-05	21200	0.000314	20600	6.39E-05
21500	0.000217	19500	9.58E-06	19300	2.17E-05	18900	3.96E-05	21200	0.000321	20600	6.39E-05
21500	0.000217	19500	9.57E-06	19300	2.17E-05	18900	3.98E-05	21200	0.000328	20600	6.39E-05
21500	0.000218	19500	9.58E-06	19300	2.17E-05	18900	4.00E-05	21200	0.000334	20600	6.40E-05
21500	0.000218	19500	9.61E-06	19300	2.17E-05	18900	4.01E-05	21200	0.000341	20600	6.40E-05
21500	0.000218	19500	9.61E-06	19300	2.17E-05	18900	4.03E-05	21200	0.000348	20600	6.40E-05
21500	0.000218	19500	9.59E-06	19300	2.18E-05	18900	4.06E-05	21200	0.000356	20600	6.41E-05
21500	0.000218	19500	9.59E-06	19300	2.18E-05	18900	4.08E-05	21200	0.000363	20600	6.41E-05
21500	0.000218	19500	9.60E-06	19300	2.18E-05	18900	4.10E-05	21200	0.000371	20600	6.41E-05
21500	0.000219	19500	9.59E-06	19300	2.18E-05	18900	4.12E-05	21200	0.000379	20600	6.41E-05
21500	0.000219	19500	9.59E-06	19300	2.18E-05	18900	4.15E-05	21200	0.000386	20600	6.42E-05
21500	0.000219	19500	9.60E-06	19300	2.18E-05	18900	4.17E-05	21200	0.000394	20600	6.42E-05
21500	0.000219	19500	9.61E-06	19300	2.18E-05	18900	4.20E-05	21200	0.000402	20600	6.42E-05

21500 0.000219 19500 9.60E-06 19300 2.18E-05 18900
4.23E-05 21200 0.000411 20600 6.43E-05
21500 0.00022 19500 9.60E-06 19300 2.18E-05 18900 4.25E-05
21200 0.000419 20600 6.43E-05
21500 0.00022 19500 9.60E-06 19300 2.18E-05 18900 4.28E-05
21200 0.000427 20600 6.43E-05
21500 0.00022 19500 9.60E-06 19300 2.18E-05 18900 4.31E-05
21200 0.000436 20600 6.44E-05
21500 0.00022 19500 9.60E-06 19300 2.18E-05 18900 4.34E-05
21200 0.000444 20600 6.44E-05
21500 0.00022 19500 9.60E-06 19300 2.18E-05 18900 4.37E-05
21200 0.000453 20600 6.44E-05
21500 0.000221 19500 9.59E-06 19300 2.19E-05 18900
4.41E-05 21200 0.000462 20600 6.44E-05
21600 0.000221 19500 9.59E-06 19300 2.19E-05 18900
4.44E-05 21200 0.00047 20600 6.45E-05
21600 0.000221 19500 9.60E-06 19300 2.19E-05 18900
4.48E-05 21200 0.000479 20600 6.45E-05
21600 0.000221 19500 9.59E-06 19300 2.19E-05 18900
4.51E-05 21200 0.000488 20600 6.45E-05
21600 0.000221 19500 9.60E-06 19300 2.19E-05 18900
4.55E-05 21200 0.000496 20600 6.46E-05
21600 0.000221 19500 9.60E-06 19300 2.19E-05 18900
4.59E-05 21200 0.000505 20600 6.46E-05
21600 0.000222 19500 9.61E-06 19300 2.19E-05 18900
4.63E-05 21200 0.000513 20600 6.46E-05
21600 0.000222 19500 9.61E-06 19300 2.19E-05 18900
4.68E-05 21200 0.000521 20600 6.47E-05
21600 0.000222 19500 9.62E-06 19300 2.19E-05 18900
4.72E-05 21200 0.000529 20600 6.47E-05
21600 0.000222 19500 9.63E-06 19300 2.19E-05 18900
4.77E-05 21200 0.000537 20600 6.47E-05
21600 0.000222 19500 9.62E-06 19300 2.19E-05 18900
4.82E-05 21200 0.000545 20700 6.48E-05
21600 0.000223 19500 9.62E-06 19300 2.20E-05 18900
4.88E-05 21200 0.000552 20700 6.48E-05
21600 0.000223 19500 9.61E-06 19300 2.20E-05 18900
4.94E-05 21200 0.000559 20700 6.48E-05
21600 0.000223 19500 9.62E-06 19300 2.20E-05 18900
5.00E-05 21200 0.000565 20700 6.48E-05
21600 0.000223 19500 9.63E-06 19300 2.20E-05 18900
5.06E-05 21200 0.000572 20700 6.49E-05
21600 0.000223 19500 9.63E-06 19300 2.20E-05 18900
5.13E-05 21200 0.000578 20700 6.49E-05
21600 0.000224 19500 9.64E-06 19300 2.20E-05 18900
5.20E-05 21200 0.000583 20700 6.50E-05
21600 0.000224 19500 9.63E-06 19300 2.20E-05 18900
5.28E-05 21200 0.000588 20700 6.50E-05
21600 0.000224 19500 9.64E-06 19300 2.20E-05 18900
5.36E-05 21200 0.000592 20700 6.50E-05
21600 0.000224 19600 9.65E-06 19300 2.20E-05 18900
5.44E-05 21200 0.000596 20700 6.50E-05
21600 0.000224 19600 9.65E-06 19300 2.20E-05 18900
5.54E-05 21200 0.000599 20700 6.51E-05
21600 0.000225 19600 9.65E-06 19400 2.21E-05 18900
5.64E-05 21200 0.000601 20700 6.51E-05
21600 0.000225 19600 9.66E-06 19400 2.21E-05 18900
5.74E-05 21200 0.000603 20700 6.51E-05
21600 0.000225 19600 9.67E-06 19400 2.21E-05 18900
5.85E-05 21200 0.000604 20700 6.52E-05
21600 0.000225 19600 9.67E-06 19400 2.21E-05 18900
5.97E-05 21200 0.000605 20700 6.52E-05
21600 0.000225 19600 9.68E-06 19400 2.21E-05 19000
6.09E-05 21200 0.000604 20700 6.52E-05
21600 0.000225 19600 9.68E-06 19400 2.21E-05 19000
6.23E-05 21200 0.000603 20700 6.52E-05
21600 0.000226 19600 9.69E-06 19400 2.21E-05 19000
6.37E-05 21200 0.000601 20700 6.53E-05

21600 0.000226 19600 9.69E-06 19400 2.21E-05 19000
6.53E-05 21200 0.000598 20700 6.53E-05
21600 0.000226 19600 9.68E-06 19400 2.21E-05 19000
6.69E-05 21200 0.000595 20700 6.53E-05
21600 0.000226 19600 9.68E-06 19400 2.21E-05 19000
6.87E-05 21200 0.000591 20700 6.54E-05
21600 0.000226 19600 9.68E-06 19400 2.21E-05 19000
7.06E-05 21200 0.000586 20700 6.54E-05
21600 0.000227 19600 9.68E-06 19400 2.21E-05 19000
7.26E-05 21200 0.00058 20700 6.54E-05
21600 0.000227 19600 9.68E-06 19400 2.21E-05 19000
7.48E-05 21200 0.000573 20700 6.55E-05
21600 0.000227 19600 9.67E-06 19400 2.22E-05 19000
7.72E-05 21200 0.000566 20700 6.55E-05
21600 0.000227 19600 9.68E-06 19400 2.22E-05 19000
7.98E-05 21200 0.000558 20700 6.55E-05
21600 0.000227 19600 9.68E-06 19400 2.22E-05 19000
8.26E-05 21300 0.00055 20700 6.55E-05
21600 0.000228 19600 9.68E-06 19400 2.22E-05 19000
8.57E-05 21300 0.000541 20700 6.56E-05
21600 0.000228 19600 9.68E-06 19400 2.22E-05 19000
8.90E-05 21300 0.000531 20700 6.56E-05
21600 0.000228 19600 9.67E-06 19400 2.22E-05 19000
9.26E-05 21300 0.000521 20700 6.56E-05
21600 0.000228 19600 9.67E-06 19400 2.22E-05 19000
9.65E-05 21300 0.000511 20700 6.57E-05
21600 0.000228 19600 9.68E-06 19400 2.22E-05 19000
0.000100714 21300 0.0005 20700 6.57E-05
21600 0.000228 19600 9.68E-06 19400 2.23E-05 19000
0.000105308 21300 0.000488 20700 6.57E-05
21600 0.000229 19600 9.70E-06 19400 2.23E-05 19000
0.000110355 21300 0.000477 20700 6.57E-05
21600 0.000229 19600 9.70E-06 19400 2.23E-05 19000
0.000115827 21300 0.000465 20700 6.58E-05
21600 0.000229 19600 9.69E-06 19400 2.23E-05 19000
0.000121787 21300 0.000453 20700 6.58E-05
21600 0.000229 19600 9.70E-06 19400 2.23E-05 19000
0.000128274 21300 0.000441 20700 6.58E-05
21600 0.000229 19600 9.69E-06 19400 2.23E-05 19000
0.000135328 21300 0.000429 20700 6.59E-05
21600 0.00023 19600 9.70E-06 19400 2.23E-05 19000
0.000142961 21300 0.000417 20700 6.59E-05
21600 0.00023 19600 9.69E-06 19400 2.23E-05 19000
0.000151258 21300 0.000405 20700 6.59E-05
21600 0.00023 19600 9.71E-06 19400 2.23E-05 19000
0.000160221 21300 0.000393 20700 6.60E-05
21600 0.00023 19600 9.71E-06 19400 2.23E-05 19000
0.000169846 21300 0.000381 20700 6.60E-05
21600 0.00023 19600 9.71E-06 19400 2.23E-05 19000
0.000180181 21300 0.00037 20700 6.61E-05
21600 0.000231 19600 9.72E-06 19400 2.24E-05 19000
0.000191181 21300 0.000358 20700 6.61E-05
21600 0.000231 19600 9.72E-06 19400 2.24E-05 19000
0.000202825 21300 0.000347 20700 6.61E-05
21600 0.000231 19600 9.72E-06 19400 2.24E-05 19000
0.000214967 21300 0.000336 20700 6.62E-05
21600 0.000231 19600 9.72E-06 19400 2.24E-05 19000
0.000227515 21300 0.000325 20700 6.62E-05
21600 0.000232 19600 9.73E-06 19400 2.24E-05 19000
0.000240238 21300 0.000315 20700 6.62E-05
21600 0.000232 19600 9.73E-06 19400 2.24E-05 19000
0.000252899 21300 0.000305 20700 6.62E-05
21600 0.000232 19600 9.73E-06 19400 2.25E-05 19000
0.000265156 21300 0.000295 20700 6.63E-05
21600 0.000232 19600 9.73E-06 19400 2.25E-05 19000
0.000276621 21300 0.000286 20700 6.63E-05
21600 0.000232 19600 9.74E-06 19400 2.25E-05 19000
0.00028685 21300 0.000276 20700 6.63E-05

21600 0.000232 19600 9.75E-06 19400 2.25E-05 19000
0.000295385 21300 0.000268 20700 6.64E-05
21600 0.000233 19600 9.75E-06 19400 2.25E-05 19000
0.000301724 21300 0.000259 20700 6.64E-05
21600 0.000233 19600 9.75E-06 19400 2.25E-05 19000
0.000305448 21300 0.000251 20700 6.64E-05
21700 0.000233 19600 9.77E-06 19400 2.25E-05 19000
0.000306196 21300 0.000244 20700 6.65E-05
21700 0.000233 19600 9.77E-06 19400 2.25E-05 19000
0.00030376 21300 0.000236 20700 6.65E-05
21700 0.000233 19600 9.78E-06 19400 2.25E-05 19000
0.000298106 21300 0.000229 20700 6.65E-05
21700 0.000234 19600 9.77E-06 19400 2.25E-05 19000
0.000289398 21300 0.000223 20700 6.66E-05
21700 0.000234 19600 9.78E-06 19400 2.25E-05 19000
0.000277982 21300 0.000216 20700 6.66E-05
21700 0.000234 19600 9.79E-06 19400 2.26E-05 19000
0.000264334 21300 0.00021 20700 6.66E-05
21700 0.000234 19600 9.79E-06 19400 2.26E-05 19000
0.000249061 21300 0.000205 20700 6.67E-05
21700 0.000234 19600 9.79E-06 19400 2.26E-05 19000
0.000232804 21300 0.000199 20700 6.67E-05
21700 0.000235 19600 9.80E-06 19400 2.26E-05 19000
0.000216172 21300 0.000194 20700 6.67E-05
21700 0.000235 19600 9.80E-06 19400 2.26E-05 19000
0.000199682 21300 0.000189 20800 6.68E-05
21700 0.000235 19600 9.81E-06 19400 2.26E-05 19000
0.000183786 21300 0.000184 20800 6.68E-05
21700 0.000235 19600 9.79E-06 19400 2.26E-05 19000
0.000168804 21300 0.00018 20800 6.68E-05
21700 0.000235 19600 9.79E-06 19400 2.26E-05 19000
0.000154966 21300 0.000176 20800 6.69E-05
21700 0.000236 19600 9.80E-06 19400 2.26E-05 19000
0.000142389 21300 0.000172 20800 6.69E-05
21700 0.000236 19600 9.80E-06 19400 2.27E-05 19000
0.0001311 21300 0.000168 20800 6.69E-05
21700 0.000236 19600 9.82E-06 19400 2.27E-05 19000
0.000121074 21300 0.000165 20800 6.70E-05
21700 0.000236 19600 9.82E-06 19400 2.27E-05 19000
0.000112285 21300 0.000161 20800 6.70E-05
21700 0.000236 19600 9.82E-06 19400 2.27E-05 19000
0.000104597 21300 0.000158 20800 6.70E-05
21700 0.000237 19700 9.83E-06 19400 2.27E-05 19000
9.79E-05 21300 0.000155 20800 6.71E-05
21700 0.000237 19700 9.85E-06 19400 2.27E-05 19000
9.21E-05 21300 0.000153 20800 6.71E-05
21700 0.000237 19700 9.86E-06 19500 2.27E-05 19000
8.71E-05 21300 0.00015 20800 6.71E-05
21700 0.000237 19700 9.86E-06 19500 2.27E-05 19000
8.28E-05 21300 0.000147 20800 6.71E-05
21700 0.000237 19700 9.86E-06 19500 2.27E-05 19000
7.91E-05 21300 0.000145 20800 6.72E-05
21700 0.000238 19700 9.86E-06 19500 2.27E-05 19000
7.58E-05 21300 0.000143 20800 6.72E-05
21700 0.000238 19700 9.87E-06 19500 2.28E-05 19100
7.30E-05 21300 0.000141 20800 6.72E-05
21700 0.000238 19700 9.88E-06 19500 2.28E-05 19100
7.06E-05 21300 0.000139 20800 6.73E-05
21700 0.000238 19700 9.87E-06 19500 2.28E-05 19100
6.84E-05 21300 0.000137 20800 6.73E-05
21700 0.000238 19700 9.88E-06 19500 2.28E-05 19100
6.66E-05 21300 0.000135 20800 6.74E-05
21700 0.000239 19700 9.89E-06 19500 2.28E-05 19100
6.49E-05 21300 0.000133 20800 6.74E-05
21700 0.000239 19700 9.89E-06 19500 2.29E-05 19100
6.35E-05 21300 0.000132 20800 6.74E-05
21700 0.000239 19700 9.89E-06 19500 2.29E-05 19100
6.22E-05 21300 0.00013 20800 6.75E-05

21700 0.000239 19700 9.89E-06 19500 2.29E-05 19100
6.10E-05 21300 0.000129 20800 6.75E-05
21700 0.00024 19700 9.89E-06 19500 2.29E-05 19100 6.00E-05
21300 0.000127 20800 6.75E-05
21700 0.00024 19700 9.89E-06 19500 2.29E-05 19100 5.91E-05
21300 0.000126 20800 6.75E-05
21700 0.00024 19700 9.90E-06 19500 2.29E-05 19100 5.82E-05
21300 0.000125 20800 6.76E-05
21700 0.00024 19700 9.90E-06 19500 2.29E-05 19100 5.75E-05
21400 0.000124 20800 6.76E-05
21700 0.00024 19700 9.91E-06 19500 2.29E-05 19100 5.68E-05
21400 0.000122 20800 6.77E-05
21700 0.000241 19700 9.91E-06 19500 2.30E-05 19100
5.62E-05 21400 0.000121 20800 6.77E-05
21700 0.000241 19700 9.92E-06 19500 2.30E-05 19100
5.56E-05 21400 0.00012 20800 6.77E-05
21700 0.000241 19700 9.93E-06 19500 2.30E-05 19100
5.51E-05 21400 0.000119 20800 6.78E-05
21700 0.000241 19700 9.93E-06 19500 2.30E-05 19100
5.47E-05 21400 0.000118 20800 6.78E-05
21700 0.000241 19700 9.92E-06 19500 2.30E-05 19100
5.42E-05 21400 0.000117 20800 6.78E-05
21700 0.000242 19700 9.92E-06 19500 2.30E-05 19100
5.39E-05 21400 0.000117 20800 6.78E-05
21700 0.000242 19700 9.94E-06 19500 2.30E-05 19100
5.35E-05 21400 0.000116 20800 6.79E-05
21700 0.000242 19700 9.93E-06 19500 2.30E-05 19100
5.32E-05 21400 0.000115 20800 6.79E-05
21700 0.000242 19700 9.93E-06 19500 2.31E-05 19100
5.29E-05 21400 0.000114 20800 6.80E-05
21700 0.000242 19700 9.93E-06 19500 2.31E-05 19100
5.26E-05 21400 0.000113 20800 6.80E-05
21700 0.000243 19700 9.94E-06 19500 2.31E-05 19100
5.23E-05 21400 0.000113 20800 6.80E-05
21700 0.000243 19700 9.94E-06 19500 2.31E-05 19100
5.21E-05 21400 0.000112 20800 6.81E-05
21700 0.000243 19700 9.94E-06 19500 2.31E-05 19100
5.18E-05 21400 0.000111 20800 6.81E-05
21700 0.000243 19700 9.93E-06 19500 2.31E-05 19100
5.16E-05 21400 0.000111 20800 6.81E-05
21700 0.000243 19700 9.93E-06 19500 2.32E-05 19100
5.14E-05 21400 0.00011 20800 6.82E-05
21700 0.000244 19700 9.94E-06 19500 2.32E-05 19100
5.12E-05 21400 0.00011 20800 6.82E-05
21700 0.000244 19700 9.95E-06 19500 2.32E-05 19100
5.10E-05 21400 0.000109 20800 6.82E-05
21700 0.000244 19700 9.95E-06 19500 2.32E-05 19100
5.09E-05 21400 0.000108 20800 6.83E-05
21700 0.000244 19700 9.95E-06 19500 2.32E-05 19100
5.07E-05 21400 0.000108 20800 6.83E-05
21700 0.000244 19700 9.95E-06 19500 2.32E-05 19100
5.05E-05 21400 0.000107 20800 6.83E-05
21700 0.000245 19700 9.96E-06 19500 2.33E-05 19100
5.04E-05 21400 0.000107 20800 6.84E-05
21700 0.000245 19700 9.97E-06 19500 2.33E-05 19100
5.03E-05 21400 0.000106 20800 6.84E-05
21700 0.000245 19700 9.97E-06 19500 2.33E-05 19100
5.01E-05 21400 0.000106 20800 6.84E-05
21700 0.000245 19700 9.97E-06 19500 2.33E-05 19100
5.00E-05 21400 0.000105 20800 6.85E-05
21700 0.000246 19700 9.97E-06 19500 2.33E-05 19100
5.00E-05 21400 0.000105 20800 6.85E-05
21700 0.000246 19700 9.98E-06 19500 2.33E-05 19100
4.99E-05 21400 0.000105 20800 6.85E-05
21700 0.000246 19700 9.99E-06 19500 2.34E-05 19100
4.98E-05 21400 0.000104 20800 6.86E-05
21800 0.000246 19700 9.99E-06 19500 2.34E-05 19100
4.97E-05 21400 0.000104 20800 6.86E-05

21800 0.000246 19700 1.00E-05 19500 2.34E-05 19100
4.96E-05 21400 0.000103 20800 6.86E-05
21800 0.000247 19700 1.00E-05 19500 2.34E-05 19100
4.96E-05 21400 0.000103 20800 6.87E-05
21800 0.000247 19700 1.00E-05 19500 2.34E-05 19100
4.95E-05 21400 0.000102 20800 6.87E-05
21800 0.000247 19700 1.00E-05 19500 2.34E-05 19100
4.95E-05 21400 0.000102 20800 6.87E-05
21800 0.000247 19700 1.00E-05 19500 2.34E-05 19100
4.94E-05 21400 0.000102 20800 6.88E-05
21800 0.000248 19700 1.00E-05 19500 2.35E-05 19100
4.94E-05 21400 0.000101 20800 6.88E-05
21800 0.000248 19700 1.00E-05 19500 2.35E-05 19100
4.93E-05 21400 0.000101 20800 6.88E-05
21800 0.000248 19700 1.00E-05 19500 2.35E-05 19100
4.93E-05 21400 0.000101 20800 6.89E-05
21800 0.000248 19700 1.00E-05 19500 2.35E-05 19100
4.93E-05 21400 0.0001 20900 6.89E-05
21800 0.000248 19700 1.00E-05 19500 2.36E-05 19100
4.92E-05 21400 0.0001 20900 6.89E-05
21800 0.000249 19700 1.00E-05 19500 2.36E-05 19100
4.92E-05 21400 9.97E-05 20900 6.90E-05
21800 0.000249 19700 1.00E-05 19500 2.36E-05 19100
4.92E-05 21400 9.94E-05 20900 6.90E-05
21800 0.000249 19700 1.00E-05 19500 2.36E-05 19100
4.92E-05 21400 9.91E-05 20900 6.90E-05
21800 0.000249 19700 1.00E-05 19500 2.37E-05 19100
4.93E-05 21400 9.88E-05 20900 6.91E-05
21800 0.00025 19700 1.00E-05 19500 2.37E-05 19100 4.93E-05
21400 9.85E-05 20900 6.91E-05
21800 0.00025 19700 1.00E-05 19500 2.37E-05 19100 4.93E-05
21400 9.82E-05 20900 6.91E-05
21800 0.00025 19700 1.00E-05 19500 2.37E-05 19100 4.93E-05
21400 9.80E-05 20900 6.92E-05
21800 0.00025 19800 1.00E-05 19500 2.37E-05 19100 4.93E-05
21400 9.77E-05 20900 6.92E-05
21800 0.000251 19800 1.00E-05 19500 2.37E-05 19100
4.93E-05 21400 9.74E-05 20900 6.92E-05
21800 0.000251 19800 1.00E-05 19600 2.38E-05 19100
4.93E-05 21400 9.72E-05 20900 6.93E-05
21800 0.000251 19800 1.00E-05 19600 2.38E-05 19100
4.93E-05 21400 9.69E-05 20900 6.93E-05
21800 0.000251 19800 1.00E-05 19600 2.38E-05 19100
4.93E-05 21400 9.67E-05 20900 6.94E-05
21800 0.000251 19800 1.00E-05 19600 2.38E-05 19100
4.93E-05 21400 9.64E-05 20900 6.94E-05
21800 0.000252 19800 1.00E-05 19600 2.39E-05 19200
4.93E-05 21400 9.62E-05 20900 6.94E-05
21800 0.000252 19800 1.00E-05 19600 2.39E-05 19200
4.94E-05 21400 9.59E-05 20900 6.95E-05
21800 0.000252 19800 1.00E-05 19600 2.39E-05 19200
4.94E-05 21400 9.57E-05 20900 6.95E-05
21800 0.000252 19800 1.00E-05 19600 2.39E-05 19200
4.94E-05 21400 9.54E-05 20900 6.95E-05
21800 0.000253 19800 1.00E-05 19600 2.39E-05 19200
4.95E-05 21400 9.52E-05 20900 6.96E-05
21800 0.000253 19800 1.00E-05 19600 2.40E-05 19200
4.95E-05 21400 9.50E-05 20900 6.96E-05
21800 0.000253 19800 1.00E-05 19600 2.40E-05 19200
4.96E-05 21400 9.48E-05 20900 6.96E-05
21800 0.000253 19800 1.01E-05 19600 2.40E-05 19200
4.96E-05 21400 9.46E-05 20900 6.97E-05
21800 0.000254 19800 1.01E-05 19600 2.40E-05 19200
4.96E-05 21400 9.43E-05 20900 6.97E-05
21800 0.000254 19800 1.01E-05 19600 2.40E-05 19200
4.97E-05 21400 9.41E-05 20900 6.97E-05
21800 0.000254 19800 1.01E-05 19600 2.40E-05 19200
4.98E-05 21400 9.39E-05 20900 6.98E-05

21800 0.000254 19800 1.01E-05 19600 2.40E-05 19200
4.98E-05 21500 9.37E-05 20900 6.98E-05
21800 0.000254 19800 1.01E-05 19600 2.41E-05 19200
4.99E-05 21500 9.35E-05 20900 6.98E-05
21800 0.000255 19800 1.01E-05 19600 2.41E-05 19200
4.99E-05 21500 9.33E-05 20900 6.99E-05
21800 0.000255 19800 1.01E-05 19600 2.41E-05 19200
5.00E-05 21500 9.31E-05 20900 6.99E-05
21800 0.000255 19800 1.01E-05 19600 2.41E-05 19200
5.00E-05 21500 9.29E-05 20900 7.00E-05
21800 0.000255 19800 1.01E-05 19600 2.41E-05 19200
5.01E-05 21500 9.28E-05 20900 7.00E-05
21800 0.000256 19800 1.01E-05 19600 2.42E-05 19200
5.01E-05 21500 9.26E-05 20900 7.00E-05
21800 0.000256 19800 1.01E-05 19600 2.42E-05 19200
5.02E-05 21500 9.24E-05 20900 7.01E-05
21800 0.000256 19800 1.01E-05 19600 2.42E-05 19200
5.02E-05 21500 9.22E-05 20900 7.01E-05
21800 0.000256 19800 1.01E-05 19600 2.42E-05 19200
5.03E-05 21500 9.21E-05 20900 7.01E-05
21800 0.000257 19800 1.01E-05 19600 2.42E-05 19200
5.04E-05 21500 9.19E-05 20900 7.02E-05
21800 0.000257 19800 1.01E-05 19600 2.42E-05 19200
5.04E-05 21500 9.18E-05 20900 7.02E-05
21800 0.000257 19800 1.01E-05 19600 2.43E-05 19200
5.05E-05 21500 9.16E-05 20900 7.02E-05
21800 0.000257 19800 1.01E-05 19600 2.43E-05 19200
5.06E-05 21500 9.14E-05 20900 7.03E-05
21800 0.000258 19800 1.01E-05 19600 2.43E-05 19200
5.06E-05 21500 9.13E-05 20900 7.03E-05
21800 0.000258 19800 1.01E-05 19600 2.43E-05 19200
5.07E-05 21500 9.11E-05 20900 7.03E-05
21800 0.000258 19800 1.01E-05 19600 2.44E-05 19200
5.07E-05 21500 9.10E-05 20900 7.04E-05
21800 0.000258 19800 1.01E-05 19600 2.44E-05 19200
5.08E-05 21500 9.08E-05 20900 7.04E-05
21800 0.000259 19800 1.01E-05 19600 2.44E-05 19200
5.09E-05 21500 9.07E-05 20900 7.04E-05
21800 0.000259 19800 1.01E-05 19600 2.44E-05 19200
5.09E-05 21500 9.05E-05 20900 7.05E-05
21800 0.000259 19800 1.01E-05 19600 2.44E-05 19200
5.10E-05 21500 9.04E-05 20900 7.05E-05
21800 0.000259 19800 1.01E-05 19600 2.45E-05 19200
5.11E-05 21500 9.02E-05 20900 7.05E-05
21800 0.00026 19800 1.01E-05 19600 2.45E-05 19200 5.12E-05
21500 9.01E-05 20900 7.06E-05
21800 0.00026 19800 1.01E-05 19600 2.45E-05 19200 5.12E-05
21500 9.00E-05 20900 7.06E-05
21800 0.00026 19800 1.01E-05 19600 2.45E-05 19200 5.13E-05
21500 8.98E-05 20900 7.06E-05
21800 0.00026 19800 1.01E-05 19600 2.45E-05 19200 5.14E-05
21500 8.97E-05 20900 7.07E-05
21800 0.000261 19800 1.01E-05 19600 2.46E-05 19200
5.15E-05 21500 8.96E-05 20900 7.07E-05
21800 0.000261 19800 1.01E-05 19600 2.46E-05 19200
5.16E-05 21500 8.95E-05 20900 7.08E-05
21800 0.000261 19800 1.01E-05 19600 2.46E-05 19200
5.17E-05 21500 8.94E-05 20900 7.08E-05
21900 0.000261 19800 1.01E-05 19600 2.46E-05 19200
5.18E-05 21500 8.92E-05 20900 7.08E-05
21900 0.000262 19800 1.02E-05 19600 2.47E-05 19200
5.18E-05 21500 8.91E-05 20900 7.08E-05
21900 0.000262 19800 1.02E-05 19600 2.47E-05 19200
5.20E-05 21500 8.90E-05 20900 7.09E-05
21900 0.000262 19800 1.02E-05 19600 2.47E-05 19200
5.20E-05 21500 8.89E-05 20900 7.09E-05
21900 0.000262 19800 1.02E-05 19600 2.47E-05 19200
5.21E-05 21500 8.88E-05 20900 7.09E-05

21900 0.000263 19800 1.02E-05 19600 2.48E-05 19200
5.22E-05 21500 8.87E-05 20900 7.10E-05
21900 0.000263 19800 1.02E-05 19600 2.48E-05 19200
5.23E-05 21500 8.86E-05 20900 7.10E-05
21900 0.000263 19800 1.02E-05 19600 2.48E-05 19200
5.24E-05 21500 8.85E-05 20900 7.10E-05
21900 0.000263 19800 1.02E-05 19600 2.48E-05 19200
5.25E-05 21500 8.84E-05 20900 7.11E-05
21900 0.000264 19800 1.02E-05 19600 2.48E-05 19200
5.26E-05 21500 8.83E-05 21000 7.11E-05
21900 0.000264 19800 1.02E-05 19600 2.49E-05 19200
5.27E-05 21500 8.82E-05 21000 7.11E-05
21900 0.000264 19800 1.02E-05 19600 2.49E-05 19200
5.28E-05 21500 8.81E-05 21000 7.12E-05
21900 0.000264 19800 1.02E-05 19600 2.49E-05 19200
5.29E-05 21500 8.80E-05 21000 7.12E-05
21900 0.000265 19800 1.02E-05 19600 2.49E-05 19200
5.30E-05 21500 8.79E-05 21000 7.13E-05
21900 0.000265 19800 1.02E-05 19600 2.50E-05 19200
5.31E-05 21500 8.78E-05 21000 7.13E-05
21900 0.000265 19800 1.02E-05 19600 2.50E-05 19200
5.32E-05 21500 8.77E-05 21000 7.13E-05
21900 0.000265 19800 1.02E-05 19600 2.50E-05 19200
5.33E-05 21500 8.76E-05 21000 7.14E-05
21900 0.000266 19800 1.02E-05 19600 2.51E-05 19200
5.34E-05 21500 8.75E-05 21000 7.14E-05
21900 0.000266 19900 1.02E-05 19600 2.51E-05 19200
5.35E-05 21500 8.74E-05 21000 7.14E-05
21900 0.000266 19900 1.02E-05 19600 2.51E-05 19200
5.36E-05 21500 8.74E-05 21000 7.15E-05
21900 0.000266 19900 1.02E-05 19700 2.51E-05 19200
5.37E-05 21500 8.73E-05 21000 7.15E-05
21900 0.000267 19900 1.02E-05 19700 2.52E-05 19200
5.38E-05 21500 8.72E-05 21000 7.15E-05
21900 0.000267 19900 1.02E-05 19700 2.52E-05 19200
5.40E-05 21500 8.71E-05 21000 7.16E-05
21900 0.000267 19900 1.02E-05 19700 2.52E-05 19200
5.41E-05 21500 8.70E-05 21000 7.16E-05
21900 0.000267 19900 1.03E-05 19700 2.52E-05 19300
5.42E-05 21500 8.70E-05 21000 7.16E-05
21900 0.000268 19900 1.03E-05 19700 2.53E-05 19300
5.43E-05 21500 8.69E-05 21000 7.17E-05
21900 0.000268 19900 1.03E-05 19700 2.53E-05 19300
5.44E-05 21500 8.68E-05 21000 7.17E-05
21900 0.000268 19900 1.03E-05 19700 2.53E-05 19300
5.45E-05 21500 8.67E-05 21000 7.18E-05
21900 0.000268 19900 1.03E-05 19700 2.54E-05 19300
5.46E-05 21500 8.66E-05 21000 7.18E-05
21900 0.000269 19900 1.03E-05 19700 2.54E-05 19300
5.47E-05 21500 8.66E-05 21000 7.18E-05
21900 0.000269 19900 1.03E-05 19700 2.54E-05 19300
5.48E-05 21500 8.65E-05 21000 7.19E-05
21900 0.000269 19900 1.03E-05 19700 2.54E-05 19300
5.49E-05 21500 8.64E-05 21000 7.19E-05
21900 0.00027 19900 1.03E-05 19700 2.55E-05 19300 5.51E-05
21500 8.64E-05 21000 7.20E-05
21900 0.00027 19900 1.03E-05 19700 2.55E-05 19300 5.52E-05
21500 8.63E-05 21000 7.20E-05
21900 0.00027 19900 1.03E-05 19700 2.55E-05 19300 5.53E-05
21500 8.62E-05 21000 7.20E-05
21900 0.00027 19900 1.03E-05 19700 2.56E-05 19300 5.55E-05
21600 8.62E-05 21000 7.21E-05
21900 0.000271 19900 1.03E-05 19700 2.56E-05 19300
5.56E-05 21600 8.61E-05 21000 7.21E-05
21900 0.000271 19900 1.03E-05 19700 2.57E-05 19300
5.58E-05 21600 8.60E-05 21000 7.21E-05
21900 0.000271 19900 1.03E-05 19700 2.57E-05 19300
5.59E-05 21600 8.60E-05 21000 7.22E-05

21900 0.000271 19900 1.03E-05 19700 2.57E-05 19300
5.60E-05 21600 8.59E-05 21000 7.22E-05
21900 0.000272 19900 1.03E-05 19700 2.58E-05 19300
5.62E-05 21600 8.59E-05 21000 7.22E-05
21900 0.000272 19900 1.03E-05 19700 2.58E-05 19300
5.63E-05 21600 8.58E-05 21000 7.23E-05
21900 0.000272 19900 1.03E-05 19700 2.58E-05 19300
5.64E-05 21600 8.58E-05 21000 7.23E-05
21900 0.000272 19900 1.03E-05 19700 2.59E-05 19300
5.66E-05 21600 8.57E-05 21000 7.24E-05
21900 0.000273 19900 1.03E-05 19700 2.59E-05 19300
5.67E-05 21600 8.57E-05 21000 7.24E-05
21900 0.000273 19900 1.03E-05 19700 2.59E-05 19300
5.68E-05 21600 8.56E-05 21000 7.24E-05
21900 0.000273 19900 1.03E-05 19700 2.60E-05 19300
5.70E-05 21600 8.56E-05 21000 7.25E-05
21900 0.000273 19900 1.03E-05 19700 2.60E-05 19300
5.72E-05 21600 8.55E-05 21000 7.25E-05
21900 0.000273 19900 1.03E-05 19700 2.60E-05 19300
5.73E-05 21600 8.55E-05 21000 7.25E-05
21900 0.000274 19900 1.03E-05 19700 2.61E-05 19300
5.75E-05 21600 8.54E-05 21000 7.26E-05
21900 0.000274 19900 1.03E-05 19700 2.61E-05 19300
5.76E-05 21600 8.54E-05 21000 7.26E-05
21900 0.000274 19900 1.03E-05 19700 2.61E-05 19300
5.78E-05 21600 8.53E-05 21000 7.26E-05
21900 0.000274 19900 1.03E-05 19700 2.62E-05 19300
5.79E-05 21600 8.53E-05 21000 7.27E-05
21900 0.000275 19900 1.03E-05 19700 2.62E-05 19300
5.81E-05 21600 8.52E-05 21000 7.27E-05
21900 0.000275 19900 1.03E-05 19700 2.63E-05 19300
5.82E-05 21600 8.52E-05 21000 7.28E-05
21900 0.000275 19900 1.04E-05 19700 2.63E-05 19300
5.84E-05 21600 8.52E-05 21000 7.28E-05
21900 0.000276 19900 1.03E-05 19700 2.64E-05 19300
5.85E-05 21600 8.51E-05 21000 7.28E-05
21900 0.000276 19900 1.04E-05 19700 2.64E-05 19300
5.86E-05 21600 8.51E-05 21000 7.29E-05
21900 0.000276 19900 1.04E-05 19700 2.64E-05 19300
5.88E-05 21600 8.50E-05 21000 7.29E-05
21900 0.000276 19900 1.04E-05 19700 2.65E-05 19300
5.89E-05 21600 8.50E-05 21000 7.29E-05
21900 0.000276 19900 1.04E-05 19700 2.66E-05 19300
5.90E-05 21600 8.49E-05 21000 7.30E-05
21900 0.000277 19900 1.04E-05 19700 2.66E-05 19300
5.92E-05 21600 8.49E-05 21000 7.30E-05
21900 0.000277 19900 1.04E-05 19700 2.66E-05 19300
5.93E-05 21600 8.49E-05 21000 7.31E-05
21900 0.000277 19900 1.04E-05 19700 2.67E-05 19300
5.94E-05 21600 8.48E-05 21000 7.31E-05
22000 0.000278 19900 1.04E-05 19700 2.68E-05 19300
5.96E-05 21600 8.48E-05 21000 7.32E-05
22000 0.000278 19900 1.04E-05 19700 2.68E-05 19300
5.97E-05 21600 8.47E-05 21000 7.32E-05
22000 0.000278 19900 1.04E-05 19700 2.68E-05 19300
5.98E-05 21600 8.47E-05 21000 7.32E-05
22000 0.000278 19900 1.04E-05 19700 2.69E-05 19300
6.00E-05 21600 8.46E-05 21000 7.33E-05
22000 0.000279 19900 1.04E-05 19700 2.70E-05 19300
6.01E-05 21600 8.46E-05 21000 7.33E-05
22000 0.000279 19900 1.04E-05 19700 2.70E-05 19300
6.02E-05 21600 8.46E-05 21000 7.34E-05
22000 0.000279 19900 1.04E-05 19700 2.71E-05 19300
6.04E-05 21600 8.45E-05 21000 7.34E-05
22000 0.000279 19900 1.04E-05 19700 2.71E-05 19300
6.06E-05 21600 8.45E-05 21000 7.34E-05
22000 0.00028 19900 1.04E-05 19700 2.72E-05 19300 6.07E-05
21600 8.45E-05 21000 7.35E-05

22000 0.00028 19900 1.04E-05 19700 2.72E-05 19300 6.08E-05
21600 8.44E-05 21100 7.35E-05
22000 0.00028 19900 1.04E-05 19700 2.74E-05 19300 6.10E-05
21600 8.44E-05 21100 7.35E-05
22000 0.00028 19900 1.04E-05 19700 2.74E-05 19300 6.12E-05
21600 8.44E-05 21100 7.36E-05
22000 0.000281 19900 1.05E-05 19700 2.75E-05 19300
6.13E-05 21600 8.43E-05 21100 7.36E-05
22000 0.000281 19900 1.04E-05 19700 2.75E-05 19300
6.14E-05 21600 8.43E-05 21100 7.36E-05
22000 0.000281 19900 1.05E-05 19700 2.76E-05 19300
6.16E-05 21600 8.42E-05 21100 7.37E-05
22000 0.000281 19900 1.05E-05 19700 2.77E-05 19300
6.18E-05 21600 8.42E-05 21100 7.37E-05
22000 0.000282 19900 1.05E-05 19700 2.77E-05 19300
6.20E-05 21600 8.42E-05 21100 7.38E-05
22000 0.000282 19900 1.05E-05 19700 2.78E-05 19300
6.21E-05 21600 8.42E-05 21100 7.38E-05
22000 0.000282 20000 1.05E-05 19700 2.79E-05 19300
6.23E-05 21600 8.41E-05 21100 7.38E-05
22000 0.000282 20000 1.05E-05 19700 2.80E-05 19300
6.25E-05 21600 8.41E-05 21100 7.39E-05
22000 0.000283 20000 1.05E-05 19800 2.80E-05 19300
6.27E-05 21600 8.41E-05 21100 7.39E-05
22000 0.000283 20000 1.05E-05 19800 2.81E-05 19300
6.29E-05 21600 8.40E-05 21100 7.39E-05
22000 0.000283 20000 1.05E-05 19800 2.82E-05 19300
6.30E-05 21600 8.40E-05 21100 7.40E-05
22000 0.000283 20000 1.05E-05 19800 2.83E-05 19300
6.32E-05 21600 8.40E-05 21100 7.40E-05
22000 0.000284 20000 1.05E-05 19800 2.84E-05 19400
6.34E-05 21600 8.39E-05 21100 7.41E-05
22000 0.000284 20000 1.05E-05 19800 2.85E-05 19400
6.36E-05 21600 8.39E-05 21100 7.41E-05
22000 0.000284 20000 1.05E-05 19800 2.86E-05 19400
6.37E-05 21600 8.39E-05 21100 7.41E-05
22000 0.000285 20000 1.05E-05 19800 2.86E-05 19400
6.39E-05 21600 8.39E-05 21100 7.42E-05
22000 0.000285 20000 1.05E-05 19800 2.87E-05 19400
6.41E-05 21600 8.38E-05 21100 7.42E-05
22000 0.000285 20000 1.05E-05 19800 2.88E-05 19400
6.43E-05 21600 8.38E-05 21100 7.42E-05
22000 0.000285 20000 1.05E-05 19800 2.89E-05 19400
6.44E-05 21600 8.38E-05 21100 7.43E-05
22000 0.000286 20000 1.05E-05 19800 2.91E-05 19400
6.46E-05 21600 8.37E-05 21100 7.43E-05
22000 0.000286 20000 1.05E-05 19800 2.92E-05 19400
6.48E-05 21600 8.37E-05 21100 7.43E-05
22000 0.000286 20000 1.05E-05 19800 2.93E-05 19400
6.50E-05 21600 8.37E-05 21100 7.44E-05
22000 0.000286 20000 1.05E-05 19800 2.94E-05 19400
6.51E-05 21600 8.37E-05 21100 7.44E-05
22000 0.000287 20000 1.05E-05 19800 2.95E-05 19400
6.53E-05 21700 8.37E-05 21100 7.44E-05
22000 0.000287 20000 1.05E-05 19800 2.96E-05 19400
6.55E-05 21700 8.36E-05 21100 7.45E-05
22000 0.000287 20000 1.05E-05 19800 2.97E-05 19400
6.57E-05 21700 8.36E-05 21100 7.45E-05
22000 0.000288 20000 1.06E-05 19800 2.98E-05 19400
6.58E-05 21700 8.36E-05 21100 7.46E-05
22000 0.000288 20000 1.06E-05 19800 3.00E-05 19400
6.60E-05 21700 8.36E-05 21100 7.46E-05
22000 0.000288 20000 1.06E-05 19800 3.01E-05 19400
6.62E-05 21700 8.36E-05 21100 7.46E-05
22000 0.000288 20000 1.06E-05 19800 3.02E-05 19400
6.64E-05 21700 8.35E-05 21100 7.47E-05
22000 0.000289 20000 1.06E-05 19800 3.03E-05 19400
6.66E-05 21700 8.35E-05 21100 7.47E-05

22000 0.000289 20000 1.06E-05 19800 3.05E-05 19400
6.68E-05 21700 8.35E-05 21100 7.48E-05
22000 0.000289 20000 1.06E-05 19800 3.06E-05 19400
6.70E-05 21700 8.35E-05 21100 7.48E-05
22000 0.000289 20000 1.06E-05 19800 3.08E-05 19400
6.72E-05 21700 8.35E-05 21100 7.48E-05
22000 0.00029 20000 1.06E-05 19800 3.09E-05 19400 6.73E-05
21700 8.35E-05 21100 7.49E-05
22000 0.00029 20000 1.06E-05 19800 3.11E-05 19400 6.75E-05
21700 8.35E-05 21100 7.49E-05
22000 0.00029 20000 1.06E-05 19800 3.12E-05 19400 6.77E-05
21700 8.35E-05 21100 7.49E-05
22000 0.000291 20000 1.06E-05 19800 3.14E-05 19400
6.79E-05 21700 8.35E-05 21100 7.49E-05
22000 0.000291 20000 1.06E-05 19800 3.16E-05 19400
6.81E-05 21700 8.34E-05 21100 7.50E-05
22000 0.000291 20000 1.06E-05 19800 3.18E-05 19400
6.83E-05 21700 8.34E-05 21100 7.50E-05
22000 0.000291 20000 1.06E-05 19800 3.19E-05 19400
6.85E-05 21700 8.34E-05 21100 7.51E-05
22000 0.000292 20000 1.06E-05 19800 3.21E-05 19400
6.87E-05 21700 8.34E-05 21100 7.51E-05
22000 0.000292 20000 1.06E-05 19800 3.23E-05 19400
6.89E-05 21700 8.34E-05 21100 7.51E-05
22000 0.000292 20000 1.06E-05 19800 3.25E-05 19400
6.91E-05 21700 8.34E-05 21100 7.52E-05
22000 0.000293 20000 1.06E-05 19800 3.27E-05 19400
6.93E-05 21700 8.34E-05 21100 7.52E-05
22000 0.000293 20000 1.06E-05 19800 3.29E-05 19400
6.95E-05 21700 8.34E-05 21100 7.52E-05
22000 0.000293 20000 1.06E-05 19800 3.31E-05 19400
6.98E-05 21700 8.34E-05 21100 7.53E-05
22000 0.000293 20000 1.06E-05 19800 3.34E-05 19400
7.00E-05 21700 8.34E-05 21100 7.53E-05
22000 0.000294 20000 1.06E-05 19800 3.36E-05 19400
7.02E-05 21700 8.34E-05 21100 7.54E-05
22000 0.000294 20000 1.06E-05 19800 3.39E-05 19400
7.04E-05 21700 8.34E-05 21100 7.54E-05
22000 0.000294 20000 1.06E-05 19800 3.41E-05 19400
7.06E-05 21700 8.34E-05 21100 7.55E-05
22000 0.000295 20000 1.06E-05 19800 3.44E-05 19400
7.09E-05 21700 8.34E-05 21100 7.55E-05
22100 0.000295 20000 1.07E-05 19800 3.46E-05 19400
7.11E-05 21700 8.34E-05 21100 7.55E-05
22100 0.000295 20000 1.07E-05 19800 3.49E-05 19400
7.13E-05 21700 8.34E-05 21100 7.56E-05
22100 0.000296 20000 1.06E-05 19800 3.52E-05 19400
7.15E-05 21700 8.34E-05 21100 7.56E-05
22100 0.000296 20000 1.07E-05 19800 3.56E-05 19400
7.17E-05 21700 8.34E-05 21100 7.56E-05
22100 0.000296 20000 1.07E-05 19800 3.59E-05 19400
7.20E-05 21700 8.34E-05 21100 7.57E-05
22100 0.000296 20000 1.07E-05 19800 3.62E-05 19400
7.22E-05 21700 8.34E-05 21100 7.57E-05
22100 0.000297 20000 1.07E-05 19800 3.66E-05 19400
7.24E-05 21700 8.34E-05 21100 7.57E-05
22100 0.000297 20000 1.07E-05 19800 3.69E-05 19400
7.26E-05 21700 8.34E-05 21100 7.58E-05
22100 0.000297 20000 1.07E-05 19800 3.73E-05 19400
7.28E-05 21700 8.34E-05 21100 7.58E-05
22100 0.000298 20000 1.07E-05 19800 3.77E-05 19400
7.30E-05 21700 8.34E-05 21200 7.59E-05
22100 0.000298 20000 1.07E-05 19800 3.81E-05 19400
7.32E-05 21700 8.34E-05 21200 7.59E-05
22100 0.000298 20000 1.07E-05 19800 3.86E-05 19400
7.35E-05 21700 8.34E-05 21200 7.59E-05
22100 0.000298 20000 1.07E-05 19800 3.90E-05 19400
7.37E-05 21700 8.34E-05 21200 7.60E-05

22100 0.000299 20000 1.07E-05 19800 3.95E-05 19400
7.39E-05 21700 8.35E-05 21200 7.60E-05
22100 0.000299 20000 1.07E-05 19800 4.00E-05 19400
7.41E-05 21700 8.35E-05 21200 7.61E-05
22100 0.000299 20000 1.07E-05 19800 4.05E-05 19400
7.44E-05 21700 8.35E-05 21200 7.61E-05
22100 0.0003 20000 1.07E-05 19800 4.10E-05 19400 7.46E-05
21700 8.35E-05 21200 7.62E-05
22100 0.0003 20000 1.07E-05 19800 4.16E-05 19400 7.48E-05
21700 8.35E-05 21200 7.62E-05
22100 0.0003 20100 1.07E-05 19800 4.22E-05 19400 7.50E-05
21700 8.35E-05 21200 7.63E-05
22100 0.000301 20100 1.07E-05 19800 4.28E-05 19400
7.53E-05 21700 8.35E-05 21200 7.63E-05
22100 0.000301 20100 1.07E-05 19900 4.35E-05 19400
7.55E-05 21700 8.35E-05 21200 7.63E-05
22100 0.000301 20100 1.07E-05 19900 4.42E-05 19400
7.58E-05 21700 8.35E-05 21200 7.64E-05
22100 0.000301 20100 1.07E-05 19900 4.49E-05 19400
7.60E-05 21700 8.35E-05 21200 7.64E-05
22100 0.000302 20100 1.07E-05 19900 4.57E-05 19400
7.63E-05 21700 8.35E-05 21200 7.64E-05
22100 0.000302 20100 1.07E-05 19900 4.65E-05 19500
7.65E-05 21700 8.35E-05 21200 7.65E-05
22100 0.000302 20100 1.07E-05 19900 4.74E-05 19500
7.67E-05 21700 8.36E-05 21200 7.65E-05
22100 0.000303 20100 1.07E-05 19900 4.82E-05 19500
7.70E-05 21700 8.36E-05 21200 7.66E-05
22100 0.000303 20100 1.08E-05 19900 4.92E-05 19500
7.73E-05 21700 8.36E-05 21200 7.66E-05
22100 0.000303 20100 1.08E-05 19900 5.02E-05 19500
7.75E-05 21700 8.36E-05 21200 7.66E-05
22100 0.000304 20100 1.08E-05 19900 5.12E-05 19500
7.77E-05 21700 8.36E-05 21200 7.67E-05
22100 0.000304 20100 1.08E-05 19900 5.24E-05 19500
7.80E-05 21700 8.36E-05 21200 7.67E-05
22100 0.000304 20100 1.08E-05 19900 5.35E-05 19500
7.82E-05 21700 8.36E-05 21200 7.68E-05
22100 0.000305 20100 1.08E-05 19900 5.47E-05 19500
7.85E-05 21700 8.37E-05 21200 7.68E-05
22100 0.000305 20100 1.08E-05 19900 5.60E-05 19500
7.87E-05 21700 8.37E-05 21200 7.69E-05
22100 0.000305 20100 1.08E-05 19900 5.74E-05 19500
7.90E-05 21700 8.37E-05 21200 7.69E-05
22100 0.000306 20100 1.08E-05 19900 5.88E-05 19500
7.93E-05 21800 8.37E-05 21200 7.69E-05
22100 0.000306 20100 1.08E-05 19900 6.03E-05 19500
7.95E-05 21800 8.37E-05 21200 7.70E-05
22100 0.000306 20100 1.08E-05 19900 6.18E-05 19500
7.98E-05 21800 8.37E-05 21200 7.70E-05
22100 0.000307 20100 1.08E-05 19900 6.35E-05 19500
8.00E-05 21800 8.37E-05 21200 7.71E-05
22100 0.000307 20100 1.08E-05 19900 6.52E-05 19500
8.03E-05 21800 8.38E-05 21200 7.71E-05
22100 0.000307 20100 1.08E-05 19900 6.70E-05 19500
8.05E-05 21800 8.38E-05 21200 7.71E-05
22100 0.000307 20100 1.08E-05 19900 6.88E-05 19500
8.08E-05 21800 8.38E-05 21200 7.72E-05
22100 0.000308 20100 1.08E-05 19900 7.08E-05 19500
8.10E-05 21800 8.38E-05 21200 7.72E-05
22100 0.000308 20100 1.08E-05 19900 7.28E-05 19500
8.13E-05 21800 8.38E-05 21200 7.72E-05
22100 0.000308 20100 1.08E-05 19900 7.48E-05 19500
8.15E-05 21800 8.39E-05 21200 7.73E-05
22100 0.000309 20100 1.08E-05 19900 7.70E-05 19500
8.18E-05 21800 8.39E-05 21200 7.73E-05
22100 0.000309 20100 1.08E-05 19900 7.91E-05 19500
8.21E-05 21800 8.39E-05 21200 7.73E-05

```
22100   0.000309        20100   1.08E-05        19900   8.13E-05        19500
8.24E-05        21800   8.39E-05        21200   7.74E-05
22100   0.00031 20100   1.09E-05        19900   8.35E-05        19500   8.27E-05
21800   8.39E-05        21200   7.74E-05
22100   0.00031 20100   1.08E-05        19900   8.58E-05        19500   8.30E-05
21800   8.40E-05        21200   7.75E-05
22100   0.00031 20100   1.09E-05        19900   8.79E-05        19500   8.33E-05
21800   8.40E-05        21200   7.75E-05
22100   0.000311        20100   1.09E-05        19900   9.01E-05        19500
8.36E-05        21800   8.40E-05        21200   7.75E-05
22100   0.000311        20100   1.09E-05        19900   9.22E-05        19500
8.39E-05        21800   8.40E-05        21200   7.76E-05
22100   0.000311        20100   1.09E-05        19900   9.42E-05        19500
8.41E-05        21800   8.40E-05        21200   7.76E-05
22100   0.000312        20100   1.09E-05        19900   9.61E-05        19500
8.44E-05        21800   8.41E-05        21200   7.77E-05
22100   0.000312        20100   1.09E-05        19900   9.78E-05        19500
8.47E-05        21800   8.41E-05        21200   7.77E-05
22100   0.000312        20100   1.09E-05        19900   9.93E-05        19500
8.50E-05        21800   8.41E-05        21200   7.77E-05
22100   0.000313        20100   1.09E-05        19900   1.01E-04        19500
8.53E-05        21800   8.41E-05        21200   7.78E-05
22100   0.000313        20100   1.09E-05        19900   1.02E-04        19500
8.56E-05        21800   8.41E-05        21200   7.78E-05
22100   0.000314        20100   1.09E-05        19900   1.02E-04        19500
8.59E-05        21800   8.42E-05        21200   7.79E-05
22100   0.000314        20100   1.09E-05        19900   1.03E-04        19500
8.62E-05        21800   8.42E-05        21200   7.79E-05
22100   0.000314        20100   1.09E-05        19900   1.03E-04        19500
8.65E-05        21800   8.42E-05        21200   7.79E-05
22100   0.000315        20100   1.09E-05        19900   1.03E-04        19500
8.68E-05        21800   8.42E-05        21200   7.80E-05
22100   0.000315        20100   1.09E-05        19900   1.03E-04        19500
8.71E-05        21800   8.43E-05        21200   7.80E-05
22200   0.000315        20100   1.09E-05        19900   1.02E-04        19500
8.75E-05        21800   8.43E-05        21200   7.81E-05
22200   0.000316        20100   1.09E-05        19900   1.01E-04        19500
8.78E-05        21800   8.43E-05        21200   7.81E-05
22200   0.000316        20100   1.09E-05        19900   9.93E-05        19500
8.81E-05        21800   8.43E-05        21200   7.81E-05
22200   0.000316        20100   1.10E-05        19900   9.77E-05        19500
8.85E-05        21800   8.43E-05        21200   7.82E-05
22200   0.000317        20100   1.10E-05        19900   9.58E-05        19500
8.88E-05        21800   8.44E-05        21200   7.82E-05
22200   0.000317        20100   1.10E-05        19900   9.38E-05        19500
8.92E-05        21800   8.44E-05        21200   7.83E-05
22200   0.000317        20100   1.10E-05        19900   9.17E-05        19500
8.95E-05        21800   8.44E-05        21200   7.83E-05
22200   0.000318        20100   1.10E-05        19900   8.94E-05        19500
8.99E-05        21800   8.44E-05        21200   7.84E-05
22200   0.000318        20100   1.10E-05        19900   8.70E-05        19500
9.02E-05        21800   8.44E-05        21200   7.84E-05
22200   0.000318        20100   1.10E-05        19900   8.46E-05        19500
9.06E-05        21800   8.45E-05        21300   7.85E-05
22200   0.000319        20100   1.10E-05        19900   8.22E-05        19500
9.09E-05        21800   8.45E-05        21300   7.85E-05
22200   0.000319        20100   1.10E-05        19900   7.98E-05        19500
9.12E-05        21800   8.45E-05        21300   7.86E-05
22200   0.000319        20100   1.10E-05        19900   7.75E-05        19500
9.16E-05        21800   8.45E-05        21300   7.86E-05
22200   0.00032 20100   1.10E-05        19900   7.52E-05        19500   9.19E-05
21800   8.46E-05        21300   7.87E-05
22200   0.00032 20100   1.10E-05        19900   7.30E-05        19500   9.23E-05
21800   8.46E-05        21300   7.87E-05
22200   0.000321        20100   1.10E-05        19900   7.08E-05        19500
9.26E-05        21800   8.46E-05        21300   7.88E-05
22200   0.000321        20100   1.10E-05        19900   6.88E-05        19500
9.30E-05        21800   8.46E-05        21300   7.88E-05
```

22200 0.000321 20100 1.10E-05 19900 6.69E-05 19500
9.34E-05 21800 8.47E-05 21300 7.89E-05
22200 0.000322 20200 1.10E-05 19900 6.50E-05 19500
9.37E-05 21800 8.47E-05 21300 7.89E-05
22200 0.000322 20200 1.10E-05 19900 6.32E-05 19500
9.41E-05 21800 8.47E-05 21300 7.89E-05
22200 0.000322 20200 1.10E-05 20000 6.15E-05 19500
9.45E-05 21800 8.47E-05 21300 7.90E-05
22200 0.000323 20200 1.10E-05 20000 5.99E-05 19500
9.48E-05 21800 8.48E-05 21300 7.90E-05
22200 0.000323 20200 1.10E-05 20000 5.84E-05 19500
9.52E-05 21800 8.48E-05 21300 7.91E-05
22200 0.000323 20200 1.10E-05 20000 5.70E-05 19500
9.56E-05 21800 8.48E-05 21300 7.91E-05
22200 0.000324 20200 1.10E-05 20000 5.57E-05 19600
9.60E-05 21800 8.48E-05 21300 7.92E-05
22200 0.000324 20200 1.10E-05 20000 5.44E-05 19600
9.64E-05 21800 8.49E-05 21300 7.92E-05
22200 0.000324 20200 1.10E-05 20000 5.33E-05 19600
9.68E-05 21800 8.49E-05 21300 7.92E-05
22200 0.000325 20200 1.10E-05 20000 5.22E-05 19600
9.72E-05 21800 8.49E-05 21300 7.93E-05
22200 0.000325 20200 1.10E-05 20000 5.12E-05 19600
9.76E-05 21800 8.49E-05 21300 7.93E-05
22200 0.000325 20200 1.10E-05 20000 5.02E-05 19600
9.79E-05 21800 8.50E-05 21300 7.94E-05
22200 0.000326 20200 1.10E-05 20000 4.93E-05 19600
9.83E-05 21800 8.50E-05 21300 7.94E-05
22200 0.000326 20200 1.10E-05 20000 4.84E-05 19600
9.87E-05 21800 8.50E-05 21300 7.95E-05
22200 0.000327 20200 1.10E-05 20000 4.76E-05 19600
9.91E-05 21800 8.51E-05 21300 7.95E-05
22200 0.000327 20200 1.10E-05 20000 4.68E-05 19600
9.95E-05 21800 8.51E-05 21300 7.96E-05
22200 0.000327 20200 1.10E-05 20000 4.61E-05 19600
9.99E-05 21800 8.51E-05 21300 7.96E-05
22200 0.000328 20200 1.10E-05 20000 4.55E-05 19600
0.000100271 21900 8.52E-05 21300 7.97E-05
22200 0.000328 20200 1.10E-05 20000 4.48E-05 19600
0.000100635 21900 8.52E-05 21300 7.97E-05
22200 0.000328 20200 1.11E-05 20000 4.42E-05 19600
0.000101007 21900 8.52E-05 21300 7.97E-05
22200 0.000329 20200 1.11E-05 20000 4.37E-05 19600
0.000101402 21900 8.53E-05 21300 7.98E-05
22200 0.000329 20200 1.11E-05 20000 4.31E-05 19600
0.000101786 21900 8.53E-05 21300 7.98E-05
22200 0.000329 20200 1.11E-05 20000 4.26E-05 19600
0.000102171 21900 8.53E-05 21300 7.98E-05
22200 0.00033 20200 1.11E-05 20000 4.22E-05 19600
0.000102571 21900 8.54E-05 21300 7.99E-05
22200 0.00033 20200 1.11E-05 20000 4.17E-05 19600
0.000102989 21900 8.54E-05 21300 7.99E-05
22200 0.000331 20200 1.11E-05 20000 4.13E-05 19600
0.000103401 21900 8.54E-05 21300 8.00E-05
22200 0.000331 20200 1.11E-05 20000 4.09E-05 19600
0.0001038 21900 8.55E-05 21300 8.00E-05
22200 0.000331 20200 1.11E-05 20000 4.05E-05 19600
0.00010423 21900 8.55E-05 21300 8.01E-05
22200 0.000332 20200 1.11E-05 20000 4.01E-05 19600
0.000104662 21900 8.55E-05 21300 8.01E-05
22200 0.000332 20200 1.11E-05 20000 3.98E-05 19600
0.000105081 21900 8.56E-05 21300 8.02E-05
22200 0.000332 20200 1.11E-05 20000 3.95E-05 19600
0.000105522 21900 8.56E-05 21300 8.02E-05
22200 0.000333 20200 1.11E-05 20000 3.92E-05 19600
0.000105965 21900 8.56E-05 21300 8.02E-05
22200 0.000333 20200 1.11E-05 20000 3.89E-05 19600
0.000106403 21900 8.57E-05 21300 8.03E-05

22200 0.000334 20200 1.11E-05 20000 3.86E-05 19600
0.000106844 21900 8.57E-05 21300 8.03E-05
22200 0.000334 20200 1.11E-05 20000 3.84E-05 19600
0.000107291 21900 8.58E-05 21300 8.04E-05
22200 0.000334 20200 1.11E-05 20000 3.81E-05 19600
0.00010776 21900 8.58E-05 21300 8.04E-05
22200 0.000335 20200 1.11E-05 20000 3.79E-05 19600
0.000108214 21900 8.58E-05 21300 8.04E-05
22200 0.000335 20200 1.11E-05 20000 3.77E-05 19600
0.000108685 21900 8.59E-05 21300 8.05E-05
22200 0.000336 20200 1.11E-05 20000 3.74E-05 19600
0.000109151 21900 8.59E-05 21300 8.05E-05
22200 0.000336 20200 1.11E-05 20000 3.72E-05 19600
0.000109605 21900 8.59E-05 21300 8.06E-05
22200 0.000336 20200 1.11E-05 20000 3.71E-05 19600
0.000110088 21900 8.60E-05 21300 8.06E-05
22200 0.000337 20200 1.11E-05 20000 3.69E-05 19600
0.000110579 21900 8.60E-05 21300 8.06E-05
22200 0.000337 20200 1.11E-05 20000 3.67E-05 19600
0.000111055 21900 8.61E-05 21300 8.07E-05
22200 0.000337 20200 1.12E-05 20000 3.65E-05 19600
0.000111576 21900 8.61E-05 21300 8.07E-05
22200 0.000338 20200 1.12E-05 20000 3.64E-05 19600
0.000112059 21900 8.61E-05 21300 8.08E-05
22200 0.000338 20200 1.12E-05 20000 3.62E-05 19600
0.000112573 21900 8.62E-05 21300 8.08E-05
22300 0.000338 20200 1.12E-05 20000 3.61E-05 19600
0.000113096 21900 8.62E-05 21300 8.08E-05
22300 0.000339 20200 1.12E-05 20000 3.59E-05 19600
0.000113621 21900 8.63E-05 21300 8.09E-05
22300 0.000339 20200 1.12E-05 20000 3.58E-05 19600
0.000114143 21900 8.63E-05 21300 8.10E-05
22300 0.00034 20200 1.12E-05 20000 3.57E-05 19600
0.000114662 21900 8.64E-05 21300 8.10E-05
22300 0.00034 20200 1.12E-05 20000 3.55E-05 19600
0.000115211 21900 8.64E-05 21300 8.10E-05
22300 0.00034 20200 1.12E-05 20000 3.54E-05 19600
0.000115749 21900 8.64E-05 21300 8.11E-05
22300 0.000341 20200 1.12E-05 20000 3.53E-05 19600
0.000116273 21900 8.65E-05 21300 8.11E-05
22300 0.000341 20200 1.12E-05 20000 3.52E-05 19600
0.000116802 21900 8.65E-05 21300 8.11E-05
22300 0.000341 20200 1.12E-05 20000 3.51E-05 19600
0.000117344 21900 8.66E-05 21300 8.12E-05
22300 0.000342 20200 1.12E-05 20000 3.50E-05 19600
0.000117876 21900 8.66E-05 21400 8.12E-05
22300 0.000342 20200 1.12E-05 20000 3.50E-05 19600
0.000118404 21900 8.67E-05 21400 8.13E-05
22300 0.000343 20200 1.12E-05 20000 3.49E-05 19600
0.000118916 21900 8.67E-05 21400 8.13E-05
22300 0.000343 20200 1.12E-05 20000 3.48E-05 19600
0.000119463 21900 8.68E-05 21400 8.14E-05
22300 0.000343 20200 1.12E-05 20000 3.47E-05 19600
0.000119985 21900 8.68E-05 21400 8.14E-05
22300 0.000344 20200 1.12E-05 20000 3.47E-05 19600
0.000120521 21900 8.69E-05 21400 8.15E-05
22300 0.000344 20200 1.12E-05 20000 3.46E-05 19600
0.000121057 21900 8.69E-05 21400 8.15E-05
22300 0.000344 20200 1.12E-05 20000 3.45E-05 19600
0.000121605 21900 8.69E-05 21400 8.15E-05
22300 0.000345 20200 1.12E-05 20000 3.45E-05 19600
0.000122148 21900 8.70E-05 21400 8.16E-05
22300 0.000345 20300 1.12E-05 20000 3.44E-05 19600
0.0001227 21900 8.70E-05 21400 8.16E-05
22300 0.000346 20300 1.12E-05 20000 3.43E-05 19600
0.000123262 21900 8.71E-05 21400 8.17E-05
22300 0.000346 20300 1.12E-05 20100 3.43E-05 19600
0.000123838 21900 8.71E-05 21400 8.17E-05

22300 0.000346 20300 1.12E-05 20100 3.43E-05 19600
0.000124406 21900 8.72E-05 21400 8.18E-05
22300 0.000347 20300 1.12E-05 20100 3.42E-05 19600
0.000124981 21900 8.72E-05 21400 8.18E-05
22300 0.000347 20300 1.12E-05 20100 3.42E-05 19600
0.000125579 21900 8.72E-05 21400 8.19E-05
22300 0.000347 20300 1.13E-05 20100 3.41E-05 19700
0.000126167 21900 8.73E-05 21400 8.19E-05
22300 0.000348 20300 1.13E-05 20100 3.41E-05 19700
0.000126746 21900 8.73E-05 21400 8.20E-05
22300 0.000348 20300 1.13E-05 20100 3.40E-05 19700
0.000127349 21900 8.74E-05 21400 8.20E-05
22300 0.000349 20300 1.13E-05 20100 3.40E-05 19700
0.000127957 21900 8.74E-05 21400 8.20E-05
22300 0.000349 20300 1.13E-05 20100 3.40E-05 19700
0.000128565 21900 8.74E-05 21400 8.21E-05
22300 0.000349 20300 1.13E-05 20100 3.40E-05 19700
0.000129194 21900 8.75E-05 21400 8.22E-05
22300 0.00035 20300 1.13E-05 20100 3.40E-05 19700
0.000129814 21900 8.75E-05 21400 8.22E-05
22300 0.00035 20300 1.13E-05 20100 3.39E-05 19700
0.000130467 21900 8.76E-05 21400 8.22E-05
22300 0.000351 20300 1.13E-05 20100 3.39E-05 19700
0.000131127 21900 8.76E-05 21400 8.23E-05
22300 0.000351 20300 1.13E-05 20100 3.39E-05 19700
0.000131775 21900 8.76E-05 21400 8.23E-05
22300 0.000351 20300 1.13E-05 20100 3.39E-05 19700
0.000132431 21900 8.77E-05 21400 8.24E-05
22300 0.000352 20300 1.13E-05 20100 3.39E-05 19700
0.000133089 22000 8.77E-05 21400 8.24E-05
22300 0.000352 20300 1.13E-05 20100 3.38E-05 19700
0.000133738 22000 8.78E-05 21400 8.25E-05
22300 0.000352 20300 1.13E-05 20100 3.38E-05 19700
0.000134385 22000 8.78E-05 21400 8.25E-05
22300 0.000353 20300 1.13E-05 20100 3.38E-05 19700
0.00013504 22000 8.79E-05 21400 8.26E-05
22300 0.000353 20300 1.13E-05 20100 3.38E-05 19700
0.000135694 22000 8.79E-05 21400 8.26E-05
22300 0.000354 20300 1.13E-05 20100 3.38E-05 19700
0.000136358 22000 8.79E-05 21400 8.27E-05
22300 0.000354 20300 1.13E-05 20100 3.38E-05 19700
0.000137008 22000 8.80E-05 21400 8.27E-05
22300 0.000354 20300 1.13E-05 20100 3.38E-05 19700
0.000137702 22000 8.80E-05 21400 8.27E-05
22300 0.000355 20300 1.13E-05 20100 3.38E-05 19700
0.00013838 22000 8.81E-05 21400 8.28E-05
22300 0.000355 20300 1.13E-05 20100 3.38E-05 19700
0.00013908 22000 8.81E-05 21400 8.29E-05
22300 0.000355 20300 1.13E-05 20100 3.38E-05 19700
0.000139788 22000 8.81E-05 21400 8.29E-05
22300 0.000356 20300 1.13E-05 20100 3.38E-05 19700
0.000140516 22000 8.82E-05 21400 8.29E-05
22300 0.000356 20300 1.13E-05 20100 3.38E-05 19700
0.000141224 22000 8.82E-05 21400 8.30E-05
22300 0.000357 20300 1.13E-05 20100 3.38E-05 19700
0.000141938 22000 8.83E-05 21400 8.30E-05
22300 0.000357 20300 1.13E-05 20100 3.38E-05 19700
0.000142632 22000 8.83E-05 21400 8.31E-05
22300 0.000357 20300 1.13E-05 20100 3.38E-05 19700
0.000143357 22000 8.83E-05 21400 8.32E-05
22300 0.000358 20300 1.13E-05 20100 3.38E-05 19700
0.000144061 22000 8.84E-05 21400 8.32E-05
22300 0.000358 20300 1.14E-05 20100 3.38E-05 19700
0.000144774 22000 8.84E-05 21400 8.32E-05
22300 0.000359 20300 1.13E-05 20100 3.38E-05 19700
0.000145494 22000 8.85E-05 21400 8.33E-05
22300 0.000359 20300 1.14E-05 20100 3.38E-05 19700
0.00014621 22000 8.85E-05 21400 8.33E-05

22300 0.000359 20300 1.14E-05 20100 3.39E-05 19700
0.000146931 22000 8.85E-05 21400 8.34E-05
22300 0.00036 20300 1.14E-05 20100 3.39E-05 19700
0.000147669 22000 8.86E-05 21400 8.34E-05
22300 0.00036 20300 1.14E-05 20100 3.39E-05 19700
0.000148423 22000 8.86E-05 21400 8.35E-05
22300 0.000361 20300 1.14E-05 20100 3.39E-05 19700
0.000149189 22000 8.87E-05 21400 8.36E-05
22300 0.000361 20300 1.14E-05 20100 3.39E-05 19700
0.000149966 22000 8.87E-05 21400 8.36E-05
22300 0.000361 20300 1.14E-05 20100 3.39E-05 19700
0.000150752 22000 8.87E-05 21400 8.37E-05
22300 0.000362 20300 1.14E-05 20100 3.39E-05 19700
0.000151568 22000 8.88E-05 21400 8.37E-05
22300 0.000362 20300 1.14E-05 20100 3.40E-05 19700
0.000152372 22000 8.88E-05 21400 8.38E-05
22300 0.000363 20300 1.14E-05 20100 3.40E-05 19700
0.000153196 22000 8.89E-05 21400 8.38E-05
22400 0.000363 20300 1.14E-05 20100 3.40E-05 19700
0.000154022 22000 8.89E-05 21400 8.39E-05
22400 0.000364 20300 1.14E-05 20100 3.40E-05 19700
0.000154869 22000 8.90E-05 21400 8.39E-05
22400 0.000364 20300 1.14E-05 20100 3.41E-05 19700
0.000155713 22000 8.90E-05 21400 8.40E-05
22400 0.000364 20300 1.14E-05 20100 3.41E-05 19700
0.000156575 22000 8.91E-05 21400 8.40E-05
22400 0.000365 20300 1.14E-05 20100 3.41E-05 19700
0.000157438 22000 8.91E-05 21400 8.41E-05
22400 0.000365 20300 1.14E-05 20100 3.41E-05 19700
0.000158314 22000 8.91E-05 21400 8.41E-05
22400 0.000366 20300 1.14E-05 20100 3.42E-05 19700
0.000159201 22000 8.92E-05 21400 8.42E-05
22400 0.000366 20300 1.15E-05 20100 3.42E-05 19700
0.000160098 22000 8.92E-05 21400 8.42E-05
22400 0.000367 20300 1.15E-05 20100 3.42E-05 19700
0.000160998 22000 8.93E-05 21400 8.43E-05
22400 0.000367 20300 1.15E-05 20100 3.42E-05 19700
0.000161905 22000 8.93E-05 21500 8.43E-05
22400 0.000367 20300 1.15E-05 20100 3.43E-05 19700
0.0001628 22000 8.94E-05 21500 8.43E-05
22400 0.000368 20300 1.15E-05 20100 3.43E-05 19700
0.000163708 22000 8.94E-05 21500 8.44E-05
22400 0.000368 20300 1.15E-05 20100 3.43E-05 19700
0.000164615 22000 8.95E-05 21500 8.45E-05
22400 0.000369 20300 1.15E-05 20100 3.44E-05 19700
0.000165539 22000 8.95E-05 21500 8.45E-05
22400 0.000369 20300 1.15E-05 20100 3.44E-05 19700
0.00016644 22000 8.96E-05 21500 8.46E-05
22400 0.00037 20300 1.15E-05 20100 3.44E-05 19700
0.000167362 22000 8.96E-05 21500 8.46E-05
22400 0.00037 20300 1.15E-05 20100 3.44E-05 19700
0.000168303 22000 8.97E-05 21500 8.47E-05
22400 0.00037 20300 1.15E-05 20100 3.45E-05 19700
0.00016923 22000 8.97E-05 21500 8.47E-05
22400 0.000371 20400 1.15E-05 20100 3.45E-05 19700
0.000170153 22000 8.98E-05 21500 8.47E-05
22400 0.000371 20400 1.15E-05 20100 3.46E-05 19700
0.00017109 22000 8.98E-05 21500 8.48E-05
22400 0.000372 20400 1.15E-05 20200 3.46E-05 19700
0.00017204 22000 8.99E-05 21500 8.48E-05
22400 0.000372 20400 1.15E-05 20200 3.46E-05 19700
0.000173001 22000 8.99E-05 21500 8.49E-05
22400 0.000373 20400 1.15E-05 20200 3.47E-05 19700
0.000173964 22000 9.00E-05 21500 8.50E-05
22400 0.000373 20400 1.15E-05 20200 3.47E-05 19700
0.000174929 22000 9.01E-05 21500 8.50E-05
22400 0.000374 20400 1.15E-05 20200 3.48E-05 19800
0.000175928 22000 9.01E-05 21500 8.50E-05

22400 0.000374 20400 1.15E-05 20200 3.48E-05 19800
0.000176943 22000 9.02E-05 21500 8.51E-05
22400 0.000374 20400 1.15E-05 20200 3.48E-05 19800
0.000177969 22000 9.02E-05 21500 8.51E-05
22400 0.000375 20400 1.15E-05 20200 3.49E-05 19800
0.000178992 22000 9.03E-05 21500 8.52E-05
22400 0.000375 20400 1.15E-05 20200 3.49E-05 19800
0.000180024 22000 9.03E-05 21500 8.52E-05
22400 0.000376 20400 1.15E-05 20200 3.49E-05 19800
0.000181055 22000 9.04E-05 21500 8.53E-05
22400 0.000376 20400 1.15E-05 20200 3.50E-05 19800
0.000182098 22000 9.04E-05 21500 8.53E-05
22400 0.000377 20400 1.15E-05 20200 3.50E-05 19800
0.000183147 22000 9.05E-05 21500 8.54E-05
22400 0.000377 20400 1.15E-05 20200 3.51E-05 19800
0.000184208 22000 9.05E-05 21500 8.55E-05
22400 0.000378 20400 1.15E-05 20200 3.51E-05 19800
0.000185267 22000 9.06E-05 21500 8.55E-05
22400 0.000378 20400 1.15E-05 20200 3.52E-05 19800
0.000186337 22000 9.06E-05 21500 8.56E-05
22400 0.000378 20400 1.16E-05 20200 3.52E-05 19800
0.000187448 22100 9.07E-05 21500 8.56E-05
22400 0.000379 20400 1.16E-05 20200 3.52E-05 19800
0.000188563 22100 9.07E-05 21500 8.57E-05
22400 0.000379 20400 1.16E-05 20200 3.53E-05 19800
0.000189692 22100 9.08E-05 21500 8.57E-05
22400 0.00038 20400 1.16E-05 20200 3.53E-05 19800
0.000190835 22100 9.09E-05 21500 8.58E-05
22400 0.00038 20400 1.16E-05 20200 3.54E-05 19800
0.000192003 22100 9.09E-05 21500 8.58E-05
22400 0.000381 20400 1.16E-05 20200 3.54E-05 19800
0.000193185 22100 9.10E-05 21500 8.59E-05
22400 0.000381 20400 1.16E-05 20200 3.55E-05 19800
0.000194376 22100 9.10E-05 21500 8.59E-05
22400 0.000382 20400 1.16E-05 20200 3.55E-05 19800
0.000195583 22100 9.11E-05 21500 8.60E-05
22400 0.000382 20400 1.16E-05 20200 3.56E-05 19800
0.000196812 22100 9.11E-05 21500 8.60E-05
22400 0.000383 20400 1.16E-05 20200 3.56E-05 19800
0.000198076 22100 9.12E-05 21500 8.61E-05
22400 0.000383 20400 1.16E-05 20200 3.57E-05 19800
0.000199347 22100 9.12E-05 21500 8.61E-05
22400 0.000383 20400 1.16E-05 20200 3.57E-05 19800
0.00020063 22100 9.13E-05 21500 8.62E-05
22400 0.000384 20400 1.16E-05 20200 3.58E-05 19800
0.000201946 22100 9.14E-05 21500 8.62E-05
22400 0.000384 20400 1.16E-05 20200 3.58E-05 19800
0.000203269 22100 9.14E-05 21500 8.63E-05
22400 0.000385 20400 1.16E-05 20200 3.59E-05 19800
0.000204581 22100 9.15E-05 21500 8.64E-05
22400 0.000385 20400 1.16E-05 20200 3.59E-05 19800
0.000205937 22100 9.16E-05 21500 8.64E-05
22400 0.000386 20400 1.16E-05 20200 3.60E-05 19800
0.0002073 22100 9.16E-05 21500 8.65E-05
22400 0.000386 20400 1.16E-05 20200 3.61E-05 19800
0.000208653 22100 9.17E-05 21500 8.65E-05
22400 0.000387 20400 1.16E-05 20200 3.61E-05 19800
0.000210014 22100 9.18E-05 21500 8.66E-05
22400 0.000387 20400 1.16E-05 20200 3.62E-05 19800
0.000211393 22100 9.18E-05 21500 8.66E-05
22400 0.000388 20400 1.16E-05 20200 3.62E-05 19800
0.00021276 22100 9.19E-05 21500 8.67E-05
22400 0.000388 20400 1.16E-05 20200 3.63E-05 19800
0.000214138 22100 9.20E-05 21500 8.68E-05
22400 0.000389 20400 1.16E-05 20200 3.63E-05 19800
0.000215518 22100 9.20E-05 21500 8.68E-05
22400 0.000389 20400 1.16E-05 20200 3.64E-05 19800
0.00021692 22100 9.21E-05 21500 8.69E-05

22400 0.00039 20400 1.16E-05 20200 3.65E-05 19800
0.000218341 22100 9.22E-05 21500 8.69E-05
22400 0.00039 20400 1.17E-05 20200 3.65E-05 19800
0.000219771 22100 9.22E-05 21500 8.70E-05
22400 0.00039 20400 1.17E-05 20200 3.66E-05 19800
0.000221204 22100 9.23E-05 21500 8.70E-05
22400 0.000391 20400 1.17E-05 20200 3.66E-05 19800
0.000222676 22100 9.24E-05 21500 8.71E-05
22400 0.000391 20400 1.17E-05 20200 3.67E-05 19800
0.000224151 22100 9.24E-05 21500 8.71E-05
22500 0.000392 20400 1.17E-05 20200 3.68E-05 19800
0.00022563 22100 9.25E-05 21500 8.72E-05
22500 0.000392 20400 1.17E-05 20200 3.68E-05 19800
0.000227133 22100 9.26E-05 21500 8.72E-05
22500 0.000393 20400 1.17E-05 20200 3.69E-05 19800
0.000228647 22100 9.26E-05 21500 8.73E-05
22500 0.000393 20400 1.17E-05 20200 3.69E-05 19800
0.000230157 22100 9.27E-05 21500 8.73E-05
22500 0.000394 20400 1.17E-05 20200 3.70E-05 19800
0.000231712 22100 9.28E-05 21500 8.74E-05
22500 0.000394 20400 1.17E-05 20200 3.71E-05 19800
0.000233278 22100 9.28E-05 21500 8.74E-05
22500 0.000395 20400 1.17E-05 20200 3.71E-05 19800
0.000234875 22100 9.29E-05 21500 8.75E-05
22500 0.000395 20400 1.17E-05 20200 3.72E-05 19800
0.000236482 22100 9.30E-05 21500 8.76E-05
22500 0.000395 20400 1.17E-05 20200 3.73E-05 19800
0.000238101 22100 9.30E-05 21500 8.76E-05
22500 0.000396 20400 1.17E-05 20200 3.73E-05 19800
0.000239764 22100 9.31E-05 21600 8.76E-05
22500 0.000396 20400 1.17E-05 20200 3.74E-05 19800
0.000241423 22100 9.32E-05 21600 8.77E-05
22500 0.000397 20400 1.17E-05 20200 3.75E-05 19800
0.000243107 22100 9.32E-05 21600 8.77E-05
22500 0.000397 20400 1.17E-05 20200 3.76E-05 19800
0.000244793 22100 9.33E-05 21600 8.78E-05
22500 0.000398 20400 1.17E-05 20200 3.76E-05 19800
0.000246507 22100 9.34E-05 21600 8.78E-05
22500 0.000398 20400 1.17E-05 20200 3.77E-05 19800
0.000248229 22100 9.35E-05 21600 8.79E-05
22500 0.000399 20400 1.17E-05 20200 3.78E-05 19800
0.000249956 22100 9.35E-05 21600 8.79E-05
22500 0.000399 20400 1.18E-05 20200 3.78E-05 19800
0.000251677 22100 9.36E-05 21600 8.80E-05
22500 0.0004 20400 1.18E-05 20200 3.79E-05 19800
0.000253442 22100 9.36E-05 21600 8.80E-05
22500 0.0004 20500 1.18E-05 20200 3.80E-05 19800
0.000255205 22100 9.37E-05 21600 8.81E-05
22500 0.000401 20500 1.18E-05 20200 3.81E-05 19800
0.000256979 22100 9.38E-05 21600 8.81E-05
22500 0.000401 20500 1.18E-05 20300 3.81E-05 19800
0.000258772 22100 9.39E-05 21600 8.82E-05
22500 0.000401 20500 1.18E-05 20300 3.82E-05 19800
0.000260582 22100 9.39E-05 21600 8.82E-05
22500 0.000402 20500 1.18E-05 20300 3.83E-05 19800
0.000262414 22100 9.40E-05 21600 8.83E-05
22500 0.000402 20500 1.18E-05 20300 3.83E-05 19800
0.000264261 22100 9.41E-05 21600 8.83E-05
22500 0.000403 20500 1.18E-05 20300 3.84E-05 19900
0.000266151 22100 9.41E-05 21600 8.84E-05
22500 0.000403 20500 1.18E-05 20300 3.85E-05 19900
0.000268064 22100 9.42E-05 21600 8.84E-05
22500 0.000404 20500 1.18E-05 20300 3.86E-05 19900
0.000269981 22100 9.43E-05 21600 8.85E-05
22500 0.000404 20500 1.18E-05 20300 3.87E-05 19900
0.000271935 22100 9.44E-05 21600 8.86E-05
22500 0.000405 20500 1.18E-05 20300 3.87E-05 19900
0.00027392 22100 9.44E-05 21600 8.86E-05

22500 0.000405 20500 1.18E-05 20300 3.88E-05 19900
0.000275913 22100 9.45E-05 21600 8.86E-05
22500 0.000406 20500 1.18E-05 20300 3.89E-05 19900
0.000277935 22100 9.46E-05 21600 8.87E-05
22500 0.000406 20500 1.18E-05 20300 3.90E-05 19900
0.000279978 22100 9.47E-05 21600 8.87E-05
22500 0.000407 20500 1.18E-05 20300 3.91E-05 19900
0.000282048 22100 9.47E-05 21600 8.88E-05
22500 0.000407 20500 1.18E-05 20300 3.92E-05 19900
0.000284141 22100 9.48E-05 21600 8.88E-05
22500 0.000408 20500 1.18E-05 20300 3.93E-05 19900
0.000286282 22100 9.49E-05 21600 8.89E-05
22500 0.000408 20500 1.18E-05 20300 3.94E-05 19900
0.000288428 22200 9.49E-05 21600 8.89E-05
22500 0.000409 20500 1.18E-05 20300 3.94E-05 19900
0.000290622 22200 9.50E-05 21600 8.90E-05
22500 0.000409 20500 1.18E-05 20300 3.95E-05 19900
0.000292846 22200 9.51E-05 21600 8.90E-05
22500 0.00041 20500 1.18E-05 20300 3.96E-05 19900
0.000295093 22200 9.52E-05 21600 8.91E-05
22500 0.00041 20500 1.18E-05 20300 3.97E-05 19900
0.000297375 22200 9.52E-05 21600 8.91E-05
22500 0.000411 20500 1.18E-05 20300 3.98E-05 19900
0.000299688 22200 9.53E-05 21600 8.91E-05
22500 0.000411 20500 1.18E-05 20300 3.99E-05 19900
0.000302015 22200 9.54E-05 21600 8.92E-05
22500 0.000412 20500 1.19E-05 20300 4.00E-05 19900
0.000304365 22200 9.55E-05 21600 8.92E-05
22500 0.000412 20500 1.19E-05 20300 4.00E-05 19900
0.000306772 22200 9.56E-05 21600 8.93E-05
22500 0.000413 20500 1.19E-05 20300 4.01E-05 19900
0.000309214 22200 9.56E-05 21600 8.93E-05
22500 0.000413 20500 1.19E-05 20300 4.02E-05 19900
0.000311671 22200 9.57E-05 21600 8.94E-05
22500 0.000414 20500 1.19E-05 20300 4.03E-05 19900
0.000314171 22200 9.58E-05 21600 8.94E-05
22500 0.000415 20500 1.19E-05 20300 4.04E-05 19900
0.000316709 22200 9.59E-05 21600 8.95E-05
22500 0.000415 20500 1.19E-05 20300 4.05E-05 19900
0.000319262 22200 9.60E-05 21600 8.95E-05
22500 0.000416 20500 1.19E-05 20300 4.06E-05 19900
0.000321844 22200 9.61E-05 21600 8.96E-05
22500 0.000416 20500 1.19E-05 20300 4.07E-05 19900
0.00032445 22200 9.61E-05 21600 8.96E-05
22500 0.000417 20500 1.19E-05 20300 4.08E-05 19900
0.000327109 22200 9.62E-05 21600 8.97E-05
22500 0.000417 20500 1.19E-05 20300 4.09E-05 19900
0.000329778 22200 9.63E-05 21600 8.97E-05
22500 0.000418 20500 1.19E-05 20300 4.10E-05 19900
0.000332477 22200 9.64E-05 21600 8.98E-05
22500 0.000418 20500 1.19E-05 20300 4.11E-05 19900
0.000335227 22200 9.65E-05 21600 8.98E-05
22500 0.000419 20500 1.20E-05 20300 4.12E-05 19900
0.000338032 22200 9.66E-05 21600 8.99E-05
22500 0.000419 20500 1.20E-05 20300 4.13E-05 19900
0.000340883 22200 9.66E-05 21600 8.99E-05
22500 0.00042 20500 1.20E-05 20300 4.14E-05 19900
0.000343785 22200 9.67E-05 21600 9.00E-05
22500 0.00042 20500 1.20E-05 20300 4.15E-05 19900
0.000346725 22200 9.68E-05 21600 9.00E-05
22500 0.000421 20500 1.20E-05 20300 4.16E-05 19900
0.000349716 22200 9.69E-05 21600 9.01E-05
22500 0.000422 20500 1.20E-05 20300 4.17E-05 19900
0.000352733 22200 9.70E-05 21600 9.01E-05
22500 0.000422 20500 1.20E-05 20300 4.18E-05 19900
0.000355792 22200 9.71E-05 21600 9.02E-05
22500 0.000423 20500 1.20E-05 20300 4.19E-05 19900
0.0003589 22200 9.72E-05 21600 9.02E-05

22500	0.000423	20500	1.20E-05	20300	4.20E-05	19900
0.000362042	22200	9.73E-05	21600	9.03E-05
22600	0.000424	20500	1.20E-05	20300	4.21E-05	19900
0.000365203	22200	9.74E-05	21600	9.04E-05
22600	0.000424	20500	1.20E-05	20300	4.22E-05	19900
0.000368402	22200	9.75E-05	21600	9.04E-05
22600	0.000425	20500	1.20E-05	20300	4.24E-05	19900
0.000371635	22200	9.75E-05	21600	9.05E-05
22600	0.000425	20500	1.20E-05	20300	4.25E-05	19900
0.000374912	22200	9.76E-05	21600	9.05E-05
22600	0.000426	20500	1.20E-05	20300	4.26E-05	19900
0.0003782	22200	9.77E-05	21600	9.06E-05
22600	0.000426	20500	1.20E-05	20300	4.27E-05	19900
0.000381531	22200	9.78E-05	21600	9.06E-05
22600	0.000427	20500	1.20E-05	20300	4.28E-05	19900
0.000384929	22200	9.79E-05	21600	9.07E-05
22600	0.000428	20500	1.20E-05	20300	4.29E-05	19900
0.000388369	22200	9.80E-05	21600	9.07E-05
22600	0.000428	20500	1.20E-05	20300	4.31E-05	19900
0.000391834	22200	9.81E-05	21600	9.08E-05
22600	0.000429	20500	1.21E-05	20300	4.32E-05	19900
0.000395363	22200	9.82E-05	21700	9.08E-05
22600	0.000429	20500	1.21E-05	20300	4.33E-05	19900
0.000398932	22200	9.83E-05	21700	9.09E-05
22600	0.00043	20500	1.21E-05	20300	4.34E-05	19900
0.000402563	22200	9.84E-05	21700	9.10E-05
22600	0.00043	20500	1.21E-05	20300	4.36E-05	19900
0.000406228	22200	9.85E-05	21700	9.10E-05
22600	0.000431	20500	1.21E-05	20300	4.37E-05	19900
0.00040994	22200	9.86E-05	21700	9.11E-05
22600	0.000431	20500	1.21E-05	20300	4.38E-05	19900
0.00041369	22200	9.87E-05	21700	9.11E-05
22600	0.000432	20500	1.21E-05	20300	4.39E-05	19900
0.000417482	22200	9.88E-05	21700	9.12E-05
22600	0.000433	20500	1.21E-05	20300	4.40E-05	19900
0.000421308	22200	9.89E-05	21700	9.12E-05
22600	0.000433	20500	1.21E-05	20300	4.42E-05	19900
0.000425201	22200	9.90E-05	21700	9.13E-05
22600	0.000434	20600	1.21E-05	20300	4.43E-05	19900
0.000429148	22200	9.91E-05	21700	9.13E-05
22600	0.000434	20600	1.21E-05	20300	4.44E-05	19900
0.00043313	22200	9.92E-05	21700	9.14E-05
22600	0.000435	20600	1.21E-05	20400	4.45E-05	19900
0.000437161	22200	9.93E-05	21700	9.15E-05
22600	0.000435	20600	1.21E-05	20400	4.47E-05	19900
0.000441265	22200	9.94E-05	21700	9.15E-05
22600	0.000436	20600	1.21E-05	20400	4.48E-05	19900
0.00044541	22200	9.96E-05	21700	9.16E-05
22600	0.000436	20600	1.21E-05	20400	4.49E-05	19900
0.000449622	22200	9.97E-05	21700	9.16E-05
22600	0.000437	20600	1.21E-05	20400	4.51E-05	20000
0.000453887	22200	9.98E-05	21700	9.17E-05
22600	0.000438	20600	1.21E-05	20400	4.52E-05	20000
0.000458233	22200	9.99E-05	21700	9.17E-05
22600	0.000438	20600	1.21E-05	20400	4.54E-05	20000
0.00046266	22200	0.0001	21700	9.18E-05
22600	0.000439	20600	1.21E-05	20400	4.55E-05	20000
0.000467169	22200	0.0001	21700	9.18E-05
22600	0.000439	20600	1.21E-05	20400	4.56E-05	20000
0.000471761	22200	0.0001	21700	9.19E-05
22600	0.00044	20600	1.21E-05	20400	4.58E-05	20000
0.00047647	22200	0.0001	21700	9.19E-05
22600	0.00044	20600	1.21E-05	20400	4.59E-05	20000
0.000481251	22200	0.0001	21700	9.20E-05
22600	0.000441	20600	1.22E-05	20400	4.61E-05	20000
0.000486116	22200	0.000101	21700	9.21E-05
22600	0.000442	20600	1.22E-05	20400	4.62E-05	20000
0.000491071	22200	0.000101	21700	9.21E-05

22600 0.000442 20600 1.22E-05 20400 4.63E-05 20000
0.000496091 22200 0.000101 21700 9.22E-05
22600 0.000443 20600 1.22E-05 20400 4.65E-05 20000
0.000501168 22200 0.000101 21700 9.23E-05
22600 0.000443 20600 1.22E-05 20400 4.66E-05 20000
0.000506329 22300 0.000101 21700 9.23E-05
22600 0.000444 20600 1.22E-05 20400 4.68E-05 20000
0.000511555 22300 0.000101 21700 9.24E-05
22600 0.000445 20600 1.22E-05 20400 4.69E-05 20000
0.000516872 22300 0.000101 21700 9.24E-05
22600 0.000445 20600 1.22E-05 20400 4.71E-05 20000
0.000522253 22300 0.000101 21700 9.25E-05
22600 0.000446 20600 1.22E-05 20400 4.73E-05 20000
0.000527724 22300 0.000102 21700 9.26E-05
22600 0.000446 20600 1.22E-05 20400 4.74E-05 20000
0.000533268 22300 0.000102 21700 9.26E-05
22600 0.000447 20600 1.22E-05 20400 4.76E-05 20000
0.00053889 22300 0.000102 21700 9.27E-05
22600 0.000447 20600 1.22E-05 20400 4.77E-05 20000
0.000544594 22300 0.000102 21700 9.28E-05
22600 0.000448 20600 1.22E-05 20400 4.79E-05 20000
0.000550378 22300 0.000102 21700 9.28E-05
22600 0.000449 20600 1.22E-05 20400 4.81E-05 20000
0.000556244 22300 0.000102 21700 9.29E-05
22600 0.000449 20600 1.22E-05 20400 4.82E-05 20000
0.000562192 22300 0.000102 21700 9.29E-05
22600 0.00045 20600 1.22E-05 20400 4.84E-05 20000
0.000568222 22300 0.000102 21700 9.30E-05
22600 0.00045 20600 1.22E-05 20400 4.86E-05 20000
0.00057435 22300 0.000103 21700 9.30E-05
22600 0.000451 20600 1.22E-05 20400 4.88E-05 20000
0.000580563 22300 0.000103 21700 9.31E-05
22600 0.000452 20600 1.22E-05 20400 4.89E-05 20000
0.000586869 22300 0.000103 21700 9.31E-05
22600 0.000452 20600 1.22E-05 20400 4.91E-05 20000
0.000593264 22300 0.000103 21700 9.32E-05
22600 0.000453 20600 1.22E-05 20400 4.93E-05 20000
0.000599765 22300 0.000103 21700 9.32E-05
22600 0.000453 20600 1.22E-05 20400 4.94E-05 20000
0.000606372 22300 0.000103 21700 9.33E-05
22600 0.000454 20600 1.23E-05 20400 4.96E-05 20000
0.000613076 22300 0.000103 21700 9.33E-05
22600 0.000455 20600 1.23E-05 20400 4.98E-05 20000
0.000619931 22300 0.000104 21700 9.34E-05
22600 0.000455 20600 1.23E-05 20400 5.00E-05 20000
0.000626892 22300 0.000104 21700 9.34E-05
22600 0.000456 20600 1.23E-05 20400 5.02E-05 20000
0.000633984 22300 0.000104 21700 9.35E-05
22600 0.000456 20600 1.23E-05 20400 5.04E-05 20000
0.000641197 22300 0.000104 21700 9.36E-05
22600 0.000457 20600 1.23E-05 20400 5.05E-05 20000
0.00064852 22300 0.000104 21700 9.36E-05
22600 0.000458 20600 1.23E-05 20400 5.07E-05 20000
0.000655983 22300 0.000104 21700 9.37E-05
22600 0.000458 20600 1.23E-05 20400 5.09E-05 20000
0.000663579 22300 0.000104 21700 9.37E-05
22600 0.000459 20600 1.23E-05 20400 5.11E-05 20000
0.000671306 22300 0.000105 21700 9.38E-05
22600 0.00046 20600 1.23E-05 20400 5.13E-05 20000
0.00067919 22300 0.000105 21700 9.38E-05
22600 0.00046 20600 1.23E-05 20400 5.15E-05 20000
0.000687246 22300 0.000105 21700 9.39E-05
22700 0.000461 20600 1.23E-05 20400 5.17E-05 20000
0.000695469 22300 0.000105 21700 9.39E-05
22700 0.000461 20600 1.23E-05 20400 5.19E-05 20000
0.000703857 22300 0.000105 21700 9.40E-05
22700 0.000462 20600 1.23E-05 20400 5.21E-05 20000
0.000712436 22300 0.000105 21700 9.41E-05

22700 0.000463 20600 1.23E-05 20400 5.24E-05 20000
0.000721175 22300 0.000106 21700 9.41E-05
22700 0.000463 20600 1.23E-05 20400 5.26E-05 20000
0.000730051 22300 0.000106 21700 9.42E-05
22700 0.000464 20600 1.23E-05 20400 5.28E-05 20000
0.000739077 22300 0.000106 21700 9.43E-05
22700 0.000465 20600 1.23E-05 20400 5.30E-05 20000
0.000748244 22300 0.000106 21700 9.43E-05
22700 0.000465 20600 1.23E-05 20400 5.32E-05 20000
0.000757539 22300 0.000106 21700 9.44E-05
22700 0.000466 20600 1.23E-05 20400 5.34E-05 20000
0.000766992 22300 0.000107 21700 9.44E-05
22700 0.000467 20600 1.24E-05 20400 5.37E-05 20000
0.000776638 22300 0.000107 21800 9.45E-05
22700 0.000467 20600 1.24E-05 20400 5.39E-05 20000
0.000786459 22300 0.000107 21800 9.46E-05
22700 0.000468 20600 1.24E-05 20400 5.41E-05 20000
0.000796479 22300 0.000107 21800 9.46E-05
22700 0.000469 20600 1.24E-05 20400 5.43E-05 20000
0.0008067 22300 0.000107 21800 9.47E-05
22700 0.000469 20600 1.24E-05 20400 5.46E-05 20000
0.000817126 22300 0.000108 21800 9.48E-05
22700 0.00047 20600 1.24E-05 20400 5.48E-05 20000
0.000827789 22300 0.000108 21800 9.49E-05
22700 0.000471 20600 1.24E-05 20400 5.51E-05 20000
0.000838656 22300 0.000108 21800 9.49E-05
22700 0.000471 20600 1.24E-05 20400 5.53E-05 20000
0.000849749 22300 0.000108 21800 9.50E-05
22700 0.000472 20600 1.24E-05 20400 5.56E-05 20000
0.000861077 22300 0.000108 21800 9.51E-05
22700 0.000473 20700 1.24E-05 20400 5.58E-05 20000
0.000872592 22300 0.000109 21800 9.51E-05
22700 0.000473 20700 1.24E-05 20400 5.61E-05 20000
0.000884354 22300 0.000109 21800 9.52E-05
22700 0.000474 20700 1.24E-05 20500 5.63E-05 20000
0.000896313 22300 0.000109 21800 9.53E-05
22700 0.000475 20700 1.24E-05 20500 5.66E-05 20000
0.000908516 22300 0.000109 21800 9.53E-05
22700 0.000475 20700 1.25E-05 20500 5.68E-05 20000
0.000920964 22300 0.00011 21800 9.54E-05
22700 0.000476 20700 1.25E-05 20500 5.71E-05 20000
0.000933662 22300 0.00011 21800 9.55E-05
22700 0.000477 20700 1.25E-05 20500 5.74E-05 20100
0.000946623 22300 0.00011 21800 9.55E-05
22700 0.000477 20700 1.25E-05 20500 5.76E-05 20100
0.000959875 22300 0.00011 21800 9.56E-05
22700 0.000478 20700 1.25E-05 20500 5.79E-05 20100
0.000973382 22300 0.00011 21800 9.57E-05
22700 0.000479 20700 1.25E-05 20500 5.82E-05 20100
0.00098717 22300 0.000111 21800 9.57E-05
22700 0.000479 20700 1.25E-05 20500 5.84E-05 20100
0.001001251 22300 0.000111 21800 9.58E-05
22700 0.00048 20700 1.25E-05 20500 5.87E-05 20100
0.001015607 22300 0.000111 21800 9.59E-05
22700 0.00048 20700 1.25E-05 20500 5.90E-05 20100
0.001030264 22300 0.000111 21800 9.60E-05
22700 0.000481 20700 1.25E-05 20500 5.93E-05 20100
0.001045237 22300 0.000112 21800 9.60E-05
22700 0.000482 20700 1.25E-05 20500 5.96E-05 20100
0.001060539 22300 0.000112 21800 9.61E-05
22700 0.000483 20700 1.25E-05 20500 5.99E-05 20100
0.001076191 22300 0.000112 21800 9.62E-05
22700 0.000483 20700 1.25E-05 20500 6.02E-05 20100
0.001092181 22300 0.000112 21800 9.63E-05
22700 0.000484 20700 1.25E-05 20500 6.05E-05 20100
0.001108533 22400 0.000113 21800 9.63E-05
22700 0.000485 20700 1.25E-05 20500 6.08E-05 20100
0.001125256 22400 0.000113 21800 9.64E-05

22700 0.000485 20700 1.25E-05 20500 6.11E-05 20100
0.001142363 22400 0.000113 21800 9.65E-05
22700 0.000486 20700 1.25E-05 20500 6.14E-05 20100
0.00115984 22400 0.000114 21800 9.66E-05
22700 0.000487 20700 1.25E-05 20500 6.18E-05 20100
0.001177734 22400 0.000114 21800 9.66E-05
22700 0.000487 20700 1.25E-05 20500 6.21E-05 20100
0.001196032 22400 0.000114 21800 9.67E-05
22700 0.000488 20700 1.25E-05 20500 6.24E-05 20100
0.00121477 22400 0.000114 21800 9.67E-05
22700 0.000489 20700 1.25E-05 20500 6.27E-05 20100
0.001233943 22400 0.000115 21800 9.68E-05
22700 0.000489 20700 1.25E-05 20500 6.31E-05 20100
0.001253564 22400 0.000115 21800 9.69E-05
22700 0.00049 20700 1.25E-05 20500 6.34E-05 20100
0.001273646 22400 0.000115 21800 9.70E-05
22700 0.000491 20700 1.25E-05 20500 6.37E-05 20100
0.001294188 22400 0.000116 21800 9.70E-05
22700 0.000492 20700 1.26E-05 20500 6.41E-05 20100
0.001315203 22400 0.000116 21800 9.71E-05
22700 0.000492 20700 1.25E-05 20500 6.44E-05 20100
0.001336745 22400 0.000116 21800 9.72E-05
22700 0.000493 20700 1.26E-05 20500 6.48E-05 20100
0.001358793 22400 0.000117 21800 9.73E-05
22700 0.000494 20700 1.26E-05 20500 6.51E-05 20100
0.001381354 22400 0.000117 21800 9.74E-05
22700 0.000494 20700 1.26E-05 20500 6.55E-05 20100
0.001404473 22400 0.000117 21800 9.74E-05
22700 0.000495 20700 1.26E-05 20500 6.59E-05 20100
0.001428127 22400 0.000118 21800 9.75E-05
22700 0.000496 20700 1.26E-05 20500 6.62E-05 20100
0.001452353 22400 0.000118 21800 9.76E-05
22700 0.000497 20700 1.26E-05 20500 6.66E-05 20100
0.001477143 22400 0.000119 21800 9.77E-05
22700 0.000497 20700 1.26E-05 20500 6.70E-05 20100
0.001502533 22400 0.000119 21800 9.78E-05
22700 0.000498 20700 1.26E-05 20500 6.74E-05 20100
0.001528534 22400 0.000119 21800 9.78E-05
22700 0.000499 20700 1.26E-05 20500 6.78E-05 20100
0.001555175 22400 0.00012 21800 9.79E-05
22700 0.0005 20700 1.26E-05 20500 6.82E-05 20100
0.001582496 22400 0.00012 21800 9.80E-05
22700 0.0005 20700 1.26E-05 20500 6.86E-05 20100
0.001610533 22400 0.000121 21800 9.80E-05
22700 0.000501 20700 1.26E-05 20500 6.90E-05 20100
0.001639299 22400 0.000121 21800 9.81E-05
22700 0.000502 20700 1.26E-05 20500 6.94E-05 20100
0.00166887 22400 0.000122 21800 9.82E-05
22700 0.000502488 20700 1.26E-05 20500 6.98E-05 20100
0.001699261 22400 0.000122 21800 9.83E-05
22700 0.000503231 20700 1.26E-05 20500 7.03E-05 20100
0.001730507 22400 0.000123 21800 9.83E-05
22700 0.000503983 20700 1.26E-05 20500 7.07E-05 20100
0.001762626 22400 0.000123 21800 9.84E-05
22800 0.000504717 20700 1.27E-05 20500 7.11E-05 20100
0.001795639 22400 0.000124 21800 9.84E-05
22800 0.000505449 20700 1.27E-05 20500 7.16E-05 20100
0.001829576 22400 0.000124 21800 9.85E-05
22800 0.000506205 20700 1.27E-05 20500 7.20E-05 20100
0.001864478 22400 0.000125 21800 9.86E-05
22800 0.000506908 20700 1.27E-05 20500 7.25E-05 20100
0.001900389 22400 0.000125 21800 9.87E-05
22800 0.000507642 20700 1.27E-05 20500 7.30E-05 20100
0.001937292 22400 0.000126 21800 9.88E-05
22800 0.000508363 20700 1.27E-05 20500 7.34E-05 20100
0.001975228 22400 0.000126 21800 9.88E-05
22800 0.0005091 20700 1.27E-05 20500 7.39E-05 20100
0.002014232 22400 0.000127 21800 9.89E-05

22800 0.000509838 20700 1.27E-05 20500 7.44E-05 20100
0.002054333 22400 0.000127 21800 9.90E-05
22800 0.000510578 20700 1.27E-05 20500 7.49E-05 20100
0.002095555 22400 0.000128067 21800 9.91E-05
22800 0.000511361 20700 1.27E-05 20500 7.54E-05 20100
0.002137951 22400 0.000128712 21900 9.92E-05
22800 0.000512132 20700 1.27E-05 20500 7.59E-05 20100
0.002181629 22400 0.000129348 21900 9.93E-05
22800 0.000512908 20700 1.27E-05 20500 7.64E-05 20100
0.002226583 22400 0.000130023 21900 9.94E-05
22800 0.000513663 20700 1.28E-05 20500 7.69E-05 20100
0.002272971 22400 0.000130703 21900 9.95E-05
22800 0.000514432 20700 1.28E-05 20500 7.75E-05 20100
0.002320804 22400 0.0001314 21900 9.96E-05
22800 0.000515167 20700 1.28E-05 20500 7.80E-05 20100
0.0023702 22400 0.000132116 21900 9.97E-05
22800 0.000515924 20700 1.28E-05 20500 7.85E-05 20100
0.002421193 22400 0.000132858 21900 9.97E-05
22800 0.000516708 20700 1.28E-05 20500 7.91E-05 20100
0.002473834 22400 0.000133616 21900 9.98E-05
22800 0.000517465 20700 1.28E-05 20500 7.97E-05 20100
0.002528146 22400 0.000134414 21900 9.99E-05
22800 0.000518245 20800 1.28E-05 20500 8.02E-05 20100
0.002584208 22400 0.000135218 21900 0.0001
22800 0.000519036 20800 1.28E-05 20500 8.08E-05 20100
0.002642047 22400 0.000136052 21900 0.00010005
22800 0.00051985 20800 1.28E-05 20600 8.14E-05 20100
0.00270174 22400 0.000136909 21900 0.000100146
22800 0.000520648 20800 1.28E-05 20600 8.20E-05 20100
0.002763407 22400 0.000137786 21900 0.000100229
22800 0.000521426 20800 1.28E-05 20600 8.26E-05 20100
0.002827134 22400 0.000138698 21900 0.000100308
22800 0.000522219 20800 1.28E-05 20600 8.32E-05 20100
0.002893004 22400 0.00013963 21900 0.000100365
22800 0.000523043 20800 1.28E-05 20600 8.38E-05 20200
0.002961048 22400 0.000140599 21900 0.000100423
22800 0.000523803 20800 1.28E-05 20600 8.45E-05 20200
0.003031417 22400 0.000141599 21900 0.0001005
22800 0.000524635 20800 1.28E-05 20600 8.51E-05 20200
0.003104175 22400 0.000142638 21900 0.000100566
22800 0.000525456 20800 1.28E-05 20600 8.58E-05 20200
0.003179463 22400 0.000143695 21900 0.000100609
22800 0.000526263 20800 1.29E-05 20600 8.64E-05 20200
0.003257409 22400 0.000144784 21900 0.000100673
22800 0.000527053 20800 1.29E-05 20600 8.71E-05 20200
0.003338173 22400 0.0001459 21900 0.000100755
22800 0.000527869 20800 1.29E-05 20600 8.78E-05 20200
0.003421917 22400 0.000147072 21900 0.000100805
22800 0.000528682 20800 1.29E-05 20600 8.85E-05 20200
0.003508796 22400 0.000148281 21900 0.000100892
22800 0.000529522 20800 1.29E-05 20600 8.92E-05 20200
0.003598961 22400 0.000149522 21900 0.000100963
22800 0.000530326 20800 1.29E-05 20600 8.99E-05 20200
0.003692574 22400 0.000150813 21900 0.000101039
22800 0.000531101 20800 1.29E-05 20600 9.07E-05 20200
0.003789791 22400 0.000152147 21900 0.000101125
22800 0.00053193 20800 1.29E-05 20600 9.14E-05 20200
0.003890778 22500 0.000153521 21900 0.000101231
22800 0.000532779 20800 1.29E-05 20600 9.22E-05 20200
0.003995704 22500 0.00015496 21900 0.000101303
22800 0.000533608 20800 1.29E-05 20600 9.29E-05 20200
0.0041047 22500 0.000156437 21900 0.000101396
22800 0.000534466 20800 1.29E-05 20600 9.37E-05 20200
0.00421795 22500 0.000157969 21900 0.000101487
22800 0.000535356 20800 1.29E-05 20600 9.45E-05 20200
0.004335651 22500 0.000159561 21900 0.000101539
22800 0.000536166 20800 1.29E-05 20600 9.53E-05 20200
0.004458042 22500 0.000161199 21900 0.000101626

22800 0.000537034 20800 1.29E-05 20600 9.62E-05 20200
0.004585329 22500 0.000162893 21900 0.000101712
22800 0.000537858 20800 1.29E-05 20600 9.70E-05 20200
0.004717758 22500 0.000164654 21900 0.00010176
22800 0.00053871 20800 1.29E-05 20600 9.79E-05 20200
0.004855588 22500 0.000166469 21900 0.000101831
22800 0.000539579 20800 1.30E-05 20600 9.87E-05 20200
0.004999131 22500 0.000168365 21900 0.000101893
22800 0.000540433 20800 1.30E-05 20600 9.96E-05 20200
0.005148687 22500 0.000170315 21900 0.00010195
22800 0.000541311 20800 1.30E-05 20600 1.00E-04 20200
0.005304608 22500 0.000172334 21900 0.000102048
22800 0.000542187 20800 1.30E-05 20600 1.01E-04 20200
0.005467278 22500 0.000174428 21900 0.000102095
22800 0.000543071 20800 1.30E-05 20600 1.02E-04 20200
0.005637131 22500 0.000176593 21900 0.000102174
22800 0.000543919 20800 1.30E-05 20600 1.03E-04 20200
0.005814553 22500 0.000178828 21900 0.000102234
22800 0.000544834 20800 1.30E-05 20600 1.04E-04 20200
0.006000057 22500 0.000181156 21900 0.000102299
22800 0.000545718 20800 1.30E-05 20600 1.05E-04 20200
0.00619408 22500 0.000183555 21900 0.000102372
22800 0.000546595 20800 1.30E-05 20600 1.06E-04 20200
0.006397124 22500 0.000186029 21900 0.000102433
22800 0.000547454 20800 1.30E-05 20600 1.07E-04 20200
0.006609729 22500 0.000188598 21900 0.000102517
22800 0.000548356 20800 1.30E-05 20600 1.08E-04 20200
0.006832436 22500 0.000191251 21900 0.000102589
22800 0.000549225 20800 1.30E-05 20600 1.09E-04 20200
0.007065835 22500 0.000193982 21900 0.000102666
22800 0.000550131 20800 1.30E-05 20600 1.10E-04 20200
0.007310537 22500 0.000196808 21900 0.000102743
22800 0.000551046 20800 1.31E-05 20600 1.12E-04 20200
0.007567196 22500 0.000199742 21900 0.00010281
22800 0.000551954 20800 1.31E-05 20600 1.13E-04 20200
0.007836518 22500 0.000202753 21900 0.000102898
22800 0.000552836 20800 1.31E-05 20600 1.14E-04 20200
0.008119282 22500 0.000205859 21900 0.000102975
22800 0.000553693 20800 1.31E-05 20600 1.15E-04 20200
0.008416309 22500 0.000209068 21900 0.000103047
22800 0.000554586 20800 1.31E-05 20600 1.16E-04 20200
0.008728553 22500 0.000212365 21900 0.000103124
22800 0.000555498 20800 1.31E-05 20600 1.17E-04 20200
0.009057007 22500 0.00021576 21900 0.000103204
22800 0.000556381 20800 1.31E-05 20600 1.19E-04 20200
0.009402806 22500 0.000219247 21900 0.000103303
22900 0.000557316 20800 1.31E-05 20600 1.20E-04 20200
0.009767096 22500 0.000222831 21900 0.00010336
22900 0.000558253 20800 1.31E-05 20600 1.21E-04 20200
0.01015107 22500 0.000226491 21900 0.000103447
22900 0.000559167 20800 1.31E-05 20600 1.22E-04 20200
0.010556 22500 0.000230232 21900 0.00010353
22900 0.000560105 20800 1.31E-05 20600 1.24E-04 20200
0.01098319 22500 0.000234036 21900 0.000103622
22900 0.000561033 20800 1.31E-05 20600 1.25E-04 20200
0.01143412 22500 0.000237909 21900 0.000103694
22900 0.000561967 20800 1.31E-05 20600 1.26E-04 20200
0.01191043 22500 0.000241841 21900 0.000103778
22900 0.000562936 20800 1.31E-05 20600 1.28E-04 20200
0.01241392 22500 0.000245817 21900 0.000103875
22900 0.00056386 20800 1.32E-05 20600 1.29E-04 20200
0.01294672 22500 0.000249831 21900 0.00010397
22900 0.000564769 20800 1.32E-05 20600 1.31E-04 20200
0.013511 22500 0.000253867 21900 0.000104052
22900 0.000565673 20800 1.32E-05 20600 1.32E-04 20200
0.01410917 22500 0.000257908 22000 0.000104148
22900 0.000566614 20800 1.32E-05 20600 1.34E-04 20200
0.01474361 22500 0.000261936 22000 0.000104231

22900 0.000567512 20800 1.32E-05 20600 1.35E-04 20200
0.01541696 22500 0.000265951 22000 0.000104312
22900 0.000568458 20800 1.32E-05 20600 1.37E-04 20200
0.01613182 22500 0.000269907 22000 0.000104405
22900 0.000569421 20800 1.32E-05 20600 1.39E-04 20200
0.01689113 22500 0.000273782 22000 0.000104494
22900 0.00057038 20800 1.32E-05 20600 1.40E-04 20200
0.01769796 22500 0.000277551 22000 0.000104602
22900 0.000571331 20800 1.32E-05 20600 1.42E-04 20200
0.01855562 22500 0.000281182 22000 0.0001047
22900 0.000572301 20800 1.32E-05 20600 1.44E-04 20200
0.01946771 22500 0.000284651 22000 0.000104804
22900 0.000573299 20800 1.32E-05 20600 1.46E-04 20200
0.02043799 22500 0.000287944 22000 0.000104885
22900 0.000574288 20900 1.32E-05 20600 1.47E-04 20200
0.02147047 22500 0.000291032 22000 0.000104989
22900 0.000575275 20900 1.33E-05 20600 1.49E-04 20200
0.02256942 22500 0.000293846 22000 0.00010507
22900 0.000576244 20900 1.33E-05 20700 1.51E-04 20200
0.02373923 22500 0.000296389 22000 0.000105167
22900 0.000577256 20900 1.33E-05 20700 1.53E-04 20200
0.02498454 22500 0.000298622 22000 0.000105264
22900 0.000578243 20900 1.33E-05 20700 1.55E-04 20200
0.02631025 22500 0.00030048 22000 0.000105344
22900 0.000579226 20900 1.33E-05 20700 1.57E-04 20200
0.02772144 22500 0.00030198 22000 0.000105438
22900 0.000580219 20900 1.33E-05 20700 1.59E-04 20300
0.02922344 22500 0.000303073 22000 0.000105525
22900 0.000581231 20900 1.33E-05 20700 1.61E-04 20300
0.03082162 22500 0.000303726 22000 0.000105618
22900 0.000582223 20900 1.33E-05 20700 1.64E-04 20300
0.03252127 22500 0.000303943 22000 0.000105727
22900 0.000583195 20900 1.33E-05 20700 1.66E-04 20300
0.03432753 22500 0.000303715 22000 0.000105829
22900 0.000584188 20900 1.33E-05 20700 1.68E-04 20300
0.03624506 22500 0.000303008 22000 0.000105928
22900 0.000585201 20900 1.33E-05 20700 1.71E-04 20300
0.03827807 22500 0.000301842 22000 0.000106017
22900 0.000586215 20900 1.33E-05 20700 1.73E-04 20300
0.04043017 22500 0.000300207 22000 0.000106113
22900 0.000587228 20900 1.33E-05 20700 1.75E-04 20300
0.04270403 22500 0.000298115 22000 0.0001062
22900 0.000588255 20900 1.34E-05 20700 1.78E-04 20300
0.04510111 22500 0.000295569 22000 0.000106307
22900 0.000589261 20900 1.34E-05 20700 1.81E-04 20300
0.04762136 22500 0.000292607 22000 0.000106406
22900 0.000590286 20900 1.34E-05 20700 1.83E-04 20300
0.05026224 22500 0.000289247 22000 0.000106514
22900 0.000591291 20900 1.34E-05 20700 1.86E-04 20300
0.05301861 22600 0.000285512 22000 0.000106604
22900 0.000592303 20900 1.34E-05 20700 1.89E-04 20300
0.05588162 22600 0.00028143 22000 0.000106712
22900 0.000593358 20900 1.34E-05 20700 1.92E-04 20300
0.05883836 22600 0.000277063 22000 0.000106821
22900 0.000594387 20900 1.34E-05 20700 1.95E-04 20300
0.06187079 22600 0.000272427 22000 0.000106928
22900 0.000595423 20900 1.34E-05 20700 1.98E-04 20300
0.06495533 22600 0.000267565 22000 0.000107017
22900 0.0005965 20900 1.35E-05 20700 2.01E-04 20300
0.06806197 22600 0.000262543 22000 0.000107129
22900 0.00059753 20900 1.35E-05 20700 2.04E-04 20300
0.07115373 22600 0.000257398 22000 0.000107231
22900 0.000598598 20900 1.35E-05 20700 2.07E-04 20300
0.07418614 22600 0.000252144 22000 0.000107337
22900 0.000599666 20900 1.35E-05 20700 2.10E-04 20300
0.07710697 22600 0.000246845 22000 0.000107443
22900 0.000600757 20900 1.35E-05 20700 2.14E-04 20300
0.07985666 22600 0.000241523 22000 0.000107565

22900 0.000601817 20900 1.35E-05 20700 2.17E-04 20300
0.08236972 22600 0.000236235 22000 0.00010767
22900 0.000602888 20900 1.35E-05 20700 2.21E-04 20300
0.0845767 22600 0.000230975 22000 0.000107789
22900 0.000603951 20900 1.35E-05 20700 2.25E-04 20300
0.08640715 22600 0.000225793 22000 0.000107903
22900 0.000605036 20900 1.35E-05 20700 2.29E-04 20300
0.08779254 22600 0.000220727 22000 0.000108025
22900 0.000606123 20900 1.36E-05 20700 2.33E-04 20300
0.08867013 22600 0.000215787 22000 0.000108139
22900 0.000607238 20900 1.36E-05 20700 2.37E-04 20300
0.08898639 22600 0.000211012 22000 0.000108249
22900 0.00060832 20900 1.36E-05 20700 2.41E-04 20300
0.0887012 22600 0.000206389 22000 0.000108381
22900 0.000609429 20900 1.36E-05 20700 2.45E-04 20300
0.0877912 22600 0.000201925 22000 0.00010849
22900 0.000610532 20900 1.36E-05 20700 2.50E-04 20300
0.08625244 22600 0.00019764 22000 0.000108602
22900 0.000611634 20900 1.36E-05 20700 2.54E-04 20300
0.08410212 22600 0.000193542 22000 0.00010872
22900 0.000612779 20900 1.36E-05 20700 2.59E-04 20300
0.08137834 22600 0.000189655 22000 0.00010884
22900 0.000613921 20900 1.36E-05 20700 2.64E-04 20300
0.07813862 22600 0.000185936 22000 0.000108964
22900 0.000615044 20900 1.36E-05 20700 2.69E-04 20300
0.07445718 22600 0.000182412 22000 0.0001091
22900 0.000616174 20900 1.37E-05 20700 2.74E-04 20300
0.07042117 22600 0.00017909 22000 0.000109223
22900 0.000617317 20900 1.37E-05 20700 2.79E-04 20300
0.06612622 22600 0.000175933 22000 0.000109331
22900 0.000618478 20900 1.37E-05 20700 2.84E-04 20300
0.06167134 22600 0.000172975 22000 0.000109457
22900 0.00061963 20900 1.37E-05 20700 2.90E-04 20300
0.05715345 22600 0.000170164 22000 0.000109582
22900 0.000620798 20900 1.37E-05 20700 2.96E-04 20300
0.05266232 22600 0.000167572 22000 0.000109685
22900 0.000621959 20900 1.37E-05 20700 3.02E-04 20300
0.04827629 22600 0.000165122 22000 0.000109807
23000 0.000623144 20900 1.37E-05 20700 3.08E-04 20300
0.04405973 22600 0.000162804 22000 0.000109933
23000 0.000624315 20900 1.37E-05 20700 3.14E-04 20300
0.04006212 22600 0.000160636 22000 0.00011004
23000 0.000625473 20900 1.37E-05 20700 3.21E-04 20300
0.03631835 22600 0.00015858 22000 0.00011018
23000 0.000626608 20900 1.37E-05 20700 3.28E-04 20300
0.03285001 22600 0.00015665 22000 0.000110291
23000 0.000627769 20900 1.38E-05 20700 3.35E-04 20300
0.02966724 22600 0.000154877 22000 0.000110422
23000 0.000628988 20900 1.38E-05 20700 3.42E-04 20300
0.02677068 22600 0.000153167 22000 0.000110521
23000 0.000630242 20900 1.38E-05 20700 3.49E-04 20300
0.02415359 22600 0.000151577 22000 0.000110649
23000 0.000631451 20900 1.38E-05 20700 3.57E-04 20300
0.02180352 22600 0.000150063 22000 0.00011078
23000 0.00063266 20900 1.38E-05 20700 3.65E-04 20300
0.01970416 22600 0.000148681 22000 0.000110909
23000 0.000633852 20900 1.38E-05 20700 3.74E-04 20300
0.01783674 22600 0.000147342 22100 0.000111045
23000 0.000635095 20900 1.38E-05 20700 3.82E-04 20300
0.01618128 22600 0.0001461 22100 0.00011119
23000 0.000636348 20900 1.38E-05 20700 3.91E-04 20300
0.01471761 22600 0.000144942 22100 0.00011132
23000 0.00063759 20900 1.38E-05 20700 4.00E-04 20300
0.01342593 22600 0.000143846 22100 0.000111472
23000 0.000638885 20900 1.38E-05 20700 4.10E-04 20300
0.01228748 22600 0.000142834 22100 0.000111589
23000 0.000640162 20900 1.38E-05 20700 4.20E-04 20300
0.01128466 22600 0.000141837 22100 0.000111718

23000 0.000641445 20900 1.38E-05 20700 4.30E-04 20300
0.01040135 22600 0.000140915 22100 0.000111843
23000 0.000642725 20900 1.39E-05 20700 4.41E-04 20300
0.009622907 22600 0.00014005 22100 0.000111984
23000 0.000644016 20900 1.39E-05 20700 4.52E-04 20300
0.00893619 22600 0.000139232 22100 0.000112106
23000 0.000645301 21000 1.39E-05 20700 4.63E-04 20300
0.008329466 22600 0.000138458 22100 0.000112252
23000 0.0006466 21000 1.39E-05 20700 4.75E-04 20300
0.007792426 22600 0.00013773 22100 0.000112393
23000 0.000647886 21000 1.39E-05 20800 4.87E-04 20300
0.007315995 22600 0.000137049 22100 0.000112535
23000 0.000649181 21000 1.39E-05 20800 5.00E-04 20300
0.006892268 22600 0.000136416 22100 0.000112683
23000 0.000650485 21000 1.39E-05 20800 5.14E-04 20300
0.006514356 22600 0.000135792 22100 0.000112822
23000 0.000651831 21000 1.40E-05 20800 5.28E-04 20300
0.006176285 22600 0.000135214 22100 0.000112973
23000 0.000653176 21000 1.40E-05 20800 5.42E-04 20400
0.005872881 22600 0.000134649 22100 0.000113112
23000 0.000654519 21000 1.40E-05 20800 5.57E-04 20400
0.005599681 22600 0.000134105 22100 0.000113249
23000 0.000655873 21000 1.40E-05 20800 5.73E-04 20400
0.005352803 22600 0.000133606 22100 0.000113394
23000 0.000657231 21000 1.40E-05 20800 5.89E-04 20400
0.005128887 22600 0.000133104 22100 0.00011354
23000 0.000658566 21000 1.40E-05 20800 6.06E-04 20400
0.004925032 22600 0.000132636 22100 0.000113685
23000 0.000659927 21000 1.40E-05 20800 6.24E-04 20400
0.004738735 22600 0.000132187 22100 0.00011384
23000 0.000661323 21000 1.40E-05 20800 6.42E-04 20400
0.004567846 22600 0.000131764 22100 0.000113999
23000 0.000662712 21000 1.41E-05 20800 6.61E-04 20400
0.004410495 22600 0.000131349 22100 0.000114151
23000 0.000664108 21000 1.41E-05 20800 6.81E-04 20400
0.004265015 22600 0.000130954 22100 0.000114297
23000 0.000665539 21000 1.41E-05 20800 7.02E-04 20400
0.00413002 22600 0.00013058 22100 0.000114452
23000 0.000666956 21000 1.41E-05 20800 7.24E-04 20400
0.004004267 22600 0.000130229 22100 0.000114589
23000 0.00066836 21000 1.41E-05 20800 7.46E-04 20400
0.003886699 22700 0.000129877 22100 0.00011474
23000 0.000669819 21000 1.41E-05 20800 7.70E-04 20400
0.003776372 22700 0.000129541 22100 0.000114899
23000 0.000671191 21000 1.42E-05 20800 7.95E-04 20400
0.003672515 22700 0.00012921 22100 0.000115052
23000 0.000672652 21000 1.42E-05 20800 8.21E-04 20400
0.003574427 22700 0.000128898 22100 0.000115201
23000 0.00067405 21000 1.42E-05 20800 8.48E-04 20400
0.003481546 22700 0.0001286 22100 0.000115362
23000 0.000675519 21000 1.42E-05 20800 8.76E-04 20400
0.003393383 22700 0.000128327 22100 0.000115532
23000 0.000676952 21000 1.42E-05 20800 9.05E-04 20400
0.003309527 22700 0.000128056 22100 0.000115696
23000 0.000678376 21000 1.42E-05 20800 9.36E-04 20400
0.00322962 22700 0.000127807 22100 0.000115851
23000 0.000679866 21000 1.43E-05 20800 9.68E-04 20400
0.003153385 22700 0.000127557 22100 0.000116015
23000 0.000681356 21000 1.43E-05 20800 1.00E-03 20400
0.003080527 22700 0.000127324 22100 0.000116177
23000 0.000682877 21000 1.43E-05 20800 1.04E-03 20400
0.003010775 22700 0.000127102 22100 0.000116344
23000 0.000684358 21000 1.43E-05 20800 1.07E-03 20400
0.002943916 22700 0.000126875 22100 0.00011652
23000 0.000685843 21000 1.43E-05 20800 1.11E-03 20400
0.002879719 22700 0.000126656 22100 0.000116707
23000 0.000687387 21000 1.43E-05 20800 1.15E-03 20400
0.002817985 22700 0.000126454 22100 0.000116867

23000 0.000688921 21000 1.43E-05 20800 1.19E-03 20400
0.002758574 22700 0.000126268 22100 0.000117062
23000 0.000690437 21000 1.44E-05 20800 1.24E-03 20400
0.002701354 22700 0.000126076 22100 0.000117254
23000 0.000692009 21000 1.44E-05 20800 1.28E-03 20400
0.002646171 22700 0.000125891 22100 0.000117418
23000 0.000693562 21000 1.44E-05 20800 1.33E-03 20400
0.0025929 22700 0.000125726 22100 0.000117597
23000 0.000695151 21000 1.44E-05 20800 1.38E-03 20400
0.002541459 22700 0.000125572 22100 0.000117786
23000 0.000696734 21000 1.45E-05 20800 1.43E-03 20400
0.002491671 22700 0.000125408 22100 0.000117976
23000 0.000698347 21000 1.45E-05 20800 1.49E-03 20400
0.00244351 22700 0.000125264 22100 0.000118171
23000 0.00069991 21000 1.45E-05 20800 1.54E-03 20400
0.002396888 22700 0.000125121 22100 0.000118351
23000 0.000701499 21000 1.46E-05 20800 1.60E-03 20400
0.002351735 22700 0.000124985 22100 0.000118544
23000 0.000703109 21000 1.46E-05 20800 1.66E-03 20400
0.002308022 22700 0.000124857 22100 0.000118735
23000 0.000704733 21000 1.46E-05 20800 1.73E-03 20400
0.002265705 22700 0.000124737 22100 0.00011894
23000 0.000706371 21000 1.46E-05 20800 1.79E-03 20400
0.002224696 22700 0.000124621 22100 0.000119148
23000 0.000708058 21000 1.46E-05 20800 1.86E-03 20400
0.002184946 22700 0.000124519 22100 0.000119346
23000 0.000709708 21000 1.47E-05 20800 1.93E-03 20400
0.002146377 22700 0.000124415 22100 0.000119553
23000 0.000711384 21000 1.47E-05 20800 2.00E-03 20400
0.002108883 22700 0.000124313 22100 0.000119771
23100 0.000713061 21000 1.47E-05 20800 2.08E-03 20400
0.002072393 22700 0.00012422 22100 0.000119993
23100 0.000714761 21000 1.47E-05 20800 2.15E-03 20400
0.002036847 22700 0.000124119 22100 0.000120197
23100 0.000716434 21000 1.48E-05 20800 2.23E-03 20400
0.002002165 22700 0.000124027 22100 0.00012043
23100 0.000718168 21000 1.48E-05 20800 2.31E-03 20400
0.00196833 22700 0.000123944 22100 0.000120653
23100 0.000719884 21000 1.48E-05 20800 2.39E-03 20400
0.001935332 22700 0.000123872 22100 0.0001209
23100 0.000721631 21000 1.49E-05 20800 2.47E-03 20400
0.00190313 22700 0.000123789 22100 0.000121139
23100 0.000723403 21000 1.49E-05 20800 2.55E-03 20400
0.001871699 22700 0.000123712 22100 0.000121358
23100 0.000725168 21000 1.49E-05 20800 2.63E-03 20400
0.001841001 22700 0.000123645 22100 0.000121602
23100 0.000726939 21000 1.49E-05 20800 2.71E-03 20400
0.001811045 22700 0.000123581 22100 0.000121836
23100 0.000728733 21000 1.50E-05 20800 2.79E-03 20400
0.001781761 22700 0.000123516 22200 0.000122085
23100 0.000730513 21000 1.50E-05 20800 2.86E-03 20400
0.001753176 22700 0.00012346 22200 0.000122331
23100 0.000732274 21000 1.50E-05 20800 2.94E-03 20400
0.001725264 22700 0.000123406 22200 0.000122578
23100 0.00073408 21000 1.51E-05 20800 3.00E-03 20400
0.001698022 22700 0.000123348 22200 0.000122833
23100 0.000735872 21000 1.51E-05 20800 3.07E-03 20400
0.001671463 22700 0.000123297 22200 0.000123091
23100 0.000737694 21000 1.51E-05 20800 3.13E-03 20400
0.001645598 22700 0.000123251 22200 0.000123347
23100 0.000739517 21000 1.52E-05 20800 3.18E-03 20400
0.001620395 22700 0.000123211 22200 0.000123614
23100 0.000741343 21000 1.52E-05 20800 3.22E-03 20400
0.001595796 22700 0.000123181 22200 0.000123881
23100 0.000743189 21000 1.53E-05 20800 3.26E-03 20400
0.001571831 22700 0.000123154 22200 0.000124134
23100 0.000745053 21100 1.53E-05 20800 3.28E-03 20400
0.001548412 22700 0.000123118 22200 0.00012441

23100 0.000746913 21100 1.53E-05 20800 3.30E-03 20400
0.001525531 22700 0.000123102 22200 0.000124678
23100 0.000748753 21100 1.54E-05 20900 3.30E-03 20400
0.001503164 22700 0.000123065 22200 0.000124978
23100 0.000750656 21100 1.54E-05 20900 3.30E-03 20400
0.001481305 22700 0.000123043 22200 0.000125274
23100 0.000752611 21100 1.54E-05 20900 3.28E-03 20400
0.001459964 22700 0.000123033 22200 0.000125553
23100 0.000754529 21100 1.55E-05 20900 3.26E-03 20400
0.001439105 22700 0.000123017 22200 0.000125855
23100 0.000756499 21100 1.55E-05 20900 3.22E-03 20500
0.001418761 22700 0.000122994 22200 0.000126166
23100 0.000758424 21100 1.56E-05 20900 3.17E-03 20500
0.001398857 22700 0.000122978 22200 0.000126461
23100 0.000760342 21100 1.56E-05 20900 3.12E-03 20500
0.001379384 22700 0.000122974 22200 0.000126768
23100 0.00076235 21100 1.57E-05 20900 3.05E-03 20500
0.001360336 22700 0.000122973 22200 0.000127064
23100 0.000764294 21100 1.57E-05 20900 2.98E-03 20500
0.001341724 22700 0.000122962 22200 0.000127382
23100 0.000766315 21100 1.57E-05 20900 2.90E-03 20500
0.00132353 22700 0.000122961 22200 0.000127717
23100 0.000768312 21100 1.58E-05 20900 2.81E-03 20500
0.001305734 22700 0.000122954 22200 0.000128037
23100 0.000770322 21100 1.58E-05 20900 2.72E-03 20500
0.001288323 22700 0.00012295 22200 0.000128383
23100 0.000772337 21100 1.59E-05 20900 2.62E-03 20500
0.001271306 22700 0.000122947 22200 0.000128712
23100 0.000774405 21100 1.59E-05 20900 2.53E-03 20500
0.001254656 22700 0.000122947 22200 0.000129069
23100 0.000776429 21100 1.60E-05 20900 2.43E-03 20500
0.001238355 22700 0.000122955 22200 0.000129434
23100 0.00077846 21100 1.61E-05 20900 2.33E-03 20500
0.001222388 22800 0.000122973 22200 0.000129787
23100 0.000780563 21100 1.61E-05 20900 2.23E-03 20500
0.001206811 22800 0.000122983 22200 0.000130152
23100 0.000782706 21100 1.62E-05 20900 2.13E-03 20500
0.00119157 22800 0.000122998 22200 0.000130521
23100 0.000784803 21100 1.62E-05 20900 2.04E-03 20500
0.001176686 22800 0.000123009 22200 0.000130888
23100 0.000786916 21100 1.63E-05 20900 1.95E-03 20500
0.001162169 22800 0.000123014 22200 0.000131276
23100 0.000789023 21100 1.64E-05 20900 1.86E-03 20500
0.00114795 22800 0.000123023 22200 0.000131656
23100 0.000791177 21100 1.65E-05 20900 1.77E-03 20500
0.001134053 22800 0.000123035 22200 0.000132053
23100 0.000793349 21100 1.65E-05 20900 1.69E-03 20500
0.001120468 22800 0.000123053 22200 0.000132471
23100 0.000795582 21100 1.66E-05 20900 1.61E-03 20500
0.001107164 22800 0.000123073 22200 0.000132886
23100 0.000797809 21100 1.67E-05 20900 1.54E-03 20500
0.001094161 22800 0.000123096 22200 0.000133309
23100 0.000800049 21100 1.68E-05 20900 1.46E-03 20500
0.001081392 22800 0.000123116 22200 0.00013373
23100 0.000802319 21100 1.69E-05 20900 1.40E-03 20500
0.001068895 22800 0.000123139 22200 0.000134177
23100 0.000804553 21100 1.70E-05 20900 1.33E-03 20500
0.001056662 22800 0.00012316 22200 0.000134629
23100 0.000806824 21100 1.71E-05 20900 1.27E-03 20500
0.001044676 22800 0.000123188 22200 0.000135074
23100 0.000809164 21100 1.72E-05 20900 1.22E-03 20500
0.00103294 22800 0.000123209 22200 0.000135542
23100 0.000811503 21100 1.73E-05 20900 1.16E-03 20500
0.001021432 22800 0.000123227 22200 0.000136026
23100 0.000813811 21100 1.74E-05 20900 1.11E-03 20500
0.001010163 22800 0.000123253 22200 0.000136523
23100 0.000816218 21100 1.75E-05 20900 1.07E-03 20500
0.000999137 22800 0.000123283 22200 0.000137017

23100 0.000818605 21100 1.77E-05 20900 1.02E-03 20500
0.000988353 22800 0.000123313 22200 0.000137528
23100 0.000821026 21100 1.78E-05 20900 9.79E-04 20500
0.000977768 22800 0.000123348 22200 0.000138046
23100 0.000823478 21100 1.79E-05 20900 9.40E-04 20500
0.000967454 22800 0.00012339 22200 0.000138561
23100 0.000825911 21100 1.80E-05 20900 9.02E-04 20500
0.000957405 22800 0.000123429 22200 0.000139101
23100 0.000828399 21100 1.82E-05 20900 8.68E-04 20500
0.000947588 22800 0.000123466 22200 0.000139657
23100 0.000830908 21100 1.83E-05 20900 8.35E-04 20500
0.000938042 22800 0.000123494 22200 0.000140209
23100 0.000833424 21100 1.85E-05 20900 8.04E-04 20500
0.000928724 22800 0.000123524 22200 0.000140783
23100 0.000835935 21100 1.86E-05 20900 7.74E-04 20500
0.000919629 22800 0.000123567 22200 0.000141359
23100 0.000838539 21100 1.88E-05 20900 7.47E-04 20500
0.000910756 22800 0.000123607 22200 0.00014197
23100 0.000841066 21100 1.90E-05 20900 7.20E-04 20500
0.00090211 22800 0.000123655 22200 0.000142564
23100 0.000843652 21100 1.92E-05 20900 6.96E-04 20500
0.000893668 22800 0.000123691 22200 0.000143194
23200 0.00084633 21100 1.94E-05 20900 6.72E-04 20500
0.000885417 22800 0.000123737 22200 0.000143851
23200 0.000848966 21100 1.96E-05 20900 6.50E-04 20500
0.000877389 22800 0.000123792 22200 0.000144485
23200 0.000851709 21100 1.98E-05 20900 6.29E-04 20500
0.000869563 22800 0.000123835 22200 0.000145162
23200 0.000854456 21100 2.00E-05 20900 6.09E-04 20500
0.000861963 22800 0.000123887 22200 0.000145828
23200 0.000857129 21100 2.02E-05 20900 5.90E-04 20500
0.000854558 22800 0.000123932 22200 0.000146515
23200 0.000859933 21100 2.05E-05 20900 5.72E-04 20500
0.000847369 22800 0.000123974 22200 0.000147218
23200 0.00086271 21100 2.08E-05 20900 5.55E-04 20500
0.000840404 22800 0.000124014 22200 0.000147943
23200 0.00086556 21100 2.10E-05 20900 5.39E-04 20500
0.000833658 22800 0.000124066 22200 0.000148684
23200 0.000868378 21100 2.13E-05 20900 5.23E-04 20500
0.000827137 22800 0.000124118 22200 0.000149453
23200 0.000871242 21100 2.17E-05 20900 5.08E-04 20500
0.000820842 22800 0.000124166 22300 0.00015022
23200 0.000874095 21100 2.20E-05 20900 4.94E-04 20500
0.000814815 22800 0.000124217 22300 0.000151001
23200 0.000877062 21100 2.24E-05 20900 4.80E-04 20500
0.000808997 22800 0.000124279 22300 0.000151823
23200 0.000879971 21100 2.27E-05 20900 4.67E-04 20500
0.000803429 22800 0.000124341 22300 0.00015266
23200 0.000882956 21100 2.31E-05 20900 4.55E-04 20500
0.0007981 22800 0.000124386 22300 0.000153511
23200 0.000885915 21100 2.36E-05 20900 4.43E-04 20500
0.000792974 22800 0.000124432 22300 0.000154384
23200 0.000888954 21100 2.40E-05 20900 4.32E-04 20500
0.000788059 22800 0.000124481 22300 0.000155284
23200 0.000892054 21100 2.45E-05 20900 4.21E-04 20500
0.000783357 22800 0.000124551 22300 0.00015622
23200 0.000895151 21100 2.51E-05 20900 4.10E-04 20500
0.000778896 22800 0.000124609 22300 0.000157152
23200 0.000898308 21200 2.56E-05 20900 4.00E-04 20500
0.000774663 22800 0.000124662 22300 0.000158097
23200 0.000901472 21200 2.62E-05 20900 3.91E-04 20500
0.000770641 22800 0.000124728 22300 0.000159098
23200 0.000904631 21200 2.69E-05 21000 3.81E-04 20500
0.000766876 22800 0.000124793 22300 0.00016011
23200 0.000907815 21200 2.76E-05 21000 3.72E-04 20500
0.000763378 22800 0.000124854 22300 0.000161161
23200 0.000911094 21200 2.83E-05 21000 3.64E-04 20500
0.000760178 22800 0.000124921 22300 0.000162228

23200 0.000914419	21200 2.92E-05	21000 3.56E-04	20500
0.00075728 22800	0.000124982 22300	0.000163322	
23200 0.000917671	21200 3.00E-05	21000 3.48E-04	20600
0.000754718 22800	0.000125043 22300	0.00016445	
23200 0.00092105	21200 3.10E-05	21000 3.40E-04	20600
0.000752533 22800	0.000125101 22300	0.000165614	
23200 0.000924409	21200 3.20E-05	21000 3.33E-04	20600
0.000750707 22800	0.000125161 22300	0.000166812	
23200 0.000927819	21200 3.32E-05	21000 3.25E-04	20600
0.00074925 22800	0.000125231 22300	0.000168046	
23200 0.000931287	21200 3.44E-05	21000 3.19E-04	20600
0.000748185 22800	0.000125301 22300	0.000169291	
23200 0.00093477	21200 3.58E-05	21000 3.12E-04	20600
0.000747522 22800	0.000125365 22300	0.000170583	
23200 0.000938319	21200 3.73E-05	21000 3.05E-04	20600
0.000747263 22800	0.000125445 22300	0.000171895	
23200 0.000941906	21200 3.89E-05	21000 2.99E-04	20600
0.000747438 22800	0.000125522 22300	0.000173272	
23200 0.000945471	21200 4.07E-05	21000 2.93E-04	20600
0.000748093 22800	0.000125577 22300	0.000174679	
23200 0.000949139	21200 4.27E-05	21000 2.87E-04	20600
0.000749262 22800	0.000125638 22300	0.000176122	
23200 0.000952872	21200 4.48E-05	21000 2.82E-04	20600
0.000750999 22800	0.000125707 22300	0.000177623	
23200 0.000956605	21200 4.72E-05	21000 2.76E-04	20600
0.000753324 22900	0.000125781 22300	0.000179165	
23200 0.000960361	21200 4.99E-05	21000 2.71E-04	20600
0.000756277 22900	0.000125845 22300	0.000180721	
23200 0.000964211	21200 5.28E-05	21000 2.66E-04	20600
0.000759882 22900	0.000125914 22300	0.000182336	
23200 0.000968097	21200 5.61E-05	21000 2.61E-04	20600
0.000764165 22900	0.000125984 22300	0.000184018	
23200 0.000972003	21200 5.97E-05	21000 2.56E-04	20600
0.000769152 22900	0.000126059 22300	0.000185724	
23200 0.000975972	21200 6.37E-05	21000 2.52E-04	20600
0.000774901 22900	0.000126133 22300	0.000187502	
23200 0.000979948	21200 6.82E-05	21000 2.47E-04	20600
0.000781437 22900	0.000126207 22300	0.000189328	
23200 0.000983915	21200 7.31E-05	21000 2.43E-04	20600
0.000788827 22900	0.000126272 22300	0.000191207	
23200 0.000988016	21200 7.86E-05	21000 2.39E-04	20600
0.000797149 22900	0.000126338 22300	0.000193149	
23200 0.000992047	21200 8.45E-05	21000 2.34E-04	20600
0.000806477 22900	0.000126399 22300	0.000195152	
23200 0.000996248	21200 9.11E-05	21000 2.30E-04	20600
0.000816901 22900	0.000126474 22300	0.00019721	
23200 0.00100042	21200 9.81E-05	21000 2.27E-04	20600
0.000828547 22900	0.000126547 22300	0.000199351	
23200 0.001004654	21200 0.000105584	21000 2.23E-04	20600
0.000841524 22900	0.000126628 22300	0.000201558	
23200 0.001008854	21200 0.000113426	21000 2.19E-04	20600
0.000855965 22900	0.000126699 22300	0.000203801	
23200 0.001013232	21200 0.000121419	21000 2.16E-04	20600
0.000871948 22900	0.000126781 22300	0.00020614	
23200 0.001017575	21200 0.000129356	21000 2.12E-04	20600
0.000889617 22900	0.000126866 22300	0.000208572	
23200 0.001021991	21200 0.000136887	21000 2.09E-04	20600
0.000909071 22900	0.000126933 22300	0.000211066	
23200 0.00102637	21200 0.000143673	21000 2.05E-04	20600
0.000930415 22900	0.000127004 22300	0.000213647	
23200 0.00103093	21200 0.000149307	21000 2.02E-04	20600
0.000953788 22900	0.000127087 22300	0.000216323	
23200 0.001035438	21200 0.000153433	21000 1.99E-04	20600
0.00097934 22900	0.000127171 22300	0.000219073	
23200 0.001040104	21200 0.000155796	21000 1.96E-04	20600
0.001007265 22900	0.00012725 22300	0.00022192	
23200 0.001044762	21200 0.000156205	21000 1.93E-04	20600
0.001037723 22900	0.000127332 22300	0.000224852	

23200 0.001049496 21200 0.000154656 21000 0.00019 20600
0.0010709 22900 0.000127425 22300 0.000227892
23200 0.001054258 21200 0.000151277 21000 0.000188 20600
0.001106999 22900 0.000127505 22300 0.000231019
23200 0.001059063 21200 0.000146323 21000 0.000185 20600
0.00114618 22900 0.000127591 22300 0.000234252
23200 0.001063946 21200 0.000140126 21000 0.000182 20600
0.001188564 22900 0.000127675 22300 0.000237596
23200 0.001068832 21200 0.000133037 21000 0.00018 20600
0.001234162 22900 0.000127752 22300 0.000241046
23200 0.001073819 21200 0.000125433 21000 0.000177 20600
0.001283014 22900 0.000127831 22300 0.000244608
23200 0.001078857 21200 0.000117629 21000 0.000175 20600
0.00133501 22900 0.000127906 22300 0.000248305
23300 0.001083989 21200 0.000109843 21000 0.000172 20600
0.001389913 22900 0.000127985 22300 0.000252102
23300 0.001089154 21200 0.000102319 21000 0.00017 20600
0.001447364 22900 0.000128075 22300 0.000256042
23300 0.001094387 21200 9.52E-05 21000 0.000168 20600
0.00150684 22900 0.000128156 22300 0.000260094
23300 0.001099694 21200 8.85E-05 21000 0.000165 20600
0.001567689 22900 0.000128225 22300 0.000264266
23300 0.00110505 21200 8.24E-05 21000 0.000163 20600
0.001629016 22900 0.00012831 22300 0.000268589
23300 0.001110512 21200 7.67E-05 21000 0.000161 20600
0.001689713 22900 0.000128392 22300 0.000273055
23300 0.001115971 21200 7.16E-05 21000 0.000159 20600
0.001748544 22900 0.000128459 22300 0.000277659
23300 0.00112152 21200 6.70E-05 21000 0.000157 20600
0.001803992 22900 0.00012853 22300 0.000282393
23300 0.001127178 21200 6.27E-05 21000 0.000155 20600
0.001854377 22900 0.000128611 22300 0.000287288
23300 0.001132929 21200 5.90E-05 21000 0.000153 20600
0.001897918 22900 0.000128698 22400 0.000292311
23300 0.001138714 21200 5.55E-05 21000 0.000151 20600
0.001932716 22900 0.000128778 22400 0.000297485
23300 0.001144531 21200 5.25E-05 21000 0.000149 20600
0.001956951 22900 0.000128861 22400 0.000302817
23300 0.001150453 21200 4.97E-05 21000 1.48E-04 20600
0.001968973 22900 0.000128944 22400 0.000308276
23300 0.001156497 21200 4.72E-05 21000 1.46E-04 20600
0.001967516 22900 0.000129024 22400 0.000313898
23300 0.001162571 21200 4.49E-05 21000 1.44E-04 20600
0.001951806 22900 0.000129111 22400 0.000319665
23300 0.001168739 21200 4.28E-05 21000 1.42E-04 20600
0.001921659 22900 0.000129191 22400 0.00032556
23300 0.001174979 21200 4.10E-05 21000 1.41E-04 20600
0.001877535 22900 0.000129274 22400 0.000331608
23300 0.001181332 21200 3.92E-05 21000 1.39E-04 20600
0.001820529 22900 0.000129352 22400 0.000337789
23300 0.001187718 21300 3.77E-05 21000 1.38E-04 20600
0.00175229 22900 0.000129432 22400 0.000344109
23300 0.001194219 21300 3.63E-05 21000 1.36E-04 20600
0.001674875 22900 0.000129517 22400 0.000350544
23300 0.00120079 21300 3.50E-05 21100 1.35E-04 20600
0.001590607 22900 0.000129601 22400 0.000357094
23300 0.00120742 21300 3.38E-05 21100 1.33E-04 20600
0.001501952 22900 0.000129696 22400 0.000363732
23300 0.001214174 21300 3.27E-05 21100 1.32E-04 20600
0.00141131 22900 0.000129774 22400 0.000370457
23300 0.001221025 21300 3.17E-05 21100 1.30E-04 20600
0.001320854 22900 0.00012987 22400 0.000377269
23300 0.001227972 21300 3.07E-05 21100 1.29E-04 20700
0.001232546 22900 0.000129973 22400 0.000384141
23300 0.001234953 21300 2.99E-05 21100 1.27E-04 20700
0.001147967 22900 0.000130055 22400 0.000391057
23300 0.001242015 21300 2.91E-05 21100 1.26E-04 20700
0.001068235 22900 0.000130134 22400 0.000397982

23300 0.001249188 21300 2.84E-05 21100 1.25E-04 20700
0.000994197 22900 0.000130229 22400 0.000404909
23300 0.001256523 21300 2.77E-05 21100 1.24E-04 20700
0.000926323 22900 0.000130327 22400 0.000411778
23300 0.001263914 21300 2.71E-05 21100 1.22E-04 20700
0.00086477 22900 0.00013041 22400 0.000418608
23300 0.00127141 21300 2.65E-05 21100 1.21E-04 20700
0.000809449 22900 0.000130505 22400 0.000425318
23300 0.00127901 21300 2.59E-05 21100 1.20E-04 20700
0.000760104 22900 0.000130603 22400 0.000431903
23300 0.001286697 21300 2.54E-05 21100 1.19E-04 20700
0.000716351 22900 0.000130699 22400 0.00043832
23300 0.001294491 21300 2.49E-05 21100 1.18E-04 20700
0.000677735 22900 0.000130796 22400 0.000444519
23300 0.001302424 21300 2.45E-05 21100 1.16E-04 20700
0.000643755 22900 0.000130894 22400 0.000450474
23300 0.001310386 21300 2.41E-05 21100 1.15E-04 20700
0.000613873 23000 0.000130992 22400 0.000456143
23300 0.001318504 21300 2.37E-05 21100 1.14E-04 20700
0.000587629 23000 0.000131082 22400 0.000461466
23300 0.001326738 21300 2.33E-05 21100 1.13E-04 20700
0.000564545 23000 0.000131179 22400 0.000466425
23300 0.001335045 21300 2.30E-05 21100 1.12E-04 20700
0.000544184 23000 0.000131278 22400 0.000470955
23300 0.001343424 21300 2.27E-05 21100 1.11E-04 20700
0.000526165 23000 0.000131376 22400 0.000475018
23300 0.001351989 21300 2.24E-05 21100 1.10E-04 20700
0.000510174 23000 0.000131485 22400 0.000478557
23300 0.00136065 21300 2.21E-05 21100 1.09E-04 20700
0.00049592 23000 0.000131578 22400 0.000481556
23300 0.001369451 21300 2.18E-05 21100 1.08E-04 20700
0.000483159 23000 0.00013168 22400 0.00048397
23300 0.001378288 21300 2.15E-05 21100 1.07E-04 20700
0.000471691 23000 0.000131793 22400 0.000485766
23300 0.001387263 21300 2.13E-05 21100 1.06E-04 20700
0.000461335 23000 0.000131878 22400 0.00048692
23300 0.001396363 21300 2.11E-05 21100 1.05E-04 20700
0.000451977 23000 0.000131976 22400 0.000487414
23300 0.001405648 21300 2.09E-05 21100 1.04E-04 20700
0.00044343 23000 0.000132079 22400 0.00048723
23300 0.001415034 21300 2.07E-05 21100 1.04E-04 20700
0.000435619 23000 0.000132186 22400 0.000486352
23300 0.001424505 21300 2.05E-05 21100 1.03E-04 20700
0.000428435 23000 0.000132283 22400 0.000484781
23300 0.00143411 21300 2.03E-05 21100 1.02E-04 20700
0.00042181 23000 0.00013239 22400 0.000482534
23300 0.001443847 21300 2.01E-05 21100 1.01E-04 20700
0.000415667 23000 0.000132496 22400 0.000479617
23300 0.001453716 21300 1.99E-05 21100 1.00E-04 20700
0.000409947 23000 0.000132591 22400 0.000476026
23300 0.001463716 21300 1.98E-05 21100 9.94E-05 20700
0.000404598 23000 0.000132695 22400 0.000471834
23300 0.001473842 21300 1.96E-05 21100 9.86E-05 20700
0.000399499 23000 0.000132801 22400 0.000467034
23300 0.001484089 21300 1.95E-05 21100 9.78E-05 20700
0.000394697 23000 0.000132899 22400 0.000461696
23300 0.001494465 21300 1.94E-05 21100 9.70E-05 20700
0.000390102 23000 0.000133005 22400 0.000455842
23300 0.001504966 21300 1.93E-05 21100 9.63E-05 20700
0.000385689 23000 0.000133108 22400 0.000449531
23300 0.001515603 21300 1.91E-05 21100 9.55E-05 20700
0.000381406 23000 0.000133207 22400 0.000442815
23300 0.001526399 21300 1.90E-05 21100 9.48E-05 20700
0.00037725 23000 0.000133306 22400 0.000435745
23300 0.001537292 21300 1.89E-05 21100 9.41E-05 20700
0.000373226 23000 0.000133422 22400 0.000428352
23300 0.001548303 21300 1.88E-05 21100 9.34E-05 20700
0.00036932 23000 0.00013352 22400 0.000420691

23300 0.001559504 21300 1.87E-05 21100 9.27E-05 20700
0.000365505 23000 0.000133635 22400 0.000412848
23300 0.001570848 21300 1.86E-05 21100 9.20E-05 20700
0.000361786 23000 0.000133741 22400 0.000404856
23300 0.001582238 21300 1.85E-05 21100 9.13E-05 20700
0.000358205 23000 0.000133837 22400 0.000396764
23400 0.001593794 21300 1.84E-05 21100 9.07E-05 20700
0.000354752 23000 0.00013394 22400 0.000388623
23400 0.001605544 21300 1.83E-05 21100 9.00E-05 20700
0.000351408 23000 0.00013404 22400 0.000380461
23400 0.001617411 21300 1.82E-05 21100 8.94E-05 20700
0.000348189 23000 0.000134148 22400 0.000372338
23400 0.001629382 21300 1.81E-05 21100 8.88E-05 20700
0.000345071 23000 0.000134251 22400 0.000364267
23400 0.001641528 21300 1.81E-05 21100 8.82E-05 20700
0.000342086 23000 0.00013435 22400 0.000356312
23400 0.001653771 21300 1.80E-05 21100 8.76E-05 20700
0.000339156 23000 0.000134454 22400 0.000348473
23400 0.001666171 21300 1.79E-05 21100 8.70E-05 20700
0.000336326 23000 0.000134556 22400 0.000340781
23400 0.001678687 21300 1.78E-05 21100 8.64E-05 20700
0.000333571 23000 0.000134657 22400 0.000333261
23400 0.001691338 21300 1.78E-05 21100 8.58E-05 20700
0.000330888 23000 0.000134758 22400 0.00032594
23400 0.001704135 21300 1.77E-05 21100 8.52E-05 20700
0.000328235 23000 0.000134857 22500 0.000318812
23400 0.001717001 21300 1.77E-05 21100 8.47E-05 20700
0.000325628 23000 0.000134958 22500 0.000311907
23400 0.001729993 21300 1.76E-05 21100 8.42E-05 20700
0.000323069 23000 0.00013506 22500 0.000305228
23400 0.001743112 21300 1.76E-05 21100 8.36E-05 20700
0.00032055 23000 0.000135159 22500 0.000298781
23400 0.001756335 21300 1.75E-05 21100 8.31E-05 20700
0.000318068 23000 0.000135257 22500 0.000292574
23400 0.001769714 21300 1.75E-05 21100 8.26E-05 20700
0.000315617 23000 0.00013537 22500 0.000286582
23400 0.001783188 21300 1.74E-05 21100 8.20E-05 20700
0.000313209 23000 0.000135459 22500 0.000280811
23400 0.001796773 21300 1.74E-05 21100 8.15E-05 20700
0.000310821 23000 0.000135572 22500 0.000275308
23400 0.00181048 21300 1.73E-05 21100 8.10E-05 20700
0.00030848 23000 0.000135674 22500 0.000270012
23400 0.001824272 21400 1.73E-05 21100 8.06E-05 20700
0.000306181 23000 0.000135765 22500 0.000264946
23400 0.001838145 21400 1.73E-05 21100 8.01E-05 20700
0.00030394 23000 0.000135866 22500 0.000260104
23400 0.001852134 21400 1.72E-05 21200 7.96E-05 20700
0.000301716 23000 0.000135964 22500 0.000255466
23400 0.001866201 21400 1.72E-05 21200 7.91E-05 20700
0.000299548 23000 0.000136068 22500 0.000251044
23400 0.001880351 21400 1.71E-05 21200 7.87E-05 20700
0.000297405 23000 0.000136166 22500 0.000246793
23400 0.00189459 21400 1.71E-05 21200 7.82E-05 20700
0.000295295 23000 0.000136267 22500 0.000242767
23400 0.001908896 21400 1.71E-05 21200 7.77E-05 20800
0.0002932 23000 0.00013637 22500 0.000238896
23400 0.001923262 21400 1.70E-05 21200 7.73E-05 20800
0.00029113 23000 0.000136472 22500 0.0002352
23400 0.001937677 21400 1.70E-05 21200 7.69E-05 20800
0.000289084 23000 0.000136573 22500 0.00023167
23400 0.001952136 21400 1.70E-05 21200 7.64E-05 20800
0.00028708 23000 0.000136672 22500 0.000228316
23400 0.001966602 21400 1.69E-05 21200 7.60E-05 20800
0.000285112 23000 0.000136778 22500 0.000225085
23400 0.001981128 21400 1.69E-05 21200 7.56E-05 20800
0.00028318 23000 0.000136882 22500 0.000222021
23400 0.001995657 21400 1.69E-05 21200 7.52E-05 20800
0.000281294 23000 0.000136982 22500 0.00021908

23400 0.00201017 21400 1.69E-05 21200 7.48E-05 20800
0.00027946 23000 0.000137086 22500 0.000216285
23400 0.002024724 21400 1.69E-05 21200 7.44E-05 20800
0.00027764 23000 0.000137192 22500 0.000213618
23400 0.00203926 21400 1.68E-05 21200 7.40E-05 20800
0.00027584 23000 0.000137307 22500 0.000211042
23400 0.00205378 21400 1.68E-05 21200 7.36E-05 20800
0.000274092 23000 0.000137404 22500 0.00020859
23400 0.00206826 21400 1.68E-05 21200 7.32E-05 20800
0.00027235 23100 0.000137513 22500 0.000206251
23400 0.002082698 21400 1.68E-05 21200 7.29E-05 20800
0.000270644 23100 0.000137632 22500 0.00020401
23400 0.002097067 21400 1.67E-05 21200 7.25E-05 20800
0.000268973 23100 0.000137723 22500 0.000201866
23400 0.002111349 21400 1.67E-05 21200 7.21E-05 20800
0.000267339 23100 0.000137829 22500 0.000199811
23400 0.002125587 21400 1.67E-05 21200 7.18E-05 20800
0.000265703 23100 0.000137934 22500 0.000197849
23400 0.002139689 21400 1.67E-05 21200 7.14E-05 20800
0.000264123 23100 0.000138038 22500 0.00019596
23400 0.00215368 21400 1.67E-05 21200 7.11E-05 20800
0.00026256 23100 0.000138139 22500 0.000194147
23400 0.002167548 21400 1.66E-05 21200 7.08E-05 20800
0.000261018 23100 0.00013824 22500 0.000192415
23400 0.002181254 21400 1.66E-05 21200 7.04E-05 20800
0.000259488 23100 0.000138345 22500 0.000190742
23400 0.002194787 21400 1.66E-05 21200 7.01E-05 20800
0.000257962 23100 0.000138454 22500 0.000189138
23400 0.002208132 21400 1.66E-05 21200 6.97E-05 20800
0.000256448 23100 0.00013855 22500 0.00018759
23400 0.002221257 21400 1.66E-05 21200 6.94E-05 20800
0.000254946 23100 0.000138653 22500 0.000186107
23400 0.002234159 21400 1.65E-05 21200 6.91E-05 20800
0.000253462 23100 0.00013876 22500 0.000184672
23400 0.00224683 21400 1.65E-05 21200 6.88E-05 20800
0.000251967 23100 0.000138859 22500 0.000183304
23400 0.002259194 21400 1.65E-05 21200 6.85E-05 20800
0.000250478 23100 0.000138964 22500 0.000181975
23400 0.002271318 21400 1.65E-05 21200 6.82E-05 20800
0.000249025 23100 0.000139056 22500 0.000180705
23400 0.002283089 21400 1.65E-05 21200 6.79E-05 20800
0.000247554 23100 0.00013917 22500 0.000179489
23400 0.002294564 21400 1.65E-05 21200 6.76E-05 20800
0.0002461 23100 0.000139277 22500 0.000178308
23400 0.002305692 21400 1.65E-05 21200 6.73E-05 20800
0.000244682 23100 0.000139383 22500 0.000177162
23400 0.002316457 21400 1.65E-05 21200 6.70E-05 20800
0.000243249 23100 0.000139491 22500 0.000176063
23400 0.002326805 21400 1.64E-05 21200 6.67E-05 20800
0.000241839 23100 0.0001396 22500 0.000174996
23400 0.002336732 21400 1.64E-05 21200 6.64E-05 20800
0.000240467 23100 0.000139693 22500 0.000173973
23400 0.002346196 21400 1.64E-05 21200 6.61E-05 20800
0.000239112 23100 0.000139799 22500 0.000172979
23400 0.002355242 21400 1.64E-05 21200 6.58E-05 20800
0.000237789 23100 0.000139899 22500 0.000172019
23400 0.002363807 21400 1.64E-05 21200 6.56E-05 20800
0.000236484 23100 0.00014001 22500 0.000171095
23400 0.002371874 21400 1.64E-05 21200 6.53E-05 20800
0.000235216 23100 0.000140114 22500 0.000170194
23400 0.002379381 21400 1.64E-05 21200 6.50E-05 20800
0.000233971 23100 0.000140219 22500 0.000169345
23400 0.002386342 21400 1.64E-05 21200 6.48E-05 20800
0.000232722 23100 0.000140322 22500 0.000168499
23400 0.002392755 21400 1.64E-05 21200 6.45E-05 20800
0.000231492 23100 0.000140439 22500 0.000167698
23500 0.002398559 21400 1.64E-05 21200 6.42E-05 20800
0.000230283 23100 0.000140537 22500 0.000166907

23500 0.002403773 21400 1.64E-05 21200 6.40E-05 20800
0.000229089 23100 0.000140648 22500 0.000166146
23500 0.002408326 21400 1.64E-05 21200 6.37E-05 20800
0.000227892 23100 0.000140758 22500 0.000165414
23500 0.002412216 21400 1.64E-05 21200 6.35E-05 20800
0.000226691 23100 0.00014086 22500 0.000164709
23500 0.002415462 21400 1.64E-05 21200 6.32E-05 20800
0.000225536 23100 0.000140973 22500 0.000164024
23500 0.002418015 21400 1.64E-05 21200 6.30E-05 20800
0.000224406 23100 0.000141071 22500 0.000163365
23500 0.002419847 21400 1.64E-05 21200 6.27E-05 20800
0.000223285 23100 0.000141176 22500 0.000162733
23500 0.002421018 21400 1.64E-05 21200 6.25E-05 20800
0.000222187 23100 0.000141286 22500 0.000162102
23500 0.002421427 21400 1.64E-05 21200 6.23E-05 20800
0.000221104 23100 0.000141402 22500 0.000161477
23500 0.002421091 21400 1.64E-05 21200 6.20E-05 20800
0.000220028 23100 0.000141532 22600 0.000160879
23500 0.002420011 21400 1.64E-05 21200 6.18E-05 20800
0.000218957 23100 0.000141635 22600 0.000160311
23500 0.002418144 21400 1.64E-05 21200 6.16E-05 20800
0.0002179 23100 0.000141742 22600 0.000159749
23500 0.002415518 21400 1.64E-05 21200 6.14E-05 20800
0.000216844 23100 0.000141849 22600 0.000159222
23500 0.002412122 21400 1.64E-05 21200 6.12E-05 20800
0.000215771 23100 0.000141974 22600 0.000158688
23500 0.002407952 21400 1.63E-05 21200 6.09E-05 20800
0.000214709 23100 0.00014209 22600 0.000158179
23500 0.002402974 21400 1.64E-05 21200 6.07E-05 20800
0.000213674 23100 0.000142194 22600 0.00015769
23500 0.00239725 21400 1.64E-05 21200 6.05E-05 20800
0.000212632 23100 0.000142308 22600 0.000157221
23500 0.002390713 21400 1.63E-05 21200 6.03E-05 20800
0.000211601 23100 0.000142443 22600 0.000156754
23500 0.002383413 21500 1.63E-05 21200 6.01E-05 20800
0.000210585 23100 0.000142553 22600 0.000156301
23500 0.002375356 21500 1.63E-05 21200 5.99E-05 20800
0.000209593 23100 0.000142661 22600 0.000155865
23500 0.002366545 21500 1.63E-05 21300 5.97E-05 20800
0.000208619 23100 0.000142775 22600 0.000155422
23500 0.00235693 21500 1.63E-05 21300 5.95E-05 20800
0.000207655 23100 0.000142903 22600 0.000155007
23500 0.002346615 21500 1.63E-05 21300 5.93E-05 20800
0.00020669 23100 0.000143035 22600 0.000154618
23500 0.002335527 21500 1.63E-05 21300 5.91E-05 20800
0.000205755 23100 0.000143144 22600 0.000154223
23500 0.002323732 21500 1.63E-05 21300 5.89E-05 20900
0.000204827 23100 0.000143258 22600 0.000153858
23500 0.002311247 21500 1.63E-05 21300 5.87E-05 20900
0.00020389 23100 0.000143373 22600 0.000153491
23500 0.0022981 21500 1.63E-05 21300 5.85E-05 20900
0.00020297 23100 0.000143498 22600 0.000153124
23500 0.002284283 21500 1.64E-05 21300 5.84E-05 20900
0.000202065 23100 0.000143611 22600 0.000152764
23500 0.002269873 21500 1.64E-05 21300 5.82E-05 20900
0.000201139 23100 0.00014374 22600 0.000152422
23500 0.002254804 21500 1.64E-05 21300 5.80E-05 20900
0.000200235 23100 0.000143858 22600 0.000152092
23500 0.002239181 21500 1.64E-05 21300 5.78E-05 20900
0.000199335 23100 0.000143975 22600 0.000151767
23500 0.002222988 21500 1.64E-05 21300 5.76E-05 20900
0.00019846 23100 0.000144084 22600 0.000151466
23500 0.002206314 21500 1.64E-05 21300 5.75E-05 20900
0.000197582 23100 0.000144204 22600 0.000151158
23500 0.002189148 21500 1.64E-05 21300 5.73E-05 20900
0.000196702 23100 0.000144331 22600 0.000150868
23500 0.002171509 21500 1.64E-05 21300 5.71E-05 20900
0.000195846 23100 0.000144429 22600 0.000150571

23500 0.002153477 21500 1.64E-05 21300 5.69E-05 20900
0.000194995 23200 0.000144559 22600 0.000150301
23500 0.002135084 21500 1.64E-05 21300 5.68E-05 20900
0.000194126 23200 0.000144679 22600 0.00015003
23500 0.002116322 21500 1.64E-05 21300 5.66E-05 20900
0.000193264 23200 0.000144815 22600 0.000149767
23500 0.002097241 21500 1.64E-05 21300 5.65E-05 20900
0.000192408 23200 0.000144909 22600 0.000149499
23500 0.002077919 21500 1.64E-05 21300 5.63E-05 20900
0.000191563 23200 0.000145044 22600 0.000149246
23500 0.00205833 21500 1.64E-05 21300 5.61E-05 20900
0.000190661 23200 0.000145172 22600 0.000149002
23500 0.002038553 21500 1.64E-05 21300 5.60E-05 20900
0.000189793 23200 0.000145303 22600 0.000148766
23500 0.002018598 21500 1.64E-05 21300 5.58E-05 20900
0.000188928 23200 0.000145412 22600 0.000148528
23500 0.001998549 21500 1.64E-05 21300 5.57E-05 20900
0.000188076 23200 0.000145534 22600 0.000148311
23500 0.001978401 21500 1.64E-05 21300 5.55E-05 20900
0.000187231 23200 0.000145672 22600 0.000148103
23500 0.00195821 21500 1.64E-05 21300 5.54E-05 20900
0.000186405 23200 0.0001458 22600 0.000147881
23500 0.001938015 21500 1.64E-05 21300 5.52E-05 20900
0.00018562 23200 0.000145934 22600 0.000147668
23500 0.001917875 21500 1.64E-05 21300 5.51E-05 20900
0.000184833 23200 0.00014607 22600 0.000147462
23500 0.001897801 21500 1.64E-05 21300 5.49E-05 20900
0.000184049 23200 0.000146205 22600 0.000147265
23500 0.001877797 21500 1.64E-05 21300 5.48E-05 20900
0.000183304 23200 0.000146336 22600 0.00014708
23500 0.001857931 21500 1.64E-05 21300 5.46E-05 20900
0.000182556 23200 0.000146467 22600 0.000146909
23500 0.001838241 21500 1.64E-05 21300 5.45E-05 20900
0.000181811 23200 0.000146588 22600 0.000146719
23500 0.001818741 21500 1.64E-05 21300 5.44E-05 20900
0.000181046 23200 0.000146724 22600 0.000146559
23500 0.00179945 21500 1.64E-05 21300 5.42E-05 20900
0.000180328 23200 0.000146857 22600 0.000146373
23500 0.001780435 21500 1.64E-05 21300 5.41E-05 20900
0.000179603 23200 0.000146977 22600 0.000146219
23500 0.001761671 21500 1.64E-05 21300 5.40E-05 20900
0.000178877 23200 0.000147116 22600 0.000146056
23500 0.001743228 21500 1.64E-05 21300 5.38E-05 20900
0.00017817 23200 0.000147249 22600 0.000145899
23500 0.001725115 21500 1.64E-05 21300 5.37E-05 20900
0.000177459 23200 0.000147386 22600 0.000145735
23500 0.001707319 21500 1.64E-05 21300 5.36E-05 20900
0.000176761 23200 0.000147517 22600 0.000145594
23500 0.001689883 21500 1.64E-05 21300 5.34E-05 20900
0.000176056 23200 0.000147652 22600 0.000145438
23500 0.00167284 21500 1.64E-05 21300 5.33E-05 20900
0.00017537 23200 0.000147783 22600 0.000145309
23500 0.001656172 21500 1.64E-05 21300 5.32E-05 20900
0.000174686 23200 0.000147904 22600 0.000145161
23500 0.001639944 21500 1.64E-05 21300 5.31E-05 20900
0.000174012 23200 0.000148037 22600 0.000145029
23500 0.00162412 21500 1.64E-05 21300 5.29E-05 20900
0.000173345 23200 0.000148174 22600 0.000144908
23600 0.001608754 21500 1.64E-05 21300 5.28E-05 20900
0.000172681 23200 0.00014831 22600 0.000144787
23600 0.001593803 21500 1.65E-05 21300 5.27E-05 20900
0.000172033 23200 0.000148449 22600 0.000144659
23600 0.001579351 21500 1.64E-05 21300 5.26E-05 20900
0.000171378 23200 0.000148588 22600 0.000144527
23600 0.001565307 21500 1.65E-05 21300 5.25E-05 20900
0.0001707 23200 0.000148726 22600 0.000144417
23600 0.001551737 21500 1.65E-05 21300 5.23E-05 20900
0.000170046 23200 0.000148845 22600 0.000144307

23600 0.001538613 21500 1.65E-05 21300 5.22E-05 20900
0.000169379 23200 0.000148976 22600 0.000144211
23600 0.001525982 21500 1.65E-05 21300 5.21E-05 20900
0.000168711 23200 0.000149102 22600 0.000144109
23600 0.001513818 21500 1.65E-05 21300 5.20E-05 20900
0.000168019 23200 0.000149231 22600 0.000143998
23600 0.001502094 21500 1.65E-05 21300 5.19E-05 20900
0.000167352 23200 0.000149364 22600 0.000143913
23600 0.00149086 21500 1.65E-05 21300 5.18E-05 20900
0.000166699 23200 0.000149493 22700 0.000143824
23600 0.001480091 21500 1.65E-05 21300 5.17E-05 20900
0.000166037 23200 0.000149618 22700 0.000143732
23600 0.001469749 21500 1.65E-05 21300 5.16E-05 20900
0.00016538 23200 0.000149745 22700 0.000143637
23600 0.001459868 21500 1.65E-05 21300 5.15E-05 20900
0.000164741 23200 0.000149854 22700 0.000143559
23600 0.001450427 21500 1.65E-05 21300 5.14E-05 20900
0.000164128 23200 0.000149974 22700 0.000143467
23600 0.001441426 21500 1.65E-05 21300 5.13E-05 20900
0.000163527 23200 0.000150092 22700 0.000143392
23600 0.001432825 21500 1.66E-05 21300 5.12E-05 20900
0.000162923 23200 0.000150232 22700 0.000143323
23600 0.001424675 21500 1.66E-05 21300 5.11E-05 20900
0.000162347 23200 0.000150347 22700 0.000143248
23600 0.001416935 21500 1.66E-05 21300 5.10E-05 20900
0.000161755 23200 0.000150475 22700 0.000143181
23600 0.001409562 21600 1.66E-05 21300 5.09E-05 20900
0.000161186 23200 0.000150593 22700 0.000143124
23600 0.001402598 21600 1.66E-05 21300 5.08E-05 20900
0.000160622 23200 0.000150722 22700 0.000143061
23600 0.001396034 21600 1.66E-05 21400 5.07E-05 20900
0.000160073 23200 0.000150854 22700 0.00014298
23600 0.001389826 21600 1.66E-05 21400 5.06E-05 20900
0.000159539 23200 0.000150979 22700 0.000142926
23600 0.001383985 21600 1.66E-05 21400 5.05E-05 20900
0.000158987 23200 0.000151096 22700 0.000142864
23600 0.001378471 21600 1.67E-05 21400 5.04E-05 20900
0.000158471 23200 0.000151223 22700 0.000142819
23600 0.00137329 21600 1.67E-05 21400 5.03E-05 21000
0.000157912 23200 0.000151346 22700 0.000142754
23600 0.001368463 21600 1.67E-05 21400 5.02E-05 21000
0.000157387 23200 0.00015146 22700 0.000142711
23600 0.001363957 21600 1.67E-05 21400 5.01E-05 21000
0.000156825 23200 0.000151597 22700 0.000142661
23600 0.001359741 21600 1.67E-05 21400 5.01E-05 21000
0.00015629 23200 0.000151706 22700 0.000142616
23600 0.001355817 21600 1.67E-05 21400 5.00E-05 21000
0.00015574 23200 0.000151833 22700 0.000142588
23600 0.00135218 21600 1.67E-05 21400 4.99E-05 21000
0.00015519 23200 0.000151951 22700 0.000142539
23600 0.001348826 21600 1.67E-05 21400 4.98E-05 21000
0.000154651 23200 0.000152074 22700 0.000142504
23600 0.001345718 21600 1.67E-05 21400 4.97E-05 21000
0.000154138 23200 0.000152191 22700 0.000142456
23600 0.001342875 21600 1.67E-05 21400 4.96E-05 21000
0.000153612 23200 0.000152309 22700 0.000142408
23600 0.001340252 21600 1.68E-05 21400 4.96E-05 21000
0.000153082 23200 0.00015244 22700 0.000142377
23600 0.001337845 21600 1.68E-05 21400 4.95E-05 21000
0.000152564 23200 0.000152568 22700 0.000142345
23600 0.001335683 21600 1.68E-05 21400 4.94E-05 21000
0.000152078 23300 0.000152681 22700 0.000142324
23600 0.001333714 21600 1.68E-05 21400 4.94E-05 21000
0.000151574 23300 0.000152809 22700 0.000142293
23600 0.001331924 21600 1.68E-05 21400 4.93E-05 21000
0.000151099 23300 0.000152928 22700 0.000142272
23600 0.001330342 21600 1.68E-05 21400 4.92E-05 21000
0.000150598 23300 0.000153067 22700 0.000142236

23600 0.00132894 21600 1.69E-05 21400 4.91E-05 21000
0.000150127 23300 0.000153195 22700 0.000142196
23600 0.001327713 21600 1.69E-05 21400 4.90E-05 21000
0.000149641 23300 0.000153332 22700 0.000142179
23600 0.001326644 21600 1.69E-05 21400 4.90E-05 21000
0.000149149 23300 0.000153466 22700 0.000142152
23600 0.001325709 21600 1.69E-05 21400 4.89E-05 21000
0.000148684 23300 0.000153606 22700 0.00014214
23600 0.001324908 21600 1.69E-05 21400 4.88E-05 21000
0.000148187 23300 0.000153748 22700 0.000142114
23600 0.00132428 21600 1.69E-05 21400 4.87E-05 21000
0.000147707 23300 0.000153882 22700 0.000142102
23600 0.001323804 21600 1.69E-05 21400 4.87E-05 21000
0.000147206 23300 0.000154017 22700 0.000142089
23600 0.001323408 21600 1.70E-05 21400 4.86E-05 21000
0.000146765 23300 0.000154155 22700 0.000142081
23600 0.00132318 21600 1.70E-05 21400 4.85E-05 21000
0.000146305 23300 0.000154295 22700 0.000142083
23600 0.001323057 21600 1.70E-05 21400 4.84E-05 21000
0.00014584 23300 0.000154434 22700 0.000142065
23600 0.001323019 21600 1.70E-05 21400 4.84E-05 21000
0.000145393 23300 0.000154581 22700 0.000142063
23600 0.00132308 21600 1.70E-05 21400 4.83E-05 21000
0.000144938 23300 0.000154716 22700 0.000142047
23600 0.001323245 21600 1.70E-05 21400 4.82E-05 21000
0.000144491 23300 0.00015485 22700 0.000142037
23600 0.001323496 21600 1.70E-05 21400 4.82E-05 21000
0.000144044 23300 0.000154998 22700 0.000142039
23600 0.001323826 21600 1.71E-05 21400 4.81E-05 21000
0.000143565 23300 0.000155134 22700 0.000142037
23600 0.001324238 21600 1.71E-05 21400 4.81E-05 21000
0.000143132 23300 0.000155264 22700 0.00014205
23600 0.001324696 21600 1.71E-05 21400 4.80E-05 21000
0.000142663 23300 0.000155418 22700 0.000142053
23600 0.00132525 21600 1.71E-05 21400 4.79E-05 21000
0.000142223 23300 0.000155552 22700 0.000142064
23600 0.001325861 21600 1.71E-05 21400 4.79E-05 21000
0.000141767 23300 0.000155699 22700 0.000142063
23600 0.001326541 21600 1.71E-05 21400 4.78E-05 21000
0.000141302 23300 0.000155843 22700 0.000142073
23600 0.001327306 21600 1.72E-05 21400 4.77E-05 21000
0.000140857 23300 0.000155984 22700 0.000142085
23600 0.001328091 21600 1.72E-05 21400 4.77E-05 21000
0.000140395 23300 0.000156125 22700 0.000142093
23600 0.001328942 21600 1.72E-05 21400 4.76E-05 21000
0.000139939 23300 0.000156256 22700 0.000142114
23600 0.001329823 21600 1.72E-05 21400 4.75E-05 21000
0.000139492 23300 0.000156394 22700 0.00014213
23600 0.001330792 21600 1.73E-05 21400 4.75E-05 21000
0.000139049 23300 0.000156546 22700 0.000142139
23700 0.001331778 21600 1.73E-05 21400 4.74E-05 21000
0.00013858 23300 0.000156686 22700 0.000142159
23700 0.001332815 21600 1.73E-05 21400 4.74E-05 21000
0.000138135 23300 0.000156808 22700 0.000142181
23700 0.0013339 21600 1.73E-05 21400 4.73E-05 21000
0.000137685 23300 0.000156966 22700 0.000142202
23700 0.001335008 21600 1.73E-05 21400 4.73E-05 21000
0.000137254 23300 0.000157091 22700 0.000142215
23700 0.001336162 21600 1.74E-05 21400 4.72E-05 21000
0.000136821 23300 0.000157232 22700 0.000142232
23700 0.001337356 21600 1.74E-05 21400 4.71E-05 21000
0.000136382 23300 0.000157377 22700 0.000142249
23700 0.001338563 21600 1.74E-05 21400 4.71E-05 21000
0.000135952 23300 0.000157518 22700 0.000142256
23700 0.001339806 21600 1.74E-05 21400 4.70E-05 21000
0.000135528 23300 0.000157663 22700 0.000142284
23700 0.00134105 21600 1.75E-05 21400 4.70E-05 21000
0.000135107 23300 0.0001578 22700 0.000142312

23700 0.001342335 21600 1.75E-05 21400 4.69E-05 21000
0.000134704 23300 0.000157931 22800 0.000142334
23700 0.001343647 21600 1.75E-05 21400 4.68E-05 21000
0.000134298 23300 0.000158082 22800 0.000142359
23700 0.001344967 21600 1.75E-05 21400 4.68E-05 21000
0.000133916 23300 0.000158219 22800 0.000142396
23700 0.001346312 21600 1.76E-05 21400 4.67E-05 21000
0.000133525 23300 0.000158366 22800 0.000142422
23700 0.001347674 21600 1.76E-05 21400 4.67E-05 21000
0.000133156 23300 0.000158517 22800 0.00014243
23700 0.001349104 21600 1.76E-05 21400 4.66E-05 21000
0.000132762 23300 0.000158663 22800 0.000142455
23700 0.001350488 21600 1.76E-05 21400 4.66E-05 21000
0.000132389 23300 0.000158817 22800 0.000142478
23700 0.001351901 21600 1.77E-05 21400 4.65E-05 21000
0.000132027 23300 0.000158946 22800 0.000142517
23700 0.001353331 21600 1.77E-05 21400 4.65E-05 21000
0.000131641 23300 0.000159092 22800 0.000142553
23700 0.001354809 21700 1.77E-05 21400 4.64E-05 21000
0.000131268 23300 0.000159237 22800 0.000142588
23700 0.001356271 21700 1.78E-05 21400 4.64E-05 21000
0.000130874 23300 0.000159377 22800 0.000142626
23700 0.001357738 21700 1.78E-05 21500 4.63E-05 21000
0.0001305 23300 0.000159524 22800 0.000142651
23700 0.001359242 21700 1.78E-05 21500 4.63E-05 21000
0.000130104 23300 0.000159675 22800 0.00014269
23700 0.001360758 21700 1.79E-05 21500 4.62E-05 21000
0.000129733 23300 0.000159816 22800 0.000142727
23700 0.001362288 21700 1.79E-05 21500 4.62E-05 21000
0.000129353 23300 0.000159971 22800 0.000142762
23700 0.001363823 21700 1.79E-05 21500 4.61E-05 21100
0.000128977 23300 0.000160118 22800 0.00014279
23700 0.001365361 21700 1.80E-05 21500 4.61E-05 21100
0.000128607 23300 0.000160261 22800 0.000142827
23700 0.001366923 21700 1.80E-05 21500 4.60E-05 21100
0.000128249 23300 0.000160417 22800 0.000142869
23700 0.001368478 21700 1.80E-05 21500 4.60E-05 21100
0.000127901 23300 0.00016056 22800 0.000142912
23700 0.001370058 21700 1.81E-05 21500 4.60E-05 21100
0.000127586 23300 0.000160703 22800 0.000142944
23700 0.001371636 21700 1.81E-05 21500 4.59E-05 21100
0.000127248 23300 0.000160863 22800 0.000142986
23700 0.001373224 21700 1.82E-05 21500 4.59E-05 21100
0.000126939 23300 0.000161006 22800 0.000143035
23700 0.001374832 21700 1.82E-05 21500 4.58E-05 21100
0.000126615 23300 0.000161174 22800 0.000143078
23700 0.001376466 21700 1.83E-05 21500 4.58E-05 21100
0.000126309 23300 0.00016132 22800 0.000143105
23700 0.001378117 21700 1.83E-05 21500 4.57E-05 21100
0.000126 23300 0.000161473 22800 0.00014315
23700 0.001379762 21700 1.84E-05 21500 4.57E-05 21100
0.000125689 23300 0.000161632 22800 0.000143188
23700 0.001381405 21700 1.84E-05 21500 4.56E-05 21100
0.000125395 23400 0.000161772 22800 0.000143241
23700 0.001383064 21700 1.85E-05 21500 4.56E-05 21100
0.000125076 23400 0.000161918 22800 0.000143284
23700 0.001384733 21700 1.85E-05 21500 4.55E-05 21100
0.00012476 23400 0.000162071 22800 0.000143342
23700 0.001386451 21700 1.86E-05 21500 4.55E-05 21100
0.000124426 23400 0.000162229 22800 0.000143387
23700 0.001388135 21700 1.86E-05 21500 4.54E-05 21100
0.000124106 23400 0.000162383 22800 0.000143432
23700 0.001389866 21700 1.87E-05 21500 4.54E-05 21100
0.000123748 23400 0.000162532 22800 0.000143476
23700 0.001391597 21700 1.87E-05 21500 4.53E-05 21100
0.000123402 23400 0.000162687 22800 0.00014352
23700 0.00139333 21700 1.88E-05 21500 4.53E-05 21100
0.000123056 23400 0.000162852 22800 0.000143559

23700 0.001395058 21700 1.88E-05 21500 4.53E-05 21100
0.000122695 23400 0.000163001 22800 0.000143605
23700 0.001396821 21700 1.89E-05 21500 4.52E-05 21100
0.000122342 23400 0.00016315 22800 0.000143646
23700 0.001398571 21700 1.90E-05 21500 4.52E-05 21100
0.000121986 23400 0.000163291 22800 0.000143695
23700 0.001400331 21700 1.90E-05 21500 4.52E-05 21100
0.000121639 23400 0.000163437 22800 0.000143728
23700 0.001402093 21700 1.91E-05 21500 4.51E-05 21100
0.000121294 23400 0.000163578 22800 0.000143774
23700 0.001403852 21700 1.92E-05 21500 4.51E-05 21100
0.000120949 23400 0.000163733 22800 0.000143819
23700 0.001405621 21700 1.92E-05 21500 4.51E-05 21100
0.000120637 23400 0.000163877 22800 0.000143871
23700 0.001407418 21700 1.93E-05 21500 4.50E-05 21100
0.00012032 23400 0.000164033 22800 0.00014392
23700 0.001409209 21700 1.94E-05 21500 4.50E-05 21100
0.000120029 23400 0.000164184 22800 0.000143955
23700 0.001411025 21700 1.95E-05 21500 4.49E-05 21100
0.00011973 23400 0.000164341 22800 0.000144011
23700 0.001412841 21700 1.95E-05 21500 4.49E-05 21100
0.000119427 23400 0.000164491 22800 0.000144053
23700 0.001414651 21700 1.96E-05 21500 4.49E-05 21100
0.000119156 23400 0.000164633 22800 0.00014411
23700 0.001416475 21700 1.97E-05 21500 4.48E-05 21100
0.000118873 23400 0.000164782 22800 0.000144152
23700 0.001418335 21700 1.98E-05 21500 4.48E-05 21100
0.000118614 23400 0.000164934 22800 0.000144209
23700 0.001420172 21700 1.99E-05 21500 4.48E-05 21100
0.000118322 23400 0.000165082 22800 0.000144256
23700 0.001422039 21700 2.00E-05 21500 4.47E-05 21100
0.000118055 23400 0.000165223 22800 0.000144301
23700 0.001423901 21700 2.01E-05 21500 4.47E-05 21100
0.000117758 23400 0.000165374 22800 0.000144341
23700 0.001425785 21700 2.02E-05 21500 4.47E-05 21100
0.00011748 23400 0.000165516 22800 0.000144388
23700 0.001427683 21700 2.03E-05 21500 4.47E-05 21100
0.000117184 23400 0.000165665 22800 0.000144432
23700 0.00142957 21700 2.04E-05 21500 4.46E-05 21100
0.000116906 23400 0.000165807 22800 0.000144497
23700 0.001431471 21700 2.05E-05 21500 4.46E-05 21100
0.000116603 23400 0.00016595 22800 0.000144538
23800 0.001433385 21700 2.07E-05 21500 4.45E-05 21100
0.0001163 23400 0.000166091 22800 0.000144591
23800 0.00143528 21700 2.08E-05 21500 4.45E-05 21100
0.00011598 23400 0.000166233 22800 0.000144649
23800 0.001437221 21700 2.10E-05 21500 4.45E-05 21100
0.000115659 23400 0.000166362 22800 0.000144698
23800 0.00143916 21700 2.11E-05 21500 4.45E-05 21100
0.000115337 23400 0.000166518 22800 0.000144745
23800 0.00144111 21700 2.13E-05 21500 4.45E-05 21100
0.000115028 23400 0.000166653 22800 0.000144801
23800 0.001443066 21700 2.14E-05 21500 4.44E-05 21100
0.000114704 23400 0.000166801 22800 0.000144856
23800 0.001445055 21700 2.16E-05 21500 4.44E-05 21100
0.000114411 23400 0.000166948 22800 0.000144899
23800 0.001447043 21700 2.18E-05 21500 4.44E-05 21100
0.000114112 23400 0.000167093 22800 0.000144946
23800 0.001449043 21700 2.19E-05 21500 4.43E-05 21100
0.000113826 23400 0.000167235 22800 0.000145002
23800 0.001451036 21700 2.21E-05 21500 4.43E-05 21100
0.000113585 23400 0.000167374 22900 0.000145062
23800 0.001453048 21700 2.23E-05 21500 4.43E-05 21100
0.000113329 23400 0.000167519 22900 0.000145117
23800 0.001455085 21700 2.26E-05 21500 4.43E-05 21100
0.000113092 23400 0.000167663 22900 0.000145181
23800 0.001457131 21700 2.28E-05 21500 4.42E-05 21100
0.000112849 23400 0.000167812 22900 0.000145245

23800	0.00145919	21700	2.30E-05	21500	4.42E-05	21100
0.000112631	23400	0.000167963	22900	0.0001453		
23800	0.00146127	21700	2.33E-05	21500	4.42E-05	21100
0.000112405	23400	0.000168116	22900	0.000145372		
23800	0.001463341	21700	2.36E-05	21500	4.41E-05	21100
0.000112179	23400	0.000168263	22900	0.000145431		
23800	0.001465445	21700	2.39E-05	21500	4.41E-05	21100
0.000111957	23400	0.00016842	22900	0.000145489		
23800	0.001467556	21700	2.42E-05	21500	4.41E-05	21100
0.000111723	23400	0.000168572	22900	0.00014554		
23800	0.001469687	21800	2.45E-05	21500	4.41E-05	21100
0.000111501	23400	0.000168724	22900	0.000145598		
23800	0.001471818	21800	2.48E-05	21500	4.40E-05	21100
0.000111274	23400	0.000168877	22900	0.000145664		
23800	0.001473956	21800	2.52E-05	21600	4.40E-05	21100
0.000111025	23400	0.000169031	22900	0.00014572		
23800	0.00147611	21800	2.56E-05	21600	4.40E-05	21100
0.000110784	23400	0.000169175	22900	0.00014579		
23800	0.001478275	21800	2.59E-05	21600	4.40E-05	21100
0.000110545	23400	0.000169332	22900	0.000145865		
23800	0.001480448	21800	2.64E-05	21600	4.39E-05	21100
0.000110311	23400	0.00016949	22900	0.000145939		
23800	0.001482652	21800	2.68E-05	21600	4.39E-05	21200
0.000110065	23400	0.000169642	22900	0.000146009		
23800	0.00148486	21800	2.73E-05	21600	4.39E-05	21200
0.000109821	23400	0.00016981	22900	0.000146062		
23800	0.001487117	21800	2.78E-05	21600	4.39E-05	21200
0.000109581	23400	0.000169972	22900	0.000146123		
23800	0.00148935	21800	2.83E-05	21600	4.38E-05	21200
0.000109344	23400	0.000170127	22900	0.000146196		
23800	0.001491605	21800	2.89E-05	21600	4.38E-05	21200
0.000109108	23400	0.000170268	22900	0.000146274		
23800	0.001493869	21800	2.95E-05	21600	4.38E-05	21200
0.000108897	23400	0.000170433	22900	0.000146345		
23800	0.001496151	21800	3.01E-05	21600	4.38E-05	21200
0.000108662	23400	0.000170596	22900	0.00014641		
23800	0.001498469	21800	3.09E-05	21600	4.37E-05	21200
0.000108416	23400	0.00017075	22900	0.000146481		
23800	0.001500766	21800	3.16E-05	21600	4.37E-05	21200
0.000108195	23400	0.000170917	22900	0.000146552		
23800	0.00150311	21800	3.25E-05	21600	4.37E-05	21200
0.000107955	23400	0.000171086	22900	0.000146613		
23800	0.001505446	21800	3.33E-05	21600	4.37E-05	21200
0.000107734	23400	0.000171248	22900	0.000146675		
23800	0.001507818	21800	3.43E-05	21600	4.36E-05	21200
0.000107505	23500	0.000171416	22900	0.000146744		
23800	0.001510207	21800	3.53E-05	21600	4.36E-05	21200
0.00010728	23500	0.000171583	22900	0.000146818		
23800	0.001512604	21800	3.63E-05	21600	4.36E-05	21200
0.000107067	23500	0.000171746	22900	0.000146873		
23800	0.001515005	21800	3.75E-05	21600	4.36E-05	21200
0.000106848	23500	0.000171908	22900	0.000146949		
23800	0.00151742	21800	3.87E-05	21600	4.36E-05	21200
0.000106618	23500	0.000172071	22900	0.000147009		
23800	0.001519848	21800	4.00E-05	21600	4.36E-05	21200
0.000106413	23500	0.000172233	22900	0.00014708		
23800	0.00152228	21800	4.14E-05	21600	4.35E-05	21200
0.000106207	23500	0.000172396	22900	0.000147161		
23800	0.001524723	21800	4.30E-05	21600	4.35E-05	21200
0.000105992	23500	0.000172567	22900	0.00014724		
23800	0.001527211	21800	4.46E-05	21600	4.35E-05	21200
0.000105778	23500	0.000172726	22900	0.000147296		
23800	0.001529706	21800	4.63E-05	21600	4.35E-05	21200
0.000105571	23500	0.000172899	22900	0.000147357		
23800	0.001532226	21800	4.81E-05	21600	4.35E-05	21200
0.000105363	23500	0.000173066	22900	0.000147421		
23800	0.001534744	21800	5.00E-05	21600	4.34E-05	21200
0.000105136	23500	0.000173234	22900	0.000147475		

23800 0.001537269 21800 5.20E-05 21600 4.34E-05 21200
0.000104919 23500 0.000173386 22900 0.000147566
23800 0.001539802 21800 5.40E-05 21600 4.34E-05 21200
0.000104712 23500 0.000173561 22900 0.000147638
23800 0.001542372 21800 5.62E-05 21600 4.34E-05 21200
0.000104478 23500 0.000173723 22900 0.000147719
23800 0.001544956 21800 5.84E-05 21600 4.34E-05 21200
0.000104234 23500 0.000173887 22900 0.000147805
23800 0.001547541 21800 6.08E-05 21600 4.34E-05 21200
0.000104006 23500 0.000174072 22900 0.000147874
23800 0.001550167 21800 6.33E-05 21600 4.33E-05 21200
0.0001038 23500 0.000174236 22900 0.000147947
23800 0.001552807 21800 6.58E-05 21600 4.33E-05 21200
0.000103583 23500 0.000174399 22900 0.000148016
23800 0.001555467 21800 6.85E-05 21600 4.33E-05 21200
0.000103371 23500 0.000174569 22900 0.00014809
23800 0.001558131 21800 7.13E-05 21600 4.33E-05 21200
0.000103167 23500 0.000174735 22900 0.000148168
23800 0.001560838 21800 7.41E-05 21600 4.33E-05 21200
0.000102969 23500 0.000174902 22900 0.000148237
23800 0.001563527 21800 7.69E-05 21600 4.33E-05 21200
0.000102758 23500 0.000175064 22900 0.000148317
23800 0.001566241 21800 7.98E-05 21600 4.32E-05 21200
0.000102559 23500 0.000175237 22900 0.000148376
23800 0.001568959 21800 8.26E-05 21600 4.32E-05 21200
0.000102377 23500 0.000175394 22900 0.000148453
23800 0.001571711 21800 8.53E-05 21600 4.32E-05 21200
0.00010219 23500 0.000175565 22900 0.000148531
23800 0.001574455 21800 8.79E-05 21600 4.32E-05 21200
0.000101993 23500 0.000175738 22900 0.00014863
23800 0.001577225 21800 9.02E-05 21600 4.32E-05 21200
0.000101797 23500 0.000175903 22900 0.0001487
23800 0.001580024 21800 9.21E-05 21600 4.32E-05 21200
0.000101604 23500 0.000176077 22900 0.000148767
23900 0.001582853 21800 9.37E-05 21600 4.32E-05 21200
0.000101395 23500 0.000176253 22900 0.000148843
23900 0.001585698 21800 9.48E-05 21600 4.31E-05 21200
0.000101185 23500 0.000176414 22900 0.000148915
23900 0.001588541 21800 9.53E-05 21600 4.31E-05 21200
0.000100975 23500 0.000176572 22900 0.000149001
23900 0.001591399 21800 9.53E-05 21600 4.31E-05 21200
0.000100786 23500 0.00017674 22900 0.000149075
23900 0.001594277 21800 9.47E-05 21600 4.31E-05 21200
0.000100582 23500 0.000176905 22900 0.000149154
23900 0.00159719 21800 9.36E-05 21600 4.31E-05 21200
0.000100361 23500 0.000177068 22900 0.00014924
23900 0.001600073 21800 9.19E-05 21600 4.31E-05 21200
0.000100165 23500 0.000177241 22900 0.00014931
23900 0.001603014 21800 8.98E-05 21600 4.31E-05 21200
0.0001 23500 0.000177416 22900 0.000149391
23900 0.001605959 21800 8.72E-05 21600 4.31E-05 21200
9.98E-05 23500 0.000177581 22900 0.00014947
23900 0.001608926 21800 8.44E-05 21600 4.31E-05 21200
9.96E-05 23500 0.000177743 23000 0.000149533
23900 0.001611899 21800 8.13E-05 21600 4.31E-05 21200
9.93E-05 23500 0.000177916 23000 0.000149612
23900 0.001614882 21800 7.81E-05 21600 4.31E-05 21200
9.92E-05 23500 0.000178075 23000 0.000149683
23900 0.00161786 21800 7.49E-05 21600 4.31E-05 21200
9.89E-05 23500 0.000178241 23000 0.000149748
23900 0.001620864 21800 7.16E-05 21600 4.31E-05 21200
9.87E-05 23500 0.000178393 23000 0.000149826
23900 0.001623866 21800 6.85E-05 21600 4.30E-05 21200
9.86E-05 23500 0.000178554 23000 0.0001499
23900 0.001626902 21800 6.54E-05 21600 4.30E-05 21200
9.84E-05 23500 0.00017872 23000 0.000149974
23900 0.001629925 21800 6.24E-05 21600 4.30E-05 21200
9.82E-05 23500 0.000178885 23000 0.000150067

23900 0.001632979 21800 5.96E-05 21600 4.30E-05 21200
9.80E-05 23500 0.000179055 23000 0.000150145
23900 0.001636032 21900 5.70E-05 21600 4.30E-05 21200
9.78E-05 23500 0.000179232 23000 0.000150207
23900 0.001639141 21900 5.45E-05 21600 4.30E-05 21200
9.77E-05 23500 0.000179405 23000 0.000150281
23900 0.001642237 21900 5.21E-05 21700 4.30E-05 21200
9.75E-05 23500 0.000179569 23000 0.000150355
23900 0.001645349 21900 5.00E-05 21700 4.30E-05 21200
9.73E-05 23500 0.000179736 23000 0.000150436
23900 0.001648451 21900 4.79E-05 21700 4.30E-05 21200
9.72E-05 23500 0.000179906 23000 0.000150512
23900 0.001651602 21900 4.60E-05 21700 4.29E-05 21200
9.70E-05 23500 0.000180078 23000 0.000150595
23900 0.001654756 21900 4.43E-05 21700 4.29E-05 21300
9.68E-05 23500 0.000180247 23000 0.000150673
23900 0.001657912 21900 4.26E-05 21700 4.29E-05 21300
9.67E-05 23500 0.00018043 23000 0.00015075
23900 0.001661119 21900 4.11E-05 21700 4.29E-05 21300
9.65E-05 23500 0.000180603 23000 0.000150836
23900 0.001664338 21900 3.98E-05 21700 4.29E-05 21300
9.63E-05 23500 0.000180779 23000 0.00015091
23900 0.001667573 21900 3.85E-05 21700 4.29E-05 21300
9.62E-05 23500 0.000180955 23000 0.000150988
23900 0.001670814 21900 3.73E-05 21700 4.29E-05 21300
9.60E-05 23500 0.000181127 23000 0.000151072
23900 0.001674082 21900 3.62E-05 21700 4.29E-05 21300
9.59E-05 23500 0.000181304 23000 0.000151138
23900 0.001677357 21900 3.52E-05 21700 4.29E-05 21300
9.57E-05 23500 0.000181481 23000 0.000151224
23900 0.001680642 21900 3.42E-05 21700 4.29E-05 21300
9.55E-05 23500 0.000181657 23000 0.000151306
23900 0.001683959 21900 3.34E-05 21700 4.29E-05 21300
9.54E-05 23500 0.00018183 23000 0.000151389
23900 0.001687302 21900 3.25E-05 21700 4.29E-05 21300
9.52E-05 23500 0.00018201 23000 0.000151479
23900 0.001690638 21900 3.18E-05 21700 4.29E-05 21300
9.50E-05 23600 0.000182203 23000 0.000151578
23900 0.001694002 21900 3.11E-05 21700 4.29E-05 21300
9.48E-05 23600 0.000182377 23000 0.000151651
23900 0.001697407 21900 3.04E-05 21700 4.29E-05 21300
9.47E-05 23600 0.000182567 23000 0.000151727
23900 0.001700834 21900 2.98E-05 21700 4.29E-05 21300
9.45E-05 23600 0.000182746 23000 0.00015182
23900 0.001704273 21900 2.92E-05 21700 4.29E-05 21300
9.43E-05 23600 0.000182919 23000 0.000151889
23900 0.001707722 21900 2.87E-05 21700 4.29E-05 21300
9.42E-05 23600 0.000183103 23000 0.000151985
23900 0.001711187 21900 2.82E-05 21700 4.29E-05 21300
9.40E-05 23600 0.000183284 23000 0.000152061
23900 0.001714692 21900 2.77E-05 21700 4.29E-05 21300
9.38E-05 23600 0.000183476 23000 0.000152158
23900 0.001718216 21900 2.73E-05 21700 4.28E-05 21300
9.37E-05 23600 0.000183656 23000 0.000152242
23900 0.001721755 21900 2.69E-05 21700 4.28E-05 21300
9.35E-05 23600 0.000183855 23000 0.000152333
23900 0.001725294 21900 2.65E-05 21700 4.28E-05 21300
9.33E-05 23600 0.000184035 23000 0.000152421
23900 0.001728867 21900 2.61E-05 21700 4.28E-05 21300
9.32E-05 23600 0.000184229 23000 0.00015251
23900 0.001732459 21900 2.58E-05 21700 4.28E-05 21300
9.30E-05 23600 0.000184401 23000 0.000152591
23900 0.001736069 21900 2.55E-05 21700 4.28E-05 21300
9.29E-05 23600 0.000184577 23000 0.000152676
23900 0.0017397 21900 2.52E-05 21700 4.28E-05 21300
9.27E-05 23600 0.000184753 23000 0.000152746
23900 0.001743352 21900 2.49E-05 21700 4.29E-05 21300
9.26E-05 23600 0.000184926 23000 0.000152842

23900 0.001747002 21900 2.46E-05 21700 4.29E-05 21300
9.24E-05 23600 0.000185095 23000 0.000152918
23900 0.001750665 21900 2.44E-05 21700 4.28E-05 21300
9.23E-05 23600 0.000185263 23000 0.000153011
23900 0.001754362 21900 2.41E-05 21700 4.29E-05 21300
9.22E-05 23600 0.000185439 23000 0.000153099
23900 0.001758058 21900 2.39E-05 21700 4.29E-05 21300
9.20E-05 23600 0.000185603 23000 0.000153204
23900 0.001761775 21900 2.37E-05 21700 4.28E-05 21300
9.18E-05 23600 0.000185773 23000 0.000153271
23900 0.001765519 21900 2.35E-05 21700 4.28E-05 21300
9.17E-05 23600 0.00018597 23000 0.000153344
23900 0.001769298 21900 2.33E-05 21700 4.28E-05 21300
9.15E-05 23600 0.000186142 23000 0.000153435
23900 0.001773086 21900 2.31E-05 21700 4.28E-05 21300
9.14E-05 23600 0.000186311 23000 0.000153507
23900 0.001776861 21900 2.29E-05 21700 4.28E-05 21300
9.12E-05 23600 0.000186476 23000 0.000153605
23900 0.001780654 21900 2.27E-05 21700 4.28E-05 21300
9.11E-05 23600 0.000186652 23000 0.000153681
23900 0.001784494 21900 2.26E-05 21700 4.28E-05 21300
9.09E-05 23600 0.000186833 23000 0.00015377
23900 0.001788357 21900 2.24E-05 21700 4.28E-05 21300
9.08E-05 23600 0.000187003 23000 0.000153852
23900 0.001792212 21900 2.23E-05 21700 4.28E-05 21300
9.06E-05 23600 0.000187198 23000 0.000153943
24000 0.001796102 21900 2.21E-05 21700 4.28E-05 21300
9.05E-05 23600 0.000187362 23000 0.000154033
24000 0.001800023 21900 2.20E-05 21700 4.28E-05 21300
9.03E-05 23600 0.000187548 23000 0.000154117
24000 0.001803965 21900 2.19E-05 21700 4.28E-05 21300
9.02E-05 23600 0.000187725 23000 0.000154196
24000 0.001807912 21900 2.18E-05 21700 4.28E-05 21300
9.00E-05 23600 0.000187895 23000 0.000154286
24000 0.001811907 21900 2.17E-05 21700 4.28E-05 21300
8.99E-05 23600 0.00018808 23000 0.000154357
24000 0.001815894 21900 2.15E-05 21700 4.28E-05 21300
8.97E-05 23600 0.000188256 23000 0.000154456
24000 0.001819899 21900 2.14E-05 21700 4.28E-05 21300
8.95E-05 23600 0.000188432 23000 0.000154536
24000 0.001823917 21900 2.13E-05 21700 4.28E-05 21300
8.94E-05 23600 0.000188605 23000 0.000154628
24000 0.001827965 21900 2.12E-05 21700 4.28E-05 21300
8.93E-05 23600 0.000188773 23000 0.000154721
24000 0.00183202 21900 2.11E-05 21700 4.28E-05 21300
8.91E-05 23600 0.000188954 23100 0.00015483
24000 0.00183613 21900 2.11E-05 21700 4.28E-05 21300
8.90E-05 23600 0.000189133 23100 0.00015491
24000 0.001840256 21900 2.10E-05 21700 4.28E-05 21300
8.89E-05 23600 0.000189328 23100 0.000154996
24000 0.001844422 21900 2.09E-05 21700 4.28E-05 21300
8.88E-05 23600 0.000189502 23100 0.000155093
24000 0.00184858 21900 2.08E-05 21700 4.28E-05 21300
8.87E-05 23600 0.000189685 23100 0.000155178
24000 0.001852773 21900 2.07E-05 21700 4.28E-05 21300
8.86E-05 23600 0.000189856 23100 0.000155269
24000 0.001856969 21900 2.07E-05 21700 4.28E-05 21300
8.85E-05 23600 0.000190039 23100 0.000155366
24000 0.00186123 21900 2.06E-05 21700 4.28E-05 21300
8.84E-05 23600 0.000190235 23100 0.000155447
24000 0.001865503 21900 2.05E-05 21700 4.28E-05 21300
8.83E-05 23600 0.000190412 23100 0.000155545
24000 0.001869786 22000 2.05E-05 21700 4.28E-05 21300
8.82E-05 23600 0.000190612 23100 0.000155631
24000 0.001874099 22000 2.04E-05 21700 4.28E-05 21300
8.81E-05 23600 0.000190799 23100 0.000155728
24000 0.001878428 22000 2.04E-05 21800 4.28E-05 21300
8.80E-05 23600 0.000190993 23100 0.00015582

24000	0.001882797	22000	2.03E-05	21800	4.28E-05	21300	8.78E-05	23600	0.00019118	23100	0.000155912
24000	0.001887186	22000	2.02E-05	21800	4.28E-05	21300	8.77E-05	23600	0.000191366	23100	0.000156007
24000	0.001891595	22000	2.02E-05	21800	4.28E-05	21300	8.76E-05	23600	0.000191552	23100	0.000156099
24000	0.001896023	22000	2.02E-05	21800	4.28E-05	21400	8.74E-05	23600	0.000191734	23100	0.000156198
24000	0.001900467	22000	2.01E-05	21800	4.28E-05	21400	8.73E-05	23600	0.000191917	23100	0.000156293
24000	0.001904927	22000	2.01E-05	21800	4.28E-05	21400	8.72E-05	23600	0.000192098	23100	0.000156399
24000	0.001909424	22000	2.00E-05	21800	4.28E-05	21400	8.70E-05	23600	0.000192276	23100	0.000156505
24000	0.001913936	22000	2.00E-05	21800	4.28E-05	21400	8.69E-05	23600	0.00019247	23100	0.00015661
24000	0.001918476	22000	1.99E-05	21800	4.28E-05	21400	8.68E-05	23600	0.000192655	23100	0.000156702
24000	0.001923039	22000	1.99E-05	21800	4.28E-05	21400	8.67E-05	23600	0.00019286	23100	0.000156779
24000	0.001927656	22000	1.99E-05	21800	4.28E-05	21400	8.66E-05	23600	0.000193046	23100	0.000156885
24000	0.00193227	22000	1.98E-05	21800	4.28E-05	21400	8.65E-05	23600	0.000193229	23100	0.000156979
24000	0.001936901	22000	1.98E-05	21800	4.28E-05	21400	8.64E-05	23600	0.00019341	23100	0.000157073
24000	0.001941538	22000	1.98E-05	21800	4.28E-05	21400	8.63E-05	23600	0.000193596	23100	0.000157182
24000	0.001946243	22000	1.97E-05	21800	4.28E-05	21400	8.61E-05	23700	0.000193796	23100	0.000157268
24000	0.001950953	22000	1.97E-05	21800	4.28E-05	21400	8.60E-05	23700	0.000193983	23100	0.000157369
24000	0.001955682	22000	1.97E-05	21800	4.28E-05	21400	8.59E-05	23700	0.000194191	23100	0.000157463
24000	0.001960479	22000	1.97E-05	21800	4.28E-05	21400	8.57E-05	23700	0.000194377	23100	0.000157564
24000	0.001965284	22000	1.96E-05	21800	4.28E-05	21400	8.56E-05	23700	0.00019457	23100	0.000157662
24000	0.001970114	22000	1.96E-05	21800	4.29E-05	21400	8.55E-05	23700	0.000194768	23100	0.000157756
24000	0.001974976	22000	1.96E-05	21800	4.29E-05	21400	8.54E-05	23700	0.000194958	23100	0.000157855
24000	0.001979855	22000	1.96E-05	21800	4.29E-05	21400	8.52E-05	23700	0.000195153	23100	0.000157949
24000	0.001984756	22000	1.95E-05	21800	4.29E-05	21400	8.51E-05	23700	0.000195343	23100	0.000158047
24000	0.001989681	22000	1.95E-05	21800	4.29E-05	21400	8.50E-05	23700	0.000195531	23100	0.000158152
24000	0.001994617	22000	1.95E-05	21800	4.29E-05	21400	8.49E-05	23700	0.000195724	23100	0.00015825
24000	0.001999587	22000	1.95E-05	21800	4.29E-05	21400	8.48E-05	23700	0.000195913	23100	0.000158358
24000	0.002004572	22000	1.95E-05	21800	4.29E-05	21400	8.47E-05	23700	0.000196116	23100	0.00015846
24000	0.002009594	22000	1.94E-05	21800	4.29E-05	21400	8.46E-05	23700	0.000196303	23100	0.00015855
24000	0.002014639	22000	1.94E-05	21800	4.29E-05	21400	8.45E-05	23700	0.000196509	23100	0.000158634
24000	0.002019727	22000	1.94E-05	21800	4.29E-05	21400	8.44E-05	23700	0.000196707	23100	0.000158741
24000	0.002024804	22000	1.94E-05	21800	4.29E-05	21400	8.43E-05	23700	0.000196901	23100	0.000158828
24000	0.002029912	22000	1.94E-05	21800	4.29E-05	21400	8.42E-05	23700	0.000197088	23100	0.000158944
24000	0.00203504	22000	1.93E-05	21800	4.29E-05	21400	8.41E-05	23700	0.000197283	23100	0.000159029
24000	0.002040199	22000	1.93E-05	21800	4.29E-05	21400	8.40E-05	23700	0.000197488	23100	0.000159129

24000 0.002045385 22000 1.93E-05 21800 4.29E-05 21400
8.39E-05 23700 0.000197685 23100 0.000159234
24000 0.002050573 22000 1.93E-05 21800 4.29E-05 21400
8.37E-05 23700 0.0001979 23100 0.000159332
24000 0.002055798 22000 1.93E-05 21800 4.29E-05 21400
8.36E-05 23700 0.000198095 23100 0.000159416
24000 0.002061039 22000 1.93E-05 21800 4.30E-05 21400
8.35E-05 23700 0.000198301 23100 0.000159515
24000 0.002066304 22000 1.93E-05 21800 4.30E-05 21400
8.34E-05 23700 0.000198499 23100 0.000159607
24000 0.002071584 22000 1.93E-05 21800 4.30E-05 21400
8.33E-05 23700 0.000198702 23100 0.000159711
24000 0.002076893 22000 1.93E-05 21800 4.30E-05 21400
8.32E-05 23700 0.000198899 23100 0.000159801
24000 0.002082227 22000 1.93E-05 21800 4.30E-05 21400
8.31E-05 23700 0.000199099 23100 0.0001599
24000 0.002087558 22000 1.93E-05 21800 4.30E-05 21400
8.30E-05 23700 0.000199289 23100 0.000159998
24100 0.002092915 22000 1.92E-05 21800 4.30E-05 21400
8.29E-05 23700 0.000199486 23100 0.000160106
24100 0.002098286 22000 1.92E-05 21800 4.30E-05 21400
8.28E-05 23700 0.000199682 23100 0.000160205
24100 0.002103691 22000 1.92E-05 21800 4.30E-05 21400
8.27E-05 23700 0.000199891 23100 0.000160309
24100 0.002109111 22000 1.92E-05 21800 4.30E-05 21400
8.26E-05 23700 0.000200082 23100 0.000160395
24100 0.002114578 22000 1.92E-05 21800 4.30E-05 21400
8.25E-05 23700 0.000200296 23100 0.000160484
24100 0.002120072 22000 1.92E-05 21800 4.30E-05 21400
8.25E-05 23700 0.0002005 23100 0.00016058
24100 0.002125598 22000 1.92E-05 21800 4.30E-05 21400
8.24E-05 23700 0.000200697 23100 0.000160671
24100 0.00213112 22000 1.92E-05 21800 4.30E-05 21400
8.23E-05 23700 0.000200889 23100 0.000160773
24100 0.002136684 22000 1.92E-05 21800 4.30E-05 21400
8.22E-05 23700 0.000201098 23100 0.000160868
24100 0.002142295 22000 1.92E-05 21800 4.31E-05 21400
8.21E-05 23700 0.00020131 23200 0.000160972
24100 0.002147912 22000 1.92E-05 21800 4.31E-05 21400
8.20E-05 23700 0.000201502 23200 0.000161078
24100 0.002153575 22000 1.92E-05 21800 4.31E-05 21400
8.19E-05 23700 0.000201713 23200 0.000161166
24100 0.00215927 22000 1.92E-05 21800 4.31E-05 21400
8.19E-05 23700 0.000201908 23200 0.000161269
24100 0.002164983 22000 1.92E-05 21800 4.31E-05 21400
8.18E-05 23700 0.000202115 23200 0.00016137
24100 0.002170742 22000 1.92E-05 21800 4.31E-05 21400
8.17E-05 23700 0.000202312 23200 0.000161466
24100 0.002176511 22000 1.91E-05 21800 4.31E-05 21400
8.16E-05 23700 0.000202511 23200 0.000161562
24100 0.002182332 22000 1.91E-05 21800 4.31E-05 21400
8.15E-05 23700 0.000202708 23200 0.000161671
24100 0.002188178 22000 1.91E-05 21800 4.31E-05 21400
8.13E-05 23700 0.000202903 23200 0.000161764
24100 0.002194039 22100 1.91E-05 21800 4.31E-05 21400
8.12E-05 23700 0.000203093 23200 0.000161854
24100 0.002199936 22100 1.91E-05 21800 4.31E-05 21400
8.11E-05 23700 0.00020329 23200 0.000161955
24100 0.002205879 22100 1.91E-05 21900 4.31E-05 21400
8.10E-05 23700 0.000203478 23200 0.000162049
24100 0.002211835 22100 1.91E-05 21900 4.32E-05 21400
8.09E-05 23700 0.000203688 23200 0.000162167
24100 0.002217826 22100 1.91E-05 21900 4.32E-05 21400
8.08E-05 23700 0.000203881 23200 0.000162263
24100 0.0022239 22100 1.91E-05 21900 4.32E-05 21400
8.07E-05 23700 0.000204086 23200 0.000162353
24100 0.002229966 22100 1.91E-05 21900 4.32E-05 21500
8.06E-05 23700 0.000204289 23200 0.000162453

24100 0.002236079 22100 1.91E-05 21900 4.32E-05 21500
8.05E-05 23700 0.00020448 23200 0.000162557
24100 0.002242211 22100 1.91E-05 21900 4.32E-05 21500
8.04E-05 23700 0.000204667 23200 0.000162669
24100 0.002248372 22100 1.91E-05 21900 4.32E-05 21500
8.03E-05 23700 0.000204866 23200 0.000162759
24100 0.002254616 22100 1.91E-05 21900 4.32E-05 21500
8.03E-05 23700 0.00020508 23200 0.000162883
24100 0.002260876 22100 1.91E-05 21900 4.32E-05 21500
8.02E-05 23700 0.000205285 23200 0.000162989
24100 0.002267184 22100 1.91E-05 21900 4.32E-05 21500
8.01E-05 23700 0.000205496 23200 0.000163093
24100 0.002273505 22100 1.91E-05 21900 4.32E-05 21500
8.00E-05 23700 0.000205693 23200 0.000163182
24100 0.002279873 22100 1.91E-05 21900 4.32E-05 21500
7.99E-05 23700 0.000205898 23200 0.000163302
24100 0.002286299 22100 1.91E-05 21900 4.33E-05 21500
7.98E-05 23700 0.000206108 23200 0.000163388
24100 0.002292752 22100 1.91E-05 21900 4.33E-05 21500
7.97E-05 23700 0.000206316 23200 0.000163506
24100 0.002299232 22100 1.91E-05 21900 4.33E-05 21500
7.96E-05 23800 0.00020652 23200 0.00016361
24100 0.002305763 22100 1.91E-05 21900 4.33E-05 21500
7.95E-05 23800 0.000206725 23200 0.000163722
24100 0.00231233 22100 1.91E-05 21900 4.33E-05 21500
7.94E-05 23800 0.000206923 23200 0.000163813
24100 0.002318918 22100 1.91E-05 21900 4.33E-05 21500
7.93E-05 23800 0.000207129 23200 0.000163941
24100 0.002325565 22100 1.90E-05 21900 4.33E-05 21500
7.92E-05 23800 0.000207332 23200 0.00016405
24100 0.002332231 22100 1.91E-05 21900 4.33E-05 21500
7.92E-05 23800 0.000207549 23200 0.000164165
24100 0.002338962 22100 1.91E-05 21900 4.33E-05 21500
7.91E-05 23800 0.000207756 23200 0.000164269
24100 0.002345726 22100 1.90E-05 21900 4.33E-05 21500
7.90E-05 23800 0.000207968 23200 0.000164364
24100 0.00235254 22100 1.90E-05 21900 4.33E-05 21500
7.89E-05 23800 0.000208186 23200 0.000164468
24100 0.002359401 22100 1.90E-05 21900 4.33E-05 21500
7.88E-05 23800 0.0002084 23200 0.000164569
24100 0.002366275 22100 1.90E-05 21900 4.34E-05 21500
7.87E-05 23800 0.000208599 23200 0.000164699
24100 0.002373177 22100 1.90E-05 21900 4.34E-05 21500
7.87E-05 23800 0.00020881 23200 0.000164812
24100 0.002380164 22100 1.90E-05 21900 4.34E-05 21500
7.86E-05 23800 0.00020904 23200 0.000164936
24100 0.002387177 22100 1.90E-05 21900 4.34E-05 21500
7.85E-05 23800 0.000209253 23200 0.000165051
24100 0.002394215 22100 1.90E-05 21900 4.34E-05 21500
7.84E-05 23800 0.000209475 23200 0.000165148
24100 0.00240131 22100 1.90E-05 21900 4.34E-05 21500
7.83E-05 23800 0.000209692 23200 0.000165264
24100 0.00240844 22100 1.90E-05 21900 4.34E-05 21500
7.83E-05 23800 0.000209916 23200 0.000165372
24100 0.002415637 22100 1.90E-05 21900 4.34E-05 21500
7.82E-05 23800 0.000210128 23200 0.000165475
24100 0.002422863 22100 1.90E-05 21900 4.35E-05 21500
7.81E-05 23800 0.000210349 23200 0.000165577
24100 0.002430129 22100 1.90E-05 21900 4.35E-05 21500
7.80E-05 23800 0.00021057 23200 0.000165689
24100 0.00243743 22100 1.90E-05 21900 4.35E-05 21500
7.80E-05 23800 0.000210787 23200 0.0001658
24100 0.002444785 22100 1.90E-05 21900 4.35E-05 21500
7.79E-05 23800 0.000210995 23200 0.000165907
24100 0.002452182 22100 1.90E-05 21900 4.35E-05 21500
7.78E-05 23800 0.000211217 23200 0.000166014
24100 0.002459596 22100 1.90E-05 21900 4.35E-05 21500
7.77E-05 23800 0.000211427 23200 0.000166122

24100 0.002467058 22100 1.90E-05 21900 4.35E-05 21500
7.76E-05 23800 0.000211656 23200 0.000166243
24100 0.002474586 22100 1.90E-05 21900 4.35E-05 21500
7.76E-05 23800 0.000211871 23200 0.000166347
24100 0.002482168 22100 1.90E-05 21900 4.35E-05 21500
7.75E-05 23800 0.000212098 23200 0.000166437
24100 0.002489818 22100 1.90E-05 21900 4.35E-05 21500
7.74E-05 23800 0.000212319 23200 0.000166536
24100 0.002497477 22100 1.90E-05 21900 4.35E-05 21500
7.74E-05 23800 0.000212539 23200 0.000166642
24200 0.002505187 22100 1.90E-05 21900 4.36E-05 21500
7.74E-05 23800 0.000212745 23200 0.000166764
24200 0.002512917 22100 1.90E-05 21900 4.36E-05 21500
7.73E-05 23800 0.00021298 23200 0.000166871
24200 0.002520747 22100 1.90E-05 21900 4.36E-05 21500
7.73E-05 23800 0.000213202 23200 0.000166977
24200 0.002528612 22100 1.90E-05 21900 4.36E-05 21500
7.72E-05 23800 0.000213422 23200 0.000167084
24200 0.002536505 22100 1.91E-05 21900 4.36E-05 21500
7.72E-05 23800 0.000213653 23200 0.000167191
24200 0.002544485 22100 1.91E-05 21900 4.36E-05 21500
7.71E-05 23800 0.000213874 23200 0.000167293
24200 0.002552504 22100 1.91E-05 21900 4.36E-05 21500
7.70E-05 23800 0.000214107 23200 0.000167408
24200 0.002560573 22100 1.91E-05 21900 4.36E-05 21500
7.70E-05 23800 0.000214335 23200 0.000167509
24200 0.002568692 22100 1.91E-05 21900 4.36E-05 21500
7.69E-05 23800 0.000214564 23200 0.000167618
24200 0.002576889 22100 1.91E-05 21900 4.37E-05 21500
7.68E-05 23800 0.000214785 23300 0.000167726
24200 0.002585116 22100 1.91E-05 21900 4.37E-05 21500
7.67E-05 23800 0.000215016 23300 0.00016784
24200 0.002593398 22100 1.91E-05 21900 4.37E-05 21500
7.66E-05 23800 0.000215237 23300 0.000167948
24200 0.002601716 22100 1.91E-05 21900 4.37E-05 21500
7.66E-05 23800 0.000215467 23300 0.000168072
24200 0.002610101 22100 1.91E-05 21900 4.37E-05 21500
7.65E-05 23800 0.000215691 23300 0.000168177
24200 0.002618553 22100 1.92E-05 21900 4.37E-05 21500
7.64E-05 23800 0.000215932 23300 0.000168298
24200 0.002627036 22100 1.92E-05 21900 4.38E-05 21500
7.63E-05 23800 0.000216156 23300 0.000168404
24200 0.002635603 22100 1.92E-05 21900 4.38E-05 21500
7.62E-05 23800 0.000216389 23300 0.00016851
24200 0.002644235 22100 1.92E-05 21900 4.38E-05 21500
7.61E-05 23800 0.000216642 23300 0.000168618
24200 0.002652916 22200 1.92E-05 21900 4.38E-05 21500
7.60E-05 23800 0.000216864 23300 0.00016873
24200 0.002661631 22200 1.92E-05 21900 4.38E-05 21500
7.59E-05 23800 0.000217089 23300 0.000168855
24200 0.002670412 22200 1.92E-05 22000 4.38E-05 21500
7.58E-05 23800 0.000217332 23300 0.000168961
24200 0.002679247 22200 1.92E-05 22000 4.38E-05 21500
7.57E-05 23800 0.000217574 23300 0.000169079
24200 0.002688155 22200 1.92E-05 22000 4.38E-05 21500
7.57E-05 23800 0.000217814 23300 0.000169197
24200 0.002697091 22200 1.92E-05 22000 4.39E-05 21500
7.56E-05 23800 0.000218063 23300 0.000169305
24200 0.002706109 22200 1.92E-05 22000 4.39E-05 21600
7.55E-05 23800 0.000218298 23300 0.000169415
24200 0.002715174 22200 1.93E-05 22000 4.39E-05 21600
7.55E-05 23800 0.000218539 23300 0.00016953
24200 0.002724316 22200 1.93E-05 22000 4.39E-05 21600
7.54E-05 23800 0.000218777 23300 0.000169637
24200 0.002733511 22200 1.93E-05 22000 4.39E-05 21600
7.53E-05 23800 0.00021901 23300 0.00016975
24200 0.002742754 22200 1.93E-05 22000 4.40E-05 21600
7.53E-05 23800 0.000219246 23300 0.000169858

24200	0.002752042	22200	1.93E-05	22000	4.40E-05	21600	7.52E-05	23800	0.000219483	23300	0.000169973
24200	0.002761409	22200	1.93E-05	22000	4.40E-05	21600	7.52E-05	23800	0.000219714	23300	0.000170097
24200	0.002770822	22200	1.93E-05	22000	4.40E-05	21600	7.51E-05	23800	0.000219951	23300	0.000170214
24200	0.002780312	22200	1.93E-05	22000	4.41E-05	21600	7.50E-05	23800	0.000220184	23300	0.000170319
24200	0.002789854	22200	1.93E-05	22000	4.41E-05	21600	7.50E-05	23800	0.000220426	23300	0.000170438
24200	0.002799463	22200	1.93E-05	22000	4.41E-05	21600	7.49E-05	23800	0.000220659	23300	0.000170548
24200	0.002809122	22200	1.93E-05	22000	4.41E-05	21600	7.48E-05	23900	0.000220896	23300	0.000170651
24200	0.002818902	22200	1.93E-05	22000	4.41E-05	21600	7.48E-05	23900	0.00022114	23300	0.000170756
24200	0.002828718	22200	1.93E-05	22000	4.41E-05	21600	7.47E-05	23900	0.000221369	23300	0.000170867
24200	0.002838597	22200	1.93E-05	22000	4.41E-05	21600	7.47E-05	23900	0.000221596	23300	0.000170991
24200	0.00284852	22200	1.93E-05	22000	4.42E-05	21600	7.46E-05	23900	0.000221841	23300	0.000171092
24200	0.002858545	22200	1.93E-05	22000	4.42E-05	21600	7.46E-05	23900	0.000222079	23300	0.00017122
24200	0.002868627	22200	1.93E-05	22000	4.42E-05	21600	7.45E-05	23900	0.000222315	23300	0.000171335
24200	0.002878779	22200	1.93E-05	22000	4.42E-05	21600	7.44E-05	23900	0.000222566	23300	0.00017144
24200	0.002889015	22200	1.93E-05	22000	4.42E-05	21600	7.44E-05	23900	0.000222803	23300	0.000171549
24200	0.00289932	22200	1.93E-05	22000	4.43E-05	21600	7.43E-05	23900	0.000223048	23300	0.00017167
24200	0.002909695	22200	1.93E-05	22000	4.43E-05	21600	7.42E-05	23900	0.000223289	23300	0.000171771
24200	0.002920133	22200	1.94E-05	22000	4.43E-05	21600	7.42E-05	23900	0.000223524	23300	0.000171888
24200	0.00293066	22200	1.94E-05	22000	4.43E-05	21600	7.41E-05	23900	0.000223765	23300	0.000172002
24200	0.002941248	22200	1.94E-05	22000	4.44E-05	21600	7.40E-05	23900	0.000223994	23300	0.000172122
24200	0.002951913	22200	1.94E-05	22000	4.44E-05	21600	7.40E-05	23900	0.000224228	23300	0.000172234
24200	0.002962632	22200	1.94E-05	22000	4.44E-05	21600	7.39E-05	23900	0.000224457	23300	0.000172358
24200	0.002973449	22200	1.94E-05	22000	4.44E-05	21600	7.38E-05	23900	0.000224688	23300	0.000172472
24200	0.002984317	22200	1.94E-05	22000	4.44E-05	21600	7.38E-05	23900	0.000224932	23300	0.000172606
24200	0.002995262	22200	1.94E-05	22000	4.45E-05	21600	7.37E-05	23900	0.00022516	23300	0.000172722
24200	0.00300629	22200	1.94E-05	22000	4.45E-05	21600	7.37E-05	23900	0.000225402	23300	0.00017283
24200	0.003017424	22200	1.94E-05	22000	4.45E-05	21600	7.36E-05	23900	0.000225653	23300	0.000172946
24200	0.003028628	22200	1.94E-05	22000	4.45E-05	21600	7.36E-05	23900	0.000225875	23300	0.000173057
24200	0.003039878	22200	1.94E-05	22000	4.45E-05	21600	7.35E-05	23900	0.000226108	23300	0.000173199
24200	0.003051222	22200	1.94E-05	22000	4.46E-05	21600	7.35E-05	23900	0.000226356	23300	0.000173317
24200	0.003062662	22200	1.94E-05	22000	4.46E-05	21600	7.34E-05	23900	0.000226594	23300	0.000173451
24200	0.003074178	22200	1.94E-05	22000	4.46E-05	21600	7.34E-05	23900	0.000226838	23300	0.000173577
24200	0.00308578	22200	1.94E-05	22000	4.46E-05	21600	7.33E-05	23900	0.000227091	23300	0.000173697
24200	0.00309744	22200	1.95E-05	22000	4.46E-05	21600	7.33E-05	23900	0.000227332	23300	0.000173817

24200 0.003109196 22200 1.95E-05 22000 4.47E-05 21600
7.32E-05 23900 0.000227582 23300 0.000173941
24300 0.003121068 22200 1.95E-05 22000 4.47E-05 21600
7.32E-05 23900 0.00022783 23300 0.000174059
24300 0.003132998 22200 1.95E-05 22000 4.47E-05 21600
7.31E-05 23900 0.00022807 23300 0.000174174
24300 0.003145027 22200 1.95E-05 22000 4.47E-05 21600
7.30E-05 23900 0.000228316 23300 0.000174293
24300 0.003157151 22200 1.95E-05 22000 4.47E-05 21600
7.29E-05 23900 0.000228564 23300 0.000174414
24300 0.003169358 22200 1.95E-05 22000 4.47E-05 21600
7.29E-05 23900 0.00022881 23300 0.000174531
24300 0.003181633 22200 1.95E-05 22000 4.48E-05 21600
7.28E-05 23900 0.000229057 23300 0.000174654
24300 0.003194043 22200 1.95E-05 22000 4.48E-05 21600
7.27E-05 23900 0.000229305 23300 0.000174775
24300 0.003206497 22200 1.95E-05 22000 4.48E-05 21600
7.27E-05 23900 0.000229572 23300 0.000174909
24300 0.003219076 22200 1.96E-05 22000 4.48E-05 21600
7.27E-05 23900 0.000229816 23300 0.000175027
24300 0.003231768 22200 1.96E-05 22000 4.48E-05 21600
7.26E-05 23900 0.000230067 23400 0.000175136
24300 0.003244528 22200 1.96E-05 22000 4.48E-05 21600
7.26E-05 23900 0.000230319 23400 0.000175255
24300 0.003257408 22200 1.96E-05 22000 4.49E-05 21600
7.26E-05 23900 0.000230559 23400 0.000175368
24300 0.003270371 22200 1.96E-05 22000 4.49E-05 21600
7.26E-05 23900 0.000230805 23400 0.000175505
24300 0.003283401 22200 1.96E-05 22000 4.49E-05 21600
7.25E-05 23900 0.000231055 23400 0.000175628
24300 0.003296577 22200 1.96E-05 22000 4.49E-05 21600
7.25E-05 23900 0.000231308 23400 0.000175757
24300 0.00330988 22200 1.96E-05 22000 4.49E-05 21600
7.25E-05 23900 0.00023156 23400 0.000175879
24300 0.00332324 22200 1.96E-05 22000 4.49E-05 21600
7.25E-05 23900 0.000231809 23400 0.000176004
24300 0.00333672 22200 1.96E-05 22000 4.49E-05 21600
7.24E-05 23900 0.000232056 23400 0.000176122
24300 0.003350318 22300 1.96E-05 22000 4.49E-05 21600
7.23E-05 23900 0.00023231 23400 0.000176243
24300 0.003364018 22300 1.96E-05 22000 4.49E-05 21600
7.23E-05 23900 0.000232561 23400 0.000176353
24300 0.003377832 22300 1.97E-05 22100 4.49E-05 21600
7.22E-05 23900 0.000232811 23400 0.000176474
24300 0.003391779 22300 1.97E-05 22100 4.50E-05 21600
7.21E-05 23900 0.000233058 23400 0.000176586
24300 0.003405772 22300 1.97E-05 22100 4.50E-05 21600
7.20E-05 23900 0.000233309 23400 0.000176708
24300 0.003419934 22300 1.97E-05 22100 4.50E-05 21600
7.19E-05 23900 0.00023355 23400 0.000176824
24300 0.003434175 22300 1.97E-05 22100 4.50E-05 21700
7.18E-05 23900 0.000233804 23400 0.000176956
24300 0.003448518 22300 1.97E-05 22100 4.50E-05 21700
7.18E-05 23900 0.000234059 23400 0.000177073
24300 0.003462986 22300 1.97E-05 22100 4.50E-05 21700
7.17E-05 23900 0.000234325 23400 0.000177209
24300 0.003477591 22300 1.97E-05 22100 4.50E-05 21700
7.16E-05 23900 0.00023459 23400 0.000177328
24300 0.003492307 22300 1.97E-05 22100 4.50E-05 21700
7.16E-05 23900 0.000234841 23400 0.000177446
24300 0.003507168 22300 1.97E-05 22100 4.51E-05 21700
7.15E-05 23900 0.000235106 23400 0.000177566
24300 0.003522122 22300 1.97E-05 22100 4.51E-05 21700
7.15E-05 23900 0.000235362 23400 0.000177688
24300 0.003537199 22300 1.97E-05 22100 4.51E-05 21700
7.15E-05 23900 0.000235611 23400 0.000177825
24300 0.003552394 22300 1.97E-05 22100 4.51E-05 21700
7.15E-05 23900 0.000235889 23400 0.000177943

24300 0.003567728 22300 1.98E-05 22100 4.51E-05 21700
7.15E-05 23900 0.000236162 23400 0.000178057
24300 0.003583182 22300 1.98E-05 22100 4.51E-05 21700
7.15E-05 23900 0.00023643 23400 0.000178178
24300 0.003598771 22300 1.98E-05 22100 4.51E-05 21700
7.15E-05 24000 0.000236705 23400 0.000178304
24300 0.003614466 22300 1.98E-05 22100 4.51E-05 21700
7.15E-05 24000 0.000236968 23400 0.000178407
24300 0.003630278 22300 1.98E-05 22100 4.52E-05 21700
7.14E-05 24000 0.000237232 23400 0.000178535
24300 0.003646257 22300 1.98E-05 22100 4.52E-05 21700
7.14E-05 24000 0.000237507 23400 0.000178652
24300 0.003662339 22300 1.98E-05 22100 4.52E-05 21700
7.14E-05 24000 0.000237781 23400 0.000178775
24300 0.003678551 22300 1.98E-05 22100 4.52E-05 21700
7.14E-05 24000 0.00023805 23400 0.000178883
24300 0.003694898 22300 1.98E-05 22100 4.52E-05 21700
7.14E-05 24000 0.000238327 23400 0.000179011
24300 0.003711398 22300 1.98E-05 22100 4.52E-05 21700
7.13E-05 24000 0.000238601 23400 0.000179128
24300 0.003727993 22300 1.98E-05 22100 4.53E-05 21700
7.13E-05 24000 0.000238874 23400 0.000179251
24300 0.003744744 22300 1.98E-05 22100 4.53E-05 21700
7.13E-05 24000 0.000239158 23400 0.000179371
24300 0.00376161 22300 1.98E-05 22100 4.53E-05 21700
7.12E-05 24000 0.000239448 23400 0.000179509
24300 0.003778646 22300 1.98E-05 22100 4.53E-05 21700
7.12E-05 24000 0.000239726 23400 0.000179625
24300 0.003795824 22300 1.98E-05 22100 4.53E-05 21700
7.11E-05 24000 0.000240002 23400 0.000179735
24300 0.003813178 22300 1.98E-05 22100 4.53E-05 21700
7.11E-05 24000 0.000240289 23400 0.000179867
24300 0.003830662 22300 1.98E-05 22100 4.53E-05 21700
7.10E-05 24000 0.000240565 23400 0.000179989
24300 0.003848268 22300 1.98E-05 22100 4.53E-05 21700
7.09E-05 24000 0.000240849 23400 0.000180128
24300 0.00386602 22300 1.98E-05 22100 4.54E-05 21700
7.09E-05 24000 0.000241138 23400 0.000180249
24300 0.003883948 22300 1.98E-05 22100 4.54E-05 21700
7.08E-05 24000 0.000241431 23400 0.000180375
24300 0.003902054 22300 1.99E-05 22100 4.54E-05 21700
7.08E-05 24000 0.000241728 23400 0.000180492
24300 0.00392031 22300 1.99E-05 22100 4.54E-05 21700
7.07E-05 24000 0.000242021 23400 0.000180628
24300 0.003938712 22300 1.99E-05 22100 4.54E-05 21700
7.07E-05 24000 0.000242297 23400 0.000180743
24300 0.003957301 22300 1.99E-05 22100 4.54E-05 21700
7.06E-05 24000 0.000242583 23400 0.000180874
24300 0.003976068 22300 1.99E-05 22100 4.55E-05 21700
7.06E-05 24000 0.000242876 23400 0.000180999
24300 0.003994988 22300 1.99E-05 22100 4.55E-05 21700
7.05E-05 24000 0.000243156 23400 0.000181124
24300 0.004014095 22300 1.99E-05 22100 4.55E-05 21700
7.04E-05 24000 0.00024344 23400 0.000181252
24300 0.004033352 22300 1.99E-05 22100 4.55E-05 21700
7.04E-05 24000 0.000243728 23400 0.000181389
24300 0.004052794 22300 1.99E-05 22100 4.55E-05 21700
7.03E-05 24000 0.000244012 23400 0.000181515
24300 0.004072414 22300 2.00E-05 22100 4.56E-05 21700
7.03E-05 24000 0.000244295 23400 0.00018165
24300 0.004092222 22300 2.00E-05 22100 4.56E-05 21700
7.02E-05 24000 0.000244583 23400 0.000181783
24400 0.004112205 22300 2.00E-05 22100 4.56E-05 21700
7.02E-05 24000 0.00024488 23400 0.000181922
24400 0.004132383 22300 2.00E-05 22100 4.56E-05 21700
7.01E-05 24000 0.000245161 23400 0.000182039
24400 0.00415275 22300 2.00E-05 22100 4.56E-05 21700
7.01E-05 24000 0.00024544 23400 0.00018216

24400 0.004173332 22300 2.00E-05 22100 4.56E-05 21700
7.00E-05 24000 0.000245726 23400 0.000182295
24400 0.004194093 22300 2.00E-05 22100 4.57E-05 21700
7.00E-05 24000 0.000245999 23400 0.000182416
24400 0.004215059 22300 2.00E-05 22100 4.57E-05 21700
6.99E-05 24000 0.000246259 23400 0.000182572
24400 0.004236201 22300 2.00E-05 22100 4.57E-05 21700
6.99E-05 24000 0.000246538 23400 0.000182689
24400 0.004257572 22300 2.00E-05 22100 4.57E-05 21700
6.98E-05 24000 0.000246816 23400 0.000182829
24400 0.004279142 22300 2.01E-05 22100 4.57E-05 21700
6.98E-05 24000 0.000247105 23400 0.000182954
24400 0.00430091 22300 2.01E-05 22100 4.57E-05 21700
6.98E-05 24000 0.000247389 23500 0.0001831
24400 0.004322909 22300 2.01E-05 22100 4.58E-05 21700
6.97E-05 24000 0.000247658 23500 0.000183223
24400 0.00434512 22300 2.01E-05 22100 4.58E-05 21700
6.97E-05 24000 0.000247932 23500 0.000183343
24400 0.004367537 22300 2.01E-05 22100 4.58E-05 21700
6.96E-05 24000 0.000248211 23500 0.000183474
24400 0.004390161 22300 2.01E-05 22100 4.58E-05 21700
6.96E-05 24000 0.000248475 23500 0.000183607
24400 0.004413009 22300 2.01E-05 22100 4.58E-05 21700
6.95E-05 24000 0.000248747 23500 0.00018374
24400 0.004436083 22300 2.01E-05 22100 4.59E-05 21700
6.95E-05 24000 0.00024902 23500 0.000183874
24400 0.004459384 22300 2.01E-05 22100 4.59E-05 21700
6.95E-05 24000 0.00024928 23500 0.000184009
24400 0.0044829 22300 2.01E-05 22100 4.59E-05 21700
6.94E-05 24000 0.000249548 23500 0.000184144
24400 0.004506657 22400 2.02E-05 22100 4.59E-05 21700
6.93E-05 24000 0.000249818 23500 0.000184277
24400 0.004530626 22400 2.02E-05 22100 4.60E-05 21700
6.93E-05 24000 0.000250093 23500 0.000184422
24400 0.004554844 22400 2.02E-05 22200 4.60E-05 21700
6.93E-05 24000 0.000250362 23500 0.000184544
24400 0.004579337 22400 2.02E-05 22200 4.60E-05 21700
6.92E-05 24000 0.000250625 23500 0.000184668
24400 0.004604058 22400 2.02E-05 22200 4.60E-05 21700
6.92E-05 24000 0.000250894 23500 0.000184813
24400 0.004629019 22400 2.02E-05 22200 4.60E-05 21700
6.91E-05 24000 0.000251155 23500 0.000184934
24400 0.00465422 22400 2.02E-05 22200 4.60E-05 21800
6.91E-05 24000 0.00025141 23500 0.000185084
24400 0.004679677 22400 2.02E-05 22200 4.61E-05 21800
6.91E-05 24000 0.000251684 23500 0.00018521
24400 0.004705393 22400 2.02E-05 22200 4.61E-05 21800
6.91E-05 24000 0.000251956 23500 0.000185358
24400 0.004731389 22400 2.02E-05 22200 4.61E-05 21800
6.90E-05 24000 0.000252229 23500 0.000185472
24400 0.004757636 22400 2.03E-05 22200 4.61E-05 21800
6.90E-05 24000 0.000252493 23500 0.000185621
24400 0.004784149 22400 2.03E-05 22200 4.61E-05 21800
6.90E-05 24000 0.000252762 23500 0.000185759
24400 0.00481092 22400 2.03E-05 22200 4.61E-05 21800
6.90E-05 24000 0.000253028 23500 0.000185892
24400 0.004837996 22400 2.03E-05 22200 4.62E-05 21800
6.90E-05 24000 0.000253292 23500 0.000186022
24400 0.00486536 22400 2.03E-05 22200 4.62E-05 21800
6.90E-05 24000 0.000253552 23500 0.000186145
24400 0.004892975 22400 2.03E-05 22200 4.62E-05 21800
6.89E-05 24000 0.000253823 23500 0.000186254
24400 0.004920891 22400 2.03E-05 22200 4.62E-05 21800
6.89E-05 24000 0.000254085 23500 0.000186402
24400 0.004949087 22400 2.04E-05 22200 4.62E-05 21800
6.89E-05 24100 0.00025435 23500 0.00018652
24400 0.004977569 22400 2.04E-05 22200 4.63E-05 21800
6.88E-05 24100 0.00025462 23500 0.000186659

24400 0.005006379 22400 2.04E-05 22200 4.63E-05 21800
6.88E-05 24100 0.000254891 23500 0.000186783
24400 0.005035457 22400 2.04E-05 22200 4.63E-05 21800
6.88E-05 24100 0.000255165 23500 0.000186922
24400 0.005064865 22400 2.04E-05 22200 4.63E-05 21800
6.87E-05 24100 0.000255445 23500 0.00018703
24400 0.005094606 22400 2.04E-05 22200 4.63E-05 21800
6.87E-05 24100 0.000255706 23500 0.000187141
24400 0.005124647 22400 2.04E-05 22200 4.63E-05 21800
6.87E-05 24100 0.000255984 23500 0.000187302
24400 0.005155 22400 2.04E-05 22200 4.64E-05 21800
6.86E-05 24100 0.000256249 23500 0.000187413
24400 0.005185683 22400 2.04E-05 22200 4.64E-05 21800
6.86E-05 24100 0.000256514 23500 0.000187551
24400 0.005216692 22400 2.04E-05 22200 4.64E-05 21800
6.86E-05 24100 0.000256791 23500 0.000187676
24400 0.005248064 22400 2.04E-05 22200 4.64E-05 21800
6.86E-05 24100 0.000257066 23500 0.000187809
24400 0.005279759 22400 2.04E-05 22200 4.64E-05 21800
6.85E-05 24100 0.000257348 23500 0.000187935
24400 0.005311834 22400 2.04E-05 22200 4.64E-05 21800
6.85E-05 24100 0.000257625 23500 0.000188091
24400 0.005344232 22400 2.04E-05 22200 4.65E-05 21800
6.85E-05 24100 0.000257905 23500 0.000188212
24400 0.005376961 22400 2.05E-05 22200 4.65E-05 21800
6.85E-05 24100 0.000258187 23500 0.000188353
24400 0.0054101 22400 2.05E-05 22200 4.65E-05 21800
6.84E-05 24100 0.000258462 23500 0.000188477
24400 0.005443565 22400 2.05E-05 22200 4.65E-05 21800
6.84E-05 24100 0.000258746 23500 0.000188627
24400 0.005477425 22400 2.05E-05 22200 4.65E-05 21800
6.84E-05 24100 0.000259036 23500 0.000188729
24400 0.005511656 22400 2.05E-05 22200 4.66E-05 21800
6.84E-05 24100 0.000259322 23500 0.000188875
24400 0.00554628 22400 2.05E-05 22200 4.66E-05 21800
6.84E-05 24100 0.000259606 23500 0.000189007
24400 0.00558127 22400 2.05E-05 22200 4.66E-05 21800
6.83E-05 24100 0.000259891 23500 0.000189167
24400 0.005616686 22400 2.06E-05 22200 4.66E-05 21800
6.83E-05 24100 0.000260183 23500 0.000189304
24400 0.005652462 22400 2.06E-05 22200 4.66E-05 21800
6.83E-05 24100 0.000260483 23500 0.000189457
24400 0.00568867 22400 2.06E-05 22200 4.66E-05 21800
6.83E-05 24100 0.000260786 23500 0.000189566
24400 0.005725307 22400 2.06E-05 22200 4.67E-05 21800
6.82E-05 24100 0.000261074 23500 0.00018969
24400 0.005762343 22400 2.06E-05 22200 4.67E-05 21800
6.82E-05 24100 0.000261378 23500 0.000189854
24400 0.005799833 22400 2.06E-05 22200 4.67E-05 21800
6.82E-05 24100 0.000261675 23500 0.000189987
24400 0.005837706 22400 2.06E-05 22200 4.67E-05 21800
6.81E-05 24100 0.000261961 23500 0.000190158
24400 0.005876021 22400 2.06E-05 22200 4.68E-05 21800
6.81E-05 24100 0.000262268 23500 0.000190286
24500 0.005914813 22400 2.06E-05 22200 4.68E-05 21800
6.81E-05 24100 0.000262569 23500 0.000190434
24500 0.005954027 22400 2.06E-05 22200 4.68E-05 21800
6.81E-05 24100 0.000262884 23500 0.000190567
24500 0.005993692 22400 2.06E-05 22200 4.68E-05 21800
6.81E-05 24100 0.000263188 23500 0.000190725
24500 0.006033855 22400 2.07E-05 22200 4.68E-05 21800
6.81E-05 24100 0.000263483 23500 0.00019086
24500 0.006074429 22400 2.07E-05 22200 4.69E-05 21800
6.81E-05 24100 0.00026379 23500 0.000190994
24500 0.006115554 22400 2.07E-05 22200 4.69E-05 21800
6.81E-05 24100 0.000264091 23500 0.000191127
24500 0.006157133 22400 2.07E-05 22200 4.69E-05 21800
6.81E-05 24100 0.000264397 23500 0.000191276

24500 0.006199205 22400 2.07E-05 22200 4.70E-05 21800
6.80E-05 24100 0.000264706 23500 0.000191407
24500 0.006241736 22400 2.07E-05 22200 4.70E-05 21800
6.80E-05 24100 0.000265007 23500 0.000191569
24500 0.006284817 22400 2.07E-05 22200 4.70E-05 21800
6.80E-05 24100 0.000265306 23600 0.000191697
24500 0.006328382 22400 2.07E-05 22200 4.70E-05 21800
6.80E-05 24100 0.000265607 23600 0.000191838
24500 0.006372454 22400 2.07E-05 22200 4.71E-05 21800
6.80E-05 24100 0.000265925 23600 0.000191979
24500 0.006417037 22400 2.07E-05 22200 4.71E-05 21800
6.79E-05 24100 0.000266235 23600 0.00019213
24500 0.006462213 22400 2.07E-05 22200 4.71E-05 21800
6.79E-05 24100 0.000266549 23600 0.000192258
24500 0.006507911 22400 2.07E-05 22200 4.71E-05 21800
6.78E-05 24100 0.000266851 23600 0.000192376
24500 0.006554183 22400 2.08E-05 22200 4.71E-05 21800
6.78E-05 24100 0.000267168 23600 0.000192534
24500 0.006601009 22400 2.08E-05 22200 4.72E-05 21800
6.77E-05 24100 0.000267476 23600 0.00019266
24500 0.006648365 22400 2.08E-05 22200 4.72E-05 21800
6.76E-05 24100 0.000267784 23600 0.000192797
24500 0.006696283 22500 2.08E-05 22200 4.72E-05 21800
6.76E-05 24100 0.0002681 23600 0.000192942
24500 0.00674483 22500 2.08E-05 22200 4.72E-05 21800
6.76E-05 24100 0.000268423 23600 0.000193081
24500 0.006793977 22500 2.08E-05 22300 4.73E-05 21800
6.75E-05 24100 0.000268751 23600 0.000193222
24500 0.006843681 22500 2.08E-05 22300 4.73E-05 21800
6.75E-05 24100 0.000269074 23600 0.000193362
24500 0.006894016 22500 2.08E-05 22300 4.73E-05 21800
6.75E-05 24100 0.000269395 23600 0.000193499
24500 0.006944957 22500 2.08E-05 22300 4.74E-05 21800
6.74E-05 24100 0.000269719 23600 0.000193642
24500 0.006996555 22500 2.08E-05 22300 4.74E-05 21900
6.74E-05 24100 0.000270032 23600 0.000193783
24500 0.007048739 22500 2.09E-05 22300 4.74E-05 21900
6.73E-05 24100 0.000270357 23600 0.000193916
24500 0.0071016 22500 2.09E-05 22300 4.74E-05 21900
6.73E-05 24100 0.000270691 23600 0.000194054
24500 0.007155101 22500 2.09E-05 22300 4.74E-05 21900
6.73E-05 24100 0.000271015 23600 0.000194218
24500 0.007209263 22500 2.09E-05 22300 4.75E-05 21900
6.73E-05 24100 0.000271344 23600 0.000194359
24500 0.007264086 22500 2.09E-05 22300 4.75E-05 21900
6.73E-05 24100 0.000271674 23600 0.000194506
24500 0.007319591 22500 2.09E-05 22300 4.75E-05 21900
6.73E-05 24100 0.00027201 23600 0.000194642
24500 0.007375768 22500 2.09E-05 22300 4.76E-05 21900
6.73E-05 24100 0.00027234 23600 0.000194791
24500 0.007432657 22500 2.10E-05 22300 4.76E-05 21900
6.73E-05 24100 0.000272689 23600 0.000194926
24500 0.007490268 22500 2.10E-05 22300 4.76E-05 21900
6.73E-05 24100 0.000273008 23600 0.000195053
24500 0.007548603 22500 2.10E-05 22300 4.76E-05 21900
6.73E-05 24100 0.00027335 23600 0.000195199
24500 0.007607653 22500 2.10E-05 22300 4.76E-05 21900
6.72E-05 24200 0.000273679 23600 0.000195348
24500 0.007667405 22500 2.10E-05 22300 4.77E-05 21900
6.72E-05 24200 0.000274004 23600 0.000195496
24500 0.007727895 22500 2.10E-05 22300 4.77E-05 21900
6.72E-05 24200 0.000274346 23600 0.000195628
24500 0.00778915 22500 2.10E-05 22300 4.77E-05 21900
6.72E-05 24200 0.000274688 23600 0.000195773
24500 0.007851185 22500 2.10E-05 22300 4.77E-05 21900
6.72E-05 24200 0.000275035 23600 0.000195922
24500 0.007913926 22500 2.10E-05 22300 4.78E-05 21900
6.72E-05 24200 0.000275376 23600 0.000196066

24500 0.007977519 22500 2.11E-05 22300 4.78E-05 21900
6.72E-05 24200 0.000275716 23600 0.000196194
24500 0.008041847 22500 2.11E-05 22300 4.78E-05 21900
6.72E-05 24200 0.000276049 23600 0.000196351
24500 0.008107004 22500 2.11E-05 22300 4.78E-05 21900
6.72E-05 24200 0.000276376 23600 0.000196471
24500 0.008172931 22500 2.11E-05 22300 4.79E-05 21900
6.72E-05 24200 0.000276724 23600 0.000196624
24500 0.008239666 22500 2.11E-05 22300 4.79E-05 21900
6.71E-05 24200 0.000277062 23600 0.000196762
24500 0.008307195 22500 2.11E-05 22300 4.79E-05 21900
6.71E-05 24200 0.000277396 23600 0.000196899
24500 0.008375619 22500 2.11E-05 22300 4.79E-05 21900
6.71E-05 24200 0.000277721 23600 0.000197061
24500 0.008444842 22500 2.11E-05 22300 4.80E-05 21900
6.71E-05 24200 0.000278057 23600 0.000197211
24500 0.008514926 22500 2.11E-05 22300 4.80E-05 21900
6.71E-05 24200 0.000278404 23600 0.000197349
24500 0.008585853 22500 2.11E-05 22300 4.80E-05 21900
6.71E-05 24200 0.000278736 23600 0.000197504
24500 0.008657664 22500 2.11E-05 22300 4.80E-05 21900
6.70E-05 24200 0.000279083 23600 0.000197623
24500 0.008730331 22500 2.11E-05 22300 4.81E-05 21900
6.70E-05 24200 0.000279402 23600 0.000197759
24500 0.00880393 22500 2.12E-05 22300 4.81E-05 21900
6.70E-05 24200 0.000279742 23600 0.000197902
24500 0.008878423 22500 2.12E-05 22300 4.81E-05 21900
6.69E-05 24200 0.000280068 23600 0.000198042
24500 0.008953803 22500 2.12E-05 22300 4.81E-05 21900
6.69E-05 24200 0.000280383 23600 0.000198212
24500 0.009030083 22500 2.12E-05 22300 4.82E-05 21900
6.69E-05 24200 0.000280721 23600 0.000198342
24500 0.009107308 22500 2.12E-05 22300 4.82E-05 21900
6.69E-05 24200 0.00028106 23600 0.000198487
24500 0.009185483 22500 2.12E-05 22300 4.82E-05 21900
6.69E-05 24200 0.000281398 23600 0.000198632
24500 0.009264587 22500 2.12E-05 22300 4.82E-05 21900
6.69E-05 24200 0.000281728 23600 0.000198783
24500 0.009344663 22500 2.12E-05 22300 4.82E-05 21900
6.69E-05 24200 0.000282069 23600 0.000198931
24500 0.009425675 22500 2.12E-05 22300 4.83E-05 21900
6.68E-05 24200 0.000282403 23600 0.00019907
24500 0.009507683 22500 2.12E-05 22300 4.83E-05 21900
6.68E-05 24200 0.000282731 23600 0.000199213
24500 0.009590618 22500 2.12E-05 22300 4.83E-05 21900
6.68E-05 24200 0.00028307 23600 0.000199375
24600 0.009674557 22500 2.13E-05 22300 4.83E-05 21900
6.68E-05 24200 0.000283402 23600 0.000199524
24600 0.009759473 22500 2.13E-05 22300 4.84E-05 21900
6.68E-05 24200 0.000283738 23600 0.000199683
24600 0.009845366 22500 2.13E-05 22300 4.84E-05 21900
6.68E-05 24200 0.000284076 23600 0.000199835
24600 0.009932267 22500 2.13E-05 22300 4.84E-05 21900
6.67E-05 24200 0.00028441 23600 0.000200004
24600 0.01002017 22500 2.13E-05 22300 4.85E-05 21900
6.67E-05 24200 0.000284757 23600 0.000200144
24600 0.01010907 22500 2.13E-05 22300 4.85E-05 21900
6.67E-05 24200 0.000285101 23600 0.000200301
24600 0.01019898 22500 2.13E-05 22300 4.85E-05 21900
6.67E-05 24200 0.000285449 23600 0.000200442
24600 0.0102899 22500 2.14E-05 22300 4.85E-05 21900
6.66E-05 24200 0.000285781 23600 0.000200573
24600 0.01038183 22500 2.14E-05 22300 4.86E-05 21900
6.66E-05 24200 0.000286138 23600 0.000200738
24600 0.0104748 22500 2.14E-05 22300 4.86E-05 21900
6.66E-05 24200 0.000286471 23700 0.00020089
24600 0.01056871 22500 2.14E-05 22300 4.86E-05 21900
6.66E-05 24200 0.000286804 23700 0.000201055

24600 0.01066365 22500 2.14E-05 22300 4.87E-05 21900
6.66E-05 24200 0.000287155 23700 0.000201198
24600 0.01075962 22500 2.14E-05 22300 4.87E-05 21900
6.66E-05 24200 0.000287497 23700 0.000201351
24600 0.0108566 22500 2.14E-05 22300 4.87E-05 21900
6.65E-05 24200 0.000287849 23700 0.000201493
24600 0.01095455 22500 2.14E-05 22300 4.87E-05 21900
6.65E-05 24200 0.000288205 23700 0.000201654
24600 0.01105351 22500 2.14E-05 22300 4.88E-05 21900
6.65E-05 24200 0.000288558 23700 0.00020179
24600 0.01115343 22500 2.14E-05 22300 4.88E-05 21900
6.65E-05 24200 0.000288901 23700 0.000201954
24600 0.01125439 22500 2.15E-05 22300 4.88E-05 21900
6.65E-05 24200 0.000289245 23700 0.000202088
24600 0.01135631 22600 2.15E-05 22300 4.88E-05 21900
6.64E-05 24200 0.000289606 23700 0.000202224
24600 0.01145917 22600 2.15E-05 22300 4.89E-05 21900
6.64E-05 24200 0.000289967 23700 0.000202367
24600 0.01156301 22600 2.15E-05 22400 4.89E-05 21900
6.64E-05 24200 0.000290316 23700 0.00020251
24600 0.0116678 22600 2.15E-05 22400 4.89E-05 21900
6.64E-05 24200 0.000290671 23700 0.00020265
24600 0.01177351 22600 2.15E-05 22400 4.90E-05 21900
6.64E-05 24200 0.000291025 23700 0.000202807
24600 0.01188015 22600 2.15E-05 22400 4.90E-05 21900
6.64E-05 24200 0.000291399 23700 0.000202949
24600 0.01198769 22600 2.15E-05 22400 4.90E-05 22000
6.63E-05 24200 0.000291767 23700 0.000203112
24600 0.01209615 22600 2.16E-05 22400 4.90E-05 22000
6.63E-05 24200 0.00029213 23700 0.00020325
24600 0.01220547 22600 2.16E-05 22400 4.91E-05 22000
6.63E-05 24200 0.000292481 23700 0.000203388
24600 0.01231562 22600 2.16E-05 22400 4.91E-05 22000
6.63E-05 24200 0.000292853 23700 0.000203537
24600 0.01242662 22600 2.16E-05 22400 4.91E-05 22000
6.63E-05 24200 0.000293197 23700 0.000203673
24600 0.01253842 22600 2.16E-05 22400 4.91E-05 22000
6.63E-05 24200 0.000293549 23700 0.000203841
24600 0.01265097 22600 2.16E-05 22400 4.92E-05 22000
6.63E-05 24200 0.000293904 23700 0.000203976
24600 0.01276432 22600 2.16E-05 22400 4.92E-05 22000
6.63E-05 24200 0.000294259 23700 0.000204119
24600 0.01287837 22600 2.16E-05 22400 4.92E-05 22000
6.63E-05 24200 0.000294619 23700 0.000204264
24600 0.0129931 22600 2.16E-05 22400 4.92E-05 22000
6.63E-05 24200 0.000294975 23700 0.000204429
24600 0.01310849 22600 2.17E-05 22400 4.93E-05 22000
6.64E-05 24200 0.000295331 23700 0.000204566
24600 0.01322451 22600 2.17E-05 22400 4.93E-05 22000
6.64E-05 24300 0.000295682 23700 0.000204712
24600 0.01334107 22600 2.17E-05 22400 4.93E-05 22000
6.64E-05 24300 0.000296024 23700 0.000204871
24600 0.01345817 22600 2.17E-05 22400 4.94E-05 22000
6.64E-05 24300 0.00029638 23700 0.000205015
24600 0.01357575 22600 2.17E-05 22400 4.94E-05 22000
6.64E-05 24300 0.000296732 23700 0.000205157
24600 0.01369371 22600 2.17E-05 22400 4.94E-05 22000
6.64E-05 24300 0.000297073 23700 0.000205315
24600 0.01381204 22600 2.17E-05 22400 4.94E-05 22000
6.64E-05 24300 0.000297419 23700 0.00020547
24600 0.01393072 22600 2.17E-05 22400 4.95E-05 22000
6.64E-05 24300 0.000297779 23700 0.000205643
24600 0.0140496 22600 2.17E-05 22400 4.95E-05 22000
6.64E-05 24300 0.000298128 23700 0.000205782
24600 0.01416866 22600 2.17E-05 22400 4.95E-05 22000
6.64E-05 24300 0.000298484 23700 0.000205961
24600 0.01428787 22600 2.18E-05 22400 4.96E-05 22000
6.64E-05 24300 0.000298844 23700 0.000206108

24600 0.0144071 22600 2.18E-05 22400 4.96E-05 22000
6.63E-05 24300 0.000299199 23700 0.00020625
24600 0.01452631 22600 2.18E-05 22400 4.96E-05 22000
6.63E-05 24300 0.000299558 23700 0.00020642
24600 0.01464539 22600 2.18E-05 22400 4.96E-05 22000
6.63E-05 24300 0.000299913 23700 0.00020657
24600 0.01476419 22600 2.18E-05 22400 4.97E-05 22000
6.62E-05 24300 0.000300252 23700 0.000206739
24600 0.01488277 22600 2.18E-05 22400 4.97E-05 22000
6.62E-05 24300 0.000300612 23700 0.000206882
24600 0.015001 22600 2.19E-05 22400 4.97E-05 22000
6.62E-05 24300 0.000300984 23700 0.000207042
24600 0.01511866 22600 2.19E-05 22400 4.98E-05 22000
6.62E-05 24300 0.000301346 23700 0.000207191
24600 0.0152358 22600 2.19E-05 22400 4.98E-05 22000
6.62E-05 24300 0.00030171 23700 0.00020734
24600 0.01535218 22600 2.19E-05 22400 4.98E-05 22000
6.61E-05 24300 0.000302081 23700 0.000207481
24600 0.0154678 22600 2.19E-05 22400 4.99E-05 22000
6.61E-05 24300 0.000302445 23700 0.000207648
24600 0.01558252 22600 2.19E-05 22400 4.99E-05 22000
6.60E-05 24300 0.000302806 23700 0.000207801
24600 0.01569616 22600 2.19E-05 22400 4.99E-05 22000
6.60E-05 24300 0.000303186 23700 0.000207952
24600 0.01580862 22600 2.19E-05 22400 5.00E-05 22000
6.60E-05 24300 0.000303562 23700 0.000208102
24600 0.01591981 22600 2.20E-05 22400 5.00E-05 22000
6.59E-05 24300 0.00030393 23700 0.000208252
24600 0.01602963 22600 2.20E-05 22400 5.00E-05 22000
6.59E-05 24300 0.000304304 23700 0.000208407
24600 0.01613789 22600 2.20E-05 22400 5.00E-05 22000
6.59E-05 24300 0.000304682 23700 0.000208576
24600 0.01624446 22600 2.20E-05 22400 5.01E-05 22000
6.59E-05 24300 0.000305065 23700 0.000208718
24600 0.01634916 22600 2.20E-05 22400 5.01E-05 22000
6.59E-05 24300 0.000305441 23700 0.000208887
24600 0.01645195 22600 2.20E-05 22400 5.01E-05 22000
6.58E-05 24300 0.000305828 23700 0.000209035
24700 0.01655258 22600 2.20E-05 22400 5.02E-05 22000
6.58E-05 24300 0.000306214 23700 0.000209169
24700 0.01665097 22600 2.20E-05 22400 5.02E-05 22000
6.58E-05 24300 0.000306608 23700 0.000209331
24700 0.01674692 22600 2.20E-05 22400 5.02E-05 22000
6.57E-05 24300 0.000306974 23700 0.000209488
24700 0.01684025 22600 2.20E-05 22400 5.02E-05 22000
6.57E-05 24300 0.000307348 23700 0.000209648
24700 0.01693085 22600 2.20E-05 22400 5.03E-05 22000
6.57E-05 24300 0.00030774 23700 0.000209798
24700 0.01701857 22600 2.20E-05 22400 5.03E-05 22000
6.57E-05 24300 0.000308132 23700 0.000209957
24700 0.01710319 22600 2.20E-05 22400 5.03E-05 22000
6.57E-05 24300 0.000308517 23700 0.000210107
24700 0.01718458 22600 2.20E-05 22400 5.03E-05 22000
6.57E-05 24300 0.000308907 23700 0.00021028
24700 0.01726255 22600 2.21E-05 22400 5.04E-05 22000
6.57E-05 24300 0.000309292 23700 0.00021042
24700 0.01733696 22600 2.21E-05 22400 5.04E-05 22000
6.57E-05 24300 0.000309684 23800 0.000210581
24700 0.01740762 22600 2.21E-05 22400 5.04E-05 22000
6.57E-05 24300 0.000310068 23800 0.00021074
24700 0.01747431 22600 2.21E-05 22400 5.05E-05 22000
6.57E-05 24300 0.000310466 23800 0.000210906
24700 0.01753689 22600 2.21E-05 22400 5.05E-05 22000
6.57E-05 24300 0.000310848 23800 0.000211039
24700 0.01759527 22600 2.21E-05 22400 5.05E-05 22000
6.57E-05 24300 0.000311241 23800 0.000211204
24700 0.01764915 22600 2.21E-05 22400 5.06E-05 22000
6.57E-05 24300 0.00031162 23800 0.00021136

24700 0.01769851 22600 2.21E-05 22400 5.06E-05 22000
6.57E-05 24300 0.000312013 23800 0.000211537
24700 0.01774303 22600 2.22E-05 22400 5.06E-05 22000
6.57E-05 24300 0.000312404 23800 0.000211703
24700 0.01778261 22600 2.22E-05 22400 5.07E-05 22000
6.57E-05 24300 0.000312801 23800 0.000211869
24700 0.01781715 22700 2.22E-05 22400 5.07E-05 22000
6.57E-05 24300 0.000313196 23800 0.000212009
24700 0.01784648 22700 2.22E-05 22400 5.07E-05 22000
6.57E-05 24300 0.000313596 23800 0.000212161
24700 0.01787041 22700 2.22E-05 22500 5.08E-05 22000
6.57E-05 24300 0.000313995 23800 0.000212326
24700 0.01788878 22700 2.22E-05 22500 5.08E-05 22000
6.56E-05 24300 0.000314384 23800 0.000212487
24700 0.01790152 22700 2.22E-05 22500 5.08E-05 22000
6.56E-05 24300 0.000314758 23800 0.000212667
24700 0.01790842 22700 2.22E-05 22500 5.08E-05 22000
6.57E-05 24300 0.000315164 23800 0.000212818
24700 0.01790945 22700 2.22E-05 22500 5.09E-05 22100
6.57E-05 24300 0.000315566 23800 0.000212969
24700 0.01790447 22700 2.23E-05 22500 5.09E-05 22100
6.57E-05 24300 0.000315967 23800 0.000213127
24700 0.01789333 22700 2.23E-05 22500 5.09E-05 22100
6.57E-05 24300 0.00031638 23800 0.000213299
24700 0.01787597 22700 2.23E-05 22500 5.09E-05 22100
6.57E-05 24300 0.000316781 23800 0.000213446
24700 0.01785229 22700 2.23E-05 22500 5.10E-05 22100
6.57E-05 24300 0.000317188 23800 0.000213607
24700 0.01782225 22700 2.23E-05 22500 5.10E-05 22100
6.58E-05 24300 0.000317591 23800 0.000213765
24700 0.01778572 22700 2.23E-05 22500 5.11E-05 22100
6.58E-05 24300 0.000318001 23800 0.000213936
24700 0.01774277 22700 2.23E-05 22500 5.11E-05 22100
6.58E-05 24300 0.000318399 23800 0.000214096
24700 0.01769325 22700 2.23E-05 22500 5.11E-05 22100
6.58E-05 24300 0.00031881 23800 0.000214259
24700 0.01763718 22700 2.24E-05 22500 5.12E-05 22100
6.58E-05 24300 0.000319202 23800 0.00021444
24700 0.01757463 22700 2.24E-05 22500 5.12E-05 22100
6.58E-05 24300 0.000319617 23800 0.000214622
24700 0.01750554 22700 2.24E-05 22500 5.12E-05 22100
6.59E-05 24400 0.000320018 23800 0.000214772
24700 0.01742988 22700 2.24E-05 22500 5.12E-05 22100
6.59E-05 24400 0.000320437 23800 0.000214938
24700 0.01734775 22700 2.24E-05 22500 5.13E-05 22100
6.59E-05 24400 0.000320848 23800 0.000215112
24700 0.01725924 22700 2.24E-05 22500 5.13E-05 22100
6.59E-05 24400 0.000321264 23800 0.000215258
24700 0.01716437 22700 2.24E-05 22500 5.13E-05 22100
6.59E-05 24400 0.000321686 23800 0.000215448
24700 0.01706325 22700 2.24E-05 22500 5.14E-05 22100
6.59E-05 24400 0.000322088 23800 0.000215607
24700 0.01695597 22700 2.25E-05 22500 5.14E-05 22100
6.59E-05 24400 0.000322487 23800 0.000215791
24700 0.0168426 22700 2.25E-05 22500 5.14E-05 22100
6.59E-05 24400 0.000322912 23800 0.000215963
24700 0.01672345 22700 2.25E-05 22500 5.15E-05 22100
6.59E-05 24400 0.000323333 23800 0.000216135
24700 0.01659854 22700 2.25E-05 22500 5.15E-05 22100
6.59E-05 24400 0.000323749 23800 0.000216281
24700 0.016468 22700 2.25E-05 22500 5.15E-05 22100
6.58E-05 24400 0.000324164 23800 0.000216475
24700 0.01633214 22700 2.25E-05 22500 5.16E-05 22100
6.58E-05 24400 0.000324581 23800 0.000216654
24700 0.01619107 22700 2.25E-05 22500 5.16E-05 22100
6.58E-05 24400 0.000325001 23800 0.000216818
24700 0.01604507 22700 2.25E-05 22500 5.16E-05 22100
6.58E-05 24400 0.000325398 23800 0.000216986

24700	0.0158943	22700	2.26E-05	22500	5.17E-05	22100	6.57E-05	24400	0.000325821	23800	0.000217153
24700	0.01573907	22700	2.26E-05	22500	5.17E-05	22100	6.57E-05	24400	0.00032624	23800	0.000217328
24700	0.01557959	22700	2.26E-05	22500	5.18E-05	22100	6.57E-05	24400	0.000326645	23800	0.000217497
24700	0.01541618	22700	2.26E-05	22500	5.18E-05	22100	6.57E-05	24400	0.00032705	23800	0.000217672
24700	0.01524904	22700	2.26E-05	22500	5.18E-05	22100	6.58E-05	24400	0.000327482	23800	0.000217867
24700	0.01507858	22700	2.26E-05	22500	5.19E-05	22100	6.58E-05	24400	0.000327888	23800	0.000218029
24700	0.01490494	22700	2.26E-05	22500	5.19E-05	22100	6.58E-05	24400	0.000328319	23800	0.000218205
24700	0.01472858	22700	2.26E-05	22500	5.19E-05	22100	6.59E-05	24400	0.000328747	23800	0.00021837
24700	0.01454977	22700	2.26E-05	22500	5.20E-05	22100	6.59E-05	24400	0.000329166	23800	0.000218527
24700	0.01436883	22700	2.26E-05	22500	5.20E-05	22100	6.59E-05	24400	0.000329604	23800	0.000218714
24700	0.014186	22700	2.26E-05	22500	5.20E-05	22100	6.59E-05	24400	0.000330014	23800	0.000218887
24700	0.01400169	22700	2.27E-05	22500	5.20E-05	22100	6.59E-05	24400	0.000330424	23800	0.000219068
24700	0.01381617	22700	2.27E-05	22500	5.21E-05	22100	6.59E-05	24400	0.000330865	23800	0.000219253
24700	0.0136299	22700	2.27E-05	22500	5.21E-05	22100	6.59E-05	24400	0.00033129	23800	0.000219409
24700	0.01344311	22700	2.27E-05	22500	5.22E-05	22100	6.59E-05	24400	0.00033173	23800	0.000219577
24800	0.01325613	22700	2.27E-05	22500	5.22E-05	22100	6.59E-05	24400	0.000332168	23800	0.000219751
24800	0.01306932	22700	2.27E-05	22500	5.22E-05	22100	6.59E-05	24400	0.000332604	23800	0.000219912
24800	0.01288286	22700	2.27E-05	22500	5.23E-05	22100	6.59E-05	24400	0.000333037	23800	0.000220099
24800	0.01269723	22700	2.28E-05	22500	5.23E-05	22100	6.59E-05	24400	0.000333476	23800	0.000220265
24800	0.01251267	22700	2.28E-05	22500	5.23E-05	22100	6.59E-05	24400	0.000333922	23800	0.000220455
24800	0.01232946	22700	2.28E-05	22500	5.24E-05	22100	6.58E-05	24400	0.000334367	23800	0.000220621
24800	0.01214789	22700	2.28E-05	22500	5.24E-05	22100	6.58E-05	24400	0.000334807	23800	0.000220784
24800	0.01196825	22700	2.28E-05	22500	5.24E-05	22100	6.58E-05	24400	0.000335237	23800	0.000220962
24800	0.01179083	22700	2.29E-05	22500	5.25E-05	22100	6.58E-05	24400	0.00033568	23800	0.000221123
24800	0.0116158	22700	2.29E-05	22500	5.25E-05	22100	6.57E-05	24400	0.000336123	23900	0.000221289
24800	0.01144344	22700	2.29E-05	22500	5.25E-05	22100	6.57E-05	24400	0.000336579	23900	0.000221457
24800	0.01127398	22700	2.29E-05	22500	5.26E-05	22100	6.57E-05	24400	0.000337027	23900	0.000221626
24800	0.01110773	22700	2.29E-05	22500	5.26E-05	22100	6.57E-05	24400	0.000337476	23900	0.000221781
24800	0.0109448	22700	2.29E-05	22500	5.26E-05	22100	6.57E-05	24400	0.000337942	23900	0.00022195
24800	0.01078528	22700	2.29E-05	22500	5.27E-05	22100	6.57E-05	24400	0.000338387	23900	0.000222119
24800	0.01062949	22700	2.29E-05	22500	5.27E-05	22100	6.57E-05	24400	0.000338821	23900	0.000222308
24800	0.0104775	22700	2.29E-05	22500	5.27E-05	22100	6.58E-05	24400	0.000339289	23900	0.000222462
24800	0.01032946	22700	2.30E-05	22500	5.28E-05	22100	6.58E-05	24400	0.000339744	23900	0.000222621
24800	0.01018555	22800	2.30E-05	22500	5.28E-05	22100	6.58E-05	24400	0.000340206	23900	0.000222755

24800 0.01004578 22800 2.30E-05 22500 5.28E-05 22100
6.58E-05 24400 0.000340666 23900 0.000222925
24800 0.009910375 22800 2.30E-05 22600 5.29E-05 22100
6.59E-05 24400 0.000341128 23900 0.000223085
24800 0.00977923 22800 2.30E-05 22600 5.29E-05 22100
6.59E-05 24400 0.000341594 23900 0.000223228
24800 0.009652632 22800 2.30E-05 22600 5.29E-05 22100
6.59E-05 24400 0.000342062 23900 0.000223391
24800 0.009530412 22800 2.30E-05 22600 5.30E-05 22100
6.59E-05 24400 0.000342533 23900 0.000223556
24800 0.009412729 22800 2.30E-05 22600 5.30E-05 22200
6.59E-05 24400 0.000343 23900 0.000223704
24800 0.009299592 22800 2.30E-05 22600 5.31E-05 22200
6.59E-05 24400 0.000343463 23900 0.000223872
24800 0.009190987 22800 2.31E-05 22600 5.31E-05 22200
6.59E-05 24400 0.000343922 23900 0.000224009
24800 0.009086973 22800 2.31E-05 22600 5.31E-05 22200
6.59E-05 24400 0.000344394 23900 0.000224173
24800 0.00898746 22800 2.31E-05 22600 5.32E-05 22200
6.58E-05 24400 0.000344871 23900 0.000224319
24800 0.008892366 22800 2.31E-05 22600 5.32E-05 22200
6.58E-05 24400 0.000345345 23900 0.000224486
24800 0.008801785 22800 2.31E-05 22600 5.32E-05 22200
6.58E-05 24400 0.000345826 23900 0.000224611
24800 0.008715617 22800 2.31E-05 22600 5.33E-05 22200
6.59E-05 24400 0.000346308 23900 0.000224773
24800 0.0086338 22800 2.31E-05 22600 5.33E-05 22200
6.59E-05 24400 0.000346798 23900 0.000224934
24800 0.008556313 22800 2.31E-05 22600 5.33E-05 22200
6.59E-05 24400 0.000347279 23900 0.000225074
24800 0.008483035 22800 2.32E-05 22600 5.34E-05 22200
6.59E-05 24400 0.00034775 23900 0.000225228
24800 0.008413851 22800 2.32E-05 22600 5.34E-05 22200
6.59E-05 24500 0.000348243 23900 0.000225406
24800 0.008348797 22800 2.32E-05 22600 5.34E-05 22200
6.60E-05 24500 0.000348733 23900 0.000225552
24800 0.008287736 22800 2.32E-05 22600 5.35E-05 22200
6.60E-05 24500 0.00034922 23900 0.000225718
24800 0.008230482 22800 2.32E-05 22600 5.35E-05 22200
6.60E-05 24500 0.000349715 23900 0.000225879
24800 0.008177035 22800 2.32E-05 22600 5.35E-05 22200
6.60E-05 24500 0.000350201 23900 0.000226029
24800 0.008127284 22800 2.32E-05 22600 5.36E-05 22200
6.60E-05 24500 0.000350705 23900 0.000226184
24800 0.008081139 22800 2.32E-05 22600 5.36E-05 22200
6.61E-05 24500 0.000351199 23900 0.000226342
24800 0.0080384 22800 2.33E-05 22600 5.36E-05 22200
6.61E-05 24500 0.000351693 23900 0.00022651
24800 0.007999019 22800 2.33E-05 22600 5.37E-05 22200
6.61E-05 24500 0.000352198 23900 0.000226673
24800 0.007962869 22800 2.33E-05 22600 5.37E-05 22200
6.61E-05 24500 0.000352692 23900 0.000226825
24800 0.007929908 22800 2.33E-05 22600 5.38E-05 22200
6.61E-05 24500 0.000353193 23900 0.000226993
24800 0.007899938 22800 2.33E-05 22600 5.38E-05 22200
6.62E-05 24500 0.000353702 23900 0.000227163
24800 0.007872819 22800 2.33E-05 22600 5.38E-05 22200
6.61E-05 24500 0.000354201 23900 0.00022733
24800 0.007848541 22800 2.33E-05 22600 5.39E-05 22200
6.61E-05 24500 0.000354715 23900 0.000227497
24800 0.007826963 22800 2.33E-05 22600 5.39E-05 22200
6.62E-05 24500 0.000355232 23900 0.000227668
24800 0.007807892 22800 2.34E-05 22600 5.40E-05 22200
6.61E-05 24500 0.000355748 23900 0.00022782
24800 0.007791281 22800 2.34E-05 22600 5.40E-05 22200
6.61E-05 24500 0.000356274 23900 0.00022801
24800 0.007777064 22800 2.34E-05 22600 5.40E-05 22200
6.61E-05 24500 0.000356786 23900 0.000228169

24800 0.007765119 22800 2.34E-05 22600 5.41E-05 22200
6.61E-05 24500 0.000357295 23900 0.000228343
24800 0.007755243 22800 2.34E-05 22600 5.41E-05 22200
6.61E-05 24500 0.000357822 23900 0.000228507
24800 0.007747359 22800 2.34E-05 22600 5.42E-05 22200
6.61E-05 24500 0.000358343 23900 0.000228695
24800 0.00774152 22800 2.34E-05 22600 5.42E-05 22200
6.61E-05 24500 0.000358867 23900 0.000228867
24800 0.00773745 22800 2.34E-05 22600 5.42E-05 22200
6.61E-05 24500 0.000359385 23900 0.00022906
24800 0.007735146 22800 2.35E-05 22600 5.43E-05 22200
6.61E-05 24500 0.000359916 23900 0.000229225
24800 0.007734546 22800 2.35E-05 22600 5.43E-05 22200
6.61E-05 24500 0.000360454 23900 0.000229403
24800 0.007735458 22800 2.35E-05 22600 5.44E-05 22200
6.61E-05 24500 0.000360969 23900 0.000229577
24800 0.00773785 22800 2.35E-05 22600 5.44E-05 22200
6.61E-05 24500 0.000361509 23900 0.000229764
24800 0.007741615 22800 2.35E-05 22600 5.45E-05 22200
6.62E-05 24500 0.00036204 23900 0.000229931
24800 0.007746691 22800 2.35E-05 22600 5.45E-05 22200
6.62E-05 24500 0.00036257 23900 0.000230117
24900 0.007753011 22800 2.35E-05 22600 5.45E-05 22200
6.62E-05 24500 0.000363092 23900 0.000230286
24900 0.007760534 22800 2.36E-05 22600 5.46E-05 22200
6.62E-05 24500 0.000363634 23900 0.000230477
24900 0.007769069 22800 2.36E-05 22600 5.46E-05 22200
6.62E-05 24500 0.000364167 23900 0.000230652
24900 0.00777859 22800 2.36E-05 22600 5.47E-05 22200
6.62E-05 24500 0.000364702 23900 0.00023084
24900 0.007789127 22800 2.36E-05 22600 5.47E-05 22200
6.62E-05 24500 0.000365254 23900 0.000231003
24900 0.007800537 22800 2.36E-05 22600 5.47E-05 22200
6.62E-05 24500 0.000365801 23900 0.000231172
24900 0.007812741 22800 2.36E-05 22600 5.48E-05 22200
6.62E-05 24500 0.00036635 23900 0.000231356
24900 0.007825722 22800 2.36E-05 22600 5.48E-05 22200
6.63E-05 24500 0.000366895 23900 0.000231528
24900 0.007839451 22800 2.36E-05 22600 5.49E-05 22200
6.63E-05 24500 0.000367429 23900 0.000231719
24900 0.007853838 22800 2.36E-05 22600 5.49E-05 22200
6.63E-05 24500 0.000367981 24000 0.000231901
24900 0.00786882 22800 2.36E-05 22600 5.49E-05 22200
6.63E-05 24500 0.000368523 24000 0.000232094
24900 0.007884343 22800 2.36E-05 22600 5.50E-05 22200
6.64E-05 24500 0.000369075 24000 0.000232287
24900 0.007900388 22800 2.37E-05 22600 5.50E-05 22200
6.64E-05 24500 0.000369618 24000 0.000232469
24900 0.007916924 22800 2.37E-05 22600 5.50E-05 22200
6.64E-05 24500 0.000370174 24000 0.000232652
24900 0.007933858 22800 2.37E-05 22600 5.51E-05 22200
6.64E-05 24500 0.000370726 24000 0.000232844
24900 0.007951153 22800 2.37E-05 22600 5.51E-05 22200
6.64E-05 24500 0.000371261 24000 0.000233029
24900 0.007968845 22800 2.37E-05 22600 5.52E-05 22200
6.64E-05 24500 0.000371808 24000 0.000233232
24900 0.007986792 22800 2.37E-05 22600 5.52E-05 22200
6.64E-05 24500 0.000372359 24000 0.000233419
24900 0.008005059 22900 2.38E-05 22600 5.53E-05 22200
6.64E-05 24500 0.000372902 24000 0.000233607
24900 0.008023534 22900 2.38E-05 22600 5.53E-05 22200
6.64E-05 24500 0.000373442 24000 0.000233795
24900 0.008042272 22900 2.38E-05 22700 5.53E-05 22200
6.64E-05 24500 0.000373987 24000 0.000234001
24900 0.008061129 22900 2.38E-05 22700 5.54E-05 22200
6.63E-05 24500 0.000374537 24000 0.000234196
24900 0.008080121 22900 2.38E-05 22700 5.54E-05 22200
6.63E-05 24500 0.000375076 24000 0.000234397

24900 0.008099332 22900 2.38E-05 22700 5.55E-05 22200
6.63E-05 24500 0.000375624 24000 0.0002346
24900 0.008118651 22900 2.38E-05 22700 5.55E-05 22300
6.63E-05 24500 0.00037618 24000 0.000234799
24900 0.00813802 22900 2.39E-05 22700 5.55E-05 22300
6.63E-05 24500 0.00037672 24000 0.000235013
24900 0.008157462 22900 2.39E-05 22700 5.56E-05 22300
6.63E-05 24500 0.000377262 24000 0.000235201
24900 0.008176925 22900 2.39E-05 22700 5.56E-05 22300
6.63E-05 24500 0.000377792 24000 0.000235416
24900 0.008196416 22900 2.39E-05 22700 5.57E-05 22300
6.63E-05 24500 0.000378337 24000 0.000235615
24900 0.008215919 22900 2.39E-05 22700 5.57E-05 22300
6.63E-05 24500 0.000378871 24000 0.000235815
24900 0.008235424 22900 2.39E-05 22700 5.57E-05 22300
6.64E-05 24500 0.000379415 24000 0.000236026
24900 0.008254915 22900 2.39E-05 22700 5.58E-05 22300
6.64E-05 24500 0.000379956 24000 0.00023624
24900 0.008274302 22900 2.40E-05 22700 5.58E-05 22300
6.64E-05 24500 0.000380496 24000 0.000236434
24900 0.008293626 22900 2.40E-05 22700 5.59E-05 22300
6.64E-05 24500 0.000381034 24000 0.000236633
24900 0.008312944 22900 2.40E-05 22700 5.59E-05 22300
6.64E-05 24500 0.000381571 24000 0.000236835
24900 0.008332019 22900 2.40E-05 22700 5.60E-05 22300
6.64E-05 24600 0.000382102 24000 0.000237036
24900 0.008351201 22900 2.40E-05 22700 5.60E-05 22300
6.65E-05 24600 0.000382631 24000 0.000237234
24900 0.008370185 22900 2.40E-05 22700 5.61E-05 22300
6.65E-05 24600 0.000383167 24000 0.000237439
24900 0.008389042 22900 2.40E-05 22700 5.61E-05 22300
6.65E-05 24600 0.000383684 24000 0.000237636
24900 0.008407809 22900 2.41E-05 22700 5.61E-05 22300
6.65E-05 24600 0.000384198 24000 0.000237833
24900 0.008426374 22900 2.41E-05 22700 5.62E-05 22300
6.65E-05 24600 0.000384718 24000 0.000238057
24900 0.008444822 22900 2.41E-05 22700 5.62E-05 22300
6.65E-05 24600 0.000385229 24000 0.000238248
24900 0.008463156 22900 2.41E-05 22700 5.63E-05 22300
6.66E-05 24600 0.000385735 24000 0.000238454
24900 0.008481357 22900 2.41E-05 22700 5.63E-05 22300
6.66E-05 24600 0.00038626 24000 0.000238656
24900 0.008499427 22900 2.41E-05 22700 5.64E-05 22300
6.66E-05 24600 0.000386763 24000 0.000238863
24900 0.00851725 22900 2.41E-05 22700 5.64E-05 22300
6.66E-05 24600 0.000387261 24000 0.000239059
24900 0.008534977 22900 2.41E-05 22700 5.64E-05 22300
6.66E-05 24600 0.000387746 24000 0.000239246
24900 0.008552476 22900 2.41E-05 22700 5.65E-05 22300
6.67E-05 24600 0.000388236 24000 0.000239454
24900 0.008569881 22900 2.42E-05 22700 5.65E-05 22300
6.67E-05 24600 0.00038872 24000 0.000239649
24900 0.008587101 22900 2.42E-05 22700 5.66E-05 22300
6.67E-05 24600 0.000389207 24000 0.000239835
24900 0.008604186 22900 2.42E-05 22700 5.66E-05 22300
6.67E-05 24600 0.000389691 24000 0.000240027
24900 0.008621127 22900 2.42E-05 22700 5.66E-05 22300
6.67E-05 24600 0.00039016 24000 0.000240218
24900 0.008637895 22900 2.42E-05 22700 5.67E-05 22300
6.67E-05 24600 0.000390614 24000 0.000240394
24900 0.00865455 22900 2.42E-05 22700 5.67E-05 22300
6.67E-05 24600 0.000391068 24000 0.000240572
24900 0.008671003 22900 2.42E-05 22700 5.68E-05 22300
6.67E-05 24600 0.000391524 24000 0.000240758
24900 0.008687351 22900 2.43E-05 22700 5.68E-05 22300
6.67E-05 24600 0.000391974 24000 0.000240934
24900 0.008703617 22900 2.43E-05 22700 5.69E-05 22300
6.67E-05 24600 0.000392412 24000 0.000241121

24900 0.008719721 22900 2.43E-05 22700 5.69E-05 22300
6.66E-05 24600 0.000392845 24000 0.000241304
24900 0.008735753 22900 2.43E-05 22700 5.70E-05 22300
6.66E-05 24600 0.000393271 24000 0.000241497
24900 0.008751659 22900 2.43E-05 22700 5.70E-05 22300
6.66E-05 24600 0.000393708 24000 0.000241698
24900 0.008767548 22900 2.43E-05 22700 5.71E-05 22300
6.66E-05 24600 0.000394136 24000 0.000241877
24900 0.008783367 22900 2.44E-05 22700 5.71E-05 22300
6.66E-05 24600 0.000394562 24000 0.000242055
24900 0.00879912 22900 2.44E-05 22700 5.71E-05 22300
6.67E-05 24600 0.00039499 24000 0.000242233
24900 0.008814871 22900 2.44E-05 22700 5.72E-05 22300
6.67E-05 24600 0.000395406 24000 0.000242437
25000 0.008830554 22900 2.44E-05 22700 5.72E-05 22300
6.67E-05 24600 0.00039582 24000 0.000242622
25000 0.008846287 22900 2.44E-05 22700 5.73E-05 22300
6.67E-05 24600 0.000396227 24000 0.000242824
25000 0.008862006 22900 2.44E-05 22700 5.73E-05 22300
6.68E-05 24600 0.000396646 24000 0.00024303
25000 0.008877829 22900 2.44E-05 22700 5.74E-05 22300
6.68E-05 24600 0.000397055 24000 0.000243227
25000 0.008893722 22900 2.44E-05 22700 5.74E-05 22300
6.69E-05 24600 0.000397478 24000 0.000243434
25000 0.008909649 22900 2.45E-05 22700 5.75E-05 22300
6.69E-05 24600 0.000397891 24000 0.000243619
25000 0.008925654 22900 2.45E-05 22700 5.75E-05 22300
6.70E-05 24600 0.000398292 24000 0.000243819
25000 0.008941803 22900 2.45E-05 22700 5.76E-05 22300
6.70E-05 24600 0.000398695 24000 0.000244006
25000 0.008958117 22900 2.45E-05 22700 5.76E-05 22300
6.70E-05 24600 0.000399092 24000 0.000244199
25000 0.008974552 22900 2.45E-05 22700 5.77E-05 22300
6.70E-05 24600 0.0003995 24100 0.000244406
25000 0.008991144 22900 2.45E-05 22700 5.77E-05 22300
6.71E-05 24600 0.00039991 24100 0.000244609
25000 0.009007948 22900 2.45E-05 22700 5.78E-05 22300
6.71E-05 24600 0.000400305 24100 0.000244802
25000 0.009024947 22900 2.46E-05 22700 5.78E-05 22300
6.71E-05 24600 0.00040071 24100 0.000245014
25000 0.009042169 22900 2.46E-05 22700 5.79E-05 22300
6.71E-05 24600 0.000401106 24100 0.000245228
25000 0.009059673 22900 2.46E-05 22700 5.79E-05 22300
6.72E-05 24600 0.000401508 24100 0.000245452
25000 0.009077404 22900 2.46E-05 22700 5.80E-05 22300
6.72E-05 24600 0.000401922 24100 0.000245662
25000 0.009095388 22900 2.46E-05 22700 5.80E-05 22300
6.72E-05 24600 0.000402323 24100 0.000245858
25000 0.009113714 22900 2.46E-05 22700 5.81E-05 22300
6.72E-05 24600 0.000402737 24100 0.000246062
25000 0.009132323 23000 2.47E-05 22700 5.81E-05 22300
6.72E-05 24600 0.00040314 24100 0.000246286
25000 0.009151261 23000 2.47E-05 22700 5.82E-05 22300
6.72E-05 24600 0.000403554 24100 0.000246501
25000 0.009170451 23000 2.47E-05 22800 5.82E-05 22300
6.72E-05 24600 0.000403957 24100 0.00024671
25000 0.009189947 23000 2.47E-05 22800 5.83E-05 22300
6.72E-05 24600 0.000404389 24100 0.000246946
25000 0.009209828 23000 2.47E-05 22800 5.83E-05 22300
6.72E-05 24600 0.00040481 24100 0.000247166
25000 0.009230022 23000 2.47E-05 22800 5.84E-05 22300
6.72E-05 24600 0.000405247 24100 0.000247387
25000 0.009250624 23000 2.48E-05 22800 5.84E-05 22400
6.73E-05 24600 0.000405683 24100 0.000247604
25000 0.009271492 23000 2.48E-05 22800 5.85E-05 22400
6.73E-05 24600 0.000406108 24100 0.000247812
25000 0.009292709 23000 2.48E-05 22800 5.86E-05 22400
6.73E-05 24600 0.000406543 24100 0.000248027

25000 0.00931432 23000 2.48E-05 22800 5.86E-05 22400
6.73E-05 24600 0.000406984 24100 0.000248229
25000 0.009336173 23000 2.48E-05 22800 5.87E-05 22400
6.73E-05 24600 0.000407426 24100 0.000248457
25000 0.009358412 23000 2.48E-05 22800 5.87E-05 22400
6.74E-05 24600 0.000407878 24100 0.000248669
25000 0.009380965 23000 2.48E-05 22800 5.88E-05 22400
6.74E-05 24600 0.000408324 24100 0.000248899
25000 0.009403801 23000 2.48E-05 22800 5.88E-05 22400
6.74E-05 24600 0.000408789 24100 0.0002491
25000 0.009426954 23000 2.48E-05 22800 5.89E-05 22400
6.74E-05 24600 0.000409238 24100 0.00024933
25000 0.009450404 23000 2.48E-05 22800 5.89E-05 22400
6.75E-05 24600 0.000409707 24100 0.00024957
25000 0.009474098 23000 2.49E-05 22800 5.90E-05 22400
6.75E-05 24600 0.000410174 24100 0.000249793
25000 0.009498142 23000 2.49E-05 22800 5.91E-05 22400
6.75E-05 24700 0.000410644 24100 0.000250017
25000 0.00952243 23000 2.49E-05 22800 5.91E-05 22400
6.75E-05 24700 0.000411128 24100 0.000250232
25000 0.009546977 23000 2.49E-05 22800 5.92E-05 22400
6.75E-05 24700 0.000411608 24100 0.000250467
25000 0.00957173 23000 2.49E-05 22800 5.92E-05 22400
6.75E-05 24700 0.000412094 24100 0.000250688
25000 0.009596781 23000 2.49E-05 22800 5.93E-05 22400
6.75E-05 24700 0.000412561 24100 0.000250919
25000 0.009621992 23000 2.50E-05 22800 5.94E-05 22400
6.76E-05 24700 0.000413055 24100 0.00025116
25000 0.009647472 23000 2.50E-05 22800 5.94E-05 22400
6.76E-05 24700 0.000413554 24100 0.000251384
25000 0.009673148 23000 2.50E-05 22800 5.95E-05 22400
6.76E-05 24700 0.000414056 24100 0.000251626
25000 0.009698978 23000 2.50E-05 22800 5.95E-05 22400
6.77E-05 24700 0.00041457 24100 0.000251859
25000 0.009724987 23000 2.50E-05 22800 5.96E-05 22400
6.77E-05 24700 0.000415082 24100 0.000252078
25000 0.00975119 23000 2.50E-05 22800 5.97E-05 22400
6.77E-05 24700 0.00041559 24100 0.000252298
25000 0.009777559 23000 2.50E-05 22800 5.97E-05 22400
6.78E-05 24700 0.000416106 24100 0.000252526
25000 0.009804076 23000 2.51E-05 22800 5.98E-05 22400
6.78E-05 24700 0.000416626 24100 0.000252758
25000 0.009830694 23000 2.51E-05 22800 5.99E-05 22400
6.78E-05 24700 0.000417145 24100 0.000252975
25000 0.00985749 23000 2.51E-05 22800 5.99E-05 22400
6.79E-05 24700 0.000417671 24100 0.000253215
25000 0.009884343 23000 2.51E-05 22800 6.00E-05 22400
6.79E-05 24700 0.00041821 24100 0.000253452
25000 0.00991136 23000 2.51E-05 22800 6.00E-05 22400
6.79E-05 24700 0.000418742 24100 0.000253674
25000 0.009938476 23000 2.51E-05 22800 6.01E-05 22400
6.79E-05 24700 0.000419288 24100 0.000253899
25000 0.009965639 23000 2.52E-05 22800 6.02E-05 22400
6.79E-05 24700 0.00041983 24100 0.00025415
25000 0.009993022 23000 2.52E-05 22800 6.02E-05 22400
6.79E-05 24700 0.000420375 24100 0.000254396
25000 0.01002046 23000 2.52E-05 22800 6.03E-05 22400
6.79E-05 24700 0.000420934 24100 0.000254611
25000 0.01004799 23000 2.52E-05 22800 6.04E-05 22400
6.79E-05 24700 0.000421484 24100 0.000254864
25000 0.01007563 23000 2.52E-05 22800 6.04E-05 22400
6.79E-05 24700 0.000422042 24100 0.000255114
25000 0.0101033 23000 2.52E-05 22800 6.05E-05 22400
6.79E-05 24700 0.000422586 24100 0.000255356
25000 0.01013108 23000 2.52E-05 22800 6.06E-05 22400
6.79E-05 24700 0.000423149 24100 0.000255607
25000 0.01015895 23000 2.52E-05 22800 6.07E-05 22400
6.80E-05 24700 0.000423722 24100 0.000255845

25000 0.01018693 23000 2.53E-05 22800 6.07E-05 22400
6.80E-05 24700 0.0004243 24100 0.000256105
25000 0.010215 23000 2.53E-05 22800 6.08E-05 22400
6.81E-05 24700 0.000424869 24100 0.000256316
25000 0.01024309 23000 2.53E-05 22800 6.08E-05 22400
6.81E-05 24700 0.000425444 24100 0.000256516
25100 0.01027133 23000 2.53E-05 22800 6.09E-05 22400
6.81E-05 24700 0.000426009 24100 0.000256746
25100 0.01029966 23000 2.53E-05 22800 6.10E-05 22400
6.81E-05 24700 0.000426575 24100 0.000256965
25100 0.01032805 23000 2.53E-05 22800 6.11E-05 22400
6.81E-05 24700 0.000427148 24100 0.000257223
25100 0.01035652 23000 2.53E-05 22800 6.11E-05 22400
6.81E-05 24700 0.000427729 24100 0.000257402
25100 0.01038511 23000 2.54E-05 22800 6.12E-05 22400
6.81E-05 24700 0.00042829 24100 0.000257623
25100 0.01041375 23000 2.54E-05 22800 6.13E-05 22400
6.81E-05 24700 0.000428884 24100 0.000257842
25100 0.01044257 23000 2.54E-05 22800 6.13E-05 22400
6.81E-05 24700 0.000429469 24100 0.000258044
25100 0.01047142 23000 2.54E-05 22800 6.14E-05 22400
6.81E-05 24700 0.000430053 24100 0.000258276
25100 0.01050041 23000 2.55E-05 22800 6.15E-05 22400
6.81E-05 24700 0.000430634 24100 0.000258454
25100 0.01052949 23000 2.55E-05 22800 6.16E-05 22400
6.81E-05 24700 0.000431225 24200 0.000258644
25100 0.0105587 23000 2.55E-05 22800 6.16E-05 22400
6.81E-05 24700 0.000431815 24200 0.000258835
25100 0.01058807 23000 2.55E-05 22800 6.17E-05 22400
6.81E-05 24700 0.000432402 24200 0.000259034
25100 0.01061747 23000 2.55E-05 22800 6.18E-05 22400
6.81E-05 24700 0.000432989 24200 0.000259255
25100 0.01064703 23000 2.55E-05 22800 6.18E-05 22400
6.81E-05 24700 0.000433562 24200 0.000259462
25100 0.01067674 23000 2.56E-05 22800 6.19E-05 22400
6.82E-05 24700 0.000434161 24200 0.000259671
25100 0.01070658 23000 2.56E-05 22800 6.20E-05 22400
6.82E-05 24700 0.000434759 24200 0.000259862
25100 0.0107366 23000 2.56E-05 22800 6.21E-05 22400
6.83E-05 24700 0.000435362 24200 0.000260071
25100 0.01076664 23000 2.56E-05 22800 6.21E-05 22400
6.83E-05 24700 0.000435947 24200 0.000260272
25100 0.01079688 23100 2.56E-05 22800 6.22E-05 22400
6.84E-05 24700 0.00043655 24200 0.000260477
25100 0.01082726 23100 2.56E-05 22800 6.23E-05 22400
6.84E-05 24700 0.000437141 24200 0.000260671
25100 0.01085781 23100 2.56E-05 22900 6.23E-05 22400
6.84E-05 24700 0.000437734 24200 0.000260841
25100 0.01088849 23100 2.56E-05 22900 6.24E-05 22400
6.85E-05 24700 0.000438322 24200 0.00026101
25100 0.01091936 23100 2.56E-05 22900 6.25E-05 22400
6.85E-05 24700 0.000438923 24200 0.000261198
25100 0.0109504 23100 2.56E-05 22900 6.26E-05 22400
6.86E-05 24700 0.000439515 24200 0.00026141
25100 0.01098162 23100 2.57E-05 22900 6.27E-05 22500
6.86E-05 24700 0.000440125 24200 0.000261599
25100 0.01101304 23100 2.57E-05 22900 6.27E-05 22500
6.86E-05 24700 0.000440726 24200 0.000261785
25100 0.01104461 23100 2.57E-05 22900 6.28E-05 22500
6.86E-05 24700 0.000441329 24200 0.000261972
25100 0.01107631 23100 2.57E-05 22900 6.29E-05 22500
6.86E-05 24700 0.000441941 24200 0.000262158
25100 0.01110827 23100 2.57E-05 22900 6.30E-05 22500
6.87E-05 24700 0.00044255 24200 0.000262338
25100 0.01114041 23100 2.57E-05 22900 6.31E-05 22500
6.87E-05 24700 0.000443168 24200 0.000262519
25100 0.01117276 23100 2.58E-05 22900 6.31E-05 22500
6.87E-05 24700 0.000443779 24200 0.000262651

25100	0.01120529	23100	2.58E-05	22900	6.32E-05	22500	6.87E-05	24700	0.000444398	24200	0.000262851
25100	0.01123806	23100	2.58E-05	22900	6.33E-05	22500	6.87E-05	24700	0.000445003	24200	0.000263021
25100	0.01127102	23100	2.58E-05	22900	6.34E-05	22500	6.88E-05	24700	0.000445618	24200	0.000263211
25100	0.0113042	23100	2.59E-05	22900	6.35E-05	22500	6.88E-05	24700	0.000446245	24200	0.0002634
25100	0.01133765	23100	2.59E-05	22900	6.36E-05	22500	6.89E-05	24800	0.000446884	24200	0.000263557
25100	0.01137131	23100	2.59E-05	22900	6.37E-05	22500	6.89E-05	24800	0.000447515	24200	0.000263737
25100	0.01140524	23100	2.59E-05	22900	6.38E-05	22500	6.89E-05	24800	0.000448154	24200	0.000263905
25100	0.01143928	23100	2.59E-05	22900	6.38E-05	22500	6.90E-05	24800	0.000448781	24200	0.000264096
25100	0.01147366	23100	2.59E-05	22900	6.39E-05	22500	6.90E-05	24800	0.000449421	24200	0.00026425
25100	0.01150826	23100	2.60E-05	22900	6.40E-05	22500	6.90E-05	24800	0.000450045	24200	0.000264408
25100	0.01154309	23100	2.60E-05	22900	6.41E-05	22500	6.90E-05	24800	0.0004507	24200	0.000264567
25100	0.01157816	23100	2.60E-05	22900	6.42E-05	22500	6.90E-05	24800	0.00045133	24200	0.000264761
25100	0.0116135	23100	2.60E-05	22900	6.43E-05	22500	6.91E-05	24800	0.000451977	24200	0.00026493
25100	0.01164908	23100	2.60E-05	22900	6.44E-05	22500	6.91E-05	24800	0.000452619	24200	0.00026509
25100	0.01168486	23100	2.60E-05	22900	6.45E-05	22500	6.91E-05	24800	0.000453275	24200	0.000265278
25100	0.01172095	23100	2.60E-05	22900	6.46E-05	22500	6.92E-05	24800	0.000453927	24200	0.000265456
25100	0.01175732	23100	2.61E-05	22900	6.47E-05	22500	6.92E-05	24800	0.000454581	24200	0.000265637
25100	0.01179395	23100	2.61E-05	22900	6.48E-05	22500	6.92E-05	24800	0.00045524	24200	0.000265795
25100	0.01183086	23100	2.61E-05	22900	6.49E-05	22500	6.92E-05	24800	0.000455904	24200	0.000265968
25100	0.01186794	23100	2.61E-05	22900	6.50E-05	22500	6.92E-05	24800	0.000456555	24200	0.000266151
25100	0.01190536	23100	2.61E-05	22900	6.51E-05	22500	6.92E-05	24800	0.000457198	24200	0.000266332
25100	0.01194306	23100	2.61E-05	22900	6.52E-05	22500	6.92E-05	24800	0.000457871	24200	0.000266534
25100	0.01198099	23100	2.61E-05	22900	6.53E-05	22500	6.92E-05	24800	0.00045853	24200	0.000266732
25100	0.0120193	23100	2.61E-05	22900	6.54E-05	22500	6.93E-05	24800	0.000459198	24200	0.000266921
25100	0.01205781	23100	2.62E-05	22900	6.55E-05	22500	6.93E-05	24800	0.000459867	24200	0.000267113
25100	0.01209663	23100	2.62E-05	22900	6.56E-05	22500	6.93E-05	24800	0.000460519	24200	0.0002673
25100	0.01213574	23100	2.62E-05	22900	6.57E-05	22500	6.93E-05	24800	0.000461173	24200	0.000267486
25100	0.01217517	23100	2.62E-05	22900	6.58E-05	22500	6.93E-05	24800	0.000461833	24200	0.000267668
25100	0.01221487	23100	2.62E-05	22900	6.59E-05	22500	6.94E-05	24800	0.000462483	24200	0.000267883
25100	0.01225481	23100	2.62E-05	22900	6.60E-05	22500	6.94E-05	24800	0.000463137	24200	0.000268082
25100	0.01229507	23100	2.62E-05	22900	6.61E-05	22500	6.94E-05	24800	0.000463791	24200	0.000268298
25100	0.01233567	23100	2.62E-05	22900	6.63E-05	22500	6.94E-05	24800	0.000464446	24200	0.000268496
25100	0.01237655	23100	2.63E-05	22900	6.64E-05	22500	6.95E-05	24800	0.0004651	24200	0.000268712
25200	0.01241774	23100	2.63E-05	22900	6.65E-05	22500	6.95E-05	24800	0.000465753	24200	0.000268927

25200	0.01245922	23100	2.63E-05	22900	6.66E-05	22500	6.96E-05	24800	0.000466418	24200	0.000269125
25200	0.01250101	23100	2.63E-05	22900	6.67E-05	22500	6.96E-05	24800	0.000467064	24200	0.000269322
25200	0.01254317	23100	2.63E-05	22900	6.68E-05	22500	6.96E-05	24800	0.000467714	24200	0.000269515
25200	0.01258569	23100	2.64E-05	22900	6.70E-05	22500	6.97E-05	24800	0.000468382	24200	0.000269724
25200	0.01262841	23100	2.64E-05	22900	6.71E-05	22500	6.97E-05	24800	0.00046903	24200	0.000269936
25200	0.01267157	23100	2.64E-05	22900	6.72E-05	22500	6.98E-05	24800	0.000469675	24200	0.000270142
25200	0.01271494	23100	2.64E-05	22900	6.73E-05	22500	6.98E-05	24800	0.00047032	24200	0.000270352
25200	0.01275874	23100	2.64E-05	22900	6.75E-05	22500	6.99E-05	24800	0.000470969	24200	0.000270568
25200	0.01280289	23100	2.65E-05	22900	6.76E-05	22500	6.99E-05	24800	0.000471626	24300	0.000270782
25200	0.0128473	23100	2.65E-05	22900	6.77E-05	22500	6.99E-05	24800	0.000472289	24300	0.000270998
25200	0.01289204	23100	2.65E-05	22900	6.79E-05	22500	7.00E-05	24800	0.000472932	24300	0.000271199
25200	0.01293722	23100	2.65E-05	22900	6.80E-05	22500	7.00E-05	24800	0.000473565	24300	0.000271401
25200	0.01298265	23100	2.65E-05	22900	6.82E-05	22500	7.00E-05	24800	0.000474215	24300	0.000271601
25200	0.01302846	23100	2.65E-05	22900	6.83E-05	22500	7.00E-05	24800	0.00047486	24300	0.000271824
25200	0.01307461	23100	2.65E-05	22900	6.85E-05	22500	7.00E-05	24800	0.000475507	24300	0.000272043
25200	0.01312109	23100	2.66E-05	22900	6.86E-05	22500	7.01E-05	24800	0.00047615	24300	0.000272254
25200	0.01316793	23100	2.66E-05	22900	6.87E-05	22500	7.01E-05	24800	0.000476802	24300	0.000272458
25200	0.01321511	23200	2.66E-05	22900	6.89E-05	22500	7.01E-05	24800	0.000477459	24300	0.000272673
25200	0.01326266	23200	2.66E-05	22900	6.90E-05	22500	7.02E-05	24800	0.000478113	24300	0.000272901
25200	0.01331059	23200	2.66E-05	23000	6.92E-05	22500	7.02E-05	24800	0.000478757	24300	0.000273109
25200	0.0133589	23200	2.66E-05	23000	6.93E-05	22500	7.02E-05	24800	0.000479421	24300	0.000273328
25200	0.01340748	23200	2.66E-05	23000	6.95E-05	22500	7.02E-05	24800	0.000480079	24300	0.000273525
25200	0.01345654	23200	2.67E-05	23000	6.97E-05	22500	7.02E-05	24800	0.000480746	24300	0.000273738
25200	0.01350596	23200	2.67E-05	23000	6.98E-05	22600	7.03E-05	24800	0.000481399	24300	0.000273948
25200	0.0135557	23200	2.67E-05	23000	7.00E-05	22600	7.03E-05	24800	0.000482055	24300	0.000274186
25200	0.0136058	23200	2.67E-05	23000	7.01E-05	22600	7.03E-05	24800	0.000482721	24300	0.000274398
25200	0.01365633	23200	2.67E-05	23000	7.03E-05	22600	7.03E-05	24800	0.000483383	24300	0.000274622
25200	0.01370725	23200	2.67E-05	23000	7.05E-05	22600	7.04E-05	24800	0.000484065	24300	0.000274856
25200	0.01375853	23200	2.67E-05	23000	7.06E-05	22600	7.04E-05	24800	0.000484738	24300	0.000275064
25200	0.01381017	23200	2.67E-05	23000	7.08E-05	22600	7.04E-05	24800	0.0004854	24300	0.000275289
25200	0.01386224	23200	2.68E-05	23000	7.10E-05	22600	7.04E-05	24800	0.000486046	24300	0.00027552
25200	0.01391474	23200	2.68E-05	23000	7.12E-05	22600	7.04E-05	24800	0.000486718	24300	0.000275732
25200	0.01396758	23200	2.68E-05	23000	7.14E-05	22600	7.04E-05	24800	0.000487357	24300	0.000275977
25200	0.01402083	23200	2.68E-05	23000	7.16E-05	22600	7.05E-05	24800	0.000488026	24300	0.000276199

25200 0.01407447 23200 2.68E-05 23000 7.18E-05 22600
7.05E-05 24900 0.000488687 24300 0.000276427
25200 0.01412846 23200 2.68E-05 23000 7.20E-05 22600
7.05E-05 24900 0.000489359 24300 0.000276659
25200 0.01418293 23200 2.68E-05 23000 7.22E-05 22600
7.05E-05 24900 0.000490023 24300 0.00027691
25200 0.01423778 23200 2.69E-05 23000 7.24E-05 22600
7.05E-05 24900 0.000490678 24300 0.000277137
25200 0.01429306 23200 2.69E-05 23000 7.26E-05 22600
7.05E-05 24900 0.000491342 24300 0.000277369
25200 0.0143488 23200 2.69E-05 23000 7.28E-05 22600
7.05E-05 24900 0.000492017 24300 0.000277603
25200 0.0144049 23200 2.69E-05 23000 7.30E-05 22600
7.05E-05 24900 0.000492699 24300 0.000277838
25200 0.01446142 23200 2.69E-05 23000 7.32E-05 22600
7.05E-05 24900 0.000493376 24300 0.000278088
25200 0.01451839 23200 2.69E-05 23000 7.35E-05 22600
7.05E-05 24900 0.000494066 24300 0.000278308
25200 0.01457571 23200 2.70E-05 23000 7.37E-05 22600
7.06E-05 24900 0.00049475 24300 0.000278572
25200 0.01463348 23200 2.70E-05 23000 7.39E-05 22600
7.06E-05 24900 0.000495432 24300 0.000278794
25200 0.01469169 23200 2.70E-05 23000 7.42E-05 22600
7.06E-05 24900 0.000496149 24300 0.000279036
25200 0.01475034 23200 2.70E-05 23000 7.44E-05 22600
7.07E-05 24900 0.000496864 24300 0.000279266
25200 0.0148094 23200 2.70E-05 23000 7.47E-05 22600
7.07E-05 24900 0.000497569 24300 0.00027952
25200 0.01486893 23200 2.70E-05 23000 7.49E-05 22600
7.08E-05 24900 0.000498272 24300 0.00027975
25200 0.01492887 23200 2.71E-05 23000 7.52E-05 22600
7.08E-05 24900 0.00049898 24300 0.000279993
25200 0.01498925 23200 2.71E-05 23000 7.55E-05 22600
7.08E-05 24900 0.000499701 24300 0.000280206
25200 0.01505006 23200 2.71E-05 23000 7.57E-05 22600
7.09E-05 24900 0.000500423 24300 0.000280434
25200 0.01511128 23200 2.71E-05 23000 7.60E-05 22600
7.10E-05 24900 0.000501157 24300 0.000280663
25200 0.01517298 23200 2.71E-05 23000 7.63E-05 22600
7.10E-05 24900 0.000501878 24300 0.000280907
25200 0.01523511 23200 2.71E-05 23000 7.66E-05 22600
7.10E-05 24900 0.00050261 24300 0.000281135
25200 0.01529768 23200 2.72E-05 23000 7.69E-05 22600
7.11E-05 24900 0.00050334 24300 0.000281382
25200 0.0153607 23200 2.72E-05 23000 7.72E-05 22600
7.11E-05 24900 0.000504105 24300 0.000281606
25200 0.01542415 23200 2.72E-05 23000 7.76E-05 22600
7.12E-05 24900 0.000504875 24300 0.000281862
25200 0.01548806 23200 2.72E-05 23000 7.79E-05 22600
7.12E-05 24900 0.000505646 24300 0.000282097
25200 0.01555244 23200 2.72E-05 23000 7.82E-05 22600
7.13E-05 24900 0.000506427 24300 0.000282309
25200 0.01561735 23200 2.73E-05 23000 7.86E-05 22600
7.13E-05 24900 0.000507217 24300 0.000282532
25200 0.0156826 23200 2.73E-05 23000 7.89E-05 22600
7.14E-05 24900 0.000508032 24300 0.000282758
25200 0.01574833 23200 2.73E-05 23000 7.93E-05 22600
7.14E-05 24900 0.000508843 24300 0.000282998
25300 0.0158145 23200 2.73E-05 23000 7.96E-05 22600
7.15E-05 24900 0.000509673 24300 0.000283239
25300 0.01588121 23200 2.73E-05 23000 8.00E-05 22600
7.15E-05 24900 0.000510505 24300 0.000283477
25300 0.01594841 23200 2.74E-05 23000 8.04E-05 22600
7.15E-05 24900 0.000511354 24300 0.00028371
25300 0.016016 23200 2.74E-05 23000 8.08E-05 22600
7.16E-05 24900 0.000512209 24300 0.00028394
25300 0.0160841 23200 2.74E-05 23000 8.12E-05 22600
7.16E-05 24900 0.000513099 24300 0.000284168

25300 0.01615266 23200 2.74E-05 23000 8.16E-05 22600
7.16E-05 24900 0.000514002 24300 0.00028439
25300 0.01622174 23200 2.74E-05 23000 8.21E-05 22600
7.17E-05 24900 0.000514843 24300 0.000284612
25300 0.01629124 23200 2.74E-05 23000 8.25E-05 22600
7.17E-05 24900 0.000515733 24300 0.000284863
25300 0.01636126 23200 2.75E-05 23000 8.30E-05 22600
7.17E-05 24900 0.000516622 24300 0.000285066
25300 0.01643179 23200 2.75E-05 23000 8.35E-05 22600
7.18E-05 24900 0.000517514 24400 0.000285298
25300 0.01650279 23200 2.75E-05 23000 8.40E-05 22600
7.18E-05 24900 0.000518412 24400 0.000285513
25300 0.0165743 23200 2.75E-05 23000 8.45E-05 22600
7.18E-05 24900 0.000519305 24400 0.000285752
25300 0.01664631 23200 2.75E-05 23000 8.50E-05 22600
7.19E-05 24900 0.000520192 24400 0.000285986
25300 0.01671879 23200 2.75E-05 23000 8.55E-05 22600
7.19E-05 24900 0.000521122 24400 0.000286217
25300 0.01679182 23200 2.76E-05 23000 8.61E-05 22600
7.19E-05 24900 0.000522011 24400 0.000286456
25300 0.01686536 23200 2.76E-05 23000 8.66E-05 22600
7.19E-05 24900 0.000522942 24400 0.00028667
25300 0.01693938 23200 2.76E-05 23000 8.72E-05 22600
7.19E-05 24900 0.000523866 24400 0.000286914
25300 0.01701397 23200 2.76E-05 23000 8.78E-05 22600
7.19E-05 24900 0.000524774 24400 0.000287157
25300 0.01708899 23300 2.76E-05 23000 8.85E-05 22600
7.19E-05 24900 0.000525654 24400 0.0002874
25300 0.01716458 23300 2.76E-05 23000 8.91E-05 22600
7.19E-05 24900 0.000526572 24400 0.000287621
25300 0.01724073 23300 2.76E-05 23100 8.98E-05 22600
7.19E-05 24900 0.000527499 24400 0.000287856
25300 0.0173174 23300 2.76E-05 23100 9.05E-05 22600
7.20E-05 24900 0.000528423 24400 0.000288116
25300 0.01739457 23300 2.77E-05 23100 9.12E-05 22600
7.20E-05 24900 0.000529354 24400 0.000288334
25300 0.01747233 23300 2.77E-05 23100 9.19E-05 22600
7.20E-05 24900 0.000530273 24400 0.000288559
25300 0.01755057 23300 2.77E-05 23100 9.27E-05 22700
7.20E-05 24900 0.000531197 24400 0.000288791
25300 0.01762941 23300 2.77E-05 23100 9.35E-05 22700
7.21E-05 24900 0.000532105 24400 0.000289026
25300 0.01770874 23300 2.77E-05 23100 9.43E-05 22700
7.21E-05 24900 0.000533026 24400 0.000289252
25300 0.01778863 23300 2.77E-05 23100 9.51E-05 22700
7.21E-05 24900 0.000533952 24400 0.000289487
25300 0.01786909 23300 2.78E-05 23100 9.60E-05 22700
7.21E-05 24900 0.000534858 24400 0.000289706
25300 0.0179501 23300 2.78E-05 23100 9.69E-05 22700
7.21E-05 24900 0.000535768 24400 0.000289958
25300 0.01803166 23300 2.78E-05 23100 9.79E-05 22700
7.22E-05 24900 0.000536689 24400 0.000290197
25300 0.01811383 23300 2.78E-05 23100 9.88E-05 22700
7.22E-05 24900 0.000537597 24400 0.000290432
25300 0.01819652 23300 2.78E-05 23100 9.99E-05 22700
7.22E-05 24900 0.000538525 24400 0.000290681
25300 0.01827982 23300 2.79E-05 23100 1.01E-04 22700
7.22E-05 24900 0.000539457 24400 0.00029091
25300 0.01836381 23300 2.79E-05 23100 1.02E-04 22700
7.23E-05 24900 0.000540394 24400 0.000291133
25300 0.01844826 23300 2.79E-05 23100 1.03E-04 22700
7.23E-05 25000 0.000541329 24400 0.000291383
25300 0.0185334 23300 2.79E-05 23100 1.04E-04 22700
7.24E-05 25000 0.000542249 24400 0.000291618
25300 0.01861904 23300 2.79E-05 23100 1.06E-04 22700
7.24E-05 25000 0.000543175 24400 0.000291859
25300 0.01870533 23300 2.79E-05 23100 1.07E-04 22700
7.25E-05 25000 0.000544113 24400 0.0002921

25300	0.01879224	23300	2.80E-05	23100	1.08E-04	22700	7.26E-05	25000	0.000545059	24400	0.000292359
25300	0.01887976	23300	2.80E-05	23100	1.10E-04	22700	7.26E-05	25000	0.000546	24400	0.000292584
25300	0.01896793	23300	2.80E-05	23100	1.11E-04	22700	7.27E-05	25000	0.000546944	24400	0.000292817
25300	0.0190567	23300	2.80E-05	23100	1.12E-04	22700	7.28E-05	25000	0.000547889	24400	0.00029304
25300	0.01914608	23300	2.80E-05	23100	1.14E-04	22700	7.28E-05	25000	0.000548843	24400	0.000293281
25300	0.01923614	23300	2.80E-05	23100	1.16E-04	22700	7.29E-05	25000	0.000549794	24400	0.000293519
25300	0.01932679	23300	2.81E-05	23100	1.17E-04	22700	7.29E-05	25000	0.000550748	24400	0.000293752
25300	0.01941814	23300	2.81E-05	23100	1.19E-04	22700	7.30E-05	25000	0.000551696	24400	0.000293993
25300	0.01951011	23300	2.81E-05	23100	1.21E-04	22700	7.30E-05	25000	0.00055264	24400	0.000294219
25300	0.01960273	23300	2.81E-05	23100	1.23E-04	22700	7.31E-05	25000	0.000553581	24400	0.000294464
25300	0.01969599	23300	2.81E-05	23100	1.25E-04	22700	7.31E-05	25000	0.000554529	24400	0.000294711
25300	0.01978997	23300	2.82E-05	23100	1.27E-04	22700	7.31E-05	25000	0.000555472	24400	0.000294969
25300	0.0198846	23300	2.82E-05	23100	1.29E-04	22700	7.32E-05	25000	0.000556423	24400	0.000295211
25300	0.01997989	23300	2.82E-05	23100	1.32E-04	22700	7.33E-05	25000	0.00055737	24400	0.000295449
25300	0.02007587	23300	2.82E-05	23100	1.34E-04	22700	7.33E-05	25000	0.000558334	24400	0.000295679
25300	0.02017252	23300	2.82E-05	23100	1.36E-04	22700	7.33E-05	25000	0.00055929	24400	0.000295933
25300	0.02026989	23300	2.83E-05	23100	1.39E-04	22700	7.34E-05	25000	0.000560238	24400	0.000296182
25300	0.02036791	23300	2.83E-05	23100	1.42E-04	22700	7.34E-05	25000	0.000561162	24400	0.000296448
25300	0.02046662	23300	2.83E-05	23100	1.45E-04	22700	7.35E-05	25000	0.000562112	24400	0.000296699
25300	0.02056609	23300	2.83E-05	23100	1.48E-04	22700	7.35E-05	25000	0.00056306	24400	0.00029695
25300	0.02066627	23300	2.83E-05	23100	1.51E-04	22700	7.35E-05	25000	0.000563996	24400	0.000297203
25300	0.02076712	23300	2.83E-05	23100	1.54E-04	22700	7.36E-05	25000	0.00056494	24400	0.000297447
25300	0.02086872	23300	2.84E-05	23100	1.57E-04	22700	7.36E-05	25000	0.000565867	24400	0.000297679
25300	0.02097103	23300	2.84E-05	23100	1.61E-04	22700	7.36E-05	25000	0.000566792	24400	0.000297922
25300	0.02107411	23300	2.84E-05	23100	1.64E-04	22700	7.37E-05	25000	0.000567701	24400	0.000298172
25400	0.02117783	23300	2.84E-05	23100	1.68E-04	22700	7.37E-05	25000	0.000568622	24400	0.000298413
25400	0.02128236	23300	2.84E-05	23100	1.72E-04	22700	7.37E-05	25000	0.000569522	24400	0.000298667
25400	0.02138762	23300	2.84E-05	23100	1.76E-04	22700	7.38E-05	25000	0.000570425	24400	0.000298917
25400	0.02149365	23300	2.84E-05	23100	1.80E-04	22700	7.38E-05	25000	0.000571301	24400	0.000299173
25400	0.02160038	23300	2.85E-05	23100	1.84E-04	22700	7.39E-05	25000	0.000572183	24400	0.000299426
25400	0.02170793	23300	2.85E-05	23100	1.88E-04	22700	7.39E-05	25000	0.000573049	24400	0.000299669
25400	0.02181621	23300	2.85E-05	23100	1.93E-04	22700	7.40E-05	25000	0.000573934	24400	0.000299926
25400	0.02192527	23300	2.85E-05	23100	1.97E-04	22700	7.41E-05	25000	0.000574804	24400	0.000300176
25400	0.02203513	23300	2.86E-05	23100	2.02E-04	22700	7.42E-05	25000	0.000575682	24400	0.000300424

25400	0.02214579	23300	2.86E-05	23100	2.06E-04	22700	7.42E-05	25000	0.000576545	24500	0.000300669
25400	0.02225723	23300	2.86E-05	23100	2.11E-04	22700	7.43E-05	25000	0.000577409	24500	0.00030092
25400	0.02236946	23300	2.86E-05	23100	2.16E-04	22700	7.43E-05	25000	0.00057826	24500	0.000301203
25400	0.02248246	23300	2.86E-05	23100	2.20E-04	22700	7.44E-05	25000	0.000579128	24500	0.00030145
25400	0.02259632	23300	2.86E-05	23100	2.25E-04	22700	7.44E-05	25000	0.000579999	24500	0.000301704
25400	0.02271101	23300	2.87E-05	23100	2.29E-04	22700	7.45E-05	25000	0.000580867	24500	0.000301966
25400	0.02282649	23300	2.87E-05	23100	2.34E-04	22700	7.45E-05	25000	0.000581739	24500	0.000302229
25400	0.02294283	23300	2.87E-05	23100	2.38E-04	22700	7.45E-05	25000	0.000582604	24500	0.000302487
25400	0.02305995	23300	2.87E-05	23100	2.42E-04	22700	7.46E-05	25000	0.000583484	24500	0.000302734
25400	0.02317795	23400	2.87E-05	23100	2.45E-04	22700	7.46E-05	25000	0.000584351	24500	0.000303001
25400	0.02329677	23400	2.87E-05	23100	2.48E-04	22700	7.46E-05	25000	0.000585239	24500	0.000303257
25400	0.02341647	23400	2.87E-05	23200	2.51E-04	22700	7.46E-05	25000	0.000586119	24500	0.000303513
25400	0.023537	23400	2.88E-05	23200	2.53E-04	22700	7.46E-05	25000	0.000587007	24500	0.000303754
25400	0.02365839	23400	2.88E-05	23200	2.55E-04	22700	7.46E-05	25000	0.000587894	24500	0.000304013
25400	0.02378064	23400	2.88E-05	23200	2.56E-04	22700	7.46E-05	25000	0.000588791	24500	0.000304293
25400	0.02390373	23400	2.88E-05	23200	2.57E-04	22800	7.46E-05	25000	0.000589681	24500	0.000304529
25400	0.02402773	23400	2.88E-05	23200	2.57E-04	22800	7.47E-05	25000	0.000590591	24500	0.000304799
25400	0.02415263	23400	2.89E-05	23200	2.56E-04	22800	7.47E-05	25000	0.000591506	24500	0.000305056
25400	0.02427842	23400	2.89E-05	23200	2.55E-04	22800	7.47E-05	25000	0.000592435	24500	0.000305323
25400	0.02440511	23400	2.89E-05	23200	2.53E-04	22800	7.47E-05	25000	0.000593362	24500	0.00030558
25400	0.02453268	23400	2.89E-05	23200	2.50E-04	22800	7.48E-05	25000	0.000594293	24500	0.000305867
25400	0.02466114	23400	2.89E-05	23200	2.47E-04	22800	7.48E-05	25000	0.00059521	24500	0.000306156
25400	0.02479046	23400	2.89E-05	23200	2.44E-04	22800	7.48E-05	25000	0.000596149	24500	0.000306407
25400	0.02492078	23400	2.89E-05	23200	2.39E-04	22800	7.49E-05	25000	0.000597111	24500	0.000306686
25400	0.02505201	23400	2.90E-05	23200	2.35E-04	22800	7.49E-05	25000	0.000598062	24500	0.000306945
25400	0.02518415	23400	2.90E-05	23200	2.30E-04	22800	7.50E-05	25000	0.00059902	24500	0.000307231
25400	0.02531725	23400	2.90E-05	23200	2.25E-04	22800	7.50E-05	25100	0.000599979	24500	0.000307525
25400	0.02545128	23400	2.90E-05	23200	2.20E-04	22800	7.51E-05	25100	0.000600945	24500	0.000307793
25400	0.02558629	23400	2.90E-05	23200	2.14E-04	22800	7.51E-05	25100	0.000601904	24500	0.000308054
25400	0.02572219	23400	2.90E-05	23200	2.09E-04	22800	7.52E-05	25100	0.000602889	24500	0.000308309
25400	0.02585912	23400	2.91E-05	23200	2.03E-04	22800	7.53E-05	25100	0.000603866	24500	0.000308579
25400	0.02599696	23400	2.91E-05	23200	1.98E-04	22800	7.53E-05	25100	0.000604844	24500	0.000308865
25400	0.02613583	23400	2.91E-05	23200	1.93E-04	22800	7.54E-05	25100	0.00060582	24500	0.000309131
25400	0.02627568	23400	2.91E-05	23200	1.87E-04	22800	7.55E-05	25100	0.000606815	24500	0.000309428

25400 0.02641654 23400 2.92E-05 23200 1.82E-04 22800
7.55E-05 25100 0.000607801 24500 0.00030969
25400 0.02655833 23400 2.92E-05 23200 1.77E-04 22800
7.56E-05 25100 0.000608802 24500 0.000309966
25400 0.02670119 23400 2.92E-05 23200 1.73E-04 22800
7.57E-05 25100 0.000609807 24500 0.000310253
25400 0.02684506 23400 2.92E-05 23200 1.68E-04 22800
7.57E-05 25100 0.00061082 24500 0.000310509
25400 0.02699001 23400 2.92E-05 23200 1.64E-04 22800
7.57E-05 25100 0.000611817 24500 0.00031079
25400 0.02713593 23400 2.92E-05 23200 1.60E-04 22800
7.58E-05 25100 0.00061283 24500 0.000311065
25400 0.02728289 23400 2.92E-05 23200 1.56E-04 22800
7.58E-05 25100 0.000613829 24500 0.000311354
25400 0.02743087 23400 2.93E-05 23200 1.53E-04 22800
7.59E-05 25100 0.000614842 24500 0.000311613
25400 0.02757998 23400 2.93E-05 23200 1.49E-04 22800
7.59E-05 25100 0.00061588 24500 0.000311904
25400 0.02773012 23400 2.93E-05 23200 1.46E-04 22800
7.59E-05 25100 0.000616906 24500 0.000312191
25400 0.02788132 23400 2.93E-05 23200 1.43E-04 22800
7.60E-05 25100 0.000617936 24500 0.000312466
25400 0.02803363 23400 2.93E-05 23200 1.40E-04 22800
7.60E-05 25100 0.000618964 24500 0.000312744
25400 0.028187 23400 2.93E-05 23200 1.38E-04 22800
7.60E-05 25100 0.000619997 24500 0.000313007
25400 0.0283415 23400 2.93E-05 23200 1.35E-04 22800
7.60E-05 25100 0.000621041 24500 0.000313287
25400 0.02849708 23400 2.93E-05 23200 1.33E-04 22800
7.60E-05 25100 0.000622074 24500 0.000313558
25400 0.02865374 23400 2.93E-05 23200 1.30E-04 22800
7.61E-05 25100 0.000623118 24500 0.000313826
25400 0.02881156 23400 2.94E-05 23200 1.28E-04 22800
7.61E-05 25100 0.000624156 24500 0.000314102
25400 0.0289705 23400 2.94E-05 23200 1.26E-04 22800
7.61E-05 25100 0.000625206 24500 0.00031437
25400 0.02913055 23400 2.94E-05 23200 1.24E-04 22800
7.62E-05 25100 0.000626255 24500 0.000314668
25400 0.02929177 23400 2.94E-05 23200 1.23E-04 22800
7.62E-05 25100 0.000627313 24500 0.000314921
25400 0.02945413 23400 2.94E-05 23200 1.21E-04 22800
7.63E-05 25100 0.000628378 24500 0.000315218
25500 0.02961769 23400 2.94E-05 23200 1.19E-04 22800
7.63E-05 25100 0.000629437 24500 0.000315479
25500 0.02978238 23400 2.95E-05 23200 1.18E-04 22800
7.63E-05 25100 0.000630516 24500 0.000315748
25500 0.02994828 23400 2.95E-05 23200 1.16E-04 22800
7.64E-05 25100 0.000631581 24500 0.000316012
25500 0.03011532 23400 2.95E-05 23200 1.15E-04 22800
7.64E-05 25100 0.000632663 24500 0.00031629
25500 0.03028352 23400 2.95E-05 23200 1.14E-04 22800
7.65E-05 25100 0.00063372 24500 0.000316575
25500 0.03045293 23400 2.95E-05 23200 1.12E-04 22800
7.65E-05 25100 0.000634803 24500 0.000316841
25500 0.03062358 23400 2.96E-05 23200 1.11E-04 22800
7.66E-05 25100 0.000635909 24500 0.000317125
25500 0.03079544 23400 2.96E-05 23200 1.10E-04 22800
7.66E-05 25100 0.000636993 24500 0.00031741
25500 0.03096853 23400 2.96E-05 23200 1.09E-04 22800
7.67E-05 25100 0.000638087 24500 0.000317694
25500 0.03114282 23400 2.97E-05 23200 1.08E-04 22800
7.68E-05 25100 0.000639179 24600 0.000317963
25500 0.03131836 23400 2.97E-05 23200 1.07E-04 22800
7.68E-05 25100 0.000640278 24600 0.000318238
25500 0.03149514 23400 2.97E-05 23200 1.06E-04 22800
7.69E-05 25100 0.00064137 24600 0.000318511
25500 0.03167317 23400 2.97E-05 23200 1.05E-04 22800
7.69E-05 25100 0.000642477 24600 0.000318791

25500 0.03185243 23400 2.97E-05 23200 1.04E-04 22800
7.70E-05 25100 0.000643576 24600 0.000319057
25500 0.03203297 23400 2.97E-05 23200 1.03E-04 22800
7.70E-05 25100 0.00064468 24600 0.000319329
25500 0.03221477 23400 2.98E-05 23200 1.02E-04 22800
7.71E-05 25100 0.000645788 24600 0.000319598
25500 0.0323979 23400 2.98E-05 23200 1.02E-04 22800
7.71E-05 25100 0.000646896 24600 0.000319891
25500 0.03258229 23400 2.98E-05 23200 1.01E-04 22800
7.71E-05 25100 0.000648001 24600 0.000320164
25500 0.03276797 23500 2.98E-05 23200 1.00E-04 22800
7.72E-05 25100 0.000649117 24600 0.00032044
25500 0.03295497 23500 2.98E-05 23200 9.95E-05 22800
7.72E-05 25100 0.000650228 24600 0.000320728
25500 0.03314331 23500 2.98E-05 23300 9.88E-05 22800
7.72E-05 25100 0.000651355 24600 0.000320985
25500 0.03333298 23500 2.99E-05 23300 9.82E-05 22800
7.72E-05 25100 0.000652471 24600 0.000321265
25500 0.03352401 23500 2.99E-05 23300 9.76E-05 22800
7.73E-05 25100 0.000653586 24600 0.000321553
25500 0.03371632 23500 2.99E-05 23300 9.70E-05 22800
7.73E-05 25100 0.000654691 24600 0.000321836
25500 0.03390998 23500 2.99E-05 23300 9.64E-05 22900
7.73E-05 25100 0.000655816 24600 0.000322102
25500 0.03410504 23500 2.99E-05 23300 9.59E-05 22900
7.74E-05 25100 0.000656958 24600 0.000322393
25500 0.03430149 23500 2.99E-05 23300 9.54E-05 22900
7.74E-05 25100 0.000658086 24600 0.000322679
25500 0.03449927 23500 2.99E-05 23300 9.49E-05 22900
7.75E-05 25100 0.000659229 24600 0.000322968
25500 0.03469842 23500 3.00E-05 23300 9.44E-05 22900
7.75E-05 25100 0.000660363 24600 0.000323243
25500 0.03489899 23500 3.00E-05 23300 9.39E-05 22900
7.76E-05 25100 0.000661503 24600 0.000323521
25500 0.03510095 23500 3.00E-05 23300 9.35E-05 22900
7.76E-05 25100 0.000662632 24600 0.000323793
25500 0.03530431 23500 3.00E-05 23300 9.30E-05 22900
7.77E-05 25100 0.000663784 24600 0.000324077
25500 0.03550908 23500 3.01E-05 23300 9.26E-05 22900
7.77E-05 25100 0.00066492 24600 0.000324344
25500 0.03571529 23500 3.01E-05 23300 9.22E-05 22900
7.78E-05 25100 0.000666078 24600 0.000324629
25500 0.03592291 23500 3.01E-05 23300 9.19E-05 22900
7.78E-05 25100 0.000667219 24600 0.000324881
25500 0.03613195 23500 3.01E-05 23300 9.15E-05 22900
7.79E-05 25200 0.000668383 24600 0.000325172
25500 0.03634246 23500 3.02E-05 23300 9.11E-05 22900
7.79E-05 25200 0.000669542 24600 0.000325453
25500 0.03655438 23500 3.02E-05 23300 9.08E-05 22900
7.80E-05 25200 0.000670712 24600 0.000325744
25500 0.03676779 23500 3.02E-05 23300 9.05E-05 22900
7.81E-05 25200 0.000671881 24600 0.000326023
25500 0.03698267 23500 3.02E-05 23300 9.01E-05 22900
7.82E-05 25200 0.000673063 24600 0.000326274
25500 0.03719901 23500 3.03E-05 23300 8.98E-05 22900
7.82E-05 25200 0.000674242 24600 0.000326561
25500 0.03741685 23500 3.03E-05 23300 8.95E-05 22900
7.83E-05 25200 0.000675419 24600 0.000326842
25500 0.03763612 23500 3.03E-05 23300 8.92E-05 22900
7.84E-05 25200 0.00067659 24600 0.000327147
25500 0.03785685 23500 3.03E-05 23300 8.90E-05 22900
7.84E-05 25200 0.000677785 24600 0.000327402
25500 0.03807914 23500 3.03E-05 23300 8.87E-05 22900
7.85E-05 25200 0.000678991 24600 0.000327708
25500 0.03830292 23500 3.04E-05 23300 8.84E-05 22900
7.86E-05 25200 0.000680185 24600 0.000328005
25500 0.03852821 23500 3.04E-05 23300 8.82E-05 22900
7.86E-05 25200 0.000681403 24600 0.000328303

25500 0.03875502 23500 3.04E-05 23300 8.79E-05 22900
7.87E-05 25200 0.000682599 24600 0.000328584
25500 0.03898333 23500 3.04E-05 23300 8.77E-05 22900
7.88E-05 25200 0.000683803 24600 0.00032887
25500 0.03921322 23500 3.04E-05 23300 8.75E-05 22900
7.88E-05 25200 0.000684995 24600 0.00032915
25500 0.03944457 23500 3.05E-05 23300 8.73E-05 22900
7.89E-05 25200 0.000686197 24600 0.000329453
25500 0.03967748 23500 3.05E-05 23300 8.71E-05 22900
7.90E-05 25200 0.000687388 24600 0.00032974
25500 0.03991194 23500 3.05E-05 23300 8.69E-05 22900
7.90E-05 25200 0.000688588 24600 0.000330028
25500 0.04014798 23500 3.05E-05 23300 8.67E-05 22900
7.91E-05 25200 0.000689771 24600 0.000330302
25500 0.04038552 23500 3.05E-05 23300 8.65E-05 22900
7.91E-05 25200 0.000690976 24600 0.000330622
25500 0.04062468 23500 3.06E-05 23300 8.63E-05 22900
7.92E-05 25200 0.000692166 24600 0.000330907
25500 0.04086534 23500 3.06E-05 23300 8.61E-05 22900
7.92E-05 25200 0.00069337 24600 0.000331211
25500 0.04110763 23500 3.06E-05 23300 8.60E-05 22900
7.93E-05 25200 0.000694568 24600 0.000331511
25500 0.04135149 23500 3.06E-05 23300 8.58E-05 22900
7.93E-05 25200 0.00069577 24600 0.000331792
25500 0.0415969 23500 3.06E-05 23300 8.56E-05 22900
7.94E-05 25200 0.000696972 24600 0.000332095
25500 0.04184395 23500 3.07E-05 23300 8.55E-05 22900
7.94E-05 25200 0.000698159 24600 0.000332396
25500 0.04209256 23500 3.07E-05 23300 8.54E-05 22900
7.95E-05 25200 0.000699346 24600 0.000332699
25500 0.04234276 23500 3.07E-05 23300 8.52E-05 22900
7.95E-05 25200 0.00070056 24600 0.000332995
25500 0.04259462 23500 3.07E-05 23300 8.51E-05 22900
7.95E-05 25200 0.000701772 24600 0.000333306
25600 0.0428481 23500 3.08E-05 23300 8.50E-05 22900
7.96E-05 25200 0.000702989 24600 0.000333603
25600 0.0431032 23500 3.08E-05 23300 8.48E-05 22900
7.96E-05 25200 0.000704217 24600 0.000333911
25600 0.04335992 23500 3.08E-05 23300 8.47E-05 22900
7.97E-05 25200 0.000705445 24600 0.000334198
25600 0.04361829 23500 3.08E-05 23300 8.46E-05 22900
7.98E-05 25200 0.000706675 24600 0.0003345
25600 0.0438783 23500 3.08E-05 23300 8.45E-05 22900
7.98E-05 25200 0.000707898 24600 0.000334793
25600 0.04413997 23500 3.09E-05 23300 8.44E-05 22900
7.99E-05 25200 0.000709138 24600 0.000335087
25600 0.0444033 23500 3.09E-05 23300 8.43E-05 22900
8.00E-05 25200 0.000710383 24600 0.000335381
25600 0.04466827 23500 3.09E-05 23300 8.42E-05 22900
8.01E-05 25200 0.000711629 24600 0.000335693
25600 0.04493492 23500 3.09E-05 23300 8.41E-05 22900
8.01E-05 25200 0.000712881 24600 0.000335966
25600 0.04520325 23500 3.10E-05 23300 8.40E-05 22900
8.02E-05 25200 0.000714139 24700 0.00033629
25600 0.04547327 23500 3.10E-05 23300 8.39E-05 22900
8.03E-05 25200 0.000715403 24700 0.000336588
25600 0.04574496 23500 3.10E-05 23300 8.38E-05 22900
8.03E-05 25200 0.000716678 24700 0.000336901
25600 0.04601837 23500 3.10E-05 23300 8.37E-05 22900
8.04E-05 25200 0.000717962 24700 0.0003372
25600 0.04629348 23500 3.10E-05 23300 8.37E-05 22900
8.04E-05 25200 0.000719242 24700 0.000337478
25600 0.04657027 23500 3.11E-05 23300 8.36E-05 22900
8.04E-05 25200 0.000720523 24700 0.000337786
25600 0.04684877 23500 3.11E-05 23300 8.35E-05 22900
8.05E-05 25200 0.0007218 24700 0.000338089
25600 0.04712886 23500 3.11E-05 23300 8.35E-05 22900
8.05E-05 25200 0.000723084 24700 0.000338399

25600 0.0474107 23500 3.11E-05 23300 8.34E-05 22900
8.06E-05 25200 0.000724375 24700 0.0003387
25600 0.0476943 23600 3.11E-05 23300 8.33E-05 22900
8.06E-05 25200 0.000725697 24700 0.000339022
25600 0.04797958 23600 3.11E-05 23300 8.33E-05 22900
8.06E-05 25200 0.00072702 24700 0.000339316
25600 0.04826656 23600 3.12E-05 23400 8.32E-05 22900
8.07E-05 25200 0.000728336 24700 0.000339637
25600 0.04855526 23600 3.12E-05 23400 8.31E-05 22900
8.07E-05 25200 0.00072965 24700 0.00033995
25600 0.04884566 23600 3.12E-05 23400 8.31E-05 22900
8.08E-05 25200 0.000730972 24700 0.000340254
25600 0.04913776 23600 3.12E-05 23400 8.30E-05 22900
8.08E-05 25200 0.000732283 24700 0.000340556
25600 0.04943156 23600 3.12E-05 23400 8.30E-05 23000
8.08E-05 25200 0.000733612 24700 0.000340855
25600 0.04972708 23600 3.13E-05 23400 8.30E-05 23000
8.09E-05 25200 0.000734928 24700 0.000341156
25600 0.05002429 23600 3.13E-05 23400 8.29E-05 23000
8.09E-05 25200 0.000736266 24700 0.000341473
25600 0.0503232 23600 3.13E-05 23400 8.29E-05 23000
8.09E-05 25200 0.000737588 24700 0.000341762
25600 0.05062383 23600 3.13E-05 23400 8.28E-05 23000
8.10E-05 25200 0.000738933 24700 0.000342091
25600 0.05092616 23600 3.14E-05 23400 8.28E-05 23000
8.10E-05 25200 0.00074027 24700 0.000342394
25600 0.05123016 23600 3.14E-05 23400 8.27E-05 23000
8.10E-05 25200 0.000741609 24700 0.000342712
25600 0.05153585 23600 3.14E-05 23400 8.27E-05 23000
8.11E-05 25200 0.000742955 24700 0.000343021
25600 0.05184324 23600 3.14E-05 23400 8.27E-05 23000
8.11E-05 25200 0.000744297 24700 0.000343322
25600 0.05215233 23600 3.15E-05 23400 8.26E-05 23000
8.11E-05 25200 0.000745635 24700 0.000343628
25600 0.05246308 23600 3.15E-05 23400 8.26E-05 23000
8.12E-05 25200 0.000746971 24700 0.000343948
25600 0.05277546 23600 3.15E-05 23400 8.26E-05 23000
8.12E-05 25300 0.000748295 24700 0.000344265
25600 0.0530895 23600 3.15E-05 23400 8.26E-05 23000
8.13E-05 25300 0.000749636 24700 0.000344559
25600 0.05340527 23600 3.15E-05 23400 8.25E-05 23000
8.13E-05 25300 0.000750995 24700 0.000344883
25600 0.05372261 23600 3.16E-05 23400 8.25E-05 23000
8.14E-05 25300 0.000752349 24700 0.000345201
25600 0.05404154 23600 3.16E-05 23400 8.25E-05 23000
8.14E-05 25300 0.000753707 24700 0.000345512
25600 0.05436211 23600 3.16E-05 23400 8.25E-05 23000
8.15E-05 25300 0.000755069 24700 0.000345821
25600 0.05468426 23600 3.16E-05 23400 8.25E-05 23000
8.16E-05 25300 0.000756418 24700 0.00034613
25600 0.05500801 23600 3.16E-05 23400 8.25E-05 23000
8.16E-05 25300 0.000757771 24700 0.000346435
25600 0.05533332 23600 3.16E-05 23400 8.24E-05 23000
8.17E-05 25300 0.00075913 24700 0.000346741
25600 0.05566022 23600 3.17E-05 23400 8.24E-05 23000
8.18E-05 25300 0.00076049 24700 0.000347046
25600 0.05598865 23600 3.17E-05 23400 8.24E-05 23000
8.19E-05 25300 0.000761858 24700 0.000347342
25600 0.05631864 23600 3.17E-05 23400 8.24E-05 23000
8.19E-05 25300 0.000763224 24700 0.000347637
25600 0.05665014 23600 3.17E-05 23400 8.24E-05 23000
8.20E-05 25300 0.000764603 24700 0.000347975
25600 0.05698319 23600 3.18E-05 23400 8.24E-05 23000
8.21E-05 25300 0.000765986 24700 0.000348273
25600 0.05731772 23600 3.18E-05 23400 8.24E-05 23000
8.21E-05 25300 0.000767378 24700 0.0003486
25600 0.05765375 23600 3.18E-05 23400 8.24E-05 23000
8.22E-05 25300 0.000768768 24700 0.000348922

25600 0.05799128 23600 3.18E-05 23400 8.24E-05 23000
8.23E-05 25300 0.00077017 24700 0.000349226
25600 0.05833023 23600 3.19E-05 23400 8.23E-05 23000
8.24E-05 25300 0.000771571 24700 0.000349532
25600 0.05867067 23600 3.19E-05 23400 8.23E-05 23000
8.24E-05 25300 0.000772966 24700 0.000349859
25600 0.05901243 23600 3.19E-05 23400 8.23E-05 23000
8.25E-05 25300 0.000774359 24700 0.000350171
25600 0.0593556 23600 3.19E-05 23400 8.23E-05 23000
8.26E-05 25300 0.000775773 24700 0.000350487
25600 0.05970027 23600 3.19E-05 23400 8.23E-05 23000
8.26E-05 25300 0.000777197 24700 0.000350818
25600 0.06004625 23600 3.20E-05 23400 8.23E-05 23000
8.27E-05 25300 0.000778618 24700 0.000351114
25600 0.06039362 23600 3.20E-05 23400 8.23E-05 23000
8.27E-05 25300 0.000780047 24700 0.000351451
25600 0.06074231 23600 3.20E-05 23400 8.23E-05 23000
8.28E-05 25300 0.000781477 24700 0.000351768
25600 0.06109234 23600 3.20E-05 23400 8.23E-05 23000
8.29E-05 25300 0.000782904 24700 0.000352077
25600 0.06144366 23600 3.20E-05 23400 8.23E-05 23000
8.30E-05 25300 0.000784328 24700 0.0003524
25600 0.06179624 23600 3.21E-05 23400 8.23E-05 23000
8.30E-05 25300 0.000785759 24700 0.000352711
25600 0.06215009 23600 3.21E-05 23400 8.24E-05 23000
8.31E-05 25300 0.00078719 24700 0.000353041
25700 0.06250518 23600 3.21E-05 23400 8.24E-05 23000
8.31E-05 25300 0.000788629 24700 0.00035337
25700 0.06286145 23600 3.21E-05 23400 8.24E-05 23000
8.32E-05 25300 0.000790057 24700 0.00035367
25700 0.0632189 23600 3.21E-05 23400 8.24E-05 23000
8.33E-05 25300 0.000791497 24700 0.000354009
25700 0.06357752 23600 3.22E-05 23400 8.24E-05 23000
8.34E-05 25300 0.000792951 24700 0.000354343
25700 0.06393726 23600 3.22E-05 23400 8.24E-05 23000
8.34E-05 25300 0.000794406 24700 0.000354658
25700 0.06429811 23600 3.22E-05 23400 8.24E-05 23000
8.35E-05 25300 0.00079586 24700 0.000354994
25700 0.06466001 23600 3.22E-05 23400 8.24E-05 23000
8.36E-05 25300 0.000797329 24700 0.000355318
25700 0.06502295 23600 3.23E-05 23400 8.24E-05 23000
8.36E-05 25300 0.000798797 24700 0.000355641
25700 0.06538688 23600 3.23E-05 23400 8.24E-05 23000
8.37E-05 25300 0.000800253 24700 0.000355971
25700 0.06575171 23600 3.23E-05 23400 8.25E-05 23000
8.37E-05 25300 0.000801702 24800 0.000356316
25700 0.06611748 23600 3.23E-05 23400 8.25E-05 23000
8.37E-05 25300 0.000803179 24800 0.000356636
25700 0.06648422 23600 3.24E-05 23400 8.25E-05 23000
8.38E-05 25300 0.000804661 24800 0.000356981
25700 0.06685178 23600 3.24E-05 23400 8.25E-05 23000
8.38E-05 25300 0.000806143 24800 0.000357308
25700 0.06722014 23600 3.24E-05 23400 8.25E-05 23000
8.39E-05 25300 0.000807646 24800 0.000357654
25700 0.06758928 23600 3.24E-05 23400 8.25E-05 23000
8.39E-05 25300 0.000809145 24800 0.000357981
25700 0.06795917 23600 3.25E-05 23400 8.25E-05 23000
8.40E-05 25300 0.000810638 24800 0.000358313
25700 0.06832974 23600 3.25E-05 23400 8.26E-05 23000
8.40E-05 25300 0.000812139 24800 0.000358649
25700 0.06870093 23600 3.25E-05 23400 8.26E-05 23000
8.41E-05 25300 0.000813647 24800 0.000358972
25700 0.06907271 23700 3.25E-05 23400 8.26E-05 23000
8.41E-05 25300 0.000815157 24800 0.000359304
25700 0.06944504 23700 3.26E-05 23400 8.26E-05 23000
8.42E-05 25300 0.000816666 24800 0.000359654
25700 0.06981787 23700 3.26E-05 23500 8.26E-05 23000
8.43E-05 25300 0.000818181 24800 0.000359962

25700 0.07019113 23700 3.26E-05 23500 8.27E-05 23000
8.43E-05 25300 0.000819697 24800 0.000360328
25700 0.0705648 23700 3.26E-05 23500 8.27E-05 23000
8.44E-05 25300 0.000821226 24800 0.000360657
25700 0.07093881 23700 3.27E-05 23500 8.27E-05 23000
8.45E-05 25300 0.000822758 24800 0.00036099
25700 0.07131309 23700 3.27E-05 23500 8.27E-05 23100
8.45E-05 25300 0.000824299 24800 0.000361334
25700 0.07168762 23700 3.27E-05 23500 8.27E-05 23100
8.46E-05 25300 0.000825842 24800 0.000361663
25700 0.07206232 23700 3.27E-05 23500 8.28E-05 23100
8.47E-05 25300 0.000827388 24800 0.00036199
25700 0.07243711 23700 3.27E-05 23500 8.28E-05 23100
8.47E-05 25300 0.000828933 24800 0.000362334
25700 0.07281192 23700 3.28E-05 23500 8.28E-05 23100
8.48E-05 25300 0.00083046 24800 0.000362684
25700 0.07318672 23700 3.28E-05 23500 8.28E-05 23100
8.49E-05 25300 0.000832019 24800 0.000363014
25700 0.07356153 23700 3.28E-05 23500 8.29E-05 23100
8.49E-05 25300 0.000833584 24800 0.000363366
25700 0.07393614 23700 3.28E-05 23500 8.29E-05 23100
8.50E-05 25300 0.000835157 24800 0.000363697
25700 0.07431056 23700 3.28E-05 23500 8.29E-05 23100
8.51E-05 25300 0.000836723 24800 0.000364043
25700 0.0746847 23700 3.29E-05 23500 8.30E-05 23100
8.52E-05 25300 0.000838302 24800 0.000364388
25700 0.07505852 23700 3.29E-05 23500 8.30E-05 23100
8.52E-05 25300 0.000839881 24800 0.000364719
25700 0.07543193 23700 3.29E-05 23500 8.30E-05 23100
8.53E-05 25400 0.000841459 24800 0.000365058
25700 0.07580484 23700 3.29E-05 23500 8.31E-05 23100
8.54E-05 25400 0.000843046 24800 0.000365402
25700 0.07617722 23700 3.30E-05 23500 8.31E-05 23100
8.55E-05 25400 0.000844642 24800 0.000365737
25700 0.07654897 23700 3.30E-05 23500 8.31E-05 23100
8.56E-05 25400 0.000846236 24800 0.000366095
25700 0.07692003 23700 3.30E-05 23500 8.32E-05 23100
8.56E-05 25400 0.000847842 24800 0.000366438
25700 0.07729032 23700 3.30E-05 23500 8.32E-05 23100
8.57E-05 25400 0.000849447 24800 0.000366787
25700 0.07765976 23700 3.31E-05 23500 8.32E-05 23100
8.58E-05 25400 0.000851068 24800 0.000367124
25700 0.07802829 23700 3.31E-05 23500 8.32E-05 23100
8.58E-05 25400 0.00085269 24800 0.000367478
25700 0.07839582 23700 3.31E-05 23500 8.33E-05 23100
8.59E-05 25400 0.000854325 24800 0.000367823
25700 0.07876225 23700 3.31E-05 23500 8.33E-05 23100
8.60E-05 25400 0.000855952 24800 0.00036816
25700 0.07912748 23700 3.31E-05 23500 8.34E-05 23100
8.60E-05 25400 0.000857606 24800 0.000368504
25700 0.0794915 23700 3.31E-05 23500 8.34E-05 23100
8.61E-05 25400 0.00085925 24800 0.00036886
25700 0.07985406 23700 3.32E-05 23500 8.35E-05 23100
8.62E-05 25400 0.000860884 24800 0.000369222
25700 0.08021521 23700 3.32E-05 23500 8.35E-05 23100
8.63E-05 25400 0.000862556 24800 0.000369568
25700 0.08057493 23700 3.32E-05 23500 8.36E-05 23100
8.63E-05 25400 0.000864226 24800 0.000369928
25700 0.08093296 23700 3.32E-05 23500 8.36E-05 23100
8.64E-05 25400 0.000865908 24800 0.00037027
25700 0.08128925 23700 3.32E-05 23500 8.37E-05 23100
8.65E-05 25400 0.00086759 24800 0.000370624
25700 0.08164372 23700 3.33E-05 23500 8.37E-05 23100
8.66E-05 25400 0.000869263 24800 0.000370982
25700 0.08199633 23700 3.33E-05 23500 8.38E-05 23100
8.66E-05 25400 0.000870958 24800 0.000371338
25700 0.08234695 23700 3.33E-05 23500 8.38E-05 23100
8.67E-05 25400 0.000872652 24800 0.000371673

25700 0.0826954 23700 3.33E-05 23500 8.39E-05 23100
8.68E-05 25400 0.000874354 24800 0.000372012
25700 0.08304165 23700 3.34E-05 23500 8.40E-05 23100
8.68E-05 25400 0.000876059 24800 0.000372366
25700 0.08338558 23700 3.34E-05 23500 8.40E-05 23100
8.69E-05 25400 0.000877763 24800 0.000372719
25700 0.08372709 23700 3.34E-05 23500 8.41E-05 23100
8.69E-05 25400 0.000879485 24800 0.000373062
25700 0.08406606 23700 3.35E-05 23500 8.42E-05 23100
8.70E-05 25400 0.000881184 24800 0.000373422
25700 0.08440245 23700 3.35E-05 23500 8.42E-05 23100
8.71E-05 25400 0.000882921 24800 0.000373774
25700 0.08473601 23700 3.35E-05 23500 8.43E-05 23100
8.71E-05 25400 0.000884661 24800 0.000374126
25700 0.08506674 23700 3.36E-05 23500 8.44E-05 23100
8.72E-05 25400 0.000886396 24800 0.000374478
25700 0.08539449 23700 3.36E-05 23500 8.45E-05 23100
8.72E-05 25400 0.000888139 24800 0.000374821
25800 0.08571916 23700 3.36E-05 23500 8.45E-05 23100
8.72E-05 25400 0.000889865 24800 0.000375174
25800 0.08604059 23700 3.37E-05 23500 8.46E-05 23100
8.73E-05 25400 0.000891603 24800 0.000375527
25800 0.08635861 23700 3.37E-05 23500 8.47E-05 23100
8.74E-05 25400 0.000893329 24800 0.00037591
25800 0.08667321 23700 3.37E-05 23500 8.48E-05 23100
8.74E-05 25400 0.000895061 24800 0.000376265
25800 0.08698427 23700 3.38E-05 23500 8.49E-05 23100
8.75E-05 25400 0.000896837 24800 0.000376628
25800 0.08729155 23700 3.38E-05 23500 8.50E-05 23100
8.75E-05 25400 0.000898591 24800 0.000376963
25800 0.08759505 23700 3.38E-05 23500 8.51E-05 23100
8.76E-05 25400 0.000900354 24800 0.000377326
25800 0.08789455 23700 3.38E-05 23500 8.51E-05 23100
8.77E-05 25400 0.000902116 24800 0.000377678
25800 0.08818996 23700 3.39E-05 23500 8.52E-05 23100
8.77E-05 25400 0.000903875 24800 0.000378032
25800 0.08848117 23700 3.39E-05 23500 8.53E-05 23100
8.78E-05 25400 0.000905646 24900 0.000378388
25800 0.08876801 23700 3.39E-05 23500 8.54E-05 23100
8.79E-05 25400 0.00090743 24900 0.000378729
25800 0.08905037 23700 3.39E-05 23500 8.55E-05 23100
8.79E-05 25400 0.000909204 24900 0.000379081
25800 0.0893281 23700 3.40E-05 23500 8.56E-05 23100
8.80E-05 25400 0.000910997 24900 0.000379445
25800 0.08960111 23700 3.40E-05 23500 8.56E-05 23100
8.81E-05 25400 0.000912771 24900 0.000379785
25800 0.08986923 23700 3.40E-05 23500 8.57E-05 23100
8.81E-05 25400 0.000914562 24900 0.000380147
25800 0.09013236 23700 3.40E-05 23500 8.58E-05 23100
8.82E-05 25400 0.000916343 24900 0.000380496
25800 0.09039032 23700 3.40E-05 23500 8.59E-05 23100
8.82E-05 25400 0.000918137 24900 0.000380846
25800 0.09064302 23700 3.40E-05 23500 8.60E-05 23100
8.83E-05 25400 0.000919952 24900 0.00038121
25800 0.0908903 23800 3.41E-05 23500 8.60E-05 23100
8.83E-05 25400 0.000921752 24900 0.000381545
25800 0.09113205 23800 3.41E-05 23500 8.61E-05 23100
8.84E-05 25400 0.000923565 24900 0.000381891
25800 0.09136807 23800 3.41E-05 23600 8.62E-05 23100
8.85E-05 25400 0.000925383 24900 0.000382251
25800 0.0915982 23800 3.41E-05 23600 8.63E-05 23100
8.85E-05 25400 0.000927189 24900 0.000382617
25800 0.09182242 23800 3.41E-05 23600 8.63E-05 23100
8.86E-05 25400 0.000929006 24900 0.000382965
25800 0.09204061 23800 3.41E-05 23600 8.64E-05 23100
8.87E-05 25400 0.000930845 24900 0.000383326
25800 0.09225252 23800 3.41E-05 23600 8.65E-05 23200
8.88E-05 25400 0.000932688 24900 0.000383673

25800	0.09245802	23800	3.41E-05	23600	8.66E-05	23200	8.88E-05	25400	0.000934545	24900	0.000384029
25800	0.09265703	23800	3.42E-05	23600	8.66E-05	23200	8.89E-05	25400	0.000936383	24900	0.000384395
25800	0.0928494	23800	3.42E-05	23600	8.67E-05	23200	8.90E-05	25400	0.00093825	24900	0.00038474
25800	0.09303499	23800	3.42E-05	23600	8.68E-05	23200	8.91E-05	25400	0.000940108	24900	0.000385094
25800	0.0932136	23800	3.42E-05	23600	8.69E-05	23200	8.92E-05	25400	0.000941976	24900	0.000385447
25800	0.0933852	23800	3.42E-05	23600	8.70E-05	23200	8.92E-05	25400	0.000943854	24900	0.000385808
25800	0.09354956	23800	3.43E-05	23600	8.70E-05	23200	8.93E-05	25400	0.000945742	24900	0.000386171
25800	0.09370662	23800	3.43E-05	23600	8.71E-05	23200	8.93E-05	25400	0.000947603	24900	0.000386522
25800	0.09385619	23800	3.43E-05	23600	8.72E-05	23200	8.94E-05	25400	0.00094949	24900	0.000386888
25800	0.09399816	23800	3.43E-05	23600	8.73E-05	23200	8.95E-05	25400	0.000951386	24900	0.000387242
25800	0.09413236	23800	3.43E-05	23600	8.73E-05	23200	8.95E-05	25500	0.000953297	24900	0.000387612
25800	0.09425867	23800	3.44E-05	23600	8.74E-05	23200	8.96E-05	25500	0.000955194	24900	0.000387977
25800	0.09437698	23800	3.44E-05	23600	8.75E-05	23200	8.97E-05	25500	0.000957111	24900	0.000388324
25800	0.09448715	23800	3.44E-05	23600	8.76E-05	23200	8.97E-05	25500	0.000959025	24900	0.000388677
25800	0.09458903	23800	3.44E-05	23600	8.76E-05	23200	8.98E-05	25500	0.000960941	24900	0.00038906
25800	0.09468243	23800	3.44E-05	23600	8.77E-05	23200	8.99E-05	25500	0.000962834	24900	0.000389431
25800	0.09476733	23800	3.45E-05	23600	8.78E-05	23200	9.00E-05	25500	0.000964759	24900	0.000389794
25800	0.09484363	23800	3.45E-05	23600	8.79E-05	23200	9.01E-05	25500	0.000966687	24900	0.000390177
25800	0.09491108	23800	3.45E-05	23600	8.79E-05	23200	9.02E-05	25500	0.000968613	24900	0.000390538
25800	0.09496959	23800	3.45E-05	23600	8.80E-05	23200	9.03E-05	25500	0.00097055	24900	0.000390896
25800	0.09501909	23800	3.45E-05	23600	8.81E-05	23200	9.04E-05	25500	0.000972491	24900	0.000391275
25800	0.09505944	23800	3.46E-05	23600	8.82E-05	23200	9.05E-05	25500	0.000974434	24900	0.000391635
25800	0.09509053	23800	3.46E-05	23600	8.82E-05	23200	9.05E-05	25500	0.000976372	24900	0.000392012
25800	0.09511223	23800	3.46E-05	23600	8.83E-05	23200	9.06E-05	25500	0.00097833	24900	0.000392373
25800	0.09512445	23800	3.46E-05	23600	8.84E-05	23200	9.07E-05	25500	0.000980278	24900	0.000392742
25800	0.09512708	23800	3.47E-05	23600	8.85E-05	23200	9.08E-05	25500	0.000982243	24900	0.000393125
25800	0.09512003	23800	3.47E-05	23600	8.85E-05	23200	9.09E-05	25500	0.000984196	24900	0.000393488
25800	0.09510313	23800	3.47E-05	23600	8.86E-05	23200	9.10E-05	25500	0.000986165	24900	0.000393873
25800	0.09507637	23800	3.47E-05	23600	8.87E-05	23200	9.10E-05	25500	0.000988145	24900	0.000394256
25800	0.09503958	23800	3.48E-05	23600	8.87E-05	23200	9.11E-05	25500	0.000990126	24900	0.000394617
25800	0.09499272	23800	3.48E-05	23600	8.88E-05	23200	9.12E-05	25500	0.000992121	24900	0.000395006
25800	0.09493569	23800	3.48E-05	23600	8.89E-05	23200	9.12E-05	25500	0.000994123	24900	0.000395362
25800	0.09486835	23800	3.49E-05	23600	8.90E-05	23200	9.13E-05	25500	0.000996145	24900	0.000395737
25800	0.09479067	23800	3.49E-05	23600	8.90E-05	23200	9.13E-05	25500	0.000998146	24900	0.000396125

25800	0.0947025	23800	3.49E-05	23600	8.91E-05	23200
9.14E-05	25500	0.001000141	24900	0.000396525		
25800	0.09460384	23800	3.49E-05	23600	8.92E-05	23200
9.14E-05	25500	0.001002158	24900	0.000396901		
25800	0.0944947	23800	3.50E-05	23600	8.92E-05	23200
9.15E-05	25500	0.001004189	24900	0.0003973		
25800	0.09437483	23800	3.50E-05	23600	8.93E-05	23200
9.16E-05	25500	0.001006232	24900	0.000397681		
25800	0.09424423	23800	3.50E-05	23600	8.94E-05	23200
9.17E-05	25500	0.001008272	24900	0.000398078		
25900	0.09410289	23800	3.50E-05	23600	8.95E-05	23200
9.18E-05	25500	0.001010325	24900	0.000398475		
25900	0.09395073	23800	3.51E-05	23600	8.95E-05	23200
9.19E-05	25500	0.001012366	24900	0.000398872		
25900	0.09378774	23800	3.51E-05	23600	8.96E-05	23200
9.20E-05	25500	0.001014427	24900	0.000399257		
25900	0.09361384	23800	3.51E-05	23600	8.97E-05	23200
9.21E-05	25500	0.001016493	24900	0.000399645		
25900	0.09342901	23800	3.51E-05	23600	8.98E-05	23200
9.22E-05	25500	0.00101856	24900	0.000400037		
25900	0.09323321	23800	3.52E-05	23600	8.98E-05	23200
9.23E-05	25500	0.001020637	24900	0.000400436		
25900	0.09302642	23800	3.52E-05	23600	8.99E-05	23200
9.23E-05	25500	0.001022726	24900	0.000400825		
25900	0.09280864	23800	3.52E-05	23600	9.00E-05	23200
9.24E-05	25500	0.001024822	24900	0.00040124		
25900	0.09257982	23800	3.53E-05	23600	9.01E-05	23200
9.25E-05	25500	0.001026932	24900	0.000401639		
25900	0.09233993	23800	3.53E-05	23600	9.01E-05	23200
9.26E-05	25500	0.001029057	25000	0.000402041		
25900	0.09208903	23800	3.53E-05	23600	9.02E-05	23200
9.27E-05	25500	0.001031178	25000	0.000402454		
25900	0.0918271	23800	3.53E-05	23600	9.03E-05	23200
9.28E-05	25500	0.001033327	25000	0.000402854		
25900	0.09155411	23800	3.54E-05	23600	9.04E-05	23200
9.29E-05	25500	0.001035484	25000	0.000403262		
25900	0.09127013	23800	3.54E-05	23600	9.05E-05	23200
9.29E-05	25500	0.001037629	25000	0.000403674		
25900	0.09097504	23800	3.54E-05	23600	9.05E-05	23200
9.30E-05	25500	0.001039762	25000	0.000404093		
25900	0.09066904	23800	3.54E-05	23600	9.06E-05	23200
9.31E-05	25500	0.001041923	25000	0.000404504		
25900	0.0903521	23800	3.55E-05	23600	9.07E-05	23200
9.32E-05	25500	0.001044106	25000	0.000404934		
25900	0.09002417	23800	3.55E-05	23600	9.08E-05	23200
9.33E-05	25500	0.001046295	25000	0.000405336		
25900	0.08968538	23900	3.55E-05	23600	9.09E-05	23200
9.34E-05	25500	0.001048502	25000	0.00040575		
25900	0.08933572	23900	3.55E-05	23600	9.10E-05	23200
9.34E-05	25500	0.001050719	25000	0.000406168		
25900	0.08897529	23900	3.55E-05	23700	9.10E-05	23200
9.35E-05	25500	0.001052923	25000	0.000406583		
25900	0.08860411	23900	3.56E-05	23700	9.11E-05	23200
9.36E-05	25500	0.001055136	25000	0.000407001		
25900	0.0882223	23900	3.56E-05	23700	9.12E-05	23200
9.37E-05	25500	0.00105733	25000	0.000407396		
25900	0.08782989	23900	3.56E-05	23700	9.13E-05	23200
9.38E-05	25500	0.001059552	25000	0.00040781		
25900	0.08742695	23900	3.56E-05	23700	9.14E-05	23300
9.39E-05	25500	0.001061795	25000	0.000408238		
25900	0.08701362	23900	3.57E-05	23700	9.15E-05	23300
9.40E-05	25500	0.001064021	25000	0.000408637		
25900	0.08658994	23900	3.57E-05	23700	9.15E-05	23300
9.40E-05	25500	0.001066272	25000	0.000409059		
25900	0.08615604	23900	3.57E-05	23700	9.17E-05	23300
9.41E-05	25500	0.001068517	25000	0.000409489		
25900	0.08571201	23900	3.58E-05	23700	9.17E-05	23300
9.42E-05	25500	0.00107077	25000	0.000409894		

25900 0.08525795 23900 3.58E-05 23700 9.18E-05 23300
9.42E-05 25500 0.001073032 25000 0.0004103
25900 0.08479402 23900 3.58E-05 23700 9.19E-05 23300
9.43E-05 25500 0.001075292 25000 0.000410695
25900 0.08432028 23900 3.58E-05 23700 9.19E-05 23300
9.43E-05 25500 0.001077582 25000 0.000411103
25900 0.0838369 23900 3.58E-05 23700 9.20E-05 23300
9.44E-05 25500 0.001079853 25000 0.000411506
25900 0.08334393 23900 3.59E-05 23700 9.21E-05 23300
9.45E-05 25500 0.001082114 25000 0.000411914
25900 0.08284166 23900 3.59E-05 23700 9.22E-05 23300
9.46E-05 25500 0.00108441 25000 0.000412309
25900 0.08233022 23900 3.59E-05 23700 9.23E-05 23300
9.47E-05 25600 0.00108671 25000 0.000412722
25900 0.08180967 23900 3.59E-05 23700 9.24E-05 23300
9.48E-05 25600 0.001089022 25000 0.000413109
25900 0.08128018 23900 3.60E-05 23700 9.25E-05 23300
9.49E-05 25600 0.001091332 25000 0.000413496
25900 0.08074199 23900 3.60E-05 23700 9.26E-05 23300
9.50E-05 25600 0.001093642 25000 0.000413889
25900 0.08019523 23900 3.60E-05 23700 9.26E-05 23300
9.51E-05 25600 0.001095966 25000 0.000414274
25900 0.07964008 23900 3.60E-05 23700 9.27E-05 23300
9.52E-05 25600 0.001098287 25000 0.000414654
25900 0.07907672 23900 3.60E-05 23700 9.28E-05 23300
9.53E-05 25600 0.001100623 25000 0.000415027
25900 0.07850537 23900 3.61E-05 23700 9.29E-05 23300
9.54E-05 25600 0.00110296 25000 0.0004154
25900 0.07792618 23900 3.61E-05 23700 9.30E-05 23300
9.54E-05 25600 0.001105305 25000 0.000415788
25900 0.07733937 23900 3.61E-05 23700 9.31E-05 23300
9.55E-05 25600 0.001107662 25000 0.000416159
25900 0.07674514 23900 3.61E-05 23700 9.32E-05 23300
9.55E-05 25600 0.001110041 25000 0.000416561
25900 0.07614371 23900 3.61E-05 23700 9.33E-05 23300
9.56E-05 25600 0.001112408 25000 0.000416947
25900 0.07553527 23900 3.62E-05 23700 9.34E-05 23300
9.57E-05 25600 0.001114793 25000 0.000417334
25900 0.07492005 23900 3.62E-05 23700 9.35E-05 23300
9.57E-05 25600 0.001117188 25000 0.000417719
25900 0.07429831 23900 3.62E-05 23700 9.36E-05 23300
9.58E-05 25600 0.001119593 25000 0.000418088
25900 0.07367021 23900 3.63E-05 23700 9.37E-05 23300
9.58E-05 25600 0.001122015 25000 0.00041847
25900 0.073036 23900 3.63E-05 23700 9.38E-05 23300
9.59E-05 25600 0.001124428 25000 0.000418862
25900 0.07239591 23900 3.63E-05 23700 9.39E-05 23300
9.60E-05 25600 0.001126827 25000 0.000419263
25900 0.07175025 23900 3.63E-05 23700 9.40E-05 23300
9.60E-05 25600 0.001129252 25000 0.000419635
25900 0.07109921 23900 3.64E-05 23700 9.41E-05 23300
9.61E-05 25600 0.001131701 25000 0.000420049
25900 0.07044294 23900 3.64E-05 23700 9.42E-05 23300
9.61E-05 25600 0.001134151 25000 0.000420426
25900 0.06978174 23900 3.64E-05 23700 9.43E-05 23300
9.62E-05 25600 0.001136613 25000 0.000420821
25900 0.0691159 23900 3.65E-05 23700 9.43E-05 23300
9.63E-05 25600 0.001139076 25000 0.000421213
25900 0.06844562 23900 3.65E-05 23700 9.44E-05 23300
9.63E-05 25600 0.001141537 25000 0.000421601
25900 0.06777121 23900 3.65E-05 23700 9.45E-05 23300
9.64E-05 25600 0.00114401 25000 0.000421986
25900 0.06709287 23900 3.65E-05 23700 9.46E-05 23300
9.65E-05 25600 0.001146483 25000 0.00042238
25900 0.06641093 23900 3.66E-05 23700 9.48E-05 23300
9.65E-05 25600 0.001148967 25000 0.000422765
25900 0.06572554 23900 3.66E-05 23700 9.48E-05 23300
9.66E-05 25600 0.001151454 25000 0.000423161

25900 0.06503704 23900 3.66E-05 23700 9.50E-05 23300
9.67E-05 25600 0.001153952 25000 0.000423547
26000 0.06434564 23900 3.66E-05 23700 9.50E-05 23300
9.69E-05 25600 0.001156456 25000 0.000423969
26000 0.06365163 23900 3.67E-05 23700 9.51E-05 23300
9.70E-05 25600 0.001158964 25000 0.000424367
26000 0.06295521 23900 3.67E-05 23700 9.52E-05 23300
9.71E-05 25600 0.001161493 25000 0.000424773
26000 0.06225669 23900 3.67E-05 23700 9.53E-05 23300
9.72E-05 25600 0.00116403 25000 0.00042518
26000 0.06155636 23900 3.67E-05 23700 9.54E-05 23300
9.74E-05 25600 0.001166565 25000 0.000425582
26000 0.06085442 23900 3.68E-05 23700 9.55E-05 23300
9.75E-05 25600 0.001169127 25000 0.00042597
26000 0.06015114 23900 3.68E-05 23700 9.56E-05 23300
9.76E-05 25600 0.001171675 25000 0.000426394
26000 0.05944674 23900 3.68E-05 23700 9.57E-05 23300
9.77E-05 25600 0.001174217 25000 0.000426814
26000 0.05874159 23900 3.68E-05 23700 9.58E-05 23300
9.78E-05 25600 0.001176778 25000 0.000427222
26000 0.05803589 23900 3.68E-05 23700 9.59E-05 23300
9.79E-05 25600 0.001179352 25100 0.00042765
26000 0.05732981 23900 3.69E-05 23700 9.59E-05 23300
9.80E-05 25600 0.001181936 25100 0.000428061
26000 0.05662365 23900 3.69E-05 23700 9.60E-05 23300
9.81E-05 25600 0.001184533 25100 0.00042848
26000 0.05591769 23900 3.69E-05 23700 9.61E-05 23300
9.82E-05 25600 0.001187131 25100 0.000428892
26000 0.05521218 23900 3.70E-05 23700 9.62E-05 23300
9.83E-05 25600 0.001189736 25100 0.000429294
26000 0.05450738 23900 3.70E-05 23700 9.63E-05 23300
9.84E-05 25600 0.001192351 25100 0.000429712
26000 0.05380351 23900 3.70E-05 23700 9.64E-05 23300
9.85E-05 25600 0.001194969 25100 0.000430124
26000 0.05310084 23900 3.70E-05 23700 9.65E-05 23300
9.86E-05 25600 0.001197596 25100 0.000430542
26000 0.05239955 23900 3.70E-05 23700 9.66E-05 23300
9.86E-05 25600 0.001200246 25100 0.00043096
26000 0.05169991 24000 3.71E-05 23700 9.67E-05 23300
9.87E-05 25600 0.001202888 25100 0.000431368
26000 0.05100214 24000 3.71E-05 23700 9.68E-05 23300
9.88E-05 25600 0.001205542 25100 0.0004318
26000 0.05030651 24000 3.71E-05 23800 9.69E-05 23300
9.89E-05 25600 0.001208214 25100 0.000432228
26000 0.04961319 24000 3.72E-05 23800 9.70E-05 23300
9.90E-05 25600 0.001210888 25100 0.000432634
26000 0.04892243 24000 3.72E-05 23800 9.71E-05 23300
9.90E-05 25600 0.001213582 25100 0.000433068
26000 0.04823446 24000 3.72E-05 23800 9.72E-05 23300
9.91E-05 25600 0.001216275 25100 0.000433484
26000 0.04754945 24000 3.73E-05 23800 9.73E-05 23400
9.92E-05 25600 0.001218987 25100 0.000433895
26000 0.04686764 24000 3.73E-05 23800 9.74E-05 23400
9.93E-05 25600 0.001221694 25100 0.000434323
26000 0.04618919 24000 3.73E-05 23800 9.75E-05 23400
9.94E-05 25600 0.001224375 25100 0.00043476
26000 0.04551437 24000 3.73E-05 23800 9.76E-05 23400
9.94E-05 25600 0.001227095 25100 0.000435176
26000 0.04484336 24000 3.73E-05 23800 9.77E-05 23400
9.95E-05 25600 0.001229828 25100 0.00043562
26000 0.0441763 24000 3.74E-05 23800 9.78E-05 23400
9.96E-05 25600 0.001232577 25100 0.000436046
26000 0.04351339 24000 3.74E-05 23800 9.79E-05 23400
9.97E-05 25600 0.001235333 25100 0.000436487
26000 0.04285481 24000 3.74E-05 23800 9.80E-05 23400
9.98E-05 25600 0.001238098 25100 0.00043692
26000 0.04220076 24000 3.75E-05 23800 9.81E-05 23400
9.99E-05 25600 0.001240864 25100 0.000437337

26000 0.04155141 24000 3.75E-05 23800 9.82E-05 23400
0.000100018 25600 0.001243652 25100 0.00043778
26000 0.04090687 24000 3.75E-05 23800 9.83E-05 23400
0.000100135 25600 0.001246439 25100 0.000438212
26000 0.04026734 24000 3.76E-05 23800 9.84E-05 23400
0.000100255 25700 0.001249239 25100 0.000438645
26000 0.03963295 24000 3.76E-05 23800 9.85E-05 23400
0.000100371 25700 0.001252053 25100 0.000439082
26000 0.03900382 24000 3.76E-05 23800 9.86E-05 23400
0.000100491 25700 0.001254866 25100 0.000439506
26000 0.03838012 24000 3.77E-05 23800 9.87E-05 23400
0.000100607 25700 0.001257696 25100 0.000439967
26000 0.03776197 24000 3.77E-05 23800 9.88E-05 23400
0.00010071 25700 0.001260544 25100 0.000440408
26000 0.03714947 24000 3.78E-05 23800 9.89E-05 23400
0.000100819 25700 0.001263396 25100 0.000440839
26000 0.0365428 24000 3.78E-05 23800 9.90E-05 23400
0.000100905 25700 0.001266281 25100 0.000441294
26000 0.03594204 24000 3.79E-05 23800 9.91E-05 23400
0.000101008 25700 0.001269151 25100 0.000441731
26000 0.03534731 24000 3.79E-05 23800 9.92E-05 23400
0.000101097 25700 0.001272052 25100 0.000442178
26000 0.0347587 24000 3.80E-05 23800 9.93E-05 23400
0.000101181 25700 0.001274946 25100 0.000442629
26000 0.03417632 24000 3.80E-05 23800 9.94E-05 23400
0.000101279 25700 0.001277822 25100 0.000443079
26000 0.0336003 24000 3.81E-05 23800 9.95E-05 23400
0.000101367 25700 0.001280724 25100 0.000443522
26000 0.03303068 24000 3.82E-05 23800 9.96E-05 23400
0.000101461 25700 0.001283641 25100 0.000443974
26000 0.03246753 24000 3.82E-05 23800 9.98E-05 23400
0.000101551 25700 0.001286568 25100 0.000444421
26000 0.03191095 24000 3.83E-05 23800 9.99E-05 23400
0.000101625 25700 0.001289481 25100 0.000444874
26000 0.03136101 24000 3.84E-05 23800 1.00E-04 23400
0.00010171 25700 0.001292422 25100 0.000445317
26000 0.03081779 24000 3.85E-05 23800 1.00E-04 23400
0.000101771 25700 0.00129536 25100 0.000445756
26000 0.03028136 24000 3.86E-05 23800 1.00E-04 23400
0.000101842 25700 0.001298299 25100 0.000446208
26000 0.02975176 24000 3.86E-05 23800 1.00E-04 23400
0.000101917 25700 0.00130125 25100 0.000446667
26000 0.02922906 24000 3.86E-05 23800 1.00E-04 23400
0.000101996 25700 0.001304209 25100 0.000447118
26000 0.02871328 24000 3.86E-05 23800 1.01E-04 23400
0.000102073 25700 0.001307173 25100 0.000447572
26000 0.02820448 24000 3.86E-05 23800 1.01E-04 23400
0.000102151 25700 0.001310148 25100 0.000448032
26000 0.02770268 24000 3.86E-05 23800 1.01E-04 23400
0.00010223 25700 0.001313119 25100 0.000448496
26000 0.02720795 24000 3.86E-05 23800 1.01E-04 23400
0.000102316 25700 0.001316117 25100 0.000448959
26000 0.02672028 24000 3.86E-05 23800 1.01E-04 23400
0.00010241 25700 0.001319119 25100 0.000449412
26000 0.02623971 24000 3.85E-05 23800 1.01E-04 23400
0.000102506 25700 0.001322152 25100 0.000449862
26000 0.02576627 24000 3.86E-05 23800 1.01E-04 23400
0.00010262 25700 0.001325182 25100 0.000450316
26000 0.02529996 24000 3.86E-05 23800 1.01E-04 23400
0.000102729 25700 0.001328239 25100 0.000450755
26000 0.02484082 24000 3.86E-05 23800 1.01E-04 23400
0.000102845 25700 0.001331283 25100 0.000451222
26100 0.02438879 24000 3.86E-05 23800 1.01E-04 23400
0.000102978 25700 0.00133432 25100 0.000451686
26100 0.02394396 24000 3.86E-05 23800 1.02E-04 23400
0.0001031 25700 0.001337396 25100 0.000452141
26100 0.02350629 24000 3.87E-05 23800 1.02E-04 23400
0.00010325 25700 0.001340484 25100 0.000452622

26100 0.02307572 24000 3.87E-05 23800 1.02E-04 23400
0.000103376 25700 0.0013436 25100 0.000453075
26100 0.02265231 24000 3.88E-05 23800 1.02E-04 23400
0.000103489 25700 0.001346727 25100 0.00045354
26100 0.02223602 24000 3.88E-05 23800 1.02E-04 23400
0.000103604 25700 0.001349867 25100 0.000454011
26100 0.02182684 24000 3.88E-05 23800 1.02E-04 23400
0.000103711 25700 0.001353003 25100 0.000454473
26100 0.02142476 24000 3.89E-05 23800 1.02E-04 23400
0.00010381 25700 0.001356174 25100 0.000454936
26100 0.02102972 24000 3.89E-05 23800 1.02E-04 23400
0.000103893 25700 0.001359345 25100 0.000455395
26100 0.02064173 24000 3.89E-05 23800 1.02E-04 23400
0.000103974 25700 0.001362517 25200 0.000455865
26100 0.02026073 24000 3.90E-05 23800 1.03E-04 23400
0.000104047 25700 0.00136571 25200 0.000456339
26100 0.01988669 24000 3.91E-05 23800 1.03E-04 23400
0.000104127 25700 0.001368912 25200 0.000456793
26100 0.01951956 24000 3.91E-05 23800 1.03E-04 23400
0.000104206 25700 0.001372118 25200 0.000457277
26100 0.01915935 24000 3.92E-05 23800 1.03E-04 23400
0.000104276 25700 0.001375343 25200 0.00045775
26100 0.01880594 24000 3.92E-05 23800 1.03E-04 23400
0.000104382 25700 0.001378588 25200 0.000458221
26100 0.01845935 24000 3.92E-05 23800 1.03E-04 23400
0.000104472 25700 0.001381845 25200 0.000458713
26100 0.01811949 24000 3.93E-05 23800 1.03E-04 23400
0.000104587 25700 0.001385114 25200 0.00045917
26100 0.0177863 24000 3.93E-05 23800 1.03E-04 23400
0.000104696 25700 0.001388396 25200 0.000459643
26100 0.01745979 24100 3.94E-05 23800 1.03E-04 23400
0.00010481 25700 0.001391678 25200 0.000460121
26100 0.01713984 24100 3.94E-05 23800 1.04E-04 23400
0.000104932 25700 0.001394944 25200 0.000460599
26100 0.01682644 24100 3.94E-05 23900 1.04E-04 23400
0.00010504 25700 0.001398245 25200 0.000461063
26100 0.01651951 24100 3.95E-05 23900 1.04E-04 23400
0.000105161 25700 0.00140155 25200 0.000461538
26100 0.01621896 24100 3.95E-05 23900 1.04E-04 23400
0.000105287 25700 0.001404892 25200 0.000462006
26100 0.01592474 24100 3.95E-05 23900 1.04E-04 23400
0.000105403 25700 0.001408225 25200 0.000462475
26100 0.0156368 24100 3.96E-05 23900 1.04E-04 23500
0.000105522 25700 0.001411568 25200 0.000462941
26100 0.01535505 24100 3.96E-05 23900 1.04E-04 23500
0.000105617 25700 0.001414928 25200 0.000463408
26100 0.01507948 24100 3.95E-05 23900 1.04E-04 23500
0.00010573 25700 0.001418287 25200 0.000463879
26100 0.01480995 24100 3.96E-05 23900 1.04E-04 23500
0.000105828 25700 0.001421672 25200 0.000464352
26100 0.01454643 24100 3.96E-05 23900 1.05E-04 23500
0.000105941 25700 0.001425051 25200 0.000464816
26100 0.01428883 24100 3.96E-05 23900 1.05E-04 23500
0.000106034 25700 0.00142844 25200 0.000465288
26100 0.01403709 24100 3.96E-05 23900 1.05E-04 23500
0.000106138 25700 0.001431838 25200 0.000465755
26100 0.01379111 24100 3.96E-05 23900 1.05E-04 23500
0.000106231 25700 0.001435258 25200 0.000466232
26100 0.01355084 24100 3.97E-05 23900 1.05E-04 23500
0.000106336 25700 0.001438687 25200 0.000466704
26100 0.01331618 24100 3.97E-05 23900 1.05E-04 23500
0.000106472 25700 0.001442137 25200 0.000467178
26100 0.01308709 24100 3.97E-05 23900 1.05E-04 23500
0.000106577 25700 0.00144559 25200 0.000467647
26100 0.01286346 24100 3.97E-05 23900 1.05E-04 23500
0.000106694 25800 0.001449057 25200 0.000468105
26100 0.01264522 24100 3.97E-05 23900 1.05E-04 23500
0.00010681 25800 0.001452549 25200 0.000468574

26100 0.01243232 24100 3.97E-05 23900 1.06E-04 23500
0.000106912 25800 0.001456041 25200 0.000469046
26100 0.01222463 24100 3.97E-05 23900 1.06E-04 23500
0.000107031 25800 0.001459517 25200 0.000469529
26100 0.01202212 24100 3.97E-05 23900 1.06E-04 23500
0.000107133 25800 0.001463028 25200 0.000469997
26100 0.01182472 24100 3.97E-05 23900 1.06E-04 23500
0.000107243 25800 0.001466546 25200 0.000470478
26100 0.01163227 24100 3.97E-05 23900 1.06E-04 23500
0.000107341 25800 0.001470096 25200 0.00047094
26100 0.01144475 24100 3.97E-05 23900 1.06E-04 23500
0.00010743 25800 0.001473643 25200 0.000471416
26100 0.01126208 24100 3.98E-05 23900 1.06E-04 23500
0.00010753 25800 0.001477198 25200 0.000471887
26100 0.01108417 24100 3.98E-05 23900 1.06E-04 23500
0.000107599 25800 0.001480779 25200 0.000472352
26100 0.01091096 24100 3.98E-05 23900 1.06E-04 23500
0.000107692 25800 0.001484351 25200 0.000472838
26100 0.0107423 24100 3.98E-05 23900 1.07E-04 23500
0.000107764 25800 0.001487948 25200 0.000473312
26100 0.01057821 24100 3.98E-05 23900 1.07E-04 23500
0.000107858 25800 0.00149155 25200 0.000473787
26100 0.01041856 24100 3.98E-05 23900 1.07E-04 23500
0.000107939 25800 0.001495173 25200 0.000474268
26100 0.01026325 24100 3.98E-05 23900 1.07E-04 23500
0.000108028 25800 0.001498807 25200 0.000474737
26100 0.01011221 24100 3.99E-05 23900 1.07E-04 23500
0.000108129 25800 0.001502458 25200 0.000475225
26100 0.009965426 24100 3.99E-05 23900 1.07E-04 23500
0.000108211 25800 0.001506122 25200 0.000475717
26100 0.009822701 24100 3.99E-05 23900 1.07E-04 23500
0.000108315 25800 0.001509806 25200 0.000476203
26100 0.009684043 24100 3.99E-05 23900 1.07E-04 23500
0.000108386 25800 0.001513508 25200 0.000476696
26100 0.009549362 24100 3.99E-05 23900 1.08E-04 23500
0.000108475 25800 0.001517221 25200 0.000477173
26100 0.009418562 24100 4.00E-05 23900 1.08E-04 23500
0.000108551 25800 0.001520949 25200 0.00047765
26100 0.0092916 24100 4.00E-05 23900 1.08E-04 23500
0.000108628 25800 0.001524673 25200 0.000478152
26100 0.009168339 24100 4.00E-05 23900 1.08E-04 23500
0.000108707 25800 0.001528394 25200 0.000478639
26100 0.009048786 24100 4.00E-05 23900 1.08E-04 23500
0.000108797 25800 0.001532141 25200 0.000479131
26100 0.008932809 24100 4.01E-05 23900 1.08E-04 23500
0.000108886 25800 0.001535909 25200 0.00047964
26100 0.008820324 24100 4.01E-05 23900 1.08E-04 23500
0.00010899 25800 0.001539687 25200 0.000480139
26100 0.008711276 24100 4.01E-05 23900 1.08E-04 23500
0.000109112 25800 0.00154348 25200 0.000480629
26100 0.008605591 24100 4.01E-05 23900 1.09E-04 23500
0.000109242 25800 0.001547286 25200 0.000481119
26100 0.008503213 24100 4.02E-05 23900 1.09E-04 23500
0.000109376 25800 0.0015511 25200 0.000481602
26200 0.008404077 24100 4.02E-05 23900 1.09E-04 23500
0.000109528 25800 0.001554909 25200 0.000482106
26200 0.008308069 24100 4.02E-05 23900 1.09E-04 23500
0.000109681 25800 0.001558751 25200 0.000482604
26200 0.008215164 24100 4.02E-05 23900 1.09E-04 23500
0.000109839 25800 0.001562595 25200 0.0004831
26200 0.00812526 24100 4.02E-05 23900 1.09E-04 23500
0.000110002 25800 0.001566451 25200 0.000483586
26200 0.008038309 24100 4.03E-05 23900 1.09E-04 23500
0.000110146 25800 0.001570311 25200 0.000484071
26200 0.007954218 24100 4.03E-05 23900 1.09E-04 23500
0.000110301 25800 0.001574198 25200 0.000484573
26200 0.007872971 24100 4.03E-05 23900 1.10E-04 23500
0.000110458 25800 0.001578103 25200 0.000485075

26200 0.007794425 24100 4.03E-05 23900 1.10E-04 23500
0.000110598 25800 0.001582013 25200 0.000485565
26200 0.007718588 24100 4.04E-05 23900 1.10E-04 23500
0.000110734 25800 0.001585951 25200 0.000486074
26200 0.007645366 24100 4.04E-05 23900 1.10E-04 23500
0.000110877 25800 0.001589904 25300 0.000486549
26200 0.007574719 24100 4.04E-05 23900 1.10E-04 23500
0.000110994 25800 0.001593866 25300 0.000487045
26200 0.007506551 24100 4.05E-05 23900 1.10E-04 23500
0.000111108 25800 0.001597841 25300 0.000487548
26200 0.007440827 24100 4.05E-05 23900 1.10E-04 23500
0.000111227 25800 0.001601805 25300 0.000488054
26200 0.007377483 24100 4.05E-05 23900 1.10E-04 23500
0.000111341 25800 0.001605806 25300 0.000488549
26200 0.007316462 24100 4.05E-05 23900 1.11E-04 23500
0.000111448 25800 0.001609829 25300 0.000489068
26200 0.007257678 24100 4.06E-05 23900 1.11E-04 23500
0.000111557 25800 0.001613862 25300 0.000489577
26200 0.007201103 24100 4.06E-05 23900 1.11E-04 23500
0.000111684 25800 0.001617905 25300 0.00049008
26200 0.007146672 24100 4.06E-05 23900 1.11E-04 23500
0.000111795 25800 0.001621961 25300 0.000490589
26200 0.00709432 24200 4.06E-05 23900 1.11E-04 23500
0.000111902 25800 0.001626032 25300 0.000491091
26200 0.007044033 24200 4.07E-05 23900 1.11E-04 23500
0.000112019 25800 0.00163011 25300 0.000491597
26200 0.006995724 24200 4.07E-05 24000 1.11E-04 23500
0.00011213 25800 0.001634211 25300 0.000492103
26200 0.006949356 24200 4.07E-05 24000 1.11E-04 23500
0.000112246 25800 0.001638342 25300 0.000492619
26200 0.006904868 24200 4.08E-05 24000 1.12E-04 23500
0.000112373 25800 0.001642484 25300 0.000493131
26200 0.006862189 24200 4.08E-05 24000 1.12E-04 23500
0.000112474 25800 0.001646651 25300 0.00049363
26200 0.006821293 24200 4.09E-05 24000 1.12E-04 23600
0.000112589 25800 0.001650819 25300 0.00049415
26200 0.006782155 24200 4.09E-05 24000 1.12E-04 23600
0.000112709 25800 0.001655013 25300 0.000494677
26200 0.006744648 24200 4.09E-05 24000 1.12E-04 23600
0.000112817 25800 0.001659235 25300 0.0004952
26200 0.006708819 24200 4.10E-05 24000 1.12E-04 23600
0.000112918 25800 0.001663458 25300 0.000495725
26200 0.006674582 24200 4.10E-05 24000 1.12E-04 23600
0.000113026 25800 0.00166771 25300 0.000496236
26200 0.006641878 24200 4.10E-05 24000 1.13E-04 23600
0.000113129 25800 0.00167198 25300 0.000496759
26200 0.006610704 24200 4.11E-05 24000 1.13E-04 23600
0.000113232 25800 0.00167624 25300 0.000497293
26200 0.00658097 24200 4.11E-05 24000 1.13E-04 23600
0.000113346 25800 0.001680508 25300 0.000497827
26200 0.00655268 24200 4.11E-05 24000 1.13E-04 23600
0.000113453 25800 0.001684816 25300 0.000498356
26200 0.006525778 24200 4.12E-05 24000 1.13E-04 23600
0.000113561 25800 0.001689137 25300 0.000498904
26200 0.006500171 24200 4.12E-05 24000 1.13E-04 23600
0.000113679 25800 0.001693476 25300 0.000499424
26200 0.006475877 24200 4.12E-05 24000 1.13E-04 23600
0.000113788 25900 0.001697842 25300 0.000499958
26200 0.006452829 24200 4.13E-05 24000 1.14E-04 23600
0.000113899 25900 0.001702201 25300 0.000500482
26200 0.006431014 24200 4.13E-05 24000 1.14E-04 23600
0.000114015 25900 0.001706563 25300 0.000501014
26200 0.006410382 24200 4.13E-05 24000 1.14E-04 23600
0.000114139 25900 0.001710942 25300 0.000501547
26200 0.006390893 24200 4.13E-05 24000 1.14E-04 23600
0.000114266 25900 0.001715338 25300 0.000502073
26200 0.00637253 24200 4.14E-05 24000 1.14E-04 23600
0.000114387 25900 0.001719763 25300 0.000502603

26200 0.006355236 24200 4.14E-05 24000 1.14E-04 23600
0.000114516 25900 0.001724183 25300 0.000503144
26200 0.006338967 24200 4.14E-05 24000 1.14E-04 23600
0.000114633 25900 0.001728599 25300 0.000503656
26200 0.006323707 24200 4.15E-05 24000 1.14E-04 23600
0.000114744 25900 0.001733045 25300 0.000504191
26200 0.006309438 24200 4.15E-05 24000 1.15E-04 23600
0.000114847 25900 0.001737523 25300 0.000504723
26200 0.006296075 24200 4.15E-05 24000 1.15E-04 23600
0.000114963 25900 0.001741979 25300 0.000505252
26200 0.00628365 24200 4.15E-05 24000 1.15E-04 23600
0.00011506 25900 0.001746466 25300 0.000505782
26200 0.006272107 24200 4.16E-05 24000 1.15E-04 23600
0.000115176 25900 0.001750996 25300 0.000506296
26200 0.006261401 24200 4.16E-05 24000 1.15E-04 23600
0.000115283 25900 0.001755536 25300 0.000506826
26200 0.006251532 24200 4.16E-05 24000 1.15E-04 23600
0.000115408 25900 0.001760071 25300 0.000507363
26200 0.006242446 24200 4.17E-05 24000 1.15E-04 23600
0.00011555 25900 0.001764616 25300 0.000507895
26200 0.006234142 24200 4.17E-05 24000 1.16E-04 23600
0.000115685 25900 0.001769171 25300 0.000508432
26200 0.006226573 24200 4.17E-05 24000 1.16E-04 23600
0.000115827 25900 0.00177375 25300 0.000508981
26200 0.006219714 24200 4.18E-05 24000 1.16E-04 23600
0.000115983 25900 0.001778362 25300 0.000509497
26200 0.006213529 24200 4.18E-05 24000 1.16E-04 23600
0.000116124 25900 0.001782982 25300 0.00051003
26200 0.00620801 24200 4.18E-05 24000 1.16E-04 23600
0.000116261 25900 0.001787607 25300 0.000510559
26200 0.006203125 24200 4.19E-05 24000 1.16E-04 23600
0.000116391 25900 0.001792269 25300 0.000511088
26200 0.006198878 24200 4.19E-05 24000 1.16E-04 23600
0.000116521 25900 0.001796935 25300 0.000511621
26200 0.006195191 24200 4.19E-05 24000 1.16E-04 23600
0.000116643 25900 0.001801618 25300 0.000512152
26200 0.006192086 24200 4.19E-05 24000 1.17E-04 23600
0.000116755 25900 0.00180633 25300 0.000512682
26200 0.006189524 24200 4.20E-05 24000 1.17E-04 23600
0.000116874 25900 0.001811062 25300 0.000513231
26200 0.006187474 24200 4.20E-05 24000 1.17E-04 23600
0.000116975 25900 0.001815805 25300 0.000513759
26200 0.006185915 24200 4.20E-05 24000 1.17E-04 23600
0.000117068 25900 0.001820562 25300 0.000514306
26200 0.006184875 24200 4.21E-05 24000 1.17E-04 23600
0.000117156 25900 0.001825352 25300 0.000514857
26300 0.006184234 24200 4.21E-05 24000 1.17E-04 23600
0.000117254 25900 0.001830166 25300 0.000515404
26300 0.006184065 24200 4.21E-05 24000 1.17E-04 23600
0.000117327 25900 0.001835015 25300 0.000515953
26300 0.006184332 24200 4.21E-05 24000 1.18E-04 23600
0.000117428 25900 0.001839882 25300 0.000516494
26300 0.006184983 24200 4.22E-05 24000 1.18E-04 23600
0.000117524 25900 0.001844757 25300 0.000517044
26300 0.006186035 24200 4.22E-05 24000 1.18E-04 23600
0.000117639 25900 0.00184967 25300 0.000517605
26300 0.006187457 24200 4.22E-05 24000 1.18E-04 23600
0.000117766 25900 0.001854572 25300 0.000518171
26300 0.006189237 24200 4.23E-05 24000 1.18E-04 23600
0.000117903 25900 0.001859514 25300 0.000518717
26300 0.006191373 24200 4.23E-05 24000 1.18E-04 23600
0.000118037 25900 0.001864477 25300 0.000519308
26300 0.006193807 24200 4.23E-05 24000 1.18E-04 23600
0.000118176 25900 0.001869479 25300 0.000519858
26300 0.00619654 24200 4.24E-05 24000 1.19E-04 23600
0.00011831 25900 0.001874503 25400 0.000520403
26300 0.006199563 24200 4.24E-05 24000 1.19E-04 23600
0.000118452 25900 0.001879538 25400 0.000520967

26300 0.006202849 24200 4.24E-05 24000 1.19E-04 23600
0.000118588 25900 0.001884598 25400 0.000521529
26300 0.00620644 24200 4.25E-05 24000 1.19E-04 23600
0.00011874 25900 0.00188967 25400 0.000522092
26300 0.006210243 24200 4.25E-05 24000 1.19E-04 23600
0.000118892 25900 0.001894772 25400 0.000522653
26300 0.006214333 24200 4.25E-05 24000 1.19E-04 23600
0.000119058 25900 0.001899901 25400 0.000523227
26300 0.006218609 24200 4.26E-05 24000 1.19E-04 23600
0.0001192 25900 0.001905039 25400 0.000523789
26300 0.006223115 24200 4.26E-05 24000 1.20E-04 23600
0.000119365 25900 0.001910202 25400 0.000524336
26300 0.006227768 24200 4.26E-05 24000 1.20E-04 23600
0.000119514 25900 0.001915387 25400 0.000524918
26300 0.006232676 24300 4.27E-05 24000 1.20E-04 23600
0.000119671 25900 0.001920612 25400 0.000525483
26300 0.006237706 24300 4.27E-05 24000 1.20E-04 23600
0.000119823 25900 0.001925863 25400 0.000526056
26300 0.00624294 24300 4.27E-05 24100 1.20E-04 23600
0.000119958 25900 0.001931129 25400 0.000526616
26300 0.006248327 24300 4.28E-05 24100 1.20E-04 23600
0.000120115 25900 0.001936433 25400 0.000527182
26300 0.006253848 24300 4.28E-05 24100 1.20E-04 23600
0.000120245 25900 0.001941755 25400 0.000527747
26300 0.006259518 24300 4.28E-05 24100 1.21E-04 23600
0.000120397 25900 0.001947094 25400 0.000528324
26300 0.00626529 24300 4.28E-05 24100 1.21E-04 23700
0.000120533 25900 0.001952436 25400 0.000528896
26300 0.006271206 24300 4.29E-05 24100 1.21E-04 23700
0.000120686 25900 0.001957816 25400 0.000529479
26300 0.006277231 24300 4.29E-05 24100 1.21E-04 23700
0.000120821 25900 0.001963223 25400 0.000530051
26300 0.006283331 24300 4.29E-05 24100 1.21E-04 23700
0.00012097 25900 0.001968676 25400 0.000530629
26300 0.006289503 24300 4.30E-05 24100 1.21E-04 23700
0.000121108 25900 0.00197414 25400 0.000531195
26300 0.006295748 24300 4.30E-05 24100 1.22E-04 23700
0.000121245 25900 0.001979611 25400 0.000531761
26300 0.006302063 24300 4.30E-05 24100 1.22E-04 23700
0.000121385 25900 0.001985115 25400 0.000532332
26300 0.006308463 24300 4.31E-05 24100 1.22E-04 23700
0.000121519 25900 0.001990641 25400 0.000532905
26300 0.006314893 24300 4.31E-05 24100 1.22E-04 23700
0.000121658 25900 0.001996181 25400 0.000533478
26300 0.00632141 24300 4.31E-05 24100 1.22E-04 23700
0.000121799 25900 0.002001758 25400 0.000534055
26300 0.006327945 24300 4.32E-05 24100 1.22E-04 23700
0.000121947 25900 0.00200734 25400 0.000534625
26300 0.006334492 24300 4.32E-05 24100 1.23E-04 23700
0.000122086 26000 0.002012955 25400 0.000535194
26300 0.006341051 24300 4.32E-05 24100 1.23E-04 23700
0.000122236 26000 0.002018588 25400 0.000535771
26300 0.006347664 24300 4.33E-05 24100 1.23E-04 23700
0.000122392 26000 0.002024254 25400 0.00053635
26300 0.006354253 24300 4.33E-05 24100 1.23E-04 23700
0.000122554 26000 0.002029933 25400 0.00053693
26300 0.006360876 24300 4.33E-05 24100 1.23E-04 23700
0.00012271 26000 0.002035631 25400 0.00053751
26300 0.006367512 24300 4.34E-05 24100 1.23E-04 23700
0.000122876 26000 0.002041351 25400 0.00053808
26300 0.00637413 24300 4.34E-05 24100 1.24E-04 23700
0.00012303 26000 0.002047086 25400 0.000538662
26300 0.006380746 24300 4.34E-05 24100 1.24E-04 23700
0.00012318 26000 0.002052841 25400 0.000539261
26300 0.006387338 24300 4.34E-05 24100 1.24E-04 23700
0.000123328 26000 0.002058574 25400 0.000539851
26300 0.006393926 24300 4.35E-05 24100 1.24E-04 23700
0.000123489 26000 0.002064354 25400 0.000540442

26300 0.006400522 24300 4.35E-05 24100 1.24E-04 23700
0.00012363 26000 0.002070159 25400 0.000541028
26300 0.006407076 24300 4.36E-05 24100 1.24E-04 23700
0.000123756 26000 0.002075989 25400 0.000541616
26300 0.006413598 24300 4.36E-05 24100 1.25E-04 23700
0.000123892 26000 0.002081825 25400 0.00054221
26300 0.006420077 24300 4.36E-05 24100 1.25E-04 23700
0.00012402 26000 0.002087675 25400 0.000542805
26300 0.006426528 24300 4.36E-05 24100 1.25E-04 23700
0.000124122 26000 0.002093539 25400 0.000543402
26300 0.006432971 24300 4.37E-05 24100 1.25E-04 23700
0.000124234 26000 0.002099423 25400 0.000543995
26300 0.006439338 24300 4.37E-05 24100 1.25E-04 23700
0.000124348 26000 0.002105319 25400 0.000544587
26300 0.006445671 24300 4.37E-05 24100 1.25E-04 23700
0.000124455 26000 0.002111253 25400 0.000545193
26300 0.00645197 24300 4.38E-05 24100 1.26E-04 23700
0.000124594 26000 0.002117186 25400 0.000545791
26300 0.006458189 24300 4.38E-05 24100 1.26E-04 23700
0.00012473 26000 0.002123154 25400 0.000546391
26300 0.006464366 24300 4.38E-05 24100 1.26E-04 23700
0.000124873 26000 0.002129148 25400 0.000546999
26300 0.006470494 24300 4.39E-05 24100 1.26E-04 23700
0.000125021 26000 0.00213516 25400 0.000547609
26300 0.006476542 24300 4.39E-05 24100 1.26E-04 23700
0.000125173 26000 0.002141208 25400 0.000548215
26300 0.006482541 24300 4.39E-05 24100 1.26E-04 23700
0.000125316 26000 0.002147277 25400 0.00054883
26300 0.006488488 24300 4.40E-05 24100 1.27E-04 23700
0.000125461 26000 0.002153369 25400 0.000549419
26300 0.006494365 24300 4.40E-05 24100 1.27E-04 23700
0.000125596 26000 0.002159489 25400 0.000550032
26300 0.006500172 24300 4.40E-05 24100 1.27E-04 23700
0.000125718 26000 0.002165626 25400 0.00055065
26300 0.006505907 24300 4.40E-05 24100 1.27E-04 23700
0.000125879 26000 0.002171765 25400 0.000551268
26300 0.006511583 24300 4.41E-05 24100 1.27E-04 23700
0.000126009 26000 0.002177944 25400 0.00055188
26400 0.006517216 24300 4.41E-05 24100 1.27E-04 23700
0.000126156 26000 0.002184162 25400 0.000552509
26400 0.006522748 24300 4.42E-05 24100 1.28E-04 23700
0.000126327 26000 0.002190399 25400 0.000553109
26400 0.006528197 24300 4.42E-05 24100 1.28E-04 23700
0.000126495 26000 0.00219665 25400 0.000553726
26400 0.006533558 24300 4.42E-05 24100 1.28E-04 23700
0.000126667 26000 0.002202932 25400 0.000554342
26400 0.006538858 24300 4.43E-05 24100 1.28E-04 23700
0.000126841 26000 0.002209231 25400 0.000554959
26400 0.006544084 24300 4.43E-05 24100 1.28E-04 23700
0.000127015 26000 0.002215551 25400 0.000555573
26400 0.006549207 24300 4.43E-05 24100 1.28E-04 23700
0.000127212 26000 0.002221899 25400 0.000556185
26400 0.006554272 24300 4.44E-05 24100 1.29E-04 23700
0.000127411 26000 0.002228271 25400 0.000556797
26400 0.00655926 24300 4.44E-05 24100 1.29E-04 23700
0.00012761 26000 0.002234657 25400 0.000557413
26400 0.006564118 24300 4.44E-05 24100 1.29E-04 23700
0.000127793 26000 0.00224108 25500 0.000558016
26400 0.006568884 24300 4.45E-05 24100 1.29E-04 23700
0.000127988 26000 0.002247532 25500 0.000558634
26400 0.006573595 24300 4.45E-05 24100 1.29E-04 23700
0.000128172 26000 0.002254007 25500 0.00055924
26400 0.006578197 24300 4.45E-05 24100 1.29E-04 23700
0.000128364 26000 0.002260523 25500 0.000559872
26400 0.006582692 24300 4.46E-05 24100 1.30E-04 23700
0.000128534 26000 0.002267066 25500 0.000560486
26400 0.006587104 24300 4.46E-05 24100 1.30E-04 23700
0.000128706 26000 0.002273637 25500 0.000561093

26400 0.006591404 24300 4.46E-05 24100 1.30E-04 23700
0.000128855 26000 0.002280238 25500 0.000561709
26400 0.006595645 24300 4.47E-05 24100 1.30E-04 23700
0.000129019 26000 0.00228685 25500 0.000562327
26400 0.00659974 24300 4.47E-05 24100 1.30E-04 23700
0.000129169 26000 0.002293472 25500 0.000562959
26400 0.006603747 24400 4.48E-05 24100 1.31E-04 23700
0.00012931 26000 0.002300153 25500 0.000563572
26400 0.006607684 24400 4.48E-05 24100 1.31E-04 23700
0.000129448 26000 0.002306858 25500 0.0005642
26400 0.006611493 24400 4.48E-05 24200 1.31E-04 23700
0.000129585 26000 0.002313599 25500 0.000564824
26400 0.006615196 24400 4.48E-05 24200 1.31E-04 23700
0.00012971 26000 0.002320375 25500 0.000565451
26400 0.006618806 24400 4.49E-05 24200 1.31E-04 23700
0.000129832 26000 0.002327185 25500 0.000566065
26400 0.006622293 24400 4.49E-05 24200 1.31E-04 23700
0.000129953 26000 0.002333997 25500 0.000566681
26400 0.006625692 24400 4.49E-05 24200 1.32E-04 23800
0.000130072 26000 0.00234085 25500 0.000567308
26400 0.006628975 24400 4.50E-05 24200 1.32E-04 23800
0.000130195 26000 0.002347739 25500 0.000567937
26400 0.006632166 24400 4.50E-05 24200 1.32E-04 23800
0.000130324 26000 0.002354657 25500 0.000568555
26400 0.006635253 24400 4.51E-05 24200 1.32E-04 23800
0.000130454 26000 0.002361595 25500 0.000569178
26400 0.006638212 24400 4.51E-05 24200 1.32E-04 23800
0.000130598 26000 0.00236857 25500 0.000569799
26400 0.006641069 24400 4.51E-05 24200 1.33E-04 23800
0.000130739 26000 0.002375579 25500 0.000570446
26400 0.006643847 24400 4.52E-05 24200 1.33E-04 23800
0.000130863 26000 0.002382634 25500 0.000571064
26400 0.00664648 24400 4.52E-05 24200 1.33E-04 23800
0.000131001 26000 0.002389704 25500 0.000571709
26400 0.006649037 24400 4.53E-05 24200 1.33E-04 23800
0.000131133 26000 0.002396819 25500 0.000572338
26400 0.006651478 24400 4.53E-05 24200 1.33E-04 23800
0.000131243 26000 0.002403967 25500 0.000572953
26400 0.006653803 24400 4.53E-05 24200 1.33E-04 23800
0.000131377 26000 0.002411148 25500 0.000573578
26400 0.006656053 24400 4.54E-05 24200 1.34E-04 23800
0.000131492 26100 0.002418346 25500 0.000574215
26400 0.006658167 24400 4.54E-05 24200 1.34E-04 23800
0.000131609 26100 0.002425548 25500 0.000574854
26400 0.006660196 24400 4.54E-05 24200 1.34E-04 23800
0.000131741 26100 0.002432819 25500 0.000575494
26400 0.006662154 24400 4.55E-05 24200 1.34E-04 23800
0.000131885 26100 0.002440112 25500 0.000576148
26400 0.006663972 24400 4.55E-05 24200 1.34E-04 23800
0.000132034 26100 0.002447454 25500 0.000576774
26400 0.006665702 24400 4.55E-05 24200 1.35E-04 23800
0.000132192 26100 0.002454812 25500 0.000577425
26400 0.006667325 24400 4.56E-05 24200 1.35E-04 23800
0.000132343 26100 0.002462205 25500 0.000578047
26400 0.006668838 24400 4.56E-05 24200 1.35E-04 23800
0.000132509 26100 0.002469626 25500 0.000578693
26400 0.006670285 24400 4.57E-05 24200 1.35E-04 23800
0.00013267 26100 0.002477074 25500 0.000579321
26400 0.006671603 24400 4.57E-05 24200 1.35E-04 23800
0.000132851 26100 0.002484559 25500 0.000579957
26400 0.006672842 24400 4.57E-05 24200 1.36E-04 23800
0.000133016 26100 0.002492073 25500 0.000580577
26400 0.006673993 24400 4.58E-05 24200 1.36E-04 23800
0.000133205 26100 0.00249961 25500 0.000581228
26400 0.006675035 24400 4.58E-05 24200 1.36E-04 23800
0.000133386 26100 0.002507189 25500 0.000581858
26400 0.006675978 24400 4.58E-05 24200 1.36E-04 23800
0.000133565 26100 0.002514806 25500 0.000582501

26400 0.006676833 24400 4.59E-05 24200 1.36E-04 23800
0.000133746 26100 0.002522461 25500 0.000583145
26400 0.006677592 24400 4.59E-05 24200 1.37E-04 23800
0.000133936 26100 0.002530142 25500 0.000583789
26400 0.006678279 24400 4.60E-05 24200 1.37E-04 23800
0.000134105 26100 0.002537866 25500 0.000584421
26400 0.006678856 24400 4.60E-05 24200 1.37E-04 23800
0.000134292 26100 0.002545618 25500 0.000585054
26400 0.006679354 24400 4.60E-05 24200 1.37E-04 23800
0.00013444 26100 0.002553413 25500 0.000585685
26400 0.006679786 24400 4.61E-05 24200 1.37E-04 23800
0.000134626 26100 0.002561224 25500 0.000586335
26400 0.006680107 24400 4.61E-05 24200 1.38E-04 23800
0.000134807 26100 0.002569044 25500 0.00058697
26400 0.006680363 24400 4.62E-05 24200 1.38E-04 23800
0.000134984 26100 0.002576929 25500 0.000587625
26400 0.006680562 24400 4.62E-05 24200 1.38E-04 23800
0.000135179 26100 0.002584852 25500 0.000588273
26400 0.006680651 24400 4.62E-05 24200 1.38E-04 23800
0.000135354 26100 0.002592824 25500 0.000588917
26400 0.006680668 24400 4.63E-05 24200 1.38E-04 23800
0.000135541 26100 0.002600823 25500 0.000589571
26400 0.006680617 24400 4.63E-05 24200 1.39E-04 23800
0.000135706 26100 0.00260886 25500 0.000590209
26400 0.006680477 24400 4.64E-05 24200 1.39E-04 23800
0.000135883 26100 0.002616929 25500 0.000590863
26400 0.006680286 24400 4.64E-05 24200 1.39E-04 23800
0.000136074 26100 0.00262504 25500 0.000591504
26400 0.006679985 24400 4.64E-05 24200 1.39E-04 23800
0.000136258 26100 0.002633194 25500 0.000592144
26500 0.006679645 24400 4.65E-05 24200 1.39E-04 23800
0.00013644 26100 0.002641387 25500 0.000592791
26500 0.006679238 24400 4.65E-05 24200 1.40E-04 23800
0.000136652 26100 0.002649613 25500 0.000593451
26500 0.006678734 24400 4.65E-05 24200 1.40E-04 23800
0.000136857 26100 0.002657883 25500 0.000594109
26500 0.006678168 24400 4.66E-05 24200 1.40E-04 23800
0.000137066 26100 0.002666196 25500 0.000594761
26500 0.006677565 24400 4.66E-05 24200 1.40E-04 23800
0.000137276 26100 0.002674557 25500 0.000595424
26500 0.006676848 24400 4.67E-05 24200 1.40E-04 23800
0.000137498 26100 0.002682965 25500 0.000596086
26500 0.006676088 24400 4.67E-05 24200 1.41E-04 23800
0.000137706 26100 0.002691416 25500 0.000596737
26500 0.006675253 24400 4.67E-05 24200 1.41E-04 23800
0.000137893 26100 0.002699907 25500 0.000597381
26500 0.006674359 24400 4.68E-05 24200 1.41E-04 23800
0.000138075 26100 0.00270845 25500 0.000598036
26500 0.006673426 24400 4.68E-05 24200 1.41E-04 23800
0.000138287 26100 0.002717014 25600 0.0005987
26500 0.006672385 24400 4.68E-05 24200 1.41E-04 23800
0.000138465 26100 0.002725608 25600 0.000599359
26500 0.006671321 24400 4.69E-05 24200 1.42E-04 23800
0.000138651 26100 0.002734269 25600 0.00060004
26500 0.006670185 24400 4.69E-05 24200 1.42E-04 23800
0.000138828 26100 0.00274296 25600 0.000600712
26500 0.006668975 24400 4.69E-05 24200 1.42E-04 23800
0.000139007 26100 0.002751702 25600 0.000601385
26500 0.006667696 24400 4.70E-05 24200 1.42E-04 23800
0.000139202 26100 0.002760481 25600 0.000602052
26500 0.006666359 24400 4.70E-05 24200 1.42E-04 23800
0.000139388 26100 0.002769301 25600 0.000602721
26500 0.006664954 24400 4.70E-05 24200 1.43E-04 23800
0.000139567 26100 0.002778142 25600 0.000603396
26500 0.00666351 24400 4.70E-05 24200 1.43E-04 23800
0.000139751 26100 0.002787021 25600 0.000604061
26500 0.006661987 24500 4.71E-05 24200 1.43E-04 23800
0.000139948 26100 0.002795937 25600 0.000604729

26500 0.006660419 24500 4.71E-05 24200 1.43E-04 23800
0.000140125 26100 0.002804889 25600 0.000605407
26500 0.006658769 24500 4.72E-05 24300 1.44E-04 23800
0.000140303 26100 0.002813877 25600 0.000606082
26500 0.006657055 24500 4.72E-05 24300 1.44E-04 23800
0.000140449 26100 0.00282291 25600 0.000606764
26500 0.006655269 24500 4.72E-05 24300 1.44E-04 23800
0.000140615 26100 0.002831962 25600 0.000607438
26500 0.006653458 24500 4.73E-05 24300 1.44E-04 23800
0.000140769 26100 0.002841079 25600 0.000608123
26500 0.006651549 24500 4.73E-05 24300 1.44E-04 23900
0.000140933 26100 0.002850227 25600 0.00060882
26500 0.006649612 24500 4.74E-05 24300 1.45E-04 23900
0.000141096 26100 0.002859425 25600 0.000609501
26500 0.006647601 24500 4.74E-05 24300 1.45E-04 23900
0.000141248 26100 0.002868662 25600 0.000610169
26500 0.006645555 24500 4.74E-05 24300 1.45E-04 23900
0.000141408 26100 0.002877945 25600 0.000610841
26500 0.006643446 24500 4.75E-05 24300 1.45E-04 23900
0.000141595 26100 0.002887254 25600 0.000611546
26500 0.00664127 24500 4.75E-05 24300 1.45E-04 23900
0.000141766 26100 0.002896574 25600 0.000612236
26500 0.006639072 24500 4.75E-05 24300 1.46E-04 23900
0.000141929 26100 0.002905973 25600 0.000612939
26500 0.006636837 24500 4.76E-05 24300 1.46E-04 23900
0.00014211 26100 0.002915434 25600 0.000613648
26500 0.006634513 24500 4.76E-05 24300 1.46E-04 23900
0.000142287 26100 0.002924945 25600 0.000614353
26500 0.00663216 24500 4.77E-05 24300 1.46E-04 23900
0.000142455 26100 0.002934509 25600 0.000615046
26500 0.006629748 24500 4.77E-05 24300 1.47E-04 23900
0.000142623 26100 0.00294411 25600 0.000615759
26500 0.006627287 24500 4.78E-05 24300 1.47E-04 23900
0.000142788 26200 0.002953748 25600 0.000616453
26500 0.00662481 24500 4.78E-05 24300 1.47E-04 23900
0.000142953 26200 0.002963427 25600 0.000617166
26500 0.006622276 24500 4.78E-05 24300 1.47E-04 23900
0.000143117 26200 0.002973163 25600 0.000617859
26500 0.006619715 24500 4.79E-05 24300 1.48E-04 23900
0.000143272 26200 0.002982936 25600 0.000618572
26500 0.006617113 24500 4.79E-05 24300 1.48E-04 23900
0.000143462 26200 0.002992747 25600 0.00061929
26500 0.006614448 24500 4.80E-05 24300 1.48E-04 23900
0.000143639 26200 0.003002614 25600 0.000619992
26500 0.00661174 24500 4.80E-05 24300 1.48E-04 23900
0.000143832 26200 0.003012534 25600 0.000620704
26500 0.006609014 24500 4.81E-05 24300 1.48E-04 23900
0.000144032 26200 0.00302252 25600 0.000621428
26500 0.006606229 24500 4.81E-05 24300 1.49E-04 23900
0.000144263 26200 0.003032572 25600 0.000622157
26500 0.006603419 24500 4.82E-05 24300 1.49E-04 23900
0.000144477 26200 0.003042672 25600 0.000622883
26500 0.006600575 24500 4.82E-05 24300 1.49E-04 23900
0.000144685 26200 0.003052815 25600 0.000623594
26500 0.006597695 24500 4.82E-05 24300 1.49E-04 23900
0.000144905 26200 0.003063002 25600 0.000624309
26500 0.006594792 24500 4.83E-05 24300 1.50E-04 23900
0.000145122 26200 0.003073253 25600 0.000625054
26500 0.006591838 24500 4.83E-05 24300 1.50E-04 23900
0.000145314 26200 0.003083529 25600 0.000625772
26500 0.006588865 24500 4.83E-05 24300 1.50E-04 23900
0.000145487 26200 0.003093876 25600 0.000626495
26500 0.006585882 24500 4.84E-05 24300 1.50E-04 23900
0.000145665 26200 0.00310428 25600 0.000627235
26500 0.00658282 24500 4.84E-05 24300 1.51E-04 23900
0.000145824 26200 0.003114749 25600 0.00062796
26500 0.006579751 24500 4.85E-05 24300 1.51E-04 23900
0.000145969 26200 0.003125274 25600 0.00062869

```
26500   0.006576643     24500   4.85E-05        24300   1.51E-04        23900
0.000146095     26200   0.003135828     25600   0.000629416
26500   0.006573512     24500   4.85E-05        24300   1.51E-04        23900
0.000146252     26200   0.003146436     25600   0.000630147
26500   0.006570345     24500   4.86E-05        24300   1.52E-04        23900
0.000146399     26200   0.003157103     25600   0.000630881
26500   0.006567131     24500   4.86E-05        24300   1.52E-04        23900
0.00014655      26200   0.003167837     25600   0.000631597
26500   0.006563924     24500   4.86E-05        24300   1.52E-04        23900
0.000146684     26200   0.003178599     25600   0.000632313
26500   0.00656069      24500   4.87E-05        24300   1.52E-04        23900
0.000146849     26200   0.003189411     25600   0.000633056
26500   0.00655739      24500   4.87E-05        24300   1.53E-04        23900
0.000147015     26200   0.003200286     25600   0.000633775
26500   0.006554062     24500   4.87E-05        24300   1.53E-04        23900
0.000147197     26200   0.003211209     25600   0.000634513
26500   0.006550761     24500   4.88E-05        24300   1.53E-04        23900
0.000147377     26200   0.003222199     25600   0.000635251
26500   0.006547399     24500   4.88E-05        24300   1.53E-04        23900
0.000147581     26200   0.00323327      25600   0.000635984
26500   0.006544006     24500   4.89E-05        24300   1.54E-04        23900
0.000147787     26200   0.003244356     25600   0.000636722
26600   0.006540608     24500   4.89E-05        24300   1.54E-04        23900
0.000148006     26200   0.003255503     25600   0.000637426
26600   0.006537173     24500   4.89E-05        24300   1.54E-04        23900
0.000148231     26200   0.00326667      25600   0.000638152
26600   0.006533741     24500   4.90E-05        24300   1.54E-04        23900
0.000148476     26200   0.003277905     25600   0.000638896
26600   0.006530249     24500   4.90E-05        24300   1.55E-04        23900
0.000148723     26200   0.003289178     25600   0.000639639
26600   0.00652676      24500   4.90E-05        24300   1.55E-04        23900
0.000148977     26200   0.003300512     25600   0.000640378
26600   0.006523281     24500   4.91E-05        24300   1.55E-04        23900
0.000149223     26200   0.003311895     25600   0.000641127
26600   0.006519762     24500   4.91E-05        24300   1.55E-04        23900
0.00014945      26200   0.003323345     25600   0.000641863
26600   0.006516216     24500   4.92E-05        24300   1.56E-04        23900
0.000149654     26200   0.003334862     25600   0.00064261
26600   0.006512672     24500   4.92E-05        24300   1.56E-04        23900
0.000149843     26200   0.00334641      25600   0.000643356
26600   0.006509101     24500   4.92E-05        24300   1.56E-04        23900
0.000150022     26200   0.003358021     25700   0.000644099
26600   0.006505552     24500   4.93E-05        24300   1.57E-04        23900
0.000150199     26200   0.003369675     25700   0.000644846
26600   0.006501949     24500   4.93E-05        24300   1.57E-04        23900
0.000150378     26200   0.003381392     25700   0.000645585
26600   0.00649836      24500   4.94E-05        24300   1.57E-04        23900
0.000150558     26200   0.003393162     25700   0.000646321
26600   0.006494758     24500   4.94E-05        24300   1.57E-04        23900
0.00015075      26200   0.003404974     25700   0.000647083
26600   0.006491123     24500   4.94E-05        24300   1.58E-04        23900
0.000150945     26200   0.003416863     25700   0.000647823
26600   0.006487479     24500   4.95E-05        24300   1.58E-04        23900
0.000151141     26200   0.003428819     25700   0.000648583
26600   0.006483867     24500   4.95E-05        24300   1.58E-04        23900
0.000151332     26200   0.003440841     25700   0.000649343
26600   0.006480189     24500   4.96E-05        24300   1.59E-04        23900
0.000151541     26200   0.003452946     25700   0.000650106
26600   0.006476546     24600   4.96E-05        24300   1.59E-04        23900
0.00015173      26200   0.003465102     25700   0.000650851
26600   0.006472886     24600   4.96E-05        24300   1.59E-04        23900
0.000151926     26200   0.00347733      25700   0.000651602
26600   0.006469216     24600   4.97E-05        24400   1.59E-04        23900
0.000152128     26200   0.003489615     25700   0.000652351
26600   0.006465567     24600   4.97E-05        24400   1.60E-04        23900
0.000152332     26200   0.00350194      25700   0.000653108
26600   0.006461889     24600   4.98E-05        24400   1.60E-04        23900
0.000152534     26200   0.003514309     25700   0.000653877
```

26600 0.006458214 24600 4.98E-05 24400 1.60E-04 23900
0.000152741 26200 0.003526781 25700 0.00065463
26600 0.006454577 24600 4.98E-05 24400 1.61E-04 24000
0.000152956 26200 0.003539324 25700 0.000655405
26600 0.006450903 24600 4.99E-05 24400 1.61E-04 24000
0.000153149 26200 0.003551926 25700 0.000656171
26600 0.006447234 24600 4.99E-05 24400 1.61E-04 24000
0.000153359 26200 0.003564609 25700 0.000656907
26600 0.006443559 24600 4.99E-05 24400 1.61E-04 24000
0.00015356 26200 0.003577336 25700 0.000657683
26600 0.00643986 24600 5.00E-05 24400 1.62E-04 24000
0.000153765 26200 0.003590137 25700 0.000658427
26600 0.006436204 24600 5.00E-05 24400 1.62E-04 24000
0.000153964 26200 0.003602989 25700 0.000659187
26600 0.006432503 24600 5.00E-05 24400 1.62E-04 24000
0.00015417 26200 0.003615925 25700 0.000659938
26600 0.006428857 24600 5.01E-05 24400 1.63E-04 24000
0.000154358 26200 0.003628905 25700 0.000660705
26600 0.006425178 24600 5.01E-05 24400 1.63E-04 24000
0.000154556 26200 0.00364196 25700 0.000661474
26600 0.006421502 24600 5.02E-05 24400 1.63E-04 24000
0.000154739 26200 0.003655079 25700 0.000662227
26600 0.006417802 24600 5.02E-05 24400 1.63E-04 24000
0.000154928 26200 0.003668258 25700 0.000662991
26600 0.006414169 24600 5.02E-05 24400 1.64E-04 24000
0.000155114 26300 0.003681525 25700 0.00066376
26600 0.006410474 24600 5.03E-05 24400 1.64E-04 24000
0.000155298 26300 0.003694862 25700 0.00066455
26600 0.006406815 24600 5.03E-05 24400 1.64E-04 24000
0.000155487 26300 0.003708256 25700 0.000665316
26600 0.006403159 24600 5.04E-05 24400 1.65E-04 24000
0.000155688 26300 0.003721737 25700 0.00066606
26600 0.006399489 24600 5.04E-05 24400 1.65E-04 24000
0.000155892 26300 0.003735284 25700 0.000666828
26600 0.006395856 24600 5.05E-05 24400 1.65E-04 24000
0.000156113 26300 0.00374887 25700 0.000667603
26600 0.006392199 24600 5.05E-05 24400 1.66E-04 24000
0.000156356 26300 0.003762525 25700 0.00066837
26600 0.006388542 24600 5.05E-05 24400 1.66E-04 24000
0.00015661 26300 0.003776277 25700 0.000669153
26600 0.006384929 24600 5.06E-05 24400 1.66E-04 24000
0.000156854 26300 0.003790093 25700 0.000669929
26600 0.006381288 24600 5.06E-05 24400 1.66E-04 24000
0.000157124 26300 0.003803986 25700 0.000670704
26600 0.006377656 24600 5.07E-05 24400 1.67E-04 24000
0.000157367 26300 0.003817964 25700 0.000671466
26600 0.006374022 24600 5.07E-05 24400 1.67E-04 24000
0.000157619 26300 0.003831994 25700 0.000672239
26600 0.006370398 24600 5.08E-05 24400 1.67E-04 24000
0.000157872 26300 0.003846104 25700 0.000673015
26600 0.006366791 24600 5.08E-05 24400 1.68E-04 24000
0.000158137 26300 0.00386028 25700 0.000673789
26600 0.006363191 24600 5.09E-05 24400 1.68E-04 24000
0.000158398 26300 0.003874513 25700 0.000674559
26600 0.006359617 24600 5.09E-05 24400 1.68E-04 24000
0.000158666 26300 0.003888844 25700 0.000675323
26600 0.006356039 24600 5.10E-05 24400 1.69E-04 24000
0.000158949 26300 0.003903244 25700 0.000676098
26600 0.006352463 24600 5.10E-05 24400 1.69E-04 24000
0.000159255 26300 0.003917734 25700 0.000676861
26600 0.00634886 24600 5.11E-05 24400 1.69E-04 24000
0.000159576 26300 0.003932283 25700 0.000677645
26600 0.00634532 24600 5.11E-05 24400 1.70E-04 24000
0.00015992 26300 0.003946925 25700 0.000678438
26600 0.006341754 24600 5.12E-05 24400 1.70E-04 24000
0.000160267 26300 0.003961658 25700 0.000679231
26600 0.006338228 24600 5.12E-05 24400 1.70E-04 24000
0.000160602 26300 0.003976469 25700 0.000680013

26600 0.00633471 24600 5.13E-05 24400 1.71E-04 24000
0.000160912 26300 0.003991371 25700 0.000680773
26600 0.006331197 24600 5.13E-05 24400 1.71E-04 24000
0.00016115 26300 0.004006342 25700 0.000681568
26600 0.006327717 24600 5.14E-05 24400 1.71E-04 24000
0.000161359 26300 0.004021395 25700 0.000682349
26600 0.006324229 24600 5.14E-05 24400 1.72E-04 24000
0.000161517 26300 0.004036479 25700 0.000683117
26600 0.006320769 24600 5.15E-05 24400 1.72E-04 24000
0.000161635 26300 0.0040517 25700 0.000683903
26600 0.006317362 24600 5.15E-05 24400 1.72E-04 24000
0.000161718 26300 0.00406701 25700 0.000684692
26600 0.006313949 24600 5.16E-05 24400 1.73E-04 24000
0.000161807 26300 0.004082409 25700 0.000685477
26700 0.006310553 24600 5.16E-05 24400 1.73E-04 24000
0.000161884 26300 0.004097903 25700 0.00068626
26700 0.006307168 24600 5.16E-05 24400 1.73E-04 24000
0.000161979 26300 0.004113457 25700 0.00068705
26700 0.006303827 24600 5.17E-05 24400 1.74E-04 24000
0.000162089 26300 0.00412911 25700 0.000687837
26700 0.006300506 24600 5.17E-05 24400 1.74E-04 24000
0.000162231 26300 0.004144834 25700 0.000688626
26700 0.00629721 24600 5.17E-05 24400 1.74E-04 24000
0.000162392 26300 0.004160638 25700 0.000689415
26700 0.006293932 24600 5.18E-05 24400 1.75E-04 24000
0.00016257 26300 0.00417654 25700 0.00069021
26700 0.006290702 24600 5.18E-05 24400 1.75E-04 24000
0.000162776 26300 0.004192525 25700 0.000691009
26700 0.00628746 24600 5.18E-05 24400 1.75E-04 24000
0.000162995 26300 0.004208597 25700 0.000691815
26700 0.006284246 24600 5.18E-05 24400 1.76E-04 24000
0.000163216 26300 0.004224751 25700 0.000692617
26700 0.006281083 24600 5.19E-05 24400 1.76E-04 24000
0.000163445 26300 0.004240994 25800 0.000693433
26700 0.006277924 24600 5.19E-05 24400 1.76E-04 24000
0.000163683 26300 0.004257341 25800 0.000694264
26700 0.006274804 24600 5.20E-05 24400 1.77E-04 24000
0.000163913 26300 0.004273779 25800 0.000695054
26700 0.006271723 24600 5.20E-05 24400 1.77E-04 24000
0.000164144 26300 0.004290322 25800 0.000695842
26700 0.006268656 24600 5.20E-05 24400 1.77E-04 24000
0.000164356 26300 0.004306949 25800 0.000696665
26700 0.006265622 24600 5.21E-05 24400 1.78E-04 24000
0.000164572 26300 0.004323666 25800 0.000697481
26700 0.006262593 24600 5.21E-05 24400 1.78E-04 24000
0.000164798 26300 0.004340442 25800 0.000698296
26700 0.006259619 24600 5.22E-05 24400 1.79E-04 24000
0.00016503 26300 0.00435736 25800 0.000699125
26700 0.006256696 24600 5.22E-05 24400 1.79E-04 24000
0.000165249 26300 0.004374389 25800 0.000699967
26700 0.006253778 24700 5.23E-05 24400 1.79E-04 24000
0.000165486 26300 0.004391507 25800 0.00070078
26700 0.006250888 24700 5.23E-05 24400 1.80E-04 24000
0.00016571 26300 0.004408731 25800 0.000701616
26700 0.006248008 24700 5.24E-05 24500 1.80E-04 24000
0.00016595 26300 0.004426053 25800 0.000702435
26700 0.006245174 24700 5.24E-05 24500 1.80E-04 24000
0.000166189 26300 0.004443462 25800 0.000703272
26700 0.00624236 24700 5.25E-05 24500 1.81E-04 24000
0.000166419 26300 0.004460971 25800 0.000704109
26700 0.006239559 24700 5.25E-05 24500 1.81E-04 24000
0.00016665 26300 0.004478582 25800 0.000704943
26700 0.006236796 24700 5.26E-05 24500 1.81E-04 24100
0.000166883 26300 0.004496292 25800 0.000705784
26700 0.006234075 24700 5.26E-05 24500 1.82E-04 24100
0.000167115 26300 0.004514098 25800 0.000706633
26700 0.006231324 24700 5.27E-05 24500 1.82E-04 24100
0.000167351 26300 0.004532021 25800 0.000707492

26700 0.006228602 24700 5.27E-05 24500 1.83E-04 24100
0.000167585 26300 0.004550025 25800 0.000708346
26700 0.006225935 24700 5.28E-05 24500 1.83E-04 24100
0.000167813 26300 0.004568135 25800 0.000709208
26700 0.00622327 24700 5.28E-05 24500 1.83E-04 24100
0.000168052 26300 0.004586369 25800 0.000710084
26700 0.006220622 24700 5.29E-05 24500 1.84E-04 24100
0.000168294 26300 0.004604687 25800 0.000710927
26700 0.006217989 24700 5.29E-05 24500 1.84E-04 24100
0.000168525 26300 0.004623129 25800 0.000711779
26700 0.006215398 24700 5.29E-05 24500 1.84E-04 24100
0.000168759 26300 0.004641667 25800 0.000712644
26700 0.00621286 24700 5.30E-05 24500 1.85E-04 24100
0.00016902 26300 0.004660291 25800 0.000713523
26700 0.006210294 24700 5.30E-05 24500 1.85E-04 24100
0.000169268 26300 0.004678979 25800 0.000714401
26700 0.006207752 24700 5.30E-05 24500 1.86E-04 24100
0.000169524 26400 0.004697835 25800 0.000715265
26700 0.006205283 24700 5.31E-05 24500 1.86E-04 24100
0.000169784 26400 0.004716793 25800 0.000716147
26700 0.006202788 24700 5.31E-05 24500 1.86E-04 24100
0.000170051 26400 0.004735864 25800 0.000717033
26700 0.006200338 24700 5.32E-05 24500 1.87E-04 24100
0.000170293 26400 0.004755058 25800 0.000717907
26700 0.006197893 24700 5.32E-05 24500 1.87E-04 24100
0.00017055 26400 0.004774339 25800 0.000718787
26700 0.006195461 24700 5.33E-05 24500 1.87E-04 24100
0.000170801 26400 0.004793732 25800 0.000719661
26700 0.006193071 24700 5.33E-05 24500 1.88E-04 24100
0.00017107 26400 0.004813244 25800 0.000720559
26700 0.006190693 24700 5.34E-05 24500 1.88E-04 24100
0.000171315 26400 0.004832861 25800 0.000721455
26700 0.006188337 24700 5.34E-05 24500 1.89E-04 24100
0.00017157 26400 0.004852619 25800 0.000722335
26700 0.006185994 24700 5.35E-05 24500 1.89E-04 24100
0.000171804 26400 0.004872465 25800 0.000723226
26700 0.00618367 24700 5.35E-05 24500 1.90E-04 24100
0.000172047 26400 0.004892448 25800 0.000724128
26700 0.006181348 24700 5.36E-05 24500 1.90E-04 24100
0.000172275 26400 0.004912547 25800 0.000725035
26700 0.006179064 24700 5.36E-05 24500 1.90E-04 24100
0.000172506 26400 0.004932771 25800 0.000725942
26700 0.006176779 24700 5.37E-05 24500 1.91E-04 24100
0.000172734 26400 0.00495314 25800 0.000726863
26700 0.006174542 24700 5.37E-05 24500 1.91E-04 24100
0.000172961 26400 0.004973622 25800 0.000727768
26700 0.006172293 24700 5.38E-05 24500 1.92E-04 24100
0.000173182 26400 0.004994262 25800 0.000728657
26700 0.006170041 24700 5.38E-05 24500 1.92E-04 24100
0.00017341 26400 0.005015018 25800 0.000729567
26700 0.006167839 24700 5.38E-05 24500 1.92E-04 24100
0.000173654 26400 0.005035886 25800 0.000730495
26700 0.006165618 24700 5.39E-05 24500 1.93E-04 24100
0.000173925 26400 0.005056859 25800 0.000731395
26700 0.0061634 24700 5.39E-05 24500 1.93E-04 24100
0.000174195 26400 0.005077997 25800 0.000732297
26700 0.006161257 24700 5.39E-05 24500 1.94E-04 24100
0.00017448 26400 0.005099261 25800 0.000733209
26700 0.006159069 24700 5.40E-05 24500 1.94E-04 24100
0.000174762 26400 0.005120671 25800 0.000734123
26700 0.006156909 24700 5.40E-05 24500 1.95E-04 24100
0.000175057 26400 0.005142209 25800 0.00073503
26700 0.00615475 24700 5.41E-05 24500 1.95E-04 24100
0.000175357 26400 0.005163856 25800 0.000735943
26700 0.006152608 24700 5.41E-05 24500 1.95E-04 24100
0.000175671 26400 0.005185649 25800 0.000736837
26700 0.006150453 24700 5.42E-05 24500 1.96E-04 24100
0.000175991 26400 0.005207547 25800 0.000737756

26700 0.006148308 24700 5.42E-05 24500 1.96E-04 24100
0.000176295 26400 0.005229584 25800 0.000738679
26700 0.006146179 24700 5.43E-05 24500 1.97E-04 24100
0.000176576 26400 0.005251765 25800 0.000739573
26700 0.006144072 24700 5.43E-05 24500 1.97E-04 24100
0.00017686 26400 0.005274058 25800 0.000740494
26800 0.00614195 24700 5.44E-05 24500 1.98E-04 24100
0.00017713 26400 0.005296478 25800 0.000741402
26800 0.006139834 24700 5.44E-05 24500 1.98E-04 24100
0.000177393 26400 0.005319044 25800 0.00074232
26800 0.006137731 24700 5.45E-05 24500 1.99E-04 24100
0.000177644 26400 0.005341741 25800 0.000743239
26800 0.006135613 24700 5.45E-05 24500 1.99E-04 24100
0.000177898 26400 0.005364605 25800 0.000744167
26800 0.00613353 24700 5.46E-05 24500 2.00E-04 24100
0.000178131 26400 0.005387592 25800 0.000745068
26800 0.006131395 24700 5.46E-05 24500 2.00E-04 24100
0.000178363 26400 0.005410731 25800 0.000745977
26800 0.006129296 24700 5.47E-05 24500 2.00E-04 24100
0.000178599 26400 0.005434006 25800 0.000746906
26800 0.006127209 24700 5.47E-05 24500 2.01E-04 24100
0.000178845 26400 0.005457429 25800 0.000747825
26800 0.006125101 24700 5.48E-05 24500 2.01E-04 24100
0.000179086 26400 0.005480947 25800 0.000748746
26800 0.006123008 24700 5.48E-05 24500 2.02E-04 24100
0.000179328 26400 0.005504663 25900 0.000749652
26800 0.006120931 24700 5.48E-05 24500 2.02E-04 24100
0.000179557 26400 0.005528541 25900 0.000750577
26800 0.006118818 24700 5.49E-05 24500 2.03E-04 24100
0.00017979 26400 0.005552583 25900 0.000751496
26800 0.006116714 24700 5.50E-05 24500 2.03E-04 24100
0.000180027 26400 0.005576775 25900 0.000752412
26800 0.006114628 24700 5.50E-05 24500 2.04E-04 24100
0.00018027 26400 0.005601108 25900 0.000753336
26800 0.006112516 24700 5.50E-05 24500 2.04E-04 24100
0.000180537 26400 0.005625597 25900 0.000754269
26800 0.006110405 24700 5.51E-05 24500 2.05E-04 24100
0.000180821 26400 0.005650238 25900 0.00075519
26800 0.006108274 24700 5.51E-05 24500 2.05E-04 24100
0.000181096 26400 0.005675044 25900 0.000756119
26800 0.00610615 24700 5.52E-05 24500 2.06E-04 24100
0.000181393 26400 0.005700009 25900 0.000757041
26800 0.006104019 24800 5.52E-05 24500 2.06E-04 24100
0.000181693 26400 0.005725126 25900 0.000757984
26800 0.006101837 24800 5.53E-05 24500 2.07E-04 24100
0.000181996 26400 0.005750418 25900 0.000758912
26800 0.00609966 24800 5.53E-05 24600 2.07E-04 24100
0.000182309 26400 0.005775855 25900 0.000759854
26800 0.00609749 24800 5.54E-05 24600 2.08E-04 24100
0.000182613 26400 0.005801473 25900 0.000760804
26800 0.006095257 24800 5.54E-05 24600 2.08E-04 24100
0.000182912 26400 0.005827266 25900 0.000761759
26800 0.006093024 24800 5.55E-05 24600 2.09E-04 24100
0.000183193 26400 0.005853218 25900 0.00076268
26800 0.00609076 24800 5.55E-05 24600 2.09E-04 24200
0.000183465 26400 0.005879351 25900 0.000763618
26800 0.006088476 24800 5.56E-05 24600 2.10E-04 24200
0.000183726 26400 0.005905649 25900 0.000764559
26800 0.006086207 24800 5.56E-05 24600 2.10E-04 24200
0.000184 26400 0.005932092 25900 0.000765502
26800 0.006083871 24800 5.57E-05 24600 2.11E-04 24200
0.000184266 26400 0.005958701 25900 0.00076646
26800 0.006081519 24800 5.57E-05 24600 2.11E-04 24200
0.000184512 26400 0.005985513 25900 0.000767397
26800 0.006079138 24800 5.58E-05 24600 2.12E-04 24200
0.000184746 26400 0.006012513 25900 0.000768358
26800 0.006076717 24800 5.58E-05 24600 2.13E-04 24200
0.000184987 26400 0.0060397 25900 0.000769311

26800 0.006074254 24800 5.59E-05 24600 2.13E-04 24200
0.000185239 26400 0.00606706 25900 0.000770252
26800 0.006071753 24800 5.59E-05 24600 2.14E-04 24200
0.0001855 26400 0.006094574 25900 0.000771193
26800 0.006069197 24800 5.60E-05 24600 2.14E-04 24200
0.000185773 26400 0.006122282 25900 0.000772153
26800 0.006066637 24800 5.60E-05 24600 2.15E-04 24200
0.000186085 26400 0.00615017 25900 0.000773081
26800 0.006064009 24800 5.61E-05 24600 2.15E-04 24200
0.000186378 26500 0.006178242 25900 0.00077407
26800 0.006061336 24800 5.61E-05 24600 2.16E-04 24200
0.000186687 26500 0.006206484 25900 0.000775007
26800 0.006058603 24800 5.62E-05 24600 2.16E-04 24200
0.000187007 26500 0.006234927 25900 0.000775958
26800 0.006055821 24800 5.62E-05 24600 2.17E-04 24200
0.000187357 26500 0.006263569 25900 0.000776907
26800 0.00605295 24800 5.62E-05 24600 2.17E-04 24200
0.000187681 26500 0.006292392 25900 0.000777879
26800 0.006050074 24800 5.63E-05 24600 2.18E-04 24200
0.000188032 26500 0.00632141 25900 0.000778847
26800 0.006047114 24800 5.63E-05 24600 2.19E-04 24200
0.00018837 26500 0.006350647 25900 0.000779821
26800 0.006044099 24800 5.64E-05 24600 2.19E-04 24200
0.000188705 26500 0.006380068 25900 0.000780782
26800 0.006041029 24800 5.64E-05 24600 2.20E-04 24200
0.000189007 26500 0.006409698 25900 0.000781726
26800 0.006037917 24800 5.65E-05 24600 2.20E-04 24200
0.000189328 26500 0.006439524 25900 0.000782708
26800 0.00603474 24800 5.65E-05 24600 2.21E-04 24200
0.000189629 26500 0.006469532 25900 0.00078368
26800 0.006031444 24800 5.65E-05 24600 2.21E-04 24200
0.000189938 26500 0.006499713 25900 0.000784668
26800 0.006028139 24800 5.66E-05 24600 2.22E-04 24200
0.000190237 26500 0.006530149 25900 0.000785632
26800 0.006024771 24800 5.66E-05 24600 2.23E-04 24200
0.000190534 26500 0.006560798 25900 0.000786628
26800 0.006021308 24800 5.67E-05 24600 2.23E-04 24200
0.000190854 26500 0.006591647 25900 0.000787611
26800 0.006017789 24800 5.67E-05 24600 2.24E-04 24200
0.000191171 26500 0.00662271 25900 0.000788579
26800 0.006014188 24800 5.68E-05 24600 2.24E-04 24200
0.000191507 26500 0.006653977 25900 0.000789579
26800 0.006010514 24800 5.68E-05 24600 2.25E-04 24200
0.000191848 26500 0.006685432 25900 0.000790554
26800 0.00600679 24800 5.69E-05 24600 2.26E-04 24200
0.000192188 26500 0.006717112 25900 0.000791546
26800 0.00600297 24800 5.69E-05 24600 2.26E-04 24200
0.000192496 26500 0.00674899 25900 0.000792529
26800 0.00599908 24800 5.70E-05 24600 2.27E-04 24200
0.000192816 26500 0.006781091 25900 0.00079351
26800 0.005995126 24800 5.71E-05 24600 2.27E-04 24200
0.000193101 26500 0.006813412 25900 0.000794506
26800 0.005991047 24800 5.71E-05 24600 2.28E-04 24200
0.000193371 26500 0.006845944 25900 0.000795509
26800 0.00598688 24800 5.72E-05 24600 2.29E-04 24200
0.00019363 26500 0.006878696 25900 0.000796522
26800 0.005982654 24800 5.72E-05 24600 2.29E-04 24200
0.00019389 26500 0.006911686 25900 0.000797513
26800 0.005978312 24800 5.73E-05 24600 2.30E-04 24200
0.000194157 26500 0.006944908 25900 0.000798539
26800 0.005973912 24800 5.74E-05 24600 2.30E-04 24200
0.000194423 26500 0.006978335 25900 0.000799525
26800 0.005969398 24800 5.74E-05 24600 2.31E-04 24200
0.000194696 26500 0.007012026 25900 0.000800512
26800 0.005964804 24800 5.75E-05 24600 2.32E-04 24200
0.00019497 26500 0.00704594 25900 0.000801515
26900 0.005960112 24800 5.75E-05 24600 2.32E-04 24200
0.000195259 26500 0.007080075 25900 0.00080253

26900 0.005955311 24800 5.76E-05 24600 2.33E-04 24200
0.000195564 26500 0.007114426 25900 0.000803539
26900 0.005950423 24800 5.76E-05 24600 2.34E-04 24200
0.000195866 26500 0.00714905 25900 0.00080454
26900 0.005945458 24800 5.77E-05 24600 2.34E-04 24200
0.000196175 26500 0.007183915 25900 0.000805553
26900 0.005940331 24800 5.78E-05 24600 2.35E-04 24200
0.000196501 26500 0.007219025 25900 0.000806574
26900 0.005935118 24800 5.78E-05 24600 2.36E-04 24200
0.000196812 26500 0.007254406 25900 0.000807581
26900 0.005929825 24800 5.79E-05 24600 2.36E-04 24200
0.000197117 26500 0.007289976 25900 0.000808584
26900 0.005924404 24800 5.79E-05 24600 2.37E-04 24200
0.000197455 26500 0.007325834 25900 0.000809593
26900 0.00591886 24800 5.80E-05 24600 2.38E-04 24200
0.000197764 26500 0.007361953 25900 0.000810612
26900 0.005913203 24800 5.80E-05 24600 2.39E-04 24200
0.000198085 26500 0.007398323 26000 0.000811618
26900 0.005907445 24800 5.81E-05 24600 2.39E-04 24200
0.00019839 26500 0.007434972 26000 0.000812621
26900 0.005901596 24800 5.82E-05 24600 2.40E-04 24200
0.000198695 26500 0.007471831 26000 0.000813633
26900 0.00589557 24800 5.82E-05 24600 2.41E-04 24200
0.000198986 26500 0.00750897 26000 0.000814648
26900 0.005889465 24800 5.82E-05 24600 2.41E-04 24200
0.000199276 26500 0.007546382 26000 0.000815699
26900 0.005883245 24800 5.83E-05 24600 2.42E-04 24200
0.000199573 26500 0.007584081 26000 0.000816717
26900 0.005876879 24800 5.83E-05 24600 2.43E-04 24200
0.000199877 26500 0.007622063 26000 0.000817749
26900 0.005870399 24800 5.84E-05 24600 2.43E-04 24200
0.000200149 26500 0.007660308 26000 0.000818768
26900 0.005863796 24800 5.84E-05 24600 2.44E-04 24200
0.000200451 26500 0.007698837 26000 0.000819774
26900 0.005857052 24900 5.85E-05 24600 2.45E-04 24200
0.000200729 26500 0.007737632 26000 0.000820808
26900 0.005850228 24900 5.85E-05 24600 2.46E-04 24200
0.000201046 26500 0.007776704 26000 0.000821837
26900 0.005843217 24900 5.86E-05 24700 2.46E-04 24200
0.000201355 26500 0.007816031 26000 0.0008229
26900 0.005836093 24900 5.86E-05 24700 2.47E-04 24200
0.000201692 26500 0.007855701 26000 0.000823929
26900 0.005828882 24900 5.87E-05 24700 2.48E-04 24200
0.000202002 26500 0.007895662 26000 0.000824975
26900 0.005821519 24900 5.87E-05 24700 2.49E-04 24200
0.000202358 26500 0.007935932 26000 0.000826029
26900 0.005814019 24900 5.88E-05 24700 2.49E-04 24300
0.000202674 26500 0.007976492 26000 0.000827075
26900 0.005806397 24900 5.88E-05 24700 2.50E-04 24300
0.00020301 26500 0.008017346 26000 0.000828124
26900 0.00579863 24900 5.89E-05 24700 2.51E-04 24300
0.000203338 26500 0.008058512 26000 0.000829175
26900 0.005790739 24900 5.89E-05 24700 2.52E-04 24300
0.000203679 26500 0.008099968 26000 0.000830248
26900 0.005782698 24900 5.90E-05 24700 2.53E-04 24300
0.000204012 26500 0.008141698 26000 0.000831313
26900 0.005774549 24900 5.91E-05 24700 2.53E-04 24300
0.00020437 26500 0.008183779 26000 0.00083236
26900 0.00576626 24900 5.91E-05 24700 2.54E-04 24300
0.000204714 26500 0.008226139 26000 0.000833436
26900 0.005757804 24900 5.92E-05 24700 2.55E-04 24300
0.00020509 26500 0.008268842 26000 0.000834512
26900 0.005749226 24900 5.92E-05 24700 2.56E-04 24300
0.000205453 26500 0.008311864 26000 0.000835593
26900 0.005740525 24900 5.93E-05 24700 2.57E-04 24300
0.000205849 26500 0.008355179 26000 0.000836673
26900 0.005731661 24900 5.93E-05 24700 2.57E-04 24300
0.000206234 26500 0.008398848 26000 0.000837784

26900 0.005722687 24900 5.94E-05 24700 2.58E-04 24300
0.000206637 26600 0.008442847 26000 0.000838851
26900 0.005713552 24900 5.94E-05 24700 2.59E-04 24300
0.000207014 26600 0.008487174 26000 0.000839945
26900 0.005704309 24900 5.95E-05 24700 2.60E-04 24300
0.000207389 26600 0.008531832 26000 0.000841024
26900 0.005694942 24900 5.95E-05 24700 2.61E-04 24300
0.000207763 26600 0.008576821 26000 0.000842118
26900 0.005685425 24900 5.96E-05 24700 2.62E-04 24300
0.00020815 26600 0.008622109 26000 0.000843241
26900 0.005675768 24900 5.96E-05 24700 2.62E-04 24300
0.000208526 26600 0.008667794 26000 0.000844314
26900 0.005666014 24900 5.97E-05 24700 2.63E-04 24300
0.000208877 26600 0.00871388 26000 0.00084543
26900 0.005656097 24900 5.97E-05 24700 2.64E-04 24300
0.000209243 26600 0.008760301 26000 0.000846528
26900 0.005646056 24900 5.98E-05 24700 2.65E-04 24300
0.000209599 26600 0.008807063 26000 0.000847621
26900 0.005635888 24900 5.99E-05 24700 2.66E-04 24300
0.000209955 26600 0.0088542 26000 0.00084873
26900 0.005625573 24900 5.99E-05 24700 2.67E-04 24300
0.000210322 26600 0.00890172 26000 0.000849821
26900 0.00561516 24900 6.00E-05 24700 2.68E-04 24300
0.000210701 26600 0.008949604 26000 0.000850949
26900 0.005604592 24900 6.00E-05 24700 2.69E-04 24300
0.000211071 26600 0.008997856 26000 0.000852057
26900 0.005593938 24900 6.01E-05 24700 2.69E-04 24300
0.000211461 26600 0.009046518 26000 0.000853149
26900 0.005583125 24900 6.01E-05 24700 2.70E-04 24300
0.000211843 26600 0.00909555 26000 0.000854264
26900 0.005572196 24900 6.01E-05 24700 2.71E-04 24300
0.00021224 26600 0.009145017 26000 0.000855385
26900 0.00556111 24900 6.02E-05 24700 2.72E-04 24300
0.000212625 26600 0.009194843 26000 0.000856542
26900 0.005549965 24900 6.02E-05 24700 2.73E-04 24300
0.000213023 26600 0.009245091 26000 0.000857651
26900 0.005538658 24900 6.03E-05 24700 2.74E-04 24300
0.000213439 26600 0.009295763 26000 0.000858793
26900 0.005527255 24900 6.03E-05 24700 2.75E-04 24300
0.000213827 26600 0.009346841 26000 0.000859917
26900 0.00551573 24900 6.04E-05 24700 2.76E-04 24300
0.000214233 26600 0.00939835 26000 0.00086102
26900 0.005504093 24900 6.04E-05 24700 2.77E-04 24300
0.000214613 26600 0.009450267 26000 0.000862143
26900 0.005492376 24900 6.05E-05 24700 2.78E-04 24300
0.000215012 26600 0.009502576 26000 0.000863284
26900 0.005480521 24900 6.05E-05 24700 2.79E-04 24300
0.000215399 26600 0.009555286 26000 0.000864426
26900 0.00546856 24900 6.06E-05 24700 2.80E-04 24300
0.000215772 26600 0.009608485 26000 0.00086556
26900 0.00545654 24900 6.06E-05 24700 2.81E-04 24300
0.000216115 26600 0.009662116 26000 0.000866698
26900 0.005444387 24900 6.07E-05 24700 2.82E-04 24300
0.000216469 26600 0.009716184 26000 0.000867848
26900 0.005432149 24900 6.08E-05 24700 2.83E-04 24300
0.000216792 26600 0.009770691 26000 0.000869002
26900 0.005419826 24900 6.08E-05 24700 2.84E-04 24300
0.000217129 26600 0.009825631 26000 0.000870142
27000 0.005407398 24900 6.09E-05 24700 2.85E-04 24300
0.000217461 26600 0.009881026 26000 0.000871292
27000 0.00539488 24900 6.10E-05 24700 2.86E-04 24300
0.000217787 26600 0.00993686 26000 0.00087244
27000 0.005382304 24900 6.10E-05 24700 2.87E-04 24300
0.000218112 26600 0.009993139 26000 0.000873601
27000 0.00536964 24900 6.11E-05 24700 2.88E-04 24300
0.000218446 26600 0.01004991 26000 0.000874733
27000 0.005356902 24900 6.12E-05 24700 2.89E-04 24300
0.000218767 26600 0.01010713 26000 0.000875881

27000 0.005344079 24900 6.12E-05 24700 2.90E-04 24300
0.000219121 26600 0.01016483 26000 0.000877046
27000 0.005331163 24900 6.13E-05 24700 2.91E-04 24300
0.00021948 26600 0.01022301 26000 0.000878222
27000 0.005318197 24900 6.14E-05 24700 2.92E-04 24300
0.000219863 26600 0.01028166 26000 0.000879388
27000 0.005305141 24900 6.14E-05 24700 2.93E-04 24300
0.000220242 26600 0.01034082 26000 0.000880579
27000 0.005292063 24900 6.15E-05 24700 2.94E-04 24300
0.000220609 26600 0.01040048 26100 0.000881736
27000 0.005278897 24900 6.15E-05 24700 2.96E-04 24300
0.000220972 26600 0.01046065 26100 0.000882889
27000 0.005265681 24900 6.16E-05 24700 2.97E-04 24300
0.000221324 26600 0.01052131 26100 0.00088405
27000 0.00525244 24900 6.16E-05 24700 2.98E-04 24300
0.000221709 26600 0.01058245 26100 0.00088522
27000 0.005239106 24900 6.17E-05 24700 2.99E-04 24300
0.000222099 26600 0.01064407 26100 0.000886424
27000 0.005225736 24900 6.17E-05 24700 3.00E-04 24300
0.000222502 26600 0.01070628 26100 0.000887597
27000 0.00521235 24900 6.18E-05 24700 3.01E-04 24300
0.00022291 26600 0.01076904 26100 0.000888795
27000 0.005198895 24900 6.19E-05 24700 3.02E-04 24300
0.000223324 26600 0.01083234 26100 0.000889977
27000 0.005185409 24900 6.19E-05 24700 3.04E-04 24300
0.00022375 26600 0.0108962 26100 0.000891158
27000 0.005171917 25000 6.20E-05 24700 3.05E-04 24300
0.000224175 26600 0.0109606 26100 0.000892357
27000 0.005158346 25000 6.20E-05 24700 3.06E-04 24300
0.000224605 26600 0.01102557 26100 0.000893541
27000 0.005144765 25000 6.21E-05 24800 3.07E-04 24300
0.000225056 26600 0.01109109 26100 0.000894725
27000 0.005131165 25000 6.22E-05 24800 3.09E-04 24300
0.000225482 26600 0.01115718 26100 0.000895945
27000 0.005117535 25000 6.22E-05 24800 3.10E-04 24300
0.000225921 26600 0.01122386 26100 0.000897129
27000 0.005103907 25000 6.23E-05 24800 3.11E-04 24300
0.000226353 26600 0.01129112 26100 0.000898335
27000 0.005090213 25000 6.24E-05 24800 3.12E-04 24400
0.000226783 26600 0.01135899 26100 0.000899537
27000 0.00507653 25000 6.24E-05 24800 3.14E-04 24400
0.0002272 26600 0.01142747 26100 0.00090078
27000 0.00506285 25000 6.25E-05 24800 3.15E-04 24400
0.000227626 26600 0.01149655 26100 0.000901978
27000 0.005049132 25000 6.25E-05 24800 3.16E-04 24400
0.000228055 26600 0.01156627 26100 0.000903216
27000 0.005035419 25000 6.26E-05 24800 3.17E-04 24400
0.000228475 26600 0.01163664 26100 0.000904438
27000 0.005021716 25000 6.27E-05 24800 3.19E-04 24400
0.0002289 26600 0.01170763 26100 0.000905646
27000 0.005008017 25000 6.27E-05 24800 3.20E-04 24400
0.000229331 26600 0.01177927 26100 0.000906879
27000 0.004994336 25000 6.28E-05 24800 3.21E-04 24400
0.000229762 26600 0.01185153 26100 0.000908089
27000 0.004980644 25000 6.29E-05 24800 3.23E-04 24400
0.000230197 26600 0.0119244 26100 0.00090934
27000 0.004966971 25000 6.30E-05 24800 3.24E-04 24400
0.000230632 26600 0.01199798 26100 0.000910551
27000 0.004953335 25000 6.31E-05 24800 3.26E-04 24400
0.000231038 26600 0.01207225 26100 0.0009118
27000 0.004939723 25000 6.32E-05 24800 3.27E-04 24400
0.000231468 26700 0.01214717 26100 0.000913036
27000 0.004926116 25000 6.33E-05 24800 3.28E-04 24400
0.000231871 26700 0.01222276 26100 0.000914255
27000 0.004912563 25000 6.34E-05 24800 3.30E-04 24400
0.00023226 26700 0.01229903 26100 0.00091551
27000 0.004899032 25000 6.35E-05 24800 3.31E-04 24400
0.000232658 26700 0.01237601 26100 0.000916729

27000 0.004885521 25000 6.36E-05 24800 3.33E-04 24400
0.000233039 26700 0.01245364 26100 0.000917971
27000 0.004872046 25000 6.37E-05 24800 3.34E-04 24400
0.000233426 26700 0.01253199 26100 0.000919223
27000 0.004858634 25000 6.38E-05 24800 3.36E-04 24400
0.000233807 26700 0.01261104 26100 0.000920453
27000 0.004845273 25000 6.39E-05 24800 3.37E-04 24400
0.000234203 26700 0.01269081 26100 0.000921684
27000 0.00483194 25000 6.41E-05 24800 3.39E-04 24400
0.000234597 26700 0.01277131 26100 0.000922927
27000 0.004818648 25000 6.42E-05 24800 3.40E-04 24400
0.000234996 26700 0.01285254 26100 0.000924203
27000 0.00480543 25000 6.43E-05 24800 3.42E-04 24400
0.000235417 26700 0.01293451 26100 0.000925426
27000 0.004792256 25000 6.43E-05 24800 3.43E-04 24400
0.000235839 26700 0.01301726 26100 0.000926719
27000 0.004779131 25000 6.43E-05 24800 3.45E-04 24400
0.000236273 26700 0.01310079 26100 0.000927943
27000 0.004766094 25000 6.43E-05 24800 3.46E-04 24400
0.000236733 26700 0.01318508 26100 0.000929178
27000 0.004753116 25000 6.43E-05 24800 3.48E-04 24400
0.000237198 26700 0.01327017 26100 0.000930445
27000 0.004740244 25000 6.43E-05 24800 3.50E-04 24400
0.000237685 26700 0.013356 26100 0.00093168
27000 0.004727394 25000 6.42E-05 24800 3.51E-04 24400
0.000238172 26700 0.01344259 26100 0.000932966
27000 0.004714637 25000 6.42E-05 24800 3.53E-04 24400
0.000238677 26700 0.01353007 26100 0.000934206
27000 0.004701989 25000 6.42E-05 24800 3.55E-04 24400
0.000239167 26700 0.01361839 26100 0.000935469
27000 0.004689417 25000 6.42E-05 24800 3.56E-04 24400
0.000239641 26700 0.01370753 26100 0.000936733
27000 0.004676912 25000 6.43E-05 24800 3.58E-04 24400
0.000240109 26700 0.01379753 26100 0.00093798
27000 0.004664502 25000 6.43E-05 24800 3.60E-04 24400
0.000240565 26700 0.01388839 26100 0.000939243
27000 0.004652149 25000 6.43E-05 24800 3.61E-04 24400
0.000241034 26700 0.0139801 26100 0.000940495
27000 0.004639939 25000 6.44E-05 24800 3.63E-04 24400
0.000241488 26700 0.01407271 26100 0.000941745
27000 0.004627777 25000 6.44E-05 24800 3.65E-04 24400
0.000241937 26700 0.0141662 26100 0.000943037
27000 0.004615735 25000 6.45E-05 24800 3.67E-04 24400
0.000242393 26700 0.0142606 26100 0.000944289
27000 0.004603815 25000 6.45E-05 24800 3.69E-04 24400
0.000242854 26700 0.01435591 26100 0.00094556
27000 0.004591931 25000 6.46E-05 24800 3.70E-04 24400
0.000243296 26700 0.01445216 26100 0.000946818
27000 0.00458019 25000 6.46E-05 24800 3.72E-04 24400
0.000243737 26700 0.01454936 26100 0.000948117
27100 0.004568544 25000 6.47E-05 24800 3.74E-04 24400
0.000244165 26700 0.01464753 26100 0.000949374
27100 0.004556988 25000 6.47E-05 24800 3.76E-04 24400
0.000244586 26700 0.01474669 26100 0.000950673
27100 0.004545552 25000 6.48E-05 24800 3.78E-04 24400
0.000245005 26700 0.01484684 26100 0.000951933
27100 0.00453421 25000 6.49E-05 24800 3.80E-04 24400
0.000245411 26700 0.01494801 26100 0.000953191
27100 0.004522985 25000 6.49E-05 24800 3.82E-04 24400
0.000245831 26700 0.0150502 26100 0.000954483
27100 0.004511865 25000 6.50E-05 24800 3.84E-04 24400
0.000246242 26700 0.01515336 26100 0.000955751
27100 0.004500847 25000 6.50E-05 24800 3.86E-04 24400
0.000246666 26700 0.01525752 26100 0.000957064
27100 0.004489926 25000 6.51E-05 24800 3.88E-04 24400
0.000247097 26700 0.01536281 26100 0.000958338
27100 0.004479155 25000 6.51E-05 24800 3.90E-04 24400
0.000247519 26700 0.01546918 26100 0.000959634

27100 0.004468449 25000 6.52E-05 24800 3.92E-04 24400
0.000247958 26700 0.01557664 26200 0.000960932
27100 0.004457866 25000 6.53E-05 24800 3.94E-04 24400
0.000248378 26700 0.01568519 26200 0.000962201
27100 0.004447428 25000 6.53E-05 24800 3.96E-04 24400
0.000248801 26700 0.01579484 26200 0.000963494
27100 0.004437079 25000 6.54E-05 24800 3.98E-04 24400
0.000249258 26700 0.0159056 26200 0.000964779
27100 0.00442685 25000 6.55E-05 24800 4.00E-04 24400
0.000249688 26700 0.01601751 26200 0.000966079
27100 0.004416694 25000 6.55E-05 24800 4.02E-04 24400
0.000250158 26700 0.01613055 26200 0.000967375
27100 0.004406692 25000 6.56E-05 24800 4.05E-04 24400
0.000250624 26700 0.01624477 26200 0.000968673
27100 0.00439677 25000 6.56E-05 24800 4.07E-04 24400
0.000251108 26700 0.01636016 26200 0.000969957
27100 0.004386965 25000 6.57E-05 24800 4.09E-04 24400
0.000251617 26700 0.01647673 26200 0.000971264
27100 0.004377273 25100 6.58E-05 24800 4.11E-04 24400
0.000252122 26700 0.01659452 26200 0.000972587
27100 0.004367724 25100 6.59E-05 24800 4.14E-04 24400
0.000252639 26700 0.01671354 26200 0.000973907
27100 0.00435825 25100 6.59E-05 24900 4.16E-04 24400
0.000253156 26700 0.01683381 26200 0.000975241
27100 0.004348899 25100 6.60E-05 24900 4.18E-04 24400
0.000253655 26700 0.01695534 26200 0.000976546
27100 0.004339651 25100 6.61E-05 24900 4.21E-04 24400
0.000254144 26700 0.01707812 26200 0.000977853
27100 0.004330497 25100 6.61E-05 24900 4.23E-04 24400
0.000254613 26700 0.01720221 26200 0.00097918
27100 0.004321498 25100 6.62E-05 24900 4.26E-04 24500
0.000255062 26700 0.01732756 26200 0.000980515
27100 0.004312593 25100 6.63E-05 24900 4.28E-04 24500
0.00025552 26700 0.01745415 26200 0.000981858
27100 0.0043038 25100 6.63E-05 24900 4.31E-04 24500
0.000255969 26700 0.01758217 26200 0.0009832
27100 0.004295134 25100 6.64E-05 24900 4.33E-04 24500
0.000256398 26700 0.01771156 26200 0.000984549
27100 0.00428655 25100 6.65E-05 24900 4.36E-04 24500
0.000256821 26700 0.01784231 26200 0.000985893
27100 0.004278092 25100 6.65E-05 24900 4.38E-04 24500
0.000257253 26700 0.01797445 26200 0.000987242
27100 0.004269768 25100 6.66E-05 24900 4.41E-04 24500
0.00025769 26700 0.018108 26200 0.000988592
27100 0.004261482 25100 6.66E-05 24900 4.44E-04 24500
0.000258145 26700 0.01824296 26200 0.000989945
27100 0.004253355 25100 6.67E-05 24900 4.46E-04 24500
0.000258584 26700 0.01837939 26200 0.000991303
27100 0.004245298 25100 6.68E-05 24900 4.49E-04 24500
0.000259038 26700 0.01851728 26200 0.000992673
27100 0.004237377 25100 6.68E-05 24900 4.52E-04 24500
0.00025951 26700 0.01865667 26200 0.000994043
27100 0.00422954 25100 6.69E-05 24900 4.55E-04 24500
0.000259985 26800 0.01879758 26200 0.000995426
27100 0.004221794 25100 6.70E-05 24900 4.58E-04 24500
0.000260462 26800 0.01894004 26200 0.000996786
27100 0.004214131 25100 6.71E-05 24900 4.61E-04 24500
0.000260975 26800 0.01908407 26200 0.000998178
27100 0.004206616 25100 6.71E-05 24900 4.63E-04 24500
0.00026148 26800 0.01922972 26200 0.000999559
27100 0.00419915 25100 6.72E-05 24900 4.66E-04 24500
0.000262024 26800 0.019377 26200 0.001000946
27100 0.004191798 25100 6.73E-05 24900 4.69E-04 24500
0.000262568 26800 0.01952595 26200 0.001002327
27100 0.004184518 25100 6.74E-05 24900 4.72E-04 24500
0.000263125 26800 0.01967657 26200 0.001003701
27100 0.004177323 25100 6.74E-05 24900 4.76E-04 24500
0.000263693 26800 0.0198289 26200 0.001005092

27100 0.00417025 25100 6.75E-05 24900 4.79E-04 24500
0.000264259 26800 0.01998291 26200 0.001006491
27100 0.004163234 25100 6.76E-05 24900 4.82E-04 24500
0.000264855 26800 0.02013862 26200 0.001007891
27100 0.004156312 25100 6.76E-05 24900 4.85E-04 24500
0.000265418 26800 0.0202962 26200 0.001009272
27100 0.004149496 25100 6.77E-05 24900 4.88E-04 24500
0.000265986 26800 0.02045561 26200 0.001010665
27100 0.004142745 25100 6.77E-05 24900 4.92E-04 24500
0.000266547 26800 0.02061686 26200 0.00101206
27100 0.004136079 25100 6.78E-05 24900 4.95E-04 24500
0.000267109 26800 0.02077997 26200 0.001013463
27100 0.004129508 25100 6.78E-05 24900 4.98E-04 24500
0.000267662 26800 0.02094498 26200 0.001014849
27100 0.00412298 25100 6.79E-05 24900 5.02E-04 24500
0.000268206 26800 0.0211119 26200 0.001016231
27100 0.004116551 25100 6.79E-05 24900 5.05E-04 24500
0.000268755 26800 0.02128078 26200 0.001017638
27100 0.004110184 25100 6.80E-05 24900 5.09E-04 24500
0.000269283 26800 0.02145164 26200 0.001019045
27100 0.004103905 25100 6.80E-05 24900 5.12E-04 24500
0.000269811 26800 0.02162453 26200 0.001020428
27100 0.004097682 25100 6.81E-05 24900 5.16E-04 24500
0.000270336 26800 0.02179945 26200 0.001021821
27100 0.004091543 25100 6.82E-05 24900 5.20E-04 24500
0.000270854 26800 0.02197647 26200 0.001023217
27100 0.004085439 25100 6.82E-05 24900 5.24E-04 24500
0.000271368 26800 0.02215558 26200 0.001024637
27100 0.004079455 25100 6.83E-05 24900 5.27E-04 24500
0.000271896 26800 0.02233686 26200 0.001026042
27100 0.004073516 25100 6.84E-05 24900 5.31E-04 24500
0.000272412 26800 0.02252033 26200 0.001027459
27100 0.004067629 25100 6.85E-05 24900 5.35E-04 24500
0.00027292 26800 0.02270603 26200 0.001028848
27100 0.004061825 25100 6.85E-05 24900 5.39E-04 24500
0.000273436 26800 0.02289397 26200 0.001030237
27100 0.004056108 25100 6.86E-05 24900 5.43E-04 24500
0.000273938 26800 0.02308417 26200 0.001031653
27100 0.00405043 25100 6.87E-05 24900 5.47E-04 24500
0.000274467 26800 0.02327665 26200 0.001033064
27100 0.004044798 25100 6.87E-05 24900 5.51E-04 24500
0.000275001 26800 0.02347143 26200 0.001034491
27200 0.004039263 25100 6.88E-05 24900 5.56E-04 24500
0.000275511 26800 0.02366867 26200 0.001035895
27200 0.004033804 25100 6.89E-05 24900 5.60E-04 24500
0.000276061 26800 0.02386835 26200 0.001037333
27200 0.004028375 25100 6.89E-05 24900 5.64E-04 24500
0.000276589 26800 0.02407049 26200 0.001038754
27200 0.004023006 25100 6.90E-05 24900 5.69E-04 24500
0.00027713 26800 0.02427511 26200 0.001040171
27200 0.0040177 25100 6.91E-05 24900 5.73E-04 24500
0.000277693 26800 0.02448228 26200 0.001041595
27200 0.004012449 25100 6.91E-05 24900 5.78E-04 24500
0.000278268 26800 0.024692 26200 0.001043016
27200 0.004007251 25100 6.92E-05 24900 5.82E-04 24500
0.000278853 26800 0.02490436 26200 0.001044442
27200 0.004002119 25100 6.93E-05 24900 5.87E-04 24500
0.000279454 26800 0.02511942 26200 0.001045908
27200 0.003997021 25100 6.93E-05 24900 5.92E-04 24500
0.000280047 26800 0.02533718 26200 0.001047336
27200 0.003992018 25100 6.94E-05 24900 5.97E-04 24500
0.00028066 26800 0.02555769 26300 0.001048778
27200 0.00398702 25100 6.95E-05 24900 6.02E-04 24500
0.000281273 26800 0.02578102 26300 0.001050226
27200 0.003982088 25100 6.95E-05 24900 6.07E-04 24500
0.000281911 26800 0.02600717 26300 0.001051699
27200 0.003977227 25100 6.96E-05 24900 6.12E-04 24500
0.000282548 26800 0.02623627 26300 0.00105315

27200 0.003972392 25100 6.97E-05 24900 6.17E-04 24500
0.000283166 26800 0.02646832 26300 0.001054628
27200 0.003967616 25100 6.97E-05 24900 6.22E-04 24500
0.000283773 26800 0.02670339 26300 0.001056086
27200 0.003962897 25100 6.98E-05 24900 6.28E-04 24500
0.000284386 26800 0.02694155 26300 0.001057541
27200 0.003958223 25100 6.98E-05 24900 6.33E-04 24500
0.000284999 26800 0.02718278 26300 0.001059021
27200 0.003953592 25100 6.99E-05 24900 6.39E-04 24500
0.000285617 26800 0.02742713 26300 0.001060479
27200 0.00394901 25200 7.00E-05 24900 6.44E-04 24500
0.000286255 26800 0.02767466 26300 0.001061981
27200 0.003944471 25200 7.00E-05 24900 6.50E-04 24500
0.000286873 26800 0.02792554 26300 0.001063468
27200 0.003940001 25200 7.01E-05 25000 6.56E-04 24500
0.000287522 26800 0.02817976 26300 0.00106496
27200 0.003935532 25200 7.01E-05 25000 6.62E-04 24500
0.000288165 26800 0.0284374 26300 0.001066461
27200 0.003931115 25200 7.02E-05 25000 6.68E-04 24500
0.000288819 26800 0.02869844 26300 0.00106795
27200 0.003926768 25200 7.03E-05 25000 6.74E-04 24500
0.000289465 26800 0.02896298 26300 0.001069432
27200 0.003922445 25200 7.04E-05 25000 6.80E-04 24600
0.000290123 26800 0.02923111 26300 0.001070929
27200 0.003918133 25200 7.04E-05 25000 6.87E-04 24600
0.000290771 26800 0.02950284 26300 0.001072423
27200 0.003913849 25200 7.05E-05 25000 6.93E-04 24600
0.000291407 26800 0.02977827 26300 0.001073937
27200 0.003909622 25200 7.05E-05 25000 7.00E-04 24600
0.000292029 26800 0.03005747 26300 0.001075429
27200 0.00390542 25200 7.06E-05 25000 7.07E-04 24600
0.000292663 26800 0.03034049 26300 0.001076941
27200 0.003901223 25200 7.07E-05 25000 7.14E-04 24600
0.000293303 26800 0.0306274 26300 0.001078451
27200 0.003897069 25200 7.08E-05 25000 7.21E-04 24600
0.00029396 26800 0.03091826 26300 0.001079981
27200 0.003892985 25200 7.09E-05 25000 7.28E-04 24600
0.000294626 26800 0.0312132 26300 0.001081491
27200 0.003888865 25200 7.09E-05 25000 7.35E-04 24600
0.000295283 26800 0.03151223 26300 0.001083037
27200 0.003884808 25200 7.10E-05 25000 7.42E-04 24600
0.000295955 26800 0.03181547 26300 0.001084559
27200 0.00388075 25200 7.11E-05 25000 7.50E-04 24600
0.000296622 26800 0.03212297 26300 0.001086075
27200 0.003876736 25200 7.12E-05 25000 7.57E-04 24600
0.000297274 26900 0.03243484 26300 0.001087616
27200 0.003872768 25200 7.12E-05 25000 7.65E-04 24600
0.000297935 26900 0.03275105 26300 0.001089143
27200 0.0038688 25200 7.13E-05 25000 7.73E-04 24600
0.000298596 26900 0.03307172 26300 0.00109072
27200 0.003864851 25200 7.14E-05 25000 7.81E-04 24600
0.00029925 26900 0.03339705 26300 0.001092269
27200 0.003860956 25200 7.14E-05 25000 7.90E-04 24600
0.000299904 26900 0.03372708 26300 0.001093818
27200 0.003857065 25200 7.15E-05 25000 7.98E-04 24600
0.000300557 26900 0.03406189 26300 0.001095382
27200 0.003853214 25200 7.16E-05 25000 8.07E-04 24600
0.000301196 26900 0.03440152 26300 0.00109694
27200 0.003849378 25200 7.17E-05 25000 8.15E-04 24600
0.000301864 26900 0.03474609 26300 0.001098487
27200 0.00384557 25200 7.17E-05 25000 8.24E-04 24600
0.000302533 26900 0.0350957 26300 0.001100059
27200 0.003841768 25200 7.18E-05 25000 8.34E-04 24600
0.000303204 26900 0.03545044 26300 0.001101625
27200 0.003837996 25200 7.19E-05 25000 8.43E-04 24600
0.000303868 26900 0.03581041 26300 0.001103205
27200 0.003834253 25200 7.20E-05 25000 8.52E-04 24600
0.000304539 26900 0.03617573 26300 0.001104768

27200 0.003830525 25200 7.20E-05 25000 8.62E-04 24600
0.000305225 26900 0.03654649 26300 0.001106357
27200 0.003826775 25200 7.21E-05 25000 8.72E-04 24600
0.000305914 26900 0.03692282 26300 0.001107938
27200 0.003823054 25200 7.22E-05 25000 8.82E-04 24600
0.000306614 26900 0.03730477 26300 0.001109537
27200 0.003819382 25200 7.23E-05 25000 8.92E-04 24600
0.000307308 26900 0.03769253 26300 0.00111114
27200 0.003815703 25200 7.23E-05 25000 9.03E-04 24600
0.000307995 26900 0.03808619 26300 0.00111276
27200 0.003812039 25200 7.24E-05 25000 9.14E-04 24600
0.000308687 26900 0.03848591 26300 0.001114351
27200 0.003808367 25200 7.25E-05 25000 9.25E-04 24600
0.000309382 26900 0.03889171 26300 0.001115951
27200 0.003804734 25200 7.26E-05 25000 9.36E-04 24600
0.00031006 26900 0.03930378 26300 0.001117576
27200 0.003801119 25200 7.26E-05 25000 9.47E-04 24600
0.000310735 26900 0.03972218 26300 0.001119185
27200 0.003797503 25200 7.27E-05 25000 9.59E-04 24600
0.000311428 26900 0.04014699 26300 0.001120811
27200 0.003793903 25200 7.28E-05 25000 9.71E-04 24600
0.000312112 26900 0.04057854 26300 0.001122445
27200 0.003790325 25200 7.29E-05 25000 9.83E-04 24600
0.00031281 26900 0.04101689 26300 0.00112406
27200 0.003786754 25200 7.29E-05 25000 9.96E-04 24600
0.000313492 26900 0.04146215 26300 0.00112569
27200 0.003783182 25200 7.30E-05 25000 1.01E-03 24600
0.000314199 26900 0.04191443 26300 0.00112731
27200 0.003779644 25200 7.31E-05 25000 1.02E-03 24600
0.000314899 26900 0.04237392 26300 0.001128947
27200 0.003776071 25200 7.32E-05 25000 1.03E-03 24600
0.000315624 26900 0.04284072 26300 0.001130562
27200 0.003772521 25200 7.32E-05 25000 1.05E-03 24600
0.000316345 26900 0.04331502 26300 0.001132186
27300 0.003768972 25200 7.33E-05 25000 1.06E-03 24600
0.000317061 26900 0.04379696 26300 0.001133843
27300 0.003765439 25200 7.34E-05 25000 1.08E-03 24600
0.000317801 26900 0.04428671 26300 0.001135455
27300 0.003761901 25200 7.35E-05 25000 1.09E-03 24600
0.000318528 26900 0.04478443 26300 0.001137087
27300 0.00375836 25200 7.36E-05 25000 1.11E-03 24600
0.00031927 26900 0.04529033 26300 0.001138747
27300 0.003754803 25200 7.37E-05 25000 1.12E-03 24600
0.000320014 26900 0.0458045 26300 0.0011404
27300 0.003751293 25200 7.38E-05 25000 1.14E-03 24600
0.000320765 26900 0.0463272 26300 0.001142055
27300 0.003747772 25200 7.38E-05 25000 1.15E-03 24600
0.000321502 26900 0.0468586 26300 0.001143709
27300 0.003744233 25200 7.39E-05 25000 1.17E-03 24600
0.000322245 26900 0.04739891 26300 0.001145369
27300 0.003740697 25200 7.40E-05 25000 1.19E-03 24600
0.000322971 26900 0.04794825 26300 0.001146993
27300 0.003737157 25200 7.41E-05 25000 1.20E-03 24600
0.000323696 26900 0.04850689 26400 0.001148652
27300 0.003733654 25200 7.42E-05 25000 1.22E-03 24600
0.00032444 26900 0.04907491 26400 0.001150314
27300 0.003730114 25200 7.42E-05 25000 1.24E-03 24600
0.000325172 26900 0.04965256 26400 0.001151986
27300 0.003726576 25200 7.43E-05 25000 1.26E-03 24600
0.000325899 26900 0.05024019 26400 0.001153643
27300 0.003723071 25200 7.43E-05 25000 1.28E-03 24600
0.000326637 26900 0.05083795 26400 0.001155317
27300 0.003719529 25200 7.44E-05 25000 1.30E-03 24600
0.000327391 26900 0.05144603 26400 0.001156976
27300 0.003716043 25200 7.44E-05 25000 1.32E-03 24600
0.000328159 26900 0.05206468 26400 0.001158659
27300 0.003712524 25200 7.45E-05 25000 1.34E-03 24600
0.000328945 26900 0.05269412 26400 0.001160321

27300 0.003709006 25200 7.45E-05 25000 1.36E-03 24600
0.000329742 26900 0.05333461 26400 0.001161991
27300 0.003705478 25300 7.46E-05 25000 1.38E-03 24600
0.00033056 26900 0.05398641 26400 0.001163682
27300 0.003701954 25300 7.47E-05 25000 1.41E-03 24600
0.000331368 26900 0.05464978 26400 0.001165354
27300 0.00369843 25300 7.47E-05 25100 1.43E-03 24600
0.00033219 26900 0.05532499 26400 0.001167016
27300 0.003694913 25300 7.48E-05 25100 1.45E-03 24600
0.000332986 26900 0.05601231 26400 0.001168714
27300 0.003691363 25300 7.49E-05 25100 1.48E-03 24600
0.000333796 26900 0.05671205 26400 0.001170391
27300 0.003687805 25300 7.50E-05 25100 1.50E-03 24600
0.000334594 26900 0.05742445 26400 0.001172095
27300 0.003684283 25300 7.51E-05 25100 1.53E-03 24700
0.000335396 26900 0.05814984 26400 0.001173821
27300 0.003680716 25300 7.51E-05 25100 1.55E-03 24700
0.000336201 26900 0.05888854 26400 0.001175533
27300 0.003677179 25300 7.52E-05 25100 1.58E-03 24700
0.000336986 26900 0.0596409 26400 0.001177237
27300 0.00367361 25300 7.53E-05 25100 1.61E-03 24700
0.000337786 26900 0.06040712 26400 0.001178941
27300 0.003670044 25300 7.54E-05 25100 1.64E-03 24700
0.00033859 26900 0.06118761 26400 0.00118065
27300 0.003666501 25300 7.54E-05 25100 1.67E-03 24700
0.000339407 26900 0.06198263 26400 0.00118237
27300 0.003662946 25300 7.55E-05 25100 1.70E-03 24700
0.000340253 26900 0.06279247 26400 0.001184107
27300 0.003659354 25300 7.56E-05 25100 1.73E-03 24700
0.000341063 26900 0.0636178 26400 0.001185853
27300 0.0036558 25300 7.56E-05 25100 1.76E-03 24700
0.000341884 26900 0.06445884 26400 0.001187599
27300 0.003652229 25300 7.57E-05 25100 1.79E-03 24700
0.000342699 26900 0.06531596 26400 0.001189322
27300 0.003648671 25300 7.58E-05 25100 1.83E-03 24700
0.000343505 26900 0.06618954 26400 0.001191072
27300 0.003645103 25300 7.58E-05 25100 1.86E-03 24700
0.000344319 27000 0.06708001 26400 0.001192813
27300 0.003641543 25300 7.59E-05 25100 1.90E-03 24700
0.000345143 27000 0.06798778 26400 0.001194571
27300 0.003637968 25300 7.60E-05 25100 1.94E-03 24700
0.00034598 27000 0.06891327 26400 0.001196322
27300 0.0036344 25300 7.61E-05 25100 1.98E-03 24700
0.000346823 27000 0.06985695 26400 0.001198105
27300 0.003630832 25300 7.61E-05 25100 2.02E-03 24700
0.000347688 27000 0.07081929 26400 0.001199845
27300 0.003627275 25300 7.62E-05 25100 2.06E-03 24700
0.000348572 27000 0.07180076 26400 0.001201608
27300 0.003623682 25300 7.63E-05 25100 2.10E-03 24700
0.000349477 27000 0.0728019 26400 0.001203426
27300 0.003620112 25300 7.64E-05 25100 2.14E-03 24700
0.000350375 27000 0.07382314 26400 0.001205202
27300 0.003616534 25300 7.65E-05 25100 2.19E-03 24700
0.000351285 27000 0.07486507 26400 0.001206962
27300 0.003612966 25300 7.66E-05 25100 2.23E-03 24700
0.000352188 27000 0.07592824 26400 0.001208759
27300 0.003609394 25300 7.66E-05 25100 2.28E-03 24700
0.000353065 27000 0.07701322 26400 0.001210552
27300 0.003605818 25300 7.67E-05 25100 2.33E-03 24700
0.000353922 27000 0.07812053 26400 0.001212342
27300 0.003602225 25300 7.68E-05 25100 2.38E-03 24700
0.000354795 27000 0.07925081 26400 0.001214141
27300 0.003598679 25300 7.69E-05 25100 2.44E-03 24700
0.000355659 27000 0.08040457 26400 0.001215955
27300 0.003595125 25300 7.70E-05 25100 2.49E-03 24700
0.000356529 27000 0.08158243 26400 0.001217795
27300 0.003591582 25300 7.71E-05 25100 2.55E-03 24700
0.000357374 27000 0.08278537 26400 0.001219605

27300 0.003588042 25300 7.71E-05 25100 2.60E-03 24700
0.000358255 27000 0.08401386 26400 0.0012214
27300 0.003584499 25300 7.72E-05 25100 2.66E-03 24700
0.00035912 27000 0.08526863 26400 0.001223249
27300 0.003580973 25300 7.73E-05 25100 2.73E-03 24700
0.000360002 27000 0.08655035 26400 0.001225052
27300 0.003577441 25300 7.74E-05 25100 2.79E-03 24700
0.000360901 27000 0.08785984 26400 0.001226892
27300 0.003573935 25300 7.75E-05 25100 2.86E-03 24700
0.000361817 27000 0.0891978 26400 0.00122867
27300 0.003570427 25300 7.76E-05 25100 2.92E-03 24700
0.000362724 27000 0.09056506 26400 0.001230508
27300 0.003566916 25300 7.76E-05 25100 3.00E-03 24700
0.000363653 27000 0.09196244 26400 0.001232362
27300 0.003563421 25300 7.77E-05 25100 3.07E-03 24700
0.000364569 27000 0.09339085 26400 0.001234233
27300 0.00355994 25300 7.78E-05 25100 3.14E-03 24700
0.000365486 27000 0.09485111 26400 0.001236071
27300 0.003556422 25300 7.79E-05 25100 3.22E-03 24700
0.000366395 27000 0.09634419 26400 0.001237896
27300 0.003552906 25300 7.80E-05 25100 3.30E-03 24700
0.00036731 27000 0.09787095 26400 0.001239802
27300 0.003549454 25300 7.81E-05 25100 3.39E-03 24700
0.000368224 27000 0.09943244 26400 0.001241665
27300 0.003545972 25300 7.82E-05 25100 3.47E-03 24700
0.00036911 27000 0.1010297 26400 0.001243521
27400 0.003542498 25300 7.83E-05 25100 3.56E-03 24700
0.000369998 27000 0.1026636 26400 0.001245392
27400 0.003539016 25300 7.83E-05 25100 3.66E-03 24700
0.000370878 27000 0.1043354 26400 0.001247248
27400 0.003535554 25300 7.84E-05 25100 3.75E-03 24700
0.000371762 27000 0.1060461 26400 0.001249097
27400 0.003532127 25300 7.85E-05 25100 3.85E-03 24700
0.000372637 27000 0.1077967 26400 0.001250977
27400 0.00352865 25300 7.86E-05 25100 3.95E-03 24700
0.000373521 27000 0.1095885 26400 0.001252876
27400 0.003525184 25300 7.87E-05 25100 4.06E-03 24700
0.000374408 27000 0.1114229 26400 0.001254799
27400 0.003521753 25300 7.88E-05 25100 4.17E-03 24700
0.000375279 27000 0.1133011 26400 0.00125671
27400 0.003518329 25300 7.89E-05 25100 4.28E-03 24700
0.000376167 27000 0.1152243 26400 0.001258636
27400 0.003514878 25300 7.90E-05 25100 4.39E-03 24700
0.000377046 27000 0.1171939 26400 0.001260531
27400 0.003511443 25300 7.90E-05 25100 4.51E-03 24700
0.000377966 27000 0.1192114 26500 0.001262481
27400 0.003508002 25300 7.91E-05 25100 4.64E-03 24700
0.000378866 27000 0.1212782 26500 0.00126442
27400 0.003504556 25300 7.92E-05 25100 4.77E-03 24700
0.000379817 27000 0.1233958 26500 0.001266315
27400 0.003501114 25300 7.93E-05 25100 4.90E-03 24700
0.000380792 27000 0.1255658 26500 0.001268252
27400 0.003497683 25300 7.93E-05 25100 5.03E-03 24700
0.000381793 27000 0.1277898 26500 0.001270175
27400 0.003494273 25300 7.94E-05 25100 5.17E-03 24700
0.000382815 27000 0.1300697 26500 0.001272122
27400 0.003490805 25300 7.95E-05 25100 5.32E-03 24700
0.00038388 27000 0.132407 26500 0.001274061
27400 0.003487376 25300 7.96E-05 25100 5.46E-03 24700
0.000384943 27000 0.1348038 26500 0.001276042
27400 0.003483969 25300 7.97E-05 25100 5.61E-03 24700
0.000385993 27000 0.1372618 26500 0.001277986
27400 0.003480528 25400 7.97E-05 25100 5.77E-03 24700
0.000387065 27000 0.139783 26500 0.001279998
27400 0.003477087 25400 7.98E-05 25100 5.93E-03 24700
0.000388112 27000 0.1423696 26500 0.001281978
27400 0.003473666 25400 7.99E-05 25200 6.09E-03 24700
0.000389178 27000 0.1450235 26500 0.00128395

27400 0.003470209 25400 8.00E-05 25200 6.26E-03 24700
0.000390218 27000 0.147747 26500 0.001285937
27400 0.003466803 25400 8.01E-05 25200 6.43E-03 24700
0.000391253 27000 0.1505421 26500 0.001287924
27400 0.003463362 25400 8.01E-05 25200 6.60E-03 24700
0.000392294 27000 0.1534114 26500 0.001289944
27400 0.003459921 25400 8.02E-05 25200 6.77E-03 24800
0.000393333 27000 0.1563576 26500 0.001291979
27400 0.003456512 25400 8.03E-05 25200 6.95E-03 24800
0.000394364 27000 0.159383 26500 0.001294012
27400 0.003453071 25400 8.04E-05 25200 7.13E-03 24800
0.000395358 27000 0.1624902 26500 0.001296066
27400 0.003449639 25400 8.05E-05 25200 7.31E-03 24800
0.000396366 27000 0.1656819 26500 0.001298076
27400 0.003446232 25400 8.06E-05 25200 7.49E-03 24800
0.000397394 27000 0.168961 26500 0.001300098
27400 0.003442825 25400 8.07E-05 25200 7.67E-03 24800
0.000398419 27000 0.1723304 26500 0.00130213
27400 0.003439402 25400 8.07E-05 25200 7.86E-03 24800
0.000399437 27000 0.1757931 26500 0.001304153
27400 0.003435988 25400 8.08E-05 25200 8.04E-03 24800
0.000400479 27000 0.1793523 26500 0.001306196
27400 0.003432555 25400 8.09E-05 25200 8.21E-03 24800
0.000401526 27000 0.1830113 26500 0.001308245
27400 0.00342914 25400 8.10E-05 25200 8.39E-03 24800
0.000402582 27000 0.1867735 26500 0.001310308
27400 0.003425685 25400 8.11E-05 25200 8.56E-03 24800
0.000403655 27000 0.1906424 26500 0.001312371
27400 0.003422238 25400 8.12E-05 25200 8.72E-03 24800
0.000404749 27100 0.1946216 26500 0.001314449
27400 0.003418832 25400 8.13E-05 25200 8.88E-03 24800
0.000405845 27100 0.1987149 26500 0.001316512
27400 0.003415401 25400 8.13E-05 25200 9.03E-03 24800
0.000406976 27100 0.2029262 26500 0.001318594
27400 0.003411951 25400 8.14E-05 25200 9.17E-03 24800
0.000408084 27100 0.2072598 26500 0.001320667
27400 0.003408505 25400 8.15E-05 25200 9.30E-03 24800
0.000409228 27100 0.2117195 26500 0.001322716
27400 0.003405023 25400 8.16E-05 25200 9.42E-03 24800
0.000410355 27100 0.2163098 26500 0.001324791
27400 0.003401597 25400 8.17E-05 25200 9.53E-03 24800
0.000411511 27100 0.2210351 26500 0.001326891
27400 0.003398126 25400 8.18E-05 25200 9.62E-03 24800
0.000412725 27100 0.2259002 26500 0.001328973
27400 0.003394679 25400 8.19E-05 25200 9.70E-03 24800
0.000413904 27100 0.2309101 26500 0.001331086
27400 0.003391227 25400 8.20E-05 25200 9.75E-03 24800
0.000415088 27100 0.2360697 26500 0.00133319
27400 0.003387788 25400 8.21E-05 25200 9.79E-03 24800
0.000416273 27100 0.241384 26500 0.001335296
27400 0.003384315 25400 8.22E-05 25200 9.81E-03 24800
0.000417458 27100 0.2468585 26500 0.001337397
27400 0.003380898 25400 8.22E-05 25200 9.81E-03 24800
0.00041863 27100 0.2524987 26500 0.00133949
27400 0.003377458 25400 8.23E-05 25200 9.79E-03 24800
0.000419782 27100 0.2583101 26500 0.001341602
27400 0.003374009 25400 8.24E-05 25200 9.75E-03 24800
0.000420949 27100 0.2642988 26500 0.001343695
27400 0.003370509 25400 8.25E-05 25200 9.69E-03 24800
0.000422119 27100 0.2704709 26500 0.001345804
27400 0.003367042 25400 8.26E-05 25200 9.60E-03 24800
0.000423299 27100 0.2768324 26500 0.001347918
27400 0.003363574 25400 8.26E-05 25200 9.50E-03 24800
0.000424491 27100 0.28339 26500 0.001350036
27400 0.003360111 25400 8.27E-05 25200 9.37E-03 24800
0.000425679 27100 0.2901502 26500 0.001352154
27400 0.003356638 25400 8.28E-05 25200 9.23E-03 24800
0.00042692 27100 0.2971197 26500 0.001354271

27400 0.003353214 25400 8.29E-05 25200 9.07E-03 24800
0.000428161 27100 0.3043056 26500 0.001356404
27400 0.003349727 25400 8.30E-05 25200 8.89E-03 24800
0.000429418 27100 0.3117148 26500 0.001358546
27400 0.003346246 25400 8.31E-05 25200 8.70E-03 24800
0.000430643 27100 0.3193547 26500 0.001360676
27400 0.00334275 25400 8.32E-05 25200 8.50E-03 24800
0.000431872 27100 0.3272325 26500 0.001362793
27400 0.003339291 25400 8.33E-05 25200 8.28E-03 24800
0.000433099 27100 0.3353559 26500 0.001364925
27400 0.003335822 25400 8.34E-05 25200 8.06E-03 24800
0.000434316 27100 0.343732 26500 0.001367062
27400 0.003332369 25400 8.34E-05 25200 7.83E-03 24800
0.00043556 27100 0.352369 26500 0.00136921
27400 0.003328894 25400 8.35E-05 25200 7.59E-03 24800
0.000436765 27100 0.361275 26500 0.001371349
27400 0.003325447 25400 8.36E-05 25200 7.36E-03 24800
0.000438016 27100 0.3704571 26500 0.001373495
27500 0.003321989 25400 8.37E-05 25200 7.12E-03 24800
0.000439279 27100 0.3799233 26500 0.001375645
27500 0.003318556 25400 8.38E-05 25200 6.89E-03 24800
0.000440564 27100 0.3896814 26500 0.001377799
27500 0.003315105 25400 8.39E-05 25200 6.66E-03 24800
0.000441869 27100 0.399739 26500 0.001379958
27500 0.003311656 25400 8.40E-05 25200 6.43E-03 24800
0.000443196 27100 0.4101036 26500 0.001382111
27500 0.003308202 25400 8.41E-05 25200 6.21E-03 24800
0.000444561 27100 0.4207823 26500 0.001384282
27500 0.003304723 25400 8.42E-05 25200 6.00E-03 24800
0.00044592 27100 0.4317824 26500 0.001386434
27500 0.003301268 25400 8.43E-05 25200 5.80E-03 24800
0.000447325 27100 0.4431104 26500 0.001388593
27500 0.003297799 25400 8.44E-05 25200 5.60E-03 24800
0.000448735 27100 0.4547725 26500 0.001390754
27500 0.003294325 25400 8.45E-05 25200 5.42E-03 24800
0.000450145 27100 0.4667745 26500 0.001392948
27500 0.003290863 25400 8.46E-05 25200 5.24E-03 24800
0.00045156 27100 0.4791211 26600 0.001395146
27500 0.003287391 25400 8.47E-05 25200 5.08E-03 24800
0.000452971 27100 0.4918165 26600 0.001397342
27500 0.003283898 25400 8.48E-05 25200 4.92E-03 24800
0.000454376 27100 0.5048638 26600 0.001399557
27500 0.003280401 25400 8.49E-05 25200 4.78E-03 24800
0.000455748 27100 0.5182654 26600 0.001401734
27500 0.003276926 25400 8.50E-05 25200 4.64E-03 24800
0.000457096 27100 0.5320217 26600 0.001403929
27500 0.003273463 25400 8.51E-05 25200 4.52E-03 24800
0.000458442 27100 0.5461322 26600 0.001406133
27500 0.003269988 25400 8.52E-05 25200 4.40E-03 24800
0.000459786 27100 0.5605938 26600 0.001408354
27500 0.00326651 25400 8.53E-05 25200 4.29E-03 24800
0.000461125 27100 0.575403 26600 0.001410572
27500 0.003263016 25400 8.54E-05 25200 4.19E-03 24800
0.000462451 27100 0.5905536 26600 0.001412795
27500 0.003259556 25500 8.55E-05 25200 4.10E-03 24800
0.000463827 27100 0.6060356 26600 0.001415041
27500 0.003256063 25500 8.56E-05 25200 4.01E-03 24800
0.000465176 27100 0.6218377 26600 0.001417283
27500 0.003252596 25500 8.57E-05 25300 3.93E-03 24800
0.000466539 27100 0.6379449 26600 0.001419529
27500 0.003249108 25500 8.58E-05 25300 3.86E-03 24800
0.000467925 27100 0.6543392 26600 0.001421776
27500 0.003245664 25500 8.59E-05 25300 3.79E-03 24800
0.000469312 27100 0.6709983 26600 0.001424013
27500 0.003242181 25500 8.60E-05 25300 3.73E-03 24800
0.000470726 27100 0.6878963 26600 0.001426274
27500 0.003238694 25500 8.61E-05 25300 3.67E-03 24900
0.000472161 27100 0.7050022 26600 0.001428525

27500 0.003235204 25500 8.62E-05 25300 3.62E-03 24900
0.000473651 27100 0.7222809 26600 0.0014308
27500 0.003231743 25500 8.63E-05 25300 3.57E-03 24900
0.00047514 27100 0.7396913 26600 0.00143306
27500 0.003228253 25500 8.64E-05 25300 3.52E-03 24900
0.000476679 27100 0.7571874 26600 0.001435353
27500 0.003224759 25500 8.65E-05 25300 3.48E-03 24900
0.000478238 27100 0.7747167 26600 0.001437647
27500 0.00322132 25500 8.66E-05 25300 3.44E-03 24900
0.000479821 27100 0.7922205 26600 0.001439935
27500 0.003217865 25500 8.67E-05 25300 3.41E-03 24900
0.000481425 27100 0.8096341 26600 0.001442258
27500 0.003214401 25500 8.68E-05 25300 3.37E-03 24900
0.000483002 27100 0.8268856 26600 0.001444551
27500 0.003210968 25500 8.69E-05 25300 3.34E-03 24900
0.000484572 27100 0.843896 26600 0.001446826
27500 0.003207524 25500 8.70E-05 25300 3.31E-03 24900
0.00048613 27100 0.8605803 26600 0.001449158
27500 0.003204065 25500 8.71E-05 25300 3.29E-03 24900
0.000487678 27100 0.876846 26600 0.00145148
27500 0.003200629 25500 8.72E-05 25300 3.26E-03 24900
0.000489231 27200 0.8925952 26600 0.001453815
27500 0.003197175 25500 8.73E-05 25300 3.24E-03 24900
0.00049076 27200 0.9077237 26600 0.001456144
27500 0.003193759 25500 8.74E-05 25300 3.21E-03 24900
0.000492296 27200 0.9221202 26600 0.001458503
27500 0.003190312 25500 8.75E-05 25300 3.19E-03 24900
0.000493835 27200 0.9356703 26600 0.001460847
27500 0.003186878 25500 8.76E-05 25300 3.17E-03 24900
0.000495353 27200 0.948256 26600 0.001463197
27500 0.003183472 25500 8.76E-05 25300 3.15E-03 24900
0.000496886 27200 0.9597564 26600 0.001465564
27500 0.003180032 25500 8.77E-05 25300 3.14E-03 24900
0.000498399 27200 0.9700494 26600 0.001467908
27500 0.003176624 25500 8.78E-05 25300 3.12E-03 24900
0.000499898 27200 0.9790144 26600 0.001470265
27500 0.003173189 25500 8.79E-05 25300 3.10E-03 24900
0.000501396 27200 0.986533 26600 0.001472621
27500 0.003169767 25500 8.80E-05 25300 3.09E-03 24900
0.000502888 27200 0.9924921 26600 0.001474996
27500 0.00316637 25500 8.81E-05 25300 3.08E-03 24900
0.000504376 27200 0.9967855 26600 0.001477362
27500 0.003162921 25500 8.82E-05 25300 3.06E-03 24900
0.000505891 27200 0.9993162 26600 0.00147976
27500 0.003159474 25500 8.83E-05 25300 3.05E-03 24900
0.000507429 27200 1 26600 0.001482183
27500 0.003156068 25500 8.84E-05 25300 3.04E-03 24900
0.000508992 27200 0.998767 26600 0.001484573
27500 0.003152618 25500 8.85E-05 25300 3.03E-03 24900
0.00051062 27200 0.9955638 26600 0.00148699
27500 0.003149171 25500 8.87E-05 25300 3.02E-03 24900
0.000512253 27200 0.990357 26600 0.001489381
27500 0.003145768 25500 8.88E-05 25300 3.01E-03 24900
0.000513932 27200 0.9831334 26600 0.001491761
27500 0.003142329 25500 8.89E-05 25300 3.01E-03 24900
0.000515649 27200 0.9739021 26600 0.001494209
27500 0.003138889 25500 8.90E-05 25300 3.00E-03 24900
0.000517383 27200 0.9626957 26600 0.001496639
27500 0.003135466 25500 8.91E-05 25300 2.99E-03 24900
0.000519137 27200 0.9495723 26600 0.001499094
27500 0.003131989 25500 8.92E-05 25300 2.99E-03 24900
0.000520882 27200 0.9346122 26600 0.001501556
27500 0.003128553 25500 8.93E-05 25300 2.98E-03 24900
0.00052265 27200 0.917918 26600 0.001504022
27500 0.003125116 25500 8.94E-05 25300 2.98E-03 24900
0.0005244 27200 0.8996151 26600 0.001506484
27500 0.003121685 25500 8.95E-05 25300 2.97E-03 24900
0.000526155 27200 0.8798481 26600 0.001508952

27500 0.003118257 25500 8.96E-05 25300 2.97E-03 24900
0.000527918 27200 0.8587788 26600 0.001511432
27500 0.00311481 25500 8.97E-05 25300 2.97E-03 24900
0.000529715 27200 0.8365823 26600 0.001513904
27500 0.003111347 25500 8.98E-05 25300 2.97E-03 24900
0.000531526 27200 0.8134444 26600 0.001516393
27500 0.003107907 25500 8.99E-05 25300 2.97E-03 24900
0.000533398 27200 0.7895573 26600 0.001518879
27500 0.003104447 25500 9.01E-05 25300 2.97E-03 24900
0.00053531 27200 0.7651161 26600 0.001521379
27600 0.003101014 25500 9.02E-05 25300 2.97E-03 24900
0.000537247 27200 0.7403147 26600 0.001523883
27600 0.003097516 25500 9.03E-05 25300 2.97E-03 24900
0.000539205 27200 0.7153423 26600 0.00152639
27600 0.00309404 25500 9.04E-05 25300 2.97E-03 24900
0.000541184 27200 0.6903794 26600 0.001528922
27600 0.003090597 25500 9.05E-05 25300 2.97E-03 24900
0.000543155 27200 0.6655962 26600 0.001531451
27600 0.003087133 25500 9.06E-05 25300 2.98E-03 24900
0.000545138 27200 0.6411479 26600 0.001533996
27600 0.003083665 25500 9.07E-05 25300 2.98E-03 24900
0.000547092 27200 0.617175 26600 0.00153653
27600 0.003080198 25500 9.08E-05 25300 2.99E-03 24900
0.000549057 27200 0.5938 26600 0.001539052
27600 0.003076722 25500 9.10E-05 25300 2.99E-03 24900
0.000551002 27200 0.5711274 26600 0.001541632
27600 0.003073268 25500 9.11E-05 25300 3.00E-03 24900
0.000552966 27200 0.549243 26600 0.0015442
27600 0.003069815 25500 9.12E-05 25300 3.01E-03 24900
0.00055492 27200 0.5282153 26700 0.001546774
27600 0.003066364 25500 9.13E-05 25300 3.01E-03 24900
0.000556902 27200 0.508094 26700 0.001549333
27600 0.003062918 25500 9.14E-05 25300 3.02E-03 24900
0.0005589 27200 0.4889117 26700 0.0015519
27600 0.003059454 25500 9.15E-05 25300 3.03E-03 24900
0.000560902 27200 0.4706869 26700 0.001554478
27600 0.003056004 25500 9.16E-05 25300 3.04E-03 24900
0.000562936 27200 0.4534239 26700 0.00155705
27600 0.003052579 25500 9.17E-05 25300 3.05E-03 24900
0.000564965 27200 0.437115 26700 0.001559631
27600 0.00304911 25500 9.18E-05 25300 3.07E-03 24900
0.000566996 27200 0.4217415 26700 0.001562211
27600 0.003045657 25500 9.19E-05 25300 3.08E-03 24900
0.000569018 27200 0.407277 26700 0.001564798
27600 0.00304219 25500 9.20E-05 25300 3.09E-03 24900
0.000571044 27200 0.3936873 26700 0.001567393
27600 0.00303875 25600 9.22E-05 25300 3.10E-03 24900
0.000573075 27200 0.3809333 26700 0.001569991
27600 0.003035296 25600 9.23E-05 25300 3.12E-03 24900
0.000575099 27200 0.3689719 26700 0.001572595
27600 0.00303184 25600 9.24E-05 25400 3.14E-03 24900
0.000577136 27200 0.3577577 26700 0.001575197
27600 0.003028372 25600 9.25E-05 25400 3.15E-03 24900
0.000579202 27200 0.3472435 26700 0.00157783
27600 0.003024966 25600 9.26E-05 25400 3.17E-03 24900
0.000581261 27200 0.3373819 26700 0.001580457
27600 0.003021543 25600 9.27E-05 25400 3.19E-03 24900
0.000583357 27200 0.3281259 26700 0.001583093
27600 0.003018099 25600 9.28E-05 25400 3.21E-03 25000
0.000585463 27200 0.3194298 26700 0.0015857
27600 0.00301469 25600 9.29E-05 25400 3.23E-03 25000
0.000587575 27200 0.3112493 26700 0.001588304
27600 0.003011241 25600 9.30E-05 25400 3.25E-03 25000
0.000589695 27200 0.3035423 26700 0.001590979
27600 0.003007843 25600 9.32E-05 25400 3.27E-03 25000
0.000591836 27200 0.2962688 26700 0.001593622
27600 0.003004435 25600 9.33E-05 25400 3.29E-03 25000
0.000593997 27200 0.2893919 26700 0.001596284

27600 0.003001029 25600 9.34E-05 25400 3.32E-03 25000
0.000596174 27200 0.2828764 26700 0.001598961
27600 0.002997645 25600 9.35E-05 25400 3.34E-03 25000
0.000598379 27200 0.27669 26700 0.001601616
27600 0.002994244 25600 9.36E-05 25400 3.37E-03 25000
0.000600623 27200 0.2708035 26700 0.001604298
27600 0.002990909 25600 9.37E-05 25400 3.39E-03 25000
0.000602888 27200 0.2651899 26700 0.001606986
27600 0.002987578 25600 9.38E-05 25400 3.42E-03 25000
0.000605189 27200 0.2598247 26700 0.00160969
27600 0.002984243 25600 9.40E-05 25400 3.45E-03 25000
0.000607546 27200 0.2546858 26700 0.001612378
27600 0.002980902 25600 9.41E-05 25400 3.48E-03 25000
0.00060995 27300 0.2497532 26700 0.001615076
27600 0.002977562 25600 9.42E-05 25400 3.51E-03 25000
0.000612386 27300 0.2450087 26700 0.001617783
27600 0.002974265 25600 9.43E-05 25400 3.54E-03 25000
0.000614871 27300 0.2404364 26700 0.001620495
27600 0.002970951 25600 9.44E-05 25400 3.57E-03 25000
0.000617412 27300 0.2360217 26700 0.001623207
27600 0.002967651 25600 9.45E-05 25400 3.61E-03 25000
0.000619998 27300 0.2317519 26700 0.00162593
27600 0.002964364 25600 9.46E-05 25400 3.64E-03 25000
0.000622632 27300 0.2276155 26700 0.001628698
27600 0.002961109 25600 9.48E-05 25400 3.68E-03 25000
0.000625302 27300 0.2236024 26700 0.001631456
27600 0.002957853 25600 9.49E-05 25400 3.72E-03 25000
0.00062801 27300 0.2197034 26700 0.001634215
27600 0.00295461 25600 9.50E-05 25400 3.76E-03 25000
0.000630714 27300 0.2159107 26700 0.001636965
27600 0.002951365 25600 9.51E-05 25400 3.80E-03 25000
0.00063342 27300 0.2122172 26700 0.001639711
27600 0.002948145 25600 9.52E-05 25400 3.84E-03 25000
0.000636131 27300 0.2086168 26700 0.001642501
27600 0.002944932 25600 9.53E-05 25400 3.88E-03 25000
0.000638851 27300 0.2051037 26700 0.001645271
27600 0.002941743 25600 9.54E-05 25400 3.93E-03 25000
0.000641597 27300 0.2016736 26700 0.001648075
27600 0.002938521 25600 9.56E-05 25400 3.97E-03 25000
0.000644333 27300 0.1983216 26700 0.001650882
27600 0.00293537 25600 9.57E-05 25400 4.02E-03 25000
0.000647122 27300 0.1950439 26700 0.00165369
27600 0.002932204 25600 9.58E-05 25400 4.07E-03 25000
0.000649929 27300 0.1918372 26700 0.001656494
27600 0.00292906 25600 9.59E-05 25400 4.12E-03 25000
0.000652723 27300 0.1886984 26700 0.0016593
27600 0.002925925 25600 9.60E-05 25400 4.17E-03 25000
0.000655484 27300 0.1856251 26700 0.00166211
27600 0.002922817 25600 9.62E-05 25400 4.22E-03 25000
0.000658172 27300 0.1826147 26700 0.001664911
27600 0.00291969 25600 9.63E-05 25400 4.27E-03 25000
0.000660813 27300 0.1796651 26700 0.001667755
27600 0.002916576 25600 9.64E-05 25400 4.33E-03 25000
0.000663381 27300 0.1767743 26700 0.00167057
27600 0.002913447 25600 9.65E-05 25400 4.39E-03 25000
0.000665905 27300 0.1739406 26700 0.001673421
27600 0.002910351 25600 9.67E-05 25400 4.45E-03 25000
0.000668386 27300 0.1711623 26700 0.001676254
27600 0.002907248 25600 9.68E-05 25400 4.51E-03 25000
0.000670914 27300 0.1684379 26700 0.001679101
27600 0.002904157 25600 9.70E-05 25400 4.57E-03 25000
0.00067348 27300 0.1657662 26700 0.001681973
27600 0.002901059 25600 9.71E-05 25400 4.63E-03 25000
0.000676136 27300 0.1631458 26700 0.001684838
27600 0.002897983 25600 9.72E-05 25400 4.70E-03 25000
0.000678852 27300 0.1605755 26700 0.001687746
27600 0.00289489 25600 9.73E-05 25400 4.76E-03 25000
0.000681572 27300 0.1580542 26700 0.001690618

27600 0.002891827 25600 9.75E-05 25400 4.83E-03 25000
0.000684388 27300 0.1555809 26700 0.001693497
27700 0.002888753 25600 9.76E-05 25400 4.90E-03 25000
0.000687224 27300 0.1531545 26700 0.001696405
27700 0.002885683 25600 9.77E-05 25400 4.98E-03 25000
0.000690059 27300 0.1507741 26700 0.001699315
27700 0.002882617 25600 9.78E-05 25400 5.05E-03 25000
0.000692962 27300 0.148439 26700 0.001702232
27700 0.002879563 25600 9.80E-05 25400 5.13E-03 25000
0.000695875 27300 0.1461481 26700 0.001705169
27700 0.002876503 25600 9.81E-05 25400 5.20E-03 25000
0.000698784 27300 0.1439005 26700 0.001708128
27700 0.002873444 25600 9.82E-05 25400 5.28E-03 25000
0.000701736 27300 0.1416954 26700 0.001711055
27700 0.002870405 25600 9.83E-05 25400 5.36E-03 25000
0.000704704 27300 0.1395321 26700 0.001714018
27700 0.002867352 25600 9.85E-05 25400 5.45E-03 25000
0.000707698 27300 0.1374097 26700 0.00171697
27700 0.002864291 25600 9.86E-05 25400 5.53E-03 25000
0.000710757 27300 0.1353274 26700 0.001719927
27700 0.00286125 25600 9.87E-05 25400 5.62E-03 25000
0.000713796 27300 0.1332846 26800 0.001722931
27700 0.002858172 25600 9.88E-05 25400 5.71E-03 25000
0.000716897 27300 0.1312804 26800 0.00172591
27700 0.002855108 25600 9.89E-05 25400 5.80E-03 25000
0.000720034 27300 0.1293141 26800 0.001728903
27700 0.002852054 25600 9.91E-05 25400 5.89E-03 25000
0.000723204 27300 0.1273849 26800 0.001731902
27700 0.002848973 25600 9.92E-05 25400 5.98E-03 25000
0.000726418 27300 0.1254922 26800 0.001734933
27700 0.002845869 25600 9.94E-05 25400 6.08E-03 25000
0.000729652 27300 0.1236353 26800 0.001737958
27700 0.002842827 25600 9.95E-05 25400 6.18E-03 25000
0.000732946 27300 0.1218133 26800 0.001740996
27700 0.002839755 25600 9.96E-05 25400 6.28E-03 25000
0.000736249 27300 0.1200258 26800 0.001744048
27700 0.002836677 25600 9.97E-05 25400 6.38E-03 25000
0.000739596 27300 0.1182719 26800 0.001747071
27700 0.002833605 25700 9.99E-05 25400 6.48E-03 25000
0.000742973 27300 0.1165511 26800 0.001750109
27700 0.002830558 25700 0.000100017 25400 6.58E-03 25000
0.000746316 27300 0.1148627 26800 0.00175319
27700 0.002827518 25700 0.000100151 25500 6.69E-03 25000
0.000749708 27300 0.1132059 26800 0.001756248
27700 0.002824422 25700 0.000100289 25500 6.79E-03 25000
0.000753111 27300 0.1115806 26800 0.001759329
27700 0.002821359 25700 0.000100423 25500 6.90E-03 25000
0.000756562 27300 0.1099856 26800 0.001762439
27700 0.002818286 25700 0.000100558 25500 7.01E-03 25000
0.000760066 27300 0.1084204 26800 0.001765545
27700 0.002815232 25700 0.000100703 25500 7.12E-03 25100
0.000763609 27300 0.1068845 26800 0.001768638
27700 0.002812177 25700 0.000100854 25500 7.22E-03 25100
0.000767188 27300 0.1053772 26800 0.00177176
27700 0.00280915 25700 0.000100998 25500 7.33E-03 25100
0.000770843 27300 0.1038981 26800 0.001774866
27700 0.002806098 25700 0.00010115 25500 7.44E-03 25100
0.000774506 27300 0.1024465 26800 0.001777986
27700 0.002803035 25700 0.000101295 25500 7.55E-03 25100
0.000778234 27300 0.101022 26800 0.001781117
27700 0.002799998 25700 0.000101431 25500 0.007663841 25100
0.000781965 27300 0.09962379 26800 0.001784251
27700 0.002796943 25700 0.000101587 25500 0.007773184 25100
0.000785752 27300 0.09825148 26800 0.001787393
27700 0.002793961 25700 0.000101728 25500 0.007881809 25100
0.000789555 27300 0.09690454 26800 0.001790553
27700 0.002790908 25700 0.000101873 25500 0.007989418 25100
0.000793423 27300 0.09558239 26800 0.001793719

27700 0.00278785 25700 0.000102007 25500 0.00809571 25100
0.000797298 27300 0.09428453 26800 0.001796888
27700 0.002784864 25700 0.000102143 25500 0.008200333 25100
0.000801228 27300 0.09301045 26800 0.001800083
27700 0.002781833 25700 0.000102273 25500 0.00830296 25100
0.000805185 27400 0.09175964 26800 0.001803301
27700 0.002778812 25700 0.000102426 25500 0.008403197 25100
0.000809126 27400 0.09053162 26800 0.001806485
27700 0.002775801 25700 0.000102556 25500 0.008500674 25100
0.000813128 27400 0.08932593 26800 0.001809665
27700 0.002772796 25700 0.000102698 25500 0.008594972 25100
0.000817145 27400 0.08814207 26800 0.001812917
27700 0.002769791 25700 0.00010285 25500 0.008685673 25100
0.000821179 27400 0.08697961 26800 0.001816127
27700 0.0027668 25700 0.000103004 25500 0.008772328 25100
0.000825293 27400 0.0858383 26800 0.00181938
27700 0.00276379 25700 0.000103149 25500 0.008854497 25100
0.000829401 27400 0.0847174 26800 0.001822635
27700 0.002760826 25700 0.000103306 25500 0.008931708 25100
0.000833524 27400 0.08361654 26800 0.001825894
27700 0.002757851 25700 0.000103451 25500 0.009003477 25100
0.000837692 27400 0.08253536 26800 0.001829155
27700 0.002754917 25700 0.000103605 25500 0.009069336 25100
0.000841866 27400 0.08147345 26800 0.001832422
27700 0.002751976 25700 0.000103756 25500 0.009128834 25100
0.000846072 27400 0.08043043 26800 0.001835713
27700 0.002749049 25700 0.000103911 25500 0.009181443 25100
0.000850331 27400 0.07940591 26800 0.001838984
27700 0.002746111 25700 0.000104043 25500 0.009226726 25100
0.000854615 27400 0.07839952 26800 0.001842273
27700 0.002743169 25700 0.000104185 25500 0.009264241 25100
0.000858937 27400 0.07741085 26800 0.001845576
27700 0.002740275 25700 0.000104326 25500 0.009293525 25100
0.000863318 27400 0.07643954 26800 0.001848895
27700 0.002737341 25700 0.00010448 25500 0.00931414 25100
0.000867744 27400 0.07548524 26800 0.00185221
27700 0.002734433 25700 0.000104631 25500 0.009325726 25100
0.000872194 27400 0.07454762 26800 0.001855533
27700 0.002731493 25700 0.000104782 25500 0.009327902 25100
0.000876712 27400 0.0736263 26800 0.001858883
27700 0.002728611 25700 0.000104928 25500 0.009320362 25100
0.000881299 27400 0.07272092 26800 0.00186223
27700 0.002725696 25700 0.000105092 25500 0.009302816 25100
0.000885932 27400 0.07183117 26800 0.001865602
27700 0.002722816 25700 0.000105248 25500 0.009275033 25100
0.000890618 27400 0.07095677 26800 0.001868938
27700 0.002719907 25700 0.000105394 25500 0.009236837 25100
0.0008954 27400 0.07009733 26800 0.001872288
27700 0.00271701 25700 0.000105556 25500 0.009188115 25100
0.000900173 27400 0.06925258 26800 0.001875678
27700 0.00271414 25700 0.000105718 25500 0.009128789 25100
0.000905014 27400 0.06842222 26800 0.00187905
27700 0.002711272 25700 0.000105872 25500 0.009058877 25100
0.000909958 27400 0.06760608 26800 0.001882465
27700 0.002708408 25700 0.000106039 25500 0.008978461 25100
0.000914911 27400 0.06680363 26800 0.001885887
27700 0.002705566 25700 0.000106205 25500 0.008887663 25100
0.000919942 27400 0.06601462 26800 0.001889306
27700 0.002702722 25700 0.000106379 25500 0.008786689 25100
0.000925061 27400 0.06523883 26800 0.001892747
27700 0.002699887 25700 0.000106562 25500 0.008675826 25100
0.000930234 27400 0.06447598 26800 0.001896178
27800 0.002697054 25700 0.000106748 25500 0.008555448 25100
0.000935499 27400 0.06372581 26800 0.001899619
27800 0.002694236 25700 0.000106935 25500 0.008425906 25100
0.000940826 27400 0.06298809 26800 0.001903048
27800 0.002691419 25700 0.000107122 25500 0.008287726 25100
0.000946198 27400 0.06226255 26800 0.001906513

27800 0.00268856 25700 0.000107298 25500 0.008141412 25100
0.000951617 27400 0.06154887 26800 0.001909989
27800 0.002685749 25700 0.00010748 25500 0.007987562 25100
0.00095709 27400 0.06084688 26800 0.00191347
27800 0.002682979 25700 0.000107673 25500 0.007826794 25100
0.0009626 27400 0.0601563 26800 0.00191696
27800 0.002680139 25700 0.000107858 25500 0.007659785 25100
0.000968169 27400 0.05947693 26800 0.001920459
27800 0.0026773 25700 0.000108037 25500 0.007487233 25100
0.000973776 27400 0.05880853 26800 0.001923978
27800 0.002674546 25700 0.000108229 25500 0.007309882 25100
0.000979468 27400 0.05815085 26800 0.001927492
27800 0.002671743 25700 0.000108404 25500 0.007128458 25100
0.000985171 27400 0.05750369 26900 0.001931051
27800 0.002668956 25700 0.000108597 25500 0.006943735 25100
0.000990949 27400 0.05686687 26900 0.001934575
27800 0.00266617 25700 0.00010877 25500 0.006756479 25100
0.000996802 27400 0.05624014 26900 0.001938109
27800 0.002663363 25700 0.000108974 25500 0.006567427 25100
0.001002725 27400 0.05562335 26900 0.001941672
27800 0.00266056 25700 0.00010915 25500 0.006377322 25100
0.001008751 27400 0.0550163 26900 0.001945237
27800 0.00265776 25700 0.000109333 25500 0.006186893 25100
0.001014855 27400 0.05441889 26900 0.001948812
27800 0.002654967 25700 0.000109523 25500 0.005996821 25100
0.001021052 27400 0.05383078 26900 0.001952417
27800 0.002652183 25700 0.000109714 25500 0.005807782 25100
0.001027327 27400 0.05325178 26900 0.001956008
27800 0.0026494 25700 0.000109908 25500 0.00562037 25100
0.001033654 27400 0.0526818 26900 0.001959622
27800 0.002646647 25800 0.000110107 25500 0.005435179 25100
0.001040038 27400 0.05212063 26900 0.001963224
27800 0.002643871 25800 0.000110305 25500 0.005252755 25100
0.0010465 27400 0.05156814 26900 0.001966843
27800 0.002641097 25800 0.000110509 25600 0.005073548 25100
0.001052988 27400 0.05102418 26900 0.001970479
27800 0.002638353 25800 0.000110711 25600 0.004898015 25100
0.001059526 27400 0.05048857 26900 0.001974098
27800 0.002635546 25800 0.00011092 25600 0.004726547 25100
0.001066081 27400 0.0499611 26900 0.001977742
27800 0.002632813 25800 0.000111129 25600 0.004559473 25100
0.001072721 27400 0.04944167 26900 0.001981396
27800 0.002630014 25800 0.000111345 25600 0.004397065 25200
0.001079423 27400 0.04893009 26900 0.001985048
27800 0.00262724 25800 0.000111555 25600 0.004239575 25200
0.001086225 27400 0.04842624 26900 0.001988725
27800 0.002624455 25800 0.000111765 25600 0.004087163 25200
0.001093098 27400 0.04792996 26900 0.001992414
27800 0.002621716 25800 0.000111983 25600 0.003939997 25200
0.001100115 27400 0.04744109 26900 0.001996098
27800 0.002618949 25800 0.000112181 25600 0.003798161 25200
0.001107211 27400 0.0469595 26900 0.001999802
27800 0.002616209 25800 0.000112391 25600 0.003661729 25200
0.001114427 27400 0.04648506 26900 0.002003483
27800 0.002613463 25800 0.000112606 25600 0.003530707 25200
0.001121728 27400 0.0460176 26900 0.002007165
27800 0.002610712 25800 0.00011282 25600 0.003405092 25200
0.001129117 27400 0.04555705 26900 0.002010879
27800 0.002607973 25800 0.000113029 25600 0.003284853 25200
0.001136608 27400 0.04510327 26900 0.0020146
27800 0.002605251 25800 0.000113242 25600 0.003169918 25200
0.001144205 27400 0.04465621 26900 0.00201833
27800 0.002602516 25800 0.000113459 25600 0.003060202 25200
0.001151873 27400 0.0442156 26900 0.002022088
27800 0.002599812 25800 0.000113665 25600 0.002955583 25200
0.00115963 27500 0.04378131 26900 0.002025838
27800 0.002597131 25800 0.000113873 25600 0.002855937 25200
0.001167465 27500 0.04335331 26900 0.002029598

27800 0.002594435 25800 0.000114092 25600 0.002761125 25200
0.001175379 27500 0.04293144 26900 0.002033367
27800 0.00259178 25800 0.000114303 25600 0.002671003 25200
0.001183426 27500 0.04251564 26900 0.002037145
27800 0.002589112 25800 0.000114506 25600 0.002585398 25200
0.001191557 27500 0.0421058 26900 0.002040907
27800 0.002586464 25800 0.000114725 25600 0.002504135 25200
0.001199811 27500 0.04170178 26900 0.002044694
27800 0.002583806 25800 0.000114932 25600 0.002427067 25200
0.001208178 27500 0.04130348 26900 0.002048482
27800 0.00258119 25800 0.000115154 25600 0.002353995 25200
0.001216662 27500 0.0409108 26900 0.002052296
27800 0.002578577 25800 0.000115384 25600 0.002284752 25200
0.001225263 27500 0.04052361 26900 0.00205611
27800 0.002575986 25800 0.000115615 25600 0.002219142 25200
0.001233993 27500 0.04014184 26900 0.002059943
27800 0.002573366 25800 0.000115841 25600 0.002156998 25200
0.001242823 27500 0.03976534 26900 0.002063785
27800 0.002570814 25800 0.000116078 25600 0.002098162 25200
0.001251756 27500 0.03939405 26900 0.002067635
27800 0.002568234 25800 0.000116309 25600 0.002042442 25200
0.001260865 27500 0.03902788 26900 0.002071507
27800 0.002565679 25800 0.000116548 25600 0.001989684 25200
0.001270035 27500 0.03866675 26900 0.002075368
27800 0.002563126 25800 0.00011678 25600 0.001939728 25200
0.001279349 27500 0.03831052 26900 0.00207922
27800 0.002560583 25800 0.00011703 25600 0.001892402 25200
0.001288748 27500 0.03795917 26900 0.002083114
27800 0.002558046 25800 0.000117274 25600 0.001847587 25200
0.00129827 27500 0.03761262 26900 0.002087014
27800 0.002555539 25800 0.000117519 25600 0.001805116 25200
0.001307933 27500 0.03727084 26900 0.002090932
27800 0.002553034 25800 0.000117768 25600 0.001764868 25200
0.001317728 27500 0.03693359 26900 0.002094849
27800 0.002550561 25800 0.000118025 25600 0.001726699 25200
0.001327635 27500 0.03660087 26900 0.002098788
27800 0.002548052 25800 0.000118286 25600 0.001690486 25200
0.001337687 27500 0.03627261 26900 0.002102713
27800 0.002545591 25800 0.000118549 25600 0.00165611 25200
0.001347912 27500 0.03594868 26900 0.002106669
27800 0.002543131 25800 0.000118823 25600 0.001623465 25200
0.001358302 27500 0.0356291 26900 0.002110623
27800 0.00254066 25800 0.000119086 25600 0.001592427 25200
0.001368834 27500 0.03531378 26900 0.002114573
27800 0.002538195 25800 0.000119335 25600 0.001562912 25200
0.001379582 27500 0.03500261 26900 0.002118555
27800 0.002535712 25800 0.000119596 25600 0.001534833 25200
0.001390475 27500 0.03469553 26900 0.002122535
27800 0.002533281 25800 0.000119863 25600 0.001508076 25200
0.001401521 27500 0.03439248 26900 0.002126523
27800 0.002530833 25800 0.000120121 25600 0.001482568 25200
0.001412752 27500 0.03409338 26900 0.002130529
27800 0.002528347 25800 0.000120393 25600 0.001458231 25200
0.001424134 27500 0.03379814 26900 0.002134545
27900 0.002525911 25800 0.000120668 25600 0.001434991 25200
0.001435644 27500 0.0335067 26900 0.002138598
27900 0.00252345 25800 0.000120965 25600 0.001412787 25200
0.001447323 27500 0.03321898 26900 0.002142636
27900 0.002520987 25800 0.000121255 25600 0.001391525 25200
0.001459172 27500 0.03293493 26900 0.002146705
27900 0.002518551 25800 0.000121553 25600 0.001371174 25200
0.001471164 27500 0.03265445 26900 0.002150751
27900 0.002516109 25800 0.000121846 25600 0.001351671 25200
0.00148336 27500 0.03237749 26900 0.00215479
27900 0.002513664 25800 0.000122142 25600 0.001332961 25200
0.001495715 27500 0.03210406 26900 0.002158882
27900 0.00251122 25800 0.000122436 25600 0.001314994 25200
0.001508275 27500 0.03183404 26900 0.002162977

27900 0.002508806 25800 0.000122732 25600 0.001297725 25200
0.001521063 27500 0.03156743 26900 0.002167105
27900 0.002506347 25800 0.000123007 25600 0.001281118 25200
0.001534 27500 0.03130409 26900 0.00217124
27900 0.002503949 25800 0.000123307 25600 0.001265127 25200
0.001547178 27500 0.03104397 27000 0.002175382
27900 0.002501527 25800 0.000123605 25600 0.001249716 25200
0.001560559 27500 0.03078699 27000 0.002179519
27900 0.002499136 25800 0.000123905 25600 0.001234835 25200
0.001574164 27500 0.03053319 27000 0.00218369
27900 0.002496743 25800 0.000124219 25600 0.00122047 25200
0.001588024 27500 0.0302825 27000 0.002187864
27900 0.002494351 25800 0.000124526 25600 0.001206585 25200
0.001602113 27500 0.03003489 27000 0.002192037
27900 0.002491939 25800 0.000124833 25600 0.001193166 25200
0.001616459 27500 0.0297903 27000 0.002196234
27900 0.002489544 25800 0.000125142 25600 0.001180173 25200
0.001631043 27500 0.02954865 27000 0.00220043
27900 0.002487179 25800 0.000125477 25600 0.001167582 25200
0.001645919 27500 0.02930991 27000 0.002204641
27900 0.002484785 25800 0.000125804 25600 0.001155367 25200
0.00166103 27500 0.02907406 27000 0.002208874
27900 0.002482376 25900 0.00012614 25600 0.001143523 25200
0.001676414 27500 0.02884105 27000 0.002213111
27900 0.002479978 25900 0.000126467 25600 0.001132017 25200
0.001692061 27500 0.02861079 27000 0.00221738
27900 0.002477568 25900 0.000126796 25700 0.001120835 25200
0.001707993 27500 0.0283833 27000 0.002221652
27900 0.002475187 25900 0.000127136 25700 0.001109945 25200
0.001724181 27500 0.02815852 27000 0.002225944
27900 0.002472815 25900 0.000127465 25700 0.001099354 25200
0.001740617 27500 0.02793642 27000 0.002230226
27900 0.002470438 25900 0.000127789 25700 0.001089036 25200
0.00175733 27500 0.02771693 27000 0.002234507
27900 0.002468057 25900 0.000128121 25700 0.001078987 25300
0.001774281 27500 0.02750007 27000 0.002238816
27900 0.002465701 25900 0.000128447 25700 0.001069179 25300
0.001791537 27500 0.02728577 27000 0.00224313
27900 0.002463329 25900 0.000128774 25700 0.001059615 25300
0.001809086 27500 0.02707408 27000 0.00224747
27900 0.002460963 25900 0.000129096 25700 0.001050296 25300
0.001826926 27500 0.02686485 27000 0.002251829
27900 0.002458633 25900 0.000129441 25700 0.001041183 25300
0.001845101 27500 0.02665799 27000 0.002256184
27900 0.002456292 25900 0.000129778 25700 0.001032292 25300
0.001863599 27500 0.02645357 27000 0.002260538
27900 0.00245395 25900 0.000130115 25700 0.001023578 25300
0.001882413 27500 0.02625152 27000 0.002264904
27900 0.002451639 25900 0.000130455 25700 0.001015046 25300
0.001901564 27500 0.02605186 27000 0.002269293
27900 0.002449278 25900 0.000130786 25700 0.001006725 25300
0.001921046 27500 0.02585453 27000 0.002273679
27900 0.002446974 25900 0.000131102 25700 0.000998562 25300
0.001940866 27500 0.0256595 27000 0.00227808
27900 0.002444639 25900 0.000131432 25700 0.000990578 25300
0.001961054 27500 0.02546671 27000 0.002282494
27900 0.002442352 25900 0.000131747 25700 0.000982749 25300
0.001981599 27600 0.02527616 27000 0.002286917
27900 0.002440018 25900 0.000132065 25700 0.000975096 25300
0.002002503 27600 0.02508778 27000 0.002291349
27900 0.002437736 25900 0.000132377 25700 0.000967583 25300
0.002023815 27600 0.02490155 27000 0.002295801
27900 0.002435422 25900 0.00013269 25700 0.000960202 25300
0.002045551 27600 0.02471747 27000 0.002300263
27900 0.002433153 25900 0.000132999 25700 0.000952974 25300
0.002067655 27600 0.02453544 27000 0.002304751
27900 0.002430868 25900 0.000133302 25700 0.000945873 25300
0.002090227 27600 0.02435546 27000 0.002309257

27900 0.002428575 25900 0.000133621 25700 0.000938906 25300
0.002113208 27600 0.02417752 27000 0.002313742
27900 0.002426298 25900 0.000133911 25700 0.000932057 25300
0.00213664 27600 0.02400153 27000 0.002318221
27900 0.002424011 25900 0.000134225 25700 0.000925336 25300
0.002160505 27600 0.02382753 27000 0.002322764
27900 0.002421771 25900 0.000134519 25700 0.000918737 25300
0.002184829 27600 0.02365545 27000 0.002327293
27900 0.002419537 25900 0.000134799 25700 0.000912255 25300
0.002209606 27600 0.02348535 27000 0.002331853
27900 0.00241725 25900 0.000135085 25700 0.000905901 25300
0.002234884 27600 0.02331705 27000 0.002336446
27900 0.002415002 25900 0.000135359 25700 0.000899642 25300
0.002260645 27600 0.02315056 27000 0.002341028
27900 0.002412777 25900 0.000135618 25700 0.0008935 25300
0.002286933 27600 0.02298586 27000 0.002345613
27900 0.002410562 25900 0.000135868 25700 0.000887443 25300
0.002313763 27600 0.02282293 27000 0.002350226
27900 0.002408361 25900 0.000136111 25700 0.000881493 25300
0.002341192 27600 0.02266178 27000 0.002354846
27900 0.002406156 25900 0.00013634 25700 0.000875642 25300
0.002369191 27600 0.02250235 27000 0.002359474
27900 0.002403948 25900 0.000136552 25700 0.000869884 25300
0.002397871 27600 0.02234462 27000 0.002364104
27900 0.002401738 25900 0.000136755 25700 0.000864227 25300
0.002427156 27600 0.02218858 27000 0.002368754
27900 0.00239955 25900 0.000136954 25700 0.000858647 25300
0.002457154 27600 0.02203416 27000 0.002373423
27900 0.002397351 25900 0.000137159 25700 0.000853161 25300
0.00248783 27600 0.02188137 27000 0.002378107
27900 0.002395182 25900 0.000137335 25700 0.000847773 25300
0.00251923 27600 0.02173018 27000 0.002382804
27900 0.002392968 25900 0.000137506 25700 0.000842452 25300
0.002551332 27600 0.02158056 27000 0.002387509
27900 0.002390792 25900 0.000137674 25700 0.000837223 25300
0.002584184 27600 0.02143249 27000 0.002392235
27900 0.002388615 25900 0.000137827 25700 0.000832058 25300
0.002617761 27600 0.02128595 27000 0.002396998
27900 0.002386453 25900 0.000137968 25700 0.000826977 25300
0.002652088 27600 0.02114093 27000 0.002401731
27900 0.002384271 25900 0.000138098 25700 0.000821967 25300
0.002687189 27600 0.0209974 27000 0.002406463
27900 0.002382138 25900 0.000138211 25700 0.000817035 25300
0.002723064 27600 0.02085534 27000 0.002411256
27900 0.002379983 25900 0.000138311 25700 0.000812173 25300
0.002759752 27600 0.02071475 27000 0.002416027
28000 0.002377848 25900 0.000138399 25700 0.000807391 25300
0.002797249 27600 0.02057568 27000 0.002420841
28000 0.002375659 25900 0.000138479 25700 0.000802685 25300
0.002835577 27600 0.02043795 27000 0.002425669
28000 0.002373554 25900 0.000138555 25700 0.000798043 25300
0.002874755 27600 0.02030161 27000 0.002430518
28000 0.002371413 25900 0.000138639 25700 0.000793479 25300
0.002914793 27600 0.02016662 27000 0.002435352
28000 0.002369317 25900 0.000138707 25700 0.000788965 25300
0.002955745 27600 0.02003297 27000 0.002440218
28000 0.002367198 25900 0.000138783 25700 0.000784533 25300
0.002997673 27600 0.01990069 27000 0.002445089
28000 0.002365095 25900 0.000138841 25700 0.000780159 25300
0.003040597 27600 0.01976974 27000 0.002449959
28000 0.002362967 25900 0.000138906 25700 0.00077585 25300
0.003084585 27600 0.01964009 27000 0.002454856
28000 0.002360851 25900 0.000138968 25700 0.000771617 25300
0.003129617 27600 0.01951171 27000 0.002459771
28000 0.002358785 25900 0.000139007 25700 0.000767439 25300
0.003175807 27600 0.01938459 27100 0.0024647
28000 0.002356673 25900 0.00013906 25700 0.000763317 25300
0.003223151 27600 0.01925873 27100 0.002469644

28000 0.002354552 25900 0.00013909 25700 0.000759269 25300
0.003271701 27600 0.01913407 27100 0.002474588
28000 0.002352435 25900 0.000139116 25700 0.000755265 25300
0.003321488 27600 0.01901064 27100 0.002479568
28000 0.002350349 25900 0.000139153 25700 0.000751339 25300
0.00337259 27600 0.01888838 27100 0.002484562
28000 0.002348267 25900 0.000139183 25700 0.000747455 25300
0.003425038 27600 0.01876729 27100 0.002489567
28000 0.002346163 25900 0.000139215 25700 0.000743638 25300
0.003478839 27600 0.01864737 27100 0.002494561
28000 0.002344087 25900 0.000139256 25700 0.000739856 25300
0.00353409 27600 0.01852858 27100 0.002499579
28000 0.002341994 25900 0.000139287 25700 0.000736144 25300
0.003590829 27600 0.01841092 27100 0.002504631
28000 0.002339891 26000 0.00013932 25700 0.000732483 25300
0.003649081 27600 0.01829438 27100 0.00250968
28000 0.002337806 26000 0.000139358 25700 0.000728873 25300
0.003708919 27600 0.018179 27100 0.002514765
28000 0.002335719 26000 0.000139391 25800 0.000725319 25300
0.003770368 27600 0.01806468 27100 0.002519862
28000 0.002333648 26000 0.000139439 25800 0.000721817 25300
0.003833474 27600 0.0179514 27100 0.002524958
28000 0.002331537 26000 0.000139504 25800 0.000718362 25300
0.003898292 27600 0.0178392 27100 0.002530062
28000 0.002329449 26000 0.000139564 25800 0.000714955 25300
0.003964918 27600 0.01772806 27100 0.002535213
28000 0.002327377 26000 0.000139646 25800 0.000711591 25400
0.004033444 27600 0.01761796 27100 0.002540356
28000 0.002325298 26000 0.000139729 25800 0.000708299 25400
0.004103933 27600 0.01750891 27100 0.002545519
28000 0.002323207 26000 0.000139823 25800 0.000705039 25400
0.004176463 27600 0.01740087 27100 0.002550691
28000 0.002321094 26000 0.000139954 25800 0.000701827 25400
0.004251132 27600 0.01729384 27100 0.002555864
28000 0.002319024 26000 0.000140084 25800 0.000698654 25400
0.004328061 27600 0.01718782 27100 0.002561058
28000 0.002316946 26000 0.000140245 25800 0.000695513 25400
0.004407302 27600 0.01708277 27100 0.002566286
28000 0.002314843 26000 0.000140401 25800 0.000692448 25400
0.004488986 27600 0.01697871 27100 0.002571547
28000 0.002312729 26000 0.000140561 25800 0.000689418 25400
0.004573195 27600 0.01687561 27100 0.002576801
28000 0.002310674 26000 0.000140743 25800 0.00068642 25400
0.004660053 27600 0.01677345 27100 0.002582078
28000 0.002308597 26000 0.00014092 25800 0.000683471 25400
0.004749675 27600 0.01667224 27100 0.002587378
28000 0.002306542 26000 0.00014111 25800 0.000680565 25400
0.004842152 27600 0.01657196 27100 0.002592669
28000 0.00230445 26000 0.000141311 25800 0.000677696 25400
0.004937645 27700 0.0164726 27100 0.002597962
28000 0.002302368 26000 0.000141515 25800 0.000674876 25400
0.005036211 27700 0.01637414 27100 0.002603294
28000 0.002300336 26000 0.000141731 25800 0.000672095 25400
0.005137995 27700 0.01627661 27100 0.002608618
28000 0.002298273 26000 0.00014197 25800 0.000669337 25400
0.005243105 27700 0.01618003 27100 0.002613988
28000 0.002296204 26000 0.000142209 25800 0.000666635 25400
0.005351637 27700 0.0160843 27100 0.002619383
28000 0.002294178 26000 0.000142478 25800 0.000663954 25400
0.005463758 27700 0.01598942 27100 0.002624774
28000 0.002292128 26000 0.000142779 25800 0.000661323 25400
0.005579571 27700 0.01589541 27100 0.002630171
28000 0.002290099 26000 0.000143081 25800 0.000658726 25400
0.005699246 27700 0.01580225 27100 0.002635598
28000 0.002288085 26000 0.000143413 25800 0.000656163 25400
0.005822936 27700 0.01570995 27100 0.002641033
28000 0.002286077 26000 0.000143753 25800 0.000653654 25400
0.005950733 27700 0.01561851 27100 0.002646487

28000 0.002284064 26000 0.000144107 25800 0.000651179 25400
0.006082865 27700 0.01552787 27100 0.002651941
28000 0.002282047 26000 0.000144471 25800 0.000648755 25400
0.006219399 27700 0.01543807 27100 0.002657418
28000 0.002280069 26000 0.000144854 25800 0.000646351 25400
0.006360598 27700 0.01534907 27100 0.00266291
28000 0.002278075 26000 0.000145261 25800 0.000643985 25400
0.006506553 27700 0.01526086 27100 0.002668429
28000 0.002276081 26000 0.000145655 25800 0.000641682 25400
0.006657545 27700 0.01517345 27100 0.002673967
28000 0.002274068 26000 0.000146074 25800 0.000639394 25400
0.006813758 27700 0.01508681 27100 0.002679517
28000 0.002272083 26000 0.000146509 25800 0.000637151 25400
0.006975446 27700 0.01500091 27100 0.002685085
28000 0.00227007 26000 0.000146945 25800 0.000634938 25400
0.007142867 27700 0.0149158 27100 0.002690702
28000 0.002268127 26000 0.000147398 25800 0.000632735 25400
0.007316192 27700 0.01483142 27100 0.002696292
28000 0.00226616 26000 0.000147862 25800 0.000630578 25400
0.007495658 27700 0.01474776 27100 0.002701873
28000 0.002264207 26000 0.000148334 25800 0.00062846 25400
0.007681469 27700 0.01466482 27100 0.0027075
28000 0.002262262 26000 0.000148811 25800 0.000626404 25400
0.007873844 27700 0.01458263 27100 0.002713138
28000 0.002260287 26000 0.000149294 25800 0.000624336 25400
0.008073006 27700 0.01450119 27100 0.002718812
28000 0.002258334 26000 0.000149803 25800 0.000622311 25400
0.008279117 27700 0.0144204 27100 0.002724494
28000 0.002256403 26000 0.000150322 25800 0.000620321 25400
0.008492463 27700 0.01434028 27100 0.002730181
28000 0.002254457 26000 0.000150853 25800 0.00061836 25400
0.00871332 27700 0.01426088 27100 0.002735877
28000 0.002252553 26000 0.0001514 25800 0.00061643 25400
0.008941906 27700 0.01418209 27100 0.002741592
28000 0.002250646 26000 0.000151949 25800 0.000614522 25400
0.009178552 27700 0.01410399 27100 0.002747348
28000 0.002248722 26000 0.00015251 25800 0.00061266 25400
0.009423525 27700 0.01402657 27100 0.002753095
28100 0.002246825 26000 0.000153067 25800 0.000610818 25400
0.00967717 27700 0.01394979 27100 0.002758839
28100 0.002244924 26000 0.000153654 25800 0.000609 25400
0.009939806 27700 0.01387364 27100 0.002764622
28100 0.002243022 26000 0.000154249 25800 0.0006072 25400
0.01021188 27700 0.01379811 27100 0.002770411
28100 0.002241154 26000 0.000154878 25800 0.00060542 25400
0.01049374 27700 0.0137232 27100 0.00277624
28100 0.002239251 26000 0.000155489 25800 0.000603688 25400
0.01078583 27700 0.0136489 27100 0.002782082
28100 0.002237343 26000 0.000156119 25800 0.000601958 25400
0.01108851 27700 0.0135752 27100 0.002787936
28100 0.002235494 26000 0.000156754 25800 0.000600274 25400
0.01140218 27700 0.01350212 27100 0.002793818
28100 0.002233609 26000 0.000157404 25800 0.000598601 25400
0.01172722 27700 0.01342961 27100 0.00279973
28100 0.002231739 26000 0.000158049 25800 0.000596945 25400
0.012064 27700 0.01335766 27100 0.002805623
28100 0.002229873 26000 0.000158697 25800 0.000595315 25400
0.01241295 27700 0.01328629 27200 0.002811521
28100 0.002228006 26000 0.000159347 25800 0.000593716 25400
0.01277449 27700 0.01321549 27200 0.002817473
28100 0.002226154 26000 0.000160002 25800 0.000592137 25400
0.01314909 27700 0.01314528 27200 0.002823433
28100 0.00222426 26000 0.00016064 25800 0.000590566 25400
0.01353717 27700 0.0130757 27200 0.002829419
28100 0.002222418 26000 0.000161296 25800 0.000589032 25400
0.0139391 27700 0.01300664 27200 0.002835431
28100 0.002220606 26000 0.000161946 25800 0.00058751 25400
0.01435521 27700 0.0129381 27200 0.002841459

28100 0.002218776 26000 0.00016259 25800 0.000586016 25400
0.01478579 27700 0.0128701 27200 0.002847502
28100 0.002216946 26000 0.000163241 25800 0.000584535 25400
0.01523108 27700 0.01280269 27200 0.002853561
28100 0.00221513 26000 0.000163889 25800 0.000583076 25400
0.01569131 27700 0.0127358 27200 0.002859627
28100 0.002213336 26100 0.000164515 25800 0.000581644 25400
0.01616661 27700 0.0126695 27200 0.002865726
28100 0.002211543 26100 0.000165143 25800 0.000580229 25400
0.01665708 27700 0.01260372 27200 0.002871827
28100 0.002209737 26100 0.000165755 25900 0.000578853 25400
0.01716273 27700 0.01253847 27200 0.002877949
28100 0.002207935 26100 0.000166356 25900 0.000577483 25400
0.01768352 27700 0.01247378 27200 0.002884105
28100 0.002206177 26100 0.000166963 25900 0.000576115 25400
0.01821925 27700 0.01240962 27200 0.00289027
28100 0.002204382 26100 0.000167544 25900 0.0005748 25400
0.01876969 27700 0.01234597 27200 0.00289646
28100 0.002202584 26100 0.0001681 25900 0.000573477 25500
0.01933438 27700 0.01228285 27200 0.002902678
28100 0.002200843 26100 0.000168652 25900 0.000572193 25500
0.0199128 27700 0.01222022 27200 0.002908917
28100 0.002199069 26100 0.000169177 25900 0.00057091 25500
0.0205042 27700 0.01215809 27200 0.002915193
28100 0.002197289 26100 0.000169688 25900 0.000569654 25500
0.02110772 27700 0.01209649 27200 0.002921454
28100 0.002195544 26100 0.000170172 25900 0.00056841 25500
0.02172218 27700 0.01203536 27200 0.002927708
28100 0.002193804 26100 0.000170637 25900 0.000567193 25500
0.02234631 27700 0.01197474 27200 0.002934019
28100 0.002192077 26100 0.000171079 25900 0.000565987 25500
0.02297845 27700 0.01191459 27200 0.00294034
28100 0.002190338 26100 0.000171496 25900 0.000564806 25500
0.02361666 27700 0.01185501 27200 0.002946692
28100 0.002188598 26100 0.000171886 25900 0.000563642 25500
0.02425863 27700 0.01179584 27200 0.00295307
28100 0.002186907 26100 0.000172255 25900 0.000562502 25500
0.02490171 27700 0.01173711 27200 0.00295946
28100 0.00218518 26100 0.000172602 25900 0.000561366 25500
0.02554294 27700 0.01167886 27200 0.00296586
28100 0.002183494 26100 0.00017292 25900 0.000560251 25500
0.02617896 27800 0.01162106 27200 0.002972271
28100 0.002181815 26100 0.000173215 25900 0.000559148 25500
0.02680614 27800 0.01156371 27200 0.002978715
28100 0.002180106 26100 0.000173502 25900 0.000558076 25500
0.02742041 27800 0.01150683 27200 0.002985182
28100 0.00217842 26100 0.000173753 25900 0.00055703 25500
0.02801741 27800 0.01145038 27200 0.002991644
28100 0.002176718 26100 0.00017399 25900 0.000555987 25500
0.02859229 27800 0.01139436 27200 0.002998127
28100 0.002175069 26100 0.000174212 25900 0.000554957 25500
0.02913998 27800 0.01133878 27200 0.003004655
28100 0.002173364 26100 0.00017441 25900 0.000553934 25500
0.02965496 27800 0.01128361 27200 0.003011192
28100 0.002171721 26100 0.000174588 25900 0.000552954 25500
0.03013148 27800 0.01122886 27200 0.003017753
28100 0.002170023 26100 0.000174732 25900 0.00055196 25500
0.03056351 27800 0.01117453 27200 0.003024339
28100 0.002168365 26100 0.000174854 25900 0.000551008 25500
0.03094488 27800 0.01112058 27200 0.00303096
28100 0.002166692 26100 0.00017496 25900 0.000550054 25500
0.03126934 27800 0.01106703 27200 0.003037609
28100 0.002165025 26100 0.000175008 25900 0.000549117 25500
0.03153077 27800 0.01101385 27200 0.003044261
28100 0.00216338 26100 0.000175066 25900 0.000548196 25500
0.03172323 27800 0.01096105 27200 0.003050907
28100 0.00216175 26100 0.000175069 25900 0.000547297 25500
0.03184119 27800 0.01090865 27200 0.0030576

28100 0.002160095 26100 0.000175067 25900 0.000546417 25500
0.03187969 27800 0.01085663 27200 0.00306433
28100 0.002158455 26100 0.000175026 25900 0.000545538 25500
0.03183436 27800 0.01080502 27200 0.003071083
28100 0.002156782 26100 0.000174959 25900 0.000544677 25500
0.03170177 27800 0.01075374 27200 0.00307786
28100 0.002155165 26100 0.000174891 25900 0.000543829 25500
0.03147948 27800 0.01070281 27200 0.003084669
28100 0.002153531 26100 0.000174779 25900 0.000543 25500
0.03116618 27800 0.01065225 27200 0.003091482
28100 0.002151911 26100 0.000174673 25900 0.000542177 25500
0.03076181 27800 0.01060204 27200 0.003098324
28100 0.002150277 26100 0.000174541 25900 0.000541373 25500
0.03026756 27800 0.01055217 27200 0.003105199
28100 0.002148675 26100 0.000174385 25900 0.000540595 25500
0.02968577 27800 0.01050266 27200 0.003112083
28100 0.002147058 26100 0.000174215 25900 0.000539824 25500
0.02902006 27800 0.01045353 27200 0.003118983
28100 0.002145417 26100 0.000174048 25900 0.000539063 25500
0.02827527 27800 0.01040469 27200 0.0031259
28100 0.002143813 26100 0.00017387 25900 0.000538317 25500
0.02745743 27800 0.01035624 27200 0.003132863
28100 0.002142205 26100 0.000173686 25900 0.000537581 25500
0.02657368 27800 0.01030812 27200 0.00313985
28100 0.002140587 26100 0.000173497 25900 0.000536865 25500
0.02563215 27800 0.01026034 27200 0.003146863
28100 0.002138934 26100 0.000173295 25900 0.00053616 25500
0.02464182 27800 0.01021289 27200 0.003153889
28100 0.00213734 26100 0.000173095 25900 0.000535461 25500
0.02361205 27800 0.01016578 27200 0.003160951
28200 0.002135703 26100 0.000172878 25900 0.000534774 25500
0.02255259 27800 0.010119 27200 0.003168051
28200 0.002134062 26100 0.00017265 25900 0.000534112 25500
0.02147318 27800 0.01007254 27200 0.00317515
28200 0.002132451 26100 0.00017241 25900 0.000533456 25500
0.02038341 27800 0.01002641 27200 0.003182236
28200 0.002130843 26100 0.000172166 25900 0.000532798 25500
0.01929256 27800 0.00998062 27200 0.00318938
28200 0.002129256 26100 0.000171914 25900 0.00053217 25500
0.01820947 27800 0.009935185 27200 0.003196539
28200 0.002127647 26100 0.000171648 25900 0.00053154 25500
0.01714232 27800 0.009890109 27200 0.003203748
28200 0.002126039 26100 0.000171385 25900 0.000530936 25500
0.01609859 27800 0.009845325 27200 0.003210947
28200 0.002124458 26100 0.000171135 25900 0.000530333 25500
0.01508487 27800 0.009800827 27200 0.003218187
28200 0.002122848 26100 0.000170883 25900 0.000529752 25500
0.0141068 27800 0.009756659 27200 0.003225425
28200 0.002121224 26100 0.000170616 25900 0.000529178 25500
0.01316899 27800 0.009712836 27300 0.003232697
28200 0.002119631 26100 0.000170374 25900 0.000528616 25500
0.01227511 27800 0.009669288 27300 0.003240007
28200 0.002118046 26100 0.000170127 25900 0.000528074 25500
0.01142782 27800 0.009626073 27300 0.003247322
28200 0.002116441 26100 0.000169874 25900 0.000527525 25500
0.01062892 27800 0.009583173 27300 0.003254649
28200 0.002114841 26100 0.000169623 25900 0.000527006 25500
0.009879358 27800 0.009540601 27300 0.003261978
28200 0.002113272 26100 0.00016937 25900 0.000526503 25500
0.00917942 27800 0.009498322 27300 0.003269355
28200 0.002111686 26100 0.00016912 25900 0.000526006 25500
0.008528738 27800 0.009456295 27300 0.003276766
28200 0.002110087 26100 0.000168866 25900 0.000525506 25500
0.007926331 27800 0.009414597 27300 0.003284176
28200 0.002108475 26100 0.000168606 25900 0.000525039 25500
0.007370826 27800 0.009373159 27300 0.003291632
28200 0.002106898 26200 0.000168365 25900 0.000524571 25500
0.006860462 27800 0.009332043 27300 0.003299098

28200 0.002105322 26200 0.00016813 25900 0.000524087 25500
0.006393095 27800 0.009291166 27300 0.003306594
28200 0.002103744 26200 0.000167889 26000 0.000523646 25500
0.005966462 27800 0.009250569 27300 0.003314099
28200 0.002102179 26200 0.000167657 26000 0.000523209 25500
0.005578123 27800 0.009210218 27300 0.003321588
28200 0.002100605 26200 0.000167418 26000 0.000522783 25500
0.005225603 27800 0.009170162 27300 0.003329143
28200 0.002099061 26200 0.000167195 26000 0.000522364 25500
0.004906348 27800 0.009130377 27300 0.003336719
28200 0.00209748 26200 0.000166958 26000 0.000521964 25600
0.004617904 27800 0.009090881 27300 0.003344334
28200 0.00209592 26200 0.000166743 26000 0.000521559 25600
0.004357774 27800 0.009051626 27300 0.003351956
28200 0.002094356 26200 0.000166549 26000 0.00052117 25600
0.004123659 27800 0.009012623 27300 0.003359617
28200 0.002092827 26200 0.000166365 26000 0.000520816 25600
0.003913236 27800 0.008973879 27300 0.003367271
28200 0.00209128 26200 0.000166184 26000 0.000520414 25600
0.003724405 27800 0.008935411 27300 0.003374985
28200 0.00208975 26200 0.000166029 26000 0.000520088 25600
0.003555155 27800 0.008897212 27300 0.003382712
28200 0.002088219 26200 0.000165867 26000 0.00051976 25600
0.003403552 27800 0.008859242 27300 0.00339046
28200 0.002086707 26200 0.000165702 26000 0.00051942 25600
0.003267918 27800 0.008821566 27300 0.00339822
28200 0.002085193 26200 0.000165546 26000 0.000519106 25600
0.003146593 27800 0.008784126 27300 0.003405996
28200 0.002083691 26200 0.000165408 26000 0.000518803 25600
0.003038127 27800 0.008746932 27300 0.00341383
28200 0.002082192 26200 0.000165268 26000 0.000518516 25600
0.002941109 27800 0.008710017 27300 0.003421697
28200 0.002080669 26200 0.000165116 26000 0.000518188 25600
0.002854333 27900 0.008673332 27300 0.003429567
28200 0.002079139 26200 0.000164977 26000 0.000517899 25600
0.002776726 27900 0.008636854 27300 0.003437497
28200 0.002077644 26200 0.000164843 26000 0.000517621 25600
0.002707228 27900 0.008600646 27300 0.003445447
28200 0.002076141 26200 0.000164717 26000 0.000517338 25600
0.002644951 27900 0.008564687 27300 0.003453432
28200 0.002074646 26200 0.000164594 26000 0.000517095 25600
0.002589046 27900 0.008528938 27300 0.003461428
28200 0.002073175 26200 0.000164473 26000 0.000516838 25600
0.002538793 27900 0.008493419 27300 0.003469439
28200 0.002071697 26200 0.000164367 26000 0.000516597 25600
0.002493531 27900 0.008458143 27300 0.003477503
28200 0.002070228 26200 0.000164267 26000 0.00051635 25600
0.002452699 27900 0.008423119 27300 0.003485589
28200 0.002068765 26200 0.000164166 26000 0.00051613 25600
0.002415776 27900 0.00838834 27300 0.003493716
28200 0.002067283 26200 0.000164082 26000 0.000515909 25600
0.0023823 27900 0.008353753 27300 0.003501857
28200 0.002065829 26200 0.00016401 26000 0.00051569 25600
0.002351885 27900 0.008319373 27300 0.003510023
28200 0.002064385 26200 0.000163947 26000 0.00051551 25600
0.002324154 27900 0.00828522 27300 0.003518213
28200 0.002062932 26200 0.00016389 26000 0.000515323 25600
0.002298793 27900 0.008251294 27300 0.003526426
28200 0.00206149 26200 0.00016385 26000 0.000515158 25600
0.002275519 27900 0.008217568 27300 0.003534666
28200 0.002060048 26200 0.000163807 26000 0.000514996 25600
0.002254084 27900 0.008184088 27300 0.003542927
28200 0.00205863 26200 0.000163761 26000 0.00051483 25600
0.002234293 27900 0.008150812 27300 0.003551211
28200 0.002057174 26200 0.000163737 26000 0.000514686 25600
0.002215956 27900 0.00811774 27300 0.003559524
28200 0.00205576 26200 0.000163707 26000 0.000514515 25600
0.002198911 27900 0.008084896 27300 0.003567879

28200 0.002054326 26200 0.000163686 26000 0.000514385 25600
0.002182986 27900 0.008052267 27300 0.00357625
28200 0.002052905 26200 0.000163677 26000 0.00051427 25600
0.002168064 27900 0.008019825 27300 0.003584652
28200 0.002051439 26200 0.000163659 26000 0.00051414 25600
0.002154048 27900 0.007987607 27300 0.003593084
28200 0.002050019 26200 0.000163652 26000 0.000514029 25600
0.00214079 27900 0.007955565 27300 0.00360155
28200 0.002048576 26200 0.00016365 26000 0.000513935 25600
0.002128255 27900 0.007923746 27300 0.00361006
28200 0.002047157 26200 0.000163642 26000 0.00051383 25600
0.002116316 27900 0.007892109 27300 0.003618545
28200 0.002045726 26200 0.000163655 26000 0.000513721 25600
0.002104944 27900 0.007860671 27300 0.00362706
28200 0.002044289 26200 0.000163671 26000 0.000513669 25600
0.002094083 27900 0.007829418 27300 0.003635638
28200 0.002042874 26200 0.000163688 26000 0.000513577 25600
0.002083692 27900 0.007798393 27300 0.003644239
28200 0.002041454 26200 0.000163721 26000 0.000513523 25600
0.002073711 27900 0.007767584 27300 0.003652879
28200 0.00204003 26200 0.000163758 26000 0.000513464 25600
0.002064123 27900 0.007736939 27300 0.003661548
28300 0.002038618 26200 0.000163775 26000 0.000513422 25600
0.002054895 27900 0.007706483 27300 0.003670253
28300 0.002037176 26200 0.00016383 26000 0.00051336 25600
0.002046012 27900 0.007676226 27300 0.00367896
28300 0.002035771 26200 0.000163855 26000 0.000513321 25600
0.002037435 27900 0.007646143 27300 0.003687717
28300 0.002034368 26200 0.000163919 26000 0.000513296 25600
0.002029169 27900 0.007616282 27300 0.003696491
28300 0.002032951 26200 0.000163958 26000 0.00051327 25600
0.002021177 27900 0.007586598 27300 0.003705282
28300 0.002031549 26200 0.000164002 26000 0.000513249 25600
0.002013476 27900 0.007557099 27300 0.003714116
28300 0.002030135 26200 0.000164055 26000 0.000513238 25600
0.002006032 27900 0.007527796 27300 0.00372298
28300 0.002028741 26200 0.000164095 26000 0.000513227 25600
0.00199885 27900 0.007498676 27300 0.003731881
28300 0.002027319 26200 0.000164146 26000 0.00051323 25600
0.001991906 27900 0.007469727 27300 0.003740828
28300 0.002025928 26200 0.000164199 26000 0.00051325 25600
0.001985216 27900 0.007440978 27400 0.003749792
28300 0.002024488 26200 0.000164255 26000 0.000513267 25600
0.001978763 27900 0.007412395 27400 0.003758798
28300 0.002023096 26200 0.000164296 26000 0.000513275 25600
0.001972569 27900 0.007383979 27400 0.003767848
28300 0.002021684 26200 0.000164356 26000 0.000513305 25600
0.001966621 27900 0.007355742 27400 0.003776932
28300 0.002020288 26200 0.00016442 26000 0.000513329 25600
0.001960897 27900 0.00732767 27400 0.003786011
28300 0.002018881 26200 0.000164476 26000 0.000513354 25600
0.00195539 27900 0.007299775 27400 0.003795131
28300 0.002017499 26200 0.000164562 26000 0.00051341 25600
0.001950068 27900 0.007272056 27400 0.003804326
28300 0.002016104 26200 0.00016463 26000 0.000513443 25600
0.001944954 27900 0.007244508 27400 0.003813522
28300 0.002014725 26200 0.000164691 26000 0.000513473 25600
0.00194004 27900 0.007217182 27400 0.003822764
28300 0.002013319 26300 0.000164786 26000 0.000513552 25600
0.001935346 27900 0.007189981 27400 0.003832033
28300 0.00201194 26300 0.00016485 26000 0.00051362 25600
0.001930884 27900 0.007162933 27400 0.003841351
28300 0.002010572 26300 0.000164944 26100 0.000513698 25600
0.001926663 27900 0.007136033 27400 0.00385068
28300 0.002009212 26300 0.000165022 26100 0.000513758 25600
0.001922721 27900 0.00710929 27400 0.003860044
28300 0.002007822 26300 0.000165102 26100 0.000513851 25600
0.001919073 27900 0.00708273 27400 0.00386943

28300 0.002006448 26300 0.000165214 26100 0.000513958 25600
0.001915736 27900 0.007056326 27400 0.003878847
28300 0.002005083 26300 0.000165298 26100 0.000514049 25700
0.0019127 27900 0.007030085 27400 0.003888288
28300 0.002003716 26300 0.000165415 26100 0.00051414 25700
0.001909921 27900 0.007004008 27400 0.003897772
28300 0.002002364 26300 0.000165504 26100 0.000514253 25700
0.001907429 27900 0.006978083 27400 0.003907288
28300 0.002000977 26300 0.000165633 26100 0.00051437 25700
0.001905167 27900 0.006952315 27400 0.003916821
28300 0.00199961 26300 0.000165746 26100 0.000514474 25700
0.001903157 27900 0.006926706 27400 0.003926401
28300 0.001998221 26300 0.000165857 26100 0.000514602 25700
0.001901383 27900 0.006901255 27400 0.003936011
28300 0.001996851 26300 0.000165993 26100 0.00051473 25700
0.00189988 27900 0.006875943 27400 0.003945673
28300 0.001995478 26300 0.000166126 26100 0.000514858 25700
0.001898651 27900 0.006850781 27400 0.003955348
28300 0.001994089 26300 0.00016625 26100 0.000514998 25700
0.001897717 27900 0.006825774 27400 0.003965028
28300 0.001992721 26300 0.00016639 26100 0.000515145 25700
0.001897072 27900 0.00680091 27400 0.003974725
28300 0.001991342 26300 0.000166518 26100 0.000515321 25700
0.001896772 27900 0.006776208 27400 0.003984503
28300 0.00198997 26300 0.00016665 26100 0.000515482 25700
0.001896767 28000 0.006751644 27400 0.003994289
28300 0.001988602 26300 0.000166793 26100 0.000515644 25700
0.001897105 28000 0.006727264 27400 0.004004105
28300 0.001987229 26300 0.000166951 26100 0.000515821 25700
0.00189779 28000 0.006703002 27400 0.004013955
28300 0.001985872 26300 0.000167097 26100 0.000516005 25700
0.001898816 28000 0.006678874 27400 0.004023835
28300 0.001984501 26300 0.000167258 26100 0.000516173 25700
0.001900195 28000 0.006654904 27400 0.004033727
28300 0.001983146 26300 0.000167413 26100 0.000516369 25700
0.001901883 28000 0.006631081 27400 0.004043663
28300 0.001981781 26300 0.000167575 26100 0.000516576 25700
0.001903896 28000 0.006607404 27400 0.004053615
28300 0.001980436 26300 0.000167744 26100 0.000516773 25700
0.001906215 28000 0.006583887 27400 0.004063578
28300 0.001979084 26300 0.000167928 26100 0.000516986 25700
0.001908858 28000 0.0065605 27400 0.004073587
28300 0.001977709 26300 0.000168108 26100 0.000517198 25700
0.001911791 28000 0.006537262 27400 0.004083626
28300 0.001976382 26300 0.000168272 26100 0.000517423 25700
0.001915065 28000 0.006514156 27400 0.004093698
28300 0.001975023 26300 0.000168456 26100 0.000517651 25700
0.001918647 28000 0.006491179 27400 0.004103825
28300 0.001973687 26300 0.000168607 26100 0.000517873 25700
0.001922567 28000 0.006468337 27400 0.004113987
28300 0.001972322 26300 0.000168756 26100 0.000518112 25700
0.001926824 28000 0.006445646 27400 0.004124179
28300 0.001970983 26300 0.000168915 26100 0.000518357 25700
0.00193142 28000 0.006423063 27400 0.004134394
28300 0.001969668 26300 0.000169044 26100 0.000518613 25700
0.001936373 28000 0.006400606 27400 0.004144644
28300 0.001968323 26300 0.000169182 26100 0.000518868 25700
0.001941678 28000 0.006378264 27400 0.004154908
28300 0.001966996 26300 0.000169334 26100 0.00051913 25700
0.001947362 28000 0.00635605 27400 0.004165167
28300 0.001965669 26300 0.000169458 26100 0.000519397 25700
0.001953437 28000 0.006333964 27400 0.004175517
28300 0.001964373 26300 0.000169567 26100 0.000519665 25700
0.001959936 28000 0.006311989 27400 0.0041859
28300 0.001963046 26300 0.000169695 26100 0.000519939 25700
0.001966859 28000 0.006290162 27400 0.004196325
28300 0.001961727 26300 0.000169829 26100 0.000520232 25700
0.001974236 28000 0.006268403 27400 0.004206778

28300 0.001960417 26300 0.000169954 26100 0.000520509 25700
0.00198205 28000 0.00624674 27400 0.004217265
28300 0.001959129 26300 0.000170115 26100 0.000520788 25700
0.001990274 28000 0.006225199 27400 0.004227778
28300 0.001957834 26300 0.000170288 26100 0.00052107 25700
0.001998923 28000 0.006203753 27400 0.004238346
28300 0.001956557 26300 0.000170468 26100 0.000521381 25700
0.002008016 28000 0.006182424 27400 0.004248965
28300 0.00195528 26300 0.000170639 26100 0.000521681 25700
0.00201755 28000 0.006161192 27400 0.004259601
28300 0.001954003 26300 0.000170813 26100 0.000521996 25700
0.002027533 28000 0.006140067 27400 0.00427029
28300 0.001952714 26300 0.000170999 26100 0.0005223 25700
0.002037974 28000 0.006119036 27400 0.004280999
28400 0.00195146 26300 0.00017119 26100 0.000522628 25700
0.002048928 28000 0.006098122 27400 0.004291772
28400 0.001950193 26300 0.000171384 26100 0.000522943 25700
0.002060384 28000 0.006077291 27400 0.004302599
28400 0.001948909 26300 0.000171601 26100 0.000523271 25700
0.002072393 28000 0.00605657 27400 0.004313469
28400 0.001947627 26300 0.000171795 26100 0.000523613 25700
0.00208498 28000 0.006035947 27400 0.004324391
28400 0.001946379 26300 0.000171984 26100 0.000523939 25700
0.002098155 28000 0.006015421 27400 0.004335345
28400 0.001945093 26300 0.000172188 26100 0.000524261 25700
0.002111954 28000 0.005994987 27400 0.004346348
28400 0.001943831 26300 0.000172419 26100 0.00052462 25700
0.002126402 28000 0.005974666 27400 0.004357378
28400 0.001942572 26300 0.000172616 26100 0.000524955 25700
0.00214151 28000 0.00595444 27400 0.004368441
28400 0.001941307 26300 0.000172819 26100 0.000525316 25700
0.00215727 28000 0.005934309 27400 0.004379565
28400 0.001940059 26300 0.000173039 26100 0.000525677 25700
0.002173694 28000 0.005914298 27500 0.004390745
28400 0.001938797 26300 0.000173232 26100 0.000526039 25700
0.002190827 28000 0.005894414 27500 0.004401973
28400 0.001937545 26300 0.000173463 26100 0.000526409 25700
0.00220861 28000 0.005874586 27500 0.00441322
28400 0.001936308 26300 0.00017372 26100 0.000526783 25700
0.00222704 28000 0.00585486 27500 0.00442451
28400 0.001935058 26300 0.000173914 26100 0.000527154 25700
0.002246122 28000 0.005835253 27500 0.00443583
28400 0.00193381 26300 0.000174084 26100 0.00052752 25700
0.002265834 28000 0.005815743 27500 0.004447214
28400 0.001932557 26300 0.000174313 26100 0.000527912 25700
0.002286207 28000 0.005796333 27500 0.004458634
28400 0.001931314 26300 0.000174534 26100 0.000528304 25700
0.002307271 28000 0.005777039 27500 0.004470101
28400 0.001930062 26300 0.000174708 26100 0.000528709 25700
0.002329075 28000 0.005757859 27500 0.004481622
28400 0.001928805 26400 0.000174907 26100 0.000529106 25700
0.002351633 28000 0.005738772 27500 0.004493188
28400 0.001927566 26400 0.000175134 26100 0.000529508 25700
0.002374991 28000 0.0057198 27500 0.004504782
28400 0.00192634 26400 0.000175337 26200 0.000529922 25700
0.002399185 28000 0.005700926 27500 0.004516442
28400 0.001925103 26400 0.00017553 26200 0.000530331 25700
0.00242423 28000 0.005682175 27500 0.004528154
28400 0.001923849 26400 0.000175758 26200 0.000530748 25700
0.00245015 28000 0.005663513 27500 0.004539902
28400 0.001922624 26400 0.000175988 26200 0.000531173 25700
0.002476951 28000 0.005644955 27500 0.004551703
28400 0.001921384 26400 0.000176205 26200 0.000531585 25800
0.002504635 28000 0.005626501 27500 0.004563565
28400 0.001920152 26400 0.000176413 26200 0.000532022 25800
0.00253321 28000 0.005608152 27500 0.00457544
28400 0.00191894 26400 0.00017664 26200 0.000532441 25800
0.002562679 28000 0.005589905 27500 0.004587349

28400 0.001917716 26400 0.000176875 26200 0.000532875 25800
0.002593009 28000 0.005571767 27500 0.004599332
28400 0.00191651 26400 0.000177095 26200 0.000533313 25800
0.002624233 28000 0.005553741 27500 0.004611371
28400 0.00191528 26400 0.000177291 26200 0.000533777 25800
0.002656344 28000 0.005535834 27500 0.004623457
28400 0.001914081 26400 0.000177527 26200 0.000534228 25800
0.002689338 28000 0.005517999 27500 0.004635597
28400 0.001912879 26400 0.000177741 26200 0.00053468 25800
0.002723202 28000 0.005500251 27500 0.00464776
28400 0.001911712 26400 0.000177963 26200 0.000535132 25800
0.002757931 28000 0.005482617 27500 0.004659994
28400 0.001910509 26400 0.000178218 26200 0.000535587 25800
0.002793519 28000 0.005465068 27500 0.004672262
28400 0.001909356 26400 0.000178463 26200 0.000536053 25800
0.00282994 28000 0.00544762 27500 0.004684577
28400 0.001908167 26400 0.000178715 26200 0.000536537 25800
0.002867182 28100 0.00543028 27500 0.004696934
28400 0.001906993 26400 0.000178951 26200 0.000537024 25800
0.002905201 28100 0.005413022 27500 0.004709348
28400 0.001905797 26400 0.000179194 26200 0.00053751 25800
0.002943949 28100 0.005395859 27500 0.004721796
28400 0.00190465 26400 0.000179442 26200 0.000538 25800
0.002983402 28100 0.005378786 27500 0.004734307
28400 0.001903495 26400 0.000179696 26200 0.000538492 25800
0.003023514 28100 0.005361798 27500 0.004746867
28400 0.00190232 26400 0.000179943 26200 0.000538988 25800
0.003064227 28100 0.005344899 27500 0.004759473
28400 0.001901139 26400 0.000180202 26200 0.000539498 25800
0.003105513 28100 0.005328089 27500 0.004772139
28400 0.001900007 26400 0.000180437 26200 0.000540015 25800
0.003147262 28100 0.005311348 27500 0.004784859
28400 0.001898856 26400 0.000180688 26200 0.00054053 25800
0.003189385 28100 0.00529469 27500 0.004797623
28400 0.001897704 26400 0.000180933 26200 0.00054105 25800
0.003231798 28100 0.005278121 27500 0.00481042
28400 0.001896562 26400 0.000181166 26200 0.000541564 25800
0.003274363 28100 0.005261634 27500 0.00482323
28400 0.001895419 26400 0.000181394 26200 0.000542094 25800
0.003316944 28100 0.005245238 27500 0.004836131
28400 0.001894279 26400 0.000181625 26200 0.000542638 25800
0.003359397 28100 0.005228939 27500 0.004849082
28400 0.001893166 26400 0.000181851 26200 0.000543179 25800
0.003401532 28100 0.005212755 27500 0.00486209
28400 0.001892017 26400 0.000182079 26200 0.00054372 25800
0.0034432 28100 0.00519662 27500 0.00487513
28400 0.001890893 26400 0.000182317 26200 0.000544268 25800
0.00348415 28100 0.005180569 27500 0.004888216
28400 0.001889781 26400 0.000182555 26200 0.000544815 25800
0.003524223 28100 0.005164607 27500 0.004901367
28400 0.001888659 26400 0.000182788 26200 0.000545376 25800
0.003563162 28100 0.005148723 27500 0.004914547
28400 0.001887567 26400 0.00018304 26200 0.000545933 25800
0.003600761 28100 0.005132946 27500 0.004927758
28400 0.001886461 26400 0.000183281 26200 0.000546502 25800
0.003636787 28100 0.005117261 27500 0.004941027
28400 0.001885364 26400 0.000183543 26200 0.000547085 25800
0.003671006 28100 0.005101664 27500 0.004954358
28400 0.00188425 26400 0.00018379 26200 0.000547668 25800
0.003703116 28100 0.005086146 27500 0.004967734
28400 0.001883158 26400 0.000184058 26200 0.00054824 25800
0.003732901 28100 0.005070713 27500 0.004981154
28400 0.001882053 26400 0.000184337 26200 0.00054882 25800
0.00376005 28100 0.005055368 27500 0.004994616
28400 0.00188096 26400 0.000184612 26200 0.000549414 25800
0.003784282 28100 0.005040101 27500 0.005008151
28400 0.001879853 26400 0.000184884 26200 0.000549998 25800
0.003805295 28100 0.005024912 27500 0.005021748

```
28400   0.001878766     26400   0.000185164     26200   0.000550596     25800
0.00382283      28100   0.005009799     27500   0.005035388
28400   0.001877688     26400   0.000185447     26200   0.000551192     25800
0.003836567     28100   0.004994761     27500   0.00504908
28400   0.001876594     26400   0.000185717     26200   0.000551794     25800
0.003846286     28100   0.004979803     27500   0.005062818
28500   0.001875518     26400   0.000186        26200   0.000552403     25800
0.003851709     28100   0.004964916     27500   0.005076568
28500   0.001874458     26400   0.000186269     26200   0.000553012     25800
0.003852604     28100   0.004950101     27500   0.005090428
28500   0.001873371     26400   0.000186548     26200   0.000553637     25800
0.003848779     28100   0.004935377     27500   0.005104341
28500   0.001872302     26400   0.000186805     26200   0.000554253     25800
0.003840029     28100   0.004920726     27500   0.005118314
28500   0.001871222     26400   0.000187074     26200   0.000554867     25800
0.003826207     28100   0.004906124     27500   0.005132325
28500   0.001870139     26400   0.000187346     26200   0.000555492     25800
0.0038072       28100   0.004891585     27500   0.005146414
28500   0.001869088     26400   0.000187625     26200   0.000556113     25800
0.003782978     28100   0.004877125     27500   0.005160555
28500   0.00186802      26400   0.00018789      26200   0.000556732     25800
0.003753606     28100   0.004862732     27500   0.005174749
28500   0.001866976     26400   0.000188155     26200   0.000557368     25800
0.00371917      28100   0.004848413     27500   0.005188991
28500   0.001865927     26400   0.000188403     26200   0.000557998     25800
0.003679879     28100   0.004834165     27600   0.005203295
28500   0.001864881     26400   0.000188633     26200   0.00055865      25800
0.003635941     28100   0.00481997      27600   0.005217664
28500   0.001863822     26400   0.000188872     26200   0.000559296     25800
0.003587619     28100   0.00480583      27600   0.005232098
28500   0.001862787     26400   0.000189117     26200   0.000559953     25800
0.003535236     28100   0.004791755     27600   0.005246584
28500   0.001861744     26400   0.000189374     26200   0.00056062      25800
0.003479114     28100   0.00477773      27600   0.005261145
28500   0.001860701     26400   0.000189632     26200   0.000561272     25800
0.003419602     28100   0.004763763     27600   0.005275772
28500   0.001859641     26400   0.000189905     26200   0.000561936     25800
0.003357108     28100   0.004749869     27600   0.005290472
28500   0.001858618     26400   0.000190174     26200   0.000562596     25800
0.00329204      28100   0.004736035     27600   0.005305241
28500   0.001857595     26400   0.000190455     26200   0.000563264     25800
0.003224857     28100   0.00472225      27600   0.005320077
28500   0.001856561     26500   0.000190731     26200   0.000563926     25800
0.003156085     28100   0.004708545     27600   0.005334954
28500   0.001855547     26500   0.000191015     26200   0.000564624     25800
0.003086227     28100   0.004694885     27600   0.005349863
28500   0.001854547     26500   0.0001913       26300   0.000565295     25800
0.003015809     28100   0.004681289     27600   0.00536489
28500   0.001853546     26500   0.000191591     26300   0.000565996     25800
0.002945352     28100   0.004667778     27600   0.005379974
28500   0.001852543     26500   0.000191875     26300   0.000566703     25800
0.002875342     28100   0.004654333     27600   0.005395139
28500   0.00185154      26500   0.000192156     26300   0.000567405     25800
0.002806218     28100   0.004640921     27600   0.005410345
28500   0.00185054      26500   0.00019243      26300   0.000568115     25900
0.0027384       28100   0.004627575     27600   0.00542562
28500   0.001849529     26500   0.000192716     26300   0.000568809     25900
0.002672286     28100   0.004614281     27600   0.005440968
28500   0.00184852      26500   0.000193        26300   0.000569519     25900
0.002608224     28100   0.004601032     27600   0.005456369
28500   0.001847519     26500   0.000193287     26300   0.000570247     25900
0.002546526     28100   0.004587872     27600   0.005471822
28500   0.001846513     26500   0.000193567     26300   0.000570985     25900
0.002487497     28100   0.004574781     27600   0.005487343
28500   0.001845493     26500   0.000193855     26300   0.000571715     25900
0.002431359     28100   0.004561744     27600   0.005502942
28500   0.001844463     26500   0.000194137     26300   0.000572463     25900
0.002378293     28100   0.004548758     27600   0.005518583
```

28500 0.001843453 26500 0.000194424 26300 0.000573195 25900
0.002328449 28100 0.004535836 27600 0.005534307
28500 0.001842407 26500 0.000194714 26300 0.00057394 25900
0.00228191 28100 0.004522972 27600 0.005550102
28500 0.001841382 26500 0.000195009 26300 0.000574689 25900
0.002238703 28100 0.004510181 27600 0.005565952
28500 0.001840311 26500 0.000195305 26300 0.000575436 25900
0.002198841 28100 0.004497436 27600 0.005581885
28500 0.00183928 26500 0.000195607 26300 0.000576193 25900
0.002162325 28200 0.004484732 27600 0.005597884
28500 0.001838222 26500 0.000195904 26300 0.000576941 25900
0.002129081 28200 0.004472135 27600 0.005613938
28500 0.00183715 26500 0.000196194 26300 0.000577705 25900
0.002099028 28200 0.004459563 27600 0.00563005
28500 0.001836115 26500 0.000196493 26300 0.000578484 25900
0.00207208 28200 0.004447017 27600 0.005646183
28500 0.001835051 26500 0.000196786 26300 0.00057925 25900
0.002048052 28200 0.004434542 27600 0.005662435
28500 0.001833987 26500 0.000197094 26300 0.000580035 25900
0.002026831 28200 0.004422136 27600 0.005678758
28500 0.001832909 26500 0.000197392 26300 0.000580818 25900
0.002008245 28200 0.004409796 27600 0.00569515
28500 0.001831834 26500 0.000197703 26300 0.000581609 25900
0.001992115 28200 0.004397507 27600 0.005711599
28500 0.00183076 26500 0.000198023 26300 0.000582401 25900
0.001978281 28200 0.004385265 27600 0.005728116
28500 0.001829711 26500 0.00019834 26300 0.000583195 25900
0.001966567 28200 0.004373067 27600 0.005744687
28500 0.001828645 26500 0.00019866 26300 0.000583976 25900
0.001956827 28200 0.004360939 27600 0.00576133
28500 0.001827592 26500 0.000198979 26300 0.000584795 25900
0.001948889 28200 0.004348878 27600 0.005778026
28500 0.001826538 26500 0.000199296 26300 0.000585609 25900
0.001942588 28200 0.004336859 27600 0.005794782
28500 0.00182548 26500 0.000199603 26300 0.00058644 25900
0.001937778 28200 0.004324908 27600 0.005811625
28500 0.001824433 26500 0.000199922 26300 0.000587275 25900
0.00193429 28200 0.004313007 27600 0.005828509
28500 0.001823394 26500 0.000200254 26300 0.000588122 25900
0.001932008 28200 0.004301163 27600 0.005845467
28500 0.00182236 26500 0.000200589 26300 0.000588965 25900
0.00193081 28200 0.004289348 27600 0.005862501
28500 0.001821308 26500 0.000200924 26300 0.000589804 25900
0.0019306 28200 0.004277618 27600 0.005879579
28500 0.001820264 26500 0.000201254 26300 0.000590655 25900
0.00193128 28200 0.004265952 27600 0.005896756
28500 0.001819233 26500 0.000201578 26300 0.000591509 25900
0.001932732 28200 0.00425434 27600 0.005913997
28500 0.001818216 26500 0.000201901 26300 0.000592365 25900
0.001934878 28200 0.004242774 27600 0.005931301
28500 0.001817203 26500 0.000202223 26300 0.000593209 25900
0.001937661 28200 0.004231256 27600 0.005948655
28500 0.001816203 26500 0.000202531 26300 0.000594093 25900
0.001940986 28200 0.004219792 27600 0.005966061
28500 0.001815207 26500 0.000202848 26300 0.000594955 25900
0.001944782 28200 0.004208396 27600 0.005983563
28500 0.001814215 26500 0.000203159 26300 0.000595842 25900
0.001948987 28200 0.004197034 27600 0.006001154
28500 0.001813235 26500 0.000203466 26300 0.000596727 25900
0.001953549 28200 0.004185762 27600 0.006018814
28500 0.001812251 26500 0.000203783 26300 0.000597612 25900
0.001958408 28200 0.004174523 27600 0.006036518
28500 0.00181129 26500 0.000204106 26300 0.000598505 25900
0.001963528 28200 0.004163327 27600 0.006054316
28500 0.001810342 26500 0.000204433 26300 0.000599396 25900
0.001968869 28200 0.004152175 27600 0.006072194
28600 0.001809394 26500 0.000204768 26300 0.000600297 25900
0.001974403 28200 0.004141062 27600 0.006090117

28600 0.001808469 26500 0.000205108 26300 0.000601203 25900
0.001980115 28200 0.004130015 27600 0.006108117
28600 0.001807527 26500 0.000205448 26300 0.000602114 25900
0.001985987 28200 0.00411903 27600 0.006126206
28600 0.00180661 26500 0.000205779 26300 0.000603043 25900
0.001992006 28200 0.004108087 27600 0.006144364
28600 0.001805662 26500 0.00020613 26300 0.000603944 25900
0.001998128 28200 0.004097187 27600 0.006162622
28600 0.001804757 26500 0.00020646 26300 0.000604869 25900
0.002004386 28200 0.004086352 27600 0.006180941
28600 0.001803846 26500 0.000206808 26300 0.000605797 25900
0.002010725 28200 0.004075543 27600 0.006199347
28600 0.001802929 26500 0.000207158 26300 0.000606714 25900
0.00201719 28200 0.004064797 27600 0.006217847
28600 0.001801994 26500 0.000207493 26300 0.00060765 25900
0.002023746 28200 0.004054088 27600 0.00623646
28600 0.001801087 26500 0.000207821 26300 0.000608582 25900
0.002030389 28200 0.004043412 27700 0.006255129
28600 0.001800186 26500 0.000208156 26300 0.000609524 25900
0.002037113 28200 0.004032782 27700 0.006273897
28600 0.001799279 26500 0.000208491 26300 0.000610447 25900
0.002043916 28200 0.004022202 27700 0.006292721
28600 0.001798375 26500 0.000208831 26300 0.000611388 25900
0.00205075 28200 0.004011652 27700 0.006311605
28600 0.001797479 26500 0.000209173 26300 0.000612319 25900
0.002057602 28200 0.004001159 27700 0.006330637
28600 0.001796586 26500 0.000209517 26300 0.000613275 25900
0.002064491 28200 0.003990711 27700 0.006349743
28600 0.001795703 26500 0.000209856 26300 0.000614227 25900
0.002071392 28200 0.003980317 27700 0.006368956
28600 0.001794796 26500 0.000210205 26300 0.000615186 25900
0.002078315 28200 0.003969944 27700 0.006388241
28600 0.001793902 26500 0.000210545 26300 0.000616167 25900
0.002085252 28200 0.003959609 27700 0.006407622
28600 0.00179303 26600 0.000210891 26300 0.000617125 25900
0.002092212 28200 0.003949311 27700 0.006427082
28600 0.001792151 26600 0.000211227 26300 0.000618075 25900
0.002099191 28200 0.003939032 27700 0.006446633
28600 0.001791291 26600 0.000211573 26400 0.000619042 25900
0.002106216 28200 0.003928836 27700 0.006466263
28600 0.001790421 26600 0.000211907 26400 0.000620017 25900
0.002113251 28200 0.003918678 27700 0.006486005
28600 0.001789554 26600 0.00021224 26400 0.000621022 25900
0.00212032 28200 0.003908559 27700 0.006505834
28600 0.001788686 26600 0.000212588 26400 0.00062201 25900
0.002127412 28200 0.003898476 27700 0.006525756
28600 0.001787818 26600 0.000212926 26400 0.000623002 26000
0.002134528 28200 0.003888448 27700 0.006545756
28600 0.00178696 26600 0.000213277 26400 0.000624015 26000
0.002141671 28200 0.00387844 27700 0.00656586
28600 0.001786092 26600 0.000213632 26400 0.000625019 26000
0.002148858 28200 0.003868498 27700 0.006586048
28600 0.001785207 26600 0.00021397 26400 0.00062603 26000
0.002156087 28200 0.003858591 27700 0.006606361
28600 0.001784347 26600 0.000214318 26400 0.000627058 26000
0.002163387 28200 0.003848719 27700 0.006626755
28600 0.001783474 26600 0.000214669 26400 0.000628069 26000
0.002170747 28200 0.003838891 27700 0.006647251
28600 0.001782609 26600 0.000215012 26400 0.000629109 26000
0.002178204 28200 0.003829121 27700 0.006667806
28600 0.001781734 26600 0.000215357 26400 0.000630144 26000
0.002185734 28200 0.003819371 27700 0.006688445
28600 0.001780866 26600 0.0002157 26400 0.000631189 26000
0.002193345 28200 0.003809683 27700 0.006709222
28600 0.001779983 26600 0.000216048 26400 0.000632225 26000
0.002201061 28200 0.003800028 27700 0.006730097
28600 0.001779138 26600 0.000216396 26400 0.00063328 26000
0.00220885 28200 0.003790452 27700 0.006751086

28600 0.001778249 26600 0.00021674 26400 0.000634345 26000
0.00221674 28300 0.003780877 27700 0.00677212
28600 0.001777373 26600 0.000217088 26400 0.000635407 26000
0.002224718 28300 0.003771338 27700 0.006793239
28600 0.001776512 26600 0.000217451 26400 0.00063647 26000
0.00223281 28300 0.003761855 27700 0.006814495
28600 0.001775642 26600 0.000217818 26400 0.000637527 26000
0.002241008 28300 0.003752387 27700 0.006835828
28600 0.001774757 26600 0.000218176 26400 0.000638612 26000
0.002249331 28300 0.003742994 27700 0.006857238
28600 0.001773871 26600 0.00021855 26400 0.000639692 26000
0.00225774 28300 0.003733635 27700 0.006878757
28600 0.001772987 26600 0.000218912 26400 0.000640787 26000
0.002266243 28300 0.003724319 27700 0.006900392
28600 0.001772085 26600 0.000219282 26400 0.00064186 26000
0.002274793 28300 0.003715042 27700 0.006922116
28600 0.001771207 26600 0.000219655 26400 0.000642976 26000
0.002283425 28300 0.003705823 27700 0.006943944
28600 0.001770318 26600 0.000220053 26400 0.000644093 26000
0.002292109 28300 0.003696628 27700 0.006965854
28600 0.001769409 26600 0.000220448 26400 0.000645208 26000
0.002300881 28300 0.003687486 27700 0.006987892
28600 0.0017685 26600 0.000220859 26400 0.000646316 26000
0.002309723 28300 0.003678371 27700 0.007010077
28600 0.001767592 26600 0.000221264 26400 0.000647446 26000
0.002318663 28300 0.003669303 27700 0.007032313
28600 0.001766703 26600 0.000221666 26400 0.000648579 26000
0.002327676 28300 0.003660264 27700 0.007054673
28600 0.001765801 26600 0.000222061 26400 0.000649699 26000
0.002336777 28300 0.003651277 27700 0.007077121
28600 0.001764906 26600 0.000222456 26400 0.000650853 26000
0.002345976 28300 0.003642312 27700 0.007099647
28600 0.001764006 26600 0.000222834 26400 0.000652001 26000
0.002355253 28300 0.003633396 27700 0.007122318
28600 0.001763105 26600 0.000223208 26400 0.000653129 26000
0.002364635 28300 0.00362451 27700 0.00714511
28600 0.001762223 26600 0.000223593 26400 0.000654295 26000
0.002374094 28300 0.003615679 27700 0.007168027
28600 0.001761319 26600 0.00022397 26400 0.000655444 26000
0.002383653 28300 0.003606854 27700 0.007191012
28600 0.001760442 26600 0.00022436 26400 0.000656598 26000
0.002393318 28300 0.003598053 27700 0.007214131
28600 0.001759573 26600 0.000224755 26400 0.000657778 26000
0.002403106 28300 0.0035893 27700 0.007237355
28600 0.001758693 26600 0.000225153 26400 0.000658954 26000
0.002413003 28300 0.003580554 27700 0.007260696
28600 0.001757823 26600 0.000225563 26400 0.000660113 26000
0.002423041 28300 0.003571845 27700 0.007284146
28600 0.001756969 26600 0.000225961 26400 0.000661285 26000
0.002433228 28300 0.003563187 27700 0.007307699
28600 0.001756113 26600 0.000226357 26400 0.000662487 26000
0.002443569 28300 0.003554552 27700 0.007331379
28600 0.001755239 26600 0.000226768 26400 0.000663675 26000
0.002454059 28300 0.003545937 27700 0.007355152
28600 0.001754409 26600 0.000227167 26400 0.000664879 26000
0.002464732 28300 0.003537363 27700 0.007379042
28600 0.001753548 26600 0.000227563 26400 0.000666067 26000
0.002475556 28300 0.003528812 27700 0.007403032
28700 0.001752704 26600 0.000227963 26400 0.000667261 26000
0.002486524 28300 0.003520283 27700 0.007427145
28700 0.001751847 26600 0.000228358 26400 0.000668478 26000
0.002497609 28300 0.003511791 27700 0.00745139
28700 0.001751007 26600 0.000228752 26400 0.000669684 26000
0.002508785 28300 0.003503323 27700 0.00747575
28700 0.001750184 26600 0.000229153 26400 0.000670894 26000
0.002520005 28300 0.003494887 27700 0.007500253
28700 0.001749341 26600 0.000229559 26400 0.000672097 26000
0.002531296 28300 0.003486498 27700 0.007524835

28700 0.001748518 26600 0.000229973 26400 0.000673348 26000
0.002542662 28300 0.003478129 27700 0.007549516
28700 0.001747709 26600 0.000230384 26400 0.000674575 26000
0.002554098 28300 0.003469801 27700 0.007574345
28700 0.001746894 26600 0.00023079 26400 0.000675831 26000
0.002565662 28300 0.003461516 27700 0.007599304
28700 0.001746067 26600 0.000231215 26400 0.000677061 26000
0.002577401 28300 0.003453292 27700 0.007624399
28700 0.001745234 26600 0.000231626 26400 0.000678309 26000
0.00258931 28300 0.003445065 27800 0.007649583
28700 0.001744417 26600 0.000232055 26400 0.000679577 26000
0.002601435 28300 0.003436871 27800 0.007674881
28700 0.001743617 26600 0.000232477 26400 0.000680835 26000
0.002613777 28300 0.003428722 27800 0.007700298
28700 0.001742787 26600 0.000232894 26400 0.000682079 26000
0.002626303 28300 0.00342059 27800 0.00772586
28700 0.00174199 26600 0.000233315 26400 0.000683361 26000
0.002639042 28300 0.003412513 27800 0.007751514
28700 0.001741185 26600 0.000233726 26400 0.000684611 26000
0.002651954 28300 0.003404467 27800 0.007777294
28700 0.001740389 26600 0.000234129 26400 0.000685895 26000
0.002665017 28300 0.003396459 27800 0.0078032
28700 0.001739563 26600 0.000234543 26400 0.000687162 26000
0.002678186 28300 0.003388477 27800 0.007829237
28700 0.001738777 26600 0.000234972 26400 0.000688454 26000
0.002691462 28300 0.003380537 27800 0.007855381
28700 0.001737968 26700 0.000235385 26400 0.000689749 26000
0.00270482 28300 0.003372619 27800 0.007881661
28700 0.001737164 26700 0.000235813 26400 0.000691039 26000
0.002718253 28300 0.003364737 27800 0.007908087
28700 0.001736348 26700 0.000236238 26500 0.000692352 26000
0.00273174 28300 0.00335688 27800 0.007934663
28700 0.001735553 26700 0.000236657 26500 0.000693662 26000
0.002745294 28300 0.003349052 27800 0.00796133
28700 0.001734737 26700 0.000237072 26500 0.000694968 26000
0.002758952 28300 0.003341255 27800 0.007988156
28700 0.001733937 26700 0.00023749 26500 0.000696268 26000
0.002772738 28300 0.003333494 27800 0.008015086
28700 0.001733125 26700 0.000237899 26500 0.000697617 26100
0.002786716 28300 0.003325758 27800 0.008042099
28700 0.001732323 26700 0.000238308 26500 0.000698952 26100
0.002800875 28300 0.003318065 27800 0.008069299
28700 0.001731496 26700 0.000238731 26500 0.000700271 26100
0.00281524 28300 0.003310394 27800 0.008096655
28700 0.001730708 26700 0.000239163 26500 0.000701631 26100
0.002829783 28300 0.003302779 27800 0.008124145
28700 0.001729895 26700 0.000239606 26500 0.000702972 26100
0.002844513 28300 0.003295171 27800 0.008151767
28700 0.001729091 26700 0.000240043 26500 0.000704335 26100
0.00285938 28300 0.003287587 27800 0.008179523
28700 0.001728288 26700 0.000240501 26500 0.000705698 26100
0.002874406 28300 0.003280047 27800 0.008207406
28700 0.001727482 26700 0.000240967 26500 0.000707075 26100
0.002889573 28300 0.003272528 27800 0.008235424
28700 0.00172668 26700 0.000241433 26500 0.00070843 26100
0.002904919 28300 0.003265054 27800 0.008263576
28700 0.001725867 26700 0.000241916 26500 0.000709805 26100
0.002920468 28300 0.003257617 27800 0.00829186
28700 0.001725059 26700 0.000242376 26500 0.000711206 26100
0.002936211 28300 0.003250219 27800 0.00832028
28700 0.001724239 26700 0.000242849 26500 0.000712614 26100
0.002952152 28400 0.003242834 27800 0.008348855
28700 0.001723423 26700 0.00024333 26500 0.00071399 26100
0.00296829 28400 0.0032355 27800 0.008377569
28700 0.001722606 26700 0.000243798 26500 0.000715402 26100
0.002984626 28400 0.003228187 27800 0.008406425
28700 0.001721785 26700 0.000244273 26500 0.000716822 26100
0.003001173 28400 0.003220916 27800 0.008435454

28700 0.001720948 26700 0.000244741 26500 0.000718241 26100
0.003017888 28400 0.003213659 27800 0.008464645
28700 0.001720138 26700 0.000245202 26500 0.000719672 26100
0.003034797 28400 0.003206442 27800 0.008493963
28700 0.001719319 26700 0.000245672 26500 0.0007211 26100
0.003051865 28400 0.003199247 27800 0.008523446
28700 0.001718491 26700 0.000246121 26500 0.000722523 26100
0.003069078 28400 0.003192089 27800 0.008553052
28700 0.001717662 26700 0.000246579 26500 0.000723982 26100
0.00308645 28400 0.00318494 27800 0.008582779
28700 0.001716836 26700 0.000247033 26500 0.000725423 26100
0.003103955 28400 0.003177844 27800 0.008612691
28700 0.001716009 26700 0.000247485 26500 0.000726871 26100
0.003121651 28400 0.003170779 27800 0.008642787
28700 0.001715192 26700 0.000247938 26500 0.000728336 26100
0.003139486 28400 0.003163758 27800 0.008673048
28700 0.00171436 26700 0.000248393 26500 0.0007298 26100
0.003157512 28400 0.003156747 27800 0.008703434
28700 0.001713539 26700 0.000248855 26500 0.000731287 26100
0.003175694 28400 0.003149747 27800 0.008733979
28700 0.001712759 26700 0.000249331 26500 0.000732783 26100
0.00319403 28400 0.003142795 27800 0.008764674
28700 0.001711947 26700 0.000249811 26500 0.000734255 26100
0.003212487 28400 0.003135847 27800 0.00879555
28700 0.001711161 26700 0.000250296 26500 0.000735748 26100
0.00323113 28400 0.003128938 27800 0.008826572
28700 0.001710365 26700 0.000250787 26500 0.00073725 26100
0.003249938 28400 0.003122069 27800 0.00885773
28700 0.001709578 26700 0.000251274 26500 0.000738771 26100
0.003268946 28400 0.003115212 27800 0.00888907
28700 0.001708752 26700 0.000251767 26500 0.000740297 26100
0.003288156 28400 0.003108369 27800 0.008920572
28700 0.001707972 26700 0.000252266 26500 0.000741837 26100
0.003307613 28400 0.003101568 27800 0.008952242
28700 0.00170719 26700 0.000252764 26500 0.000743356 26100
0.003327274 28400 0.003094778 27800 0.008984078
28700 0.001706396 26700 0.000253248 26500 0.000744879 26100
0.00334721 28400 0.003088024 27800 0.009016088
28700 0.001705592 26700 0.000253735 26500 0.000746419 26100
0.003367389 28400 0.003081284 27800 0.009048287
28700 0.001704827 26700 0.000254224 26500 0.000747954 26100
0.003387763 28400 0.003074556 27800 0.009080633
28700 0.001704042 26700 0.000254719 26500 0.000749501 26100
0.003408345 28400 0.003067844 27800 0.009113161
28700 0.001703268 26700 0.000255209 26500 0.000751059 26100
0.003429165 28400 0.003061166 27800 0.009145838
28700 0.00170248 26700 0.000255712 26500 0.000752629 26100
0.003450184 28400 0.003054488 27800 0.009178645
28700 0.001701718 26700 0.000256206 26500 0.000754205 26100
0.003471443 28400 0.003047838 27800 0.009211683
28800 0.001700935 26700 0.000256706 26500 0.00075578 26100
0.003492977 28400 0.003041205 27800 0.009244906
28800 0.001700174 26700 0.000257217 26500 0.000757362 26100
0.003514784 28400 0.003034613 27800 0.00927831
28800 0.001699404 26700 0.000257728 26500 0.000758975 26100
0.003536877 28400 0.003028022 27800 0.009311871
28800 0.001698636 26700 0.00025823 26500 0.000760574 26100
0.003559232 28400 0.003021433 27800 0.009345617
28800 0.001697892 26700 0.000258732 26500 0.000762179 26100
0.003581899 28400 0.003014887 27800 0.009379526
28800 0.001697131 26700 0.000259222 26500 0.000763787 26100
0.003604845 28400 0.003008332 27800 0.009413622
28800 0.001696406 26700 0.000259708 26500 0.000765414 26100
0.003628093 28400 0.003001822 27800 0.009447894
28800 0.001695656 26700 0.00026019 26500 0.000767049 26100
0.003651604 28400 0.002995333 27800 0.009482342
28800 0.001694916 26700 0.000260669 26500 0.000768696 26100
0.003675369 28400 0.002988872 27800 0.009516978

28800 0.001694154 26700 0.000261156 26500 0.000770342 26100
0.003699362 28400 0.002982435 27900 0.009551798
28800 0.001693407 26700 0.000261654 26500 0.000771999 26100
0.003723573 28400 0.002976023 27900 0.00958681
28800 0.001692646 26700 0.000262164 26500 0.000773637 26100
0.003747978 28400 0.002969636 27900 0.009622009
28800 0.001691901 26700 0.000262676 26500 0.000775303 26100
0.003772578 28400 0.002963269 27900 0.009657411
28800 0.001691144 26700 0.000263187 26500 0.000776961 26100
0.003797374 28400 0.002956938 27900 0.009693027
28800 0.001690403 26700 0.000263701 26500 0.000778653 26100
0.003822379 28400 0.00295063 27900 0.009728799
28800 0.001689659 26700 0.000264207 26500 0.000780315 26100
0.003847564 28400 0.002944339 27900 0.009764783
28800 0.001688923 26700 0.00026471 26500 0.000782001 26100
0.003873035 28400 0.002938088 27900 0.009800933
28800 0.00168817 26700 0.000265224 26500 0.000783679 26100
0.003898781 28400 0.002931851 27900 0.009837226
28800 0.001687449 26800 0.000265724 26500 0.000785363 26100
0.003924845 28400 0.002925656 27900 0.009873789
28800 0.001686701 26800 0.000266217 26500 0.000787067 26100
0.003951277 28400 0.002919486 27900 0.009910559
28800 0.001685972 26800 0.000266713 26600 0.000788791 26100
0.003978035 28400 0.002913367 27900 0.009947537
28800 0.001685217 26800 0.000267207 26600 0.000790495 26100
0.004005134 28400 0.002907249 27900 0.009984676
28800 0.001684478 26800 0.000267697 26600 0.000792215 26100
0.004032576 28400 0.002901156 27900 0.01002205
28800 0.001683782 26800 0.000268189 26600 0.00079392 26100
0.004060286 28400 0.002895099 27900 0.0100596
28800 0.001683047 26800 0.000268671 26600 0.000795677 26200
0.004088284 28400 0.002889046 27900 0.01009737
28800 0.001682368 26800 0.00026916 26600 0.00079741 26200
0.004116558 28400 0.002883024 27900 0.01013534
28800 0.001681649 26800 0.000269639 26600 0.000799163 26200
0.004145064 28400 0.002877052 27900 0.0101735
28800 0.001680938 26800 0.000270114 26600 0.000800907 26200
0.004173802 28400 0.002871096 27900 0.01021188
28800 0.001680208 26800 0.000270592 26600 0.000802657 26200
0.004202791 28400 0.002865166 27900 0.01025046
28800 0.001679502 26800 0.000271083 26600 0.00080445 26200
0.004232063 28400 0.002859272 27900 0.01028925
28800 0.001678785 26800 0.000271578 26600 0.000806256 26200
0.004261652 28400 0.002853397 27900 0.01032826
28800 0.001678091 26800 0.000272076 26600 0.000808024 26200
0.004291615 28400 0.002847552 27900 0.0103675
28800 0.001677369 26800 0.000272573 26600 0.000809815 26200
0.004321963 28400 0.002841723 27900 0.01040697
28800 0.001676668 26800 0.000273082 26600 0.00081162 26200
0.004352711 28400 0.002835894 27900 0.01044664
28800 0.001675971 26800 0.000273586 26600 0.000813424 26200
0.004383862 28400 0.002830088 27900 0.01048654
28800 0.001675269 26800 0.000274094 26600 0.000815233 26200
0.004415457 28500 0.002824296 27900 0.01052663
28800 0.001674553 26800 0.000274607 26600 0.000817057 26200
0.004447433 28500 0.002818528 27900 0.01056692
28800 0.00167387 26800 0.000275117 26600 0.000818882 26200
0.004479818 28500 0.002812786 27900 0.0106075
28800 0.001673165 26800 0.00027562 26600 0.000820715 26200
0.004512615 28500 0.002807062 27900 0.01064827
28800 0.001672479 26800 0.000276134 26600 0.000822554 26200
0.004545795 28500 0.00280137 27900 0.01068928
28800 0.001671775 26800 0.000276638 26600 0.000824425 26200
0.004579374 28500 0.002795692 27900 0.01073049
28800 0.001671086 26800 0.000277143 26600 0.000826288 26200
0.004613334 28500 0.002790025 27900 0.01077193
28800 0.001670392 26800 0.000277652 26600 0.000828159 26200
0.004647706 28500 0.00278438 27900 0.0108136

28800 0.001669687 26800 0.00027814 26600 0.000830041 26200
0.00468246 28500 0.002778742 27900 0.0108555
28800 0.001669004 26800 0.000278638 26600 0.000831953 26200
0.00471763 28500 0.002773135 27900 0.01089762
28800 0.001668311 26800 0.000279136 26600 0.000833849 26200
0.004753208 28500 0.002767561 27900 0.01093998
28800 0.001667616 26800 0.000279628 26600 0.000835758 26200
0.004789184 28500 0.002761994 27900 0.01098259
28800 0.001666907 26800 0.000280127 26600 0.000837663 26200
0.00482552 28500 0.002756437 27900 0.01102541
28800 0.001666214 26800 0.000280644 26600 0.000839588 26200
0.004862245 28500 0.002750925 27900 0.0110685
28800 0.001665508 26800 0.000281152 26600 0.000841538 26200
0.00489933 28500 0.002745414 27900 0.01111183
28800 0.001664797 26800 0.000281668 26600 0.000843485 26200
0.004936798 28500 0.002739926 27900 0.01115541
28800 0.001664068 26800 0.000282189 26600 0.000845459 26200
0.00497462 28500 0.002734439 27900 0.01119927
28800 0.001663374 26800 0.000282714 26600 0.000847421 26200
0.005012846 28500 0.002728962 27900 0.01124335
28800 0.001662655 26800 0.000283231 26600 0.000849372 26200
0.00505144 28500 0.002723491 27900 0.01128771
28800 0.00166193 26800 0.000283737 26600 0.00085135 26200
0.005090457 28500 0.00271805 27900 0.01133228
28800 0.001661232 26800 0.000284254 26600 0.00085334 26200
0.005129883 28500 0.002712625 27900 0.01137705
28800 0.001660524 26800 0.00028475 26600 0.000855325 26200
0.005169744 28500 0.002707235 27900 0.01142217
28800 0.001659811 26800 0.000285239 26600 0.000857327 26200
0.005210038 28500 0.002701849 27900 0.01146754
28800 0.001659101 26800 0.00028575 26600 0.000859335 26200
0.005250748 28500 0.002696483 27900 0.01151321
28800 0.001658384 26800 0.000286225 26600 0.000861349 26200
0.005291907 28500 0.002691143 27900 0.01155912
28800 0.001657673 26800 0.00028671 26600 0.000863383 26200
0.005333503 28500 0.002685793 27900 0.0116053
28800 0.001656988 26800 0.000287197 26600 0.000865408 26200
0.005375595 28500 0.002680479 27900 0.01165176
28800 0.001656295 26800 0.00028767 26600 0.000867444 26200
0.005418201 28500 0.002675177 27900 0.01169848
28800 0.001655627 26800 0.000288138 26600 0.000869509 26200
0.005461384 28500 0.002669887 27900 0.01174548
28900 0.001654933 26800 0.000288596 26600 0.000871582 26200
0.005505148 28500 0.00266464 27900 0.01179276
28900 0.001654254 26800 0.00028904 26600 0.00087364 26200
0.005549522 28500 0.002659427 27900 0.01184032
28900 0.001653567 26800 0.000289503 26600 0.000875718 26200
0.00559451 28500 0.002654221 27900 0.01188814
28900 0.001652896 26800 0.000289957 26600 0.000877819 26200
0.00564014 28500 0.002649043 27900 0.01193628
28900 0.001652225 26800 0.000290425 26600 0.000879923 26200
0.005686395 28500 0.002643903 27900 0.01198469
28900 0.001651548 26800 0.000290889 26600 0.000882038 26200
0.005733301 28500 0.00263878 27900 0.01203339
28900 0.001650869 26800 0.000291367 26600 0.000884181 26200
0.005780845 28500 0.002633665 27900 0.01208242
28900 0.001650205 26800 0.000291844 26600 0.0008863 26200
0.005829029 28500 0.002628566 27900 0.01213172
28900 0.001649554 26800 0.000292336 26600 0.000888431 26200
0.005877823 28500 0.002623493 27900 0.01218134
28900 0.001648892 26800 0.000292829 26600 0.000890579 26200
0.005927265 28500 0.002618446 28000 0.01223123
28900 0.00164823 26800 0.000293325 26600 0.000892732 26200
0.005977349 28500 0.002613415 28000 0.01228139
28900 0.001647574 26800 0.000293817 26600 0.000894904 26200
0.006028046 28500 0.002608417 28000 0.0123319
28900 0.00164692 26800 0.000294302 26600 0.000897082 26200
0.006079395 28500 0.002603439 28000 0.01238275

28900 0.001646277 26800 0.000294789 26600 0.000899272 26200
0.00613136 28500 0.002598488 28000 0.01243391
28900 0.001645613 26800 0.000295263 26600 0.000901485 26200
0.006183944 28500 0.002593553 28000 0.01248535
28900 0.001644975 26800 0.000295751 26600 0.000903711 26200
0.006237127 28500 0.002588613 28000 0.01253713
28900 0.00164434 26800 0.000296242 26600 0.000905915 26200
0.006290939 28500 0.00258371 28000 0.0125892
28900 0.001643694 26800 0.000296708 26600 0.000908142 26200
0.006345401 28500 0.002578809 28000 0.01264159
28900 0.001643058 26900 0.000297193 26600 0.000910398 26200
0.00640054 28500 0.002573924 28000 0.01269431
28900 0.001642409 26900 0.000297665 26600 0.000912662 26200
0.006456359 28500 0.002569079 28000 0.01274733
28900 0.001641777 26900 0.000298133 26700 0.000914934 26200
0.006512867 28500 0.002564241 28000 0.01280068
28900 0.001641097 26900 0.000298584 26700 0.000917201 26200
0.006570085 28500 0.002559403 28000 0.01285436
28900 0.001640451 26900 0.000299053 26700 0.000919499 26200
0.006628044 28500 0.002554589 28000 0.0129084
28900 0.001639797 26900 0.000299525 26700 0.00092181 26200
0.006686728 28500 0.002549777 28000 0.01296275
28900 0.001639125 26900 0.000300009 26700 0.000924108 26300
0.006746195 28500 0.002544978 28000 0.01301745
28900 0.001638449 26900 0.000300484 26700 0.000926438 26300
0.006806436 28500 0.002540186 28000 0.01307253
28900 0.001637787 26900 0.000300976 26700 0.000928757 26300
0.006867491 28500 0.002535386 28000 0.0131279
28900 0.001637112 26900 0.00030148 26700 0.0009311 26300
0.006929357 28500 0.002530606 28000 0.01318364
28900 0.00163643 26900 0.000301977 26700 0.000933449 26300
0.006992104 28500 0.002525832 28000 0.01323968
28900 0.001635736 26900 0.000302494 26700 0.000935812 26300
0.007055736 28500 0.002521051 28000 0.01329603
28900 0.001635047 26900 0.000303006 26700 0.000938178 26300
0.007120281 28500 0.00251629 28000 0.01335279
28900 0.001634367 26900 0.000303515 26700 0.000940552 26300
0.007185781 28500 0.00251153 28000 0.01340991
28900 0.001633681 26900 0.000304019 26700 0.000942948 26300
0.007252226 28500 0.002506786 28000 0.01346741
28900 0.001632969 26900 0.000304515 26700 0.000945357 26300
0.007319606 28500 0.00250204 28000 0.01352526
28900 0.001632279 26900 0.000305008 26700 0.000947772 26300
0.007387893 28500 0.002497289 28000 0.01358347
28900 0.001631592 26900 0.000305483 26700 0.000950185 26300
0.007457113 28600 0.002492568 28000 0.01364201
28900 0.001630895 26900 0.000305956 26700 0.000952603 26300
0.007527216 28600 0.00248784 28000 0.01370096
28900 0.001630215 26900 0.00030644 26700 0.000955055 26300
0.007598247 28600 0.002483138 28000 0.01376026
28900 0.001629525 26900 0.000306918 26700 0.000957513 26300
0.00767017 28600 0.002478456 28000 0.01381992
28900 0.00162885 26900 0.00030739 26700 0.000959987 26300
0.00774301 28600 0.002473796 28000 0.01387999
28900 0.001628141 26900 0.000307873 26700 0.00096246 26300
0.007816788 28600 0.002469146 28000 0.01394041
28900 0.001627459 26900 0.000308371 26700 0.000964945 26300
0.007891531 28600 0.002464518 28000 0.01400124
28900 0.001626783 26900 0.000308872 26700 0.000967435 26300
0.007967274 28600 0.002459893 28000 0.01406245
28900 0.001626107 26900 0.000309365 26700 0.000969942 26300
0.008044085 28600 0.002455303 28000 0.01412407
28900 0.001625414 26900 0.000309851 26700 0.000972463 26300
0.008121966 28600 0.002450719 28000 0.01418608
28900 0.00162476 26900 0.000310356 26700 0.000974971 26300
0.008200978 28600 0.002446147 28000 0.0142485
28900 0.001624079 26900 0.000310855 26700 0.000977508 26300
0.008281098 28600 0.002441605 28000 0.01431132

28900 0.001623422 26900 0.000311362 26700 0.000980046 26300
0.008362336 28600 0.00243709 28000 0.01437452
28900 0.001622744 26900 0.000311885 26700 0.00098259 26300
0.008444722 28600 0.002432562 28000 0.0144381
28900 0.001622094 26900 0.000312395 26700 0.000985155 26300
0.008528226 28600 0.002428078 28000 0.01450213
28900 0.001621425 26900 0.000312932 26700 0.000987731 26300
0.00861296 28600 0.002423609 28000 0.01456659
28900 0.001620799 26900 0.000313466 26700 0.000990326 26300
0.008698906 28600 0.00241917 28000 0.01463146
28900 0.001620146 26900 0.000313995 26700 0.000992924 26300
0.008786046 28600 0.002414717 28000 0.01469676
28900 0.001619493 26900 0.000314539 26700 0.000995541 26300
0.008874387 28600 0.002410267 28000 0.01476248
28900 0.001618872 26900 0.000315074 26700 0.000998163 26300
0.00896394 28600 0.002405847 28000 0.01482858
28900 0.001618234 26900 0.000315601 26700 0.001000782 26300
0.009054689 28600 0.002401416 28000 0.01489515
28900 0.001617605 26900 0.000316129 26700 0.00100344 26300
0.009146716 28600 0.002397016 28000 0.01496216
28900 0.001616966 26900 0.00031665 26700 0.0010061 26300
0.009240032 28600 0.002392627 28000 0.01502959
28900 0.001616333 26900 0.000317157 26700 0.001008771 26300
0.009334711 28600 0.002388269 28000 0.01509748
28900 0.001615694 26900 0.000317679 26700 0.001011469 26300
0.00943077 28600 0.002383902 28000 0.01516578
28900 0.001615073 26900 0.000318207 26700 0.001014175 26300
0.009528276 28600 0.002379557 28000 0.01523458
28900 0.001614446 26900 0.000318747 26700 0.001016892 26300
0.009627193 28600 0.002375217 28000 0.01530383
28900 0.001613791 26900 0.000319293 26700 0.001019617 26300
0.009727585 28600 0.002370897 28000 0.01537356
28900 0.001613156 26900 0.000319848 26700 0.00102236 26300
0.009829431 28600 0.002366588 28000 0.01544375
29000 0.001612508 26900 0.000320422 26700 0.001025107 26300
0.009932795 28600 0.002362295 28000 0.01551442
29000 0.001611882 26900 0.000320994 26700 0.001027876 26300
0.01003768 28600 0.002358022 28000 0.01558558
29000 0.001611246 26900 0.000321574 26700 0.001030647 26300
0.01014417 28600 0.002353773 28000 0.01565715
29000 0.001610616 26900 0.00032217 26700 0.001033435 26300
0.01025232 28600 0.002349516 28000 0.01572917
29000 0.001609975 26900 0.00032275 26700 0.001036245 26300
0.01036218 28600 0.002345301 28000 0.01580174
29000 0.001609335 26900 0.000323336 26700 0.001039057 26300
0.01047382 28600 0.002341108 28000 0.01587479
29000 0.001608724 26900 0.000323917 26700 0.001041891 26300
0.01058725 28600 0.002336941 28000 0.01594838
29000 0.001608071 26900 0.000324503 26700 0.001044747 26300
0.01070248 28600 0.002332776 28000 0.01602243
29000 0.00160744 26900 0.000325087 26700 0.00104761 26300
0.01081953 28600 0.002328617 28000 0.01609701
29000 0.001606803 26900 0.000325689 26700 0.001050471 26300
0.01093846 28600 0.002324498 28100 0.01617207
29000 0.001606173 26900 0.000326288 26700 0.001053357 26300
0.01105931 28600 0.002320378 28100 0.01624765
29000 0.001605551 26900 0.000326898 26700 0.001056281 26300
0.01118211 28600 0.002316294 28100 0.01632375
29000 0.001604936 26900 0.000327498 26700 0.001059198 26300
0.01130695 28600 0.002312229 28100 0.01640036
29000 0.00160432 26900 0.000328086 26700 0.001062133 26300
0.01143389 28600 0.002308198 28100 0.01647749
29000 0.0016037 26900 0.0003287 26700 0.001065086 26300
0.01156292 28600 0.002304169 28100 0.0165552
29000 0.001603084 26900 0.000329297 26700 0.001068048 26300
0.01169408 28600 0.002300163 28100 0.01663341
29000 0.001602462 26900 0.000329904 26700 0.001071021 26300
0.01182734 28600 0.00229617 28100 0.01671219

29000 0.001601844 26900 0.000330495 26700 0.001074011 26300
0.01196268 28600 0.002292188 28100 0.01679153
29000 0.001601209 27000 0.000331079 26700 0.001077015 26300
0.01210012 28600 0.002288223 28100 0.01687146
29000 0.001600587 27000 0.000331668 26700 0.001080013 26300
0.0122397 28600 0.002284263 28100 0.01695194
29000 0.001599984 27000 0.000332246 26800 0.001083053 26300
0.0123814 28600 0.002280321 28100 0.01703299
29000 0.001599354 27000 0.000332818 26800 0.001086093 26300
0.0125253 28600 0.002276414 28100 0.01711459
29000 0.001598745 27000 0.000333404 26800 0.001089145 26300
0.01267144 28600 0.002272496 28100 0.01719673
29000 0.001598135 27000 0.000333983 26800 0.001092234 26300
0.01281986 28600 0.002268603 28100 0.01727952
29000 0.001597522 27000 0.000334567 26800 0.001095331 26400
0.01297068 28600 0.002264743 28100 0.01736293
29000 0.001596908 27000 0.000335155 26800 0.001098455 26400
0.01312394 28600 0.002260908 28100 0.01744696
29000 0.00159629 27000 0.000335741 26800 0.001101582 26400
0.01327973 28600 0.002257062 28100 0.01753156
29000 0.001595676 27000 0.000336347 26800 0.001104733 26400
0.01343811 28600 0.002253231 28100 0.01761676
29000 0.00159508 27000 0.000336951 26800 0.001107876 26400
0.01359919 28600 0.002249423 28100 0.01770256
29000 0.001594475 27000 0.000337565 26800 0.001111033 26400
0.01376304 28600 0.002245615 28100 0.01778901
29000 0.001593893 27000 0.000338186 26800 0.001114235 26400
0.01392974 28600 0.002241838 28100 0.01787608
29000 0.001593288 27000 0.000338812 26800 0.001117436 26400
0.01409931 28600 0.00223808 28100 0.01796376
29000 0.001592688 27000 0.000339438 26800 0.001120647 26400
0.01427183 28600 0.002234344 28100 0.01805209
29000 0.001592075 27000 0.000340069 26800 0.001123886 26400
0.01444733 28600 0.002230613 28100 0.01814106
29000 0.001591476 27000 0.000340721 26800 0.001127145 26400
0.01462591 28600 0.002226898 28100 0.01823067
29000 0.001590889 27000 0.000341382 26800 0.001130408 26400
0.01480757 28700 0.002223197 28100 0.01832095
29000 0.001590289 27000 0.000342036 26800 0.001133683 26400
0.01499242 28700 0.002219507 28100 0.0184119
29000 0.001589671 27000 0.000342706 26800 0.001137007 26400
0.01518052 28700 0.002215823 28100 0.01850354
29000 0.00158909 27000 0.00034338 26800 0.001140305 26400
0.01537191 28700 0.002212145 28100 0.01859581
29000 0.001588494 27000 0.000344038 26800 0.001143646 26400
0.01556661 28700 0.002208481 28100 0.01868878
29000 0.001587901 27000 0.000344711 26800 0.00114698 26400
0.01576466 28700 0.002204836 28100 0.0187824
29000 0.001587326 27000 0.000345381 26800 0.001150376 26400
0.0159661 28700 0.002201182 28100 0.01887665
29000 0.001586747 27000 0.000346047 26800 0.001153772 26400
0.01617096 28700 0.002197542 28100 0.01897166
29000 0.001586149 27000 0.000346722 26800 0.001157175 26400
0.01637932 28700 0.002193917 28100 0.01906737
29000 0.001585581 27000 0.000347377 26800 0.001160627 26400
0.01659125 28700 0.002190311 28100 0.01916378
29000 0.001584994 27000 0.000348046 26800 0.001164077 26400
0.01680683 28700 0.002186681 28100 0.0192609
29000 0.001584409 27000 0.000348724 26800 0.001167545 26400
0.01702621 28700 0.00218306 28100 0.01935876
29000 0.001583835 27000 0.000349403 26800 0.001171035 26400
0.0172495 28700 0.002179446 28100 0.01945728
29000 0.001583284 27000 0.000350093 26800 0.001174546 26400
0.01747683 28700 0.002175821 28100 0.01955657
29000 0.001582729 27000 0.00035079 26800 0.001178079 26400
0.01770835 28700 0.002172226 28100 0.01965655
29000 0.00158216 27000 0.000351471 26800 0.001181643 26400
0.01794413 28700 0.002168628 28100 0.0197573

29000 0.001581622 27000 0.000352137 26800 0.001185209 26400
0.01818428 28700 0.00216505 28100 0.0198588
29000 0.001581041 27000 0.000352829 26800 0.001188792 26400
0.01842884 28700 0.002161462 28100 0.01996104
29000 0.001580485 27000 0.000353516 26800 0.001192399 26400
0.0186779 28700 0.002157887 28100 0.02006406
29000 0.001579943 27000 0.000354209 26800 0.001196039 26400
0.01893153 28700 0.002154302 28100 0.02016784
29000 0.001579394 27000 0.000354899 26800 0.001199694 26400
0.01918975 28700 0.00215073 28100 0.02027242
29000 0.00157882 27000 0.000355593 26800 0.00120336 26400
0.01945266 28700 0.002147156 28100 0.02037781
29000 0.001578277 27000 0.000356284 26800 0.001207044 26400
0.01972037 28700 0.002143585 28100 0.02048399
29000 0.001577717 27000 0.000356987 26800 0.001210742 26400
0.01999295 28700 0.002140025 28100 0.02059099
29000 0.001577162 27000 0.000357681 26800 0.001214453 26400
0.02027056 28700 0.002136478 28100 0.02069876
29000 0.001576604 27000 0.000358388 26800 0.001218183 26400
0.02055329 28700 0.002132913 28100 0.02080731
29000 0.001576046 27000 0.000359098 26800 0.001221942 26400
0.02084119 28700 0.002129367 28100 0.02091675
29000 0.001575508 27000 0.000359792 26800 0.001225714 26400
0.02113445 28700 0.002125841 28100 0.02102704
29000 0.001574962 27000 0.000360493 26800 0.001229509 26400
0.02143313 28700 0.00212233 28100 0.02113817
29100 0.001574395 27000 0.000361186 26800 0.001233314 26400
0.02173739 28700 0.002118821 28100 0.02125016
29100 0.001573852 27000 0.000361875 26800 0.001237154 26400
0.02204742 28700 0.002115307 28100 0.02136302
29100 0.001573348 27000 0.000362568 26800 0.001240996 26400
0.02236338 28700 0.002111798 28100 0.02147673
29100 0.00157282 27000 0.000363248 26800 0.001244852 26400
0.02268546 28700 0.002108289 28100 0.02159135
29100 0.001572295 27000 0.000363943 26800 0.001248728 26400
0.02301376 28700 0.002104816 28100 0.02170684
29100 0.001571766 27000 0.000364631 26800 0.001252633 26400
0.02334841 28700 0.002101338 28100 0.02182322
29100 0.001571244 27000 0.000365326 26800 0.001256562 26400
0.0236895 28700 0.002097886 28100 0.02194053
29100 0.001570702 27000 0.000366034 26800 0.001260506 26400
0.02403713 28700 0.002094441 28100 0.02205873
29100 0.00157018 27000 0.000366757 26800 0.001264476 26400
0.02439148 28700 0.002090999 28100 0.02217788
29100 0.001569669 27000 0.000367493 26800 0.001268464 26400
0.02475267 28700 0.00208757 28200 0.02229797
29100 0.001569138 27000 0.000368233 26800 0.001272467 26400
0.02512086 28700 0.002084141 28200 0.02241898
29100 0.001568603 27000 0.000368971 26800 0.001276487 26400
0.02549624 28700 0.002080723 28200 0.02254099
29100 0.001568092 27000 0.000369725 26800 0.001280532 26400
0.02587897 28700 0.002077317 28200 0.02266396
29100 0.001567581 27000 0.000370473 26800 0.001284606 26400
0.02626924 28700 0.002073904 28200 0.02278787
29100 0.001567062 27000 0.000371208 26800 0.001288692 26400
0.02666722 28700 0.00207052 28200 0.02291272
29100 0.00156657 27000 0.00037196 26800 0.001292804 26400
0.02707312 28700 0.002067115 28200 0.0230385
29100 0.001566066 27000 0.000372698 26800 0.001296936 26400
0.02748711 28700 0.002063714 28200 0.02316534
29100 0.00156554 27000 0.00037345 26800 0.001301086 26400
0.02790941 28700 0.002060326 28200 0.02329323
29100 0.001565046 27100 0.000374199 26800 0.001305275 26400
0.02834014 28700 0.00205695 28200 0.0234221
29100 0.00156455 27100 0.000374951 26800 0.001309481 26400
0.02877943 28700 0.002053568 28200 0.02355199
29100 0.001564054 27100 0.000375711 26900 0.00131372 26400
0.0292274 28700 0.002050183 28200 0.02368292

29100 0.001563579 27100 0.000376471 26900 0.00131797 26400
0.02968422 28700 0.002046794 28200 0.02381485
29100 0.001563092 27100 0.000377226 26900 0.001322231 26400
0.03014997 28700 0.002043403 28200 0.02394785
29100 0.001562648 27100 0.000377981 26900 0.001326529 26400
0.03062484 28700 0.002040035 28200 0.02408187
29100 0.001562182 27100 0.000378726 26900 0.001330851 26500
0.03110895 28700 0.00203666 28200 0.02421697
29100 0.00156172 27100 0.000379464 26900 0.001335207 26500
0.03160245 28700 0.002033308 28200 0.02435317
29100 0.001561243 27100 0.000380209 26900 0.001339558 26500
0.03210547 28700 0.002029953 28200 0.02449048
29100 0.001560777 27100 0.000380956 26900 0.00134395 26500
0.03261821 28700 0.002026598 28200 0.02462887
29100 0.001560306 27100 0.000381708 26900 0.001348367 26500
0.03314084 28700 0.002023256 28200 0.02476839
29100 0.001559833 27100 0.000382465 26900 0.00135281 26500
0.03367359 28700 0.00201992 28200 0.02490903
29100 0.001559359 27100 0.000383227 26900 0.001357253 26500
0.03421669 28700 0.00201659 28200 0.02505084
29100 0.001558897 27100 0.000384014 26900 0.001361726 26500
0.03477039 28700 0.002013273 28200 0.02519379
29100 0.001558416 27100 0.000384794 26900 0.001366237 26500
0.03533494 28700 0.002009953 28200 0.02533788
29100 0.001557974 27100 0.000385582 26900 0.001370752 26500
0.03591063 28700 0.002006662 28200 0.02548316
29100 0.001557501 27100 0.000386387 26900 0.001375286 26500
0.03649777 28700 0.002003365 28200 0.02562951
29100 0.001557042 27100 0.000387183 26900 0.001379855 26500
0.03709668 28800 0.002000075 28200 0.02577716
29100 0.001556572 27100 0.000387986 26900 0.001384442 26500
0.03770759 28800 0.001996809 28200 0.02592603
29100 0.001556118 27100 0.000388787 26900 0.001389077 26500
0.03833065 28800 0.001993569 28200 0.02607614
29100 0.001555633 27100 0.000389583 26900 0.001393727 26500
0.03896592 28800 0.001990319 28200 0.02622744
29100 0.001555165 27100 0.000390396 26900 0.001398408 26500
0.03961348 28800 0.00198708 28200 0.02638003
29100 0.001554725 27100 0.000391218 26900 0.001403114 26500
0.0402734 28800 0.001983852 28200 0.02653384
29100 0.001554266 27100 0.000392046 26900 0.001407829 26500
0.04094576 28800 0.001980632 28200 0.02668894
29100 0.001553842 27100 0.000392888 26900 0.001412581 26500
0.04163079 28800 0.001977438 28200 0.0268453
29100 0.001553387 27100 0.000393728 26900 0.001417367 26500
0.04232875 28800 0.001974257 28200 0.02700297
29100 0.00155294 27100 0.000394566 26900 0.001422189 26500
0.04303993 28800 0.0019711 28200 0.02716194
29100 0.001552466 27100 0.000395417 26900 0.001427034 26500
0.04376463 28800 0.001967956 28200 0.02732226
29100 0.001552012 27100 0.00039627 26900 0.001431917 26500
0.0445031 28800 0.001964816 28200 0.02748387
29100 0.001551564 27100 0.000397124 26900 0.001436818 26500
0.04525556 28800 0.001961692 28200 0.02764686
29100 0.001551109 27100 0.00039797 26900 0.001441748 26500
0.04602215 28800 0.001958591 28200 0.02781121
29100 0.001550648 27100 0.000398807 26900 0.001446698 26500
0.04680303 28800 0.001955492 28200 0.02797695
29100 0.001550192 27100 0.000399655 26900 0.001451674 26500
0.0475983 28800 0.001952419 28200 0.0281441
29100 0.001549756 27100 0.00040049 26900 0.001456695 26500
0.04840794 28800 0.001949349 28200 0.02831266
29100 0.001549311 27100 0.000401334 26900 0.001461735 26500
0.04923189 28800 0.001946317 28200 0.02848256
29100 0.00154885 27100 0.00040219 26900 0.001466804 26500
0.05007003 28800 0.00194327 28200 0.02865383
29100 0.001548412 27100 0.000403039 26900 0.001471895 26500
0.05092235 28800 0.001940233 28200 0.02882666

29100 0.00154797 27100 0.000403902 26900 0.001477018 26500
0.05178882 28800 0.001937223 28200 0.02900091
29100 0.001547532 27100 0.000404761 26900 0.001482188 26500
0.05266936 28800 0.001934223 28200 0.02917663
29100 0.001547093 27100 0.00040562 26900 0.001487374 26500
0.05356397 28800 0.001931233 28200 0.02935382
29100 0.00154666 27100 0.000406492 26900 0.001492604 26500
0.05447262 28800 0.001928254 28200 0.0295325
29100 0.001546226 27100 0.000407371 26900 0.001497851 26500
0.05539526 28800 0.001925286 28200 0.02971267
29100 0.001545792 27100 0.000408247 26900 0.001503115 26500
0.05633177 28800 0.001922316 28200 0.02989438
29100 0.001545366 27100 0.000409138 26900 0.001508414 26500
0.05728198 28800 0.00191938 28200 0.0300776
29100 0.001544937 27100 0.000410022 26900 0.001513764 26500
0.05824571 28800 0.001916444 28200 0.03026239
29100 0.001544503 27100 0.000410889 26900 0.001519128 26500
0.05922263 28800 0.001913523 28200 0.03044878
29200 0.001544071 27100 0.000411772 26900 0.001524517 26500
0.06021246 28800 0.001910599 28200 0.03063673
29200 0.001543645 27100 0.000412656 26900 0.001529941 26500
0.06121486 28800 0.001907681 28200 0.03082631
29200 0.001543217 27100 0.000413536 26900 0.001535392 26500
0.06222941 28800 0.001904777 28200 0.03101751
29200 0.001542765 27100 0.000414417 26900 0.001540883 26500
0.06325569 28800 0.001901887 28200 0.03121036
29200 0.001542318 27100 0.000415299 26900 0.001546396 26500
0.06429321 28800 0.001898999 28200 0.03140493
29200 0.001541861 27100 0.000416186 26900 0.001551932 26500
0.06534148 28800 0.001896121 28200 0.03160115
29200 0.001541419 27100 0.000417079 26900 0.001557509 26500
0.06639988 28800 0.00189325 28200 0.03179907
29200 0.001540962 27100 0.000417982 26900 0.001563097 26500
0.06746776 28800 0.001890391 28200 0.03199867
29200 0.001540531 27100 0.000418893 26900 0.001568736 26500
0.06854428 28800 0.001887534 28200 0.03219989
29200 0.001540076 27100 0.000419805 26900 0.001574387 26500
0.0696286 28800 0.001884694 28300 0.03240301
29200 0.001539637 27100 0.000420738 26900 0.001580078 26500
0.07071964 28800 0.001881876 28300 0.03260791
29200 0.001539178 27100 0.000421662 26900 0.001585809 26500
0.07181623 28800 0.001879071 28300 0.03281459
29200 0.001538708 27100 0.000422587 26900 0.001591565 26500
0.07291722 28800 0.001876275 28300 0.03302309
29200 0.001538241 27100 0.000423527 26900 0.001597356 26500
0.07402146 28800 0.001873475 28300 0.03323345
29200 0.001537804 27100 0.000424463 26900 0.001603176 26500
0.07512777 28800 0.001870669 28300 0.03344563
29200 0.001537348 27100 0.000425403 26900 0.001609009 26500
0.07623489 28800 0.001867888 28300 0.03365967
29200 0.001536896 27100 0.00042634 26900 0.001614881 26500
0.07734146 28800 0.001865122 28300 0.03387564
29200 0.001536453 27100 0.000427263 26900 0.001620815 26500
0.07844599 28800 0.001862368 28300 0.03409352
29200 0.001535991 27200 0.000428172 26900 0.001626774 26500
0.0795469 28800 0.001859626 28300 0.03431336
29200 0.001535516 27200 0.000429099 26900 0.001632758 26500
0.08064241 28800 0.001856908 28300 0.03453517
29200 0.001535062 27200 0.000430037 27000 0.001638785 26500
0.08173053 28800 0.001854185 28300 0.03475899
29200 0.001534603 27200 0.000430973 27000 0.001644843 26500
0.08280895 28800 0.001851465 28300 0.03498484
29200 0.001534119 27200 0.000431921 27000 0.001650932 26500
0.08387511 28800 0.001848764 28300 0.03521275
29200 0.001533635 27200 0.000432878 27000 0.001657056 26500
0.08492624 28800 0.001846071 28300 0.03544278
29200 0.001533169 27200 0.000433843 27000 0.001663199 26600
0.08595958 28800 0.001843401 28300 0.03567492

29200 0.001532691 27200 0.000434813 27000 0.001669402 26600
0.08697237 28800 0.001840728 28300 0.03590918
29200 0.001532206 27200 0.000435786 27000 0.001675612 26600
0.08796208 28800 0.001838068 28300 0.03614557
29200 0.001531731 27200 0.000436767 27000 0.001681855 26600
0.08892611 28800 0.001835426 28300 0.03638409
29200 0.001531249 27200 0.00043774 27000 0.001688148 26600
0.08986187 28800 0.001832781 28300 0.03662492
29200 0.001530784 27200 0.000438716 27000 0.001694464 26600
0.09076656 28800 0.001830148 28300 0.03686806
29200 0.001530312 27200 0.00043968 27000 0.001700833 26600
0.091637 28800 0.001827549 28300 0.03711348
29200 0.001529802 27200 0.000440649 27000 0.001707227 26600
0.09246989 28800 0.001824944 28300 0.03736119
29200 0.001529326 27200 0.000441614 27000 0.001713651 26600
0.09326184 28800 0.001822332 28300 0.03761131
29200 0.001528864 27200 0.000442584 27000 0.0017201 26600
0.09400923 28800 0.001819737 28300 0.03786378
29200 0.001528388 27200 0.000443555 27000 0.001726578 26600
0.09470849 28800 0.001817149 28300 0.03811869
29200 0.001527946 27200 0.000444526 27000 0.001733098 26600
0.09535591 28900 0.001814579 28300 0.03837609
29200 0.001527471 27200 0.000445491 27000 0.001739645 26600
0.09594776 28900 0.001812016 28300 0.03863598
29200 0.001527012 27200 0.000446441 27000 0.001746246 26600
0.09648033 28900 0.001809473 28300 0.03889843
29200 0.001526519 27200 0.000447429 27000 0.001752848 26600
0.09695002 28900 0.00180693 28300 0.03916348
29200 0.001526074 27200 0.000448415 27000 0.001759503 26600
0.09735329 28900 0.001804392 28300 0.03943117
29200 0.001525608 27200 0.000449405 27000 0.001766184 26600
0.09768666 28900 0.001801859 28300 0.03970155
29200 0.001525141 27200 0.000450411 27000 0.001772898 26600
0.09794667 28900 0.001799325 28300 0.03997465
29200 0.001524672 27200 0.000451429 27000 0.001779633 26600
0.0981298 28900 0.001796819 28300 0.04025055
29200 0.001524218 27200 0.00045246 27000 0.001786399 26600
0.09823259 28900 0.001794308 28300 0.04052925
29200 0.001523753 27200 0.000453493 27000 0.001793212 26600
0.09825163 28900 0.001791811 28300 0.04081081
29200 0.001523308 27200 0.00045455 27000 0.001800046 26600
0.09818382 28900 0.001789326 28300 0.04109518
29200 0.001522859 27200 0.000455602 27000 0.001806908 26600
0.09802642 28900 0.001786839 28300 0.04138243
29200 0.001522426 27200 0.00045667 27000 0.001813795 26600
0.09777732 28900 0.001784354 28300 0.04167278
29200 0.001521986 27200 0.000457739 27000 0.001820726 26600
0.09743477 28900 0.001781898 28300 0.04196618
29200 0.001521539 27200 0.000458808 27000 0.001827696 26600
0.09699743 28900 0.001779445 28300 0.04226268
29200 0.001521086 27200 0.000459877 27000 0.001834689 26600
0.09646452 28900 0.001776994 28300 0.04256227
29200 0.001520647 27200 0.000460957 27000 0.001841727 26600
0.0958356 28900 0.00177455 28300 0.04286506
29200 0.001520219 27200 0.00046203 27000 0.001848762 26600
0.09511061 28900 0.001772106 28300 0.04317105
29200 0.0015198 27200 0.000463114 27000 0.00185583 26600
0.09428995 28900 0.001769678 28300 0.04348035
29200 0.00151939 27200 0.000464206 27000 0.001862944 26600
0.09337432 28900 0.001767262 28300 0.04379295
29200 0.001518961 27200 0.000465286 27000 0.001870082 26600
0.09236465 28900 0.001764854 28300 0.04410894
29200 0.001518552 27200 0.000466362 27000 0.001877262 26600
0.09126208 28900 0.001762467 28300 0.04442839
29200 0.001518127 27200 0.000467456 27000 0.001884456 26600
0.0900679 28900 0.001760076 28300 0.04475131
29200 0.001517694 27200 0.000468562 27000 0.001891686 26600
0.0887836 28900 0.001757696 28300 0.04507779

29200 0.001517279 27200 0.000469672 27000 0.001898936 26600
0.08741111 28900 0.001755315 28300 0.04540788
29200 0.001516833 27200 0.000470785 27000 0.001906221 26600
0.08595283 28900 0.001752947 28300 0.04574164
29200 0.001516389 27200 0.000471887 27000 0.001913524 26600
0.08441184 28900 0.001750578 28300 0.04607915
29200 0.001515978 27200 0.000472993 27000 0.001920855 26600
0.08279195 28900 0.001748214 28300 0.04642046
29200 0.001515543 27200 0.000474089 27000 0.001928221 26600
0.08109767 28900 0.001745866 28300 0.04676557
29300 0.001515112 27200 0.000475197 27000 0.001935609 26600
0.07933426 28900 0.001743514 28300 0.04711451
29300 0.001514681 27200 0.000476298 27000 0.001943016 26600
0.07750738 28900 0.001741167 28300 0.04746733
29300 0.001514255 27200 0.000477418 27000 0.001950459 26600
0.07562312 28900 0.001738814 28300 0.04782429
29300 0.001513811 27200 0.000478529 27000 0.001957928 26600
0.07368744 28900 0.001736474 28300 0.04818532
29300 0.001513389 27200 0.00047965 27000 0.001965439 26600
0.07170631 28900 0.001734151 28300 0.04855048
29300 0.001512954 27200 0.000480767 27000 0.001972957 26600
0.06968582 28900 0.001731811 28300 0.04891984
29300 0.001512502 27200 0.000481899 27000 0.001980521 26600
0.06763206 28900 0.001729464 28300 0.04929343
29300 0.001512096 27200 0.00048303 27000 0.001988084 26600
0.06555127 28900 0.001727109 28300 0.04967132
29300 0.001511674 27200 0.000484163 27000 0.001995667 26600
0.0634498 28900 0.001724745 28300 0.05005361
29300 0.001511252 27200 0.000485307 27000 0.002003285 26600
0.06133415 28900 0.001722384 28400 0.05044033
29300 0.001510821 27200 0.000486431 27000 0.002010949 26600
0.05921099 28900 0.001720034 28400 0.05083153
29300 0.001510397 27200 0.000487548 27000 0.002018637 26600
0.05708697 28900 0.001717682 28400 0.05122732
29300 0.001509959 27200 0.000488688 27000 0.002026328 26600
0.05496876 28900 0.001715314 28400 0.05162777
29300 0.001509532 27200 0.000489814 27000 0.002034044 26600
0.05286272 28900 0.001712954 28400 0.05203292
29300 0.001509113 27200 0.000490958 27000 0.002041798 26600
0.05077497 28900 0.001710583 28400 0.05244287
29300 0.001508668 27200 0.000492094 27000 0.002049562 26600
0.04871114 28900 0.001708217 28400 0.05285766
29300 0.001508244 27200 0.000493235 27000 0.002057348 26600
0.04667651 28900 0.001705847 28400 0.05327738
29300 0.00150782 27200 0.000494384 27000 0.002065166 26600
0.04467587 28900 0.001703478 28400 0.05370209
29300 0.001507409 27300 0.000495534 27000 0.002073003 26600
0.04271364 28900 0.001701123 28400 0.05413182
29300 0.001506985 27300 0.000496696 27000 0.002080865 26600
0.04079391 28900 0.001698772 28400 0.05456664
29300 0.001506555 27300 0.000497866 27100 0.002088754 26600
0.03892047 28900 0.001696418 28400 0.05500657
29300 0.001506144 27300 0.000499046 27100 0.002096651 26600
0.03709675 28900 0.001694069 28400 0.05545193
29300 0.001505727 27300 0.000500219 27100 0.00210458 26600
0.03532585 28900 0.001691737 28400 0.05590261
29300 0.001505325 27300 0.000501404 27100 0.002112552 26600
0.03361047 28900 0.00168943 28400 0.05635872
29300 0.00150491 27300 0.000502584 27100 0.002120525 26700
0.03195294 28900 0.001687129 28400 0.05682036
29300 0.001504488 27300 0.000503766 27100 0.002128518 26700
0.03035513 28900 0.001684826 28400 0.0572876
29300 0.001504095 27300 0.00050495 27100 0.002136529 26700
0.02881852 28900 0.001682539 28400 0.05776043
29300 0.001503692 27300 0.000506138 27100 0.002144545 26700
0.02734416 28900 0.001680261 28400 0.05823909
29300 0.001503301 27300 0.000507337 27100 0.002152592 26700
0.02593274 28900 0.001677997 28400 0.05872357

29300 0.001502912 27300 0.000508527 27100 0.002160662 26700
0.02458464 28900 0.001675767 28400 0.05921399
29300 0.001502523 27300 0.000509721 27100 0.002168756 26700
0.0232999 28900 0.001673542 28400 0.05971041
29300 0.00150215 27300 0.00051095 27100 0.002176857 26700
0.02207821 28900 0.001671325 28400 0.06021298
29300 0.001501778 27300 0.000512174 27100 0.002184973 26700
0.0209189 28900 0.001669124 28400 0.06072177
29300 0.001501412 27300 0.00051342 27100 0.002193107 26700
0.01982105 28900 0.001666929 28400 0.06123696
29300 0.001501025 27300 0.000514673 27100 0.002201269 26700
0.01878342 28900 0.001664751 28400 0.06175854
29300 0.001500655 27300 0.000515925 27100 0.002209431 26700
0.0178047 29000 0.00166258 28400 0.06228669
29300 0.001500274 27300 0.00051719 27100 0.002217619 26700
0.01688339 29000 0.001660408 28400 0.06282146
29300 0.001499918 27300 0.000518442 27100 0.002225824 26700
0.01601793 29000 0.001658254 28400 0.06336299
29300 0.001499549 27300 0.000519702 27100 0.002234045 26700
0.01520664 29000 0.001656115 28400 0.06391125
29300 0.001499208 27300 0.000520964 27100 0.002242278 26700
0.01444776 29000 0.001653973 28400 0.06446639
29300 0.001498865 27300 0.000522239 27100 0.002250521 26700
0.01373943 29000 0.001651825 28400 0.06502876
29300 0.001498515 27300 0.000523501 27100 0.002258794 26700
0.01307965 29000 0.001649705 28400 0.06559829
29300 0.001498185 27300 0.000524785 27100 0.002267084 26700
0.01246644 29000 0.001647601 28400 0.06617513
29300 0.001497821 27300 0.000526062 27100 0.002275405 26700
0.01189776 29000 0.001645492 28400 0.06675937
29300 0.001497486 27300 0.000527354 27100 0.002283754 26700
0.01137158 29000 0.001643378 28400 0.06735113
29300 0.001497163 27300 0.000528656 27100 0.002292103 26700
0.01088578 29000 0.001641271 28400 0.06795052
29300 0.001496851 27300 0.000529971 27100 0.002300464 26700
0.01043826 29000 0.001639172 28400 0.06855773
29300 0.001496544 27300 0.00053129 27100 0.002308864 26700
0.01002693 29000 0.00163707 28400 0.06917284
29300 0.00149625 27300 0.000532622 27100 0.002317285 26700
0.009649656 29000 0.001634996 28400 0.06979601
29300 0.001495928 27300 0.000533965 27100 0.002325728 26700
0.009304452 29000 0.001632911 28400 0.07042734
29300 0.001495617 27300 0.000535304 27100 0.002334191 26700
0.008989383 29000 0.001630828 28400 0.07106704
29300 0.001495327 27300 0.000536664 27100 0.002342663 26700
0.008702598 29000 0.001628752 28400 0.07171518
29300 0.001495032 27300 0.000538008 27100 0.002351175 26700
0.008442294 29000 0.00162668 28400 0.07237196
29300 0.001494739 27300 0.000539352 27100 0.002359693 26700
0.00820672 29000 0.001624611 28400 0.07303749
29300 0.001494419 27300 0.000540687 27100 0.002368225 26700
0.007994225 29000 0.001622548 28400 0.07371198
29300 0.001494125 27300 0.000542034 27100 0.00237677 26700
0.007803133 29000 0.001620469 28400 0.07439552
29300 0.00149384 27300 0.000543352 27100 0.002385345 26700
0.007631896 29000 0.001618408 28400 0.07508828
29300 0.001493561 27300 0.000544681 27100 0.002393931 26700
0.007479016 29000 0.00161635 28400 0.07579033
29300 0.001493259 27300 0.000546012 27100 0.002402545 26700
0.007343087 29000 0.001614287 28400 0.07650184
29300 0.001492994 27300 0.000547365 27100 0.002411152 26700
0.0072228 29000 0.001612224 28400 0.07722323
29300 0.00149268 27300 0.000548736 27100 0.00241981 26700
0.007116904 29000 0.001610169 28400 0.07795447
29300 0.001492425 27300 0.000550117 27100 0.002428479 26700
0.007024208 29000 0.00160813 28400 0.07869579
29300 0.001492098 27300 0.000551499 27100 0.002437164 26700
0.006943607 29000 0.001606085 28400 0.07944732

29300 0.001491824 27300 0.00055291 27100 0.002445871 26700
0.006874046 29000 0.001604038 28400 0.08020933
29400 0.001491534 27300 0.000554307 27100 0.002454584 26700
0.0068145 29000 0.001602002 28400 0.08098189
29400 0.001491253 27300 0.000555719 27100 0.002463304 26700
0.006764051 29000 0.001599966 28400 0.08176526
29400 0.001490973 27300 0.000557138 27100 0.002472064 26700
0.006721791 29000 0.001597933 28400 0.08255968
29400 0.001490688 27300 0.000558539 27100 0.002480838 26700
0.006686893 29000 0.001595919 28400 0.08336531
29400 0.001490407 27300 0.000559928 27100 0.002489625 26700
0.006658553 29000 0.001593901 28400 0.08418234
29400 0.001490106 27300 0.000561327 27100 0.002498443 26700
0.00663604 29000 0.001591884 28400 0.08501096
29400 0.001489817 27300 0.000562732 27100 0.002507277 26700
0.00661868 29000 0.001589879 28400 0.08585147
29400 0.001489518 27300 0.000564152 27100 0.002516123 26700
0.006605896 29000 0.001587876 28400 0.08670405
29400 0.001489211 27300 0.000565583 27100 0.002524989 26700
0.006597131 29000 0.001585886 28400 0.08756892
29400 0.001488879 27300 0.000567013 27100 0.002533858 26700
0.006591939 29000 0.001583894 28500 0.08844632
29400 0.001488574 27300 0.00056844 27100 0.002542745 26700
0.006589869 29000 0.001581897 28500 0.0893365
29400 0.001488255 27300 0.000569862 27100 0.002551643 26700
0.006590554 29000 0.001579919 28500 0.09023959
29400 0.001487954 27300 0.000571282 27100 0.00256056 26700
0.00659362 29000 0.001577939 28500 0.0911558
29400 0.001487652 27300 0.000572695 27100 0.002569495 26700
0.006598732 29000 0.001575963 28500 0.09208538
29400 0.001487329 27300 0.000574152 27100 0.002578435 26700
0.006605586 29000 0.001573989 28500 0.09302887
29400 0.001486999 27300 0.00057561 27100 0.002587402 26700
0.006613832 29000 0.001572035 28500 0.09398628
29400 0.001486689 27300 0.000577087 27100 0.002596386 26700
0.006623218 29000 0.0015701 28500 0.09495793
29400 0.001486358 27300 0.000578571 27100 0.002605374 26700
0.006633476 29000 0.001568159 28500 0.09594404
29400 0.001486029 27400 0.000580065 27100 0.002614387 26700
0.006644396 29000 0.001566209 28500 0.09694489
29400 0.001485746 27400 0.000581548 27100 0.002623389 26700
0.006655732 29000 0.001564268 28500 0.09796073
29400 0.001485415 27400 0.000583046 27200 0.002632397 26700
0.006667274 29000 0.00156234 28500 0.09899191
29400 0.001485099 27400 0.000584564 27200 0.002641435 26700
0.006678829 29000 0.001560412 28500 0.1000387
29400 0.001484822 27400 0.000586054 27200 0.002650475 26700
0.006690186 29000 0.001558506 28500 0.1011013
29400 0.00148451 27400 0.000587574 27200 0.00265954 26700
0.006701218 29000 0.001556595 28500 0.1021802
29400 0.001484186 27400 0.000589115 27200 0.002668605 26800
0.006711824 29000 0.001554692 28500 0.1032757
29400 0.001483877 27400 0.000590662 27200 0.002677679 26800
0.006721903 29000 0.001552794 28500 0.1043879
29400 0.001483568 27400 0.00059222 27200 0.002686768 26800
0.00673139 29000 0.001550901 28500 0.1055174
29400 0.001483232 27400 0.000593781 27200 0.002695862 26800
0.006740239 29000 0.001549014 28500 0.1066644
29400 0.001482892 27400 0.000595346 27200 0.002704964 26800
0.006748413 29000 0.001547126 28500 0.1078292
29400 0.001482585 27400 0.000596902 27200 0.002714081 26800
0.006755855 29000 0.001545245 28500 0.1090123
29400 0.001482253 27400 0.000598455 27200 0.0027232 26800
0.006762522 29000 0.001543368 28500 0.1102139
29400 0.001481931 27400 0.000600008 27200 0.002732334 26800
0.006768376 29000 0.001541479 28500 0.1114344
29400 0.001481619 27400 0.000601562 27200 0.002741486 26800
0.0067734 29000 0.001539602 28500 0.1126741

29400 0.001481332 27400 0.000603148 27200 0.002750638 26800
0.006777551 29000 0.001537722 28500 0.1139338
29400 0.001480985 27400 0.000604742 27200 0.002759807 26800
0.006780826 29000 0.001535851 28500 0.1152135
29400 0.001480682 27400 0.000606349 27200 0.002769003 26800
0.006783193 29100 0.001533992 28500 0.1165137
29400 0.001480359 27400 0.000607964 27200 0.002778178 26800
0.006784655 29100 0.001532118 28500 0.1178349
29400 0.001480032 27400 0.000609574 27200 0.002787385 26800
0.006785196 29100 0.001530253 28500 0.1191774
29400 0.00147974 27400 0.000611181 27200 0.002796583 26800
0.006784758 29100 0.001528379 28500 0.1205417
29400 0.001479448 27400 0.000612787 27200 0.002805787 26800
0.006783344 29100 0.001526519 28500 0.1219283
29400 0.00147917 27400 0.000614388 27200 0.002815005 26800
0.006780873 29100 0.001524648 28500 0.1233376
29400 0.001478855 27400 0.000615989 27200 0.002824225 26800
0.006777323 29100 0.001522794 28500 0.1247701
29400 0.00147856 27400 0.000617584 27200 0.002833464 26800
0.006772678 29100 0.001520941 28500 0.1262263
29400 0.001478281 27400 0.00061921 27200 0.002842701 26800
0.006766945 29100 0.001519099 28500 0.1277068
29400 0.001477998 27400 0.000620855 27200 0.002851949 26800
0.006760172 29100 0.001517251 28500 0.1292119
29400 0.001477682 27400 0.000622516 27200 0.002861191 26800
0.006752407 29100 0.001515422 28500 0.1307423
29400 0.001477386 27400 0.000624193 27200 0.00287044 26800
0.006743721 29100 0.001513596 28500 0.1322985
29400 0.001477074 27400 0.000625873 27200 0.002879677 26800
0.0067342 29100 0.001511779 28500 0.133881
29400 0.001476777 27400 0.000627556 27200 0.002888913 26800
0.00672386 29100 0.001509971 28500 0.1354904
29400 0.001476486 27400 0.000629234 27200 0.002898154 26800
0.006712765 29100 0.001508177 28500 0.1371273
29400 0.001476197 27400 0.000630922 27200 0.002907395 26800
0.006700951 29100 0.001506373 28500 0.138792
29400 0.00147591 27400 0.000632597 27200 0.002916635 26800
0.006688411 29100 0.001504599 28500 0.1404853
29400 0.001475626 27400 0.000634288 27200 0.002925864 26800
0.006675195 29100 0.001502812 28500 0.1422081
29400 0.001475323 27400 0.000635973 27200 0.002935094 26800
0.006661258 29100 0.001501049 28500 0.1439608
29400 0.001475027 27400 0.00063766 27200 0.002944345 26800
0.006646621 29100 0.001499305 28500 0.1457441
29400 0.001474724 27400 0.000639341 27200 0.002953567 26800
0.006631252 29100 0.001497555 28500 0.1475585
29400 0.001474395 27400 0.00064103 27200 0.0029628 26800
0.006615153 29100 0.001495812 28500 0.1494049
29400 0.00147414 27400 0.000642726 27200 0.002972015 26800
0.006598298 29100 0.001494062 28500 0.1512838
29400 0.001473832 27400 0.000644439 27200 0.002981207 26800
0.006580715 29100 0.001492339 28500 0.1531961
29400 0.001473579 27400 0.000646176 27200 0.002990426 26800
0.006562446 29100 0.001490595 28500 0.1551424
29400 0.001473284 27400 0.000647906 27200 0.002999638 26800
0.006543503 29100 0.00148888 28500 0.1571236
29400 0.001472999 27400 0.000649637 27200 0.003008843 26800
0.006523971 29100 0.001487146 28500 0.1591404
29400 0.001472705 27400 0.000651396 27200 0.003018046 26800
0.006503916 29100 0.00148541 28500 0.1611935
29400 0.001472417 27400 0.000653149 27200 0.003027252 26800
0.00648339 29100 0.001483688 28500 0.163284
29500 0.001472153 27400 0.000654916 27200 0.003036438 26800
0.006462388 29100 0.001481957 28500 0.1654125
29500 0.001471867 27400 0.000656688 27200 0.003045632 26800
0.006440977 29100 0.001480235 28500 0.1675799
29500 0.001471567 27400 0.000658477 27200 0.003054807 26800
0.006419124 29100 0.001478521 28500 0.1697872

29500 0.001471284 27400 0.000660281 27200 0.003063979 26800
0.006396861 29100 0.001476791 28500 0.1720352
29500 0.001471004 27400 0.000662092 27200 0.003073154 26800
0.006374196 29100 0.001475082 28500 0.1743248
29500 0.001470743 27400 0.000663929 27200 0.003082332 26800
0.006351151 29100 0.001473364 28500 0.1766569
29500 0.001470475 27400 0.000665768 27200 0.003091488 26800
0.006327779 29100 0.001471654 28500 0.1790324
29500 0.001470188 27400 0.000667623 27200 0.003100641 26800
0.006304127 29100 0.00146994 28500 0.1814528
29500 0.00146992 27400 0.00066948 27200 0.003109786 26800
0.006280235 29100 0.001468243 28500 0.1839186
29500 0.001469658 27400 0.00067136 27200 0.003118961 26800
0.006256115 29100 0.001466559 28600 0.1864309
29500 0.0014694 27400 0.000673245 27200 0.003128102 26800
0.006231777 29100 0.001464864 28600 0.1889909
29500 0.001469133 27400 0.00067514 27200 0.003137258 26800
0.006207239 29100 0.001463178 28600 0.1915997
29500 0.001468898 27400 0.000677053 27200 0.003146395 26800
0.006182458 29100 0.001461498 28600 0.1942583
29500 0.001468651 27400 0.000678969 27200 0.00315552 26800
0.006157444 29100 0.00145982 28600 0.1969679
29500 0.001468413 27400 0.000680908 27200 0.003164665 26800
0.006132217 29100 0.001458132 28600 0.1997298
29500 0.001468181 27400 0.000682835 27200 0.003173792 26800
0.006106787 29100 0.001456456 28600 0.2025451
29500 0.001467942 27400 0.000684747 27200 0.003182923 26800
0.006081185 29100 0.001454786 28600 0.2054152
29500 0.001467732 27400 0.000686687 27200 0.003192049 26800
0.006055456 29100 0.001453102 28600 0.2083412
29500 0.00146749 27500 0.000688617 27200 0.003201166 26800
0.006029611 29100 0.001451425 28600 0.2113246
29500 0.001467275 27500 0.000690549 27200 0.003210276 26800
0.00600361 29100 0.001449742 28600 0.2143665
29500 0.001467034 27500 0.000692483 27300 0.003219386 26800
0.00597749 29100 0.001448047 28600 0.2174685
29500 0.0014668 27500 0.000694429 27300 0.00322848 26800
0.005951219 29100 0.001446361 28600 0.2206319
29500 0.001466559 27500 0.00069637 27300 0.003237589 26800
0.005924789 29100 0.001444679 28600 0.2238581
29500 0.001466354 27500 0.000698308 27300 0.003246669 26800
0.005898235 29100 0.001442999 28600 0.2271486
29500 0.001466137 27500 0.000700247 27300 0.003255761 26900
0.005871581 29100 0.001441298 28600 0.2305046
29500 0.001465958 27500 0.00070219 27300 0.00326485 26900
0.005844824 29100 0.001439609 28600 0.2339278
29500 0.001465768 27500 0.000704142 27300 0.00327393 26900
0.005818001 29100 0.001437912 28600 0.2374204
29500 0.001465541 27500 0.000706101 27300 0.003283009 26900
0.005791084 29100 0.001436232 28600 0.2409834
29500 0.001465364 27500 0.000708086 27300 0.003292099 26900
0.005764083 29100 0.001434555 28600 0.2446185
29500 0.001465136 27500 0.000710078 27300 0.003301169 26900
0.005736971 29100 0.001432879 28600 0.2483276
29500 0.001464909 27500 0.00071209 27300 0.00331026 26900
0.005709797 29100 0.001431205 28600 0.2521122
29500 0.001464727 27500 0.000714129 27300 0.003319333 26900
0.005682529 29100 0.001429528 28600 0.2559742
29500 0.00146452 27500 0.000716182 27300 0.003328376 26900
0.005655246 29100 0.001427857 28600 0.2599154
29500 0.001464328 27500 0.000718264 27300 0.003337463 26900
0.005627975 29100 0.001426193 28600 0.2639377
29500 0.001464126 27500 0.000720342 27300 0.00334654 26900
0.005600709 29100 0.001424542 28600 0.2680428
29500 0.001463909 27500 0.000722412 27300 0.003355634 26900
0.005573496 29200 0.001422893 28600 0.2722329
29500 0.001463697 27500 0.00072451 27300 0.003364731 26900
0.005546355 29200 0.001421248 28600 0.2765098

29500 0.001463488 27500 0.000726615 27300 0.003373834 26900
0.00551928 29200 0.001419612 28600 0.2808757
29500 0.001463292 27500 0.000728721 27300 0.003382941 26900
0.00549225 29200 0.001417971 28600 0.2853325
29500 0.001463047 27500 0.00073084 27300 0.003392051 26900
0.005465318 29200 0.001416342 28600 0.2898823
29500 0.001462811 27500 0.000732969 27300 0.00340117 26900
0.005438463 29200 0.00141472 28600 0.2945274
29500 0.001462579 27500 0.000735097 27300 0.00341031 26900
0.005411683 29200 0.001413084 28600 0.2992699
29500 0.001462341 27500 0.000737242 27300 0.00341945 26900
0.005384983 29200 0.00141147 28600 0.3041119
29500 0.001462096 27500 0.000739389 27300 0.003428605 26900
0.005358331 29200 0.001409847 28600 0.3090554
29500 0.001461852 27500 0.000741531 27300 0.003437781 26900
0.005331724 29200 0.001408241 28600 0.3141029
29500 0.001461604 27500 0.000743677 27300 0.003446983 26900
0.005305169 29200 0.001406637 28600 0.3192574
29500 0.001461338 27500 0.000745811 27300 0.003456194 26900
0.005278656 29200 0.001405044 28600 0.3245207
29500 0.001461099 27500 0.000747959 27300 0.003465453 26900
0.005252203 29200 0.001403468 28600 0.3298953
29500 0.001460822 27500 0.000750102 27300 0.003474725 26900
0.005225817 29200 0.001401895 28600 0.3353834
29500 0.001460562 27500 0.000752246 27300 0.003484029 26900
0.005199568 29200 0.001400325 28600 0.3409879
29500 0.001460303 27500 0.0007544 27300 0.003493352 26900
0.005173414 29200 0.00139876 28600 0.3467109
29500 0.001460048 27500 0.00075656 27300 0.003502694 26900
0.005147397 29200 0.001397208 28600 0.352555
29500 0.001459795 27500 0.000758733 27300 0.003512082 26900
0.005121513 29200 0.001395657 28600 0.358523
29500 0.001459543 27500 0.000760908 27300 0.003521518 26900
0.005095762 29200 0.00139412 28600 0.3646171
29500 0.001459282 27500 0.000763084 27300 0.003530989 26900
0.005070142 29200 0.0013926 28600 0.3708402
29500 0.001459 27500 0.000765316 27300 0.003540473 26900
0.005044692 29200 0.001391073 28600 0.3771945
29500 0.001458735 27500 0.000767559 27300 0.003550011 26900
0.005019398 29200 0.001389544 28600 0.3836829
29500 0.001458472 27500 0.000769821 27300 0.003559587 26900
0.004994284 29200 0.001388024 28600 0.3903078
29500 0.001458193 27500 0.000772103 27300 0.003569194 26900
0.004969337 29200 0.001386518 28600 0.3970716
29500 0.001457903 27500 0.00077439 27300 0.003578844 26900
0.004944552 29200 0.001385024 28600 0.4039771
29500 0.001457621 27500 0.000776671 27300 0.003588545 26900
0.00491986 29200 0.001383513 28600 0.4110265
29500 0.001457328 27500 0.000778956 27300 0.003598284 26900
0.004895286 29200 0.001382008 28600 0.4182223
29500 0.001457059 27500 0.000781246 27300 0.003608066 26900
0.004870813 29200 0.001380508 28600 0.4255666
29500 0.001456792 27500 0.000783537 27300 0.003617906 26900
0.004846433 29200 0.001379002 28600 0.433062
29600 0.001456497 27500 0.000785828 27300 0.003627802 26900
0.004822173 29200 0.001377489 28600 0.4407113
29600 0.00145619 27500 0.000788132 27300 0.003637748 26900
0.004798021 29200 0.001375992 28600 0.4485159
29600 0.00145591 27500 0.000790464 27300 0.003647771 26900
0.004773988 29200 0.00137452 28600 0.4564779
29600 0.001455598 27500 0.000792793 27300 0.003657827 26900
0.00475009 29200 0.001373016 28600 0.4645994
29600 0.001455297 27500 0.000795136 27300 0.003667961 26900
0.004726331 29200 0.001371518 28600 0.4728822
29600 0.001455028 27500 0.000797491 27300 0.003678145 26900
0.004702701 29200 0.001370016 28600 0.4813278
29600 0.001454744 27500 0.000799856 27300 0.003688365 26900
0.004679191 29200 0.001368523 28600 0.4899378

29600 0.001454462 27500 0.000802228 27300 0.003698675 26900
0.004655861 29200 0.001367026 28600 0.4987135
29600 0.001454192 27500 0.000804603 27300 0.003709062 26900
0.004632676 29200 0.001365534 28600 0.5076558
29600 0.001453904 27500 0.000806963 27300 0.003719521 26900
0.004609663 29200 0.00136406 28700 0.5167657
29600 0.001453592 27500 0.000809353 27300 0.003730048 26900
0.004586818 29200 0.001362562 28700 0.5260437
29600 0.001453317 27500 0.000811736 27300 0.003740647 26900
0.004564139 29200 0.001361069 28700 0.5354899
29600 0.001453022 27500 0.000814132 27300 0.003751318 26900
0.004541624 29200 0.001359593 28700 0.5451042
29600 0.001452735 27500 0.000816546 27300 0.003762068 26900
0.004519292 29200 0.001358095 28700 0.5548863
29600 0.001452423 27500 0.00081897 27300 0.003772898 26900
0.004497123 29200 0.001356621 28700 0.5648353
29600 0.001452146 27500 0.000821405 27300 0.003783805 26900
0.004475138 29200 0.00135514 28700 0.5749496
29600 0.001451854 27500 0.000823853 27300 0.003794794 26900
0.004453323 29200 0.001353683 28700 0.5852276
29600 0.001451564 27500 0.000826321 27300 0.00380587 26900
0.004431702 29200 0.00135221 28700 0.5956663
29600 0.001451291 27600 0.000828796 27300 0.003817037 26900
0.004410278 29200 0.001350754 28700 0.6062638
29600 0.001451008 27600 0.000831287 27300 0.003828303 26900
0.004389036 29200 0.001349296 28700 0.6170167
29600 0.001450716 27600 0.000833786 27400 0.003839649 26900
0.004367975 29200 0.001347869 28700 0.6279199
29600 0.001450444 27600 0.00083632 27400 0.003851109 26900
0.004347101 29200 0.001346454 28700 0.6389689
29600 0.001450137 27600 0.000838864 27400 0.003862646 26900
0.004326373 29200 0.001345031 28700 0.650158
29600 0.001449843 27600 0.00084142 27400 0.003874298 26900
0.004305812 29200 0.0013436 28700 0.6614809
29600 0.001449594 27600 0.000843981 27400 0.003886043 27000
0.004285379 29200 0.001342182 28700 0.6729299
29600 0.001449315 27600 0.00084657 27400 0.003897874 27000
0.004265083 29200 0.001340776 28700 0.6844966
29600 0.001449058 27600 0.000849179 27400 0.003909833 27000
0.004244896 29200 0.001339372 28700 0.6961718
29600 0.001448786 27600 0.000851785 27400 0.003921902 27000
0.00422483 29200 0.001337983 28700 0.7079449
29600 0.001448517 27600 0.000854388 27400 0.003934088 27000
0.004204871 29200 0.001336574 28700 0.7198045
29600 0.001448254 27600 0.000857023 27400 0.00394637 27000
0.004185008 29200 0.001335185 28700 0.7317374
29600 0.001447997 27600 0.000859651 27400 0.003958774 27000
0.004165262 29200 0.001333789 28700 0.7437296
29600 0.001447736 27600 0.00086229 27400 0.003971279 27000
0.004145634 29200 0.001332405 28700 0.7557656
29600 0.001447477 27600 0.000864927 27400 0.003983915 27000
0.004126101 29200 0.001330999 28700 0.7678285
29600 0.001447224 27600 0.000867572 27400 0.003996656 27000
0.004106717 29200 0.001329615 28700 0.7799
29600 0.00144699 27600 0.000870229 27400 0.004009538 27000
0.00408745 29200 0.001328224 28700 0.7919598
29600 0.001446765 27600 0.000872899 27400 0.004022527 27000
0.004068344 29300 0.001326833 28700 0.8039867
29600 0.001446534 27600 0.000875585 27400 0.004035639 27000
0.004049401 29300 0.001325453 28700 0.815957
29600 0.001446327 27600 0.000878275 27400 0.004048888 27000
0.004030629 29300 0.001324073 28700 0.8278466
29600 0.001446123 27600 0.000880982 27400 0.004062281 27000
0.004012073 29300 0.001322702 28700 0.8396296
29600 0.001445879 27600 0.000883678 27400 0.004075797 27000
0.003993719 29300 0.001321321 28700 0.8512766
29600 0.001445694 27600 0.000886402 27400 0.004089448 27000
0.003975585 29300 0.001319981 28700 0.8627584

29600 0.001445492 27600 0.000889119 27400 0.004103225 27000
0.003957636 29300 0.001318618 28700 0.8740429
29600 0.001445292 27600 0.000891844 27400 0.004117146 27000
0.003939873 29300 0.001317254 28700 0.8850974
29600 0.001445134 27600 0.000894593 27400 0.004131188 27000
0.003922263 29300 0.001315911 28700 0.8958871
29600 0.001444943 27600 0.000897361 27400 0.004145341 27000
0.003904785 29300 0.00131457 28700 0.9063753
29600 0.001444782 27600 0.000900155 27400 0.004159651 27000
0.00388743 29300 0.001313221 28700 0.9165244
29600 0.001444617 27600 0.000902971 27400 0.004174128 27000
0.003870192 29300 0.001311895 28700 0.9262952
29600 0.00144445 27600 0.000905786 27400 0.004188744 27000
0.003853068 29300 0.001310583 28700 0.9356477
29600 0.001444298 27600 0.00090864 27400 0.004203501 27000
0.003836086 29300 0.00130926 28700 0.9445403
29600 0.001444138 27600 0.000911504 27400 0.004218399 27000
0.003819237 29300 0.001307951 28700 0.9529305
29600 0.001444013 27600 0.000914378 27400 0.004233429 27000
0.003802534 29300 0.001306621 28700 0.9607757
29600 0.001443847 27600 0.00091725 27400 0.004248615 27000
0.003786042 29300 0.00130532 28700 0.9680328
29600 0.001443707 27600 0.00092013 27400 0.004263944 27000
0.003769713 29300 0.001304015 28700 0.9746584
29600 0.001443553 27600 0.000923012 27400 0.004279433 27000
0.003753532 29300 0.001302712 28700 0.9806097
29600 0.001443437 27600 0.000925905 27400 0.004295055 27000
0.003737539 29300 0.001301404 28700 0.9858442
29600 0.001443282 27600 0.000928798 27400 0.004310871 27000
0.003721702 29300 0.001300114 28700 0.9903198
29600 0.001443146 27600 0.00093171 27400 0.004326816 27000
0.003706053 29300 0.001298808 28700 0.9939977
29600 0.001443031 27600 0.000934645 27400 0.004342943 27000
0.003690563 29300 0.001297505 28700 0.9968399
29600 0.001442868 27600 0.000937576 27400 0.004359234 27000
0.00367523 29300 0.001296214 28700 0.9988091
29600 0.001442741 27600 0.000940543 27400 0.004375691 27000
0.003660036 29300 0.001294913 28700 0.9998726
29600 0.001442565 27600 0.000943529 27400 0.004392308 27000
0.003644973 29300 0.001293629 28700 0.9999999
29600 0.001442411 27600 0.000946517 27400 0.004409093 27000
0.003630031 29300 0.001292328 28700 0.9991652
29600 0.001442285 27600 0.000949557 27400 0.004426059 27000
0.003615176 29300 0.001291023 28700 0.9973455
29600 0.00144215 27600 0.000952627 27400 0.004443168 27000
0.003600433 29300 0.001289718 28700 0.994523
29700 0.001442028 27600 0.00095572 27400 0.004460467 27000
0.003585802 29300 0.00128841 28700 0.9906845
29700 0.00144193 27600 0.000958816 27400 0.004477978 27000
0.003571273 29300 0.001287099 28700 0.9858223
29700 0.001441802 27600 0.000961942 27400 0.004495643 27000
0.003556872 29300 0.001285778 28700 0.9799343
29700 0.001441653 27600 0.000965098 27400 0.004513497 27000
0.003542607 29300 0.001284464 28700 0.9730241
29700 0.001441544 27600 0.000968281 27400 0.004531515 27000
0.003528485 29300 0.001283162 28700 0.9651019
29700 0.001441419 27600 0.000971528 27400 0.004549721 27000
0.003514507 29300 0.001281854 28700 0.9561836
29700 0.001441264 27600 0.000974736 27400 0.004568109 27000
0.003500702 29300 0.001280532 28700 0.946292
29700 0.001441135 27600 0.000978004 27400 0.004586683 27000
0.003487095 29300 0.001279216 28700 0.9354565
29700 0.001441007 27600 0.000981255 27400 0.004605438 27000
0.003473658 29300 0.001277899 28700 0.9237126
29700 0.001440893 27600 0.00098449 27400 0.004624378 27000
0.003460422 29300 0.001276599 28800 0.9111021
29700 0.001440774 27600 0.000987715 27400 0.004643503 27000
0.003447341 29300 0.001275274 28800 0.8976721

29700 0.001440636 27600 0.000990956 27400 0.004662827 27000
0.003434425 29300 0.00127395 28800 0.8834779
29700 0.001440551 27600 0.0009942 27400 0.004682343 27000
0.003421626 29300 0.001272629 28800 0.8685773
29700 0.001440404 27600 0.000997471 27400 0.004702067 27000
0.003408945 29300 0.001271329 28800 0.8530331
29700 0.00144027 27600 0.001000746 27400 0.004721973 27000
0.003396397 29300 0.001270046 28800 0.8369134
29700 0.001440144 27600 0.001004071 27400 0.004742075 27000
0.003383944 29300 0.001268741 28800 0.8202894
29700 0.001439998 27600 0.001007425 27400 0.004762363 27000
0.003371599 29300 0.001267438 28800 0.8032347
29700 0.001439882 27600 0.001010805 27400 0.004782857 27000
0.003359356 29300 0.001266163 28800 0.7858251
29700 0.001439769 27700 0.001014206 27400 0.004803518 27000
0.003347225 29300 0.001264898 28800 0.7681375
29700 0.001439619 27700 0.00101763 27400 0.004824391 27000
0.003335213 29300 0.00126361 28800 0.7502491
29700 0.001439494 27700 0.001021042 27500 0.004845495 27000
0.003323332 29300 0.001262348 28800 0.7322367
29700 0.001439355 27700 0.001024474 27500 0.004866793 27000
0.003311577 29300 0.001261097 28800 0.7141759
29700 0.001439224 27700 0.001027981 27500 0.004888294 27000
0.003299949 29300 0.001259839 28800 0.6961396
29700 0.001439099 27700 0.001031453 27500 0.00491 27000
0.003288446 29300 0.001258594 28800 0.6781988
29700 0.001438944 27700 0.001034934 27500 0.004931899 27100
0.003277032 29300 0.001257329 28800 0.6604201
29700 0.001438811 27700 0.001038434 27500 0.004954018 27100
0.003265725 29300 0.001256082 28800 0.6428668
29700 0.001438634 27700 0.001041924 27500 0.004976334 27100
0.003254507 29300 0.00125484 28800 0.6255972
29700 0.001438463 27700 0.001045409 27500 0.004998872 27100
0.003243386 29300 0.001253604 28800 0.6086647
29700 0.001438329 27700 0.001048902 27500 0.005021615 27100
0.003232369 29300 0.001252375 28800 0.5921172
29700 0.001438159 27700 0.001052438 27500 0.005044566 27100
0.003221389 29300 0.001251115 28800 0.5759969
29700 0.001438051 27700 0.00105602 27500 0.005067742 27100
0.003210529 29300 0.001249874 28800 0.5603411
29700 0.001437914 27700 0.00105962 27500 0.005091143 27100
0.003199803 29300 0.001248629 28800 0.5451804
29700 0.001437738 27700 0.001063257 27500 0.00511476 27100
0.003189156 29300 0.001247394 28800 0.5305391
29700 0.001437607 27700 0.001066918 27500 0.005138602 27100
0.003178647 29300 0.001246165 28800 0.5164372
29700 0.001437426 27700 0.001070628 27500 0.005162654 27100
0.003168268 29300 0.001244938 28800 0.5028885
29700 0.001437239 27700 0.001074328 27500 0.00518695 27100
0.00315808 29400 0.001243703 28800 0.489902
29700 0.001437145 27700 0.001078062 27500 0.005211459 27100
0.003148026 29400 0.001242481 28800 0.4774813
29700 0.001437 27700 0.001081808 27500 0.005236171 27100
0.00313817 29400 0.001241253 28800 0.4656259
29700 0.001436885 27700 0.001085554 27500 0.005261131 27100
0.003128502 29400 0.00124003 28800 0.454331
29700 0.001436742 27700 0.001089292 27500 0.005286322 27100
0.003118969 29400 0.001238818 28800 0.4435878
29700 0.001436595 27700 0.001093033 27500 0.005311755 27100
0.003109595 29400 0.001237599 28800 0.433385
29700 0.001436448 27700 0.001096806 27500 0.005337417 27100
0.003100318 29400 0.001236385 28800 0.4237076
29700 0.001436336 27700 0.001100606 27500 0.005363323 27100
0.003091189 29400 0.001235208 28800 0.4145386
29700 0.001436215 27700 0.001104433 27500 0.005389456 27100
0.003082104 29400 0.001234015 28800 0.405859
29700 0.001436072 27700 0.00110828 27500 0.005415835 27100
0.003073122 29400 0.001232824 28800 0.3976479

29700 0.001435924 27700 0.001112156 27500 0.005442461 27100
0.003064226 29400 0.001231653 28800 0.3898837
29700 0.001435802 27700 0.00111607 27500 0.005469322 27100
0.003055389 29400 0.001230482 28800 0.3825434
29700 0.001435688 27700 0.001120014 27500 0.005496421 27100
0.003046589 29400 0.001229307 28800 0.375604
29700 0.001435582 27700 0.001123984 27500 0.005523779 27100
0.003037847 29400 0.001228153 28800 0.3690417
29700 0.001435459 27700 0.001127996 27500 0.005551381 27100
0.003029156 29400 0.001226979 28800 0.3628336
29700 0.001435363 27700 0.001132034 27500 0.005579251 27100
0.00302053 29400 0.001225841 28800 0.356956
29700 0.001435201 27700 0.001136097 27500 0.005607376 27100
0.003012 29400 0.001224706 28800 0.3513857
29700 0.001435123 27700 0.001140165 27500 0.005635772 27100
0.003003607 29400 0.001223594 28800 0.3461007
29700 0.00143502 27700 0.001144241 27500 0.005664404 27100
0.002995349 29400 0.001222458 28800 0.3410798
29700 0.001434895 27700 0.00114831 27500 0.005693319 27100
0.002987245 29400 0.001221326 28800 0.3363023
29700 0.001434824 27700 0.001152395 27500 0.005722475 27100
0.002979276 29400 0.001220199 28800 0.3317483
29700 0.001434733 27700 0.00115649 27500 0.005751873 27100
0.002971428 29400 0.001219097 28800 0.3273995
29700 0.001434616 27700 0.001160601 27500 0.005781563 27100
0.002963686 29400 0.001217971 28800 0.323238
29700 0.001434549 27700 0.001164737 27500 0.00581152 27100
0.002956058 29400 0.001216825 28800 0.3192473
29700 0.001434448 27700 0.001168894 27500 0.005841766 27100
0.002948536 29400 0.00121574 28800 0.315412
29700 0.001434373 27700 0.001173116 27500 0.005872267 27100
0.0029411 29400 0.00121462 28800 0.3117177
29700 0.001434313 27700 0.00117738 27500 0.005903045 27100
0.002933788 29400 0.001213509 28800 0.308151
29700 0.001434203 27700 0.001181695 27500 0.005934096 27100
0.002926582 29400 0.001212394 28800 0.3046997
29700 0.001434132 27700 0.001186032 27500 0.005965434 27100
0.002919498 29400 0.001211274 28800 0.3013522
29800 0.001434024 27700 0.001190418 27500 0.005997035 27100
0.002912541 29400 0.001210157 28800 0.2980986
29800 0.001433922 27700 0.001194796 27500 0.006028926 27100
0.002905727 29400 0.001209059 28800 0.2949293
29800 0.001433851 27700 0.001199188 27500 0.006061094 27100
0.00289903 29400 0.001207916 28800 0.2918357
29800 0.00143376 27700 0.001203576 27500 0.006093556 27100
0.002892474 29400 0.001206805 28800 0.2888102
29800 0.001433719 27700 0.001207971 27500 0.006126298 27100
0.002886072 29400 0.001205698 28800 0.2858461
29800 0.001433646 27700 0.001212362 27500 0.006159341 27100
0.00287977 29400 0.001204576 28800 0.2829371
29800 0.001433547 27700 0.001216774 27500 0.006192678 27100
0.002873588 29400 0.001203459 28800 0.2800773
29800 0.001433475 27700 0.001221188 27500 0.006226327 27100
0.002867503 29400 0.001202371 28800 0.277262
29800 0.001433366 27700 0.001225621 27500 0.006260244 27100
0.002861495 29400 0.001201261 28800 0.2744869
29800 0.0014333 27700 0.001230077 27500 0.006294493 27100
0.002855562 29400 0.00120015 28900 0.2717484
29800 0.001433253 27700 0.00123459 27500 0.006329022 27100
0.002849681 29400 0.001199069 28900 0.269043
29800 0.001433167 27700 0.001239128 27500 0.006363827 27100
0.002843856 29400 0.00119798 28900 0.2663679
29800 0.001433116 27700 0.001243715 27500 0.006398978 27100
0.002838107 29400 0.001196883 28900 0.2637205
29800 0.001433046 27700 0.001248328 27500 0.006434451 27100
0.002832402 29400 0.001195781 28900 0.2610987
29800 0.001432968 27700 0.001252955 27500 0.006470237 27100
0.002826802 29400 0.001194707 28900 0.2585008

29800 0.001432893 27700 0.001257642 27500 0.006506342 27100
0.002821311 29400 0.001193615 28900 0.2559252
29800 0.001432832 27700 0.001262357 27500 0.006542773 27100
0.002815899 29400 0.001192552 28900 0.2533707
29800 0.001432786 27700 0.001267112 27500 0.006579526 27100
0.002810548 29400 0.00119148 28900 0.2508361
29800 0.001432713 27800 0.001271881 27500 0.006616605 27100
0.002805257 29400 0.001190442 28900 0.2483206
29800 0.00143263 27800 0.001276693 27500 0.006654026 27100
0.00280005 29400 0.001189393 28900 0.2458237
29800 0.001432581 27800 0.001281514 27600 0.006691781 27100
0.002794906 29400 0.001188352 28900 0.2433448
29800 0.001432516 27800 0.001286369 27600 0.006729887 27100
0.002789879 29400 0.001187308 28900 0.2408835
29800 0.001432441 27800 0.001291235 27600 0.006768331 27100
0.002784966 29400 0.001186272 28900 0.2384394
29800 0.001432395 27800 0.001296133 27600 0.006807137 27100
0.002780171 29400 0.001185243 28900 0.2360129
29800 0.001432356 27800 0.001301055 27600 0.006846292 27200
0.002775496 29400 0.001184239 28900 0.2336034
29800 0.001432284 27800 0.00130602 27600 0.006885814 27200
0.002770943 29400 0.001183241 28900 0.2312108
29800 0.001432241 27800 0.001311 27600 0.006925701 27200
0.0027665 29400 0.001182282 28900 0.2288352
29800 0.001432195 27800 0.001316007 27600 0.006965942 27200
0.002762113 29400 0.001181262 28900 0.226477
29800 0.001432131 27800 0.001321025 27600 0.007006565 27200
0.002757846 29400 0.001180268 28900 0.2241362
29800 0.001432102 27800 0.001326076 27600 0.007047534 27200
0.002753653 29400 0.001179285 28900 0.2218129
29800 0.001432033 27800 0.001331117 27600 0.007088863 27200
0.002749518 29400 0.001178296 28900 0.2195074
29800 0.001431989 27800 0.001336184 27600 0.00713059 27200
0.002745474 29400 0.001177307 28900 0.2172199
29800 0.001431943 27800 0.00134126 27600 0.00717269 27200
0.002741484 29400 0.001176324 28900 0.2149505
29800 0.001431845 27800 0.001346341 27600 0.00721518 27200
0.002737559 29400 0.00117534 28900 0.2126996
29800 0.001431769 27800 0.001351474 27600 0.007258046 27200
0.002733697 29400 0.001174343 28900 0.2104672
29800 0.001431681 27800 0.001356642 27600 0.007301297 27200
0.002729906 29500 0.001173336 28900 0.2082536
29800 0.00143162 27800 0.001361857 27600 0.007344945 27200
0.002726213 29500 0.001172358 28900 0.206059
29800 0.001431503 27800 0.001367093 27600 0.00738898 27200
0.002722617 29500 0.001171335 28900 0.2038836
29800 0.001431419 27800 0.001372381 27600 0.007433404 27200
0.002719135 29500 0.001170335 28900 0.2017277
29800 0.00143132 27800 0.001377708 27600 0.007478236 27200
0.002715757 29500 0.001169347 28900 0.1995913
29800 0.001431232 27800 0.001383071 27600 0.007523482 27200
0.002712493 29500 0.001168328 28900 0.1974747
29800 0.001431148 27800 0.001388483 27600 0.007569127 27200
0.002709296 29500 0.001167329 28900 0.195378
29800 0.001431065 27800 0.001393942 27600 0.0076152 27200
0.002706188 29500 0.001166307 28900 0.1933017
29800 0.001431001 27800 0.001399445 27600 0.007661705 27200
0.002703169 29500 0.001165287 28900 0.1912455
29800 0.001430854 27800 0.001404991 27600 0.007708635 27200
0.002700179 29500 0.001164295 28900 0.1892095
29800 0.001430763 27800 0.001410563 27600 0.007756026 27200
0.00269726 29500 0.001163267 28900 0.1871939
29800 0.001430629 27800 0.001416155 27600 0.007803857 27200
0.002694367 29500 0.001162239 28900 0.1851989
29800 0.001430504 27800 0.001421769 27600 0.007852127 27200
0.002691524 29500 0.00116122 28900 0.1832245
29800 0.001430435 27800 0.001427433 27600 0.00790084 27200
0.002688696 29500 0.001160179 28900 0.1812708

29800 0.001430328 27800 0.00143309 27600 0.007949973 27200
0.002685922 29500 0.001159178 28900 0.1793378
29800 0.001430226 27800 0.001438784 27600 0.007999639 27200
0.002683207 29500 0.001158166 28900 0.1774257
29800 0.001430132 27800 0.001444478 27600 0.008049758 27200
0.00268056 29500 0.001157145 28900 0.1755342
29800 0.001430019 27800 0.001450167 27600 0.00810036 27200
0.002677993 29500 0.001156112 28900 0.1736636
29800 0.001429906 27800 0.001455906 27600 0.008151457 27200
0.002675562 29500 0.001155067 28900 0.1718137
29800 0.001429806 27800 0.001461689 27600 0.00820305 27200
0.002673223 29500 0.001154039 28900 0.1699846
29800 0.001429707 27800 0.001467528 27600 0.008255134 27200
0.002670985 29500 0.001152996 28900 0.1681761
29800 0.001429585 27800 0.001473423 27600 0.008307715 27200
0.002668857 29500 0.001151962 28900 0.1663883
29800 0.00142947 27800 0.001479361 27600 0.00836081 27200
0.002666823 29500 0.001150932 28900 0.1646211
29800 0.001429351 27800 0.001485338 27600 0.008414426 27200
0.002664838 29500 0.0011499 28900 0.1628744
29800 0.00142924 27800 0.001491357 27600 0.008468552 27200
0.002662922 29500 0.001148876 28900 0.1611482
29800 0.001429135 27800 0.001497405 27600 0.00852322 27200
0.002661059 29500 0.001147836 28900 0.1594422
29800 0.00142902 27800 0.001503475 27600 0.008578433 27200
0.002659266 29500 0.001146823 28900 0.1577567
29800 0.001428931 27800 0.001509588 27600 0.008634195 27200
0.002657507 29500 0.001145823 28900 0.1560914
29800 0.001428811 27800 0.001515742 27600 0.008690506 27200
0.002655824 29500 0.001144803 28900 0.154446
29900 0.001428707 27800 0.001521938 27600 0.008747384 27200
0.0026542 29500 0.001143796 28900 0.1528206
29900 0.001428572 27800 0.001528162 27600 0.008804831 27200
0.002652602 29500 0.001142787 28900 0.1512151
29900 0.001428457 27800 0.001534435 27600 0.008862851 27200
0.002651057 29500 0.001141776 28900 0.1496293
29900 0.001428348 27800 0.001540739 27600 0.008921435 27200
0.002649552 29500 0.001140762 28900 0.148063
29900 0.001428236 27800 0.001547072 27600 0.008980575 27200
0.002648074 29500 0.001139776 28900 0.1465161
29900 0.001428112 27800 0.001553443 27600 0.009040354 27200
0.002646635 29500 0.001138796 28900 0.1449885
29900 0.001427999 27800 0.001559836 27600 0.009100733 27200
0.002645265 29500 0.001137799 28900 0.1434799
29900 0.001427869 27800 0.001566244 27600 0.009161726 27200
0.002643945 29500 0.001136837 28900 0.1419902
29900 0.001427725 27800 0.001572711 27600 0.009223321 27200
0.002642694 29500 0.001135859 28900 0.1405192
29900 0.001427609 27800 0.001579221 27600 0.009285552 27200
0.00264152 29500 0.001134913 29000 0.1390668
29900 0.001427476 27800 0.001585772 27600 0.009348412 27200
0.002640422 29500 0.001133944 29000 0.1376327
29900 0.001427345 27800 0.001592371 27600 0.009411887 27200
0.002639404 29500 0.001132989 29000 0.1362168
29900 0.001427186 27800 0.001599024 27600 0.00947604 27200
0.002638463 29500 0.001132039 29000 0.1348189
29900 0.001427073 27800 0.001605722 27600 0.009540839 27200
0.00263758 29500 0.001131092 29000 0.1334387
29900 0.001426949 27800 0.00161247 27600 0.009606311 27200
0.002636782 29500 0.001130164 29000 0.1320761
29900 0.001426816 27800 0.001619273 27600 0.009672449 27200
0.002636003 29500 0.00112924 29000 0.1307308
29900 0.001426709 27800 0.001626113 27600 0.00973929 27200
0.002635286 29500 0.001128313 29000 0.129403
29900 0.001426608 27800 0.001632969 27600 0.009806841 27200
0.002634608 29500 0.001127403 29000 0.1280921
29900 0.001426457 27900 0.001639839 27600 0.009875096 27200
0.002633955 29500 0.00112648 29000 0.1267979

29900 0.001426346 27900 0.001646754 27600 0.009944101 27200
0.002633334 29500 0.001125574 29000 0.1255202
29900 0.001426199 27900 0.001653688 27700 0.01001383 27200
0.002632756 29500 0.001124664 29000 0.124259
29900 0.001426067 27900 0.001660668 27700 0.0100843 27200
0.002632234 29500 0.001123748 29000 0.1230141
29900 0.001425977 27900 0.001667713 27700 0.01015552 27200
0.002631754 29500 0.001122836 29000 0.1217852
29900 0.001425892 27900 0.001674797 27700 0.01022746 27200
0.002631329 29500 0.001121926 29000 0.120572
29900 0.001425776 27900 0.001681948 27700 0.01030024 27300
0.002630973 29500 0.00112103 29000 0.1193744
29900 0.001425674 27900 0.001689144 27700 0.01037381 27300
0.002630674 29500 0.00112013 29000 0.1181923
29900 0.001425563 27900 0.001696364 27700 0.01044818 27300
0.002630417 29500 0.001119243 29000 0.1170253
29900 0.001425446 27900 0.00170366 27700 0.01052336 27300
0.002630229 29500 0.00111834 29000 0.1158734
29900 0.001425344 27900 0.001711008 27700 0.01059937 27300
0.002630096 29500 0.001117443 29000 0.1147362
29900 0.00142525 27900 0.001718391 27700 0.0106762 27300
0.002630034 29500 0.001116533 29000 0.1136137
29900 0.001425139 27900 0.001725815 27700 0.01075388 27300
0.002630048 29500 0.001115633 29000 0.1125055
29900 0.001425028 27900 0.001733284 27700 0.01083243 27300
0.002630122 29500 0.001114725 29000 0.1114115
29900 0.001424936 27900 0.001740785 27700 0.01091184 27300
0.002630236 29500 0.001113822 29000 0.1103316
29900 0.001424851 27900 0.001748355 27700 0.01099214 27300
0.002630405 29500 0.001112912 29000 0.1092655
29900 0.001424774 27900 0.001756003 27700 0.01107334 27300
0.002630558 29500 0.001112004 29000 0.108213
29900 0.001424707 27900 0.001763732 27700 0.01115545 27300
0.002630714 29600 0.001111095 29000 0.1071742
29900 0.001424647 27900 0.001771534 27700 0.01123851 27300
0.002630869 29600 0.001110196 29000 0.1061485
29900 0.001424547 27900 0.001779402 27700 0.0113225 27300
0.002631005 29600 0.001109298 29000 0.1051359
29900 0.001424494 27900 0.001787328 27700 0.01140746 27300
0.002631126 29600 0.001108406 29000 0.1041362
29900 0.001424401 27900 0.001795304 27700 0.0114934 27300
0.002631257 29600 0.001107506 29000 0.1031493
29900 0.001424328 27900 0.001803318 27700 0.01158032 27300
0.00263139 29600 0.001106596 29000 0.102175
29900 0.001424298 27900 0.001811374 27700 0.01166823 27300
0.002631533 29600 0.001105698 29000 0.1012131
29900 0.001424237 27900 0.001819469 27700 0.01175713 27300
0.0026317 29600 0.001104798 29000 0.1002634
29900 0.001424205 27900 0.001827622 27700 0.01184711 27300
0.002631876 29600 0.001103908 29000 0.0993259
29900 0.001424158 27900 0.001835792 27700 0.01193814 27300
0.002632069 29600 0.00110301 29000 0.09840024
29900 0.001424097 27900 0.001844001 27700 0.01203027 27300
0.002632274 29600 0.001102126 29000 0.09748631
29900 0.001424039 27900 0.001852273 27700 0.01212346 27300
0.00263249 29600 0.001101238 29000 0.09658394
29900 0.001423999 27900 0.001860601 27700 0.01221776 27300
0.002632719 29600 0.001100374 29000 0.095693
29900 0.001423971 27900 0.001868989 27700 0.01231318 27300
0.002632904 29600 0.001099497 29000 0.09481323
29900 0.001423915 27900 0.00187743 27700 0.01240973 27300
0.002633103 29600 0.001098624 29000 0.0939446
29900 0.001423853 27900 0.001885927 27700 0.01250745 27300
0.002633282 29600 0.001097756 29000 0.09308682
29900 0.001423815 27900 0.001894482 27700 0.01260635 27300
0.002633467 29600 0.00109689 29000 0.09223983
29900 0.001423798 27900 0.00190309 27700 0.01270644 27300
0.002633657 29600 0.001096025 29000 0.09140341

29900 0.001423784 27900 0.001911754 27700 0.01280774 27300
0.002633863 29600 0.001095171 29000 0.09057743
29900 0.00142375 27900 0.001920473 27700 0.01291029 27300
0.002634085 29600 0.001094307 29000 0.08976191
29900 0.001423766 27900 0.001929237 27700 0.0130141 27300
0.002634324 29600 0.001093466 29000 0.08895652
29900 0.001423709 27900 0.001938027 27700 0.01311918 27300
0.002634611 29600 0.001092628 29000 0.088161
29900 0.001423693 27900 0.001946894 27700 0.01322558 27300
0.002634921 29600 0.001091796 29000 0.08737531
29900 0.001423656 27900 0.001955812 27700 0.01333326 27300
0.002635218 29600 0.00109096 29000 0.08659938
29900 0.001423616 27900 0.001964791 27700 0.01344228 27300
0.002635502 29600 0.00109011 29000 0.08583306
29900 0.001423631 27900 0.001973862 27700 0.01355265 27300
0.002635737 29600 0.001089278 29000 0.08507621
29900 0.001423615 27900 0.001983012 27700 0.01366436 27300
0.00263595 29600 0.001088442 29000 0.08432864
29900 0.001423603 27900 0.001992234 27700 0.01377751 27300
0.00263611 29600 0.001087614 29000 0.08359025
29900 0.001423609 27900 0.002001541 27700 0.01389208 27300
0.002636267 29600 0.001086781 29000 0.08286092
30000 0.001423594 27900 0.002010876 27700 0.01400811 27300
0.002636403 29600 0.001085959 29000 0.08214049
30000 0.001423585 27900 0.002020324 27700 0.0141256 27300
0.002636551 29600 0.001085128 29000 0.0814288
30000 0.001423595 27900 0.00202984 27700 0.01424458 27300
0.002636706 29600 0.00108431 29000 0.08072581
30000 0.001423612 27900 0.002039429 27700 0.01436509 27300
0.002636881 29600 0.001083475 29000 0.08003138
30000 0.00142359 27900 0.002049098 27700 0.01448713 27300
0.002637063 29600 0.001082649 29000 0.07934528
30000 0.00142358 27900 0.002058816 27700 0.01461074 27300
0.002637241 29600 0.001081813 29000 0.07866751
30000 0.001423553 27900 0.002068614 27700 0.01473595 27300
0.002637446 29600 0.001080981 29000 0.07799787
30000 0.001423554 27900 0.002078484 27700 0.01486279 27300
0.002637648 29600 0.001080137 29000 0.07733622
30000 0.001423545 27900 0.002088443 27700 0.0149913 27300
0.002637819 29600 0.0010793 29000 0.07668249
30000 0.00142355 27900 0.00209849 27700 0.01512149 27300
0.002637947 29600 0.001078452 29100 0.07603667
30000 0.00142357 27900 0.002108605 27700 0.01525342 27300
0.002638067 29600 0.001077629 29100 0.0753985
30000 0.001423547 27900 0.002118769 27700 0.0153871 27300
0.002638147 29600 0.001076797 29100 0.07476782
30000 0.001423564 27900 0.002129008 27700 0.01552257 27300
0.002638179 29600 0.001075973 29100 0.07414463
30000 0.001423516 27900 0.002139283 27700 0.01565986 27300
0.002638179 29600 0.001075144 29100 0.07352871
30000 0.001423495 27900 0.002149597 27700 0.01579898 27300
0.002638124 29600 0.001074305 29100 0.07292012
30000 0.001423509 27900 0.002160008 27700 0.01593996 27300
0.002638017 29600 0.001073475 29100 0.07231868
30000 0.001423512 27900 0.002170465 27700 0.01608281 27300
0.002637861 29600 0.001072649 29100 0.07172429
30000 0.001423515 27900 0.002181001 27700 0.01622764 27300
0.002637621 29600 0.001071838 29100 0.07113681
30000 0.00142351 28000 0.002191609 27700 0.01637446 27300
0.00263735 29600 0.001071018 29100 0.07055619
30000 0.001423511 28000 0.002202265 27700 0.0165233 27300
0.002637021 29600 0.001070202 29100 0.0699823
30000 0.001423502 28000 0.002212992 27800 0.01667418 27300
0.002636625 29600 0.001069405 29100 0.06941506
30000 0.001423522 28000 0.002223808 27800 0.01682712 27300
0.002636214 29600 0.001068605 29100 0.06885434
30000 0.001423517 28000 0.002234684 27800 0.01698219 27300
0.002635772 29600 0.001067798 29100 0.06830006

30000 0.001423473 28000 0.002245657 27800 0.01713941 27300
0.002635316 29600 0.001067002 29100 0.06775214
30000 0.001423478 28000 0.002256702 27800 0.01729883 27400
0.002634873 29600 0.001066204 29100 0.0672105
30000 0.001423467 28000 0.00226785 27800 0.01746047 27400
0.002634424 29600 0.001065417 29100 0.06667498
30000 0.001423489 28000 0.002279059 27800 0.01762441 27400
0.002633971 29600 0.001064617 29100 0.06614559
30000 0.001423478 28000 0.002290417 27800 0.01779068 27400
0.002633509 29600 0.001063833 29100 0.06562217
30000 0.001423495 28000 0.002301833 27800 0.01795928 27400
0.002633059 29600 0.001063034 29100 0.06510485
30000 0.00142353 28000 0.002313325 27800 0.01813032 27400
0.002632587 29600 0.001062247 29100 0.0645933
30000 0.00142351 28000 0.002324937 27800 0.01830383 27400
0.002632115 29600 0.001061467 29100 0.06408741
30000 0.00142353 28000 0.002336633 27800 0.01847984 27400
0.002631621 29600 0.001060691 29100 0.06358721
30000 0.001423527 28000 0.002348351 27800 0.01865839 27400
0.0026311 29600 0.001059895 29100 0.06309259
30000 0.001423515 28000 0.002360217 27800 0.01883955 27400
0.002630522 29600 0.001059101 29100 0.06260354
30000 0.001423559 28000 0.002372134 27800 0.01902334 27400
0.002629922 29600 0.001058318 29100 0.06211994
30000 0.00142357 28000 0.002384174 27800 0.01920979 27400
0.002629242 29700 0.001057528 29100 0.06164173
30000 0.001423608 28000 0.00239634 27800 0.01939904 27400
0.002628528 29700 0.001056743 29100 0.06116884
30000 0.00142361 28000 0.002408587 27800 0.01959108 27400
0.002627752 29700 0.001055956 29100 0.0607012
30000 0.001423638 28000 0.002420851 27800 0.019786 27400
0.002626952 29700 0.001055167 29100 0.06023867
30000 0.001423617 28000 0.002433266 27800 0.01998383 27400
0.002626143 29700 0.001054388 29100 0.05978127
30000 0.001423668 28000 0.002445815 27800 0.02018463 27400
0.002625301 29700 0.001053603 29100 0.05932885
30000 0.001423653 28000 0.002458452 27800 0.02038845 27400
0.002624425 29700 0.00105281 29100 0.05888137
30000 0.001423687 28000 0.002471213 27800 0.02059536 27400
0.002623534 29700 0.001052021 29100 0.05843877
30000 0.00142366 28000 0.00248405 27800 0.02080543 27400
0.002622592 29700 0.001051239 29100 0.058001
30000 0.001423664 28000 0.00249697 27800 0.02101872 27400
0.002621617 29700 0.001050456 29100 0.05756794
30000 0.001423665 28000 0.002510026 27800 0.02123529 27400
0.002620606 29700 0.001049683 29100 0.05713959
30000 0.001423703 28000 0.002523174 27800 0.0214552 27400
0.002619546 29700 0.0010489 29100 0.05671585
30000 0.00142372 28000 0.002536394 27800 0.02167853 27400
0.002618475 29700 0.001048132 29100 0.0562968
30000 0.001423754 28000 0.002549767 27800 0.02190534 27400
0.002617372 29700 0.001047359 29100 0.05588219
30000 0.001423752 28000 0.002563239 27800 0.02213572 27400
0.002616243 29700 0.001046605 29100 0.05547195
30000 0.001423779 28000 0.002576816 27800 0.02236973 27400
0.002615097 29700 0.001045848 29100 0.05506609
30000 0.001423767 28000 0.002590519 27800 0.02260744 27400
0.002613951 29700 0.001045085 29100 0.05466453
30000 0.001423785 28000 0.002604282 27800 0.02284892 27400
0.002612808 29700 0.001044327 29100 0.05426728
30000 0.001423836 28000 0.002618157 27800 0.02309423 27400
0.002611666 29700 0.00104357 29100 0.05387422
30000 0.001423861 28000 0.002632163 27800 0.02334342 27400
0.002610524 29700 0.00104282 29100 0.05348537
30000 0.001423878 28000 0.002646259 27800 0.0235967 27400
0.002609416 29700 0.001042069 29100 0.05310061
30000 0.001423921 28000 0.002660468 27800 0.02385407 27400
0.002608301 29700 0.001041329 29100 0.05271988

30000 0.001423936 28000 0.002674774 27800 0.02411563 27400
0.002607177 29700 0.001040598 29100 0.05234315
30000 0.00142397 28000 0.002689218 27800 0.02438144 27400
0.002606048 29700 0.001039861 29100 0.05197034
30000 0.001424004 28000 0.002703791 27800 0.02465161 27400
0.002604929 29700 0.001039137 29100 0.05160141
30000 0.001424064 28000 0.002718485 27800 0.02492623 27400
0.002603764 29700 0.001038397 29100 0.05123631
30000 0.001424075 28000 0.002733299 27800 0.02520541 27400
0.0026026 29700 0.001037673 29100 0.05087497
30000 0.001424109 28000 0.002748234 27800 0.02548922 27400
0.002601423 29700 0.001036956 29100 0.05051734
30000 0.001424136 28000 0.002763269 27800 0.02577779 27400
0.002600207 29700 0.00103623 29100 0.05016341
30100 0.001424179 28000 0.002778446 27800 0.02607121 27400
0.002598959 29700 0.001035515 29100 0.04981306
30100 0.001424244 28000 0.002793724 27800 0.0263696 27400
0.002597691 29700 0.001034799 29100 0.04946631
30100 0.001424304 28000 0.002809134 27800 0.02667306 27400
0.002596396 29700 0.001034089 29100 0.04912319
30100 0.001424412 28000 0.002824683 27800 0.02698171 27400
0.002595113 29700 0.001033386 29100 0.04878351
30100 0.001424459 28000 0.00284034 27800 0.02729566 27400
0.002593852 29700 0.001032692 29100 0.0484472
30100 0.001424544 28000 0.002856133 27800 0.02761504 27400
0.002592644 29700 0.001032002 29100 0.04811425
30100 0.001424604 28000 0.002872042 27800 0.02793997 27400
0.002591467 29700 0.001031298 29100 0.04778467
30100 0.001424707 28000 0.002888083 27800 0.02827058 27400
0.002590346 29700 0.001030596 29100 0.04745843
30100 0.001424824 28000 0.002904291 27800 0.02860692 27400
0.002589281 29700 0.001029898 29100 0.04713547
30100 0.001424925 28000 0.00292064 27800 0.02894916 27400
0.002588267 29700 0.001029199 29200 0.04681575
30100 0.001425059 28000 0.002937164 27800 0.02929754 27400
0.002587295 29700 0.001028496 29200 0.04649919
30100 0.001425166 28000 0.002953866 27800 0.02965211 27400
0.002586399 29700 0.001027797 29200 0.04618579
30100 0.001425283 28000 0.002970672 27800 0.03001302 27400
0.002585553 29700 0.001027104 29200 0.04587549
30100 0.001425412 28000 0.002987623 27800 0.03038042 27400
0.002584797 29700 0.001026399 29200 0.04556827
30100 0.001425566 28000 0.003004729 27800 0.03075447 27400
0.002584119 29700 0.001025697 29200 0.04526405
30100 0.001425673 28000 0.003021951 27800 0.03113531 27400
0.002583529 29700 0.00102498 29200 0.04496283
30100 0.00142581 28000 0.003039286 27800 0.0315231 27400
0.002583016 29700 0.001024264 29200 0.04466453
30100 0.001425903 28000 0.003056766 27800 0.03191801 27400
0.002582565 29700 0.001023542 29200 0.04436918
30100 0.001426063 28100 0.003074367 27800 0.03232023 27400
0.002582175 29700 0.001022811 29200 0.04407668
30100 0.001426162 28100 0.003092102 27800 0.03272992 27400
0.002581814 29700 0.001022084 29200 0.04378702
30100 0.001426295 28100 0.003109997 27900 0.03314727 27400
0.002581472 29700 0.001021349 29200 0.04350019
30100 0.001426434 28100 0.003128059 27900 0.03357244 27400
0.002581163 29700 0.001020601 29200 0.04321625
30100 0.001426606 28100 0.003146291 27900 0.03400566 27400
0.00258089 29700 0.00101986 29200 0.04293496
30100 0.001426711 28100 0.003164676 27900 0.03444713 27400
0.002580686 29700 0.001019114 29200 0.04265633
30100 0.001426869 28100 0.003183239 27900 0.03489701 27500
0.002580533 29700 0.001018368 29200 0.04238037
30100 0.001426974 28100 0.003202018 27900 0.03535555 27500
0.002580457 29700 0.001017612 29200 0.04210704
30100 0.001427123 28100 0.003220916 27900 0.03582293 27500
0.002580474 29700 0.001016854 29200 0.04183633

30100 0.001427293 28100 0.003240004 27900 0.03629934 27500
0.002580541 29700 0.001016091 29200 0.04156822
30100 0.001427441 28100 0.003259292 27900 0.03678501 27500
0.002580682 29700 0.001015339 29200 0.04130266
30100 0.001427592 28100 0.003278763 27900 0.03728029 27500
0.002580885 29700 0.00101459 29200 0.04103961
30100 0.001427758 28100 0.003298401 27900 0.03778536 27500
0.002581145 29700 0.001013839 29200 0.04077905
30100 0.001427911 28100 0.003318172 27900 0.03830044 27500
0.002581497 29700 0.001013105 29200 0.04052093
30100 0.001428074 28100 0.003338133 27900 0.03882581 27500
0.002581937 29700 0.001012355 29200 0.04026521
30100 0.001428233 28100 0.003358279 27900 0.03936173 27500
0.002582463 29700 0.001011625 29200 0.04001189
30100 0.00142842 28100 0.003378609 27900 0.03990846 27500
0.002583063 29700 0.001010886 29200 0.03976092
30100 0.001428566 28100 0.003399149 27900 0.04046629 27500
0.002583751 29800 0.001010164 29200 0.03951227
30100 0.001428722 28100 0.003419884 27900 0.04103551 27500
0.002584509 29800 0.001009429 29200 0.03926592
30100 0.001428902 28100 0.003440803 27900 0.04161645 27500
0.002585328 29800 0.001008706 29200 0.03902182
30100 0.001429058 28100 0.003461945 27900 0.04220939 27500
0.002586221 29800 0.001007987 29200 0.03877997
30100 0.00142924 28100 0.003483259 27900 0.04281468 27500
0.002587195 29800 0.001007279 29200 0.03854033
30100 0.001429429 28100 0.00350481 27900 0.04343262 27500
0.002588237 29800 0.001006564 29200 0.03830301
30100 0.001429601 28100 0.003526588 27900 0.04406356 27500
0.002589399 29800 0.001005858 29200 0.03806777
30100 0.001429772 28100 0.003548594 27900 0.04470788 27500
0.002590673 29800 0.001005164 29200 0.03783463
30100 0.001429954 28100 0.00357078 27900 0.04536594 27500
0.002592095 29800 0.001004469 29200 0.03760361
30100 0.001430138 28100 0.003593157 27900 0.04603811 27500
0.002593659 29800 0.001003759 29200 0.0373747
30100 0.001430311 28100 0.003615722 27900 0.04672476 27500
0.002595387 29800 0.001003064 29200 0.03714787
30100 0.001430516 28100 0.003638477 27900 0.04742628 27500
0.002597245 29800 0.001002356 29200 0.03692311
30100 0.001430704 28100 0.003661435 27900 0.04814306 27500
0.002599235 29800 0.001001661 29200 0.03670041
30100 0.001430904 28100 0.003684608 27900 0.04887567 27500
0.00260134 29800 0.001000964 29200 0.03647969
30100 0.001431113 28100 0.003708 27900 0.04962445 27500
0.002603547 29800 0.001000269 29200 0.03626097
30100 0.001431309 28100 0.003731578 27900 0.05038983 27500
0.002605848 29800 0.00099959 29200 0.0360442
30100 0.001431507 28100 0.003755466 27900 0.05117233 27500
0.002608211 29800 0.000998899 29200 0.03582938
30100 0.001431699 28100 0.003779609 27900 0.05197243 27500
0.002610669 29800 0.000998217 29200 0.03561646
30100 0.001431923 28100 0.003803999 27900 0.05279059 27500
0.002613162 29800 0.000997527 29200 0.03540543
30100 0.001432102 28100 0.003828627 27900 0.05362741 27500
0.002615712 29800 0.000996835 29200 0.03519627
30100 0.001432295 28100 0.00385347 27900 0.05448338 27500
0.002618305 29800 0.000996146 29200 0.03498897
30100 0.001432502 28100 0.003878535 27900 0.05535907 27500
0.00262098 29800 0.000995448 29200 0.03478348
30100 0.00143271 28100 0.003903822 27900 0.05625508 27500
0.002623704 29800 0.000994762 29200 0.03457982
30100 0.001432919 28100 0.003929357 27900 0.05717199 27500
0.002626518 29800 0.000994086 29200 0.03437795
30100 0.001433163 28100 0.003955152 27900 0.05811042 27500
0.002629382 29800 0.000993399 29200 0.03417797
30100 0.001433417 28100 0.00398121 27900 0.05907102 27500
0.002632349 29800 0.000992734 29200 0.03397965

30100 0.001433605 28100 0.004007553 27900 0.06005448 27500
0.002635386 29800 0.000992068 29200 0.033783
30100 0.001433846 28100 0.004034154 27900 0.06106146 27500
0.002638484 29800 0.000991398 29200 0.03358807
30100 0.001434044 28100 0.004061042 27900 0.06209266 27500
0.002641638 29800 0.000990739 29200 0.03339481
30200 0.001434243 28100 0.004088216 27900 0.06314883 27500
0.002644815 29800 0.000990072 29200 0.03320324
30200 0.001434486 28100 0.004115669 27900 0.06423066 27500
0.002648005 29800 0.000989403 29200 0.03301333
30200 0.001434702 28100 0.004143406 27900 0.06533893 27500
0.00265121 29800 0.000988739 29200 0.03282505
30200 0.001434944 28100 0.004171426 27900 0.06647468 27500
0.002654402 29800 0.00098809 29200 0.03263836
30200 0.001435204 28100 0.004199721 27900 0.06763863 27500
0.002657586 29800 0.000987445 29200 0.03245327
30200 0.00143541 28100 0.004228245 27900 0.06883163 27500
0.002660769 29800 0.00098681 29200 0.03226971
30200 0.001435654 28100 0.004257094 27900 0.07005459 27500
0.002663927 29800 0.000986175 29200 0.03208773
30200 0.001435911 28100 0.004286232 27900 0.07130849 27500
0.002667086 29800 0.000985542 29200 0.03190723
30200 0.001436162 28100 0.004315653 27900 0.07259428 27500
0.002670252 29800 0.000984915 29200 0.03172826
30200 0.001436377 28100 0.004345384 27900 0.07391296 27500
0.002673423 29800 0.000984281 29300 0.03155077
30200 0.001436636 28100 0.004375426 27900 0.07526562 27500
0.002676614 29800 0.000983654 29300 0.03137474
30200 0.001436879 28100 0.004405778 27900 0.07665327 27500
0.002679839 29800 0.000983025 29300 0.03120016
30200 0.001437114 28100 0.004436471 27900 0.07807713 27500
0.002683066 29800 0.000982399 29300 0.03102701
30200 0.001437351 28100 0.00446751 27900 0.07953829 27500
0.00268633 29800 0.000981793 29300 0.03085527
30200 0.001437592 28100 0.004498864 27900 0.08103798 27500
0.002689584 29800 0.000981177 29300 0.03068503
30200 0.001437877 28100 0.004530579 27900 0.08257747 27500
0.002692824 29800 0.000980574 29300 0.03051615
30200 0.001438116 28100 0.004562595 27900 0.08415803 27500
0.002696037 29800 0.00097996 29300 0.0303486
30200 0.00143839 28100 0.00459495 27900 0.08578102 27500
0.002699192 29800 0.000979361 29300 0.03018244
30200 0.00143863 28200 0.004627601 27900 0.08744781 27500
0.002702285 29800 0.000978765 29300 0.03001764
30200 0.001438896 28200 0.004660556 27900 0.08915983 27500
0.002705262 29800 0.000978144 29300 0.02985418
30200 0.001439161 28200 0.00469381 28000 0.0909185 27500
0.002708149 29800 0.000977533 29300 0.02969206
30200 0.001439439 28200 0.004727378 28000 0.09272541 27500
0.00271093 29800 0.000976908 29300 0.02953129
30200 0.001439719 28200 0.004761273 28000 0.09458233 27500
0.002713554 29800 0.000976298 29300 0.02937178
30200 0.001440003 28200 0.004795483 28000 0.0964908 27500
0.002716104 29800 0.000975694 29300 0.02921358
30200 0.001440277 28200 0.00483 28000 0.0984525 27600
0.00271853 29800 0.000975079 29300 0.02905666
30200 0.001440557 28200 0.004864916 28000 0.1004692 27600
0.00272084 29800 0.000974465 29300 0.028901
30200 0.00144086 28200 0.004900199 28000 0.1025429 27600
0.002723033 29800 0.000973852 29300 0.02874658
30200 0.001441175 28200 0.004935864 28000 0.1046753 27600
0.002725068 29800 0.000973213 29300 0.02859337
30200 0.001441472 28200 0.004971946 28000 0.1068685 27600
0.002726959 29800 0.000972582 29300 0.0284414
30200 0.001441764 28200 0.005008452 28000 0.1091246 27600
0.002728701 29800 0.000971943 29300 0.02829063
30200 0.001442094 28200 0.005045356 28000 0.1114455 27600
0.002730283 29800 0.000971296 29300 0.02814104

30200 0.001442418 28200 0.005082707 28000 0.1138336 27600
0.002731676 29800 0.000970651 29300 0.02799263
30200 0.001442759 28200 0.005120477 28000 0.1162911 27600
0.002732855 29800 0.000970014 29300 0.02784541
30200 0.001443129 28200 0.00515868 28000 0.1188203 27600
0.002733843 29800 0.000969366 29300 0.02769944
30200 0.001443491 28200 0.005197299 28000 0.1214237 27600
0.002734592 29800 0.000968723 29300 0.02755455
30200 0.001443828 28200 0.005236338 28000 0.1241037 27600
0.002735112 29900 0.000968078 29300 0.02741076
30200 0.001444193 28200 0.005275808 28000 0.1268629 27600
0.002735373 29900 0.000967442 29300 0.02726811
30200 0.001444529 28200 0.005315731 28000 0.1297039 27600
0.002735412 29900 0.000966804 29300 0.02712654
30200 0.001444914 28200 0.005356079 28000 0.1326295 27600
0.002735204 29900 0.000966145 29300 0.0269861
30200 0.001445294 28200 0.005396864 28000 0.1356422 27600
0.002734775 29900 0.000965501 29300 0.02684678
30200 0.001445711 28200 0.005438076 28000 0.1387451 27600
0.002734107 29900 0.000964853 29300 0.02670852
30200 0.001446119 28200 0.005479709 28000 0.1419413 27600
0.002733241 29900 0.000964216 29300 0.02657134
30200 0.00144654 28200 0.005521773 28000 0.1452335 27600
0.002732109 29900 0.000963595 29300 0.02643523
30200 0.001446973 28200 0.00556422 28000 0.1486249 27600
0.00273077 29900 0.00096297 29300 0.02630015
30200 0.001447394 28200 0.005607187 28000 0.1521185 27600
0.002729164 29900 0.000962351 29300 0.02616614
30200 0.001447842 28200 0.005650667 28000 0.1557177 27600
0.002727318 29900 0.000961735 29300 0.02603314
30200 0.001448293 28200 0.005694648 28000 0.1594255 27600
0.002725146 29900 0.000961112 29300 0.02590115
30200 0.001448759 28200 0.00573912 28000 0.1632452 27600
0.002722654 29900 0.000960502 29300 0.02577016
30200 0.001449197 28200 0.005784116 28000 0.1671803 27600
0.002719799 29900 0.000959884 29300 0.02564017
30200 0.001449689 28200 0.005829579 28000 0.1712339 27600
0.002716566 29900 0.000959264 29300 0.02551117
30200 0.001450158 28200 0.005875539 28000 0.1754095 27600
0.002712942 29900 0.000958661 29300 0.02538315
30200 0.001450685 28200 0.005921982 28000 0.1797103 27600
0.002708938 29900 0.000958065 29300 0.02525611
30200 0.001451199 28200 0.005968885 28000 0.1841398 27600
0.00270452 29900 0.000957467 29300 0.02513011
30200 0.001451732 28200 0.006016268 28000 0.1887012 27600
0.002699726 29900 0.000956884 29300 0.02500503
30200 0.001452246 28200 0.006064141 28000 0.1933976 27600
0.002694574 29900 0.000956298 29300 0.02488084
30200 0.001452779 28200 0.006112528 28000 0.1982324 27600
0.002689041 29900 0.000955725 29300 0.02475757
30200 0.001453319 28200 0.00616147 28000 0.2032083 27600
0.002683166 29900 0.000955151 29300 0.02463526
30200 0.001453892 28200 0.006210946 28000 0.2083283 27600
0.002676946 29900 0.000954562 29300 0.02451386
30200 0.001454459 28200 0.006261013 28000 0.2135949 27600
0.002670345 29900 0.00095399 29300 0.02439335
30200 0.001455043 28200 0.006311651 28000 0.2190108 27600
0.00266338 29900 0.000953409 29300 0.02427379
30200 0.001455646 28200 0.006362879 28000 0.2245783 27600
0.002656013 29900 0.000952844 29300 0.02415512
30200 0.001456261 28200 0.006414669 28000 0.230299 27600
0.002648216 29900 0.00095229 29300 0.02403733
30200 0.001456887 28200 0.006467041 28000 0.2361743 27600
0.002640016 29900 0.000951736 29300 0.02392038
30200 0.001457498 28200 0.006520031 28000 0.2422053 27600
0.002631374 29900 0.000951185 29300 0.02380432
30300 0.001458155 28200 0.00657359 28000 0.2483924 27600
0.002622324 29900 0.00095063 29300 0.0236891

30300 0.001458815 28200 0.006627727 28000 0.2547354 27600
0.002612837 29900 0.000950075 29300 0.02357474
30300 0.001459469 28200 0.006682428 28000 0.2612335 27600
0.002602948 29900 0.000949522 29300 0.02346123
30300 0.001460127 28200 0.006737697 28000 0.267885 27600
0.002592665 29900 0.000948964 29300 0.0233485
30300 0.001460817 28200 0.006793555 28000 0.2746872 27600
0.002582006 29900 0.000948407 29300 0.02323661
30300 0.001461522 28200 0.006849988 28000 0.2816364 27600
0.002570913 29900 0.000947851 29300 0.02312555
30300 0.001462233 28200 0.006907032 28000 0.288728 27600
0.002559444 29900 0.000947308 29300 0.02301527
30300 0.001462964 28200 0.006964698 28000 0.2959556 27600
0.002547562 29900 0.000946746 29300 0.02290586
30300 0.001463724 28200 0.007023021 28000 0.3033117 27600
0.002535268 29900 0.000946196 29300 0.02279721
30300 0.001464468 28200 0.007082007 28000 0.3107869 27600
0.00252258 29900 0.00094564 29400 0.02268928
30300 0.001465218 28200 0.007141706 28000 0.3183704 27600
0.002509471 29900 0.000945098 29400 0.02258215
30300 0.001465976 28200 0.007202109 28000 0.3260492 27600
0.002496004 29900 0.000944557 29400 0.02247578
30300 0.001466753 28200 0.007263233 28000 0.333808 27600
0.002482152 29900 0.000943996 29400 0.02237018
30300 0.001467571 28200 0.007325094 28000 0.3416293 27600
0.002467937 29900 0.000943444 29400 0.02226534
30300 0.001468372 28200 0.007387627 28000 0.3494937 27600
0.002453401 29900 0.000942888 29400 0.02216125
30300 0.001469177 28200 0.00745079 28000 0.3573787 27600
0.00243851 29900 0.000942331 29400 0.02205791
30300 0.00147002 28200 0.007514588 28000 0.3652586 27600
0.002423289 29900 0.000941786 29400 0.02195526
30300 0.001470836 28200 0.007578965 28000 0.3731054 27600
0.002407723 29900 0.000941242 29400 0.02185336
30300 0.001471693 28300 0.007644044 28000 0.3808878 27600
0.002391834 29900 0.000940701 29400 0.02175219
30300 0.001472545 28300 0.007709793 28000 0.3885714 27600
0.002375619 29900 0.000940163 29400 0.02165169
30300 0.001473411 28300 0.007776269 28100 0.3961186 27600
0.002359091 29900 0.000939617 29400 0.02155192
30300 0.00147426 28300 0.007843516 28100 0.4034887 27600
0.002342284 29900 0.000939072 29400 0.02145281
30300 0.001475121 28300 0.007911568 28100 0.4106377 27600
0.002325201 29900 0.000938526 29400 0.02135443
30300 0.00147601 28300 0.007980457 28100 0.4175191 27600
0.002307861 29900 0.000937978 29400 0.0212567
30300 0.00147691 28300 0.00805017 28100 0.4240831 27700
0.002290261 29900 0.000937439 29400 0.02115966
30300 0.001477806 28300 0.008120721 28100 0.4302778 27700
0.002272439 29900 0.000936911 29400 0.02106329
30300 0.001478739 28300 0.008192118 28100 0.4360492 27700
0.002254378 29900 0.000936372 29400 0.02096767
30300 0.001479683 28300 0.008264361 28100 0.4413417 27700
0.002236095 29900 0.000935827 29400 0.02087267
30300 0.001480608 28300 0.00833742 28100 0.446099 27700
0.002217612 29900 0.000935298 29400 0.02077829
30300 0.001481554 28300 0.008411324 28100 0.4502648 27700
0.002198901 29900 0.00093479 29400 0.02068456
30300 0.001482494 28300 0.008486046 28100 0.4537836 27700
0.002179989 29900 0.000934265 29400 0.02059146
30300 0.001483454 28300 0.008561604 28100 0.4566017 27700
0.002160901 29900 0.000933732 29400 0.02049902
30300 0.001484447 28300 0.008638026 28100 0.458668 27700
0.00214167 29900 0.000933214 29400 0.02040721
30300 0.001485434 28300 0.008715334 28100 0.4599363 27700
0.002122319 29900 0.000932674 29400 0.02031608
30300 0.001486449 28300 0.008793506 28100 0.4603655 27700
0.002102885 29900 0.000932159 29400 0.02022554

30300 0.001487473 28300 0.008872594 28100 0.4599201 27700
0.002083434 30000 0.00093165 29400 0.02013561
30300 0.001488497 28300 0.008952599 28100 0.4585734 27700
0.002063981 30000 0.000931128 29400 0.0200463
30300 0.001489547 28300 0.009033574 28100 0.4563075 27700
0.002044584 30000 0.000930625 29400 0.0199576
30300 0.00149062 28300 0.009115512 28100 0.4531142 27700
0.002025233 30000 0.00093012 29400 0.01986946
30300 0.001491699 28300 0.009198454 28100 0.4489962 27700
0.002005921 30000 0.000929608 29400 0.01978195
30300 0.00149278 28300 0.009282372 28100 0.4439677 27700
0.001986631 30000 0.000929096 29400 0.01969502
30300 0.001493863 28300 0.009367336 28100 0.4380543 27700
0.001967383 30000 0.000928574 29400 0.01960868
30300 0.001494993 28300 0.009453311 28100 0.4312936 27700
0.001948204 30000 0.000928061 29400 0.0195229
30300 0.001496138 28300 0.009540292 28100 0.4237349 27700
0.001929075 30000 0.000927561 29400 0.01943769
30300 0.001497302 28300 0.00962832 28100 0.415438 27700
0.001910072 30000 0.000927052 29400 0.01935306
30300 0.001498486 28300 0.009717353 28100 0.4064725 27700
0.001891191 30000 0.000926547 29400 0.01926906
30300 0.001499711 28300 0.00980743 28100 0.3969169 27700
0.001872481 30000 0.00092604 29400 0.01918561
30300 0.001500924 28300 0.009898573 28100 0.3868566 27700
0.001853952 30000 0.000925533 29400 0.01910264
30300 0.001502152 28300 0.009990774 28100 0.3763822 27700
0.001835605 30000 0.000925034 29400 0.01902026
30300 0.001503395 28300 0.01008411 28100 0.3655875 27700
0.001817529 30000 0.000924537 29400 0.01893841
30300 0.001504675 28300 0.01017854 28100 0.3545678 27700
0.001799697 30000 0.00092402 29400 0.01885711
30300 0.001505969 28300 0.01027411 28100 0.343417 27700
0.001782145 30000 0.000923537 29400 0.01877634
30300 0.00150727 28300 0.01037082 28100 0.3322274 27700
0.001764849 30000 0.000923033 29400 0.01869615
30300 0.001508609 28300 0.01046869 28100 0.3210857 27700
0.001747867 30000 0.000922511 29400 0.01861643
30300 0.001509943 28300 0.01056763 28100 0.3100723 27700
0.001731185 30000 0.000922032 29400 0.01853725
30300 0.00151132 28300 0.01066775 28100 0.2992605 27700
0.001714826 30000 0.000921555 29400 0.01845858
30300 0.001512678 28300 0.01076914 28100 0.2887152 27700
0.001698787 30000 0.000921058 29400 0.01838041
30300 0.001514097 28300 0.01087179 28100 0.2784918 27700
0.001683102 30000 0.000920559 29400 0.01830273
30300 0.0015155 28300 0.01097571 28100 0.2686361 27700
0.00166775 30000 0.000920058 29400 0.01822556
30300 0.001516934 28300 0.01108093 28100 0.2591841 27700
0.00165275 30000 0.000919572 29400 0.01814886
30300 0.001518354 28300 0.01118749 28100 0.2501625 27700
0.001638126 30000 0.000919063 29400 0.01807265
30300 0.001519821 28300 0.01129544 28100 0.2415885 27700
0.001623906 30000 0.000918582 29400 0.01799691
30300 0.001521266 28300 0.01140484 28100 0.2334714 27700
0.001610118 30000 0.000918084 29400 0.01792167
30300 0.001522759 28300 0.0115157 28100 0.2258127 27700
0.001596754 30000 0.000917604 29400 0.01784689
30400 0.001524253 28300 0.01162809 28100 0.2186074 27700
0.00158384 30000 0.000917128 29400 0.01777266
30400 0.001525776 28300 0.01174206 28100 0.2118445 27700
0.001571368 30000 0.000916642 29400 0.01769886
30400 0.001527287 28300 0.0118576 28100 0.2055088 27700
0.001559369 30000 0.00091617 29400 0.01762547
30400 0.001528803 28300 0.01197472 28100 0.199581 27700
0.001547822 30000 0.000915703 29400 0.01755255
30400 0.001530326 28300 0.01209345 28100 0.1940393 27700
0.001536785 30000 0.000915239 29400 0.0174801

30400 0.001531882 28300 0.01221381 28100 0.1888598 27700
0.001526187 30000 0.00091475 29400 0.01740809
30400 0.001533466 28300 0.01233582 28100 0.1840175 27700
0.001516122 30000 0.00091427 29400 0.01733656
30400 0.001535053 28300 0.01245949 28100 0.1794872 27700
0.001506535 30000 0.000913791 29400 0.01726548
30400 0.001536616 28300 0.01258484 28100 0.1752434 27700
0.001497478 30000 0.000913307 29400 0.01719485
30400 0.00153822 28300 0.01271185 28100 0.1712609 27700
0.001488928 30000 0.00091285 29500 0.01712466
30400 0.00153982 28300 0.01284059 28100 0.167516 27700
0.001480909 30000 0.000912374 29500 0.01705488
30400 0.00154143 28300 0.01297116 28100 0.1639858 27700
0.001473396 30000 0.000911906 29500 0.01698556
30400 0.001543076 28300 0.01310356 28100 0.160649 27700
0.001466405 30000 0.000911448 29500 0.01691664
30400 0.001544726 28300 0.01323787 28100 0.1574858 27700
0.001459896 30000 0.000910977 29500 0.01684816
30400 0.001546345 28300 0.01337412 28100 0.1544777 27700
0.001453853 30000 0.000910501 29500 0.01678008
30400 0.001547974 28300 0.01351233 28100 0.151608 27700
0.001448262 30000 0.000910032 29500 0.01671243
30400 0.001549629 28300 0.01365259 28100 0.1488616 27700
0.001443127 30000 0.00090956 29500 0.01664517
30400 0.001551309 28300 0.01379487 28100 0.1462251 27700
0.001438452 30000 0.000909099 29500 0.01657834
30400 0.001552981 28400 0.01393921 28100 0.1436862 27700
0.001434249 30000 0.000908635 29500 0.0165119
30400 0.001554684 28400 0.01408564 28100 0.1412343 27700
0.001430512 30000 0.000908173 29500 0.01644594
30400 0.001556395 28400 0.01423422 28200 0.13886 27700
0.001427206 30000 0.000907719 29500 0.01638034
30400 0.001558083 28400 0.01438502 28200 0.136555 27700
0.001424324 30000 0.00090727 29500 0.01631511
30400 0.001559808 28400 0.0145381 28200 0.1343123 27700
0.001421867 30000 0.000906825 29500 0.01625028
30400 0.001561522 28400 0.01469359 28200 0.1321257 27700
0.001419834 30000 0.000906394 29500 0.01618585
30400 0.001563267 28400 0.01485162 28200 0.1299899 27800
0.001418219 30000 0.000905942 29500 0.01612183
30400 0.001565018 28400 0.01501228 28200 0.1279003 27800
0.001416991 30000 0.0009055 29500 0.01605819
30400 0.001566792 28400 0.01517567 28200 0.1258534 27800
0.00141617 30000 0.000905043 29500 0.01599496
30400 0.001568573 28400 0.01534189 28200 0.1238458 27800
0.001415755 30000 0.000904598 29500 0.01593211
30400 0.001570338 28400 0.01551095 28200 0.1218747 27800
0.001415726 30000 0.000904168 29500 0.01586963
30400 0.001572142 28400 0.01568292 28200 0.119938 27800
0.00141606 30000 0.000903728 29500 0.01580754
30400 0.001573915 28400 0.01585795 28200 0.1180338 27800
0.001416754 30000 0.0009033 29500 0.01574583
30400 0.001575758 28400 0.01603607 28200 0.1161606 27800
0.001417813 30000 0.00090287 29500 0.01568446
30400 0.001577536 28400 0.0162173 28200 0.1143171 27800
0.001419218 30000 0.000902433 29500 0.01562347
30400 0.001579355 28400 0.01640172 28200 0.1125025 27800
0.001420986 30000 0.000901991 29500 0.01556286
30400 0.001581131 28400 0.01658937 28200 0.1107158 27800
0.0014231 30000 0.000901567 29500 0.0155026
30400 0.001582975 28400 0.01678035 28200 0.1089563 27800
0.001425549 30100 0.000901135 29500 0.0154427
30400 0.001584794 28400 0.01697475 28200 0.1072237 27800
0.001428349 30100 0.000900708 29500 0.01538315
30400 0.001586635 28400 0.01717263 28200 0.1055173 27800
0.001431455 30100 0.000900279 29500 0.01532395
30400 0.001588471 28400 0.01737414 28200 0.1038371 27800
0.00143484 30100 0.00089985 29500 0.01526517

30400 0.001590347 28400 0.01757938 28200 0.1021825 27800
0.00143849 30100 0.000899423 29500 0.01520669
30400 0.001592205 28400 0.01778847 28200 0.1005534 27800
0.00144237 30100 0.000899001 29500 0.01514851
30400 0.001594044 28400 0.01800153 28200 0.09894972 27800
0.00144646 30100 0.000898588 29500 0.0150907
30400 0.001595883 28400 0.01821868 28200 0.09737117 27800
0.001450767 30100 0.000898172 29500 0.01503318
30400 0.00159776 28400 0.01844004 28200 0.09581765 27800
0.001455247 30100 0.000897752 29500 0.01497602
30400 0.001599644 28400 0.01866561 28200 0.094289 27800
0.001459882 30100 0.000897343 29500 0.01491921
30400 0.001601515 28400 0.01889549 28200 0.09278526 27800
0.00146467 30100 0.000896905 29500 0.01486271
30400 0.00160341 28400 0.01912969 28200 0.09130616 27800
0.001469573 30100 0.000896497 29500 0.01480655
30400 0.001605286 28400 0.01936832 28200 0.08985148 27800
0.001474583 30100 0.000896083 29500 0.01475071
30400 0.001607185 28400 0.01961147 28200 0.08842117 27800
0.001479691 30100 0.000895672 29500 0.01469518
30400 0.001609062 28400 0.01985941 28200 0.08701507 27800
0.001484916 30100 0.000895263 29500 0.01463997
30400 0.001610974 28400 0.02011229 28200 0.08563302 27800
0.001490255 30100 0.000894857 29500 0.01458509
30400 0.001612883 28400 0.02037024 28200 0.08427485 27800
0.001495698 30100 0.000894446 29500 0.01453049
30400 0.001614769 28400 0.02063349 28200 0.08294038 27800
0.001501248 30100 0.000894023 29500 0.01447621
30400 0.001616654 28400 0.02090217 28200 0.08162934 27800
0.001506916 30100 0.000893604 29500 0.01442224
30400 0.001618543 28400 0.02117653 28200 0.08034156 27800
0.001512686 30100 0.000893185 29500 0.01436857
30400 0.001620439 28400 0.02145669 28200 0.07907677 27800
0.001518538 30100 0.00089277 29500 0.0143152
30400 0.001622338 28400 0.02174289 28200 0.0778347 27800
0.001524458 30100 0.00089236 29500 0.01426216
30400 0.001624229 28400 0.02203536 28200 0.07661514 27800
0.001530405 30100 0.000891943 29500 0.01420947
30400 0.001626129 28400 0.02233432 28200 0.07541776 27800
0.001536392 30100 0.000891532 29500 0.01415703
30400 0.001628009 28400 0.02264003 28200 0.07424228 27800
0.001542417 30100 0.000891116 29500 0.01410487
30400 0.001629876 28400 0.02295266 28200 0.07308844 27800
0.00154848 30100 0.000890714 29500 0.014053
30400 0.001631738 28400 0.02327235 28200 0.07195593 27800
0.001554593 30100 0.000890297 29500 0.01400142
30400 0.001633628 28400 0.02359933 28200 0.07084441 27800
0.001560703 30100 0.000889874 29500 0.01395016
30400 0.0016355 28400 0.02393367 28200 0.06975362 27800
0.001566806 30100 0.000889468 29500 0.01389919
30500 0.001637366 28400 0.02427554 28200 0.06868334 27800
0.001572868 30100 0.000889049 29500 0.01384849
30500 0.001639222 28400 0.0246251 28200 0.06763308 27800
0.001578846 30100 0.00088863 29500 0.0137981
30500 0.001641069 28400 0.0249825 28200 0.06660254 27800
0.00158473 30100 0.000888187 29500 0.01374797
30500 0.001642885 28400 0.02534794 28200 0.06559142 27800
0.001590559 30100 0.000887763 29500 0.01369811
30500 0.001644702 28400 0.0257219 28200 0.06459939 27800
0.001596292 30100 0.000887364 29500 0.01364854
30500 0.001646536 28400 0.02610455 28200 0.06362617 27800
0.001601969 30100 0.000886936 29500 0.01359924
30500 0.00164835 28400 0.02649613 28200 0.06267143 27800
0.001607584 30100 0.000886504 29500 0.01355018
30500 0.001650134 28400 0.02689699 28200 0.06173484 27800
0.00161314 30100 0.000886068 29500 0.0135014
30500 0.001651906 28400 0.02730739 28200 0.06081606 27800
0.00161867 30100 0.000885626 29500 0.01345289

30500 0.001653678 28400 0.0277277 28200 0.05991478 27800
0.00162419 30100 0.00088519 29600 0.01340464
30500 0.001655418 28400 0.02815827 28200 0.05903066 27800
0.001629673 30100 0.000884735 29600 0.01335665
30500 0.001657146 28400 0.02859949 28200 0.05816338 27800
0.001635106 30100 0.000884293 29600 0.01330892
30500 0.001658877 28400 0.02905175 28200 0.05731263 27800
0.001640453 30100 0.000883836 29600 0.01326153
30500 0.001660593 28400 0.02951544 28200 0.05647807 27800
0.001645704 30100 0.000883393 29600 0.01321433
30500 0.00166225 28400 0.02999094 28200 0.05565937 27800
0.001650861 30100 0.000882949 29600 0.01316737
30500 0.001663915 28400 0.03047863 28200 0.05485627 27800
0.00165589 30100 0.00088251 29600 0.01312065
30500 0.001665539 28400 0.03097891 28200 0.05406841 27800
0.001660815 30100 0.000882057 29600 0.01307417
30500 0.001667148 28400 0.03149222 28200 0.05329547 27800
0.001665623 30100 0.000881612 29600 0.01302797
30500 0.001668747 28500 0.03201883 28200 0.05253723 27800
0.001670333 30100 0.00088117 29600 0.01298202
30500 0.001670313 28500 0.03255913 28200 0.05179341 27800
0.001674922 30100 0.000880717 29600 0.01293631
30500 0.00167188 28500 0.03311349 28300 0.05106359 27800
0.001679426 30100 0.000880297 29600 0.01289086
30500 0.001673418 28500 0.03368232 28300 0.05034752 27800
0.001683848 30100 0.00087985 29600 0.01284564
30500 0.001674937 28500 0.03426617 28300 0.0496449 27800
0.001688189 30100 0.000879412 29600 0.01280065
30500 0.001676429 28500 0.03486595 28300 0.0489555 27800
0.001692465 30100 0.000878977 29600 0.01275591
30500 0.001677906 28500 0.03548217 28300 0.04827901 27900
0.001696632 30100 0.000878547 29600 0.0127114
30500 0.001679361 28500 0.03611552 28300 0.04761518 27900
0.001700691 30100 0.000878119 29600 0.01266711
30500 0.001680755 28500 0.0367665 28300 0.04696377 27900
0.001704595 30100 0.000877676 29600 0.01262305
30500 0.001682144 28500 0.03743568 28300 0.04632448 27900
0.001708357 30100 0.000877257 29600 0.01257925
30500 0.00168353 28500 0.03812359 28300 0.04569701 27900
0.001711962 30100 0.000876831 29600 0.01253564
30500 0.001684868 28500 0.03883083 28300 0.04508122 27900
0.001715412 30100 0.000876391 29600 0.01249228
30500 0.00168618 28500 0.03955806 28300 0.04447674 27900
0.001718691 30100 0.000875971 29600 0.01244915
30500 0.001687484 28500 0.04030599 28300 0.04388342 27900
0.001721799 30100 0.000875552 29600 0.01240628
30500 0.001688777 28500 0.04107541 28300 0.04330095 27900
0.001724805 30100 0.000875123 29600 0.01236364
30500 0.001690056 28500 0.04186718 28300 0.04272914 27900
0.001727685 30100 0.000874707 29600 0.01232118
30500 0.001691272 28500 0.04268221 28300 0.04216773 27900
0.001730458 30100 0.000874302 29600 0.01227896
30500 0.001692467 28500 0.04352147 28300 0.04161655 27900
0.00173319 30200 0.000873886 29600 0.01223696
30500 0.001693648 28500 0.044386 28300 0.04107533 27900
0.001735827 30200 0.00087347 29600 0.01219521
30500 0.001694797 28500 0.04527677 28300 0.04054387 27900
0.001738384 30200 0.000873065 29600 0.01215369
30500 0.001695897 28500 0.0461949 28300 0.04002207 27900
0.001740827 30200 0.000872652 29600 0.01211241
30500 0.001696999 28500 0.04714158 28300 0.03950958 27900
0.001743156 30200 0.00087225 29600 0.01207135
30500 0.001698048 28500 0.04811797 28300 0.03900624 27900
0.001745335 30200 0.000871834 29600 0.01203051
30500 0.001699093 28500 0.04912543 28300 0.03851188 27900
0.001747403 30200 0.000871436 29600 0.0119899
30500 0.001700083 28500 0.05016555 28300 0.03802629 27900
0.001749337 30200 0.000871028 29600 0.0119495

30500 0.001701076 28500 0.05123946 28300 0.0375493 27900
0.001751152 30200 0.000870645 29600 0.01190932
30500 0.001702018 28500 0.05234857 28300 0.03708073 27900
0.001752802 30200 0.000870249 29600 0.01186934
30500 0.001702918 28500 0.05349418 28300 0.0366204 27900
0.001754308 30200 0.000869856 29600 0.01182957
30500 0.001703765 28500 0.05467768 28300 0.03616811 27900
0.001755657 30200 0.00086947 29600 0.01179003
30500 0.001704628 28500 0.05590043 28300 0.03572373 27900
0.001756844 30200 0.000869094 29600 0.0117507
30500 0.00170545 28500 0.05716395 28300 0.03528703 27900
0.001757875 30200 0.000868703 29600 0.01171157
30500 0.001706235 28500 0.05846992 28300 0.03485791 27900
0.001758752 30200 0.000868322 29600 0.01167266
30500 0.001707028 28500 0.05982018 28300 0.03443616 27900
0.001759484 30200 0.000867962 29600 0.01163402
30500 0.001707773 28500 0.06121685 28300 0.0340216 27900
0.001760083 30200 0.000867592 29600 0.01159554
30500 0.001708484 28500 0.06266223 28300 0.03361414 27900
0.001760568 30200 0.000867236 29600 0.01155723
30500 0.001709157 28500 0.06415866 28300 0.03321361 27900
0.00176093 30200 0.000866888 29600 0.01151914
30500 0.001709792 28500 0.06570857 28300 0.03281987 27900
0.001761207 30200 0.000866545 29600 0.01148122
30500 0.001710422 28500 0.06731453 28300 0.03243274 27900
0.001761347 30200 0.000866205 29600 0.01144351
30500 0.001711027 28500 0.06897909 28300 0.03205215 27900
0.001761447 30200 0.000865869 29600 0.01140602
30500 0.001711625 28500 0.07070507 28300 0.03167799 27900
0.001761465 30200 0.000865528 29600 0.01136871
30500 0.001712174 28500 0.07249545 28300 0.03131 27900
0.001761402 30200 0.000865211 29600 0.0113316
30500 0.001712715 28500 0.07435317 28300 0.03094813 27900
0.001761274 30200 0.000864893 29600 0.01129468
30500 0.001713236 28500 0.07628158 28300 0.03059222 27900
0.001761068 30200 0.000864583 29600 0.01125794
30500 0.001713715 28500 0.07828454 28300 0.03024218 27900
0.001760814 30200 0.000864276 29600 0.01122139
30500 0.001714197 28500 0.08036567 28300 0.02989787 27900
0.001760506 30200 0.00086398 29600 0.01118501
30500 0.001714662 28500 0.08252905 28300 0.02955922 27900
0.00176013 30200 0.00086367 29600 0.0111488
30600 0.001715078 28500 0.08477906 28300 0.02922607 27900
0.001759704 30200 0.000863373 29600 0.01111277
30600 0.00171548 28500 0.08712042 28300 0.02889831 27900
0.001759226 30200 0.000863067 29600 0.01107693
30600 0.001715891 28500 0.08955803 28300 0.02857583 27900
0.001758732 30200 0.000862768 29600 0.01104125
30600 0.001716251 28500 0.0920971 28300 0.02825855 27900
0.001758168 30200 0.000862458 29600 0.01100575
30600 0.001716609 28500 0.09474312 28300 0.02794634 27900
0.001757537 30200 0.000862159 29600 0.01097043
30600 0.001716965 28500 0.09750159 28300 0.0276391 27900
0.001756812 30200 0.000861866 29600 0.01093533
30600 0.0017173 28500 0.1003784 28300 0.02733668 27900
0.001755992 30200 0.000861571 29600 0.01090037
30600 0.001717603 28500 0.1033799 28300 0.02703906 27900
0.001755096 30200 0.000861277 29600 0.01086557
30600 0.001717881 28500 0.1065127 28300 0.02674612 27900
0.001754091 30200 0.000860995 29600 0.01083093
30600 0.001718138 28500 0.1097843 28300 0.02645778 27900
0.001753025 30200 0.00086071 29700 0.01079644
30600 0.001718397 28500 0.1132027 28300 0.02617389 27900
0.001751846 30200 0.000860414 29700 0.01076212
30600 0.001718646 28500 0.1167766 28300 0.02589443 27900
0.001750604 30200 0.000860125 29700 0.01072798
30600 0.001718879 28500 0.1205155 28300 0.02561934 27900
0.001749267 30200 0.000859823 29700 0.01069403

30600 0.001719077 28500 0.1244296 28300 0.02534844 27900
0.001747823 30200 0.000859526 29700 0.01066023
30600 0.001719267 28500 0.1285296 28300 0.02508169 27900
0.001746263 30200 0.000859226 29700 0.01062662
30600 0.001719469 28500 0.132827 28300 0.02481901 27900
0.00174459 30200 0.000858934 29700 0.01059315
30600 0.001719619 28500 0.1373333 28300 0.02456033 27900
0.001742816 30200 0.000858626 29700 0.01055984
30600 0.001719781 28500 0.1420605 28300 0.02430556 27900
0.001740944 30200 0.000858325 29700 0.01052669
30600 0.001719936 28600 0.1470218 28300 0.02405468 27900
0.001738971 30200 0.000858012 29700 0.01049369
30600 0.001720045 28600 0.1522315 28300 0.02380755 27900
0.001736887 30200 0.000857701 29700 0.01046088
30600 0.001720142 28600 0.1577052 28400 0.02356414 27900
0.001734717 30200 0.000857379 29700 0.01042821
30600 0.001720257 28600 0.1634596 28400 0.02332435 27900
0.001732474 30200 0.000857051 29700 0.01039565
30600 0.001720341 28600 0.169513 28400 0.02308813 27900
0.001730137 30200 0.000856722 29700 0.01036328
30600 0.00172042 28600 0.1758847 28400 0.02285543 27900
0.001727725 30200 0.000856387 29700 0.01033107
30600 0.001720504 28600 0.1825955 28400 0.02262614 28000
0.001725256 30200 0.000856055 29700 0.01029906
30600 0.001720582 28600 0.1896673 28400 0.0224002 28000
0.001722737 30200 0.000855714 29700 0.01026714
30600 0.001720651 28600 0.1971234 28400 0.0221776 28000
0.001720207 30200 0.000855372 29700 0.01023537
30600 0.001720698 28600 0.2049887 28400 0.02195819 28000
0.001717675 30200 0.000855036 29700 0.01020375
30600 0.001720715 28600 0.2132903 28400 0.02174199 28000
0.001715121 30200 0.000854692 29700 0.01017226
30600 0.001720767 28600 0.2220574 28400 0.02152888 28000
0.001712579 30200 0.000854346 29700 0.01014094
30600 0.001720798 28600 0.2313217 28400 0.02131888 28000
0.001710038 30200 0.000853993 29700 0.01010977
30600 0.001720831 28600 0.2411163 28400 0.02111192 28000
0.001707476 30200 0.000853634 29700 0.01007875
30600 0.00172085 28600 0.2514762 28400 0.02090789 28000
0.001704917 30200 0.000853275 29700 0.01004789
30600 0.00172087 28600 0.2624376 28400 0.02070674 28000
0.001702356 30200 0.000852916 29700 0.01001717
30600 0.001720873 28600 0.2740395 28400 0.02050841 28000
0.001699776 30200 0.000852564 29700 0.009986575
30600 0.001720858 28600 0.2863236 28400 0.02031291 28000
0.001697161 30300 0.000852212 29700 0.009956115
30600 0.001720855 28600 0.2993327 28400 0.02012015 28000
0.001694492 30300 0.000851849 29700 0.0099258
30600 0.001720847 28600 0.3131118 28400 0.01993011 28000
0.001691748 30300 0.000851494 29700 0.009895607
30600 0.001720812 28600 0.3277072 28400 0.01974274 28000
0.001688915 30300 0.00085113 29700 0.009865553
30600 0.001720779 28600 0.3431661 28400 0.01955795 28000
0.001686 30300 0.000850767 29700 0.009835652
30600 0.001720762 28600 0.3595364 28400 0.01937573 28000
0.001683001 30300 0.000850406 29700 0.009805874
30600 0.001720725 28600 0.3768661 28400 0.019196 28000
0.001679964 30300 0.000850035 29700 0.009776257
30600 0.001720685 28600 0.3952031 28400 0.01901875 28000
0.00167689 30300 0.000849671 29700 0.009746799
30600 0.001720657 28600 0.4145931 28400 0.01884395 28000
0.00167382 30300 0.00084931 29700 0.009717518
30600 0.001720633 28600 0.4350787 28400 0.01867152 28000
0.001670762 30300 0.000848937 29700 0.009688333
30600 0.0017206 28600 0.4566957 28400 0.01850142 28000
0.00166772 30300 0.000848577 29700 0.009659269
30600 0.001720554 28600 0.4794712 28400 0.01833363 28000
0.00166469 30300 0.000848216 29700 0.009630338

30600 0.001720491 28600 0.5034214 28400 0.01816808 28000
0.001661655 30300 0.00084786 29700 0.009601521
30600 0.001720447 28600 0.5285472 28400 0.01800474 28000
0.001658638 30300 0.000847497 29700 0.009572871
30600 0.001720406 28600 0.5548332 28400 0.01784359 28000
0.0016556 30300 0.000847144 29700 0.009544345
30600 0.001720359 28600 0.5822415 28400 0.0176846 28000
0.001652565 30300 0.000846778 29700 0.009515976
30600 0.001720324 28600 0.6107082 28400 0.0175277 28000
0.00164952 30300 0.000846412 29700 0.009487715
30600 0.001720286 28600 0.6401363 28400 0.01737285 28000
0.00164648 30300 0.000846063 29700 0.009459591
30600 0.001720236 28600 0.6703911 28400 0.01722001 28000
0.001643452 30300 0.000845713 29700 0.009431596
30600 0.001720182 28600 0.7012935 28400 0.01706916 28000
0.001640445 30300 0.000845372 29700 0.009403731
30600 0.001720172 28600 0.7326152 28400 0.01692027 28000
0.001637422 30300 0.000845031 29700 0.009375968
30600 0.001720145 28600 0.7640782 28400 0.01677332 28000
0.00163438 30300 0.000844694 29700 0.009348354
30600 0.001720086 28600 0.795353 28400 0.01662828 28000
0.001631281 30300 0.000844351 29700 0.009320848
30600 0.001720044 28600 0.8260612 28400 0.01648508 28000
0.001628135 30300 0.000844014 29700 0.00929348
30600 0.001720028 28600 0.855776 28400 0.01634373 28000
0.001624919 30300 0.000843676 29700 0.009266219
30600 0.001719999 28600 0.8840261 28400 0.01620416 28000
0.001621653 30300 0.000843341 29700 0.009239082
30600 0.001719962 28600 0.910306 28400 0.01606636 28000
0.001618326 30300 0.000843005 29700 0.009212084
30600 0.001719948 28600 0.9340941 28400 0.01593031 28000
0.00161499 30300 0.000842675 29700 0.00918524
30600 0.00171994 28600 0.9548804 28400 0.01579595 28000
0.001611658 30300 0.000842339 29700 0.009158489
30700 0.001719933 28600 0.9721958 28400 0.01566329 28000
0.001608339 30300 0.000842013 29700 0.009131836
30700 0.001719909 28600 0.9856362 28400 0.01553227 28000
0.001605026 30300 0.000841694 29700 0.009105327
30700 0.001719917 28600 0.9948813 28400 0.01540287 28000
0.001601724 30300 0.000841381 29700 0.009078934
30700 0.001719914 28600 0.9997079 28400 0.01527508 28000
0.001598413 30300 0.000841067 29700 0.009052681
30700 0.001719944 28600 1 28400 0.01514887 28000
0.001595097 30300 0.000840755 29700 0.009026546
30700 0.001719975 28600 0.9957556 28400 0.01502425 28000
0.001591758 30300 0.000840443 29700 0.009000524
30700 0.001720008 28600 0.9870883 28400 0.01490114 28000
0.001588383 30300 0.000840122 29700 0.008974619
30700 0.00172004 28600 0.974225 28400 0.01477951 28000
0.001584974 30300 0.000839816 29700 0.008948824
30700 0.001720087 28600 0.9574961 28400 0.01465933 28000
0.001581549 30300 0.000839521 29700 0.008923143
30700 0.001720125 28600 0.9373151 28400 0.01454064 28000
0.001578122 30300 0.000839227 29800 0.008897556
30700 0.001720204 28600 0.9141513 28400 0.01442338 28000
0.001574689 30300 0.000838926 29800 0.008872117
30700 0.001720284 28600 0.8885024 28400 0.01430753 28000
0.001571262 30300 0.000838628 29800 0.008846772
30700 0.001720341 28600 0.8608662 28400 0.01419308 28000
0.001567842 30300 0.000838334 29800 0.008821539
30700 0.001720417 28600 0.8317225 28400 0.01408001 28000
0.00156443 30300 0.000838042 29800 0.008796444
30700 0.001720504 28600 0.8015214 28400 0.01396829 28000
0.001561047 30300 0.000837745 29800 0.008771437
30700 0.001720586 28600 0.7706782 28400 0.01385789 28000
0.00155767 30300 0.000837456 29800 0.008746549
30700 0.001720688 28600 0.7395669 28400 0.01374877 28000
0.001554287 30300 0.000837155 29800 0.008721788

30700 0.0017208 28600 0.7085174 28400 0.01364095 28000
0.001550932 30300 0.000836857 29800 0.008697174
30700 0.001720936 28700 0.6778116 28400 0.01353438 28000
0.001547597 30300 0.000836561 29800 0.008672647
30700 0.001721058 28700 0.647683 28400 0.01342909 28000
0.001544295 30300 0.000836268 29800 0.008648223
30700 0.001721181 28700 0.6183192 28500 0.01332497 28000
0.001541006 30300 0.000835983 29800 0.008623904
30700 0.001721309 28700 0.5898643 28500 0.01322208 28000
0.001537716 30300 0.0008357 29800 0.008599683
30700 0.001721463 28700 0.5624246 28500 0.01312036 28000
0.001534417 30300 0.000835406 29800 0.0085756
30700 0.00172162 28700 0.5360728 28500 0.01301983 28000
0.001531079 30300 0.000835117 29800 0.00855164
30700 0.001721754 28700 0.5108528 28500 0.01292049 28100
0.001527695 30300 0.000834818 29800 0.008527797
30700 0.001721938 28700 0.4867846 28500 0.01282224 28100
0.001524262 30300 0.00083452 29800 0.008504065
30700 0.001722116 28700 0.4638692 28500 0.01272511 28100
0.001520792 30300 0.000834223 29800 0.008480431
30700 0.001722279 28700 0.4420935 28500 0.01262906 28100
0.001517281 30300 0.000833934 29800 0.008456915
30700 0.001722439 28700 0.4214331 28500 0.01253411 28100
0.001513799 30300 0.000833644 29800 0.008433498
30700 0.001722632 28700 0.4018551 28500 0.01244022 28100
0.001510329 30300 0.000833353 29800 0.008410188
30700 0.001722813 28700 0.3833224 28500 0.0123474 28100
0.001506912 30300 0.00083306 29800 0.008386976
30700 0.001722987 28700 0.3657939 28500 0.01225562 28100
0.001503554 30300 0.000832774 29800 0.008363887
30700 0.00172315 28700 0.349226 28500 0.01216487 28100
0.001500273 30300 0.000832485 29800 0.008340907
30700 0.001723338 28700 0.333573 28500 0.01207514 28100
0.001497056 30300 0.000832187 29800 0.008318001
30700 0.001723525 28700 0.3187889 28500 0.0119864 28100
0.001493888 30300 0.000831908 29800 0.008295202
30700 0.0017237 28700 0.304827 28500 0.01189864 28100
0.001490763 30400 0.000831627 29800 0.0082725
30700 0.001723879 28700 0.291641 28500 0.01181186 28100
0.001487668 30400 0.000831354 29800 0.008249953
30700 0.001724094 28700 0.2791851 28500 0.01172602 28100
0.001484612 30400 0.000831074 29800 0.008227488
30700 0.001724273 28700 0.2674156 28500 0.01164113 28100
0.001481591 30400 0.000830808 29800 0.008205092
30700 0.00172445 28700 0.2562907 28500 0.01155716 28100
0.00147859 30400 0.000830547 29800 0.008182788
30700 0.001724633 28700 0.2457712 28500 0.0114741 28100
0.001475598 30400 0.000830304 29800 0.008160577
30700 0.001724821 28700 0.2358199 28500 0.01139198 28100
0.0014726 30400 0.000830053 29800 0.008138481
30700 0.001725022 28700 0.2264022 28500 0.01131072 28100
0.001469618 30400 0.00082981 29800 0.008116484
30700 0.001725201 28700 0.2174859 28500 0.0112304 28100
0.001466641 30400 0.000829567 29800 0.00809459
30700 0.001725385 28700 0.2090411 28500 0.01115091 28100
0.001463674 30400 0.000829324 29800 0.008072786
30700 0.00172556 28700 0.2010399 28500 0.01107223 28100
0.001460712 30400 0.000829093 29800 0.008051071
30700 0.00172574 28700 0.1934561 28500 0.0109944 28100
0.001457773 30400 0.000828861 29800 0.008029458
30700 0.00172592 28700 0.186264 28500 0.01091742 28100
0.00145486 30400 0.000828633 29800 0.008007918
30700 0.001726089 28700 0.1794389 28500 0.01084122 28100
0.001451974 30400 0.000828398 29800 0.007986467
30700 0.001726253 28700 0.1729583 28500 0.01076585 28100
0.001449109 30400 0.00082817 29800 0.007965103
30700 0.001726399 28700 0.1668008 28500 0.01069128 28100
0.001446285 30400 0.000827944 29800 0.007943834

30700 0.001726543 28700 0.160947 28500 0.01061748 28100
0.001443497 30400 0.000827718 29800 0.00792265
30700 0.001726708 28700 0.1553788 28500 0.01054444 28100
0.001440755 30400 0.000827473 29800 0.007901531
30700 0.001726841 28700 0.1500794 28500 0.01047215 28100
0.001438032 30400 0.000827243 29800 0.007880511
30700 0.001726983 28700 0.145033 28500 0.01040061 28100
0.001435317 30400 0.000827005 29800 0.007859557
30700 0.001727115 28700 0.1402248 28500 0.01032982 28100
0.001432622 30400 0.00082676 29800 0.007838759
30700 0.001727272 28700 0.135641 28500 0.01025973 28100
0.001429944 30400 0.000826507 29800 0.007817993
30700 0.001727389 28700 0.1312685 28500 0.01019038 28100
0.001427301 30400 0.000826248 29800 0.00779729
30700 0.001727501 28700 0.1270955 28500 0.01012172 28100
0.001424647 30400 0.000826009 29800 0.007776659
30700 0.001727588 28700 0.1231108 28500 0.01005376 28100
0.00142198 30400 0.000825755 29800 0.007756105
30700 0.001727716 28700 0.1193038 28500 0.009986506 28100
0.001419322 30400 0.000825499 29800 0.007735629
30700 0.001727843 28700 0.1156648 28500 0.009919927 28100
0.001416643 30400 0.000825233 29800 0.007715258
30700 0.00172793 28700 0.1121841 28500 0.009854059 28100
0.001413966 30400 0.000824974 29800 0.007694963
30700 0.001728041 28700 0.1088531 28500 0.009788834 28100
0.001411272 30400 0.000824709 29800 0.007674736
30800 0.001728144 28700 0.1056631 28500 0.00972423 28100
0.001408595 30400 0.000824453 29800 0.00765458
30800 0.001728227 28700 0.1026065 28500 0.009660298 28100
0.001405906 30400 0.000824191 29800 0.007634502
30800 0.001728293 28700 0.09967562 28500 0.00959702 28100
0.001403244 30400 0.000823927 29800 0.007614488
30800 0.001728372 28700 0.09686372 28500 0.009534352 28100
0.001400596 30400 0.00082366 29800 0.007594567
30800 0.001728446 28700 0.09416468 28500 0.009472335 28100
0.001397975 30400 0.000823388 29800 0.007574687
30800 0.001728498 28700 0.09157273 28500 0.009410932 28100
0.001395399 30400 0.000823125 29800 0.007554895
30800 0.001728537 28700 0.0890828 28500 0.009350132 28100
0.001392878 30400 0.000822859 29800 0.007535184
30800 0.001728609 28700 0.08669008 28500 0.009289915 28100
0.001390396 30400 0.000822588 29800 0.007515533
30800 0.00172863 28700 0.08439016 28500 0.009230312 28100
0.001387963 30400 0.000822329 29800 0.00749596
30800 0.001728656 28700 0.08217864 28500 0.009171277 28100
0.001385551 30400 0.000822065 29900 0.007476456
30800 0.001728678 28700 0.0800513 28500 0.009112831 28100
0.001383162 30400 0.000821811 29900 0.007457061
30800 0.001728709 28700 0.07800399 28500 0.00905494 28100
0.001380801 30400 0.000821543 29900 0.007437724
30800 0.001728717 28700 0.0760327 28500 0.008997607 28100
0.001378465 30400 0.00082128 29900 0.007418437
30800 0.00172874 28700 0.07413368 28500 0.008940832 28100
0.001376177 30400 0.000821029 29900 0.007399215
30800 0.001728728 28700 0.07230339 28500 0.008884601 28100
0.0013739 30400 0.000820771 29900 0.007380078
30800 0.001728719 28700 0.07053848 28500 0.008828912 28100
0.001371679 30400 0.000820512 29900 0.007361034
30800 0.001728697 28700 0.06883594 28500 0.008773752 28100
0.0013695 30400 0.000820242 29900 0.007342074
30800 0.001728678 28700 0.06719303 28500 0.008719139 28100
0.001367367 30400 0.000819981 29900 0.007323188
30800 0.001728653 28800 0.06560718 28500 0.008665031 28100
0.001365281 30400 0.000819712 29900 0.007304389
30800 0.0017286 28800 0.06407591 28500 0.008611419 28100
0.00136322 30400 0.000819451 29900 0.00728565
30800 0.001728563 28800 0.06259705 28600 0.008558317 28100
0.001361176 30400 0.000819181 29900 0.007267002

30800 0.001728469 28800 0.06116799 28600 0.008505709 28100
0.001359132 30400 0.000818909 29900 0.007248428
30800 0.001728403 28800 0.05978646 28600 0.00845359 28100
0.001357092 30400 0.000818628 29900 0.007229915
30800 0.001728331 28800 0.05845039 28600 0.008401981 28100
0.00135503 30400 0.000818346 29900 0.00721148
30800 0.001728217 28800 0.05715774 28600 0.008350865 28200
0.001352962 30400 0.000818075 29900 0.007193127
30800 0.001728114 28800 0.05590663 28600 0.008300193 28200
0.001350905 30400 0.000817794 29900 0.007174867
30800 0.001728006 28800 0.05469536 28600 0.008250017 28200
0.001348856 30400 0.000817506 29900 0.007156674
30800 0.001727864 28800 0.05352238 28600 0.008200279 28200
0.001346834 30400 0.000817232 29900 0.007138563
30800 0.001727728 28800 0.0523862 28600 0.008151035 28200
0.001344823 30400 0.000816953 29900 0.007120515
30800 0.001727591 28800 0.05128548 28600 0.008102208 28200
0.001342867 30400 0.000816676 29900 0.007102599
30800 0.001727461 28800 0.05021891 28600 0.008053822 28200
0.001340932 30400 0.00081638 29900 0.007084745
30800 0.001727291 28800 0.04918517 28600 0.00800589 28200
0.001339057 30400 0.000816103 29900 0.007066926
30800 0.001727117 28800 0.0481829 28600 0.007958386 28200
0.001337227 30400 0.000815832 29900 0.007049194
30800 0.001726899 28800 0.04721076 28600 0.007911315 28200
0.001335421 30400 0.000815571 29900 0.007031526
30800 0.001726718 28800 0.0462675 28600 0.007864663 28200
0.001333685 30400 0.000815315 29900 0.007013954
30800 0.001726531 28800 0.04535198 28600 0.007818438 28200
0.001331987 30500 0.000815048 29900 0.006996463
30800 0.001726309 28800 0.04446323 28600 0.007772665 28200
0.001330395 30500 0.000814793 29900 0.006979039
30800 0.001726103 28800 0.04360023 28600 0.007727273 28200
0.001328868 30500 0.000814562 29900 0.006961711
30800 0.001725883 28800 0.04276199 28600 0.007682262 28200
0.001327455 30500 0.000814307 29900 0.006944431
30800 0.001725652 28800 0.0419477 28600 0.007637644 28200
0.001326138 30500 0.000814073 29900 0.006927231
30800 0.001725415 28800 0.04115623 28600 0.007593423 28200
0.001324899 30500 0.00081385 29900 0.006910093
30800 0.001725172 28800 0.04038674 28600 0.007549591 28200
0.001323738 30500 0.000813629 29900 0.006893018
30800 0.001724936 28800 0.03963853 28600 0.007506161 28200
0.001322607 30500 0.000813403 29900 0.006875997
30800 0.00172467 28800 0.03891095 28600 0.007463113 28200
0.001321509 30500 0.000813181 29900 0.006859057
30800 0.001724384 28800 0.03820324 28600 0.007420416 28200
0.001320463 30500 0.000812976 29900 0.006842184
30800 0.001724129 28800 0.03751473 28600 0.007378104 28200
0.001319409 30500 0.000812774 29900 0.006825362
30800 0.001723849 28800 0.03684465 28600 0.007336155 28200
0.001318406 30500 0.000812572 29900 0.006808619
30800 0.001723565 28800 0.0361923 28600 0.007294569 28200
0.001317403 30500 0.000812377 29900 0.006791923
30800 0.001723276 28800 0.03555702 28600 0.007253326 28200
0.001316403 30500 0.000812187 29900 0.006775318
30800 0.001723002 28800 0.03493819 28600 0.007212433 28200
0.00131541 30500 0.000811993 29900 0.006758752
30800 0.001722703 28800 0.03433521 28600 0.007171889 28200
0.001314426 30500 0.000811804 29900 0.006742233
30800 0.001722384 28800 0.03374757 28600 0.007131711 28200
0.001313433 30500 0.000811626 29900 0.006725775
30800 0.001722077 28800 0.0331747 28600 0.007091842 28200
0.001312469 30500 0.000811441 29900 0.006709389
30800 0.001721765 28800 0.0326162 28600 0.007052325 28200
0.00131155 30500 0.000811263 29900 0.006693046
30800 0.00172145 28800 0.03207159 28600 0.007013129 28200
0.001310646 30500 0.000811067 29900 0.006676793

30800 0.001721092 28800 0.03154047 28600 0.006974316 28200
0.001309792 30500 0.000810887 29900 0.006660606
30800 0.001720773 28800 0.03102242 28600 0.006935804 28200
0.001308961 30500 0.000810702 29900 0.006644472
30800 0.001720418 28800 0.030517 28600 0.006897606 28200
0.001308166 30500 0.000810523 29900 0.006628399
30800 0.001720082 28800 0.03002387 28600 0.006859744 28200
0.001307389 30500 0.000810324 29900 0.006612393
30800 0.001719703 28800 0.02954235 28600 0.006822199 28200
0.00130662 30500 0.000810146 29900 0.006596433
30800 0.001719341 28800 0.02907204 28600 0.006784967 28200
0.001305884 30500 0.000809951 29900 0.006580527
30800 0.001718959 28800 0.02861265 28600 0.006748062 28200
0.00130516 30500 0.000809748 29900 0.006564674
30800 0.00171857 28800 0.02816383 28600 0.006711455 28200
0.001304477 30500 0.00080955 29900 0.006548887
30800 0.001718154 28800 0.0277253 28600 0.006675186 28200
0.00130383 30500 0.00080935 29900 0.006533148
30900 0.001717768 28800 0.0272968 28600 0.006639172 28200
0.001303199 30500 0.000809141 29900 0.006517482
30900 0.001717356 28800 0.02687808 28600 0.006603502 28200
0.001302591 30500 0.000808929 29900 0.006501862
30900 0.001716938 28800 0.02646888 28600 0.0065681 28200
0.001301996 30500 0.000808718 29900 0.006486315
30900 0.001716511 28800 0.02606904 28600 0.006533009 28200
0.001301377 30500 0.000808504 29900 0.006470853
30900 0.001716116 28800 0.02567835 28600 0.006498173 28200
0.001300744 30500 0.000808286 29900 0.00645541
30900 0.001715669 28800 0.02529659 28600 0.006463656 28200
0.001300095 30500 0.000808076 29900 0.006440023
30900 0.001715209 28800 0.02492355 28600 0.006429404 28200
0.00129943 30500 0.000807869 29900 0.006424696
30900 0.001714721 28800 0.02455888 28600 0.006395439 28200
0.001298763 30500 0.000807654 29900 0.006409406
30900 0.001714271 28800 0.02420229 28600 0.006361752 28200
0.001298121 30500 0.000807438 29900 0.006394177
30900 0.001713814 28800 0.02385347 28600 0.006328343 28200
0.001297461 30500 0.000807217 30000 0.006379035
30900 0.001713337 28800 0.02351215 28600 0.006295228 28200
0.001296807 30500 0.000807011 30000 0.006363941
30900 0.001712865 28800 0.02317811 28600 0.006262346 28200
0.001296138 30500 0.000806802 30000 0.006348884
30900 0.001712378 28800 0.02285118 28600 0.006229719 28200
0.001295428 30500 0.000806603 30000 0.006333878
30900 0.001711907 28800 0.02253128 28600 0.006197363 28200
0.001294661 30500 0.000806394 30000 0.00631893
30900 0.001711397 28800 0.02221808 28600 0.006165284 28200
0.001293808 30500 0.000806207 30000 0.006304041
30900 0.001710912 28800 0.02191142 28600 0.00613344 28200
0.001292879 30500 0.00080601 30000 0.006289199
30900 0.001710408 28800 0.02161114 28600 0.006101849 28200
0.001291814 30500 0.000805809 30000 0.006274377
30900 0.001709906 28800 0.02131703 28600 0.006070526 28200
0.00129064 30500 0.000805609 30000 0.006259642
30900 0.00170939 28900 0.02102888 28600 0.00603945 28200
0.001289369 30500 0.000805411 30000 0.006244957
30900 0.001708894 28900 0.02074652 28600 0.006008595 28200
0.001287964 30500 0.000805205 30000 0.006230306
30900 0.001708371 28900 0.02046976 28700 0.005977997 28200
0.001286442 30500 0.000805007 30000 0.006215721
30900 0.001707866 28900 0.02019849 28700 0.00594764 28200
0.001284797 30500 0.000804806 30000 0.006201183
30900 0.001707341 28900 0.0199326 28700 0.005917518 28200
0.001283007 30500 0.000804615 30000 0.00618673
30900 0.001706854 28900 0.01967189 28700 0.005887619 28200
0.001281066 30500 0.000804421 30000 0.006172305
30900 0.001706327 28900 0.01941622 28700 0.005857954 28300
0.001278989 30500 0.000804231 30000 0.006157917

30900 0.001705815 28900 0.0191654 28700 0.005828505 28300
0.001276749 30500 0.000804043 30000 0.006143573
30900 0.001705292 28900 0.01891926 28700 0.005799276 28300
0.001274369 30500 0.000803837 30000 0.006129315
30900 0.001704781 28900 0.01867775 28700 0.005770287 28300
0.001271852 30500 0.000803639 30000 0.006115105
30900 0.00170428 28900 0.01844073 28700 0.005741511 28300
0.001269187 30500 0.000803426 30000 0.006100945
30900 0.00170377 28900 0.01820819 28700 0.005712945 28300
0.001266403 30500 0.000803225 30000 0.006086842
30900 0.001703256 28900 0.01798006 28700 0.005684604 28300
0.001263501 30500 0.000803017 30000 0.006072811
30900 0.001702747 28900 0.01775628 28700 0.005656452 28300
0.001260458 30500 0.000802814 30000 0.006058803
30900 0.001702265 28900 0.01753681 28700 0.005628513 28300
0.0012573 30500 0.000802598 30000 0.006044875
30900 0.001701745 28900 0.01732144 28700 0.005600779 28300
0.001253978 30500 0.000802397 30000 0.006030969
30900 0.00170125 28900 0.01711005 28700 0.005573252 28300
0.001250501 30500 0.000802189 30000 0.006017123
30900 0.001700755 28900 0.01690255 28700 0.005545934 28300
0.001246814 30600 0.000801967 30000 0.006003325
30900 0.001700254 28900 0.0166988 28700 0.005518851 28300
0.00124293 30600 0.000801755 30000 0.005989593
30900 0.00169975 28900 0.01649866 28700 0.00549193 28300
0.00123883 30600 0.000801539 30000 0.005975888
30900 0.001699276 28900 0.01630204 28700 0.005465247 28300
0.001234511 30600 0.000801309 30000 0.005962227
30900 0.001698772 28900 0.01610887 28700 0.005438724 28300
0.001229993 30600 0.000801093 30000 0.005948642
30900 0.001698295 28900 0.01591907 28700 0.005412426 28300
0.001225239 30600 0.000800872 30000 0.005935107
30900 0.001697816 28900 0.01573263 28700 0.005386342 28300
0.001220295 30600 0.000800657 30000 0.00592165
30900 0.001697354 28900 0.01554947 28700 0.005360431 28300
0.001215141 30600 0.000800439 30000 0.005908238
30900 0.001696884 28900 0.01536959 28700 0.005334748 28300
0.001209814 30600 0.000800222 30000 0.005894834
30900 0.001696399 28900 0.0151929 28700 0.005309205 28300
0.001204324 30600 0.000800009 30000 0.005881498
30900 0.001695915 28900 0.01501929 28700 0.005283893 28300
0.00119868 30600 0.000799796 30000 0.005868212
30900 0.001695462 28900 0.0148487 28700 0.00525876 28300
0.001192889 30600 0.000799586 30000 0.005854971
30900 0.001695017 28900 0.01468096 28700 0.005233813 28300
0.001186966 30600 0.000799371 30000 0.005841787
30900 0.001694549 28900 0.014516 28700 0.005209095 28300
0.001180915 30600 0.000799175 30000 0.005828677
30900 0.001694128 28900 0.01435377 28700 0.005184541 28300
0.001174765 30600 0.000798968 30000 0.005815578
30900 0.001693668 28900 0.0141942 28700 0.005160156 28300
0.001168531 30600 0.000798767 30000 0.005802535
30900 0.001693232 28900 0.01403735 28700 0.00513596 28300
0.001162175 30600 0.000798566 30000 0.005789554
30900 0.001692765 28900 0.0138831 28700 0.00511194 28300
0.001155764 30600 0.000798386 30000 0.005776626
30900 0.001692324 28900 0.01373144 28700 0.005088113 28300
0.001149276 30600 0.000798194 30000 0.005763739
30900 0.001691875 28900 0.01358236 28700 0.005064448 28300
0.001142736 30600 0.000798008 30000 0.005750895
30900 0.001691436 28900 0.0134358 28700 0.005040998 28300
0.001136179 30600 0.000797821 30000 0.005738107
30900 0.001690983 28900 0.01329172 28700 0.005017678 28300
0.001129609 30600 0.000797637 30000 0.005725353
30900 0.001690558 28900 0.01315006 28700 0.004994559 28300
0.001123055 30600 0.000797454 30000 0.005712638
30900 0.001690096 28900 0.01301074 28700 0.004971584 28300
0.001116582 30600 0.000797276 30000 0.005699948

30900 0.001689653 28900 0.01287367 28700 0.004948791 28300
0.001110146 30600 0.000797102 30000 0.005687315
30900 0.001689198 28900 0.01273878 28700 0.004926149 28300
0.001103788 30600 0.000796942 30000 0.005674763
30900 0.001688769 28900 0.01260598 28700 0.004903679 28300
0.001097518 30600 0.000796774 30000 0.005662237
30900 0.001688314 28900 0.01247524 28700 0.004881334 28300
0.001091324 30600 0.000796607 30000 0.005649751
30900 0.001687871 28900 0.0123465 28700 0.004859189 28300
0.001085192 30600 0.000796454 30000 0.005637283
31000 0.001687411 28900 0.01221969 28700 0.004837188 28300
0.001079156 30600 0.000796298 30000 0.005624882
31000 0.001686956 28900 0.01209482 28700 0.004815327 28300
0.001073203 30600 0.000796152 30000 0.00561252
31000 0.001686518 28900 0.0119718 28700 0.004793643 28300
0.001067348 30600 0.000795992 30000 0.005600194
31000 0.001686082 28900 0.01185062 28700 0.00477212 28300
0.001061613 30600 0.000795845 30000 0.005587925
31000 0.001685628 28900 0.01173126 28700 0.004750747 28300
0.001056013 30600 0.000795694 30000 0.005575729
31000 0.001685197 28900 0.0116137 28700 0.004729528 28300
0.001050571 30600 0.000795557 30000 0.005563544
31000 0.001684755 28900 0.01149794 28700 0.004708458 28300
0.001045288 30600 0.000795417 30000 0.00555141
31000 0.001684288 28900 0.01138392 28700 0.004687527 28300
0.001040162 30600 0.000795295 30000 0.00553929
31000 0.00168386 28900 0.01127156 28700 0.004666748 28300
0.00103521 30600 0.000795161 30000 0.005527233
31000 0.001683421 28900 0.0111609 28700 0.004646129 28300
0.001030426 30600 0.000795026 30100 0.005515225
31000 0.001682966 28900 0.01105193 28700 0.004625653 28300
0.001025781 30600 0.000794891 30100 0.005503242
31000 0.001682515 28900 0.01094457 28700 0.004605305 28300
0.001021312 30600 0.000794764 30100 0.005491295
31000 0.001682075 28900 0.01083878 28700 0.004585109 28300
0.001016974 30600 0.000794634 30100 0.005479402
31000 0.001681623 28900 0.01073455 28700 0.00456505 28300
0.001012795 30600 0.000794508 30100 0.005467529
31000 0.001681175 28900 0.01063174 28700 0.004545112 28300
0.001008755 30600 0.000794376 30100 0.0054557
31000 0.001680718 28900 0.01053039 28700 0.00452532 28300
0.001004877 30600 0.000794256 30100 0.005443962
31000 0.001680294 28900 0.01043048 28700 0.00450565 28300
0.001001147 30600 0.00079412 30100 0.005432235
31000 0.001679829 28900 0.01033196 28700 0.004486124 28300
0.000997579 30600 0.000793994 30100 0.005420527
31000 0.00167938 29000 0.01023489 28700 0.004466739 28300
0.000994174 30600 0.000793869 30100 0.005408851
31000 0.001678937 29000 0.01013924 28700 0.004447469 28300
0.000990929 30600 0.00079374 30100 0.005397219
31000 0.001678483 29000 0.010045 28800 0.004428323 28300
0.000987808 30600 0.000793623 30100 0.005385608
31000 0.001678043 29000 0.009952142 28800 0.004409329 28300
0.000984799 30600 0.000793485 30100 0.005374065
31000 0.001677577 29000 0.009860601 28800 0.004390484 28300
0.0009819 30600 0.000793363 30100 0.005362543
31000 0.001677141 29000 0.009770333 28800 0.00437175 28300
0.000979084 30600 0.000793241 30100 0.005351063
31000 0.001676676 29000 0.009681308 28800 0.004353135 28400
0.000976352 30600 0.000793106 30100 0.005339626
31000 0.001676232 29000 0.009593495 28800 0.004334643 28400
0.000973696 30600 0.000792971 30100 0.005328238
31000 0.001675741 29000 0.009506792 28800 0.004316254 28400
0.000971127 30600 0.000792845 30100 0.005316876
31000 0.001675286 29000 0.009421213 28800 0.004298007 28400
0.000968644 30600 0.000792703 30100 0.005305538
31000 0.001674825 29000 0.009336757 28800 0.004279867 28400
0.000966239 30600 0.000792565 30100 0.005294238

31000 0.001674343 29000 0.009253382 28800 0.004261867 28400
0.000963888 30600 0.000792417 30100 0.005282968
31000 0.0016739 29000 0.009171104 28800 0.004243975 28400
0.000961615 30600 0.000792277 30100 0.005271732
31000 0.001673424 29000 0.00908991 28800 0.004226188 28400
0.000959386 30600 0.000792127 30100 0.00526053
31000 0.001672936 29000 0.009009812 28800 0.004208535 28400
0.00095723 30600 0.000791974 30100 0.00524937
31000 0.001672472 29000 0.008930789 28800 0.004190987 28400
0.000955103 30600 0.00079181 30100 0.00523823
31000 0.001671988 29000 0.00885284 28800 0.00417355 28400
0.000953061 30600 0.000791659 30100 0.005227176
31000 0.001671528 29000 0.008775954 28800 0.004156223 28400
0.000951062 30700 0.000791502 30100 0.005216128
31000 0.00167106 29000 0.008700098 28800 0.004139001 28400
0.000949128 30700 0.000791352 30100 0.005205108
31000 0.001670594 29000 0.008625233 28800 0.004121885 28400
0.000947264 30700 0.000791199 30100 0.005194122
31000 0.001670113 29000 0.008551315 28800 0.004104877 28400
0.000945466 30700 0.000791049 30100 0.005183181
31000 0.001669647 29000 0.008478329 28800 0.004087988 28400
0.000943705 30700 0.000790901 30100 0.005172272
31000 0.001669202 29000 0.008406233 28800 0.004071201 28400
0.000941983 30700 0.000790739 30100 0.005161407
31000 0.001668723 29000 0.008335028 28800 0.004054552 28400
0.000940309 30700 0.000790605 30100 0.005150572
31000 0.001668297 29000 0.008264757 28800 0.004037992 28400
0.000938646 30700 0.000790452 30100 0.005139787
31000 0.001667852 29000 0.008195404 28800 0.004021512 28400
0.000936994 30700 0.000790309 30100 0.005129037
31000 0.001667414 29000 0.008126996 28800 0.00400515 28400
0.000935346 30700 0.000790171 30100 0.005118322
31000 0.001666951 29000 0.008059475 28800 0.003988909 28400
0.000933705 30700 0.000790034 30100 0.005107633
31000 0.001666513 29000 0.007992803 28800 0.003972742 28400
0.000932077 30700 0.000789897 30100 0.005096994
31000 0.001666074 29000 0.007926978 28800 0.00395668 28400
0.000930456 30700 0.000789759 30100 0.005086378
31000 0.001665638 29000 0.007862015 28800 0.003940728 28400
0.000928861 30700 0.000789622 30100 0.005075802
31000 0.001665206 29000 0.007797856 28800 0.003924878 28400
0.000927324 30700 0.000789486 30100 0.005065256
31000 0.001664792 29000 0.007734492 28800 0.003909129 28400
0.0009258 30700 0.000789347 30100 0.00505476
31000 0.001664368 29000 0.007671905 28800 0.003893482 28400
0.000924324 30700 0.000789235 30100 0.005044302
31000 0.001663945 29000 0.007610078 28800 0.003877922 28400
0.000922853 30700 0.000789098 30100 0.005033875
31000 0.00166351 29000 0.007548992 28800 0.003862471 28400
0.000921379 30700 0.000788973 30100 0.005023521
31000 0.001663135 29000 0.007488637 28800 0.003847118 28400
0.00091988 30700 0.000788848 30100 0.005013183
31000 0.001662734 29000 0.007429016 28800 0.003831857 28400
0.000918357 30700 0.000788726 30100 0.005002856
31000 0.001662335 29000 0.007370113 28800 0.003816685 28400
0.000916807 30700 0.000788611 30100 0.004992574
31000 0.001661908 29000 0.007311889 28800 0.003801617 28400
0.000915248 30700 0.000788495 30100 0.004982346
31000 0.00166152 29000 0.007254364 28800 0.003786641 28400
0.000913681 30700 0.000788384 30100 0.004972138
31000 0.001661148 29000 0.007197499 28800 0.003771754 28400
0.000912103 30700 0.000788267 30100 0.004961973
31000 0.001660769 29000 0.007141298 28800 0.003756983 28400
0.000910526 30700 0.000788169 30100 0.004951832
31000 0.001660411 29000 0.007085759 28800 0.00374228 28400
0.000908964 30700 0.000788064 30100 0.004941742
31000 0.00166003 29000 0.007030888 28800 0.003727648 28400
0.00090737 30700 0.00078797 30100 0.004931674

31000 0.001659661 29000 0.006976735 28800 0.003713124 28400
0.000905795 30700 0.000787874 30100 0.004921648
31100 0.001659287 29000 0.006923209 28800 0.003698696 28400
0.000904193 30700 0.000787792 30100 0.004911636
31100 0.001658906 29000 0.006870316 28800 0.003684343 28400
0.000902613 30700 0.00078771 30100 0.004901662
31100 0.001658534 29000 0.00681806 28800 0.003670071 28400
0.000900994 30700 0.000787622 30100 0.004891708
31100 0.001658157 29000 0.006766471 28800 0.003655899 28400
0.00089938 30700 0.000787542 30100 0.004881782
31100 0.001657791 29000 0.006715524 28800 0.003641789 28400
0.000897763 30700 0.000787469 30100 0.004871886
31100 0.001657433 29000 0.00666518 28800 0.003627786 28400
0.000896155 30700 0.000787386 30100 0.004862025
31100 0.001657051 29000 0.00661541 28800 0.003613854 28400
0.000894555 30700 0.000787314 30100 0.004852196
31100 0.001656689 29000 0.006566154 28800 0.003599995 28400
0.000892974 30700 0.000787231 30100 0.004842396
31100 0.001656306 29000 0.006517388 28800 0.003586229 28400
0.000891401 30700 0.000787154 30100 0.004832674
31100 0.001655977 29000 0.006469068 28800 0.00357253 28400
0.000889815 30700 0.000787083 30200 0.004822952
31100 0.001655616 29000 0.006421217 28800 0.00355892 28400
0.000888235 30700 0.000787009 30200 0.00481323
31100 0.001655262 29000 0.006373852 28800 0.003545381 28400
0.000886625 30700 0.000786942 30200 0.00480356
31100 0.001654885 29000 0.006326988 28800 0.003531926 28400
0.00088499 30700 0.000786857 30200 0.004793936
31100 0.001654534 29000 0.006280654 28800 0.003518551 28400
0.000883324 30700 0.000786778 30200 0.004784314
31100 0.001654213 29000 0.006234872 28800 0.003505245 28400
0.000881621 30700 0.000786693 30200 0.004774751
31100 0.00165387 29000 0.006189626 28800 0.003492047 28400
0.00087991 30700 0.000786609 30200 0.004765209
31100 0.001653559 29000 0.006144914 28800 0.003478916 28400
0.000878174 30700 0.000786508 30200 0.004755705
31100 0.001653233 29000 0.006100728 28800 0.003465879 28400
0.000876421 30700 0.000786416 30200 0.004746234
31100 0.001652931 29100 0.00605709 28800 0.003452898 28400
0.000874673 30700 0.000786331 30200 0.004736792
31100 0.001652607 29100 0.006013903 28800 0.003439994 28400
0.00087295 30700 0.00078624 30200 0.004727363
31100 0.001652296 29100 0.005971182 28900 0.003427176 28400
0.000871248 30700 0.000786148 30200 0.00471799
31100 0.001651991 29100 0.005928915 28900 0.00341444 28400
0.000869569 30700 0.000786053 30200 0.004708628
31100 0.001651679 29100 0.005887078 28900 0.003401788 28400
0.000867899 30700 0.000785961 30200 0.004699317
31100 0.001651372 29100 0.005845669 28900 0.003389181 28400
0.00086625 30700 0.000785859 30200 0.00469002
31100 0.001651099 29100 0.005804683 28900 0.003376672 28500
0.000864598 30700 0.000785756 30200 0.004680751
31100 0.001650801 29100 0.005764148 28900 0.003364241 28500
0.000862923 30700 0.000785661 30200 0.004671524
31100 0.001650527 29100 0.005724076 28900 0.003351885 28500
0.000861214 30700 0.000785553 30200 0.004662336
31100 0.001650251 29100 0.005684502 28900 0.003339586 28500
0.000859475 30700 0.000785463 30200 0.004653187
31100 0.001650002 29100 0.005645406 28900 0.003327369 28500
0.000857727 30700 0.000785364 30200 0.00464405
31100 0.00164974 29100 0.005606794 28900 0.003315196 28500
0.00085599 30700 0.000785275 30200 0.004634913
31100 0.001649505 29100 0.005568646 28900 0.003303111 28500
0.000854284 30700 0.000785188 30200 0.004625842
31100 0.001649243 29100 0.005530929 28900 0.003291091 28500
0.000852598 30700 0.000785107 30200 0.004616801
31100 0.001649012 29100 0.005493591 28900 0.003279149 28500
0.00085094 30700 0.000785027 30200 0.004607778

31100 0.001648794 29100 0.005456625 28900 0.003267259 28500
0.000849287 30700 0.000784947 30200 0.004598803
31100 0.001648572 29100 0.005419988 28900 0.003255463 28500
0.000847632 30700 0.000784864 30200 0.004589832
31100 0.00164837 29100 0.005383688 28900 0.003243745 28500
0.000845961 30800 0.000784788 30200 0.004580939
31100 0.001648139 29100 0.005347736 28900 0.003232055 28500
0.000844264 30800 0.000784716 30200 0.004572049
31100 0.001647943 29100 0.005312157 28900 0.003220443 28500
0.000842551 30800 0.000784649 30200 0.004563197
31100 0.001647709 29100 0.005276898 28900 0.003208888 28500
0.000840846 30800 0.000784571 30200 0.00455435
31100 0.001647501 29100 0.005241977 28900 0.00319741 28500
0.000839162 30800 0.000784506 30200 0.004545556
31100 0.001647293 29100 0.005207414 28900 0.003186016 28500
0.000837527 30800 0.000784438 30200 0.004536777
31100 0.001647087 29100 0.005173174 28900 0.003174648 28500
0.000835942 30800 0.000784373 30200 0.004528034
31100 0.001646871 29100 0.005139215 28900 0.003163379 28500
0.000834414 30800 0.000784304 30200 0.004519308
31100 0.001646694 29100 0.005105544 28900 0.003152177 28500
0.000832956 30800 0.000784235 30200 0.004510608
31100 0.001646483 29100 0.005072184 28900 0.003140989 28500
0.000831523 30800 0.000784162 30200 0.004501969
31100 0.001646294 29100 0.005039135 28900 0.003129898 28500
0.000830095 30800 0.000784088 30200 0.004493319
31100 0.001646074 29100 0.005006433 28900 0.003118855 28500
0.000828654 30800 0.000784019 30200 0.004484761
31100 0.001645917 29100 0.004974026 28900 0.003107882 28500
0.000827198 30800 0.00078395 30200 0.004476201
31100 0.00164571 29100 0.004941988 28900 0.003096965 28500
0.000825721 30800 0.000783882 30200 0.004467626
31100 0.001645524 29100 0.004910264 28900 0.003086117 28500
0.000824229 30800 0.000783824 30200 0.004459097
31100 0.0016453 29100 0.004878855 28900 0.003075319 28500
0.000822731 30800 0.000783751 30200 0.004450587
31100 0.001645117 29100 0.004847742 28900 0.003064559 28500
0.000821239 30800 0.000783689 30200 0.004442099
31100 0.001644936 29100 0.004816933 28900 0.003053877 28500
0.000819741 30800 0.00078362 30200 0.00443365
31100 0.001644751 29100 0.004786419 28900 0.003043274 28500
0.000818286 30800 0.000783545 30200 0.004425235
31100 0.001644574 29100 0.004756202 28900 0.003032729 28500
0.000816842 30800 0.000783465 30200 0.004416842
31100 0.0016444 29100 0.004726285 28900 0.003022224 28500
0.000815416 30800 0.000783397 30200 0.004408481
31100 0.001644232 29100 0.004696728 28900 0.00301177 28500
0.000813995 30800 0.000783335 30200 0.004400129
31100 0.001644048 29100 0.004667452 28900 0.003001363 28500
0.00081258 30800 0.000783262 30200 0.004391811
31100 0.001643881 29100 0.004638462 28900 0.00299102 28500
0.000811181 30800 0.000783185 30200 0.004383526
31100 0.001643695 29100 0.004609783 28900 0.002980743 28500
0.000809778 30800 0.000783112 30200 0.004375238
31100 0.001643516 29100 0.004581382 28900 0.002970517 28500
0.000808364 30800 0.000783032 30200 0.004367006
31100 0.001643331 29100 0.004553241 28900 0.002960365 28500
0.000806957 30800 0.00078295 30200 0.004358762
31100 0.001643161 29100 0.004525373 28900 0.002950247 28500
0.000805526 30800 0.000782871 30200 0.004350555
31100 0.00164298 29100 0.004497766 28900 0.002940197 28500
0.000804056 30800 0.000782787 30200 0.004342378
31200 0.001642811 29100 0.004470406 28900 0.002930185 28500
0.000802554 30800 0.000782703 30200 0.004334233
31200 0.001642633 29100 0.004443286 28900 0.002920246 28500
0.000801039 30800 0.000782624 30200 0.004326121
31200 0.001642486 29100 0.004416393 28900 0.002910353 28500
0.000799501 30800 0.000782538 30200 0.004318031

31200 0.001642322 29100 0.004389759 28900 0.002900533 28500
0.000797968 30800 0.000782462 30200 0.004309941
31200 0.001642161 29100 0.004363384 28900 0.002890758 28500
0.000796441 30800 0.000782385 30200 0.00430188
31200 0.001641999 29100 0.00433726 28900 0.002881032 28500
0.000794913 30800 0.000782308 30200 0.00429386
31200 0.001641845 29100 0.004311398 28900 0.002871371 28500
0.000793384 30800 0.000782226 30200 0.004285857
31200 0.001641701 29100 0.004285768 28900 0.002861751 28500
0.000791841 30800 0.000782148 30200 0.0042779
31200 0.001641527 29100 0.004260376 28900 0.002852216 28500
0.0007903 30800 0.000782061 30200 0.004269967
31200 0.00164139 29100 0.004235205 28900 0.002842727 28500
0.000788761 30800 0.000781986 30300 0.004262082
31200 0.001641231 29100 0.004210272 28900 0.002833281 28500
0.000787231 30800 0.000781901 30300 0.004254223
31200 0.001641106 29100 0.004185614 28900 0.002823889 28500
0.000785727 30800 0.000781831 30300 0.004246363
31200 0.00164095 29100 0.004161133 28900 0.002814549 28500
0.000784243 30800 0.00078175 30300 0.004238527
31200 0.001640789 29100 0.004136849 28900 0.002805254 28500
0.000782806 30800 0.000781674 30300 0.004230731
31200 0.001640641 29100 0.00411277 28900 0.002796027 28500
0.000781407 30800 0.000781604 30300 0.004222946
31200 0.001640498 29100 0.004088889 28900 0.002786843 28500
0.000780031 30800 0.000781531 30300 0.004215192
31200 0.001640343 29100 0.004065183 28900 0.002777709 28500
0.000778707 30800 0.000781458 30300 0.00420745
31200 0.001640206 29100 0.004041659 28900 0.002768625 28500
0.0007774 30800 0.000781393 30300 0.004199758
31200 0.001640045 29200 0.004018317 28900 0.002759592 28500
0.000776122 30800 0.000781317 30300 0.004192074
31200 0.001639901 29200 0.003995168 28900 0.002750593 28500
0.00077486 30800 0.000781252 30300 0.004184418
31200 0.001639741 29200 0.00397223 29000 0.002741651 28500
0.000773632 30800 0.000781181 30300 0.004176823
31200 0.001639641 29200 0.003949493 29000 0.002732753 28500
0.000772405 30800 0.000781116 30300 0.004169228
31200 0.001639506 29200 0.003926968 29000 0.002723886 28500
0.000771208 30800 0.000781041 30300 0.004161629
31200 0.001639359 29200 0.003904634 29000 0.00271507 28500
0.000770023 30800 0.000780979 30300 0.004154075
31200 0.001639197 29200 0.003882502 29000 0.002706303 28600
0.000768823 30800 0.000780915 30300 0.004146507
31200 0.001639065 29200 0.003860554 29000 0.002697561 28600
0.000767643 30800 0.000780855 30300 0.004138987
31200 0.001638933 29200 0.003838793 29000 0.002688869 28600
0.000766426 30800 0.000780789 30300 0.004131504
31200 0.001638795 29200 0.003817237 29000 0.00268023 28600
0.000765209 30800 0.000780729 30300 0.004124014
31200 0.001638698 29200 0.003795863 29000 0.002671627 28600
0.00076397 30800 0.000780674 30300 0.004116538
31200 0.001638555 29200 0.003774684 29000 0.002663061 28600
0.0007627 30800 0.000780615 30300 0.004109131
31200 0.001638456 29200 0.003753708 29000 0.002654533 28600
0.000761393 30800 0.000780569 30300 0.004101673
31200 0.001638335 29200 0.003732871 29000 0.002646042 28600
0.000760053 30800 0.000780512 30300 0.004094269
31200 0.001638234 29200 0.003712192 29000 0.002637591 28600
0.000758713 30800 0.000780461 30300 0.00408689
31200 0.001638114 29200 0.003691711 29000 0.002629183 28600
0.000757359 30800 0.000780407 30300 0.004079485
31200 0.001638014 29200 0.003671425 29000 0.002620817 28600
0.000756007 30800 0.000780355 30300 0.004072146
31200 0.001637898 29200 0.003651317 29000 0.00261249 28600
0.000754677 30900 0.000780306 30300 0.004064818
31200 0.001637813 29200 0.003631391 29000 0.002604218 28600
0.000753364 30900 0.000780252 30300 0.00405748

31200 0.001637685 29200 0.003611627 29000 0.002595979 28600
0.000752069 30900 0.000780197 30300 0.004050188
31200 0.001637619 29200 0.00359202 29000 0.002587785 28600
0.00075077 30900 0.000780147 30300 0.004042882
31200 0.001637524 29200 0.003572564 29000 0.002579627 28600
0.00074949 30900 0.00078009 30300 0.004035639
31200 0.001637467 29200 0.003553236 29000 0.002571515 28600
0.000748191 30900 0.000780047 30300 0.004028395
31200 0.001637396 29200 0.003534066 29000 0.002563434 28600
0.000746881 30900 0.000779995 30300 0.004021168
31200 0.00163732 29200 0.003515072 29000 0.002555385 28600
0.000745556 30900 0.000779949 30300 0.004013942
31200 0.001637243 29200 0.003496253 29000 0.002547395 28600
0.000744203 30900 0.0007799 30300 0.004006736
31200 0.001637196 29200 0.003477636 29000 0.002539437 28600
0.000742831 30900 0.000779847 30300 0.003999575
31200 0.001637152 29200 0.003459185 29000 0.002531529 28600
0.000741457 30900 0.000779797 30300 0.003992438
31200 0.001637109 29200 0.003440905 29000 0.002523659 28600
0.000740106 30900 0.000779748 30300 0.003985325
31200 0.001637064 29200 0.00342275 29000 0.002515837 28600
0.000738789 30900 0.000779708 30300 0.0039782
31200 0.001637027 29200 0.003404736 29000 0.002508032 28600
0.000737482 30900 0.000779658 30300 0.003971132
31200 0.001637009 29200 0.003386857 29000 0.002500276 28600
0.000736232 30900 0.000779621 30300 0.00396407
31200 0.001636962 29200 0.003369092 29000 0.002492556 28600
0.000735011 30900 0.000779574 30300 0.003957003
31200 0.001636931 29200 0.00335147 29000 0.002484858 28600
0.000733826 30900 0.000779533 30300 0.003949992
31200 0.001636895 29200 0.003334005 29000 0.002477222 28600
0.000732676 30900 0.000779482 30300 0.003942976
31200 0.001636863 29200 0.003316708 29000 0.002469614 28600
0.000731549 30900 0.000779436 30300 0.003935988
31200 0.001636839 29200 0.00329957 29000 0.002462046 28600
0.000730423 30900 0.000779386 30300 0.003928995
31200 0.001636816 29200 0.003282584 29000 0.002454535 28600
0.000729308 30900 0.000779334 30300 0.003922051
31200 0.001636778 29200 0.003265722 29000 0.002447055 28600
0.0007282 30900 0.000779285 30300 0.003915117
31200 0.00163677 29200 0.003249011 29000 0.002439607 28600
0.000727068 30900 0.000779232 30300 0.003908197
31200 0.001636747 29200 0.003232429 29000 0.002432204 28600
0.000725937 30900 0.000779174 30300 0.003901312
31200 0.001636743 29200 0.003215971 29000 0.002424839 28600
0.000724798 30900 0.000779129 30300 0.003894443
31200 0.001636741 29200 0.003199658 29000 0.002417516 28600
0.000723648 30900 0.000779066 30300 0.003887546
31200 0.001636706 29200 0.00318348 29000 0.002410211 28600
0.000722508 30900 0.000779014 30300 0.003880655
31200 0.001636691 29200 0.003167396 29000 0.002402969 28600
0.000721359 30900 0.000778959 30300 0.003873802
31200 0.001636688 29200 0.003151436 29000 0.002395753 28600
0.000720217 30900 0.000778897 30300 0.003866979
31300 0.001636699 29200 0.00313556 29000 0.002388572 28600
0.000719094 30900 0.000778837 30300 0.003860168
31300 0.001636686 29200 0.003119795 29000 0.002381438 28600
0.000717983 30900 0.000778776 30300 0.003853408
31300 0.001636707 29200 0.003104147 29000 0.002374338 28600
0.000716893 30900 0.000778719 30300 0.003846617
31300 0.001636713 29200 0.003088601 29000 0.002367262 28600
0.000715813 30900 0.000778668 30300 0.00383988
31300 0.001636724 29200 0.003073196 29000 0.002360233 28600
0.000714744 30900 0.000778623 30300 0.003833141
31300 0.001636721 29200 0.003057874 29000 0.002353227 28600
0.000713692 30900 0.000778574 30300 0.003826373
31300 0.001636723 29200 0.003042652 29000 0.00234626 28600
0.000712645 30900 0.000778537 30300 0.003819674

31300 0.001636723 29200 0.003027528 29000 0.002339339 28600
0.000711619 30900 0.000778488 30300 0.003812965
31300 0.001636728 29200 0.003012512 29000 0.002332446 28600
0.000710582 30900 0.000778451 30300 0.003806286
31300 0.001636727 29200 0.002997621 29000 0.002325593 28600
0.000709552 30900 0.00077842 30400 0.003799614
31300 0.001636747 29200 0.002982844 29000 0.002318772 28600
0.00070852 30900 0.000778396 30400 0.003792968
31300 0.001636742 29200 0.002968176 29000 0.002311969 28600
0.00070749 30900 0.000778367 30400 0.003786313
31300 0.001636764 29200 0.002953642 29000 0.002305221 28600
0.00070643 30900 0.000778353 30400 0.003779693
31300 0.001636753 29200 0.002939203 29000 0.002298485 28600
0.000705375 30900 0.000778333 30400 0.003773079
31300 0.001636778 29200 0.002924874 29000 0.002291778 28600
0.000704299 30900 0.000778327 30400 0.003766517
31300 0.00163679 29200 0.00291068 29000 0.002285095 28600
0.000703232 30900 0.000778313 30400 0.003759899
31300 0.001636801 29200 0.002896622 29000 0.002278446 28600
0.000702155 30900 0.000778313 30400 0.003753334
31300 0.001636792 29200 0.00288268 29000 0.002271851 28600
0.000701064 30900 0.000778318 30400 0.003746805
31300 0.001636801 29300 0.002868888 29000 0.002265276 28600
0.00069998 30900 0.000778311 30400 0.003740279
31300 0.00163683 29300 0.002855231 29000 0.002258731 28600
0.000698896 30900 0.000778314 30400 0.003733789
31300 0.00163686 29300 0.002841684 29100 0.00225223 28600
0.000697819 30900 0.000778317 30400 0.003727332
31300 0.001636894 29300 0.002828267 29100 0.002245765 28600
0.00069676 30900 0.000778324 30400 0.003720878
31300 0.001636917 29300 0.002814929 29100 0.002239311 28600
0.000695699 30900 0.000778334 30400 0.003714443
31300 0.001636959 29300 0.002801699 29100 0.002232893 28600
0.000694665 30900 0.000778355 30400 0.003708041
31300 0.00163697 29300 0.00278856 29100 0.002226508 28700
0.000693641 30900 0.000778377 30400 0.003701638
31300 0.001636995 29300 0.002775455 29100 0.002220141 28700
0.000692618 30900 0.000778403 30400 0.003695228
31300 0.001637031 29300 0.00276242 29100 0.002213825 28700
0.000691607 30900 0.000778424 30400 0.003688873
31300 0.001637067 29300 0.002749486 29100 0.00220753 28700
0.000690578 30900 0.000778452 30400 0.003682526
31300 0.001637078 29300 0.002736593 29100 0.002201259 28700
0.000689551 30900 0.000778475 30400 0.003676192
31300 0.001637114 29300 0.002723768 29100 0.002195017 28700
0.000688502 30900 0.000778512 30400 0.003669874
31300 0.001637138 29300 0.002711028 29100 0.002188805 28700
0.000687455 30900 0.000778548 30400 0.003663605
31300 0.001637194 29300 0.002698322 29100 0.002182625 28700
0.000686394 30900 0.00077858 30400 0.003657359
31300 0.001637229 29300 0.002685746 29100 0.002176458 28700
0.000685348 30900 0.000778617 30400 0.003651137
31300 0.001637284 29300 0.002673258 29100 0.002170328 28700
0.000684321 30900 0.00077867 30400 0.003644949
31300 0.001637317 29300 0.0026609 29100 0.002164226 28700
0.00068334 30900 0.0007787 30400 0.003638725
31300 0.001637355 29300 0.002648694 29100 0.002158144 28700
0.000682415 31000 0.000778759 30400 0.003632548
31300 0.001637397 29300 0.002636577 29100 0.002152099 28700
0.000681545 31000 0.000778815 30400 0.00362634
31300 0.001637434 29300 0.002624579 29100 0.002146071 28700
0.00068073 31000 0.000778854 30400 0.003620196
31300 0.001637491 29300 0.002612693 29100 0.002140074 28700
0.000679949 31000 0.000778901 30400 0.003614058
31300 0.001637542 29300 0.002600871 29100 0.002134119 28700
0.000679193 31000 0.000778955 30400 0.003607943
31300 0.001637614 29300 0.002589109 29100 0.002128185 28700
0.000678449 31000 0.000779013 30400 0.003601866

31300 0.00163766 29300 0.002577448 29100 0.002122275 28700
0.000677693 31000 0.000779061 30400 0.003595787
31300 0.001637726 29300 0.002565846 29100 0.002116398 28700
0.000676911 31000 0.000779115 30400 0.0035897
31300 0.001637774 29300 0.002554271 29100 0.002110557 28700
0.000676112 31000 0.000779165 30400 0.003583651
31300 0.001637823 29300 0.002542758 29100 0.002104732 28700
0.00067526 31000 0.000779225 30400 0.003577625
31300 0.001637874 29300 0.002531298 29100 0.002098952 28700
0.000674381 31000 0.000779261 30400 0.00357163
31300 0.001637925 29300 0.002519906 29100 0.002093202 28700
0.000673465 31000 0.000779311 30400 0.003565637
31300 0.001637979 29300 0.002508598 29100 0.002087464 28700
0.000672513 31000 0.000779346 30400 0.003559652
31300 0.001638028 29300 0.002497381 29100 0.002081765 28700
0.000671522 31000 0.000779389 30400 0.00355364
31300 0.001638038 29300 0.002486233 29100 0.002076083 28700
0.000670526 31000 0.000779421 30400 0.003547717
31300 0.001638106 29300 0.002475194 29100 0.002070429 28700
0.000669478 31000 0.000779452 30400 0.00354179
31300 0.001638134 29300 0.002464252 29100 0.002064802 28700
0.000668422 31000 0.000779482 30400 0.003535897
31300 0.001638188 29300 0.00245337 29100 0.002059193 28700
0.000667354 31000 0.000779522 30400 0.003530024
31300 0.001638234 29300 0.002442606 29100 0.002053625 28700
0.000666268 31000 0.00077954 30400 0.003524079
31300 0.001638278 29300 0.002431914 29100 0.002048077 28700
0.000665189 31000 0.000779573 30400 0.003518198
31300 0.001638312 29300 0.002421297 29100 0.002042549 28700
0.000664082 31000 0.000779592 30400 0.003512343
31300 0.001638349 29300 0.002410734 29100 0.002037041 28700
0.000662981 31000 0.000779601 30400 0.003506504
31300 0.0016384 29300 0.002400247 29100 0.002031562 28700
0.000661878 31000 0.000779621 30400 0.003500743
31300 0.001638426 29300 0.002389808 29100 0.002026126 28700
0.000660795 31000 0.00077963 30400 0.003494971
31300 0.001638494 29300 0.002379443 29100 0.002020692 28700
0.000659722 31000 0.000779649 30400 0.003489188
31300 0.001638524 29300 0.002369153 29100 0.002015279 28700
0.000658654 31000 0.000779653 30400 0.003483423
31300 0.001638595 29300 0.002358962 29100 0.002009886 28700
0.000657643 31000 0.000779656 30400 0.003477693
31300 0.001638632 29300 0.002348862 29100 0.002004524 28700
0.000656646 31000 0.00077966 30400 0.003471949
31300 0.001638662 29300 0.002338839 29100 0.001999182 28700
0.000655725 31000 0.000779669 30400 0.003466248
31400 0.001638704 29300 0.002328898 29100 0.001993867 28700
0.000654821 31000 0.000779664 30400 0.003460538
31400 0.001638756 29300 0.002319035 29100 0.001988563 28700
0.000653953 31000 0.000779658 30400 0.00345485
31400 0.001638813 29300 0.002309239 29100 0.001983293 28700
0.000653131 31000 0.000779652 30400 0.003449172
31400 0.00163888 29300 0.002299475 29100 0.001978037 28700
0.000652295 31000 0.000779641 30400 0.003443525
31400 0.001638901 29300 0.002289758 29100 0.001972808 28700
0.000651481 31000 0.000779627 30400 0.003437918
31400 0.001638974 29300 0.002280061 29100 0.001967597 28700
0.000650665 31000 0.000779604 30400 0.003432296
31400 0.001639029 29300 0.00227041 29100 0.001962415 28700
0.000649872 31000 0.000779571 30400 0.003426675
31400 0.001639126 29300 0.002260796 29100 0.001957245 28700
0.000649071 31000 0.000779552 30400 0.003421092
31400 0.001639216 29300 0.002251247 29100 0.001952093 28700
0.000648274 31000 0.000779522 30400 0.003415546
31400 0.001639293 29300 0.002241775 29100 0.001946964 28700
0.000647489 31000 0.000779497 30500 0.003410006
31400 0.001639357 29300 0.002232384 29100 0.001941866 28700
0.000646675 31000 0.000779461 30500 0.003404418

31400 0.001639447 29300 0.002223066 29100 0.001936778 28700
0.000645882 31000 0.000779425 30500 0.003398914
31400 0.001639557 29300 0.002213826 29100 0.001931712 28700
0.00064504 31000 0.000779384 30500 0.003393426
31400 0.00163967 29300 0.002204655 29100 0.001926674 28700
0.000644198 31000 0.000779348 30500 0.003387916
31400 0.0016398 29300 0.002195565 29100 0.001921654 28700
0.000643349 31000 0.000779316 30500 0.003382416
31400 0.001639916 29300 0.002186531 29100 0.001916649 28700
0.00064248 31000 0.000779267 30500 0.003376983
31400 0.00164007 29300 0.002177575 29100 0.001911668 28700
0.000641633 31000 0.000779226 30500 0.003371556
31400 0.001640184 29300 0.002168654 29100 0.001906701 28700
0.000640771 31000 0.000779197 30500 0.003366107
31400 0.001640321 29400 0.00215975 29100 0.001901768 28700
0.000639914 31000 0.000779159 30500 0.003360674
31400 0.001640446 29400 0.002150893 29100 0.001896849 28700
0.000639056 31000 0.000779121 30500 0.003355268
31400 0.001640594 29400 0.002142091 29200 0.00189196 28700
0.000638204 31000 0.000779081 30500 0.00334987
31400 0.001640732 29400 0.00213335 29200 0.001887088 28700
0.000637368 31000 0.000779055 30500 0.003344453
31400 0.001640867 29400 0.002124663 29200 0.001882235 28700
0.000636527 31000 0.00077903 30500 0.003339086
31400 0.001640995 29400 0.002116031 29200 0.001877402 28700
0.00063574 31000 0.000778994 30500 0.003333756
31400 0.001641143 29400 0.002107439 29200 0.001872591 28800
0.000634971 31000 0.000778969 30500 0.003328371
31400 0.001641282 29400 0.002098898 29200 0.0018678 28800
0.000634246 31000 0.00077895 30500 0.003323047
31400 0.00164145 29400 0.002090382 29200 0.001863043 28800
0.000633531 31000 0.000778935 30500 0.003317806
31400 0.001641609 29400 0.002081906 29200 0.00185828 28800
0.000632829 31000 0.000778925 30500 0.003312439
31400 0.001641746 29400 0.002073479 29200 0.001853555 28800
0.000632143 31000 0.000778919 30500 0.003307147
31400 0.00164188 29400 0.002065106 29200 0.001848845 28800
0.000631434 31000 0.000778918 30500 0.003301884
31400 0.001642032 29400 0.002056792 29200 0.001844157 28800
0.000630739 31000 0.000778913 30500 0.003296639
31400 0.001642209 29400 0.002048559 29200 0.001839474 28800
0.000629983 31000 0.000778925 30500 0.00329137
31400 0.001642371 29400 0.002040396 29200 0.001834838 28800
0.000629226 31000 0.00077894 30500 0.003286174
31400 0.001642558 29400 0.002032306 29200 0.001830215 28800
0.000628457 31000 0.000778946 30500 0.003280989
31400 0.001642713 29400 0.00202428 29200 0.001825596 28800
0.000627645 31000 0.000778965 30500 0.003275785
31400 0.001642914 29400 0.00201631 29200 0.001821002 28800
0.000626837 31100 0.000778983 30500 0.003270614
31400 0.001643111 29400 0.002008376 29200 0.00181643 28800
0.000626005 31100 0.000779011 30500 0.003265474
31400 0.001643284 29400 0.00200045 29200 0.001811882 28800
0.000625191 31100 0.000779044 30500 0.003260301
31400 0.001643469 29400 0.001992577 29200 0.001807357 28800
0.000624378 31100 0.000779063 30500 0.003255154
31400 0.001643647 29400 0.001984742 29200 0.001802842 28800
0.000623558 31100 0.000779096 30500 0.003250044
31400 0.001643846 29400 0.001976957 29200 0.001798358 28800
0.000622777 31100 0.000779134 30500 0.003244926
31400 0.001644052 29400 0.001969244 29200 0.001793894 28800
0.000621992 31100 0.000779171 30500 0.003239849
31400 0.001644247 29400 0.001961577 29200 0.001789445 28800
0.000621247 31100 0.000779213 30500 0.003234749
31400 0.001644482 29400 0.00195397 29200 0.00178501 28800
0.000620491 31100 0.000779268 30500 0.003229695
31400 0.001644704 29400 0.001946413 29200 0.001780608 28800
0.00061974 31100 0.000779308 30500 0.003224635

31400 0.001644957 29400 0.001938894 29200 0.001776214 28800
0.000618997 31100 0.000779365 30500 0.0032196
31400 0.001645202 29400 0.00193141 29200 0.001771833 28800
0.000618274 31100 0.000779421 30500 0.003214529
31400 0.00164546 29400 0.001923949 29200 0.001767475 28800
0.000617546 31100 0.000779474 30500 0.003209463
31400 0.001645701 29400 0.001916506 29200 0.001763146 28800
0.000616813 31100 0.000779525 30500 0.003204441
31400 0.001645966 29400 0.001909089 29200 0.001758825 28800
0.000616104 31100 0.000779582 30500 0.003199439
31400 0.001646244 29400 0.001901699 29200 0.001754518 28800
0.000615375 31100 0.000779634 30500 0.003194411
31400 0.001646536 29400 0.001894344 29200 0.001750239 28800
0.000614626 31100 0.000779687 30500 0.00318944
31400 0.001646825 29400 0.001887044 29200 0.00174597 28800
0.000613849 31100 0.000779757 30500 0.003184488
31400 0.001647096 29400 0.001879793 29200 0.001741719 28800
0.000613056 31100 0.000779813 30500 0.00317952
31400 0.001647399 29400 0.001872626 29200 0.001737463 28800
0.00061225 31100 0.00077987 30500 0.003174588
31400 0.001647679 29400 0.00186549 29200 0.00173324 28800
0.000611444 31100 0.000779939 30500 0.003169651
31400 0.001647965 29400 0.001858399 29200 0.001729025 28800
0.00061064 31100 0.000779997 30500 0.003164715
31400 0.001648258 29400 0.001851375 29200 0.001724836 28800
0.000609883 31100 0.000780058 30500 0.003159807
31400 0.001648534 29400 0.001844419 29200 0.001720645 28800
0.000609136 31100 0.000780114 30500 0.003154932
31400 0.00164882 29400 0.001837517 29200 0.001716494 28800
0.000608419 31100 0.000780181 30500 0.003150071
31400 0.001649114 29400 0.001830668 29200 0.001712342 28800
0.000607718 31100 0.00078024 30500 0.003145174
31400 0.001649377 29400 0.001823855 29200 0.00170821 28800
0.000607031 31100 0.000780299 30500 0.003140316
31400 0.001649681 29400 0.001817083 29200 0.001704074 28800
0.000606335 31100 0.000780377 30500 0.003135446
31400 0.001649956 29400 0.00181034 29200 0.001699978 28800
0.000605636 31100 0.000780442 30500 0.003130649
31500 0.001650262 29400 0.001803629 29200 0.001695871 28800
0.000604904 31100 0.000780527 30500 0.003125839
31500 0.001650528 29400 0.001796959 29200 0.001691802 28800
0.000604142 31100 0.000780603 30500 0.003121027
31500 0.001650796 29400 0.001790316 29200 0.001687739 28800
0.000603377 31100 0.000780686 30500 0.003116217
31500 0.001651023 29400 0.001783721 29200 0.001683695 28800
0.000602564 31100 0.000780782 30500 0.003111427
31500 0.001651293 29400 0.001777147 29200 0.001679669 28800
0.000601757 31100 0.000780872 30500 0.003106639
31500 0.00165157 29400 0.001770609 29200 0.001675647 28800
0.000600909 31100 0.000780963 30500 0.00310189
31500 0.001651831 29400 0.001764113 29200 0.001671657 28800
0.00060007 31100 0.000781059 30500 0.003097142
31500 0.0016521 29400 0.001757646 29200 0.001667672 28800
0.000599236 31100 0.000781157 30500 0.003092419
31500 0.001652358 29400 0.001751215 29200 0.001663708 28800
0.000598428 31100 0.000781262 30500 0.003087674
31500 0.001652631 29400 0.001744853 29200 0.001659747 28800
0.000597657 31100 0.000781364 30600 0.003082952
31500 0.001652875 29400 0.001738514 29200 0.001655811 28800
0.00059693 31100 0.000781483 30600 0.003078226
31500 0.001653132 29400 0.001732208 29200 0.001651883 28800
0.000596252 31100 0.000781591 30600 0.00307353
31500 0.001653366 29400 0.001725954 29200 0.001647989 28800
0.000595612 31100 0.000781697 30600 0.003068829
31500 0.001653612 29400 0.001719742 29200 0.001644107 28800
0.000595016 31100 0.000781804 30600 0.003064116
31500 0.001653852 29400 0.001713559 29200 0.001640246 28800
0.000594447 31100 0.000781917 30600 0.003059437

31500 0.001654076 29400 0.001707409 29200 0.001636383 28800
0.000593859 31100 0.000782031 30600 0.003054763
31500 0.001654304 29400 0.001701262 29200 0.001632553 28800
0.000593291 31100 0.000782143 30600 0.003050134
31500 0.001654535 29400 0.001695142 29200 0.001628718 28800
0.000592686 31100 0.000782257 30600 0.003045456
31500 0.001654772 29500 0.001689038 29200 0.001624908 28800
0.00059205 31100 0.000782366 30600 0.003040837
31500 0.001655023 29500 0.001682957 29200 0.001621104 28800
0.000591388 31100 0.000782483 30600 0.003036194
31500 0.001655265 29500 0.001676924 29300 0.001617335 28800
0.000590678 31100 0.000782577 30600 0.003031553
31500 0.001655497 29500 0.001670923 29300 0.001613567 28800
0.000589954 31100 0.000782687 30600 0.003026937
31500 0.001655731 29500 0.001664967 29300 0.001609822 28800
0.000589192 31100 0.000782801 30600 0.003022355
31500 0.00165598 29500 0.001659065 29300 0.001606077 28800
0.000588441 31100 0.000782908 30600 0.003017755
31500 0.001656218 29500 0.00165322 29300 0.001602366 28900
0.000587673 31100 0.000783009 30600 0.003013192
31500 0.001656458 29500 0.001647419 29300 0.001598665 28900
0.000586927 31100 0.000783111 30600 0.003008654
31500 0.00165671 29500 0.001641662 29300 0.001594978 28900
0.000586197 31100 0.000783219 30600 0.003004114
31500 0.001656952 29500 0.001635952 29300 0.001591302 28900
0.000585467 31100 0.000783322 30600 0.0029996
31500 0.001657219 29500 0.001630294 29300 0.001587633 28900
0.000584727 31100 0.000783428 30600 0.0029951
31500 0.001657452 29500 0.001624633 29300 0.001583986 28900
0.000584007 31100 0.000783535 30600 0.002990628
31500 0.001657685 29500 0.001618986 29300 0.001580359 28900
0.000583277 31100 0.000783642 30600 0.002986129
31500 0.001657916 29500 0.001613365 29300 0.001576754 28900
0.000582544 31100 0.000783746 30600 0.002981654
31500 0.001658165 29500 0.001607762 29300 0.001573168 28900
0.000581809 31100 0.000783843 30600 0.00297719
31500 0.001658402 29500 0.001602179 29300 0.001569584 28900
0.000581099 31100 0.000783954 30600 0.002972752
31500 0.001658644 29500 0.00159664 29300 0.001566008 28900
0.000580426 31100 0.00078406 30600 0.002968311
31500 0.00165886 29500 0.001591113 29300 0.001562456 28900
0.000579785 31200 0.000784157 30600 0.002963914
31500 0.001659102 29500 0.00158562 29300 0.001558908 28900
0.000579163 31200 0.000784266 30600 0.002959519
31500 0.001659335 29500 0.001580169 29300 0.001555369 28900
0.000578584 31200 0.000784362 30600 0.002955167
31500 0.001659605 29500 0.001574743 29300 0.001551838 28900
0.000578021 31200 0.000784467 30600 0.002950802
31500 0.001659864 29500 0.001569367 29300 0.001548331 28900
0.000577492 31200 0.000784556 30600 0.002946431
31500 0.001660091 29500 0.001564031 29300 0.00154484 28900
0.000576975 31200 0.000784658 30600 0.002942065
31500 0.001660304 29500 0.001558714 29300 0.001541369 28900
0.000576449 31200 0.000784759 30600 0.002937735
31500 0.001660569 29500 0.00155343 29300 0.001537903 28900
0.000575947 31200 0.000784856 30600 0.002933406
31500 0.001660799 29500 0.001548157 29300 0.001534448 28900
0.000575427 31200 0.000784941 30600 0.002929094
31500 0.001661061 29500 0.001542885 29300 0.001531025 28900
0.00057492 31200 0.000785034 30600 0.002924799
31500 0.001661343 29500 0.001537618 29300 0.001527603 28900
0.000574381 31200 0.000785129 30600 0.002920486
31500 0.001661586 29500 0.001532362 29300 0.0015242 28900
0.000573831 31200 0.000785222 30600 0.00291621
31500 0.001661882 29500 0.001527157 29300 0.001520806 28900
0.000573232 31200 0.000785308 30600 0.002911926
31500 0.001662144 29500 0.001521971 29300 0.001517414 28900
0.000572625 31200 0.000785402 30600 0.002907664

31500 0.00166241 29500 0.001516825 29300 0.001514065 28900
0.000571997 31200 0.000785488 30600 0.002903405
31500 0.001662683 29500 0.001511732 29300 0.001510721 28900
0.000571356 31200 0.000785571 30600 0.002899124
31500 0.001662958 29500 0.001506688 29300 0.001507407 28900
0.000570705 31200 0.000785657 30600 0.002894884
31500 0.00166324 29500 0.001501682 29300 0.001504098 28900
0.000570059 31200 0.000785743 30600 0.002890602
31500 0.001663531 29500 0.001496739 29300 0.001500797 28900
0.000569396 31200 0.000785825 30600 0.002886375
31500 0.001663789 29500 0.001491804 29300 0.001497522 28900
0.000568736 31200 0.000785903 30600 0.002882146
31500 0.001664081 29500 0.001486888 29300 0.001494254 28900
0.000568055 31200 0.000785982 30600 0.002877914
31500 0.001664369 29500 0.001481995 29300 0.001490998 28900
0.000567373 31200 0.000786067 30600 0.002873714
31500 0.001664665 29500 0.001477097 29300 0.001487761 28900
0.000566696 31200 0.000786149 30600 0.002869516
31500 0.001664972 29500 0.001472217 29300 0.001484535 28900
0.000566031 31200 0.000786224 30600 0.002865292
31500 0.001665249 29500 0.001467352 29300 0.001481343 28900
0.000565394 31200 0.000786308 30600 0.002861091
31500 0.001665517 29500 0.001462483 29300 0.001478161 28900
0.000564763 31200 0.0007864 30600 0.002856907
31500 0.001665817 29500 0.001457625 29300 0.001474984 28900
0.000564152 31200 0.00078648 30600 0.002852715
31500 0.001666121 29500 0.001452779 29300 0.001471833 28900
0.000563548 31200 0.000786555 30600 0.002848555
31500 0.001666407 29500 0.001447942 29300 0.001468698 28900
0.000562944 31200 0.000786642 30600 0.002844381
31600 0.001666704 29500 0.001443118 29300 0.001465578 28900
0.000562342 31200 0.000786736 30600 0.002840227
31600 0.001666983 29500 0.00143833 29300 0.001462457 28900
0.000561741 31200 0.000786816 30600 0.002836073
31600 0.001667288 29500 0.001433598 29300 0.001459353 28900
0.000561141 31200 0.000786912 30600 0.002831923
31600 0.001667552 29500 0.001428901 29300 0.001456263 28900
0.000560559 31200 0.000787006 30600 0.002827794
31600 0.001667844 29500 0.001424245 29300 0.001453201 28900
0.000559981 31200 0.000787089 30600 0.00282366
31600 0.001668115 29500 0.001419629 29300 0.001450134 28900
0.000559407 31200 0.000787184 30600 0.002819517
31600 0.001668385 29500 0.001415063 29300 0.001447083 28900
0.000558812 31200 0.000787275 30600 0.002815392
31600 0.00166866 29500 0.001410524 29300 0.001444048 28900
0.000558224 31200 0.000787375 30600 0.002811272
31600 0.001668935 29500 0.001406023 29300 0.00144102 28900
0.000557647 31200 0.00078747 30600 0.002807159
31600 0.001669183 29500 0.001401522 29300 0.001437992 28900
0.000557064 31200 0.000787566 30700 0.002803073
31600 0.001669452 29500 0.001397044 29300 0.001434972 28900
0.000556457 31200 0.000787667 30700 0.002798958
31600 0.00166973 29500 0.001392581 29300 0.00143196 28900
0.000555853 31200 0.000787773 30700 0.002794912
31600 0.001670004 29500 0.001388131 29300 0.001428963 28900
0.000555262 31200 0.000787878 30700 0.002790851
31600 0.001670304 29500 0.00138372 29300 0.00142596 28900
0.000554686 31200 0.000787975 30700 0.002786774
31600 0.001670559 29500 0.001379343 29300 0.00142297 28900
0.000554118 31200 0.000788097 30700 0.002782712
31600 0.001670827 29500 0.001374987 29300 0.001419991 28900
0.000553558 31200 0.000788215 30700 0.002778685
31600 0.001671137 29500 0.001370676 29300 0.001417021 28900
0.000553008 31200 0.000788332 30700 0.002774637
31600 0.001671437 29500 0.001366379 29300 0.001414055 28900
0.000552444 31200 0.000788449 30700 0.002770625
31600 0.001671728 29600 0.001362094 29300 0.001411104 28900
0.000551904 31200 0.000788569 30700 0.00276663

31600 0.001672058 29600 0.001357828 29300 0.001408158 28900
0.000551348 31200 0.000788705 30700 0.00276265
31600 0.001672381 29600 0.001353565 29400 0.001405209 28900
0.000550802 31200 0.000788838 30700 0.002758688
31600 0.001672712 29600 0.001349331 29400 0.001402276 28900
0.000550246 31200 0.000788974 30700 0.002754716
31600 0.001673041 29600 0.001345089 29400 0.001399339 28900
0.000549703 31200 0.000789114 30700 0.002750769
31600 0.001673361 29600 0.001340857 29400 0.001396418 28900
0.000549167 31200 0.000789264 30700 0.002746818
31600 0.001673692 29600 0.001336639 29400 0.001393512 29000
0.000548645 31200 0.000789407 30700 0.002742861
31600 0.001674039 29600 0.001332452 29400 0.001390612 29000
0.000548099 31200 0.000789556 30700 0.002738952
31600 0.001674395 29600 0.00132829 29400 0.001387713 29000
0.000547578 31200 0.000789719 30700 0.002735028
31600 0.00167475 29600 0.001324163 29400 0.00138483 29000
0.000547035 31200 0.000789876 30700 0.002731136
31600 0.00167508 29600 0.001320048 29400 0.001381953 29000
0.000546472 31200 0.000790029 30700 0.002727263
31600 0.001675425 29600 0.001315972 29400 0.001379078 29000
0.000545878 31200 0.000790199 30700 0.002723351
31600 0.001675804 29600 0.001311899 29400 0.001376221 29000
0.000545274 31200 0.000790361 30700 0.00271951
31600 0.001676176 29600 0.001307826 29400 0.001373371 29000
0.000544641 31200 0.000790534 30700 0.002715662
31600 0.001676537 29600 0.001303775 29400 0.001370529 29000
0.000544018 31200 0.000790697 30700 0.002711804
31600 0.001676897 29600 0.001299731 29400 0.001367708 29000
0.000543391 31200 0.000790865 30700 0.00270796
31600 0.00167724 29600 0.001295693 29400 0.001364893 29000
0.000542784 31200 0.000791041 30700 0.002704149
31600 0.001677621 29600 0.001291682 29400 0.001362081 29000
0.000542204 31300 0.000791207 30700 0.002700327
31600 0.001677996 29600 0.001287673 29400 0.001359283 29000
0.000541616 31300 0.000791376 30700 0.002696515
31600 0.001678382 29600 0.001283687 29400 0.001356495 29000
0.000541053 31300 0.000791543 30700 0.002692729
31600 0.001678749 29600 0.001279743 29400 0.001353728 29000
0.000540491 31300 0.000791725 30700 0.002688927
31600 0.001679116 29600 0.001275799 29400 0.001350967 29000
0.000539931 31300 0.000791888 30700 0.002685161
31600 0.00167948 29600 0.001271888 29400 0.001348207 29000
0.000539391 31300 0.000792056 30700 0.002681407
31600 0.001679851 29600 0.001268036 29400 0.001345469 29000
0.000538859 31300 0.000792232 30700 0.002677669
31600 0.001680195 29600 0.001264186 29400 0.001342754 29000
0.000538338 31300 0.000792408 30700 0.002673916
31600 0.001680548 29600 0.001260393 29400 0.001340046 29000
0.000537835 31300 0.000792574 30700 0.002670178
31600 0.001680912 29600 0.001256646 29400 0.001337343 29000
0.000537327 31300 0.000792747 30700 0.002666439
31600 0.001681257 29600 0.001252904 29400 0.001334673 29000
0.000536832 31300 0.000792925 30700 0.002662706
31600 0.001681607 29600 0.001249197 29400 0.001331993 29000
0.000536345 31300 0.000793098 30700 0.00265902
31600 0.001681958 29600 0.001245519 29400 0.001329345 29000
0.000535883 31300 0.000793272 30700 0.002655327
31600 0.001682331 29600 0.001241873 29400 0.001326676 29000
0.000535421 31300 0.000793453 30700 0.00265165
31600 0.001682663 29600 0.001238239 29400 0.001324044 29000
0.000534955 31300 0.000793625 30700 0.002647985
31600 0.001683045 29600 0.001234633 29400 0.001321415 29000
0.000534496 31300 0.000793807 30700 0.002644307
31600 0.001683398 29600 0.001231021 29400 0.00131879 29000
0.00053404 31300 0.000793982 30700 0.002640647
31600 0.001683737 29600 0.001227452 29400 0.001316182 29000
0.000533557 31300 0.00079416 30700 0.002636971

31600 0.001684091 29600 0.001223889 29400 0.001313592 29000
0.000533094 31300 0.000794354 30700 0.002633353
31600 0.00168443 29600 0.001220351 29400 0.001311002 29000
0.000532616 31300 0.000794536 30700 0.002629734
31600 0.001684804 29600 0.001216794 29400 0.001308423 29000
0.000532131 31300 0.000794718 30700 0.002626132
31600 0.001685152 29600 0.001213263 29400 0.001305871 29000
0.000531657 31300 0.000794899 30700 0.002622561
31600 0.001685525 29600 0.001209731 29400 0.001303311 29000
0.000531174 31300 0.000795096 30700 0.002618972
31600 0.001685885 29600 0.001206194 29400 0.001300756 29000
0.000530702 31300 0.000795281 30700 0.002615391
31600 0.001686245 29600 0.001202682 29400 0.001298213 29000
0.000530236 31300 0.000795484 30700 0.002611791
31600 0.001686622 29600 0.001199193 29400 0.001295664 29000
0.00052978 31300 0.000795679 30700 0.002608233
31600 0.001686974 29600 0.001195689 29400 0.001293135 29000
0.000529324 31300 0.000795875 30700 0.002604674
31600 0.001687342 29600 0.001192197 29400 0.001290616 29000
0.000528877 31300 0.000796071 30700 0.00260113
31600 0.001687699 29600 0.001188708 29400 0.001288102 29000
0.00052843 31300 0.000796266 30700 0.002597584
31700 0.001688086 29600 0.001185224 29400 0.0012856 29000
0.000527995 31300 0.000796463 30700 0.002594027
31700 0.001688451 29600 0.001181768 29400 0.001283102 29000
0.000527567 31300 0.000796665 30700 0.002590528
31700 0.001688796 29600 0.00117832 29400 0.001280619 29000
0.000527168 31300 0.000796861 30700 0.002586987
31700 0.001689176 29600 0.001174913 29400 0.001278126 29000
0.000526755 31300 0.000797072 30700 0.002583478
31700 0.00168957 29600 0.001171549 29400 0.001275653 29000
0.000526353 31300 0.000797273 30700 0.002580018
31700 0.001689952 29600 0.001168186 29400 0.001273182 29000
0.000525931 31300 0.000797475 30700 0.00257654
31700 0.001690322 29600 0.001164845 29400 0.001270719 29000
0.000525511 31300 0.000797676 30700 0.002573034
31700 0.001690685 29600 0.001161533 29400 0.00126828 29000
0.000525095 31300 0.00079788 30700 0.002569573
31700 0.001691053 29600 0.001158226 29400 0.001265828 29000
0.000524662 31300 0.000798092 30700 0.002566098
31700 0.001691444 29600 0.001154933 29400 0.001263398 29000
0.000524226 31300 0.000798286 30800 0.002562641
31700 0.001691821 29600 0.001151642 29400 0.001260975 29000
0.000523757 31300 0.000798496 30800 0.00255918
31700 0.001692197 29600 0.001148365 29400 0.001258563 29000
0.000523296 31300 0.000798694 30800 0.002555754
31700 0.00169258 29600 0.001145115 29400 0.00125616 29000
0.000522818 31300 0.000798908 30800 0.002552299
31700 0.001692959 29600 0.001141889 29400 0.001253774 29000
0.000522333 31300 0.000799119 30800 0.002548882
31700 0.001693368 29600 0.001138689 29400 0.001251387 29000
0.00052186 31300 0.000799328 30800 0.00254545
31700 0.001693749 29600 0.001135511 29400 0.001249006 29000
0.000521383 31300 0.000799545 30800 0.002542036
31700 0.001694124 29600 0.001132356 29400 0.001246645 29000
0.00052092 31300 0.000799762 30800 0.002538628
31700 0.001694522 29600 0.001129198 29400 0.001244295 29000
0.000520463 31300 0.000799986 30800 0.00253521
31700 0.001694913 29700 0.001126057 29400 0.00124195 29000
0.000520004 31300 0.0008002 30800 0.00253183
31700 0.001695293 29700 0.001122908 29400 0.001239616 29000
0.00051956 31300 0.000800439 30800 0.002528438
31700 0.001695686 29700 0.001119789 29500 0.001237286 29000
0.00051911 31300 0.000800664 30800 0.002525065
31700 0.001696058 29700 0.001116673 29500 0.001234971 29000
0.000518677 31300 0.000800893 30800 0.002521696
31700 0.00169647 29700 0.001113574 29500 0.00123265 29000
0.000518248 31300 0.000801133 30800 0.002518353

31700 0.001696873 29700 0.001110509 29500 0.001230348 29000
0.000517812 31300 0.000801362 30800 0.002515001
31700 0.001697315 29700 0.001107451 29500 0.001228046 29100
0.000517399 31300 0.000801599 30800 0.002511676
31700 0.001697741 29700 0.00110442 29500 0.001225752 29100
0.00051697 31300 0.000801834 30800 0.002508336
31700 0.001698142 29700 0.001101408 29500 0.001223471 29100
0.000516565 31300 0.000802077 30800 0.002505008
31700 0.001698541 29700 0.001098414 29500 0.001221198 29100
0.000516144 31300 0.000802307 30800 0.002501705
31700 0.001698993 29700 0.001095428 29500 0.001218933 29100
0.000515704 31300 0.000802548 30800 0.002498397
31700 0.001699419 29700 0.001092449 29500 0.001216677 29100
0.000515238 31300 0.000802778 30800 0.002495114
31700 0.001699874 29700 0.001089487 29500 0.00121444 29100
0.000514738 31300 0.000803004 30800 0.00249183
31700 0.001700332 29700 0.00108653 29500 0.001212199 29100
0.000514208 31300 0.000803252 30800 0.002488576
31700 0.001700775 29700 0.001083588 29500 0.001209974 29100
0.00051365 31300 0.000803478 30800 0.002485321
31700 0.001701246 29700 0.001080664 29500 0.001207763 29100
0.000513094 31300 0.000803707 30800 0.002482082
31700 0.001701715 29700 0.00107774 29500 0.001205534 29100
0.000512537 31300 0.00080394 30800 0.002478831
31700 0.00170217 29700 0.001074808 29500 0.001203341 29100
0.000512009 31400 0.000804169 30800 0.002475591
31700 0.00170264 29700 0.001071876 29500 0.001201142 29100
0.00051148 31400 0.00080439 30800 0.002472342
31700 0.001703122 29700 0.00106896 29500 0.00119896 29100
0.000510985 31400 0.000804611 30800 0.002469119
31700 0.001703607 29700 0.001066044 29500 0.00119677 29100
0.000510543 31400 0.000804839 30800 0.002465897
31700 0.001704064 29700 0.001063155 29500 0.001194595 29100
0.000510115 31400 0.000805063 30800 0.002462669
31700 0.001704533 29700 0.00106027 29500 0.001192428 29100
0.000509736 31400 0.000805277 30800 0.002459469
31700 0.001705024 29700 0.001057402 29500 0.001190265 29100
0.000509345 31400 0.000805505 30800 0.002456262
31700 0.001705503 29700 0.001054566 29500 0.001188105 29100
0.000508961 31400 0.000805733 30800 0.002453107
31700 0.001706009 29700 0.001051735 29500 0.001185959 29100
0.000508531 31400 0.000805951 30800 0.002449931
31700 0.001706504 29700 0.001048939 29500 0.001183795 29100
0.000508103 31400 0.000806181 30800 0.002446743
31700 0.001706982 29700 0.001046166 29500 0.001181663 29100
0.000507667 31400 0.000806405 30800 0.002443563
31700 0.001707448 29700 0.001043411 29500 0.001179528 29100
0.000507203 31400 0.000806631 30800 0.002440415
31700 0.001707941 29700 0.001040675 29500 0.001177388 29100
0.000506765 31400 0.000806856 30800 0.002437223
31700 0.001708444 29700 0.00103795 29500 0.001175275 29100
0.000506329 31400 0.000807083 30800 0.002434078
31700 0.00170892 29700 0.001035246 29500 0.001173163 29100
0.000505892 31400 0.000807303 30800 0.002430954
31700 0.001709445 29700 0.001032557 29500 0.001171054 29100
0.000505473 31400 0.000807526 30800 0.002427797
31700 0.001709909 29700 0.001029881 29500 0.001168955 29100
0.000505058 31400 0.000807759 30800 0.002424673
31700 0.001710415 29700 0.001027247 29500 0.001166856 29100
0.000504662 31400 0.000807975 30800 0.00242154
31700 0.001710888 29700 0.001024617 29500 0.001164768 29100
0.000504278 31400 0.000808202 30800 0.002418407
31700 0.001711358 29700 0.001021981 29500 0.001162689 29100
0.000503944 31400 0.000808421 30800 0.002415282
31700 0.001711822 29700 0.001019365 29500 0.001160621 29100
0.000503608 31400 0.00080864 30800 0.002412143
31700 0.001712309 29700 0.00101675 29500 0.001158559 29100
0.000503305 31400 0.000808859 30800 0.002409047

31700 0.001712794 29700 0.001014137 29500 0.001156495 29100
0.000502979 31400 0.00080909 30800 0.002405907
31700 0.001713273 29700 0.001011554 29500 0.001154449 29100
0.000502668 31400 0.000809305 30800 0.002402798
31700 0.001713736 29700 0.001008968 29500 0.001152412 29100
0.000502337 31400 0.000809533 30800 0.002399701
31700 0.001714208 29700 0.001006408 29500 0.001150383 29100
0.000501991 31400 0.000809763 30800 0.002396592
31700 0.001714689 29700 0.001003855 29500 0.001148357 29100
0.000501601 31400 0.00080998 30800 0.00239351
31700 0.001715153 29700 0.001001295 29500 0.00114633 29100
0.000501225 31400 0.00081021 30800 0.002390431
31700 0.001715643 29700 0.000998736 29500 0.001144303 29100
0.00050079 31400 0.000810438 30800 0.002387336
31800 0.001716091 29700 0.000996174 29500 0.00114231 29100
0.00050036 31400 0.000810669 30800 0.002384244
31800 0.001716547 29700 0.0009936 29500 0.001140309 29100
0.000499911 31400 0.000810898 30800 0.002381179
31800 0.001717043 29700 0.000991039 29500 0.00113831 29100
0.000499478 31400 0.000811123 30800 0.002378091
31800 0.001717516 29700 0.000988476 29500 0.001136322 29100
0.000499013 31400 0.000811349 30800 0.002375028
31800 0.001718002 29700 0.000985923 29500 0.001134347 29100
0.000498532 31400 0.000811577 30800 0.002371984
31800 0.001718475 29700 0.000983414 29500 0.001132365 29100
0.000498045 31400 0.000811826 30800 0.002368939
31800 0.001718953 29700 0.000980922 29500 0.001130409 29100
0.000497585 31400 0.000812061 30800 0.002365893
31800 0.001719411 29700 0.000978478 29500 0.00112845 29100
0.00049717 31400 0.000812291 30800 0.002362859
31800 0.001719885 29700 0.000976049 29500 0.001126498 29100
0.000496753 31400 0.000812535 30800 0.002359832
31800 0.001720337 29700 0.000973627 29500 0.001124558 29100
0.000496371 31400 0.000812772 30900 0.002356789
31800 0.001720812 29700 0.000971228 29500 0.001122615 29100
0.000496025 31400 0.000812999 30900 0.002353759
31800 0.001721274 29700 0.000968845 29500 0.001120693 29100
0.000495681 31400 0.000813219 30900 0.002350755
31800 0.001721743 29700 0.000966458 29500 0.001118771 29100
0.000495354 31400 0.000813449 30900 0.002347718
31800 0.001722206 29700 0.00096408 29500 0.001116861 29100
0.000495015 31400 0.000813666 30900 0.002344697
31800 0.001722643 29700 0.000961695 29500 0.001114951 29100
0.00049467 31400 0.000813895 30900 0.002341699
31800 0.001723091 29700 0.000959323 29500 0.001113058 29100
0.000494323 31400 0.00081412 30900 0.002338697
31800 0.001723549 29700 0.000956949 29500 0.001111165 29100
0.000493941 31400 0.000814328 30900 0.002335734
31800 0.001724033 29700 0.000954566 29500 0.001109284 29100
0.000493529 31400 0.000814541 30900 0.002332766
31800 0.00172448 29800 0.000952186 29500 0.0011074 29100
0.000493094 31400 0.00081475 30900 0.002329791
31800 0.001724907 29800 0.000949803 29500 0.001105536 29100
0.000492663 31400 0.000814961 30900 0.002326824
31800 0.001725315 29800 0.000947424 29600 0.001103673 29100
0.000492251 31400 0.000815157 30900 0.002323872
31800 0.001725783 29800 0.000945054 29600 0.001101813 29100
0.000491846 31400 0.000815365 30900 0.002320915
31800 0.00172624 29800 0.000942694 29600 0.001099964 29100
0.000491457 31400 0.000815556 30900 0.002317961
31800 0.001726711 29800 0.000940372 29600 0.001098123 29100
0.000491087 31400 0.000815751 30900 0.002315051
31800 0.001727156 29800 0.000938084 29600 0.001096287 29200
0.000490729 31400 0.000815945 30900 0.002312139
31800 0.001727635 29800 0.000935818 29600 0.001094467 29200
0.000490393 31400 0.000816131 30900 0.002309227
31800 0.001728087 29800 0.000933595 29600 0.001092643 29200
0.0004901 31400 0.000816314 30900 0.002306318

31800 0.001728528 29800 0.000931376 29600 0.001090822 29200
0.000489821 31400 0.000816505 30900 0.002303411
31800 0.001728977 29800 0.00092916 29600 0.001089025 29200
0.000489563 31400 0.000816684 30900 0.002300528
31800 0.001729436 29800 0.000926943 29600 0.001087221 29200
0.000489287 31400 0.000816861 30900 0.002297619
31800 0.001729875 29800 0.000924727 29600 0.001085432 29200
0.000489019 31400 0.00081704 30900 0.002294743
31800 0.001730348 29800 0.000922501 29600 0.001083635 29200
0.000488735 31400 0.000817227 30900 0.002291847
31800 0.001730783 29800 0.000920274 29600 0.001081846 29200
0.000488397 31400 0.000817399 30900 0.00228897
31800 0.001731226 29800 0.000918051 29600 0.001080072 29200
0.000488095 31400 0.000817566 30900 0.002286116
31800 0.00173168 29800 0.00091585 29600 0.0010783 29200
0.000487747 31400 0.000817739 30900 0.002283249
31800 0.001732152 29800 0.000913652 29600 0.00107652 29200
0.000487378 31500 0.000817907 30900 0.002280418
31800 0.00173262 29800 0.000911465 29600 0.001074763 29200
0.000486992 31500 0.000818064 30900 0.002277572
31800 0.001733087 29800 0.000909297 29600 0.001072998 29200
0.000486615 31500 0.000818224 30900 0.002274733
31800 0.001733553 29800 0.000907131 29600 0.001071242 29200
0.000486233 31500 0.000818389 30900 0.002271908
31800 0.001734035 29800 0.000904986 29600 0.001069494 29200
0.000485858 31500 0.00081855 30900 0.002269076
31800 0.001734526 29800 0.000902854 29600 0.001067758 29200
0.000485506 31500 0.000818696 30900 0.002266262
31800 0.001735009 29800 0.000900727 29600 0.001066015 29200
0.000485151 31500 0.000818845 30900 0.002263466
31800 0.001735515 29800 0.000898613 29600 0.001064301 29200
0.000484783 31500 0.000818986 30900 0.002260685
31800 0.001736019 29800 0.000896522 29600 0.001062576 29200
0.000484406 31500 0.000819138 30900 0.002257891
31800 0.001736508 29800 0.000894436 29600 0.001060854 29200
0.000484064 31500 0.000819277 30900 0.002255116
31800 0.001737019 29800 0.000892387 29600 0.001059141 29200
0.000483705 31500 0.000819405 30900 0.002252345
31800 0.001737495 29800 0.000890342 29600 0.001057425 29200
0.000483331 31500 0.000819553 30900 0.00224957
31800 0.001737976 29800 0.000888287 29600 0.001055734 29200
0.000482941 31500 0.000819693 30900 0.002246814
31800 0.001738492 29800 0.000886251 29600 0.00105404 29200
0.000482593 31500 0.000819827 30900 0.002244046
31800 0.001738999 29800 0.00088422 29600 0.001052367 29200
0.000482219 31500 0.000819956 30900 0.002241292
31800 0.001739499 29800 0.000882185 29600 0.001050676 29200
0.000481832 31500 0.00082011 30900 0.002238546
31800 0.001740002 29800 0.00088017 29600 0.001048993 29200
0.000481447 31500 0.000820242 30900 0.002235812
31800 0.001740506 29800 0.00087816 29600 0.001047321 29200
0.000481068 31500 0.000820381 30900 0.00223308
31800 0.001741026 29800 0.000876188 29600 0.001045657 29200
0.000480676 31500 0.000820528 30900 0.00223033
31800 0.001741539 29800 0.000874226 29600 0.001043992 29200
0.00048028 31500 0.000820672 30900 0.002227644
31800 0.001742064 29800 0.000872293 29600 0.001042334 29200
0.000479906 31500 0.000820816 30900 0.002224938
31800 0.001742593 29800 0.000870372 29600 0.001040676 29200
0.00047951 31500 0.000820964 30900 0.002222212
31800 0.001743089 29800 0.000868464 29600 0.001039038 29200
0.000479133 31500 0.000821124 30900 0.00221953
31800 0.001743576 29800 0.000866563 29600 0.00103739 29200
0.000478766 31500 0.000821271 30900 0.002216832
31800 0.001744117 29800 0.000864656 29600 0.00103574 29200
0.0004784 31500 0.000821423 30900 0.002214148
31800 0.001744634 29800 0.000862746 29600 0.00103411 29200
0.000478034 31500 0.00082158 30900 0.002211462

31800 0.001745177 29800 0.000860838 29600 0.001032484 29200
0.000477655 31500 0.000821737 30900 0.002208791
31800 0.001745719 29800 0.000858951 29600 0.001030859 29200
0.000477265 31500 0.00082189 30900 0.002206114
31800 0.001746253 29800 0.000857073 29600 0.001029245 29200
0.000476843 31500 0.000822035 30900 0.002203429
31900 0.001746825 29800 0.000855225 29600 0.001027617 29200
0.000476454 31500 0.000822181 30900 0.002200785
31900 0.001747354 29800 0.000853383 29600 0.001026006 29200
0.000476029 31500 0.000822345 30900 0.002198134
31900 0.001747883 29800 0.000851532 29600 0.001024404 29200
0.000475614 31500 0.000822484 30900 0.002195486
31900 0.001748417 29800 0.000849697 29600 0.001022799 29200
0.000475273 31500 0.000822633 30900 0.00219281
31900 0.001748955 29800 0.000847884 29600 0.001021203 29200
0.000474933 31500 0.000822773 30900 0.002190181
31900 0.001749522 29800 0.000846051 29600 0.001019592 29200
0.000474638 31500 0.000822906 30900 0.002187541
31900 0.001750062 29800 0.000844235 29600 0.001017999 29200
0.000474355 31500 0.000823053 30900 0.002184901
31900 0.001750599 29800 0.000842408 29600 0.001016396 29200
0.000474156 31500 0.000823185 30900 0.002182288
31900 0.001751171 29800 0.000840602 29600 0.00101482 29200
0.000473915 31500 0.000823322 30900 0.002179679
31900 0.001751756 29800 0.000838807 29600 0.001013229 29200
0.000473687 31500 0.000823458 31000 0.002177063
31900 0.001752335 29800 0.000837035 29600 0.00101165 29200
0.000473438 31500 0.000823605 31000 0.002174479
31900 0.001752908 29800 0.000835267 29600 0.00101007 29200
0.000473122 31500 0.000823747 31000 0.002171885
31900 0.001753483 29800 0.000833536 29600 0.001008506 29200
0.000472827 31500 0.000823892 31000 0.002169303
31900 0.001754043 29800 0.000831798 29600 0.001006929 29200
0.000472507 31500 0.000824042 31000 0.00216669
31900 0.001754668 29800 0.000830087 29600 0.001005364 29200
0.000472194 31500 0.000824195 31000 0.002164108
31900 0.001755284 29800 0.000828383 29600 0.001003803 29200
0.000471896 31500 0.000824348 31000 0.002161544
31900 0.001755898 29800 0.000826707 29600 0.001002265 29200
0.000471597 31500 0.000824516 31000 0.002158946
31900 0.001756507 29800 0.000825057 29600 0.001000714 29200
0.000471343 31500 0.000824687 31000 0.002156385
31900 0.001757129 29900 0.000823402 29600 0.00099917 29200
0.000471084 31500 0.000824855 31000 0.002153819
31900 0.001757754 29900 0.000821752 29600 0.000997635 29200
0.000470814 31500 0.000825041 31000 0.002151266
31900 0.001758378 29900 0.000820092 29700 0.000996103 29200
0.000470539 31500 0.000825236 31000 0.002148694
31900 0.001758998 29900 0.000818415 29700 0.000994582 29200
0.000470231 31500 0.000825426 31000 0.00214614
31900 0.001759619 29900 0.000816732 29700 0.000993067 29200
0.000469937 31500 0.000825628 31000 0.002143564
31900 0.001760249 29900 0.000815069 29700 0.00099156 29200
0.000469668 31500 0.000825837 31000 0.002141003
31900 0.001760896 29900 0.000813388 29700 0.000990051 29300
0.000469365 31500 0.000826052 31000 0.00213845
31900 0.00176153 29900 0.000811728 29700 0.000988547 29300
0.00046908 31500 0.000826283 31000 0.002135902
31900 0.001762166 29900 0.000810064 29700 0.000987049 29300
0.00046879 31500 0.000826506 31000 0.00213332
31900 0.001762822 29900 0.000808431 29700 0.000985559 29300
0.000468493 31500 0.000826743 31000 0.002130772
31900 0.001763478 29900 0.000806819 29700 0.000984069 29300
0.000468176 31500 0.000826981 31000 0.002128255
31900 0.001764146 29900 0.000805236 29700 0.000982586 29300
0.000467826 31500 0.000827224 31000 0.002125752
31900 0.001764812 29900 0.000803682 29700 0.000981116 29300
0.0004675 31500 0.000827474 31000 0.002123194

31900 0.001765439 29900 0.000802154 29700 0.000979656 29300
0.000467174 31500 0.000827734 31000 0.002120678
31900 0.001766089 29900 0.00080064 29700 0.000978189 29300
0.000466823 31500 0.000827981 31000 0.002118125
31900 0.00176677 29900 0.000799155 29700 0.000976738 29300
0.000466526 31500 0.000828233 31000 0.002115622
31900 0.00176742 29900 0.00079766 29700 0.000975293 29300
0.000466204 31500 0.000828492 31000 0.002113115
31900 0.001768101 29900 0.000796176 29700 0.000973862 29300
0.000465918 31600 0.000828756 31000 0.002110606
31900 0.001768784 29900 0.000794709 29700 0.000972424 29300
0.000465631 31600 0.000829013 31000 0.002108092
31900 0.001769465 29900 0.000793238 29700 0.000971003 29300
0.0004654 31600 0.000829279 31000 0.002105574
31900 0.001770158 29900 0.000791788 29700 0.000969576 29300
0.000465175 31600 0.000829545 31000 0.002103077
31900 0.001770836 29900 0.000790337 29700 0.000968162 29300
0.000464981 31600 0.00082982 31000 0.002100556
31900 0.00177146 29900 0.000788891 29700 0.000966751 29300
0.000464832 31600 0.000830094 31000 0.002098107
31900 0.001772136 29900 0.000787453 29700 0.000965349 29300
0.000464727 31600 0.000830364 31000 0.002095609
31900 0.001772797 29900 0.000786043 29700 0.000963955 29300
0.000464622 31600 0.000830635 31000 0.002093124
31900 0.001773472 29900 0.000784617 29700 0.000962558 29300
0.000464519 31600 0.000830902 31000 0.002090634
31900 0.001774111 29900 0.000783209 29700 0.00096117 29300
0.000464466 31600 0.000831166 31000 0.00208814
31900 0.001774758 29900 0.000781794 29700 0.000959783 29300
0.000464384 31600 0.000831441 31000 0.002085689
31900 0.001775411 29900 0.000780404 29700 0.000958396 29300
0.000464256 31600 0.000831713 31000 0.00208322
31900 0.001776072 29900 0.000779015 29700 0.000957024 29300
0.000464095 31600 0.00083198 31000 0.002080774
31900 0.001776726 29900 0.000777635 29700 0.00095565 29300
0.000463832 31600 0.000832251 31000 0.002078335
31900 0.001777409 29900 0.000776274 29700 0.000954283 29300
0.000463538 31600 0.000832514 31000 0.002075845
31900 0.001778036 29900 0.000774915 29700 0.000952923 29300
0.000463209 31600 0.000832786 31000 0.002073396
31900 0.001778655 29900 0.000773541 29700 0.000951558 29300
0.000462821 31600 0.000833061 31000 0.002070932
31900 0.001779341 29900 0.000772192 29700 0.000950203 29300
0.000462486 31600 0.000833319 31000 0.00206851
31900 0.001780002 29900 0.000770818 29700 0.000948859 29300
0.000462098 31600 0.000833584 31000 0.002066023
31900 0.001780651 29900 0.000769449 29700 0.000947521 29300
0.000461732 31600 0.000833842 31000 0.002063635
31900 0.001781273 29900 0.000768101 29700 0.000946177 29300
0.000461357 31600 0.000834099 31000 0.002061211
31900 0.0017819 29900 0.000766769 29700 0.000944833 29300
0.000461039 31600 0.000834364 31000 0.002058777
31900 0.001782543 29900 0.000765455 29700 0.000943504 29300
0.000460716 31600 0.000834632 31000 0.002056349
31900 0.001783176 29900 0.000764148 29700 0.000942176 29300
0.000460405 31600 0.000834909 31000 0.002053895
31900 0.001783797 29900 0.000762848 29700 0.000940864 29300
0.000460111 31600 0.000835182 31000 0.00205148
31900 0.001784431 29900 0.00076157 29700 0.000939554 29300
0.000459797 31600 0.000835448 31000 0.002049061
31900 0.001785054 29900 0.000760342 29700 0.000938245 29300
0.000459514 31600 0.000835708 31000 0.002046617
31900 0.001785699 29900 0.000759099 29700 0.000936942 29300
0.000459266 31600 0.000835984 31000 0.002044216
31900 0.001786306 29900 0.000757886 29700 0.000935638 29300
0.000458977 31600 0.000836265 31000 0.002041792
32000 0.001786909 29900 0.000756681 29700 0.000934343 29300
0.000458673 31600 0.000836547 31000 0.002039425

32000 0.001787529 29900 0.000755473 29700 0.000933049 29300
0.000458365 31600 0.000836841 31000 0.002037021
32000 0.001788132 29900 0.000754247 29700 0.000931768 29300
0.000458038 31600 0.000837132 31000 0.002034677
32000 0.001788769 29900 0.000753022 29700 0.000930479 29300
0.000457654 31600 0.000837428 31000 0.002032325
32000 0.0017894 29900 0.000751783 29700 0.000929198 29300
0.00045734 31600 0.000837728 31000 0.00202993
32000 0.001790002 29900 0.00075054 29700 0.00092793 29300
0.000456975 31600 0.000838042 31000 0.002027563
32000 0.001790619 29900 0.000749257 29700 0.000926655 29300
0.000456661 31600 0.000838358 31000 0.002025211
32000 0.001791263 29900 0.000747973 29700 0.000925401 29300
0.000456336 31600 0.000838683 31000 0.002022885
32000 0.001791897 29900 0.000746666 29700 0.000924145 29300
0.000456039 31600 0.000838982 31000 0.002020545
32000 0.001792518 29900 0.000745358 29700 0.000922893 29300
0.000455756 31600 0.000839306 31100 0.002018184
32000 0.001793186 29900 0.000744071 29700 0.00092163 29300
0.0004555 31600 0.000839625 31100 0.002015859
32000 0.00179381 29900 0.000742778 29700 0.000920383 29300
0.000455238 31600 0.000839963 31100 0.002013531
32000 0.001794458 29900 0.000741482 29700 0.000919141 29300
0.000455054 31600 0.000840276 31100 0.00201121
32000 0.001795097 29900 0.000740155 29700 0.000917896 29300
0.000454853 31600 0.000840616 31100 0.002008874
32000 0.001795722 29900 0.000738826 29700 0.00091666 29300
0.000454656 31600 0.00084095 31100 0.002006565
32000 0.001796377 29900 0.000737484 29700 0.000915428 29300
0.000454475 31600 0.000841268 31100 0.002004267
32000 0.001796997 29900 0.000736141 29700 0.000914194 29300
0.000454228 31600 0.000841592 31100 0.002001957
32000 0.001797651 29900 0.000734774 29700 0.000912971 29300
0.000454024 31600 0.00084193 31100 0.001999661
32000 0.001798284 30000 0.000733404 29700 0.000911746 29300
0.000453789 31600 0.000842253 31100 0.001997345
32000 0.00179892 30000 0.000732026 29700 0.000910524 29300
0.000453542 31600 0.000842573 31100 0.001995096
32000 0.001799563 30000 0.000730631 29800 0.000909307 29300
0.000453328 31600 0.00084291 31100 0.00199279
32000 0.001800203 30000 0.000729214 29800 0.000908097 29300
0.000453086 31600 0.000843238 31100 0.001990539
32000 0.001800877 30000 0.000727805 29800 0.000906877 29300
0.000452819 31600 0.000843564 31100 0.0019883
32000 0.001801555 30000 0.00072639 29800 0.000905672 29300
0.000452605 31600 0.000843903 31100 0.001986017
32000 0.001802164 30000 0.000724979 29800 0.000904472 29400
0.000452357 31600 0.000844245 31100 0.001983777
32000 0.001802803 30000 0.000723526 29800 0.000903263 29400
0.000452208 31600 0.000844598 31100 0.001981512
32000 0.001803489 30000 0.000722087 29800 0.000902071 29400
0.000451972 31600 0.000844945 31100 0.001979281
32000 0.001804131 30000 0.000720617 29800 0.000900878 29400
0.000451817 31600 0.000845297 31100 0.001977047
32000 0.001804796 30000 0.000719136 29800 0.000899695 29400
0.000451595 31600 0.000845645 31100 0.00197483
32000 0.001805435 30000 0.000717661 29800 0.000898503 29400
0.000451404 31600 0.000846022 31100 0.001972601
32000 0.001806142 30000 0.000716157 29800 0.000897324 29400
0.000451131 31600 0.000846389 31100 0.001970383
32000 0.001806835 30000 0.00071464 29800 0.000896151 29400
0.000450908 31600 0.000846756 31100 0.001968194
32000 0.001807511 30000 0.000713108 29800 0.000894977 29400
0.00045068 31600 0.000847133 31100 0.001965979
32000 0.001808197 30000 0.000711542 29800 0.000893812 29400
0.000450398 31600 0.000847505 31100 0.001963781
32000 0.001808885 30000 0.000709966 29800 0.00089266 29400
0.000450084 31600 0.000847888 31100 0.001961584

32000 0.001809557 30000 0.000708383 29800 0.000891503 29400
0.000449822 31700 0.000848257 31100 0.00195939
32000 0.001810237 30000 0.000706794 29800 0.000890363 29400
0.000449509 31700 0.000848645 31100 0.001957188
32000 0.001810922 30000 0.000705185 29800 0.000889217 29400
0.000449259 31700 0.000849035 31100 0.001955001
32000 0.001811591 30000 0.00070357 29800 0.000888073 29400
0.000448947 31700 0.000849425 31100 0.001952842
32000 0.001812256 30000 0.000701943 29800 0.000886933 29400
0.000448683 31700 0.000849838 31100 0.001950662
32000 0.001812996 30000 0.000700294 29800 0.000885803 29400
0.00044842 31700 0.00085025 31100 0.001948509
32000 0.001813711 30000 0.000698625 29800 0.000884673 29400
0.0004482 31700 0.000850656 31100 0.001946363
32000 0.001814449 30000 0.000696935 29800 0.000883553 29400
0.000448027 31700 0.000851053 31100 0.001944185
32000 0.001815125 30000 0.000695238 29800 0.000882437 29400
0.000447853 31700 0.00085146 31100 0.001942029
32000 0.001815811 30000 0.00069352 29800 0.000881313 29400
0.000447697 31700 0.000851866 31100 0.001939855
32000 0.001816567 30000 0.0006918 29800 0.00088021 29400
0.000447539 31700 0.000852284 31100 0.001937706
32000 0.001817257 30000 0.000690069 29800 0.000879103 29400
0.000447373 31700 0.000852692 31100 0.001935545
32000 0.001817958 30000 0.000688348 29800 0.000878004 29400
0.000447239 31700 0.000853118 31100 0.001933406
32000 0.001818699 30000 0.000686632 29800 0.000876906 29400
0.000447094 31700 0.000853538 31100 0.001931271
32000 0.001819417 30000 0.000684921 29800 0.000875818 29400
0.000446881 31700 0.000853969 31100 0.001929121
32000 0.001820144 30000 0.000683235 29800 0.000874737 29400
0.000446692 31700 0.000854385 31100 0.001926985
32000 0.001820847 30000 0.00068155 29800 0.000873649 29400
0.000446457 31700 0.000854825 31100 0.001924847
32000 0.001821548 30000 0.000679858 29800 0.000872571 29400
0.000446228 31700 0.000855227 31100 0.001922715
32000 0.001822256 30000 0.00067818 29800 0.000871499 29400
0.000445961 31700 0.000855635 31100 0.001920562
32000 0.001822953 30000 0.000676498 29800 0.000870422 29400
0.000445687 31700 0.000856027 31100 0.001918427
32000 0.001823674 30000 0.000674813 29800 0.000869362 29400
0.000445431 31700 0.000856453 31100 0.001916272
32000 0.001824368 30000 0.000673138 29800 0.000868306 29400
0.00044525 31700 0.000856847 31100 0.001914138
32000 0.001825044 30000 0.000671463 29800 0.000867247 29400
0.000444983 31700 0.000857253 31100 0.001912012
32000 0.001825751 30000 0.000669784 29800 0.000866195 29400
0.000444733 31700 0.000857651 31100 0.00190988
32000 0.001826413 30000 0.000668112 29800 0.000865153 29400
0.000444569 31700 0.000858051 31100 0.001907778
32000 0.001827126 30000 0.000666446 29800 0.000864108 29400
0.000444372 31700 0.000858456 31100 0.00190567
32000 0.001827823 30000 0.000664787 29800 0.000863068 29400
0.000444187 31700 0.000858857 31100 0.001903533
32000 0.001828469 30000 0.000663142 29800 0.00086203 29400
0.000444008 31700 0.000859258 31100 0.001901403
32000 0.00182913 30000 0.000661484 29800 0.000860999 29400
0.00044388 31700 0.000859667 31100 0.001899282
32100 0.001829816 30000 0.000659821 29800 0.000859978 29400
0.000443676 31700 0.000860064 31100 0.001897174
32100 0.001830495 30000 0.000658145 29800 0.000858957 29400
0.000443488 31700 0.000860463 31100 0.001895064
32100 0.001831186 30000 0.000656461 29800 0.000857952 29400
0.000443301 31700 0.000860877 31100 0.001892973
32100 0.001831885 30000 0.000654781 29800 0.000856933 29400
0.000443104 31700 0.000861271 31100 0.001890855
32100 0.001832574 30000 0.000653096 29800 0.000855934 29400
0.00044288 31700 0.000861669 31100 0.001888752

32100 0.001833261 30000 0.000651433 29800 0.000854939 29400
0.000442647 31700 0.000862089 31100 0.001886648
32100 0.001833941 30000 0.000649786 29800 0.00085394 29400
0.000442406 31700 0.000862532 31100 0.001884543
32100 0.001834582 30000 0.000648138 29800 0.000852948 29400
0.000442163 31700 0.000862947 31100 0.001882448
32100 0.001835248 30000 0.000646514 29800 0.000851971 29400
0.00044194 31700 0.000863358 31100 0.001880337
32100 0.001835904 30000 0.000644904 29800 0.000850976 29400
0.000441708 31700 0.000863786 31200 0.001878244
32100 0.001836578 30000 0.000643315 29800 0.000850009 29400
0.000441483 31700 0.000864201 31200 0.001876152
32100 0.001837241 30000 0.000641734 29800 0.00084903 29400
0.000441283 31700 0.000864593 31200 0.001874058
32100 0.0018379 30000 0.000640165 29800 0.000848076 29400
0.000441078 31700 0.000865037 31200 0.001871986
32100 0.001838568 30000 0.000638603 29800 0.000847099 29400
0.000440858 31700 0.000865475 31200 0.001869912
32100 0.001839271 30000 0.000637047 29800 0.000846146 29400
0.00044063 31700 0.000865911 31200 0.001867855
32100 0.001839953 30000 0.000635485 29800 0.000845182 29400
0.000440419 31700 0.000866344 31200 0.0018658
32100 0.001840612 30000 0.000633929 29800 0.000844233 29400
0.000440224 31700 0.000866786 31200 0.001863729
32100 0.001841235 30000 0.000632394 29800 0.000843287 29400
0.000439977 31700 0.000867225 31200 0.001861673
32100 0.001841892 30100 0.000630854 29800 0.000842329 29400
0.000439756 31700 0.000867669 31200 0.001859625
32100 0.001842593 30100 0.000629323 29800 0.000841387 29400
0.000439515 31700 0.000868111 31200 0.001857577
32100 0.001843255 30100 0.000627803 29900 0.000840451 29400
0.000439277 31700 0.000868559 31200 0.001855564
32100 0.001843955 30100 0.000626308 29900 0.000839507 29400
0.000439001 31700 0.000869009 31200 0.001853549
32100 0.001844649 30100 0.000624832 29900 0.000838568 29400
0.000438728 31700 0.000869482 31200 0.001851537
32100 0.001845341 30100 0.00062336 29900 0.000837634 29400
0.000438465 31700 0.000869951 31200 0.001849521
32100 0.001846045 30100 0.000621922 29900 0.000836699 29500
0.000438218 31700 0.000870424 31200 0.001847523
32100 0.001846749 30100 0.000620483 29900 0.000835778 29500
0.000437962 31700 0.000870903 31200 0.001845529
32100 0.001847439 30100 0.00061903 29900 0.000834848 29500
0.000437746 31700 0.00087141 31200 0.001843529
32100 0.001848133 30100 0.000617598 29900 0.00083392 29500
0.00043753 31700 0.000871922 31200 0.001841525
32100 0.001848818 30100 0.000616165 29900 0.000832992 29500
0.000437336 31700 0.000872418 31200 0.001839545
32100 0.001849546 30100 0.000614745 29900 0.000832082 29500
0.000437168 31700 0.000872921 31200 0.001837579
32100 0.001850238 30100 0.000613333 29900 0.000831172 29500
0.000437004 31700 0.000873385 31200 0.001835603
32100 0.00185091 30100 0.000611946 29900 0.000830253 29500
0.000436869 31700 0.000873886 31200 0.001833633
32100 0.001851598 30100 0.000610567 29900 0.000829345 29500
0.000436734 31700 0.000874391 31200 0.001831669
32100 0.001852307 30100 0.000609209 29900 0.000828444 29500
0.000436597 31700 0.000874899 31200 0.001829734
32100 0.001853008 30100 0.000607845 29900 0.000827541 29500
0.000436471 31700 0.000875388 31200 0.001827786
32100 0.001853731 30100 0.000606493 29900 0.00082665 29500
0.000436373 31800 0.000875869 31200 0.001825838
32100 0.00185441 30100 0.000605144 29900 0.000825738 29500
0.00043629 31800 0.000876403 31200 0.001823902
32100 0.00185508 30100 0.000603783 29900 0.00082485 29500
0.000436204 31800 0.000876908 31200 0.001821956
32100 0.001855804 30100 0.000602426 29900 0.000823952 29500
0.000436102 31800 0.000877435 31200 0.001820033

32100 0.001856501 30100 0.000601077 29900 0.000823062 29500
0.000435966 31800 0.000877943 31200 0.001818097
32100 0.001857211 30100 0.000599735 29900 0.000822183 29500
0.000435828 31800 0.000878463 31200 0.001816196
32100 0.001857931 30100 0.000598423 29900 0.000821306 29500
0.000435678 31800 0.000878981 31200 0.001814292
32100 0.001858652 30100 0.000597107 29900 0.000820433 29500
0.000435527 31800 0.000879493 31200 0.001812385
32100 0.001859376 30100 0.000595817 29900 0.000819552 29500
0.000435364 31800 0.000880029 31200 0.001810482
32100 0.001860086 30100 0.000594536 29900 0.000818691 29500
0.000435214 31800 0.000880569 31200 0.001808589
32100 0.001860805 30100 0.000593255 29900 0.000817816 29500
0.000435075 31800 0.00088109 31200 0.001806697
32100 0.001861518 30100 0.000591985 29900 0.000816951 29500
0.000434931 31800 0.000881633 31200 0.001804817
32100 0.001862249 30100 0.000590716 29900 0.000816083 29500
0.00043479 31800 0.000882188 31200 0.001802922
32100 0.001863001 30100 0.000589458 29900 0.000815236 29500
0.000434641 31800 0.000882722 31200 0.001801036
32100 0.001863721 30100 0.000588205 29900 0.000814395 29500
0.000434496 31800 0.000883309 31200 0.001799157
32100 0.001864447 30100 0.000586984 29900 0.000813534 29500
0.000434345 31800 0.000883876 31200 0.001797316
32100 0.001865195 30100 0.000585786 29900 0.000812694 29500
0.000434215 31800 0.000884434 31200 0.001795444
32100 0.001865971 30100 0.000584616 29900 0.000811862 29500
0.000434087 31800 0.000884995 31200 0.001793613
32100 0.001866731 30100 0.000583451 29900 0.000811041 29500
0.000433924 31800 0.000885557 31200 0.001791786
32100 0.001867482 30100 0.000582308 29900 0.000810207 29500
0.000433774 31800 0.000886124 31200 0.001789923
32100 0.001868224 30100 0.000581179 29900 0.000809372 29500
0.000433629 31800 0.000886694 31200 0.00178809
32100 0.001869007 30100 0.000580053 29900 0.000808561 29500
0.000433493 31800 0.000887281 31200 0.001786247
32100 0.001869786 30100 0.000578912 29900 0.000807741 29500
0.000433339 31800 0.00088785 31200 0.001784434
32100 0.001870577 30100 0.000577766 29900 0.000806932 29500
0.000433179 31800 0.000888423 31200 0.00178262
32100 0.001871386 30100 0.000576609 29900 0.000806144 29500
0.000433009 31800 0.000889004 31200 0.001780831
32100 0.001872174 30100 0.000575446 29900 0.000805336 29500
0.000432821 31800 0.000889581 31200 0.001779028
32100 0.001872975 30100 0.000574268 29900 0.000804548 29500
0.000432652 31800 0.000890177 31200 0.001777231
32100 0.001873794 30100 0.000573102 29900 0.000803754 29500
0.000432491 31800 0.000890766 31200 0.001775426
32100 0.001874604 30100 0.000571938 29900 0.000802955 29500
0.00043234 31800 0.000891348 31200 0.001773635
32200 0.0018754 30100 0.000570772 29900 0.000802172 29500
0.000432192 31800 0.000891924 31200 0.00177185
32200 0.00187622 30100 0.000569608 29900 0.000801392 29500
0.00043208 31800 0.000892496 31200 0.001770045
32200 0.001877031 30100 0.000568464 29900 0.000800602 29500
0.000432001 31800 0.000893068 31200 0.001768238
32200 0.001877841 30100 0.000567334 29900 0.000799832 29500
0.000431942 31800 0.000893648 31200 0.001766462
32200 0.001878655 30100 0.000566207 29900 0.000799063 29500
0.000431892 31800 0.000894222 31200 0.001764679
32200 0.00187945 30100 0.0005651 29900 0.000798291 29500
0.000431853 31800 0.000894808 31200 0.001762893
32200 0.001880272 30100 0.00056399 29900 0.000797521 29500
0.00043181 31800 0.000895375 31200 0.001761114
32200 0.00188111 30100 0.000562887 29900 0.000796774 29500
0.000431744 31800 0.000895953 31200 0.001759356
32200 0.001881934 30100 0.000561777 29900 0.000796017 29500
0.000431655 31800 0.000896525 31200 0.001757571

32200 0.001882776 30100 0.00056069 29900 0.000795253 29500
0.000431543 31800 0.000897095 31300 0.001755794
32200 0.001883603 30100 0.000559591 29900 0.000794507 29500
0.000431416 31800 0.000897666 31300 0.001753994
32200 0.001884417 30100 0.000558502 29900 0.000793757 29500
0.000431304 31800 0.000898238 31300 0.001752208
32200 0.001885264 30100 0.000557419 29900 0.000793013 29500
0.00043111 31800 0.000898813 31300 0.001750429
32200 0.001886098 30100 0.000556335 29900 0.000792277 29500
0.000430941 31800 0.000899382 31300 0.001748648
32200 0.001886958 30100 0.000555265 29900 0.000791539 29500
0.000430761 31800 0.000899968 31300 0.001746885
32200 0.001887837 30100 0.00055419 29900 0.000790819 29500
0.000430591 31800 0.000900542 31300 0.001745106
32200 0.001888693 30100 0.000553103 29900 0.000790085 29500
0.000430437 31800 0.00090113 31300 0.001743317
32200 0.001889563 30100 0.000552038 29900 0.000789364 29500
0.000430302 31800 0.00090172 31300 0.001741559
32200 0.001890418 30200 0.000550973 29900 0.000788657 29500
0.000430189 31800 0.000902308 31300 0.001739781
32200 0.001891291 30200 0.000549894 29900 0.000787941 29500
0.000430102 31800 0.000902904 31300 0.001737996
32200 0.001892145 30200 0.000548827 30000 0.000787223 29500
0.000430033 31800 0.000903499 31300 0.00173621
32200 0.001893014 30200 0.000547771 30000 0.000786512 29500
0.000429963 31800 0.0009041 31300 0.001734419
32200 0.001893892 30200 0.000546726 30000 0.000785816 29500
0.000429898 31800 0.000904703 31300 0.001732628
32200 0.001894798 30200 0.000545696 30000 0.000785138 29500
0.000429838 31800 0.00090531 31300 0.001730846
32200 0.001895663 30200 0.000544681 30000 0.000784436 29600
0.000429718 31800 0.000905921 31300 0.001729075
32200 0.001896568 30200 0.000543675 30000 0.000783753 29600
0.000429589 31800 0.000906532 31300 0.001727301
32200 0.001897461 30200 0.000542675 30000 0.000783078 29600
0.000429463 31800 0.000907155 31300 0.001725547
32200 0.001898338 30200 0.00054168 30000 0.000782404 29600
0.000429285 31800 0.000907765 31300 0.001723781
32200 0.001899251 30200 0.00054069 30000 0.000781732 29600
0.000429135 31800 0.000908391 31300 0.001722009
32200 0.001900139 30200 0.000539696 30000 0.00078106 29600
0.000428958 31800 0.000909014 31300 0.001720226
32200 0.001901008 30200 0.000538684 30000 0.000780382 29600
0.000428794 31800 0.000909637 31300 0.00171845
32200 0.001901943 30200 0.000537667 30000 0.000779728 29600
0.000428616 31800 0.000910267 31300 0.001716699
32200 0.001902837 30200 0.000536645 30000 0.000779071 29600
0.000428449 31800 0.000910887 31300 0.001714952
32200 0.001903749 30200 0.000535626 30000 0.000778429 29600
0.000428281 31800 0.000911517 31300 0.001713202
32200 0.001904651 30200 0.000534619 30000 0.000777779 29600
0.000428137 31800 0.00091214 31300 0.001711466
32200 0.001905585 30200 0.000533604 30000 0.000777135 29600
0.000428023 31900 0.000912768 31300 0.001709728
32200 0.001906535 30200 0.000532599 30000 0.000776495 29600
0.000427868 31900 0.000913398 31300 0.001707998
32200 0.001907433 30200 0.000531606 30000 0.000775856 29600
0.000427767 31900 0.000914033 31300 0.001706269
32200 0.001908339 30200 0.000530597 30000 0.000775229 29600
0.000427611 31900 0.000914662 31300 0.001704537
32200 0.001909257 30200 0.000529613 30000 0.000774591 29600
0.000427492 31900 0.000915296 31300 0.001702799
32200 0.001910166 30200 0.00052863 30000 0.000773965 29600
0.000427348 31900 0.000915924 31300 0.001701075
32200 0.001911104 30200 0.000527652 30000 0.000773339 29600
0.000427195 31900 0.000916555 31300 0.001699354
32200 0.001912024 30200 0.000526676 30000 0.000772735 29600
0.000427048 31900 0.000917185 31300 0.001697647

32200 0.001912954 30200 0.000525704 30000 0.000772116 29600
0.000426883 31900 0.000917818 31300 0.001695939
32200 0.001913872 30200 0.000524722 30000 0.000771505 29600
0.000426741 31900 0.00091845 31300 0.001694251
32200 0.001914801 30200 0.000523749 30000 0.000770908 29600
0.000426596 31900 0.000919089 31300 0.001692579
32200 0.001915755 30200 0.00052279 30000 0.000770304 29600
0.000426454 31900 0.000919719 31300 0.001690885
32200 0.001916701 30200 0.000521848 30000 0.000769707 29600
0.000426325 31900 0.000920365 31300 0.001689205
32200 0.001917617 30200 0.000520922 30000 0.000769111 29600
0.000426144 31900 0.000921012 31300 0.001687501
32200 0.001918511 30200 0.000519999 30000 0.000768525 29600
0.000426028 31900 0.000921658 31300 0.00168581
32200 0.001919473 30200 0.000519084 30000 0.000767946 29600
0.000425874 31900 0.000922312 31300 0.001684129
32200 0.001920408 30200 0.000518163 30000 0.000767364 29600
0.000425748 31900 0.000922956 31300 0.001682466
32200 0.001921368 30200 0.000517241 30000 0.000766799 29600
0.000425629 31900 0.000923612 31300 0.001680815
32200 0.001922309 30200 0.000516323 30000 0.000766231 29600
0.000425511 31900 0.000924263 31300 0.001679152
32200 0.001923278 30200 0.00051539 30000 0.000765672 29600
0.000425433 31900 0.000924929 31300 0.00167749
32200 0.001924251 30200 0.000514466 30000 0.000765108 29600
0.000425337 31900 0.000925593 31300 0.001675819
32200 0.001925197 30200 0.000513542 30000 0.000764557 29600
0.000425241 31900 0.000926253 31300 0.001674169
32200 0.001926158 30200 0.000512606 30000 0.000764007 29600
0.000425163 31900 0.000926916 31300 0.001672508
32200 0.00192709 30200 0.000511685 30000 0.000763456 29600
0.000425072 31900 0.000927574 31300 0.001670857
32200 0.001928015 30200 0.000510759 30000 0.000762914 29600
0.000424988 31900 0.000928238 31300 0.00166919
32200 0.001928976 30200 0.000509852 30000 0.000762381 29600
0.000424914 31900 0.000928908 31300 0.001667525
32200 0.001929922 30200 0.000508941 30000 0.000761856 29600
0.000424828 31900 0.000929573 31300 0.001665864
32200 0.001930887 30200 0.000508033 30000 0.000761327 29600
0.000424771 31900 0.000930251 31300 0.001664233
32200 0.001931845 30200 0.000507121 30000 0.000760812 29600
0.000424694 31900 0.000930931 31300 0.001662574
32300 0.001932827 30200 0.0005062 30000 0.000760302 29600
0.000424623 31900 0.000931624 31300 0.001660939
32300 0.001933801 30200 0.000505282 30000 0.000759797 29600
0.000424565 31900 0.000932306 31300 0.001659307
32300 0.001934808 30200 0.000504365 30000 0.000759282 29600
0.000424527 31900 0.000933001 31300 0.001657642
32300 0.001935738 30200 0.000503436 30000 0.00075877 29600
0.000424478 31900 0.000933712 31300 0.001655988
32300 0.001936713 30200 0.000502503 30000 0.000758268 29600
0.000424458 31900 0.000934432 31300 0.001654349
32300 0.001937723 30200 0.000501575 30000 0.00075777 29600
0.000424409 31900 0.000935161 31300 0.001652718
32300 0.001938711 30200 0.000500651 30000 0.000757287 29600
0.00042438 31900 0.000935872 31300 0.00165108
32300 0.001939718 30200 0.000499739 30000 0.000756806 29600
0.00042434 31900 0.000936618 31300 0.001649452
32300 0.001940724 30200 0.000498857 30000 0.000756325 29600
0.0004243 31900 0.000937363 31300 0.001647819
32300 0.001941736 30200 0.000497972 30000 0.000755849 29600
0.000424218 31900 0.000938114 31400 0.001646197
32300 0.001942739 30200 0.000497115 30000 0.000755373 29600
0.000424151 31900 0.000938875 31400 0.001644572
32300 0.001943726 30200 0.000496276 30000 0.000754906 29600
0.000424065 31900 0.000939638 31400 0.001642963
32300 0.001944723 30200 0.000495414 30000 0.000754446 29600
0.000423953 31900 0.000940414 31400 0.001641341

32300 0.001945735 30200 0.000494584 30000 0.000753982 29600
0.00042382 31900 0.000941187 31400 0.00163972
32300 0.001946769 30200 0.00049375 30000 0.000753528 29600
0.000423667 31900 0.000941973 31400 0.001638075
32300 0.001947795 30200 0.000492933 30000 0.000753086 29600
0.000423497 31900 0.000942757 31400 0.001636456
32300 0.001948822 30200 0.000492112 30000 0.000752644 29600
0.000423323 31900 0.000943547 31400 0.001634842
32300 0.001949843 30200 0.000491296 30000 0.000752209 29600
0.000423163 31900 0.000944336 31400 0.001633237
32300 0.00195084 30300 0.000490457 30000 0.000751775 29600
0.000422993 31900 0.000945129 31400 0.001631633
32300 0.001951866 30300 0.000489626 30000 0.000751362 29600
0.000422835 31900 0.000945935 31400 0.001630057
32300 0.00195292 30300 0.000488799 30100 0.00075094 29600
0.000422672 31900 0.000946731 31400 0.001628463
32300 0.001953959 30300 0.000487972 30100 0.000750521 29600
0.000422521 31900 0.000947521 31400 0.001626853
32300 0.001954939 30300 0.000487138 30100 0.000750103 29600
0.000422373 31900 0.000948327 31400 0.001625245
32300 0.001955947 30300 0.000486333 30100 0.000749704 29600
0.000422243 31900 0.000949137 31400 0.001623642
32300 0.00195698 30300 0.000485515 30100 0.000749307 29700
0.000422132 31900 0.000949942 31400 0.001622055
32300 0.001958011 30300 0.000484733 30100 0.000748918 29700
0.000422057 31900 0.000950731 31400 0.001620454
32300 0.001959009 30300 0.000483925 30100 0.000748541 29700
0.000421999 31900 0.000951544 31400 0.001618886
32300 0.001960041 30300 0.000483124 30100 0.000748152 29700
0.000421935 31900 0.000952355 31400 0.001617305
32300 0.001961045 30300 0.000482323 30100 0.000747784 29700
0.00042188 31900 0.000953164 31400 0.001615732
32300 0.001962077 30300 0.000481526 30100 0.000747415 29700
0.000421826 31900 0.00095398 31400 0.001614158
32300 0.001963066 30300 0.000480732 30100 0.000747053 29700
0.000421753 31900 0.000954798 31400 0.001612571
32300 0.001964081 30300 0.000479901 30100 0.000746698 29700
0.000421701 31900 0.00095561 31400 0.001611011
32300 0.001965086 30300 0.000479061 30100 0.000746337 29700
0.000421622 31900 0.000956433 31400 0.001609434
32300 0.001966068 30300 0.000478213 30100 0.000745984 29700
0.000421567 31900 0.000957248 31400 0.00160786
32300 0.001967077 30300 0.000477385 30100 0.000745643 29700
0.000421494 31900 0.000958078 31400 0.001606292
32300 0.001968072 30300 0.000476543 30100 0.000745308 29700
0.00042145 32000 0.000958904 31400 0.001604731
32300 0.001969051 30300 0.000475691 30100 0.000744977 29700
0.000421395 32000 0.000959734 31400 0.001603171
32300 0.001970051 30300 0.000474862 30100 0.000744639 29700
0.000421362 32000 0.000960561 31400 0.001601618
32300 0.001971015 30300 0.000474038 30100 0.000744331 29700
0.000421299 32000 0.000961415 31400 0.001600074
32300 0.001972021 30300 0.000473215 30100 0.00074401 29700
0.000421229 32000 0.000962259 31400 0.001598531
32300 0.001973013 30300 0.000472407 30100 0.000743693 29700
0.000421164 32000 0.000963115 31400 0.001596978
32300 0.001973986 30300 0.000471615 30100 0.000743389 29700
0.000421087 32000 0.00096397 31400 0.001595432
32300 0.001974957 30300 0.000470821 30100 0.000743088 29700
0.00042101 32000 0.000964836 31400 0.001593876
32300 0.001975928 30300 0.000470041 30100 0.000742786 29700
0.000420909 32000 0.0009657 31400 0.001592334
32300 0.001976898 30300 0.000469278 30100 0.000742503 29700
0.00042082 32000 0.000966558 31400 0.001590797
32300 0.001977877 30300 0.000468504 30100 0.000742227 29700
0.00042073 32000 0.000967439 31400 0.001589268
32300 0.001978875 30300 0.000467736 30100 0.000741951 29700
0.000420627 32000 0.000968313 31400 0.001587747

32300 0.00197985 30300 0.000466972 30100 0.000741684 29700
0.000420538 32000 0.000969184 31400 0.00158622
32300 0.001980829 30300 0.000466221 30100 0.000741423 29700
0.000420457 32000 0.000970066 31400 0.001584703
32300 0.00198177 30300 0.000465468 30100 0.000741159 29700
0.00042037 32000 0.000970943 31400 0.001583164
32300 0.001982743 30300 0.000464708 30100 0.000740913 29700
0.000420318 32000 0.000971832 31400 0.001581653
32300 0.00198369 30300 0.000463952 30100 0.000740664 29700
0.000420263 32000 0.000972704 31400 0.001580147
32300 0.001984657 30300 0.0004632 30100 0.000740442 29700
0.000420231 32000 0.000973577 31400 0.001578609
32300 0.001985613 30300 0.000462452 30100 0.000740205 29700
0.000420214 32000 0.00097445 31400 0.001577105
32300 0.001986594 30300 0.000461709 30100 0.000739997 29700
0.000420206 32000 0.000975323 31400 0.00157559
32300 0.001987546 30300 0.00046098 30100 0.000739779 29700
0.000420193 32000 0.000976202 31400 0.001574075
32300 0.001988485 30300 0.000460239 30100 0.000739584 29700
0.000420187 32000 0.000977068 31400 0.001572572
32300 0.001989468 30300 0.000459499 30100 0.000739397 29700
0.00042017 32000 0.000977956 31400 0.001571088
32300 0.001990431 30300 0.000458767 30100 0.000739211 29700
0.000420155 32000 0.000978833 31400 0.001569589
32300 0.001991378 30300 0.00045802 30100 0.000739032 29700
0.000420154 32000 0.000979709 31400 0.001568093
32300 0.001992341 30300 0.000457271 30100 0.000738865 29700
0.000420166 32000 0.000980593 31400 0.001566584
32300 0.001993268 30300 0.00045652 30100 0.000738711 29700
0.000420171 32000 0.000981474 31400 0.001565095
32300 0.00199422 30300 0.000455773 30100 0.000738563 29700
0.000420178 32000 0.00098236 31400 0.001563601
32300 0.001995182 30300 0.000455033 30100 0.000738428 29700
0.000420193 32000 0.000983231 31400 0.001562111
32400 0.00199614 30300 0.000454303 30100 0.000738297 29700
0.000420207 32000 0.00098412 31400 0.001560648
32400 0.00199711 30300 0.000453581 30100 0.00073818 29700
0.000420225 32000 0.000985009 31400 0.001559143
32400 0.001998064 30300 0.000452854 30100 0.000738061 29700
0.000420227 32000 0.000985897 31400 0.001557662
32400 0.001999065 30300 0.000452159 30100 0.000737971 29700
0.000420241 32000 0.000986792 31400 0.001556176
32400 0.002000011 30300 0.000451461 30100 0.000737869 29700
0.000420229 32000 0.000987688 31400 0.001554695
32400 0.002000954 30300 0.000450746 30100 0.000737786 29700
0.000420235 32000 0.000988587 31400 0.001553212
32400 0.0020019 30300 0.000450032 30100 0.000737708 29700
0.00042022 32000 0.000989482 31400 0.001551729
32400 0.002002883 30300 0.000449318 30100 0.000737641 29700
0.000420221 32000 0.000990383 31400 0.001550238
32400 0.002003853 30300 0.000448604 30100 0.000737572 29700
0.000420219 32000 0.000991281 31400 0.001548752
32400 0.002004862 30300 0.000447893 30100 0.000737525 29700
0.000420218 32000 0.000992193 31500 0.001547266
32400 0.002005834 30300 0.000447183 30100 0.000737477 29700
0.000420196 32000 0.000993102 31500 0.001545794
32400 0.002006867 30300 0.000446467 30100 0.000737435 29700
0.000420159 32000 0.000994019 31500 0.001544318
32400 0.002007873 30300 0.000445757 30100 0.00073742 29700
0.00042011 32000 0.000994949 31500 0.001542858
32400 0.002008931 30300 0.000445041 30100 0.000737398 29700
0.000420039 32000 0.000995875 31500 0.001541415
32400 0.002009944 30300 0.000444326 30100 0.000737384 29700
0.000419988 32000 0.000996808 31500 0.001539944
32400 0.002010974 30300 0.000443624 30100 0.000737375 29700
0.000419935 32000 0.000997741 31500 0.001538473
32400 0.002011966 30300 0.000442918 30100 0.000737376 29700
0.000419888 32000 0.000998682 31500 0.001537005

32400 0.002013028 30300 0.000442207 30100 0.000737388 29700
0.000419849 32000 0.000999627 31500 0.001535557
32400 0.002014093 30400 0.000441514 30100 0.0007374 29700
0.000419818 32000 0.001000565 31500 0.001534087
32400 0.002015172 30400 0.00044082 30100 0.000737436 29700
0.000419798 32000 0.001001521 31500 0.001532645
32400 0.002016241 30400 0.000440128 30200 0.000737473 29700
0.000419769 32000 0.001002469 31500 0.001531199
32400 0.002017305 30400 0.000439427 30200 0.000737513 29700
0.000419732 32000 0.00100342 31500 0.001529756
32400 0.002018381 30400 0.000438742 30200 0.000737576 29700
0.000419708 32000 0.001004379 31500 0.001528315
32400 0.002019453 30400 0.000438039 30200 0.000737639 29700
0.000419655 32000 0.001005341 31500 0.001526867
32400 0.002020538 30400 0.000437338 30200 0.000737712 29800
0.000419604 32000 0.001006302 31500 0.001525433
32400 0.002021605 30400 0.000436647 30200 0.000737796 29800
0.000419549 32000 0.001007251 31500 0.001523996
32400 0.002022696 30400 0.000435954 30200 0.000737891 29800
0.000419476 32000 0.001008212 31500 0.00152254
32400 0.002023787 30400 0.000435273 30200 0.000737989 29800
0.000419426 32000 0.001009163 31500 0.001521107
32400 0.002024895 30400 0.000434596 30200 0.000738108 29800
0.000419373 32000 0.001010135 31500 0.00151967
32400 0.002025987 30400 0.00043394 30200 0.000738227 29800
0.000419312 32000 0.001011105 31500 0.001518243
32400 0.002027099 30400 0.000433291 30200 0.000738358 29800
0.00041928 32000 0.001012075 31500 0.001516802
32400 0.002028219 30400 0.000432638 30200 0.000738506 29800
0.000419251 32000 0.001013054 31500 0.001515397
32400 0.002029314 30400 0.000431996 30200 0.00073866 29800
0.000419228 32000 0.00101403 31500 0.001513986
32400 0.002030442 30400 0.000431346 30200 0.000738823 29800
0.000419227 32000 0.001015023 31500 0.001512569
32400 0.002031522 30400 0.000430691 30200 0.00073899 29800
0.000419258 32000 0.001016015 31500 0.001511153
32400 0.002032595 30400 0.000430037 30200 0.000739174 29800
0.000419303 32100 0.00101701 31500 0.00150974
32400 0.00203371 30400 0.000429373 30200 0.000739362 29800
0.000419368 32100 0.001018012 31500 0.001508349
32400 0.002034802 30400 0.000428712 30200 0.000739569 29800
0.000419435 32100 0.001019003 31500 0.001506945
32400 0.002035953 30400 0.000428048 30200 0.000739797 29800
0.000419496 32100 0.00102002 31500 0.001505551
32400 0.002037077 30400 0.000427386 30200 0.000740018 29800
0.000419563 32100 0.001021026 31500 0.00150416
32400 0.002038212 30400 0.000426736 30200 0.000740238 29800
0.000419592 32100 0.001022039 31500 0.001502777
32400 0.002039363 30400 0.000426089 30200 0.000740506 29800
0.000419621 32100 0.001023058 31500 0.001501394
32400 0.002040465 30400 0.000425451 30200 0.000740762 29800
0.000419611 32100 0.001024095 31500 0.001500011
32400 0.002041587 30400 0.000424783 30200 0.000741045 29800
0.00041956 32100 0.001025118 31500 0.001498636
32400 0.002042723 30400 0.000424129 30200 0.000741324 29800
0.0004195 32100 0.001026157 31500 0.001497262
32400 0.002043853 30400 0.000423475 30200 0.000741611 29800
0.000419396 32100 0.001027183 31500 0.00149587
32400 0.002044989 30400 0.000422809 30200 0.000741919 29800
0.000419298 32100 0.001028219 31500 0.001494497
32400 0.002046143 30400 0.00042215 30200 0.000742248 29800
0.000419192 32100 0.00102927 31500 0.001493129
32400 0.002047264 30400 0.000421492 30200 0.000742562 29800
0.000419105 32100 0.00103032 31500 0.001491753
32400 0.002048426 30400 0.000420834 30200 0.0007429 29800
0.000419012 32100 0.001031375 31500 0.001490384
32400 0.002049594 30400 0.000420177 30200 0.000743274 29800
0.000418937 32100 0.001032441 31500 0.00148904

32400 0.002050774 30400 0.00041951 30200 0.000743638 29800
0.000418897 32100 0.001033509 31500 0.001487693
32400 0.002051977 30400 0.000418838 30200 0.000744025 29800
0.000418847 32100 0.001034583 31500 0.001486331
32400 0.002053143 30400 0.000418171 30200 0.000744423 29800
0.000418824 32100 0.001035667 31500 0.001484969
32400 0.002054335 30400 0.000417492 30200 0.000744827 29800
0.000418806 32100 0.00103675 31500 0.001483604
32400 0.002055541 30400 0.000416818 30200 0.000745256 29800
0.000418794 32100 0.001037838 31500 0.001482254
32400 0.002056759 30400 0.00041614 30200 0.000745712 29800
0.000418762 32100 0.001038924 31500 0.001480891
32400 0.002058014 30400 0.00041547 30200 0.000746172 29800
0.000418755 32100 0.001040017 31500 0.001479546
32400 0.00205924 30400 0.000414797 30200 0.000746636 29800
0.000418742 32100 0.001041124 31500 0.001478205
32400 0.002060493 30400 0.000414142 30200 0.00074712 29800
0.000418753 32100 0.001042225 31500 0.001476864
32400 0.002061768 30400 0.0004135 30200 0.000747638 29800
0.000418784 32100 0.001043338 31500 0.001475531
32400 0.002062988 30400 0.000412861 30200 0.000748162 29800
0.000418831 32100 0.001044452 31500 0.001474192
32400 0.002064222 30400 0.000412234 30200 0.0007487 29800
0.000418899 32100 0.001045577 31500 0.001472862
32400 0.002065472 30400 0.000411609 30200 0.000749262 29800
0.000418952 32100 0.00104669 31500 0.001471539
32500 0.002066714 30400 0.000411015 30200 0.000749834 29800
0.000419019 32100 0.001047817 31500 0.001470197
32500 0.002067978 30400 0.000410401 30200 0.00075042 29800
0.000419112 32100 0.001048943 31500 0.001468868
32500 0.00206922 30400 0.000409816 30200 0.000751035 29800
0.000419203 32100 0.001050083 31500 0.001467549
32500 0.002070461 30400 0.000409227 30200 0.000751659 29800
0.000419309 32100 0.001051218 31500 0.001466234
32500 0.002071742 30400 0.00040865 30200 0.000752297 29800
0.000419417 32100 0.001052359 31500 0.001464918
32500 0.002073037 30400 0.00040806 30200 0.000752967 29800
0.000419522 32100 0.001053518 31500 0.001463611
32500 0.002074323 30400 0.000407483 30200 0.000753657 29800
0.000419618 32100 0.001054672 31500 0.001462321
32500 0.002075619 30400 0.000406903 30200 0.000754351 29800
0.000419669 32100 0.001055834 31500 0.001461013
32500 0.002076866 30400 0.000406328 30200 0.000755073 29800
0.00041972 32100 0.001056992 31500 0.00145971
32500 0.002078158 30400 0.000405756 30200 0.000755813 29800
0.000419763 32100 0.001058159 31600 0.001458413
32500 0.002079451 30400 0.00040518 30200 0.000756574 29800
0.000419843 32100 0.001059333 31600 0.001457134
32500 0.002080733 30400 0.000404597 30200 0.00075737 29800
0.000419851 32100 0.001060504 31600 0.001455841
32500 0.002082051 30400 0.00040401 30200 0.000758191 29800
0.000419891 32100 0.001061681 31600 0.001454572
32500 0.002083354 30400 0.000403401 30200 0.00075901 29800
0.000419924 32100 0.001062864 31600 0.001453296
32500 0.002084642 30400 0.000402802 30200 0.000759871 29800
0.000419898 32100 0.001064045 31600 0.001452028
32500 0.002085991 30400 0.0004022 30200 0.000760746 29800
0.000419859 32100 0.001065229 31600 0.001450763
32500 0.002087268 30400 0.000401591 30200 0.000761645 29800
0.000419822 32100 0.001066423 31600 0.001449495
32500 0.002088581 30400 0.000400988 30200 0.000762582 29800
0.000419792 32100 0.001067613 31600 0.001448227
32500 0.002089916 30500 0.000400381 30200 0.000763532 29800
0.000419781 32100 0.001068815 31600 0.001446964
32500 0.002091228 30500 0.000399802 30200 0.000764506 29800
0.000419721 32100 0.001070012 31600 0.001445684
32500 0.002092534 30500 0.000399211 30300 0.000765495 29800
0.000419734 32100 0.001071215 31600 0.001444406

32500 0.002093881 30500 0.000398632 30300 0.000766536 29800
0.000419713 32100 0.001072425 31600 0.001443152
32500 0.002095197 30500 0.000398056 30300 0.000767574 29800
0.000419643 32100 0.001073635 31600 0.001441884
32500 0.002096527 30500 0.000397477 30300 0.000768656 29800
0.000419648 32100 0.001074852 31600 0.001440616
32500 0.002097855 30500 0.000396883 30300 0.000769762 29900
0.000419544 32100 0.001076079 31600 0.001439366
32500 0.00209918 30500 0.0003963 30300 0.000770891 29900
0.000419539 32100 0.001077312 31600 0.001438096
32500 0.002100539 30500 0.000395696 30300 0.000772044 29900
0.0004195 32100 0.001078554 31600 0.001436817
32500 0.00210188 30500 0.000395094 30300 0.000773219 29900
0.000419432 32100 0.0010798 31600 0.001435539
32500 0.002103186 30500 0.000394493 30300 0.000774441 29900
0.000419458 32100 0.001081057 31600 0.001434273
32500 0.002104521 30500 0.000393889 30300 0.000775672 29900
0.000419475 32100 0.001082312 31600 0.001432998
32500 0.002105859 30500 0.000393288 30300 0.000776948 29900
0.000419553 32100 0.00108358 31600 0.001431721
32500 0.002107215 30500 0.000392707 30300 0.000778248 29900
0.000419624 32100 0.001084854 31600 0.00143045
32500 0.00210858 30500 0.000392125 30300 0.000779582 29900
0.000419701 32100 0.001086139 31600 0.001429182
32500 0.002109913 30500 0.000391574 30300 0.000780951 29900
0.000419818 32100 0.001087427 31600 0.001427921
32500 0.002111289 30500 0.000391041 30300 0.000782349 29900
0.000419942 32100 0.001088717 31600 0.001426653
32500 0.002112603 30500 0.000390515 30300 0.000783781 29900
0.000420034 32200 0.00109002 31600 0.001425386
32500 0.002113961 30500 0.000390002 30300 0.00078525 29900
0.000420125 32200 0.001091323 31600 0.001424116
32500 0.002115298 30500 0.000389488 30300 0.000786748 29900
0.000420203 32200 0.001092635 31600 0.001422851
32500 0.002116631 30500 0.000388988 30300 0.000788288 29900
0.00042029 32200 0.001093945 31600 0.001421572
32500 0.002117979 30500 0.000388459 30300 0.000789847 29900
0.000420358 32200 0.001095261 31600 0.001420288
32500 0.002119336 30500 0.000387946 30300 0.000791456 29900
0.000420394 32200 0.001096589 31600 0.001419035
32500 0.002120646 30500 0.00038742 30300 0.000793104 29900
0.000420479 32200 0.00109792 31600 0.001417777
32500 0.002121998 30500 0.000386886 30300 0.000794793 29900
0.000420563 32200 0.001099258 31600 0.001416514
32500 0.00212333 30500 0.000386355 30300 0.000796497 29900
0.000420659 32200 0.001100601 31600 0.001415273
32500 0.002124677 30500 0.000385819 30300 0.000798257 29900
0.000420767 32200 0.001101962 31600 0.001414029
32500 0.002126045 30500 0.000385278 30300 0.000800042 29900
0.000420853 32200 0.001103322 31600 0.001412757
32500 0.002127376 30500 0.000384722 30300 0.000801868 29900
0.000420979 32200 0.001104691 31600 0.001411493
32500 0.00212869 30500 0.000384179 30300 0.000803736 29900
0.000421089 32200 0.001106073 31600 0.001410244
32500 0.002130045 30500 0.000383625 30300 0.000805641 29900
0.000421216 32200 0.001107447 31600 0.001408994
32500 0.002131381 30500 0.000383059 30300 0.000807587 29900
0.000421308 32200 0.001108842 31600 0.001407761
32500 0.002132726 30500 0.000382503 30300 0.00080959 29900
0.000421437 32200 0.001110244 31600 0.001406531
32500 0.002134056 30500 0.000381949 30300 0.000811624 29900
0.000421544 32200 0.001111656 31600 0.001405302
32500 0.002135403 30500 0.000381398 30300 0.000813676 29900
0.000421618 32200 0.001113066 31600 0.00140409
32500 0.00213675 30500 0.000380845 30300 0.000815799 29900
0.000421713 32200 0.00111449 31600 0.001402856
32500 0.002138068 30500 0.000380304 30300 0.000817958 29900
0.000421812 32200 0.001115926 31600 0.001401644

32500 0.002139396 30500 0.00037975 30300 0.000820161 29900
0.000421908 32200 0.00111736 31600 0.001400433
32500 0.002140742 30500 0.000379199 30300 0.000822404 29900
0.00042203 32200 0.001118801 31600 0.001399213
32500 0.002142058 30500 0.000378681 30300 0.0008247 29900
0.000422122 32200 0.001120242 31600 0.001397996
32500 0.002143397 30500 0.000378132 30300 0.000827034 29900
0.000422235 32200 0.001121686 31600 0.00139677
32500 0.002144735 30500 0.000377593 30300 0.000829411 29900
0.000422354 32200 0.001123145 31600 0.001395587
32500 0.002146044 30500 0.00037706 30300 0.000831844 29900
0.000422451 32200 0.0011246 31600 0.001394388
32500 0.002147386 30500 0.000376532 30300 0.000834327 29900
0.00042253 32200 0.00112607 31600 0.001393173
32500 0.00214873 30500 0.000375978 30300 0.000836854 29900
0.000422597 32200 0.001127533 31600 0.001391978
32500 0.0021501 30500 0.000375435 30300 0.000839414 29900
0.000422621 32200 0.001129009 31600 0.001390796
32600 0.002151446 30500 0.000374886 30300 0.000842044 29900
0.000422647 32200 0.00113049 31600 0.001389591
32600 0.002152785 30500 0.000374343 30300 0.000844712 29900
0.000422655 32200 0.001131972 31600 0.001388381
32600 0.002154101 30500 0.000373819 30300 0.000847459 29900
0.000422641 32200 0.001133449 31600 0.001387196
32600 0.002155469 30500 0.000373311 30300 0.000850255 29900
0.000422669 32200 0.001134947 31600 0.001386004
32600 0.002156806 30500 0.000372782 30300 0.000853081 29900
0.000422694 32200 0.001136451 31600 0.001384795
32600 0.002158198 30500 0.000372284 30300 0.000855984 29900
0.000422724 32200 0.001137953 31600 0.001383609
32600 0.002159582 30500 0.000371786 30300 0.000858956 29900
0.000422762 32200 0.001139477 31600 0.001382419
32600 0.002160957 30500 0.000371303 30300 0.000861963 29900
0.000422817 32200 0.001140987 31600 0.001381234
32600 0.002162362 30500 0.00037083 30300 0.000865048 29900
0.000422857 32200 0.00114252 31600 0.001380044
32600 0.002163741 30500 0.000370321 30300 0.000868186 29900
0.000422891 32200 0.001144059 31700 0.001378853
32600 0.002165134 30500 0.000369818 30300 0.000871393 29900
0.000422928 32200 0.001145595 31700 0.001377673
32600 0.002166541 30500 0.000369315 30300 0.00087465 29900
0.000422975 32200 0.00114714 31700 0.001376488
32600 0.002167935 30500 0.000368827 30300 0.000878004 29900
0.000423015 32200 0.001148688 31700 0.001375277
32600 0.002169368 30500 0.000368318 30300 0.000881407 29900
0.000423115 32200 0.001150245 31700 0.001374084
32600 0.002170788 30500 0.000367795 30300 0.000884869 29900
0.0004232 32200 0.001151806 31700 0.00137291
32600 0.002172202 30500 0.000367271 30300 0.000888409 29900
0.000423297 32200 0.001153384 31700 0.001371727
32600 0.002173645 30500 0.000366743 30300 0.000892008 29900
0.000423412 32200 0.001154965 31700 0.001370527
32600 0.002175109 30500 0.000366209 30300 0.00089567 29900
0.000423516 32200 0.001156548 31700 0.001369356
32600 0.002176545 30600 0.000365701 30300 0.000899416 29900
0.000423594 32200 0.001158125 31700 0.001368182
32600 0.002178033 30600 0.000365173 30300 0.000903226 29900
0.000423668 32200 0.001159727 31700 0.001367002
32600 0.002179474 30600 0.000364664 30400 0.000907084 29900
0.000423691 32200 0.001161343 31700 0.001365823
32600 0.002180932 30600 0.000364141 30400 0.000911041 29900
0.000423699 32200 0.001162946 31700 0.001364646
32600 0.00218242 30600 0.000363638 30400 0.000915061 29900
0.000423698 32200 0.001164565 31700 0.001363485
32600 0.002183894 30600 0.000363104 30400 0.000919135 29900
0.000423646 32200 0.001166178 31700 0.001362319
32600 0.002185422 30600 0.000362581 30400 0.000923301 30000
0.000423617 32200 0.001167811 31700 0.001361156

32600 0.002186945 30600 0.000362058 30400 0.000927552 30000
0.000423578 32200 0.001169458 31700 0.001359999
32600 0.002188459 30600 0.000361534 30400 0.000931851 30000
0.000423575 32200 0.001171104 31700 0.001358842
32600 0.002190025 30600 0.000361026 30400 0.000936248 30000
0.000423591 32200 0.00117277 31700 0.001357693
32600 0.002191541 30600 0.000360508 30400 0.000940733 30000
0.000423638 32200 0.001174445 31700 0.001356537
32600 0.002193073 30600 0.000360007 30400 0.000945272 30000
0.000423688 32200 0.001176127 31700 0.001355394
32600 0.002194614 30600 0.000359508 30400 0.000949914 30000
0.000423798 32200 0.001177824 31700 0.001354243
32600 0.002196155 30600 0.000359035 30400 0.00095463 30000
0.000423904 32200 0.001179523 31700 0.001353078
32600 0.00219771 30600 0.00035855 30400 0.000959404 30000
0.000424063 32200 0.001181224 31700 0.00135193
32600 0.002199281 30600 0.000358044 30400 0.000964266 30000
0.000424217 32200 0.001182947 31700 0.001350797
32600 0.002200828 30600 0.000357571 30400 0.000969187 30000
0.000424386 32200 0.001184669 31700 0.001349649
32600 0.002202412 30600 0.000357072 30400 0.000974182 30000
0.00042457 32300 0.001186428 31700 0.001348503
32600 0.002204014 30600 0.000356589 30400 0.000979235 30000
0.000424747 32300 0.001188196 31700 0.001347377
32600 0.002205614 30600 0.000356104 30400 0.000984401 30000
0.000424907 32300 0.001189964 31700 0.001346258
32600 0.002207231 30600 0.000355623 30400 0.000989635 30000
0.000425037 32300 0.001191757 31700 0.001345104
32600 0.00220881 30600 0.000355122 30400 0.000994918 30000
0.000425153 32300 0.00119353 31700 0.001343964
32600 0.002210402 30600 0.000354626 30400 0.001000275 30000
0.000425232 32300 0.001195319 31700 0.00134282
32600 0.00221203 30600 0.000354133 30400 0.001005701 30000
0.000425309 32300 0.001197136 31700 0.001341708
32600 0.002213605 30600 0.000353633 30400 0.001011214 30000
0.000425359 32300 0.001198946 31700 0.001340571
32600 0.002215259 30600 0.00035318 30400 0.001016782 30000
0.00042542 32300 0.001200765 31700 0.00133944
32600 0.002216888 30600 0.000352707 30400 0.001022408 30000
0.000425457 32300 0.001202597 31700 0.001338319
32600 0.002218517 30600 0.000352269 30400 0.001028098 30000
0.000425532 32300 0.001204428 31700 0.001337209
32600 0.002220185 30600 0.000351816 30400 0.001033832 30000
0.000425596 32300 0.001206286 31700 0.001336074
32600 0.002221789 30600 0.000351373 30400 0.001039644 30000
0.000425693 32300 0.001208152 31700 0.001334945
32600 0.00222344 30600 0.000350915 30400 0.00104549 30000
0.000425782 32300 0.00121 31700 0.001333835
32600 0.002225068 30600 0.000350452 30400 0.001051376 30000
0.000425905 32300 0.001211876 31700 0.001332717
32600 0.002226725 30600 0.000350014 30400 0.001057313 30000
0.000426023 32300 0.001213762 31700 0.001331579
32600 0.002228364 30600 0.000349554 30400 0.001063289 30000
0.000426153 32300 0.001215638 31700 0.001330457
32600 0.00223002 30600 0.000349098 30400 0.001069295 30000
0.000426307 32300 0.001217554 31700 0.001329335
32600 0.002231658 30600 0.000348645 30400 0.00107531 30000
0.000426421 32300 0.001219442 31700 0.001328225
32600 0.002233332 30600 0.000348207 30400 0.001081353 30000
0.000426584 32300 0.00122136 31700 0.001327092
32600 0.00223501 30600 0.000347767 30400 0.001087393 30000
0.000426729 32300 0.001223286 31700 0.001325993
32600 0.002236677 30600 0.000347342 30400 0.001093445 30000
0.000426853 32300 0.001225237 31700 0.00132488
32600 0.002238384 30600 0.000346929 30400 0.001099507 30000
0.00042699 32300 0.001227176 31700 0.00132374
32600 0.002240036 30600 0.000346519 30400 0.001105537 30000
0.000427116 32300 0.001229136 31700 0.001322603

32600 0.002241697 30600 0.000346094 30400 0.001111555 30000
0.000427244 32300 0.001231104 31700 0.001321486
32600 0.002243391 30600 0.000345661 30400 0.001117538 30000
0.000427363 32300 0.001233075 31700 0.001320383
32600 0.002245084 30600 0.000345228 30400 0.001123497 30000
0.000427452 32300 0.00123506 31700 0.001319257
32600 0.002246787 30600 0.000344818 30400 0.00112939 30000
0.00042758 32300 0.001237049 31700 0.001318155
32600 0.002248495 30600 0.000344368 30400 0.001135208 30000
0.000427697 32300 0.001239066 31700 0.001317044
32700 0.002250211 30600 0.00034392 30400 0.001140959 30000
0.000427815 32300 0.001241053 31700 0.001315943
32700 0.002251946 30600 0.00034348 30400 0.001146614 30000
0.000427943 32300 0.001243089 31700 0.001314831
32700 0.002253619 30600 0.000343044 30400 0.001152169 30000
0.000428067 32300 0.001245107 31700 0.001313723
32700 0.002255322 30600 0.000342604 30400 0.001157602 30000
0.000428209 32300 0.001247148 31700 0.001312619
32700 0.002257027 30600 0.000342161 30400 0.0011629 30000
0.000428359 32300 0.001249182 31700 0.00131151
32700 0.002258738 30600 0.000341726 30400 0.001168051 30000
0.000428499 32300 0.001251238 31700 0.001310392
32700 0.002260468 30600 0.000341288 30400 0.001173026 30000
0.000428685 32300 0.001253281 31700 0.001309293
32700 0.002262204 30600 0.00034086 30400 0.001177821 30000
0.000428806 32300 0.001255355 31700 0.001308203
32700 0.00226391 30600 0.00034043 30400 0.001182398 30000
0.00042894 32300 0.001257434 31700 0.001307114
32700 0.002265644 30600 0.000340002 30400 0.001186761 30000
0.00042912 32300 0.001259509 31800 0.001306011
32700 0.002267377 30600 0.00033958 30400 0.001190861 30000
0.000429266 32300 0.001261606 31800 0.001304926
32700 0.00226912 30600 0.000339157 30400 0.001194714 30000
0.000429458 32300 0.00126371 31800 0.00130384
32700 0.002270875 30600 0.000338724 30400 0.001198286 30000
0.000429587 32300 0.001265827 31800 0.001302732
32700 0.002272598 30600 0.000338266 30400 0.00120155 30000
0.00042976 32300 0.001267956 31800 0.001301625
32700 0.002274319 30600 0.000337838 30400 0.001204484 30000
0.000429933 32300 0.001270087 31800 0.001300541
32700 0.002276108 30600 0.000337391 30400 0.001207071 30000
0.000430115 32300 0.001272225 31800 0.001299466
32700 0.002277849 30600 0.000336944 30400 0.001209309 30000
0.000430285 32300 0.001274391 31800 0.001298368
32700 0.002279613 30600 0.000336491 30400 0.001211139 30000
0.000430448 32300 0.001276554 31800 0.00129729
32700 0.002281397 30700 0.000336038 30400 0.001212551 30000
0.000430626 32300 0.001278746 31800 0.001296217
32700 0.002283156 30700 0.000335585 30400 0.001213549 30000
0.000430802 32300 0.001280934 31800 0.001295141
32700 0.002284964 30700 0.000335127 30500 0.001214102 30000
0.000430964 32300 0.001283139 31800 0.001294062
32700 0.002286717 30700 0.000334691 30500 0.001214193 30000
0.000431118 32300 0.001285362 31800 0.001292985
32700 0.002288495 30700 0.000334229 30500 0.001213788 30000
0.000431278 32300 0.001287578 31800 0.001291915
32700 0.002290282 30700 0.00033378 30500 0.001212887 30000
0.000431445 32300 0.001289807 31800 0.001290852
32700 0.00229207 30700 0.000333315 30500 0.001211483 30100
0.000431637 32300 0.001292045 31800 0.001289773
32700 0.002293876 30700 0.000332859 30500 0.001209545 30100
0.000431823 32300 0.001294283 31800 0.001288694
32700 0.002295704 30700 0.000332397 30500 0.001207064 30100
0.000432008 32300 0.00129654 31800 0.001287641
32700 0.002297502 30700 0.000331948 30500 0.001204042 30100
0.000432214 32300 0.001298801 31800 0.001286575
32700 0.002299337 30700 0.000331501 30500 0.001200461 30100
0.000432436 32300 0.001301069 31800 0.00128551

32700 0.002301182 30700 0.000331087 30500 0.001196329 30100
0.000432654 32300 0.001303355 31800 0.001284465
32700 0.002303011 30700 0.000330664 30500 0.001191637 30100
0.000432847 32300 0.001305648 31800 0.001283413
32700 0.002304886 30700 0.000330257 30500 0.001186397 30100
0.000433083 32300 0.001307947 31800 0.001282336
32700 0.002306712 30700 0.000329868 30500 0.001180603 30100
0.000433325 32300 0.00131025 31800 0.001281269
32700 0.002308532 30700 0.000329464 30500 0.001174274 30100
0.000433546 32300 0.001312569 31800 0.001280218
32700 0.002310419 30700 0.000329092 30500 0.001167406 30100
0.000433825 32300 0.001314897 31800 0.001279176
32700 0.002312276 30700 0.000328682 30500 0.001160046 30100
0.000434029 32400 0.001317229 31800 0.001278121
32700 0.002314169 30700 0.000328301 30500 0.001152192 30100
0.000434282 32400 0.001319577 31800 0.001277075
32700 0.002316042 30700 0.000327921 30500 0.001143848 30100
0.00043449 32400 0.001321938 31800 0.001276044
32700 0.002317938 30700 0.000327537 30500 0.00113509 30100
0.000434705 32400 0.001324309 31800 0.001275004
32700 0.002319861 30700 0.000327157 30500 0.001125901 30100
0.000434883 32400 0.001326716 31800 0.001273961
32700 0.002321739 30700 0.000326785 30500 0.001116329 30100
0.000435015 32400 0.001329103 31800 0.001272923
32700 0.002323629 30700 0.000326405 30500 0.001106418 30100
0.000435152 32400 0.001331526 31800 0.001271887
32700 0.002325531 30700 0.00032604 30500 0.001096195 30100
0.000435261 32400 0.001333969 31800 0.001270854
32700 0.002327421 30700 0.000325646 30500 0.001085713 30100
0.000435383 32400 0.001336417 31800 0.001269798
32700 0.002329352 30700 0.000325256 30500 0.001075 30100
0.00043556 32400 0.001338889 31800 0.001268769
32700 0.00233129 30700 0.000324837 30500 0.001064107 30100
0.000435735 32400 0.001341361 31800 0.001267743
32700 0.002333197 30700 0.000324409 30500 0.001053074 30100
0.000435926 32400 0.001343859 31800 0.00126672
32700 0.00233514 30700 0.000323993 30500 0.001041962 30100
0.000436163 32400 0.001346368 31800 0.001265688
32700 0.002337088 30700 0.000323571 30500 0.001030787 30100
0.000436402 32400 0.001348898 31800 0.001264679
32700 0.002339041 30700 0.000323153 30500 0.00101965 30100
0.000436661 32400 0.001351443 31800 0.001263657
32700 0.002341019 30700 0.000322744 30500 0.001008551 30100
0.00043694 32400 0.001353994 31800 0.001262631
32700 0.00234295 30700 0.000322341 30500 0.000997546 30100
0.000437205 32400 0.001356577 31800 0.001261601
32700 0.00234489 30700 0.000321934 30500 0.000986703 30100
0.000437463 32400 0.001359152 31800 0.001260581
32700 0.002346862 30700 0.000321534 30500 0.000976019 30100
0.000437747 32400 0.001361737 31800 0.00125958
32700 0.00234881 30700 0.000321158 30500 0.000965584 30100
0.000437994 32400 0.001364333 31800 0.001258556
32700 0.002350815 30700 0.00032077 30500 0.000955417 30100
0.000438233 32400 0.001366948 31800 0.001257543
32700 0.002352813 30700 0.000320375 30500 0.000945524 30100
0.000438466 32400 0.001369591 31800 0.001256542
32700 0.002354795 30700 0.000319976 30500 0.00093601 30100
0.000438712 32400 0.001372221 31800 0.00125554
32700 0.002356826 30700 0.000319575 30500 0.000926823 30100
0.000438923 32400 0.001374881 31800 0.001254519
32700 0.00235879 30700 0.000319161 30500 0.000918031 30100
0.000439134 32400 0.001377551 31800 0.001253517
32700 0.002360771 30700 0.000318756 30500 0.000909644 30100
0.000439346 32400 0.001380227 31800 0.001252511
32700 0.002362803 30700 0.000318335 30500 0.00090168 30100
0.00043955 32400 0.001382901 31800 0.001251503
32700 0.002364765 30700 0.000317947 30500 0.000894161 30100
0.000439751 32400 0.001385596 31800 0.001250477

32700 0.002366761 30700 0.000317551 30500 0.000887103 30100
0.000440002 32400 0.001388302 31800 0.001249469
32800 0.002368774 30700 0.000317174 30500 0.000880495 30100
0.00044022 32400 0.00139102 31800 0.00124847
32800 0.002370772 30700 0.000316803 30500 0.000874349 30100
0.00044045 32400 0.001393747 31800 0.001247461
32800 0.002372798 30700 0.000316412 30500 0.000868638 30100
0.000440716 32400 0.001396499 31800 0.001246444
32800 0.002374839 30700 0.000316056 30500 0.000863404 30100
0.000440972 32400 0.001399253 31800 0.001245448
32800 0.002376864 30700 0.000315699 30500 0.000858624 30100
0.000441225 32400 0.001402036 31800 0.001244452
32800 0.002378937 30700 0.000315316 30500 0.000854257 30100
0.000441474 32400 0.001404829 31800 0.001243441
32800 0.002380958 30700 0.000314956 30500 0.000850341 30100
0.00044173 32400 0.001407628 31800 0.001242425
32800 0.002382973 30700 0.00031457 30500 0.000846823 30100
0.000441968 32400 0.001410435 31800 0.001241412
32800 0.002385038 30700 0.000314203 30500 0.000843691 30100
0.000442205 32400 0.001413264 31800 0.001240418
32800 0.002387102 30700 0.000313843 30500 0.000840966 30100
0.000442451 32400 0.001416104 31900 0.001239411
32800 0.002389182 30700 0.000313439 30500 0.000838593 30100
0.000442684 32400 0.001418963 31900 0.001238408
32800 0.002391293 30700 0.000313054 30500 0.000836543 30100
0.0004429 32400 0.001421826 31900 0.001237422
32800 0.0023934 30700 0.000312676 30500 0.00083483 30100
0.000443144 32400 0.001424715 31900 0.001236434
32800 0.002395533 30700 0.000312304 30500 0.000833407 30100
0.000443362 32400 0.001427614 31900 0.001235425
32800 0.002397646 30700 0.000311891 30500 0.000832263 30100
0.000443601 32400 0.001430536 31900 0.001234426
32800 0.002399769 30700 0.000311485 30500 0.000831368 30100
0.000443819 32400 0.001433465 31900 0.001233436
32800 0.002401905 30700 0.000311095 30500 0.000830707 30100
0.000444069 32400 0.00143641 31900 0.001232451
32800 0.00240404 30700 0.000310722 30500 0.000830262 30100
0.000444347 32400 0.001439361 31900 0.001231438
32800 0.002406209 30800 0.000310325 30500 0.000830031 30100
0.000444629 32400 0.001442321 31900 0.001230441
32800 0.002408386 30800 0.000309963 30500 0.000829959 30100
0.000444903 32400 0.001445299 31900 0.001229455
32800 0.002410543 30800 0.000309592 30600 0.000830046 30100
0.000445195 32400 0.001448292 31900 0.001228463
32800 0.002412737 30800 0.00030923 30600 0.000830264 30100
0.000445489 32400 0.001451303 31900 0.00122746
32800 0.002414955 30800 0.00030888 30600 0.000830609 30100
0.000445769 32400 0.001454317 31900 0.001226479
32800 0.002417177 30800 0.000308527 30600 0.000831055 30100
0.000446052 32400 0.001457369 31900 0.001225493
32800 0.002419423 30800 0.000308181 30600 0.000831585 30200
0.00044635 32400 0.001460434 31900 0.001224494
32800 0.002421635 30800 0.000307818 30600 0.000832188 30200
0.000446676 32400 0.001463503 31900 0.001223494
32800 0.002423872 30800 0.000307449 30600 0.000832823 30200
0.000447001 32400 0.001466577 31900 0.001222506
32800 0.002426131 30800 0.000307119 30600 0.00083353 30200
0.000447356 32400 0.001469702 31900 0.001221534
32800 0.00242837 30800 0.000306746 30600 0.000834278 30200
0.000447687 32400 0.001472818 31900 0.001220546
32800 0.002430642 30800 0.000306408 30600 0.000835076 30200
0.000448037 32400 0.001475959 31900 0.001219571
32800 0.002432934 30800 0.00030602 30600 0.000835828 30200
0.000448361 32400 0.001479119 31900 0.001218605
32800 0.002435199 30800 0.000305662 30600 0.000836627 30200
0.000448672 32400 0.001482315 31900 0.00121764
32800 0.00243752 30800 0.000305304 30600 0.000837419 30200
0.000448945 32400 0.00148551 31900 0.001216667

32800 0.002439773 30800 0.00030494 30600 0.000838207 30200
0.000449217 32400 0.001488742 31900 0.001215694
32800 0.002442063 30800 0.000304572 30600 0.000838997 30200
0.000449483 32400 0.001491996 31900 0.001214736
32800 0.002444376 30800 0.00030422 30600 0.000839762 30200
0.000449743 32500 0.00149526 31900 0.001213782
32800 0.002446673 30800 0.00030387 30600 0.000840497 30200
0.000450005 32500 0.001498549 31900 0.001212797
32800 0.002448972 30800 0.000303489 30600 0.000841206 30200
0.000450307 32500 0.001501849 31900 0.001211846
32800 0.002451312 30800 0.000303154 30600 0.00084189 30200
0.000450575 32500 0.001505167 31900 0.001210907
32800 0.002453616 30800 0.000302765 30600 0.000842532 30200
0.000450897 32500 0.001508508 31900 0.001209961
32800 0.00245595 30800 0.000302426 30600 0.000843163 30200
0.000451236 32500 0.001511868 31900 0.001209027
32800 0.002458287 30800 0.000302033 30600 0.00084373 30200
0.000451563 32500 0.001515252 31900 0.001208111
32800 0.002460633 30800 0.000301707 30600 0.000844266 30200
0.000451885 32500 0.001518657 31900 0.001207185
32800 0.002462992 30800 0.00030132 30600 0.000844777 30200
0.000452219 32500 0.001522084 31900 0.001206253
32800 0.002465341 30800 0.000300969 30600 0.000845236 30200
0.000452556 32500 0.001525524 31900 0.001205319
32800 0.002467667 30800 0.000300628 30600 0.000845683 30200
0.000452862 32500 0.00152898 31900 0.001204398
32800 0.002470028 30800 0.000300281 30600 0.000846053 30200
0.000453172 32500 0.001532461 31900 0.001203498
32800 0.002472369 30800 0.000299931 30600 0.000846418 30200
0.000453455 32500 0.001535957 31900 0.001202582
32800 0.002474759 30800 0.0002996 30600 0.000846718 30200
0.000453735 32500 0.001539473 31900 0.001201682
32800 0.002477159 30800 0.000299266 30600 0.000846972 30200
0.000454006 32500 0.001543014 31900 0.001200794
32800 0.002479528 30800 0.000298938 30600 0.00084724 30200
0.000454259 32500 0.001546583 31900 0.001199912
32800 0.002481933 30800 0.00029861 30600 0.000847458 30200
0.000454538 32500 0.001550169 31900 0.001199026
32800 0.002484299 30800 0.000298282 30600 0.000847633 30200
0.000454783 32500 0.001553775 31900 0.001198137
32800 0.002486693 30800 0.000297944 30600 0.00084778 30200
0.000455055 32500 0.001557406 31900 0.001197259
32800 0.002489083 30800 0.000297591 30600 0.000847907 30200
0.000455325 32500 0.001561057 31900 0.001196378
32800 0.002491471 30800 0.000297256 30600 0.000847996 30200
0.000455608 32500 0.001564724 31900 0.001195482
32800 0.002493871 30800 0.000296925 30600 0.000848066 30200
0.000455914 32500 0.001568412 31900 0.001194595
32800 0.002496308 30800 0.000296614 30600 0.000848093 30200
0.000456241 32500 0.001572117 31900 0.001193733
32800 0.002498689 30800 0.000296262 30600 0.000848112 30200
0.000456583 32500 0.001575861 31900 0.001192861
32800 0.002501114 30800 0.000295903 30600 0.000848096 30200
0.000456937 32500 0.001579619 31900 0.001191994
32800 0.002503536 30800 0.000295543 30600 0.000848046 30200
0.000457303 32500 0.001583405 31900 0.00119113
32800 0.002505973 30800 0.000295193 30600 0.000848024 30200
0.000457673 32500 0.001587217 31900 0.001190264
32800 0.002508433 30800 0.000294823 30600 0.000847928 30200
0.000458069 32500 0.001591049 31900 0.001189392
32800 0.002510867 30800 0.000294466 30600 0.000847849 30200
0.000458501 32500 0.001594905 31900 0.00118851
32900 0.002513284 30800 0.000294118 30600 0.000847766 30200
0.000458922 32500 0.001598774 31900 0.001187631
32900 0.002515751 30800 0.00029378 30600 0.000847665 30200
0.000459393 32500 0.001602671 31900 0.001186776
32900 0.002518202 30800 0.000293426 30600 0.000847541 30200
0.000459823 32500 0.001606592 31900 0.001185902

32900 0.002520675 30800 0.000293115 30600 0.000847429 30200
0.000460273 32500 0.001610533 31900 0.001185038
32900 0.002523142 30800 0.000292789 30600 0.000847282 30200
0.000460697 32500 0.001614498 31900 0.001184181
32900 0.002525622 30800 0.000292471 30600 0.000847139 30200
0.000461096 32500 0.001618492 31900 0.001183315
32900 0.002528116 30800 0.000292165 30600 0.000847005 30200
0.000461502 32500 0.001622502 31900 0.001182447
32900 0.002530573 30800 0.000291848 30600 0.000846856 30200
0.000461864 32500 0.001626545 31900 0.001181571
32900 0.002533067 30800 0.000291525 30600 0.000846722 30200
0.000462246 32500 0.00163061 31900 0.001180702
32900 0.002535547 30800 0.000291225 30600 0.000846581 30200
0.00046262 32500 0.001634697 32000 0.001179834
32900 0.002538024 30800 0.000290921 30600 0.000846425 30200
0.000462998 32500 0.001638805 32000 0.001178946
32900 0.002540547 30800 0.000290616 30600 0.000846288 30200
0.000463388 32500 0.001642938 32000 0.001178079
32900 0.002543058 30800 0.000290312 30600 0.000846159 30200
0.000463785 32500 0.001647099 32000 0.001177209
32900 0.002545553 30800 0.000289982 30600 0.00084603 30200
0.000464181 32500 0.001651285 32000 0.001176349
32900 0.002548095 30800 0.000289676 30600 0.000845882 30200
0.00046456 32500 0.0016555 32000 0.001175488
32900 0.002550628 30800 0.000289356 30600 0.00084576 30200
0.00046495 32500 0.00165974 32000 0.00117463
32900 0.00255318 30800 0.000289013 30600 0.000845642 30200
0.000465343 32500 0.001664009 32000 0.001173762
32900 0.002555748 30800 0.000288681 30600 0.000845533 30200
0.000465758 32500 0.001668305 32000 0.001172889
32900 0.002558291 30900 0.000288359 30600 0.000845445 30200
0.000466197 32500 0.001672626 32000 0.001172017
32900 0.002560835 30900 0.000287999 30600 0.000845356 30200
0.000466639 32500 0.001676967 32000 0.001171144
32900 0.002563428 30900 0.000287674 30700 0.000845257 30200
0.000467089 32500 0.001681324 32000 0.001170288
32900 0.002566001 30900 0.000287327 30700 0.000845192 30200
0.000467541 32500 0.001685712 32000 0.001169418
32900 0.002568616 30900 0.000286976 30700 0.000845168 30200
0.000468008 32500 0.001690118 32000 0.001168562
32900 0.002571235 30900 0.000286648 30700 0.000845117 30200
0.000468467 32500 0.001694553 32000 0.0011677
32900 0.002573851 30900 0.000286319 30700 0.000845099 30300
0.000468935 32500 0.001699015 32000 0.001166849
32900 0.002576504 30900 0.000285957 30700 0.000845093 30300
0.000469376 32500 0.001703492 32000 0.001165975
32900 0.002579121 30900 0.000285624 30700 0.000845102 30300
0.000469836 32500 0.001707984 32000 0.001165119
32900 0.002581761 30900 0.000285309 30700 0.000845145 30300
0.000470289 32500 0.001712505 32000 0.001164248
32900 0.002584416 30900 0.000284983 30700 0.000845163 30300
0.000470736 32500 0.001717047 32000 0.001163391
32900 0.00258705 30900 0.00028468 30700 0.000845241 30300
0.000471157 32500 0.001721606 32000 0.0011625
32900 0.002589711 30900 0.000284359 30700 0.000845331 30300
0.000471586 32500 0.00172618 32000 0.001161631
32900 0.002592376 30900 0.000284034 30700 0.000845448 30300
0.000471987 32500 0.001730777 32000 0.001160772
32900 0.002595041 30900 0.000283692 30700 0.000845541 30300
0.000472384 32500 0.001735394 32000 0.001159911
32900 0.00259774 30900 0.000283381 30700 0.000845707 30300
0.000472762 32500 0.001740041 32000 0.001159065
32900 0.00260043 30900 0.000283063 30700 0.000845849 30300
0.000473137 32500 0.001744711 32000 0.001158219
32900 0.002603121 30900 0.000282718 30700 0.000846048 30300
0.000473514 32600 0.001749405 32000 0.001157372
32900 0.00260587 30900 0.000282386 30700 0.000846275 30300
0.000473895 32600 0.001754127 32000 0.001156498

32900 0.002608557 30900 0.000282085 30700 0.000846493 30300
0.000474292 32600 0.001758878 32000 0.001155644
32900 0.002611256 30900 0.000281743 30700 0.000846733 30300
0.000474687 32600 0.001763651 32000 0.001154781
32900 0.002614022 30900 0.000281433 30700 0.000847021 30300
0.000475092 32600 0.001768457 32000 0.001153951
32900 0.002616748 30900 0.000281114 30700 0.000847331 30300
0.000475474 32600 0.00177331 32000 0.001153094
32900 0.002619521 30900 0.000280797 30700 0.000847664 30300
0.00047586 32600 0.001778179 32000 0.001152254
32900 0.002622281 30900 0.000280495 30700 0.000848032 30300
0.000476236 32600 0.001783094 32000 0.001151409
32900 0.002625073 30900 0.000280191 30700 0.000848438 30300
0.000476582 32600 0.001788043 32000 0.001150567
32900 0.002627897 30900 0.000279899 30700 0.000848856 30300
0.000476931 32600 0.001793032 32000 0.00114972
32900 0.002630677 30900 0.000279591 30700 0.000849305 30300
0.000477282 32600 0.001798055 32000 0.00114888
32900 0.002633481 30900 0.000279308 30700 0.000849786 30300
0.000477646 32600 0.001803124 32000 0.001148033
32900 0.002636307 30900 0.000279033 30700 0.000850273 30300
0.000478022 32600 0.001808228 32000 0.001147195
32900 0.002639117 30900 0.000278705 30700 0.000850809 30300
0.000478411 32600 0.001813371 32000 0.00114633
32900 0.002641967 30900 0.000278422 30700 0.00085137 30300
0.000478843 32600 0.001818542 32000 0.001145495
32900 0.002644832 30900 0.00027811 30700 0.00085199 30300
0.000479274 32600 0.00182375 32000 0.001144657
32900 0.002647668 30900 0.000277817 30700 0.000852582 30300
0.000479728 32600 0.001829002 32000 0.001143837
32900 0.002650548 30900 0.000277474 30700 0.000853228 30300
0.000480207 32600 0.001834283 32000 0.001143009
32900 0.002653457 30900 0.000277153 30700 0.000853906 30300
0.000480708 32600 0.001839605 32000 0.00114219
32900 0.002656365 30900 0.000276841 30700 0.000854606 30300
0.000481237 32600 0.001844962 32000 0.001141363
32900 0.002659306 30900 0.000276493 30700 0.000855357 30300
0.000481779 32600 0.001850361 32000 0.001140534
32900 0.002662219 30900 0.000276187 30700 0.000856129 30300
0.000482352 32600 0.001855783 32000 0.001139711
32900 0.002665136 30900 0.000275914 30700 0.000856925 30300
0.000482945 32600 0.001861235 32000 0.00113889
32900 0.002668097 30900 0.000275572 30700 0.000857743 30300
0.000483522 32600 0.00186673 32000 0.0011381
32900 0.00267104 30900 0.000275275 30700 0.000858592 30300
0.000484086 32600 0.001872267 32000 0.001137276
32900 0.002674039 30900 0.000274994 30700 0.000859473 30300
0.000484637 32600 0.001877826 32000 0.001136473
32900 0.002677039 30900 0.000274718 30700 0.000860393 30300
0.000485187 32600 0.001883437 32000 0.001135661
32900 0.002680029 30900 0.000274425 30700 0.000861322 30300
0.000485678 32600 0.00188908 32000 0.001134859
32900 0.002683071 30900 0.000274153 30700 0.000862244 30300
0.000486194 32600 0.001894759 32000 0.001134039
33000 0.002686076 30900 0.000273898 30700 0.000863232 30300
0.000486712 32600 0.001900473 32000 0.001133227
33000 0.00268909 30900 0.000273588 30700 0.000864268 30300
0.000487237 32600 0.001906242 32000 0.00113242
33000 0.002692118 30900 0.000273304 30700 0.000865306 30300
0.000487755 32600 0.001912038 32000 0.001131611
33000 0.002695171 30900 0.000273011 30700 0.000866397 30300
0.000488284 32600 0.001917883 32000 0.001130779
33000 0.002698228 30900 0.000272738 30700 0.000867496 30300
0.000488811 32600 0.001923759 32000 0.001129977
33000 0.002701301 30900 0.000272447 30700 0.00086864 30300
0.000489355 32600 0.001929682 32000 0.001129159
33000 0.002704358 30900 0.000272167 30700 0.00086979 30300
0.000489887 32600 0.001935643 32000 0.001128354

33000 0.002707484 30900 0.000271868 30700 0.000870994 30300
0.00049041 32600 0.001941656 32000 0.001127551
33000 0.002710595 30900 0.000271568 30700 0.000872233 30300
0.000490925 32600 0.001947704 32000 0.001126748
33000 0.002713705 30900 0.000271308 30700 0.000873497 30300
0.000491429 32600 0.001953815 32100 0.001125941
33000 0.002716845 30900 0.000271026 30700 0.000874776 30300
0.000491924 32600 0.001959965 32100 0.001125124
33000 0.00271996 30900 0.000270724 30700 0.000876103 30300
0.000492448 32600 0.001966159 32100 0.001124317
33000 0.00272309 30900 0.000270436 30700 0.000877475 30300
0.000492974 32600 0.001972397 32100 0.001123493
33000 0.002726253 30900 0.000270148 30700 0.000878858 30300
0.000493512 32600 0.001978682 32100 0.001122708
33000 0.002729403 30900 0.000269857 30700 0.000880301 30300
0.000494023 32600 0.001985012 32100 0.00112189
33000 0.002732605 30900 0.000269575 30700 0.000881765 30300
0.000494587 32600 0.001991389 32100 0.001121089
33000 0.002735792 30900 0.000269306 30700 0.000883233 30300
0.000495145 32600 0.001997814 32100 0.001120291
33000 0.002738994 30900 0.000269043 30700 0.000884776 30300
0.000495699 32600 0.002004281 32100 0.001119493
33000 0.002742254 31000 0.000268771 30700 0.000886337 30300
0.00049625 32600 0.002010805 32100 0.001118694
33000 0.002745446 31000 0.000268505 30700 0.000887939 30300
0.00049682 32600 0.00201736 32100 0.001117894
33000 0.002748662 31000 0.000268256 30800 0.000889574 30300
0.000497387 32600 0.002023984 32100 0.00111709
33000 0.002751883 31000 0.000267995 30800 0.000891248 30300
0.000497947 32600 0.002030632 32100 0.001116297
33000 0.002755114 31000 0.000267714 30800 0.000892972 30300
0.000498501 32600 0.002037344 32100 0.001115476
33000 0.002758369 31000 0.000267431 30800 0.000894729 30300
0.00049907 32600 0.002044087 32100 0.001114688
33000 0.002761634 31000 0.00026718 30800 0.000896522 30400
0.000499594 32600 0.00205088 32100 0.001113895
33000 0.002764855 31000 0.000266897 30800 0.000898377 30400
0.000500085 32600 0.002057717 32100 0.001113106
33000 0.002768134 31000 0.000266641 30800 0.000900228 30400
0.000500576 32600 0.002064602 32100 0.001112315
33000 0.002771442 31000 0.000266372 30800 0.000902141 30400
0.000501043 32600 0.002071532 32100 0.001111543
33000 0.002774731 31000 0.000266101 30800 0.000904095 30400
0.000501522 32600 0.002078512 32100 0.001110766
33000 0.002778062 31000 0.000265812 30800 0.000906092 30400
0.000502003 32600 0.002085544 32100 0.001109985
33000 0.002781346 31000 0.000265531 30800 0.00090813 30400
0.000502519 32600 0.002092619 32100 0.001109195
33000 0.002784658 31000 0.000265243 30800 0.00091022 30400
0.000503062 32600 0.002099728 32100 0.001108414
33000 0.00278799 31000 0.00026497 30800 0.000912315 30400
0.000503643 32600 0.002106891 32100 0.001107658
33000 0.002791336 31000 0.000264687 30800 0.000914472 30400
0.000504256 32600 0.002114102 32100 0.001106881
33000 0.002794693 31000 0.000264405 30800 0.000916682 30400
0.000504901 32600 0.00212135 32100 0.001106116
33000 0.002798072 31000 0.000264134 30800 0.000918925 30400
0.000505577 32700 0.002128645 32100 0.001105359
33000 0.002801433 31000 0.000263855 30800 0.000921213 30400
0.000506213 32700 0.002135989 32100 0.001104585
33000 0.002804851 31000 0.0002636 30800 0.000923539 30400
0.000506879 32700 0.002143388 32100 0.00110382
33000 0.002808226 31000 0.000263341 30800 0.00092589 30400
0.000507524 32700 0.002150821 32100 0.001103051
33000 0.00281163 31000 0.000263073 30800 0.000928307 30400
0.000508191 32700 0.002158314 32100 0.001102291
33000 0.002815034 31000 0.000262815 30800 0.000930756 30400
0.000508828 32700 0.00216585 32100 0.001101529

33000 0.002818467 31000 0.000262556 30800 0.000933284 30400
0.000509496 32700 0.002173443 32100 0.001100746
33000 0.0028219 31000 0.000262283 30800 0.000935833 30400
0.000510168 32700 0.002181077 32100 0.001099991
33000 0.002825367 31000 0.000262017 30800 0.000938463 30400
0.000510845 32700 0.002188762 32100 0.001099233
33000 0.002828829 31000 0.000261729 30800 0.000941107 30400
0.000511523 32700 0.002196505 32100 0.00109846
33000 0.00283234 31000 0.000261453 30800 0.00094382 30400
0.0005122 32700 0.002204303 32100 0.001097709
33000 0.002835855 31000 0.00026116 30800 0.000946543 30400
0.000512879 32700 0.002212147 32100 0.001096955
33000 0.002839374 31000 0.000260869 30800 0.000949347 30400
0.00051355 32700 0.002220057 32100 0.001096213
33000 0.002842928 31000 0.000260586 30800 0.000952168 30400
0.00051424 32700 0.002228028 32100 0.001095451
33000 0.002846467 31000 0.000260312 30800 0.000955051 30400
0.00051493 32700 0.002236051 32100 0.001094686
33000 0.002850011 31000 0.000260046 30800 0.000957993 30400
0.000515619 32700 0.002244135 32100 0.001093925
33000 0.002853608 31000 0.000259788 30800 0.000960969 30400
0.000516305 32700 0.002252273 32100 0.001093193
33000 0.0028572 31000 0.00025951 30800 0.000964025 30400
0.000516971 32700 0.002260475 32100 0.001092434
33000 0.002860821 31000 0.000259233 30800 0.000967147 30400
0.000517648 32700 0.002268744 32100 0.001091688
33000 0.002864461 31000 0.000258971 30800 0.000970303 30400
0.000518353 32700 0.002277077 32100 0.001090948
33000 0.002868125 31000 0.000258701 30800 0.000973514 30400
0.000519024 32700 0.002285472 32100 0.00109021
33000 0.002871827 31000 0.000258453 30800 0.000976769 30400
0.000519725 32700 0.002293945 32100 0.001089468
33000 0.00287551 31000 0.000258202 30800 0.000980094 30400
0.000520428 32700 0.002302474 32100 0.001088725
33000 0.002879209 31000 0.000257937 30800 0.000983466 30400
0.000521149 32700 0.00231108 32100 0.001087983
33000 0.00288293 31000 0.000257691 30800 0.000986891 30400
0.000521851 32700 0.002319747 32100 0.001087249
33000 0.002886666 31000 0.000257438 30800 0.000990394 30400
0.000522584 32700 0.002328484 32100 0.001086488
33000 0.002890413 31000 0.000257169 30800 0.00099394 30400
0.000523315 32700 0.002337297 32100 0.001085753
33000 0.002894207 31000 0.000256908 30800 0.000997545 30400
0.000524042 32700 0.002346181 32100 0.001085023
33000 0.002897978 31000 0.000256648 30800 0.001001214 30400
0.000524768 32700 0.00235514 32100 0.001084295
33100 0.002901801 31000 0.000256384 30800 0.001004935 30400
0.000525489 32700 0.00236418 32100 0.001083563
33100 0.002905667 31000 0.000256114 30800 0.001008728 30400
0.000526226 32700 0.002373296 32100 0.001082845
33100 0.002909524 31000 0.000255845 30800 0.001012559 30400
0.000526963 32700 0.002382483 32100 0.001082119
33100 0.002913434 31000 0.000255565 30800 0.001016458 30400
0.000527723 32700 0.002391752 32100 0.0010814
33100 0.002917329 31000 0.00025529 30800 0.001020409 30400
0.000528496 32700 0.002401094 32100 0.00108068
33100 0.002921238 31000 0.00025503 30800 0.001024453 30400
0.000529289 32700 0.002410513 32100 0.00107996
33100 0.002925192 31000 0.000254761 30800 0.001028548 30400
0.000530098 32700 0.002420012 32100 0.001079252
33100 0.00292913 31000 0.000254482 30800 0.001032692 30400
0.000530908 32700 0.002429588 32100 0.001078526
33100 0.00293315 31000 0.000254214 30800 0.001036915 30400
0.000531727 32700 0.002439249 32100 0.001077811
33100 0.002937166 31000 0.000253965 30800 0.001041225 30400
0.000532574 32700 0.002448984 32200 0.001077105
33100 0.002941186 31000 0.000253705 30800 0.001045585 30400
0.000533395 32700 0.002458792 32200 0.001076405

33100 0.002945254 31000 0.000253465 30800 0.001050021 30400
0.000534252 32700 0.002468677 32200 0.00107568
33100 0.002949299 31000 0.000253223 30800 0.001054512 30400
0.000535109 32700 0.002478649 32200 0.001074962
33100 0.002953376 31000 0.000252973 30800 0.001059075 30400
0.000535983 32700 0.002488701 32200 0.001074254
33100 0.002957488 31000 0.000252742 30800 0.001063735 30400
0.000536815 32700 0.002498824 32200 0.001073549
33100 0.002961572 31000 0.00025251 30800 0.001068464 30400
0.000537682 32700 0.002509017 32200 0.001072816
33100 0.002965703 31000 0.000252267 30800 0.001073249 30400
0.000538518 32700 0.00251928 32200 0.001072105
33100 0.002969868 31000 0.000252027 30800 0.001078121 30400
0.000539342 32700 0.002529628 32200 0.001071392
33100 0.002974028 31100 0.000251783 30800 0.001083046 30400
0.000540142 32700 0.002540053 32200 0.001070673
33100 0.002978217 31100 0.000251529 30800 0.001088073 30400
0.000540925 32700 0.002550559 32200 0.001069964
33100 0.002982459 31100 0.000251273 30900 0.001093168 30400
0.000541685 32700 0.002561132 32200 0.001069274
33100 0.002986673 31100 0.000251011 30900 0.001098348 30400
0.000542399 32700 0.002571791 32200 0.001068569
33100 0.002990967 31100 0.000250754 30900 0.00110359 30400
0.000543117 32700 0.002582531 32200 0.001067867
33100 0.002995219 31100 0.000250501 30900 0.001108937 30400
0.000543809 32700 0.002593365 32200 0.001067157
33100 0.002999456 31100 0.00025025 30900 0.001114343 30500
0.000544489 32700 0.002604277 32200 0.00106645
33100 0.00300377 31100 0.000249998 30900 0.00111983 30500
0.000545173 32700 0.002615272 32200 0.001065766
33100 0.003008104 31100 0.000249749 30900 0.0011254 30500
0.000545848 32700 0.002626342 32200 0.001065051
33100 0.003012437 31100 0.000249511 30900 0.001131062 30500
0.000546559 32700 0.002637511 32200 0.001064336
33100 0.003016814 31100 0.000249277 30900 0.001136825 30500
0.00054729 32700 0.002648765 32200 0.001063638
33100 0.003021209 31100 0.000249043 30900 0.001142674 30500
0.000548021 32700 0.002660101 32200 0.001062945
33100 0.003025634 31100 0.000248821 30900 0.001148631 30500
0.000548797 32700 0.002671539 32200 0.001062231
33100 0.00303004 31100 0.000248597 30900 0.00115465 30500
0.000549566 32700 0.002683061 32200 0.001061516
33100 0.003034479 31100 0.000248358 30900 0.00116079 30500
0.000550383 32700 0.002694672 32200 0.001060814
33100 0.003038928 31100 0.000248126 30900 0.001167036 30500
0.000551193 32700 0.002706374 32200 0.001060111
33100 0.003043405 31100 0.000247882 30900 0.00117335 30500
0.000552035 32700 0.002718173 32200 0.001059382
33100 0.003047898 31100 0.000247627 30900 0.001179784 30500
0.000552905 32800 0.002730064 32200 0.001058674
33100 0.003052419 31100 0.000247375 30900 0.001186315 30500
0.000553786 32800 0.002742061 32200 0.001057953
33100 0.003056931 31100 0.000247114 30900 0.001192938 30500
0.000554683 32800 0.002754141 32200 0.001057237
33100 0.0030615 31100 0.000246858 30900 0.001199671 30500
0.000555585 32800 0.002766319 32200 0.001056525
33100 0.003066089 31100 0.000246601 30900 0.001206512 30500
0.000556484 32800 0.002778599 32200 0.001055823
33100 0.003070692 31100 0.000246339 30900 0.001213467 30500
0.000557386 32800 0.002790982 32200 0.001055116
33100 0.003075339 31100 0.000246088 30900 0.001220534 30500
0.0005583 32800 0.002803448 32200 0.00105439
33100 0.003079961 31100 0.000245836 30900 0.001227703 30500
0.00055922 32800 0.002816028 32200 0.001053677
33100 0.003084631 31100 0.000245588 30900 0.001234983 30500
0.000560153 32800 0.002828704 32200 0.001052958
33100 0.003089331 31100 0.000245337 30900 0.001242402 30500
0.000561062 32800 0.002841497 32200 0.001052258

33100 0.00309404 31100 0.000245074 30900 0.001249897 30500
0.000561972 32800 0.002854377 32200 0.001051537
33100 0.003098787 31100 0.000244805 30900 0.001257544 30500
0.000562911 32800 0.002867363 32200 0.001050833
33100 0.003103559 31100 0.00024455 30900 0.001265308 30500
0.000563848 32800 0.002880459 32200 0.001050135
33100 0.003108325 31100 0.000244296 30900 0.001273202 30500
0.000564784 32800 0.002893661 32200 0.001049426
33100 0.003113169 31100 0.000244056 30900 0.0012812 30500
0.000565746 32800 0.002906978 32200 0.00104872
33100 0.003117974 31100 0.000243844 30900 0.00128936 30500
0.000566698 32800 0.002920404 32200 0.001047998
33100 0.003122808 31100 0.000243615 30900 0.001297631 30500
0.000567672 32800 0.00293394 32200 0.001047292
33100 0.003127661 31100 0.000243404 30900 0.001306025 30500
0.000568602 32800 0.002947582 32200 0.001046573
33100 0.003132548 31100 0.000243208 30900 0.001314575 30500
0.000569541 32800 0.002961339 32200 0.00104585
33100 0.003137464 31100 0.000243002 30900 0.001323247 30500
0.000570485 32800 0.002975215 32200 0.001045149
33100 0.003142395 31100 0.000242809 30900 0.001332048 30500
0.000571413 32800 0.002989205 32200 0.001044435
33100 0.003147325 31100 0.000242619 30900 0.001340991 30500
0.000572351 32800 0.003003309 32200 0.001043733
33100 0.00315229 31100 0.000242418 30900 0.001350083 30500
0.000573296 32800 0.003017548 32200 0.001043034
33100 0.003157283 31100 0.000242216 30900 0.001359327 30500
0.000574261 32800 0.003031898 32200 0.001042349
33100 0.003162291 31100 0.000241998 30900 0.001368703 30500
0.00057523 32800 0.003046357 32200 0.001041642
33100 0.003167322 31100 0.000241769 30900 0.001378241 30500
0.000576222 32800 0.00306095 32200 0.001040941
33100 0.003172348 31100 0.000241536 30900 0.001387911 30500
0.000577253 32800 0.003075658 32200 0.001040237
33100 0.003177351 31100 0.000241302 30900 0.001397757 30500
0.000578293 32800 0.003090495 32200 0.001039529
33100 0.003182431 31100 0.000241057 30900 0.001407746 30500
0.00057935 32800 0.003105452 32200 0.001038842
33200 0.003187491 31100 0.000240805 30900 0.001417889 30500
0.000580433 32800 0.003120523 32200 0.001038153
33200 0.003192624 31100 0.000240557 30900 0.001428213 30500
0.000581565 32800 0.003135722 32200 0.00103746
33200 0.003197733 31100 0.000240319 30900 0.001438677 30500
0.000582702 32800 0.003151061 32200 0.001036781
33200 0.00320285 31100 0.000240063 30900 0.00144934 30500
0.00058385 32800 0.003166509 32200 0.001036092
33200 0.003208036 31100 0.00023984 30900 0.00146017 30500
0.000585013 32800 0.003182098 32200 0.001035412
33200 0.003213188 31100 0.000239608 30900 0.00147115 30500
0.000586172 32800 0.003197814 32200 0.00103472
33200 0.00321834 31100 0.000239377 30900 0.001482323 30500
0.00058734 32800 0.003213663 32200 0.001034036
33200 0.003223525 31100 0.000239152 30900 0.001493659 30500
0.000588501 32800 0.003229635 32200 0.00103334
33200 0.00322873 31100 0.000238936 30900 0.001505194 30500
0.000589658 32800 0.003245754 32200 0.00103264
33200 0.00323394 31100 0.000238738 30900 0.001516889 30500
0.000590881 32800 0.003261994 32300 0.001031955
33200 0.003239213 31100 0.000238514 30900 0.001528784 30500
0.000592078 32800 0.003278382 32300 0.00103128
33200 0.003244461 31100 0.000238306 30900 0.00154085 30500
0.000593303 32800 0.003294904 32300 0.001030602
33200 0.003249753 31100 0.000238102 30900 0.001553138 30500
0.000594519 32800 0.003311583 32300 0.001029935
33200 0.003255069 31100 0.000237892 30900 0.001565598 30500
0.000595734 32800 0.003328396 32300 0.001029269
33200 0.003260413 31100 0.000237684 30900 0.001578259 30500
0.00059692 32800 0.003345351 32300 0.001028595

33200 0.003265768 31100 0.000237473 30900 0.001591134 30500
0.000598068 32800 0.003362451 32300 0.001027932
33200 0.003271126 31100 0.000237271 30900 0.001604212 30500
0.000599213 32800 0.0033797 32300 0.001027262
33200 0.0032765 31100 0.000237074 30900 0.001617499 30500
0.000600318 32800 0.0033971 32300 0.001026592
33200 0.003281916 31200 0.000236865 30900 0.001630996 30500
0.000601393 32800 0.003414642 32300 0.001025957
33200 0.003287348 31200 0.000236656 30900 0.001644695 30500
0.000602451 32800 0.003432335 32300 0.001025302
33200 0.003292813 31200 0.000236453 31000 0.001658641 30500
0.000603527 32800 0.003450179 32300 0.001024651
33200 0.003298292 31200 0.000236243 31000 0.001672794 30500
0.000604614 32800 0.003468183 32300 0.001024015
33200 0.0033038 31200 0.000236025 31000 0.001687192 30500
0.000605701 32800 0.003486324 32300 0.001023382
33200 0.003309347 31200 0.000235817 31000 0.001701801 30500
0.000606825 32800 0.003504632 32300 0.001022738
33200 0.003314879 31200 0.000235594 31000 0.001716657 30600
0.000607961 32800 0.003523093 32300 0.001022101
33200 0.003320454 31200 0.000235368 31000 0.001731738 30600
0.00060914 32800 0.003541707 32300 0.001021464
33200 0.003326049 31200 0.000235138 31000 0.001747069 30600
0.000610332 32800 0.003560462 32300 0.001020838
33200 0.003331663 31200 0.000234893 31000 0.001762642 30600
0.000611534 32800 0.003579388 32300 0.001020186
33200 0.00333734 31200 0.000234655 31000 0.001778459 30600
0.000612754 32800 0.003598462 32300 0.001019565
33200 0.003343017 31200 0.000234413 31000 0.001794508 30600
0.000613994 32800 0.003617699 32300 0.001018927
33200 0.003348718 31200 0.000234163 31000 0.001810816 30600
0.000615214 32800 0.003637093 32300 0.001018302
33200 0.003354481 31200 0.00023394 31000 0.001827388 30600
0.000616425 32800 0.003656662 32300 0.001017682
33200 0.003360279 31200 0.000233708 31000 0.001844208 30600
0.000617661 32800 0.00367638 32300 0.001017073
33200 0.003366109 31200 0.00023348 31000 0.001861299 30600
0.00061889 32800 0.003696258 32300 0.001016455
33200 0.00337195 31200 0.000233254 31000 0.001878667 30600
0.000620112 32800 0.0037163 32300 0.001015844
33200 0.003377829 31200 0.000233034 31000 0.001896268 30600
0.000621371 32900 0.003736511 32300 0.00101522
33200 0.003383712 31200 0.000232818 31000 0.001914169 30600
0.000622651 32900 0.003756881 32300 0.001014596
33200 0.003389682 31200 0.000232597 31000 0.001932319 30600
0.000623952 32900 0.003777413 32300 0.001013981
33200 0.003395648 31200 0.000232372 31000 0.001950759 30600
0.000625263 32900 0.003798114 32300 0.001013374
33200 0.003401696 31200 0.000232156 31000 0.00196947 30600
0.000626596 32900 0.003818978 32300 0.001012761
33200 0.003407757 31200 0.000231944 31000 0.001988464 30600
0.000627937 32900 0.003840007 32300 0.001012154
33200 0.003413829 31200 0.000231722 31000 0.002007741 30600
0.000629258 32900 0.003861187 32300 0.001011541
33200 0.003419963 31200 0.000231514 31000 0.002027304 30600
0.000630583 32900 0.003882547 32300 0.001010933
33200 0.003426111 31200 0.000231307 31000 0.00204716 30600
0.000631891 32900 0.003904064 32300 0.001010317
33200 0.003432313 31200 0.000231096 31000 0.002067291 30600
0.000633208 32900 0.003925751 32300 0.001009722
33200 0.003438519 31200 0.000230889 31000 0.002087723 30600
0.000634486 32900 0.00394759 32300 0.001009115
33200 0.003444768 31200 0.000230679 31000 0.002108448 30600
0.000635798 32900 0.003969595 32300 0.001008497
33200 0.003451033 31200 0.000230491 31000 0.00212947 30600
0.000637131 32900 0.003991762 32300 0.001007896
33200 0.00345736 31200 0.000230303 31000 0.002150781 30600
0.000638473 32900 0.004014101 32300 0.001007286

33200 0.003463706 31200 0.000230119 31000 0.002172391 30600
0.000639844 32900 0.004036604 32300 0.001006662
33200 0.003470077 31200 0.000229936 31000 0.002194311 30600
0.000641234 32900 0.004059275 32300 0.001006059
33200 0.0034765 31200 0.00022975 31000 0.002216516 30600
0.000642657 32900 0.004082098 32300 0.001005464
33200 0.003482918 31200 0.000229557 31000 0.002239034 30600
0.00064407 32900 0.004105089 32300 0.001004838
33200 0.003489428 31200 0.000229355 31000 0.002261853 30600
0.000645509 32900 0.004128244 32300 0.001004234
33200 0.003495919 31200 0.000229157 31000 0.002284976 30600
0.00064695 32900 0.004151569 32300 0.001003621
33200 0.003502414 31200 0.000228951 31000 0.002308391 30600
0.000648405 32900 0.004175054 32300 0.001003
33200 0.003509016 31200 0.000228739 31000 0.002332099 30600
0.000649866 32900 0.004198712 32300 0.001002408
33200 0.003515602 31200 0.000228531 31000 0.002356105 30600
0.000651314 32900 0.00422253 32300 0.001001793
33200 0.003522252 31200 0.000228323 31000 0.002380411 30600
0.000652758 32900 0.004246521 32300 0.001001184
33200 0.00352892 31200 0.000228132 31000 0.00240501 30600
0.000654206 32900 0.004270689 32300 0.001000594
33200 0.003535623 31200 0.000227931 31000 0.00242991 30600
0.000655632 32900 0.004295008 32300 0.000999985
33200 0.003542402 31200 0.000227745 31000 0.002455096 30600
0.000657076 32900 0.00431951 32300 0.000999389
33200 0.003549147 31200 0.00022756 31000 0.002480556 30600
0.0006585 32900 0.004344185 32300 0.000998768
33200 0.003555951 31200 0.000227366 31000 0.002506286 30600
0.000659953 32900 0.004369035 32300 0.000998162
33300 0.003562807 31200 0.000227182 31000 0.002532288 30600
0.000661407 32900 0.004394051 32300 0.000997556
33300 0.003569663 31200 0.000226991 31000 0.002558543 30600
0.000662875 32900 0.00441924 32300 0.000996925
33300 0.003576597 31200 0.000226806 31000 0.002585053 30600
0.000664363 32900 0.004444595 32300 0.000996321
33300 0.003583545 31200 0.000226619 31000 0.002611807 30600
0.000665886 32900 0.004470136 32300 0.000995707
33300 0.003590512 31200 0.000226426 31000 0.002638783 30600
0.000667444 32900 0.004495835 32300 0.000995083
33300 0.003597576 31200 0.000226227 31000 0.002665978 30600
0.000669033 32900 0.004521719 32300 0.000994488
33300 0.003604649 31200 0.000226034 31000 0.002693372 30600
0.000670655 32900 0.004547764 32300 0.000993883
33300 0.00361177 31200 0.000225822 31000 0.002720957 30600
0.000672286 32900 0.004573982 32300 0.000993271
33300 0.003618959 31200 0.000225619 31000 0.002748718 30600
0.000673943 32900 0.004600371 32300 0.000992668
33300 0.003626143 31200 0.000225401 31000 0.002776624 30600
0.000675628 32900 0.004626922 32400 0.000992041
33300 0.003633374 31200 0.000225197 31000 0.00280468 30600
0.00067732 32900 0.004653646 32400 0.00099142
33300 0.003640674 31200 0.000224974 31000 0.002832849 30600
0.000679033 32900 0.004680531 32400 0.000990807
33300 0.003648007 31200 0.000224759 31000 0.002861111 30600
0.000680743 32900 0.004707573 32400 0.000990183
33300 0.003655402 31200 0.000224545 31000 0.002889463 30600
0.000682445 32900 0.004734777 32400 0.000989564
33300 0.003662837 31200 0.000224336 31000 0.002917852 30600
0.000684152 32900 0.004762144 32400 0.000988956
33300 0.003670295 31200 0.000224126 31000 0.002946277 30600
0.000685813 32900 0.004789652 32400 0.000988347
33300 0.003677844 31200 0.000223918 31000 0.002974706 30600
0.000687505 32900 0.004817321 32400 0.000987748
33300 0.003685399 31200 0.00022372 31000 0.003003092 30600
0.000689169 32900 0.004845151 32400 0.000987132
33300 0.003693002 31300 0.000223513 31000 0.003031417 30600
0.000690867 32900 0.004873119 32400 0.000986517

33300 0.003700667 31300 0.00022332 31000 0.003059645 30600
0.000692537 32900 0.004901238 32400 0.000985903
33300 0.003708361 31300 0.000223101 31100 0.003087747 30600
0.000694235 32900 0.004929487 32400 0.00098529
33300 0.003716109 31300 0.000222916 31100 0.003115676 30600
0.000695954 32900 0.004957888 32400 0.000984686
33300 0.00372388 31300 0.00022271 31100 0.003143406 30600
0.0006977 32900 0.004986436 32400 0.000984075
33300 0.00373169 31300 0.000222516 31100 0.003170891 30600
0.000699463 32900 0.005015113 32400 0.000983451
33300 0.003739587 31300 0.000222307 31100 0.003198088 30700
0.000701252 32900 0.005043924 32400 0.000982857
33300 0.003747508 31300 0.000222105 31100 0.003224938 30700
0.000703068 32900 0.005072858 32400 0.000982258
33300 0.003755462 31300 0.000221914 31100 0.00325142 30700
0.000704899 32900 0.005101918 32400 0.000981654
33300 0.003763494 31300 0.000221722 31100 0.003277455 30700
0.000706771 32900 0.005131108 32400 0.000981063
33300 0.003771511 31300 0.000221529 31100 0.003302998 30700
0.000708658 32900 0.005160414 32400 0.000980458
33300 0.003779571 31300 0.000221353 31100 0.003328007 30700
0.000710578 32900 0.00518984 32400 0.000979857
33300 0.003787694 31300 0.000221167 31100 0.003352414 30700
0.000712517 32900 0.005219358 32400 0.000979279
33300 0.003795846 31300 0.000220987 31100 0.003376159 30700
0.000714459 32900 0.005248982 32400 0.000978675
33300 0.00380407 31300 0.000220809 31100 0.003399193 30700
0.000716435 32900 0.005278711 32400 0.000978096
33300 0.003812304 31300 0.000220632 31100 0.003421431 30700
0.000718419 32900 0.00530852 32400 0.000977504
33300 0.003820595 31300 0.00022044 31100 0.003442827 30700
0.000720377 32900 0.005338411 32400 0.000976941
33300 0.003828968 31300 0.000220253 31100 0.003463309 30700
0.000722352 33000 0.005368388 32400 0.000976361
33300 0.003837322 31300 0.000220069 31100 0.003482801 30700
0.000724301 33000 0.005398423 32400 0.00097579
33300 0.003845729 31300 0.000219865 31100 0.00350122 30700
0.000726283 33000 0.005428517 32400 0.000975209
33300 0.003854174 31300 0.000219666 31100 0.003518516 30700
0.000728233 33000 0.005458651 32400 0.000974646
33300 0.003862649 31300 0.000219456 31100 0.003534605 30700
0.00073016 33000 0.005488818 32400 0.000974067
33300 0.003871205 31300 0.000219262 31100 0.003549409 30700
0.000732098 33000 0.005519016 32400 0.000973509
33300 0.003879786 31300 0.000219056 31100 0.003562852 30700
0.000734045 33000 0.005549231 32400 0.000972947
33300 0.003888375 31300 0.000218862 31100 0.003574863 30700
0.000735964 33000 0.00557946 32400 0.00097238
33300 0.003897057 31300 0.000218678 31100 0.003585355 30700
0.000737896 33000 0.005609682 32400 0.000971838
33300 0.003905791 31300 0.000218498 31100 0.003594264 30700
0.000739815 33000 0.005639876 32400 0.000971287
33300 0.003914559 31300 0.00021833 31100 0.003601489 30700
0.000741742 33000 0.005670049 32400 0.000970743
33300 0.003923399 31300 0.00021816 31100 0.003606989 30700
0.000743685 33000 0.005700179 32400 0.000970202
33300 0.003932259 31300 0.000217992 31100 0.003610661 30700
0.000745653 33000 0.005730255 32400 0.000969644
33300 0.003941167 31300 0.000217835 31100 0.003612462 30700
0.000747624 33000 0.005760272 32400 0.000969106
33300 0.003950155 31300 0.000217662 31100 0.003612296 30700
0.000749636 33000 0.005790215 32400 0.000968579
33300 0.003959179 31300 0.000217492 31100 0.003610095 30700
0.000751661 33000 0.005820071 32400 0.000968038
33300 0.003968274 31300 0.000217323 31100 0.003605816 30700
0.000753747 33000 0.005849829 32400 0.000967505
33300 0.003977393 31300 0.000217146 31100 0.003599385 30700
0.00075585 33000 0.005879477 32400 0.000966969

33300 0.003986576 31300 0.00021696 31100 0.003590738 30700
0.000757954 33000 0.005909 32400 0.000966448
33300 0.00399584 31300 0.000216779 31100 0.003579847 30700
0.000760122 33000 0.005938379 32400 0.000965911
33300 0.004005127 31300 0.000216598 31100 0.003566646 30700
0.000762294 33000 0.005967609 32400 0.000965387
33300 0.004014463 31300 0.000216403 31100 0.003551098 30700
0.000764525 33000 0.005996669 32400 0.000964849
33300 0.004023867 31300 0.000216209 31100 0.003533213 30700
0.000766747 33000 0.006025537 32400 0.000964329
33300 0.004033319 31300 0.000216001 31100 0.003512914 30700
0.00076896 33000 0.006054194 32400 0.000963779
33300 0.004042838 31300 0.000215807 31100 0.0034902 30700
0.000771188 33000 0.006082631 32400 0.00096325
33300 0.004052395 31300 0.000215603 31100 0.003465087 30700
0.000773386 33000 0.006110827 32400 0.000962722
33300 0.004062006 31300 0.000215411 31100 0.003437569 30700
0.00077557 33000 0.006138757 32400 0.000962185
33300 0.004071699 31300 0.000215223 31100 0.003407652 30700
0.000777735 33000 0.006166405 32400 0.000961666
33300 0.004081449 31300 0.000215046 31100 0.003375365 30700
0.000779906 33000 0.006193746 32400 0.000961144
33400 0.004091253 31300 0.000214864 31100 0.003340726 30700
0.000782063 33000 0.006220754 32400 0.000960615
33400 0.004101105 31300 0.000214689 31100 0.003303818 30700
0.000784255 33000 0.006247409 32400 0.000960079
33400 0.004110999 31300 0.000214513 31100 0.003264669 30700
0.000786457 33000 0.006273686 32400 0.00095955
33400 0.004120913 31300 0.000214342 31100 0.003223384 30700
0.000788679 33000 0.006299559 32400 0.000959019
33400 0.004130933 31300 0.000214155 31100 0.003179989 30700
0.000790923 33000 0.006325 32400 0.000958485
33400 0.004140982 31300 0.000213975 31100 0.003134604 30700
0.000793199 33000 0.006349992 32400 0.000957954
33400 0.004151109 31300 0.000213795 31100 0.003087355 30700
0.000795523 33000 0.006374503 32400 0.000957422
33400 0.004161292 31300 0.000213613 31100 0.003038341 30700
0.000797901 33000 0.006398494 32400 0.000956883
33400 0.004171495 31300 0.00021343 31100 0.002987687 30700
0.000800301 33000 0.006421963 32400 0.000956358
33400 0.004181779 31300 0.000213265 31100 0.002935492 30700
0.000802779 33000 0.006444865 32500 0.000955811
33400 0.004192098 31300 0.00021308 31100 0.002881948 30700
0.000805289 33000 0.006467181 32500 0.000955282
33400 0.004202474 31300 0.000212901 31100 0.002827144 30700
0.000807854 33000 0.006488867 32500 0.000954736
33400 0.004212899 31300 0.000212719 31100 0.002771281 30700
0.000810439 33000 0.006509909 32500 0.000954205
33400 0.004223371 31300 0.000212535 31100 0.002714508 30700
0.00081304 33000 0.006530264 32500 0.000953646
33400 0.004233901 31300 0.000212363 31100 0.00265699 30700
0.000815674 33000 0.006549902 32500 0.000953105
33400 0.004244476 31300 0.000212184 31100 0.002598917 30700
0.000818307 33000 0.006568792 32500 0.000952553
33400 0.004255112 31300 0.00021202 31100 0.002540433 30700
0.000820968 33000 0.006586907 32500 0.000952004
33400 0.004265807 31300 0.000211856 31100 0.002481741 30700
0.000823651 33000 0.006604204 32500 0.000951466
33400 0.004276568 31400 0.000211696 31100 0.002422978 30700
0.000826365 33000 0.006620665 32500 0.000950931
33400 0.004287383 31400 0.000211523 31100 0.002364346 30700
0.000829107 33000 0.006636238 32500 0.000950387
33400 0.00429825 31400 0.000211355 31200 0.002306025 30700
0.000831879 33000 0.006650895 32500 0.000949844
33400 0.004309151 31400 0.000211179 31200 0.002248155 30700
0.000834685 33000 0.006664603 32500 0.000949302
33400 0.00432011 31400 0.000211012 31200 0.002190948 30700
0.000837514 33000 0.006677353 32500 0.000948752

33400 0.00433115 31400 0.000210827 31200 0.002134503 30700
0.000840382 33000 0.006689079 32500 0.000948227
33400 0.00434224 31400 0.000210649 31200 0.002079013 30800
0.000843224 33000 0.006699735 32500 0.000947685
33400 0.004353434 31400 0.000210465 31200 0.002024587 30800
0.000846109 33000 0.006709344 32500 0.000947155
33400 0.004364658 31400 0.000210282 31200 0.0019714 30800
0.000849014 33000 0.006717826 32500 0.000946627
33400 0.004375953 31400 0.000210095 31200 0.001919539 30800
0.000851929 33000 0.006725152 32500 0.000946107
33400 0.004387327 31400 0.000209907 31200 0.001869118 30800
0.000854869 33000 0.006731292 32500 0.000945571
33400 0.004398738 31400 0.000209731 31200 0.001820267 30800
0.000857799 33000 0.006736208 32500 0.000945053
33400 0.00441024 31400 0.000209551 31200 0.00177305 30800
0.000860739 33000 0.006739867 32500 0.000944517
33400 0.004421796 31400 0.000209378 31200 0.001727557 30800
0.000863642 33000 0.006742237 32500 0.000944004
33400 0.00443341 31400 0.000209211 31200 0.00168385 30800
0.00086653 33000 0.006743263 32500 0.000943465
33400 0.004445077 31400 0.000209052 31200 0.001641983 30800
0.000869418 33000 0.006742923 32500 0.000942942
33400 0.004456865 31400 0.000208879 31200 0.001601993 30800
0.000872295 33000 0.006741186 32500 0.000942424
33400 0.0044687 31400 0.000208711 31200 0.00156391 30800
0.000875218 33100 0.006738002 32500 0.000941893
33400 0.004480636 31400 0.000208547 31200 0.001527768 30800
0.000878163 33100 0.006733355 32500 0.000941377
33400 0.004492657 31400 0.000208377 31200 0.001493548 30800
0.000881145 33100 0.006727211 32500 0.00094087
33400 0.004504726 31400 0.000208212 31200 0.001461258 30800
0.000884154 33100 0.006719517 32500 0.000940355
33400 0.00451691 31400 0.000208051 31200 0.001430911 30800
0.000887202 33100 0.006710241 32500 0.00093984
33400 0.004529121 31400 0.000207882 31200 0.001402431 30800
0.000890273 33100 0.006699377 32500 0.000939325
33400 0.004541397 31400 0.000207732 31200 0.001375841 30800
0.000893346 33100 0.006686883 32500 0.000938796
33400 0.004553784 31400 0.000207561 31200 0.001351021 30800
0.000896453 33100 0.006672699 32500 0.000938289
33400 0.004566282 31400 0.000207397 31200 0.001327997 30800
0.000899556 33100 0.006656835 32500 0.000937755
33400 0.004578826 31400 0.000207226 31200 0.00130669 30800
0.000902679 33100 0.006639268 32500 0.000937229
33400 0.004591497 31400 0.000207035 31200 0.001287034 30800
0.000905824 33100 0.006619931 32500 0.000936696
33400 0.004604242 31400 0.000206852 31200 0.001268984 30800
0.000908997 33100 0.006598841 32500 0.00093618
33400 0.004617083 31400 0.000206671 31200 0.001252447 30800
0.000912212 33100 0.006575961 32500 0.000935646
33400 0.004629983 31400 0.00020648 31200 0.001237364 30800
0.000915477 33100 0.006551262 32500 0.000935124
33400 0.004642957 31400 0.000206298 31200 0.001223646 30800
0.000918762 33100 0.006524739 32500 0.000934582
33400 0.004656025 31400 0.000206114 31200 0.001211236 30800
0.00092208 33100 0.006496375 32500 0.000934063
33400 0.004669179 31400 0.000205933 31200 0.001200061 30800
0.000925396 33100 0.006466162 32500 0.000933514
33400 0.004682404 31400 0.000205746 31200 0.00119004 30800
0.00092876 33100 0.006434061 32500 0.000932979
33400 0.004695718 31400 0.000205576 31200 0.001181087 30800
0.000932134 33100 0.006400085 32500 0.000932436
33400 0.004709125 31400 0.000205423 31200 0.001173115 30800
0.000935548 33100 0.006364223 32500 0.000931887
33400 0.004722653 31400 0.000205267 31200 0.001166073 30800
0.000939004 33100 0.006326452 32500 0.000931343
33400 0.004736248 31400 0.000205113 31200 0.001159868 30800
0.00094251 33100 0.006286804 32500 0.000930823

33400 0.004749943 31400 0.000204954 31200 0.001154442 30800
0.000946056 33100 0.006245258 32500 0.000930281
33400 0.004763745 31400 0.000204806 31200 0.001149723 30800
0.000949651 33100 0.006201821 32500 0.000929745
33400 0.004777612 31400 0.000204662 31200 0.001145636 30800
0.000953276 33100 0.006156507 32500 0.000929211
33400 0.004791568 31400 0.00020453 31200 0.001142124 30800
0.000956941 33100 0.006109337 32500 0.000928683
33400 0.004805643 31400 0.000204387 31200 0.001139124 30800
0.000960637 33100 0.006060293 32500 0.000928146
33400 0.004819799 31400 0.000204252 31200 0.001136572 30800
0.000964341 33100 0.006009426 32500 0.000927608
33400 0.004834047 31400 0.000204127 31200 0.00113442 30800
0.000968081 33100 0.005956742 32500 0.000927085
33500 0.004848388 31400 0.000203994 31200 0.001132614 30800
0.000971856 33100 0.005902261 32500 0.000926553
33500 0.004862813 31400 0.000203874 31200 0.001131115 30800
0.00097564 33100 0.005846035 32500 0.000926034
33500 0.004877362 31400 0.000203745 31200 0.00112985 30800
0.00097948 33100 0.005788062 32500 0.000925516
33500 0.004891961 31400 0.000203608 31200 0.001128796 30800
0.000983347 33100 0.005728392 32500 0.000924992
33500 0.004906656 31400 0.00020347 31200 0.001127889 30800
0.000987266 33100 0.005667065 32500 0.000924459
33500 0.004921455 31400 0.000203318 31200 0.001127129 30800
0.000991185 33100 0.00560412 32500 0.000923937
33500 0.004936346 31400 0.00020316 31200 0.001126467 30800
0.000995133 33100 0.005539609 32500 0.0009234
33500 0.004951323 31400 0.000202994 31200 0.001125858 30800
0.000999144 33100 0.005473568 32500 0.000922878
33500 0.004966423 31400 0.000202824 31200 0.001125279 30800
0.001003165 33100 0.005406036 32500 0.000922356
33500 0.00498159 31400 0.000202643 31200 0.001124712 30800
0.001007239 33100 0.005337105 32600 0.000921823
33500 0.004996888 31400 0.000202481 31200 0.00112413 30800
0.001011356 33100 0.005266803 32600 0.000921306
33500 0.005012291 31400 0.000202303 31200 0.001123508 30800
0.001015522 33100 0.005195203 32600 0.000920803
33500 0.005027784 31400 0.000202139 31200 0.001122826 30800
0.001019707 33100 0.005122364 32600 0.000920287
33500 0.005043394 31400 0.000201979 31200 0.001122091 30800
0.001023928 33100 0.005048361 32600 0.000919781
33500 0.005059081 31400 0.000201814 31200 0.001121258 30800
0.001028181 33100 0.004973268 32600 0.000919259
33500 0.005074828 31400 0.00020166 31200 0.001120327 30800
0.00103246 33100 0.004897145 32600 0.000918758
33500 0.005090747 31400 0.000201513 31200 0.001119283 30800
0.001036766 33100 0.004820062 32600 0.00091826
33500 0.00510675 31400 0.000201362 31200 0.001118123 30800
0.00104108 33100 0.004742114 32600 0.000917745
33500 0.005122895 31500 0.000201223 31200 0.001116847 30800
0.001045406 33100 0.004663376 32600 0.000917248
33500 0.005139143 31500 0.000201067 31200 0.001115435 30800
0.001049755 33100 0.004583921 32600 0.000916756
33500 0.005155479 31500 0.000200924 31300 0.001113896 30800
0.001054099 33100 0.004503847 32600 0.000916272
33500 0.005171976 31500 0.000200789 31300 0.001112206 30800
0.001058494 33100 0.004423228 32600 0.00091578
33500 0.00518854 31500 0.000200643 31300 0.001110384 30800
0.001062913 33100 0.004342154 32600 0.000915301
33500 0.005205233 31500 0.000200489 31300 0.00110841 30800
0.001067381 33100 0.004260714 32600 0.000914801
33500 0.005222037 31500 0.000200327 31300 0.001106304 30900
0.001071884 33100 0.004179002 32600 0.000914325
33500 0.005238962 31500 0.000200174 31300 0.001104058 30900
0.001076418 33100 0.004097104 32600 0.00091383
33500 0.005255995 31500 0.000200018 31300 0.001101655 30900
0.001081012 33100 0.004015103 32600 0.000913332

33500 0.005273184 31500 0.000199869 31300 0.001099124 30900
0.00108563 33100 0.003933105 32600 0.000912858
33500 0.005290454 31500 0.000199707 31300 0.001096442 30900
0.001090275 33100 0.00385119 32600 0.000912363
33500 0.005307897 31500 0.000199551 31300 0.001093638 30900
0.001094921 33100 0.003769451 32600 0.00091188
33500 0.005325479 31500 0.000199398 31300 0.001090682 30900
0.001099589 33100 0.003687982 32600 0.000911415
33500 0.005343168 31500 0.000199248 31300 0.001087597 30900
0.001104268 33100 0.003606869 32600 0.000910936
33500 0.005360996 31500 0.000199098 31300 0.001084396 30900
0.00110897 33100 0.003526193 32600 0.000910478
33500 0.005378952 31500 0.000198961 31300 0.001081074 30900
0.001113721 33100 0.003446045 32600 0.000909992
33500 0.005397015 31500 0.000198827 31300 0.001077628 30900
0.00111851 33100 0.003366505 32600 0.000909521
33500 0.00541524 31500 0.000198685 31300 0.001074064 30900
0.001123357 33200 0.003287647 32600 0.00090905
33500 0.005433619 31500 0.000198549 31300 0.001070384 30900
0.001128247 33200 0.003209564 32600 0.000908585
33500 0.005452132 31500 0.000198418 31300 0.001066607 30900
0.00113321 33200 0.003132327 32600 0.000908114
33500 0.005470759 31500 0.000198275 31300 0.001062719 30900
0.001138226 33200 0.003056005 32600 0.000907642
33500 0.005489531 31500 0.000198137 31300 0.001058746 30900
0.001143281 33200 0.002980671 32600 0.000907192
33500 0.00550845 31500 0.000197994 31300 0.001054685 30900
0.001148405 33200 0.002906389 32600 0.000906725
33500 0.005527483 31500 0.000197844 31300 0.001050531 30900
0.00115357 33200 0.002833228 32600 0.000906258
33500 0.005546664 31500 0.000197689 31300 0.001046278 30900
0.001158814 33200 0.002761245 32600 0.000905788
33500 0.005565964 31500 0.000197524 31300 0.001041965 30900
0.001164086 33200 0.002690496 32600 0.000905329
33500 0.005585401 31500 0.000197366 31300 0.001037583 30900
0.001169411 33200 0.002621036 32600 0.000904861
33500 0.005605002 31500 0.000197207 31300 0.001033104 30900
0.001174798 33200 0.002552918 32600 0.000904395
33500 0.005624713 31500 0.000197059 31300 0.001028584 30900
0.001180222 33200 0.002486194 32600 0.000903927
33500 0.005644554 31500 0.000196902 31300 0.001023994 30900
0.001185691 33200 0.002420894 32600 0.000903457
33500 0.005664563 31500 0.000196767 31300 0.001019353 30900
0.001191182 33200 0.002357062 32600 0.000902988
33500 0.005684706 31500 0.000196621 31300 0.001014655 30900
0.001196743 33200 0.002294736 32600 0.000902545
33500 0.005704986 31500 0.000196471 31300 0.001009913 30900
0.001202333 33200 0.002233951 32600 0.000902072
33500 0.005725401 31500 0.000196314 31300 0.001005132 30900
0.001207967 33200 0.002174731 32600 0.000901615
33500 0.005745952 31500 0.000196157 31300 0.001000301 30900
0.001213647 33200 0.002117097 32600 0.000901136
33500 0.005766603 31500 0.000195998 31300 0.000995438 30900
0.00121939 33200 0.002061073 32600 0.000900677
33500 0.005787442 31500 0.000195831 31300 0.000990539 30900
0.001225198 33200 0.002006663 32600 0.000900206
33500 0.005808402 31500 0.000195672 31300 0.000985618 30900
0.001231005 33200 0.001953895 32600 0.000899738
33500 0.005829533 31500 0.000195513 31300 0.000980663 30900
0.001236883 33200 0.001902772 32600 0.000899288
33500 0.00585078 31500 0.000195349 31300 0.000975687 30900
0.001242781 33200 0.001853278 32600 0.000898822
33500 0.005872182 31500 0.000195185 31300 0.0009707 30900
0.001248726 33200 0.001805451 32600 0.000898374
33500 0.005893728 31500 0.000195035 31300 0.000965694 30900
0.00125473 33200 0.00175926 32600 0.000897918
33500 0.005915414 31500 0.00019489 31300 0.00096068 30900
0.001260804 33200 0.001714711 32600 0.000897463

33500 0.005937261 31500 0.000194757 31300 0.000955634 30900
0.001266977 33200 0.001671794 32600 0.000896983
33500 0.005959226 31500 0.000194619 31300 0.0009506 30900
0.001273212 33200 0.001630487 32600 0.000896543
33500 0.005981373 31500 0.000194485 31300 0.000945565 30900
0.001279525 33200 0.001590792 32600 0.000896087
33600 0.006003645 31500 0.000194368 31300 0.000940505 30900
0.001285933 33200 0.001552677 32600 0.000895638
33600 0.006026089 31500 0.000194247 31300 0.000935463 30900
0.001292375 33200 0.001516117 32600 0.000895188
33600 0.006048678 31500 0.00019412 31300 0.000930415 30900
0.001298905 33200 0.001481115 32600 0.000894725
33600 0.006071438 31500 0.000194004 31300 0.000925384 30900
0.001305497 33200 0.001447615 32600 0.000894291
33600 0.006094362 31500 0.000193878 31300 0.000920354 30900
0.00131217 33200 0.001415608 32600 0.000893844
33600 0.006117417 31500 0.000193755 31300 0.00091532 30900
0.001318905 33200 0.001385066 32600 0.000893418
33600 0.006140672 31500 0.00019363 31300 0.000910305 30900
0.001325764 33200 0.001355958 32600 0.000892982
33600 0.006164057 31500 0.000193507 31300 0.000905302 30900
0.001332661 33200 0.001328246 32600 0.000892529
33600 0.006187579 31500 0.000193376 31300 0.000900313 30900
0.001339646 33200 0.001301904 32600 0.000892101
33600 0.006211312 31500 0.000193233 31300 0.000895338 30900
0.001346696 33200 0.001276884 32700 0.000891695
33600 0.006235204 31500 0.000193099 31300 0.000890387 30900
0.001353779 33200 0.00125316 32700 0.000891243
33600 0.006259284 31500 0.000192958 31300 0.000885451 30900
0.001360933 33200 0.001230697 32700 0.000890831
33600 0.006283518 31500 0.000192808 31300 0.000880536 30900
0.001368127 33200 0.001209431 32700 0.000890417
33600 0.006307906 31500 0.000192653 31300 0.000875636 30900
0.001375372 33200 0.001189373 32700 0.000890007
33600 0.006332506 31500 0.000192503 31300 0.000870767 30900
0.00138267 33200 0.001170447 32700 0.000889577
33600 0.006357247 31500 0.000192349 31300 0.000865927 30900
0.001390017 33200 0.001152609 32700 0.000889164
33600 0.006382164 31500 0.000192199 31300 0.000861094 30900
0.001397431 33200 0.001135851 32700 0.000888744
33600 0.006407253 31500 0.000192027 31300 0.000856285 30900
0.001404883 33200 0.001120111 32700 0.000888349
33600 0.006432527 31600 0.00019187 31300 0.000851522 30900
0.001412414 33200 0.001105369 32700 0.000887919
33600 0.006457955 31600 0.000191715 31300 0.000846758 30900
0.00142004 33200 0.001091562 32700 0.000887518
33600 0.006483582 31600 0.00019155 31400 0.000842043 30900
0.001427757 33200 0.001078656 32700 0.000887114
33600 0.00650937 31600 0.000191399 31400 0.00083733 30900
0.001435567 33200 0.00106663 32700 0.00088672
33600 0.006535381 31600 0.000191252 31400 0.000832666 30900
0.001443486 33200 0.001055419 32700 0.000886303
33600 0.006561572 31600 0.000191113 31400 0.00082803 30900
0.001451497 33200 0.001044999 32700 0.000885908
33600 0.006587937 31600 0.000190983 31400 0.000823418 31000
0.001459592 33200 0.001035335 32700 0.000885533
33600 0.00661452 31600 0.000190856 31400 0.000818847 31000
0.001467798 33200 0.001026378 32700 0.000885138
33600 0.006641238 31600 0.000190734 31400 0.000814277 31000
0.001476055 33200 0.001018115 32700 0.00088474
33600 0.006668143 31600 0.000190612 31400 0.000809766 31000
0.001484388 33200 0.001010492 32700 0.000884344
33600 0.006695254 31600 0.00019049 31400 0.000805273 31000
0.001492843 33200 0.001003473 32700 0.000883937
33600 0.006722558 31600 0.000190362 31400 0.000800823 31000
0.001501344 33200 0.000997037 32700 0.00088354
33600 0.006750052 31600 0.000190241 31400 0.000796402 31000
0.001509958 33200 0.000991139 32700 0.000883169

33600 0.006777754 31600 0.000190102 31400 0.000792014 31000
0.001518662 33200 0.000985749 32700 0.000882777
33600 0.006805606 31600 0.000189979 31400 0.000787663 31000
0.001527456 33200 0.000980843 32700 0.00088239
33600 0.006833696 31600 0.000189833 31400 0.000783336 31000
0.001536376 33200 0.000976385 32700 0.00088201
33600 0.00686194 31600 0.00018969 31400 0.000779066 31000
0.001545381 33200 0.000972338 32700 0.000881619
33600 0.006890373 31600 0.000189541 31400 0.00077482 31000
0.001554453 33300 0.000968693 32700 0.000881218
33600 0.006919006 31600 0.00018938 31400 0.000770599 31000
0.001563577 33300 0.000965414 32700 0.000880831
33600 0.00694781 31600 0.000189229 31400 0.000766414 31000
0.001572744 33300 0.000962474 32700 0.00088044
33600 0.006976847 31600 0.000189083 31400 0.000762253 31000
0.001581972 33300 0.000959852 32700 0.000880048
33600 0.007006037 31600 0.000188947 31400 0.000758134 31000
0.001591244 33300 0.000957498 32700 0.000879664
33600 0.007035435 31600 0.000188815 31400 0.000754043 31000
0.001600588 33300 0.000955425 32700 0.00087925
33600 0.00706505 31600 0.000188697 31400 0.000749996 31000
0.00161002 33300 0.000953585 32700 0.000878856
33600 0.007094857 31600 0.000188571 31400 0.000745988 31000
0.001619564 33300 0.000951964 32700 0.00087846
33600 0.007124856 31600 0.000188463 31400 0.000741992 31000
0.001629218 33300 0.000950556 32700 0.000878069
33600 0.007155073 31600 0.000188361 31400 0.000738056 31000
0.00163902 33300 0.000949319 32700 0.000877686
33600 0.007185458 31600 0.000188255 31400 0.000734137 31000
0.001648967 33300 0.000948253 32700 0.000877283
33600 0.007216031 31600 0.000188159 31400 0.000730249 31000
0.001659067 33300 0.000947333 32700 0.000876873
33600 0.007246834 31600 0.000188054 31400 0.000726392 31000
0.001669338 33300 0.000946537 32700 0.00087649
33600 0.007277841 31600 0.000187938 31400 0.000722582 31000
0.001679755 33300 0.000945861 32700 0.000876087
33600 0.007309092 31600 0.000187824 31400 0.000718799 31000
0.001690326 33300 0.000945275 32700 0.000875687
33600 0.007340499 31600 0.00018769 31400 0.000715047 31000
0.001700979 33300 0.000944769 32700 0.000875296
33600 0.007372139 31600 0.000187553 31400 0.00071134 31000
0.001711759 33300 0.000944355 32700 0.000874911
33600 0.00740401 31600 0.000187413 31400 0.000707663 31000
0.001722632 33300 0.000943986 32700 0.000874503
33600 0.007436068 31600 0.000187269 31400 0.000704023 31000
0.001733568 33300 0.000943657 32700 0.000874102
33600 0.007468307 31600 0.000187131 31400 0.000700426 31000
0.001744609 33300 0.000943379 32700 0.000873703
33600 0.007500793 31600 0.000186987 31400 0.000696852 31000
0.001755676 33300 0.000943133 32700 0.000873303
33600 0.007533458 31600 0.000186857 31400 0.000693324 31000
0.001766876 33300 0.000942904 32700 0.000872881
33600 0.007566373 31600 0.000186736 31400 0.000689817 31000
0.001778159 33300 0.00094268 32700 0.000872498
33600 0.007599489 31600 0.000186615 31400 0.000686347 31000
0.001789527 33300 0.000942452 32700 0.000872086
33600 0.007632799 31600 0.000186499 31400 0.000682907 31000
0.001801011 33300 0.000942227 32700 0.000871671
33600 0.007666367 31600 0.000186401 31400 0.000679511 31000
0.00181262 33300 0.00094199 32700 0.000871266
33600 0.007700171 31600 0.000186284 31400 0.00067614 31000
0.001824384 33300 0.000941725 32700 0.000870871
33600 0.007734155 31600 0.00018617 31400 0.00067281 31000
0.001836293 33300 0.00094145 32700 0.00087049
33600 0.00776839 31600 0.000186037 31400 0.000669514 31000
0.001848391 33300 0.000941142 32700 0.000870091
33700 0.007802846 31600 0.000185915 31400 0.000666254 31000
0.001860523 33300 0.000940798 32700 0.00086969

33700 0.007837475 31600 0.000185792 31400 0.000663013 31000
0.001872796 33300 0.000940428 32700 0.000869288
33700 0.007872391 31600 0.000185658 31400 0.000659816 31000
0.001885193 33300 0.000939999 32700 0.000868915
33700 0.007907502 31600 0.000185521 31400 0.000656652 31000
0.001897692 33300 0.000939527 32700 0.000868509
33700 0.007942851 31600 0.000185394 31400 0.000653516 31000
0.001910345 33300 0.000939006 32700 0.00086813
33700 0.007978419 31600 0.000185247 31400 0.000650409 31000
0.001923141 33300 0.000938432 32700 0.000867726
33700 0.00801421 31600 0.00018511 31400 0.000647324 31000
0.001936103 33300 0.000937805 32700 0.000867335
33700 0.008050224 31600 0.000184987 31400 0.000644282 31000
0.001949247 33300 0.000937108 32700 0.00086695
33700 0.008086456 31600 0.000184856 31400 0.000641275 31000
0.00196253 33300 0.000936344 32700 0.000866556
33700 0.008122906 31600 0.000184736 31400 0.00063829 31000
0.001976059 33300 0.000935521 32800 0.000866159
33700 0.008159571 31600 0.000184597 31400 0.000635339 31000
0.001989753 33300 0.000934627 32800 0.000865786
33700 0.008196457 31600 0.000184476 31400 0.000632421 31000
0.002003645 33300 0.000933673 32800 0.0008654
33700 0.008233565 31600 0.00018435 31400 0.000629501 31000
0.002017708 33300 0.000932641 32800 0.000865024
33700 0.008270895 31600 0.000184221 31400 0.000626626 31000
0.002031952 33300 0.000931532 32800 0.000864657
33700 0.008308405 31600 0.00018408 31400 0.000623775 31000
0.002046309 33300 0.000930346 32800 0.000864269
33700 0.008346198 31600 0.000183963 31400 0.000620953 31000
0.002060875 33300 0.000929083 32800 0.000863893
33700 0.008384188 31600 0.000183834 31400 0.000618155 31000
0.002075544 33300 0.000927759 32800 0.000863522
33700 0.008422391 31600 0.000183702 31400 0.000615381 31000
0.002090293 33300 0.000926358 32800 0.000863145
33700 0.008460824 31700 0.000183559 31400 0.000612623 31000
0.002105254 33300 0.000924883 32800 0.000862776
33700 0.008499444 31700 0.000183439 31400 0.000609912 31000
0.002120389 33300 0.000923335 32800 0.000862406
33700 0.008538267 31700 0.000183305 31500 0.000607217 31000
0.002135719 33300 0.000921721 32800 0.000862046
33700 0.008577319 31700 0.000183169 31500 0.00060454 31000
0.002151264 33300 0.000920023 32800 0.000861678
33700 0.008616584 31700 0.000183033 31500 0.000601895 31000
0.002167018 33300 0.000918269 32800 0.000861343
33700 0.00865609 31700 0.000182907 31500 0.000599277 31000
0.002182969 33300 0.00091643 32800 0.000860972
33700 0.0086958 31700 0.00018277 31500 0.000596672 31100
0.002199132 33300 0.000914526 32800 0.000860603
33700 0.008735695 31700 0.000182649 31500 0.0005941 31100
0.002215486 33300 0.000912558 32800 0.000860262
33700 0.008775821 31700 0.000182527 31500 0.000591543 31100
0.002232033 33300 0.000910523 32800 0.000859887
33700 0.008816154 31700 0.000182394 31500 0.000589023 31100
0.002248785 33300 0.00090842 32800 0.000859518
33700 0.008856696 31700 0.000182273 31500 0.000586531 31100
0.002265733 33300 0.000906249 32800 0.000859155
33700 0.008897416 31700 0.000182143 31500 0.000584065 31100
0.002282842 33300 0.000904017 32800 0.000858787
33700 0.008938332 31700 0.000182024 31500 0.00058162 31100
0.002300128 33300 0.000901737 32800 0.00085844
33700 0.008979449 31700 0.000181911 31500 0.000579179 31100
0.002317593 33300 0.000899376 32800 0.000858072
33700 0.009020768 31700 0.000181792 31500 0.000576767 31100
0.00233526 33300 0.000896962 32800 0.000857712
33700 0.009062248 31700 0.000181668 31500 0.000574379 31100
0.002353059 33300 0.00089449 32800 0.00085735
33700 0.009103952 31700 0.000181565 31500 0.000572023 31100
0.002371097 33300 0.00089197 32800 0.000856998

33700 0.00914587 31700 0.000181452 31500 0.000569675 31100
0.002389339 33400 0.000889384 32800 0.000856645
33700 0.009187937 31700 0.000181344 31500 0.000567351 31100
0.002407867 33400 0.000886758 32800 0.000856308
33700 0.009230172 31700 0.000181228 31500 0.000565047 31100
0.002426637 33400 0.000884071 32800 0.000855951
33700 0.009272591 31700 0.000181122 31500 0.000562766 31100
0.002445698 33400 0.000881344 32800 0.000855608
33700 0.009315152 31700 0.000181014 31500 0.000560523 31100
0.002465072 33400 0.00087859 32800 0.000855253
33700 0.009357892 31700 0.000180896 31500 0.00055828 31100
0.00248476 33400 0.000875773 32800 0.000854911
33700 0.009400803 31700 0.000180784 31500 0.000556074 31100
0.002504697 33400 0.000872919 32800 0.000854572
33700 0.009443889 31700 0.000180673 31500 0.000553895 31100
0.002524926 33400 0.00087002 32800 0.000854244
33700 0.009487112 31700 0.000180551 31500 0.00055171 31100
0.002545341 33400 0.000867089 32800 0.000853907
33700 0.009530459 31700 0.000180436 31500 0.000549561 31100
0.002566043 33400 0.00086411 32800 0.000853575
33700 0.009573963 31700 0.000180317 31500 0.000547425 31100
0.002586958 33400 0.000861111 32800 0.000853233
33700 0.009617587 31700 0.00018018 31500 0.000545324 31100
0.002608118 33400 0.000858062 32800 0.000852885
33700 0.009661351 31700 0.00018005 31500 0.000543232 31100
0.002629575 33400 0.000854984 32800 0.000852554
33700 0.009705221 31700 0.000179912 31500 0.000541172 31100
0.002651319 33400 0.00085187 32800 0.000852205
33700 0.00974921 31700 0.000179789 31500 0.000539116 31100
0.002673359 33400 0.000848748 32800 0.000851875
33700 0.009793296 31700 0.000179667 31500 0.000537094 31100
0.002695774 33400 0.00084557 32800 0.000851539
33700 0.009837507 31700 0.000179556 31500 0.000535076 31100
0.002718519 33400 0.000842381 32800 0.000851201
33700 0.009881801 31700 0.000179441 31500 0.000533085 31100
0.002741624 33400 0.000839148 32800 0.000850853
33700 0.009926197 31700 0.000179343 31500 0.000531113 31100
0.002765069 33400 0.000835904 32800 0.000850517
33700 0.009970691 31700 0.000179244 31500 0.000529163 31100
0.002788811 33400 0.000832637 32800 0.000850186
33700 0.01001525 31700 0.00017914 31500 0.000527212 31100
0.002812852 33400 0.000829348 32800 0.000849858
33700 0.01005988 31700 0.000179033 31500 0.000525299 31100
0.002837248 33400 0.000826035 32800 0.000849534
33700 0.01010456 31700 0.00017893 31500 0.000523398 31100
0.002861899 33400 0.000822712 32800 0.0008492
33700 0.01014925 31700 0.000178824 31500 0.000521528 31100
0.002886821 33400 0.000819375 32800 0.000848852
33700 0.01019401 31700 0.00017871 31500 0.000519655 31100
0.002912021 33400 0.000816002 32800 0.000848517
33700 0.01023885 31700 0.000178593 31500 0.000517831 31100
0.002937515 33400 0.000812622 32800 0.000848183
33700 0.01028368 31700 0.000178485 31500 0.000516003 31100
0.002963308 33400 0.000809232 32800 0.000847872
33700 0.01032854 31700 0.000178365 31500 0.00051418 31100
0.002989437 33400 0.000805818 32800 0.000847537
33700 0.01037339 31700 0.000178257 31500 0.000512396 31100
0.003015892 33400 0.000802402 32800 0.000847211
33800 0.01041825 31700 0.00017815 31500 0.000510616 31100
0.003042717 33400 0.000798979 32800 0.000846886
33800 0.01046307 31700 0.000178045 31500 0.000508864 31100
0.003069887 33400 0.000795538 32800 0.000846538
33800 0.01050786 31700 0.000177944 31500 0.000507117 31100
0.003097449 33400 0.000792087 32800 0.000846199
33800 0.0105526 31700 0.000177839 31500 0.000505403 31100
0.003125347 33400 0.00078862 32800 0.000845855
33800 0.01059727 31700 0.000177744 31500 0.000503696 31100
0.00315365 33400 0.000785164 32800 0.000845521

33800 0.01064187 31700 0.000177654 31500 0.000501997 31100
0.003182339 33400 0.000781682 32800 0.000845213
33800 0.01068639 31700 0.000177556 31500 0.000500321 31100
0.003211423 33400 0.000778202 32800 0.000844884
33800 0.01073079 31700 0.000177454 31500 0.000498647 31100
0.003240917 33400 0.000774716 32800 0.000844551
33800 0.01077512 31700 0.000177348 31500 0.000497001 31100
0.003270817 33400 0.000771231 32800 0.000844219
33800 0.01081932 31700 0.000177242 31500 0.000495355 31100
0.003301162 33400 0.000767745 32900 0.000843909
33800 0.01086339 31700 0.000177125 31500 0.000493738 31100
0.003331927 33400 0.000764253 32900 0.000843591
33800 0.0109073 31700 0.000177009 31500 0.000492126 31100
0.003363126 33400 0.000760771 32900 0.000843269
33800 0.010951 31700 0.000176893 31500 0.000490529 31100
0.003394723 33400 0.000757278 32900 0.000842955
33800 0.01099453 31700 0.000176779 31500 0.000488949 31100
0.003426804 33400 0.000753799 32900 0.000842649
33800 0.01103788 31700 0.000176664 31500 0.000487366 31100
0.003459342 33400 0.000750308 32900 0.000842323
33800 0.011081 31700 0.00017656 31500 0.000485825 31100
0.003492342 33400 0.000746827 32900 0.000842026
33800 0.01112387 31700 0.000176457 31500 0.000484275 31100
0.003525884 33400 0.000743353 32900 0.000841735
33800 0.01116648 31700 0.00017634 31500 0.000482756 31100
0.003559957 33400 0.000739874 32900 0.000841431
33800 0.01120881 31800 0.000176227 31500 0.000481241 31100
0.003594536 33400 0.000736412 32900 0.000841141
33800 0.01125087 31800 0.000176121 31500 0.000479745 31100
0.003629694 33400 0.000732947 32900 0.000840858
33800 0.01129258 31800 0.000176006 31600 0.000478256 31100
0.003665413 33400 0.000729484 32900 0.000840562
33800 0.01133396 31800 0.000175895 31600 0.000476789 31100
0.003701726 33400 0.00072603 32900 0.000840277
33800 0.01137496 31800 0.000175775 31600 0.000475332 31100
0.003738582 33400 0.000722572 32900 0.000839988
33800 0.0114156 31800 0.000175655 31600 0.000473895 31100
0.003776026 33400 0.000719139 32900 0.000839708
33800 0.01145581 31800 0.00017555 31600 0.000472455 31200
0.003814051 33400 0.000715695 32900 0.000839431
33800 0.01149566 31800 0.000175455 31600 0.000471035 31200
0.00385266 33400 0.000712256 32900 0.000839158
33800 0.01153501 31800 0.000175358 31600 0.000469618 31200
0.003891882 33400 0.000708836 32900 0.000838889
33800 0.01157393 31800 0.000175258 31600 0.000468225 31200
0.003931721 33400 0.000705412 32900 0.000838612
33800 0.01161237 31800 0.000175161 31600 0.000466833 31200
0.003972158 33400 0.000702006 32900 0.000838371
33800 0.01165029 31800 0.000175053 31600 0.000465453 31200
0.004013166 33400 0.000698607 32900 0.000838106
33800 0.01168769 31800 0.000174941 31600 0.000464086 31200
0.004054762 33400 0.000695232 32900 0.000837847
33800 0.01172449 31800 0.000174836 31600 0.000462734 31200
0.004096955 33400 0.00069186 32900 0.000837592
33800 0.01176068 31800 0.000174723 31600 0.0004614 31200
0.004139672 33400 0.000688505 32900 0.000837326
33800 0.01179633 31800 0.000174606 31600 0.000460061 31200
0.004183009 33400 0.000685152 32900 0.000837074
33800 0.01183132 31800 0.000174499 31600 0.000458748 31200
0.004226892 33400 0.000681814 32900 0.00083682
33800 0.01186567 31800 0.000174388 31600 0.000457448 31200
0.004271383 33500 0.0006785 32900 0.000836572
33800 0.01189933 31800 0.000174264 31600 0.000456151 31200
0.004316487 33500 0.000675184 32900 0.000836325
33800 0.01193222 31800 0.000174154 31600 0.000454867 31200
0.004362212 33500 0.000671903 32900 0.000836068
33800 0.01196442 31800 0.000174051 31600 0.000453596 31200
0.004408658 33500 0.000668626 32900 0.000835825

33800 0.01199581 31800 0.000173947 31600 0.000452339 31200
0.004455827 33500 0.000665372 32900 0.000835568
33800 0.01202639 31800 0.000173857 31600 0.000451088 31200
0.004503808 33500 0.000662142 32900 0.000835295
33800 0.01205614 31800 0.000173758 31600 0.000449843 31200
0.004552559 33500 0.000658925 32900 0.000835044
33800 0.012085 31800 0.000173671 31600 0.000448618 31200
0.00460214 33500 0.000655734 32900 0.000834784
33800 0.01211295 31800 0.000173582 31600 0.000447391 31200
0.00465259 33500 0.000652554 32900 0.000834528
33800 0.01213997 31800 0.000173481 31600 0.000446181 31200
0.004703853 33500 0.0006494 32900 0.000834263
33800 0.01216598 31800 0.000173383 31600 0.000444969 31200
0.00475593 33500 0.000646266 32900 0.000833987
33800 0.01219102 31800 0.000173288 31600 0.000443774 31200
0.00480876 33500 0.000643144 32900 0.000833719
33800 0.01221505 31800 0.000173183 31600 0.000442583 31200
0.004862342 33500 0.000640052 32900 0.000833475
33800 0.01223798 31800 0.000173072 31600 0.000441399 31200
0.004916639 33500 0.00063699 32900 0.000833218
33800 0.01225981 31800 0.00017296 31600 0.000440223 31200
0.004971686 33500 0.000633939 32900 0.000832955
33800 0.01228047 31800 0.00017286 31600 0.000439063 31200
0.00502746 33500 0.000630914 32900 0.000832679
33800 0.01229994 31800 0.00017276 31600 0.000437915 31200
0.005083976 33500 0.000627918 32900 0.000832415
33800 0.0123182 31800 0.000172657 31600 0.000436759 31200
0.005141296 33500 0.000624927 32900 0.000832144
33800 0.01233525 31800 0.00017256 31600 0.000435631 31200
0.005199388 33500 0.000621971 32900 0.000831881
33800 0.01235102 31800 0.000172471 31600 0.000434503 31200
0.00525837 33500 0.000619032 32900 0.000831616
33800 0.01236542 31800 0.000172371 31600 0.000433391 31200
0.005318217 33500 0.000616104 32900 0.000831359
33800 0.01237848 31800 0.000172273 31600 0.00043228 31200
0.005378967 33500 0.000613205 32900 0.000831098
33800 0.01239016 31800 0.000172175 31600 0.000431184 31200
0.005440732 33500 0.000610329 32900 0.000830844
33800 0.01240037 31800 0.000172083 31600 0.000430105 31200
0.005503489 33500 0.000607471 32900 0.000830568
33800 0.01240916 31800 0.000171991 31600 0.000429039 31200
0.005567365 33500 0.000604637 32900 0.00083031
33800 0.01241642 31800 0.000171898 31600 0.00042796 31200
0.005632358 33500 0.000601817 32900 0.000830038
33800 0.01242214 31800 0.000171818 31600 0.000426906 31200
0.005698508 33500 0.000599026 32900 0.000829789
33800 0.01242631 31800 0.000171739 31600 0.000425859 31200
0.005765827 33500 0.000596251 32900 0.000829534
33800 0.01242888 31800 0.000171657 31600 0.00042483 31200
0.005834327 33500 0.000593494 32900 0.000829289
33900 0.01242978 31800 0.000171574 31600 0.000423789 31200
0.005903974 33500 0.000590757 32900 0.000829021
33900 0.01242906 31800 0.000171494 31600 0.000422774 31200
0.005974717 33500 0.000588041 32900 0.000828775
33900 0.01242663 31800 0.00017141 31600 0.00042176 31200
0.006046575 33500 0.000585337 32900 0.000828531
33900 0.01242245 31800 0.000171318 31600 0.00042077 31200
0.006119532 33500 0.000582662 32900 0.000828301
33900 0.0124165 31800 0.00017123 31600 0.000419771 31200
0.006193585 33500 0.000580011 32900 0.000828051
33900 0.01240875 31800 0.00017113 31600 0.000418785 31200
0.006268802 33500 0.000577375 32900 0.000827814
33900 0.01239914 31800 0.000171033 31600 0.000417814 31200
0.006345171 33500 0.000574754 32900 0.000827575
33900 0.01238773 31800 0.000170929 31600 0.000416841 31200
0.006422794 33500 0.000572155 32900 0.000827361
33900 0.0123744 31800 0.000170823 31600 0.000415882 31200
0.006501656 33500 0.000569574 32900 0.000827126

33900	0.0123592	31800	0.000170726	31600	0.000414926	31200
0.006581797	33500	0.000567014	33000	0.000826912
33900	0.01234204	31800	0.000170621	31600	0.00041398	31200
0.006663178	33500	0.000564473	33000	0.000826692
33900	0.0123229	31800	0.000170519	31600	0.000413046	31200
0.006745825	33500	0.000561952	33000	0.000826477
33900	0.0123018	31800	0.000170424	31600	0.000412115	31200
0.006829666	33500	0.000559453	33000	0.000826269
33900	0.01227869	31800	0.000170328	31600	0.000411198	31200
0.006914712	33500	0.000556972	33000	0.000826053
33900	0.01225353	31800	0.000170227	31600	0.000410283	31200
0.007000981	33500	0.000554505	33000	0.000825835
33900	0.01222632	31800	0.000170123	31600	0.000409369	31200
0.00708849	33500	0.000552065	33000	0.000825632
33900	0.01219708	31800	0.000170026	31600	0.000408467	31200
0.007177236	33500	0.000549652	33000	0.000825417
33900	0.01216573	31800	0.000169931	31600	0.000407566	31200
0.007267295	33500	0.000547251	33000	0.000825221
33900	0.01213229	31900	0.000169836	31600	0.000406691	31200
0.007358627	33500	0.000544872	33000	0.000825014
33900	0.01209673	31900	0.000169743	31600	0.0004058	31200
0.007451226	33500	0.000542517	33000	0.000824799
33900	0.01205909	31900	0.00016965	31700	0.000404931	31200
0.007545085	33500	0.000540171	33000	0.000824599
33900	0.01201934	31900	0.000169558	31700	0.000404067	31200
0.007640209	33500	0.000537852	33000	0.000824414
33900	0.01197744	31900	0.000169461	31700	0.000403225	31200
0.007736619	33500	0.000535561	33000	0.000824209
33900	0.01193344	31900	0.000169375	31700	0.000402367	31200
0.007834389	33500	0.000533287	33000	0.000824013
33900	0.01188727	31900	0.000169285	31700	0.000401524	31300
0.007933528	33500	0.000531039	33000	0.000823824
33900	0.01183898	31900	0.0001692	31700	0.000400695	31300
0.008034043	33500	0.000528805	33000	0.000823603
33900	0.01178859	31900	0.000169111	31700	0.000399859	31300
0.008135971	33500	0.000526584	33000	0.000823404
33900	0.01173607	31900	0.000169033	31700	0.000399045	31300
0.008239263	33500	0.000524378	33000	0.000823199
33900	0.01168148	31900	0.000168963	31700	0.000398232	31300
0.008343957	33500	0.000522204	33000	0.000823013
33900	0.01162478	31900	0.000168887	31700	0.000397425	31300
0.008450067	33500	0.00052003	33000	0.000822822
33900	0.01156602	31900	0.000168805	31700	0.000396645	31300
0.008557603	33500	0.000517885	33000	0.00082265
33900	0.01150519	31900	0.000168725	31700	0.000395857	31300
0.008666589	33500	0.00051576	33000	0.000822453
33900	0.01144234	31900	0.000168641	31700	0.00039509	31300
0.00877698	33500	0.000513649	33000	0.000822248
33900	0.01137746	31900	0.000168539	31700	0.000394324	31300
0.008888808	33500	0.000511559	33000	0.000822038
33900	0.01131064	31900	0.000168436	31700	0.000393562	31300
0.009001918	33500	0.000509476	33000	0.000821854
33900	0.01124186	31900	0.00016834	31700	0.000392815	31300
0.009116309	33600	0.000507413	33000	0.000821664
33900	0.01117116	31900	0.000168245	31700	0.000392072	31300
0.009231932	33600	0.000505364	33000	0.000821487
33900	0.01109863	31900	0.000168138	31700	0.000391348	31300
0.009348793	33600	0.000503333	33000	0.000821288
33900	0.01102427	31900	0.000168052	31700	0.00039062	31300
0.009466928	33600	0.000501328	33000	0.00082112
33900	0.01094815	31900	0.000167968	31700	0.000389902	31300
0.009586288	33600	0.000499332	33000	0.000820927
33900	0.01087034	31900	0.000167886	31700	0.000389195	31300
0.009706872	33600	0.000497351	33000	0.000820752
33900	0.01079084	31900	0.000167803	31700	0.000388502	31300
0.009828599	33600	0.000495394	33000	0.000820586
33900	0.01070978	31900	0.000167713	31700	0.000387809	31300
0.009951408	33600	0.000493445	33000	0.000820416

33900 0.01062714 31900 0.000167634 31700 0.000387121 31300
0.01007519 33600 0.000491522 33000 0.000820247
33900 0.01054301 31900 0.000167553 31700 0.00038645 31300
0.01019986 33600 0.00048962 33000 0.000820064
33900 0.01045753 31900 0.000167468 31700 0.000385767 31300
0.01032535 33600 0.000487732 33000 0.000819892
33900 0.0103707 31900 0.000167387 31700 0.000385119 31300
0.01045159 33600 0.000485855 33000 0.000819722
33900 0.01028264 31900 0.000167316 31700 0.000384465 31300
0.01057851 33600 0.000483994 33000 0.000819562
33900 0.01019341 31900 0.000167234 31700 0.000383819 31300
0.01070602 33600 0.000482157 33000 0.000819393
33900 0.01010308 31900 0.000167173 31700 0.000383192 31300
0.01083409 33600 0.000480336 33000 0.000819231
33900 0.01001176 31900 0.0001671 31700 0.000382568 31300
0.01096262 33600 0.000478538 33000 0.000819056
33900 0.009919536 31900 0.000167044 31700 0.000381947 31300
0.01109155 33600 0.000476754 33000 0.00081889
33900 0.009826488 31900 0.000166968 31700 0.000381337 31300
0.01122081 33600 0.000474985 33000 0.000818705
33900 0.009732709 31900 0.000166895 31700 0.000380722 31300
0.01135026 33600 0.000473233 33000 0.000818542
33900 0.009638309 31900 0.000166815 31700 0.000380129 31300
0.0114798 33600 0.000471498 33000 0.000818367
33900 0.009543418 31900 0.000166729 31700 0.000379541 31300
0.01160929 33600 0.000469775 33000 0.000818213
33900 0.0094481 31900 0.00016664 31700 0.000378974 31300
0.01173859 33600 0.000468069 33000 0.00081804
33900 0.009352476 31900 0.000166553 31700 0.000378399 31300
0.0118675 33600 0.000466383 33000 0.000817872
33900 0.009256658 31900 0.00016647 31700 0.000377839 31300
0.01199581 33600 0.000464709 33000 0.000817686
33900 0.009160772 31900 0.000166392 31700 0.000377293 31300
0.01212333 33600 0.000463052 33000 0.000817532
33900 0.009064905 31900 0.000166303 31700 0.000376759 31300
0.01224982 33600 0.000461404 33000 0.000817361
33900 0.008969197 31900 0.000166211 31700 0.000376224 31300
0.01237507 33600 0.000459777 33000 0.000817199
33900 0.008873706 31900 0.000166134 31700 0.000375706 31300
0.01249885 33600 0.000458167 33000 0.000817025
33900 0.008778604 31900 0.000166051 31700 0.000375185 31300
0.01262098 33600 0.000456582 33000 0.000816846
34000 0.008684013 31900 0.00016597 31700 0.000374674 31300
0.01274127 33600 0.000454997 33000 0.000816668
34000 0.008590041 31900 0.000165893 31700 0.00037419 31300
0.01285952 33600 0.000453435 33000 0.000816492
34000 0.008496794 31900 0.000165814 31700 0.000373694 31300
0.01297549 33600 0.000451887 33000 0.00081633
34000 0.008404385 31900 0.000165731 31700 0.000373223 31300
0.0130889 33600 0.000450351 33000 0.00081615
34000 0.008312965 31900 0.000165656 31700 0.00037276 31300
0.01319946 33600 0.000448833 33000 0.000815984
34000 0.008222646 31900 0.000165578 31700 0.000372303 31300
0.01330683 33600 0.000447342 33000 0.000815813
34000 0.008133478 31900 0.000165493 31700 0.000371856 31300
0.01341072 33600 0.000445862 33000 0.000815639
34000 0.008045666 31900 0.000165424 31700 0.000371428 31300
0.01351073 33600 0.000444396 33000 0.000815449
34000 0.007959281 31900 0.000165349 31700 0.000371004 31300
0.01360656 33600 0.000442946 33000 0.00081527
34000 0.007874421 31900 0.000165268 31700 0.0003706 31300
0.01369783 33600 0.000441519 33100 0.000815094
34000 0.007791226 31900 0.00016519 31700 0.00037019 31300
0.01378428 33600 0.000440088 33100 0.000814927
34000 0.007709829 31900 0.000165109 31700 0.000369809 31300
0.01386557 33600 0.000438691 33100 0.000814746
34000 0.007630247 31900 0.000165032 31700 0.000369423 31300
0.01394138 33600 0.000437304 33100 0.000814569

34000	0.007552697	31900	0.000164961	31700	0.000369063	31300	0.0140114	33600	0.000435924	33100	0.000814381
34000	0.007477211	31900	0.000164886	31700	0.000368708	31300	0.01407522	33600	0.000434564	33100	0.000814219
34000	0.007403897	31900	0.000164813	31700	0.000368376	31300	0.01413244	33600	0.000433223	33100	0.00081405
34000	0.007332849	31900	0.000164731	31700	0.00036805	31300	0.01418262	33600	0.000431889	33100	0.00081388
34000	0.007264129	31900	0.000164662	31700	0.000367739	31300	0.01422532	33600	0.000430576	33100	0.00081372
34000	0.007197853	32000	0.000164601	31700	0.000367445	31300	0.01426006	33600	0.000429273	33100	0.000813522
34000	0.007134116	32000	0.000164532	31700	0.000367169	31300	0.01428638	33600	0.00042798	33100	0.000813366
34000	0.007072981	32000	0.000164462	31800	0.000366906	31300	0.01430388	33600	0.000426703	33100	0.000813187
34000	0.00701451	32000	0.000164388	31800	0.000366658	31300	0.01431215	33600	0.000425439	33100	0.000813035
34000	0.006958755	32000	0.000164305	31800	0.000366427	31300	0.01431084	33600	0.000424192	33100	0.000812868
34000	0.006905796	32000	0.000164225	31800	0.000366218	31300	0.0142997	33600	0.000422956	33100	0.000812713
34000	0.006855702	32000	0.000164148	31800	0.000366022	31400	0.01427845	33600	0.000421731	33100	0.000812553
34000	0.006808483	32000	0.000164052	31800	0.000365851	31400	0.01424683	33600	0.000420524	33100	0.000812407
34000	0.006764211	32000	0.000163976	31800	0.000365695	31400	0.0142046	33600	0.000419321	33100	0.000812228
34000	0.0067229	32000	0.000163885	31800	0.000365568	31400	0.01415142	33600	0.000418141	33100	0.000812068
34000	0.006684627	32000	0.000163809	31800	0.00036546	31400	0.01408694	33600	0.00041697	33100	0.000811906
34000	0.006649374	32000	0.000163736	31800	0.00036536	31400	0.01401086	33600	0.000415807	33100	0.00081174
34000	0.006617174	32000	0.000163659	31800	0.000365294	31400	0.01392285	33600	0.00041466	33100	0.000811569
34000	0.006588035	32000	0.000163585	31800	0.000365226	31400	0.01382264	33600	0.000413519	33100	0.000811409
34000	0.006562004	32000	0.000163514	31800	0.000365203	31400	0.01371011	33600	0.000412398	33100	0.000811242
34000	0.006539071	32000	0.000163449	31800	0.000365184	31400	0.0135852	33600	0.000411288	33100	0.000811077
34000	0.006519224	32000	0.000163371	31800	0.000365183	31400	0.01344792	33600	0.000410201	33100	0.000810929
34000	0.006502433	32000	0.000163294	31800	0.000365211	31400	0.01329838	33700	0.000409111	33100	0.000810752
34000	0.006488691	32000	0.000163221	31800	0.000365255	31400	0.01313666	33700	0.000408046	33100	0.000810577
34000	0.006477964	32000	0.000163154	31800	0.000365329	31400	0.01296287	33700	0.000406992	33100	0.000810398
34000	0.006470281	32000	0.000163071	31800	0.000365405	31400	0.0127772	33700	0.000405942	33100	0.000810225
34000	0.006465598	32000	0.000162992	31800	0.000365517	31400	0.01257982	33700	0.000404909	33100	0.000810058
34000	0.006463878	32000	0.00016292	31800	0.000365629	31400	0.01237101	33700	0.000403888	33100	0.000809879
34000	0.006465038	32000	0.000162841	31800	0.000365765	31400	0.01215109	33700	0.000402881	33100	0.0008097
34000	0.006469054	32000	0.00016278	31800	0.000365911	31400	0.01192046	33700	0.00040188	33100	0.000809532
34000	0.006475887	32000	0.000162723	31800	0.000366064	31400	0.01167953	33700	0.000400896	33100	0.000809355
34000	0.006485501	32000	0.000162657	31800	0.000366238	31400	0.01142884	33700	0.000399917	33100	0.000809179
34000	0.006497798	32000	0.0001626	31800	0.000366407	31400	0.01116893	33700	0.000398946	33100	0.000808987
34000	0.006512729	32000	0.000162535	31800	0.000366581	31400	0.01090031	33700	0.000397982	33100	0.000808792

34000 0.006530209 32000 0.00016247 31800 0.00036675 31400
0.01062361 33700 0.000397037 33100 0.000808611
34000 0.006550178 32000 0.000162395 31800 0.000366903 31400
0.01033953 33700 0.000396085 33100 0.000808419
34000 0.006572559 32000 0.000162316 31800 0.00036706 31400
0.01004883 33700 0.00039516 33100 0.000808228
34000 0.006597271 32000 0.000162226 31800 0.000367185 31400
0.00975241 33700 0.000394232 33100 0.000808042
34000 0.006624245 32000 0.000162139 31800 0.000367301 31400
0.00945126 33700 0.000393329 33100 0.000807836
34000 0.006653424 32000 0.00016205 31800 0.000367383 31400
0.009146318 33700 0.000392429 33100 0.000807645
34000 0.00668466 32000 0.000161957 31800 0.000367438 31400
0.008838543 33700 0.000391543 33100 0.000807453
34000 0.006717906 32000 0.000161865 31800 0.000367455 31400
0.008528871 33700 0.000390659 33100 0.000807262
34000 0.006753039 32000 0.000161785 31800 0.000367405 31400
0.008218147 33700 0.000389795 33100 0.000807044
34000 0.00678997 32000 0.000161715 31800 0.000367334 31400
0.007907317 33700 0.000388928 33100 0.000806852
34000 0.006828664 32000 0.000161649 31800 0.00036719 31400
0.00759728 33700 0.000388079 33100 0.000806628
34000 0.00686897 32000 0.000161586 31800 0.000366992 31400
0.007289055 33700 0.000387232 33100 0.000806428
34000 0.006910827 32000 0.000161531 31800 0.000366718 31400
0.006983584 33700 0.000386405 33100 0.00080622
34000 0.006954099 32000 0.000161478 31800 0.000366387 31400
0.00668186 33700 0.000385584 33100 0.000806014
34000 0.006998674 32000 0.00016143 31800 0.000365977 31400
0.006384796 33700 0.000384768 33100 0.00080581
34000 0.007044551 32000 0.00016138 31800 0.000365495 31400
0.006093309 33700 0.000383968 33100 0.000805597
34000 0.00709151 32000 0.000161323 31800 0.000364961 31400
0.005808174 33700 0.000383179 33100 0.0008054
34100 0.007139544 32000 0.000161266 31800 0.000364336 31400
0.005530178 33700 0.000382398 33100 0.000805172
34100 0.007188498 32000 0.000161204 31800 0.000363641 31400
0.005259996 33700 0.000381618 33100 0.00080495
34100 0.007238308 32000 0.000161139 31800 0.000362889 31400
0.004998289 33700 0.000380851 33100 0.000804747
34100 0.007288871 32000 0.000161074 31800 0.000362073 31400
0.004745682 33700 0.000380079 33100 0.000804519
34100 0.007340117 32000 0.000161001 31800 0.000361211 31400
0.004502784 33700 0.000379326 33100 0.000804305
34100 0.007391891 32000 0.000160936 31800 0.000360276 31400
0.004270086 33700 0.000378577 33100 0.000804075
34100 0.007444192 32000 0.000160869 31800 0.000359304 31400
0.004048048 33700 0.000377831 33100 0.000803843
34100 0.007496898 32000 0.000160803 31800 0.000358297 31400
0.003837062 33700 0.000377111 33100 0.000803633
34100 0.007549873 32000 0.000160727 31800 0.000357267 31400
0.003637437 33700 0.000376397 33100 0.000803413
34100 0.007603072 32000 0.000160653 31800 0.000356209 31400
0.00344944 33700 0.000375679 33200 0.000803183
34100 0.007656406 32000 0.000160571 31800 0.000355128 31400
0.003273234 33700 0.000374984 33200 0.000802934
34100 0.007709745 32000 0.000160498 31800 0.000354052 31400
0.003108954 33700 0.000374285 33200 0.000802689
34100 0.007763111 32000 0.000160413 31800 0.000352975 31400
0.002956659 33700 0.000373597 33200 0.000802417
34100 0.007816394 32000 0.00016033 31800 0.000351896 31400
0.002816371 33700 0.000372915 33200 0.000802167
34100 0.007869493 32000 0.000160255 31800 0.000350826 31400
0.002688027 33700 0.000372254 33200 0.000801913
34100 0.007922338 32000 0.000160182 31800 0.000349783 31400
0.002571561 33700 0.00037159 33200 0.000801628
34100 0.007974872 32000 0.000160127 31800 0.000348744 31400
0.0024668 33700 0.000370933 33200 0.000801359

34100 0.008027074 32000 0.000160079 31800 0.00034774 31400
0.002373561 33700 0.000370292 33200 0.000801074
34100 0.008078792 32100 0.000160036 31800 0.00034677 31400
0.002291548 33700 0.00036966 33200 0.000800818
34100 0.008130026 32100 0.00016001 31800 0.00034583 31400
0.002220493 33700 0.00036904 33200 0.000800525
34100 0.008180683 32100 0.000159982 31900 0.000344901 31400
0.002160003 33700 0.000368424 33200 0.00080021
34100 0.008230739 32100 0.000159955 31900 0.000344014 31400
0.002109675 33700 0.000367827 33200 0.000799934
34100 0.008280115 32100 0.000159934 31900 0.000343143 31400
0.002069089 33700 0.000367228 33200 0.00079961
34100 0.0083288 32100 0.000159897 31900 0.000342323 31400
0.002037817 33700 0.000366646 33200 0.000799304
34100 0.008376676 32100 0.00015986 31900 0.000341494 31500
0.002015324 33700 0.000366073 33200 0.000798972
34100 0.008423821 32100 0.00015981 31900 0.000340718 31500
0.002001161 33700 0.000365498 33200 0.000798632
34100 0.008470099 32100 0.000159755 31900 0.000339962 31500
0.001994829 33700 0.000364944 33200 0.000798289
34100 0.008515499 32100 0.000159695 31900 0.000339221 31500
0.0019958 33700 0.000364397 33200 0.000797937
34100 0.00855998 32100 0.000159638 31900 0.000338507 31500
0.002003571 33700 0.000363855 33200 0.000797593
34100 0.008603484 32100 0.000159568 31900 0.000337812 31500
0.00201762 33700 0.000363319 33200 0.000797223
34100 0.008646007 32100 0.000159512 31900 0.000337141 31500
0.002037453 33700 0.000362799 33200 0.000796845
34100 0.008687542 32100 0.000159449 31900 0.000336476 31500
0.002062514 33700 0.000362284 33200 0.000796464
34100 0.008728028 32100 0.000159396 31900 0.000335822 31500
0.002092328 33700 0.000361771 33200 0.000796095
34100 0.008767441 32100 0.000159341 31900 0.000335203 31500
0.002126374 33700 0.00036127 33200 0.0007957
34100 0.008805767 32100 0.000159287 31900 0.000334574 31500
0.002164158 33700 0.000360777 33200 0.000795282
34100 0.008842971 32100 0.000159241 31900 0.000333964 31500
0.002205236 33800 0.000360286 33200 0.000794882
34100 0.008879082 32100 0.000159187 31900 0.00033336 31500
0.002249151 33800 0.000359803 33200 0.000794475
34100 0.008914004 32100 0.000159129 31900 0.000332773 31500
0.002295514 33800 0.000359331 33200 0.000794061
34100 0.008947785 32100 0.000159073 31900 0.0003322 31500
0.002343971 33800 0.000358861 33200 0.000793649
34100 0.008980416 32100 0.000159016 31900 0.000331629 31500
0.002394133 33800 0.000358398 33200 0.000793204
34100 0.009011833 32100 0.000158962 31900 0.00033106 31500
0.002445652 33800 0.000357939 33200 0.000792772
34100 0.009042057 32100 0.000158918 31900 0.000330505 31500
0.002498177 33800 0.000357476 33200 0.00079235
34100 0.009071136 32100 0.000158877 31900 0.00032996 31500
0.002551411 33800 0.000357027 33200 0.000791896
34100 0.009098958 32100 0.00015884 31900 0.00032941 31500
0.002605056 33800 0.000356588 33200 0.000791461
34100 0.009125623 32100 0.000158797 31900 0.000328881 31500
0.002658893 33800 0.000356148 33200 0.000790971
34100 0.009151099 32100 0.000158765 31900 0.00032835 31500
0.002712687 33800 0.000355713 33200 0.000790502
34100 0.009175356 32100 0.000158725 31900 0.000327835 31500
0.002766215 33800 0.000355296 33200 0.000790041
34100 0.009198429 32100 0.000158688 31900 0.000327312 31500
0.002819262 33800 0.000354876 33200 0.000789546
34100 0.009220286 32100 0.000158639 31900 0.000326788 31500
0.002871568 33800 0.000354456 33200 0.000789066
34100 0.00924092 32100 0.000158602 31900 0.000326293 31500
0.002922977 33800 0.00035405 33200 0.000788569
34100 0.009260432 32100 0.000158562 31900 0.000325787 31500
0.002973251 33800 0.00035364 33200 0.000788073

34100 0.009278769 32100 0.000158523 31900 0.000325288 31500
0.003022307 33800 0.000353243 33200 0.000787567
34100 0.009295953 32100 0.000158481 31900 0.000324799 31500
0.003070007 33800 0.000352857 33200 0.00078705
34100 0.009311981 32100 0.000158438 31900 0.000324316 31500
0.003116266 33800 0.000352464 33200 0.000786507
34100 0.009326835 32100 0.000158402 31900 0.000323822 31500
0.003160961 33800 0.000352089 33200 0.000785974
34100 0.009340598 32100 0.000158369 31900 0.000323346 31500
0.003204003 33800 0.000351707 33200 0.000785443
34100 0.009353224 32100 0.000158324 31900 0.000322866 31500
0.003245306 33800 0.000351333 33200 0.000784918
34100 0.009364756 32100 0.000158291 31900 0.000322394 31500
0.003284774 33800 0.00035097 33200 0.000784374
34100 0.009375213 32100 0.000158253 31900 0.000321935 31500
0.003322356 33800 0.000350605 33200 0.00078382
34100 0.00938459 32100 0.000158222 31900 0.000321473 31500
0.003358027 33800 0.000350249 33200 0.000783261
34100 0.009392922 32100 0.000158204 31900 0.000321002 31500
0.003391785 33800 0.000349897 33200 0.000782711
34100 0.009400244 32100 0.000158168 31900 0.000320557 31500
0.003423682 33800 0.000349539 33200 0.000782131
34100 0.009406488 32100 0.000158138 31900 0.000320094 31500
0.003453709 33800 0.000349196 33200 0.00078155
34100 0.009411788 32100 0.0001581 31900 0.000319633 31500
0.003481866 33800 0.000348859 33200 0.00078096
34200 0.009416139 32100 0.000158073 31900 0.000319188 31500
0.003508166 33800 0.000348523 33200 0.000780345
34200 0.009419488 32100 0.000158035 31900 0.000318752 31500
0.003532603 33800 0.000348202 33200 0.000779763
34200 0.009421906 32100 0.000158004 31900 0.000318299 31500
0.003555176 33800 0.000347868 33200 0.000779168
34200 0.009423397 32100 0.000157974 31900 0.000317868 31500
0.003575922 33800 0.000347561 33200 0.000778542
34200 0.009423953 32100 0.000157948 31900 0.000317444 31500
0.003594847 33800 0.000347251 33200 0.0007779
34200 0.009423678 32100 0.000157918 31900 0.00031701 31500
0.003611961 33800 0.000346945 33200 0.000777299
34200 0.009422564 32100 0.0001579 31900 0.000316573 31500
0.003627331 33800 0.000346639 33200 0.000776685
34200 0.009420592 32100 0.000157883 31900 0.000316149 31500
0.003641002 33800 0.000346355 33200 0.000776038
34200 0.00941778 32100 0.000157864 31900 0.000315724 31500
0.003653031 33800 0.000346056 33200 0.00077541
34200 0.009414166 32100 0.000157849 31900 0.000315298 31500
0.003663457 33800 0.000345775 33300 0.000774783
34200 0.009409806 32100 0.000157824 31900 0.000314906 31500
0.003672346 33800 0.000345501 33300 0.000774171
34200 0.009404635 32100 0.000157805 31900 0.000314481 31500
0.003679714 33800 0.000345235 33300 0.00077351
34200 0.009398763 32100 0.000157772 31900 0.000314075 31500
0.00368563 33800 0.000344963 33300 0.000772884
34200 0.009392149 32100 0.000157731 31900 0.000313686 31500
0.003690098 33800 0.000344691 33300 0.000772252
34200 0.009384836 32100 0.00015769 31900 0.000313279 31500
0.003693214 33800 0.000344434 33300 0.000771608
34200 0.009376863 32100 0.000157652 31900 0.000312877 31500
0.003694989 33800 0.000344171 33300 0.000770953
34200 0.00936823 32100 0.000157613 31900 0.000312485 31500
0.003695519 33800 0.000343915 33300 0.000770359
34200 0.009358924 32100 0.000157576 31900 0.000312093 31500
0.003694857 33800 0.000343678 33300 0.000769719
34200 0.009349052 32200 0.000157542 31900 0.000311707 31500
0.003693064 33800 0.000343433 33300 0.000769072
34200 0.009338558 32200 0.000157515 31900 0.000311317 31500
0.003690196 33800 0.000343195 33300 0.000768467
34200 0.009327474 32200 0.000157484 32000 0.000310939 31500
0.003686308 33800 0.000342973 33300 0.000767867

34200 0.009315818 32200 0.000157459 32000 0.000310562 31500
0.003681439 33800 0.000342734 33300 0.000767208
34200 0.009303597 32200 0.000157433 32000 0.000310203 31500
0.003675649 33800 0.000342516 33300 0.000766588
34200 0.009290827 32200 0.00015741 32000 0.000309835 31500
0.003668994 33800 0.000342289 33300 0.000765978
34200 0.009277595 32200 0.000157384 32000 0.00030946 31600
0.003661507 33800 0.000342078 33300 0.000765353
34200 0.00926384 32200 0.000157364 32000 0.000309092 31600
0.003653264 33800 0.00034185 33300 0.000764728
34200 0.00924963 32200 0.000157338 32000 0.000308741 31600
0.003644346 33800 0.000341649 33300 0.000764117
34200 0.009234924 32200 0.000157323 32000 0.000308378 31600
0.003634791 33800 0.000341444 33300 0.000763468
34200 0.009219728 32200 0.000157296 32000 0.000308027 31600
0.003624598 33800 0.000341251 33300 0.000762879
34200 0.009204158 32200 0.000157279 32000 0.000307684 31600
0.003613792 33800 0.000341053 33300 0.000762283
34200 0.009188135 32200 0.000157261 32000 0.000307339 31600
0.003602348 33800 0.000340858 33300 0.00076168
34200 0.009171718 32200 0.000157233 32000 0.000306989 31600
0.003590285 33800 0.000340668 33300 0.000761092
34200 0.009154879 32200 0.000157215 32000 0.000306641 31600
0.003577555 33800 0.000340487 33300 0.000760469
34200 0.009137684 32200 0.000157189 32000 0.000306302 31600
0.003564159 33800 0.000340307 33300 0.000759894
34200 0.009120105 32200 0.000157168 32000 0.000305969 31600
0.00355013 33800 0.00034012 33300 0.000759304
34200 0.00910219 32200 0.000157147 32000 0.000305617 31600
0.00353552 33900 0.000339954 33300 0.000758702
34200 0.009083905 32200 0.000157131 32000 0.000305275 31600
0.003520363 33900 0.000339791 33300 0.00075812
34200 0.009065339 32200 0.00015711 32000 0.000304928 31600
0.003504717 33900 0.000339625 33300 0.000757557
34200 0.009046473 32200 0.000157101 32000 0.000304582 31600
0.003488629 33900 0.000339482 33300 0.000756972
34200 0.009027255 32200 0.000157089 32000 0.000304233 31600
0.003472142 33900 0.000339336 33300 0.000756416
34200 0.009007794 32200 0.000157085 32000 0.000303907 31600
0.003455317 33900 0.000339189 33300 0.000755824
34200 0.008988013 32200 0.000157079 32000 0.000303555 31600
0.00343814 33900 0.00033906 33300 0.000755278
34200 0.008967969 32200 0.000157077 32000 0.00030323 31600
0.003420673 33900 0.000338935 33300 0.000754722
34200 0.008947717 32200 0.000157073 32000 0.000302888 31600
0.003402924 33900 0.00033881 33300 0.000754191
34200 0.008927216 32200 0.000157076 32000 0.000302555 31600
0.003384972 33900 0.000338692 33300 0.000753624
34200 0.008906511 32200 0.000157075 32000 0.00030223 31600
0.003366802 33900 0.000338588 33300 0.000753101
34200 0.00888555 32200 0.00015708 32000 0.000301903 31600
0.003348518 33900 0.000338492 33300 0.00075259
34200 0.008864402 32200 0.000157084 32000 0.000301576 31600
0.003330066 33900 0.000338409 33300 0.00075204
34200 0.008843067 32200 0.00015709 32000 0.000301254 31600
0.003311478 33900 0.000338312 33300 0.000751526
34200 0.008821516 32200 0.000157094 32000 0.000300939 31600
0.003292767 33900 0.000338239 33300 0.000750998
34200 0.008799808 32200 0.000157089 32000 0.000300612 31600
0.003273878 33900 0.000338165 33300 0.000750491
34200 0.008777939 32200 0.000157082 32000 0.000300296 31600
0.003254833 33900 0.000338093 33300 0.000749975
34200 0.008755888 32200 0.000157093 32000 0.000299974 31600
0.003235629 33900 0.000338027 33300 0.000749474
34200 0.008733685 32200 0.0001571 32000 0.000299672 31600
0.003216324 33900 0.000337965 33300 0.00074899
34200 0.008711387 32200 0.0001571 32000 0.000299362 31600
0.003196866 33900 0.000337911 33300 0.00074847

34200 0.008688919 32200 0.000157112 32000 0.000299047 31600
0.003177335 33900 0.000337866 33300 0.000747986
34200 0.008666369 32200 0.000157113 32000 0.000298731 31600
0.003157729 33900 0.000337804 33300 0.000747469
34200 0.008643718 32200 0.000157115 32000 0.000298431 31600
0.003138059 33900 0.000337778 33300 0.000747015
34200 0.008620939 32200 0.000157116 32000 0.000298127 31600
0.003118364 33900 0.000337733 33300 0.000746543
34200 0.008598091 32200 0.000157127 32000 0.000297833 31600
0.003098687 33900 0.000337708 33300 0.00074607
34200 0.008575127 32200 0.00015712 32000 0.000297529 31600
0.003079026 33900 0.000337666 33300 0.000745585
34200 0.008552084 32200 0.000157138 32000 0.000297232 31600
0.003059429 33900 0.000337633 33300 0.000745123
34200 0.008529 32200 0.000157135 32000 0.000296936 31600
0.003039879 33900 0.000337608 33300 0.000744617
34200 0.008505868 32200 0.000157145 32000 0.000296644 31600
0.003020407 33900 0.000337586 33300 0.000744131
34300 0.008482694 32200 0.00015716 32000 0.000296351 31600
0.003000972 33900 0.000337571 33300 0.000743655
34300 0.00845944 32200 0.000157187 32000 0.00029607 31600
0.002981576 33900 0.000337549 33300 0.000743183
34300 0.008436132 32200 0.000157206 32000 0.000295786 31600
0.002962195 33900 0.000337551 33300 0.000742693
34300 0.008412821 32200 0.00015723 32000 0.000295497 31600
0.002942821 33900 0.00033753 33300 0.000742246
34300 0.008389449 32200 0.000157254 32000 0.000295222 31600
0.002923454 33900 0.000337529 33300 0.000741774
34300 0.008366093 32200 0.000157276 32000 0.000294933 31600
0.002904122 33900 0.000337511 33300 0.000741286
34300 0.008342707 32200 0.000157314 32000 0.000294657 31600
0.002884815 33900 0.000337513 33300 0.000740803
34300 0.008319292 32200 0.000157334 32000 0.000294379 31600
0.00286554 33900 0.000337503 33300 0.000740332
34300 0.008295905 32200 0.000157363 32000 0.000294104 31600
0.002846331 33900 0.0003375 33300 0.000739875
34300 0.008272508 32200 0.000157389 32000 0.000293823 31600
0.002827196 33900 0.000337501 33400 0.000739391
34300 0.008249079 32200 0.000157406 32000 0.000293553 31600
0.002808094 33900 0.000337502 33400 0.000738942
34300 0.008225727 32200 0.000157415 32000 0.000293272 31600
0.002789072 33900 0.000337497 33400 0.000738464
34300 0.008202394 32200 0.000157419 32000 0.000292995 31600
0.002770148 33900 0.000337498 33400 0.000738015
34300 0.008179064 32200 0.000157421 32000 0.000292728 31600
0.002751327 33900 0.000337495 33400 0.000737565
34300 0.008155772 32200 0.00015743 32000 0.000292452 31600
0.002732632 33900 0.000337493 33400 0.000737106
34300 0.0081325 32200 0.000157443 32000 0.000292189 31600
0.002714085 33900 0.000337505 33400 0.000736667
34300 0.008109242 32200 0.000157463 32000 0.000291911 31600
0.002695681 33900 0.000337525 33400 0.000736213
34300 0.008086022 32200 0.000157491 32000 0.000291646 31600
0.002677397 33900 0.000337532 33400 0.000735743
34300 0.008062859 32300 0.000157517 32000 0.000291386 31600
0.002659243 33900 0.000337539 33400 0.000735277
34300 0.008039756 32300 0.00015754 32000 0.000291123 31600
0.00264123 33900 0.000337555 33400 0.000734811
34300 0.008016689 32300 0.000157572 32100 0.000290885 31600
0.002623356 33900 0.000337549 33400 0.000734349
34300 0.007993682 32300 0.0001576 32100 0.00029063 31600
0.00260565 33900 0.000337563 33400 0.000733888
34300 0.007970736 32300 0.000157628 32100 0.000290383 31600
0.002588087 33900 0.000337586 33400 0.000733431
34300 0.007947824 32300 0.000157669 32100 0.000290129 31600
0.002570657 33900 0.000337608 33400 0.000732974
34300 0.007925006 32300 0.000157697 32100 0.000289884 31700
0.002553375 33900 0.000337619 33400 0.000732513

34300 0.007902252 32300 0.000157729 32100 0.000289636 31700
0.002536211 33900 0.000337654 33400 0.000732039
34300 0.007879533 32300 0.000157758 32100 0.000289406 31700
0.002519159 33900 0.000337688 33400 0.000731585
34300 0.007856897 32300 0.000157796 32100 0.000289168 31700
0.002502271 33900 0.000337717 33400 0.000731104
34300 0.007834359 32300 0.000157824 32100 0.000288932 31700
0.0024855 33900 0.00033775 33400 0.000730627
34300 0.007811864 32300 0.000157848 32100 0.000288696 31700
0.002468826 33900 0.000337778 33400 0.000730136
34300 0.007789466 32300 0.000157891 32100 0.000288468 31700
0.002452298 33900 0.000337817 33400 0.000729643
34300 0.007767173 32300 0.000157932 32100 0.000288239 31700
0.002435853 33900 0.000337856 33400 0.000729154
34300 0.007744947 32300 0.00015798 32100 0.000288001 31700
0.002419538 33900 0.000337902 33400 0.000728683
34300 0.007722816 32300 0.000158044 32100 0.000287771 31700
0.002403309 33900 0.000337944 33400 0.00072818
34300 0.00770078 32300 0.000158119 32100 0.000287555 31700
0.002387156 33900 0.000337988 33400 0.000727681
34300 0.007678761 32300 0.000158201 32100 0.000287306 31700
0.002371087 34000 0.000338036 33400 0.000727169
34300 0.007656888 32300 0.000158275 32100 0.000287082 31700
0.00235512 34000 0.000338094 33400 0.000726658
34300 0.007635102 32300 0.000158358 32100 0.000286852 31700
0.002339293 34000 0.000338141 33400 0.00072615
34300 0.007613421 32300 0.000158435 32100 0.000286633 31700
0.002323539 34000 0.00033821 33400 0.000725632
34300 0.007591816 32300 0.000158526 32100 0.000286417 31700
0.002307914 34000 0.000338266 33400 0.000725097
34300 0.007570313 32300 0.000158614 32100 0.00028619 31700
0.002292404 34000 0.00033834 33400 0.000724562
34300 0.00754895 32300 0.000158713 32100 0.000285969 31700
0.002277004 34000 0.000338413 33400 0.000724025
34300 0.00752764 32300 0.000158816 32100 0.000285739 31700
0.002261705 34000 0.000338473 33400 0.000723507
34300 0.007506439 32300 0.000158906 32100 0.000285513 31700
0.002246541 34000 0.000338547 33400 0.000722955
34300 0.007485353 32300 0.000158996 32100 0.000285279 31700
0.002231465 34000 0.000338631 33400 0.000722411
34300 0.007464362 32300 0.000159081 32100 0.000285061 31700
0.002216506 34000 0.00033872 33400 0.000721852
34300 0.007443452 32300 0.000159169 32100 0.000284848 31700
0.002201671 34000 0.000338789 33400 0.000721277
34300 0.007422687 32300 0.000159245 32100 0.000284641 31700
0.002186987 34000 0.000338887 33400 0.000720706
34300 0.00740198 32300 0.000159321 32100 0.000284409 31700
0.002172432 34000 0.000338981 33400 0.000720125
34300 0.007381429 32300 0.000159409 32100 0.000284207 31700
0.002158007 34000 0.000339071 33400 0.000719543
34300 0.007360977 32300 0.000159505 32100 0.000283977 31700
0.002143697 34000 0.000339177 33400 0.000718954
34300 0.007340607 32300 0.000159586 32100 0.000283758 31700
0.002129532 34000 0.000339287 33400 0.000718385
34300 0.00732033 32300 0.000159684 32100 0.000283557 31700
0.002115523 34000 0.000339401 33400 0.000717751
34300 0.007300157 32300 0.000159784 32100 0.000283349 31700
0.002101643 34000 0.000339494 33400 0.000717152
34300 0.007280073 32300 0.000159897 32100 0.000283128 31700
0.002087919 34000 0.000339622 33400 0.000716529
34300 0.007260152 32300 0.000159995 32100 0.000282921 31700
0.002074335 34000 0.000339728 33400 0.000715894
34300 0.007240316 32300 0.000160103 32100 0.000282706 31700
0.002060891 34000 0.000339848 33400 0.000715283
34300 0.007220606 32300 0.000160217 32100 0.000282502 31700
0.002047602 34000 0.000339986 33400 0.000714687
34300 0.007200995 32300 0.000160331 32100 0.000282297 31700
0.002034466 34000 0.000340121 33400 0.000714052

34300 0.007181472 32300 0.000160428 32100 0.000282086 31700
0.002021478 34000 0.000340251 33400 0.000713405
34300 0.007162073 32300 0.000160536 32100 0.000281876 31700
0.002008643 34000 0.000340386 33400 0.000712763
34300 0.007142765 32300 0.000160639 32100 0.000281682 31700
0.00199595 34000 0.000340513 33400 0.000712135
34300 0.007123605 32300 0.000160725 32100 0.000281463 31700
0.001983375 34000 0.000340642 33400 0.000711456
34300 0.00710453 32300 0.000160812 32100 0.000281263 31700
0.001970973 34000 0.000340776 33400 0.000710785
34400 0.007085557 32300 0.000160908 32100 0.000281057 31700
0.001958653 34000 0.000340927 33400 0.000710135
34400 0.007066693 32300 0.000161013 32100 0.000280855 31700
0.001946443 34000 0.000341064 33400 0.000709456
34400 0.007047948 32300 0.000161117 32100 0.000280664 31700
0.001934336 34000 0.000341214 33400 0.000708779
34400 0.007029288 32300 0.000161225 32100 0.000280461 31700
0.001922267 34000 0.000341383 33400 0.00070809
34400 0.007010759 32300 0.000161347 32100 0.000280262 31700
0.00191027 34000 0.00034153 33400 0.000707421
34400 0.006992355 32300 0.000161471 32100 0.000280074 31700
0.001898336 34000 0.000341702 33400 0.000706723
34400 0.006974052 32300 0.000161597 32100 0.000279868 31700
0.001886492 34000 0.000341883 33400 0.000706019
34400 0.00695587 32300 0.000161728 32100 0.000279676 31700
0.001874735 34000 0.000342039 33400 0.000705294
34400 0.006937769 32300 0.000161865 32100 0.000279488 31700
0.001863074 34000 0.000342219 33400 0.000704598
34400 0.006919753 32300 0.000162006 32100 0.000279289 31700
0.001851458 34000 0.000342395 33500 0.000703865
34400 0.006901938 32300 0.000162141 32100 0.000279096 31700
0.001839957 34000 0.00034257 33500 0.000703149
34400 0.00688418 32300 0.000162283 32100 0.000278905 31700
0.001828501 34000 0.000342756 33500 0.00070245
34400 0.006866574 32300 0.000162434 32100 0.000278711 31700
0.001817145 34000 0.000342947 33500 0.000701733
34400 0.006849022 32300 0.000162595 32100 0.000278525 31700
0.001805864 34000 0.00034316 33500 0.000700995
34400 0.006831593 32300 0.000162749 32100 0.000278332 31700
0.001794658 34000 0.00034337 33500 0.00070027
34400 0.006814293 32300 0.000162911 32100 0.000278142 31700
0.00178353 34000 0.000343544 33500 0.000699542
34400 0.006797068 32300 0.000163092 32100 0.000277963 31700
0.001772477 34000 0.000343738 33500 0.000698809
34400 0.00677997 32300 0.000163266 32100 0.000277755 31700
0.001761527 34000 0.000343934 33500 0.000698072
34400 0.006762969 32400 0.000163441 32100 0.000277576 31700
0.001750677 34000 0.000344146 33500 0.000697325
34400 0.006746091 32400 0.000163631 32100 0.000277382 31700
0.001739947 34000 0.000344343 33500 0.000696614
34400 0.006729305 32400 0.000163822 32200 0.0002772 31700
0.001729369 34000 0.000344558 33500 0.000695864
34400 0.006712636 32400 0.000163999 32200 0.000277022 31700
0.001718972 34000 0.000344772 33500 0.000695144
34400 0.006696049 32400 0.000164189 32200 0.000276839 31700
0.001708733 34000 0.000344969 33500 0.000694411
34400 0.006679652 32400 0.000164384 32200 0.000276658 31700
0.001698646 34000 0.000345172 33500 0.000693681
34400 0.006663329 32400 0.000164578 32200 0.000276474 31800
0.001688735 34000 0.000345426 33500 0.000692995
34400 0.006647109 32400 0.000164778 32200 0.000276301 31800
0.001678959 34000 0.000345635 33500 0.000692304
34400 0.006631021 32400 0.000164976 32200 0.000276138 31800
0.001669288 34000 0.000345846 33500 0.000691608
34400 0.006615014 32400 0.000165176 32200 0.000275942 31800
0.00165973 34000 0.000346079 33500 0.000690924
34400 0.006599109 32400 0.000165388 32200 0.000275768 31800
0.001650249 34000 0.000346303 33500 0.000690184

34400 0.006583366 32400 0.000165588 32200 0.000275597 31800
0.001640828 34000 0.000346525 33500 0.000689454
34400 0.006567736 32400 0.000165797 32200 0.000275431 31800
0.00163145 34000 0.000346748 33500 0.000688747
34400 0.006552211 32400 0.000166023 32200 0.000275262 31800
0.001622162 34000 0.000346984 33500 0.000688071
34400 0.006536816 32400 0.000166248 32200 0.000275082 31800
0.001612926 34000 0.000347226 33500 0.000687389
34400 0.006521514 32400 0.000166476 32200 0.000274914 31800
0.001603764 34000 0.000347451 33500 0.000686742
34400 0.006506361 32400 0.000166711 32200 0.000274736 31800
0.001594671 34000 0.000347673 33500 0.000686065
34400 0.006491273 32400 0.000166956 32200 0.000274579 31800
0.001585668 34100 0.000347923 33500 0.000685409
34400 0.006476312 32400 0.000167207 32200 0.000274386 31800
0.001576766 34100 0.000348148 33500 0.000684768
34400 0.006461485 32400 0.00016746 32200 0.00027421 31800
0.001567898 34100 0.000348421 33500 0.000684133
34400 0.006446733 32400 0.000167725 32200 0.000274035 31800
0.001559126 34100 0.000348653 33500 0.000683527
34400 0.006432107 32400 0.00016799 32200 0.000273873 31800
0.001550434 34100 0.000348912 33500 0.000682887
34400 0.0064176 32400 0.000168263 32200 0.000273704 31800
0.001541807 34100 0.000349161 33500 0.000682269
34400 0.006403133 32400 0.000168543 32200 0.000273532 31800
0.001533265 34100 0.000349401 33500 0.000681681
34400 0.00638883 32400 0.000168833 32200 0.000273355 31800
0.001524823 34100 0.000349653 33500 0.000681053
34400 0.006374639 32400 0.000169123 32200 0.000273191 31800
0.001516482 34100 0.000349909 33500 0.000680464
34400 0.006360532 32400 0.00016941 32200 0.000273027 31800
0.001508195 34100 0.000350167 33500 0.000679899
34400 0.006346521 32400 0.000169697 32200 0.000272861 31800
0.001500009 34100 0.000350413 33500 0.000679352
34400 0.006332584 32400 0.000169972 32200 0.000272681 31800
0.001491872 34100 0.000350677 33500 0.000678771
34400 0.00631875 32400 0.000170249 32200 0.000272523 31800
0.001483811 34100 0.000350943 33500 0.000678248
34400 0.006305046 32400 0.000170532 32200 0.000272351 31800
0.001475795 34100 0.000351201 33500 0.000677706
34400 0.006291409 32400 0.000170816 32200 0.000272193 31800
0.001467843 34100 0.000351462 33500 0.000677212
34400 0.006277876 32400 0.000171122 32200 0.000272035 31800
0.001459967 34100 0.000351734 33500 0.000676716
34400 0.006264417 32400 0.000171427 32200 0.00027186 31800
0.001452127 34100 0.000351996 33500 0.000676199
34400 0.006251034 32400 0.00017175 32200 0.000271703 31800
0.001444321 34100 0.00035226 33500 0.000675722
34400 0.006237793 32400 0.000172077 32200 0.000271529 31800
0.001436535 34100 0.000352519 33500 0.000675295
34400 0.006224573 32400 0.000172416 32200 0.000271369 31800
0.001428749 34100 0.000352786 33500 0.000674794
34400 0.006211467 32400 0.000172749 32200 0.000271195 31800
0.001421003 34100 0.000353058 33500 0.000674357
34400 0.006198444 32400 0.000173085 32200 0.000271036 31800
0.001413252 34100 0.000353318 33500 0.000673945
34400 0.006185495 32400 0.000173423 32200 0.000270877 31800
0.001405571 34100 0.000353589 33500 0.000673536
34400 0.006172634 32400 0.000173772 32200 0.000270714 31800
0.001397961 34100 0.000353863 33500 0.000673141
34400 0.006159868 32400 0.000174125 32200 0.000270559 31800
0.001390455 34100 0.000354127 33500 0.000672747
34400 0.006147147 32400 0.000174489 32200 0.00027039 31800
0.00138305 34100 0.000354402 33500 0.000672403
34400 0.006134551 32400 0.000174873 32200 0.000270223 31800
0.00137573 34100 0.00035468 33500 0.000671995
34400 0.00612204 32400 0.000175253 32200 0.000270067 31800
0.00136854 34100 0.00035496 33500 0.000671658

34400 0.006109595 32400 0.000175652 32200 0.000269904 31800
0.001361445 34100 0.000355246 33500 0.00067136
34500 0.006097217 32400 0.000176062 32200 0.000269748 31800
0.00135448 34100 0.000355518 33500 0.000671001
34500 0.006084927 32400 0.000176476 32200 0.000269595 31800
0.001347575 34100 0.000355803 33500 0.000670677
34500 0.006072662 32400 0.000176904 32200 0.000269433 31800
0.001340721 34100 0.000356091 33500 0.000670373
34500 0.006060562 32400 0.000177336 32200 0.000269275 31800
0.001333916 34100 0.00035638 33500 0.000670048
34500 0.006048488 32400 0.000177781 32200 0.000269121 31800
0.001327182 34100 0.000356684 33500 0.000669761
34500 0.006036547 32400 0.00017823 32200 0.000268958 31800
0.001320487 34100 0.000356975 33500 0.000669486
34500 0.006024667 32400 0.000178678 32200 0.000268819 31800
0.001313843 34100 0.000357259 33500 0.000669222
34500 0.006012846 32400 0.000179139 32200 0.000268671 31800
0.001307289 34100 0.000357557 33500 0.000668934
34500 0.006001147 32400 0.000179598 32200 0.000268521 31800
0.001300765 34100 0.000357849 33500 0.000668686
34500 0.005989478 32400 0.000180066 32200 0.000268375 31800
0.001294326 34100 0.000358143 33600 0.000668451
34500 0.005977932 32400 0.000180531 32200 0.000268212 31800
0.001287907 34100 0.000358451 33600 0.000668225
34500 0.005966454 32400 0.000181003 32200 0.000268068 31800
0.001281538 34100 0.00035875 33600 0.000667962
34500 0.005955033 32400 0.000181493 32200 0.000267929 31800
0.001275189 34100 0.000359042 33600 0.000667737
34500 0.005943724 32400 0.000181992 32200 0.000267775 31800
0.00126887 34100 0.000359347 33600 0.000667559
34500 0.005932502 32400 0.000182486 32200 0.000267642 31800
0.001262601 34100 0.000359641 33600 0.000667329
34500 0.005921316 32400 0.00018299 32200 0.000267488 31800
0.001256323 34100 0.000359944 33600 0.000667109
34500 0.005910274 32400 0.000183505 32200 0.000267348 31800
0.001250054 34100 0.000360246 33600 0.00066692
34500 0.005899304 32400 0.000184028 32200 0.000267198 31800
0.001243865 34100 0.000360551 33600 0.000666793
34500 0.005888422 32500 0.000184554 32200 0.000267042 31800
0.001237697 34100 0.000360846 33600 0.000666623
34500 0.005877613 32500 0.000185095 32200 0.000266902 31800
0.001231618 34100 0.000361156 33600 0.000666426
34500 0.005866891 32500 0.00018565 32300 0.000266753 31800
0.00122563 34100 0.000361454 33600 0.00066628
34500 0.005856199 32500 0.000186214 32300 0.000266603 31800
0.001219691 34100 0.000361754 33600 0.000666126
34500 0.005845662 32500 0.000186783 32300 0.000266453 31800
0.001213883 34100 0.000362056 33600 0.00066597
34500 0.005835166 32500 0.000187352 32300 0.000266304 31800
0.001208135 34100 0.000362357 33600 0.00066584
34500 0.005824772 32500 0.000187944 32300 0.000266158 31900
0.001202537 34100 0.000362662 33600 0.000665707
34500 0.005814462 32500 0.000188547 32300 0.000266032 31900
0.00119698 34100 0.000362958 33600 0.000665614
34500 0.005804194 32500 0.000189132 32300 0.000265888 31900
0.001191532 34100 0.000363271 33600 0.000665457
34500 0.005794032 32500 0.000189721 32300 0.000265741 31900
0.001186155 34100 0.000363574 33600 0.000665354
34500 0.005783918 32500 0.000190311 32300 0.000265596 31900
0.001180827 34100 0.000363871 33600 0.000665275
34500 0.005773929 32500 0.000190888 32300 0.00026545 31900
0.00117558 34100 0.000364183 33600 0.00066515
34500 0.005764006 32500 0.000191481 32300 0.000265308 31900
0.001170353 34100 0.000364482 33600 0.000665015
34500 0.005754116 32500 0.000192086 32300 0.000265165 31900
0.001165115 34100 0.000364786 33600 0.000664925
34500 0.005744329 32500 0.000192711 32300 0.000265024 31900
0.001159968 34100 0.000365088 33600 0.000664846

34500 0.005734599 32500 0.000193333 32300 0.000264889 31900
0.001154841 34100 0.000365385 33600 0.000664787
34500 0.005724926 32500 0.000193978 32300 0.00026475 31900
0.001149794 34100 0.000365695 33600 0.000664745
34500 0.005715361 32500 0.000194646 32300 0.000264605 31900
0.001144747 34200 0.000366006 33600 0.000664673
34500 0.005705866 32500 0.000195324 32300 0.000264464 31900
0.00113972 34200 0.000366305 33600 0.000664602
34500 0.005696448 32500 0.000196027 32300 0.00026431 31900
0.001134783 34200 0.00036661 33600 0.000664548
34500 0.005687086 32500 0.000196742 32300 0.000264172 31900
0.001129836 34200 0.000366912 33600 0.000664446
34500 0.005677782 32500 0.000197478 32300 0.000264022 31900
0.001124949 34200 0.000367218 33600 0.000664429
34500 0.005668527 32500 0.00019823 32300 0.000263888 31900
0.001120032 34200 0.000367526 33600 0.000664352
34500 0.0056594 32500 0.000198984 32300 0.000263751 31900
0.001115176 34200 0.000367836 33600 0.000664298
34500 0.005650317 32500 0.000199736 32300 0.000263601 31900
0.001110359 34200 0.000368138 33600 0.000664239
34500 0.005641327 32500 0.000200494 32300 0.000263465 31900
0.001105592 34200 0.000368438 33600 0.000664204
34500 0.005632364 32500 0.000201256 32300 0.000263332 31900
0.001100845 34200 0.000368742 33600 0.000664161
34500 0.005623483 32500 0.000202002 32300 0.000263205 31900
0.001096139 34200 0.000369049 33600 0.000664119
34500 0.005614666 32500 0.000202746 32300 0.000263067 31900
0.001091432 34200 0.000369355 33600 0.000664083
34500 0.005605918 32500 0.000203493 32300 0.000262939 31900
0.001086766 34200 0.000369649 33600 0.000664032
34500 0.005597264 32500 0.000204235 32300 0.000262796 31900
0.001082099 34200 0.000369954 33600 0.000663998
34500 0.005588643 32500 0.00020498 32300 0.000262657 31900
0.001077473 34200 0.00037025 33600 0.000663912
34500 0.005580103 32500 0.00020574 32300 0.000262526 31900
0.001072866 34200 0.000370546 33600 0.000663843
34500 0.00557162 32500 0.000206505 32300 0.000262394 31900
0.00106834 34200 0.000370844 33600 0.000663816
34500 0.005563208 32500 0.000207261 32300 0.000262272 31900
0.001063794 34200 0.000371127 33600 0.000663755
34500 0.005554825 32500 0.000208031 32300 0.00026214 31900
0.001059247 34200 0.000371422 33600 0.000663674
34500 0.005546572 32500 0.000208807 32300 0.000262006 31900
0.001054741 34200 0.000371721 33600 0.000663613
34500 0.005538397 32500 0.000209578 32300 0.000261884 31900
0.001050255 34200 0.000372014 33600 0.00066354
34500 0.005530242 32500 0.000210359 32300 0.00026175 31900
0.001045809 34200 0.000372313 33600 0.000663463
34500 0.005522191 32500 0.000211132 32300 0.000261626 31900
0.001041362 34200 0.000372595 33600 0.00066341
34500 0.005514158 32500 0.00021191 32300 0.000261488 31900
0.001036936 34200 0.000372885 33600 0.000663358
34500 0.005506177 32500 0.00021269 32300 0.000261359 31900
0.00103251 34200 0.000373181 33600 0.000663287
34500 0.005498311 32500 0.000213454 32300 0.000261227 31900
0.001028204 34200 0.000373475 33600 0.00066322
34500 0.0054905 32500 0.000214212 32300 0.000261096 31900
0.001023858 34200 0.000373764 33600 0.000663198
34500 0.005482758 32500 0.000214984 32300 0.000260972 31900
0.001019532 34200 0.00037406 33600 0.000663159
34500 0.005475065 32500 0.000215744 32300 0.000260836 31900
0.001015246 34200 0.000374361 33600 0.000663115
34600 0.005467433 32500 0.000216489 32300 0.000260721 31900
0.001011 34200 0.000374661 33600 0.000663029
34600 0.005459883 32500 0.000217228 32300 0.000260583 31900
0.001006854 34200 0.000374958 33600 0.000662988
34600 0.00545235 32500 0.000217973 32300 0.000260476 31900
0.001002688 34200 0.000375257 33600 0.000662937

34600 0.00544491 32500 0.000218702 32300 0.000260344 31900
0.000998583 34200 0.000375559 33600 0.000662868
34600 0.005437519 32500 0.000219415 32300 0.00026022 31900
0.000994477 34200 0.000375865 33600 0.000662796
34600 0.005430163 32500 0.000220134 32300 0.000260089 31900
0.000990451 34200 0.000376168 33600 0.000662745
34600 0.005422894 32500 0.000220845 32300 0.000259971 31900
0.000986526 34200 0.000376481 33600 0.000662707
34600 0.005415665 32500 0.000221525 32300 0.000259852 31900
0.00098254 34200 0.000376788 33600 0.000662649
34600 0.005408515 32500 0.000222212 32300 0.000259719 31900
0.000978635 34200 0.000377113 33600 0.000662583
34600 0.005401433 32500 0.000222879 32300 0.000259591 31900
0.000974729 34200 0.000377426 33700 0.000662519
34600 0.005394449 32500 0.000223521 32300 0.000259477 31900
0.000970844 34200 0.000377759 33700 0.000662487
34600 0.005387496 32500 0.000224156 32300 0.000259345 31900
0.000967019 34200 0.000378081 33700 0.000662422
34600 0.005380595 32500 0.000224776 32300 0.000259226 31900
0.000963213 34200 0.000378407 33700 0.000662392
34600 0.005373751 32500 0.000225374 32300 0.000259077 31900
0.000959428 34200 0.000378743 33700 0.000662316
34600 0.005366924 32500 0.000225977 32300 0.000258967 31900
0.000955663 34200 0.000379078 33700 0.000662225
34600 0.005360188 32500 0.000226539 32300 0.000258836 31900
0.000951918 34200 0.000379418 33700 0.000662142
34600 0.005353498 32500 0.000227084 32300 0.000258718 31900
0.000948232 34200 0.000379747 33700 0.000662093
34600 0.005346854 32500 0.000227605 32300 0.000258588 31900
0.000944587 34200 0.00038009 33700 0.000662005
34600 0.005340279 32600 0.0002281 32300 0.000258466 31900
0.000940962 34200 0.00038042 33700 0.000661939
34600 0.00533371 32600 0.000228553 32300 0.000258347 31900
0.000937397 34200 0.000380771 33700 0.000661843
34600 0.005327264 32600 0.000228987 32400 0.000258223 31900
0.000933872 34200 0.000381112 33700 0.000661758
34600 0.005320806 32600 0.000229383 32400 0.000258104 31900
0.000930307 34200 0.000381449 33700 0.000661646
34600 0.005314402 32600 0.000229755 32400 0.000257982 31900
0.000926822 34200 0.000381792 33700 0.000661545
34600 0.005308065 32600 0.000230087 32400 0.00025785 31900
0.000923398 34200 0.000382136 33700 0.000661425
34600 0.005301717 32600 0.000230401 32400 0.000257728 32000
0.000919973 34200 0.000382478 33700 0.00066131
34600 0.005295442 32600 0.000230694 32400 0.000257602 32000
0.000916588 34200 0.000382833 33700 0.000661212
34600 0.005289211 32600 0.000230936 32400 0.000257479 32000
0.000913294 34200 0.000383171 33700 0.000661086
34600 0.005283033 32600 0.000231161 32400 0.000257351 32000
0.000909999 34200 0.000383516 33700 0.000660987
34600 0.00527694 32600 0.000231358 32400 0.000257228 32000
0.000906674 34200 0.000383857 33700 0.000660855
34600 0.005270855 32600 0.000231516 32400 0.000257102 32000
0.00090338 34200 0.000384206 33700 0.000660763
34600 0.005264824 32600 0.000231648 32400 0.000256987 32000
0.000900055 34200 0.000384562 33700 0.000660637
34600 0.005258836 32600 0.000231741 32400 0.000256865 32000
0.000896771 34200 0.00038491 33700 0.000660502
34600 0.005252886 32600 0.000231814 32400 0.000256734 32000
0.000893476 34200 0.000385269 33700 0.000660356
34600 0.005246944 32600 0.000231862 32400 0.00025662 32000
0.000890151 34200 0.000385627 33700 0.00066023
34600 0.005241082 32600 0.000231874 32400 0.000256496 32000
0.000886847 34200 0.00038599 33700 0.000660091
34600 0.00523525 32600 0.000231846 32400 0.000256378 32000
0.000883562 34300 0.000386342 33700 0.000659941
34600 0.005229474 32600 0.000231801 32400 0.000256269 32000
0.000880283 34300 0.000386717 33700 0.000659803

34600 0.005223738 32600 0.000231714 32400 0.000256141 32000
0.000877008 34300 0.000387087 33700 0.000659703
34600 0.005218017 32600 0.000231593 32400 0.000256046 32000
0.000873744 34300 0.000387456 33700 0.000659541
34600 0.005212404 32600 0.000231439 32400 0.000255925 32000
0.000870474 34300 0.000387831 33700 0.000659394
34600 0.005206746 32600 0.000231258 32400 0.000255824 32000
0.000867239 34300 0.000388199 33700 0.000659267
34600 0.0052012 32600 0.000231042 32400 0.000255711 32000
0.00086403 34300 0.000388582 33700 0.00065911
34600 0.005195693 32600 0.000230792 32400 0.000255601 32000
0.000860876 34300 0.000388957 33700 0.000658949
34600 0.005190168 32600 0.000230515 32400 0.000255502 32000
0.000857761 34300 0.000389341 33700 0.0006588
34600 0.005184707 32600 0.000230217 32400 0.000255389 32000
0.000854722 34300 0.000389726 33700 0.000658651
34600 0.00517928 32600 0.000229871 32400 0.000255277 32000
0.000851733 34300 0.000390107 33700 0.000658511
34600 0.005173868 32600 0.000229504 32400 0.000255167 32000
0.000848771 34300 0.000390493 33700 0.000658326
34600 0.00516853 32600 0.000229097 32400 0.000255062 32000
0.000845842 34300 0.000390881 33700 0.000658174
34600 0.005163273 32600 0.000228659 32400 0.000254955 32000
0.000842926 34300 0.000391271 33700 0.000658016
34600 0.005158047 32600 0.000228183 32400 0.000254849 32000
0.000840029 34300 0.000391662 33700 0.000657874
34600 0.005152882 32600 0.000227673 32400 0.00025474 32000
0.000837148 34300 0.00039204 33700 0.000657682
34600 0.005147755 32600 0.000227136 32400 0.000254622 32000
0.000834309 34300 0.000392423 33700 0.000657529
34600 0.005142598 32600 0.000226565 32400 0.000254524 32000
0.000831472 34300 0.000392815 33700 0.000657337
34600 0.005137567 32600 0.000225972 32400 0.000254418 32000
0.000828646 34300 0.000393202 33700 0.000657142
34600 0.005132555 32600 0.000225358 32400 0.000254313 32000
0.000825832 34300 0.000393578 33700 0.000656983
34600 0.00512759 32600 0.000224733 32400 0.000254208 32000
0.000823013 34300 0.000393964 33700 0.000656803
34600 0.005122646 32600 0.000224086 32400 0.0002541 32000
0.000820237 34300 0.000394343 33700 0.000656614
34600 0.005117774 32600 0.00022342 32400 0.000254008 32000
0.000817478 34300 0.000394734 33700 0.000656435
34600 0.005112948 32600 0.000222744 32400 0.000253902 32000
0.000814739 34300 0.000395117 33700 0.000656244
34600 0.005108134 32600 0.000222056 32400 0.000253808 32000
0.000812038 34300 0.00039549 33700 0.000656069
34600 0.005103403 32600 0.000221352 32400 0.000253712 32000
0.000809366 34300 0.000395877 33700 0.000655889
34600 0.005098694 32600 0.000220638 32400 0.000253614 32000
0.00080672 34300 0.000396251 33700 0.000655683
34600 0.005094003 32600 0.000219907 32400 0.00025352 32000
0.000804114 34300 0.000396637 33700 0.000655466
34600 0.005089379 32600 0.000219152 32400 0.000253414 32000
0.000801546 34300 0.000397022 33700 0.000655298
34700 0.00508478 32600 0.000218372 32400 0.000253328 32000
0.000798999 34300 0.000397393 33700 0.00065509
34700 0.005080202 32600 0.000217577 32400 0.000253222 32000
0.000796481 34300 0.00039778 33700 0.000654888
34700 0.005075657 32600 0.000216773 32400 0.000253125 32000
0.000793988 34300 0.000398166 33700 0.000654668
34700 0.005071184 32600 0.000215933 32400 0.00025303 32000
0.000791539 34300 0.000398548 33700 0.000654468
34700 0.005066742 32600 0.000215088 32400 0.000252934 32000
0.000789106 34300 0.000398934 33700 0.000654283
34700 0.005062344 32600 0.000214223 32400 0.000252838 32000
0.000786692 34300 0.000399309 33700 0.000654062
34700 0.005057977 32600 0.000213358 32400 0.000252733 32000
0.000784301 34300 0.000399697 33700 0.000653851

34700	0.005053624	32600	0.000212478	32400	0.000252645	32000	0.000781906	34300	0.000400088	33700	0.000653629
34700	0.005049386	32600	0.000211586	32400	0.000252549	32000	0.000779497	34300	0.000400473	33700	0.000653411
34700	0.005045138	32600	0.0002107	32400	0.000252451	32000	0.000777094	34300	0.000400858	33800	0.000653202
34700	0.005040972	32600	0.000209829	32400	0.000252357	32000	0.000774681	34300	0.000401252	33800	0.000652995
34700	0.005036805	32600	0.000208953	32400	0.00025226	32000	0.000772267	34300	0.000401641	33800	0.000652757
34700	0.00503268	32600	0.000208078	32400	0.00025217	32000	0.000769874	34300	0.000402036	33800	0.000652518
34700	0.005028598	32600	0.000207208	32400	0.000252084	32000	0.000767455	34300	0.000402428	33800	0.000652294
34700	0.005024549	32600	0.00020633	32400	0.000251993	32000	0.000765067	34300	0.000402816	33800	0.00065206
34700	0.005020545	32600	0.00020545	32400	0.0002519	32000	0.000762724	34300	0.000403222	33800	0.000651818
34700	0.005016574	32600	0.000204558	32400	0.000251809	32000	0.000760376	34300	0.000403613	33800	0.00065158
34700	0.005012592	32600	0.000203673	32400	0.000251727	32000	0.000758077	34300	0.000404028	33800	0.000651333
34700	0.005008676	32700	0.000202788	32400	0.000251634	32000	0.000755834	34300	0.000404437	33800	0.000651096
34700	0.005004824	32700	0.000201888	32400	0.000251551	32000	0.000753641	34300	0.000404851	33800	0.000650851
34700	0.005000941	32700	0.000200988	32500	0.000251457	32000	0.000751483	34300	0.000405271	33800	0.00065059
34700	0.004997119	32700	0.000200098	32500	0.000251375	32000	0.00074933	34300	0.000405692	33800	0.000650336
34700	0.004993338	32700	0.000199206	32500	0.000251284	32000	0.000747207	34300	0.000406111	33800	0.000650092
34700	0.004989596	32700	0.00019832	32500	0.000251183	32000	0.000745119	34300	0.000406547	33800	0.000649831
34700	0.004985892	32700	0.000197445	32500	0.000251104	32100	0.000743026	34300	0.000406982	33800	0.000649568
34700	0.004982217	32700	0.000196582	32500	0.000251011	32100	0.000740979	34300	0.000407416	33800	0.0006493
34700	0.004978513	32700	0.000195723	32500	0.00025092	32100	0.000738906	34300	0.000407861	33800	0.000649028
34700	0.004974934	32700	0.000194867	32500	0.000250836	32100	0.000736833	34300	0.000408303	33800	0.000648743
34700	0.004971332	32700	0.00019402	32500	0.000250737	32100	0.000734775	34300	0.000408756	33800	0.000648496
34700	0.004967816	32700	0.000193202	32500	0.000250653	32100	0.000732737	34300	0.000409217	33800	0.00064821
34700	0.004964274	32700	0.000192372	32500	0.000250563	32100	0.000730644	34300	0.000409673	33800	0.000647923
34700	0.004960812	32700	0.000191554	32500	0.000250478	32100	0.000728581	34300	0.000410139	33800	0.000647649
34700	0.004957385	32700	0.000190744	32500	0.000250379	32100	0.000726498	34300	0.000410597	33800	0.000647379
34700	0.004953947	32700	0.000189923	32500	0.000250293	32100	0.000724375	34300	0.00041105	33800	0.000647098
34700	0.004950541	32700	0.000189105	32500	0.000250195	32100	0.000722283	34300	0.000411514	33800	0.000646813
34700	0.004947193	32700	0.000188295	32500	0.000250109	32100	0.000720155	34400	0.000411977	33800	0.000646519
34700	0.004943823	32700	0.000187496	32500	0.000250024	32100	0.000718042	34400	0.000412452	33800	0.000646235
34700	0.004940514	32700	0.000186714	32500	0.00024993	32100	0.000715919	34400	0.000412922	33800	0.000645959
34700	0.004937227	32700	0.000185923	32500	0.000249846	32100	0.000713846	34400	0.000413388	33800	0.00064567
34700	0.004933937	32700	0.000185144	32500	0.000249745	32100	0.000711728	34400	0.000413867	33800	0.000645379
34700	0.004930724	32700	0.000184385	32500	0.000249659	32100	0.00070961	34400	0.000414335	33800	0.00064509

34700 0.004927516 32700 0.000183635 32500 0.000249572 32100
0.000707522 34400 0.000414787 33800 0.000644813
34700 0.004924355 32700 0.000182892 32500 0.000249464 32100
0.000705444 34400 0.000415251 33800 0.000644532
34700 0.004921232 32700 0.000182155 32500 0.00024938 32100
0.000703381 34400 0.000415716 33800 0.00064423
34700 0.004918088 32700 0.000181431 32500 0.000249282 32100
0.000701409 34400 0.000416171 33800 0.000643948
34700 0.004914945 32700 0.000180708 32500 0.000249189 32100
0.000699436 34400 0.000416637 33800 0.000643645
34700 0.004911905 32700 0.000179981 32500 0.000249094 32100
0.000697493 34400 0.000417097 33800 0.000643354
34700 0.004908888 32700 0.00017926 32500 0.000249005 32100
0.00069554 34400 0.00041754 33800 0.000643069
34700 0.004905924 32700 0.00017856 32500 0.000248906 32100
0.000693658 34400 0.000417994 33800 0.000642775
34700 0.004902969 32700 0.00017786 32500 0.000248816 32100
0.00069178 34400 0.000418437 33800 0.000642513
34700 0.004900059 32700 0.000177177 32500 0.000248722 32100
0.000689853 34400 0.00041889 33800 0.000642213
34700 0.004897193 32700 0.000176499 32500 0.000248636 32100
0.00068796 34400 0.000419336 33800 0.000641949
34700 0.004894335 32700 0.00017583 32500 0.000248558 32100
0.000686007 34400 0.00041977 33800 0.000641668
34700 0.004891533 32700 0.000175167 32500 0.000248456 32100
0.000684075 34400 0.000420215 33800 0.000641391
34700 0.00488877 32700 0.000174517 32500 0.000248377 32100
0.000682207 34400 0.000420649 33800 0.000641097
34700 0.004885998 32700 0.000173882 32500 0.000248283 32100
0.000680289 34400 0.000421078 33800 0.000640828
34700 0.004883268 32700 0.000173257 32500 0.000248201 32100
0.000678422 34400 0.000421511 33800 0.000640545
34700 0.004880584 32700 0.000172635 32500 0.000248118 32100
0.000676584 34400 0.000421938 33800 0.000640264
34700 0.00487791 32700 0.000172029 32500 0.000248029 32100
0.000674797 34400 0.000422369 33800 0.000639986
34700 0.004875317 32700 0.000171429 32500 0.000247935 32100
0.000673054 34400 0.0004228 33800 0.0006397
34700 0.004872744 32700 0.000170837 32500 0.000247856 32100
0.000671337 34400 0.000423227 33800 0.000639424
34700 0.004870215 32700 0.000170244 32500 0.000247762 32100
0.000669625 34400 0.000423646 33800 0.000639135
34700 0.004867733 32700 0.000169667 32500 0.000247687 32100
0.000667977 34400 0.000424074 33800 0.000638838
34700 0.004865248 32700 0.0001691 32500 0.000247597 32100
0.000666365 34400 0.000424496 33800 0.00063856
34800 0.004862778 32700 0.000168553 32500 0.000247522 32100
0.000664783 34400 0.000424938 33800 0.000638277
34800 0.004860374 32700 0.000167997 32500 0.000247433 32100
0.000663221 34400 0.000425371 33800 0.000637979
34800 0.004857992 32700 0.000167447 32500 0.00024736 32100
0.000661688 34400 0.000425802 33800 0.000637704
34800 0.004855603 32700 0.000166923 32500 0.000247279 32100
0.000660131 34400 0.000426244 33800 0.000637423
34800 0.00485327 32700 0.000166397 32500 0.000247199 32100
0.000658589 34400 0.000426675 33800 0.000637133
34800 0.004850965 32700 0.00016589 32500 0.000247129 32100
0.000657002 34400 0.000427124 33800 0.000636838
34800 0.004848694 32700 0.000165388 32500 0.000247045 32100
0.00065544 34400 0.000427568 33800 0.000636555
34800 0.004846425 32700 0.000164883 32500 0.000246974 32100
0.000653802 34400 0.00042801 33800 0.000636263
34800 0.004844187 32700 0.000164382 32500 0.000246893 32100
0.00065214 34400 0.000428461 33800 0.00063597
34800 0.004841979 32700 0.000163868 32500 0.00024683 32100
0.000650448 34400 0.000428907 33900 0.000635663
34800 0.004839783 32700 0.000163382 32500 0.000246757 32100
0.000648761 34400 0.000429359 33900 0.000635383

34800 0.004837583 32700 0.000162893 32500 0.000246689 32100
0.000647008 34400 0.000429812 33900 0.000635079
34800 0.004835427 32700 0.000162405 32500 0.000246626 32100
0.000645276 34400 0.000430264 33900 0.000634773
34800 0.004833264 32700 0.000161932 32500 0.000246554 32100
0.000643543 34400 0.000430724 33900 0.000634471
34800 0.004831147 32700 0.000161483 32500 0.000246477 32100
0.000641821 34400 0.000431181 33900 0.000634172
34800 0.004829087 32700 0.000161022 32500 0.000246421 32100
0.000640103 34400 0.000431632 33900 0.000633872
34800 0.004827018 32700 0.000160583 32500 0.000246348 32100
0.000638426 34400 0.00043209 33900 0.000633587
34800 0.004824991 32700 0.000160144 32500 0.000246292 32100
0.000636784 34400 0.000432541 33900 0.000633265
34800 0.004822969 32800 0.000159725 32500 0.00024622 32100
0.000635212 34400 0.000433001 33900 0.000632959
34800 0.004820896 32800 0.000159303 32500 0.000246159 32100
0.000633614 34400 0.000433468 33900 0.000632653
34800 0.004818958 32800 0.000158885 32600 0.000246093 32100
0.000632057 34400 0.000433933 33900 0.000632338
34800 0.00481702 32800 0.00015846 32600 0.000246033 32100
0.000630515 34400 0.000434392 33900 0.000632041
34800 0.004815115 32800 0.000158061 32600 0.000245976 32100
0.000629008 34400 0.000434858 33900 0.000631728
34800 0.004813251 32800 0.000157651 32600 0.000245917 32100
0.000627466 34400 0.000435314 33900 0.000631418
34800 0.004811394 32800 0.000157262 32600 0.000245868 32200
0.000625979 34400 0.000435783 33900 0.0006311
34800 0.004809574 32800 0.000156869 32600 0.000245802 32200
0.000624512 34400 0.00043625 33900 0.000630793
34800 0.004807772 32800 0.000156489 32600 0.000245763 32200
0.000623075 34400 0.00043672 33900 0.000630476
34800 0.004806018 32800 0.000156115 32600 0.000245697 32200
0.000621658 34400 0.000437186 33900 0.000630166
34800 0.00480426 32800 0.000155734 32600 0.00024564 32200
0.000620326 34400 0.000437652 33900 0.000629841
34800 0.004802529 32800 0.000155373 32600 0.000245593 32200
0.000618959 34400 0.000438113 33900 0.000629536
34800 0.004800824 32800 0.000155004 32600 0.00024554 32200
0.000617647 34400 0.000438585 33900 0.000629212
34800 0.004799144 32800 0.000154626 32600 0.000245486 32200
0.000616336 34400 0.000439057 33900 0.000628893
34800 0.004797472 32800 0.000154264 32600 0.000245434 32200
0.000615014 34400 0.000439543 33900 0.000628586
34800 0.004795855 32800 0.000153893 32600 0.000245371 32200
0.000613682 34400 0.000440024 33900 0.000628266
34800 0.004794283 32800 0.000153529 32600 0.00024532 32200
0.000612325 34400 0.000440505 33900 0.000627958
34800 0.004792719 32800 0.000153154 32600 0.000245254 32200
0.000610933 34500 0.000440983 33900 0.000627674
34800 0.004791193 32800 0.00015279 32600 0.000245185 32200
0.000609486 34500 0.000441474 33900 0.000627359
34800 0.004789667 32800 0.000152424 32600 0.000245128 32200
0.000608104 34500 0.000441961 33900 0.00062705
34800 0.004788146 32800 0.000152073 32600 0.000245068 32200
0.000606707 34500 0.000442459 33900 0.000626754
34800 0.004786706 32800 0.000151733 32600 0.000244993 32200
0.00060536 34500 0.000442967 33900 0.000626444
34800 0.004785254 32800 0.000151377 32600 0.000244926 32200
0.000604053 34500 0.000443466 33900 0.000626147
34800 0.004783846 32800 0.00015105 32600 0.000244871 32200
0.000602782 34500 0.000443979 33900 0.000625837
34800 0.004782464 32800 0.000150711 32600 0.000244797 32200
0.00060155 34500 0.000444486 33900 0.000625545
34800 0.004781097 32800 0.00015038 32600 0.000244728 32200
0.000600328 34500 0.000445001 33900 0.000625245
34800 0.004779763 32800 0.000150061 32600 0.000244647 32200
0.000599112 34500 0.000445514 33900 0.000624927

34800	0.004778414	32800	0.000149754	32600	0.000244576	32200	0.000597915	34500	0.00044603	33900	0.000624634
34800	0.004777115	32800	0.000149455	32600	0.000244502	32200	0.000596683	34500	0.000446547	33900	0.000624336
34800	0.004775849	32800	0.000149162	32600	0.000244437	32200	0.000595452	34500	0.000447058	33900	0.000624035
34800	0.004774562	32800	0.000148884	32600	0.000244358	32200	0.00059421	34500	0.000447563	33900	0.000623744
34800	0.004773317	32800	0.000148605	32600	0.000244293	32200	0.000592953	34500	0.000448076	33900	0.000623442
34800	0.004772092	32800	0.000148325	32600	0.000244212	32200	0.000591671	34500	0.000448584	33900	0.000623142
34800	0.004770854	32800	0.000148051	32600	0.000244137	32200	0.000590395	34500	0.000449094	33900	0.000622842
34800	0.004769687	32800	0.000147789	32600	0.000244049	32200	0.000589113	34500	0.000449606	33900	0.00062253
34800	0.004768536	32800	0.000147511	32600	0.00024397	32200	0.000587836	34500	0.000450114	33900	0.000622249
34800	0.004767403	32800	0.000147236	32600	0.000243887	32200	0.000586579	34500	0.00045062	33900	0.000621975
34800	0.004766299	32800	0.000146958	32600	0.000243807	32200	0.000585313	34500	0.000451125	33900	0.00062166
34800	0.004765175	32800	0.000146684	32600	0.000243728	32200	0.000584091	34500	0.000451628	33900	0.000621375
34800	0.004764026	32800	0.000146405	32600	0.000243643	32200	0.000582839	34500	0.00045214	33900	0.000621081
34800	0.004762991	32800	0.000146131	32600	0.000243566	32200	0.000581618	34500	0.000452641	33900	0.000620789
34800	0.004761937	32800	0.000145845	32600	0.000243489	32200	0.000580388	34500	0.000453153	33900	0.000620502
34800	0.004760934	32800	0.000145579	32600	0.000243413	32200	0.000579182	34500	0.000453657	33900	0.000620221
34800	0.004759925	32800	0.000145309	32600	0.000243335	32200	0.000577988	34500	0.000454159	33900	0.000619924
34800	0.004758939	32800	0.000145048	32600	0.000243258	32200	0.000576806	34500	0.000454667	33900	0.000619641
34800	0.004757948	32800	0.000144786	32600	0.000243179	32200	0.000575619	34500	0.000455159	33900	0.000619348
34900	0.004756995	32800	0.000144535	32600	0.000243093	32200	0.000574445	34500	0.000455659	33900	0.000619065
34900	0.004756054	32800	0.000144284	32600	0.000243035	32200	0.000573266	34500	0.000456154	33900	0.000618776
34900	0.00475511	32800	0.000144037	32600	0.000242963	32200	0.000572077	34500	0.000456658	33900	0.000618477
34900	0.004754175	32800	0.000143808	32600	0.000242878	32200	0.00057088	34500	0.000457147	33900	0.000618188
34900	0.004753278	32800	0.000143569	32600	0.00024281	32200	0.000569689	34500	0.000457656	33900	0.000617905
34900	0.004752377	32800	0.000143337	32600	0.000242731	32200	0.000568487	34500	0.000458161	33900	0.00061761
34900	0.004751499	32800	0.000143104	32600	0.000242663	32200	0.000567275	34500	0.000458664	33900	0.000617328
34900	0.004750641	32800	0.00014289	32600	0.000242587	32200	0.000566034	34500	0.000459171	33900	0.00061704
34900	0.004749832	32800	0.000142676	32600	0.000242525	32200	0.000564799	34500	0.000459683	33900	0.000616759
34900	0.004749013	32800	0.000142476	32600	0.000242455	32200	0.000563528	34500	0.000460183	34000	0.000616489
34900	0.004748245	32800	0.00014227	32600	0.000242384	32200	0.000562273	34500	0.000460673	34000	0.000616209
34900	0.004747482	32800	0.000142089	32600	0.000242322	32200	0.000561049	34500	0.000461186	34000	0.000615932
34900	0.004746686	32800	0.000141905	32600	0.000242267	32200	0.000559805	34500	0.000461691	34000	0.000615653
34900	0.004745981	32800	0.000141715	32600	0.000242201	32200	0.000558621	34500	0.000462204	34000	0.000615372
34900	0.004745261	32800	0.000141514	32600	0.000242144	32200	0.000557442	34500	0.00046272	34000	0.00061511

34900	0.00474458	32800	0.000141327	32600	0.000242086	32200
0.00055628	34500	0.000463245	34000	0.000614842		
34900	0.00474393	32800	0.000141136	32600	0.00024203	32200
0.000555166	34500	0.000463768	34000	0.000614563		
34900	0.004743296	32800	0.000140946	32600	0.000241948	32200
0.000554037	34500	0.000464285	34000	0.000614292		
34900	0.004742673	32900	0.000140755	32600	0.000241885	32200
0.000552898	34500	0.0004648	34000	0.000614016		
34900	0.004742051	32900	0.000140566	32600	0.000241822	32200
0.000551776	34500	0.000465331	34000	0.000613753		
34900	0.004741479	32900	0.00014038	32700	0.000241769	32200
0.000550655	34500	0.00046585	34000	0.00061347		
34900	0.004740905	32900	0.000140184	32700	0.000241717	32200
0.000549501	34500	0.000466377	34000	0.000613182		
34900	0.004740324	32900	0.000140002	32700	0.000241647	32200
0.000548367	34500	0.000466906	34000	0.00061292		
34900	0.004739787	32900	0.000139822	32700	0.000241588	32200
0.00054727	34500	0.000467433	34000	0.00061264		
34900	0.004739249	32900	0.000139635	32700	0.000241533	32300
0.000546176	34500	0.000467981	34000	0.000612364		
34900	0.004738729	32900	0.000139462	32700	0.000241465	32300
0.000545125	34500	0.000468501	34000	0.000612084		
34900	0.004738237	32900	0.000139291	32700	0.000241404	32300
0.000544088	34500	0.000469031	34000	0.000611819		
34900	0.004737776	32900	0.000139113	32700	0.000241361	32300
0.000543097	34500	0.000469558	34000	0.000611553		
34900	0.004737333	32900	0.000138924	32700	0.000241297	32300
0.000542088	34500	0.000470098	34000	0.000611294		
34900	0.004736924	32900	0.000138743	32700	0.000241242	32300
0.000541089	34500	0.000470604	34000	0.000611026		
34900	0.004736498	32900	0.000138558	32700	0.000241188	32300
0.000540148	34500	0.000471144	34000	0.000610758		
34900	0.004736057	32900	0.000138374	32700	0.000241125	32300
0.000539174	34500	0.000471691	34000	0.000610488		
34900	0.004735696	32900	0.000138186	32700	0.000241089	32300
0.000538248	34500	0.000472225	34000	0.000610224		
34900	0.004735304	32900	0.000138012	32700	0.000241034	32300
0.000537304	34500	0.000472747	34000	0.00060996		
34900	0.004734968	32900	0.000137846	32700	0.000240978	32300
0.000536377	34500	0.000473281	34000	0.000609686		
34900	0.004734623	32900	0.000137683	32700	0.000240929	32300
0.000535419	34600	0.000473818	34000	0.000609421		
34900	0.004734312	32900	0.000137531	32700	0.000240877	32300
0.000534485	34600	0.000474346	34000	0.000609147		
34900	0.004734002	32900	0.000137371	32700	0.000240821	32300
0.000533521	34600	0.000474863	34000	0.000608878		
34900	0.004733673	32900	0.000137213	32700	0.000240759	32300
0.00053253	34600	0.000475388	34000	0.000608602		
34900	0.004733386	32900	0.000137039	32700	0.000240722	32300
0.000531543	34600	0.000475936	34000	0.000608335		
34900	0.004733106	32900	0.000136878	32700	0.000240684	32300
0.000530537	34600	0.000476467	34000	0.000608061		
34900	0.004732823	32900	0.000136719	32700	0.000240629	32300
0.000529543	34600	0.000477003	34000	0.00060777		
34900	0.004732582	32900	0.000136568	32700	0.000240581	32300
0.000528569	34600	0.000477552	34000	0.000607507		
34900	0.004732342	32900	0.000136402	32700	0.000240527	32300
0.00052759	34600	0.000478072	34000	0.000607227		
34900	0.004732086	32900	0.000136238	32700	0.000240471	32300
0.000526649	34600	0.000478624	34000	0.000606957		
34900	0.004731881	32900	0.000136089	32700	0.000240422	32300
0.000525728	34600	0.000479157	34000	0.000606681		
34900	0.004731688	32900	0.000135928	32700	0.000240375	32300
0.000524776	34600	0.000479701	34000	0.000606405		
34900	0.004731503	32900	0.000135773	32700	0.000240312	32300
0.000523883	34600	0.000480237	34000	0.000606148		
34900	0.004731344	32900	0.000135625	32700	0.000240257	32300
0.000522999	34600	0.000480794	34000	0.000605863		

34900 0.00473116 32900 0.000135485 32700 0.000240202 32300
0.00052216 34600 0.000481341 34000 0.000605601
34900 0.004730976 32900 0.000135338 32700 0.000240148 32300
0.000521317 34600 0.000481899 34000 0.00060532
34900 0.004730827 32900 0.0001352 32700 0.000240102 32300
0.000520533 34600 0.000482454 34000 0.000605058
34900 0.004730702 32900 0.000135057 32700 0.000240058 32300
0.000519744 34600 0.000483006 34000 0.000604784
34900 0.00473059 32900 0.000134924 32700 0.000240013 32300
0.000518991 34600 0.000483582 34000 0.00060452
34900 0.004730475 32900 0.000134793 32700 0.000239951 32300
0.000518238 34600 0.000484154 34000 0.000604256
34900 0.004730402 32900 0.000134679 32700 0.000239918 32300
0.000517489 34600 0.000484719 34000 0.000603991
34900 0.004730313 32900 0.000134562 32700 0.00023987 32300
0.000516693 34600 0.000485279 34000 0.000603725
34900 0.004730226 32900 0.000134455 32700 0.00023983 32300
0.000515889 34600 0.000485858 34000 0.000603451
34900 0.00473017 32900 0.000134345 32700 0.000239785 32300
0.000515076 34600 0.000486437 34000 0.000603199
34900 0.00473012 32900 0.000134243 32700 0.000239733 32300
0.000514239 34600 0.000487017 34000 0.000602929
34900 0.004730046 32900 0.000134133 32700 0.000239695 32300
0.000513401 34600 0.000487595 34000 0.000602657
34900 0.004730013 32900 0.000134024 32700 0.000239659 32300
0.000512567 34600 0.000488176 34000 0.000602395
34900 0.004730002 32900 0.000133901 32700 0.000239621 32300
0.000511719 34600 0.000488772 34000 0.000602124
34900 0.004729955 32900 0.000133791 32700 0.000239565 32300
0.00051085 34600 0.000489366 34000 0.000601847
35000 0.00472995 32900 0.000133679 32700 0.00023951 32300
0.000509976 34600 0.00048996 34000 0.000601585
35000 0.004729985 32900 0.00013357 32700 0.000239471 32300
0.000509102 34600 0.000490556 34000 0.000601319
35000 0.004730043 32900 0.000133449 32700 0.000239413 32300
0.000508226 34600 0.000491152 34000 0.000601055
35000 0.00473012 32900 0.000133333 32700 0.00023936 32300
0.000507325 34600 0.000491753 34000 0.000600767
35000 0.004730199 32900 0.000133206 32700 0.000239317 32300
0.000506439 34600 0.000492344 34000 0.000600502
35000 0.004730257 32900 0.000133083 32700 0.000239267 32300
0.000505558 34600 0.000492945 34000 0.000600231
35000 0.004730394 32900 0.000132956 32700 0.000239215 32300
0.000504636 34600 0.000493552 34000 0.000599968
35000 0.004730545 32900 0.000132829 32700 0.000239181 32300
0.000503735 34600 0.000494155 34000 0.000599693
35000 0.004730719 32900 0.000132698 32700 0.000239131 32300
0.000502841 34600 0.000494754 34000 0.000599427
35000 0.004730907 32900 0.000132567 32700 0.000239077 32300
0.000501973 34600 0.000495359 34100 0.000599142
35000 0.004731127 32900 0.000132439 32700 0.000239037 32300
0.000501086 34600 0.000495965 34100 0.000598876
35000 0.004731361 32900 0.000132312 32700 0.000238992 32300
0.000500243 34600 0.000496569 34100 0.000598589
35000 0.004731594 32900 0.00013219 32700 0.000238946 32300
0.000499404 34600 0.000497172 34100 0.000598325
35000 0.00473186 32900 0.000132066 32700 0.000238888 32300
0.000498575 34600 0.000497783 34100 0.000598044
35000 0.004732146 32900 0.000131947 32700 0.000238849 32300
0.000497774 34600 0.000498385 34100 0.000597765
35000 0.004732439 32900 0.000131832 32700 0.0002388 32300
0.000497003 34600 0.000498989 34100 0.000597483
35000 0.004732776 32900 0.000131723 32700 0.000238754 32300
0.000496225 34600 0.000499583 34100 0.000597197
35000 0.004733145 32900 0.000131625 32700 0.000238702 32300
0.000495456 34600 0.000500202 34100 0.000596925
35000 0.004733498 33000 0.000131521 32700 0.000238658 32300
0.000494698 34600 0.000500807 34100 0.000596646

35000 0.004733896 33000 0.000131431 32700 0.000238606 32300
0.000493969 34600 0.000501416 34100 0.00059637
35000 0.00473435 33000 0.00013134 32800 0.000238561 32300
0.000493233 34600 0.00050202 34100 0.000596099
35000 0.004734817 33000 0.000131234 32800 0.000238506 32300
0.000492492 34600 0.000502619 34100 0.000595845
35000 0.004735299 33000 0.000131127 32800 0.000238463 32300
0.000491734 34600 0.000503208 34100 0.000595568
35000 0.004735794 33000 0.000131033 32800 0.000238407 32300
0.000490978 34600 0.000503815 34100 0.000595294
35000 0.004736254 33000 0.000130932 32800 0.000238361 32400
0.000490219 34600 0.00050441 34100 0.000595032
35000 0.004736804 33000 0.00013084 32800 0.000238312 32400
0.000489446 34600 0.000505014 34100 0.000594769
35000 0.004737366 33000 0.000130736 32800 0.000238272 32400
0.000488667 34600 0.000505613 34100 0.000594509
35000 0.004737964 33000 0.000130652 32800 0.000238237 32400
0.000487888 34600 0.000506215 34100 0.000594251
35000 0.004738581 33000 0.000130572 32800 0.000238181 32400
0.000487145 34600 0.000506828 34100 0.000593986
35000 0.004739212 33000 0.000130479 32800 0.000238133 32400
0.000486389 34600 0.000507425 34100 0.000593724
35000 0.004739861 33000 0.000130395 32800 0.000238096 32400
0.000485645 34600 0.000508031 34100 0.000593462
35000 0.004740512 33000 0.000130303 32800 0.000238061 32400
0.000484917 34600 0.000508632 34100 0.000593211
35000 0.004741191 33000 0.000130212 32800 0.000238018 32400
0.000484201 34600 0.000509227 34100 0.000592945
35000 0.004741889 33000 0.000130115 32800 0.00023797 32400
0.000483487 34600 0.000509824 34100 0.000592692
35000 0.004742578 33000 0.000130025 32800 0.000237931 32400
0.000482759 34600 0.000510418 34100 0.000592427
35000 0.004743288 33000 0.000129932 32800 0.000237896 32400
0.00048206 34700 0.000511031 34100 0.000592169
35000 0.004744026 33000 0.000129826 32800 0.00023785 32400
0.000481339 34700 0.000511633 34100 0.000591917
35000 0.004744763 33000 0.000129721 32800 0.000237804 32400
0.000480619 34700 0.000512258 34100 0.000591671
35000 0.004745512 33000 0.000129628 32800 0.000237762 32400
0.000479882 34700 0.00051287 34100 0.000591415
35000 0.004746293 33000 0.000129511 32800 0.000237739 32400
0.000479136 34700 0.000513453 34100 0.000591175
35000 0.004747089 33000 0.000129414 32800 0.000237684 32400
0.000478355 34700 0.000514081 34100 0.000590934
35000 0.004747906 33000 0.000129305 32800 0.000237641 32400
0.000477577 34700 0.000514694 34100 0.000590678
35000 0.004748704 33000 0.00012921 32800 0.000237589 32400
0.000476821 34700 0.000515296 34100 0.000590433
35000 0.004749486 33000 0.000129129 32800 0.00023754 32400
0.000476052 34700 0.000515931 34100 0.000590192
35000 0.00475034 33000 0.000129039 32800 0.000237508 32400
0.000475294 34700 0.000516554 34100 0.000589963
35000 0.004751176 33000 0.000128951 32800 0.000237465 32400
0.00047457 34700 0.00051717 34100 0.00058973
35000 0.004752052 33000 0.000128869 32800 0.000237428 32400
0.000473862 34700 0.000517812 34100 0.000589508
35000 0.004752904 33000 0.000128792 32800 0.000237384 32400
0.000473191 34700 0.00051842 34100 0.000589269
35000 0.004753787 33000 0.000128717 32800 0.000237329 32400
0.000472535 34700 0.000519036 34100 0.000589033
35000 0.00475467 33000 0.000128646 32800 0.000237299 32400
0.000471896 34700 0.000519657 34100 0.000588805
35000 0.004755541 33000 0.000128578 32800 0.000237268 32400
0.000471255 34700 0.000520289 34100 0.00058857
35000 0.004756424 33000 0.000128509 32800 0.00023721 32400
0.000470617 34700 0.000520912 34100 0.000588339
35000 0.004757297 33000 0.000128431 32800 0.000237176 32400
0.000469981 34700 0.000521519 34100 0.000588104

35000 0.00475818 33000 0.00012837 32800 0.000237128 32400
0.000469353 34700 0.000522125 34100 0.000587867
35000 0.004759072 33000 0.000128284 32800 0.00023709 32400
0.000468737 34700 0.000522738 34100 0.000587635
35000 0.004759981 33000 0.00012819 32800 0.000237066 32400
0.000468111 34700 0.000523356 34100 0.000587404
35000 0.004760882 33000 0.000128107 32800 0.000237021 32400
0.000467476 34700 0.000523982 34100 0.000587187
35000 0.00476178 33000 0.000128022 32800 0.000236978 32400
0.000466837 34700 0.000524587 34100 0.000586943
35000 0.004762727 33000 0.000127918 32800 0.00023695 32400
0.000466204 34700 0.000525183 34100 0.000586721
35000 0.004763664 33000 0.000127823 32800 0.000236907 32400
0.00046558 34700 0.000525799 34100 0.000586506
35000 0.004764625 33000 0.000127732 32800 0.000236883 32400
0.000464944 34700 0.000526405 34100 0.000586265
35000 0.004765569 33000 0.000127645 32800 0.000236851 32400
0.000464321 34700 0.00052701 34100 0.000586037
35000 0.004766483 33000 0.000127539 32800 0.000236811 32400
0.000463698 34700 0.000527621 34100 0.000585798
35000 0.004767453 33000 0.000127459 32800 0.000236783 32400
0.000463102 34700 0.000528227 34100 0.000585581
35100 0.004768443 33000 0.000127356 32800 0.000236749 32400
0.000462494 34700 0.000528844 34100 0.000585349
35100 0.004769436 33000 0.000127287 32800 0.000236715 32400
0.000461913 34700 0.000529469 34100 0.000585133
35100 0.004770443 33000 0.000127187 32800 0.000236679 32400
0.000461367 34700 0.000530065 34100 0.000584895
35100 0.00477148 33000 0.000127109 32800 0.000236627 32400
0.000460816 34700 0.000530669 34100 0.000584663
35100 0.004772514 33000 0.000127029 32800 0.000236603 32400
0.000460288 34700 0.000531283 34100 0.000584434
35100 0.00477355 33000 0.000126963 32800 0.000236562 32400
0.000459772 34700 0.000531897 34100 0.00058421
35100 0.004774609 33000 0.000126887 32800 0.000236517 32400
0.000459284 34700 0.000532488 34100 0.000583976
35100 0.004775658 33000 0.000126822 32800 0.000236491 32400
0.000458769 34700 0.000533081 34100 0.000583739
35100 0.004776697 33000 0.000126764 32800 0.000236454 32400
0.000458293 34700 0.000533696 34100 0.000583499
35100 0.004777757 33000 0.000126693 32800 0.000236428 32400
0.000457792 34700 0.000534316 34200 0.000583275
35100 0.004778834 33000 0.000126633 32800 0.000236381 32400
0.000457287 34700 0.00053494 34200 0.000583046
35100 0.004779902 33000 0.000126581 32800 0.000236336 32400
0.000456748 34700 0.000535581 34200 0.00058283
35100 0.004780992 33000 0.000126535 32800 0.00023629 32400
0.000456183 34700 0.000536177 34200 0.000582584
35100 0.004782111 33000 0.000126457 32800 0.000236269 32400
0.000455584 34700 0.000536795 34200 0.000582362
35100 0.004783245 33000 0.000126387 32800 0.000236212 32400
0.000454956 34700 0.000537421 34200 0.000582143
35100 0.004784393 33000 0.000126299 32800 0.000236197 32400
0.000454305 34700 0.000538029 34200 0.000581906
35100 0.004785524 33000 0.000126221 32800 0.000236152 32400
0.000453657 34700 0.000538662 34200 0.000581681
35100 0.004786635 33000 0.000126158 32800 0.000236112 32400
0.000453013 34700 0.000539289 34200 0.000581448
35100 0.004787805 33100 0.000126078 32800 0.000236089 32400
0.00045239 34700 0.000539922 34200 0.000581239
35100 0.004788956 33100 0.00012599 32800 0.000236045 32400
0.000451752 34700 0.000540571 34200 0.00058101
35100 0.004790133 33100 0.000125912 32900 0.000236004 32400
0.000451118 34700 0.000541204 34200 0.000580789
35100 0.004791312 33100 0.000125834 32900 0.000235983 32400
0.0004505 34700 0.000541844 34200 0.000580554
35100 0.0047925 33100 0.000125764 32900 0.000235935 32400
0.000449854 34700 0.000542482 34200 0.000580336

35100 0.004793698 33100 0.000125715 32900 0.000235912 32400
0.000449201 34700 0.000543128 34200 0.000580101
35100 0.00479488 33100 0.000125651 32900 0.000235883 32500
0.000448537 34700 0.000543776 34200 0.000579878
35100 0.004796085 33100 0.000125589 32900 0.000235852 32500
0.000447899 34700 0.000544432 34200 0.000579654
35100 0.004797271 33100 0.000125537 32900 0.000235831 32500
0.000447235 34700 0.000545095 34200 0.000579422
35100 0.004798475 33100 0.000125486 32900 0.000235787 32500
0.00044663 34700 0.00054574 34200 0.000579173
35100 0.004799661 33100 0.000125441 32900 0.000235763 32500
0.000446056 34700 0.000546396 34200 0.000578946
35100 0.004800888 33100 0.000125379 32900 0.000235727 32500
0.000445498 34700 0.000547064 34200 0.000578717
35100 0.004802086 33100 0.000125332 32900 0.000235698 32500
0.00044497 34700 0.000547736 34200 0.000578487
35100 0.004803304 33100 0.000125279 32900 0.000235667 32500
0.000444467 34700 0.000548393 34200 0.000578257
35100 0.00480456 33100 0.000125225 32900 0.000235638 32500
0.000443979 34700 0.000549055 34200 0.000578032
35100 0.004805818 33100 0.000125181 32900 0.000235618 32500
0.000443485 34700 0.000549742 34200 0.000577799
35100 0.004807081 33100 0.00012511 32900 0.000235594 32500
0.000443 34700 0.000550397 34200 0.000577551
35100 0.004808326 33100 0.000125058 32900 0.000235558 32500
0.000442507 34800 0.00055107 34200 0.000577327
35100 0.004809521 33100 0.000125015 32900 0.000235528 32500
0.000441991 34800 0.000551762 34200 0.000577098
35100 0.004810808 33100 0.000124941 32900 0.000235511 32500
0.000441478 34800 0.000552437 34200 0.000576879
35100 0.004812087 33100 0.000124891 32900 0.000235478 32500
0.000440912 34800 0.000553111 34200 0.000576644
35100 0.004813378 33100 0.000124848 32900 0.000235454 32500
0.000440331 34800 0.000553808 34200 0.000576424
35100 0.004814694 33100 0.00012479 32900 0.00023543 32500
0.000439773 34800 0.000554492 34200 0.000576189
35100 0.004815997 33100 0.000124742 32900 0.000235407 32500
0.000439179 34800 0.000555191 34200 0.000575975
35100 0.004817326 33100 0.000124702 32900 0.000235383 32500
0.000438589 34800 0.000555869 34200 0.000575735
35100 0.004818644 33100 0.000124628 32900 0.000235368 32500
0.000438048 34800 0.000556556 34200 0.000575513
35100 0.004819998 33100 0.000124589 32900 0.000235326 32500
0.00043751 34800 0.000557234 34200 0.000575292
35100 0.004821332 33100 0.000124526 32900 0.000235313 32500
0.000437001 34800 0.00055793 34200 0.00057506
35100 0.004822669 33100 0.000124455 32900 0.000235287 32500
0.000436516 34800 0.000558615 34200 0.000574828
35100 0.004824012 33100 0.000124397 32900 0.000235273 32500
0.000436078 34800 0.000559306 34200 0.000574605
35100 0.004825375 33100 0.000124317 32900 0.000235251 32500
0.000435625 34800 0.000559986 34200 0.000574379
35100 0.004826728 33100 0.000124278 32900 0.000235236 32500
0.000435219 34800 0.000560675 34200 0.000574172
35100 0.004828108 33100 0.000124191 32900 0.000235216 32500
0.000434786 34800 0.000561353 34200 0.000573946
35100 0.004829522 33100 0.000124143 32900 0.000235187 32500
0.00043439 34800 0.000562045 34200 0.000573726
35100 0.004830944 33100 0.000124082 32900 0.000235169 32500
0.000433955 34800 0.000562739 34200 0.000573501
35100 0.004832375 33100 0.000124012 32900 0.000235147 32500
0.000433522 34800 0.00056341 34200 0.000573274
35100 0.004833806 33100 0.00012396 32900 0.000235114 32500
0.000433073 34800 0.000564078 34200 0.000573057
35100 0.004835224 33100 0.000123888 32900 0.000235107 32500
0.000432588 34800 0.000564771 34200 0.000572832
35100 0.00483668 33100 0.000123818 32900 0.000235082 32500
0.0004321 34800 0.000565445 34200 0.000572624

35100 0.004838152 33100 0.000123745 32900 0.000235055 32500
0.000431579 34800 0.000566127 34200 0.00057241
35100 0.004839648 33100 0.000123669 32900 0.000235038 32500
0.000431056 34800 0.000566821 34200 0.000572187
35100 0.004841144 33100 0.000123601 32900 0.000235023 32500
0.000430538 34800 0.000567492 34200 0.000571972
35100 0.004842655 33100 0.00012354 32900 0.000235007 32500
0.000430037 34800 0.000568173 34200 0.000571752
35100 0.004844173 33100 0.000123463 32900 0.000234989 32500
0.000429549 34800 0.000568833 34200 0.000571525
35100 0.004845672 33100 0.000123388 32900 0.000234983 32500
0.000429078 34800 0.00056951 34200 0.000571308
35100 0.004847222 33100 0.000123335 32900 0.000234953 32500
0.00042865 34800 0.000570183 34200 0.00057109
35200 0.004848758 33100 0.000123264 32900 0.00023494 32500
0.000428224 34800 0.000570856 34200 0.000570859
35200 0.004850292 33100 0.0001232 32900 0.000234908 32500
0.000427856 34800 0.000571524 34200 0.000570624
35200 0.004851852 33100 0.000123161 32900 0.0002349 32500
0.000427503 34800 0.00057219 34200 0.0005704
35200 0.004853405 33100 0.000123118 32900 0.000234882 32500
0.000427168 34800 0.000572868 34200 0.000570175
35200 0.004854971 33100 0.000123084 32900 0.00023486 32500
0.000426845 34800 0.000573552 34200 0.000569956
35200 0.004856571 33100 0.000123046 32900 0.000234833 32500
0.000426475 34800 0.000574222 34200 0.000569734
35200 0.004858193 33100 0.000123 32900 0.000234829 32500
0.000426119 34800 0.000574899 34200 0.000569525
35200 0.004859815 33100 0.000122976 32900 0.000234817 32500
0.000425719 34800 0.00057559 34200 0.000569304
35200 0.004861488 33100 0.000122928 32900 0.000234801 32500
0.0004253 34800 0.000576278 34200 0.000569072
35200 0.004863114 33100 0.000122901 32900 0.000234776 32500
0.000424855 34800 0.00057696 34300 0.000568844
35200 0.004864733 33100 0.000122844 32900 0.000234755 32500
0.000424414 34800 0.000577658 34300 0.000568617
35200 0.004866413 33100 0.000122786 32900 0.000234724 32500
0.000423946 34800 0.000578358 34300 0.000568395
35200 0.004868099 33100 0.000122728 32900 0.000234713 32500
0.000423471 34800 0.000579068 34300 0.000568181
35200 0.004869815 33100 0.000122682 32900 0.000234703 32500
0.000422985 34800 0.000579784 34300 0.00056796
35200 0.004871543 33100 0.000122612 32900 0.000234689 32500
0.000422522 34800 0.000580496 34300 0.00056774
35200 0.004873291 33100 0.000122566 32900 0.000234677 32500
0.000422074 34800 0.000581213 34300 0.000567507
35200 0.004875015 33100 0.000122545 32900 0.000234658 32500
0.000421643 34800 0.000581904 34300 0.00056728
35200 0.004876768 33100 0.000122487 32900 0.000234653 32500
0.000421205 34800 0.000582622 34300 0.000567058
35200 0.004878539 33200 0.000122435 32900 0.000234631 32500
0.000420797 34800 0.000583355 34300 0.000566825
35200 0.004880296 33200 0.00012237 32900 0.000234611 32500
0.000420376 34800 0.000584083 34300 0.000566586
35200 0.004882088 33200 0.000122329 33000 0.000234583 32500
0.000419976 34800 0.000584821 34300 0.000566351
35200 0.004883874 33200 0.000122266 33000 0.000234573 32500
0.00041958 34800 0.000585549 34300 0.00056611
35200 0.004885679 33200 0.000122219 33000 0.000234563 32500
0.000419172 34800 0.000586291 34300 0.000565897
35200 0.004887495 33200 0.000122155 33000 0.000234538 32500
0.000418782 34800 0.000587053 34300 0.00056568
35200 0.004889322 33200 0.000122097 33000 0.000234512 32600
0.000418379 34800 0.000587792 34300 0.000565457
35200 0.004891191 33200 0.000122022 33000 0.000234508 32600
0.000417988 34800 0.000588531 34300 0.000565246
35200 0.004893072 33200 0.000121981 33000 0.000234492 32600
0.00041761 34800 0.000589288 34300 0.000565033

35200 0.004894983 33200 0.000121953 33000 0.000234489 32600
0.000417245 34800 0.000590049 34300 0.000564805
35200 0.004896876 33200 0.000121916 33000 0.000234467 32600
0.000416899 34800 0.0005908 34300 0.000564596
35200 0.004898746 33200 0.000121857 33000 0.000234458 32600
0.000416536 34800 0.000591565 34300 0.00056437
35200 0.00490068 33200 0.000121826 33000 0.00023444 32600
0.000416163 34800 0.000592316 34300 0.000564175
35200 0.004902605 33200 0.000121791 33000 0.000234436 32600
0.000415753 34800 0.000593081 34300 0.00056396
35200 0.004904589 33200 0.000121743 33000 0.000234413 32600
0.000415319 34800 0.000593852 34300 0.000563765
35200 0.004906573 33200 0.00012171 33000 0.00023441 32600
0.000414904 34800 0.000594612 34300 0.000563546
35200 0.004908565 33200 0.000121674 33000 0.000234397 32600
0.000414488 34800 0.000595386 34300 0.000563343
35200 0.004910565 33200 0.000121635 33000 0.000234386 32600
0.000414068 34900 0.000596147 34300 0.000563134
35200 0.004912558 33200 0.000121604 33000 0.000234391 32600
0.000413675 34900 0.000596923 34300 0.000562943
35200 0.004914587 33200 0.000121547 33000 0.000234385 32600
0.000413297 34900 0.000597688 34300 0.000562731
35200 0.004916602 33200 0.000121527 33000 0.000234367 32600
0.000412916 34900 0.00059845 34300 0.000562528
35200 0.004918621 33200 0.000121491 33000 0.000234348 32600
0.000412543 34900 0.00059922 34300 0.000562302
35200 0.004920661 33200 0.000121441 33000 0.000234371 32600
0.000412168 34900 0.000599995 34300 0.000562101
35200 0.00492271 33200 0.000121415 33000 0.000234359 32600
0.000411775 34900 0.000600762 34300 0.000561897
35200 0.004924753 33200 0.000121365 33000 0.000234353 32600
0.000411382 34900 0.000601552 34300 0.000561692
35200 0.004926821 33200 0.000121298 33000 0.00023433 32600
0.000410976 34900 0.000602329 34300 0.000561483
35200 0.004928914 33200 0.000121237 33000 0.000234329 32600
0.000410558 34900 0.000603112 34300 0.000561276
35200 0.004930995 33200 0.000121218 33000 0.000234326 32600
0.000410153 34900 0.000603898 34300 0.000561072
35200 0.004933133 33200 0.000121206 33000 0.000234322 32600
0.000409737 34900 0.000604682 34300 0.000560864
35200 0.004935229 33200 0.000121177 33000 0.0002343 32600
0.000409346 34900 0.000605452 34300 0.000560649
35200 0.004937331 33200 0.000121111 33000 0.000234298 32600
0.000408943 34900 0.000606238 34300 0.00056045
35200 0.004939482 33200 0.000121035 33000 0.000234291 32600
0.00040854 34900 0.000607029 34300 0.000560241
35200 0.004941627 33200 0.000120987 33000 0.000234285 32600
0.00040812 34900 0.000607815 34300 0.000560044
35200 0.004943809 33200 0.000120937 33000 0.000234288 32600
0.000407679 34900 0.000608612 34300 0.000559837
35200 0.004945998 33200 0.00012089 33000 0.000234279 32600
0.000407259 34900 0.000609392 34300 0.000559634
35200 0.004948196 33200 0.000120808 33000 0.000234269 32600
0.000406808 34900 0.000610174 34300 0.000559432
35200 0.004950404 33200 0.000120758 33000 0.000234263 32600
0.000406367 34900 0.000610963 34300 0.000559218
35200 0.004952615 33200 0.000120704 33000 0.000234254 32600
0.000405937 34900 0.000611757 34300 0.000559003
35200 0.004954858 33200 0.000120649 33000 0.000234251 32600
0.000405504 34900 0.000612546 34300 0.000558798
35200 0.004957108 33200 0.000120591 33000 0.000234241 32600
0.000405063 34900 0.000613326 34300 0.000558569
35200 0.00495936 33200 0.00012056 33000 0.00023423 32600
0.000404643 34900 0.000614111 34300 0.000558361
35200 0.004961623 33200 0.00012052 33000 0.000234229 32600
0.00040424 34900 0.000614893 34300 0.000558147
35200 0.004963933 33200 0.00012047 33000 0.000234233 32600
0.000403864 34900 0.000615669 34300 0.000557949

35200 0.00496622 33200 0.000120406 33000 0.000234221 32600
0.000403529 34900 0.000616467 34300 0.000557738
35200 0.004968557 33200 0.000120364 33000 0.000234211 32600
0.000403178 34900 0.000617232 34300 0.000557531
35200 0.004970903 33200 0.000120274 33000 0.000234208 32600
0.00040287 34900 0.00061801 34300 0.000557329
35300 0.004973243 33200 0.000120228 33000 0.000234192 32600
0.000402577 34900 0.000618784 34300 0.000557131
35300 0.004975635 33200 0.000120175 33000 0.000234178 32600
0.000402284 34900 0.000619552 34300 0.000556928
35300 0.004977987 33200 0.000120159 33000 0.000234163 32600
0.000401997 34900 0.000620334 34300 0.000556735
35300 0.004980335 33200 0.0001201 33000 0.000234147 32600
0.000401689 34900 0.000621104 34300 0.000556529
35300 0.004982725 33200 0.000120082 33000 0.000234136 32600
0.000401381 34900 0.000621879 34300 0.000556331
35300 0.004985143 33200 0.00012004 33000 0.00023414 32600
0.00040104 34900 0.000622661 34300 0.000556146
35300 0.004987551 33200 0.00012 33000 0.000234135 32600
0.000400692 34900 0.000623453 34300 0.00055595
35300 0.004989977 33200 0.00011995 33000 0.000234118 32600
0.000400364 34900 0.000624239 34300 0.000555758
35300 0.004992425 33200 0.000119896 33000 0.000234109 32600
0.000400004 34900 0.000625025 34300 0.000555565
35300 0.004994862 33200 0.000119834 33000 0.000234095 32600
0.000399661 34900 0.000625818 34400 0.000555373
35300 0.004997287 33200 0.000119788 33000 0.000234096 32600
0.000399318 34900 0.000626616 34400 0.000555174
35300 0.00499976 33200 0.000119742 33000 0.000234086 32600
0.000399002 34900 0.000627415 34400 0.000554988
35300 0.005002208 33200 0.000119653 33000 0.000234065 32600
0.000398672 34900 0.000628207 34400 0.00055477
35300 0.005004658 33200 0.000119577 33000 0.000234052 32600
0.000398387 34900 0.000629019 34400 0.000554576
35300 0.005007089 33200 0.00011951 33000 0.000234056 32600
0.000398139 34900 0.000629844 34400 0.000554379
35300 0.005009564 33200 0.000119451 33000 0.000234054 32600
0.000397889 34900 0.000630653 34400 0.000554181
35300 0.005012026 33200 0.000119385 33000 0.000234047 32600
0.000397656 34900 0.000631479 34400 0.000553977
35300 0.0050145 33200 0.000119332 33000 0.000234044 32600
0.000397411 34900 0.000632302 34400 0.000553784
35300 0.005017004 33300 0.000119278 33000 0.000234064 32600
0.000397185 34900 0.000633136 34400 0.000553587
35300 0.005019511 33300 0.000119227 33000 0.000234055 32600
0.00039692 34900 0.000633964 34400 0.000553395
35300 0.005022019 33300 0.000119158 33100 0.000234057 32600
0.000396665 34900 0.000634806 34400 0.000553185
35300 0.005024525 33300 0.00011913 33100 0.000234047 32600
0.000396404 34900 0.000635642 34400 0.000552996
35300 0.00502698 33300 0.000119092 33100 0.000234063 32600
0.000396114 34900 0.00063648 34400 0.000552804
35300 0.005029487 33300 0.000119056 33100 0.000234042 32600
0.000395803 34900 0.000637332 34400 0.000552594
35300 0.005032008 33300 0.000119016 33100 0.000234059 32700
0.000395503 34900 0.000638175 34400 0.000552407
35300 0.00503453 33300 0.000118985 33100 0.000234056 32700
0.000395203 34900 0.000639034 34400 0.000552216
35300 0.005037066 33300 0.000118943 33100 0.000234058 32700
0.000394912 34900 0.000639884 34400 0.000552014
35300 0.005039597 33300 0.000118918 33100 0.000234072 32700
0.000394607 34900 0.000640725 34400 0.000551831
35300 0.005042139 33300 0.000118855 33100 0.000234071 32700
0.000394296 34900 0.000641569 34400 0.000551625
35300 0.005044645 33300 0.000118814 33100 0.000234078 32700
0.000393991 34900 0.000642418 34400 0.000551438
35300 0.005047192 33300 0.000118775 33100 0.000234082 32700
0.000393641 34900 0.000643264 34400 0.00055124

35300 0.005049725 33300 0.000118746 33100 0.00023407 32700
0.00039328 34900 0.000644113 34400 0.000551036
35300 0.005052248 33300 0.000118696 33100 0.000234084 32700
0.00039292 34900 0.000644957 34400 0.00055084
35300 0.005054783 33300 0.000118652 33100 0.000234085 32700
0.000392524 34900 0.000645808 34400 0.000550643
35300 0.005057332 33300 0.000118614 33100 0.00023409 32700
0.000392149 34900 0.000646666 34400 0.00055044
35300 0.005059863 33300 0.000118559 33100 0.000234097 32700
0.000391773 35000 0.000647515 34400 0.000550249
35300 0.005062417 33300 0.000118555 33100 0.000234097 32700
0.000391358 35000 0.000648354 34400 0.000550052
35300 0.005064978 33300 0.000118534 33100 0.000234099 32700
0.000390992 35000 0.000649189 34400 0.000549857
35300 0.005067542 33300 0.000118504 33100 0.000234093 32700
0.000390666 35000 0.000650027 34400 0.000549675
35300 0.005070114 33300 0.000118498 33100 0.000234113 32700
0.000390331 35000 0.000650878 34400 0.000549473
35300 0.005072669 33300 0.000118474 33100 0.000234123 32700
0.000390051 35000 0.000651713 34400 0.000549275
35300 0.005075165 33300 0.00011846 33100 0.000234126 32700
0.000389755 35000 0.000652539 34400 0.00054908
35300 0.005077725 33300 0.000118421 33100 0.000234124 32700
0.000389465 35000 0.000653373 34400 0.000548886
35300 0.005080288 33300 0.00011839 33100 0.00023413 32700
0.000389189 35000 0.000654209 34400 0.000548701
35300 0.005082868 33300 0.000118374 33100 0.000234141 32700
0.000388919 35000 0.000655054 34400 0.000548511
35300 0.005085464 33300 0.000118347 33100 0.000234157 32700
0.000388664 35000 0.000655895 34400 0.000548314
35300 0.005088057 33300 0.000118326 33100 0.000234182 32700
0.000388383 35000 0.000656727 34400 0.000548124
35300 0.005090651 33300 0.000118301 33100 0.00023418 32700
0.000388093 35000 0.000657559 34400 0.000547916
35300 0.005093227 33300 0.000118289 33100 0.000234205 32700
0.000387798 35000 0.000658386 34400 0.000547732
35300 0.005095825 33300 0.000118258 33100 0.000234224 32700
0.000387477 35000 0.000659219 34400 0.000547525
35300 0.005098431 33300 0.000118232 33100 0.000234238 32700
0.000387137 35000 0.000660056 34400 0.000547324
35300 0.005101027 33300 0.000118185 33100 0.000234242 32700
0.000386806 35000 0.000660893 34400 0.00054713
35300 0.00510363 33300 0.000118155 33100 0.000234261 32700
0.000386476 35000 0.000661721 34400 0.000546935
35300 0.005106265 33300 0.000118125 33100 0.000234286 32700
0.00038614 35000 0.000662561 34400 0.000546744
35300 0.005108898 33300 0.000118078 33100 0.000234303 32700
0.00038583 35000 0.00066339 34400 0.000546548
35300 0.005111552 33300 0.000118042 33100 0.000234304 32700
0.00038552 35000 0.000664223 34400 0.000546339
35300 0.005114233 33300 0.000118003 33100 0.000234335 32700
0.000385224 35000 0.000665058 34400 0.000546151
35300 0.00511693 33300 0.00011797 33100 0.000234371 32700
0.000384934 35000 0.0006659 34400 0.000545967
35300 0.005119652 33300 0.000117918 33100 0.000234381 32700
0.000384643 35000 0.000666736 34400 0.000545767
35300 0.005122385 33300 0.000117891 33100 0.000234387 32700
0.000384383 35000 0.000667565 34400 0.000545586
35300 0.005125083 33300 0.000117859 33100 0.00023441 32700
0.000384108 35000 0.000668395 34400 0.000545391
35300 0.005127835 33300 0.000117819 33100 0.000234417 32700
0.000383832 35000 0.00066923 34400 0.000545193
35300 0.005130597 33300 0.000117778 33100 0.000234422 32700
0.000383557 35000 0.000670059 34400 0.000545016
35300 0.005133386 33300 0.000117721 33100 0.000234462 32700
0.000383251 35000 0.000670918 34400 0.000544837
35400 0.005136196 33300 0.000117673 33100 0.000234495 32700
0.000382971 35000 0.00067175 34400 0.000544654

35400 0.00513901 33300 0.000117615 33100 0.000234494 32700
0.00038264 35000 0.000672598 34400 0.00054448
35400 0.005141844 33300 0.000117586 33100 0.000234527 32700
0.000382315 35000 0.000673433 34400 0.000544298
35400 0.005144684 33300 0.000117541 33100 0.000234532 32700
0.000382015 35000 0.000674281 34400 0.000544115
35400 0.005147537 33300 0.000117502 33100 0.000234532 32700
0.000381689 35000 0.000675123 34400 0.000543931
35400 0.005150388 33300 0.000117465 33100 0.000234531 32700
0.000381389 35000 0.000675968 34400 0.000543746
35400 0.005153265 33300 0.000117435 33100 0.000234549 32700
0.000381104 35000 0.000676811 34400 0.000543568
35400 0.005156141 33300 0.000117378 33100 0.000234572 32700
0.000380843 35000 0.000677672 34400 0.000543397
35400 0.005159038 33300 0.000117351 33100 0.000234594 32700
0.000380583 35000 0.00067853 34400 0.000543221
35400 0.005161942 33300 0.000117285 33100 0.000234609 32700
0.000380353 35000 0.000679401 34500 0.000543036
35400 0.005164848 33300 0.000117244 33100 0.000234606 32700
0.000380107 35000 0.000680256 34500 0.000542857
35400 0.005167799 33300 0.000117201 33100 0.000234629 32700
0.000379892 35000 0.000681127 34500 0.000542689
35400 0.005170752 33300 0.000117149 33100 0.000234638 32700
0.000379662 35000 0.000682001 34500 0.000542524
35400 0.005173714 33300 0.000117102 33100 0.000234648 32700
0.000379431 35000 0.000682879 34500 0.000542357
35400 0.005176683 33300 0.000117062 33100 0.000234677 32700
0.000379211 35000 0.000683759 34500 0.00054219
35400 0.005179595 33300 0.000117025 33100 0.000234673 32700
0.000378976 35000 0.000684644 34500 0.000542015
35400 0.005182579 33300 0.000116993 33100 0.000234675 32700
0.000378735 35000 0.000685531 34500 0.00054184
35400 0.005185564 33300 0.000116958 33100 0.000234692 32700
0.000378505 35000 0.000686431 34500 0.000541678
35400 0.005188575 33400 0.000116913 33100 0.000234687 32700
0.000378255 35000 0.000687334 34500 0.000541511
35400 0.005191588 33400 0.000116877 33100 0.000234675 32700
0.000378034 35000 0.000688233 34500 0.000541349
35400 0.005194632 33400 0.000116842 33200 0.000234712 32700
0.000377809 35000 0.000689133 34500 0.000541187
35400 0.005197671 33400 0.000116813 33200 0.000234721 32700
0.000377594 35000 0.000690028 34500 0.000541011
35400 0.005200706 33400 0.00011678 33200 0.000234727 32700
0.000377403 35000 0.000690946 34500 0.000540838
35400 0.00520376 33400 0.000116751 33200 0.000234727 32700
0.000377203 35000 0.000691853 34500 0.000540668
35400 0.005206799 33400 0.000116715 33200 0.000234717 32800
0.000377033 35000 0.000692758 34500 0.000540496
35400 0.005209849 33400 0.00011669 33200 0.000234715 32800
0.000376873 35000 0.00069367 34500 0.000540327
35400 0.005212897 33400 0.000116655 33200 0.000234721 32800
0.000376708 35000 0.00069459 34500 0.000540161
35400 0.005215978 33400 0.000116636 33200 0.000234741 32800
0.000376542 35000 0.000695512 34500 0.000539987
35400 0.005219042 33400 0.000116622 33200 0.000234733 32800
0.000376367 35000 0.00069643 34500 0.000539824
35400 0.005222146 33400 0.000116615 33200 0.000234725 32800
0.000376172 35000 0.000697351 34500 0.000539657
35400 0.00522529 33400 0.000116594 33200 0.000234716 32800
0.000375967 35000 0.000698273 34500 0.000539499
35400 0.005228435 33400 0.000116575 33200 0.000234734 32800
0.000375706 35000 0.000699212 34500 0.00053934
35400 0.005231601 33400 0.000116549 33200 0.000234748 32800
0.000375461 35000 0.000700143 34500 0.000539176
35400 0.005234759 33400 0.000116527 33200 0.000234749 32800
0.000375221 35000 0.000701071 34500 0.000539017
35400 0.005237891 33400 0.000116486 33200 0.000234763 32800
0.00037497 35000 0.000702001 34500 0.000538851

35400 0.005241087 33400 0.000116458 33200 0.000234778 32800
0.00037472 35100 0.000702938 34500 0.000538695
35400 0.005244275 33400 0.000116416 33200 0.000234785 32800
0.00037444 35100 0.000703892 34500 0.000538539
35400 0.005247489 33400 0.000116385 33200 0.000234803 32800
0.000374149 35100 0.000704843 34500 0.000538387
35400 0.005250717 33400 0.000116338 33200 0.000234814 32800
0.000373854 35100 0.00070579 34500 0.000538237
35400 0.005253935 33400 0.000116301 33200 0.000234815 32800
0.000373543 35100 0.000706745 34500 0.000538082
35400 0.00525718 33400 0.000116269 33200 0.000234823 32800
0.000373223 35100 0.000707686 34500 0.000537919
35400 0.005260402 33400 0.000116251 33200 0.000234834 32800
0.000372888 35100 0.000708647 34500 0.000537763
35400 0.005263634 33400 0.000116221 33200 0.000234839 32800
0.000372547 35100 0.000709607 34500 0.000537599
35400 0.005266847 33400 0.000116184 33200 0.000234848 32800
0.000372227 35100 0.000710567 34500 0.000537436
35400 0.005270098 33400 0.000116154 33200 0.000234844 32800
0.000371932 35100 0.00071154 34500 0.000537271
35400 0.005273294 33400 0.000116121 33200 0.000234863 32800
0.000371681 35100 0.000712501 34500 0.000537108
35400 0.005276549 33400 0.000116096 33200 0.000234871 32800
0.000371471 35100 0.000713469 34500 0.000536946
35400 0.005279765 33400 0.000116063 33200 0.000234891 32800
0.000371281 35100 0.000714438 34500 0.000536789
35400 0.005282999 33400 0.000116028 33200 0.000234885 32800
0.00037112 35100 0.000715407 34500 0.000536621
35400 0.005286262 33400 0.000116004 33200 0.000234891 32800
0.000370975 35100 0.000716367 34500 0.000536467
35400 0.005289521 33400 0.000115986 33200 0.00023491 32800
0.000370825 35100 0.000717339 34500 0.00053631
35400 0.005292768 33400 0.000115957 33200 0.000234938 32800
0.000370705 35100 0.000718306 34500 0.000536132
35400 0.005295991 33400 0.000115934 33200 0.000234946 32800
0.000370605 35100 0.000719268 34500 0.000535973
35400 0.00529915 33400 0.000115891 33200 0.000234959 32800
0.000370484 35100 0.000720232 34500 0.000535807
35400 0.005302393 33400 0.000115875 33200 0.00023498 32800
0.000370354 35100 0.000721197 34500 0.000535643
35400 0.005305625 33400 0.000115845 33200 0.000234995 32800
0.000370224 35100 0.000722156 34500 0.000535482
35400 0.005308886 33400 0.000115807 33200 0.000235024 32800
0.000370084 35100 0.000723118 34500 0.000535315
35400 0.005312145 33400 0.000115783 33200 0.000235025 32800
0.000369954 35100 0.000724061 34500 0.000535151
35400 0.005315396 33400 0.00011576 33200 0.000235049 32800
0.000369763 35100 0.000725016 34500 0.000534992
35400 0.005318684 33400 0.00011576 33200 0.000235095 32800
0.000369593 35100 0.000725957 34500 0.00053482
35400 0.005321939 33400 0.000115752 33200 0.000235154 32800
0.000369373 35100 0.000726896 34500 0.000534645
35400 0.005325238 33400 0.000115738 33200 0.000235173 32800
0.000369153 35100 0.00072784 34500 0.00053447
35400 0.005328501 33400 0.000115731 33200 0.000235201 32800
0.000368913 35100 0.000728776 34500 0.000534299
35400 0.005331817 33400 0.000115723 33200 0.00023525 32800
0.000368662 35100 0.000729717 34500 0.000534121
35500 0.005335066 33400 0.00011571 33200 0.000235276 32800
0.000368382 35100 0.000730658 34500 0.00053395
35500 0.005338399 33400 0.000115676 33200 0.000235312 32800
0.000368141 35100 0.000731597 34500 0.000533772
35500 0.005341708 33400 0.000115638 33200 0.000235312 32800
0.000367891 35100 0.00073254 34500 0.000533609
35500 0.005345045 33400 0.000115617 33200 0.000235341 32800
0.000367641 35100 0.000733472 34500 0.000533426
35500 0.005348419 33400 0.000115575 33200 0.000235366 32800
0.000367391 35100 0.000734413 34500 0.000533252

35500 0.005351811 33400 0.000115552 33200 0.000235425 32800
0.00036717 35100 0.00073535 34500 0.000533092
35500 0.005355202 33400 0.000115522 33200 0.000235451 32800
0.00036699 35100 0.000736289 34500 0.000532917
35500 0.00535858 33400 0.00011549 33200 0.000235492 32800
0.00036683 35100 0.000737228 34500 0.000532748
35500 0.005361962 33400 0.000115458 33200 0.000235513 32800
0.000366659 35100 0.000738187 34500 0.000532578
35500 0.005365376 33400 0.000115412 33200 0.000235547 32800
0.00036653 35100 0.000739149 34600 0.000532412
35500 0.005368813 33400 0.000115369 33200 0.000235575 32800
0.000366369 35100 0.000740105 34600 0.00053226
35500 0.005372282 33400 0.000115324 33200 0.000235614 32800
0.000366259 35100 0.000741077 34600 0.000532085
35500 0.00537574 33400 0.000115288 33200 0.000235635 32800
0.000366129 35100 0.000742041 34600 0.000531934
35500 0.00537922 33400 0.000115251 33200 0.000235684 32800
0.000365999 35100 0.000743021 34600 0.000531771
35500 0.005382723 33400 0.000115202 33200 0.000235706 32800
0.000365899 35100 0.000743998 34600 0.000531605
35500 0.005386188 33400 0.000115172 33200 0.000235728 32800
0.000365788 35100 0.000744986 34600 0.000531443
35500 0.005389703 33400 0.000115135 33200 0.000235763 32800
0.000365689 35100 0.000745978 34600 0.000531284
35500 0.005393197 33400 0.000115119 33200 0.000235791 32800
0.000365578 35100 0.000746977 34600 0.00053111
35500 0.005396697 33500 0.000115092 33200 0.000235812 32800
0.000365438 35100 0.000747989 34600 0.000530945
35500 0.005400219 33500 0.000115072 33200 0.000235854 32800
0.000365268 35100 0.000749 34600 0.00053078
35500 0.00540375 33500 0.000115056 33300 0.000235889 32800
0.000365108 35100 0.00075001 34600 0.000530614
35500 0.00540728 33500 0.000115037 33300 0.000235918 32800
0.000364898 35100 0.000751046 34600 0.000530466
35500 0.00541085 33500 0.000115018 33300 0.000235944 32800
0.000364677 35100 0.000752066 34600 0.000530303
35500 0.005414447 33500 0.000114998 33300 0.000235991 32800
0.000364417 35100 0.0007531 34600 0.000530156
35500 0.005418022 33500 0.000114981 33300 0.000236029 32900
0.000364136 35100 0.000754136 34600 0.000530015
35500 0.005421645 33500 0.000114963 33300 0.000236082 32900
0.000363866 35100 0.000755171 34600 0.000529863
35500 0.00542525 33500 0.00011494 33300 0.000236098 32900
0.000363596 35100 0.000756208 34600 0.000529714
35500 0.005428824 33500 0.00011492 33300 0.000236137 32900
0.000363315 35100 0.000757249 34600 0.000529559
35500 0.00543246 33500 0.000114896 33300 0.000236183 32900
0.000363025 35100 0.000758298 34600 0.000529413
35500 0.005436109 33500 0.000114881 33300 0.000236234 32900
0.000362735 35100 0.000759343 34600 0.000529263
35500 0.005439782 33500 0.000114855 33300 0.000236277 32900
0.000362445 35100 0.000760395 34600 0.000529118
35500 0.005443472 33500 0.000114835 33300 0.000236288 32900
0.000362164 35100 0.000761435 34600 0.000528979
35500 0.00544717 33500 0.000114814 33300 0.000236314 32900
0.000361894 35100 0.000762474 34600 0.000528836
35500 0.005450863 33500 0.000114799 33300 0.000236356 32900
0.000361603 35100 0.000763515 34600 0.000528686
35500 0.005454557 33500 0.000114774 33300 0.000236374 32900
0.000361343 35100 0.000764546 34600 0.000528539
35500 0.005458267 33500 0.000114764 33300 0.000236416 32900
0.000361083 35200 0.000765583 34600 0.000528397
35500 0.005461953 33500 0.000114755 33300 0.000236449 32900
0.000360862 35200 0.000766611 34600 0.000528256
35500 0.005465684 33500 0.000114717 33300 0.000236452 32900
0.000360602 35200 0.000767649 34600 0.000528108
35500 0.00546939 33500 0.000114701 33300 0.000236496 32900
0.000360392 35200 0.000768668 34600 0.000527969

35500 0.005473114 33500 0.000114673 33300 0.000236534 32900
0.000360191 35200 0.000769685 34600 0.000527825
35500 0.005476849 33500 0.000114646 33300 0.000236551 32900
0.000360001 35200 0.000770723 34600 0.000527686
35500 0.005480584 33500 0.000114614 33300 0.000236579 32900
0.000359891 35200 0.000771741 34600 0.000527534
35500 0.005484357 33500 0.000114581 33300 0.000236614 32900
0.000359741 35200 0.000772771 34600 0.000527385
35500 0.005488126 33500 0.000114549 33300 0.000236651 32900
0.000359611 35200 0.000773804 34600 0.000527246
35500 0.005491931 33500 0.000114516 33300 0.000236677 32900
0.000359541 35200 0.00077483 34600 0.000527094
35500 0.005495718 33500 0.000114504 33300 0.000236696 32900
0.000359421 35200 0.000775857 34600 0.000526948
35500 0.005499451 33500 0.000114463 33300 0.000236735 32900
0.0003593 35200 0.000776888 34600 0.000526792
35500 0.005503264 33500 0.000114438 33300 0.000236796 32900
0.00035919 35200 0.000777928 34600 0.000526655
35500 0.005507047 33500 0.000114415 33300 0.000236805 32900
0.00035907 35200 0.000778969 34600 0.00052651
35500 0.005510907 33500 0.000114375 33300 0.000236841 32900
0.00035895 35200 0.000780026 34600 0.000526346
35500 0.005514744 33500 0.00011434 33300 0.000236885 32900
0.000358809 35200 0.000781072 34600 0.000526204
35500 0.005518565 33500 0.00011431 33300 0.000236892 32900
0.000358649 35200 0.000782125 34600 0.000526053
35500 0.005522434 33500 0.00011427 33300 0.000236935 32900
0.000358499 35200 0.000783182 34600 0.000525902
35500 0.00552626 33500 0.00011424 33300 0.000236955 32900
0.000358359 35200 0.000784248 34600 0.00052574
35500 0.005530121 33500 0.000114217 33300 0.000236982 32900
0.000358219 35200 0.00078531 34600 0.000525595
35500 0.005533974 33500 0.00011419 33300 0.000237019 32900
0.000358058 35200 0.000786376 34600 0.000525436
35500 0.005537821 33500 0.00011416 33300 0.000237039 32900
0.000357918 35200 0.000787446 34600 0.000525265
35500 0.005541666 33500 0.000114142 33300 0.000237108 32900
0.000357818 35200 0.000788528 34600 0.000525121
35500 0.005545545 33500 0.000114134 33300 0.000237164 32900
0.000357698 35200 0.000789599 34600 0.000524961
35500 0.005549392 33500 0.000114102 33300 0.000237181 32900
0.000357558 35200 0.000790685 34600 0.000524816
35500 0.005553286 33500 0.00011407 33300 0.00023722 32900
0.000357438 35200 0.000791767 34600 0.000524656
35500 0.005557169 33500 0.000114021 33300 0.000237277 32900
0.000357257 35200 0.000792853 34600 0.000524513
35500 0.005561041 33500 0.000113986 33300 0.000237339 32900
0.000357137 35200 0.000793948 34600 0.000524365
35500 0.005564958 33500 0.000113944 33300 0.000237396 32900
0.000357037 35200 0.000795038 34600 0.000524212
35600 0.005568844 33500 0.000113904 33300 0.000237431 32900
0.000356897 35200 0.000796114 34600 0.000524057
35600 0.005572699 33500 0.000113864 33300 0.000237474 32900
0.000356717 35200 0.000797207 34600 0.0005239
35600 0.005576604 33500 0.000113826 33300 0.000237521 32900
0.000356537 35200 0.000798309 34600 0.000523747
35600 0.005580483 33500 0.000113818 33300 0.000237563 32900
0.000356396 35200 0.000799418 34600 0.00052359
35600 0.005584394 33500 0.000113795 33300 0.000237617 32900
0.000356196 35200 0.00080052 34600 0.000523438
35600 0.00558831 33500 0.000113805 33300 0.000237656 32900
0.000356016 35200 0.000801625 34600 0.000523293
35600 0.005592215 33500 0.000113797 33300 0.000237695 32900
0.000355775 35200 0.000802733 34600 0.000523141
35600 0.005596133 33500 0.000113793 33300 0.000237787 32900
0.000355555 35200 0.000803851 34600 0.000522987
35600 0.005600019 33500 0.000113787 33300 0.000237852 32900
0.000355315 35200 0.000804955 34600 0.000522832

35600 0.005603926 33500 0.000113783 33300 0.000237891 32900
0.000355074 35200 0.000806072 34700 0.000522677
35600 0.005607835 33500 0.000113775 33300 0.000237932 32900
0.000354834 35200 0.00080718 34700 0.000522516
35600 0.005611737 33500 0.000113758 33300 0.000237981 32900
0.000354634 35200 0.000808309 34700 0.000522358
35600 0.005615637 33500 0.000113732 33300 0.000238024 32900
0.000354434 35200 0.000809423 34700 0.000522201
35600 0.005619561 33500 0.000113718 33300 0.000238094 32900
0.000354274 35200 0.000810541 34700 0.000522044
35600 0.005623473 33500 0.000113689 33300 0.000238119 32900
0.000354073 35200 0.000811679 34700 0.00052189
35600 0.00562742 33500 0.000113669 33300 0.000238171 32900
0.000353873 35200 0.000812803 34700 0.000521724
35600 0.005631373 33500 0.000113632 33300 0.000238223 32900
0.000353713 35200 0.000813928 34700 0.000521569
35600 0.00563533 33500 0.000113605 33300 0.000238307 32900
0.000353532 35200 0.000815069 34700 0.000521415
35600 0.005639309 33600 0.000113571 33300 0.000238337 32900
0.000353412 35200 0.000816205 34700 0.000521253
35600 0.005643268 33600 0.000113538 33300 0.000238398 32900
0.000353292 35200 0.000817339 34700 0.000521095
35600 0.00564722 33600 0.000113499 33400 0.00023844 32900
0.000353232 35200 0.000818486 34700 0.00052093
35600 0.005651195 33600 0.000113471 33400 0.000238512 32900
0.000353212 35200 0.000819623 34700 0.000520768
35600 0.005655233 33600 0.00011344 33400 0.000238579 32900
0.000353152 35200 0.000820788 34700 0.00052061
35600 0.005659256 33600 0.00011341 33400 0.000238611 32900
0.000353112 35200 0.000821944 34700 0.000520443
35600 0.005663295 33600 0.000113388 33400 0.000238647 33000
0.000353052 35200 0.000823095 34700 0.000520294
35600 0.005667353 33600 0.000113367 33400 0.000238721 33000
0.000352992 35200 0.000824256 34700 0.000520132
35600 0.005671434 33600 0.000113348 33400 0.000238781 33000
0.000352951 35200 0.000825422 34700 0.000519975
35600 0.005675482 33600 0.000113322 33400 0.000238838 33000
0.000352871 35200 0.000826585 34700 0.000519808
35600 0.005679603 33600 0.000113303 33400 0.000238911 33000
0.000352812 35200 0.000827752 34700 0.000519645
35600 0.005683702 33600 0.000113285 33400 0.00023896 33000
0.000352751 35200 0.00082892 34700 0.000519488
35600 0.005687802 33600 0.000113268 33400 0.000239018 33000
0.000352651 35200 0.000830094 34700 0.000519326
35600 0.005691887 33600 0.000113247 33400 0.000239088 33000
0.000352491 35200 0.000831263 34700 0.000519174
35600 0.005696049 33600 0.000113237 33400 0.000239137 33000
0.000352331 35200 0.000832431 34700 0.000519015
35600 0.005700191 33600 0.000113216 33400 0.000239172 33000
0.000352171 35200 0.000833615 34700 0.000518862
35600 0.005704364 33600 0.000113197 33400 0.000239241 33000
0.000352071 35200 0.000834791 34700 0.000518703
35600 0.005708585 33600 0.000113176 33400 0.000239304 33000
0.000351911 35300 0.000835962 34700 0.000518553
35600 0.005712812 33600 0.000113151 33400 0.000239369 33000
0.00035181 35300 0.000837152 34700 0.000518412
35600 0.005717037 33600 0.00011314 33400 0.000239415 33000
0.00035171 35300 0.000838339 34700 0.000518252
35600 0.005721289 33600 0.000113123 33400 0.000239475 33000
0.00035163 35300 0.000839522 34700 0.000518098
35600 0.005725475 33600 0.000113105 33400 0.000239536 33000
0.00035151 35300 0.000840703 34700 0.000517943
35600 0.005729741 33600 0.000113099 33400 0.0002396 33000
0.00035141 35300 0.000841897 34700 0.000517799
35600 0.005734023 33600 0.000113082 33400 0.000239686 33000
0.00035129 35300 0.000843092 34700 0.000517641
35600 0.005738327 33600 0.000113062 33400 0.000239736 33000
0.000351149 35300 0.000844282 34700 0.000517492

35600 0.005742637 33600 0.000113062 33400 0.000239807 33000
0.000351029 35300 0.00084548 34700 0.000517347
35600 0.005746951 33600 0.000113043 33400 0.000239879 33000
0.000350949 35300 0.000846682 34700 0.000517195
35600 0.005751295 33600 0.000113022 33400 0.000239967 33000
0.000350869 35300 0.000847879 34700 0.000517053
35600 0.005755609 33600 0.000113009 33400 0.000240032 33000
0.000350729 35300 0.00084908 34700 0.000516893
35600 0.005759958 33600 0.000112988 33400 0.000240125 33000
0.000350669 35300 0.000850278 34700 0.00051675
35600 0.005764281 33600 0.000112968 33400 0.00024016 33000
0.000350569 35300 0.000851482 34700 0.000516603
35600 0.005768613 33600 0.000112949 33400 0.00024026 33000
0.000350449 35300 0.000852694 34700 0.000516448
35600 0.005772955 33600 0.000112934 33400 0.000240308 33000
0.000350349 35300 0.000853895 34700 0.000516297
35600 0.005777339 33600 0.000112907 33400 0.000240384 33000
0.000350268 35300 0.000855107 34700 0.000516149
35600 0.005781699 33600 0.000112876 33400 0.000240442 33000
0.000350188 35300 0.00085632 34700 0.000516012
35600 0.005786092 33600 0.000112849 33400 0.000240514 33000
0.000350088 35300 0.000857539 34700 0.000515844
35600 0.005790489 33600 0.000112804 33400 0.000240563 33000
0.000349968 35300 0.000858761 34700 0.000515707
35600 0.005794894 33600 0.000112778 33400 0.000240628 33000
0.000349908 35300 0.000859981 34700 0.000515575
35600 0.005799322 33600 0.000112739 33400 0.000240696 33000
0.000349788 35300 0.000861218 34700 0.00051542
35600 0.005803717 33600 0.000112711 33400 0.000240753 33000
0.000349667 35300 0.000862442 34700 0.000515273
35600 0.005808089 33600 0.000112684 33400 0.00024082 33000
0.000349567 35300 0.000863677 34700 0.000515134
35600 0.005812523 33600 0.000112662 33400 0.000240887 33000
0.000349487 35300 0.000864915 34700 0.000514979
35600 0.005816943 33600 0.000112654 33400 0.000240959 33000
0.000349327 35300 0.000866162 34700 0.000514843
35600 0.0058214 33600 0.000112645 33400 0.000241033 33000
0.000349187 35300 0.000867408 34700 0.000514703
35600 0.005825892 33600 0.000112647 33400 0.000241078 33000
0.000349086 35300 0.000868652 34700 0.00051455
35600 0.005830344 33600 0.000112638 33400 0.000241176 33000
0.000348986 35300 0.000869906 34700 0.000514413
35700 0.005834831 33600 0.000112636 33400 0.000241229 33000
0.000348866 35300 0.000871159 34700 0.000514259
35700 0.005839299 33600 0.000112627 33400 0.000241302 33000
0.000348706 35300 0.000872405 34700 0.000514122
35700 0.005843807 33600 0.000112611 33400 0.000241388 33000
0.000348626 35300 0.000873661 34700 0.000513966
35700 0.005848293 33600 0.000112606 33400 0.000241449 33000
0.000348526 35300 0.000874915 34700 0.000513813
35700 0.005852787 33600 0.00011258 33400 0.000241525 33000
0.000348406 35300 0.000876183 34700 0.000513665
35700 0.00585726 33600 0.000112564 33400 0.000241582 33000
0.000348305 35300 0.000877436 34700 0.000513518
35700 0.005861801 33600 0.000112559 33400 0.000241651 33000
0.000348226 35300 0.000878694 34700 0.000513374
35700 0.005866322 33600 0.000112538 33400 0.000241713 33000
0.000348145 35300 0.000879951 34700 0.000513221
35700 0.005870876 33600 0.000112521 33400 0.000241792 33000
0.000348085 35300 0.00088121 34700 0.000513056
35700 0.005875421 33600 0.000112497 33400 0.000241857 33000
0.000348016 35300 0.000882469 34800 0.00051291
35700 0.005879981 33600 0.000112488 33400 0.000241948 33000
0.000347935 35300 0.000883733 34800 0.000512764
35700 0.005884573 33600 0.00011247 33400 0.000241994 33000
0.000347905 35300 0.000884991 34800 0.000512602
35700 0.005889162 33600 0.000112458 33400 0.000242064 33000
0.000347805 35300 0.000886236 34800 0.000512458

35700 0.005893703 33600 0.000112445 33400 0.000242142 33000
0.000347735 35300 0.00088749 34800 0.000512295
35700 0.005898301 33600 0.000112428 33400 0.000242197 33000
0.000347645 35300 0.000888754 34800 0.000512137
35700 0.005902898 33600 0.000112423 33400 0.000242294 33000
0.000347505 35300 0.000890006 34800 0.00051197
35700 0.005907531 33600 0.000112404 33400 0.000242357 33000
0.000347395 35300 0.000891265 34800 0.000511827
35700 0.00591218 33600 0.000112401 33400 0.000242428 33000
0.000347274 35300 0.000892533 34800 0.000511661
35700 0.005916795 33700 0.000112385 33400 0.000242495 33000
0.000347124 35300 0.000893791 34800 0.000511499
35700 0.005921443 33700 0.000112372 33400 0.000242597 33000
0.000347044 35300 0.000895059 34800 0.00051134
35700 0.005926099 33700 0.000112362 33500 0.000242659 33000
0.000346984 35300 0.000896315 34800 0.000511177
35700 0.005930759 33700 0.000112325 33500 0.000242735 33000
0.000346934 35300 0.000897577 34800 0.000510994
35700 0.005935412 33700 0.000112307 33500 0.000242801 33000
0.000346874 35300 0.000898839 34800 0.000510835
35700 0.005940089 33700 0.000112264 33500 0.0002429 33000
0.000346814 35300 0.000900126 34800 0.000510659
35700 0.005944753 33700 0.000112228 33500 0.000242968 33100
0.000346684 35300 0.000901387 34800 0.000510494
35700 0.005949469 33700 0.000112196 33500 0.000243048 33100
0.000346554 35300 0.000902665 34800 0.000510318
35700 0.005954165 33700 0.000112158 33500 0.000243117 33100
0.000346414 35300 0.000903945 34800 0.000510152
35700 0.005958898 33700 0.000112126 33500 0.000243184 33100
0.000346249 35300 0.000905232 34800 0.00050997
35700 0.005963654 33700 0.000112092 33500 0.000243245 33100
0.000346068 35300 0.000906529 34800 0.000509796
35700 0.005968408 33700 0.000112059 33500 0.000243347 33100
0.000345878 35300 0.00090783 34800 0.000509633
35700 0.005973192 33700 0.000112031 33500 0.000243407 33100
0.000345698 35300 0.000909137 34800 0.000509453
35700 0.005977984 33700 0.000112007 33500 0.000243492 33100
0.000345507 35300 0.000910455 34800 0.000509276
35700 0.00598272 33700 0.000111987 33500 0.000243549 33100
0.000345377 35300 0.000911772 34800 0.000509092
35700 0.005987537 33700 0.000111963 33500 0.000243626 33100
0.000345257 35300 0.000913089 34800 0.000508902
35700 0.005992375 33700 0.000111937 33500 0.000243713 33100
0.000345162 35300 0.000914425 34800 0.000508718
35700 0.005997241 33700 0.000111917 33500 0.000243793 33100
0.000345082 35400 0.000915749 34800 0.000508545
35700 0.006002136 33700 0.000111918 33500 0.000243859 33100
0.000345007 35400 0.00091709 34800 0.000508366
35700 0.006007033 33700 0.000111888 33500 0.000243938 33100
0.000344947 35400 0.000918434 34800 0.000508191
35700 0.006011944 33700 0.000111863 33500 0.000244043 33100
0.000344871 35400 0.000919791 34800 0.000508011
35700 0.006016853 33700 0.000111852 33500 0.000244123 33100
0.000344796 35400 0.000921136 34800 0.000507824
35700 0.006021801 33700 0.000111838 33500 0.000244216 33100
0.000344734 35400 0.000922482 34800 0.000507624
35700 0.006026743 33700 0.000111833 33500 0.000244283 33100
0.000344674 35400 0.000923852 34800 0.000507457
35700 0.006031706 33700 0.000111819 33500 0.000244359 33100
0.000344604 35400 0.000925224 34800 0.000507276
35700 0.006036656 33700 0.0001118 33500 0.000244446 33100
0.000344536 35400 0.000926583 34800 0.0005071
35700 0.006041639 33700 0.000111785 33500 0.000244534 33100
0.000344486 35400 0.000927956 34800 0.000506913
35700 0.006046627 33700 0.000111764 33500 0.000244608 33100
0.000344411 35400 0.000929338 34800 0.000506746
35700 0.006051674 33700 0.000111751 33500 0.000244691 33100
0.000344341 35400 0.000930724 34800 0.000506564

35700 0.006056734 33700 0.000111735 33500 0.000244754 33100
0.000344271 35400 0.000932103 34800 0.000506377
35700 0.006061807 33700 0.000111728 33500 0.000244845 33100
0.000344178 35400 0.000933496 34800 0.000506213
35700 0.006066907 33700 0.000111722 33500 0.000244913 33100
0.000344071 35400 0.000934894 34800 0.000506019
35700 0.006071997 33700 0.000111726 33500 0.000244997 33100
0.000343951 35400 0.00093628 34800 0.000505857
35700 0.006077061 33700 0.000111728 33500 0.000245069 33100
0.000343813 35400 0.000937681 34800 0.000505676
35700 0.006082181 33700 0.000111735 33500 0.000245142 33100
0.00034367 35400 0.000939094 34800 0.000505486
35700 0.006087304 33700 0.00011174 33500 0.000245227 33100
0.000343515 35400 0.000940509 34800 0.000505314
35700 0.006092433 33700 0.000111742 33500 0.000245302 33100
0.000343352 35400 0.000941929 34800 0.000505147
35700 0.006097586 33700 0.000111741 33500 0.000245387 33100
0.000343196 35400 0.000943352 34800 0.000504977
35700 0.006102739 33700 0.000111726 33500 0.000245489 33100
0.000343043 35400 0.000944786 34800 0.000504819
35700 0.006107907 33700 0.000111708 33500 0.000245567 33100
0.000342902 35400 0.000946219 34800 0.000504644
35700 0.006113044 33700 0.000111689 33500 0.000245639 33100
0.000342749 35400 0.000947657 34800 0.000504483
35700 0.006118204 33700 0.000111659 33500 0.000245737 33100
0.000342619 35400 0.000949106 34800 0.000504302
35700 0.006123338 33700 0.000111632 33500 0.000245816 33100
0.00034247 35400 0.000950551 34800 0.000504146
35700 0.006128533 33700 0.000111599 33500 0.00024591 33100
0.00034231 35400 0.000952018 34800 0.000503975
35700 0.006133649 33700 0.000111569 33500 0.000245989 33100
0.000342157 35400 0.000953477 34800 0.000503815
35700 0.00613884 33700 0.000111548 33500 0.000246074 33100
0.000341994 35400 0.000954938 34800 0.000503657
35800 0.006144002 33700 0.000111511 33500 0.000246168 33100
0.000341827 35400 0.000956415 34800 0.000503508
35800 0.00614919 33700 0.000111497 33500 0.00024625 33100
0.000341646 35400 0.000957896 34800 0.00050337
35800 0.006154395 33700 0.000111466 33500 0.000246337 33100
0.000341484 35400 0.000959376 34800 0.000503209
35800 0.006159605 33700 0.000111438 33500 0.000246415 33100
0.000341299 35400 0.00096086 34800 0.000503061
35800 0.006164839 33700 0.000111408 33500 0.000246502 33100
0.000341141 35400 0.000962348 34800 0.000502906
35800 0.006170037 33700 0.000111396 33500 0.000246596 33100
0.000340979 35400 0.000963836 34800 0.00050276
35800 0.006175159 33700 0.000111384 33500 0.000246688 33100
0.000340836 35400 0.000965325 34800 0.000502625
35800 0.006180404 33700 0.000111364 33500 0.000246781 33100
0.000340691 35400 0.000966816 34800 0.000502474
35800 0.006185633 33700 0.000111349 33500 0.000246879 33100
0.00034055 35400 0.000968312 34800 0.000502321
35800 0.006190885 33700 0.000111327 33500 0.000246966 33100
0.00034041 35400 0.000969795 34900 0.000502181
35800 0.006196166 33700 0.000111307 33500 0.000247076 33100
0.000340281 35400 0.000971283 34900 0.000502056
35800 0.006201423 33700 0.00011128 33500 0.000247186 33100
0.000340136 35400 0.000972779 34900 0.000501924
35800 0.006206709 33700 0.000111259 33500 0.000247292 33100
0.000340025 35400 0.000974268 34900 0.0005018
35800 0.006211972 33700 0.000111247 33500 0.000247386 33100
0.000339945 35400 0.000975762 34900 0.000501668
35800 0.006217283 33700 0.000111219 33500 0.000247497 33100
0.000339893 35400 0.000977253 34900 0.000501535
35800 0.006222535 33700 0.000111206 33500 0.000247601 33100
0.000339873 35400 0.000978734 34900 0.000501423
35800 0.006227859 33700 0.000111187 33500 0.000247716 33100
0.000339863 35400 0.000980232 34900 0.000501304

35800 0.006233117 33700 0.000111188 33500 0.000247818 33100
0.000339883 35400 0.000981716 34900 0.000501194
35800 0.006238459 33800 0.000111172 33500 0.000247935 33100
0.000339925 35400 0.000983212 34900 0.00050108
35800 0.006243765 33800 0.00011116 33500 0.000248029 33100
0.000339938 35400 0.000984709 34900 0.000500975
35800 0.0062491 33800 0.000111144 33600 0.000248155 33100
0.000339963 35400 0.000986206 34900 0.000500865
35800 0.006254476 33800 0.000111124 33600 0.000248248 33100
0.000339978 35400 0.00098771 34900 0.000500741
35800 0.006259869 33800 0.000111103 33600 0.000248365 33100
0.000339977 35400 0.00098921 34900 0.000500643
35800 0.006265248 33800 0.000111073 33600 0.000248471 33100
0.000339985 35400 0.000990716 34900 0.000500518
35800 0.006270644 33800 0.000111045 33600 0.000248583 33200
0.00033998 35400 0.000992206 34900 0.000500419
35800 0.00627601 33800 0.000111026 33600 0.000248693 33200
0.000339982 35400 0.000993703 34900 0.000500306
35800 0.006281413 33800 0.000110988 33600 0.000248794 33200
0.000339952 35400 0.00099521 34900 0.000500193
35800 0.006286845 33800 0.000110966 33600 0.000248903 33200
0.00033991 35400 0.000996718 34900 0.000500094
35800 0.006292284 33800 0.000110936 33600 0.000249007 33200
0.000339845 35400 0.000998227 34900 0.000499974
35800 0.006297723 33800 0.000110921 33600 0.000249123 33200
0.000339767 35400 0.000999738 34900 0.000499878
35800 0.006303173 33800 0.000110888 33600 0.00024925 33200
0.000339655 35400 0.001001254 34900 0.000499783
35800 0.006308652 33800 0.000110867 33600 0.000249367 33200
0.000339549 35400 0.001002768 34900 0.000499663
35800 0.006314077 33800 0.000110837 33600 0.000249485 33200
0.000339452 35400 0.001004277 34900 0.000499574
35800 0.006319571 33800 0.000110809 33600 0.000249601 33200
0.000339357 35400 0.001005805 34900 0.000499453
35800 0.006325029 33800 0.000110786 33600 0.000249719 33200
0.000339282 35400 0.00100732 34900 0.000499372
35800 0.006330495 33800 0.000110746 33600 0.000249837 33200
0.000339209 35500 0.001008849 34900 0.000499257
35800 0.006335961 33800 0.00011073 33600 0.000249947 33200
0.000339159 35500 0.001010379 34900 0.000499162
35800 0.006341444 33800 0.00011072 33600 0.000250071 33200
0.000339149 35500 0.001011911 34900 0.000499052
35800 0.006346945 33800 0.00011069 33600 0.00025018 33200
0.000339114 35500 0.001013452 34900 0.000498942
35800 0.006352465 33800 0.000110679 33600 0.000250317 33200
0.000339074 35500 0.001015004 34900 0.000498839
35800 0.006358001 33800 0.000110673 33600 0.000250425 33200
0.000339026 35500 0.001016554 34900 0.000498751
35800 0.006363547 33800 0.000110661 33600 0.00025055 33200
0.000338979 35500 0.001018113 34900 0.000498642
35800 0.006369111 33800 0.000110655 33600 0.000250675 33200
0.000338916 35500 0.001019673 34900 0.000498531
35800 0.00637468 33800 0.000110652 33600 0.000250803 33200
0.000338859 35500 0.001021233 34900 0.000498428
35800 0.006380164 33800 0.000110645 33600 0.000250919 33200
0.000338794 35500 0.001022805 34900 0.000498319
35800 0.006385761 33800 0.000110627 33600 0.000251037 33200
0.000338736 35500 0.001024385 34900 0.000498215
35800 0.006391357 33800 0.000110627 33600 0.00025116 33200
0.000338679 35500 0.001025967 34900 0.000498109
35800 0.006396982 33800 0.000110619 33600 0.000251288 33200
0.000338626 35500 0.001027548 34900 0.000497999
35800 0.006402635 33800 0.000110616 33600 0.000251421 33200
0.000338586 35500 0.001029137 34900 0.000497903
35800 0.006408253 33800 0.000110613 33600 0.000251565 33200
0.000338544 35500 0.001030726 34900 0.000497798
35800 0.006413905 33800 0.000110622 33600 0.000251691 33200
0.000338488 35500 0.001032321 34900 0.000497688

35800 0.006419541 33800 0.000110636 33600 0.000251831 33200
0.000338431 35500 0.001033914 34900 0.000497595
35800 0.006425201 33800 0.000110647 33600 0.000251965 33200
0.000338361 35500 0.001035512 34900 0.000497485
35800 0.006430865 33800 0.000110665 33600 0.000252084 33200
0.000338301 35500 0.001037112 34900 0.000497371
35800 0.006436542 33800 0.000110674 33600 0.000252237 33200
0.000338226 35500 0.001038724 34900 0.000497255
35800 0.006442214 33800 0.000110688 33600 0.000252359 33200
0.000338173 35500 0.001040322 34900 0.000497159
35800 0.006447899 33800 0.000110695 33600 0.000252496 33200
0.000338126 35500 0.001041928 34900 0.000497061
35800 0.006453624 33800 0.000110676 33600 0.000252626 33200
0.00033804 35500 0.001043532 34900 0.000496946
35800 0.006459336 33800 0.000110662 33600 0.000252763 33200
0.000337968 35500 0.001045149 34900 0.000496827
35800 0.006465072 33800 0.000110633 33600 0.000252896 33200
0.000337883 35500 0.001046761 34900 0.000496696
35800 0.006470856 33800 0.0001106 33600 0.000253028 33200
0.00033778 35500 0.001048376 34900 0.000496603
35800 0.00647663 33800 0.000110562 33600 0.000253168 33200
0.000337695 35500 0.001049996 34900 0.000496472
35800 0.00648241 33800 0.000110541 33600 0.000253298 33200
0.000337607 35500 0.001051615 34900 0.000496353
35800 0.006488164 33800 0.000110506 33600 0.000253433 33200
0.000337545 35500 0.001053242 34900 0.000496228
35900 0.006493985 33800 0.000110466 33600 0.000253556 33200
0.00033749 35500 0.001054879 34900 0.000496118
35900 0.006499788 33800 0.000110453 33600 0.000253677 33200
0.000337457 35500 0.001056513 34900 0.000496011
35900 0.006505681 33800 0.000110429 33600 0.000253816 33200
0.000337422 35500 0.001058151 34900 0.000495887
35900 0.006511582 33800 0.000110412 33600 0.000253948 33200
0.000337389 35500 0.001059803 34900 0.000495755
35900 0.006517434 33800 0.000110396 33600 0.000254092 33200
0.000337364 35500 0.001061452 34900 0.000495652
35900 0.006523347 33800 0.00011039 33600 0.000254224 33200
0.000337322 35500 0.001063115 34900 0.000495507
35900 0.006529249 33800 0.00011038 33600 0.000254372 33200
0.000337307 35500 0.001064771 34900 0.000495392
35900 0.006535205 33800 0.000110364 33600 0.000254506 33200
0.000337272 35500 0.00106644 34900 0.000495268
35900 0.006541156 33800 0.000110354 33600 0.000254645 33200
0.000337277 35500 0.001068127 34900 0.000495149
35900 0.00654709 33800 0.000110338 33600 0.000254784 33200
0.000337262 35500 0.00106981 35000 0.000495022
35900 0.006553036 33800 0.000110326 33600 0.000254913 33200
0.000337267 35500 0.001071499 35000 0.000494902
35900 0.006559049 33800 0.000110298 33600 0.000255041 33200
0.000337277 35500 0.001073197 35000 0.000494776
35900 0.006565051 33800 0.000110267 33600 0.000255181 33200
0.000337267 35500 0.001074903 35000 0.000494658
35900 0.006571128 33800 0.000110243 33600 0.000255328 33200
0.000337247 35500 0.001076616 35000 0.000494524
35900 0.00657719 33800 0.000110217 33600 0.000255462 33200
0.000337189 35500 0.001078326 35000 0.000494406
35900 0.006583279 33800 0.000110189 33600 0.000255593 33200
0.000337104 35500 0.001080051 35000 0.000494288
35900 0.00658941 33800 0.00011016 33600 0.000255733 33200
0.000337002 35500 0.00108178 35000 0.000494152
35900 0.006595521 33800 0.000110148 33600 0.000255863 33200
0.000336884 35500 0.001083501 35000 0.000494022
35900 0.006601612 33900 0.000110134 33600 0.000256019 33200
0.000336766 35500 0.001085238 35000 0.000493864
35900 0.006607781 33900 0.00011013 33600 0.000256137 33200
0.000336654 35500 0.001086979 35000 0.000493752
35900 0.006613937 33900 0.000110132 33700 0.000256293 33200
0.000336556 35500 0.001088722 35000 0.000493618

35900 0.006620131	33900 0.000110137	33700 0.000256438	33200
0.000336481 35500	0.001090476 35000	0.000493494	
35900 0.006626372	33900 0.000110134	33700 0.000256591	33200
0.000336421 35500	0.001092225 35000	0.00049337	
35900 0.006632592	33900 0.000110138	33700 0.000256752	33200
0.000336371 35500	0.001093978 35000	0.000493243	
35900 0.006638821	33900 0.000110136	33700 0.000256903	33300
0.000336326 35500	0.001095737 35000	0.000493104	
35900 0.006645056	33900 0.00011013	33700 0.000257082	33300
0.000336301 35500	0.001097477 35000	0.000492986	
35900 0.00665133	33900 0.000110128	33700 0.000257229	33300
0.000336278 35500	0.001099225 35000	0.000492851	
35900 0.006657595	33900 0.000110124	33700 0.000257402	33300
0.000336268 35500	0.001100974 35000	0.000492716	
35900 0.006663862	33900 0.000110124	33700 0.000257559	33300
0.000336221 35500	0.001102729 35000	0.000492579	
35900 0.006670113	33900 0.000110118	33700 0.00025772	33300
0.000336201 35500	0.001104474 35000	0.000492453	
35900 0.006676403	33900 0.000110118	33700 0.000257885	33300
0.000336201 35500	0.001106219 35000	0.00049233	
35900 0.006682694	33900 0.000110101	33700 0.000258038	33300
0.000336173 35500	0.001107967 35000	0.000492202	
35900 0.006689044	33900 0.000110101	33700 0.000258215	33300
0.000336158 35500	0.001109708 35000	0.000492056	
35900 0.006695354	33900 0.000110088	33700 0.000258375	33300
0.000336146 35500	0.001111457 35000	0.000491918	
35900 0.006701692	33900 0.000110078	33700 0.000258535	33300
0.000336095 35500	0.001113198 35000	0.000491808	
35900 0.006708106	33900 0.000110061	33700 0.000258713	33300
0.000336043 35600	0.001114937 35000	0.000491654	
35900 0.006714436	33900 0.000110037	33700 0.000258875	33300
0.000335983 35600	0.001116669 35000	0.000491541	
35900 0.006720753	33900 0.000110022	33700 0.000259046	33300
0.00033592 35600	0.001118408 35000	0.00049138	
35900 0.006727167	33900 0.000110002	33700 0.000259212	33300
0.000335853 35600	0.001120141 35000	0.000491253	
35900 0.006733577	33900 0.000109992	33700 0.000259383	33300
0.000335803 35600	0.001121881 35000	0.000491145	
35900 0.006740022	33900 0.000109986	33700 0.000259548	33300
0.000335763 35600	0.001123617 35000	0.000490987	
35900 0.00674646	33900 0.000109987	33700 0.000259728	33300
0.000335725 35600	0.001125344 35000	0.000490852	
35900 0.006752934	33900 0.000109984	33700 0.000259911	33300
0.00033569 35600	0.001127081 35000	0.000490729	
35900 0.006759404	33900 0.000109978	33700 0.000260093	33300
0.00033569 35600	0.001128827 35000	0.000490578	
35900 0.00676585	33900 0.000109976	33700 0.000260276	33300
0.000335708 35600	0.001130549 35000	0.000490456	
35900 0.00677236	33900 0.000109982	33700 0.000260463	33300
0.000335735 35600	0.001132286 35000	0.000490319	
35900 0.006778856	33900 0.000109974	33700 0.000260636	33300
0.000335763 35600	0.001134024 35000	0.000490181	
35900 0.006785334	33900 0.000109975	33700 0.000260825	33300
0.0003358 35600	0.001135772 35000	0.000490049	
35900 0.006791855	33900 0.000109974	33700 0.000261009	33300
0.000335833 35600	0.001137507 35000	0.000489902	
35900 0.006798385	33900 0.000109977	33700 0.00026119	33300
0.000335855 35600	0.001139252 35000	0.000489764	
35900 0.006804903	33900 0.000109977	33700 0.000261358	33300
0.000335848 35600	0.001141014 35000	0.000489655	
35900 0.006811463	33900 0.000109981	33700 0.000261544	33300
0.000335846 35600	0.001142783 35000	0.000489514	
35900 0.006818055	33900 0.000109974	33700 0.000261731	33300
0.000335833 35600	0.001144543 35000	0.000489388	
35900 0.006824669	33900 0.000109968	33700 0.000261915	33300
0.000335775 35600	0.00114632 35000	0.000489266	
35900 0.006831318	33900 0.000109948	33700 0.000262097	33300
0.000335733 35600	0.00114811 35000	0.000489123	

35900 0.006837924 33900 0.000109931 33700 0.000262287 33300
0.000335665 35600 0.001149904 35000 0.00048901
35900 0.006844483 33900 0.000109913 33700 0.000262474 33300
0.000335595 35600 0.001151707 35000 0.000488867
35900 0.006851193 33900 0.000109891 33700 0.000262646 33300
0.000335518 35600 0.00115353 35000 0.000488762
35900 0.00685787 33900 0.000109875 33700 0.000262833 33300
0.000335425 35600 0.001155362 35000 0.000488638
35900 0.006864562 33900 0.000109855 33700 0.000263034 33300
0.000335332 35600 0.001157208 35000 0.000488509
35900 0.006871274 33900 0.000109836 33700 0.000263231 33300
0.000335215 35600 0.001159063 35000 0.00048838
35900 0.006878001 33900 0.000109815 33700 0.000263425 33300
0.0003351 35600 0.001160929 35000 0.00048827
35900 0.00688473 33900 0.000109789 33700 0.000263617 33300
0.000334987 35600 0.001162805 35000 0.000488142
35900 0.00689143 33900 0.000109757 33700 0.000263822 33300
0.000334874 35600 0.001164683 35000 0.000488036
36000 0.006898168 33900 0.000109742 33700 0.000264014 33300
0.000334769 35600 0.001166571 35000 0.000487914
36000 0.006904891 33900 0.000109716 33700 0.000264205 33300
0.000334664 35600 0.001168468 35000 0.000487757
36000 0.0069116 33900 0.000109683 33700 0.000264414 33300
0.000334559 35600 0.001170383 35000 0.000487643
36000 0.006918359 33900 0.000109666 33700 0.000264601 33300
0.000334469 35600 0.001172293 35000 0.000487528
36000 0.006925112 33900 0.000109639 33700 0.000264793 33300
0.000334401 35600 0.001174204 35000 0.000487398
36000 0.006931863 33900 0.000109619 33700 0.000264979 33300
0.000334299 35600 0.001176127 35000 0.000487271
36000 0.006938661 33900 0.000109608 33700 0.000265187 33300
0.000334198 35600 0.001178056 35000 0.000487158
36000 0.006945461 33900 0.000109591 33700 0.000265361 33300
0.000334088 35600 0.001179989 35000 0.000487032
36000 0.006952268 33900 0.000109585 33700 0.000265552 33300
0.000333983 35600 0.001181928 35000 0.000486935
36000 0.006959102 33900 0.000109578 33700 0.000265744 33300
0.000333888 35600 0.001183872 35100 0.000486782
36000 0.00696592 33900 0.000109574 33700 0.000265947 33300
0.000333815 35600 0.001185812 35100 0.000486667
36000 0.00697272 33900 0.00010958 33700 0.000266143 33300
0.000333763 35600 0.001187771 35100 0.000486533
36000 0.006979593 33900 0.000109572 33700 0.000266324 33300
0.00033374 35600 0.001189725 35100 0.00048641
36000 0.006986452 33900 0.000109577 33700 0.000266521 33300
0.000333753 35600 0.001191694 35100 0.00048629
36000 0.006993342 33900 0.00010959 33700 0.000266729 33300
0.000333783 35600 0.001193664 35100 0.00048618
36000 0.007000301 33900 0.000109588 33700 0.000266926 33300
0.00033385 35600 0.001195644 35100 0.000486061
36000 0.00700717 33900 0.000109605 33700 0.000267124 33300
0.000333928 35600 0.001197631 35100 0.000485945
36000 0.00701414 33900 0.000109603 33700 0.000267327 33300
0.000333983 35600 0.001199635 35100 0.000485828
36000 0.00702109 34000 0.000109603 33700 0.00026754 33300
0.000334053 35600 0.001201623 35100 0.000485735
36000 0.007028092 34000 0.000109602 33700 0.000267742 33300
0.000334074 35600 0.001203626 35100 0.000485585
36000 0.007035035 34000 0.00010959 33800 0.000267949 33300
0.000334096 35600 0.001205628 35100 0.000485474
36000 0.007042022 34000 0.000109565 33800 0.000268144 33300
0.000334068 35600 0.001207654 35100 0.000485372
36000 0.007048976 34000 0.000109548 33800 0.000268348 33300
0.000334037 35600 0.00120967 35100 0.000485236
36000 0.007056006 34000 0.000109529 33800 0.000268556 33300
0.000334008 35600 0.001211698 35100 0.000485125
36000 0.007063051 34000 0.000109502 33800 0.000268755 33400
0.000333972 35600 0.001213729 35100 0.00048503

36000 0.007070139 34000 0.000109485 33800 0.000268977 33400
0.000333953 35600 0.001215777 35100 0.00048488
36000 0.007077209 34000 0.000109458 33800 0.000269179 33400
0.000333952 35600 0.001217814 35100 0.000484771
36000 0.007084283 34000 0.000109441 33800 0.000269379 33400
0.000333939 35600 0.001219864 35100 0.000484649
36000 0.007091418 34000 0.000109416 33800 0.000269587 33400
0.000333946 35600 0.001221912 35100 0.000484531
36000 0.007098538 34000 0.0001094 33800 0.000269807 33400
0.00033397 35600 0.001223951 35100 0.000484419
36000 0.007105606 34000 0.000109381 33800 0.00027002 33400
0.000333972 35600 0.001226002 35100 0.000484301
36000 0.007112746 34000 0.000109357 33800 0.000270226 33400
0.000333984 35600 0.001228061 35100 0.000484189
36000 0.007119892 34000 0.000109347 33800 0.00027044 33400
0.000334005 35600 0.001230119 35100 0.000484066
36000 0.007127082 34000 0.000109333 33800 0.000270648 33400
0.000334028 35600 0.00123218 35100 0.000483949
36000 0.00713434 34000 0.000109316 33800 0.000270873 33400
0.000334039 35600 0.001234246 35100 0.000483833
36000 0.007141496 34000 0.000109315 33800 0.000271102 33400
0.000334066 35700 0.001236315 35100 0.000483729
36000 0.007148707 34000 0.000109319 33800 0.000271315 33400
0.000334101 35700 0.001238388 35100 0.000483602
36000 0.007155887 34000 0.000109315 33800 0.000271547 33400
0.000334132 35700 0.001240457 35100 0.000483521
36000 0.007163117 34000 0.000109326 33800 0.000271766 33400
0.000334159 35700 0.001242528 35100 0.000483377
36000 0.007170309 34000 0.000109326 33800 0.000271992 33400
0.000334151 35700 0.001244616 35100 0.000483271
36000 0.007177505 34000 0.000109323 33800 0.000272209 33400
0.000334131 35700 0.00124671 35100 0.000483136
36000 0.007184705 34000 0.000109325 33800 0.00027244 33400
0.000334105 35700 0.001248804 35100 0.000482992
36000 0.007191959 34000 0.000109315 33800 0.000272663 33400
0.000334099 35700 0.001250906 35100 0.000482883
36000 0.007199181 34000 0.000109304 33800 0.000272889 33400
0.00033408 35700 0.001253013 35100 0.000482748
36000 0.007206453 34000 0.000109296 33800 0.000273115 33400
0.000334086 35700 0.001255139 35100 0.000482609
36000 0.007213694 34000 0.000109282 33800 0.000273345 33400
0.00033411 35700 0.001257272 35100 0.000482491
36000 0.007220997 34000 0.000109278 33800 0.000273584 33400
0.000334126 35700 0.001259414 35100 0.000482377
36000 0.007228291 34000 0.000109274 33800 0.000273807 33400
0.000334159 35700 0.001261562 35100 0.000482254
36000 0.007235582 34000 0.000109261 33800 0.000274039 33400
0.000334211 35700 0.001263713 35100 0.000482114
36000 0.007242798 34000 0.000109268 33800 0.000274268 33400
0.000334248 35700 0.001265867 35100 0.000481966
36000 0.007250119 34000 0.000109264 33800 0.0002745 33400
0.000334288 35700 0.001268047 35100 0.000481854
36000 0.00725745 34000 0.00010927 33800 0.000274741 33400
0.000334324 35700 0.001270222 35100 0.000481733
36000 0.007264825 34000 0.000109279 33800 0.000274975 33400
0.000334357 35700 0.001272401 35100 0.000481607
36000 0.007272205 34000 0.000109279 33800 0.000275216 33400
0.000334379 35700 0.001274573 35100 0.000481471
36000 0.007279575 34000 0.000109282 33800 0.000275465 33400
0.000334393 35700 0.001276765 35100 0.000481358
36000 0.00728697 34000 0.000109278 33800 0.000275709 33400
0.000334409 35700 0.001278947 35100 0.000481217
36000 0.007294368 34000 0.000109276 33800 0.000275958 33400
0.000334419 35700 0.001281141 35100 0.000481088
36000 0.007301803 34000 0.000109282 33800 0.000276209 33400
0.000334426 35700 0.001283328 35100 0.000480961
36000 0.007309244 34000 0.000109281 33800 0.000276461 33400
0.000334422 35700 0.001285522 35100 0.000480833

36000 0.007316724 34000 0.00010927 33800 0.000276704 33400
0.00033441 35700 0.001287721 35100 0.000480693
36000 0.007324179 34000 0.000109277 33800 0.000276956 33400
0.000334413 35700 0.001289916 35100 0.000480554
36000 0.007331675 34000 0.000109272 33800 0.000277207 33400
0.000334428 35700 0.00129211 35100 0.000480438
36000 0.007339181 34000 0.000109262 33800 0.000277463 33400
0.000334444 35700 0.001294326 35100 0.000480335
36000 0.007346746 34000 0.000109244 33800 0.000277713 33400
0.000334457 35700 0.00129654 35100 0.00048019
36100 0.007354327 34000 0.000109214 33800 0.000277966 33400
0.000334504 35700 0.001298756 35100 0.000480048
36100 0.007361907 34000 0.00010919 33800 0.000278224 33400
0.000334519 35700 0.001300968 35100 0.000479942
36100 0.007369529 34000 0.000109163 33800 0.000278477 33400
0.000334554 35700 0.001303199 35100 0.000479817
36100 0.007377167 34000 0.000109129 33800 0.000278737 33400
0.000334581 35700 0.001305423 35100 0.000479689
36100 0.007384802 34000 0.000109105 33800 0.000279005 33400
0.000334587 35700 0.001307661 35100 0.00047957
36100 0.007392484 34000 0.000109078 33800 0.000279264 33400
0.000334592 35700 0.001309923 35100 0.000479457
36100 0.007400199 34000 0.000109069 33800 0.000279523 33400
0.000334596 35700 0.001312188 35100 0.000479334
36100 0.007407908 34000 0.000109056 33800 0.000279791 33400
0.000334551 35700 0.001314467 35100 0.000479226
36100 0.007415672 34000 0.000109028 33800 0.000280056 33400
0.000334494 35700 0.001316747 35100 0.000479104
36100 0.007423416 34000 0.000109006 33800 0.000280334 33400
0.000334442 35700 0.001319038 35200 0.000478998
36100 0.0074312 34000 0.000108984 33800 0.000280609 33400
0.000334363 35700 0.001321341 35200 0.00047888
36100 0.007438977 34000 0.000108972 33800 0.000280878 33400
0.000334315 35700 0.001323657 35200 0.000478791
36100 0.007446804 34000 0.000108962 33800 0.000281147 33400
0.000334278 35700 0.001325968 35200 0.000478662
36100 0.007454606 34000 0.000108951 33800 0.00028143 33400
0.000334246 35700 0.001328299 35200 0.000478558
36100 0.007462473 34000 0.00010894 33800 0.000281706 33400
0.000334234 35700 0.001330634 35200 0.000478435
36100 0.007470272 34000 0.000108936 33800 0.000281986 33400
0.00033423 35700 0.001332979 35200 0.000478338
36100 0.007478192 34000 0.000108934 33800 0.000282257 33400
0.000334264 35700 0.001335324 35200 0.000478249
36100 0.00748607 34000 0.000108921 33800 0.000282541 33400
0.000334294 35700 0.001337696 35200 0.000478153
36100 0.007493982 34100 0.000108908 33800 0.000282821 33400
0.000334319 35700 0.001340073 35200 0.000478044
36100 0.007501916 34100 0.000108892 33800 0.0002831 33400
0.000334329 35700 0.001342453 35200 0.000477945
36100 0.00750989 34100 0.000108886 33900 0.000283375 33400
0.00033433 35700 0.001344836 35200 0.000477826
36100 0.007517867 34100 0.000108883 33900 0.00028367 33400
0.000334338 35700 0.001347238 35200 0.000477727
36100 0.007525851 34100 0.000108876 33900 0.000283956 33400
0.000334328 35700 0.001349625 35200 0.000477616
36100 0.007533797 34100 0.000108874 33900 0.000284247 33400
0.000334329 35700 0.00135202 35200 0.000477501
36100 0.007541834 34100 0.00010886 33900 0.000284538 33500
0.000334308 35700 0.001354433 35200 0.000477381
36100 0.007549887 34100 0.000108858 33900 0.000284842 33500
0.000334309 35700 0.001356847 35200 0.000477306
36100 0.007557965 34100 0.00010886 33900 0.000285133 33500
0.000334283 35700 0.001359269 35200 0.00047721
36100 0.007566116 34100 0.000108856 33900 0.000285429 33500
0.000334242 35700 0.001361687 35200 0.000477089
36100 0.007574239 34100 0.000108851 33900 0.000285738 33500
0.000334197 35700 0.001364115 35200 0.000477013

36100 0.007582393 34100 0.000108855 33900 0.000286038 33500
0.000334144 35700 0.001366547 35200 0.000476882
36100 0.007590521 34100 0.000108863 33900 0.000286339 33500
0.000334089 35700 0.001368978 35200 0.000476771
36100 0.007598696 34100 0.000108866 33900 0.00028665 33500
0.00033404 35700 0.001371411 35200 0.000476681
36100 0.007606885 34100 0.000108871 33900 0.000286967 33500
0.00033399 35700 0.001373853 35200 0.000476562
36100 0.007615093 34100 0.000108871 33900 0.000287296 33500
0.000333978 35700 0.001376293 35200 0.000476473
36100 0.007623286 34100 0.000108876 33900 0.000287612 33500
0.000333963 35700 0.001378735 35200 0.000476387
36100 0.007631553 34100 0.000108875 33900 0.000287919 33500
0.000333979 35800 0.001381178 35200 0.000476292
36100 0.007639802 34100 0.000108866 33900 0.000288235 33500
0.000333985 35800 0.00138363 35200 0.000476184
36100 0.00764809 34100 0.000108846 33900 0.000288544 33500
0.000333985 35800 0.001386093 35200 0.000476108
36100 0.007656376 34100 0.000108819 33900 0.00028885 33500
0.000333964 35800 0.001388552 35200 0.000475993
36100 0.007664714 34100 0.000108793 33900 0.000289171 33500
0.000333921 35800 0.001391011 35200 0.000475913
36100 0.007673052 34100 0.000108767 33900 0.000289485 33500
0.000333876 35800 0.00139349 35200 0.000475772
36100 0.007681398 34100 0.000108741 33900 0.000289806 33500
0.000333826 35800 0.001395955 35200 0.000475681
36100 0.007689739 34100 0.000108723 33900 0.000290129 33500
0.000333771 35800 0.001398423 35200 0.000475598
36100 0.007698112 34100 0.000108702 33900 0.000290446 33500
0.000333722 35800 0.00140092 35200 0.000475484
36100 0.007706523 34100 0.000108704 33900 0.000290777 33500
0.000333697 35800 0.001403421 35200 0.000475411
36100 0.007714957 34100 0.000108708 33900 0.000291094 33500
0.000333679 35800 0.001405927 35200 0.000475328
36100 0.007723441 34100 0.000108708 33900 0.000291423 33500
0.00033369 35800 0.00140844 35200 0.000475246
36100 0.007731887 34100 0.000108707 33900 0.000291749 33500
0.000333711 35800 0.001410973 35200 0.000475163
36100 0.007740365 34100 0.000108699 33900 0.000292085 33500
0.00033374 35800 0.001413507 35200 0.000475042
36100 0.007748848 34100 0.000108701 33900 0.000292413 33500
0.000333772 35800 0.001416051 35200 0.000474957
36100 0.007757369 34100 0.000108697 33900 0.000292758 33500
0.000333801 35800 0.001418608 35200 0.000474884
36100 0.007765892 34100 0.000108691 33900 0.000293095 33500
0.000333795 35800 0.001421175 35200 0.000474738
36100 0.007774412 34100 0.000108678 33900 0.000293438 33500
0.000333781 35800 0.001423752 35200 0.000474647
36100 0.007782931 34100 0.000108672 33900 0.00029378 33500
0.000333725 35800 0.001426333 35200 0.000474573
36100 0.007791539 34100 0.000108667 33900 0.000294119 33500
0.000333666 35800 0.001428916 35200 0.000474479
36100 0.007800114 34100 0.000108656 33900 0.000294459 33500
0.000333597 35800 0.001431517 35200 0.000474356
36100 0.007808759 34100 0.000108653 33900 0.000294797 33500
0.00033351 35800 0.001434137 35200 0.000474284
36100 0.007817395 34100 0.000108639 33900 0.000295136 33500
0.000333437 35800 0.001436752 35200 0.00047417
36100 0.007826075 34100 0.000108638 33900 0.000295483 33500
0.00033337 35800 0.001439369 35200 0.000474094
36100 0.007834798 34100 0.000108636 33900 0.000295833 33500
0.000333318 35800 0.00144201 35200 0.000474
36100 0.007843524 34100 0.000108628 33900 0.000296184 33500
0.000333275 35800 0.001444643 35200 0.000473898
36100 0.0078522 34100 0.000108622 33900 0.000296536 33500
0.00033326 35800 0.001447272 35200 0.000473823
36100 0.007860959 34100 0.000108605 33900 0.000296894 33500
0.000333247 35800 0.001449923 35200 0.000473678

36100 0.00786976 34100 0.000108601 33900 0.000297254 33500
0.000333266 35800 0.001452577 35200 0.000473606
36200 0.007878605 34100 0.000108586 33900 0.000297618 33500
0.00033328 35800 0.001455218 35200 0.000473482
36200 0.00788751 34100 0.000108577 33900 0.000297981 33500
0.000333258 35800 0.001457865 35200 0.000473421
36200 0.007896352 34100 0.00010857 33900 0.000298348 33500
0.000333277 35800 0.001460525 35200 0.000473287
36200 0.00790527 34100 0.000108559 33900 0.000298722 33500
0.000333286 35800 0.001463182 35200 0.00047317
36200 0.0079142 34100 0.000108554 33900 0.000299089 33500
0.000333306 35800 0.001465843 35200 0.000473096
36200 0.007923125 34100 0.000108553 33900 0.000299467 33500
0.00033335 35800 0.001468492 35200 0.000472985
36200 0.007932114 34100 0.00010855 33900 0.000299839 33500
0.000333439 35800 0.001471159 35200 0.000472891
36200 0.007941104 34100 0.000108545 33900 0.00030024 33500
0.000333543 35800 0.001473822 35200 0.000472732
36200 0.007950089 34100 0.00010855 33900 0.000300616 33500
0.000333672 35800 0.001476481 35200 0.000472659
36200 0.007959138 34100 0.000108547 33900 0.000300995 33500
0.000333826 35800 0.001479135 35300 0.000472507
36200 0.007968202 34100 0.000108533 33900 0.000301381 33500
0.000333988 35800 0.001481805 35300 0.00047247
36200 0.007977312 34100 0.000108521 33900 0.00030177 33500
0.000334139 35800 0.001484495 35300 0.000472378
36200 0.007986436 34100 0.000108502 33900 0.000302147 33500
0.000334291 35800 0.001487166 35300 0.000472301
36200 0.007995589 34100 0.000108498 33900 0.000302541 33500
0.000334409 35800 0.001489851 35300 0.000472186
36200 0.008004826 34100 0.000108491 33900 0.000302933 33500
0.000334528 35800 0.001492552 35300 0.000472109
36200 0.008014035 34100 0.000108488 33900 0.00030333 33500
0.000334642 35800 0.001495243 35300 0.000471957
36200 0.008023244 34100 0.000108487 33900 0.000303723 33500
0.000334752 35800 0.001497953 35300 0.000471835
36200 0.00803251 34100 0.000108484 33900 0.000304128 33500
0.000334837 35800 0.001500682 35300 0.000471737
36200 0.008041836 34200 0.000108494 33900 0.000304535 33500
0.000334926 35800 0.001503404 35300 0.000471661
36200 0.008051203 34200 0.000108493 33900 0.000304934 33500
0.000334988 35800 0.001506146 35300 0.000471542
36200 0.008060637 34200 0.000108493 34000 0.000305345 33500
0.000335014 35800 0.00150889 35300 0.000471467
36200 0.00807001 34200 0.000108484 34000 0.000305757 33500
0.000335037 35800 0.001511655 35300 0.000471371
36200 0.008079478 34200 0.000108471 34000 0.000306166 33500
0.000335042 35800 0.001514428 35300 0.000471311
36200 0.008088942 34200 0.000108456 34000 0.000306591 33500
0.00033502 35800 0.001517206 35300 0.000471196
36200 0.00809846 34200 0.000108441 34000 0.000307013 33600
0.000334966 35800 0.001519995 35300 0.000471036
36200 0.008107956 34200 0.000108435 34000 0.000307437 33600
0.000334893 35800 0.001522803 35300 0.000471
36200 0.008117515 34200 0.000108415 34000 0.000307872 33600
0.000334782 35800 0.001525613 35300 0.000470902
36200 0.008127061 34200 0.00010841 34000 0.0003083 33600
0.000334673 35800 0.001528435 35300 0.000470762
36200 0.008136682 34200 0.000108394 34000 0.000308724 33600
0.00033457 35800 0.001531262 35300 0.000470688
36200 0.008146299 34200 0.000108387 34000 0.000309166 33600
0.000334475 35800 0.001534114 35300 0.000470555
36200 0.008156005 34200 0.000108383 34000 0.000309594 33600
0.000334377 35800 0.001536958 35300 0.000470473
36200 0.008165676 34200 0.000108383 34000 0.000310035 33600
0.000334335 35800 0.001539812 35300 0.000470357
36200 0.008175428 34200 0.000108373 34000 0.000310472 33600
0.000334296 35800 0.001542671 35300 0.000470297

36200 0.008185166 34200 0.000108368 34000 0.000310917 33600
0.000334268 35800 0.001545545 35300 0.000470179
36200 0.00819494 34200 0.00010836 34000 0.000311355 33600
0.000334258 35800 0.00154842 35300 0.00047012
36200 0.00820467 34200 0.000108357 34000 0.000311805 33600
0.000334258 35900 0.0015513 35300 0.000470021
36200 0.008214514 34200 0.000108351 34000 0.000312261 33600
0.000334247 35900 0.001554197 35300 0.000469942
36200 0.008224337 34200 0.000108348 34000 0.000312714 33600
0.000334236 35900 0.0015571 35300 0.000469902
36200 0.008234241 34200 0.000108339 34000 0.000313177 33600
0.000334233 35900 0.001560016 35300 0.000469797
36200 0.008244161 34200 0.000108323 34000 0.000313637 33600
0.000334222 35900 0.001562927 35300 0.000469663
36200 0.008254076 34200 0.000108311 34000 0.000314099 33600
0.000334232 35900 0.001565849 35300 0.000469566
36200 0.008264004 34200 0.00010829 34000 0.000314563 33600
0.000334252 35900 0.001568776 35300 0.000469468
36200 0.008273989 34200 0.000108269 34000 0.000315026 33600
0.000334282 35900 0.001571719 35300 0.000469366
36200 0.008283969 34200 0.000108263 34000 0.000315497 33600
0.000334335 35900 0.001574655 35300 0.000469289
36200 0.008294039 34200 0.000108257 34000 0.000315969 33600
0.00033439 35900 0.001577609 35300 0.000469173
36200 0.008304067 34200 0.000108257 34000 0.000316458 33600
0.000334438 35900 0.001580575 35300 0.000469144
36200 0.008314099 34200 0.000108261 34000 0.000316934 33600
0.000334515 35900 0.001583542 35300 0.000469071
36200 0.008324234 34200 0.000108274 34000 0.000317415 33600
0.000334599 35900 0.00158651 35300 0.000468952
36200 0.008334341 34200 0.000108286 34000 0.000317905 33600
0.000334689 35900 0.001589503 35300 0.00046887
36200 0.008344513 34200 0.000108298 34000 0.000318382 33600
0.000334747 35900 0.001592488 35300 0.000468771
36200 0.008354695 34200 0.000108314 34000 0.000318873 33600
0.000334802 35900 0.001595491 35300 0.000468726
36200 0.008364922 34200 0.000108319 34000 0.000319363 33600
0.000334815 35900 0.001598501 35300 0.000468591
36200 0.008375163 34200 0.000108325 34000 0.000319861 33600
0.000334816 35900 0.001601522 35300 0.000468513
36200 0.008385421 34200 0.000108328 34000 0.000320366 33600
0.000334806 35900 0.001604547 35300 0.000468416
36200 0.008395651 34200 0.000108316 34000 0.000320853 33600
0.000334788 35900 0.001607574 35300 0.000468338
36200 0.008405951 34200 0.000108325 34000 0.000321362 33600
0.000334778 35900 0.001610621 35300 0.000468197
36200 0.008416299 34200 0.000108322 34000 0.000321878 33600
0.00033479 35900 0.001613669 35300 0.00046814
36200 0.008426689 34200 0.000108324 34000 0.000322389 33600
0.00033482 35900 0.001616736 35300 0.000468076
36200 0.008437104 34200 0.000108317 34000 0.000322902 33600
0.000334886 35900 0.001619802 35300 0.000467949
36200 0.008447486 34200 0.000108323 34000 0.000323427 33600
0.000334983 35900 0.001622887 35300 0.000467847
36200 0.008457904 34200 0.000108324 34000 0.000323939 33600
0.000335088 35900 0.001625985 35300 0.000467736
36200 0.008468306 34200 0.000108327 34000 0.000324467 33600
0.000335208 35900 0.001629103 35300 0.000467654
36200 0.008478791 34200 0.000108347 34000 0.000324997 33600
0.000335335 35900 0.001632221 35300 0.000467585
36200 0.008489268 34200 0.000108357 34000 0.000325529 33600
0.000335408 35900 0.001635355 35300 0.000467462
36300 0.008499734 34200 0.000108357 34000 0.000326079 33600
0.000335463 35900 0.001638492 35300 0.000467392
36300 0.0085102 34200 0.000108358 34000 0.000326619 33600
0.000335511 35900 0.001641646 35300 0.00046728
36300 0.008520751 34200 0.000108354 34000 0.000327166 33600
0.000335518 35900 0.001644815 35300 0.000467167

36300 0.0085313 34200 0.000108355 34000 0.00032771 33600
0.000335523 35900 0.001648008 35300 0.000467059
36300 0.008541904 34200 0.000108349 34000 0.000328258 33600
0.000335501 35900 0.001651198 35300 0.00046695
36300 0.008552522 34200 0.000108341 34000 0.000328815 33600
0.00033549 35900 0.001654394 35300 0.000466871
36300 0.008563184 34200 0.000108341 34000 0.000329371 33600
0.000335464 35900 0.001657606 35300 0.000466779
36300 0.008573844 34200 0.00010833 34000 0.000329933 33600
0.00033546 35900 0.001660827 35300 0.000466667
36300 0.008584536 34200 0.000108323 34000 0.000330503 33600
0.000335453 35900 0.001664042 35300 0.000466586
36300 0.008595194 34200 0.000108324 34000 0.000331071 33600
0.000335466 35900 0.001667272 35400 0.000466476
36300 0.008605953 34200 0.000108321 34000 0.000331654 33600
0.000335457 35900 0.00167052 35400 0.000466365
36300 0.008616742 34200 0.000108326 34000 0.000332235 33600
0.00033548 35900 0.001673771 35400 0.000466263
36300 0.00862757 34200 0.000108329 34000 0.00033282 33600
0.0003355 35900 0.00167704 35400 0.000466181
36300 0.008638451 34200 0.000108329 34000 0.000333408 33600
0.000335554 35900 0.001680308 35400 0.000466069
36300 0.008649327 34200 0.000108331 34000 0.000334011 33600
0.000335636 35900 0.001683583 35400 0.00046598
36300 0.008660227 34200 0.000108338 34000 0.000334601 33600
0.000335737 35900 0.001686882 35400 0.000465868
36300 0.008671158 34200 0.000108323 34000 0.000335207 33600
0.000335866 35900 0.001690183 35400 0.000465745
36300 0.008682142 34200 0.000108327 34000 0.000335811 33600
0.00033601 35900 0.001693492 35400 0.000465654
36300 0.00869312 34300 0.000108316 34000 0.000336416 33600
0.000336126 35900 0.001696812 35400 0.000465531
36300 0.008704129 34300 0.00010831 34000 0.000337041 33600
0.000336226 35900 0.001700149 35400 0.000465429
36300 0.008715188 34300 0.000108305 34100 0.000337656 33600
0.000336281 35900 0.001703487 35400 0.000465294
36300 0.008726297 34300 0.000108297 34100 0.000338271 33600
0.000336327 35900 0.00170684 35400 0.000465174
36300 0.008737445 34300 0.000108304 34100 0.000338905 33600
0.000336317 35900 0.001710208 35400 0.000465063
36300 0.008748637 34300 0.000108305 34100 0.000339528 33600
0.000336288 35900 0.001713585 35400 0.000464954
36300 0.008759876 34300 0.000108309 34100 0.000340153 33700
0.000336232 35900 0.001716979 35400 0.000464842
36300 0.008771142 34300 0.000108313 34100 0.000340787 33700
0.00033615 35900 0.001720378 35400 0.00046474
36300 0.008782508 34300 0.000108312 34100 0.00034143 33700
0.000336095 35900 0.001723796 35400 0.000464607
36300 0.008793832 34300 0.00010831 34100 0.000342069 33700
0.000336047 35900 0.001727216 35400 0.000464494
36300 0.00880519 34300 0.000108314 34100 0.000342727 33700
0.000335992 35900 0.001730649 35400 0.000464383
36300 0.008816615 34300 0.000108314 34100 0.000343372 33700
0.00033597 35900 0.001734091 35400 0.000464259
36300 0.008828107 34300 0.000108318 34100 0.000344039 33700
0.000335972 35900 0.001737542 35400 0.000464155
36300 0.008839637 34300 0.000108335 34100 0.000344696 33700
0.000335996 35900 0.001741016 35400 0.000464034
36300 0.008851225 34300 0.000108335 34100 0.000345359 33700
0.000336075 35900 0.001744479 35400 0.000463931
36300 0.008862807 34300 0.000108357 34100 0.000346039 33700
0.000336181 35900 0.001747956 35400 0.000463839
36300 0.008874448 34300 0.000108363 34100 0.000346695 33700
0.000336306 35900 0.001751445 35400 0.000463681
36300 0.008886121 34300 0.000108366 34100 0.00034738 33700
0.000336482 36000 0.001754946 35400 0.000463583
36300 0.008897827 34300 0.000108371 34100 0.000348061 33700
0.000336657 36000 0.001758451 35400 0.000463444

36300 0.008909576 34300 0.000108369 34100 0.000348756 33700
0.000336824 36000 0.001761963 35400 0.000463351
36300 0.008921337 34300 0.000108354 34100 0.000349449 33700
0.000336981 36000 0.001765484 35400 0.000463219
36300 0.008933135 34300 0.000108353 34100 0.00035015 33700
0.00033713 36000 0.001769007 35400 0.000463125
36300 0.008944992 34300 0.000108339 34100 0.000350845 33700
0.000337245 36000 0.001772542 35400 0.000463021
36300 0.008956893 34300 0.000108334 34100 0.000351559 33700
0.000337354 36000 0.001776096 35400 0.000462912
36300 0.008968851 34300 0.00010832 34100 0.000352266 33700
0.000337405 36000 0.001779643 35400 0.000462799
36300 0.008980817 34300 0.000108318 34100 0.000352987 33700
0.000337454 36000 0.001783211 35400 0.00046268
36300 0.008992837 34300 0.000108311 34100 0.000353697 33700
0.000337448 36000 0.001786782 35400 0.000462596
36300 0.00900492 34300 0.000108311 34100 0.000354418 33700
0.00033746 36000 0.001790355 35400 0.000462483
36300 0.009017024 34300 0.000108303 34100 0.000355143 33700
0.00033749 36000 0.001793933 35400 0.000462389
36300 0.009029097 34300 0.0001083 34100 0.000355889 33700
0.000337517 36000 0.001797533 35400 0.000462286
36300 0.009041247 34300 0.000108293 34100 0.000356624 33700
0.000337562 36000 0.00180114 35400 0.00046217
36300 0.009053434 34300 0.000108304 34100 0.00035736 33700
0.000337616 36000 0.001804757 35400 0.000462064
36300 0.009065677 34300 0.00010831 34100 0.000358101 33700
0.000337663 36000 0.001808398 35400 0.000461972
36300 0.00907795 34300 0.000108312 34100 0.000358846 33700
0.000337695 36000 0.001812044 35400 0.000461882
36300 0.009090264 34300 0.000108316 34100 0.000359605 33700
0.000337722 36000 0.001815694 35400 0.000461802
36300 0.009102604 34300 0.00010832 34100 0.000360349 33700
0.000337737 36000 0.001819364 35400 0.000461697
36300 0.009114947 34300 0.000108308 34100 0.000361115 33700
0.000337716 36000 0.001823043 35400 0.000461602
36300 0.009127341 34300 0.000108316 34100 0.000361884 33700
0.000337702 36000 0.001826733 35400 0.000461519
36300 0.009139767 34300 0.000108311 34100 0.000362655 33700
0.000337658 36000 0.001830443 35400 0.000461412
36300 0.009152202 34300 0.000108297 34100 0.000363432 33700
0.000337627 36000 0.001834161 35400 0.000461314
36300 0.009164671 34300 0.000108292 34100 0.000364204 33700
0.000337608 36000 0.001837895 35400 0.000461243
36300 0.009177189 34300 0.000108274 34100 0.000364995 33700
0.0003376 36000 0.001841645 35400 0.000461153
36300 0.009189719 34300 0.00010826 34100 0.000365794 33700
0.000337609 36000 0.001845402 35400 0.000461076
36300 0.009202317 34300 0.000108239 34100 0.000366594 33700
0.000337605 36000 0.001849168 35400 0.000460973
36300 0.009214957 34300 0.000108232 34100 0.000367402 33700
0.000337628 36000 0.001852957 35400 0.000460922
36300 0.009227632 34300 0.000108212 34100 0.000368216 33700
0.000337624 36000 0.001856753 35400 0.000460851
36400 0.009240347 34300 0.000108209 34100 0.000369036 33700
0.000337604 36000 0.001860557 35400 0.000460756
36400 0.009253103 34300 0.000108195 34100 0.000369856 33700
0.000337614 36000 0.001864377 35400 0.000460674
36400 0.009265785 34300 0.000108187 34100 0.000370703 33700
0.000337615 36000 0.00186822 35400 0.000460599
36400 0.009278584 34300 0.000108172 34100 0.000371531 33700
0.000337604 36000 0.001872058 35400 0.000460516
36400 0.009291465 34300 0.00010817 34100 0.000372383 33700
0.000337591 36000 0.0018759 35400 0.000460441
36400 0.009304394 34300 0.000108158 34100 0.000373221 33700
0.000337589 36000 0.001879773 35400 0.000460351
36400 0.009317356 34300 0.000108146 34100 0.000374075 33700
0.000337579 36000 0.001883636 35400 0.000460269

36400 0.009330376 34300 0.000108142 34100 0.000374938 33700
0.000337597 36000 0.001887501 35400 0.000460204
36400 0.009343437 34300 0.00010814 34100 0.000375787 33700
0.000337607 36000 0.001891388 35400 0.00046012
36400 0.009356514 34300 0.000108136 34100 0.000376663 33700
0.000337644 36000 0.00189528 35500 0.000460045
36400 0.009369645 34300 0.000108145 34100 0.000377541 33700
0.000337701 36000 0.00189917 35500 0.000459966
36400 0.009382843 34300 0.000108143 34100 0.000378426 33700
0.000337781 36000 0.001903067 35500 0.000459892
36400 0.009396069 34300 0.000108138 34100 0.000379312 33700
0.000337878 36000 0.001906984 35500 0.000459814
36400 0.009409325 34300 0.000108142 34100 0.000380202 33700
0.000338003 36000 0.001910899 35500 0.000459726
36400 0.009422644 34300 0.000108145 34100 0.000381103 33700
0.000338154 36000 0.001914814 35500 0.000459661
36400 0.009436018 34300 0.000108144 34100 0.000382003 33700
0.00033831 36000 0.001918743 35500 0.000459578
36400 0.009449473 34300 0.000108145 34100 0.000382932 33700
0.000338446 36000 0.001922689 35500 0.000459497
36400 0.009462973 34300 0.000108149 34100 0.000383846 33700
0.000338588 36000 0.00192665 35500 0.000459429
36400 0.009476569 34400 0.000108147 34100 0.000384774 33700
0.000338685 36000 0.001930624 35500 0.000459357
36400 0.00949021 34400 0.000108155 34100 0.0003857 33700
0.000338757 36000 0.001934595 35500 0.00045927
36400 0.009503883 34400 0.000108156 34200 0.00038664 33700
0.000338836 36000 0.001938585 35500 0.000459188
36400 0.009517583 34400 0.000108164 34200 0.000387596 33700
0.000338878 36000 0.001942599 35500 0.000459114
36400 0.009531385 34400 0.000108165 34200 0.000388547 33700
0.000338927 36000 0.001946624 35500 0.000459032
36400 0.009545229 34400 0.000108182 34200 0.000389512 33700
0.000338972 36000 0.00195067 35500 0.000458939
36400 0.009559185 34400 0.000108187 34200 0.000390478 33800
0.000339032 36000 0.00195475 35500 0.000458855
36400 0.009573178 34400 0.000108183 34200 0.000391459 33800
0.000339074 36000 0.001958835 35500 0.000458786
36400 0.009587211 34400 0.000108187 34200 0.000392449 33800
0.000339164 36000 0.001962948 35500 0.000458697
36400 0.009601329 34400 0.000108203 34200 0.00039343 33800
0.000339228 36000 0.001967079 35500 0.000458609
36400 0.009615459 34400 0.000108203 34200 0.000394422 33800
0.000339312 36000 0.001971225 35500 0.00045854
36400 0.009629638 34400 0.000108221 34200 0.000395429 33800
0.000339382 36000 0.001975403 35500 0.000458466
36400 0.009643895 34400 0.000108225 34200 0.000396449 33800
0.000339447 36000 0.001979603 35500 0.000458379
36400 0.009658187 34400 0.000108223 34200 0.000397468 33800
0.000339524 36000 0.001983818 35500 0.000458299
36400 0.009672507 34400 0.000108226 34200 0.000398496 33800
0.000339627 36000 0.00198806 35500 0.000458212
36400 0.009686928 34400 0.000108217 34200 0.000399526 33800
0.000339741 36000 0.001992319 35500 0.000458119
36400 0.00970141 34400 0.00010821 34200 0.00040057 33800
0.000339888 36000 0.001996613 35500 0.000458028
36400 0.009715924 34400 0.000108201 34200 0.000401627 33800
0.000340039 36100 0.002000921 35500 0.000457948
36400 0.009730497 34400 0.0001082 34200 0.000402685 33800
0.000340197 36100 0.002005264 35500 0.000457849
36400 0.009745154 34400 0.000108188 34200 0.000403748 33800
0.000340353 36100 0.002009617 35500 0.000457763
36400 0.009759875 34400 0.000108189 34200 0.000404809 33800
0.000340487 36100 0.002013992 35500 0.000457679
36400 0.009774619 34400 0.000108185 34200 0.000405887 33800
0.000340622 36100 0.002018388 35500 0.000457571
36400 0.009789333 34400 0.000108197 34200 0.000406992 33800
0.000340739 36100 0.002022806 35500 0.000457471

36400 0.009804209 34400 0.000108192 34200 0.000408073 33800
0.00034083 36100 0.002027231 35500 0.000457381
36400 0.009819116 34400 0.0001082 34200 0.000409188 33800
0.000340924 36100 0.002031662 35500 0.000457295
36400 0.009834121 34400 0.000108217 34200 0.000410298 33800
0.000340989 36100 0.00203612 35500 0.000457219
36400 0.009849169 34400 0.000108216 34200 0.000411432 33800
0.000341079 36100 0.002040594 35500 0.000457134
36400 0.009864249 34400 0.000108225 34200 0.00041256 33800
0.000341158 36100 0.00204508 35500 0.000457062
36400 0.009879373 34400 0.000108231 34200 0.000413698 33800
0.000341236 36100 0.00204957 35500 0.000456967
36400 0.009894534 34400 0.000108224 34200 0.000414857 33800
0.000341337 36100 0.002054078 35500 0.00045688
36400 0.009909745 34400 0.000108244 34200 0.000416015 33800
0.000341434 36100 0.002058589 35500 0.000456789
36400 0.009925017 34400 0.00010825 34200 0.000417186 33800
0.000341494 36100 0.002063124 35500 0.000456699
36400 0.009940346 34400 0.00010826 34200 0.000418366 33800
0.000341558 36100 0.002067658 35500 0.000456624
36400 0.009955677 34400 0.00010828 34200 0.000419557 33800
0.000341614 36100 0.002072208 35500 0.000456515
36400 0.009971092 34400 0.000108281 34200 0.000420756 33800
0.000341661 36100 0.00207677 35500 0.000456436
36400 0.009986562 34400 0.000108282 34200 0.000421968 33800
0.000341731 36100 0.002081343 35500 0.00045635
36400 0.01000209 34400 0.000108261 34200 0.00042319 33800
0.000341802 36100 0.002085933 35500 0.000456263
36400 0.01001767 34400 0.000108252 34200 0.00042442 33800
0.000341883 36100 0.002090534 35500 0.000456197
36400 0.01003335 34400 0.000108227 34200 0.000425664 33800
0.000341966 36100 0.00209514 35500 0.000456114
36400 0.01004908 34400 0.000108212 34200 0.000426916 33800
0.000342075 36100 0.002099763 35500 0.000456024
36400 0.01006485 34400 0.000108186 34200 0.000428176 33800
0.000342229 36100 0.002104379 35500 0.00045595
36400 0.01008065 34400 0.00010817 34200 0.000429464 33800
0.00034239 36100 0.002109006 35500 0.000455868
36400 0.01009654 34400 0.000108152 34200 0.000430745 33800
0.000342562 36100 0.002113655 35500 0.000455778
36400 0.01011253 34400 0.000108147 34200 0.000432045 33800
0.000342731 36100 0.002118308 35500 0.000455688
36400 0.01012861 34400 0.000108148 34200 0.000433353 33800
0.00034288 36100 0.002122976 35500 0.000455618
36400 0.01014474 34400 0.000108148 34200 0.00043467 33800
0.000343049 36100 0.002127655 35500 0.000455535
36500 0.01016092 34400 0.00010816 34200 0.000436006 33800
0.000343166 36100 0.002132349 35500 0.000455442
36500 0.0101772 34400 0.00010816 34200 0.000437351 33800
0.000343265 36100 0.002137037 35500 0.000455325
36500 0.01019352 34400 0.000108165 34200 0.000438701 33800
0.00034334 36100 0.002141734 35500 0.000455264
36500 0.01020997 34400 0.000108173 34200 0.000440077 33800
0.000343399 36100 0.002146454 35500 0.000455156
36500 0.01022649 34400 0.000108178 34200 0.000441454 33800
0.000343435 36100 0.002151181 35500 0.000455043
36500 0.01024307 34400 0.000108187 34200 0.000442844 33800
0.000343473 36100 0.002155921 35500 0.000454948
36500 0.01025973 34400 0.000108198 34200 0.000444247 33800
0.000343494 36100 0.002160679 35500 0.000454872
36500 0.01027648 34400 0.000108217 34200 0.000445662 33800
0.000343548 36100 0.002165447 35500 0.000454785
36500 0.01029333 34400 0.000108234 34200 0.000447086 33800
0.00034357 36100 0.002170236 35500 0.000454706
36500 0.01031031 34400 0.000108237 34200 0.000448529 33800
0.000343589 36100 0.00217505 35600 0.000454584
36500 0.01032735 34400 0.000108254 34200 0.000449974 33800
0.000343638 36100 0.00217988 35600 0.000454499

36500 0.01034447 34400 0.000108256 34200 0.000451434 33800
0.000343636 36100 0.002184737 35600 0.000454384
36500 0.01036174 34400 0.000108265 34200 0.00045291 33800
0.000343643 36100 0.002189603 35600 0.000454299
36500 0.01037905 34400 0.000108246 34200 0.000454393 33800
0.000343652 36100 0.002194485 35600 0.000454195
36500 0.01039637 34400 0.000108226 34200 0.000455892 33800
0.000343667 36100 0.002199383 35600 0.0004541
36500 0.01041385 34400 0.000108203 34200 0.000457385 33800
0.000343702 36100 0.002204304 35600 0.000454006
36500 0.01043146 34400 0.000108181 34200 0.000458908 33800
0.000343721 36100 0.00220925 35600 0.000453921
36500 0.01044916 34400 0.000108163 34200 0.000460439 33800
0.000343768 36100 0.002214201 35600 0.000453839
36500 0.01046697 34500 0.000108148 34200 0.000461971 33800
0.000343827 36100 0.002219159 35600 0.00045374
36500 0.01048486 34500 0.00010814 34200 0.000463527 33800
0.000343914 36100 0.002224157 35600 0.000453671
36500 0.01050285 34500 0.000108133 34300 0.000465087 33800
0.000344012 36100 0.002229148 35600 0.000453599
36500 0.01052092 34500 0.000108126 34300 0.00046667 33800
0.000344146 36100 0.002234163 35600 0.000453492
36500 0.01053909 34500 0.000108129 34300 0.000468254 33800
0.0003443 36100 0.002239183 35600 0.000453404
36500 0.01055736 34500 0.000108137 34300 0.000469845 33800
0.000344432 36100 0.00224422 35600 0.000453329
36500 0.01057571 34500 0.000108144 34300 0.000471453 33900
0.000344558 36100 0.002249274 35600 0.000453207
36500 0.01059416 34500 0.000108153 34300 0.00047308 33900
0.000344731 36100 0.002254336 35600 0.00045311
36500 0.01061272 34500 0.000108152 34300 0.000474711 33900
0.000344885 36100 0.002259403 35600 0.000453025
36500 0.01063135 34500 0.000108155 34300 0.000476354 33900
0.000345056 36100 0.002264509 35600 0.000452927
36500 0.01065013 34500 0.000108135 34300 0.000478013 33900
0.000345244 36100 0.002269613 35600 0.00045287
36500 0.010669 34500 0.000108127 34300 0.00047968 33900
0.000345439 36100 0.002274741 35600 0.000452753
36500 0.01068799 34500 0.000108119 34300 0.000481367 33900
0.000345632 36100 0.002279874 35600 0.000452679
36500 0.01070707 34500 0.000108124 34300 0.000483073 33900
0.000345839 36100 0.00228502 35600 0.000452578
36500 0.01072624 34500 0.00010812 34300 0.000484771 33900
0.000346063 36100 0.002290181 35600 0.000452454
36500 0.01074546 34500 0.00010813 34300 0.000486508 33900
0.000346269 36100 0.002295347 35600 0.000452332
36500 0.01076487 34500 0.000108138 34300 0.00048823 33900
0.000346478 36100 0.00230055 35600 0.000452219
36500 0.01078437 34500 0.000108155 34300 0.00048999 33900
0.000346659 36200 0.002305758 35600 0.000452124
36500 0.01080403 34500 0.000108171 34300 0.000491751 33900
0.000346805 36200 0.002310993 35600 0.000452049
36500 0.01082378 34500 0.000108191 34300 0.000493523 33900
0.00034695 36200 0.002316249 35600 0.000451908
36500 0.0108436 34500 0.00010821 34300 0.000495317 33900
0.000347056 36200 0.002321524 35600 0.000451814
36500 0.01086355 34500 0.000108233 34300 0.000497123 33900
0.000347148 36200 0.002326814 35600 0.000451715
36500 0.01088362 34500 0.000108243 34300 0.000498954 33900
0.00034725 36200 0.002332115 35600 0.000451624
36500 0.01090378 34500 0.000108266 34300 0.00050079 33900
0.000347359 36200 0.002337426 35600 0.000451531
36500 0.01092403 34500 0.000108295 34300 0.00050264 33900
0.000347482 36200 0.002342769 35600 0.000451431
36500 0.01094441 34500 0.000108321 34300 0.000504504 33900
0.000347608 36200 0.002348136 35600 0.000451339
36500 0.01096493 34500 0.00010834 34300 0.000506396 33900
0.000347777 36200 0.002353505 35600 0.000451242

36500	0.01098556	34500	0.000108357	34300	0.000508303	33900
0.000348	36200	0.002358885	35600	0.000451195
36500	0.01100633	34500	0.000108376	34300	0.000510214	33900
0.000348233	36200	0.002364296	35600	0.000451108
36500	0.01102724	34500	0.00010838	34300	0.000512143	33900
0.00034845	36200	0.002369739	35600	0.000451054
36500	0.01104825	34500	0.000108383	34300	0.0005141	33900
0.000348688	36200	0.002375188	35600	0.000450978
36500	0.01106942	34500	0.000108388	34300	0.000516068	33900
0.000348872	36200	0.002380636	35600	0.000450933
36500	0.01109067	34500	0.000108401	34300	0.000518056	33900
0.000349042	36200	0.002386103	35600	0.000450866
36500	0.01111206	34500	0.000108402	34300	0.000520047	33900
0.000349162	36200	0.002391571	35600	0.000450795
36500	0.01113351	34500	0.000108404	34300	0.000522074	33900
0.000349295	36200	0.002397072	35600	0.0004507
36500	0.01115519	34500	0.000108399	34300	0.000524109	33900
0.000349397	36200	0.002402602	35600	0.000450604
36500	0.01117695	34500	0.000108386	34300	0.000526175	33900
0.00034946	36200	0.002408143	35600	0.000450528
36500	0.01119889	34500	0.000108374	34300	0.000528246	33900
0.000349541	36200	0.002413708	35600	0.000450437
36500	0.01122099	34500	0.00010836	34300	0.000530334	33900
0.000349609	36200	0.002419278	35600	0.000450389
36500	0.01124318	34500	0.000108349	34300	0.000532457	33900
0.000349685	36200	0.002424897	35600	0.000450313
36500	0.01126552	34500	0.00010833	34300	0.000534575	33900
0.000349785	36200	0.002430522	35600	0.000450226
36500	0.01128798	34500	0.000108313	34300	0.000536726	33900
0.000349904	36200	0.002436166	35600	0.000450165
36500	0.01131061	34500	0.000108318	34300	0.000538887	33900
0.000350032	36200	0.002441847	35600	0.000450076
36500	0.01133338	34500	0.000108314	34300	0.000541057	33900
0.000350194	36200	0.002447544	35600	0.000450011
36500	0.01135626	34500	0.000108323	34300	0.00054326	33900
0.000350349	36200	0.002453285	35600	0.000449934
36500	0.01137929	34500	0.000108335	34300	0.00054548	33900
0.000350484	36200	0.002459039	35600	0.00044985
36600	0.01140252	34500	0.000108344	34300	0.000547713	33900
0.000350598	36200	0.002464825	35600	0.00044979
36600	0.0114259	34500	0.000108364	34300	0.000549976	33900
0.000350721	36200	0.00247065	35600	0.000449718
36600	0.01144947	34500	0.000108371	34300	0.000552239	33900
0.00035083	36200	0.0024765	35600	0.000449661
36600	0.01147315	34500	0.000108377	34300	0.000554539	33900
0.00035094	36200	0.002482393	35600	0.000449602
36600	0.01149702	34500	0.00010839	34300	0.000556853	33900
0.000351002	36200	0.00248831	35600	0.00044955
36600	0.01152107	34500	0.000108401	34300	0.000559189	33900
0.000351067	36200	0.002494247	35600	0.000449495
36600	0.01154523	34500	0.000108404	34300	0.00056154	33900
0.000351119	36200	0.002500219	35600	0.000449438
36600	0.01156952	34500	0.000108414	34300	0.000563913	33900
0.000351166	36200	0.002506216	35600	0.000449383
36600	0.01159404	34500	0.000108419	34300	0.000566301	33900
0.000351199	36200	0.002512257	35600	0.000449313
36600	0.01161876	34500	0.000108424	34300	0.000568736	33900
0.000351238	36200	0.002518318	35700	0.00044925
36600	0.01164366	34500	0.000108429	34300	0.000571166	33900
0.000351277	36200	0.002524415	35700	0.000449194
36600	0.01166876	34500	0.000108433	34300	0.000573631	33900
0.000351343	36200	0.002530537	35700	0.000449121
36600	0.01169399	34500	0.000108435	34300	0.000576117	33900
0.000351438	36200	0.00253669	35700	0.000449074
36600	0.01171946	34500	0.000108449	34300	0.00057863	33900
0.000351546	36200	0.002542854	35700	0.000449004
36600	0.01174507	34500	0.000108455	34300	0.000581158	33900
0.000351677	36200	0.002549045	35700	0.000448952

36600 0.01177088 34500 0.000108478 34300 0.000583715 33900
0.000351833 36200 0.002555242 35700 0.000448889
36600 0.0117969 34500 0.000108497 34300 0.000586286 33900
0.000351985 36200 0.002561474 35700 0.000448825
36600 0.0118231 34500 0.000108512 34300 0.000588889 33900
0.000352127 36200 0.002567717 35700 0.00044877
36600 0.01184949 34600 0.000108539 34300 0.000591505 33900
0.000352314 36200 0.002573998 35700 0.000448698
36600 0.01187614 34600 0.00010855 34300 0.000594154 33900
0.000352448 36200 0.00258028 35700 0.000448638
36600 0.01190295 34600 0.000108564 34400 0.000596812 33900
0.000352594 36200 0.002586596 35700 0.00044857
36600 0.01193004 34600 0.000108575 34400 0.000599512 33900
0.000352729 36200 0.002592924 35700 0.00044851
36600 0.01195731 34600 0.000108588 34400 0.000602224 33900
0.000352838 36200 0.002599295 35700 0.000448443
36600 0.01198482 34600 0.000108591 34400 0.000604959 33900
0.000352926 36200 0.002605655 35700 0.000448381
36600 0.01201256 34600 0.000108593 34400 0.000607709 34000
0.000353038 36200 0.002612049 35700 0.000448325
36600 0.0120405 34600 0.000108579 34400 0.000610502 34000
0.000353142 36200 0.002618445 35700 0.000448258
36600 0.01206859 34600 0.000108582 34400 0.000613302 34000
0.000353232 36200 0.002624868 35700 0.000448199
36600 0.012097 34600 0.000108561 34400 0.000616127 34000
0.000353358 36200 0.002631331 35700 0.000448129
36600 0.01212565 34600 0.000108562 34400 0.000618986 34000
0.000353483 36200 0.002637804 35700 0.000448042
36600 0.01215455 34600 0.000108565 34400 0.000621851 34000
0.000353653 36200 0.002644304 35700 0.000447988
36600 0.0121837 34600 0.000108553 34400 0.000624758 34000
0.000353835 36200 0.002650822 35700 0.000447916
36600 0.01221304 34600 0.000108553 34400 0.000627676 34000
0.000354069 36200 0.002657375 35700 0.000447848
36600 0.01224265 34600 0.000108556 34400 0.000630614 34000
0.000354317 36200 0.002663932 35700 0.000447766
36600 0.01227246 34600 0.000108555 34400 0.000633582 34000
0.00035461 36200 0.002670537 35700 0.000447688
36600 0.01230256 34600 0.000108561 34400 0.000636568 34000
0.000354907 36200 0.002677169 35700 0.000447611
36600 0.01233295 34600 0.00010856 34400 0.000639574 34000
0.000355189 36300 0.002683808 35700 0.000447529
36600 0.01236355 34600 0.000108565 34400 0.000642618 34000
0.000355441 36300 0.002690486 35700 0.000447459
36600 0.01239439 34600 0.000108566 34400 0.000645682 34000
0.000355694 36300 0.002697196 35700 0.000447379
36600 0.01242554 34600 0.000108553 34400 0.000648773 34000
0.000355884 36300 0.002703931 35700 0.000447296
36600 0.01245695 34600 0.00010855 34400 0.000651884 34000
0.000356077 36300 0.002710689 35700 0.000447217
36600 0.01248867 34600 0.000108538 34400 0.000655032 34000
0.000356207 36300 0.002717494 35700 0.000447125
36600 0.01252064 34600 0.000108527 34400 0.000658183 34000
0.000356311 36300 0.002724305 35700 0.000447057
36600 0.01255293 34600 0.000108516 34400 0.000661377 34000
0.000356375 36300 0.002731142 35700 0.00044698
36600 0.01258551 34600 0.00010851 34400 0.000664598 34000
0.000356466 36300 0.002737994 35700 0.000446891
36600 0.01261831 34600 0.000108501 34400 0.000667838 34000
0.000356508 36300 0.002744858 35700 0.000446802
36600 0.01265138 34600 0.00010852 34400 0.000671108 34000
0.000356581 36300 0.00275176 35700 0.000446713
36600 0.01268481 34600 0.00010852 34400 0.000674405 34000
0.000356658 36300 0.002758676 35700 0.000446613
36600 0.0127186 34600 0.000108537 34400 0.000677715 34000
0.000356743 36300 0.002765607 35700 0.000446535
36600 0.01275266 34600 0.000108561 34400 0.000681068 34000
0.000356868 36300 0.002772561 35700 0.000446454

36600 0.01278707 34600 0.000108579 34400 0.000684433 34000
0.000357035 36300 0.002779539 35700 0.000446367
36600 0.01282174 34600 0.000108601 34400 0.000687831 34000
0.000357183 36300 0.002786533 35700 0.000446286
36600 0.01285678 34600 0.00010862 34400 0.000691253 34000
0.000357357 36300 0.002793549 35700 0.000446193
36600 0.01289211 34600 0.000108638 34400 0.000694704 34000
0.000357559 36300 0.002800567 35700 0.000446104
36600 0.01292777 34600 0.000108664 34400 0.000698177 34000
0.000357743 36300 0.002807613 35700 0.000446014
36600 0.01296381 34600 0.000108679 34400 0.000701668 34000
0.000357919 36300 0.002814665 35700 0.00044592
36600 0.01300021 34600 0.000108698 34400 0.000705196 34000
0.000358078 36300 0.002821747 35700 0.000445847
36600 0.01303694 34600 0.000108718 34400 0.000708733 34000
0.000358264 36300 0.002828857 35700 0.000445736
36600 0.01307402 34600 0.000108714 34400 0.000712307 34000
0.000358431 36300 0.002835952 35700 0.000445647
36600 0.01311151 34600 0.000108718 34400 0.000715899 34000
0.000358615 36300 0.002843098 35700 0.000445553
36600 0.01314939 34600 0.000108712 34400 0.000719517 34000
0.000358775 36300 0.002850273 35700 0.000445464
36600 0.01318765 34600 0.000108702 34400 0.000723163 34000
0.000358912 36300 0.002857443 35700 0.000445384
36600 0.0132263 34600 0.000108692 34400 0.000726835 34000
0.000359062 36300 0.002864634 35700 0.000445299
36600 0.01326539 34600 0.000108675 34400 0.000730522 34000
0.000359228 36300 0.002871867 35700 0.000445213
36600 0.01330486 34600 0.000108656 34400 0.000734243 34000
0.000359398 36300 0.002879109 35700 0.000445134
36700 0.01334469 34600 0.000108655 34400 0.000737982 34000
0.000359563 36300 0.002886377 35700 0.000445041
36700 0.01338503 34600 0.000108638 34400 0.000741744 34000
0.000359808 36300 0.002893668 35700 0.000444961
36700 0.01342584 34600 0.000108637 34400 0.000745529 34000
0.000360008 36300 0.002901014 35700 0.000444875
36700 0.01346709 34600 0.00010865 34400 0.000749346 34000
0.000360228 36300 0.002908367 35700 0.000444797
36700 0.0135088 34600 0.00010866 34400 0.000753189 34000
0.000360496 36300 0.002915755 35700 0.000444735
36700 0.01355095 34600 0.00010868 34400 0.000757052 34000
0.000360751 36300 0.002923164 35700 0.000444652
36700 0.01359362 34600 0.000108701 34400 0.000760941 34000
0.000361012 36300 0.002930616 35700 0.000444567
36700 0.01363672 34600 0.000108721 34400 0.000764858 34000
0.000361315 36300 0.002938089 35700 0.000444482
36700 0.01368033 34600 0.000108753 34400 0.000768788 34000
0.000361587 36300 0.002945585 35700 0.000444395
36700 0.01372444 34600 0.000108774 34400 0.000772734 34000
0.000361885 36300 0.00295313 35800 0.000444314
36700 0.01376906 34600 0.000108802 34400 0.000776736 34000
0.000362128 36300 0.002960674 35800 0.000444249
36700 0.0138142 34600 0.000108821 34400 0.000780735 34000
0.000362406 36300 0.002968256 35800 0.000444164
36700 0.01385991 34600 0.000108823 34400 0.000784768 34000
0.000362627 36300 0.002975859 35800 0.000444084
36700 0.01390617 34600 0.000108828 34400 0.000788831 34000
0.000362858 36300 0.002983547 35800 0.000444015
36700 0.013953 34600 0.000108821 34400 0.000792912 34000
0.000363058 36300 0.00299122 35800 0.000443927
36700 0.01400043 34600 0.000108809 34400 0.000797011 34000
0.000363286 36300 0.002998947 35800 0.000443861
36700 0.01404845 34600 0.000108799 34400 0.000801133 34000
0.00036349 36300 0.003006688 35800 0.000443782
36700 0.01409704 34600 0.000108778 34400 0.000805289 34000
0.000363724 36300 0.003014498 35800 0.000443719
36700 0.01414624 34700 0.000108764 34400 0.000809453 34000
0.000363993 36300 0.003022305 35800 0.000443647

36700	0.014196	34700	0.000108752	34400	0.00081365	34000
0.000364216	36300	0.003030165	35800	0.000443592		
36700	0.01424645	34700	0.00010874	34500	0.000817861	34000
0.000364493	36300	0.003038032	35800	0.000443524		
36700	0.01429754	34700	0.000108725	34500	0.000822109	34000
0.000364746	36300	0.003045956	35800	0.000443458		
36700	0.01434939	34700	0.000108731	34500	0.000826369	34000
0.000365004	36300	0.003053902	35800	0.000443386		
36700	0.0144019	34700	0.000108732	34500	0.00083066	34000
0.000365244	36300	0.003061886	35800	0.000443342		
36700	0.01445506	34700	0.000108744	34500	0.000834983	34100
0.00036549	36300	0.003069905	35800	0.000443262		
36700	0.01450898	34700	0.000108757	34500	0.000839317	34100
0.000365714	36300	0.003077967	35800	0.000443202		
36700	0.0145636	34700	0.000108779	34500	0.000843686	34100
0.000365976	36300	0.00308601	35800	0.00044313		
36700	0.01461896	34700	0.000108813	34500	0.000848064	34100
0.000366229	36300	0.003094107	35800	0.000443075		
36700	0.01467509	34700	0.000108838	34500	0.000852471	34100
0.000366507	36300	0.003102215	35800	0.000443012		
36700	0.01473198	34700	0.000108876	34500	0.000856925	34100
0.000366765	36300	0.003110373	35800	0.000442933		
36700	0.01478966	34700	0.000108905	34500	0.000861402	34100
0.000367058	36300	0.003118552	35800	0.000442863		
36700	0.0148482	34700	0.000108924	34500	0.000865902	34100
0.000367327	36300	0.003126775	35800	0.000442814		
36700	0.01490756	34700	0.00010894	34500	0.000870433	34100
0.000367619	36300	0.003135014	35800	0.000442751		
36700	0.01496784	34700	0.000108958	34500	0.000875004	34100
0.000367885	36300	0.003143316	35800	0.000442672		
36700	0.01502898	34700	0.000108967	34500	0.000879596	34100
0.000368156	36300	0.003151626	35800	0.00044262		
36700	0.01509103	34700	0.000108966	34500	0.000884237	34100
0.000368422	36400	0.00315998	35800	0.000442564		
36700	0.01515403	34700	0.000108961	34500	0.000888905	34100
0.00036868	36400	0.003168358	35800	0.000442498		
36700	0.0152179	34700	0.000108965	34500	0.000893602	34100
0.000368958	36400	0.003176763	35800	0.000442434		
36700	0.01528268	34700	0.00010897	34500	0.000898347	34100
0.000369208	36400	0.003185242	35800	0.000442388		
36700	0.01534855	34700	0.000108964	34500	0.000903118	34100
0.000369483	36400	0.003193727	35800	0.000442296		
36700	0.01541539	34700	0.000108961	34500	0.000907962	34100
0.000369727	36400	0.003202255	35800	0.000442233		
36700	0.01548328	34700	0.000108958	34500	0.000912837	34100
0.000369961	36400	0.003210833	35800	0.000442166		
36700	0.01555225	34700	0.000108942	34500	0.000917761	34100
0.000370206	36400	0.003219431	35800	0.000442109		
36700	0.01562224	34700	0.000108933	34500	0.000922739	34100
0.000370419	36400	0.003228092	35800	0.000442054		
36700	0.01569338	34700	0.000108927	34500	0.000927763	34100
0.00037062	36400	0.003236764	35800	0.000441981		
36700	0.0157656	34700	0.000108928	34500	0.00093284	34100
0.000370837	36400	0.003245486	35800	0.000441929		
36700	0.01583899	34700	0.00010894	34500	0.000937974	34100
0.000371059	36400	0.003254252	35800	0.000441864		
36700	0.01591356	34700	0.000108942	34500	0.000943171	34100
0.000371307	36400	0.003263061	35800	0.000441792		
36700	0.01598936	34700	0.000108955	34500	0.000948442	34100
0.000371563	36400	0.003271905	35800	0.000441724		
36700	0.01606638	34700	0.000108965	34500	0.000953769	34100
0.000371859	36400	0.003280797	35800	0.000441635		
36700	0.01614473	34700	0.000108974	34500	0.000959165	34100
0.000372153	36400	0.003289741	35800	0.000441572		
36700	0.0162244	34700	0.000108984	34500	0.000964642	34100
0.000372504	36400	0.003298736	35800	0.000441499		
36700	0.01630542	34700	0.000108997	34500	0.000970204	34100
0.000372851	36400	0.003307765	35800	0.000441432		

36700 0.01638787 34700 0.000109001 34500 0.000975841 34100
0.000373246 36400 0.003316854 35800 0.000441373
36700 0.01647173 34700 0.000108999 34500 0.000981569 34100
0.000373648 36400 0.003325985 35800 0.0004413
36700 0.01655706 34700 0.000108996 34500 0.000987384 34100
0.000374086 36400 0.003335139 35800 0.000441217
36700 0.01664383 34700 0.000108997 34500 0.000993296 34100
0.000374549 36400 0.003344355 35800 0.000441144
36700 0.01673209 34700 0.000109 34500 0.0009993 34100
0.000374991 36400 0.003353626 35800 0.000441069
36700 0.01682204 34700 0.000108995 34500 0.001005424 34100
0.000375441 36400 0.003362934 35800 0.00044098
36700 0.01691361 34700 0.000108988 34500 0.001011664 34100
0.00037583 36400 0.003372274 35800 0.000440893
36700 0.01700687 34700 0.000108994 34500 0.001017996 34100
0.000376224 36400 0.003381676 35800 0.000440815
36700 0.01710189 34700 0.000108974 34500 0.001024465 34100
0.00037658 36400 0.0033911 35800 0.000440749
36700 0.01719864 34700 0.000108966 34500 0.001031043 34100
0.000376903 36400 0.003400593 35800 0.000440661
36700 0.0172972 34700 0.00010896 34500 0.001037757 34100
0.000377193 36400 0.003410105 35800 0.000440584
36800 0.0173976 34700 0.000108958 34500 0.001044605 34100
0.000377531 36400 0.003419653 35800 0.000440495
36800 0.01749994 34700 0.000108965 34500 0.001051582 34100
0.000377835 36400 0.003429239 35800 0.0004404
36800 0.01760425 34700 0.000108966 34500 0.001058694 34100
0.000378138 36400 0.003438872 35800 0.000440308
36800 0.01771056 34700 0.000108984 34500 0.001065975 34100
0.000378458 36400 0.003448521 35800 0.000440223
36800 0.01781894 34700 0.00010902 34500 0.001073382 34100
0.000378789 36400 0.003458224 35800 0.00044012
36800 0.01792947 34700 0.000109052 34500 0.001080938 34100
0.00037911 36400 0.003467945 35800 0.000440034
36800 0.01804222 34700 0.000109088 34500 0.001088672 34100
0.000379451 36400 0.003477712 35800 0.000439935
36800 0.01815724 34700 0.000109124 34500 0.001096549 34100
0.000379737 36400 0.003487505 35800 0.000439849
36800 0.01827464 34700 0.000109162 34500 0.001104578 34100
0.00038005 36400 0.00349734 35800 0.000439763
36800 0.01839443 34700 0.000109192 34500 0.001112794 34100
0.000380328 36400 0.003507199 35900 0.000439662
36800 0.0185167 34700 0.000109218 34500 0.001121168 34100
0.000380588 36400 0.003517078 35900 0.000439576
36800 0.01864149 34700 0.000109232 34500 0.001129706 34100
0.000380841 36400 0.003526975 35900 0.000439493
36800 0.01876882 34700 0.000109255 34500 0.001138418 34100
0.00038107 36400 0.003536926 35900 0.000439401
36800 0.01889897 34700 0.000109259 34500 0.001147308 34100
0.000381315 36400 0.003546886 35900 0.000439304
36800 0.01903186 34700 0.000109261 34500 0.001156368 34100
0.000381556 36400 0.003556883 35900 0.000439232
36800 0.01916762 34700 0.000109278 34500 0.001165606 34100
0.000381856 36400 0.003566909 35900 0.000439133
36800 0.01930637 34700 0.00010928 34500 0.00117501 34100
0.000382156 36400 0.00357696 35900 0.000439062
36800 0.01944814 34700 0.000109278 34500 0.001184587 34100
0.000382512 36400 0.003587051 35900 0.000438976
36800 0.01959308 34800 0.000109282 34500 0.001194346 34100
0.000382861 36400 0.003597153 35900 0.00043889
36800 0.01974121 34800 0.000109291 34500 0.00120427 34100
0.000383326 36400 0.003607288 35900 0.000438807
36800 0.01989272 34800 0.000109289 34600 0.001214369 34100
0.000383788 36400 0.003617463 35900 0.000438735
36800 0.02004766 34800 0.000109295 34600 0.001224627 34100
0.000384286 36400 0.003627671 35900 0.000438648
36800 0.0202061 34800 0.000109297 34600 0.001235062 34100
0.000384712 36400 0.003637906 35900 0.000438579

36800 0.02036824 34800 0.000109306 34600 0.001245657 34100
0.00038516 36400 0.003648194 35900 0.000438494
36800 0.02053417 34800 0.000109311 34600 0.001256413 34200
0.000385571 36400 0.003658508 35900 0.000438423
36800 0.02070405 34800 0.000109318 34600 0.001267325 34200
0.000385931 36400 0.003668868 35900 0.000438348
36800 0.02087798 34800 0.000109313 34600 0.001278393 34200
0.00038629 36400 0.003679281 35900 0.000438275
36800 0.02105607 34800 0.000109317 34600 0.001289607 34200
0.000386647 36400 0.003689749 35900 0.000438221
36800 0.02123853 34800 0.000109321 34600 0.001300958 34200
0.000387043 36400 0.003700264 35900 0.000438134
36800 0.02142543 34800 0.000109329 34600 0.00131246 34200
0.000387383 36400 0.003710826 35900 0.000438068
36800 0.0216169 34800 0.000109334 34600 0.001324097 34200
0.000387834 36400 0.003721432 35900 0.000437992
36800 0.02181307 34800 0.000109339 34600 0.00133585 34200
0.000388263 36400 0.003732112 35900 0.000437919
36800 0.02201428 34800 0.000109345 34600 0.001347742 34200
0.000388709 36400 0.003742838 35900 0.000437848
36800 0.02222052 34800 0.000109359 34600 0.001359751 34200
0.000389174 36400 0.003753624 35900 0.000437783
36800 0.02243208 34800 0.000109366 34600 0.001371864 34200
0.000389612 36400 0.003764471 35900 0.000437723
36800 0.02264911 34800 0.000109374 34600 0.001384086 34200
0.000390089 36500 0.003775373 35900 0.00043766
36800 0.02287171 34800 0.000109378 34600 0.001396414 34200
0.000390556 36500 0.003786345 35900 0.000437597
36800 0.0231002 34800 0.000109385 34600 0.001408825 34200
0.000390987 36500 0.003797353 35900 0.00043753
36800 0.0233347 34800 0.000109401 34600 0.001421316 34200
0.000391384 36500 0.003808438 35900 0.000437454
36800 0.02357544 34800 0.000109415 34600 0.001433884 34200
0.000391724 36500 0.003819575 35900 0.000437383
36800 0.02382265 34800 0.000109433 34600 0.001446509 34200
0.00039211 36500 0.003830771 35900 0.000437337
36800 0.02407654 34800 0.000109445 34600 0.001459209 34200
0.000392449 36500 0.00384203 35900 0.000437279
36800 0.02433736 34800 0.000109463 34600 0.001471939 34200
0.000392854 36500 0.003853358 35900 0.000437216
36800 0.02460539 34800 0.000109474 34600 0.001484711 34200
0.000393346 36500 0.003864731 35900 0.000437175
36800 0.02488083 34800 0.000109484 34600 0.00149752 34200
0.000393859 36500 0.003876186 35900 0.000437099
36800 0.025164 34800 0.000109477 34600 0.001510345 34200
0.000394365 36500 0.00388769 35900 0.000437043
36800 0.02545515 34800 0.000109477 34600 0.001523166 34200
0.00039493 36500 0.003899254 35900 0.000436975
36800 0.02575465 34800 0.000109476 34600 0.001535999 34200
0.000395508 36500 0.003910873 35900 0.000436926
36800 0.02606265 34800 0.000109478 34600 0.001548824 34200
0.000396117 36500 0.003922534 35900 0.000436849
36800 0.02637954 34800 0.000109467 34600 0.001561627 34200
0.000396694 36500 0.003934242 35900 0.0004368
36800 0.02670552 34800 0.000109471 34600 0.001574402 34200
0.000397271 36500 0.003946032 35900 0.000436733
36800 0.02704114 34800 0.00010947 34600 0.001587143 34200
0.000397851 36500 0.003957852 35900 0.000436676
36800 0.02738664 34800 0.000109483 34600 0.001599838 34200
0.000398409 36500 0.003969721 35900 0.000436601
36800 0.02774238 34800 0.000109488 34600 0.001612485 34200
0.000398956 36500 0.003981647 35900 0.000436541
36800 0.02810875 34800 0.000109494 34600 0.001625053 34200
0.000399496 36500 0.00399361 35900 0.000436503
36800 0.02848604 34800 0.000109497 34600 0.00163757 34200
0.000400049 36500 0.004005629 35900 0.000436451
36800 0.02887472 34800 0.000109509 34600 0.001649993 34200
0.000400593 36500 0.004017679 35900 0.000436393

36800 0.02927516 34800 0.000109517 34600 0.001662328 34200
0.000401163 36500 0.004029783 35900 0.000436324
36800 0.02968783 34800 0.000109535 34600 0.001674563 34200
0.000401734 36500 0.004041949 35900 0.000436287
36800 0.03011316 34800 0.000109542 34600 0.001686674 34200
0.000402292 36500 0.00405415 35900 0.000436241
36800 0.03055156 34800 0.000109569 34600 0.001698686 34200
0.000402874 36500 0.004066403 35900 0.000436183
36800 0.03100349 34800 0.000109592 34600 0.001710573 34200
0.000403463 36500 0.004078716 35900 0.00043612
36800 0.03146949 34800 0.000109606 34600 0.001722297 34200
0.000404001 36500 0.004091074 35900 0.000436077
36800 0.03194998 34800 0.000109639 34600 0.001733905 34200
0.000404569 36500 0.004103499 35900 0.000436035
36900 0.0324455 34800 0.000109648 34600 0.001745333 34200
0.000405094 36500 0.004115979 35900 0.000435993
36900 0.03295656 34800 0.000109675 34600 0.001756599 34200
0.000405643 36500 0.004128538 35900 0.00043595
36900 0.03348369 34800 0.000109699 34600 0.001767694 34200
0.000406197 36500 0.004141154 35900 0.000435898
36900 0.03402738 34800 0.000109722 34600 0.001778595 34200
0.000406769 36500 0.004153815 35900 0.000435853
36900 0.03458814 34800 0.000109732 34600 0.001789293 34200
0.000407379 36500 0.004166543 35900 0.000435808
36900 0.03516646 34800 0.000109755 34600 0.001799781 34200
0.000408024 36500 0.004179351 35900 0.00043575
36900 0.03576313 34800 0.000109767 34600 0.001810044 34200
0.000408689 36500 0.004192225 35900 0.000435711
36900 0.0363785 34800 0.000109781 34600 0.001820084 34200
0.000409366 36500 0.004205158 35900 0.000435643
36900 0.03701325 34800 0.000109798 34600 0.001829856 34200
0.00041005 36500 0.004218162 35900 0.000435597
36900 0.03766781 34800 0.000109806 34600 0.001839374 34200
0.000410744 36500 0.004231224 36000 0.000435561
36900 0.03834279 34800 0.000109801 34600 0.001848633 34200
0.000411445 36500 0.004244361 36000 0.000435518
36900 0.03903867 34800 0.000109791 34600 0.001857603 34200
0.000412136 36500 0.00425755 36000 0.000435466
36900 0.03975596 34800 0.000109786 34600 0.001866266 34200
0.000412817 36500 0.004270801 36000 0.000435412
36900 0.04049518 34800 0.000109775 34600 0.001874615 34200
0.000413476 36500 0.004284115 36000 0.000435377
36900 0.04125684 34800 0.000109766 34600 0.001882635 34200
0.000414122 36500 0.004297489 36000 0.000435319
36900 0.04204127 34800 0.000109769 34600 0.001890318 34200
0.00041477 36500 0.004310931 36000 0.000435276
36900 0.04284893 34800 0.000109774 34600 0.001897638 34200
0.000415405 36500 0.004324438 36000 0.000435227
36900 0.04368014 34800 0.000109797 34600 0.001904587 34200
0.000416025 36500 0.004338004 36000 0.00043518
36900 0.04453522 34900 0.000109806 34600 0.001911155 34200
0.000416703 36500 0.004351639 36000 0.000435121
36900 0.04541435 34900 0.000109822 34600 0.001917324 34200
0.000417404 36500 0.004365352 36000 0.000435069
36900 0.04631763 34900 0.000109831 34700 0.00192308 34200
0.000418139 36500 0.004379133 36000 0.000435013
36900 0.0472452 34900 0.000109849 34700 0.001928398 34200
0.000418871 36500 0.004392965 36000 0.000434945
36900 0.048197 34900 0.000109851 34700 0.001933287 34200
0.000419625 36500 0.004406863 36000 0.000434886
36900 0.04917275 34900 0.000109866 34700 0.001937713 34200
0.00042038 36500 0.004420823 36000 0.000434834
36900 0.05017219 34900 0.000109867 34700 0.00194166 34300
0.000421153 36500 0.004434856 36000 0.000434776
36900 0.05119513 34900 0.000109867 34700 0.001945129 34300
0.000421917 36500 0.004448958 36000 0.000434722
36900 0.05224076 34900 0.000109875 34700 0.001948096 34300
0.000422648 36500 0.004463126 36000 0.000434655

36900 0.05330842 34900 0.000109882 34700 0.001950536 34300
0.000423415 36500 0.004477353 36000 0.000434581
36900 0.0543972 34900 0.000109884 34700 0.001952439 34300
0.000424151 36500 0.004491653 36000 0.000434534
36900 0.05550598 34900 0.000109878 34700 0.00195381 34300
0.000424898 36500 0.004506019 36000 0.000434474
36900 0.05663339 34900 0.000109873 34700 0.001954605 34300
0.000425626 36500 0.004520425 36000 0.00043441
36900 0.05777796 34900 0.000109882 34700 0.001954816 34300
0.000426382 36500 0.004534908 36000 0.000434329
36900 0.058938 34900 0.00010989 34700 0.001954427 34300
0.000427134 36500 0.004549454 36000 0.000434284
36900 0.06011149 34900 0.000109896 34700 0.001953426 34300
0.000427909 36500 0.00456406 36000 0.000434212
36900 0.06129626 34900 0.000109925 34700 0.001951806 34300
0.000428708 36500 0.004578737 36000 0.000434146
36900 0.06248989 34900 0.000109941 34700 0.001949533 34300
0.000429556 36600 0.004593475 36000 0.000434069
36900 0.06368973 34900 0.000109975 34700 0.001946597 34300
0.000430404 36600 0.004608264 36000 0.000434
36900 0.06489294 34900 0.000110001 34700 0.001943005 34300
0.000431327 36600 0.00462313 36000 0.000433921
36900 0.06609642 34900 0.00011003 34700 0.001938723 34300
0.00043227 36600 0.00463806 36000 0.000433872
36900 0.06729686 34900 0.00011005 34700 0.001933754 34300
0.000433214 36600 0.004653069 36000 0.0004338
36900 0.06849074 34900 0.000110083 34700 0.001928081 34300
0.000434177 36600 0.004668136 36000 0.000433723
36900 0.06967451 34900 0.000110103 34700 0.001921698 34300
0.000435154 36600 0.004683265 36000 0.000433656
36900 0.07084421 34900 0.000110133 34700 0.001914585 34300
0.000436128 36600 0.004698468 36000 0.00043357
36900 0.07199597 34900 0.000110157 34700 0.001906747 34300
0.000437118 36600 0.004713753 36000 0.000433507
36900 0.07312588 34900 0.000110168 34700 0.00189817 34300
0.000438135 36600 0.004729115 36000 0.000433424
36900 0.07422964 34900 0.000110183 34700 0.001888859 34300
0.000439157 36600 0.004744546 36000 0.00043335
36900 0.07530316 34900 0.000110206 34700 0.0018788 34300
0.000440204 36600 0.004760049 36000 0.00043326
36900 0.07634222 34900 0.000110224 34700 0.001867978 34300
0.000441264 36600 0.004775627 36000 0.000433193
36900 0.07734255 34900 0.000110245 34700 0.00185642 34300
0.000442346 36600 0.004791284 36000 0.000433111
36900 0.07829989 34900 0.000110257 34700 0.001844118 34300
0.000443428 36600 0.004807002 36000 0.000433045
36900 0.0792101 34900 0.000110275 34700 0.001831058 34300
0.000444526 36600 0.004822802 36000 0.000432952
36900 0.08006909 34900 0.000110266 34700 0.001817262 34300
0.000445608 36600 0.004838677 36000 0.000432874
36900 0.08087278 34900 0.000110264 34700 0.001802727 34300
0.000446683 36600 0.004854627 36000 0.00043279
36900 0.08161725 34900 0.000110276 34700 0.00178747 34300
0.000447726 36600 0.004870648 36000 0.000432713
36900 0.08229876 34900 0.000110276 34700 0.001771492 34300
0.000448771 36600 0.004886746 36000 0.000432613
36900 0.08291369 34900 0.00011029 34700 0.001754804 34300
0.000449816 36600 0.004902911 36000 0.000432546
36900 0.08345856 34900 0.000110307 34700 0.00173743 34300
0.000450888 36600 0.004919157 36000 0.000432456
36900 0.08393018 34900 0.000110342 34700 0.001719384 34300
0.000451968 36600 0.004935489 36000 0.000432393
36900 0.08432563 34900 0.000110357 34700 0.001700693 34300
0.00045306 36600 0.004951889 36000 0.000432312
36900 0.08464228 34900 0.00011038 34700 0.001681355 34300
0.00045415 36600 0.004968363 36000 0.000432229
36900 0.0848778 34900 0.000110388 34700 0.001661428 34300
0.00045528 36600 0.004984907 36000 0.000432142

36900 0.08503016 34900 0.000110391 34700 0.00164092 34300
0.000456394 36600 0.005001521 36000 0.000432072
36900 0.08509797 34900 0.000110398 34700 0.00161984 34300
0.00045753 36600 0.005018239 36000 0.00043199
36900 0.08508039 34900 0.000110408 34700 0.001598256 34300
0.000458701 36600 0.005035032 36000 0.000431928
37000 0.08497671 34900 0.000110411 34700 0.001576185 34300
0.000459855 36600 0.005051907 36000 0.000431863
37000 0.084787 34900 0.000110419 34700 0.001553662 34300
0.000461036 36600 0.005068866 36000 0.000431773
37000 0.08451188 34900 0.000110437 34700 0.001530708 34300
0.00046222 36600 0.005085907 36000 0.00043171
37000 0.08415247 34900 0.000110453 34700 0.001507401 34300
0.000463423 36600 0.005103039 36000 0.00043164
37000 0.08371054 34900 0.000110444 34700 0.001483747 34300
0.000464636 36600 0.005120234 36000 0.000431572
37000 0.08318838 34900 0.000110453 34700 0.001459816 34300
0.000465895 36600 0.005137525 36000 0.000431485
37000 0.08258884 34900 0.000110452 34700 0.001435638 34300
0.000467176 36600 0.005154906 36000 0.000431427
37000 0.08191533 34900 0.000110452 34700 0.001411252 34300
0.000468494 36600 0.005172376 36000 0.000431351
37000 0.08117171 34900 0.000110459 34700 0.001386745 34300
0.000469817 36600 0.005189923 36000 0.000431281
37000 0.08036222 34900 0.000110466 34700 0.001362128 34300
0.000471224 36600 0.005207576 36100 0.000431202
37000 0.07949159 34900 0.000110482 34700 0.00133748 34300
0.000472664 36600 0.005225321 36100 0.000431144
37000 0.07856466 34900 0.000110494 34700 0.001312843 34300
0.00047415 36600 0.005243166 36100 0.000431072
37000 0.07758676 34900 0.000110526 34700 0.001288271 34300
0.000475659 36600 0.005261112 36100 0.000431009
37000 0.0765631 34900 0.000110544 34700 0.001263841 34300
0.000477191 36600 0.00527916 36100 0.00043094
37000 0.07549921 34900 0.000110573 34700 0.001239576 34300
0.000478709 36600 0.005297311 36100 0.000430878
37000 0.07440054 34900 0.000110593 34700 0.001215551 34300
0.000480234 36600 0.00531556 36100 0.000430814
37000 0.0732724 34900 0.000110625 34700 0.001191816 34300
0.000481762 36600 0.005333919 36100 0.000430751
37000 0.07212014 34900 0.000110645 34700 0.001168443 34300
0.000483302 36600 0.005352397 36100 0.0004307
37000 0.07094911 35000 0.000110674 34700 0.001145463 34300
0.000484876 36600 0.005370978 36100 0.000430639
37000 0.06976406 35000 0.000110688 34700 0.001122954 34300
0.00048643 36600 0.005389696 36100 0.000430585
37000 0.06856976 35000 0.000110717 34800 0.001100947 34300
0.000487995 36600 0.005408519 36100 0.000430527
37000 0.06737065 35000 0.000110724 34800 0.001079487 34300
0.000489598 36600 0.005427466 36100 0.000430471
37000 0.06617084 35000 0.000110736 34800 0.001058644 34300
0.000491203 36600 0.005446537 36100 0.000430409
37000 0.06497411 35000 0.000110737 34800 0.001038444 34300
0.000492836 36600 0.005465717 36100 0.000430361
37000 0.06378397 35000 0.00011073 34800 0.00101896 34400
0.000494479 36600 0.005485016 36100 0.000430305
37000 0.06260359 35000 0.000110725 34800 0.001000191 34400
0.000496168 36600 0.005504436 36100 0.00043025
37000 0.06143575 35000 0.000110717 34800 0.000982192 34400
0.000497864 36600 0.005523973 36100 0.000430182
37000 0.06028292 35000 0.00011072 34800 0.000965022 34400
0.000499576 36600 0.005543623 36100 0.000430132
37000 0.05914734 35000 0.000110716 34800 0.000948664 34400
0.000501319 36600 0.005563392 36100 0.00043007
37000 0.05803086 35000 0.000110709 34800 0.000933187 34400
0.000503064 36600 0.005583269 36100 0.000430016
37000 0.05693508 35000 0.000110715 34800 0.000918597 34400
0.000504833 36600 0.005603272 36100 0.00042995

37000 0.05586141 35000 0.000110716 34800 0.000904929 34400
0.000506607 36600 0.005623393 36100 0.000429902
37000 0.05481088 35000 0.000110727 34800 0.00089218 34400
0.000508366 36600 0.005643618 36100 0.000429856
37000 0.05378437 35000 0.000110745 34800 0.000880386 34400
0.000510117 36600 0.005663956 36100 0.000429794
37000 0.05278251 35000 0.000110772 34800 0.000869541 34400
0.000511878 36600 0.0056844 36100 0.000429748
37000 0.05180579 35000 0.000110797 34800 0.000859634 34400
0.000513652 36700 0.005704944 36100 0.000429695
37000 0.05085451 35000 0.000110834 34800 0.000850714 34400
0.000515426 36700 0.005725615 36100 0.000429646
37000 0.04992901 35000 0.00011087 34800 0.000842754 34400
0.000517239 36700 0.005746395 36100 0.000429594
37000 0.04902923 35000 0.000110901 34800 0.000835755 34400
0.00051905 36700 0.005767284 36100 0.000429541
37000 0.04815504 35000 0.000110945 34800 0.000829699 34400
0.000520899 36700 0.005788289 36100 0.000429484
37000 0.04730634 35000 0.000110973 34800 0.00082457 34400
0.000522774 36700 0.005809399 36100 0.000429427
37000 0.04648285 35000 0.000111008 34800 0.000820371 34400
0.000524648 36700 0.005830615 36100 0.000429372
37000 0.04568424 35000 0.000111034 34800 0.000817075 34400
0.000526557 36700 0.005851933 36100 0.000429319
37000 0.0449101 35000 0.000111059 34800 0.000814666 34400
0.000528495 36700 0.005873364 36100 0.000429262
37000 0.04416 35000 0.000111091 34800 0.000813108 34400
0.0005305 36700 0.005894908 36100 0.000429207
37000 0.04343347 35000 0.000111104 34800 0.000812366 34400
0.000532556 36700 0.005916553 36100 0.000429138
37000 0.04272997 35000 0.000111134 34800 0.000812436 34400
0.000534679 36700 0.005938317 36100 0.000429079
37000 0.04204895 35000 0.000111148 34800 0.000813265 34400
0.000536866 36700 0.005960202 36100 0.000429015
37000 0.04138989 35000 0.000111162 34800 0.000814826 34400
0.000539135 36700 0.005982184 36100 0.00042896
37000 0.04075219 35000 0.000111157 34800 0.000817099 34400
0.000541456 36700 0.006004282 36100 0.000428893
37000 0.04013531 35000 0.00011116 34800 0.000820029 34400
0.000543819 36700 0.006026495 36100 0.000428839
37000 0.03953859 35000 0.000111149 34800 0.000823575 34400
0.000546205 36700 0.006048829 36100 0.000428786
37000 0.03896146 35000 0.000111144 34800 0.000827714 34400
0.000548642 36700 0.006071274 36100 0.000428725
37000 0.03840334 35000 0.000111137 34800 0.00083239 34400
0.000551124 36700 0.006093814 36100 0.000428667
37000 0.03786357 35000 0.000111132 34800 0.000837572 34400
0.000553654 36700 0.006116468 36100 0.000428608
37000 0.03734161 35000 0.000111131 34800 0.000843232 34400
0.000556202 36700 0.006139261 36100 0.000428538
37000 0.03683693 35000 0.000111118 34800 0.000849313 34400
0.000558801 36700 0.006162164 36100 0.000428483
37000 0.03634892 35000 0.000111117 34800 0.000855804 34400
0.000561423 36700 0.006185182 36100 0.000428431
37000 0.03587703 35000 0.000111125 34800 0.000862625 34400
0.000564057 36700 0.006208319 36100 0.000428378
37000 0.03542075 35000 0.000111123 34800 0.000869736 34400
0.000566682 36700 0.006231569 36100 0.000428325
37000 0.03497949 35000 0.000111127 34800 0.000877144 34400
0.00056933 36700 0.006254941 36100 0.000428279
37000 0.03455278 35000 0.00011113 34800 0.000884764 34400
0.000571947 36700 0.006278415 36100 0.000428221
37000 0.03414007 35000 0.000111132 34800 0.000892595 34400
0.000574581 36700 0.006302026 36100 0.000428177
37000 0.03374095 35000 0.000111139 34800 0.00090057 34400
0.000577248 36700 0.006325733 36100 0.000428129
37100 0.0333549 35000 0.000111132 34800 0.000908669 34400
0.00057993 36700 0.006349593 36100 0.000428067

37100 0.03298148 35000 0.000111129 34800 0.000916868 34400
0.000582673 36700 0.006373553 36100 0.000428015
37100 0.03262023 35000 0.000111146 34800 0.000925109 34400
0.000585429 36700 0.006397638 36100 0.000427969
37100 0.03227079 35000 0.000111145 34800 0.000933376 34400
0.0005882 36700 0.006421859 36100 0.000427924
37100 0.03193268 35000 0.000111147 34800 0.000941648 34400
0.000591028 36700 0.006446208 36100 0.000427875
37100 0.03160554 35000 0.000111161 34800 0.000949876 34400
0.000593817 36700 0.006470674 36100 0.000427843
37100 0.03128899 35000 0.00011117 34800 0.000958054 34400
0.000596639 36700 0.006495283 36100 0.000427801
37100 0.03098267 35000 0.000111176 34800 0.00096615 34400
0.000599514 36700 0.00652002 36100 0.000427753
37100 0.03068615 35000 0.000111187 34800 0.000974134 34400
0.000602424 36700 0.006544889 36100 0.000427714
37100 0.03039909 35000 0.000111204 34800 0.000981987 34400
0.00060538 36700 0.0065699 36200 0.000427663
37100 0.03012113 35000 0.000111231 34800 0.00098971 34400
0.000608405 36700 0.006595066 36200 0.000427616
37100 0.02985213 35000 0.000111238 34800 0.000997254 34400
0.000611476 36700 0.00662037 36200 0.000427569
37100 0.02959166 35000 0.000111261 34800 0.001004639 34400
0.000614627 36700 0.006645824 36200 0.000427526
37100 0.02933945 35000 0.000111282 34800 0.001011807 34400
0.000617854 36700 0.006671425 36200 0.000427486
37100 0.02909524 35000 0.000111304 34800 0.001018756 34400
0.000621123 36700 0.006697181 36200 0.000427441
37100 0.0288587 35000 0.00011132 34800 0.001025499 34400
0.000624395 36700 0.006723091 36200 0.0004274
37100 0.02862959 35000 0.000111348 34800 0.001032002 34400
0.000627713 36700 0.006749141 36200 0.000427353
37100 0.02840764 35000 0.000111367 34800 0.001038263 34400
0.000631046 36700 0.006775349 36200 0.000427306
37100 0.02819264 35100 0.000111397 34800 0.001044269 34400
0.000634393 36700 0.006801737 36200 0.00042726
37100 0.02798436 35100 0.0001114 34800 0.00105001 34400
0.000637703 36700 0.006828288 36200 0.000427198
37100 0.02778257 35100 0.00011142 34900 0.001055491 34400
0.00064103 36700 0.00685499 36200 0.000427149
37100 0.02758699 35100 0.000111444 34900 0.001060692 34400
0.000644389 36700 0.00688187 36200 0.000427098
37100 0.02739752 35100 0.000111457 34900 0.001065624 34400
0.000647805 36700 0.00690892 36200 0.000427048
37100 0.02721388 35100 0.000111471 34900 0.001070272 34400
0.000651273 36700 0.006936124 36200 0.000426999
37100 0.02703592 35100 0.000111481 34900 0.001074644 34500
0.000654804 36700 0.006963512 36200 0.000426953
37100 0.02686347 35100 0.000111487 34900 0.001078725 34500
0.000658375 36700 0.006991056 36200 0.000426904
37100 0.02669635 35100 0.000111488 34900 0.001082522 34500
0.000661979 36700 0.007018778 36200 0.000426854
37100 0.02653432 35100 0.000111487 34900 0.001086036 34500
0.000665617 36700 0.007046663 36200 0.000426811
37100 0.02637728 35100 0.000111477 34900 0.00108928 34500
0.000669272 36700 0.00707469 36200 0.000426748
37100 0.02622497 35100 0.000111478 34900 0.001092233 34500
0.000672961 36700 0.00710293 36200 0.0004267
37100 0.02607736 35100 0.000111468 34900 0.001094898 34500
0.000676608 36700 0.00713132 36200 0.000426639
37100 0.02593431 35100 0.00011147 34900 0.001097317 34500
0.000680264 36700 0.007159889 36200 0.0004266
37100 0.02579559 35100 0.000111485 34900 0.001099434 34500
0.000683923 36700 0.00718864 36200 0.000426545
37100 0.02566112 35100 0.000111495 34900 0.001101281 34500
0.0006876 36700 0.007217559 36200 0.000426493
37100 0.02553072 35100 0.000111513 34900 0.00110288 34500
0.000691285 36700 0.007246651 36200 0.000426448

37100 0.02540435 35100 0.000111537 34900 0.001104211 34500
0.00069503 36800 0.007275911 36200 0.000426399
37100 0.02528173 35100 0.000111569 34900 0.001105285 34500
0.000698796 36800 0.00730535 36200 0.000426356
37100 0.02516289 35100 0.000111601 34900 0.001106108 34500
0.000702642 36800 0.00733497 36200 0.000426311
37100 0.02504764 35100 0.000111632 34900 0.001106686 34500
0.000706484 36800 0.00736477 36200 0.000426255
37100 0.02493589 35100 0.000111664 34900 0.001107022 34500
0.000710377 36800 0.007394735 36200 0.000426213
37100 0.02482753 35100 0.000111699 34900 0.001107123 34500
0.000714325 36800 0.007424907 36200 0.000426165
37100 0.02472247 35100 0.00011173 34900 0.001106997 34500
0.000718329 36800 0.007455262 36200 0.000426115
37100 0.02462061 35100 0.000111762 34900 0.001106631 34500
0.000722359 36800 0.007485791 36200 0.000426068
37100 0.02452187 35100 0.000111778 34900 0.001106063 34500
0.000726398 36800 0.007516522 36200 0.000426035
37100 0.02442611 35100 0.0001118 34900 0.001105266 34500
0.000730488 36800 0.007547445 36200 0.000425993
37100 0.02433331 35100 0.000111825 34900 0.001104278 34500
0.000734575 36800 0.007578537 36200 0.000425946
37100 0.02424334 35100 0.000111844 34900 0.001103094 34500
0.000738673 36800 0.007609826 36200 0.000425911
37100 0.02415605 35100 0.000111872 34900 0.001101722 34500
0.000742794 36800 0.007641285 36200 0.000425863
37100 0.02407138 35100 0.000111887 34900 0.00110016 34500
0.000746881 36800 0.007672947 36200 0.000425822
37100 0.02398941 35100 0.0001119 34900 0.001098421 34500
0.000750942 36800 0.007704807 36200 0.000425788
37100 0.02390992 35100 0.000111914 34900 0.001096524 34500
0.00075494 36800 0.007736858 36200 0.00042575
37100 0.02383287 35100 0.000111924 34900 0.001094459 34500
0.000758876 36800 0.007769112 36200 0.000425728
37100 0.02375823 35100 0.000111929 34900 0.001092225 34500
0.000762752 36800 0.007801541 36200 0.000425688
37100 0.02368588 35100 0.00011193 34900 0.001089856 34500
0.000766575 36800 0.007834176 36200 0.000425651
37100 0.02361577 35100 0.000111934 34900 0.00108733 34500
0.000770358 36800 0.00786699 36200 0.000425622
37100 0.02354784 35100 0.000111948 34900 0.001084666 34500
0.000774104 36800 0.007900001 36200 0.000425594
37100 0.02348203 35100 0.000111965 34900 0.00108187 34500
0.000777812 36800 0.00793324 36200 0.000425562
37100 0.02341832 35100 0.000111977 34900 0.001078938 34500
0.000781441 36800 0.007966683 36200 0.000425514
37100 0.0233566 35100 0.000111987 34900 0.001075897 34500
0.000784958 36800 0.008000331 36200 0.000425483
37100 0.02329685 35100 0.000112007 34900 0.001072718 34500
0.000788351 36800 0.008034203 36200 0.000425442
37100 0.02323902 35100 0.000112035 34900 0.001069447 34500
0.00079161 36800 0.008068256 36200 0.000425411
37100 0.02318306 35100 0.000112047 34900 0.001066049 34500
0.000794721 36800 0.008102564 36200 0.000425387
37100 0.02312895 35100 0.000112065 34900 0.001062558 34500
0.000797674 36800 0.008137071 36200 0.000425362
37100 0.02307658 35100 0.000112078 34900 0.001058956 34500
0.000800505 36800 0.008171805 36200 0.000425339
37200 0.02302599 35100 0.00011209 34900 0.001055262 34500
0.000803206 36800 0.008206758 36200 0.000425308
37200 0.02297706 35100 0.000112102 34900 0.001051508 34500
0.000805829 36800 0.008241937 36200 0.000425282
37200 0.02292973 35100 0.00011211 34900 0.001047654 34500
0.00080835 36800 0.008277335 36200 0.00042526
37200 0.02288395 35100 0.000112117 34900 0.001043715 34500
0.000810844 36800 0.008312931 36200 0.000425234
37200 0.02283981 35100 0.000112114 34900 0.00103972 34500
0.000813309 36800 0.008348785 36200 0.000425197

37200 0.02279721 35100 0.000112133 34900 0.001035661 34500
0.000815674 36800 0.008384866 36200 0.000425181
37200 0.0227561 35100 0.000112147 34900 0.001031522 34500
0.000817954 36800 0.008421164 36200 0.000425167
37200 0.02271644 35100 0.000112171 34900 0.001027299 34500
0.000820087 36800 0.00845767 36200 0.000425145
37200 0.02267818 35100 0.000112184 34900 0.001023071 34500
0.000822067 36800 0.008494473 36200 0.000425118
37200 0.02264133 35100 0.000112221 34900 0.001018748 34500
0.000823776 36800 0.008531394 36300 0.000425105
37200 0.02260578 35100 0.000112256 34900 0.00101441 34500
0.000825279 36800 0.008568592 36300 0.000425086
37200 0.02257157 35100 0.000112293 34900 0.001009985 34500
0.000826453 36800 0.008606001 36300 0.000425061
37200 0.02253867 35100 0.000112308 34900 0.001005523 34500
0.000827306 36800 0.008643667 36300 0.000425028
37200 0.02250699 35100 0.000112343 34900 0.001001027 34500
0.000827786 36800 0.008681572 36300 0.000425021
37200 0.02247652 35100 0.000112364 34900 0.00099648 34500
0.000827918 36800 0.008719711 36300 0.000424998
37200 0.02244728 35100 0.000112377 34900 0.00099192 34500
0.000827705 36800 0.008758151 36300 0.000424968
37200 0.02241922 35100 0.000112413 34900 0.000987323 34500
0.000827179 36800 0.008796765 36300 0.000424956
37200 0.02239231 35100 0.000112436 34900 0.000982701 34500
0.000826382 36800 0.008835698 36300 0.000424943
37200 0.02236653 35200 0.000112442 34900 0.000978072 34500
0.000825291 36800 0.008874751 36300 0.00042492
37200 0.02234187 35200 0.000112456 34900 0.000973389 34500
0.00082396 36800 0.008914137 36300 0.000424898
37200 0.02231827 35200 0.000112467 35000 0.000968724 34500
0.000822392 36800 0.00895384 36300 0.000424877
37200 0.02229567 35200 0.000112491 35000 0.000964025 34500
0.000820503 36800 0.008993683 36300 0.000424853
37200 0.02227404 35200 0.000112492 35000 0.000959308 34500
0.000818308 36800 0.009033876 36300 0.00042483
37200 0.0222535 35200 0.000112494 35000 0.000954582 34500
0.000815828 36800 0.009074297 36300 0.000424805
37200 0.02223393 35200 0.000112488 35000 0.000949867 34600
0.000813013 36800 0.009114991 36300 0.000424781
37200 0.02221531 35200 0.000112504 35000 0.000945122 34600
0.000809851 36800 0.009155961 36300 0.000424754
37200 0.02219764 35200 0.000112503 35000 0.000940383 34600
0.000806375 36800 0.009197231 36300 0.000424729
37200 0.02218089 35200 0.000112515 35000 0.000935653 34600
0.000802569 36800 0.009238777 36300 0.000424695
37200 0.02216504 35200 0.000112511 35000 0.000930919 34600
0.000798448 36800 0.0092806 36300 0.000424666
37200 0.02215002 35200 0.000112512 35000 0.000926189 34600
0.000794032 36800 0.009322718 36300 0.000424638
37200 0.0221359 35200 0.000112531 35000 0.000921452 34600
0.000789316 36800 0.009365134 36300 0.000424604
37200 0.02212265 35200 0.000112518 35000 0.000916736 34600
0.000784279 36800 0.009407788 36300 0.000424564
37200 0.0221102 35200 0.000112519 35000 0.000912033 34600
0.00077899 36800 0.009450776 36300 0.000424522
37200 0.02209854 35200 0.000112522 35000 0.000907326 34600
0.000773337 36800 0.009494108 36300 0.000424484
37200 0.02208769 35200 0.000112509 35000 0.000902639 34600
0.000767389 36800 0.009537715 36300 0.000424449
37200 0.02207764 35200 0.000112499 35000 0.000897959 34600
0.000761155 36900 0.009581679 36300 0.00042441
37200 0.02206837 35200 0.000112501 35000 0.000893286 34600
0.000754534 36900 0.009625876 36300 0.000424382
37200 0.02205984 35200 0.0001125 35000 0.000888626 34600
0.000747596 36900 0.009670455 36300 0.000424356
37200 0.02205208 35200 0.000112509 35000 0.000883987 34600
0.000740281 36900 0.009715364 36300 0.000424306

37200 0.02204502 35200 0.000112525 35000 0.000879365 34600
0.000732725 36900 0.009760552 36300 0.000424278
37200 0.02203863 35200 0.000112552 35000 0.000874757 34600
0.000724919 36900 0.009806121 36300 0.000424242
[truncated: 338,780 more chars]
