# Supplementary material for: Understanding nonlinear vibration behaviours in high-power ultrasonic surgical devices
Source: Proc Math Phys Eng Sci. 2015 Apr 8;471(2176):20140906. doi: 10.1098/rspa.2014.0906 (PMC4991263; doi:10.1098/rspa.2014.0906)
Supplement: Power Spectrum Data [file rspa20140906supp2.pdf]

PowerSpectrumData
Power Spectrum of devices when excited at close to resonance at 50Vrms.

I1 I2 I3 I4 OT7
BI
Frequency Responses (um) Frequency Responses (um) Frequency
Responses (um) Frequency Responses (um) Frequency Responses (um)
Frequency Responses (um)
64 0.198421345 64 0.157098446 64 0.044434793 64
0.039393708 64 0.05306914 64 0.051076593
72 0.096083408 72 0.070159149 68 0.062570827 68
0.048841957 72 0.119996723 72 0.065149594
80 0.025888539 80 0.168990213 72 0.052383519 72
0.094108516 80 0.191411018 80 0.08566292
88 0.102716389 88 0.2887999 76 0.068869769 76
0.128815038 88 0.2250775 88 0.093919727
96 0.240041146 96 0.2609152 80 0.113742477 80
0.09037993 96 0.15314683 96 0.04111731
104 0.247610121 104 0.131283435 84 0.11861643 84
0.051889569 104 0.129752153 104 0.029614177
112 0.092479582 112 0.051433919 88 0.086310967 88
0.044843389 112 0.113847236 112 0.049989252
120 0.087172659 120 0.113999173 92 0.049290462 92
0.021444104 120 0.143902944 120 0.032651802
128 0.148982873 128 0.044846438 96 0.013522754 96
0.039343926 128 0.199013419 128 0.01975228
136 0.081579463 136 0.211578066 100 0.071600334 100
0.06037904 136 0.170635875 136 0.095425734
144 0.103029852 144 0.257078936 104 0.051495586 104
0.036398269 144 0.095291987 144 0.091940354
152 0.041099735 152 0.180262753 108 0.015524696 108
0.048824477 152 0.096643351 152 0.015835245
160 0.055893193 160 0.174584348 112 0.038366899 112
0.070398601 160 0.157947521 160 0.054198754
168 0.064722117 168 0.169832186 116 0.042935993 116
0.050483413 168 0.178420393 168 0.030901534
176 0.063148997 176 0.119014614 120 0.038305974 120
0.02345376 176 0.178503644 176 0.023635083
184 0.119996868 184 0.109515546 124 0.004475076 124
0.026889515 184 0.127616455 184 0.039219474
192 0.1770573 192 0.171849446 128 0.03722285 128
0.027019061 192 0.092781505 192 0.068358037
200 0.163690508 200 0.278696185 132 0.013490844 132
0.022718936 200 0.093014089 200 0.057396184
208 0.139521319 208 0.301293738 136 0.040672472 136
0.031792326 208 0.110444751 208 0.039500857
216 0.135408394 216 0.22094007 140 0.054274311 140
0.065431355 216 0.135841051 216 0.045587916
224 0.140444565 224 0.169524181 144 0.069657443 144
0.077648583 224 0.149593223 224 0.017932123
232 0.11705354 232 0.170628628 148 0.062506144 148
0.074273972 232 0.114764516 232 0.056180197
240 0.056259371 240 0.129195774 152 0.022497517 152
0.046114612 240 0.044210046 240 0.040936939
248 0.025064886 248 0.063231528 156 0.05799186 156
0.03655587 248 0.076846336 248 0.040166964
256 0.100637139 256 0.048497448 160 0.034054752 160
0.04646229 256 0.081068371 256 0.034383938
264 0.150721185 264 0.086332606 164 0.014137451 164
0.057517849 264 0.035768484 264 0.019013834
272 0.132521265 272 0.112259266 168 0.043951735 168
0.123720165 272 0.053164135 272 0.028410896
280 0.092926719 280 0.107576576 172 0.05079756 172
0.135130278 280 0.071671231 280 0.060867864
288 0.082611514 288 0.037290571 176 0.022374092 176
0.121258236 288 0.09033773 288 0.077199846
296 0.049319304 296 0.054531403 180 0.016952599 180
0.123084508 296 0.040680825 296 0.08283517
304 0.006697949 304 0.057865462 184 0.031319003 184

0.09679934 304 0.064638036 304 0.093902578
312 0.076369746 312 0.05742137 188 0.070932962 188
0.030702478 312 0.020436562 312 0.091138092
320 0.157183168 320 0.013140277 192 0.088426205 192
0.029210443 320 0.133414316 320 0.069947535
328 0.139765718 328 0.066146415 196 0.090322865 196
0.03897179 328 0.164365848 328 0.047119622
336 0.039545204 336 0.009874475 200 0.09353584 200
0.030328212 336 0.095548538 336 0.041954616
344 0.049481223 344 0.090300542 204 0.132385656 204
0.048351998 344 0.070165326 344 0.035124776
352 0.104878207 352 0.111157635 208 0.120368393 208
0.089068257 352 0.055224195 352 0.026848189
360 0.137309719 360 0.085139676 212 0.090734844 212
0.111435409 360 0.094551178 360 0.025494706
368 0.126658474 368 0.05706009 216 0.085031541 216
0.084050313 368 0.112706475 368 0.064237713
376 0.099440011 376 0.014970045 220 0.035531448 220
0.035536152 376 0.015337193 376 0.052900574
384 0.100333375 384 0.05609329 224 0.050959377 224
0.114542636 384 0.07464151 384 0.018713938
392 0.096387528 392 0.076728349 228 0.042033327 228
0.124102735 392 0.05862015 392 0.010578055
400 0.070992268 400 0.092890055 232 0.044699416 232
0.091755988 400 0.121602505 400 0.017186038
408 0.007699378 408 0.117467098 236 0.059780174 236
0.065562774 408 0.072911855 408 0.029206984
416 0.072134382 416 0.093243936 240 0.0791044 240
0.112668036 416 0.048411646 416 0.026286818
424 0.06443412 424 0.036179576 244 0.035712244 244
0.090716763 424 0.12235991 424 0.006960143
432 0.034866389 432 0.04934274 248 0.055415363 248
0.040499235 432 0.162584314 432 0.022398737
440 0.084722706 440 0.090144982 252 0.044173801 252
0.042659387 440 0.146428633 440 0.074635798
448 0.067593028 448 0.137032621 256 0.045097237 256
0.021933918 448 0.102615384 448 0.092462862
456 0.01180151 456 0.10529843 260 0.086605418 260
0.09688874 456 0.046085817 456 0.047278907
464 0.032615069 464 0.045460078 264 0.079723337 264
0.096368829 464 0.03084723 464 0.010737724
472 0.054900374 472 0.083903491 268 0.044571669 268
0.061220875 472 0.078028243 472 0.032603864
480 0.114534581 480 0.052911444 272 0.019664203 272
0.042483389 480 0.12573009 480 0.023883051
488 0.099527591 488 0.128453205 276 0.016993272 276
0.032892178 488 0.087396482 488 0.063880856
496 0.076890392 496 0.270206103 280 0.014836503 280
0.022939292 496 0.066230023 496 0.064592299
504 0.10616464 504 0.24824412 284 0.029907618 284
0.07150638 504 0.017312907 504 0.053124666
512 0.123568694 512 0.155593996 288 0.056014767 288
0.065125809 512 0.095234842 512 0.070713992
520 0.111876238 520 0.16991829 292 0.05911347 292
0.039552619 520 0.154092224 520 0.069267735
528 0.079038277 528 0.163463905 296 0.061141676 296
0.031350977 528 0.153952671 528 0.037973474
536 0.031116237 536 0.057481804 300 0.035554625 300
0.079981757 536 0.074507661 536 0.015793019
544 0.125561943 544 0.058964524 304 0.02805232 304
0.124645623 544 0.019277901 544 0.006953504
552 0.145769868 552 0.071839793 308 0.050829141 308
0.130537941 552 0.055332654 552 0.037979051
560 0.07493126 560 0.03823312 312 0.032911001 312
0.051753152 560 0.099502242 560 0.057336154
568 0.008252534 568 0.058060716 316 0.064921871 316
0.033522625 568 0.100049343 568 0.052465883
576 0.052811927 576 0.158101918 320 0.069621136 320

0.041331878 576 0.030569918 576 0.017946382
584 0.076171156 584 0.198629801 324 0.045053606 324
0.017720398 584 0.075530588 584 0.029006491
592 0.092259812 592 0.14194331 328 0.050340037 328
0.044170141 592 0.041614847 592 0.064959837
600 0.043325181 600 0.0417872 332 0.070519636 332
0.044515389 600 0.042029216 600 0.072526302
608 0.042483411 608 0.058541868 336 0.048752776 336
0.011371045 608 0.094341624 608 0.055627279
616 0.035058991 616 0.126128449 340 0.019153722 340
0.003366533 616 0.117446121 616 0.054616023
624 0.047630525 624 0.157700838 344 0.076016659 344
0.027422142 624 0.050714636 624 0.062471612
632 0.090539652 632 0.148292791 348 0.099688114 348
0.057553156 632 0.085233856 632 0.03768611
640 0.130519766 640 0.128046653 352 0.077329853 352
0.06782572 640 0.114626193 640 0.00526681
648 0.197370683 648 0.118029915 356 0.071084483 356
0.078387013 648 0.126245388 648 0.022577331
656 0.212424566 656 0.116313771 360 0.064750799 360
0.077153993 656 0.109619527 656 0.039885366
664 0.1491203 664 0.063106374 364 0.033179382 364
0.039339473 664 0.094665564 664 0.04430694
672 0.071281131 672 0.063845204 368 0.01741155 368
0.03657092 672 0.082662649 672 0.03970644
680 0.031528336 680 0.165903286 372 0.014540938 372
0.01921062 680 0.061584797 680 0.027684137
688 0.063556196 688 0.17498793 376 0.04660961 376
0.06634165 688 0.069130903 688 0.022723161
696 0.117207608 696 0.136779884 380 0.074168456 380
0.099290926 696 0.035493529 696 0.020086913
704 0.115518975 704 0.084941428 384 0.037863327 384
0.094506177 704 0.071319853 704 0.037830694
712 0.092339171 712 0.101559272 388 0.046557936 388
0.09934893 712 0.074693555 712 0.056338122
720 0.08488902 720 0.089741116 392 0.059165763 392
0.115700823 720 0.011037993 720 0.038261154
728 0.054438671 728 0.074620657 396 0.027264357 396
0.077444565 728 0.056657438 728 0.026979591
736 0.107386542 736 0.076928838 400 0.014909924 400
0.052920943 736 0.047992737 736 0.084422172
744 0.107798442 744 0.089531051 404 0.036310554 404
0.078871111 744 0.056255001 744 0.083103914
752 0.065140339 752 0.048762882 408 0.067394969 408
0.030757732 752 0.05340256 752 0.062397332
760 0.067563007 760 0.053934011 412 0.069910937 412
0.052035321 760 0.072012692 760 0.053410229
768 0.103285049 768 0.105310151 416 0.062631996 416
0.079684287 768 0.095610929 768 0.04811179
776 0.152093475 776 0.149774133 420 0.056732857 420
0.065926484 776 0.155704999 776 0.032827677
784 0.16366315 784 0.092933653 424 0.043386532 424
0.082888386 784 0.153975226 784 0.007496153
792 0.164398385 792 0.011586852 428 0.038780519 428
0.092461094 792 0.119644974 792 0.022473847
800 0.147527433 800 0.089834633 432 0.023555285 432
0.091780028 800 0.106622865 800 0.012509918
808 0.121918434 808 0.093720162 436 0.016780618 436
0.072996721 808 0.060643564 808 0.024863934
816 0.103250764 816 0.091140428 440 0.045938479 440
0.029905714 816 0.064358515 816 0.047710142
824 0.095460469 824 0.165655976 444 0.032131113 444
0.020773781 824 0.123309321 824 0.040953102
832 0.088680732 832 0.127320585 448 0.023415058 448
0.038140312 832 0.108201297 832 0.005053357
840 0.058298436 840 0.101268641 452 0.041278785 452
0.015889264 840 0.076395627 840 0.02403548
848 0.06198512 848 0.107870255 456 0.085831445 456

0.008018358 848 0.095735668 848 0.042684627
856 0.085431464 856 0.074636242 460 0.090777481 460
0.008677872 856 0.100215431 856 0.057756239
864 0.032569864 864 0.092790804 464 0.045366596 464
0.024612278 864 0.071767616 864 0.060367562
872 0.037915343 872 0.058334434 468 0.062642306 468
0.025812231 872 0.070098788 872 0.056608769
880 0.033036584 880 0.113531118 472 0.052746727 472
0.037096972 880 0.141229626 880 0.060204129
888 0.068592053 888 0.127642139 476 0.038956663 476
0.059128161 888 0.165552978 888 0.061162631
896 0.066820896 896 0.049315848 480 0.033375305 480
0.039311173 896 0.125178631 896 0.029211164
904 0.117902688 904 0.161705946 484 0.017525785 484
0.011464406 904 0.057555626 904 0.049497525
912 0.130966466 912 0.156013703 488 0.035220262 488
0.038441984 912 0.069747999 912 0.067304878
920 0.054753677 920 0.107726788 492 0.064746615 492
0.060559047 920 0.087049033 920 0.041851439
928 0.018841212 928 0.102040489 496 0.084753745 496
0.037304671 928 0.110291076 928 0.02060961
936 0.076149314 936 0.136824339 500 0.088759938 500
0.056152876 936 0.109923065 936 0.015408436
944 0.115069444 944 0.140914083 504 0.065362532 504
0.054997818 944 0.094549432 944 0.029012377
952 0.063948632 952 0.072546165 508 0.028752744 508
0.043053828 952 0.126760744 952 0.062555009
960 0.037506365 960 0.018809014 512 0.047306803 512
0.039896611 960 0.151341243 960 0.056164969
968 0.114726317 968 0.04563609 516 0.047213769 516
0.02864405 968 0.130660075 968 0.056846813
976 0.146731298 976 0.014983387 520 0.047782411 520
0.013498436 976 0.077379482 976 0.06927747
984 0.12180231 984 0.069370159 524 0.051593099 524
0.028922117 984 0.152994108 984 0.062105028
992 0.050715018 992 0.122684534 528 0.055163771 528
0.040728981 992 0.186470541 992 0.037755446
1000 0.095798998 1000 0.14075324 532 0.055420482 532
0.012023859 1000 0.169376348 1000 0.02905494
1010 0.157678268 1010 0.107176675 536 0.045488279 536
0.009283042 1008 0.125844875 1008 0.036323258
1020 0.110591071 1020 0.070668517 540 0.039404073 540
0.045655081 1016 0.08616507 1016 0.030119372
1020 0.147535568 1020 0.106550353 544 0.048352886 544
0.071747541 1024 0.048832979 1024 0.040741896
1030 0.181917378 1030 0.14200971 548 0.053363001 548
0.0410715 1032 0.043791737 1032 0.058743568
1040 0.137826908 1040 0.146133709 552 0.060228522 552
0.023125582 1040 0.102682614 1040 0.047657239
1050 0.11189907 1050 0.14585293 556 0.056713412 556
0.01458848 1048 0.063274682 1048 0.010278802
1060 0.097426979 1060 0.118073309 560 0.042077842 560
0.072590439 1056 0.122424783 1056 0.04069438
1060 0.029921912 1060 0.052754858 564 0.027991002 564
0.086568893 1064 0.18006601 1064 0.044402277
1070 0.120948433 1070 0.017765049 568 0.009213373 568
0.03829199 1072 0.136459115 1072 0.02497773
1080 0.158089999 1080 0.040185743 572 0.026382408 572
0.02712284 1080 0.096096097 1080 0.028568265
1090 0.204972879 1090 0.051968415 576 0.027815238 576
0.05348336 1088 0.106675921 1088 0.05159845
1100 0.185175376 1100 0.065368789 580 0.114638227 580
0.047009173 1096 0.073822077 1096 0.063995183
1100 0.069558708 1100 0.116243624 584 0.116451782 584
0.0293216 1104 0.045244098 1104 0.035528334
1110 0.1666912 1110 0.126251558 588 0.021142212 588
0.031321855 1112 0.107854023 1112 0.004174266
1120 0.158971598 1120 0.076161807 592 0.040524916 592

0.040829349	1120	0.089102883	1120	0.019879331		
1130	0.128625936	1130	0.108688437	596	0.044287528	596
0.069290865	1128	0.103051032	1128	0.029681311		
1140	0.177784678	1140	0.129952329	600	0.026098825	600
0.091783026	1136	0.113974231	1136	0.041309941		
1140	0.219942013	1140	0.091020447	604	0.019096262	604
0.076315766	1144	0.035391644	1144	0.051031602		
1150	0.245565229	1150	0.02453472	608	0.060725703	608
0.044097549	1152	0.023618306	1152	0.040918403		
1160	0.272475765	1160	0.068951173	612	0.079615631	612
0.014110025	1160	0.030307328	1160	0.047403573		
1170	0.241626928	1170	0.107153566	616	0.085075437	616
0.007036727	1168	0.036311885	1168	0.049153641		
1180	0.094294424	1180	0.10429028	620	0.066359949	620
0.02482614	1176	0.043281216	1176	0.053845939		
1180	0.085608677	1180	0.166353043	624	0.015574773	624
0.029302237	1184	0.04588965	1184	0.066140201		
1190	0.157023401	1190	0.188409744	628	0.095084717	628
0.020084854	1192	0.067549619	1192	0.059808983		
1200	0.124110855	1200	0.114341274	632	0.107949345	632
0.002106385	1200	0.0528153	1200	0.031714211		
1210	0.077146935	1210	0.016953938	636	0.09784702	636
0.011750722	1208	0.066118191	1208	0.021562175		
1220	0.021607273	1220	0.055623841	640	0.080438447	640
0.047346683	1216	0.070476686	1216	0.065165521		
1220	0.112393638	1220	0.080366524	644	0.069786598	644
0.045669542	1224	0.148599385	1224	0.087738015		
1230	0.140628778	1230	0.083028332	648	0.054467069	648
0.008192912	1232	0.168107669	1232	0.080872684		
1240	0.082079321	1240	0.024305949	652	0.037474889	652
0.058647231	1240	0.12788389	1240	0.04763381		
1250	0.058615329	1250	0.043103006	656	0.031217638	656
0.086669214	1248	0.137358016	1248	0.006836435		
1260	0.138475661	1260	0.084221101	660	0.053686639	660
0.064921616	1256	0.144949693	1256	0.052467705		
1260	0.106223852	1260	0.082544873	664	0.060779872	664
0.028716973	1264	0.087453685	1264	0.070583737		
1270	0.078653968	1270	0.053664036	668	0.028116228	668
0.064451568	1272	0.147045895	1272	0.053113919		
1280	0.111552268	1280	0.046632846	672	0.059830811	672
0.093559218	1280	0.186251505	1280	0.036140005		
1290	0.077531848	1290	0.106008556	676	0.07369883	676
0.100450183	1288	0.163358244	1288	0.030546202		
1300	0.03932554	1300	0.177434616	680	0.07156143	680
0.08511186	1296	0.155766465	1296	0.004004144		
1300	0.090821231	1300	0.155077185	684	0.072956063	684
0.046489768	1304	0.112673457	1304	0.030515779		
1310	0.075582197	1310	0.110464949	688	0.055371467	688
0.024142526	1312	0.120898709	1312	0.02593518		
1320	0.046262616	1320	0.122912577	692	0.067020046	692
0.04557777	1320	0.17714304	1320	0.02643235		
1330	0.067377128	1330	0.110030378	696	0.064048902	696
0.061169681	1328	0.116353353	1328	0.050909792		
1340	0.063151529	1340	0.034619388	700	0.042629308	700
0.044768207	1336	0.063355568	1336	0.039645864		
1340	0.074808959	1340	0.113895781	704	0.054703953	704
0.009831657	1344	0.033868619	1344	0.037083544		
1350	0.07640398	1350	0.150031149	708	0.058066398	708
0.054996526	1352	0.04996128	1352	0.033280459		
1360	0.055366822	1360	0.142657984	712	0.012959395	712
0.083438419	1360	0.059675807	1360	0.018004592		
1370	0.103280734	1370	0.123986174	716	0.076436176	716
0.052593263	1368	0.050903913	1368	0.058773141		
1380	0.151504763	1380	0.077166958	720	0.08297975	720
0.05817975	1376	0.0535041	1376	0.060217531		
1380	0.174152141	1380	0.064980355	724	0.057012214	724
0.081346596	1384	0.01972584	1384	0.05044158		
1390	0.228546181	1390	0.112128219	728	0.037384249	728

0.06675833	1392	0.04243834	1392	0.051208557		
1400	0.169698455	1400	0.219545939	732	0.013792369	732
0.042130563	1400	0.01197715	1400	0.029547515		
1410	0.049539362	1410	0.224764051	736	0.008300875	736
0.045274825	1408	0.10683859	1408	0.009627739		
1420	0.029794888	1420	0.083617131	740	0.018745841	740
0.025698642	1416	0.13431847	1416	0.015900951		
1420	0.049638395	1420	0.077730954	744	0.037274625	744
0.015615548	1424	0.106289132	1424	0.023008297		
1430	0.109660119	1430	0.117245771	748	0.054606302	748
0.039299182	1432	0.068812973	1432	0.012990974		
1440	0.118728916	1440	0.106922176	752	0.049239166	752
0.086702443	1440	0.099300407	1440	0.023754179		
1450	0.109261506	1450	0.096791526	756	0.025248717	756
0.121007659	1448	0.095358104	1448	0.011079661		
1460	0.090074202	1460	0.07198409	760	0.016805681	760
0.103520884	1456	0.065433829	1456	0.036573889		
1460	0.064940869	1460	0.066326509	764	0.033625565	764
0.032402433	1464	0.031785246	1464	0.012673387		
1470	0.089356923	1470	0.125362654	768	0.047880247	768
0.032099386	1472	0.025090787	1472	0.03765172		
1480	0.066056149	1480	0.14361144	772	0.04775187	772
0.042345571	1480	0.022400975	1480	0.058126385		
1490	0.03975529	1490	0.095502524	776	0.035773413	776
0.032763317	1488	0.094509567	1488	0.033766053		
1500	0.015474461	1500	0.064504493	780	0.035881923	780
0.028007278	1496	0.097376928	1496	0.009568354		
1500	0.069923161	1500	0.090452653	784	0.042487336	784
0.014175199	1504	0.062621184	1504	0.023691497		
1510	0.073912263	1510	0.091444657	788	0.044790286	788
0.020131463	1512	0.02619401	1512	0.023112383		
1520	0.06780991	1520	0.058565689	792	0.049604169	792
0.011257271	1520	0.040360548	1520	0.031697618		
1530	0.076887991	1530	0.107767715	796	0.011817314	796
0.015433485	1528	0.116422547	1528	0.031654839		
1540	0.110075198	1540	0.166459649	800	0.070544411	800
0.01180641	1536	0.19838415	1536	0.025596955		
1540	0.157989984	1540	0.147199913	804	0.077775789	804
0.072355826	1544	0.196816225	1544	0.058348236		
1550	0.091467693	1550	0.111200767	808	0.059551243	808
0.103372819	1552	0.142583464	1552	0.063342028		
1560	0.088715999	1560	0.185847705	812	0.07455382	812
0.085953208	1560	0.078211793	1560	0.041325166		
1570	0.111403453	1570	0.192660271	816	0.080119527	816
0.0548563	1568	0.100545869	1568	0.024586227		
1580	0.059359769	1580	0.087088025	820	0.061116094	820
0.029600451	1576	0.131723195	1576	0.018384524		
1580	0.019112606	1580	0.035765952	824	0.057686415	824
0.06835549	1584	0.089021458	1584	0.013063658		
1590	0.09001304	1590	0.035825073	828	0.066319983	828
0.127218198	1592	0.038618575	1592	0.014199718		
1600	0.095870273	1600	0.032600892	832	0.0647544	832
0.127709864	1600	0.053824453	1600	0.013818502		
1610	0.135556373	1610	0.094895957	836	0.030728887	836
0.072124974	1608	0.042256077	1608	0.010236953		
1620	0.145514146	1620	0.198771479	840	0.037828078	840
0.03660905	1616	0.063280517	1616	0.028861021		
1620	0.06603841	1620	0.216402274	844	0.108838431	844
0.029430892	1624	0.146290185	1624	0.070419905		
1630	0.015212749	1630	0.131861045	848	0.133167894	848
0.035756533	1632	0.151624249	1632	0.058578149		
1640	0.030184852	1640	0.057098376	852	0.072182185	852
0.043762528	1640	0.136593939	1640	0.013297688		
1650	0.014489494	1650	0.020007943	856	0.048956859	856
0.0488798	1648	0.142194288	1648	0.046605714		
1660	0.032690012	1660	0.055826829	860	0.034546811	860
0.064681415	1656	0.128959568	1656	0.05044687		
1660	0.03305521	1660	0.027773405	864	0.102197744	864

0.099115649	1664	0.103167884	1664	0.033884895		
1670	0.041208532	1670	0.083805047	868	0.111853726	868
0.099355668	1672	0.104420877	1672	0.030296627		
1680	0.094895389	1680	0.057147339	872	0.051121748	872
0.082103907	1680	0.118531018	1680	0.032575761		
1690	0.143403144	1690	0.071106719	876	0.002482397	876
0.089693276	1688	0.11225116	1688	0.008765924		
1700	0.11511457	1700	0.17573795	880	0.017778932	880
0.100318059	1696	0.089900801	1696	0.027214717		
1700	0.101469348	1700	0.200233728	884	0.026020598	884
0.095828014	1704	0.090894915	1704	0.056222376		
1710	0.130896443	1710	0.070270828	888	0.041913394	888
0.091924405	1712	0.073838332	1712	0.058750684		
1720	0.114901297	1720	0.062642037	892	0.066294851	892
0.085412641	1720	0.053168795	1720	0.043222637		
1730	0.075163422	1730	0.037364327	896	0.048917991	896
0.058044825	1728	0.11499619	1728	0.037352271		
1740	0.070552575	1740	0.10123181	900	0.014873708	900
0.046912071	1736	0.166310099	1736	0.025861198		
1740	0.079065125	1740	0.134138609	904	0.024330082	904
0.037681519	1744	0.163379111	1744	0.023956247		
1750	0.034999121	1750	0.142849328	908	0.05132104	908
0.041683659	1752	0.098780656	1752	0.021196687		
1760	0.046962301	1760	0.092787348	912	0.04521442	912
0.068833411	1760	0.024731866	1760	0.027013562		
1770	0.084610168	1770	0.032419674	916	0.023689132	916
0.07482995	1768	0.033375898	1768	0.06726112		
1780	0.089634785	1780	0.005755067	920	0.017360959	920
0.075813528	1776	0.041015821	1776	0.037011199		
1780	0.097257464	1780	0.045784622	924	0.024493975	924
0.047677411	1784	0.013086545	1784	0.032300977		
1790	0.079471836	1790	0.065484659	928	0.009258353	928
0.040871219	1792	0.0227814	1792	0.058650694		
1800	0.111773959	1800	0.037630816	932	0.052034586	932
0.070471164	1800	0.043847926	1800	0.035063487		
1810	0.129596243	1810	0.059268394	936	0.046773326	936
0.086967921	1808	0.064184009	1808	0.013964693		
1820	0.144275691	1820	0.106336651	940	0.00171776	940
0.0736945	1816	0.06063364	1816	0.027770635		
1820	0.124537095	1820	0.123542297	944	0.029949379	944
0.03526314	1824	0.053800213	1824	0.010589513		
1830	0.076817669	1830	0.096406264	948	0.051684536	948
0.032403048	1832	0.037298109	1832	0.035515779		
1840	0.093848219	1840	0.026700418	952	0.03778141	952
0.066336557	1840	0.015623889	1840	0.047633708		
1850	0.139646698	1850	0.059314294	956	0.018674879	956
0.06848466	1848	0.020825431	1848	0.036396807		
1860	0.099535981	1860	0.082475228	960	0.051932544	960
0.058182595	1856	0.020921883	1856	0.029833273		
1860	0.060861305	1860	0.092150061	964	0.06329899	964
0.006728834	1864	0.032275941	1864	0.027865106		
1870	0.083714112	1870	0.0982977	968	0.028860754	968
0.069751433	1872	0.10285469	1872	0.045408029		
1880	0.080805177	1880	0.057481615	972	0.031043765	972
0.062245781	1880	0.176209753	1880	0.072160794		
1890	0.099015641	1890	0.043639015	976	0.007702802	976
0.030965166	1888	0.129700653	1888	0.071082897		
1900	0.077974146	1900	0.07717737	980	0.017002714	980
0.020157644	1896	0.058283062	1896	0.04616502		
1900	0.089034329	1900	0.01058896	984	0.016487526	984
0.036429676	1904	0.056739536	1904	0.027146018		
1910	0.061795807	1910	0.077761739	988	0.024526271	988
0.044742013	1912	0.045399949	1912	0.043633798		
1920	0.048575206	1920	0.120980629	992	0.01387168	992
0.055882367	1920	0.123155682	1920	0.07064204		
1930	0.040913878	1930	0.113544789	996	0.0484671	996
0.086261738	1928	0.100651123	1928	0.09139644		
1940	0.048472641	1940	0.047940281	1000	0.064219981	1000

```
0.0719858       1936    0.045716195     1936    0.07995221
1940    0.066099143     1940    0.052316747     1000    0.044004224     1004
0.005437524     1944    0.055447377     1944    0.059573991
1950    0.089476802     1950    0.050081082     1010    0.066230212     1008
0.056233392     1952    0.074090203     1952    0.049317994
1960    0.09620878      1960    0.053226169     1010    0.073187737     1012
0.050266423     1960    0.030438245     1960    0.019008308
1970    0.053220494     1970    0.163991455     1020    0.054142118     1016
0.034693985     1968    0.076984703     1968    0.016658873
1980    0.01204044      1980    0.272118545     1020    0.003628917     1020
0.062772982     1976    0.140854609     1976    0.005822575
1980    0.050525108     1980    0.292112585     1020    0.06199006      1024
0.084606392     1984    0.117152558     1984    0.0508993
1990    0.072526258     1990    0.188118458     1030    0.064330016     1028
0.065390879     1992    0.074017888     1992    0.056670186
2000    0.076601631     2000    0.051385785     1030    0.031032945     1032
0.033400695     2000    0.054431293     2000    0.040909246
2010    0.101511876     2010    0.040823037     1040    0.043339343     1036
0.016525693     2008    0.060674145     2008    0.038015471
2020    0.08148131      2020    0.049659611     1040    0.043808446     1040
0.013208382     2016    0.092446666     2016    0.034004679
2020    0.058631027     2020    0.08376209      1040    0.037689653     1044
0.074104559     2024    0.089925728     2024    0.030331967
2030    0.053196833     2030    0.127187115     1050    0.019288285     1048
0.08237255      2032    0.093741459     2032    0.054864893
2040    0.021809241     2040    0.142599369     1050    0.020073725     1052
0.029696133     2040    0.09991839      2040    0.054311415
2050    0.044832763     2050    0.117561373     1060    0.028843895     1056
0.040491348     2048    0.026996559     2048    0.046167948
2060    0.044434535     2060    0.053609969     1060    0.029118401     1060
0.069990194     2056    0.081481339     2056    0.038418883
2060    0.03479821      2060    0.042001517     1060    0.015488144     1064
0.084009233     2064    0.109204113     2064    0.044875706
2070    0.100570462     2070    0.092362825     1070    0.031972744     1068
0.093915063     2072    0.094018426     2072    0.059019254
2080    0.147145431     2080    0.096168355     1070    0.060033148     1072
0.080330508     2080    0.062853811     2080    0.029906709
2090    0.09582311      2090    0.040551604     1080    0.065102911     1076
0.076052536     2088    0.022452663     2088    0.025645277
2100    0.119852899     2100    0.041149429     1080    0.083825129     1080
0.041388474     2096    0.019079636     2096    0.0315201
2100    0.133547903     2100    0.012014347     1080    0.056900797     1084
0.045155615     2104    0.105093721     2104    0.011979545
2110    0.064002314     2110    0.081622151     1090    0.027911507     1088
0.078386584     2112    0.176066679     2112    0.012693969
2120    0.036612728     2120    0.073693176     1090    0.018722116     1092
0.056598219     2120    0.144296151     2120    0.032540429
2130    0.05284285      2130    0.082387924     1100    0.034012621     1096
0.031895314     2128    0.023739687     2128    0.046536556
2140    0.078192577     2140    0.096719545     1100    0.082417028     1100
0.054265896     2136    0.099998433     2136    0.032257998
2140    0.174547924     2140    0.053681328     1100    0.097889613     1104
0.089442248     2144    0.147799059     2144    0.029293207
2150    0.257485779     2150    0.068778347     1110    0.082594066     1108
0.101440492     2152    0.125923354     2152    0.076194556
2160    0.256029045     2160    0.105716754     1110    0.047887937     1112
0.086400767     2160    0.070728929     2160    0.09186731
2170    0.220932343     2170    0.071332608     1120    0.012227069     1116
0.034901805     2168    0.006126756     2168    0.055389661
2180    0.144232151     2180    0.024378645     1120    0.037904923     1120
0.039654628     2176    0.035786201     2176    0.026188651
2180    0.07073662      2180    0.091583032     1120    0.068193171     1124
0.056346427     2184    0.0737722       2184    0.049155377
2190    0.119067823     2190    0.129197754     1130    0.050945197     1128
0.039192444     2192    0.054971093     2192    0.051750667
2200    0.1061648       2200    0.126048151     1130    0.025703936     1132
0.049983897     2200    0.025031939     2200    0.035895439
2210    0.094574476     2210    0.118356176     1140    0.037494901     1136
```

0.095823663 2208 0.032455289 2208 0.018770426
2220 0.095001458 2220 0.072198083 1140 0.002534557 1140
0.111529349 2216 0.058812111 2216 0.047910024
2220 0.05955794 2220 0.012786267 1140 0.045204852 1144
0.090189191 2224 0.079810023 2224 0.035903719
2230 0.096752257 2230 0.074132448 1150 0.068142406 1148
0.07613872 2232 0.069661524 2232 0.033219531
2240 0.118243719 2240 0.144929698 1150 0.071406488 1152
0.053568871 2240 0.081709768 2240 0.078577432
2250 0.08014981 2250 0.155806294 1160 0.062934436 1156
0.037759561 2248 0.148142746 2248 0.057512385
2260 0.014847402 2260 0.11008908 1160 0.057049078 1160
0.041635412 2256 0.142569974 2256 0.057959071
2260 0.094481751 2260 0.083074032 1160 0.031846765 1164
0.043733639 2264 0.092427079 2264 0.08974794
2270 0.087360808 2270 0.096366559 1170 0.017232034 1168
0.034032084 2272 0.044736898 2272 0.096463875
2280 0.022649911 2280 0.127104635 1170 0.020985892 1172
0.008161395 2280 0.076439945 2280 0.049569495
2290 0.031160285 2290 0.145039419 1180 0.009639767 1176
0.029336638 2288 0.14426418 2288 0.014456135
2300 0.051432664 2300 0.087414162 1180 0.038270438 1180
0.077662742 2296 0.119404045 2296 0.02891341
2300 0.131800189 2300 0.03275655 1180 0.077103927 1184
0.090457594 2304 0.103106664 2304 0.043246801
2310 0.122654761 2310 0.087105233 1190 0.039351296 1188
0.063043306 2312 0.11685591 2312 0.034831028
2320 0.05591282 2320 0.125523788 1190 0.043876695 1192
0.024287214 2320 0.089411828 2320 0.043637258
2330 0.031406333 2330 0.166436818 1200 0.061512357 1196
0.070100512 2328 0.045793986 2328 0.046293611
2340 0.098514276 2340 0.13042723 1200 0.026642829 1200
0.086713582 2336 0.031009411 2336 0.041078845
2340 0.17444606 2340 0.08456401 1200 0.026575099 1204
0.064948523 2344 0.020177651 2344 0.026588285
2350 0.136656599 2350 0.04573026 1210 0.045695862 1208
0.040078921 2352 0.04370428 2352 0.064923719
2360 0.067890833 2360 0.018491723 1210 0.057932557 1212
0.013195112 2360 0.104064813 2360 0.09051527
2370 0.055965884 2370 0.071260933 1220 0.056320801 1216
0.03602829 2368 0.120568264 2368 0.060561684
2380 0.015273497 2380 0.08893296 1220 0.045923938 1220
0.027947153 2376 0.03384268 2376 0.02536385
2380 0.086559339 2380 0.079664482 1220 0.030853902 1224
0.018550069 2384 0.042816635 2384 0.032640619
2390 0.101726851 2390 0.091229194 1230 0.028555896 1228
0.054305419 2392 0.013334179 2392 0.073078918
2400 0.076723562 2400 0.190066029 1230 0.014991271 1232
0.087971414 2400 0.093165509 2400 0.078475328
2410 0.077133242 2410 0.264775299 1240 0.030462186 1236
0.040925588 2408 0.095246425 2408 0.046945967
2420 0.040506297 2420 0.191945758 1240 0.042282405 1240
0.045829584 2416 0.051089523 2416 0.055367375
2420 0.052860964 2420 0.076891221 1240 0.030123749 1244
0.051288589 2424 0.030234791 2424 0.064198976
2430 0.114074763 2430 0.038946895 1250 0.007525561 1248
0.026772295 2432 0.106195184 2432 0.042216878
2440 0.150484935 2440 0.016541435 1250 0.006006468 1252
0.03973431 2440 0.091256305 2440 0.008520583
2450 0.148145628 2450 0.062008767 1260 0.012860664 1256
0.020350948 2448 0.03418803 2448 0.033940651
2460 0.12074034 2460 0.055218698 1260 0.056799065 1260
0.036747661 2456 0.065113389 2456 0.035351437
2460 0.145117345 2460 0.025982994 1260 0.073053729 1264
0.01036191 2464 0.099944409 2464 0.04152508
2470 0.124333325 2470 0.114920498 1270 0.047549977 1268
0.017609893 2472 0.054079195 2472 0.027717024
2480 0.056606976 2480 0.104029889 1270 0.029683504 1272

0.059021993	2480	0.027552525	2480	0.004008322		
2490	0.061469116	2490	0.019078694	1280	0.039402472	1276
0.10318237	2488	0.028866221	2488	0.008076387		
2500	0.057387912	2500	0.072436014	1280	0.042295065	1280
0.089327245	2496	0.080157814	2496	0.016414093		
2500	0.051419793	2500	0.063531516	1280	0.026023836	1284
0.017142142	2504	0.087130284	2504	0.024502479		
2510	0.111246765	2510	0.040565392	1290	0.037450049	1288
0.04163594	2512	0.061323473	2512	0.04703836		
2520	0.102473867	2520	0.089677385	1290	0.063014886	1292
0.025685074	2520	0.058927624	2520	0.055352844		
2530	0.026886917	2530	0.075103293	1300	0.041501353	1296
0.020097003	2528	0.120903016	2528	0.045087927		
2540	0.054259544	2540	0.066606328	1300	0.010295851	1300
0.004255352	2536	0.109660083	2536	0.044721324		
2540	0.042825526	2540	0.054554184	1300	0.027726286	1304
0.024368874	2544	0.041223317	2544	0.051360708		
2550	0.066712761	2550	0.047654477	1310	0.009717744	1308
0.063292726	2552	0.016380605	2552	0.050754821		
2560	0.164760903	2560	0.060013004	1310	0.022316643	1312
0.104054787	2560	0.053766456	2560	0.055660017		
2570	0.187736573	2570	0.070955342	1320	0.048601625	1316
0.120090212	2568	0.088978937	2568	0.054822536		
2580	0.16008137	2580	0.086324762	1320	0.062310028	1320
0.084974963	2576	0.102951555	2576	0.058029473		
2580	0.094222996	2580	0.057749745	1320	0.037546917	1324
0.027516231	2584	0.055805987	2584	0.05612777		
2590	0.086494998	2590	0.044367371	1330	0.041337378	1328
0.055496417	2592	0.030403569	2592	0.031671127		
2600	0.154017253	2600	0.078576901	1330	0.036957332	1332
0.065002772	2600	0.010471692	2600	0.01580716		
2610	0.190974315	2610	0.120734447	1340	0.063641965	1336
0.053622207	2608	0.081183491	2608	0.049097951		
2620	0.147901519	2620	0.088457549	1340	0.064871027	1340
0.047031459	2616	0.11257147	2616	0.073383817		
2620	0.066764464	2620	0.008363862	1340	0.027959626	1344
0.033100067	2624	0.102648628	2624	0.041541625		
2630	0.057873735	2630	0.085102947	1350	0.014036177	1348
0.03111468	2632	0.040802283	2632	0.026916865		
2640	0.114601338	2640	0.131413283	1350	0.044211876	1352
0.037256428	2640	0.020186086	2640	0.048722217		
2650	0.157459058	2650	0.102063284	1360	0.094374729	1356
0.017202498	2648	0.023034389	2648	0.067207875		
2660	0.21306779	2660	0.045995585	1360	0.075001961	1360
0.053091055	2656	0.054644803	2656	0.067278743		
2660	0.121891288	2660	0.146885359	1360	0.03273525	1364
0.102378828	2664	0.036634123	2664	0.048294583		
2670	0.046640143	2670	0.130808476	1370	0.047112317	1368
0.085004991	2672	0.024676514	2672	0.048132457		
2680	0.099132449	2680	0.007529704	1370	0.051325162	1372
0.049056496	2680	0.051107651	2680	0.048187627		
2690	0.093378658	2690	0.123099555	1380	0.024766598	1376
0.059816553	2688	0.007731311	2688	0.033030952		
2700	0.082973405	2700	0.166346014	1380	0.006036485	1380
0.062030282	2696	0.065010638	2696	0.018956014		
2700	0.033138284	2700	0.127832944	1380	0.020422647	1384
0.027647291	2704	0.098556942	2704	0.033382454		
2710	0.053079879	2710	0.043163898	1390	0.036034013	1388
0.040142622	2712	0.079584133	2712	0.024806668		
2720	0.091834969	2720	0.044121138	1390	0.033737211	1392
0.050235467	2720	0.011086901	2720	0.045799658		
2730	0.04705185	2730	0.057532307	1400	0.019286808	1396
0.064220927	2728	0.10207919	2728	0.092187351		
2740	0.022908916	2740	0.043314652	1400	0.024363781	1400
0.047399804	2736	0.14881976	2736	0.106828964		
2740	0.042852062	2740	0.011574951	1400	0.057331945	1404
0.016667516	2744	0.084269414	2744	0.083139385		
2750	0.101159494	2750	0.064120475	1410	0.076621502	1408

0.07014149	2752	0.09471341	2752	0.048920461		
2760	0.14170607	2760	0.067153749	1410	0.079781639	1412
0.079221092	2760	0.057793033	2760	0.041771498		
2770	0.107054635	2770	0.050348739	1420	0.084433668	1416
0.053552052	2768	0.109512883	2768	0.037053436		
2780	0.071660812	2780	0.022032198	1420	0.043132324	1420
0.064858818	2776	0.071037284	2776	0.039317994		
2780	0.060965893	2780	0.020719139	1420	0.040731862	1424
0.080357677	2784	0.073575764	2784	0.029071514		
2790	0.047950587	2790	0.098828023	1430	0.083067142	1428
0.055638106	2792	0.128521453	2792	0.022060372		
2800	0.051343566	2800	0.176813424	1430	0.100065823	1432
0.028345779	2800	0.101827762	2800	0.033303095		
2810	0.066445144	2810	0.108681539	1440	0.093988274	1436
0.050930823	2808	0.152359309	2808	0.029907173		
2820	0.032643955	2820	0.05150801	1440	0.058721475	1440
0.050124192	2816	0.149053594	2816	0.015370633		
2820	0.020147996	2820	0.122110781	1440	0.02023137	1444
0.039677081	2824	0.05966971	2824	0.016850354		
2830	0.057820431	2830	0.150444554	1450	0.030969284	1448
0.032724362	2832	0.030516299	2832	0.055453635		
2840	0.102827318	2840	0.10843081	1450	0.057464884	1452
0.003718189	2840	0.050316459	2840	0.091642716		
2850	0.136010451	2850	0.022555943	1460	0.041652624	1456
0.040725921	2848	0.052009567	2848	0.094229828		
2860	0.130389934	2860	0.130300643	1460	0.006402652	1460
0.063552274	2856	0.07781537	2856	0.068141708		
2860	0.158280658	2860	0.131401714	1460	0.036400114	1464
0.039047995	2864	0.041435102	2864	0.05001912		
2870	0.185862926	2870	0.061829669	1470	0.041019011	1468
0.028302302	2872	0.00578729	2872	0.024489413		
2880	0.150958033	2880	0.037518152	1470	0.045686848	1472
0.061981024	2880	0.04143142	2880	0.026658727		
2890	0.184089425	2890	0.023272551	1480	0.038347298	1476
0.050967909	2888	0.108766551	2888	0.034475226		
2900	0.108128195	2900	0.023233788	1480	0.035352252	1480
0.025959431	2896	0.114829883	2896	0.030684249		
2900	0.031464293	2900	0.073032163	1480	0.028895382	1484
0.035375371	2904	0.036377209	2904	0.034966106		
2910	0.075560041	2910	0.158377355	1490	0.019568992	1488
0.06010916	2912	0.078462057	2912	0.043516855		
2920	0.143517959	2920	0.150299195	1490	0.047890047	1492
0.047554153	2920	0.105258674	2920	0.041593481		
2930	0.20269955	2930	0.073150368	1500	0.062952495	1496
0.073649942	2928	0.124532729	2928	0.027194275		
2940	0.227376019	2940	0.017084964	1500	0.062350184	1500
0.118015785	2936	0.130954999	2936	0.009975346		
2940	0.140696691	2940	0.055467292	1500	0.059783619	1504
0.124820159	2944	0.057727084	2944	0.020386246		
2950	0.133727925	2950	0.086860011	1510	0.067853653	1508
0.086142747	2952	0.074491174	2952	0.03276546		
2960	0.182327058	2960	0.082249142	1510	0.073326046	1512
0.05045396	2960	0.126356695	2960	0.042039585		
2970	0.099961668	2970	0.065806838	1520	0.042047836	1516
0.031846961	2968	0.101638201	2968	0.050891074		
2980	0.034605495	2980	0.040386778	1520	0.026805428	1520
0.015387806	2976	0.061338884	2976	0.045080542		
2980	0.05720109	2980	0.019731957	1520	0.028064951	1524
0.026087522	2984	0.072526593	2984	0.036850506		
2990	0.057594607	2990	0.029420196	1530	0.022675136	1528
0.042123134	2992	0.029850573	2992	0.042820462		
3000	0.151227926	3000	0.027945742	1530	0.055971344	1532
0.027342532	3000	0.075385069	3000	0.052214931		
3010	0.217542241	3010	0.063404616	1540	0.074991323	1536
0.025764231	3008	0.095656404	3008	0.060127863		
3020	0.168190833	3020	0.128172513	1540	0.053331263	1540
0.063093896	3016	0.05062301	3016	0.054768487		
3020	0.09705795	3020	0.143026001	1540	0.032383676	1544

0.057074652 3024 0.02534506 3024 0.034905239
3030 0.030023895 3030 0.121349069 1550 0.026424748 1548
0.064413063 3032 0.078382276 3032 0.03530908
3040 0.036542744 3040 0.094110794 1550 0.010075477 1552
0.089422581 3040 0.165525111 3040 0.046465415
3050 0.098953802 3050 0.047884965 1560 0.003692382 1556
0.074862772 3048 0.178071918 3048 0.033918521
3060 0.152584209 3060 0.060159713 1560 0.005152329 1560
0.042910622 3056 0.12013095 3056 0.020627251
3060 0.122821351 3060 0.067059104 1560 0.022297423 1564
0.051565847 3064 0.091630638 3064 0.036178619
3070 0.004892568 3070 0.051659412 1570 0.057138419 1568
0.061380357 3072 0.104228944 3072 0.044073317
3080 0.076220029 3080 0.058332378 1570 0.044667762 1572
0.031361866 3080 0.079034624 3080 0.037218335
3090 0.022124854 3090 0.08776757 1580 0.047070069 1576
0.026022704 3088 0.0572438 3088 0.014235775
3100 0.112273432 3100 0.074049509 1580 0.096298012 1580
0.026324826 3096 0.056446927 3096 0.030217927
3100 0.095080955 3100 0.079410471 1580 0.081196158 1584
0.031582807 3104 0.082047038 3104 0.032481959
3110 0.063064137 3110 0.109756256 1590 0.057351968 1588
0.082595376 3112 0.101656398 3112 0.045054658
3120 0.148241394 3120 0.15076196 1590 0.053962343 1592
0.077384742 3120 0.10637947 3120 0.071483395
3130 0.117899166 3130 0.204517055 1600 0.071100702 1596
0.087076667 3128 0.074244999 3128 0.060926774
3140 0.170780579 3140 0.190315666 1600 0.084948137 1600
0.12803456 3136 0.095541953 3136 0.031663123
3140 0.202089679 3140 0.098084631 1600 0.060583134 1604
0.157763541 3144 0.117665091 3144 0.055994355
3150 0.101579972 3150 0.07272105 1610 0.031116 1608
0.168860468 3152 0.094036332 3152 0.036102181
3160 0.030834275 3160 0.043895605 1610 0.028078639 1612
0.17722651 3160 0.181541124 3160 0.046156652
3170 0.067320427 3170 0.053248201 1620 0.020106789 1616
0.146280086 3168 0.102012047 3168 0.059614744
3180 0.070596485 3180 0.068359863 1620 0.03095291 1620
0.080581493 3176 0.070627546 3176 0.045221266
3180 0.081196493 3180 0.014044396 1620 0.075455326 1624
0.029254054 3184 0.120777135 3184 0.040147992
3190 0.117258795 3190 0.063486397 1630 0.085350148 1628
0.05462323 3192 0.052174411 3192 0.031613574
3200 0.109044049 3200 0.054740609 1630 0.046134377 1632
0.068843961 3200 0.13564447 3200 0.011910856
3210 0.036433856 3210 0.098141572 1640 0.016918313 1636
0.076021577 3208 0.149670391 3208 0.042618282
3220 0.109334251 3220 0.13733466 1640 0.031800333 1640
0.057577589 3216 0.111886599 3216 0.029838966
3220 0.141010548 3220 0.13826204 1640 0.013446776 1644
0.046010133 3224 0.134852161 3224 0.037729355
3230 0.050959112 3230 0.090498084 1650 0.066952809 1648
0.04490398 3232 0.113693786 3232 0.059211889
3240 0.111967187 3240 0.057032699 1650 0.087850327 1652
0.034210385 3240 0.055733755 3240 0.03935341
3250 0.127204301 3250 0.082177292 1660 0.074501477 1656
0.05989784 3248 0.058493999 3248 0.023142415
3260 0.112147929 3260 0.070341128 1660 0.040952476 1660
0.080372702 3256 0.077120138 3256 0.019207973
3260 0.142106306 3260 0.063069136 1660 0.022290946 1664
0.051992414 3264 0.111475994 3264 0.021712307
3270 0.134514645 3270 0.071554248 1670 0.027134878 1668
0.011583805 3272 0.116603966 3272 0.054065997
3280 0.122486177 3280 0.0566335 1670 0.028327964 1672
0.019411444 3280 0.091301095 3280 0.075905875
3290 0.122936486 3290 0.103362567 1680 0.039722036 1676
0.045215227 3288 0.099986428 3288 0.037498918
3300 0.1276611 3300 0.138789678 1680 0.045010678 1680

0.057704703 3296 0.051526586 3296 0.01115649
3300 0.146614926 3300 0.091096037 1680 0.018456245 1684
0.05423167 3304 0.07943932 3304 0.020135371
3310 0.151980479 3310 0.105112988 1690 0.019037469 1688
0.044253411 3312 0.057221623 3312 0.069102862
3320 0.099242643 3320 0.144168939 1690 0.04405811 1692
0.010982464 3320 0.046075376 3320 0.082734179
3330 0.053021988 3330 0.109998749 1700 0.035970468 1696
0.052451669 3328 0.021950236 3328 0.066862907
3340 0.058364454 3340 0.070639995 1700 0.028403914 1700
0.078282348 3336 0.026010253 3336 0.048818944
3340 0.06415214 3340 0.068307265 1700 0.028919607 1704
0.05192664 3344 0.044194701 3344 0.030078403
3350 0.110717592 3350 0.047060792 1710 0.062397659 1708
0.05027587 3352 0.083172483 3352 0.03600412
3360 0.178662464 3360 0.004999389 1710 0.059790404 1712
0.079601588 3360 0.093786228 3360 0.050431729
3370 0.188253689 3370 0.102054139 1720 0.027845377 1716
0.071761053 3368 0.05465623 3368 0.040629126
3380 0.127876396 3380 0.157056085 1720 0.009026451 1720
0.057424692 3376 0.054936805 3376 0.032255623
3380 0.069547095 3380 0.115209397 1720 0.031939249 1724
0.092098118 3384 0.05182331 3384 0.030728865
3390 0.093482369 3390 0.04923626 1730 0.036181202 1728
0.097770135 3392 0.040654053 3392 0.017878669
3400 0.129246851 3400 0.025389736 1730 0.027577748 1732
0.047088397 3400 0.014252296 3400 0.011549372
3410 0.092136317 3410 0.099623772 1740 0.074507414 1736
0.034366727 3408 0.034587309 3408 0.043207605
3420 0.054818309 3420 0.098008866 1740 0.102972867 1740
0.046028752 3416 0.059766026 3416 0.041648498
3420 0.104481427 3420 0.08937277 1740 0.054614411 1744
0.03044483 3424 0.088284978 3424 0.037973579
3430 0.142934616 3430 0.151536384 1750 0.039575782 1748
0.083062769 3432 0.125465478 3432 0.034433753
3440 0.137421579 3440 0.165317514 1750 0.060165952 1752
0.135950395 3440 0.126882849 3440 0.021373924
3450 0.081163089 3450 0.096705415 1760 0.042242125 1756
0.155602058 3448 0.117340802 3448 0.050495364
3460 0.032847591 3460 0.06393771 1760 0.036176662 1760
0.140073623 3456 0.125129066 3456 0.034512035
3460 0.090062335 3460 0.026251153 1760 0.046829031 1764
0.109840665 3464 0.13631901 3464 0.047009497
3470 0.12400336 3470 0.101895814 1770 0.05840656 1768
0.082780251 3472 0.152917885 3472 0.069108726
3480 0.050089315 3480 0.124880797 1770 0.054252199 1772
0.071039685 3480 0.128878674 3480 0.055383574
3490 0.05449 3490 0.050263549 1780 0.045454402 1776
0.075098789 3488 0.085477674 3488 0.029115414
3500 0.047744863 3500 0.050839819 1780 0.033330878 1780
0.078728932 3496 0.086091401 3496 0.015899805
3500 0.076609322 3500 0.054943179 1780 0.028807514 1784
0.072557275 3504 0.078962054 3504 0.015426827
3510 0.107465385 3510 0.055266988 1790 0.027879705 1788
0.026616855 3512 0.105555868 3512 0.023200113
3520 0.079213154 3520 0.031821121 1790 0.012745185 1792
0.049130496 3520 0.198062408 3520 0.024826039
3530 0.064712025 3530 0.050301642 1800 0.003657147 1796
0.081303326 3528 0.182937321 3528 0.007857799
3540 0.123301783 3540 0.070006485 1800 0.012559081 1800
0.069728194 3536 0.213655425 3536 0.052250798
3540 0.165392819 3540 0.008755713 1800 0.024712321 1804
0.059333557 3544 0.222509465 3544 0.061095678
3550 0.207390665 3550 0.052692773 1810 0.04383038 1808
0.090886773 3552 0.127823005 3552 0.022290376
3560 0.151042914 3560 0.073791722 1810 0.022535514 1812
0.098206161 3560 0.133823924 3560 0.025052374
3570 0.08000798 3570 0.151571789 1820 0.031296884 1816

0.076233089 3568 0.150519466 3568 0.034783312
3580 0.082899809 3580 0.218027213 1820 0.056553232 1820
0.091632777 3576 0.190642575 3576 0.02669566
3580 0.07564079 3580 0.196099485 1820 0.045409986 1824
0.101352613 3584 0.197785558 3584 0.041845553
3590 0.024850097 3590 0.115732437 1830 0.023662795 1828
0.057887668 3592 0.158738724 3592 0.013510988
3600 0.040154544 3600 0.054216162 1830 0.028770335 1832
0.029254445 3600 0.164330966 3600 0.04472601
3610 0.087524539 3610 0.040386858 1840 0.030598851 1836
0.032918564 3608 0.132051093 3608 0.041058484
3620 0.140901379 3620 0.084154126 1840 0.033640783 1840
0.052204854 3616 0.094631854 3616 0.011952427
3620 0.131300985 3620 0.113346257 1840 0.043710574 1844
0.056182911 3624 0.101317819 3624 0.032506068
3630 0.071670642 3630 0.026467758 1850 0.056120702 1848
0.028252978 3632 0.093588744 3632 0.003840017
3640 0.044139975 3640 0.093246425 1850 0.033461893 1852
0.017028644 3640 0.091864931 3640 0.020402911
3650 0.064562009 3650 0.097003118 1860 0.008941001 1856
0.035616926 3648 0.131916023 3648 0.014269279
3660 0.070194088 3660 0.007647554 1860 0.017518449 1860
0.010980653 3656 0.150394844 3656 0.01645333
3660 0.133867856 3660 0.120471348 1860 0.030254363 1864
0.076758239 3664 0.102361533 3664 0.031775075
3670 0.192945169 3670 0.170850938 1870 0.045488276 1868
0.090415982 3672 0.051968553 3672 0.026815138
3680 0.105125044 3680 0.106237269 1870 0.05347393 1872
0.065048895 3680 0.079946418 3680 0.032898821
3690 0.046537469 3690 0.005265729 1880 0.048147344 1876
0.026759479 3688 0.123533726 3688 0.03592567
3700 0.091946866 3700 0.028902181 1880 0.022273922 1880
0.046931698 3696 0.172129178 3696 0.02880508
3700 0.086152242 3700 0.04275639 1880 0.028629358 1884
0.032021941 3704 0.153010464 3704 0.019973531
3710 0.061466264 3710 0.026399614 1890 0.031160646 1888
0.050229955 3712 0.0769486 3712 0.064225023
3720 0.043530559 3720 0.135109818 1890 0.036044476 1892
0.061085535 3720 0.057705853 3720 0.076395052
3730 0.16393399 3730 0.177614784 1900 0.036144735 1896
0.063251202 3728 0.072347059 3728 0.061499748
3740 0.160258598 3740 0.088745939 1900 0.031632721 1900
0.067820634 3736 0.08778838 3736 0.044914461
3740 0.061619183 3740 0.032123327 1900 0.036373662 1904
0.068699774 3744 0.051178446 3744 0.028557741
3750 0.058286449 3750 0.120802943 1910 0.037860958 1908
0.049287832 3752 0.089392968 3752 0.010471153
3760 0.096236858 3760 0.140685588 1910 0.020544432 1912
0.041986565 3760 0.066265428 3760 0.024610739
3770 0.12183575 3770 0.099091471 1920 0.010727152 1916
0.064804161 3768 0.004117468 3768 0.008392047
3780 0.074937663 3780 0.065081666 1920 0.013601943 1920
0.063139894 3776 0.054786171 3776 0.033734883
3780 0.020773798 3780 0.033784912 1920 0.054626227 1924
0.036603586 3784 0.059574024 3784 0.024145613
3790 0.060206876 3790 0.050736828 1930 0.077136137 1928
0.057233265 3792 0.037836642 3792 0.062979605
3800 0.101671794 3800 0.081075596 1930 0.069673661 1932
0.061666782 3800 0.045660225 3800 0.051646668
3810 0.093831455 3810 0.146574341 1940 0.07278171 1936
0.042643285 3808 0.097552365 3808 0.013206698
3820 0.054012817 3820 0.220199159 1940 0.063937594 1940
0.020538999 3816 0.13556912 3816 0.025492271
3820 0.055328517 3820 0.175439578 1940 0.022461114 1944
0.032374752 3824 0.151371569 3824 0.062985244
3830 0.136457384 3830 0.03731353 1950 0.066053879 1948
0.047004083 3832 0.116206007 3832 0.074679527
3840 0.126628468 3840 0.143595869 1950 0.062175786 1952

0.033809149 3840 0.016249789 3840 0.039302053
3850 0.100390367 3850 0.168648753 1960 0.035969599 1956
0.020768879 3848 0.103932187 3848 0.002989642
3860 0.107528103 3860 0.151745626 1960 0.028045371 1960
0.027964759 3856 0.181063588 3856 0.023284634
3860 0.053515105 3860 0.09129812 1960 0.042758002 1964
0.045196466 3864 0.188136838 3864 0.048026737
3870 0.048087619 3870 0.068436224 1970 0.044237913 1968
0.035291676 3872 0.137373529 3872 0.061548788
3880 0.068777343 3880 0.148206061 1970 0.033528198 1972
0.058519123 3880 0.093283336 3880 0.060485349
3890 0.060750957 3890 0.161298289 1980 0.097798133 1976
0.07462041 3888 0.076137076 3888 0.03988075
3900 0.101799727 3900 0.175094393 1980 0.105901556 1980
0.066684625 3896 0.072940035 3896 0.018233637
3900 0.124003331 3900 0.201693794 1980 0.088662826 1984
0.054400996 3904 0.108254542 3904 0.021972523
3910 0.125698396 3910 0.145139915 1990 0.05940432 1988
0.041400068 3912 0.14027649 3912 0.035233919
3920 0.180125775 3920 0.039859722 1990 0.041837495 1992
0.013134636 3920 0.072717354 3920 0.024346609
3930 0.159120551 3930 0.170232874 2000 0.039276623 1996
0.054604876 3928 0.030042094 3928 0.010151633
3940 0.046981983 3940 0.153637491 2000 0.053143132 2000
0.067142013 3936 0.06672863 3936 0.027817805
3940 0.026745676 3940 0.103602528 2000 0.05147935 2004
0.052224033 3944 0.106401247 3944 0.02184587
3950 0.023298611 3950 0.072110866 2010 0.076035693 2008
0.044858967 3952 0.153709741 3952 0.020288475
3960 0.044088585 3960 0.034622746 2010 0.084848813 2012
0.019359799 3960 0.13428407 3960 0.04584192
3970 0.080455102 3970 0.071130089 2020 0.051916464 2016
0.037193044 3968 0.05444212 3968 0.034381385
3980 0.080493708 3980 0.098819677 2020 0.059508569 2020
0.063503227 3976 0.095359173 3976 0.012135373
3980 0.084107385 3980 0.105122592 2020 0.104565661 2024
0.057659268 3984 0.144547695 3984 0.014865953
3990 0.090892128 3990 0.092143921 2030 0.122217753 2028
0.037973015 3992 0.07744995 3992 0.020073439
4000 0.077140612 4000 0.066244 2030 0.103580351 2032
0.022773847 4000 0.053334316 4000 0.020742431
4010 0.1016233 4010 0.048518163 2040 0.067353198 2036
0.046676451 4008 0.117786804 4008 0.03707529
4020 0.1283936 4020 0.082697872 2040 0.064972897 2040
0.08033705 4016 0.148460749 4016 0.014721695
4020 0.126236584 4020 0.125902225 2040 0.066382199 2044
0.091077716 4024 0.188046019 4024 0.029413544
4030 0.071534167 4030 0.087640867 2050 0.048499485 2048
0.111045862 4032 0.119094868 4032 0.041258354
4040 0.047423593 4040 0.087785433 2050 0.019130404 2052
0.115440678 4040 0.103516548 4040 0.019135248
4050 0.142234741 4050 0.124805505 2060 0.018867018 2056
0.087239685 4048 0.173759006 4048 0.054643879
4060 0.170098458 4060 0.115943549 2060 0.048625381 2060
0.061225881 4056 0.188208985 4056 0.048236205
4060 0.183611119 4060 0.169078689 2060 0.060847251 2064
0.033296921 4064 0.129182314 4064 0.032172815
4070 0.142191886 4070 0.16498813 2070 0.041885218 2068
0.051801795 4072 0.1379147 4072 0.088305271
4080 0.046315785 4080 0.153252128 2070 0.030761268 2072
0.055196084 4080 0.213997336 4080 0.106946936
4090 0.045833891 4090 0.140984906 2080 0.043362972 2076
0.057417925 4088 0.187782993 4088 0.080742328
4100 0.043389322 4100 0.088238085 2080 0.02822205 2080
0.03198738 4096 0.096665121 4096 0.038140141
4100 0.084298597 4100 0.043262626 2080 0.032869913 2084
0.03261368 4104 0.069759117 4104 0.048073387
4110 0.108961845 4110 0.067758039 2090 0.05568855 2088

0.052068237 4112 0.096261196 4112 0.07370473
4120 0.152598034 4120 0.158165975 2090 0.027146771 2092
0.034744033 4120 0.139736119 4120 0.065716675
4130 0.154679685 4130 0.115998715 2100 0.015428152 2096
0.037626007 4128 0.112610855 4128 0.052562991
4140 0.16018044 4140 0.036079022 2100 0.013868063 2100
0.064576714 4136 0.031217733 4136 0.052419389
4140 0.138103336 4140 0.023640769 2100 0.027551134 2104
0.058392627 4144 0.08899378 4144 0.053265714
4150 0.059579259 4150 0.034096356 2110 0.040880084 2108
0.049961651 4152 0.112434769 4152 0.05766088
4160 0.025039171 4160 0.066323824 2110 0.044396027 2112
0.060535509 4160 0.115588249 4160 0.044166784
4170 0.052266172 4170 0.039958894 2120 0.056920009 2116
0.057639903 4168 0.122471989 4168 0.032393324
4180 0.108959815 4180 0.175562775 2120 0.079076424 2120
0.044494918 4176 0.166227954 4176 0.020479773
4180 0.110497822 4180 0.229816316 2120 0.080068843 2124
0.06491119 4184 0.102856822 4184 0.005144113
4190 0.080632875 4190 0.157138479 2130 0.033101118 2128
0.093369861 4192 0.099009099 4192 0.01327651
4200 0.116226271 4200 0.111512803 2130 0.032340642 2132
0.090925176 4200 0.165788414 4200 0.043460215
4210 0.148829539 4210 0.144870603 2140 0.016655586 2136
0.092638496 4208 0.157605813 4208 0.057920406
4220 0.128683533 4220 0.115552168 2140 0.098916768 2140
0.083237508 4216 0.131843684 4216 0.044852859
4220 0.060426133 4220 0.105241357 2140 0.121788973 2144
0.06155231 4224 0.058995363 4224 0.030545478
4230 0.019010824 4230 0.149873973 2150 0.071141578 2148
0.088727793 4232 0.057537272 4232 0.038103579
4240 0.025593321 4240 0.128874075 2150 0.044813092 2152
0.063933207 4240 0.054197008 4240 0.051279851
4250 0.0688582 4250 0.118760632 2160 0.065431574 2156
0.007970979 4248 0.081905128 4248 0.03115413
4260 0.121524674 4260 0.143633064 2160 0.04354132 2160
0.031546846 4256 0.112913935 4256 0.030742227
4260 0.102419115 4260 0.10119428 2160 0.032203268 2164
0.047763711 4264 0.094024894 4264 0.065470085
4270 0.126300816 4270 0.04001569 2170 0.042631109 2168
0.101055339 4272 0.09201542 4272 0.053119198
4280 0.147561994 4280 0.046686673 2170 0.023688617 2172
0.109911147 4280 0.117355899 4280 0.038736176
4290 0.122195459 4290 0.066203196 2180 0.050935127 2176
0.086950618 4288 0.110850378 4288 0.064606888
4300 0.052272633 4300 0.032637458 2180 0.024834142 2180
0.070570146 4296 0.085926447 4296 0.05905631
4300 0.0610551 4300 0.025551948 2180 0.03207746 2184
0.051578434 4304 0.050996499 4304 0.033445864
4310 0.052850042 4310 0.055534005 2190 0.05902936 2188
0.056082878 4312 0.131847803 4312 0.044265264
4320 0.057327776 4320 0.078598991 2190 0.069417183 2192
0.053565942 4320 0.183225683 4320 0.091831891
4330 0.060352013 4330 0.050727769 2200 0.076595214 2196
0.006678047 4328 0.150115331 4328 0.099174533
4340 0.070169357 4340 0.024531693 2200 0.05101788 2200
0.044048076 4336 0.077359917 4336 0.059926137
4340 0.035033056 4340 0.026336458 2200 0.020897787 2204
0.064322849 4344 0.083603474 4344 0.02636446
4350 0.059485545 4350 0.052896303 2210 0.079928621 2208
0.057249745 4352 0.077858051 4352 0.046762598
4360 0.082998507 4360 0.116815616 2210 0.070873823 2212
0.060770453 4360 0.018513627 4360 0.032989017
4370 0.126742714 4370 0.094142211 2220 0.02405928 2216
0.036352391 4368 0.036929585 4368 0.059946899
4380 0.135896756 4380 0.067333225 2220 0.047837628 2220
0.006602885 4376 0.056208144 4376 0.103572391
4380 0.175064619 4380 0.107204854 2220 0.022177615 2224

0.039979044	4384	0.061877901	4384	0.095899406		
4390	0.2219991	4390	0.179544164	2230	0.024292936	2228
0.078553654	4392	0.148664054	4392	0.072156094		
4400	0.205687029	4400	0.187535058	2230	0.011382376	2232
0.078031924	4400	0.173220585	4400	0.055905206		
4410	0.169601728	4410	0.093694798	2240	0.009646954	2236
0.063325671	4408	0.109678615	4408	0.042866926		
4420	0.149641463	4420	0.009446932	2240	0.024868483	2240
0.051708936	4416	0.095384457	4416	0.049544156		
4420	0.085677028	4420	0.034061246	2240	0.052279265	2244
0.054078733	4424	0.094476054	4424	0.053120602		
4430	0.041374955	4430	0.064044718	2250	0.043701773	2248
0.073963573	4432	0.141006006	4432	0.04740819		
4440	0.118378339	4440	0.1618159	2250	0.037799928	2252
0.107370055	4440	0.182682575	4440	0.054301094		
4450	0.106372158	4450	0.189587139	2260	0.034305791	2256
0.090377915	4448	0.235699946	4448	0.0711139		
4460	0.088107605	4460	0.138607808	2260	0.020540947	2260
0.045399298	4456	0.264678209	4456	0.079773548		
4460	0.07317985	4460	0.081039805	2260	0.02974465	2264
0.018758612	4464	0.23051136	4464	0.073421994		
4470	0.066982102	4470	0.031365467	2270	0.046806315	2268
0.024528659	4472	0.141944198	4472	0.040077484		
4480	0.091122594	4480	0.06314864	2270	0.048396232	2272
0.033624816	4480	0.033033532	4480	0.03289049		
4490	0.100009478	4490	0.091378293	2280	0.054865635	2276
0.024995597	4488	0.10232049	4488	0.075372314		
4500	0.061500119	4500	0.034897443	2280	0.064310567	2280
0.032967892	4496	0.077393357	4496	0.074258394		
4500	0.075157215	4500	0.066043613	2280	0.066883898	2284
0.038613103	4504	0.117633143	4504	0.04115545		
4510	0.083339975	4510	0.118545613	2290	0.095282892	2288
0.024047628	4512	0.113857001	4512	0.046561097		
4520	0.093550734	4520	0.073785137	2290	0.110120338	2292
0.08180365	4520	0.101425641	4520	0.07031062		
4530	0.117640891	4530	0.045697852	2300	0.090871014	2296
0.097301811	4528	0.1372158	4528	0.056122568		
4540	0.13523722	4540	0.061612365	2300	0.054822136	2300
0.06252186	4536	0.121873767	4536	0.014698185		
4540	0.089941997	4540	0.023915929	2300	0.054700391	2304
0.008084333	4544	0.076850578	4544	0.030321027		
4550	0.089163696	4550	0.090275025	2310	0.06966589	2308
0.035078076	4552	0.105567313	4552	0.048661364		
4560	0.137810945	4560	0.134248709	2310	0.047818474	2312
0.039210605	4560	0.126133208	4560	0.047736216		
4570	0.11852484	4570	0.115679075	2320	0.02840806	2316
0.047771249	4568	0.103905244	4568	0.065906286		
4580	0.1324529	4580	0.088567635	2320	0.024828454	2320
0.049687147	4576	0.118093412	4576	0.087753804		
4580	0.127864201	4580	0.023350049	2320	0.013275627	2324
0.047459511	4584	0.106659245	4584	0.074678035		
4590	0.06297636	4590	0.127530366	2330	0.041938685	2328
0.033904107	4592	0.084863968	4592	0.039016417		
4600	0.015396508	4600	0.151562955	2330	0.067648114	2332
0.002953709	4600	0.107410459	4600	0.027562155		
4610	0.066671804	4610	0.025822857	2340	0.059889128	2336
0.055313722	4608	0.084957515	4608	0.041151983		
4620	0.099609024	4620	0.082037674	2340	0.034839839	2340
0.068777161	4616	0.079000216	4616	0.049434322		
4620	0.086949316	4620	0.018451112	2340	0.023770244	2344
0.03346815	4624	0.106184714	4624	0.042764539		
4630	0.111885529	4630	0.07749621	2350	0.058449987	2348
0.028459353	4632	0.079502912	4632	0.030735555		
4640	0.102425664	4640	0.048917405	2350	0.053043048	2352
0.03989082	4640	0.036699854	4640	0.027453871		
4650	0.092626331	4650	0.031646399	2360	0.021025559	2356
0.018691975	4648	0.029644034	4648	0.041351312		
4660	0.099528901	4660	0.059237449	2360	0.043837612	2360

0.021189171 4656 0.036988691 4656 0.050402454
4660 0.085845131 4660 0.058035414 2360 0.045843681 2364
0.040463616 4664 0.083885308 4664 0.064073924
4670 0.068690839 4670 0.041931744 2370 0.029208828 2368
0.065027853 4672 0.059338483 4672 0.05288727
4680 0.071487157 4680 0.05664293 2370 0.049189126 2372
0.05269489 4680 0.009639117 4680 0.020021931
4690 0.131223481 4690 0.084549058 2380 0.048838421 2376
0.032125881 4688 0.015581221 4688 0.010668623
4700 0.20488177 4700 0.038913306 2380 0.027402537 2380
0.035972735 4696 0.027866534 4696 0.021920339
4700 0.2208808 4700 0.056251207 2380 0.010287577 2384
0.034786033 4704 0.076877041 4704 0.018801822
4710 0.17381132 4710 0.058518526 2390 0.04428054 2388
0.046646288 4712 0.156487731 4712 0.028295568
4720 0.079276731 4720 0.145429193 2390 0.052629675 2392
0.027298063 4720 0.174516055 4720 0.014738723
4730 0.015982714 4730 0.106157102 2400 0.049246562 2396
0.021250347 4728 0.088375731 4728 0.018023475
4740 0.031322852 4740 0.057506899 2400 0.063920459 2400
0.055851 4736 0.054541368 4736 0.062338862
4740 0.093725961 4740 0.102289472 2400 0.064250649 2404
0.063481559 4744 0.094574745 4744 0.08164929
4750 0.114957154 4750 0.121502366 2410 0.053804313 2408
0.080026694 4752 0.056264169 4752 0.030997202
4760 0.102263563 4760 0.0351993 2410 0.053285105 2412
0.084104511 4760 0.060870545 4760 0.022531498
4770 0.102714716 4770 0.147078565 2420 0.053848449 2416
0.062357998 4768 0.182775184 4768 0.04724065
4780 0.071364913 4780 0.211532416 2420 0.031619104 2420
0.02831711 4776 0.22380134 4776 0.065047876
4780 0.003358385 4780 0.181633339 2420 0.003430759 2424
0.034484245 4784 0.21082435 4784 0.071111266
4790 0.027505916 4790 0.121948368 2430 0.020847534 2428
0.083711777 4792 0.188274396 4792 0.043794949
4800 0.08865499 4800 0.052156134 2430 0.04016377 2432
0.085121836 4800 0.113952359 4800 0.01023461
4810 0.114506183 4810 0.075680822 2440 0.03046824 2436
0.063977685 4808 0.032338892 4808 0.020396794
4820 0.007216621 4820 0.077174191 2440 0.018365843 2440
0.054258147 4816 0.040579063 4816 0.048156224
4820 0.115672483 4820 0.009028275 2440 0.009802266 2444
0.024522386 4824 0.073963274 4824 0.050253912
4830 0.100569137 4830 0.028725917 2450 0.055614735 2448
0.043978034 4832 0.07682111 4832 0.042359799
4840 0.041505165 4840 0.015911353 2450 0.042896769 2452
0.070966402 4840 0.069493275 4840 0.025283558
4850 0.023316134 4850 0.048576298 2460 0.018781688 2456
0.097262142 4848 0.09157262 4848 0.012780431
4860 0.093770235 4860 0.04083649 2460 0.029125416 2460
0.069520007 4856 0.08120593 4856 0.028562134
4860 0.127625346 4860 0.03379428 2460 0.025206691 2464
0.030775969 4864 0.126136773 4864 0.060635644
4870 0.082552324 4870 0.110066569 2470 0.029701072 2468
0.073730706 4872 0.164002283 4872 0.079457772
4880 0.016251406 4880 0.149724293 2470 0.055633842 2472
0.07006446 4880 0.168009894 4880 0.064126529
4890 0.042428208 4890 0.177823385 2480 0.06422514 2476
0.065554086 4888 0.116556526 4888 0.051185605
4900 0.068340101 4900 0.193385859 2480 0.056802757 2480
0.056211593 4896 0.085669803 4896 0.019139314
4900 0.037866008 4900 0.10334819 2480 0.067794834 2484
0.041963966 4904 0.04740011 4904 0.014047599
4910 0.017995248 4910 0.079669175 2490 0.079625563 2488
0.017835748 4912 0.055184948 4912 0.046782883
4920 0.04211365 4920 0.090809408 2490 0.074678719 2492
0.019109119 4920 0.116391908 4920 0.07217635
4930 0.010442755 4930 0.09439281 2500 0.074636519 2496

0.039248032	4928	0.136023883	4928	0.065792185		
4940	0.091187889	4940	0.093541901	2500	0.088739434	2500
0.016364276	4936	0.076681419	4936	0.057660393		
4940	0.138801581	4940	0.16538192	2500	0.074708602	2504
0.037404676	4944	0.013089835	4944	0.055546821		
4950	0.067178102	4950	0.182215343	2510	0.037928679	2508
0.055005272	4952	0.060691637	4952	0.021061476		
4960	0.018938919	4960	0.135125534	2510	0.057909332	2512
0.037128633	4960	0.19434467	4960	0.043537813		
4970	0.02035274	4970	0.097731725	2520	0.059390612	2516
0.031018251	4968	0.246126729	4968	0.067398105		
4980	0.079228747	4980	0.049038823	2520	0.028448754	2520
0.080447848	4976	0.174846151	4976	0.045563615		
4980	0.114870425	4980	0.028897015	2520	0.027508208	2524
0.067666952	4984	0.095191499	4984	0.030634994		
4990	0.104264807	4990	0.102761325	2530	0.059011811	2528
0.046103367	4992	0.049012055	4992	0.032923195		
5000	0.026957543	5000	0.103139493	2530	0.057218946	2532
0.081477345	5000	0.080022539	5000	0.032887001		
5010	0.070713737	5010	0.049458973	2540	0.025496	2536
0.088492976	5008	0.107440355	5008	0.030623571		
5020	0.091052825	5020	0.144864549	2540	0.040362171	2540
0.057552828	5016	0.053828459	5016	0.029285533		
5020	0.04409493	5020	0.068157635	2540	0.008404878	2544
0.057211539	5024	0.024700214	5024	0.045735225		
5030	0.042996013	5030	0.089306959	2550	0.04969896	2548
0.051981169	5032	0.046719633	5032	0.046388319		
5040	0.081980048	5040	0.113052782	2550	0.070225295	2552
0.004852327	5040	0.097861972	5040	0.019524568		
5050	0.14684668	5050	0.058016365	2560	0.05519122	2556
0.044393597	5048	0.079096615	5048	0.061022613		
5060	0.142655874	5060	0.086993503	2560	0.041967294	2560
0.023310313	5056	0.063925087	5056	0.043330969		
5060	0.086570275	5060	0.043553242	2560	0.043952008	2564
0.022183735	5064	0.092864408	5064	0.01157242		
5070	0.092368129	5070	0.126338811	2570	0.044350207	2568
0.051313258	5072	0.100891557	5072	0.015038704		
5080	0.052861095	5080	0.192745545	2570	0.045312721	2572
0.058378788	5080	0.090238267	5080	0.033821514		
5090	0.016392061	5090	0.186234044	2580	0.029047513	2576
0.062703635	5088	0.101117745	5088	0.04784621		
5100	0.064687214	5100	0.130379369	2580	0.066197616	2580
0.081009712	5096	0.061845596	5096	0.039074705		
5100	0.105691433	5100	0.118287389	2580	0.085857864	2584
0.081650207	5104	0.030169282	5104	0.014018075		
5110	0.091410155	5110	0.09914254	2590	0.076008393	2588
0.075128221	5112	0.056397119	5112	0.022129618		
5120	0.077033627	5120	0.059971451	2590	0.066915287	2592
0.091311493	5120	0.009766366	5120	0.03325193		
5130	0.087218839	5130	0.026926136	2600	0.068022018	2596
0.12303656	5128	0.014782703	5128	0.032699405		
5140	0.109868233	5140	0.046550886	2600	0.063600935	2600
0.084600062	5136	0.071951923	5136	0.046146626		
5140	0.09476878	5140	0.134927831	2600	0.040716124	2604
0.02537611	5144	0.123940787	5144	0.065940585		
5150	0.0926141	5150	0.169397609	2610	0.033813856	2608
0.010310588	5152	0.072598792	5152	0.049230977		
5160	0.080545418	5160	0.102087397	2610	0.023762397	2612
0.033741351	5160	0.102672231	5160	0.017362399		
5170	0.044611104	5170	0.009311093	2620	0.031546781	2616
0.053187876	5168	0.167365171	5168	0.013967896		
5180	0.049789283	5180	0.078689402	2620	0.016273829	2620
0.049487724	5176	0.234043998	5176	0.02955048		
5180	0.039375249	5180	0.129231238	2620	0.044063614	2624
0.032752854	5184	0.198957249	5184	0.025381241		
5190	0.068293441	5190	0.167292746	2630	0.067352696	2628
0.032391119	5192	0.045913443	5192	0.029422639		
5200	0.144840131	5200	0.120414894	2630	0.069211907	2632

0.053019427 5200 0.167500679 5200 0.031677089
5210 0.137071635 5210 0.069289716 2640 0.03240646 2636
0.070255133 5208 0.163425823 5208 0.003276326
5220 0.062507723 5220 0.05600127 2640 0.020260988 2640
0.062375781 5216 0.087560191 5216 0.031933611
5220 0.061330029 5220 0.075655356 2640 0.031495671 2644
0.034755612 5224 0.075956501 5224 0.044842465
5230 0.05902733 5230 0.086592569 2650 0.019447663 2648
0.021416423 5232 0.111347341 5232 0.060406303
5240 0.026310921 5240 0.056709589 2650 0.044295517 2652
0.079954589 5240 0.102049729 5240 0.068457266
5250 0.067015411 5250 0.0730838 2660 0.047398713 2656
0.07361461 5248 0.097762197 5248 0.043156058
5260 0.067510955 5260 0.118475677 2660 0.080797989 2660
0.025867368 5256 0.100438585 5256 0.017984459
5260 0.079472135 5260 0.153626461 2660 0.084453634 2664
0.007544067 5264 0.116686475 5264 0.043785734
5270 0.092359907 5270 0.090500318 2670 0.033588563 2668
0.008624635 5272 0.128515239 5272 0.040020408
5280 0.078978374 5280 0.062300482 2670 0.080027043 2672
0.011799461 5280 0.057803143 5280 0.032188964
5290 0.051851814 5290 0.059672591 2680 0.101810088 2676
0.023978866 5288 0.055532772 5288 0.08511528
5300 0.033313732 5300 0.026023019 2680 0.057770518 2680
0.008686318 5296 0.014334582 5296 0.097274606
5300 0.014943432 5300 0.062504369 2680 0.056498411 2684
0.049278671 5304 0.050056133 5304 0.047495636
5310 0.049380389 5310 0.046297166 2690 0.071695351 2688
0.040225943 5312 0.068163812 5312 0.05818325
5320 0.026363447 5320 0.055551907 2690 0.041390053 2692
0.019455543 5320 0.07603853 5320 0.051859282
5330 0.038080292 5330 0.066727967 2700 0.02929288 2696
0.043914301 5328 0.082821214 5328 0.025101932
5340 0.087262568 5340 0.052936059 2700 0.041470936 2700
0.074421529 5336 0.086803142 5336 0.006780392
5340 0.140470729 5340 0.057929352 2700 0.04491608 2704
0.054398792 5344 0.152024979 5344 0.013027221
5350 0.185850964 5350 0.056011057 2710 0.054671582 2708
0.052317682 5352 0.131729801 5352 0.014232433
5360 0.198731709 5360 0.110849898 2710 0.05857593 2712
0.043755103 5360 0.043952528 5360 0.04504704
5370 0.149265295 5370 0.084008112 2720 0.039146093 2716
0.045960547 5368 0.097567412 5368 0.043444219
5380 0.062155566 5380 0.054481461 2720 0.023928486 2720
0.053179123 5376 0.133929934 5376 0.021450704
5380 0.119651195 5380 0.043271673 2720 0.044240289 2724
0.041376778 5384 0.124876853 5384 0.02092974
5390 0.133915775 5390 0.186629099 2730 0.073250623 2728
0.047011366 5392 0.167690189 5392 0.050640068
5400 0.067899935 5400 0.208799378 2730 0.068650967 2732
0.030226482 5400 0.147970306 5400 0.071483904
5410 0.057910587 5410 0.118875469 2740 0.067963367 2736
0.021704887 5408 0.059592247 5408 0.062821469
5420 0.124178056 5420 0.087114364 2740 0.05055785 2740
0.023931296 5416 0.023881848 5416 0.037593585
5420 0.118351411 5420 0.143883575 2740 0.015648677 2744
0.047232439 5424 0.051234205 5424 0.011590654
5430 0.069257556 5430 0.166665705 2750 0.013937123 2748
0.051292052 5432 0.066156492 5432 0.024438412
5440 0.062355321 5440 0.108258544 2750 0.044390279 2752
0.031409138 5440 0.037601425 5440 0.049302795
5450 0.014071014 5450 0.062371139 2760 0.040981529 2756
0.011202255 5448 0.028854385 5448 0.040905761
5460 0.05218084 5460 0.08826665 2760 0.056618246 2760
0.011212576 5456 0.037271868 5456 0.011373853
5460 0.097448319 5460 0.080047947 2760 0.069470836 2764
0.014561309 5464 0.071777016 5464 0.00770995
5470 0.045368637 5470 0.02256251 2770 0.057668796 2768

0.057422472 5472 0.097585937 5472 0.014316882
5480 0.048017359 5480 0.117575284 2770 0.074833792 2772
0.062823587 5480 0.09139625 5480 0.026287888
5490 0.053501586 5490 0.17044376 2780 0.07359335 2776
0.040772124 5488 0.099305114 5488 0.035254812
5500 0.089971596 5500 0.098462551 2780 0.048096827 2780
0.041563755 5496 0.169123189 5496 0.035936504
5500 0.05851519 5500 0.013771705 2780 0.042375472 2784
0.037664515 5504 0.17844142 5504 0.014787876
5510 0.044484285 5510 0.024328887 2790 0.016604212 2788
0.005437398 5512 0.156091366 5512 0.015106809
5520 0.059258273 5520 0.031311745 2790 0.007035251 2792
0.044824137 5520 0.149542815 5520 0.023420729
5530 0.029376295 5530 0.093953371 2800 0.022981494 2796
0.045276178 5528 0.135982715 5528 0.023016795
5540 0.098347729 5540 0.081039216 2800 0.003963456 2800
0.00296063 5536 0.119351404 5536 0.037933853
5540 0.066271292 5540 0.026875006 2800 0.036843612 2804
0.030735824 5544 0.030735984 5544 0.037198952
5550 0.065670538 5550 0.038924038 2810 0.035198176 2808
0.03007403 5552 0.084309184 5552 0.025913267
5560 0.10677395 5560 0.09236273 2810 0.049325645 2812
0.07403244 5560 0.11194258 5560 0.038802329
5570 0.057649206 5570 0.158610638 2820 0.060660615 2816
0.058528593 5568 0.034080251 5568 0.059716156
5580 0.106662548 5580 0.106431042 2820 0.037393129 2820
0.023247871 5576 0.109151974 5576 0.041564901
5580 0.154966866 5580 0.07709604 2820 0.025414916 2824
0.075476928 5584 0.13393948 5584 0.019225474
5590 0.156590511 5590 0.123432386 2830 0.026516818 2828
0.063110718 5592 0.07281547 5592 0.064250693
5600 0.16098535 5600 0.066489505 2830 0.040190305 2832
0.041693533 5600 0.059289403 5600 0.068715883
5610 0.090180918 5610 0.015065442 2840 0.061447987 2836
0.059414793 5608 0.111601541 5608 0.040562321
5620 0.014225429 5620 0.042577176 2840 0.043014752 2840
0.039723222 5616 0.14928884 5616 0.005549328
5620 0.034820267 5620 0.049240665 2840 0.018773324 2844
0.021007578 5624 0.168029655 5624 0.038121823
5630 0.035140816 5630 0.027793776 2850 0.049578921 2848
0.035832713 5632 0.159729578 5632 0.03984509
5640 0.086754357 5640 0.11236767 2850 0.042827905 2852
0.045594792 5640 0.129933324 5640 0.042816359
5650 0.120434881 5650 0.069665395 2860 0.071319635 2856
0.039668943 5648 0.093935953 5648 0.054555709
5660 0.119973265 5660 0.020881711 2860 0.073355506 2860
0.028034418 5656 0.066901615 5656 0.051903331
5660 0.102459795 5660 0.036192203 2860 0.03074254 2864
0.058214762 5664 0.02667646 5664 0.045024182
5670 0.053952419 5670 0.018603965 2870 0.020708278 2868
0.085948472 5672 0.062674713 5672 0.040254137
5680 0.025263402 5680 0.060065107 2870 0.02066757 2872
0.069749338 5680 0.08277125 5680 0.021922682
5690 0.039374561 5690 0.086255728 2880 0.001256853 2876
0.048791131 5688 0.096805263 5688 0.015824478
5700 0.03172267 5700 0.097495402 2880 0.012475315 2880
0.07677175 5696 0.080675396 5696 0.020922864
5700 0.089997215 5700 0.159149568 2880 0.037703547 2884
0.061798441 5704 0.045609555 5704 0.015533182
5710 0.046195659 5710 0.145867365 2890 0.070843962 2888
0.034554785 5712 0.182618024 5712 0.015604799
5720 0.050115341 5720 0.051168485 2890 0.078698111 2892
0.047836678 5720 0.262909161 5720 0.043821357
5730 0.127236694 5730 0.065817796 2900 0.062730251 2896
0.054870827 5728 0.189603409 5728 0.063613581
5740 0.148376901 5740 0.148804175 2900 0.02958669 2900
0.023727598 5736 0.083132582 5736 0.056049903
5740 0.08599936 5740 0.185989135 2900 0.026646592 2904

0.039080616 5744 0.08869623 5744 0.050014602
5750 0.079964062 5750 0.131877925 2910 0.059016362 2908
0.039357245 5752 0.115246861 5752 0.051690317
5760 0.140507501 5760 0.039915281 2910 0.063799125 2912
0.05487465 5760 0.06560269 5760 0.037661845
5770 0.168780126 5770 0.043879962 2920 0.038926646 2916
0.069254413 5768 0.02451299 5768 0.025085314
5780 0.163059201 5780 0.076577897 2920 0.076790922 2920
0.04595656 5776 0.11391181 5776 0.045318331
5780 0.125725885 5780 0.042896852 2920 0.099750672 2924
0.019104793 5784 0.15591133 5784 0.047912021
5790 0.075206925 5790 0.056162873 2930 0.059906884 2928
0.029199899 5792 0.110214794 5792 0.027534743
5800 0.022523251 5800 0.038535021 2930 0.01435456 2932
0.036365869 5800 0.089814166 5800 0.035430196
5810 0.080650578 5810 0.02802342 2940 0.063371997 2936
0.03521661 5808 0.122943369 5808 0.054618726
5820 0.086972665 5820 0.032184158 2940 0.059316721 2940
0.032850898 5816 0.092760689 5816 0.055703844
5820 0.077933932 5820 0.075609518 2940 0.062855077 2944
0.026374806 5824 0.062104191 5824 0.073033712
5830 0.050189508 5830 0.104305043 2950 0.099873701 2948
0.013575838 5832 0.122825804 5832 0.094696028
5840 0.029672068 5840 0.063583095 2950 0.09422224 2952
0.02485257 5840 0.11031983 5840 0.093043214
5850 0.059145332 5850 0.055874229 2960 0.024515572 2956
0.046619025 5848 0.10181086 5848 0.077430603
5860 0.036087105 5860 0.067172863 2960 0.070745002 2960
0.046363748 5856 0.137721945 5856 0.079078869
5860 0.050687413 5860 0.040577364 2960 0.065655149 2964
0.030437566 5864 0.162695651 5864 0.05768321
5870 0.12958741 5870 0.069508598 2970 0.029864003 2968
0.034016302 5872 0.145958548 5872 0.025119949
5880 0.092681155 5880 0.02757845 2970 0.049080045 2972
0.019474283 5880 0.058109945 5880 0.029734783
5890 0.090277623 5890 0.107843189 2980 0.048410046 2976
0.032626496 5888 0.069752765 5888 0.03202217
5900 0.077977864 5900 0.166229816 2980 0.055061708 2980
0.06759313 5896 0.064421241 5896 0.069536764
5900 0.050664523 5900 0.11480035 2980 0.050806873 2984
0.059291713 5904 0.028098448 5904 0.077871147
5910 0.083470266 5910 0.083528168 2990 0.011799821 2988
0.035262259 5912 0.066257417 5912 0.040792296
5920 0.102654325 5920 0.061619714 2990 0.053940948 2992
0.045019242 5920 0.099564568 5920 0.044903387
5930 0.101854072 5930 0.026999551 3000 0.064386077 2996
0.033342058 5928 0.133081063 5928 0.048925838
5940 0.102563099 5940 0.075182499 3000 0.034321634 3000
0.023570528 5936 0.136566669 5936 0.025899435
5940 0.021976808 5940 0.010007549 3000 0.008573979 3004
0.046637564 5944 0.073368072 5944 0.011673837
5950 0.097287979 5950 0.082047896 3010 0.014040378 3008
0.057680165 5952 0.104167433 5952 0.017765755
5960 0.074757518 5960 0.091372516 3010 0.02173843 3012
0.024638484 5960 0.14379152 5960 0.033722128
5970 0.027527263 5970 0.07316019 3020 0.051661991 3016
0.031322656 5968 0.119333919 5968 0.038849194
5980 0.069651811 5980 0.060030121 3020 0.030973792 3020
0.059504248 5976 0.073595526 5976 0.045885619
5980 0.105070496 5980 0.040932711 3020 0.058535163 3024
0.073382384 5984 0.048712875 5984 0.071101007
5990 0.128888991 5990 0.024499221 3030 0.072037452 3028
0.099672157 5992 0.056028395 5992 0.059098322
6000 0.113123067 6000 0.035464989 3030 0.05542667 3032
0.087514432 6000 0.075542921 6000 0.040709929
6010 0.12803571 6010 0.088819295 3040 0.048978742 3036
0.083670981 6008 0.075479286 6008 0.036932142
6020 0.125146806 6020 0.060792285 3040 0.044127501 3040

0.085585256 6016 0.112562921 6016 0.056318368
6020 0.067551271 6020 0.059787388 3040 0.04988692 3044
0.042490505 6024 0.145129889 6024 0.079486687
6030 0.08312727 6030 0.077201927 3050 0.063266787 3048
0.058007732 6032 0.08549045 6032 0.056864774
6040 0.061116887 6040 0.119640834 3050 0.047956542 3052
0.067471759 6040 0.080307509 6040 0.018156381
6050 0.068105452 6050 0.086021828 3060 0.059338127 3056
0.055263517 6048 0.05317399 6048 0.005229436
6060 0.144040183 6060 0.057819649 3060 0.060421658 3060
0.037488597 6056 0.118214273 6056 0.011561367
6060 0.154507419 6060 0.185244586 3060 0.031607466 3064
0.020894329 6064 0.101707563 6064 0.022808345
6070 0.05805316 6070 0.211273145 3070 0.017227072 3068
0.040451992 6072 0.031779116 6072 0.035852088
6080 0.100238394 6080 0.1986586 3070 0.034626024 3072
0.047901638 6080 0.026085836 6080 0.049360762
6090 0.138866904 6090 0.182971576 3080 0.121480953 3076
0.03424575 6088 0.085325053 6088 0.055149973
6100 0.063879226 6100 0.158136405 3080 0.137848896 3080
0.021755652 6096 0.160709009 6096 0.042783966
6100 0.102761442 6100 0.10759908 3080 0.063352927 3084
0.051704094 6104 0.19262021 6104 0.032960357
6110 0.172546294 6110 0.065309556 3090 0.026794716 3088
0.031723608 6112 0.155828239 6112 0.046526115
6120 0.164740064 6120 0.058160807 3090 0.027978991 3092
0.055142213 6120 0.092358088 6120 0.056658806
6130 0.091876391 6130 0.056060912 3100 0.032305514 3096
0.07102493 6128 0.091016744 6128 0.05493368
6140 0.052076357 6140 0.038104685 3100 0.014136252 3100
0.049216098 6136 0.135931405 6136 0.035959405
6140 0.089009474 6140 0.029433169 3100 0.011609339 3104
0.051713079 6144 0.100804376 6144 0.025902049
6150 0.105435494 6150 0.048126509 3110 0.03441333 3108
0.063797997 6152 0.033133831 6152 0.019298495
6160 0.127030231 6160 0.097449818 3110 0.060791514 3112
0.093935581 6160 0.025265408 6160 0.032874294
6170 0.110533409 6170 0.098960678 3120 0.06568859 3116
0.065773224 6168 0.075779564 6168 0.017901986
6180 0.090223577 6180 0.117443793 3120 0.079055768 3120
0.00524956 6176 0.136564283 6176 0.038067617
6180 0.070932074 6180 0.110195513 3120 0.100597601 3124
0.046853973 6184 0.125691571 6184 0.068239635
6190 0.066140638 6190 0.089405374 3130 0.098613571 3128
0.045046385 6192 0.068177978 6192 0.076437849
6200 0.084111394 6200 0.082528459 3130 0.057779282 3132
0.03319891 6200 0.055934739 6200 0.059916562
6210 0.110301269 6210 0.109231871 3140 0.012768694 3136
0.043597633 6208 0.072100869 6208 0.022115795
6220 0.101712736 6220 0.178287068 3140 0.028118186 3140
0.028995846 6216 0.093949187 6216 0.044334487
6220 0.070124821 6220 0.177337774 3140 0.071096889 3144
0.006195752 6224 0.125707899 6224 0.020804651
6230 0.045631332 6230 0.095024188 3150 0.078057994 3148
0.011341511 6232 0.117241732 6232 0.033544242
6240 0.090399903 6240 0.020719152 3150 0.038862818 3152
0.046269859 6240 0.072625488 6240 0.02402742
6250 0.067795518 6250 0.021658121 3160 0.024951738 3156
0.061255727 6248 0.027212422 6248 0.028546192
6260 0.02334061 6260 0.04361368 3160 0.038972365 3160
0.056410063 6256 0.058526035 6256 0.021903134
6260 0.054561682 6260 0.061147352 3160 0.018449557 3164
0.04246991 6264 0.075333614 6264 0.01079182
6270 0.048005462 6270 0.094964875 3170 0.016311698 3168
0.044159591 6272 0.06735346 6272 0.041985648
6280 0.050901526 6280 0.14294732 3170 0.036505386 3172
0.05860539 6280 0.11783732 6280 0.066733177
6290 0.066275949 6290 0.128531916 3180 0.037078797 3176

0.086963417 6288 0.098311131 6288 0.05199499
6300 0.087034321 6300 0.041170537 3180 0.029995808 3180
0.101061836 6296 0.046448509 6296 0.031020769
6300 0.164039884 6300 0.092899238 3180 0.004376451 3184
0.059572009 6304 0.002538253 6304 0.017458464
6310 0.16434968 6310 0.13133543 3190 0.02981479 3188
0.028046972 6312 0.045935489 6312 0.019925928
6320 0.09599964 6320 0.078862817 3190 0.028300439 3192
0.03930433 6320 0.048289803 6320 0.040878804
6330 0.012379011 6330 0.057162662 3200 0.038263184 3196
0.04913524 6328 0.07122237 6328 0.053313652
6340 0.066019347 6340 0.116493553 3200 0.017370881 3200
0.098087192 6336 0.119693927 6336 0.023299604
6340 0.083776322 6340 0.153779023 3200 0.028491291 3204
0.101934187 6344 0.060213886 6344 0.019689076
6350 0.061829858 6350 0.084277723 3210 0.062727253 3208
0.069951078 6352 0.013686463 6352 0.041969186
6360 0.10543727 6360 0.045602821 3210 0.081200436 3212
0.085195519 6360 0.079610509 6360 0.063460502
6370 0.122323443 6370 0.090610047 3220 0.074802767 3216
0.069091562 6368 0.170919666 6368 0.055397915
6380 0.08308523 6380 0.077953315 3220 0.052855558 3220
0.044774894 6376 0.171029373 6376 0.041542964
6380 0.041617153 6380 0.095767398 3220 0.04198527 3224
0.014886809 6384 0.11037707 6384 0.026330965
6390 0.013538473 6390 0.119152763 3230 0.024353225 3228
0.036620462 6392 0.089208792 6392 0.026995425
6400 0.033566597 6400 0.068865105 3230 0.002107201 3232
0.05718365 6400 0.099174737 6400 0.021980042
6410 0.038628747 6410 0.057247686 3240 0.013490231 3236
0.070090049 6408 0.116285177 6408 0.01420864
6420 0.071389863 6420 0.074117234 3240 0.005111547 3240
0.076622375 6416 0.079332873 6416 0.040323292
6420 0.130168003 6420 0.077348224 3240 0.036709822 3244
0.03824526 6424 0.030834483 6424 0.037824964
6430 0.117639262 6430 0.080457932 3250 0.068475856 3248
0.020998559 6432 0.063398904 6432 0.020400437
6440 0.082712679 6440 0.064312524 3250 0.065929547 3252
0.049681028 6440 0.12800406 6440 0.015692731
6450 0.111938563 6450 0.012321503 3260 0.037618618 3256
0.017906754 6448 0.117823547 6448 0.015354373
6460 0.121995727 6460 0.115290321 3260 0.062248044 3260
0.081107784 6456 0.0518335 6456 0.01672767
6460 0.098782519 6460 0.114377617 3260 0.065464701 3264
0.11369009 6464 0.111287111 6464 0.032499091
6470 0.099360659 6470 0.079578567 3270 0.047503483 3268
0.078098841 6472 0.003122203 6472 0.047838203
6480 0.130284578 6480 0.094950825 3270 0.040838411 3272
0.050726223 6480 0.096656986 6480 0.059468846
6490 0.168744024 6490 0.065870132 3280 0.022380827 3276
0.078207115 6488 0.088556051 6488 0.068906884
6500 0.172578104 6500 0.035400335 3280 0.035347865 3280
0.086857792 6496 0.066370842 6496 0.063909283
6500 0.147557119 6500 0.031969386 3280 0.035602534 3284
0.08469366 6504 0.085818669 6504 0.050111838
6510 0.130793328 6510 0.076672157 3290 0.039908089 3288
0.091698355 6512 0.115251336 6512 0.034598186
6520 0.112558169 6520 0.139796524 3290 0.066583925 3292
0.070552313 6520 0.085252206 6520 0.020821655
6530 0.08207978 6530 0.148494786 3300 0.074375254 3296
0.033128708 6528 0.043463973 6528 0.047786976
6540 0.045308749 6540 0.078748453 3300 0.041844287 3300
0.04517052 6536 0.011603301 6536 0.044197921
6540 0.058560356 6540 0.072867711 3300 0.01490518 3304
0.040031911 6544 0.095345924 6544 0.04510078
6550 0.069485956 6550 0.090072594 3310 0.015382613 3308
0.0398505 6552 0.152914509 6552 0.04862262
6560 0.072183888 6560 0.06969965 3310 0.013860878 3312

0.045925473	6560	0.112421178	6560	0.061333034		
6570	0.067641748	6570	0.04422749	3320	0.007466062	3316
0.055392928	6568	0.026123104	6568	0.034831068		
6580	0.024667903	6580	0.059213864	3320	0.018112556	3320
0.055812554	6576	0.084112202	6576	0.025009453		
6580	0.049291	6580	0.114137722	3320	0.017498167	3324
0.045428005	6584	0.171487146	6584	0.031170857		
6590	0.06038951	6590	0.127569146	3330	0.01445652	3328
0.065872431	6592	0.175345049	6592	0.018432458		
6600	0.038273243	6600	0.127982115	3330	0.001999953	3332
0.066505381	6600	0.096530268	6600	0.013674696		
6610	0.111383015	6610	0.102461156	3340	0.015635689	3336
0.057042733	6608	0.15759893	6608	0.028035442		
6620	0.140163364	6620	0.030336118	3340	0.02475719	3340
0.06880505	6616	0.20323573	6616	0.020178452		
6620	0.138500589	6620	0.029805049	3340	0.012518445	3344
0.038969309	6624	0.164834971	6624	0.018754266		
6630	0.153287824	6630	0.02308727	3350	0.02900389	3348
0.026947921	6632	0.155302973	6632	0.026757109		
6640	0.168574203	6640	0.008373531	3350	0.079101257	3352
0.051342468	6640	0.100544836	6640	0.035756391		
6650	0.181296709	6650	0.037980099	3360	0.094755109	3356
0.033220385	6648	0.065830885	6648	0.039831226		
6660	0.165978534	6660	0.079314727	3360	0.067125206	3360
0.06335017	6656	0.108612156	6656	0.026495887		
6660	0.11430007	6660	0.088239111	3360	0.035841582	3364
0.06419752	6664	0.107580476	6664	0.003371562		
6670	0.103373895	6670	0.066650922	3370	0.016437462	3368
0.063662919	6672	0.045287008	6672	0.010152783		
6680	0.135078794	6680	0.045875557	3370	0.016800495	3372
0.049969971	6680	0.10268436	6680	0.0137638		
6690	0.131694775	6690	0.06217121	3380	0.02300246	3376
0.019719597	6688	0.182850155	6688	0.030079771		
6700	0.18878236	6700	0.11446284	3380	0.016613954	3380
0.053013322	6696	0.164984725	6696	0.045580859		
6700	0.227123368	6700	0.148674008	3380	0.02587864	3384
0.064276268	6704	0.109946042	6704	0.050147275		
6710	0.158904804	6710	0.194956359	3390	0.035629098	3388
0.072606956	6712	0.086561944	6712	0.065938242		
6720	0.082667604	6720	0.214764746	3390	0.028173743	3392
0.06421822	6720	0.062732972	6720	0.065054992		
6730	0.083242376	6730	0.162479016	3400	0.043281878	3396
0.063221116	6728	0.053592103	6728	0.052335723		
6740	0.077040953	6740	0.075322518	3400	0.04111887	3400
0.040669158	6736	0.083929372	6736	0.055939087		
6740	0.068190762	6740	0.118100761	3400	0.017347958	3404
0.00848607	6744	0.069931761	6744	0.05518421		
6750	0.07932428	6750	0.137810698	3410	0.029876841	3408
0.021882706	6752	0.050924347	6752	0.030612129		
6760	0.064365035	6760	0.094828851	3410	0.049642185	3412
0.031254163	6760	0.098655146	6760	0.009454168		
6770	0.038677994	6770	0.079684141	3420	0.068826252	3416
0.032490636	6768	0.077500459	6768	0.002457663		
6780	0.033284938	6780	0.109168643	3420	0.072172006	3420
0.071988114	6776	0.018616453	6776	0.008874096		
6780	0.04074366	6780	0.099284844	3420	0.019071098	3424
0.069786154	6784	0.114151459	6784	0.014650173		
6790	0.026319629	6790	0.107003485	3430	0.04253292	3428
0.076188109	6792	0.078782869	6792	0.050735045		
6800	0.030536954	6800	0.091707341	3430	0.048341284	3432
0.056334404	6800	0.052403761	6800	0.101953541		
6810	0.095196308	6810	0.017817649	3440	0.010506451	3436
0.045892859	6808	0.061687104	6808	0.125751365		
6820	0.158573559	6820	0.039178147	3440	0.034002231	3440
0.089222878	6816	0.025195952	6816	0.096807016		
6820	0.154302194	6820	0.034829176	3440	0.043349002	3444
0.071545088	6824	0.065359556	6824	0.040718685		
6830	0.107144973	6830	0.076924247	3450	0.045543104	3448

0.065242864 6832 0.056768102 6832 0.013916735
6840 0.115869094 6840 0.072875504 3450 0.039824303 3452
0.067767869 6840 0.149774161 6840 0.01363602
6850 0.134743693 6850 0.063347412 3460 0.02783725 3456
0.022993321 6848 0.124919461 6848 0.048415208
6860 0.101840276 6860 0.101773396 3460 0.031837772 3460
0.030496471 6856 0.018614455 6856 0.075353506
6860 0.024310786 6860 0.113486298 3460 0.023272254 3464
0.052764088 6864 0.048134043 6864 0.080538739
6870 0.069454123 6870 0.089433721 3470 0.018495466 3468
0.069443144 6872 0.063092943 6872 0.067017434
6880 0.083180195 6880 0.070611 3470 0.037002403 3472
0.081663347 6880 0.131576235 6880 0.057309964
6890 0.06874399 6890 0.045355911 3480 0.028183767 3476
0.05356249 6888 0.150806911 6888 0.029718203
6900 0.043575288 6900 0.057858091 3480 0.008259756 3480
0.015509098 6896 0.145182305 6896 0.034226876
6900 0.068923735 6900 0.115737923 3480 0.035018187 3484
0.027194084 6904 0.096377087 6904 0.041409792
6910 0.143023368 6910 0.141492841 3490 0.036525871 3488
0.045163812 6912 0.113104274 6912 0.029380388
6920 0.237879388 6920 0.08395363 3490 0.037640904 3492
0.016326991 6920 0.133317735 6920 0.038539765
6930 0.243744551 6930 0.092688504 3500 0.051936055 3496
0.04818432 6928 0.12954943 6928 0.05542277
6940 0.191541185 6940 0.09146805 3500 0.058522594 3500
0.091048925 6936 0.162434051 6936 0.05278473
6940 0.128030471 6940 0.049866991 3500 0.048709422 3504
0.10099959 6944 0.126705753 6944 0.046918936
6950 0.093293085 6950 0.033667911 3510 0.032608681 3508
0.062256724 6952 0.051748797 6952 0.051980471
6960 0.090799353 6960 0.053894895 3510 0.012803165 3512
0.081248203 6960 0.032804783 6960 0.035660232
6970 0.138063551 6970 0.082574617 3520 0.045542143 3516
0.117220501 6968 0.014317512 6968 0.019901172
6980 0.189253188 6980 0.113027672 3520 0.061714098 3520
0.131872978 6976 0.077126802 6976 0.03154152
6980 0.173487599 6980 0.136682458 3520 0.057249661 3524
0.136080911 6984 0.12670063 6984 0.045980294
6990 0.124209037 6990 0.106293781 3530 0.042688538 3528
0.088290202 6992 0.117482116 6992 0.030778898
7000 0.170751329 7000 0.088482098 3530 0.03278174 3532
0.056572575 7000 0.087499029 7000 0.004952838
7010 0.208519574 7010 0.141969053 3540 0.04587217 3536
0.099336139 7008 0.039403716 7008 0.019998095
7020 0.137972108 7020 0.17774239 3540 0.038021124 3540
0.141830795 7016 0.076720258 7016 0.0290074
7020 0.192493754 7020 0.17456463 3540 0.058401118 3544
0.140929085 7024 0.124834973 7024 0.05854245
7030 0.193323853 7030 0.150110893 3550 0.029431676 3548
0.077652061 7032 0.105649539 7032 0.06444256
7040 0.103381135 7040 0.117040079 3550 0.048255835 3552
0.050066879 7040 0.100414465 7040 0.03956784
7050 0.080861209 7050 0.094207106 3560 0.061369974 3556
0.103905106 7048 0.140396485 7048 0.005976804
7060 0.056169811 7060 0.072020092 3560 0.049791728 3560
0.091298338 7056 0.139969969 7056 0.030115492
7060 0.071812661 7060 0.087361368 3560 0.053800919 3564
0.099871577 7064 0.141844459 7064 0.02466214
7070 0.094672912 7070 0.108364373 3570 0.038129929 3568
0.106820509 7072 0.171639593 7072 0.010929324
7080 0.034902525 7080 0.109787165 3570 0.053330688 3572
0.103593455 7080 0.064551336 7080 0.036092042
7090 0.073923497 7090 0.123391932 3580 0.063148393 3576
0.129484514 7088 0.097552409 7088 0.042956031
7100 0.094877651 7100 0.117993841 3580 0.048508584 3580
0.129363019 7096 0.137172726 7096 0.047361387
7100 0.054318622 7100 0.088265675 3580 0.048909777 3584

0.116058036 7104 0.102116115 7104 0.04245466
7110 0.10286535 7110 0.116967771 3590 0.052081123 3588
0.104756502 7112 0.059408721 7112 0.012867839
7120 0.04018208 7120 0.141370678 3590 0.04669846 3592
0.087850836 7120 0.074734657 7120 0.023633489
7130 0.122051511 7130 0.195314788 3600 0.050707062 3596
0.096916869 7128 0.09321273 7128 0.032711781
7140 0.101365607 7140 0.199895149 3600 0.054854845 3600
0.116673669 7136 0.069415582 7136 0.033695491
7140 0.040004292 7140 0.124711412 3600 0.052753901 3604
0.121151068 7144 0.026368267 7144 0.039570776
7150 0.088868961 7150 0.073475567 3610 0.074444288 3608
0.106360945 7152 0.013469958 7152 0.027229262
7160 0.066802277 7160 0.067184272 3610 0.061254505 3612
0.060159026 7160 0.089875393 7160 0.038752758
7170 0.039607337 7170 0.024309156 3620 0.020596497 3616
0.071926814 7168 0.134181654 7168 0.051434876
7180 0.061803759 7180 0.080303871 3620 0.027995584 3620
0.099049576 7176 0.031538486 7176 0.062816129
7180 0.126705796 7180 0.131458059 3620 0.030578576 3624
0.11366597 7184 0.07987408 7184 0.056800916
7190 0.081238395 7190 0.081109247 3630 0.020926718 3628
0.140287375 7192 0.054082695 7192 0.042055286
7200 0.022013768 7200 0.040808296 3630 0.061681501 3632
0.116970055 7200 0.054034939 7200 0.050743627
7210 0.023215209 7210 0.089594054 3640 0.108535722 3636
0.104093873 7208 0.048440619 7208 0.059386981
7220 0.040534313 7220 0.076129756 3640 0.072163348 3640
0.122035868 7216 0.034846133 7216 0.067459398
7220 0.09297247 7220 0.080078273 3640 0.010854863 3644
0.09342502 7224 0.082625164 7224 0.040532668
7230 0.092582115 7230 0.059612426 3650 0.01253946 3648
0.089125802 7232 0.095784359 7232 0.008201778
7240 0.039618975 7240 0.038063252 3650 0.027454615 3652
0.124327547 7240 0.082903942 7240 0.009283332
7250 0.091593756 7250 0.04350597 3660 0.010946163 3656
0.092913746 7248 0.067802459 7248 0.018333894
7260 0.136835384 7260 0.12171649 3660 0.039041075 3660
0.069654154 7256 0.048498798 7256 0.046150111
7260 0.106253581 7260 0.156183873 3660 0.042234999 3664
0.077012111 7264 0.060135186 7264 0.055564182
7270 0.060326423 7270 0.068235146 3670 0.028360249 3668
0.072789218 7272 0.089610498 7272 0.062456253
7280 0.057211913 7280 0.073174408 3670 0.020945076 3672
0.099440331 7280 0.15465729 7280 0.054642405
7290 0.076337958 7290 0.11189255 3680 0.008044584 3676
0.105544626 7288 0.176379355 7288 0.023913874
7300 0.079472775 7300 0.064525259 3680 0.027961305 3680
0.115902665 7296 0.121829784 7296 0.022956087
7300 0.038065118 7300 0.028620852 3680 0.070702874 3684
0.122769285 7304 0.02483177 7304 0.067633606
7310 0.032061591 7310 0.066913301 3690 0.093240124 3688
0.096911492 7312 0.089983594 7312 0.067523521
7320 0.049041817 7320 0.068963847 3690 0.075001197 3692
0.077935001 7320 0.083883766 7320 0.037648679
7330 0.105310115 7330 0.051364703 3700 0.073456911 3696
0.081227037 7328 0.082634499 7328 0.031154039
7340 0.089614063 7340 0.075851458 3700 0.042687079 3700
0.093444185 7336 0.050796731 7336 0.019540616
7340 0.092275215 7340 0.089030516 3700 0.036950227 3704
0.093046889 7344 0.035348312 7344 0.047268244
7350 0.134527421 7350 0.045119152 3710 0.005710129 3708
0.097648612 7352 0.065473141 7352 0.066346016
7360 0.172435728 7360 0.032075499 3710 0.020289306 3712
0.111169262 7360 0.041972577 7360 0.041147308
7370 0.15331294 7370 0.069185917 3720 0.036268 3716
0.084443156 7368 0.037893024 7368 0.048024165
7380 0.172104643 7380 0.10176246 3720 0.083327628 3720

0.019704829 7376 0.060087648 7376 0.097743316
7380 0.172067579 7380 0.165585109 3720 0.093811344 3724
0.075368269 7384 0.064470769 7384 0.104151368
7390 0.137822106 7390 0.135631563 3730 0.061906663 3728
0.072141709 7392 0.076185315 7392 0.076581448
7400 0.120694036 7400 0.05129493 3730 0.02810952 3732
0.05768432 7400 0.126988656 7400 0.048542868
7410 0.084723426 7410 0.084945968 3740 0.021505472 3736
0.085128457 7408 0.160263895 7408 0.014508992
7420 0.044432069 7420 0.021554963 3740 0.032630542 3740
0.100229125 7416 0.154690978 7416 0.021504768
7420 0.107611348 7420 0.068433168 3740 0.09446335 3744
0.058421858 7424 0.1759437 7424 0.020368385
7430 0.178806484 7430 0.086290012 3750 0.089563262 3748
0.006316278 7432 0.196524838 7432 0.009942926
7440 0.131152221 7440 0.048662361 3750 0.042870244 3752
0.06000857 7440 0.175199093 7440 0.017352892
7450 0.09988089 7450 0.059550072 3760 0.036168836 3756
0.067170848 7448 0.14829167 7448 0.013543459
7460 0.115933894 7460 0.063583197 3760 0.023811619 3760
0.034914538 7456 0.055582357 7456 0.014971262
7460 0.177346271 7460 0.103624552 3760 0.039959388 3764
0.043106848 7464 0.093024508 7464 0.014973742
7470 0.111300571 7470 0.095577707 3770 0.084785912 3768
0.05163626 7472 0.094313997 7472 0.025549542
7480 0.129018459 7480 0.048130856 3770 0.087139822 3772
0.039711049 7480 0.01740203 7480 0.059108108
7490 0.131135035 7490 0.047083911 3780 0.069641566 3776
0.045780082 7488 0.072937466 7488 0.06411082
7500 0.064241947 7500 0.042603137 3780 0.07257035 3780
0.059880036 7496 0.044730361 7496 0.051942996
7500 0.090972659 7500 0.049526236 3780 0.06957521 3784
0.085164204 7504 0.068869857 7504 0.052891755
7510 0.123960228 7510 0.0817568 3790 0.040321054 3788
0.086677159 7512 0.086656 7512 0.042006614
7520 0.111917922 7520 0.103330145 3790 0.034415018 3792
0.073989693 7520 0.062544306 7520 0.024394212
7530 0.110755478 7530 0.121676734 3800 0.063380801 3796
0.087912798 7528 0.08730674 7528 0.011341283
7540 0.148441498 7540 0.078545825 3800 0.04411392 3800
0.080201106 7536 0.075552474 7536 0.020626456
7540 0.15432309 7540 0.092488146 3800 0.039787781 3804
0.038462622 7544 0.092659691 7544 0.020296528
7550 0.098345176 7550 0.1456225 3810 0.08093239 3808
0.04657992 7552 0.044795779 7552 0.006666431
7560 0.067931338 7560 0.129996959 3810 0.083006227 3812
0.060647515 7560 0.032220698 7560 0.021579243
7570 0.083882638 7570 0.131989888 3820 0.050947896 3816
0.062209314 7568 0.065040622 7568 0.016192244
7580 0.061149018 7580 0.090377864 3820 0.005616476 3820
0.048777696 7576 0.068913199 7576 0.018300518
7580 0.070489557 7580 0.064241322 3820 0.043430424 3824
0.030243715 7584 0.07733133 7584 0.031876032
7590 0.05721752 7590 0.04283716 3830 0.055567125 3828
0.050213184 7592 0.102006998 7592 0.035532034
7600 0.070392991 7600 0.09543771 3830 0.033255787 3832
0.080172998 7600 0.121266821 7600 0.009002609
7610 0.091461254 7610 0.053136417 3840 0.061542654 3836
0.091177702 7608 0.07427104 7608 0.029911515
7620 0.070980721 7620 0.061187981 3840 0.07287191 3840
0.0671292 7616 0.049609487 7616 0.020992982
7620 0.049103754 7620 0.119312754 3840 0.057641526 3844
0.031229498 7624 0.085373577 7624 0.032542073
7630 0.005685637 7630 0.149729764 3850 0.014715875 3848
0.017846116 7632 0.084207968 7632 0.058667552
7640 0.051941894 7640 0.132307061 3850 0.036073267 3852
0.008274975 7640 0.087990418 7640 0.056056932
7650 0.045376019 7650 0.085764747 3860 0.053484113 3856

0.021892427 7648 0.0959545 7648 0.062949861
7660 0.027541641 7660 0.024101448 3860 0.039897328 3860
0.031445117 7656 0.077684104 7656 0.074660355
7660 0.033383432 7660 0.085669795 3860 0.028086677 3864
0.031224896 7664 0.051373136 7664 0.071217874
7670 0.018380335 7670 0.145315425 3870 0.03081232 3868
0.059298331 7672 0.048698992 7672 0.054300785
7680 0.040235089 7680 0.080083606 3870 0.057428264 3872
0.070666174 7680 0.056419354 7680 0.033148222
7690 0.077177967 7690 0.004776267 3880 0.075152122 3876
0.038786358 7688 0.081271413 7688 0.024728404
7700 0.088328918 7700 0.012330663 3880 0.043849424 3880
0.016096812 7696 0.110083071 7696 0.010466999
7700 0.016998576 7700 0.086352498 3880 0.026174686 3884
0.032430948 7704 0.06925231 7704 0.023469966
7710 0.113851689 7710 0.149450018 3890 0.045358687 3888
0.063192871 7712 0.031481784 7712 0.039649065
7720 0.170651096 7720 0.151021391 3890 0.046882324 3892
0.06058488 7720 0.036291502 7720 0.018370371
7730 0.084272855 7730 0.113140268 3900 0.020379219 3896
0.038354719 7728 0.024738922 7728 0.02962052
7740 0.087474553 7740 0.104492974 3900 0.039480899 3900
0.098481731 7736 0.10747982 7736 0.045781031
7740 0.130727436 7740 0.15123871 3900 0.062037201 3904
0.11442924 7744 0.137737399 7744 0.019737348
7750 0.10172162 7750 0.135097668 3910 0.049304144 3908
0.060118782 7752 0.097330318 7752 0.017489148
7760 0.11729265 7760 0.110259083 3910 0.047128844 3912
0.017725264 7760 0.017200942 7760 0.008570441
7770 0.13852879 7770 0.105065912 3920 0.061749466 3916
0.048795941 7768 0.113202543 7768 0.034003926
7780 0.100704696 7780 0.066818728 3920 0.056798115 3920
0.08799108 7776 0.152640961 7776 0.016975502
7780 0.04928503 7780 0.048758677 3920 0.067096771 3924
0.113755174 7784 0.118458433 7784 0.031763528
7790 0.089120717 7790 0.119547287 3930 0.073362855 3928
0.084247586 7792 0.10846799 7792 0.051390147
7800 0.162820041 7800 0.127163745 3930 0.031512191 3932
0.084176194 7800 0.146629885 7800 0.047387286
7810 0.173458553 7810 0.139214069 3940 0.022274981 3936
0.037114816 7808 0.166629499 7808 0.02949145
7820 0.134860239 7820 0.080493221 3940 0.004564045 3940
0.004116228 7816 0.160224561 7816 0.014325069
7820 0.082713836 7820 0.081877515 3940 0.011581408 3944
0.031723717 7824 0.120196957 7824 0.009382899
7830 0.070119029 7830 0.153811445 3950 0.019014458 3948
0.040882027 7832 0.045832436 7832 0.008821547
7840 0.023267747 7840 0.161495671 3950 0.040858311 3952
0.053058069 7840 0.060955466 7840 0.017715818
7850 0.028509398 7850 0.191132829 3960 0.043260734 3956
0.053872998 7848 0.133296693 7848 0.019590003
7860 0.067320711 7860 0.171789055 3960 0.047832979 3960
0.053505759 7856 0.197888309 7856 0.007872658
7860 0.046823698 7860 0.088846944 3960 0.056135905 3964
0.067836256 7864 0.205583274 7864 0.024988472
7870 0.053164764 7870 0.028644236 3970 0.06237044 3968
0.044399913 7872 0.149330691 7872 0.043511122
7880 0.068475754 7880 0.109196633 3970 0.047566467 3972
0.013792247 7880 0.05155211 7880 0.051617921
7890 0.017506407 7890 0.060479255 3980 0.030683998 3976
0.038925631 7888 0.10179713 7888 0.042474003
7900 0.102912563 7900 0.06392994 3980 0.029956265 3980
0.049647235 7896 0.120849705 7896 0.02371695
7900 0.093063638 7900 0.062705418 3980 0.044981563 3984
0.009450691 7904 0.06833372 7904 0.015226128
7910 0.074028321 7910 0.027676058 3990 0.027577578 3988
0.04125858 7912 0.047170048 7912 0.006303081
7920 0.028053823 7920 0.056653455 3990 0.030694133 3992

0.078348377 7920 0.12002814 7920 0.027169092
7930 0.062507141 7930 0.059933216 4000 0.058061694 3996
0.107598302 7928 0.16423178 7928 0.026605718
7940 0.065248481 7940 0.054292766 4000 0.071194416 4000
0.090169735 7936 0.152051813 7936 0.030521744
7940 0.046691748 7940 0.157134098 4000 0.070079928 4004
0.065495529 7944 0.114782881 7944 0.054584329
7950 0.043805354 7950 0.176700996 4010 0.055905057 4008
0.057120298 7952 0.109196379 7952 0.065216693
7960 0.060615275 7960 0.110566092 4010 0.039542778 4012
0.046350211 7960 0.139488111 7960 0.03865359
7970 0.056078046 7970 0.066205983 4020 0.054654862 4016
0.068946909 7968 0.122860831 7968 0.016407077
7980 0.045355337 7980 0.107232292 4020 0.038866492 4020
0.040775914 7976 0.038526065 7976 0.039330513
7980 0.025661542 7980 0.145501443 4020 0.021116444 4024
0.03664699 7984 0.05098051 7984 0.049444454
7990 0.066864021 7990 0.147875355 4030 0.05628826 4028
0.069389818 7992 0.094179974 7992 0.069936032
8000 0.127792475 8000 0.125919003 4030 0.049792256 4032
0.087752465 8000 0.132649293 8000 0.038460457
8010 0.132712696 8010 0.103757411 4040 0.030873958 4036
0.095640535 8008 0.109700122 8008 0.046962301
8020 0.138012576 8020 0.102267455 4040 0.005818483 4040
0.079873949 8016 0.052425818 8016 0.107314882
8020 0.147702158 8020 0.080810991 4040 0.06132741 4044
0.062675164 8024 0.089066416 8024 0.105319101
8030 0.083610627 8030 0.047550024 4050 0.070366303 4048
0.045404384 8032 0.055110664 8032 0.068200752
8040 0.057052042 8040 0.033764842 4050 0.037560778 4052
0.034211102 8040 0.0421359 8040 0.025216123
8050 0.161460775 8050 0.054309159 4060 0.078748599 4056
0.017193919 8048 0.090253772 8048 0.019525354
8060 0.144663893 8060 0.117310869 4060 0.092506118 4060
0.023995108 8056 0.05585462 8056 0.029200521
8060 0.055314245 8060 0.155108254 4060 0.064188404 4064
0.046164711 8064 0.043879416 8064 0.041120191
8070 0.103708982 8070 0.149520915 4070 0.083723833 4068
0.081463841 8072 0.132073139 8072 0.049092367
8080 0.182238189 8080 0.129501786 4070 0.09635149 4072
0.109412446 8080 0.153268396 8080 0.035918991
8090 0.188298858 8090 0.092549737 4080 0.048253289 4076
0.085515974 8088 0.1183317 8088 0.046510846
8100 0.138857766 8100 0.05122897 4080 0.005325199 4080
0.061686369 8096 0.083004292 8096 0.034513207
8100 0.104334766 8100 0.122541285 4080 0.010388483 4084
0.071473063 8104 0.127489388 8104 0.015273814
8110 0.061400984 8110 0.134857954 4090 0.028124383 4088
0.050084371 8112 0.100091362 8112 0.067104535
8120 0.039774343 8120 0.121472185 4090 0.034777466 4092
0.038027512 8120 0.096884585 8120 0.080440193
8130 0.164743105 8130 0.090239766 4100 0.012595029 4096
0.051486881 8128 0.102955324 8128 0.027609569
8140 0.20722374 8140 0.009510566 4100 0.047956957 4100
0.05434686 8136 0.100530429 8136 0.081303486
8140 0.107502208 8140 0.053601463 4100 0.082627426 4104
0.034843877 8144 0.09858331 8144 0.083453662
8150 0.095090705 8150 0.092767899 4110 0.084299827 4108
0.056689423 8152 0.070092894 8152 0.048317736
8160 0.131298206 8160 0.124004379 4110 0.085173138 4112
0.059437742 8160 0.030019033 8160 0.053574022
8170 0.108373519 8170 0.078336954 4120 0.062641477 4116
0.058267462 8168 0.078066769 8168 0.063032399
8180 0.118558062 8180 0.028801849 4120 0.030302625 4120
0.04725279 8176 0.087477944 8176 0.029387569
8180 0.132386034 8180 0.070132948 4120 0.09662415 4124
0.032018026 8184 0.084141393 8184 0.04466861
8190 0.08515206 8190 0.127846958 4130 0.088338267 4128

0.026562526 8192 0.066324399 8192 0.040368585
8200 0.037455666 8200 0.103384751 4130 0.015761734 4132
0.032273554 8200 0.178115458 8200 0.023977889
8210 0.098841345 8210 0.039135288 4140 0.044081178 4136
0.00153773 8208 0.178618095 8208 0.009802805
8220 0.13021419 8220 0.040003542 4140 0.019348921 4140
0.006208894 8216 0.059405924 8216 0.01735586
8220 0.074878939 8220 0.077160323 4140 0.031972926 4144
0.024021638 8224 0.010185759 8224 0.0209313
8230 0.040999414 8230 0.038286384 4150 0.0268084 4148
0.062923173 8232 0.058430138 8232 0.039044775
8240 0.067990251 8240 0.106662541 4150 0.033652748 4152
0.097359334 8240 0.079364727 8240 0.046961683
8250 0.102094193 8250 0.132865753 4160 0.047553538 4156
0.120621036 8248 0.070786373 8248 0.072378069
8260 0.117564487 8260 0.035553319 4160 0.042478325 4160
0.079370933 8256 0.070067865 8256 0.052995521
8260 0.129394103 8260 0.040514333 4160 0.03782033 4164
0.02388143 8264 0.05913922 8264 0.040935542
8270 0.123352787 8270 0.048300673 4170 0.025443587 4168
0.023982448 8272 0.081573875 8272 0.070115093
8280 0.086404587 8280 0.058925285 4170 0.022744545 4172
0.022403125 8280 0.070580551 8280 0.06591955
8290 0.093251976 8290 0.048347887 4180 0.04207544 4176
0.024808078 8288 0.091471535 8288 0.048561033
8300 0.155716116 8300 0.011280906 4180 0.049819555 4180
0.070431801 8296 0.140804885 8296 0.034933019
8300 0.129174805 8300 0.004522848 4180 0.060724891 4184
0.062775624 8304 0.225775424 8304 0.04388193
8310 0.023406037 8310 0.021771304 4190 0.068604022 4188
0.031823718 8312 0.227621379 8312 0.014108644
8320 0.088695757 8320 0.035525911 4190 0.042382377 4192
0.041903943 8320 0.112574409 8320 0.037088885
8330 0.110097935 8330 0.068936315 4200 0.021135025 4196
0.063197011 8328 0.060306942 8328 0.039385373
8340 0.094244024 8340 0.076938835 4200 0.042290438 4200
0.062049068 8336 0.091081907 8336 0.021059059
8340 0.15502992 8340 0.093815434 4200 0.045094406 4204
0.016208434 8344 0.093711809 8344 0.01397664
8350 0.220240341 8350 0.09609382 4210 0.018930254 4208
0.031638359 8352 0.077118217 8352 0.050507235
8360 0.160164433 8360 0.033668519 4210 0.033879587 4212
0.027427084 8360 0.031687334 8360 0.092877359
8370 0.007041281 8370 0.082182349 4220 0.062153755 4216
0.036345766 8368 0.064375694 8368 0.11751175
8380 0.091553906 8380 0.090793263 4220 0.071261369 4220
0.057765123 8376 0.036726135 8376 0.12233945
8380 0.05146828 8380 0.035086931 4220 0.077947581 4224
0.073174306 8384 0.051358864 8384 0.102157763
8390 0.021287855 8390 0.05358443 4230 0.080540958 4228
0.060324655 8392 0.081792205 8392 0.059623009
8400 0.070199407 8400 0.08903844 4230 0.081720595 4232
0.033167318 8400 0.069728463 8400 0.026737467
8410 0.107156768 8410 0.045716613 4240 0.097219418 4236
0.040817005 8408 0.076141849 8408 0.049521459
8420 0.110483445 8420 0.092823968 4240 0.08822266 4240
0.046278314 8416 0.024440806 8416 0.070923394
8420 0.071011309 8420 0.067291469 4240 0.073851581 4244
0.015856798 8424 0.069291265 8424 0.069193287
8430 0.072741321 8430 0.021374335 4250 0.045095065 4248
0.100898615 8432 0.09096376 8432 0.03604564
8440 0.115337884 8440 0.055259399 4250 0.026448757 4252
0.117243435 8440 0.058706504 8440 0.042546704
8450 0.070438488 8450 0.022012806 4260 0.049823135 4256
0.086898646 8448 0.054822922 8448 0.057315199
8460 0.068175752 8460 0.028686018 4260 0.067385758 4260
0.069240057 8456 0.0916235 8456 0.067433379
8460 0.024649244 8460 0.05964499 4260 0.070349852 4264

0.057203764 8464 0.10041202 8464 0.059601931
8470 0.065674627 8470 0.089897207 4270 0.083146042 4268
0.004657028 8472 0.120586505 8472 0.027578759
8480 0.111145411 8480 0.097863347 4270 0.086447726 4272
0.024378142 8480 0.069635993 8480 0.011194136
8490 0.084647334 8490 0.070297218 4280 0.079405436 4276
0.010914699 8488 0.024384819 8488 0.008451239
8500 0.062037623 8500 0.121800993 4280 0.060843864 4280
0.047354253 8496 0.039388637 8496 0.009891443
8500 0.146069069 8500 0.140552831 4280 0.026131165 4284
0.063404768 8504 0.018544637 8504 0.012086287
8510 0.217638051 8510 0.108238783 4290 0.032676486 4288
0.049708164 8512 0.030125957 8512 0.009832257
8520 0.176303554 8520 0.092081551 4290 0.064553286 4292
0.070002185 8520 0.041769126 8520 0.02492736
8530 0.090416994 8530 0.082469145 4300 0.062370033 4296
0.089479057 8528 0.098565739 8528 0.034446664
8540 0.059755472 8540 0.079452955 4300 0.027718863 4300
0.050223105 8536 0.130199493 8536 0.075654672
8540 0.061119346 8540 0.083312647 4300 0.030519961 4304
0.026053123 8544 0.064310087 8544 0.106250649
8550 0.059831404 8550 0.053561627 4310 0.035629077 4308
0.046942561 8552 0.101345511 8552 0.087122317
8560 0.135797046 8560 0.052441785 4310 0.034552977 4312
0.037051101 8560 0.103605991 8560 0.056891255
8570 0.13313668 8570 0.073740055 4320 0.065274871 4316
0.033420507 8568 0.064722568 8568 0.038634589
8580 0.052029314 8580 0.078552184 4320 0.062651619 4320
0.073058072 8576 0.080882397 8576 0.025951304
8580 0.077147997 8580 0.034997192 4320 0.03764479 4324
0.089898182 8584 0.122814628 8584 0.031702981
8590 0.147569008 8590 0.043797787 4330 0.044378918 4328
0.090022899 8592 0.031631615 8592 0.037225513
8600 0.168678161 8600 0.046764886 4330 0.026494205 4332
0.034281904 8600 0.122641519 8600 0.07077308
8610 0.121046185 8610 0.03064004 4340 0.047667702 4336
0.052394022 8608 0.133839945 8608 0.085234657
8620 0.057769164 8620 0.03617732 4340 0.042801465 4340
0.085647844 8616 0.033520995 8616 0.065226064
8620 0.023773353 8620 0.034780955 4340 0.023396551 4344
0.06696205 8624 0.013915864 8624 0.043540156
8630 0.045486133 8630 0.105478313 4350 0.021243925 4348
0.047601752 8632 0.091859423 8632 0.035986479
8640 0.110143505 8640 0.158187788 4350 0.038213908 4352
0.054095235 8640 0.119575678 8640 0.02579771
8650 0.141191325 8650 0.179059993 4360 0.056553847 4356
0.081533995 8648 0.068672394 8648 0.029651732
8660 0.104739076 8660 0.117321091 4360 0.047326328 4360
0.074637726 8656 0.053993932 8656 0.017276509
8660 0.068260633 8660 0.032210654 4360 0.061968844 4364
0.059695496 8664 0.04495931 8664 0.011156206
8670 0.054803622 8670 0.046463385 4370 0.063746717 4368
0.056001423 8672 0.015648644 8672 0.016052765
8680 0.04059514 8680 0.101254009 4370 0.019803349 4372
0.055819819 8680 0.079407408 8680 0.0183169
8690 0.143693688 8690 0.15957185 4380 0.052037725 4376
0.035354322 8688 0.088560519 8688 0.030278885
8700 0.152042965 8700 0.178905743 4380 0.061449762 4380
0.039398223 8696 0.032062457 8696 0.040512019
8700 0.05510643 8700 0.156409224 4380 0.043785989 4384
0.086825698 8704 0.084228443 8704 0.05437477
8710 0.104195366 8710 0.088576773 4390 0.019988262 4388
0.091812348 8712 0.152841326 8712 0.061858838
8720 0.150644454 8720 0.150355948 4390 0.009358142 4392
0.042889387 8720 0.165862788 8720 0.044674682
8730 0.095424271 8730 0.169001126 4400 0.028544569 4396
0.02203156 8728 0.165761798 8728 0.019020446
8740 0.052759569 8740 0.116913296 4400 0.024692292 4400

0.046416237 8736 0.144358318 8736 0.014863106
8740 0.076758093 8740 0.078285761 4400 0.014947488 4404
0.044048833 8744 0.024274326 8744 0.031741416
8750 0.050462262 8750 0.036032638 4410 0.030539253 4408
0.007816376 8752 0.124473445 8752 0.038573126
8760 0.052201751 8760 0.02310558 4410 0.034253706 4412
0.058394919 8760 0.131206384 8760 0.044663149
8770 0.032007032 8770 0.084381696 4420 0.020555261 4416
0.066393739 8768 0.049139315 8768 0.051796826
8780 0.058918799 8780 0.129125009 4420 0.030441444 4420
0.038815564 8776 0.025196559 8776 0.041824325
8780 0.085069696 8780 0.112254333 4420 0.043451142 4424
0.037850925 8784 0.022020598 8784 0.05688941
8790 0.076507553 8790 0.032630218 4430 0.037487764 4428
0.046375859 8792 0.034737339 8792 0.035951867
8800 0.137273368 8800 0.040946947 4430 0.051246632 4432
0.064113025 8800 0.058986079 8800 0.029448718
8810 0.158743641 8810 0.017682998 4440 0.066435474 4436
0.047164969 8808 0.079231002 8808 0.04257798
8820 0.121520257 8820 0.0054548 4440 0.067507943 4440
0.034828223 8816 0.023619221 8816 0.033734759
8820 0.046707406 8820 0.042199463 4440 0.042207328 4444
0.078270459 8824 0.084935615 8824 0.028731536
8830 0.0868891 8830 0.048533959 4450 0.038895461 4448
0.061928899 8832 0.06997466 8832 0.017344873
8840 0.11296817 8840 0.070500006 4450 0.054522756 4452
0.046727666 8840 0.089929534 8840 0.021525288
8850 0.057416517 8850 0.067199973 4460 0.030628657 4456
0.049460319 8848 0.081183687 8848 0.046801102
8860 0.026050235 8860 0.055848082 4460 0.086989312 4460
0.059207599 8856 0.026390411 8856 0.054196891
8860 0.079182537 8860 0.098267061 4460 0.086854379 4464
0.050873594 8864 0.107937922 8864 0.050988478
8870 0.140072676 8870 0.128248008 4470 0.070283262 4468
0.022314571 8872 0.115921394 8872 0.030815878
8880 0.117434029 8880 0.131370194 4470 0.032520999 4472
0.042018401 8880 0.101424608 8880 0.018274914
8890 0.058592301 8890 0.06549542 4480 0.010028996 4476
0.043070038 8888 0.117024472 8888 0.020096806
8900 0.101077829 8900 0.126562998 4480 0.055910859 4480
0.021303698 8896 0.08447178 8896 0.01930894
8900 0.104886844 8900 0.231102371 4480 0.070687122 4484
0.055061039 8904 0.019628153 8904 0.01140694
8910 0.025320655 8910 0.207530815 4490 0.054296092 4488
0.039419643 8912 0.027287062 8912 0.002418625
8920 0.084465079 8920 0.102222162 4490 0.055411587 4492
0.015871712 8920 0.040178889 8920 0.019336458
8930 0.108098349 8930 0.055705681 4500 0.04060959 4496
0.007369628 8928 0.075289841 8928 0.045466542
8940 0.03609046 8940 0.156086055 4500 0.043321332 4500
0.020456864 8936 0.038603641 8936 0.036310219
8940 0.032528707 8940 0.172779066 4500 0.026827791 4504
0.049418512 8944 0.018668952 8944 0.005246856
8950 0.038488586 8950 0.145380589 4510 0.068142908 4508
0.079818892 8952 0.021390706 8952 0.033185264
8960 0.068549474 8960 0.113787501 4510 0.106307773 4512
0.115402458 8960 0.014963868 8960 0.038006743
8970 0.048950653 8970 0.102589329 4520 0.078436679 4516
0.106454027 8968 0.037867711 8968 0.008808534
8980 0.03671171 8980 0.076334727 4520 0.019098425 4520
0.055002798 8976 0.067681591 8976 0.039677809
8980 0.044759716 8980 0.11333602 4520 0.021994601 4524
0.046948637 8984 0.033881708 8984 0.02933191
8990 0.058776044 8990 0.103234721 4530 0.033352302 4528
0.03034918 8992 0.11470457 8992 0.010549269
9000 0.07302373 9000 0.054815908 4530 0.017344724 4532
0.041760788 9000 0.149382803 9000 0.038414066
9010 0.098464421 9010 0.03222363 4540 0.054871242 4536

0.092838425 9008 0.126465456 9008 0.043450829
9020 0.080678263 9020 0.087473018 4540 0.105923355 4540
0.092686467 9016 0.081029852 9016 0.048235917
9020 0.026708847 9020 0.065673979 4540 0.099837416 4544
0.039427574 9024 0.074655232 9024 0.03752876
9030 0.067551991 9030 0.046052574 4550 0.066514396 4548
0.044989072 9032 0.084564526 9032 0.02889323
9040 0.172419983 9040 0.118232478 4550 0.052179068 4552
0.016818543 9040 0.101984791 9040 0.012279234
9050 0.215281558 9050 0.163232093 4560 0.028127066 4556
0.026767952 9048 0.112552923 9048 0.028313039
9060 0.198257446 9060 0.133769485 4560 0.067009365 4560
0.026169522 9056 0.112296351 9056 0.046327401
9060 0.171135296 9060 0.035732257 4560 0.072874253 4564
0.087001332 9064 0.109138578 9064 0.043620621
9070 0.127042425 9070 0.043790646 4570 0.055601868 4568
0.083432584 9072 0.131367168 9072 0.047443027
9080 0.034720062 9080 0.09857001 4570 0.055642457 4572
0.032978911 9080 0.167139966 9080 0.040342475
9090 0.065215769 9090 0.165500024 4580 0.066928871 4576
0.068399386 9088 0.157309478 9088 0.013498083
9100 0.090396257 9100 0.160051394 4580 0.056784087 4580
0.064894244 9096 0.114048002 9096 0.006080308
9100 0.079146608 9100 0.080750891 4580 0.04971823 4584
0.032757234 9104 0.078627432 9104 0.036198449
9110 0.050701674 9110 0.073147712 4590 0.052429376 4588
0.011904253 9112 0.033749133 9112 0.055780023
9120 0.028229806 9120 0.128500731 4590 0.077054487 4592
0.026656302 9120 0.0267826 9120 0.05299105
9130 0.083209292 9130 0.131272391 4600 0.08647448 4596
0.035014509 9128 0.074754527 9128 0.044624332
9140 0.046742393 9140 0.067591805 4600 0.054337368 4600
0.0609948 9136 0.107102271 9136 0.036157489
9140 0.058506346 9140 0.092532944 4600 0.041854499 4604
0.058355112 9144 0.10000851 9144 0.010334165
9150 0.161718839 9150 0.079773512 4610 0.023990102 4608
0.027226388 9152 0.102811377 9152 0.039910268
9160 0.193744199 9160 0.135050869 4610 0.067901448 4612
0.041679105 9160 0.1078887 9160 0.036439054
9170 0.117619093 9170 0.101514983 4620 0.059796737 4616
0.057599089 9168 0.070370508 9168 0.044046628
9180 0.031606654 9180 0.02689423 4620 0.024532053 4620
0.040993025 9176 0.040789797 9176 0.064955093
9180 0.071224691 9180 0.026304419 4620 0.078787765 4624
0.072350893 9184 0.064861335 9184 0.05952159
9190 0.116644325 9190 0.063372718 4630 0.064633146 4628
0.059672311 9192 0.115408664 9192 0.030923839
9200 0.068366651 9200 0.125632433 4630 0.082066574 4632
0.00796105 9200 0.131629844 9200 0.02135923
9210 0.039088391 9210 0.141864308 4640 0.088262503 4636
0.016621487 9208 0.073525349 9208 0.043090124
9220 0.045839075 9220 0.110961242 4640 0.051160627 4640
0.01328814 9216 0.021559252 9216 0.071953902
9220 0.085152933 9220 0.023126011 4640 0.043377255 4644
0.037281843 9224 0.111101486 9224 0.060142687
9230 0.069158712 9230 0.086303757 4650 0.041479896 4648
0.053655753 9232 0.094986273 9232 0.011587311
9240 0.056815996 9240 0.112725589 4650 0.041378266 4652
0.050495735 9240 0.011357703 9240 0.021312872
9250 0.101668928 9250 0.068269867 4660 0.056878635 4656
0.058603091 9248 0.043158059 9248 0.007865392
9260 0.07799326 9260 0.077474397 4660 0.070262613 4660
0.067655346 9256 0.028728506 9256 0.052043703
9260 0.061607781 9260 0.046746158 4660 0.045224031 4664
0.091974638 9264 0.060725459 9264 0.087862623
9270 0.111512047 9270 0.099356941 4670 0.048854072 4668
0.119576871 9272 0.112133079 9272 0.075435513
9280 0.079930054 9280 0.145242754 4670 0.062289721 4672

0.126506159 9280 0.18788832 9280 0.029742401
9290 0.081962033 9290 0.125934865 4680 0.068912632 4676
0.077417899 9288 0.195783111 9288 0.002704702
9300 0.050981817 9300 0.068379333 4680 0.056862355 4680
0.015641823 9296 0.156999988 9296 0.027502587
9300 0.076575416 9300 0.028080145 4680 0.023326424 4684
0.030467638 9304 0.074189396 9304 0.069703609
9310 0.103622755 9310 0.053685693 4690 0.020693857 4688
0.044597397 9312 0.046212164 9312 0.069610942
9320 0.049793001 9320 0.045004999 4690 0.044990353 4692
0.032548203 9320 0.085179345 9320 0.025760568
9330 0.091338676 9330 0.074511052 4700 0.031104872 4696
0.076946359 9328 0.061247818 9328 0.018774274
9340 0.107584579 9340 0.039823848 4700 0.039981416 4700
0.103200706 9336 0.086785331 9336 0.008132283
9340 0.034487915 9340 0.162442914 4700 0.08408969 4704
0.092071015 9344 0.10933265 9344 0.01757292
9350 0.116544245 9350 0.201900315 4710 0.050523293 4708
0.047710342 9352 0.152485955 9352 0.010635786
9360 0.086364198 9360 0.170602405 4710 0.062099782 4712
0.01445094 9360 0.194402891 9360 0.047823349
9370 0.063232779 9370 0.165145306 4720 0.063873165 4716
0.041220563 9368 0.157332805 9368 0.07048867
9380 0.095372612 9380 0.13666712 4720 0.032512129 4720
0.078374316 9376 0.073524228 9376 0.048635826
9380 0.092469461 9380 0.013394536 4720 0.048622813 4724
0.081505597 9384 0.061315361 9384 0.045158649
9390 0.095213814 9390 0.058388629 4730 0.035117195 4728
0.049041279 9392 0.129087974 9392 0.069941249
9400 0.110679161 9400 0.064768814 4730 0.02994488 4732
0.014161806 9400 0.143486949 9400 0.038179896
9410 0.112624592 9410 0.152211651 4740 0.011712425 4736
0.044942615 9408 0.068466638 9408 0.012561633
9420 0.124032653 9420 0.069826347 4740 0.008221719 4740
0.064711756 9416 0.016044853 9416 0.023856848
9420 0.120024088 9420 0.085427899 4740 0.026545067 4744
0.080652753 9424 0.005109665 9424 0.029927702
9430 0.066622917 9430 0.076581986 4750 0.039611397 4748
0.092282404 9432 0.059215556 9432 0.014640767
9440 0.050953775 9440 0.053872744 4750 0.038870312 4752
0.118687647 9440 0.056508226 9440 0.013207534
9450 0.077736695 9450 0.034854234 4760 0.05807959 4756
0.109450215 9448 0.093427792 9448 0.024734434
9460 0.037699138 9460 0.071772854 4760 0.044840868 4760
0.067161331 9456 0.15140597 9456 0.015561256
9460 0.117025702 9460 0.177118884 4760 0.030389014 4764
0.022250975 9464 0.169679319 9464 0.008769652
9470 0.116094721 9470 0.151222455 4770 0.032698994 4768
0.059397273 9472 0.140774108 9472 0.039716586
9480 0.055149561 9480 0.074064279 4770 0.071030976 4772
0.085885149 9480 0.076476652 9480 0.063645792
9490 0.077318611 9490 0.10954601 4780 0.05511639 4776
0.090399968 9488 0.018219052 9488 0.060849041
9500 0.1165957 9500 0.116823729 4780 0.027741728 4780
0.099515368 9496 0.094754447 9496 0.070534341
9500 0.12250914 9500 0.1009525 4780 0.0300462 4784
0.081119964 9504 0.148292645 9504 0.057435711
9510 0.096336313 9510 0.10047722 4790 0.032615095 4788
0.026326952 9512 0.185633224 9512 0.052832293
9520 0.06455437 9520 0.110895446 4790 0.055394405 4792
0.025184463 9520 0.168694736 9520 0.045220691
9530 0.082727995 9530 0.059080168 4800 0.05614194 4796
0.046535315 9528 0.081405306 9528 0.03678642
9540 0.152618551 9540 0.067003122 4800 0.022040782 4800
0.059347291 9536 0.087033237 9536 0.068933834
9540 0.221936003 9540 0.111161673 4800 0.059306534 4804
0.096958596 9544 0.151554268 9544 0.081850812
9550 0.192352353 9550 0.064034954 4810 0.026642116 4808

0.140470801 9552 0.122055521 9552 0.069387599
9560 0.078208694 9560 0.060258983 4810 0.0599915 4812
0.104831204 9560 0.066534711 9560 0.04352981
9570 0.074792253 9570 0.092155038 4820 0.053143831 4816
0.049790124 9568 0.083597501 9568 0.017926945
9580 0.094730836 9580 0.123578124 4820 0.008879031 4820
0.091630966 9576 0.100758261 9576 0.024536208
9580 0.041243795 9580 0.038995673 4820 0.013101447 4824
0.104917956 9584 0.025891079 9584 0.060457176
9590 0.044932669 9590 0.089994333 4830 0.010721536 4828
0.062919324 9592 0.071707429 9592 0.050624203
9600 0.051369923 9600 0.136346541 4830 0.019616147 4832
0.018333623 9600 0.058196707 9600 0.017435092
9610 0.09348568 9610 0.078379591 4840 0.036903497 4836
0.060734776 9608 0.057452351 9608 0.048307506
9620 0.125566454 9620 0.019351644 4840 0.036233771 4840
0.083632993 9616 0.147822066 9616 0.041062394
9620 0.090003669 9620 0.07513311 4840 0.047629328 4844
0.084686704 9624 0.147786138 9624 0.026497202
9630 0.035296675 9630 0.068548223 4850 0.04481028 4848
0.076148266 9632 0.095519848 9632 0.028141365
9640 0.056266341 9640 0.145704689 4850 0.01978171 4852
0.067398614 9640 0.065879111 9640 0.016513603
9650 0.140749763 9650 0.149169064 4860 0.044126624 4856
0.074773081 9648 0.05596238 9648 0.049855382
9660 0.181436684 9660 0.065996137 4860 0.061933977 4860
0.086046523 9656 0.020575491 9656 0.071580704
9660 0.119955701 9660 0.092476985 4860 0.037544251 4864
0.086398104 9664 0.011408487 9664 0.073032046
9670 0.005194143 9670 0.137247145 4870 0.040918068 4868
0.076869015 9672 0.028477491 9672 0.062297258
9680 0.078131954 9680 0.127586711 4870 0.034008637 4872
0.043429365 9680 0.068022295 9680 0.032160177
9690 0.127844469 9690 0.085353095 4880 0.003756725 4876
0.079933612 9688 0.13471162 9688 0.00835766
9700 0.175752444 9700 0.088234141 4880 0.037024842 4880
0.10402059 9696 0.136968374 9696 0.004950527
9700 0.200399838 9700 0.131708643 4880 0.061282684 4884
0.090091548 9704 0.108832894 9704 0.039137678
9710 0.186656413 9710 0.172922868 4890 0.06163001 4888
0.044584416 9712 0.081006132 9712 0.054265733
9720 0.150787746 9720 0.136703995 4890 0.036465564 4892
0.095541815 9720 0.018520193 9720 0.027158723
9730 0.102331222 9730 0.067217698 4900 0.013849171 4896
0.125673818 9728 0.062313782 9728 0.011872762
9740 0.023372528 9740 0.088731635 4900 0.014144981 4900
0.105009596 9736 0.116021271 9736 0.008908614
9740 0.0363281 9740 0.143456971 4900 0.021130269 4904
0.072941584 9744 0.099411809 9744 0.013292395
9750 0.033764238 9750 0.143425234 4910 0.023883597 4908
0.047136444 9752 0.023616232 9752 0.027907343
9760 0.020712721 9760 0.111447 4910 0.030801293 4912
0.047716443 9760 0.069279369 9760 0.055079319
9770 0.026753784 9770 0.094224088 4920 0.065587054 4916
0.055630168 9768 0.084079424 9768 0.061732812
9780 0.065641216 9780 0.071623334 4920 0.097662181 4920
0.074376461 9776 0.022391447 9776 0.050471903
9780 0.059585731 9780 0.06717322 4920 0.081423459 4924
0.07826888 9784 0.051960538 9784 0.022833376
9790 0.093200026 9790 0.108939232 4930 0.033598499 4928
0.106225627 9792 0.054807813 9792 0.021611253
9800 0.16019016 9800 0.113935523 4930 0.030647381 4932
0.142679811 9800 0.111876732 9800 0.05412801
9810 0.16817842 9810 0.094183139 4940 0.068095651 4936
0.119372235 9808 0.225412426 9808 0.065318818
9820 0.150398934 9820 0.101355145 4940 0.06683774 4940
0.047712023 9816 0.170770974 9816 0.054525001
9820 0.154639289 9820 0.134878835 4940 0.053755706 4944

0.100130332 9824 0.055932065 9824 0.058195259
9830 0.140771939 9830 0.076499135 4950 0.029321613 4948
0.124933285 9832 0.031253236 9832 0.063518281
9840 0.08410601 9840 0.007212934 4950 0.033996887 4952
0.101276055 9840 0.06800508 9840 0.044040382
9850 0.056116198 9850 0.008787094 4960 0.041925199 4956
0.08326488 9848 0.140834498 9848 0.042880693
9860 0.089816429 9860 0.009860174 4960 0.020318101 4960
0.055519915 9856 0.113562594 9856 0.044579774
9860 0.084527252 9860 0.036561214 4960 0.015565236 4964
0.045069199 9864 0.046237179 9864 0.029085133
9870 0.078581295 9870 0.101753212 4970 0.031468946 4968
0.081518083 9872 0.032266566 9872 0.038597646
9880 0.042330164 9880 0.113167938 4970 0.056053919 4972
0.086892324 9880 0.068570771 9880 0.066962661
9890 0.056169716 9890 0.067843728 4980 0.039783099 4976
0.061676976 9888 0.105315514 9888 0.068626956
9900 0.109615743 9900 0.091346323 4980 0.022442931 4980
0.055053966 9896 0.08723553 9896 0.066306486
9900 0.175684298 9900 0.136812945 4980 0.023170589 4984
0.08418443 9904 0.060283412 9904 0.062870327
9910 0.182881616 9910 0.112334477 4990 0.014429576 4988
0.107071617 9912 0.165944483 9912 0.047325313
9920 0.088492685 9920 0.087828383 4990 0.024657893 4992
0.093808252 9920 0.24062254 9920 0.036538862
9930 0.034449335 9930 0.109293011 5000 0.022262393 4996
0.084812891 9928 0.180705931 9928 0.043790256
9940 0.015487622 9940 0.086560984 5000 0.01839051 5000
0.06827194 9936 0.061929553 9936 0.054640779
9940 0.100652775 9940 0.03805045 5000 0.026820902 5004
0.055779539 9944 0.059313683 9944 0.05883451
9950 0.134706031 9950 0.023276802 5010 0.008201855 5008
0.074446412 9952 0.039157367 9952 0.049529779
9960 0.086107044 9960 0.007014474 5010 0.035879501 5012
0.093362512 9960 0.053273936 9960 0.019068804
9970 0.062869003 9970 0.009742856 5020 0.048828071 5016
0.055077937 9968 0.055696975 9968 0.075321957
9980 0.120609991 9980 0.079072292 5020 0.077708028 5020
0.014940617 9976 0.022347247 9976 0.067072113
9980 0.17044702 9980 0.115060779 5020 0.093912066 5024
0.054247605 9984 0.042477877 9984 0.010281678
9990 0.136270624 9990 0.094076524 5030 0.075206699 5028
0.043392633 9992 0.077020421 9992 0.03058599
10000 0.068059089 10000 0.066763336 5030 0.038676128 5032
0.032525382 10000 0.078603705 10000 0.009751899
10000 0.080485908 10000 0.041238152 5040 0.037623104 5036
0.04899612 10008 0.032443004 10008 0.032169097
10000 0.113691152 10000 0.036588899 5040 0.030768566 5040
0.071774673 10016 0.067815621 10016 0.039548173
10000 0.15160309 10000 0.075383105 5040 0.008973209 5044
0.088131965 10024 0.095564123 10024 0.012832968
10000 0.150195993 10000 0.063035703 5050 0.051162399 5048
0.094541516 10032 0.08325118 10032 0.017793975
10000 0.10124554 10000 0.087139102 5050 0.079316451 5052
0.083834122 10040 0.072504496 10040 0.014483225
10000 0.039417373 10000 0.106704829 5060 0.06050292 5056
0.054770488 10048 0.079580823 10048 0.026963387
10100 0.091561524 10100 0.132590634 5060 0.03872758 5060
0.042486539 10056 0.06635076 10056 0.045212953
10100 0.144325503 10100 0.107185544 5060 0.058646619 5064
0.043587163 10064 0.094910654 10064 0.025091138
10100 0.088268287 10100 0.086926222 5070 0.087607034 5068
0.0146341 10072 0.080784252 10072 0.021898282
10100 0.076321972 10100 0.121130994 5070 0.086918139 5072
0.038938466 10080 0.074862575 10080 0.044594093
10100 0.083341118 10100 0.090201844 5080 0.035393285 5076
0.049951708 10088 0.123087622 10088 0.052767326
10100 0.07085484 10100 0.095402051 5080 0.038190043 5080

0.075143653 10096 0.102203791 10096 0.033742163
10100 0.06732015 10100 0.113899972 5080 0.068625152 5084
0.070010217 10104 0.095788542 10104 0.043663094
10100 0.010583193 10100 0.122118552 5090 0.056309404 5088
0.080761165 10112 0.158125476 10112 0.018284392
10100 0.076668322 10100 0.073622432 5090 0.032991105 5092
0.068030342 10120 0.119379933 10120 0.0340056
10100 0.126588406 10100 0.009534842 5100 0.041778327 5096
0.044988985 10128 0.05827938 10128 0.056807938
10100 0.10820121 10100 0.02583007 5100 0.036916314 5100
0.083156752 10136 0.127424734 10136 0.05820327
10100 0.086758628 10100 0.036005706 5100 0.023409302 5104
0.050523096 10144 0.11765011 10144 0.039743809
10200 0.148646621 10200 0.119758988 5110 0.005966513 5108
0.015365216 10152 0.084948792 10152 0.05619014
10200 0.124512517 10200 0.162522454 5110 0.020913743 5112
0.007466986 10160 0.010855341 10160 0.096704396
10200 0.045572324 10200 0.128670014 5120 0.018131133 5116
0.030723379 10168 0.098944125 10168 0.07493142
10200 0.043940487 10200 0.040754785 5120 0.021025329 5120
0.046567289 10176 0.124838989 10176 0.04428349
10200 0.037425718 10200 0.053924257 5120 0.039081693 5124
0.060155897 10184 0.112107948 10184 0.079639125
10200 0.114581744 10200 0.096884549 5130 0.051510455 5128
0.063706655 10192 0.072215597 10192 0.094234973
10200 0.1093627 10200 0.110029381 5130 0.057745361 5132
0.026906735 10200 0.05718218 10200 0.075031378
10200 0.060140879 10200 0.147029481 5140 0.043093307 5136
0.045316865 10208 0.066523768 10208 0.051816416
10200 0.023326506 10200 0.093999712 5140 0.022866205 5140
0.077193487 10216 0.013457474 10216 0.05489909
10200 0.08855699 10200 0.131606765 5140 0.041682266 5144
0.091346978 10224 0.055381002 10224 0.03564588
10200 0.031851472 10200 0.235670915 5150 0.025751682 5148
0.049130318 10232 0.049179795 10232 0.014159185
10200 0.112736365 10200 0.237400688 5150 0.01264455 5152
0.05244033 10240 0.115061463 10240 0.045475183
10200 0.095255113 10200 0.13665286 5160 0.039655384 5156
0.100693666 10248 0.159375748 10248 0.043518056
10300 0.049539634 10300 0.067546898 5160 0.04148838 5160
0.085405889 10256 0.16023498 10256 0.033195902
10300 0.085280262 10300 0.069009664 5160 0.036145033 5164
0.047038484 10264 0.15531393 10264 0.017465631
10300 0.103297352 10300 0.079108228 5170 0.025272288 5168
0.020630361 10272 0.133481691 10272 0.007626186
10300 0.119771357 10300 0.075676515 5170 0.029599321 5172
0.015075898 10280 0.12239076 10280 0.016323244
10300 0.141424884 10300 0.05604823 5180 0.0710186 5176
0.031765885 10288 0.141646 10288 0.027085569
10300 0.109522502 10300 0.123963924 5180 0.084980013 5180
0.038876442 10296 0.151463188 10296 0.025922269
10300 0.057446679 10300 0.083338266 5180 0.076234632 5184
0.067437526 10304 0.142942285 10304 0.023283961
10300 0.06384973 10300 0.06955029 5190 0.058585767 5188
0.065490051 10312 0.11189247 10312 0.038458034
10300 0.015590786 10300 0.057401205 5190 0.043380111 5192
0.055302124 10320 0.100650621 10320 0.049685928
10300 0.038319853 10300 0.095467767 5200 0.050056984 5196
0.061921513 10328 0.14489259 10328 0.060429393
10300 0.059294205 10300 0.070855873 5200 0.045416884 5200
0.06483429 10336 0.117917945 10336 0.068389119
10300 0.051608949 10300 0.049117949 5200 0.020232614 5204
0.052053456 10344 0.051202569 10344 0.060685805
10400 0.04123522 10400 0.083056351 5210 0.019742487 5208
0.044744327 10352 0.08835968 10352 0.072550582
10400 0.069899317 10400 0.054196047 5210 0.06105208 5212
0.057834837 10360 0.145752274 10360 0.064399443
10400 0.095204072 10400 0.020458154 5220 0.086295971 5216

0.080006554	10368	0.172721208	10368	0.058973263		
10400	0.035142293	10400	0.065432934	5220	0.063979547	5220
0.103557242	10376	0.157605523	10376	0.05673011		
10400	0.106157146	10400	0.046350251	5220	0.00905556	5224
0.09769488	10384	0.098196651	10384	0.037597161		
10400	0.11998057	10400	0.007630818	5230	0.019666237	5228
0.056979563	10392	0.025729863	10392	0.0082092		
10400	0.080846548	10400	0.033570435	5230	0.009893293	5232
0.030155603	10400	0.068235378	10400	0.019153453		
10400	0.117463431	10400	0.040192212	5240	0.040680101	5236
0.032663618	10408	0.090036978	10408	0.032508215		
10400	0.104760875	10400	0.100033576	5240	0.045863028	5240
0.024745754	10416	0.078807178	10416	0.030728257		
10400	0.060034054	10400	0.2194548	5240	0.045401059	5244
0.029432307	10424	0.015989892	10424	0.024055762		
10400	0.057578014	10400	0.242492126	5250	0.076170232	5248
0.030593983	10432	0.085808257	10432	0.018141174		
10400	0.108380758	10400	0.162449404	5250	0.102987804	5252
0.024811683	10440	0.116152587	10440	0.03437827		
10400	0.082387844	10400	0.078507277	5260	0.083290463	5256
0.03773547	10448	0.068919093	10448	0.032722441		
10500	0.027242626	10500	0.050444251	5260	0.038273513	5260
0.06745357	10456	0.044264831	10456	0.015715034		
10500	0.024363328	10500	0.014684642	5260	0.040054358	5264
0.062719315	10464	0.048459744	10464	0.034057928		
10500	0.083094994	10500	0.025470661	5270	0.074415053	5268
0.040677747	10472	0.040990286	10472	0.002319886		
10500	0.143254481	10500	0.030837764	5270	0.046917998	5272
0.028776529	10480	0.046548481	10480	0.033048436		
10500	0.173678941	10500	0.020425932	5280	0.011470219	5276
0.031951942	10488	0.029809284	10488	0.037726539		
10500	0.176671573	10500	0.031038009	5280	0.00892806	5280
0.016069635	10496	0.038637467	10496	0.04656642		
10500	0.139504235	10500	0.072780094	5280	0.027378621	5284
0.018809902	10504	0.065779888	10504	0.066974099		
10500	0.12775576	10500	0.044612254	5290	0.034127032	5288
0.030478792	10512	0.069788715	10512	0.069898684		
10500	0.172672735	10500	0.082549974	5290	0.037916991	5292
0.070963513	10520	0.05607542	10520	0.062230669		
10500	0.193147513	10500	0.118707889	5300	0.05505473	5296
0.121670499	10528	0.064051696	10528	0.05094364		
10500	0.151315675	10500	0.036688947	5300	0.041148211	5300
0.107094529	10536	0.073191128	10536	0.048204005		
10500	0.059846061	10500	0.161581876	5300	0.040683903	5304
0.04536011	10544	0.075567114	10544	0.061952989		
10600	0.039823579	10600	0.160570664	5310	0.048150174	5308
0.025594731	10552	0.05650687	10552	0.033649809		
10600	0.027753886	10600	0.07217277	5310	0.053347798	5312
0.048660837	10560	0.007288142	10560	0.028310453		
10600	0.075936005	10600	0.047231577	5320	0.05373728	5316
0.077487355	10568	0.072361298	10568	0.047578196		
10600	0.132019618	10600	0.114562819	5320	0.052733089	5320
0.114455354	10576	0.116720767	10576	0.046828936		
10600	0.09169463	10600	0.108453911	5320	0.043555821	5324
0.121211022	10584	0.130987654	10584	0.037101741		
10600	0.042742027	10600	0.075341901	5330	0.036444904	5328
0.086214277	10592	0.086383421	10592	0.010305654		
10600	0.034022229	10600	0.088630412	5330	0.018740775	5332
0.054256801	10600	0.0280438	10600	0.018095325		
10600	0.075223841	10600	0.085237531	5340	0.039642077	5336
0.055998524	10608	0.134101894	10608	0.005666584		
10600	0.049592356	10600	0.065710636	5340	0.055258326	5340
0.072484145	10616	0.157260801	10616	0.022540044		
10600	0.037308124	10600	0.118305135	5340	0.039713592	5344
0.106884232	10624	0.108924447	10624	0.005635493		
10600	0.033899152	10600	0.139589305	5350	0.023757775	5348
0.104026287	10632	0.071004797	10632	0.049988135		
10600	0.00956508	10600	0.105260806	5350	0.022886243	5352

0.052068583 10640 0.114514704 10640 0.057201425
10600 0.043440483 10600 0.030730938 5360 0.039688901 5356
0.034340967 10648 0.164183671 10648 0.053783286
10700 0.060476497 10700 0.041007483 5360 0.02564299 5360
0.10233889 10656 0.127810068 10656 0.092736467
10700 0.023246599 10700 0.071872171 5360 0.006087899 5364
0.098081771 10664 0.107326174 10664 0.075801952
10700 0.038463204 10700 0.096208991 5370 0.029101679 5368
0.043273343 10672 0.158132505 10672 0.033690136
10700 0.130568413 10700 0.07128204 5370 0.02776701 5372
0.088759618 10680 0.041993026 10680 0.020157113
10700 0.134981616 10700 0.077408346 5380 0.024424198 5376
0.108023356 10688 0.110641406 10688 0.051516123
10700 0.072491683 10700 0.106043248 5380 0.042405463 5380
0.07178363 10696 0.125498278 10696 0.044357916
10700 0.146224251 10700 0.035369008 5380 0.045162771 5384
0.051096158 10704 0.118433018 10704 0.04725808
10700 0.220034418 10700 0.06573608 5390 0.038215036 5388
0.098198951 10712 0.094447045 10712 0.045951496
10700 0.217990644 10700 0.026592012 5390 0.040553958 5392
0.13833995 10720 0.039741895 10720 0.030041103
10700 0.15226625 10700 0.063602864 5400 0.061778541 5396
0.115828989 10728 0.032067088 10728 0.035517354
10700 0.112147609 10700 0.178000875 5400 0.054839231 5400
0.044037821 10736 0.031580628 10736 0.021992293
10700 0.107683671 10700 0.276848034 5400 0.029875797 5404
0.050703977 10744 0.106684835 10744 0.011561395
10800 0.081893981 10800 0.249715843 5410 0.006798387 5408
0.070246249 10752 0.128379645 10752 0.018957095
10800 0.039320392 10800 0.154142894 5410 0.036132518 5412
0.039440736 10760 0.146902749 10760 0.025057772
10800 0.05003604 10800 0.081636019 5420 0.072203067 5416
0.012279563 10768 0.153197049 10768 0.01934697
10800 0.069631955 10800 0.050432213 5420 0.06270242 5420
0.050697443 10776 0.05535407 10776 0.020209873
10800 0.03810005 10800 0.085133623 5420 0.053746582 5424
0.075120304 10784 0.065065018 10784 0.014178583
10800 0.113482434 10800 0.049751667 5430 0.062574807 5428
0.110434958 10792 0.124440849 10792 0.00488291
10800 0.13068851 10800 0.027922202 5430 0.027603202 5432
0.1335813 10800 0.175433845 10800 0.006649207
10800 0.108617678 10800 0.029083245 5440 0.019469953 5436
0.0784472 10808 0.139038951 10808 0.006279203
10800 0.089759386 10800 0.040395134 5440 0.036633308 5440
0.023366951 10816 0.072440598 10816 0.022962488
10800 0.083768799 10800 0.0953938 5440 0.02720321 5444
0.045698871 10824 0.134760266 10824 0.042613214
10800 0.069708185 10800 0.143034588 5450 0.011367454 5448
0.045041528 10832 0.049502181 10832 0.044987784
10800 0.043194083 10800 0.112465692 5450 0.02690058 5452
0.045027577 10840 0.077989236 10840 0.032557629
10800 0.086790737 10800 0.061516985 5460 0.051460647 5456
0.069231319 10848 0.050346178 10848 0.015588996
10900 0.060743118 10900 0.060705759 5460 0.041019866 5460
0.08964233 10856 0.060239334 10856 0.019685373
10900 0.020363936 10900 0.129431006 5460 0.020929188 5464
0.08248524 10864 0.104138308 10864 0.056051216
10900 0.059653408 10900 0.149025145 5470 0.012028536 5468
0.033040105 10872 0.087412504 10872 0.071468574
10900 0.035010628 10900 0.106427709 5470 0.035787696 5472
0.032509011 10880 0.096192263 10880 0.037748705
10900 0.042397744 10900 0.144350023 5480 0.046743833 5476
0.081832346 10888 0.14021268 10888 0.009304252
10900 0.039107265 10900 0.167453313 5480 0.03449486 5480
0.104382358 10896 0.164005848 10896 0.028254868
10900 0.016439401 10900 0.122204743 5480 0.029089017 5484
0.094174808 10904 0.138830248 10904 0.031624088
10900 0.025554346 10900 0.155865491 5490 0.015563301 5488

0.10289951	10912	0.079617792	10912	0.049393271		
10900	0.06719854	10900	0.17062659	5490	0.031816959	5492
0.086736385	10920	0.104940256	10920	0.062870677		
10900	0.062937499	10900	0.118242286	5500	0.050170864	5496
0.053201351	10928	0.136023184	10928	0.038373255		
10900	0.07600199	10900	0.131345106	5500	0.037461377	5500
0.067331028	10936	0.135123206	10936	0.035478235		
10900	0.105375191	10900	0.111222842	5500	0.031615993	5504
0.070074704	10944	0.085645501	10944	0.075562806		
11000	0.093700008	11000	0.091701309	5510	0.06585852	5508
0.056605026	10952	0.036654572	10952	0.082674822		
11000	0.008101766	11000	0.087202607	5510	0.095896554	5512
0.059003913	10960	0.011250044	10960	0.058327529		
11000	0.026973597	11000	0.051463929	5520	0.088381348	5516
0.056503395	10968	0.026255841	10968	0.033075117		
11000	0.04955172	11000	0.028237904	5520	0.041792271	5520
0.041375373	10976	0.04337899	10976	0.033637276		
11000	0.091586531	11000	0.023762863	5520	0.02592782	5524
0.029081977	10984	0.054587719	10984	0.032759101		
11000	0.099969875	11000	0.086809043	5530	0.054359131	5528
0.078794772	10992	0.129857435	10992	0.030145817		
11000	0.093563074	11000	0.07680001	5530	0.071961164	5532
0.090430112	11000	0.202813244	11000	0.079718855		
11000	0.08565715	11000	0.052927389	5540	0.045671619	5536
0.071130839	11008	0.157523551	11008	0.054613949		
11000	0.09913207	11000	0.066178225	5540	0.004251118	5540
0.051451825	11016	0.038708677	11016	0.013000744		
11000	0.066555833	11000	0.044928544	5540	0.007417968	5544
0.019372506	11024	0.069715374	11024	0.040579223		
11000	0.020182535	11000	0.034840487	5550	0.014540357	5548
0.050444847	11032	0.040843323	11032	0.037626196		
11000	0.041154966	11000	0.076865224	5550	0.035435791	5552
0.114112772	11040	0.075173804	11040	0.014632868		
11000	0.06391271	11000	0.053761254	5560	0.061777239	5556
0.108366272	11048	0.103382146	11048	0.012955286		
11100	0.045066798	11100	0.032604301	5560	0.049563972	5560
0.062359868	11056	0.065965258	11056	0.009479499		
11100	0.073128373	11100	0.056183206	5560	0.043012351	5564
0.055828503	11064	0.104014609	11064	0.034052155		
11100	0.079017853	11100	0.043529606	5570	0.057519512	5568
0.034327353	11072	0.096671356	11072	0.063726482		
11100	0.085712571	11100	0.115660449	5570	0.044857818	5572
0.017002036	11080	0.123216116	11080	0.04487162		
11100	0.112404268	11100	0.193845859	5580	0.020399264	5576
0.050863731	11088	0.194882726	11088	0.022751961		
11100	0.139239971	11100	0.18714754	5580	0.015619164	5580
0.047322596	11096	0.180951276	11096	0.038455582		
11100	0.11964664	11100	0.066575332	5580	0.042940988	5584
0.049065107	11104	0.130593923	11104	0.03317224		
11100	0.065500477	11100	0.008016143	5590	0.054766839	5588
0.042926098	11112	0.134976421	11112	0.026829646		
11100	0.086429158	11100	0.044486762	5590	0.061850464	5592
0.01281233	11120	0.050033355	11120	0.020477792		
11100	0.111349138	11100	0.087173503	5600	0.049767281	5596
0.013992029	11128	0.062567306	11128	0.020183374		
11100	0.133068955	11100	0.105345745	5600	0.051864823	5600
0.044761793	11136	0.039352828	11136	0.045607605		
11100	0.097821328	11100	0.163749995	5600	0.032917375	5604
0.050334798	11144	0.054166856	11144	0.027073364		
11200	0.058022713	11200	0.176450908	5610	0.035284535	5608
0.051155283	11152	0.083427505	11152	0.016233928		
11200	0.058449412	11200	0.114921641	5610	0.030730953	5612
0.059574348	11160	0.129646884	11160	0.00466748		
11200	0.10323571	11200	0.068942303	5620	0.016972712	5616
0.053603224	11168	0.169877676	11168	0.031649099		
11200	0.123841601	11200	0.047381775	5620	0.030283482	5620
0.04572362	11176	0.154459878	11176	0.033264863		
11200	0.077171833	11200	0.045770365	5620	0.02707469	5624

0.029953975	11184	0.162430646	11184	0.027561406		
11200	0.009934723	11200	0.056863239	5630	0.030663749	5628
0.032651326	11192	0.202569834	11192	0.032958757		
11200	0.013365543	11200	0.027886288	5630	0.062606261	5632
0.044100263	11200	0.15460173	11200	0.021584056		
11200	0.059096838	11200	0.033301778	5640	0.067543937	5636
0.06753963	11208	0.083134553	11208	0.015791587		
11200	0.075507276	11200	0.037473274	5640	0.025413927	5640
0.080729478	11216	0.080366481	11216	0.018400244		
11200	0.064222877	11200	0.040296076	5640	0.053959793	5644
0.083050269	11224	0.132143526	11224	0.015046111		
11200	0.013784805	11200	0.05484738	5650	0.073414296	5648
0.0835174	11232	0.125055813	11232	0.021838458		
11200	0.075385739	11200	0.1152244	5650	0.06790278	5652
0.085427811	11240	0.095445976	11240	0.007038049		
11200	0.064680266	11200	0.14411546	5660	0.033875473	5656
0.071020251	11248	0.09132328	11248	0.019986046		
11300	0.055181623	11300	0.100665457	5660	0.025430303	5660
0.038142876	11256	0.082833336	11256	0.023526705		
11300	0.097828946	11300	0.025341773	5660	0.03936547	5664
0.025182109	11264	0.089285473	11264	0.044972887		
11300	0.037795489	11300	0.024419969	5670	0.003177767	5668
0.045033063	11272	0.09891799	11272	0.065071676		
11300	0.125845225	11300	0.047920628	5670	0.028150045	5672
0.043633558	11280	0.08781012	11280	0.057260324		
11300	0.18718747	11300	0.055761033	5680	0.044265373	5676
0.028869612	11288	0.105866559	11288	0.030343423		
11300	0.108526299	11300	0.061015177	5680	0.061723338	5680
0.017693445	11296	0.047672278	11296	0.004652763		
11300	0.011508774	11300	0.03125205	5680	0.045828008	5684
0.038186252	11304	0.037997263	11304	0.028518756		
11300	0.029480403	11300	0.043098669	5690	0.043784618	5688
0.066612593	11312	0.142879654	11312	0.054859909		
11300	0.056577781	11300	0.055924556	5690	0.049342798	5692
0.018334798	11320	0.2627098	11320	0.06955881		
11300	0.109013883	11300	0.159388918	5700	0.049862327	5696
0.047400572	11328	0.281909481	11328	0.06512471		
11300	0.093980598	11300	0.186408914	5700	0.040130235	5700
0.075038188	11336	0.209904378	11336	0.027885995		
11300	0.107169457	11300	0.122924248	5700	0.047347254	5704
0.074391624	11344	0.234956519	11344	0.030897994		
11400	0.112988368	11400	0.190313265	5710	0.049356728	5708
0.046441121	11352	0.190241466	11352	0.062990053		
11400	0.052433225	11400	0.223934665	5710	0.037965558	5712
0.064628395	11360	0.113413495	11360	0.045830337		
11400	0.036409696	11400	0.153043904	5720	0.04162472	5716
0.070935748	11368	0.061692117	11368	0.037148402		
11400	0.08387932	11400	0.13151436	5720	0.046130601	5720
0.095715899	11376	0.27201476	11376	0.044448858		
11400	0.110101704	11400	0.148786479	5720	0.031440126	5724
0.114326511	11384	0.370653695	11384	0.028383764		
11400	0.073406452	11400	0.126510756	5730	0.024342482	5728
0.077164768	11392	0.271122263	11392	0.048493297		
11400	0.122990852	11400	0.065726599	5730	0.022844488	5732
0.052351767	11400	0.130452114	11400	0.074719079		
11400	0.126799234	11400	0.023901468	5740	0.028051927	5736
0.102556056	11408	0.095620337	11408	0.074476629		
11400	0.034803681	11400	0.067731919	5740	0.02348365	5740
0.106716921	11416	0.089791865	11416	0.069840287		
11400	0.013385089	11400	0.163399571	5740	0.041742551	5744
0.037638249	11424	0.056101901	11424	0.025345657		
11400	0.038413149	11400	0.226390126	5750	0.040984021	5748
0.044653389	11432	0.039271181	11432	0.03052592		
11400	0.082020786	11400	0.180953284	5750	0.045110206	5752
0.093467395	11440	0.114197908	11440	0.05765582		
11400	0.087284345	11400	0.043931301	5760	0.058653412	5756
0.112163936	11448	0.088861649	11448	0.059676717		
11500	0.035585625	11500	0.098279241	5760	0.089592359	5760

0.078036093	11456	0.025580806	11456	0.019843617		
11500	0.066855071	11500	0.118400829	5760	0.062929066	5764
0.032110009	11464	0.122431113	11464	0.023432847		
11500	0.121061523	11500	0.066343091	5770	0.022092994	5768
0.021345935	11472	0.101689795	11472	0.0398993		
11500	0.147828716	11500	0.074104195	5770	0.056555284	5772
0.041755771	11480	0.077016921	11480	0.047853919		
11500	0.146284613	11500	0.093790644	5780	0.062967549	5776
0.041475203	11488	0.174850021	11488	0.042766307		
11500	0.142506223	11500	0.05937021	5780	0.068404974	5780
0.004088041	11496	0.209714184	11496	0.006953216		
11500	0.09157441	11500	0.037903796	5780	0.056553949	5784
0.029745415	11504	0.126634506	11504	0.039845218		
11500	0.057665853	11500	0.086987224	5790	0.019914889	5788
0.080047365	11512	0.03614524	11512	0.061683582		
11500	0.091374815	11500	0.067906374	5790	0.034334862	5792
0.082046005	11520	0.075552795	11520	0.057852172		
11500	0.13813244	11500	0.148250998	5800	0.036357858	5796
0.025240939	11528	0.087224616	11528	0.039341743		
11500	0.120015706	11500	0.169780164	5800	0.043279801	5800
0.045934317	11536	0.084549793	11536	0.032502456		
11500	0.054615921	11500	0.1932421	5800	0.058608079	5804
0.054164641	11544	0.070881339	11544	0.032714805		
11600	0.06797535	11600	0.17143575	5810	0.083850711	5808
0.069436923	11552	0.053630676	11552	0.027313678		
11600	0.078351521	11600	0.070806927	5810	0.089460547	5812
0.072370604	11560	0.033468376	11560	0.0520137		
11600	0.049866878	11600	0.062335181	5820	0.029171268	5816
0.049666709	11568	0.102135367	11568	0.038906113		
11600	0.02523201	11600	0.063379288	5820	0.060700859	5820
0.045320376	11576	0.134263494	11576	0.019906338		
11600	0.056241312	11600	0.014953876	5820	0.087897628	5824
0.069124435	11584	0.132265646	11584	0.022613755		
11600	0.122187339	11600	0.043130389	5830	0.067747809	5828
0.07464282	11592	0.092664937	11592	0.007628694		
11600	0.146662801	11600	0.001457188	5830	0.065221248	5832
0.03470368	11600	0.122789541	11600	0.004983314		
11600	0.137544019	11600	0.038547114	5840	0.032931672	5836
0.025261479	11608	0.121758829	11608	0.010821366		
11600	0.166605809	11600	0.088241934	5840	0.005478079	5840
0.044681936	11616	0.037327161	11616	0.025185318		
11600	0.205334189	11600	0.0949682	5840	0.011024978	5844
0.068035552	11624	0.073080788	11624	0.031128391		
11600	0.194390421	11600	0.103026025	5850	0.025535086	5848
0.09135007	11632	0.076233591	11632	0.005625653		
11600	0.157981224	11600	0.057937981	5850	0.038458402	5852
0.070796683	11640	0.03883645	11640	0.051609077		
11600	0.119842123	11600	0.015405714	5860	0.017114962	5856
0.049688675	11648	0.112068796	11648	0.074914227		
11700	0.098301345	11700	0.02946446	5860	0.048556245	5860
0.058783629	11656	0.144154678	11656	0.041397729		
11700	0.097757235	11700	0.020229039	5860	0.050339004	5864
0.051754287	11664	0.113711947	11664	0.012785824		
11700	0.065297907	11700	0.036699021	5870	0.020623791	5868
0.05273784	11672	0.057504876	11672	0.030613632		
11700	0.071155642	11700	0.054916138	5870	0.006834672	5872
0.073850562	11680	0.058525136	11680	0.030462245		
11700	0.029384621	11700	0.038096416	5880	0.051011019	5876
0.03738442	11688	0.083628249	11688	0.02338465		
11700	0.083415813	11700	0.028575269	5880	0.056291017	5880
0.060716579	11696	0.083470841	11696	0.017881695		
11700	0.148563326	11700	0.095547483	5880	0.019030118	5884
0.110488603	11704	0.157399904	11704	0.009811836		
11700	0.180629053	11700	0.085554515	5890	0.023303403	5888
0.092695176	11712	0.164835903	11712	0.026290496		
11700	0.148530715	11700	0.062381405	5890	0.041884905	5892
0.088699897	11720	0.110433852	11720	0.04437906		
11700	0.13031128	11700	0.014823764	5900	0.02513845	5896

0.074280208 11728 0.086484855 11728 0.039013914
11700 0.116671654 11700 0.027537473 5900 0.025994099 5900
0.040867384 11736 0.034935947 11736 0.025838113
11700 0.096747128 11700 0.042674623 5900 0.025081368 5904
0.033243137 11744 0.020301046 11744 0.033936503
11800 0.103818667 11800 0.101308287 5910 0.035027148 5908
0.072161914 11752 0.110140252 11752 0.060836079
11800 0.13281827 11800 0.090245871 5910 0.054819699 5912
0.104661485 11760 0.16899874 11760 0.064106833
11800 0.173938054 11800 0.03068689 5920 0.054347544 5916
0.088129476 11768 0.144411898 11768 0.026154665
11800 0.212449231 11800 0.130965316 5920 0.054891421 5920
0.027326778 11776 0.095187861 11776 0.005378771
11800 0.21021557 11800 0.124606333 5920 0.075997377 5924
0.036655452 11784 0.028626202 11784 0.020512398
11800 0.132875474 11800 0.069138259 5930 0.076819117 5928
0.058266171 11792 0.037126716 11792 0.04536644
11800 0.066350083 11800 0.065746448 5930 0.036239318 5932
0.069374 11800 0.014890707 11800 0.041718107
11800 0.077319921 11800 0.124650775 5940 0.055831075 5936
0.065382446 11808 0.058552385 11808 0.026907865
11800 0.101903905 11800 0.153474466 5940 0.067438741 5940
0.061699357 11816 0.060347036 11816 0.034624078
11800 0.092309223 11800 0.109984627 5940 0.039904211 5944
0.075305114 11824 0.078287296 11824 0.062763691
11800 0.067014917 11800 0.10545909 5950 0.009683106 5948
0.061223647 11832 0.101743019 11832 0.075073884
11800 0.045530069 11800 0.104633546 5950 0.010297265 5952
0.049359351 11840 0.078175261 11840 0.061532388
11800 0.097227647 11800 0.097552264 5960 0.057128527 5956
0.058179205 11848 0.034521654 11848 0.02046681
11900 0.149496656 11900 0.084700536 5960 0.081955579 5960
0.05446557 11856 0.06982412 11856 0.014197101
11900 0.176720613 11900 0.054121567 5960 0.032930231 5964
0.053778527 11864 0.14243371 11864 0.048449805
11900 0.157340314 11900 0.057919158 5970 0.04888271 5968
0.09344735 11872 0.157659655 11872 0.056482135
11900 0.082975952 11900 0.045873807 5970 0.086044631 5972
0.085235843 11880 0.131275039 11880 0.00883438
11900 0.031399552 11900 0.057799698 5980 0.06993171 5976
0.071389251 11888 0.090545771 11888 0.033148735
11900 0.038072994 11900 0.091600297 5980 0.036643334 5980
0.079615464 11896 0.064341715 11896 0.020186883
11900 0.015001675 11900 0.137794501 5980 0.030084992 5984
0.031445932 11904 0.054703764 11904 0.022016677
11900 0.013681535 11900 0.168894505 5990 0.071273295 5988
0.018947138 11912 0.06593256 11912 0.048903086
11900 0.108989654 11900 0.118732343 5990 0.07012923 5992
0.028227014 11920 0.088454763 11920 0.054351825
11900 0.172114014 11900 0.046034656 6000 0.029594703 5996
0.032358581 11928 0.050971306 11928 0.060850678
11900 0.144071135 11900 0.044024338 6000 0.007489272 6000
0.045300618 11936 0.021616508 11936 0.045502195
11900 0.094139388 11900 0.083711471 6000 0.044426506 6004
0.063644293 11944 0.013231948 11944 0.010494118
12000 0.137667274 12000 0.11468742 6010 0.063268904 6008
0.084882326 11952 0.05391059 11952 0.034569475
12000 0.134973365 12000 0.082181694 6010 0.055453584 6012
0.081249418 11960 0.10751091 11960 0.075517099
12000 0.119389486 12000 0.033260141 6020 0.043756023 6016
0.030781077 11968 0.099422694 11968 0.074080286
12000 0.136297269 12000 0.031853953 6020 0.01872611 6020
0.052067604 11976 0.034690049 11976 0.02046165
12000 0.165680656 12000 0.052380194 6020 0.01007642 6024
0.069994349 11984 0.099331483 11984 0.046265177
12000 0.125631428 12000 0.12471278 6030 0.025762163 6028
0.099013931 11992 0.12627065 11992 0.042296189
12000 0.066386259 12000 0.105527659 6030 0.041873758 6032

0.112075322 12000 0.097621683 12000 0.035778929
12000 0.148047795 12000 0.044250708 6040 0.039918145 6036
0.097249824 12008 0.027702386 12008 0.044749748
12000 0.17791416 12000 0.105488805 6040 0.049216847 6040
0.067738067 12016 0.072106937 12016 0.049024682
12000 0.183634228 12000 0.127369116 6040 0.039215564 6044
0.030657662 12024 0.058703638 12024 0.019633391
12000 0.177896844 12000 0.116638788 6050 0.011577509 6048
0.064640379 12032 0.067849855 12032 0.045425193
12000 0.14105966 12000 0.076105862 6050 0.039074563 6052
0.089422807 12040 0.130689645 12040 0.067637368
12000 0.159823889 12000 0.044158831 6060 0.056711062 6056
0.078392041 12048 0.114500472 12048 0.009037061
12100 0.237645888 12100 0.056768236 6060 0.043141019 6060
0.064945954 12056 0.070358845 12056 0.066322529
12100 0.258170039 12100 0.086291911 6060 0.009421483 6064
0.06037554 12064 0.074275609 12064 0.076129938
12100 0.203379649 12100 0.104946819 6070 0.01109759 6068
0.032911808 12072 0.081403014 12072 0.070552706
12100 0.154182286 12100 0.097746975 6070 0.014558161 6072
0.01537692 12080 0.118174947 12080 0.097465716
12100 0.134747665 12100 0.081493105 6080 0.046568679 6076
0.040104067 12088 0.112093163 12088 0.088982859
12100 0.100516292 12100 0.09955409 6080 0.060066494 6080
0.064345622 12096 0.028617591 12096 0.071831724
12100 0.028023482 12100 0.128128435 6080 0.031096293 6084
0.055132321 12104 0.049463484 12104 0.077936005
12100 0.062395367 12100 0.121286154 6090 0.004978038 6088
0.043816119 12112 0.05802375 12112 0.071449962
12100 0.064380249 12100 0.062362451 6090 0.026594214 6092
0.064084336 12120 0.01790965 12120 0.036648427
12100 0.071513663 12100 0.052309493 6100 0.039841452 6096
0.082480161 12128 0.02583996 12128 0.027573826
12100 0.07641939 12100 0.070350448 6100 0.044562967 6100
0.064733147 12136 0.05453475 12136 0.020206529
12100 0.076645003 12100 0.035111938 6100 0.056625173 6104
0.074344593 12144 0.101614824 12144 0.01812015
12200 0.076020449 12200 0.082297163 6110 0.053132986 6108
0.063569765 12152 0.073117219 12152 0.035105317
12200 0.078398967 12200 0.024040111 6110 0.036210331 6112
0.050943781 12160 0.091342146 12160 0.037622169
12200 0.06041097 12200 0.110112778 6120 0.036748028 6116
0.066344386 12168 0.161708173 12168 0.032966145
12200 0.024886938 12200 0.111964095 6120 0.038286333 6120
0.047967722 12176 0.175616806 12176 0.022586854
12200 0.04442659 12200 0.10981792 6120 0.028370703 6124
0.00106515 12184 0.108168671 12184 0.019339799
12200 0.044911321 12200 0.105224193 6130 0.02863971 6128
0.035409827 12192 0.045383116 12192 0.019679961
12200 0.073593023 12200 0.076924225 6130 0.015045665 6132
0.060881404 12200 0.0839317 12200 0.027881548
12200 0.091647387 12200 0.124243816 6140 0.022220314 6136
0.038712296 12208 0.125882696 12208 0.053763721
12200 0.064322128 12200 0.121846671 6140 0.023361185 6140
0.030313644 12216 0.107578861 12216 0.061846295
12200 0.036338905 12200 0.058588437 6140 0.057639667 6144
0.076527198 12224 0.067353569 12224 0.040043451
12200 0.090558482 12200 0.026710073 6150 0.075383425 6148
0.077650773 12232 0.058299589 12232 0.029106155
12200 0.151633678 12200 0.0887024 6150 0.073483461 6152
0.000218797 12240 0.064177664 12240 0.024775867
12200 0.154555804 12200 0.110138935 6160 0.120608667 6156
0.021467975 12248 0.046598671 12248 0.079134487
12300 0.120874902 12300 0.106352069 6160 0.093422474 6160
0.039595441 12256 0.036984147 12256 0.073657262
12300 0.096537311 12300 0.126803643 6160 0.006870418 6164
0.069401598 12264 0.072850955 12264 0.035725607
12300 0.098299504 12300 0.160519165 6170 0.035138935 6168

0.079013524	12272	0.109877205	12272	0.008417398		
12300	0.131447989	12300	0.18369162	6170	0.016747459	6172
0.064321692	12280	0.151921428	12280	0.021124732		
12300	0.162148921	12300	0.164772923	6180	0.019950032	6176
0.070628274	12288	0.081353486	12288	0.020529597		
12300	0.204471624	12300	0.098217861	6180	0.037207243	6180
0.052952273	12296	0.057100078	12296	0.030335015		
12300	0.187569196	12300	0.039771669	6180	0.076817894	6184
0.059001475	12304	0.095492062	12304	0.010430176		
12300	0.062450476	12300	0.043292319	6190	0.09187933	6188
0.048714101	12312	0.053970616	12312	0.029245888		
12300	0.11252983	12300	0.07329927	6190	0.087920518	6192
0.02934308	12320	0.074846808	12320	0.054629109		
12300	0.155790098	12300	0.094199721	6200	0.057973364	6196
0.049619848	12328	0.151510889	12328	0.051225561		
12300	0.101588419	12300	0.023860714	6200	0.046534264	6200
0.041937619	12336	0.201251765	12336	0.025264624		
12300	0.047365837	12300	0.047424728	6200	0.047643676	6204
0.008768609	12344	0.184431498	12344	0.024944706		
12400	0.052374344	12400	0.010457478	6210	0.01622184	6208
0.038865132	12352	0.179793016	12352	0.045227975		
12400	0.023514667	12400	0.111921327	6210	0.045856494	6212
0.081319347	12360	0.229574755	12360	0.032934542		
12400	0.053806933	12400	0.175239053	6220	0.06399204	6216
0.101921018	12368	0.251222896	12368	0.032719632		
12400	0.072188421	12400	0.116490497	6220	0.036313224	6220
0.097503464	12376	0.186914636	12376	0.064116364		
12400	0.101565027	12400	0.047919581	6220	0.036358091	6224
0.076389384	12384	0.060183036	12384	0.053631247		
12400	0.124109516	12400	0.168331506	6230	0.032538417	6228
0.038127411	12392	0.106705687	12392	0.034305042		
12400	0.109217115	12400	0.138908246	6230	0.02306615	6232
0.021476099	12400	0.156368536	12400	0.030525378		
12400	0.093979179	12400	0.07051367	6240	0.016413958	6236
0.028659777	12408	0.10546553	12408	0.056718869		
12400	0.137797556	12400	0.060263483	6240	0.022625027	6240
0.019906711	12416	0.051482755	12416	0.099599631		
12400	0.17138847	12400	0.017153938	6240	0.043695476	6244
0.024004978	12424	0.082819832	12424	0.11243773		
12400	0.090628324	12400	0.069169575	6250	0.036298334	6248
0.04404744	12432	0.153518224	12432	0.104611871		
12400	0.080485152	12400	0.157011906	6250	0.021819938	6252
0.071452072	12440	0.147943225	12440	0.094434436		
12400	0.174201298	12400	0.194695124	6260	0.022483891	6256
0.076772631	12448	0.058584323	12448	0.065894637		
12500	0.142935489	12500	0.159443284	6260	0.016214433	6260
0.067681795	12456	0.040030744	12456	0.03291083		
12500	0.016779886	12500	0.061358398	6260	0.06546903	6264
0.034068471	12464	0.046273901	12464	0.030982556		
12500	0.137782379	12500	0.144751175	6270	0.087685243	6268
0.054815806	12472	0.124585291	12472	0.022791573		
12500	0.147495594	12500	0.2075558	6270	0.077157922	6272
0.044356755	12480	0.10532514	12480	0.027485799		
12500	0.066582092	12500	0.169558349	6280	0.072826246	6276
0.0628946	12488	0.07360155	12488	0.040573177		
12500	0.011710375	12500	0.085805732	6280	0.044864213	6280
0.078780584	12496	0.04278139	12496	0.039395553		
12500	0.099151468	12500	0.011159403	6280	0.034928318	6284
0.05854835	12504	0.096616765	12504	0.057248279		
12500	0.16535219	12500	0.077652672	6290	0.038149134	6288
0.056312289	12512	0.137615905	12512	0.05329003		
12500	0.161609496	12500	0.026150849	6290	0.074290234	6292
0.077195757	12520	0.162579876	12520	0.018784567		
12500	0.185950091	12500	0.099052071	6300	0.083652456	6296
0.099891324	12528	0.109755194	12528	0.02442342		
12500	0.228947509	12500	0.093016926	6300	0.064313004	6300
0.112526352	12536	0.036367484	12536	0.029790335		
12500	0.183279175	12500	0.013283866	6300	0.051910356	6304

0.106509571	12544	0.109375687	12544	0.030313742		
12600	0.094895426	12600	0.049952909	6310	0.0569363	6308
0.072062336	12552	0.030286607	12552	0.039093015		
12600	0.043466622	12600	0.031174546	6310	0.052422314	6312
0.04978904	12560	0.135794616	12560	0.040901014		
12600	0.03377995	12600	0.031701897	6320	0.020316662	6316
0.055457997	12568	0.144478545	12568	0.023334364		
12600	0.014737957	12600	0.046225407	6320	0.031814234	6320
0.044481982	12576	0.091037102	12576	0.0133286		
12600	0.042882657	12600	0.045526285	6320	0.063400665	6324
0.017560811	12584	0.102350328	12584	0.029601415		
12600	0.040067018	12600	0.040722007	6330	0.042193733	6328
0.026461325	12592	0.047330876	12592	0.024473404		
12600	0.024375366	12600	0.000909617	6330	0.017214377	6332
0.049784634	12600	0.039812083	12600	0.043938835		
12600	0.021788092	12600	0.058919195	6340	0.055382396	6336
0.061631152	12608	0.046826466	12608	0.070329239		
12600	0.064945278	12600	0.023441126	6340	0.054875818	6340
0.041171381	12616	0.112057038	12616	0.063323932		
12600	0.08318515	12600	0.074537078	6340	0.026487214	6344
0.061357787	12624	0.140902543	12624	0.035565485		
12600	0.092292787	12600	0.087123459	6350	0.028289045	6348
0.074589414	12632	0.128724773	12632	0.022432349		
12600	0.034801939	12600	0.012265488	6350	0.033926161	6352
0.069548631	12640	0.095995485	12640	0.022179545		
12600	0.09875105	12600	0.096119526	6360	0.048943173	6356
0.060226565	12648	0.09409112	12648	0.02080872		
12700	0.13048586	12700	0.11542681	6360	0.050641924	6360
0.040461771	12656	0.126416576	12656	0.031780401		
12700	0.061731866	12700	0.093888593	6360	0.03762409	6364
0.03962249	12664	0.106288797	12664	0.025399402		
12700	0.099411795	12700	0.093439274	6370	0.063925865	6368
0.030628289	12672	0.083917243	12672	0.02180451		
12700	0.141198921	12700	0.107704887	6370	0.073772179	6372
0.027136581	12680	0.081352671	12680	0.028425973		
12700	0.120713339	12700	0.133574563	6380	0.042767839	6376
0.046092118	12688	0.045644014	12688	0.018829167		
12700	0.16384157	12700	0.133473179	6380	0.022955008	6380
0.039754894	12696	0.08283856	12696	0.044321547		
12700	0.202255236	12700	0.105563842	6380	0.016171762	6384
0.037838316	12704	0.176662055	12704	0.064643173		
12700	0.196454261	12700	0.076395438	6390	0.031454842	6388
0.027111206	12712	0.169994731	12712	0.0550515		
12700	0.144385965	12700	0.087474888	6390	0.044419066	6392
0.015451442	12720	0.09291101	12720	0.037009893		
12700	0.203391551	12700	0.072240269	6400	0.052623909	6396
0.054210959	12728	0.045053657	12728	0.054602129		
12700	0.411069603	12700	0.066004875	6400	0.037737613	6400
0.086489075	12736	0.101579193	12736	0.062408828		
12700	0.555698935	12700	0.058793197	6400	0.016875607	6404
0.051428553	12744	0.14233889	12744	0.05422486		
12800	0.438526215	12800	0.082174454	6410	0.028195173	6408
0.047814996	12752	0.107895292	12752	0.021577127		
12800	0.168154001	12800	0.102412778	6410	0.042924861	6412
0.059750099	12760	0.054860379	12760	0.007534319		
12800	0.02551326	12800	0.00662077	6420	0.071832481	6416
0.039901857	12768	0.021692616	12768	0.011770011		
12800	0.114139446	12800	0.130998393	6420	0.099156845	6420
0.006784651	12776	0.038568978	12776	0.006671183		
12800	0.126049243	12800	0.192604654	6420	0.083281935	6424
0.024228328	12784	0.027392032	12784	0.044466753		
12800	0.090911628	12800	0.177891154	6430	0.055232868	6428
0.06417813	12792	0.025904483	12792	0.070342212		
12800	0.075486554	12800	0.11191138	6430	0.077187389	6432
0.061920167	12800	0.037832127	12800	0.061661289		
12800	0.103648446	12800	0.061497376	6440	0.090917594	6436
0.074580654	12808	0.0139855	12808	0.028518445		
12800	0.123531267	12800	0.073347925	6440	0.049056736	6440

0.063753614 12816 0.029786841 12816 0.01951579
12800 0.155025671 12800 0.069810114 6440 0.013305396 6444
0.043195898 12824 0.024075975 12824 0.049141418
12800 0.146629188 12800 0.054393386 6450 0.008907443 6448
0.018732522 12832 0.021443077 12832 0.033212135
12800 0.046001165 12800 0.122323763 6450 0.015990479 6452
0.026464864 12840 0.068070804 12840 0.020078865
12800 0.169453269 12800 0.142676319 6460 0.001323456 6456
0.072480303 12848 0.09051578 12848 0.014447336
12900 0.243711184 12900 0.112158479 6460 0.016873355 6460
0.046890986 12856 0.062575433 12856 0.01663669
12900 0.189319239 12900 0.091398855 6460 0.045085253 6464
0.051690015 12864 0.02083933 12864 0.036073958
12900 0.031666841 12900 0.040127747 6470 0.052999974 6468
0.082256345 12872 0.043348719 12872 0.023379835
12900 0.1058579 12900 0.009162552 6470 0.076060453 6472
0.088363107 12880 0.110878937 12880 0.030804364
12900 0.113576294 12900 0.015881864 6480 0.059010785 6476
0.068307942 12888 0.154409893 12888 0.08330142
12900 0.155353584 12900 0.032168107 6480 0.003403799 6480
0.008307416 12896 0.126400978 12896 0.094346804
12900 0.174332774 12900 0.06831887 6480 0.053907072 6484
0.028154487 12904 0.070211732 12904 0.075897042
12900 0.204726064 12900 0.025251306 6490 0.063555126 6488
0.006590254 12912 0.05212436 12912 0.044594282
12900 0.248492899 12900 0.067805937 6490 0.041262603 6492
0.048473765 12920 0.025195055 12920 0.031633055
12900 0.231907179 12900 0.091661663 6500 0.012199632 6496
0.063928143 12928 0.047071673 12928 0.023989105
12900 0.189234503 12900 0.098311881 6500 0.028250048 6500
0.059937021 12936 0.063417123 12936 0.00890611
12900 0.264582283 12900 0.037197089 6500 0.02761973 6504
0.054029599 12944 0.06067213 12944 0.037053735
13000 0.307206269 13000 0.092460206 6510 0.040939573 6508
0.116741787 12952 0.073088289 12952 0.063311854
13000 0.321665691 13000 0.160679221 6510 0.064648149 6512
0.147266925 12960 0.12858602 12960 0.066746135
13000 0.487612968 13000 0.194258391 6520 0.049611899 6516
0.109771339 12968 0.177863796 12968 0.050180821
13000 1.245252905 13000 0.147717249 6520 0.036138234 6520
0.043927019 12976 0.19722234 12976 0.037780621
13000 1.075559296 13000 0.103063336 6520 0.054592907 6524
0.055289162 12984 0.194052555 12984 0.060683433
13000 0.603676018 13000 0.092671653 6530 0.055644272 6528
0.021478041 12992 0.158724448 12992 0.090363377
13000 4.554029088 13000 0.027091091 6530 0.036300597 6532
0.015065302 13000 0.116404212 13000 0.050363695
13000 12.76010463 13000 0.049578637 6540 0.005368249 6536
0.031750831 13008 0.100310033 13008 0.037294289
13000 17.996911 13000 0.05355629 6540 0.016153172 6540
0.046151596 13016 0.081419181 13016 0.094091323
13000 14.43381325 13000 0.028999502 6540 0.014619082 6544
0.019399182 13024 0.087062304 13024 0.100373611
13000 6.127174013 13000 0.11161311 6550 0.011053679 6548
0.037723945 13032 0.09102004 13032 0.076123273
13000 4.356775438 13000 0.154835129 6550 0.021834167 6552
0.060116294 13040 0.119625525 13040 0.050950905
13000 3.212098963 13000 0.139144351 6560 0.033388616 6556
0.081951672 13048 0.076816265 13048 0.027586941
13100 0.740253541 13100 0.07278852 6560 0.025628327 6560
0.076112687 13056 0.006623325 13056 0.012404283
13100 1.413620538 13100 0.106847809 6560 0.048820646 6564
0.058327907 13064 0.097739569 13064 0.044136261
13100 1.247140695 13100 0.181760625 6570 0.073607116 6568
0.032757929 13072 0.248509343 13072 0.030250381
13100 1.190624316 13100 0.202632088 6570 0.050814386 6572
0.010421653 13080 0.250647135 13080 0.022542368
13100 1.196986298 13100 0.153339511 6580 0.072694973 6576

0.013260095 13088 0.200679759 13088 0.011997493
13100 1.082317904 13100 0.062116647 6580 0.067440378 6580
0.055253517 13096 0.16586257 13096 0.048801328
13100 0.958441466 13100 0.070894486 6580 0.05362483 6584
0.062999476 13104 0.138402 13104 0.038362108
13100 0.989794848 13100 0.076526034 6590 0.019641275 6588
0.067171997 13112 0.071791401 13112 0.007376966
13100 1.032594009 13100 0.076694749 6590 0.0588148 6592
0.114140567 13120 0.058268899 13120 0.03979167
13100 0.975571456 13100 0.069760637 6600 0.069178444 6596
0.114742674 13128 0.121540266 13128 0.04121747
13100 1.032549539 13100 0.095945936 6600 0.043360742 6600
0.062980173 13136 0.109262837 13136 0.032680324
13100 1.221233514 13100 0.064868866 6600 0.042286371 6604
0.022228476 13144 0.063094412 13144 0.020769783
13200 1.331396863 13200 0.031423857 6610 0.055018983 6608
0.06630092 13152 0.010903223 13152 0.0088357
13200 1.559748425 13200 0.077993573 6610 0.037553975 6612
0.046479414 13160 0.04974048 13160 0.016438806
13200 1.794027863 13200 0.055730565 6620 0.030168394 6616
0.014950193 13168 0.119929813 13168 0.046175243
13200 2.045444913 13200 0.02939437 6620 0.033221688 6620
0.01489234 13176 0.105017876 13176 0.073388263
13200 2.211711838 13200 0.073471849 6620 0.042527929 6624
0.035073917 13184 0.10747878 13184 0.079663645
13200 2.225962238 13200 0.112370326 6630 0.029713385 6628
0.050174043 13192 0.176160596 13192 0.05156342
13200 2.148821488 13200 0.084360261 6630 0.023348248 6632
0.02790424 13200 0.186183403 13200 0.008409306
13200 1.862384025 13200 0.067512403 6640 0.035610869 6636
0.031596403 13208 0.106784384 13208 0.023103257
13200 1.427830775 13200 0.061068909 6640 0.054840864 6640
0.022731862 13216 0.049442562 13216 0.03360277
13200 0.815967448 13200 0.113054135 6640 0.05747172 6644
0.031204236 13224 0.046776539 13224 0.008634478
13200 0.567111828 13200 0.162331664 6650 0.024380712 6648
0.020690644 13232 0.058368758 13232 0.03110334
13200 0.629754446 13200 0.123490492 6650 0.016430247 6652
0.011975429 13240 0.113228911 13240 0.034892211
13200 1.262748038 13200 0.052106239 6660 0.054684664 6656
0.015104495 13248 0.111307847 13248 0.033789507
13300 3.226312575 13300 0.10494727 6660 0.05643726 6660
0.015582613 13256 0.107099775 13256 0.031818141
13300 3.335596063 13300 0.191237953 6660 0.0300878 6664
0.004780563 13264 0.139176016 13264 0.015512633
13300 9.079438638 13300 0.132227753 6670 0.080615762 6668
0.0216915 13272 0.146877239 13272 0.027841044
13300 18.70898525 13300 0.081640559 6670 0.084597421 6672
0.029231163 13280 0.139864496 13280 0.039029772
13300 20.68980225 13300 0.092053531 6680 0.051765601 6676
0.005903109 13288 0.120717203 13288 0.072245239
13300 12.94639525 13300 0.081973092 6680 0.027403645 6680
0.037795257 13296 0.132408299 13296 0.076738157
13300 3.835540263 13300 0.061367835 6680 0.033446569 6684
0.063325169 13304 0.104950203 13304 0.037794504
13300 1.243376173 13300 0.044975652 6690 0.015395597 6688
0.066729685 13312 0.125006554 13312 0.018206612
13300 1.469235863 13300 0.068353351 6690 0.015471022 6692
0.027557422 13320 0.183610493 13320 0.025962003
13300 1.693290663 13300 0.040603154 6700 0.042277225 6696
0.015977988 13328 0.217353213 13328 0.035016354
13300 0.968399749 13300 0.085070977 6700 0.069036469 6700
0.020217945 13336 0.193314118 13336 0.040208241
13300 0.84285019 13300 0.007390473 6700 0.062289393 6704
0.047524201 13344 0.195157758 13344 0.028930263
13400 0.588292198 13400 0.151614266 6710 0.040316714 6708
0.054395216 13352 0.375133328 13352 0.018674331
13400 0.532433274 13400 0.175589769 6710 0.057242069 6712

0.025138426	13360	0.572806806	13360	0.010201784		
13400	0.363265601	13400	0.129151201	6720	0.077735575	6716
0.001861535	13368	0.712768175	13368	0.022937331		
13400	0.290687225	13400	0.051736268	6720	0.081071157	6720
0.031928856	13376	1.033568523	13376	0.036418242		
13400	0.261659763	13400	0.025312536	6720	0.085957705	6724
0.070585018	13384	1.197465929	13384	0.024201514		
13400	0.149318599	13400	0.090040281	6730	0.103478305	6728
0.071836577	13392	1.22198998	13392	0.024731082		
13400	0.193070766	13400	0.117049494	6730	0.110420224	6732
0.03439666	13400	1.07226288	13400	0.022749491		
13400	0.180927324	13400	0.064635569	6740	0.101049431	6736
0.020759668	13408	0.994076603	13408	0.02829442		
13400	0.129035005	13400	0.088342255	6740	0.063279033	6740
0.037225927	13416	1.840482113	13416	0.025308886		
13400	0.058838701	13400	0.121565754	6740	0.047229743	6744
0.047400066	13424	3.458524825	13424	0.013419291		
13400	0.062554966	13400	0.065529755	6750	0.055355253	6748
0.033389406	13432	5.319816063	13432	0.020464499		
13400	0.038453047	13400	0.033081218	6750	0.0518007	6752
0.007889515	13440	7.158330638	13440	0.045774301		
13400	0.067203044	13400	0.06065291	6760	0.061884246	6756
0.064419153	13448	8.5413577	13448	0.052539439		
13500	0.079579892	13500	0.086429856	6760	0.079514328	6760
0.094990668	13456	9.42949765	13456	0.067030116		
13500	0.07010431	13500	0.135780793	6760	0.039496736	6764
0.078976489	13464	9.60471015	13464	0.052264531		
13500	0.166361599	13500	0.09925809	6770	0.02469022	6768
0.041650059	13472	9.666142988	13472	0.024402765		
13500	0.164279176	13500	0.056947181	6770	0.036376776	6772
0.023192526	13480	10.5184326	13480	0.017383502		
13500	0.208166559	13500	0.108813387	6780	0.048075217	6776
0.003792663	13488	11.68596651	13488	0.015206691		
13500	0.192093809	13500	0.106913438	6780	0.050806055	6780
0.016581053	13496	0	13496	0.006444325		
13500	0.032448985	13500	0.047102716	6780	0.026574358	6784
0.003365539	13504	12.00326719	13504	0.051357813		
13500	0.056341403	13500	0.139641735	6790	0.025058989	6788
0.030445503	13512	10.51369124	13512	0.081693972		
13500	0.007915287	13500	0.142413016	6790	0.046671099	6792
0.083334177	13520	8.601785638	13520	0.06801654		
13500	0.083440595	13500	0.0981876	6800	0.056163757	6796
0.106942105	13528	6.616862488	13528	0.07824997		
13500	0.1122869	13500	0.134996168	6800	0.021574415	6800
0.08847045	13536	4.968894175	13536	0.070072383		
13500	0.189632366	13500	0.021425909	6800	0.020002644	6804
0.050285224	13544	4.193968138	13544	0.034962544		
13600	0.301959314	13600	0.115160387	6810	0.033460878	6808
0.019261712	13552	4.078931638	13552	0.022658292		
13600	0.250295038	13600	0.055178916	6810	0.058088819	6812
0.063795102	13560	4.109911625	13560	0.022922093		
13600	0.158968876	13600	0.07940302	6820	0.062126273	6816
0.075090524	13568	4.068805838	13568	0.029340485		
13600	0.105503597	13600	0.046831341	6820	0.048713366	6820
0.035713507	13576	3.678802863	13576	0.076129887		
13600	0.070897579	13600	0.069132111	6820	0.039951803	6824
0.016412405	13584	2.919926538	13584	0.059490976		
13600	0.141670375	13600	0.083973027	6830	0.030610168	6828
0.044849774	13592	2.114222613	13592	0.0185873		
13600	0.165362231	13600	0.007621099	6830	0.018748462	6832
0.064478249	13600	1.48647255	13600	0.032489312		
13600	0.137099865	13600	0.116876072	6840	0.040999996	6836
0.065059096	13608	1.046749414	13608	0.066236629		
13600	0.101370155	13600	0.15428981	6840	0.06386375	6840
0.038362483	13616	0.947525259	13616	0.05462387		
13600	0.056233716	13600	0.110863257	6840	0.065840883	6844
0.019877703	13624	0.867204683	13624	0.037278711		
13600	0.063335472	13600	0.04876321	6850	0.047657686	6848

0.052518644 13632 0.776094559 13632 0.042114029
13600 0.048060898 13600 0.013996551 6850 0.030844811 6852
0.088586254 13640 0.733160938 13640 0.033856974
13600 0.023814931 13600 0.035338078 6860 0.044517841 6856
0.074419768 13648 0.630656025 13648 0.054902434
13700 0.069633577 13700 0.110856548 6860 0.085527267 6860
0.021733757 13656 0.424402009 13656 0.053706302
13700 0.028436554 13700 0.134324583 6860 0.077526209 6864
0.007098881 13664 0.147162281 13664 0.005409186
13700 0.068293732 13700 0.102323509 6870 0.051531824 6868
0.016941709 13672 0.186169476 13672 0.062941974
13700 0.099032543 13700 0.057859739 6870 0.053874028 6872
0.028229159 13680 0.218118963 13680 0.079692392
13700 0.09251602 13700 0.081604601 6880 0.070050497 6876
0.047887341 13688 0.265281124 13688 0.05209048
13700 0.085217733 13700 0.120250683 6880 0.066403816 6880
0.045649507 13696 0.09445678 13696 0.034988614
13700 0.073523232 13700 0.066435663 6880 0.040407522 6884
0.042947409 13704 0.141785116 13704 0.055787947
13700 0.109245229 13700 0.068135276 6890 0.032174106 6888
0.049432954 13712 0.079431244 13712 0.083630177
13700 0.15158685 13700 0.081722421 6890 0.036279238 6892
0.051684925 13720 0.12179296 13720 0.082536004
13700 0.10538019 13700 0.071295428 6900 0.042839925 6896
0.04873419 13728 0.15762652 13728 0.062665422
13700 0.088687033 13700 0.103350809 6900 0.046732836 6900
0.028007367 13736 0.087672568 13736 0.029365139
13700 0.077772136 13700 0.094764517 6900 0.074232085 6904
0.042540913 13744 0.115876312 13744 0.010763936
13800 0.055288849 13800 0.077289362 6910 0.083138722 6908
0.055737797 13752 0.159769246 13752 0.013561886
13800 0.05758573 13800 0.042116059 6910 0.050899063 6912
0.043278305 13760 0.135537091 13760 0.020617366
13800 0.069565562 13800 0.066761139 6920 0.010540001 6916
0.063651103 13768 0.114735871 13768 0.017864368
13800 0.102493694 13800 0.108516666 6920 0.029774252 6920
0.071243179 13776 0.085017331 13776 0.040729592
13800 0.09150766 13800 0.128101689 6920 0.028401562 6924
0.044242679 13784 0.119233861 13784 0.057879835
13800 0.184850956 13800 0.106975487 6930 0.037818925 6928
0.105769017 13792 0.182052376 13792 0.05225333
13800 0.182877499 13800 0.046901689 6930 0.055867371 6932
0.086412059 13800 0.168476486 13800 0.029723362
13800 0.06144659 13800 0.070446193 6940 0.062651081 6936
0.046149839 13808 0.065875422 13808 0.036027257
13800 0.05492691 13800 0.106716718 6940 0.05870215 6940
0.038435839 13816 0.093273244 13816 0.034983612
13800 0.091951632 13800 0.130606844 6940 0.048630627 6944
0.059442013 13824 0.122174562 13824 0.015627986
13800 0.12228312 13800 0.157559829 6950 0.042709969 6948
0.074277516 13832 0.027040369 13832 0.029862147
13800 0.123257007 13800 0.100557641 6950 0.034690063 6952
0.049831026 13840 0.102822705 13840 0.050928004
13800 0.116226293 13800 0.026040794 6960 0.025250161 6956
0.04576776 13848 0.124812417 13848 0.04943406
13900 0.089481764 13900 0.07695792 6960 0.061541054 6960
0.063496336 13856 0.036838708 13856 0.036977221
13900 0.007615554 13900 0.027409602 6960 0.068516114 6964
0.070318383 13864 0.080616017 13864 0.032824195
13900 0.090007969 13900 0.005156382 6970 0.028703864 6968
0.045469435 13872 0.127187566 13872 0.030164327
13900 0.058450514 13900 0.059549639 6970 0.013976187 6972
0.013412613 13880 0.085206004 13880 0.024646253
13900 0.005597207 13900 0.118138494 6980 0.034473451 6976
0.036723559 13888 0.057166446 13888 0.027029502
13900 0.022673552 13900 0.143372149 6980 0.034121262 6980
0.047702481 13896 0.074137097 13896 0.043258759
13900 0.041442578 13900 0.097799501 6980 0.039677972 6984

0.056596604 13904 0.130333021 13904 0.047223381
13900 0.079620411 13900 0.109019333 6990 0.054037919 6988
0.073338728 13912 0.20853433 13912 0.026291747
13900 0.099380537 13900 0.151929751 6990 0.017230746 6992
0.066661996 13920 0.187814774 13920 0.030753276
13900 0.024904641 13900 0.130118816 7000 0.039149843 6996
0.029153798 13928 0.055950794 13928 0.060639901
13900 0.110932007 13900 0.115579664 7000 0.054719913 7000
0.016045167 13936 0.066470486 13936 0.0674861
13900 0.115149902 13900 0.137036055 7000 0.057119287 7004
0.032016873 13944 0.026803946 13944 0.043103901
14000 0.096052652 14000 0.144485631 7010 0.035771504 7008
0.044738321 13952 0.111335095 13952 0.007561322
14000 0.14197659 14000 0.153495669 7010 0.017776712 7012
0.031659394 13960 0.062605468 13960 0.033990153
14000 0.150402556 14000 0.119366654 7020 0.022795672 7016
0.011517895 13968 0.027202908 13968 0.051000443
14000 0.098138298 14000 0.048396101 7020 0.035250516 7020
0.034162258 13976 0.073148789 13976 0.041378866
14000 0.050202565 14000 0.024395446 7020 0.017295555 7024
0.01657398 13984 0.100719713 13984 0.033777003
14000 0.083366831 14000 0.036299029 7030 0.029695419 7028
0.0408095 13992 0.089968729 13992 0.039866434
14000 0.08261812 14000 0.057270321 7030 0.062285602 7032
0.060621082 14000 0.051252311 14000 0.032885891
14000 0.024612446 14000 0.058747199 7040 0.084790358 7036
0.050516945 14008 0.02466094 14008 0.019901181
14000 0.103999337 14000 0.055336084 7040 0.093049654 7040
0.06142597 14016 0.146620019 14016 0.014943174
14000 0.198265349 14000 0.052077805 7040 0.060412407 7044
0.049365459 14024 0.223743453 14024 0.024815612
14000 0.195105036 14000 0.108581255 7050 0.024051687 7048
0.027503958 14032 0.153183414 14032 0.041714
14000 0.077778903 14000 0.118494827 7050 0.069663569 7052
0.010817839 14040 0.071843526 14040 0.046565885
14000 0.092085938 14000 0.070347924 7060 0.061634644 7056
0.05296935 14048 0.098366509 14048 0.053546995
14100 0.145229875 14100 0.049385955 7060 0.087113665 7060
0.079799218 14056 0.106841355 14056 0.056927773
14100 0.161884178 14100 0.121362762 7060 0.102743798 7064
0.027556907 14064 0.020879112 14064 0.045867488
14100 0.161833246 14100 0.114304552 7070 0.092855691 7068
0.034177567 14072 0.042663782 14072 0.027177061
14100 0.13418142 14100 0.054504089 7070 0.093856586 7072
0.035460376 14080 0.037535745 14080 0.060114689
14100 0.131808366 14100 0.101204438 7080 0.103725746 7076
0.019780169 14088 0.069852678 14088 0.087125438
14100 0.146549224 14100 0.186595324 7080 0.07220413 7080
0.027854847 14096 0.096108117 14096 0.062844192
14100 0.114603783 14100 0.176611895 7080 0.034886925 7084
0.032937413 14104 0.058767404 14104 0.033293203
14100 0.045586428 14100 0.123269492 7090 0.032017084 7088
0.021032091 14112 0.068667687 14112 0.034225916
14100 0.03602534 14100 0.044964661 7090 0.018017056 7092
0.045853263 14120 0.122524318 14120 0.033473647
14100 0.10658626 14100 0.035780908 7100 0.024492803 7096
0.074763761 14128 0.123305392 14128 0.04770458
14100 0.154737908 14100 0.121664059 7100 0.06656567 7100
0.044378016 14136 0.093697468 14136 0.053694042
14100 0.102410508 14100 0.169565523 7100 0.050405273 7104
0.039859118 14144 0.083840118 14144 0.040745097
14200 0.072576819 14200 0.138482589 7110 0.017299999 7108
0.048061975 14152 0.091138245 14152 0.013130538
14200 0.083831197 14200 0.054925193 7110 0.074035765 7112
0.053587137 14160 0.14766815 14160 0.021384698
14200 0.146483974 14200 0.045472738 7120 0.078826372 7116
0.051246945 14168 0.12982577 14168 0.053271582
14200 0.113119786 14200 0.008208407 7120 0.024274288 7120

0.042236869 14176 0.068151523 14176 0.074545795
14200 0.069226044 14200 0.008467314 7120 0.01250567 7124
0.029385745 14184 0.111815258 14184 0.054179418
14200 0.069341804 14200 0.128447646 7130 0.038277725 7128
0.022671837 14192 0.210864513 14192 0.013414864
14200 0.107319742 14200 0.191603016 7130 0.054628879 7132
0.021747535 14200 0.273797778 14200 0.007635057
14200 0.155899819 14200 0.117857606 7140 0.048344573 7136
0.008555528 14208 0.262886955 14208 0.012115433
14200 0.149906395 14200 0.063961365 7140 0.028843942 7140
0.020583819 14216 0.207360514 14216 0.044976627
14200 0.119015887 14200 0.16729928 7140 0.043309752 7144
0.017787736 14224 0.155326198 14224 0.072094597
14200 0.04442476 14200 0.219970549 7150 0.022731319 7148
0.031270611 14232 0.094086769 14232 0.050470539
14200 0.032662294 14200 0.171340828 7150 0.072853603 7152
0.052633503 14240 0.041390278 14240 0.011069318
14200 0.007956872 14200 0.115590876 7160 0.107683336 7156
0.050988158 14248 0.016221657 14248 0.007096339
14300 0.034451063 14300 0.109017215 7160 0.12043411 7160
0.055169399 14256 0.047820253 14256 0.022182505
14300 0.028279705 14300 0.107488988 7160 0.107940701 7164
0.070189846 14264 0.068000642 14264 0.040270592
14300 0.013571863 14300 0.137533688 7170 0.075837052 7168
0.072870025 14272 0.100526195 14272 0.007640564
14300 0.029070016 14300 0.135240204 7170 0.0319625 7172
0.05543133 14280 0.068849629 14280 0.031274798
14300 0.034460969 14300 0.053145952 7180 0.022386894 7176
0.060890092 14288 0.036656911 14288 0.018888135
14300 0.031394066 14300 0.047270303 7180 0.06576645 7180
0.050174156 14296 0.036831825 14296 0.018888208
14300 0.048544222 14300 0.044094366 7180 0.066879322 7184
0.008871545 14304 0.045347562 14304 0.039741994
14300 0.089965361 14300 0.178181799 7190 0.039372862 7188
0.043481847 14312 0.052865471 14312 0.042455511
14300 0.170357641 14300 0.24788949 7190 0.009715114 7192
0.027749175 14320 0.077528814 14320 0.037290458
14300 0.261694775 14300 0.215180393 7200 0.030775795 7196
0.025597294 14328 0.126189581 14328 0.059514641
14300 0.240565788 14300 0.161370219 7200 0.053502616 7200
0.042611322 14336 0.125766281 14336 0.072820607
14300 0.104717787 14300 0.078605066 7200 0.054052965 7204
0.044553432 14344 0.072008246 14344 0.061807477
14400 0.041513249 14400 0.08892105 7210 0.044012395 7208
0.053208503 14352 0.054239554 14352 0.059927061
14400 0.055409775 14400 0.148108128 7210 0.045060078 7212
0.068022775 14360 0.040162937 14360 0.05582883
14400 0.085471351 14400 0.126895175 7220 0.050622293 7216
0.063004176 14368 0.077385717 14368 0.047197475
14400 0.175364258 14400 0.056793953 7220 0.023506293 7220
0.028077173 14376 0.102964645 14376 0.066876659
14400 0.182441406 14400 0.13540902 7220 0.056371275 7224
0.017026916 14384 0.153697125 14384 0.053311454
14400 0.085452048 14400 0.093345625 7230 0.064886182 7228
0.018322253 14392 0.157822855 14392 0.015823391
14400 0.119404998 14400 0.071258655 7230 0.074773154 7232
0.033478471 14400 0.095636708 14400 0.06441414
14400 0.168535553 14400 0.059489885 7240 0.095746465 7236
0.050760122 14408 0.064651147 14408 0.039704974
14400 0.218331785 14400 0.025948442 7240 0.091039328 7240
0.06854385 14416 0.031930991 14416 0.005358454
14400 0.183348268 14400 0.105288658 7240 0.081222177 7244
0.059173184 14424 0.040394833 14424 0.003718821
14400 0.113952141 14400 0.16512096 7250 0.074268595 7248
0.060855979 14432 0.065365995 14432 0.032997872
14400 0.11307541 14400 0.123806211 7250 0.048092992 7252
0.089946159 14440 0.144517355 14440 0.037543672
14400 0.083267187 14400 0.015413507 7260 0.018966393 7256

```
0.106194733	14448	0.205904115	14448	0.023016344
14500	0.019464893	14500	0.089865607	7260	0.029715884	7260
0.081271006	14456	0.146577149	14456	0.038721366
14500	0.016894035	14500	0.073079209	7260	0.063461463	7264
0.027802931	14464	0.046200668	14464	0.010042253
14500	0.040575615	14500	0.031499043	7270	0.115290197	7268
0.044534285	14472	0.139778436	14472	0.052476487
14500	0.075806653	14500	0.040290943	7270	0.140223609	7272
0.061720108	14480	0.095940239	14480	0.065488479
14500	0.077049583	14500	0.042905231	7280	0.123637787	7276
0.066456836	14488	0.027438091	14488	0.021711834
14500	0.037502072	14500	0.078013043	7280	0.119256874	7280
0.079301477	14496	0.094385934	14496	0.02268704
14500	0.071109331	14500	0.045363096	7280	0.128196378	7284
0.090964008	14504	0.074758311	14504	0.043638291
14500	0.026624315	14500	0.075387616	7290	0.105170293	7288
0.074524389	14512	0.032195902	14512	0.060725899
14500	0.13209341	14500	0.168565938	7290	0.043569831	7292
0.040444764	14520	0.021458916	14520	0.052564057
14500	0.153140951	14500	0.122033969	7300	0.017372975	7296
0.023977469	14528	0.03647502	14528	0.041558466
14500	0.102723141	14500	0.042494758	7300	0.030360277	7300
0.033532317	14536	0.016840997	14536	0.066531313
14500	0.112255184	14500	0.044187895	7300	0.011658055	7304
0.026696498	14544	0.121062578	14544	0.05346095
14600	0.106387568	14600	0.067432804	7310	0.052034375	7308
0.029937839	14552	0.210667175	14552	0.062610103
14600	0.085335036	14600	0.06527581	7310	0.058014139	7312
0.036325448	14560	0.203567383	14560	0.069765993
14600	0.03984481	14600	0.033074415	7320	0.059718412	7316
0.066125074	14568	0.088180917	14568	0.076670716
14600	0.048334681	14600	0.076000761	7320	0.049357706	7320
0.053145537	14576	0.008340115	14576	0.095565207
14600	0.109511275	14600	0.068585337	7320	0.031674772	7324
0.006863341	14584	0.068656424	14584	0.072149916
14600	0.082673745	14600	0.061952014	7330	0.012193748	7328
0.059608054	14592	0.147398053	14592	0.022992555
14600	0.02386541	14600	0.079272606	7330	0.027103519	7332
0.059534679	14600	0.104106992	14600	0.018676356
14600	0.069085181	14600	0.103287996	7340	0.019845744	7336
0.023233944	14608	0.030514477	14608	0.054141765
14600	0.096906857	14600	0.118441283	7340	0.058502803	7340
0.014145557	14616	0.051922052	14616	0.065
14600	0.090168695	14600	0.095058917	7340	0.081629289	7344
0.067388231	14624	0.097322241	14624	0.032970464
14600	0.098562312	14600	0.043067794	7350	0.070258961	7348
0.094024719	14632	0.070156973	14632	0.029829775
14600	0.113526607	14600	0.094459669	7350	0.046472469	7352
0.107037129	14640	0.079158141	14640	0.055248449
14600	0.077326353	14600	0.158493873	7360	0.05926278	7356
0.06285678	14648	0.091167378	14648	0.036662354
14700	0.077515106	14700	0.123259568	7360	0.054535791	7360
0.060789123	14656	0.077223303	14656	0.014568022
14700	0.130606306	14700	0.031354295	7360	0.047060501	7364
0.095253512	14664	0.10202374	14664	0.021589258
14700	0.09907916	14700	0.122799276	7370	0.043490767	7368
0.066850684	14672	0.149920946	14672	0.026100126
14700	0.051809748	14700	0.073698699	7370	0.026344347	7372
0.037755654	14680	0.133723254	14680	0.04474468
14700	0.061299237	14700	0.020070638	7380	0.073388677	7376
0.024617195	14688	0.069064285	14688	0.06382406
14700	0.142786114	14700	0.021241058	7380	0.110963098	7380
0.083685955	14696	0.062446976	14696	0.064859363
14700	0.115944706	14700	0.058243932	7380	0.117669275	7384
0.049471495	14704	0.077656187	14704	0.053899752
14700	0.033044089	14700	0.017033868	7390	0.111432491	7388
0.022022437	14712	0.070040129	14712	0.042447409
14700	0.052592619	14700	0.066374792	7390	0.10460987	7392
```

0.044491666 14720 0.068057772 14720 0.038259055
14700 0.037350124 14700 0.133930501 7400 0.082190083 7396
0.063872802 14728 0.079018064 14728 0.027511835
14700 0.104986641 14700 0.143367318 7400 0.051953262 7400
0.081043727 14736 0.095251613 14736 0.03231761
14700 0.16196756 14700 0.106921361 7400 0.03458533 7404
0.034011238 14744 0.122355996 14744 0.066001609
14800 0.186747115 14800 0.081429178 7410 0.013212689 7408
0.099195895 14752 0.098137498 14752 0.079445621
14800 0.204588593 14800 0.068444097 7410 0.02026113 7412
0.140655771 14760 0.013027697 14760 0.067792847
14800 0.105060179 14800 0.051366449 7420 0.043555279 7416
0.083215469 14768 0.042989068 14768 0.048968221
14800 0.094146126 14800 0.041166513 7420 0.072466581 7420
0.045835408 14776 0.050311679 14776 0.036767364
14800 0.185792625 14800 0.045901797 7420 0.067146808 7424
0.074657364 14784 0.089614172 14784 0.051943578
14800 0.144781356 14800 0.034942812 7430 0.045075129 7428
0.064990956 14792 0.138163669 14792 0.074848853
14800 0.034634151 14800 0.033907716 7430 0.022837588 7432
0.049394028 14800 0.16972229 14800 0.081359067
14800 0.088636152 14800 0.026371874 7440 0.02346634 7436
0.046597645 14808 0.150408654 14808 0.079165635
14800 0.076268763 14800 0.024588775 7440 0.06990986 7440
0.021120879 14816 0.147642903 14816 0.080376129
14800 0.053397602 14800 0.028330876 7440 0.080555466 7444
0.032647491 14824 0.1639736 14824 0.081017926
14800 0.09010559 14800 0.007329448 7450 0.064847773 7448
0.032955308 14832 0.116201125 14832 0.067276793
14800 0.092570292 14800 0.038857459 7450 0.050916879 7452
0.034378511 14840 0.045654462 14840 0.04838717
14800 0.070163442 14800 0.052771586 7460 0.0426804 7456
0.061674728 14848 0.013255713 14848 0.075787641
14900 0.007768371 14900 0.060797847 7460 0.033560646 7460
0.068485257 14856 0.062445834 14856 0.099368081
14900 0.091126181 14900 0.047549922 7460 0.018476063 7464
0.061470717 14864 0.107576634 14864 0.081333557
14900 0.146311591 14900 0.028115595 7470 0.010401311 7468
0.047887555 14872 0.115764691 14872 0.035453431
14900 0.150720749 14900 0.057557143 7470 0.054849978 7472
0.071975403 14880 0.087492233 14880 0.015509082
14900 0.135183538 14900 0.078737532 7480 0.095029187 7476
0.091260568 14888 0.044045508 14888 0.021347427
14900 0.107607571 14900 0.044550729 7480 0.09430902 7480
0.07462105 14896 0.041820073 14896 0.010427443
14900 0.124434781 14900 0.084410072 7480 0.064782791 7484
0.058084079 14904 0.03945284 14904 0.00048693
14900 0.150800871 14900 0.109681052 7490 0.035741945 7488
0.041521133 14912 0.032171312 14912 0.025929046
14900 0.09783807 14900 0.060891038 7490 0.023665249 7492
0.054944096 14920 0.055606546 14920 0.052499137
14900 0.075514749 14900 0.018343895 7500 0.021224871 7496
0.088600107 14928 0.067959212 14928 0.043103581
14900 0.02442276 14900 0.093585244 7500 0.033628949 7500
0.079694037 14936 0.112681635 14936 0.033696248
14900 0.097843236 14900 0.182729141 7500 0.049214559 7504
0.057159858 14944 0.070219969 14944 0.027892036
15000 0.089133842 15000 0.124719532 7510 0.063887928 7508
0.03842104 14952 0.060439881 14952 0.016829423
15000 0.031595326 15000 0.056723657 7510 0.050221403 7512
0.051470423 14960 0.104502797 14960 0.029070112
15000 0.09567706 15000 0.114388276 7520 0.044506804 7516
0.073080417 14968 0.039023234 14968 0.022470254
15000 0.088728986 15000 0.103389997 7520 0.042646992 7520
0.07316238 14976 0.088489192 14976 0.015153453
15000 0.073479198 15000 0.101605074 7520 0.047966241 7524
0.069456044 14984 0.11100094 14984 0.020737025
15000 0.10175143 15000 0.114856681 7530 0.094124538 7528

0.029983938 14992 0.106739601 14992 0.010811849
15000 0.084914958 15000 0.108005319 7530 0.093703449 7532
0.057715435 15000 0.0972856 15000 0.009432537
15000 0.035646692 15000 0.139694079 7540 0.059302907 7536
0.111921087 15008 0.082233099 15008 0.0129036
15000 0.023993431 15000 0.166731435 7540 0.030217309 7540
0.097164506 15016 0.179216513 15016 0.023505438
15000 0.104709696 15000 0.115131887 7540 0.057648071 7544
0.040861713 15024 0.226266464 15024 0.053330608
15000 0.135165756 15000 0.082439554 7550 0.092517796 7548
0.086427157 15032 0.182543038 15032 0.074241638
15000 0.027273387 15000 0.087046232 7550 0.060169092 7552
0.123523438 15040 0.118064447 15040 0.08464772
15000 0.120800367 15000 0.123371137 7560 0.013250228 7556
0.121441641 15048 0.126360698 15048 0.061711711
15100 0.092432594 15100 0.13113259 7560 0.046424408 7560
0.107910819 15056 0.075253192 15056 0.030064814
15100 0.074300304 15100 0.056519468 7560 0.055482829 7564
0.09137809 15064 0.01451833 15064 0.016304017
15100 0.081184524 15100 0.032000313 7570 0.070997536 7568
0.065906796 15072 0.048788992 15072 0.026513324
15100 0.08703192 15100 0.06453463 7570 0.077608274 7572
0.033904937 15080 0.058797152 15080 0.046631751
15100 0.115268398 15100 0.054791261 7580 0.057087793 7576
0.071205141 15088 0.058133031 15088 0.047756035
15100 0.149138578 15100 0.077724239 7580 0.057116165 7580
0.091714152 15096 0.040621559 15096 0.046452329
15100 0.160116906 15100 0.103589577 7580 0.061945517 7584
0.06222626 15104 0.049690571 15104 0.044293993
15100 0.108946508 15100 0.122149999 7590 0.055154917 7588
0.050451963 15112 0.065332926 15112 0.046081426
15100 0.064418222 15100 0.140845979 7590 0.053996999 7592
0.072540534 15120 0.014008331 15120 0.035351495
15100 0.045609922 15100 0.181140421 7600 0.04805189 7596
0.059974467 15128 0.032397948 15128 0.029558869
15100 0.063260071 15100 0.187346711 7600 0.062717365 7600
0.061805673 15136 0.087137712 15136 0.050864644
15100 0.10291997 15100 0.126045998 7600 0.05083924 7604
0.099586425 15144 0.109777167 15144 0.030383721
15200 0.078401688 15200 0.092239316 7610 0.038026221 7608
0.083442697 15152 0.083933453 15152 0.013473925
15200 0.114213741 15200 0.081952901 7610 0.020017023 7612
0.056864235 15160 0.147526138 15160 0.029320976
15200 0.113520895 15200 0.059827522 7620 0.010234391 7616
0.105952735 15168 0.156515816 15168 0.011279969
15200 0.170745101 15200 0.059839957 7620 0.025536307 7620
0.116601259 15176 0.163300298 15176 0.042081145
15200 0.172822081 15200 0.014494081 7620 0.027672004 7624
0.095047304 15184 0.188655104 15184 0.064169668
15200 0.11794555 15200 0.011467494 7630 0.02009886 7628
0.076533892 15192 0.137477503 15192 0.043504595
15200 0.063924046 15200 0.07390276 7630 0.013878207 7632
0.062257983 15200 0.084102248 15200 0.018882254
15200 0.033403314 15200 0.164210869 7640 0.014528775 7636
0.050375395 15208 0.082869126 15208 0.029503512
15200 0.073310745 15200 0.189212733 7640 0.028633813 7640
0.054736604 15216 0.120470206 15216 0.041311017
15200 0.047795671 15200 0.142337173 7640 0.029151552 7644
0.05968531 15224 0.108428481 15224 0.059778769
15200 0.049716648 15200 0.040309024 7650 0.032742664 7648
0.061100865 15232 0.044133125 15232 0.041159961
15200 0.0431927 15200 0.04576529 7650 0.013558266 7652
0.071140719 15240 0.017307417 15240 0.028005858
15200 0.019323532 15200 0.062342151 7660 0.047842128 7656
0.103088227 15248 0.044702796 15248 0.049688631
15300 0.051381794 15300 0.074489602 7660 0.079606449 7660
0.115959396 15256 0.022665523 15256 0.05928172
15300 0.06108993 15300 0.093410752 7660 0.053485055 7664

0.093809489	15264	0.038062583	15264	0.046082885		
15300	0.066558176	15300	0.038404498	7670	0.016688158	7668
0.064179498	15272	0.111298708	15272	0.02692911		
15300	0.078980171	15300	0.144251724	7670	0.025123976	7672
0.072558054	15280	0.195330431	15280	0.020370868		
15300	0.104689279	15300	0.214032945	7680	0.031873329	7676
0.09007354	15288	0.197495553	15288	0.040516228		
15300	0.114761628	15300	0.152421446	7680	0.018068809	7680
0.070901748	15296	0.119069548	15296	0.036391371		
15300	0.150236926	15300	0.144999474	7680	0.008433504	7684
0.078798206	15304	0.066793837	15304	0.032788408		
15300	0.21437346	15300	0.099292789	7690	0.018718098	7688
0.077724159	15312	0.051549836	15312	0.042893065		
15300	0.145913088	15300	0.131315886	7690	0.029413672	7692
0.096860196	15320	0.043678334	15320	0.039015078		
15300	0.080824248	15300	0.16055505	7700	0.018816732	7696
0.11093045	15328	0.068758935	15328	0.024677242		
15300	0.13348987	15300	0.106598513	7700	0.038009755	7700
0.151391476	15336	0.045353263	15336	0.018738967		
15300	0.17635744	15300	0.085597829	7700	0.025728097	7704
0.183429104	15344	0.022630442	15344	0.006686228		
15400	0.141986616	15400	0.068718444	7710	0.037369719	7708
0.141671233	15352	0.028525728	15352	0.024303969		
15400	0.100698169	15400	0.141029858	7710	0.037401635	7712
0.047465761	15360	0.084322142	15360	0.054670876		
15400	0.04086379	15400	0.187998143	7720	0.023231263	7716
0.100306796	15368	0.141472629	15368	0.054631193		
15400	0.038407576	15400	0.129788329	7720	0.023152097	7720
0.109214554	15376	0.107921558	15376	0.010223554		
15400	0.029640693	15400	0.09325517	7720	0.033165761	7724
0.073594507	15384	0.030382114	15384	0.036261561		
15400	0.087678556	15400	0.064797685	7730	0.0351899	7728
0.082964289	15392	0.016582477	15392	0.060787024		
15400	0.170129715	15400	0.030434852	7730	0.013943506	7732
0.129188149	15400	0.056490972	15400	0.084167754		
15400	0.183730299	15400	0.031870139	7740	0.021438022	7736
0.141518598	15408	0.080223392	15408	0.084376137		
15400	0.141240278	15400	0.038465547	7740	0.019241208	7740
0.110432004	15416	0.124290324	15416	0.051534538		
15400	0.095092655	15400	0.027053604	7740	0.040576659	7744
0.074020209	15424	0.159215036	15424	0.029481127		
15400	0.108588443	15400	0.096219214	7750	0.03745345	7748
0.084033905	15432	0.088680237	15432	0.027701		
15400	0.107153144	15400	0.176439061	7750	0.018763914	7752
0.111749389	15440	0.083806255	15440	0.010057945		
15400	0.078631936	15400	0.156143549	7760	0.039533967	7756
0.106293752	15448	0.122010286	15448	0.026894386		
15500	0.111452733	15500	0.105300278	7760	0.027438744	7760
0.109318353	15456	0.020675607	15456	0.036400052		
15500	0.129003398	15500	0.084025654	7760	0.010772333	7764
0.12238443	15464	0.089970934	15464	0.027147573		
15500	0.139430908	15500	0.073280266	7770	0.039864564	7768
0.096848067	15472	0.074971104	15472	0.038244903		
15500	0.078765457	15500	0.058977155	7770	0.061176514	7772
0.099708115	15480	0.094187948	15480	0.044399803		
15500	0.040420408	15500	0.022269027	7780	0.052654457	7776
0.102063539	15488	0.089308494	15488	0.031563995		
15500	0.015819584	15500	0.030053418	7780	0.049932874	7780
0.087618304	15496	0.093530915	15496	0.021535621		
15500	0.067727131	15500	0.013074189	7780	0.035048852	7784
0.084095955	15504	0.17312073	15504	0.023396724		
15500	0.111226509	15500	0.051799976	7790	0.012282137	7788
0.05059805	15512	0.111521928	15512	0.055923152		
15500	0.122065379	15500	0.107982138	7790	0.016139757	7792
0.088333931	15520	0.02542418	15520	0.073164665		
15500	0.11404368	15500	0.196013891	7800	0.01078947	7796
0.129783308	15528	0.061490377	15528	0.053083797		
15500	0.067241352	15500	0.119832621	7800	0.018832614	7800

0.093974151 15536 0.065935965 15536 0.024724433
15500 0.025766762 15500 0.132104804 7800 0.030322455 7804
0.044640794 15544 0.068956026 15544 0.035701989
15600 0.055157445 15600 0.074162686 7810 0.021454813 7808
0.10204289 15552 0.140275879 15552 0.055377739
15600 0.044224493 15600 0.010833066 7810 0.037332207 7812
0.079416663 15560 0.219179986 15560 0.066423265
15600 0.075490199 15600 0.112594724 7820 0.043627959 7816
0.047398298 15568 0.213596083 15568 0.052699503
15600 0.140401113 15600 0.205809716 7820 0.029520606 7820
0.041972631 15576 0.134253234 15576 0.02924675
15600 0.171665204 15600 0.213957058 7820 0.037888105 7824
0.053580367 15584 0.068732254 15584 0.027529914
15600 0.124631319 15600 0.176428323 7830 0.08126058 7828
0.062312007 15592 0.043889799 15592 0.030314763
15600 0.053346881 15600 0.113343514 7830 0.096081494 7832
0.074417359 15600 0.040703188 15600 0.044779346
15600 0.101415055 15600 0.026183805 7840 0.063490166 7836
0.089558111 15608 0.065500331 15608 0.039209361
15600 0.162929064 15600 0.08163914 7840 0.052911779 7840
0.097187702 15616 0.101770354 15616 0.038179016
15600 0.218614689 15600 0.029036901 7840 0.066211156 7844
0.056501936 15624 0.187903614 15624 0.053871532
15600 0.260256959 15600 0.096883814 7850 0.03713233 7848
0.030674622 15632 0.128382235 15632 0.036254551
15600 0.215597756 15600 0.134205359 7850 0.049161838 7852
0.057205685 15640 0.051293348 15640 0.018636991
15600 0.109788598 15600 0.133912981 7860 0.081050486 7856
0.080109494 15648 0.0118208 15648 0.034209006
15700 0.109530847 15700 0.120981327 7860 0.069367205 7860
0.100707592 15656 0.034282333 15656 0.05914205
15700 0.094419163 15700 0.053883312 7860 0.050125229 7864
0.104958694 15664 0.080239217 15664 0.052126045
15700 0.069887632 15700 0.055279004 7870 0.055933946 7868
0.094460731 15672 0.131230525 15672 0.022399552
15700 0.047211346 15700 0.141607743 7870 0.092102091 7872
0.062170358 15680 0.147440515 15680 0.038193579
15700 0.028934242 15700 0.193382104 7880 0.096810298 7876
0.025320451 15688 0.109425113 15688 0.060544058
15700 0.096360243 15700 0.144990496 7880 0.043609096 7880
0.045683708 15696 0.045646953 15696 0.091692455
15700 0.063555439 15700 0.05642245 7880 0.068720867 7884
0.073907308 15704 0.129145833 15704 0.082560568
15700 0.087348664 15700 0.048639002 7890 0.10504415 7888
0.057335728 15712 0.173999506 15712 0.053943793
15700 0.07614222 15700 0.083919375 7890 0.080952021 7892
0.039312407 15720 0.108362088 15720 0.092859948
15700 0.089994945 15700 0.095842996 7900 0.045681474 7896
0.028228518 15728 0.085982501 15728 0.069043228
15700 0.176547401 15700 0.074548159 7900 0.046650817 7900
0.034156001 15736 0.074380732 15736 0.032223845
15700 0.202810683 15700 0.0754496 7900 0.08837114 7904
0.041424439 15744 0.101446953 15744 0.057482808
15800 0.183605356 15800 0.073175514 7910 0.114423972 7908
0.061181556 15752 0.031410036 15752 0.04535335
15800 0.124194819 15800 0.056853933 7910 0.098729644 7912
0.071184673 15760 0.105436739 15760 0.05991228
15800 0.072722061 15800 0.076092918 7920 0.063144857 7916
0.01312311 15768 0.148096675 15768 0.048257283
15800 0.07391582 15800 0.122306912 7920 0.037016976 7920
0.081151993 15776 0.126051368 15776 0.023826526
15800 0.082936211 15800 0.063418789 7920 0.039486538 7924
0.129082225 15784 0.087635563 15784 0.016585171
15800 0.091430797 15800 0.061883198 7930 0.091771181 7928
0.119384065 15792 0.089056957 15792 0.037201964
15800 0.148962965 15800 0.103226288 7930 0.110375942 7932
0.077401339 15800 0.095182244 15800 0.047953206
15800 0.169755659 15800 0.093639967 7940 0.08311501 7936

0.026100292 15808 0.075915981 15808 0.029987334
15800 0.092892638 15800 0.061058883 7940 0.043441891 7940
0.01804241 15816 0.122444049 15816 0.020517909
15800 0.042365686 15800 0.087140637 7940 0.033054243 7944
0.013679854 15824 0.201454226 15824 0.015132559
15800 0.098563738 15800 0.055070319 7950 0.061936102 7948
0.068044705 15832 0.151248998 15832 0.020597958
15800 0.061021656 15800 0.058845868 7950 0.040435789 7952
0.130018015 15840 0.059132632 15840 0.043277967
15800 0.017459233 15800 0.108864304 7960 0.049669339 7956
0.123914841 15848 0.058924823 15848 0.062330903
15900 0.047735568 15900 0.07995074 7960 0.080120182 7960
0.084333013 15856 0.076543089 15856 0.0821305
15900 0.039665447 15900 0.04042987 7960 0.065333399 7964
0.064033979 15864 0.088856024 15864 0.114630529
15900 0.045893554 15900 0.037206544 7970 0.040743573 7968
0.052959265 15872 0.066320776 15872 0.125731705
15900 0.15748803 15900 0.041967203 7970 0.048890743 7972
0.051140825 15880 0.064915796 15880 0.090599169
15900 0.28990701 15900 0.092387192 7980 0.053844531 7976
0.081932405 15888 0.092360002 15888 0.04599529
15900 0.27619992 15900 0.155618168 7980 0.033650198 7980
0.126546293 15896 0.080558428 15896 0.014358878
15900 0.150235953 15900 0.15742892 7980 0.019575236 7984
0.122893849 15904 0.029329016 15904 0.010754004
15900 0.048790062 15900 0.097507196 7990 0.031283937 7988
0.082699808 15912 0.046337347 15912 0.0187965
15900 0.020805306 15900 0.124050144 7990 0.03565181 7992
0.07708872 15920 0.071598406 15920 0.032059041
15900 0.041834053 15900 0.10250901 8000 0.031307991 7996
0.066466535 15928 0.047052185 15928 0.046402671
15900 0.006751504 15900 0.115209215 8000 0.04149527 8000
0.092751958 15936 0.093476388 15936 0.036117392
15900 0.075488781 15900 0.143826503 8000 0.06838009 8004
0.131564419 15944 0.14339597 15944 0.008379739
16000 0.057732043 16000 0.108208253 8010 0.067532012 8008
0.155781366 15952 0.154674155 15952 0.049815368
16000 0.061163984 16000 0.073359384 8010 0.037257622 8012
0.130547545 15960 0.169855223 15960 0.072412899
16000 0.1500207 16000 0.060021765 8020 0.020622983 8016
0.065362045 15968 0.208292069 15968 0.066421599
16000 0.170730301 16000 0.076658223 8020 0.017191678 8020
0.03467434 15976 0.206073979 15976 0.060769653
16000 0.113884307 16000 0.146829698 8020 0.030483589 8024
0.055936696 15984 0.142073318 15984 0.069156049
16000 0.054554534 16000 0.143873811 8030 0.044915883 8028
0.058616497 15992 0.119620221 15992 0.066447836
16000 0.061606268 16000 0.049309067 8030 0.040762974 8032
0.058228856 16000 0.099989105 16000 0.029156117
16000 0.071377894 16000 0.094481067 8040 0.078720921 8036
0.058127229 16008 0.049256523 16008 0.017348353
16000 0.110509194 16000 0.13796032 8040 0.072709292 8040
0.025754216 16016 0.067436078 16016 0.043888475
16000 0.133295965 16000 0.071948947 8040 0.050976421 8044
0.030745403 16024 0.104723498 16024 0.057267604
16000 0.096888442 16000 0.017799344 8050 0.048918562 8048
0.060112117 16032 0.135316994 16032 0.063706677
16000 0.039030594 16000 0.137461815 8050 0.061918523 8052
0.070624607 16040 0.125901643 16040 0.044596589
16000 0.063482527 16000 0.184760633 8060 0.09609024 8056
0.075847129 16048 0.083911073 16048 0.026893767
16100 0.048660539 16100 0.077994613 8060 0.095662421 8060
0.062979569 16056 0.058509144 16056 0.047135593
16100 0.016769101 16100 0.064642794 8060 0.084352963 8064
0.049746403 16064 0.050099814 16064 0.044567445
16100 0.048594317 16100 0.107729662 8070 0.059410028 8068
0.048206424 16072 0.037619637 16072 0.036885864
16100 0.123441554 16100 0.050788796 8070 0.030666612 8072

0.048742521 16080 0.12355826 16080 0.012839197
16100 0.145116894 16100 0.061823237 8080 0.024372683 8076
0.034285906 16088 0.156980925 16088 0.012374811
16100 0.099956866 16100 0.082652601 8080 0.008927036 8080
0.036683097 16096 0.143838494 16096 0.011631682
16100 0.046242734 16100 0.115289513 8080 0.030008407 8084
0.052523294 16104 0.134310365 16104 0.029874862
16100 0.016331609 16100 0.087859589 8090 0.043004067 8088
0.06809659 16112 0.079820136 16112 0.028495726
16100 0.018499326 16100 0.050858882 8090 0.053983873 8092
0.096625801 16120 0.073890238 16120 0.013860247
16100 0.035572037 16100 0.061974184 8100 0.058769492 8096
0.112862399 16128 0.16395314 16128 0.026930367
16100 0.057677076 16100 0.052528736 8100 0.031244024 8100
0.100388883 16136 0.120447912 16136 0.052961983
16100 0.081112688 16100 0.091051581 8100 0.054525866 8104
0.067594854 16144 0.03024505 16144 0.056552392
16200 0.09334369 16200 0.062393912 8110 0.059942886 8108
0.069917107 16152 0.03960597 16152 0.027084199
16200 0.129782683 16200 0.03061293 8110 0.004062308 8112
0.080397957 16160 0.059887399 16160 0.017437627
16200 0.13576429 16200 0.036486734 8120 0.051564493 8116
0.073149786 16168 0.083679748 16168 0.020734888
16200 0.097770892 16200 0.088745139 8120 0.077309895 8120
0.059057042 16176 0.110795409 16176 0.022091635
16200 0.04807776 16200 0.148303894 8120 0.09656691 8124
0.060579623 16184 0.097477008 16184 0.052066855
16200 0.030849737 16200 0.143589773 8130 0.077806326 8128
0.06351903 16192 0.110494038 16192 0.087498018
16200 0.102822516 16200 0.181941243 8130 0.048214653 8132
0.029520439 16200 0.135278883 16200 0.082649589
16200 0.092772752 16200 0.11505188 8140 0.044265678 8136
0.024036146 16208 0.070524264 16208 0.048426886
16200 0.040764233 16200 0.061325794 8140 0.042476924 8140
0.034210269 16216 0.084328909 16216 0.044609024
16200 0.112373229 16200 0.07882592 8140 0.023674054 8144
0.025369835 16224 0.124142869 16224 0.049982144
16200 0.05140523 16200 0.126080704 8150 0.016602551 8148
0.021106525 16232 0.110719338 16232 0.014027274
16200 0.064856562 16200 0.117419404 8150 0.063011263 8152
0.035455545 16240 0.097059528 16240 0.014493916
16200 0.084823165 16200 0.106021624 8160 0.085146618 8156
0.055774603 16248 0.086209926 16248 0.006556745
16300 0.006383287 16300 0.143592216 8160 0.079410427 8160
0.097583594 16256 0.051210449 16256 0.019117477
16300 0.096425087 16300 0.200895374 8160 0.054569555 8164
0.085520311 16264 0.053826207 16264 0.048800677
16300 0.11413559 16300 0.220677655 8170 0.025746072 8168
0.041382133 16272 0.108396882 16272 0.059085778
16300 0.07985178 16300 0.164867103 8170 0.048207901 8172
0.060523507 16280 0.113866008 16280 0.045117438
16300 0.035328671 16300 0.101629506 8180 0.075238662 8176
0.070431161 16288 0.086148066 16288 0.032920514
16300 0.02669312 16300 0.081055812 8180 0.053375687 8180
0.03647519 16296 0.05447818 16296 0.041302432
16300 0.04893903 16300 0.059915863 8180 0.036430276 8184
0.013006411 16304 0.054707842 16304 0.071743962
16300 0.061129489 16300 0.045540575 8190 0.06878742 8188
0.016242251 16312 0.059378814 16312 0.085577616
16300 0.072126008 16300 0.04563688 8190 0.054392029 8192
0.015467651 16320 0.099287616 16320 0.058330206
16300 0.072784795 16300 0.07121855 8200 0.028013765 8196
0.020561 16328 0.093110299 16328 0.039212609
16300 0.017092129 16300 0.126293715 8200 0.059313872 8200
0.041048195 16336 0.048254489 16336 0.044627748
16300 0.100479665 16300 0.161546151 8200 0.05282147 8204
0.053208605 16344 0.066619541 16344 0.029069443
16400 0.156941504 16400 0.116617164 8210 0.042475022 8208

0.025833651 16352 0.027320497 16352 0.005296286
16400 0.105862135 16400 0.07692221 8210 0.138097471 8212
0.045193061 16360 0.051800496 16360 0.014009787
16400 0.090346053 16400 0.105224848 8220 0.148595601 8216
0.061678882 16368 0.100722878 16368 0.023258221
16400 0.108113643 16400 0.116112693 8220 0.096199765 8220
0.092260118 16376 0.074070682 16376 0.028139632
16400 0.094093914 16400 0.11167831 8220 0.044287113 8224
0.057535694 16384 0.026755073 16384 0.044255001
16400 0.092889073 16400 0.162248936 8230 0.016845519 8228
0.0506098 16392 0.091844086 16392 0.052868636
16400 0.051042665 16400 0.172990214 8230 0.027106764 8232
0.097542907 16400 0.084088446 16400 0.009391019
16400 0.11467914 16400 0.150553896 8240 0.026103351 8236
0.097304997 16408 0.041347998 16408 0.063666506
16400 0.122042518 16400 0.134290458 8240 0.017830738 8240
0.083886931 16416 0.075153388 16416 0.088077526
16400 0.084018167 16400 0.086179185 8240 0.064406973 8244
0.065642656 16424 0.126575295 16424 0.051010353
16400 0.105124229 16400 0.085512926 8250 0.084061474 8248
0.067025365 16432 0.093886447 16432 0.019092009
16400 0.079352918 16400 0.098428238 8250 0.056830391 8252
0.062740444 16440 0.0209562 16440 0.036515135
16400 0.108402637 16400 0.132681599 8260 0.030408573 8256
0.028986793 16448 0.047506088 16448 0.042631935
16500 0.090126356 16500 0.064503234 8260 0.029297982 8260
0.004784609 16456 0.022655931 16456 0.075931028
16500 0.0310982 16500 0.035312172 8260 0.038088943 8264
0.01523328 16464 0.096829055 16464 0.063311032
16500 0.055730638 16500 0.052837193 8270 0.038766142 8268
0.062320003 16472 0.136667324 16472 0.029845507
16500 0.070442213 16500 0.041515563 8270 0.041219824 8272
0.10579059 16480 0.10859059 16480 0.022684857
16500 0.054487078 16500 0.044620188 8280 0.042892072 8276
0.069794027 16488 0.077930294 16488 0.022666349
16500 0.035917346 16500 0.045848785 8280 0.054702861 8280
0.008614184 16496 0.078475663 16496 0.029673207
16500 0.039852337 16500 0.040487543 8280 0.054361735 8284
0.027091082 16504 0.097844924 16504 0.028871395
16500 0.133719281 16500 0.076467346 8290 0.026975429 8288
0.009624315 16512 0.125161868 16512 0.009056642
16500 0.175717286 16500 0.096208205 8290 0.049754595 8292
0.042189509 16520 0.139481825 16520 0.061206978
16500 0.119387281 16500 0.080071237 8300 0.060937775 8296
0.064909305 16528 0.133160793 16528 0.070206523
16500 0.032592219 16500 0.052137271 8300 0.069013237 8300
0.045147466 16536 0.068318856 16536 0.048566781
16500 0.103253638 16500 0.026769067 8300 0.098429438 8304
0.02857634 16544 0.053332944 16544 0.044538978
16600 0.174986738 16600 0.051859504 8310 0.079882557 8308
0.037742662 16552 0.11991978 16552 0.043377921
16600 0.185082449 16600 0.082772815 8310 0.044551263 8312
0.043213095 16560 0.118947821 16560 0.016020804
16600 0.133230846 16600 0.085621112 8320 0.075054297 8316
0.049306087 16568 0.089650319 16568 0.013740587
16600 0.038307906 16600 0.074038871 8320 0.065169661 8320
0.028192768 16576 0.09225429 16576 0.0295347
16600 0.05167054 16600 0.00582527 8320 0.081383056 8324
0.022222945 16584 0.115331699 16584 0.042705578
16600 0.046159836 16600 0.020258411 8330 0.076432334 8328
0.015390382 16592 0.076867545 16592 0.039608407
16600 0.054328986 16600 0.063063817 8330 0.104202241 8332
0.042579792 16600 0.064693435 16600 0.026449527
16600 0.079573598 16600 0.083064391 8340 0.127556283 8336
0.070963484 16608 0.098027107 16608 0.056498113
16600 0.037458638 16600 0.049612379 8340 0.092933748 8340
0.038301336 16616 0.082439205 16616 0.066538109
16600 0.092974457 16600 0.028531129 8340 0.038830138 8344

0.059811697	16624	0.073735944	16624	0.065819069		
16600	0.118798729	16600	0.058211634	8350	0.006280165	8348
0.076617725	16632	0.180843694	16632	0.056709738		
16600	0.044240464	16600	0.086111933	8350	0.00818256	8352
0.061976483	16640	0.228482706	16640	0.039993502		
16600	0.033584234	16600	0.074299584	8360	0.018972103	8356
0.018577917	16648	0.186795224	16648	0.03885799		
16700	0.059864553	16700	0.087944638	8360	0.027493878	8360
0.072584473	16656	0.104632869	16656	0.058005757		
16700	0.087107044	16700	0.072994582	8360	0.014814852	8364
0.102618717	16664	0.055254473	16664	0.038969076		
16700	0.119991018	16700	0.057027617	8370	0.032171563	8368
0.104566083	16672	0.10473052	16672	0.027616623		
16700	0.102263766	16700	0.138887335	8370	0.046328125	8372
0.111355672	16680	0.169854859	16680	0.059960563		
16700	0.056311565	16700	0.125649444	8380	0.069944166	8376
0.082485516	16688	0.191481799	16688	0.056243927		
16700	0.040915656	16700	0.051554758	8380	0.058021618	8380
0.039147886	16696	0.114374743	16696	0.027885739		
16700	0.032471151	16700	0.068943198	8380	0.016722073	8384
0.072773342	16704	0.015668915	16704	0.049529088		
16700	0.048966002	16700	0.125988561	8390	0.035032495	8388
0.093354669	16712	0.010243467	16712	0.048949511		
16700	0.112304231	16700	0.092345523	8390	0.028956603	8392
0.099851917	16720	0.067324931	16720	0.058876911		
16700	0.171233434	16700	0.031032974	8400	0.029274601	8396
0.086754932	16728	0.156407914	16728	0.068495836		
16700	0.179454655	16700	0.078886478	8400	0.041005591	8400
0.049323171	16736	0.186270641	16736	0.043285465		
16700	0.207307283	16700	0.126036364	8400	0.042115662	8404
0.021662301	16744	0.14245999	16744	0.006562435		
16800	0.216026761	16800	0.098519064	8410	0.049310111	8408
0.025865898	16752	0.064670108	16752	0.02218963		
16800	0.14135227	16800	0.059222057	8410	0.040349336	8412
0.033653741	16760	0.042720672	16760	0.030948933		
16800	0.024946834	16800	0.080838261	8420	0.016899396	8416
0.022246743	16768	0.129181921	16768	0.029621395		
16800	0.097889817	16800	0.060492865	8420	0.024717261	8420
0.080025071	16776	0.138043324	16776	0.007844079		
16800	0.123201215	16800	0.035156303	8420	0.031027394	8424
0.107269007	16784	0.08049528	16784	0.04727026		
16800	0.088415007	16800	0.022818007	8430	0.04012679	8428
0.102942147	16792	0.040463161	16792	0.056463483		
16800	0.059341986	16800	0.063895364	8430	0.038023081	8432
0.087279499	16800	0.068388566	16800	0.045535424		
16800	0.088989131	16800	0.057702477	8440	0.035790192	8436
0.061123814	16808	0.072093244	16808	0.048685521		
16800	0.078873149	16800	0.043245433	8440	0.043046195	8440
0.050039966	16816	0.108840002	16816	0.036644		
16800	0.091580703	16800	0.055110748	8440	0.029209361	8444
0.043574532	16824	0.118822725	16824	0.010115973		
16800	0.096619835	16800	0.022198843	8450	0.033520973	8448
0.031939544	16832	0.056562032	16832	0.024869218		
16800	0.092809249	16800	0.062062783	8450	0.036951809	8452
0.096912459	16840	0.101298276	16840	0.02278863		
16800	0.096063755	16800	0.069210844	8460	0.010974328	8456
0.089312824	16848	0.140992066	16848	0.015203828		
16900	0.085514483	16900	0.115153933	8460	0.047734626	8460
0.029095414	16856	0.156594804	16856	0.005966264		
16900	0.097028264	16900	0.088822562	8460	0.061356775	8464
0.037448001	16864	0.132206725	16864	0.036151581		
16900	0.084739055	16900	0.08569775	8470	0.036178066	8468
0.062997191	16872	0.082738501	16872	0.059249447		
16900	0.059632042	16900	0.13126593	8470	0.046844172	8472
0.078398458	16880	0.052553482	16880	0.031749954		
16900	0.080060388	16900	0.132193658	8480	0.055344295	8476
0.08385587	16888	0.06938912	16888	0.041806583		
16900	0.137250506	16900	0.101300284	8480	0.049019116	8480

```
0.084008585	16896	0.060246792	16896	0.051938412
16900	0.15705399	16900	0.082594044	8480	0.056267742	8484
0.0775925	16904	0.037947022	16904	0.059265058
16900	0.124454236	16900	0.054127817	8490	0.054269753	8488
0.070445036	16912	0.055015236	16912	0.069327034
16900	0.115135095	16900	0.039344523	8490	0.04278393	8492
0.088384884	16920	0.109154426	16920	0.071470066
16900	0.106142441	16900	0.053130145	8500	0.014679722	8496
0.064659689	16928	0.130260261	16928	0.034540852
16900	0.072942421	16900	0.057346228	8500	0.04482835	8500
0.050958315	16936	0.063194108	16936	0.054707212
16900	0.045669098	16900	0.084545558	8500	0.065928871	8504
0.108112945	16944	0.013874231	16944	0.099174715
17000	0.05437091	17000	0.069447167	8510	0.03960279	8508
0.09601286	16952	0.087210756	16952	0.094943353
17000	0.048521764	17000	0.110003508	8510	0.04910295	8512
0.069326721	16960	0.191755505	16960	0.049449176
17000	0.071306437	17000	0.135457653	8520	0.072420575	8516
0.064720189	16968	0.158020703	16968	0.018227451
17000	0.141192955	17000	0.056505309	8520	0.087510591	8520
0.042814278	16976	0.062176492	16976	0.031908887
17000	0.126172934	17000	0.046578596	8520	0.063707121	8524
0.066708359	16984	0.053101267	16984	0.036310757
17000	0.125792853	17000	0.016287209	8530	0.054704513	8528
0.118964934	16992	0.022325519	16992	0.034147775
17000	0.15932195	17000	0.073071809	8530	0.071996867	8532
0.137901589	17000	0.075220189	17000	0.024629429
17000	0.12858909	17000	0.072797149	8540	0.061283652	8536
0.1165391	17008	0.067916408	17008	0.006671748
17000	0.054723823	17000	0.078195524	8540	0.027172422	8540
0.050200477	17016	0.118229014	17016	0.02777115
17000	0.051690928	17000	0.148925174	8540	0.018773922	8544
0.109478518	17024	0.169491614	17024	0.041668365
17000	0.089119283	17000	0.151177068	8550	0.008137721	8548
0.111914465	17032	0.140775359	17032	0.037977745
17000	0.102465092	17000	0.048653663	8550	0.014657399	8552
0.072849347	17040	0.046542846	17040	0.037377035
17000	0.085227235	17000	0.048346719	8560	0.028440101	8556
0.073113777	17048	0.035354413	17048	0.055718294
17100	0.039559098	17100	0.054789725	8560	0.018403558	8560
0.088558096	17056	0.110837777	17056	0.078289973
17100	0.063924766	17100	0.064302651	8560	0.026512596	8564
0.08906595	17064	0.161188276	17064	0.059954546
17100	0.100308251	17100	0.090672853	8570	0.042888329	8568
0.07756774	17072	0.190739476	17072	0.009126174
17100	0.081082704	17100	0.048621529	8570	0.056049175	8572
0.053427015	17080	0.159596763	17080	0.021759877
17100	0.035890102	17100	0.08334747	8580	0.045917223	8576
0.031489901	17088	0.056827834	17088	0.016131446
17100	0.038668233	17100	0.067812027	8580	0.024081121	8580
0.044984801	17096	0.112087415	17096	0.015739393
17100	0.06763485	17100	0.120730671	8580	0.050953684	8584
0.047371668	17104	0.079095349	17104	0.043486023
17100	0.108123379	17100	0.083172497	8590	0.082313898	8588
0.049828312	17112	0.034733213	17112	0.036177822
17100	0.064549604	17100	0.033805423	8590	0.064303953	8592
0.086114917	17120	0.104307386	17120	0.012116122
17100	0.070975148	17100	0.048708756	8600	0.053590513	8596
0.089239773	17128	0.141399636	17128	0.047862344
17100	0.097161581	17100	0.06072241	8600	0.047070294	8600
0.074322801	17136	0.129132808	17136	0.052447704
17100	0.083704072	17100	0.063992753	8600	0.098823504	8604
0.091726615	17144	0.116620409	17144	0.03428731
17200	0.028186583	17200	0.068496891	8610	0.119228396	8608
0.088558001	17152	0.108066219	17152	0.003480946
17200	0.021101592	17200	0.175386755	8610	0.080604732	8612
0.033695967	17160	0.077905839	17160	0.042163152
17200	0.026603348	17200	0.197083776	8620	0.051098916	8616
```

0.092487178 17168 0.095134143 17168 0.064884589
17200 0.003511007 17200 0.115827512 8620 0.010325659 8620
0.122368641 17176 0.116089963 17176 0.04727914
17200 0.023649851 17200 0.148173611 8620 0.042417629 8624
0.105229919 17184 0.074660842 17184 0.020252421
17200 0.053301825 17200 0.140857431 8630 0.05992184 8628
0.081411265 17192 0.020324445 17192 0.018388055
17200 0.034330584 17200 0.029268485 8630 0.080518192 8632
0.057424142 17200 0.038092381 17200 0.024060906
17200 0.052939082 17200 0.109312925 8640 0.087463683 8636
0.076453129 17208 0.074266493 17208 0.012417749
17200 0.077252356 17200 0.130143424 8640 0.062697407 8640
0.110928268 17216 0.101474281 17216 0.033709879
17200 0.033986598 17200 0.07870885 8640 0.042608554 8644
0.116674491 17224 0.165270569 17224 0.05351481
17200 0.036400528 17200 0.05456233 8650 0.025992567 8648
0.10928222 17232 0.169707666 17232 0.061396044
17200 0.086353393 17200 0.034915083 8650 0.015443606 8652
0.112097718 17240 0.122998361 17240 0.043626016
17200 0.114758026 17200 0.006876811 8660 0.021630622 8656
0.120146913 17248 0.128470449 17248 0.042014253
17300 0.065033331 17300 0.058614522 8660 0.024801362 8660
0.105549094 17256 0.12005469 17256 0.051161736
17300 0.052263637 17300 0.070977418 8660 0.058572336 8664
0.105956387 17264 0.060278628 17264 0.024791472
17300 0.035910227 17300 0.047637146 8670 0.058411337 8668
0.133694703 17272 0.04275524 17272 0.013694345
17300 0.027586215 17300 0.075482931 8670 0.033859138 8672
0.114956027 17280 0.091488953 17280 0.016079026
17300 0.066221815 17300 0.15544807 8680 0.050553186 8676
0.103965314 17288 0.094916431 17288 0.023091145
17300 0.092748531 17300 0.146500403 8680 0.082301252 8680
0.070995193 17296 0.065893983 17296 0.029679995
17300 0.07300896 17300 0.081153914 8680 0.093465438 8684
0.038059119 17304 0.016989285 17304 0.028884169
17300 0.020986594 17300 0.064391687 8690 0.077944096 8688
0.068269408 17312 0.080037775 17312 0.052222084
17300 0.068256908 17300 0.02300799 8690 0.056585195 8692
0.088789624 17320 0.076591234 17320 0.042839034
17300 0.056655255 17300 0.119019467 8700 0.053324475 8696
0.04908647 17328 0.047549944 17328 0.006800597
17300 0.016288001 17300 0.165885344 8700 0.059921309 8700
0.049562579 17336 0.083533836 17336 0.055465902
17300 0.083671497 17300 0.180849719 8700 0.058063146 8704
0.076855045 17344 0.139888318 17344 0.071969174
17400 0.068970716 17400 0.162836251 8710 0.038581511 8708
0.09084741 17352 0.084209089 17352 0.060052782
17400 0.103865255 17400 0.120633318 8710 0.010313654 8712
0.134849441 17360 0.122212194 17360 0.034594603
17400 0.148753854 17400 0.133597583 8720 0.014747874 8716
0.110553818 17368 0.197081215 17368 0.052844909
17400 0.14646379 17400 0.123028178 8720 0.023570687 8720
0.022290222 17376 0.116255265 17376 0.058343772
17400 0.096418247 17400 0.055109049 8720 0.028179273 8724
0.039432722 17384 0.029338353 17384 0.021265629
17400 0.026827991 17400 0.112575144 8730 0.034398829 8728
0.077095727 17392 0.050900999 17392 0.044469125
17400 0.049470997 17400 0.207357734 8730 0.020209391 8732
0.081070175 17400 0.049799386 17400 0.065564731
17400 0.013299764 17400 0.123736347 8740 0.010715594 8736
0.078330377 17408 0.064280081 17408 0.084004983
17400 0.067007517 17400 0.066482178 8740 0.00451878 8740
0.09417814 17416 0.110596717 17416 0.059716935
17400 0.054515629 17400 0.102641818 8740 0.00962725 8744
0.110730085 17424 0.087200962 17424 0.01428698
17400 0.036571459 17400 0.068028749 8750 0.032310931 8748
0.081634615 17432 0.121341676 17432 0.075610129
17400 0.048725855 17400 0.009831865 8750 0.052231997 8752

0.026247906	17440	0.157962684	17440	0.090068184		
17400	0.050439863	17400	0.099843426	8760	0.034031033	8756
0.02621682	17448	0.089908659	17448	0.054318411		
17500	0.06268538	17500	0.156046299	8760	0.022026523	8760
0.055210749	17456	0.064165033	17456	0.04762127		
17500	0.038720133	17500	0.104441409	8760	0.031179203	8764
0.054939577	17464	0.094921641	17464	0.074990217		
17500	0.109735163	17500	0.035853609	8770	0.028125913	8768
0.058501177	17472	0.081312886	17472	0.062515719		
17500	0.129121633	17500	0.040154824	8770	0.014792387	8772
0.085316591	17480	0.045507684	17480	0.020561014		
17500	0.108178887	17500	0.087155058	8780	0.020005407	8776
0.078496545	17488	0.101497528	17488	0.034697303		
17500	0.062186089	17500	0.106671694	8780	0.020219764	8780
0.091374022	17496	0.043346965	17496	0.025871603		
17500	0.043945376	17500	0.054267279	8780	0.037529597	8784
0.050075145	17504	0.023233055	17504	0.022006383		
17500	0.039848063	17500	0.043843156	8790	0.059959653	8788
0.058268834	17512	0.011859646	17512	0.019610885		
17500	0.022667571	17500	0.024318457	8790	0.058919373	8792
0.104546387	17520	0.037106151	17520	0.031489581		
17500	0.044447468	17500	0.121069796	8800	0.062265208	8796
0.08413176	17528	0.099107609	17528	0.021181009		
17500	0.074155141	17500	0.165312216	8800	0.043432516	8800
0.069406873	17536	0.148285355	17536	0.018517842		
17500	0.080598227	17500	0.146390856	8800	0.009514336	8804
0.085066437	17544	0.103762512	17544	0.046028494		
17600	0.054899494	17600	0.137929935	8810	0.034506495	8808
0.100307334	17552	0.002616083	17552	0.050085229		
17600	0.036177666	17600	0.108664717	8810	0.054166427	8812
0.124257436	17560	0.068707974	17560	0.040491166		
17600	0.03224353	17600	0.071145609	8820	0.026184667	8816
0.093205061	17568	0.090676535	17568	0.025401972		
17600	0.069390917	17600	0.072547045	8820	0.015301064	8820
0.005735896	17576	0.074898824	17576	0.040779316		
17600	0.150806911	17600	0.064916072	8820	0.015331316	8824
0.058299429	17584	0.072430521	17584	0.083003033		
17600	0.111536829	17600	0.03980855	8830	0.009286097	8828
0.049010556	17592	0.09202728	17592	0.102784958		
17600	0.070156006	17600	0.02801421	8830	0.035423193	8832
0.068030859	17600	0.057191854	17600	0.068719121		
17600	0.044019973	17600	0.066290617	8840	0.044939494	8836
0.058233189	17608	0.102040482	17608	0.027275512		
17600	0.045273297	17600	0.094391529	8840	0.033290591	8840
0.023858776	17616	0.151519445	17616	0.057496007		
17600	0.03243444	17600	0.085489657	8840	0.028783357	8844
0.020513582	17624	0.0708359	17624	0.077564058		
17600	0.036621197	17600	0.127641514	8850	0.030859286	8848
0.054472435	17632	0.068951391	17632	0.050864015		
17600	0.10507027	17600	0.16559819	8850	0.03162722	8852
0.097877644	17640	0.120166966	17640	0.017086892		
17600	0.131425564	17600	0.089178706	8860	0.028167664	8856
0.073288444	17648	0.10245444	17648	0.010848036		
17700	0.111885907	17700	0.039327719	8860	0.07016537	8860
0.03029375	17656	0.070871771	17656	0.034589404		
17700	0.108239605	17700	0.023541321	8860	0.051284646	8864
0.045956582	17664	0.076678291	17664	0.071684255		
17700	0.097694101	17700	0.012889441	8870	0.004257146	8868
0.089216832	17672	0.130431305	17672	0.08874169		
17700	0.089794965	17700	0.082537859	8870	0.025838115	8872
0.087263921	17680	0.10686952	17680	0.078954596		
17700	0.111070549	17700	0.122314974	8880	0.025334959	8876
0.022716084	17688	0.074596552	17688	0.062956387		
17700	0.082418417	17700	0.089678375	8880	0.038975024	8880
0.035440928	17696	0.123916398	17696	0.075689102		
17700	0.088409543	17700	0.022823211	8880	0.028652035	8884
0.098837583	17704	0.107319684	17704	0.091867922		
17700	0.080672937	17700	0.050928135	8890	0.019103249	8888

0.122268917 17712 0.06903035 17712 0.066754656
17700 0.083590741 17700 0.069288857 8890 0.033927827 8892
0.081305123 17720 0.113821508 17720 0.028288876
17700 0.14100477 17700 0.024182407 8900 0.037451937 8896
0.021645064 17728 0.158307434 17728 0.044913009
17700 0.175733629 17700 0.042021668 8900 0.039818129 8900
0.027172409 17736 0.179181734 17736 0.019855388
17700 0.127038729 17700 0.068182279 8900 0.038974315 8904
0.073982585 17744 0.154928043 17744 0.024633066
17800 0.054139538 17800 0.128422645 8910 0.027089507 8908
0.080885184 17752 0.101506055 17752 0.013141812
17800 0.05519297 17800 0.121197663 8910 0.038888753 8912
0.066252076 17760 0.041952302 17760 0.024580533
17800 0.029707147 17800 0.066073779 8920 0.04275937 8916
0.032877186 17768 0.031230582 17768 0.054958557
17800 0.094189301 17800 0.018109206 8920 0.013471197 8920
0.037026584 17776 0.07383449 17776 0.092399387
17800 0.097558128 17800 0.10133817 8920 0.023881292 8924
0.045977107 17784 0.073462012 17784 0.088196983
17800 0.129915439 17800 0.092414251 8930 0.045589884 8928
0.060930885 17792 0.010127546 17792 0.053055683
17800 0.091367721 17800 0.047194197 8930 0.043541139 8932
0.077335724 17800 0.040447496 17800 0.044796459
17800 0.039367686 17800 0.141734636 8940 0.018748842 8936
0.119776247 17808 0.021543288 17808 0.049305414
17800 0.007293064 17800 0.078462181 8940 0.047790905 8940
0.151293046 17816 0.023862971 17816 0.057709567
17800 0.031756565 17800 0.044361288 8940 0.107475295 8944
0.06382 17824 0.053940021 17824 0.035548514
17800 0.032070664 17800 0.07951157 8950 0.101248108 8948
0.031787273 17832 0.099799378 17832 0.012800468
17800 0.151821645 17800 0.121409612 8950 0.039845254 8952
0.075118842 17840 0.099647958 17840 0.036150002
17800 0.212827028 17800 0.065722037 8960 0.037444104 8956
0.092979208 17848 0.100218618 17848 0.039634579
17900 0.198442184 17900 0.06778488 8960 0.038517304 8960
0.003841068 17856 0.104317704 17856 0.0533114
17900 0.141018536 17900 0.135003705 8960 0.039355615 8964
0.071352224 17864 0.128320259 17864 0.050469971
17900 0.096483178 17900 0.148980936 8970 0.058051723 8968
0.076279612 17872 0.11209341 17872 0.044537072
17900 0.093854796 17900 0.160502489 8970 0.072269861 8972
0.060129492 17880 0.069746551 17880 0.036228696
17900 0.044569839 17900 0.18916151 8980 0.084233361 8976
0.047397305 17888 0.075839409 17888 0.02251793
17900 0.137169685 17900 0.18162503 8980 0.095211195 8980
0.082103063 17896 0.09238386 17896 0.023800032
17900 0.174956469 17900 0.170631756 8980 0.1109691 8984
0.068043039 17904 0.09236364 17904 0.035845082
17900 0.179311304 17900 0.197134999 8990 0.097071272 8988
0.062297215 17912 0.145611164 17912 0.02089639
17900 0.125974664 17900 0.178391318 8990 0.070194918 8992
0.00863634 17920 0.13750649 17920 0.02567833
17900 0.019932813 17900 0.101193589 9000 0.067323286 8996
0.053934775 17928 0.109446548 17928 0.063399159
17900 0.049306585 17900 0.13363389 9000 0.061322229 9000
0.032126744 17936 0.073649033 17936 0.05905248
17900 0.102982645 17900 0.123217047 9000 0.056951547 9004
0.02629341 17944 0.020627644 17944 0.049200327
18000 0.221309063 18000 0.084183135 9010 0.0529108 9008
0.036427111 17952 0.04587981 17952 0.049430855
18000 0.195013229 18000 0.088324217 9010 0.015256578 9012
0.084844032 17960 0.049914852 17960 0.017796125
18000 0.068108457 18000 0.125378065 9020 0.013699659 9016
0.063366177 17968 0.044479191 17968 0.020755706
18000 0.11622742 18000 0.18800229 9020 0.027089092 9020
0.050375176 17976 0.058291378 17976 0.018682491
18000 0.064747961 18000 0.211296545 9020 0.042342639 9024

0.061092862 17984 0.05959241 17984 0.028792576
18000 0.050683582 18000 0.176880101 9030 0.008313332 9028
0.043000775 17992 0.064052299 17992 0.049763108
18000 0.040809537 18000 0.14107069 9030 0.057134861 9032
0.018590528 18000 0.114316725 18000 0.044173303
18000 0.032671378 18000 0.11294437 9040 0.054476634 9036
0.033492568 18008 0.163564226 18008 0.013974525
18000 0.018586925 18000 0.051382991 9040 0.029090919 9040
0.048897036 18016 0.142909499 18016 0.015557333
18000 0.086147738 18000 0.067999936 9040 0.018909384 9044
0.031762535 18024 0.04484089 18024 0.021129603
18000 0.179122508 18000 0.05196338 9050 0.046287052 9048
0.028166678 18032 0.026533466 18032 0.016259442
18000 0.163489735 18000 0.037735499 9050 0.022447937 9052
0.007316249 18040 0.01989141 18040 0.011358016
18000 0.122933154 18000 0.078314341 9060 0.009689433 9056
0.031798347 18048 0.012367389 18048 0.016461885
18100 0.152183493 18100 0.12353067 9060 0.024192794 9060
0.027321981 18056 0.065996668 18056 0.010421627
18100 0.121770987 18100 0.136409538 9060 0.011070631 9064
0.007697859 18064 0.116672331 18064 0.048491365
18100 0.057934209 18100 0.072774368 9070 0.050570161 9068
0.013513797 18072 0.11033885 18072 0.037095892
18100 0.063616171 18100 0.020890522 9070 0.058020236 9072
0.012159732 18080 0.083428524 18080 0.044958684
18100 0.068585738 18100 0.047305151 9080 0.029216719 9076
0.033444921 18088 0.082364895 18088 0.068796937
18100 0.015132822 18100 0.096325151 9080 0.034404005 9080
0.050154449 18096 0.068375259 18096 0.019439918
18100 0.05711363 18100 0.091164751 9080 0.022043221 9084
0.042420619 18104 0.072194758 18104 0.039934497
18100 0.052996889 18100 0.024382962 9090 0.00395372 9088
0.019494491 18112 0.088505869 18112 0.060776612
18100 0.052480878 18100 0.070521506 9090 0.016885429 9092
0.035624107 18120 0.085067411 18120 0.052777676
18100 0.083444633 18100 0.127269901 9100 0.030102679 9096
0.066406712 18128 0.039360439 18128 0.029421837
18100 0.08875833 18100 0.14481532 9100 0.027752925 9100
0.077740479 18136 0.045290613 18136 0.025920987
18100 0.114553892 18100 0.102705562 9100 0.080134727 9104
0.011878329 18144 0.033816832 18144 0.02220277
18200 0.133011825 18200 0.042803491 9110 0.110366491 9108
0.079541875 18152 0.042789718 18152 0.022822675
18200 0.098085649 18200 0.046714107 9110 0.061513201 9112
0.10583597 18160 0.122628189 18160 0.023891425
18200 0.128094558 18200 0.056994453 9120 0.018550816 9116
0.101043675 18168 0.172024461 18168 0.01496601
18200 0.165144069 18200 0.140258475 9120 0.064198772 9120
0.071415874 18176 0.174617555 18176 0.023372637
18200 0.133546695 18200 0.102681624 9120 0.071848066 9124
0.015191034 18184 0.126462095 18184 0.021676837
18200 0.130290616 18200 0.072230017 9130 0.065216991 9128
0.034571487 18192 0.099307981 18192 0.034262987
18200 0.130851768 18200 0.114496826 9130 0.043434564 9132
0.020806177 18200 0.08667996 18200 0.058262504
18200 0.086941967 18200 0.14109732 9140 0.046327263 9136
0.015776828 18208 0.057562611 18208 0.058323436
18200 0.130848363 18200 0.123335543 9140 0.037742851 9140
0.059193557 18216 0.100624064 18216 0.03269326
18200 0.077683457 18200 0.092070768 9140 0.069467838 9144
0.077456127 18224 0.092169517 18224 0.052883956
18200 0.04180094 18200 0.108998858 9150 0.105849329 9148
0.069962109 18232 0.062829065 18232 0.039138333
18200 0.041556588 18200 0.092429385 9150 0.079501959 9152
0.066952103 18240 0.101646219 18240 0.006257143
18200 0.038511829 18200 0.046106277 9160 0.039357885 9156
0.051864987 18248 0.126620843 18248 0.047696227
18300 0.133685273 18300 0.039437757 9160 0.014019774 9160

0.109054723 18256 0.049079441 18256 0.075510368
18300 0.197036876 18300 0.040046281 9160 0.041280098 9164
0.140132775 18264 0.043686956 18264 0.077472585
18300 0.172782399 18300 0.069130387 9170 0.066145418 9168
0.173719541 18272 0.144712598 18272 0.063227504
18300 0.041027812 18300 0.069223541 9170 0.033176868 9172
0.167416613 18280 0.233362531 18280 0.060963117
18300 0.098036027 18300 0.02256277 9180 0.049525817 9176
0.099098936 18288 0.184041666 18288 0.069862064
18300 0.136559684 18300 0.004392135 9180 0.078685538 9180
0.056525692 18296 0.025895717 18296 0.050026167
18300 0.097894677 18300 0.043641543 9180 0.063338033 9184
0.021124246 18304 0.134381204 18304 0.014386735
18300 0.072692441 18300 0.097934295 9190 0.039587048 9188
0.058758284 18312 0.117087511 18312 0.04946817
18300 0.102636688 18300 0.10608005 9190 0.02296118 9192
0.074269949 18320 0.076469056 18320 0.032165874
18300 0.092932824 18300 0.105518651 9200 0.039802053 9196
0.038185073 18328 0.094524192 18328 0.019432366
18300 0.076119439 18300 0.071680181 9200 0.026508098 9200
0.015216508 18336 0.070462324 18336 0.040425894
18300 0.094888266 18300 0.058689257 9200 0.056688987 9204
0.043310967 18344 0.053310138 18344 0.081340848
18400 0.100113372 18400 0.095716918 9210 0.041154606 9208
0.072969953 18352 0.020667247 18352 0.064183565
18400 0.105701969 18400 0.108146618 9210 0.058588212 9212
0.069263377 18360 0.036346835 18360 0.02612738
18400 0.094298499 18400 0.097562865 9220 0.053507371 9216
0.04998911 18368 0.105180625 18368 0.019352025
18400 0.113234069 18400 0.095319694 9220 0.030042198 9220
0.054891385 18376 0.197833113 18376 0.005169753
18400 0.151271845 18400 0.093570467 9220 0.029271507 9224
0.060910588 18384 0.242743729 18384 0.045915695
18400 0.150923938 18400 0.102575767 9230 0.032827298 9228
0.028000441 18392 0.242190378 18392 0.067267807
18400 0.117213924 18400 0.056458557 9230 0.0460339 9232
0.035617559 18400 0.194689128 18400 0.057110061
18400 0.113297843 18400 0.021841845 9240 0.059793889 9236
0.079763573 18408 0.081936196 18408 0.056106874
18400 0.131449764 18400 0.081621081 9240 0.060404818 9240
0.062433952 18416 0.022513033 18416 0.057805479
18400 0.103347025 18400 0.132784335 9240 0.041291212 9244
0.033992794 18424 0.060127572 18424 0.048633236
18400 0.090848393 18400 0.18829151 9250 0.025020476 9248
0.053740067 18432 0.092101065 18432 0.033266446
18400 0.086853121 18400 0.211912324 9250 0.011675134 9252
0.039177809 18440 0.13054187 18440 0.053440283
18400 0.030892832 18400 0.161717413 9260 0.057908645 9256
0.047025074 18448 0.139306401 18448 0.040808096
18500 0.095171876 18500 0.128475819 9260 0.070890928 9260
0.053150172 18456 0.145537255 18456 0.024858644
18500 0.114252754 18500 0.125352875 9260 0.041830579 9264
0.034001849 18464 0.108225766 18464 0.044989913
18500 0.124755999 18500 0.089587222 9270 0.025364998 9268
0.026849404 18472 0.051415769 18472 0.068564994
18500 0.140617616 18500 0.083193336 9270 0.044124376 9272
0.048547325 18480 0.060409759 18480 0.071923714
18500 0.027427168 18500 0.071018032 9280 0.063903623 9276
0.074532443 18488 0.078608951 18488 0.051261264
18500 0.133398396 18500 0.092216156 9280 0.066616718 9280
0.096436641 18496 0.072359515 18496 0.026241929
18500 0.137075389 18500 0.138448711 9280 0.056034118 9284
0.069325441 18504 0.01828363 18504 0.028904173
18500 0.097671495 18500 0.120440192 9290 0.028246332 9288
0.02132893 18512 0.046645797 18512 0.030560212
18500 0.089947498 18500 0.048329079 9290 0.029767894 9292
0.021982787 18520 0.026019319 18520 0.040581734
18500 0.056316563 18500 0.027827369 9300 0.037427955 9296

```
0.013761471	18528	0.094477378	18528	0.066632609
18500	0.068429188	18500	0.062773317	9300	0.057198009	9300
0.061165782	18536	0.061244493	18536	0.031527448
18500	0.091379334	18500	0.071644914	9300	0.0314354	9304
0.046885394	18544	0.011551589	18544	0.045254124
18600	0.078595083	18600	0.040711941	9310	0.049490933	9308
0.034060853	18552	0.014082079	18552	0.04028406
18600	0.094487943	18600	0.036748465	9310	0.036906949	9312
0.067623048	18560	0.08703761	18560	0.017435426
18600	0.13548191	18600	0.060070961	9320	0.037269088	9316
0.072972245	18568	0.11207979	18568	0.036750167
18600	0.060878087	18600	0.04722676	9320	0.053318723	9320
0.009698898	18576	0.161911695	18576	0.035861329
18600	0.112770242	18600	0.135858718	9320	0.034312241	9324
0.053246451	18584	0.118607844	18584	0.038137077
18600	0.153983813	18600	0.11100198	9330	0.033079879	9328
0.075726952	18592	0.057586487	18592	0.02261153
18600	0.05375336	18600	0.029195899	9330	0.076593824	9332
0.095617455	18600	0.1449423	18600	0.038227216
18600	0.065718487	18600	0.032064541	9340	0.078756377	9336
0.099949379	18608	0.100068879	18608	0.036822818
18600	0.061108978	18600	0.064082975	9340	0.066514564	9340
0.076479824	18616	0.174451329	18616	0.014267406
18600	0.014238824	18600	0.060114886	9340	0.064025822	9344
0.038122526	18624	0.200773836	18624	0.022390794
18600	0.024004867	18600	0.043890515	9350	0.065997454	9348
0.061262443	18632	0.147017199	18632	0.038671518
18600	0.06376407	18600	0.118993601	9350	0.03941244	9352
0.088277637	18640	0.088965986	18640	0.042496329
18600	0.106036663	18600	0.079649028	9360	0.034163451	9356
0.070624839	18648	0.126891595	18648	0.041616499
18700	0.133664275	18700	0.066240398	9360	0.045691806	9360
0.020970785	18656	0.174868198	18656	0.05864503
18700	0.119875214	18700	0.131063629	9360	0.046111876	9364
0.01748767	18664	0.12145134	18664	0.07227841
18700	0.068206551	18700	0.097215838	9370	0.030748248	9368
0.038971641	18672	0.08036552	18672	0.062565647
18700	0.01413192	18700	0.083339808	9370	0.017320566	9372
0.03995821	18680	0.07550681	18680	0.027363642
18700	0.035494126	18700	0.06462491	9380	0.018437077	9376
0.038703362	18688	0.062542771	18688	0.024663474
18700	0.110654233	18700	0.051346262	9380	0.038992788	9380
0.117639262	18696	0.163231496	18696	0.033744436
18700	0.17170512	18700	0.05363371	9380	0.056034922	9384
0.136993971	18704	0.138014075	18704	0.03487572
18700	0.199045316	18700	0.064207059	9390	0.052440719	9388
0.095752956	18712	0.068823581	18712	0.013502063
18700	0.205105855	18700	0.173097709	9390	0.061770661	9392
0.053014028	18720	0.087948458	18720	0.029254134
18700	0.186496385	18700	0.209969031	9400	0.057345435	9396
0.01334856	18728	0.112250797	18728	0.037641537
18700	0.175293259	18700	0.185032244	9400	0.061342893	9400
0.043150187	18736	0.094836491	18736	0.021355951
18700	0.130492583	18700	0.149637883	9400	0.06656078	9404
0.048389247	18744	0.057428701	18744	0.029675955
18800	0.062854619	18800	0.053188283	9410	0.03099789	9408
0.081775332	18752	0.043812306	18752	0.054144242
18800	0.107376269	18800	0.084478917	9410	0.033282733	9412
0.096636955	18760	0.02779442	18760	0.044796878
18800	0.124115861	18800	0.10993769	9420	0.039609055	9416
0.090011512	18768	0.044238273	18768	0.022461863
18800	0.06994258	18800	0.032716416	9420	0.036603477	9420
0.088134169	18776	0.051567327	18776	0.037992228
18800	0.016057278	18800	0.126385421	9420	0.04920382	9424
0.095501287	18784	0.052090669	18784	0.035386358
18800	0.044248653	18800	0.142327903	9430	0.057929716	9428
0.055115066	18792	0.105484447	18792	0.032486263
18800	0.095737341	18800	0.147834333	9430	0.075077245	9432
```

0.029284947 18800 0.07566897 18800 0.023247245
18800 0.145206795 18800 0.1613684 9440 0.087731642 9436
0.055566168 18808 0.113353934 18808 0.041298394
18800 0.119882789 18800 0.087883876 9440 0.072671734 9440
0.027172888 18816 0.194889203 18816 0.070776543
18800 0.031694493 18800 0.124597107 9440 0.033098091 9444
0.018504987 18824 0.131507316 18824 0.066269837
18800 0.076536642 18800 0.153075133 9450 0.029350989 9448
0.04713512 18832 0.070139744 18832 0.054762128
18800 0.089227062 18800 0.097063828 9450 0.039345636 9452
0.059621172 18840 0.072184557 18840 0.050450613
18800 0.054798689 18800 0.067740686 9460 0.046864192 9456
0.057045443 18848 0.105201114 18848 0.027729608
18900 0.038248636 18900 0.069465474 9460 0.046812533 9460
0.082952523 18856 0.154668916 18856 0.029533765
18900 0.016103186 18900 0.069005546 9460 0.027953653 9464
0.083062296 18864 0.153952686 18864 0.047529386
18900 0.022985396 18900 0.068798021 9470 0.030292737 9468
0.034917528 18872 0.074344382 18872 0.017254655
18900 0.100332683 18900 0.030042023 9470 0.081974984 9472
0.028651946 18880 0.047571499 18880 0.015461912
18900 0.146698964 18900 0.013955796 9480 0.074243595 9476
0.076274839 18888 0.081264356 18888 0.007698191
18900 0.051675503 18900 0.02229977 9480 0.067901179 9480
0.089377703 18896 0.122598111 18896 0.020908508
18900 0.035039368 18900 0.068761954 9480 0.071697185 9484
0.058897105 18904 0.088812274 18904 0.022698119
18900 0.026167158 18900 0.097493881 9490 0.028477043 9488
0.054608299 18912 0.045337474 18912 0.030960779
18900 0.03335184 18900 0.068511501 9490 0.022095146 9492
0.057232726 18920 0.064706568 18920 0.016562468
18900 0.063258056 18900 0.034608893 9500 0.06356099 9496
0.058473634 18928 0.06226571 18928 0.050111885
18900 0.070036156 18900 0.048064685 9500 0.091728682 9500
0.066428176 18936 0.022766559 18936 0.066771419
18900 0.087446708 18900 0.026412066 9500 0.105716521 9504
0.045534205 18944 0.042952008 18944 0.034794244
19000 0.111741065 19000 0.072854244 9510 0.078206838 9508
0.074606884 18952 0.11488652 18952 0.052702821
19000 0.139478594 19000 0.036486246 9510 0.031681342 9512
0.098560551 18960 0.153621819 18960 0.076960983
19000 0.176449918 19000 0.082659601 9520 0.038677536 9516
0.059145637 18968 0.116560877 18968 0.055075092
19000 0.124593396 19000 0.131948124 9520 0.033736247 9520
0.053753553 18976 0.09185703 18976 0.03065382
19000 0.094926319 19000 0.106799656 9520 0.024147255 9524
0.045997738 18984 0.140542106 18984 0.025577485
19000 0.130533415 19000 0.0283836 9530 0.017698898 9528
0.054780154 18992 0.175083551 18992 0.036648071
19000 0.097755605 19000 0.060099967 9530 0.015938027 9532
0.099601733 19000 0.078997793 19000 0.066798821
19000 0.041411899 19000 0.043999859 9540 0.023307717 9536
0.108475986 19008 0.111124347 19008 0.071682887
19000 0.038945727 19000 0.086052234 9540 0.05815093 9540
0.116961957 19016 0.225043841 19016 0.043674703
19000 0.06176535 19000 0.113358721 9540 0.071298055 9544
0.143912155 19024 0.192513733 19024 0.027276021
19000 0.098112025 19000 0.076472825 9550 0.059388876 9548
0.137411596 19032 0.132499481 19032 0.018995577
19000 0.085794585 19000 0.065009277 9550 0.062395353 9552
0.074005846 19040 0.103909777 19040 0.009995312
19000 0.043179836 19000 0.02322 9560 0.07177602 9556
0.049230901 19048 0.072979041 19048 0.032922526
19100 0.130063054 19100 0.085704771 9560 0.043124946 9560
0.066204746 19056 0.021809321 19056 0.034537901
19100 0.125865496 19100 0.112922215 9560 0.017697839 9564
0.057815749 19064 0.099741985 19064 0.035592391
19100 0.097168406 19100 0.137701733 9570 0.020337293 9568

0.071064736 19072 0.085104082 19072 0.038020695
19100 0.112198592 19100 0.155386951 9570 0.026098416 9572
0.131790483 19080 0.037480819 19080 0.030470601
19100 0.147126775 19100 0.145496335 9580 0.057953635 9576
0.153422924 19088 0.043021089 19088 0.023655435
19100 0.169775841 19100 0.107381842 9580 0.085366919 9580
0.134124698 19096 0.052374362 19096 0.007253808
19100 0.19571172 19100 0.030499388 9580 0.0909804 9584
0.101273123 19104 0.066741319 19104 0.004937021
19100 0.138805918 19100 0.103922663 9590 0.082248203 9588
0.052538955 19112 0.055785436 19112 0.02374645
19100 0.008828933 19100 0.181493219 9590 0.061580504 9592
0.022928607 19120 0.027897635 19120 0.044794993
19100 0.035354136 19100 0.160653945 9600 0.056121673 9596
0.079206409 19128 0.060308186 19128 0.062792475
19100 0.157177798 19100 0.056584166 9600 0.069302507 9600
0.090991671 19136 0.131213558 19136 0.07182204
19100 0.240065623 19100 0.065734923 9600 0.058003425 9604
0.052809257 19144 0.154622205 19144 0.065892142
19200 0.214807078 19200 0.115566232 9610 0.055577577 9608
0.030330521 19152 0.079364421 19152 0.064355991
19200 0.125785984 19200 0.112131027 9610 0.091977105 9612
0.05125512 19160 0.041023792 19160 0.053498006
19200 0.051017538 19200 0.068094603 9620 0.101323836 9616
0.076240867 19168 0.09660318 19168 0.037089863
19200 0.034191588 19200 0.018565759 9620 0.069733942 9620
0.125411229 19176 0.134390678 19176 0.030074931
19200 0.007074962 19200 0.028926799 9620 0.026345231 9624
0.124103957 19184 0.143935511 19184 0.024600007
19200 0.055056113 19200 0.076128563 9630 0.062038685 9628
0.07047534 19192 0.094733143 19192 0.018599425
19200 0.117079435 19200 0.091233022 9630 0.09813386 9632
0.01736871 19200 0.048483435 19200 0.014772346
19200 0.109419816 19200 0.100484613 9640 0.10979276 9636
0.030942891 19208 0.032318443 19208 0.023847329
19200 0.032328935 19200 0.121087593 9640 0.105874293 9640
0.063755622 19216 0.046701738 19216 0.037688078
19200 0.021746915 19200 0.081434708 9640 0.072241084 9644
0.08407062 19224 0.119548902 19224 0.042750147
19200 0.017997571 19200 0.040392402 9650 0.047931655 9648
0.087972716 19232 0.119654491 19232 0.044732373
19200 0.018645766 19200 0.085745516 9650 0.051310963 9652
0.055172397 19240 0.08982273 19240 0.050139952
19200 0.076507386 19200 0.065245957 9660 0.059010286 9656
0.025268388 19248 0.058135243 19248 0.054412569
19300 0.186227874 19300 0.093292394 9660 0.044265435 9660
0.105839732 19256 0.041369804 19256 0.043737618
19300 0.24570874 19300 0.098972501 9660 0.036997364 9664
0.14465471 19264 0.127882071 19264 0.020071957
19300 0.133179653 19300 0.085953878 9670 0.067938105 9668
0.129487045 19272 0.124154118 19272 0.027268063
19300 0.090401663 19300 0.113634589 9670 0.092422626 9672
0.085114531 19280 0.083840227 19280 0.059325936
19300 0.167374863 19300 0.066884262 9680 0.072308059 9676
0.030466084 19288 0.085564308 19288 0.042252646
19300 0.130354573 19300 0.032044223 9680 0.041594514 9680
0.060100236 19296 0.113269874 19296 0.007418119
19300 0.088409652 19300 0.064228574 9680 0.052489668 9684
0.063443433 19304 0.108478831 19304 0.011998701
19300 0.034608864 19300 0.082444734 9690 0.069521477 9688
0.040055333 19312 0.089499779 19312 0.032087304
19300 0.026735028 19300 0.048795948 9690 0.0758061 9692
0.018078004 19320 0.046607907 19320 0.056852186
19300 0.068254667 19300 0.06628647 9700 0.061570157 9696
0.025526149 19328 0.029495992 19328 0.066795714
19300 0.054135799 19300 0.068157395 9700 0.03667405 9700
0.025594007 19336 0.041679141 19336 0.051349911
19300 0.026534655 19300 0.064559616 9700 0.047738533 9704

0.01153651	19344	0.017899958	19344	0.052991731		
19400	0.053386295	19400	0.079411409	9710	0.081039347	9708
0.0441144	19352	0.046038404	19352	0.070274116		
19400	0.108703243	19400	0.112988128	9710	0.077212622	9712
0.033050419	19360	0.079593854	19360	0.034819215		
19400	0.164872151	19400	0.126890911	9720	0.042059241	9716
0.013125309	19368	0.02738227	19368	0.02638023		
19400	0.171498993	19400	0.078554898	9720	0.027468168	9720
0.021141654	19376	0.067943678	19376	0.059719932		
19400	0.115129682	19400	0.07403858	9720	0.073926734	9724
0.005357312	19384	0.098229932	19384	0.037208181		
19400	0.093861811	19400	0.120759607	9730	0.081364065	9728
0.019301511	19392	0.084971151	19392	0.039603194		
19400	0.148228544	19400	0.109653527	9730	0.053571279	9732
0.029546773	19400	0.067870409	19400	0.03415639		
19400	0.10426544	19400	0.067670684	9740	0.040781473	9736
0.032470583	19408	0.114498318	19408	0.020658217		
19400	0.081097649	19400	0.089674075	9740	0.029368688	9740
0.046173209	19416	0.19287612	19416	0.052767726		
19400	0.122431025	19400	0.11600864	9740	0.032184875	9744
0.071568473	19424	0.197541216	19424	0.045862547		
19400	0.102910373	19400	0.072882074	9750	0.052450592	9748
0.049647457	19432	0.159413699	19432	0.023625917		
19400	0.087080873	19400	0.018527082	9750	0.070810282	9752
0.039940343	19440	0.09133594	19440	0.00635815		
19400	0.169251624	19400	0.093845323	9760	0.084989959	9756
0.069775866	19448	0.095788179	19448	0.044035249		
19500	0.098254852	19500	0.092842252	9760	0.068751921	9760
0.062322572	19456	0.060327282	19456	0.052988919		
19500	0.040366391	19500	0.035013811	9760	0.045247198	9764
0.081368395	19464	0.051355677	19464	0.004261431		
19500	0.064722764	19500	0.021630476	9770	0.060454051	9768
0.09477089	19472	0.075241871	19472	0.035467645		
19500	0.077232624	19500	0.065184788	9770	0.075922966	9772
0.058286758	19480	0.084054496	19480	0.022530152		
19500	0.120017132	19500	0.071769049	9780	0.073267591	9776
0.042358384	19488	0.092656366	19488	0.019365332		
19500	0.102548678	19500	0.053409025	9780	0.058186059	9780
0.030046811	19496	0.075016702	19496	0.04236896		
19500	0.088766574	19500	0.037969312	9780	0.051997446	9784
0.048030663	19504	0.040437273	19504	0.058250469		
19500	0.050411043	19500	0.057363348	9790	0.069467053	9788
0.094971438	19512	0.11175689	19512	0.076823199		
19500	0.027252951	19500	0.060095303	9790	0.073615993	9792
0.091476315	19520	0.160762458	19520	0.067893503		
19500	0.04908086	19500	0.109887915	9800	0.042376134	9796
0.072393399	19528	0.228523073	19528	0.027411617		
19500	0.059874372	19500	0.165927311	9800	0.027963641	9800
0.021176767	19536	0.221796494	19536	0.03368366		
19500	0.07282658	19500	0.182458753	9800	0.040088289	9804
0.028038128	19544	0.131029883	19544	0.070514681		
19600	0.036454294	19600	0.153136891	9810	0.052813699	9808
0.022451919	19552	0.149064901	19552	0.059685062		
19600	0.099150806	19600	0.081130413	9810	0.071804556	9812
0.019581501	19560	0.161164819	19560	0.030116638		
19600	0.180275441	19600	0.014748052	9820	0.074185722	9816
0.049448543	19568	0.064529835	19568	0.037552974		
19600	0.110251305	19600	0.00472615	9820	0.063996165	9820
0.07446199	19576	0.032432654	19576	0.0416488		
19600	0.067680121	19600	0.022075459	9820	0.054603799	9824
0.060371775	19584	0.051642099	19584	0.043854387		
19600	0.058418562	19600	0.051673571	9830	0.047132522	9828
0.040553368	19592	0.042112566	19592	0.041569118		
19600	0.047734367	19600	0.038103255	9830	0.031450109	9832
0.027263101	19600	0.024039753	19600	0.023172948		
19600	0.075027609	19600	0.042845652	9840	0.013371915	9836
0.035543257	19608	0.042869611	19608	0.041602241		
19600	0.075117285	19600	0.062238243	9840	0.025012732	9840

0.027767603 19616 0.086745007 19616 0.063427906
19600 0.073722549 19600 0.025083222 9840 0.026343483 9844
0.090516078 19624 0.137208583 19624 0.028514578
19600 0.048139838 19600 0.049163842 9850 0.032467393 9848
0.121704667 19632 0.165136968 19632 0.053787961
19600 0.068213463 19600 0.042673528 9850 0.028414341 9852
0.095326832 19640 0.135905503 19640 0.0681116
19600 0.135022666 19600 0.030873358 9860 0.030689123 9856
0.057045585 19648 0.096951575 19648 0.020499316
19700 0.139010925 19700 0.042895645 9860 0.035425132 9860
0.070157716 19656 0.0882729 19656 0.063587933
19700 0.055043904 19700 0.067623711 9860 0.022507848 9864
0.106399166 19664 0.072638373 19664 0.048646623
19700 0.014496593 19700 0.079793266 9870 0.02719694 9868
0.090555979 19672 0.094339033 19672 0.014365474
19700 0.077765515 19700 0.030064332 9870 0.008379164 9872
0.028849534 19680 0.099090321 19680 0.042692856
19700 0.114539776 19700 0.083068197 9880 0.040998813 9876
0.035505094 19688 0.058001755 19688 0.043757449
19700 0.097537006 19700 0.099882978 9880 0.02611341 9880
0.065660046 19696 0.016005863 19696 0.00739251
19700 0.101162506 19700 0.069415488 9880 0.032259108 9884
0.045089088 19704 0.044993958 19704 0.029447172
19700 0.084529565 19700 0.120854515 9890 0.064065163 9888
0.065787557 19712 0.118191594 19712 0.015098069
19700 0.028676994 19700 0.142067801 9890 0.058762842 9892
0.094032446 19720 0.164880941 19720 0.02356263
19700 0.051720861 19700 0.117741314 9900 0.036077232 9896
0.074663039 19728 0.123707723 19728 0.03046004
19700 0.076342723 19700 0.03824876 9900 0.051864376 9900
0.060053968 19736 0.064729466 19736 0.022203261
19700 0.077343721 19700 0.020310421 9900 0.086687804 9904
0.052321815 19744 0.115169511 19744 0.00606254
19800 0.097167002 19800 0.006811612 9910 0.084817337 9908
0.056541059 19752 0.092285023 19752 0.058506241
19800 0.118319775 19800 0.056296012 9910 0.038704919 9912
0.072185329 19760 0.047722322 19760 0.072905241
19800 0.112001289 19800 0.089617715 9920 0.038027985 9916
0.074300217 19768 0.044580425 19768 0.028542238
19800 0.123672653 19800 0.124589293 9920 0.036496367 9920
0.070284812 19776 0.098063938 19776 0.041316631
19800 0.157077448 19800 0.16372076 9920 0.016698637 9924
0.058583439 19784 0.141120793 19784 0.047475929
19800 0.139900701 19800 0.171429885 9930 0.032470733 9928
0.054488828 19792 0.13315244 19792 0.031520693
19800 0.08411723 19800 0.143045808 9930 0.031754833 9932
0.051082127 19800 0.104711944 19800 0.014057708
19800 0.112949325 19800 0.094163093 9940 0.024358822 9936
0.032583994 19808 0.072466508 19808 0.021378024
19800 0.178082148 19800 0.130977904 9940 0.057610574 9940
0.045668767 19816 0.041233481 19816 0.030799809
19800 0.194990833 19800 0.147920583 9940 0.036502352 9944
0.06269075 19824 0.052696501 19824 0.030955431
19800 0.164343815 19800 0.115670446 9950 0.012769586 9948
0.044275599 19832 0.06630837 19832 0.034033641
19800 0.100285419 19800 0.118313335 9950 0.012005722 9952
0.072463939 19840 0.134350834 19840 0.017048724
19800 0.037356786 19800 0.087842884 9960 0.028626009 9956
0.099978635 19848 0.187351499 19848 0.024149929
19900 0.018443956 19900 0.055784669 9960 0.003841212 9960
0.082591374 19856 0.12467122 19856 0.047473983
19900 0.052401694 19900 0.092446971 9960 0.074668751 9964
0.056222052 19864 0.028290218 19864 0.061427098
19900 0.024333012 19900 0.117331896 9970 0.11712662 9968
0.043293432 19872 0.102910832 19872 0.066398417
19900 0.037542519 19900 0.107558357 9970 0.086702828 9972
0.04733857 19880 0.111099653 19880 0.073560637
19900 0.046052115 19900 0.170677711 9980 0.046937403 9976

0.051224717 19888 0.123202175 19888 0.073903248
19900 0.073483665 19900 0.111466106 9980 0.056667388 9980
0.077962308 19896 0.135520983 19896 0.054139702
19900 0.182754608 19900 0.060453152 9980 0.048847087 9984
0.096837939 19904 0.148617343 19904 0.03206464
19900 0.204081633 19900 0.018748633 9990 0.023823362 9988
0.095248033 19912 0.152234469 19912 0.049210954
19900 0.117239411 19900 0.029225434 9990 0.028991492 9992
0.070578491 19920 0.066218468 19920 0.062805448
19900 0.054140219 19900 0.048426882 10000 0.034040899 9996
0.041955293 19928 0.064495405 19928 0.046051486
19900 0.056146993 19900 0.156116366 10000 0.018515802 10000
0.035014531 19936 0.101948266 19936 0.017744744
19900 0.065525346 19900 0.151921428 10000 0.033972541 10004
0.039308921 19944 0.098462944 19944 0.039127775
20000 0.11512205 20000 0.059058504 10000 0.056385525 10008
0.036244568 19952 0.105184765 19952 0.067851855
20000 0.087181761 20000 0.024965948 10000 0.081943079 10012
0.017114358 19960 0.087096712 19960 0.060576094
20000 0.08309228 20000 0.040841849 10000 0.101540034 10016
0.024919036 19968 0.053197349 19968 0.017680193
20000 0.079906931 20000 0.015724563 10000 0.085717948 10020
0.045298169 19976 0.064031614 19976 0.03494872
20000 0.06771319 20000 0.036685866 10000 0.056984696 10024
0.064102445 19984 0.045774916 19984 0.034711025
20000 0.041626277 20000 0.036732414 10000 0.055611821 10028
0.057898418 19992 0.055459532 19992 0.043687629
20000 0.054252407 20000 0.001460894 10000 0.048586859 10032
0.023727542 20000 0.098621495 20000 0.062688137
20000 0.052392919 20000 0.049843999 10000 0.057962276 10036
0.020597265 20008 0.121113197 20008 0.078465739
20000 0.045763951 20000 0.107119653 10000 0.082367442 10040
0.023300254 20016 0.087749446 20016 0.09231412
20000 0.077550874 20000 0.165205769 10000 0.084413267 10044
0.014141253 20024 0.049970575 20024 0.073848321
20000 0.04098874 20000 0.19872062 10000 0.047450631 10048
0.02886043 20032 0.084061852 20032 0.030928219
20000 0.082912702 20000 0.192883265 10100 0.065349654 10052
0.016976064 20040 0.096626274 20040 0.019488207
20000 0.09451068 20000 0.168949089 10100 0.055854427 10056
0.070769696 20048 0.158969619 20048 0.019730805
20100 0.08779965 20100 0.145447354 10100 0.039947514 10060
0.090114336 20056 0.169448176 20056 0.01969876
20100 0.055619636 20100 0.091613249 10100 0.010134447 10064
0.063889216 20064 0.093219052 20064 0.033961656
20100 0.099506935 20100 0.025159176 10100 0.054923654 10068
0.040906383 20072 0.079339254 20072 0.010408955
20100 0.207499761 20100 0.010370946 10100 0.085025342 10072
0.047606445 20080 0.145277474 20080 0.033183886
20100 0.219286245 20100 0.047424514 10100 0.107761502 10076
0.049925053 20088 0.167716949 20088 0.047548649
20100 0.145278449 20100 0.057798436 10100 0.106802858 10080
0.019382078 20096 0.095768388 20096 0.051769537
20100 0.097114666 20100 0.030181203 10100 0.066039669 10084
0.050136856 20104 0.044159566 20104 0.038211489
20100 0.074880285 20100 0.042640131 10100 0.025531273 10088
0.07110554 20112 0.028898796 20112 0.027382193
20100 0.029390023 20100 0.045628509 10100 0.03801419 10092
0.048815899 20120 0.05938235 20120 0.008411104
20100 0.04339248 20100 0.089862711 10100 0.042234224 10096
0.010922335 20128 0.1514904 20128 0.022400667
20100 0.079846715 20100 0.17680446 10100 0.033571458 10100
0.048701339 20136 0.163335906 20136 0.028291015
20100 0.120612152 20100 0.151961664 10100 0.038009686 10104
0.095347379 20144 0.090336296 20144 0.043673856
20200 0.113584378 20200 0.053000898 10100 0.045712004 10108
0.076249569 20152 0.088860812 20152 0.047507194
20200 0.084853986 20200 0.064234708 10100 0.025946805 10112

0.038282917 20160 0.16347703 20160 0.035552002
20200 0.076191347 20200 0.117043586 10100 0.011820915 10116
0.024339483 20168 0.163570629 20168 0.034085268
20200 0.093661824 20200 0.118650918 10100 0.010287173 10120
0.016843322 20176 0.094027084 20176 0.050315128
20200 0.161315329 20200 0.094030896 10100 0.013729162 10124
0.022272643 20184 0.049240629 20184 0.048922299
20200 0.184825083 20200 0.048964688 10100 0.009779545 10128
0.023020612 20192 0.113400194 20192 0.003235834
20200 0.118409116 20200 0.031011648 10100 0.004095353 10132
0.041295007 20200 0.119332101 20200 0.046197987
20200 0.080931546 20200 0.061269755 10100 0.041869294 10136
0.045135392 20208 0.086466222 20208 0.043153137
20200 0.082556457 20200 0.079969708 10100 0.050710984 10140
0.011140962 20216 0.086945423 20216 0.010946496
20200 0.089688365 20200 0.150008215 10100 0.010297696 10144
0.011538046 20224 0.126608313 20224 0.028042266
20200 0.084974425 20200 0.173801876 10100 0.033616176 10148
0.016139436 20232 0.120591991 20232 0.01368181
20200 0.065141627 20200 0.131325491 10200 0.035736648 10152
0.010414142 20240 0.042368192 20240 0.02766585
20200 0.085848609 20200 0.061173319 10200 0.035732155 10156
0.070476126 20248 0.073922907 20248 0.031948424
20300 0.121895471 20300 0.093420516 10200 0.054731696 10160
0.082822444 20256 0.098086697 20256 0.03440736
20300 0.086384112 20300 0.120367047 10200 0.034291021 10164
0.063800893 20264 0.125269799 20264 0.05098011
20300 0.025549007 20300 0.097951575 10200 0.028647817 10168
0.084330306 20272 0.118510325 20272 0.082813655
20300 0.056102192 20300 0.023321061 10200 0.035358891 10172
0.101367681 20280 0.004500889 20280 0.086287641
20300 0.044604185 20300 0.107769927 10200 0.038628434 10176
0.070901479 20288 0.108743843 20288 0.029359157
20300 0.025422063 20300 0.216278015 10200 0.030597996 10180
0.034621895 20296 0.07692098 20296 0.028537028
20300 0.036643505 20300 0.207559656 10200 0.020080923 10184
0.016823728 20304 0.036194622 20304 0.007319506
20300 0.047863567 20300 0.153743153 10200 0.049211965 10188
0.040262439 20312 0.05767656 20312 0.046964204
20300 0.153742396 20300 0.138149276 10200 0.068254893 10192
0.010716465 20320 0.05836174 20320 0.062331412
20300 0.191318206 20300 0.105991181 10200 0.051922005 10196
0.059875398 20328 0.081425562 20328 0.041384676
20300 0.135492854 20300 0.073292242 10200 0.041590589 10200
0.05391593 20336 0.06065657 20336 0.025683017
20300 0.07587421 20300 0.064751162 10200 0.007637359 10204
0.031201595 20344 0.01120368 20344 0.017774148
20400 0.065321503 20400 0.078393648 10200 0.057990201 10208
0.027739287 20352 0.037686346 20352 0.025436157
20400 0.034898789 20400 0.11145469 10200 0.063650055 10212
0.005736065 20360 0.054250169 20360 0.046060326
20400 0.047873316 20400 0.166683123 10200 0.057721256 10216
0.018922277 20368 0.092335053 20368 0.016950786
20400 0.12636013 20400 0.162632278 10200 0.070970047 10220
0.029395435 20376 0.128932501 20376 0.0417144
20400 0.170303814 20400 0.106962929 10200 0.108605957 10224
0.003486527 20384 0.139521071 20384 0.069107831
20400 0.1023234 20400 0.05598896 10200 0.108020468 10228
0.038555099 20392 0.119599499 20392 0.052951687
20400 0.056012261 20400 0.043179796 10200 0.048669972 10232
0.088730107 20400 0.145161889 20400 0.034119676
20400 0.137415496 20400 0.099556957 10200 0.027299815 10236
0.088766305 20408 0.147703816 20408 0.046451678
20400 0.142437551 20400 0.137823314 10200 0.016928239 10240
0.094062729 20416 0.115422554 20416 0.056540604
20400 0.060100301 20400 0.09010471 10200 0.037097936 10244
0.074198011 20424 0.109363857 20424 0.04503359
20400 0.065340515 20400 0.119089957 10200 0.032943921 10248

0.007133528 20432 0.122077821 20432 0.05511006
20400 0.093938179 20400 0.116545212 10300 0.029490244 10252
0.036433688 20440 0.146824051 20440 0.094154937
20400 0.061317485 20400 0.100241821 10300 0.034030672 10256
0.088531684 20448 0.082674385 20448 0.107113112
20500 0.082072795 20500 0.089437635 10300 0.034087028 10260
0.083350584 20456 0.063209875 20456 0.069931892
20500 0.121330282 20500 0.123916179 10300 0.024601057 10264
0.05311118 20464 0.138691685 20464 0.027797609
20500 0.093837465 20500 0.123555292 10300 0.057967191 10268
0.031993244 20472 0.144837351 20472 0.017744844
20500 0.057560006 20500 0.100861158 10300 0.010921034 10272
0.016982467 20480 0.091887356 20480 0.009726332
20500 0.067225861 20500 0.117113319 10300 0.077617246 10276
0.043896103 20488 0.042760799 20488 0.031517924
20500 0.051378258 20500 0.148284904 10300 0.08898916 10280
0.074579948 20496 0.128749089 20496 0.05266193
20500 0.091316048 20500 0.161849909 10300 0.050373776 10284
0.012801885 20504 0.18776457 20504 0.042844855
20500 0.10161219 20500 0.12161777 10300 0.054725922 10288
0.055550041 20512 0.121721532 20512 0.020435176
20500 0.037965445 20500 0.109973691 10300 0.040846582 10292
0.045881952 20520 0.031523679 20520 0.010292047
20500 0.141863333 20500 0.169323641 10300 0.051026214 10296
0.05018133 20528 0.019277461 20528 0.023396682
20500 0.16864491 20500 0.189973056 10300 0.056758028 10300
0.064215448 20536 0.026657215 20536 0.02529516
20500 0.107578584 20500 0.134442656 10300 0.038948718 10304
0.045102876 20544 0.062274376 20544 0.026894271
20600 0.102525606 20600 0.098442964 10300 0.042693449 10308
0.07031687 20552 0.142060686 20552 0.017288367
20600 0.082736478 20600 0.078675614 10300 0.0630625 10312
0.100093406 20560 0.12165965 20560 0.028866507
20600 0.024045346 20600 0.049244263 10300 0.070318885 10316
0.131749534 20568 0.063703359 20568 0.060885031
20600 0.090070156 20600 0.105264859 10300 0.059289643 10320
0.141649144 20576 0.06335631 20576 0.065942077
20600 0.088794121 20600 0.039143306 10300 0.061432496 10324
0.105846681 20584 0.081795479 20584 0.053722106
20600 0.10648898 20600 0.096037496 10300 0.059005917 10328
0.060197788 20592 0.058683541 20592 0.049720999
20600 0.10478581 20600 0.154246154 10300 0.052256564 10332
0.037901878 20600 0.067465968 20600 0.04428604
20600 0.0936336 20600 0.141137265 10300 0.053818083 10336
0.078968689 20608 0.062371284 20608 0.033203069
20600 0.090505004 20600 0.156333044 10300 0.055673532 10340
0.059511494 20616 0.056204612 20616 0.016873857
20600 0.042985677 20600 0.100182399 10300 0.048577502 10344
0.01779465 20624 0.023095079 20624 0.065312408
20600 0.080082027 20600 0.009592311 10300 0.034008623 10348
0.021438524 20632 0.066439949 20632 0.078356708
20600 0.079867037 20600 0.032835749 10400 0.027131717 10352
0.018422106 20640 0.103504368 20640 0.046109733
20600 0.126475119 20600 0.055880399 10400 0.021277243 10356
0.071325587 20648 0.135813214 20648 0.023302833
20700 0.163220655 20700 0.06365298 10400 0.050183367 10360
0.082003833 20656 0.162605328 20656 0.013000338
20700 0.114503455 20700 0.047574959 10400 0.039885174 10364
0.063034982 20664 0.081227576 20664 0.036554447
20700 0.058712118 20700 0.060315259 10400 0.016294916 10368
0.091044967 20672 0.053018961 20672 0.043695683
20700 0.079862395 20700 0.062746549 10400 0.024834111 10372
0.128367843 20680 0.056641242 20680 0.033001405
20700 0.099788966 20700 0.037360518 10400 0.026506574 10376
0.106917243 20688 0.04667549 20688 0.039934057
20700 0.064943379 20700 0.047937498 10400 0.046839508 10380
0.068575224 20696 0.095502473 20696 0.021877453
20700 0.083612176 20700 0.07243998 10400 0.010957899 10384

0.042472886	20704	0.097206837	20704	0.015264903		
20700	0.075583484	20700	0.067922156	10400	0.059485206	10388
0.056365385	20712	0.031862961	20712	0.015525151		
20700	0.051370971	20700	0.033650707	10400	0.089925306	10392
0.092390263	20720	0.021564081	20720	0.03465694		
20700	0.116458927	20700	0.079796831	10400	0.064977809	10396
0.090474554	20728	0.069301648	20728	0.027119992		
20700	0.098011791	20700	0.084010528	10400	0.004611012	10400
0.046376732	20736	0.14672536	20736	0.031649001		
20700	0.108240172	20700	0.025720428	10400	0.030692307	10404
0.072195144	20744	0.083253821	20744	0.064223139		
20800	0.09334059	20800	0.072946707	10400	0.009963943	10408
0.069696667	20752	0.045371413	20752	0.061886552		
20800	0.049913615	20800	0.107155109	10400	0.036711925	10412
0.023040595	20760	0.049470753	20760	0.02579572		
20800	0.120489385	20800	0.074442985	10400	0.042344414	10416
0.008426408	20768	0.050782077	20768	0.04180929		
20800	0.088509652	20800	0.014914461	10400	0.020570657	10420
0.04573695	20776	0.102428101	20776	0.042577987		
20800	0.101095124	20800	0.062376814	10400	0.033353124	10424
0.077156641	20784	0.055088091	20784	0.047260975		
20800	0.08899645	20800	0.094821517	10400	0.051464456	10428
0.088026274	20792	0.086239445	20792	0.056373436		
20800	0.044687124	20800	0.060508159	10400	0.00751927	10432
0.09675113	20800	0.085836146	20800	0.051106465		
20800	0.053561016	20800	0.065836	10400	0.081556173	10436
0.081102655	20808	0.033415592	20808	0.041707557		
20800	0.07868166	20800	0.055300498	10400	0.055404984	10440
0.032026575	20816	0.038371141	20816	0.024850679		
20800	0.052150444	20800	0.026472235	10400	0.05079732	10444
0.039201004	20824	0.020187034	20824	0.010949005		
20800	0.01723107	20800	0.103640014	10400	0.105786749	10448
0.092543494	20832	0.088001412	20832	0.007097109		
20800	0.023907684	20800	0.107929947	10500	0.065446926	10452
0.112269365	20840	0.097680793	20840	0.012159873		
20800	0.023383005	20800	0.071151349	10500	0.014564621	10456
0.101454272	20848	0.053759075	20848	0.00855091		
20900	0.02411162	20900	0.012702597	10500	0.02468689	10460
0.079143021	20856	0.059299069	20856	0.046393736		
20900	0.037079361	20900	0.105478597	10500	0.009405769	10464
0.101296886	20864	0.117846394	20864	0.074103977		
20900	0.021794705	20900	0.170361541	10500	0.039379553	10468
0.140958596	20872	0.104429062	20872	0.059243499		
20900	0.024761665	20900	0.191890693	10500	0.023122624	10472
0.116427487	20880	0.036521826	20880	0.032693224		
20900	0.03792988	20900	0.129425796	10500	0.051442206	10476
0.016171796	20888	0.128473155	20888	0.050198942		
20900	0.021310681	20900	0.088284993	10500	0.07221596	10480
0.056014804	20896	0.190801788	20896	0.026868942		
20900	0.033498989	20900	0.091013411	10500	0.021179685	10484
0.073956937	20904	0.162355311	20904	0.018819079		
20900	0.032281827	20900	0.059619397	10500	0.014538997	10488
0.091571194	20912	0.093850184	20912	0.025040545		
20900	0.096770316	20900	0.024502293	10500	0.0515186	10492
0.115285562	20920	0.063883686	20920	0.062307117		
20900	0.111772875	20900	0.059031328	10500	0.064652522	10496
0.096974953	20928	0.062976615	20928	0.058899856		
20900	0.073159405	20900	0.135024763	10500	0.023536992	10500
0.048077487	20936	0.045202745	20936	0.036070851		
20900	0.060587703	20900	0.184185003	10500	0.009154124	10504
0.005141857	20944	0.046474535	20944	0.057727662		
21000	0.059053633	21000	0.205391465	10500	0.020621721	10508
0.043686978	20952	0.08204631	20952	0.072334915		
21000	0.036503756	21000	0.14809941	10500	0.021181391	10512
0.05800225	20960	0.075711687	20960	0.04537289		
21000	0.109645655	21000	0.079825586	10500	0.038654998	10516
0.054404922	20968	0.037334699	20968	0.047605277		
21000	0.0964262	21000	0.075807409	10500	0.046768611	10520

0.058082551 20976 0.052950596 20976 0.020284759
21000 0.047039932 21000 0.054785072 10500 0.044420736 10524
0.046943904 20984 0.086690663 20984 0.04565579
21000 0.070487717 21000 0.018041279 10500 0.023167284 10528
0.020059571 20992 0.051380681 20992 0.04550516
21000 0.114626688 21000 0.094175084 10500 0.028309767 10532
0.09047168 21000 0.059296344 21000 0.020500607
21000 0.11205687 21000 0.127006599 10500 0.043538446 10536
0.10222735 21008 0.123448845 21008 0.052817908
21000 0.077184093 21000 0.13403775 10500 0.011566949 10540
0.078782723 21016 0.125662569 21016 0.080121972
21000 0.028772511 21000 0.051851253 10500 0.032033127 10544
0.093129274 21024 0.194778448 21024 0.060705737
21000 0.030236894 21000 0.087677865 10500 0.034498098 10548
0.098835262 21032 0.201878909 21032 0.025544108
21000 0.071780007 21000 0.126270839 10600 0.036424877 10552
0.067894136 21040 0.121799247 21040 0.010826518
21000 0.095388867 21000 0.116606039 10600 0.008661306 10556
0.030750169 21048 0.084232968 21048 0.033186447
21100 0.041364572 21100 0.104502949 10600 0.036842765 10560
0.034091925 21056 0.04275555 21056 0.059662409
21100 0.095359959 21100 0.019814906 10600 0.033437314 10564
0.059704835 21064 0.021430054 21064 0.073075615
21100 0.063035885 21100 0.148190113 10600 0.025259575 10568
0.046299836 21072 0.032726039 21072 0.057939833
21100 0.031158619 21100 0.219648835 10600 0.023658578 10572
0.015979231 21080 0.01636128 21080 0.024482928
21100 0.045762743 21100 0.136420989 10600 0.051025822 10576
0.040241717 21088 0.082709499 21088 0.032928823
21100 0.085277236 21100 0.037071113 10600 0.045204004 10580
0.090697708 21096 0.083397295 21096 0.038550319
21100 0.091701179 21100 0.13197439 10600 0.008306523 10584
0.106018466 21104 0.091867383 21104 0.041812833
21100 0.02777887 21100 0.196977155 10600 0.016238962 10588
0.093907591 21112 0.081708822 21112 0.024061079
21100 0.018252726 21100 0.157559509 10600 0.008627864 10592
0.046294754 21120 0.036230398 21120 0.032733315
21100 0.032686145 21100 0.153910078 10600 0.016111078 10596
0.040454506 21128 0.054539625 21128 0.061578983
21100 0.015652509 21100 0.190091726 10600 0.018829513 10600
0.063121959 21136 0.038329261 21136 0.106700158
21100 0.064230597 21100 0.158577531 10600 0.034484914 10604
0.038274258 21144 0.082674327 21144 0.11511449
21200 0.085791398 21200 0.084443505 10600 0.061952291 10608
0.035464 21152 0.062141153 21152 0.070984897
21200 0.118491109 21200 0.089245878 10600 0.031339045 10612
0.031726435 21160 0.048784819 21160 0.056811517
21200 0.19852395 21200 0.124405051 10600 0.027395754 10616
0.015777478 21168 0.100108424 21168 0.058371854
21200 0.197119865 21200 0.077818993 10600 0.044867189 10620
0.036390436 21176 0.14634199 21176 0.042847241
21200 0.127221501 21200 0.042122894 10600 0.036749534 10624
0.017117956 21184 0.124385959 21184 0.028410781
21200 0.081412138 21200 0.156452355 10600 0.054264681 10628
0.047453337 21192 0.072950337 21192 0.031526892
21200 0.059808554 21200 0.134935981 10600 0.084456144 10632
0.037446142 21200 0.064772816 21200 0.055019762
21200 0.072333009 21200 0.015892778 10600 0.067174442 10636
0.031931209 21208 0.08730308 21208 0.072672978
21200 0.133931171 21200 0.040245508 10600 0.042450745 10640
0.020454063 21216 0.10217246 21216 0.074605239
21200 0.153447981 21200 0.032003562 10600 0.044651486 10644
0.043166881 21224 0.128227854 21224 0.057598019
21200 0.114312017 21200 0.04533328 10600 0.04029991 10648
0.073682553 21232 0.128144253 21232 0.04646098
21200 0.080310711 21200 0.065705644 10700 0.016452421 10652
0.045671775 21240 0.08882086 21240 0.037806978
21200 0.042613148 21200 0.110056855 10700 0.029966583 10656

0.036895795 21248 0.071091097 21248 0.046095072
21300 0.040959159 21300 0.094246774 10700 0.059433027 10660
0.09097458 21256 0.099730969 21256 0.058590471
21300 0.036281585 21300 0.064216773 10700 0.036700625 10664
0.110771107 21264 0.124436119 21264 0.054798762
21300 0.032767712 21300 0.065985078 10700 0.050929728 10668
0.058910078 21272 0.104324558 21272 0.076259319
21300 0.102140162 21300 0.08823524 10700 0.107046879 10672
0.060183447 21280 0.033481494 21280 0.081078819
21300 0.083813349 21300 0.087034547 10700 0.106546992 10676
0.068528651 21288 0.019536457 21288 0.049614439
21300 0.045263427 21300 0.096241303 10700 0.089353125 10680
0.043263037 21296 0.029665695 21296 0.010955016
21300 0.052370251 21300 0.145941391 10700 0.085924723 10684
0.027622484 21304 0.074175216 21304 0.023696595
21300 0.045558536 21300 0.119547098 10700 0.054487689 10688
0.065984772 21312 0.14211201 21312 0.027077498
21300 0.087361856 21300 0.038190821 10700 0.003878268 10692
0.083297164 21320 0.269927405 21320 0.024000216
21300 0.153746136 21300 0.136937684 10700 0.017121425 10696
0.051578409 21328 0.300484099 21328 0.038290731
21300 0.075785167 21300 0.127935215 10700 0.005029819 10700
0.047847811 21336 0.174005734 21336 0.039463088
21300 0.09340675 21300 0.05293014 10700 0.014169176 10704
0.078732614 21344 0.065423403 21344 0.036785968
21400 0.082015053 21400 0.061125073 10700 0.024144767 10708
0.057484591 21352 0.063563079 21352 0.015448071
21400 0.026855523 21400 0.047476144 10700 0.064984481 10712
0.05866199 21360 0.076996148 21360 0.018843146
21400 0.019931364 21400 0.152254434 10700 0.067356079 10716
0.074302552 21368 0.075702483 21368 0.031500931
21400 0.077253717 21400 0.116352807 10700 0.014793888 10720
0.08294265 21376 0.090385482 21376 0.036385383
21400 0.044959437 21400 0.08186567 10700 0.086817308 10724
0.08312241 21384 0.134670933 21384 0.040669045
21400 0.048191319 21400 0.131530789 10700 0.091428825 10728
0.061110462 21392 0.155473609 21392 0.048193931
21400 0.091304384 21400 0.108215158 10700 0.041519132 10732
0.070188864 21400 0.133328009 21400 0.046839727
21400 0.061321385 21400 0.158583178 10700 0.003327058 10736
0.078493948 21408 0.107202774 21408 0.021721931
21400 0.038591021 21400 0.220828995 10700 0.02242836 10740
0.082301354 21416 0.091680988 21416 0.004700881
21400 0.113771857 21400 0.232257968 10700 0.03076272 10744
0.087748333 21424 0.08866685 21424 0.028584218
21400 0.153541609 21400 0.174751331 10700 0.010874848 10748
0.092325936 21432 0.116535484 21432 0.064239575
21400 0.15526131 21400 0.091957867 10800 0.026053067 10752
0.110538211 21440 0.087801804 21440 0.057389094
21400 0.147916828 21400 0.053359916 10800 0.029789517 10756
0.097298595 21448 0.028387369 21448 0.01556184
21500 0.14370009 21500 0.045523644 10800 0.023292339 10760
0.065264263 21456 0.043719992 21456 0.018686853
21500 0.139303229 21500 0.060887451 10800 0.078156634 10764
0.049224658 21464 0.033896024 21464 0.020663694
21500 0.159797971 21500 0.049456365 10800 0.061538063 10768
0.071469571 21472 0.020561005 21472 0.026501284
21500 0.172063709 21500 0.070014568 10800 0.013456109 10772
0.094617717 21480 0.056886893 21480 0.038341968
21500 0.137258219 21500 0.093609677 10800 0.028238208 10776
0.067727109 21488 0.036867918 21488 0.045851932
21500 0.108126544 21500 0.071570656 10800 0.039217277 10780
0.060968723 21496 0.03902465 21496 0.048492606
21500 0.116680807 21500 0.101524725 10800 0.059255675 10784
0.073602212 21504 0.065358683 21504 0.059822382
21500 0.132602538 21500 0.072635747 10800 0.03693203 10788
0.027594933 21512 0.119996003 21512 0.05824956
21500 0.109690751 21500 0.014705484 10800 0.002734535 10792

0.045827917 21520 0.126939588 21520 0.040031162
21500 0.078894802 21500 0.022989165 10800 0.035420639 10796
0.101392252 21528 0.066699264 21528 0.023924184
21500 0.090415553 21500 0.047168607 10800 0.03014896 10800
0.109812245 21536 0.083635983 21536 0.026305117
21500 0.12162506 21500 0.093009337 10800 0.018106748 10804
0.034662731 21544 0.073070041 21544 0.02521772
21600 0.09714601 21600 0.090235844 10800 0.048979913 10808
0.04306926 21552 0.037305304 21552 0.046427125
21600 0.02893871 21600 0.053100874 10800 0.095501891 10812
0.026677051 21560 0.065781584 21560 0.024293842
21600 0.004269782 21600 0.169867213 10800 0.113387621 10816
0.020681731 21568 0.028109151 21568 0.046024856
21600 0.078002107 21600 0.177173846 10800 0.064384331 10820
0.04896351 21576 0.049095455 21576 0.064526481
21600 0.107727057 21600 0.183672674 10800 0.016346567 10824
0.074457617 21584 0.100500205 21584 0.059002879
21600 0.081090191 21600 0.246143114 10800 0.046573641 10828
0.069925001 21592 0.174855363 21592 0.040191746
21600 0.13284506 21600 0.204765281 10800 0.036477555 10832
0.052395822 21600 0.173486929 21600 0.027813432
21600 0.144146266 21600 0.092920323 10800 0.036669604 10836
0.034865985 21608 0.125798906 21608 0.009164436
21600 0.057055579 21600 0.132963265 10800 0.050655624 10840
0.111975809 21616 0.086862994 21616 0.018862953
21600 0.056464756 21600 0.179736293 10800 0.044829303 10844
0.093063369 21624 0.080090154 21624 0.014578801
21600 0.071719172 21600 0.170563944 10800 0.008853332 10848
0.046163084 21632 0.114566276 21632 0.013857958
21600 0.081747683 21600 0.109018292 10900 0.01630678 10852
0.039948172 21640 0.143434706 21640 0.035759651
21600 0.084756335 21600 0.128312021 10900 0.02611194 10856
0.084629777 21648 0.105121173 21648 0.024166591
21700 0.078700228 21700 0.18994385 10900 0.050182935 10860
0.058694004 21656 0.061962142 21656 0.015034915
21700 0.076068449 21700 0.147921645 10900 0.052865922 10864
0.056610799 21664 0.077324468 21664 0.019566742
21700 0.035282032 21700 0.07145515 10900 0.031535772 10868
0.085179723 21672 0.058205089 21672 0.030459141
21700 0.04745039 21700 0.06034163 10900 0.040021831 10872
0.064209562 21680 0.123552352 21680 0.053928601
21700 0.033204447 21700 0.069845039 10900 0.033062326 10876
0.036224967 21688 0.078350342 21688 0.048462174
21700 0.028377323 21700 0.075047232 10900 0.037422651 10880
0.04212794 21696 0.088028261 21696 0.028462086
21700 0.018101524 21700 0.081064674 10900 0.056951121 10884
0.033130324 21704 0.200144525 21704 0.006942068
21700 0.085123451 21700 0.067589201 10900 0.061053215 10888
0.05880091 21712 0.142258315 21712 0.02874128
21700 0.137306546 21700 0.066051405 10900 0.087446388 10892
0.083099658 21720 0.065704015 21720 0.055454169
21700 0.103231236 21700 0.166606639 10900 0.11332865 10896
0.065981054 21728 0.12765023 21728 0.044502234
21700 0.026505446 21700 0.162068449 10900 0.09349794 10900
0.047921076 21736 0.040089239 21736 0.003296194
21700 0.04004109 21700 0.064579457 10900 0.068861635 10904
0.05776768 21744 0.018156394 21744 0.038304606
21800 0.065410342 21800 0.0417669 10900 0.040440431 10908
0.052866435 21752 0.0842152 21752 0.06085194
21800 0.086199732 21800 0.005980985 10900 0.02457568 10912
0.02225149 21760 0.119629323 21760 0.088428365
21800 0.055340544 21800 0.079327299 10900 0.045408571 10916
0.081592509 21768 0.149878746 21768 0.10826315
21800 0.062546547 21800 0.110622954 10900 0.069220077 10920
0.078030505 21776 0.136862014 21776 0.102835591
21800 0.149499989 21800 0.062500956 10900 0.071830036 10924
0.088575544 21784 0.091349633 21784 0.075982469
21800 0.21812976 21800 0.061976745 10900 0.036290297 10928

0.049348957	21792	0.042689826	21792	0.041972598		
21800	0.228900463	21800	0.05139695	10900	0.028932402	10932
0.055895092	21800	0.103381259	21800	0.013212064		
21800	0.185012803	21800	0.080315738	10900	0.04968945	10936
0.098785728	21808	0.056830137	21808	0.028121991		
21800	0.136285991	21800	0.061878403	10900	0.017893737	10940
0.110309477	21816	0.056332236	21816	0.073015261		
21800	0.15026085	21800	0.09991094	10900	0.07040015	10944
0.083724197	21824	0.048677626	21824	0.102568578		
21800	0.184080753	21800	0.189169223	10900	0.089510933	10948
0.015931608	21832	0.029444062	21832	0.098053271		
21800	0.182773714	21800	0.174519898	11000	0.08419095	10952
0.044350025	21840	0.124407365	21840	0.06465285		
21800	0.13123492	21800	0.107605927	11000	0.077484823	10956
0.065941051	21848	0.181749506	21848	0.033526216		
21900	0.071779221	21900	0.049524275	11000	0.076233504	10960
0.108443324	21856	0.158892698	21856	0.034309014		
21900	0.038180238	21900	0.052393643	11000	0.061250838	10964
0.099026329	21864	0.106315361	21864	0.031651951		
21900	0.043868684	21900	0.059884078	11000	0.023523904	10968
0.042942917	21872	0.104435421	21872	0.007461298		
21900	0.040675543	21900	0.067919544	11000	0.052234082	10972
0.022859043	21880	0.103643142	21880	0.034875549		
21900	0.049311937	21900	0.131608293	11000	0.113320682	10976
0.016312773	21888	0.084601044	21888	0.06094958		
21900	0.038979619	21900	0.188638281	11000	0.119603377	10980
0.035774479	21896	0.072963871	21896	0.046050118		
21900	0.094384632	21900	0.197423025	11000	0.0965078	10984
0.084832842	21904	0.048051639	21904	0.011976133		
21900	0.140829826	21900	0.162051146	11000	0.066006141	10988
0.093983865	21912	0.076273318	21912	0.034260014		
21900	0.134369563	21900	0.115304167	11000	0.014603432	10992
0.079009245	21920	0.03953831	21920	0.058595426		
21900	0.080662452	21900	0.091198468	11000	0.047153662	10996
0.068363152	21928	0.060892828	21928	0.040565614		
21900	0.038502541	21900	0.0941322	11000	0.066260793	11000
0.084325089	21936	0.094027993	21936	0.02936017		
21900	0.049296876	21900	0.102255319	11000	0.039003124	11004
0.07863449	21944	0.070528775	21944	0.061786421		
22000	0.077867473	22000	0.107994485	11000	0.030441321	11008
0.070259419	21952	0.102411541	21952	0.069025118		
22000	0.102429309	22000	0.073357405	11000	0.071350747	11012
0.091039328	21960	0.117032614	21960	0.056354296		
22000	0.095927368	22000	0.07250964	11000	0.070718255	11016
0.047735615	21968	0.04743864	21968	0.025243258		
22000	0.087511791	22000	0.047395515	11000	0.039946863	11020
0.053900574	21976	0.042032501	21976	0.009789476		
22000	0.111215872	22000	0.14477536	11000	0.030339546	11024
0.079109683	21984	0.059697697	21984	0.019591613		
22000	0.098540302	22000	0.146629769	11000	0.065511486	11028
0.083404615	21992	0.072893665	21992	0.011103269		
22000	0.034224773	22000	0.101286758	11000	0.082593455	11032
0.061560619	22000	0.110373279	22000	0.040482548		
22000	0.079589314	22000	0.11998284	11000	0.068266352	11036
0.011278683	22008	0.114782219	22008	0.053024334		
22000	0.191341561	22000	0.103773891	11000	0.046702127	11040
0.056614641	22016	0.107085914	22016	0.030033059		
22000	0.213233201	22000	0.047500736	11000	0.054999564	11044
0.04346923	22024	0.149631916	22024	0.062553692		
22000	0.18258224	22000	0.027420681	11000	0.06448834	11048
0.074625074	22032	0.204779398	22032	0.070553717		
22000	0.169377366	22000	0.022961653	11100	0.065340566	11052
0.121721692	22040	0.170936124	22040	0.044406967		
22000	0.11241818	22000	0.032919619	11100	0.052357991	11056
0.094797746	22048	0.088064087	22048	0.048863174		
22100	0.074005569	22100	0.049136364	11100	0.057120768	11060
0.08233796	22056	0.031879707	22056	0.037404585		
22100	0.122904239	22100	0.016652368	11100	0.072322458	11064

0.099884637 22064 0.05637536 22064 0.03782157
22100 0.111922433 22100 0.049655268 11100 0.027687271 11068
0.079293888 22072 0.07712844 22072 0.048877093
22100 0.048052563 22100 0.068693931 11100 0.052352403 11072
0.044366861 22080 0.066825269 22080 0.047996651
22100 0.021382297 22100 0.035305693 11100 0.043057327 11076
0.06990847 22088 0.087132823 22088 0.030327175
22100 0.00949273 22100 0.06281105 11100 0.004879596 11080
0.055212368 22096 0.096421944 22096 0.010508797
22100 0.012377471 22100 0.177870621 11100 0.008296584 11084
0.042371656 22104 0.057704718 22104 0.036406374
22100 0.019239538 22100 0.193372268 11100 0.003282805 11088
0.072771152 22112 0.043750344 22112 0.065079017
22100 0.068227804 22100 0.0599903 11100 0.02351323 11092
0.126669169 22120 0.084428008 22120 0.056686724
22100 0.074549884 22100 0.090409929 11100 0.057077745 11096
0.127051695 22128 0.085784734 22128 0.021759754
22100 0.101207013 22100 0.091786984 11100 0.059759928 11100
0.087683773 22136 0.060622951 22136 0.072720868
22100 0.114821574 22100 0.091502028 11100 0.041055148 11104
0.042990174 22144 0.031410753 22144 0.065117733
22200 0.070139788 22200 0.165256671 11100 0.050160459 11108
0.030232874 22152 0.049093065 22152 0.015890712
22200 0.007215728 22200 0.209276638 11100 0.022558819 11112
0.06312679 22160 0.110502049 22160 0.022758983
22200 0.060971393 22200 0.209459439 11100 0.015947422 11116
0.046596397 22168 0.16207232 22168 0.012777159
22200 0.073377174 22200 0.125902254 11100 0.010253983 11120
0.052369702 22176 0.153300629 22176 0.004535596
22200 0.067908783 22200 0.079118516 11100 0.012936819 11124
0.106335894 22184 0.103673519 22184 0.018113431
22200 0.096399206 22200 0.094695388 11100 0.03321383 11128
0.127293068 22192 0.077044177 22192 0.043581844
22200 0.103996528 22200 0.092626426 11100 0.067781977 11132
0.138045841 22200 0.057915579 22200 0.054969263
22200 0.0575667 22200 0.045212513 11100 0.087995511 11136
0.101525875 22208 0.081177364 22208 0.057241334
22200 0.051459865 22200 0.084671097 11100 0.098049597 11140
0.052153089 22216 0.115946728 22216 0.055249046
22200 0.117480646 22200 0.174375513 11100 0.061906489 11144
0.085972402 22224 0.103252321 22224 0.042199936
22200 0.080795333 22200 0.18812572 11100 0.064123859 11148
0.102977727 22232 0.099797573 22232 0.029558119
22200 0.066391243 22200 0.165102713 11200 0.057636953 11152
0.103594153 22240 0.024364887 22240 0.042101703
22200 0.101014666 22200 0.096781914 11200 0.06753546 11156
0.105162835 22248 0.089880137 22248 0.048507711
22300 0.124700309 22300 0.032946511 11200 0.094608084 11160
0.089365145 22256 0.082134386 22256 0.03006384
22300 0.115299103 22300 0.045454137 11200 0.075959732 11164
0.05479197 22264 0.061220118 22264 0.021373062
22300 0.120389472 22300 0.162183976 11200 0.04870195 11168
0.034851433 22272 0.119170989 22272 0.02015676
22300 0.114630122 22300 0.23413061 11200 0.058728685 11172
0.016117214 22280 0.121977682 22280 0.05666204
22300 0.066373308 22300 0.131780268 11200 0.019909205 11176
0.089145062 22288 0.105586107 22288 0.087597451
22300 0.050258877 22300 0.038379279 11200 0.044869408 11180
0.133258233 22296 0.054194406 22296 0.068934925
22300 0.095574877 22300 0.082804916 11200 0.029745426 11184
0.133315538 22304 0.063921085 22304 0.028091714
22300 0.084225168 22300 0.082955907 11200 0.038053095 11188
0.151352229 22312 0.012345601 22312 0.019048677
22300 0.0595856 22300 0.132401095 11200 0.042670657 11192
0.158285416 22320 0.114865652 22320 0.03701889
22300 0.141398618 22300 0.102478458 11200 0.042165819 11196
0.114266586 22328 0.149885585 22328 0.051421612
22300 0.101244012 22300 0.046160087 11200 0.038860409 11200

0.095023745 22336 0.124868137 22336 0.068144123
22300 0.046118381 22300 0.024332236 11200 0.019042413 11204
0.120751647 22344 0.086988395 22344 0.073296847
22400 0.026391621 22400 0.086003587 11200 0.06117257 11208
0.139120704 22352 0.094119096 22352 0.082756211
22400 0.105764906 22400 0.118816308 11200 0.068360045 11212
0.137217299 22360 0.085218213 22360 0.100920443
22400 0.13720023 22400 0.071274684 11200 0.045829336 11216
0.125787221 22368 0.070248178 22368 0.098195102
22400 0.148180014 22400 0.08019626 11200 0.023645374 11220
0.153368251 22376 0.122078534 22376 0.070324408
22400 0.146609906 22400 0.051975942 11200 0.019972615 11224
0.207538673 22384 0.153543544 22384 0.060605747
22400 0.092657174 22400 0.05494403 11200 0.015893314 11228
0.209207953 22392 0.126771658 22392 0.071085815
22400 0.154869908 22400 0.095847521 11200 0.004283035 11232
0.102338156 22400 0.037242826 22400 0.064766253
22400 0.191223343 22400 0.101343321 11200 0.024027529 11236
0.050723862 22408 0.04851813 22408 0.058748898
22400 0.149787171 22400 0.052295636 11200 0.040085644 11240
0.099352248 22416 0.036912894 22416 0.046595313
22400 0.048629372 22400 0.120240482 11200 0.06458642 11244
0.05312089 22424 0.045716646 22424 0.024039491
22400 0.047721373 22400 0.208708123 11200 0.069630019 11248
0.02557556 22432 0.094725008 22432 0.035322231
22400 0.003493254 22400 0.274117745 11300 0.061042614 11252
0.077160476 22440 0.066097935 22440 0.054852775
22400 0.075839009 22400 0.264408213 11300 0.057358167 11256
0.12828855 22448 0.01165067 22448 0.052798107
22500 0.066833782 22500 0.172165004 11300 0.055603461 11260
0.138390416 22456 0.076435295 22456 0.059587863
22500 0.036989932 22500 0.061046747 11300 0.052711912 11264
0.105345847 22464 0.109365203 22464 0.072497256
22500 0.136533025 22500 0.040235518 11300 0.015006477 11268
0.100214202 22472 0.086660701 22472 0.053311298
22500 0.143522033 22500 0.039894821 11300 0.027123491 11272
0.141647761 22480 0.061950435 22480 0.019350002
22500 0.064465858 22500 0.032931468 11300 0.034483412 11276
0.11924906 22488 0.106913649 22488 0.039024246
22500 0.036342724 22500 0.052536085 11300 0.028303075 11280
0.054801043 22496 0.122407204 22496 0.030580199
22500 0.034098885 22500 0.011976839 11300 0.059270831 11284
0.06890684 22504 0.113635295 22504 0.026073363
22500 0.041513336 22500 0.054035601 11300 0.065295775 11288
0.138565956 22512 0.110818204 22512 0.023890081
22500 0.066024113 22500 0.063954954 11300 0.054707569 11292
0.188215766 22520 0.074851494 22520 0.019159441
22500 0.055434877 22500 0.119675759 11300 0.049403247 11296
0.219089066 22528 0.085201456 22528 0.03892004
22500 0.041699415 22500 0.113710274 11300 0.062274936 11300
0.202618219 22536 0.140804375 22536 0.031502728
22500 0.049589369 22500 0.064628468 11300 0.052145082 11304
0.086126085 22544 0.180807954 22544 0.052124451
22600 0.032982393 22600 0.05408729 11300 0.03707283 11308
0.094580057 22552 0.182888165 22552 0.037479771
22600 0.049620012 22600 0.131542518 11300 0.044054428 11312
0.130889108 22560 0.182562494 22560 0.014706293
22600 0.065586355 22600 0.093765229 11300 0.040087653 11316
0.065204054 22568 0.16622126 22568 0.022039916
22600 0.048804199 22600 0.06387398 11300 0.038402457 11320
0.060305272 22576 0.070369453 22576 0.062481704
22600 0.126420099 22600 0.073180629 11300 0.020468313 11324
0.162415964 22584 0.066482557 22584 0.076721211
22600 0.085000655 22600 0.0452737 11300 0.053006195 11328
0.188028368 22592 0.059475977 22592 0.072622417
22600 0.035357069 22600 0.129540261 11300 0.098914657 11332
0.143760918 22600 0.091733004 22600 0.069490969
22600 0.050196464 22600 0.179547773 11300 0.085737149 11336

0.075799777 22608 0.088709006 22608 0.059828155
22600 0.025234018 22600 0.157327784 11300 0.06593368 11340
0.066428889 22616 0.045862715 22616 0.0345728
22600 0.060607465 22600 0.119947727 11300 0.074559983 11344
0.022222206 22624 0.024363266 22624 0.039227849
22600 0.052009058 22600 0.081901279 11300 0.070872011 11348
0.140589488 22632 0.048850383 22632 0.04298897
22600 0.092241426 22600 0.051249597 11400 0.076856813 11352
0.204793353 22640 0.015733016 22640 0.024459649
22600 0.137622715 22600 0.106392065 11400 0.055690733 11356
0.175002715 22648 0.072679854 22648 0.061594619
22700 0.140420845 22700 0.112987749 11400 0.015716436 11360
0.102461359 22656 0.127296619 22656 0.065509979
22700 0.072206858 22700 0.135602153 11400 0.035283618 11364
0.03937108 22664 0.175610956 22664 0.056160763
22700 0.039750154 22700 0.178215924 11400 0.069301561 11368
0.076176177 22672 0.126763655 22672 0.0446185
22700 0.059619382 22700 0.19600548 11400 0.070172544 11372
0.125391788 22680 0.00786129 22680 0.06189369
22700 0.095377436 22700 0.232092891 11400 0.03600214 11376
0.125978448 22688 0.103773673 22688 0.067465742
22700 0.102420883 22700 0.202891854 11400 0.025151112 11380
0.114188086 22696 0.113719463 22696 0.0377414
22700 0.056989124 22700 0.114900773 11400 0.037131205 11384
0.110534813 22704 0.064704582 22704 0.014271574
22700 0.132246628 22700 0.093965457 11400 0.059471237 11388
0.117164287 22712 0.024770161 22712 0.016427262
22700 0.10684444 22700 0.075075455 11400 0.080670805 11392
0.13845849 22720 0.008857277 22720 0.035160967
22700 0.080103586 22700 0.044435426 11400 0.031426429 11396
0.12873231 22728 0.043047003 22728 0.03177896
22700 0.141365818 22700 0.045763303 11400 0.052968644 11400
0.079027472 22736 0.030617306 22736 0.003189415
22700 0.085666965 22700 0.042317402 11400 0.028873965 11404
0.090854701 22744 0.055505625 22744 0.024867364
22800 0.095676551 22800 0.053841839 11400 0.012996507 11408
0.119888129 22752 0.093327464 22752 0.032906948
22800 0.113920869 22800 0.103968952 11400 0.056437748 11412
0.09757901 22760 0.042544954 22760 0.032622073
22800 0.076825003 22800 0.069945323 11400 0.072961208 11416
0.086465399 22768 0.039348644 22768 0.018158877
22800 0.026019816 22800 0.037178696 11400 0.036419624 11420
0.074507065 22776 0.065759785 22776 0.012413922
22800 0.049084352 22800 0.02285064 11400 0.02057886 11424
0.070711802 22784 0.052035251 22784 0.042984197
22800 0.10228841 22800 0.101961479 11400 0.004402106 11428
0.107214073 22792 0.045752713 22792 0.038766139
22800 0.107755637 22800 0.075134405 11400 0.027621733 11432
0.098854573 22800 0.156380294 22800 0.018736906
22800 0.088317371 22800 0.065841705 11400 0.023564804 11436
0.103630737 22808 0.245068863 22808 0.065830107
22800 0.040689924 22800 0.113590904 11400 0.016497517 11440
0.131669804 22816 0.235670654 22816 0.080422586
22800 0.030408353 22800 0.050314382 11400 0.029266865 11444
0.127877604 22824 0.169981416 22824 0.05539749
22800 0.058611178 22800 0.012987509 11400 0.021832069 11448
0.093190836 22832 0.124435988 22832 0.041467618
22800 0.052972726 22800 0.068612477 11500 0.009462016 11452
0.060962062 22840 0.096066222 22840 0.044963701
22800 0.059167065 22800 0.127329128 11500 0.013156398 11456
0.043988755 22848 0.039861727 22848 0.034506313
22900 0.047680267 22900 0.076905002 11500 0.016753838 11460
0.061062521 22856 0.032637276 22856 0.019004541
22900 0.025128262 22900 0.056495966 11500 0.057512556 11464
0.086673659 22864 0.074352545 22864 0.036985082
22900 0.101208447 22900 0.037445785 11500 0.074258664 11468
0.066643268 22872 0.08432629 22872 0.049325277
22900 0.190926178 22900 0.049994276 11500 0.048143626 11472

0.024497635	22880	0.070227558	22880	0.041733492						
22900	0.201301373	22900	0.099987912	11500	0.04927619	11476				
0.085532192	22888	0.02165711	22888	0.025460895						
22900	0.12131266	22900	0.165223566	11500	0.034347049	11480				
0.13610904	22896	0.116467592	22896	0.029143319						
22900	0.062012623	22900	0.219627225	11500	0.011455082	11484				
0.12334816	22904	0.156910348	22904	0.048018115						
22900	0.088361565	22900	0.192515814	11500	0.034979854	11488				
0.06921951	22912	0.11134916	22912	0.050935771						
22900	0.150166889	22900	0.153683054	11500	0.061133476	11492				
0.077332588	22920	0.045722365	22920	0.049489583						
22900	0.131361666	22900	0.171529799	11500	0.058341619	11496				
0.099858793	22928	0.041849195	22928	0.028343555						
22900	0.060188617	22900	0.154055364	11500	0.011632393	11500				
0.090635273	22936	0.043467571	22936	0.062953201						
22900	0.071904178	22900	0.113771093	11500	0.040836887	11504				
0.059605591	22944	0.018179131	22944	0.077566416						
23000	0.118792799	23000	0.094394622	11500	0.062071114	11508				
0.053607135	22952	0.033139248	22952	0.07735884						
23000	0.0944122	23000	0.040385323	11500	0.055135766	11512				
0.093782321	22960	0.097099444	22960	0.094205585						
23000	0.023148972	23000	0.08077074	11500	0.054512158	11516				
0.139566066	22968	0.136367933	22968	0.074829761						
23000	0.061589955	23000	0.085373526	11500	0.078319084	11520				
0.142128046	22976	0.073518706	22976	0.025409265						
23000	0.089034787	23000	0.078679877	11500	0.074821102	11524				
0.101778212	22984	0.036784895	22984	0.029614579						
23000	0.103674101	23000	0.073438285	11500	0.036598147	11528				
0.046977926	22992	0.075840791	22992	0.048197457						
23000	0.08217647	23000	0.098697863	11500	0.061992716	11532				
0.082868057	23000	0.074000171	23000	0.067298322						
23000	0.024180468	23000	0.098219258	11500	0.069143389	11536				
0.117707634	23008	0.060531987	23008	0.060943879						
23000	0.11130458	23000	0.063385385	11500	0.04864646	11540				
0.07111529	23016	0.138020333	23016	0.020414382						
23000	0.090657566	23000	0.074679891	11500	0.011217941	11544				
0.072163079	23024	0.182944714	23024	0.033024287						
23000	0.047076759	23000	0.119450539	11500	0.02180684	11548				
0.115988674	23032	0.184871883	23032	0.041753519						
23000	0.086117361	23000	0.106208114	11600	0.028213861	11552				
0.100028556	23040	0.147499494	23040	0.052114821						
23000	0.127920444	23000	0.081128732	11600	0.018433055	11556				
0.103931925	23048	0.037283182	23048	0.051285493						
23100	0.130750458	23100	0.053574924	11600	0.011259208	11560				
0.147119411	23056	0.140837409	23056	0.02231388						
23100	0.076134231	23100	0.019539722	11600	0.006868158	11564				
0.168117461	23064	0.174183776	23064	0.027037448						
23100	0.04336776	23100	0.111895322	11600	0.022184491	11568				
0.150274645	23072	0.098935278	23072	0.032896667						
23100	0.022290504	23100	0.164004494	11600	0.071974369	11572				
0.089484413	23080	0.105269581	23080	0.00769297						
23100	0.064318578	23100	0.136515068	11600	0.106353364	11576				
0.100618141	23088	0.108236192	23088	0.014500442						
23100	0.079194673	23100	0.090845984	11600	0.087705943	11580				
0.126798026	23096	0.12486284	23096	0.005620788						
23100	0.161597069	23100	0.108869848	11600	0.052859174	11584				
0.11544329	23104	0.128357483	23104	0.045571789						
23100	0.150250169	23100	0.11122771	11600	0.045459859	11588				
0.104641498	23112	0.136960225	23112	0.08237327						
23100	0.12571321	23100	0.080709193	11600	0.054285636	11592				
0.092726485	23120	0.091406677	23120	0.077041368						
23100	0.091957998	23100	0.15504616	11600	0.053107702	11596				
0.054299453	23128	0.023551133	23128	0.035094225						
23100	0.049347327	23100	0.102205893	11600	0.041322488	11600				
0.050366347	23136	0.076762801	23136	0.020022699						
23100	0.121190926	23100	0.081935039	11600	0.025285339	11604				
0.0858598	23144	0.110099783	23144	0.055317465						
23200	0.135018374	23200	0.091701302	11600	0.035029039	11608				

0.081249069 23152 0.075943193 23152 0.060518178
23200 0.115903589 23200 0.069616224 11600 0.043764372 11612
0.055491357 23160 0.030495899 23160 0.050526818
23200 0.139574331 23200 0.088999987 11600 0.019582727 11616
0.034952518 23168 0.02455384 23168 0.05169211
23200 0.110507353 23200 0.052575619 11600 0.034792593 11620
0.082474515 23176 0.03910024 23176 0.058461959
23200 0.08410919 23200 0.074913463 11600 0.059653783 11624
0.080216545 23184 0.070745387 23184 0.036456408
23200 0.144270554 23200 0.088188026 11600 0.057585025 11628
0.057147052 23192 0.194162436 23192 0.02683743
23200 0.1159811 23200 0.041545143 11600 0.043939494 11632
0.016195592 23200 0.178101298 23200 0.080927028
23200 0.023331832 23200 0.002522843 11600 0.052914678 11636
0.038876042 23208 0.061446197 23208 0.078382771
23200 0.05718715 23200 0.058348556 11600 0.057284698 11640
0.072871226 23216 0.110084366 23216 0.026515772
23200 0.061921979 23200 0.107139786 11600 0.019752915 11644
0.085976055 23224 0.089433219 23224 0.029314677
23200 0.001860444 23200 0.037441325 11600 0.032804819 11648
0.052190848 23232 0.02021969 23232 0.059803871
23200 0.089443551 23200 0.096598975 11700 0.056412249 11652
0.037460763 23240 0.063713371 23240 0.05266014
23200 0.147717495 23200 0.128741638 11700 0.050134346 11656
0.012549197 23248 0.107128442 23248 0.024847366
23300 0.170058819 23300 0.099859099 11700 0.030857209 11660
0.039054557 23256 0.043811397 23256 0.036241967
23300 0.167436098 23300 0.089569832 11700 0.025376065 11664
0.052483447 23264 0.080891441 23264 0.027053404
23300 0.130964909 23300 0.09709024 11700 0.022697281 11668
0.050524552 23272 0.107004504 23272 0.022473301
23300 0.083667335 23300 0.092248207 11700 0.032757132 11672
0.047273381 23280 0.079248079 23280 0.024372268
23300 0.01894792 23300 0.067343608 11700 0.028628821 11676
0.094893032 23288 0.065048582 23288 0.009312695
23300 0.087196742 23300 0.105789412 11700 0.013307102 11680
0.121537923 23296 0.025929448 23296 0.010118686
23300 0.113737347 23300 0.228392688 11700 0.05559282 11684
0.082814004 23304 0.038822771 23304 0.010103136
23300 0.068381887 23300 0.244636001 11700 0.07047884 11688
0.003987787 23312 0.023146036 23312 0.043376527
23300 0.080778562 23300 0.203434465 11700 0.06097446 11692
0.042500826 23320 0.072336545 23320 0.044990895
23300 0.151433196 23300 0.127786319 11700 0.046142657 11696
0.050193852 23328 0.09202282 23328 0.029887116
23300 0.184070785 23300 0.08160463 11700 0.017519184 11700
0.070403061 23336 0.142730889 23336 0.080381513
23300 0.144277365 23300 0.145848055 11700 0.021884507 11704
0.078660116 23344 0.126100218 23344 0.098118835
23400 0.135368726 23400 0.125387465 11700 0.037856855 11708
0.043843735 23352 0.036562095 23352 0.033713062
23400 0.086875101 23400 0.05727902 11700 0.027617691 11712
0.02881784 23360 0.058085385 23360 0.06264144
23400 0.032039843 23400 0.094112191 11700 0.017843866 11716
0.076086079 23368 0.068590744 23368 0.072130257
23400 0.059980015 23400 0.097638105 11700 0.027733604 11720
0.055524473 23376 0.059977425 23376 0.016595535
23400 0.063253006 23400 0.088565997 11700 0.040582399 11724
0.050665574 23384 0.132197834 23384 0.03032603
23400 0.053913795 23400 0.067801477 11700 0.05036384 11728
0.082529674 23392 0.132868518 23392 0.065935717
23400 0.09815221 23400 0.09232531 11700 0.025856023 11732
0.087867942 23400 0.07303727 23400 0.082002458
23400 0.076185752 23400 0.147822183 11700 0.040857591 11736
0.106164953 23408 0.096397271 23408 0.062381616
23400 0.054798755 23400 0.128117914 11700 0.05628926 11740
0.125265463 23416 0.055590885 23416 0.055870547
23400 0.047939637 23400 0.044183613 11700 0.038289574 11744

0.093339753 23424 0.001934409 23424 0.053672658
23400 0.021183694 23400 0.075530887 11700 0.020059304 11748
0.043498934 23432 0.044774875 23432 0.013579665
23400 0.097424141 23400 0.12081513 11800 0.045935911 11752
0.034893561 23440 0.094038747 23440 0.034420933
23400 0.088250388 23400 0.037480215 11800 0.062889638 11756
0.03865872 23448 0.12433168 23448 0.030098281
23500 0.072449278 23500 0.156466281 11800 0.014052183 11760
0.019514873 23456 0.154085108 23456 0.020718531
23500 0.059105878 23500 0.208080179 11800 0.044411219 11764
0.032498367 23464 0.136849384 23464 0.053089112
23500 0.061815874 23500 0.150704728 11800 0.053787295 11768
0.036282152 23472 0.080388076 23472 0.044706332
23500 0.103910948 23500 0.0934118 11800 0.030884716 11772
0.040872033 23480 0.066070636 23480 0.038784045
23500 0.148083098 23500 0.048418202 11800 0.030439507 11776
0.097490709 23488 0.08270648 23488 0.008946307
23500 0.123846141 23500 0.088315333 11800 0.038918086 11780
0.102649858 23496 0.058817052 23496 0.025343068
23500 0.099803227 23500 0.093340874 11800 0.040705883 11784
0.084273008 23504 0.091689406 23504 0.039149894
23500 0.130873159 23500 0.015115752 11800 0.043957392 11788
0.056915247 23512 0.16755586 23512 0.032273139
23500 0.091728995 23500 0.150803273 11800 0.058187201 11792
0.040622788 23520 0.179387076 23520 0.022039112
23500 0.0153331 23500 0.148396998 11800 0.063569576 11796
0.07906172 23528 0.107668893 23528 0.02480986
23500 0.027427501 23500 0.044036766 11800 0.033450779 11800
0.103714789 23536 0.058889298 23536 0.049757924
23500 0.066686131 23500 0.004605965 11800 0.041545434 11804
0.084326763 23544 0.074882264 23544 0.063425105
23600 0.117084594 23600 0.032388569 11800 0.052347419 11808
0.065346096 23552 0.045402452 23552 0.060175626
23600 0.055565892 23600 0.080050799 11800 0.035062167 11812
0.075551303 23560 0.042723856 23560 0.045364272
23600 0.099358244 23600 0.126490456 11800 0.002131394 11816
0.109771383 23568 0.082638879 23568 0.051013281
23600 0.149356609 23600 0.147051411 11800 0.02728933 11820
0.11821073 23576 0.068420181 23576 0.034012144
23600 0.101776728 23600 0.148612875 11800 0.027446014 11824
0.08912621 23584 0.115957213 23584 0.085202155
23600 0.121622892 23600 0.108351167 11800 0.007634232 11828
0.041210205 23592 0.164057099 23592 0.10594706
23600 0.034915662 23600 0.082759929 11800 0.019689434 11832
0.051752057 23600 0.105843159 23600 0.04746607
23600 0.177348381 23600 0.020586473 11800 0.020012072 11836
0.063471423 23608 0.062329338 23608 0.037290934
23600 0.158391805 23600 0.075619973 11800 0.036578065 11840
0.049190978 23616 0.054334836 23616 0.037433736
23600 0.04040814 23600 0.093479917 11800 0.021102329 11844
0.046969155 23624 0.027573266 23624 0.004428663
23600 0.064132139 23600 0.068611829 11800 0.022673019 11848
0.051286763 23632 0.044194669 23632 0.015606591
23600 0.101287827 23600 0.038915536 11900 0.061847146 11852
0.025408193 23640 0.040501771 23640 0.02260522
23600 0.134640635 23600 0.03655945 11900 0.038151087 11856
0.023702518 23648 0.045898702 23648 0.054987613
23700 0.071942828 23700 0.048314752 11900 0.021745902 11860
0.05231411 23656 0.091851551 23656 0.024933452
23700 0.020833906 23700 0.017309041 11900 0.051866307 11864
0.04374834 23664 0.069251546 23664 0.032304652
23700 0.02675639 23700 0.063459906 11900 0.04241908 11868
0.005518775 23672 0.026417763 23672 0.055430344
23700 0.08353372 23700 0.048497343 11900 0.018943119 11872
0.019227049 23680 0.024907517 23680 0.070863629
23700 0.187103899 23700 0.050291412 11900 0.023939179 11876
0.012088872 23688 0.014374841 23688 0.060401075
23700 0.23713446 23700 0.041916504 11900 0.016751532 11880

0.016827107 23696 0.08470309 23696 0.003796573
23700 0.195952074 23700 0.100626603 11900 0.016512779 11884
0.053245753 23704 0.104562016 23704 0.027948863
23700 0.081862803 23700 0.115394585 11900 0.002495479 11888
0.08321357 23712 0.037583995 23712 0.03218561
23700 0.019181884 23700 0.051669835 11900 0.011703159 11892
0.094024079 23720 0.08626272 23720 0.072873336
23700 0.079185629 23700 0.035067184 11900 0.019799745 11896
0.08659895 23728 0.045022189 23728 0.050559378
23700 0.166268015 23700 0.056225639 11900 0.027659498 11900
0.075195159 23736 0.038420312 23736 0.032440963
23700 0.171074033 23700 0.066789682 11900 0.03949524 11904
0.060902901 23744 0.029738654 23744 0.077909179
23800 0.093869261 23800 0.055483924 11900 0.041217398 11908
0.013724256 23752 0.05014948 23752 0.058388359
23800 0.072599243 23800 0.029298479 11900 0.045909594 11912
0.046770507 23760 0.07701639 23760 0.022659237
23800 0.089441004 23800 0.095219941 11900 0.071378636 11916
0.089172005 23768 0.080952639 23768 0.024257299
23800 0.063223066 23800 0.087059554 11900 0.042136711 11920
0.095428608 23776 0.029425195 23776 0.0262411
23800 0.012902932 23800 0.04263379 11900 0.019183397 11924
0.074750271 23784 0.096855154 23784 0.0421037
23800 0.044428904 23800 0.082265011 11900 0.052587751 11928
0.032718468 23792 0.074804346 23792 0.027857004
23800 0.125069564 23800 0.147722764 11900 0.079104721 11932
0.019914074 23800 0.019450865 23800 0.005166177
23800 0.118106131 23800 0.171090273 11900 0.046917303 11936
0.049446193 23808 0.022048331 23808 0.023037022
23800 0.063288491 23800 0.1150729 11900 0.006182536 11940
0.078731566 23816 0.033847409 23816 0.063014421
23800 0.06238481 23800 0.055539287 11900 0.040217616 11944
0.075504184 23824 0.044434797 23824 0.07776828
23800 0.110441099 23800 0.072597861 11900 0.075674238 11948
0.080456652 23832 0.059916161 23832 0.053547403
23800 0.105986597 23800 0.058708018 12000 0.047121579 11952
0.120631077 23840 0.116296549 23840 0.02567228
23800 0.075081021 23800 0.169840278 12000 0.014069841 11956
0.13058992 23848 0.100440026 23848 0.020498774
23900 0.08685761 23900 0.236631676 12000 0.029871766 11960
0.099230558 23856 0.049209513 23856 0.031003106
23900 0.111175737 23900 0.210100189 12000 0.019737034 11964
0.046266217 23864 0.020127929 23864 0.033747201
23900 0.108688902 23900 0.113040667 12000 0.019792675 11968
0.025612668 23872 0.013616283 23872 0.024232477
23900 0.080855287 23900 0.070536713 12000 0.033348508 11972
0.022830995 23880 0.037354064 23880 0.05369071
23900 0.043936881 23900 0.065803506 12000 0.016206865 11976
0.021484262 23888 0.069665191 23888 0.035490844
23900 0.076453958 23900 0.049382154 12000 0.03784163 11980
0.054148124 23896 0.029104644 23896 0.010264318
23900 0.049409104 23900 0.082597224 12000 0.047165326 11984
0.081495535 23904 0.052408745 23904 0.023687919
23900 0.015721307 23900 0.102845166 12000 0.022100903 11988
0.082802486 23912 0.026691405 23912 0.055594675
23900 0.036204256 23900 0.144270788 12000 0.008621421 11992
0.060817602 23920 0.013778022 23920 0.064646134
23900 0.045226781 23900 0.131901121 12000 0.027222417 11996
0.052125386 23928 0.059914855 23928 0.062456806
23900 0.026028622 23900 0.119453303 12000 0.035775553 12000
0.050301296 23936 0.118588432 23936 0.063695712
23900 0.125854101 23900 0.058523619 12000 0.059918297 12004
0.042381984 23944 0.136506191 23944 0.051275052
24000 0.123148479 24000 0.106623709 12000 0.045831821 12008
0.042872969 23952 0.144274353 23952 0.029011932
24000 0.050882591 24000 0.099024801 12000 0.05939159 12012
0.061976447 23960 0.119668002 23960 0.022778993
24000 0.030636784 24000 0.020087618 12000 0.087011489 12016

0.067541165		23968	0.087077198		23968	0.019560226		
24000	0.070672424		24000	0.062852698		12000	0.078785146	12020
0.0491627		23976	0.079058606		23976	0.050853767		
24000	0.111004985		24000	0.02103602		12000	0.046880574	12024
0.061725456		23984	0.081869803		23984	0.05489984		
24000	0.190959924		24000	0.073021984		12000	0.023973036	12028
0.055550219		23992	0.13332798		23992	0.049364782		
24000	0.226032499		24000	0.086456108		12000	0.045292421	12032
0.034295634		24000	0.169168663		24000	0.009491969		
24000	0.195145825		24000	0.085408814		12000	0.057330632	12036
0.027521517		24008	0.133443085		24008	0.063038475		
24000	0.114829963		24000	0.103320228		12000	0.006732779	12040
0.058884569		24016	0.054267857		24016	0.042770262		
24000	0.048351423		24000	0.054213542		12000	0.072899398	12044
0.072930809		24024	0.021515192		24024	0.025018775		
24000	0.105040563		24000	0.07918082		12000	0.108959888	12048
0.055016739		24032	0.058522503		24032	0.055547498		
24000	0.10992121		24000	0.110218498		12100	0.088650369	12052
0.031250977		24040	0.095820054		24040	0.059437294		
24000	0.050345403		24000	0.105068917		12100	0.060599134	12056
0.075982141		24048	0.113793103		24048	0.056726756		
24100	0.12622423		24100	0.137125448		12100	0.051157946	12060
0.062548621		24056	0.10048609		24056	0.020342646		
24100	0.172692511		24100	0.170700281		12100	0.02986339	12064
0.052375148		24064	0.123556922		24064	0.064051637		
24100	0.13229606		24100	0.125897438		12100	0.02584948	12068
0.069901333		24072	0.165457255		24072	0.063265456		
24100	0.068089772		24100	0.085507505		12100	0.028382774	12072
0.065122702		24080	0.111083486		24080	0.043310451		
24100	0.132346176		24100	0.0701155		12100	0.062666673	12076
0.088309927		24088	0.040923358		24088	0.048253871		
24100	0.133086273		24100	0.044672557		12100	0.052676027	12080
0.091779046		24096	0.096743454		24096	0.070278322		
24100	0.117215786		24100	0.080138198		12100	0.030471238	12084
0.093564646		24104	0.13727363		24104	0.051517443		
24100	0.100426914		24100	0.095915275		12100	0.07425814	12088
0.096998352		24112	0.14000175		24112	0.019186546		
24100	0.006876697		24100	0.041837338		12100	0.056076897	12092
0.11301192		24120	0.100035737		24120	0.024984232		
24100	0.139838536		24100	0.067693531		12100	0.019666322	12096
0.104830979		24128	0.088140325		24128	0.070581351		
24100	0.174828179		24100	0.122336205		12100	0.064803273	12100
0.051485757		24136	0.089947986		24136	0.074478645		
24100	0.150609994		24100	0.082274877		12100	0.10695104	12104
0.007552705		24144	0.066999673		24144	0.035188623		
24200	0.148510183		24200	0.095786527		12100	0.122518264	12108
0.050046801		24152	0.050559713		24152	0.061819686		
24200	0.131264068		24200	0.117988493		12100	0.091534748	12112
0.059497143		24160	0.045298548		24160	0.064896609		
24200	0.056730874		24200	0.074587624		12100	0.032033029	12116
0.040419076		24168	0.030992807		24168	0.047211579		
24200	0.041999974		24200	0.035322009		12100	0.053139382	12120
0.107780092		24176	0.081659768		24176	0.071859293		
24200	0.086248329		24200	0.07589977		12100	0.064203327	12124
0.152987239		24184	0.113539507		24184	0.067573761		
24200	0.056258246		24200	0.040248349		12100	0.050719031	12128
0.123999169		24192	0.136617484		24192	0.076069933		
24200	0.131848734		24200	0.042038868		12100	0.044958786	12132
0.098019773		24200	0.173680484		24200	0.088295565		
24200	0.155222879		24200	0.023944926		12100	0.048297865	12136
0.066952889		24208	0.205818855		24208	0.067203786		
24200	0.08900879		24200	0.080669954		12100	0.045404242	12140
0.047210098		24216	0.210342129		24216	0.03409692		
24200	0.056111498		24200	0.083444924		12100	0.049490682	12144
0.088938476		24224	0.159868593		24224	0.032359429		
24200	0.041999123		24200	0.037526985		12100	0.054551165	12148
0.117723081		24232	0.064168686		24232	0.056190223		
24200	0.069012669		24200	0.017749184		12200	0.05622943	12152

0.14296129 24240 0.049706818 24240 0.037785096
24200 0.095042058 24200 0.033875473 12200 0.03783988 12156
0.133533148 24248 0.036457408 24248 0.02242624
24300 0.03743215 24300 0.09329008 12200 0.001003152 12160
0.103685787 24256 0.0952146 24256 0.04194029
24300 0.028468134 24300 0.134086339 12200 0.030587697 12164
0.075504635 24264 0.072690833 24264 0.069142712
24300 0.07866192 24300 0.113229806 12200 0.030527997 12168
0.054192591 24272 0.052985583 24272 0.069271351
24300 0.156716138 24300 0.058417332 12200 0.067212299 12172
0.040642339 24280 0.065703338 24280 0.019567109
24300 0.157111455 24300 0.035379439 12200 0.11734906 12176
0.013435946 24288 0.138990218 24288 0.044740169
24300 0.092700124 24300 0.044230994 12200 0.11560975 12180
0.041989832 24296 0.158225623 24296 0.056824825
24300 0.033220935 24300 0.063961299 12200 0.064120599 12184
0.060928349 24304 0.144151956 24304 0.024778183
24300 0.029954845 24300 0.035955811 12200 0.00669713 12188
0.067061446 24312 0.119746343 24312 0.04745647
24300 0.04421343 24300 0.082176186 12200 0.040244839 12192
0.058442107 24320 0.068894464 24320 0.083068917
24300 0.055678687 24300 0.162137905 12200 0.068722977 12196
0.020001049 24328 0.065094115 24328 0.072221847
24300 0.089285008 24300 0.203100659 12200 0.090624715 12200
0.047360773 24336 0.097811491 24336 0.058092177
24300 0.116609059 24300 0.175056295 12200 0.11268066 12204
0.057896694 24344 0.123203558 24344 0.049453243
24400 0.09978372 24400 0.119993987 12200 0.095559743 12208
0.060245588 24352 0.103343082 24352 0.05075413
24400 0.077322002 24400 0.048090169 12200 0.047971833 12212
0.043835415 24360 0.055934077 24360 0.051802588
24400 0.046553163 24400 0.029745046 12200 0.022369852 12216
0.02095908 24368 0.065583423 24368 0.033495959
24400 0.143494791 24400 0.063319567 12200 0.02771294 12220
0.039596413 24376 0.126765255 24376 0.069645663
24400 0.203932431 24400 0.075910524 12200 0.031680305 12224
0.023589773 24384 0.164051323 24384 0.068550282
24400 0.178756789 24400 0.033484506 12200 0.022689052 12228
0.015516946 24392 0.14528344 24392 0.049543069
24400 0.106639265 24400 0.109553934 12200 0.030207084 12232
0.00445796 24400 0.064697262 24400 0.040135896
24400 0.038590242 24400 0.160435593 12200 0.083540028 12236
0.047428039 24408 0.055101486 24408 0.023875597
24400 0.060667262 24400 0.156065711 12200 0.109465524 12240
0.07069901 24416 0.106461877 24416 0.018364604
24400 0.160235374 24400 0.126871863 12200 0.085245752 12244
0.042932708 24424 0.137359311 24424 0.049100218
24400 0.14071347 24400 0.079323181 12200 0.060928796 12248
0.022950608 24432 0.151435161 24432 0.04172351
24400 0.102466358 24400 0.089108304 12300 0.017334422 12252
0.023541674 24440 0.14252153 24440 0.029688014
24400 0.129889828 24400 0.12125606 12300 0.034127686 12256
0.056251167 24448 0.027737095 24448 0.038679875
24500 0.131540495 24500 0.132941611 12300 0.062824598 12260
0.087060049 24456 0.063954445 24456 0.0491381
24500 0.084029547 24500 0.093143935 12300 0.068063309 12264
0.097227814 24464 0.060535422 24464 0.048791637
24500 0.054007825 24500 0.099657198 12300 0.068227309 12268
0.068253379 24472 0.065327811 24472 0.045830355
24500 0.064543499 24500 0.209158374 12300 0.05075779 12272
0.041368934 24480 0.015482299 24480 0.0342403
24500 0.089901398 24500 0.140360789 12300 0.030981966 12276
0.043008146 24488 0.084135369 24488 0.008421677
24500 0.090416623 24500 0.051322069 12300 0.04324142 12280
0.063547916 24496 0.081240221 24496 0.038118295
24500 0.085397696 24500 0.009667883 12300 0.025413412 12284
0.043554679 24504 0.033662192 24504 0.030660067
24500 0.067743153 24500 0.068357396 12300 0.04085957 12288

0.019309799 24512 0.121529585 24512 0.028230696
24500 0.035581481 24500 0.091526505 12300 0.053324977 12292
0.025189833 24520 0.121037789 24520 0.054085907
24500 0.115710449 24500 0.101818034 12300 0.03249865 12296
0.038858903 24528 0.072534545 24528 0.03912301
24500 0.087738299 24500 0.093734292 12300 0.055923836 12300
0.081183483 24536 0.058659651 24536 0.033218166
24500 0.041788229 24500 0.025265808 12300 0.08303536 12304
0.091268848 24544 0.081620834 24544 0.04194662
24600 0.10654323 24600 0.083956191 12300 0.0772602 12308
0.075610245 24552 0.064182867 24552 0.010035973
24600 0.055694385 24600 0.113982438 12300 0.058812006 12312
0.083636573 24560 0.134747679 24560 0.041718526
24600 0.057075624 24600 0.087798202 12300 0.081587044 12316
0.079024496 24568 0.218005211 24568 0.028593137
24600 0.050232924 24600 0.045400549 12300 0.074410724 12320
0.047975416 24576 0.161450531 24576 0.005371789
24600 0.028773069 24600 0.035866684 12300 0.053555337 12324
0.026254844 24584 0.030822164 24584 0.007966264
24600 0.092323004 24600 0.05187104 12300 0.039572929 12328
0.016815555 24592 0.057515958 24592 0.008133483
24600 0.097258278 24600 0.038258419 12300 0.032481199 12332
0.028467623 24600 0.080217702 24600 0.036469373
24600 0.054339693 24600 0.076125616 12300 0.062403211 12336
0.029670178 24608 0.076887642 24608 0.052400723
24600 0.024610103 24600 0.131990834 12300 0.093854309 12340
0.040130988 24616 0.087224129 24616 0.046766996
24600 0.048744147 24600 0.11540149 12300 0.098419696 12344
0.043662887 24624 0.125050618 24624 0.00309391
24600 0.049230574 24600 0.068343303 12300 0.08300467 12348
0.021639526 24632 0.157946518 24632 0.059864171
24600 0.080462902 24600 0.091238144 12400 0.068489309 12352
0.006595187 24640 0.152998356 24640 0.095061943
24600 0.08709987 24600 0.142224016 12400 0.045940265 12356
0.017995808 24648 0.139610944 24648 0.080256454
24700 0.057079113 24700 0.137520314 12400 0.0231861 12360
0.030207304 24656 0.130784436 24656 0.030504132
24700 0.047529 24700 0.039412778 12400 0.034633642 12364
0.036989473 24664 0.107583372 24664 0.024176627
24700 0.042271135 24700 0.038926199 12400 0.053091877 12368
0.04503805 24672 0.091004156 24672 0.04548884
24700 0.138889591 24700 0.054670389 12400 0.051930776 12372
0.053853535 24680 0.139973723 24680 0.066298337
24700 0.136173476 24700 0.052775853 12400 0.031900505 12376
0.033040342 24688 0.134220508 24688 0.097731274
24700 0.105717423 24700 0.023594455 12400 0.027942328 12380
0.007805741 24696 0.086270055 24696 0.083832383
24700 0.100082711 24700 0.067549532 12400 0.03383193 12384
0.037788017 24704 0.11340381 24704 0.032239736
24700 0.045025561 24700 0.05234026 12400 0.026951877 12388
0.049665076 24712 0.118609583 24712 0.017046161
24700 0.049596361 24700 0.017178578 12400 0.009341023 12392
0.044433593 24720 0.111886009 24720 0.023201072
24700 0.05659246 24700 0.051601375 12400 0.029159199 12396
0.06904186 24728 0.125273756 24728 0.000562619
24700 0.093222319 24700 0.076312404 12400 0.030513022 12400
0.05882499 24736 0.08462595 24736 0.030607622
24700 0.162635071 24700 0.052913751 12400 0.06417733 12404
0.037035268 24744 0.107870816 24744 0.038176167
24800 0.15719817 24800 0.039428556 12400 0.090232716 12408
0.043175667 24752 0.108858854 24752 0.02221158
24800 0.132192435 24800 0.09576678 12400 0.045911769 12412
0.011632644 24760 0.040251933 24760 0.047594462
24800 0.137220574 24800 0.130758591 12400 0.050405561 12416
0.031218759 24768 0.116007192 24768 0.062825842
24800 0.097333577 24800 0.13730627 12400 0.110607303 12420
0.060153219 24776 0.126120285 24776 0.03133326
24800 0.088685592 24800 0.075760137 12400 0.084305808 12424

0.0675747 24784 0.173172331 24784 0.017919516
24800 0.11315669 24800 0.014111565 12400 0.021592932 12428
0.045563284 24792 0.161293195 24792 0.002470826
24800 0.127133971 24800 0.040391726 12400 0.064050786 12432
0.015139807 24800 0.093727882 24800 0.036241258
24800 0.135271315 24800 0.082843726 12400 0.065066713 12436
0.037231555 24808 0.060791124 24808 0.062099833
24800 0.021791217 24800 0.116851887 12400 0.024426166 12440
0.055322289 24816 0.02597021 24816 0.060806498
24800 0.104498293 24800 0.094920513 12400 0.048680086 12444
0.06885433 24824 0.14301455 24824 0.030671941
24800 0.05751093 24800 0.060630893 12400 0.024217417 12448
0.073272408 24832 0.158600379 24832 0.015743495
24800 0.047209578 24800 0.06185293 12500 0.033370994 12452
0.037545331 24840 0.059538535 24840 0.004501188
24800 0.063593805 24800 0.108287852 12500 0.039161238 12456
0.020416044 24848 0.02060192 24848 0.029921894
24900 0.044654556 24900 0.178023634 12500 0.004116223 12460
0.040078892 24856 0.053076459 24856 0.075555596
24900 0.135460271 24900 0.154759779 12500 0.040599334 12464
0.033933004 24864 0.15185478 24864 0.111886191
24900 0.16325264 24900 0.03742666 12500 0.035180612 12468
0.034248515 24872 0.167153266 24872 0.102233767
24900 0.136425093 24900 0.1071109 12500 0.009780066 12472
0.048573111 24880 0.10259886 24880 0.059154656
24900 0.09965097 24900 0.055250039 12500 0.05740688 12476
0.026316862 24888 0.085451342 24888 0.015470674
24900 0.102192091 24900 0.039425424 12500 0.05824407 12480
0.021809188 24896 0.088386449 24896 0.013749262
24900 0.14937442 24900 0.037088412 12500 0.015021015 12484
0.031457115 24904 0.08103525 24904 0.01483392
24900 0.189176339 24900 0.091608097 12500 0.041757827 12488
0.033162196 24912 0.072187111 24912 0.029528063
24900 0.140584874 24900 0.12857675 12500 0.037557653 12492
0.033941175 24920 0.1042308 24920 0.028417891
24900 0.02285365 24900 0.086294611 12500 0.020339205 12496
0.080355312 24928 0.14181438 24928 0.011659336
24900 0.134516988 24900 0.032448541 12500 0.045049241 12500
0.088399276 24936 0.162766068 24936 0.057714264
24900 0.090396294 24900 0.076401462 12500 0.075478936 12504
0.052320898 24944 0.165575738 24944 0.087329368
25000 0.065182379 25000 0.079638208 12500 0.062799751 12508
0.060893584 24952 0.08677732 24952 0.061244857
25000 0.080697319 25000 0.079228514 12500 0.02651503 12512
0.077692581 24960 0.100652149 24960 0.01164478
25000 0.050294108 25000 0.090799331 12500 0.011876667 12516
0.09597293 24968 0.168528219 24968 0.06342875
25000 0.064650922 25000 0.051760944 12500 0.023155504 12520
0.106405409 24976 0.095776122 24976 0.057126075
25000 0.1022457 25000 0.01596941 12500 0.021029149 12524
0.0766253 24984 0.048856637 24984 0.013029877
25000 0.13748814 25000 0.042193598 12500 0.025575284 12528
0.018535322 24992 0.096855736 24992 0.050752078
25000 0.115403083 25000 0.074941818 12500 0.078223937 12532
0.052332223 25000 0.122765312 25000 0.043113982
25000 0.051894265 25000 0.079127087 12500 0.13020914 12536
0.062341605 25008 0.139084135 25008 0.016478391
25000 0.028192748 25000 0.093372604 12500 0.10979073 12540
0.025044359 25016 0.077828721 25016 0.012705494
25000 0.103196195 25000 0.162298544 12500 0.055117227 12544
0.019668767 25024 0.043121647 25024 0.008945425
25000 0.166702026 25000 0.170871834 12500 0.028368429 12548
0.035016456 25032 0.115501367 25032 0.005142074
25000 0.101103542 25000 0.111118272 12600 0.012061755 12552
0.058411941 25040 0.080685575 25040 0.009439483
25000 0.010403751 25000 0.05127996 12600 0.016300934 12556
0.047523252 25048 0.054675889 25048 0.027529444
25100 0.022812938 25100 0.019317185 12600 0.015087584 12560

0.048006448	25056	0.057939506	25056	0.025744583		
25100	0.023459801	25100	0.089690169	12600	0.039226885	12564
0.086560664	25064	0.095828473	25064	0.022733064		
25100	0.01994068	25100	0.100240322	12600	0.068590227	12568
0.086085755	25072	0.110538655	25072	0.029674202		
25100	0.071881987	25100	0.051377556	12600	0.06538436	12572
0.020608768	25080	0.097985561	25080	0.059526828		
25100	0.058330905	25100	0.025891461	12600	0.055792996	12576
0.092198963	25088	0.085783271	25088	0.058541511		
25100	0.086139895	25100	0.032654774	12600	0.061921899	12580
0.14896359	25096	0.062097613	25096	0.032410644		
25100	0.091559916	25100	0.01347919	12600	0.055217526	12584
0.113852519	25104	0.037911574	25104	0.001103799		
25100	0.081189413	25100	0.026361495	12600	0.017517792	12588
0.035908459	25112	0.035197412	25112	0.028752507		
25100	0.12403063	25100	0.025267289	12600	0.018055074	12592
0.035032677	25120	0.079733647	25120	0.046179132		
25100	0.133167924	25100	0.077911718	12600	0.035995101	12596
0.057644549	25128	0.117732598	25128	0.049860206		
25100	0.080703518	25100	0.07605963	12600	0.0396746	12600
0.077550641	25136	0.081100778	25136	0.048487957		
25100	0.036786914	25100	0.093528834	12600	0.038528975	12604
0.08581554	25144	0.093032046	25144	0.049157952		
25200	0.032409964	25200	0.103251201	12600	0.064396998	12608
0.098717028	25152	0.134114991	25152	0.034051031		
25200	0.101379847	25200	0.032097978	12600	0.095224692	12612
0.10321055	25160	0.170766754	25160	0.016934358		
25200	0.135447321	25200	0.095881864	12600	0.115602416	12616
0.040012255	25168	0.136513904	25168	0.050247218		
25200	0.109114837	25200	0.064574502	12600	0.111343674	12620
0.033888322	25176	0.056642213	25176	0.072011222		
25200	0.07769576	25200	0.123082893	12600	0.097581244	12624
0.057626286	25184	0.144466598	25184	0.081342572		
25200	0.228028016	25200	0.086271997	12600	0.084739324	12628
0.041402454	25192	0.12233037	25192	0.068577938		
25200	0.316126796	25200	0.017669845	12600	0.055135472	12632
0.012272228	25200	0.066023051	25200	0.046453959		
25200	0.216194661	25200	0.059464168	12600	0.036566482	12636
0.020267176	25208	0.03307487	25208	0.039689396		
25200	0.063148837	25200	0.06468931	12600	0.034407367	12640
0.048304442	25216	0.041216197	25216	0.036101497		
25200	0.083276136	25200	0.138114119	12600	0.02393984	12644
0.076460136	25224	0.091625007	25224	0.042812244		
25200	0.110611247	25200	0.110075955	12600	0.030167428	12648
0.074697986	25232	0.140988254	25232	0.061315601		
25200	0.074825577	25200	0.015901293	12700	0.066338464	12652
0.065071661	25240	0.142516699	25240	0.056857451		
25200	0.03373505	25200	0.075018012	12700	0.066987108	12656
0.086000473	25248	0.104043313	25248	0.025795225		
25300	0.055338791	25300	0.13899096	12700	0.015120661	12660
0.091273818	25256	0.093771305	25256	0.042540403		
25300	0.143529891	25300	0.132092319	12700	0.066499873	12664
0.032777007	25264	0.056546945	25264	0.029186036		
25300	0.209330886	25300	0.084658379	12700	0.071528695	12668
0.051488427	25272	0.047303933	25272	0.031978914		
25300	0.194975743	25300	0.102287399	12700	0.032047199	12672
0.088905676	25280	0.09576483	25280	0.024722685		
25300	0.126329571	25300	0.066251443	12700	0.065664739	12676
0.082124803	25288	0.112900045	25288	0.030490388		
25300	0.062267289	25300	0.024236102	12700	0.091183661	12680
0.045047571	25296	0.08779834	25296	0.058946913		
25300	0.026866661	25300	0.04607243	12700	0.136635179	12684
0.051249266	25304	0.089707828	25304	0.04865279		
25300	0.041824722	25300	0.116432471	12700	0.075706572	12688
0.07878719	25312	0.064545086	25312	0.023021039		
25300	0.059825434	25300	0.142577803	12700	0.044604065	12692
0.087201712	25320	0.026623822	25320	0.043891192		
25300	0.136753719	25300	0.131875626	12700	0.068978596	12696

0.055201337 25328 0.073117721 25328 0.039041406
25300 0.211876468 25300 0.106555774 12700 0.025210946 12700
0.041503379 25336 0.136546544 25336 0.0262052
25300 0.22079151 25300 0.058639573 12700 0.063791245 12704
0.063560518 25344 0.143269179 25344 0.003076509
25400 0.175958893 25400 0.02629825 12700 0.070461356 12708
0.077450051 25352 0.096276766 25352 0.033243116
25400 0.136419286 25400 0.066873734 12700 0.024759795 12712
0.063365485 25360 0.088603025 25360 0.04749724
25400 0.086205517 25400 0.031935269 12700 0.066103916 12716
0.021730686 25368 0.022679022 25368 0.034948338
25400 0.051253181 25400 0.021817197 12700 0.069083748 12720
0.03159155 25376 0.111597175 25376 0.022815004
25400 0.121074249 25400 0.034497214 12700 0.036596808 12724
0.067796034 25384 0.181394033 25384 0.005903646
25400 0.143658646 25400 0.088043598 12700 0.032137963 12728
0.046009034 25392 0.143700483 25392 0.024295388
25400 0.070485199 25400 0.114767827 12700 0.035769936 12732
0.070969938 25400 0.044515076 25400 0.028533688
25400 0.142517165 25400 0.103433449 12700 0.031005631 12736
0.069838832 25408 0.045053661 25408 0.009477671
25400 0.098201374 25400 0.096690972 12700 0.062294479 12740
0.024736226 25416 0.087982036 25416 0.014299645
25400 0.076179873 25400 0.066124943 12700 0.098074474 12744
0.021167796 25424 0.093583418 25424 0.002388709
25400 0.073904106 25400 0.029747944 12700 0.095757896 12748
0.049860617 25432 0.07512467 25432 0.021459739
25400 0.032196356 25400 0.160666881 12800 0.052672913 12752
0.02718995 25440 0.051156785 25440 0.018577308
25400 0.056771587 25400 0.208001759 12800 0.00466565 12756
0.013928763 25448 0.031168213 25448 0.007151411
25500 0.092140253 25500 0.129210821 12800 0.029603792 12760
0.061026192 25456 0.035259422 25456 0.009653163
25500 0.124084167 25500 0.032308057 12800 0.057499983 12764
0.093681658 25464 0.078424717 25464 0.026609068
25500 0.139939235 25500 0.020914024 12800 0.064062508 12768
0.054744109 25472 0.11213839 25472 0.034244804
25500 0.146137668 25500 0.03864793 12800 0.052001851 12772
0.006426671 25480 0.089334186 25480 0.13725867
25500 0.073078947 25500 0.097756754 12800 0.027389555 12776
0.047168011 25488 0.023969751 25488 0.195295434
25500 0.059676135 25500 0.102069942 12800 0.020558578 12780
0.100108344 25496 0.038380153 25496 0.150970228
25500 0.070379669 25500 0.055539826 12800 0.051238963 12784
0.093487579 25504 0.039089428 25504 0.055487315
25500 0.090046662 25500 0.060332113 12800 0.05459418 12788
0.070127884 25512 0.037964124 25512 0.011168938
25500 0.154792796 25500 0.048601487 12800 0.060792623 12792
0.084924075 25520 0.040670875 25520 0.029140885
25500 0.163939374 25500 0.006302756 12800 0.072393777 12796
0.095986514 25528 0.036938287 25528 0.032534801
25500 0.179466326 25500 0.040864405 12800 0.074255368 12800
0.122839134 25536 0.030503525 25536 0.030162013
25500 0.102985658 25500 0.032206768 12800 0.069492489 12804
0.095477182 25544 0.052001407 25544 0.048368365
25600 0.108246808 25600 0.105991028 12800 0.055093402 12808
0.040340477 25552 0.104864979 25552 0.07645032
25600 0.111921348 25600 0.111923648 12800 0.033090772 12812
0.03439869 25560 0.160543495 25560 0.072196803
25600 0.055091601 25600 0.046443929 12800 0.044969009 12816
0.044246688 25568 0.168971048 25568 0.061519924
25600 0.039353949 25600 0.052225954 12800 0.055702549 12820
0.071989503 25576 0.127472799 25576 0.058377053
25600 0.031039886 25600 0.081228893 12800 0.024599911 12824
0.108120054 25584 0.038608705 25584 0.06453773
25600 0.041134994 25600 0.130017026 12800 0.023114541 12828
0.106361542 25592 0.034788052 25592 0.063120948
25600 0.11124782 25600 0.124240047 12800 0.024496187 12832

0.08091957 25600 0.05432278 25600 0.035961504
25600 0.16261694 25600 0.108827757 12800 0.043182867 12836
0.062306994 25608 0.098246346 25608 0.012938112
25600 0.153219764 25600 0.09467575 12800 0.034893634 12840
0.053059022 25616 0.087103239 25616 0.014782002
25600 0.139117503 25600 0.031149579 12800 0.027617514 12844
0.027977534 25624 0.046887642 25624 0.016780963
25600 0.121529309 25600 0.091832269 12800 0.033312746 12848
0.002411766 25632 0.021311094 25632 0.013922787
25600 0.054430118 25600 0.14057789 12900 0.0128923 12852
0.033218745 25640 0.074106356 25640 0.019920504
25600 0.043320801 25600 0.098876211 12900 0.04650011 12856
0.044824672 25648 0.063139727 25648 0.027510314
25700 0.073646515 25700 0.046336078 12900 0.06790577 12860
0.053343611 25656 0.068349473 25656 0.026413107
25700 0.043502281 25700 0.033377542 12900 0.088949761 12864
0.090104964 25664 0.070741589 25664 0.01330445
25700 0.101098216 25700 0.114074974 12900 0.058356109 12868
0.095226889 25672 0.049921673 25672 0.029252884
25700 0.047862915 25700 0.153527988 12900 0.041895746 12872
0.025149797 25680 0.078395759 25680 0.048411279
25700 0.033224224 25700 0.122333193 12900 0.043412922 12876
0.051191437 25688 0.118782285 25688 0.053442516
25700 0.033437373 25700 0.062251937 12900 0.02538483 12880
0.050546434 25696 0.110922971 25696 0.03492408
25700 0.07208372 25700 0.053489734 12900 0.037668342 12884
0.035828409 25704 0.047760383 25704 0.004403492
25700 0.078200363 25700 0.141187061 12900 0.046153869 12888
0.054180313 25712 0.095866213 25712 0.023154193
25700 0.11674971 25700 0.181484663 12900 0.062820567 12892
0.049803162 25720 0.154415975 25720 0.049638104
25700 0.182166143 25700 0.151037559 12900 0.057693287 12896
0.034695389 25728 0.127812688 25728 0.062094965
25700 0.211237319 25700 0.120297889 12900 0.019404602 12900
0.047810983 25736 0.036435326 25736 0.052159889
25700 0.195144909 25700 0.116944015 12900 0.028620219 12904
0.047260597 25744 0.068500347 25744 0.027325203
25800 0.219201174 25800 0.129356893 12900 0.031121996 12908
0.064202606 25752 0.100773425 25752 0.045136894
25800 0.37052756 25800 0.134092595 12900 0.016690665 12912
0.069762209 25760 0.122817728 25760 0.071323535
25800 0.378635654 25800 0.121294041 12900 0.050594896 12916
0.040266605 25768 0.049323156 25768 0.103512066
25800 0.225971336 25800 0.070518552 12900 0.054519234 12920
0.070261463 25776 0.091769936 25776 0.102214501
25800 0.253719161 25800 0.034183984 12900 0.041618005 12924
0.080178426 25784 0.139538461 25784 0.076629498
25800 0.215256776 25800 0.105424173 12900 0.026086003 12928
0.054813045 25792 0.08327698 25792 0.053088799
25800 0.142436256 25800 0.134714254 12900 0.037063092 12932
0.039727471 25800 0.128224419 25800 0.024021394
25800 0.220606803 25800 0.10572881 12900 0.044527238 12936
0.055916091 25808 0.124525745 25808 0.046269095
25800 0.118044067 25800 0.058584468 12900 0.027882219 12940
0.092883944 25816 0.120789911 25816 0.050959025
25800 0.071221904 25800 0.011076741 12900 0.038657738 12944
0.12242813 25824 0.103574494 25824 0.054435906
25800 0.072119437 25800 0.046521069 12900 0.058315804 12948
0.086939865 25832 0.080721897 25832 0.041422896
25800 0.210502069 25800 0.083779793 13000 0.040835235 12952
0.004695805 25840 0.07683313 25840 0.006099665
25800 0.283276895 25800 0.047459398 13000 0.032050331 12956
0.061044317 25848 0.073265925 25848 0.031804586
25900 0.205451114 25900 0.098826859 13000 0.038741338 12960
0.086698645 25856 0.166794955 25856 0.021841372
25900 0.076324504 25900 0.128658183 13000 0.019848263 12964
0.072533323 25864 0.160298543 25864 0.012780594
25900 0.096017837 25900 0.078062018 13000 0.020612724 12968

0.062182699	25872	0.026540054	25872	0.007401305		
25900	0.16540871	25900	0.054011962	13000	0.050520608	12972
0.035178644	25880	0.116043251	25880	0.005882569		
25900	0.22920144	25900	0.104089537	13000	0.056726636	12976
0.032581924	25888	0.189963131	25888	0.035429424		
25900	0.253531878	25900	0.157690505	13000	0.047819041	12980
0.059328773	25896	0.167344915	25896	0.061163199		
25900	0.23189593	25900	0.116768199	13000	0.074673932	12984
0.079984158	25904	0.121973098	25904	0.051236675		
25900	0.209944599	25900	0.067810361	13000	0.079642792	12988
0.07355641	25912	0.100286241	25912	0.045272544		
25900	0.111844689	25900	0.017210103	13000	0.075897944	12992
0.030117595	25920	0.043171305	25920	0.065051405		
25900	0.121689751	25900	0.07285062	13000	0.099545847	12996
0.029825365	25928	0.009251198	25928	0.062788982		
25900	0.103837461	25900	0.106212341	13000	0.07876032	13000
0.042555781	25936	0.047818652	25936	0.049543905		
25900	0.141056138	25900	0.118511904	13000	0.02356028	13004
0.017287226	25944	0.039727886	25944	0.067887537		
26000	0.281614106	26000	0.169593885	13000	0.014406137	13008
0.032162629	25952	0.173319029	25952	0.062405517		
26000	0.335791934	26000	0.120696801	13000	0.030118059	13012
0.015119498	25960	0.174763904	25960	0.054200868		
26000	0.241521004	26000	0.018105628	13000	0.031138916	13016
0.016474489	25968	0.089893205	25968	0.027877004		
26000	0.279244938	26000	0.019395378	13000	0.019964593	13020
0.023881697	25976	0.070066642	25976	0.044601449		
26000	0.343724765	26000	0.049405691	13000	0.027749706	13024
0.041548839	25984	0.108866159	25984	0.037249982		
26000	0.478932518	26000	0.079595789	13000	0.040186071	13028
0.087808134	25992	0.117097319	25992	0.024928804		
26000	0.51614159	26000	0.073469251	13000	0.036461344	13032
0.07997208	26000	0.092591734	26000	0.033507233		
26000	0.253498205	26000	0.011901136	13000	0.028738077	13036
0.046240493	26008	0.077832236	26008	0.010644516		
26000	0.705211016	26000	0.095377465	13000	0.016178032	13040
0.052788324	26016	0.106692402	26016	0.030175357		
26000	2.323335038	26000	0.093954397	13000	0.027560996	13044
0.05818082	26024	0.119174132	26024	0.059536546		
26000	3.603070513	26000	0.063324551	13000	0.034673183	13048
0.044033062	26032	0.14706052	26032	0.06449785		
26000	3.081803438	26000	0.125672319	13100	0.047307349	13052
0.015265725	26040	0.145047293	26040	0.053369498		
26000	1.39963755	26000	0.174956396	13100	0.061785308	13056
0.039519637	26048	0.093835712	26048	0.059685302		
26100	1.317128538	26100	0.156743293	13100	0.036559472	13060
0.07110701	26056	0.086153188	26056	0.064387321		
26100	0.924149295	26100	0.12006273	13100	0.016241742	13064
0.092537775	26064	0.02074797	26064	0.050474821		
26100	0.567290933	26100	0.085563392	13100	0.029067731	13068
0.064153377	26072	0.085967848	26072	0.023497802		
26100	0.900483225	26100	0.027551218	13100	0.044238994	13072
0.034674311	26080	0.047684978	26080	0.036752455		
26100	0.802319614	26100	0.026367234	13100	0.038767259	13076
0.032024716	26088	0.084015548	26088	0.046522906		
26100	0.783120806	26100	0.066464359	13100	0.023679095	13080
0.015706797	26096	0.085409782	26096	0.027754164		
26100	0.811338018	26100	0.170128799	13100	0.002553522	13084
0.035822824	26104	0.068633162	26104	0.024988729		
26100	1.107447199	26100	0.20643977	13100	0.01446747	13088
0.061819766	26112	0.092498798	26112	0.0453551		
26100	1.167542069	26100	0.124292463	13100	0.042348165	13092
0.063387066	26120	0.093846895	26120	0.066891662		
26100	1.058106078	26100	0.016080479	13100	0.073650692	13096
0.031865202	26128	0.027872924	26128	0.044950837		
26100	1.102081849	26100	0.035917536	13100	0.0496387	13100
0.070281829	26136	0.056714274	26136	0.027221238		
26100	1.3021183	26100	0.03202078	13100	0.05594996	13104

0.057077792 26144 0.071201859 26144 0.03664539
26200 1.458215525 26200 0.088050147 13100 0.070013615 13108
0.058625949 26152 0.03486696 26152 0.014920982
26200 1.566023338 26200 0.087920176 13100 0.028284207 13112
0.062413892 26160 0.029799194 26160 0.021030006
26200 1.650157963 26200 0.01049502 13100 0.123186474 13116
0.049784805 26168 0.099086647 26168 0.04396134
26200 1.861865638 26200 0.128525513 13100 0.132350324 13120
0.025799469 26176 0.123130041 26176 0.054674783
26200 2.136107538 26200 0.190215884 13100 0.072652743 13124
0.003441681 26184 0.050825162 26184 0.061960047
26200 2.250387563 26200 0.153748915 13100 0.020417161 13128
0.045851757 26192 0.100875768 26192 0.083442239
26200 2.503407888 26200 0.044980963 13100 0.031196258 13132
0.082201332 26200 0.107768545 26200 0.099621466
26200 2.8372826 26200 0.031834086 13100 0.060820436 13136
0.066823479 26208 0.018449038 26208 0.087783097
26200 3.453040263 26200 0.015546375 13100 0.06215254 13140
0.061534513 26216 0.063039923 26216 0.05845389
26200 3.816122425 26200 0.082050414 13100 0.059751081 13144
0.051388477 26224 0.032565426 26224 0.026599873
26200 4.678344825 26200 0.103157552 13100 0.064141575 13148
0.032597505 26232 0.10383466 26232 0.014541225
26200 5.40011795 26200 0.067933397 13200 0.058312508 13152
0.083626888 26240 0.145979023 26240 0.032422558
26200 6.708449213 26200 0.019241657 13200 0.045655928 13156
0.050900395 26248 0.109152141 26248 0.017748051
26300 9.645686488 26300 0.035286284 13200 0.048644928 13160
0.031325169 26256 0.112354632 26256 0.028067065
26300 13.86415488 26300 0.020861377 13200 0.056038414 13164
0.071677132 26264 0.119963603 26264 0.021212452
26300 14.63045925 26300 0.093693023 13200 0.040721527 13168
0.079407197 26272 0.092282207 26272 0.016836726
26300 15.58769588 26300 0.122758676 13200 0.01590148 13172
0.073446907 26280 0.148135485 26280 0.025167125
26300 85.12227988 26300 0.066120301 13200 0.030641681 13176
0.104347586 26288 0.089353707 26288 0.036223362
26300 188.6407138 26300 0.025762714 13200 0.051830368 13180
0.105182648 26296 0.029704006 26296 0.044920045
26300 216.697455 26300 0.018200688 13200 0.027026144 13184
0.03960562 26304 0.09393091 26304 0.052079828
26300 134.3213325 26300 0.03012309 13200 0.03020755 13188
0.028712493 26312 0.110303539 26312 0.056746932
26300 35.05374488 26300 0.051868905 13200 0.029675986 13192
0.058682705 26320 0.100405821 26320 0.055197612
26300 19.84744888 26300 0.16009841 13200 0.041587136 13196
0.06708486 26328 0.058079175 26328 0.04234966
26300 10.91304143 26300 0.148002 13200 0.058143414 13200
0.070292816 26336 0.066224995 26336 0.018951074
26300 9.6470248 26300 0.080121878 13200 0.050708597 13204
0.096080592 26344 0.140584831 26344 0.005608907
26400 5.561814638 26400 0.078875521 13200 0.047919049 13208
0.088386383 26352 0.154446854 26352 0.011835105
26400 3.578190463 26400 0.076924618 13200 0.070631228 13212
0.090880589 26360 0.130184068 26360 0.017108721
26400 1.908993463 26400 0.08611376 13200 0.058850012 13216
0.048339545 26368 0.081008795 26368 0.020297301
26400 0.532322446 26400 0.102780025 13200 0.051550833 13220
0.064559987 26376 0.083277744 26376 0.020615464
26400 0.203701201 26400 0.098716271 13200 0.025104053 13224
0.109495341 26384 0.109214874 26384 0.021919123
26400 0.754641428 26400 0.156653463 13200 0.024491488 13228
0.097037941 26392 0.070729351 26392 0.02794536
26400 0.988156185 26400 0.140537203 13200 0.028864091 13232
0.080710524 26400 0.045383433 26400 0.038424445
26400 1.017008443 26400 0.064365828 13200 0.031957832 13236
0.104109917 26408 0.107869375 26408 0.047682352
26400 0.895547215 26400 0.085859691 13200 0.057224035 13240

0.089299159 26416 0.168995394 26416 0.044650784
26400 0.856851694 26400 0.155997841 13200 0.059071026 13244
0.074891679 26424 0.148260195 26424 0.0300022
26400 0.924265245 26400 0.155029048 13200 0.053674572 13248
0.096282936 26432 0.11595268 26432 0.036253194
26400 1.0166805 26400 0.106976884 13300 0.03842037 13252
0.09069364 26440 0.157350238 26440 0.024607396
26400 1.131444588 26400 0.11472762 13300 0.015623471 13256
0.098478362 26448 0.16136936 26448 0.021861477
26500 1.126500661 26500 0.088902008 13300 0.025308011 13260
0.088291832 26456 0.084715262 26456 0.005887525
26500 1.142789843 26500 0.0697379 13300 0.052129726 13264
0.07177575 26464 0.056157933 26464 0.055329787
26500 1.066013473 26500 0.081983548 13300 0.029261266 13268
0.062097017 26472 0.04512431 26472 0.081651029
26500 1.109172241 26500 0.082413986 13300 0.011051616 13272
0.085269378 26480 0.074461124 26480 0.046490139
26500 1.064458979 26500 0.074878604 13300 0.009150434 13276
0.115721021 26488 0.048201819 26488 0.010992682
26500 1.012325286 26500 0.122586163 13300 0.031862623 13280
0.103266211 26496 0.078415564 26496 0.04297472
26500 1.061833114 26500 0.13900864 13300 0.019154773 13284
0.037758415 26504 0.138393734 26504 0.051814135
26500 0.969768909 26500 0.267235911 13300 0.015521613 13288
0.076948716 26512 0.088526489 26512 0.055115437
26500 1.00212323 26500 0.299942621 13300 0.00085051 13292
0.089625617 26520 0.075175674 26520 0.058975071
26500 0.628115376 26500 0.182885269 13300 0.040713683 13296
0.06275779 26528 0.068693487 26528 0.050325551
26500 0.986596919 26500 0.081176549 13300 0.038498813 13300
0.078321464 26536 0.049935123 26536 0.061259954
26500 1.887665825 26500 0.084186118 13300 0.057513877 13304
0.105703955 26544 0.071562077 26544 0.064001877
26600 1.803588938 26600 0.126063125 13300 0.068513946 13308
0.076194308 26552 0.062968495 26552 0.042984633
26600 4.247468425 26600 0.140192336 13300 0.023575229 13312
0.085549014 26560 0.116407231 26560 0.019617934
26600 6.509923838 26600 0.065579763 13300 0.03972929 13316
0.095194664 26568 0.091854337 26568 0.010608347
26600 5.57667855 26600 0.087944696 13300 0.054196022 13320
0.053942164 26576 0.007305693 26576 0.01079487
26600 2.611215225 26600 0.14738286 13300 0.03130448 13324
0.059591184 26584 0.033100761 26584 0.005149524
26600 0.415578251 26600 0.072158444 13300 0.01807933 13328
0.064255175 26592 0.062348008 26592 0.007029242
26600 0.33318362 26600 0.084583349 13300 0.038901319 13332
0.053573167 26600 0.111743604 26600 0.010017027
26600 0.670299166 26600 0.075116965 13300 0.045545974 13336
0.116141535 26608 0.06884693 26608 0.030670733
26600 0.448372011 26600 0.09751664 13300 0.016582739 13340
0.156033363 26616 0.01898765 26616 0.023606202
26600 0.108255561 26600 0.150250911 13300 0.034164965 13344
0.099758989 26624 0.042691318 26624 0.035313478
26600 0.225095108 26600 0.167343256 13300 0.051415256 13348
0.053722895 26632 0.073092931 26632 0.083929517
26600 0.261807406 26600 0.14166518 13400 0.015872047 13352
0.072585084 26640 0.005171447 26640 0.072469738
26600 0.109588807 26600 0.073188872 13400 0.020506692 13356
0.055326651 26648 0.088275192 26648 0.036234251
26700 0.154217836 26700 0.089697394 13400 0.011878089 13360
0.032922235 26656 0.096580036 26656 0.026022921
26700 0.19803422 26700 0.091926922 13400 0.042036936 13364
0.055764824 26664 0.019697749 26664 0.011948291
26700 0.096149612 26700 0.098357778 13400 0.042474949 13368
0.060587121 26672 0.092146853 26672 0.018436975
26700 0.048519978 26700 0.069134687 13400 0.017577937 13372
0.085069267 26680 0.089229136 26680 0.039521048
26700 0.064966516 26700 0.046493638 13400 0.024432051 13376

0.07708199 26688 0.171264851 26688 0.045026878
26700 0.19335316 26700 0.044737237 13400 0.037504968 13380
0.053137454 26696 0.215435546 26696 0.025243637
26700 0.221133654 26700 0.074607357 13400 0.011526844 13384
0.046791756 26704 0.099993828 26704 0.030436208
26700 0.138422081 26700 0.088595312 13400 0.019611453 13388
0.105547639 26712 0.037215115 26712 0.04480781
26700 0.035166548 26700 0.070511931 13400 0.036290043 13392
0.078420511 26720 0.06645031 26720 0.031386819
26700 0.124807921 26700 0.049945771 13400 0.053612057 13396
0.007615014 26728 0.134646616 26728 0.022024215
26700 0.167052553 26700 0.041038373 13400 0.048654314 13400
0.042681251 26736 0.159157164 26736 0.046154586
26700 0.087432018 26700 0.09436332 13400 0.024909024 13404
0.055720924 26744 0.193454631 26744 0.059594066
26800 0.053908356 26800 0.115220166 13400 0.04041307 13408
0.075538643 26752 0.240273323 26752 0.040642713
26800 0.10378001 26800 0.089333225 13400 0.053617809 13412
0.104108229 26760 0.531598344 26760 0.029239509
26800 0.099542602 26800 0.106258332 13400 0.05634781 13416
0.037009737 26768 0.353053533 26768 0.057640515
26800 0.093073781 26800 0.102157319 13400 0.059715512 13420
0.069360001 26776 0.742820906 26776 0.037968806
26800 0.049753926 26800 0.049149672 13400 0.04875541 13424
0.119667064 26784 1.152366168 26784 0.05465611
26800 0.006616511 26800 0.052547704 13400 0.020252801 13428
0.086254542 26792 0.730087399 26792 0.060333183
26800 0.03640803 26800 0.046924419 13400 0.078891382 13432
0.035762849 26800 1.583383538 26800 0.031667183
26800 0.126159925 26800 0.036796515 13400 0.107441534 13436
0.058665341 26808 2.369671825 26808 0.010472825
26800 0.158999465 26800 0.068192458 13400 0.095922012 13440
0.089692374 26816 1.390988013 26816 0.06306982
26800 0.17607359 26800 0.066570137 13400 0.055624263 13444
0.080060599 26824 4.065858663 26824 0.080846898
26800 0.263263791 26800 0.056331373 13400 0.018316981 13448
0.034688343 26832 3.34472465 26832 0.052064806
26800 0.245541101 26800 0.196819339 13500 0.056410245 13452
0.017434655 26840 5.253801125 26840 0.038517919
26800 0.146390579 26800 0.189028651 13500 0.051564122 13456
0.05269585 26848 5.916972175 26848 0.067325622
26900 0.090270994 26900 0.116603318 13500 0.040146046 13460
0.074784541 26856 11.40374225 26856 0.085759588
26900 0.102934631 26900 0.116561074 13500 0.02633901 13464
0.044838613 26864 9.211692963 26864 0.076033808
26900 0.125833933 26900 0.114674236 13500 0.013093442 13468
0.014756344 26872 19.48071275 26872 0.049551734
26900 0.105050378 26900 0.09705234 13500 0.014710106 13472
0.006615011 26880 28.81314975 26880 0.024135841
26900 0.065751439 26900 0.078010839 13500 0.040312607 13476
0.042277468 26888 14.05436638 26888 0.030376721
26900 0.041720236 26900 0.130201035 13500 0.051771323 13480
0.067634021 26896 44.06674575 26896 0.021192798
26900 0.025900225 26900 0.077587894 13500 0.055412242 13484
0.03493877 26904 31.03897538 26904 0.005150032
26900 0.031402262 26900 0.138535455 13500 0.058366299 13488
0.032816261 26912 35.1926535 26912 0.037245212
26900 0.054891269 26900 0.160288619 13500 0.019947376 13492
0.074736832 26920 57.50915413 26920 0.073671756
26900 0.03399549 26900 0.087063505 13500 0.044930141 13496
0.073942567 26928 42.36235475 26928 0.060915245
26900 0.058955451 26900 0.079442987 13500 0.063112348 13500
0.020318537 26936 100.2039909 26936 0.018376773
26900 0.109698915 26900 0.112495378 13500 0.071678107 13504
0.031850625 26944 263.0189363 26944 0.010712086
27000 0.099298712 27000 0.065974833 13500 0.078763413 13508
0.049037488 26952 0 26952 0.013394416
27000 0.067999776 27000 0.010311659 13500 0.07944063 13512

0.054883825 26960 244.7362538 26960 0.06273295
27000 0.091058573 27000 0.035092544 13500 0.074644042 13516
0.043188051 26968 112.2758016 26968 0.097130236
27000 0.145039666 27000 0.078117999 13500 0.050544302 13520
0.029574572 26976 31.06392175 26976 0.053861346
27000 0.175126886 27000 0.04486453 13500 0.023258222 13524
0.043089534 26984 20.57187638 26984 0.00848531
27000 0.142450415 27000 0.040653547 13500 0.031608059 13528
0.0517675 26992 14.714214 26992 0.004527717
27000 0.075730597 27000 0.111321773 13500 0.043402448 13532
0.047683632 27000 14.45883238 27000 0.035820656
27000 0.051879219 27000 0.099812787 13500 0.070640359 13536
0.012371535 27008 8.012688713 27008 0.015033998
27000 0.142397418 27000 0.042041254 13500 0.080159167 13540
0.027647244 27016 6.363377438 27016 0.049902224
27000 0.171914915 27000 0.110219859 13500 0.040971896 13544
0.058185317 27024 9.331484325 27024 0.045313998
27000 0.167363775 27000 0.115098497 13500 0.027349333 13548
0.061098101 27032 5.081063138 27032 0.032355638
27000 0.137531155 27000 0.058332662 13600 0.053546337 13552
0.064529013 27040 1.501034475 27040 0.066405875
27000 0.021788277 27000 0.070184375 13600 0.07241132 13556
0.059535967 27048 4.485651388 27048 0.05340526
27100 0.09736955 27100 0.089159279 13600 0.088620283 13560
0.037001057 27056 2.654014388 27056 0.020794558
27100 0.101346799 27100 0.041729087 13600 0.072190182 13564
0.017187114 27064 1.641871413 27064 0.032144675
27100 0.028050967 27100 0.072476389 13600 0.024993868 13568
0.046019053 27072 2.65242695 27072 0.04190057
27100 0.090145986 27100 0.107665292 13600 0.015556489 13572
0.077178323 27080 1.864085438 27080 0.051026676
27100 0.064555832 27100 0.078515659 13600 0.027652071 13576
0.060540639 27088 1.595749175 27088 0.05027272
27100 0.015292866 27100 0.02314503 13600 0.037173479 13580
0.066953617 27096 1.415356875 27096 0.057205689
27100 0.055926645 27100 0.07495789 13600 0.047126177 13584
0.077246928 27104 0.876723381 27104 0.03859603
27100 0.037803151 27100 0.080930957 13600 0.061852828 13588
0.04826481 27112 0.614365098 27112 0.022861626
27100 0.0521976 27100 0.06162806 13600 0.041974399 13592
0.020001222 27120 0.415080401 27120 0.040649444
27100 0.070091039 27100 0.154997411 13600 0.017775203 13596
0.007138437 27128 0.425396545 27128 0.052066604
27100 0.081305254 27100 0.166141734 13600 0.054897697 13600
0.054696808 27136 0.647418085 27136 0.012338159
27100 0.090650436 27100 0.114973496 13600 0.072599483 13604
0.119530982 27144 0.448964304 27144 0.044483935
27200 0.099462821 27200 0.068220121 13600 0.068118694 13608
0.093728551 27152 0.412405149 27152 0.044271816
27200 0.144111836 27200 0.040872779 13600 0.040715284 13612
0.04273219 27160 0.294437806 27160 0.068926602
27200 0.151953398 27200 0.046461948 13600 0.010956638 13616
0.062643121 27168 0.211872263 27168 0.077784498
27200 0.072812378 27200 0.02094366 13600 0.01663229 13620
0.054938613 27176 0.110197805 27176 0.056223733
27200 0.065529959 27200 0.059853501 13600 0.00960841 13624
0.051697589 27184 0.138125266 27184 0.077653087
27200 0.163062694 27200 0.097417113 13600 0.028764076 13628
0.036669902 27192 0.236979569 27192 0.047192323
27200 0.162742755 27200 0.115175943 13600 0.050798743 13632
0.006757401 27200 0.167764156 27200 0.043716824
27200 0.039335384 27200 0.101791622 13600 0.046745376 13636
0.049763894 27208 0.064524531 27208 0.014331724
27200 0.06669767 27200 0.082413164 13600 0.054049524 13640
0.077090408 27216 0.025403462 27216 0.019352057
27200 0.096187025 27200 0.091955211 13600 0.060679442 13644
0.075740951 27224 0.079541649 27224 0.031873558
27200 0.113670059 27200 0.099520519 13600 0.063812819 13648

0.049551276 27232 0.068944559 27232 0.054586213
27200 0.080170532 27200 0.089351546 13700 0.035776906 13652
0.048772999 27240 0.092286427 27240 0.048340542
27200 0.063618361 27200 0.05463593 13700 0.064526736 13656
0.1043292 27248 0.115315888 27248 0.028842529
27300 0.149268351 27300 0.028357112 13700 0.102987069 13660
0.134282178 27256 0.124851562 27256 0.074003168
27300 0.160413401 27300 0.061441839 13700 0.056971279 13664
0.100886864 27264 0.069767462 27264 0.083548606
27300 0.123899867 27300 0.060145121 13700 0.036046033 13668
0.047880025 27272 0.066535911 27272 0.048139122
27300 0.142706485 27300 0.050825514 13700 0.058685469 13672
0.04518031 27280 0.105543637 27280 0.026918089
27300 0.13462588 27300 0.020327809 13700 0.057781319 13676
0.048987811 27288 0.040374933 27288 0.01413974
27300 0.069369642 27300 0.032256477 13700 0.065307853 13680
0.064913482 27296 0.082839179 27296 0.048454662
27300 0.046153658 27300 0.073685747 13700 0.06567128 13684
0.082725252 27304 0.114207891 27304 0.061320192
27300 0.04285682 27300 0.087000211 13700 0.052607018 13688
0.078406098 27312 0.066706591 27312 0.02892295
27300 0.160811425 27300 0.031687265 13700 0.026863505 13692
0.04666199 27320 0.046164729 27320 0.041767271
27300 0.208185229 27300 0.067497073 13700 0.019542898 13696
0.052405612 27328 0.11414101 27328 0.066298577
27300 0.204876153 27300 0.058432659 13700 0.046672525 13700
0.056961137 27336 0.150069798 27336 0.06232028
27300 0.179460825 27300 0.034683151 13700 0.030332731 13704
0.046533216 27344 0.131610795 27344 0.020988458
27400 0.117324489 27400 0.105004729 13700 0.067396191 13708
0.079508383 27352 0.109187808 27352 0.05568588
27400 0.121536708 27400 0.131639826 13700 0.116184208 13712
0.106596424 27360 0.068746471 27360 0.09080382
27400 0.104223007 27400 0.122823854 13700 0.115869014 13716
0.138131043 27368 0.078015983 27368 0.073008159
27400 0.11975543 27400 0.142544391 13700 0.069395566 13720
0.143719634 27376 0.101041005 27376 0.022502793
27400 0.101724036 27400 0.098156881 13700 0.060183622 13724
0.106158441 27384 0.084942636 27384 0.040776555
27400 0.053402357 27400 0.045748573 13700 0.060090024 13728
0.065228996 27392 0.078555495 27392 0.059983493
27400 0.037606526 27400 0.087812259 13700 0.035967023 13732
0.063631218 27400 0.037738148 27400 0.082963743
27400 0.038135037 27400 0.075137934 13700 0.022997805 13736
0.054210694 27408 0.048956768 27408 0.054505865
27400 0.034795252 27400 0.112643837 13700 0.038265531 13740
0.045559973 27416 0.045067427 27416 0.011012903
27400 0.036416848 27400 0.08199914 13700 0.057345413 13744
0.071129332 27424 0.043572411 27424 0.022166943
27400 0.056398658 27400 0.048157315 13700 0.06448663 13748
0.090613386 27432 0.116920164 27432 0.018228529
27400 0.078811609 27400 0.073082549 13800 0.101859776 13752
0.075092183 27440 0.110317094 27440 0.041895779
27400 0.023679673 27400 0.084092229 13800 0.152194349 13756
0.049964558 27448 0.009920906 27448 0.052428692
27500 0.048821858 27500 0.045079451 13800 0.139268523 13760
0.049228373 27456 0.081320097 27456 0.037199829
27500 0.100813049 27500 0.094250696 13800 0.05205904 13764
0.062193933 27464 0.099615099 27464 0.069039452
27500 0.169546213 27500 0.167159248 13800 0.03772747 13768
0.041488373 27472 0.082937608 27472 0.058456484
27500 0.129998313 27500 0.17071364 13800 0.097493154 13772
0.02091307 27480 0.069161557 27480 0.025225954
27500 0.053941774 27500 0.072220995 13800 0.160665514 13776
0.085787622 27488 0.05607725 27488 0.046709964
27500 0.038038274 27500 0.101938873 13800 0.133125431 13780
0.137489653 27496 0.055216846 27496 0.064541513
27500 0.008860175 27500 0.079980608 13800 0.033906301 13784

0.139439231 27504 0.118540571 27504 0.042418884
27500 0.053323591 27500 0.047796366 13800 0.071937953 13788
0.089905683 27512 0.125227118 27512 0.002183273
27500 0.056311332 27500 0.14291679 13800 0.10441996 13792
0.064354121 27520 0.11090278 27520 0.003562709
27500 0.016123207 27500 0.181515439 13800 0.130888409 13796
0.04008702 27528 0.08144274 27528 0.040415755
27500 0.033887802 27500 0.196902024 13800 0.127884138 13800
0.026806614 27536 0.059176546 27536 0.066132445
27500 0.099495002 27500 0.179739655 13800 0.026095027 13804
0.063188309 27544 0.064295811 27544 0.061494749
27600 0.125148916 27600 0.080480662 13800 0.11318054 13808
0.049886086 27552 0.104939732 27552 0.079424666
27600 0.033411132 27600 0.039390772 13800 0.16720235 13812
0.053392905 27560 0.097988646 27560 0.074348456
27600 0.094370626 27600 0.069651142 13800 0.157269911 13816
0.094938958 27568 0.077947181 27568 0.054798729
27600 0.130217901 27600 0.03031776 13800 0.117957614 13820
0.078107267 27576 0.065573942 27576 0.030002544
27600 0.133912836 27600 0.01987967 13800 0.088020264 13824
0.062593397 27584 0.049200757 27584 0.046667039
27600 0.105492305 27600 0.02487299 13800 0.196012378 13828
0.036877646 27592 0.05972174 27592 0.031942036
27600 0.032639488 27600 0.069780857 13800 0.182290809 13832
0.05122977 27600 0.01286691 27600 0.074284246
27600 0.007152365 27600 0.077318684 13800 0.384264189 13836
0.081568207 27608 0.111931215 27608 0.147671468
27600 0.125553881 27600 0.126964296 13800 0.54529577 13840
0.038853021 27616 0.106793035 27616 0.121755962
27600 0.143746321 27600 0.192206513 13800 0.370346621 13844
0.030753057 27624 0.058134734 27624 0.026601205
27600 0.024483181 27600 0.149886211 13800 0.18705093 13848
0.043196418 27632 0.116585128 27632 0.073969546
27600 0.146785271 27600 0.066818 13900 0.171550259 13852
0.060556475 27640 0.10510267 27640 0.101348596
27600 0.230234364 27600 0.042147571 13900 0.222598275 13856
0.056338813 27648 0.118113683 27648 0.103641178
27700 0.173445325 27700 0.119664372 13900 0.211376755 13860
0.016822967 27656 0.076531993 27656 0.081541373
27700 0.095710762 27700 0.199460833 13900 0.027280581 13864
0.032554635 27664 0.033762648 27664 0.073646428
27700 0.09590877 27700 0.188527556 13900 0.221959315 13868
0.043933418 27672 0.093280265 27672 0.092058632
27700 0.089176429 27700 0.12197937 13900 0.259782334 13872
0.041330648 27680 0.030366951 27680 0.057995916
27700 0.051229574 27700 0.068489702 13900 0.210644649 13876
0.031387142 27688 0.067710804 27688 0.120032193
27700 0.020810794 27700 0.003822631 13900 0.523840369 13880
0.031430362 27696 0.099227829 27696 0.137706011
27700 0.084993248 27700 0.074866977 13900 0.417174596 13884
0.030018462 27704 0.086772561 27704 0.073877614
27700 0.146874066 27700 0.094459909 13900 1.198361628 13888
0.071979797 27712 0.056232024 27712 0.080351987
27700 0.154011431 27700 0.107054933 13900 2.689580438 13892
0.05270142 27720 0.018678153 27720 0.082238257
27700 0.110425041 27700 0.114328919 13900 2.960168525 13896
0.090325331 27728 0.072939904 27728 0.070573864
27700 0.049376176 27700 0.079258105 13900 1.699007463 13900
0.155832546 27736 0.124535698 27736 0.118932534
27700 0.077385972 27700 0.059702463 13900 0.310851378 13904
0.13280334 27744 0.141962446 27744 0.15072382
27800 0.116665389 27800 0.072338669 13900 0.06035269 13908
0.067842346 27752 0.134534538 27752 0.090946553
27800 0.097562857 27800 0.048366459 13900 0.3191875 13912
0.05135635 27760 0.128196596 27760 0.04956669
27800 0.075984739 27800 0.033800145 13900 0.648417743 13916
0.054224332 27768 0.056082623 27768 0.113397691
27800 0.152028166 27800 0.094308838 13900 2.745861888 13920

0.069491689 27776 0.062972627 27776 0.12366868
27800 0.186140125 27800 0.084352541 13900 4.213151525 13924
0.082179322 27784 0.088949273 27784 0.060157567
27800 0.148413281 27800 0.044240838 13900 3.5319291 13928
0.070985348 27792 0.109339359 27792 0.07171958
27800 0.119356475 27800 0.085530082 13900 1.441172675 13932
0.054788259 27800 0.248293684 27800 0.096371237
27800 0.122854137 27800 0.104752959 13900 1.044684323 13936
0.057223715 27808 0.263173016 27808 0.099530844
27800 0.097208256 27800 0.079493984 13900 1.759081263 13940
0.114056828 27816 0.136479881 27816 0.122491154
27800 0.119514734 27800 0.030714051 13900 2.12292815 13944
0.133215698 27824 0.019165151 27824 0.149783184
27800 0.19552093 27800 0.12056288 13900 6.7204698 13948
0.073200827 27832 0.05795815 27832 0.187898636
27800 0.189189421 27800 0.191332118 14000 10.29975991 13952
0.021735097 27840 0.121269142 27840 0.132654459
27800 0.120964047 27800 0.160265743 14000 8.991337388 13956
0.051783856 27848 0.112473383 27848 0.107573957
27900 0.112624643 27900 0.13174802 14000 4.166206813 13960
0.071617353 27856 0.092219605 27856 0.038134258
27900 0.097163538 27900 0.177888709 14000 0.550112164 13964
0.048233804 27864 0.091381335 27864 0.10617196
27900 0.033097706 27900 0.098571865 14000 0.983375009 13968
0.022134156 27872 0.083803061 27872 0.154061039
27900 0.041426709 27900 0.034755842 14000 0.740752555 13972
0.039594357 27880 0.094873685 27880 0.138271396
27900 0.063497275 27900 0.11598815 14000 5.185612475 13976
0.037632617 27888 0.079828264 27888 0.147507439
27900 0.094646508 27900 0.155669798 14000 10.56399663 13980
0.017971848 27896 0.088590998 27896 0.155300368
27900 0.119526732 27900 0.133654379 14000 11.62159815 13984
0.029206693 27904 0.083047104 27904 0.203526491
27900 0.068241519 27900 0.08221867 14000 7.2168787 13988
0.043253418 27912 0.023190038 27912 0.150172855
27900 0.071931172 27900 0.145667553 14000 2.178832888 13992
0.019700172 27920 0.111925387 27920 0.161499309
27900 0.191144034 27900 0.12225777 14000 2.0220438 13996
0.011023639 27928 0.171840161 27928 0.144348494
27900 0.147818355 27900 0.05837857 14000 1.048573759 14000
0.032633423 27936 0.135753363 27936 0.134702655
27900 0.032117521 27900 0.078697987 14000 2.250343325 14004
0.045176439 27944 0.089881003 27944 0.228574485
28000 0.105678126 28000 0.095077019 14000 5.2090222 14008
0.050780422 27952 0.044014731 27952 0.222239498
28000 0.070791073 28000 0.068344729 14000 6.151428913 14012
0.04918205 27960 0.057754551 27960 0.167591338
28000 0.013171532 28000 0.051193234 14000 3.96843255 14016
0.040160383 27968 0.128829386 27968 0.15979982
28000 0.01088633 28000 0.103526654 14000 0.903479814 14020
0.040224688 27976 0.090358873 27976 0.227943253
28000 0.096179618 28000 0.123776146 14000 0.413034868 14024
0.03336063 27984 0.028916049 27984 0.208937926
28000 0.134555215 28000 0.103964187 14000 0.129462336 14028
0.012670841 27992 0.034693181 27992 0.18473524
28000 0.119123564 28000 0.03775291 14000 1.009926084 14032
0.05726215 28000 0.034447545 28000 0.240300709
28000 0.129772773 28000 0.076736789 14000 3.85196135 14036
0.088318142 28008 0.031952164 28008 0.198830909
28000 0.153245011 28000 0.114319286 14000 6.213337188 14040
0.066620909 28016 0.057860019 28016 0.249382894
28000 0.15312573 28000 0.102746955 14000 5.576224538 14044
0.058931342 28024 0.112013768 28024 0.272593635
28000 0.103699706 28000 0.023547229 14000 2.562471438 14048
0.033863438 28032 0.167314633 28032 0.245963165
28000 0.028080773 28000 0.10494372 14100 1.059660223 14052
0.023567931 28040 0.145234531 28040 0.242457725
28000 0.037921287 28000 0.164529353 14100 1.224630046 14056

0.05585275	28048	0.0704833	28048	0.244753203
28100	0.080072488	28100	0.172442873	14100	0.4869322	14060
0.080283229	28056	0.056254874	28056	0.283778994
28100	0.1298438	28100	0.111195099	14100	1.069774036	14064
0.080887694	28064	0.145658341	28064	0.299891108
28100	0.160587879	28100	0.044446544	14100	1.2501271	14068
0.05885632	28072	0.15524849	28072	0.337190694
28100	0.107933629	28100	0.130472166	14100	0.779401278	14072
0.042677169	28080	0.088613379	28080	0.293499238
28100	0.064269458	28100	0.101441794	14100	0.085671483	14076
0.024435714	28088	0.072894	28088	0.279814849
28100	0.078584213	28100	0.057892907	14100	0.543176779	14080
0.029516647	28096	0.100720732	28096	0.348670321
28100	0.08960066	28100	0.09176162	14100	0.524675183	14084
0.055265155	28104	0.066156397	28104	0.469969703
28100	0.06587791	28100	0.090316295	14100	0.441365526	14088
0.055945166	28112	0.070968454	28112	0.460334296
28100	0.017531163	28100	0.060631763	14100	0.220118294	14092
0.056712052	28120	0.054331878	28120	0.3801157
28100	0.107487555	28100	0.008143006	14100	1.185026719	14096
0.044118413	28128	0.080731923	28128	0.397703785
28100	0.133996786	28100	0.089906949	14100	1.559966825	14100
0.025675305	28136	0.089827037	28136	0.459550124
28100	0.097615419	28100	0.121583697	14100	1.004396356	14104
0.030212479	28144	0.067185698	28144	0.516586296
28200	0.048475136	28200	0.118117161	14100	0.456328184	14108
0.035294634	28152	0.112502137	28152	0.516803935
28200	0.090552516	28200	0.087680019	14100	0.559648674	14112
0.050189156	28160	0.096023345	28160	0.446139281
28200	0.113094451	28200	0.023465889	14100	0.423646329	14116
0.033788718	28168	0.128306289	28168	0.507455901
28200	0.041784544	28200	0.097024044	14100	0.528958276	14120
0.012960476	28176	0.09160101	28176	0.581425615
28200	0.050449067	28200	0.103021121	14100	0.500707421	14124
0.020684511	28184	0.036775156	28184	0.542108435
28200	0.088296598	28200	0.068658519	14100	0.363236264	14128
0.037871898	28192	0.056945857	28192	0.644903689
28200	0.072616203	28200	0.046449251	14100	0.471763895	14132
0.061432307	28200	0.005340797	28200	0.646441826
28200	0.029499752	28200	0.024396064	14100	0.596072816	14136
0.059367809	28208	0.071109047	28208	0.657498779
28200	0.085413158	28200	0.138657153	14100	0.635283883	14140
0.080329512	28216	0.083683903	28216	0.700596021
28200	0.060564686	28200	0.247164571	14100	0.547698116	14144
0.079180114	28224	0.060662733	28224	0.731019594
28200	0.07785896	28200	0.219145761	14100	0.464720099	14148
0.081595208	28232	0.028188119	28232	0.780561998
28200	0.079438527	28200	0.095328316	14200	0.52614382	14152
0.140286051	28240	0.029696192	28240	0.792538514
28200	0.095263524	28200	0.077432742	14200	0.718295865	14156
0.171239328	28248	0.044518463	28248	0.966859109
28300	0.0631515	28300	0.152582725	14200	0.713355374	14160
0.128963904	28256	0.041289804	28256	0.974324823
28300	0.058175112	28300	0.269085198	14200	0.573750469	14164
0.060386094	28264	0.0945953	28264	0.989005551
28300	0.07851077	28300	0.245860894	14200	0.5507319	14168
0.118675809	28272	0.150626773	28272	1.048591686
28300	0.116312425	28300	0.194568841	14200	0.50304184	14172
0.140295975	28280	0.07289763	28280	1.130112796
28300	0.056266796	28300	0.253824634	14200	0.58976264	14176
0.076307122	28288	0.089688503	28288	1.237633173
28300	0.027517106	28300	0.261895184	14200	0.567354669	14180
0.053786876	28296	0.041523239	28296	1.320828688
28300	0.034623095	28300	0.227184559	14200	0.529876735	14184
0.079728365	28304	0.066520799	28304	1.396971638
28300	0.062746127	28300	0.226527496	14200	0.532163831	14188
0.051605246	28312	0.063956904	28312	1.490066763
28300	0.070054521	28300	0.225947471	14200	0.596611878	14192

0.049829167 28320 0.020501278 28320 1.612552088
28300 0.036374171 28300 0.232393563 14200 0.620778475 14196
0.042613134 28328 0.036269958 28328 1.824638338
28300 0.040562176 28300 0.297984079 14200 0.472666493 14200
0.033846147 28336 0.035760182 28336 1.91087125
28300 0.020494783 28300 0.35109953 14200 0.316620979 14204
0.066410474 28344 0.022046492 28344 2.087451513
28400 0.032864384 28400 0.374529773 14200 0.369037298 14208
0.071930175 28352 0.085805979 28352 2.3784556
28400 0.094964889 28400 0.396363933 14200 0.42374563 14212
0.046943387 28360 0.085717147 28360 2.561907288
28400 0.111934649 28400 0.304004119 14200 0.284410228 14216
0.066434252 28368 0.04367486 28368 2.859469038
28400 0.064902459 28400 0.299449428 14200 0.105060724 14220
0.086060463 28376 0.057562775 28376 3.1233707
28400 0.036167647 28400 0.282937951 14200 0.115749826 14224
0.076341174 28384 0.114806207 28384 3.5047133
28400 0.046392008 28400 0.322947774 14200 0.12024177 14228
0.080290716 28392 0.201734089 28392 3.92600475
28400 0.048646121 28400 0.437419163 14200 0.111097223 14232
0.077215838 28400 0.209663921 28400 4.347701088
28400 0.057932866 28400 0.411792396 14200 0.092479022 14236
0.041316976 28408 0.180566305 28408 5.19550685
28400 0.108250104 28400 0.494822628 14200 0.055743043 14240
0.064740125 28416 0.132841568 28416 5.7026567
28400 0.163742065 28400 0.538064691 14200 0.040264709 14244
0.085868574 28424 0.08243951 28424 6.752348038
28400 0.141930606 28400 0.507492106 14200 0.0907621 14248
0.047950161 28432 0.090510039 28432 8.240148425
28400 0.039900144 28400 0.756405294 14300 0.131620909 14252
0.07764389 28440 0.087046508 28440 9.066242725
28400 0.039161991 28400 0.983350678 14300 0.104477847 14256
0.10271887 28448 0.051562387 28448 13.0946655
28500 0.019629244 28500 0.84059994 14300 0.048212023 14260
0.05992526 28456 0.028990278 28456 17.14085225
28500 0.011833772 28500 0.329327159 14300 0.006827083 14264
0.028911727 28464 0.081418322 28464 19.23473738
28500 0.043459608 28500 0.750906184 14300 0.031973341 14268
0.023676787 28472 0.08871717 28472 112.0444313
28500 0.071855124 28500 2.125629463 14300 0.053385706 14272
0.04465225 28480 0.09510044 28480 290.5434075
28500 0.079502977 28500 3.877234175 14300 0.051053128 14276
0.06713027 28488 0.11827721 28488 336.9504513
28500 0.04554887 28500 5.816216113 14300 0.031651685 14280
0.085964217 28496 0.07665679 28496 190.791875
28500 0.134033443 28500 7.205428088 14300 0.05085185 14284
0.088292974 28504 0.013573614 28504 32.44033088
28500 0.109525456 28500 8.960111063 14300 0.126998086 14288
0.037152247 28512 0.01463993 28512 24.21929125
28500 0.056251676 28500 12.37390656 14300 0.118149117 14292
0.048754249 28520 0.040044059 28520 13.98289488
28500 0.050245919 28500 20.27430013 14300 0.052591378 14296
0.072552357 28528 0.063479347 28528 11.84323896
28500 0.059725247 28500 35.75468063 14300 0.042801275 14300
0.07061354 28536 0.057692541 28536 10.13691258
28500 0.147453669 28500 55.00623213 14300 0.056615809 14304
0.060634298 28544 0.050004022 28544 7.6913368
28600 0.079608362 28600 74.14521275 14300 0.032510881 14308
0.062482759 28552 0.050552055 28552 6.854699925
28600 0.089207046 28600 87.39878988 14300 0.004995328 14312
0.068066518 28560 0.125415696 28560 5.900647025
28600 0.085912579 28600 97.98949963 14300 0.048444999 14316
0.056016314 28568 0.160204014 28568 4.890383688
28600 0.066944303 28600 157.9193475 14300 0.098014985 14320
0.084767256 28576 0.064651511 28576 4.257267338
28600 0.170069601 28600 224.9104375 14300 0.08513908 14324
0.113223512 28584 0.087471693 28584 3.667728738
28600 0.212141298 28600 215.2934375 14300 0.034444784 14328

0.095422045	28592	0.110985013	28592	3.061720638
28600	0.157831223	28600	129.229665	14300	0.005011555	14332
0.061851788	28600	0.064249281	28600	2.759661063
28600	0.079382837	28600	41.31366313	14300	0.033108827	14336
0.018380564	28608	0.01491143	28608	2.430192663
28600	0.046117541	28600	21.10536775	14300	0.055659974	14340
0.036964571	28616	0.067703877	28616	2.1861752
28600	0.014380142	28600	21.41021	14300	0.051556348	14344
0.091767419	28624	0.036277772	28624	1.99000375
28600	0.026439167	28600	22.07436038	14300	0.04061755	14348
0.115241608	28632	0.049033599	28632	1.785092525
28600	0.022049404	28600	14.71473275	14400	0.052501422	14352
0.107581844	28640	0.088424495	28640	1.575032488
28600	0.043379605	28600	6.837181288	14400	0.052399486	14356
0.086852109	28648	0.074863157	28648	1.441266388
28700	0.094241979	28700	3.458060788	14400	0.037383183	14360
0.032974709	28656	0.097864679	28656	1.219443395
28700	0.077752513	28700	4.443789363	14400	0.046042369	14364
0.041916981	28664	0.093698858	28664	0.994865318
28700	0.057672605	28700	4.277858888	14400	0.031158794	14368
0.093497241	28672	0.066735869	28672	0.865228881
28700	0.137253403	28700	3.778558463	14400	0.015797525	14372
0.079372701	28680	0.036944985	28680	0.728340586
28700	0.211703081	28700	2.984119113	14400	0.022973014	14376
0.086923727	28688	0.058865477	28688	0.655643176
28700	0.125183564	28700	2.500279338	14400	0.00685889	14380
0.079308324	28696	0.107459782	28696	0.577722968
28700	0.076612814	28700	2.072755475	14400	0.051534647	14384
0.043076256	28704	0.033422941	28704	0.533578219
28700	0.161850098	28700	2.106222788	14400	0.066973582	14388
0.051923125	28712	0.08733273	28712	0.519129156
28700	0.109598339	28700	1.737132788	14400	0.02621501	14392
0.124751314	28720	0.113268012	28720	0.444104546
28700	0.038817987	28700	1.452261813	14400	0.064074768	14396
0.125777311	28728	0.08983833	28728	0.454795285
28700	0.086535692	28700	1.513773813	14400	0.109154971	14400
0.130136351	28736	0.059011421	28736	0.451262371
28700	0.140866323	28700	1.231288654	14400	0.071165225	14404
0.161093238	28744	0.01946892	28744	0.406502339
28800	0.153081783	28800	0.835733489	14400	0.019302062	14408
0.117346572	28752	0.05919933	28752	0.356507691
28800	0.123130012	28800	0.758760143	14400	0.006547008	14412
0.032317654	28760	0.063706189	28760	0.295986393
28800	0.085231543	28800	0.795704895	14400	0.035534238	14416
0.026276508	28768	0.028020249	28768	0.286497059
28800	0.051118939	28800	0.785368903	14400	0.062185536	14420
0.0717484	28776	0.076583427	28776	0.323898159
28800	0.099195335	28800	0.51510986	14400	0.070277725	14424
0.120136239	28784	0.079442383	28784	0.332086696
28800	0.103366474	28800	0.555496779	14400	0.047690814	14428
0.10924285	28792	0.093625422	28792	0.304295245
28800	0.068340756	28800	0.803246396	14400	0.033039258	14432
0.071293158	28800	0.08540992	28800	0.288837823
28800	0.068844624	28800	0.73907274	14400	0.035063062	14436
0.08179908	28808	0.042424548	28808	0.332514406
28800	0.083353167	28800	0.501362724	14400	0.025730167	14440
0.084441803	28816	0.048986662	28816	0.317202328
28800	0.05305024	28800	0.36010376	14400	0.019658854	14444
0.06809186	28824	0.08196092	28824	0.264939619
28800	0.049970804	28800	0.419906515	14400	0.021732332	14448
0.089024194	28832	0.111708527	28832	0.231253944
28800	0.096558055	28800	0.50451234	14500	0.011036266	14452
0.086392465	28840	0.060749047	28840	0.21109145
28800	0.105909559	28800	0.496210764	14500	0.057746122	14456
0.026738067	28848	0.060833954	28848	0.266134128
28900	0.047018883	28900	0.326804409	14500	0.071438786	14460
0.040416693	28856	0.10463479	28856	0.315707323
28900	0.108131324	28900	0.204684285	14500	0.075252261	14464

0.059705573 28864 0.084349143 28864 0.220493719
28900 0.121548233 28900 0.2264762 14500 0.076695316 14468
0.061153696 28872 0.043109536 28872 0.187394005
28900 0.124130835 28900 0.21346964 14500 0.05895962 14472
0.045684821 28880 0.126175743 28880 0.176759655
28900 0.097628028 28900 0.361745246 14500 0.032522708 14476
0.026328708 28888 0.091202805 28888 0.167441554
28900 0.1586703 28900 0.41462807 14500 0.055099721 14480
0.034077115 28896 0.040855332 28896 0.149522515
28900 0.275230268 28900 0.361798593 14500 0.062053521 14484
0.043663942 28904 0.119799312 28904 0.107262385
28900 0.278194173 28900 0.28699747 14500 0.033247714 14488
0.026848018 28912 0.143026176 28912 0.169973616
28900 0.172976638 28900 0.261335401 14500 0.033315446 14492
0.052501706 28920 0.126686209 28920 0.156862516
28900 0.074547468 28900 0.266786956 14500 0.062567713 14496
0.050404637 28928 0.069354923 28928 0.19448626
28900 0.054545995 28900 0.186494639 14500 0.067984642 14500
0.059113398 28936 0.00690675 28936 0.199548871
28900 0.015079428 28900 0.075752185 14500 0.033056542 14504
0.069650356 28944 0.045165354 28944 0.154004549
29000 0.062975581 29000 0.173056863 14500 0.047648046 14508
0.062633437 28952 0.061498991 28952 0.119744342
29000 0.071315764 29000 0.252536236 14500 0.067505862 14512
0.039185168 28960 0.072579089 28960 0.125690596
29000 0.072702715 29000 0.177358786 14500 0.065999251 14516
0.045265599 28968 0.092543145 28968 0.187194848
29000 0.07761499 29000 0.102416823 14500 0.087003973 14520
0.074673575 28976 0.069101414 28976 0.141016644
29000 0.048165162 29000 0.127335574 14500 0.088203255 14524
0.068139729 28984 0.05599828 28984 0.087735643
29000 0.027360948 29000 0.082488215 14500 0.046676232 14528
0.026967056 28992 0.025447504 28992 0.123863923
29000 0.067206696 29000 0.078777724 14500 0.024606292 14532
0.042891068 29000 0.067473367 29000 0.09650179
29000 0.062116546 29000 0.103463637 14500 0.016040585 14536
0.05953743 29008 0.091233065 29008 0.071808572
29000 0.10089064 29000 0.103278675 14500 0.028438812 14540
0.044806006 29016 0.10113194 29016 0.120337048
29000 0.136105241 29000 0.126487794 14500 0.035707642 14544
0.021014275 29024 0.133360169 29024 0.158865223
29000 0.112554611 29000 0.118858319 14500 0.029168397 14548
0.040891064 29032 0.134921371 29032 0.127053179
29000 0.062455729 29000 0.195156769 14600 0.030008039 14552
0.04054861 29040 0.078851095 29040 0.111150868
29000 0.139470824 29000 0.210774859 14600 0.024889705 14556
0.016433349 29048 0.105413681 29048 0.105711828
29100 0.180700081 29100 0.118113501 14600 0.049321112 14560
0.037535501 29056 0.085664156 29056 0.116260904
29100 0.182901233 29100 0.038859438 14600 0.052063744 14564
0.013950952 29064 0.061409453 29064 0.104419145
29100 0.139340468 29100 0.056221823 14600 0.050088576 14568
0.040533301 29072 0.01197573 29072 0.095190917
29100 0.0960217 29100 0.030809282 14600 0.060651975 14572
0.032834596 29080 0.079133402 29080 0.099372141
29100 0.137704046 29100 0.156483264 14600 0.056458666 14576
0.012131386 29088 0.078464647 29088 0.056018031
29100 0.104971296 29100 0.155090529 14600 0.024925721 14580
0.024721518 29096 0.076329452 29096 0.115747171
29100 0.026774767 29100 0.073280746 14600 0.009082523 14584
0.036553807 29104 0.121637742 29104 0.119480137
29100 0.057473837 29100 0.038014423 14600 0.028090224 14588
0.059180169 29112 0.116052965 29112 0.106946645
29100 0.121272562 29100 0.022327713 14600 0.038727871 14592
0.044361063 29120 0.050264553 29120 0.094566887
29100 0.122331301 29100 0.124327082 14600 0.029321573 14596
0.008623387 29128 0.004967177 29128 0.080158556
29100 0.070907096 29100 0.167409831 14600 0.035260619 14600

0.047528953	29136	0.021401398	29136	0.067046574
29100	0.15099693	29100	0.145241473	14600	0.045855184	14604
0.070152601	29144	0.026486421	29144	0.09131994
29200	0.178358721	29200	0.102416583	14600	0.058130445	14608
0.081599348	29152	0.053702283	29152	0.095683259
29200	0.112213405	29200	0.083358784	14600	0.058662023	14612
0.089389017	29160	0.106522188	29160	0.096109558
29200	0.092357535	29200	0.079199468	14600	0.034818677	14616
0.082121325	29168	0.117965625	29168	0.122201062
29200	0.138322226	29200	0.101980135	14600	0.01997807	14620
0.055170305	29176	0.090862719	29176	0.046294535
29200	0.115803923	29200	0.157229923	14600	0.00266446	14624
0.048169939	29184	0.09704016	29184	0.085169057
29200	0.071402683	29200	0.190934953	14600	0.054450105	14628
0.054236894	29192	0.092284063	29192	0.11213086
29200	0.097084769	29200	0.13575 14600	0.093516603	14632
0.045343946	29200	0.056337383	29200	0.098651108
29200	0.139022115	29200	0.097004297	14600	0.07350657	14636
0.013359207	29208	0.016554153	29208	0.129631444
29200	0.14482408	29200	0.114405724	14600	0.030295805	14640
0.023468589	29216	0.091233575	29216	0.100251767
29200	0.112004105	29200	0.113823298	14600	0.056736131	14644
0.046032299	29224	0.14754555	29224	0.056881334
29200	0.083686762	29200	0.058866648	14600	0.067142173	14648
0.036219946	29232	0.106704312	29232	0.068814559
29200	0.079700956	29200	0.006507762	14700	0.055796623	14652
0.085694846	29240	0.077144294	29240	0.059003502
29200	0.078539386	29200	0.037967213	14700	0.035545741	14656
0.149360829	29248	0.101696787	29248	0.039485327
29300	0.082174185	29300	0.073044095	14700	0.026990103	14660
0.134822098	29256	0.024772504	29256	0.03628209
29300	0.087069697	29300	0.076278389	14700	0.006388642	14664
0.099339057	29264	0.027859298	29264	0.028296045
29300	0.040389266	29300	0.059583043	14700	0.029488301	14668
0.058195736	29272	0.069887297	29272	0.064902881
29300	0.099326971	29300	0.042764987	14700	0.038851977	14672
0.037116966	29280	0.132630041	29280	0.104795232
29300	0.141288998	29300	0.009067389	14700	0.03861852	14676
0.027548487	29288	0.12415259	29288	0.086838685
29300	0.104074148	29300	0.021927097	14700	0.045714722	14680
0.037774553	29296	0.100192956	29296	0.061970823
29300	0.088512526	29300	0.044769797	14700	0.042170959	14684
0.019975219	29304	0.050879473	29304	0.050812359
29300	0.127534469	29300	0.033089309	14700	0.018992785	14688
0.037966958	29312	0.086894266	29312	0.063674357
29300	0.133295645	29300	0.049799477	14700	0.009491885	14692
0.055186501	29320	0.073751362	29320	0.078876801
29300	0.108845801	29300	0.105850995	14700	0.027513683	14696
0.080041122	29328	0.067448098	29328	0.021506308
29300	0.101087921	29300	0.053284999	14700	0.034093715	14700
0.10271911	29336	0.099694094	29336	0.052328665
29300	0.105180247	29300	0.040098144	14700	0.022364135	14704
0.115121307	29344	0.083329847	29344	0.042660027
29400	0.153273664	29400	0.056941819	14700	0.04004499	14708
0.08330775	29352	0.139565905	29352	0.037024562
29400	0.201225805	29400	0.045107452	14700	0.080207174	14712
0.040834642	29360	0.197690256	29360	0.064948581
29400	0.171109946	29400	0.070186972	14700	0.080820784	14716
0.065201813	29368	0.157416929	29368	0.075824173
29400	0.105547857	29400	0.09939653	14700	0.077811172	14720
0.123310616	29376	0.071029914	29376	0.076818025
29400	0.024642022	29400	0.071349692	14700	0.072689581	14724
0.127609368	29384	0.077140139	29384	0.053510365
29400	0.057656496	29400	0.056263623	14700	0.054515971	14728
0.103294784	29392	0.054999044	29392	0.03180937
29400	0.100476827	29400	0.114712952	14700	0.038420545	14732
0.094459596	29400	0.034327186	29400	0.060800558
29400	0.195762114	29400	0.144260106	14700	0.011133478	14736

0.092038165 29408 0.112016969 29408 0.030626077
29400 0.129030319 29400 0.14923625 14700 0.035639074 14740
0.068737892 29416 0.177110168 29416 0.037541919
29400 0.026792679 29400 0.037924343 14700 0.047810656 14744
0.083359664 29424 0.140598276 29424 0.048975471
29400 0.083397121 29400 0.118411961 14700 0.038945102 14748
0.101439131 29432 0.069304333 29432 0.046255795
29400 0.077992474 29400 0.141082914 14800 0.023297043 14752
0.076209879 29440 0.032731234 29440 0.074643009
29400 0.049772967 29400 0.064945671 14800 0.025348165 14756
0.024152689 29448 0.091130401 29448 0.028356002
29500 0.02216826 29500 0.050565228 14800 0.035313817 14760
0.024105737 29456 0.163321761 29456 0.057475361
29500 0.021665561 29500 0.057670186 14800 0.019589157 14764
0.043441371 29464 0.159370044 29464 0.091102556
29500 0.063980246 29500 0.125142746 14800 0.028175367 14768
0.039015537 29472 0.093014896 29472 0.071646035
29500 0.030208861 29500 0.203084739 14800 0.047998783 14772
0.045137731 29480 0.036572332 29480 0.020546631
29500 0.085477703 29500 0.173249005 14800 0.034296008 14776
0.076609103 29488 0.067020737 29488 0.05994576
29500 0.143994913 29500 0.080290782 14800 0.034358815 14780
0.048013284 29496 0.069588663 29496 0.045352244
29500 0.090122208 29500 0.03872002 14800 0.040687839 14784
0.044162556 29504 0.047600992 29504 0.036195383
29500 0.034320092 29500 0.12415 14800 0.039981624 14788
0.087934299 29512 0.08194425 29512 0.081618018
29500 0.011655215 29500 0.158156836 14800 0.01502113 14792
0.0771919 29520 0.117218442 29520 0.05937547
29500 0.062250692 29500 0.135155205 14800 0.048000886 14796
0.051875963 29528 0.136812101 29528 0.047566344
29500 0.129584864 29500 0.151492291 14800 0.070935283 14800
0.075096425 29536 0.115054267 29536 0.111333218
29500 0.115145784 29500 0.181721509 14800 0.043692584 14804
0.096440694 29544 0.06256035 29544 0.115424795
29600 0.074063704 29600 0.15511339 14800 0.044163186 14808
0.08306864 29552 0.065673718 29552 0.055585129
29600 0.075401258 29600 0.104902261 14800 0.081899627 14812
0.076733821 29560 0.102776212 29560 0.035490324
29600 0.038817194 29600 0.105799627 14800 0.074003234 14816
0.077336343 29568 0.102638151 29568 0.015815394
29600 0.055167111 29600 0.112124231 14800 0.010090111 14820
0.052363801 29576 0.090920381 29576 0.065897359
29600 0.072313662 29600 0.087986533 14800 0.047633363 14824
0.0647165 29584 0.111794405 29584 0.058658174
29600 0.030561027 29600 0.046993991 14800 0.037202084 14828
0.140431366 29592 0.1268856 29592 0.070840055
29600 0.12193033 29600 0.114881841 14800 0.055003096 14832
0.153778499 29600 0.119881159 29600 0.075354154
29600 0.151997665 29600 0.073566596 14800 0.092138274 14836
0.0886989 29608 0.067341127 29608 0.048943071
29600 0.095231284 29600 0.019007406 14800 0.051752981 14840
0.010234767 29616 0.033282602 29616 0.039594321
29600 0.039331695 29600 0.09049938 14800 0.021589587 14844
0.043637097 29624 0.101501471 29624 0.010323552
29600 0.05281244 29600 0.114701419 14800 0.03260724 14848
0.087598179 29632 0.109241315 29632 0.033504493
29600 0.027426244 29600 0.084130319 14900 0.038552855 14852
0.107087355 29640 0.043205171 29640 0.050262643
29600 0.085552107 29600 0.049654016 14900 0.068598267 14856
0.138580799 29648 0.060162132 29648 0.051989489
29700 0.141070865 29700 0.080479018 14900 0.056461686 14860
0.139226424 29656 0.099371384 29656 0.021465885
29700 0.110829693 29700 0.085407468 14900 0.046873221 14864
0.052308442 29664 0.107152773 29664 0.057313184
29700 0.143341124 29700 0.111824309 14900 0.044944613 14868
0.062111059 29672 0.127919251 29672 0.077240708
29700 0.184187956 29700 0.148197433 14900 0.024571866 14872

0.07922946 29680 0.143593846 29680 0.05281642
29700 0.170172716 29700 0.100219149 14900 0.022692286 14876
0.061031806 29688 0.094518953 29688 0.011199858
29700 0.169922641 29700 0.08179967 14900 0.078789613 14880
0.053608314 29696 0.092637973 29696 0.024014149
29700 0.169604508 29700 0.075295 14900 0.104955805 14884
0.061149629 29704 0.124192491 29704 0.025769678
29700 0.109081717 29700 0.046152294 14900 0.086222521 14888
0.09270673 29712 0.158161508 29712 0.007805254
29700 0.030806266 29700 0.011853714 14900 0.056406327 14892
0.117853982 29720 0.189778846 29720 0.01949756
29700 0.033631444 29700 0.032074924 14900 0.056204222 14896
0.086406006 29728 0.15536409 29728 0.03764503
29700 0.077746299 29700 0.115164832 14900 0.042142023 14900
0.028957509 29736 0.073879935 29736 0.009963331
29700 0.140209391 29700 0.168769751 14900 0.012665905 14904
0.051192455 29744 0.067306064 29744 0.050578499
29800 0.173692475 29800 0.129601249 14900 0.031468899 14908
0.084514861 29752 0.055140092 29752 0.060902767
29800 0.141930475 29800 0.0605842 14900 0.045043427 14912
0.11402378 29760 0.100913378 29760 0.050847888
29800 0.117593751 29800 0.088082314 14900 0.032795168 14916
0.135567214 29768 0.16592977 29768 0.054721833
29800 0.13659893 29800 0.164226206 14900 0.041872801 14920
0.108226021 29776 0.177925641 29776 0.024054576
29800 0.15670486 29800 0.165518446 14900 0.047084104 14924
0.081005528 29784 0.136449759 29784 0.020409747
29800 0.116533542 29800 0.072503695 14900 0.054172655 14928
0.085766144 29792 0.032492135 29792 0.043143235
29800 0.035765334 29800 0.052263495 14900 0.100428435 14932
0.11041369 29800 0.063235508 29800 0.037264428
29800 0.042566302 29800 0.09376902 14900 0.104456536 14936
0.118610784 29808 0.09058039 29808 0.010891782
29800 0.050216164 29800 0.09568761 14900 0.066801717 14940
0.085832609 29816 0.066187124 29816 0.024610343
29800 0.058151669 29800 0.104563667 14900 0.032518983 14944
0.044213059 29824 0.04644641 29824 0.02251679
29800 0.075826909 29800 0.105624516 14900 0.007066838 14948
0.062560561 29832 0.065153632 29832 0.019807605
29800 0.088489745 29800 0.137540993 15000 0.017184575 14952
0.069415517 29840 0.084597552 29840 0.05349268
29800 0.118095297 29800 0.151092594 15000 0.061823383 14956
0.07650903 29848 0.024180041 29848 0.089269968
29900 0.172698929 29900 0.103732105 15000 0.067893197 14960
0.076192839 29856 0.077171222 29856 0.080644109
29900 0.160966404 29900 0.055052355 15000 0.01744876 14964
0.05171074 29864 0.108203181 29864 0.073418785
29900 0.028308685 29900 0.017564515 15000 0.027106726 14968
0.024345633 29872 0.078329067 29872 0.039811934
29900 0.065448148 29900 0.055158525 15000 0.060189072 14972
0.052929492 29880 0.058073612 29880 0.013677412
29900 0.004847549 29900 0.064561144 15000 0.052630858 14976
0.078159901 29888 0.096738637 29888 0.018082288
29900 0.077876277 29900 0.056654681 15000 0.022470844 14980
0.040431867 29896 0.080231781 29896 0.020783731
29900 0.027561788 29900 0.055949484 15000 0.032353615 14984
0.048566075 29904 0.064672066 29904 0.030311046
29900 0.053882013 29900 0.067468747 15000 0.059113929 14988
0.093801311 29912 0.120971061 29912 0.053389395
29900 0.024851141 29900 0.131329551 15000 0.095805539 14992
0.099589379 29920 0.177278591 29920 0.060403338
29900 0.072224546 29900 0.081062899 15000 0.111806876 14996
0.092196518 29928 0.164303346 29928 0.055981684
29900 0.023391092 29900 0.089249632 15000 0.108203894 15000
0.09021983 29936 0.112985603 29936 0.059919821
29900 0.057672492 29900 0.084838313 15000 0.087376924 15004
0.065850298 29944 0.156314286 29944 0.051499454
30000 0.070556227 30000 0.033207802 15000 0.051388499 15008

0.035101635 29952 0.17819136 29952 0.014804225
30000 0.011583054 30000 0.02631469 15000 0.035528479 15012
0.028871957 29960 0.104932406 29960 0.034364315
30000 0.051188024 30000 0.060006594 15000 0.042354113 15016
0.017515247 29968 0.034963654 29968 0.037009682
30000 0.051459967 30000 0.052346335 15000 0.04946371 15020
0.037119029 29976 0.147247454 29976 0.011112147
30000 0.025355561 30000 0.047044439 15000 0.057326597 15024
0.008660793 29984 0.147180894 29984 0.012861895
30000 0.088428838 30000 0.09775396 15000 0.052941348 15028
0.047807484 29992 0.075973338 29992 0.020358733
30000 0.099330362 30000 0.054731747 15000 0.034208712 15032
0.078585181 30000 0.054084314 30000 0.009658843
30000 0.060105085 30000 0.070066635 15000 0.024990537 15036
0.072362149 30008 0.066282351 30008 0.004575329
30000 0.053201376 30000 0.085142609 15000 0.027062784 15040
0.040388743 30016 0.069782007 30016 0.009337899
30000 0.095725278 30000 0.109062014 15000 0.039984061 15044
0.054915225 30024 0.061233666 30024 0.026122434
30000 0.067844994 30000 0.086031709 15000 0.049806913 15048
0.051663836 30032 0.055451736 30032 0.040244373
30000 0.080087652 30000 0.033894892 15100 0.033237968 15052
0.039467926 30040 0.066177927 30040 0.029158622
30000 0.124450948 30000 0.126507788 15100 0.04322124 15056
0.058548565 30048 0.045000757 30048 0.028919387
30100 0.103780061 30100 0.150897321 15100 0.05996001 15060
0.060418006 30056 0.034250119 30056 0.061642648
30100 0.062331099 30100 0.075938609 15100 0.075018535 15064
0.043944903 30064 0.081170249 30064 0.05185551
30100 0.026641006 30100 0.040449071 15100 0.092744296 15068
0.04497706 30072 0.108745269 30072 0.05021524
30100 0.098197503 30100 0.09873099 15100 0.098733064 15072
0.07293086 30080 0.039894367 30080 0.052017571
30100 0.112033558 30100 0.136880583 15100 0.07760786 15076
0.057501566 30088 0.113048904 30088 0.077874254
30100 0.101619909 30100 0.170120329 15100 0.034091707 15080
0.043572767 30096 0.169252613 30096 0.056217814
30100 0.139433934 30100 0.150122855 15100 0.025160922 15084
0.055836121 30104 0.111365334 30104 0.018783992
30100 0.098632801 30100 0.080701429 15100 0.045244171 15088
0.035331454 30112 0.035417517 30112 0.081307662
30100 0.055260156 30100 0.082840794 15100 0.072506664 15092
0.025261692 30120 0.012437413 30120 0.086095737
30100 0.031955071 30100 0.083049068 15100 0.089357571 15096
0.053474316 30128 0.076777375 30128 0.034751498
30100 0.030645802 30100 0.030541301 15100 0.095692652 15100
0.063072374 30136 0.137308744 30136 0.045892812
30100 0.067875117 30100 0.017477272 15100 0.119726101 15104
0.091627408 30144 0.181391704 30144 0.076922304
30200 0.120860226 30200 0.016692879 15100 0.110740235 15108
0.096959571 30152 0.185234429 30152 0.055911249
30200 0.115100957 30200 0.031890686 15100 0.070025053 15112
0.087422981 30160 0.139360753 30160 0.056707275
30200 0.018370869 30200 0.067143381 15100 0.048455673 15116
0.083623352 30168 0.075860604 30168 0.068171226
30200 0.072392555 30200 0.147315994 15100 0.029566561 15120
0.069374291 30176 0.035249625 30176 0.077868812
30200 0.067818051 30200 0.156353504 15100 0.036716527 15124
0.032314754 30184 0.039470833 30184 0.062684405
30200 0.047998026 30200 0.076410521 15100 0.052012831 15128
0.05389837 30192 0.060724029 30192 0.024304767
30200 0.043718428 30200 0.028906756 15100 0.045966688 15132
0.070662383 30200 0.029623512 30200 0.024187964
30200 0.065631197 30200 0.08317022 15100 0.058938753 15136
0.06612359 30208 0.016134392 30208 0.047919682
30200 0.094632174 30200 0.067376488 15100 0.06555333 15140
0.071056886 30216 0.051251816 30216 0.042854033
30200 0.072522154 30200 0.133862166 15100 0.050168819 15144

0.018693727 30224 0.094474104 30224 0.033491386
30200 0.091242378 30200 0.178434551 15100 0.04127697 15148
0.059381597 30232 0.07758884 30232 0.021721909
30200 0.095734773 30200 0.132553629 15200 0.042145442 15152
0.082610335 30240 0.011772203 30240 0.03840265
30200 0.089473171 30200 0.105917257 15200 0.074937816 15156
0.078396501 30248 0.081056969 30248 0.068454283
30300 0.059686758 30300 0.106517189 15200 0.085059677 15160
0.067679554 30256 0.116737196 30256 0.100754856
30300 0.039696231 30300 0.093271672 15200 0.074136347 15164
0.066155109 30264 0.069353315 30264 0.06589156
30300 0.032101623 30300 0.090260044 15200 0.048615297 15168
0.09145731 30272 0.015466509 30272 0.025983634
30300 0.053657805 30300 0.134078044 15200 0.042894379 15172
0.111591158 30280 0.075803539 30280 0.054811004
30300 0.051076902 30300 0.106931133 15200 0.047270998 15176
0.073792049 30288 0.07854982 30288 0.026719425
30300 0.018933732 30300 0.049874623 15200 0.014981156 15180
0.053743719 30296 0.081127342 30296 0.044526267
30300 0.0327214 30300 0.071931776 15200 0.036667778 15184
0.062912426 30304 0.087820175 30304 0.027313296
30300 0.082120685 30300 0.068010479 15200 0.064441243 15188
0.060180872 30312 0.057604138 30312 0.033252287
30300 0.088565968 30300 0.024253586 15200 0.050817391 15192
0.043107855 30320 0.042680262 30320 0.070846501
30300 0.059825674 30300 0.047184385 15200 0.04033012 15196
0.020180245 30328 0.074072726 30328 0.024323821
30300 0.119539836 30300 0.100209152 15200 0.063746738 15200
0.048849244 30336 0.177741545 30336 0.067946327
30300 0.145023544 30300 0.145477665 15200 0.045954035 15204
0.055050085 30344 0.15821938 30344 0.082111365
30400 0.123767328 30400 0.13132517 15200 0.016015052 15208
0.042015392 30352 0.060330385 30352 0.030534582
30400 0.084080049 30400 0.049456794 15200 0.020513784 15212
0.013735097 30360 0.056787671 30360 0.027520515
30400 0.079063568 30400 0.069642905 15200 0.060777384 15216
0.062406863 30368 0.066365064 30368 0.057567446
30400 0.061876621 30400 0.11404125 15200 0.07378171 15220
0.096956297 30376 0.001589291 30376 0.067040928
30400 0.07718378 30400 0.164605401 15200 0.048532187 15224
0.093593779 30384 0.110015317 30384 0.051529485
30400 0.063303625 30400 0.098157951 15200 0.042673692 15228
0.057959296 30392 0.128062733 30392 0.007296093
30400 0.097081378 30400 0.079979582 15200 0.071032846 15232
0.052807402 30400 0.028384564 30400 0.055039378
30400 0.130531378 30400 0.022687102 15200 0.096404925 15236
0.046390102 30408 0.136831848 30408 0.088222085
30400 0.071754119 30400 0.105420782 15200 0.088404871 15240
0.0424637 30416 0.1227662 30416 0.088414359
30400 0.003154954 30400 0.133565671 15200 0.048643808 15244
0.075660159 30424 0.066902823 30424 0.080477556
30400 0.082516381 30400 0.062546773 15200 0.063130196 15248
0.101790909 30432 0.103802238 30432 0.054481316
30400 0.132446439 30400 0.07311019 15300 0.090786372 15252
0.106307554 30440 0.117643067 30440 0.025000243
30400 0.124019876 30400 0.144896724 15300 0.118637036 15256
0.076523247 30448 0.112890964 30448 0.030866104
30500 0.112243331 30500 0.175766574 15300 0.129153311 15260
0.131718596 30456 0.106661391 30456 0.05818249
30500 0.111162728 30500 0.120677447 15300 0.096339689 15264
0.147387313 30464 0.101096935 30464 0.063592976
30500 0.077790915 30500 0.029640485 15300 0.046289955 15268
0.066227585 30472 0.061390958 30472 0.049501272
30500 0.082146449 30500 0.114150244 15300 0.014321751 15272
0.02310235 30480 0.044478366 30480 0.067239642
30500 0.094770934 30500 0.143480488 15300 0.054875374 15276
0.042956617 30488 0.059900798 30488 0.078160549
30500 0.058397825 30500 0.135153503 15300 0.09402314 15280

0.065300606 30496 0.123596066 30496 0.073459501
30500 0.027575048 30500 0.112617061 15300 0.08113643 15284
0.081700768 30504 0.136088231 30504 0.049125094
30500 0.094654788 30500 0.112401933 15300 0.038432601 15288
0.105779276 30512 0.050329763 30512 0.036610993
30500 0.148453371 30500 0.156164664 15300 0.007943981 15292
0.097572032 30520 0.052418054 30520 0.03571665
30500 0.140663396 30500 0.122927362 15300 0.020503987 15296
0.101840837 30528 0.118818491 30528 0.016011736
30500 0.043329237 30500 0.04244881 15300 0.010054134 15300
0.103722043 30536 0.122495607 30536 0.045585293
30500 0.101114783 30500 0.082093269 15300 0.045899436 15304
0.057298476 30544 0.10131715 30544 0.042026215
30600 0.094662471 30600 0.13325637 15300 0.053529497 15308
0.046223075 30552 0.117960735 30552 0.035083955
30600 0.034877747 30600 0.195614354 15300 0.059086335 15312
0.059412625 30560 0.105601474 30560 0.04926051
30600 0.077973789 30600 0.209453166 15300 0.067415953 15316
0.050254232 30568 0.055439523 30568 0.039427683
30600 0.148234554 30600 0.126321611 15300 0.048884329 15320
0.040210103 30576 0.098484794 30576 0.038266706
30600 0.097551783 30600 0.038520728 15300 0.016318418 15324
0.05213737 30584 0.091869799 30584 0.036464615
30600 0.046635709 30600 0.043987904 15300 0.04374329 15328
0.06790895 30592 0.076522563 30592 0.015678577
30600 0.053062711 30600 0.088165958 15300 0.075302349 15332
0.095832285 30600 0.121014251 30600 0.01801536
30600 0.123734964 30600 0.092852584 15300 0.089504225 15336
0.134562841 30608 0.041823612 30608 0.010091486
30600 0.163361358 30600 0.042292308 15300 0.097057724 15340
0.120808953 30616 0.051594205 30616 0.037827882
30600 0.087590772 30600 0.031442862 15300 0.05505524 15344
0.049554692 30624 0.009925242 30624 0.045321274
30600 0.032102467 30600 0.049694038 15300 0.028514638 15348
0.020772881 30632 0.112679722 30632 0.038595725
30600 0.075384181 30600 0.025766716 15400 0.043220014 15352
0.090420253 30640 0.092056987 30640 0.038013459
30600 0.053652824 30600 0.046095327 15400 0.039109658 15356
0.133941256 30648 0.079141915 30648 0.005800825
30700 0.05804043 30700 0.064842854 15400 0.048785034 15360
0.117487005 30656 0.115643066 30656 0.062198487
30700 0.115375784 30700 0.05925 15400 0.059257225 15364
0.091064365 30664 0.037264294 30664 0.085786138
30700 0.089001849 30700 0.042770244 15400 0.041893076 15368
0.115535266 30672 0.059216563 30672 0.060793595
30700 0.027684882 30700 0.030549461 15400 0.02274597 15372
0.115874922 30680 0.098600824 30680 0.030264302
30700 0.095357405 30700 0.121431556 15400 0.021848051 15376
0.070775714 30688 0.093331313 30688 0.041507665
30700 0.111369242 30700 0.191574378 15400 0.010132019 15380
0.07914599 30696 0.076834207 30696 0.085961307
30700 0.062265339 30700 0.193207801 15400 0.04162615 15384
0.085014224 30704 0.120230026 30704 0.091046437
30700 0.046298326 30700 0.150325431 15400 0.041845469 15388
0.062293257 30712 0.121101883 30712 0.042718479
30700 0.087903725 30700 0.065752938 15400 0.04091562 15392
0.059731297 30720 0.076091623 30720 0.057923549
30700 0.088655201 30700 0.050072642 15400 0.084025887 15396
0.049055529 30728 0.064024178 30728 0.062029569
30700 0.12657883 30700 0.045334757 15400 0.079681995 15400
0.104701612 30736 0.105428473 30736 0.058907026
30700 0.101563048 30700 0.095404525 15400 0.036678554 15404
0.165202844 30744 0.086882727 30744 0.060343027
30800 0.045829878 30800 0.122138779 15400 0.034177389 15408
0.126524218 30752 0.034950885 30752 0.049351591
30800 0.047400008 30800 0.104745952 15400 0.027975362 15412
0.043303411 30760 0.111402136 30760 0.040475014
30800 0.088681911 30800 0.094014904 15400 0.027150933 15416

0.049026276 30768 0.133881651 30768 0.05251055
30800 0.067439461 30800 0.090000329 15400 0.046220554 15420
0.123401987 30776 0.104120052 30776 0.043964672
30800 0.126609899 30800 0.145154321 15400 0.05124466 15424
0.139412659 30784 0.109349145 30784 0.02415911
30800 0.126590153 30800 0.170946864 15400 0.02919593 15428
0.096201613 30792 0.07644765 30792 0.054343349
30800 0.045375473 30800 0.089147179 15400 0.024325258 15432
0.069683963 30800 0.044548589 30800 0.045184028
30800 0.016686227 30800 0.062873027 15400 0.055051627 15436
0.052700045 30808 0.042707769 30808 0.04001092
30800 0.012837279 30800 0.11889053 15400 0.063224405 15440
0.034370944 30816 0.032725078 30816 0.040641884
30800 0.014757235 30800 0.115762865 15400 0.08122826 15444
0.021623297 30824 0.083915053 30824 0.035624631
30800 0.093504219 30800 0.077944234 15400 0.067567235 15448
0.034550019 30832 0.124794955 30832 0.028887584
30800 0.142306934 30800 0.077016965 15500 0.023391627 15452
0.072069161 30840 0.070280614 30840 0.0295804
30800 0.104960942 30800 0.094632131 15500 0.043607844 15456
0.076016426 30848 0.082859915 30848 0.028116743
30900 0.102148464 30900 0.065167966 15500 0.047950336 15460
0.0584011 30856 0.094845396 30856 0.026230293
30900 0.097851742 30900 0.096982541 15500 0.029711986 15464
0.035053112 30864 0.042301337 30864 0.040571787
30900 0.061000017 30900 0.130175176 15500 0.010928421 15468
0.014372344 30872 0.110417706 30872 0.044632379
30900 0.07217985 30900 0.123251113 15500 0.032367181 15472
0.014162995 30880 0.14445839 30880 0.021222468
30900 0.077685341 30900 0.12735436 15500 0.063168591 15476
0.035119469 30888 0.130741988 30888 0.029334198
30900 0.112930771 30900 0.073728268 15500 0.051117986 15480
0.085194537 30896 0.109156114 30896 0.039229573
30900 0.128200394 30900 0.032364387 15500 0.034439523 15484
0.113206894 30904 0.056255409 30904 0.058494468
30900 0.126065818 30900 0.06602794 15500 0.073396725 15488
0.113970076 30912 0.08830789 30912 0.054125736
30900 0.061243067 30900 0.133025431 15500 0.053634543 15492
0.0969096 30920 0.144349309 30920 0.011573678
30900 0.051933777 30900 0.108969311 15500 0.020049172 15496
0.086014392 30928 0.095866191 30928 0.023808216
30900 0.088843422 30900 0.100499834 15500 0.020762982 15500
0.064544656 30936 0.027539425 30936 0.020484089
30900 0.090745598 30900 0.092150949 15500 0.018205567 15504
0.033978802 30944 0.048947601 30944 0.006058146
31000 0.100016703 31000 0.082873994 15500 0.010431739 15508
0.031397933 30952 0.056552985 30952 0.008341901
31000 0.103836363 31000 0.059440823 15500 0.012548734 15512
0.031153795 30960 0.038369042 30960 0.011462741
31000 0.171745385 31000 0.037435839 15500 0.029403798 15516
0.010944702 30968 0.058691156 30968 0.020180271
31000 0.157601666 31000 0.072700183 15500 0.02216816 15520
0.017507622 30976 0.061556064 30976 0.025942576
31000 0.101220016 31000 0.0879816 15500 0.017290522 15524
0.030590476 30984 0.036670823 30984 0.058258189
31000 0.054077151 31000 0.110606845 15500 0.03550656 15528
0.04239774 30992 0.124464685 30992 0.081254519
31000 0.04901403 31000 0.106473417 15500 0.060623421 15532
0.048315607 31000 0.069356458 31000 0.078928919
31000 0.102972226 31000 0.021159049 15500 0.084141539 15536
0.040746509 31008 0.050609142 31008 0.044474713
31000 0.158226626 31000 0.097065102 15500 0.05610154 15540
0.012054786 31016 0.031860713 31016 0.016368511
31000 0.148105639 31000 0.09312616 15500 0.012184176 15544
0.040917108 31024 0.087839748 31024 0.040995783
31000 0.107725013 31000 0.051987066 15500 0.054363387 15548
0.051902109 31032 0.104196945 31032 0.04575292
31000 0.057218884 31000 0.082541752 15600 0.087133143 15552

0.05571168 31040 0.064441294 31040 0.03260282
31000 0.091037822 31000 0.0956644 15600 0.09419916 15556
0.040441701 31048 0.078451361 31048 0.020958507
31100 0.097321456 31100 0.09401569 15600 0.063762556 15560
0.057793779 31056 0.08579206 31056 0.053244527
31100 0.08841986 31100 0.061291379 15600 0.038671209 15564
0.0680784 31064 0.131880064 31064 0.073233903
31100 0.086832937 31100 0.031343821 15600 0.040658499 15568
0.063886379 31072 0.1104189 31072 0.06393544
31100 0.065860142 31100 0.059704289 15600 0.050118451 15572
0.107341133 31080 0.03750778 31080 0.006315237
31100 0.03843569 31100 0.127860505 15600 0.042327181 15576
0.143010576 31088 0.030864347 31088 0.049058712
31100 0.075901218 31100 0.127794309 15600 0.045068166 15580
0.099744582 31096 0.103322251 31096 0.040459836
31100 0.138299161 31100 0.050462153 15600 0.047560137 15584
0.045443423 31104 0.189639643 31104 0.033580796
31100 0.156820388 31100 0.009505736 15600 0.033784974 15588
0.060459846 31112 0.192301203 31112 0.067111469
31100 0.145363374 31100 0.093954688 15600 0.031210202 15592
0.104701379 31120 0.173544351 31120 0.056968707
31100 0.120875411 31100 0.191972401 15600 0.04953731 15596
0.136812625 31128 0.141112309 31128 0.033370554
31100 0.039731633 31100 0.199751011 15600 0.067433364 15600
0.126111933 31136 0.13744329 31136 0.078411773
31100 0.090800131 31100 0.136515243 15600 0.076621247 15604
0.101374084 31144 0.180398988 31144 0.092423012
31200 0.140749239 31200 0.059335856 15600 0.079086291 15608
0.102608094 31152 0.17057333 31152 0.076986187
31200 0.062646897 31200 0.030563104 15600 0.06618059 15612
0.12273308 31160 0.147742133 31160 0.039979128
31200 0.018625538 31200 0.094117146 15600 0.036900394 15616
0.098975797 31168 0.118343021 31168 0.02697285
31200 0.02189971 31200 0.097004508 15600 0.040273393 15620
0.04801061 31176 0.126586165 31176 0.025002635
31200 0.059912709 31200 0.0830516 15600 0.049862556 15624
0.038693637 31184 0.135682509 31184 0.014477424
31200 0.104740815 31200 0.123875929 15600 0.047214482 15628
0.065106171 31192 0.107761218 31192 0.040849383
31200 0.115900497 31200 0.103881175 15600 0.054120879 15632
0.079264828 31200 0.04385064 31200 0.062900312
31200 0.054253949 31200 0.091111528 15600 0.036589911 15636
0.079144877 31208 0.069850452 31208 0.062342704
31200 0.03482194 31200 0.092831862 15600 0.016599674 15640
0.090262663 31216 0.032389089 31216 0.06776127
31200 0.048365535 31200 0.038128404 15600 0.033607899 15644
0.113624687 31224 0.069663656 31224 0.083332627
31200 0.128537315 31200 0.014658197 15600 0.030670166 15648
0.080254373 31232 0.088094355 31232 0.089981273
31200 0.121016674 31200 0.06366743 15700 0.028793829 15652
0.031701111 31240 0.054336131 31240 0.081630351
31200 0.027867432 31200 0.091508147 15700 0.03596121 15656
0.094587063 31248 0.044627268 31248 0.068171859
31300 0.138015806 31300 0.122268451 15700 0.062625186 15660
0.129271154 31256 0.035700818 31256 0.033905246
31300 0.140181583 31300 0.131771201 15700 0.067411151 15664
0.127765365 31264 0.076687764 31264 0.025643978
31300 0.08865774 31300 0.079341524 15700 0.052475501 15668
0.134981179 31272 0.078387042 31272 0.033154745
31300 0.050269991 31300 0.055319197 15700 0.04525706 15672
0.118521733 31280 0.073274518 31280 0.023993309
31300 0.052965144 31300 0.022204704 15700 0.050578674 15676
0.090404486 31288 0.027133365 31288 0.014616313
31300 0.059696802 31300 0.059178121 15700 0.02409763 15680
0.112404363 31296 0.051273837 31296 0.048447713
31300 0.090302987 31300 0.059151171 15700 0.047757487 15684
0.131283115 31304 0.082271967 31304 0.07184947
31300 0.191797691 31300 0.054541953 15700 0.0806967 15688

0.132308153 31312 0.102356848 31312 0.060937979
31300 0.226160978 31300 0.041635776 15700 0.050229584 15692
0.131744571 31320 0.085512846 31320 0.031574815
31300 0.149951229 31300 0.046851921 15700 0.017983708 15696
0.127766588 31328 0.04566331 31328 0.035390654
31300 0.150841108 31300 0.123331076 15700 0.036726702 15700
0.142921083 31336 0.135953975 31336 0.051643197
31300 0.142744568 31300 0.151897999 15700 0.017753195 15704
0.15678929 31344 0.137159455 31344 0.042075142
31400 0.009902516 31400 0.114355331 15700 0.043443433 15708
0.155011789 31352 0.067798326 31352 0.074741744
31400 0.084749489 31400 0.035227378 15700 0.082833511 15712
0.139293858 31360 0.115921699 31360 0.092373186
31400 0.079446429 31400 0.04858401 15700 0.090957037 15716
0.102880593 31368 0.128671528 31368 0.077196681
31400 0.024257472 31400 0.072840063 15700 0.072230811 15720
0.064706088 31376 0.04172901 31376 0.011849254
31400 0.050714371 31400 0.087381413 15700 0.048383387 15724
0.057984933 31384 0.078222613 31384 0.060689159
31400 0.069821937 31400 0.095726246 15700 0.014335633 15728
0.067262379 31392 0.01430412 31392 0.042191132
31400 0.039136816 31400 0.107860353 15700 0.025110396 15732
0.043330776 31400 0.129050764 31400 0.015865186
31400 0.035228462 31400 0.13861488 15700 0.006961527 15736
0.193829241 31408 0.200782598 31408 0.011758667
31400 0.073667667 31400 0.186465476 15700 0.030274729 15740
0.219113601 31416 0.170067273 31416 0.01993132
31400 0.072174844 31400 0.193848071 15700 0.039263297 15744
0.13716938 31424 0.119324985 31424 0.001196536
31400 0.050826151 31400 0.161921329 15700 0.013955517 15748
0.090099362 31432 0.110433161 31432 0.032206055
31400 0.027679193 31400 0.140253659 15800 0.040710602 15752
0.051491188 31440 0.119167569 31440 0.051607156
31400 0.041092415 31400 0.134643516 15800 0.078400241 15756
0.057041063 31448 0.066160559 31448 0.056593708
31500 0.011144548 31500 0.100251331 15800 0.05965765 15760
0.114500283 31456 0.072803639 31456 0.031629119
31500 0.108495842 31500 0.120497993 15800 0.053390999 15764
0.117279691 31464 0.094116447 31464 0.045858025
31500 0.150561609 31500 0.144960431 15800 0.025148682 15768
0.12878637 31472 0.08244074 31472 0.050008828
31500 0.117251431 31500 0.096232441 15800 0.021208134 15772
0.1629498 31480 0.079662168 31480 0.072823699
31500 0.060747032 31500 0.086624423 15800 0.050656297 15776
0.170258398 31488 0.040382693 31488 0.094913739
31500 0.040785733 31500 0.119974604 15800 0.042503369 15780
0.139041659 31496 0.059154132 31496 0.088239372
31500 0.072383431 31500 0.105214953 15800 0.014184201 15784
0.097150733 31504 0.156546958 31504 0.066747816
31500 0.083208484 31500 0.022182294 15800 0.037349269 15788
0.074857875 31512 0.149506494 31512 0.028116987
31500 0.058294376 31500 0.028247976 15800 0.051395451 15792
0.095087482 31520 0.080780497 31520 0.030904266
31500 0.052472704 31500 0.088378605 15800 0.055486682 15796
0.098471493 31528 0.062449159 31528 0.060911014
31500 0.107830056 31500 0.111181209 15800 0.071059389 15800
0.078768106 31536 0.064664317 31536 0.066439519
31500 0.10336702 31500 0.075595985 15800 0.068831098 15804
0.057855858 31544 0.132891815 31544 0.059403301
31600 0.034801746 31600 0.083106206 15800 0.029446394 15808
0.062769155 31552 0.209337246 31552 0.044214758
31600 0.069455695 31600 0.094993833 15800 0.041935549 15812
0.075296106 31560 0.164222641 31560 0.005286866
31600 0.12048988 31600 0.01123648 15800 0.071975141 15816
0.085080515 31568 0.024197845 31568 0.049905673
31600 0.064723783 31600 0.061116502 15800 0.055188131 15820
0.075333985 31576 0.09424879 31576 0.034695422
31600 0.062502964 31600 0.04716307 15800 0.022957001 15824

0.085547195 31584 0.055461809 31584 0.016696098
31600 0.094253832 31600 0.045221503 15800 0.0117895 15828
0.090448775 31592 0.106548199 31592 0.048709459
31600 0.109828892 31600 0.089955778 15800 0.02858858 15832
0.061393359 31600 0.169845981 31600 0.075011441
31600 0.13536666 31600 0.032556072 15800 0.033692861 15836
0.056759698 31608 0.122722326 31608 0.047865786
31600 0.164321376 31600 0.026751137 15800 0.029477982 15840
0.044428547 31616 0.181730065 31616 0.05837276
31600 0.207402161 31600 0.130778775 15800 0.032374559 15844
0.055662946 31624 0.176291156 31624 0.064082356
31600 0.150929511 31600 0.241968723 15800 0.011828593 15848
0.108740846 31632 0.113007533 31632 0.031025305
31600 0.078291421 31600 0.210510349 15900 0.023579371 15852
0.102295577 31640 0.172170694 31640 0.050474231
31600 0.101447447 31600 0.165879901 15900 0.026391455 15856
0.060369679 31648 0.105602499 31648 0.036313075
31700 0.094197116 31700 0.148338251 15900 0.039983966 15860
0.082545892 31656 0.051125204 31656 0.012763549
31700 0.077840479 31700 0.058374109 15900 0.072062139 15864
0.10540642 31664 0.072936906 31664 0.024389534
31700 0.129572189 31700 0.085088177 15900 0.067613517 15868
0.091711328 31672 0.152884968 31672 0.044678658
31700 0.146373568 31700 0.093307113 15900 0.054684006 15872
0.079105696 31680 0.17619392 31680 0.049958515
31700 0.034677985 31700 0.084514504 15900 0.081980354 15876
0.119120712 31688 0.080847582 31688 0.050130464
31700 0.12474398 31700 0.109021647 15900 0.065168068 15880
0.118650612 31696 0.1022125 31696 0.060169907
31700 0.165127086 31700 0.11942166 15900 0.039332994 15884
0.076380973 31704 0.164418933 31704 0.046603229
31700 0.091038579 31700 0.058593822 15900 0.051865136 15888
0.061849176 31712 0.171282241 31712 0.012559734
31700 0.045592045 31700 0.032204764 15900 0.061932689 15892
0.074907715 31720 0.126978601 31720 0.056082365
31700 0.119043099 31700 0.09744626 15900 0.016631753 15896
0.052458414 31728 0.009891652 31728 0.058631027
31700 0.143138735 31700 0.161072195 15900 0.029047324 15900
0.081728605 31736 0.071929448 31736 0.022600818
31700 0.100878067 31700 0.110886904 15900 0.015578831 15904
0.169373889 31744 0.066802881 31744 0.008191878
31800 0.096436961 31800 0.076466153 15900 0.026115116 15908
0.179214869 31752 0.124071041 31752 0.034326899
31800 0.164732381 31800 0.077256351 15900 0.054915778 15912
0.103122518 31760 0.051545925 31760 0.060398779
31800 0.195733635 31800 0.100058271 15900 0.056259152 15916
0.00288234 31768 0.070058974 31768 0.050267478
31800 0.184379678 31800 0.099806071 15900 0.054614135 15920
0.045803347 31776 0.071827664 31776 0.055976674
31800 0.16071061 31800 0.068308444 15900 0.071951174 15924
0.045645975 31784 0.042592539 31784 0.046889811
31800 0.119872755 31800 0.053344797 15900 0.073239469 15928
0.078069286 31792 0.060376657 31792 0.017613633
31800 0.054281347 31800 0.013089725 15900 0.124616825 15932
0.123969017 31800 0.053182153 31800 0.040878447
31800 0.040521059 31800 0.013937446 15900 0.109323119 15936
0.108489017 31808 0.082466322 31808 0.014037727
31800 0.04963201 31800 0.013134535 15900 0.051303563 15940
0.075026845 31816 0.120186815 31816 0.027619488
31800 0.069830006 31800 0.037648355 15900 0.043313587 15944
0.062396772 31824 0.120450946 31824 0.048231792
31800 0.143055556 31800 0.037353046 15900 0.043988697 15948
0.006584454 31832 0.094670664 31832 0.066725123
31800 0.184496094 31800 0.026307336 16000 0.034663608 15952
0.060966868 31840 0.087173161 31840 0.065664579
31800 0.164821656 31800 0.043181732 16000 0.019793622 15956
0.091124777 31848 0.065225519 31848 0.029216664
31900 0.115715578 31900 0.039170951 16000 0.068564448 15960

0.102226361 31856 0.012895061 31856 0.028859289
31900 0.123174803 31900 0.070999551 16000 0.096961267 15964
0.125547041 31864 0.12309356 31864 0.038957514
31900 0.141034179 31900 0.16464213 16000 0.061282 15968
0.124061815 31872 0.176122746 31872 0.022985629
31900 0.105274601 31900 0.135302689 16000 0.019779043 15972
0.070840179 31880 0.097170414 31880 0.047632235
31900 0.06381538 31900 0.018094645 16000 0.041599313 15976
0.075568351 31888 0.036139991 31888 0.025810157
31900 0.010688624 31900 0.070787239 16000 0.020784781 15980
0.104821469 31896 0.080156031 31896 0.034671466
31900 0.090611167 31900 0.07167364 16000 0.016872822 15984
0.086437081 31904 0.099355675 31904 0.046557736
31900 0.096012904 31900 0.108714237 16000 0.043986838 15988
0.089132394 31912 0.120166937 31912 0.038922291
31900 0.07531415 31900 0.100354788 16000 0.057094887 15992
0.080332487 31920 0.108324282 31920 0.026457807
31900 0.074348653 31900 0.079944039 16000 0.031937085 15996
0.033681008 31928 0.074219526 31928 0.031913914
31900 0.065901433 31900 0.031929983 16000 0.011237647 16000
0.029422412 31936 0.058115929 31936 0.024049095
31900 0.082378509 31900 0.048611961 16000 0.029781519 16004
0.066250934 31944 0.041601128 31944 0.008682437
32000 0.067371831 32000 0.057183515 16000 0.046061956 16008
0.083237668 31952 0.009293079 31952 0.008777903
32000 0.042425112 32000 0.067104891 16000 0.070058995 16012
0.094568248 31960 0.019665915 31960 0.019935029
32000 0.025101372 32000 0.074998199 16000 0.070381779 16016
0.104724291 31968 0.011679226 31968 0.020507057
32000 0.041406009 32000 0.048750124 16000 0.05413175 16020
0.085673106 31976 0.010107381 31976 0.016479336
32000 0.06973814 32000 0.020795931 16000 0.042050077 16024
0.049214636 31984 0.030253192 31984 0.045390196
32000 0.050063136 32000 0.004367751 16000 0.014439626 16028
0.062105042 31992 0.05855682 31992 0.015195802
32000 0.026459136 32000 0.062772073 16000 0.031145788 16032
0.071898598 32000 0.044563276 32000 0.036707756
32000 0.061622763 32000 0.084500964 16000 0.036152444 16036
0.087216227 32008 0.02783578 32008 0.0384653
32000 0.051106588 32000 0.079231686 16000 0.017061855 16040
0.109947767 32016 0.037827838 32016 0.039634622
32000 0.013190074 32000 0.073108764 16000 0.036568494 16044
0.107686392 32024 0.07111254 32024 0.041182047
32000 0.086471322 32000 0.070302143 16000 0.021386391 16048
0.08560811 32032 0.1041715 32032 0.014651108
32000 0.110020912 32000 0.045921726 16100 0.035997069 16052
0.042466385 32040 0.146012884 32040 0.006477886
32000 0.085541884 32000 0.030974738 16100 0.050929761 16056
0.059636455 32048 0.118099277 32048 0.029040704
32100 0.087763176 32100 0.079011727 16100 0.043068958 16060
0.113207359 32056 0.0869543 32056 0.04245936
32100 0.101693666 32100 0.067766909 16100 0.056260768 16064
0.108762164 32064 0.115879862 32064 0.025893804
32100 0.087376 32100 0.055878787 16100 0.067994755 16068
0.103295206 32072 0.015890575 32072 0.007174008
32100 0.065855886 32100 0.080337333 16100 0.046438294 16072
0.069589747 32080 0.140297605 32080 0.004379838
32100 0.062225299 32100 0.088187328 16100 0.016842296 16076
0.022880893 32088 0.161334756 32088 0.010693343
32100 0.075129807 32100 0.048782633 16100 0.006358692 16080
0.07556982 32096 0.113195754 32096 0.010975183
32100 0.054259523 32100 0.063771819 16100 0.014071107 16084
0.102107093 32104 0.079535472 32104 0.020504611
32100 0.059216629 32100 0.095729105 16100 0.016477503 16088
0.088763045 32112 0.050928709 32112 0.057234916
32100 0.081250248 32100 0.058021102 16100 0.032997868 16092
0.05599048 32120 0.03413067 32120 0.086046006
32100 0.104732048 32100 0.054765824 16100 0.040493374 16096

0.033285778 32128 0.083082152 32128 0.076341377
32100 0.095874071 32100 0.070942217 16100 0.021263493 16100
0.039826904 32136 0.090574235 32136 0.042307111
32100 0.060160037 32100 0.058370399 16100 0.038494458 16104
0.090547525 32144 0.09729006 32144 0.024537929
32200 0.023566588 32200 0.045026405 16100 0.022284285 16108
0.085459542 32152 0.115917464 32152 0.040173363
32200 0.05472983 32200 0.012958032 16100 0.052857547 16112
0.040214611 32160 0.141959899 32160 0.066274217
32200 0.080133519 32200 0.048735703 16100 0.062471168 16116
0.029475788 32168 0.164377416 32168 0.074045085
32200 0.06493635 32200 0.051478335 16100 0.025403 16120
0.045940142 32176 0.139005206 32176 0.042055002
32200 0.075782766 32200 0.043586522 16100 0.03282 16124
0.055589058 32184 0.068679488 32184 0.04782623
32200 0.042996268 32200 0.069688344 16100 0.050304709 16128
0.057571186 32192 0.067660199 32192 0.078156416
32200 0.035636007 32200 0.089614514 16100 0.032285283 16132
0.038723847 32200 0.075066186 32200 0.061749764
32200 0.082237348 32200 0.074428979 16100 0.023744513 16136
0.055670447 32208 0.06497217 32208 0.036121215
32200 0.077060038 32200 0.086277491 16100 0.03131029 16140
0.113005175 32216 0.074485943 32216 0.030834879
32200 0.093069793 32200 0.038082915 16100 0.057325826 16144
0.111824935 32224 0.087141933 32224 0.062230502
32200 0.13937593 32200 0.097955272 16100 0.076878823 16148
0.039982675 32232 0.032688917 32232 0.048709269
32200 0.098721786 32200 0.090928443 16200 0.070785762 16152
0.049942031 32240 0.093935647 32240 0.003742042
32200 0.030323119 32200 0.058804359 16200 0.053007061 16156
0.069029542 32248 0.12139733 32248 0.041286992
32300 0.083770952 32300 0.10970002 16200 0.060842012 16160
0.035763093 32256 0.067311317 32256 0.048569742
32300 0.0478291 32300 0.125019913 16200 0.057645106 16164
0.074922267 32264 0.022737724 32264 0.019131199
32300 0.015974514 32300 0.102986785 16200 0.047104819 16168
0.099327852 32272 0.163784309 32272 0.019285746
32300 0.039061593 32300 0.08235799 16200 0.057605783 16172
0.088666377 32280 0.200331196 32280 0.040644918
32300 0.09436487 32300 0.047101585 16200 0.067394038 16176
0.118331227 32288 0.109939931 32288 0.053508677
32300 0.137170966 32300 0.048201313 16200 0.081410733 16180
0.143815648 32296 0.054492481 32296 0.03091255
32300 0.156027949 32300 0.071310184 16200 0.037295296 16184
0.146810416 32304 0.053786822 32304 0.011100849
32300 0.086440261 32300 0.062928928 16200 0.050606435 16188
0.152105815 32312 0.045473425 32312 0.03183524
32300 0.009109042 32300 0.02061385 16200 0.102918442 16192
0.128063591 32320 0.080366583 32320 0.022236049
32300 0.00823209 32300 0.016104768 16200 0.095582269 16196
0.076554679 32328 0.161504416 32328 0.026972153
32300 0.055499557 32300 0.09552118 16200 0.049935923 16200
0.08448134 32336 0.18764766 32336 0.052949941
32300 0.172077228 32300 0.103404571 16200 0.024462785 16204
0.109386296 32344 0.201754446 32344 0.071869188
32400 0.207311693 32400 0.022378008 16200 0.028196524 16208
0.134646224 32352 0.153597488 32352 0.060757477
32400 0.113993257 32400 0.092145478 16200 0.056686615 16212
0.135029171 32360 0.115680763 32360 0.043629643
32400 0.070346097 32400 0.117547315 16200 0.081230173 16216
0.105693682 32368 0.117429874 32368 0.03680569
32400 0.089547648 32400 0.145494894 16200 0.086314212 16220
0.095239266 32376 0.097749362 32376 0.012044148
32400 0.060909952 32400 0.210133061 16200 0.095175157 16224
0.103705424 32384 0.068876696 32384 0.040761992
32400 0.029952033 32400 0.237971618 16200 0.097761193 16228
0.103116494 32392 0.062594379 32392 0.069836322
32400 0.073386866 32400 0.16026599 16200 0.067505476 16232

0.093352552 32400 0.059493708 32400 0.064518237
32400 0.070098358 32400 0.105194507 16200 0.032633543 16236
0.123457838 32408 0.043528504 32408 0.062119827
32400 0.061726125 32400 0.165210455 16200 0.047482285 16240
0.121041318 32416 0.007030703 32416 0.066720786
32400 0.075909651 32400 0.189689555 16200 0.021669901 16244
0.081334678 32424 0.049410501 32424 0.043804452
32400 0.080721504 32400 0.140581775 16200 0.035788617 16248
0.056999252 32432 0.021563108 32432 0.007755258
32400 0.123528997 32400 0.07934821 16300 0.047754489 16252
0.055822402 32440 0.059715232 32440 0.031453274
32400 0.170710424 32400 0.040601233 16300 0.014736925 16256
0.093636663 32448 0.078113975 32448 0.011353816
32500 0.146747043 32500 0.061675295 16300 0.021550235 16260
0.077652287 32456 0.177319001 32456 0.033809909
32500 0.132402725 32500 0.086718384 16300 0.031926084 16264
0.016310523 32464 0.204602111 32464 0.04490267
32500 0.175707174 32500 0.026479509 16300 0.049499111 16268
0.065928871 32472 0.156276685 32472 0.011152523
32500 0.146093459 32500 0.049200215 16300 0.040665487 16272
0.093494848 32480 0.18045769 32480 0.02681918
32500 0.013139631 32500 0.106735744 16300 0.026687943 16276
0.096097341 32488 0.181134746 32488 0.027271526
32500 0.129963868 32500 0.163979741 16300 0.024486944 16280
0.132806759 32496 0.141290686 32496 0.020930398
32500 0.182436779 32500 0.105776882 16300 0.043480904 16284
0.126501516 32504 0.106279571 32504 0.058066358
32500 0.160577125 32500 0.115958894 16300 0.06067671 16288
0.051051717 32512 0.051768755 32512 0.055993794
32500 0.132683141 32500 0.149288971 16300 0.03737642 16292
0.03239539 32520 0.018178527 32520 0.053878903
32500 0.095653391 32500 0.114598603 16300 0.033057622 16296
0.084012347 32528 0.051331666 32528 0.048155987
32500 0.052630538 32500 0.067251225 16300 0.042109827 16300
0.089781934 32536 0.086676366 32536 0.02674357
32500 0.05949496 32500 0.081882725 16300 0.066789195 16304
0.081923092 32544 0.079150392 32544 0.052487536
32600 0.077809702 32600 0.128335174 16300 0.046711575 16308
0.058739344 32552 0.075539254 32552 0.064795349
32600 0.071592018 32600 0.153246496 16300 0.016673861 16312
0.047671336 32560 0.114758339 32560 0.030793417
32600 0.091996066 32600 0.180192976 16300 0.009664517 16316
0.091045811 32568 0.13033126 32568 0.013042341
32600 0.166512895 32600 0.139250843 16300 0.026969752 16320
0.074078635 32576 0.088845642 32576 0.033331271
32600 0.176693299 32600 0.015135473 16300 0.033897402 16324
0.021994541 32584 0.030057932 32584 0.025226265
32600 0.098590084 32600 0.110921217 16300 0.024841553 16328
0.055282955 32592 0.03025106 32592 0.044460834
32600 0.053439424 32600 0.1774681 16300 0.041705698 16332
0.108379412 32600 0.112216185 32600 0.060058857
32600 0.106925945 32600 0.176510585 16300 0.045809989 16336
0.121351593 32608 0.115434632 32608 0.04437926
32600 0.111121779 32600 0.124993079 16300 0.007289365 16340
0.083412539 32616 0.043608841 32616 0.075585333
32600 0.109276465 32600 0.073595096 16300 0.019045563 16344
0.088443063 32624 0.034300323 32624 0.087351997
32600 0.093655297 32600 0.072526134 16300 0.065941124 16348
0.074910917 32632 0.049312497 32632 0.066892993
32600 0.083569546 32600 0.076290431 16400 0.069180089 16352
0.088624678 32640 0.105389721 32640 0.03328154
32600 0.090897469 32600 0.018273047 16400 0.051810104 16356
0.152257896 32648 0.086814951 32648 0.013872277
32700 0.061753759 32700 0.055258443 16400 0.058989146 16360
0.110288464 32656 0.038094313 32656 0.042805146
32700 0.024360019 32700 0.020186186 16400 0.026325586 16364
0.102880062 32664 0.068222027 32664 0.024496756
32700 0.05756137 32700 0.064789936 16400 0.029571216 16368

0.108563276 32672 0.110682719 32672 0.037110625
32700 0.01679954 32700 0.104902108 16400 0.053818039 16372
0.077072087 32680 0.040434738 32680 0.038130747
32700 0.058565882 32700 0.079818601 16400 0.036340498 16376
0.142759644 32688 0.040995885 32688 0.01420149
32700 0.098335819 32700 0.024924708 16400 0.020242516 16380
0.185062309 32696 0.018008004 32696 0.041155923
32700 0.067063993 32700 0.014819266 16400 0.050506777 16384
0.191664964 32704 0.00774542 32704 0.047909773
32700 0.020401112 32700 0.051545321 16400 0.075543634 16388
0.153882516 32712 0.077012388 32712 0.04382187
32700 0.065589877 32700 0.052669922 16400 0.065089713 16392
0.071945222 32720 0.100441779 32720 0.04253893
32700 0.076102377 32700 0.063380809 16400 0.030310248 16396
0.071389841 32728 0.058461908 32728 0.053162417
32700 0.093045266 32700 0.091381124 16400 0.091002141 16400
0.104829313 32736 0.052620417 32736 0.062963009
32700 0.112556598 32700 0.067702495 16400 0.101669881 16404
0.133342095 32744 0.110718742 32744 0.042852818
32800 0.051881703 32800 0.065250148 16400 0.056173816 16408
0.128766318 32752 0.210561223 32752 0.017812963
32800 0.04040714 32800 0.17086012 16400 0.013756663 16412
0.084905041 32760 0.206549681 32760 0.075985045
32800 0.015524429 32800 0.177051901 16400 0.020989603 16416
0.036028061 32768 0.120378922 32768 0.080659898
32800 0.025697316 32800 0.121181271 16400 0.033278033 16420
0.050561012 32776 0.114492475 32776 0.054927547
32800 0.06355915 32800 0.133444235 16400 0.048113474 16424
0.102445105 32784 0.226255783 32784 0.041535968
32800 0.123136852 32800 0.177651411 16400 0.031500022 16428
0.093014773 32792 0.256275933 32792 0.03549756
32800 0.116267635 32800 0.231245184 16400 0.023779927 16432
0.041008836 32800 0.172924861 32800 0.030201773
32800 0.044638153 32800 0.226726014 16400 0.0085977 16436
0.050791714 32808 0.073061783 32808 0.024675079
32800 0.034353023 32800 0.155026865 16400 0.046813868 16440
0.095724587 32816 0.031227417 32816 0.04594533
32800 0.057928668 32800 0.062028332 16400 0.080148631 16444
0.073542498 32824 0.077698678 32824 0.045755376
32800 0.059569251 32800 0.083595231 16400 0.061711377 16448
0.055739725 32832 0.092338574 32832 0.027473783
32800 0.050039838 32800 0.168335173 16500 0.029244042 16452
0.069541988 32840 0.031693606 32840 0.037053665
32800 0.050083705 32800 0.129566979 16500 0.036283003 16456
0.086451335 32848 0.025384659 32848 0.074478841
32900 0.034553403 32900 0.130165179 16500 0.059721329 16460
0.097185352 32856 0.016147158 32856 0.087614077
32900 0.051197363 32900 0.14357151 16500 0.048755737 16464
0.089744048 32864 0.066305365 32864 0.061024144
32900 0.129125866 32900 0.088360612 16500 0.034332392 16468
0.082121798 32872 0.069576301 32872 0.046673427
32900 0.1528555 32900 0.102075901 16500 0.018946421 16472
0.118297692 32880 0.030325391 32880 0.05396381
32900 0.121431222 32900 0.040142149 16500 0.026883514 16476
0.135473936 32888 0.057493635 32888 0.038581155
32900 0.107490334 32900 0.037717127 16500 0.064227614 16480
0.084303771 32896 0.088537927 32896 0.023833294
32900 0.12454894 32900 0.09923365 16500 0.05455716 16484
0.050663333 32904 0.054869324 32904 0.020848216
32900 0.101875907 32900 0.13034408 16500 0.021206262 16488
0.065789282 32912 0.076514785 32912 0.007168306
32900 0.06644446 32900 0.139433046 16500 0.019991834 16492
0.044654003 32920 0.089687695 32920 0.037887265
32900 0.145533646 32900 0.170658663 16500 0.007792264 16496
0.046797064 32928 0.051094579 32928 0.057722034
32900 0.19362419 32900 0.14534546 16500 0.015287948 16500
0.056746078 32936 0.009214803 32936 0.104205334
32900 0.140453136 32900 0.097882978 16500 0.036939029 16504

0.043938086 32944 0.049368129 32944 0.108188877
33000 0.058802845 33000 0.071374227 16500 0.083176768 16508
0.110554895 32952 0.071761751 32952 0.091337031
33000 0.064022039 33000 0.104161671 16500 0.070477152 16512
0.152082066 32960 0.015277303 32960 0.047346963
33000 0.059542042 33000 0.141704091 16500 0.042633208 16516
0.12639178 32968 0.098439254 32968 0.035049401
33000 0.056968365 33000 0.131477805 16500 0.062112311 16520
0.090816597 32976 0.134896138 32976 0.045963974
33000 0.102902923 33000 0.128833431 16500 0.049325849 16524
0.071035633 32984 0.10438358 32984 0.045778987
33000 0.151417261 33000 0.160748794 16500 0.045677269 16528
0.076251592 32992 0.110675508 32992 0.061236766
33000 0.148581749 33000 0.112995615 16500 0.073953219 16532
0.090150003 33000 0.130973771 33000 0.109391251
33000 0.138481003 33000 0.065545362 16500 0.04021002 16536
0.075090218 33008 0.118119031 33008 0.166592596
33000 0.144308025 33000 0.079320416 16500 0.043695683 16540
0.063809122 33016 0.087725326 33016 0.142478806
33000 0.160947951 33000 0.080223399 16500 0.027264347 16544
0.083816609 33024 0.026506605 33024 0.044361688
33000 0.169112028 33000 0.035265573 16500 0.057038276 16548
0.083977895 33032 0.023472125 33032 0.033012468
33000 0.180972493 33000 0.064929438 16600 0.065293018 16552
0.073946932 33040 0.030008283 33040 0.030848605
33000 0.105487161 33000 0.062724488 16600 0.055748049 16556
0.111172536 33048 0.051992527 33048 0.011700834
33100 0.066524655 33100 0.061218307 16600 0.025835434 16560
0.100417572 33056 0.054841163 33056 0.042923617
33100 0.157801056 33100 0.051423805 16600 0.035385459 16564
0.062500316 33064 0.063943255 33064 0.065729968
33100 0.141579119 33100 0.033922162 16600 0.069847301 16568
0.081888182 33072 0.049800841 33072 0.048093669
33100 0.151803019 33100 0.059931219 16600 0.066630782 16572
0.074255928 33080 0.08119164 33080 0.03887045
33100 0.154697641 33100 0.08496557 16600 0.068730165 16576
0.075905671 33088 0.101243764 33088 0.04607769
33100 0.08087395 33100 0.043771692 16600 0.101515725 16580
0.142553574 33096 0.092018156 33096 0.048412596
33100 0.10609976 33100 0.087649212 16600 0.083538413 16584
0.142201519 33104 0.09929364 33104 0.057327012
33100 0.09349511 33100 0.151026893 16600 0.027384083 16588
0.111311114 33112 0.07236348 33112 0.061878534
33100 0.074072523 33100 0.120730794 16600 0.055562116 16592
0.112275149 33120 0.030455238 33120 0.041899952
33100 0.112573747 33100 0.044041561 16600 0.049371753 16596
0.109794142 33128 0.12275757 33128 0.015540576
33100 0.168709666 33100 0.028588373 16600 0.059779366 16600
0.084024898 33136 0.126378684 33136 0.023998875
33100 0.183257725 33100 0.038483722 16600 0.062061867 16604
0.061292252 33144 0.116914926 33144 0.024422418
33200 0.147076876 33200 0.077175777 16600 0.055265049 16608
0.053926855 33152 0.158685186 33152 0.024310442
33200 0.092300936 33200 0.138115065 16600 0.044859418 16612
0.078972407 33160 0.128420681 33160 0.068761008
33200 0.017893615 33200 0.097228207 16600 0.03887053 16616
0.07922716 33168 0.18701081 33168 0.062824998
33200 0.108461209 33200 0.080615726 16600 0.031980937 16620
0.098920493 33176 0.232157239 33176 0.043946264
33200 0.13880126 33200 0.102852559 16600 0.043756441 16624
0.112841575 33184 0.176323185 33184 0.00960096
33200 0.087933477 33200 0.146325634 16600 0.056581994 16628
0.071780654 33192 0.105443272 33192 0.044886387
33200 0.062020525 33200 0.155295361 16600 0.068131187 16632
0.042049986 33200 0.101845617 33200 0.007411157
33200 0.097194235 33200 0.101559817 16600 0.043513221 16636
0.105317544 33208 0.087334047 33208 0.038891707
33200 0.099912191 33200 0.063128107 16600 0.026789405 16640

0.082440456 33216 0.076980083 33216 0.020543945
33200 0.042808864 33200 0.088905596 16600 0.059165341 16644
0.065433953 33224 0.060948591 33224 0.013452923
33200 0.105769585 33200 0.090744965 16600 0.039579296 16648
0.058901245 33232 0.00732298 33232 0.028863193
33200 0.116094758 33200 0.043928445 16700 0.017924107 16652
0.03856566 33240 0.081487611 33240 0.051215109
33200 0.034366505 33200 0.078614517 16700 0.030679472 16656
0.02130306 33248 0.111386522 33248 0.058531889
33300 0.090543646 33300 0.133380556 16700 0.045039007 16660
0.052915068 33256 0.08583851 33256 0.042295502
33300 0.126294791 33300 0.112322938 16700 0.040849976 16664
0.033282766 33264 0.050045961 33264 0.007836926
33300 0.078851314 33300 0.030542873 16700 0.017236231 16668
0.023986799 33272 0.167268329 33272 0.008686716
33300 0.050013136 33300 0.022075945 16700 0.019942316 16672
0.038519927 33280 0.18671903 33280 0.019117861
33300 0.135709371 33300 0.057361336 16700 0.049526239 16676
0.056239907 33288 0.177480223 33288 0.045591787
33300 0.118590804 33300 0.071269344 16700 0.06544916 16680
0.07012047 33296 0.161681238 33296 0.049136917
33300 0.006378611 33300 0.12419805 16700 0.051366063 16684
0.0766335 33304 0.08065733 33304 0.04464309
33300 0.074095893 33300 0.157150571 16700 0.02212111 16688
0.082442108 33312 0.035551129 33312 0.022761038
33300 0.041893341 33300 0.094126597 16700 0.037565693 16692
0.052385294 33320 0.066636982 33320 0.013814357
33300 0.031845488 33300 0.148359526 16700 0.076760261 16696
0.046250814 33328 0.06558594 33328 0.02319064
33300 0.100955134 33300 0.149572633 16700 0.066446381 16700
0.102216349 33336 0.051325154 33336 0.021538801
33300 0.121880708 33300 0.049740214 16700 0.028996612 16704
0.104349769 33344 0.071090988 33344 0.020758462
33400 0.067434674 33400 0.122185666 16700 0.049153456 16708
0.061479477 33352 0.045821074 33352 0.021560089
33400 0.040335519 33400 0.152706998 16700 0.035192003 16712
0.036765046 33360 0.097437027 33360 0.022984554
33400 0.088478177 33400 0.123825361 16700 0.004928434 16716
0.033383076 33368 0.14578944 33368 0.045294266
33400 0.070863389 33400 0.055759479 16700 0.015842496 16720
0.016105605 33376 0.102132442 33376 0.03241601
33400 0.068266942 33400 0.036563633 16700 0.026763019 16724
0.028595232 33384 0.033150227 33384 0.011575141
33400 0.113814356 33400 0.027565864 16700 0.05210828 16728
0.023528286 33392 0.022888826 33392 0.013348612
33400 0.114117647 33400 0.035960868 16700 0.035113495 16732
0.045208028 33400 0.095648837 33400 0.022329661
33400 0.059064172 33400 0.043797405 16700 0.024149422 16736
0.01853589 33408 0.099591787 33408 0.046042129
33400 0.066875728 33400 0.101038568 16700 0.015228679 16740
0.002210408 33416 0.063860163 33416 0.063331936
33400 0.097947355 33400 0.088706926 16700 0.020991531 16744
0.013036002 33424 0.020140678 33424 0.057378489
33400 0.074182572 33400 0.048321377 16700 0.032190106 16748
0.012909922 33432 0.030841271 33432 0.031657641
33400 0.083922329 33400 0.094446616 16800 0.033412613 16752
0.032055075 33440 0.00341568 33440 0.050612398
33400 0.070972259 33400 0.104444196 16800 0.065909044 16756
0.044637793 33448 0.088871835 33448 0.084092855
33500 0.036665122 33500 0.112724876 16800 0.074488351 16760
0.058740847 33456 0.082223458 33456 0.081298109
33500 0.07390057 33500 0.120398712 16800 0.023326982 16764
0.016479929 33464 0.056165569 33464 0.057478053
33500 0.135178634 33500 0.098325472 16800 0.009491946 16768
0.052571166 33472 0.104882442 33472 0.065936467
33500 0.103278158 33500 0.076520475 16800 0.016505846 16772
0.049944803 33480 0.117491312 33480 0.063317333
33500 0.06428588 33500 0.073062729 16800 0.03789493 16776

0.033682001 33488 0.069097565 33488 0.052893436
33500 0.055298409 33500 0.054267777 16800 0.040473566 16780
0.028609424 33496 0.075800774 33496 0.056213405
33500 0.077194294 33500 0.093992283 16800 0.046559642 16784
0.073307281 33504 0.160760726 33504 0.036736674
33500 0.109933353 33500 0.056976987 16800 0.043392756 16788
0.093261275 33512 0.170298503 33512 0.00619345
33500 0.096190765 33500 0.062442436 16800 0.019115347 16792
0.072893046 33520 0.137796743 33520 0.026162759
33500 0.052254683 33500 0.096106938 16800 0.02917022 16796
0.060215807 33528 0.068954643 33528 0.018121704
33500 0.030396373 33500 0.09795832 16800 0.050548741 16800
0.043785469 33536 0.049933991 33536 0.020433796
33500 0.093867304 33500 0.107530817 16800 0.055959041 16804
0.008905919 33544 0.091574751 33544 0.018319233
33600 0.111139052 33600 0.097597753 16800 0.049725542 16808
0.03900646 33552 0.023896077 33552 0.021715505
33600 0.044268058 33600 0.074204261 16800 0.043593733 16812
0.079712707 33560 0.034588247 33560 0.026740772
33600 0.022596723 33600 0.124432525 16800 0.043052649 16816
0.069431022 33568 0.060812887 33568 0.010737303
33600 0.019170926 33600 0.132809393 16800 0.038033206 16820
0.020629161 33576 0.0949108 33576 0.02634583
33600 0.014919438 33600 0.132687841 16800 0.063784304 16824
0.046487214 33584 0.13700097 33584 0.015484095
33600 0.030660256 33600 0.146096369 16800 0.060141265 16828
0.058251404 33592 0.123998296 33592 0.028434361
33600 0.122329671 33600 0.131996305 16800 0.018121769 16832
0.062833155 33600 0.057693986 33600 0.040314902
33600 0.17966346 33600 0.09136548 16800 0.047683614 16836
0.050115024 33608 0.06818938 33608 0.022925682
33600 0.124295388 33600 0.090435642 16800 0.085886342 16840
0.024862409 33616 0.020187707 33616 0.033228811
33600 0.096003809 33600 0.121187441 16800 0.082464481 16844
0.011264787 33624 0.130220171 33624 0.028091996
33600 0.089830326 33600 0.178701419 16800 0.047514775 16848
0.033408083 33632 0.168793719 33632 0.009798074
33600 0.098210199 33600 0.166647601 16900 0.011706431 16852
0.080706166 33640 0.118698685 33640 0.03760658
33600 0.046588124 33600 0.098401826 16900 0.015921491 16856
0.093048271 33648 0.073573021 33648 0.0582461
33700 0.127826949 33700 0.091790891 16900 0.044606037 16860
0.096805386 33656 0.073213705 33656 0.070621987
33700 0.213761305 33700 0.097410673 16900 0.065905064 16864
0.076716278 33664 0.101347818 33664 0.071643997
33700 0.129108754 33700 0.033894306 16900 0.055883829 16868
0.041706026 33672 0.090733731 33672 0.045279354
33700 0.029340768 33700 0.045549688 16900 0.046563746 16872
0.115658841 33680 0.076518947 33680 0.026120177
33700 0.031663789 33700 0.029005807 16900 0.0608314 16876
0.12272496 33688 0.047417387 33688 0.056333513
33700 0.06777719 33700 0.071766626 16900 0.045695044 16880
0.063345324 33696 0.049277984 33696 0.056590827
33700 0.017445862 33700 0.112758877 16900 0.056291192 16884
0.047271824 33704 0.037359041 33704 0.036605867
33700 0.076537435 33700 0.108977401 16900 0.073362069 16888
0.060573759 33712 0.046968333 33712 0.040674342
33700 0.089464404 33700 0.099343655 16900 0.088720328 16892
0.029513971 33720 0.084932064 33720 0.040274033
33700 0.087221109 33700 0.154303474 16900 0.090552727 16896
0.04273673 33728 0.10923708 33728 0.035587946
33700 0.143493205 33700 0.177225244 16900 0.069140151 16900
0.072411167 33736 0.09121222 33736 0.023209217
33700 0.132577843 33700 0.118649703 16900 0.051802683 16904
0.059880687 33744 0.067088578 33744 0.045588062
33800 0.053173997 33800 0.072507413 16900 0.059889117 16908
0.111526089 33752 0.010034267 33752 0.070955241
33800 0.070983093 33800 0.129652719 16900 0.057512705 16912

0.16266611 33760 0.075704556 33760 0.036565318
33800 0.074835763 33800 0.184688528 16900 0.026694208 16916
0.127991545 33768 0.083741434 33768 0.03206468
33800 0.044855893 33800 0.137759853 16900 0.02989542 16920
0.059706457 33776 0.096457508 33776 0.042235377
33800 0.037715305 33800 0.049643222 16900 0.072669536 16924
0.029976103 33784 0.116695876 33784 0.019244695
33800 0.052153715 33800 0.088350222 16900 0.093207775 16928
0.060707334 33792 0.084068262 33792 0.062219166
33800 0.03627373 33800 0.078387435 16900 0.085079286 16932
0.078282843 33800 0.034926965 33800 0.063742147
33800 0.094609895 33800 0.060618586 16900 0.066430584 16936
0.068236594 33808 0.060559589 33808 0.039144492
33800 0.15886451 33800 0.057810801 16900 0.049325565 16940
0.029284369 33816 0.054409225 33816 0.021035932
33800 0.141118508 33800 0.009504538 16900 0.029720983 16944
0.029593726 33824 0.038935701 33824 0.017824907
33800 0.08752702 33800 0.037369631 16900 0.040387822 16948
0.074780677 33832 0.08125684 33832 0.01843728
33800 0.143459329 33800 0.050146828 17000 0.046852849 16952
0.101345955 33840 0.097911849 33840 0.005604503
33800 0.108390152 33800 0.045012275 17000 0.036369595 16956
0.089034453 33848 0.106023894 33848 0.021531929
33900 0.041476629 33900 0.023977802 17000 0.021757041 16960
0.034499051 33856 0.084674393 33856 0.015124209
33900 0.01183017 33900 0.038780538 17000 0.016768312 16964
0.092260758 33864 0.039408293 33864 0.036514339
33900 0.083681211 33900 0.030432711 17000 0.037758251 16968
0.08104906 33872 0.15661199 33872 0.067359018
33900 0.140607051 33900 0.076225071 17000 0.020661306 16972
0.056123172 33880 0.173815759 33880 0.049638198
33900 0.131624809 33900 0.105747808 17000 0.030434378 16976
0.096025644 33888 0.098375684 33888 0.06859206
33900 0.109448221 33900 0.104571751 17000 0.040270712 16980
0.089251633 33896 0.045461278 33896 0.061612351
33900 0.048600803 33900 0.077986042 17000 0.019568526 16984
0.052760846 33904 0.097296259 33904 0.003870508
33900 0.083466519 33900 0.074444361 17000 0.019580934 16988
0.040144969 33912 0.089835041 33912 0.059864226
33900 0.14519121 33900 0.017359931 17000 0.009229368 16992
0.051001873 33920 0.060285209 33920 0.066861823
33900 0.063613559 33900 0.054403929 17000 0.005019549 16996
0.070149334 33928 0.071722003 33928 0.035543988
33900 0.053268621 33900 0.080345213 17000 0.027055796 17000
0.123990685 33936 0.060747367 33936 0.026300062
33900 0.084815452 33900 0.075017422 17000 0.071534974 17004
0.165581325 33944 0.042793599 33944 0.010011448
34000 0.070884475 34000 0.092326263 17000 0.096133903 17008
0.165636331 33952 0.037083253 33952 0.045252637
34000 0.040588722 34000 0.058260805 17000 0.073577008 17012
0.10465907 33960 0.086963824 33960 0.047266669
34000 0.056245102 34000 0.026058819 17000 0.036885158 17016
0.069297552 33968 0.097748431 33968 0.030161666
34000 0.093657574 34000 0.088868052 17000 0.045071294 17020
0.1118293 33976 0.089497225 33976 0.028590006
34000 0.119674398 34000 0.116296229 17000 0.067830581 17024
0.131182344 33984 0.100798119 33984 0.038179085
34000 0.102984959 34000 0.092808681 17000 0.03418027 17028
0.142296718 33992 0.20023188 33992 0.05198866
34000 0.03273545 34000 0.084498432 17000 0.030240344 17032
0.142424149 34000 0.189547718 34000 0.059118545
34000 0.068889262 34000 0.149522763 17000 0.068209432 17036
0.120539095 34008 0.082620725 34008 0.041044808
34000 0.074380776 34000 0.143337689 17000 0.055672979 17040
0.074349315 34016 0.107142223 34016 0.02128555
34000 0.077783399 34000 0.075556251 17000 0.043597731 17044
0.026943004 34024 0.130129861 34024 0.029914972
34000 0.090245325 34000 0.049381284 17000 0.043219941 17048

0.04017145 34032 0.082119055 34032 0.020693366
34000 0.069128582 34000 0.016316331 17100 0.066287212 17052
0.051931893 34040 0.063068714 34040 0.025908115
34000 0.093071823 34000 0.060477498 17100 0.093749288 17056
0.057619651 34048 0.076153207 34048 0.042007156
34100 0.07152919 34100 0.082600534 17100 0.088694891 17060
0.047436235 34056 0.031889071 34056 0.05927571
34100 0.042003849 34100 0.057184796 17100 0.05827679 17064
0.026307636 34064 0.07852025 34064 0.060852086
34100 0.040424238 34100 0.120604214 17100 0.014435787 17068
0.039634553 34072 0.056984231 34072 0.055293927
34100 0.044506334 34100 0.134021189 17100 0.019650883 17072
0.064599029 34080 0.036214002 34080 0.054686359
34100 0.093989256 34100 0.134829418 17100 0.031498053 17076
0.051007901 34088 0.039347262 34088 0.055606623
34100 0.10594225 34100 0.098471966 17100 0.038263184 17080
0.035010555 34096 0.067461726 34096 0.047129695
34100 0.066616332 34100 0.058604885 17100 0.032489108 17084
0.013555291 34104 0.187111116 34104 0.048264035
34100 0.025344436 34100 0.060292772 17100 0.034101144 17088
0.011610787 34112 0.246811629 34112 0.042746458
34100 0.055417553 34100 0.055321914 17100 0.038474962 17092
0.038823124 34120 0.237703905 34120 0.037288195
34100 0.124026847 34100 0.05665435 17100 0.048420283 17096
0.08830052 34128 0.210741636 34128 0.022817048
34100 0.191839659 34100 0.070057766 17100 0.050668197 17100
0.082127757 34136 0.154083959 34136 0.024076197
34100 0.171364765 34100 0.051719198 17100 0.051811934 17104
0.037183818 34144 0.107712127 34144 0.073244621
34200 0.08186671 34200 0.089730602 17100 0.054282897 17108
0.01849422 34152 0.059712489 34152 0.061527833
34200 0.109065579 34200 0.16581206 17100 0.043848249 17112
0.057882262 34160 0.03490728 34160 0.067665955
34200 0.066667963 34200 0.171885884 17100 0.051601277 17116
0.043394499 34168 0.073677584 34168 0.086332628
34200 0.037690574 34200 0.086290231 17100 0.044658907 17120
0.023113656 34176 0.057194786 34176 0.048061098
34200 0.069234164 34200 0.05464533 17100 0.047410445 17124
0.034127126 34184 0.090929381 34184 0.01672787
34200 0.08962657 34200 0.108059103 17100 0.046550202 17128
0.071873314 34192 0.11371672 34192 0.021691816
34200 0.037871483 34200 0.121510369 17100 0.016754599 17132
0.060959836 34200 0.04612756 34200 0.032446234
34200 0.089600806 34200 0.069385002 17100 0.023361747 17136
0.024518198 34208 0.076696808 34208 0.049965576
34200 0.173643013 34200 0.052712716 17100 0.051079547 17140
0.009037843 34216 0.140658841 34216 0.052815674
34200 0.145179729 34200 0.141340555 17100 0.050225768 17144
0.025539825 34224 0.136291783 34224 0.044045592
34200 0.070915041 34200 0.149527754 17100 0.015213632 17148
0.052361666 34232 0.138413481 34232 0.025458634
34200 0.035918863 34200 0.111910995 17200 0.033284254 17152
0.080303231 34240 0.12830265 34240 0.036137299
34200 0.12302812 34200 0.074129326 17200 0.038192935 17156
0.091093156 34248 0.098162178 34248 0.04599517
34300 0.132736168 34300 0.0508302 17200 0.014895557 17160
0.108133216 34256 0.12720903 34256 0.038529604
34300 0.088355715 34300 0.012688239 17200 0.018772036 17164
0.099242316 34264 0.126688873 34264 0.04978948
34300 0.100854893 34300 0.092477574 17200 0.041676271 17168
0.036375397 34272 0.085887099 34272 0.031976004
34300 0.119670884 34300 0.145453625 17200 0.094521514 17172
0.021183741 34280 0.059985905 34280 0.024897856
34300 0.176579474 34300 0.101993603 17200 0.084177045 17176
0.015388805 34288 0.045196499 34288 0.037707352
34300 0.206688463 34300 0.018162084 17200 0.025980815 17180
0.012526684 34296 0.020391708 34296 0.041836487
34300 0.122395053 34300 0.041335421 17200 0.02813961 17184

0.00721645 34304 0.016550837 34304 0.039207134
34300 0.053209613 34300 0.051657709 17200 0.023489476 17188
0.020397602 34312 0.122946352 34312 0.054421831
34300 0.041268722 34300 0.14585456 17200 0.010097719 17192
0.029710509 34320 0.179383205 34320 0.088414759
34300 0.053677286 34300 0.134541755 17200 0.020199144 17196
0.079733087 34328 0.114186834 34328 0.101542421
34300 0.071662049 34300 0.033972436 17200 0.028643777 17200
0.06254355 34336 0.040565512 34336 0.074069198
34300 0.034764642 34300 0.080490747 17200 0.059148581 17204
0.029623618 34344 0.084335814 34344 0.044257413
34400 0.062448606 34400 0.113401889 17200 0.064260144 17208
0.065376262 34352 0.124926242 34352 0.018243976
34400 0.096742995 34400 0.136925155 17200 0.013334798 17212
0.082142156 34360 0.126953426 34360 0.026774915
34400 0.084923202 34400 0.148514416 17200 0.061637817 17216
0.026782769 34368 0.109814573 34368 0.032269287
34400 0.123220336 34400 0.076672397 17200 0.043366741 17220
0.10624923 34376 0.117126212 34376 0.013622388
34400 0.141938901 34400 0.031971726 17200 0.018425211 17224
0.107250278 34384 0.136804884 34384 0.013749954
34400 0.09935641 34400 0.029630472 17200 0.049075938 17228
0.028534763 34392 0.103612176 34392 0.060726994
34400 0.036604888 34400 0.070033813 17200 0.046324731 17232
0.047686983 34400 0.007707023 34400 0.073505893
34400 0.069817244 34400 0.111784924 17200 0.006962442 17236
0.076292774 34408 0.080240621 34408 0.041746484
34400 0.106453903 34400 0.051607167 17200 0.035736288 17240
0.072135132 34416 0.074096999 34416 0.0231985
34400 0.101922924 34400 0.03823658 17200 0.01652012 17244
0.057436919 34424 0.064232932 34424 0.040877407
34400 0.088846631 34400 0.082883424 17200 0.05382505 17248
0.041050884 34432 0.082389801 34432 0.025417412
34400 0.050520546 34400 0.095456737 17300 0.074072392 17252
0.104562176 34440 0.108356777 34440 0.013181351
34400 0.070193681 34400 0.063877436 17300 0.062460946 17256
0.124481332 34448 0.128127504 34448 0.018039697
34500 0.074718017 34500 0.037427475 17300 0.0386988 17260
0.096160395 34456 0.102681508 34456 0.028080334
34500 0.027572049 34500 0.087563436 17300 0.022583796 17264
0.082993684 34464 0.068173635 34464 0.034973378
34500 0.105342988 34500 0.13058589 17300 0.06037702 17268
0.093819341 34472 0.149742744 34472 0.021120293
34500 0.090085268 34500 0.142763965 17300 0.073168136 17272
0.117416952 34480 0.134710921 34480 0.018063758
34500 0.047258509 34500 0.10337554 17300 0.054763506 17276
0.080845493 34488 0.069001762 34488 0.035520967
34500 0.096001735 34500 0.103329199 17300 0.023381379 17280
0.048796122 34496 0.021329484 34496 0.026157801
34500 0.181848605 34500 0.174630143 17300 0.071852264 17284
0.085299478 34504 0.018340455 34504 0.020377582
34500 0.176204704 34500 0.206769313 17300 0.047979771 17288
0.079413214 34512 0.09682402 34512 0.047759473
34500 0.067775924 34500 0.159005489 17300 0.018255661 17292
0.063253327 34520 0.161082339 34520 0.045836106
34500 0.025604841 34500 0.097702818 17300 0.023979796 17296
0.052844433 34528 0.138347998 34528 0.028498895
34500 0.01980106 34500 0.082631326 17300 0.052960226 17300
0.012321238 34536 0.098022647 34536 0.023582999
34500 0.035995603 34500 0.087648186 17300 0.03872973 17304
0.075228287 34544 0.056511526 34544 0.00984688
34600 0.029148932 34600 0.055573266 17300 0.037710495 17308
0.132477726 34552 0.018068664 34552 0.016511534
34600 0.075346907 34600 0.047125701 17300 0.027795224 17312
0.128036729 34560 0.052330575 34560 0.027166145
34600 0.094529118 34600 0.109265078 17300 0.018943816 17316
0.108633241 34568 0.138831558 34568 0.05001422
34600 0.045551737 34600 0.118205026 17300 0.031333311 17320

0.098227516 34576 0.151188025 34576 0.042602769
34600 0.034871671 34600 0.12955429 17300 0.049207592 17324
0.070065231 34584 0.107951717 34584 0.037698839
34600 0.099059078 34600 0.12571612 17300 0.036111553 17328
0.022299893 34592 0.032255757 34592 0.015489703
34600 0.175017805 34600 0.093053423 17300 0.011110137 17332
0.017059541 34600 0.014074602 34600 0.02665845
34600 0.164644764 34600 0.015625641 17300 0.041465686 17336
0.047217931 34608 0.140087898 34608 0.005039584
34600 0.068226567 34600 0.059349106 17300 0.061644896 17340
0.06271941 34616 0.25561318 34616 0.045620276
34600 0.069727212 34600 0.062437757 17300 0.085927211 17344
0.047736765 34624 0.207728371 34624 0.04507865
34600 0.127360793 34600 0.040435865 17300 0.081040533 17348
0.061091771 34632 0.186345511 34632 0.006792849
34600 0.149796586 34600 0.052060321 17400 0.065878929 17352
0.093519921 34640 0.244826108 34640 0.016062766
34600 0.172195286 34600 0.108274951 17400 0.03034654 17356
0.118555072 34648 0.234482461 34648 0.038283248
34700 0.187897501 34700 0.08516293 17400 0.031085536 17360
0.120039556 34656 0.169028746 34656 0.068240908
34700 0.152417444 34700 0.074896052 17400 0.067008718 17364
0.064769716 34664 0.146721228 34664 0.076398952
34700 0.119096469 34700 0.09821073 17400 0.053943481 17368
0.042018939 34672 0.111524023 34672 0.05486828
34700 0.074931973 34700 0.020697826 17400 0.046917598 17372
0.030869964 34680 0.128310858 34680 0.052995962
34700 0.036041165 34700 0.086712265 17400 0.046796467 17376
0.021334246 34688 0.19245212 34688 0.061577062
34700 0.058174399 34700 0.09307739 17400 0.036079317 17380
0.030692074 34696 0.181032796 34696 0.0498277
34700 0.12261275 34700 0.141070603 17400 0.070137787 17384
0.014557226 34704 0.134624425 34704 0.027165183
34700 0.132624365 34700 0.053285461 17400 0.081097744 17388
0.049996299 34712 0.08104874 34712 0.043967426
34700 0.12114978 34700 0.077934696 17400 0.065847962 17392
0.108331718 34720 0.011769926 34720 0.06165426
34700 0.076168515 34700 0.038411563 17400 0.04211174 17396
0.117851909 34728 0.113266739 34728 0.070147326
34700 0.0117062 34700 0.087530971 17400 0.032466513 17400
0.082876941 34736 0.160920085 34736 0.097877703
34700 0.029972758 34700 0.079831014 17400 0.032411492 17404
0.101544567 34744 0.13187436 34744 0.113261092
34800 0.096443968 34800 0.044758013 17400 0.035670237 17408
0.14299342 34752 0.076908429 34752 0.091852307
34800 0.122115525 34800 0.085161984 17400 0.036871228 17412
0.146951279 34760 0.084486572 34760 0.079558493
34800 0.050261482 34800 0.113247712 17400 0.05772003 17416
0.123278878 34768 0.112024318 34768 0.093368981
34800 0.092256065 34800 0.114204835 17400 0.104085535 17420
0.1193465 34776 0.090834154 34776 0.048575836
34800 0.100160556 34800 0.111551948 17400 0.113473529 17424
0.141625481 34784 0.070102462 34784 0.015939031
34800 0.016832766 34800 0.070290509 17400 0.058816415 17428
0.121064877 34792 0.076094897 34792 0.017707776
34800 0.048679052 34800 0.029283425 17400 0.049529568 17432
0.039814058 34800 0.088198307 34800 0.039510716
34800 0.054981472 34800 0.04463016 17400 0.075509961 17436
0.07457639 34808 0.111293259 34808 0.076524746
34800 0.086343374 34800 0.019485848 17400 0.062647407 17440
0.109983383 34816 0.072771771 34816 0.091013579
34800 0.160540964 34800 0.077433586 17400 0.067255867 17444
0.061506049 34824 0.048082442 34824 0.055041568
34800 0.178173926 34800 0.108585715 17400 0.06460576 17448
0.02195414 34832 0.026934258 34832 0.02069123
34800 0.112074522 34800 0.070761147 17500 0.023051764 17452
0.035399586 34840 0.057870835 34840 0.065701213
34800 0.047655987 34800 0.121770092 17500 0.04075791 17456

0.036732243	34848	0.094453841	34848	0.06040221		
34900	0.016691052	34900	0.154687558	17500	0.06082093	17460
0.039535564	34856	0.076939366	34856	0.022945364		
34900	0.01745078	34900	0.14216626	17500	0.035073503	17464
0.027352375	34864	0.035927169	34864	0.032807206		
34900	0.031400196	34900	0.072838746	17500	0.021076487	17468
0.053620861	34872	0.082473147	34872	0.058010559		
34900	0.125347914	34900	0.030788084	17500	0.029536894	17472
0.105402643	34880	0.113950868	34880	0.044056495		
34900	0.194083331	34900	0.052642234	17500	0.024678981	17476
0.16133269	34888	0.074010604	34888	0.015107815		
34900	0.264513918	34900	0.092224575	17500	0.014504733	17480
0.173370339	34896	0.054707514	34896	0.031711032		
34900	0.242693946	34900	0.068506204	17500	0.045586239	17484
0.145428261	34904	0.045523146	34904	0.068780479		
34900	0.15683574	34900	0.091598384	17500	0.073097304	17488
0.139906231	34912	0.051617528	34912	0.081374208		
34900	0.099978293	34900	0.155630013	17500	0.061397332	17492
0.119846547	34920	0.034799512	34920	0.069315785		
34900	0.046076486	34900	0.189684724	17500	0.049956499	17496
0.036874972	34928	0.126143641	34928	0.051258165		
34900	0.027974507	34900	0.137936223	17500	0.02698597	17500
0.05977551	34936	0.153807458	34936	0.032812019		
34900	0.036043199	34900	0.017756593	17500	0.013921696	17504
0.105395578	34944	0.114867165	34944	0.015428759		
35000	0.105666957	35000	0.05258113	17500	0.01046027	17508
0.115441188	34952	0.079491481	34952	0.017938721		
35000	0.136333998	35000	0.041232281	17500	0.061725543	17512
0.100831756	34960	0.112049034	34960	0.013860867		
35000	0.078374287	35000	0.088507826	17500	0.08772649	17516
0.045164823	34968	0.134300775	34968	0.015817268		
35000	0.012205213	35000	0.099532488	17500	0.049698363	17520
0.080456441	34976	0.063942061	34976	0.030219091		
35000	0.057859041	35000	0.049224265	17500	0.010096491	17524
0.104254752	34984	0.134842776	34984	0.022418055		
35000	0.075671844	35000	0.025166522	17500	0.035905967	17528
0.097061726	34992	0.156108566	34992	0.01069982		
35000	0.071073511	35000	0.023135432	17500	0.02509582	17532
0.062499181	35000	0.127526291	35000	0.026505937		
35000	0.033309072	35000	0.095693817	17500	0.011527095	17536
0.019306226	35008	0.089292022	35008	0.042999058		
35000	0.023660223	35000	0.135624926	17500	0.017644248	17540
0.065752414	35016	0.036170572	35016	0.039990962		
35000	0.037918966	35000	0.089103269	17500	0.010885194	17544
0.06600943	35024	0.079030695	35024	0.048379076		
35000	0.040614574	35000	0.024485265	17500	0.011877813	17548
0.033660606	35032	0.086297296	35032	0.063194486		
35000	0.053004784	35000	0.015982621	17600	0.013141447	17552
0.011744194	35040	0.127638195	35040	0.078212557		
35000	0.054503817	35000	0.04958349	17600	0.040275518	17556
0.02964009	35048	0.110003406	35048	0.091834329		
35100	0.031209653	35100	0.048332811	17600	0.036359877	17560
0.035483798	35056	0.071062808	35056	0.097213822		
35100	0.061165069	35100	0.061743747	17600	0.003534762	17564
0.04574459	35064	0.050888211	35064	0.09007279		
35100	0.098370016	35100	0.049279403	17600	0.036902588	17568
0.058130936	35072	0.125916296	35072	0.057962036		
35100	0.072415132	35100	0.020465184	17600	0.073529744	17572
0.064589614	35080	0.075480435	35080	0.016440265		
35100	0.036779922	35100	0.096254713	17600	0.064862252	17576
0.074106094	35088	0.056137658	35088	0.009976878		
35100	0.082793937	35100	0.123780657	17600	0.04880242	17580
0.074949312	35096	0.101661492	35096	0.027616194		
35100	0.047300295	35100	0.070126378	17600	0.0869699	17584
0.053642474	35104	0.072648203	35104	0.042725096		
35100	0.02022154	35100	0.05515961	17600	0.062744119	17588
0.021766291	35112	0.055348068	35112	0.059457529		
35100	0.05092537	35100	0.179078663	17600	0.031912387	17592

0.014820368 35120 0.112286289 35120 0.034784607
35100 0.032941112 35100 0.282734516 17600 0.050933759 17596
0.030908668 35128 0.151054861 35128 0.004319171
35100 0.100573852 35100 0.301898283 17600 0.034645294 17600
0.042299154 35136 0.082640923 35136 0.021768996
35100 0.129441993 35100 0.237845306 17600 0.010198865 17604
0.043199358 35144 0.02028776 35144 0.028448536
35200 0.053550277 35200 0.169913983 17600 0.006347374 17608
0.019600631 35152 0.080516635 35152 0.040642095
35200 0.03409768 35200 0.173081236 17600 0.022813494 17612
0.040150906 35160 0.090130656 35160 0.054851484
35200 0.051880932 35200 0.18191355 17600 0.028325949 17616
0.018871027 35168 0.022921804 35168 0.045279518
35200 0.123466263 35200 0.078541052 17600 0.022273214 17620
0.038103884 35176 0.082318067 35176 0.026681808
35200 0.121686448 35200 0.070703536 17600 0.064255866 17624
0.074524192 35184 0.086656168 35184 0.06554617
35200 0.120129269 35200 0.09290989 17600 0.092500071 17628
0.09283275 35192 0.070795861 35192 0.050194984
35200 0.170006169 35200 0.051145307 17600 0.083292878 17632
0.100282254 35200 0.098911631 35200 0.028473154
35200 0.215688516 35200 0.020871346 17600 0.048269423 17636
0.078392994 35208 0.160585026 35208 0.056349818
35200 0.18250673 35200 0.046031611 17600 0.06708708 17640
0.046406411 35216 0.171285661 35216 0.0575718
35200 0.137809548 35200 0.087217552 17600 0.088262488 17644
0.042731546 35224 0.123127815 35224 0.019578842
35200 0.087346525 35200 0.041253283 17600 0.064289066 17648
0.049406364 35232 0.065003354 35232 0.028685659
35200 0.004641426 35200 0.031446867 17700 0.086213746 17652
0.076279146 35240 0.018656763 35240 0.009685857
35200 0.037113507 35200 0.068093155 17700 0.091833492 17656
0.123236328 35248 0.039185645 35248 0.047452559
35300 0.036967238 35300 0.051852192 17700 0.042637974 17660
0.106737563 35256 0.095514151 35256 0.0590782
35300 0.039794741 35300 0.112243382 17700 0.006516704 17664
0.045836117 35264 0.139364594 35264 0.029093068
35300 0.014152859 35300 0.185807701 17700 0.017493505 17668
0.057847876 35272 0.162836731 35272 0.016441851
35300 0.075254815 35300 0.107655724 17700 0.04979736 17672
0.046079582 35280 0.158701965 35280 0.012536834
35300 0.108450746 35300 0.0877286 17700 0.07904724 17676
0.018291825 35288 0.13787458 35288 0.030107454
35300 0.075375232 35300 0.106550833 17700 0.066561246 17680
0.062608619 35296 0.081767947 35296 0.059362952
35300 0.063960826 35300 0.04564082 17700 0.053668806 17684
0.048592527 35304 0.07070625 35304 0.050696257
35300 0.044731332 35300 0.007277658 17700 0.069423652 17688
0.034821525 35312 0.159062824 35312 0.012765229
35300 0.073703464 35300 0.010463044 17700 0.070241062 17692
0.013305134 35320 0.200509763 35320 0.040987081
35300 0.165942125 35300 0.075534408 17700 0.043147877 17696
0.029445324 35328 0.140037126 35328 0.02981688
35300 0.168795581 35300 0.115563737 17700 0.020250898 17700
0.048798196 35336 0.09124315 35336 0.024663807
35300 0.088926943 35300 0.063354506 17700 0.077067409 17704
0.050467301 35344 0.146386563 35344 0.040826264
35400 0.18211217 35400 0.050755974 17700 0.11579803 17708
0.061318533 35352 0.213935055 35352 0.02719146
35400 0.178214934 35400 0.01928279 17700 0.103521801 17712
0.044024215 35360 0.225804135 35360 0.032770149
35400 0.080639315 35400 0.045619381 17700 0.063799867 17716
0.031073763 35368 0.127663298 35368 0.035541634
35400 0.110720168 35400 0.071763818 17700 0.047748785 17720
0.104664556 35376 0.054412187 35376 0.019086421
35400 0.125538586 35400 0.096250908 17700 0.044049139 17724
0.133518479 35384 0.092171424 35384 0.020751639
35400 0.03090614 35400 0.131881243 17700 0.043757569 17728

0.096219133 35392 0.059556645 35392 0.019421981
35400 0.079144338 35400 0.112618342 17700 0.035077432 17732
0.066005574 35400 0.10974484 35400 0.007689187
35400 0.081629441 35400 0.060527393 17700 0.018278688 17736
0.065341126 35408 0.12069349 35408 0.011090651
35400 0.027610866 35400 0.102432583 17700 0.008715405 17740
0.069122951 35416 0.036887046 35416 0.029352032
35400 0.008551011 35400 0.035129855 17700 0.008059541 17744
0.07320322 35424 0.088394161 35424 0.069434143
35400 0.085216088 35400 0.043594882 17700 0.015358777 17748
0.079199366 35432 0.148819265 35432 0.096656651
35400 0.150748921 35400 0.070791408 17800 0.045246594 17752
0.077711076 35440 0.164851939 35440 0.090527741
35400 0.114691291 35400 0.133080423 17800 0.056179139 17756
0.089126152 35448 0.161385004 35448 0.082707367
35500 0.044728113 35500 0.11005791 17800 0.077685421 17760
0.101685233 35456 0.093505412 35456 0.078122539
35500 0.066647459 35500 0.138621953 17800 0.081153652 17764
0.079280981 35464 0.07461058 35464 0.051733245
35500 0.060311919 35500 0.133824141 17800 0.058518577 17768
0.051594274 35472 0.136185758 35472 0.040590461
35500 0.013592006 35500 0.044209832 17800 0.03268505 17772
0.037469148 35480 0.098193443 35480 0.052460815
35500 0.044433895 35500 0.07308861 17800 0.004948867 17776
0.036912785 35488 0.010259791 35488 0.073635732
35500 0.086264969 35500 0.152318884 17800 0.021155966 17780
0.077953104 35496 0.05129334 35496 0.08899028
35500 0.085524378 35500 0.170227446 17800 0.013936989 17784
0.080378188 35504 0.079690522 35504 0.078304001
35500 0.067473455 35500 0.149314335 17800 0.029005565 17788
0.06665531 35512 0.079393925 35512 0.04998115
35500 0.102393147 35500 0.129839275 17800 0.057183948 17792
0.061735576 35520 0.069511028 35520 0.030613959
35500 0.11925312 35500 0.132273883 17800 0.056711342 17796
0.051772615 35528 0.06343193 35528 0.011587781
35500 0.128014698 35500 0.098393437 17800 0.059529219 17800
0.009632572 35536 0.074148418 35536 0.030940471
35500 0.137334471 35500 0.003807845 17800 0.047728852 17804
0.055838147 35544 0.116156392 35544 0.040247771
35600 0.153363871 35600 0.092654896 17800 0.025691152 17808
0.095211282 35552 0.128783388 35552 0.044362267
35600 0.150718115 35600 0.097464785 17800 0.035499183 17812
0.094510288 35560 0.15866023 35560 0.044869674
35600 0.154247435 35600 0.082997394 17800 0.045015124 17816
0.065110377 35568 0.202621334 35568 0.028286062
35600 0.141864701 35600 0.115157251 17800 0.046163757 17820
0.040971063 35576 0.17970326 35576 0.026454638
35600 0.104968611 35600 0.116116891 17800 0.052786709 17824
0.043866432 35584 0.119832097 35584 0.031060445
35600 0.10981282 35600 0.070775161 17800 0.070415728 17828
0.068474903 35592 0.082944178 35592 0.011159285
35600 0.153646034 35600 0.103805774 17800 0.073565228 17832
0.044099081 35600 0.022403854 35600 0.036121703
35600 0.164091544 35600 0.011812102 17800 0.061386199 17836
0.028615516 35608 0.098146222 35608 0.073241368
35600 0.162879965 35600 0.171675434 17800 0.054820681 17840
0.057782389 35616 0.091360998 35616 0.063027801
35600 0.164062193 35600 0.258208689 17800 0.053951764 17844
0.082678918 35624 0.062137646 35624 0.018845603
35600 0.1115618 35600 0.18950821 17800 0.054558121 17848
0.051646006 35632 0.081663631 35632 0.010775919
35600 0.085857944 35600 0.049233779 17900 0.062808402 17852
0.024484298 35640 0.051493629 35640 0.041039981
35600 0.019570363 35600 0.079124024 17900 0.077429308 17856
0.094628085 35648 0.137083 35648 0.063020889
35700 0.168536703 35700 0.078551959 17900 0.043793065 17860
0.10678242 35656 0.132345391 35656 0.052475294
35700 0.199302944 35700 0.040065585 17900 0.036668462 17864

0.051704126 35664 0.097334953 35664 0.018861951
35700 0.095721072 35700 0.04902959 17900 0.047969868 17868
0.029949444 35672 0.127313528 35672 0.029205476
35700 0.01364873 35700 0.053618584 17900 0.027731612 17872
0.093654373 35680 0.145519778 35680 0.059958948
35700 0.025135816 35700 0.043325694 17900 0.009427243 17876
0.124511018 35688 0.074019779 35688 0.040286766
35700 0.054832508 35700 0.037990798 17900 0.027798058 17880
0.095931995 35696 0.07391761 35696 0.008465869
35700 0.083486964 35700 0.016068398 17900 0.052147221 17884
0.065226101 35704 0.128473578 35704 0.043034997
35700 0.065547647 35700 0.117772797 17900 0.049211336 17888
0.060673167 35712 0.117575088 35712 0.04710706
35700 0.009753853 35700 0.173934998 17900 0.035868365 17892
0.025355228 35720 0.114434224 35720 0.044336084
35700 0.021174346 35700 0.143506186 17900 0.024456618 17896
0.037333619 35728 0.150895561 35728 0.035725159
35700 0.027275484 35700 0.092795643 17900 0.049940656 17900
0.069984439 35736 0.158365729 35736 0.028357506
35700 0.046799436 35700 0.078827579 17900 0.079728605 17904
0.067025183 35744 0.147079554 35744 0.039406285
35800 0.101813377 35800 0.072332339 17900 0.060816012 17908
0.033379973 35752 0.104459505 35752 0.061440776
35800 0.198477151 35800 0.094465009 17900 0.0103754 17912
0.035004687 35760 0.030888219 35760 0.04910482
35800 0.189443861 35800 0.178386806 17900 0.014511645 17916
0.085336847 35768 0.028167209 35768 0.031997271
35800 0.095791424 35800 0.225727431 17900 0.045582845 17920
0.067665038 35776 0.032653148 35776 0.059906015
35800 0.052559812 35800 0.195071974 17900 0.059241389 17924
0.010924686 35784 0.037138107 35784 0.059231352
35800 0.04251939 35800 0.133834838 17900 0.033820477 17928
0.044031949 35792 0.024532006 35792 0.027569315
35800 0.06463319 35800 0.131510344 17900 0.01360358 17932
0.063641142 35800 0.032537206 35800 0.008606567
35800 0.050275969 35800 0.111305984 17900 0.020823371 17936
0.056206012 35808 0.062924926 35808 0.013358572
35800 0.097616721 35800 0.051900737 17900 0.008492225 17940
0.057926045 35816 0.047869529 35816 0.033978089
35800 0.071166614 35800 0.112001566 17900 0.027065566 17944
0.071123664 35824 0.003166093 35824 0.079446349
35800 0.023355757 35800 0.15425653 17900 0.05733209 17948
0.069680093 35832 0.071891453 35832 0.072301569
35800 0.055274635 35800 0.12950215 18000 0.043946711 17952
0.044005232 35840 0.122977668 35840 0.051740226
35800 0.079202429 35800 0.108165594 18000 0.01997081 17956
0.049290509 35848 0.121640311 35848 0.050290484
35900 0.057733818 35900 0.058050013 18000 0.027604201 17960
0.049570332 35856 0.080224061 35856 0.048105514
35900 0.042774336 35900 0.042074149 18000 0.026318325 17964
0.048346992 35864 0.079749872 35864 0.048037866
35900 0.07110434 35900 0.052634296 18000 0.057490332 17968
0.043530734 35872 0.073005118 35872 0.050944851
35900 0.156681606 35900 0.089272333 18000 0.080133221 17972
0.025220701 35880 0.036623384 35880 0.021661474
35900 0.109623725 35900 0.112862879 18000 0.081223916 17976
0.025930967 35888 0.0542003 35888 0.01585588
35900 0.112882444 35900 0.094611576 18000 0.056771165 17980
0.012636966 35896 0.071064744 35896 0.015922116
35900 0.132842571 35900 0.038168128 18000 0.012009325 17984
0.046945523 35904 0.050197792 35904 0.024321023
35900 0.026486043 35900 0.025376008 18000 0.0093587 17988
0.077961136 35912 0.111897381 35912 0.015699916
35900 0.135329683 35900 0.107909888 18000 0.038764047 17992
0.052519659 35920 0.121827427 35920 0.029902329
35900 0.22040632 35900 0.152600668 18000 0.043004679 17996
0.023685127 35928 0.082900973 35928 0.05025087
35900 0.187074649 35900 0.076376258 18000 0.009994041 18000

0.029728781	35936	0.033788972	35936	0.040677125		
35900	0.094588468	35900	0.098721066	18000	0.044200078	18004
0.067113215	35944	0.037545633	35944	0.027743015		
36000	0.037403293	36000	0.156071969	18000	0.070787406	18008
0.064279229	35952	0.111920555	35952	0.03104275		
36000	0.018936595	36000	0.153058078	18000	0.057483696	18012
0.028441134	35960	0.12726913	35960	0.030350948		
36000	0.068370711	36000	0.093975905	18000	0.010978463	18016
0.064874752	35968	0.06077822	35968	0.036401743		
36000	0.121494188	36000	0.093443916	18000	0.032371758	18020
0.052234005	35976	0.039362752	35976	0.04395673		
36000	0.127431719	36000	0.090103909	18000	0.050513307	18024
0.056714755	35984	0.09641231	35984	0.041982734		
36000	0.106197105	36000	0.074047675	18000	0.057688405	18028
0.047092868	35992	0.142264719	35992	0.028418675		
36000	0.138602321	36000	0.05021496	18000	0.040164781	18032
0.04745417	36000	0.156603943	36000	0.011954611		
36000	0.130023821	36000	0.126581989	18000	0.022812108	18036
0.056903398	36008	0.075890705	36008	0.030494008		
36000	0.077868557	36000	0.106577529	18000	0.028843431	18040
0.097119468	36016	0.072288858	36016	0.034612618		
36000	0.09776024	36000	0.042956715	18000	0.046866713	18044
0.096480108	36024	0.117204196	36024	0.031845437		
36000	0.079431746	36000	0.067725552	18000	0.052944801	18048
0.057838264	36032	0.082508792	36032	0.058707843		
36000	0.05276694	36000	0.13654893	18100	0.052796822	18052
0.037184189	36040	0.142748744	36040	0.044378056		
36000	0.015610904	36000	0.110396912	18100	0.062449915	18056
0.047247741	36048	0.091215414	36048	0.039828152		
36100	0.056806715	36100	0.025631849	18100	0.054743636	18060
0.019642866	36056	0.03755841	36056	0.057406694		
36100	0.085641761	36100	0.064662127	18100	0.022223627	18064
0.077915	36064	0.130070694	36064	0.042638127		
36100	0.035613462	36100	0.134423288	18100	0.025073157	18068
0.095485928	36072	0.152853099	36072	0.033486424		
36100	0.056984707	36100	0.180679999	18100	0.02064844	18072
0.068095236	36080	0.120979101	36080	0.02700046		
36100	0.099787198	36100	0.180958115	18100	0.024042322	18076
0.064728825	36088	0.081714454	36088	0.011542487		
36100	0.126335566	36100	0.143520185	18100	0.022476372	18080
0.009676792	36096	0.071540075	36096	0.027356013		
36100	0.134428031	36100	0.120213445	18100	0.058510341	18084
0.019518195	36104	0.090307614	36104	0.009653115		
36100	0.108818626	36100	0.117276679	18100	0.067558009	18088
0.009610823	36112	0.079092977	36112	0.013099144		
36100	0.144688456	36100	0.146711871	18100	0.038009519	18092
0.041930744	36120	0.02792315	36120	0.01146542		
36100	0.16331824	36100	0.141777535	18100	0.059012029	18096
0.090439018	36128	0.038267412	36128	0.021596854		
36100	0.125009639	36100	0.070583868	18100	0.067876892	18100
0.033165885	36136	0.008987941	36136	0.012236984		
36100	0.157684874	36100	0.016219452	18100	0.047342277	18104
0.027668964	36144	0.005277453	36144	0.062334351		
36200	0.167188483	36200	0.030028685	18100	0.015387161	18108
0.070916685	36152	0.010412784	36152	0.116798641		
36200	0.159935866	36200	0.050907711	18100	0.009608337	18112
0.094966032	36160	0.025240361	36160	0.08894685		
36200	0.101753845	36200	0.12840578	18100	0.023120138	18116
0.020200445	36168	0.064477383	36168	0.042162712		
36200	0.144651785	36200	0.225965399	18100	0.042101117	18120
0.062630934	36176	0.156555834	36176	0.049669056		
36200	0.232366045	36200	0.274416583	18100	0.048457427	18124
0.075717129	36184	0.169420193	36184	0.028019835		
36200	0.178196308	36200	0.254256098	18100	0.053041687	18128
0.099369892	36192	0.107865169	36192	0.028048338		
36200	0.07575065	36200	0.224835356	18100	0.023941213	18132
0.060088365	36200	0.068067035	36200	0.046289195		
36200	0.034897763	36200	0.198805675	18100	0.056621702	18136

0.040032923 36208 0.065741733 36208 0.041731164
36200 0.02564444 36200 0.141063981 18100 0.091985246 18140
0.104509687 36216 0.044492859 36216 0.061307212
36200 0.015572527 36200 0.082396036 18100 0.058281548 18144
0.114003407 36224 0.035091423 36224 0.024364217
36200 0.045638 36200 0.063709835 18100 0.018934943 18148
0.074619209 36232 0.091273607 36232 0.047301764
36200 0.071686773 36200 0.072012328 18200 0.073070871 18152
0.045618395 36240 0.112672802 36240 0.043910324
36200 0.045871864 36200 0.08196976 18200 0.068209876 18156
0.039714185 36248 0.08071604 36248 0.039612391
36300 0.080347068 36300 0.062497893 18200 0.03166906 18160
0.063820597 36256 0.053038217 36256 0.035132976
36300 0.1230325 36300 0.077275596 18200 0.009966353 18164
0.021546375 36264 0.043902681 36264 0.016675822
36300 0.085357606 36300 0.159016111 18200 0.012887792 18168
0.082556398 36272 0.047732352 36272 0.033771259
36300 0.089171175 36300 0.129973618 18200 0.011784861 18172
0.114134855 36280 0.115201263 36280 0.06296637
36300 0.114944785 36300 0.03140733 18200 0.001899186 18176
0.087089429 36288 0.14489585 36288 0.051005503
36300 0.115441886 36300 0.034910747 18200 0.031114825 18180
0.05025162 36296 0.125822583 36296 0.064980646
36300 0.047724094 36300 0.04563437 18200 0.070690294 18184
0.110372923 36304 0.131912616 36304 0.074491982
36300 0.051702737 36300 0.126892831 18200 0.074254895 18188
0.076355645 36312 0.128022978 36312 0.079492274
36300 0.087560817 36300 0.163255449 18200 0.07046861 18192
0.041665138 36320 0.11979702 36320 0.06047836
36300 0.070538976 36300 0.144166523 18200 0.068819514 18196
0.043557015 36328 0.180169969 36328 0.051622283
36300 0.039000181 36300 0.026304251 18200 0.057797992 18200
0.038766902 36336 0.194721914 36336 0.067704175
36300 0.062063926 36300 0.130468703 18200 0.066859779 18204
0.05531111 36344 0.158820156 36344 0.063373605
36400 0.088273453 36400 0.164441998 18200 0.066849359 18208
0.046568403 36352 0.106829604 36352 0.041267609
36400 0.078544559 36400 0.114244838 18200 0.038722588 18212
0.039008795 36360 0.057380999 36360 0.022004657
36400 0.138618998 36400 0.061101186 18200 0.013394258 18216
0.069906295 36368 0.038692731 36368 0.016200902
36400 0.16499951 36400 0.196190493 18200 0.008862807 18220
0.0643733 36376 0.078781624 36376 0.019288631
36400 0.117248899 36400 0.151913846 18200 0.031712189 18224
0.033352171 36384 0.091535068 36384 0.014076437
36400 0.065717264 36400 0.058565154 18200 0.053974582 18228
0.07889275 36392 0.104728868 36392 0.042365839
36400 0.019984156 36400 0.1071839 18200 0.03515595 18232
0.126430998 36400 0.08780331 36400 0.028151342
36400 0.088968656 36400 0.132713453 18200 0.018285393 18236
0.134831775 36408 0.063098203 36408 0.018610401
36400 0.185996484 36400 0.102343693 18200 0.051379942 18240
0.071587099 36416 0.094729498 36416 0.030062751
36400 0.18308741 36400 0.064418673 18200 0.063870648 18244
0.059869039 36424 0.087977067 36424 0.014041876
36400 0.098160344 36400 0.12307179 18200 0.037391648 18248
0.112365655 36432 0.093413197 36432 0.019228306
36400 0.037743164 36400 0.09794665 18300 0.017490374 18252
0.107929431 36440 0.066681801 36440 0.02554341
36400 0.058997441 36400 0.060006256 18300 0.038535643 18256
0.059148122 36448 0.123824109 36448 0.05961402
36500 0.087587599 36500 0.0615143 18300 0.064977074 18260
0.028414099 36456 0.140965524 36456 0.078792968
36500 0.029670995 36500 0.064896529 18300 0.057911999 18264
0.034246328 36464 0.074302305 36464 0.077229801
36500 0.065597458 36500 0.06337103 18300 0.048515318 18268
0.060415277 36472 0.027478112 36472 0.080781458
36500 0.105355444 36500 0.047955822 18300 0.045467688 18272

0.07913712	36480	0.074758471	36480	0.070805341		
36500	0.096382428	36500	0.028677774	18300	0.035425095	18276
0.079185003	36488	0.136242495	36488	0.042193522		
36500	0.058951584	36500	0.069734335	18300	0.033130866	18280
0.055882698	36496	0.165911028	36496	0.005239176		
36500	0.051890547	36500	0.11256709	18300	0.023325034	18284
0.034885321	36504	0.135105438	36504	0.018100762		
36500	0.091023307	36500	0.133436406	18300	0.012758593	18288
0.072632887	36512	0.051262752	36512	0.022176506		
36500	0.102250502	36500	0.198250113	18300	0.014398128	18292
0.070481445	36520	0.083026032	36520	0.048960636		
36500	0.150513414	36500	0.20667669	18300	0.038317568	18296
0.060567119	36528	0.026743035	36528	0.0728495		
36500	0.163852048	36500	0.133166308	18300	0.030654646	18300
0.063295331	36536	0.07345817	36536	0.054286822		
36500	0.100235411	36500	0.090109352	18300	0.021125805	18304
0.081543964	36544	0.08767016	36544	0.012574502		
36600	0.040069284	36600	0.058415932	18300	0.047803278	18308
0.071313487	36552	0.124824976	36552	0.039255367		
36600	0.136971241	36600	0.01508257	18300	0.04436665	18312
0.052411469	36560	0.077167046	36560	0.069550544		
36600	0.219601803	36600	0.036131842	18300	0.01945399	18316
0.086191256	36568	0.065984415	36568	0.073830532		
36600	0.188855571	36600	0.053435972	18300	0.018198558	18320
0.083461229	36576	0.130827554	36576	0.046517998		
36600	0.106668165	36600	0.086605665	18300	0.03077916	18324
0.021428121	36584	0.103191807	36584	0.047936574		
36600	0.08834972	36600	0.018325342	18300	0.050174946	18328
0.023249426	36592	0.116469433	36592	0.054488053		
36600	0.064410939	36600	0.090685513	18300	0.042505748	18332
0.037201175	36600	0.140714663	36600	0.024020294		
36600	0.026313916	36600	0.125779378	18300	0.031785847	18336
0.048380589	36608	0.090057554	36608	0.02059751		
36600	0.104003993	36600	0.139629978	18300	0.047753052	18340
0.044280791	36616	0.050807845	36616	0.045794044		
36600	0.077426244	36600	0.180601404	18300	0.064673848	18344
0.064630534	36624	0.032860153	36624	0.041270349		
36600	0.042912201	36600	0.173671899	18300	0.052936513	18348
0.079024649	36632	0.009128943	36632	0.043040407		
36600	0.098957295	36600	0.134416885	18400	0.041149404	18352
0.077796343	36640	0.063095722	36640	0.056019693		
36600	0.093353017	36600	0.093653071	18400	0.052810421	18356
0.069489295	36648	0.123549806	36648	0.038179354		
36700	0.068492693	36700	0.059130613	18400	0.054565317	18360
0.052169718	36656	0.10706624	36656	0.023021106		
36700	0.035858411	36700	0.069753281	18400	0.032502827	18364
0.074645745	36664	0.054857275	36664	0.052913114		
36700	0.090986563	36700	0.05685167	18400	0.086490392	18368
0.119351629	36672	0.110772111	36672	0.064627391		
36700	0.073444244	36700	0.006867506	18400	0.155048139	18372
0.129289066	36680	0.153293571	36680	0.049521277		
36700	0.096049029	36700	0.037995571	18400	0.149363986	18376
0.090671361	36688	0.03770458	36688	0.05451417		
36700	0.102789571	36700	0.090069705	18400	0.091103146	18380
0.061841463	36696	0.119111399	36696	0.070802424		
36700	0.098467361	36700	0.088318731	18400	0.022389422	18384
0.063482628	36704	0.149722531	36704	0.080653968		
36700	0.192544176	36700	0.137692215	18400	0.045636989	18388
0.094871699	36712	0.100428937	36712	0.083891777		
36700	0.269802258	36700	0.157854098	18400	0.057935627	18392
0.108686036	36720	0.086963439	36720	0.070750139		
36700	0.204402895	36700	0.213986961	18400	0.026129113	18396
0.070943861	36728	0.084037791	36728	0.053819422		
36700	0.116527881	36700	0.217848545	18400	0.095627111	18400
0.032854194	36736	0.056310673	36736	0.039804862		
36700	0.108602515	36700	0.123483522	18400	0.128470041	18404
0.036029225	36744	0.024911911	36744	0.020684416		
36800	0.187675716	36800	0.082982231	18400	0.103360988	18408

0.086578781	36752	0.036479709	36752	0.022031338
36800	0.29334682	36800	0.078954414	18400	0.082485844	18412
0.093822193	36760	0.032215874	36760	0.02748203
36800	0.305781636	36800	0.087814784	18400	0.06587313	18416
0.068587382	36768	0.048338283	36768	0.019797435
36800	0.207467456	36800	0.133967129	18400	0.07289042	18420
0.076186538	36776	0.020263658	36776	0.004223439
36800	0.127558	36800	0.094656483	18400	0.0682479	18424
0.067365269	36784	0.118059295	36784	0.019895193
36800	0.14265129	36800	0.143498546	18400	0.040825096	18428
0.029916142	36792	0.141455661	36792	0.045754547
36800	0.099999728	36800	0.104293824	18400	0.022717268	18432
0.037189726	36800	0.080369042	36800	0.048391259
36800	0.032399905	36800	0.043898359	18400	0.049597074	18436
0.0588148	36808	0.041267449	36808	0.042073389
36800	0.109909408	36800	0.105446066	18400	0.087356748	18440
0.058951649	36816	0.142473044	36816	0.034488097
36800	0.114477334	36800	0.155866786	18400	0.091224472	18444
0.065265347	36824	0.198314126	36824	0.01759378
36800	0.089215304	36800	0.129202018	18400	0.054255947	18448
0.051436728	36832	0.193562999	36832	0.030958745
36800	0.115746567	36800	0.077556491	18500	0.024073155	18452
0.019928095	36840	0.19367761	36840	0.021477201
36800	0.141457174	36800	0.041705938	18500	0.061992774	18456
0.073203642	36848	0.179097173	36848	0.047284138
36900	0.122884958	36900	0.054912212	18500	0.072275383	18460
0.115770243	36856	0.105107982	36856	0.052819327
36900	0.09241196	36900	0.11192853	18500	0.047073758	18464
0.090125512	36864	0.091852577	36864	0.027795604
36900	0.094002156	36900	0.118198652	18500	0.090131012	18468
0.055599889	36872	0.130567278	36872	0.037642269
36900	0.128432511	36900	0.073503499	18500	0.087134824	18472
0.015393142	36880	0.109519802	36880	0.041278458
36900	0.128294749	36900	0.147202984	18500	0.04815478	18476
0.075409022	36888	0.028812732	36888	0.038798149
36900	0.098068987	36900	0.198453403	18500	0.01504243	18480
0.128505446	36896	0.133776106	36896	0.050177212
36900	0.142367309	36900	0.126022045	18500	0.055159755	18484
0.093948496	36904	0.072684801	36904	0.069726717
36900	0.193891538	36900	0.02842506	18500	0.085562468	18488
0.026633466	36912	0.03982224	36912	0.076159682
36900	0.110266672	36900	0.036195859	18500	0.05938625	18492
0.047429963	36920	0.035614245	36920	0.053529493
36900	0.085423963	36900	0.03018475	18500	0.019329571	18496
0.072043076	36928	0.03991065	36928	0.039441027
36900	0.11928827	36900	0.035699788	18500	0.049078397	18500
0.057522277	36936	0.085459818	36936	0.074137977
36900	0.120865007	36900	0.036184407	18500	0.096918077	18504
0.051012332	36944	0.086929642	36944	0.068803653
37000	0.199251605	37000	0.074511845	18500	0.108566652	18508
0.067265784	36952	0.110464745	36952	0.019514464
37000	0.152934255	37000	0.072641837	18500	0.063540632	18512
0.06242322	36960	0.133344744	36960	0.0368849
37000	0.072868577	37000	0.019009685	18500	0.016290398	18516
0.049572715	36968	0.13802253	36968	0.049072936
37000	0.152803506	37000	0.028008626	18500	0.01282957	18520
0.062760235	36976	0.072541872	36976	0.06108854
37000	0.261810638	37000	0.070714777	18500	0.017870225	18524
0.067020315	36984	0.021344369	36984	0.064780143
37000	0.268857751	37000	0.09848459	18500	0.033080312	18528
0.038835409	36992	0.062468404	36992	0.050567738
37000	0.154278634	37000	0.058138867	18500	0.038240549	18532
0.00173217	37000	0.083515137	37000	0.02831326
37000	0.024649336	37000	0.070969443	18500	0.045539826	18536
0.037108286	37008	0.049543858	37008	0.032683067
37000	0.093480987	37000	0.128577594	18500	0.041837422	18540
0.06093521	37016	0.037288533	37016	0.05001195
37000	0.11402004	37000	0.067243862	18500	0.035165114	18544

0.064727254 37024 0.090999616 37024 0.044220997
37000 0.114530667 37000 0.070191971 18500 0.065701577 18548
0.045294579 37032 0.089908288 37032 0.021780776
37000 0.131256195 37000 0.153731075 18600 0.092320035 18552
0.007855326 37040 0.103192622 37040 0.003701474
37000 0.103653772 37000 0.142635035 18600 0.066619541 18556
0.061837593 37048 0.089509187 37048 0.01684935
37100 0.065036671 37100 0.06566268 18600 0.031183739 18560
0.079537989 37056 0.050181261 37056 0.036943045
37100 0.056858116 37100 0.039980267 18600 0.008222005 18564
0.050199833 37064 0.013782305 37064 0.023084271
37100 0.14342193 37100 0.079478385 18600 0.048051679 18568
0.011086974 37072 0.015056919 37072 0.013841223
37100 0.21403408 37100 0.061622683 18600 0.072447867 18572
0.033310524 37080 0.016858821 37080 0.023767436
37100 0.172507294 37100 0.047323847 18600 0.095787982 18576
0.053899657 37088 0.049424074 37088 0.055818335
37100 0.09796604 37100 0.048356225 18600 0.109363209 18580
0.021058055 37096 0.04515221 37096 0.07007963
37100 0.030659172 37100 0.021991367 18600 0.094942785 18584
0.039661165 37104 0.027916512 37104 0.08232781
37100 0.058423499 37100 0.089863061 18600 0.074607873 18588
0.058997637 37112 0.022055891 37112 0.119817421
37100 0.08278367 37100 0.096084616 18600 0.043443844 18592
0.043464042 37120 0.076008502 37120 0.105621431
37100 0.082455059 37100 0.068343754 18600 0.032632135 18596
0.057376637 37128 0.102232043 37128 0.066505665
37100 0.030936681 37100 0.078152225 18600 0.06736067 18600
0.072004856 37136 0.086571548 37136 0.056211229
37100 0.106983964 37100 0.171345543 18600 0.08888833 18604
0.069197049 37144 0.066326291 37144 0.047446494
37200 0.152182664 37200 0.114950068 18600 0.065111301 18608
0.047443406 37152 0.103030907 37152 0.019238236
37200 0.094507173 37200 0.014717052 18600 0.032899006 18612
0.033026376 37160 0.076230426 37160 0.0221639
37200 0.026793709 37200 0.053571785 18600 0.004346268 18616
0.050521008 37168 0.13210086 37168 0.021045955
37200 0.106793392 37200 0.043957378 18600 0.039138176 18620
0.061311468 37176 0.205359975 37176 0.013993464
37200 0.141242578 37200 0.096001626 18600 0.052028463 18624
0.040983901 37184 0.17417426 37184 0.028589549
37200 0.139327414 37200 0.15920031 18600 0.048885988 18628
0.021467544 37192 0.106042666 37192 0.056783661
37200 0.119185788 37200 0.091185219 18600 0.035546535 18632
0.07120036 37200 0.106462969 37200 0.056785786
37200 0.06888686 37200 0.035021392 18600 0.048983176 18636
0.081980725 37208 0.146818361 37208 0.035223551
37200 0.062232415 37200 0.051319723 18600 0.031595238 18640
0.03987725 37216 0.121778212 37216 0.033239357
37200 0.099686396 37200 0.120114128 18600 0.061773055 18644
0.041519263 37224 0.050532606 37224 0.024092728
37200 0.150295543 37200 0.15770593 18600 0.072553085 18648
0.08358965 37232 0.093998839 37232 0.01907982
37200 0.124819169 37200 0.230948238 18700 0.055422559 18652
0.108708249 37240 0.108641769 37240 0.048182759
37200 0.054771175 37200 0.236849271 18700 0.046132802 18656
0.088120316 37248 0.083072948 37248 0.056064549
37300 0.14425056 37300 0.150846099 18700 0.033299104 18660
0.051724477 37256 0.054730535 37256 0.018300823
37300 0.134817383 37300 0.12413472 18700 0.062239196 18664
0.021983682 37264 0.028763023 37264 0.0479349
37300 0.05220504 37300 0.152456676 18700 0.079694815 18668
0.090125308 37272 0.014780118 37272 0.07988124
37300 0.019489 37300 0.112238471 18700 0.041538962 18672
0.172428118 37280 0.088732799 37280 0.068331712
37300 0.046527894 37300 0.081137448 18700 0.040881681 18676
0.161249961 37288 0.193352273 37288 0.052763164
37300 0.054056374 37300 0.085938584 18700 0.050872073 18680

0.086017135 37296 0.188093618 37296 0.048431288
37300 0.086853324 37300 0.025799347 18700 0.058131071 18684
0.04270878 37304 0.082768354 37304 0.070923357
37300 0.137157476 37300 0.043176769 18700 0.047406251 18688
0.048700087 37312 0.005898253 37312 0.088282039
37300 0.135560899 37300 0.047264628 18700 0.035330479 18692
0.05020867 37320 0.041169471 37320 0.064983091
37300 0.04575463 37300 0.021624332 18700 0.005287618 18696
0.071619281 37328 0.0588125 37328 0.034500234
37300 0.057769339 37300 0.029313142 18700 0.037731377 18700
0.095591051 37336 0.074691539 37336 0.045943136
37300 0.138886375 37300 0.079622609 18700 0.026750173 18704
0.099820798 37344 0.046084741 37344 0.040105701
37400 0.160867509 37400 0.130175628 18700 0.00645693 18708
0.054612676 37352 0.058127447 37352 0.015051088
37400 0.126841536 37400 0.14331004 18700 0.015977837 18712
0.052058593 37360 0.012312064 37360 0.028820476
37400 0.141365469 37400 0.083620107 18700 0.03059908 18716
0.069728652 37368 0.038754373 37368 0.04251557
37400 0.197172369 37400 0.012781038 18700 0.037294944 18720
0.032610129 37376 0.071905291 37376 0.025722782
37400 0.191947853 37400 0.055326429 18700 0.027211821 18724
0.027541675 37384 0.070603812 37384 0.014964904
37400 0.140181394 37400 0.076656463 18700 0.012241865 18728
0.06464358 37392 0.048156177 37392 0.033495628
37400 0.18082108 37400 0.097520562 18700 0.030327499 18732
0.081350423 37400 0.038940394 37400 0.036787522
37400 0.159744464 37400 0.126388331 18700 0.03526784 18736
0.083620849 37408 0.046473335 37408 0.054592387
37400 0.029250639 37400 0.096865682 18700 0.05773524 18740
0.051798521 37416 0.051190753 37416 0.077818935
37400 0.097600743 37400 0.046150399 18700 0.060385206 18744
0.060724564 37424 0.047692854 37424 0.071066941
37400 0.106434483 37400 0.061283194 18700 0.048569218 18748
0.064300431 37432 0.048295522 37432 0.036902209
37400 0.041608626 37400 0.10923158 18800 0.059776583 18752
0.104782943 37440 0.050904924 37440 0.011727111
37400 0.06885457 37400 0.131077861 18800 0.051521503 18756
0.115444032 37448 0.007968297 37448 0.037331334
37500 0.069867114 37500 0.097854587 18800 0.030365467 18760
0.0651335 37456 0.073808922 37456 0.08036002
37500 0.031205767 37500 0.06918094 18800 0.007473478 18764
0.055222503 37464 0.091893897 37464 0.099897508
37500 0.114549526 37500 0.147155588 18800 0.014376139 18768
0.034903314 37472 0.032290496 37472 0.073890486
37500 0.097168791 37500 0.108754335 18800 0.025430174 18772
0.007507474 37480 0.094493749 37480 0.054735516
37500 0.063380103 37500 0.090243528 18800 0.022612649 18776
0.028910279 37488 0.095821211 37488 0.05601699
37500 0.065240769 37500 0.110353205 18800 0.024353389 18780
0.040102015 37496 0.084751278 37496 0.050909373
37500 0.097166936 37500 0.12239565 18800 0.054946417 18784
0.047117352 37504 0.137911804 37504 0.020693437
37500 0.159324278 37500 0.090690883 18800 0.047663107 18788
0.043007949 37512 0.076142307 37512 0.02454021
37500 0.203783216 37500 0.059685815 18800 0.014674657 18792
0.056047051 37520 0.03112519 37520 0.042209773
37500 0.093826355 37500 0.159803065 18800 0.048285852 18796
0.050317798 37528 0.1270379 37528 0.030538282
37500 0.09337606 37500 0.125713138 18800 0.038457161 18800
0.032393815 37536 0.126159634 37536 0.012016392
37500 0.098737095 37500 0.027267281 18800 0.030084046 18804
0.010383792 37544 0.039027953 37544 0.005629817
37600 0.04083163 37600 0.053945918 18800 0.052550437 18808
0.031986911 37552 0.079276833 37552 0.013708002
37600 0.103104612 37600 0.146598875 18800 0.035586894 18812
0.01622293 37560 0.131731533 37560 0.030603336
37600 0.079074511 37600 0.203929361 18800 0.024567959 18816

0.008161086 37568 0.114840164 37568 0.063373074
37600 0.054054093 37600 0.142958015 18800 0.03199674 18820
0.024360774 37576 0.072945775 37576 0.063167558
37600 0.082278726 37600 0.032858676 18800 0.025087576 18824
0.046969308 37584 0.051018393 37584 0.029651663
37600 0.100470563 37600 0.090617941 18800 0.005509508 18828
0.046101366 37592 0.059593422 37592 0.007445127
37600 0.156557653 37600 0.166920203 18800 0.021008509 18832
0.066320448 37600 0.08182451 37600 0.01579426
37600 0.131701294 37600 0.169955238 18800 0.049509265 18836
0.023196182 37608 0.09429871 37608 0.002962648
37600 0.094480194 37600 0.112430847 18800 0.068338341 18840
0.022688791 37616 0.098207071 37616 0.051196708
37600 0.012144828 37600 0.070006892 18800 0.096184223 18844
0.05400267 37624 0.127618885 37624 0.071918577
37600 0.126370098 37600 0.039940594 18800 0.138529955 18848
0.065630666 37632 0.130107786 37632 0.076078133
37600 0.174633315 37600 0.031258955 18900 0.130746368 18852
0.055829831 37640 0.122325771 37640 0.09061554
37600 0.151192974 37600 0.097834731 18900 0.071770519 18856
0.046771027 37648 0.071738868 37648 0.07421471
37700 0.109249661 37700 0.070106304 18900 0.072242816 18860
0.062677507 37656 0.03807326 37656 0.045211622
37700 0.077044526 37700 0.076706841 18900 0.067304485 18864
0.086175511 37664 0.110049979 37664 0.04509684
37700 0.063723957 37700 0.104396015 18900 0.04077343 18868
0.07867247 37672 0.109087079 37672 0.023323344
37700 0.053327854 37700 0.02764788 18900 0.060396156 18872
0.056643869 37680 0.059072936 37680 0.041831496
37700 0.012667299 37700 0.078233265 18900 0.045889836 18876
0.082676626 37688 0.070241898 37688 0.040465431
37700 0.045854893 37700 0.137114913 18900 0.016264952 18880
0.103582926 37696 0.091606584 37696 0.026183472
37700 0.112731417 37700 0.128666333 18900 0.0343619 18884
0.049342401 37704 0.071837574 37704 0.022408516
37700 0.143867933 37700 0.088282315 18900 0.062974548 18888
0.024609384 37712 0.085583073 37712 0.040895509
37700 0.05020525 37700 0.096858763 18900 0.041203664 18892
0.044136592 37720 0.115076429 37720 0.05456285
37700 0.093891053 37700 0.158428244 18900 0.020369078 18896
0.035816069 37728 0.163988633 37728 0.022543738
37700 0.095857053 37700 0.162437559 18900 0.015113028 18900
0.105179912 37736 0.181783791 37736 0.02052456
37700 0.125950581 37700 0.03175512 18900 0.033005028 18904
0.108906213 37744 0.106024847 37744 0.027503158
37800 0.18247102 37800 0.110537636 18900 0.026367241 18908
0.069802452 37752 0.108331333 37752 0.023588667
37800 0.156383074 37800 0.146811595 18900 0.006412488 18912
0.058862064 37760 0.146035498 37760 0.021602145
37800 0.113610542 37800 0.117135009 18900 0.013900636 18916
0.048302427 37768 0.107633059 37768 0.046669764
37800 0.058294536 37800 0.03376418 18900 0.04829864 18920
0.087448745 37776 0.02986951 37776 0.064448112
37800 0.05654039 37800 0.200351453 18900 0.065159969 18924
0.082889652 37784 0.032246568 37784 0.048798764
37800 0.058932372 37800 0.211053834 18900 0.029095923 18928
0.069543537 37792 0.014525732 37792 0.033650867
37800 0.088885347 37800 0.036162503 18900 0.032469412 18932
0.015201097 37800 0.039524148 37800 0.023573597
37800 0.145587183 37800 0.148706691 18900 0.038964299 18936
0.095411924 37808 0.060026508 37808 0.005676055
37800 0.112088841 37800 0.18061453 18900 0.025452318 18940
0.073689087 37816 0.085709464 37816 0.026599102
37800 0.013796445 37800 0.113836686 18900 0.013508184 18944
0.121312929 37824 0.122654121 37824 0.028890088
37800 0.075943623 37800 0.104270832 18900 0.040464438 18948
0.171919295 37832 0.131217283 37832 0.022486915
37800 0.062821142 37800 0.12699682 19000 0.042379787 18952

0.143063488 37840 0.100109093 37840 0.007998214
37800 0.066022425 37800 0.087925553 19000 0.022525703 18956
0.10183479 37848 0.083023937 37848 0.02856092
37900 0.046965299 37900 0.047462065 19000 0.035509114 18960
0.086392065 37856 0.047587411 37856 0.041000018
37900 0.029390641 37900 0.040584484 19000 0.042284035 18964
0.034178578 37864 0.048115977 37864 0.021069811
37900 0.03618492 37900 0.033122335 19000 0.048378286 18968
0.080615799 37872 0.065846391 37872 0.014740349
37900 0.083175597 37900 0.058756144 19000 0.054566757 18972
0.145667465 37880 0.074996176 37880 0.027599772
37900 0.118317905 37900 0.089711539 19000 0.049481259 18976
0.1209162 37888 0.100334059 37888 0.011211226
37900 0.042426735 37900 0.093729592 19000 0.028908684 18980
0.053924137 37896 0.084005093 37896 0.041987911
37900 0.071983122 37900 0.095703712 19000 0.023912879 18984
0.047863083 37904 0.090057809 37904 0.074903037
37900 0.095875686 37900 0.147461251 19000 0.060268932 18988
0.072154158 37912 0.097996039 37912 0.062484745
37900 0.079043035 37900 0.125817125 19000 0.077132529 18992
0.051495907 37920 0.0771985 37920 0.053071777
37900 0.083920233 37900 0.050649858 19000 0.041448104 18996
0.03767915 37928 0.1551139 37928 0.023023296
37900 0.077242083 37900 0.035873407 19000 0.028760531 19000
0.038670758 37936 0.203637523 37936 0.018239633
37900 0.121898644 37900 0.094962263 19000 0.061749932 19004
0.075269803 37944 0.150865803 37944 0.030240688
38000 0.135225143 38000 0.139509779 19000 0.048621976 19008
0.073096766 37952 0.067993533 37952 0.036710586
38000 0.071226808 38000 0.152439126 19000 0.028881545 19012
0.030669831 37960 0.069341782 37960 0.027721728
38000 0.098502234 38000 0.09644778 19000 0.085276472 19016
0.008685993 37968 0.119903409 37968 0.00913775
38000 0.121543388 38000 0.044412722 19000 0.085606007 19020
0.017015813 37976 0.146147475 37976 0.012818538
38000 0.089810303 38000 0.051079544 19000 0.055113993 19024
0.035895846 37984 0.10903029 37984 0.043788459
38000 0.075302793 38000 0.038092599 19000 0.045169087 19028
0.07195722 37992 0.067089386 37992 0.045261284
38000 0.137283991 38000 0.024245741 19000 0.048628514 19032
0.088458473 38000 0.086884815 38000 0.025961072
38000 0.164273458 38000 0.115924209 19000 0.047213904 19036
0.066185596 38008 0.168298968 38008 0.061847139
38000 0.086784188 38000 0.157134855 19000 0.037366233 19040
0.055736797 38016 0.163448844 38016 0.047009813
38000 0.002409786 38000 0.122571422 19000 0.016050293 19044
0.048829414 38024 0.076327284 38024 0.040455576
38000 0.007534201 38000 0.080460646 19000 0.009786815 19048
0.001229594 38032 0.019467334 38032 0.056152327
38000 0.04794358 38000 0.018413273 19100 0.022431505 19052
0.067301422 38040 0.071704453 38040 0.075113188
38000 0.075002543 38000 0.124973827 19100 0.043059328 19056
0.110106106 38048 0.097252887 38048 0.055278917
38100 0.138134769 38100 0.113257382 19100 0.046098459 19060
0.087796667 38056 0.120248929 38056 0.01805484
38100 0.133689406 38100 0.103202139 19100 0.030146737 19064
0.015894853 38064 0.109465094 38064 0.044885048
38100 0.06426467 38100 0.109693217 19100 0.02561172 19068
0.047654467 38072 0.096433381 38072 0.066480294
38100 0.084257939 38100 0.069798378 19100 0.068239184 19072
0.063200576 38080 0.085797648 38080 0.069245893
38100 0.116223615 38100 0.080699545 19100 0.042384407 19076
0.043159274 38088 0.050666637 38088 0.041629588
38100 0.084860207 38100 0.086246757 19100 0.058123736 19080
0.02813778 38096 0.07967883 38096 0.022287813
38100 0.033941902 38100 0.076840377 19100 0.059094997 19084
0.040634182 38104 0.095017153 38104 0.027024744
38100 0.023507246 38100 0.054424407 19100 0.04779275 19088

0.02644525 38112 0.087613495 38112 0.040410887
38100 0.068650814 38100 0.076052013 19100 0.034668228 19092
0.024356687 38120 0.082084058 38120 0.051065163
38100 0.117549214 38100 0.091883194 19100 0.020378409 19096
0.023434997 38128 0.045685931 38128 0.055822402
38100 0.13750288 38100 0.05700544 19100 0.066090848 19100
0.063353051 38136 0.064788474 38136 0.029348181
38100 0.08990708 38100 0.047295456 19100 0.0874541 19104
0.0707156 38144 0.035465408 38144 0.020791871
38200 0.095821364 38200 0.056953079 19100 0.046542748 19108
0.044670498 38152 0.090013862 38152 0.049558861
38200 0.19046836 38200 0.079745558 19100 0.035117395 19112
0.038342823 38160 0.125966471 38160 0.048984246
38200 0.178130009 38200 0.087060347 19100 0.054363387 19116
0.078393401 38168 0.064271153 38168 0.043054046
38200 0.125682899 38200 0.093514937 19100 0.038639275 19120
0.10999287 38176 0.005875436 38176 0.043882275
38200 0.088930101 38200 0.113868489 19100 0.02343843 19124
0.108848028 38184 0.06755245 38184 0.043189117
38200 0.02360261 38200 0.142418489 19100 0.050341881 19128
0.108207845 38192 0.108414388 38192 0.024710655
38200 0.101420592 38200 0.108104789 19100 0.060939088 19132
0.121970115 38200 0.102982442 38200 0.032082615
38200 0.111453301 38200 0.088564477 19100 0.053666707 19136
0.11915106 38208 0.099489866 38208 0.050429731
38200 0.073005402 38200 0.157329079 19100 0.055058961 19140
0.083568266 38216 0.126613654 38216 0.05296967
38200 0.109302033 38200 0.18304518 19100 0.050476116 19144
0.050814415 38224 0.068396723 38224 0.03456475
38200 0.109775261 38200 0.171957945 19100 0.037436661 19148
0.078282021 38232 0.039893377 38232 0.003303984
38200 0.069039932 38200 0.126858431 19200 0.022639131 19152
0.054478336 38240 0.096872565 38240 0.011355022
38200 0.093585702 38200 0.043676264 19200 0.005023376 19156
0.045486992 38248 0.100255958 38248 0.06656177
38300 0.204438256 38300 0.057880436 19200 0.049278922 19160
0.041696516 38256 0.10022091 38256 0.092196977
38300 0.218924761 38300 0.086061191 19200 0.075180484 19164
0.012968129 38264 0.164766054 38264 0.032983975
38300 0.127185965 38300 0.10135604 19200 0.047795245 19168
0.063971849 38272 0.211678024 38272 0.070183465
38300 0.077660327 38300 0.170461688 19200 0.028564431 19172
0.069489885 38280 0.175143083 38280 0.0966433
38300 0.086301661 38300 0.097391865 19200 0.084719788 19176
0.083003346 38288 0.149835789 38288 0.049106511
38300 0.107628373 38300 0.064531429 19200 0.082356331 19180
0.127029358 38296 0.123533901 38296 0.058047299
38300 0.164620185 38300 0.114762785 19200 0.047610611 19184
0.113426919 38304 0.058705231 38304 0.065063403
38300 0.189172163 38300 0.114207942 19200 0.044043823 19188
0.042323612 38312 0.09089266 38312 0.054842014
38300 0.170041036 38300 0.098284254 19200 0.016835396 19192
0.013129851 38320 0.179730166 38320 0.027126274
38300 0.169672159 38300 0.039064777 19200 0.027119962 19196
0.032737113 38328 0.178620089 38328 0.019851106
38300 0.151815344 38300 0.04901992 19200 0.035383207 19200
0.055398523 38336 0.027772327 38336 0.049175073
38300 0.101796766 38300 0.059177921 19200 0.022776421 19204
0.071379451 38344 0.15705984 38344 0.045597164
38400 0.059623748 38400 0.067074463 19200 0.031158565 19208
0.08381081 38352 0.116021023 38352 0.013685893
38400 0.093947405 38400 0.141428813 19200 0.04465981 19212
0.07249802 38360 0.017218148 38360 0.027369379
38400 0.174793328 38400 0.158164883 19200 0.067609632 19216
0.038235612 38368 0.063529013 38368 0.050459079
38400 0.230553284 38400 0.16464862 19200 0.053061787 19220
0.022221548 38376 0.070689348 38376 0.046131048
38400 0.195309665 38400 0.148743523 19200 0.013568832 19224

0.036341142 38384 0.100919264 38384 0.014954487
38400 0.143579586 38400 0.115202791 19200 0.056496079 19228
0.075737189 38392 0.145216501 38392 0.022085005
38400 0.157892675 38400 0.117726748 19200 0.063448373 19232
0.112818285 38400 0.106814674 38400 0.0295021
38400 0.174229674 38400 0.136479779 19200 0.035291236 19236
0.100049227 38408 0.072908224 38408 0.03230678
38400 0.109154302 38400 0.129711349 19200 0.031381969 19240
0.07703626 38416 0.11499515 38416 0.061183593
38400 0.052166859 38400 0.105504478 19200 0.039138569 19244
0.075228003 38424 0.116522228 38424 0.078564328
38400 0.126715516 38400 0.0556048 19200 0.019772246 19248
0.059337657 38432 0.072855248 38432 0.053137923
38400 0.226147255 38400 0.139750163 19300 0.010549257 19252
0.020644768 38440 0.041098807 38440 0.015212262
38400 0.22053445 38400 0.109397319 19300 0.014164481 19256
0.021990529 38448 0.042105152 38448 0.043535638
38500 0.113551731 38500 0.046450492 19300 0.031123967 19260
0.042085114 38456 0.059489408 38456 0.045331162
38500 0.03033732 38500 0.124858023 19300 0.027754211 19264
0.061248618 38464 0.082704384 38464 0.029661886
38500 0.13632713 38500 0.131014589 19300 0.009041963 19268
0.043417858 38472 0.0309647 38472 0.019156752
38500 0.219725364 38500 0.094757954 19300 0.003839034 19272
0.017569124 38480 0.054138713 38480 0.028551671
38500 0.204412864 38500 0.063982385 19300 0.022167724 19276
0.039864568 38488 0.041049745 38488 0.05018184
38500 0.105007515 38500 0.074069918 19300 0.043079228 19280
0.023500405 38496 0.020396579 38496 0.043927634
38500 0.09053079 38500 0.079676298 19300 0.047109315 19284
0.032767632 38504 0.032622545 38504 0.021694876
38500 0.088185589 38500 0.066615241 19300 0.085835563 19288
0.077171033 38512 0.037818703 38512 0.017329139
38500 0.093038972 38500 0.022922222 19300 0.120900884 19292
0.109714827 38520 0.121369951 38520 0.022610979
38500 0.059672715 38500 0.018179722 19300 0.092052032 19296
0.076703334 38528 0.19821708 38528 0.033206481
38500 0.16266777 38500 0.081017453 19300 0.025470679 19300
0.021059908 38536 0.214224361 38536 0.049250666
38500 0.194247375 38500 0.092626353 19300 0.027000628 19304
0.032232281 38544 0.135138296 38544 0.0466569
38600 0.071697687 38600 0.043222928 19300 0.052147712 19308
0.053206702 38552 0.040501851 38552 0.061152641
38600 0.07029315 38600 0.03736468 19300 0.045739133 19312
0.02302006 38560 0.144287755 38560 0.089408561
38600 0.143073048 38600 0.084054802 19300 0.012848861 19316
0.024857905 38568 0.12110098 38568 0.068088302
38600 0.161538833 38600 0.109285145 19300 0.026575841 19320
0.061239756 38576 0.08005467 38576 0.020201578
38600 0.104514511 38600 0.088287125 19300 0.025142959 19324
0.055548604 38584 0.071288203 38584 0.00986234
38600 0.020541664 38600 0.040526596 19300 0.096715106 19328
0.057833178 38592 0.073607764 38592 0.016360547
38600 0.040137431 38600 0.040954303 19300 0.09264789 19332
0.060677892 38600 0.111558948 38600 0.02564098
38600 0.075106334 38600 0.114480426 19300 0.02721371 19336
0.092877141 38608 0.123434671 38608 0.073789182
38600 0.113448092 38600 0.070761947 19300 0.053960608 19340
0.110466594 38616 0.165285411 38616 0.070313785
38600 0.119316945 38600 0.085521184 19300 0.080107457 19344
0.08053684 38624 0.213104403 38624 0.035196223
38600 0.113027723 38600 0.128730331 19300 0.084320847 19348
0.047259473 38632 0.152620298 38632 0.034653182
38600 0.085924628 38600 0.040676561 19400 0.071333001 19352
0.032679916 38640 0.109976347 38640 0.044371096
38600 0.126183891 38600 0.059228063 19400 0.072608425 19356
0.011009096 38648 0.148898034 38648 0.013162792
38700 0.204843178 38700 0.111844478 19400 0.061786661 19360

0.036006328	38656	0.134755668	38656	0.036743648		
38700	0.167896505	38700	0.148065985	19400	0.035723984	19364
0.09636677	38664	0.057624566	38664	0.040102546		
38700	0.105623069	38700	0.074545336	19400	0.045716664	19368
0.108843822	38672	0.05059121	38672	0.012925531		
38700	0.051157153	38700	0.072557101	19400	0.059967351	19372
0.059281261	38680	0.023323659	38680	0.026269823		
38700	0.060463368	38700	0.100060963	19400	0.057794186	19376
0.013093626	38688	0.062109219	38688	0.054150023		
38700	0.135079405	38700	0.043279651	19400	0.028210923	19380
0.020333964	38696	0.07671686	38696	0.053539516		
38700	0.122211757	38700	0.056702065	19400	0.021465728	19384
0.04153858	38704	0.048575341	38704	0.061119616		
38700	0.107099542	38700	0.087612432	19400	0.018332783	19388
0.020715126	38712	0.01947778	38712	0.064031076		
38700	0.197657123	38700	0.071110866	19400	0.03750842	19392
0.035781279	38720	0.045922661	38720	0.05483938		
38700	0.210686936	38700	0.052693202	19400	0.052117957	19396
0.045063804	38728	0.065166336	38728	0.036718917		
38700	0.12892377	38700	0.083463179	19400	0.02677613	19400
0.010212269	38736	0.04344212	38736	0.015920245		
38700	0.023970066	38700	0.058502301	19400	0.019809811	19404
0.048882324	38744	0.085265419	38744	0.031956177		
38800	0.045896497	38800	0.06556134	19400	0.073106283	19408
0.055476263	38752	0.085889005	38752	0.024615651		
38800	0.094632043	38800	0.143187164	19400	0.081046645	19412
0.055998309	38760	0.091936323	38760	0.020088161		
38800	0.149739761	38800	0.164329613	19400	0.044671499	19416
0.042558386	38768	0.119602963	38768	0.034396493		
38800	0.268798438	38800	0.152638604	19400	0.045127446	19420
0.015468653	38776	0.080680009	38776	0.022039456		
38800	0.332652563	38800	0.178597693	19400	0.067415567	19424
0.049216469	38784	0.174605303	38784	0.020709767		
38800	0.195777335	38800	0.187449449	19400	0.025884538	19428
0.037049715	38792	0.196508103	38792	0.048540213		
38800	0.203854491	38800	0.131887689	19400	0.05377844	19432
0.02965028	38800	0.12461748	38800	0.068592875		
38800	0.247481978	38800	0.064636908	19400	0.071935589	19436
0.010472344	38808	0.067797446	38808	0.065382788		
38800	0.128021624	38800	0.087852211	19400	0.061907907	19440
0.030875723	38816	0.066652472	38816	0.047774378		
38800	0.024548221	38800	0.19731796	19400	0.058169662	19444
0.041431355	38824	0.093134717	38824	0.044576002		
38800	0.072955634	38800	0.222061514	19400	0.042079086	19448
0.082859027	38832	0.040835301	38832	0.047396286		
38800	0.027968123	38800	0.128960091	19500	0.020621368	19452
0.089398913	38840	0.049620558	38840	0.0131181		
38800	0.087139451	38800	0.084832376	19500	0.005198569	19456
0.043135875	38848	0.039819359	38848	0.022762331		
38900	0.138081334	38900	0.084714106	19500	0.021427257	19460
0.011374073	38856	0.02566896	38856	0.030485282		
38900	0.132002679	38900	0.21310577	19500	0.032751188	19464
0.024837927	38864	0.08625917	38864	0.04730012		
38900	0.119806406	38900	0.212824466	19500	0.057936955	19468
0.033755088	38872	0.137799536	38872	0.054156666		
38900	0.137606549	38900	0.109919747	19500	0.088409157	19472
0.031424479	38880	0.140876408	38880	0.047998925		
38900	0.187630853	38900	0.059073791	19500	0.086315587	19476
0.020077961	38888	0.09090871	38888	0.069727008		
38900	0.120760073	38900	0.024172714	19500	0.068316069	19480
0.0258368	38896	0.072567978	38896	0.072037212		
38900	0.046984653	38900	0.041698258	19500	0.063994572	19484
0.035649791	38904	0.067690198	38904	0.047925092		
38900	0.161039294	38900	0.093009039	19500	0.051568168	19488
0.051388455	38912	0.056274119	38912	0.019903022		
38900	0.106238564	38900	0.114952083	19500	0.031185973	19492
0.046402296	38920	0.095690935	38920	0.021045487		
38900	0.068894246	38900	0.076187112	19500	0.011365885	19496

0.048525457 38928 0.100632708 38928 0.02901532
38900 0.09487752 38900 0.065469707 19500 0.05168482 19500
0.067365545 38936 0.033043249 38936 0.05936504
38900 0.151009838 38900 0.112451991 19500 0.045188885 19504
0.084208361 38944 0.083096013 38944 0.049044753
39000 0.126281796 39000 0.160712894 19500 0.003910484 19508
0.057805119 38952 0.112954673 38952 0.039128492
39000 0.052036001 39000 0.142772304 19500 0.026698113 19512
0.007045885 38960 0.055033903 38960 0.059081427
39000 0.142351798 39000 0.145100553 19500 0.024450186 19516
0.009939287 38968 0.010671037 38968 0.045075343
39000 0.244918483 39000 0.164417099 19500 0.027296914 19520
0.033714503 38976 0.024735851 38976 0.034310819
39000 0.23885406 39000 0.128273853 19500 0.01098032 19524
0.057813817 38984 0.021547658 38984 0.039646988
39000 0.090962341 39000 0.120931261 19500 0.009941794 19528
0.085146312 38992 0.04233923 38992 0.033849276
39000 0.264685834 39000 0.110272922 19500 0.028256596 19532
0.041954878 39000 0.103979881 39000 0.027278151
39000 0.591637159 39000 0.083194304 19500 0.04823479 19536
0.031506919 39008 0.104983308 39008 0.035477544
39000 0.523384136 39000 0.096520402 19500 0.053122796 19540
0.013805279 39016 0.085951891 39016 0.047738606
39000 0.234813036 39000 0.082979001 19500 0.036829461 19544
0.028801736 39024 0.074762131 39024 0.048699847
39000 0.926197739 39000 0.060498085 19500 0.02714173 19548
0.022930432 39032 0.024047953 39032 0.046736306
39000 2.343190838 39000 0.052632273 19600 0.042689651 19552
0.028731896 39040 0.099761732 39040 0.055057142
39000 3.196078813 39000 0.044636236 19600 0.022107581 19556
0.076315962 39048 0.120004588 39048 0.073906347
39100 2.494232963 39100 0.040225918 19600 0.005839428 19560
0.087872417 39056 0.054449061 39056 0.087483488
39100 1.050608465 39100 0.086621709 19600 0.035363959 19564
0.057437108 39064 0.066721368 39064 0.072058356
39100 0.932639581 39100 0.090785805 19600 0.048893238 19568
0.053065018 39072 0.027647593 39072 0.06116867
39100 0.826406525 39100 0.03591281 19600 0.033019565 19572
0.113422721 39080 0.07846595 39080 0.058410002
39100 0.262721761 39100 0.164904778 19600 0.051190229 19576
0.150549065 39088 0.129846769 39088 0.045427594
39100 0.120775498 39100 0.176996851 19600 0.045546451 19580
0.09326337 39096 0.131931461 39096 0.052721051
39100 0.101076221 39100 0.052235024 19600 0.00634779 19584
0.025596381 39104 0.081154343 39104 0.062527019
39100 0.075614473 39100 0.051645293 19600 0.03841018 19588
0.010222928 39112 0.048259739 39112 0.077525168
39100 0.189140993 39100 0.067615118 19600 0.042972879 19592
0.031744337 39120 0.067514608 39120 0.080417449
39100 0.234870021 39100 0.152706169 19600 0.049219991 19596
0.080069687 39128 0.02716183 39128 0.055429948
39100 0.177427311 39100 0.200594775 19600 0.043750162 19600
0.063051142 39136 0.052626932 39136 0.045228702
39100 0.132998889 39100 0.155386355 19600 0.056565063 19604
0.029156334 39144 0.046328529 39144 0.049336453
39200 0.212556741 39200 0.088206165 19600 0.033719167 19608
0.002584181 39152 0.046446952 39152 0.033460565
39200 0.305414258 39200 0.052027986 19600 0.046407578 19612
0.039891624 39160 0.107266555 39160 0.029153396
39200 0.299694163 39200 0.150050444 19600 0.092769784 19616
0.032413162 39168 0.110049485 39168 0.046956058
39200 0.384519255 39200 0.192097694 19600 0.091839051 19620
0.018691913 39176 0.063644409 39176 0.069153539
39200 0.457943766 39200 0.130766464 19600 0.07352652 19624
0.048322439 39184 0.027048636 39184 0.080556492
39200 0.433887791 39200 0.07780418 19600 0.053834003 19628
0.066087225 39192 0.03056588 39192 0.063791675
39200 0.412068854 39200 0.137843338 19600 0.050803137 19632

0.068408757 39200 0.080951169 39200 0.026098203
39200 0.505119563 39200 0.150708584 19600 0.086211447 19636
0.026704542 39208 0.123427948 39208 0.014504025
39200 0.633353019 39200 0.108414388 19600 0.090939582 19640
0.065818043 39216 0.122733545 39216 0.009148079
39200 0.633072399 39200 0.126041734 19600 0.05985222 19644
0.069838316 39224 0.135514914 39224 0.022257931
39200 0.836973079 39200 0.163398465 19600 0.040675423 19648
0.024242589 39232 0.151759509 39232 0.058573198
39200 1.016749535 39200 0.089189365 19700 0.038684571 19652
0.006378616 39240 0.08171409 39240 0.085002495
39200 1.231409609 39200 0.008052166 19700 0.030660136 19656
0.014652625 39248 0.016655262 39248 0.058948768
39300 1.343205225 39300 0.028406374 19700 0.014842114 19660
0.029153984 39256 0.01792362 39256 0.01535113
39300 1.60870445 39300 0.065830951 19700 0.055140281 19664
0.06149344 39264 0.061987084 39264 0.004847662
39300 3.022291925 39300 0.037866208 19700 0.076728742 19668
0.059219223 39272 0.041732132 39272 0.013378875
39300 4.560913425 39300 0.032573833 19700 0.060232786 19672
0.064941327 39280 0.058418686 39280 0.031315783
39300 2.734903013 39300 0.034256165 19700 0.028074099 19676
0.072638919 39288 0.128818283 39288 0.039544731
39300 2.96704495 39300 0.045752513 19700 0.037451246 19680
0.064609645 39296 0.12098912 39296 0.020295174
39300 18.33882375 39300 0.067619483 19700 0.068954665 19684
0.04625188 39304 0.049195023 39304 0.033616943
39300 39.17833788 39300 0.092786511 19700 0.11810664 19688
0.019988216 39312 0.074202115 39312 0.044186425
39300 45.7128175 39300 0.089447611 19700 0.127622858 19692
0.031303545 39320 0.103071674 39320 0.024939911
39300 30.0543625 39300 0.099499121 19700 0.086049571 19696
0.04279237 39328 0.068789319 39328 0.02148241
39300 10.64306964 39300 0.081725033 19700 0.033374803 19700
0.06931345 39336 0.06174951 39336 0.013940086
39300 9.974494575 39300 0.067238594 19700 0.051035717 19704
0.084833089 39344 0.089895511 39344 0.049138365
39400 5.6599942 39400 0.124115308 19700 0.066432185 19708
0.045929857 39352 0.064520784 39352 0.045685305
39400 2.005000138 39400 0.169891529 19700 0.051516399 19712
0.026040518 39360 0.043563272 39360 0.005856325
39400 3.026659138 39400 0.169877414 19700 0.032009542 19716
0.053291227 39368 0.071887997 39368 0.053272819
39400 2.551069488 39400 0.154474314 19700 0.019611682 19720
0.04427445 39376 0.074693999 39376 0.044951707
39400 2.696130663 39400 0.150709734 19700 0.030273879 19724
0.036332258 39384 0.007158625 39384 0.014702137
39400 2.418053575 39400 0.129472551 19700 0.029984216 19728
0.07940732 39392 0.093676732 39392 0.02145024
39400 2.252936363 39400 0.085150001 19700 0.019103447 19732
0.085804961 39400 0.109386972 39400 0.018834006
39400 2.170832825 39400 0.07726848 19700 0.048354297 19736
0.065907145 39408 0.09502504 39408 0.039687646
39400 1.96182495 39400 0.160378476 19700 0.075436969 19740
0.028622468 39416 0.071916642 39416 0.059885493
39400 1.941505938 39400 0.225412194 19700 0.063056192 19744
0.017290653 39424 0.053600601 39424 0.058014201
39400 1.944908875 39400 0.185634839 19700 0.042456155 19748
0.045157965 39432 0.093125644 39432 0.085680877
39400 1.88579445 39400 0.128628366 19800 0.042381271 19752
0.049621292 39440 0.07334954 39440 0.081835628
39400 1.926655538 39400 0.138296775 19800 0.026626945 19756
0.029685896 39448 0.02058361 39448 0.036245059
39500 1.90887845 39500 0.072837342 19800 0.001208315 19760
0.021754733 39456 0.087775181 39456 0.010497703
39500 1.86583015 39500 0.148133069 19800 0.010814534 19764
0.03403431 39464 0.130579633 39464 0.032225158
39500 1.753913938 39500 0.228208883 19800 0.032805867 19768

0.025592812 39472 0.177521316 39472 0.044926823
39500 1.652985813 39500 0.15146911 19800 0.075501783 19772
0.045387853 39480 0.168602288 39480 0.047654627
39500 1.654620288 39500 0.068280911 19800 0.068606663 19776
0.061375802 39488 0.110853951 39488 0.061267063
39500 1.636316538 39500 0.083037754 19800 0.062165353 19780
0.058048674 39496 0.122603742 39496 0.061111998
39500 1.739485538 39500 0.036386802 19800 0.113670736 19784
0.066939567 39504 0.134966438 39504 0.04499247
39500 1.784730938 39500 0.055406817 19800 0.120472854 19788
0.031604133 39512 0.115280804 39512 0.02939037
39500 1.821677438 39500 0.129585241 19800 0.083620958 19792
0.021998794 39520 0.075523887 39520 0.030529518
39500 2.02944875 39500 0.167055085 19800 0.045806228 19796
0.018863308 39528 0.060140945 39528 0.027354352
39500 2.669814738 39500 0.121241064 19800 0.015863385 19800
0.047675047 39536 0.055322365 39536 0.02178552
39500 2.112171375 39500 0.027739943 19800 0.018240353 19804
0.054345423 39544 0.063851694 39544 0.043094704
39600 4.025846725 39600 0.034934052 19800 0.033282755 19808
0.03439375 39552 0.12593143 39552 0.042135809
39600 8.663984013 39600 0.092094837 19800 0.026484244 19812
0.055472985 39560 0.178656585 39560 0.022267715
39600 7.894025188 39600 0.16157495 19800 0.00960914 19816
0.076083925 39568 0.159275558 39568 0.039184204
39600 28.96919663 39600 0.236412161 19800 0.03830947 19820
0.06954695 39576 0.12775042 39576 0.043621789
39600 51.28319563 39600 0.291248521 19800 0.043039876 19824
0.058458878 39584 0.062986612 39584 0.024166613
39600 48.11550675 39600 0.235369836 19800 0.043058524 19828
0.030349584 39592 0.048142509 39592 0.016830862
39600 23.54056575 39600 0.112134323 19800 0.049259856 19832
0.027583683 39600 0.038593702 39600 0.013727686
39600 4.8337034 39600 0.111706511 19800 0.033917517 19836
0.060259426 39608 0.027736356 39608 0.043099895
39600 3.242098488 39600 0.121229641 19800 0.015946405 19840
0.068226029 39616 0.111677742 39616 0.07396398
39600 3.9075017 39600 0.058075424 19800 0.015428226 19844
0.074216863 39624 0.175674548 39624 0.099634213
39600 3.179517575 39600 0.090495654 19800 0.040941297 19848
0.06490243 39632 0.192943219 39632 0.092730283
39600 2.059865275 39600 0.161795819 19900 0.033745044 19852
0.036158552 39640 0.153065194 39640 0.047742225
39600 1.818440388 39600 0.138684685 19900 0.027668248 19856
0.051687217 39648 0.066806126 39648 0.013262036
39700 1.742657275 39700 0.11014269 19900 0.031898664 19860
0.031238262 39656 0.057860423 39656 0.055624627
39700 1.4561644 39700 0.083452163 19900 0.028275263 19864
0.087691507 39664 0.111852758 39664 0.064177613
39700 1.336947663 39700 0.082854225 19900 0.030011664 19868
0.080575112 39672 0.13461498 39672 0.04931798
39700 1.290874213 39700 0.077190045 19900 0.045020282 19872
0.024634726 39680 0.150880965 39680 0.056159523
39700 1.221372863 39700 0.092217335 19900 0.04919809 19876
0.047804631 39688 0.125250721 39688 0.06205267
39700 1.013263361 39700 0.061656101 19900 0.047869165 19880
0.019139994 39696 0.13562107 39696 0.039706265
39700 0.980955316 39700 0.134447008 19900 0.015687729 19884
0.062674706 39704 0.114668932 39704 0.02697007
39700 0.91830414 39700 0.127995066 19900 0.009954008 19888
0.040048704 39712 0.037609618 39712 0.038458416
39700 0.728633604 39700 0.054193861 19900 0.029053448 19892
0.039338014 39720 0.038524457 39720 0.030515625
39700 0.625811809 39700 0.01609054 19900 0.025461155 19896
0.05880577 39728 0.05492321 39728 0.008445705
39700 0.591955671 39700 0.01386959 19900 0.021803329 19900
0.0799603 39736 0.028653161 39736 0.03711267
39700 0.594736543 39700 0.041332318 19900 0.028451437 19904

0.09916984 39744 0.012288081 39744 0.038170711
39800 0.541846094 39800 0.027745695 19900 0.041184947 19908
0.118085394 39752 0.037894861 39752 0.044281092
39800 0.322974084 39800 0.027511585 19900 0.050384344 19912
0.109869732 39760 0.107829415 39760 0.046799411
39800 0.331061135 39800 0.058039805 19900 0.073823954 19916
0.064128137 39768 0.118098302 39768 0.038888091
39800 0.411193673 39800 0.038058723 19900 0.100231358 19920
0.055230419 39776 0.090763366 39776 0.057593108
39800 0.368140958 39800 0.02537804 19900 0.081971615 19924
0.067159752 39784 0.095729563 39784 0.087145978
39800 0.248122844 39800 0.051868989 19900 0.04213387 19928
0.069269569 39792 0.09122464 39792 0.087000713
39800 0.01905904 39800 0.056394118 19900 0.044315588 19932
0.078565099 39800 0.093705516 39800 0.0718558
39800 0.205556543 39800 0.041531584 19900 0.063109706 19936
0.041922045 39808 0.121776422 39808 0.036882026
39800 0.217455134 39800 0.022885515 19900 0.036212969 19940
0.037362992 39816 0.107957145 39816 0.015526668
39800 0.565875963 39800 0.032670836 19900 0.045917444 19944
0.035738962 39824 0.080923484 39824 0.027991348
39800 0.738553761 39800 0.021700013 19900 0.089184126 19948
0.029492421 39832 0.064250366 39832 0.023283383
39800 0.87230379 39800 0.0290545 20000 0.066563618 19952
0.041779942 39840 0.093172122 39840 0.025940717
39800 1.833293 39800 0.017859513 20000 0.051074389 19956
0.061681938 39848 0.128363798 39848 0.074263611
39900 2.2723584 39900 0.035739333 20000 0.041171508 19960
0.078222198 39856 0.12512879 39856 0.101385616
39900 1.673770838 39900 0.072560295 20000 0.035001405 19964
0.0545937 39864 0.089744288 39864 0.0478701
39900 0.708951906 39900 0.051128562 20000 0.047642046 19968
0.057145142 39872 0.103689577 39872 0.040560066
39900 0.171611479 39900 0.075466975 20000 0.04153698 19972
0.084847125 39880 0.123064892 39880 0.058492675
39900 0.130757384 39900 0.146756036 20000 0.026322918 19976
0.085369022 39888 0.104647545 39888 0.031184271
39900 0.307630515 39900 0.152575624 20000 0.012963023 19980
0.068907932 39896 0.075029981 39896 0.008888578
39900 0.218024986 39900 0.097754841 20000 0.003566763 19984
0.078400626 39904 0.118469368 39904 0.026429287
39900 0.095069874 39900 0.038070641 20000 0.020374468 19988
0.080706355 39912 0.079561571 39912 0.052411502
39900 0.088578941 39900 0.096132753 20000 0.03349283 19992
0.042062868 39920 0.116832336 39920 0.061314015
39900 0.09017167 39900 0.1081269 20000 0.051629304 19996
0.016228445 39928 0.224476636 39928 0.051796465
39900 0.126955333 39900 0.089500129 20000 0.07762311 20000
0.053854197 39936 0.225983779 39936 0.029292096
39900 0.093651994 39900 0.09099266 20000 0.072732706 20004
0.048564984 39944 0.151916524 39944 0.006731978
40000 0.082575549 40000 0.124916536 20000 0.031276664 20008
0.039081631 39952 0.095333555 39952 0.011263986
40000 0.126159328 40000 0.136749164 20000 0.024247938 20012
0.060805483 39960 0.02825254 39960 0.028812539
40000 0.126934058 40000 0.10249357 20000 0.008573395 20016
0.054231579 39968 0.062940126 39968 0.044224766
40000 0.095065123 40000 0.078530924 20000 0.010197495 20020
0.010358242 39976 0.060928149 39976 0.033402703
40000 0.120253309 40000 0.043765595 20000 0.014916507 20024
0.05627604 39984 0.067204288 39984 0.007645031
40000 0.152240639 40000 0.003465693 20000 0.023296401 20028
0.055318466 39992 0.092671886 39992 0.017227472
40000 0.087912478 40000 0.05405545 20000 0.032011431 20032
0.026385713 40000 0.084733285 40000 0.047153437
40000 0.054141958 40000 0.085424792 20000 0.021651298 20036
0.020203099 40008 0.058756672 40008 0.042486605
40000 0.022992703 40000 0.055797507 20000 0.048027407 20040

0.027209522 40016 0.08996456 40016 0.015066824
40000 0.06906582 40000 0.118322496 20000 0.050860617 20044
0.006521867 40024 0.094168019 40024 0.034608263
40000 0.071163348 40000 0.161317046 20000 0.012410896 20048
0.0383921 40032 0.08145524 40032 0.034289951
40000 0.032606891 40000 0.145385974 20100 0.019038411 20052
0.092000832 40040 0.112060487 40040 0.022246779
40000 0.012421694 40000 0.10918855 20100 0.030628096 20056
0.103837279 40048 0.139715121 40048 0.021331374
40100 0.050544852 40100 0.065767461 20100 0.055487719 20060
0.066083783 40056 0.083423365 40056 0.01152192
40100 0.077266101 40100 0.029429957 20100 0.080876052 20064
0.048382659 40064 0.029090112 40064 0.011134208
40100 0.03635939 40100 0.088636967 20100 0.081622304 20068
0.047911974 40072 0.077076809 40072 0.0064202
40100 0.064163505 40100 0.15283408 20100 0.060585153 20072
0.049431612 40080 0.150403168 40080 0.013233645
40100 0.12307422 40100 0.139459691 20100 0.034160756 20076
0.073707139 40088 0.138464763 40088 0.014219841
40100 0.109311361 40100 0.039865372 20100 0.01498623 20080
0.056131172 40096 0.064875276 40096 0.024183366
40100 0.086917215 40100 0.101094629 20100 0.014247543 20084
0.044466018 40104 0.039593146 40104 0.010263658
40100 0.047452286 40100 0.124380036 20100 0.03189456 20088
0.042640131 40112 0.065870598 40112 0.016029115
40100 0.052751995 40100 0.09674544 20100 0.038356993 20092
0.020054014 40120 0.027254209 40120 0.033233413
40100 0.098769378 40100 0.108625296 20100 0.035662692 20096
0.02904935 40128 0.066538982 40128 0.034715886
40100 0.10951794 40100 0.089865687 20100 0.079076774 20100
0.068010471 40136 0.157762901 40136 0.030990897
40100 0.079956924 40100 0.078856916 20100 0.06254363 20104
0.07684133 40144 0.173602239 40144 0.036183286
40200 0.042720643 40200 0.050568309 20100 0.064338157 20108
0.059213729 40152 0.099678793 40152 0.035450907
40200 0.048674512 40200 0.058635611 20100 0.11042902 20112
0.057371832 40160 0.028858845 40160 0.021224634
40200 0.034058063 40200 0.069630522 20100 0.090013273 20116
0.043936147 40168 0.139681958 40168 0.016031463
40200 0.03742801 40200 0.069548878 20100 0.031161366 20120
0.014722044 40176 0.171975465 40176 0.045967983
40200 0.034024168 40200 0.018801464 20100 0.063743428 20124
0.047386995 40184 0.185675978 40184 0.055979439
40200 0.067754249 40200 0.069036629 20100 0.06304367 20128
0.052207877 40192 0.243491654 40192 0.019731768
40200 0.121425503 40200 0.063252672 20100 0.047842579 20132
0.00974932 40200 0.120929231 40200 0.025227979
40200 0.115693052 40200 0.108270273 20100 0.054824883 20136
0.065021668 40208 0.115533396 40208 0.040412149
40200 0.082036619 40200 0.129519146 20100 0.038046659 20140
0.077642042 40216 0.13159086 40216 0.03980858
40200 0.025940773 40200 0.093529321 20100 0.036416594 20144
0.063936823 40224 0.084581436 40224 0.033750854
40200 0.029768537 40200 0.075019765 20100 0.039903429 20148
0.01224942 40232 0.097115757 40232 0.026039412
40200 0.014360807 40200 0.054392218 20200 0.034773366 20152
0.05177909 40240 0.083178617 40240 0.034690951
40200 0.03299972 40200 0.024585133 20200 0.062039297 20156
0.066909655 40248 0.07320958 40248 0.036652855
40300 0.084158623 40300 0.017836128 20200 0.074502757 20160
0.059571688 40256 0.087266722 40256 0.032081342
40300 0.170746396 40300 0.062436382 20200 0.048185131 20164
0.060642957 40264 0.124271115 40264 0.032369324
40300 0.213138221 40300 0.069337395 20200 0.01931684 20168
0.046337638 40272 0.250020355 40272 0.022864495
40300 0.179744559 40300 0.039954819 20200 0.020004858 20172
0.021746624 40280 0.128325628 40280 0.034484452
40300 0.10672355 40300 0.061922176 20200 0.03893879 20176

0.03359796	40288	0.103466817	40288	0.047614241		
40300	0.060583101	40300	0.126770159	20200	0.025119452	20180
0.041355379	40296	0.169220744	40296	0.034360783		
40300	0.068097528	40300	0.15484386	20200	0.035975336	20184
0.004268335	40304	0.215339285	40304	0.029453435		
40300	0.090455862	40300	0.084596592	20200	0.036169233	20188
0.015122607	40312	0.258738408	40312	0.052973239		
40300	0.069036418	40300	0.023132132	20200	0.017608019	20192
0.016043523	40320	0.507193909	40320	0.055300308		
40300	0.05819965	40300	0.108532862	20200	0.04990902	20196
0.036879585	40328	0.740169955	40328	0.03646985		
40300	0.066017521	40300	0.16560893	20200	0.008895748	20200
0.049292619	40336	0.757598144	40336	0.027612188		
40300	0.072502429	40300	0.125537495	20200	0.050601298	20204
0.039689901	40344	0	40344	0.030789568		
40400	0.061674793	40400	0.021088499	20200	0.062399566	20208
0.052301522	40352	0.610537304	40352	0.017370097		
40400	0.046161516	40400	0.077506098	20200	0.037612939	20212
0.050944946	40360	0.430869899	40360	0.007447228		
40400	0.066737317	40400	0.101126134	20200	0.04746913	20216
0.052967698	40368	0.58905984	40368	0.022220103		
40400	0.086081171	40400	0.078825135	20200	0.075983968	20220
0.01418459	40376	1.108604134	40376	0.038825569		
40400	0.057872214	40400	0.09149823	20200	0.067521636	20224
0.07168654	40384	1.716466975	40384	0.041377542		
40400	0.055015335	40400	0.09058346	20200	0.048823196	20228
0.08525882	40392	2.376881663	40392	0.042006432		
40400	0.007719687	40400	0.006981655	20200	0.027838525	20232
0.075437907	40400	2.999929475	40400	0.050038456		
40400	0.071391842	40400	0.101850936	20200	0.013355441	20236
0.070108057	40408	3.232714488	40408	0.016517064		
40400	0.075869175	40400	0.133391135	20200	0.035769055	20240
0.037631875	40416	3.149026313	40416	0.028500674		
40400	0.046808633	40400	0.091407361	20200	0.060899696	20244
0.062441111	40424	3.101387988	40424	0.029389385		
40400	0.032507953	40400	0.072190633	20200	0.060402486	20248
0.035328911	40432	3.27079045	40432	0.023399533		
40400	0.021243292	40400	0.05307237	20300	0.030365851	20252
0.041789583	40440	3.607331775	40440	0.015513801		
40400	0.053098742	40400	0.058804071	20300	0.036608264	20256
0.097079734	40448	3.655170788	40448	0.04058101		
40500	0.057295169	40500	0.065916567	20300	0.004861358	20260
0.099545781	40456	3.36663495	40456	0.062300554		
40500	0.065364715	40500	0.043627711	20300	0.033921049	20264
0.05998238	40464	3.052582738	40464	0.029301304		
40500	0.008619291	40500	0.096426833	20300	0.037219455	20268
0.05842153	40472	2.4452426	40472	0.029655053		
40500	0.128439191	40500	0.075887227	20300	0.027007583	20272
0.072017181	40480	1.784357588	40480	0.028325681		
40500	0.154019915	40500	0.031204334	20300	0.02435016	20276
0.066533452	40488	1.52780255	40488	0.026501506		
40500	0.065720582	40500	0.042082695	20300	0.05552674	20280
0.036460566	40496	1.302493388	40496	0.061554703		
40500	0.057702891	40500	0.043497719	20300	0.085369662	20284
0.023729186	40504	1.135414583	40504	0.073438401		
40500	0.097633318	40500	0.036588921	20300	0.099222074	20288
0.051848281	40512	1.189869479	40512	0.05582302		
40500	0.136170871	40500	0.034741348	20300	0.118315198	20292
0.080292244	40520	1.153225545	40520	0.04042959		
40500	0.182115473	40500	0.079412464	20300	0.098420089	20296
0.053470274	40528	0.93851029	40528	0.035246827		
40500	0.212704195	40500	0.079813945	20300	0.051128762	20300
0.011987477	40536	0.931713031	40536	0.02942251		
40500	0.179259921	40500	0.016680027	20300	0.039980594	20304
0.06437907	40544	0.855368213	40544	0.019320267		
40600	0.146965598	40600	0.054583081	20300	0.041146581	20308
0.073179734	40552	0.544707989	40552	0.013251018		
40600	0.179775336	40600	0.059976992	20300	0.031481952	20312

0.018668743 40560 0.417082221 40560 0.046000172
40600 0.245922361 40600 0.095932941 20300 0.034691246 20316
0.038015511 40568 0.346888264 40568 0.037795267
40600 0.223252515 40600 0.137821436 20300 0.016435943 20320
0.055202265 40576 0.425733073 40576 0.037840782
40600 0.144412829 40600 0.103092512 20300 0.013422407 20324
0.048103524 40584 0.419935328 40584 0.044315046
40600 0.09974718 40600 0.038504109 20300 0.004402458 20328
0.040516025 40592 0.353109878 40592 0.045716097
40600 0.093091134 40600 0.083602477 20300 0.046384088 20332
0.071278417 40600 0.413139148 40600 0.045796223
40600 0.136190094 40600 0.091011694 20300 0.044882519 20336
0.058908536 40608 0.370128023 40608 0.053813783
40600 0.160614684 40600 0.105116094 20300 0.02108473 20340
0.054406599 40616 0.178129106 40616 0.061817809
40600 0.096841322 40600 0.147200626 20300 0.025539972 20344
0.041832565 40624 0.107712302 40624 0.056473444
40600 0.057125966 40600 0.1636261 20300 0.054791963 20348
0.038362185 40632 0.246698531 40632 0.04720733
40600 0.095610754 40600 0.148872001 20400 0.050361105 20352
0.065403408 40640 0.301287423 40640 0.041757474
40600 0.0916871 40600 0.108080931 20400 0.015417125 20356
0.07293719 40648 0.26459925 40648 0.064123175
40700 0.110113469 40700 0.090755209 20400 0.023078923 20360
0.06169502 40656 0.160854558 40656 0.046281461
40700 0.06583699 40700 0.121464866 20400 0.0562719 20364
0.037955175 40664 0.04681596 40664 0.010790931
40700 0.130095446 40700 0.129148029 20400 0.071943432 20368
0.024305506 40672 0.122146375 40672 0.016707121
40700 0.157770875 40700 0.221438851 20400 0.039718245 20372
0.044121905 40680 0.186142075 40680 0.018869852
40700 0.187735815 40700 0.240731781 20400 0.030816394 20376
0.051084917 40688 0.110084562 40688 0.00367777
40700 0.183765559 40700 0.120466226 20400 0.085803898 20380
0.029696514 40696 0.029364714 40696 0.005479922
40700 0.122757672 40700 0.050161925 20400 0.084415071 20384
0.031033105 40704 0.137807889 40704 0.01855994
40700 0.066286906 40700 0.123400896 20400 0.060549111 20388
0.075202013 40712 0.163165474 40712 0.034036584
40700 0.076301039 40700 0.132700313 20400 0.026091964 20392
0.12860357 40720 0.078769182 40720 0.045469747
40700 0.083066094 40700 0.034275199 20400 0.041746469 20396
0.11877135 40728 0.068210422 40728 0.024891426
40700 0.077309356 40700 0.053909851 20400 0.073077841 20400
0.070013346 40736 0.153378758 40736 0.039717299
40700 0.06860296 40700 0.071731636 20400 0.068613968 20404
0.028883056 40744 0.195763918 40744 0.013637694
40800 0.027721026 40800 0.048947793 20400 0.052004492 20408
0.034430639 40752 0.118960648 40752 0.037229002
40800 0.130686676 40800 0.043038668 20400 0.048233171 20412
0.056265704 40760 0.047792662 40760 0.07103337
40800 0.219994559 40800 0.069035792 20400 0.062880587 20416
0.065437649 40768 0.070366819 40768 0.087458619
40800 0.206865749 40800 0.052273055 20400 0.042870222 20420
0.095564603 40776 0.121074525 40776 0.062311345
40800 0.149774685 40800 0.023765191 20400 0.049517552 20424
0.078179524 40784 0.066410677 40784 0.023757331
40800 0.143535246 40800 0.018792431 20400 0.041321597 20428
0.077248071 40792 0.048627215 40792 0.033929013
40800 0.146638224 40800 0.049203649 20400 0.040711042 20432
0.09462201 40800 0.073235067 40800 0.034053388
40800 0.141830563 40800 0.077759396 20400 0.041463703 20436
0.078840116 40808 0.125390011 40808 0.01792189
40800 0.074289055 40800 0.121847021 20400 0.011936555 20440
0.05701173 40816 0.178179588 40816 0.019173269
40800 0.037026475 40800 0.148952575 20400 0.046334295 20444
0.023189359 40824 0.159686548 40824 0.022888509
40800 0.047436872 40800 0.094542338 20400 0.057241748 20448

0.037951879 40832 0.128106621 40832 0.02298789
40800 0.053545533 40800 0.062979125 20500 0.06625105 20452
0.022629167 40840 0.136769668 40840 0.033920638
40800 0.081199389 40800 0.141309488 20500 0.055686636 20456
0.020602847 40848 0.153738045 40848 0.049531529
40900 0.03116245 40900 0.195121888 20500 0.020223683 20460
0.068059235 40856 0.110605193 40856 0.039391623
40900 0.043027194 40900 0.153156988 20500 0.013793884 20464
0.056575766 40864 0.072994306 40864 0.008710834
40900 0.031628431 40900 0.119377801 20500 0.019387086 20468
0.012154893 40872 0.060194227 40872 0.033253298
40900 0.080468322 40900 0.122614365 20500 0.013867507 20472
0.013708738 40880 0.055556171 40880 0.029847122
40900 0.045972898 40900 0.12876514 20500 0.031033811 20476
0.028940844 40888 0.098284669 40888 0.01540198
40900 0.057137782 40900 0.099258934 20500 0.054301549 20480
0.023427459 40896 0.121611447 40896 0.044252287
40900 0.10807634 40900 0.018446288 20500 0.054110373 20484
0.024408713 40904 0.020268899 40904 0.049515365
40900 0.06356767 40900 0.080541227 20500 0.023454939 20488
0.025557742 40912 0.094654191 40912 0.052301792
40900 0.046702706 40900 0.065916509 20500 0.011009935 20492
0.041721894 40920 0.047787093 40920 0.043716707
40900 0.107516375 40900 0.048910741 20500 0.049952927 20496
0.010583108 40928 0.105785017 40928 0.031308286
40900 0.104767372 40900 0.075780255 20500 0.089371672 20500
0.051924697 40936 0.141224445 40936 0.03792686
40900 0.100793244 40900 0.07009897 20500 0.10016431 20504
0.080992162 40944 0.141367636 40944 0.040582094
41000 0.089581765 41000 0.051965497 20500 0.08238884 20508
0.084082029 40952 0.048614722 40952 0.028591539
41000 0.081689177 41000 0.094498326 20500 0.051939456 20512
0.044960754 40960 0.057241989 40960 0.005761026
41000 0.1038509 41000 0.142811274 20500 0.024187106 20516
0.031190568 40968 0.106154046 40968 0.020065914
41000 0.135149153 41000 0.175106121 20500 0.005683801 20520
0.035185789 40976 0.097586511 40976 0.035750672
41000 0.123323815 41000 0.17171813 20500 0.001526343 20524
0.011996291 40984 0.057407422 40984 0.040858307
41000 0.074174226 41000 0.133611465 20500 0.007896329 20528
0.022337901 40992 0.047589449 40992 0.018923596
41000 0.078913297 41000 0.062616069 20500 0.009276071 20532
0.020124155 41000 0.020647867 41000 0.029847784
41000 0.144764738 41000 0.1348277 20500 0.028973287 20536
0.02665572 41008 0.038942097 41008 0.047043515
41000 0.119240765 41000 0.181992436 20500 0.062635227 20540
0.062640858 41016 0.075582073 41016 0.030985026
41000 0.077882709 41000 0.159661533 20500 0.038377046 20544
0.048814964 41024 0.06719488 41024 0.092286333
41000 0.039789164 41000 0.108343404 20500 0.036293553 20548
0.044193937 41032 0.0277444 41032 0.097446413
41000 0.033795655 41000 0.08243895 20600 0.060068578 20552
0.04603677 41040 0.021900589 41040 0.05847049
41000 0.072394978 41000 0.099190023 20600 0.027057122 20556
0.03533961 41048 0.016243923 41048 0.043630949
41100 0.100024539 41100 0.107774533 20600 0.051176721 20560
0.009463973 41056 0.066696171 41056 0.024716757
41100 0.10730842 41100 0.060565501 20600 0.076589 20564
0.032867512 41064 0.09428629 41064 0.033542645
41100 0.068030626 41100 0.058628044 20600 0.05787241 20568
0.023289094 41072 0.058951704 41072 0.048187463
41100 0.055114091 41100 0.144162011 20600 0.049891285 20572
0.032054457 41080 0.133708848 41080 0.045490786
41100 0.120358425 41100 0.166370199 20600 0.05563529 20576
0.098913617 41088 0.095247626 41088 0.024720797
41100 0.095546231 41100 0.112448652 20600 0.032830605 20580
0.123862395 41096 0.051918356 41096 0.038395607
41100 0.059694285 41100 0.097575277 20600 0.012854176 20584

0.103035658 41104 0.142770106 41104 0.055420114
41100 0.064426429 41100 0.141269339 20600 0.011968299 20588
0.086668872 41112 0.136534844 41112 0.051135223
41100 0.057726451 41100 0.1778062 20600 0.02571874 20592
0.091427872 41120 0.069693102 41120 0.046839665
41100 0.067425484 41100 0.180337461 20600 0.028688857 20596
0.080243081 41128 0.06979637 41128 0.039656748
41100 0.058995793 41100 0.172386048 20600 0.0347635 20600
0.027971728 41136 0.138888906 41136 0.040571747
41100 0.059630962 41100 0.139095283 20600 0.011132456 20604
0.039236933 41144 0.149667234 41144 0.035192326
41200 0.05448698 41200 0.048854505 20600 0.013611882 20608
0.042567092 41152 0.100344878 41152 0.02941285
41200 0.111810579 41200 0.054243217 20600 0.021263417 20612
0.026798591 41160 0.069224829 41160 0.060203616
41200 0.163153265 41200 0.059891954 20600 0.038420807 20616
0.078147379 41168 0.053123804 41168 0.046734545
41200 0.159145159 41200 0.039855659 20600 0.039432052 20620
0.074597483 41176 0.077422672 41176 0.048465096
41200 0.130251443 41200 0.066266017 20600 0.021091282 20624
0.069614856 41184 0.104768144 41184 0.044812725
41200 0.087531444 41200 0.109911838 20600 0.007667627 20628
0.039744922 41192 0.115572082 41192 0.013945937
41200 0.063529427 41200 0.145479833 20600 0.013682949 20632
0.004627514 41200 0.09926824 41200 0.039322888
41200 0.045977253 41200 0.081536324 20600 0.04372573 20636
0.011590992 41208 0.060960159 41208 0.056460838
41200 0.041773048 41200 0.10002256 20600 0.07545908 20640
0.025751428 41216 0.111874651 41216 0.047384147
41200 0.062695544 41200 0.044209028 20600 0.064340566 20644
0.034300574 41224 0.112449139 41224 0.010306328
41200 0.168493848 41200 0.102355538 20600 0.046637117 20648
0.034727116 41232 0.033469427 41232 0.035162295
41200 0.179243813 41200 0.101017467 20700 0.033665849 20652
0.042074473 41240 0.091721377 41240 0.035150501
41200 0.109587287 41200 0.176507834 20700 0.058440379 20656
0.035168854 41248 0.100608806 41248 0.007953588
41300 0.026064567 41300 0.189791739 20700 0.082814535 20660
0.015073193 41256 0.042030453 41256 0.042604119
41300 0.025014901 41300 0.143794809 20700 0.07105017 20664
0.023528663 41264 0.066165725 41264 0.062472143
41300 0.05557821 41300 0.049507973 20700 0.019717427 20668
0.021152002 41272 0.087017885 41272 0.064493848
41300 0.105068051 41300 0.071565031 20700 0.055314686 20672
0.033014596 41280 0.10036082 41280 0.042908105
41300 0.154604451 41300 0.018721279 20700 0.073311363 20676
0.049973787 41288 0.140845106 41288 0.055077315
41300 0.149711631 41300 0.1014726 20700 0.047820275 20680
0.040662555 41296 0.167258739 41296 0.051586751
41300 0.126610379 41300 0.05557327 20700 0.02335607 20684
0.06805388 41304 0.137375319 41304 0.023752562
41300 0.095520234 41300 0.05678301 20700 0.012897431 20688
0.058207254 41312 0.063522799 41312 0.023457918
41300 0.069188274 41300 0.020822856 20700 0.024320854 20692
0.044239547 41320 0.034015116 41320 0.013048498
41300 0.034901241 41300 0.074877331 20700 0.052244115 20696
0.059826201 41328 0.054497199 41328 0.01914227
41300 0.083736064 41300 0.074669661 20700 0.060644135 20700
0.082138548 41336 0.010492925 41336 0.017400851
41300 0.176489368 41300 0.140611854 20700 0.041254149 20704
0.060495091 41344 0.043379307 41344 0.032114549
41400 0.160937765 41400 0.214521803 20700 0.050937117 20708
0.009050853 41352 0.012249004 41352 0.017470711
41400 0.106638909 41400 0.121274912 20700 0.072119823 20712
0.020189802 41360 0.040391336 41360 0.024411167
41400 0.104438033 41400 0.107828782 20700 0.053779088 20716
0.034335451 41368 0.016906948 41368 0.020410886
41400 0.122061414 41400 0.153332629 20700 0.037268317 20720

0.065482112	41376	0.061850784	41376	0.019267665		
41400	0.090700021	41400	0.10370768	20700	0.029342078	20724
0.059954105	41384	0.053309672	41384	0.010562588		
41400	0.043399294	41400	0.069222057	20700	0.016456286	20728
0.042454463	41392	0.079581427	41392	0.00929817		
41400	0.043437514	41400	0.081685968	20700	0.015779568	20732
0.07832898	41400	0.155982896	41400	0.02925264		
41400	0.08366775	41400	0.087616871	20700	0.023661583	20736
0.108486333	41408	0.213767984	41408	0.030261374		
41400	0.049443766	41400	0.10979631	20700	0.036919893	20740
0.095096373	41416	0.228776903	41416	0.003287093		
41400	0.030416757	41400	0.124146201	20700	0.078851088	20744
0.089189191	41424	0.167097228	41424	0.013575721		
41400	0.079214849	41400	0.110100773	20700	0.103580598	20748
0.039844981	41432	0.052279222	41432	0.027802056		
41400	0.081168742	41400	0.062372434	20800	0.06941281	20752
0.055963446	41440	0.123905847	41440	0.035167297		
41400	0.045371922	41400	0.068179033	20800	0.033635024	20756
0.068537214	41448	0.055548801	41448	0.0227532		
41500	0.031668082	41500	0.045873498	20800	0.051148578	20760
0.049655781	41456	0.043972617	41456	0.055315755		
41500	0.049244041	41500	0.077760727	20800	0.053593241	20764
0.02932387	41464	0.078807512	41464	0.058487935		
41500	0.0896684	41500	0.19070413	20800	0.00608829	20768
0.032788528	41472	0.203311138	41472	0.023411383		
41500	0.059212194	41500	0.186498278	20800	0.03693852	20772
0.027222062	41480	0.223148716	41480	0.018521751		
41500	0.025000851	41500	0.124446146	20800	0.019584015	20776
0.029296434	41488	0.176734786	41488	0.02124715		
41500	0.048309827	41500	0.085453299	20800	0.033015451	20780
0.030604875	41496	0.118267722	41496	0.02009512		
41500	0.054820346	41500	0.058380101	20800	0.019056522	20784
0.07397114	41504	0.045922116	41504	0.017473258		
41500	0.023028379	41500	0.118246324	20800	0.034071702	20788
0.066729561	41512	0.10132324	41512	0.048375117		
41500	0.034207558	41500	0.134212198	20800	0.058468122	20792
0.042040603	41520	0.107349108	41520	0.072388015		
41500	0.026927268	41500	0.14009398	20800	0.090170383	20796
0.049658691	41528	0.078653604	41528	0.039247821		
41500	0.069662259	41500	0.195967281	20800	0.081528473	20800
0.008010486	41536	0.064350046	41536	0.016227134		
41500	0.121076613	41500	0.196784953	20800	0.013394892	20804
0.041612995	41544	0.043281918	41544	0.024377569		
41600	0.172478904	41600	0.13467703	20800	0.052622952	20808
0.090352514	41552	0.028019569	41552	0.020517196		
41600	0.14163871	41600	0.04552742	20800	0.051165142	20812
0.096033487	41560	0.119537406	41560	0.031250915		
41600	0.036836158	41600	0.080324782	20800	0.024650533	20816
0.046887064	41568	0.154480251	41568	0.018501925		
41600	0.085201646	41600	0.121733538	20800	0.04673583	20820
0.025133588	41576	0.120671255	41576	0.01290452		
41600	0.148605701	41600	0.177175753	20800	0.045295292	20824
0.041246007	41584	0.079418664	41584	0.01948556		
41600	0.178852818	41600	0.137403301	20800	0.050518593	20828
0.059837821	41592	0.053704549	41592	0.004462283		
41600	0.186454578	41600	0.059369733	20800	0.03381844	20832
0.067439352	41600	0.072103961	41600	0.029559858		
41600	0.16286115	41600	0.090769667	20800	0.045780918	20836
0.028006054	41608	0.108124434	41608	0.050080642		
41600	0.104960804	41600	0.111111614	20800	0.09445021	20840
0.034659963	41616	0.09697464	41616	0.045593835		
41600	0.052716863	41600	0.043528598	20800	0.069371854	20844
0.036465648	41624	0.020727055	41624	0.036125177		
41600	0.047779529	41600	0.068915659	20800	0.037757225	20848
0.031200329	41632	0.082946564	41632	0.043580589		
41600	0.073516938	41600	0.086936772	20900	0.05360867	20852
0.046013051	41640	0.119525197	41640	0.027304848		
41600	0.086976725	41600	0.021587126	20900	0.091998074	20856

0.044932091 41648 0.097639866 41648 0.022340928
41700 0.081643382 41700 0.030850657 20900 0.095816278 20860
0.032820131 41656 0.09723931 41656 0.038466318
41700 0.111813788 41700 0.032775228 20900 0.048547656 20864
0.051192837 41664 0.055554327 41664 0.042693766
41700 0.12911357 41700 0.125952225 20900 0.001221376 20868
0.103323881 41672 0.03890563 41672 0.043841137
41700 0.069192443 41700 0.163262114 20900 0.019878751 20872
0.11814625 41680 0.070217167 41680 0.038123166
41700 0.00337822 41700 0.137240059 20900 0.012408184 20876
0.070548216 41688 0.074575015 41688 0.022081349
41700 0.069799768 41700 0.133432564 20900 0.013627613 20880
0.06693192 41696 0.122702171 41696 0.007502465
41700 0.084804378 41700 0.093427298 20900 0.014358358 20884
0.08130401 41704 0.144411199 41704 0.01861847
41700 0.020637861 41700 0.050333638 20900 0.024107421 20888
0.059459202 41712 0.081253085 41712 0.025104664
41700 0.057509398 41700 0.129994616 20900 0.050148243 20892
0.041920026 41720 0.058414888 41720 0.008330749
41700 0.082902312 41700 0.151226355 20900 0.080973296 20896
0.038864651 41728 0.104493476 41728 0.022520686
41700 0.086004482 41700 0.132708433 20900 0.071428105 20900
0.02831665 41736 0.10585288 41736 0.046857163
41700 0.119629076 41700 0.046637804 20900 0.028834997 20904
0.037717899 41744 0.06956709 41744 0.032915861
41800 0.150489999 41800 0.098987905 20900 0.018437522 20908
0.078378296 41752 0.013099093 41752 0.020290159
41800 0.146527688 41800 0.118572149 20900 0.03101143 20912
0.075228651 41760 0.046314588 41760 0.016192569
41800 0.140388641 41800 0.07465027 20900 0.024320365 20916
0.054281958 41768 0.094236079 41768 0.050386592
41800 0.083556486 41800 0.174737885 20900 0.030393048 20920
0.05260812 41776 0.107572065 41776 0.06269083
41800 0.058856815 41800 0.161338656 20900 0.04504863 20924
0.062027793 41784 0.125023551 41784 0.025247342
41800 0.010876141 41800 0.094419076 20900 0.034194229 20928
0.078780315 41792 0.123909893 41792 0.010331585
41800 0.11677009 41800 0.051547937 20900 0.023240384 20932
0.068222958 41800 0.146249979 41800 0.014141391
41800 0.090014946 41800 0.060048395 20900 0.033028849 20936
0.049708164 41808 0.157110364 41808 0.031459062
41800 0.053490432 41800 0.015772916 20900 0.030500787 20940
0.034765195 41816 0.077451725 41816 0.052931639
41800 0.098957105 41800 0.066156652 20900 0.044925164 20944
0.034887195 41824 0.021617021 41824 0.051957581
41800 0.049258808 41800 0.134118105 20900 0.02372149 20948
0.055918161 41832 0.015346752 41832 0.025769341
41800 0.039775183 41800 0.141909011 21000 0.040131363 20952
0.057163372 41840 0.076282566 41840 0.016273834
41800 0.125867591 41800 0.098839395 21000 0.093205126 20956
0.118784556 41848 0.109343724 41848 0.033602286
41900 0.106442552 41900 0.0933107 21000 0.077723496 20960
0.143396566 41856 0.097200129 41856 0.030360887
41900 0.041153846 41900 0.131438631 21000 0.029100886 20964
0.088357621 41864 0.100827936 41864 0.030358471
41900 0.070907059 41900 0.084703563 21000 0.023898645 20968
0.043307202 41872 0.112009635 41872 0.026613587
41900 0.054187836 41900 0.061012976 21000 0.025909822 20972
0.027402566 41880 0.087086504 41880 0.042041338
41900 0.071850795 41900 0.056601693 21000 0.041480791 20976
0.011193291 41888 0.069450027 41888 0.056117384
41900 0.049952003 41900 0.107364693 21000 0.057649206 20980
0.040291303 41896 0.131401729 41896 0.051775518
41900 0.091387628 41900 0.115031646 21000 0.069193207 20984
0.048201535 41904 0.104114493 41904 0.022894115
41900 0.222572475 41900 0.068507558 21000 0.063780673 20988
0.033181892 41912 0.01407414 41912 0.027502654
41900 0.33010781 41900 0.075114462 21000 0.053679603 20992

0.023078546 41920 0.064434673 41920 0.047342852
41900 0.288916111 41900 0.050363036 21000 0.051654795 20996
0.059262791 41928 0.110277368 41928 0.050336123
41900 0.154542446 41900 0.071919894 21000 0.074506577 21000
0.064271015 41936 0.074364834 41936 0.062518207
41900 0.050723633 41900 0.074929791 21000 0.087042616 21004
0.087031796 41944 0.027191103 41944 0.042773598
42000 0.09693508 42000 0.06414298 21000 0.073113224 21008
0.077578217 41952 0.054178116 41952 0.024105999
42000 0.151273955 42000 0.063545136 21000 0.051250081 21012
0.017446497 41960 0.072234645 41960 0.025837651
42000 0.105256615 42000 0.140145566 21000 0.029994042 21016
0.094205243 41968 0.021431661 41968 0.019847023
42000 0.017216224 42000 0.163806951 21000 0.0185764 21020
0.138234304 41976 0.060998875 41976 0.024648512
42000 0.039610815 42000 0.101253951 21000 0.024375511 21024
0.114261362 41984 0.022877503 41984 0.021149745
42000 0.037137415 42000 0.012492354 21000 0.018344757 21028
0.130711851 41992 0.051825031 41992 0.005152828
42000 0.040592368 42000 0.138992604 21000 0.013639886 21032
0.143159399 42000 0.074177668 42000 0.01579554
42000 0.104573453 42000 0.166433281 21000 0.02481642 21036
0.098956036 42008 0.048091188 42008 0.024395475
42000 0.172424654 42000 0.207914505 21000 0.043185028 21040
0.055943074 42016 0.062550695 42016 0.057155157
42000 0.084097148 42000 0.25747309 21000 0.045587625 21044
0.04682476 42024 0.078576704 42024 0.0537206
42000 0.03655989 42000 0.202635289 21000 0.050047525 21048
0.019803001 42032 0.065066553 42032 0.029631034
42000 0.023594539 42000 0.112447349 21100 0.058456961 21052
0.109853914 42040 0.07992523 42040 0.014855095
42000 0.09971526 42000 0.036337482 21100 0.023862456 21056
0.109367298 42048 0.128447355 42048 0.043263783
42100 0.120763034 42100 0.046263853 21100 0.027253978 21060
0.071846771 42056 0.127560211 42056 0.043408269
42100 0.132949878 42100 0.068682268 21100 0.031693046 21064
0.083532148 42064 0.074678123 42064 0.029215215
42100 0.177017326 42100 0.023291816 21100 0.028729704 21068
0.092955568 42072 0.077089266 42072 0.016513748
42100 0.150515364 42100 0.022309709 21100 0.053860371 21072
0.085016727 42080 0.142404606 42080 0.015982936
42100 0.056047833 42100 0.042851869 21100 0.041690862 21076
0.088475972 42088 0.114702656 42088 0.039842274
42100 0.110191621 42100 0.124764949 21100 0.029976918 21080
0.093118564 42096 0.052490195 42096 0.042782824
42100 0.104204519 42100 0.11869934 21100 0.032399606 21084
0.074608928 42104 0.076395372 42104 0.05693264
42100 0.022842667 42100 0.067880676 21100 0.065339293 21088
0.066158093 42112 0.085850988 42112 0.059363971
42100 0.080751837 42100 0.052351516 21100 0.071291303 21092
0.052884887 42120 0.020180312 42120 0.017198989
42100 0.044102984 42100 0.083762403 21100 0.082113118 21096
0.072459807 42128 0.140108881 42128 0.045660974
42100 0.077232566 42100 0.078804143 21100 0.084162173 21100
0.124963743 42136 0.146336534 42136 0.038127313
42100 0.151570901 42100 0.075796816 21100 0.051677231 21104
0.126985251 42144 0.041161162 42144 0.025643807
42200 0.167723833 42200 0.10562814 21100 0.007442897 21108
0.090231442 42152 0.069450405 42152 0.020439595
42200 0.105641841 42200 0.152537818 21100 0.079014651 21112
0.061415805 42160 0.052962365 42160 0.006569264
42200 0.156495181 42200 0.157638511 21100 0.11907857 21116
0.059197726 42168 0.04959487 42168 0.037901282
42200 0.084573687 42200 0.122255369 21100 0.090993963 21120
0.07282498 42176 0.074160205 42176 0.051853491
42200 0.334857673 42200 0.072712697 21100 0.063584637 21124
0.112341142 42184 0.062714258 42184 0.035967511
42200 0.514125044 42200 0.075304946 21100 0.060931427 21128

0.154715818 42192 0.157719361 42192 0.036225603
42200 0.494722859 42200 0.077920551 21100 0.046928646 21132
0.148759121 42200 0.124493148 42200 0.022148977
42200 0.310239935 42200 0.052766274 21100 0.051227726 21136
0.157010509 42208 0.04540963 42208 0.035004392
42200 0.10645212 42200 0.00672954 21100 0.025718449 21140
0.147650295 42216 0.048160095 42216 0.058577964
42200 0.02043689 42200 0.04522469 21100 0.030676587 21144
0.095004187 42224 0.08483007 42224 0.059330152
42200 0.053631673 42200 0.035168538 21100 0.070103255 21148
0.058120473 42232 0.08705521 42232 0.038645896
42200 0.044067798 42200 0.051076393 21200 0.095539268 21152
0.064971544 42240 0.052786716 42240 0.023475493
42200 0.109358887 42200 0.12551673 21200 0.101590696 21156
0.064888889 42248 0.077509991 42248 0.067111549
42300 0.09298537 42300 0.184560355 21200 0.06474604 21160
0.064096137 42256 0.082168634 42256 0.074141499
42300 0.085708976 42300 0.21226742 21200 0.009347877 21164
0.05754719 42264 0.058299764 42264 0.043578362
42300 0.095833915 42300 0.215225285 21200 0.020032627 21168
0.061287239 42272 0.079426085 42272 0.022871307
42300 0.106995532 42300 0.221183364 21200 0.032979082 21172
0.047561698 42280 0.106437336 42280 0.040055871
42300 0.106142747 42300 0.200193973 21200 0.060268118 21176
0.023150293 42288 0.104021921 42288 0.039681312
42300 0.091131959 42300 0.150517415 21200 0.082278704 21180
0.080799655 42296 0.181662079 42296 0.041566284
42300 0.087152832 42300 0.104313374 21200 0.081431615 21184
0.086338536 42304 0.156298876 42304 0.057692701
42300 0.119525488 42300 0.083821695 21200 0.065167114 21188
0.04831003 42312 0.050891449 42312 0.028514724
42300 0.155984483 42300 0.066986555 21200 0.076220968 21192
0.040950385 42320 0.080233695 42320 0.041195941
42300 0.163748409 42300 0.066477129 21200 0.091584887 21196
0.045886136 42328 0.025043313 42328 0.060563361
42300 0.123948834 42300 0.116975018 21200 0.086756358 21200
0.064687476 42336 0.114228154 42336 0.049100312
42300 0.0616619 42300 0.09151519 21200 0.052312669 21204
0.098721808 42344 0.182183533 42344 0.030117572
42400 0.083290775 42400 0.023349448 21200 0.047321682 21208
0.079300058 42352 0.241916946 42352 0.009527837
42400 0.11848638 42400 0.007175799 21200 0.065689557 21212
0.037663958 42360 0.175091525 42360 0.043542354
42400 0.105988678 42400 0.027424732 21200 0.055461071 21216
0.076567092 42368 0.058824393 42368 0.022982349
42400 0.119481418 42400 0.036448946 21200 0.04597746 21220
0.084939151 42376 0.064240157 42376 0.042367028
42400 0.098166478 42400 0.047320165 21200 0.023447597 21224
0.083194573 42384 0.010961259 42384 0.02917965
42400 0.059344533 42400 0.058829894 21200 0.005706202 21228
0.107574684 42392 0.093810901 42392 0.026363159
42400 0.047473179 42400 0.042087169 21200 0.025351246 21232
0.124016267 42400 0.206024968 42400 0.045862511
42400 0.094903597 42400 0.145836908 21200 0.052206124 21236
0.089023357 42408 0.263847993 42408 0.023378419
42400 0.17252739 42400 0.131776659 21200 0.047192378 21240
0.038001937 42416 0.159446049 42416 0.026711537
42400 0.190024511 42400 0.058920065 21200 0.018389523 21244
0.059864637 42424 0.008988086 42424 0.061104307
42400 0.142749749 42400 0.009300746 21200 0.041488631 21248
0.076108096 42432 0.054868411 42432 0.080738173
42400 0.094329116 42400 0.093455135 21300 0.06779041 21252
0.061146406 42440 0.010641698 42440 0.068650341
42400 0.109018089 42400 0.16236867 21300 0.0753805 21256
0.020985492 42448 0.020842179 42448 0.055224937
42500 0.175898531 42500 0.139242548 21300 0.071528586 21260
0.04855937 42456 0.017561684 42456 0.024816593
42500 0.191775748 42500 0.079494334 21300 0.031903041 21264

0.100776219 42464 0.096146825 42464 0.014085785
42500 0.149979795 42500 0.059182872 21300 0.018326291 21268
0.118014388 42472 0.135503389 42472 0.010005718
42500 0.105081657 42500 0.052448628 21300 0.045536115 21272
0.114497831 42480 0.037120622 42480 0.02392265
42500 0.042297972 42500 0.088118904 21300 0.057447396 21276
0.092901508 42488 0.103207836 42488 0.041542739
42500 0.044575449 42500 0.112454734 21300 0.032300923 21280
0.067360154 42496 0.116676252 42496 0.038452621
42500 0.172025204 42500 0.069567279 21300 0.027829439 21284
0.046860721 42504 0.160914875 42504 0.016046124
42500 0.213864856 42500 0.022077782 21300 0.043480177 21288
0.055162491 42512 0.266234216 42512 0.046049205
42500 0.128262924 42500 0.025972689 21300 0.068026806 21292
0.041279753 42520 0.350069429 42520 0.088237262
42500 0.018280907 42500 0.099638543 21300 0.047096288 21296
0.001269476 42528 0.313285593 42528 0.096678654
42500 0.05035791 42500 0.11940398 21300 0.024000556 21300
0.059100028 42536 0.159474541 42536 0.077793848
42500 0.071063179 42500 0.061114442 21300 0.018539036 21304
0.075049349 42544 0.077220095 42544 0.043190372
42600 0.026396874 42600 0.066310735 21300 0.027638203 21308
0.061857201 42552 0.042552823 42552 0.025846473
42600 0.094049392 42600 0.133847221 21300 0.04388969 21312
0.085338033 42560 0.082660212 42560 0.023545537
42600 0.157040005 42600 0.123676553 21300 0.027787704 21316
0.12038424 42568 0.177258233 42568 0.024919544
42600 0.135138616 42600 0.044530316 21300 0.03087876 21320
0.114691356 42576 0.19981066 42576 0.066340923
42600 0.10280592 42600 0.050196468 21300 0.047386217 21324
0.081678263 42584 0.229539845 42584 0.058081172
42600 0.088034889 42600 0.039018665 21300 0.028806799 21328
0.065825407 42592 0.267301599 42592 0.005261317
42600 0.047220397 42600 0.027761222 21300 0.029106957 21332
0.057071109 42600 0.258430285 42600 0.025511306
42600 0.036619265 42600 0.03705034 21300 0.021476586 21336
0.042800028 42608 0.178283154 42608 0.015085321
42600 0.045295386 42600 0.023643517 21300 0.004473811 21340
0.038641771 42616 0.013026191 42616 0.010986643
42600 0.053503103 42600 0.086566797 21300 0.0133541 21344
0.049694594 42624 0.16972411 42624 0.056548361
42600 0.046678772 42600 0.106221931 21300 0.057558329 21348
0.062963132 42632 0.259378051 42632 0.055062526
42600 0.02956155 42600 0.078845711 21400 0.059916758 21352
0.047397778 42640 0.267275056 42640 0.02812484
42600 0.057664161 42600 0.068460264 21400 0.010381292 21356
0.052448115 42648 0.272835343 42648 0.088199719
42700 0.097837285 42700 0.099065626 21400 0.044509849 21360
0.087195665 42656 0.239175963 42656 0.080684258
42700 0.08461449 42700 0.086132379 21400 0.062998042 21364
0.103701539 42664 0.147026731 42664 0.058581907
42700 0.068709502 42700 0.062284817 21400 0.048239344 21368
0.092500246 42672 0.062287298 42672 0.03658087
42700 0.098153869 42700 0.139213298 21400 0.027327256 21372
0.058492984 42680 0.047734164 42680 0.026495472
42700 0.13865158 42700 0.237146509 21400 0.078612284 21376
0.022636572 42688 0.065006374 42688 0.048941813
42700 0.163154269 42700 0.246432028 21400 0.071637012 21380
0.025149451 42696 0.073575371 42696 0.087768953
42700 0.124978236 42700 0.133934388 21400 0.031405587 21384
0.035838832 42704 0.074065581 42704 0.076269622
42700 0.097642529 42700 0.031569249 21400 0.011399942 21388
0.065868066 42712 0.008130371 42712 0.061542349
42700 0.045890112 42700 0.072843977 21400 0.005499251 21392
0.071067618 42720 0.103271108 42720 0.069797708
42700 0.013971747 42700 0.109334767 21400 0.011488439 21396
0.048214512 42728 0.089738096 42728 0.052910797
42700 0.070550363 42700 0.165657766 21400 0.03180635 21400

0.03528368 42736 0.069529924 42736 0.073743155
42700 0.139695913 42700 0.190756196 21400 0.053243395 21404
0.028323171 42744 0.127193401 42744 0.077605109
42800 0.140422883 42800 0.171792024 21400 0.057928231 21408
0.035791294 42752 0.085662447 42752 0.06579038
42800 0.10119284 42800 0.117763244 21400 0.047072219 21412
0.10380063 42760 0.029628034 42760 0.026488679
42800 0.098363351 42800 0.07709644 21400 0.09005468 21416
0.138139673 42768 0.023387109 42768 0.022057246
42800 0.048861439 42800 0.076322765 21400 0.06210088 21420
0.121309873 42776 0.066273307 42776 0.037400572
42800 0.114560186 42800 0.083878134 21400 0.024118979 21424
0.094191128 42784 0.059950333 42784 0.050022969
42800 0.123763224 42800 0.09023981 21400 0.059083544 21428
0.062025312 42792 0.067610265 42792 0.041982326
42800 0.124783313 42800 0.113292022 21400 0.044487078 21432
0.052262418 42800 0.122310914 42800 0.027981145
42800 0.146732651 42800 0.080243401 21400 0.044010823 21436
0.060368991 42808 0.157553194 42808 0.028009514
42800 0.122096972 42800 0.054190721 21400 0.068860507 21440
0.04504457 42816 0.159589326 42816 0.023414061
42800 0.051791925 42800 0.061269675 21400 0.063483363 21444
0.017791979 42824 0.135568741 42824 0.010852979
42800 0.043397609 42800 0.033928431 21400 0.031930347 21448
0.037188896 42832 0.088890585 42832 0.011991108
42800 0.054519951 42800 0.102081278 21500 0.030159919 21452
0.073371601 42840 0.049360016 42840 0.017506365
42800 0.068641188 42800 0.117251679 21500 0.053344578 21456
0.064174492 42848 0.063547581 42848 0.042094587
42900 0.092838607 42900 0.151079294 21500 0.049005204 21460
0.071129973 42856 0.037452934 42856 0.055715111
42900 0.092563445 42900 0.12051277 21500 0.034733927 21464
0.100628138 42864 0.031387812 42864 0.045177319
42900 0.159856645 42900 0.017748984 21500 0.038894184 21468
0.083746505 42872 0.016242491 42872 0.021349471
42900 0.154521084 42900 0.074686941 21500 0.046592697 21472
0.098404642 42880 0.111923997 42880 0.014747355
42900 0.079316429 42900 0.100840545 21500 0.04033995 21476
0.146284714 42888 0.149955886 42888 0.026233582
42900 0.024299905 42900 0.114031987 21500 0.046276178 21480
0.154646463 42896 0.106182175 42896 0.021090757
42900 0.082013292 42900 0.061320934 21500 0.035849356 21484
0.129819528 42904 0.060835937 42904 0.018836205
42900 0.099293036 42900 0.065653556 21500 0.023702038 21488
0.071169692 42912 0.067740686 42912 0.017693537
42900 0.105363404 42900 0.11795458 21500 0.030173875 21492
0.024095083 42920 0.082007486 42920 0.019017443
42900 0.116992822 42900 0.075309574 21500 0.048158694 21496
0.093208109 42928 0.082842962 42928 0.043656579
42900 0.093792129 42900 0.056585341 21500 0.041718562 21500
0.114764742 42936 0.097619959 42936 0.04311918
42900 0.089488378 42900 0.09990135 21500 0.042514053 21504
0.114963885 42944 0.090905131 42944 0.028772778
43000 0.070016016 43000 0.110040557 21500 0.03001577 21508
0.130096581 42952 0.059853257 42952 0.033786848
43000 0.091278489 43000 0.117709933 21500 0.007065491 21512
0.105870946 42960 0.095376381 42960 0.063247921
43000 0.189781495 43000 0.117555479 21500 0.025460444 21516
0.005554257 42968 0.091736503 42968 0.074226598
43000 0.146376609 43000 0.079976904 21500 0.044823479 21520
0.093811701 42976 0.084382475 42976 0.054411175
43000 0.094405106 43000 0.074803233 21500 0.073391209 21524
0.11191707 42984 0.096425509 42984 0.036832174
43000 0.11244182 43000 0.052155792 21500 0.094011586 21528
0.105942723 42992 0.056778881 42992 0.049614839
43000 0.164611833 43000 0.030408302 21500 0.073780953 21532
0.117438445 43000 0.107715816 43000 0.051797681
43000 0.15126288 43000 0.10362122 21500 0.007896091 21536

0.097393277 43008 0.11267935 43008 0.018869447
43000 0.070157941 43000 0.08487016 21500 0.048152171 21540
0.072683986 43016 0.090235604 43016 0.024123299
43000 0.058955706 43000 0.045042081 21500 0.022929209 21544
0.067206289 43024 0.096720643 43024 0.03160911
43000 0.144303601 43000 0.150198118 21500 0.01765329 21548
0.077044977 43032 0.154851238 43032 0.033411321
43000 0.125771286 43000 0.125019913 21600 0.016960905 21552
0.068454618 43040 0.185081939 43040 0.061244289
43000 0.031804448 43000 0.051229967 21600 0.025600526 21556
0.025390616 43048 0.088012523 43048 0.061524268
43100 0.057956859 43100 0.086411492 21600 0.01926496 21560
0.071487972 43056 0.03687049 43056 0.045058321
43100 0.069685004 43100 0.070835595 21600 0.042400101 21564
0.090899484 43064 0.081684593 43064 0.034948891
43100 0.031292937 43100 0.111016911 21600 0.049851402 21568
0.061252686 43072 0.121719153 43072 0.055569963
43100 0.120438832 43100 0.085306907 21600 0.067314657 21572
0.054078344 43080 0.167404126 43080 0.074133764
43100 0.141391909 43100 0.019697149 21600 0.073114927 21576
0.048581493 43088 0.153819943 43088 0.061554034
43100 0.026452814 43100 0.019335055 21600 0.056741254 21580
0.023670467 43096 0.110178502 43096 0.053063712
43100 0.107708576 43100 0.041491119 21600 0.042905871 21584
0.034345369 43104 0.104449748 43104 0.08532589
43100 0.146363629 43100 0.076499273 21600 0.019763329 21588
0.072874813 43112 0.132284956 43112 0.069535323
43100 0.096241922 43100 0.054598208 21600 0.045847246 21592
0.099598852 43120 0.087800319 43120 0.024437561
43100 0.019259092 43100 0.054475269 21600 0.055909466 21596
0.151355984 43128 0.057917892 43128 0.052245094
43100 0.060260212 43100 0.036434562 21600 0.030575306 21600
0.173891341 43136 0.063353393 43136 0.0616748
43100 0.088541026 43100 0.046990026 21600 0.044932 21604
0.102311395 43144 0.00401854 43144 0.026623806
43200 0.041349984 43200 0.090048314 21600 0.041614596 21608
0.066045788 43152 0.04867504 43152 0.017909461
43200 0.039761519 43200 0.084854786 21600 0.059030281 21612
0.117446238 43160 0.097046235 43160 0.026611531
43200 0.040618932 43200 0.08141221 21600 0.111268557 21616
0.138809526 43168 0.127757405 43168 0.025292855
43200 0.073228584 43200 0.045609246 21600 0.116657255 21620
0.132929898 43176 0.115125979 43176 0.033702272
43200 0.116290008 43200 0.028429913 21600 0.064015716 21624
0.081989412 43184 0.068154091 43184 0.055031349
43200 0.109368608 43200 0.082219696 21600 0.021295546 21628
0.019398471 43192 0.044090168 43192 0.054061602
43200 0.094155526 43200 0.104730629 21600 0.062578038 21632
0.016002718 43200 0.128805798 43200 0.020737438
43200 0.067774832 43200 0.095966105 21600 0.108166321 21636
0.028681548 43208 0.163840886 43208 0.010729009
43200 0.081911319 43200 0.090567926 21600 0.08995652 21640
0.036364691 43216 0.150302964 43216 0.018293375
43200 0.095996882 43200 0.085640568 21600 0.02921715 21644
0.039067643 43224 0.113160037 43224 0.047323923
43200 0.040976058 43200 0.068370267 21600 0.023332554 21648
0.04260839 43232 0.087046894 43232 0.046695994
43200 0.074589232 43200 0.063083251 21700 0.030389103 21652
0.039801151 43240 0.092419985 43240 0.021111871
43200 0.112270645 43200 0.036131158 21700 0.034819463 21656
0.027925938 43248 0.096397365 43248 0.020987869
43300 0.104306426 43300 0.048576654 21700 0.042979464 21660
0.046344951 43256 0.051197178 43256 0.035423404
43300 0.107512322 43300 0.085742373 21700 0.061419778 21664
0.053904423 43264 0.064470478 43264 0.016644441
43300 0.113194779 43300 0.063742518 21700 0.077926656 21668
0.065560453 43272 0.113648152 43272 0.026366726
43300 0.122341284 43300 0.102631224 21700 0.071794391 21672

0.09129072	43280	0.085118896	43280	0.060173683
43300	0.147390718	43300	0.132712419	21700	0.065705026	21676
0.083738538	43288	0.018798706	43288	0.081324521
43300	0.123339865	43300	0.078724603	21700	0.051959662	21680
0.06186348	43296	0.090145157	43296	0.073753487
43300	0.086147215	43300	0.097610595	21700	0.0213402	21684
0.052074378	43304	0.166271391	43304	0.079734164
43300	0.158472729	43300	0.124276834	21700	0.006644275	21688
0.032224107	43312	0.194626511	43312	0.093024341
43300	0.21922549	43300	0.105973348	21700	0.030033529	21692
0.035998601	43320	0.183307711	43320	0.07009157
43300	0.181059615	43300	0.0469064	21700	0.034536271	21696
0.049844522	43328	0.131106251	43328	0.032219421
43300	0.100132289	43300	0.085648695	21700	0.016541664	21700
0.061769402	43336	0.04736835	43336	0.035364446
43300	0.056257733	43300	0.063859348	21700	0.008063987	21704
0.057183479	43344	0.033969467	43344	0.053166437
43400	0.08818091	43400	0.037154478	21700	0.016458374	21708
0.028730179	43352	0.122455749	43352	0.054010572
43400	0.111817273	43400	0.015747603	21700	0.0305094	21712
0.028438768	43360	0.184686971	43360	0.0406402
43400	0.09180334	43400	0.052914693	21700	0.024754167	21716
0.073569732	43368	0.220866438	43368	0.030870942
43400	0.064499909	43400	0.112453454	21700	0.011489049	21720
0.070835791	43376	0.184554345	43376	0.03364947
43400	0.054521286	43400	0.158528491	21700	0.018492798	21724
0.050294395	43384	0.098744873	43384	0.047352551
43400	0.066120891	43400	0.184618635	21700	0.074136253	21728
0.015545809	43392	0.017133882	43392	0.079127589
43400	0.05579909	43400	0.156131966	21700	0.109147753	21732
0.04173748	43400	0.056421817	43400	0.082247738
43400	0.051226667	43400	0.08009046	21700	0.088149223	21736
0.040871899	43408	0.111782181	43408	0.048822298
43400	0.042065964	43400	0.087670655	21700	0.041361185	21740
0.037274607	43416	0.152079243	43416	0.040523944
43400	0.024468825	43400	0.107486412	21700	0.108509754	21744
0.010635143	43424	0.107918306	43424	0.044724016
43400	0.077431418	43400	0.113082366	21700	0.141383178	21748
0.022859395	43432	0.036808833	43432	0.034192657
43400	0.118177035	43400	0.081564125	21800	0.097441225	21752
0.026672264	43440	0.044262724	43440	0.048992089
43400	0.107540036	43400	0.054332111	21800	0.042982803	21756
0.004295667	43448	0.080953374	43448	0.070787159
43500	0.00640367	43500	0.083010855	21800	0.044132568	21760
0.058426551	43456	0.050669474	43456	0.057088932
43500	0.065303379	43500	0.098106626	21800	0.045160599	21764
0.104605584	43464	0.05340299	43464	0.044254532
43500	0.083135674	43500	0.043882203	21800	0.061899002	21768
0.075245174	43472	0.102821396	43472	0.053381362
43500	0.039566672	43500	0.098764787	21800	0.104635357	21772
0.039208171	43480	0.115916511	43480	0.060637391
43500	0.093487375	43500	0.131616908	21800	0.116383511	21776
0.027089775	43488	0.105752595	43488	0.049686747
43500	0.140128439	43500	0.05399291	21800	0.075392643	21780
0.005686064	43496	0.090522175	43496	0.033641711
43500	0.126554325	43500	0.109238994	21800	0.035789588	21784
0.044654622	43504	0.030981075	43504	0.012276098
43500	0.084835592	43500	0.188300124	21800	0.039636914	21788
0.059259495	43512	0.011840136	43512	0.021657659
43500	0.011433859	43500	0.133200941	21800	0.049292019	21792
0.044660897	43520	0.061615858	43520	0.047766152
43500	0.024159921	43500	0.059247017	21800	0.014219196	21796
0.060197006	43528	0.053419044	43528	0.061391969
43500	0.045676319	43500	0.006351943	21800	0.042626707	21800
0.103410472	43536	0.086616805	43536	0.061331899
43500	0.122669473	43500	0.060678329	21800	0.047599991	21804
0.099569719	43544	0.10377005	43544	0.064560838
43600	0.123632854	43600	0.076910568	21800	0.043859531	21808

0.035962989	43552	0.059120936	43552	0.087599488		
43600	0.116961921	43600	0.017824579	21800	0.059950315	21812
0.017652175	43560	0.087395616	43560	0.096889242		
43600	0.129229463	43600	0.008510781	21800	0.045202618	21816
0.039451992	43568	0.193405911	43568	0.076442695		
43600	0.098833261	43600	0.058733505	21800	0.028841841	21820
0.04930791	43576	0.214029613	43576	0.054121279		
43600	0.062500454	43600	0.122027705	21800	0.034470806	21824
0.050119077	43584	0.116669151	43584	0.040589366		
43600	0.032504191	43600	0.150811393	21800	0.040490559	21828
0.051938459	43592	0.054180509	43592	0.034133649		
43600	0.104652121	43600	0.11436201	21800	0.043216842	21832
0.063123669	43600	0.060550319	43600	0.049015773		
43600	0.199855611	43600	0.021608676	21800	0.049016344	21836
0.078632562	43608	0.099884885	43608	0.083121435		
43600	0.230544233	43600	0.101516911	21800	0.042781096	21840
0.080010032	43616	0.125847436	43616	0.081379854		
43600	0.16708154	43600	0.095129864	21800	0.053009997	21844
0.080975297	43624	0.135384616	43624	0.041605003		
43600	0.13655296	43600	0.011299416	21800	0.073228577	21848
0.117192991	43632	0.096236829	43632	0.011068812		
43600	0.147057479	43600	0.092026749	21900	0.043420609	21852
0.132835703	43640	0.099352292	43640	0.018141494		
43600	0.040892603	43600	0.117515898	21900	0.019798319	21856
0.081759972	43648	0.099745834	43648	0.041478466		
43700	0.15159788	43700	0.043397969	21900	0.007468739	21860
0.10659582	43656	0.061552237	43656	0.024119298		
43700	0.126493701	43700	0.088383837	21900	0.016949225	21864
0.138451913	43664	0.044553017	43664	0.021230486		
43700	0.022144906	43700	0.128571338	21900	0.049120521	21868
0.122247002	43672	0.078960969	43672	0.032846576		
43700	0.060630875	43700	0.11987277	21900	0.043010248	21872
0.135248658	43680	0.173548615	43680	0.02547594		
43700	0.083655323	43700	0.106806918	21900	0.043472461	21876
0.096243661	43688	0.176183778	43688	0.020892669		
43700	0.157706673	43700	0.060281138	21900	0.040066621	21880
0.077047211	43696	0.104481835	43696	0.062444378		
43700	0.177897004	43700	0.06600748	21900	0.020772273	21884
0.104881365	43704	0.05612821	43704	0.08126672		
43700	0.166069236	43700	0.105677587	21900	0.031885767	21888
0.094173796	43712	0.010789591	43712	0.046029221		
43700	0.158133058	43700	0.072184812	21900	0.056705994	21892
0.102194688	43720	0.056074685	43720	0.011068336		
43700	0.080441998	43700	0.024489689	21900	0.046674162	21896
0.127017396	43728	0.105346371	43728	0.028811952		
43700	0.077995181	43700	0.039851773	21900	0.018739729	21900
0.113560505	43736	0.10839793	43736	0.024675279		
43700	0.126796381	43700	0.050112441	21900	0.060698818	21904
0.08201423	43744	0.046072266	43744	0.013090456		
43800	0.079036814	43800	0.123789607	21900	0.043052387	21908
0.06257034	43752	0.041767173	43752	0.018728526		
43800	0.030291898	43800	0.120273166	21900	0.032896693	21912
0.032355889	43760	0.065298504	43760	0.046970195		
43800	0.11398099	43800	0.064028725	21900	0.017614777	21916
0.034702749	43768	0.089671987	43768	0.044714394		
43800	0.129681575	43800	0.052660773	21900	0.009934357	21920
0.110079629	43776	0.105945786	43776	0.016421187		
43800	0.14877312	43800	0.044610439	21900	0.009994281	21924
0.131305133	43784	0.08761252	43784	0.034900819		
43800	0.116908603	43800	0.03447467	21900	0.019288451	21928
0.072177434	43792	0.043333443	43792	0.052780768		
43800	0.10314594	43800	0.025162332	21900	0.031162552	21932
0.035020996	43800	0.062664542	43800	0.069322727		
43800	0.122298952	43800	0.067199755	21900	0.077460223	21936
0.060513045	43808	0.153188331	43808	0.069182032		
43800	0.058503076	43800	0.08393771	21900	0.102313788	21940
0.089467365	43816	0.14893357	43816	0.027935605		
43800	0.043422595	43800	0.061103681	21900	0.081817518	21944

0.125435414 43824 0.075896038 43824 0.052917771
43800 0.026442767 43800 0.101920523 21900 0.052132673 21948
0.153562098 43832 0.080379337 43832 0.0667278
43800 0.049005183 43800 0.157427363 22000 0.027565158 21952
0.123403486 43840 0.113935566 43840 0.034940247
43800 0.079719845 43800 0.136990216 22000 0.004203409 21956
0.1186515 43848 0.060920676 43848 0.017972703
43900 0.097084259 43900 0.040626877 22000 0.01499146 21960
0.163535748 43856 0.034315071 43856 0.025102843
43900 0.051454343 43900 0.060820756 22000 0.02750971 21964
0.109244742 43864 0.12783613 43864 0.044435383
43900 0.028204171 43900 0.106482061 22000 0.040193347 21968
0.081579747 43872 0.163699093 43872 0.057213983
43900 0.051814011 43900 0.090129324 22000 0.047373793 21972
0.145202124 43880 0.090896683 43880 0.04444136
43900 0.116718751 43900 0.042735795 22000 0.038887949 21976
0.173911613 43888 0.051077434 43888 0.02319695
43900 0.121107223 43900 0.086075736 22000 0.013844395 21980
0.125060076 43896 0.097995006 43896 0.038149959
43900 0.072687981 43900 0.143470301 22000 0.004867995 21984
0.103363593 43904 0.131362801 43904 0.078211866
43900 0.0409975 43900 0.170353393 22000 0.027890903 21988
0.144420643 43912 0.104773884 43912 0.074231684
43900 0.077802368 43900 0.177837981 22000 0.052332252 21992
0.134459974 43920 0.039684575 43920 0.028154989
43900 0.101253303 43900 0.147157233 22000 0.044120086 21996
0.117162519 43928 0.067394103 43928 0.021465701
43900 0.104529579 43900 0.062035069 22000 0.040833995 22000
0.15791053 43936 0.105087878 43936 0.037547237
43900 0.060000642 43900 0.074508876 22000 0.038507271 22004
0.161325821 43944 0.045090415 43944 0.049422291
44000 0.039805393 44000 0.169576699 22000 0.042865544 22008
0.133251276 43952 0.136309144 43952 0.030649273
44000 0.045925892 44000 0.174685701 22000 0.054958196 22012
0.114322364 43960 0.154431545 43960 0.009913936
44000 0.047926791 44000 0.114071903 22000 0.055965109 22016
0.091900271 43968 0.080823716 43968 0.022743368
44000 0.031629181 44000 0.059912469 22000 0.038525555 22020
0.09786454 43976 0.040841725 43976 0.037536243
44000 0.138449498 44000 0.048898182 22000 0.010582828 22024
0.146080493 43984 0.052949257 43984 0.02857718
44000 0.197328234 44000 0.00537041 22000 0.031595679 22028
0.140249351 43992 0.05590203 43992 0.017057637
44000 0.131143985 44000 0.059472393 22000 0.053907388 22032
0.088609479 44000 0.034951303 44000 0.00844259
44000 0.051090927 44000 0.114268602 22000 0.055581797 22036
0.047028658 44008 0.014673049 44008 0.018118155
44000 0.071586852 44000 0.122301062 22000 0.026646374 22040
0.036849739 44016 0.04298694 44016 0.011341227
44000 0.083324616 44000 0.056299272 22000 0.021342365 22044
0.073379313 44024 0.103201412 44024 0.019708554
44000 0.153145331 44000 0.025124258 22000 0.06391402 22048
0.123883277 44032 0.134276866 44032 0.067376415
44000 0.125250328 44000 0.070725953 22100 0.092847768 22052
0.215630848 44040 0.08279211 44040 0.073823081
44000 0.109014851 44000 0.11163616 22100 0.05743745 22056
0.252480153 44048 0.061714287 44048 0.033715183
44100 0.165834863 44100 0.092337818 22100 0.043463228 22060
0.212600731 44056 0.131834779 44056 0.040599571
44100 0.163596924 44100 0.020571662 22100 0.069299043 22064
0.134875504 44064 0.061322702 44064 0.028033797
44100 0.102728518 44100 0.062342813 22100 0.064138578 22068
0.090689006 44072 0.013098855 44072 0.055368782
44100 0.032429489 44100 0.084440784 22100 0.033738026 22072
0.094890391 44080 0.030843923 44080 0.066216948
44100 0.110446592 44100 0.115222967 22100 0.033908531 22076
0.112891838 44088 0.077272649 44088 0.057815993
44100 0.180231408 44100 0.025451478 22100 0.05269844 22080

0.114725677 44096 0.07506369 44096 0.052652827
44100 0.13396093 44100 0.122993602 22100 0.057562742 22084
0.089443223 44104 0.129258376 44104 0.039007002
44100 0.02336593 44100 0.091143127 22100 0.093514769 22088
0.098838063 44112 0.151379266 44112 0.027958422
44100 0.068835812 44100 0.058301339 22100 0.107865657 22092
0.125207444 44120 0.07617721 44120 0.050454197
44100 0.093200848 44100 0.057946083 22100 0.07473903 22096
0.111063382 44128 0.055832363 44128 0.048986382
44100 0.150534354 44100 0.096291296 22100 0.052260839 22100
0.06473597 44136 0.107197528 44136 0.010856181
44100 0.136163566 44100 0.145413025 22100 0.083952975 22104
0.09587971 44144 0.104628598 44144 0.019553998
44200 0.048898848 44200 0.127339371 22100 0.091912232 22108
0.128053245 44152 0.088343302 44152 0.040475577
44200 0.072871357 44200 0.152099689 22100 0.057812729 22112
0.155451256 44160 0.034038447 44160 0.045701418
44200 0.043608063 44200 0.229298326 22100 0.040668772 22116
0.139825003 44168 0.037537604 44168 0.007827082
44200 0.029342829 44200 0.257660256 22100 0.079674719 22120
0.113366274 44176 0.034132401 44176 0.044366389
44200 0.058134068 44200 0.159304371 22100 0.076415337 22124
0.13716433 44184 0.02780063 44184 0.049932001
44200 0.056530993 44200 0.063902109 22100 0.040288913 22128
0.171557448 44192 0.050053171 44192 0.028761249
44200 0.110336798 44200 0.095079588 22100 0.050878181 22132
0.203274278 44200 0.0920893 44200 0.018974875
44200 0.183715209 44200 0.077673038 22100 0.024998379 22136
0.179676645 44208 0.137415176 44208 0.021431511
44200 0.189477083 44200 0.057697762 22100 0.021390491 22140
0.162408411 44216 0.109740126 44216 0.038159054
44200 0.116169897 44200 0.103846141 22100 0.053698306 22144
0.163730685 44224 0.09980394 44224 0.008143789
44200 0.118631004 44200 0.056994497 22100 0.064565262 22148
0.112971458 44232 0.14305026 44232 0.020366713
44200 0.138657939 44200 0.098831901 22200 0.047940699 22152
0.07218204 44240 0.130523273 44240 0.005132349
44200 0.04191629 44200 0.087408094 22200 0.033165194 22156
0.039284234 44248 0.131685738 44248 0.023269589
44300 0.115866096 44300 0.053888678 22200 0.048688813 22160
0.055261418 44256 0.145086844 44256 0.027117199
44300 0.110742934 44300 0.10166 22200 0.031907242 22164
0.124655213 44264 0.103534207 44264 0.039882991
44300 0.043889326 44300 0.083945706 22200 0.036660025 22168
0.170161468 44272 0.049354356 44272 0.058167381
44300 0.030392112 44300 0.016193719 22200 0.034524041 22172
0.129590015 44280 0.031735497 44280 0.051689462
44300 0.041132633 44300 0.039747185 22200 0.028711027 22176
0.076790646 44288 0.11478064 44288 0.028384791
44300 0.072601928 44300 0.011824724 22200 0.042581589 22180
0.06533743 44296 0.181117765 44296 0.020172558
44300 0.191574378 44300 0.072450945 22200 0.043331464 22184
0.067596651 44304 0.177699534 44304 0.026624959
44300 0.18890317 44300 0.099363475 22200 0.033153247 22188
0.117950222 44312 0.144404913 44312 0.044129221
44300 0.158665324 44300 0.08033809 22200 0.027584898 22192
0.144392965 44320 0.077082164 44320 0.070730072
44300 0.110099645 44300 0.079138059 22200 0.045352572 22196
0.094995536 44328 0.144532708 44328 0.069309252
44300 0.024778763 44300 0.08314904 22200 0.041403142 22200
0.058237405 44336 0.169014224 44336 0.024698018
44300 0.070202354 44300 0.070659313 22200 0.03405313 22204
0.124838334 44344 0.092920702 44344 0.041703112
44400 0.047045043 44400 0.02238689 22200 0.022120623 22208
0.12366187 44352 0.115315175 44352 0.084115149
44400 0.050845301 44400 0.079068159 22200 0.065052074 22212
0.070997056 44360 0.192012929 44360 0.084302337
44400 0.05931751 44400 0.152648078 22200 0.07649562 22216

0.088141445 44368 0.134534435 44368 0.080159574
44400 0.114667841 44400 0.11429756 22200 0.052505802 22220
0.081160477 44376 0.01657123 44376 0.091468428
44400 0.140786724 44400 0.025582105 22200 0.031359734 22224
0.03487158 44384 0.076686381 44384 0.075909571
44400 0.074637348 44400 0.065391498 22200 0.028085138 22228
0.133558176 44392 0.083411811 44392 0.046533503
44400 0.018777533 44400 0.171253545 22200 0.018478206 22232
0.18144444 44400 0.020333397 44400 0.038050148
44400 0.024489082 44400 0.224206495 22200 0.012423558 22236
0.138694304 44408 0.056507033 44408 0.032112643
44400 0.028795041 44400 0.10028109 22200 0.018083953 22240
0.109021326 44416 0.067870831 44416 0.021637736
44400 0.084800493 44400 0.034274424 22200 0.034182245 22244
0.149644009 44424 0.125620849 44424 0.02010746
44400 0.097604716 44400 0.077266886 22200 0.023999017 22248
0.124751372 44432 0.158848038 44432 0.041056803
44400 0.052702264 44400 0.099548961 22300 0.035025885 22252
0.035641428 44440 0.115074356 44440 0.017970047
44400 0.043182452 44400 0.060973824 22300 0.056850502 22256
0.064531771 44448 0.079803525 44448 0.025283063
44500 0.100162382 44500 0.081643833 22300 0.078912635 22260
0.110953042 44456 0.071832088 44456 0.051532636
44500 0.094122101 44500 0.07258587 22300 0.090612462 22264
0.101943289 44464 0.08723815 44464 0.081964216
44500 0.098610268 44500 0.058724472 22300 0.067152956 22268
0.084349573 44472 0.07311895 44472 0.082945233
44500 0.096072203 44500 0.042509149 22300 0.046994323 22272
0.080813705 44480 0.069657152 44480 0.052341176
44500 0.011500834 44500 0.015544791 22300 0.053429874 22276
0.071198345 44488 0.071363844 44488 0.027487395
44500 0.085944848 44500 0.027528417 22300 0.018649171 22280
0.03492496 44496 0.081903367 44496 0.019336472
44500 0.082166509 44500 0.041884225 22300 0.040382245 22284
0.042117703 44504 0.0916235 44504 0.01529849
44500 0.036535974 44500 0.055312357 22300 0.067658039 22288
0.082128798 44512 0.112751353 44512 0.004276812
44500 0.085712556 44500 0.058279216 22300 0.061683466 22292
0.106555817 44520 0.1680274 44520 0.028063416
44500 0.140082819 44500 0.026325339 22300 0.058568439 22296
0.095928648 44528 0.190184103 44528 0.028728959
44500 0.119118122 44500 0.056250108 22300 0.07719038 22300
0.05034358 44536 0.161121745 44536 0.01345073
44500 0.07437943 44500 0.052055093 22300 0.087424858 22304
0.066789107 44544 0.114663264 44544 0.039138704
44600 0.090746507 44600 0.018379236 22300 0.063339809 22308
0.106870386 44552 0.065194763 44552 0.053758729
44600 0.052798001 44600 0.030948708 22300 0.044701381 22312
0.123318838 44560 0.072857212 44560 0.046461672
44600 0.073835225 44600 0.090965747 22300 0.03553959 22316
0.093107919 44568 0.148565436 44568 0.023452167
44600 0.086177141 44600 0.155897593 22300 0.038700699 22320
0.037866572 44576 0.116126292 44576 0.023414194
44600 0.059590278 44600 0.141661309 22300 0.061077808 22324
0.041528969 44584 0.045190285 44584 0.045554243
44600 0.098298762 44600 0.089564994 22300 0.042666987 22328
0.097626937 44592 0.039197028 44592 0.049826813
44600 0.143521305 44600 0.171443899 22300 0.02524005 22332
0.135996655 44600 0.039596445 44600 0.052074898
44600 0.130857646 44600 0.188230654 22300 0.022910199 22336
0.083011684 44608 0.112684444 44608 0.071035873
44600 0.063920335 44600 0.119692275 22300 0.02218553 22340
0.048897935 44616 0.16394908 44616 0.071377595
44600 0.005940746 44600 0.069903115 22300 0.031780612 22344
0.078910824 44624 0.116011834 44624 0.028888051
44600 0.018904661 44600 0.091256188 22300 0.034951179 22348
0.07386144 44632 0.078318757 44632 0.032672837
44600 0.033931414 44600 0.047149817 22400 0.00907659 22352

0.061528852 44640 0.054953347 44640 0.055296056
44600 0.083142229 44600 0.057111582 22400 0.041821193 22356
0.058520996 44648 0.039574083 44648 0.049285813
44700 0.113104114 44700 0.117582873 22400 0.013347022 22360
0.072196519 44656 0.086079061 44656 0.038379207
44700 0.108827931 44700 0.149188971 22400 0.021327529 22364
0.093639064 44664 0.06519704 44664 0.048644517
44700 0.101728023 44700 0.10268436 22400 0.05742388 22368
0.127221516 44672 0.018992761 44672 0.087050692
44700 0.059578255 44700 0.022867063 22400 0.09822291 22372
0.137245064 44680 0.027209055 44680 0.077164426
44700 0.031897071 44700 0.060772567 22400 0.095548137 22376
0.123894119 44688 0.100654688 44688 0.045851128
44700 0.078112978 44700 0.0766101 22400 0.058713857 22380
0.083321851 44696 0.084460553 44696 0.077597688
44700 0.062701118 44700 0.048922833 22400 0.044379653 22384
0.077609868 44704 0.110352587 44704 0.061700797
44700 0.037646114 44700 0.030349822 22400 0.018925543 22388
0.062776933 44712 0.150702966 44712 0.04294483
44700 0.060263123 44700 0.058112444 22400 0.03517517 22392
0.020448784 44720 0.100167148 44720 0.009261424
44700 0.076117809 44700 0.096964534 22400 0.081040314 22396
0.034233413 44728 0.03735174 44728 0.050706072
44700 0.056334837 44700 0.101384758 22400 0.068163099 22400
0.07234903 44736 0.047309397 44736 0.04969829
44700 0.040839783 44700 0.103633982 22400 0.01485459 22404
0.077165722 44744 0.049917075 44744 0.025807351
44800 0.077284785 44800 0.11317747 22400 0.037118363 22408
0.059387363 44752 0.061764848 44752 0.060572482
44800 0.033983954 44800 0.062479667 22400 0.034892357 22412
0.058303482 44760 0.122108191 44760 0.068971436
44800 0.026422547 44800 0.04663405 22400 0.031640106 22416
0.050795854 44768 0.139966593 44768 0.068586953
44800 0.05323744 44800 0.104335646 22400 0.036636866 22420
0.024015099 44776 0.083619976 44776 0.022620425
44800 0.101291887 44800 0.113961083 22400 0.031056094 22424
0.027734886 44784 0.09119296 44784 0.043698667
44800 0.077822639 44800 0.076894568 22400 0.048481721 22428
0.060644106 44792 0.053976128 44792 0.055220688
44800 0.046768506 44800 0.113225658 22400 0.042749409 22432
0.090655121 44800 0.071467977 44800 0.063048916
44800 0.019879177 44800 0.06843821 22400 0.021925471 22436
0.074584612 44808 0.100893201 44808 0.038458322
44800 0.043489716 44800 0.038545968 22400 0.014781444 22440
0.038055601 44816 0.046558467 44816 0.070741196
44800 0.083184721 44800 0.054817738 22400 0.023751545 22444
0.019870351 44824 0.073056006 44824 0.089879875
44800 0.101772435 44800 0.048904927 22400 0.031316453 22448
0.083680716 44832 0.117718446 44832 0.059976559
44800 0.087425098 44800 0.04994337 22500 0.049105551 22452
0.123470105 44840 0.068484362 44840 0.026489446
44800 0.05692364 44800 0.011663668 22500 0.054420209 22456
0.122714133 44848 0.043498181 44848 0.038982289
44900 0.079347403 44900 0.047269194 22500 0.039480216 22460
0.111649941 44856 0.084645653 44856 0.051488187
44900 0.053957017 44900 0.005727399 22500 0.016907201 22464
0.07200538 44864 0.050221403 44864 0.026591195
44900 0.00983928 44900 0.01707509 22500 0.019922627 22468
0.01071491 44872 0.091819486 44872 0.019258296
44900 0.062091596 44900 0.069984788 22500 0.038199552 22472
0.05018805 44880 0.128970525 44880 0.020719992
44900 0.085668158 44900 0.138203934 22500 0.026003696 22476
0.091492759 44888 0.070640934 44888 0.018683226
44900 0.085708009 44900 0.0992594 22500 0.03251779 22480
0.069332898 44896 0.080954313 44896 0.03105303
44900 0.131577865 44900 0.06789904 22500 0.042220807 22484
0.047118803 44904 0.040122541 44904 0.022482673
44900 0.124293903 44900 0.116828116 22500 0.02081266 22488

0.072837895 44912 0.024073375 44912 0.028908788
44900 0.086552551 44900 0.134804985 22500 0.006969862 22492
0.110594599 44920 0.012423169 44920 0.019217723
44900 0.045195273 44900 0.155713453 22500 0.009821127 22496
0.093958734 44928 0.082806982 44928 0.00967342
44900 0.031176402 44900 0.125637293 22500 0.038837894 22500
0.040766758 44936 0.08734734 44936 0.009902773
44900 0.016560658 44900 0.068305213 22500 0.054751345 22504
0.054152308 44944 0.055617195 44944 0.02323718
45000 0.125024991 45000 0.078425313 22500 0.048659553 22508
0.087807486 44952 0.078567384 44952 0.052069176
45000 0.198007925 45000 0.106812586 22500 0.062892686 22512
0.090900132 44960 0.134438931 44960 0.027955128
45000 0.206296434 45000 0.127629043 22500 0.070217597 22516
0.060958497 44968 0.109960864 44968 0.027650116
45000 0.154316819 45000 0.091858856 22500 0.047190864 22520
0.028064818 44976 0.059713777 44976 0.05566443
45000 0.064976048 45000 0.069836904 22500 0.045323803 22524
0.061888437 44984 0.063585721 44984 0.048604587
45000 0.049469101 45000 0.136981908 22500 0.021060267 22528
0.057828678 44992 0.102541941 44992 0.031981996
45000 0.130829169 45000 0.14337711 22500 0.039117786 22532
0.044022439 45000 0.095445954 45000 0.078498582
45000 0.177362658 45000 0.067303008 22500 0.031478183 22536
0.044748642 45008 0.069279115 45008 0.053861029
45000 0.128160653 45000 0.133146386 22500 0.030182953 22540
0.027512209 45016 0.090332207 45016 0.018210869
45000 0.036778249 45000 0.202144293 22500 0.043343414 22544
0.006462541 45024 0.059932459 45024 0.061452651
45000 0.071680297 45000 0.136642659 22500 0.06137001 22548
0.043463795 45032 0.086354092 45032 0.033223751
45000 0.105424217 45000 0.039772247 22600 0.090190755 22552
0.057897978 45040 0.085103951 45040 0.030149397
45000 0.161104589 45000 0.062828905 22600 0.069785223 22556
0.069475478 45048 0.042995187 45048 0.058044105
45100 0.119052813 45100 0.101414378 22600 0.019732239 22560
0.089255751 45056 0.026700416 45056 0.04248981
45100 0.053163931 45100 0.105277322 22600 0.052754858 22564
0.085354332 45064 0.07113193 45064 0.019410858
45100 0.133305046 45100 0.06021349 22600 0.029282986 22568
0.069864349 45072 0.126800849 45072 0.019050109
45100 0.052029918 45100 0.022017734 22600 0.015383434 22572
0.067998117 45080 0.140860269 45080 0.027128948
45100 0.208275276 45100 0.038026872 22600 0.028813283 22576
0.03039567 45088 0.110767127 45088 0.029208139
45100 0.256080123 45100 0.035499008 22600 0.030296034 22580
0.0407683 45096 0.032878914 45096 0.022577935
45100 0.156917449 45100 0.12596653 22600 0.026383705 22584
0.071979979 45104 0.060388851 45104 0.046672296
45100 0.08259774 45100 0.216246394 22600 0.023655959 22588
0.04163733 45112 0.077131765 45112 0.012845788
45100 0.060713657 45100 0.180081246 22600 0.016871009 22592
0.014103453 45120 0.007136896 45120 0.04616443
45100 0.036000267 45100 0.089926798 22600 0.019736564 22596
0.022753964 45128 0.091153801 45128 0.071692964
45100 0.017165383 45100 0.152610606 22600 0.030781259 22600
0.030046265 45136 0.130462766 45136 0.034702935
45100 0.024938443 45100 0.157858944 22600 0.027864126 22604
0.041463401 45144 0.083899337 45144 0.047368743
45200 0.060604289 45200 0.075818003 22600 0.034339751 22608
0.05355342 45152 0.054253698 45152 0.082401406
45200 0.018651204 45200 0.023640352 22600 0.03990018 22612
0.043420703 45160 0.094713025 45160 0.0685497
45200 0.116196716 45200 0.051683364 22600 0.038170689 22616
0.075386888 45168 0.114130766 45168 0.048196809
45200 0.168820639 45200 0.070703209 22600 0.029979858 22620
0.129432476 45176 0.110152301 45176 0.034088113
45200 0.108021173 45200 0.040071172 22600 0.026550571 22624

0.131508205 45184 0.113959752 45184 0.038799561
45200 0.085701315 45200 0.135513838 22600 0.008214636 22628
0.07382976 45192 0.086105327 45192 0.050108785
45200 0.136169881 45200 0.161287288 22600 0.046286881 22632
0.008202829 45200 0.009398482 45200 0.016696506
45200 0.103733022 45200 0.098975717 22600 0.078411671 22636
0.064547625 45208 0.045483041 45208 0.057689689
45200 0.039107919 45200 0.013732007 22600 0.036758487 22640
0.089540365 45216 0.020791425 45216 0.093206836
45200 0.056006426 45200 0.04474504 22600 0.053689128 22644
0.085995634 45224 0.085060448 45224 0.078862431
45200 0.103522209 45200 0.078669982 22600 0.086153552 22648
0.063802174 45232 0.059503364 45232 0.03584932
45200 0.129278575 45200 0.087155982 22700 0.058734306 22652
0.058820802 45240 0.031405267 45240 0.024543244
45200 0.104910061 45200 0.06256847 22700 0.043694021 22656
0.066108303 45248 0.008381071 45248 0.039887687
45300 0.06369756 45300 0.014743782 22700 0.078442317 22660
0.052474454 45256 0.014504914 45256 0.028055525
45300 0.084042746 45300 0.072151401 22700 0.069829883 22664
0.075393546 45264 0.016462047 45264 0.038934115
45300 0.118137919 45300 0.099101773 22700 0.004801141 22668
0.101512676 45272 0.055021239 45272 0.058449845
45300 0.121252124 45300 0.083524654 22700 0.043661239 22672
0.095760435 45280 0.107478736 45280 0.069695831
45300 0.11724775 45300 0.029422163 22700 0.028201252 22676
0.065904947 45288 0.113019276 45288 0.053337648
45300 0.094652023 45300 0.038239643 22700 0.018326175 22680
0.040277835 45296 0.130394765 45296 0.032351531
45300 0.061289167 45300 0.066544802 22700 0.041665317 22684
0.02345662 45304 0.066792396 45304 0.031583109
45300 0.052251635 45300 0.045628527 22700 0.07937842 22688
0.012883693 45312 0.026748337 45312 0.037578164
45300 0.034113105 45300 0.117033247 22700 0.074617601 22692
0.025654521 45320 0.032066222 45320 0.025837149
45300 0.040480169 45300 0.093828778 22700 0.044624776 22696
0.036334597 45328 0.091841175 45328 0.027131053
45300 0.050343169 45300 0.191286599 22700 0.040512023 22700
0.039060236 45336 0.153081331 45336 0.028235849
45300 0.072321949 45300 0.262553541 22700 0.06678277 22704
0.032718359 45344 0.166322178 45344 0.033013777
45400 0.147739731 45400 0.203572606 22700 0.073197596 22708
0.032556549 45352 0.15063236 45352 0.049018199
45400 0.152659079 45400 0.130286383 22700 0.082275932 22712
0.088315865 45360 0.136151838 45360 0.032041491
45400 0.119886645 45400 0.025481224 22700 0.07226949 22716
0.128875661 45368 0.112252586 45368 0.029035944
45400 0.056843295 45400 0.052101841 22700 0.014550555 22720
0.112193637 45376 0.056511402 45376 0.014761843
45400 0.04383667 45400 0.069605907 22700 0.011768954 22724
0.051374409 45384 0.059148963 45384 0.042573032
45400 0.06180643 45400 0.056312554 22700 0.02801827 22728
0.01061713 45392 0.040317238 45392 0.065390465
45400 0.034046181 45400 0.07635849 22700 0.063289437 22732
0.02079034 45400 0.054324872 45400 0.022189744
45400 0.102107573 45400 0.118247524 22700 0.079455713 22736
0.007480284 45408 0.033974677 45408 0.01079668
45400 0.197070753 45400 0.126444545 22700 0.096848336 22740
0.017185081 45416 0.045116722 45416 0.014306421
45400 0.174989168 45400 0.116700911 22700 0.101543847 22744
0.054167591 45424 0.023250532 45424 0.045454002
45400 0.108400112 45400 0.167525911 22700 0.072972289 22748
0.077328557 45432 0.01473915 45432 0.040062885
45400 0.084133935 45400 0.140757911 22800 0.030746011 22752
0.060510851 45440 0.022109571 45440 0.06391768
45400 0.071846189 45400 0.07024347 22800 0.019847928 22756
0.036080124 45448 0.037202739 45448 0.094984964
45500 0.097246186 45500 0.089313762 22800 0.024799456 22760

0.039884239 45456 0.03226777 45456 0.091672824
45500 0.094623611 45500 0.116592593 22800 0.020073672 22764
0.106025647 45464 0.045740348 45464 0.059849066
45500 0.04231745 45500 0.058340185 22800 0.021418375 22768
0.083078077 45472 0.0253286 45472 0.037775484
45500 0.055732235 45500 0.047936137 22800 0.050057217 22772
0.020847201 45480 0.056022829 45480 0.077569079
45500 0.074940857 45500 0.100830039 22800 0.019319621 22776
0.032023203 45488 0.074787451 45488 0.062015366
45500 0.021071486 45500 0.053957865 22800 0.026768556 22780
0.074704803 45496 0.095862401 45496 0.018250268
45500 0.131662876 45500 0.047442118 22800 0.010775164 22784
0.085813073 45504 0.15483232 45504 0.023143181
45500 0.138191899 45500 0.114060837 22800 0.044507855 22788
0.07637035 45512 0.17698828 45512 0.029088247
45500 0.030424151 45500 0.131381879 22800 0.042287753 22792
0.066354056 45520 0.141253433 45520 0.025758343
45500 0.053359207 45500 0.122129102 22800 0.030539199 22796
0.046315417 45528 0.132960384 45528 0.056497393
45500 0.025639436 45500 0.085068736 22800 0.039476141 22800
0.050758103 45536 0.08423617 45536 0.07394539
45500 0.012432155 45500 0.048364058 22800 0.043358032 22804
0.041473373 45544 0.072538984 45544 0.052005242
45600 0.061947809 45600 0.031124513 22800 0.050206818 22808
0.044088927 45552 0.099383055 45552 0.057284549
45600 0.090082518 45600 0.043873104 22800 0.060883409 22812
0.035084719 45560 0.030391761 45560 0.080039477
45600 0.077039207 45600 0.05235627 22800 0.057106925 22816
0.019781617 45568 0.119074743 45568 0.076662305
45600 0.093968432 45600 0.016149283 22800 0.032068518 22820
0.054143777 45576 0.134292015 45576 0.056902445
45600 0.096796699 45600 0.08065853 22800 0.010382957 22824
0.048747123 45584 0.053425458 45584 0.041750453
45600 0.066390159 45600 0.063928463 22800 0.048548034 22828
0.060813996 45592 0.061736275 45592 0.045653418
45600 0.038826511 45600 0.040631298 22800 0.108028595 22832
0.056350746 45600 0.069852511 45600 0.073591589
45600 0.106697167 45600 0.097398515 22800 0.074759744 22836
0.04413259 45608 0.064867629 45608 0.082153034
45600 0.125500883 45600 0.115158706 22800 0.01905574 22840
0.010010483 45616 0.063379514 45616 0.03235885
45600 0.099880839 45600 0.096589902 22800 0.045856719 22844
0.059438651 45624 0.049583527 45624 0.031062737
45600 0.075578289 45600 0.027277682 22800 0.014633114 22848
0.066700843 45632 0.033484768 45632 0.036986308
45600 0.068258873 45600 0.123978694 22900 0.025124718 22852
0.030556723 45640 0.067103174 45640 0.061028793
45600 0.071144779 45600 0.161095959 22900 0.036363843 22856
0.021587111 45648 0.14051779 45648 0.070374306
45700 0.078506026 45700 0.121817917 22900 0.02999662 22860
0.016950307 45656 0.127067469 45656 0.05555986
45700 0.092812625 45700 0.06860018 22900 0.04780438 22864
0.016458531 45664 0.048581806 45664 0.034376342
45700 0.08785681 45700 0.092466471 22900 0.065153748 22868
0.040111376 45672 0.041588781 45672 0.027186206
45700 0.070445931 45700 0.125393941 22900 0.04133007 22872
0.010423926 45680 0.089776957 45680 0.050994288
45700 0.105127372 45700 0.114939641 22900 0.006511998 22876
0.023155164 45688 0.092111921 45688 0.051285995
45700 0.102891594 45700 0.07075879 22900 0.024434219 22880
0.045563542 45696 0.051471045 45696 0.040509574
45700 0.046451147 45700 0.02879057 22900 0.058010708 22884
0.068952344 45704 0.082895313 45704 0.063525498
45700 0.030612591 45700 0.018901621 22900 0.057011243 22888
0.065962391 45712 0.085665299 45712 0.028552138
45700 0.06904592 45700 0.02282972 22900 0.038137398 22892
0.037620219 45720 0.03568146 45720 0.005799029
45700 0.107823813 45700 0.09808832 22900 0.014827875 22896

0.04453753 45728 0.025675876 45728 0.006029119
45700 0.153111993 45700 0.157088653 22900 0.03529555 22900
0.049080667 45736 0.080404636 45736 0.027871156
45700 0.116663425 45700 0.155458213 22900 0.011540524 22904
0.026058815 45744 0.123669917 45744 0.045128585
45800 0.030739666 45800 0.087009867 22900 0.030687043 22908
0.052650823 45752 0.139105701 45752 0.034543962
45800 0.051476945 45800 0.075107884 22900 0.026586407 22912
0.041165327 45760 0.132562855 45760 0.018726823
45800 0.017676926 45800 0.136918111 22900 0.080564416 22916
0.025512971 45768 0.094292409 45768 0.026998985
45800 0.07056594 45800 0.166311875 22900 0.083687199 22920
0.108277403 45776 0.034427711 45776 0.045958903
45800 0.115001902 45800 0.114597955 22900 0.06209172 22924
0.083048384 45784 0.06767951 45784 0.061646511
45800 0.089877671 45800 0.013778041 22900 0.031056821 22928
0.05733376 45792 0.131823399 45792 0.060665774
45800 0.077804391 45800 0.106136373 22900 0.076778866 22932
0.012016743 45800 0.152661189 45800 0.043694585
45800 0.082958381 45800 0.131579771 22900 0.067355148 22936
0.113474001 45808 0.090289555 45808 0.029385004
45800 0.093205519 45800 0.132101603 22900 0.003460067 22940
0.128160158 45816 0.101052123 45816 0.031570446
45800 0.111404617 45800 0.106629435 22900 0.035685047 22944
0.06783841 45824 0.050966555 45824 0.020521895
45800 0.159920398 45800 0.040821687 22900 0.04762859 22948
0.048695118 45832 0.097948701 45832 0.025649157
45800 0.201235308 45800 0.026335609 23000 0.056756457 22952
0.045668185 45840 0.160160969 45840 0.031126263
45800 0.173871479 45800 0.038821905 23000 0.059524195 22956
0.035401146 45848 0.10952718 45848 0.008389108
45900 0.144800811 45900 0.031918349 23000 0.042061445 22960
0.0610438 45856 0.062331921 45856 0.033953016
45900 0.115309878 45900 0.101491482 23000 0.037124893 22964
0.054703167 45864 0.053685442 45864 0.039890158
45900 0.037754886 45900 0.128718559 23000 0.034914938 22968
0.066095156 45872 0.047840524 45872 0.031579159
45900 0.071831702 45900 0.096273339 23000 0.022849232 22972
0.06653116 45880 0.043075284 45880 0.048877137
45900 0.139384225 45900 0.034923851 23000 0.018694576 22976
0.073226896 45888 0.037308542 45888 0.026908892
45900 0.13958069 45900 0.033659591 23000 0.029976267 22980
0.043475422 45896 0.025006506 45896 0.030562776
45900 0.137146446 45900 0.06837718 23000 0.025726653 22984
0.040012397 45904 0.048652557 45904 0.030683921
45900 0.147989689 45900 0.04118427 23000 0.022254671 22988
0.050716211 45912 0.082558116 45912 0.025470501
45900 0.073219853 45900 0.0408407 23000 0.055697659 22992
0.007534779 45920 0.051910214 45920 0.062636005
45900 0.071856463 45900 0.032587144 23000 0.043467488 22996
0.056549594 45928 0.065421664 45928 0.070872666
45900 0.136475341 45900 0.08537402 23000 0.017153066 23000
0.043283781 45936 0.075320699 45936 0.047167254
45900 0.114484727 45900 0.087249006 23000 0.010459808 23004
0.054154552 45944 0.038825932 45944 0.03022722
46000 0.047391532 46000 0.069291593 23000 0.036267025 23008
0.03564106 45952 0.066821587 45952 0.037356058
46000 0.061145904 46000 0.069310256 23000 0.049079081 23012
0.046043555 45960 0.078873178 45960 0.038878203
46000 0.139152209 46000 0.028341745 23000 0.041533011 23016
0.081813625 45968 0.114052171 45968 0.014035697
46000 0.148424108 46000 0.04556654 23000 0.024029732 23020
0.114046648 45976 0.042086336 45976 0.053966625
46000 0.081320941 46000 0.111367779 23000 0.046583387 23024
0.063699787 45984 0.036705384 45984 0.072458905
46000 0.066434594 46000 0.124937433 23000 0.038696409 23028
0.030093004 45992 0.033514061 45992 0.035601221
46000 0.164948855 46000 0.076127086 23000 0.044660959 23032

0.04117631 46000 0.079322737 46000 0.026495867
46000 0.115466399 46000 0.027325094 23000 0.050659295 23036
0.061103594 46008 0.151777844 46008 0.031225358
46000 0.010762656 46000 0.09418602 23000 0.026005751 23040
0.079333397 46016 0.105886844 46016 0.012240753
46000 0.093784562 46000 0.130836474 23000 0.023341745 23044
0.068512243 46024 0.075038144 46024 0.015456884
46000 0.160525248 46000 0.101488273 23000 0.033880966 23048
0.046178211 46032 0.154176101 46032 0.023381394
46000 0.14120125 46000 0.04427238 23100 0.045105266 23052
0.066091699 46040 0.095101786 46040 0.028832334
46000 0.109419365 46000 0.068075264 23100 0.023511057 23056
0.075174547 46048 0.043259512 46048 0.051389325
46100 0.123501231 46100 0.073497402 23100 0.021245302 23060
0.063985564 46056 0.091784619 46056 0.045465877
46100 0.089184629 46100 0.054177814 23100 0.016060227 23064
0.047094763 46064 0.077291501 46064 0.070764872
46100 0.052709002 46100 0.109355904 23100 0.039319963 23068
0.033396565 46072 0.061151448 46072 0.083379287
46100 0.075263823 46100 0.205751916 23100 0.053762396 23072
0.023085684 46080 0.057818092 46080 0.069665337
46100 0.062470521 46100 0.202813346 23100 0.041412739 23076
0.012291434 46088 0.040492425 46088 0.069527108
46100 0.075151227 46100 0.12515529 23100 0.050557363 23080
0.066831883 46096 0.052310825 46096 0.031864835
46100 0.072923067 46100 0.043506461 23100 0.036708618 23084
0.065302906 46104 0.154099559 46104 0.027019016
46100 0.181224445 46100 0.101291691 23100 0.031559583 23088
0.042429412 46112 0.176023415 46112 0.050240706
46100 0.147859828 46100 0.212607193 23100 0.04085551 23092
0.040263862 46120 0.091941423 46120 0.035155812
46100 0.024714494 46100 0.171161403 23100 0.035685727 23096
0.051630483 46128 0.00993772 46128 0.00253863
46100 0.122337398 46100 0.083992556 23100 0.030279143 23100
0.056619734 46136 0.023655919 46136 0.043912551
46100 0.135252674 46100 0.145147031 23100 0.058329148 23104
0.068185495 46144 0.029066368 46144 0.064114785
46200 0.062735242 46200 0.156638795 23100 0.044414708 23108
0.055408582 46152 0.080026017 46152 0.07851587
46200 0.189967163 46200 0.149572385 23100 0.027923605 23112
0.071635783 46160 0.061596482 46160 0.085791849
46200 0.185498444 46200 0.138850504 23100 0.075704636 23116
0.058154441 46168 0.072522191 46168 0.062667787
46200 0.082623788 46200 0.052539541 23100 0.07259383 23120
0.057968362 46176 0.177984926 46176 0.010076988
46200 0.047413345 46200 0.024190098 23100 0.059748909 23124
0.069617207 46184 0.181027354 46184 0.043530705
46200 0.133817478 46200 0.039296119 23100 0.048377384 23128
0.051844887 46192 0.078562749 46192 0.044689681
46200 0.188436475 46200 0.111724774 23100 0.041369913 23132
0.025334708 46200 0.095232688 46200 0.024016303
46200 0.13774012 46200 0.110830057 23100 0.042437685 23136
0.093137038 46208 0.098776349 46208 0.026809143
46200 0.066674707 46200 0.180585863 23100 0.033386932 23140
0.104609033 46216 0.05313279 46216 0.032662305
46200 0.072311283 46200 0.207733741 23100 0.012676306 23144
0.068851805 46224 0.028768825 46224 0.02000211
46200 0.054041117 46200 0.172282963 23100 0.052204207 23148
0.049229311 46232 0.049867795 46232 0.013826804
46200 0.08433374 46200 0.140730845 23200 0.048098915 23152
0.076151824 46240 0.079680467 46240 0.035565543
46200 0.093247305 46200 0.119091666 23200 0.014923461 23156
0.077258803 46248 0.038268125 46248 0.085772248
46300 0.059566919 46300 0.130388391 23200 0.0090859 23160
0.045943016 46256 0.078448553 46256 0.096242955
46300 0.106134859 46300 0.103723643 23200 0.038367001 23164
0.035135294 46264 0.141592828 46264 0.068091904
46300 0.115274517 46300 0.182916993 23200 0.080111953 23168

0.099538891 46272 0.172835615 46272 0.027726592
46300 0.106500484 46300 0.198921145 23200 0.08725415 23172
0.108776076 46280 0.093967668 46280 0.016196442
46300 0.119456287 46300 0.168087413 23200 0.056909128 23176
0.08603419 46288 0.095643154 46288 0.033831086
46300 0.092509494 46300 0.130332934 23200 0.056742007 23180
0.104182778 46296 0.080656544 46296 0.015438634
46300 0.032406897 46300 0.038341179 23200 0.052973966 23184
0.084974912 46304 0.033531294 46304 0.014672013
46300 0.032257711 46300 0.087172535 23200 0.042992971 23188
0.068673595 46312 0.09794427 46312 0.020643429
46300 0.073626587 46300 0.088790839 23200 0.031471336 23192
0.116236464 46320 0.059949689 46320 0.043665055
46300 0.075106138 46300 0.159234274 23200 0.014191589 23196
0.10024105 46328 0.044621593 46328 0.062184685
46300 0.034578396 46300 0.16908432 23200 0.025242987 23200
0.015755557 46336 0.128318031 46336 0.064981534
46300 0.00646547 46300 0.112371759 23200 0.047691839 23204
0.074104028 46344 0.169563835 46344 0.043751679
46400 0.023341567 46400 0.082140869 23200 0.017353124 23208
0.131728316 46352 0.152335546 46352 0.031154243
46400 0.014522759 46400 0.12746657 23200 0.04660834 23212
0.169350009 46360 0.110569468 46360 0.025904485
46400 0.094695286 46400 0.123297592 23200 0.077911318 23216
0.18152874 46368 0.074410426 46368 0.017832976
46400 0.120526565 46400 0.046082867 23200 0.059667698 23220
0.125234946 46376 0.073692441 46376 0.032837048
46400 0.12322866 46400 0.065190929 23200 0.035817611 23224
0.049095583 46384 0.077586396 46384 0.047275411
46400 0.122523547 46400 0.038185259 23200 0.035517169 23228
0.024307918 46392 0.052383224 46392 0.069963084
46400 0.060906485 46400 0.035650312 23200 0.041987063 23232
0.056852823 46400 0.038630802 46400 0.090292269
46400 0.033372751 46400 0.090197813 23200 0.025726937 23236
0.0437448 46408 0.048944356 46408 0.075442586
46400 0.030058143 46400 0.109823835 23200 0.030118243 23240
0.038552542 46416 0.055233311 46416 0.046838075
46400 0.042827825 46400 0.093483788 23200 0.05917329 23244
0.058279205 46424 0.042243166 46424 0.044901168
46400 0.142604419 46400 0.1101456 23200 0.054355151 23248
0.034365563 46432 0.032823991 46432 0.035503977
46400 0.17464615 46400 0.159210001 23300 0.038267677 23252
0.038150702 46440 0.088629859 46440 0.057807549
46400 0.064449938 46400 0.174669578 23300 0.014762386 23256
0.110200497 46448 0.084010622 46448 0.048089267
46500 0.098791745 46500 0.13565633 23300 0.095734831 23260
0.146412844 46456 0.08902626 46456 0.003587364
46500 0.155766495 46500 0.10672104 23300 0.130640649 23264
0.128533168 46464 0.092509748 46464 0.046108173
46500 0.112062633 46500 0.109009459 23300 0.095226256 23268
0.071739109 46472 0.04670976 46472 0.038072511
46500 0.045186858 46500 0.118570461 23300 0.053440621 23272
0.040724492 46480 0.073190466 46480 0.014109955
46500 0.056456043 46500 0.030764062 23300 0.046371177 23276
0.079220175 46488 0.057797861 46488 0.022226081
46500 0.088356472 46500 0.087086257 23300 0.03848784 23280
0.085432861 46496 0.073683259 46496 0.035198576
46500 0.093043047 46500 0.105056068 23300 0.031326941 23284
0.04161859 46504 0.040005321 46504 0.037427086
46500 0.098549797 46500 0.058040678 23300 0.018592227 23288
0.05703617 46512 0.052832656 46512 0.033076889
46500 0.088764879 46500 0.020737847 23300 0.00684869 23292
0.071008639 46520 0.04831425 46520 0.049264862
46500 0.059264155 46500 0.01262802 23300 0.037864978 23296
0.102575556 46528 0.015156933 46528 0.073973191
46500 0.080081969 46500 0.024572983 23300 0.05044428 23300
0.126107393 46536 0.037226288 46536 0.071584938
46500 0.072759576 46500 0.023088443 23300 0.0096287 23304

0.120265606 46544 0.064406748 46544 0.050412604
46600 0.09889451 46600 0.030666677 23300 0.051162675 23308
0.105083149 46552 0.04412857 46552 0.016345492
46600 0.121159479 46600 0.092931579 23300 0.062190527 23312
0.106328116 46560 0.013940156 46560 0.037523325
46600 0.131693218 46600 0.10501119 23300 0.046071586 23316
0.149160391 46568 0.070736394 46568 0.073744894
46600 0.125803854 46600 0.06642942 23300 0.026079395 23320
0.143680169 46576 0.16747856 46576 0.068354195
46600 0.110820351 46600 0.051814426 23300 0.043766941 23324
0.090816728 46584 0.145380385 46584 0.064942949
46600 0.050880593 46600 0.137806346 23300 0.065658926 23328
0.087675981 46592 0.058234815 46592 0.100733378
46600 0.013738559 46600 0.185147118 23300 0.058748658 23332
0.090100053 46600 0.042122167 46600 0.097835611
46600 0.040558021 46600 0.13871245 23300 0.063035928 23336
0.096022115 46608 0.068437716 46608 0.045218621
46600 0.062565152 46600 0.087926965 23300 0.100196441 23340
0.099175755 46616 0.10756743 46616 0.005198588
46600 0.038138591 46600 0.149775908 23300 0.085741922 23344
0.107784472 46624 0.092701579 46624 0.016527718
46600 0.062703824 46600 0.180293413 23300 0.063738626 23348
0.164097845 46632 0.061805797 46632 0.012967998
46600 0.121278463 46600 0.156684168 23400 0.031360294 23352
0.166091675 46640 0.052740692 46640 0.035556597
46600 0.114269606 46600 0.12338681 23400 0.024908597 23356
0.123236401 46648 0.122364654 46648 0.061071602
46700 0.039848885 46700 0.142090765 23400 0.05540032 23360
0.121591031 46656 0.074095653 46656 0.050281215
46700 0.070647388 46700 0.122303522 23400 0.081010287 23364
0.144646241 46664 0.056602014 46664 0.044249504
46700 0.113522736 46700 0.049172748 23400 0.103767779 23368
0.132835703 46672 0.089891793 46672 0.022914715
46700 0.106489213 46700 0.070046539 23400 0.116058647 23372
0.076298806 46680 0.131472728 46680 0.045124889
46700 0.06286399 46700 0.135030335 23400 0.097365293 23376
0.078235426 46688 0.164816244 46688 0.04595955
46700 0.006283914 46700 0.156452486 23400 0.039698203 23380
0.115532392 46696 0.122466867 46696 0.006262643
46700 0.018249084 46700 0.117626136 23400 0.006830777 23384
0.13346094 46704 0.127195861 46704 0.032102937
46700 0.128533575 46700 0.095649863 23400 0.026433341 23388
0.14145668 46712 0.074401782 46712 0.029505165
46700 0.201064991 46700 0.063763182 23400 0.093776922 23392
0.108627115 46720 0.089761677 46720 0.008183026
46700 0.128174259 46700 0.051839139 23400 0.092799957 23396
0.101107929 46728 0.14867248 46728 0.033054919
46700 0.029343542 46700 0.069792062 23400 0.02424412 23400
0.119442899 46736 0.130308515 46736 0.081323909
46700 0.07404809 46700 0.051681305 23400 0.038320148 23404
0.106664709 46744 0.090108537 46744 0.090447822
46800 0.061284656 46800 0.077049372 23400 0.048214468 23408
0.098013865 46752 0.090140718 46752 0.060408722
46800 0.086243439 46800 0.153388668 23400 0.04458156 23412
0.092068069 46760 0.122180194 46760 0.05240785
46800 0.071795941 46800 0.145793034 23400 0.003302007 23416
0.071071612 46768 0.139526106 46768 0.054413515
46800 0.071981347 46800 0.133244466 23400 0.064896725 23420
0.051157222 46776 0.18920649 46776 0.039981289
46800 0.132149363 46800 0.070021706 23400 0.114293427 23424
0.086961605 46784 0.141461481 46784 0.079627971
46800 0.137630661 46800 0.010426521 23400 0.109809618 23428
0.110663794 46792 0.063046427 46792 0.116584488
46800 0.090196867 46800 0.091407412 23400 0.046392634 23432
0.102868158 46800 0.07214948 46800 0.089234141
46800 0.142984588 46800 0.157600865 23400 0.050410759 23436
0.104009101 46808 0.115784555 46808 0.048097336
46800 0.161282923 46800 0.160394688 23400 0.06535853 23440

0.120158518 46816 0.102381346 46816 0.019800933
46800 0.106000334 46800 0.170466054 23400 0.034749985 23444
0.12929675 46824 0.066993802 46824 0.021603666
46800 0.08377785 46800 0.177007736 23400 0.023431301 23448
0.093681483 46832 0.112363996 46832 0.013453443
46800 0.093113071 46800 0.149089043 23500 0.043170352 23452
0.049431455 46840 0.15309111 46840 0.008085828
46800 0.059612059 46800 0.143473459 23500 0.085110136 23456
0.06768843 46848 0.17187414 46848 0.012628663
46900 0.072804636 46900 0.14470458 23500 0.091010916 23460
0.091150367 46856 0.164949903 46856 0.041619089
46900 0.041578423 46900 0.105676336 23500 0.066681518 23464
0.076710203 46864 0.121827718 46864 0.05406313
46900 0.044446777 46900 0.048635702 23500 0.08403157 23468
0.079193196 46872 0.065911263 46872 0.024462885
46900 0.088569927 46900 0.036679936 23500 0.063477957 23472
0.087079265 46880 0.050191706 46880 0.021811409
46900 0.078876183 46900 0.063072577 23500 0.030009731 23476
0.082732891 46888 0.054972425 46888 0.036489324
46900 0.06416464 46900 0.086246961 23500 0.066137094 23480
0.089423891 46896 0.0463008 46896 0.003264783
46900 0.06580646 46900 0.06984808 23500 0.073746196 23484
0.103059778 46904 0.046142668 46904 0.03991491
46900 0.054089134 46900 0.100893187 23500 0.065383727 23488
0.073616036 46912 0.041495488 46912 0.05125867
46900 0.102668717 46900 0.122284881 23500 0.056702345 23492
0.013074217 46920 0.0425078 46920 0.041969459
46900 0.150490159 46900 0.073109513 23500 0.066324879 23496
0.08561783 46928 0.044380755 46928 0.030550178
46900 0.193723055 46900 0.021550681 23500 0.06176086 23500
0.135881244 46936 0.10175052 46936 0.030636889
46900 0.173920256 46900 0.026019321 23500 0.023804305 23504
0.103256454 46944 0.168372383 46944 0.028858227
47000 0.075458047 47000 0.069311362 23500 0.032720236 23508
0.048483329 46952 0.111307447 46952 0.027379781
47000 0.063030304 47000 0.126168409 23500 0.074219148 23512
0.087282228 46960 0.040932882 46960 0.022066342
47000 0.099554505 47000 0.137693089 23500 0.10120443 23516
0.06775574 46968 0.104676088 46968 0.034529843
47000 0.072572533 47000 0.10326827 23500 0.104025137 23520
0.040592666 46976 0.077332494 46976 0.057316793
47000 0.078707031 47000 0.077914425 23500 0.088077628 23524
0.065319087 46984 0.034388282 46984 0.080205711
47000 0.063011772 47000 0.065814973 23500 0.081288839 23528
0.101150108 46992 0.004208892 46992 0.091291397
47000 0.089015528 47000 0.021976202 23500 0.054246106 23532
0.087702021 47000 0.044219611 47000 0.093942465
47000 0.049652874 47000 0.063398038 23500 0.018800449 23536
0.052205785 47008 0.093748597 47008 0.095725525
47000 0.033406621 47000 0.053982327 23500 0.076310411 23540
0.032919772 47016 0.123506485 47016 0.074324176
47000 0.060414164 47000 0.024315074 23500 0.082388491 23544
0.02392535 47024 0.081905215 47024 0.03218167
47000 0.070309237 47000 0.066076449 23500 0.058675378 23548
0.07943005 47032 0.055300643 47032 0.024026735
47000 0.071275666 47000 0.050082257 23600 0.043478951 23552
0.109407512 47040 0.087512221 47040 0.013637949
47000 0.142939244 47000 0.015313442 23600 0.02785244 23556
0.098707336 47048 0.083764273 47048 0.034750672
47100 0.229287238 47100 0.01525779 23600 0.021395128 23560
0.049677343 47056 0.053929576 47056 0.04802645
47100 0.207992431 47100 0.045774228 23600 0.038692771 23564
0.0846931 47064 0.053968823 47064 0.034544391
47100 0.156971306 47100 0.072954899 23600 0.077201701 23568
0.143781523 47072 0.067198853 47072 0.026843225
47100 0.19112615 47100 0.060465805 23600 0.100335288 23572
0.13570879 47080 0.112459093 47080 0.039917188
47100 0.200711205 47100 0.063392501 23600 0.09328359 23576

0.071083065 47088 0.078018944 47088 0.051204715
47100 0.13407314 47100 0.110125788 23600 0.081368438 23580
0.043196531 47096 0.112939764 47096 0.055992907
47100 0.041197691 47100 0.151617351 23600 0.070589456 23584
0.021583137 47104 0.150693639 47104 0.053605465
47100 0.103148901 47100 0.158861076 23600 0.052170275 23588
0.077941782 47112 0.151346714 47112 0.05240766
47100 0.143888231 47100 0.136566086 23600 0.028935823 23592
0.089995803 47120 0.150559819 47120 0.045586268
47100 0.108729248 47100 0.116587158 23600 0.025170795 23596
0.065323009 47128 0.146774518 47128 0.034979032
47100 0.048895356 47100 0.107547428 23600 0.041472562 23600
0.053122982 47136 0.053639367 47136 0.039522216
47100 0.052133324 47100 0.050492148 23600 0.044583503 23604
0.075869779 47144 0.044751749 47144 0.033434633
47200 0.047976777 47200 0.061824736 23600 0.065049848 23608
0.084139872 47152 0.020664733 47152 0.018780409
47200 0.069691749 47200 0.138559234 23600 0.07948713 23612
0.113331138 47160 0.059926446 47160 0.025551215
47200 0.154052715 47200 0.160008669 23600 0.051318279 23616
0.119644152 47168 0.075212374 47168 0.035739267
47200 0.12858097 47200 0.13428001 23600 0.068423418 23620
0.066814209 47176 0.067950234 47176 0.036072288
47200 0.085959873 47200 0.130848363 23600 0.09946739 23624
0.062293562 47184 0.078853111 47184 0.03692972
47200 0.156173031 47200 0.16104168 23600 0.088349938 23628
0.071942304 47192 0.12510248 47192 0.040356379
47200 0.211636405 47200 0.123610938 23600 0.050209957 23632
0.049100996 47200 0.07079629 47200 0.062217812
47200 0.173241656 47200 0.068326866 23600 0.021926067 23636
0.024551844 47208 0.072326773 47208 0.065264372
47200 0.073620853 47200 0.032971057 23600 0.029387657 23640
0.01066281 47216 0.166650949 47216 0.036044192
47200 0.072025847 47200 0.106295098 23600 0.04688952 23644
0.062641688 47224 0.148888954 47224 0.016366093
47200 0.12145182 47200 0.124884624 23600 0.036563288 23648
0.105528736 47232 0.079422243 47232 0.040808816
47200 0.101402009 47200 0.078467325 23700 0.004639383 23652
0.087635774 47240 0.004004403 47240 0.055453817
47200 0.04858672 47200 0.055701767 23700 0.033527784 23656
0.053449905 47248 0.044838092 47248 0.050440631
47300 0.097823402 47300 0.086626984 23700 0.042096977 23660
0.058537935 47256 0.047354595 47256 0.034233308
47300 0.036357669 47300 0.056831472 23700 0.026659289 23664
0.051818661 47264 0.080746329 47264 0.045789868
47300 0.080757411 47300 0.034285647 23700 0.033500066 23668
0.031884218 47272 0.14689735 47272 0.046403809
47300 0.074067553 47300 0.062185995 23700 0.067698813 23672
0.026127782 47280 0.127854248 47280 0.01268
47300 0.059052727 47300 0.065259694 23700 0.051808158 23676
0.026637272 47288 0.064624641 47288 0.037399212
47300 0.119975572 47300 0.048817627 23700 0.040923835 23680
0.044989054 47296 0.109069195 47296 0.052304753
47300 0.123717473 47300 0.051698724 23700 0.039841729 23684
0.06542178 47304 0.104872081 47304 0.0296783
47300 0.054854481 47300 0.023569186 23700 0.01237353 23688
0.05882089 47312 0.118802913 47312 0.001509513
47300 0.01658869 47300 0.07675885 23700 0.018506937 23692
0.067255962 47320 0.085321808 47320 0.013627147
47300 0.02446478 47300 0.139536598 23700 0.034921035 23696
0.051785686 47328 0.041978685 47328 0.022690099
47300 0.015774169 47300 0.148077175 23700 0.049492792 23700
0.019583853 47336 0.063455133 47336 0.012215158
47300 0.025629081 47300 0.115003633 23700 0.009280971 23704
0.051498122 47344 0.094029405 47344 0.017824301
47400 0.065155386 47400 0.090662979 23700 0.031037656 23708
0.021657826 47352 0.091300746 47352 0.032527565
47400 0.106491054 47400 0.061789651 23700 0.036764304 23712

0.035298064	47360	0.12522981	47360	0.046550958
47400	0.099732919	47400	0.034945417	23700	0.060282044	23716
0.069924361	47368	0.133751266	47368	0.03661741
47400	0.03649345	47400	0.09666456	23700	0.035073466	23720
0.067684923	47376	0.071854454	47376	0.022315429
47400	0.042569409	47400	0.089622918	23700	0.030968655	23724
0.064030544	47384	0.040899002	47384	0.037620772
47400	0.085869724	47400	0.055891964	23700	0.068561669	23728
0.09345905	47392	0.076068514	47392	0.047744088
47400	0.119589844	47400	0.004865732	23700	0.079677338	23732
0.098700031	47400	0.072452487	47400	0.015604705
47400	0.122924306	47400	0.100899822	23700	0.074179239	23736
0.052116018	47408	0.048427748	47408	0.028410235
47400	0.093270792	47400	0.11378387	23700	0.060792074	23740
0.030633324	47416	0.044969023	47416	0.038611874
47400	0.052137159	47400	0.060068593	23700	0.051703279	23744
0.036886249	47424	0.145791971	47424	0.045147357
47400	0.027144792	47400	0.070474307	23700	0.068553934	23748
0.035305278	47432	0.148439693	47432	0.04698896
47400	0.002994345	47400	0.061594699	23800	0.067629102	23752
0.024189216	47440	0.050586037	47440	0.034417924
47400	0.006923644	47400	0.050138118	23800	0.017124554	23756
0.017880244	47448	0.029306593	47448	0.034546389
47500	0.049702994	47500	0.03387871	23800	0.02300379	23760
0.014823706	47456	0.090275564	47456	0.022415134
47500	0.092691334	47500	0.025363996	23800	0.008893323	23764
0.020153824	47464	0.178658054	47464	0.023930954
47500	0.06651185	47500	0.0444709	23800	0.036890848	23768
0.05056199	47472	0.150344261	47472	0.053871496
47500	0.112501439	47500	0.046994508	23800	0.036333895	23772
0.081224978	47480	0.041297997	47480	0.080135527
47500	0.166279715	47500	0.013668665	23800	0.029956331	23776
0.086877342	47488	0.112790309	47488	0.052662828
47500	0.094717521	47500	0.029873918	23800	0.029178282	23780
0.067542955	47496	0.153029993	47496	0.033952965
47500	0.020900601	47500	0.047346879	23800	0.037480368	23784
0.039760103	47504	0.110682267	47504	0.033190245
47500	0.039250455	47500	0.040187497	23800	0.034943932	23788
0.057023171	47512	0.04283118	47512	0.013305285
47500	0.008806972	47500	0.075323769	23800	0.01702458	23792
0.060493916	47520	0.077903249	47520	0.046515117
47500	0.023765757	47500	0.097018128	23800	0.03762724	23796
0.01681613	47528	0.100468737	47528	0.053516196
47500	0.037944632	47500	0.096677017	23800	0.057696117	23800
0.050052753	47536	0.089991823	47536	0.052435469
47500	0.065851011	47500	0.146287711	23800	0.07236746	23804
0.056036537	47544	0.021728916	47544	0.058370439
47600	0.092603194	47600	0.15591635	23800	0.058359616	23808
0.033286095	47552	0.074549113	47552	0.031220483
47600	0.031675081	47600	0.085338681	23800	0.01913762	23812
0.021460863	47560	0.065791181	47560	0.027628807
47600	0.098056393	47600	0.02362214	23800	0.036735764	23816
0.018413179	47568	0.087737528	47568	0.053325624
47600	0.109665605	47600	0.083717445	23800	0.066797424	23820
0.024334406	47576	0.097123681	47576	0.059058712
47600	0.045328667	47600	0.121357785	23800	0.04345044	23824
0.021189235	47584	0.083751133	47584	0.056548219
47600	0.112289963	47600	0.084857056	23800	0.023802266	23828
0.044334352	47592	0.169105216	47592	0.049352515
47600	0.146408521	47600	0.067862791	23800	0.048497797	23832
0.058924285	47600	0.20961804	47600	0.029325083
47600	0.124267448	47600	0.036334768	23800	0.062211548	23836
0.045591914	47608	0.154364956	47608	0.04481868
47600	0.110846937	47600	0.093063536	23800	0.055249431	23840
0.05715547	47616	0.091936126	47616	0.017806013
47600	0.093545663	47600	0.168112019	23800	0.050417693	23844
0.080927166	47624	0.08446502	47624	0.047079888
47600	0.061525643	47600	0.171448934	23800	0.046669262	23848

```
0.059483562	47632	0.075394048	47632	0.059204293
47600	0.112220303	47600	0.127036634	23900	0.049795628	23852
0.005852984	47640	0.056444598	47640	0.019888827
47600	0.116624331	47600	0.074090342	23900	0.05575343	23856
0.04764344	47648	0.016115484	47648	0.020658501
47700	0.032178272	47700	0.055814446	23900	0.061968109	23860
0.035042129	47656	0.119252116	47656	0.02425672
47700	0.020479303	47700	0.09076923	23900	0.050154984	23864
0.017247627	47664	0.171841268	47664	0.036262136
47700	0.061789498	47700	0.11504352	23900	0.038146627	23868
0.027032538	47672	0.122481288	47672	0.053794975
47700	0.1200841	47700	0.09959608	23900	0.030878313	23872
0.035342036	47680	0.114473602	47680	0.04024199
47700	0.114227194	47700	0.075061325	23900	0.007404904	23876
0.046591791	47688	0.136463466	47688	0.02267504
47700	0.124506478	47700	0.102203725	23900	0.016764947	23880
0.048143651	47696	0.092746996	47696	0.009940029
47700	0.103188613	47700	0.119601282	23900	0.06377315	23884
0.065523229	47704	0.065952394	47704	0.038660841
47700	0.092867289	47700	0.088251072	23900	0.075381278	23888
0.068414156	47712	0.027294289	47712	0.035619971
47700	0.1256455	47700	0.043410171	23900	0.05530272	23892
0.069135385	47720	0.040993622	47720	0.023271508
47700	0.118715259	47700	0.061723418	23900	0.050467283	23896
0.045972061	47728	0.047800269	47728	0.027824553
47700	0.140952019	47700	0.13217672	23900	0.053667194	23900
0.026425687	47736	0.026540667	47736	0.042538693
47700	0.159713498	47700	0.200243186	23900	0.031010641	23904
0.081090853	47744	0.07406118	47744	0.046841236
47800	0.131093446	47800	0.165821315	23900	0.027577855	23908
0.070223294	47752	0.122850383	47752	0.030744126
47800	0.083210143	47800	0.097398712	23900	0.061317202	23912
0.024827687	47760	0.113744129	47760	0.02362454
47800	0.087646418	47800	0.052243966	23900	0.072833725	23916
0.100283891	47768	0.094270909	47768	0.033520097
47800	0.081190046	47800	0.042444946	23900	0.04881814	23920
0.105957974	47776	0.008088458	47776	0.044008652
47800	0.01111352	47800	0.040676583	23900	0.012423207	23924
0.06117655	47784	0.106939973	47784	0.043646694
47800	0.095229829	47800	0.028609198	23900	0.038509097	23928
0.023355171	47792	0.153661036	47792	0.029572819
47800	0.079870581	47800	0.014446531	23900	0.07481833	23932
0.069801055	47800	0.10518013	47800	0.010081524
47800	0.068386158	47800	0.01518559	23900	0.073740077	23936
0.089861183	47808	0.009874389	47808	0.021662165
47800	0.028118939	47800	0.060804483	23900	0.051203584	23940
0.088597328	47816	0.071207913	47816	0.029476914
47800	0.055094599	47800	0.110482441	23900	0.045022469	23944
0.071718794	47824	0.132552915	47824	0.038203711
47800	0.077556862	47800	0.138567135	23900	0.021196827	23948
0.028182392	47832	0.134613481	47832	0.030178861
47800	0.119513527	47800	0.117354939	24000	0.014866006	23952
0.032978282	47840	0.122878264	47840	0.016222426
47800	0.15587789	47800	0.04167742	24000	0.04886512	23956
0.058399975	47848	0.138708463	47848	0.042513831
47900	0.122817961	47900	0.022865943	24000	0.05084174	23960
0.060828388	47856	0.155375398	47856	0.03471673
47900	0.043626937	47900	0.033623554	24000	0.032683569	23964
0.053461034	47864	0.111793299	47864	0.011432551
47900	0.091367285	47900	0.097814191	24000	0.008017535	23968
0.06880099	47872	0.022253269	47872	0.038876216
47900	0.118119831	47900	0.072144067	24000	0.02256824	23972
0.055609085	47880	0.031565105	47880	0.046650115
47900	0.089861358	47900	0.045198689	24000	0.054090666	23976
0.041284984	47888	0.115315605	47888	0.050546412
47900	0.072537121	47900	0.073578238	24000	0.087658962	23980
0.045231798	47896	0.149257394	47896	0.018023055
47900	0.044443405	47900	0.067376801	24000	0.091858143	23984
```

0.048395075 47904 0.14728168 47904 0.044601329
47900 0.016639137 47900 0.058447553 24000 0.072166149 23988
0.081064463 47912 0.145844111 47912 0.069381618
47900 0.023315819 47900 0.048391292 24000 0.032394528 23992
0.10748818 47920 0.084572857 47920 0.063359985
47900 0.057558296 47900 0.100356396 24000 0.057174766 23996
0.05462112 47928 0.038300444 47928 0.049545448
47900 0.012943871 47900 0.044120319 24000 0.078026125 24000
0.044741555 47936 0.052331005 47936 0.037031099
47900 0.094035262 47900 0.068925961 24000 0.060181435 24004
0.085336469 47944 0.071402079 47944 0.033876637
48000 0.152542518 48000 0.098096643 24000 0.038483617 24008
0.049706949 47952 0.080657381 47952 0.019083384
48000 0.15333241 48000 0.114575261 24000 0.055596887 24012
0.006334008 47960 0.08709541 47960 0.008672675
48000 0.149093481 48000 0.114237977 24000 0.069783229 24016
0.022055281 47968 0.089248475 47968 0.009877544
48000 0.125972626 48000 0.112659625 24000 0.047986279 24020
0.056765111 47976 0.11543509 47976 0.046496134
48000 0.096746764 48000 0.110018293 24000 0.040117389 24024
0.085389809 47984 0.110405512 47984 0.044422497
48000 0.120613331 48000 0.057772788 24000 0.034746179 24028
0.086019703 47992 0.02666121 47992 0.020367519
48000 0.105107793 48000 0.02683152 24000 0.005561515 24032
0.086738633 48000 0.045825171 48000 0.077547484
48000 0.084663254 48000 0.074986172 24000 0.048222002 24036
0.088090485 48008 0.063624648 48008 0.071477269
48000 0.083157254 48000 0.12279526 24000 0.067390363 24040
0.075403586 48016 0.099950514 48016 0.044614873
48000 0.053948916 48000 0.118898322 24000 0.045319361 24044
0.047152378 48024 0.127789419 48024 0.019748393
48000 0.028061084 48000 0.086969259 24000 0.041410942 24048
0.034283658 48032 0.13343629 48032 0.008563159
48000 0.033302265 48000 0.084078216 24100 0.054020213 24052
0.035633319 48040 0.106573687 48040 0.015185421
48000 0.038887105 48000 0.097804797 24100 0.04417136 24056
0.011694733 48048 0.011441381 48048 0.025756393
48100 0.061330175 48100 0.167755789 24100 0.054397213 24060
0.040201008 48056 0.124617858 48056 0.023051434
48100 0.042525822 48100 0.144293925 24100 0.061853549 24064
0.048227394 48064 0.162790456 48064 0.025915599
48100 0.077032972 48100 0.041359082 24100 0.051903153 24068
0.035317065 48072 0.09625731 48072 0.033671455
48100 0.047445159 48100 0.05898394 24100 0.011609979 24072
0.040533487 48080 0.037922411 48080 0.025060112
48100 0.051315561 48100 0.104049796 24100 0.035678306 24076
0.035419103 48088 0.067045599 48088 0.040755491
48100 0.083853425 48100 0.115706505 24100 0.043269331 24080
0.018817837 48096 0.113154267 48096 0.020133881
48100 0.073016534 48100 0.077350291 24100 0.022252492 24084
0.013474539 48104 0.134156363 48104 0.013867257
48100 0.086079686 48100 0.038419799 24100 0.030462925 24088
0.012892556 48112 0.107138294 48112 0.014631756
48100 0.13418324 48100 0.082398918 24100 0.03592006 24092
0.031151038 48120 0.081611586 48120 0.020821684
48100 0.14485493 48100 0.076320568 24100 0.03268896 24096
0.069337155 48128 0.094583025 48128 0.069452406
48100 0.097894321 48100 0.166987753 24100 0.070092152 24100
0.077818222 48136 0.073172858 48136 0.084672836
48100 0.004154782 48100 0.247940043 24100 0.069124981 24104
0.043612978 48144 0.085263557 48144 0.056218687
48200 0.089523812 48200 0.141034209 24100 0.033746386 24108
0.060143084 48152 0.124565151 48152 0.014313057
48200 0.118904362 48200 0.058173373 24100 0.008952457 24112
0.069695787 48160 0.135624723 48160 0.003881838
48200 0.129213105 48200 0.139510885 24100 0.015096581 24116
0.056077421 48168 0.13335915 48168 0.017205104
48200 0.149141983 48200 0.18750818 24100 0.041235777 24120

0.061576546 48176 0.087281005 48176 0.030377716
48200 0.12087619 48200 0.195686414 24100 0.04084735 24124
0.081928105 48184 0.038177106 48184 0.016288195
48200 0.043105585 48200 0.106288208 24100 0.018767047 24128
0.067790294 48192 0.061818246 48192 0.030448544
48200 0.026129717 48200 0.102710525 24100 0.028264438 24132
0.032590142 48200 0.10097769 48200 0.034490371
48200 0.067388886 48200 0.130016051 24100 0.034896879 24136
0.011334004 48208 0.150175678 48208 0.006673884
48200 0.087337314 48200 0.079422687 24100 0.054807366 24140
0.003714817 48216 0.160799028 48216 0.011586458
48200 0.08205718 48200 0.021631075 24100 0.064183056 24144
0.017039196 48224 0.140132761 48224 0.022542576
48200 0.101320009 48200 0.072528761 24100 0.056876583 24148
0.043885382 48232 0.187372526 48232 0.026386773
48200 0.020233618 48200 0.128309911 24200 0.043409982 24152
0.056326171 48240 0.220483795 48240 0.045339788
48200 0.131386944 48200 0.09581136 24200 0.007737294 24156
0.03114498 48248 0.176270186 48248 0.038105496
48300 0.151763335 48300 0.054096156 24200 0.025243591 24160
0.002637148 48256 0.088227906 48256 0.008762865
48300 0.086532629 48300 0.116634277 24200 0.035646528 24164
0.037006779 48264 0.018390767 48264 0.044299719
48300 0.089314504 48300 0.112016154 24200 0.019449239 24168
0.069798887 48272 0.084857951 48272 0.067993933
48300 0.110124973 48300 0.114055998 24200 0.046435591 24172
0.083022977 48280 0.102867605 48280 0.060074843
48300 0.131057401 48300 0.069810812 24200 0.066515102 24176
0.078534817 48288 0.067102672 48288 0.039322709
48300 0.117825264 48300 0.073860036 24200 0.091725808 24180
0.064195025 48296 0.020156955 48296 0.031400472
48300 0.169614024 48300 0.111082503 24200 0.093152099 24184
0.034925037 48304 0.063719846 48304 0.033385186
48300 0.243341914 48300 0.096082549 24200 0.050616869 24188
0.064836277 48312 0.038194994 48312 0.034463024
48300 0.202183815 48300 0.088628854 24200 0.014438399 24192
0.053340391 48320 0.039365845 48320 0.025813675
48300 0.152325025 48300 0.073636402 24200 0.019422954 24196
0.035039007 48328 0.083189705 48328 0.008589233
48300 0.115807714 48300 0.084664978 24200 0.050140738 24200
0.042846128 48336 0.151096945 48336 0.016071293
48300 0.065054075 48300 0.051679417 24200 0.068363668 24204
0.061628154 48344 0.151319051 48344 0.038606217
48400 0.045407251 48400 0.056474564 24200 0.058524111 24208
0.049487258 48352 0.148175284 48352 0.075225224
48400 0.05081746 48400 0.100650461 24200 0.053686148 24212
0.03904906 48360 0.123794729 48360 0.077258723
48400 0.08517081 48400 0.098747529 24200 0.026427286 24216
0.022354643 48368 0.075565884 48368 0.043307493
48400 0.106367792 48400 0.07160478 24200 0.029521398 24220
0.021257121 48376 0.049712613 48376 0.020298729
48400 0.052224867 48400 0.035234385 24200 0.066533416 24224
0.044146607 48384 0.026145792 48384 0.034016299
48400 0.038860155 48400 0.056610465 24200 0.060489936 24228
0.045379908 48392 0.039978422 48392 0.019501624
48400 0.034140172 48400 0.051907206 24200 0.052174528 24232
0.021100812 48400 0.038181053 48400 0.045667981
48400 0.09641677 48400 0.037996895 24200 0.071888295 24236
0.060974711 48408 0.056356483 48408 0.037652982
48400 0.113480826 48400 0.033247328 24200 0.060859587 24240
0.092561772 48416 0.082497943 48416 0.044478285
48400 0.080279337 48400 0.059470032 24200 0.036674559 24244
0.06995631 48424 0.086783781 48424 0.036596459
48400 0.078436286 48400 0.072303075 24200 0.047594636 24248
0.040572642 48432 0.024089728 48432 0.013531457
48400 0.094718998 48400 0.051234078 24300 0.035343073 24252
0.021367232 48440 0.041410327 48440 0.035738129
48400 0.046913985 48400 0.100548474 24300 0.028422508 24256

0.017798573 48448 0.05946387 48448 0.032454536
48500 0.029328792 48500 0.135424125 24300 0.053770407 24260
0.055919147 48456 0.084721491 48456 0.045302335
48500 0.101163554 48500 0.174586181 24300 0.036686815 24264
0.039674051 48464 0.04032996 48464 0.080829399
48500 0.185806391 48500 0.188358638 24300 0.015792179 24268
0.026304851 48472 0.064965272 48472 0.095801152
48500 0.173173933 48500 0.166895086 24300 0.02091494 24272
0.069471083 48480 0.057957223 48480 0.077209006
48500 0.119203345 48500 0.100084195 24300 0.020068903 24276
0.043885902 48488 0.17335682 48488 0.045102064
48500 0.13434948 48500 0.030988023 24300 0.034517758 24280
0.02461936 48496 0.124601444 48496 0.026091271
48500 0.150807668 48500 0.023286972 24300 0.027738955 24284
0.037855203 48504 0.047436563 48504 0.017531238
48500 0.094538496 48500 0.098785698 24300 0.00773161 24288
0.054039807 48512 0.064213818 48512 0.039947619
48500 0.021112788 48500 0.090161811 24300 0.039510382 24292
0.074716569 48520 0.174639863 48520 0.049361104
48500 0.042936183 48500 0.056700734 24300 0.028216908 24296
0.090138979 48528 0.103863436 48528 0.025334435
48500 0.033932443 48500 0.180969684 24300 0.027213882 24300
0.049214094 48536 0.053733431 48536 0.03188719
48500 0.070682014 48500 0.165074656 24300 0.028131648 24304
0.037818212 48544 0.044960834 48544 0.032395306
48600 0.066216169 48600 0.094823845 24300 0.010735756 24308
0.063350599 48552 0.02970531 48552 0.030490024
48600 0.058465634 48600 0.116301744 24300 0.024062381 24312
0.038527913 48560 0.034636367 48560 0.026541156
48600 0.079236379 48600 0.060924249 24300 0.05250645 24316
0.018127537 48568 0.092846378 48568 0.035795638
48600 0.104006162 48600 0.022022468 24300 0.071340386 24320
0.062708801 48576 0.163449556 48576 0.037088215
48600 0.057353562 48600 0.015830063 24300 0.050787035 24324
0.085487263 48584 0.152830719 48584 0.034286684
48600 0.07576005 48600 0.033658893 24300 0.020399271 24328
0.0542321 48592 0.065296612 48592 0.012749509
48600 0.111642592 48600 0.028148064 24300 0.010173499 24332
0.050554449 48600 0.095638476 48600 0.02803311
48600 0.111665366 48600 0.08297043 24300 0.016754233 24336
0.04333114 48608 0.109827553 48608 0.023123073
48600 0.1466986 48600 0.06767382 24300 0.043876007 24340
0.006248464 48616 0.148416658 48616 0.002269905
48600 0.146452236 48600 0.009115542 24300 0.058636819 24344
0.032707991 48624 0.182208765 48624 0.035075722
48600 0.087708642 48600 0.052900265 24300 0.051895167 24348
0.037008795 48632 0.162986078 48632 0.065981476
48600 0.073438525 48600 0.048043628 24400 0.086406682 24352
0.021244294 48640 0.129778345 48640 0.048665563
48600 0.114002665 48600 0.03960999 24400 0.063432264 24356
0.020198493 48648 0.114268791 48648 0.005166014
48700 0.135428404 48700 0.082911807 24400 0.008227209 24360
0.036222446 48656 0.04780549 48656 0.045054876
48700 0.076275479 48700 0.079672856 24400 0.040279603 24364
0.026047137 48664 0.03087024 48664 0.062009807
48700 0.044860401 48700 0.062926658 24400 0.044214463 24368
0.039203842 48672 0.066334527 48672 0.042649965
48700 0.082243911 48700 0.072235591 24400 0.075160075 24372
0.061810657 48680 0.101369755 48680 0.050336053
48700 0.042758962 48700 0.056590488 24400 0.0975039 24376
0.054913391 48688 0.17853509 48688 0.068674672
48700 0.061561739 48700 0.048050559 24400 0.075342119 24380
0.034940204 48696 0.204555326 48696 0.044050332
48700 0.080269085 48700 0.163802571 24400 0.011939705 24384
0.036282967 48704 0.13964648 48704 0.074187119
48700 0.018655092 48700 0.155982954 24400 0.028049681 24388
0.013743148 48712 0.171107764 48712 0.124640545
48700 0.118663069 48700 0.067037159 24400 0.027108985 24392

0.070533461 48720 0.278115768 48720 0.095950949
48700 0.114749819 48700 0.029097451 24400 0.026728712 24396
0.123805337 48728 0.28066244 48728 0.051439085
48700 0.075891381 48700 0.080346516 24400 0.029428957 24400
0.118134805 48736 0.147100058 48736 0.050205017
48700 0.10056435 48700 0.110224122 24400 0.080091617 24404
0.0902428 48744 0.035418805 48744 0.072398085
48800 0.055375189 48800 0.047583188 24400 0.086549248 24408
0.070245507 48752 0.00466866 48752 0.089162189
48800 0.062021733 48800 0.039322724 24400 0.072520023 24412
0.038497903 48760 0.03657673 48760 0.06205996
48800 0.084625426 48800 0.03120162 24400 0.062950989 24416
0.00962043 48768 0.086280372 48768 0.026079169
48800 0.103253777 48800 0.050527884 24400 0.062529027 24420
0.041989446 48776 0.118633427 48776 0.030252224
48800 0.154000838 48800 0.087417306 24400 0.034742774 24424
0.063500665 48784 0.094451229 48784 0.033299697
48800 0.154116365 48800 0.147808241 24400 0.038937225 24428
0.048352256 48792 0.116628711 48792 0.006350518
48800 0.100097503 48800 0.174888191 24400 0.014090428 24432
0.03331347 48800 0.147453815 48800 0.036048899
48800 0.110027475 48800 0.095520532 24400 0.043888769 24436
0.045524732 48808 0.138060131 48808 0.039588202
48800 0.164497541 48800 0.115173236 24400 0.016030552 24440
0.057911369 48816 0.092461414 48816 0.046737107
48800 0.121756741 48800 0.148434003 24400 0.069269336 24444
0.053475938 48824 0.083477382 48824 0.066958135
48800 0.078527039 48800 0.105409919 24400 0.066302338 24448
0.101785059 48832 0.081400642 48832 0.063310064
48800 0.122944039 48800 0.141453856 24500 0.0652081 24452
0.13529291 48840 0.070147813 48840 0.044688084
48800 0.115577997 48800 0.159935793 24500 0.048111266 24456
0.111495378 48848 0.074889438 48848 0.050462506
48900 0.102581973 48900 0.091041446 24500 0.00345181 24460
0.066780893 48856 0.046198125 48856 0.035460253
48900 0.075014345 48900 0.037946098 24500 0.058658414 24464
0.042307285 48864 0.072594652 48864 0.029689725
48900 0.062036525 48900 0.077246346 24500 0.087314606 24468
0.056168596 48872 0.12646128 48872 0.014114654
48900 0.092434188 48900 0.127756691 24500 0.061870138 24472
0.082013045 48880 0.090701913 48880 0.020951351
48900 0.097864548 48900 0.074794218 24500 0.013423877 24476
0.056003544 48888 0.044679808 48888 0.009765392
48900 0.108396089 48900 0.04256098 24500 0.029234905 24480
0.06136833 48896 0.073793621 48896 0.026076512
48900 0.078625555 48900 0.099346704 24500 0.033181488 24484
0.098289485 48904 0.083054467 48904 0.014572041
48900 0.090708731 48900 0.089820169 24500 0.04578595 24488
0.105555904 48912 0.043541299 48912 0.004349351
48900 0.127007501 48900 0.084010717 24500 0.068065674 24492
0.060698869 48920 0.033040196 48920 0.008410322
48900 0.10005611 48900 0.081017221 24500 0.047624424 24496
0.026029651 48928 0.100738129 48928 0.01688969
48900 0.125211809 48900 0.033293032 24500 0.023595518 24500
0.058531172 48936 0.150247754 48936 0.027566703
48900 0.140973789 48900 0.032471056 24500 0.032805372 24504
0.049943763 48944 0.134517774 48944 0.032358807
49000 0.078108111 49000 0.039718976 24500 0.033765242 24508
0.060207709 48952 0.076041433 48952 0.0202541
49000 0.045640863 49000 0.047293277 24500 0.042790205 24512
0.081841747 48960 0.045675679 48960 0.022939457
49000 0.071248491 49000 0.079433979 24500 0.044810124 24516
0.076874043 48968 0.026117912 48968 0.026224743
49000 0.117229174 49000 0.060343435 24500 0.035830482 24520
0.049572183 48976 0.077896526 48976 0.010105691
49000 0.083222636 49000 0.117468575 24500 0.051812025 24524
0.027691964 48984 0.080147634 48984 0.046187084
49000 0.038736642 49000 0.098966528 24500 0.087984867 24528

0.018355055 48992 0.021786074 48992 0.051535611
49000 0.034892608 49000 0.059727558 24500 0.098354569 24532
0.017648385 49000 0.095235606 49000 0.048372069
49000 0.09814043 49000 0.046149464 24500 0.075136995 24536
0.074881806 49008 0.129606575 49008 0.053234555
49000 0.15339606 49000 0.040075745 24500 0.06316325 24540
0.081131002 49016 0.120929581 49016 0.055701745
49000 0.109609136 49000 0.058778027 24500 0.061866624 24544
0.04344642 49024 0.117285497 49024 0.036631209
49000 0.07428063 49000 0.03563194 24500 0.03964403 24548
0.048897731 49032 0.107242886 49032 0.019083467
49000 0.095744959 49000 0.089415007 24600 0.019722924 24552
0.064672437 49040 0.064600004 49040 0.025078705
49000 0.142235221 49000 0.120163044 24600 0.055673867 24556
0.094092109 49048 0.046818408 49048 0.039258543
49100 0.103402017 49100 0.049821691 24600 0.074531265 24560
0.102094578 49056 0.054417131 49056 0.048028775
49100 0.07680335 49100 0.088471687 24600 0.044511664 24564
0.066698063 49064 0.057493897 49064 0.040290892
49100 0.115525261 49100 0.146484745 24600 0.013247157 24568
0.089522451 49072 0.038075883 49072 0.025823025
49100 0.15778844 49100 0.121265563 24600 0.045106332 24572
0.09385946 49080 0.082482999 49080 0.042470885
49100 0.15765462 49100 0.044358094 24600 0.059342769 24576
0.052109532 49088 0.088612556 49088 0.043311273
49100 0.123519989 49100 0.059772676 24600 0.038902414 24580
0.056618148 49096 0.060657148 49096 0.01684356
49100 0.076308723 49100 0.099941179 24600 0.024824372 24584
0.158937415 49104 0.065612869 49104 0.036323148
49100 0.056555564 49100 0.084411819 24600 0.033322653 24588
0.153288609 49112 0.059908685 49112 0.070801762
49100 0.039242881 49100 0.042775639 24600 0.012636623 24592
0.084436375 49120 0.142018253 49120 0.057457721
49100 0.054044471 49100 0.016588141 24600 0.048573587 24596
0.032968146 49128 0.170560059 49128 0.043873362
49100 0.115116673 49100 0.064753731 24600 0.087235436 24600
0.032048032 49136 0.079705205 49136 0.039502942
49100 0.105682513 49100 0.09213802 24600 0.120317185 24604
0.065340668 49144 0.102655948 49144 0.01715709
49200 0.035250705 49200 0.061359126 24600 0.109336193 24608
0.086136795 49152 0.147900471 49152 0.03092851
49200 0.067942776 49200 0.093453535 24600 0.060787323 24612
0.083916275 49160 0.147771294 49160 0.041348641
49200 0.118558084 49200 0.133449678 24600 0.017530894 24616
0.062037914 49168 0.146870981 49168 0.027515158
49200 0.14928641 49200 0.167341423 24600 0.019426736 24620
0.029401259 49176 0.128674045 49176 0.038749004
49200 0.156569993 49200 0.13298326 24600 0.010806278 24624
0.019251564 49184 0.100229205 49184 0.06742472
49200 0.079959922 49200 0.092327791 24600 0.027029819 24628
0.034444893 49192 0.064459338 49192 0.055225231
49200 0.109504552 49200 0.193163738 24600 0.042829805 24632
0.044226599 49200 0.096213218 49200 0.048641003
49200 0.234073959 49200 0.228863108 24600 0.029387453 24636
0.022322271 49208 0.128025684 49208 0.02178353
49200 0.20732719 49200 0.181837343 24600 0.007782962 24640
0.040079398 49216 0.064442145 49216 0.042717311
49200 0.049639977 49200 0.074175274 24600 0.008258626 24644
0.089632958 49224 0.095865711 49224 0.057021141
49200 0.046897741 49200 0.053604319 24600 0.013322208 24648
0.093473907 49232 0.195964808 49232 0.031684831
49200 0.056772256 49200 0.125850158 24700 0.043975218 24652
0.062656269 49240 0.152933179 49240 0.018227649
49200 0.154647845 49200 0.151592598 24700 0.049722628 24656
0.006895545 49248 0.019436468 49248 0.024689583
49300 0.153571396 49300 0.125419669 24700 0.045817913 24660
0.053176245 49256 0.125291291 49256 0.0303629
49300 0.092676608 49300 0.091472022 24700 0.049384238 24664

0.065475186	49264	0.190523948	49264	0.041628773		
49300	0.087686734	49300	0.054625365	24700	0.050987121	24668
0.078433914	49272	0.21190953	49272	0.047917696		
49300	0.078899997	49300	0.06345124	24700	0.082279228	24672
0.09999828	49280	0.107277709	49280	0.040298572		
49300	0.015130796	49300	0.084575775	24700	0.091401693	24676
0.086371176	49288	0.056250006	49288	0.014888432		
49300	0.077523422	49300	0.128902553	24700	0.060123846	24680
0.059609964	49296	0.068603004	49296	0.006324393		
49300	0.045903129	49300	0.12314746	24700	0.067659872	24684
0.045468441	49304	0.091722723	49304	0.028097273		
49300	0.01984164	49300	0.040519575	24700	0.104360377	24688
0.046261004	49312	0.128958796	49312	0.039383645		
49300	0.031073869	49300	0.04445804	24700	0.092182294	24692
0.034516575	49320	0.063547246	49320	0.03857151		
49300	0.005858768	49300	0.07824794	24700	0.028003948	24696
0.007270665	49328	0.015454421	49328	0.043599415		
49300	0.049143906	49300	0.048976464	24700	0.050834424	24700
0.056287547	49336	0.063868742	49336	0.024392541		
49300	0.048246115	49300	0.0520377	24700	0.044526179	24704
0.094444709	49344	0.028951512	49344	0.029990004		
49400	0.04493105	49400	0.079337384	24700	0.036971538	24708
0.116168099	49352	0.053623895	49352	0.056332097		
49400	0.060249593	49400	0.090912756	24700	0.037893999	24712
0.11091277	49360	0.036938724	49360	0.037042715		
49400	0.066205575	49400	0.091283051	24700	0.015996737	24716
0.064246553	49368	0.016489141	49368	0.034852805		
49400	0.07573983	49400	0.063758394	24700	0.014688046	24720
0.061947707	49376	0.020144556	49376	0.04660201		
49400	0.07774499	49400	0.048699054	24700	0.023216375	24724
0.052401243	49384	0.060188188	49384	0.007268902		
49400	0.080135869	49400	0.051438616	24700	0.03409139	24728
0.023558579	49392	0.099317091	49392	0.048864382		
49400	0.096241529	49400	0.021499667	24700	0.03683546	24732
0.052422893	49400	0.076001998	49400	0.059766637		
49400	0.115003735	49400	0.053936212	24700	0.067122041	24736
0.073702024	49408	0.013890074	49408	0.036269834		
49400	0.100614787	49400	0.06563498	24700	0.081389131	24740
0.087545508	49416	0.030893662	49416	0.049596292		
49400	0.04646102	49400	0.046563499	24700	0.065740991	24744
0.092166054	49424	0.052511725	49424	0.083585066		
49400	0.029571296	49400	0.029497312	24700	0.054818705	24748
0.075965159	49432	0.063149535	49432	0.062513769		
49400	0.055575314	49400	0.069356946	24800	0.041900115	24752
0.096198761	49440	0.044152665	49440	0.028338516		
49400	0.097513344	49400	0.069318288	24800	0.0107423	24756
0.112303984	49448	0.041447122	49448	0.004643595		
49500	0.108300759	49500	0.09030247	24800	0.076430137	24760
0.076849086	49456	0.122656929	49456	0.038218888		
49500	0.12591861	49500	0.169565349	24800	0.111911977	24764
0.080019265	49464	0.161687553	49464	0.042582404		
49500	0.126252896	49500	0.108163564	24800	0.081962171	24768
0.111123853	49472	0.11997667	49472	0.016142316		
49500	0.072501323	49500	0.067416375	24800	0.034322435	24772
0.096606651	49480	0.014107392	49480	0.018984114		
49500	0.10076812	49500	0.110088411	24800	0.007394837	24776
0.049614679	49488	0.080176207	49488	0.03447272		
49500	0.113249436	49500	0.076503689	24800	0.03486035	24780
0.016657135	49496	0.087592358	49496	0.018883657		
49500	0.080862599	49500	0.039912811	24800	0.083854822	24784
0.032938136	49504	0.078717683	49504	0.019974719		
49500	0.016451113	49500	0.069102018	24800	0.052850031	24788
0.05007337	49512	0.110776273	49512	0.032709118		
49500	0.079263147	49500	0.040173836	24800	0.034680998	24792
0.042292188	49520	0.08990943	49520	0.02118468		
49500	0.045695608	49500	0.125031234	24800	0.058812562	24796
0.043579577	49528	0.050512645	49528	0.024044875		
49500	0.091420356	49500	0.121849	24800	0.035044963	24800

0.058981379	49536	0.031001411	49536	0.044501452		
49500	0.141257304	49500	0.10918561	24800	0.023535755	24804
0.085196378	49544	0.08611862	49544	0.073456482		
49600	0.113187292	49600	0.107878135	24800	0.040769395	24808
0.123752281	49552	0.093133051	49552	0.05892765		
49600	0.135685695	49600	0.10366978	24800	0.064377404	24812
0.126233354	49560	0.069048045	49560	0.028013443		
49600	0.140683813	49600	0.156834358	24800	0.036705522	24816
0.083238374	49568	0.052671105	49568	0.00263492		
49600	0.081301252	49600	0.15915696	24800	0.022802098	24820
0.047830366	49576	0.035800331	49576	0.012516003		
49600	0.03791113	49600	0.068554953	24800	0.050025326	24824
0.052496722	49584	0.057386766	49584	0.025846521		
49600	0.103400882	49600	0.050528222	24800	0.073073512	24828
0.046773021	49592	0.071474518	49592	0.053711639		
49600	0.138534103	49600	0.040088737	24800	0.080017439	24832
0.065925931	49600	0.072369956	49600	0.06217909		
49600	0.093715775	49600	0.034236957	24800	0.058942565	24836
0.055275843	49608	0.07176667	49608	0.045443852		
49600	0.043868171	49600	0.077229757	24800	0.0187151	24840
0.046563418	49616	0.073662704	49616	0.023919656		
49600	0.062385559	49600	0.115673181	24800	0.034072447	24844
0.082765168	49624	0.064680557	49624	0.032183751		
49600	0.116105046	49600	0.105736122	24800	0.035223777	24848
0.120969249	49632	0.109706889	49632	0.038011811		
49600	0.148654071	49600	0.073408861	24900	0.021458636	24852
0.091801216	49640	0.165469218	49640	0.02266919		
49600	0.15422325	49600	0.052480675	24900	0.058233709	24856
0.035722387	49648	0.150195323	49648	0.012060148		
49700	0.179094641	49700	0.072876479	24900	0.061292631	24860
0.086217042	49656	0.123005986	49656	0.02940671		
49700	0.174907604	49700	0.1114509	24900	0.058003188	24864
0.082683233	49664	0.1260922	49664	0.029456649		
49700	0.116086805	49700	0.07892018	24900	0.071513641	24868
0.062119798	49672	0.135868453	49672	0.032433058		
49700	0.099903315	49700	0.022606289	24900	0.070452719	24872
0.079184021	49680	0.08156868	49680	0.023662595		
49700	0.123157864	49700	0.046466623	24900	0.048836693	24876
0.093564864	49688	0.029763129	49688	0.013711247		
49700	0.111149464	49700	0.009083259	24900	0.026665881	24880
0.081912673	49696	0.058668149	49696	0.032491316		
49700	0.076285498	49700	0.073828131	24900	0.029804764	24884
0.063639804	49704	0.111320856	49704	0.007615537		
49700	0.107720189	49700	0.055549783	24900	0.077639299	24888
0.045505916	49712	0.083568863	49712	0.031039395		
49700	0.196260983	49700	0.116178795	24900	0.05861832	24892
0.021185322	49720	0.043269418	49720	0.022551316		
49700	0.228747449	49700	0.160748983	24900	0.04329723	24896
0.028426277	49728	0.090451045	49728	0.038445553		
49700	0.170989224	49700	0.075139942	24900	0.03937148	24900
0.046760899	49736	0.089113702	49736	0.060290986		
49700	0.068970774	49700	0.076800294	24900	0.015068263	24904
0.058539892	49744	0.016749455	49744	0.02937862		
49800	0.150938024	49800	0.114576949	24900	0.029725628	24908
0.053421325	49752	0.100846904	49752	0.011977156		
49800	0.18095292	49800	0.08468797	24900	0.045455025	24912
0.05084393	49760	0.13511497	49760	0.003966653		
49800	0.105588726	49800	0.048662645	24900	0.053292828	24916
0.04356341	49768	0.112586364	49768	0.013584521		
49800	0.10494404	49800	0.048293074	24900	0.057208799	24920
0.003896671	49776	0.142894758	49776	0.010491084		
49800	0.136358228	49800	0.091984664	24900	0.055110573	24924
0.038124377	49784	0.15803898	49784	0.0254592		
49800	0.12627551	49800	0.093627066	24900	0.05790361	24928
0.051873263	49792	0.108354456	49792	0.05779076		
49800	0.066466375	49800	0.034762426	24900	0.078181191	24932
0.053039923	49800	0.001446165	49800	0.086559041		
49800	0.054924982	49800	0.130142245	24900	0.091907234	24936

0.051699553 49808 0.083644292 49808 0.044350098
49800 0.121879479 49800 0.143091923 24900 0.069870708 24940
0.041427971 49816 0.091866306 49816 0.057153837
49800 0.155144356 49800 0.202384835 24900 0.023204413 24944
0.044958222 49824 0.068323025 49824 0.081255181
49800 0.098546807 49800 0.266095536 24900 0.032687101 24948
0.05479758 49832 0.07519652 49832 0.087409193
49800 0.03657609 49800 0.217829598 25000 0.056574201 24952
0.097012366 49840 0.072886825 49840 0.087204178
49800 0.097596967 49800 0.115701041 25000 0.04953477 24956
0.09015164 49848 0.083763393 49848 0.074340263
49900 0.080895545 49900 0.080555998 25000 0.058283498 24960
0.041932239 49856 0.099712153 49856 0.043836339
49900 0.065575478 49900 0.073613068 25000 0.054217624 24964
0.029263272 49864 0.072113537 49864 0.007683007
49900 0.1091089 49900 0.052623382 25000 0.043574179 24968
0.053990279 49872 0.052071504 49872 0.025242383
49900 0.168613769 49900 0.076076387 25000 0.027574024 24972
0.056738114 49880 0.062133659 49880 0.042043939
49900 0.135665846 49900 0.143751866 25000 0.037218721 24976
0.085491935 49888 0.021475034 49888 0.09236575
49900 0.071778697 49900 0.143318598 25000 0.055652708 24980
0.048651775 49896 0.075130112 49896 0.08989768
49900 0.030591189 49900 0.049616287 25000 0.053034415 24984
0.019586605 49904 0.155213173 49904 0.043585424
49900 0.030030135 49900 0.070111732 25000 0.047891364 24988
0.052482541 49912 0.189546234 49912 0.040495928
49900 0.099733334 49900 0.120327553 25000 0.044283363 24992
0.056905417 49920 0.129661159 49920 0.021978531
49900 0.103328966 49900 0.075139578 25000 0.079750607 24996
0.036195299 49928 0.110090135 49928 0.008991887
49900 0.054756838 49900 0.017487826 25000 0.083775965 25000
0.028156659 49936 0.133039954 49936 0.007169554
49900 0.10729675 49900 0.059042508 25000 0.050834726 25004
0.067500791 49944 0.090335794 49944 0.02678387
50000 0.123817445 50000 0.099976831 25000 0.055863515 25008
0.095974174 49952 0.015041204 49952 0.030035175
50000 0.116550109 50000 0.068864873 25000 0.06556313 25012
0.103201179 49960 0.071254624 49960 0.009951439
50000 0.093899464 50000 0.066275199 25000 0.060742379 25016
0.070948408 49968 0.103215949 49968 0.054312681
50000 0.111589674 50000 0.071780531 25000 0.059056703 25020
0.052820313 49976 0.100444791 49976 0.068372792
50000 0.112673522 50000 0.081808976 25000 0.045171171 25024
0.041786934 49984 0.060903563 49984 0.059591537
50000 0.058293299 50000 0.127842024 25000 0.046041849 25028
0.037389182 49992 0.037789087 49992 0.055091063
50000 0.068352376 50000 0.132639601 25000 0.063466956 25032
0.037343049 50000 0.093639981 50000 0.055886241
50000 0.024694235 50000 0.088113418 25000 0.052688207 25036
0.054247143 50008 0.049125756 50008 0.056904104
50000 0.042805892 50000 0.036320638 25000 0.028917717 25040
0.067744484 50016 0.045055887 50016 0.045443754
50000 0.017446922 50000 0.039168386 25000 0.035097921 25044
0.091963215 50024 0.081203645 50024 0.022653854
50000 0.067375942 50000 0.082420047 25000 0.035160883 25048
0.077078345 50032 0.0618181 50032 0.03670546
50000 0.094416362 50000 0.076332093 25100 0.037142756 25052
0.02320784 50040 0.076722754 50040 0.050185783
50000 0.056258468 50000 0.035482808 25100 0.051098032 25056
0.035814843 50048 0.016715463 50048 0.065115084
50100 0.045142937 50100 0.086863707 25100 0.058944945 25060
0.062837215 50056 0.095158954 50056 0.062387167
50100 0.046777179 50100 0.175543595 25100 0.050345738 25064
0.03859607 50064 0.121044381 50064 0.038067596
50100 0.101682395 50100 0.157246119 25100 0.043156571 25068
0.031682623 50072 0.125464053 50072 0.045362565
50100 0.122457015 50100 0.080852406 25100 0.025082767 25072

0.072040362 50080 0.132539339 50080 0.072336014
50100 0.145964106 50100 0.05398599 25100 0.041158757 25076
0.06814173 50088 0.09483668 50088 0.07752479
50100 0.15705965 50100 0.090441194 25100 0.085639309 25080
0.027366114 50096 0.100860641 50096 0.071463292
50100 0.1038552 50100 0.17229763 25100 0.082233062 25084
0.057665311 50104 0.112561414 50104 0.049453342
50100 0.019228623 50100 0.193569358 25100 0.07041682 25088
0.040124876 50112 0.118358308 50112 0.036021411
50100 0.054898577 50100 0.125802835 25100 0.049512044 25092
0.001627966 50120 0.098482502 50120 0.037095379
50100 0.099903722 50100 0.059051461 25100 0.052990654 25096
0.022614067 50128 0.049143338 50128 0.036156718
50100 0.050567858 50100 0.018210028 25100 0.11089183 25100
0.014824989 50136 0.070913156 50136 0.080177408
50100 0.101382764 50100 0.090118498 25100 0.124502927 25104
0.005435385 50144 0.143239159 50144 0.067758665
50200 0.203677293 50200 0.144123769 25100 0.070361173 25108
0.009609332 50152 0.180086834 50152 0.009881202
50200 0.146099221 50200 0.077382967 25100 0.015386566 25112
0.029778501 50160 0.155609553 50160 0.048140617
50200 0.067286557 50200 0.098490607 25100 0.024712088 25116
0.049257975 50168 0.108140099 50168 0.058932863
50200 0.115823314 50200 0.044151606 25100 0.030383373 25120
0.057987098 50176 0.110390858 50176 0.038917122
50200 0.102393053 50200 0.036414524 25100 0.042653923 25124
0.079813457 50184 0.100752353 50184 0.012285584
50200 0.107984168 50200 0.078280478 25100 0.060617331 25128
0.054937409 50192 0.090291462 50192 0.011041911
50200 0.132308488 50200 0.046418241 25100 0.079655052 25132
0.019034615 50200 0.097623793 50200 0.014053284
50200 0.119417549 50200 0.026477372 25100 0.085706794 25136
0.071019284 50208 0.077592587 50208 0.031884771
50200 0.096437769 50200 0.07356704 25100 0.069599642 25140
0.0952228 50216 0.052308253 50216 0.053558251
50200 0.105965053 50200 0.089039932 25100 0.044259585 25144
0.074733158 50224 0.041537336 50224 0.070200418
50200 0.132236891 50200 0.0829407 25100 0.045177516 25148
0.044015 50232 0.032601307 50232 0.056136698
50200 0.117997282 50200 0.138020463 25200 0.061916449 25152
0.068107882 50240 0.030860298 50240 0.042709296
50200 0.104622021 50200 0.108068496 25200 0.056792844 25156
0.07107608 50248 0.039812847 50248 0.032790034
50300 0.090589136 50300 0.099662502 25200 0.024636938 25160
0.046154626 50256 0.115909468 50256 0.015951004
50300 0.078166966 50300 0.122348763 25200 0.041969914 25164
0.015583772 50264 0.147263563 50264 0.014757114
50300 0.061520746 50300 0.112108457 25200 0.059254628 25168
0.039725874 50272 0.170673986 50272 0.021061056
50300 0.037915099 50300 0.113353955 25200 0.050108447 25172
0.04453415 50280 0.168809828 50280 0.03060438
50300 0.120511351 50300 0.103454819 25200 0.062846455 25176
0.040145384 50288 0.191384839 50288 0.02039697
50300 0.121514262 50300 0.046934649 25200 0.024207226 25180
0.042601896 50296 0.231396873 50296 0.010922121
50300 0.070525151 50300 0.153546528 25200 0.038230715 25184
0.048050402 50304 0.170559463 50304 0.015234287
50300 0.04753768 50300 0.200768234 25200 0.074707808 25188
0.06472502 50312 0.132508576 50312 0.062609433
50300 0.08118587 50300 0.118233402 25200 0.096430653 25192
0.054985758 50320 0.155675633 50320 0.092810202
50300 0.075645614 50300 0.042931319 25200 0.094447161 25196
0.031362404 50328 0.102887592 50328 0.069245594
50300 0.043921787 50300 0.053618529 25200 0.061250852 25200
0.01767223 50336 0.043532768 50336 0.03065923
50300 0.084515079 50300 0.057141166 25200 0.02833876 25204
0.027901915 50344 0.047672045 50344 0.015704221
50400 0.092539434 50400 0.118882235 25200 0.066445995 25208

0.05555449 50352 0.069004214 50352 0.019548881
50400 0.115965333 50400 0.190121268 25200 0.06912183 25212
0.072570249 50360 0.072284172 50360 0.034260615
50400 0.102685699 50400 0.155357295 25200 0.052693013 25216
0.05738443 50368 0.065993161 50368 0.039922867
50400 0.014131818 50400 0.036700072 25200 0.025457186 25220
0.049861283 50376 0.098619603 50376 0.054258617
50400 0.074648626 50400 0.065167937 25200 0.062993931 25224
0.054142271 50384 0.0869125 50384 0.063653824
50400 0.10912701 50400 0.089425957 25200 0.09295877 25228
0.044202614 50392 0.029777759 50392 0.061926578
50400 0.135900016 50400 0.075411394 25200 0.072240371 25232
0.017475657 50400 0.073330259 50400 0.056917139
50400 0.190323466 50400 0.089160531 25200 0.066034998 25236
0.035066074 50408 0.113424903 50408 0.042670192
50400 0.224246993 50400 0.115824354 25200 0.055263972 25240
0.060058512 50416 0.121152611 50416 0.032414057
50400 0.179185066 50400 0.115094576 25200 0.04236693 25244
0.05658923 50424 0.129382999 50424 0.029128076
50400 0.088102199 50400 0.055658558 25200 0.072485331 25248
0.030367011 50432 0.151204149 50432 0.019867704
50400 0.071826071 50400 0.064518557 25300 0.075724805 25252
0.011398581 50440 0.098233781 50440 0.023648581
50400 0.072778697 50400 0.119896307 25300 0.058745543 25256
0.034881487 50448 0.089074558 50448 0.037752154
50500 0.079119251 50500 0.089615358 25300 0.028560236 25260
0.037243437 50456 0.214028333 50456 0.060521208
50500 0.072616342 50500 0.050423769 25300 0.026911463 25264
0.05918559 50464 0.200434951 50464 0.071008428
50500 0.028532628 50500 0.081053389 25300 0.067697336 25268
0.09435937 50472 0.14424957 50472 0.034008695
50500 0.021643567 50500 0.047892117 25300 0.064946078 25272
0.089251778 50480 0.08793734 50480 0.022278502
50500 0.030938689 50500 0.024531364 25300 0.031592968 25276
0.075244338 50488 0.041767085 50488 0.010510626
50500 0.07036535 50500 0.026227262 25300 0.018575653 25280
0.104386476 50496 0.017044476 50496 0.035621579
50500 0.134698551 50500 0.066374123 25300 0.002708127 25284
0.120247525 50504 0.050319402 50504 0.059125763
50500 0.108053311 50500 0.127624531 25300 0.037580165 25288
0.103393781 50512 0.109877918 50512 0.044496526
50500 0.069202768 50500 0.085276901 25300 0.059068247 25292
0.055359244 50520 0.087102184 50520 0.029803312
50500 0.112151065 50500 0.06088549 25300 0.063653963 25296
0.075272066 50528 0.03643482 50528 0.01993711
50500 0.105166284 50500 0.157253773 25300 0.066900975 25300
0.062325642 50536 0.082627863 50536 0.028461185
50500 0.076529454 50500 0.193959728 25300 0.062064173 25304
0.046571284 50544 0.083538325 50544 0.064645006
50600 0.035957692 50600 0.188017744 25300 0.061761974 25308
0.05397006 50552 0.133201029 50552 0.055386168
50600 0.080977981 50600 0.108531269 25300 0.03620817 25312
0.053140026 50560 0.148680804 50560 0.017247512
50600 0.087018081 50600 0.074046177 25300 0.036033733 25316
0.034353521 50568 0.120928467 50568 0.043167995
50600 0.029057173 50600 0.084654537 25300 0.052127507 25320
0.031923628 50576 0.042843825 50576 0.048609098
50600 0.060034934 50600 0.109274384 25300 0.004331262 25324
0.057049088 50584 0.098215569 50584 0.048043603
50600 0.038654107 50600 0.119316945 25300 0.042605036 25328
0.041672884 50592 0.028161141 50592 0.057368536
50600 0.113458147 50600 0.103473612 25300 0.005875176 25332
0.047231122 50600 0.139978045 50600 0.054862292
50600 0.178077025 50600 0.120586556 25300 0.039916384 25336
0.039899838 50608 0.176776725 50608 0.030578078
50600 0.101303776 50600 0.112092712 25300 0.03900997 25340
0.008977659 50616 0.098902099 50616 0.019830332
50600 0.00468982 50600 0.098405988 25300 0.036445799 25344

0.032235599 50624 0.005257359 50624 0.017348921
50600 0.020057714 50600 0.111258334 25300 0.031152667 25348
0.04233258 50632 0.087607186 50632 0.017589855
50600 0.087580571 50600 0.080811151 25400 0.023427499 25352
0.044504479 50640 0.165527483 50640 0.02296811
50600 0.076221899 50600 0.032031046 25400 0.024112875 25356
0.045640642 50648 0.161977965 50648 0.03871449
50700 0.085739172 50700 0.089458663 25400 0.027378854 25360
0.050496528 50656 0.101793907 50656 0.04623516
50700 0.060293318 50700 0.138867545 25400 0.035902667 25364
0.057686306 50664 0.058780701 50664 0.051364172
50700 0.023695371 50700 0.17994488 25400 0.01798702 25368
0.026442138 50672 0.098663295 50672 0.061820261
50700 0.093744617 50700 0.138378383 25400 0.021384116 25372
0.035178549 50680 0.072508621 50680 0.053117681
50700 0.13258052 50700 0.113613234 25400 0.030219984 25376
0.04906713 50688 0.074885546 50688 0.036256817
50700 0.145945741 50700 0.111644993 25400 0.034329536 25380
0.049927992 50696 0.172281973 50696 0.042138632
50700 0.17599827 50700 0.077830082 25400 0.061387364 25384
0.033106364 50704 0.107423039 50704 0.069322647
50700 0.105425381 50700 0.049498158 25400 0.039398219 25388
0.054759505 50712 0.096195057 50712 0.070541188
50700 0.034684712 50700 0.105138955 25400 0.03299928 25392
0.0594597 50720 0.047667716 50720 0.046112866
50700 0.046657689 50700 0.082016995 25400 0.047424543 25396
0.021364744 50728 0.082000552 50728 0.020620044
50700 0.062424406 50700 0.156914438 25400 0.041517498 25400
0.029467834 50736 0.093394941 50736 0.018686533
50700 0.086348548 50700 0.18545345 25400 0.018085568 25404
0.020258698 50744 0.103012419 50744 0.059283633
50800 0.086994296 50800 0.112849295 25400 0.028628338 25408
0.004772923 50752 0.098821387 50752 0.074225296
50800 0.032960856 50800 0.035853595 25400 0.051664647 25412
0.005080295 50760 0.129725493 50760 0.052825591
50800 0.057036235 50800 0.097807708 25400 0.052380659 25416
0.00675229 50768 0.158017326 50768 0.011477699
50800 0.085363136 50800 0.063637082 25400 0.03612462 25420
0.020119911 50776 0.125134655 50776 0.016992795
50800 0.10026824 50800 0.033115091 25400 0.003460685 25424
0.049735168 50784 0.051731673 50784 0.033153276
50800 0.099077399 50800 0.099240177 25400 0.026932441 25428
0.105872321 50792 0.025929778 50792 0.064759537
50800 0.055246845 50800 0.119361648 25400 0.038796923 25432
0.116241434 50800 0.043146822 50800 0.085384505
50800 0.04686162 50800 0.118994249 25400 0.038030583 25436
0.075742144 50808 0.015226826 50808 0.055580968
50800 0.112251582 50800 0.107809661 25400 0.034373377 25440
0.055898803 50816 0.048850725 50816 0.034709592
50800 0.136232411 50800 0.086885128 25400 0.046911893 25444
0.027897952 50824 0.020160247 50824 0.039991242
50800 0.078905883 50800 0.047537251 25400 0.068174792 25448
0.042938435 50832 0.090124995 50832 0.063753534
50800 0.101449681 50800 0.028201219 25500 0.074626209 25452
0.097720898 50840 0.051225859 50840 0.080787781
50800 0.211162114 50800 0.084256382 25500 0.066689681 25456
0.0751076 50848 0.039982893 50848 0.049710601
50900 0.268839533 50900 0.130430985 25500 0.055303066 25460
0.030944975 50856 0.077723766 50856 0.017679331
50900 0.179268871 50900 0.045962366 25500 0.056289126 25464
0.030878251 50864 0.062295032 50864 0.004680001
50900 0.044683708 50900 0.144369391 25500 0.047333437 25468
0.033149328 50872 0.045220033 50872 0.04327557
50900 0.066714085 50900 0.180304181 25500 0.003625743 25472
0.033327531 50880 0.039176575 50880 0.07933141
50900 0.049713886 50900 0.052371092 25500 0.05332661 25476
0.039301962 50888 0.047222144 50888 0.063810963
50900 0.057990626 50900 0.092905888 25500 0.080606907 25480

0.058217513 50896 0.029099605 50896 0.046893096
50900 0.075444106 50900 0.027658731 25500 0.058868449 25484
0.053559961 50904 0.047503123 50904 0.055505243
50900 0.032688538 50900 0.12394879 25500 0.014490726 25488
0.018514218 50912 0.033079912 50912 0.060853181
50900 0.053134696 50900 0.123306149 25500 0.018477364 25492
0.012067091 50920 0.069209447 50920 0.046475765
50900 0.135971438 50900 0.136765899 25500 0.026477239 25496
0.018630273 50928 0.076372206 50928 0.005339514
50900 0.217172201 50900 0.090193826 25500 0.00326057 25500
0.022556244 50936 0.08953597 50936 0.035986468
50900 0.200940748 50900 0.076059987 25500 0.048961741 25504
0.03135244 50944 0.084430954 50944 0.041273048
51000 0.09222563 51000 0.099472985 25500 0.070267888 25508
0.055059511 50952 0.056187007 50952 0.059153284
51000 0.040707204 51000 0.026747526 25500 0.067083711 25512
0.082867184 50960 0.039444531 50960 0.086037333
51000 0.063709646 51000 0.017284347 25500 0.076012511 25516
0.073143245 50968 0.115568902 50968 0.041228497
51000 0.078161516 51000 0.024649078 25500 0.089115616 25520
0.023083692 50976 0.094548312 50976 0.037918631
51000 0.074149837 51000 0.052362972 25500 0.064442589 25524
0.017165197 50984 0.04435701 50984 0.019958308
51000 0.035570905 51000 0.042948777 25500 0.032813703 25528
0.030842577 50992 0.096365533 50992 0.033008942
51000 0.098246404 51000 0.052104024 25500 0.010772973 25532
0.06688101 51000 0.126468178 51000 0.046970883
51000 0.19762518 51000 0.099971469 25500 0.014707336 25536
0.080238897 51008 0.081849343 51008 0.041580228
51000 0.16818475 51000 0.070399881 25500 0.030746025 25540
0.071701732 51016 0.034636956 51016 0.047949965
51000 0.066865687 51000 0.060949289 25500 0.034737965 25544
0.051154493 51024 0.027998463 51024 0.046251786
51000 0.052932821 51000 0.149166503 25500 0.035259291 25548
0.027061744 51032 0.048259091 51032 0.021310174
51000 0.058241603 51000 0.146900624 25600 0.049739054 25552
0.057776008 51040 0.045830475 51040 0.012470652
51000 0.023509709 51000 0.112796668 25600 0.017143211 25556
0.04571729 51048 0.037823589 51048 0.021500162
51100 0.016868282 51100 0.122049234 25600 0.049492268 25560
0.011686133 51056 0.003590085 51056 0.016848462
51100 0.073616531 51100 0.138796023 25600 0.053863758 25564
0.037709706 51064 0.085647473 51064 0.033014738
51100 0.130983375 51100 0.116943607 25600 0.037036043 25568
0.041422365 51072 0.126442654 51072 0.063628191
51100 0.146504725 51100 0.085666172 25600 0.03535446 25572
0.020671994 51080 0.087910892 51080 0.069184702
51100 0.111941852 51100 0.061367529 25600 0.041110416 25576
0.021675603 51088 0.006017529 51088 0.039141673
51100 0.125121384 51100 0.057255507 25600 0.033514018 25580
0.056989175 51096 0.074810858 51096 0.012814651
51100 0.11818768 51100 0.031366304 25600 0.019367497 25584
0.09403492 51104 0.048438404 51104 0.036232897
51100 0.036654248 51100 0.107374624 25600 0.030327004 25588
0.074586773 51112 0.076778917 51112 0.055353201
51100 0.108629211 51100 0.187512429 25600 0.025680678 25592
0.037583181 51120 0.17055645 51120 0.023873887
51100 0.159326344 51100 0.216157874 25600 0.05345183 25596
0.035358189 51128 0.145489918 51128 0.026408219
51100 0.12084272 51100 0.189602055 25600 0.077081568 25600
0.043453529 51136 0.092947186 51136 0.03414767
51100 0.071068476 51100 0.12022625 25600 0.065498367 25604
0.047058733 51144 0.070393086 51144 0.038875747
51200 0.131806228 51200 0.038442551 25600 0.007604081 25608
0.029976724 51152 0.068486101 51152 0.046624162
51200 0.163481556 51200 0.0537448 25600 0.06101267 25612
0.007895627 51160 0.084621461 51160 0.038369235
51200 0.154636815 51200 0.071411778 25600 0.041768264 25616

0.03702482 51168 0.112743459 51168 0.046805701
51200 0.127578591 51200 0.127727443 25600 0.049842398 25620
0.021435517 51176 0.133129404 51176 0.067248322
51200 0.092446615 51200 0.176111659 25600 0.078194898 25624
0.026417909 51184 0.092922113 51184 0.063284861
51200 0.063589119 51200 0.14672066 25600 0.049971881 25628
0.024320705 51192 0.079060883 51192 0.040666128
51200 0.059655558 51200 0.089295216 25600 0.023940696 25632
0.03338452 51200 0.107437714 51200 0.010399609
51200 0.079115605 51200 0.094307208 25600 0.036540834 25636
0.060157297 51208 0.099241355 51208 0.029113764
51200 0.085028201 51200 0.071689188 25600 0.023656859 25640
0.044947014 51216 0.08269574 51216 0.037001031
51200 0.04351656 51200 0.098587902 25600 0.032488642 25644
0.023913519 51224 0.106913954 51224 0.054009946
51200 0.135049574 51200 0.113344038 25600 0.06815049 25648
0.032787273 51232 0.168759013 51232 0.062391082
51200 0.102287355 51200 0.036436824 25700 0.067796733 25652
0.046632526 51240 0.141489189 51240 0.063155996
51200 0.02607788 51200 0.049559523 25700 0.051318562 25656
0.057381418 51248 0.028886941 51248 0.057868485
51300 0.076408054 51300 0.077599405 25700 0.060578452 25660
0.064518041 51256 0.139771961 51256 0.06407826
51300 0.057419919 51300 0.067751687 25700 0.086530978 25664
0.048624213 51264 0.173129505 51264 0.091350798
51300 0.044956749 51300 0.056888875 25700 0.084123087 25668
0.029659557 51272 0.102766855 51272 0.118091477
51300 0.122655649 51300 0.120114055 25700 0.066331049 25672
0.013267989 51280 0.089289861 51280 0.118745425
51300 0.217224544 51300 0.123360078 25700 0.039743361 25676
0.018042563 51288 0.120807752 51288 0.086673907
51300 0.22974213 51300 0.06522295 25700 0.035276687 25680
0.049511411 51296 0.125732288 51296 0.04492914
51300 0.182663134 51300 0.04477445 25700 0.045965233 25684
0.028365104 51304 0.118396478 51304 0.027341672
51300 0.096806718 51300 0.014601044 25700 0.041996609 25688
0.012569144 51312 0.070271482 51312 0.026870512
51300 0.074978903 51300 0.077073783 25700 0.02617724 25692
0.04592953 51320 0.046516747 51320 0.029850737
51300 0.127945969 51300 0.139410099 25700 0.008914254 25696
0.085412423 51328 0.075172698 51328 0.036530892
51300 0.081726452 51300 0.143932994 25700 0.022775783 25700
0.060860413 51336 0.144889709 51336 0.029525345
51300 0.044508975 51300 0.074548691 25700 0.038359176 25704
0.023588895 51344 0.196691254 51344 0.026554841
51400 0.132851986 51400 0.026377129 25700 0.033748263 25708
0.007525861 51352 0.210797938 51352 0.036846304
51400 0.145982965 51400 0.04755217 25700 0.022141252 25712
0.037164871 51360 0.149085449 51360 0.029942836
51400 0.04115573 51400 0.067222682 25700 0.043442306 25716
0.051884486 51368 0.03743339 51368 0.036397374
51400 0.057608806 51400 0.106830004 25700 0.068751324 25720
0.068286157 51376 0.050633487 51376 0.064135282
51400 0.049985829 51400 0.149670741 25700 0.062317609 25724
0.066903784 51384 0.0145853 51384 0.057261961
51400 0.11974098 51400 0.12038221 25700 0.043532004 25728
0.023599254 51392 0.098179029 51392 0.024869367
51400 0.182688716 51400 0.099440062 25700 0.058074063 25732
0.045884834 51400 0.048166538 51400 0.020620788
51400 0.129428866 51400 0.150926571 25700 0.078238292 25736
0.064426364 51408 0.101487196 51408 0.008915697
51400 0.046375975 51400 0.16948428 25700 0.078266727 25740
0.073957308 51416 0.157586925 51416 0.024428091
51400 0.078502279 51400 0.142011456 25700 0.051682509 25744
0.061454528 51424 0.069674614 51424 0.03354091
51400 0.100645528 51400 0.11396104 25700 0.018479279 25748
0.041756914 51432 0.084735912 51432 0.025547623
51400 0.088184861 51400 0.089610803 25800 0.004925962 25752

0.070831564	51440	0.116526055	51440	0.013640512		
51400	0.063123182	51400	0.073400901	25800	0.006546777	25756
0.096051037	51448	0.059758586	51448	0.034097473		
51500	0.055733486	51500	0.051625564	25800	0.024390158	25760
0.091413218	51456	0.028226767	51456	0.017528613		
51500	0.076742639	51500	0.063832689	25800	0.036160116	25764
0.063108702	51464	0.060921277	51464	0.033471675		
51500	0.087162218	51500	0.093362556	25800	0.044819408	25768
0.023023042	51472	0.088965295	51472	0.041108466		
51500	0.057319874	51500	0.066706205	25800	0.066261593	25772
0.010349497	51480	0.093346389	51480	0.007081848		
51500	0.091887952	51500	0.074574775	25800	0.065397398	25776
0.057060577	51488	0.094507785	51488	0.0266918		
51500	0.075344266	51500	0.070620081	25800	0.056639943	25780
0.069862843	51496	0.109936867	51496	0.050537907		
51500	0.037460541	51500	0.062488871	25800	0.05385327	25784
0.052641386	51504	0.095411553	51504	0.070777576		
51500	0.076141638	51500	0.021827174	25800	0.049385501	25788
0.039286406	51512	0.057409972	51512	0.063642314		
51500	0.066777415	51500	0.045939909	25800	0.055484125	25792
0.040773306	51520	0.032921649	51520	0.057483841		
51500	0.138632313	51500	0.093651383	25800	0.063629319	25796
0.035719491	51528	0.064551576	51528	0.05619177		
51500	0.122491882	51500	0.089736204	25800	0.043103148	25800
0.026828684	51536	0.101468409	51536	0.024376621		
51500	0.073817362	51500	0.065596505	25800	0.018332014	25804
0.052298827	51544	0.065102009	51544	0.03584705		
51600	0.040251078	51600	0.058740781	25800	0.019845993	25808
0.082442915	51552	0.079639933	51552	0.056708308		
51600	0.024892235	51600	0.10595686	25800	0.012843581	25812
0.099407866	51560	0.058137972	51560	0.057733148		
51600	0.010531002	51600	0.153951026	25800	0.050297666	25816
0.083009516	51568	0.049132432	51568	0.029030256		
51600	0.045977973	51600	0.149771455	25800	0.051232182	25820
0.035403999	51576	0.100507008	51576	0.006531259		
51600	0.117879608	51600	0.132231595	25800	0.023182571	25824
0.008199255	51584	0.061170242	51584	0.009345176		
51600	0.144550839	51600	0.134352856	25800	0.051590607	25828
0.006683809	51592	0.052763524	51592	0.042081567		
51600	0.129257344	51600	0.127911918	25800	0.07010626	25832
0.013090615	51600	0.111443849	51600	0.058090755		
51600	0.076694116	51600	0.131943714	25800	0.063542488	25836
0.015661133	51608	0.081071099	51608	0.014199841		
51600	0.012735584	51600	0.129216118	25800	0.039861316	25840
0.019608178	51616	0.037537244	51616	0.078145626		
51600	0.053174663	51600	0.075522934	25800	0.037447269	25844
0.030562434	51624	0.098271499	51624	0.059913298		
51600	0.071796465	51600	0.065269502	25800	0.045900662	25848
0.025418774	51632	0.106192565	51632	0.003138665		
51600	0.101238962	51600	0.124948798	25900	0.044849567	25852
0.044605902	51640	0.101579972	51640	0.047689166		
51600	0.103506514	51600	0.148182168	25900	0.026485483	25856
0.046610196	51648	0.08893534	51648	0.061450141		
51700	0.079726102	51700	0.086435488	25900	0.036159865	25860
0.021438658	51656	0.071808019	51656	0.03891439		
51700	0.101429818	51700	0.031398406	25900	0.047575144	25864
0.015870604	51664	0.051064199	51664	0.005802591		
51700	0.105233514	51700	0.029105249	25900	0.024128533	25868
0.03363641	51672	0.040802246	51672	0.022645749		
51700	0.07522016	51700	0.043919776	25900	0.077454039	25872
0.050729374	51680	0.013659253	51680	0.01094908		
51700	0.046007772	51700	0.011512991	25900	0.067101464	25876
0.033582615	51688	0.032585376	51688	0.026820251		
51700	0.057982408	51700	0.058161244	25900	0.066229841	25880
0.038370657	51696	0.049158531	51696	0.032813474		
51700	0.124113096	51700	0.076582204	25900	0.076042459	25884
0.070482405	51704	0.007944106	51704	0.020195867		
51700	0.142745645	51700	0.033462435	25900	0.065854263	25888

0.07378617 51712 0.057745183 51712 0.005476146
51700 0.108569308 51700 0.005654298 25900 0.043001335 25892
0.072158226 51720 0.055392706 51720 0.008764145
51700 0.079497237 51700 0.032709431 25900 0.0332696 25896
0.060626851 51728 0.035726091 51728 0.016668082
51700 0.105388477 51700 0.142285906 25900 0.016330403 25900
0.07235528 51736 0.087163593 51736 0.04445446
51700 0.156986105 51700 0.241969945 25900 0.074247255 25904
0.068522066 51744 0.169625288 51744 0.060878669
51800 0.146003251 51800 0.2541953 25900 0.102980725 25908
0.056839275 51752 0.196113964 51752 0.060670231
51800 0.100633864 51800 0.206484969 25900 0.062353342 25912
0.053966534 51760 0.16822276 51760 0.03146844
51800 0.053343549 51800 0.121972524 25900 0.025516858 25916
0.049007766 51768 0.103585611 51768 0.009927594
51800 0.043549553 51800 0.082573191 25900 0.034789286 25920
0.086183543 51776 0.056653818 51776 0.0126167
51800 0.019370587 51800 0.138785181 25900 0.027532586 25924
0.095456046 51784 0.095216143 51784 0.022563105
51800 0.073580275 51800 0.18792499 25900 0.037058013 25928
0.073481446 51792 0.088720997 51792 0.0594753
51800 0.111763722 51800 0.198665308 25900 0.052970056 25932
0.066440858 51800 0.037386559 51800 0.072535426
51800 0.128535946 51800 0.170519809 25900 0.058897666 25936
0.070234033 51808 0.056246921 51808 0.052213814
51800 0.144694364 51800 0.143654368 25900 0.055391305 25940
0.068924011 51816 0.136384814 51816 0.020322324
51800 0.107729502 51800 0.093121329 25900 0.065768749 25944
0.04004849 51824 0.150332213 51824 0.014696167
51800 0.026168296 51800 0.027386312 25900 0.047637052 25948
0.008300633 51832 0.090552494 51832 0.023477644
51800 0.081010905 51800 0.045702906 26000 0.005688591 25952
0.015842754 51840 0.093474861 51840 0.024685734
51800 0.068480607 51800 0.057512832 26000 0.026746948 25956
0.060645478 51848 0.141133336 51848 0.0297309
51900 0.073787516 51900 0.09124662 26000 0.049230748 25960
0.081015598 51856 0.116491749 51856 0.040927949
51900 0.117126554 51900 0.167424106 26000 0.057852984 25964
0.061451552 51864 0.099802652 51864 0.054795062
51900 0.116622003 51900 0.206655721 26000 0.060522907 25968
0.017946371 51872 0.13300289 51872 0.052401596
51900 0.057275789 51900 0.136039118 26000 0.042360749 25972
0.085663109 51880 0.098921497 51880 0.051406994
51900 0.026906342 51900 0.122662517 26000 0.013275436 25976
0.076013675 51888 0.001423456 51888 0.06826203
51900 0.039117873 51900 0.110225919 26000 0.020117359 25980
0.057278146 51896 0.11718375 51896 0.06627314
51900 0.047749662 51900 0.12004171 26000 0.05440968 25984
0.07275882 51904 0.183187861 51904 0.045646659
51900 0.085060674 51900 0.138312621 26000 0.085602689 25988
0.034878918 51912 0.179705006 51912 0.050101629
51900 0.105875311 51900 0.125604959 26000 0.078463621 25992
0.05199607 51920 0.093203475 51920 0.043787361
51900 0.108623164 51900 0.079407721 26000 0.010626714 25996
0.027069151 51928 0.052778596 51928 0.021344911
51900 0.14830142 51900 0.072975585 26000 0.07201553 26000
0.064488202 51936 0.116390518 51936 0.023836608
51900 0.121661367 51900 0.105665669 26000 0.095498363 26004
0.117847565 51944 0.081432467 51944 0.063514177
52000 0.101147583 52000 0.086581218 26000 0.056326673 26008
0.106697982 51952 0.099118275 51952 0.097700257
52000 0.0977194 52000 0.108670101 26000 0.030568423 26012
0.04887722 51960 0.081278995 51960 0.092954673
52000 0.029176557 52000 0.115464798 26000 0.022126857 26016
0.02904732 51968 0.031650467 51968 0.058427944
52000 0.019622645 52000 0.033907832 26000 0.004014043 26020
0.02744393 51976 0.028308339 51976 0.022934992
52000 0.036509173 52000 0.0955172 26000 0.010703966 26024

0.076228665 51984 0.019603716 51984 0.030376657
52000 0.115373092 52000 0.108199412 26000 0.028032851 26028
0.140920428 51992 0.086121392 51992 0.062540377
52000 0.164271988 52000 0.033181685 26000 0.043864609 26032
0.121793695 52000 0.09070841 52000 0.080730715
52000 0.112199145 52000 0.128510569 26000 0.04842188 26036
0.109487533 52008 0.095119518 52008 0.088180204
52000 0.063924214 52000 0.082813676 26000 0.043989126 26040
0.144102509 52016 0.056692643 52016 0.084283987
52000 0.083004736 52000 0.034947196 26000 0.030418325 26044
0.126524231 52024 0.062423438 52024 0.066795408
52000 0.060341365 52000 0.028550141 26000 0.022303553 26048
0.086198379 52032 0.118838631 52032 0.055572014
52000 0.036584224 52000 0.063343883 26100 0.055808803 26052
0.095227479 52040 0.050313545 52040 0.057791225
52000 0.042619777 52000 0.079121142 26100 0.08280894 26056
0.096528078 52048 0.138530348 52048 0.054625507
52100 0.08555664 52100 0.074716576 26100 0.064387103 26060
0.050978761 52056 0.183832498 52056 0.04185177
52100 0.119573575 52100 0.039642971 26100 0.019878979 26064
0.068350855 52064 0.139518408 52064 0.017177814
52100 0.101327605 52100 0.041242973 26100 0.051527117 26068
0.084091415 52072 0.112654278 52072 0.028810124
52100 0.118627548 52100 0.029671219 26100 0.029597808 26072
0.090507077 52080 0.068094421 52080 0.021381276
52100 0.116347903 52100 0.037365666 26100 0.018981031 26076
0.083378858 52088 0.032810203 52088 0.049813636
52100 0.090774382 52100 0.084101841 26100 0.028801836 26080
0.038633843 52096 0.130536355 52096 0.085444983
52100 0.054599983 52100 0.057832531 26100 0.060506965 26084
0.075206364 52104 0.157313568 52104 0.072205279
52100 0.020534155 52100 0.056736069 26100 0.0791848 26088
0.074929834 52112 0.121199795 52112 0.036278318
52100 0.076589473 52100 0.057857847 26100 0.063276762 26092
0.010685124 52120 0.092625087 52120 0.032604308
52100 0.125431703 52100 0.015205019 26100 0.022524997 26096
0.098410259 52128 0.11461533 52128 0.044896384
52100 0.097068732 52100 0.04870219 26100 0.010424652 26100
0.105223946 52136 0.08958426 52136 0.030366582
52100 0.061477796 52100 0.056190318 26100 0.023567629 26104
0.03769427 52144 0.173003603 52144 0.033372264
52200 0.062112864 52200 0.005624869 26100 0.0628255 26108
0.047551661 52152 0.265608658 52152 0.053042964
52200 0.043697277 52200 0.057109701 26100 0.07877918 26112
0.055500928 52160 0.236254855 52160 0.079932033
52200 0.037584054 52200 0.051408544 26100 0.071216979 26116
0.106687003 52168 0.119079312 52168 0.08848033
52200 0.058610785 52200 0.011130409 26100 0.042874752 26120
0.128899003 52176 0.033620894 52176 0.063772204
52200 0.089294568 52200 0.071707924 26100 0.010224633 26124
0.085910666 52184 0.051632189 52184 0.037326932
52200 0.12614255 52200 0.091235706 26100 0.020223279 26128
0.107393506 52192 0.059915394 52192 0.017996848
52200 0.11412966 52200 0.087260734 26100 0.034440287 26132
0.098737328 52200 0.045483499 52200 0.039900369
52200 0.094154319 52200 0.096286058 26100 0.043512537 26136
0.047798862 52208 0.023094099 52208 0.076677068
52200 0.09946902 52200 0.105638108 26100 0.046836714 26140
0.021930046 52216 0.07952442 52216 0.077796845
52200 0.093988383 52200 0.056297726 26100 0.053053373 26144
0.04790146 52224 0.118581629 52224 0.063685759
52200 0.086101703 52200 0.029564204 26100 0.080699843 26148
0.061091159 52232 0.135753523 52232 0.035806534
52200 0.0758877 52200 0.095790689 26200 0.075841599 26152
0.031171425 52240 0.094495073 52240 0.016764036
52200 0.083117004 52200 0.125274514 26200 0.067560381 26156
0.064273823 52248 0.015497501 52248 0.023373714
52300 0.154133334 52300 0.121006226 26200 0.07397122 26160

0.072909614 52256 0.056468893 52256 0.032062562
52300 0.167084159 52300 0.087886714 26200 0.06562232 26164
0.039759529 52264 0.120086181 52264 0.022578173
52300 0.092799637 52300 0.066082866 26200 0.030253852 26168
0.021119118 52272 0.123613339 52272 0.042816133
52300 0.045974874 52300 0.060156996 26200 0.00479357 26172
0.030918491 52280 0.079764461 52280 0.03853182
52300 0.10654526 52300 0.040061514 26200 0.019026771 26176
0.094383402 52288 0.088098073 52288 0.03196195
52300 0.134608403 52300 0.089436195 26200 0.016960075 26180
0.091344213 52296 0.057812926 52296 0.026550637
52300 0.082400344 52300 0.127654275 26200 0.018963792 26184
0.022906608 52304 0.132154135 52304 0.054038417
52300 0.276533654 52300 0.063582782 26200 0.052615771 26188
0.025715064 52312 0.136938775 52312 0.052251988
52300 0.815248408 52300 0.031148364 26200 0.043450724 26192
0.033227934 52320 0.056759734 52320 0.016044862
52300 1.59564405 52300 0.01511721 26200 0.032247808 26196
0.067124449 52328 0.072850009 52328 0.035216061
52300 1.893583688 52300 0.032132506 26200 0.06664078 26200
0.067844558 52336 0.163169941 52336 0.045133787
52300 1.31739955 52300 0.06589594 26200 0.059796093 26204
0.055283217 52344 0.197718095 52344 0.030897434
52400 0.580436143 52400 0.088323948 26200 0.042257161 26208
0.049109323 52352 0.103091166 52352 0.051826406
52400 0.63325098 52400 0.011022996 26200 0.0346241 26212
0.088589863 52360 0.110970643 52360 0.068523077
52400 0.435518945 52400 0.111482208 26200 0.049206999 26216
0.075108612 52368 0.146458523 52368 0.06841452
52400 0.073698117 52400 0.101318379 26200 0.078302415 26220
0.034322253 52376 0.059571117 52376 0.057635883
52400 0.080181395 52400 0.052541353 26200 0.072732401 26224
0.06950464 52384 0.077045232 52384 0.040554769
52400 0.088698289 52400 0.072566661 26200 0.059000533 26228
0.075299897 52392 0.091794922 52392 0.024937528
52400 0.201239644 52400 0.035003126 26200 0.051167917 26232
0.027041071 52400 0.074471886 52400 0.011641599
52400 0.164967584 52400 0.030534451 26200 0.045323461 26236
0.043591488 52408 0.06249563 52408 0.02212855
52400 0.068162583 52400 0.083584178 26200 0.053443979 26240
0.082202576 52416 0.141671583 52416 0.02619378
52400 0.085328458 52400 0.194596083 26200 0.059855582 26244
0.094306932 52424 0.149106985 52424 0.035256118
52400 0.068987167 52400 0.174417466 26200 0.05279787 26248
0.071337992 52432 0.093772804 52432 0.038648599
52400 0.082836894 52400 0.09008783 26300 0.018250852 26252
0.031937758 52440 0.018379202 52440 0.027964228
52400 0.169966559 52400 0.095905263 26300 0.003576186 26256
0.066202803 52448 0.13220559 52448 0.007208348
52500 0.201688919 52500 0.112177309 26300 0.013938958 26260
0.027947628 52456 0.219361813 52456 0.030412044
52500 0.191834551 52500 0.100829326 26300 0.037358062 26264
0.017886443 52464 0.160720025 52464 0.040132873
52500 0.152714303 52500 0.082952538 26300 0.030843366 26268
0.036883808 52472 0.028253349 52472 0.035784513
52500 0.118974895 52500 0.088358414 26300 0.018772429 26272
0.019134017 52480 0.073244359 52480 0.032765784
52500 0.205076634 52500 0.156870359 26300 0.055424993 26276
0.025792826 52488 0.093544899 52488 0.046405756
52500 0.248118595 52500 0.201347983 26300 0.081604419 26280
0.032226129 52496 0.062964013 52496 0.070151946
52500 0.192589753 52500 0.116935509 26300 0.051459643 26284
0.055383025 52504 0.032838678 52504 0.060192946
52500 0.100792888 52500 0.082968538 26300 0.06295825 26288
0.075104785 52512 0.052049709 52512 0.01874308
52500 0.174528949 52500 0.129264169 26300 0.100245365 26292
0.107272477 52520 0.039445607 52520 0.005984728
52500 0.227609068 52500 0.123375707 26300 0.083203486 26296

0.051633091 52528 0.075531862 52528 0.008625369
52500 0.138042699 52500 0.093369548 26300 0.048898019 26300
0.069789814 52536 0.163708435 52536 0.018618825
52500 0.193359214 52500 0.076332814 26300 0.03025896 26304
0.112516289 52544 0.204682496 52544 0.046410583
52600 0.319687738 52600 0.095684547 26300 0.043810549 26308
0.073735071 52552 0.178993258 52552 0.044827255
52600 0.708061794 52600 0.085514221 26300 0.043023145 26312
0.081234161 52560 0.110379478 52560 0.009131753
52600 0.928697176 52600 0.056484048 26300 0.01496915 26316
0.078854391 52568 0.042803047 52568 0.017669854
52600 0.631293283 52600 0.057890145 26300 0.041008352 26320
0.034152949 52576 0.028040719 52576 0.017683211
52600 1.017741278 52600 0.084855645 26300 0.017078088 26324
0.045401022 52584 0.083815365 52584 0.031587879
52600 4.529012838 52600 0.08384815 26300 0.032889457 26328
0.042456595 52592 0.124020473 52592 0.027191842
52600 7.842751225 52600 0.067083252 26300 0.022685648 26332
0.034893499 52600 0.13785249 52600 0.014130208
52600 7.457137113 52600 0.064278189 26300 0.037700236 26336
0.038566926 52608 0.122445839 52608 0.017225435
52600 3.907899838 52600 0.113740549 26300 0.024701518 26340
0.018026489 52616 0.108434862 52616 0.031119012
52600 1.102441689 52600 0.152037595 26300 0.025856596 26344
0.052892792 52624 0.129878215 52624 0.029085062
52600 0.367375789 52600 0.103039231 26300 0.043061911 26348
0.068876558 52632 0.174312329 52632 0.036226451
52600 0.277106418 52600 0.078201163 26400 0.030473113 26352
0.045820692 52640 0.184203381 52640 0.035494195
52600 0.347566704 52600 0.135156399 26400 0.021520888 26356
0.072754803 52648 0.106871754 52648 0.008459016
52700 0.155276604 52700 0.13724716 26400 0.011525703 26360
0.069971175 52656 0.015349564 52656 0.016303
52700 0.206918238 52700 0.149125728 26400 0.027768423 26364
0.045497902 52664 0.087709384 52664 0.031720862
52700 0.240962705 52700 0.130950023 26400 0.080173726 26368
0.052230778 52672 0.128606741 52672 0.078130921
52700 0.226804739 52700 0.080813552 26400 0.085682281 26372
0.02989806 52680 0.053924734 52680 0.094146963
52700 0.190908483 52700 0.082828738 26400 0.038046452 26376
0.019060008 52688 0.166518308 52688 0.077421217
52700 0.300627523 52700 0.089624693 26400 0.036262103 26380
0.024856021 52696 0.185508718 52696 0.051287792
52700 0.346651126 52700 0.06585224 26400 0.045595338 26384
0.068850532 52704 0.111105226 52704 0.022476463
52700 0.220606846 52700 0.059541617 26400 0.060610146 26388
0.089345078 52712 0.069559006 52712 0.002424827
52700 0.186197664 52700 0.102658676 26400 0.074215568 26392
0.043401986 52720 0.065343134 52720 0.017415276
52700 0.245583535 52700 0.123240912 26400 0.069724236 26396
0.036670277 52728 0.089838155 52728 0.017479662
52700 0.152741151 52700 0.094213981 26400 0.051859992 26400
0.035385972 52736 0.099085206 52736 0.008081701
52700 0.151579151 52700 0.062171173 26400 0.017056689 26404
0.041311731 52744 0.058330821 52744 0.038297967
52800 0.207227611 52800 0.040954772 26400 0.037391965 26408
0.059375387 52752 0.036162706 52752 0.048530194
52800 0.196195048 52800 0.039309027 26400 0.040949966 26412
0.021176742 52760 0.102108679 52760 0.05508876
52800 0.190591745 52800 0.049366237 26400 0.042642823 26416
0.019824096 52768 0.103268998 52768 0.079850506
52800 0.149521453 52800 0.153284884 26400 0.076453609 26420
0.011039037 52776 0.04230786 52776 0.059244696
52800 0.110078829 52800 0.102302052 26400 0.06207003 26424
0.003931631 52784 0.074333417 52784 0.038187958
52800 0.148319595 52800 0.03276689 26400 0.053293352 26428
0.016322505 52792 0.076503486 52792 0.088532164
52800 0.194525958 52800 0.051850297 26400 0.064828644 26432

0.044215281 52800 0.02670058 52800 0.059976039
52800 0.108938199 52800 0.023245084 26400 0.035922672 26436
0.055880486 52808 0.027181905 52808 0.005748128
52800 0.162666533 52800 0.036331076 26400 0.025002315 26440
0.057170317 52816 0.073506359 52816 0.005017507
52800 0.23520636 52800 0.045808287 26400 0.039739942 26444
0.054852964 52824 0.091547256 52824 0.036183668
52800 0.171933513 52800 0.07868676 26400 0.032229731 26448
0.05617808 52832 0.073358155 52832 0.072987539
52800 0.613926328 52800 0.152302615 26500 0.07553905 26452
0.036198646 52840 0.086218432 52840 0.080557024
52800 0.670054695 52800 0.120300763 26500 0.05985465 26456
0.018834477 52848 0.103928607 52848 0.06209658
52900 0.599223189 52900 0.079895785 26500 0.037035799 26460
0.029310355 52856 0.050624356 52856 0.032410291
52900 1.907767263 52900 0.171507928 26500 0.058998856 26464
0.050465045 52864 0.074828553 52864 0.050809038
52900 2.611408938 52900 0.164764119 26500 0.041849002 26468
0.042553955 52872 0.072637929 52872 0.053403997
52900 2.025322988 52900 0.073813062 26500 0.053320913 26472
0.011355561 52880 0.033817945 52880 0.033333814
52900 0.85913029 52900 0.026837342 26500 0.066649605 26476
0.05912382 52888 0.072663133 52888 0.006782041
52900 0.173418754 52900 0.025129932 26500 0.029788535 26480
0.039933853 52896 0.105571853 52896 0.022688482
52900 0.082607236 52900 0.041611424 26500 0.055338114 26484
0.014796976 52904 0.091427741 52904 0.041437183
52900 0.147397891 52900 0.077382349 26500 0.074653173 26488
0.045055789 52912 0.017163295 52912 0.03470738
52900 0.058592341 52900 0.078430196 26500 0.098273049 26492
0.068276771 52920 0.046439327 52920 0.01821106
52900 0.096475087 52900 0.051150222 26500 0.101827674 26496
0.04774909 52928 0.101053192 52928 0.030340763
52900 0.039739567 52900 0.05694593 26500 0.056113666 26500
0.027443717 52936 0.089896399 52936 0.053902975
52900 0.006014136 52900 0.021291859 26500 0.029517814 26504
0.029837027 52944 0.031285632 52944 0.036472175
53000 0.028032462 53000 0.091567752 26500 0.038248902 26508
0.026727226 52952 0.038083665 52952 0.023006684
53000 0.125934035 53000 0.139084834 26500 0.019821318 26512
0.006088964 52960 0.055780045 52960 0.044511624
53000 0.19072635 53000 0.125134116 26500 0.023520755 26516
0.011140452 52968 0.0477127 52968 0.043951859
53000 0.155545261 53000 0.131052468 26500 0.045628938 26520
0.028250337 52976 0.046388534 52976 0.015700127
53000 0.062029132 53000 0.076082026 26500 0.040765339 26524
0.063569125 52984 0.081865423 52984 0.01684525
53000 0.044479482 53000 0.072309427 26500 0.021485854 26528
0.049195343 52992 0.06670216 52992 0.043724933
53000 0.049450544 53000 0.138350443 26500 0.01076959 26532
0.004965207 53000 0.075306547 53000 0.048809117
53000 0.042594587 53000 0.109811641 26500 0.034466713 26536
0.0016608 53008 0.139220574 53008 0.018255596
53000 0.063247957 53000 0.090731846 26500 0.043262451 26540
0.033455664 53016 0.124074402 53016 0.017117052
53000 0.107619133 53000 0.065114997 26500 0.045910645 26544
0.031036685 53024 0.060753635 53024 0.043064349
53000 0.127205858 53000 0.061796593 26500 0.049119932 26548
0.032620552 53032 0.053382675 53032 0.04339105
53000 0.137680894 53000 0.082086015 26600 0.055711225 26552
0.048453538 53040 0.0690948 53040 0.012812918
53000 0.151467029 53000 0.072134273 26600 0.021828397 26556
0.049570201 53048 0.075181401 53048 0.029418372
53100 0.192166946 53100 0.059173253 26600 0.041511183 26560
0.015834658 53056 0.072187257 53056 0.036754744
53100 0.161043078 53100 0.053084808 26600 0.034154546 26564
0.035915015 53064 0.076409386 53064 0.034871369
53100 0.108595174 53100 0.103609382 26600 0.013146769 26568

0.062817017 53072 0.090919493 53072 0.064795539
53100 0.125399325 53100 0.13451057 26600 0.013918513 26572
0.071166942 53080 0.128977291 53080 0.051617219
53100 0.105767394 53100 0.133497655 26600 0.003837818 26576
0.068134163 53088 0.169803985 53088 0.020031786
53100 0.069339723 53100 0.114059963 26600 0.03510762 26580
0.064497654 53096 0.221378869 53096 0.027983022
53100 0.094441261 53100 0.029335257 26600 0.081469108 26584
0.057235637 53104 0.276383158 53104 0.053438744
53100 0.18152289 53100 0.12873084 26600 0.095122479 26588
0.038403145 53112 0.188104328 53112 0.051937601
53100 0.20393332 53100 0.156376569 26600 0.071714094 26592
0.031839405 53120 0.083472376 53120 0.06508438
53100 0.232570194 53100 0.087697132 26600 0.031429812 26596
0.007653162 53128 0.079747515 53128 0.086521708
53100 0.29135641 53100 0.073158466 26600 0.028767991 26600
0.041174935 53136 0.017296354 53136 0.053289721
53100 0.2724691 53100 0.105892985 26600 0.021087326 26604
0.03752414 53144 0.031041014 53144 0.028329325
53200 0.194842476 53200 0.081532715 26600 0.014849768 26608
0.020356038 53152 0.062293846 53152 0.042905456
53200 0.117828553 53200 0.05012771 26600 0.010974276 26612
0.024227804 53160 0.102123529 53160 0.051430296
53200 0.053627027 53200 0.03369751 26600 0.02433155 26616
0.017452465 53168 0.097710901 53168 0.043077856
53200 0.008339211 53200 0.046551562 26600 0.030930958 26620
0.047591973 53176 0.024190733 53176 0.029198771
53200 0.026889356 53200 0.124250379 26600 0.029646717 26624
0.041636089 53184 0.035840647 53184 0.025901341
53200 0.037892056 53200 0.101416357 26600 0.021143998 26628
0.061083832 53192 0.066356748 53192 0.044318913
53200 0.072316572 53200 0.023635023 26600 0.048758579 26632
0.106576525 53200 0.087593748 53200 0.051820607
53200 0.104681451 53200 0.106222833 26600 0.063098785 26636
0.070877555 53208 0.096201795 53208 0.024191728
53200 0.086380089 53200 0.149908286 26600 0.040098836 26640
0.031092972 53216 0.106110092 53216 0.01885064
53200 0.024876514 53200 0.179359748 26600 0.046408051 26644
0.011045451 53224 0.092603164 53224 0.038552593
53200 0.077395598 53200 0.127245323 26600 0.051494404 26648
0.026354774 53232 0.099465426 53232 0.043817607
53200 0.201536809 53200 0.075247976 26700 0.050176681 26652
0.032917651 53240 0.125200648 53240 0.061952836
53200 0.217041321 53200 0.132982415 26700 0.083818202 26656
0.025972602 53248 0.093814269 53248 0.065626336
53300 0.113977549 53300 0.15736869 26700 0.10327308 26660
0.039592833 53256 0.022516464 53256 0.035154069
53300 0.117566429 53300 0.07848868 26700 0.078964193 26664
0.056171222 53264 0.04546707 53264 0.023346622
53300 0.15835285 53300 0.063075975 26700 0.061222789 26668
0.062986714 53272 0.062544132 53272 0.053376411
53300 0.110101057 53300 0.124030732 26700 0.064925909 26672
0.088421599 53280 0.077972669 53280 0.047891153
53300 0.035676763 53300 0.109876564 26700 0.064609194 26676
0.070178969 53288 0.052026218 53288 0.034215729
53300 0.095765005 53300 0.062900253 26700 0.044934353 26680
0.033529381 53296 0.043397304 53296 0.019326883
53300 0.126118466 53300 0.075011216 26700 0.037110156 26684
0.038472692 53304 0.057118392 53304 0.010591918
53300 0.057277095 53300 0.117962896 26700 0.052013318 26688
0.035617763 53312 0.069378315 53312 0.020215175
53300 0.078514699 53300 0.093681207 26700 0.048598438 26692
0.018220455 53320 0.074556541 53320 0.04733662
53300 0.060594357 53300 0.076094671 26700 0.054909317 26696
0.055349119 53328 0.033401746 53328 0.073912474
53300 0.10743126 53300 0.033153094 26700 0.043222673 26700
0.077970821 53336 0.118142103 53336 0.058360056
53300 0.143861108 53300 0.0411902 26700 0.017800374 26704

0.064213753 53344 0.173306471 53344 0.036691658
53400 0.145943603 53400 0.026747322 26700 0.060466176 26708
0.122133846 53352 0.175780268 53352 0.051205501
53400 0.093863688 53400 0.130467931 26700 0.060862825 26712
0.128908854 53360 0.161464311 53360 0.052929994
53400 0.053459695 53400 0.190499035 26700 0.027655235 26716
0.122526952 53368 0.163221645 53368 0.051492469
53400 0.073862779 53400 0.139069816 26700 0.048019607 26720
0.119359487 53376 0.154177716 53376 0.033872209
53400 0.070271955 53400 0.061323335 26700 0.064639928 26724
0.056142711 53384 0.109137523 53384 0.037906393
53400 0.070910573 53400 0.03691874 26700 0.054056014 26728
0.086549393 53392 0.055439901 53392 0.074754753
53400 0.110460765 53400 0.016054561 26700 0.03587321 26732
0.100127283 53400 0.010682932 53400 0.053950844
53400 0.150605701 53400 0.019814381 26700 0.015240798 26736
0.109341818 53408 0.033599503 53408 0.022576543
53400 0.141889468 53400 0.042698237 26700 0.029893481 26740
0.11541487 53416 0.027895172 53416 0.023081004
53400 0.077308767 53400 0.081308652 26700 0.020861047 26744
0.082469436 53424 0.051550753 53424 0.037833055
53400 0.073015952 53400 0.093531475 26700 0.041804105 26748
0.04500962 53432 0.068230787 53432 0.04901677
53400 0.113014736 53400 0.03938549 26800 0.063505468 26752
0.081137201 53440 0.015085666 53440 0.031517757
53400 0.199792645 53400 0.098708464 26800 0.01923898 26756
0.131071036 53448 0.031873049 53448 0.036685829
53500 0.208328871 53500 0.149911183 26800 0.028984894 26760
0.062210762 53456 0.111501802 53456 0.034584518
53500 0.085789085 53500 0.129332199 26800 0.008740849 26764
0.050245293 53464 0.238254361 53464 0.051869938
53500 0.109095177 53500 0.088429442 26800 0.070298971 26768
0.102565304 53472 0.194863475 53472 0.04679526
53500 0.247202203 53500 0.133686234 26800 0.083449122 26772
0.062895626 53480 0.065067907 53480 0.012809042
53500 0.222422969 53500 0.130046698 26800 0.020109394 26776
0.114395094 53488 0.014698167 53488 0.035157544
53500 0.097976597 53500 0.103227445 26800 0.028623781 26780
0.13797007 53496 0.046425335 53496 0.066358654
53500 0.028361925 53500 0.100308047 26800 0.010782156 26784
0.062484978 53504 0.076009354 53504 0.065568391
53500 0.119964861 53500 0.094697622 26800 0.00628699 26788
0.117841148 53512 0.074001408 53512 0.031130283
53500 0.1509305 53500 0.101863181 26800 0.015490006 26792
0.189475511 53520 0.112127949 53520 0.014106909
53500 0.152527951 53500 0.076408243 26800 0.014289544 26796
0.106795291 53528 0.046743935 53528 0.02922973
53500 0.160117139 53500 0.084620122 26800 0.062519779 26800
0.087962821 53536 0.106510161 53536 0.031286603
53500 0.15844329 53500 0.089094967 26800 0.056611945 26804
0.070408896 53544 0.165763616 53544 0.058307658
53600 0.080203696 53600 0.14270324 26800 0.047095557 26808
0.070087503 53552 0.117661439 53552 0.075005162
53600 0.064967157 53600 0.220838804 26800 0.064934546 26812
0.200962531 53560 0.068199952 53560 0.044523953
53600 0.08424143 53600 0.182518226 26800 0.046491183 26816
0.174626184 53568 0.082732411 53568 0.03833848
53600 0.105004976 53600 0.072096089 26800 0.059905331 26820
0.103769293 53576 0.031965818 53576 0.079729121
53600 0.152848603 53600 0.014323968 26800 0.064528904 26824
0.17904643 53584 0.091353788 53584 0.072614275
53600 0.143461991 53600 0.027336648 26800 0.037266837 26828
0.193025364 53592 0.087187953 53592 0.045586647
53600 0.072524272 53600 0.086023021 26800 0.035339734 26832
0.064285589 53600 0.057154797 53600 0.027481223
53600 0.077488847 53600 0.109116692 26800 0.026651289 26836
0.141321013 53608 0.072523275 53608 0.008432272
53600 0.122735844 53600 0.067532084 26800 0.00825083 26840

0.038327238 53616 0.078917903 53616 0.030191086
53600 0.117196345 53600 0.043742948 26800 0.028627055 26844
0.115066483 53624 0.075118645 53624 0.05160585
53600 0.09122918 53600 0.066009598 26800 0.060287657 26848
0.20603335 53632 0.052475767 53632 0.070205017
53600 0.115174022 53600 0.11626097 26900 0.071206814 26852
0.040795847 53640 0.047467351 53640 0.079061647
53600 0.123643389 53600 0.157634975 26900 0.051319759 26856
0.151215674 53648 0.086156462 53648 0.072639887
53700 0.082036953 53700 0.085040178 26900 0.029656034 26860
0.350791 53656 0.111053982 53656 0.050794159
53700 0.0730584 53700 0.086483364 26900 0.012496961 26864
0.10924747 53664 0.188966879 53664 0.041092873
53700 0.066557972 53700 0.044616536 26900 0.049998267 26868
0.288571784 53672 0.156492694 53672 0.063700703
53700 0.179437891 53700 0.043534066 26900 0.081230755 26872
0.174213245 53680 0.017769653 53680 0.082455445
53700 0.213028049 53700 0.053209347 26900 0.095279844 26876
0.187023354 53688 0.099790465 53688 0.068815491
53700 0.129585198 53700 0.0456782 26900 0.066620625 26880
0.217934721 53696 0.088512403 53696 0.046560457
53700 0.084232197 53700 0.005385346 26900 0.05524762 26884
0.071562266 53704 0.078014862 53704 0.038037531
53700 0.050894621 53700 0.024108669 26900 0.059116021 26888
0.138558345 53712 0.078553909 53712 0.043678028
53700 0.033213044 53700 0.102762017 26900 0.017442841 26892
0.414837181 53720 0.093770112 53720 0.038655704
53700 0.091972703 53700 0.087383378 26900 0.031460342 26896
0.129337903 53728 0.193555796 53728 0.011757534
53700 0.108440858 53700 0.042389049 26900 0.037727583 26900
0.844153401 53736 0.15775126 53736 0.041433956
53700 0.005017707 53700 0.056474677 26900 0.04928108 26904
1.084640623 53744 0.155734335 53744 0.054002296
53800 0.082347513 53800 0.052871812 26900 0.095857402 26908
1.60289835 53752 0.424907834 53752 0.066973691
53800 0.086317778 53800 0.086965847 26900 0.103966297 26912
2.359237288 53760 0.626465714 53760 0.08584957
53800 0.087739783 53800 0.135004419 26900 0.109691478 26916
3.464030338 53768 0.161784861 53768 0.083923071
53800 0.036512742 53800 0.141887081 26900 0.077105091 26920
4.455518438 53776 0.9387022 53776 0.042802374
53800 0.098625656 53800 0.129840176 26900 0.051825831 26924
5.331406838 53784 0.674017123 53784 0.007147173
53800 0.126689789 53800 0.151778659 26900 0.074965523 26928
7.325905375 53792 0.823878101 53792 0.008919361
53800 0.060654704 53800 0.13309154 26900 0.038479684 26932
8.113224063 53800 1.015574555 53800 0.008010271
53800 0.03841535 53800 0.046327266 26900 0.025597188 26936
9.792630563 53808 1.2740537 53808 0.01018993
53800 0.05711618 53800 0.069354581 26900 0.024898149 26940
11.30019966 53816 1.421560538 53816 0.031501513
53800 0.01520603 53800 0.078336991 26900 0.008802815 26944
12.31461205 53824 1.89841865 53824 0.025595884
53800 0.054553988 53800 0.105455918 26900 0.034160563 26948
14.506734 53832 2.744391788 53832 0.045907356
53800 0.063407912 53800 0.10727156 27000 0.04556905 26952
16.1853805 53840 1.157519408 53840 0.044159882
53800 0.065842323 53800 0.074597934 27000 0.035859775 26956
21.40484188 53848 3.290641588 53848 0.020420119
53900 0.110226007 53900 0.024040977 27000 0.009466863 26960
51.75878113 53856 2.35564075 53856 0.052789736
53900 0.022631646 53900 0.085279717 27000 0.041670108 26964
155.0139338 53864 2.017772525 53864 0.067820947
53900 0.150549924 53900 0.130947258 27000 0.048298047 26968
193.6024125 53872 3.683504188 53872 0.081498336
53900 0.191691971 53900 0.127020904 27000 0.011599092 26972
121.2721318 53880 1.807352988 53880 0.063651853
53900 0.079800244 53900 0.150508088 27000 0.094792798 26976

28.11937963	53888	4.273507275	53888	0.029830331		
53900	0.061646919	53900	0.190773556	27000	0.093696057	26980
7.848418313	53896	9.783586488	53896	0.050421008		
53900	0.043440447	53900	0.164158016	27000	0.040753344	26984
2.054950225	53904	12.27216609	53904	0.02711315		
53900	0.047281352	53900	0.10171788	27000	0.052379906	26988
0.996421324	53912	10.40691789	53912	0.037371865		
53900	0.077299148	53900	0.052382962	27000	0.057291589	26992
1.114342361	53920	5.931915713	53920	0.062515268		
53900	0.067320681	53900	0.051909829	27000	0.07687309	26996
1.005351776	53928	1.893758075	53928	0.078953046		
53900	0.02763924	53900	0.056123579	27000	0.066466069	27000
1.371614175	53936	1.5462765	53936	0.06375472		
53900	0.042103875	53900	0.071392919	27000	0.041143179	27004
1.622472188	53944	0.636129931	53944	0.03344248		
54000	0.046703568	54000	0.14388714	27000	0.034484074	27008
1.260296325	53952	0.82612905	53952	0.082001752		
54000	0.1039696	54000	0.123048303	27000	0.02777579	27012
1.538061188	53960	1.225843094	53960	0.120410659		
54000	0.128391563	54000	0.045910685	27000	0.024956818	27016
0.938851969	53968	0.475642824	53968	0.121499943		
54000	0.108918728	54000	0.15550165	27000	0.024082918	27020
0.740052958	53976	0.641912804	53976	0.088228509		
54000	0.076987446	54000	0.176137983	27000	0.03527775	27024
1.118797344	53984	0.840731024	53984	0.034540946		
54000	0.057890517	54000	0.109423716	27000	0.033062686	27028
0.705997344	53992	0.528806123	53992	0.019340765		
54000	0.035896803	54000	0.079038291	27000	0.032902975	27032
0.697214855	54000	0.138866395	54000	0.028030039		
54000	0.046920432	54000	0.075228993	27000	0.048894599	27036
0.624812325	54008	0.360605976	54008	0.004258552		
54000	0.122257014	54000	0.039807321	27000	0.046032419	27040
0.526177523	54016	0.382949685	54016	0.049710005		
54000	0.107446111	54000	0.090691327	27000	0.054375822	27044
0.419775578	54024	0.31815347	54024	0.070429553		
54000	0.08271238	54000	0.048514638	27000	0.067566485	27048
0.381771359	54032	0.45338404	54032	0.052132204		
54000	0.067102468	54000	0.046958929	27100	0.032679069	27052
0.479870766	54040	0.351659954	54040	0.03138301		
54000	0.052689393	54000	0.084067906	27100	0.051287025	27056
0.365669258	54048	0.15391635	54048	0.033559667		
54100	0.06999461	54100	0.144664999	27100	0.08108456	27060
0.43324093	54056	0.073059033	54056	0.03972384		
54100	0.080214275	54100	0.108427223	27100	0.060104849	27064
0.343842229	54064	0.071633273	54064	0.033603294		
54100	0.11737649	54100	0.053486008	27100	0.026384387	27068
0.305229798	54072	0.144096543	54072	0.036902184		
54100	0.106897736	54100	0.064652661	27100	0.013784546	27072
0.321995409	54080	0.160591851	54080	0.062378174		
54100	0.104854436	54100	0.08917458	27100	0.045160301	27076
0.253201346	54088	0.132606773	54088	0.084496649		
54100	0.141317113	54100	0.121186029	27100	0.065203931	27080
0.311591196	54096	0.065750559	54096	0.065645429		
54100	0.124800135	54100	0.071295581	27100	0.074476804	27084
0.179195413	54104	0.149018786	54104	0.040029474		
54100	0.082287894	54100	0.040010051	27100	0.088519562	27088
0.217209716	54112	0.157825693	54112	0.037256232		
54100	0.012835635	54100	0.081956292	27100	0.073855386	27092
0.254874496	54120	0.022574161	54120	0.045963345		
54100	0.100284487	54100	0.053570111	27100	0.047849771	27096
0.137856565	54128	0.141213823	54128	0.032761531		
54100	0.089774338	54100	0.082498191	27100	0.054440399	27100
0.223286261	54136	0.136827264	54136	0.048688027		
54100	0.053634234	54100	0.145898768	27100	0.075156844	27104
0.083502047	54144	0.084549618	54144	0.061897023		
54200	0.09355801	54200	0.189766798	27100	0.056669895	27108
0.143854981	54152	0.213980646	54152	0.069495516		
54200	0.081001344	54200	0.16862851	27100	0.019694749	27112

0.216707253 54160 0.245844975 54160 0.051085146
54200 0.015600435 54200 0.112870446 27100 0.063169871 27116
0.148499298 54168 0.137077863 54168 0.02770223
54200 0.049529266 54200 0.128037456 27100 0.064099259 27120
0.248868076 54176 0.042005828 54176 0.044887103
54200 0.067789253 54200 0.1478669 27100 0.080895501 27124
0.118264223 54184 0.088648754 54184 0.042382559
54200 0.101742728 54200 0.095354735 27100 0.057249181 27128
0.107134503 54192 0.107477659 54192 0.025244875
54200 0.134660979 54200 0.098828117 27100 0.028226494 27132
0.135049908 54200 0.102242084 54200 0.037648373
54200 0.112294227 54200 0.171826789 27100 0.029274303 27136
0.096929558 54208 0.12669203 54208 0.061164181
54200 0.08610123 54200 0.097199794 27100 0.026728596 27140
0.166772589 54216 0.090204972 54216 0.058742819
54200 0.066710956 54200 0.045292156 27100 0.046441001 27144
0.095172276 54224 0.050508497 54224 0.022161599
54200 0.032090306 54200 0.074421703 27100 0.046659738 27148
0.099958117 54232 0.101561098 54232 0.021560281
54200 0.019850171 54200 0.088833629 27200 0.0760361 27152
0.160746873 54240 0.096718701 54240 0.030739193
54200 0.04747904 54200 0.155976304 27200 0.104092738 27156
0.216099026 54248 0.075307842 54248 0.023071649
54300 0.085301559 54300 0.151682034 27200 0.102743776 27160
0.135985086 54256 0.0672108 54256 0.046355079
54300 0.062442567 54300 0.091485556 27200 0.062189451 27164
0.005069817 54264 0.041822419 54264 0.067234621
54300 0.048867467 54300 0.08944302 27200 0.032561391 27168
0.127281238 54272 0.043354146 54272 0.054479948
54300 0.110036512 54300 0.095219701 27200 0.028612431 27172
0.115769712 54280 0.049539791 54280 0.012985883
54300 0.116302224 54300 0.122339814 27200 0.014312913 27176
0.069930466 54288 0.06527736 54288 0.060733433
54300 0.056430981 54300 0.133433613 27200 0.046927609 27180
0.094808413 54296 0.053053009 54296 0.061271843
54300 0.079476558 54300 0.093538853 27200 0.079058787 27184
0.089552195 54304 0.053040771 54304 0.046528203
54300 0.119013545 54300 0.065137916 27200 0.05778213 27188
0.084839245 54312 0.109980785 54312 0.040505263
54300 0.06211079 54300 0.079527054 27200 0.057941412 27192
0.067331057 54320 0.108908927 54320 0.036681322
54300 0.097838805 54300 0.103137994 27200 0.062240179 27196
0.067688605 54328 0.079699705 54328 0.020181078
54300 0.126944433 54300 0.115405579 27200 0.023514782 27200
0.06896587 54336 0.131293826 54336 0.037713679
54300 0.098309138 54300 0.079331454 27200 0.006555112 27204
0.059688708 54344 0.138197589 54344 0.060580765
54400 0.089153655 54400 0.06698088 27200 0.050789931 27208
0.098743294 54352 0.085476619 54352 0.051616433
54400 0.092367685 54400 0.114332608 27200 0.062890431 27212
0.146334496 54360 0.109241657 54360 0.025298152
54400 0.082293307 54400 0.119305529 27200 0.028658849 27216
0.043012158 54368 0.15432098 54368 0.024284382
54400 0.108089771 54400 0.126877189 27200 0.046144622 27220
0.1042851 54376 0.181095093 54376 0.038342467
54400 0.197134301 54400 0.10902041 27200 0.041940013 27224
0.100438971 54384 0.144155725 54384 0.028586448
54400 0.187597543 54400 0.045808058 27200 0.095454874 27228
0.034354966 54392 0.094037852 54392 0.029073979
54400 0.079292047 54400 0.056455618 27200 0.082604107 27232
0.039332801 54400 0.083395898 54400 0.05759038
54400 0.082852654 54400 0.110440888 27200 0.023337369 27236
0.072811439 54408 0.018792815 54408 0.093962335
54400 0.138692398 54400 0.155069313 27200 0.038479531 27240
0.04689916 54416 0.108944572 54416 0.095981435
54400 0.11917486 54400 0.182704054 27200 0.050149913 27244
0.054512813 54424 0.15914788 54424 0.043077482
54400 0.066805289 54400 0.244189229 27200 0.073301962 27248

0.087350578 54432 0.094225972 54432 0.036545112
54400 0.03735855 54400 0.265908835 27300 0.059234335 27252
0.051715833 54440 0.079367783 54440 0.071250215
54400 0.099572622 54400 0.201242816 27300 0.05115836 27256
0.015524349 54448 0.111025845 54448 0.055151617
54500 0.083322477 54500 0.097006363 27300 0.068058282 27260
0.06722664 54456 0.103134211 54456 0.034842014
54500 0.044567387 54500 0.046969904 27300 0.090564208 27264
0.028041575 54464 0.045651024 54464 0.042820731
54500 0.026106132 54500 0.116110925 27300 0.089372079 27268
0.025959935 54472 0.130014246 54472 0.036359059
54500 0.03649561 54500 0.075526587 27300 0.11319013 27272
0.019879395 54480 0.156464521 54480 0.006349769
54500 0.080963473 54500 0.043133183 27300 0.11982704 27276
0.059112128 54488 0.065030748 54488 0.014049934
54500 0.126356594 54500 0.026281048 27300 0.097997741 27280
0.13095056 54496 0.033814111 54496 0.054174278
54500 0.14162474 54500 0.05721438 27300 0.120388853 27284
0.057878006 54504 0.034776709 54504 0.084300882
54500 0.115353607 54500 0.059968857 27300 0.156025403 27288
0.071960079 54512 0.039347578 54512 0.031306587
54500 0.167516759 54500 0.033521468 27300 0.111738453 27292
0.088910114 54520 0.055264616 54520 0.065093147
54500 0.178309769 54500 0.095211683 27300 0.063744257 27296
0.080200574 54528 0.080981023 54528 0.062827028
54500 0.116992604 54500 0.107558743 27300 0.024901239 27300
0.023010274 54536 0.101002639 54536 0.032507109
54500 0.112652 54500 0.097504853 27300 0.047366688 27304
0.061387218 54544 0.125892184 54544 0.033729772
54600 0.14248518 54600 0.130437038 27300 0.082779756 27308
0.042790525 54552 0.143810248 54552 0.039499388
54600 0.110279128 54600 0.087405351 27300 0.069375987 27312
0.077789642 54560 0.130806468 54560 0.092012881
54600 0.053721895 54600 0.093327049 27300 0.037476286 27316
0.09091472 54568 0.105844862 54568 0.111829169
54600 0.058425936 54600 0.186091565 27300 0.023474175 27320
0.042527896 54576 0.069517235 54576 0.084729451
54600 0.070714908 54600 0.112747424 27300 0.048155678 27324
0.081686427 54584 0.053168384 54584 0.066618697
54600 0.021260472 54600 0.043353139 27300 0.043656615 27328
0.138068455 54592 0.058594393 54592 0.072265953
54600 0.062151099 54600 0.073966978 27300 0.030899995 27332
0.0667227 54600 0.056670211 54600 0.054985652
54600 0.044660192 54600 0.081075923 27300 0.018624538 27336
0.055453944 54608 0.123419828 54608 0.059593644
54600 0.022116765 54600 0.079907957 27300 0.033020326 27340
0.080686557 54616 0.11772976 54616 0.069429603
54600 0.12109222 54600 0.084096224 27300 0.043144817 27344
0.065291017 54624 0.046263249 54624 0.044753673
54600 0.182580378 54600 0.060104223 27300 0.060355378 27348
0.077065379 54632 0.025837901 54632 0.03956581
54600 0.10564203 54600 0.031452189 27400 0.092627037 27352
0.05243894 54640 0.049434169 54640 0.071879083
54600 0.017054628 54600 0.051738804 27400 0.061098304 27356
0.059925391 54648 0.015073266 54648 0.091107169
54700 0.10761273 54700 0.058811707 27400 0.022943072 27360
0.115650408 54656 0.070561735 54656 0.084441832
54700 0.161949924 54700 0.015054795 27400 0.019516876 27364
0.081490951 54664 0.103286169 54664 0.044739369
54700 0.130285088 54700 0.056551053 27400 0.088007124 27368
0.04531 54672 0.067015273 54672 0.039256403
54700 0.079900012 54700 0.102946709 27400 0.101658821 27372
0.120416305 54680 0.075836309 54680 0.050899838
54700 0.124791142 54700 0.122839818 27400 0.078924895 27376
0.107459193 54688 0.143735204 54688 0.022622115
54700 0.157952528 54700 0.126863758 27400 0.064599728 27380
0.088097331 54696 0.149043786 54696 0.006066785
54700 0.071547627 54700 0.126937506 27400 0.01282304 27384

0.113111892 54704 0.116357856 54704 0.013226398
54700 0.068451591 54700 0.083326464 27400 0.044659388 27388
0.061414241 54712 0.125935374 54712 0.039068149
54700 0.058803271 54700 0.078522651 27400 0.008006917 27392
0.040161773 54720 0.081250008 54720 0.065433364
54700 0.064203297 54700 0.031869768 27400 0.041453543 27396
0.057637953 54728 0.022449922 54728 0.077182427
54700 0.067473535 54700 0.173570719 27400 0.060792423 27400
0.027035134 54736 0.035687066 54736 0.064365966
54700 0.072308547 54700 0.15856653 27400 0.086451342 27404
0.035179503 54744 0.078079625 54744 0.027304175
54800 0.029213203 54800 0.069761263 27400 0.050961509 27408
0.05135229 54752 0.097104254 54752 0.030526178
54800 0.062316125 54800 0.101967948 27400 0.068285364 27412
0.10125534 54760 0.065852124 54760 0.038805447
54800 0.066313616 54800 0.110814239 27400 0.031792977 27416
0.116152172 54768 0.096483214 54768 0.038379385
54800 0.035116715 54800 0.139907511 27400 0.075454635 27420
0.044861099 54776 0.108366498 54776 0.021331183
54800 0.02397688 54800 0.148217078 27400 0.122470359 27424
0.081130202 54784 0.018827484 54784 0.037539725
54800 0.060156846 54800 0.178186703 27400 0.12270399 27428
0.123041711 54792 0.080403886 54792 0.048382026
54800 0.092838469 54800 0.198734604 27400 0.085480795 27432
0.09894956 54800 0.090750844 54800 0.03942868
54800 0.102819082 54800 0.140542805 27400 0.057391921 27436
0.10107764 54808 0.014285704 54808 0.058970116
54800 0.071626593 54800 0.088203655 27400 0.032658965 27440
0.099848134 54816 0.075900687 54816 0.078820653
54800 0.036585778 54800 0.093146817 27400 0.107422471 27444
0.028443881 54824 0.153222165 54824 0.047845148
54800 0.016812208 54800 0.09327328 27400 0.155986593 27448
0.069552676 54832 0.180036819 54832 0.014336981
54800 0.064610205 54800 0.069556685 27500 0.10772398 27452
0.073218427 54840 0.101770238 54840 0.01811005
54800 0.102747588 54800 0.041874984 27500 0.048974176 27456
0.051719897 54848 0.017556964 54848 0.028033081
54900 0.069051952 54900 0.078442572 27500 0.030339759 27460
0.079777943 54856 0.060510211 54856 0.008655285
54900 0.039688624 54900 0.176952163 27500 0.100676756 27464
0.085440246 54864 0.059799346 54864 0.031711676
54900 0.069425747 54900 0.19771191 27500 0.116981843 27468
0.018562212 54872 0.110774046 54872 0.007307905
54900 0.039484967 54900 0.13610709 27500 0.12775707 27472
0.024170326 54880 0.067592126 54880 0.05036953
54900 0.036053003 54900 0.073586947 27500 0.063961401 27476
0.030499312 54888 0.059811988 54888 0.071858551
54900 0.124584607 54900 0.019204996 27500 0.056621389 27480
0.032240965 54896 0.127687555 54896 0.070243288
54900 0.179337425 54900 0.043445667 27500 0.040982483 27484
0.091626687 54904 0.110230321 54904 0.045230667
54900 0.142484118 54900 0.051464016 27500 0.056918791 27488
0.091152724 54912 0.040351242 54912 0.013708212
54900 0.07771216 54900 0.086393782 27500 0.119558754 27492
0.075361495 54920 0.053625528 54920 0.010767887
54900 0.112351823 54900 0.086926739 27500 0.08127802 27496
0.062599873 54928 0.094559204 54928 0.031080355
54900 0.193564396 54900 0.071684932 27500 0.060662886 27500
0.017486022 54936 0.070745649 54936 0.041854142
54900 0.174740199 54900 0.053611253 27500 0.060118797 27504
0.0620992 54944 0.08325441 54944 0.058321744
55000 0.087362816 55000 0.039438037 27500 0.14936665 27508
0.093429357 54952 0.022868248 54952 0.070286129
55000 0.139367825 55000 0.019315048 27500 0.184022138 27512
0.098418866 54960 0.06141502 54960 0.045410168
55000 0.151765125 55000 0.002432657 27500 0.18581911 27516
0.08719763 54968 0.082530874 54968 0.045122535
55000 0.094709452 55000 0.034219924 27500 0.073319272 27520

0.073475203	54976	0.062794512	54976	0.053239404		
55000	0.13192094	55000	0.081826496	27500	0.056597037	27524
0.069240843	54984	0.129622101	54984	0.017649854		
55000	0.151277185	55000	0.117337411	27500	0.08822963	27528
0.080291276	54992	0.133977563	54992	0.075957978		
55000	0.057259473	55000	0.125171603	27500	0.099223813	27532
0.083014238	55000	0.061933737	55000	0.057228648		
55000	0.070531401	55000	0.112493741	27500	0.08497102	27536
0.046168934	55008	0.04739374	55008	0.013766336		
55000	0.085392945	55000	0.053992899	27500	0.107743887	27540
0.023580775	55016	0.0531521	55016	0.019334124		
55000	0.054578901	55000	0.05561217	27500	0.11509312	27544
0.034186258	55024	0.078965095	55024	0.048483627		
55000	0.041640324	55000	0.117874188	27500	0.034836259	27548
0.006661606	55032	0.169774809	55032	0.053423308		
55000	0.032009131	55000	0.107469081	27600	0.055808967	27552
0.067578665	55040	0.217310066	55040	0.029960529		
55000	0.027460706	55000	0.039775805	27600	0.055020708	27556
0.095570998	55048	0.187846206	55048	0.01439679		
55100	0.044620541	55100	0.049946902	27600	0.102198283	27560
0.091779235	55056	0.114447736	55056	0.012619443		
55100	0.037510923	55100	0.101497666	27600	0.089368667	27564
0.089290144	55064	0.021982225	55064	0.010492899		
55100	0.081965773	55100	0.101329759	27600	0.118679178	27568
0.060441958	55072	0.083860577	55072	0.023052666		
55100	0.147530169	55100	0.074149386	27600	0.129600173	27572
0.021045926	55080	0.159074771	55080	0.044936263		
55100	0.132012006	55100	0.061678664	27600	0.176833914	27576
0.065817396	55088	0.154914611	55088	0.061827377		
55100	0.051504965	55100	0.08727485	27600	0.119517026	27580
0.085656218	55096	0.083182917	55096	0.058745205		
55100	0.038100414	55100	0.091076581	27600	0.111820235	27584
0.088463501	55104	0.01499489	55104	0.05472709		
55100	0.104161256	55100	0.120518213	27600	0.111169124	27588
0.137929121	55112	0.013105056	55112	0.043513406		
55100	0.140662945	55100	0.093547351	27600	0.089072601	27592
0.128659959	55120	0.038843362	55120	0.033240674		
55100	0.105952786	55100	0.085628046	27600	0.155922084	27596
0.073082476	55128	0.027841195	55128	0.035291338		
55100	0.072196606	55100	0.153946021	27600	0.084761166	27600
0.024646546	55136	0.078354322	55136	0.010324243		
55100	0.175914581	55100	0.137437354	27600	0.12051886	27604
0.089736008	55144	0.14992521	55144	0.017165061		
55200	0.153430359	55200	0.122139332	27600	0.149966901	27608
0.105322804	55152	0.119660304	55152	0.022095142		
55200	0.054209584	55200	0.110423753	27600	0.157100061	27612
0.088067667	55160	0.12247634	55160	0.032723208		
55200	0.0799794	55200	0.090645699	27600	0.101474114	27616
0.053979249	55168	0.096696371	55168	0.043412092		
55200	0.093272152	55200	0.087641813	27600	0.087566892	27620
0.014085773	55176	0.041280131	55176	0.052652977		
55200	0.094101561	55200	0.074906937	27600	0.015588323	27624
0.03678213	55184	0.03970712	55184	0.062362793		
55200	0.089028297	55200	0.048870268	27600	0.138966585	27628
0.05487035	55192	0.03970923	55192	0.065605331		
55200	0.136875315	55200	0.028701628	27600	0.182269141	27632
0.071786562	55200	0.071228446	55200	0.060512255		
55200	0.125844614	55200	0.075808224	27600	0.182143893	27636
0.062476625	55208	0.084119936	55208	0.037975369		
55200	0.037912312	55200	0.066870918	27600	0.158673124	27640
0.037719558	55216	0.010942142	55216	0.055082935		
55200	0.051436666	55200	0.064513006	27600	0.103723483	27644
0.06920017	55224	0.099676465	55224	0.071469345		
55200	0.059073049	55200	0.075636104	27600	0.157479925	27648
0.063211759	55232	0.098323901	55232	0.024698578		
55200	0.007759741	55200	0.009386362	27700	0.143714525	27652
0.076844073	55240	0.002838168	55240	0.012356025		
55200	0.051737537	55200	0.082104358	27700	0.125906896	27656

0.098368815 55248 0.044584151 55248 0.031479453
55300 0.07199547 55300 0.142349644 27700 0.14138408 27660
0.070614115 55256 0.056349451 55256 0.070011673
55300 0.062023035 55300 0.19995225 27700 0.194151523 27664
0.010415572 55264 0.126987041 55264 0.089677946
55300 0.072729337 55300 0.195330911 27700 0.181720155 27668
0.050945047 55272 0.131355991 55272 0.07816271
55300 0.111075417 55300 0.116433475 27700 0.206012628 27672
0.090233792 55280 0.056115248 55280 0.04193182
55300 0.108551845 55300 0.03920043 27700 0.129700115 27676
0.092284441 55288 0.017993369 55288 0.017650333
55300 0.072002542 55300 0.056247056 27700 0.093552801 27680
0.068344081 55296 0.089912806 55296 0.031598778
55300 0.155022398 55300 0.060707898 27700 0.153591944 27684
0.042624644 55304 0.1427565 55304 0.049591923
55300 0.172921849 55300 0.040109364 27700 0.157744158 27688
0.063209816 55312 0.116020885 55312 0.036501726
55300 0.121468132 55300 0.061959625 27700 0.134871138 27692
0.072283496 55320 0.050411658 55320 0.021626518
55300 0.034071094 55300 0.08772123 27700 0.172963759 27696
0.086893386 55328 0.057490473 55328 0.032281434
55300 0.04284898 55300 0.11687728 27700 0.222441304 27700
0.095578238 55336 0.063461026 55336 0.045089131
55300 0.047228747 55300 0.102690145 27700 0.248597731 27704
0.053928005 55344 0.093028451 55344 0.039893017
55400 0.023494135 55400 0.06971824 27700 0.284840556 27708
0.028317383 55352 0.12699941 55352 0.013963876
55400 0.107181048 55400 0.066579538 27700 0.226329896 27712
0.014536397 55360 0.118851327 55360 0.013002745
55400 0.138513148 55400 0.057154943 27700 0.304322748 27716
0.041405932 55368 0.148905194 55368 0.009457252
55400 0.104627543 55400 0.101701618 27700 0.260556146 27720
0.063662388 55376 0.169798761 55376 0.003972773
55400 0.026253232 55400 0.08300085 27700 0.341622741 27724
0.062249223 55384 0.182642296 55384 0.013092831
55400 0.052860862 55400 0.116957446 27700 0.333124714 27728
0.043438831 55392 0.142666409 55392 0.017961125
55400 0.083145715 55400 0.069799367 27700 0.149880653 27732
0.014199431 55400 0.053947872 55400 0.038175473
55400 0.092720919 55400 0.092805189 27700 0.101269397 27736
0.068776244 55408 0.05540526 55408 0.052371684
55400 0.03587102 55400 0.099612116 27700 0.239314016 27740
0.080284262 55416 0.153985675 55416 0.053735155
55400 0.086353088 55400 0.117144642 27700 0.278113061 27744
0.08920893 55424 0.166642916 55424 0.055466335
55400 0.04965542 55400 0.15606216 27700 0.261437701 27748
0.092477756 55432 0.086788663 55432 0.038184244
55400 0.058681137 55400 0.102331396 27800 0.190694205 27752
0.070889772 55440 0.066366316 55440 0.021326396
55400 0.113747963 55400 0.033254775 27800 0.378305588 27756
0.022156088 55448 0.096069096 55448 0.021099799
55500 0.098846467 55500 0.07852741 27800 0.555708655 27760
0.015952814 55456 0.072155359 55456 0.015186068
55500 0.121342731 55500 0.079226171 27800 0.398504403 27764
0.025754909 55464 0.049397975 55464 0.035587778
55500 0.129467663 55500 0.062170715 27800 0.356798176 27768
0.042601962 55472 0.039158786 55472 0.011761539
55500 0.074855205 55500 0.081982427 27800 0.514638843 27772
0.071085517 55480 0.027777342 55480 0.019352601
55500 0.109256143 55500 0.080831371 27800 0.480372604 27776
0.076246863 55488 0.051181702 55488 0.031711006
55500 0.077991041 55500 0.085835294 27800 0.329472533 27780
0.081383318 55496 0.110961424 55496 0.067644593
55500 0.10880523 55500 0.073779127 27800 0.536680979 27784
0.080481448 55504 0.1713534 55504 0.079997866
55500 0.041611747 55500 0.051892774 27800 0.387885579 27788
0.046006171 55512 0.197431364 55512 0.056096636
55500 0.073885043 55500 0.033608794 27800 0.583612301 27792

0.04991304 55520 0.135270864 55520 0.034248282
55500 0.093435621 55500 0.023062134 27800 0.7624508 27796
0.066361317 55528 0.053298005 55528 0.029638319
55500 0.035606528 55500 0.071753944 27800 0.51903812 27800
0.043831376 55536 0.055303772 55536 0.02440929
55500 0.014629698 55500 0.112000307 27800 0.666056644 27804
0.02885948 55544 0.146521358 55544 0.009850181
55600 0.037007038 55600 0.12342853 27800 0.631717906 27808
0.057367372 55552 0.231171565 55552 0.020545593
55600 0.153174639 55600 0.153992645 27800 1.551553725 27812
0.062543899 55560 0.186935896 55560 0.043447599
55600 0.202774784 55600 0.160084441 27800 2.117819388 27816
0.017176853 55568 0.117539741 55568 0.043278586
55600 0.115193907 55600 0.094008523 27800 1.8878039 27820
0.071339724 55576 0.126179671 55576 0.050384737
55600 0.049223243 55600 0.131817549 27800 1.605115363 27824
0.086514039 55584 0.138309043 55584 0.079916274
55600 0.074945521 55600 0.152295659 27800 1.207918744 27828
0.108512082 55592 0.113085167 55592 0.094695948
55600 0.112459711 55600 0.111349429 27800 0.440065429 27832
0.108492939 55600 0.106622116 55600 0.096500793
55600 0.129966444 55600 0.069721624 27800 0.091143644 27836
0.081623759 55608 0.126702041 55608 0.063716012
55600 0.162815224 55600 0.060989638 27800 1.021019533 27840
0.093622017 55616 0.085785141 55616 0.006552069
55600 0.18298658 55600 0.040937448 27800 3.999905663 27844
0.09562209 55624 0.016471384 55624 0.027882517
55600 0.141196215 55600 0.12991023 27800 7.496767213 27848
0.059155889 55632 0.064543347 55632 0.009464214
55600 0.077442302 55600 0.216159083 27900 7.364087738 27852
0.080058089 55640 0.090668218 55640 0.036547481
55600 0.057634097 55600 0.167863153 27900 3.357288425 27856
0.055806242 55648 0.06974936 55648 0.041793053
55700 0.038164923 55700 0.022839682 27900 4.107781688 27860
0.051378826 55656 0.043736654 55656 0.012103129
55700 0.061734012 55700 0.112873866 27900 3.271449125 27864
0.107306754 55664 0.064105421 55664 0.055447395
55700 0.100913385 55700 0.140532051 27900 6.285376375 27868
0.099918478 55672 0.056207187 55672 0.108275774
55700 0.064315573 55700 0.11982931 27900 20.18173788 27872
0.073610077 55680 0.093090115 55680 0.079065147
55700 0.012995459 55700 0.065835528 27900 31.6547155 27876
0.072204864 55688 0.037432488 55688 0.025143343
55700 0.040482475 55700 0.027994523 27900 30.84068 27880
0.041250769 55696 0.05171269 55696 0.050041792
55700 0.097722848 55700 0.055940862 27900 18.50674488 27884
0.027552629 55704 0.027282329 55704 0.067276997
55700 0.078779201 55700 0.105461353 27900 6.4893025 27888
0.063011838 55712 0.0590491 55712 0.054831806
55700 0.080926606 55700 0.09420525 27900 5.498113113 27892
0.067018555 55720 0.071844115 55720 0.032880678
55700 0.101658217 55700 0.080702404 27900 5.0358125 27896
0.077638164 55728 0.049752547 55728 0.019391193
55700 0.07325942 55700 0.031804186 27900 6.065422675 27900
0.086259017 55736 0.087849825 55736 0.070032569
55700 0.041385076 55700 0.079796191 27900 10.9946467 27904
0.076975703 55744 0.064259759 55744 0.084612031
55800 0.061356652 55800 0.122261059 27900 16.23819213 27908
0.096251461 55752 0.058430811 55752 0.036337195
55800 0.128258398 55800 0.138351593 27900 11.6599733 27912
0.123974984 55760 0.065346678 55760 0.016595501
55800 0.147879823 55800 0.137949043 27900 11.76579669 27916
0.088065033 55768 0.090103109 55768 0.023392535
55800 0.085470863 55800 0.196299385 27900 15.5756595 27920
0.01808486 55776 0.14554715 55776 0.038215691
55800 0.012008313 55800 0.231548635 27900 18.9418755 27924
0.035319943 55784 0.09549228 55784 0.058178215
55800 0.106305466 55800 0.13760323 27900 64.8088455 27928

0.042629716 55792 0.039316514 55792 0.069282309
55800 0.186022996 55800 0.086645407 27900 174.8615063 27932
0.038772785 55800 0.036812799 55800 0.060463044
55800 0.152776745 55800 0.057679052 27900 226.3939238 27936
0.016414017 55808 0.045736182 55808 0.049953615
55800 0.022588198 55800 0.042720003 27900 158.197 27940
0.024823035 55816 0.089307519 55816 0.050249688
55800 0.113513488 55800 0.061959378 27900 48.5425815 27944
0.038018916 55824 0.094314622 55824 0.039265764
55800 0.124665094 55800 0.117631535 27900 21.54052263 27948
0.06443468 55832 0.144652048 55832 0.026650847
55800 0.109421802 55800 0.187409998 28000 13.76822588 27952
0.04930039 55840 0.171406456 55840 0.026028007
55800 0.070456801 55800 0.182436051 28000 12.18455099 27956
0.049162394 55848 0.15698692 55848 0.014853324
55900 0.014248962 55900 0.108702552 28000 8.376587188 27960
0.046260779 55856 0.137566094 55856 0.035693527
55900 0.061231782 55900 0.067770729 28000 8.495552463 27964
0.029872845 55864 0.103659048 55864 0.030085128
55900 0.11308011 55900 0.067827896 28000 6.021459588 27968
0.041368621 55872 0.062733292 55872 0.039517468
55900 0.058081219 55900 0.082236416 28000 4.616377413 27972
0.035490939 55880 0.062055682 55880 0.03128664
55900 0.033396766 55900 0.108661974 28000 3.7556286 27976
0.010843133 55888 0.04644601 55888 0.020233187
55900 0.097720578 55900 0.06332442 28000 3.098192863 27980
0.058077927 55896 0.051254407 55896 0.018312938
55900 0.149315601 55900 0.037901922 28000 2.011950363 27984
0.086425949 55904 0.088690962 55904 0.020625776
55900 0.170090119 55900 0.079566773 28000 4.394881888 27988
0.076251701 55912 0.079988371 55912 0.026422013
55900 0.138769509 55900 0.058547928 28000 8.2511194 27992
0.07075652 55920 0.050693663 55920 0.047399433
55900 0.094168005 55900 0.07412149 28000 6.268193013 27996
0.053958003 55928 0.038890099 55928 0.041298576
55900 0.069406568 55900 0.097114498 28000 2.7747273 28000
0.063470245 55936 0.034136087 55936 0.01802384
55900 0.048722333 55900 0.081689635 28000 1.125447103 28004
0.100577432 55944 0.024585905 55944 0.024894016
56000 0.05326801 56000 0.163739911 28000 1.074430533 28008
0.084611296 55952 0.065222172 55952 0.031881915
56000 0.06903035 56000 0.150306121 28000 1.385883075 28012
0.061987536 55960 0.150360909 55960 0.022357601
56000 0.07275532 56000 0.097279815 28000 1.824375713 28016
0.039009483 55968 0.162175595 55968 0.005061472
56000 0.077361299 56000 0.033989098 28000 3.793321313 28020
0.023453846 55976 0.097716613 55976 0.00747455
56000 0.043553446 56000 0.079705176 28000 3.674046838 28024
0.037537964 55984 0.052751831 55984 0.004794209
56000 0.014117312 56000 0.152896406 28000 1.929041 28028
0.045381868 55992 0.02467167 55992 0.020528987
56000 0.032277676 56000 0.195499568 28000 0.35998848 28032
0.067119538 56000 0.043958389 56000 0.036354544
56000 0.082411207 56000 0.181752766 28000 0.470086466 28036
0.103692517 56008 0.088388049 56008 0.025502211
56000 0.121792669 56000 0.156375056 28000 0.348072703 28040
0.079565725 56016 0.173835914 56016 0.038054659
56000 0.117226045 56000 0.06608558 28000 1.136545325 28044
0.04622015 56024 0.237789849 56024 0.044437154
56000 0.073988231 56000 0.066436696 28000 2.2888165 28048
0.027177539 56032 0.179923721 56032 0.044802215
56000 0.025633108 56000 0.057039066 28100 2.864496788 28052
0.035460569 56040 0.098558878 56040 0.034882258
56000 0.055809444 56000 0.074951 28100 2.4249847 28056
0.077384029 56048 0.032427262 56048 0.026052523
56100 0.082661856 56100 0.099627607 28100 1.224307693 28060
0.0389046 56056 0.137024443 56056 0.090189729
56100 0.072228948 56100 0.079631624 28100 0.315503275 28064

0.059111459	56064	0.170322935	56064	0.09822235		
56100	0.065099048	56100	0.078828904	28100	0.689030741	28068
0.075605152	56072	0.17335701	56072	0.065763838		
56100	0.064473548	56100	0.034117948	28100	0.534257503	28072
0.077990488	56080	0.193047555	56080	0.0415375		
56100	0.103177583	56100	0.073614334	28100	1.024257391	28076
0.057410754	56088	0.158403156	56088	0.039530158		
56100	0.109498418	56100	0.083470739	28100	1.396200388	28080
0.026632319	56096	0.101379999	56096	0.039822626		
56100	0.047198129	56100	0.055692322	28100	1.113216743	28084
0.040302137	56104	0.058541933	56104	0.031426109		
56100	0.073439296	56100	0.021380092	28100	0.499451999	28088
0.085593674	56112	0.082625396	56112	0.043918979		
56100	0.090783447	56100	0.019920526	28100	0.039040351	28092
0.096718672	56120	0.103556311	56120	0.070863665		
56100	0.058168629	56100	0.053333559	28100	0.225535536	28096
0.069377289	56128	0.090103415	56128	0.063271473		
56100	0.087166889	56100	0.121914804	28100	0.240832204	28100
0.071339295	56136	0.084570282	56136	0.027296635		
56100	0.109662913	56100	0.105436964	28100	0.188947524	28104
0.088848261	56144	0.084633633	56144	0.036257381		
56200	0.084167041	56200	0.039354502	28100	0.279575121	28108
0.079286525	56152	0.05859364	56152	0.048907448		
56200	0.069014168	56200	0.0368976	28100	0.452418055	28112
0.060243026	56160	0.028757921	56160	0.048628124		
56200	0.02360351	56200	0.114521659	28100	0.429831736	28116
0.080229285	56168	0.033287499	56168	0.048594608		
56200	0.052258154	56200	0.154741153	28100	0.234362044	28120
0.070440176	56176	0.091931252	56176	0.055388628		
56200	0.115403433	56200	0.08623171	28100	0.260782021	28124
0.080234306	56184	0.071758819	56184	0.050302868		
56200	0.126825908	56200	0.022518721	28100	0.277125451	28128
0.095122872	56192	0.007136487	56192	0.04511467		
56200	0.09562428	56200	0.034960423	28100	0.297624648	28132
0.091291731	56200	0.03022722	56200	0.056194098		
56200	0.084777072	56200	0.067907851	28100	0.465434453	28136
0.060963361	56208	0.004961973	56208	0.051388524		
56200	0.036364138	56200	0.044724326	28100	0.440035365	28140
0.050710951	56216	0.016015287	56216	0.008253573		
56200	0.110750953	56200	0.081799924	28100	0.17715487	28144
0.073549731	56224	0.024132414	56224	0.050990228		
56200	0.089506866	56200	0.153060188	28100	0.092163078	28148
0.06062102	56232	0.039497012	56232	0.067697416		
56200	0.033979588	56200	0.077730838	28200	0.137908559	28152
0.039075094	56240	0.048292168	56240	0.039080733		
56200	0.098964963	56200	0.099607401	28200	0.099264878	28156
0.044479504	56248	0.062613312	56248	0.037997888		
56300	0.054314307	56300	0.118867174	28200	0.14286561	28160
0.02297539	56256	0.076832861	56256	0.040829717		
56300	0.045815541	56300	0.136887465	28200	0.099418867	28164
0.067270274	56264	0.072853007	56264	0.059219572		
56300	0.123642632	56300	0.133316396	28200	0.119312936	28168
0.090514805	56272	0.109294706	56272	0.053373835		
56300	0.157305098	56300	0.020852709	28200	0.152989975	28172
0.096234529	56280	0.038516955	56280	0.011780996		
56300	0.145353216	56300	0.101543192	28200	0.194275054	28176
0.088703942	56288	0.132722839	56288	0.026939215		
56300	0.121235491	56300	0.093195711	28200	0.193836095	28180
0.078180761	56296	0.190639126	56296	0.034571513		
56300	0.112469206	56300	0.043130927	28200	0.144578866	28184
0.075836848	56304	0.123234204	56304	0.028364942		
56300	0.111513247	56300	0.122471538	28200	0.097314711	28188
0.058803893	56312	0.082529521	56312	0.014480018		
56300	0.061453233	56300	0.071017217	28200	0.173351379	28192
0.061250481	56320	0.071601739	56320	0.012696393		
56300	0.033593686	56300	0.012106888	28200	0.168331316	28196
0.072739589	56328	0.01003919	56328	0.031932606		
56300	0.107315093	56300	0.063150706	28200	0.082579274	28200

0.054639364 56336 0.054011958 56336 0.043682769
56300 0.152023436 56300 0.0612394 28200 0.04554195 28204
0.058950092 56344 0.091392511 56344 0.052669842
56400 0.113249145 56400 0.09297215 28200 0.095632728 28208
0.078068173 56352 0.085196392 56352 0.040627543
56400 0.073793592 56400 0.124503655 28200 0.089539113 28212
0.08508972 56360 0.023750325 56360 0.019889465
56400 0.122284575 56400 0.093755007 28200 0.092246453 28216
0.060256731 56368 0.073340852 56368 0.02930528
56400 0.104376013 56400 0.087120927 28200 0.12155679 28220
0.020724485 56376 0.107682041 56376 0.002024824
56400 0.110398258 56400 0.06880716 28200 0.089769244 28224
0.021023328 56384 0.062323132 56384 0.033926343
56400 0.157492759 56400 0.06656794 28200 0.081439226 28228
0.038394031 56392 0.037360111 56392 0.03042515
56400 0.150923166 56400 0.039336697 28200 0.052883952 28232
0.061676139 56400 0.081950522 56400 0.018230339
56400 0.115697651 56400 0.071452792 28200 0.077185388 28236
0.064317872 56408 0.078497142 56408 0.052556159
56400 0.084475505 56400 0.128927626 28200 0.104354091 28240
0.073467651 56416 0.064577165 56416 0.061503641
56400 0.049156621 56400 0.10252824 28200 0.133447436 28244
0.096729295 56424 0.094948577 56424 0.039444036
56400 0.068029651 56400 0.062197854 28200 0.146292383 28248
0.06900508 56432 0.100128214 56432 0.0243253
56400 0.103661441 56400 0.194521286 28300 0.182998309 28252
0.06690147 56440 0.039384777 56440 0.042458534
56400 0.066912493 56400 0.175533656 28300 0.131930806 28256
0.062843225 56448 0.105703286 56448 0.058517304
56500 0.043815238 56500 0.093101728 28300 0.074849195 28260
0.021484251 56456 0.143535668 56456 0.073928924
56500 0.080184262 56500 0.063161417 28300 0.142980688 28264
0.068997622 56464 0.08497628 56464 0.06640939
56500 0.047335223 56500 0.110210393 28300 0.125513253 28268
0.128856803 56472 0.046325724 56472 0.03641333
56500 0.015928201 56500 0.071392162 28300 0.097357035 28272
0.170212326 56480 0.104847211 56480 0.017285354
56500 0.047434911 56500 0.09595829 28300 0.06806833 28276
0.166929298 56488 0.09414825 56488 0.025425366
56500 0.02324936 56500 0.136852541 28300 0.042144959 28280
0.14189542 56496 0.048998638 56496 0.023091989
56500 0.085987966 56500 0.107060383 28300 0.085912179 28284
0.111426896 56504 0.083324398 56504 0.058928014
56500 0.076116674 56500 0.128456784 28300 0.127322244 28288
0.085973297 56512 0.056511784 56512 0.05940174
56500 0.058658508 56500 0.151074841 28300 0.102575228 28292
0.066325556 56520 0.080262813 56520 0.019692956
56500 0.169596751 56500 0.098759774 28300 0.077297918 28296
0.056760055 56528 0.069654336 56528 0.026789421
56500 0.161251446 56500 0.055205412 28300 0.05677369 28300
0.080527585 56536 0.017807979 56536 0.050357896
56500 0.085211519 56500 0.044979806 28300 0.075439944 28304
0.092139446 56544 0.076091725 56544 0.042267562
56600 0.092114009 56600 0.039931172 28300 0.066680135 28308
0.087484143 56552 0.0761669 56552 0.048953294
56600 0.065546861 56600 0.076917495 28300 0.090640999 28312
0.086168591 56560 0.070042537 56560 0.108017928
56600 0.02966112 56600 0.074739539 28300 0.072469091 28316
0.095588643 56568 0.055041677 56568 0.091108828
56600 0.00939376 56600 0.007705434 28300 0.116283271 28320
0.089879082 56576 0.052139138 56576 0.026854164
56600 0.013520373 56600 0.099996018 28300 0.169868479 28324
0.062160369 56584 0.066779721 56584 0.091315385
56600 0.055031982 56600 0.13277815 28300 0.124043552 28328
0.093119117 56592 0.055583674 56592 0.075689146
56600 0.111093192 56600 0.051067575 28300 0.068826157 28332
0.088005443 56600 0.065164219 56600 0.04016182
56600 0.110400564 56600 0.046708716 28300 0.039222199 28336

0.078694189 56608 0.094345669 56608 0.091827213
56600 0.050682404 56600 0.104913612 28300 0.058440073 28340
0.115868301 56616 0.136710791 56616 0.104958621
56600 0.078275727 56600 0.129899549 28300 0.075878663 28344
0.115885065 56624 0.133480891 56624 0.075478936
56600 0.126198138 56600 0.077076715 28300 0.061765291 28348
0.091332462 56632 0.070689785 56632 0.081368744
56600 0.152359105 56600 0.051119707 28400 0.031096253 28352
0.064713517 56640 0.117891599 56640 0.110249072
56600 0.085642896 56600 0.085246931 28400 0.040485506 28356
0.078369514 56648 0.13986387 56648 0.11687068
56700 0.026828588 56700 0.109779307 28400 0.103790851 28360
0.047484504 56656 0.079635996 56656 0.069239461
56700 0.045351437 56700 0.097182296 28400 0.146236984 28364
0.038787061 56664 0.037451304 56664 0.001816644
56700 0.065598208 56700 0.041951429 28400 0.105060724 28368
0.043785887 56672 0.085700456 56672 0.075507742
56700 0.084625957 56700 0.065457316 28400 0.043713892 28372
0.006470147 56680 0.117281838 56680 0.080335543
56700 0.052486634 56700 0.10742103 28400 0.076064796 28376
0.093347677 56688 0.11241679 56688 0.081892846
56700 0.128442073 56700 0.12313528 28400 0.057034224 28380
0.141884971 56696 0.086978245 56696 0.12275987
56700 0.134570699 56700 0.120175202 28400 0.050923161 28384
0.104711733 56704 0.094756433 56704 0.061090199
56700 0.076089586 56700 0.090222384 28400 0.040273844 28388
0.007318819 56712 0.081686267 56712 0.084282765
56700 0.101915379 56700 0.101640078 28400 0.028093182 28392
0.074674179 56720 0.10926363 56720 0.142656354
56700 0.127936204 56700 0.182288291 28400 0.016102151 28396
0.082693601 56728 0.079957375 56728 0.089178
56700 0.140828838 56700 0.198395618 28400 0.020961385 28400
0.089424015 56736 0.067490626 56736 0.073003474
56700 0.170555403 56700 0.082805971 28400 0.037194754 28404
0.072153358 56744 0.106076084 56744 0.101235499
56800 0.179921248 56800 0.054224871 28400 0.043876546 28408
0.04573182 56752 0.127977415 56752 0.158078343
56800 0.118615761 56800 0.075611584 28400 0.013572894 28412
0.106081345 56760 0.120219746 56760 0.145770115
56800 0.124757411 56800 0.033061911 28400 0.040068517 28416
0.134233153 56768 0.098074066 56768 0.12851847
56800 0.112672315 56800 0.061492581 28400 0.038966991 28420
0.092450646 56776 0.083719329 56776 0.145257334
56800 0.083429557 56800 0.115346964 28400 0.105332554 28424
0.069971422 56784 0.101005862 56784 0.081617116
56800 0.084773179 56800 0.118087344 28400 0.14150847 28428
0.105645544 56792 0.110780551 56792 0.153251021
56800 0.066992107 56800 0.056625304 28400 0.121771664 28432
0.100852449 56800 0.065614921 56800 0.176577873
56800 0.085760083 56800 0.048241163 28400 0.054621756 28436
0.085801126 56808 0.037430062 56808 0.121200741
56800 0.093857525 56800 0.038323022 28400 0.057420748 28440
0.084295985 56816 0.064907115 56816 0.166649174
56800 0.023801189 56800 0.045778706 28400 0.072185692 28444
0.079290985 56824 0.048437702 56824 0.193173895
56800 0.075026153 56800 0.095714742 28400 0.020838132 28448
0.056565379 56832 0.008728986 56832 0.189874626
56800 0.068420959 56800 0.153393441 28500 0.034975572 28452
0.075779302 56840 0.006912828 56840 0.253486825
56800 0.053600634 56800 0.0778787 28500 0.037557536 28456
0.086301254 56848 0.08341502 56848 0.193250948
56900 0.173050649 56900 0.10226592 28500 0.053820084 28460
0.081497441 56856 0.12671428 56856 0.163390083
56900 0.19345693 56900 0.203610325 28500 0.07799155 28464
0.074612479 56864 0.133527938 56864 0.34520941
56900 0.166329075 56900 0.161129458 28500 0.075869866 28468
0.067393914 56872 0.126234009 56872 0.36766287
56900 0.17338105 56900 0.023021979 28500 0.033959692 28472

0.091872149 56880 0.080024962 56880 0.349814916
56900 0.175688023 56900 0.113791117 28500 0.021743415 28476
0.076429169 56888 0.129271008 56888 0.339449441
56900 0.119796416 56900 0.162628683 28500 0.038451155 28480
0.055007255 56896 0.141226279 56896 0.345188048
56900 0.036841906 56900 0.112855079 28500 0.055386008 28484
0.060022056 56904 0.077854304 56904 0.48430005
56900 0.046545596 56900 0.068165515 28500 0.081127633 28488
0.063867519 56912 0.060694594 56912 0.50445745
56900 0.10068898 56900 0.079705365 28500 0.070817208 28492
0.081389866 56920 0.097215285 56920 0.576396764
56900 0.119425022 56900 0.064086678 28500 0.034977267 28496
0.080766469 56928 0.076765777 56928 0.662394799
56900 0.125542138 56900 0.161057979 28500 0.031573942 28500
0.061908431 56936 0.051029587 56936 0.841223984
56900 0.121629768 56900 0.232094506 28500 0.065579247 28504
0.071917064 56944 0.093890027 56944 1.383777363
57000 0.162952493 57000 0.236120846 28500 0.040042887 28508
0.082673687 56952 0.123815989 56952 1.461434763
57000 0.143175538 57000 0.146666556 28500 0.024017969 28512
0.082576917 56960 0.085557083 56960 9.095722813
57000 0.058692549 57000 0.067442867 28500 0.008116102 28516
0.07421495 56968 0.064985201 56968 17.46592113
57000 0.109062261 57000 0.049563059 28500 0.058143196 28520
0.073585441 56976 0.057739628 56976 16.42822663
57000 0.093389877 57000 0.07998701 28500 0.098448873 28524
0.100173143 56984 0.031774038 56984 7.296406663
57000 0.037811311 57000 0.210690035 28500 0.098012424 28528
0.100862802 56992 0.026118616 56992 1.02727022
57000 0.048845239 57000 0.284212263 28500 0.066994922 28532
0.06154085 57000 0.01054052 57000 1.09641999
57000 0.062281419 57000 0.268592529 28500 0.054402135 28536
0.044773649 57008 0.025851314 57008 0.819150475
57000 0.126141124 57000 0.318280159 28500 0.059924347 28540
0.062550789 57016 0.076005548 57016 0.511400285
57000 0.115445553 57000 0.368291861 28500 0.053051037 28544
0.070610724 57024 0.100874437 57024 0.480381103
57000 0.088131237 57000 0.23065164 28500 0.026497695 28548
0.060291124 57032 0.106420208 57032 0.381984603
57000 0.023745275 57000 0.120843855 28600 0.04021118 28552
0.02396423 57040 0.155360234 57040 0.296383369
57000 0.066049317 57000 0.303895999 28600 0.081116566 28556
0.0788502 57048 0.139207215 57048 0.28837999
57100 0.139391719 57100 0.267298135 28600 0.065782253 28560
0.141747601 57056 0.065890839 57056 0.238930501
57100 0.179519979 57100 0.263596158 28600 0.054531185 28564
0.146745005 57064 0.01568779 57064 0.179806434
57100 0.140481469 57100 0.440521778 28600 0.081047736 28568
0.121711601 57072 0.089800029 57072 0.121914833
57100 0.072707626 57100 0.655124314 28600 0.081139959 28572
0.088364213 57080 0.192727631 57080 0.122879166
57100 0.06181642 57100 0.905197929 28600 0.063813081 28576
0.04951896 57088 0.191897023 57088 0.037166152
57100 0.086768101 57100 1.224174514 28600 0.045666562 28580
0.026243233 57096 0.116832074 57096 0.052877647
57100 0.096926662 57100 1.608291288 28600 0.032998429 28584
0.009998636 57104 0.113958544 57104 0.056632631
57100 0.096425181 57100 1.996338363 28600 0.018908002 28588
0.03073128 57112 0.183336175 57112 0.056233916
57100 0.047882444 57100 2.5148706 28600 0.015987331 28592
0.044722652 57120 0.192489854 57120 0.021493426
57100 0.053996333 57100 3.220423825 28600 0.032229636 28596
0.063157582 57128 0.134127099 57128 0.020174264
57100 0.055652214 57100 3.798387475 28600 0.057446727 28600
0.124127415 57136 0.081665661 57136 0.041756
57100 0.093195413 57100 4.016715563 28600 0.045257351 28604
0.115186551 57144 0.059065431 57144 0.061753555
57200 0.159849485 57200 3.763719925 28600 0.020393187 28608

0.047049649 57152 0.071375325 57152 0.089917725
57200 0.083221799 57200 3.76598165 28600 0.035028086 28612
0.037313486 57160 0.076777375 57160 0.111254522
57200 0.049507755 57200 5.279264875 28600 0.049526276 28616
0.103865015 57168 0.072277398 57168 0.092005932
57200 0.044643584 57200 6.375493488 28600 0.03912958 28620
0.11461557 57176 0.068555477 57176 0.015700325
57200 0.055437951 57200 5.409816275 28600 0.043968721 28624
0.060388535 57184 0.084686748 57184 0.06284046
57200 0.080196092 57200 3.397803288 28600 0.032687491 28628
0.042961627 57192 0.084179716 57192 0.096459364
57200 0.12531878 57200 2.284134738 28600 0.018140845 28632
0.073614778 57200 0.089409143 57200 0.148362713
57200 0.138412288 57200 1.617129188 28600 0.03850957 28636
0.06836078 57208 0.08732768 57208 0.116281743
57200 0.095352138 57200 1.148995245 28600 0.06161465 28640
0.069860405 57216 0.052531683 57216 0.062082247
57200 0.056522047 57200 0.817018095 28600 0.067484158 28644
0.050983734 57224 0.013020701 57224 0.099013487
57200 0.052880372 57200 0.3323376 28600 0.054011976 28648
0.008623671 57232 0.050682749 57232 0.133682378
57200 0.081464867 57200 0.113012982 28700 0.03987092 28652
0.049872637 57240 0.103733124 57240 0.061068837
57200 0.09387896 57200 0.15052197 28700 0.008704391 28656
0.091386981 57248 0.129235806 57248 0.069676193
57300 0.140360629 57300 0.026701242 28700 0.006790614 28660
0.089309739 57256 0.12637653 57256 0.086927241
57300 0.181422133 57300 0.115079172 28700 0.02409101 28664
0.073157185 57264 0.099763922 57264 0.040364947
57300 0.121638303 57300 0.124863145 28700 0.039832165 28668
0.080327693 57272 0.114913637 57272 0.027778848
57300 0.028093142 57300 0.139417534 28700 0.043106949 28672
0.060499089 57280 0.140647796 57280 0.014865238
57300 0.033703102 57300 0.197847199 28700 0.032254819 28676
0.049081529 57288 0.069814923 57288 0.042114203
57300 0.043207005 57300 0.238076711 28700 0.006726571 28680
0.081612263 57296 0.065531582 57296 0.084890889
57300 0.067731497 57300 0.2085538 28700 0.043758457 28684
0.074097094 57304 0.070164722 57304 0.054261505
57300 0.142413599 57300 0.072152943 28700 0.050348241 28688
0.073585114 57312 0.103697093 57312 0.03169366
57300 0.158679846 57300 0.096778931 28700 0.042457763 28692
0.100830112 57320 0.116886156 57320 0.085020154
57300 0.16198869 57300 0.171298773 28700 0.0248818 28696
0.084146137 57328 0.091067814 57328 0.102541686
57300 0.132880421 57300 0.143251193 28700 0.028683171 28700
0.047974976 57336 0.039397441 57336 0.058282774
57300 0.093031929 57300 0.07919527 28700 0.041322546 28704
0.04959025 57344 0.145616258 57344 0.020157711
57400 0.056480873 57400 0.049813021 28700 0.052909305 28708
0.096180702 57352 0.184309261 57352 0.064415384
57400 0.106605163 57400 0.057551351 28700 0.031126088 28712
0.100593003 57360 0.092771865 57360 0.062387393
57400 0.168146508 57400 0.138267729 28700 0.041558564 28716
0.076746292 57368 0.04130711 57368 0.038532868
57400 0.171476903 57400 0.164395679 28700 0.030802297 28720
0.087485183 57376 0.090660586 57376 0.030366276
57400 0.107883818 57400 0.1128316 28700 0.053410819 28724
0.103237224 57384 0.032075393 57384 0.059858277
57400 0.097163938 57400 0.046126246 28700 0.070369242 28728
0.085364874 57392 0.06721 57392 0.098561308
57400 0.120157136 57400 0.0160911 28700 0.081142185 28732
0.084982268 57400 0.056885692 57400 0.070962771
57400 0.087247994 57400 0.093190065 28700 0.064126638 28736
0.10095469 57408 0.117140444 57408 0.010391344
57400 0.033807857 57400 0.184978751 28700 0.041294199 28740
0.08554916 57416 0.156751339 57416 0.02340967
57400 0.02373552 57400 0.263572263 28700 0.027824844 28744

0.066535758 57424 0.109828936 57424 0.009856373
57400 0.081517224 57400 0.26139582 28700 0.029520759 28748
0.092789582 57432 0.070638816 57432 0.040000843
57400 0.085427782 57400 0.12518531 28800 0.04812788 28752
0.094554947 57440 0.133108523 57440 0.032631917
57400 0.049890568 57400 0.080280981 28800 0.046039513 28756
0.099433935 57448 0.098512232 57448 0.025748612
57500 0.107746986 57500 0.1597074 28800 0.018264465 28760
0.092604678 57456 0.030127209 57456 0.055664295
57500 0.085264757 57500 0.16700766 28800 0.041652904 28764
0.028821572 57464 0.176977745 57464 0.042221305
57500 0.043603635 57500 0.259620429 28800 0.049074093 28768
0.048750786 57472 0.258995336 57472 0.016913327
57500 0.1009638 57500 0.224420394 28800 0.03783084 28772
0.090164635 57480 0.201190138 57480 0.057468613
57500 0.081907238 57500 0.084854983 28800 0.014355132 28776
0.110886547 57488 0.080550308 57488 0.089467947
57500 0.138181859 57500 0.097447934 28800 0.033383469 28780
0.116498661 57496 0.081870909 57496 0.075919277
57500 0.186987149 57500 0.14367605 28800 0.047355152 28784
0.089509274 57504 0.03999981 57504 0.039117364
57500 0.179069714 57500 0.15910744 28800 0.065208777 28788
0.05296 57512 0.13946378 57512 0.028753208
57500 0.110857058 57500 0.118461117 28800 0.115682262 28792
0.103308514 57520 0.086742373 57520 0.052264059
57500 0.076910859 57500 0.042789106 28800 0.121760742 28796
0.161167773 57528 0.087994275 57528 0.070749833
57500 0.085065265 57500 0.072093484 28800 0.084846237 28800
0.125444073 57536 0.162205718 57536 0.037508406
57500 0.034450677 57500 0.070542614 28800 0.056038713 28804
0.037395072 57544 0.12858375 57544 0.024335092
57600 0.058301408 57600 0.099104778 28800 0.036925092 28808
0.128718239 57552 0.109037072 57552 0.047615395
57600 0.100839134 57600 0.172271495 28800 0.047433874 28812
0.197870439 57560 0.119171338 57560 0.030433044
57600 0.128067958 57600 0.121155106 28800 0.092793576 28816
0.188054023 57568 0.142036298 57568 0.042791751
57600 0.127877545 57600 0.055004417 28800 0.087392393 28820
0.118580094 57576 0.091334761 57576 0.05843179
57600 0.104569583 57600 0.112621929 28800 0.060271337 28824
0.042953489 57584 0.022914366 57584 0.065233289
57600 0.06533612 57600 0.130466141 28800 0.067145338 28828
0.120509511 57592 0.066992681 57592 0.08170939
57600 0.019676663 57600 0.106239029 28800 0.046894475 28832
0.148671941 57600 0.080599952 57600 0.099141209
57600 0.115381285 57600 0.155482529 28800 0.039882976 28836
0.094409144 57608 0.07404996 57608 0.08840712
57600 0.141080396 57600 0.159637624 28800 0.030462612 28840
0.095359384 57616 0.034809713 57616 0.057706158
57600 0.15210954 57600 0.120841549 28800 0.021136862 28844
0.099002602 57624 0.093542316 57624 0.028889659
57600 0.175949973 57600 0.087153858 28800 0.033505072 28848
0.096243355 57632 0.149636923 57632 0.024281679
57600 0.143083889 57600 0.115831419 28900 0.065245105 28852
0.064268606 57640 0.11252908 57640 0.04063328
57600 0.074195821 57600 0.127744614 28900 0.071324022 28856
0.043811364 57648 0.057213052 57648 0.063830776
57700 0.015898328 57700 0.068993191 28900 0.055537803 28860
0.033432199 57656 0.156687268 57656 0.068843146
57700 0.046482397 57700 0.043945383 28900 0.040915504 28864
0.025155869 57664 0.163671546 57664 0.057312762
57700 0.054833159 57700 0.071368886 28900 0.024300163 28868
0.078412202 57672 0.120866156 57672 0.046474659
57700 0.055412442 57700 0.086173575 28900 0.008557536 28872
0.081349084 57680 0.09615803 57680 0.046317127
57700 0.091302391 57700 0.057906535 28900 0.033807672 28876
0.044083066 57688 0.036652742 57688 0.034955159
57700 0.088393717 57700 0.027742424 28900 0.044160726 28880

0.040381703 57696 0.017924143 57696 0.016580571
57700 0.097387158 57700 0.023338651 28900 0.017812394 28884
0.080377722 57704 0.059185135 57704 0.044447545
57700 0.137251176 57700 0.096613127 28900 0.016080789 28888
0.075781922 57712 0.131626439 57712 0.059974289
57700 0.121802732 57700 0.1310402 28900 0.018593877 28892
0.053277236 57720 0.064903819 57720 0.090984126
57700 0.073542258 57700 0.081643622 28900 0.008895436 28896
0.02190641 57728 0.050999846 57728 0.101183643
57700 0.107407934 57700 0.063228807 28900 0.025100213 28900
0.059576272 57736 0.051470779 57736 0.06271981
57700 0.136152346 57700 0.075787022 28900 0.059162569 28904
0.07282212 57744 0.032524822 57744 0.014523531
57800 0.132222529 57800 0.072960298 28900 0.047898808 28908
0.057329828 57752 0.055563782 57752 0.011522054
57800 0.140065808 57800 0.084651874 28900 0.024943765 28912
0.041479922 57760 0.018150364 57760 0.027226812
57800 0.100654201 57800 0.083459549 28900 0.03878829 28916
0.011720635 57768 0.096055373 57768 0.049336402
57800 0.051731244 57800 0.043221524 28900 0.033582597 28920
0.065299821 57776 0.136394316 57776 0.052692438
57800 0.071858405 57800 0.144602629 28900 0.015981581 28924
0.081977967 57784 0.094343501 57784 0.022094468
57800 0.089279478 57800 0.198364476 28900 0.027797394 28928
0.065653294 57792 0.03208487 57792 0.029530404
57800 0.09032002 57800 0.138022544 28900 0.031688818 28932
0.050607523 57800 0.090884321 57800 0.021130869
57800 0.111831192 57800 0.052162799 28900 0.012043394 28936
0.061632221 57808 0.117141302 57808 0.034227611
57800 0.072211362 57800 0.121989215 28900 0.028225442 28940
0.046992027 57816 0.105020692 57816 0.057767324
57800 0.022606713 57800 0.089709196 28900 0.026183765 28944
0.03279157 57824 0.132081579 57824 0.066761575
57800 0.060231883 57800 0.102645783 28900 0.049212784 28948
0.05915431 57832 0.13208283 57832 0.032269316
57800 0.07290097 57800 0.141638346 29000 0.08492545 28952
0.065252534 57840 0.066218869 57840 0.028654882
57800 0.106730389 57800 0.080108483 29000 0.104379164 28956
0.034122808 57848 0.040890933 57848 0.069008602
57900 0.061464147 57900 0.019940808 29000 0.107388005 28960
0.038402406 57856 0.061147672 57856 0.065791355
57900 0.133446418 57900 0.1310847 29000 0.087387613 28964
0.08962139 57864 0.024906134 57864 0.038972328
57900 0.172405241 57900 0.154615889 29000 0.069673079 28968
0.119270211 57872 0.013780335 57872 0.02698997
57900 0.059753544 57900 0.137860785 29000 0.050098566 28972
0.105101572 57880 0.016904865 57880 0.02164778
57900 0.122195925 57900 0.1347098 29000 0.011698628 28976
0.052952892 57888 0.005781959 57888 0.010876974
57900 0.148339925 57900 0.027585229 29000 0.027554386 28980
0.017832295 57896 0.020840542 57896 0.053864456
57900 0.063037478 57900 0.106408341 29000 0.034387496 28984
0.031717067 57904 0.013576515 57904 0.061370127
57900 0.016195834 57900 0.106756626 29000 0.022096587 28988
0.069670459 57912 0.056182835 57912 0.041233019
57900 0.03536437 57900 0.067989247 29000 0.056921454 28992
0.091450755 57920 0.082294188 57920 0.011616473
57900 0.099264056 57900 0.060784878 29000 0.067184454 28996
0.107223343 57928 0.193427638 57928 0.01707873
57900 0.125564896 57900 0.10627426 29000 0.050786162 29000
0.109519438 57936 0.279463856 57936 0.0328102
57900 0.082950683 57900 0.103982042 29000 0.058742739 29004
0.066887515 57944 0.196802969 57944 0.057794023
58000 0.060448947 58000 0.0791106 29000 0.052382169 29008
0.018134268 57952 0.069588801 57952 0.039861836
58000 0.163406978 58000 0.076645803 29000 0.027115621 29012
0.090167399 57960 0.03884836 57960 0.017941227
58000 0.105011124 58000 0.058175421 29000 0.009696878 29016

0.113714901	57968	0.061577812	57968	0.037982918
58000	0.015951548	58000	0.058778162	29000	0.038724658	29020
0.114701259	57976	0.048524911	57976	0.017106348
58000	0.011027183	58000	0.065964319	29000	0.031687978	29024
0.105862753	57984	0.085541426	57984	0.013695091
58000	0.10968728	58000	0.021972392	29000	0.013212572	29028
0.057615616	57992	0.147096201	57992	0.027865144
58000	0.164592043	58000	0.137259485	29000	0.048610233	29032
0.03368923	58000	0.117744501	58000	0.029930257
58000	0.182107935	58000	0.161233664	29000	0.061544008	29036
0.036053079	58008	0.078160498	58008	0.026329373
58000	0.176326459	58000	0.086795277	29000	0.045746783	29040
0.021484706	58016	0.100054131	58016	0.023212211
58000	0.155287125	58000	0.030248137	29000	0.043930999	29044
0.036858557	58024	0.086792032	58024	0.009702108
58000	0.140367134	58000	0.061530096	29000	0.071727955	29048
0.076258941	58032	0.044223998	58032	0.024227036
58000	0.042723474	58000	0.14967761	29100	0.086070235	29052
0.08204185	58040	0.117639807	58040	0.013645266
58000	0.093920797	58000	0.182022268	29100	0.057256966	29056
0.049463677	58048	0.14609423	58048	0.028159386
58100	0.127879466	58100	0.112950176	29100	0.042839034	29060
0.018217188	58056	0.111086942	58056	0.034453649
58100	0.057377831	58100	0.048460297	29100	0.036012963	29064
0.082992432	58064	0.069574999	58064	0.053414657
58100	0.062731604	58100	0.07612379	29100	0.031983087	29068
0.093609655	58072	0.060428403	58072	0.063990672
58100	0.039904106	58100	0.148624313	29100	0.036493297	29072
0.036726622	58080	0.12499404	58080	0.061265491
58100	0.021923654	58100	0.174522836	29100	0.048748967	29076
0.031995722	58088	0.113175076	58088	0.058014968
58100	0.050109378	58100	0.143725949	29100	0.064816748	29080
0.076011413	58096	0.122947298	58096	0.054761571
58100	0.078369027	58100	0.109640139	29100	0.057542005	29084
0.061804552	58104	0.17369719	58104	0.045393925
58100	0.069283291	58100	0.048936883	29100	0.057413261	29088
0.010733559	58112	0.166618629	58112	0.03203933
58100	0.0702416	58100	0.056815527	29100	0.035549318	29092
0.00997 58120	0.059546837	58120	0.023868304
58100	0.06868787	58100	0.11841664	29100	0.016214852	29096
0.025146317	58128	0.10179024	58128	0.02255895
58100	0.051424438	58100	0.121054261	29100	0.025459742	29100
0.047011909	58136	0.196824578	58136	0.023066652
58100	0.047810787	58100	0.094205832	29100	0.042976844	29104
0.054856126	58144	0.16811039	58144	0.024153729
58200	0.055597353	58200	0.096159223	29100	0.050811577	29108
0.042604217	58152	0.089282039	58152	0.052098705
58200	0.070675567	58200	0.11340058	29100	0.043290165	29112
0.063317319	58160	0.034211764	58160	0.067810528
58200	0.097822347	58200	0.065081578	29100	0.058647118	29116
0.0615174	58168	0.034859368	58168	0.079346159
58200	0.102973354	58200	0.039901846	29100	0.058361264	29120
0.03359855	58176	0.038118695	58176	0.080392791
58200	0.062619132	58200	0.048078331	29100	0.008617528	29124
0.06776378	58184	0.109810426	58184	0.072848161
58200	0.055691013	58200	0.041955813	29100	0.052007392	29128
0.067812485	58192	0.15586373	58192	0.031705597
58200	0.06286901	58200	0.117186166	29100	0.082690342	29132
0.036512236	58200	0.1359288	58200	0.034254314
58200	0.028954644	58200	0.131761758	29100	0.083476552	29136
0.013724944	58208	0.106069456	58208	0.033678527
58200	0.037946167	58200	0.124455139	29100	0.069710623	29140
0.005169437	58216	0.046280624	58216	0.042798074
58200	0.007296865	58200	0.088672437	29100	0.0495391	29144
0.019291518	58224	0.077599623	58224	0.062145431
58200	0.080522754	58200	0.018271609	29100	0.011714596	29148
0.038492068	58232	0.117927753	58232	0.052413954
58200	0.168768791	58200	0.028512048	29200	0.022734734	29152

0.033452401 58240 0.053443488 58240 0.069047026
58200 0.140978053 58200 0.035151195 29200 0.03331136 29156
0.049215534 58248 0.05747666 58248 0.066108172
58300 0.074271688 58300 0.092007511 29200 0.05916856 29160
0.064814492 58256 0.17274193 58256 0.053130814
58300 0.110129826 58300 0.074261167 29200 0.062038322 29164
0.060680934 58264 0.187796875 58264 0.037295962
58300 0.100188059 58300 0.084452229 29200 0.053558437 29168
0.0332237 58272 0.110204055 58272 0.010061123
58300 0.054175962 58300 0.06580248 29200 0.045886267 29172
0.091950256 58280 0.095018513 58280 0.047821894
58300 0.029279858 58300 0.003062903 29200 0.020793958 29176
0.110001412 58288 0.146110556 58288 0.065214073
58300 0.075269505 58300 0.052176583 29200 0.05129788 29180
0.078817604 58296 0.113732865 58296 0.054556367
58300 0.079863814 58300 0.032540924 29200 0.064356267 29184
0.026271337 58304 0.050735474 58304 0.040140396
58300 0.104636914 58300 0.068311572 29200 0.029432911 29188
0.050846378 58312 0.068580652 58312 0.02988266
58300 0.162483819 58300 0.100594189 29200 0.037757145 29192
0.048938644 58320 0.068685804 58320 0.022855169
58300 0.095440875 58300 0.012503582 29200 0.046642592 29196
0.033195829 58328 0.109031891 58328 0.049404738
58300 0.014988957 58300 0.090476257 29200 0.120963297 29200
0.037861177 58336 0.126910366 58336 0.083462248
58300 0.045653447 58300 0.112929207 29200 0.1439037 29204
0.033074499 58344 0.107444343 58344 0.095769901
58400 0.108092092 58400 0.110795591 29200 0.086013875 29208
0.015322952 58352 0.136079776 58352 0.074493932
58400 0.17051911 58400 0.122259924 29200 0.036794048 29212
0.047887439 58360 0.131485068 58360 0.041108266
58400 0.212904036 58400 0.096186537 29200 0.073555872 29216
0.088924215 58368 0.131775116 58368 0.033490658
58400 0.16303439 58400 0.055343986 29200 0.089954272 29220
0.104323372 58376 0.114283314 58376 0.054726083
58400 0.060242481 58400 0.130876491 29200 0.065180502 29224
0.065756132 58384 0.129916284 58384 0.064116262
58400 0.019114217 58400 0.170210973 29200 0.012841058 29228
0.029532166 58392 0.158883894 58392 0.042086904
58400 0.01750931 58400 0.148403328 29200 0.039875864 29232
0.042693144 58400 0.141050201 58400 0.041468491
58400 0.02727398 58400 0.03779582 29200 0.049508733 29236
0.046900834 58408 0.09310466 58408 0.035379741
58400 0.067379748 58400 0.122036756 29200 0.0496824 29240
0.034020886 58416 0.048687409 58416 0.040108298
58400 0.070971561 58400 0.157475443 29200 0.047166384 29244
0.019276926 58424 0.038828657 58424 0.050955678
58400 0.041098461 58400 0.105592379 29200 0.057810543 29248
0.03918881 58432 0.102144972 58432 0.069662878
58400 0.029290135 58400 0.037567228 29300 0.035189467 29252
0.056068824 58440 0.127675769 58440 0.066224537
58400 0.081121376 58400 0.058015554 29300 0.022344033 29256
0.08378589 58448 0.089509631 58448 0.03452957
58500 0.133425099 58500 0.061153565 29300 0.026071084 29260
0.076710319 58456 0.053284017 58456 0.04775486
58500 0.116436866 58500 0.095175215 29300 0.032128126 29264
0.051518618 58464 0.039688173 58464 0.059196002
58500 0.046722231 58500 0.06002868 29300 0.021672429 29268
0.029502582 58472 0.057817859 58472 0.034019358
58500 0.074837786 58500 0.119482604 29300 0.017903098 29272
0.051973155 58480 0.043568864 58480 0.016570622
58500 0.098200595 58500 0.126571816 29300 0.023420507 29276
0.034112138 58488 0.056370463 58488 0.037972553
58500 0.043746029 58500 0.052812884 29300 0.011539827 29280
0.02053243 58496 0.025833058 58496 0.047928035
58500 0.067371351 58500 0.057486552 29300 0.030518357 29284
0.013258143 58504 0.063394495 58504 0.039058123
58500 0.068661415 58500 0.056270961 29300 0.0221223 29288

0.031405165 58512 0.085346699 58512 0.065861408
58500 0.020319183 58500 0.017255126 29300 0.022959643 29292
0.056715639 58520 0.14910764 58520 0.058328391
58500 0.032505886 58500 0.029199922 29300 0.050444807 29296
0.069801448 58528 0.093377326 58528 0.057903777
58500 0.04532182 58500 0.0637903 29300 0.075992386 29300
0.066902532 58536 0.073122908 58536 0.069279166
58500 0.118837445 58500 0.077534438 29300 0.078474259 29304
0.042411612 58544 0.081705599 58544 0.048615631
58600 0.21654561 58600 0.06610305 29300 0.062526626 29308
0.00557915 58552 0.055602795 58552 0.017807288
58600 0.183654033 58600 0.030264062 29300 0.034843077 29312
0.021448312 58560 0.034205401 58560 0.026352549
58600 0.055366352 58600 0.041865416 29300 0.033917902 29316
0.029107725 58568 0.05634471 58568 0.028038436
58600 0.110628025 58600 0.103632738 29300 0.031186926 29320
0.026202328 58576 0.100745499 58576 0.062557221
58600 0.131164619 58600 0.138762276 29300 0.013830165 29324
0.040999512 58584 0.12932453 58584 0.054863394
58600 0.133462221 58600 0.137693991 29300 0.015008174 29328
0.064881533 58592 0.130197455 58592 0.040656418
58600 0.194907858 58600 0.13672965 29300 0.05136041 29332
0.091796843 58600 0.094525181 58600 0.037534705
58600 0.174256683 58600 0.169047199 29300 0.092486858 29336
0.102129234 58608 0.078079407 58608 0.023751672
58600 0.092795985 58600 0.190696315 29300 0.064720167 29340
0.067745183 58616 0.075717027 58616 0.03402177
58600 0.09577108 58600 0.15182406 29300 0.052174113 29344
0.020715908 58624 0.02508051 58624 0.047064346
58600 0.127316948 58600 0.082917177 29300 0.08269868 29348
0.011072087 58632 0.043167569 58632 0.047374138
58600 0.130498244 58600 0.066447792 29400 0.071898357 29352
0.014898278 58640 0.098521268 58640 0.042744592
58600 0.107901869 58600 0.112214526 29400 0.054727334 29356
0.026907273 58648 0.103149076 58648 0.033853517
58700 0.038247523 58700 0.102797676 29400 0.032552744 29360
0.059488568 58656 0.052229785 58656 0.02100768
58700 0.031437805 58700 0.033428201 29400 0.041260693 29364
0.040028564 58664 0.054876928 58664 0.018219911
58700 0.071215705 58700 0.051555449 29400 0.062350606 29368
0.009419731 58672 0.069756963 58672 0.033767756
58700 0.098818549 58700 0.107909094 29400 0.081028025 29372
0.061575309 58680 0.047958794 58680 0.033512584
58700 0.140282631 58700 0.11123289 29400 0.048596216 29376
0.088085071 58688 0.014894995 58688 0.039454732
58700 0.159677963 58700 0.03597212 29400 0.016121709 29380
0.067213965 58696 0.079787969 58696 0.058973907
58700 0.149702595 58700 0.047195739 29400 0.044266461 29384
0.074176853 58704 0.095868905 58704 0.048775186
58700 0.128912754 58700 0.117974916 29400 0.047770693 29388
0.090689588 58712 0.114296949 58712 0.039404713
58700 0.102098682 58700 0.153170375 29400 0.019061457 29392
0.084280619 58720 0.112132344 58720 0.057776884
58700 0.086458771 58700 0.080478145 29400 0.0290836 29396
0.061007082 58728 0.065144086 58728 0.081263155
58700 0.05547663 58700 0.056185698 29400 0.021748983 29400
0.033322012 58736 0.057578818 58736 0.074949479
58700 0.038787493 58700 0.096486321 29400 0.019845284 29404
0.034614637 58744 0.092657501 58744 0.036767917
58800 0.038350114 58800 0.147433791 29400 0.033755816 29408
0.069965747 58752 0.081691484 58752 0.017830744
58800 0.029583393 58800 0.160713549 29400 0.029243516 29412
0.080909478 58760 0.029160688 58760 0.044512573
58800 0.063895386 58800 0.102080303 29400 0.017260118 29416
0.066011322 58768 0.015726328 58768 0.05281304
58800 0.127201085 58800 0.03512295 29400 0.060410897 29420
0.075463002 58776 0.068021109 58776 0.036224981
58800 0.139620606 58800 0.043342869 29400 0.075240685 29424

0.052527623 58784 0.139869779 58784 0.036357025
58800 0.071866984 58800 0.077971054 29400 0.058122743 29428
0.054354023 58792 0.164194236 58792 0.034476168
58800 0.047686604 58800 0.046065365 29400 0.064336033 29432
0.097273056 58800 0.148016319 58800 0.029661293
58800 0.0908941 58800 0.048879996 29400 0.07334815 29436
0.058100603 58808 0.11731124 58808 0.03232788
58800 0.083193154 58800 0.080826852 29400 0.060552975 29440
0.060786304 58816 0.082615363 58816 0.048718543
58800 0.046607325 58800 0.049593069 29400 0.05814336 29444
0.053969092 58824 0.121951118 58824 0.054979173
58800 0.022081178 58800 0.04750106 29400 0.046349011 29448
0.057908106 58832 0.170916304 58832 0.047289315
58800 0.038172155 58800 0.054708096 29500 0.05709266 29452
0.059337344 58840 0.124226062 58840 0.060088791
58800 0.066649489 58800 0.051666491 29500 0.07880215 29456
0.024596966 58848 0.093585077 58848 0.050126564
58900 0.125820153 58900 0.081809674 29500 0.050002091 29460
0.030405485 58856 0.096761585 58856 0.044413551
58900 0.15016731 58900 0.128944769 29500 0.022697781 29464
0.053836069 58864 0.10175183 58864 0.067986752
58900 0.108661086 58900 0.170791376 29500 0.054426422 29468
0.075536955 58872 0.110524139 58872 0.08184107
58900 0.086000226 58900 0.147830535 29500 0.043634082 29472
0.039171599 58880 0.10721825 58880 0.065750828
58900 0.117889467 58900 0.091606344 29500 0.018811581 29476
0.025409894 58888 0.076657321 58888 0.00855193
58900 0.159441843 58900 0.018631859 29500 0.018363866 29480
0.039215072 58896 0.025311649 58896 0.038225273
58900 0.147120533 58900 0.063571053 29500 0.043490527 29484
0.042155945 58904 0.041955584 58904 0.042382468
58900 0.100134734 58900 0.045244298 29500 0.054919849 29488
0.04564496 58912 0.100932681 58912 0.025334499
58900 0.063719395 58900 0.041672229 29500 0.037482929 29492
0.066595487 58920 0.144900638 58920 0.02796676
58900 0.077780751 58900 0.047823436 29500 0.031818116 29496
0.051910331 58928 0.116704126 58928 0.047991562
58900 0.14714885 58900 0.103159677 29500 0.061835875 29500
0.039910967 58936 0.053512071 58936 0.059686245
58900 0.161984354 58900 0.142127538 29500 0.079055557 29504
0.104253115 58944 0.143499041 58944 0.041737636
59000 0.116054405 59000 0.106554508 29500 0.087093686 29508
0.122765428 58952 0.167535298 58952 0.024897458
59000 0.110056491 59000 0.043206612 29500 0.100140511 29512
0.071269802 58960 0.147853556 58960 0.052509597
59000 0.120551864 59000 0.098528915 29500 0.086594264 29516
0.026702499 58968 0.089444977 58968 0.042307773
59000 0.062371648 59000 0.065788699 29500 0.031582815 29520
0.040565985 58976 0.034600613 58976 0.03544585
59000 0.012620328 59000 0.140256656 29500 0.053194664 29524
0.084100226 58984 0.070319788 58984 0.04900646
59000 0.001140629 59000 0.153444736 29500 0.032914373 29528
0.062037187 58992 0.10162437 58992 0.023359735
59000 0.032273551 59000 0.065014989 29500 0.018854356 29532
0.023791579 59000 0.106233449 59000 0.021273429
59000 0.062049556 59000 0.049926279 29500 0.006607727 29536
0.035114725 59008 0.109330678 59008 0.052215168
59000 0.147953528 59000 0.056709876 29500 0.021030664 29540
0.06917963 59016 0.156010589 59016 0.050054103
59000 0.183739394 59000 0.023374612 29500 0.020806017 29544
0.089179113 59024 0.135567549 59024 0.045034816
59000 0.173183536 59000 0.04115401 29500 0.031945587 29548
0.085300271 59032 0.086937769 59032 0.058856938
59000 0.127830368 59000 0.058774102 29600 0.026507627 29552
0.098781929 59040 0.078038989 59040 0.056070727
59000 0.105370891 59000 0.109784152 29600 0.032601733 29556
0.090908368 59048 0.121107776 59048 0.022422812
59100 0.108508619 59100 0.088508525 29600 0.02541384 29560

0.055129101	59056	0.121759549	59056	0.007365616		
59100	0.09867725	59100	0.042972042	29600	0.016646434	29564
0.012831852	59064	0.115564035	59064	0.045739456		
59100	0.087751316	59100	0.073068331	29600	0.054258242	29568
0.027666303	59072	0.071574374	59072	0.063529864		
59100	0.05489634	59100	0.084925065	29600	0.055541452	29572
0.050397222	59080	0.082001599	59080	0.019565017		
59100	0.019591016	59100	0.043323053	29600	0.024742916	29576
0.051887066	59088	0.13068442	59088	0.038512852		
59100	0.05802156	59100	0.043483062	29600	0.01732111	29580
0.068368994	59096	0.076215831	59096	0.073603544		
59100	0.138787393	59100	0.080915022	29600	0.033585096	29584
0.086147658	59104	0.136199524	59104	0.085074215		
59100	0.16146322	59100	0.122331127	29600	0.04443475	29588
0.078388774	59112	0.127423831	59112	0.062070772		
59100	0.114088602	59100	0.119705954	29600	0.03647853	29592
0.075991258	59120	0.034561417	59120	0.029457517		
59100	0.075254015	59100	0.099809382	29600	0.014792872	29596
0.067242043	59128	0.016394635	59128	0.026918568		
59100	0.076730212	59100	0.080937716	29600	0.022553932	29600
0.015809421	59136	0.030261474	59136	0.064998858		
59100	0.074792784	59100	0.056841182	29600	0.042903441	29604
0.024957401	59144	0.059409504	59144	0.08140094		
59200	0.043009626	59200	0.012632684	29600	0.032880038	29608
0.031872856	59152	0.089775538	59152	0.069042319		
59200	0.077564408	59200	0.005815971	29600	0.025496776	29612
0.029815237	59160	0.17462291	59160	0.062204948		
59200	0.075577475	59200	0.061150211	29600	0.034384982	29616
0.036898528	59168	0.15849428	59168	0.07801687		
59200	0.047634025	59200	0.100079255	29600	0.031510273	29620
0.046265333	59176	0.051251416	59176	0.081727492		
59200	0.04006692	59200	0.126277431	29600	0.014378509	29624
0.02770929	59184	0.119014003	59184	0.048117287		
59200	0.033770622	59200	0.120809433	29600	0.031679632	29628
0.067112291	59192	0.136810603	59192	0.017325283		
59200	0.055698645	59200	0.116184579	29600	0.05510791	29632
0.113206072	59200	0.06258244	59200	0.018067616		
59200	0.074136719	59200	0.104453182	29600	0.084237465	29636
0.075325355	59208	0.057789108	59208	0.01752256		
59200	0.084383748	59200	0.095132375	29600	0.125027116	29640
0.05433994	59216	0.116464333	59216	0.033543052		
59200	0.012709111	59200	0.04386836	29600	0.13541324	29644
0.057056059	59224	0.074006421	59224	0.030308678		
59200	0.059106456	59200	0.029219036	29600	0.106277628	29648
0.009342069	59232	0.00788425	59232	0.013514674		
59200	0.046543206	59200	0.063341933	29700	0.083689687	29652
0.024811512	59240	0.035898349	59240	0.029358043		
59200	0.043977205	59200	0.117447489	29700	0.029207655	29656
0.048956361	59248	0.020986285	59248	0.036825615		
59300	0.038354105	59300	0.098276418	29700	0.057187797	29660
0.050415241	59256	0.031385229	59256	0.022608552		
59300	0.050292874	59300	0.051047762	29700	0.08890163	29664
0.049415408	59264	0.074557764	59264	0.040533192		
59300	0.161022501	59300	0.025860201	29700	0.066011846	29668
0.049474438	59272	0.130752073	59272	0.058824582		
59300	0.177677531	59300	0.02082949	29700	0.049571907	29672
0.050772811	59280	0.094888885	59280	0.075669399		
59300	0.094721305	59300	0.071474475	29700	0.035219855	29676
0.07469704	59288	0.033408018	59288	0.075027747		
59300	0.080373655	59300	0.066094952	29700	0.045378969	29680
0.042889864	59296	0.119065961	59296	0.053673037		
59300	0.109951012	59300	0.010342721	29700	0.037219474	29684
0.02246821	59304	0.126801591	59304	0.020247289		
59300	0.127490421	59300	0.064493484	29700	0.034177403	29688
0.024256318	59312	0.088930232	59312	0.038536218		
59300	0.105499516	59300	0.154621826	29700	0.007338466	29692
0.028482675	59320	0.129217515	59320	0.033444238		
59300	0.049681657	59300	0.20762434	29700	0.009904559	29696

0.035441612 59328 0.098086624 59328 0.016532866
59300 0.074945667 59300 0.175210749 29700 0.027888302 29700
0.015935113 59336 0.07836373 59336 0.022692617
59300 0.09147964 59300 0.142226286 29700 0.062008257 29704
0.01764106 59344 0.067020934 59344 0.024679961
59400 0.062046383 59400 0.139363226 29700 0.077484241 29708
0.05318792 59352 0.046201549 59352 0.036662492
59400 0.075583921 59400 0.090867485 29700 0.076501136 29712
0.056274254 59360 0.044538854 59360 0.049770406
59400 0.097088348 59400 0.036425096 29700 0.057474405 29716
0.053305033 59368 0.041509622 59368 0.068333684
59400 0.094913441 59400 0.025924761 29700 0.045752451 29720
0.058773712 59376 0.036996979 59376 0.062297222
59400 0.099675657 59400 0.020129754 29700 0.022592212 29724
0.037265221 59384 0.060727947 59384 0.029549277
59400 0.057566223 59400 0.013742743 29700 0.016877144 29728
0.012300574 59392 0.097965174 59392 0.053652133
59400 0.051397434 59400 0.035060009 29700 0.025193731 29732
0.039788425 59400 0.139314303 59400 0.078182435
59400 0.070665228 59400 0.06687588 29700 0.040431885 29736
0.029390423 59408 0.126193219 59408 0.062598956
59400 0.092696282 59400 0.080667465 29700 0.02853032 29740
0.044491189 59416 0.120843746 59416 0.066698478
59400 0.041506657 59400 0.114276758 29700 0.002043064 29744
0.068700341 59424 0.085361586 59424 0.079102269
59400 0.014928946 59400 0.126323343 29700 0.043080105 29748
0.04884076 59432 0.064026899 59432 0.059830854
59400 0.006551621 59400 0.039234612 29800 0.090874702 29752
0.037376114 59440 0.120669633 59440 0.025473653
59400 0.019973755 59400 0.05408196 29800 0.089269612 29756
0.034500165 59448 0.162193043 59448 0.040434105
59500 0.076197022 59500 0.044543875 29800 0.043458193 29760
0.023899085 59456 0.13217621 59456 0.028014863
59500 0.152207315 59500 0.083087929 29800 0.034501449 29764
0.025679306 59464 0.068685644 59464 0.010372754
59500 0.114475748 59500 0.133348745 29800 0.032941014 29768
0.017978091 59472 0.017305325 59472 0.02289668
59500 0.054839195 59500 0.16252682 29800 0.026850628 29772
0.050130875 59480 0.073328156 59480 0.018163504
59500 0.088163128 59500 0.149840358 29800 0.012198629 29776
0.079391117 59488 0.173755093 59488 0.01081442
59500 0.104466126 59500 0.070182068 29800 0.06271743 29780
0.091191643 59496 0.246091251 59496 0.024973682
59500 0.067200701 59500 0.021787933 29800 0.076456097 29784
0.08867358 59504 0.20867445 59504 0.040311399
59500 0.064626969 59500 0.013845745 29800 0.037121983 29788
0.078582329 59512 0.097950622 59512 0.03930091
59500 0.096176707 59500 0.047182111 29800 0.006573603 29792
0.077886027 59520 0.026497753 59520 0.043714572
59500 0.103884711 59500 0.073052099 29800 0.011462568 29796
0.056519719 59528 0.022627341 59528 0.076343211
59500 0.050602084 59500 0.075534663 29800 0.01841009 29800
0.045519591 59536 0.042379605 59536 0.084425767
59500 0.181013224 59500 0.068789195 29800 0.026116295 29804
0.031092215 59544 0.033629029 59544 0.064038963
59600 0.177838199 59600 0.056046752 29800 0.013618501 29808
0.037695601 59552 0.035034966 59552 0.03699644
59600 0.039199749 59600 0.110835325 29800 0.031565451 29812
0.064217922 59560 0.070790826 59560 0.030846743
59600 0.069339047 59600 0.109956563 29800 0.050141371 29816
0.070559865 59568 0.106334461 59568 0.037911843
59600 0.042848038 59600 0.086122585 29800 0.066870176 29820
0.042323813 59576 0.142444681 59576 0.044026161
59600 0.057236128 59600 0.145569909 29800 0.075106378 29824
0.012209928 59584 0.11548648 59584 0.031985746
59600 0.130757195 59600 0.113694827 29800 0.080225567 29828
0.058538422 59592 0.042519991 59592 0.023364557
59600 0.157230228 59600 0.05533774 29800 0.098546865 29832

0.077587363 59600 0.116347379 59600 0.039113951
59600 0.123875405 59600 0.010362389 29800 0.110212539 29836
0.069363807 59608 0.063841966 59608 0.070685448
59600 0.043626602 59600 0.102744576 29800 0.076533943 29840
0.026384712 59616 0.083043524 59616 0.076104931
59600 0.083029241 59600 0.159140604 29800 0.055477187 29844
0.020301281 59624 0.083270796 59624 0.056515957
59600 0.10561321 59600 0.153018293 29800 0.06724587 29848
0.044270433 59632 0.048850565 59632 0.050483886
59600 0.093032701 59600 0.127679334 29900 0.040696861 29852
0.059760012 59640 0.075236174 59640 0.058088688
59600 0.112425041 59600 0.117531134 29900 0.019187613 29856
0.056174558 59648 0.116204428 59648 0.054837052
59700 0.121103047 59700 0.131909358 29900 0.032661956 29860
0.063960564 59656 0.126091458 59656 0.058818292
59700 0.054087337 59700 0.145078433 29900 0.018542925 29864
0.070307273 59664 0.12508218 59664 0.065461216
59700 0.1013164 59700 0.14653233 29900 0.016191549 29868
0.069137292 59672 0.139986893 59672 0.054817687
59700 0.147778715 59700 0.086330219 29900 0.018550445 29872
0.071273316 59680 0.112894966 59680 0.030279372
59700 0.125387728 59700 0.09199528 29900 0.040364608 29876
0.068800524 59688 0.099408455 59688 0.016933664
59700 0.112259222 59700 0.12923844 29900 0.061314422 29880
0.065292152 59696 0.078535093 59696 0.031168711
59700 0.138231 59700 0.087200155 29900 0.050808092 29884
0.072024537 59704 0.07678938 59704 0.04045559
59700 0.169359715 59700 0.048571375 29900 0.016273625 29888
0.061812061 59712 0.079277219 59712 0.007830788
59700 0.148426654 59700 0.075618569 29900 0.049379065 29892
0.045337489 59720 0.05129294 59720 0.051012848
59700 0.141955679 59700 0.095181706 29900 0.037102505 29896
0.048255926 59728 0.133333714 59728 0.088169967
59700 0.177177804 59700 0.08278749 29900 0.013471279 29900
0.048230973 59736 0.144944715 59736 0.098296667
59700 0.162369368 59700 0.023479084 29900 0.040667921 29904
0.056620964 59744 0.073651878 59744 0.070092909
59800 0.113660572 59800 0.037026573 29900 0.027391785 29908
0.044823377 59752 0.031288535 59752 0.012934033
59800 0.102949125 59800 0.008516694 29900 0.003104382 29912
0.012876525 59760 0.091694536 59760 0.034584526
59800 0.087103566 59800 0.068289948 29900 0.031872234 29916
0.016992823 59768 0.170153056 59768 0.038750699
59800 0.063075 59800 0.125833205 29900 0.046793746 29920
0.003299161 59776 0.158228083 59776 0.064284177
59800 0.119777986 59800 0.116868454 29900 0.065794506 29924
0.034123234 59784 0.147422965 59784 0.061165527
59800 0.122256213 59800 0.050893352 29900 0.063598884 29928
0.041622472 59792 0.139640964 59792 0.051650044
59800 0.027438617 59800 0.013252388 29900 0.028833743 29932
0.04755165 59800 0.082814382 59800 0.055508135
59800 0.07330723 59800 0.017287799 29900 0.050327151 29936
0.07530953 59808 0.050805451 59808 0.05021835
59800 0.085065622 59800 0.033489203 29900 0.057595844 29940
0.063922438 59816 0.079196478 59816 0.042556068
59800 0.018964471 59800 0.07134636 29900 0.038672526 29944
0.057341564 59824 0.13248423 59824 0.044682423
59800 0.079134537 59800 0.129732391 29900 0.005180258 29948
0.04969351 59832 0.11692803 59832 0.025226304
59800 0.083682862 59800 0.109254543 30000 0.062094994 29952
0.043895718 59840 0.014453026 59840 0.034760702
59800 0.085078937 59800 0.065356515 30000 0.095487987 29956
0.052326086 59848 0.095324424 59848 0.058422662
59900 0.128477826 59900 0.06199802 30000 0.097117605 29960
0.037900383 59856 0.122929778 59856 0.06231069
59900 0.10360586 59900 0.015072056 30000 0.082576888 29964
0.022265758 59864 0.108190921 59864 0.05845138
59900 0.01671365 59900 0.072707808 30000 0.050218736 29968

0.034871337 59872 0.093070943 59872 0.030170984
59900 0.104138992 59900 0.053081989 30000 0.008644505 29972
0.052408683 59880 0.078924612 59880 0.029795303
59900 0.138061266 59900 0.1320835 30000 0.022144983 29976
0.042859694 59888 0.061947234 59888 0.064977481
59900 0.08159002 59900 0.174508241 30000 0.03175957 29980
0.031427862 59896 0.040922245 59896 0.068315727
59900 0.066356144 59900 0.119504271 30000 0.03214963 29984
0.042784668 59904 0.05397818 59904 0.048146932
59900 0.123537669 59900 0.130830348 30000 0.055664827 29988
0.045286502 59912 0.046696656 59912 0.036153931
59900 0.067018431 59900 0.128190004 30000 0.074760414 29992
0.076990029 59920 0.071442701 59920 0.011257006
59900 0.104196952 59900 0.111060494 30000 0.052485775 29996
0.095147443 59928 0.129226334 59928 0.021823089
59900 0.078330653 59900 0.103996543 30000 0.02499151 30000
0.085913685 59936 0.098841978 59936 0.023742985
59900 0.076143362 59900 0.093099283 30000 0.012973733 30004
0.077342374 59944 0.073687304 59944 0.026491965
60000 0.168375308 60000 0.08543134 30000 0.010093947 30008
0.057268593 59952 0.141613608 59952 0.042664691
60000 0.209145758 60000 0.072563082 30000 0.013310606 30012
0.024600102 59960 0.111815542 59960 0.042929049
60000 0.139504555 60000 0.047640067 30000 0.02946247 30016
0.075720425 59968 0.064610358 59968 0.076923199
60000 0.05790557 60000 0.065150794 30000 0.088455054 30020
0.107815227 59976 0.064456261 59976 0.074459342
60000 0.046502351 60000 0.167744919 30000 0.103380378 30024
0.068720881 59984 0.01284834 59984 0.048602782
60000 0.081497135 60000 0.206923781 30000 0.060033923 30028
0.053709497 59992 0.037808783 59992 0.059328912
60000 0.083500352 60000 0.155607456 30000 0.040448762 30032
0.05861339 60000 0.033305743 60000 0.022540904
60000 0.043468208 60000 0.126824365 30000 0.058823196 30036
0.045943823 60008 0.054996475 60008 0.030960557
60000 0.058429996 60000 0.124376078 30000 0.037772301 30040
0.013564268 60016 0.041362546 60016 0.060322735
60000 0.070097631 60000 0.037411857 30000 0.009169863 30044
0.022361079 60024 0.09905803 60024 0.0508921
60000 0.061294399 60000 0.110277368 30000 0.030577754 30048
0.039227511 60032 0.16936475 60032 0.02040897
60000 0.023741002 60000 0.100904945 30100 0.025615507 30052
0.048979189 60040 0.15271944 60040 0.002923219
60000 0.039863764 60000 0.007677011 30100 0.051920088 30056
0.05258443 60048 0.086727894 60048 0.021269134
60100 0.089959729 60100 0.078260324 30100 0.073728821 30060
0.030757245 60056 0.055190867 60056 0.026603742
60100 0.118107426 60100 0.126464321 30100 0.091487913 30064
0.026180698 60064 0.081712489 60064 0.029301291
60100 0.124484359 60100 0.117041702 30100 0.084381005 30068
0.035739398 60072 0.0927293 60072 0.040449704
60100 0.069469825 60100 0.071953306 30100 0.056036934 30072
0.032344837 60080 0.082611317 60080 0.050582756
60100 0.050710005 60100 0.09079741 30100 0.034907836 30076
0.016519085 60088 0.104493032 60088 0.056413745
60100 0.088998873 60100 0.038079397 30100 0.04301337 30080
0.010679288 60096 0.15727029 60096 0.045728062
60100 0.09683855 60100 0.097200013 30100 0.042860411 30084
0.0554127 60104 0.152619366 60104 0.027146569
60100 0.056587687 60100 0.131381646 30100 0.02515017 30088
0.097436641 60112 0.127952895 60112 0.016766375
60100 0.08483533 60100 0.089002497 30100 0.053163127 30092
0.086207809 60120 0.107186294 60120 0.027541182
60100 0.072346331 60100 0.078523888 30100 0.063723092 30096
0.048072714 60128 0.157094488 60128 0.051739724
60100 0.043145465 60100 0.125955295 30100 0.0652245 30100
0.016901244 60136 0.166244746 60136 0.056009809
60100 0.034556324 60100 0.114385264 30100 0.062741136 30104

0.026009 60144 0.14412895 60144 0.042036438
60200 0.035950197 60200 0.106343105 30100 0.05193289 30108
0.020562586 60152 0.126064726 60152 0.012253751
60200 0.071899274 60200 0.086870117 30100 0.015547006 30112
0.040310111 60160 0.069382993 60160 0.021394591
60200 0.053971049 60200 0.071292547 30100 0.020235622 30116
0.078720485 60168 0.036821508 60168 0.015607899
60200 0.077584271 60200 0.114422299 30100 0.037669408 30120
0.102767081 60176 0.040104835 60176 0.03769941
60200 0.087128326 60200 0.148680993 30100 0.048236394 30124
0.061218023 60184 0.014914177 60184 0.052114978
60200 0.065314038 60200 0.100440106 30100 0.074550328 30128
0.009853995 60192 0.076156241 60192 0.033429275
60200 0.099253681 60200 0.035738685 30100 0.059626356 30132
0.05282159 60200 0.124779952 60200 0.004533739
60200 0.078201905 60200 0.108131746 30100 0.029493087 30136
0.106815583 60208 0.087674933 60208 0.018323683
60200 0.02243871 60200 0.125773426 30100 0.099746372 30140
0.098533703 60216 0.041292671 60216 0.031976251
60200 0.091932845 60200 0.0876985 30100 0.089765032 30144
0.089474721 60224 0.018552544 60224 0.025455314
60200 0.1005093 60200 0.020430811 30100 0.039231389 30148
0.05721135 60232 0.066371256 60232 0.038569993
60200 0.058434671 60200 0.038594219 30200 0.00752095 30152
0.055652745 60240 0.124187092 60240 0.073896707
60200 0.014405026 60200 0.030231984 30200 0.033555018 30156
0.090316215 60248 0.122339858 60248 0.083478364
60300 0.055904744 60300 0.072310511 30200 0.071735361 30160
0.075396893 60256 0.109955661 60256 0.07645481
60300 0.071812116 60300 0.166517523 30200 0.074307311 30164
0.04979091 60264 0.103871491 60264 0.051556424
60300 0.094317016 60300 0.092103684 30200 0.04999918 30168
0.057689238 60272 0.061238592 60272 0.004923824
60300 0.057938047 60300 0.070146518 30200 0.056063906 30172
0.027982636 60280 0.008223775 60280 0.032261454
60300 0.052235893 60300 0.040901698 30200 0.071978458 30176
0.054723354 60288 0.007395362 60288 0.056667788
60300 0.073143317 60300 0.109602588 30200 0.060898434 30180
0.0664084 60296 0.084871586 60296 0.045993849
60300 0.081722465 60300 0.116176263 30200 0.023762133 30184
0.055102235 60304 0.118546916 60304 0.018601159
60300 0.148437306 60300 0.081599006 30200 0.027899794 30188
0.068283691 60312 0.068568617 60312 0.061748324
60300 0.159188538 60300 0.071196228 30200 0.052896976 30192
0.052893611 60320 0.13697239 60320 0.045943951
60300 0.10690176 60300 0.030076297 30200 0.0278895 30196
0.036858415 60328 0.107535918 60328 0.005237909
60300 0.039003582 60300 0.062292304 30200 0.046227386 30200
0.018687086 60336 0.105024497 60336 0.024963198
60300 0.064014108 60300 0.033188528 30200 0.060326838 30204
0.003076973 60344 0.132343078 60344 0.008199607
60400 0.067388246 60400 0.03224325 30200 0.027614633 30208
0.035131314 60352 0.112533417 60352 0.046614408
60400 0.115386101 60400 0.071763396 30200 0.007237943 30212
0.05488082 60360 0.15775433 60360 0.056102479
60400 0.140232224 60400 0.107653112 30200 0.023081115 30216
0.030833191 60368 0.138239455 60368 0.049479233
60400 0.055240536 60400 0.127085689 30200 0.050603623 30220
0.025338864 60376 0.097629309 60376 0.055866098
60400 0.049231378 60400 0.129450433 30200 0.066379849 30224
0.023169534 60384 0.110225607 60384 0.07511866
60400 0.084571104 60400 0.076907119 30200 0.042917374 30228
0.017049868 60392 0.08767697 60392 0.066329107
60400 0.089742367 60400 0.063384032 30200 0.039873528 30232
0.040828028 60400 0.019946179 60400 0.035562254
60400 0.03777352 60400 0.094691284 30200 0.055455683 30236
0.008237656 60408 0.029657036 60408 0.020714073
60400 0.0911014 60400 0.113185335 30200 0.038269889 30240

0.02135891 60416 0.03143022 60416 0.03104709
60400 0.12243916 60400 0.069381087 30200 0.027928332 30244
0.034484081 60424 0.07193163 60424 0.038878734
60400 0.023445035 60400 0.046504265 30200 0.025730647 30248
0.038746919 60432 0.094016032 60432 0.023683635
60400 0.061899125 60400 0.050904971 30300 0.016084465 30252
0.003066574 60440 0.070145034 60440 0.006352842
60400 0.045532703 60400 0.054290627 30300 0.020706237 30256
0.021174637 60448 0.021957703 60448 0.017269402
60500 0.110273395 60500 0.101401842 30300 0.012910538 30260
0.028066441 60456 0.025052137 60456 0.027531891
60500 0.118785873 60500 0.154846711 30300 0.049796221 30264
0.079218138 60464 0.11553552 60464 0.041460888
60500 0.054303288 60500 0.133984868 30300 0.058464131 30268
0.057153309 60472 0.165820296 60472 0.064379157
60500 0.020386155 60500 0.099144258 30300 0.054523538 30272
0.020628319 60480 0.114172464 60480 0.067093941
60500 0.024450366 60500 0.116684365 30300 0.047300106 30276
0.01389302 60488 0.072148629 60488 0.036742724
60500 0.027506088 60500 0.118595948 30300 0.048993396 30280
0.011181364 60496 0.090814276 60496 0.033488439
60500 0.096432988 60500 0.061928113 30300 0.054560253 30284
0.008129652 60504 0.075757802 60504 0.051950807
60500 0.158531199 60500 0.072763272 30300 0.035293313 30288
0.042029773 60512 0.010369095 60512 0.0790173
60500 0.131229811 60500 0.114213945 30300 0.006693655 30292
0.018965428 60520 0.043123113 60520 0.071842282
60500 0.086438144 60500 0.113166731 30300 0.006123038 30296
0.039758172 60528 0.072197407 60528 0.049284612
60500 0.090362104 60500 0.044951306 30300 0.042200776 30300
0.04286319 60536 0.103449471 60536 0.035602938
60500 0.123145437 60500 0.096285286 30300 0.042944401 30304
0.004911676 60544 0.118282238 60544 0.025132402
60600 0.155280024 60600 0.160505355 30300 0.015021239 30308
0.058396243 60552 0.079751146 60552 0.005538446
60600 0.109398301 60600 0.162494645 30300 0.037298316 30312
0.055021064 60560 0.017384647 60560 0.03264489
60600 0.045958452 60600 0.096599375 30300 0.011791677 30316
0.047079211 60568 0.022387192 60568 0.0365202
60600 0.106977503 60600 0.094881914 30300 0.014215102 30320
0.036422014 60576 0.121482008 60576 0.038752773
60600 0.147820145 60600 0.105999534 30300 0.050835941 30324
0.009382745 60584 0.125962848 60584 0.066287437
60600 0.145761253 60600 0.071897477 30300 0.078048688 30328
0.040729803 60592 0.031935317 60592 0.04943243
60600 0.104033468 60600 0.103217484 30300 0.077117838 30332
0.027895178 60600 0.079228776 60600 0.042124073
60600 0.056919605 60600 0.126525993 30300 0.041802923 30336
0.028386117 60608 0.120942837 60608 0.041465366
60600 0.10483653 60600 0.135106936 30300 0.027151393 30340
0.024972647 60616 0.117936696 60616 0.034983244
60600 0.071476803 60600 0.158631141 30300 0.079364349 30344
0.059974853 60624 0.10998267 60624 0.08615043
60600 0.026352436 60600 0.151277549 30300 0.084441665 30348
0.073224393 60632 0.065856279 60632 0.086022985
60600 0.081024038 60600 0.071030161 30400 0.048739661 30352
0.096955111 60640 0.082885017 60640 0.045713434
60600 0.17459497 60600 0.057560901 30400 0.011892403 30356
0.088527173 60648 0.089793473 60648 0.032034182
60700 0.166578771 60700 0.052656713 30400 0.019518739 30360
0.061032402 60656 0.031770374 60656 0.028962555
60700 0.161962845 60700 0.032976474 30400 0.034043816 30364
0.052404153 60664 0.019945786 60664 0.023683666
60700 0.214265441 60700 0.070980532 30400 0.024237243 30368
0.036499012 60672 0.055133245 60672 0.064394299
60700 0.145004756 60700 0.023593719 30400 0.012571762 30372
0.071445589 60680 0.090724599 60680 0.07270951
60700 0.184676581 60700 0.017387882 30400 0.036085741 30376

0.029253137 60688 0.095660726 60688 0.078087614
60700 0.225880591 60700 0.007794019 30400 0.052868651 30380
0.060590122 60696 0.095021067 60696 0.092210568
60700 0.165199148 60700 0.066421315 30400 0.052136969 30384
0.096645847 60704 0.0825237 60704 0.067570138
60700 0.098856348 60700 0.145041471 30400 0.041396121 30388
0.060554798 60712 0.049224775 60712 0.063674968
60700 0.016624292 60700 0.116884708 30400 0.029409221 30392
0.090929316 60720 0.049856699 60720 0.03525256
60700 0.070984948 60700 0.073579424 30400 0.019116898 30396
0.08036552 60728 0.032130127 60728 0.030472987
60700 0.099004494 60700 0.140359873 30400 0.020592928 30400
0.058662823 60736 0.03176348 60736 0.050129922
60700 0.024916282 60700 0.094924188 30400 0.013292549 30404
0.029682589 60744 0.05940584 60744 0.05549959
60800 0.066041139 60800 0.053944299 30400 0.010465133 30408
0.020336895 60752 0.100225487 60752 0.044490251
60800 0.041186577 60800 0.02534564 30400 0.051640061 30412
0.049357732 60760 0.099259807 60760 0.041666204
60800 0.094685063 60800 0.080315607 30400 0.091738861 30416
0.085660649 60768 0.033306784 60768 0.046817881
60800 0.202742725 60800 0.067005669 30400 0.073179042 30420
0.066108958 60776 0.045669745 60776 0.068516762
60800 0.199716261 60800 0.033847235 30400 0.018299323 30424
0.0252589 60784 0.043607415 60784 0.047394795
60800 0.147594459 60800 0.073059804 30400 0.038288108 30428
0.060402723 60792 0.038909049 60792 0.024606079
60800 0.116061259 60800 0.072114366 30400 0.033680142 30432
0.106456122 60800 0.02986075 60800 0.059759561
60800 0.094443152 60800 0.094656411 30400 0.015174722 30436
0.11475369 60808 0.0552948 60808 0.053184336
60800 0.053111049 60800 0.110371359 30400 0.030230673 30440
0.086518616 60816 0.114621529 60816 0.03149236
60800 0.048517279 60800 0.081698461 30400 0.035206351 30444
0.05777739 60824 0.163601333 60824 0.024827461
60800 0.081724611 60800 0.045274803 30400 0.036854173 30448
0.057764799 60832 0.170576765 60832 0.03688658
60800 0.12754499 60800 0.054211949 30500 0.051224884 30452
0.087325476 60840 0.137313676 60840 0.071648326
60800 0.164315934 60800 0.041130385 30500 0.064701904 30456
0.078117177 60848 0.084459629 60848 0.063709289
60900 0.083343897 60900 0.047635254 30500 0.061281666 30460
0.07768051 60856 0.046975434 60856 0.011510215
60900 0.009634304 60900 0.061183782 30500 0.056787059 30464
0.078610086 60864 0.036645735 60864 0.038500279
60900 0.081571299 60900 0.119560929 30500 0.039129227 30468
0.070638162 60872 0.026799291 60872 0.025099973
60900 0.095470517 60900 0.180318791 30500 0.040646104 30472
0.079188765 60880 0.059361861 60880 0.009562801
60900 0.044761888 60900 0.196588633 30500 0.086492764 30476
0.048657079 60888 0.092840099 60888 0.008893198
60900 0.029155222 60900 0.147192318 30500 0.073934207 30480
0.04998923 60896 0.073450989 60896 0.020860322
60900 0.050105093 60900 0.08656014 30500 0.043131782 30484
0.03634052 60904 0.100684236 60904 0.011663587
60900 0.084812426 60900 0.066123153 30500 0.05167448 30488
0.065321707 60912 0.132057685 60912 0.034573903
60900 0.166773898 60900 0.073650728 30500 0.050177896 30492
0.083455576 60920 0.120975899 60920 0.058631347
60900 0.249891484 60900 0.06101765 30500 0.051507603 30496
0.087666071 60928 0.099691199 60928 0.055175307
60900 0.227026613 60900 0.040860268 30500 0.032593933 30500
0.069589514 60936 0.085082626 60936 0.05464744
60900 0.132351998 60900 0.046110221 30500 0.027871356 30504
0.038737017 60944 0.104748069 60944 0.041641062
61000 0.130436398 61000 0.038797101 30500 0.023927385 30508
0.022838 60952 0.078756959 60952 0.026901929
61000 0.106147258 61000 0.099086923 30500 0.055336386 30512

0.024001505 60960 0.030940373 60960 0.027864788
61000 0.063572967 61000 0.054736618 30500 0.06317089 30516
0.098760014 60968 0.040378163 60968 0.009836029
61000 0.115048337 61000 0.024143918 30500 0.063948108 30520
0.142320278 60976 0.037966267 60976 0.044768134
61000 0.202854891 61000 0.046556859 30500 0.030553052 30524
0.095539384 60984 0.011212611 60984 0.05243815
61000 0.192116743 61000 0.106808722 30500 0.02520299 30528
0.017100721 60992 0.06760197 60992 0.053436881
61000 0.127401174 61000 0.10314586 30500 0.032646214 30532
0.025805788 61000 0.125253093 61000 0.053324773
61000 0.096265299 61000 0.052989126 30500 0.045036493 30536
0.05755949 61008 0.113765535 61008 0.024554611
61000 0.053622345 61000 0.018688315 30500 0.058582929 30540
0.04986209 61016 0.061195031 61016 0.050014405
61000 0.008700093 61000 0.045199085 30500 0.042967051 30544
0.037935017 61024 0.065758693 61024 0.029794808
61000 0.030360048 61000 0.115043811 30500 0.009880156 30548
0.073753596 61032 0.110599111 61032 0.016731316
61000 0.052398194 61000 0.145078084 30600 0.018101686 30552
0.062323394 61040 0.116606694 61040 0.017330143
61000 0.040497002 61000 0.104366773 30600 0.029918096 30556
0.036621888 61048 0.048245787 61048 0.013546473
61100 0.054941815 61100 0.117064374 30600 0.060724506 30560
0.023952345 61056 0.055903445 61056 0.03089344
61100 0.059894246 61100 0.111436304 30600 0.074424104 30564
0.074155054 61064 0.035269921 61064 0.047406582
61100 0.060928975 61100 0.071219016 30600 0.028862087 30568
0.098290249 61072 0.07663585 61072 0.06868926
61100 0.085078136 61100 0.038283139 30600 0.059192978 30572
0.071074152 61080 0.09941461 61080 0.078170197
61100 0.102044163 61100 0.021886439 30600 0.098505065 30576
0.005718723 61088 0.090510803 61088 0.066573732
61100 0.105722669 61100 0.042401309 30600 0.05240129 30580
0.062359759 61096 0.090137684 61096 0.030351503
61100 0.081391801 61100 0.117223885 30600 0.038730374 30584
0.051990977 61104 0.075265889 61104 0.026718202
61100 0.084850624 61100 0.143839673 30600 0.084636835 30588
0.080371145 61112 0.093570641 61112 0.060120379
61100 0.108191591 61100 0.081968625 30600 0.08731951 30592
0.118931246 61120 0.150033956 61120 0.063152969
61100 0.090819827 61100 0.105063708 30600 0.053814318 30596
0.13192145 61128 0.20767287 61128 0.042474894
61100 0.060512142 61100 0.111117486 30600 0.032331849 30600
0.118497665 61136 0.13400722 61136 0.057090911
61100 0.073620598 61100 0.050029921 30600 0.040420004 30604
0.071634859 61144 0.035783833 61144 0.038677477
61200 0.112387628 61200 0.048886963 30600 0.003895021 30608
0.071547656 61152 0.061441759 61152 0.020646869
61200 0.089621157 61200 0.075623721 30600 0.026092053 30612
0.068189511 61160 0.039383667 61160 0.044691195
61200 0.102463382 61200 0.102305814 30600 0.013389188 30616
0.041395699 61168 0.007963974 61168 0.026070278
61200 0.155239483 61200 0.111260779 30600 0.020378189 30620
0.07514644 61176 0.061187515 61176 0.016545022
61200 0.132493151 61200 0.063244756 30600 0.027677135 30624
0.103217535 61184 0.189213766 61184 0.03356747
61200 0.09017509 61200 0.07280962 30600 0.045151595 30628
0.095239506 61192 0.224448595 61192 0.066640459
61200 0.084278632 61200 0.07377466 30600 0.077525205 30632
0.077623372 61200 0.119638156 61200 0.066830449
61200 0.102006554 61200 0.020050798 30600 0.087130487 30636
0.06089091 61208 0.028229568 61208 0.029829922
61200 0.084950189 61200 0.091787224 30600 0.054868553 30640
0.031769829 61216 0.092579008 61216 0.043043045
61200 0.048817346 61200 0.089769637 30600 0.049085928 30644
0.027520044 61224 0.136765259 61224 0.082632418
61200 0.019116616 61200 0.016656419 30600 0.076298988 30648

0.033149889 61232 0.086125234 61232 0.056419904
61200 0.040339368 61200 0.077877696 30700 0.069066351 30652
0.061150582 61240 0.027077889 61240 0.026962534
61200 0.077860554 61200 0.158582625 30700 0.040237352 30656
0.085704669 61248 0.069708425 61248 0.01935549
61300 0.082495135 61300 0.12889212 30700 0.040906747 30660
0.069816408 61256 0.090175548 61256 0.038413637
61300 0.057672216 61300 0.095665535 30700 0.043889402 30664
0.027684546 61264 0.081819067 61264 0.033426299
61300 0.061281971 61300 0.078839068 30700 0.032414901 30668
0.015493026 61272 0.019847639 61272 0.021351407
61300 0.099480843 61300 0.119477205 30700 0.031090072 30672
0.052758351 61280 0.040012743 61280 0.027066833
61300 0.092342147 61300 0.135934578 30700 0.053744101 30676
0.04947554 61288 0.01344757 61288 0.023861045
61300 0.097581156 61300 0.147244165 30700 0.049213835 30680
0.018718327 61296 0.060388655 61296 0.044837016
61300 0.138938296 61300 0.138490286 30700 0.031205018 30684
0.040826049 61304 0.069418828 61304 0.028016679
61300 0.110395406 61300 0.110768211 30700 0.029940171 30688
0.091771683 61312 0.019852361 61312 0.034155753
61300 0.064653803 61300 0.058674905 30700 0.027094242 30692
0.09993698 61320 0.084233281 61320 0.067614114
61300 0.036506976 61300 0.026545993 30700 0.02397848 30696
0.065872679 61328 0.090295427 61328 0.063758344
61300 0.049519586 61300 0.095931333 30700 0.021626644 30700
0.074408235 61336 0.119581229 61336 0.025502392
61300 0.046833284 61300 0.07700664 30700 0.033917026 30704
0.076236633 61344 0.149145926 61344 0.017079914
61400 0.052162246 61400 0.014983508 30700 0.04783451 30708
0.033054628 61352 0.121075536 61352 0.038896465
61400 0.083079998 61400 0.053773481 30700 0.03736197 30712
0.049457791 61360 0.141904588 61360 0.03674483
61400 0.111281777 61400 0.045097841 30700 0.024259762 30716
0.093804592 61368 0.179809358 61368 0.04875115
61400 0.039716964 61400 0.039735045 30700 0.048956423 30720
0.088751076 61376 0.116274168 61376 0.038597013
61400 0.117038479 61400 0.053874544 30700 0.081439626 30724
0.073299467 61384 0.027281283 61384 0.01492291
61400 0.089713889 61400 0.072043098 30700 0.051131301 30728
0.053849391 61392 0.057132907 61392 0.050236478
61400 0.060954179 61400 0.082632832 30700 0.008957535 30732
0.029847786 61400 0.106378582 61400 0.043204269
61400 0.10839176 61400 0.105544103 30700 0.028984554 30736
0.035215038 61408 0.070417904 61408 0.036770518
61400 0.102720987 61400 0.108187654 30700 0.05921481 30740
0.07368614 61416 0.032141968 61416 0.03728905
61400 0.059402326 61400 0.097690536 30700 0.059994887 30744
0.096764416 61424 0.056381628 61424 0.027466231
61400 0.05520253 61400 0.107417094 30700 0.063963489 30748
0.07257174 61432 0.052535015 61432 0.039013779
61400 0.08947547 61400 0.10367603 30800 0.069850132 30752
0.050897175 61440 0.059211761 61440 0.037048721
61400 0.151479283 61400 0.064489723 30800 0.053537333 30756
0.024309857 61448 0.083995161 61448 0.018440096
61500 0.187542159 61500 0.04680569 30800 0.037313497 30760
0.035430439 61456 0.098442572 61456 0.014605504
61500 0.196191323 61500 0.111300207 30800 0.026836382 30764
0.060447121 61464 0.066077009 61464 0.033803597
61500 0.139471536 61500 0.137113136 30800 0.012991103 30768
0.050859097 61472 0.0433106 61472 0.051805528
61500 0.031463635 61500 0.106806583 30800 0.044927445 30772
0.064805696 61480 0.148995794 61480 0.050876166
61500 0.028128921 61500 0.089629546 30800 0.045533972 30776
0.080791499 61488 0.180304385 61488 0.035993278
61500 0.05853811 61500 0.062765386 30800 0.054028878 30780
0.062642917 61496 0.143825484 61496 0.017698698
61500 0.119214827 61500 0.109703455 30800 0.068772686 30784

0.070620772 61504 0.167568651 61504 0.014607284
61500 0.110487694 61500 0.130439221 30800 0.076991797 30788
0.082291976 61512 0.195563408 61512 0.025087955
61500 0.073328658 61500 0.07528576 30800 0.072403665 30792
0.043462806 61520 0.098930861 61520 0.013131613
61500 0.052074367 61500 0.126986036 30800 0.055672226 30796
0.00192632 61528 0.059668426 61528 0.016643851
61500 0.051445484 61500 0.112314388 30800 0.01992977 30800
0.00712758 61536 0.13571883 61536 0.038731221
61500 0.142345306 61500 0.16165679 30800 0.001447775 30804
0.008086847 61544 0.129654814 61544 0.042743272
61600 0.164489756 61600 0.190769483 30800 0.021788668 30808
0.007678216 61552 0.073930685 61552 0.026322925
61600 0.147133368 61600 0.151989793 30800 0.048688486 30812
0.014228472 61560 0.040833751 61560 0.009968042
61600 0.139714379 61600 0.080399637 30800 0.043437274 30816
0.057242069 61568 0.168732644 61568 0.055259719
61600 0.14578327 61600 0.053779786 30800 0.040537281 30820
0.113806236 61576 0.223948359 61576 0.058099391
61600 0.130094618 61600 0.027628152 30800 0.046517744 30824
0.085725551 61584 0.185940851 61584 0.01497816
61600 0.117551295 61600 0.0219951 30800 0.00747772 30828
0.00988485 61592 0.151303364 61592 0.049028135
61600 0.13104314 61600 0.042520434 30800 0.053748747 30832
0.009894491 61600 0.191212181 61600 0.052814925
61600 0.07547289 61600 0.071595416 30800 0.043957745 30836
0.02729364 61608 0.19066791 61608 0.023272191
61600 0.016996803 61600 0.092838869 30800 0.03527242 30840
0.036886144 61616 0.133789669 61616 0.008830136
61600 0.091556358 61600 0.068319889 30800 0.084401785 30844
0.060384977 61624 0.048305108 61624 0.017345801
61600 0.1534357 61600 0.067686844 30800 0.089543348 30848
0.025256166 61632 0.03673699 61632 0.010758441
61600 0.160934564 61600 0.153762754 30900 0.051951156 30852
0.080853832 61640 0.092583032 61640 0.027005575
61600 0.142609031 61600 0.205671691 30900 0.03480219 30856
0.128569241 61648 0.091544804 61648 0.048177444
61700 0.173078195 61700 0.129126929 30900 0.04274226 30860
0.055036744 61656 0.040305633 61656 0.048528062
61700 0.223588824 61700 0.052064497 30900 0.039055569 30864
0.065941356 61664 0.029573519 61664 0.01777666
61700 0.181281488 61700 0.045200621 30900 0.033524793 30868
0.0975597 61672 0.049476614 61672 0.040042778
61700 0.069791589 61700 0.143643513 30900 0.035978999 30872
0.071655537 61680 0.125062289 61680 0.046587826
61700 0.076694509 61700 0.193607688 30900 0.038831622 30876
0.040859202 61688 0.19369756 61688 0.023616685
61700 0.043926601 61700 0.148700783 30900 0.046188285 30880
0.031345426 61696 0.14547199 61696 0.0160493
61700 0.086796477 61700 0.120648911 30900 0.068149537 30884
0.018092955 61704 0.016582868 61704 0.051348336
61700 0.075163851 61700 0.121146448 30900 0.062059415 30888
0.022174239 61712 0.133588211 61712 0.073710253
61700 0.04333608 61700 0.112550413 30900 0.027695767 30892
0.02040136 61720 0.245823613 61720 0.046882997
61700 0.128589513 61700 0.109256725 30900 0.051888423 30896
0.023710354 61728 0.246102631 61728 0.014118281
61700 0.109930006 61700 0.075420518 30900 0.05699728 30900
0.039909119 61736 0.206317621 61736 0.002993623
61700 0.052873053 61700 0.008586058 30900 0.044889428 30904
0.046749621 61744 0.225203243 61744 0.01185962
61800 0.051962496 61800 0.101206475 30900 0.04132112 30908
0.050073053 61752 0.236709515 61752 0.041873584
61800 0.109714987 61800 0.187681974 30900 0.062050327 30912
0.073135074 61760 0.204353971 61760 0.064159365
61800 0.176280679 61800 0.126059793 30900 0.029382672 30916
0.079853715 61768 0.145872444 61768 0.059425831
61800 0.20406874 61800 0.016323378 30900 0.031598651 30920

0.051060211 61776 0.084197134 61776 0.051024697
61800 0.153394226 61800 0.034216115 30900 0.022976659 30924
0.012721128 61784 0.039717532 61784 0.040848088
61800 0.112287846 61800 0.068775109 30900 0.007082605 30928
0.058423451 61792 0.063035535 61792 0.017893177
61800 0.091516798 61800 0.123137012 30900 0.03163351 30932
0.052295622 61800 0.09125776 61800 0.030319152
61800 0.058626472 61800 0.154337569 30900 0.06271126 30936
0.021480595 61808 0.013977138 61808 0.069837864
61800 0.115845425 61800 0.208819721 30900 0.067718152 30940
0.060733575 61816 0.111749323 61816 0.042326377
61800 0.129301145 61800 0.248547673 30900 0.061439328 30944
0.062820829 61824 0.13940534 61824 0.029495293
61800 0.040282179 61800 0.202523763 30900 0.045667628 30948
0.049250226 61832 0.09795004 61832 0.03594836
61800 0.055470857 61800 0.141248689 31000 0.014695861 30952
0.057280209 61840 0.069459304 61840 0.055254299
61800 0.025323465 61800 0.107923144 31000 0.020361751 30956
0.081154423 61848 0.049626742 61848 0.082829807
61900 0.109740555 61900 0.03157802 31000 0.028332346 30960
0.110560475 61856 0.059238806 61856 0.05871406
61900 0.138666204 61900 0.116343574 31000 0.034707606 30964
0.090677189 61864 0.064723165 61864 0.018044746
61900 0.04799779 61900 0.145995903 31000 0.030507703 30968
0.044056113 61872 0.044163327 61872 0.008821087
61900 0.044055694 61900 0.086474189 31000 0.024159692 30972
0.014992638 61880 0.044262593 61880 0.01607312
61900 0.040212621 61900 0.086517932 31000 0.034219644 30976
0.031759795 61888 0.092066046 61888 0.033540408
61900 0.073927848 61900 0.081202685 31000 0.047768939 30980
0.041266398 61896 0.152425971 61896 0.064302469
61900 0.098593911 61900 0.057323872 31000 0.033312703 30984
0.040827727 61904 0.168015133 61904 0.071343377
61900 0.194142078 61900 0.099914629 31000 0.045498709 30988
0.044515098 61912 0.155240021 61912 0.041190335
61900 0.154066161 61900 0.046054109 31000 0.069476977 30992
0.033809527 61920 0.142954159 61920 0.011838503
61900 0.12161176 61900 0.097253782 31000 0.073964831 30996
0.074479503 61928 0.122133701 61928 0.010125294
61900 0.147993254 61900 0.117499367 31000 0.058506925 31000
0.059527778 61936 0.042442589 61936 0.006494733
61900 0.108735279 61900 0.060172635 31000 0.032141001 31004
0.055665678 61944 0.046845569 61944 0.017380726
62000 0.048400383 62000 0.025339565 31000 0.008467116 31008
0.044847995 61952 0.06428903 61952 0.042743053
62000 0.090093745 62000 0.07080035 31000 0.031358446 31012
0.034713223 61960 0.024586445 61960 0.040715499
62000 0.188683014 62000 0.019939353 31000 0.044892571 31016
0.051397557 61968 0.090353831 61968 0.043204113
62000 0.182369055 62000 0.150359789 31000 0.071427152 31020
0.056345842 61976 0.177463575 61976 0.057843452
62000 0.068584755 62000 0.255775144 31000 0.095560987 31024
0.081087237 61984 0.172578686 61984 0.041011721
62000 0.12167215 62000 0.222882009 31000 0.08877767 31028
0.100080855 61992 0.157868534 61992 0.029902902
62000 0.188625054 62000 0.13378034 31000 0.062042338 31032
0.082712133 62000 0.082219536 62000 0.066949622
62000 0.213241161 62000 0.081929837 31000 0.049579918 31036
0.068485373 62008 0.055341141 62008 0.065611443
62000 0.181992553 62000 0.087269131 31000 0.055820441 31040
0.070925431 62016 0.09420971 62016 0.027518207
62000 0.105296836 62000 0.092432274 31000 0.061113722 31044
0.080467638 62024 0.067771667 62024 0.012453204
62000 0.072502226 62000 0.100118552 31000 0.062716215 31048
0.07730478 62032 0.066291439 62032 0.031080541
62000 0.113541049 62000 0.080503538 31100 0.06072734 31052
0.061356237 62040 0.040328516 62040 0.048338818
62000 0.135456285 62000 0.08366425 31100 0.040464751 31056

0.046079847 62048 0.068300149 62048 0.033786342
62100 0.107938911 62100 0.079464662 31100 0.047252579 31060
0.052937994 62056 0.07612678 62056 0.039577943
62100 0.03936221 62100 0.045949208 31100 0.055279146 31064
0.070753777 62064 0.056309451 62064 0.04914873
62100 0.126652638 62100 0.06609202 31100 0.059602011 31068
0.032522239 62072 0.110612979 62072 0.061841151
62100 0.169460341 62100 0.032021977 31100 0.030742474 31072
0.014575455 62080 0.111764137 62080 0.041193605
62100 0.164684694 62100 0.097259013 31100 0.026437536 31076
0.032606316 62088 0.084577165 62088 0.019382609
62100 0.193176428 62100 0.114908209 31100 0.020901549 31080
0.048695216 62096 0.073456446 62096 0.035456105
62100 0.213429158 62100 0.06465854 31100 0.017221295 31084
0.084699947 62104 0.090564543 62104 0.025809537
62100 0.176356276 62100 0.043404725 31100 0.02661788 31088
0.105921019 62112 0.069767164 62112 0.005642005
62100 0.09899673 62100 0.072153751 31100 0.065351203 31092
0.104550731 62120 0.060493745 62120 0.02403402
62100 0.055131863 62100 0.097374476 31100 0.088427994 31096
0.066845139 62128 0.067436136 62128 0.034345634
62100 0.043479584 62100 0.101447709 31100 0.067864436 31100
0.001849338 62136 0.063919877 62136 0.040220748
62100 0.085090724 62100 0.022224305 31100 0.080179372 31104
0.039178525 62144 0.080821264 62144 0.061252438
62200 0.078155419 62200 0.04992355 31100 0.085553416 31108
0.052127107 62152 0.08249293 62152 0.059112048
62200 0.045994344 62200 0.025071489 31100 0.056766152 31112
0.030286024 62160 0.049245038 62160 0.031799405
62200 0.074425734 62200 0.014335405 31100 0.003320963 31116
0.031330939 62168 0.055428995 62168 0.018812392
62200 0.149104366 62200 0.030838983 31100 0.035886475 31120
0.018034789 62176 0.047143381 62176 0.017752163
62200 0.129898806 62200 0.074407137 31100 0.011816008 31124
0.002910621 62184 0.071290575 62184 0.029243816
62200 0.049813149 62200 0.06579412 31100 0.037904261 31128
0.018151621 62192 0.1399561 62192 0.025701535
62200 0.097876655 62200 0.072538634 31100 0.032403139 31132
0.025867395 62200 0.14963164 62200 0.010142015
62200 0.132029861 62200 0.080510501 31100 0.021096574 31136
0.012812692 62208 0.126641724 62208 0.004801602
62200 0.097406475 62200 0.078328842 31100 0.051329476 31140
0.003089494 62216 0.126709929 62216 0.005182405
62200 0.077972938 62200 0.16991797 31100 0.065748754 31144
0.009823472 62224 0.165766673 62224 0.021853126
62200 0.078379482 62200 0.153355403 31100 0.040540046 31148
0.020944946 62232 0.217912151 62232 0.022794178
62200 0.052284951 62200 0.065573819 31200 0.03369003 31152
0.018930803 62240 0.196624125 62240 0.014858688
62200 0.121595891 62200 0.050619154 31200 0.062348438 31156
0.024748188 62248 0.131104825 62248 0.008969801
62300 0.122995582 62300 0.060064813 31200 0.057466576 31160
0.088204477 62256 0.094516385 62256 0.042947591
62300 0.083595107 62300 0.066802306 31200 0.035233785 31164
0.104899773 62264 0.096892967 62264 0.077772005
62300 0.04521044 62300 0.046268295 31200 0.018376695 31168
0.065533335 62272 0.083397485 62272 0.076195211
62300 0.098489778 62300 0.048659913 31200 0.019432597 31172
0.050918385 62280 0.024542345 62280 0.034222117
62300 0.142321995 62300 0.068173453 31200 0.052347583 31176
0.069086091 62288 0.050147337 62288 0.048182523
62300 0.098526391 62300 0.070848219 31200 0.067833084 31180
0.043811742 62296 0.062210609 62296 0.102123588
62300 0.065050888 62300 0.08659657 31200 0.053855612 31184
0.026779611 62304 0.078460711 62304 0.112979498
62300 0.134497328 62300 0.122961792 31200 0.0240829 31188
0.07324091 62312 0.068536065 62312 0.083132152
62300 0.094244264 62300 0.141899044 31200 0.04567804 31192

0.077122306 62320 0.032960874 62320 0.045677782
62300 0.070373353 62300 0.123343954 31200 0.110024419 31196
0.068455614 62328 0.056109555 62328 0.031194733
62300 0.102401726 62300 0.104942861 31200 0.114648829 31200
0.063257969 62336 0.079318852 62336 0.03403849
62300 0.060867376 62300 0.094505238 31200 0.038894563 31204
0.050516715 62344 0.024617984 62344 0.043569587
62400 0.042592834 62400 0.099102537 31200 0.050246035 31208
0.072788185 62352 0.121939469 62352 0.044526096
62400 0.061652689 62400 0.091293681 31200 0.073954754 31212
0.088352565 62360 0.117851872 62360 0.048155653
62400 0.062651423 62400 0.051275401 31200 0.083097752 31216
0.076154349 62368 0.022839353 62368 0.053616695
62400 0.008261702 62400 0.04943652 31200 0.08134872 31220
0.056593392 62376 0.14530505 62376 0.037576327
62400 0.115653893 62400 0.130395274 31200 0.083985309 31224
0.051977146 62384 0.165390971 62384 0.012938363
62400 0.139710304 62400 0.160022551 31200 0.100172882 31228
0.075746211 62392 0.129908221 62392 0.021606755
62400 0.095490439 62400 0.152173089 31200 0.092144059 31232
0.082296654 62400 0.129701468 62400 0.032886263
62400 0.100708872 62400 0.09207776 31200 0.083273342 31236
0.035263864 62408 0.15109143 62408 0.054812746
62400 0.074597439 62400 0.074784395 31200 0.054629927 31240
0.040760573 62416 0.139610245 62416 0.067039277
62400 0.080833743 62400 0.183683879 31200 0.031154687 31244
0.05981783 62424 0.09993554 62424 0.048436385
62400 0.103505074 62400 0.150401888 31200 0.044177723 31248
0.045675846 62432 0.032834618 62432 0.039623479
62400 0.102900813 62400 0.078885321 31300 0.049660634 31252
0.014259507 62440 0.029942577 62440 0.039324361
62400 0.094511175 62400 0.08485507 31300 0.04521968 31256
0.020716299 62448 0.025217516 62448 0.032992153
62500 0.055559583 62500 0.125978448 31300 0.030551462 31260
0.015586562 62456 0.138422038 62456 0.027353797
62500 0.046148642 62500 0.109760527 31300 0.044444416 31264
0.043002823 62464 0.175682391 62464 0.038870239
62500 0.113948809 62500 0.043131691 31300 0.03986985 31268
0.070188966 62472 0.136453309 62472 0.053131607
62500 0.156247654 62500 0.029940709 31300 0.002010129 31272
0.072020273 62480 0.087492859 62480 0.07751707
62500 0.125448816 62500 0.003039983 31300 0.026771615 31276
0.060917788 62488 0.050803272 62488 0.091859387
62500 0.032727185 62500 0.059761973 31300 0.011477928 31280
0.075639349 62496 0.05071938 62496 0.08677016
62500 0.067393303 62500 0.083483043 31300 0.069074733 31284
0.080913502 62504 0.088818277 62504 0.053196407
62500 0.18008714 62500 0.016135575 31300 0.057646368 31288
0.061300918 62512 0.090611298 62512 0.01248217
62500 0.217256238 62500 0.162875528 31300 0.01045586 31292
0.041144522 62520 0.099143777 62520 0.015125094
62500 0.13226051 62500 0.224939723 31300 0.047344973 31296
0.028596272 62528 0.040959785 62528 0.050298837
62500 0.065088891 62500 0.163995675 31300 0.03798378 31300
0.044510678 62536 0.061234634 62536 0.059952476
62500 0.122564772 62500 0.153254645 31300 0.022865565 31304
0.070165712 62544 0.089675647 62544 0.024720157
62600 0.168264334 62600 0.1464034 31300 0.026802227 31308
0.029107496 62552 0.018874674 62552 0.003048406
62600 0.102424412 62600 0.053988981 31300 0.045861558 31312
0.03169947 62560 0.111827387 62560 0.017864117
62600 0.064587192 62600 0.13469522 31300 0.061072788 31316
0.067859124 62568 0.125241961 62568 0.022227223
62600 0.098874487 62600 0.134566784 31300 0.056533791 31320
0.093193281 62576 0.056986366 62576 0.026211583
62600 0.083841675 62600 0.040226358 31300 0.045810797 31324
0.072520612 62584 0.114802533 62584 0.045709548
62600 0.076334734 62600 0.036997415 31300 0.034728233 31328

0.048413192 62592 0.084179534 62592 0.031882155
62600 0.043780819 62600 0.016061704 31300 0.02701598 31332
0.033747347 62600 0.044665947 62600 0.024611745
62600 0.06647659 62600 0.032426742 31300 0.034969475 31336
0.028719074 62608 0.116317802 62608 0.029966539
62600 0.042776614 62600 0.040835836 31300 0.043004566 31340
0.056340345 62616 0.12039033 62616 0.026357024
62600 0.073535441 62600 0.065712746 31300 0.034867877 31344
0.071100294 62624 0.061125778 62624 0.038455484
62600 0.181121708 62600 0.025282843 31300 0.066059016 31348
0.04965685 62632 0.093760114 62632 0.038833081
62600 0.163713063 62600 0.095559837 31400 0.054422682 31352
0.051188952 62640 0.073005285 62640 0.00899642
62600 0.078527264 62600 0.183174576 31400 0.031473632 31356
0.076294862 62648 0.040477826 62648 0.04289547
62700 0.0565218 62700 0.245742645 31400 0.045465775 31360
0.082266852 62656 0.09958489 62656 0.028471741
62700 0.01868789 62700 0.203488613 31400 0.069895665 31364
0.081845967 62664 0.119146534 62664 0.004986704
62700 0.066810251 62700 0.088544846 31400 0.048787842 31368
0.085573745 62672 0.174357381 62672 0.016797312
62700 0.13337929 62700 0.008340041 31400 0.037061051 31372
0.075520533 62680 0.212291576 62680 0.026215519
62700 0.100121513 62700 0.01788382 31400 0.062125757 31376
0.076668475 62688 0.150448075 62688 0.014877486
62700 0.028031756 62700 0.020823332 31400 0.023490946 31380
0.091107831 62696 0.041840591 62696 0.013237921
62700 0.026749511 62700 0.019623745 31400 0.030266547 31384
0.070129085 62704 0.112354006 62704 0.015263549
62700 0.085642911 62700 0.054228378 31400 0.027752447 31388
0.027175302 62712 0.084848944 62712 0.004086569
62700 0.052935517 62700 0.065204113 31400 0.017958699 31392
0.009163364 62720 0.08166393 62720 0.012193052
62700 0.05743323 62700 0.109285218 31400 0.01017788 31396
0.006641874 62728 0.10311143 62728 0.003241452
62700 0.131846333 62700 0.125290564 31400 0.044870347 31400
0.021045433 62736 0.09089899 62736 0.028819382
62700 0.152091946 62700 0.120389072 31400 0.016565544 31404
0.030720075 62744 0.080579375 62744 0.039776856
62800 0.115898962 62800 0.109933877 31400 0.048811067 31408
0.035597779 62752 0.082608516 62752 0.055978198
62800 0.078286932 62800 0.116417861 31400 0.095023177 31412
0.066528563 62760 0.128351545 62760 0.056789577
62800 0.112693488 62800 0.112557151 31400 0.118709082 31416
0.088713437 62768 0.170206943 62768 0.04623882
62800 0.088706351 62800 0.034152174 31400 0.077722805 31420
0.072939991 62776 0.169717154 62776 0.048172704
62800 0.032283966 62800 0.164920391 31400 0.004458988 31424
0.06504247 62784 0.110611196 62784 0.071330374
62800 0.012149687 62800 0.165054298 31400 0.042970936 31428
0.052444022 62792 0.006208901 62792 0.092534807
62800 0.07209352 62800 0.110345442 31400 0.059789272 31432
0.023462262 62800 0.063563122 62800 0.084976215
62800 0.120217643 62800 0.051186089 31400 0.075692442 31436
0.078016186 62808 0.04787141 62808 0.06046248
62800 0.060421484 62800 0.012720983 31400 0.08249325 31440
0.109346918 62816 0.039176473 62816 0.038630173
62800 0.068587848 62800 0.052646657 31400 0.087833054 31444
0.111586065 62824 0.083903091 62824 0.020355086
62800 0.135665905 62800 0.063620639 31400 0.080883758 31448
0.101015699 62832 0.052817268 62832 0.035261699
62800 0.109189379 62800 0.076553588 31500 0.02792596 31452
0.099519428 62840 0.026829668 62840 0.060241717
62800 0.0377705 62800 0.142940568 31500 0.07439113 31456
0.050834289 62848 0.106035448 62848 0.07963433
62900 0.090301495 62900 0.108259941 31500 0.093866191 31460
0.033188095 62856 0.132310059 62856 0.077786244
62900 0.077748846 62900 0.034220913 31500 0.034633777 31464

0.07913779 62864 0.047267058 62864 0.055080684
62900 0.016761351 62900 0.034671077 31500 0.054766511 31468
0.075674077 62872 0.084368403 62872 0.028279814
62900 0.062213927 62900 0.034344932 31500 0.038252601 31472
0.017006452 62880 0.115861978 62880 0.009824258
62900 0.106181527 62900 0.041255371 31500 0.001347078 31476
0.01732889 62888 0.128945205 62888 0.015165107
62900 0.123204314 62900 0.109991997 31500 0.017461172 31480
0.048395479 62896 0.135676659 62896 0.021885686
62900 0.115551091 62900 0.077571225 31500 0.029548894 31484
0.042771684 62904 0.094786396 62904 0.035796675
62900 0.11245416 62900 0.060696784 31500 0.046009835 31488
0.024644782 62912 0.04931615 62912 0.058695609
62900 0.116605494 62900 0.08389623 31500 0.058730926 31492
0.062336658 62920 0.055606764 62920 0.042253469
62900 0.096895019 62900 0.011223462 31500 0.039789506 31496
0.036101839 62928 0.142964825 62928 0.011437301
62900 0.047557856 62900 0.062390667 31500 0.038887301 31500
0.042342632 62936 0.162502969 62936 0.034900531
62900 0.044080298 62900 0.040451698 31500 0.037939128 31504
0.086200416 62944 0.077140481 62944 0.037520415
63000 0.087476641 63000 0.054175707 31500 0.024575476 31508
0.06224517 62952 0.021919692 62952 0.02300626
63000 0.101578626 63000 0.072508672 31500 0.033456261 31512
0.044244807 62960 0.027764369 62960 0.025636322
63000 0.040780469 63000 0.065013133 31500 0.075254342 31516
0.088056026 62968 0.039878858 62968 0.074714451
63000 0.069088877 63000 0.043521784 31500 0.066918452 31520
0.061399631 62976 0.05044054 62976 0.111973954
63000 0.059102335 63000 0.090601148 31500 0.056658853 31524
0.023525723 62984 0.073471085 62984 0.077757548
63000 0.033207965 63000 0.139662574 31500 0.084717322 31528
0.020952215 62992 0.08183621 62992 0.025023712
63000 0.048153423 63000 0.096494841 31500 0.050231174 31532
0.019321524 63000 0.046838006 63000 0.030376887
63000 0.115952294 63000 0.057263118 31500 0.012529467 31536
0.012745547 63008 0.013202495 63008 0.020728705
63000 0.142294055 63000 0.171593245 31500 0.016023349 31540
0.041634539 63016 0.09990824 63016 0.02115972
63000 0.067049303 63000 0.191725281 31500 0.019320138 31544
0.057250527 63024 0.187164508 63024 0.037591944
63000 0.047358641 63000 0.118492586 31500 0.010505238 31548
0.027316197 63032 0.150861 63032 0.029397163
63000 0.085268162 63000 0.107134612 31600 0.023358647 31552
0.036609086 63040 0.006310239 63040 0.018256991
63000 0.156895578 63000 0.08549307 31600 0.037234666 31556
0.053868313 63048 0.088608525 63048 0.044729262
63100 0.177669266 63100 0.06894485 31600 0.079142425 31560
0.042518132 63056 0.109212393 63056 0.063455678
63100 0.121692014 63100 0.055353266 31600 0.097046017 31564
0.022074752 63064 0.112009184 63064 0.066171902
63100 0.039216062 63100 0.06778098 31600 0.052778283 31568
0.01655399 63072 0.051945535 63072 0.042652457
63100 0.038709579 63100 0.071700102 31600 0.03563146 31572
0.028973374 63080 0.072999181 63080 0.045378794
63100 0.060166269 63100 0.060978873 31600 0.030774005 31576
0.018169179 63088 0.11172759 63088 0.018937557
63100 0.033289864 63100 0.073076008 31600 0.023198787 31580
0.035897334 63096 0.097856784 63096 0.078771962
63100 0.038557013 63100 0.077406134 31600 0.053648517 31584
0.025491676 63104 0.091205504 63104 0.099547411
63100 0.050511979 63100 0.032622902 31600 0.06595254 31588
0.031897689 63112 0.06976357 63112 0.07064773
63100 0.094788426 63100 0.035068151 31600 0.065563356 31592
0.059602324 63120 0.047391808 63120 0.038285234
63100 0.166556754 63100 0.020223011 31600 0.051762556 31596
0.02991821 63128 0.045706016 63128 0.019931094
63100 0.132239001 63100 0.029167763 31600 0.00953405 31600

0.042735923 63136 0.037692706 63136 0.012235197
63100 0.12838839 63100 0.058558417 31600 0.052071042 31604
0.047501486 63144 0.109138317 63144 0.031777767
63200 0.140352524 63200 0.045565394 31600 0.088394379 31608
0.006682551 63152 0.127656298 63152 0.039601284
63200 0.127756735 63200 0.014627494 31600 0.068342059 31612
0.031078969 63160 0.178619666 63160 0.030054442
63200 0.162353855 63200 0.053739674 31600 0.026535914 31616
0.068013243 63168 0.173475128 63168 0.008361427
63200 0.036615213 63200 0.113681948 31600 0.014171528 31620
0.106535714 63176 0.087862471 63176 0.008613176
63200 0.085944143 63200 0.049572358 31600 0.020835625 31624
0.110935296 63184 0.014912947 63184 0.014313651
63200 0.092168673 63200 0.029419511 31600 0.043679247 31628
0.110159941 63192 0.033224369 63192 0.046111869
63200 0.053817064 63200 0.065864602 31600 0.055741773 31632
0.075708274 63200 0.031183536 63200 0.059394028
63200 0.057878675 63200 0.156882074 31600 0.037829603 31636
0.020270549 63208 0.038561833 63208 0.061852188
63200 0.09814003 63200 0.103900835 31600 0.038482773 31640
0.011598942 63216 0.051661526 63216 0.066817709
63200 0.072438605 63200 0.056644774 31600 0.076306511 31644
0.038331229 63224 0.1217752 63224 0.074760479
63200 0.035413781 63200 0.067554574 31600 0.092700502 31648
0.007438484 63232 0.150126478 63232 0.052275598
63200 0.019348418 63200 0.078311452 31700 0.078542122 31652
0.05057664 63240 0.111889291 63240 0.021717382
63200 0.068377856 63200 0.059573231 31700 0.040852126 31656
0.02077797 63248 0.197706643 63248 0.049761529
63300 0.036133697 63300 0.096114018 31700 0.008123012 31660
0.00907093 63256 0.289194228 63256 0.053895699
63300 0.136741626 63300 0.115036222 31700 0.022684999 31664
0.071514449 63264 0.278588443 63264 0.053102293
63300 0.157811665 63300 0.071196439 31700 0.01608055 31668
0.082439496 63272 0.194925465 63272 0.033023542
63300 0.175922338 63300 0.043111693 31700 0.018293298 31672
0.052073265 63280 0.100575227 63280 0.01766797
63300 0.160361393 63300 0.067119072 31700 0.036165406 31676
0.079652753 63288 0.015925722 63288 0.013395778
63300 0.151053173 63300 0.164088343 31700 0.048677157 31680
0.073268813 63296 0.023963716 63296 0.013638345
63300 0.079309721 63300 0.193847081 31700 0.047242858 31684
0.024074421 63304 0.082131526 63304 0.037022521
63300 0.055927871 63300 0.147419298 31700 0.025605517 31688
0.002840146 63312 0.142519959 63312 0.060812192
63300 0.050634666 63300 0.118268297 31700 0.017578315 31692
0.062578074 63320 0.10691068 63320 0.057106379
63300 0.092236114 63300 0.131463298 31700 0.009548953 31696
0.071021648 63328 0.105509484 63328 0.041288669
63300 0.19982773 63300 0.097352458 31700 0.028561241 31700
0.048388833 63336 0.064790744 63336 0.054835011
63300 0.150761581 63300 0.045379566 31700 0.060577288 31704
0.027758233 63344 0.077620367 63344 0.088227353
63400 0.07559557 63400 0.077474848 31700 0.043645872 31708
0.004402575 63352 0.154656896 63352 0.089460446
63400 0.0469966 63400 0.129759631 31700 0.018513983 31712
0.017981278 63360 0.155023226 63360 0.039329243
63400 0.069087626 63400 0.154937225 31700 0.033797416 31716
0.01254135 63368 0.068636138 63368 0.042362539
63400 0.104242638 63400 0.131688693 31700 0.068289868 31720
0.025811309 63376 0.059450016 63376 0.070392998
63400 0.11887624 63400 0.109758592 31700 0.101251382 31724
0.036405083 63384 0.084163796 63384 0.052822823
63400 0.090427224 63400 0.08077269 31700 0.079010737 31728
0.038212409 63392 0.097902419 63392 0.029107521
63400 0.121513018 63400 0.059179834 31700 0.046986304 31732
0.0485602 63400 0.0857786 63400 0.03557769
63400 0.10195197 63400 0.108603192 31700 0.044739467 31736

0.056759684 63408 0.118697128 63408 0.00837473
63400 0.091321192 63400 0.102515405 31700 0.043874796 31740
0.045268498 63416 0.073010255 63416 0.023241111
63400 0.105543761 63400 0.091193215 31700 0.03357543 31744
0.023789198 63424 0.047281345 63424 0.059047856
63400 0.095384909 63400 0.029009369 31700 0.031098171 31748
0.071169976 63432 0.115185911 63432 0.086649634
63400 0.065040782 63400 0.089596601 31800 0.030971118 31752
0.070595692 63440 0.072861629 63440 0.078318131
63400 0.119154785 63400 0.149372121 31800 0.021512535 31756
0.053572963 63448 0.093223709 63448 0.045530047
63500 0.142790785 63500 0.115233459 31800 0.010225777 31760
0.040580235 63456 0.11182404 63456 0.02706665
63500 0.079679754 63500 0.028269569 31800 0.021549226 31764
0.033072614 63464 0.072890507 63464 0.035239678
63500 0.048213038 63500 0.027697985 31800 0.028116732 31768
0.055183191 63472 0.089901529 63472 0.038068054
63500 0.118416341 63500 0.064564891 31800 0.070855465 31772
0.070460672 63480 0.172190733 63480 0.03294335
63500 0.058950518 63500 0.147846076 31800 0.091254085 31776
0.050903946 63488 0.191784028 63488 0.017795865
63500 0.056950619 63500 0.20966235 31800 0.056349447 31780
0.041551561 63496 0.126856074 63496 0.018528572
63500 0.053490057 63500 0.197139336 31800 0.020024187 31784
0.004040559 63504 0.082684543 63504 0.034333043
63500 0.056938796 63500 0.091051181 31800 0.063974709 31788
0.07350638 63512 0.123397957 63512 0.033060911
63500 0.127166524 63500 0.028660974 31800 0.083003411 31792
0.106898093 63520 0.168176076 63520 0.032805321
63500 0.080903286 63500 0.02940808 31800 0.080118727 31796
0.065591332 63528 0.191755665 63528 0.054406304
63500 0.049316357 63500 0.031584568 31800 0.063993728 31800
0.023635656 63536 0.12420486 63536 0.03933148
63500 0.132696529 63500 0.091981856 31800 0.047783182 31804
0.038798582 63544 0.029845212 63544 0.018786464
63600 0.109880668 63600 0.13836706 31800 0.048317023 31808
0.044677217 63552 0.077227909 63552 0.014614995
63600 0.082494909 63600 0.164386496 31800 0.042273965 31812
0.032035874 63560 0.033464046 63560 0.021686541
63600 0.104836421 63600 0.188692343 31800 0.045159344 31816
0.028532433 63568 0.080377322 63568 0.013944108
63600 0.059284565 63600 0.167458275 31800 0.037687805 31820
0.005482174 63576 0.079554469 63576 0.04915347
63600 0.054350938 63600 0.100582161 31800 0.028917555 31824
0.058092701 63584 0.054456188 63584 0.075063836
63600 0.026690306 63600 0.061552557 31800 0.027559772 31828
0.059555201 63592 0.02912944 63592 0.058251404
63600 0.039098359 63600 0.064623317 31800 0.02699371 31832
0.052757368 63600 0.047340702 63600 0.035870915
63600 0.036579262 63600 0.045167421 31800 0.063279666 31836
0.066577988 63608 0.052206022 63608 0.048042381
63600 0.026189087 63600 0.053431664 31800 0.059452064 31840
0.0459601 63616 0.040964864 63616 0.063673462
63600 0.067094501 63600 0.035564335 31800 0.049579481 31844
0.057633562 63624 0.150415406 63624 0.060092447
63600 0.129687673 63600 0.010149822 31800 0.053676791 31848
0.093960742 63632 0.195789064 63632 0.046139499
63600 0.190486768 63600 0.012137424 31900 0.025367777 31852
0.096554337 63640 0.152712878 63640 0.013511705
63600 0.152171211 63600 0.009520573 31900 0.047603695 31856
0.105702551 63648 0.069865309 63648 0.049758848
63700 0.122256213 63700 0.056806723 31900 0.039594106 31860
0.11827748 63656 0.060690036 63656 0.067947054
63700 0.135885304 63700 0.113394068 31900 0.032282933 31864
0.08981151 63664 0.063578082 63664 0.049534712
63700 0.089897105 63700 0.156640774 31900 0.059794893 31868
0.049917457 63672 0.02401826 63672 0.046115336
63700 0.02197675 63700 0.113065122 31900 0.056777361 31872

0.036215752 63680 0.042400683 63680 0.070678558
63700 0.093846909 63700 0.011064572 31900 0.06436868 31876
0.045344048 63688 0.078175872 63688 0.064660962
63700 0.129130815 63700 0.063422674 31900 0.062127947 31880
0.056801426 63696 0.099178898 63696 0.03789808
63700 0.115754643 63700 0.163868171 31900 0.059701601 31884
0.067825677 63704 0.084507403 63704 0.030686661
63700 0.050015311 63700 0.21521443 31900 0.072248091 31888
0.073984928 63712 0.103575345 63712 0.030084484
63700 0.023202234 63700 0.156923154 31900 0.066768967 31892
0.048554939 63720 0.148802151 63720 0.039458344
63700 0.037880676 63700 0.087570996 31900 0.040529263 31896
0.015516856 63728 0.097698059 63728 0.034370722
63700 0.044902998 63700 0.074851079 31900 0.015712967 31900
0.066092383 63736 0.011485079 63736 0.003109251
63700 0.055151068 63700 0.087408786 31900 0.062592386 31904
0.090157599 63744 0.035473033 63744 0.022520828
63800 0.059835504 63800 0.088973684 31900 0.095425843 31908
0.103270795 63752 0.050178234 63752 0.028582197
63800 0.075126176 63800 0.12228766 31900 0.062610016 31912
0.076899611 63760 0.016835138 63760 0.023477516
63800 0.070744522 63800 0.137562268 31900 0.063245956 31916
0.02454503 63768 0.033996148 63768 0.008945589
63800 0.094715731 63800 0.099189761 31900 0.074404423 31920
0.039457475 63776 0.046329882 63776 0.023443685
63800 0.104590923 63800 0.046013622 31900 0.065274355 31924
0.060664948 63784 0.077419798 63784 0.028466706
63800 0.134398476 63800 0.049245788 31900 0.055214146 31928
0.089671688 63792 0.066116096 63792 0.020269601
63800 0.114661612 63800 0.024502466 31900 0.022700562 31932
0.114618182 63800 0.043607302 63800 0.037000562
63800 0.048008042 63800 0.060670282 31900 0.034534154 31936
0.1084998 63808 0.081644714 63808 0.040959058
63800 0.036603226 63800 0.08947571 31900 0.048976075 31940
0.065902466 63816 0.054298758 63816 0.066745146
63800 0.123923557 63800 0.119240205 31900 0.035407196 31944
0.061223567 63824 0.021416587 63824 0.056430174
63800 0.148682636 63800 0.107785876 31900 0.055508426 31948
0.081102793 63832 0.038411192 63832 0.043002743
63800 0.105675084 63800 0.07356565 32000 0.07852789 31952
0.05760766 63840 0.037915543 63840 0.039976228
63800 0.045394972 63800 0.095865631 32000 0.082627259 31956
0.031039443 63848 0.068775618 63848 0.036655663
63900 0.045551384 63900 0.067588699 32000 0.076010721 31960
0.053562031 63856 0.091791335 63856 0.070856702
63900 0.128356158 63900 0.015479762 32000 0.069807189 31964
0.063815693 63864 0.078701283 63864 0.080599661
63900 0.144922204 63900 0.02019236 32000 0.041783449 31968
0.073182186 63872 0.07418669 63872 0.052657106
63900 0.109840148 63900 0.082897379 32000 0.052554464 31972
0.059221195 63880 0.095425443 63880 0.033559973
63900 0.085449887 63900 0.126848011 32000 0.049311526 31976
0.048800132 63888 0.143433238 63888 0.03817011
63900 0.083460036 63900 0.118997348 32000 0.038665752 31980
0.055264645 63896 0.148913605 63896 0.039642713
63900 0.039514849 63900 0.085117383 32000 0.030915442 31984
0.053970751 63904 0.165775069 63904 0.02818091
63900 0.093885064 63900 0.051625528 32000 0.021051337 31988
0.045488636 63912 0.213671549 63912 0.028478815
63900 0.087247136 63900 0.057165205 32000 0.024311514 31992
0.031525549 63920 0.187825994 63920 0.031416188
63900 0.03138823 63900 0.110373876 32000 0.063031315 31996
0.01287065 63928 0.111112015 63928 0.033710749
63900 0.097557597 63900 0.101934384 32000 0.073655967 32000
0.017786922 63936 0.133941824 63936 0.054515429
63900 0.070863025 63900 0.039289778 32000 0.052883155 32004
0.049618728 63944 0.087452616 63944 0.055407199
64000 0.040195595 64000 0.1685647 32000 0.041689888 32008

0.074960844 63952 0.015024532 63952 0.042998625
64000 0.053050389 64000 0.182754623 32000 0.046038909 32012
0.084812935 63960 0.014596733 63960 0.062699452
64000 0.160634489 64000 0.104959836 32000 0.069918599 32016
0.100771584 63968 0.092312039 63968 0.068027359
64000 0.192418608 64000 0.080294412 32000 0.070458438 32020
0.127113104 63976 0.150871696 63976 0.0314723
64000 0.157541203 64000 0.13864573 32000 0.051528179 32024
0.123350648 63984 0.112015099 63984 0.013101123
64000 0.088188019 64000 0.112097616 32000 0.035280711 32028
0.084970219 63992 0.028108847 63992 0.027640017
64000 0.052972046 64000 0.030677948 32000 0.035326255 32032
0.043863471 64000 0.059546896 64000 0.029212391
64000 0.186963473 64000 0.054414519 32000 0.07563136 32036
0.027238786 64008 0.099764227 64008 0.027042248
64000 0.179306939 64000 0.143883095 32000 0.072109731 32040
0.025103171 64016 0.035291865 64016 0.029200479
64000 0.115586161 64000 0.236914828 32000 0.035342786 32044
0.007873648 64024 0.028020268 64024 0.036173213
64000 0.081086844 64000 0.27896036 32000 0.030083247 32048
0.02816843 64032 0.047222224 64032 0.020619502
64000 0.119150609 64000 0.146683873 32100 0.024761253 32052
0.034354813 64040 0.14592502 64040 0.054117161
64000 0.111454945 64000 0.030119672 32100 0.048159764 32056
0.049426308 64048 0.149715954 64048 0.070186979
64100 0.043759992 64100 0.072992174 32100 0.083589563 32060
0.065638691 64056 0.089727408 64056 0.043467084
64100 0.218924179 64100 0.161716089 32100 0.069922004 32064
0.061699546 64064 0.018684579 64064 0.037855723
64100 0.233576488 64100 0.179492694 32100 0.050271134 32068
0.044504006 64072 0.09373315 64072 0.039999864
64100 0.123207879 64100 0.114123264 32100 0.067445646 32072
0.055654095 64080 0.124671438 64080 0.029065552
64100 0.09203636 64100 0.067390181 32100 0.041828742 32076
0.051832569 64088 0.115985786 64088 0.016881048
64100 0.131982291 64100 0.088007211 32100 0.035860354 32080
0.025165266 64096 0.072751347 64096 0.025653702
64100 0.106919892 64100 0.121831356 32100 0.03001348 32084
0.041298561 64104 0.045945064 64104 0.030574713
64100 0.093621973 64100 0.134059781 32100 0.020344698 32088
0.073387972 64112 0.088573084 64112 0.037926417
64100 0.121811339 64100 0.129830354 32100 0.021491655 32092
0.059859467 64120 0.122706464 64120 0.024960344
64100 0.106466301 64100 0.0626201 32100 0.037352373 32096
0.02626672 64128 0.085249601 64128 0.007777231
64100 0.0565722 64100 0.026342112 32100 0.061326777 32100
0.01259901 64136 0.058996524 64136 0.01975009
64100 0.089876659 64100 0.060148668 32100 0.085303662 32104
0.053851632 64144 0.119239463 64144 0.049282804
64200 0.157299335 64200 0.061938568 32100 0.08215414 32108
0.073574018 64152 0.13489883 64152 0.065372158
64200 0.155583054 64200 0.035630488 32100 0.043876731 32112
0.051339339 64160 0.084898995 64160 0.071662566
64200 0.159879943 64200 0.014966551 32100 0.037128531 32116
0.050772975 64168 0.05788124 64168 0.094697345
64200 0.085343818 64200 0.046688841 32100 0.026909411 32120
0.100627673 64176 0.026854756 64176 0.087826615
64200 0.063225831 64200 0.082953149 32100 0.015494066 32124
0.080313454 64184 0.031867076 64184 0.056805042
64200 0.156711569 64200 0.087299923 32100 0.007596033 32128
0.02558192 64192 0.108106979 64192 0.044877408
64200 0.174165718 64200 0.008041656 32100 0.032376374 32132
0.043026455 64200 0.085718239 64200 0.043729484
64200 0.118267875 64200 0.120440876 32100 0.053070857 32136
0.095971205 64208 0.08872358 64208 0.023205661
64200 0.076442688 64200 0.154662208 32100 0.044290089 32140
0.094599083 64216 0.19330607 64216 0.013781675
64200 0.017729095 64200 0.093851668 32100 0.040339848 32144

0.062117775	64224	0.198499765	64224	0.028575889
64200	0.108698376	64200	0.051547813	32100	0.032732889	32148
0.064444219	64232	0.163952209	64232	0.015333493
64200	0.105632869	64200	0.049712631	32200	0.019319385	32152
0.06343946	64240	0.121803278	64240	0.012595246
64200	0.065703142	64200	0.077070123	32200	0.01888206	32156
0.069295573	64248	0.097566859	64248	0.027557848
64300	0.066533263	64300	0.12683995	32200	0.052702853	32160
0.097220713	64256	0.10094553	64256	0.012964731
64300	0.011613027	64300	0.129591048	32200	0.07783901	32164
0.09005452	64264	0.032474571	64264	0.036783589
64300	0.021281596	64300	0.121784433	32200	0.090772141	32168
0.027878463	64272	0.052993157	64272	0.061210485
64300	0.027897204	64300	0.074823831	32200	0.112269285	32172
0.028835388	64280	0.049565486	64280	0.051096253
64300	0.036683861	64300	0.021015208	32200	0.127710213	32176
0.027335118	64288	0.027188817	64288	0.040295254
64300	0.026629938	64300	0.014175213	32200	0.090700152	32180
0.043000528	64296	0.095163596	64296	0.030471159
64300	0.059834219	64300	0.07495944	32200	0.01230531	32184
0.037046764	64304	0.078202364	64304	0.025071453
64300	0.041744112	64300	0.106900203	32200	0.054420914	32188
0.04878861	64312	0.107440377	64312	0.034136836
64300	0.022023001	64300	0.062759173	32200	0.050794559	32192
0.072314615	64320	0.075753211	64320	0.010224441
64300	0.028961742	64300	0.035656205	32200	0.006051901	32196
0.063705586	64328	0.007710457	64328	0.056570891
64300	0.066678214	64300	0.031170974	32200	0.050456241	32200
0.028203089	64336	0.00790351	64336	0.051732728
64300	0.109024957	64300	0.012663201	32200	0.077610021	32204
0.018376346	64344	0.007090355	64344	0.016873697
64400	0.067425193	64400	0.056366258	32200	0.096889802	32208
0.026393425	64352	0.067531699	64352	0.058269354
64400	0.042428866	64400	0.131874273	32200	0.081083344	32212
0.024313291	64360	0.085335392	64360	0.064052649
64400	0.105080493	64400	0.110384288	32200	0.058415393	32216
0.020097063	64368	0.077959943	64368	0.040218376
64400	0.123131104	64400	0.022965527	32200	0.063099877	32220
0.017617935	64376	0.061319617	64376	0.011428473
64400	0.138892836	64400	0.022829578	32200	0.062508327	32224
0.052620424	64384	0.03422322	64384	0.019463609
64400	0.107926491	64400	0.041317075	32200	0.053055672	32228
0.076677992	64392	0.0640336	64392	0.043284937
64400	0.064664331	64400	0.059603706	32200	0.026370704	32232
0.086795524	64400	0.03299215	64400	0.038709732
64400	0.049664861	64400	0.057058813	32200	0.025440653	32236
0.093480012	64408	0.036948768	64408	0.024292727
64400	0.033719873	64400	0.131384295	32200	0.067669891	32240
0.064210952	64416	0.05554736	64416	0.02656817
64400	0.019184261	64400	0.13516414	32200	0.072089315	32244
0.011332389	64424	0.024886354	64424	0.042331205
64400	0.063117717	64400	0.155163346	32200	0.037508959	32248
0.054534965	64432	0.117738178	64432	0.047748999
64400	0.125028076	64400	0.134950766	32300	0.018566207	32252
0.035499885	64440	0.216453395	64440	0.043386794
64400	0.147731495	64400	0.069607973	32300	0.04527616	32256
0.012286705	64448	0.284235575	64448	0.050525829
64500	0.143371581	64500	0.054698601	32300	0.060030157	32260
0.010313623	64456	0.241660251	64456	0.053972177
64500	0.160404495	64500	0.04398153	32300	0.043844775	32264
0.020776215	64464	0.109557084	64464	0.024825611
64500	0.168447834	64500	0.050190676	32300	0.133535781	32268
0.001252318	64472	0.039517505	64472	0.020096786
64500	0.136690461	64500	0.084079642	32300	0.158955474	32272
0.018636265	64480	0.099651639	64480	0.063000887
64500	0.094798197	64500	0.114530034	32300	0.08418542	32276
0.020795207	64488	0.167703561	64488	0.09322659
64500	0.062964864	64500	0.096895776	32300	0.030541556	32280

0.037978141 64496 0.12788111 64496 0.062263949
64500 0.029381467 64500 0.032788939 32300 0.072508592 32284
0.071696923 64504 0.022365413 64504 0.03653848
64500 0.023910028 64500 0.009652798 32300 0.097162127 32288
0.080699108 64512 0.063201791 64512 0.032426113
64500 0.03695886 64500 0.056390185 32300 0.086312939 32292
0.041836738 64520 0.072619645 64520 0.042795869
64500 0.029593462 64500 0.088473382 32300 0.051940318 32296
0.037366626 64528 0.047846399 64528 0.057693666
64500 0.114704562 64500 0.078196281 32300 0.035622779 32300
0.01659418 64536 0.064329128 64536 0.026637383
64500 0.184466786 64500 0.105622006 32300 0.033974822 32304
0.025965632 64544 0.157338844 64544 0.020756172
64600 0.081342245 64600 0.101828817 32300 0.030873522 32308
0.042031385 64552 0.149199783 64552 0.037128535
64600 0.115100789 64600 0.0788568 32300 0.009942449 32312
0.05398993 64560 0.085794076 64560 0.05958671
64600 0.192026419 64600 0.08639595 32300 0.008208433 32316
0.063323976 64568 0.042315714 64568 0.034364373
64600 0.140086064 64600 0.083022314 32300 0.038185739 32320
0.086858192 64576 0.064776024 64576 0.018082246
64600 0.066561326 64600 0.126931569 32300 0.089678295 32324
0.075200653 64584 0.081575505 64584 0.03467533
64600 0.076444143 64600 0.144413018 32300 0.085796659 32328
0.009727799 64592 0.018291812 64592 0.037107038
64600 0.117453237 64600 0.099522498 32300 0.040488441 32332
0.050898128 64600 0.082368664 64600 0.053910237
64600 0.07023284 64600 0.065314205 32300 0.032886204 32336
0.034538793 64608 0.075673917 64608 0.064096894
64600 0.081064434 64600 0.101584701 32300 0.048061404 32340
0.02057149 64616 0.063024025 64616 0.067219109
64600 0.071438539 64600 0.082600527 32300 0.027379088 32344
0.0151158 64624 0.113115682 64624 0.061055682
64600 0.070751521 64600 0.07890581 32300 0.019761843 32348
0.005653913 64632 0.118910415 64632 0.02731024
64600 0.210900281 64600 0.140908815 32400 0.046717538 32352
0.052913416 64640 0.022441211 64640 0.023700319
64600 0.263286376 64600 0.081311417 32400 0.075766897 32356
0.061822517 64648 0.124187369 64648 0.02525434
64700 0.170957646 64700 0.005399566 32400 0.055919729 32360
0.007383346 64656 0.145090016 64656 0.026199725
64700 0.039855455 64700 0.054177763 32400 0.00890256 32364
0.04141 64664 0.121485144 64664 0.019530602
64700 0.029786412 64700 0.074633848 32400 0.018494759 32368
0.073338408 64672 0.090288791 64672 0.025277785
64700 0.097471209 64700 0.051303046 32400 0.009408035 32372
0.089845635 64680 0.048304635 64680 0.033350567
64700 0.135425041 64700 0.068762725 32400 0.022136994 32376
0.089138281 64688 0.026206797 64688 0.015767566
64700 0.120981567 64700 0.043207881 32400 0.06583643 32380
0.05756419 64696 0.078251862 64696 0.015278592
64700 0.095327319 64700 0.054703003 32400 0.152983121 32384
0.045262554 64704 0.13337862 64704 0.064102242
64700 0.084320513 64700 0.115802199 32400 0.131030014 32388
0.052226958 64712 0.122268291 64712 0.091792099
64700 0.091289447 64700 0.133541428 32400 0.029621406 32392
0.064148189 64720 0.059749797 64720 0.067870678
64700 0.08645255 64700 0.1438029 32400 0.051574461 32396
0.049664999 64728 0.085871136 64728 0.023511289
64700 0.065979497 64700 0.049930106 32400 0.047649493 32400
0.039016097 64736 0.08036173 64736 0.010399975
64700 0.038736718 64700 0.030778505 32400 0.042823369 32404
0.052807583 64744 0.096364041 64744 0.026239015
64800 0.01166541 64800 0.045635417 32400 0.0062485 32408
0.049966242 64752 0.094337898 64752 0.052217751
64800 0.068169604 64800 0.15209183 32400 0.06923113 32412
0.022176851 64760 0.096788201 64760 0.06010761
64800 0.106549036 64800 0.121168654 32400 0.091655369 32416

0.028593808 64768 0.123013815 64768 0.065762215
64800 0.075889497 64800 0.042423832 32400 0.008416991 32420
0.036559497 64776 0.106421823 64776 0.068868561
64800 0.06919817 64800 0.083403014 32400 0.110687579 32424
0.016818674 64784 0.039127364 64784 0.058577723
64800 0.08690331 64800 0.122779238 32400 0.135781928 32428
0.058678197 64792 0.020499409 64792 0.031849409
64800 0.079980375 64800 0.126341226 32400 0.070735245 32432
0.075645825 64800 0.006468553 64800 0.014746961
64800 0.045367026 64800 0.091262788 32400 0.056337241 32436
0.060195926 64808 0.02745597 64808 0.020277079
64800 0.036106783 64800 0.133740031 32400 0.191647486 32440
0.053800875 64816 0.059753045 64816 0.059112026
64800 0.035588801 64800 0.155376213 32400 0.279356231 32444
0.024812583 64824 0.172193991 64824 0.0719858
64800 0.029829012 64800 0.039893297 32400 0.240072521 32448
0.012768212 64832 0.222542206 64832 0.065101987
64800 0.097593569 64800 0.094867894 32500 0.122289246 32452
0.011578693 64840 0.186890291 64840 0.058591722
64800 0.136871328 64800 0.062843552 32500 0.057937425 32456
0.033767788 64848 0.111896115 64848 0.045115456
64900 0.149102969 64900 0.103427177 32500 0.036777521 32460
0.039597624 64856 0.059777442 64856 0.035643952
64900 0.1487798 64900 0.125022518 32500 0.027372133 32464
0.036677579 64864 0.044905821 64864 0.038197333
64900 0.100838617 64900 0.053844229 32500 0.026930949 32468
0.018443338 64872 0.055387194 64872 0.023099074
64900 0.028118062 64900 0.02000398 32500 0.021440848 32472
0.021904656 64880 0.09717267 64880 0.022671649
64900 0.110156339 64900 0.01444719 32500 0.018473835 32476
0.033960689 64888 0.113902468 64888 0.050360326
64900 0.080823877 64900 0.071695278 32500 0.029467476 32480
0.068709713 64896 0.093226394 64896 0.042283104
64900 0.041801901 64900 0.061419232 32500 0.071649432 32484
0.071988608 64904 0.062077561 64904 0.018869228
64900 0.030765994 64900 0.047072543 32500 0.094313757 32488
0.076224431 64912 0.048814462 64912 0.01772854
64900 0.027373104 64900 0.097167002 32500 0.070087037 32492
0.087248183 64920 0.068476118 64920 0.023489942
64900 0.052310628 64900 0.132631466 32500 0.035213325 32496
0.080728598 64928 0.127104563 64928 0.015396761
64900 0.098151759 64900 0.134870715 32500 0.038248079 32500
0.060344104 64936 0.134043163 64936 0.050248262
64900 0.111599831 64900 0.130602901 32500 0.060770559 32504
0.032143416 64944 0.090899986 64944 0.046060704
65000 0.105306623 65000 0.106498774 32500 0.050322131 32508
0.03419946 64952 0.039156032 64952 0.027075108
65000 0.103948703 65000 0.097646633 32500 0.016012098 32512
0.037098784 64960 0.085617176 64960 0.010349298
65000 0.080556667 65000 0.067687688 32500 0.040854946 32516
0.048428916 64968 0.074011208 64968 0.045367335
65000 0.057599966 65000 0.044390876 32500 0.056994581 32520
0.085884996 64976 0.047462712 64976 0.064063337
65000 0.183011929 65000 0.130667453 32500 0.018852654 32524
0.040439067 64984 0.056592518 64984 0.063575899
65000 0.181904194 65000 0.084972242 32500 0.033182238 32528
0.057451103 64992 0.115435985 64992 0.046516023
65000 0.059933416 65000 0.035985719 32500 0.032941087 32532
0.07302517 65000 0.146725811 65000 0.061622159
65000 0.13720154 65000 0.052409494 32500 0.01522399 32536
0.056036039 65008 0.138102405 65008 0.062027735
65000 0.199389703 65000 0.044597204 32500 0.011013572 32540
0.081195656 65016 0.113765294 65016 0.021972292
65000 0.100238663 65000 0.04349924 32500 0.015147642 32544
0.063529995 65024 0.115170835 65024 0.029845933
65000 0.022341485 65000 0.063337204 32500 0.017828867 32548
0.044022429 65032 0.099698635 65032 0.060365317
65000 0.08434349 65000 0.16789962 32600 0.040198531 32552

0.036805592 65040 0.180068848 65040 0.046145924
65000 0.110707711 65000 0.210079918 32600 0.067864334 32556
0.062745465 65048 0.167843013 65048 0.020428406
65100 0.079762889 65100 0.155506933 32600 0.049785536 32560
0.055682194 65056 0.035465044 65056 0.011310455
65100 0.08566209 65100 0.047884707 32600 0.073207419 32564
0.041702526 65064 0.065161054 65064 0.016388194
65100 0.07556715 65100 0.050239902 32600 0.093023555 32568
0.040910054 65072 0.060045444 65072 0.046322464
65100 0.073773976 65100 0.09866897 32600 0.078878926 32572
0.023628307 65080 0.050500585 65080 0.064900545
65100 0.069549038 65100 0.119230193 32600 0.041534633 32576
0.033846856 65088 0.10099783 65088 0.034337645
65100 0.065314831 65100 0.119178549 32600 0.023428567 32580
0.0235727 65096 0.165276055 65096 0.005350103
65100 0.074490112 65100 0.113239723 32600 0.026170021 32584
0.009532875 65104 0.215480119 65104 0.040571664
65100 0.051031981 65100 0.077118741 32600 0.038334718 32588
0.051514457 65112 0.145692699 65112 0.051009476
65100 0.149465923 65100 0.015845821 32600 0.048981085 32592
0.106178668 65120 0.049264643 65120 0.017787605
65100 0.119140947 65100 0.045883255 32600 0.037722941 32596
0.084408872 65128 0.114316819 65128 0.041967935
65100 0.028172242 65100 0.013528897 32600 0.013516182 32600
0.060144655 65136 0.012513532 65136 0.025584268
65100 0.076382879 65100 0.056754263 32600 0.063778411 32604
0.066501132 65144 0.033827688 65144 0.022796474
65200 0.043488093 65200 0.069628295 32600 0.063848049 32608
0.074634641 65152 0.029856255 65152 0.018031322
65200 0.062273248 65200 0.061884341 32600 0.040204395 32612
0.078111007 65160 0.067992347 65160 0.018894165
65200 0.098075252 65200 0.090877453 32600 0.05711654 32616
0.071846327 65168 0.01822091 65168 0.038563485
65200 0.118532473 65200 0.09617897 32600 0.043968346 32620
0.023275819 65176 0.115741343 65176 0.044531051
65200 0.120142635 65200 0.052024134 32600 0.06641353 32624
0.050556348 65184 0.180089338 65184 0.013873808
65200 0.084739819 65200 0.026284768 32600 0.104423125 32628
0.064429289 65192 0.082570317 65192 0.03737503
65200 0.044053126 65200 0.051605006 32600 0.069446738 32632
0.043381737 65200 0.162499549 65200 0.065356326
65200 0.030380115 65200 0.060694045 32600 0.054620788 32636
0.060581915 65208 0.227635669 65208 0.077537588
65200 0.029922127 65200 0.040723349 32600 0.056976227 32640
0.073354575 65216 0.198644295 65216 0.076232376
65200 0.01084745 65200 0.031066436 32600 0.014375953 32644
0.051676376 65224 0.225396624 65224 0.058345595
65200 0.058689057 65200 0.073908748 32600 0.012582459 32648
0.056694385 65232 0.31930898 65232 0.031962587
65200 0.072194845 65200 0.098611228 32700 0.046398545 32652
0.044592795 65240 0.352011674 65240 0.013806443
65200 0.020725434 65200 0.056882123 32700 0.070919647 32656
0.059637539 65248 0.234836 65248 0.033267115
65300 0.040136521 65300 0.045793004 32700 0.086650885 32660
0.066531648 65256 0.054352004 65256 0.037586156
65300 0.097245909 65300 0.091035392 32700 0.065155364 32664
0.042623746 65264 0.070022659 65264 0.017033464
65300 0.127349601 65300 0.066344961 32700 0.007677975 32668
0.055378943 65272 0.102692109 65272 0.034680998
65300 0.091096052 65300 0.054481781 32700 0.044754703 32672
0.084897649 65280 0.05660392 65280 0.051348048
65300 0.115503521 65300 0.066345994 32700 0.047318921 32676
0.069740228 65288 0.059881986 65288 0.053185482
65300 0.197226633 65300 0.103580926 32700 0.064603402 32680
0.025323638 65296 0.08157047 65296 0.052129934
65300 0.19311106 65300 0.157935341 32700 0.058349469 32684
0.015698419 65304 0.150183769 65304 0.034514127
65300 0.105173596 65300 0.217965834 32700 0.035350848 32688

0.011618135	65312	0.159243806	65312	0.034130331
65300	0.054570224	65300	0.183461918	32700	0.04343396	32692
0.035533514	65320	0.189747909	65320	0.037602531
65300	0.05296895	65300	0.094687952	32700	0.022886852	32696
0.070618749	65328	0.194920431	65328	0.030197027
65300	0.107042084	65300	0.026527936	32700	0.055739158	32700
0.061202787	65336	0.031789721	65336	0.022174232
65300	0.139990661	65300	0.065532484	32700	0.07394627	32704
0.02906263	65344	0.133750771	65344	0.035788682
65400	0.162946439	65400	0.149153013	32700	0.070368413	32708
0.001423652	65352	0.064966007	65352	0.045379296
65400	0.139077194	65400	0.210433398	32700	0.055347926	32712
0.02527393	65360	0.14283089	65360	0.025747258
65400	0.066867906	65400	0.203955628	32700	0.06588555	32716
0.009999017	65368	0.152177265	65368	0.01789483
65400	0.056967016	65400	0.127064778	32700	0.046305755	32720
0.053031348	65376	0.037569706	65376	0.040071882
65400	0.125219871	65400	0.117180927	32700	0.044793807	32724
0.045038465	65384	0.062140338	65384	0.056344699
65400	0.08275087	65400	0.13616764	32700	0.049825132	32728
0.033868841	65392	0.059973801	65392	0.04690198
65400	0.062735315	65400	0.134827293	32700	0.032594311	32732
0.01932192	65400	0.08016442	65400	0.027073462
65400	0.083242194	65400	0.095925745	32700	0.063664244	32736
0.050150167	65408	0.097500102	65408	0.020484942
65400	0.068320158	65400	0.086482076	32700	0.065164699	32740
0.056685924	65416	0.077390694	65416	0.034143512
65400	0.102518578	65400	0.069729766	32700	0.039692699	32744
0.056361194	65424	0.064917287	65424	0.05456763
65400	0.121468213	65400	0.024690575	32700	0.018481733	32748
0.040820803	65432	0.122951664	65432	0.072574316
65400	0.056749163	65400	0.064347987	32800	0.011639266	32752
0.061721919	65440	0.195761735	65440	0.059882048
65400	0.034299119	65400	0.042773121	32800	0.020262558	32756
0.086419321	65448	0.216382235	65448	0.026349011
65500	0.069831585	65500	0.024730358	32800	0.052153686	32760
0.041288324	65456	0.172989589	65456	0.014060698
65500	0.087871318	65500	0.052462954	32800	0.08227292	32764
0.069268324	65464	0.094527539	65464	0.01089678
65500	0.104273757	65500	0.038322702	32800	0.101413985	32768
0.092070237	65472	0.137412091	65472	0.036214049
65500	0.053040792	65500	0.062088875	32800	0.096256459	32772
0.056709123	65480	0.181645184	65480	0.05317499
65500	0.076351047	65500	0.11386694	32800	0.056647146	32776
0.059655373	65488	0.11547008	65488	0.046113983
65500	0.036962796	65500	0.145439219	32800	0.005182229	32780
0.026740298	65496	0.081330465	65496	0.051285151
65500	0.053196814	65500	0.143087644	32800	0.014678863	32784
0.044768451	65504	0.052313084	65504	0.074571879
65500	0.026657801	65500	0.056824476	32800	0.019758076	32788
0.070560869	65512	0.046204845	65512	0.067988687
65500	0.066310204	65500	0.040595653	32800	0.029903666	32792
0.03303905	65520	0.103542348	65520	0.030950112
65500	0.095404226	65500	0.05532421	32800	0.042638603	32796
0.011779784	65528	0.086593391	65528	0.006738001
65500	0.099964105	65500	0.03132088	32800	0.040220275	32800
0.03980707	65536	0.029362424	65536	0.02493727
65500	0.099169592	65500	0.013178063	32800	0.045188837	32804
0.037857539	65544	0.029638006	65544	0.036518486
65600	0.075071752	65600	0.02618794	32800	0.072552233	32808
0.042737447	65552	0.032030075	65552	0.019172578
65600	0.039865678	65600	0.015952064	32800	0.044407461	32812
0.046253321	65560	0.115532632	65560	0.018466804
65600	0.052388499	65600	0.046342422	32800	0.002981394	32816
0.03496568	65568	0.154753841	65568	0.031207215
65600	0.067758483	65600	0.048481372	32800	0.028362381	32820
0.028831175	65576	0.162450335	65576	0.028478073
65600	0.083177976	65600	0.101773498	32800	0.05319959	32824

0.014243938	65584	0.162316414	65584	0.061691455		
65600	0.142132194	65600	0.094507843	32800	0.061839295	32828
0.03376383	65592	0.162783254	65592	0.074102522		
65600	0.172870743	65600	0.043115259	32800	0.076630648	32832
0.06605783	65600	0.152302556	65600	0.04192979		
65600	0.131825364	65600	0.041296433	32800	0.063344858	32836
0.071533992	65608	0.160245414	65608	0.045092922		
65600	0.235434505	65600	0.069052971	32800	0.041217441	32840
0.023978995	65616	0.201489601	65616	0.053562086		
65600	0.221225099	65600	0.063056388	32800	0.046605452	32844
0.043157419	65624	0.229790414	65624	0.030476054		
65600	0.064029096	65600	0.045205521	32800	0.043186639	32848
0.02964701	65632	0.185432931	65632	0.015388576		
65600	0.065721812	65600	0.036210196	32900	0.073133626	32852
0.029960465	65640	0.127143881	65640	0.023587401		
65600	0.093010698	65600	0.03135905	32900	0.077896439	32856
0.03248085	65648	0.191293235	65648	0.044546516		
65700	0.13707584	65700	0.108193024	32900	0.043095079	32860
0.012243263	65656	0.227506054	65656	0.048302009		
65700	0.186987658	65700	0.125848586	32900	0.044845481	32864
0.017308243	65664	0.19027527	65664	0.044349876		
65700	0.187157056	65700	0.105040774	32900	0.061511913	32868
0.029552453	65672	0.093965507	65672	0.054320306		
65700	0.198390029	65700	0.091246489	32900	0.083241539	32872
0.064881555	65680	0.050216571	65680	0.036110796		
65700	0.199547969	65700	0.078606441	32900	0.070858296	32876
0.043879689	65688	0.09557556	65688	0.019566622		
65700	0.118327407	65700	0.079864374	32900	0.040256524	32880
0.024433333	65696	0.07910042	65696	0.021137015		
65700	0.017469491	65700	0.113492715	32900	0.037008798	32884
0.037138634	65704	0.022354032	65704	0.060116323		
65700	0.061915744	65700	0.178028175	32900	0.05059559	32888
0.05283043	65712	0.060750925	65712	0.050471193		
65700	0.027404274	65700	0.224999894	32900	0.028964245	32892
0.067971028	65720	0.057320958	65720	0.043349697		
65700	0.021135887	65700	0.208456666	32900	0.012060164	32896
0.075028016	65728	0.066960267	65728	0.06278386		
65700	0.078031015	65700	0.174050744	32900	0.039377526	32900
0.087613393	65736	0.085711079	65736	0.024796944		
65700	0.091569782	65700	0.157089875	32900	0.067469628	32904
0.095759264	65744	0.024794621	65744	0.051179759		
65800	0.024162508	65800	0.117373493	32900	0.072040915	32908
0.076547956	65752	0.074536285	65752	0.056993456		
65800	0.042985041	65800	0.062260551	32900	0.062541258	32912
0.059121212	65760	0.069946305	65760	0.016088061		
65800	0.062910498	65800	0.051391733	32900	0.050175946	32916
0.070923008	65768	0.011650587	65768	0.029413695		
65800	0.083482591	65800	0.117342934	32900	0.04897609	32920
0.067070898	65776	0.048814189	65776	0.018919267		
65800	0.097330652	65800	0.101395039	32900	0.059922873	32924
0.023893781	65784	0.037600399	65784	0.010321809		
65800	0.067855122	65800	0.061042258	32900	0.066911242	32928
0.031932068	65792	0.017053444	65792	0.029225255		
65800	0.06449817	65800	0.118788965	32900	0.073921547	32932
0.05356341	65800	0.042830525	65800	0.045027216		
65800	0.123062899	65800	0.15573435	32900	0.073568896	32936
0.056820187	65808	0.080011399	65808	0.013561821		
65800	0.226286168	65800	0.150878346	32900	0.064403706	32940
0.059412498	65816	0.139359239	65816	0.013226828		
65800	0.2743199	65800	0.163576523	32900	0.064419772	32944
0.03791038	65824	0.192100634	65824	0.027234664		
65800	0.185697398	65800	0.130741988	32900	0.068981506	32948
0.01040491	65832	0.198772846	65832	0.053013508		
65800	0.048560734	65800	0.04999516	33000	0.064145497	32952
0.050532166	65840	0.127543143	65840	0.016589485		
65800	0.183361146	65800	0.139822485	33000	0.033217995	32956
0.079024459	65848	0.045223475	65848	0.035185385		
65900	0.186376026	65900	0.185026555	33000	0.024575164	32960

0.081999962	65856	0.104451974	65856	0.01375882		
65900	0.13218903	65900	0.10922183	33000	0.054832246	32964
0.052787324	65864	0.106124928	65864	0.057176294		
65900	0.177346549	65900	0.069591166	33000	0.09664995	32968
0.052486277	65872	0.044344826	65872	0.070267655		
65900	0.240855283	65900	0.108218039	33000	0.091400281	32972
0.076002318	65880	0.076539764	65880	0.053725893		
65900	0.296680723	65900	0.128297935	33000	0.060247134	32976
0.072343522	65888	0.014524792	65888	0.033809189		
65900	0.295574108	65900	0.106877065	33000	0.045072575	32980
0.055703677	65896	0.101401492	65896	0.007913892		
65900	0.22443934	65900	0.051038489	33000	0.065367422	32984
0.041325253	65904	0.14854352	65904	0.005345723		
65900	0.197632675	65900	0.047668236	33000	0.09979344	32988
0.03531971	65912	0.119201082	65912	0.026030424		
65900	0.143655663	65900	0.098379322	33000	0.103021419	32992
0.044177068	65920	0.148578081	65920	0.046770674		
65900	0.092560644	65900	0.126626211	33000	0.072350791	32996
0.026373447	65928	0.079066995	65928	0.043687829		
65900	0.095091455	65900	0.079519872	33000	0.044635643	33000
0.024255483	65936	0.076936296	65936	0.032734417		
65900	0.19398563	65900	0.065817199	33000	0.016467542	33004
0.030300702	65944	0.095524156	65944	0.003011336		
66000	0.240328896	66000	0.015318779	33000	0.01562897	33008
0.010860326	65952	0.08627939	65952	0.03791041		
66000	0.131938126	66000	0.05324516	33000	0.042692882	33012
0.016432079	65960	0.122477359	65960	0.047372163		
66000	0.076344608	66000	0.085619933	33000	0.051614905	33016
0.016454827	65968	0.149621075	65968	0.042457574		
66000	0.112214635	66000	0.054346878	33000	0.055265919	33020
0.032899727	65976	0.107509899	65976	0.055344008		
66000	0.047927311	66000	0.050912262	33000	0.056513545	33024
0.066831242	65984	0.026073003	65984	0.055725392		
66000	0.101799582	66000	0.146711449	33000	0.075221746	33028
0.067345798	65992	0.04415643	65992	0.044780285		
66000	0.136065428	66000	0.173957568	33000	0.081801263	33032
0.027364415	66000	0.071876464	66000	0.034349749		
66000	0.073960015	66000	0.055515844	33000	0.064561209	33036
0.058748748	66008	0.056453635	66008	0.035202636		
66000	0.124100261	66000	0.079748032	33000	0.047889727	33040
0.060292525	66016	0.03001738	66016	0.043188622		
66000	0.133973358	66000	0.067547851	33000	0.035513527	33044
0.007530799	66024	0.070846545	66024	0.04006004		
66000	0.099753779	66000	0.083635925	33000	0.017418681	33048
0.061860461	66032	0.143626646	66032	0.036593985		
66000	0.137829179	66000	0.135634706	33100	0.020042142	33052
0.072216644	66040	0.162712371	66040	0.046849586		
66000	0.098234494	66000	0.220003378	33100	0.042430595	33056
0.05167189	66048	0.114818184	66048	0.059241065		
66100	0.01150798	66100	0.225844836	33100	0.055877394	33060
0.037516256	66056	0.115610143	66056	0.043156633		
66100	0.057501293	66100	0.108825887	33100	0.041381485	33064
0.020740068	66064	0.130717628	66064	0.021977581		
66100	0.062116451	66100	0.084251777	33100	0.01553517	33068
0.022477356	66072	0.064282838	66072	0.015411146		
66100	0.105744693	66100	0.082264465	33100	0.038415903	33072
0.041149393	66080	0.064392945	66080	0.028488754		
66100	0.127764695	66100	0.04413259	33100	0.04780374	33076
0.073998024	66088	0.126821993	66088	0.049222017		
66100	0.103285427	66100	0.132531451	33100	0.063285457	33080
0.071824456	66096	0.05308344	66096	0.076378405		
66100	0.105204992	66100	0.114893599	33100	0.070089569	33084
0.075659678	66104	0.092408467	66104	0.093234405		
66100	0.14321458	66100	0.09561031	33100	0.058540041	33088
0.105440318	66112	0.113017668	66112	0.082901264		
66100	0.127527121	66100	0.126895219	33100	0.028575094	33092
0.087172091	66120	0.094085182	66120	0.033059037		
66100	0.109201057	66100	0.181636323	33100	0.030290139	33096

0.03322756 66128 0.143184778 66128 0.034362776
66100 0.103820436 66100 0.186575606 33100 0.072354109 33100
0.034395394 66136 0.119130855 66136 0.061502273
66100 0.060978451 66100 0.04831186 33100 0.075942699 33104
0.070335373 66144 0.042510666 66144 0.041802286
66200 0.252974481 66200 0.101740799 33100 0.087502827 33108
0.081741069 66152 0.087333712 66152 0.03045584
66200 0.313318044 66200 0.165557518 33100 0.063089297 33112
0.083690844 66160 0.069407186 66160 0.040221672
66200 0.225308643 66200 0.129063905 33100 0.042886575 33116
0.062334926 66168 0.075774318 66168 0.025142947
66200 0.098127333 66200 0.035684196 33100 0.038814607 33120
0.05710913 66176 0.156444949 66176 0.006023936
66200 0.024857485 66200 0.061052037 33100 0.04918989 33124
0.065962813 66184 0.178971518 66184 0.033083117
66200 0.072322328 66200 0.142658959 33100 0.024486773 33128
0.045438301 66192 0.15324926 66192 0.050673494
66200 0.120066288 66200 0.207752921 33100 0.02064101 33132
0.006337354 66200 0.077077573 66200 0.041392403
66200 0.127874635 66200 0.245755393 33100 0.029327206 33136
0.009023549 66208 0.039847328 66208 0.03619149
66200 0.076793243 66200 0.207954901 33100 0.037299946 33140
0.014984906 66216 0.063778396 66216 0.025105299
66200 0.021230713 66200 0.201458199 33100 0.071611612 33144
0.021669936 66224 0.03682185 66224 0.006250546
66200 0.060393697 66200 0.250627694 33100 0.101980702 33148
0.025627584 66232 0.044518893 66232 0.031809752
66200 0.061604522 66200 0.146861916 33200 0.085631487 33152
0.074596312 66240 0.067262437 66240 0.031403157
66200 0.023254264 66200 0.038016533 33200 0.05755488 33156
0.090664697 66248 0.05947765 66248 0.026121423
66300 0.052808911 66300 0.102205973 33200 0.071129769 33160
0.080479716 66256 0.052088755 66256 0.066514607
66300 0.142954523 66300 0.10067557 33200 0.070075344 33164
0.070945382 66264 0.0685247 66264 0.067286208
66300 0.216990855 66300 0.094869494 33200 0.062454499 33168
0.035879912 66272 0.020908967 66272 0.033122717
66300 0.218324771 66300 0.103660241 33200 0.067140813 33172
0.009866075 66280 0.117391719 66280 0.020856907
66300 0.15929724 66300 0.141580851 33200 0.052697535 33176
0.049815204 66288 0.123155929 66288 0.07469079
66300 0.117672884 66300 0.171399791 33200 0.02084542 33180
0.077874327 66296 0.08332701 66296 0.095809592
66300 0.109990244 66300 0.132466375 33200 0.018657182 33184
0.075144468 66304 0.071255781 66304 0.063325744
66300 0.086976659 66300 0.132230474 33200 0.02212001 33188
0.042772837 66312 0.085144005 66312 0.029470766
66300 0.061738247 66300 0.120117024 33200 0.021220056 33192
0.028519396 66320 0.094544572 66320 0.008557566
66300 0.055727949 66300 0.088047178 33200 0.061944775 33196
0.031255768 66328 0.072355397 66328 0.044325374
66300 0.055232744 66300 0.057909765 33200 0.082479237 33200
0.027299429 66336 0.032499018 66336 0.07768342
66300 0.088413181 66300 0.058047692 33200 0.086700922 33204
0.013790017 66344 0.066067834 66344 0.08303587
66400 0.145558151 66400 0.076424716 33200 0.070195682 33208
0.009174696 66352 0.099731144 66352 0.064467306
66400 0.159452699 66400 0.085583219 33200 0.016445985 33212
0.03280654 66360 0.148926861 66360 0.043562985
66400 0.095278061 66400 0.088795183 33200 0.021374159 33216
0.05840819 66368 0.167732476 66368 0.041257456
66400 0.049244096 66400 0.098284312 33200 0.009649424 33220
0.02898067 66376 0.104605053 66376 0.059915255
66400 0.133215711 66400 0.071362876 33200 0.022043501 33224
0.021108182 66384 0.12996122 66384 0.079459722
66400 0.166670798 66400 0.090351845 33200 0.039492017 33228
0.062890038 66392 0.16288631 66392 0.081422593
66400 0.116620417 66400 0.100468555 33200 0.012787186 33232

0.081317965 66400 0.094930554 66400 0.078990888
66400 0.1085478 66400 0.024772344 33200 0.029251321 33236
0.036316957 66408 0.04984266 66408 0.077726669
66400 0.094048111 66400 0.057484078 33200 0.060495833 33240
0.108768894 66416 0.104380473 66416 0.05796774
66400 0.012512403 66400 0.079593083 33200 0.078752972 33244
0.143669284 66424 0.067358407 66424 0.021140542
66400 0.064697539 66400 0.08296912 33200 0.079034871 33248
0.095989592 66432 0.038256341 66432 0.029047616
66400 0.016723221 66400 0.028738481 33300 0.068465364 33252
0.025712832 66440 0.063951193 66440 0.001423629
66400 0.056149922 66400 0.030959793 33300 0.063101084 33256
0.06236558 66448 0.101223195 66448 0.058992588
66500 0.082920327 66500 0.007926144 33300 0.048053295 33260
0.065177221 66456 0.116873278 66456 0.070679045
66500 0.115157891 66500 0.061685998 33300 0.042750642 33264
0.102169979 66464 0.090775422 66464 0.028304248
66500 0.109146087 66500 0.052324951 33300 0.057638532 33268
0.107906963 66472 0.063479558 66472 0.053104825
66500 0.1290941 66500 0.058479607 33300 0.024986908 33272
0.035124162 66480 0.018919576 66480 0.071341427
66500 0.128349449 66500 0.051764528 33300 0.016405995 33276
0.01763356 66488 0.028644756 66488 0.066046654
66500 0.058771144 66500 0.058290258 33300 0.055270393 33280
0.050035018 66496 0.039973202 66496 0.053882908
66500 0.088338595 66500 0.070618175 33300 0.059285947 33284
0.045525791 66504 0.028871829 66504 0.050358125
66500 0.110333931 66500 0.059304035 33300 0.04991889 33288
0.035886926 66512 0.062495179 66512 0.059459246
66500 0.121967954 66500 0.083305211 33300 0.05539642 33292
0.064124652 66520 0.045616383 66520 0.0464882
66500 0.127860928 66500 0.07340523 33300 0.048175873 33296
0.084249899 66528 0.038119146 66528 0.033156677
66500 0.058377504 66500 0.076100281 33300 0.039402563 33300
0.089716741 66536 0.120724028 66536 0.071525625
66500 0.102822451 66500 0.133007969 33300 0.047135618 33304
0.078649537 66544 0.18509131 66544 0.066729626
66600 0.198702314 66600 0.134851536 33300 0.074114621 33308
0.103986371 66552 0.146405146 66552 0.042041884
66600 0.172889778 66600 0.038683062 33300 0.067347792 33312
0.100115125 66560 0.050350351 66560 0.037100534
66600 0.04948895 66600 0.071226881 33300 0.023315533 33316
0.075684184 66568 0.032736934 66568 0.033308847
66600 0.029167213 66600 0.042442462 33300 0.024486535 33320
0.033151715 66576 0.066604473 66576 0.015942767
66600 0.014438476 66600 0.057579007 33300 0.017443752 33324
0.057803609 66584 0.107833017 66584 0.07581769
66600 0.081759281 66600 0.087223634 33300 0.059657083 33328
0.117178599 66592 0.110705769 66592 0.066844645
66600 0.091097529 66600 0.077652774 33300 0.131289664 33332
0.136650328 66600 0.062919658 66600 0.030562671
66600 0.054034168 66600 0.055444725 33300 0.119923338 33336
0.132758928 66608 0.084073443 66608 0.055615114
66600 0.042393811 66600 0.049721442 33300 0.060515809 33340
0.101514372 66616 0.1187124 66616 0.027978458
66600 0.045411667 66600 0.065597655 33300 0.032966833 33344
0.087911685 66624 0.181991534 66624 0.041320069
66600 0.08559609 66600 0.106437714 33300 0.035472684 33348
0.093170034 66632 0.184622346 66632 0.076391552
66600 0.104115257 66600 0.09944637 33400 0.026111127 33352
0.080943617 66640 0.133621201 66640 0.064754684
66600 0.134854025 66600 0.064008789 33400 0.033047541 33356
0.041943429 66648 0.089432455 66648 0.068558671
66700 0.175735623 66700 0.057426081 33400 0.049577877 33360
0.013184537 66656 0.065420602 66656 0.085069805
66700 0.16304935 66700 0.095047712 33400 0.05813711 33364
0.053344618 66664 0.020608726 66664 0.088953057
66700 0.129972178 66700 0.031603846 33400 0.032069929 33368

0.032974702 66672 0.056189838 66672 0.073781725
66700 0.099692763 66700 0.045435925 33400 0.027495276 33372
0.064564636 66680 0.090874266 66680 0.042627838
66700 0.030188303 66700 0.046588226 33400 0.057758472 33376
0.109567889 66688 0.080403472 66688 0.024297087
66700 0.064794156 66700 0.02306494 33400 0.052934644 33380
0.074524091 66696 0.027525914 66696 0.029029136
66700 0.125256848 66700 0.086976579 33400 0.063059662 33384
0.052758409 66704 0.086783111 66704 0.047332789
66700 0.154436435 66700 0.0799388 33400 0.083705141 33388
0.095727919 66712 0.11548464 66712 0.049613052
66700 0.140119606 66700 0.084239116 33400 0.112256181 33392
0.101731414 66720 0.057102952 66720 0.050463794
66700 0.110100256 66700 0.139004231 33400 0.090875372 33396
0.076435325 66728 0.124855986 66728 0.057637473
66700 0.117741292 66700 0.10417444 33400 0.049785343 33400
0.066047076 66736 0.142948193 66736 0.083133738
66700 0.17890826 66700 0.027104599 33400 0.052372066 33404
0.097060343 66744 0.108992135 66744 0.08535121
66800 0.214236846 66800 0.025990463 33400 0.084697786 33408
0.06906459 66752 0.031470248 66752 0.041540756
66800 0.163783145 66800 0.059081278 33400 0.085491847 33412
0.007039158 66760 0.10930562 66760 0.020028298
66800 0.119130767 66800 0.097760152 33400 0.080131373 33416
0.034044355 66768 0.143189536 66768 0.036541267
66800 0.13511641 66800 0.101233381 33400 0.078929683 33420
0.046892128 66776 0.081998995 66776 0.01834664
66800 0.084743733 66800 0.14225475 33400 0.057103549 33424
0.073813128 66784 0.047013618 66784 0.005074046
66800 0.05176567 66800 0.21077707 33400 0.041775154 33428
0.09704104 66792 0.158256284 66792 0.025450028
66800 0.103061822 66800 0.210616374 33400 0.059809707 33432
0.087316061 66800 0.156743801 66800 0.050835151
66800 0.101458827 66800 0.131469525 33400 0.043080283 33436
0.05936416 66808 0.070349313 66808 0.02718999
66800 0.047983322 66800 0.077109326 33400 0.029807827 33440
0.014554339 66816 0.127299965 66816 0.028785431
66800 0.015316547 66800 0.092904949 33400 0.053079413 33444
0.023167702 66824 0.16149247 66824 0.052677377
66800 0.050498969 66800 0.059012371 33400 0.065389941 33448
0.055865523 66832 0.152185865 66832 0.04758079
66800 0.085919564 66800 0.060665247 33500 0.047150687 33452
0.131859183 66840 0.09677752 66840 0.034301713
66800 0.070845759 66800 0.130782879 33500 0.04600258 33456
0.174274391 66848 0.062828563 66848 0.052740117
66900 0.067373156 66900 0.084381354 33500 0.072406627 33460
0.151640939 66856 0.11253883 66856 0.056844408
66900 0.063154599 66900 0.058719063 33500 0.064386055 33464
0.114823037 66864 0.170891639 66864 0.041104297
66900 0.025855954 66900 0.076045821 33500 0.037777994 33468
0.089497269 66872 0.093874289 66872 0.016352158
66900 0.039474926 66900 0.115263763 33500 0.031894699 33472
0.108393339 66880 0.051688494 66880 0.025728668
66900 0.059829043 66900 0.138805481 33500 0.03719499 33476
0.116225601 66888 0.046213514 66888 0.038189581
66900 0.074288946 66900 0.127885768 33500 0.078448749 33480
0.077598263 66896 0.038544484 66896 0.036033518
66900 0.094574483 66900 0.11127066 33500 0.105055493 33484
0.065795488 66904 0.032005155 66904 0.057009493
66900 0.129100518 66900 0.119230921 33500 0.093952418 33488
0.074687494 66912 0.04786273 66912 0.068205431
66900 0.043478289 66900 0.102443846 33500 0.068926529 33492
0.090236732 66920 0.07388604 66920 0.040914405
66900 0.09272428 66900 0.051852563 33500 0.047364079 33496
0.084643929 66928 0.093142211 66928 0.010264748
66900 0.122110272 66900 0.072479677 33500 0.023313383 33500
0.096021053 66936 0.098452525 66936 0.016014317
66900 0.114481154 66900 0.084890708 33500 0.027201237 33504

0.146032413 66944 0.060735802 66944 0.022471289
67000 0.140025878 67000 0.153748304 33500 0.048260434 33508
0.137714364 66952 0.007789627 66952 0.040261446
67000 0.200628346 67000 0.178904476 33500 0.038766233 33512
0.052139803 66960 0.020386484 66960 0.05042689
67000 0.196954818 67000 0.126580096 33500 0.022548624 33516
0.028475601 66968 0.030004436 66968 0.030989872
67000 0.133369656 67000 0.070759306 33500 0.081528728 33520
0.030503128 66976 0.043049706 66976 0.032794345
67000 0.047313559 67000 0.040950054 33500 0.074561802 33524
0.022762888 66984 0.051710555 66984 0.049097303
67000 0.070161856 67000 0.020271542 33500 0.049434704 33528
0.018259585 66992 0.063319989 66992 0.022285478
67000 0.093004768 67000 0.03257193 33500 0.037003752 33532
0.027648512 67000 0.097020347 67000 0.035335314
67000 0.070826609 67000 0.033539604 33500 0.021717331 33536
0.050839295 67008 0.082892664 67008 0.044316577
67000 0.053422569 67000 0.052603151 33500 0.043532626 33540
0.068923924 67016 0.070246104 67016 0.054969762
67000 0.00486512 67000 0.065308588 33500 0.023125675 33544
0.077157078 67024 0.097658107 67024 0.059085287
67000 0.095035277 67000 0.061321312 33500 0.042592827 33548
0.057108566 67032 0.103251521 67032 0.033850072
67000 0.13320407 67000 0.044388533 33600 0.059719103 33552
0.060590392 67040 0.103913306 67040 0.005550611
67000 0.069939022 67000 0.039274179 33600 0.059320006 33556
0.094946372 67048 0.081288614 67048 0.020859394
67100 0.043966731 67100 0.094824456 33600 0.079194309 33560
0.076618191 67056 0.06490454 67056 0.041733889
67100 0.112950664 67100 0.078953861 33600 0.108534332 33564
0.046377591 67064 0.11245138 67064 0.029846435
67100 0.158333321 67100 0.12802599 33600 0.095781557 33568
0.064025255 67072 0.103159167 67072 0.007799406
67100 0.115248899 67100 0.131518683 33600 0.049544193 33572
0.065280867 67080 0.072811818 67080 0.032452252
67100 0.064598113 67100 0.075599455 33600 0.035519031 33576
0.029910065 67088 0.12054963 67088 0.05959977
67100 0.061871251 67100 0.06501015 33600 0.047417518 33580
0.082592851 67096 0.130482324 67096 0.050674105
67100 0.110764893 67100 0.016730412 33600 0.064188949 33584
0.087854227 67104 0.121960933 67104 0.02372338
67100 0.128951433 67100 0.078882426 33600 0.055424418 33588
0.060764534 67112 0.102808117 67112 0.003167611
67100 0.084236592 67100 0.104871331 33600 0.052132444 33592
0.076783392 67120 0.095613301 67120 0.016092137
67100 0.045611003 67100 0.037316651 33600 0.071035145 33596
0.09725448 67128 0.055429344 67128 0.033983706
67100 0.141660814 67100 0.0257375 33600 0.052790692 33600
0.067357665 67136 0.07133402 67136 0.02185126
67100 0.101125981 67100 0.06020096 33600 0.037825641 33604
0.020257741 67144 0.101251862 67144 0.007541945
67200 0.038009919 67200 0.079111378 33600 0.061868486 33608
0.05362483 67152 0.045577155 67152 0.027247557
67200 0.128435844 67200 0.100946912 33600 0.052671432 33612
0.119545352 67160 0.023717477 67160 0.035326284
67200 0.154444774 67200 0.09036548 33600 0.039014514 33616
0.127052495 67168 0.104044892 67168 0.019162433
67200 0.09037392 67200 0.042391181 33600 0.060426348 33620
0.077474935 67176 0.132430083 67176 0.037904902
67200 0.016331089 67200 0.0480899 33600 0.086887063 33624
0.011435102 67184 0.051849438 67184 0.01586584
67200 0.020914124 67200 0.101584883 33600 0.088763045 33628
0.061090424 67192 0.028352928 67192 0.029134773
67200 0.053021002 67200 0.097197561 33600 0.050228602 33632
0.09507798 67200 0.13538271 67200 0.012210191
67200 0.078166901 67200 0.06128152 33600 0.00924171 33636
0.124111 67208 0.278055173 67208 0.017440554
67200 0.081589824 67200 0.067606721 33600 0.031342162 33640

0.144068225 67216 0.189959945 67216 0.043533673
67200 0.105627427 67200 0.101807898 33600 0.019063407 33644
0.112101676 67224 0.102897087 67224 0.054794477
67200 0.088895133 67200 0.091858681 33600 0.065207474 33648
0.048885784 67232 0.141038676 67232 0.042834512
67200 0.045148754 67200 0.05964037 33700 0.079860976 33652
0.013459247 67240 0.006742443 67240 0.032333257
67200 0.037193622 67200 0.065296576 33700 0.035691119 33656
0.006590345 67248 0.046200028 67248 0.038795148
67300 0.039229748 67300 0.104960192 33700 0.025189303 33660
0.042283569 67256 0.105518746 67256 0.025281401
67300 0.082433646 67300 0.142430625 33700 0.01960677 33664
0.069040863 67264 0.112652298 67264 0.021429936
67300 0.105307343 67300 0.097133576 33700 0.02742779 33668
0.056637597 67272 0.240318143 67272 0.023595118
67300 0.105518288 67300 0.073952724 33700 0.047642763 33672
0.034040368 67280 0.428110186 67280 0.027711678
67300 0.104104314 67300 0.054603617 33700 0.047578513 33676
0.032897038 67288 0.542209193 67288 0.062358973
67300 0.087554865 67300 0.037567752 33700 0.012231591 33680
0.039390237 67296 0.387385343 67296 0.064078682
67300 0.101918413 67300 0.060048751 33700 0.025426909 33684
0.032110194 67304 0.480408751 67304 0.049399932
67300 0.067408357 67300 0.076811215 33700 0.022647069 33688
0.048945094 67312 0.511009886 67312 0.027936865
67300 0.10245607 67300 0.116351694 33700 0.023808534 33692
0.11303588 67320 0.446798396 67320 0.033475706
67300 0.162226308 67300 0.079936595 33700 0.039635128 33696
0.081806284 67328 0.507735939 67328 0.046309506
67300 0.105281564 67300 0.066245862 33700 0.052909214 33700
0.032223619 67336 0.717905409 67336 0.070246315
67300 0.057896847 67300 0.121261008 33700 0.037809568 33704
0.050237748 67344 0.985018444 67344 0.067710767
67400 0.031537064 67400 0.124044673 33700 0.00665497 33708
0.107796266 67352 1.146111754 67352 0.041520332
67400 0.091060785 67400 0.074289543 33700 0.043945231 33712
0.132850211 67360 1.145098009 67360 0.025959706
67400 0.124410668 67400 0.070843555 33700 0.060243725 33716
0.081488179 67368 0 67368 0.036329431
67400 0.081657985 67400 0.099777812 33700 0.043790213 33720
0.030925516 67376 1.079964103 67376 0.041006173
67400 0.031355699 67400 0.072926574 33700 0.029755693 33724
0.058811904 67384 1.07437151 67384 0.050124614
67400 0.026070577 67400 0.083403793 33700 0.027680368 33728
0.061806604 67392 1.1736179 67392 0.056574074
67400 0.039413553 67400 0.068710266 33700 0.008858948 33732
0.049080849 67400 1.165577094 67400 0.034079458
67400 0.046992009 67400 0.0409569 33700 0.061943661 33736
0.031266922 67408 0.976857613 67408 0.010924726
67400 0.116331779 67400 0.097449949 33700 0.060410286 33740
0.02762174 67416 0.886332303 67416 0.007629057
67400 0.165030055 67400 0.099205274 33700 0.035608828 33744
0.074755517 67424 0.854521175 67424 0.019729119
67400 0.144112346 67400 0.059414957 33700 0.070720191 33748
0.090407702 67432 0.74261392 67432 0.01706174
67400 0.07746245 67400 0.068164882 33800 0.076862023 33752
0.050309598 67440 0.533050741 67440 0.026307771
67400 0.054008644 67400 0.161173594 33800 0.05506544 33756
0.045646462 67448 0.48095698 67448 0.033259697
67500 0.066383844 67500 0.186267149 33800 0.065004759 33760
0.035400553 67456 0.412246969 67456 0.028623381
67500 0.089240064 67500 0.089576577 33800 0.054789594 33764
0.02134595 67464 0.352865026 67464 0.023583403
67500 0.112853821 67500 0.047598882 33800 0.039805498 33768
0.042478307 67472 0.236427354 67472 0.0250431
67500 0.118304321 67500 0.118398675 33800 0.074140997 33772
0.083739142 67480 0.24416644 67480 0.045876495
67500 0.051234754 67500 0.066838933 33800 0.047787009 33776

0.085186293 67488 0.310764124 67488 0.043198907
67500 0.090456189 67500 0.07000201 33800 0.028133101 33780
0.075956297 67496 0.283780246 67496 0.022193448
67500 0.140655088 67500 0.143952493 33800 0.037286642 33784
0.088836707 67504 0.25774003 67504 0.028787084
67500 0.136147108 67500 0.140575489 33800 0.006481339 33788
0.086334927 67512 0.162991608 67512 0.060836752
67500 0.113745417 67500 0.073192234 33800 0.050784096 33792
0.092031725 67520 0.149240878 67520 0.051399205
67500 0.07978981 67500 0.027115875 33800 0.07164335 33796
0.100479148 67528 0.277157989 67528 0.010495528
67500 0.083729603 67500 0.089297755 33800 0.029647161 33800
0.05111604 67536 0.311036304 67536 0.034386063
67500 0.074345466 67500 0.085596301 33800 0.049921011 33804
0.03671736 67544 0.24919433 67544 0.04004885
67600 0.004377141 67600 0.065677756 33800 0.035936042 33808
0.077623809 67552 0.146896899 67552 0.034350316
67600 0.119801422 67600 0.126810016 33800 0.034564095 33812
0.097564116 67560 0.115873285 67560 0.013000053
67600 0.183601689 67600 0.173944384 33800 0.047953072 33816
0.091449372 67568 0.139600626 67568 0.022075858
67600 0.12809693 67600 0.133912021 33800 0.026881256 33820
0.071014503 67576 0.120143312 67576 0.066572
67600 0.038879854 67600 0.103285529 33800 0.015904032 33824
0.08075629 67584 0.05738642 67584 0.091374845
67600 0.120517834 67600 0.060246446 33800 0.040624382 33828
0.101528836 67592 0.128569329 67592 0.079747173
67600 0.192886699 67600 0.047047568 33800 0.062830353 33832
0.065203247 67600 0.164490703 67600 0.068599751
67600 0.218625603 67600 0.13279033 33800 0.06033606 33836
0.059513401 67608 0.157527204 67608 0.042189346
67600 0.12591602 67600 0.107154156 33800 0.050074115 33840
0.088967361 67616 0.091815724 67616 0.019039822
67600 0.030071587 67600 0.008207193 33800 0.051451087 33844
0.093476672 67624 0.071179966 67624 0.038376937
67600 0.067307265 67600 0.016084741 33800 0.051224881 33848
0.104068007 67632 0.008681979 67632 0.045630884
67600 0.041826756 67600 0.057830555 33900 0.041435767 33852
0.098275858 67640 0.052909436 67640 0.034189208
67600 0.030257677 67600 0.072544026 33900 0.037823807 33856
0.075020733 67648 0.015980335 67648 0.011926215
67700 0.026363565 67700 0.032881948 33900 0.050009669 33860
0.065334702 67656 0.011912501 67656 0.038094331
67700 0.058082358 67700 0.117604635 33900 0.051209619 33864
0.048119862 67664 0.044438999 67664 0.034878482
67700 0.063004867 67700 0.137473515 33900 0.028967206 33868
0.019923018 67672 0.023669645 67672 0.015649588
67700 0.086940163 67700 0.160689975 33900 0.079476806 33872
0.069855283 67680 0.040610961 67680 0.011096708
67700 0.097915479 67700 0.166866506 33900 0.112069516 33876
0.058864713 67688 0.053647389 67688 0.009512046
67700 0.057932008 67700 0.101337988 33900 0.061193437 33880
0.066873865 67696 0.073973868 67696 0.010570076
67700 0.027898075 67700 0.016556625 33900 0.071359958 33884
0.085683707 67704 0.066017958 67704 0.010414239
67700 0.017401413 67700 0.017321896 33900 0.095733565 33888
0.068332127 67712 0.045493562 67712 0.047371203
67700 0.036696874 67700 0.044055243 33900 0.070144146 33892
0.041112278 67720 0.026885798 67720 0.056986955
67700 0.123356556 67700 0.077944183 33900 0.026742986 33896
0.018855835 67728 0.057398709 67728 0.042543165
67700 0.129204141 67700 0.073355419 33900 0.030661689 33900
0.063512423 67736 0.056556899 67736 0.032436037
67700 0.059305978 67700 0.165974984 33900 0.016818351 33904
0.095375464 67744 0.110120091 67744 0.026848797
67800 0.169510866 67800 0.239805653 33900 0.038127153 33908
0.053026128 67752 0.20643913 67752 0.037596121
67800 0.124794489 67800 0.150054083 33900 0.027548593 33912

0.047922964 67760 0.194376364 67760 0.03047815
67800 0.051592328 67800 0.030724241 33900 0.015890319 33916
0.05823579 67768 0.104158709 67768 0.011343971
67800 0.068893358 67800 0.096367628 33900 0.027084398 33920
0.06637843 67776 0.079790443 67776 0.042551099
67800 0.072874245 67800 0.081347185 33900 0.038375623 33924
0.089544883 67784 0.056341301 67784 0.086971115
67800 0.047048055 67800 0.011867157 33900 0.062072621 33928
0.072577241 67792 0.086453438 67792 0.07105423
67800 0.088191555 67800 0.049252558 33900 0.088979869 33932
0.064699518 67800 0.104262785 67800 0.015388989
67800 0.037101905 67800 0.040213763 33900 0.074766096 33936
0.072471878 67808 0.034253335 67808 0.022689927
67800 0.084010389 67800 0.103008475 33900 0.057259353 33940
0.05591254 67816 0.028855247 67816 0.033630709
67800 0.082259139 67800 0.051742754 33900 0.071308459 33944
0.08329573 67824 0.044842469 67824 0.030656123
67800 0.048700651 67800 0.048368314 33900 0.063930042 33948
0.045538312 67832 0.02591309 67832 0.051480678
67800 0.048060232 67800 0.053176445 34000 0.020371728 33952
0.01489377 67840 0.125962906 67840 0.055564389
67800 0.034326033 67800 0.105864012 34000 0.040225139 33956
0.065892091 67848 0.183408018 67848 0.023303757
67900 0.036669047 67900 0.104428742 34000 0.051485233 33960
0.088212699 67856 0.118032855 67856 0.055666966
67900 0.117916228 67900 0.10018154 34000 0.026532003 33964
0.072858726 67864 0.078328609 67864 0.070621885
67900 0.147669868 67900 0.081321232 34000 0.012083426 33968
0.057232956 67872 0.140889315 67872 0.065101798
67900 0.116238618 67900 0.058626996 34000 0.04824479 33972
0.031192692 67880 0.133294205 67880 0.057483157
67900 0.054739689 67900 0.095848183 34000 0.067801659 33976
0.042953579 67888 0.058067286 67888 0.039711191
67900 0.048347047 67900 0.144802325 34000 0.042680833 33980
0.040601321 67896 0.059040904 67896 0.023917333
67900 0.046646102 67900 0.089944282 34000 0.031001324 33984
0.039595361 67904 0.107683591 67904 0.023556569
67900 0.054316846 67900 0.071460672 34000 0.059312824 33988
0.048072521 67912 0.119829136 67912 0.011514658
67900 0.08974658 67900 0.050888095 34000 0.048152029 33992
0.039984065 67920 0.041949203 67920 0.041271029
67900 0.104649109 67900 0.068151981 34000 0.069503018 33996
0.050388659 67928 0.095229632 67928 0.051631756
67900 0.092379312 67900 0.084092404 34000 0.033687553 34000
0.034790013 67936 0.118473385 67936 0.032250115
67900 0.019968395 67900 0.060208637 34000 0.008262768 34004
0.01663534 67944 0.168613129 67944 0.020525558
68000 0.022397104 68000 0.03639426 34000 0.010607396 34008
0.009714636 67952 0.180411065 67952 0.007588174
68000 0.030062827 68000 0.043619035 34000 0.042867712 34012
0.015514295 67960 0.086720844 67960 0.008358604
68000 0.02430407 68000 0.008830111 34000 0.060732524 34016
0.051082156 67968 0.076528166 67968 0.005924712
68000 0.083807703 68000 0.062632062 34000 0.036771678 34020
0.062718493 67976 0.104629457 67976 0.0214054
68000 0.143772443 68000 0.095887765 34000 0.022809349 34024
0.051526797 67984 0.09060969 67984 0.044091627
68000 0.084555984 68000 0.106856314 34000 0.030517844 34028
0.036237136 67992 0.091206523 67992 0.043514145
68000 0.070020942 68000 0.120593264 34000 0.077623728 34032
0.017320361 68000 0.105824503 68000 0.021149957
68000 0.10592847 68000 0.058929629 34000 0.087169763 34036
0.064623135 68008 0.079738224 68008 0.032778691
68000 0.090304769 68000 0.052689131 34000 0.060936058 34040
0.061727806 68016 0.013794166 68016 0.059767568
68000 0.049655428 68000 0.022726839 34000 0.04475153 34044
0.037644815 68024 0.08820081 68024 0.084781146
68000 0.082328494 68000 0.086899556 34000 0.07412897 34048

0.030733314 68032 0.129803535 68032 0.051871259
68000 0.107356602 68000 0.062166124 34100 0.098691664 34052
0.04893075 68040 0.132758461 68040 0.03363454
68000 0.083666826 68000 0.02027701 34100 0.040248113 34056
0.055841254 68048 0.146013481 68048 0.077141966
68100 0.053125328 68100 0.032082277 34100 0.052704651 34060
0.02867486 68056 0.17201608 68056 0.066332294
68100 0.072875089 68100 0.032850447 34100 0.079157806 34064
0.03372342 68064 0.131622263 68064 0.046540583
68100 0.085772132 68100 0.006455478 34100 0.051268402 34068
0.049526891 68072 0.029238228 68072 0.053882286
68100 0.05166942 68100 0.027293609 34100 0.040618463 34072
0.027406439 68080 0.053091539 68080 0.068211455
68100 0.058713238 68100 0.046421304 34100 0.015390699 34076
0.007289416 68088 0.080432226 68088 0.062668565
68100 0.065997687 68100 0.04048882 34100 0.011335186 34080
0.018509649 68096 0.087187298 68096 0.047225534
68100 0.048432907 68100 0.068071975 34100 0.029065428 34084
0.051268758 68104 0.07195089 68104 0.055673823
68100 0.041752912 68100 0.122637663 34100 0.033855653 34088
0.056840476 68112 0.057175166 68112 0.051800591
68100 0.08359769 68100 0.137571333 34100 0.01560182 34092
0.03912333 68120 0.203028409 68120 0.038515111
68100 0.105738625 68100 0.105272833 34100 0.035599704 34096
0.039928378 68128 0.238918263 68128 0.019854455
68100 0.078802921 68100 0.056860466 34100 0.039045455 34100
0.044706234 68136 0.172485453 68136 0.024121839
68100 0.108615634 68100 0.029819079 34100 0.039623545 34104
0.024894458 68144 0.126629035 68144 0.010188851
68200 0.129585125 68200 0.040100826 34100 0.026541269 34108
0.028632794 68152 0.119842516 68152 0.0166181
68200 0.082173872 68200 0.035855879 34100 0.046854275 34112
0.033654509 68160 0.120513381 68160 0.030500845
68200 0.046335488 68200 0.006991912 34100 0.057487596 34116
0.044201046 68168 0.08090862 68168 0.029956626
68200 0.05584152 68200 0.064506981 34100 0.04363008 34120
0.07594614 68176 0.079304075 68176 0.018181248
68200 0.074816642 68200 0.099749115 34100 0.030987125 34124
0.050454055 68184 0.057093686 68184 0.008162066
68200 0.033917106 68200 0.071334274 34100 0.047158817 34128
0.020802489 68192 0.011789082 68192 0.015315814
68200 0.13001627 68200 0.038792441 34100 0.070726375 34132
0.039004535 68200 0.042288848 68200 0.032377731
68200 0.111381211 68200 0.009589921 34100 0.043038293 34136
0.03122395 68208 0.062093597 68208 0.063407839
68200 0.236714478 68200 0.068413538 34100 0.053735988 34140
0.066653156 68216 0.13405901 68216 0.09816714
68200 0.360652484 68200 0.060951654 34100 0.083913765 34144
0.066796871 68224 0.168240993 68224 0.082672559
68200 0.28587741 68200 0.133616151 34100 0.096779862 34148
0.018534674 68232 0.112787566 68232 0.042159885
68200 0.099652534 68200 0.207771081 34200 0.090575704 34152
0.027805103 68240 0.069095331 68240 0.02273831
68200 0.069803878 68200 0.121749799 34200 0.071994298 34156
0.055293964 68248 0.091944312 68248 0.030602361
68300 0.059308059 68300 0.101410362 34200 0.038295511 34160
0.043929376 68256 0.106216874 68256 0.068191373
68300 0.066550223 68300 0.146716804 34200 0.053917964 34164
0.038571547 68264 0.146188468 68264 0.089122965
68300 0.020007448 68300 0.100987825 34200 0.050572857 34168
0.051028794 68272 0.154193448 68272 0.057214133
68300 0.054682987 68300 0.037158097 34200 0.047597987 34172
0.012122021 68280 0.143789658 68280 0.024723791
68300 0.094957242 68300 0.109421861 34200 0.079641162 34176
0.04822472 68288 0.110740468 68288 0.019568479
68300 0.119043805 68300 0.091353024 34200 0.076160926 34180
0.06768616 68296 0.065138898 68296 0.017007527
68300 0.083294879 68300 0.083491032 34200 0.032483942 34184

0.080378952	68304	0.0267257	68304	0.015978834
68300	0.050296298	68300	0.11598508	34200	0.023010925	34188
0.101018675	68312	0.055483775	68312	0.02180336
68300	0.076922399	68300	0.134763338	34200	0.019119789	34192
0.084189807	68320	0.02748756	68320	0.051839066
68300	0.040270326	68300	0.089257585	34200	0.02924452	34196
0.05784665	68328	0.015895726	68328	0.030669744
68300	0.094667019	68300	0.005448717	34200	0.035223704	34200
0.04550512	68336	0.075307115	68336	0.04941654
68300	0.11903033	68300	0.069213813	34200	0.05551317	34204
0.024815325	68344	0.099631667	68344	0.045312856
68400	0.031866111	68400	0.098731383	34200	0.055221259	34208
0.060104118	68352	0.092602444	68352	0.048130318
68400	0.093960167	68400	0.123371574	34200	0.06153786	34212
0.06207516	68360	0.102355734	68360	0.074270596
68400	0.178066766	68400	0.155295856	34200	0.084020234	34216
0.055397468	68368	0.093056297	68368	0.054669421
68400	0.241641188	68400	0.185635596	34200	0.087260116	34220
0.026416217	68376	0.087288565	68376	0.036259527
68400	0.17944054	68400	0.167548613	34200	0.080338155	34224
0.062830484	68384	0.07333258	68384	0.036999725
68400	0.091943861	68400	0.141209894	34200	0.070574206	34228
0.088742454	68392	0.076027391	68392	0.043612708
68400	0.130258574	68400	0.113011767	34200	0.03746368	34232
0.058766334	68400	0.1053171	68400	0.037940397
68400	0.064640924	68400	0.12215614	34200	0.047251408	34236
0.044513221	68408	0.105824009	68408	0.030174069
68400	0.060593102	68400	0.153850211	34200	0.072696581	34240
0.067110246	68416	0.064046464	68416	0.051767383
68400	0.096981785	68400	0.149891871	34200	0.081892533	34244
0.0682764	68424	0.052421103	68424	0.078080608
68400	0.116875388	68400	0.124274215	34200	0.044664354	34248
0.039674702	68432	0.087871595	68432	0.061922947
68400	0.14617003	68400	0.11936776	34300	0.028479686	34252
0.019077264	68440	0.102898848	68440	0.047461173
68400	0.133650756	68400	0.118834432	34300	0.028913888	34256
0.008407866	68448	0.087826316	68448	0.055741046
68500	0.101738275	68500	0.108942382	34300	0.046942045	34260
0.054139324	68456	0.059862625	68456	0.025732132
68500	0.14544971	68500	0.080717124	34300	0.050289807	34264
0.076986828	68464	0.03417084	68464	0.060027152
68500	0.226822028	68500	0.054252083	34300	0.03915255	34268
0.06302831	68472	0.059595954	68472	0.056951896
68500	0.311526208	68500	0.057932532	34300	0.010716955	34272
0.039893675	68480	0.067504014	68480	0.019790276
68500	0.327484129	68500	0.031465876	34300	0.050179027	34276
0.074863718	68488	0.090111244	68488	0.033291093
68500	0.225096723	68500	0.018378074	34300	0.074571173	34280
0.125747975	68496	0.111751215	68496	0.069288391
68500	0.121886209	68500	0.032949982	34300	0.067200737	34284
0.102449252	68504	0.151938133	68504	0.06409386
68500	0.127734733	68500	0.099709294	34300	0.043723372	34288
0.043914613	68512	0.133033624	68512	0.043368644
68500	0.108482942	68500	0.10794311	34300	0.031409716	34292
0.060164777	68520	0.15297615	68520	0.037472561
68500	0.145894534	68500	0.056049736	34300	0.03493853	34296
0.068765192	68528	0.09288382	68528	0.036895679
68500	0.200695533	68500	0.055534623	34300	0.011310958	34300
0.072973242	68536	0.053930784	68536	0.027597653
68500	0.147701489	68500	0.016756767	34300	0.038355447	34304
0.072911993	68544	0.094465395	68544	0.015860092
68600	0.056169101	68600	0.113086273	34300	0.077718534	34308
0.079141755	68552	0.123599428	68552	0.033475164
68600	0.106818246	68600	0.132128408	34300	0.106209867	34312
0.094334711	68560	0.129348613	68560	0.033803302
68600	0.094671465	68600	0.073904928	34300	0.094171483	34316
0.080318423	68568	0.041848827	68568	0.033473676
68600	0.032909207	68600	0.021194021	34300	0.057129997	34320

0.014155228 68576 0.111261761 68576 0.017608078
68600 0.031642019 68600 0.018607403 34300 0.013360335 34324
0.061926316 68584 0.16069178 68584 0.022942895
68600 0.084034356 68600 0.059982092 34300 0.05288974 34328
0.095177275 68592 0.086334199 68592 0.027750695
68600 0.103158403 68600 0.072704112 34300 0.075609991 34332
0.093821815 68600 0.030373003 68600 0.00859197
68600 0.04562645 68600 0.033385579 34300 0.056435223 34336
0.041850017 68608 0.059302936 68608 0.042789347
68600 0.025541865 68600 0.040025057 34300 0.009429047 34340
0.029599405 68616 0.025899639 68616 0.071085655
68600 0.057409969 68600 0.078650613 34300 0.021334932 34344
0.069895199 68624 0.092714974 68624 0.073561583
68600 0.028939328 68600 0.172181943 34300 0.017741619 34348
0.079954276 68632 0.099194862 68632 0.052015748
68600 0.046118908 68600 0.186426668 34400 0.01993813 34352
0.051724906 68640 0.065617627 68640 0.059541661
68600 0.125931226 68600 0.101223697 34400 0.026884482 34356
0.043302633 68648 0.0177027 68648 0.038949285
68700 0.186513979 68700 0.026296157 34400 0.034529123 34360
0.052109022 68656 0.026912285 68656 0.043313008
68700 0.215006818 68700 0.042716765 34400 0.038103393 34364
0.058170037 68664 0.028949373 68664 0.068318193
68700 0.17866031 68700 0.046666119 34400 0.044484772 34368
0.055432462 68672 0.029634184 68672 0.056737306
68700 0.119099554 68700 0.073232674 34400 0.066932043 34372
0.024233661 68680 0.065355162 68680 0.017543038
68700 0.059709608 68700 0.13417736 34400 0.076539349 34376
0.020945974 68688 0.091237307 68688 0.028045595
68700 0.084180167 68700 0.173480381 34400 0.035933648 34380
0.018103709 68696 0.052672196 68696 0.035804551
68700 0.083189239 68700 0.112129324 34400 0.054554865 34384
0.036831978 68704 0.041693817 68704 0.041943968
68700 0.057453985 68700 0.05061811 34400 0.049357674 34388
0.04188688 68712 0.126729646 68712 0.024021476
68700 0.059809652 68700 0.095787007 34400 0.021651034 34392
0.047657879 68720 0.110129586 68720 0.01662336
68700 0.240290058 68700 0.128926084 34400 0.035337111 34396
0.02800865 68728 0.020212323 68728 0.020005369
68700 0.396494986 68700 0.129799708 34400 0.043254655 34400
0.007944253 68736 0.057962476 68736 0.022577526
68700 0.315123558 68700 0.068844136 34400 0.039331917 34404
0.047392503 68744 0.038759477 68744 0.019421877
68800 0.44680867 68800 0.034369907 34400 0.05399041 34408
0.072502597 68752 0.065082735 68752 0.028035629
68800 0.604356115 68800 0.043786971 34400 0.052997682 34412
0.047118385 68760 0.068468929 68760 0.042085798
68800 0.462420081 68800 0.030463849 34400 0.067920693 34416
0.033039316 68768 0.080719867 68768 0.022508079
68800 0.215204418 68800 0.119246637 34400 0.058664926 34420
0.017990917 68776 0.106837462 68776 0.015795797
68800 0.097961194 68800 0.097788245 34400 0.020304284 34424
0.028299519 68784 0.09702368 68784 0.039904753
68800 0.127697495 68800 0.052514468 34400 0.010142899 34428
0.053233995 68792 0.057109475 68792 0.047724672
68800 0.09503225 68800 0.104806524 34400 0.013750278 34432
0.055691497 68800 0.086243395 68800 0.024059695
68800 0.068104841 68800 0.081752776 34400 0.01638834 34436
0.072847281 68808 0.142702134 68808 0.006925057
68800 0.122594458 68800 0.058835278 34400 0.019892807 34440
0.058477086 68816 0.172053041 68816 0.012447993
68800 0.104127124 68800 0.099536861 34400 0.049506103 34444
0.043693264 68824 0.156069218 68824 0.028249317
68800 0.048879338 68800 0.144363978 34400 0.07461707 34448
0.05033179 68832 0.085640655 68832 0.047006608
68800 0.099908073 68800 0.133790716 34500 0.075517935 34452
0.043018077 68840 0.033285447 68840 0.050353214
68800 0.084765918 68800 0.136876698 34500 0.042947664 34456

0.077184639 68848 0.047420359 68848 0.02509703
68900 0.057602956 68900 0.220303904 34500 0.027776672 34460
0.063470732 68856 0.0358166 68856 0.018802295
68900 0.040210252 68900 0.217392604 34500 0.085566506 34464
0.043474436 68864 0.111499408 68864 0.033457043
68900 0.030832493 68900 0.137799973 34500 0.090928334 34468
0.051670457 68872 0.139870958 68872 0.021844475
68900 0.054411576 68900 0.12707054 34500 0.061164166 34472
0.06302759 68880 0.072135721 68880 0.029380348
68900 0.044674307 68900 0.141944344 34500 0.059202965 34476
0.07742412 68888 0.023154276 68888 0.0597894
68900 0.038082042 68900 0.044504886 34500 0.079621248 34480
0.083943596 68896 0.034464662 68896 0.077158009
68900 0.084040956 68900 0.06213921 34500 0.060927643 34484
0.078926802 68904 0.032323133 68904 0.054679549
68900 0.022942288 68900 0.070232833 34500 0.030251929 34488
0.081919236 68912 0.046244673 68912 0.017007087
68900 0.112284884 68900 0.011056301 34500 0.01088739 34492
0.077749362 68920 0.006018552 68920 0.017441424
68900 0.122728277 68900 0.100750876 34500 0.020946147 34496
0.053880802 68928 0.044377146 68928 0.039518389
68900 0.082461877 68900 0.113092494 34500 0.050404353 34500
0.055617667 68936 0.005673076 68936 0.044515757
68900 0.072847215 68900 0.001403654 34500 0.074495205 34504
0.017732753 68944 0.04466383 68944 0.037577342
69000 0.082565784 69000 0.072093535 34500 0.057406138 34508
0.076410921 68952 0.049849536 68952 0.033948563
69000 0.113023991 69000 0.018871611 34500 0.027367865 34512
0.090481335 68960 0.09387303 68960 0.034649478
69000 0.092615846 69000 0.094759729 34500 0.032172568 34516
0.047749545 68968 0.110054643 68968 0.031176271
69000 0.026379465 69000 0.110633329 34500 0.034515862 34520
0.042444182 68976 0.07159041 68976 0.019243242
69000 0.151560438 69000 0.012900382 34500 0.031677741 34524
0.029671464 68984 0.069876282 68984 0.047756446
69000 0.164671874 69000 0.060413382 34500 0.034089957 34528
0.004292077 68992 0.098727833 68992 0.084302737
69000 0.067360939 69000 0.091927584 34500 0.054737335 34532
0.038202943 69000 0.099137389 69000 0.075250049
69000 0.069309986 69000 0.096145777 34500 0.07526749 34536
0.076971999 69008 0.12429709 69008 0.018101957
69000 0.093154951 69000 0.041559833 34500 0.081118917 34540
0.101358477 69016 0.167298771 69016 0.038675535
69000 0.085890824 69000 0.048853803 34500 0.032113167 34544
0.088036817 69024 0.167814345 69024 0.036739868
69000 0.103966042 69000 0.03839819 34500 0.049692782 34548
0.032561722 69032 0.146042876 69032 0.01507646
69000 0.033464909 69000 0.063042768 34600 0.104167666 34552
0.035727822 69040 0.152250039 69040 0.035786819
69000 0.108324166 69000 0.051079911 34600 0.092926137 34556
0.077171135 69048 0.094342155 69048 0.048473368
69100 0.189489773 69100 0.03469341 34600 0.037426078 34560
0.084212414 69056 0.072195464 69056 0.051375391
69100 0.164781246 69100 0.05234209 34600 0.005721624 34564
0.073757728 69064 0.213397841 69064 0.064169872
69100 0.085435466 69100 0.061485902 34600 0.00322813 34568
0.06818556 69072 0.230591803 69072 0.076278768
69100 0.01817348 69100 0.088516405 34600 0.043594631 34572
0.040213487 69080 0.17194188 69080 0.053776443
69100 0.095290248 69100 0.091792004 34600 0.07502496 34576
0.084411659 69088 0.109696506 69088 0.026861053
69100 0.07493094 69100 0.055896853 34600 0.082979073 34580
0.135301481 69096 0.083567291 69096 0.031895859
69100 0.049899238 69100 0.011483124 34600 0.078836718 34584
0.125131861 69104 0.140875505 69104 0.026467251
69100 0.138205913 69100 0.076403863 34600 0.068926216 34588
0.113621056 69112 0.139334065 69112 0.017060627
69100 0.113059527 69100 0.090879657 34600 0.044325865 34592

0.109941473 69120 0.028581166 69120 0.01558584
69100 0.02597196 69100 0.024080609 34600 0.012668415 34596
0.070075337 69128 0.086698652 69128 0.028685498
69100 0.037616988 69100 0.030962823 34600 0.022606553 34600
0.06585518 69136 0.11490292 69136 0.066910012
69100 0.014123732 69100 0.039400155 34600 0.054612709 34604
0.089372967 69144 0.068490699 69144 0.087161767
69200 0.073189054 69200 0.075724507 34600 0.082887738 34608
0.099923906 69152 0.079017875 69152 0.059904818
69200 0.060628794 69200 0.138448115 34600 0.079778743 34612
0.120207071 69160 0.119477845 69160 0.017592263
69200 0.02610608 69200 0.199633636 34600 0.059721682 34616
0.111350819 69168 0.111558038 69168 0.010854288
69200 0.017969831 69200 0.230105986 34600 0.045549343 34620
0.072307557 69176 0.090666275 69176 0.033771325
69200 0.049296894 69200 0.221370486 34600 0.047199017 34624
0.040871164 69184 0.047326677 69184 0.035762463
69200 0.111940433 69200 0.155937553 34600 0.039313429 34628
0.068368718 69192 0.043507935 69192 0.029232389
69200 0.126194456 69200 0.05822274 34600 0.017015078 34632
0.130765839 69200 0.052616248 69200 0.04084863
69200 0.037857815 69200 0.022294253 34600 0.008581304 34636
0.147768748 69208 0.115741634 69208 0.070516879
69200 0.073083458 69200 0.007826039 34600 0.007718704 34640
0.094755684 69216 0.1839998 69216 0.088114539
69200 0.087234534 69200 0.06985345 34600 0.032838812 34644
0.057606285 69224 0.204494281 69224 0.077100565
69200 0.041810159 69200 0.11931749 34600 0.069563728 34648
0.111732283 69232 0.185373806 69232 0.048876191
69200 0.111405883 69200 0.128021639 34700 0.064581662 34652
0.13390019 69240 0.15480895 69240 0.025728192
69200 0.175216323 69200 0.09806201 34700 0.033001274 34656
0.112373724 69248 0.107376072 69248 0.012124436
69300 0.192615291 69300 0.087132263 34700 0.015880441 34660
0.086001557 69256 0.020324744 69256 0.021035707
69300 0.146437873 69300 0.079684854 34700 0.039177361 34664
0.063470536 69264 0.064992993 69264 0.032863463
69300 0.112405585 69300 0.066199609 34700 0.056824145 34668
0.065747285 69272 0.112332949 69272 0.05117245
69300 0.170527783 69300 0.146335806 34700 0.073571297 34672
0.088487119 69280 0.128256011 69280 0.033431588
69300 0.201422649 69300 0.179627489 34700 0.061170191 34676
0.050676368 69288 0.127897874 69288 0.030755142
69300 0.178561008 69300 0.147947954 34700 0.013855225 34680
0.028441862 69296 0.053924032 69296 0.053165721
69300 0.129113876 69300 0.084862593 34700 0.063947962 34684
0.084121275 69304 0.049088809 69304 0.031224518
69300 0.093747309 69300 0.034140514 34700 0.101409867 34688
0.10516253 69312 0.05662968 69312 0.022651509
69300 0.050892213 69300 0.075262309 34700 0.103073529 34692
0.041505409 69320 0.021526295 69320 0.03092676
69300 0.052632709 69300 0.175779264 34700 0.073372641 34696
0.043686796 69328 0.069364382 69328 0.023165536
69300 0.043465076 69300 0.17418548 34700 0.036302266 34700
0.079007514 69336 0.111314745 69336 0.053190543
69300 0.076807432 69300 0.086403161 34700 0.02192019 34704
0.05733213 69344 0.113736576 69344 0.054171484
69400 0.080443286 69400 0.134941336 34700 0.029980447 34708
0.052211286 69352 0.100142628 69352 0.027637414
69400 0.051557559 69400 0.067737259 34700 0.04401679 34712
0.069682668 69360 0.0711959 69360 0.041724834
69400 0.131498076 69400 0.048434718 34700 0.051949079 34716
0.084141015 69368 0.046734611 69368 0.028933209
69400 0.228022575 69400 0.091732218 34700 0.054238004 34720
0.080386337 69376 0.078008918 69376 0.012072823
69400 0.229504324 69400 0.123817692 34700 0.104413688 34724
0.053010277 69384 0.099922218 69384 0.046662921
69400 0.146062011 69400 0.131676031 34700 0.127451174 34728

0.081199221 69392 0.118429554 69392 0.097255055
69400 0.061592509 69400 0.086424632 34700 0.089894471 34732
0.104198472 69400 0.135157636 69400 0.104466155
69400 0.083484003 69400 0.007032567 34700 0.037560811 34736
0.13712795 69408 0.097106859 69408 0.050844996
69400 0.072215466 69400 0.038492042 34700 0.01168568 34740
0.120970697 69416 0.087470748 69416 0.028686243
69400 0.030484802 69400 0.03347309 34700 0.034681882 34744
0.017588047 69424 0.112575137 69424 0.036252292
69400 0.057743826 69400 0.004317496 34700 0.042895732 34748
0.071492461 69432 0.113822614 69432 0.017355033
69400 0.072547126 69400 0.026351665 34800 0.029019901 34752
0.085666827 69440 0.107498468 69440 0.06159742
69400 0.024273439 69400 0.013401295 34800 0.032967433 34756
0.089627894 69448 0.073860589 69448 0.067445384
69500 0.069509959 69500 0.086125321 34800 0.036141846 34760
0.138800243 69456 0.039082348 69456 0.053494339
69500 0.086137196 69500 0.139096781 34800 0.041752646 34764
0.145801183 69464 0.043442196 69464 0.038398244
69500 0.061813247 69500 0.145062164 34800 0.043433305 34768
0.117017851 69472 0.067990266 69472 0.02408103
69500 0.068181849 69500 0.113065573 34800 0.046297326 34772
0.113800183 69480 0.103402672 69480 0.018392713
69500 0.010812892 69500 0.083203566 34800 0.050600622 34776
0.119495227 69488 0.065268177 69488 0.014798632
69500 0.097887838 69500 0.085197891 34800 0.051237563 34780
0.084076186 69496 0.033611286 69496 0.03140554
69500 0.151243833 69500 0.076589822 34800 0.041288873 34784
0.022920833 69504 0.076627679 69504 0.049319955
69500 0.138491073 69500 0.097907665 34800 0.031882923 34788
0.047934431 69512 0.052849126 69512 0.046736939
69500 0.09024921 69500 0.157539209 34800 0.0144523 34792
0.076547003 69520 0.050161852 69520 0.039057715
69500 0.063369829 69500 0.15107468 34800 0.012387053 34796
0.06426069 69528 0.16449565 69528 0.051726296
69500 0.111066736 69500 0.076097756 34800 0.018681454 34800
0.019956935 69536 0.28436046 69536 0.042167849
69500 0.17194559 69500 0.040985055 34800 0.037371392 34804
0.045483033 69544 0.285356771 69544 0.010192692
69600 0.1560893 69600 0.017857208 34800 0.03870629 34808
0.097273434 69552 0.223195414 69552 0.045450153
69600 0.089191191 69600 0.065224514 34800 0.016579863 34812
0.078013531 69560 0.16680028 69560 0.062337538
69600 0.07509724 69600 0.063232612 34800 0.028874809 34816
0.026504562 69568 0.101397382 69568 0.053842843
69600 0.120734112 69600 0.05563489 34800 0.062322739 34820
0.096491953 69576 0.052633903 69576 0.055365625
69600 0.13908789 69600 0.034094541 34800 0.083090337 34824
0.113068782 69584 0.024919385 69584 0.017533832
69600 0.115257259 69600 0.05422609 34800 0.10658399 34828
0.092942675 69592 0.085955435 69592 0.031792439
69600 0.076986165 69600 0.03465468 34800 0.09491 34832
0.063587409 69600 0.104075676 69600 0.045837431
69600 0.070134687 69600 0.079387915 34800 0.027617423 34836
0.066700086 69608 0.090204405 69608 0.04865952
69600 0.083442297 69600 0.117631789 34800 0.065902743 34840
0.091335729 69616 0.15961047 69616 0.056254252
69600 0.11654124 69600 0.145070764 34800 0.073063049 34844
0.096426244 69624 0.217284324 69624 0.060870229
69600 0.175109395 69600 0.107626314 34800 0.05772576 34848
0.036387548 69632 0.220723145 69632 0.045875815
69600 0.126276049 69600 0.112755886 34900 0.021475109 34852
0.080717538 69640 0.128992266 69640 0.0236219
69600 0.065094231 69600 0.121333018 34900 0.028040151 34856
0.114051589 69648 0.011659934 69648 0.038879556
69700 0.028637289 69700 0.090538699 34900 0.024169036 34860
0.086764252 69656 0.102347163 69656 0.077247889
69700 0.089773945 69700 0.045600024 34900 0.012304087 34864

0.070017995 69664 0.079705627 69664 0.062142302
69700 0.117806034 69700 0.034595611 34900 0.02169746 34868
0.047110836 69672 0.014253949 69672 0.025347277
69700 0.101046848 69700 0.10638903 34900 0.01848586 34872
0.033560275 69680 0.082200575 69680 0.028632705
69700 0.072077055 69700 0.148499966 34900 0.056912333 34876
0.045044249 69688 0.08584655 69688 0.02885631
69700 0.07205861 69700 0.103658698 34900 0.0915087 34880
0.055518707 69696 0.036012887 69696 0.014253431
69700 0.053712789 69700 0.069540212 34900 0.087899352 34884
0.064976877 69704 0.014327393 69704 0.029936093
69700 0.048131908 69700 0.100466852 34900 0.037420086 34888
0.063256928 69712 0.030343806 69712 0.001607476
69700 0.124923885 69700 0.122516532 34900 0.030149568 34892
0.063487292 69720 0.03453239 69720 0.028631204
69700 0.138935488 69700 0.071724957 34900 0.044494285 34896
0.041544794 69728 0.020996135 69728 0.027272623
69700 0.101806916 69700 0.106178086 34900 0.02260827 34900
0.037139114 69736 0.043760207 69736 0.00443844
69700 0.046577792 69700 0.134255766 34900 0.062578307 34904
0.099809222 69744 0.04152552 69744 0.036674512
69800 0.068341818 69800 0.082296407 34900 0.056857818 34908
0.103598337 69752 0.017863502 69752 0.032861473
69800 0.057841666 69800 0.033033233 34900 0.030289668 34912
0.038308241 69760 0.022150754 69760 0.033567238
69800 0.029582057 69800 0.056887871 34900 0.033554305 34916
0.030525596 69768 0.055283741 69768 0.062534913
69800 0.027219219 69800 0.095268748 34900 0.017581953 34920
0.065046108 69776 0.062905143 69776 0.050316568
69800 0.045406396 69800 0.066249384 34900 0.047775397 34924
0.080870836 69784 0.06955619 69784 0.026461683
69800 0.04790035 69800 0.018786144 34900 0.045959609 34928
0.05820868 69792 0.049323506 69792 0.022320886
69800 0.029181396 69800 0.050777784 34900 0.025961228 34932
0.021411028 69800 0.043458796 69800 0.023732617
69800 0.027996286 69800 0.048747748 34900 0.021230511 34936
0.018033625 69808 0.12424271 69808 0.002813125
69800 0.073539188 69800 0.086289896 34900 0.05299136 34940
0.03153467 69816 0.224564094 69816 0.042740685
69800 0.121887933 69800 0.138656876 34900 0.057288416 34944
0.057626447 69824 0.270572404 69824 0.05304932
69800 0.14628256 69800 0.063268512 34900 0.03295701 34948
0.082266568 69832 0.161005723 69832 0.032460241
69800 0.122264697 69800 0.063354637 35000 0.006898963 34952
0.055741035 69840 0.025625106 69840 0.019213569
69800 0.116031974 69800 0.121462486 35000 0.018951152 34956
0.044562883 69848 0.140937351 69848 0.011017472
69900 0.13214843 69900 0.10910078 35000 0.019408597 34960
0.088980924 69856 0.145156883 69856 0.015307953
69900 0.091141563 69900 0.082829334 35000 0.013469473 34964
0.083572231 69864 0.111708039 69864 0.012664124
69900 0.051149378 69900 0.086266628 35000 0.05152297 34968
0.062914216 69872 0.055265438 69872 0.015207191
69900 0.106780943 69900 0.137068113 35000 0.07189429 34972
0.043021431 69880 0.077651042 69880 0.010254711
69900 0.100834273 69900 0.130157918 35000 0.062542291 34976
0.070651215 69888 0.15491643 69888 0.005549614
69900 0.08420719 69900 0.099819459 35000 0.041135805 34980
0.082894687 69896 0.104971812 69896 0.007878904
69900 0.089249727 69900 0.115938259 35000 0.032621698 34984
0.040748273 69904 0.063500527 69904 0.033603144
69900 0.108359571 69900 0.064728985 35000 0.048117388 34988
0.043424894 69912 0.118058721 69912 0.066760265
69900 0.14371326 69900 0.05847069 35000 0.056921421 34992
0.060061826 69920 0.046922251 69920 0.07166584
69900 0.150263878 69900 0.072258074 35000 0.055361954 34996
0.023984054 69928 0.037718419 69928 0.024354982
69900 0.112400754 69900 0.065029315 35000 0.072691903 35000

0.057256606 69936 0.063335378 69936 0.038019301
69900 0.028577459 69900 0.085492669 35000 0.074717165 35004
0.091612776 69944 0.065745357 69944 0.045882709
70000 0.136445393 70000 0.134804548 35000 0.052926211 35008
0.049337599 69952 0.110839035 69952 0.060858572
70000 0.15413409 70000 0.14263166 35000 0.039206836 35012
0.013002143 69960 0.156019698 69960 0.049537812
70000 0.069030102 70000 0.12820118 35000 0.018620809 35016
0.046129728 69968 0.141090278 69968 0.029019378
70000 0.054446104 70000 0.070874587 35000 0.035315457 35020
0.066411769 69976 0.096940625 69976 0.014029007
70000 0.089777204 70000 0.005704079 35000 0.08284548 35024
0.058851034 69984 0.03813201 69984 0.021964492
70000 0.111637179 70000 0.064686152 35000 0.100534693 35028
0.075640412 69992 0.095577125 69992 0.034869507
70000 0.101030382 70000 0.148990671 35000 0.055779572 35032
0.066723042 70000 0.098111508 70000 0.036804046
70000 0.142603313 70000 0.154961861 35000 0.027694061 35036
0.056580531 70008 0.045777117 70008 0.039052036
70000 0.162692435 70000 0.059677375 35000 0.052693788 35040
0.074680815 70016 0.10107486 70016 0.043021792
70000 0.092707545 70000 0.01810201 35000 0.061721985 35044
0.07481371 70024 0.079267069 70024 0.052545973
70000 0.052332329 70000 0.127282401 35000 0.066799832 35048
0.085066989 70032 0.084119092 70032 0.061207917
70000 0.034248376 70000 0.172298009 35100 0.076774551 35052
0.067166067 70040 0.090466499 70040 0.042141986
70000 0.164828118 70000 0.105963343 35100 0.084542655 35056
0.024596593 70048 0.129229724 70048 0.03590584
70100 0.161154646 70100 0.139351818 35100 0.079664424 35060
0.047785037 70056 0.189905274 70056 0.03522071
70100 0.065730717 70100 0.160559895 35100 0.069673042 35064
0.100645841 70064 0.129355889 70064 0.027701088
70100 0.096564276 70100 0.072186726 35100 0.061372164 35068
0.117803975 70072 0.052738687 70072 0.029918972
70100 0.082542414 70100 0.131671346 35100 0.053031385 35072
0.081500242 70080 0.092914903 70080 0.028737919
70100 0.094152769 70100 0.232083519 35100 0.037065704 35076
0.055506982 70088 0.098614866 70088 0.01965579
70100 0.158110925 70100 0.190633699 35100 0.034438832 35080
0.065193126 70096 0.132200861 70096 0.024137553
70100 0.171833541 70100 0.080409925 35100 0.035732141 35084
0.058233851 70104 0.130082146 70104 0.022371012
70100 0.094768198 70100 0.083606574 35100 0.03210142 35088
0.003346296 70112 0.087026958 70112 0.010620566
70100 0.058206453 70100 0.09272332 35100 0.014734307 35092
0.06720455 70120 0.06563207 70120 0.017068234
70100 0.089288289 70100 0.010514326 35100 0.009393581 35096
0.070136164 70128 0.101286081 70128 0.035076882
70100 0.092105627 70100 0.036156282 35100 0.027016902 35100
0.063113454 70136 0.139913245 70136 0.043290969
70100 0.087137036 70100 0.048498274 35100 0.035709272 35104
0.072234929 70144 0.11143797 70144 0.054670578
70200 0.052710897 70200 0.050089657 35100 0.030737789 35108
0.050737377 70152 0.042089498 70152 0.053381689
70200 0.111514099 70200 0.091671907 35100 0.054817792 35112
0.002386258 70160 0.028532431 70160 0.037085483
70200 0.134560353 70200 0.067137589 35100 0.039238843 35116
0.077939847 70168 0.092789953 70168 0.02584464
70200 0.057870992 70200 0.030873052 35100 0.038680551 35120
0.136840259 70176 0.121416131 70176 0.011628397
70200 0.115337723 70200 0.049347003 35100 0.082167964 35124
0.086865664 70184 0.088335663 70184 0.021281376
70200 0.232576131 70200 0.119343815 35100 0.087588815 35128
0.036012152 70192 0.036717182 70192 0.048832088
70200 0.249534816 70200 0.119995937 35100 0.061641411 35132
0.058381156 70200 0.054766406 70200 0.049526399
70200 0.145486324 70200 0.111125904 35100 0.033118176 35136

0.078487952 70208 0.109335146 70208 0.010833844
70200 0.028735285 70200 0.09653598 35100 0.007351193 35140
0.097446871 70216 0.0631117 70216 0.023696681
70200 0.043152908 70200 0.090755195 35100 0.033249973 35144
0.072497853 70224 0.038307841 70224 0.006311137
70200 0.038806182 70200 0.134510265 35100 0.071808165 35148
0.022831451 70232 0.124079117 70232 0.040686053
70200 0.022310323 70200 0.148153603 35200 0.060431663 35152
0.062046449 70240 0.157981748 70240 0.054493565
70200 0.072928946 70200 0.119079952 35200 0.058153368 35156
0.118238895 70248 0.119592958 70248 0.033335244
70300 0.108092201 70300 0.062700688 35200 0.040598665 35160
0.114512761 70256 0.097215328 70256 0.033491146
70300 0.048695521 70300 0.044665798 35200 0.007991053 35164
0.084258121 70264 0.064769709 70264 0.038786693
70300 0.041374406 70300 0.026689173 35200 0.032222473 35168
0.058208916 70272 0.012981845 70272 0.037741203
70300 0.013610278 70300 0.118143777 35200 0.045704092 35172
0.069042333 70280 0.057742262 70280 0.027574775
70300 0.131056834 70300 0.084017687 35200 0.024621259 35176
0.064999171 70288 0.0917824 70288 0.023922243
70300 0.178587856 70300 0.080837534 35200 0.033256834 35180
0.070947353 70296 0.110150417 70296 0.033893342
70300 0.117297175 70300 0.068879417 35200 0.037837344 35184
0.114105096 70304 0.063164276 70304 0.030641113
70300 0.025168964 70300 0.041860953 35200 0.05051526 35188
0.12921993 70312 0.049234761 70312 0.020479733
70300 0.031328727 70300 0.08008429 35200 0.065645792 35192
0.117537595 70320 0.037204212 70320 0.018349963
70300 0.031427371 70300 0.035567206 35200 0.039563121 35196
0.095700656 70328 0.047072765 70328 0.024877241
70300 0.074770018 70300 0.012227882 35200 0.010510775 35200
0.070358903 70336 0.021165997 70336 0.037408987
70300 0.072029223 70300 0.020769245 35200 0.000545281 35204
0.059559599 70344 0.067875 70344 0.043606739
70400 0.035507619 70400 0.01792345 35200 0.011437777 35208
0.093054594 70352 0.108177839 70352 0.037661106
70400 0.047054808 70400 0.074788368 35200 0.025494601 35212
0.108714514 70360 0.062071631 70360 0.028337516
70400 0.049452712 70400 0.102229213 35200 0.046336892 35216
0.077766053 70368 0.045945279 70368 0.01674632
70400 0.029294442 70400 0.051215848 35200 0.065358931 35220
0.054486984 70376 0.129367444 70376 0.014998798
70400 0.110464105 70400 0.084044921 35200 0.054589978 35224
0.051023355 70384 0.175838868 70384 0.046774068
70400 0.124652361 70400 0.138263596 35200 0.051761854 35228
0.046975212 70392 0.129512003 70392 0.078341989
70400 0.014105445 70400 0.061816034 35200 0.0419713 35232
0.059028993 70400 0.11880324 70400 0.053563123
70400 0.132647634 70400 0.079739104 35200 0.040563598 35236
0.050125316 70408 0.139298718 70408 0.006496031
70400 0.14886698 70400 0.089749927 35200 0.039796065 35240
0.048116257 70416 0.113249618 70416 0.006647925
70400 0.071028066 70400 0.060101567 35200 0.059928498 35244
0.084325511 70424 0.052960953 70424 0.022645165
70400 0.120180557 70400 0.059041853 35200 0.080238162 35248
0.089557536 70432 0.042815787 70432 0.02126127
70400 0.080909951 70400 0.041553092 35300 0.065474662 35252
0.072308365 70440 0.083943152 70440 0.013423182
70400 0.071525748 70400 0.010182488 35300 0.066744724 35256
0.064404718 70448 0.108331682 70448 0.029203584
70500 0.050944858 70500 0.028308281 35300 0.095243951 35260
0.08287657 70456 0.088884983 70456 0.039957333
70500 0.062274492 70500 0.022605147 35300 0.10378335 35264
0.08819138 70464 0.036773829 70464 0.051109109
70500 0.106162406 70500 0.085780703 35300 0.074362702 35268
0.057151585 70472 0.021088701 70472 0.024500125
70500 0.157052608 70500 0.11347777 35300 0.039187646 35272

0.037193422	70480	0.038588682	70480	0.068856396		
70500	0.119762961	70500	0.103188773	35300	0.007079477	35276
0.072855008	70488	0.131290639	70488	0.093082897		
70500	0.043910568	70500	0.166295373	35300	0.03306181	35280
0.073628209	70496	0.202144613	70496	0.074958196		
70500	0.103211649	70500	0.145693775	35300	0.055167981	35284
0.022924472	70504	0.213622479	70504	0.037667935		
70500	0.113904913	70500	0.069925947	35300	0.039190938	35288
0.044197954	70512	0.158262846	70512	0.010305134		
70500	0.006599627	70500	0.068077075	35300	0.027578717	35292
0.070530878	70520	0.096299074	70520	0.029599645		
70500	0.089846268	70500	0.070015572	35300	0.066308545	35296
0.066715154	70528	0.066917513	70528	0.017573062		
70500	0.029216153	70500	0.071708571	35300	0.052586478	35300
0.057677513	70536	0.034954755	70536	0.013616989		
70500	0.084305517	70500	0.116444651	35300	0.037401096	35304
0.073008814	70544	0.043541444	70544	0.035999023		
70600	0.109130546	70600	0.106822525	35300	0.011937639	35308
0.088108674	70552	0.017835095	70552	0.044894878		
70600	0.067199413	70600	0.079467056	35300	0.045548979	35312
0.0461524	70560	0.095932082	70560	0.024360961		
70600	0.0111747	70600	0.066341214	35300	0.067272289	35316
0.034918889	70568	0.119328732	70568	0.034981407		
70600	0.095353382	70600	0.040550578	35300	0.027631737	35320
0.035452285	70576	0.074394498	70576	0.037775459		
70600	0.126982675	70600	0.052445525	35300	0.022158287	35324
0.038736031	70584	0.132868489	70584	0.032738128		
70600	0.11744714	70600	0.033058579	35300	0.011309071	35328
0.111911439	70592	0.155025511	70592	0.023616711		
70600	0.135590831	70600	0.113254668	35300	0.026486992	35332
0.100269783	70600	0.1015405	70600	0.048802409		
70600	0.131450143	70600	0.15462104	35300	0.048846327	35336
0.076449083	70608	0.05440896	70608	0.086374494		
70600	0.054469016	70600	0.154854235	35300	0.060932234	35340
0.11519924	70616	0.02827927	70616	0.088370798		
70600	0.114180148	70600	0.098072909	35300	0.05764233	35344
0.131857844	70624	0.057371995	70624	0.057924386		
70600	0.20932834	70600	0.055657918	35300	0.0446382	35348
0.083964755	70632	0.033741606	70632	0.028537559		
70600	0.184367833	70600	0.125892446	35400	0.042527692	35352
0.031846372	70640	0.052045642	70640	0.023398139		
70600	0.043644657	70600	0.092662289	35400	0.029287525	35356
0.027709359	70648	0.067071072	70648	0.056498684		
70700	0.088582463	70700	0.012240539	35400	0.014818397	35360
0.028015376	70656	0.030734726	70656	0.075427692		
70700	0.127529013	70700	0.058224654	35400	0.0190219	35364
0.017749628	70664	0.075471711	70664	0.058952926		
70700	0.124593062	70700	0.09578951	35400	0.043511685	35368
0.053542448	70672	0.169118808	70672	0.02150394		
70700	0.083879044	70700	0.074559772	35400	0.075591037	35372
0.082071594	70680	0.209178543	70680	0.052641091		
70700	0.07863433	70700	0.012368117	35400	0.090362213	35376
0.077498422	70688	0.132473958	70688	0.070421069		
70700	0.118751552	70700	0.001803515	35400	0.069779504	35380
0.074081516	70696	0.068739588	70696	0.047488713		
70700	0.142637408	70700	0.04785477	35400	0.012090435	35384
0.060486127	70704	0.066399851	70704	0.023004121		
70700	0.121563695	70700	0.046495723	35400	0.013034423	35388
0.066224151	70712	0.025050818	70712	0.022034694		
70700	0.047987509	70700	0.060843453	35400	0.027742586	35392
0.080319471	70720	0.074361167	70720	0.016482827		
70700	0.023377968	70700	0.084125517	35400	0.027902865	35396
0.095757285	70728	0.10832792	70728	0.038019753		
70700	0.069654794	70700	0.104087485	35400	0.027167491	35400
0.094475319	70736	0.143030964	70736	0.04434539		
70700	0.055860481	70700	0.106117819	35400	0.048757906	35404
0.058408474	70744	0.055713252	70744	0.020974885		
70800	0.068380337	70800	0.067756068	35400	0.018616325	35408

0.043524287 70752 0.102356666 70752 0.010288853
70800 0.113924987 70800 0.066929672 35400 0.018223074 35412
0.034446483 70760 0.14051031 70760 0.024445682
70800 0.098910146 70800 0.077885939 35400 0.015536351 35416
0.032422147 70768 0.133571244 70768 0.054249264
70800 0.089686517 70800 0.093150586 35400 0.047385624 35420
0.025030184 70776 0.103251849 70776 0.059133057
70800 0.094896117 70800 0.12624485 35400 0.043230546 35424
0.054851949 70784 0.012958494 70784 0.05134365
70800 0.148797219 70800 0.165307501 35400 0.013756669 35428
0.065856431 70792 0.070090289 70792 0.048091944
70800 0.227897108 70800 0.209052989 35400 0.007916476 35432
0.067109191 70800 0.0991177 70800 0.023980785
70800 0.213863735 70800 0.236341206 35400 0.02322081 35436
0.049870883 70808 0.107883912 70808 0.006181658
70800 0.124774946 70800 0.188024904 35400 0.01267449 35440
0.057119327 70816 0.11830443 70816 0.012735813
70800 0.047696431 70800 0.084700565 35400 0.00928341 35444
0.064595057 70824 0.125478939 70824 0.019461277
70800 0.097287062 70800 0.072581177 35400 0.017536142 35448
0.095382951 70832 0.101306359 70832 0.023389875
70800 0.090773356 70800 0.049667138 35500 0.059145521 35452
0.132522356 70840 0.046850597 70840 0.007666762
70800 0.025596659 70800 0.081175786 35500 0.065355765 35456
0.135261129 70848 0.085311425 70848 0.021802427
70900 0.068338362 70900 0.108659915 35500 0.054259705 35460
0.084120919 70856 0.205872566 70856 0.05694334
70900 0.049025359 70900 0.114501156 35500 0.046808338 35464
0.031645705 70864 0.185759724 70864 0.050854178
70900 0.121187259 70900 0.098095276 35500 0.014092712 35468
0.057431746 70872 0.073225761 70872 0.025428435
70900 0.134945513 70900 0.05821131 35500 0.020814201 35472
0.088342269 70880 0.063565101 70880 0.013203458
70900 0.064481435 70900 0.145185419 35500 0.026224974 35476
0.102668848 70888 0.074357478 70888 0.017425105
70900 0.071033988 70900 0.145156403 35500 0.063631691 35480
0.052357638 70896 0.120980294 70896 0.030984771
70900 0.047860543 70900 0.046479414 35500 0.078301295 35484
0.04045566 70904 0.182022123 70904 0.048137394
70900 0.0549164 70900 0.032259479 35500 0.062566316 35488
0.108884146 70912 0.178459871 70912 0.036890651
70900 0.038490965 70900 0.039797931 35500 0.034063905 35492
0.1301757 70920 0.112855007 70920 0.021265418
70900 0.037776124 70900 0.021967211 35500 0.008962546 35496
0.098413351 70928 0.090563626 70928 0.023819812
70900 0.088604305 70900 0.029202736 35500 0.010694099 35500
0.081958613 70936 0.091601789 70936 0.02927443
70900 0.140260149 70900 0.070252238 35500 0.013754419 35504
0.09593997 70944 0.09107915 70944 0.010980043
71000 0.112427908 71000 0.044398577 35500 0.032522436 35508
0.107915701 70952 0.050968272 70952 0.017470327
71000 0.025268995 71000 0.0334978 35500 0.044315384 35512
0.102104634 70960 0.092793365 70960 0.031536536
71000 0.107256521 71000 0.055818913 35500 0.046818441 35516
0.068570233 70968 0.129918116 70968 0.042048847
71000 0.105626976 71000 0.070008086 35500 0.04540227 35520
0.067623987 70976 0.062444335 70976 0.025994405
71000 0.096535514 71000 0.083891144 35500 0.039060469 35524
0.071735754 70984 0.02906894 70984 0.011268279
71000 0.139274576 71000 0.15611347 35500 0.028159258 35528
0.050988692 70992 0.044911001 70992 0.03593936
71000 0.129596229 71000 0.214797095 35500 0.029267712 35532
0.039111379 71000 0.018944844 71000 0.065713204
71000 0.151098866 71000 0.218918285 35500 0.020537364 35536
0.03985002 71008 0.113861686 71008 0.047880785
71000 0.128823595 71000 0.168673738 35500 0.035522731 35540
0.032015578 71016 0.167451595 71016 0.017386306
71000 0.086016087 71000 0.075062351 35500 0.044525816 35544

0.061580257 71024 0.131280205 71024 0.004809572
71000 0.187753744 71000 0.035720568 35500 0.043287826 35548
0.056786477 71032 0.035232111 71032 0.029096218
71000 0.201982184 71000 0.120353514 35600 0.05609556 35552
0.042017538 71040 0.063443535 71040 0.04465261
71000 0.091217997 71000 0.12924755 35600 0.091721435 35556
0.038901395 71048 0.080167054 71048 0.052747113
71100 0.066471723 71100 0.048849015 35600 0.090624577 35560
0.060146875 71056 0.054168311 71056 0.045562869
71100 0.151926318 71100 0.05616865 35600 0.058048234 35564
0.085103558 71064 0.045880232 71064 0.032833283
71100 0.111492693 71100 0.158543305 35600 0.043605654 35568
0.113199509 71072 0.074546661 71072 0.021096843
71100 0.095864278 71100 0.129340013 35600 0.053353393 35572
0.111762893 71080 0.068100519 71080 0.029885117
71100 0.165397243 71100 0.050215087 35600 0.057673817 35576
0.084958396 71088 0.05263411 71088 0.04661232
71100 0.148400388 71100 0.112398804 35600 0.043212967 35580
0.092736773 71096 0.065143031 71096 0.037973714
71100 0.200431211 71100 0.037119287 35600 0.022879945 35584
0.073585994 71104 0.052743035 71104 0.02748885
71100 0.18625379 71100 0.12487841 35600 0.039047289 35588
0.036239646 71112 0.009477432 71112 0.01667463
71100 0.096190561 71100 0.179680865 35600 0.035443274 35592
0.062316576 71120 0.035050234 71120 0.028320668
71100 0.033325741 71100 0.160690659 35600 0.02114786 35596
0.074768992 71128 0.092460134 71128 0.020843614
71100 0.048823822 71100 0.154045585 35600 0.040188417 35600
0.065287088 71136 0.083021092 71136 0.024857111
71100 0.111492329 71100 0.14152372 35600 0.021184676 35604
0.057504192 71144 0.021151576 71144 0.038796334
71200 0.11274367 71200 0.104709456 35600 0.017018983 35608
0.055209053 71152 0.093232105 71152 0.049714792
71200 0.113875947 71200 0.034922014 35600 0.003265226 35612
0.044906017 71160 0.064519692 71160 0.043516142
71200 0.169335791 71200 0.082652623 35600 0.034339435 35616
0.032326065 71168 0.014968443 71168 0.031451895
71200 0.153299435 71200 0.164704906 35600 0.040935091 35620
0.007294576 71176 0.065551882 71176 0.015420934
71200 0.09807061 71200 0.139562835 35600 0.025681062 35624
0.05939059 71184 0.084722182 71184 0.015315736
71200 0.066553184 71200 0.098947407 35600 0.060183829 35628
0.115349569 71192 0.017770175 71192 0.011260473
71200 0.065362445 71200 0.11449452 35600 0.120766294 35632
0.113528709 71200 0.061022111 71200 0.050889732
71200 0.081902595 71200 0.146531209 35600 0.093327581 35636
0.041702053 71208 0.060127073 71208 0.066672052
71200 0.113063128 71200 0.104648811 35600 0.037142665 35640
0.056995879 71216 0.091086266 71216 0.015284028
71200 0.116930991 71200 0.025746342 35600 0.034605939 35644
0.120237433 71224 0.128724626 71224 0.040687031
71200 0.112625996 71200 0.049445855 35600 0.02807493 35648
0.129773078 71232 0.165687729 71232 0.03659731
71200 0.054432574 71200 0.082065591 35700 0.037089285 35652
0.088178334 71240 0.165727076 71240 0.017467462
71200 0.082745952 71200 0.079890247 35700 0.04960351 35656
0.034139022 71248 0.131005305 71248 0.012728471
71300 0.108211556 71300 0.071724484 35700 0.013974436 35660
0.04542651 71256 0.085684631 71256 0.008596107
71300 0.016904931 71300 0.058807087 35700 0.035586218 35664
0.067198977 71264 0.041280968 71264 0.024468898
71300 0.055862733 71300 0.012306818 35700 0.050744264 35668
0.051643707 71272 0.112439673 71272 0.028686076
71300 0.035306839 71300 0.078771162 35700 0.032945442 35672
0.020202559 71280 0.082552491 71280 0.030709354
71300 0.059876558 71300 0.122621015 35700 0.014521423 35676
0.049808572 71288 0.040892213 71288 0.020856354
71300 0.080224956 71300 0.094472161 35700 0.026759441 35680

0.066292661 71296 0.085520005 71296 0.012462081
71300 0.088743247 71300 0.058162168 35700 0.040346637 35684
0.078954799 71304 0.165526595 71304 0.022261986
71300 0.117720134 71300 0.083817424 35700 0.031924006 35688
0.069669339 71312 0.188041784 71312 0.010538487
71300 0.142821519 71300 0.038912509 35700 0.016570491 35692
0.030237005 71320 0.153344445 71320 0.006999598
71300 0.092815724 71300 0.082223938 35700 0.02915629 35696
0.03709647 71328 0.09733106 71328 0.036616337
71300 0.051592473 71300 0.090624402 35700 0.053736552 35700
0.038501876 71336 0.029515049 71336 0.067000103
71300 0.106675303 71300 0.080374273 35700 0.064817934 35704
0.055842909 71344 0.049370683 71344 0.060325354
71400 0.08177149 71400 0.127625404 35700 0.059580467 35708
0.059535454 71352 0.036926147 71352 0.030296891
71400 0.040156017 71400 0.15727077 35700 0.034114117 35712
0.030100287 71360 0.046308102 71360 0.006085294
71400 0.095207055 71400 0.1489641 35700 0.015366417 35716
0.062066079 71368 0.046100777 71368 0.039619554
71400 0.094859926 71400 0.078011886 35700 0.005535564 35720
0.056256446 71376 0.036733531 71376 0.041374031
71400 0.064444037 71400 0.011182894 35700 0.053794604 35724
0.004164449 71384 0.036239526 71384 0.018229903
71400 0.081802886 71400 0.110215617 35700 0.086340777 35728
0.082541184 71392 0.115167692 71392 0.023411883
71400 0.123231381 71400 0.147394035 35700 0.075393647 35732
0.108412271 71400 0.159769595 71400 0.027904907
71400 0.137717536 71400 0.02217574 35700 0.057998896 35736
0.06106669 71408 0.106082705 71408 0.032593594
71400 0.107704203 71400 0.184690508 35700 0.052971227 35740
0.005253453 71416 0.018636922 71416 0.03058173
71400 0.109151384 71400 0.194193694 35700 0.036174763 35744
0.04316483 71424 0.060047354 71424 0.01748773
71400 0.142268823 71400 0.099487072 35700 0.027876227 35748
0.07887531 71432 0.108768858 71432 0.01553322
71400 0.085655811 71400 0.047850877 35800 0.056692559 35752
0.10597278 71440 0.18010239 71440 0.04085989
71400 0.035517212 71400 0.050121795 35800 0.039082352 35756
0.097071919 71448 0.166097816 71448 0.074590273
71500 0.114819362 71500 0.054582266 35800 0.030736472 35760
0.027494596 71456 0.136620714 71456 0.073517222
71500 0.203732066 71500 0.017574921 35800 0.074770665 35764
0.099925448 71464 0.090442176 71464 0.063303574
71500 0.174132306 71500 0.036619156 35800 0.103422601 35768
0.127215055 71472 0.082769017 71472 0.082151695
71500 0.114604314 71500 0.091327151 35800 0.079534497 35772
0.081417122 71480 0.079508769 71480 0.096684686
71500 0.109330009 71500 0.126549014 35800 0.038136383 35776
0.024900981 71488 0.01125272 71488 0.085155931
71500 0.060202587 71500 0.07126623 35800 0.043566928 35780
0.033771365 71496 0.069079979 71496 0.023549063
71500 0.067271285 71500 0.084165382 35800 0.039597813 35784
0.07138394 71504 0.089210051 71504 0.052074254
71500 0.106076928 71500 0.118058175 35800 0.033058095 35788
0.088329551 71512 0.121571124 71512 0.047977177
71500 0.109547545 71500 0.136695919 35800 0.02264525 35792
0.084184954 71520 0.142999619 71520 0.023721177
71500 0.089245936 71500 0.143034209 35800 0.013942949 35796
0.083003833 71528 0.16048523 71528 0.030386176
71500 0.09825734 71500 0.105650142 35800 0.032234333 35800
0.074535885 71536 0.168517479 71536 0.019231584
71500 0.128004656 71500 0.17746634 35800 0.05620833 35804
0.055150245 71544 0.102016158 71544 0.045862969
71600 0.126434286 71600 0.203593678 35800 0.048114725 35808
0.033335276 71552 0.021414027 71552 0.063729422
71600 0.128298765 71600 0.119042852 35800 0.040610281 35812
0.048385424 71560 0.054351742 71560 0.067435933
71600 0.146610735 71600 0.031729473 35800 0.117663229 35816

0.048386879 71568 0.031081137 71568 0.065086286
71600 0.097234013 71600 0.056546738 35800 0.099781471 35820
0.051232528 71576 0.049080685 71576 0.05918858
71600 0.069056267 71600 0.056822333 35800 0.025974257 35824
0.05068467 71584 0.078301368 71584 0.032712123
71600 0.107457716 71600 0.025750935 35800 0.074164367 35828
0.070679875 71592 0.069188551 71592 0.041453099
71600 0.10039255 71600 0.060116414 35800 0.073437528 35832
0.058281919 71600 0.037370621 71600 0.056873898
71600 0.103537001 71600 0.028419741 35800 0.036518784 35836
0.091139998 71608 0.088123488 71608 0.039632483
71600 0.088132103 71600 0.112610971 35800 0.048419661 35840
0.115881514 71616 0.171618013 71616 0.020533989
71600 0.105382329 71600 0.105383013 35800 0.053885986 35844
0.131395108 71624 0.145426369 71624 0.020205689
71600 0.110968205 71600 0.029718438 35800 0.048654474 35848
0.109803368 71632 0.054709806 71632 0.009684657
71600 0.112329006 71600 0.042964937 35900 0.052978769 35852
0.016471859 71640 0.070787828 71640 0.024997746
71600 0.094551338 71600 0.029890145 35900 0.072504008 35856
0.074199532 71648 0.102300553 71648 0.034467772
71700 0.091523521 71700 0.094622708 35900 0.097747041 35860
0.093617979 71656 0.058668396 71656 0.008475632
71700 0.098154014 71700 0.126887506 35900 0.062062354 35864
0.071964874 71664 0.016898806 71664 0.010879378
71700 0.075354939 71700 0.157074901 35900 0.013081266 35868
0.031127485 71672 0.084070038 71672 0.04063487
71700 0.093406379 71700 0.150820881 35900 0.004082309 35872
0.007468307 71680 0.155738904 71680 0.05861534
71700 0.124469036 71700 0.111074733 35900 0.014250704 35876
0.017554658 71688 0.091030175 71688 0.062498701
71700 0.065118024 71700 0.105727442 35900 0.015687863 35880
0.047477668 71696 0.054902121 71696 0.054220534
71700 0.065201588 71700 0.088287066 35900 0.025213682 35884
0.096068216 71704 0.026461974 71704 0.031054515
71700 0.094264695 71700 0.03456555 35900 0.045308956 35888
0.122516183 71712 0.068812158 71712 0.036809219
71700 0.079602949 71700 0.093454291 35900 0.02656222 35892
0.102011305 71720 0.089576512 71720 0.051206978
71700 0.05593073 71700 0.090309732 35900 0.05286856 35896
0.050089497 71728 0.065425687 71728 0.041735719
71700 0.059266189 71700 0.081337952 35900 0.054779754 35900
0.038887672 71736 0.030751697 71736 0.033060907
71700 0.079059748 71700 0.11872621 35900 0.04726816 35904
0.08438831 71744 0.071276016 71744 0.072720148
71800 0.039187933 71800 0.138692588 35900 0.046993806 35908
0.07381248 71752 0.117293261 71752 0.107890002
71800 0.114223694 71800 0.074987627 35900 0.041612599 35912
0.050739145 71760 0.139386961 71760 0.074950462
71800 0.160513563 71800 0.030203004 35900 0.016149019 35916
0.094350537 71768 0.127951381 71768 0.023345716
71800 0.127064254 71800 0.035239798 35900 0.033484474 35920
0.137122858 71776 0.072957497 71776 0.011414821
71800 0.051921808 71800 0.075306642 35900 0.026998678 35924
0.109781511 71784 0.022804325 71784 0.030736708
71800 0.013479905 71800 0.101704274 35900 0.016738066 35928
0.0682627 71792 0.10388143 71792 0.047913745
71800 0.043460881 71800 0.112610062 35900 0.014817928 35932
0.04989286 71800 0.132746936 71800 0.043673696
71800 0.077854515 71800 0.19087577 35900 0.023891065 35936
0.052901538 71808 0.10378271 71808 0.062555751
71800 0.100040306 71800 0.252701139 35900 0.035139623 35940
0.037767677 71816 0.079015183 71816 0.080851467
71800 0.123006641 71800 0.179048089 35900 0.060237122 35944
0.02661609 71824 0.11605693 71824 0.107996093
71800 0.067663095 71800 0.092443348 35900 0.072769282 35948
0.049746828 71832 0.089395755 71832 0.082514074
71800 0.019144667 71800 0.07887117 36000 0.066545756 35952

0.072235009 71840 0.011038082 71840 0.034679502
71800 0.039846829 71800 0.065878703 36000 0.088309455 35956
0.077702265 71848 0.042368349 71848 0.040463994
71900 0.06607351 71900 0.171864376 36000 0.067703069 35960
0.055058798 71856 0.021703823 71856 0.058608384
71900 0.06406883 71900 0.211896171 36000 0.021708886 35964
0.024131492 71864 0.047737689 71864 0.044373559
71900 0.109503541 71900 0.192929525 36000 0.010248184 35968
0.00566441 71872 0.084171596 71872 0.030361878
71900 0.175747235 71900 0.13236329 36000 0.018482777 35972
0.036815331 71880 0.075409742 71880 0.026526184
71900 0.130513014 71900 0.101014564 36000 0.027047976 35976
0.057539983 71888 0.130232336 71888 0.02704337
71900 0.014328542 71900 0.07685789 36000 0.004083248 35980
0.037430658 71896 0.16046084 71896 0.0674927
71900 0.040269304 71900 0.051051862 36000 0.028741628 35984
0.023542425 71904 0.103238766 71904 0.049357896
71900 0.113641509 71900 0.071564915 36000 0.018790363 35988
0.013307215 71912 0.027279344 71912 0.027866985
71900 0.1409049 71900 0.097448225 36000 0.06623425 35992
0.042025815 71920 0.09070008 71920 0.022234641
71900 0.067705427 71900 0.100901372 36000 0.069932205 35996
0.015301668 71928 0.10519963 71928 0.011491518
71900 0.031690212 71900 0.109015346 36000 0.052943047 36000
0.010243888 71936 0.163922509 71936 0.019269059
71900 0.101876787 71900 0.101564387 36000 0.079317397 36004
0.045325895 71944 0.131845838 71944 0.022847335
72000 0.102547128 72000 0.075757904 36000 0.121663565 36008
0.084954656 71952 0.01908428 71952 0.034869001
72000 0.059298593 72000 0.04341203 36000 0.128205371 36012
0.083647581 71960 0.044037483 71960 0.044484106
72000 0.065614564 72000 0.030435465 36000 0.099941724 36016
0.080223428 71968 0.014682505 71968 0.037232217
72000 0.05824781 72000 0.083780076 36000 0.073505413 36020
0.077079392 71976 0.083112645 71976 0.011023134
72000 0.031934629 72000 0.158862065 36000 0.06607949 36024
0.072857598 71984 0.127319974 71984 0.009641608
72000 0.073763265 72000 0.197509159 36000 0.037757938 36028
0.07742962 71992 0.114394767 71992 0.014378439
72000 0.164368409 72000 0.16737105 36000 0.01538072 36032
0.071024027 72000 0.093926661 72000 0.053771044
72000 0.175213746 72000 0.070453069 36000 0.020324638 36036
0.079103076 72008 0.045818837 72008 0.061481973
72000 0.103598344 72000 0.079610472 36000 0.009270512 36040
0.101188023 72016 0.039962764 72016 0.04188891
72000 0.07282976 72000 0.096883974 36000 0.025506632 36044
0.089293433 72024 0.106294901 72024 0.021620426
72000 0.107504297 72000 0.020957146 36000 0.032500055 36048
0.059507373 72032 0.128848813 72032 0.019129915
72000 0.08460063 72000 0.08618772 36100 0.014742659 36052
0.068289752 72040 0.087282453 72040 0.034846689
72000 0.030205831 72000 0.079515718 36100 0.049581853 36056
0.08812856 72048 0.085179214 72048 0.031383934
72100 0.091738584 72100 0.123675287 36100 0.056221277 36060
0.071960931 72056 0.100052275 72056 0.034252509
72100 0.120316225 72100 0.094690273 36100 0.068643421 36064
0.045541234 72064 0.059935472 72064 0.038393955
72100 0.039545925 72100 0.076248369 36100 0.046075296 36068
0.019049676 72072 0.06726195 72072 0.037734077
72100 0.139989265 72100 0.165817764 36100 0.023964216 36072
0.017407981 72080 0.110501445 72080 0.050356186
72100 0.214417218 72100 0.155915084 36100 0.050404789 36076
0.007499102 72088 0.116406227 72088 0.067560744
72100 0.145740153 72100 0.0781216 36100 0.057262194 36080
0.005550242 72096 0.09139425 72096 0.059468464
72100 0.066774861 72100 0.063706822 36100 0.054376011 36084
0.024146159 72104 0.036739097 72104 0.019504922
72100 0.053365027 72100 0.060147035 36100 0.036367674 36088

0.025030042 72112 0.014134746 72112 0.009426418
72100 0.017057604 72100 0.094817988 36100 0.031311531 36092
0.01315263 72120 0.009806366 72120 0.013110468
72100 0.047589947 72100 0.099646 36100 0.040940176 36096
0.013578203 72128 0.025727812 72128 0.026377633
72100 0.067457942 72100 0.081134815 36100 0.078721554 36100
0.055859906 72136 0.121628313 72136 0.022608027
72100 0.063622385 72100 0.103194332 36100 0.095144962 36104
0.051499927 72144 0.128497675 72144 0.012309029
72200 0.085965708 72200 0.13482284 36100 0.070082329 36108
0.032992612 72152 0.047423666 72152 0.014250553
72200 0.121028199 72200 0.14329479 36100 0.061897634 36112
0.022975901 72160 0.048912181 72160 0.029868847
72200 0.112274785 72200 0.122932164 36100 0.06323561 36116
0.021205135 72168 0.06559414 72168 0.038738799
72200 0.108473534 72200 0.069437483 36100 0.041863572 36120
0.04317634 72176 0.087554174 72176 0.03710917
72200 0.116423551 72200 0.044540844 36100 0.057567559 36124
0.0764621 72184 0.150956519 72184 0.044757955
72200 0.102159771 72200 0.103907303 36100 0.103504375 36128
0.044082539 72192 0.192087551 72192 0.072679948
72200 0.03651364 72200 0.140168734 36100 0.078061072 36132
0.03344471 72200 0.135225375 72200 0.057360405
72200 0.048114871 72200 0.050271145 36100 0.045425397 36136
0.084650601 72208 0.054363391 72208 0.029077854
72200 0.086863453 72200 0.13271319 36100 0.051705851 36140
0.084922504 72216 0.027002399 72216 0.022018312
72200 0.072603158 72200 0.208864033 36100 0.027134842 36144
0.078001554 72224 0.095185409 72224 0.035030596
72200 0.089121371 72200 0.138113625 36100 0.01488857 36148
0.069346439 72232 0.133758193 72232 0.05492305
72200 0.069967769 72200 0.044097353 36200 0.011726747 36152
0.052945074 72240 0.150258813 72240 0.034989374
72200 0.018036277 72200 0.036607777 36200 0.024660794 36156
0.039262526 72248 0.163911915 72248 0.059687365
72300 0.046625915 72300 0.007423272 36200 0.045507204 36160
0.022552242 72256 0.15625867 72256 0.07753032
72300 0.066355351 72300 0.038724542 36200 0.053144337 36164
0.031326108 72264 0.150107663 72264 0.051014475
72300 0.009436199 72300 0.023077513 36200 0.037646092 36168
0.032633881 72272 0.106152133 72272 0.022058115
72300 0.068578323 72300 0.070242044 36200 0.031255138 36172
0.039715516 72280 0.059240851 72280 0.024289937
72300 0.08116768 72300 0.039992978 36200 0.066942164 36176
0.027261607 72288 0.072754679 72288 0.048467573
72300 0.073901283 72300 0.039246166 36200 0.05399785 36180
0.016990716 72296 0.06730391 72296 0.022164752
72300 0.089664871 72300 0.053839554 36200 0.019438418 36184
0.050427338 72304 0.046447705 72304 0.022770499
72300 0.114599308 72300 0.053090862 36200 0.055105957 36188
0.067295106 72312 0.155825401 72312 0.014974536
72300 0.098777637 72300 0.102101039 36200 0.048518341 36192
0.043200205 72320 0.144487553 72320 0.010590295
72300 0.015798991 72300 0.103596824 36200 0.006772207 36196
0.01599011 72328 0.06282179 72328 0.01529578
72300 0.111050984 72300 0.061997307 36200 0.042388456 36200
0.010071583 72336 0.018733803 72336 0.060160419
72300 0.167179373 72300 0.069981579 36200 0.058650541 36204
0.063228792 72344 0.08694399 72344 0.088662455
72400 0.135830983 72400 0.119691096 36200 0.080412116 36208
0.062251456 72352 0.142059813 72352 0.087322165
72400 0.053977128 72400 0.112584494 36200 0.095260264 36212
0.030674975 72360 0.131193301 72360 0.083330022
72400 0.02504783 72400 0.058747988 36200 0.078194738 36216
0.080612022 72368 0.04107432 72368 0.073115407
72400 0.034144057 72400 0.098743061 36200 0.018667968 36220
0.081903767 72376 0.012262368 72376 0.064591732
72400 0.096066957 72400 0.111220266 36200 0.05510966 36224

0.058960475	72384	0.032421682	72384	0.045487635
72400	0.038118775	72400	0.103695777	36200	0.05894628	36228
0.008950623	72392	0.071932431	72392	0.039843937
72400	0.162681143	72400	0.078978992	36200	0.03752486	36232
0.036364403	72400	0.046652291	72400	0.054637119
72400	0.234159001	72400	0.068528214	36200	0.052429496	36236
0.032653908	72408	0.078346289	72408	0.051282816
72400	0.171256266	72400	0.053564421	36200	0.041877065	36240
0.01815872	72416	0.141924771	72416	0.031461135
72400	0.105245264	72400	0.038796901	36200	0.052555413	36244
0.027349403	72424	0.148107385	72424	0.039206421
72400	0.034064935	72400	0.078741068	36200	0.060671831	36248
0.041783434	72432	0.101439342	72432	0.025810898
72400	0.069874623	72400	0.07251557	36300	0.036146055	36252
0.0509608	72440	0.052799613	72440	0.025232239
72400	0.100356418	72400	0.061983272	36300	0.020029003	36256
0.075997166	72448	0.024444027	72448	0.026049536
72500	0.063071399	72500	0.03307814	36300	0.054317425	36260
0.089200854	72456	0.032093765	72456	0.020662787
72500	0.053996326	72500	0.065290347	36300	0.057532234	36264
0.070098111	72464	0.018965166	72464	0.035574951
72500	0.086085507	72500	0.098070188	36300	0.021841299	36268
0.02642545	72472	0.072602314	72472	0.041082811
72500	0.024047491	72500	0.119668577	36300	0.024008668	36272
0.021524664	72480	0.084653802	72480	0.019825389
72500	0.140637625	72500	0.069860689	36300	0.012355562	36276
0.026424144	72488	0.070887727	72488	0.018076333
72500	0.084270723	72500	0.03626632	36300	0.050980751	36280
0.006003538	72496	0.089825291	72496	0.040649702
72500	0.083586572	72500	0.064935361	36300	0.034755696	36284
0.048976082	72504	0.050777195	72504	0.060479491
72500	0.141859783	72500	0.046542034	36300	0.02281563	36288
0.06769281	72512	0.109048509	72512	0.04357467
72500	0.061811399	72500	0.084044375	36300	0.034852008	36292
0.058293335	72520	0.144371821	72520	0.016583674
72500	0.122989979	72500	0.163985053	36300	0.028991673	36296
0.030337065	72528	0.114229952	72528	0.052372943
72500	0.175036999	72500	0.164974248	36300	0.03706697	36300
0.071697301	72536	0.136460454	72536	0.04923726
72500	0.144279926	72500	0.087876746	36300	0.021525877	36304
0.059303173	72544	0.158337614	72544	0.054103708
72600	0.16622286	72600	0.032326625	36300	0.04867946	36308
0.01409227	72552	0.073263153	72552	0.081415732
72600	0.187729558	72600	0.095737771	36300	0.095350988	36312
0.013730418	72560	0.079901307	72560	0.059560887
72600	0.145548358	72600	0.123212027	36300	0.088694091	36316
0.016421296	72568	0.10727944	72568	0.080002239
72600	0.037664271	72600	0.056287987	36300	0.05645354	36320
0.02797436	72576	0.071936956	72576	0.096424075
72600	0.034918598	72600	0.01023634	36300	0.054976012	36324
0.024615743	72584	0.086703883	72584	0.036664584
72600	0.037400398	72600	0.039696501	36300	0.056377365	36328
0.035342175	72592	0.081094215	72592	0.039348779
72600	0.054980468	72600	0.094180708	36300	0.044238463	36332
0.036320384	72600	0.041070354	72600	0.052568797
72600	0.034165139	72600	0.070893388	36300	0.048402602	36336
0.004959152	72608	0.05575268	72608	0.023885414
72600	0.085535168	72600	0.036071055	36300	0.078147721	36340
0.057547903	72616	0.10823302	72616	0.008473991
72600	0.130522938	72600	0.124543702	36300	0.098589058	36344
0.092598682	72624	0.129751191	72624	0.016127382
72600	0.082036553	72600	0.130573055	36300	0.091777161	36348
0.081163744	72632	0.128138694	72632	0.049317921
72600	0.010138211	72600	0.087550769	36400	0.07617307	36352
0.062109211	72640	0.124658589	72640	0.070278133
72600	0.047016627	72600	0.047201458	36400	0.064712738	36356
0.06944182	72648	0.102651669	72648	0.040491657
72700	0.053595271	72700	0.035100351	36400	0.049280992	36360

0.107182059	72656	0.07479357	72656	0.021642463		
72700	0.0660804	72700	0.03891049	36400	0.023161889	36364
0.087010332	72664	0.074826814	72664	0.037880389		
72700	0.092686802	72700	0.046944591	36400	0.017806144	36368
0.016565937	72672	0.082094775	72672	0.043181852		
72700	0.078682308	72700	0.093937386	36400	0.031715263	36372
0.029401052	72680	0.053542473	72680	0.04470109		
72700	0.070311719	72700	0.118357864	36400	0.041923249	36376
0.020909616	72688	0.043669836	72688	0.05187112		
72700	0.079566707	72700	0.062698688	36400	0.041218067	36380
0.039974209	72696	0.077131328	72696	0.054883883		
72700	0.115219955	72700	0.06685828	36400	0.01955162	36384
0.092658898	72704	0.057180041	72704	0.050849052		
72700	0.094874878	72700	0.117997057	36400	0.015988951	36388
0.094565672	72712	0.042890253	72712	0.109281675		
72700	0.020854337	72700	0.077793418	36400	0.043404689	36392
0.066796187	72720	0.056211073	72720	0.144335805		
72700	0.039966606	72700	0.053855023	36400	0.052528234	36396
0.059048856	72728	0.070640206	72728	0.110799927		
72700	0.066851586	72700	0.097226795	36400	0.048179783	36400
0.052294614	72736	0.073625	72736	0.058528422		
72700	0.055052038	72700	0.10319666	36400	0.063008723	36404
0.037231839	72744	0.109562301	72744	0.014138715		
72800	0.091017559	72800	0.09495514	36400	0.035047975	36408
0.053515305	72752	0.077912853	72752	0.033515189		
72800	0.085251937	72800	0.104336228	36400	0.036543421	36412
0.066878914	72760	0.060061724	72760	0.04065047		
72800	0.096558972	72800	0.087729648	36400	0.020437619	36416
0.035119909	72768	0.141570593	72768	0.01861288		
72800	0.127924955	72800	0.036787576	36400	0.005625965	36420
0.023847362	72776	0.124766099	72776	0.018242683		
72800	0.08371575	72800	0.014609631	36400	0.017268068	36424
0.051135954	72784	0.118565899	72784	0.01061718		
72800	0.01393078	72800	0.020288921	36400	0.017478969	36428
0.088411674	72792	0.091651229	72792	0.03586278		
72800	0.032781845	72800	0.091776194	36400	0.004208633	36432
0.086152599	72800	0.048337734	72800	0.057955658		
72800	0.042550848	72800	0.124529703	36400	0.032023698	36436
0.028426428	72808	0.047612284	72808	0.06622904		
72800	0.073380761	72800	0.090701287	36400	0.078452256	36440
0.056609657	72816	0.015065931	72816	0.040835552		
72800	0.068167523	72800	0.084019943	36400	0.083795894	36444
0.098823446	72824	0.031852669	72824	0.028643226		
72800	0.026318236	72800	0.100567988	36400	0.058261925	36448
0.102955848	72832	0.040780684	72832	0.030715455		
72800	0.061639075	72800	0.079829923	36500	0.04616411	36452
0.061664767	72840	0.019275709	72840	0.031303302		
72800	0.066176486	72800	0.068773043	36500	0.027343576	36456
0.028196	72848	0.042508029	72848	0.028534001		
72900	0.088251603	72900	0.09328044	36500	0.03708314	36460
0.017070215	72856	0.060693405	72856	0.033224718		
72900	0.123884543	72900	0.160347685	36500	0.023903822	36464
0.04304398	72864	0.155931398	72864	0.054083652		
72900	0.125266438	72900	0.199946706	36500	0.0157906	36468
0.04694991	72872	0.197970105	72872	0.069224938		
72900	0.045965433	72900	0.145548896	36500	0.038250524	36472
0.024928866	72880	0.116849536	72880	0.075851443		
72900	0.05752164	72900	0.046964062	36500	0.049970284	36476
0.02591846	72888	0.030468096	72888	0.044302975		
72900	0.023534962	72900	0.02266776	36500	0.070890346	36480
0.036287074	72896	0.096639815	72896	0.031106723		
72900	0.066355242	72900	0.036124566	36500	0.052412866	36484
0.019411133	72904	0.156700145	72904	0.045935172		
72900	0.142461795	72900	0.156562688	36500	0.029166624	36488
0.00654255	72912	0.190934719	72912	0.049074763		
72900	0.16445505	72900	0.22741541	36500	0.022293994	36492
0.009738301	72920	0.18642981	72920	0.04846674		
72900	0.123167076	72900	0.1959972	36500	0.062955653	36496

0.013124421	72928	0.192969426	72928	0.03687547		
72900	0.15015922	72900	0.140340999	36500	0.087586246	36500
0.020409156	72936	0.176898539	72936	0.015859483		
72900	0.175981535	72900	0.088736357	36500	0.061090141	36504
0.035812402	72944	0.098081204	72944	0.021008373		
73000	0.133955823	73000	0.054980606	36500	0.028251177	36508
0.042675896	72952	0.092354116	72952	0.032188953		
73000	0.05612621	73000	0.116286101	36500	0.051659412	36512
0.024353076	72960	0.047425387	72960	0.021109867		
73000	0.06339189	73000	0.102404985	36500	0.048215286	36516
0.027338756	72968	0.045210236	72968	0.011171921		
73000	0.082971092	73000	0.075035823	36500	0.032224751	36520
0.007569886	72976	0.087574656	72976	0.017174521		
73000	0.140713644	73000	0.11541733	36500	0.051637064	36524
0.045885503	72984	0.07516973	72984	0.046370922		
73000	0.131206733	73000	0.097518423	36500	0.062866922	36528
0.090319059	72992	0.105581057	72992	0.041119492		
73000	0.072395233	73000	0.040219238	36500	0.063081265	36532
0.113064416	73000	0.157632313	73000	0.01776595		
73000	0.041750092	73000	0.067314562	36500	0.045863428	36536
0.104203944	73008	0.136106231	73008	0.020358162		
73000	0.084790416	73000	0.076189688	36500	0.043635089	36540
0.068867899	73016	0.102595324	73016	0.049587114		
73000	0.136335206	73000	0.037704696	36500	0.052091247	36544
0.034802568	73024	0.075766264	73024	0.042269578		
73000	0.097607735	73000	0.017874889	36500	0.034679935	36548
0.026088965	73032	0.01056699	73032	0.017325447		
73000	0.034354449	73000	0.099228448	36600	0.008054181	36552
0.039667433	73040	0.056739886	73040	0.050088565		
73000	0.003843596	73000	0.176602771	36600	0.038130831	36556
0.049602662	73048	0.052408646	73048	0.058519272		
73100	0.048270045	73100	0.197905756	36600	0.057883124	36560
0.050059651	73056	0.006411544	73056	0.044765446		
73100	0.060660274	73100	0.147424639	36600	0.064173044	36564
0.013758022	73064	0.026705189	73064	0.047585145		
73100	0.079212899	73100	0.031559568	36600	0.055437635	36568
0.047858866	73072	0.042347361	73072	0.047038542		
73100	0.140734818	73100	0.055502311	36600	0.04627294	36572
0.070049173	73080	0.045027882	73080	0.043162992		
73100	0.185617173	73100	0.046288082	36600	0.054073029	36576
0.064254731	73088	0.044687451	73088	0.024063293		
73100	0.179662064	73100	0.081788778	36600	0.054738102	36580
0.05701718	73096	0.085349791	73096	0.043201293		
73100	0.07911658	73100	0.045390734	36600	0.056818295	36584
0.057413326	73104	0.18378433	73104	0.065514563		
73100	0.045359087	73100	0.018249102	36600	0.060383089	36588
0.048539241	73112	0.157979638	73112	0.043356591		
73100	0.067306246	73100	0.044456829	36600	0.028650688	36592
0.02457263	73120	0.067583351	73120	0.034269244		
73100	0.011467014	73100	0.082561106	36600	0.041473664	36596
0.018407571	73128	0.074196491	73128	0.054439803		
73100	0.024294683	73100	0.072264404	36600	0.05124959	36600
0.0298003	73136	0.058483929	73136	0.036360692		
73100	0.145168103	73100	0.058762496	36600	0.065924833	36604
0.025717278	73144	0.033138385	73144	0.023609067		
73200	0.230150151	73200	0.085371612	36600	0.097018085	36608
0.008595091	73152	0.07616818	73152	0.035840712		
73200	0.16899925	73200	0.0602767	36600	0.127101943	36612
0.018977444	73160	0.093482631	73160	0.055468303		
73200	0.046489746	73200	0.079213911	36600	0.119704404	36616
0.036287711	73168	0.067153887	73168	0.061245555		
73200	0.076587647	73200	0.130969013	36600	0.088481538	36620
0.072363779	73176	0.081989078	73176	0.0396951		
73200	0.14349939	73200	0.083013249	36600	0.070948743	36624
0.09760492	73184	0.064698106	73184	0.014510767		
73200	0.155998059	73200	0.024061967	36600	0.076903081	36628
0.103527069	73192	0.038601451	73192	0.014872071		
73200	0.13385991	73200	0.072675233	36600	0.074132709	36632

0.072614319 73200 0.046951485 73200 0.030103032
73200 0.113073962 73200 0.065201522 36600 0.062469786 36636
0.053437412 73208 0.086039101 73208 0.034573717
73200 0.07874547 73200 0.032203239 36600 0.070911141 36640
0.058112109 73216 0.045830017 73216 0.022981662
73200 0.030425039 73200 0.078695557 36600 0.101051897 36644
0.068073641 73224 0.060595085 73224 0.026585545
73200 0.056723147 73200 0.040847477 36600 0.062619518 36648
0.07652989 73232 0.050990293 73232 0.046443496
73200 0.035788617 73200 0.077910285 36700 0.06825044 36652
0.074043521 73240 0.025927648 73240 0.036326761
73200 0.067993875 73200 0.097280965 36700 0.039173512 36656
0.055061628 73248 0.010386272 73248 0.026592834
73300 0.050436251 73300 0.050296221 36700 0.040900253 36660
0.076876655 73256 0.045119272 73256 0.05731373
73300 0.08217385 73300 0.055175002 36700 0.081369035 36664
0.135897251 73264 0.062233019 73264 0.084199848
73300 0.108467422 73300 0.086468215 36700 0.064813728 36668
0.117405027 73272 0.027139849 73272 0.069220354
73300 0.117287753 73300 0.063973879 36700 0.05890261 36672
0.063474086 73280 0.124862694 73280 0.020305715
73300 0.074489697 73300 0.043599259 36700 0.071888921 36676
0.026485133 73288 0.17198459 73288 0.00649363
73300 0.052721778 73300 0.121380712 36700 0.07398492 36680
0.020859199 73296 0.11614494 73296 0.010436516
73300 0.066344801 73300 0.084624022 36700 0.042675401 36684
0.048882455 73304 0.053010655 73304 0.033311539
73300 0.031073767 73300 0.116324722 36700 0.018436496 36688
0.064314794 73312 0.046379912 73312 0.046029902
73300 0.046494562 73300 0.1650216 36700 0.028545985 36692
0.072054943 73320 0.057840356 73320 0.045391662
73300 0.056558994 73300 0.14802131 36700 0.035633959 36696
0.057841095 73328 0.02694395 73328 0.013621083
73300 0.065062486 73300 0.049581784 36700 0.04205967 36700
0.038538175 73336 0.039711063 73336 0.046513411
73300 0.084305932 73300 0.072034396 36700 0.071148468 36704
0.049143524 73344 0.100708516 73344 0.067310066
73400 0.061709099 73400 0.083113002 36700 0.075092867 36708
0.053956395 73352 0.129502441 73352 0.043895987
73400 0.005583894 73400 0.026821344 36700 0.046598696 36712
0.037457128 73360 0.090628382 73360 0.031715332
73400 0.068196896 73400 0.070769813 36700 0.023389288 36716
0.053022373 73368 0.034130346 73368 0.035173238
73400 0.10264945 73400 0.037540322 36700 0.045570501 36720
0.051892355 73376 0.102144586 73376 0.031566229
73400 0.072246097 73400 0.083759427 36700 0.050015733 36724
0.033921897 73384 0.168745988 73384 0.012837977
73400 0.052333966 73400 0.12081716 36700 0.054539116 36728
0.002904998 73392 0.192836 73392 0.027512066
73400 0.074420153 73400 0.086054613 36700 0.067677829 36732
0.010556404 73400 0.132279049 73400 0.054936554
73400 0.037681701 73400 0.117151445 36700 0.061487961 36736
0.012739927 73408 0.035691523 73408 0.060379334
73400 0.022407461 73400 0.161038523 36700 0.015234264 36740
0.04795643 73416 0.015599742 73416 0.07300642
73400 0.076260243 73400 0.118156044 36700 0.029761513 36744
0.07610974 73424 0.020559242 73424 0.069654248
73400 0.145999176 73400 0.020248706 36700 0.062067098 36748
0.059368067 73432 0.057266418 73432 0.048586502
73400 0.224319621 73400 0.07190257 36800 0.069687514 36752
0.05483106 73440 0.071759707 73440 0.031906871
73400 0.21895078 73400 0.059475471 36800 0.012047766 36756
0.044097691 73448 0.075960794 73448 0.019088448
73500 0.145820028 73500 0.085679414 36800 0.080832535 36760
0.054406835 73456 0.104066923 73456 0.028336146
73500 0.092590875 73500 0.071115726 36800 0.124999395 36764
0.096303775 73464 0.122214202 73464 0.018248711
73500 0.057644335 73500 0.004787886 36800 0.113800867 36768

0.04321383 73472 0.084853076 73472 0.020166179
73500 0.021141048 73500 0.074118892 36800 0.065181164 36772
0.036849535 73480 0.109030443 73480 0.01602665
73500 0.059042563 73500 0.096658281 36800 0.007314923 36776
0.060695584 73488 0.111590976 73488 0.032024338
73500 0.054533717 73500 0.122789657 36800 0.028570205 36780
0.043296641 73496 0.013065618 73496 0.01963811
73500 0.017071609 73500 0.095078387 36800 0.047284968 36784
0.019062558 73504 0.123375896 73504 0.019689405
73500 0.013782443 73500 0.021095106 36800 0.077672339 36788
0.030466114 73512 0.189308659 73512 0.044205739
73500 0.010471886 73500 0.022553113 36800 0.087276312 36792
0.020549576 73520 0.145966449 73520 0.047689042
73500 0.054854867 73500 0.101156176 36800 0.026605523 36796
0.043457545 73528 0.095692914 73528 0.007049637
73500 0.135112423 73500 0.161769568 36800 0.058116413 36800
0.057432582 73536 0.072099596 73536 0.036154906
73500 0.103459439 73500 0.098641191 36800 0.060584258 36804
0.032254618 73544 0.088015666 73544 0.033334716
73600 0.077086785 73600 0.06459788 36800 0.042231961 36808
0.019921952 73552 0.208334634 73552 0.02878787
73600 0.107645545 73600 0.122967991 36800 0.049595641 36812
0.044430817 73560 0.252739963 73560 0.020429772
73600 0.088595669 73600 0.08256625 36800 0.026493666 36816
0.058115194 73568 0.159428164 73568 0.018072822
73600 0.080434329 73600 0.114784387 36800 0.018343886 36820
0.057452242 73576 0.019232548 73576 0.03956955
73600 0.077355835 73600 0.14440903 36800 0.029574352 36824
0.064445048 73584 0.053251086 73584 0.054886739
73600 0.067906192 73600 0.10489596 36800 0.030244117 36828
0.043967521 73592 0.069580274 73592 0.058849419
73600 0.074244876 73600 0.061339626 36800 0.064310574 36832
0.027201833 73600 0.069913142 73600 0.064120643
73600 0.085510415 73600 0.048187027 36800 0.070833463 36836
0.080633312 73608 0.074352247 73608 0.042147149
73600 0.068037305 73600 0.059765775 36800 0.068384732 36840
0.08979706 73616 0.082223618 73616 0.023856142
73600 0.021285952 73600 0.072580908 36800 0.074604548 36844
0.070708295 73624 0.130687229 73624 0.013060026
73600 0.075859935 73600 0.058575763 36800 0.066305824 36848
0.056912897 73632 0.157706149 73632 0.042711516
73600 0.083432293 73600 0.065509172 36900 0.044363904 36852
0.038414717 73640 0.103440172 73640 0.058215799
73600 0.065328895 73600 0.119409531 36900 0.012708674 36856
0.014911833 73648 0.048743714 73648 0.049794417
73700 0.06316934 73700 0.063519583 36900 0.05924881 36860
0.030502997 73656 0.086692744 73656 0.052114738
73700 0.083533822 73700 0.039106599 36900 0.087673099 36864
0.051778225 73664 0.116825693 73664 0.048228136
73700 0.086833788 73700 0.04623325 36900 0.068945912 36868
0.062518346 73672 0.060961633 73672 0.040547384
73700 0.071607756 73700 0.019001624 36900 0.046541416 36872
0.073237148 73680 0.043144315 73680 0.063788299
73700 0.027277678 73700 0.082529346 36900 0.011335676 36876
0.065049942 73688 0.093051625 73688 0.068603222
73700 0.078530517 73700 0.127990585 36900 0.019222849 36880
0.075379052 73696 0.067580535 73696 0.047986061
73700 0.073479147 73700 0.093664268 36900 0.03664197 36884
0.061920968 73704 0.060207123 73704 0.024833727
73700 0.048918089 73700 0.05875279 36900 0.05248404 36888
0.012823071 73712 0.042948068 73712 0.013457891
73700 0.071566028 73700 0.082436585 36900 0.036210557 36892
0.032534179 73720 0.0625963 73720 0.016266906
73700 0.105609273 73700 0.096289157 36900 0.051312589 36896
0.054959983 73728 0.07110068 73728 0.033676384
73700 0.115019066 73700 0.053472712 36900 0.009196123 36900
0.084235209 73736 0.073098541 73736 0.087941546
73700 0.122673635 73700 0.02831124 36900 0.046221412 36904

0.079243597 73744 0.081618986 73744 0.106326654
73800 0.142860066 73800 0.027635344 36900 0.048810238 36908
0.072039627 73752 0.074503092 73752 0.091614493
73800 0.119204859 73800 0.060711966 36900 0.019727884 36912
0.095295116 73760 0.035408226 73760 0.052401276
73800 0.033098884 73800 0.103922306 36900 0.033792669 36916
0.099035875 73768 0.018181447 73768 0.026429214
73800 0.05554135 73800 0.125431354 36900 0.044525303 36920
0.083069455 73776 0.063250278 73776 0.039691226
73800 0.07151044 73800 0.082171166 36900 0.037704213 36924
0.035456855 73784 0.132318499 73784 0.028817918
73800 0.06682278 73800 0.074606091 36900 0.073331874 36928
0.027710425 73792 0.168713873 73792 0.018099519
73800 0.042110591 73800 0.136718984 36900 0.087507593 36932
0.041093859 73800 0.200081136 73800 0.042468731
73800 0.048387228 73800 0.136686955 36900 0.086038846 36936
0.048102589 73808 0.18314374 73808 0.042354215
73800 0.061777442 73800 0.094825984 36900 0.064482636 36940
0.055184857 73816 0.165585079 73816 0.023642837
73800 0.096426782 73800 0.04402893 36900 0.013198393 36944
0.081583865 73824 0.129675348 73824 0.020330406
73800 0.10307302 73800 0.118461867 36900 0.072243565 36948
0.080873411 73832 0.08914074 73832 0.019431098
73800 0.032385433 73800 0.133206603 37000 0.094055926 36952
0.065846471 73840 0.117042859 73840 0.007047805
73800 0.060089922 73800 0.093338829 37000 0.053552241 36956
0.029510655 73848 0.153669476 73848 0.026675025
73900 0.072529241 73900 0.073120762 37000 0.041221894 36960
0.087359535 73856 0.138809075 73856 0.022771204
73900 0.035584977 73900 0.028391367 37000 0.051760442 36964
0.121344725 73864 0.098322555 73864 0.023974666
73900 0.015176579 73900 0.116559902 37000 0.056215074 36968
0.084507476 73872 0.028849634 73872 0.033660399
73900 0.014035009 73900 0.146283419 37000 0.080031066 36972
0.046176563 73880 0.051931242 73880 0.035028734
73900 0.026719421 73900 0.128533808 37000 0.0800243 36976
0.057283531 73888 0.075605931 73888 0.031736832
73900 0.085903092 73900 0.14353088 37000 0.054673201 36980
0.078705598 73896 0.092062765 73896 0.083536987
73900 0.120256475 73900 0.148964508 37000 0.008690076 36984
0.089091867 73904 0.137102746 73904 0.073990974
73900 0.10342271 73900 0.094365729 37000 0.023529137 36988
0.092129005 73912 0.143938553 73912 0.052118783
73900 0.036822785 73900 0.030560514 37000 0.036414898 36992
0.10736382 73920 0.076748234 73920 0.020140607
73900 0.044690423 73900 0.041264164 37000 0.049584451 36996
0.108357512 73928 0.080582962 73928 0.057089528
73900 0.104110601 73900 0.052881405 37000 0.043934098 37000
0.091925642 73936 0.026314392 73936 0.053125517
73900 0.128094879 73900 0.046924753 37000 0.025862591 37004
0.058396585 73944 0.075569311 73944 0.037842285
74000 0.077902747 74000 0.018021932 37000 0.007538059 37008
0.05060414 73952 0.082390179 73952 0.056866731
74000 0.063005165 74000 0.026455686 37000 0.05210306 37012
0.085331725 73960 0.137836484 73960 0.064587577
74000 0.050622111 74000 0.046031601 37000 0.089704925 37016
0.060578303 73968 0.095309122 73968 0.054748914
74000 0.00997467 74000 0.040132396 37000 0.076327429 37020
0.103285594 73976 0.058780675 73976 0.023039049
74000 0.07955046 74000 0.006399543 37000 0.029923953 37024
0.120388548 73984 0.065530068 73984 0.02292478
74000 0.08743097 74000 0.057269583 37000 0.041292307 37028
0.088645691 73992 0.094414732 73992 0.038625913
74000 0.08042829 74000 0.065049368 37000 0.077226709 37032
0.047164598 74000 0.101173973 74000 0.033347682
74000 0.089238143 74000 0.051507195 37000 0.067951121 37036
0.024704772 74008 0.087093547 74008 0.011508058
74000 0.138396135 74000 0.07076445 37000 0.045559889 37040

0.045206056 74016 0.150821434 74016 0.041082862
74000 0.181304844 74000 0.137118914 37000 0.041385836 37044
0.079905469 74024 0.094334937 74024 0.061635285
74000 0.084647167 74000 0.197040135 37000 0.049827449 37048
0.076745724 74032 0.093997449 74032 0.055553192
74000 0.055731798 74000 0.174475994 37100 0.025540177 37052
0.046818081 74040 0.113594375 74040 0.039741466
74000 0.061646759 74000 0.092907641 37100 0.035141555 37056
0.104244668 74048 0.130250104 74048 0.022589422
74100 0.03738446 74100 0.074726697 37100 0.054806824 37060
0.13173213 74056 0.084473904 74056 0.049247756
74100 0.049196719 74100 0.102811006 37100 0.052893352 37064
0.144488629 74064 0.056418088 74064 0.06084143
74100 0.05686321 74100 0.12749352 37100 0.017402415 37068
0.129593959 74072 0.037514736 74072 0.032518317
74100 0.067875997 74100 0.11775639 37100 0.060236194 37072
0.069972368 74080 0.060526487 74080 0.022127864
74100 0.126372688 74100 0.108801745 37100 0.095974356 37076
0.050046008 74088 0.100289173 74088 0.018728446
74100 0.12140027 74100 0.083916712 37100 0.091028 37080
0.054013672 74096 0.085839034 74096 0.02392129
74100 0.055261571 74100 0.087288012 37100 0.093733564 37084
0.024665749 74104 0.076157732 74104 0.075877157
74100 0.084394669 74100 0.061237784 37100 0.108534885 37088
0.012655999 74112 0.058151225 74112 0.082171035
74100 0.092135386 74100 0.021536438 37100 0.098463948 37092
0.037341673 74120 0.068726469 74120 0.057253703
74100 0.062341809 74100 0.073013143 37100 0.071053801 37096
0.050125087 74128 0.071563401 74128 0.034802259
74100 0.137629831 74100 0.137408148 37100 0.045990477 37100
0.052923566 74136 0.063570813 74136 0.020585016
74100 0.108405038 74100 0.148881794 37100 0.025956657 37104
0.052246014 74144 0.054210512 74144 0.02997939
74200 0.111041409 74200 0.088567431 37100 0.035178447 37108
0.089847781 74152 0.064631888 74152 0.05354705
74200 0.096941643 74200 0.064256135 37100 0.037279493 37112
0.078092446 74160 0.055955879 74160 0.041490643
74200 0.029037312 74200 0.12882342 37100 0.038547172 37116
0.047369264 74168 0.037460526 74168 0.047247784
74200 0.007525583 74200 0.173052001 37100 0.059535629 37120
0.032300544 74176 0.081177546 74176 0.046893016
74200 0.079665471 74200 0.152537919 37100 0.072888855 37124
0.018663252 74184 0.084694038 74184 0.051792755
74200 0.136568225 74200 0.118103388 37100 0.034124798 37128
0.072809256 74192 0.027959548 74192 0.051268053
74200 0.101577505 74200 0.12241285 37100 0.032607866 37132
0.094355652 74200 0.059709637 74200 0.041859566
74200 0.049730075 74200 0.147524435 37100 0.021369471 37136
0.096491407 74208 0.123507052 74208 0.026161204
74200 0.094190131 74200 0.137549738 37100 0.038223501 37140
0.072747265 74216 0.10090552 74216 0.001864648
74200 0.069675916 74200 0.08140719 37100 0.04574467 37144
0.079211503 74224 0.051687963 74224 0.033627213
74200 0.126195083 74200 0.018079967 37100 0.078668491 37148
0.072125695 74232 0.037198668 74232 0.042528893
74200 0.17871747 74200 0.053072854 37200 0.058753496 37152
0.055089615 74240 0.064492917 74240 0.008731687
74200 0.15470208 74200 0.096993426 37200 0.015853084 37156
0.030236453 74248 0.093693896 74248 0.023037448
74300 0.065726104 74300 0.098868375 37200 0.03246401 37160
0.046462195 74256 0.079668986 74256 0.018725343
74300 0.156196009 74300 0.087257351 37200 0.044922152 37164
0.065238295 74264 0.074761178 74264 0.011751153
74300 0.192653039 74300 0.091362133 37200 0.017285489 37168
0.080702826 74272 0.039010487 74272 0.013445985
74300 0.137032955 74300 0.056552632 37200 0.004281344 37172
0.083513638 74280 0.063015199 74280 0.014778801
74300 0.10255586 74300 0.051302301 37200 0.025722906 37176

0.035662517 74288 0.105747269 74288 0.061652594
74300 0.106056767 74300 0.035917637 37200 0.021226748 37180
0.051808533 74296 0.084519073 74296 0.104847306
74300 0.10491279 74300 0.027485945 37200 0.019259996 37184
0.073384334 74304 0.057776182 74304 0.092441078
74300 0.060718074 74300 0.057008914 37200 0.046774389 37188
0.045496952 74312 0.035037509 74312 0.026447229
74300 0.016463542 74300 0.086753389 37200 0.057582452 37192
0.057781388 74320 0.019418685 74320 0.024483979
74300 0.039476901 74300 0.106009167 37200 0.057193272 37196
0.065965898 74328 0.027981099 74328 0.022831906
74300 0.070332055 74300 0.052529271 37200 0.054300039 37200
0.019169405 74336 0.048630838 74336 0.04186614
74300 0.052397019 74300 0.015774986 37200 0.043023523 37204
0.057780631 74344 0.048788974 74344 0.040291125
74400 0.045236728 74400 0.051961877 37200 0.046751538 37208
0.034970526 74352 0.036294045 74352 0.019113846
74400 0.108172892 74400 0.070548005 37200 0.048426253 37212
0.020242205 74360 0.064282154 74360 0.046894573
74400 0.151252316 74400 0.05985544 37200 0.052196272 37216
0.021694563 74368 0.105893043 74368 0.065804605
74400 0.116792093 74400 0.005925079 37200 0.051152929 37220
0.019039777 74376 0.16410515 74376 0.073802359
74400 0.066908593 74400 0.078322628 37200 0.02644575 37224
0.017634171 74384 0.123786274 74384 0.089348418
74400 0.074368058 74400 0.112981892 37200 0.041842654 37228
0.05376479 74392 0.03237246 74392 0.079012345
74400 0.050817707 74400 0.128590575 37200 0.028543933 37232
0.099585268 74400 0.065646833 74400 0.019034857
74400 0.064177883 74400 0.157105009 37200 0.012046542 37236
0.104849816 74408 0.064848748 74408 0.049412029
74400 0.045961162 74400 0.150753054 37200 0.02912803 37240
0.121514349 74416 0.062742693 74416 0.074950105
74400 0.035864294 74400 0.172449654 37200 0.02975347 37244
0.128293351 74424 0.046869143 74424 0.066318709
74400 0.081443111 74400 0.155739603 37200 0.02622713 37248
0.104365143 74432 0.056578014 74432 0.053601649
74400 0.153796515 74400 0.08572355 37300 0.027258584 37252
0.063000174 74440 0.087079076 74440 0.035467681
74400 0.185400204 74400 0.139348791 37300 0.038498067 37256
0.084776984 74448 0.076646902 74448 0.031116048
74500 0.165732549 74500 0.159135118 37300 0.039674855 37260
0.098496508 74456 0.074610609 74456 0.030250149
74500 0.111719492 74500 0.136198549 37300 0.028759187 37264
0.098080323 74464 0.107924454 74464 0.025609412
74500 0.040454535 74500 0.100364377 37300 0.043740241 37268
0.066115128 74472 0.082887898 74472 0.038766677
74500 0.124811006 74500 0.063797612 37300 0.06339547 37272
0.016372187 74480 0.049347887 74480 0.034836354
74500 0.102715188 74500 0.080416634 37300 0.049797083 37276
0.024169234 74488 0.080177037 74488 0.008339991
74500 0.053932123 74500 0.049547296 37300 0.016734473 37280
0.013767115 74496 0.059946866 74496 0.023978224
74500 0.093058792 74500 0.05459075 37300 0.023667664 37284
0.055657612 74504 0.068397036 74504 0.044858873
74500 0.071235059 74500 0.097458549 37300 0.082885934 37288
0.071408947 74512 0.070612594 74512 0.046478217
74500 0.141927434 74500 0.062747218 37300 0.079173689 37292
0.048780621 74520 0.079669277 74520 0.033330838
74500 0.152165798 74500 0.026776586 37300 0.048216596 37296
0.047566027 74528 0.088346016 74528 0.01375139
74500 0.096212127 74500 0.057344183 37300 0.048248483 37300
0.137611875 74536 0.067170804 74536 0.003802255
74500 0.065865854 74500 0.104864164 37300 0.015210352 37304
0.155403351 74544 0.06113459 74544 0.035793859
74600 0.028507955 74600 0.08204038 37300 0.045224038 37308
0.097798016 74552 0.050706574 74552 0.063877225
74600 0.017912653 74600 0.059625003 37300 0.042562228 37312

0.078306046 74560 0.131687863 74560 0.048786213
74600 0.039120296 74600 0.049427457 37300 0.04718823 37316
0.069219597 74568 0.10674353 74568 0.024748821
74600 0.083739113 74600 0.093572395 37300 0.09857821 37320
0.044319946 74576 0.07587752 74576 0.062242063
74600 0.093617542 74600 0.09574103 37300 0.108689383 37324
0.062849824 74584 0.048028633 74584 0.076418859
74600 0.059684819 74600 0.080550846 37300 0.084381354 37328
0.111695808 74592 0.013585469 74592 0.072503433
74600 0.061451538 74600 0.093573413 37300 0.054562544 37332
0.113901871 74600 0.038389888 74600 0.056585297
74600 0.123413047 74600 0.121602308 37300 0.052022951 37336
0.066681001 74608 0.039596234 74608 0.039038812
74600 0.142538033 74600 0.116232935 37300 0.067540226 37340
0.022193961 74616 0.031002437 74616 0.041800824
74600 0.05603263 74600 0.09994991 37300 0.066278779 37344
0.091290633 74624 0.034522265 74624 0.052899486
74600 0.046770612 74600 0.082254672 37300 0.037199239 37348
0.125559018 74632 0.049194016 74632 0.047930735
74600 0.096838361 74600 0.071218099 37400 0.01172577 37352
0.120897843 74640 0.056443652 74640 0.030418447
74600 0.109482069 74600 0.073535412 37400 0.013841386 37356
0.083322622 74648 0.043859152 74648 0.028143897
74700 0.102016376 74700 0.118575401 37400 0.007725726 37360
0.022483258 74656 0.044800916 74656 0.0251897
74700 0.094078925 74700 0.103127488 37400 0.017693357 37364
0.038544782 74664 0.066247325 74664 0.036181606
74700 0.097685312 74700 0.030697316 37400 0.011812574 37368
0.063906875 74672 0.05883518 74672 0.061167229
74700 0.071004128 74700 0.054545166 37400 0.011043522 37372
0.049540351 74680 0.029238521 74680 0.062639396
74700 0.087264445 74700 0.039029579 37400 0.030761356 37376
0.118618242 74688 0.150496504 74688 0.049553517
74700 0.119188051 74700 0.057584843 37400 0.04218753 37380
0.150751599 74696 0.181817071 74696 0.02552556
74700 0.09613132 74700 0.010147966 37400 0.02779092 37384
0.111675086 74704 0.10537175 74704 0.025405554
74700 0.089889538 74700 0.083670406 37400 0.026761494 37388
0.060264396 74712 0.028886401 74712 0.015459435
74700 0.135911673 74700 0.129559689 37400 0.045599576 37392
0.009692454 74720 0.041955136 74720 0.019609848
74700 0.115526869 74700 0.13626102 37400 0.055907425 37396
0.043065626 74728 0.089675479 74728 0.029633993
74700 0.031653497 74700 0.084325278 37400 0.044275101 37400
0.053694243 74736 0.11864314 74736 0.006122422
74700 0.06003719 74700 0.01712638 37400 0.043727305 37404
0.068513531 74744 0.112859045 74744 0.031833955
74800 0.154909533 74800 0.060803679 37400 0.057371057 37408
0.079086283 74752 0.113850467 74752 0.04335634
74800 0.199246759 74800 0.097018659 37400 0.070447088 37412
0.069562797 74760 0.12404978 74760 0.050926406
74800 0.115141556 74800 0.099855395 37400 0.073304182 37416
0.085449559 74768 0.120873738 74768 0.022465723
74800 0.075582277 74800 0.086043416 37400 0.045606623 37420
0.101165228 74776 0.115997842 74776 0.022591483
74800 0.044061308 74800 0.06604223 37400 0.024563698 37424
0.093316536 74784 0.112571448 74784 0.036854643
74800 0.031759657 74800 0.04079262 37400 0.026052494 37428
0.083389248 74792 0.156220965 74792 0.035678717
74800 0.103394639 74800 0.01965182 37400 0.044572884 37432
0.067871231 74800 0.12175359 74800 0.050576098
74800 0.11109317 74800 0.053400901 37400 0.066798675 37436
0.061971645 74808 0.081320293 74808 0.041560474
74800 0.134604489 74800 0.056918427 37400 0.055755379 37440
0.104069251 74816 0.088496745 74816 0.025005325
74800 0.1899333 74800 0.019709867 37400 0.024366411 37444
0.127231891 74824 0.16188965 74824 0.023142095
74800 0.19726035 74800 0.037965252 37400 0.030896903 37448

0.081727543 74832 0.138587363 74832 0.020572026
74800 0.163582241 74800 0.095478914 37500 0.033994689 37452
0.079689213 74840 0.031643609 74840 0.014805779
74800 0.120343022 74800 0.124315644 37500 0.027697721 37456
0.098505603 74848 0.076136523 74848 0.019301939
74900 0.078750149 74900 0.082634957 37500 0.024338116 37460
0.062131527 74856 0.067786736 74856 0.051033148
74900 0.043975611 74900 0.02997109 37500 0.083190294 37464
0.103541119 74864 0.04155323 74864 0.038965161
74900 0.099620593 74900 0.052412248 37500 0.089393987 37468
0.105599582 74872 0.06353105 74872 0.010690838
74900 0.112559428 74900 0.131471635 37500 0.05089106 37472
0.052795745 74880 0.039402403 74880 0.021392956
74900 0.095063595 74900 0.173730746 37500 0.016527931 37476
0.064807733 74888 0.01793833 74888 0.028305394
74900 0.117291929 74900 0.118325341 37500 0.036513055 37480
0.114700008 74896 0.040150459 74896 0.035426358
74900 0.142418765 74900 0.139155396 37500 0.04817461 37484
0.099221892 74904 0.07440721 74904 0.042610478
74900 0.114084214 74900 0.192066116 37500 0.024711773 37488
0.059653892 74912 0.108414322 74912 0.044309723
74900 0.096896009 74900 0.135185051 37500 0.049320708 37492
0.016053617 74920 0.097222284 74920 0.024052606
74900 0.085748463 74900 0.036507347 37500 0.073304982 37496
0.064415617 74928 0.015302898 74928 0.020230118
74900 0.041919258 74900 0.044048222 37500 0.074296091 37500
0.118296484 74936 0.108429282 74936 0.030026949
74900 0.01994231 74900 0.107875429 37500 0.07137849 37504
0.136565738 74944 0.116250951 74944 0.016972856
75000 0.075584096 75000 0.071009767 37500 0.045256307 37508
0.11765481 74952 0.133398673 74952 0.021530113
75000 0.120808763 75000 0.065090411 37500 0.021884373 37512
0.126165993 74960 0.155382878 74960 0.034834724
75000 0.054257063 75000 0.093723684 37500 0.068810157 37516
0.110621943 74968 0.081449958 74968 0.065030225
75000 0.144966864 75000 0.046709123 37500 0.081977669 37520
0.106385312 74976 0.026332686 74976 0.049785249
75000 0.119838973 75000 0.059484955 37500 0.065581931 37524
0.115243049 74984 0.055563163 74984 0.018418883
75000 0.029336008 75000 0.070838076 37500 0.025914374 37528
0.084293868 74992 0.083301333 74992 0.04985679
75000 0.123314036 75000 0.070678019 37500 0.005801032 37532
0.024739416 75000 0.111195906 75000 0.048843296
75000 0.093006733 75000 0.086347238 37500 0.029782015 37536
0.020473815 75008 0.052667867 75008 0.032695651
75000 0.030860956 75000 0.135794559 37500 0.046606889 37540
0.031799562 75016 0.127665524 75016 0.010966609
75000 0.076668024 75000 0.179056923 37500 0.025220359 37544
0.069232105 75024 0.24917291 75024 0.021388107
75000 0.143155558 75000 0.147127678 37500 0.054508186 37548
0.059695365 75032 0.204096054 75032 0.01937856
75000 0.192801075 75000 0.09852634 37600 0.067852729 37552
0.040645806 75040 0.073402065 75040 0.046131514
75000 0.16948588 75000 0.133744863 37600 0.047486235 37556
0.047856625 75048 0.042443917 75048 0.045283377
75100 0.129413369 75100 0.116759657 37600 0.038339193 37560
0.055213033 75056 0.057492314 75056 0.001788775
75100 0.106929569 75100 0.039550923 37600 0.053123393 37564
0.048423288 75064 0.049121045 75064 0.025737456
75100 0.073691968 75100 0.222708055 37600 0.080715356 37568
0.029037954 75072 0.030296125 75072 0.023202096
75100 0.077789671 75100 0.241054716 37600 0.066926659 37572
0.054324475 75080 0.089647423 75080 0.017143368
75100 0.113504953 75100 0.123762773 37600 0.01590301 37576
0.089350273 75088 0.059858594 75088 0.023894154
75100 0.126815125 75100 0.06997196 37600 0.060458395 37580
0.091484428 75096 0.085799817 75096 0.071271534
75100 0.114769136 75100 0.089089175 37600 0.074268799 37584

0.033084885 75104 0.131440713 75104 0.0699999
75100 0.077851655 75100 0.105154759 37600 0.037679962 37588
0.015682646 75112 0.147039508 75112 0.045524052
75100 0.016817776 75100 0.042310272 37600 0.010416397 37592
0.04099909 75120 0.080383601 75120 0.050007893
75100 0.107550855 75100 0.143955418 37600 0.032408872 37596
0.059742837 75128 0.076822202 75128 0.060903167
75100 0.109259665 75100 0.155967544 37600 0.041127889 37600
0.073925527 75136 0.051604086 75136 0.039169278
75100 0.035570814 75100 0.07419871 37600 0.021876349 37604
0.054539833 75144 0.030828374 75144 0.018493691
75200 0.016673803 75200 0.032940989 37600 0.027624457 37608
0.028032957 75152 0.041184168 75152 0.008895066
75200 0.075222124 75200 0.014850642 37600 0.041466123 37612
0.038637598 75160 0.065626489 75160 0.003375261
75200 0.114098242 75200 0.049925227 37600 0.059490056 37616
0.029243689 75168 0.121361831 75168 0.018782135
75200 0.13296942 75200 0.105348045 37600 0.089393601 37620
0.024438539 75176 0.125425854 75176 0.043050757
75200 0.164733269 75200 0.128751926 37600 0.069663423 37624
0.032739688 75184 0.153789079 75184 0.048125374
75200 0.138996068 75200 0.126191008 37600 0.029003772 37628
0.061995881 75192 0.194198569 75192 0.036134497
75200 0.082852224 75200 0.086254106 37600 0.028054228 37632
0.052634016 75200 0.201608593 75200 0.029864923
75200 0.01159314 75200 0.049488488 37600 0.04285582 37636
0.03439271 75208 0.145068043 75208 0.024271507
75200 0.021379157 75200 0.052222524 37600 0.055885426 37640
0.045035744 75216 0.075667587 75216 0.009949095
75200 0.055108714 75200 0.044482051 37600 0.102508246 37644
0.039519335 75224 0.037654463 75224 0.022888746
75200 0.110166184 75200 0.050950788 37600 0.092467082 37648
0.053029911 75232 0.036396385 75232 0.025550588
75200 0.087747088 75200 0.039702682 37700 0.037819966 37652
0.047339494 75240 0.069765425 75240 0.006337557
75200 0.090560927 75200 0.052623353 37700 0.040483563 37656
0.046639336 75248 0.09816949 75248 0.025330861
75300 0.09524799 75300 0.075524906 37700 0.047048838 37660
0.107503482 75256 0.060697003 75256 0.029566967
75300 0.108826862 75300 0.051183575 37700 0.040539031 37664
0.101079371 75264 0.033499797 75264 0.057093988
75300 0.088946064 75300 0.020001344 37700 0.025731362 37668
0.062455583 75272 0.027297057 75272 0.053218966
75300 0.123590115 75300 0.084683044 37700 0.025873373 37672
0.029738951 75280 0.029459126 75280 0.036275989
75300 0.109985791 75300 0.140634671 37700 0.077594137 37676
0.048870588 75288 0.050475261 75288 0.027895529
75300 0.071825023 75300 0.156094291 37700 0.095797717 37680
0.068543392 75296 0.073837575 75296 0.020452342
75300 0.036441947 75300 0.102540456 37700 0.086698798 37684
0.074671269 75304 0.067248417 75304 0.028045693
75300 0.08420158 75300 0.029819343 37700 0.078816054 37688
0.08383314 75312 0.10676284 75312 0.05746408
75300 0.110553076 75300 0.018068062 37700 0.05757076 37692
0.0727109 75320 0.10280242 75320 0.082036102
75300 0.109463043 75300 0.045017285 37700 0.030463067 37696
0.100549267 75328 0.09039892 75328 0.053518015
75300 0.134514906 75300 0.088805966 37700 0.025547621 37700
0.12186938 75336 0.119552788 75336 0.043240292
75300 0.153512258 75300 0.084975145 37700 0.028364102 37704
0.072453251 75344 0.044092281 75344 0.058224094
75400 0.158942189 75400 0.013984832 37700 0.023805411 37708
0.046159814 75352 0.125188235 75352 0.025605325
75400 0.150937805 75400 0.044627996 37700 0.039611459 37712
0.047207566 75360 0.156854018 75360 0.020013007
75400 0.104308048 75400 0.044819593 37700 0.049841572 37716
0.015687692 75368 0.115495255 75368 0.034515586
75400 0.081415499 75400 0.042001164 37700 0.065133179 37720

0.033316122	75376	0.037519283	75376	0.038887763				
75400	0.076969445	75400	0.008353368	37700	0.06934534	37724		
0.078371151	75384	0.013553782	75384	0.046468889				
75400	0.055379864	75400	0.052196865	37700	0.042665812	37728		
0.079014702	75392	0.072717543	75392	0.024041934				
75400	0.033724562	75400	0.017112707	37700	0.030809897	37732		
0.032254189	75400	0.19113363	75400	0.032754866				
75400	0.045951088	75400	0.071542658	37700	0.030450323	37736		
0.058695732	75408	0.245702831	75408	0.001459394				
75400	0.009824848	75400	0.058352591	37700	0.016828186	37740		
0.064282467	75416	0.221220253	75416	0.03951657				
75400	0.062211453	75400	0.027147484	37700	0.05723182	37744		
0.060289029	75424	0.202261115	75424	0.04997179				
75400	0.061311454	75400	0.079470367	37700	0.064288273	37748		
0.05613294	75432	0.193927451	75432	0.030816809				
75400	0.054530403	75400	0.12938214	37800	0.042367035	37752		
0.032756838	75440	0.127931256	75440	0.014734905				
75400	0.079437945	75400	0.068687754	37800	0.057199708	37756		
0.02055888	75448	0.143063153	75448	0.02663375				
75500	0.100020268	75500	0.10544424	37800	0.076104429	37760		
0.011364451	75456	0.14995363	75456	0.048154943				
75500	0.070799732	75500	0.080123733	37800	0.071705086	37764		
0.065507862	75464	0.081314051	75464	0.052986379				
75500	0.026359066	75500	0.0337891	37800	0.047588041	37768		
0.091860755	75472	0.073616982	75472	0.010952705				
75500	0.040829116	75500	0.074228105	37800	0.01364387	37772		
0.071840193	75480	0.063125619	75480	0.046916666				
75500	0.015535154	75500	0.089544497	37800	0.003169323	37776		
0.041305895	75488	0.031138803	75488	0.054467022				
75500	0.107991473	75500	0.077059114	37800	0.016500349	37780		
0.020227375	75496	0.029267503	75496	0.038906401				
75500	0.161145305	75500	0.045967037	37800	0.036533878	37784		
0.055934244	75504	0.042672396	75504	0.015759217				
75500	0.18566307	75500	0.043976499	37800	0.032877862	37788		
0.033994405	75512	0.124742466	75512	0.024835919				
75500	0.162452343	75500	0.085393767	37800	0.047855188	37792		
0.038493025	75520	0.176695074	75520	0.042281798				
75500	0.108650078	75500	0.128523476	37800	0.0417224	37796		
0.042624615	75528	0.117590069	75528	0.03087084				
75500	0.04240045	75500	0.101869155	37800	0.03008193	37800		
0.051357463	75536	0.071205184	75536	0.041710689				
75500	0.098182049	75500	0.105995525	37800	0.046899633	37804		
0.078716337	75544	0.141916943	75544	0.061461673				
75600	0.167706458	75600	0.127179621	37800	0.098082834	37808		
0.074857002	75552	0.148257401	75552	0.044387802				
75600	0.128327258	75600	0.096242584	37800	0.126134678	37812		
0.053967237	75560	0.091430833	75560	0.01153012				
75600	0.056507648	75600	0.084067011	37800	0.109166133	37816		
0.048320151	75568	0.087561843	75568	0.022548389				
75600	0.027848933	75600	0.072366318	37800	0.077483644	37820		
0.034834091	75576	0.129595035	75576	0.014921567				
75600	0.048516798	75600	0.082424987	37800	0.036236164	37824		
0.073980598	75584	0.081903141	75584	0.02552723				
75600	0.063109474	75600	0.08881581	37800	0.024506382	37828		
0.076205746	75592	0.047135618	75592	0.026429912				
75600	0.072498471	75600	0.015371204	37800	0.038727798	37832		
0.056500157	75600	0.143077909	75600	0.014196088				
75600	0.080017162	75600	0.058929734	37800	0.025902775	37836		
0.075142853	75608	0.164704078	75608	0.058974008				
75600	0.037357371	75600	0.025126174	37800	0.02708427	37840		
0.090362861	75616	0.125687759	75616	0.068776353				
75600	0.104615756	75600	0.063008156	37800	0.044102268	37844		
0.049442693	75624	0.096233183	75624	0.039083181				
75600	0.152596128	75600	0.110294619	37800	0.026523909	37848		
0.01577166	75632	0.077252022	75632	0.04878316				
75600	0.10557405	75600	0.096376483	37900	0.00931325	37852		
0.015709002	75640	0.039714818	75640	0.069425412				
75600	0.054171167	75600	0.091508118	37900	0.018183635	37856		

0.054715471 75648 0.076742013 75648 0.057994639
75700 0.019392763 75700 0.174209796 37900 0.028243325 37860
0.11361052 75656 0.07134401 75656 0.028526807
75700 0.020737349 75700 0.169656981 37900 0.05893187 37864
0.119673772 75664 0.064774795 75664 0.047652906
75700 0.040936335 75700 0.067948131 37900 0.081824081 37868
0.10841179 75672 0.119470511 75672 0.0266728
75700 0.022578315 75700 0.059929946 37900 0.064282322 37872
0.078170087 75680 0.149809304 75680 0.031662894
75700 0.056728168 75700 0.099195931 37900 0.053072843 37876
0.05166023 75688 0.113842776 75688 0.053143031
75700 0.032629461 75700 0.092345195 37900 0.077733908 37880
0.062169405 75696 0.074872274 75696 0.054441338
75700 0.100064208 75700 0.102497223 37900 0.067496047 37884
0.029877627 75704 0.088925721 75704 0.040793482
75700 0.047548885 75700 0.192164981 37900 0.044254772 37888
0.038064856 75712 0.079790378 75712 0.039879451
75700 0.082759689 75700 0.17691344 37900 0.063402164 37892
0.064537839 75720 0.068711983 75720 0.009318524
75700 0.067004177 75700 0.037419392 37900 0.068327121 37896
0.076203352 75728 0.099304234 75728 0.048711627
75700 0.062048115 75700 0.116314295 37900 0.062855288 37900
0.097970369 75736 0.02708104 75736 0.039890056
75700 0.067600253 75700 0.156713024 37900 0.073733536 37904
0.122470461 75744 0.051647927 75744 0.061105027
75800 0.039408467 75800 0.117403855 37900 0.040068775 37908
0.111760368 75752 0.049647963 75752 0.054320644
75800 0.060815361 75800 0.058422498 37900 0.024447843 37912
0.086129076 75760 0.054448541 75760 0.023248898
75800 0.091493523 75800 0.084976331 37900 0.045830286 37916
0.069694012 75768 0.087724664 75768 0.06911275
75800 0.120274708 75800 0.056253524 37900 0.037139507 37920
0.046115216 75776 0.097857716 75776 0.07864178
75800 0.116670752 75800 0.064280277 37900 0.039525319 37924
0.049564504 75784 0.1139663 75784 0.05266018
75800 0.102637576 75800 0.065267152 37900 0.040940653 37928
0.027458826 75792 0.154711758 75792 0.018294953
75800 0.101959027 75800 0.070405338 37900 0.051809555 37932
0.059629118 75800 0.162910365 75800 0.017351165
75800 0.060135157 75800 0.107276588 37900 0.062065097 37936
0.125429288 75808 0.112356313 75808 0.021722357
75800 0.016935626 75800 0.122733341 37900 0.04741818 37940
0.095360083 75816 0.068764217 75816 0.038285667
75800 0.029770934 75800 0.115463117 37900 0.011236418 37944
0.027372085 75824 0.071393719 75824 0.05721223
75800 0.038143888 75800 0.079062702 37900 0.025955562 37948
0.06316205 75832 0.0562223 75832 0.059727165
75800 0.014033524 75800 0.044012737 38000 0.047471025 37952
0.075722026 75840 0.018135173 75840 0.042555017
75800 0.105165964 75800 0.058767822 38000 0.0402312 37956
0.064422435 75848 0.076718643 75848 0.031595522
75900 0.117943913 75900 0.091971655 38000 0.019727664 37960
0.030912401 75856 0.040498719 75856 0.014426624
75900 0.039979186 75900 0.145132886 38000 0.018419891 37964
0.069008631 75864 0.078440164 75864 0.022805498
75900 0.061676343 75900 0.164220314 38000 0.031525968 37968
0.03344701 75872 0.155156231 75872 0.056597044
75900 0.148207343 75900 0.127987878 38000 0.024244993 37972
0.042893553 75880 0.102709004 75880 0.075063399
75900 0.197444431 75900 0.088411027 38000 0.038481609 37976
0.077236626 75888 0.01413535 75888 0.063642954
75900 0.127341075 75900 0.072666728 38000 0.037017893 37980
0.085296386 75896 0.021724072 75896 0.052779004
75900 0.053852938 75900 0.080502694 38000 0.046778136 37984
0.065003835 75904 0.092713177 75904 0.056586174
75900 0.06507009 75900 0.09476718 38000 0.068910566 37988
0.05455775 75912 0.134141199 75912 0.049195947
75900 0.005396777 75900 0.086914355 38000 0.066571651 37992

0.074818767 75920 0.098019904 75920 0.02272707
75900 0.068511232 75900 0.03923005 38000 0.070375339 37996
0.062084888 75928 0.072097144 75928 0.012024241
75900 0.02909447 75900 0.043971788 38000 0.069960406 38000
0.035949113 75936 0.095336036 75936 0.012061431
75900 0.11795068 75900 0.119230492 38000 0.066161912 38004
0.068755602 75944 0.160317898 75944 0.014275106
76000 0.19834176 76000 0.12326728 38000 0.039295432 38008
0.058819227 75952 0.17255111 75952 0.017824412
76000 0.16605889 76000 0.072607683 38000 0.038084836 38012
0.048191439 75960 0.125031569 75960 0.03706065
76000 0.104408078 76000 0.037568621 38000 0.044127817 38016
0.033393164 75968 0.18072252 75968 0.03756387
76000 0.130563509 76000 0.038731654 38000 0.074472846 38020
0.04881556 75976 0.247238844 75976 0.016995336
76000 0.146130973 76000 0.029133553 38000 0.09714708 38024
0.083383304 75984 0.158880838 75984 0.008775564
76000 0.109489047 76000 0.119273544 38000 0.045636702 38028
0.077326979 75992 0.073125477 75992 0.012857799
76000 0.084397703 76000 0.12288084 38000 0.027719583 38032
0.082400693 76000 0.018053601 76000 0.01638185
76000 0.10281134 76000 0.068914211 38000 0.03330797 38036
0.110781781 76008 0.06941703 76008 0.033543678
76000 0.077358323 76000 0.088119545 38000 0.016715949 38040
0.147545215 76016 0.113728165 76016 0.046120644
76000 0.065674984 76000 0.03008236 38000 0.04838319 38044
0.116626943 76024 0.18584085 76024 0.046148551
76000 0.111716945 76000 0.046079847 38000 0.048216178 38048
0.043532567 76032 0.11899323 76032 0.055516728
76000 0.080412385 76000 0.033368295 38100 0.022719391 38052
0.069149457 76040 0.043632808 76040 0.061093073
76000 0.066162204 76000 0.055313718 38100 0.030873645 38056
0.109401386 76048 0.066877874 76048 0.038345115
76100 0.093779767 76100 0.011924531 38100 0.061986626 38060
0.088635497 76056 0.024034023 76056 0.029759662
76100 0.050753552 76100 0.0981583 38100 0.043036158 38064
0.040497274 76064 0.042129523 76064 0.028302417
76100 0.013170786 76100 0.105140927 38100 0.042153472 38068
0.02532783 76072 0.066577959 76072 0.030255334
76100 0.04608537 76100 0.083662249 38100 0.041358497 38072
0.041000894 76080 0.128833911 76080 0.031232885
76100 0.055386186 76100 0.15213895 38100 0.013608784 38076
0.062056242 76088 0.020701204 76088 0.025792167
76100 0.048768688 76100 0.202964351 38100 0.009510442 38080
0.085299907 76096 0.11986115 76096 0.016088094
76100 0.076875382 76100 0.110198547 38100 0.023032913 38084
0.068084417 76104 0.109050292 76104 0.025638014
76100 0.050330509 76100 0.077415149 38100 0.042854797 38088
0.03409007 76112 0.078331628 76112 0.071035684
76100 0.117751384 76100 0.151233428 38100 0.027225904 38092
0.033791348 76120 0.128826578 76120 0.083794519
76100 0.163508958 76100 0.098800279 38100 0.050910374 38096
0.016826041 76128 0.094141353 76128 0.046984864
76100 0.13526468 76100 0.066971916 38100 0.056672605 38100
0.049652379 76136 0.016963675 76136 0.00360846
76100 0.094389856 76100 0.120924116 38100 0.05503622 38104
0.103346029 76144 0.06322602 76144 0.012200863
76200 0.099384371 76200 0.07138963 38100 0.02891913 38108
0.110609864 76152 0.052602387 76152 0.019027626
76200 0.126459753 76200 0.049429276 38100 0.008345928 38112
0.048364331 76160 0.080002217 76160 0.037739472
76200 0.129914683 76200 0.089816378 38100 0.024958285 38116
0.045535693 76168 0.188351173 76168 0.053434855
76200 0.104805004 76200 0.086213884 38100 0.065656692 38120
0.083890445 76176 0.254603219 76176 0.071369534
76200 0.085085972 76200 0.039836305 38100 0.108579356 38124
0.091709786 76184 0.216561894 76184 0.084368563
76200 0.085393316 76200 0.040209314 38100 0.132657005 38128

0.062255946 76192 0.053631018 76192 0.064056825
76200 0.094920906 76200 0.095590847 38100 0.100016478 38132
0.057027992 76200 0.124168786 76200 0.015189846
76200 0.126558996 76200 0.158051248 38100 0.041564908 38136
0.040497067 76208 0.107618704 76208 0.05549207
76200 0.134781018 76200 0.192926251 38100 0.015688138 38140
0.057023932 76216 0.132848524 76216 0.057769328
76200 0.044673929 76200 0.114872812 38100 0.044878958 38144
0.081614533 76224 0.159378964 76224 0.026798018
76200 0.071316528 76200 0.079550875 38100 0.112550159 38148
0.047664253 76232 0.09117715 76232 0.039797498
76200 0.085147571 76200 0.125210906 38200 0.116385098 38152
0.076409007 76240 0.040926716 76240 0.015677062
76200 0.086145279 76200 0.065414635 38200 0.050299375 38156
0.08739614 76248 0.073432377 76248 0.009529752
76300 0.108293614 76300 0.057119767 38200 0.019100531 38160
0.059347949 76256 0.114498711 76256 0.027592299
76300 0.143651604 76300 0.096028605 38200 0.039720821 38164
0.106008374 76264 0.12338355 76264 0.040100324
76300 0.149752305 76300 0.113733455 38200 0.036108704 38168
0.129514229 76272 0.105296189 76272 0.040769181
76300 0.104334016 76300 0.126160681 38200 0.052720214 38172
0.111843459 76280 0.035050343 76280 0.029639408
76300 0.160457698 76300 0.084520165 38200 0.062984807 38176
0.059511171 76288 0.100599013 76288 0.024899629
76300 0.176112168 76300 0.062086663 38200 0.02715661 38180
0.014604588 76296 0.142422359 76296 0.033990586
76300 0.08148756 76300 0.062142339 38200 0.027647071 38184
0.060938604 76304 0.083120547 76304 0.024222498
76300 0.080453574 76300 0.045386521 38200 0.034876797 38188
0.211882828 76312 0.099350153 76312 0.027568032
76300 0.058703303 76300 0.062598578 38200 0.030993826 38192
0.384229585 76320 0.042941247 76320 0.060974246
76300 0.049294307 76300 0.070319649 38200 0.035249264 38196
0.413586269 76328 0.07017301 76328 0.054865817
76300 0.05603112 76300 0.088003006 38200 0.033413115 38200
0.242272669 76336 0.031631313 76336 0.057705514
76300 0.077193567 76300 0.042783027 38200 0.015461284 38204
0.085507847 76344 0.114468021 76344 0.057203615
76400 0.106564708 76400 0.073379277 38200 0.024970101 38208
0.15314309 76352 0.158421943 76352 0.040762083
76400 0.049924172 76400 0.088052781 38200 0.031753491 38212
0.1619049 76360 0.126958185 76360 0.057813806
76400 0.043634991 76400 0.055045715 38200 0.053551044 38216
0.110750414 76368 0.082965031 76368 0.09792253
76400 0.06745031 76400 0.027326143 38200 0.069872738 38220
0.113450078 76376 0.078151781 76376 0.104724422
76400 0.098155506 76400 0.038393326 38200 0.043463217 38224
0.161402175 76384 0.111200694 76384 0.063098734
76400 0.088151821 76400 0.051580882 38200 0.039976232 38228
0.184363904 76392 0.113151276 76392 0.045765512
76400 0.019031873 76400 0.05359251 38200 0.062187974 38232
0.132495174 76400 0.090861679 76400 0.045108809
76400 0.083322586 76400 0.114966679 38200 0.052141531 38236
0.022246533 76408 0.092568975 76408 0.028336021
76400 0.141028664 76400 0.142442266 38200 0.021601185 38240
0.114792951 76416 0.085997664 76416 0.052681378
76400 0.101108919 76400 0.110296518 38200 0.007747602 38244
0.119372642 76424 0.071946422 76424 0.041730073
76400 0.031828367 76400 0.084708809 38200 0.028543833 38248
0.055458477 76432 0.011348135 76432 0.024165129
76400 0.011718309 76400 0.134902031 38300 0.040298663 38252
0.046842652 76440 0.02693575 76440 0.048123678
76400 0.021932023 76400 0.210131373 38300 0.047241352 38256
0.073392155 76448 0.039774888 76448 0.059493359
76500 0.017785756 76500 0.201309348 38300 0.064829139 38260
0.054043623 76456 0.127744119 76456 0.058023536
76500 0.017607565 76500 0.110594687 38300 0.063718253 38264

0.07754039 76464 0.123808975 76464 0.037431535
76500 0.050991188 76500 0.092538503 38300 0.029715759 38268
0.134215254 76472 0.103720369 76472 0.011257152
76500 0.094547642 76500 0.1194773 38300 0.011083492 38272
0.085926062 76480 0.114624047 76480 0.020872847
76500 0.038847109 76500 0.199414004 38300 0.033116223 38276
0.033318331 76488 0.094966425 76488 0.058451926
76500 0.103637161 76500 0.183452169 38300 0.035842808 38280
0.10393998 76496 0.03331052 76496 0.084153209
76500 0.148610416 76500 0.078109741 38300 0.049481365 38284
0.109641827 76504 0.041472573 76504 0.077887293
76500 0.115675925 76500 0.067335568 38300 0.088443281 38288
0.043855682 76512 0.035286907 76512 0.041572544
76500 0.072068186 76500 0.09704972 38300 0.057940208 38292
0.079668542 76520 0.020318998 76520 0.023705967
76500 0.072682618 76500 0.067010347 38300 0.004965192 38296
0.070261121 76528 0.054978988 76528 0.049688148
76500 0.093383591 76500 0.064497101 38300 0.011602729 38300
0.062759405 76536 0.039353745 76536 0.038015562
76500 0.116505908 76500 0.068754787 38300 0.02655764 38304
0.090964029 76544 0.084032916 76544 0.022442015
76600 0.081526618 76600 0.096609692 38300 0.042392207 38308
0.084032479 76552 0.147223109 76552 0.022117363
76600 0.034395773 76600 0.132828965 38300 0.034887497 38312
0.048763959 76560 0.222055329 76560 0.030719195
76600 0.087957124 76600 0.140727076 38300 0.04845914 38316
0.047591544 76568 0.204611483 76568 0.026214326
76600 0.168548606 76600 0.067136149 38300 0.072807343 38320
0.069591144 76576 0.102991522 76576 0.023528817
76600 0.13910426 76600 0.038321239 38300 0.05403344 38324
0.079056481 76584 0.021939581 76584 0.040274284
76600 0.110081783 76600 0.040086183 38300 0.022331726 38328
0.08102617 76592 0.044530574 76592 0.059016438
76600 0.069346599 76600 0.040533869 38300 0.014005096 38332
0.068225476 76600 0.025554376 76600 0.05613613
76600 0.062318803 76600 0.075330136 38300 0.035650581 38336
0.031533826 76608 0.028053884 76608 0.023269155
76600 0.0871308 76600 0.062922591 38300 0.042849541 38340
0.087085151 76616 0.096575539 76616 0.001143373
76600 0.073405427 76600 0.059010552 38300 0.019856419 38344
0.11374805 76624 0.144004181 76624 0.024095361
76600 0.04419675 76600 0.118385062 38300 0.080636324 38348
0.164065583 76632 0.117480289 76632 0.040673935
76600 0.048899601 76600 0.214809959 38400 0.08868103 38352
0.178634844 76640 0.059547809 76640 0.044780696
76600 0.087116205 76600 0.229445431 38400 0.083897219 38356
0.131299749 76648 0.032883359 76648 0.04221909
76700 0.078325 76700 0.186385936 38400 0.064031541 38360
0.060354862 76656 0.071610928 76656 0.017877554
76700 0.046393143 76700 0.159931529 38400 0.012306858 38364
0.035850855 76664 0.063579857 76664 0.009045851
76700 0.060329603 76700 0.092437178 38400 0.035276727 38368
0.120683537 76672 0.090901973 76672 0.035177316
76700 0.071685616 76700 0.024851262 38400 0.030121248 38372
0.145598926 76680 0.133750903 76680 0.045873076
76700 0.047480626 76700 0.138695701 38400 0.022386987 38376
0.082211947 76688 0.123909951 76688 0.0641855
76700 0.020550888 76700 0.126138243 38400 0.042016891 38380
0.0477045 76696 0.100960191 76696 0.065332562
76700 0.019360328 76700 0.021606762 38400 0.076526703 38384
0.064533197 76704 0.087234112 76704 0.059365662
76700 0.078041165 76700 0.035232584 38400 0.10273169 38388
0.083986 76712 0.122205122 76712 0.046512734
76700 0.12463842 76700 0.085668675 38400 0.089874615 38392
0.074216565 76720 0.177184295 76720 0.048625287
76700 0.111888723 76700 0.144412406 38400 0.050665793 38396
0.072959309 76728 0.204793396 76728 0.08034394
76700 0.024924126 76700 0.13642176 38400 0.014961537 38400

0.116172414 76736 0.143458994 76736 0.069813017
76700 0.087521949 76700 0.050894691 38400 0.035870417 38404
0.131886801 76744 0.082619532 76744 0.041184063
76800 0.125177074 76800 0.040225783 38400 0.074662217 38408
0.101577411 76752 0.13859621 76752 0.018669371
76800 0.115685769 76800 0.049163147 38400 0.069823589 38412
0.115939649 76760 0.173271678 76760 0.014105445
76800 0.113764043 76800 0.042567506 38400 0.045844787 38416
0.146847583 76768 0.143926489 76768 0.034514837
76800 0.142014964 76800 0.045173576 38400 0.039297371 38420
0.105703344 76776 0.134102011 76776 0.029074005
76800 0.136852235 76800 0.046060435 38400 0.042198102 38424
0.050642815 76784 0.134142553 76784 0.022118496
76800 0.092020775 76800 0.056129731 38400 0.047512454 38428
0.105022948 76792 0.117471835 76792 0.030723906
76800 0.043927201 76800 0.015578664 38400 0.051757044 38432
0.137019436 76800 0.163493693 76800 0.012507687
76800 0.16253331 76800 0.075688047 38400 0.055151108 38436
0.132416914 76808 0.141892743 76808 0.028164841
76800 0.153444926 76800 0.09216764 38400 0.046991303 38440
0.100495468 76816 0.094860945 76816 0.049796763
76800 0.021250919 76800 0.010341583 38400 0.037611611 38444
0.097878052 76824 0.100096389 76824 0.046766414
76800 0.089387722 76800 0.045780293 38400 0.031939417 38448
0.099205296 76832 0.077371733 76832 0.02687913
76800 0.111501264 76800 0.021862938 38500 0.0603074 38452
0.093778479 76840 0.101805308 76840 0.020394258
76800 0.157918753 76800 0.035690067 38500 0.081562728 38456
0.10974866 76848 0.130468849 76848 0.021432876
76900 0.180164686 76900 0.069657384 38500 0.04208463 38460
0.128739295 76856 0.088420915 76856 0.040554838
76900 0.143304045 76900 0.034171015 38500 0.04345538 38464
0.112674818 76864 0.105296524 76864 0.039477378
76900 0.080220634 76900 0.032599153 38500 0.043484652 38468
0.037009799 76872 0.15439812 76872 0.062461419
76900 0.053253247 76900 0.019702742 38500 0.021031459 38472
0.082482315 76880 0.141889468 76880 0.076141936
76900 0.06101828 76900 0.030346915 38500 0.012846016 38476
0.139417898 76888 0.070671689 76888 0.048189031
76900 0.055356028 76900 0.037674494 38500 0.049368424 38480
0.155607108 76896 0.037350823 76896 0.016605738
76900 0.077518896 76900 0.063271997 38500 0.049620736 38484
0.125774648 76904 0.110818313 76904 0.026938111
76900 0.12386599 76900 0.024430146 38500 0.020047757 38488
0.062657113 76912 0.150835928 76912 0.032608965
76900 0.051647177 76900 0.086729269 38500 0.039933337 38492
0.061724488 76920 0.104411876 76920 0.028351526
76900 0.108419852 76900 0.088766057 38500 0.076239441 38496
0.116156261 76928 0.05642913 76928 0.015238403
76900 0.140573524 76900 0.04568317 38500 0.06708486 38500
0.164964543 76936 0.05715189 76936 0.049514878
76900 0.09424024 76900 0.159450021 38500 0.020550437 38504
0.142885198 76944 0.034461038 76944 0.068555841
77000 0.048589118 77000 0.168585568 38500 0.029069075 38508
0.085319305 76952 0.01776516 76952 0.029021394
77000 0.031188047 77000 0.122157144 38500 0.029975157 38512
0.09904828 76960 0.051608837 76960 0.013963331
77000 0.056006757 77000 0.080580488 38500 0.008423863 38516
0.108967179 76968 0.0269454 76968 0.009801107
77000 0.077080025 77000 0.033485027 38500 0.033688899 38520
0.058779457 76976 0.087998167 76976 0.028049428
77000 0.074734075 77000 0.015492697 38500 0.071680806 38524
0.061194849 76984 0.115868781 76984 0.069002548
77000 0.069413429 77000 0.041069572 38500 0.078185738 38528
0.085648804 76992 0.060230803 76992 0.069865724
77000 0.147903425 77000 0.040816751 38500 0.030967825 38532
0.089125395 77000 0.161619435 77000 0.009752841
77000 0.130820306 77000 0.086387496 38500 0.014736916 38536

0.052603198 77008 0.130235436 77008 0.052175336
77000 0.013157623 77000 0.140523116 38500 0.058275036 38540
0.023219936 77016 0.109822067 77016 0.050204591
77000 0.125022853 77000 0.117847725 38500 0.07291665 38544
0.044087061 77024 0.15599042 77024 0.048034737
77000 0.117042786 77000 0.067770423 38500 0.035042078 38548
0.059092661 77032 0.144131234 77032 0.047892696
77000 0.033412714 77000 0.023774615 38600 0.00948779 38552
0.061717285 77040 0.104735766 77040 0.044648325
77000 0.063859799 77000 0.052808264 38600 0.059155929 38556
0.031460575 77048 0.091932088 77048 0.051645824
77100 0.036837391 77100 0.07992184 38600 0.097727258 38560
0.037132584 77056 0.069894682 77056 0.033378135
77100 0.05862067 77100 0.089612549 38600 0.075749835 38564
0.076107863 77064 0.032200238 77064 0.029635414
77100 0.05899512 77100 0.068029556 38600 0.035469624 38568
0.087072229 77072 0.1022578 77072 0.052260621
77100 0.029321824 77100 0.015904474 38600 0.022953576 38572
0.081450584 77080 0.181664945 77080 0.03669232
77100 0.042163341 77100 0.062655963 38600 0.011100351 38576
0.068027461 77088 0.151349144 77088 0.017129481
77100 0.048039583 77100 0.049035152 38600 0.034196484 38580
0.078563324 77096 0.016546572 77096 0.041793603
77100 0.087331544 77100 0.053667787 38600 0.046070392 38584
0.078433841 77104 0.119595556 77104 0.078674311
77100 0.075960554 77100 0.0142854 38600 0.062981533 38588
0.047421672 77112 0.118869677 77112 0.088076798
77100 0.11409117 77100 0.021122945 38600 0.058527461 38592
0.051088533 77120 0.148384046 77120 0.051941395
77100 0.104417552 77100 0.019328716 38600 0.015011178 38596
0.037643938 77128 0.115116724 77128 0.028994691
77100 0.036966485 77100 0.014875695 38600 0.026684245 38600
0.061701998 77136 0.039305262 77136 0.040668223
77100 0.136973656 77100 0.088046116 38600 0.031249008 38604
0.070357251 77144 0.04517474 77144 0.035409186
77200 0.139831929 77200 0.172275803 38600 0.015798007 38608
0.034192406 77152 0.093887087 77152 0.045870693
77200 0.112964743 77200 0.124343729 38600 0.039039147 38612
0.080005797 77160 0.139959884 77160 0.038675902
77200 0.068755799 77200 0.01020218 38600 0.049712897 38616
0.098778422 77168 0.171266264 77168 0.007220029
77200 0.037970774 77200 0.029732893 38600 0.0243739 38620
0.083875821 77176 0.197851201 77176 0.041401745
77200 0.095993564 77200 0.027990793 38600 0.020495145 38624
0.070565562 77184 0.19193333 77184 0.052024407
77200 0.069539223 77200 0.06674468 38600 0.031876716 38628
0.075897631 77192 0.150256601 77192 0.071134011
77200 0.074603275 77200 0.117600961 38600 0.029646915 38632
0.069978487 77200 0.120773926 77200 0.06889701
77200 0.091066919 77200 0.122955811 38600 0.025795087 38636
0.077584475 77208 0.05560337 77208 0.041314965
77200 0.127738109 77200 0.050683357 38600 0.013305828 38640
0.079646619 77216 0.10617818 77216 0.020514171
77200 0.135190785 77200 0.063475185 38600 0.025312444 38644
0.045538891 77224 0.120535449 77224 0.024199442
77200 0.07481494 77200 0.109108376 38600 0.027158492 38648
0.057785044 77232 0.087352106 77232 0.019341427
77200 0.079084581 77200 0.085142128 38700 0.047182155 38652
0.086367116 77240 0.074443451 77240 0.02411738
77200 0.135532639 77200 0.033643803 38700 0.048365102 38656
0.061741383 77248 0.068819856 77248 0.022637152
77300 0.132436486 77300 0.047091871 38700 0.007771637 38660
0.029770406 77256 0.106575921 77256 0.01701938
77300 0.092159979 77300 0.10528251 38700 0.03769546 38664
0.056440527 77264 0.037242793 77264 0.038296097
77300 0.014865652 77300 0.104171435 38700 0.058279344 38668
0.056965801 77272 0.04621478 77272 0.053725304
77300 0.077674638 77300 0.07105471 38700 0.078498393 38672

0.065493492 77280 0.012641724 77280 0.038721235
77300 0.082976869 77300 0.066151224 38700 0.068425419 38676
0.071141127 77288 0.092795126 77288 0.028204257
77300 0.059370694 77300 0.100998943 38700 0.032210406 38680
0.030990199 77296 0.171852501 77296 0.04320603
77300 0.06465066 77300 0.052367668 38700 0.026319627 38684
0.032399468 77304 0.204805765 77304 0.020416152
77300 0.077174474 77300 0.047107325 38700 0.079970647 38688
0.072417475 77312 0.125987834 77312 0.021791097
77300 0.05487234 77300 0.038740127 38700 0.129218228 38692
0.08227877 77320 0.053041338 77320 0.043040756
77300 0.084711268 77300 0.082911785 38700 0.125392428 38696
0.069326197 77328 0.080618374 77328 0.038350492
77300 0.078518773 77300 0.13916906 38700 0.077451463 38700
0.067879628 77336 0.094569165 77336 0.015411817
77300 0.030973803 77300 0.099930257 38700 0.014785576 38704
0.07974459 77344 0.107253261 77344 0.035095716
77400 0.063223357 77400 0.04104944 38700 0.052866468 38708
0.106671876 77352 0.102931117 77352 0.059971666
77400 0.071383875 77400 0.135190393 38700 0.059925835 38712
0.095914009 77360 0.070165137 77360 0.058385922
77400 0.020541793 77400 0.109989298 38700 0.045326837 38716
0.036478621 77368 0.005889781 77368 0.027339553
77400 0.085728279 77400 0.092569462 38700 0.048564485 38720
0.092506707 77376 0.029641426 77376 0.022307646
77400 0.137904426 77400 0.099699362 38700 0.075186341 38724
0.133798254 77384 0.041186791 77384 0.044681594
77400 0.11573176 77400 0.080192447 38700 0.102533682 38728
0.121848607 77392 0.096696509 77392 0.043940941
77400 0.081246384 77400 0.066042492 38700 0.091136935 38732
0.102444887 77400 0.060785191 77400 0.048267819
77400 0.075604658 77400 0.06575408 38700 0.071663257 38736
0.114032402 77408 0.077054516 77408 0.058072419
77400 0.09026107 77400 0.115849463 38700 0.038378934 38740
0.076958742 77416 0.128319749 77416 0.02910361
77400 0.048951173 77400 0.152981214 38700 0.013309002 38744
0.027739112 77424 0.144646954 77424 0.012471919
77400 0.022526256 77400 0.133505426 38700 0.01707403 38748
0.033398552 77432 0.059486065 77432 0.041476356
77400 0.013654747 77400 0.09193521 38800 0.017610808 38752
0.051142863 77440 0.022401362 77440 0.070006085
77400 0.027479515 77400 0.074232004 38800 0.046945399 38756
0.043235683 77448 0.039603423 77448 0.07996046
77500 0.051347332 77500 0.076656637 38800 0.059411301 38760
0.028205255 77456 0.081681108 77456 0.067298439
77500 0.026283284 77500 0.074051146 38800 0.04536999 38764
0.074511416 77464 0.085135209 77464 0.037598471
77500 0.052301173 77500 0.079958605 38800 0.04589223 38768
0.099383164 77472 0.087588051 77472 0.02269747
77500 0.046523877 77500 0.080973543 38800 0.07231169 38772
0.0825143 77480 0.069970141 77480 0.041119001
77500 0.111754904 77500 0.071028429 38800 0.042968673 38776
0.069403352 77488 0.070876151 77488 0.032625339
77500 0.062449755 77500 0.022776663 38800 0.027612588 38780
0.07759357 77496 0.100168581 77496 0.020785859
77500 0.015001202 77500 0.110779576 38800 0.017773824 38784
0.093744689 77504 0.11738998 77504 0.045732719
77500 0.022310056 77500 0.181495721 38800 0.024081344 38788
0.098786673 77512 0.093159513 77512 0.051984775
77500 0.021146789 77500 0.155591508 38800 0.052068575 38792
0.092741582 77520 0.039051523 77520 0.026538299
77500 0.053032083 77500 0.052635118 38800 0.061937884 38796
0.083823106 77528 0.098481964 77528 0.011473648
77500 0.068565983 77500 0.084039821 38800 0.054735316 38800
0.10874468 77536 0.105845822 77536 0.010668557
77500 0.094558163 77500 0.13754837 38800 0.032086449 38804
0.097861412 77544 0.018311843 77544 0.007844518
77600 0.128255676 77600 0.084515516 38800 0.016362306 38808

```
0.057682868	77552	0.05925525	77552	0.018607916
77600	0.140507589	77600	0.158276933	38800	0.015398407	38812
0.093553121	77560	0.091549526	77560	0.045756122
77600	0.135855444	77600	0.226004515	38800	0.027833656	38816
0.126114421	77568	0.121345125	77568	0.033696182
77600	0.087001521	77600	0.117531636	38800	0.033825734	38820
0.155627248	77576	0.125726306	77576	0.011577072
77600	0.022240552	77600	0.088933419	38800	0.031008047	38824
0.166976621	77584	0.061298852	77584	0.032761825
77600	0.026626427	77600	0.098205179	38800	0.036574544	38828
0.137493873	77592	0.089034693	77592	0.027226501
77600	0.026142565	77600	0.047932172	38800	0.021943915	38832
0.129584878	77600	0.106059582	77600	0.038415521
77600	0.01851633	77600	0.069793721	38800	0.026346355	38836
0.109128836	77608	0.095118703	77608	0.077739707
77600	0.01698453	77600	0.149320724	38800	0.040941992	38840
0.046621175	77616	0.086382963	77616	0.092042661
77600	0.079468082	77600	0.199328293	38800	0.066227352	38844
0.068554888	77624	0.064419604	77624	0.047593592
77600	0.114123148	77600	0.119523276	38800	0.045698434	38848
0.070751696	77632	0.046826161	77632	0.034893768
77600	0.090609778	77600	0.01997363	38900	0.018960584	38852
0.024743456	77640	0.10943204	77640	0.044210508
77600	0.057041569	77600	0.086346117	38900	0.028738958	38856
0.049013357	77648	0.103247126	77648	0.020783922
77700	0.082229009	77700	0.145648854	38900	0.016031801	38860
0.029714163	77656	0.075622156	77656	0.019441281
77700	0.12930487	77700	0.170683095	38900	0.01220226	38864
0.03391692	77664	0.059775804	77664	0.023891933
77700	0.164775018	77700	0.209189224	38900	0.038129725	38868
0.087522043	77672	0.060247727	77672	0.020539937
77700	0.158851879	77700	0.231213256	38900	0.02389365	38872
0.106512111	77680	0.037331418	77680	0.003036337
77700	0.117687101	77700	0.183379146	38900	0.045113684	38876
0.042382726	77688	0.063670625	77688	0.028831661
77700	0.047518148	77700	0.10311386	38900	0.099514451	38880
0.042391832	77696	0.048412083	77696	0.051724081
77700	0.072897325	77700	0.020383988	38900	0.070852868	38884
0.10654574	77704	0.073811149	77704	0.056318797
77700	0.163991674	77700	0.025914946	38900	0.002479782	38888
0.162680459	77712	0.081588965	77712	0.051612489
77700	0.125831196	77700	0.028748827	38900	0.052749318	38892
0.15245394	77720	0.039697843	77720	0.071298462
77700	0.091603724	77700	0.076984288	38900	0.080973281	38896
0.107825232	77728	0.027652433	77728	0.069938775
77700	0.132785499	77700	0.093197974	38900	0.076485158	38900
0.122801706	77736	0.162110955	77736	0.031369164
77700	0.161109099	77700	0.033053584	38900	0.043111937	38904
0.109093591	77744	0.201265516	77744	0.019618205
77800	0.145282844	77800	0.069491325	38900	0.050774808	38908
0.092847266	77752	0.079864774	77752	0.031115491
77800	0.121948033	77800	0.132767251	38900	0.052707077	38912
0.078625853	77760	0.077719524	77760	0.032348457
77800	0.12571596	77800	0.088892259	38900	0.027404509	38916
0.023229712	77768	0.088136279	77768	0.041644311
77800	0.04866852	77800	0.002294345	38900	0.011195863	38920
0.087713183	77776	0.01095191	77776	0.054035147
77800	0.073168565	77800	0.032721302	38900	0.048621172	38924
0.097767617	77784	0.060308037	77784	0.054072087
77800	0.108699009	77800	0.074935415	38900	0.080881131	38928
0.066955181	77792	0.049816699	77792	0.044445282
77800	0.116819385	77800	0.107657623	38900	0.045721637	38932
0.074032381	77800	0.085924112	77800	0.039990548
77800	0.10195018	77800	0.111321257	38900	0.084188192	38936
0.080113248	77808	0.089679132	77808	0.029732104
77800	0.130602988	77800	0.083554376	38900	0.121630226	38940
0.07715507	77816	0.110884903	77816	0.010593032
77800	0.173848938	77800	0.059590926	38900	0.090281901	38944
```

0.105554689 77824 0.068551104 77824 0.017421377
77800 0.157272356 77800 0.126639105 38900 0.078688907 38948
0.119742661 77832 0.067992187 77832 0.049568025
77800 0.088200628 77800 0.176799221 39000 0.063150095 38952
0.097667136 77840 0.158102849 77840 0.065721484
77800 0.06772139 77800 0.159265284 39000 0.027775754 38956
0.095357209 77848 0.167765699 77848 0.029465069
77900 0.056549725 77900 0.093628267 39000 0.005952109 38960
0.118913944 77856 0.103720719 77856 0.034321271
77900 0.055945453 77900 0.074328949 39000 0.029584249 38964
0.082738705 77864 0.045760498 77864 0.055031382
77900 0.03596273 77900 0.11287829 39000 0.052136791 38968
0.038676681 77872 0.082671832 77872 0.037352249
77900 0.026017717 77900 0.173291671 39000 0.060674622 38972
0.089529385 77880 0.161387754 77880 0.015048192
77900 0.047666741 77900 0.171046413 39000 0.060202041 38976
0.095640346 77888 0.228440345 77888 0.050035371
77900 0.084857973 77900 0.074576878 39000 0.053162967 38980
0.08848263 77896 0.213947643 77896 0.059329537
77900 0.064150554 77900 0.014530795 39000 0.051593175 38984
0.123260383 77904 0.102762584 77904 0.044609627
77900 0.132262693 77900 0.067890389 39000 0.065308603 38988
0.119439748 77912 0.030264424 77912 0.069429378
77900 0.161205899 77900 0.119405835 39000 0.070235656 38992
0.10226787 77920 0.105993044 77920 0.052762076
77900 0.114941569 77900 0.096390264 39000 0.051976149 38996
0.09760345 77928 0.09456306 77928 0.031068215
77900 0.097371209 77900 0.035594261 39000 0.02543628 39000
0.072386421 77936 0.03216002 77936 0.025774967
77900 0.058934558 77900 0.053298281 39000 0.021061893 39004
0.088082132 77944 0.064552245 77944 0.039903232
78000 0.126776068 78000 0.053051415 39000 0.029579545 39008
0.124777056 77952 0.066337925 77952 0.062441919
78000 0.090969064 78000 0.046558671 39000 0.023804212 39012
0.096401775 77960 0.041873478 77960 0.072115407
78000 0.10450722 78000 0.063876913 39000 0.067516594 39016
0.022856022 77968 0.079672675 77968 0.070225549
78000 0.083303719 78000 0.055600445 39000 0.07350136 39020
0.058880862 77976 0.1034733 77976 0.050726769
78000 0.075524476 78000 0.089522233 39000 0.021561316 39024
0.086533095 77984 0.130026485 77984 0.026743956
78000 0.123760285 78000 0.146725259 39000 0.038156755 39028
0.062776453 77992 0.152599736 77992 0.019634617
78000 0.104771512 78000 0.132796588 39000 0.046420715 39032
0.052710067 78000 0.14447495 78000 0.022242984
78000 0.066516004 78000 0.122168116 39000 0.028643024 39036
0.054364773 78008 0.08707463 78008 0.039254344
78000 0.078995996 78000 0.03353279 39000 0.035468205 39040
0.053594005 78016 0.023825713 78016 0.061590457
78000 0.110097666 78000 0.068489149 39000 0.036574329 39044
0.072550218 78024 0.003144889 78024 0.075594537
78000 0.106200496 78000 0.071208931 39000 0.013411341 39048
0.046010569 78032 0.067488538 78032 0.082712213
78000 0.11650777 78000 0.02297575 39100 0.046005498 39052
0.037863021 78040 0.08693547 78040 0.068296962
78000 0.076206714 78000 0.07392525 39100 0.05109753 39056
0.078302903 78048 0.057748614 78048 0.04532007
78100 0.018350665 78100 0.039775507 39100 0.040786476 39060
0.100692145 78056 0.094677322 78056 0.047811347
78100 0.053772896 78100 0.015287744 39100 0.050767427 39064
0.111039452 78064 0.135628666 78064 0.048945378
78100 0.069428897 78100 0.047532532 39100 0.029240924 39068
0.095503048 78072 0.13900669 78072 0.031350501
78100 0.097423937 78100 0.064026906 39100 0.010479427 39072
0.065555221 78080 0.177095878 78080 0.025740916
78100 0.12695133 78100 0.034534838 39100 0.029059407 39076
0.05507638 78088 0.197652553 78088 0.032763543
78100 0.121558878 78100 0.041005809 39100 0.03759687 39080

0.073350871	78096	0.168423925	78096	0.033500095
78100	0.071757793	78100	0.094176699	39100	0.010797437	39084
0.091512869	78104	0.14788931	78104	0.024120933
78100	0.014709672	78100	0.126779749	39100	0.050961971	39088
0.115485025	78112	0.178783215	78112	0.064207598
78100	0.0456292	78100	0.075112585	39100	0.026626294	39092
0.103658371	78120	0.207639721	78120	0.049116305
78100	0.030710897	78100	0.025301184	39100	0.026808231	39096
0.03989871	78128	0.138956544	78128	0.034257737
78100	0.012650203	78100	0.044756776	39100	0.038703896	39100
0.04760881	78136	0.051622392	78136	0.057842412
78100	0.044326047	78100	0.070423899	39100	0.018635245	39104
0.057923342	78144	0.072713105	78144	0.031790336
78200	0.105735584	78200	0.05383974	39100	0.010475858	39108
0.086005312	78152	0.106410647	78152	0.021967322
78200	0.139434371	78200	0.020789417	39100	0.02142369	39112
0.100282756	78160	0.167572216	78160	0.020761347
78200	0.102409584	78200	0.006993184	39100	0.047224567	39116
0.077071782	78168	0.183402459	78168	0.004067594
78200	0.070680355	78200	0.050274313	39100	0.074595126	39120
0.076871438	78176	0.097148019	78176	0.019124302
78200	0.022602577	78200	0.076185497	39100	0.082334278	39124
0.072169263	78184	0.056114343	78184	0.042166274
78200	0.056537454	78200	0.076177872	39100	0.045095028	39128
0.070648719	78192	0.109448338	78192	0.049542145
78200	0.078264537	78200	0.047323214	39100	0.027094045	39132
0.070206472	78200	0.144042919	78200	0.039030481
78200	0.099638492	78200	0.090346024	39100	0.062868399	39136
0.047156926	78208	0.128004481	78208	0.04961975
78200	0.084315172	78200	0.146530103	39100	0.087387707	39140
0.052176256	78216	0.030123087	78216	0.050859137
78200	0.06549796	78200	0.103118124	39100	0.094494222	39144
0.025409825	78224	0.137058741	78224	0.07149476
78200	0.058625108	78200	0.0259808	39100	0.066637927	39148
0.029587747	78232	0.154137743	78232	0.057784586
78200	0.081509854	78200	0.076237389	39200	0.044744935	39152
0.079074613	78240	0.132365691	78240	0.051087434
78200	0.073506817	78200	0.118304437	39200	0.024954865	39156
0.077731886	78248	0.073576768	78248	0.053391072
78300	0.024741767	78300	0.173705019	39200	0.011153002	39160
0.056511395	78256	0.037780377	78256	0.038970844
78300	0.075446718	78300	0.168609084	39200	0.014670555	39164
0.089363035	78264	0.105387531	78264	0.046023615
78300	0.069688365	78300	0.055495126	39200	0.013665147	39168
0.131440728	78272	0.088146939	78272	0.071479837
78300	0.05529066	78300	0.040988285	39200	0.024745017	39172
0.142917154	78280	0.071096234	78280	0.065847919
78300	0.039966733	78300	0.022070011	39200	0.047370602	39176
0.081511527	78288	0.051324743	78288	0.037887654
78300	0.046965557	78300	0.033801007	39200	0.042611624	39180
0.014399356	78296	0.027992286	78296	0.023333621
78300	0.099371413	78300	0.019680332	39200	0.026001693	39184
0.060127492	78304	0.028958391	78304	0.024100586
78300	0.136253133	78300	0.063711712	39200	0.065607419	39188
0.049110884	78312	0.039638031	78312	0.008813183
78300	0.128208136	78300	0.116056253	39200	0.063376348	39192
0.031565891	78320	0.111595968	78320	0.027599961
78300	0.112420268	78300	0.077774217	39200	0.028769573	39196
0.003785471	78328	0.207613295	78328	0.067003508
78300	0.143093203	78300	0.047733745	39200	0.026965838	39200
0.036115464	78336	0.187218916	78336	0.05602989
78300	0.146109364	78300	0.065865716	39200	0.068082314	39204
0.091759204	78344	0.071820447	78344	0.010769883
78400	0.117986827	78400	0.08849585	39200	0.072482952	39208
0.139715616	78352	0.104854495	78352	0.024573727
78400	0.24707985	78400	0.134243819	39200	0.043064461	39212
0.125604361	78360	0.104248677	78360	0.035458725
78400	0.39833074	78400	0.10631216	39200	0.013559823	39216

0.061876228 78368 0.026754806 78368 0.033294655
78400 0.279620144 78400 0.079261408 39200 0.018479834 39220
0.02041504 78376 0.088406683 78376 0.033815613
78400 0.236281063 78400 0.052649502 39200 0.039373048 39224
0.044720946 78384 0.054857683 78384 0.032222757
78400 0.316740566 78400 0.118985736 39200 0.035490866 39228
0.064205968 78392 0.013590899 78392 0.020328547
78400 0.189611979 78400 0.097050433 39200 0.057545105 39232
0.090373062 78400 0.011316128 78400 0.011176246
78400 0.047304577 78400 0.00377243 39200 0.071095034 39236
0.110273388 78408 0.040558422 78408 0.017782302
78400 0.069354246 78400 0.006310901 39200 0.087722649 39240
0.117016549 78416 0.127294858 78416 0.036627203
78400 0.071968156 78400 0.049273214 39200 0.046676487 39244
0.079593716 78424 0.166570143 78424 0.064367981
78400 0.075027383 78400 0.052080228 39200 0.040470492 39248
0.017649474 78432 0.12283049 78432 0.06489559
78400 0.086322689 78400 0.041188756 39300 0.080859456 39252
0.047409241 78440 0.066263478 78440 0.038699731
78400 0.148914929 78400 0.06268773 39300 0.060177496 39256
0.054023156 78448 0.030993662 78448 0.014122578
78500 0.151014654 78500 0.069377646 39300 0.064617481 39260
0.042356798 78456 0.012592376 78456 0.0220727
78500 0.090843001 78500 0.044904053 39300 0.099212812 39264
0.046229328 78464 0.02017069 78464 0.057927795
78500 0.023812769 78500 0.087485278 39300 0.090116584 39268
0.055148455 78472 0.040855775 78472 0.079542304
78500 0.055174671 78500 0.120490251 39300 0.04511766 39272
0.048227434 78480 0.024487059 78480 0.053832358
78500 0.112547838 78500 0.121064404 39300 0.032454467 39276
0.021378139 78488 0.07352078 78488 0.04412288
78500 0.094609401 78500 0.090791771 39300 0.066759167 39280
0.018628669 78496 0.061313316 78496 0.01969285
78500 0.132478963 78500 0.082907631 39300 0.070453352 39284
0.045574743 78504 0.080254547 78504 0.029412522
78500 0.183033146 78500 0.053969889 39300 0.051960018 39288
0.070237802 78512 0.136738513 78512 0.032861539
78500 0.098174169 78500 0.031033484 39300 0.04156266 39292
0.064739819 78520 0.057944526 78520 0.039897317
78500 0.090282971 78500 0.051772662 39300 0.050465151 39296
0.029009161 78528 0.052669064 78528 0.032572079
78500 0.174376808 78500 0.053947711 39300 0.06410107 39300
0.095957119 78536 0.114218812 78536 0.01904895
78500 0.125157778 78500 0.116444426 39300 0.059605936 39304
0.104310326 78544 0.131043708 78544 0.017965398
78600 0.138735894 78600 0.095799485 39300 0.054262717 39308
0.071496834 78552 0.107898304 78552 0.033192202
78600 0.265365175 78600 0.07506198 39300 0.048470327 39312
0.045476852 78560 0.014054618 78560 0.045466375
78600 0.280959561 78600 0.158983633 39300 0.03458927 39316
0.018090743 78568 0.108624947 78568 0.035528028
78600 0.229337064 78600 0.171436798 39300 0.015220019 39320
0.057607409 78576 0.103686012 78576 0.009153077
78600 0.23503766 78600 0.048656577 39300 0.013370949 39324
0.067213405 78584 0.050214127 78584 0.019599582
78600 0.777175475 78600 0.030445361 39300 0.018578952 39328
0.036921909 78592 0.081670733 78592 0.036729991
78600 0.952798699 78600 0.077585282 39300 0.0318284 39332
0.040642641 78600 0.176029979 78600 0.058582096
78600 0.364892273 78600 0.143213954 39300 0.059827798 39336
0.041541891 78608 0.184264616 78608 0.069945854
78600 0.896429178 78600 0.084710919 39300 0.062972758 39340
0.045772555 78616 0.126637169 78616 0.056211175
78600 3.44270305 78600 0.127803985 39300 0.061690342 39344
0.055035413 78624 0.060152106 78624 0.035045679
78600 6.038608963 78600 0.12929368 39300 0.06228155 39348
0.059124639 78632 0.150645399 78632 0.017859376
78600 5.9887059 78600 0.107059605 39400 0.030480582 39352

0.067788067 78640 0.184644436 78640 0.015916983
78600 3.24326125 78600 0.128437984 39400 0.037633909 39356
0.045609741 78648 0.10932533 78648 0.027283615
78700 1.569031625 78700 0.110744237 39400 0.07230843 39360
0.034278615 78656 0.101020567 78656 0.02666971
78700 1.675154663 78700 0.027914053 39400 0.056647292 39364
0.043875021 78664 0.077911303 78664 0.016101647
78700 0.778155344 78700 0.092016409 39400 0.010937524 39368
0.051214287 78672 0.07221775 78672 0.024653267
78700 0.081203914 78700 0.088118533 39400 0.06976111 39372
0.044891323 78680 0.042830776 78680 0.025522688
78700 0.285504036 78700 0.2249807 39400 0.096075943 39376
0.051684441 78688 0.093970666 78688 0.017237582
78700 0.322409993 78700 0.229621946 39400 0.075939184 39380
0.051091454 78696 0.192708424 78696 0.012348379
78700 0.272377103 78700 0.108847358 39400 0.032757384 39384
0.038763494 78704 0.274723425 78704 0.051810948
78700 0.179395269 78700 0.027900698 39400 0.028175669 39388
0.012618755 78712 0.159624368 78712 0.06629344
78700 0.252936414 78700 0.08166238 39400 0.047862995 39392
0.083571205 78720 0.092122595 78720 0.065900153
78700 0.250317419 78700 0.078965604 39400 0.054139415 39396
0.138964359 78728 0.20809668 78728 0.042895728
78700 0.163545599 78700 0.094992392 39400 0.041735726 39400
0.123817896 78736 0.164409809 78736 0.055341563
78700 0.026535543 78700 0.132684756 39400 0.021183685 39404
0.070197071 78744 0.113808572 78744 0.06218296
78800 0.132813279 78800 0.16874446 39400 0.044730336 39408
0.050766761 78752 0.170049913 78752 0.044796463
78800 0.1391806 78800 0.162085563 39400 0.048343627 39412
0.066088636 78760 0.146179256 78760 0.015784361
78800 0.105979212 78800 0.171630701 39400 0.031229163 39416
0.043095828 78768 0.116789925 78768 0.038866248
78800 0.113221649 78800 0.141202313 39400 0.023009045 39420
0.080769612 78776 0.104211926 78776 0.061958446
78800 0.214355445 78800 0.088256988 39400 0.018985413 39424
0.105407962 78784 0.097484168 78784 0.056907342
78800 0.199656614 78800 0.099402969 39400 0.073578944 39428
0.092099894 78792 0.07460879 78792 0.057781319
78800 0.164471174 78800 0.148721098 39400 0.115692637 39432
0.040640793 78800 0.048428057 78800 0.054785607
78800 0.402004953 78800 0.160382188 39400 0.101552563 39436
0.033742079 78808 0.123159014 78808 0.050933832
78800 0.444943959 78800 0.125687715 39400 0.067806926 39440
0.065339875 78816 0.078971723 78816 0.026044902
78800 0.295159349 78800 0.095338794 39400 0.023504057 39444
0.05479858 78824 0.061593382 78824 0.020233019
78800 0.479537063 78800 0.042638578 39400 0.025869975 39448
0.037230409 78832 0.059388618 78832 0.038237347
78800 0.518485148 78800 0.069912327 39500 0.047981142 39452
0.040584237 78840 0.13393737 78840 0.02895873
78800 0.59008121 78800 0.167904975 39500 0.026423802 39456
0.049209844 78848 0.155951843 78848 0.028241662
78900 0.705603743 78900 0.165356876 39500 0.006684991 39460
0.041876247 78856 0.059413651 78856 0.032550517
78900 1.3744731 78900 0.087669694 39500 0.029607143 39464
0.030527179 78864 0.069810289 78864 0.00450004
78900 1.465631188 78900 0.146251813 39500 0.069502334 39468
0.02946514 78872 0.139601034 78872 0.049055307
78900 1.3080301 78900 0.152773704 39500 0.078366815 39472
0.018508559 78880 0.063716354 78880 0.058826048
78900 4.23459755 78900 0.119035889 39500 0.042386255 39476
0.046896515 78888 0.14445631 78888 0.028233937
78900 12.58094613 78900 0.120554949 39500 0.020707699 39480
0.032281005 78896 0.083196406 78896 0.014945035
78900 18.23271438 78900 0.138562304 39500 0.052107931 39484
0.03316786 78904 0.037360594 78904 0.019609392
78900 15.00190325 78900 0.122057863 39500 0.026482978 39488

0.032116146	78912	0.056898683	78912	0.022316499		
78900	6.438763813	78900	0.082093866	39500	0.030425721	39492
0.034312598	78920	0.05651687	78920	0.07935326		
78900	3.281128363	78900	0.161553966	39500	0.050789102	39496
0.054876578	78928	0.093969393	78928	0.116050644		
78900	2.471275625	78900	0.160287935	39500	0.052395357	39500
0.074630727	78936	0.084475425	78936	0.104214167		
78900	0.697594194	78900	0.014701938	39500	0.045884495	39504
0.073575691	78944	0.039809078	78944	0.065894521		
79000	1.277155238	79000	0.141636178	39500	0.05551261	39508
0.042577354	78952	0.050340907	78952	0.050733834		
79000	1.025019913	79000	0.150022446	39500	0.036366637	39512
0.033145447	78960	0.112498281	78960	0.053822176		
79000	0.891642761	79000	0.073637391	39500	0.030457108	39516
0.024704536	78968	0.099700075	78968	0.053333108		
79000	0.820628076	79000	0.026021919	39500	0.041679054	39520
0.023888888	78976	0.043020773	78976	0.038812359		
79000	0.723791309	79000	0.100400721	39500	0.032513442	39524
0.035723559	78984	0.116906027	78984	0.049930262		
79000	0.584711554	79000	0.188298553	39500	0.021628131	39528
0.023259754	78992	0.10153441	78992	0.05362989		
79000	0.661410159	79000	0.194084306	39500	0.02416855	39532
0.036561429	79000	0.038837334	79000	0.034445711		
79000	0.614345481	79000	0.140671371	39500	0.047755031	39536
0.047440826	79008	0.111028974	79008	0.03642411		
79000	0.648026646	79000	0.11093606	39500	0.057026649	39540
0.034783003	79016	0.053431395	79016	0.021416216		
79000	0.678061974	79000	0.127158128	39500	0.036519748	39544
0.049624283	79024	0.053675216	79024	0.007071564		
79000	0.518184854	79000	0.093437535	39500	0.031402404	39548
0.049556908	79032	0.07337096	79032	0.024718474		
79000	0.43790511	79000	0.015851136	39600	0.037065478	39552
0.013789379	79040	0.002820387	79040	0.030043562		
79000	0.429155189	79000	0.074471886	39600	0.025114692	39556
0.04368442	79048	0.015290147	79048	0.01460454		
79100	0.452681358	79100	0.084290332	39600	0.016901568	39560
0.085763975	79056	0.09051487	79056	0.012027493		
79100	0.420807308	79100	0.107446576	39600	0.038134378	39564
0.070536691	79064	0.171953173	79064	0.034848115		
79100	0.342711865	79100	0.072429095	39600	0.048234611	39568
0.036434627	79072	0.199883478	79072	0.040012867		
79100	0.358628604	79100	0.043615175	39600	0.064210224	39572
0.028258623	79080	0.135654321	79080	0.026941167		
79100	0.452809909	79100	0.05428154	39600	0.066811415	39576
0.042803033	79088	0.055823512	79088	0.050858944		
79100	0.335341174	79100	0.046533911	39600	0.05266337	39580
0.074489151	79096	0.073036994	79096	0.075851916		
79100	0.210506478	79100	0.078928475	39600	0.046605092	39584
0.105822684	79104	0.11984208	79104	0.07280297		
79100	0.320847583	79100	0.070599468	39600	0.03602248	39588
0.082685365	79112	0.102893988	79112	0.062001724		
79100	0.195308035	79100	0.029122646	39600	0.06108301	39592
0.022206294	79120	0.083194769	79120	0.055152428		
79100	0.197654838	79100	0.045204248	39600	0.084591411	39596
0.010938517	79128	0.112009897	79128	0.05170525		
79100	0.149520478	79100	0.113676346	39600	0.071154151	39600
0.045194694	79136	0.088956513	79136	0.042224121		
79100	0.69478422	79100	0.11030231	39600	0.052487205	39604
0.081027974	79144	0.054093758	79144	0.018256957		
79200	0.686841493	79200	0.048374866	39600	0.042551143	39608
0.081336832	79152	0.073949836	79152	0.008105224		
79200	1.32393465	79200	0.017998555	39600	0.050105777	39612
0.043536391	79160	0.058963906	79160	0.018345498		
79200	3.81831475	79200	0.020406806	39600	0.069302951	39616
0.028238696	79168	0.028536251	79168	0.020377227		
79200	4.851269538	79200	0.044999324	39600	0.069075861	39620
0.087422435	79176	0.055377648	79176	0.026770191		
79200	3.26228095	79200	0.092278846	39600	0.051709401	39624

0.127733306 79184 0.133372974 79184 0.047697886
79200 0.994106289 79200 0.095658965 39600 0.047591722 39628
0.107880129 79192 0.187407394 79192 0.080927559
79200 0.572274381 79200 0.012410255 39600 0.049787366 39632
0.031411448 79200 0.194762063 79200 0.08202203
79200 0.54321991 79200 0.079419944 39600 0.041901996 39636
0.031540785 79208 0.099525561 79208 0.053737615
79200 0.64502313 79200 0.083647719 39600 0.042036274 39640
0.048024413 79216 0.086788015 79216 0.027314593
79200 0.321009633 79200 0.07181905 39600 0.058060017 39644
0.067715315 79224 0.115315073 79224 0.020660727
79200 0.313876314 79200 0.034113004 39600 0.044984139 39648
0.043426633 79232 0.103951927 79232 0.029650535
79200 0.421078643 79200 0.001411648 39700 0.006172384 39652
0.023279736 79240 0.104214858 79240 0.01494548
79200 0.517426116 79200 0.08258006 39700 0.010265835 39656
0.02148732 79248 0.077408404 79248 0.012250081
79300 0.417443341 79300 0.15129945 39700 0.0072197 39660
0.037841823 79256 0.057613321 79256 0.033818571
79300 0.275819766 79300 0.112856636 39700 0.026566093 39664
0.117842414 79264 0.023500232 79264 0.033261011
79300 0.255035994 79300 0.0378931 39700 0.04581394 39668
0.134406408 79272 0.070843875 79272 0.013535768
79300 0.257463573 79300 0.062043218 39700 0.060392238 39672
0.077302771 79280 0.10097109 79280 0.040788516
79300 0.356453733 79300 0.089193389 39700 0.035971712 39676
0.043306132 79288 0.074921685 79288 0.046184297
79300 0.23379414 79300 0.032660097 39700 0.027908878 39680
0.106348183 79296 0.083758707 79296 0.016682685
79300 0.218537665 79300 0.05728316 39700 0.062574967 39684
0.115461633 79304 0.03786973 79304 0.020439691
79300 0.279248546 79300 0.097754608 39700 0.034740511 39688
0.078792378 79312 0.076297903 79312 0.021733418
79300 0.151245229 79300 0.062422507 39700 0.024785915 39692
0.023291254 79320 0.060961695 79320 0.003311925
79300 0.158874201 79300 0.018904888 39700 0.052067051 39696
0.025895897 79328 0.099377183 79328 0.020525547
79300 0.189824889 79300 0.070608941 39700 0.0663053 39700
0.022763365 79336 0.151503889 79336 0.041965905
79300 0.196257504 79300 0.059285321 39700 0.049503095 39704
0.051373794 79344 0.075329008 79344 0.046406974
79400 0.135491936 79400 0.037404283 39700 0.034696725 39708
0.058887057 79352 0.086937755 79352 0.034561679
79400 0.162137993 79400 0.046915957 39700 0.038257109 39712
0.041351006 79360 0.234237595 79360 0.03299633
79400 0.106289896 79400 0.05586111 39700 0.041587329 39716
0.02721784 79368 0.293152581 79368 0.039367951
79400 0.100816185 79400 0.055235465 39700 0.025757818 39720
0.052375905 79376 0.216013781 79376 0.041482046
79400 0.165705803 79400 0.057364941 39700 0.02549037 39724
0.030456749 79384 0.084796899 79384 0.069488924
79400 0.047331054 79400 0.05983733 39700 0.030254974 39728
0.021158112 79392 0.005855523 79392 0.059431739
79400 0.101881393 79400 0.087406777 39700 0.02404829 39732
0.070667629 79400 0.044434852 79400 0.064058972
79400 0.009249784 79400 0.110321176 39700 0.041007959 39736
0.073155643 79408 0.077304634 79408 0.05542333
79400 0.197177069 79400 0.077671139 39700 0.056017707 39740
0.038930371 79416 0.057923408 79416 0.020628124
79400 0.171566426 79400 0.043572101 39700 0.035684221 39744
0.054829528 79424 0.045447276 79424 0.02202555
79400 0.203703836 79400 0.09313511 39700 0.031219192 39748
0.0542378 79432 0.078926401 79432 0.035564262
79400 0.613373355 79400 0.059976315 39800 0.034206805 39752
0.041205523 79440 0.021602793 79440 0.04261504
79400 0.61154447 79400 0.047744379 39800 0.029500543 39756
0.046845005 79448 0.026578655 79448 0.046593457
79500 0.237698944 79500 0.109405657 39800 0.021058573 39760

0.030483687 79456 0.059100432 79456 0.032160031
79500 0.052775751 79500 0.121362849 39800 0.022545239 39764
0.051482493 79464 0.120081226 79464 0.005982316
79500 0.086270877 79500 0.090318587 39800 0.051382864 39768
0.035217501 79472 0.114559385 79472 0.017080223
79500 0.070824906 79500 0.025915379 39800 0.038562968 39772
0.037188136 79480 0.135892711 79480 0.034818218
79500 0.06548049 79500 0.081037884 39800 0.014714807 39776
0.050015569 79488 0.148026913 79488 0.059824008
79500 0.037473663 79500 0.146243925 39800 0.033880395 39780
0.053013351 79496 0.104598039 79496 0.061986131
79500 0.048437971 79500 0.173083434 39800 0.023655101 39784
0.053291322 79504 0.032837928 79504 0.044010285
79500 0.077329147 79500 0.15387802 39800 0.000896054 39788
0.04286307 79512 0.056054014 79512 0.03022065
79500 0.087273089 79500 0.114402043 39800 0.035739024 39792
0.023830116 79520 0.0753196 79520 0.009776326
79500 0.084063351 79500 0.103816659 39800 0.06627867 39796
0.072324358 79528 0.042855252 79528 0.039470378
79500 0.063053034 79500 0.063368483 39800 0.066529443 39800
0.062023268 79536 0.003289218 79536 0.067068715
79500 0.032185319 79500 0.041069518 39800 0.036503894 39804
0.034093788 79544 0.082108651 79544 0.058628109
79600 0.04959001 79600 0.037619215 39800 0.058302219 39808
0.028431248 79552 0.10662678 79552 0.035396632
79600 0.097056356 79600 0.152436464 39800 0.044756678 39812
0.02359149 79560 0.082880164 79560 0.052435717
79600 0.101865015 79600 0.200015536 39800 0.035766352 39816
0.096369106 79568 0.120509641 79568 0.037343212
79600 0.105872648 79600 0.145054219 39800 0.056496658 39820
0.121230463 79576 0.083862833 79576 0.018992301
79600 0.098164448 79600 0.063642809 39800 0.07143175 39824
0.079907244 79584 0.077153971 79584 0.031352181
79600 0.031845117 79600 0.06793192 39800 0.069126298 39828
0.081131584 79592 0.073847674 79592 0.031995594
79600 0.012252381 79600 0.03139838 39800 0.040957264 39832
0.108143977 79600 0.03041438 79600 0.050334311
79600 0.082649931 79600 0.037955699 39800 0.012240746 39836
0.098605065 79608 0.079087171 79608 0.077851539
79600 0.120559598 79600 0.061518353 39800 0.004236005 39840
0.056407174 79616 0.063597225 79616 0.066757675
79600 0.050189552 79600 0.074247815 39800 0.051184838 39844
0.052205131 79624 0.030181658 79624 0.017705868
79600 0.124028811 79600 0.090485934 39800 0.092254028 39848
0.080650236 79632 0.100852951 79632 0.009962768
79600 0.206213081 79600 0.088116278 39900 0.071830589 39852
0.065066321 79640 0.141161886 79640 0.058210579
79600 0.186041624 79600 0.117672862 39900 0.002229352 39856
0.027891158 79648 0.102647413 79648 0.072205308
79700 0.026389982 79700 0.082630228 39900 0.043689423 39860
0.027679795 79656 0.136737115 79656 0.038604358
79700 0.168629195 79700 0.022891889 39900 0.033733515 39864
0.042965672 79664 0.138058415 79664 0.006534939
79700 0.211319698 79700 0.1016475 39900 0.033844463 39868
0.055359702 79672 0.151004323 79672 0.041629282
79700 0.124577535 79700 0.161216828 39900 0.039173359 39872
0.040722782 79680 0.102952414 79680 0.035595545
79700 0.076150463 79700 0.140106829 39900 0.055928409 39876
0.014622933 79688 0.031153228 79688 0.011251454
79700 0.124390499 79700 0.04035866 39900 0.031830758 39880
0.080219434 79696 0.070428876 79696 0.00588779
79700 0.142179924 79700 0.023915283 39900 0.023953031 39884
0.107957974 79704 0.079412355 79704 0.033832501
79700 0.069781854 79700 0.046415378 39900 0.038962437 39888
0.072187017 79712 0.105590487 79712 0.028478689
79700 0.024330842 79700 0.088861845 39900 0.01925246 39892
0.062424922 79720 0.06143131 79720 0.028061564
79700 0.009545478 79700 0.069319598 39900 0.028225333 39896

0.070497474 79728 0.120431534 79728 0.035828631
79700 0.032480017 79700 0.022019254 39900 0.047205755 39900
0.033712222 79736 0.166663973 79736 0.022484352
79700 0.085474938 79700 0.020549793 39900 0.068016561 39904
0.015962738 79744 0.131788533 79744 0.012177286
79800 0.098074932 79800 0.012213748 39900 0.068414011 39908
0.002903896 79752 0.088430388 79752 0.04641297
79800 0.061304098 79800 0.091423623 39900 0.049761358 39912
0.021009739 79760 0.055864239 79760 0.063512394
79800 0.097067103 79800 0.134657181 39900 0.026404316 39916
0.019862682 79768 0.072334391 79768 0.054136184
79800 0.059441318 79800 0.10817196 39900 0.025269814 39920
0.04457009 79776 0.090341702 79776 0.005495262
79800 0.032599182 79800 0.077605029 39900 0.041222589 39924
0.07966633 79784 0.045113353 79784 0.05394268
79800 0.074474636 79800 0.068370864 39900 0.054738928 39928
0.084616557 79792 0.054187083 79792 0.043899741
79800 0.097655124 79800 0.062773521 39900 0.04961712 39932
0.037232698 79800 0.060217368 79800 0.029789491
79800 0.056866062 79800 0.095459785 39900 0.044767508 39936
0.022754941 79808 0.116006631 79808 0.06312412
79800 0.019733587 79800 0.105535983 39900 0.038458613 39940
0.044699849 79816 0.096258526 79816 0.050967319
79800 0.021117759 79800 0.145704238 39900 0.016048274 39944
0.051859486 79824 0.027067663 79824 0.026609392
79800 0.014374454 79800 0.124316735 39900 0.048992875 39948
0.019096473 79832 0.046488352 79832 0.016893784
79800 0.008043626 79800 0.098073149 40000 0.061619358 39952
0.022522572 79840 0.055496344 79840 0.009960228
79800 0.057999871 79800 0.086446045 40000 0.047515889 39956
0.056523844 79848 0.096013137 79848 0.021420332
79900 0.133505994 79900 0.070993854 40000 0.035395522 39960
0.075466465 79856 0.147262355 79856 0.041123538
79900 0.172699306 79900 0.039935752 40000 0.042262371 39964
0.072540643 79864 0.167512684 79864 0.038917035
79900 0.155483591 79900 0.104914398 40000 0.035386074 39968
0.057101774 79872 0.137079885 79872 0.021142238
79900 0.125567734 79900 0.165281264 40000 0.022877759 39972
0.056870307 79880 0.072642288 79880 0.00915593
79900 0.098500146 79900 0.153114291 40000 0.032150303 39976
0.026374497 79888 0.110276385 79888 0.016497017
79900 0.0470864 79900 0.169680846 40000 0.041132731 39980
0.048353177 79896 0.176672911 79896 0.026450083
79900 0.039499682 79900 0.133947528 40000 0.0273447 39984
0.086861648 79904 0.222441609 79904 0.030135407
79900 0.058855854 79900 0.09753427 40000 0.008062963 39988
0.063007959 79912 0.16340903 79912 0.016430293
79900 0.053562817 79900 0.155253249 40000 0.037901467 39992
0.030257534 79920 0.063600855 79920 0.015442063
79900 0.11716719 79900 0.085959422 40000 0.057933285 39996
0.037445047 79928 0.07267834 79928 0.040196352
79900 0.160653319 79900 0.054160835 40000 0.058591068 40000
0.0252397 79936 0.156578448 79936 0.040835843
79900 0.049752882 79900 0.062938671 40000 0.031008593 40004
0.035006091 79944 0.1634012 79944 0.0351985
80000 0.15152173 80000 0.030616873 40000 0.03941037 40008
0.040160805 79952 0.123785954 79952 0.061453517
80000 0.20450131 80000 0.146655818 40000 0.045164459 40012
0.014236553 79960 0.131996349 79960 0.075131124
80000 0.142666308 80000 0.183746568 40000 0.029278665 40016
0.043257245 79968 0.178421688 79968 0.064899607
80000 0.112244299 80000 0.111185669 40000 0.039311941 40020
0.06079123 79976 0.135203285 79976 0.030616902
80000 0.149287735 80000 0.035476402 40000 0.059221562 40024
0.065858512 79984 0.061242659 79984 0.044881592
80000 0.160475218 80000 0.0771013 40000 0.070661292 40028
0.031335789 79992 0.056832087 79992 0.04824662
80000 0.107384876 80000 0.098483964 40000 0.079351601 40032

0.078832534 80000 0.10447234 80000 0.035085901
80000 0.075973905 80000 0.061722676 40000 0.077421879 40036
0.080483769 80008 0.084274259 80008 0.021053265
80000 0.070127207 80000 0.027945644 40000 0.044172983 40040
0.058568159 80016 0.043202719 80016 0.014733754
80000 0.019238189 80000 0.0433881 40000 0.017533172 40044
0.062843312 80024 0.0617552 80024 0.021291804
80000 0.108752189 80000 0.085546344 40000 0.049699109 40048
0.047244434 80032 0.088111701 80032 0.015392399
80000 0.147554776 80000 0.134116985 40100 0.059865735 40052
0.007238712 80040 0.035667967 80040 0.009654402
80000 0.117114323 80000 0.132266753 40100 0.071281917 40056
0.021517186 80048 0.056216384 80048 0.019500145
80100 0.048723821 80100 0.075796423 40100 0.082220664 40060
0.031699798 80056 0.092421098 80056 0.047728187
80100 0.032491353 80100 0.033985376 40100 0.080151687 40064
0.034056644 80064 0.101623351 80064 0.064999542
80100 0.034910823 80100 0.042366013 40100 0.029720497 40068
0.017841974 80072 0.072061812 80072 0.046723686
80100 0.069581693 80100 0.065917564 40100 0.02142089 40072
0.005271695 80080 0.06477828 80080 0.003014479
80100 0.027586642 80100 0.073861025 40100 0.025751915 40076
0.021993847 80088 0.064596999 80088 0.057304544
80100 0.066856606 80100 0.115129406 40100 0.027550947 40080
0.055350203 80096 0.034888151 80096 0.088415174
80100 0.07029235 80100 0.142704885 40100 0.036767196 40084
0.087687651 80104 0.111435205 80104 0.092666298
80100 0.076546901 80100 0.096948046 40100 0.027708531 40088
0.089169727 80112 0.132528178 80112 0.070100723
80100 0.052895426 80100 0.091176335 40100 0.02441482 40092
0.048786762 80120 0.101248508 80120 0.045655856
80100 0.044468641 80100 0.133727415 40100 0.04154944 40096
0.038805269 80128 0.092382208 80128 0.043930857
80100 0.061971507 80100 0.104822895 40100 0.036479327 40100
0.067633926 80136 0.102817641 80136 0.047120862
80100 0.03438662 80100 0.041215069 40100 0.060727216 40104
0.041978397 80144 0.104033956 80144 0.039744769
80200 0.046421341 80200 0.040449555 40100 0.078642472 40108
0.009377562 80152 0.073109419 80152 0.051355837
80200 0.027255099 80200 0.071573268 40100 0.058435198 40112
0.040949184 80160 0.067451751 80160 0.055462995
80200 0.055264784 80200 0.110255634 40100 0.017621276 40116
0.072024115 80168 0.087706831 80168 0.043224576
80200 0.051887229 80200 0.122035453 40100 0.018494293 40120
0.083886283 80176 0.036888738 80176 0.018662538
80200 0.062145948 80200 0.096011499 40100 0.02981159 40124
0.08274732 80184 0.029689443 80184 0.014824083
80200 0.097849486 80200 0.135380149 40100 0.044063138 40128
0.067154171 80192 0.095501928 80192 0.022256674
80200 0.079858139 80200 0.194562759 40100 0.06384265 40132
0.08104572 80200 0.147016778 80200 0.02937725
80200 0.0932244 80200 0.208911399 40100 0.062478102 40136
0.11480353 80208 0.114915929 80208 0.047594578
80200 0.103559483 80200 0.164160113 40100 0.047899495 40140
0.130448665 80216 0.091528345 80216 0.042724394
80200 0.022529501 80200 0.042818905 40100 0.026617192 40144
0.107044107 80224 0.090845526 80224 0.033454417
80200 0.097575154 80200 0.073563526 40100 0.023924677 40148
0.038380436 80232 0.082810759 80232 0.041002921
80200 0.151259374 80200 0.043329241 40200 0.039268241 40152
0.101060628 80240 0.1092366 80240 0.070445974
80200 0.120924451 80200 0.011058071 40200 0.027341715 40156
0.141100798 80248 0.098421304 80248 0.081288141
80300 0.066807959 80300 0.017272643 40200 0.025643065 40160
0.12762862 80256 0.054003122 80256 0.070972878
80300 0.034572477 80300 0.040690353 40200 0.013236488 40164
0.105787825 80264 0.059744009 80264 0.0588535
80300 0.010634376 80300 0.043978103 40200 0.028473625 40168

0.06840557 80272 0.060909355 80272 0.026716052
80300 0.031373631 80300 0.087654073 40200 0.035253623 40172
0.03763357 80280 0.068907772 80280 0.027462667
80300 0.033930064 80300 0.054768636 40200 0.035609872 40176
0.045843874 80288 0.146634411 80288 0.049151458
80300 0.045986813 80300 0.090928334 40200 0.057100002 40180
0.063902924 80296 0.17616585 80296 0.071649476
80300 0.02916325 80300 0.10438219 40200 0.069593065 40184
0.106434149 80304 0.097836928 80304 0.081188227
80300 0.041513722 80300 0.030246356 40200 0.063526975 40188
0.149318498 80312 0.055943528 80312 0.065474647
80300 0.042431562 80300 0.020776008 40200 0.079050333 40192
0.165327801 80320 0.124904604 80320 0.049087197
80300 0.110470057 80300 0.030753214 40200 0.077015742 40196
0.151733984 80328 0.088380475 80328 0.034415662
80300 0.12113413 80300 0.060128903 40200 0.040758783 40200
0.119675642 80336 0.052370258 80336 0.042629727
80300 0.051470332 80300 0.050783667 40200 0.006481946 40204
0.072566312 80344 0.105636995 80344 0.052367577
80400 0.006146397 80400 0.039663803 40200 0.005803531 40208
0.078291516 80352 0.149308748 80352 0.049611928
80400 0.020781543 80400 0.024245808 40200 0.018775856 40212
0.102991631 80360 0.084734165 80360 0.049078684
80400 0.006513332 80400 0.108931141 40200 0.049331189 40216
0.091389047 80368 0.018956238 80368 0.01773921
80400 0.012419366 80400 0.17177347 40200 0.066075998 40220
0.0887848 80376 0.072454954 80376 0.025092912
80400 0.097684781 80400 0.139678261 40200 0.068484951 40224
0.100902493 80384 0.078571728 80384 0.050281338
80400 0.192858861 80400 0.059756876 40200 0.075397613 40228
0.124555096 80392 0.076082368 80392 0.062644271
80400 0.202268915 80400 0.052553365 40200 0.052265692 40232
0.141463214 80400 0.073014991 80400 0.045550998
80400 0.140444958 80400 0.02450106 40200 0.013961091 40236
0.097416938 80408 0.010477558 80408 0.018525456
80400 0.057317669 80400 0.027957889 40200 0.046302041 40240
0.0077565 80416 0.109792418 80416 0.014179964
80400 0.029529816 80400 0.0814737 40200 0.053571868 40244
0.069546644 80424 0.13504806 80424 0.018428133
80400 0.082850085 80400 0.135613765 40200 0.060112096 40248
0.088710796 80432 0.06623377 80432 0.029466899
80400 0.184150456 80400 0.125186373 40300 0.08073845 40252
0.064607899 80440 0.089407295 80440 0.020538801
80400 0.20567546 80400 0.051218853 40300 0.05539232 40256
0.023138178 80448 0.07454431 80448 0.033271204
80500 0.127864813 80500 0.04076734 40300 0.051140491 40260
0.057141868 80456 0.025320056 80456 0.051000185
80500 0.058493664 80500 0.03679232 40300 0.076977398 40264
0.104938285 80464 0.080207436 80464 0.024543995
80500 0.060120958 80500 0.024958563 40300 0.045918496 40268
0.134491158 80472 0.092102047 80472 0.045730369
80500 0.133855326 80500 0.077092547 40300 0.062035295 40272
0.113458198 80480 0.175887778 80480 0.070803915
80500 0.165569218 80500 0.120217454 40300 0.076140692 40276
0.051448558 80488 0.206978294 80488 0.036645655
80500 0.106921361 80500 0.191980725 40300 0.052340944 40280
0.068630186 80496 0.122750134 80496 0.009684672
80500 0.045311717 80500 0.196504873 40300 0.028835029 40284
0.116569943 80504 0.046820758 80504 0.032247939
80500 0.167349398 80500 0.121564604 40300 0.032273605 40288
0.127391628 80512 0.012228502 80512 0.044712226
80500 0.177519221 80500 0.044347777 40300 0.041172254 40292
0.087068358 80520 0.084549633 80520 0.040984953
80500 0.071596318 80500 0.089344176 40300 0.033528493 40296
0.064928521 80528 0.114262941 80528 0.018748564
80500 0.033978762 80500 0.128097774 40300 0.007792654 40300
0.089174246 80536 0.112971102 80536 0.013364349
80500 0.024361347 80500 0.098082084 40300 0.067398898 40304

0.096242242	80544	0.110931964	80544	0.033242464		
80600	0.056212131	80600	0.067359622	40300	0.093475836	40308
0.084202577	80552	0.065772358	80552	0.039339549		
80600	0.106310916	80600	0.041780782	40300	0.051697538	40312
0.080817983	80560	0.051224673	80560	0.038395865		
80600	0.217366193	80600	0.096889969	40300	0.036529047	40316
0.104114544	80568	0.066689361	80568	0.021729402		
80600	0.316106831	80600	0.157549511	40300	0.081219994	40320
0.128862331	80576	0.064040454	80576	0.001763985		
80600	0.248341501	80600	0.150924578	40300	0.084520478	40324
0.090272792	80584	0.072133269	80584	0.021993628		
80600	0.114444942	80600	0.093078204	40300	0.062615298	40328
0.045815945	80592	0.058141792	80592	0.015543648		
80600	0.161881456	80600	0.074978932	40300	0.058117024	40332
0.076119584	80600	0.13082537	80600	0.032032098		
80600	0.198938709	80600	0.084234038	40300	0.051887979	40336
0.083992469	80608	0.148718551	80608	0.059780236		
80600	0.183971294	80600	0.089014917	40300	0.054614706	40340
0.106666608	80616	0.129223801	80616	0.04667219		
80600	0.154559835	80600	0.059122343	40300	0.047687299	40344
0.091029651	80624	0.215273918	80624	0.05714237		
80600	0.13642195	80600	0.020951831	40300	0.051959069	40348
0.061537947	80632	0.413814181	80632	0.038775372		
80600	0.106993211	80600	0.029731427	40400	0.054373912	40352
0.0711619	80640	0.455354399	80640	0.03821712		
80600	0.070722446	80600	0.008859744	40400	0.066392793	40356
0.068519716	80648	0.197720335	80648	0.057805071		
80700	0.042316853	80700	0.099464363	40400	0.064830878	40360
0.067578425	80656	0.35018439	80656	0.08151657		
80700	0.062083105	80700	0.16254907	40400	0.045902671	40364
0.055943776	80664	0.283347326	80664	0.06758338		
80700	0.077703182	80700	0.136233691	40400	0.062053601	40368
0.053661337	80672	0.352017203	80672	0.02210495		
80700	0.168828541	80700	0.084084604	40400	0.080103186	40372
0.072887167	80680	0.528096164	80680	0.025071251		
80700	0.24313711	80700	0.068565663	40400	0.108855944	40376
0.096626056	80688	0.637007063	80688	0.043438391		
80700	0.200895855	80700	0.096406126	40400	0.082966828	40380
0.101937738	80696	0.728010259	80696	0.063380227		
80700	0.155512621	80700	0.123644466	40400	0.034194825	40384
0.07771016	80704	0.83865429	80704	0.044983899		
80700	0.188244085	80700	0.098165721	40400	0.03594812	40388
0.064229404	80712	1.334865575	80712	0.01274202		
80700	0.271327561	80700	0.091838032	40400	0.035555	40392
0.076852521	80720	0.874370569	80720	0.012003355		
80700	0.254064769	80700	0.175192646	40400	0.045307243	40396
0.064174172	80728	1.847968325	80728	0.043353055		
80700	0.145317011	80700	0.165037156	40400	0.072637173	40400
0.035382265	80736	1.305431013	80736	0.050622071		
80700	0.098340293	80700	0.115635776	40400	0.067595589	40404
0.02999475	80744	2.021107363	80744	0.01902751		
80800	0.087286418	80800	0.115011972	40400	0.056147776	40408
0.020923617	80752	2.345012963	80752	0.037920643		
80800	0.035249119	80800	0.10345097	40400	0.045293975	40412
0.037815167	80760	2.110301288	80760	0.092338087		
80800	0.039399638	80800	0.053182463	40400	0.024848943	40416
0.054693599	80768	2.964973913	80768	0.100076504		
80800	0.062082159	80800	0.054733999	40400	0.045141336	40420
0.071322684	80776	3.13003175	80776	0.049924463		
80800	0.062281804	80800	0.060377475	40400	0.074649972	40424
0.085197171	80784	3.87948495	80784	0.007090241		
80800	0.088381305	80800	0.023223327	40400	0.044619166	40428
0.065044878	80792	2.408030675	80792	0.011374653		
80800	0.14180546	80800	0.024519704	40400	0.028289838	40432
0.021836326	80800	4.241897263	80800	0.009117949		
80800	0.16765813	80800	0.052517022	40400	0.028468743	40436
0.041673127	80808	2.630658675	80808	0.034344444		
80800	0.106757419	80800	0.06242653	40400	0.019055671	40440

0.085120613 80816 2.660446563 80816 0.031692667
80800 0.089276306 80800 0.032905296 40400 0.025734527 40444
0.103642211 80824 3.968403675 80824 0.004179768
80800 0.088600486 80800 0.039902418 40400 0.033547443 40448
0.099066943 80832 0.836899563 80832 0.025788622
80800 0.075285352 80800 0.068461704 40500 0.041909654 40452
0.080395606 80840 4.742173013 80840 0.020983249
80800 0.066256456 80800 0.058934766 40500 0.033717312 40456
0.067350658 80848 8.440966713 80848 0.034479501
80900 0.054565142 80900 0.035731733 40500 0.00864608 40460
0.042845517 80856 0 80856 0.02907568
80900 0.111278401 80900 0.060988368 40500 0.051901392 40464
0.081269682 80864 12.00084481 80864 0.014476682
80900 0.112600566 80900 0.108914974 40500 0.044701657 40468
0.118392403 80872 8.428025063 80872 0.051920808
80900 0.024947434 80900 0.133567431 40500 0.009389078 40472
0.123114092 80880 3.3071395 80880 0.072612602
80900 0.041112409 80900 0.133909373 40500 0.026834954 40476
0.067083958 80888 1.311235125 80888 0.072819705
80900 0.022751689 80900 0.090769419 40500 0.042053762 40480
0.008945732 80896 1.241241116 80896 0.040150968
80900 0.052808326 80900 0.00972163 40500 0.056640361 40484
0.060565977 80904 0.845564239 80904 0.012427099
80900 0.093879309 80900 0.063709937 40500 0.082804865 40488
0.077639401 80912 1.398750113 80912 0.042030264
80900 0.124470302 80900 0.104370927 40500 0.070385853 40492
0.068621317 80920 0.878245279 80920 0.073571158
80900 0.138723684 80900 0.118699405 40500 0.042085667 40496
0.051460502 80928 0.340832805 80928 0.048819176
80900 0.113310794 80900 0.072066388 40500 0.033439912 40500
0.069185306 80936 0.94556046 80936 0.009593005
80900 0.057495552 80900 0.028078248 40500 0.015739915 40504
0.093296796 80944 0.701636658 80944 0.028194803
81000 0.089483001 81000 0.097594137 40500 0.020516487 40508
0.094709314 80952 0.02768585 80952 0.047138859
81000 0.166621626 81000 0.107159576 40500 0.042075033 40512
0.083603001 80960 0.605251931 80960 0.037543046
81000 0.210035273 81000 0.100509504 40500 0.052116437 40516
0.081855847 80968 0.616736303 80968 0.015616954
81000 0.222852963 81000 0.032303637 40500 0.039728118 40520
0.121631303 80976 0.163312303 80976 0.038114995
81000 0.135849535 81000 0.105769344 40500 0.06480523 40524
0.101174737 80984 0.245513889 80984 0.029610683
81000 0.025996287 81000 0.090358037 40500 0.063353546 40528
0.044937835 80992 0.263533643 80992 0.036707963
81000 0.026577869 81000 0.122498939 40500 0.059944709 40532
0.028287937 81000 0.207038044 81000 0.036125672
81000 0.027055934 81000 0.108447413 40500 0.039905757 40536
0.075874799 81008 0.233340251 81008 0.029756231
81000 0.116822601 81000 0.01802163 40500 0.051682906 40540
0.090886504 81016 0.15074671 81016 0.027122344
81000 0.1030657 81000 0.12515625 40500 0.085650201 40544
0.079134523 81024 0.07318757 81024 0.015926464
81000 0.018975079 81000 0.112328162 40500 0.074978801 40548
0.057381782 81032 0.061321603 81032 0.012111746
81000 0.073236777 81000 0.070658272 40600 0.030844658 40552
0.03047077 81040 0.044879205 81040 0.020235753
81000 0.048298622 81000 0.137668744 40600 0.054145563 40556
0.095029514 81048 0.120702171 81048 0.018873794
81100 0.087135901 81100 0.219319758 40600 0.085710919 40560
0.143083321 81056 0.162746874 81056 0.01425339
81100 0.116228017 81100 0.150408654 40600 0.084143547 40564
0.116291223 81064 0.142132616 81064 0.037911246
81100 0.112654794 81100 0.06875414 40600 0.082852559 40568
0.05707247 81072 0.087475943 81072 0.035221015
81100 0.047211961 81100 0.074850424 40600 0.074509204 40572
0.029532403 81080 0.030936746 81080 0.006925212
81100 0.051043244 81100 0.183087308 40600 0.038627106 40576

0.084258012		81088	0.074454343		81088	0.015463156		
81100	0.036351343		81100	0.155837989		40600	0.033036864	40580
0.075176082		81096	0.07599259		81096	0.034203469		
81100	0.064952124		81100	0.092051036		40600	0.056766992	40584
0.049869719		81104	0.093601011		81104	0.052687719		
81100	0.089348912		81100	0.077397584		40600	0.040183091	40588
0.019296163		81112	0.050033592		81112	0.062954721		
81100	0.065108921		81100	0.108880573		40600	0.009365149	40592
0.031698983		81120	0.112904054		81120	0.071634531		
81100	0.055523302		81100	0.17372756		40600	0.035356501	40596
0.069189766		81128	0.180920223		81128	0.066187007		
81100	0.047313646		81100	0.139860451		40600	0.020884821	40600
0.096646632		81136	0.057836111		81136	0.035250374		
81100	0.017148681		81100	0.036303627		40600	0.035591937	40604
0.10084406		81144	0.102933824		81144	0.02439389		
81200	0.137138428		81200	0.17118112		40600	0.055339682	40608
0.099857978		81152	0.137004041		81152	0.033887143		
81200	0.26617886		81200	0.176706163		40600	0.024026873	40612
0.088934394		81160	0.145867045		81160	0.019845611		
81200	0.245837553		81200	0.069233895		40600	0.009905356	40616
0.105080158		81168	0.108748573		81168	0.024719944		
81200	0.117809446		81200	0.051720832		40600	0.012838557	40620
0.137215175		81176	0.039790157		81176	0.045157318		
81200	0.093933966		81200	0.058685222		40600	0.024965711	40624
0.151475164		81184	0.055506767		81184	0.060214567		
81200	0.230579695		81200	0.025574145		40600	0.012925118	40628
0.12719346		81192	0.084450403		81192	0.050313334		
81200	0.233487531		81200	0.027029751		40600	0.016403285	40632
0.066670538		81200	0.127425869		81200	0.046001489		
81200	0.02711729		81200	0.046950816		40600	0.023108429	40636
0.008512941		81208	0.160801589		81208	0.036779005		
81200	0.173451118		81200	0.018308221		40600	0.034099983	40640
0.055476474		81216	0.130165893		81216	0.026042506		
81200	0.524498988		81200	0.117176467		40600	0.042821299	40644
0.077043573		81224	0.095810421		81224	0.010679575		
81200	1.168598188		81200	0.125238861		40600	0.038663009	40648
0.072556817		81232	0.094516487		81232	0.02329259		
81200	1.359844		81200	0.092621784		40700	0.042627064	40652
0.058850597		81240	0.085522603		81240	0.036619189		
81200	0.773958688		81200	0.136669725		40700	0.038571041	40656
0.050724724		81248	0.076244171		81248	0.006647821		
81300	0.073706629		81300	0.12192646		40700	0.019314164	40660
0.044764067		81256	0.049297771		81256	0.055275708		
81300	0.294171769		81300	0.083594503		40700	0.033617365	40664
0.072585921		81264	0.087320666		81264	0.055768833		
81300	0.223539144		81300	0.034358262		40700	0.050396779	40668
0.088248868		81272	0.186289995		81272	0.039473536		
81300	0.172770073		81300	0.044263688		40700	0.035290537	40672
0.073193121		81280	0.22792237		81280	0.06031496		
81300	0.178802161		81300	0.15494856		40700	0.017288679	40676
0.071618903		81288	0.174736356		81288	0.102977276		
81300	0.18204916		81300	0.224216899		40700	0.05298348	40680
0.079709382		81296	0.057914571		81296	0.100497447		
81300	0.128137719		81300	0.19197943		40700	0.069861737	40684
0.075693934		81304	0.041668685		81304	0.061815626		
81300	0.072917057		81300	0.101897051		40700	0.060293289	40688
0.129033143		81312	0.029675495		81312	0.037972965		
81300	0.084032559		81300	0.045899065		40700	0.045822937	40692
0.143832018		81320	0.029460731		81320	0.04855818		
81300	0.087592431		81300	0.07561074		40700	0.024678613	40696
0.142377306		81328	0.040324398		81328	0.071475246		
81300	0.052613414		81300	0.141634359		40700	0.030517365	40700
0.137771538		81336	0.019851479		81336	0.068270674		
81300	0.032384898		81300	0.122312573		40700	0.050318075	40704
0.10740944		81344	0.119729579		81344	0.062569721		
81400	0.100301033		81400	0.052230338		40700	0.039597609	40708
0.077720019		81352	0.146861101		81352	0.070313727		
81400	0.198416165		81400	0.066643428		40700	0.028223654	40712

0.076973956 81360 0.089161003 81360 0.065958258
81400 0.290517404 81400 0.125714229 40700 0.028985633 40716
0.07663064 81368 0.068557216 81368 0.032507865
81400 0.249824806 81400 0.077484583 40700 0.016523674 40720
0.072268682 81376 0.115964518 81376 0.041923846
81400 0.200781943 81400 0.061201899 40700 0.024844903 40724
0.105618005 81384 0.117224423 81384 0.068285204
81400 0.243092901 81400 0.133350026 40700 0.034242501 40728
0.102789723 81392 0.109863453 81392 0.038236423
81400 0.156515845 81400 0.091979782 40700 0.051441894 40732
0.078685764 81400 0.069037327 81400 0.018067374
81400 0.219029273 81400 0.09907702 40700 0.055730547 40736
0.122459605 81408 0.107958738 81408 0.039585495
81400 0.25857825 81400 0.142234756 40700 0.059038015 40740
0.125439343 81416 0.055672121 81416 0.055341883
81400 0.19073594 81400 0.122991143 40700 0.062996594 40744
0.075362026 81424 0.072381481 81424 0.077093049
81400 0.207280216 81400 0.066420354 40700 0.029654719 40748
0.04734426 81432 0.162501005 81432 0.063618267
81400 0.282362773 81400 0.112124195 40800 0.022099788 40752
0.068491499 81440 0.205026489 81440 0.025831307
81400 0.325915665 81400 0.142167584 40800 0.029482553 40756
0.074033305 81448 0.144873076 81448 0.022551181
81500 0.484552816 81500 0.158522991 40800 0.002673627 40760
0.090693073 81456 0.082637969 81456 0.05223725
81500 0.599725929 81500 0.127968276 40800 0.030763924 40764
0.045752768 81464 0.107816464 81464 0.059638729
81500 0.264633273 81500 0.09748694 40800 0.03473269 40768
0.06032232 81472 0.106688458 81472 0.03029939
81500 0.71322266 81500 0.047244281 40800 0.046342604 40772
0.122489524 81480 0.046340578 81480 0.034385306
81500 1.114879269 81500 0.07993693 40800 0.0296502 40776
0.11948654 81488 0.101088575 81488 0.027110562
81500 3.590708363 81500 0.138051488 40800 0.030776213 40780
0.067981433 81496 0.190054038 81496 0.016215516
81500 6.134561725 81500 0.13268787 40800 0.068087291 40784
0.075832279 81504 0.185531113 81504 0.046933696
81500 5.883643875 81500 0.077528792 40800 0.026551034 40788
0.073950934 81512 0.093597904 81512 0.075650554
81500 3.1490731 81500 0.088838511 40800 0.04841877 40792
0.034891345 81520 0.04425531 81520 0.055056207
81500 0.675113988 81500 0.117836637 40800 0.058123576 40796
0.081675607 81528 0.060652212 81528 0.028743856
81500 0.651695649 81500 0.048887097 40800 0.013756881 40800
0.124188635 81536 0.117607626 81536 0.016706248
81500 0.635378645 81500 0.065935463 40800 0.023882973 40804
0.117473144 81544 0.167645921 81544 0.005373242
81600 0.307718816 81600 0.100508856 40800 0.046737001 40808
0.094231058 81552 0.154372159 81552 0.011748829
81600 0.151630331 81600 0.039205264 40800 0.053471162 40812
0.073613046 81560 0.094788375 81560 0.016390661
81600 0.075813878 81600 0.017558949 40800 0.043562322 40816
0.046461955 81568 0.036650257 81568 0.028015282
81600 0.106406144 81600 0.00935579 40800 0.025868034 40820
0.043856879 81576 0.022831024 81576 0.039245959
81600 0.105197774 81600 0.083066574 40800 0.014623501 40824
0.03892717 81584 0.025910522 81584 0.03473433
81600 0.050304905 81600 0.124656872 40800 0.021277685 40828
0.042425476 81592 0.058405112 81592 0.038064754
81600 0.086772241 81600 0.140645163 40800 0.020020112 40832
0.063098436 81600 0.056201934 81600 0.068315785
81600 0.03956669 81600 0.188850769 40800 0.012456219 40836
0.072527386 81608 0.028076303 81608 0.069562892
81600 0.057586523 81600 0.199252638 40800 0.015333717 40840
0.104094288 81616 0.008961012 81616 0.028682307
81600 0.060627557 81600 0.102987942 40800 0.028628812 40844
0.094266841 81624 0.026520744 81624 0.04064188
81600 0.053043237 81600 0.084204934 40800 0.029911991 40848

0.045603691 81632 0.017950806 81632 0.045186334
81600 0.142196761 81600 0.11842313 40900 0.039743693 40852
0.164042868 81640 0.085860651 81640 0.016354796
81600 0.175033173 81600 0.113376009 40900 0.051010971 40856
0.14078556 81648 0.073792937 81648 0.052485113
81700 0.143143086 81700 0.122848287 40900 0.048964121 40860
0.037957481 81656 0.021744585 81656 0.085956708
81700 0.093776776 81700 0.084406325 40900 0.093705181 40864
0.052737287 81664 0.030094467 81664 0.097107666
81700 0.039790699 81700 0.046557972 40900 0.114260431 40868
0.07093203 81672 0.050861301 81672 0.079452686
81700 0.058455105 81700 0.064856642 40900 0.061661398 40872
0.059049569 81680 0.069869115 81680 0.038539442
81700 0.090204747 81700 0.091953712 40900 0.011406421 40876
0.023100301 81688 0.144286344 81688 0.040254963
81700 0.104555023 81700 0.043243785 40900 0.043751286 40880
0.016376243 81696 0.159006799 81696 0.064508276
81700 0.327248446 81700 0.067164401 40900 0.009109064 40884
0.025133189 81704 0.089069923 81704 0.082008119
81700 0.371853646 81700 0.123106671 40900 0.059865153 40888
0.019732606 81712 0.099239493 81712 0.063774562
81700 0.194288674 81700 0.157907474 40900 0.076693519 40892
0.041026797 81720 0.125833628 81720 0.008477764
81700 0.236441934 81700 0.152986308 40900 0.06823783 40896
0.079719321 81728 0.101052647 81728 0.042968506
81700 0.220816771 81700 0.121406018 40900 0.038782644 40900
0.102644095 81736 0.08638791 81736 0.03780983
81700 1.081758295 81700 0.142770558 40900 0.033627581 40904
0.096098302 81744 0.079603582 81744 0.044004632
81800 1.403200438 81800 0.172801694 40900 0.074557771 40908
0.03730243 81752 0.107301457 81752 0.064011641
81800 1.387128375 81800 0.146711304 40900 0.110515401 40912
0.036379493 81760 0.144828649 81760 0.048614114
81800 4.004955763 81800 0.125034406 40900 0.101930426 40916
0.017291286 81768 0.146136634 81768 0.0121975
81800 5.680392963 81800 0.14028103 40900 0.053069663 40920
0.013216656 81776 0.078341087 81776 0.070896313
81800 4.557211888 81800 0.11178189 40900 0.003910045 40924
0.05340936 81784 0.017576358 81784 0.084874897
81800 2.0160554 81800 0.040451396 40900 0.055882352 40928
0.058135574 81792 0.059014226 81792 0.088477093
81800 0.380911486 81800 0.059163045 40900 0.090531357 40932
0.029900713 81800 0.05599701 81800 0.087777728
81800 0.658463221 81800 0.061178565 40900 0.084803804 40936
0.03450724 81808 0.052141561 81808 0.049337003
81800 0.876811216 81800 0.021650309 40900 0.059752918 40940
0.049045881 81816 0.084142077 81816 0.032566964
81800 0.553359103 81800 0.02989211 40900 0.056975106 40944
0.06089473 81824 0.042752858 81824 0.055322522
81800 0.236967128 81800 0.038334601 40900 0.062132829 40948
0.061710314 81832 0.05476327 81832 0.061038401
81800 0.019545109 81800 0.063750718 41000 0.071088842 40952
0.101780628 81840 0.091971167 81840 0.040864474
81800 0.200530194 81800 0.09600701 41000 0.066133456 40956
0.110850429 81848 0.080943653 81848 0.076569842
81900 0.204660668 81900 0.073812509 41000 0.044418579 40960
0.060925813 81856 0.113941482 81856 0.113647388
81900 0.171811684 81900 0.004422032 41000 0.029867024 40964
0.012594464 81864 0.150674663 81864 0.105865882
81900 0.0719124 81900 0.090959373 41000 0.029829575 40968
0.012080366 81872 0.150573745 81872 0.068055771
81900 0.192024149 81900 0.156632669 41000 0.025719761 40972
0.010659793 81880 0.133302805 81880 0.052217849
81900 0.206637255 81900 0.184665493 41000 0.019193523 40976
0.029339561 81888 0.104848034 81888 0.040295214
81900 0.113645503 81900 0.17508032 41000 0.018655563 40980
0.048410606 81896 0.116426556 81896 0.025960229
81900 0.13581125 81900 0.128114086 41000 0.011938641 40984

0.073964831 81904 0.138394389 81904 0.038172009
81900 0.145403275 81900 0.07469541 41000 0.04139074 40988
0.10050417 81912 0.089770867 81912 0.063460422
81900 0.154347799 81900 0.042364074 41000 0.054205044 40992
0.104294486 81920 0.055750748 81920 0.058813392
81900 0.172361804 81900 0.035003963 41000 0.045067485 40996
0.035640522 81928 0.064148226 81928 0.029806755
81900 0.13667201 81900 0.020339257 41000 0.028199067 41000
0.054733355 81936 0.155831585 81936 0.013035869
81900 0.100850477 81900 0.019997475 41000 0.025262465 41004
0.06773002 81944 0.162701646 81944 0.040954819
82000 0.038875642 82000 0.078224453 41000 0.050471339 41008
0.032420485 81952 0.063873602 81952 0.041700951
82000 0.054640601 82000 0.070331698 41000 0.028206448 41012
0.035615034 81960 0.047067995 81960 0.022374556
82000 0.054435997 82000 0.026780615 41000 0.069242378 41016
0.082551283 81968 0.057362238 81968 0.008990753
82000 0.163842385 82000 0.063885993 41000 0.043900112 41020
0.10371011 81976 0.082851977 81976 0.006865158
82000 0.179756724 82000 0.124810351 41000 0.058635331 41024
0.101225065 81984 0.080754864 81984 0.025714966
82000 0.089005545 82000 0.133692781 41000 0.060067057 41028
0.061240156 81992 0.034761568 81992 0.041230447
82000 0.045488901 82000 0.132403191 41000 0.011052352 41032
0.009618589 82000 0.018406274 82000 0.010504922
82000 0.078038851 82000 0.103221646 41000 0.026547386 41036
0.03572975 82008 0.046850248 82008 0.026208792
82000 0.198721843 82000 0.039793842 41000 0.024302182 41040
0.07953828 82016 0.090351285 82016 0.028914288
82000 0.278194668 82000 0.077878518 41000 0.049580187 41044
0.027934922 82024 0.072291652 82024 0.021748318
82000 0.363681581 82000 0.096420452 41000 0.018415225 41048
0.070072201 82032 0.062035142 82032 0.03644641
82000 0.734317815 82000 0.031036339 41100 0.038091446 41052
0.053162359 82040 0.024922088 82040 0.018569113
82000 0.773747859 82000 0.081839513 41100 0.039095547 41056
0.00648415 82048 0.04241514 82048 0.017230343
82100 0.417218108 82100 0.137919124 41100 0.032898533 41060
0.04068773 82056 0.030361684 82056 0.036561276
82100 0.106130974 82100 0.131657973 41100 0.017502884 41064
0.049088871 82064 0.062087675 82064 0.036146568
82100 0.145330458 82100 0.093961862 41100 0.024175895 41068
0.032444779 82072 0.057716843 82072 0.033460288
82100 0.251796533 82100 0.089755289 41100 0.048365349 41072
0.04671705 82080 0.0425621 82080 0.027606669
82100 0.233684594 82100 0.130533168 41100 0.042490519 41076
0.064341199 82088 0.044927332 82088 0.051125535
82100 0.071623108 82100 0.153816509 41100 0.018275861 41080
0.025327938 82096 0.103509563 82096 0.068062
82100 0.074624768 82100 0.101904749 41100 0.053580079 41084
0.027762982 82104 0.175619528 82104 0.058151516
82100 0.036500009 82100 0.093455252 41100 0.098998688 41088
0.057862308 82112 0.146039893 82112 0.02206379
82100 0.086930202 82100 0.118099197 41100 0.093361487 41092
0.04875382 82120 0.053154563 82120 0.028871797
82100 0.144110585 82100 0.089913898 41100 0.058127382 41096
0.030965337 82128 0.040492549 82128 0.035146371
82100 0.143820245 82100 0.069299837 41100 0.025627514 41100
0.019686935 82136 0.149851054 82136 0.019820294
82100 0.157529474 82100 0.117847456 41100 0.022210657 41104
0.029810404 82144 0.156708571 82144 0.044017947
82200 0.132660556 82200 0.14211185 41100 0.017258804 41108
0.048972732 82152 0.06663856 82152 0.016474607
82200 0.071083348 82200 0.124220664 41100 0.020422065 41112
0.080502767 82160 0.062159568 82160 0.060603161
82200 0.001554698 82200 0.130169793 41100 0.0402273 41116
0.0786167 82168 0.032099499 82168 0.045036286
82200 0.033517747 82200 0.086200882 41100 0.037426518 41120

0.062626124 82176 0.062586325 82176 0.015931672
82200 0.038009046 82200 0.030158171 41100 0.043949975 41124
0.050383205 82184 0.10949438 82184 0.034665372
82200 0.032886266 82200 0.110828325 41100 0.075648641 41128
0.043753364 82192 0.080092483 82192 0.071608236
82200 0.027733941 82200 0.101670441 41100 0.08387374 41132
0.051381754 82200 0.014350008 82200 0.058923077
82200 0.018219838 82200 0.107675478 41100 0.050453706 41136
0.069878879 82208 0.072714305 82208 0.020836382
82200 0.035682373 82200 0.166345751 41100 0.034342364 41140
0.048501206 82216 0.1299164 82216 0.027500159
82200 0.056923178 82200 0.163864883 41100 0.06987813 41144
0.032294469 82224 0.147614279 82224 0.019543435
82200 0.052460902 82200 0.075563068 41100 0.09754119 41148
0.085921703 82232 0.104288607 82232 0.065248052
82200 0.085945176 82200 0.021284326 41200 0.074967175 41152
0.109917339 82240 0.055153931 82240 0.098936514
82200 0.053854124 82200 0.037993978 41200 0.034449651 41156
0.100071316 82248 0.051660099 82248 0.068926835
82300 0.068734822 82300 0.067770794 41200 0.011469192 41160
0.040236242 82256 0.015409958 82256 0.039543767
82300 0.140105796 82300 0.025285417 41200 0.011903042 41164
0.03654966 82264 0.068742251 82264 0.038675637
82300 0.121499499 82300 0.040611347 41200 0.013755048 41168
0.060209266 82272 0.11749901 82272 0.038983966
82300 0.072442163 82300 0.063239488 41200 0.018644181 41172
0.067158915 82280 0.081701866 82280 0.016694323
82300 0.069382637 82300 0.046276553 41200 0.036795409 41176
0.050557756 82288 0.070191964 82288 0.011720192
82300 0.034634126 82300 0.023866287 41200 0.045749395 41180
0.029918119 82296 0.076819881 82296 0.023530927
82300 0.172618559 82300 0.079170422 41200 0.037724141 41184
0.045396097 82304 0.047596397 82304 0.026765789
82300 0.274029299 82300 0.048880404 41200 0.035200337 41188
0.067693385 82312 0.075276294 82312 0.015996595
82300 0.27644771 82300 0.038683324 41200 0.037245336 41192
0.038011473 82320 0.066717752 82320 0.007838111
82300 0.163653953 82300 0.092485301 41200 0.019975612 41196
0.027732394 82328 0.035853762 82328 0.003956921
82300 0.041922289 82300 0.114815375 41200 0.053407319 41200
0.052130275 82336 0.021199359 82336 0.022382668
82300 0.107316177 82300 0.118401615 41200 0.040116465 41204
0.070277827 82344 0.113659742 82344 0.035232733
82400 0.168710133 82400 0.121025347 41200 0.049940623 41208
0.084849831 82352 0.121671255 82352 0.039526967
82400 0.128483633 82400 0.096587261 41200 0.053826207 41212
0.053976932 82360 0.046908106 82360 0.043350072
82400 0.043565542 82400 0.149736793 41200 0.023016351 41216
0.04177021 82368 0.054339111 82368 0.036197627
82400 0.042873457 82400 0.140316363 41200 0.047071586 41220
0.049014285 82376 0.142787656 82376 0.047695317
82400 0.087333494 82400 0.086539636 41200 0.037760128 41224
0.044031644 82384 0.12011909 82384 0.060904404
82400 0.069339745 82400 0.05668552 41200 0.017336548 41228
0.049150367 82392 0.02002277 82392 0.052743926
82400 0.043960041 82400 0.117914991 41200 0.020401338 41232
0.076238262 82400 0.103259168 82400 0.017655577
82400 0.073971118 82400 0.192594948 41200 0.020182684 41236
0.049352035 82408 0.076640841 82408 0.025471301
82400 0.082590748 82400 0.205524455 41200 0.059247843 41240
0.025998163 82416 0.027813348 82416 0.022344704
82400 0.050462117 82400 0.133797031 41200 0.048067013 41244
0.048620463 82424 0.089115747 82424 0.039471361
82400 0.042323853 82400 0.058682566 41200 0.049804348 41248
0.028686882 82432 0.095787407 82432 0.073281
82400 0.070535505 82400 0.068943758 41300 0.085782682 41252
0.01564933 82440 0.025226282 82440 0.045754212
82400 0.056400422 82400 0.09123348 41300 0.067451874 41256

0.044343618 82448 0.071550028 82448 0.056342025
82500 0.026276499 82500 0.070951137 41300 0.045665813 41260
0.037812533 82456 0.090150374 82456 0.091707239
82500 0.116469935 82500 0.034246077 41300 0.037656173 41264
0.027732016 82464 0.063003965 82464 0.063085812
82500 0.15039921 82500 0.046050071 41300 0.044599001 41268
0.063375082 82472 0.07994099 82472 0.033848955
82500 0.131636189 82500 0.077774443 41300 0.122797588 41272
0.072307084 82480 0.08817386 82480 0.03051045
82500 0.122492391 82500 0.124505794 41300 0.115182986 41276
0.039629009 82488 0.074885837 82488 0.016218342
82500 0.063128966 82500 0.166064638 41300 0.055328994 41280
0.001242124 82496 0.111403388 82496 0.029407142
82500 0.031607913 82500 0.191466315 41300 0.038781265 41284
0.035583584 82504 0.092916256 82504 0.050126288
82500 0.109320288 82500 0.209924838 41300 0.048296814 41288
0.063224354 82512 0.011613533 82512 0.026903741
82500 0.132529065 82500 0.132626985 41300 0.025431558 41292
0.057926496 82520 0.075774144 82520 0.009481429
82500 0.085400927 82500 0.058263653 41300 0.063235384 41296
0.052715885 82528 0.102692902 82528 0.023113638
82500 0.073680596 82500 0.132504079 41300 0.110867266 41300
0.04380558 82536 0.095233183 82536 0.033304394
82500 0.10462918 82500 0.051182167 41300 0.143852594 41304
0.074247677 82544 0.083277053 82544 0.024334986
82600 0.101251171 82600 0.047996997 41300 0.129112974 41308
0.100819831 82552 0.056226523 82552 0.013763359
82600 0.062687039 82600 0.082072285 41300 0.054724853 41312
0.059941936 82560 0.016948785 82560 0.014493237
82600 0.025912861 82600 0.102258178 41300 0.032098011 41316
0.052698695 82568 0.035064724 82568 0.014367042
82600 0.053155738 82600 0.034817298 41300 0.093696443 41320
0.016855105 82576 0.022838458 82576 0.017787101
82600 0.024793793 82600 0.110280496 41300 0.096553617 41324
0.024380011 82584 0.045451034 82584 0.01723736
82600 0.047603277 82600 0.167365011 41300 0.041224186 41328
0.031752079 82592 0.070379858 82592 0.030372406
82600 0.081520055 82600 0.123540827 41300 0.073445539 41332
0.016236756 82600 0.090724985 82600 0.033683373
82600 0.086648142 82600 0.100289763 41300 0.107667445 41336
0.050548708 82608 0.075262498 82608 0.017663257
82600 0.077530021 82600 0.080135469 41300 0.079365069 41340
0.073994859 82616 0.054322194 82616 0.01102157
82600 0.071919705 82600 0.068842935 41300 0.075947479 41344
0.09249671 82624 0.098870274 82624 0.024975019
82600 0.075843345 82600 0.076757264 41300 0.078582758 41348
0.105412633 82632 0.130318964 82632 0.032730332
82600 0.070500348 82600 0.045016172 41400 0.026823884 41352
0.080117337 82640 0.082375511 82640 0.042500182
82600 0.042967797 82600 0.009221004 41400 0.052146817 41356
0.030996736 82648 0.013590921 82648 0.059048063
82700 0.107532644 82700 0.103674254 41400 0.07620862 41360
0.037252288 82656 0.03484471 82656 0.045168701
82700 0.183064345 82700 0.183152763 41400 0.082366249 41364
0.038612918 82664 0.091322043 82664 0.026362131
82700 0.183336451 82700 0.129859094 41400 0.063266081 41368
0.020209138 82672 0.100739067 82672 0.03378444
82700 0.085533749 82700 0.041135354 41400 0.028282004 41372
0.015883821 82680 0.066889239 82680 0.061799663
82700 0.066314147 82700 0.029833183 41400 0.058394853 41376
0.043494438 82688 0.058664016 82688 0.057142373
82700 0.116290823 82700 0.103390521 41400 0.017051809 41380
0.061434599 82696 0.074496275 82696 0.061352905
82700 0.074872573 82700 0.139135955 41400 0.020660067 41384
0.073419637 82704 0.112849601 82704 0.057368554
82700 0.069755704 82700 0.108290442 41400 0.031373696 41388
0.059029058 82712 0.163854973 82712 0.041724634
82700 0.08326117 82700 0.099457968 41400 0.029802497 41392

0.031018641	82720	0.121773468	82720	0.039766557		
82700	0.088236666	82700	0.106813241	41400	0.048069596	41396
0.047270001	82728	0.052011546	82728	0.026931159		
82700	0.039623788	82700	0.065277622	41400	0.08022297	41400
0.064764477	82736	0.065155691	82736	0.016992526		
82700	0.130080981	82700	0.045473105	41400	0.057833084	41404
0.070194976	82744	0.053770884	82744	0.024298257		
82800	0.127790306	82800	0.072860741	41400	0.02815536	41408
0.054424108	82752	0.028304074	82752	0.040255989		
82800	0.055506047	82800	0.09724954	41400	0.017759236	41412
0.052089737	82760	0.048689297	82760	0.050247942		
82800	0.160864584	82800	0.118331831	41400	0.068298723	41416
0.026252528	82768	0.111252652	82768	0.071557872		
82800	0.19383886	82800	0.131244465	41400	0.047942984	41420
0.029701021	82776	0.126663901	82776	0.076975819		
82800	0.101885773	82800	0.124967992	41400	0.019026969	41424
0.056057335	82784	0.097962387	82784	0.052499519		
82800	0.085160362	82800	0.085373918	41400	0.036468184	41428
0.050998977	82792	0.076241326	82792	0.024015215		
82800	0.102609651	82800	0.026290481	41400	0.033183369	41432
0.075598568	82800	0.122225567	82800	0.027369073		
82800	0.046485955	82800	0.048546866	41400	0.035967099	41436
0.098824101	82808	0.085724678	82808	0.024049452		
82800	0.085921049	82800	0.027262333	41400	0.010000152	41440
0.081179969	82816	0.142241188	82816	0.025906571		
82800	0.101485544	82800	0.055059532	41400	0.059843311	41444
0.05291892	82824	0.19256727	82824	0.019840389		
82800	0.063668158	82800	0.053179145	41400	0.073867574	41448
0.062529478	82832	0.176242465	82832	0.010365433		
82800	0.042316235	82800	0.027895376	41500	0.068060508	41452
0.091906812	82840	0.110670357	82840	0.02301211		
82800	0.125658728	82800	0.03668663	41500	0.055771605	41456
0.067501722	82848	0.033550114	82848	0.01846928		
82900	0.10876514	82900	0.086273598	41500	0.03873387	41460
0.018832368	82856	0.087101937	82856	0.031220527		
82900	0.034439021	82900	0.068733731	41500	0.028978309	41464
0.039777064	82864	0.0977194	82864	0.054038803		
82900	0.0547696	82900	0.05694414	41500	0.040649084	41468
0.064006228	82872	0.111358306	82872	0.02845659		
82900	0.087613626	82900	0.056867022	41500	0.026614383	41472
0.059347935	82880	0.169860359	82880	0.006082172		
82900	0.10533238	82900	0.015208184	41500	0.018761484	41476
0.038873481	82888	0.186710779	82888	0.0296855		
82900	0.113428738	82900	0.052632218	41500	0.045409506	41480
0.028655821	82896	0.136097268	82896	0.059373982		
82900	0.110376663	82900	0.012765486	41500	0.059790946	41484
0.097932702	82904	0.073079493	82904	0.052505919		
82900	0.103189166	82900	0.082455801	41500	0.047729052	41488
0.10940287	82912	0.070364142	82912	0.012680117		
82900	0.134529356	82900	0.085431937	41500	0.033264758	41492
0.050311734	82920	0.095705967	82920	0.004721643		
82900	0.166663318	82900	0.108794207	41500	0.070207105	41496
0.06567096	82928	0.1236924	82928	0.032653323		
82900	0.13857297	82900	0.040882984	41500	0.042423053	41500
0.066725283	82936	0.184826859	82936	0.051454721		
82900	0.141658078	82900	0.031196538	41500	0.042970736	41504
0.012197682	82944	0.144458856	82944	0.034268433		
83000	0.121658079	83000	0.02544879	41500	0.079390105	41508
0.041129173	82952	0.073157775	82952	0.010152253		
83000	0.090388596	83000	0.057863901	41500	0.110109664	41512
0.079404461	82960	0.0473734	82960	0.036722708		
83000	0.104602012	83000	0.013019816	41500	0.095367228	41516
0.085739303	82968	0.054922013	82968	0.057603531		
83000	0.096522563	83000	0.010245815	41500	0.040117964	41520
0.077906931	82976	0.063313091	82976	0.060477087		
83000	0.07231698	83000	0.071271847	41500	0.031259282	41524
0.058945541	82984	0.109990848	82984	0.037140675		
83000	0.069858579	83000	0.098355871	41500	0.065592343	41528

0.034235727	82992	0.15901307	82992	0.015475751		
83000	0.050376846	83000	0.070474533	41500	0.069553615	41532
0.052087416	83000	0.099356206	83000	0.012206944		
83000	0.041650841	83000	0.020090112	41500	0.061930739	41536
0.079097023	83008	0.135269191	83008	0.020318508		
83000	0.116233634	83000	0.051782139	41500	0.011387662	41540
0.066019748	83016	0.18213359	83016	0.065762608		
83000	0.129452703	83000	0.047532354	41500	0.092990835	41544
0.034140521	83024	0.108145134	83024	0.081044607		
83000	0.051200765	83000	0.018893019	41500	0.103752187	41548
0.02571878	83032	0.076062992	83032	0.068687165		
83000	0.064893429	83000	0.048840971	41600	0.036176789	41552
0.051004445	83040	0.123047445	83040	0.053817654		
83000	0.108879336	83000	0.050641476	41600	0.032483174	41556
0.068077607	83048	0.034406978	83048	0.015187063		
83100	0.150761728	83100	0.087206681	41600	0.057828478	41560
0.060106355	83056	0.07939012	83056	0.033019369		
83100	0.232368475	83100	0.142743054	41600	0.096608332	41564
0.066426015	83064	0.13201304	83064	0.038137714		
83100	0.235936328	83100	0.114709554	41600	0.132285175	41568
0.093031304	83072	0.129307708	83072	0.063261126		
83100	0.128484011	83100	0.040165127	41600	0.145951286	41572
0.048850237	83080	0.065361521	83080	0.048863349		
83100	0.088051289	83100	0.071833616	41600	0.092770184	41576
0.034925961	83088	0.113625814	83088	0.032403717		
83100	0.031487209	83100	0.048944567	41600	0.018065	41580
0.101619815	83096	0.112583104	83096	0.048167909		
83100	0.064628104	83100	0.11541936	41600	0.053598866	41584
0.124764978	83104	0.062309642	83104	0.051151419		
83100	0.104344457	83100	0.166707556	41600	0.100918056	41588
0.096253571	83112	0.063597814	83112	0.039603187		
83100	0.109748435	83100	0.158174328	41600	0.09723589	41592
0.066455985	83120	0.112499656	83120	0.038728431		
83100	0.074881675	83100	0.129616689	41600	0.04097991	41596
0.088109096	83128	0.110110574	83128	0.027943364		
83100	0.012350623	83100	0.085342304	41600	0.044332202	41600
0.110775516	83136	0.144995968	83136	0.020217245		
83100	0.050700583	83100	0.069126603	41600	0.075201504	41604
0.089245215	83144	0.152866691	83144	0.033984492		
83200	0.076593635	83200	0.029464618	41600	0.143396218	41608
0.086357046	83152	0.073698742	83152	0.036300313		
83200	0.063673026	83200	0.136633549	41600	0.129901455	41612
0.087655382	83160	0.046831388	83160	0.047543541		
83200	0.031368971	83200	0.087934983	41600	0.067890207	41616
0.05107835	83168	0.084191859	83168	0.065469598		
83200	0.044544671	83200	0.108002023	41600	0.042235151	41620
0.035742738	83176	0.077205827	83176	0.056290275		
83200	0.147734333	83200	0.162442346	41600	0.117888296	41624
0.07195631	83184	0.083314313	83184	0.041189811		
83200	0.183683034	83200	0.106709973	41600	0.120922137	41628
0.050344221	83192	0.087284418	83192	0.019757623		
83200	0.148703096	83200	0.03218096	41600	0.028572282	41632
0.057026235	83200	0.084201296	83200	0.022067359		
83200	0.080107573	83200	0.079634374	41600	0.041131221	41636
0.067112815	83208	0.070724658	83208	0.062554827		
83200	0.069907648	83200	0.100430654	41600	0.060495626	41640
0.065673274	83216	0.053378812	83216	0.057993551		
83200	0.020104288	83200	0.052475814	41600	0.061594401	41644
0.096165604	83224	0.043482603	83224	0.016064634		
83200	0.121711128	83200	0.017245253	41600	0.059241338	41648
0.131505033	83232	0.068423869	83232	0.032125474		
83200	0.14068083	83200	0.03694386	41700	0.182578283	41652
0.112275178	83240	0.072753879	83240	0.021305901		
83200	0.086996908	83200	0.082902756	41700	0.255635765	41656
0.052366217	83248	0.048291215	83248	0.005977271		
83300	0.056043544	83300	0.137991199	41700	0.229245503	41660
0.055271168	83256	0.0214435	83256	0.018862435		
83300	0.066058259	83300	0.092019494	41700	0.129846289	41664

0.064029417 83264 0.091789429 83264 0.036662965
83300 0.016758471 83300 0.102060847 41700 0.089062371 41668
0.035588215 83272 0.113836119 83272 0.06869786
83300 0.054699485 83300 0.149347529 41700 0.167412888 41672
0.040130584 83280 0.06211998 83280 0.077958219
83300 0.088056557 83300 0.071779228 41700 0.20112998 41676
0.054117361 83288 0.106052044 83288 0.041475571
83300 0.11227082 83300 0.011590565 41700 0.26223145 41680
0.06707725 83296 0.109088345 83296 0.014648719
83300 0.141945289 83300 0.022285918 41700 0.223324445 41684
0.113313792 83304 0.036725756 83304 0.01697721
83300 0.146449631 83300 0.065816195 41700 0.130582034 41688
0.089740272 83312 0.078788362 83312 0.02491876
83300 0.079773541 83300 0.076025412 41700 0.148566105 41692
0.020071786 83320 0.100417936 83320 0.031861266
83300 0.041453845 83300 0.083564089 41700 0.124879487 41696
0.039383525 83328 0.060993985 83328 0.04847562
83300 0.077908553 83300 0.078878744 41700 0.248129829 41700
0.078529694 83336 0.041999065 83336 0.053792428
83300 0.06483293 83300 0.13421505 41700 0.167415449 41704
0.10858546 83344 0.047372334 83344 0.027008058
83400 0.073185613 83400 0.190889725 41700 0.564537069 41708
0.097249635 83352 0.072067174 83352 0.027934948
83400 0.12216279 83400 0.152138891 41700 0.93932444 41712
0.055691144 83360 0.103159568 83360 0.020717522
83400 0.136339615 83400 0.066977038 41700 0.813466904 41716
0.029099629 83368 0.081368933 83368 0.029325904
83400 0.130357003 83400 0.028256414 41700 0.338825223 41720
0.043469168 83376 0.049044222 83376 0.048855192
83400 0.105251987 83400 0.066758279 41700 0.167697843 41724
0.095106501 83384 0.018502056 83384 0.036443711
83400 0.051104562 83400 0.052041098 41700 0.280672306 41728
0.090172776 83392 0.079772311 83392 0.012179402
83400 0.076767894 83400 0.019982394 41700 0.235308005 41732
0.083973704 83400 0.125699823 83400 0.016594513
83400 0.085694133 83400 0.031258729 41700 0.219530543 41736
0.038105347 83408 0.090103917 83408 0.011189317
83400 0.048161281 83400 0.081387923 41700 0.33317387 41740
0.081402402 83416 0.034203506 83416 0.017490316
83400 0.090131725 83400 0.088466732 41700 0.563510111 41744
0.118164833 83424 0.119511336 83424 0.034166711
83400 0.124177866 83400 0.081674036 41700 0.605362816 41748
0.095279815 83432 0.134365516 83432 0.029667706
83400 0.106262356 83400 0.072931078 41800 0.358981371 41752
0.108126435 83440 0.115178125 83440 0.025256446
83400 0.115255272 83400 0.0322384 41800 0.616583449 41756
0.137978889 83448 0.119392731 83448 0.043609583
83500 0.176415779 83500 0.015287775 41800 0.449786283 41760
0.14809877 83456 0.072528434 83456 0.039904458
83500 0.138787539 83500 0.092021444 41800 1.028856495 41764
0.105006649 83464 0.073777104 83464 0.013500589
83500 0.071480565 83500 0.127753709 41800 2.718325238 41768
0.041619125 83472 0.144298726 83472 0.025311256
83500 0.096891068 83500 0.066145767 41800 3.7048643 41772
0.058367117 83480 0.151153494 83480 0.008423898
83500 0.003720837 83500 0.058306898 41800 3.152653338 41776
0.079004254 83488 0.182785778 83488 0.028478187
83500 0.141903628 83500 0.147183396 41800 1.4953271 41780
0.062093772 83496 0.201406481 83496 0.052760501
83500 0.148416148 83500 0.131477893 41800 0.419198943 41784
0.047462847 83504 0.175897964 83504 0.069537295
83500 0.070496004 83500 0.099499936 41800 0.366448193 41788
0.031016203 83512 0.165237493 83512 0.077072931
83500 0.070241353 83500 0.142128076 41800 0.807996781 41792
0.072660958 83520 0.123240257 83520 0.058383917
83500 0.111463065 83500 0.156430906 41800 1.374337475 41796
0.087906272 83528 0.024688847 83528 0.038131704
83500 0.102287319 83500 0.106023115 41800 1.397813438 41800

0.080617807 83536 0.08850664 83536 0.040039377
83500 0.057207755 83500 0.132045753 41800 1.27138325 41804
0.049343649 83544 0.030265856 83544 0.048224658
83600 0.045661171 83600 0.117089003 41800 0.977112446 41808
0.07371993 83552 0.066908775 83552 0.058490321
83600 0.036778009 83600 0.074990065 41800 0.22121174 41812
0.104785417 83560 0.102298203 83560 0.069286281
83600 0.031351563 83600 0.071021714 41800 1.612596675 41816
0.075439137 83568 0.161458331 83568 0.072528143
83600 0.013429047 83600 0.068530819 41800 1.046267571 41820
0.109920729 83576 0.164318859 83576 0.034009943
83600 0.067638066 83600 0.088154455 41800 4.42652265 41824
0.100107514 83584 0.113674709 83584 0.048242135
83600 0.094373441 83600 0.134043934 41800 9.949425238 41828
0.065753382 83592 0.118791591 83592 0.06962489
83600 0.053481195 83600 0.150273744 41800 11.15560904 41832
0.053744057 83600 0.116314091 83600 0.068838504
83600 0.035883957 83600 0.118957178 41800 6.681466938 41836
0.068611364 83608 0.064332286 83608 0.048468392
83600 0.062107763 83600 0.080354526 41800 1.643654775 41840
0.100738631 83616 0.044938552 83616 0.028380495
83600 0.058855345 83600 0.072272531 41800 1.045644749 41844
0.090676178 83624 0.073590876 83624 0.023008466
83600 0.049894945 83600 0.096285898 41800 0.731276231 41848
0.069878392 83632 0.107774147 83632 0.008932532
83600 0.051805844 83600 0.156539784 41900 2.183857838 41852
0.054755044 83640 0.083835243 83640 0.01962404
83600 0.061142862 83600 0.144713151 41900 6.828073413 41856
0.068529109 83648 0.054784952 83648 0.020119471
83700 0.040521569 83700 0.118154429 41900 10.9128654 41860
0.08818254 83656 0.079471298 83656 0.034380362
83700 0.042171658 83700 0.097287331 41900 9.6249599 41864
0.08357082 83664 0.087132452 83664 0.052467029
83700 0.055019358 83700 0.084813277 41900 4.476235713 41868
0.121006349 83672 0.025772157 83672 0.037167003
83700 0.051715786 83700 0.049715727 41900 3.897723738 41872
0.133828304 83680 0.062568732 83680 0.035805424
83700 0.093681156 83700 0.069111287 41900 5.589268175 41876
0.103369159 83688 0.082487837 83688 0.042507185
83700 0.07760711 83700 0.131149573 41900 7.123955525 41880
0.100320161 83696 0.039487237 83696 0.046970021
83700 0.009036183 83700 0.142370904 41900 20.92492025 41884
0.091452559 83704 0.059059017 83704 0.05081527
83700 0.066228844 83700 0.146355713 41900 30.15429713 41888
0.096800854 83712 0.104308856 83712 0.020604854
83700 0.051198349 83700 0.107341482 41900 24.5366395 41892
0.098990953 83720 0.06527661 83720 0.039908642
83700 0.123664679 83700 0.074791577 41900 10.14355384 41896
0.0824893 83728 0.038619557 83728 0.057943853
83700 0.192851061 83700 0.143502519 41900 1.828963175 41900
0.09124379 83736 0.086788605 83736 0.006358853
83700 0.218419736 83700 0.130463755 41900 3.529942825 41904
0.105827377 83744 0.125856678 83744 0.047358517
83800 0.209114631 83800 0.096451935 41900 2.325732725 41908
0.068478745 83752 0.092815462 83752 0.052868349
83800 0.193445798 83800 0.083137988 41900 16.496405 41912
0.030942054 83760 0.05672194 83760 0.063104133
83800 0.181980024 83800 0.059002734 41900 32.02547138 41916
0.052813826 83768 0.059902759 83768 0.040245563
83800 0.126903105 83800 0.145314073 41900 33.18229313 41920
0.041751519 83776 0.075920616 83776 0.007033983
83800 0.05717589 83800 0.098733137 41900 19.027371 41924
0.070920527 83784 0.06951885 83784 0.021795477
83800 0.062119259 83800 0.048841932 41900 5.10950945 41928
0.090602232 83792 0.144577673 83792 0.034673529
83800 0.112009686 83800 0.032640102 41900 5.103787875 41932
0.076523909 83800 0.092614544 83800 0.049083948
83800 0.064156317 83800 0.043069627 41900 2.321695675 41936

0.087575027 83808 0.055315406 83808 0.03823535
83800 0.075052107 83800 0.071362149 41900 8.162278688 41940
0.098588134 83816 0.087143759 83816 0.013056069
83800 0.131989625 83800 0.06098513 41900 18.48755775 41944
0.082140243 83824 0.095625706 83824 0.026516998
83800 0.06081217 83800 0.05211914 41900 21.504961 41948
0.063700296 83832 0.145903483 83832 0.030529966
83800 0.072136783 83800 0.062962863 42000 13.73670625 41952
0.077298792 83840 0.118171978 83840 0.004064772
83800 0.15329133 83800 0.066111723 42000 3.04703275 41956
0.087219727 83848 0.046791531 83848 0.02504212
83900 0.155787216 83900 0.113096117 42000 1.870064188 41960
0.09533186 83856 0.055966604 83856 0.044324726
83900 0.12378383 83900 0.129284323 42000 1.613834525 41964
0.116320189 83864 0.078015204 83864 0.056822541
83900 0.076923847 83900 0.046272278 42000 5.579802675 41968
0.131964741 83872 0.100743746 83872 0.05694478
83900 0.043583568 83900 0.031310461 42000 16.6250635 41972
0.120735807 83880 0.132462024 83880 0.052598585
83900 0.102906342 83900 0.044134024 42000 24.558926 41976
0.067247238 83888 0.113027869 83888 0.006809159
83900 0.083180334 83900 0.036462752 42000 21.01433463 41980
0.063787207 83896 0.076630189 83896 0.046474892
83900 0.044879467 83900 0.043249536 42000 8.986486125 41984
0.069122951 83904 0.040710696 83904 0.047773418
83900 0.06439662 83900 0.086126158 42000 3.789548525 41988
0.071820599 83912 0.010118355 83912 0.023813886
83900 0.118333264 83900 0.053042117 42000 4.619311538 41992
0.076357101 83920 0.048334157 83920 0.02692455
83900 0.111621383 83900 0.079329337 42000 1.415782025 41996
0.051106643 83928 0.119833385 83928 0.027894024
83900 0.083320018 83900 0.165475358 42000 4.425954075 42000
0.110984234 83936 0.134948015 83936 0.023931607
83900 0.10169109 83900 0.126314073 42000 5.892033688 42004
0.139198368 83944 0.127672771 83944 0.051541116
84000 0.090316047 84000 0.014991098 42000 4.519590175 42008
0.129336535 83952 0.112727779 83952 0.053377677
84000 0.054377069 84000 0.070741895 42000 1.635568913 42012
0.106558371 83960 0.110281129 83960 0.02699648
84000 0.077235869 84000 0.093376249 42000 1.42553635 42016
0.048980441 83968 0.144745268 83968 0.036554371
84000 0.094054281 84000 0.136444098 42000 0.774865679 42020
0.051973646 83976 0.131329871 83976 0.066780332
84000 0.085217245 84000 0.20298321 42000 0.535278465 42024
0.079734746 83984 0.107884604 83984 0.063139429
84000 0.038398397 84000 0.246509619 42000 4.0623704 42028
0.087175722 83992 0.119172619 83992 0.024915516
84000 0.04119298 84000 0.267603959 42000 9.766641075 42032
0.063876083 84000 0.161202086 84000 0.011470115
84000 0.077402416 84000 0.204042531 42000 11.92345843 42036
0.053548629 84008 0.156606853 84008 0.0162824
84000 0.080472688 84000 0.148090316 42000 7.8938799 42040
0.069044712 84016 0.0748893 84016 0.037359168
84000 0.104871368 84000 0.137893804 42000 1.8481448 42044
0.08020355 84024 0.040990697 84024 0.064248561
84000 0.105726955 84000 0.090696129 42000 2.84772785 42048
0.095300427 84032 0.04168809 84032 0.043933993
84000 0.03407272 84000 0.122851881 42100 1.824821113 42052
0.090597081 84040 0.040115268 84040 0.042645548
84000 0.10086321 84000 0.161784774 42100 1.526980425 42056
0.065296226 84048 0.081284052 84048 0.026581491
84100 0.12858781 84100 0.10550407 42100 1.820216188 42060
0.047592497 84056 0.160652548 84056 0.02451489
84100 0.11015185 84100 0.067918933 42100 0.815019769 42064
0.045381894 84064 0.153033441 84064 0.029069019
84100 0.108068154 84100 0.124037295 42100 1.278744775 42068
0.037432921 84072 0.013317741 84072 0.030467225
84100 0.073273172 84100 0.146294144 42100 1.525492413 42072

0.012163587	84080	0.138970354	84080	0.064220847		
84100	0.037567312	84100	0.10891493	42100	1.38320925	42076
0.047753274	84088	0.077490615	84088	0.051412659		
84100	0.103138787	84100	0.065169086	42100	1.096698921	42080
0.071751849	84096	0.052037416	84096	0.053352756		
84100	0.12799869	84100	0.04846368	42100	0.350814138	42084
0.055899858	84104	0.020214075	84104	0.03581612		
84100	0.066467357	84100	0.048128415	42100	3.129220338	42088
0.052760683	84112	0.040136911	84112	0.029787407		
84100	0.129991619	84100	0.011685868	42100	5.263672213	42092
0.057168305	84120	0.085648448	84120	0.035933899		
84100	0.188462741	84100	0.042455115	42100	4.60641365	42096
0.047727401	84128	0.206744429	84128	0.02388909		
84100	0.156499664	84100	0.047848211	42100	1.732041713	42100
0.035769146	84136	0.260040746	84136	0.04416384		
84100	0.13555841	84100	0.018690691	42100	0.747475191	42104
0.016396049	84144	0.175926601	84144	0.058887996		
84200	0.139840253	84200	0.115411174	42100	0.91705384	42108
0.050492392	84152	0.098211465	84152	0.039182338		
84200	0.089581976	84200	0.140698976	42100	0.353119453	42112
0.070196591	84160	0.083076404	84160	0.024416237		
84200	0.08391367	84200	0.083303065	42100	0.49366639	42116
0.064522399	84168	0.058652819	84168	0.039280265		
84200	0.049153961	84200	0.046041765	42100	1.022662501	42120
0.05641071	84176	0.097152653	84176	0.057193196		
84200	0.035877718	84200	0.047770405	42100	1.760368125	42124
0.011818052	84184	0.14429717	84184	0.085503816		
84200	0.041261348	84200	0.065590248	42100	1.478861788	42128
0.029979867	84192	0.062249135	84192	0.081527527		
84200	0.049494527	84200	0.098929886	42100	0.894403144	42132
0.032877331	84200	0.096366137	84200	0.049395007		
84200	0.056905457	84200	0.068758563	42100	0.857286098	42136
0.04611041	84208	0.149778046	84208	0.051637671		
84200	0.054896467	84200	0.013653453	42100	0.416367315	42140
0.058564296	84216	0.119482269	84216	0.05584805		
84200	0.050810355	84200	0.034877918	42100	0.826351345	42144
0.077841702	84224	0.103067461	84224	0.07798108		
84200	0.026477332	84200	0.111246038	42100	1.6984822	42148
0.051964151	84232	0.137652868	84232	0.089524394		
84200	0.070894515	84200	0.169011619	42200	1.693427213	42152
0.040912502	84240	0.066309665	84240	0.063625957		
84200	0.123601218	84200	0.085205407	42200	0.871737371	42156
0.086717773	84248	0.063856976	84248	0.028848555		
84300	0.12405985	84300	0.086133827	42200	0.216100176	42160
0.095830597	84256	0.059180693	84256	0.019487558		
84300	0.075914453	84300	0.089141446	42200	0.304979446	42164
0.090352958	84264	0.057483812	84264	0.018699075		
84300	0.013999905	84300	0.062738094	42200	0.139126801	42168
0.083861138	84272	0.126639061	84272	0.018450679		
84300	0.083905215	84300	0.142784338	42200	0.158052193	42172
0.092186674	84280	0.1258642	84280	0.02061729		
84300	0.040014678	84300	0.136771094	42200	0.312360615	42176
0.104609397	84288	0.07014849	84288	0.007229849		
84300	0.046644669	84300	0.079136502	42200	0.866854389	42180
0.11322293	84296	0.038902075	84296	0.032488191		
84300	0.023418841	84300	0.034657125	42200	0.917055528	42184
0.095433665	84304	0.086691143	84304	0.04402499		
84300	0.039987004	84300	0.097439159	42200	0.512134459	42188
0.047658708	84312	0.036912374	84312	0.023371827		
84300	0.062141473	84300	0.10180808	42200	0.446835853	42192
0.075197189	84320	0.060366845	84320	0.034175355		
84300	0.040622861	84300	0.087877277	42200	0.410367706	42196
0.070836082	84328	0.094236253	84328	0.021411257		
84300	0.029469517	84300	0.070855371	42200	0.173939145	42200
0.060966078	84336	0.088195091	84336	0.02565373		
84300	0.022968352	84300	0.068203881	42200	0.546773779	42204
0.079557496	84344	0.140318036	84344	0.04985643		
84400	0.054254182	84400	0.127836728	42200	0.616734149	42208

0.079819474	84352	0.135943265	84352	0.059689814
84400	0.089456087	84400	0.066124318	42200	0.286803261	42212
0.063752981	84360	0.03520622	84360	0.054566441
84400	0.092849135	84400	0.103188089	42200	0.168949133	42216
0.077672048	84368	0.062556683	84368	0.057138335
84400	0.077352568	84400	0.145192593	42200	0.328365219	42220
0.072159899	84376	0.080620521	84376	0.050982533
84400	0.05744772	84400	0.061974381	42200	0.23912602	42224
0.032179454	84384	0.09920093	84384	0.029915775
84400	0.042763379	84400	0.030576306	42200	0.222291055	42228
0.080860395	84392	0.139408846	84392	0.043632059
84400	0.057013527	84400	0.059505881	42200	0.148858511	42232
0.090225782	84400	0.167171601	84400	0.05008424
84400	0.069538801	84400	0.065285276	42200	0.361044396	42236
0.061233506	84408	0.159612915	84408	0.039808394
84400	0.043161388	84400	0.054054912	42200	0.544824346	42240
0.068257818	84416	0.095628588	84416	0.027779999
84400	0.056909481	84400	0.062572304	42200	0.428024418	42244
0.071703893	84424	0.053849552	84424	0.009769302
84400	0.103570572	84400	0.122985803	42200	0.305529131	42248
0.057157758	84432	0.06308264	84432	0.019525041
84400	0.07826947	84400	0.159493691	42300	0.312422694	42252
0.038503149	84440	0.082013372	84440	0.046350433
84400	0.108644847	84400	0.082057355	42300	0.249117321	42256
0.015136944	84448	0.116933399	84448	0.05142569
84500	0.142683566	84500	0.03582154	42300	0.275475119	42260
0.041022984	84456	0.138656353	84456	0.030113801
84500	0.08639913	84500	0.106573665	42300	0.275550846	42264
0.038218019	84464	0.066108201	84464	0.024147583
84500	0.079105281	84500	0.11884531	42300	0.219944646	42268
0.022731107	84472	0.029598026	84472	0.043130687
84500	0.048720867	84500	0.074290088	42300	0.138194751	42272
0.009679497	84480	0.051265772	84480	0.05988944
84500	0.031033327	84500	0.015094893	42300	0.23065669	42276
0.005723778	84488	0.076351971	84488	0.069426504
84500	0.068575435	84500	0.06768429	42300	0.245773321	42280
0.013005619	84496	0.088989116	84496	0.07363582
84500	0.133346039	84500	0.15236708	42300	0.230122038	42284
0.039197035	84504	0.081973187	84504	0.051454623
84500	0.144829538	84500	0.16938668	42300	0.21728847	42288
0.058524438	84512	0.050723167	84512	0.038359711
84500	0.103367514	84500	0.073451593	42300	0.217780893	42292
0.095450341	84520	0.002605642	84520	0.041392155
84500	0.070091039	84500	0.03255612	42300	0.299135834	42296
0.105965206	84528	0.055673339	84528	0.029198416
84500	0.039678886	84500	0.062893843	42300	0.322030886	42300
0.078448189	84536	0.086454966	84536	0.043905584
84500	0.007579781	84500	0.146838371	42300	0.235447151	42304
0.019057156	84544	0.113286056	84544	0.022232991
84600	0.046940106	84600	0.144050136	42300	0.203140211	42308
0.035337085	84552	0.140819611	84552	0.022201557
84600	0.051705712	84600	0.079574536	42300	0.186868754	42312
0.038018468	84560	0.095002681	84560	0.019199215
84600	0.077638884	84600	0.085946915	42300	0.158920404	42316
0.027851116	84568	0.155190355	84568	0.003857976
84600	0.06762195	84600	0.109718167	42300	0.187937214	42320
0.050970684	84576	0.156862254	84576	0.017589364
84600	0.042867596	84600	0.123464575	42300	0.170006315	42324
0.084440348	84584	0.143313708	84584	0.03416399
84600	0.060429029	84600	0.118158605	42300	0.224921881	42328
0.101908649	84592	0.120194149	84592	0.030330955
84600	0.141206736	84600	0.050349561	42300	0.141360651	42332
0.065436841	84600	0.080727943	84600	0.021793358
84600	0.186067336	84600	0.071275586	42300	0.136296323	42336
0.034337474	84608	0.086679851	84608	0.036808029
84600	0.126266183	84600	0.130457251	42300	0.227913188	42340
0.030634226	84616	0.126103653	84616	0.026106731
84600	0.116725409	84600	0.081388069	42300	0.181598734	42344

0.01063768 84624 0.105256688 84624 0.028084418
84600 0.137911455 84600 0.081141887 42300 0.097509263 42348
0.050057217 84632 0.029031253 84632 0.008352255
84600 0.113283109 84600 0.082805476 42400 0.097052012 42352
0.038885894 84640 0.088773086 84640 0.031065247
84600 0.051545649 84600 0.079492646 42400 0.169815306 42356
0.036250087 84648 0.099506971 84648 0.055274391
84700 0.014732196 84700 0.044994191 42400 0.176278453 42360
0.017803452 84656 0.148904379 84656 0.054514767
84700 0.008918187 84700 0.008478696 42400 0.111535206 42364
0.023757613 84664 0.19311368 84664 0.033681801
84700 0.020807514 84700 0.037292626 42400 0.066507884 42368
0.050514627 84672 0.139987388 84672 0.006690311
84700 0.025402385 84700 0.048583832 42400 0.10525441 42372
0.081233084 84680 0.079882586 84680 0.028370374
84700 0.036528796 84700 0.065084736 42400 0.142614474 42376
0.09563526 84688 0.021439382 84688 0.060715764
84700 0.085011925 84700 0.082608618 42400 0.184148754 42380
0.061583211 84696 0.056086879 84696 0.084130108
84700 0.169992694 84700 0.158763374 42400 0.128687781 42384
0.034368513 84704 0.031070154 84704 0.079463447
84700 0.171322594 84700 0.186600955 42400 0.077620571 42388
0.07916669 84712 0.06685569 84712 0.040439678
84700 0.111092304 84700 0.137364681 42400 0.158919575 42392
0.086468972 84720 0.040093495 84720 0.017339962
84700 0.089393267 84700 0.072793759 42400 0.139244061 42396
0.077067904 84728 0.056763915 84728 0.038755698
84700 0.07929252 84700 0.015880663 42400 0.072613977 42400
0.068436224 84736 0.113447626 84736 0.055445133
84700 0.015602927 84700 0.052515952 42400 0.083592291 42404
0.049091974 84744 0.12406295 84744 0.068067006
84800 0.017183045 84800 0.088076122 42400 0.072130504 42408
0.035187713 84752 0.130370681 84752 0.055974673
84800 0.02247126 84800 0.165605539 42400 0.040949963 42412
0.030878051 84760 0.191865009 84760 0.016390228
84800 0.038368689 84800 0.220330185 42400 0.07069204 42416
0.0742423 84768 0.246754091 84768 0.0264915
84800 0.004526077 84800 0.196627094 42400 0.048966678 42420
0.101186721 84776 0.244135634 84776 0.048836188
84800 0.043454777 84800 0.151059314 42400 0.040408646 42424
0.082993131 84784 0.200568393 84784 0.071667288
84800 0.03678726 84800 0.060793038 42400 0.106574058 42428
0.050222225 84792 0.134503745 84792 0.078851394
84800 0.053362444 84800 0.038273483 42400 0.145420898 42432
0.018618812 84800 0.045566372 84800 0.032316828
84800 0.057518333 84800 0.043436528 42400 0.11357246 42436
0.009397279 84808 0.037021771 84808 0.029681798
84800 0.03766501 84800 0.081497252 42400 0.046840116 42440
0.02272128 84816 0.038464881 84816 0.039416525
84800 0.092276008 84800 0.101036756 42400 0.021783801 42444
0.034759272 84824 0.035495166 84824 0.030379742
84800 0.113229835 84800 0.109334411 42400 0.034149623 42448
0.057354027 84832 0.052355888 84832 0.054102628
84800 0.07700939 84800 0.123219739 42500 0.038318089 42452
0.076378135 84840 0.062574072 84840 0.02892325
84800 0.080201804 84800 0.116044823 42500 0.08824766 42456
0.067809546 84848 0.138062009 84848 0.033106277
84900 0.085728316 84900 0.107940534 42500 0.059130529 42460
0.037664981 84856 0.115151081 84856 0.040415544
84900 0.166491024 84900 0.098669087 42500 0.032610806 42464
0.036060774 84864 0.06141355 84864 0.007939931
84900 0.107396932 84900 0.137375231 42500 0.0216307 42468
0.015950456 84872 0.066276982 84872 0.017055691
84900 0.058196303 84900 0.139232856 42500 0.012776249 42472
0.019647559 84880 0.023003004 84880 0.04116297
84900 0.084796913 84900 0.037220711 42500 0.010513334 42476
0.040525192 84888 0.070077957 84888 0.064977699
84900 0.058282185 84900 0.119813893 42500 0.034111043 42480

0.058168902	84896	0.151399741	84896	0.068938032
84900	0.015327622	84900	0.101598824	42500	0.066744753	42484
0.094255491	84904	0.175447844	84904	0.070220536
84900	0.023872435	84900	0.186859733	42500	0.065812448	42488
0.083731946	84912	0.128730855	84912	0.078000812
84900	0.012345368	84900	0.194177366	42500	0.061270039	42492
0.049039347	84920	0.058851365	84920	0.068457368
84900	0.010052083	84900	0.071339309	42500	0.053221596	42496
0.030292866	84928	0.022177113	84928	0.036657883
84900	0.057256089	84900	0.048796981	42500	0.030060061	42500
0.027819133	84936	0.006509609	84936	0.0380653
84900	0.043069344	84900	0.058239173	42500	0.041218689	42504
0.017260978	84944	0.066758912	84944	0.035676698
85000	0.050297502	85000	0.063506246	42500	0.053431428	42508
0.01394771	84952	0.097815384	84952	0.036936392
85000	0.100960366	85000	0.141616445	42500	0.027590235	42512
0.018910218	84960	0.073123767	84960	0.045207307
85000	0.113560302	85000	0.127168984	42500	0.043731692	42516
0.06023566	84968	0.029729163	84968	0.036432244
85000	0.128062646	85000	0.032197608	42500	0.069270915	42520
0.070450631	84976	0.089092915	84976	0.040211806
85000	0.101208847	85000	0.062913117	42500	0.063600695	42524
0.062500578	84984	0.076119686	84984	0.055203389
85000	0.05919375	85000	0.044681732	42500	0.032999746	42528
0.030015124	84992	0.027810162	84992	0.036578778
85000	0.050544488	85000	0.021308106	42500	0.025514435	42532
0.039220071	85000	0.040222723	85000	0.018272605
85000	0.092100512	85000	0.054087042	42500	0.05773372	42536
0.020265021	85008	0.061444851	85008	0.029181107
85000	0.148026244	85000	0.090444242	42500	0.029135546	42540
0.04389777	85016	0.063542335	85016	0.018422476
85000	0.136223025	85000	0.078823003	42500	0.032002197	42544
0.049052786	85024	0.017486305	85024	0.044333589
85000	0.075893993	85000	0.046402132	42500	0.043845081	42548
0.035339042	85032	0.087267326	85032	0.034893488
85000	0.058862475	85000	0.047024703	42600	0.040721428	42552
0.020352241	85040	0.118321914	85040	0.018030891
85000	0.095935422	85000	0.113561175	42600	0.035367473	42556
0.016914133	85048	0.06512284	85048	0.019840842
85100	0.140232266	85100	0.171369261	42600	0.042173579	42560
0.025628222	85056	0.068809313	85056	0.0128068
85100	0.139179858	85100	0.130708068	42600	0.058223974	42564
0.049576036	85064	0.155969071	85064	0.017443414
85100	0.100681136	85100	0.093552808	42600	0.019804791	42568
0.06256732	85072	0.091964073	85072	0.018023009
85100	0.049609345	85100	0.036186906	42600	0.017005454	42572
0.092953276	85080	0.009324624	85080	0.02168382
85100	0.034592766	85100	0.106099149	42600	0.031119795	42576
0.100815807	85088	0.045564695	85088	0.039068305
85100	0.094802366	85100	0.124958693	42600	0.028454586	42580
0.069061309	85096	0.100776721	85096	0.042898959
85100	0.072027462	85100	0.055946635	42600	0.016817157	42584
0.040007544	85104	0.155083778	85104	0.051841129
85100	0.016341935	85100	0.007453759	42600	0.026203798	42588
0.057126857	85112	0.180662376	85112	0.043836462
85100	0.062873733	85100	0.061613137	42600	0.028800572	42592
0.025225398	85120	0.091042828	85120	0.009449322
85100	0.008723917	85100	0.085478656	42600	0.025065479	42596
0.096553064	85128	0.049289512	85128	0.052177064
85100	0.103977232	85100	0.039651419	42600	0.046280882	42600
0.137263836	85136	0.130804794	85136	0.076982367
85100	0.136327391	85100	0.044487188	42600	0.074177646	42604
0.119604418	85144	0.139501048	85144	0.071704199
85200	0.043712698	85200	0.092691502	42600	0.068622416	42608
0.075694581	85152	0.146412698	85152	0.03645361
85200	0.122527737	85200	0.168890605	42600	0.045222289	42612
0.067329922	85160	0.189928556	85160	0.012043445
85200	0.167586739	85200	0.141059223	42600	0.032968441	42616

0.045294823 85168 0.217528591 85168 0.036597845
85200 0.144037621 85200 0.060433391 42600 0.0390118 42620
0.015827698 85176 0.190899256 85176 0.029115201
85200 0.092896837 85200 0.045533154 42600 0.044587159 42624
0.045734931 85184 0.153840636 85184 0.007766988
85200 0.037254358 85200 0.014365454 42600 0.048903436 42628
0.02020896 85192 0.156393129 85192 0.019295576
85200 0.01432354 85200 0.037241622 42600 0.039361643 42632
0.008259408 85200 0.1210142 85200 0.049099868
85200 0.023164408 85200 0.045788685 42600 0.040553612 42636
0.049492752 85208 0.045375116 85208 0.066845896
85200 0.086653185 85200 0.020977435 42600 0.052191081 42640
0.098812219 85216 0.029916115 85216 0.055338092
85200 0.109681285 85200 0.04273386 42600 0.030182808 42644
0.099014316 85224 0.011003925 85224 0.029568832
85200 0.060156628 85200 0.080386875 42600 0.017276758 42648
0.075782831 85232 0.086182721 85232 0.031897318
85200 0.014915248 85200 0.100969715 42700 0.021326505 42652
0.079750309 85240 0.108268156 85240 0.049842423
85200 0.073228737 85200 0.086894339 42700 0.028072775 42656
0.067263172 85248 0.080490172 85248 0.104023842
85300 0.111769638 85300 0.042979595 42700 0.043887354 42660
0.038666542 85256 0.019354284 85256 0.089919289
85300 0.121548212 85300 0.112988208 42700 0.019618025 42664
0.026256519 85264 0.073673444 85264 0.015664384
85300 0.067168847 85300 0.112518224 42700 0.04538288 42668
0.045426576 85272 0.105814892 85272 0.067526918
85300 0.043125801 85300 0.065491746 42700 0.06828487 42672
0.074584066 85280 0.111254332 85280 0.088417604
85300 0.020987762 85300 0.021391985 42700 0.046706504 42676
0.07115741 85288 0.102055383 85288 0.094120856
85300 0.105685016 85300 0.092672832 42700 0.040876912 42680
0.064511231 85296 0.03747347 85296 0.058576377
85300 0.104593186 85300 0.101880934 42700 0.040807579 42684
0.05882316 85304 0.037250575 85304 0.06781899
85300 0.08740971 85300 0.043965643 42700 0.033264289 42688
0.069167996 85312 0.110872628 85312 0.066935929
85300 0.097365417 85300 0.148180275 42700 0.033538279 42692
0.072938092 85320 0.14578308 85320 0.070706483
85300 0.093148454 85300 0.19398563 42700 0.039564999 42696
0.089541893 85328 0.060054732 85328 0.142485005
85300 0.060767095 85300 0.159896765 42700 0.020237611 42700
0.120872457 85336 0.061504783 85336 0.142419521
85300 0.021769651 85300 0.128610496 42700 0.05838056 42704
0.173551685 85344 0.073057148 85344 0.057371028
85400 0.044645007 85400 0.096675954 42700 0.052910844 42708
0.186995196 85352 0.045152068 85352 0.099342695
85400 0.079248261 85400 0.06508598 42700 0.019655814 42712
0.132537433 85360 0.045368885 85360 0.138673248
85400 0.11491467 85400 0.142897726 42700 0.010451784 42716
0.075655247 85368 0.080286678 85368 0.156240684
85400 0.1463347 85400 0.062441242 42700 0.005717323 42720
0.05558609 85376 0.076283985 85376 0.164857745
85400 0.16758377 85400 0.100465477 42700 0.025293704 42724
0.06948298 85384 0.060383842 85384 0.128290325
85400 0.150331078 85400 0.209428545 42700 0.031089392 42728
0.032513675 85392 0.036321329 85392 0.130294385
85400 0.079778743 85400 0.231070284 42700 0.023660283 42732
0.037472273 85400 0.089809284 85400 0.12723048
85400 0.039252904 85400 0.146317921 42700 0.05459895 42736
0.066762223 85408 0.138259944 85408 0.233750135
85400 0.026529275 85400 0.006434612 42700 0.063030188 42740
0.101810409 85416 0.058481393 85416 0.300700194
85400 0.026826316 85400 0.090373316 42700 0.039026589 42744
0.084445412 85424 0.015026644 85424 0.290127035
85400 0.015797114 85400 0.120832621 42700 0.025483625 42748
0.026956246 85432 0.051361334 85432 0.536975276
85400 0.07752145 85400 0.140871256 42800 0.045781439 42752

0.082464183 85440 0.0887719 85440 0.868042524
85400 0.111230751 85400 0.172917615 42800 0.068018919 42756
0.103952632 85448 0.067380148 85448 3.767517863
85500 0.07720475 85500 0.1496068 42800 0.071452589 42760
0.045024 85456 0.031609841 85456 5.821175413
85500 0.044337943 85500 0.067124245 42800 0.066615103 42764
0.061690611 85464 0.03651475 85464 4.496112463
85500 0.0697172 85500 0.055873348 42800 0.041841951 42768
0.105781597 85472 0.090837333 85472 1.45618525
85500 0.10949342 85500 0.151657266 42800 0.024822557 42772
0.117450334 85480 0.102907776 85480 0.423163845
85500 0.109076173 85500 0.19051123 42800 0.036033369 42776
0.072159848 85488 0.050586044 85488 0.295960141
85500 0.053589178 85500 0.139717784 42800 0.030057729 42780
0.065586391 85496 0.093566436 85496 0.222188174
85500 0.0882397 85500 0.061042651 42800 0.038015016 42784
0.099765304 85504 0.094724011 85504 0.147460785
85500 0.153349334 85500 0.066759581 42800 0.043028325 42788
0.066309694 85512 0.040858777 85512 0.106889689
85500 0.162985176 85500 0.109056382 42800 0.060840412 42792
0.031922966 85520 0.120877528 85520 0.13790137
85500 0.127269755 85500 0.126215876 42800 0.039707542 42796
0.107914115 85528 0.070578666 85528 0.116617688
85500 0.07476085 85500 0.065337197 42800 0.013075589 42800
0.127709776 85536 0.067041663 85536 0.065376102
85500 0.058052221 85500 0.090572132 42800 0.024493515 42804
0.108434419 85544 0.068828798 85544 0.102280472
85600 0.070160328 85600 0.14628275 42800 0.022795715 42808
0.064583161 85552 0.100005986 85552 0.059169201
85600 0.083884559 85600 0.196994923 42800 0.013182688 42812
0.069585556 85560 0.034540113 85560 0.075868295
85600 0.074552234 85600 0.222583113 42800 0.052451862 42816
0.102289661 85568 0.108492874 85568 0.105296356
85600 0.076352364 85600 0.175340233 42800 0.038642425 42820
0.148609076 85576 0.077577628 85576 0.058132388
85600 0.10671543 85600 0.092941096 42800 0.021953401 42824
0.169047068 85584 0.069015616 85584 0.013356632
85600 0.101826408 85600 0.037188056 42800 0.050022354 42828
0.14276462 85592 0.101107602 85592 0.033612978
85600 0.030666051 85600 0.108234388 42800 0.072281531 42832
0.096610762 85600 0.084525818 85600 0.03839238
85600 0.053070548 85600 0.094380681 42800 0.082270359 42836
0.072367395 85608 0.058572165 85608 0.009212689
85600 0.021191328 85600 0.070008777 42800 0.075807417 42840
0.041942923 85616 0.023393524 85616 0.018361203
85600 0.109513618 85600 0.169491643 42800 0.070276132 42844
0.048472375 85624 0.032053984 85624 0.02391518
85600 0.15070953 85600 0.287471573 42800 0.017864346 42848
0.086971319 85632 0.092297087 85632 0.032044605
85600 0.112378999 85600 0.249375153 42900 0.067110988 42852
0.101208876 85640 0.12896648 85640 0.021542797
85600 0.047021978 85600 0.189802974 42900 0.091954695 42856
0.103450169 85648 0.077334989 85648 0.037755344
85700 0.064261833 85700 0.296992483 42900 0.077999161 42860
0.068992747 85656 0.029099903 85656 0.043418138
85700 0.125890874 85700 0.501001894 42900 0.049782342 42864
0.041546893 85664 0.030146754 85664 0.046228091
85700 0.121084449 85700 0.679581659 42900 0.031435044 42868
0.093644543 85672 0.04347078 85672 0.03406599
85700 0.065288245 85700 0.835188781 42900 0.042553529 42872
0.1340241 85680 0.111887232 85680 0.033577995
85700 0.04581473 85700 0.929811853 42900 0.04588734 42876
0.131411929 85688 0.095308635 85688 0.023878138
85700 0.117225332 85700 0.929469126 42900 0.054897217 42880
0.074734693 85696 0.037642894 85696 0.010695458
85700 0.16778511 85700 0.96719648 42900 0.045092416 42884
0.028739843 85704 0.102563805 85704 0.024439547
85700 0.123806924 85700 1.135007595 42900 0.05391975 42888

0.067854053 85712 0.064143496 85712 0.04612987
85700 0.070738301 85700 1.303517488 42900 0.071198869 42892
0.087155546 85720 0.028534922 85720 0.013565366
85700 0.157505885 85700 1.292880863 42900 0.063796157 42896
0.053194439 85728 0.03355206 85728 0.055515964
85700 0.183494573 85700 1.10385404 42900 0.042380667 42900
0.06216421 85736 0.041839336 85736 0.018014423
85700 0.109713452 85700 0.84931025 42900 0.010534211 42904
0.098355726 85744 0.092800772 85744 0.065414148
85800 0.050625808 85800 0.789210084 42900 0.052871448 42908
0.091370705 85752 0.070579321 85752 0.089158282
85800 0.048899874 85800 1.103935764 42900 0.062861698 42912
0.04699768 85760 0.040895706 85760 0.09668315
85800 0.061331004 85800 1.287219813 42900 0.064904321 42916
0.034580498 85768 0.050922383 85768 0.098546989
85800 0.100633079 85800 1.119329478 42900 0.076871525 42920
0.116659467 85776 0.081600083 85776 0.062427185
85800 0.090177724 85800 0.826233125 42900 0.05467681 42924
0.15459719 85784 0.190358536 85784 0.026151514
85800 0.032867421 85800 0.686102081 42900 0.022482838 42928
0.108319007 85792 0.191373183 85792 0.03997565
85800 0.054352175 85800 0.485066615 42900 0.030832318 42932
0.099111545 85800 0.015893405 85800 0.084476407
85800 0.090152178 85800 0.340059808 42900 0.055341734 42936
0.067093082 85808 0.161193923 85808 0.071674993
85800 0.129274281 85800 0.280219334 42900 0.081902821 42940
0.019485269 85816 0.146730395 85816 0.022695587
85800 0.112726666 85800 0.254808925 42900 0.023294366 42944
0.099566554 85824 0.091264978 85824 0.055227782
85800 0.045446865 85800 0.241563598 42900 0.048566755 42948
0.113630282 85832 0.147219369 85832 0.081203027
85800 0.040366173 85800 0.204126176 43000 0.071513685 42952
0.080565587 85840 0.102307669 85840 0.062882442
85800 0.083901949 85800 0.161715318 43000 0.064430948 42956
0.053710348 85848 0.103350634 85848 0.05355199
85900 0.089164707 85900 0.170063039 43000 0.037054393 42960
0.044374352 85856 0.129753869 85856 0.101947946
85900 0.071253875 85900 0.193457353 43000 0.04112213 42964
0.060773411 85864 0.108738961 85864 0.096651471
85900 0.144015736 85900 0.148990585 43000 0.052456289 42968
0.048287144 85872 0.096612828 85872 0.080644335
85900 0.132582558 85900 0.160854091 43000 0.038064332 42972
0.035876947 85880 0.119778481 85880 0.072571085
85900 0.067094203 85900 0.193495361 43000 0.008218404 42976
0.054228596 85888 0.080118523 85888 0.0308524
85900 0.050453098 85900 0.14612927 43000 0.025946418 42980
0.07839586 85896 0.125572289 85896 0.018749679
85900 0.09067457 85900 0.056691752 43000 0.038394908 42984
0.074102965 85904 0.22197442 85904 0.039764735
85900 0.148139326 85900 0.105730709 43000 0.016161288 42988
0.061444982 85912 0.151826214 85912 0.027170101
85900 0.170200453 85900 0.17417781 43000 0.05137804 42992
0.070842398 85920 0.035759342 85920 0.032337481
85900 0.120447883 85900 0.141964978 43000 0.07369044 42996
0.075506971 85928 0.109474189 85928 0.049473048
85900 0.057018882 85900 0.162452066 43000 0.041567684 43000
0.041368658 85936 0.070827271 85936 0.024625177
85900 0.012195481 85900 0.216915593 43000 0.028664463 43004
0.036627716 85944 0.115905023 85944 0.022680921
86000 0.088758476 86000 0.211041538 43000 0.035963658 43008
0.054504777 85952 0.121758159 85952 0.027199303
86000 0.134745599 86000 0.188501566 43000 0.007691637 43012
0.071140246 85960 0.051445873 85960 0.004434161
86000 0.100428588 86000 0.159811796 43000 0.007989771 43016
0.077954479 85968 0.06412583 85968 0.043405697
86000 0.08082149 86000 0.115974384 43000 0.042316609 43020
0.078024219 85976 0.05653195 85976 0.089760251
86000 0.096698328 86000 0.043767475 43000 0.067659807 43024

0.070633316 85984 0.061150568 85984 0.070992915
86000 0.064610562 86000 0.039975232 43000 0.057684265 43028
0.070578382 85992 0.103451464 85992 0.036771216
86000 0.085426975 86000 0.081281643 43000 0.045346744 43032
0.090295318 86000 0.121808931 86000 0.057057787
86000 0.116283307 86000 0.058514484 43000 0.051438667 43036
0.106373802 86008 0.090284819 86008 0.045922076
86000 0.04520045 86000 0.019051189 43000 0.037756981 43040
0.072398965 86016 0.02948462 86016 0.047810809
86000 0.061749175 86000 0.084534549 43000 0.02171578 43044
0.051560983 86024 0.039132734 86024 0.064683809
86000 0.054755274 86000 0.057804846 43000 0.018810926 43048
0.057632737 86032 0.059472546 86032 0.086864588
86000 0.053435957 86000 0.026685057 43100 0.023334382 43052
0.055379118 86040 0.057684742 86040 0.065251385
86000 0.010707632 86000 0.035836707 43100 0.032051699 43056
0.053607069 86048 0.001963557 86048 0.045672299
86100 0.08592516 86100 0.056037676 43100 0.05909862 43060
0.011042395 86056 0.05385447 86056 0.044115986
86100 0.143714933 86100 0.05661144 43100 0.057580724 43064
0.043179796 86064 0.0646115 86064 0.03843477
86100 0.12736785 86100 0.028299148 43100 0.04365373 43068
0.100339341 86072 0.054053031 86072 0.026007378
86100 0.055327168 86100 0.048846163 43100 0.038182756 43072
0.095839365 86080 0.021929422 86080 0.042773118
86100 0.100996156 86100 0.083053245 43100 0.060862843 43076
0.06861415 86088 0.023193343 86088 0.061364757
86100 0.147141705 86100 0.062568179 43100 0.091765956 43080
0.066979359 86096 0.030651929 86096 0.056188746
86100 0.144968508 86100 0.088097302 43100 0.088978166 43084
0.063111234 86104 0.106943626 86104 0.02188074
86100 0.061622821 86100 0.060619685 43100 0.042785727 43088
0.037894202 86112 0.113719958 86112 0.056073968
86100 0.033080596 86100 0.029058338 43100 0.082558683 43092
0.036455342 86120 0.065789354 86120 0.085403219
86100 0.082331164 86100 0.050041756 43100 0.110771704 43096
0.038738373 86128 0.05221894 86128 0.06797949
86100 0.123391132 86100 0.106235333 43100 0.066060165 43100
0.039016264 86136 0.09152748 86136 0.032933334
86100 0.140398945 86100 0.112469381 43100 0.017055892 43104
0.058511447 86144 0.148168576 86144 0.033234035
86200 0.120497869 86200 0.043297223 43100 0.023334565 43108
0.080124162 86152 0.164149723 86152 0.049880568
86200 0.099182777 86200 0.075289303 43100 0.035195644 43112
0.054312855 86160 0.097551441 86160 0.026626585
86200 0.081342099 86200 0.095648633 43100 0.036315607 43116
0.062387895 86168 0.043475095 86168 0.025347094
86200 0.063417632 86200 0.056842921 43100 0.043437998 43120
0.115157884 86176 0.064485139 86176 0.036421756
86200 0.05932105 86200 0.091754751 43100 0.057314333 43124
0.144613266 86184 0.063489235 86184 0.013172608
86200 0.015908023 86200 0.085925633 43100 0.047196663 43128
0.089405126 86192 0.065453372 86192 0.000872393
86200 0.046558445 86200 0.088411442 43100 0.018231936 43132
0.021255113 86200 0.081859973 86200 0.014096174
86200 0.068032561 86200 0.088193832 43100 0.030717987 43136
0.025287811 86208 0.018721357 86208 0.031654108
86200 0.061662868 86200 0.036636589 43100 0.080943231 43140
0.067200534 86216 0.066733963 86216 0.024861844
86200 0.051872507 86200 0.032349366 43100 0.08754903 43144
0.06620591 86224 0.115520947 86224 0.022438166
86200 0.079578051 86200 0.061882543 43100 0.059340633 43148
0.004911153 86232 0.104744606 86232 0.074073621
86200 0.0984582 86200 0.158406081 43200 0.039026927 43152
0.035623289 86240 0.05354821 86240 0.077968703
86200 0.007774105 86200 0.122017918 43200 0.027599577 43156
0.070201502 86248 0.046996214 86248 0.063560998
86300 0.050430928 86300 0.025903013 43200 0.022208027 43160

0.072569994	86256	0.033137443	86256	0.057061021		
86300	0.047927599	86300	0.030056426	43200	0.030459793	43164
0.078652505	86264	0.063793312	86264	0.020267115		
86300	0.073673276	86300	0.039590628	43200	0.042305128	43168
0.104606443	86272	0.025275238	86272	0.03039287		
86300	0.036589059	86300	0.050345734	43200	0.042334898	43172
0.086024069	86280	0.061469553	86280	0.034829998		
86300	0.082186343	86300	0.112317568	43200	0.046980382	43176
0.041369734	86288	0.068979847	86288	0.018861309		
86300	0.141770463	86300	0.145175086	43200	0.051468334	43180
0.06280854	86296	0.121066601	86296	0.014231818		
86300	0.120959776	86300	0.080821861	43200	0.075522534	43184
0.073030118	86304	0.228625576	86304	0.0421152		
86300	0.021392201	86300	0.036899557	43200	0.093576462	43188
0.084050647	86312	0.246109441	86312	0.045006174		
86300	0.056260837	86300	0.06680841	43200	0.080240599	43192
0.101063306	86320	0.18209836	86320	0.011608314		
86300	0.053426033	86300	0.104764818	43200	0.04447758	43196
0.07432128	86328	0.068847272	86328	0.043315467		
86300	0.006215445	86300	0.076898818	43200	0.019644031	43200
0.006724079	86336	0.047285987	86336	0.064505635		
86300	0.094628944	86300	0.040319425	43200	0.037330898	43204
0.048466121	86344	0.053048421	86344	0.045622946		
86400	0.115312891	86400	0.082437713	43200	0.047790694	43208
0.069934344	86352	0.054528468	86352	0.035170975		
86400	0.090908325	86400	0.054214983	43200	0.054788954	43212
0.075878612	86360	0.006766848	86360	0.039677285		
86400	0.114075847	86400	0.038604765	43200	0.057806919	43216
0.064356194	86368	0.057906334	86368	0.040546522		
86400	0.088195484	86400	0.102298283	43200	0.044245728	43220
0.050930281	86376	0.087706343	86376	0.027005805		
86400	0.066000415	86400	0.101965023	43200	0.024204232	43224
0.086299457	86384	0.063057079	86384	0.011548349		
86400	0.084552579	86400	0.038609716	43200	0.017678045	43228
0.101606187	86392	0.035464564	86392	0.048239763		
86400	0.083386622	86400	0.066677094	43200	0.042542433	43232
0.06182857	86400	0.041429343	86400	0.046373782		
86400	0.069331443	86400	0.026085827	43200	0.054987882	43236
0.085448715	86408	0.081086502	86408	0.02507592		
86400	0.073807729	86400	0.103982136	43200	0.024769895	43240
0.091823502	86416	0.081257495	86416	0.023136516		
86400	0.067224559	86400	0.094553616	43200	0.03436306	43244
0.094232637	86424	0.026862492	86424	0.030962936		
86400	0.084659179	86400	0.032722881	43200	0.048202015	43248
0.086888598	86432	0.032426069	86432	0.028558216		
86400	0.10293939	86400	0.046175563	43300	0.021686308	43252
0.078624202	86440	0.040634201	86440	0.016690165		
86400	0.085432112	86400	0.040619456	43300	0.029758085	43256
0.064269487	86448	0.039581046	86448	0.043801138		
86500	0.040590829	86500	0.076962373	43300	0.065207969	43260
0.094410127	86456	0.062560968	86456	0.04414923		
86500	0.04502618	86500	0.07590116	43300	0.060405921	43264
0.134418748	86464	0.038749233	86464	0.019637453		
86500	0.054021282	86500	0.060971586	43300	0.022628636	43268
0.09668812	86472	0.037064019	86472	0.030537467		
86500	0.043381948	86500	0.081682847	43300	0.016599381	43272
0.046468289	86480	0.064471882	86480	0.03370296		
86500	0.038520881	86500	0.134137343	43300	0.024004092	43276
0.05727019	86488	0.161585485	86488	0.033985201		
86500	0.053548465	86500	0.12337057	43300	0.024151164	43280
0.039849565	86496	0.153229514	86496	0.045439196		
86500	0.109007429	86500	0.071891664	43300	0.014731487	43284
0.047039197	86504	0.081953462	86504	0.037664431		
86500	0.047138223	86500	0.031866726	43300	0.036478879	43288
0.067781271	86512	0.138616263	86512	0.028632703		
86500	0.10027751	86500	0.055420289	43300	0.033084103	43292
0.085805586	86520	0.15504226	86520	0.039304745		
86500	0.114567891	86500	0.088215616	43300	0.012065876	43296

0.1171654 86528 0.101165206 86528 0.043557411
86500 0.039850151 86500 0.089387679 43300 0.067284978 43300
0.162832818 86536 0.133589245 86536 0.051246949
86500 0.142780191 86500 0.08954831 43300 0.099451405 43304
0.137282114 86544 0.168862084 86544 0.05639892
86600 0.227901371 86600 0.057368106 43300 0.082154707 43308
0.029783656 86552 0.146372011 86552 0.05426769
86600 0.214624859 86600 0.047164194 43300 0.039398809 43312
0.058218408 86560 0.078421668 86560 0.088119603
86600 0.158365118 86600 0.078432517 43300 0.031669686 43316
0.102458078 86568 0.066182256 86568 0.078043457
86600 0.099945632 86600 0.094287505 43300 0.053486096 43320
0.122789279 86576 0.079538702 86576 0.042959717
86600 0.084218627 86600 0.130652363 43300 0.051273084 43324
0.075659606 86584 0.065947388 86584 0.033544508
86600 0.059346381 86600 0.090735914 43300 0.027526032 43328
0.067882364 86592 0.059357735 86592 0.046347141
86600 0.048588972 86600 0.011043058 43300 0.04217678 43332
0.089644491 86600 0.084061743 86600 0.047763391
86600 0.084240739 86600 0.014422387 43300 0.045788031 43336
0.101935271 86608 0.144211546 86608 0.017989409
86600 0.143418584 86600 0.068755115 43300 0.048072587 43340
0.088696441 86616 0.082159459 86616 0.010987045
86600 0.192359323 86600 0.060058414 43300 0.06156147 43344
0.085741303 86624 0.077738318 86624 0.007131049
86600 0.159473479 86600 0.065112719 43300 0.054301276 43348
0.071933027 86632 0.096372656 86632 0.006036566
86600 0.074910509 86600 0.104883606 43400 0.046487134 43352
0.057290126 86640 0.095631767 86640 0.029504756
86600 0.039210954 86600 0.050364182 43400 0.043281125 43356
0.045877539 86648 0.033706092 86648 0.040746738
86700 0.021163742 86700 0.05419532 43400 0.042167092 43360
0.088533598 86656 0.086717584 86656 0.034893215
86700 0.083822612 86700 0.062713065 43400 0.052110881 43364
0.1091339 86664 0.10821475 86664 0.034083754
86700 0.089804351 86700 0.07099611 43400 0.056565827 43368
0.082041574 86672 0.045161898 86672 0.036974183
86700 0.043450185 86700 0.215246764 43400 0.05736974 43372
0.065500615 86680 0.112457201 86680 0.015464164
86700 0.032729735 86700 0.226186458 43400 0.036734222 43376
0.049942588 86688 0.168192491 86688 0.025944992
86700 0.063178501 86700 0.14155863 43400 0.032606928 43380
0.086366941 86696 0.185935045 86696 0.049378094
86700 0.09590626 86700 0.072425952 43400 0.027869988 43384
0.056846926 86704 0.149152053 86704 0.073281859
86700 0.109089713 86700 0.033590906 43400 0.04487345 43388
0.039982453 86712 0.084368818 86712 0.070571885
86700 0.065118707 86700 0.04655201 43400 0.047197573 43392
0.042990312 86720 0.102734339 86720 0.04240745
86700 0.013821956 86700 0.128265965 43400 0.040124112 43396
0.031082673 86728 0.13429219 86728 0.033190594
86700 0.003011728 86700 0.164648868 43400 0.034994533 43400
0.046656413 86736 0.151603191 86736 0.01901472
86700 0.034225941 86700 0.150386316 43400 0.023305473 43404
0.065400818 86744 0.159603441 86744 0.030236197
86800 0.043972112 86800 0.080778613 43400 0.030705451 43408
0.10239744 86752 0.085921376 86752 0.027055658
86800 0.075024007 86800 0.105302614 43400 0.035095814 43412
0.129666761 86760 0.030651008 86760 0.020937943
86800 0.100240664 86800 0.093611634 43400 0.01671604 43416
0.087238383 86768 0.044049488 86768 0.027586213
86800 0.039919192 86800 0.046503683 43400 0.029381114 43420
0.109046989 86776 0.071321716 86776 0.033056113
86800 0.051425526 86800 0.076789831 43400 0.035513036 43424
0.193064581 86784 0.15364126 86784 0.040412375
86800 0.05148779 86800 0.110319626 43400 0.025858941 43428
0.171910404 86792 0.148035819 86792 0.030275251
86800 0.031261578 86800 0.144531121 43400 0.026061505 43432

0.102816703 86800 0.105070169 86800 0.03148559
86800 0.042462543 86800 0.14467175 43400 0.039523147 43436
0.068482666 86808 0.07547962 86808 0.02755949
86800 0.049145347 86800 0.131332505 43400 0.026063784 43440
0.045709978 86816 0.042894895 86816 0.007489287
86800 0.056368011 86800 0.064528766 43400 0.003716447 43444
0.067148321 86824 0.076532866 86824 0.02540654
86800 0.06022424 86800 0.035206769 43400 0.027673154 43448
0.082241953 86832 0.160766664 86832 0.02699031
86800 0.044599387 86800 0.060226044 43500 0.026340138 43452
0.092786169 86840 0.071779105 86840 0.030595664
86800 0.021268523 86800 0.061255851 43500 0.023108716 43456
0.108524619 86848 0.109974106 86848 0.026730573
86900 0.048110349 86900 0.067490706 43500 0.023135588 43460
0.136849748 86856 0.189753599 86856 0.051526087
86900 0.057014298 86900 0.052612548 43500 0.04293432 43464
0.167219129 86864 0.170896761 86864 0.046475012
86900 0.062851737 86900 0.037450071 43500 0.039648538 43468
0.157976654 86872 0.176808506 86872 0.030693354
86900 0.066744251 86900 0.055325872 43500 0.011718153 43472
0.107500753 86880 0.148501116 86880 0.02326428
86900 0.042410378 86900 0.027220625 43500 0.010465093 43476
0.047978392 86888 0.101431273 86888 0.057439444
86900 0.043470187 86900 0.045035737 43500 0.029438601 43480
0.029715558 86896 0.074601776 86896 0.094899966
86900 0.066530462 86900 0.130584434 43500 0.060165978 43484
0.007828674 86904 0.0409809 86904 0.075839256
86900 0.039205919 86900 0.163168763 43500 0.059288163 43488
0.034752942 86912 0.045594548 86912 0.029937706
86900 0.074394309 86900 0.057604109 43500 0.022951299 43492
0.060028309 86920 0.06170792 86920 0.072066061
86900 0.124092898 86900 0.055860011 43500 0.057210109 43496
0.058841652 86928 0.036197951 86928 0.118349832
86900 0.095600561 86900 0.065621229 43500 0.047780111 43500
0.078020603 86936 0.11147753 86936 0.111945621
86900 0.055680081 86900 0.024884859 43500 0.030716099 43504
0.069463815 86944 0.137058698 86944 0.053028991
87000 0.176750153 87000 0.020412268 43500 0.038858194 43508
0.052343483 86952 0.087680986 86952 0.043426953
87000 0.216392815 87000 0.06879124 43500 0.05082192 43512
0.081580409 86960 0.021529453 86960 0.075237578
87000 0.119581258 87000 0.084197302 43500 0.031890551 43516
0.10332001 86968 0.097727214 86968 0.050967381
87000 0.067565772 87000 0.006781061 43500 0.013422454 43520
0.089471148 86976 0.136208895 86976 0.008365525
87000 0.149883898 87000 0.079347388 43500 0.020404088 43524
0.109864479 86984 0.085244647 86984 0.029663137
87000 0.106912834 87000 0.033366516 43500 0.044689241 43528
0.116678632 86992 0.020376005 86992 0.002562877
87000 0.078123114 87000 0.05505596 43500 0.033273202 43532
0.095977128 87000 0.102243095 87000 0.028020759
87000 0.072729141 87000 0.051936004 43500 0.025896765 43536
0.101895828 87008 0.138309784 87008 0.015925871
87000 0.04014598 87000 0.023245053 43500 0.042404863 43540
0.111683752 87016 0.076770572 87016 0.02274914
87000 0.047830436 87000 0.030261977 43500 0.050522642 43544
0.118616837 87024 0.144725273 87024 0.022064392
87000 0.109914414 87000 0.026837593 43500 0.055402426 43548
0.082098617 87032 0.220389324 87032 0.049556413
87000 0.108160784 87000 0.140925986 43600 0.055780773 43552
0.046623954 87040 0.209678445 87040 0.053879379
87000 0.039863124 87000 0.151265601 43600 0.02998034 43556
0.044178589 87048 0.150116481 87048 0.023872553
87100 0.048677084 87100 0.077225384 43600 0.022419914 43560
0.034839282 87056 0.138571806 87056 0.012740178
87100 0.073219722 87100 0.096003983 43600 0.01065006 43564
0.067345893 87064 0.114984483 87064 0.020195492
87100 0.125575665 87100 0.127116931 43600 0.003025827 43568

0.096365577 87072 0.048443861 87072 0.047397487
87100 0.202839685 87100 0.143529615 43600 0.01325052 43572
0.096732438 87080 0.051986448 87080 0.048214195
87100 0.201889009 87100 0.128577668 43600 0.040775954 43576
0.083280451 87088 0.075380915 87088 0.026982496
87100 0.080851714 87100 0.147749278 43600 0.058407619 43580
0.103495891 87096 0.088620342 87096 0.033250602
87100 0.039368242 87100 0.16295875 43600 0.041887262 43584
0.077335266 87104 0.119475088 87104 0.037370737
87100 0.074014839 87100 0.107615393 43600 0.052478528 43588
0.009066282 87112 0.065504231 87112 0.024357079
87100 0.038111284 87100 0.064253443 43600 0.083208295 43592
0.075065414 87120 0.109317269 87120 0.011674648
87100 0.04632329 87100 0.041663083 43600 0.071383998 43596
0.100124838 87128 0.09091309 87128 0.042973687
87100 0.102745791 87100 0.012745825 43600 0.029262079 43600
0.069229762 87136 0.02900113 87136 0.036384201
87100 0.111562869 87100 0.070028611 43600 0.033844015 43604
0.062674611 87144 0.084923115 87144 0.011784619
87200 0.088737041 87200 0.10139287 43600 0.04222264 43608
0.074704381 87152 0.147061088 87152 0.025184851
87200 0.057496796 87200 0.094468815 43600 0.060389208 43612
0.06501576 87160 0.138765099 87160 0.018967287
87200 0.047868369 87200 0.050846418 43600 0.037956412 43616
0.06975609 87168 0.101114034 87168 0.029462148
87200 0.055885987 87200 0.088951085 43600 0.031750285 43620
0.064039821 87176 0.016074429 87176 0.065272994
87200 0.109385735 87200 0.087738648 43600 0.062825609 43624
0.057278481 87184 0.120325203 87184 0.080289821
87200 0.13023385 87200 0.021491065 43600 0.069906761 43628
0.084745057 87192 0.129023581 87192 0.053822823
87200 0.114590926 87200 0.113752605 43600 0.080313475 43632
0.096709489 87200 0.102000056 87200 0.039417701
87200 0.114708826 87200 0.164707279 43600 0.051815645 43636
0.056957935 87208 0.123427191 87208 0.044504381
87200 0.114374088 87200 0.078736528 43600 0.013721969 43640
0.029345059 87216 0.086776934 87216 0.043910484
87200 0.03999886 87200 0.016428932 43600 0.034282861 43644
0.038600781 87224 0.029846719 87224 0.029332598
87200 0.038594506 87200 0.037390058 43600 0.002980534 43648
0.027518971 87232 0.056026456 87232 0.018518033
87200 0.030711999 87200 0.070431503 43700 0.036343667 43652
0.077727229 87240 0.049450842 87240 0.037431499
87200 0.027438568 87200 0.024376501 43700 0.054380809 43656
0.135472946 87248 0.054741628 87248 0.064935775
87300 0.039324623 87300 0.018759583 43700 0.059956186 43660
0.133175359 87256 0.074824624 87256 0.069890884
87300 0.060400897 87300 0.019853 43700 0.037189231 43664
0.055262764 87264 0.091836519 87264 0.063794818
87300 0.059062088 87300 0.032927241 43700 0.006223615 43668
0.050851828 87272 0.120105266 87272 0.069017464
87300 0.057030869 87300 0.057611007 43700 0.022389044 43672
0.031907439 87280 0.094674098 87280 0.061598337
87300 0.033325956 87300 0.03766541 43700 0.033746499 43676
0.019666735 87288 0.13146193 87288 0.049028207
87300 0.062577623 87300 0.007444625 43700 0.044575332 43680
0.011183487 87296 0.10242439 87296 0.04546213
87300 0.042759453 87300 0.014188556 43700 0.02599477 43684
0.02195094 87304 0.128438609 87304 0.039388844
87300 0.032232194 87300 0.040866602 43700 0.017277145 43688
0.043932068 87312 0.192918495 87312 0.037772879
87300 0.056496738 87300 0.063177657 43700 0.02701214 43692
0.039094208 87320 0.228305144 87320 0.05701997
87300 0.128710818 87300 0.075861681 43700 0.013283956 43696
0.017894807 87328 0.197779285 87328 0.076432007
87300 0.203021511 87300 0.093061259 43700 0.018434812 43700
0.02788741 87336 0.115537499 87336 0.063173669
87300 0.146220031 87300 0.127495383 43700 0.046825368 43704

0.058404199 87344 0.044767319 87344 0.04210258
87400 0.08966191 87400 0.085355532 43700 0.03722934 43708
0.060811657 87352 0.028314069 87352 0.03722126
87400 0.1636136 87400 0.022632718 43700 0.053747903 43712
0.054826731 87360 0.085395957 87360 0.052983363
87400 0.143317113 87400 0.048214442 43700 0.078406672 43716
0.064476953 87368 0.079762474 87368 0.070647802
87400 0.07060435 87400 0.10344632 43700 0.068559835 43720
0.070189424 87376 0.020459824 87376 0.054127675
87400 0.079771919 87400 0.094702271 43700 0.056267832 43724
0.071091112 87384 0.044066092 87384 0.020313966
87400 0.120916448 87400 0.088109744 43700 0.050151044 43728
0.079275102 87392 0.020575657 87392 0.046204244
87400 0.093364564 87400 0.099387078 43700 0.039281069 43732
0.08229036 87400 0.028604041 87400 0.04630486
87400 0.102052378 87400 0.134170594 43700 0.04068992 43736
0.047573416 87408 0.050769155 87408 0.012518412
87400 0.175613066 87400 0.183066906 43700 0.023407481 43740
0.023641554 87416 0.060744787 87416 0.007179266
87400 0.180264906 87400 0.187921513 43700 0.044154123 43744
0.026637756 87424 0.075102253 87424 0.016157834
87400 0.150567518 87400 0.111060115 43700 0.069666101 43748
0.008556127 87432 0.080495905 87432 0.021974956
87400 0.123458056 87400 0.122826983 43800 0.046201189 43752
0.028877537 87440 0.065809749 87440 0.050473955
87400 0.074906413 87400 0.103539824 43800 0.044825454 43756
0.038889793 87448 0.029610934 87448 0.053974767
87500 0.056598226 87500 0.055540095 43800 0.051064755 43760
0.028792267 87456 0.034481858 87456 0.023894087
87500 0.063936641 87500 0.053162159 43800 0.030134755 43764
0.073075855 87464 0.058384914 87464 0.007171911
87500 0.015741904 87500 0.051016483 43800 0.006070415 43768
0.112164526 87472 0.060258368 87472 0.010434334
87500 0.095115473 87500 0.008424337 43800 0.030060091 43772
0.114522547 87480 0.083137471 87480 0.023900091
87500 0.082362734 87500 0.05294981 43800 0.032234424 43776
0.129824883 87488 0.136712654 87488 0.043759847
87500 0.022252873 87500 0.042257903 43800 0.052897944 43780
0.087095446 87496 0.167559134 87496 0.058064226
87500 0.043591463 87500 0.065860819 43800 0.067801535 43784
0.016656275 87504 0.083136831 87504 0.04841806
87500 0.090671405 87500 0.103592109 43800 0.071705887 43788
0.073291463 87512 0.096614662 87512 0.032267017
87500 0.095895797 87500 0.110283458 43800 0.051993771 43792
0.122838334 87520 0.172769374 87520 0.020456209
87500 0.067256646 87500 0.06305764 43800 0.019674055 43796
0.108110413 87528 0.090600239 87528 0.034293949
87500 0.09601393 87500 0.036984504 43800 0.026089147 43800
0.077756558 87536 0.011158721 87536 0.047107762
87500 0.105665524 87500 0.078082339 43800 0.011104478 43804
0.06072846 87544 0.064842454 87544 0.052300838
87600 0.042161024 87600 0.022152664 43800 0.015044171 43808
0.068449088 87552 0.082548228 87552 0.063579348
87600 0.076355987 87600 0.052878222 43800 0.011473295 43812
0.076994118 87560 0.06646572 87560 0.078701356
87600 0.121374018 87600 0.044058415 43800 0.012043107 43816
0.101683269 87568 0.093566923 87568 0.072759111
87600 0.015920083 87600 0.037501519 43800 0.022532608 43820
0.133196053 87576 0.122423749 87576 0.052314754
87600 0.113946633 87600 0.063760948 43800 0.028293767 43824
0.134867864 87584 0.121226411 87584 0.038003793
87600 0.114047674 87600 0.097458833 43800 0.049564642 43828
0.087451947 87592 0.084959225 87592 0.034980356
87600 0.026098822 87600 0.095456533 43800 0.041461142 43832
0.039377577 87600 0.065000342 87600 0.025503507
87600 0.077601093 87600 0.118667675 43800 0.031072072 43836
0.016630167 87608 0.147895015 87608 0.030453919
87600 0.136024435 87600 0.145934159 43800 0.058260837 43840

0.02006541	87616	0.136935661	87616	0.078141799		
87600	0.132148809	87600	0.102848186	43800	0.092036331	43844
0.05523027	87624	0.074110983	87624	0.086028173		
87600	0.052325358	87600	0.085920969	43800	0.089127745	43848
0.080720987	87632	0.030989122	87632	0.056084944		
87600	0.068015128	87600	0.130983011	43900	0.042060059	43852
0.07342911	87640	0.010315733	87640	0.046296042		
87600	0.016331394	87600	0.103263621	43900	0.019475696	43856
0.043811782	87648	0.068251473	87648	0.054139022		
87700	0.076452197	87700	0.053732623	43900	0.053764103	43860
0.058532285	87656	0.112648209	87656	0.051583644		
87700	0.034666573	87700	0.010782413	43900	0.071277165	43864
0.09344858	87664	0.118895507	87664	0.06177507		
87700	0.098578115	87700	0.062983207	43900	0.061798128	43868
0.081381615	87672	0.091626607	87672	0.097504744		
87700	0.06154328	87700	0.103510138	43900	0.060008508	43872
0.083363964	87680	0.080303085	87680	0.102235179		
87700	0.063926782	87700	0.167488848	43900	0.063111962	43876
0.068243186	87688	0.124913655	87688	0.066691726		
87700	0.016357799	87700	0.197129455	43900	0.046218644	43880
0.021313041	87696	0.109898334	87696	0.087654182		
87700	0.131693873	87700	0.101478283	43900	0.035829573	43884
0.044647779	87704	0.020978241	87704	0.100968464		
87700	0.179290918	87700	0.17060239	43900	0.036760761	43888
0.049396553	87712	0.060223156	87712	0.081990802		
87700	0.12907898	87700	0.202403055	43900	0.032501539	43892
0.051326289	87720	0.043826203	87720	0.072296331		
87700	0.06108715	87700	0.140099975	43900	0.045821169	43896
0.071970375	87728	0.103812854	87728	0.045302317		
87700	0.054356005	87700	0.100632446	43900	0.040620929	43900
0.095494659	87736	0.1064229	87736	0.025969319		
87700	0.105376341	87700	0.05982419	43900	0.020805968	43904
0.07990934	87744	0.045301134	87744	0.062570245		
87800	0.139717915	87800	0.056523608	43900	0.024076349	43908
0.049117429	87752	0.05031165	87752	0.044700817		
87800	0.092610615	87800	0.122175377	43900	0.037466376	43912
0.081132173	87760	0.104113344	87760	0.024643669		
87800	0.112873036	87800	0.151678105	43900	0.061540035	43916
0.080862308	87768	0.139046766	87768	0.039694816		
87800	0.150876935	87800	0.09025992	43900	0.052636417	43920
0.057401958	87776	0.161424469	87776	0.049697672		
87800	0.064738633	87800	0.014500894	43900	0.096658136	43924
0.124107653	87784	0.146885883	87784	0.042201034		
87800	0.054609853	87800	0.080930193	43900	0.149062209	43928
0.136022281	87792	0.054735985	87792	0.038212991		
87800	0.080823498	87800	0.088100424	43900	0.106743493	43932
0.098081735	87800	0.089824775	87800	0.020534284		
87800	0.046111221	87800	0.035747446	43900	0.009345497	43936
0.046554913	87808	0.103942475	87808	0.025817773		
87800	0.041767849	87800	0.064612832	43900	0.029520099	43940
0.034991826	87816	0.023446284	87816	0.04132072		
87800	0.018151692	87800	0.09694005	43900	0.032962387	43944
0.086575761	87824	0.055649955	87824	0.043065491		
87800	0.041303651	87800	0.106660526	43900	0.060556646	43948
0.083809566	87832	0.064279753	87832	0.043921988		
87800	0.077563964	87800	0.079776728	44000	0.045062054	43952
0.056511373	87840	0.075083299	87840	0.030443127		
87800	0.087294669	87800	0.028251881	44000	0.027858476	43956
0.029738576	87848	0.092961418	87848	0.021019929		
87900	0.053857628	87900	0.023617573	44000	0.077978351	43960
0.020394529	87856	0.082334438	87856	0.021614193		
87900	0.038450544	87900	0.023804567	44000	0.05531804	43964
0.024683062	87864	0.061417683	87864	0.022016115		
87900	0.006542572	87900	0.05521598	44000	0.028322042	43968
0.008525757	87872	0.031966694	87872	0.023143955		
87900	0.016695625	87900	0.030103965	44000	0.050701881	43972
0.02362751	87880	0.047621426	87880	0.032186465		
87900	0.076755161	87900	0.042040687	44000	0.041539617	43976

0.064611806	87888	0.06287513	87888	0.040235969
87900	0.10837632	87900	0.024101733	44000	0.024662966	43980
0.093990399	87896	0.0860855	87896	0.028967183
87900	0.107708038	87900	0.133199966	44000	0.032033535	43984
0.077021825	87904	0.106944048	87904	0.004245993
87900	0.080954313	87900	0.130753543	44000	0.091628492	43988
0.054523869	87912	0.072541327	87912	0.042482461
87900	0.082049832	87900	0.055708268	44000	0.112031274	43992
0.030112684	87920	0.059628859	87920	0.069412214
87900	0.105656749	87900	0.06332952	44000	0.089811452	43996
0.064240907	87928	0.167607228	87928	0.062808213
87900	0.058730773	87900	0.119177079	44000	0.060376762	44000
0.078444769	87936	0.158306968	87936	0.010522294
87900	0.070599985	87900	0.124964325	44000	0.032933782	44004
0.100496909	87944	0.092117494	87944	0.062992411
88000	0.13983497	88000	0.101174141	44000	0.038425529	44008
0.082867708	87952	0.068948895	87952	0.082331681
88000	0.154910988	88000	0.080364553	44000	0.069778194	44012
0.027726463	87960	0.028723081	87960	0.095444244
88000	0.151247615	88000	0.113451868	44000	0.077876561	44016
0.027575705	87968	0.022652896	87968	0.091738009
88000	0.094610892	88000	0.114093695	44000	0.075435717	44020
0.065822344	87976	0.046327321	87976	0.049126815
88000	0.053026204	88000	0.11738632	44000	0.048667578	44024
0.086652523	87984	0.092405302	87984	0.006855683
88000	0.068806134	88000	0.041028434	44000	0.015481917	44028
0.07631597	87992	0.073057759	87992	0.040688239
88000	0.094236028	88000	0.081460494	44000	0.058467376	44032
0.040436818	88000	0.012406666	88000	0.054750097
88000	0.069779206	88000	0.060709175	44000	0.044018303	44036
0.013969887	88008	0.034518438	88008	0.048153626
88000	0.048757673	88000	0.064576176	44000	0.016270196	44040
0.040447543	88016	0.027848804	88016	0.03958133
88000	0.042864878	88000	0.063583873	44000	0.047560021	44044
0.106673448	88024	0.087529363	88024	0.016385719
88000	0.060729657	88000	0.033232573	44000	0.070474227	44048
0.118670498	88032	0.119373486	88032	0.01190154
88000	0.10342661	88000	0.096353579	44100	0.077848228	44052
0.060583952	88040	0.083070176	88040	0.016093289
88000	0.123745645	88000	0.087459615	44100	0.046145793	44056
0.105146799	88048	0.059242768	88048	0.020706437
88100	0.156819123	88100	0.092966424	44100	0.024181212	44060
0.093320472	88056	0.078002093	88056	0.027838714
88100	0.157870033	88100	0.123335747	44100	0.021252063	44064
0.043667351	88064	0.107149863	88064	0.040174669
88100	0.117749994	88100	0.077890494	44100	0.024351843	44068
0.026244772	88072	0.057826484	88072	0.020749707
88100	0.062732273	88100	0.059838185	44100	0.02236842	44072
0.069960632	88080	0.094123483	88080	0.033898949
88100	0.030230482	88100	0.149715546	44100	0.03047357	44076
0.096006988	88088	0.090479982	88088	0.029879429
88100	0.101702244	88100	0.155471891	44100	0.033404831	44080
0.067776433	88096	0.079283418	88096	0.044064647
88100	0.236480279	88100	0.090185073	44100	0.032504566	44084
0.01480008	88104	0.06594442	88104	0.037655795
88100	0.240880196	88100	0.123533013	44100	0.039555984	44088
0.045838955	88112	0.037302478	88112	0.011073209
88100	0.170941509	88100	0.128718574	44100	0.05390614	44092
0.043270909	88120	0.105318439	88120	0.032553147
88100	0.094476054	88100	0.16623197	44100	0.032160959	44096
0.051978142	88128	0.163014411	88128	0.043507571
88100	0.090179681	88100	0.148853608	44100	0.020977361	44100
0.141489669	88136	0.160359633	88136	0.038056816
88100	0.085436703	88100	0.036398731	44100	0.034731227	44104
0.161128016	88144	0.158994459	88144	0.028353454
88200	0.051078314	88200	0.061510153	44100	0.010909725	44108
0.101535166	88152	0.12177293	88152	0.036099802
88200	0.075126249	88200	0.086909844	44100	0.019497213	44112

0.033174045 88160 0.022953795 88160 0.050913594
88200 0.085951338 88200 0.140000543 44100 0.009736304 44116
0.018505984 88168 0.129271706 88168 0.04218574
88200 0.018629909 88200 0.173656285 44100 0.059817994 44120
0.069512542 88176 0.160038763 88176 0.023829953
88200 0.023549659 88200 0.161308955 44100 0.073387571 44124
0.090523652 88184 0.069153241 88184 0.024953106
88200 0.058023125 88200 0.075688899 44100 0.039467293 44128
0.086785803 88192 0.072959301 88192 0.03220416
88200 0.083484003 88200 0.058047215 44100 0.011420778 44132
0.076226446 88200 0.101295933 88200 0.039318271
88200 0.114296323 88200 0.078054254 44100 0.043923737 44136
0.039518953 88208 0.055711971 88208 0.053453328
88200 0.134808535 88200 0.054371572 44100 0.053079486 44140
0.091896669 88216 0.041893898 88216 0.077641518
88200 0.091751746 88200 0.054378335 44100 0.042045544 44144
0.124002138 88224 0.033777364 88224 0.06513649
88200 0.025909409 88200 0.116418225 44100 0.069171299 44148
0.072813004 88232 0.086481778 88232 0.026023616
88200 0.034900553 88200 0.117125528 44200 0.105998144 44152
0.008578046 88240 0.105557163 88240 0.024330368
88200 0.037987487 88200 0.03573564 44200 0.068464971 44156
0.050855058 88248 0.02747649 88248 0.059856928
88300 0.02635401 88300 0.106148073 44200 0.02644192 44160
0.052917843 88256 0.039121893 88256 0.065796594
88300 0.02777207 88300 0.141317665 44200 0.076646851 44164
0.047877737 88264 0.130203888 88264 0.049300739
88300 0.035109741 88300 0.136694289 44200 0.064411106 44168
0.050323579 88272 0.160999815 88272 0.030286512
88300 0.056802295 88300 0.062501778 44200 0.031623255 44172
0.062392821 88280 0.072658251 88280 0.027164422
88300 0.094028721 88300 0.070169073 44200 0.032498887 44176
0.073104187 88288 0.034403602 88288 0.022199713
88300 0.128632354 88300 0.134351794 44200 0.029525478 44180
0.071636234 88296 0.08310991 88296 0.081612525
88300 0.11331174 88300 0.076991033 44200 0.043116754 44184
0.062080057 88304 0.102117476 88304 0.092880677
88300 0.059091435 88300 0.04647101 44200 0.062655752 44188
0.05352384 88312 0.116000731 88312 0.099487501
88300 0.109145185 88300 0.122812329 44200 0.059559439 44192
0.046398178 88320 0.07307277 88320 0.122100755
88300 0.158632101 88300 0.144520324 44200 0.038501217 44196
0.080013124 88328 0.045423309 88328 0.102119026
88300 0.07120581 88300 0.092550028 44200 0.03678901 44200
0.090860631 88336 0.104283659 88336 0.061094223
88300 0.036411118 88300 0.025962616 44200 0.066348388 44204
0.035093181 88344 0.106186359 88344 0.032975429
88400 0.083562482 88400 0.022633869 44200 0.041432992 44208
0.047062538 88352 0.087047774 88352 0.012973619
88400 0.098886783 88400 0.034200395 44200 0.035691621 44212
0.061155392 88360 0.094472831 88360 0.027431392
88400 0.131429536 88400 0.010102402 44200 0.049335555 44216
0.050787901 88368 0.188214719 88368 0.066790526
88400 0.088955269 88400 0.077502358 44200 0.026894048 44220
0.048988186 88376 0.219097653 88376 0.07455967
88400 0.076855155 88400 0.117459349 44200 0.007920765 44224
0.030300807 88384 0.140079458 88384 0.043992419
88400 0.052306878 88400 0.07151132 44200 0.023989589 44228
0.047116129 88392 0.038732262 88392 0.027345366
88400 0.11465713 88400 0.056007095 44200 0.005902687 44232
0.106603489 88400 0.069726921 88400 0.02879244
88400 0.095257557 88400 0.077964949 44200 0.036546549 44236
0.092376991 88408 0.077341887 88408 0.052475734
88400 0.036209793 88400 0.117093674 44200 0.074376563 44240
0.034019548 88416 0.068581678 88416 0.051029143
88400 0.076989854 88400 0.069044116 44200 0.101320045 44244
0.040235573 88424 0.075192751 88424 0.04377275
88400 0.104544801 88400 0.03593972 44200 0.078540579 44248

0.054910779 88432 0.108192013 88432 0.029713949
88400 0.125841194 88400 0.039986495 44300 0.034019053 44252
0.062177329 88440 0.094924006 88440 0.048913869
88400 0.133810173 88400 0.073111594 44300 0.027995464 44256
0.073019168 88448 0.060355294 88448 0.089340087
88500 0.097660784 88500 0.103376093 44300 0.039500163 44260
0.070700095 88456 0.095437499 88456 0.126067418
88500 0.049882878 88500 0.19493181 44300 0.022939716 44264
0.049182505 88464 0.169088483 88464 0.097541823
88500 0.009094433 88500 0.230591744 44300 0.011657634 44268
0.0658543 88472 0.15940603 88472 0.01917961
88500 0.051378222 88500 0.167848659 44300 0.032417098 44272
0.086251821 88480 0.053234722 88480 0.05523051
88500 0.091438946 88500 0.081094571 44300 0.039081871 44276
0.064926513 88488 0.106585314 88488 0.076871846
88500 0.111955655 88500 0.034570658 44300 0.03011156 44280
0.052884061 88496 0.157941831 88496 0.07581729
88500 0.109813591 88500 0.023883775 44300 0.050180002 44284
0.086342778 88504 0.119165983 88504 0.074265256
88500 0.084478103 88500 0.046880796 44300 0.066204826 44288
0.064812666 88512 0.129299399 88512 0.064151027
88500 0.085403306 88500 0.081260878 44300 0.066889646 44292
0.047488706 88520 0.198035966 88520 0.028925597
88500 0.08263348 88500 0.019499708 44300 0.073061172 44296
0.076107834 88528 0.192449151 88528 0.026134165
88500 0.099530866 88500 0.049946215 44300 0.084594292 44300
0.069809888 88536 0.142378601 88536 0.049618262
88500 0.118441305 88500 0.070536422 44300 0.056251483 44304
0.060699193 88544 0.11943774 88544 0.059114351
88600 0.099015917 88600 0.087584107 44300 0.007838848 44308
0.061843697 88552 0.132657195 88552 0.060464379
88600 0.108424676 88600 0.088628316 44300 0.016793834 44312
0.019627963 88560 0.10696947 88560 0.070541362
88600 0.109079847 88600 0.063825566 44300 0.01134025 44316
0.061637678 88568 0.09726682 88568 0.059446433
88600 0.084920946 88600 0.043579334 44300 0.03234906 44320
0.08468279 88576 0.09062935 88576 0.028711369
88600 0.046888406 88600 0.031190961 44300 0.037793769 44324
0.067085362 88584 0.059695743 88584 0.020598869
88600 0.049853861 88600 0.057534602 44300 0.013695738 44328
0.049958326 88592 0.010301214 88592 0.03368191
88600 0.070469003 88600 0.123064936 44300 0.047532391 44332
0.066525005 88600 0.027997265 88600 0.052454252
88600 0.127454405 88600 0.187205311 44300 0.080370737 44336
0.085489563 88608 0.047790603 88608 0.052479241
88600 0.132976013 88600 0.178486008 44300 0.103373015 44340
0.084510575 88616 0.067117049 88616 0.047744725
88600 0.122985599 88600 0.110860834 44300 0.096960437 44344
0.040591407 88624 0.041806354 88624 0.039000941
88600 0.098764947 88600 0.125941239 44300 0.049484166 44348
0.020062966 88632 0.04933574 88632 0.015622081
88600 0.044705048 88600 0.138964576 44400 0.025504687 44352
0.028587207 88640 0.081026279 88640 0.016255595
88600 0.022719161 88600 0.103000406 44400 0.053117241 44356
0.047227753 88648 0.066443477 88648 0.027137408
88700 0.052114145 88700 0.086689019 44400 0.066629662 44360
0.049246264 88656 0.042945289 88656 0.042349268
88700 0.138163683 88700 0.098258497 44400 0.056814144 44364
0.033110337 88664 0.059580994 88664 0.040846269
88700 0.216728091 88700 0.090931644 44400 0.047598038 44368
0.045551431 88672 0.083302555 88672 0.014249059
88700 0.161502816 88700 0.057410016 44400 0.028795839 44372
0.056569676 88680 0.065637381 88680 0.01140436
88700 0.009914061 88700 0.099312958 44400 0.039792249 44376
0.068581961 88688 0.047290956 88688 0.024248557
88700 0.085869047 88700 0.146280538 44400 0.05644876 44380
0.062606945 88696 0.045037654 88696 0.026233282
88700 0.075662414 88700 0.101012221 44400 0.043216787 44384

0.029814511 88704 0.018150449 88704 0.010546397
88700 0.118029377 88700 0.042339892 44400 0.034106372 44388
0.009822144 88712 0.0176591 88712 0.045049139
88700 0.104292325 88700 0.099795077 44400 0.033831584 44392
0.006977944 88720 0.032579974 88720 0.086157474
88700 0.053621316 88700 0.060633283 44400 0.033851287 44396
0.023999503 88728 0.032913824 88728 0.063575018
88700 0.082183455 88700 0.042078533 44400 0.04231697 44400
0.029213094 88736 0.084817184 88736 0.029655172
88700 0.144121048 88700 0.080737817 44400 0.052581636 44404
0.042972144 88744 0.10232169 88744 0.069519439
88800 0.162875949 88800 0.042488529 44400 0.057208286 44408
0.088174362 88752 0.14460423 88752 0.098797529
88800 0.142464109 88800 0.049063798 44400 0.044930028 44412
0.09613367 88760 0.188569219 88760 0.103263425
88800 0.147516519 88800 0.121129546 44400 0.013093167 44416
0.076197372 88768 0.14015341 88768 0.047329049
88800 0.120417717 88800 0.162653829 44400 0.01731143 44420
0.082551153 88776 0.069774644 88776 0.020032829
88800 0.096646181 88800 0.14962435 44400 0.021833603 44424
0.061862229 88784 0.020018335 88784 0.021065347
88800 0.03971632 88800 0.110443943 44400 0.005882268 44428
0.041722928 88792 0.019901432 88792 0.027921198
88800 0.108601424 88800 0.079567384 44400 0.030935626 44432
0.045350127 88800 0.03170994 88800 0.048374674
88800 0.039027713 88800 0.09138156 44400 0.041965133 44436
0.080615122 88808 0.018449684 88808 0.047095098
88800 0.110431902 88800 0.07881481 44400 0.031581585 44440
0.052596752 88816 0.065661603 88816 0.017242432
88800 0.121287929 88800 0.043255855 44400 0.045732388 44444
0.011123787 88824 0.091232127 88824 0.019482943
88800 0.042098713 88800 0.095087497 44400 0.055063774 44448
0.031097872 88832 0.100640675 88832 0.046538771
88800 0.037687943 88800 0.053048807 44500 0.037680209 44452
0.077504359 88840 0.145076556 88840 0.059024911
88800 0.104824394 88800 0.026102107 44500 0.029355746 44456
0.095512893 88848 0.142336373 88848 0.067882364
88900 0.147414598 88900 0.042552856 44500 0.045517034 44460
0.088347304 88856 0.070377973 88856 0.050490482
88900 0.158182884 88900 0.083769999 44500 0.040955518 44464
0.044699991 88864 0.056207588 88864 0.012890771
88900 0.12279478 88900 0.06805922 44500 0.00997694 44468
0.049574312 88872 0.10128002 88872 0.024931018
88900 0.07205375 88900 0.009759671 44500 0.008010821 44472
0.081468119 88880 0.1659689 88880 0.02997744
88900 0.103184029 88900 0.057235469 44500 0.025271309 44476
0.083766594 88888 0.183804543 88888 0.038955121
88900 0.107736225 88900 0.035810757 44500 0.020485672 44480
0.08776781 88896 0.14411642 88896 0.032845514
88900 0.096043666 88900 0.016145128 44500 0.018264836 44484
0.055146669 88904 0.036324709 88904 0.033381533
88900 0.088117631 88900 0.021113146 44500 0.054551041 44488
0.062918945 88912 0.11663785 88912 0.027478174
88900 0.118295247 88900 0.039150622 44500 0.086005908 44492
0.097921511 88920 0.110758549 88920 0.015288613
88900 0.128694199 88900 0.072624673 44500 0.079001511 44496
0.099433797 88928 0.023709787 88928 0.029584868
88900 0.092604198 88900 0.099412951 44500 0.030910036 44500
0.091362315 88936 0.141590295 88936 0.025598654
88900 0.043979635 88900 0.117783049 44500 0.044277374 44504
0.074204625 88944 0.120282501 88944 0.036115864
89000 0.061940838 89000 0.060520946 44500 0.035429261 44508
0.055003977 88952 0.091253613 88952 0.029150599
89000 0.122912767 89000 0.018611445 44500 0.059326987 44512
0.026579748 88960 0.121593861 88960 0.024999716
89000 0.167166014 89000 0.110779714 44500 0.069612099 44516
0.012991014 88968 0.088609071 88968 0.004293981
89000 0.206331344 89000 0.151137661 44500 0.054046475 44520

0.051200401 88976 0.089797984 88976 0.04505938
89000 0.200151903 89000 0.112611386 44500 0.009833857 44524
0.074710282 88984 0.138429241 88984 0.071056238
89000 0.110120942 89000 0.059698657 44500 0.061589984 44528
0.050191775 88992 0.105459156 88992 0.067208021
89000 0.142059158 89000 0.056756278 44500 0.065486856 44532
0.028930324 89000 0.03514217 89000 0.0506629
89000 0.186322504 89000 0.066446919 44500 0.001408744 44536
0.058353424 89008 0.020900352 89008 0.03352038
89000 0.199621514 89000 0.022492397 44500 0.049351485 44540
0.107055006 89016 0.056000481 89016 0.012682056
89000 0.224736301 89000 0.068871872 44500 0.035670353 44544
0.079995327 89024 0.076609838 89024 0.016188858
89000 0.138682998 89000 0.079265672 44500 0.010135433 44548
0.018615929 89032 0.11187105 89032 0.032989989
89000 0.022559992 89000 0.035568734 44600 0.035454315 44552
0.062608015 89040 0.15712592 89040 0.051353338
89000 0.133111825 89000 0.095002804 44600 0.066961322 44556
0.075863616 89048 0.138248128 89048 0.043612905
89100 0.131602225 89100 0.068217378 44600 0.060020448 44560
0.052665455 89056 0.078051606 89056 0.032179163
89100 0.08840581 89100 0.053189717 44600 0.029834844 44564
0.025144142 89064 0.025796775 89064 0.089804416
89100 0.082690669 89100 0.154519861 44600 0.022099683 44568
0.044720731 89072 0.035619807 89072 0.103623293
89100 0.043021511 89100 0.175211608 44600 0.029063076 44572
0.084969295 89080 0.017735056 89080 0.081165446
89100 0.1161447 89100 0.08110093 44600 0.024829409 44576
0.064347027 89088 0.059441674 89088 0.045912158
89100 0.132896966 89100 0.123070451 44600 0.028099048 44580
0.015250307 89096 0.103187042 89096 0.008575737
89100 0.098358862 89100 0.202461466 44600 0.040857878 44584
0.061399915 89104 0.099206787 89104 0.046146452
89100 0.035649129 89100 0.147030805 44600 0.041943647 44588
0.069094655 89112 0.09893992 89112 0.072581017
89100 0.052623891 89100 0.049985694 44600 0.061550949 44592
0.093696945 89120 0.098278513 89120 0.056684494
89100 0.085072163 89100 0.032981847 44600 0.077487406 44596
0.126604923 89128 0.073555326 89128 0.030100364
89100 0.078138954 89100 0.042889635 44600 0.088335102 44600
0.132713933 89136 0.056382996 89136 0.022536444
89100 0.046517391 89100 0.10087838 44600 0.104933577 44604
0.12789933 89144 0.074363423 89144 0.053015763
89200 0.053398147 89200 0.123172271 44600 0.091578593 44608
0.102116901 89152 0.111226626 89152 0.064274551
89200 0.025289153 89200 0.141503289 44600 0.04933203 44612
0.092872186 89160 0.062286184 89160 0.043109958
89200 0.03536795 89200 0.125167353 44600 0.044552307 44616
0.108046857 89168 0.045893994 89168 0.045394521
89200 0.088487381 89200 0.110985187 44600 0.049631577 44620
0.08639141 89176 0.098292905 89176 0.056798454
89200 0.158418814 89200 0.074021671 44600 0.083967781 44624
0.062907573 89184 0.087348686 89184 0.029827694
89200 0.148289503 89200 0.08341705 44600 0.086824774 44628
0.005487271 89192 0.068442663 89192 0.03281765
89200 0.124614249 89200 0.071207433 44600 0.054155411 44632
0.066371264 89200 0.068450892 89200 0.064702952
89200 0.150376524 89200 0.008571591 44600 0.048513779 44636
0.079327969 89208 0.110378744 89208 0.052862677
89200 0.157721326 89200 0.032236945 44600 0.054302022 44640
0.059097689 89216 0.160318465 89216 0.039122897
89200 0.132135509 89200 0.037239315 44600 0.064935673 44644
0.03559118 89224 0.134737653 89224 0.03730262
89200 0.129692009 89200 0.036028581 44600 0.078608799 44648
0.085210842 89232 0.077610443 89232 0.017320526
89200 0.156866255 89200 0.041451298 44700 0.095596028 44652
0.045959481 89240 0.056693276 89240 0.019836425
89200 0.15035941 89200 0.099352074 44700 0.082913553 44656

0.057393099 89248 0.050729192 89248 0.031015225
89300 0.142742734 89300 0.102386439 44700 0.044841236 44660
0.131238165 89256 0.081230726 89256 0.043494612
89300 0.099466524 89300 0.064991727 44700 0.056363104 44664
0.162359545 89264 0.165408274 89264 0.071521907
89300 0.091201691 89300 0.04040392 44700 0.066594468 44668
0.138942968 89272 0.119950761 89272 0.075263379
89300 0.159710791 89300 0.124683371 44700 0.040718842 44672
0.121771351 89280 0.04575534 89280 0.053085441
89300 0.115131836 89300 0.156688635 44700 0.018679652 44676
0.090546761 89288 0.057595218 89288 0.019733989
89300 0.074206466 89300 0.135368085 44700 0.019692287 44680
0.044685792 89296 0.052942087 89296 0.03544697
89300 0.135808746 89300 0.094023431 44700 0.017643608 44684
0.063098873 89304 0.147721003 89304 0.039370127
89300 0.156640511 89300 0.055841512 44700 0.048921229 44688
0.090056914 89312 0.128618631 89312 0.048162005
89300 0.073174451 89300 0.060065293 44700 0.053083939 44692
0.087200162 89320 0.078205179 89320 0.04586736
89300 0.048632272 89300 0.07974296 44700 0.059843118 44696
0.037968326 89328 0.035432127 89328 0.060548729
89300 0.094782627 89300 0.064373955 44700 0.063739259 44700
0.039589846 89336 0.038230846 89336 0.046106463
89300 0.066033128 89300 0.054314223 44700 0.028165512 44704
0.091672242 89344 0.114965907 89344 0.025281654
89400 0.028815763 89400 0.08603288 44700 0.035181754 44708
0.137683718 89352 0.141535683 89352 0.037222857
89400 0.063050975 89400 0.039381543 44700 0.014035217 44712
0.141338184 89360 0.071605551 89360 0.045135217
89400 0.074136049 89400 0.126415951 44700 0.063395826 44716
0.062521278 89368 0.043815402 89368 0.042392785
89400 0.118949109 89400 0.06974482 44700 0.084557913 44720
0.086016262 89376 0.057180281 89376 0.02255351
89400 0.15645496 89400 0.094189243 44700 0.07933117 44724
0.094226365 89384 0.061642197 89384 0.0142025
89400 0.161180113 89400 0.138311909 44700 0.099265802 44728
0.084377527 89392 0.105893611 89392 0.016575243
89400 0.135448426 89400 0.047521688 44700 0.109858651 44732
0.08532378 89400 0.087984226 89400 0.018123978
89400 0.084798136 89400 0.022372304 44700 0.094189556 44736
0.076088902 89408 0.078743942 89408 0.034887544
89400 0.074077667 89400 0.073734896 44700 0.075405274 44740
0.046628706 89416 0.086506429 89416 0.038805098
89400 0.074791424 89400 0.130911009 44700 0.052825912 44744
0.014189222 89424 0.068654852 89424 0.027012136
89400 0.112632457 89400 0.141081371 44700 0.019464962 44748
0.089173293 89432 0.045721652 89432 0.013838943
89400 0.147666069 89400 0.127889813 44800 0.030689906 44752
0.126893966 89440 0.039143171 89440 0.013367261
89400 0.126417538 89400 0.109557805 44800 0.052107516 44756
0.092448252 89448 0.053404685 89448 0.001460284
89500 0.074306488 89500 0.080085716 44800 0.035448204 44760
0.032550266 89456 0.055636236 89456 0.046719666
89500 0.02564444 89500 0.008212454 44800 0.017029064 44764
0.095220203 89464 0.070684677 89464 0.067286412
89500 0.050729293 89500 0.056569625 44800 0.032248892 44768
0.167171413 89472 0.094271265 89472 0.074719304
89500 0.104718296 89500 0.11406705 44800 0.033662826 44772
0.162099139 89480 0.083203056 89480 0.065365137
89500 0.124844053 89500 0.18000952 44800 0.026007108 44776
0.129995431 89488 0.055729426 89488 0.058847923
89500 0.093906419 89500 0.177573529 44800 0.037819507 44780
0.078930098 89496 0.044227789 89496 0.076527387
89500 0.053791082 89500 0.16642973 44800 0.007208056 44784
0.028268583 89504 0.078870355 89504 0.093948125
89500 0.040762352 89500 0.231882689 44800 0.045978446 44788
0.048777736 89512 0.095786585 89512 0.082551735
89500 0.100608333 89500 0.200025563 44800 0.028667671 44792

0.060621238 89520 0.068114299 89520 0.040648352
89500 0.197818386 89500 0.054707307 44800 0.030964642 44796
0.070061455 89528 0.05504705 89528 0.011212021
89500 0.212594343 89500 0.067923429 44800 0.062178136 44800
0.055435005 89536 0.074523458 89536 0.025655761
89500 0.127837309 89500 0.112730108 44800 0.046357203 44804
0.00360535 89544 0.079601719 89544 0.043686796
89600 0.101778023 89600 0.0933267 44800 0.013321474 44808
0.049695667 89552 0.088625537 89552 0.027568713
89600 0.045791534 89600 0.121005876 44800 0.024601523 44812
0.044301574 89560 0.097479773 89560 0.035454803
89600 0.00291859 89600 0.145218219 44800 0.040724204 44816
0.057400011 89568 0.087999943 89568 0.085314401
89600 0.052682655 89600 0.104181563 44800 0.018555737 44820
0.048699378 89576 0.11578689 89576 0.065183529
89600 0.104077466 89600 0.050323899 44800 0.041407071 44824
0.091147216 89584 0.146893246 89584 0.035519159
89600 0.108494904 89600 0.119752396 44800 0.08401533 44828
0.105343381 89592 0.150309134 89592 0.040007315
89600 0.094536947 89600 0.087612105 44800 0.068321213 44832
0.043279986 89600 0.095923009 89600 0.02292343
89600 0.061745872 89600 0.007466688 44800 0.025149751 44836
0.084791929 89608 0.130793823 89608 0.044994489
89600 0.100027144 89600 0.09879603 44800 0.013597163 44840
0.109089713 89616 0.120102217 89616 0.081335507
89600 0.180252856 89600 0.143958706 44800 0.039893803 44844
0.054280627 89624 0.026277052 89624 0.053685453
89600 0.147164071 89600 0.123211168 44800 0.040430918 44848
0.022474536 89632 0.030099616 89632 0.0218018
89600 0.037897367 89600 0.041813117 44900 0.00752238 44852
0.010803967 89640 0.001809847 89640 0.028347837
89600 0.075201409 89600 0.023362936 44900 0.027850316 44856
0.023102462 89648 0.075805991 89648 0.026299329
89700 0.11415418 89700 0.061855622 44900 0.019898513 44860
0.03069266 89656 0.084379251 89656 0.018823217
89700 0.142315883 89700 0.072044692 44900 0.014586216 44864
0.031080799 89664 0.044831777 89664 0.03295414
89700 0.146467908 89700 0.055797453 44900 0.020867223 44868
0.016060816 89672 0.129820328 89672 0.053887146
89700 0.109599343 89700 0.086335982 44900 0.022886414 44872
0.021543619 89680 0.132189321 89680 0.066897614
89700 0.035642857 89700 0.100899975 44900 0.054791082 44876
0.044136097 89688 0.092909038 89688 0.038340197
89700 0.032178486 89700 0.09203043 44900 0.07557292 44880
0.044978759 89696 0.091511822 89696 0.022087417
89700 0.057774621 89700 0.086159147 44900 0.061531908 44884
0.032490003 89704 0.082910861 89704 0.046085697
89700 0.064253727 89700 0.074256866 44900 0.049127189 44888
0.046262685 89712 0.037578127 89712 0.028497892
89700 0.09897865 89700 0.093400406 44900 0.053892993 44892
0.033715143 89720 0.017303322 89720 0.017765371
89700 0.124947837 89700 0.164172103 44900 0.050307153 44896
0.024809569 89728 0.048957892 89728 0.022176655
89700 0.079056699 89700 0.160317096 44900 0.056005836 44900
0.037716553 89736 0.072280673 89736 0.045309531
89700 0.005833409 89700 0.12897508 44900 0.063046035 44904
0.028772962 89744 0.065264489 89744 0.089063775
89800 0.061638442 89800 0.129608219 44900 0.028184009 44908
0.066644316 89752 0.111754591 89752 0.106048319
89800 0.104588151 89800 0.118057098 44900 0.019109199 44912
0.055768505 89760 0.160014781 89760 0.074844727
89800 0.116121453 89800 0.039653169 44900 0.04184237 44916
0.060580536 89768 0.144873265 89768 0.044496883
89800 0.191906321 89800 0.119148877 44900 0.051845575 44920
0.044603923 89776 0.126676489 89776 0.039330473
89800 0.228150864 89800 0.182500021 44900 0.064214532 44924
0.007494813 89784 0.124610451 89784 0.030788706
89800 0.147983199 89800 0.143725891 44900 0.060622544 44928

0.009470172	89792	0.104723848	89792	0.04017141
89800	0.053166885	89800	0.052578631	44900	0.03232895	44932
0.025740246	89800	0.102855243	89800	0.038464721
89800	0.058761925	89800	0.025584053	44900	0.033518645	44936
0.006771303	89808	0.17644219	89808	0.008511444
89800	0.084071049	89800	0.064309854	44900	0.033913668	44940
0.029374116	89816	0.164516474	89816	0.01783386
89800	0.061019855	89800	0.054967335	44900	0.038573624	44944
0.049895887	89824	0.063211373	89824	0.026896521
89800	0.156502458	89800	0.025790811	44900	0.040252304	44948
0.048746751	89832	0.037499496	89832	0.002652535
89800	0.146921811	89800	0.044227203	45000	0.042806751	44952
0.057665566	89840	0.050290342	89840	0.038331225
89800	0.106114036	89800	0.035275421	45000	0.057075173	44956
0.039620703	89848	0.015136888	89848	0.042779528
89900	0.093674091	89900	0.020492711	45000	0.07627593	44960
0.026797157	89856	0.024423798	89856	0.042385054
89900	0.069288777	89900	0.020742638	45000	0.070945323	44964
0.051968054	89864	0.082270308	89864	0.05891392
89900	0.05432043	89900	0.076409408	45000	0.073367868	44968
0.084594722	89872	0.141460186	89872	0.049079281
89900	0.051851042	89900	0.102633014	45000	0.056025645	44972
0.039405411	89880	0.073913594	89880	0.047198315
89900	0.119256336	89900	0.070292335	45000	0.021222764	44976
0.028637129	89888	0.083049097	89888	0.06896913
89900	0.061556602	89900	0.025744652	45000	0.010793058	44980
0.106054111	89896	0.10931131	89896	0.051927058
89900	0.044233719	89900	0.041566236	45000	0.01686627	44984
0.120075711	89904	0.084527572	89904	0.026837015
89900	0.103909195	89900	0.068341571	45000	0.006044305	44988
0.090598369	89912	0.099871439	89912	0.015952172
89900	0.096609387	89900	0.067774774	45000	0.02349233	44992
0.072386902	89920	0.180964839	89920	0.026711883
89900	0.096103824	89900	0.084920379	45000	0.043906573	44996
0.025162766	89928	0.167333914	89928	0.042334967
89900	0.081745377	89900	0.05583464	45000	0.060928163	45000
0.013575692	89936	0.094291674	89936	0.012191655
89900	0.06589081	89900	0.136248695	45000	0.098283577	45004
0.050657713	89944	0.046645251	89944	0.032944619
90000	0.104537925	90000	0.16410093	45000	0.10715823	45008
0.082364713	89952	0.019654834	89952	0.040604784
90000	0.152728215	90000	0.08549622	45000	0.073906595	45012
0.058028982	89960	0.036014324	89960	0.033827531
90000	0.131906884	90000	0.12790662	45000	0.003833275	45016
0.051509782	89968	0.057566671	89968	0.0254726
90000	0.078220866	90000	0.175082329	45000	0.07245149	45020
0.066815264	89976	0.084802334	89976	0.032062675
90000	0.048356786	90000	0.141089375	45000	0.067477842	45024
0.052699019	89984	0.080256061	89984	0.024516618
90000	0.084093896	90000	0.11511099	45000	0.045027089	45028
0.03592969	89992	0.029106961	89992	0.060115934
90000	0.021972928	90000	0.052147338	45000	0.038720471	45032
0.085325315	90000	0.133977898	90000	0.068025671
90000	0.136027331	90000	0.003771326	45000	0.020111758	45036
0.100135789	90008	0.190355145	90008	0.044512977
90000	0.253374979	90000	0.018625324	45000	0.035318877	45040
0.058387454	90016	0.167742023	90016	0.010388716
90000	0.226189266	90000	0.022527811	45000	0.020052219	45044
0.009338927	90024	0.082227467	90024	0.024485751
90000	0.068456036	90000	0.004519339	45000	0.015661584	45048
0.044028897	90032	0.050343457	90032	0.042846896
90000	0.087710767	90000	0.053720072	45100	0.041542688	45052
0.051457038	90040	0.036480044	90040	0.0494942
90000	0.125156191	90000	0.104377934	45100	0.039181741	45056
0.028820587	90048	0.038667255	90048	0.045600416
90100	0.072963369	90100	0.14767937	45100	0.024596429	45060
0.024268358	90056	0.076162847	90056	0.040300929
90100	0.053866334	90100	0.180432384	45100	0.054397577	45064

0.069696129 90064 0.111924856 90064 0.020386133
90100 0.03915076 90100 0.189305225 45100 0.070744798 45068
0.092851427 90072 0.084819811 90072 0.016412398
90100 0.108874468 90100 0.109516404 45100 0.056723933 45072
0.080613158 90080 0.11371122 90080 0.052382999
90100 0.146397143 90100 0.033629152 45100 0.064356456 45076
0.079908648 90088 0.152497698 90088 0.049254675
90100 0.164269324 90100 0.015532263 45100 0.067443973 45080
0.083226027 90096 0.114107497 90096 0.022926159
90100 0.163986944 90100 0.089818132 45100 0.017315284 45084
0.057768131 90104 0.039499031 90104 0.025133828
90100 0.095683943 90100 0.188170946 45100 0.081718463 45088
0.08537979 90112 0.063175612 90112 0.010951678
90100 0.048417911 90100 0.22273435 45100 0.099368721 45092
0.112071888 90120 0.134836446 90120 0.020954072
90100 0.085405263 90100 0.169124208 45100 0.049970444 45096
0.0859305 90128 0.163059114 90128 0.02164411
90100 0.101945487 90100 0.107837805 45100 0.03073725 45100
0.047729121 90136 0.140507255 90136 0.037377853
90100 0.073721632 90100 0.108254033 45100 0.058079895 45104
0.047816338 90144 0.108819324 90144 0.045168254
90200 0.034174671 90200 0.069551177 45100 0.03539343 45108
0.107488217 90152 0.084297295 90152 0.047412512
90200 0.099880002 90200 0.037706475 45100 0.050141818 45112
0.13920851 90160 0.040437673 90160 0.041910942
90200 0.094272538 90200 0.064107124 45100 0.097759628 45116
0.122079247 90168 0.042973617 90168 0.033701464
90200 0.11140664 90200 0.049508479 45100 0.093049843 45120
0.102065671 90176 0.129545995 90176 0.035224104
90200 0.062921288 90200 0.015847361 45100 0.061559877 45124
0.09947332 90184 0.159010029 90184 0.03642328
90200 0.075363081 90200 0.035514488 45100 0.034576897 45128
0.072534036 90192 0.102972022 90192 0.041365824
90200 0.06860889 90200 0.067078909 45100 0.013987116 45132
0.019245812 90200 0.037884202 90200 0.055944529
90200 0.080717728 90200 0.040568542 45100 0.015084344 45136
0.055379245 90208 0.059853868 90208 0.061176914
90200 0.138653981 90200 0.033463679 45100 0.058975376 45140
0.075981181 90216 0.053753567 90216 0.039599912
90200 0.095576725 90200 0.010491818 45100 0.082148996 45144
0.048300102 90224 0.042775166 90224 0.011066192
90200 0.053962711 90200 0.096401956 45100 0.048477181 45148
0.025033403 90232 0.10617829 90232 0.010442295
90200 0.142555713 90200 0.120427445 45200 0.04199582 45152
0.085879328 90240 0.170599248 90240 0.034928748
90200 0.105333391 90200 0.047951759 45200 0.08338911 45156
0.087119071 90248 0.149767991 90248 0.065298962
90300 0.067247034 90300 0.060471899 45200 0.054025946 45160
0.042151398 90256 0.118391174 90256 0.074995653
90300 0.063897365 90300 0.052898355 45200 0.00761279 45164
0.013072693 90264 0.080500024 90264 0.044653283
90300 0.066186003 90300 0.037055954 45200 0.022567992 45168
0.022265575 90272 0.02027353 90272 0.0212056
90300 0.054957305 90300 0.052268078 45200 0.038061411 45172
0.025068655 90280 0.064756147 90280 0.0274168
90300 0.058034093 90300 0.0905813 45200 0.037459271 45176
0.053803207 90288 0.086546352 90288 0.074466763
90300 0.100394609 90300 0.063766391 45200 0.047658723 45180
0.077993762 90296 0.085047177 90296 0.06989477
90300 0.057909841 90300 0.010576659 45200 0.068093163 45184
0.081583188 90304 0.07377562 90304 0.037838421
90300 0.023248796 90300 0.040759514 45200 0.02644338 45188
0.091996713 90312 0.092325128 90312 0.033390621
90300 0.037807946 90300 0.079776808 45200 0.041781328 45192
0.095255746 90320 0.012104601 90320 0.021186075
90300 0.076800854 90300 0.144525329 45200 0.053305084 45196
0.05256943 90328 0.11838608 90328 0.058903828
90300 0.086447755 90300 0.127698091 45200 0.022604927 45200

0.044127886 90336 0.140788135 90336 0.053213847
90300 0.076382508 90300 0.063722793 45200 0.063233354 45204
0.069745183 90344 0.115004055 90344 0.018132332
90400 0.099946941 90400 0.153778645 45200 0.066666376 45208
0.054063228 90352 0.121967431 90352 0.044268829
90400 0.106466141 90400 0.155899078 45200 0.046899895 45212
0.056003108 90360 0.14332628 90360 0.04013451
90400 0.060170874 90400 0.130145985 45200 0.049933136 45216
0.106254083 90368 0.098033976 90368 0.0245581
90400 0.015815998 90400 0.129022665 45200 0.090865695 45220
0.096028212 90376 0.052585405 90376 0.032559303
90400 0.052645512 90400 0.173623558 45200 0.099439909 45224
0.037858281 90384 0.085402673 90384 0.037604183
90400 0.062673062 90400 0.199943854 45200 0.062624 45228
0.072623763 90392 0.175420966 90392 0.010412913
90400 0.121913377 90400 0.168347716 45200 0.038957402 45232
0.072606104 90400 0.239082626 90400 0.028613027
90400 0.13373587 90400 0.137217853 45200 0.035049183 45236
0.014528684 90408 0.196132009 90408 0.033945697
90400 0.045156936 90400 0.115811257 45200 0.024313564 45240
0.052732848 90416 0.076419041 90416 0.035040783
90400 0.05145041 90400 0.118629658 45200 0.034528232 45244
0.061782637 90424 0.095426723 90424 0.055838002
90400 0.120905126 90400 0.155773509 45200 0.039038227 45248
0.065316956 90432 0.168224244 90432 0.067217552
90400 0.132084009 90400 0.142418605 45300 0.079806203 45252
0.045812842 90440 0.190568724 90440 0.057458474
90400 0.056077421 90400 0.097701057 45300 0.054011518 45256
0.060520641 90448 0.115114643 90448 0.051987281
90500 0.056410547 90500 0.091106784 45300 0.043143606 45260
0.096369695 90456 0.084813102 90456 0.063651467
90500 0.097701653 90500 0.078630445 45300 0.05919196 45264
0.091576309 90464 0.076487842 90464 0.058757742
90500 0.061063787 90500 0.086368142 45300 0.038763617 45268
0.068759022 90472 0.118768097 90472 0.042494634
90500 0.055031949 90500 0.04159586 45300 0.041954787 45272
0.042442433 90480 0.072536735 90480 0.06985895
90500 0.043534266 90500 0.028054439 45300 0.034084529 45276
0.070007489 90488 0.090258844 90488 0.05997108
90500 0.068672292 90500 0.074163261 45300 0.047061385 45280
0.075160417 90496 0.120552475 90496 0.061149309
90500 0.094744879 90500 0.188766688 45300 0.061565646 45284
0.05188684 90504 0.119269811 90504 0.037247508
90500 0.060183185 90500 0.182206204 45300 0.037003898 45288
0.0388564 90512 0.161301854 90512 0.002256919
90500 0.10711846 90500 0.107215579 45300 0.036401376 45292
0.041473322 90520 0.21829184 90520 0.043678207
90500 0.105634434 90500 0.102491809 45300 0.051275914 45296
0.023819364 90528 0.206330733 90528 0.080392652
90500 0.078484074 90500 0.122506812 45300 0.024566436 45300
0.059610964 90536 0.097847078 90536 0.06590999
90500 0.104756131 90500 0.03584036 45300 0.013309771 45304
0.104202976 90544 0.08174558 90544 0.027764485
90600 0.091203285 90600 0.132381334 45300 0.023495308 45308
0.072472016 90552 0.114446943 90552 0.049492363
90600 0.183718381 90600 0.209853475 45300 0.052808053 45312
0.02379762 90560 0.128599088 90560 0.044079698
90600 0.150116451 90600 0.175309688 45300 0.087694796 45316
0.031366126 90568 0.095647716 90568 0.014346887
90600 0.113279122 90600 0.143406869 45300 0.098445904 45320
0.035871442 90576 0.060945498 90576 0.001774333
90600 0.136178293 90600 0.150935215 45300 0.060022554 45324
0.055847384 90584 0.053958102 90584 0.01772175
90600 0.121515463 90600 0.15572751 45300 0.03644254 45328
0.104757848 90592 0.042045038 90592 0.040213636
90600 0.107449596 90600 0.16755033 45300 0.030760541 45332
0.133488371 90600 0.023066186 90600 0.052191048
90600 0.106930464 90600 0.204214623 45300 0.044408898 45336

0.102840175 90608 0.026059512 90608 0.045040219
90600 0.185902653 90600 0.177572438 45300 0.044298646 45340
0.011120637 90616 0.019449806 90616 0.018160086
90600 0.174757778 90600 0.08469035 45300 0.067056943 45344
0.11542315 90624 0.024402338 90624 0.025591389
90600 0.103730148 90600 0.093686664 45300 0.093197392 45348
0.125041989 90632 0.0380743 90632 0.054477907
90600 0.100669298 90600 0.090834205 45400 0.099723322 45352
0.093659561 90640 0.07393217 90640 0.04684474
90600 0.059218688 90600 0.074989497 45400 0.075034826 45356
0.062510109 90648 0.078476711 90648 0.007445745
90700 0.027001732 90700 0.034029665 45400 0.037122161 45360
0.014499486 90656 0.074319389 90656 0.027107311
90700 0.064108841 90700 0.029281962 45400 0.034661847 45364
0.037234597 90664 0.04182482 90664 0.019969515
90700 0.075130738 90700 0.014469822 45400 0.059258982 45368
0.066991372 90672 0.024684116 90672 0.054568711
90700 0.060026137 90700 0.090897462 45400 0.06988606 45372
0.062979292 90680 0.039390176 90680 0.059268197
90700 0.079421232 90700 0.143832324 45400 0.05996125 45376
0.026986154 90688 0.063599917 90688 0.038814407
90700 0.154936526 90700 0.124720798 45400 0.076988428 45380
0.028108092 90696 0.069674781 90696 0.03957326
90700 0.202610754 90700 0.049669456 45400 0.096894451 45384
0.043753975 90704 0.023312086 90704 0.030354573
90700 0.135215436 90700 0.023132647 45400 0.099610377 45388
0.040338899 90712 0.066776222 90712 0.045582863
90700 0.120288307 90700 0.038375998 45400 0.06947689 45392
0.046987971 90720 0.119682554 90720 0.068267225
90700 0.126936546 90700 0.069919101 45400 0.079913058 45396
0.065987049 90728 0.086061475 90728 0.095461779
90700 0.089190959 90700 0.014069013 45400 0.085008702 45400
0.035629139 90736 0.01839263 90736 0.088584129
90700 0.046998215 90700 0.068083282 45400 0.048990405 45404
0.003633195 90744 0.023899111 90744 0.053902371
90800 0.089142857 90800 0.093471477 45400 0.028556831 45408
0.028313176 90752 0.028614297 90752 0.057761477
90800 0.081402461 90800 0.086005653 45400 0.073205323 45412
0.070522088 90760 0.136009738 90760 0.066402034
90800 0.024739013 90800 0.011588485 45400 0.083402571 45416
0.079433878 90768 0.164867845 90768 0.070604736
90800 0.097302167 90800 0.050825445 45400 0.042762371 45420
0.052763131 90776 0.068712288 90776 0.058200614
90800 0.09069618 90800 0.096368131 45400 0.039027342 45424
0.017811868 90784 0.030564603 90784 0.040083898
90800 0.055340093 90800 0.150064486 45400 0.072202922 45428
0.027767133 90792 0.008447833 90792 0.048659251
90800 0.083395345 90800 0.082539271 45400 0.042159853 45432
0.048107573 90800 0.072988267 90800 0.02783914
90800 0.095517753 90800 0.043829656 45400 0.007450562 45436
0.043424501 90808 0.115896808 90808 0.017396371
90800 0.137933923 90800 0.072077011 45400 0.006581578 45440
0.025248222 90816 0.120393917 90816 0.029746425
90800 0.124351966 90800 0.06145049 45400 0.007207667 45444
0.03902394 90824 0.046192559 90824 0.021937027
90800 0.056903504 90800 0.137034644 45400 0.043870168 45448
0.04092338 90832 0.034477682 90832 0.066807348
90800 0.084190899 90800 0.174220739 45500 0.087257307 45452
0.039690149 90840 0.071047485 90840 0.093642411
90800 0.205532095 90800 0.113679635 45500 0.066751803 45456
0.026190483 90848 0.14085295 90848 0.099653917
90900 0.191157088 90900 0.029642557 45500 0.022808816 45460
0.006763243 90856 0.198410068 90856 0.081863596
90900 0.060714297 90900 0.05326953 45500 0.04291609 45464
0.018246665 90864 0.175462905 90864 0.042774085
90900 0.070621383 90900 0.039822437 45500 0.078970603 45468
0.059050963 90872 0.130279426 90872 0.014999048
90900 0.089580004 90900 0.058338741 45500 0.068626287 45472

0.091387257 90880 0.139952943 90880 0.047967413
90900 0.030290479 90900 0.045657089 45500 0.045063869 45476
0.099557547 90888 0.166329016 90888 0.065101143
90900 0.04741798 90900 0.052571861 45500 0.028566841 45480
0.066520268 90896 0.173916779 90896 0.046765745
90900 0.081697224 90900 0.047308691 45500 0.011676872 45484
0.035714667 90904 0.163101489 90904 0.023053926
90900 0.099193378 90900 0.019828474 45500 0.010582427 45488
0.071000075 90912 0.14398928 90912 0.027064651
90900 0.084145846 90900 0.095799158 45500 0.027419857 45492
0.108334018 90920 0.089777888 90920 0.044944467
90900 0.01604685 90900 0.111867252 45500 0.044627828 45496
0.125418184 90928 0.009835222 90928 0.061991188
90900 0.057977017 90900 0.086850414 45500 0.027896849 45500
0.098564531 90936 0.041061478 90936 0.052656516
90900 0.079077639 90900 0.076790479 45500 0.056795452 45504
0.066234439 90944 0.007825537 90944 0.026132877
91000 0.114633294 91000 0.083274215 45500 0.08353759 45508
0.053160231 90952 0.064538246 90952 0.060009246
91000 0.142487188 91000 0.05353272 45500 0.082245359 45512
0.032551448 90960 0.10509295 90960 0.040869585
91000 0.146968261 91000 0.026733327 45500 0.069509079 45516
0.039508996 90968 0.086837477 90968 0.027821747
91000 0.091140551 91000 0.070385693 45500 0.087787979 45520
0.040263181 90976 0.050631661 90976 0.061475563
91000 0.046303016 91000 0.095776733 45500 0.097650278 45524
0.030340432 90984 0.035156325 90984 0.02832224
91000 0.093663104 91000 0.16848174 45500 0.072526716 45528
0.072975934 90992 0.036000944 90992 0.016092779
91000 0.06810038 91000 0.140313146 45500 0.042075917 45532
0.108223256 91000 0.023868903 91000 0.009880036
91000 0.065717439 91000 0.040538387 45500 0.040991192 45536
0.105407329 91008 0.033138163 91008 0.013595677
91000 0.083203478 91000 0.02828467 45500 0.054498938 45540
0.070846509 91016 0.037233393 91016 0.019210531
91000 0.084246225 91000 0.051765921 45500 0.062040657 45544
0.026538424 91024 0.081478596 91024 0.014678103
91000 0.087152788 91000 0.045182194 45500 0.055685767 45548
0.028119279 91032 0.140292279 91032 0.01500284
91000 0.065244793 91000 0.023657833 45600 0.055884819 45552
0.034733828 91040 0.118849021 91040 0.018705079
91000 0.032679953 91000 0.073208765 45600 0.051800613 45556
0.03830744 91048 0.032264677 91048 0.017425673
91100 0.074838899 91100 0.070278649 45600 0.033530141 45560
0.056077155 91056 0.022620814 91056 0.042186177
91100 0.047170321 91100 0.031742547 45600 0.01712932 45564
0.090673311 91064 0.043943681 91064 0.05804262
91100 0.053418247 91100 0.016817838 45600 0.039499799 45568
0.093566509 91072 0.078441335 91072 0.081341183
91100 0.082362574 91100 0.026036547 45600 0.066451226 45572
0.049196427 91080 0.116421521 91080 0.089142101
91100 0.043849868 91100 0.102843151 45600 0.046493919 45576
0.049191447 91088 0.083864405 91088 0.055929744
91100 0.102354097 91100 0.160302326 45600 0.026643727 45580
0.051540868 91096 0.008992867 91096 0.059036465
91100 0.168655591 91100 0.134030925 45600 0.044913366 45584
0.031436597 91104 0.080643804 91104 0.087031083
91100 0.11139641 91100 0.094262046 45600 0.074134601 45588
0.020866541 91112 0.127741136 91112 0.083799059
91100 0.050650742 91100 0.05571606 45600 0.065592554 45592
0.040952891 91120 0.098467783 91120 0.057395893
91100 0.075464006 91100 0.048243983 45600 0.086988534 45596
0.041130505 91128 0.058743954 91128 0.048065718
91100 0.078723788 91100 0.038045029 45600 0.070537812 45600
0.021509737 91136 0.057295965 91136 0.044822216
91100 0.04805209 91100 0.052371164 45600 0.043587188 45604
0.022993974 91144 0.062251704 91144 0.032355183
91200 0.025112426 91200 0.112702677 45600 0.07988765 45608

0.03721246 91152 0.068654466 91152 0.042154883
91200 0.070020418 91200 0.160815616 45600 0.079757432 45612
0.053991571 91160 0.137817595 91160 0.031428164
91200 0.055636891 91200 0.150121443 45600 0.043276494 45616
0.068763264 91168 0.207055783 91168 0.029272958
91200 0.058387232 91200 0.115253039 45600 0.034960416 45620
0.091857859 91176 0.198898183 91176 0.032014123
91200 0.111118272 91200 0.094831834 45600 0.046238009 45624
0.089218607 91184 0.08451537 91184 0.01761193
91200 0.109649023 91200 0.056476325 45600 0.052359341 45628
0.044903423 91192 0.059575719 91192 0.023755254
91200 0.090190653 91200 0.105653999 45600 0.039007682 45632
0.006183056 91200 0.118249867 91200 0.056782763
91200 0.10147116 91200 0.187241313 45600 0.022300254 45636
0.067711022 91208 0.153382513 91208 0.066884255
91200 0.056792997 91200 0.177357026 45600 0.039980165 45640
0.083218874 91216 0.188788603 91216 0.030497642
91200 0.069932787 91200 0.116441654 45600 0.012542462 45644
0.057518748 91224 0.157727933 91224 0.011622577
91200 0.108198634 91200 0.091897811 45600 0.044554065 45648
0.065833221 91232 0.051310068 91232 0.018095965
91200 0.093836869 91200 0.030749998 45700 0.040193467 45652
0.058744765 91240 0.088188877 91240 0.032654833
91200 0.141679979 91200 0.095070485 45700 0.044655379 45656
0.044353481 91248 0.091422553 91248 0.015133766
91300 0.127581458 91300 0.106979722 45700 0.040871011 45660
0.051896488 91256 0.056418594 91256 0.032662174
91300 0.055796638 91300 0.082955106 45700 0.02933314 45664
0.03342456 91264 0.085065069 91264 0.023313703
91300 0.063754305 91300 0.069750422 45700 0.0423895 45668
0.023389643 91272 0.086969973 91272 0.016875056
91300 0.062067848 91300 0.119235207 45700 0.027259694 45672
0.052921594 91280 0.051364641 91280 0.038962266
91300 0.042442956 91300 0.107467226 45700 0.012370353 45676
0.071959941 91288 0.0632226 91288 0.028122942
91300 0.130124129 91300 0.038997881 45700 0.032008869 45680
0.074947719 91296 0.06002099 91296 0.01982816
91300 0.147298109 91300 0.020213038 45700 0.049451086 45684
0.078367724 91304 0.042549971 91304 0.041257452
91300 0.03943011 91300 0.069413742 45700 0.034821194 45688
0.107547952 91312 0.028741655 91312 0.023573401
91300 0.086830238 91300 0.16028843 45700 0.019146695 45692
0.136449744 91320 0.090561021 91320 0.012101114
91300 0.132962989 91300 0.174262735 45700 0.027073726 45696
0.11296782 91328 0.11948232 91328 0.042721356
91300 0.092508708 91300 0.116371171 45700 0.050821625 45700
0.063499429 91336 0.086782005 91336 0.061077495
91300 0.029134446 91300 0.092004368 45700 0.050771712 45704
0.030882842 91344 0.072637646 91344 0.046280766
91400 0.119549826 91400 0.069886279 45700 0.084270985 45708
0.045945584 91352 0.05591441 91352 0.025641582
91400 0.125225779 91400 0.025968961 45700 0.065971231 45712
0.066193097 91360 0.060624163 91360 0.026621465
91400 0.069467373 91400 0.02093669 45700 0.031534757 45716
0.085772474 91368 0.147502971 91368 0.009182596
91400 0.089653593 91400 0.038951705 45700 0.05428067 45720
0.087793269 91376 0.106899439 91376 0.016692096
91400 0.070601935 91400 0.0085276 45700 0.048949751 45724
0.05467964 91384 0.055503238 91384 0.051340019
91400 0.048161379 91400 0.05889525 45700 0.014242531 45728
0.054637014 91392 0.133445094 91392 0.054239925
91400 0.046560206 91400 0.068549402 45700 0.044670902 45732
0.062090971 91400 0.111326917 91400 0.053359549
91400 0.097098549 91400 0.040168587 45700 0.064028391 45736
0.062181098 91408 0.057950376 91408 0.044217184
91400 0.09045763 91400 0.051756357 45700 0.053814074 45740
0.065752451 91416 0.039829865 91416 0.010200713
91400 0.05838044 91400 0.096734817 45700 0.031100652 45744

0.048466609 91424 0.054423472 91424 0.03238193
91400 0.043301185 91400 0.031238342 45700 0.017375509 45748
0.059169604 91432 0.082275758 91432 0.029287017
91400 0.08107318 91400 0.026863761 45800 0.012180521 45752
0.060678729 91440 0.097128352 91440 0.032943448
91400 0.099239609 91400 0.004138508 45800 0.025951336 45756
0.037779948 91448 0.13179428 91448 0.047639758
91500 0.037990736 91500 0.043804859 45800 0.034277058 45760
0.064619999 91456 0.087900822 91456 0.054249598
91500 0.054748765 91500 0.046786608 45800 0.035940375 45764
0.117113988 91464 0.010493637 91464 0.064166081
91500 0.056494748 91500 0.008463074 45800 0.036629899 45768
0.09382025 91472 0.017291293 91472 0.057655077
91500 0.021732396 91500 0.022113243 45800 0.025589767 45772
0.073933283 91480 0.019893965 91480 0.019813877
91500 0.034737568 91500 0.047028207 45800 0.018387507 45776
0.022861837 91488 0.075788084 91488 0.030728181
91500 0.034959328 91500 0.05618334 45800 0.016263777 45780
0.081201986 91496 0.104363186 91496 0.053923264
91500 0.013477868 91500 0.044898548 45800 0.01241855 45784
0.110318622 91504 0.085260792 91504 0.058459718
91500 0.062694686 91500 0.099778881 45800 0.033836037 45788
0.060351293 91512 0.091534021 91512 0.052687057
91500 0.090373258 91500 0.194957524 45800 0.046435249 45792
0.071216404 91520 0.060059301 91520 0.020726933
91500 0.07221655 91500 0.222776304 45800 0.029692952 45796
0.151568194 91528 0.067882473 91528 0.011765603
91500 0.027241968 91500 0.103258622 45800 0.029247603 45800
0.157362461 91536 0.041879895 91536 0.036129732
91500 0.13725739 91500 0.017699191 45800 0.033669086 45804
0.093367562 91544 0.025423877 91544 0.063825246
91600 0.237238724 91600 0.044529315 45800 0.045718094 45808
0.031054631 91552 0.030931078 91552 0.049940132
91600 0.184764765 91600 0.08656038 45800 0.050335104 45812
0.065228516 91560 0.064078711 91560 0.043878987
91600 0.063007938 91600 0.075591437 45800 0.04219338 45816
0.068842543 91568 0.093888506 91568 0.041741099
91600 0.097625809 91600 0.019026085 45800 0.033112468 45820
0.065010296 91576 0.122581245 91576 0.023090961
91600 0.051077819 91600 0.022492954 45800 0.017140499 45824
0.093480092 91584 0.146293241 91584 0.040013554
91600 0.058153666 91600 0.019624991 45800 0.016860025 45828
0.118935037 91592 0.084499174 91592 0.061168146
91600 0.028518274 91600 0.046983696 45800 0.060933468 45832
0.113194205 91600 0.039845018 91600 0.051710013
91600 0.061272833 91600 0.079415222 45800 0.083953186 45836
0.096285046 91608 0.087638335 91608 0.047934092
91600 0.137930358 91600 0.091821079 45800 0.074741547 45840
0.109841843 91616 0.06814878 91616 0.052230938
91600 0.075088705 91600 0.066590954 45800 0.093583658 45844
0.112277252 91624 0.082555926 91624 0.027107841
91600 0.176789225 91600 0.071101764 45800 0.08618609 45848
0.093896713 91632 0.091271584 91632 0.013309431
91600 0.487919839 91600 0.06200857 45900 0.069305475 45852
0.104092796 91640 0.093372641 91640 0.021046701
91600 0.636913523 91600 0.04285097 45900 0.037867885 45856
0.075705844 91648 0.085466949 91648 0.031530322
91700 0.516392524 91700 0.075084601 45900 0.031701195 45860
0.036619906 91656 0.088256536 91656 0.047991445
91700 0.232879349 91700 0.120710065 45900 0.049398786 45864
0.028848466 91664 0.062748724 91664 0.06066389
91700 0.135319016 91700 0.144830075 45900 0.084155116 45868
0.010400057 91672 0.042010801 91672 0.053407184
91700 0.170928746 91700 0.11215925 45900 0.097894161 45872
0.026154105 91680 0.024694753 91680 0.034296809
91700 0.170732165 91700 0.058359194 45900 0.091590991 45876
0.047731199 91688 0.098989178 91688 0.030917443
91700 0.144419566 91700 0.051346036 45900 0.07620499 45880

0.097950848 91696 0.171039545 91696 0.021642625
91700 0.158115538 91700 0.116791896 45900 0.070694703 45884
0.131665641 91704 0.199166214 91704 0.064750057
91700 0.202245953 91700 0.165078891 45900 0.068434929 45888
0.097221644 91712 0.156417445 91712 0.098580167
91700 0.155278394 91700 0.145107013 45900 0.049714425 45892
0.039273866 91720 0.098475393 91720 0.070738621
91700 0.033603588 91700 0.08250893 45900 0.006385119 45896
0.033215765 91728 0.072021445 91728 0.007805707
91700 0.108196524 91700 0.071177063 45900 0.020853346 45900
0.0378285 91736 0.048406229 91736 0.025681879
91700 0.03487994 91700 0.042959793 45900 0.036008532 45904
0.055653763 91744 0.015491738 91744 0.005846587
91800 0.174253248 91800 0.010592246 45900 0.069136149 45908
0.092817383 91752 0.008632383 91752 0.020317886
91800 0.16431036 91800 0.084685758 45900 0.072350835 45912
0.077267294 91760 0.019966385 91760 0.019698511
91800 0.096302327 91800 0.115277129 45900 0.034231376 45916
0.028703194 91768 0.086773755 91768 0.01600428
91800 0.10130211 91800 0.091616668 45900 0.020263782 45920
0.037551283 91776 0.147379688 91776 0.032401225
91800 0.090717767 91800 0.13795348 45900 0.015244751 45924
0.063271742 91784 0.170079525 91784 0.036146023
91800 0.024897396 91800 0.180656644 45900 0.038021059 45928
0.066480112 91792 0.189454019 91792 0.005856495
91800 0.068648544 91800 0.164663085 45900 0.048072798 45932
0.059517603 91800 0.162113953 91800 0.037037517
91800 0.14880771 91800 0.113233618 45900 0.019796617 45936
0.037161299 91808 0.11375895 91808 0.044738477
91800 0.078277546 91800 0.109328168 45900 0.026853206 45940
0.032149452 91816 0.110705594 91816 0.033876957
91800 0.026460948 91800 0.093137351 45900 0.014875215 45944
0.075917335 91824 0.099402896 91824 0.03184038
91800 0.064922562 91800 0.050673614 45900 0.015987225 45948
0.080350408 91832 0.105916894 91832 0.02803896
91800 0.209115315 91800 0.050534414 46000 0.004722448 45952
0.053340464 91840 0.084638617 91840 0.028220999
91800 0.198503956 91800 0.073901989 46000 0.029694242 45956
0.071694769 91848 0.042297321 91848 0.06538129
91900 0.124813596 91900 0.059220387 46000 0.018258099 45960
0.088303226 91856 0.069964808 91856 0.071142968
91900 0.289326709 91900 0.059012746 46000 0.032815882 45964
0.060206545 91864 0.105216132 91864 0.051072351
91900 0.361200219 91900 0.100958481 46000 0.023066898 45968
0.024682266 91872 0.107795968 91872 0.029056275
91900 0.557074381 91900 0.090086505 46000 0.026755071 45972
0.051755484 91880 0.074608019 91880 0.025126952
91900 0.526206801 91900 0.129434179 46000 0.054860011 45976
0.052739983 91888 0.058274043 91888 0.034563771
91900 0.096297932 91900 0.138940216 46000 0.048768667 45980
0.048496549 91896 0.067862115 91896 0.024549294
91900 0.916556339 91900 0.051540286 46000 0.023061195 45984
0.054761935 91904 0.10818593 91904 0.049620063
91900 2.610413825 91900 0.105571817 46000 0.044990185 45988
0.04996817 91912 0.08502011 91912 0.059060356
91900 3.855088025 91900 0.101190177 46000 0.055674787 45992
0.034505127 91920 0.090953181 91920 0.032871427
91900 3.276010275 91900 0.061123072 46000 0.059830196 45996
0.033349777 91928 0.06290678 91928 0.017766817
91900 1.559508263 91900 0.036276411 46000 0.075704855 46000
0.035226618 91936 0.040401064 91936 0.025441561
91900 1.129487646 91900 0.063552041 46000 0.070105198 46004
0.013947039 91944 0.037495996 91944 0.036182755
92000 0.975075644 92000 0.071695002 46000 0.033526787 46008
0.024738345 91952 0.082005616 91952 0.025519643
92000 0.326307025 92000 0.075563046 46000 0.029556792 46012
0.032665743 91960 0.073245399 91960 0.024478066
92000 0.139520548 92000 0.094540868 46000 0.035638303 46016

0.014901758 91968 0.059662198 91968 0.067013745
92000 0.143769401 92000 0.137379175 46000 0.054338325 46020
0.031725707 91976 0.072267743 91976 0.061177081
92000 0.143943456 92000 0.142975914 46000 0.054311895 46024
0.031642456 91984 0.016656069 91984 0.03159052
92000 0.233909421 92000 0.102674458 46000 0.038850874 46028
0.0094451 91992 0.028673894 91992 0.034545978
92000 0.270015124 92000 0.058582617 46000 0.024807008 46032
0.036125312 92000 0.035362402 92000 0.029733585
92000 0.201792588 92000 0.033096003 46000 0.038136044 46036
0.06121138 92008 0.03694768 92008 0.037203972
92000 0.16428536 92000 0.059182639 46000 0.042413256 46040
0.097582764 92016 0.081393904 92016 0.041514984
92000 0.146573089 92000 0.063108935 46000 0.058559373 46044
0.078828285 92024 0.121008256 92024 0.018967226
92000 0.199071495 92000 0.047022626 46000 0.096399876 46048
0.046663783 92032 0.13624574 92032 0.047053629
92000 0.305116934 92000 0.10328932 46100 0.056193407 46052
0.043280175 92040 0.078202902 92040 0.07050117
92000 0.254449464 92000 0.115801646 46100 0.02439331 46056
0.035414079 92048 0.131933558 92048 0.070408336
92100 0.158205178 92100 0.067420944 46100 0.067419031 46060
0.019600197 92056 0.122771293 92056 0.052537624
92100 0.208490878 92100 0.057746976 46100 0.092249567 46064
0.043784858 92064 0.015835482 92064 0.017637496
92100 0.17350004 92100 0.073561059 46100 0.097139891 46068
0.059609571 92072 0.092684437 92072 0.029788429
92100 0.013653244 92100 0.097840559 46100 0.098577613 46072
0.029957524 92080 0.114362651 92080 0.062612402
92100 0.106101521 92100 0.134901726 46100 0.090176247 46076
0.00775822 92088 0.065096894 92088 0.057093341
92100 0.028080205 92100 0.184799035 46100 0.045146033 46080
0.030506542 92096 0.126183484 92096 0.045223009
92100 0.091595262 92100 0.146372273 46100 0.012230788 46084
0.046389298 92104 0.161004791 92104 0.063061794
92100 0.109442248 92100 0.064137588 46100 0.021434877 46088
0.059001257 92112 0.039834333 92112 0.050259019
92100 0.15856963 92100 0.057331028 46100 0.015381222 46092
0.055762415 92120 0.058780497 92120 0.021084847
92100 0.151800821 92100 0.091626636 46100 0.017142196 46096
0.050405419 92128 0.084566891 92128 0.031762946
92100 0.164074241 92100 0.07705074 46100 0.028990471 46100
0.044093878 92136 0.175488821 92136 0.049030212
92100 0.136723 92100 0.063432119 46100 0.04925497 46104
0.038962258 92144 0.088325018 92144 0.048034046
92200 0.074694064 92200 0.098402932 46100 0.071782066 46108
0.042047392 92152 0.216329165 92152 0.035230612
92200 0.444702309 92200 0.104375358 46100 0.099147321 46112
0.044929562 92160 0.202272524 92160 0.025456911
92200 0.446039776 92200 0.051552324 46100 0.09825208 46116
0.063081643 92168 0.146108389 92168 0.010871969
92200 1.242997125 92200 0.032978482 46100 0.06815829 46120
0.080002712 92176 0.185343946 92176 0.009726235
92200 3.51643725 92200 0.059212442 46100 0.06459828 46124
0.084134706 92184 0.261404814 92184 0.025351172
92200 4.531541838 92200 0.087597269 46100 0.057107092 46128
0.070828857 92192 0.424278668 92192 0.034640896
92200 3.1895407 92200 0.120528021 46100 0.058652455 46132
0.045528559 92200 0.331546936 92200 0.023152565
92200 1.056459383 92200 0.103080296 46100 0.075081975 46136
0.042956457 92208 0.110008565 92208 0.02330853
92200 0.6726461 92200 0.066116707 46100 0.07675989 46140
0.029433029 92216 0.072188166 92216 0.043245629
92200 0.422212586 92200 0.0475794 46100 0.068409725 46144
0.040709831 92224 0.234876498 92224 0.050029503
92200 0.274750084 92200 0.072711351 46100 0.058139547 46148
0.080893005 92232 0.232419319 92232 0.04565953
92200 0.243406845 92200 0.029126115 46200 0.073190255 46152

0.080636783 92240 0.05868926 92240 0.034401728
92200 0.306739298 92200 0.094381976 46200 0.068386529 46156
0.037536989 92248 0.105128478 92248 0.013362855
92300 0.285013259 92300 0.151801025 46200 0.121750687 46160
0.011979804 92256 0.096023876 92256 0.010116645
92300 0.186543155 92300 0.141765646 46200 0.160937634 46164
0.031885378 92264 0.077837314 92264 0.005275558
92300 0.261723035 92300 0.112305061 46200 0.116683208 46168
0.113337541 92272 0.093853094 92272 0.022857408
92300 0.308770483 92300 0.11065221 46200 0.0432542 46172
0.139501775 92280 0.132190733 92280 0.037494057
92300 0.330327865 92300 0.042879874 46200 0.01206335 46176
0.079715734 92288 0.057122579 92288 0.048805396
92300 0.282664668 92300 0.012973674 46200 0.027303806 46180
0.090395319 92296 0.085116022 92296 0.040321891
92300 0.103510989 92300 0.08326587 46200 0.057952442 46184
0.132321511 92304 0.045152083 92304 0.031155632
92300 0.114383947 92300 0.095817522 46200 0.048671533 46188
0.12047901 92312 0.164784463 92312 0.031562457
92300 0.259998604 92300 0.071493319 46200 0.023706494 46192
0.08956153 92320 0.225466065 92320 0.016591483
92300 0.271557365 92300 0.085173757 46200 0.01327261 46196
0.066719658 92328 0.124007129 92328 0.039693419
92300 0.140922726 92300 0.099932273 46200 0.011335976 46200
0.046807094 92336 0.186065998 92336 0.056172794
92300 0.093794355 92300 0.120694458 46200 0.045886252 46204
0.038881037 92344 0.150747 92344 0.034360641
92400 0.185579061 92400 0.033297059 46200 0.080489772 46208
0.032948592 92352 0.071269416 92352 0.041381925
92400 0.175156514 92400 0.12043368 46200 0.020452108 46212
0.041170471 92360 0.108405198 92360 0.071787115
92400 0.20508244 92400 0.161207121 46200 0.082163075 46216
0.054820332 92368 0.156377123 92368 0.049840986
92400 0.207716498 92400 0.096567615 46200 0.144674603 46220
0.063677187 92376 0.089670699 92376 0.005286711
92400 0.110757595 92400 0.046458066 46200 0.167989463 46224
0.03392169 92384 0.055519256 92384 0.037877475
92400 0.07431228 92400 0.037346727 46200 0.107639993 46228
0.016822667 92392 0.091223548 92392 0.022285121
92400 0.072203991 92400 0.0625545 46200 0.01785621 46232
0.010364825 92400 0.060739134 92400 0.029511593
92400 0.120688179 92400 0.057342491 46200 0.047190501 46236
0.055113589 92408 0.067243811 92408 0.062529463
92400 0.133763911 92400 0.133480935 46200 0.070056376 46240
0.07559199 92416 0.066767723 92416 0.040916093
92400 0.044429511 92400 0.137712458 46200 0.099765384 46244
0.073347095 92424 0.087029657 92424 0.01063547
92400 0.1771854 92400 0.132719463 46200 0.137004681 46248
0.028887249 92432 0.109144981 92432 0.028478111
92400 0.183172408 92400 0.070905378 46300 0.149127809 46252
0.082634011 92440 0.063374835 92440 0.036435697
92400 0.132046829 92400 0.080833517 46300 0.090342343 46256
0.081163496 92448 0.06890361 92448 0.016935843
92500 0.442196266 92500 0.119469187 46300 0.055399014 46260
0.017584203 92456 0.100438672 92456 0.028099101
92500 0.500913476 92500 0.117269228 46300 0.082164523 46264
0.023103574 92464 0.016432807 92464 0.023881681
92500 0.250176701 92500 0.074820076 46300 0.103847036 46268
0.032047443 92472 0.115821582 92472 0.03000224
92500 0.108588843 92500 0.050424198 46300 0.120179509 46272
0.012974546 92480 0.156676543 92480 0.034246641
92500 0.212536746 92500 0.151567714 46300 0.182735138 46276
0.025319738 92488 0.089949775 92488 0.02407813
92500 0.19635346 92500 0.234013961 46300 0.227606811 46280
0.028588907 92496 0.033619665 92496 0.020470756
92500 0.059493097 92500 0.261959358 46300 0.196615976 46284
0.073000265 92504 0.008818929 92504 0.016405587
92500 0.096720396 92500 0.167079866 46300 0.138353556 46288

0.088150955 92512 0.07173606 92512 0.028895025
92500 0.143642261 92500 0.06687695 46300 0.091085116 46292
0.053757263 92520 0.103479761 92520 0.01870992
92500 0.126967833 92500 0.053149968 46300 0.058009329 46296
0.059551829 92528 0.083301813 92528 0.021189329
92500 0.109045992 92500 0.102653816 46300 0.023736586 46300
0.068827481 92536 0.086816923 92536 0.021325304
92500 0.078085162 92500 0.143911776 46300 0.142909411 46304
0.04475957 92544 0.081392049 92544 0.005970596
92600 0.207918493 92600 0.10280266 46300 0.254003128 46308
0.045002944 92552 0.054169937 92552 0.032264663
92600 0.209758088 92600 0.009055359 46300 0.241919581 46312
0.044109664 92560 0.024732693 92560 0.046778416
92600 0.099986799 92600 0.1136016 46300 0.131868786 46316
0.032786305 92568 0.128773041 92568 0.063180007
92600 0.064779488 92600 0.136108065 46300 0.045351131 46320
0.029142302 92576 0.18739936 92576 0.065633773
92600 0.133635476 92600 0.089918824 46300 0.074079719 46324
0.071474533 92584 0.171068954 92584 0.04134492
92600 0.120744626 92600 0.024456092 46300 0.06926967 46328
0.044630851 92592 0.158842566 92592 0.024729307
92600 0.014423824 92600 0.048508544 46300 0.033542117 46332
0.051022977 92600 0.090804642 92600 0.055116409
92600 0.0626529 92600 0.038749106 46300 0.016535127 46336
0.05310664 92608 0.017766948 92608 0.060306953
92600 0.04353949 92600 0.030412399 46300 0.057921796 46340
0.062662715 92616 0.030592171 92616 0.031641084
92600 0.050658895 92600 0.050983355 46300 0.063801628 46344
0.068206151 92624 0.039170871 92624 0.041589712
92600 0.105011292 92600 0.021572378 46300 0.048071361 46348
0.041535688 92632 0.062578234 92632 0.051475698
92600 0.179352574 92600 0.019577115 46400 0.065792912 46352
0.028334347 92640 0.150308755 92640 0.033716493
92600 0.140791686 92600 0.016230053 46400 0.080878264 46356
0.019355231 92648 0.237709174 92648 0.059799455
92700 0.066146247 92700 0.046598256 46400 0.032686206 46360
0.029960982 92656 0.20983917 92656 0.103184284
92700 0.152628854 92700 0.088519271 46400 0.136597416 46364
0.045947068 92664 0.080665261 92664 0.100870588
92700 0.157459479 92700 0.083629406 46400 0.187367289 46368
0.065581007 92672 0.09551784 92672 0.065066073
92700 0.115381015 92700 0.056731315 46400 0.161504169 46372
0.067995839 92680 0.105415005 92680 0.018920269
92700 0.069414899 92700 0.048304999 46400 0.083218067 46376
0.033791406 92688 0.021576212 92688 0.017021564
92700 0.060275936 92700 0.049775845 46400 0.022134851 46380
0.014319709 92696 0.131520618 92696 0.006756399
92700 0.105572028 92700 0.039997562 46400 0.055379605 46384
0.052101936 92704 0.142373319 92704 0.017372562
92700 0.144665538 92700 0.08893646 46400 0.02667722 46388
0.064301647 92712 0.088482811 92712 0.01337256
92700 0.106443644 92700 0.106113985 46400 0.087164466 46392
0.055900859 92720 0.075381242 92720 0.020272941
92700 0.048172053 92700 0.105964915 46400 0.121966186 46396
0.048803056 92728 0.084383202 92728 0.05009654
92700 0.137915239 92700 0.114946117 46400 0.09063301 46400
0.027072087 92736 0.07166915 92736 0.061755789
92700 0.171442574 92700 0.089794092 46400 0.021078728 46404
0.040927727 92744 0.147700078 92744 0.035872799
92800 0.173068635 92800 0.043050721 46400 0.036192701 46408
0.073905765 92752 0.156927985 92752 0.043869481
92800 0.145575905 92800 0.033903376 46400 0.028641402 46412
0.080431593 92760 0.062851374 92760 0.082475126
92800 0.087350258 92800 0.110058179 46400 0.022908049 46416
0.050015777 92768 0.042322823 92768 0.071990944
92800 0.045178203 92800 0.155256858 46400 0.105077816 46420
0.083498337 92776 0.067330315 92776 0.036178721
92800 0.029075865 92800 0.15709638 46400 0.260475529 46424

```
0.145938553	92784	0.033108168	92784	0.037061014
92800	0.073919793	92800	0.132959146	46400	0.31170802	46428
0.143727928	92792	0.159635864	92792	0.012429942
92800	0.082362974	92800	0.119431119	46400	0.209141523	46432
0.098883313	92800	0.146350081	92800	0.014115577
92800	0.121666148	92800	0.141390686	46400	0.096037773	46436
0.065205386	92808	0.068997644	92808	0.015521619
92800	0.15550993	92800	0.157195959	46400	0.105427847	46440
0.02019109	92816	0.109445085	92816	0.045813355
92800	0.135576949	92800	0.080843674	46400	0.049411858	46444
0.043804641	92824	0.100459998	92824	0.057436944
92800	0.112095302	92800	0.046196154	46400	0.04175886	46448
0.059901766	92832	0.076343597	92832	0.04599812
92800	0.079666366	92800	0.063251311	46500	0.079116391	46452
0.045831606	92840	0.03664894	92840	0.042360654
92800	0.026561986	92800	0.061026909	46500	0.111604677	46456
0.070484923	92848	0.077468343	92848	0.059625269
92900	0.022623895	92900	0.044745786	46500	0.112433751	46460
0.113752227	92856	0.084771658	92856	0.058635633
92900	0.044537592	92900	0.039587176	46500	0.077531302	46464
0.120655612	92864	0.072569448	92864	0.056493205
92900	0.062140673	92900	0.040379444	46500	0.065322274	46468
0.079740174	92872	0.046381756	92872	0.042603493
92900	0.058849055	92900	0.101418649	46500	0.042202672	46472
0.059976355	92880	0.018356168	92880	0.037627113
92900	0.089765927	92900	0.131483219	46500	0.056902307	46476
0.081923747	92888	0.051170679	92888	0.020832044
92900	0.079767619	92900	0.104620624	46500	0.12629015	46480
0.072842682	92896	0.078728292	92896	0.048984763
92900	0.015936441	92900	0.102389524	46500	0.139329554	46484
0.048321977	92904	0.094494688	92904	0.07841429
92900	0.077439763	92900	0.038718168	46500	0.079680816	46488
0.071763236	92912	0.053578609	92912	0.074510521
92900	0.069524933	92900	0.072355833	46500	0.035353103	46492
0.0623085	92920	0.017547125	92920	0.030756379
92900	0.037898917	92900	0.090133552	46500	0.011032469	46496
0.010002657	92928	0.002697687	92928	0.039563409
92900	0.099776487	92900	0.098530698	46500	0.028277218	46500
0.075175172	92936	0.0348102	92936	0.048628412
92900	0.114037852	92900	0.096504518	46500	0.03920537	46504
0.10197016	92944	0.056761837	92944	0.04158797
93000	0.047650261	93000	0.053581651	46500	0.025947918	46508
0.094897572	92952	0.075971373	92952	0.043716256
93000	0.045133129	93000	0.058453708	46500	0.026267848	46512
0.06063818	92960	0.07831792	92960	0.039452847
93000	0.123246791	93000	0.024693247	46500	0.045280074	46516
0.00835947	92968	0.050891758	92968	0.018178693
93000	0.132383183	93000	0.119052929	46500	0.027152148	46520
0.016753196	92976	0.093553608	92976	0.014457619
93000	0.052344836	93000	0.203152056	46500	0.03960506	46524
0.02763152	92984	0.099551719	92984	0.027321517
93000	0.052511292	93000	0.198961876	46500	0.040565668	46528
0.065458517	92992	0.101481317	92992	0.033038949
93000	0.109787223	93000	0.129758555	46500	0.021462609	46532
0.087351669	93000	0.144200166	93000	0.028681363
93000	0.14322612	93000	0.037681879	46500	0.04539566	46536
0.060064507	93008	0.178401475	93008	0.033434946
93000	0.132386253	93000	0.104252969	46500	0.073621792	46540
0.021652271	93016	0.117479161	93016	0.059452563
93000	0.083437844	93000	0.16453951	46500	0.093223724	46544
0.046695011	93024	0.059044076	93024	0.061341671
93000	0.047080983	93000	0.184511285	46500	0.080689715	46548
0.088787667	93032	0.051085535	93032	0.026919008
93000	0.024336045	93000	0.178964066	46600	0.074071308	46552
0.081756385	93040	0.064555665	93040	0.017886843
93000	0.044821063	93000	0.131766326	46600	0.096214499	46556
0.060408456	93048	0.11366736	93048	0.068098685
93100	0.048543392	93100	0.081881161	46600	0.05875693	46560
```

0.049025799	93056	0.083752238	93056	0.106504202		
93100	0.026239461	93100	0.073953786	46600	0.01483878	46564
0.033839417	93064	0.022654111	93064	0.116053714		
93100	0.061907551	93100	0.075747863	46600	0.059931168	46568
0.087445878	93072	0.028172104	93072	0.095067611		
93100	0.103216749	93100	0.104107086	46600	0.068947818	46572
0.073574214	93080	0.053511201	93080	0.059532649		
93100	0.108800268	93100	0.146258535	46600	0.055481538	46576
0.066880471	93088	0.035854053	93088	0.011198729		
93100	0.061705337	93100	0.089506058	46600	0.059572409	46580
0.068266403	93096	0.015649934	93096	0.029230037		
93100	0.037690166	93100	0.060000777	46600	0.078023892	46584
0.087337867	93104	0.054377662	93104	0.019505129		
93100	0.050957835	93100	0.113962386	46600	0.047322883	46588
0.10459662	93112	0.104783598	93112	0.032869757		
93100	0.073040261	93100	0.059182337	46600	0.02440342	46592
0.087899207	93120	0.103126869	93120	0.033228724		
93100	0.058695146	93100	0.102703292	46600	0.054999418	46596
0.065377142	93128	0.060023027	93128	0.03835875		
93100	0.134856149	93100	0.10732621	46600	0.036258509	46600
0.058942616	93136	0.086477936	93136	0.044697248		
93100	0.182826785	93100	0.108133565	46600	0.022405397	46604
0.040127252	93144	0.109684981	93144	0.034653625		
93200	0.097400327	93200	0.127403691	46600	0.010796158	46608
0.031541367	93152	0.076766628	93152	0.017382132		
93200	0.034123765	93200	0.132126923	46600	0.00292416	46612
0.045431698	93160	0.103674691	93160	0.005235517		
93200	0.132954505	93200	0.143628029	46600	0.008033754	46616
0.03534725	93168	0.121515033	93168	0.005928687		
93200	0.165592966	93200	0.101196405	46600	0.019720334	46620
0.011361754	93176	0.03820195	93176	0.014644391		
93200	0.131653724	93200	0.052106301	46600	0.02647226	46624
0.057996709	93184	0.103336264	93184	0.01706526		
93200	0.11553699	93200	0.099487879	46600	0.050442268	46628
0.093396076	93192	0.107581109	93192	0.028227969		
93200	0.103317565	93200	0.167573249	46600	0.083754596	46632
0.103655402	93200	0.058986901	93200	0.020702539		
93200	0.123999067	93200	0.178026035	46600	0.105340259	46636
0.086725348	93208	0.053878466	93208	0.007270194		
93200	0.114851187	93200	0.107735337	46600	0.068132402	46640
0.036971989	93216	0.068786467	93216	0.017545412		
93200	0.105837724	93200	0.033857778	46600	0.019183446	46644
0.029740819	93224	0.082517101	93224	0.021139609		
93200	0.145683953	93200	0.024270808	46600	0.059123217	46648
0.0274265	93232	0.082474347	93232	0.01133954		
93200	0.165169185	93200	0.037758029	46700	0.085160595	46652
0.054078355	93240	0.045719255	93240	0.015438887		
93200	0.092558294	93200	0.029932782	46700	0.047038397	46656
0.103850027	93248	0.044243909	93248	0.031506414		
93300	0.059859958	93300	0.089233334	46700	0.008001728	46660
0.081387101	93256	0.097601944	93256	0.028018518		
93300	0.075801458	93300	0.13495583	46700	0.039732146	46664
0.043268297	93264	0.068555775	93264	0.046678724		
93300	0.102389073	93300	0.151473403	46700	0.05616988	46668
0.065881774	93272	0.036050118	93272	0.068906011		
93300	0.074590498	93300	0.10616974	46700	0.036080583	46672
0.067136338	93280	0.070827329	93280	0.034203735		
93300	0.073550196	93300	0.074499803	46700	0.033888995	46676
0.039216477	93288	0.087195265	93288	0.008317607		
93300	0.146415521	93300	0.089686451	46700	0.022577649	46680
0.056416498	93296	0.144128834	93296	0.017802026		
93300	0.132442874	93300	0.064845342	46700	0.044145858	46684
0.084702915	93304	0.218989343	93304	0.022210308		
93300	0.089232824	93300	0.069855989	46700	0.100718098	46688
0.094975432	93312	0.176227055	93312	0.018801062		
93300	0.17369815	93300	0.082387967	46700	0.119685908	46692
0.105154446	93320	0.035151432	93320	0.039180741		
93300	0.199927163	93300	0.101831938	46700	0.064061635	46696

0.089815476 93328 0.072374372 93328 0.060182381
93300 0.161305375 93300 0.071122806 46700 0.019433262 46700
0.049754908 93336 0.082611841 93336 0.065487759
93300 0.078659505 93300 0.034132918 46700 0.020111929 46704
0.018582328 93344 0.082257444 93344 0.052310981
93400 0.074857795 93400 0.060998944 46700 0.061511841 46708
0.035763016 93352 0.091884278 93352 0.029010254
93400 0.146783131 93400 0.100620659 46700 0.076331133 46712
0.071670787 93360 0.116159477 93360 0.033713139
93400 0.092123832 93400 0.133348309 46700 0.065782275 46716
0.058688926 93368 0.137148701 93368 0.069645706
93400 0.011626941 93400 0.148372986 46700 0.058332251 46720
0.039227456 93376 0.111785972 93376 0.093026036
93400 0.023283141 93400 0.111223963 46700 0.047573409 46724
0.05339873 93384 0.076535158 93384 0.079442987
93400 0.024576828 93400 0.064962558 46700 0.047995392 46728
0.061127757 93392 0.092025184 93392 0.059525861
93400 0.022096494 93400 0.085181637 46700 0.064198255 46732
0.057158064 93400 0.101457699 93400 0.037273407
93400 0.035263711 93400 0.035287663 46700 0.057810386 46736
0.045543151 93408 0.082237275 93408 0.020793743
93400 0.107389235 93400 0.011382329 46700 0.023464905 46740
0.032166379 93416 0.068746289 93416 0.029484021
93400 0.095688083 93400 0.037953148 46700 0.02419235 46744
0.060528513 93424 0.047411348 93424 0.029556606
93400 0.007566292 93400 0.062666033 46700 0.038318951 46748
0.069044872 93432 0.044732336 93432 0.026233904
93400 0.065154171 93400 0.044855151 46800 0.036864145 46752
0.040405343 93440 0.08290085 93440 0.017889188
93400 0.049088263 93400 0.019555888 46800 0.048555088 46756
0.007886149 93448 0.063381318 93448 0.032898235
93500 0.057933579 93500 0.045910045 46800 0.025929949 46760
0.024053106 93456 0.052872183 93456 0.055371991
93500 0.025944071 93500 0.062286024 46800 0.003110407 46764
0.042294043 93464 0.095908072 93464 0.035486315
93500 0.054838984 93500 0.083967643 46800 0.017557029 46768
0.035999205 93472 0.04550791 93472 0.018058478
93500 0.089180183 93500 0.084780957 46800 0.004373174 46772
0.060350743 93480 0.073478303 93480 0.034176504
93500 0.0417394 93500 0.089360976 46800 0.022741482 46776
0.075133969 93488 0.115635579 93488 0.057726789
93500 0.010356848 93500 0.115179493 46800 0.009475808 46780
0.038425609 93496 0.158401934 93496 0.070922084
93500 0.04468123 93500 0.098560158 46800 0.028393732 46784
0.044587225 93504 0.159061586 93504 0.060174374
93500 0.090891284 93500 0.089248955 46800 0.038113922 46788
0.065398592 93512 0.077192417 93512 0.012835924
93500 0.112462913 93500 0.124753977 46800 0.025479914 46792
0.072649935 93520 0.045337714 93520 0.035638728
93500 0.100515041 93500 0.148003528 46800 0.041964358 46796
0.059028302 93528 0.110712557 93528 0.043485627
93500 0.062178668 93500 0.10644483 46800 0.031914329 46800
0.045010911 93536 0.104690596 93536 0.033085777
93500 0.115542782 93500 0.04122232 46800 0.004600942 46804
0.061298546 93544 0.118005482 93544 0.051123043
93600 0.173680179 93600 0.097228367 46800 0.04803441 46808
0.079163845 93552 0.097330158 93552 0.054415938
93600 0.174148706 93600 0.135200273 46800 0.087908491 46812
0.061028142 93560 0.017344915 93560 0.056833287
93600 0.101734127 93600 0.096017662 46800 0.08905014 46816
0.035737598 93568 0.105641113 93568 0.055129203
93600 0.051814528 93600 0.043575103 46800 0.03081765 46820
0.050783088 93576 0.143191006 93576 0.02003058
93600 0.110780347 93600 0.036363548 46800 0.060053848 46824
0.039377235 93584 0.14939929 93584 0.020087127
93600 0.127619671 93600 0.085891603 46800 0.105084488 46828
0.022143653 93592 0.14399887 93592 0.037266218
93600 0.059191207 93600 0.05762095 46800 0.089105946 46832

0.028939525 93600 0.021303584 93600 0.024202574
93600 0.142354883 93600 0.057725745 46800 0.066284716 46836
0.059460221 93608 0.183555894 93608 0.018716828
93600 0.124386323 93600 0.07213153 46800 0.061751474 46840
0.072419083 93616 0.15251954 93616 0.051758067
93600 0.053264255 93600 0.162376411 46800 0.034408156 46844
0.036551228 93624 0.017883245 93624 0.046488749
93600 0.029743891 93600 0.176883114 46800 0.015067485 46848
0.050336032 93632 0.095238072 93632 0.006715454
93600 0.026950476 93600 0.105610328 46900 0.041557731 46852
0.094299307 93640 0.161739474 93640 0.038162914
93600 0.031012391 93600 0.070860355 46900 0.040995612 46856
0.085962973 93648 0.146037651 93648 0.019060517
93700 0.062697509 93700 0.087081826 46900 0.040582629 46860
0.055857643 93656 0.120545825 93656 0.030166499
93700 0.092042457 93700 0.103758037 46900 0.043430198 46864
0.039461109 93664 0.130259126 93664 0.026934858
93700 0.165810765 93700 0.14658719 46900 0.062330371 46868
0.021997299 93672 0.101357364 93672 0.063735752
93700 0.202266906 93700 0.197312213 46900 0.056674446 46872
0.012962215 93680 0.119721866 93680 0.075005359
93700 0.137540934 93700 0.168431609 46900 0.04787205 46876
0.018994462 93688 0.127932144 93688 0.044039723
93700 0.053052423 93700 0.098811361 46900 0.056342549 46880
0.033474531 93696 0.146706313 93696 0.042198048
93700 0.085723295 93700 0.182915378 46900 0.055432396 46884
0.026471955 93704 0.154286434 93704 0.03420714
93700 0.074174459 93700 0.228577163 46900 0.059223581 46888
0.017606524 93712 0.133066948 93712 0.015777969
93700 0.088067907 93700 0.161716234 46900 0.052638443 46892
0.039451323 93720 0.140665463 93720 0.030358595
93700 0.080537706 93700 0.079014339 46900 0.008018335 46896
0.07016203 93728 0.142009303 93728 0.027445509
93700 0.066571491 93700 0.121961704 46900 0.025196796 46900
0.068825451 93736 0.129711058 93736 0.035937792
93700 0.060517825 93700 0.123329228 46900 0.026500951 46904
0.040152379 93744 0.137830721 93744 0.041489784
93800 0.092221017 93800 0.070594317 46900 0.034861187 46908
0.035336718 93752 0.126023428 93752 0.056130532
93800 0.054867822 93800 0.054670167 46900 0.0415424 46912
0.026075326 93760 0.082332976 93760 0.074625721
93800 0.072244184 93800 0.050064355 46900 0.056461988 46916
0.036619567 93768 0.121602985 93768 0.05180938
93800 0.058890677 93800 0.019960671 46900 0.041357343 46920
0.037764829 93776 0.136679199 93776 0.062238833
93800 0.034620716 93800 0.057251175 46900 0.030218489 46924
0.021596146 93784 0.081203871 93784 0.082859107
93800 0.037958806 93800 0.100915961 46900 0.030695264 46928
0.03531 93792 0.053066979 93792 0.072480776
93800 0.038670412 93800 0.119984165 46900 0.029696734 46932
0.024068369 93800 0.035813224 93800 0.067789988
93800 0.058679409 93800 0.182752905 46900 0.047402598 46936
0.05183505 93808 0.007583724 93808 0.072825431
93800 0.037404323 93800 0.157520059 46900 0.071238676 46940
0.090219255 93816 0.060548984 93816 0.078863617
93800 0.028890006 93800 0.130360758 46900 0.05025477 46944
0.072853385 93824 0.096713935 93824 0.067329849
93800 0.054464017 93800 0.142450474 46900 0.025364016 46948
0.058460686 93832 0.053726959 93832 0.029394181
93800 0.084479849 93800 0.142523029 47000 0.04784562 46952
0.050317209 93840 0.065394554 93840 0.012438133
93800 0.071628187 93800 0.107734952 47000 0.056473644 46956
0.03443089 93848 0.151115586 93848 0.010721918
93900 0.109971465 93900 0.092440561 47000 0.081581144 46960
0.017710972 93856 0.208971876 93856 0.019947247
93900 0.126477884 93900 0.088594738 47000 0.067405832 46964
0.009294407 93864 0.171344014 93864 0.034132241
93900 0.138987525 93900 0.093308459 47000 0.048448397 46968

0.042872038 93872 0.105795938 93872 0.051707735
93900 0.111503468 93900 0.052019037 47000 0.05575176 46972
0.063066662 93880 0.06859834 93880 0.07270967
93900 0.067736532 93900 0.03870199 47000 0.030335523 46976
0.073117524 93888 0.009134327 93888 0.061896884
93900 0.077199242 93900 0.068447014 47000 0.037339381 46980
0.064240951 93896 0.035029294 93896 0.029904655
93900 0.060478411 93900 0.042178064 47000 0.052283373 46984
0.02630639 93904 0.063312982 93904 0.095781063
93900 0.016300333 93900 0.057437133 47000 0.064909596 46988
0.025672516 93912 0.071025061 93912 0.128068365
93900 0.036252073 93900 0.045229219 47000 0.043206637 46992
0.038464419 93920 0.060694754 93920 0.076592842
93900 0.04783523 93900 0.078224119 47000 0.008802266 46996
0.048531209 93928 0.020470197 93928 0.010575503
93900 0.09230923 93900 0.142987454 47000 0.029098696 47000
0.071606366 93936 0.047037058 93936 0.01916636
93900 0.016492437 93900 0.194679204 47000 0.018297171 47004
0.087047702 93944 0.047032903 93944 0.04145395
94000 0.097429867 94000 0.137146664 47000 0.052102227 47008
0.082133607 93952 0.040182746 93952 0.033151595
94000 0.102455277 94000 0.090588837 47000 0.061374798 47012
0.054773878 93960 0.066063345 93960 0.023024502
94000 0.031902047 94000 0.108349901 47000 0.039650382 47016
0.048202517 93968 0.033083248 93968 0.025352225
94000 0.145510246 94000 0.102462065 47000 0.012430803 47020
0.078795463 93976 0.084498701 93976 0.026147101
94000 0.208171478 94000 0.101320977 47000 0.051533043 47024
0.09151212 93984 0.123071615 93984 0.041892072
94000 0.143203375 94000 0.09471173 47000 0.062920852 47028
0.067317051 93992 0.097088465 93992 0.047695412
94000 0.055478853 94000 0.064087617 47000 0.075594748 47032
0.026410162 94000 0.124244936 94000 0.022056416
94000 0.030786127 94000 0.078624573 47000 0.091166949 47036
0.036823793 94008 0.227570505 94008 0.041790128
94000 0.037193699 94000 0.069286762 47000 0.072070747 47040
0.072663868 94016 0.168323764 94016 0.065446773
94000 0.058006215 94000 0.04313681 47000 0.034446999 47044
0.066508117 94024 0.088066889 94024 0.057369816
94000 0.103541235 94000 0.052748444 47000 0.060886374 47048
0.049900311 94032 0.099633071 94032 0.055480938
94000 0.092565606 94000 0.05832448 47100 0.058875077 47052
0.031977532 94040 0.066271146 94040 0.040132425
94000 0.059221213 94000 0.010469895 47100 0.018199129 47056
0.010688018 94048 0.0308573 94048 0.03795098
94100 0.066649875 94100 0.066106572 47100 0.059846734 47060
0.028978508 94056 0.094353512 94056 0.052989377
94100 0.088407345 94100 0.077354176 47100 0.027851642 47064
0.051620529 94064 0.098017117 94064 0.027955677
94100 0.090727386 94100 0.073093906 47100 0.035130532 47068
0.040014395 94072 0.031419291 94072 0.028190296
94100 0.095793053 94100 0.067821937 47100 0.053053951 47072
0.035713292 94080 0.034040637 94080 0.068486697
94100 0.097536904 94100 0.029978311 47100 0.04572413 47076
0.050139464 94088 0.030620526 94088 0.044712819
94100 0.075882665 94100 0.05624535 47100 0.032627413 47080
0.027858881 94096 0.058909962 94096 0.014240765
94100 0.017830886 94100 0.095346717 47100 0.00784348 47084
0.020318208 94104 0.12576209 94104 0.036703339
94100 0.045884404 94100 0.10924341 47100 0.023570516 47088
0.081189246 94112 0.106749794 94112 0.027485794
94100 0.028782943 94100 0.057310612 47100 0.03236765 47092
0.093740666 94120 0.030749226 94120 0.025790241
94100 0.042844604 94100 0.055759974 47100 0.00925953 47096
0.066163797 94128 0.062944127 94128 0.044267981
94100 0.038657821 94100 0.04016258 47100 0.028435303 47100
0.049065377 94136 0.067994013 94136 0.067534922
94100 0.051845527 94100 0.118225718 47100 0.037889815 47104

0.057796315 94144 0.098458113 94144 0.058612572
94200 0.056604062 94200 0.065823784 47100 0.04192811 47108
0.04317025 94152 0.103287617 94152 0.029260256
94200 0.040627128 94200 0.03919964 47100 0.047755544 47112
0.02175573 94160 0.06446171 94160 0.013232595
94200 0.053541753 94200 0.11031521 47100 0.056352998 47116
0.051242056 94168 0.120710458 94168 0.019037187
94200 0.031371601 94200 0.157175149 47100 0.068105721 47120
0.064796041 94176 0.118207012 94176 0.039204537
94200 0.029796296 94200 0.16903007 47100 0.062406274 47124
0.041585587 94184 0.062147286 94184 0.028166274
94200 0.008998181 94200 0.178668139 47100 0.054012482 47128
0.00368359 94192 0.056674875 94192 0.048481972
94200 0.085307409 94200 0.174607165 47100 0.094643532 47132
0.055452507 94200 0.097189179 94200 0.030426018
94200 0.108682194 94200 0.12555797 47100 0.048194113 47136
0.085059757 94208 0.014292267 94208 0.015739517
94200 0.083845305 94200 0.112192087 47100 0.046898898 47140
0.058686175 94216 0.121436089 94216 0.04318673
94200 0.096935633 94200 0.101253951 47100 0.044812332 47144
0.029833387 94224 0.087846165 94224 0.05831708
94200 0.024111634 94200 0.024728364 47100 0.016108028 47148
0.048111218 94232 0.04259207 94232 0.032658005
94200 0.136458824 94200 0.119810648 47200 0.047310252 47152
0.05636087 94240 0.088981207 94240 0.044356759
94200 0.205354984 94200 0.126777959 47200 0.077125158 47156
0.051581399 94248 0.068633795 94248 0.074452451
94300 0.22176902 94300 0.108106353 47200 0.06782866 47160
0.037932816 94256 0.106596657 94256 0.050363775
94300 0.185733836 94300 0.088494977 47200 0.048991085 47164
0.020158472 94264 0.142835706 94264 0.04619734
94300 0.123849022 94300 0.087506371 47200 0.047070647 47168
0.042201966 94272 0.213695428 94272 0.079690035
94300 0.052489242 94300 0.128793719 47200 0.053373802 47172
0.039886774 94280 0.17932459 94280 0.069071619
94300 0.018965729 94300 0.179303315 47200 0.066145753 47176
0.056378161 94288 0.128166371 94288 0.01766576
94300 0.00801371 94300 0.195835398 47200 0.088936751 47180
0.063075488 94296 0.329895819 94296 0.05350322
94300 0.018950337 94300 0.14926579 47200 0.097923403 47184
0.074414274 94304 0.356208388 94304 0.052825435
94300 0.086472835 94300 0.052962929 47200 0.078490091 47188
0.040000283 94312 0.245560571 94312 0.043827826
94300 0.176023794 94300 0.061861858 47200 0.029678138 47192
0.058587917 94320 0.284115144 94320 0.056258738
94300 0.136409538 94300 0.099950608 47200 0.046824898 47196
0.098801029 94328 0.36216696 94328 0.063738298
94300 0.080086997 94300 0.00294842 47200 0.08295782 47200
0.060694743 94336 0.299573381 94336 0.044838322
94300 0.139815311 94300 0.124878483 47200 0.095694057 47204
0.056012155 94344 0.297602499 94344 0.02645484
94400 0.160891214 94400 0.146396341 47200 0.095300013 47208
0.06227025 94352 0.282390189 94352 0.030849136
94400 0.138388816 94400 0.061258354 47200 0.05827548 47212
0.076048302 94360 0.072530245 94360 0.043653603
94400 0.11630213 94400 0.046654506 47200 0.027191725 47216
0.092554677 94368 0.229495883 94368 0.053759883
94400 0.080001737 94400 0.086337423 47200 0.018978833 47220
0.091880123 94376 0.351547933 94376 0.047756297
94400 0.065435706 94400 0.059556205 47200 0.026395344 47224
0.043945303 94384 0.234540479 94384 0.035850302
94400 0.087094464 94400 0.04946825 47200 0.057316636 47228
0.068716501 94392 0.092085589 94392 0.021110123
94400 0.09582581 94400 0.01332485 47200 0.089448695 47232
0.064958622 94400 0.069338203 94400 0.013457186
94400 0.085004089 94400 0.027605163 47200 0.066965265 47236
0.045632482 94408 0.175052831 94408 0.029736253
94400 0.082010578 94400 0.019256906 47200 0.004110095 47240

0.049277172 94416 0.256301806 94416 0.039423539
94400 0.10367416 94400 0.026265565 47200 0.044254564 47244
0.034233181 94424 0.127267268 94424 0.039558679
94400 0.144954538 94400 0.062595573 47200 0.054990065 47248
0.012650836 94432 0.107238411 94432 0.056011864
94400 0.144691774 94400 0.109721586 47300 0.087643115 47252
0.0388974 94440 0.163098303 94440 0.043019689
94400 0.079553822 94400 0.146136866 47300 0.088554662 47256
0.072887575 94448 0.102947772 94448 0.035587855
94500 0.049255126 94500 0.141699886 47300 0.05369405 47260
0.073780473 94456 0.063087733 94456 0.034856883
94500 0.125150968 94500 0.056834873 47300 0.028126025 47264
0.080245016 94464 0.015789037 94464 0.018883653
94500 0.076852826 94500 0.045634311 47300 0.015892809 47268
0.063784959 94472 0.017995519 94472 0.034579331
94500 0.046508096 94500 0.071857554 47300 0.007785384 47272
0.054676235 94480 0.056782847 94480 0.056285582
94500 0.0841566 94500 0.058107016 47300 0.010608677 47276
0.051821415 94488 0.151007233 94488 0.055480105
94500 0.07717681 94500 0.109579232 47300 0.044881312 47280
0.012206214 94496 0.192311781 94496 0.035599609
94500 0.096626071 94500 0.161777061 47300 0.068202142 47284
0.03166567 94504 0.1609061 94504 0.008217285
94500 0.302021829 94500 0.090874921 47300 0.038943505 47288
0.046293957 94512 0.111428199 94512 0.026637272
94500 0.704810896 94500 0.010047618 47300 0.022195845 47292
0.057744779 94520 0.066673863 94520 0.01689732
94500 0.759142451 94500 0.038115624 47300 0.011183556 47296
0.052287112 94528 0.041151961 94528 0.036843605
94500 0.358830585 94500 0.083955376 47300 0.015415779 47300
0.057754511 94536 0.054863867 94536 0.102320904
94500 0.125367413 94500 0.057449808 47300 0.042280277 47304
0.05920717 94544 0.01314768 94544 0.125820035
94600 0.188743623 94600 0.125693405 47300 0.049540682 47308
0.064605156 94552 0.066147703 94552 0.085825901
94600 0.170915388 94600 0.134746064 47300 0.024344276 47312
0.090224603 94560 0.048135637 94560 0.04493224
94600 0.160963726 94600 0.11122787 47300 0.014907542 47316
0.05994274 94568 0.072317838 94568 0.045638568
94600 0.180175135 94600 0.062113766 47300 0.025770689 47320
0.032808344 94576 0.097325246 94576 0.051198076
94600 0.175079418 94600 0.03672492 47300 0.025523517 47324
0.045498135 94584 0.076267286 94584 0.03842896
94600 0.231451283 94600 0.124546554 47300 0.011786102 47328
0.047625148 94592 0.041770683 94592 0.035990583
94600 0.274343561 94600 0.144420833 47300 0.026402429 47332
0.052930205 94600 0.047303685 94600 0.024789553
94600 0.192008244 94600 0.085401327 47300 0.019949577 47336
0.067893285 94608 0.102695405 94608 0.029254163
94600 0.103011567 94600 0.009041615 47300 0.03628237 47340
0.049492221 94616 0.097243617 94616 0.050439889
94600 0.139404946 94600 0.035615834 47300 0.037473907 47344
0.004097947 94624 0.099831283 94624 0.033299071
94600 0.162999175 94600 0.046815047 47300 0.039186893 47348
0.035164416 94632 0.089153065 94632 0.020082141
94600 0.104560815 94600 0.035101857 47400 0.050578867 47352
0.044162425 94640 0.076989221 94640 0.058974252
94600 0.057602392 94600 0.024144132 47400 0.031682859 47356
0.063339845 94648 0.100484002 94648 0.064599677
94700 0.045320678 94700 0.026314718 47400 0.040914263 47360
0.097692886 94656 0.076678305 94656 0.041610823
94700 0.090891233 94700 0.106027706 47400 0.040633495 47364
0.112382171 94664 0.049137747 94664 0.035498171
94700 0.079581703 94700 0.156406851 47400 0.031550109 47368
0.08480229 94672 0.078849465 94672 0.039733535
94700 0.156174153 94700 0.177915354 47400 0.051431711 47372
0.023427616 94680 0.153587943 94680 0.042721109
94700 0.177499256 94700 0.17833797 47400 0.063241118 47376

0.014759999 94688 0.177639231 94688 0.02642944
94700 0.122675265 94700 0.12602593 47400 0.055134718 47380
0.060407354 94696 0.13591905 94696 0.028278362
94700 0.111529829 94700 0.045544217 47400 0.03508724 47384
0.078941172 94704 0.086131724 94704 0.025003927
94700 0.114185095 94700 0.075552111 47400 0.002234485 47388
0.056216566 94712 0.053422627 94712 0.030499386
94700 0.133426278 94700 0.136369578 47400 0.041159441 47392
0.055819211 94720 0.065791821 94720 0.036717873
94700 0.09201259 94700 0.120448553 47400 0.053919208 47396
0.016458263 94728 0.097893484 94728 0.024717803
94700 0.102657919 94700 0.072462906 47400 0.036007386 47400
0.061819636 94736 0.070933078 94736 0.041179865
94700 0.105729872 94700 0.120759163 47400 0.013500597 47404
0.099600526 94744 0.06717514 94744 0.044101773
94800 0.045288849 94800 0.048873724 47400 0.006827892 47408
0.092695329 94752 0.07569875 94752 0.029316983
94800 0.165209654 94800 0.066384651 47400 0.033854878 47412
0.075550634 94760 0.073619565 94760 0.025323496
94800 0.183785858 94800 0.061123377 47400 0.052437783 47416
0.057904032 94768 0.052770058 94768 0.092753719
94800 0.205402306 94800 0.113571201 47400 0.056400553 47420
0.030449393 94776 0.0748452 94776 0.096371034
94800 0.965772895 94800 0.113147653 47400 0.049986054 47424
0.010723058 94784 0.111873604 94784 0.047879552
94800 1.5548605 94800 0.071492628 47400 0.040895775 47428
0.032731037 94792 0.113606628 94792 0.014821246
94800 1.363205613 94800 0.054562337 47400 0.048550013 47432
0.074110561 94800 0.133893963 94800 0.006508081
94800 0.671508605 94800 0.018955372 47400 0.0599462 47436
0.121052581 94808 0.150753273 94808 0.018887475
94800 0.185418613 94800 0.118174932 47400 0.065006832 47440
0.115613082 94816 0.114892544 94816 0.035063407
94800 0.07265708 94800 0.101075078 47400 0.05129863 47444
0.095831507 94824 0.077662429 94824 0.056181387
94800 0.170978135 94800 0.046322781 47400 0.04208766 47448
0.073175826 94832 0.020813746 94832 0.034962734
94800 0.150220338 94800 0.095704214 47500 0.064599037 47452
0.020039894 94840 0.068603178 94840 0.020846625
94800 0.127085805 94800 0.102310019 47500 0.061788756 47456
0.011308261 94848 0.091783164 94848 0.034482106
94900 0.077662007 94900 0.047636906 47500 0.056771653 47460
0.002390009 94856 0.038280916 94856 0.021838194
94900 0.072790812 94900 0.036192087 47500 0.046842866 47464
0.015728685 94864 0.078827841 94864 0.045661476
94900 0.088447807 94900 0.076073586 47500 0.076271848 47468
0.06889848 94872 0.167662001 94872 0.055862074
94900 0.042324227 94900 0.103296938 47500 0.102142047 47472
0.080800252 94880 0.170252606 94880 0.041070536
94900 0.025213469 94900 0.191478175 47500 0.068737929 47476
0.053369735 94888 0.10877286 94888 0.011497227
94900 0.027495722 94900 0.222894931 47500 0.043577024 47480
0.060344144 94896 0.11376114 94896 0.054424938
94900 0.099433324 94900 0.16179301 47500 0.039154948 47484
0.086376822 94904 0.106218773 94904 0.054111475
94900 0.154799374 94900 0.151596484 47500 0.032739881 47488
0.054710308 94912 0.046253539 94912 0.016995915
94900 0.161521414 94900 0.1712175 47500 0.04288552 47492
0.026102434 94920 0.10202836 94920 0.036103716
94900 0.116754767 94900 0.09921423 47500 0.041535117 47496
0.033455741 94928 0.087165521 94928 0.046052624
94900 0.096615811 94900 0.071311195 47500 0.020407673 47500
0.04459573 94936 0.030906944 94936 0.027529059
94900 0.095163101 94900 0.065212684 47500 0.034934954 47504
0.043502794 94944 0.105321393 94944 0.057200872
95000 0.101929953 95000 0.005829702 47500 0.076301854 47508
0.039172661 94952 0.08241355 94952 0.062345222
95000 0.088078436 95000 0.032896678 47500 0.06563621 47512

```
0.043795317	94960	0.047982267	94960	0.047151723
95000	0.064881693	95000	0.053925873	47500	0.01722414	47516
0.056716064	94968	0.048291229	94968	0.048082209
95000	0.100838399	95000	0.14391348	47500	0.006691402	47520
0.051808664	94976	0.041108084	94976	0.045449742
95000	0.11769338	95000	0.167613936	47500	0.014765429	47524
0.032984688	94984	0.141010066	94984	0.049462815
95000	0.057955553	95000	0.147882588	47500	0.027707714	47528
0.029741401	94992	0.183983488	94992	0.04469186
95000	0.075173724	95000	0.133045309	47500	0.06083147	47532
0.024589888	95000	0.146635211	95000	0.050414725
95000	0.058195408	95000	0.116692056	47500	0.082206854	47536
0.013823736	95008	0.086163389	95008	0.023556067
95000	0.041020605	95000	0.110242712	47500	0.073150732	47540
0.017013623	95016	0.051398954	95016	0.024820645
95000	0.062799911	95000	0.15026571	47500	0.092685637	47544
0.018360701	95024	0.024180035	95024	0.02038397
95000	0.138410048	95000	0.172136176	47500	0.100400641	47548
0.013798891	95032	0.075433833	95032	0.055478518
95000	0.166983199	95000	0.075432305	47600	0.056255503	47552
0.023346403	95040	0.044761942	95040	0.108360058
95000	0.258942543	95000	0.047077596	47600	0.008760211	47556
0.02352577	95048	0.060435934	95048	0.107512649
95100	0.57363027	95100	0.12375752	47600	0.025350946	47560
0.023373374	95056	0.046558616	95056	0.058627502
95100	0.678794516	95100	0.137444891	47600	0.019556579	47564
0.077447352	95064	0.062402185	95064	0.021635915
95100	0.469641149	95100	0.078340723	47600	0.034439981	47568
0.11139163	95072	0.119955847	95072	0.054088996
95100	0.163395598	95100	0.075284268	47600	0.053146432	47572
0.070841903	95080	0.111557143	95080	0.03699314
95100	0.007064874	95100	0.047139805	47600	0.057507725	47576
0.019289193	95088	0.095901167	95088	0.04878347
95100	0.10068815	95100	0.101874415	47600	0.043794564	47580
0.040897852	95096	0.122035781	95096	0.067633257
95100	0.136961244	95100	0.131151391	47600	0.037808772	47584
0.022766491	95104	0.075221324	95104	0.058542129
95100	0.108870379	95100	0.093159673	47600	0.055820299	47588
0.038711918	95112	0.012500808	95112	0.050143426
95100	0.09265446	95100	0.0167114	47600	0.052657793	47592
0.012019156	95120	0.028127473	95120	0.0493226
95100	0.128814965	95100	0.101661455	47600	0.025999183	47596
0.028175391	95128	0.048489339	95128	0.04201187
95100	0.216955043	95100	0.171464839	47600	0.020223706	47600
0.020233882	95136	0.029810082	95136	0.034604349
95100	0.26458138	95100	0.152089939	47600	0.027907388	47604
0.033826593	95144	0.033484495	95144	0.030469044
95200	0.248564408	95200	0.110993751	47600	0.035123499	47608
0.03476191	95152	0.044857952	95152	0.017944829
95200	0.166587371	95200	0.097805671	47600	0.033451299	47612
0.057690493	95160	0.056659326	95160	0.00298377
95200	0.070723087	95200	0.053379157	47600	0.053320579	47616
0.069382157	95168	0.035324803	95168	0.005761811
95200	0.05756766	95200	0.007497987	47600	0.064711901	47620
0.08726793	95176	0.053918113	95176	0.015925651
95200	0.098775934	95200	0.012587588	47600	0.043433567	47624
0.087033368	95184	0.085324056	95184	0.036594825
95200	0.132086934	95200	0.033585147	47600	0.038351976	47628
0.073525422	95192	0.03446409	95192	0.036952515
95200	0.122253725	95200	0.023108951	47600	0.05674456	47632
0.044880708	95200	0.0718925	95200	0.035017383
95200	0.090999281	95200	0.10223448	47600	0.050470324	47636
0.061664876	95208	0.100189209	95208	0.072448114
95200	0.115618554	95200	0.173335546	47600	0.037005157	47640
0.045179677	95216	0.051951764	95216	0.088221743
95200	0.052678726	95200	0.097859978	47600	0.021923039	47644
0.015742055	95224	0.035359055	95224	0.064622996
95200	0.078763951	95200	0.040177554	47600	0.005861535	47648
```

0.016949818 95232 0.062507548 95232 0.046218043
95200 0.034370598 95200 0.09319622 47700 0.053316493 47652
0.027727876 95240 0.067553825 95240 0.056197649
95200 0.117971518 95200 0.0799271 47700 0.083374805 47656
0.017571092 95248 0.053006941 95248 0.043024771
95300 0.152003588 95300 0.079954545 47700 0.057937185 47660
0.024753084 95256 0.051110044 95256 0.051300605
95300 0.162894154 95300 0.144887018 47700 0.027717581 47664
0.024314699 95264 0.025882375 95264 0.069499765
95300 0.148103631 95300 0.128344444 47700 0.035056106 47668
0.023006091 95272 0.039329058 95272 0.05400935
95300 0.034428224 95300 0.035031615 47700 0.026030639 47672
0.047112011 95280 0.074554308 95280 0.021546121
95300 0.169191219 95300 0.086232467 47700 0.017822573 47676
0.087012173 95288 0.05836771 95288 0.036688063
95300 0.18416089 95300 0.132701985 47700 0.012161619 47680
0.09607934 95296 0.057699155 95296 0.073646632
95300 0.093091825 95300 0.048977392 47700 0.018302297 47684
0.05105542 95304 0.066286149 95304 0.072963536
95300 0.130973014 95300 0.043542223 47700 0.008303116 47688
0.036850855 95312 0.032158136 95312 0.064325199
95300 0.149270461 95300 0.058306378 47700 0.035392091 47692
0.069073831 95320 0.108357534 95320 0.057140602
95300 0.166636878 95300 0.102791666 47700 0.042101176 47696
0.072364041 95328 0.218425078 95328 0.023622073
95300 0.156939204 95300 0.103327926 47700 0.060764538 47700
0.055328892 95336 0.234141015 95336 0.008400327
95300 0.06199428 95300 0.027468881 47700 0.066053057 47704
0.036121881 95344 0.167226666 95344 0.029406878
95400 0.085989006 95400 0.044651999 47700 0.020061476 47708
0.080172227 95352 0.082120583 95352 0.079982216
95400 0.063548287 95400 0.06044775 47700 0.047649912 47712
0.097106 95360 0.053230826 95360 0.11069543
95400 0.036979214 95400 0.107379186 47700 0.062498373 47716
0.063723674 95368 0.113002629 95368 0.082471081
95400 0.030094714 95400 0.066662215 47700 0.041812509 47720
0.026250282 95376 0.062772277 95376 0.027890994
95400 0.097473836 95400 0.037791324 47700 0.044251861 47724
0.020302288 95384 0.056060446 95384 0.032413915
95400 0.093449555 95400 0.087179367 47700 0.016600909 47728
0.039404335 95392 0.068934402 95392 0.046491772
95400 0.032409116 95400 0.0950588 47700 0.042577271 47732
0.036885253 95400 0.073340489 95400 0.032263968
95400 0.107619242 95400 0.123566701 47700 0.063582585 47736
0.017556693 95408 0.172592161 95408 0.017523522
95400 0.055922483 95400 0.126253698 47700 0.054897912 47740
0.040238909 95416 0.173357446 95416 0.021393278
95400 0.061436578 95400 0.066107103 47700 0.041252966 47744
0.092357106 95424 0.123155187 95424 0.024333072
95400 0.126306855 95400 0.041708318 47700 0.040872052 47748
0.082550563 95432 0.075396223 95432 0.008626451
95400 0.120825287 95400 0.079555408 47800 0.025467882 47752
0.04753429 95440 0.047257221 95440 0.021766464
95400 0.106536107 95400 0.112311325 47800 0.021663331 47756
0.076993274 95448 0.021289021 95448 0.044001492
95500 0.100074714 95500 0.154078633 47800 0.013943821 47760
0.111760804 95456 0.035383775 95456 0.044240554
95500 0.051474901 95500 0.177225491 47800 0.014500606 47764
0.103658735 95464 0.085619948 95464 0.029113322
95500 0.06002334 95500 0.119772456 47800 0.025296658 47768
0.058659545 95472 0.115908937 95472 0.039643626
95500 0.138512041 95500 0.097572192 47800 0.027046428 47772
0.027722887 95480 0.142692458 95480 0.067717258
95500 0.116148265 95500 0.191273183 47800 0.022607104 47776
0.056503861 95488 0.149151325 95488 0.092467242
95500 0.059992526 95500 0.177717636 47800 0.040560404 47780
0.070726281 95496 0.147073675 95496 0.070737064
95500 0.112641537 95500 0.099778699 47800 0.044434419 47784

0.083660125 95504 0.158344061 95504 0.018459741
95500 0.178184143 95500 0.057054109 47800 0.042786673 47788
0.078122153 95512 0.20528525 95512 0.075993979
95500 0.16160318 95500 0.051194289 47800 0.059514227 47792
0.066870962 95520 0.224420481 95520 0.076472599
95500 0.119080869 95500 0.062603671 47800 0.043030988 47796
0.060250812 95528 0.145070051 95528 0.056896592
95500 0.124537823 95500 0.077596822 47800 0.01847083 47800
0.062264189 95536 0.070648181 95536 0.060942559
95500 0.161599718 95500 0.129684355 47800 0.064778069 47804
0.109270273 95544 0.071505208 95544 0.037153641
95600 0.122192651 95600 0.12153366 47800 0.077314136 47808
0.117240154 95552 0.039942159 95552 0.03066663
95600 0.032146105 95600 0.068820671 47800 0.079954538 47812
0.062362371 95560 0.10640236 95560 0.056486824
95600 0.048266775 95600 0.136704111 47800 0.061366503 47816
0.011284859 95568 0.19481995 95568 0.053615091
95600 0.145083351 95600 0.143072306 47800 0.03003969 47820
0.032992382 95576 0.148262538 95576 0.054250289
95600 0.146069783 95600 0.082759259 47800 0.042965883 47824
0.031052925 95584 0.068424546 95584 0.0446261
95600 0.09893778 95600 0.144923863 47800 0.022510843 47828
0.039262533 95592 0.043554057 95592 0.025362291
95600 0.084931737 95600 0.177097565 47800 0.021976548 47832
0.05222128 95600 0.054838347 95600 0.030427173
95600 0.071340735 95600 0.131351714 47800 0.041812502 47836
0.055918612 95608 0.052410869 95608 0.017312586
95600 0.080729398 95600 0.138128671 47800 0.050185463 47840
0.067833113 95616 0.053435921 95616 0.040022718
95600 0.084152009 95600 0.162521625 47800 0.043949 47844
0.062332627 95624 0.060958137 95624 0.034490724
95600 0.066340464 95600 0.137006763 47800 0.045772438 47848
0.046366935 95632 0.069783746 95632 0.045258257
95600 0.076399752 95600 0.100673067 47900 0.047534828 47852
0.041579118 95640 0.099084042 95640 0.048027498
95600 0.109533961 95600 0.12119599 47900 0.037795398 47856
0.022909968 95648 0.110924259 95648 0.02261109
95700 0.04255633 95700 0.092947979 47900 0.030093916 47860
0.074506475 95656 0.090299058 95656 0.021655176
95700 0.059810929 95700 0.066638808 47900 0.02363322 47864
0.094782983 95664 0.076849552 95664 0.0357959
95700 0.065105 95700 0.078921963 47900 0.031409705 47868
0.088686567 95672 0.07825809 95672 0.044076678
95700 0.017271492 95700 0.021333659 47900 0.074424817 47872
0.088944515 95680 0.082594568 95680 0.020185176
95700 0.020758067 95700 0.095559764 47900 0.082378167 47876
0.084305502 95688 0.108772343 95688 0.028736045
95700 0.037002934 95700 0.141233846 47900 0.02357122 47880
0.071882219 95696 0.124971484 95696 0.023895975
95700 0.03516996 95700 0.095383948 47900 0.031543765 47884
0.051675586 95704 0.102824313 95704 0.005209248
95700 0.030595496 95700 0.047429952 47900 0.020441365 47888
0.062215251 95712 0.026177242 95712 0.019907278
95700 0.051540505 95700 0.074420852 47900 0.021650039 47892
0.112328373 95720 0.078742844 95720 0.028461591
95700 0.073608033 95700 0.053595795 47900 0.029217894 47896
0.125115999 95728 0.09579389 95728 0.037844569
95700 0.082439408 95700 0.162649405 47900 0.046819801 47900
0.095734402 95736 0.072932831 95736 0.037207388
95700 0.073289724 95700 0.107917163 47900 0.0339824 47904
0.086592467 95744 0.033659308 95744 0.021647871
95800 0.032941847 95800 0.131541921 47900 0.035527693 47908
0.097689888 95752 0.061948005 95752 0.045304354
95800 0.050205697 95800 0.15190593 47900 0.086551605 47912
0.083867926 95760 0.074940035 95760 0.035362085
95800 0.08992511 95800 0.117526601 47900 0.08971314 47916
0.028010913 95768 0.043105218 95768 0.022211083
95800 0.175821959 95800 0.113862669 47900 0.057244066 47920

0.055903849	95776	0.026205229	95776	0.023420422		
95800	0.194761116	95800	0.179423994	47900	0.030742449	47924
0.078074831	95784	0.06686258	95784	0.041305877		
95800	0.122444675	95800	0.178733244	47900	0.024406096	47928
0.062030777	95792	0.094439813	95792	0.052605436		
95800	0.053260985	95800	0.104296632	47900	0.030358129	47932
0.089927336	95800	0.10671073	95800	0.038590966		
95800	0.041914835	95800	0.030919196	47900	0.071785871	47936
0.095031806	95808	0.113646303	95808	0.036103043		
95800	0.065734428	95800	0.007158926	47900	0.106064319	47940
0.067447953	95816	0.100302474	95816	0.038920418		
95800	0.046205514	95800	0.041744119	47900	0.1073686	47944
0.045183359	95824	0.048241054	95824	0.040065468		
95800	0.065396598	95800	0.089957757	47900	0.085831212	47948
0.024316323	95832	0.045778426	95832	0.026017113		
95800	0.065846616	95800	0.051831594	48000	0.034430457	47952
0.07324091	95840	0.128228058	95840	0.025500438		
95800	0.067719957	95800	0.132825255	48000	0.029530458	47956
0.104589883	95848	0.152317021	95848	0.042059292		
95900	0.117815594	95900	0.177964495	48000	0.068035362	47960
0.067126348	95856	0.090700793	95856	0.027274526		
95900	0.055366043	95900	0.167981576	48000	0.046602334	47964
0.020326941	95864	0.025065163	95864	0.019300735		
95900	0.064846041	95900	0.134064438	48000	0.021622658	47968
0.07932102	95872	0.117174139	95872	0.031601554		
95900	0.142706908	95900	0.069700574	48000	0.04481712	47972
0.086836095	95880	0.112155591	95880	0.05273724		
95900	0.131977081	95900	0.106935877	48000	0.035172099	47976
0.081865299	95888	0.079077494	95888	0.048451359		
95900	0.084185274	95900	0.120747296	48000	0.028906425	47980
0.062911553	95896	0.018199929	95896	0.023480183		
95900	0.057501693	95900	0.110789806	48000	0.049757698	47984
0.05336629	95904	0.097100128	95904	0.021127766		
95900	0.107336004	95900	0.11636089	48000	0.069954105	47988
0.089817833	95912	0.12399783	95912	0.050566647		
95900	0.092456648	95900	0.107532811	48000	0.078869911	47992
0.100708123	95920	0.043746804	95920	0.04177534		
95900	0.047200065	95900	0.032796248	48000	0.093120369	47996
0.079165431	95928	0.081562583	95928	0.003154589		
95900	0.064070489	95900	0.107037595	48000	0.085288666	48000
0.031926727	95936	0.086706081	95936	0.038810573		
95900	0.108967834	95900	0.074993259	48000	0.030714455	48004
0.041337324	95944	0.049640759	95944	0.046727884		
96000	0.05797618	96000	0.006344858	48000	0.040249281	48008
0.059705242	95952	0.078256962	95952	0.054252992		
96000	0.047896676	96000	0.022602284	48000	0.065082742	48012
0.037968221	95960	0.047255831	95960	0.068498703		
96000	0.058345813	96000	0.093308845	48000	0.014656694	48016
0.092928262	95968	0.02355628	95968	0.086675835		
96000	0.038385224	96000	0.161510776	48000	0.065290034	48020
0.104303668	95976	0.066421453	95976	0.100397985		
96000	0.022001565	96000	0.174230329	48000	0.090135218	48024
0.045966979	95984	0.108199078	95984	0.079711113		
96000	0.050149585	96000	0.154274225	48000	0.073356154	48028
0.021332564	95992	0.068187663	95992	0.055604367		
96000	0.12774585	96000	0.111745889	48000	0.093129573	48032
0.007622824	96000	0.043818898	96000	0.05447966		
96000	0.157906034	96000	0.016911674	48000	0.109180575	48036
0.015736274	96008	0.046187361	96008	0.035328179		
96000	0.105511579	96000	0.118759468	48000	0.083268264	48040
0.056226694	96016	0.034408597	96016	0.005235032		
96000	0.062599334	96000	0.185638274	48000	0.034971126	48044
0.121605284	96024	0.025653711	96024	0.023791734		
96000	0.106506857	96000	0.186467921	48000	0.047449114	48048
0.138394928	96032	0.009746696	96032	0.005696623		
96000	0.081564169	96000	0.156537688	48100	0.057187615	48052
0.106532185	96040	0.057946225	96040	0.017545037		
96000	0.051039875	96000	0.114879265	48100	0.045178771	48056

0.117745811 96048 0.047688012 96048 0.019231902
96100 0.091058653 96100 0.118480275 48100 0.045705583 48060
0.108117572 96056 0.042571403 96056 0.016921782
96100 0.140490214 96100 0.155301198 48100 0.08824504 48064
0.08162875 96064 0.100566576 96064 0.04952581
96100 0.111112757 96100 0.140419201 48100 0.094480842 48068
0.081099701 96072 0.101862206 96072 0.062756801
96100 0.039097417 96100 0.060186001 48100 0.044643341 48072
0.062269974 96080 0.099002107 96080 0.04439662
96100 0.072244759 96100 0.063636726 48100 0.045435765 48076
0.027247803 96088 0.05514572 96088 0.040442068
96100 0.095505951 96100 0.126124288 48100 0.034065259 48080
0.022790571 96096 0.02684275 96096 0.048956994
96100 0.094306502 96100 0.1831064 48100 0.01548386 48084
0.063931504 96104 0.083080755 96104 0.039512379
96100 0.12762571 96100 0.199569433 48100 0.049646795 48088
0.082026927 96112 0.096527474 96112 0.031428866
96100 0.147900311 96100 0.191247688 48100 0.048125396 48092
0.044162185 96120 0.036990216 96120 0.041649353
96100 0.083273779 96100 0.162898098 48100 0.025979947 48096
0.010854365 96128 0.068975569 96128 0.038138125
96100 0.046247838 96100 0.087730448 48100 0.023290491 48100
0.033879922 96136 0.132965114 96136 0.053357817
96100 0.107703512 96100 0.051686122 48100 0.049042636 48104
0.064112821 96144 0.116525378 96144 0.051739862
96200 0.030602485 96200 0.025595493 48100 0.06538818 48108
0.075756427 96152 0.085925312 96152 0.023423965
96200 0.079836376 96200 0.115921954 48100 0.047060115 48112
0.072028575 96160 0.058732872 96160 0.02672019
96200 0.065380104 96200 0.186779013 48100 0.035713933 48116
0.091142334 96168 0.080264137 96168 0.022579245
96200 0.084110376 96200 0.160853655 48100 0.069642687 48120
0.081383922 96176 0.161330158 96176 0.015502932
96200 0.106756961 96200 0.111681315 48100 0.093446222 48124
0.048252448 96184 0.15434144 96184 0.041924315
96200 0.057275658 96200 0.075511591 48100 0.041099745 48128
0.070276452 96192 0.130506086 96192 0.034409863
96200 0.038250211 96200 0.050237439 48100 0.034120905 48132
0.051492916 96200 0.142909106 96200 0.022014285
96200 0.101514561 96200 0.068554706 48100 0.068001667 48136
0.053949167 96208 0.071647693 96208 0.015869728
96200 0.145043058 96200 0.104912746 48100 0.085747088 48140
0.116704359 96216 0.066906156 96216 0.03834852
96200 0.111044319 96200 0.055306871 48100 0.101547383 48144
0.145517639 96224 0.128171378 96224 0.045249668
96200 0.02643643 96200 0.101496829 48100 0.078895471 48148
0.09902889 96232 0.06639721 96232 0.02205497
96200 0.113657705 96200 0.155348753 48200 0.032640513 48152
0.046964258 96240 0.02394757 96240 0.039764378
96200 0.18038576 96200 0.141218101 48200 0.040908763 48156
0.049405957 96248 0.142647041 96248 0.073219257
96300 0.1440989 96300 0.078162637 48200 0.029560277 48160
0.03164219 96256 0.221102076 96256 0.059405906
96300 0.074454838 96300 0.054818007 48200 0.025426696 48164
0.018714123 96264 0.101919686 96264 0.028505107
96300 0.093210219 96300 0.071885734 48200 0.050417282 48168
0.036397065 96272 0.106444059 96272 0.023017841
96300 0.137512965 96300 0.035857516 48200 0.074643365 48172
0.025448175 96280 0.177134003 96280 0.030823496
96300 0.121825397 96300 0.053385775 48200 0.08697818 48176
0.037388047 96288 0.087271517 96288 0.04759755
96300 0.075769938 96300 0.058203932 48200 0.099447396 48180
0.066540742 96296 0.040302635 96296 0.071311144
96300 0.042301133 96300 0.029256435 48200 0.136436254 48184
0.049027618 96304 0.086573993 96304 0.065252018
96300 0.042766893 96300 0.084954154 48200 0.147314874 48188
0.008927656 96312 0.029911935 96312 0.016337403
96300 0.019174342 96300 0.075089141 48200 0.096855583 48192

0.0556796 96320 0.072665629 96320 0.041021747
96300 0.02630798 96300 0.029650471 48200 0.072180155 48196
0.069733229 96328 0.083785701 96328 0.0750576
96300 0.083319552 96300 0.042056341 48200 0.121623896 48200
0.069505411 96336 0.08914221 96336 0.073913696
96300 0.149681583 96300 0.071379138 48200 0.149803048 48204
0.043699463 96344 0.076141078 96344 0.057816658
96400 0.15982971 96400 0.038128455 48200 0.116658579 48208
0.064807289 96352 0.047155081 96352 0.041944029
96400 0.142826539 96400 0.053025997 48200 0.077334102 48212
0.095074822 96360 0.058668353 96360 0.022870849
96400 0.121857083 96400 0.102939404 48200 0.048087979 48216
0.079361773 96368 0.056983168 96368 0.024460404
96400 0.077049459 96400 0.124789003 48200 0.0380686 48220
0.014162763 96376 0.082305422 96376 0.013719511
96400 0.041436149 96400 0.124091865 48200 0.027608927 48224
0.10357795 96384 0.037695627 96384 0.03574674
96400 0.104771811 96400 0.183408585 48200 0.021863132 48228
0.097281634 96392 0.077512312 96392 0.030847914
96400 0.197083631 96400 0.210823986 48200 0.037344245 48232
0.044138178 96400 0.145725208 96400 0.036721322
96400 0.206754165 96400 0.151145898 48200 0.045886587 48236
0.020252186 96408 0.080505866 96408 0.029087756
96400 0.169496838 96400 0.077181372 48200 0.052690026 48240
0.052074411 96416 0.095961048 96416 0.011058173
96400 0.143888305 96400 0.053212261 48200 0.062175481 48244
0.091904934 96424 0.166362865 96424 0.016104734
96400 0.113813061 96400 0.016740311 48200 0.069600006 48248
0.075403361 96432 0.085164014 96432 0.038725037
96400 0.063312538 96400 0.079385391 48300 0.066955938 48252
0.027722113 96440 0.079302517 96440 0.045817076
96400 0.041965708 96400 0.131164983 48300 0.057664431 48256
0.032921485 96448 0.101220656 96448 0.040652121
96500 0.087436158 96500 0.100083053 48300 0.054812041 48260
0.041329149 96456 0.007745223 96456 0.028381028
96500 0.138283125 96500 0.064789456 48300 0.045090226 48264
0.031357329 96464 0.106493448 96464 0.021643331
96500 0.108251836 96500 0.09445873 48300 0.046965924 48268
0.042087348 96472 0.083065053 96472 0.024006444
96500 0.083190898 96500 0.078478392 48300 0.091433154 48272
0.029163004 96480 0.041951233 96480 0.027866394
96500 0.112173781 96500 0.039279337 48300 0.124365746 48276
0.015334597 96488 0.057521596 96488 0.028691089
96500 0.085376494 96500 0.045345292 48300 0.115659277 48280
0.046047731 96496 0.072324263 96496 0.019079842
96500 0.045262303 96500 0.11890458 48300 0.086209839 48284
0.093717354 96504 0.128621949 96504 0.019846515
96500 0.033445354 96500 0.143366444 48300 0.059773818 48288
0.10818676 96512 0.211404579 96512 0.038601516
96500 0.02785584 96500 0.115386902 48300 0.036967154 48292
0.063945576 96520 0.194995984 96520 0.05182832
96500 0.017467926 96500 0.1265631 48300 0.027873226 48296
0.043980723 96528 0.092307062 96528 0.071811242
96500 0.017829629 96500 0.119504191 48300 0.025970097 48300
0.064445012 96536 0.054296394 96536 0.056180139
96500 0.034152272 96500 0.069831185 48300 0.026688669 48304
0.082086801 96544 0.050691098 96544 0.027384564
96600 0.01289129 96600 0.004851984 48300 0.028724187 48308
0.080486134 96552 0.030462154 96552 0.032569467
96600 0.038520167 96600 0.020325289 48300 0.033422279 48312
0.115600349 96560 0.050238265 96560 0.025671348
96600 0.024088291 96600 0.049884027 48300 0.067609122 48316
0.122106168 96568 0.083161511 96568 0.048281734
96600 0.071060902 96600 0.062950581 48300 0.06527175 48320
0.086557338 96576 0.087273111 96576 0.03206281
96600 0.093853763 96600 0.098375371 48300 0.040365139 48324
0.089775967 96584 0.138861419 96584 0.019332425
96600 0.091391099 96600 0.111895475 48300 0.065552878 48328

0.096203199 96592 0.135496478 96592 0.049347076
96600 0.041237636 96600 0.073288407 48300 0.135646536 48332
0.084686028 96600 0.076898592 96600 0.061611907
96600 0.03636449 96600 0.070217415 48300 0.141476426 48336
0.078273937 96608 0.018294593 96608 0.067864428
96600 0.08873194 96600 0.142527934 48300 0.092880066 48340
0.070612863 96616 0.071459188 96616 0.064538777
96600 0.072455368 96600 0.178124349 48300 0.050244151 48344
0.07780442 96624 0.105790292 96624 0.030368163
96600 0.037433267 96600 0.166831436 48300 0.024429886 48348
0.074594442 96632 0.103420927 96632 0.042504064
96600 0.005971166 96600 0.151425295 48400 0.048378199 48352
0.09044772 96640 0.054691642 96640 0.090576272
96600 0.106393789 96600 0.124235783 48400 0.061512903 48356
0.14575824 96648 0.030410445 96648 0.064381507
96700 0.121474091 96700 0.055943292 48400 0.068671252 48360
0.152387598 96656 0.048309219 96656 0.027644346
96700 0.03703505 96700 0.08117035 48400 0.043709966 48364
0.110326895 96664 0.014890848 96664 0.054004573
96700 0.0311039 96700 0.131724824 48400 0.030144598 48368
0.088703768 96672 0.019568111 96672 0.056232402
96700 0.025683883 96700 0.169825871 48400 0.037049274 48372
0.068148976 96680 0.042843956 96680 0.018425506
96700 0.015540729 96700 0.188367733 48400 0.037358972 48376
0.004721944 96688 0.005670952 96688 0.018820436
96700 0.078745077 96700 0.134544971 48400 0.016591304 48380
0.076537013 96696 0.055708624 96696 0.004886369
96700 0.099958532 96700 0.065194239 48400 0.011122062 48384
0.076968128 96704 0.078313584 96704 0.067049376
96700 0.070657559 96700 0.029346627 48400 0.039669645 48388
0.047376139 96712 0.072048984 96712 0.080561782
96700 0.059630176 96700 0.11293876 48400 0.053600925 48392
0.049567789 96720 0.028630762 96720 0.021127036
96700 0.089591464 96700 0.062662592 48400 0.046486697 48396
0.114830415 96728 0.058566748 96728 0.028564824
96700 0.07527382 96700 0.09146121 48400 0.028975363 48400
0.13074161 96736 0.026456597 96736 0.01834427
96700 0.072662333 96700 0.185382931 48400 0.030304784 48404
0.102105485 96744 0.064161723 96744 0.053043055
96800 0.16374806 96800 0.182564574 48400 0.019143061 48408
0.049909486 96752 0.073062016 96752 0.064850203
96800 0.134932678 96800 0.102378246 48400 0.040564872 48412
0.038937971 96760 0.071603281 96760 0.034916087
96800 0.029451938 96800 0.009021845 48400 0.065852604 48416
0.086112217 96768 0.056079825 96768 0.025469357
96800 0.14398538 96800 0.071824543 48400 0.075231699 48420
0.073249947 96776 0.070479364 96776 0.023515286
96800 0.132504953 96800 0.068286019 48400 0.080300335 48424
0.039522511 96784 0.117203868 96784 0.02285413
96800 0.089980735 96800 0.043280212 48400 0.047664118 48428
0.035977453 96792 0.104446408 96792 0.037255668
96800 0.066623004 96800 0.067101588 48400 0.032450556 48432
0.012418184 96800 0.062850362 96800 0.010891099
96800 0.076054326 96800 0.078822253 48400 0.065844644 48436
0.064297041 96808 0.054183885 96808 0.040199499
96800 0.097586228 96800 0.070002796 48400 0.076387514 48440
0.108176755 96816 0.150610926 96816 0.034014338
96800 0.063906271 96800 0.140191624 48400 0.078322788 48444
0.10985991 96824 0.119637625 96824 0.023093935
96800 0.014177658 96800 0.17064769 48400 0.050397146 48448
0.141215205 96832 0.015828031 96832 0.005866642
96800 0.053345761 96800 0.099235142 48500 0.017514518 48452
0.16025141 96840 0.034146691 96840 0.009528431
96800 0.019214738 96800 0.054383592 48500 0.017268581 48456
0.122584679 96848 0.002984072 96848 0.014733222
96900 0.056393441 96900 0.143488956 48500 0.039773695 48460
0.097515382 96856 0.079785736 96856 0.026370737
96900 0.091950897 96900 0.136650008 48500 0.048032369 48464

0.100859703 96864 0.09521388 96864 0.035765268
96900 0.113339062 96900 0.088499444 48500 0.036786038 48468
0.05845357 96872 0.032588519 96872 0.019036794
96900 0.12958757 96900 0.016936838 48500 0.042270764 48472
0.045058267 96880 0.143820348 96880 0.033114138
96900 0.097201242 96900 0.051199855 48500 0.047610665 48476
0.088962333 96888 0.190659819 96888 0.065268512
96900 0.093920426 96900 0.062529754 48500 0.039683713 48480
0.10751684 96896 0.158272625 96896 0.074800053
96900 0.162123354 96900 0.039508737 48500 0.050384158 48484
0.10130032 96904 0.115979448 96904 0.076686112
96900 0.169918654 96900 0.036199188 48500 0.057099245 48488
0.08332303 96912 0.100992722 96912 0.047822061
96900 0.052668838 96900 0.0833288 48500 0.055199715 48492
0.118574237 96920 0.091336646 96920 0.015176945
96900 0.058529415 96900 0.1224968 48500 0.053825745 48496
0.126775384 96928 0.08631462 96928 0.030384774
96900 0.042364722 96900 0.131842113 48500 0.019290093 48500
0.066544235 96936 0.099262055 96936 0.033359505
96900 0.063741623 96900 0.120249308 48500 0.015391641 48504
0.057606776 96944 0.104296596 96944 0.02806012
97000 0.110686706 97000 0.071443319 48500 0.024397863 48508
0.076833298 96952 0.066071734 96952 0.053132742
97000 0.066610657 97000 0.03673667 48500 0.03555217 48512
0.087298104 96960 0.012393341 96960 0.04109033
97000 0.024175011 97000 0.043403165 48500 0.042614385 48516
0.11958892 96968 0.036428493 96968 0.011137619
97000 0.052002328 97000 0.046550817 48500 0.047836031 48520
0.143260448 96976 0.07508049 96976 0.021831003
97000 0.066895285 97000 0.1124868 48500 0.040903095 48524
0.126693791 96984 0.125513689 96984 0.010898141
97000 0.063510219 97000 0.153585119 48500 0.017396504 48528
0.063340369 96992 0.103195554 96992 0.016493517
97000 0.112141708 97000 0.097099292 48500 0.059913848 48532
0.037992988 97000 0.01233559 97000 0.020929336
97000 0.071514201 97000 0.018869669 48500 0.102762082 48536
0.06750926 97008 0.069283611 97008 0.012733279
97000 0.048472957 97000 0.045638342 48500 0.072018709 48540
0.061587787 97016 0.058803566 97016 0.055915389
97000 0.135147959 97000 0.089084089 48500 0.019962883 48544
0.049396862 97024 0.036186939 97024 0.053625034
97000 0.192546169 97000 0.14752199 48500 0.040838957 48548
0.079400299 97032 0.07968054 97032 0.017725401
97000 0.185742553 97000 0.178592366 48600 0.085749045 48552
0.090796799 97040 0.072970564 97040 0.062424268
97000 0.155872273 97000 0.155560119 48600 0.07468347 48556
0.056684745 97048 0.040404142 97048 0.057408339
97100 0.135142764 97100 0.09896238 48600 0.051065774 48560
0.03153105 97056 0.157253744 97056 0.046951027
97100 0.110852299 97100 0.058016296 48600 0.031537529 48564
0.006331109 97064 0.251483813 97064 0.039498566
97100 0.080496793 97100 0.042792173 48600 0.043957993 48568
0.027309377 97072 0.197983318 97072 0.038212165
97100 0.042315107 97100 0.032232994 48600 0.033346372 48572
0.028865981 97080 0.095520729 97080 0.033746594
97100 0.03213614 97100 0.040800249 48600 0.070059025 48576
0.021720574 97088 0.093937459 97088 0.025726431
97100 0.101129313 97100 0.069412607 48600 0.068203284 48580
0.063832966 97096 0.100910867 97096 0.031595144
97100 0.10936756 97100 0.057623609 48600 0.013820821 48584
0.074731732 97104 0.114547038 97104 0.072460563
97100 0.062761552 97100 0.030362367 48600 0.067915971 48588
0.049859711 97112 0.100373436 97112 0.087189277
97100 0.041707881 97100 0.039487812 48600 0.056372337 48592
0.047478443 97120 0.087866203 97120 0.070624024
97100 0.060888189 97100 0.053377429 48600 0.048195518 48596
0.081573846 97128 0.062095314 97128 0.04631139
97100 0.134926726 97100 0.046080812 48600 0.062354033 48600

0.059658662 97136 0.021772163 97136 0.028088029
97100 0.096222138 97100 0.042952157 48600 0.055760535 48604
0.023207125 97144 0.048019363 97144 0.039548784
97200 0.058046917 97200 0.039893552 48600 0.048069775 48608
0.020863568 97152 0.067983754 97152 0.048183305
97200 0.051592539 97200 0.017830536 48600 0.042373817 48612
0.05612836 97160 0.078428107 97160 0.04358741
97200 0.086026535 97200 0.037065987 48600 0.034309385 48616
0.043334891 97168 0.05717087 97168 0.034869077
97200 0.08871199 97200 0.075613934 48600 0.028347775 48620
0.002004906 97176 0.073833217 97176 0.006245915
97200 0.02844194 97200 0.111454734 48600 0.022806773 48624
0.02527078 97184 0.100604586 97184 0.042360152
97200 0.040799696 97200 0.149156476 48600 0.013087409 48628
0.034047949 97192 0.072604009 97192 0.059200644
97200 0.097319171 97200 0.144572434 48600 0.024468081 48632
0.034035016 97200 0.072783005 97200 0.060521492
97200 0.098277902 97200 0.09896799 48600 0.046159978 48636
0.025149626 97208 0.116967596 97208 0.042170959
97200 0.024146484 97200 0.040620056 48600 0.049271195 48640
0.035318164 97216 0.147114828 97216 0.011271
97200 0.08142415 97200 0.035827412 48600 0.045552097 48644
0.037139478 97224 0.07688097 97224 0.021074855
97200 0.09462965 97200 0.08069272 48600 0.041209059 48648
0.006748742 97232 0.072270741 97232 0.022043483
97200 0.010417856 97200 0.064134707 48700 0.050639417 48652
0.020826759 97240 0.093216739 97240 0.045280907
97200 0.075401018 97200 0.024306683 48700 0.070230672 48656
0.020658172 97248 0.045002329 97248 0.05789322
97300 0.099949328 97300 0.024918723 48700 0.06278637 48660
0.016968837 97256 0.101813428 97256 0.031241449
97300 0.112631758 97300 0.077306133 48700 0.053051299 48664
0.047074107 97264 0.114223229 97264 0.038451486
97300 0.119432938 97300 0.095543292 48700 0.046279838 48668
0.085285567 97272 0.082720369 97272 0.005802975
97300 0.114688977 97300 0.041036707 48700 0.016785634 48672
0.090216068 97280 0.084777625 97280 0.04393264
97300 0.094952862 97300 0.065964756 48700 0.028805343 48676
0.046614016 97288 0.06028287 97288 0.047861755
97300 0.059725608 97300 0.045896231 48700 0.01010274 48680
0.024051555 97296 0.047415149 97296 0.018671952
97300 0.071212126 97300 0.082745079 48700 0.028169126 48684
0.079299425 97304 0.011033569 97304 0.038908431
97300 0.153340559 97300 0.16160449 48700 0.013447891 48688
0.101555772 97312 0.037194484 97312 0.061738181
97300 0.168804764 97300 0.133480396 48700 0.027179982 48692
0.080756836 97320 0.110365938 97320 0.061729719
97300 0.10419781 97300 0.031903743 48700 0.058262744 48696
0.076559212 97328 0.143581536 97328 0.053694057
97300 0.116220501 97300 0.072124276 48700 0.069753602 48700
0.103804588 97336 0.128249376 97336 0.053197644
97300 0.172325511 97300 0.046773908 48700 0.057620357 48704
0.071803945 97344 0.116807576 97344 0.040724812
97400 0.112906651 97400 0.040960156 48700 0.020190388 48708
0.068542628 97352 0.118833828 97352 0.048383761
97400 0.017321043 97400 0.096658914 48700 0.026204945 48712
0.061091989 97360 0.11594659 97360 0.035992991
97400 0.086035281 97400 0.101750957 48700 0.025503956 48716
0.052136504 97368 0.116511292 97368 0.023690562
97400 0.147632265 97400 0.06946089 48700 0.041219915 48720
0.016071581 97376 0.142500183 97376 0.00779379
97400 0.189792889 97400 0.045687299 48700 0.039816208 48724
0.010708541 97384 0.190171515 97384 0.029357434
97400 0.161559059 97400 0.058367943 48700 0.010137279 48728
0.029226834 97392 0.16712569 97392 0.046725916
97400 0.093415445 97400 0.064109816 48700 0.009194353 48732
0.04261098 97400 0.07876422 97400 0.067739813
97400 0.084117492 97400 0.069695154 48700 0.026627149 48736

0.072633397	97408	0.036361606	97408	0.047306523
97400	0.086708627	97400	0.057005713	48700	0.044650366	48740
0.050272018	97416	0.094891555	97416	0.023640821
97400	0.068845184	97400	0.113013615	48700	0.018264524	48744
0.035980815	97424	0.073587871	97424	0.043825679
97400	0.064488828	97400	0.127451349	48700	0.038406688	48748
0.068413829	97432	0.035211248	97432	0.063647072
97400	0.065669177	97400	0.10994781	48800	0.048119149	48752
0.075594151	97440	0.038875282	97440	0.065366054
97400	0.048689304	97400	0.069285372	48800	0.025440984	48756
0.007604273	97448	0.085260661	97448	0.043180575
97500	0.002942129	97500	0.098228287	48800	0.008673996	48760
0.039782797	97456	0.110355701	97456	0.020827169
97500	0.085768195	97500	0.094915849	48800	0.010880776	48764
0.051016956	97464	0.080976206	97464	0.025773561
97500	0.16891399	97500	0.022171644	48800	0.022014929	48768
0.04663779	97472	0.035710644	97472	0.017313687
97500	0.172931498	97500	0.043541491	48800	0.035980662	48772
0.018590299	97480	0.047777568	97480	0.014626754
97500	0.084297957	97500	0.023807012	48800	0.030228197	48776
0.04870311	97488	0.044725028	97488	0.028904842
97500	0.061592225	97500	0.048598678	48800	0.030308616	48780
0.049887196	97496	0.016385848	97496	0.043739106
97500	0.068611007	97500	0.050859999	48800	0.031125899	48784
0.041071169	97504	0.069421738	97504	0.053966189
97500	0.056598456	97500	0.052290186	48800	0.012235855	48788
0.040997347	97512	0.149181695	97512	0.035233603
97500	0.090687077	97500	0.054149357	48800	0.016018694	48792
0.034750159	97520	0.107818181	97520	0.032412369
97500	0.143800396	97500	0.040360039	48800	0.037393438	48796
0.092946386	97528	0.029497105	97528	0.07477482
97500	0.13412883	97500	0.01177673	48800	0.051324183	48800
0.111423899	97536	0.010554849	97536	0.056604476
97500	0.095985241	97500	0.035833851	48800	0.043658711	48804
0.058386257	97544	0.032151875	97544	0.019201965
97600	0.06312476	97600	0.050100422	48800	0.038410493	48808
0.034422978	97552	0.050121194	97552	0.039510665
97600	0.096265409	97600	0.037068592	48800	0.054024062	48812
0.044417477	97560	0.136344286	97560	0.004992586
97600	0.161714226	97600	0.142847711	48800	0.064971668	48816
0.048304257	97568	0.110990324	97568	0.037240854
97600	0.134241608	97600	0.183680183	48800	0.070699753	48820
0.031656102	97576	0.059382084	97576	0.02608186
97600	0.02459697	97600	0.168575061	48800	0.06455688	48824
0.063049629	97584	0.214315485	97584	0.023936191
97600	0.042762742	97600	0.133829555	48800	0.057574183	48828
0.092365735	97592	0.207890553	97592	0.018681187
97600	0.038141974	97600	0.065709457	48800	0.04213025	48832
0.087816712	97600	0.074945965	97600	0.019767145
97600	0.112306334	97600	0.01184253	48800	0.06012883	48836
0.07735267	97608	0.042116935	97608	0.037202728
97600	0.136374438	97600	0.018225943	48800	0.078081488	48840
0.043127628	97616	0.09448643	97616	0.049185823
97600	0.094466319	97600	0.044340468	48800	0.048348404	48844
0.011325829	97624	0.13056121	97624	0.040901945
97600	0.081106613	97600	0.053766158	48800	0.036211342	48848
0.019514229	97632	0.109949906	97632	0.015951951
97600	0.10119626	97600	0.017529437	48900	0.055205834	48852
0.032940276	97640	0.14524073	97640	0.019220828
97600	0.12525382	97600	0.026801023	48900	0.019226443	48856
0.088566056	97648	0.14737359	97648	0.052460335
97700	0.149290921	97700	0.024099834	48900	0.043010674	48860
0.095892552	97656	0.067664463	97656	0.061735955
97700	0.093460803	97700	0.096648968	48900	0.089737958	48864
0.061773142	97664	0.030882544	97664	0.04755428
97700	0.021852826	97700	0.169020234	48900	0.079654165	48868
0.075700584	97672	0.10846019	97672	0.051655916
97700	0.076282086	97700	0.159882039	48900	0.010994429	48872

0.09506302	97680	0.159803153	97680	0.0579429		
97700	0.093520663	97700	0.106452033	48900	0.058981827	48876
0.085849293	97688	0.108865097	97688	0.028291415		
97700	0.064327483	97700	0.096555901	48900	0.054386419	48880
0.052711592	97696	0.047624879	97696	0.00564594		
97700	0.074434298	97700	0.079958219	48900	0.045201039	48884
0.068243084	97704	0.06325289	97704	0.026722511		
97700	0.046738845	97700	0.054532149	48900	0.041738713	48888
0.097050623	97712	0.083645748	97712	0.030033902		
97700	0.084952291	97700	0.035154677	48900	0.038268936	48892
0.117855139	97720	0.10699618	97720	0.024088553		
97700	0.120781013	97700	0.092459559	48900	0.103457795	48896
0.118594471	97728	0.05768596	97728	0.068853595		
97700	0.07803741	97700	0.089974186	48900	0.1104451	48900
0.094774463	97736	0.049158567	97736	0.064811982		
97700	0.001489029	97700	0.039060433	48900	0.086539898	48904
0.122111072	97744	0.081304628	97744	0.062294443		
97800	0.070854723	97800	0.019880934	48900	0.060899834	48908
0.147027968	97752	0.099274417	97752	0.067481153		
97800	0.041815769	97800	0.062616971	48900	0.057108355	48912
0.106463762	97760	0.073469957	97760	0.054731125		
97800	0.022018563	97800	0.061790168	48900	0.077489865	48916
0.057168625	97768	0.034662255	97768	0.041375501		
97800	0.029630888	97800	0.060585542	48900	0.073622774	48920
0.028463035	97776	0.047784732	97776	0.025462035		
97800	0.069840709	97800	0.075157688	48900	0.03519675	48924
0.046404948	97784	0.0356618	97784	0.018294266		
97800	0.126477623	97800	0.073198804	48900	0.027769458	48928
0.060418592	97792	0.026216774	97792	0.021517935		
97800	0.18621693	97800	0.050485698	48900	0.057856341	48932
0.044525124	97800	0.020714626	97800	0.017839115		
97800	0.18100247	97800	0.056435471	48900	0.062175714	48936
0.040327104	97808	0.017569315	97808	0.028059991		
97800	0.109787754	97800	0.129519161	48900	0.05900368	48940
0.03202635	97816	0.050256407	97816	0.058280064		
97800	0.047553927	97800	0.190874445	48900	0.056411231	48944
0.04458577	97824	0.083889958	97824	0.082805236		
97800	0.098814286	97800	0.18144722	48900	0.030418858	48948
0.058154456	97832	0.090470487	97832	0.036637674		
97800	0.081361512	97800	0.127665044	49000	0.03733511	48952
0.051004579	97840	0.079326623	97840	0.078369158		
97800	0.023753935	97800	0.070859242	49000	0.059044938	48956
0.078404039	97848	0.051392817	97848	0.079296711		
97900	0.105310341	97900	0.060690389	49000	0.048181842	48960
0.094382049	97856	0.038708458	97856	0.016530996		
97900	0.145649654	97900	0.052190182	49000	0.029830375	48964
0.071289345	97864	0.042710377	97864	0.039711922		
97900	0.108809829	97900	0.01807329	49000	0.060430182	48968
0.093329225	97872	0.031556516	97872	0.053680036		
97900	0.101085112	97900	0.013113629	49000	0.045378743	48972
0.095240073	97880	0.024145886	97880	0.016340535		
97900	0.073881274	97900	0.061115919	49000	0.0087573	48976
0.095422693	97888	0.03357367	97888	0.041937004		
97900	0.049169423	97900	0.106641513	49000	0.040789109	48980
0.144175035	97896	0.085630323	97896	0.049636921		
97900	0.035306759	97900	0.094823292	49000	0.054208322	48984
0.163097328	97904	0.127686013	97904	0.023261478		
97900	0.098195422	97900	0.090990478	49000	0.043584223	48988
0.14066344	97912	0.096960481	97912	0.047347949		
97900	0.117691569	97900	0.106573971	49000	0.012763026	48992
0.115623589	97920	0.094136631	97920	0.057521775		
97900	0.107165601	97900	0.046954508	49000	0.050591683	48996
0.123134145	97928	0.098313416	97928	0.024660421		
97900	0.092232338	97900	0.056430286	49000	0.06239309	49000
0.095118914	97936	0.069873851	97936	0.027444447		
97900	0.066287517	97900	0.110094617	49000	0.076079203	49004
0.06430439	97944	0.103065402	97944	0.038553728		
98000	0.022005414	98000	0.089618603	49000	0.080464837	49008

0.11249516 97952 0.161456831 97952 0.02305471
98000 0.069334383 98000 0.085167041 49000 0.066785382 49012
0.133102265 97960 0.155903981 97960 0.025032299
98000 0.031705062 98000 0.076272794 49000 0.051542396 49016
0.076208991 97968 0.12819795 97968 0.047404032
98000 0.098628159 98000 0.058192003 49000 0.046945836 49020
0.059763257 97976 0.094759249 97976 0.027510243
98000 0.150468841 98000 0.053559783 49000 0.057834717 49024
0.112500435 97984 0.111122965 97984 0.018007953
98000 0.134711241 98000 0.06999504 49000 0.065008382 49028
0.081851009 97992 0.118461161 97992 0.017029805
98000 0.108790548 98000 0.064672815 49000 0.059981245 49032
0.013357967 98000 0.105329578 98000 0.039351657
98000 0.017674964 98000 0.049079954 49000 0.037606413 49036
0.051283816 98008 0.114626128 98008 0.02912932
98000 0.126665531 98000 0.03614759 49000 0.030365662 49040
0.078244157 98016 0.147872895 98016 0.010658859
98000 0.168362458 98000 0.031250245 49000 0.028050836 49044
0.103281825 98024 0.180258386 98024 0.044271172
98000 0.132305271 98000 0.055789067 49000 0.024538967 49048
0.106175699 98032 0.13868908 98032 0.037767681
98000 0.095672665 98000 0.049452126 49100 0.052754418 49052
0.088607179 98040 0.049696238 98040 0.009879414
98000 0.104011982 98000 0.065373468 49100 0.046138994 49056
0.089356567 98048 0.129342385 98048 0.004676127
98100 0.068637251 98100 0.062585255 49100 0.030174781 49060
0.074654548 98056 0.077779987 98056 0.012398829
98100 0.041083877 98100 0.071041999 49100 0.046154491 49064
0.097708675 98064 0.069312409 98064 0.012770991
98100 0.026265119 98100 0.108998545 49100 0.067994515 49068
0.150020628 98072 0.097967401 98072 0.041522548
98100 0.102842518 98100 0.100660094 49100 0.075267155 49072
0.133217808 98080 0.066789726 98080 0.056857203
98100 0.149930929 98100 0.043530785 49100 0.054526408 49076
0.088101166 98088 0.10189067 98088 0.062082334
98100 0.128443411 98100 0.060886127 49100 0.058535596 49080
0.123667487 98096 0.06604322 98096 0.062495165
98100 0.070754824 98100 0.051183852 49100 0.019739297 49084
0.161670221 98104 0.113661386 98104 0.053940985
98100 0.0308989 98100 0.041426669 49100 0.030653304 49088
0.159969364 98112 0.104207618 98112 0.063590618
98100 0.112405847 98100 0.044123492 49100 0.055808683 49092
0.149930551 98120 0.043099801 98120 0.079387733
98100 0.163563251 98100 0.092519549 49100 0.0449769 49096
0.156647904 98128 0.141651414 98128 0.092072769
98100 0.120656194 98100 0.09201029 49100 0.005677555 49100
0.16736766 98136 0.098709978 98136 0.095121424
98100 0.022642227 98100 0.04032148 49100 0.036548259 49104
0.138963093 98144 0.038359707 98144 0.087644097
98200 0.05785696 98200 0.039612995 49100 0.065045009 49108
0.090927722 98152 0.111923109 98152 0.06316667
98200 0.097946344 98200 0.090141926 49100 0.062542349 49112
0.105379033 98160 0.135522816 98160 0.060112016
98200 0.053444281 98200 0.163172198 49100 0.025492858 49116
0.117404539 98168 0.108289423 98168 0.081472215
98200 0.066276545 98200 0.196897396 49100 0.048359656 49120
0.101393816 98176 0.074409029 98176 0.069425871
98200 0.148218925 98200 0.20386056 49100 0.05204467 49124
0.091164009 98184 0.11358755 98184 0.021017684
98200 0.179728784 98200 0.154688765 49100 0.037860027 49128
0.066105888 98192 0.074766962 98192 0.039326507
98200 0.17813324 98200 0.080474914 49100 0.041946911 49132
0.062551218 98200 0.041695177 98200 0.072546674
98200 0.121517267 98200 0.020682392 49100 0.040190094 49136
0.083189676 98208 0.080254868 98208 0.066913563
98200 0.022551985 98200 0.018572533 49100 0.039253995 49140
0.055000543 98216 0.048693943 98216 0.044868662
98200 0.042684042 98200 0.016739532 49100 0.021103724 49144

0.045479825 98224 0.045728539 98224 0.043688728
98200 0.040994124 98200 0.071740848 49100 0.041356459 49148
0.067119632 98232 0.076844597 98232 0.049899074
98200 0.073098381 98200 0.051299368 49200 0.03904189 49152
0.048407299 98240 0.061434679 98240 0.04542846
98200 0.12495472 98200 0.084528576 49200 0.046601799 49156
0.090401023 98248 0.1415351 98248 0.038331498
98300 0.124887709 98300 0.144124119 49200 0.120196077 49160
0.105269493 98256 0.23976904 98256 0.02349671
98300 0.137907133 98300 0.140541524 49200 0.127312756 49164
0.087093133 98264 0.216455199 98264 0.018484758
98300 0.155488524 98300 0.119911056 49200 0.081487211 49168
0.076147706 98272 0.145063619 98272 0.058406484
98300 0.142937948 98300 0.124958722 49200 0.05198042 49172
0.072407915 98280 0.095372488 98280 0.042691758
98300 0.125132298 98300 0.097737342 49200 0.012821688 49176
0.06794509 98288 0.071542898 98288 0.023015309
98300 0.089824818 98300 0.089719652 49200 0.020432384 49180
0.074050804 98296 0.081609251 98296 0.073020812
98300 0.112488197 98300 0.092200033 49200 0.035218807 49184
0.08084008 98304 0.043933243 98304 0.060007198
98300 0.129183841 98300 0.115878538 49200 0.042330015 49188
0.084498752 98312 0.08222273 98312 0.020546922
98300 0.111354464 98300 0.135098439 49200 0.063879656 49192
0.102028549 98320 0.137954878 98320 0.086226268
98300 0.040166775 98300 0.109621564 49200 0.094262789 49196
0.113914146 98328 0.118985816 98328 0.104368468
98300 0.065197099 98300 0.044819874 49200 0.08703475 49200
0.077479643 98336 0.091453694 98336 0.07783671
98300 0.083505984 98300 0.131196518 49200 0.054936947 49204
0.015659061 98344 0.131565773 98344 0.025581812
98400 0.077348268 98400 0.146149178 49200 0.014313247 49208
0.038335813 98352 0.161584336 98352 0.033865192
98400 0.143521785 98400 0.114077448 49200 0.04412065 49212
0.079351245 98360 0.121680329 98360 0.052789939
98400 0.173796013 98400 0.122147249 49200 0.052683841 49216
0.134489078 98368 0.047364523 98368 0.050685441
98400 0.123622755 98400 0.074821634 49200 0.054960518 49220
0.166192331 98376 0.057176017 98376 0.065264714
98400 0.086322754 98400 0.00889147 49200 0.045103352 49224
0.143603495 98384 0.104983905 98384 0.083406776
98400 0.111646259 98400 0.008095059 49200 0.033652388 49228
0.131618014 98392 0.057388552 98392 0.078180936
98400 0.097099663 98400 0.037372342 49200 0.015815014 49232
0.125724851 98400 0.05924299 98400 0.050732255
98400 0.054517735 98400 0.078566591 49200 0.015624406 49236
0.092133072 98408 0.048771635 98408 0.018288582
98400 0.050719704 98400 0.050192706 49200 0.051886527 49240
0.082704013 98416 0.014399057 98416 0.041245585
98400 0.087620487 98400 0.043353586 49200 0.054898817 49244
0.104169689 98424 0.044437798 98424 0.023698514
98400 0.080306112 98400 0.064865715 49200 0.008021853 49248
0.086800188 98432 0.072309056 98432 0.024059969
98400 0.134189978 98400 0.130801113 49300 0.047226436 49252
0.066128225 98440 0.099695615 98440 0.054364868
98400 0.153059241 98400 0.121700738 49300 0.059968519 49256
0.068393143 98448 0.048661252 98448 0.060509392
98500 0.111151756 98500 0.053642645 49300 0.036852747 49260
0.07981708 98456 0.033241391 98456 0.053086744
98500 0.053173662 98500 0.040632403 49300 0.010547374 49264
0.088198722 98464 0.06768469 98464 0.080041617
98500 0.019107447 98500 0.090220143 49300 0.041153136 49268
0.087615532 98472 0.098080025 98472 0.076251236
98500 0.035573365 98500 0.095978379 49300 0.068541573 49272
0.065288972 98480 0.074521471 98480 0.034281096
98500 0.033579716 98500 0.095967451 49300 0.048520356 49276
0.032208987 98488 0.074509058 98488 0.007299775
98500 0.015581634 98500 0.130223096 49300 0.035898989 49280

0.029759083 98496 0.058313402 98496 0.040905441
98500 0.065999178 98500 0.144235834 49300 0.030271058 49284
0.0400553 98504 0.041869538 98504 0.058213966
98500 0.096984622 98500 0.097708318 49300 0.041352068 49288
0.063477113 98512 0.055376921 98512 0.061266961
98500 0.085785003 98500 0.036846741 49300 0.02541976 49292
0.121152181 98520 0.087011489 98520 0.055927958
98500 0.08967561 98500 0.078138786 49300 0.019279158 49296
0.132805479 98528 0.116508076 98528 0.036336707
98500 0.095561823 98500 0.121767487 49300 0.033932636 49300
0.055837441 98536 0.071545459 98536 0.011060533
98500 0.070384689 98500 0.102197191 49300 0.020771226 49304
0.018613591 98544 0.068083049 98544 0.029857698
98600 0.085225001 98600 0.00361376 49300 0.02556857 49308
0.02731886 98552 0.071301132 98552 0.028870585
98600 0.120232209 98600 0.088209265 49300 0.018761995 49312
0.037945716 98560 0.09670436 98560 0.036033889
98600 0.117054864 98600 0.132976988 49300 0.042241307 49316
0.052898893 98568 0.103334569 98568 0.046219971
98600 0.089460096 98600 0.118761753 49300 0.081985163 49320
0.074606876 98576 0.101264857 98576 0.016898348
98600 0.054952965 98600 0.052518124 49300 0.085170257 49324
0.095899166 98584 0.086841341 98584 0.055321849
98600 0.037077476 98600 0.063201849 49300 0.032119668 49328
0.069990689 98592 0.084975698 98592 0.094811258
98600 0.136706033 98600 0.023425071 49300 0.045689489 49332
0.093006609 98600 0.120749777 98600 0.055028533
98600 0.110299952 98600 0.057371475 49300 0.07364191 49336
0.113928611 98608 0.104579784 98608 0.031452466
98600 0.021395526 98600 0.070027963 49300 0.059079172 49340
0.085033185 98616 0.069901667 98616 0.033089087
98600 0.033442779 98600 0.034888013 49300 0.05927675 49344
0.075650642 98624 0.0283349 98624 0.042157488
98600 0.044383382 98600 0.044146709 49300 0.076065793 49348
0.068233145 98632 0.110615336 98632 0.060891427
98600 0.069024936 98600 0.056341527 49400 0.071513132 49352
0.047108268 98640 0.155017988 98640 0.071154282
98600 0.108707434 98600 0.076549637 49400 0.043195443 49356
0.041343959 98648 0.147590458 98648 0.096861302
98700 0.10617543 98700 0.142168515 49400 0.017472505 49360
0.037681195 98656 0.084446947 98656 0.090469112
98700 0.045064629 98700 0.10777243 49400 0.049858369 49364
0.053472548 98664 0.045026824 98664 0.050269227
98700 0.040347492 98700 0.051439292 49400 0.03548697 49368
0.091662128 98672 0.064097003 98672 0.034983106
98700 0.101983918 98700 0.089233246 49400 0.016667967 49372
0.099723155 98680 0.078387893 98680 0.033057757
98700 0.089262816 98700 0.15427341 49400 0.031368876 49376
0.101890415 98688 0.060088467 98688 0.020097024
98700 0.067543639 98700 0.044147197 49400 0.040883941 49380
0.058929334 98696 0.053082695 98696 0.018141071
98700 0.087793036 98700 0.086227068 49400 0.045337169 49384
0.025527736 98704 0.121868143 98704 0.018678547
98700 0.014380482 98700 0.092499919 49400 0.061627485 49388
0.030322284 98712 0.138726319 98712 0.01784433
98700 0.089231777 98700 0.080809135 49400 0.035161094 49392
0.071831157 98720 0.067863279 98720 0.008231928
98700 0.072200834 98700 0.098551784 49400 0.031639953 49396
0.109003617 98728 0.015495578 98728 0.024198111
98700 0.062859072 98700 0.041489748 49400 0.046077617 49400
0.073404059 98736 0.02595027 98736 0.023844556
98700 0.087294218 98700 0.033057131 49400 0.040930714 49404
0.070135859 98744 0.061936808 98744 0.028212384
98800 0.123342092 98800 0.009172068 49400 0.064622043 49408
0.102832833 98752 0.057412875 98752 0.037938269
98800 0.140464806 98800 0.058252826 49400 0.064234104 49412
0.083993909 98760 0.102759994 98760 0.011229849
98800 0.119336582 98800 0.044145338 49400 0.025950294 49416

0.087145541		98768	0.126462488		98768	0.029985977			
98800	0.107013075		98800	0.048561225		49400	0.019783358		49420
0.145265149		98776	0.094667914		98776	0.024464467			
98800	0.13438001		98800	0.058892205		49400	0.028269735		49424
0.139388183		98784	0.067701411		98784	0.020858204			
98800	0.125756036		98800	0.060410202		49400	0.019153891		49428
0.08207489		98792	0.086129694		98792	0.03486537			
98800	0.067708483		98800	0.159684176		49400	0.020457046		49432
0.107093743		98800	0.121314195		98800	0.01218311			
98800	0.035835798		98800	0.221304319		49400	0.029474037		49436
0.114239781		98808	0.131766559		98808	0.013787842			
98800	0.057693742		98800	0.096266405		49400	0.023480512		49440
0.11170729		98816	0.1118937		98816	0.034708934			
98800	0.017960445		98800	0.098808516		49400	0.025433272		49444
0.066128931		98824	0.070105365		98824	0.063208368			
98800	0.020735186		98800	0.089487607		49400	0.053337582		49448
0.055426899		98832	0.046676385		98832	0.058444264			
98800	0.015299924		98800	0.125796694		49500	0.064409171		49452
0.131759996		98840	0.071842245		98840	0.013179385			
98800	0.052739768		98800	0.179390453		49500	0.043021733		49456
0.12773987		98848	0.102488957		98848	0.030577299			
98900	0.059841292		98900	0.109609544		49500	0.044513716		49460
0.086795342		98856	0.129174921		98856	0.038682618			
98900	0.063434971		98900	0.031369935		49500	0.058033726		49464
0.059664049		98864	0.158876239		98864	0.053662236			
98900	0.064639717		98900	0.081818595		49500	0.046336234		49468
0.040840787		98872	0.140020201		98872	0.046116405			
98900	0.042346954		98900	0.129493666		49500	0.070609021		49472
0.019646355		98880	0.108696338		98880	0.026926449			
98900	0.05160235		98900	0.106307532		49500	0.064184555		49476
0.043093365		98888	0.086709908		98888	0.043245967			
98900	0.083207648		98900	0.02211947		49500	0.054565659		49480
0.083881823		98896	0.04394447		98896	0.034243811			
98900	0.091703449		98900	0.022615921		49500	0.041636617		49484
0.091071255		98904	0.03332935		98904	0.037300637			
98900	0.137822731		98900	0.073232201		49500	0.045538083		49488
0.056575525		98912	0.078375924		98912	0.00829773			
98900	0.102520666		98900	0.16212865		49500	0.04753207		49492
0.017274488		98920	0.092207374		98920	0.020373624			
98900	0.058104008		98900	0.130927176		49500	0.02339871		49496
0.02001837		98928	0.042191241		98928	0.031210453			
98900	0.092982111		98900	0.029779627		49500	0.03863977		49500
0.027566817		98936	0.034870944		98936	0.056439523			
98900	0.038624927		98900	0.172666056		49500	0.046086712		49504
0.049116268		98944	0.046333465		98944	0.038387469			
99000	0.04133895		99000	0.184965568		49500	0.048327336		49508
0.064485117		98952	0.068534813		98952	0.040784005			
99000	0.046953959		99000	0.098214354		49500	0.040851526		49512
0.068742818		98960	0.101588674		98960	0.052616815			
99000	0.172781801		99000	0.048107871		49500	0.034526343		49516
0.088032823		98968	0.047593399		98968	0.024472269			
99000	0.237908781		99000	0.03412236		49500	0.040540021		49520
0.085224849		98976	0.041866373		98976	0.043601245			
99000	0.163410325		99000	0.036679128		49500	0.060927407		49524
0.046002595		98984	0.055210334		98984	0.057334713			
99000	0.032823784		99000	0.035211586		49500	0.080710219		49528
0.011771403		98992	0.059146994		98992	0.060058756			
99000	0.023883635		99000	0.021941258		49500	0.071152092		49532
0.053703589		99000	0.023171766		99000	0.046688601			
99000	0.03903504		99000	0.035661455		49500	0.057551573		49536
0.087178174		99008	0.092564616		99008	0.013794666			
99000	0.091394788		99000	0.091013506		49500	0.05958106		49540
0.075250609		99016	0.095385352		99016	0.038257738			
99000	0.074063646		99000	0.162190133		49500	0.044979748		49544
0.040367755		99024	0.120206758		99024	0.047282225			
99000	0.063417239		99000	0.145627958		49500	0.022015234		49548
0.042403663		99032	0.211128864		99032	0.049400907			
99000	0.063496525		99000	0.067935893		49600	0.013155886		49552

0.062434905 99040 0.143473313 99040 0.028334132
99000 0.024170447 99000 0.011916746 49600 0.032791184 49556
0.053181993 99048 0.022247579 99048 0.021539367
99100 0.037200705 99100 0.043911558 49600 0.059740662 49560
0.033433356 99056 0.071678231 99056 0.028164153
99100 0.063608997 99100 0.105815176 49600 0.068942427 49564
0.028920018 99064 0.151901316 99064 0.009419129
99100 0.068163798 99100 0.119199591 49600 0.035267236 49568
0.043267621 99072 0.190194013 99072 0.033856111
99100 0.0460579 99100 0.07579504 49600 0.011658822 49572
0.05830036 99080 0.102286453 99080 0.069776725
99100 0.081896993 99100 0.061840947 49600 0.018876162 49576
0.058564805 99088 0.027767927 99088 0.083219602
99100 0.126297208 99100 0.057095873 49600 0.051373703 49580
0.059254257 99096 0.054846729 99096 0.06116047
99100 0.026191276 99100 0.067267349 49600 0.052395833 49584
0.042257332 99104 0.078245357 99104 0.033571185
99100 0.098270117 99100 0.075218122 49600 0.047066566 49588
0.017510878 99112 0.062748899 99112 0.031947995
99100 0.106951535 99100 0.072816751 49600 0.076012068 49592
0.063412415 99120 0.021674465 99120 0.039103743
99100 0.065345521 99100 0.072737566 49600 0.091091322 49596
0.041483821 99128 0.035570884 99128 0.014551288
99100 0.066959656 99100 0.038733291 49600 0.086041131 49600
0.068722431 99136 0.053940083 99136 0.020951955
99100 0.148951673 99100 0.089039459 49600 0.072921546 49604
0.098483797 99144 0.05034616 99144 0.020196521
99200 0.221596289 99200 0.07300669 49600 0.057712263 49608
0.097271513 99152 0.01514358 99152 0.016204991
99200 0.210740109 99200 0.08190253 49600 0.038961513 49612
0.057631547 99160 0.039427003 99160 0.027583072
99200 0.12659069 99200 0.0779111 49600 0.018384442 49616
0.002612836 99168 0.071710696 99168 0.01868722
99200 0.07833659 99200 0.105887863 49600 0.034454955 49620
0.020246811 99176 0.080602629 99176 0.005545636
99200 0.095001655 99200 0.083650877 49600 0.023650218 49624
0.048464048 99184 0.123276055 99184 0.012091197
99200 0.091164446 99200 0.070567578 49600 0.036009922 49628
0.036245627 99192 0.129020031 99192 0.021315951
99200 0.100909201 99200 0.149606145 49600 0.023866436 49632
0.025602338 99200 0.120259283 99200 0.011005063
99200 0.12362127 99200 0.073039424 49600 0.038345454 49636
0.031042968 99208 0.138821008 99208 0.009232529
99200 0.126797488 99200 0.131406633 49600 0.007508964 49640
0.028562745 99216 0.15389212 99216 0.02871845
99200 0.064726897 99200 0.135327835 49600 0.052714939 49644
0.087710781 99224 0.146584454 99224 0.077632169
99200 0.021422489 99200 0.0911588 49600 0.069082722 49648
0.080238599 99232 0.111201873 99232 0.092534785
99200 0.094280993 99200 0.057247988 49700 0.036515645 49652
0.064685482 99240 0.070685062 99240 0.057810914
99200 0.127135834 99200 0.018291317 49700 0.014634108 49656
0.073343545 99248 0.119814358 99248 0.019065768
99300 0.040793282 99300 0.067376379 49700 0.028032324 49660
0.029212948 99256 0.091237453 99256 0.001060047
99300 0.095522999 99300 0.091951995 49700 0.024790334 49664
0.032739823 99264 0.019658035 99264 0.004898098
99300 0.129819556 99300 0.078113983 49700 0.002844396 49668
0.022497548 99272 0.085432417 99272 0.006521087
99300 0.13573248 99300 0.044031363 49700 0.046982816 49672
0.025000769 99280 0.076337419 99280 0.006547065
99300 0.108847409 99300 0.104847917 49700 0.049406983 49676
0.0333602 99288 0.071012037 99288 0.014833766
99300 0.079109181 99300 0.114346512 49700 0.015401327 49680
0.026503938 99296 0.107062981 99296 0.008902475
99300 0.07568724 99300 0.138016199 49700 0.041783289 49684
0.008183826 99304 0.121357683 99304 0.028368653
99300 0.044580196 99300 0.093978459 49700 0.029738729 49688

0.020790836 99312 0.058306574 99312 0.047399215
99300 0.015524094 99300 0.13501705 49700 0.041569172 49692
0.03020403 99320 0.088869172 99320 0.049641916
99300 0.06328748 99300 0.128900938 49700 0.045292312 49696
0.025087631 99328 0.086177104 99328 0.058249625
99300 0.103223945 99300 0.081952683 49700 0.033369473 49700
0.07216628 99336 0.066171422 99336 0.069174457
99300 0.166926199 99300 0.030669195 49700 0.009974311 49704
0.098916746 99344 0.065127482 99344 0.060027254
99400 0.203525546 99400 0.085758933 49700 0.02012987 49708
0.093687864 99352 0.094579213 99352 0.077950797
99400 0.144626174 99400 0.040687392 49700 0.027040072 49712
0.066266322 99360 0.164524245 99360 0.096012278
99400 0.051731196 99400 0.081141596 49700 0.034628542 49716
0.092218063 99368 0.148710504 99368 0.100040481
99400 0.019415593 99400 0.088991779 49700 0.033016338 49720
0.112935428 99376 0.129390144 99376 0.0861054
99400 0.064923937 99400 0.033823926 49700 0.027264292 49724
0.090793248 99384 0.127123916 99384 0.047280413
99400 0.092984497 99400 0.114820476 49700 0.029392199 49728
0.049226874 99392 0.114752547 99392 0.043590218
99400 0.09055026 99400 0.162445984 49700 0.040338928 49732
0.039204588 99400 0.117531905 99400 0.053613436
99400 0.111196139 99400 0.140391654 49700 0.069861148 49736
0.050385857 99408 0.120510405 99408 0.03364429
99400 0.123386824 99400 0.052220195 49700 0.09200073 49740
0.030252239 99416 0.122060592 99416 0.046882593
99400 0.083552171 99400 0.037604645 49700 0.077631521 49744
0.03196043 99424 0.053210049 99424 0.024622381
99400 0.020751379 99400 0.123395133 49700 0.03703625 49748
0.04731003 99432 0.096771073 99432 0.039365095
99400 0.036121594 99400 0.132343455 49800 0.02534349 49752
0.033579519 99440 0.160793949 99440 0.057309328
99400 0.020983816 99400 0.082614788 49800 0.070128881 49756
0.031874937 99448 0.056997425 99448 0.074748394
99500 0.130363493 99500 0.043010492 49800 0.096127791 49760
0.049429127 99456 0.111081638 99456 0.070683695
99500 0.238103501 99500 0.099935343 49800 0.093917042 49764
0.054583303 99464 0.090911533 99464 0.056742221
99500 0.226568751 99500 0.098308738 49800 0.07321524 49768
0.04467295 99472 0.039060604 99472 0.057537742
99500 0.134963543 99500 0.085160675 49800 0.0516615 49772
0.040049345 99480 0.020020752 99480 0.061030492
99500 0.203802484 99500 0.088002402 49800 0.047903886 49776
0.011190878 99488 0.077140248 99488 0.051147104
99500 0.208576021 99500 0.084030013 49800 0.027651351 49780
0.048424488 99496 0.120249722 99496 0.056887966
99500 0.125062958 99500 0.094777861 49800 0.009101905 49784
0.048870967 99504 0.079415069 99504 0.083551735
99500 0.04511463 99500 0.097896082 49800 0.015067704 49788
0.043855413 99512 0.019809075 99512 0.065591368
99500 0.088639077 99500 0.104020037 49800 0.021087726 49792
0.069885704 99520 0.045342229 99520 0.034620993
99500 0.188004356 99500 0.120663819 49800 0.039650051 49796
0.095427815 99528 0.060926646 99528 0.021297083
99500 0.16710718 99500 0.088386303 49800 0.039700022 49800
0.094041679 99536 0.096338976 99536 0.017931547
99500 0.082756364 99500 0.033270437 49800 0.011472873 49804
0.059609614 99544 0.120866112 99544 0.040847102
99600 0.038862105 99600 0.074886913 49800 0.023520155 49808
0.02609048 99552 0.08054058 99552 0.032061307
99600 0.097984688 99600 0.109960711 49800 0.027402941 49812
0.017938482 99560 0.094144751 99560 0.023304758
99600 0.147501385 99600 0.130097236 49800 0.030016727 49816
0.071326322 99568 0.121867764 99568 0.050312741
99600 0.159596879 99600 0.098297976 49800 0.027127817 49820
0.091851427 99576 0.054118413 99576 0.062178959
99600 0.155518151 99600 0.041615844 49800 0.012408363 49824

0.053679385	99584	0.050049246	99584	0.050914448
99600	0.133674083	99600	0.09797846	49800	0.021215508	49828
0.02319164	99592	0.053130036	99592	0.037172915
99600	0.14292814	99600	0.128277243	49800	0.068588619	49832
0.028121393	99600	0.133182795	99600	0.021498177
99600	0.190370701	99600	0.086338463	49800	0.100124809	49836
0.009699118	99608	0.236012828	99608	0.013949191
99600	0.175994865	99600	0.092583803	49800	0.065230102	49840
0.029208286	99616	0.212401311	99616	0.05358646
99600	0.146676611	99600	0.090218899	49800	0.050690105	49844
0.052500469	99624	0.134402858	99624	0.069385314
99600	0.120045479	99600	0.082983257	49800	0.061366489	49848
0.025351335	99632	0.134121481	99632	0.046961977
99600	0.066481189	99600	0.112116999	49900	0.028762122	49852
0.045859557	99640	0.071069735	99640	0.023599912
99600	0.12224984	99600	0.120553348	49900	0.02135646	49856
0.077099023	99648	0.032970704	99648	0.028975343
99700	0.170628161	99700	0.104945459	49900	0.062819883	49860
0.083345432	99656	0.066599794	99656	0.036251342
99700	0.087013323	99700	0.105994412	49900	0.066900779	49864
0.059167513	99664	0.068443456	99664	0.025893289
99700	0.046557751	99700	0.078284815	49900	0.04940651	49868
0.058264603	99672	0.090826499	99672	0.023440576
99700	0.019586916	99700	0.010605741	49900	0.034655241	49872
0.044977853	99680	0.103527753	99680	0.034759491
99700	0.120311161	99700	0.07223546	49900	0.041764266	49876
0.046901434	99688	0.070571085	99688	0.040303527
99700	0.15026379	99700	0.080102262	49900	0.06482195	49880
0.068838635	99696	0.093041192	99696	0.037406102
99700	0.06909512	99700	0.030845302	49900	0.068734946	49884
0.066714107	99704	0.057606296	99704	0.024308769
99700	0.060098857	99700	0.061118059	49900	0.074144264	49888
0.031538617	99712	0.029391871	99712	0.03813162
99700	0.117388045	99700	0.094980504	49900	0.070796123	49892
0.04536378	99720	0.020684083	99720	0.044043689
99700	0.109462133	99700	0.082277853	49900	0.035103018	49896
0.073274801	99728	0.048611346	99728	0.034088349
99700	0.052625248	99700	0.067155052	49900	0.007682111	49900
0.102967599	99736	0.052997802	99736	0.023117662
99700	0.023544462	99700	0.072361727	49900	0.004815087	49904
0.093137402	99744	0.052387091	99744	0.064567699
99800	0.062541054	99800	0.078084588	49900	0.024890813	49908
0.056536912	99752	0.099554985	99752	0.092159149
99800	0.038433631	99800	0.044786444	49900	0.041955645	49912
0.027138471	99760	0.070457973	99760	0.079382357
99800	0.026897276	99800	0.077065168	49900	0.061663261	49916
0.017898301	99768	0.024626239	99768	0.077251832
99800	0.029301158	99800	0.052514628	49900	0.067224704	49920
0.017763281	99776	0.036162834	99776	0.084606705
99800	0.043273682	99800	0.078082187	49900	0.038239894	49924
0.061479484	99784	0.065431064	99784	0.051793784
99800	0.050047842	99800	0.067110632	49900	0.016527258	49928
0.076862467	99792	0.099026162	99792	0.013655892
99800	0.0452848	99800	0.025010588	49900	0.048971411	49932
0.082498198	99800	0.083944622	99800	0.030591978
99800	0.069359121	99800	0.009940685	49900	0.049140235	49936
0.086170265	99808	0.040399551	99808	0.02488893
99800	0.082859406	99800	0.044347104	49900	0.022232218	49940
0.061044433	99816	0.036217101	99816	0.046364268
99800	0.04054582	99800	0.05497137	49900	0.035009271	49944
0.054320961	99824	0.078248886	99824	0.037746773
99800	0.04572306	99800	0.101450285	49900	0.041715823	49948
0.059227707	99832	0.092368027	99832	0.026427862
99800	0.05869153	99800	0.12023712	50000	0.010132685	49952
0.057642534	99840	0.062439314	99840	0.028661258
99800	0.060199189	99800	0.016935965	50000	0.024299499	49956
0.099123514	99848	0.077765581	99848	0.0301332
99900	0.073630217	99900	0.115277908	50000	0.017984012	49960

0.072107243 99856 0.054093642 99856 0.034058925
99900 0.061120729 99900 0.138974501 50000 0.042843691 49964
0.01273997 99864 0.094430812 99864 0.077836587
99900 0.021311857 99900 0.088032932 50000 0.039219318 49968
0.049535018 99872 0.16928275 99872 0.062114174
99900 0.100817764 99900 0.0697094 50000 0.015559101 49972
0.053449181 99880 0.163719145 99880 0.005389325
99900 0.130727858 99900 0.12348358 50000 0.030859304 49976
0.007474394 99888 0.101647398 99888 0.0118603
99900 0.156685434 99900 0.146007195 50000 0.070260714 49980
0.043682379 99896 0.058872978 99896 0.016947157
99900 0.177568873 99900 0.04719826 50000 0.068010471 49984
0.040520667 99904 0.030380659 99904 0.040262821
99900 0.105778738 99900 0.099661833 50000 0.008307153 49988
0.031248866 99912 0.030509691 99912 0.053130741
99900 0.050353781 99900 0.126224564 50000 0.077327262 49992
0.041317704 99920 0.032151951 99920 0.051945881
99900 0.122718702 99900 0.060039562 50000 0.063115862 49996
0.02808261 99928 0.120196877 99928 0.028861472
99900 0.117590913 99900 0.048994982 50000 0.010352569 50000
0.0117823 99936 0.126469168 99936 0.017675533
99900 0.101445483 99900 0.065614142 50000 0.030301429 50004
0.031357489 99944 0.081762337 99944 0.03588125
100000 0.108706197 100000 0.060722538 50000 0.054482902 50008
0.077264551 99952 0.027480717 99952 0.051074174
100000 0.153972258 100000 0.063014893 50000 0.051136423 50012
0.097058299 99960 0.03351886 99960 0.03950618
100000 0.126490296 100000 0.043760934 50000 0.064110252 50016
0.066944915 99968 0.125172024 99968 0.019847195
100000 0.074428652 100000 0.082644619 50000 0.079236837 50020
0.051875286 99976 0.143586046 99976 0.03737396
100000 0.079685466 100000 0.13746279 50000 0.064648179 50024
0.071678987 99984 0.083749699 99984 0.048271617
100000 0.131972833 100000 0.260301866 50000 0.035953428 50028
0.047522895 99992 0.210606668 99992 0.036540871
50000 0.053163061 50032 0.059132777

50000 0.06300005 50036 0.070005634

50000 0.061882696 50040 0.064490887

50000 0.062658379 50044 0.085800966

50000 0.064640968 50048 0.092687056

50100 0.059746199 50052 0.069092697

50100 0.019160587 50056 0.064246357

50100 0.021404343 50060 0.063891173

50100 0.037549373 50064 0.036245638

50100 0.012242391 50068 0.01285957

50100 0.043999098 50072 0.014651943

50100 0.077122757 50076 0.010155659

50100 0.078816833 50080 0.02961451

50100 0.056749032 50084 0.050338182

50100 0.046091675 50088 0.07168451

50100 0.041742296 50092 0.084817453

50100 0.027815078 50096 0.052507159

PowerSpectrumData

50100	0.068679328	50100	0.012128604
50100	0.035291545	50104	0.030433603
50100	0.038829428	50108	0.036315509
50100	0.015619036	50112	0.067470792
50100	0.00870531	50116	0.092254275
50100	0.01189541	50120	0.063191612
50100	0.04502364	50124	0.034108816
50100	0.093085859	50128	0.043882665
50100	0.108401364	50132	0.060528961
50100	0.078308738	50136	0.043230251
50100	0.059414477	50140	0.054865042
50100	0.058675723	50144	0.064434513
50100	0.044311171	50148	0.083409686
50200	0.024958657	50152	0.08343393
50200	0.030800064	50156	0.041428775
50200	0.042531105	50160	0.06802741
50200	0.043953183	50164	0.111668967
50200	0.04599334	50168	0.114056275
50200	0.041276864	50172	0.058216829
50200	0.020618372	50176	0.064257722
50200	0.011316278	50180	0.083129817
50200	0.060596405	50184	0.099014331
50200	0.101065532	50188	0.125042425
50200	0.072911811	50192	0.121111669
50200	0.017275037	50196	0.130237676
50200	0.047707963	50200	0.150771156
50200	0.055093169	50204	0.120962002
50200	0.075246535	50208	0.093359224
50200	0.085853615	50212	0.09521687
50200	0.116110023	50216	0.108624459
50200	0.110725188	50220	0.152323046
50200	0.059396272	50224	0.132295828
50200	0.03027767	50228	0.049354054
50200	0.031049873	50232	0.040317951

PowerSpectrumData

50200	0.022920503	50236	0.07188251
50200	0.036684909	50240	0.084299521
50200	0.078736761	50244	0.124860468
50200	0.062473686	50248	0.16895053
50300	0.024530053	50252	0.15815717
50300	0.016975149	50256	0.121397614
50300	0.026850645	50260	0.110005472
50300	0.024425053	50264	0.097029646
50300	0.040591123	50268	0.094099021
50300	0.054939075	50272	0.090418747
50300	0.019249908	50276	0.125746461
50300	0.047866204	50280	0.106263629
50300	0.044775708	50284	0.061714418
50300	0.036423546	50288	0.057794485
50300	0.08461751	50292	0.054445594
50300	0.095490352	50296	0.083616178
50300	0.061568841	50300	0.099863282
50300	0.039954834	50304	0.099634111
50300	0.07535523	50308	0.093975599
50300	0.077589706	50312	0.098223361
50300	0.03914049	50316	0.110423753
50300	0.013286427	50320	0.075216223
50300	0.047106314	50324	0.052893636
50300	0.063298605	50328	0.075943019
50300	0.053019561	50332	0.107167871
50300	0.011374067	50336	0.128966858
50300	0.036880716	50340	0.114111506
50300	0.049702834	50344	0.081957027
50300	0.042999582	50348	0.143810365
50400	0.057406349	50352	0.177957103
50400	0.081535931	50356	0.137299518
50400	0.053089287	50360	0.079897385
50400	0.045876128	50364	0.127478051
50400	0.017451537	50368	0.162251716

PowerSpectrumData

50400	0.040805815	50372	0.147350963
50400	0.057566958	50376	0.139548808
50400	0.047302845	50380	0.124242346
50400	0.033235519	50384	0.132371016
50400	0.039905572	50388	0.132098169
50400	0.066981083	50392	0.079038851
50400	0.070756112	50396	0.110359397
50400	0.046319889	50400	0.145173355
50400	0.026047583	50404	0.113758055
50400	0.040144321	50408	0.12551689
50400	0.050313953	50412	0.141128199
50400	0.028412302	50416	0.120725119
50400	0.017196764	50420	0.095995849
50400	0.043780518	50424	0.113457012
50400	0.063214386	50428	0.107222586
50400	0.031835218	50432	0.114015544
50400	0.027193009	50436	0.13369837
50400	0.077770128	50440	0.131498528
50400	0.062537743	50444	0.146459788
50400	0.004456437	50448	0.155219445
50500	0.040609572	50452	0.126452128
50500	0.056055538	50456	0.133350899
50500	0.039596936	50460	0.167427119
50500	0.018597293	50464	0.125744351
50500	0.023139253	50468	0.086087996
50500	0.042677617	50472	0.123985868
50500	0.089576635	50476	0.148539344
50500	0.072066025	50480	0.168386905
50500	0.032190779	50484	0.189982136
50500	0.042366824	50488	0.187585218
50500	0.051428273	50492	0.165608348
50500	0.023015964	50496	0.13994769
50500	0.03591333	50500	0.107769971
50500	0.013134722	50504	0.102940816

PowerSpectrumData

50500	0.073492425	50508	0.134784685
50500	0.107987835	50512	0.12855031
50500	0.080840102	50516	0.084562904
50500	0.064862776	50520	0.074183226
50500	0.090110807	50524	0.100206773
50500	0.082526094	50528	0.130469344
50500	0.043599157	50532	0.149521031
50500	0.005228108	50536	0.161412885
50500	0.027546139	50540	0.178529691
50500	0.038535516	50544	0.153725254
50500	0.033059419	50548	0.08950544
50600	0.046796307	50552	0.071374634
50600	0.070015791	50556	0.073680916
50600	0.051970197	50560	0.115635725
50600	0.057079597	50564	0.17594792
50600	0.10539878	50568	0.197125643
50600	0.103703133	50572	0.181014359
50600	0.057629612	50576	0.170711254
50600	0.053864016	50580	0.124676313
50600	0.061714345	50584	0.059208389
50600	0.017494769	50588	0.022326662
50600	0.08353249	50592	0.018994324
50600	0.093748407	50596	0.113715396
50600	0.062750507	50600	0.181719646
50600	0.03923172	50604	0.172693646
50600	0.03180141	50608	0.117400683
50600	0.029237595	50612	0.04716999
50600	0.037431535	50616	0.00746659
50600	0.054776978	50620	0.034458466
50600	0.040185143	50624	0.069729846
50600	0.025343939	50628	0.114955466
50600	0.037057202	50632	0.121342215
50600	0.05104431	50636	0.149710366
50600	0.046446865	50640	0.145006853

PowerSpectrumData

50600	0.025152234	50644	0.069627582
50600	0.02226118	50648	0.100339443
50700	0.044932764	50652	0.138863223
50700	0.01699745	50656	0.125004794
50700	0.0572684	50660	0.097245669
50700	0.080996426	50664	0.050060287
50700	0.054794571	50668	0.009427366
50700	0.019854202	50672	0.051183266
50700	0.056019246	50676	0.071634153
50700	0.035555495	50680	0.07334914
50700	0.00590642	50684	0.05574969
50700	0.02668006	50688	0.029151226
50700	0.070226037	50692	0.032290001
50700	0.069131849	50696	0.060537532
50700	0.034053643	50700	0.090683949
50700	0.013726089	50704	0.075745338
50700	0.030623192	50708	0.075903779
50700	0.030381387	50712	0.0766175
50700	0.015286045	50716	0.071146096
50700	0.025128098	50720	0.047309692
50700	0.063691252	50724	0.037638238
50700	0.072322757	50728	0.046551009
50700	0.057060013	50732	0.037949078
50700	0.035150461	50736	0.055180499
50700	0.009289623	50740	0.043351742
50700	0.024904201	50744	0.026883638
50700	0.046602509	50748	0.05580148
50800	0.011671649	50752	0.077384488
50800	0.087042994	50756	0.05621016
50800	0.10387443	50760	0.031137348
50800	0.064426437	50764	0.04413522
50800	0.058301986	50768	0.031413878
50800	0.072231189	50772	0.02161655
50800	0.046154033	50776	0.02995312

PowerSpectrumData

50800	0.012730658	50780	0.021699083
50800	0.021067723	50784	0.0229997
50800	0.040466959	50788	0.034060249
50800	0.060026552	50792	0.047156293
50800	0.043916254	50796	0.050591636
50800	0.038779923	50800	0.028454739
50800	0.020244956	50804	0.003619117
50800	0.008983719	50808	0.020159678
50800	0.031152827	50812	0.053396234
50800	0.086824948	50816	0.089579749
50800	0.103277896	50820	0.104018793
50800	0.085868691	50824	0.085903368
50800	0.080683742	50828	0.069874688
50800	0.047554058	50832	0.042843851
50800	0.017942723	50836	0.020159499
50800	0.020990772	50840	0.057208818
50800	0.023871391	50844	0.080442391
50800	0.019682144	50848	0.088422938
50900	0.029187631	50852	0.056667668
50900	0.02511387	50856	0.002703896
50900	0.044109856	50860	0.026517788
50900	0.038274484	50864	0.036898589
50900	0.019207912	50868	0.045372923
50900	0.04521132	50872	0.032687389
50900	0.079901329	50876	0.079899146
50900	0.097274591	50880	0.098573233
50900	0.054238211	50884	0.06534248
50900	0.005957761	50888	0.040191259
50900	0.010767987	50892	0.043942906
50900	0.036591311	50896	0.061072227
50900	0.038858492	50900	0.052872922
50900	0.015197072	50904	0.016780403
50900	0.019769399	50908	0.03007753
50900	0.038242542	50912	0.035942208

PowerSpectrumData

50900	0.050683815	50916	0.016819828
50900	0.027542708	50920	0.046675257
50900	0.033558947	50924	0.075965887
50900	0.013158536	50928	0.078341167
50900	0.026342994	50932	0.040417668
50900	0.025482332	50936	0.039729643
50900	0.037333353	50940	0.095650263
50900	0.015371097	50944	0.102174876
50900	0.013429747	50948	0.030427513
51000	0.026170075	50952	0.032674681
51000	0.033275363	50956	0.050246476
51000	0.029982604	50960	0.047018719
51000	0.01751769	50964	0.044706791
51000	0.026325213	50968	0.057320878
51000	0.030483034	50972	0.062108084
51000	0.057236252	50976	0.075518925
51000	0.045224242	50980	0.076369513
51000	0.035184075	50984	0.068780573
51000	0.038838487	50988	0.055335888
51000	0.024305808	50992	0.041540039
51000	0.013133636	50996	0.020176234
51000	0.005917801	51000	0.016871814
51000	0.015109653	51004	0.037119022
51000	0.029162879	51008	0.028460006
51000	0.028961891	51012	0.040198731
51000	0.019658608	51016	0.041432657
51000	0.039918446	51020	0.033675096
51000	0.0706703	51024	0.029813071
51000	0.083664745	51028	0.022515646
51000	0.075652512	51032	0.026240479
51000	0.057690602	51036	0.027951031
51000	0.051946376	51040	0.019770427
51000	0.041869538	51044	0.008688162
51000	0.024424388	51048	0.023754094

PowerSpectrumData

51100	0.023572484	51052	0.041956591
51100	0.026661595	51056	0.032385764
51100	0.011871402	51060	0.007012332
51100	0.045918252	51064	0.019758485
51100	0.061767416	51068	0.039180712
51100	0.055610926	51072	0.06183696
51100	0.038274928	51076	0.057532914
51100	0.057588339	51080	0.056984747
51100	0.056188601	51084	0.035292505
51100	0.075755692	51088	0.0227623
51100	0.081687744	51092	0.027176322
51100	0.037334656	51096	0.043363514
51100	0.024909376	51100	0.036936956
51100	0.056277688	51104	0.061099847
51100	0.058202502	51108	0.058081219
51100	0.059198981	51112	0.040512212
51100	0.046763518	51116	0.048333459
51100	0.04714762	51120	0.040378061
51100	0.070466202	51124	0.016095402
51100	0.09320792	51128	0.031449599
51100	0.080555052	51132	0.046834186
51100	0.072930605	51136	0.009333892
51100	0.090349924	51140	0.027372291
51100	0.064540109	51144	0.016891527
51100	0.032613421	51148	0.015870854
51200	0.047460235	51152	0.025843243
51200	0.032077096	51156	0.029799721
51200	0.013540695	51160	0.013990766
51200	0.045438857	51164	0.011597968
51200	0.060352017	51168	0.042188189
51200	0.049872131	51172	0.061567662
51200	0.034498673	51176	0.028925862
51200	0.027297889	51180	0.027071223
51200	0.03313299	51184	0.011905877

PowerSpectrumData

51200	0.050674611	51188	0.01870575
51200	0.059355803	51192	0.050273156
51200	0.064224805	51196	0.030986066
51200	0.057302994	51200	0.023572897
51200	0.031541007	51204	0.056517572
51200	0.015720238	51208	0.061321691
51200	0.041583331	51212	0.059963422
51200	0.067380301	51216	0.024431809
51200	0.109653069	51220	0.042894393
51200	0.08351999	51224	0.075261742
51200	0.037901573	51228	0.071407136
51200	0.017272034	51232	0.042434545
51200	0.07235822	51236	0.036404941
51200	0.06029302	51240	0.057007037
51200	0.021722932	51244	0.055417662
51200	0.085949134	51248	0.0355505
51300	0.077085817	51252	0.053991684
51300	0.030440442	51256	0.047234411
51300	0.006860264	51260	0.03433782
51300	0.005563235	51264	0.108487046
51300	0.017271757	51268	0.096533004
51300	0.017425367	51272	0.043000768
51300	0.042378506	51276	0.011993739
51300	0.043547138	51280	0.003893003
51300	0.02687117	51284	0.032668817
51300	0.035634581	51288	0.07409371
51300	0.049425242	51292	0.071723393
51300	0.066474029	51296	0.040754596
51300	0.045441877	51300	0.02893083
51300	0.037356895	51304	0.026107637
51300	0.06230484	51308	0.049897048
51300	0.045359673	51312	0.031929951
51300	0.016649468	51316	0.015774152
51300	0.010922727	51320	0.048042213

PowerSpectrumData

51300	0.041222349	51324	0.05665519
51300	0.050239214	51328	0.057814585
51300	0.031272422	51332	0.057458707
51300	0.036522026	51336	0.027519855
51300	0.017798258	51340	0.019743988
51300	0.014189275	51344	0.055570432
51300	0.050732164	51348	0.049637078
51400	0.077831865	51352	0.002618279
51400	0.073985102	51356	0.030650521
51400	0.040621861	51360	0.062011204
51400	0.009762471	51364	0.09184151
51400	0.058684753	51368	0.078696663
51400	0.096520504	51372	0.051045063
51400	0.081666476	51376	0.018003077
51400	0.018559989	51380	0.013905936
51400	0.04966266	51384	0.019777033
51400	0.033889362	51388	0.089676803
51400	0.025491867	51392	0.114783419
51400	0.014568938	51396	0.062550818
51400	0.010064834	51400	0.062101804
51400	0.02482438	51404	0.092004622
51400	0.039668295	51408	0.13769712
51400	0.039134349	51412	0.149363841
51400	0.039243714	51416	0.098587596
51400	0.020930025	51420	0.035082172
51400	0.024543273	51424	0.030172794
51400	0.041687792	51428	0.034755456
51400	0.047477068	51432	0.018117749
51400	0.027418237	51436	0.033962569
51400	0.036954039	51440	0.072848707
51400	0.058134785	51444	0.095664807
51400	0.047177204	51448	0.081999002
51500	0.027829927	51452	0.049223014
51500	0.042524796	51456	0.054082178

PowerSpectrumData

51500	0.049603499	51460	0.075799297
51500	0.047994254	51464	0.087549764
51500	0.028524877	51468	0.089570211
51500	0.009775353	51472	0.105910709
51500	0.026207496	51476	0.083712039
51500	0.067619702	51480	0.057132664
51500	0.091156959	51484	0.100204379
51500	0.040444924	51488	0.076416109
51500	0.042076394	51492	0.033298009
51500	0.05732775	51496	0.064227548
51500	0.012858983	51500	0.084014413
51500	0.023220644	51504	0.058105088
51500	0.029739795	51508	0.063049942
51500	0.013196524	51512	0.125190156
51500	0.033366603	51516	0.13107076
51500	0.05987148	51520	0.088986781
51500	0.048369468	51524	0.056157191
51500	0.015363559	51528	0.062240208
51500	0.016931015	51532	0.072277609
51500	0.022117391	51536	0.061104118
51500	0.025346182	51540	0.047606794
51500	0.027669294	51544	0.096842712
51500	0.016573031	51548	0.123024511
51600	0.021401413	51552	0.091636815
51600	0.017116548	51556	0.054795281
51600	0.007477766	51560	0.045215391
51600	0.040427822	51564	0.083985062
51600	0.066566332	51568	0.09327368
51600	0.082423772	51572	0.075357188
51600	0.080235455	51576	0.049475268
51600	0.065446162	51580	0.024649622
51600	0.069108231	51584	0.043839267
51600	0.083695057	51588	0.050937764
51600	0.068851121	51592	0.046126632

PowerSpectrumData

51600	0.046237215	51596	0.029079338
51600	0.037990318	51600	0.027022223
51600	0.061707113	51604	0.030501535
51600	0.077650686	51608	0.031562402
51600	0.054619701	51612	0.068217203
51600	0.031830616	51616	0.090360241
51600	0.006302059	51620	0.057659065
51600	0.0048742	51624	0.021875801
51600	0.031898635	51628	0.0535039
51600	0.047937578	51632	0.090650181
51600	0.051303181	51636	0.130190746
51600	0.055504115	51640	0.124329687
51600	0.053426677	51644	0.049118633
51600	0.050456114	51648	0.082566905
51700	0.052485892	51652	0.098843229
51700	0.028890725	51656	0.058753813
51700	0.031908323	51660	0.064292966
51700	0.082542283	51664	0.079740566
51700	0.091713577	51668	0.072414579
51700	0.0534508	51672	0.081913837
51700	0.017221739	51676	0.090939771
51700	0.034896075	51680	0.084985048
51700	0.041874169	51684	0.078403202
51700	0.040258557	51688	0.081181948
51700	0.022623943	51692	0.068700094
51700	0.036937468	51696	0.064272063
51700	0.047388781	51700	0.055371271
51700	0.031116892	51704	0.034949604
51700	0.033933284	51708	0.03815279
51700	0.044001357	51712	0.019703708
51700	0.033102424	51716	0.013795721
51700	0.024570536	51720	0.029453631
51700	0.015144374	51724	0.030758984
51700	0.011537352	51728	0.064999396

PowerSpectrumData

51700	0.032132757	51732	0.066205343
51700	0.029699688	51736	0.055805911
51700	0.021968623	51740	0.054882512
51700	0.025515243	51744	0.038018581
51700	0.002031882	51748	0.05785545
51800	0.015720796	51752	0.114852017
51800	0.034645993	51756	0.105304374
51800	0.043206641	51760	0.071757015
51800	0.047915644	51764	0.075120559
51800	0.036362948	51768	0.052823212
51800	0.037437138	51772	0.05107596
51800	0.049453614	51776	0.046752779
51800	0.03907346	51780	0.033926695
51800	0.043958677	51784	0.035586796
51800	0.058269608	51788	0.011087775
51800	0.056603116	51792	0.03993151
51800	0.026588532	51796	0.046927766
51800	0.023480081	51800	0.030146968
51800	0.031941454	51804	0.02824786
51800	0.031367028	51808	0.069301001
51800	0.055354787	51812	0.072723458
51800	0.079033518	51816	0.034743705
51800	0.058284117	51820	0.021099852
51800	0.04621645	51824	0.044658198
51800	0.030158364	51828	0.066840541
51800	0.022155955	51832	0.058745802
51800	0.076454024	51836	0.07220736
51800	0.092414644	51840	0.045576813
51800	0.071768722	51844	0.041875483
51800	0.046736583	51848	0.022112898
51900	0.032284246	51852	0.034134977
51900	0.03173187	51856	0.044020126
51900	0.032351691	51860	0.030468307
51900	0.042854506	51864	0.04335092

PowerSpectrumData

51900	0.060011185	51868	0.057662823
51900	0.07357233	51872	0.022886339
51900	0.074689415	51876	0.01107757
51900	0.080900863	51880	0.010762945
51900	0.08380074	51884	0.065047563
51900	0.049787181	51888	0.096590855
51900	0.022476406	51892	0.041416581
51900	0.037235397	51896	0.017505385
51900	0.049470986	51900	0.024496321
51900	0.026752936	51904	0.052825802
51900	0.030087871	51908	0.024987478
51900	0.052391541	51912	0.046246008
51900	0.044771281	51916	0.050613006
51900	0.049260867	51920	0.050745446
51900	0.073900577	51924	0.103791921
51900	0.084739368	51928	0.114069713
51900	0.073414609	51932	0.075777811
51900	0.031662861	51936	0.052327836
51900	0.037037844	51940	0.078683886
51900	0.067885048	51944	0.06592198
51900	0.085130516	51948	0.073429845
52000	0.070469017	51952	0.113232884
52000	0.032849359	51956	0.081689803
52000	0.021605303	51960	0.007929942
52000	0.0219717	51964	0.03214573
52000	0.024511999	51968	0.04027399
52000	0.015718797	51972	0.049811577
52000	0.013715797	51976	0.055597629
52000	0.032392913	51980	0.048516802
52000	0.050370607	51984	0.039655912
52000	0.066078297	51988	0.042008694
52000	0.100156816	51992	0.056468292
52000	0.09097845	51996	0.043189437
52000	0.032695316	52000	0.041904186

52000	0.056540255	52004	0.030825129
52000	0.086328219	52008	0.078282239
52000	0.078409066	52012	0.078095647
52000	0.019488947	52016	0.025604835
52000	0.052647356	52020	0.014400862
52000	0.040253457	52024	0.019485837
52000	0.036166213	52028	0.024815279
52000	0.05060227	52032	0.07674488
52000	0.056844765	52036	0.060969622
52000	0.063393825	52040	0.067707173
52000	0.036257396	52044	0.10421536
52000	0.013580056	52048	0.078183366
52100	0.015860522	52052	0.043240867
52100	0.039934774	52056	0.060030136
52100	0.05081014	52060	0.026689808
52100	0.031445037	52064	0.06151251
52100	0.003078848	52068	0.106168001
52100	0.034034296	52072	0.114325645
52100	0.051552917	52076	0.082581646
52100	0.033327458	52080	0.022055856
52100	0.052465843	52084	0.023653451
52100	0.054211367	52088	0.029091258
52100	0.031515971	52092	0.03706268
52100	0.034658075	52096	0.047267375
52100	0.066663793	52100	0.049126516
52100	0.073935989	52104	0.035567526
52100	0.052996107	52108	0.022042486
52100	0.042837633	52112	0.071918905
52100	0.011900425	52116	0.100235448
52100	0.015597963	52120	0.104775936
52100	0.021050488	52124	0.070031412
52100	0.039889997	52128	0.031369669
52100	0.066544606	52132	0.065205837
52100	0.102164762	52136	0.0685423

PowerSpectrumData

52100	0.091968112	52140	0.067574336
52100	0.034271816	52144	0.076601551
52100	0.01361399	52148	0.071268027
52200	0.035850924	52152	0.067110756
52200	0.03087501	52156	0.068623325
52200	0.032238189	52160	0.037045109
52200	0.015704705	52164	0.068760797
52200	0.033700017	52168	0.107677115
52200	0.076683304	52172	0.110078763
52200	0.089248002	52176	0.078660778
52200	0.066673871	52180	0.031897631
52200	0.068057387	52184	0.023235692
52200	0.073277726	52188	0.017785091
52200	0.049124476	52192	0.045899924
52200	0.030993579	52196	0.078718338
52200	0.02970782	52200	0.04470965
52200	0.043702548	52204	0.008267696
52200	0.051002779	52208	0.015686454
52200	0.05048186	52212	0.02133703
52200	0.034620687	52216	0.038375642
52200	0.014130285	52220	0.040777821
52200	0.030205581	52224	0.003517267
52200	0.027942369	52228	0.033015771
52200	0.023625431	52232	0.038061211
52200	0.014054504	52236	0.018988463
52200	0.009685056	52240	0.025991043
52200	0.018563056	52244	0.067721412
52200	0.005680126	52248	0.093640287
52300	0.01392334	52252	0.051349671
52300	0.047849069	52256	0.037666832
52300	0.059397669	52260	0.063566069
52300	0.023246437	52264	0.056645433
52300	0.040733292	52268	0.067518384
52300	0.070982424	52272	0.079271638

PowerSpectrumData

52300	0.056949673	52276	0.047574529
52300	0.037677826	52280	0.041759526
52300	0.035620335	52284	0.017596021
52300	0.045216373	52288	0.031594223
52300	0.047810216	52292	0.028457604
52300	0.024351664	52296	0.004894852
52300	0.020928346	52300	0.023312296
52300	0.081927297	52304	0.018168632
52300	0.105044564	52308	0.039092454
52300	0.047940481	52312	0.033117805
52300	0.03960984	52316	0.044143209
52300	0.055909946	52320	0.045745383
52300	0.046699603	52324	0.042416374
52300	0.086977401	52328	0.025241701
52300	0.103870341	52332	0.049801689
52300	0.102519916	52336	0.057118483
52300	0.054006527	52340	0.048628648
52300	0.038366816	52344	0.028809733
52300	0.052419284	52348	0.056257159
52400	0.022049095	52352	0.053329681
52400	0.097941869	52356	0.026380529
52400	0.124253129	52360	0.048074715
52400	0.090654365	52364	0.09425031
52400	0.026404365	52368	0.092075694
52400	0.038370141	52372	0.065302746
52400	0.035733076	52376	0.072520743
52400	0.006534515	52380	0.092729082
52400	0.041454434	52384	0.074671181
52400	0.061497281	52388	0.070507114
52400	0.046630059	52392	0.076874552
52400	0.021172047	52396	0.031865609
52400	0.024392975	52400	0.024548705
52400	0.033645054	52404	0.019174422
52400	0.057777656	52408	0.034960562

PowerSpectrumData

52400	0.069324822	52412	0.071143171
52400	0.051450348	52416	0.088796653
52400	0.047026279	52420	0.077779943
52400	0.053804615	52424	0.033304896
52400	0.058860245	52428	0.040250288
52400	0.02945465	52432	0.059787366
52400	0.012996247	52436	0.015656347
52400	0.0262221	52440	0.039763621
52400	0.013868652	52444	0.081111837
52400	0.036989379	52448	0.104281258
52500	0.078229678	52452	0.05588519
52500	0.06249085	52456	0.046543959
52500	0.057699916	52460	0.069036811
52500	0.084130908	52464	0.048289603
52500	0.089550209	52468	0.04626263
52500	0.07946993	52472	0.056869161
52500	0.048614253	52476	0.077436955
52500	0.01606976	52480	0.07247179
52500	0.061834253	52484	0.017294631
52500	0.068589463	52488	0.056171073
52500	0.069977163	52492	0.08122775
52500	0.071615708	52496	0.037250829
52500	0.069707669	52500	0.045034656
52500	0.068939487	52504	0.042917847
52500	0.041093001	52508	0.046960584
52500	0.03107739	52512	0.09299977
52500	0.043798602	52516	0.092831244
52500	0.059956696	52520	0.050600058
52500	0.077396391	52524	0.035904839
52500	0.054843102	52528	0.05181885
52500	0.03457897	52532	0.050170544
52500	0.044626962	52536	0.044292159
52500	0.059089438	52540	0.026397664
52500	0.054044613	52544	0.025170975

PowerSpectrumData

52500	0.022362932	52548	0.020764715
52600	0.011600356	52552	0.040143026
52600	0.033697292	52556	0.053955337
52600	0.057366284	52560	0.037298778
52600	0.051613817	52564	0.03210342
52600	0.012332583	52568	0.070244045
52600	0.018225737	52572	0.091247675
52600	0.02198193	52576	0.10353588
52600	0.019545338	52580	0.101882259
52600	0.036554731	52584	0.066979817
52600	0.052767384	52588	0.033617362
52600	0.058564332	52592	0.047631827
52600	0.086136257	52596	0.061872997
52600	0.07745286	52600	0.019356943
52600	0.022786695	52604	0.067396635
52600	0.030497473	52608	0.053567794
52600	0.023931323	52612	0.010590253
52600	0.038094833	52616	0.023276174
52600	0.066784967	52620	0.044083026
52600	0.032930933	52624	0.105243875
52600	0.057726014	52628	0.129109249
52600	0.070687682	52632	0.105619707
52600	0.055198689	52636	0.047973634
52600	0.036531106	52640	0.010116432
52600	0.019353322	52644	0.034641282
52600	0.011282198	52648	0.041746353
52700	0.018738077	52652	0.032439039
52700	0.046787067	52656	0.037741451
52700	0.052130064	52660	0.075415403
52700	0.048423219	52664	0.088954563
52700	0.053992822	52668	0.070131755
52700	0.055593602	52672	0.056494675
52700	0.024919476	52676	0.014728924
52700	0.09552436	52680	0.050936582

52700	0.114335431	52684	0.058021422
52700	0.070728987	52688	0.042584346
52700	0.076665645	52692	0.031412699
52700	0.072587536	52696	0.02929714
52700	0.062206273	52700	0.06660154
52700	0.068574227	52704	0.055485998
52700	0.051896845	52708	0.025505377
52700	0.035749381	52712	0.037482121
52700	0.046113582	52716	0.042659576
52700	0.049294424	52720	0.038967213
52700	0.014461649	52724	0.043422861
52700	0.022340275	52728	0.041248823
52700	0.070910544	52732	0.061311344
52700	0.089519155	52736	0.066002074
52700	0.061218983	52740	0.059598882
52700	0.022167025	52744	0.098597862
52700	0.019768098	52748	0.124187296
52800	0.052058611	52752	0.082832441
52800	0.041043069	52756	0.032287055
52800	0.009615395	52760	0.010641147
52800	0.027594822	52764	0.022149648
52800	0.032714834	52768	0.025680845
52800	0.071499198	52772	0.00757261
52800	0.055968514	52776	0.055682212
52800	0.038676029	52780	0.069482499
52800	0.040950341	52784	0.06893769
52800	0.031869571	52788	0.032672258
52800	0.023893565	52792	0.034848305
52800	0.016664704	52796	0.044034019
52800	0.027639839	52800	0.003530614
52800	0.053705193	52804	0.016081136
52800	0.031856216	52808	0.045801204
52800	0.015229331	52812	0.08989207
52800	0.032927565	52816	0.104053055

PowerSpectrumData

52800	0.041052692	52820	0.076835182
52800	0.048489637	52824	0.029221779
52800	0.042327392	52828	0.02304886
52800	0.056447818	52832	0.033470988
52800	0.075361655	52836	0.046676691
52800	0.08475162	52840	0.010839808
52800	0.057293491	52844	0.011829397
52800	0.058219102	52848	0.036176036
52900	0.064340908	52852	0.056619865
52900	0.051503041	52856	0.053184358
52900	0.032942575	52860	0.026768861
52900	0.024204599	52864	0.043710177
52900	0.032866599	52868	0.075422053
52900	0.045608453	52872	0.07764488
52900	0.055255569	52876	0.039392482
52900	0.044335327	52880	0.057514997
52900	0.032413234	52884	0.060239021
52900	0.051038984	52888	0.026754429
52900	0.055825694	52892	0.029400457
52900	0.042414005	52896	0.044318662
52900	0.075225151	52900	0.040938452
52900	0.066497654	52904	0.052163803
52900	0.032599415	52908	0.043855001
52900	0.053644617	52912	0.044728899
52900	0.072578761	52916	0.067529662
52900	0.079431135	52920	0.072441413
52900	0.083054081	52924	0.084239204
52900	0.068190122	52928	0.058373145
52900	0.037344857	52932	0.023365097
52900	0.007280477	52936	0.044044908
52900	0.052138064	52940	0.083768915
52900	0.038998733	52944	0.088854678
52900	0.04006258	52948	0.055082619
53000	0.059150279	52952	0.061312872

PowerSpectrumData

53000	0.032642431	52956	0.118116077
53000	0.021232081	52960	0.128844943
53000	0.029830842	52964	0.09502194
53000	0.051733685	52968	0.04813687
53000	0.038620394	52972	0.024518537
53000	0.023092516	52976	0.04151848
53000	0.021800359	52980	0.022345572
53000	0.050304971	52984	0.042661744
53000	0.066878958	52988	0.060917759
53000	0.04975171	52992	0.093871335
53000	0.028801158	52996	0.124216254
53000	0.05242505	53000	0.109066816
53000	0.054364504	53004	0.061388724
53000	0.003913961	53008	0.01354222
53000	0.070640388	53012	0.069817746
53000	0.072450312	53016	0.064653745
53000	0.036186364	53020	0.020231433
53000	0.039688479	53024	0.0190872
53000	0.024199149	53028	0.04043568
53000	0.04299494	53032	0.057388901
53000	0.040206662	53036	0.056214369
53000	0.019162348	53040	0.043789212
53000	0.045419671	53044	0.055177145
53000	0.033290518	53048	0.068006193
53100	0.024045496	53052	0.036119629
53100	0.024710553	53056	0.005119212
53100	0.034747623	53060	0.027348804
53100	0.03361766	53064	0.044838085
53100	0.038547274	53068	0.018818057
53100	0.074344251	53072	0.026594616
53100	0.084369742	53076	0.021936674
53100	0.066897781	53080	0.013033822
53100	0.044825938	53084	0.047258658
53100	0.012173441	53088	0.080159894

PowerSpectrumData

53100	0.028961586	53092	0.08003226
53100	0.034619239	53096	0.063678956
53100	0.016604323	53100	0.026866588
53100	0.041142066	53104	0.010921065
53100	0.085034764	53108	0.010648825
53100	0.082669307	53112	0.028420151
53100	0.053103806	53116	0.085565262
53100	0.042758129	53120	0.105044142
53100	0.071083021	53124	0.045270357
53100	0.085244508	53128	0.034882407
53100	0.050392846	53132	0.064265536
53100	0.012570203	53136	0.05898995
53100	0.037225142	53140	0.040822622
53100	0.054689295	53144	0.05374389
53100	0.071923416	53148	0.070763388
53200	0.043124172	53152	0.080120452
53200	0.043099964	53156	0.090034504
53200	0.089964451	53160	0.099016557
53200	0.084682077	53164	0.064396358
53200	0.060168492	53168	0.082508515
53200	0.027314389	53172	0.093657291
53200	0.032708143	53176	0.013706849
53200	0.07659819	53180	0.086050633
53200	0.062173414	53184	0.071594019
53200	0.025920695	53188	0.019644385
53200	0.051823558	53192	0.076432451
53200	0.033589826	53196	0.056697318
53200	0.03304669	53200	0.053550124
53200	0.012203985	53204	0.033914428
53200	0.035692803	53208	0.014956067
53200	0.046317808	53212	0.027324519
53200	0.027746013	53216	0.020746507
53200	0.037059843	53220	0.03449139
53200	0.075663025	53224	0.02920686

PowerSpectrumData

53200	0.083689978	53228	0.026433248
53200	0.045603239	53232	0.062879277
53200	0.009913249	53236	0.071123388
53200	0.027931208	53240	0.047136015
53200	0.02885645	53244	0.021631959
53200	0.021980855	53248	0.032746273
53300	0.045756904	53252	0.052681491
53300	0.065840133	53256	0.049684568
53300	0.067587862	53260	0.056031859
53300	0.043814689	53264	0.06987933
53300	0.014060269	53268	0.084360945
53300	0.032552114	53272	0.053453216
53300	0.020097752	53276	0.029764631
53300	0.048743354	53280	0.054409466
53300	0.052068495	53284	0.032265343
53300	0.030731553	53288	0.041461983
53300	0.035937916	53292	0.056230765
53300	0.03073876	53296	0.079937738
53300	0.031597068	53300	0.049764338
53300	0.064930144	53304	0.031740823
53300	0.071661947	53308	0.075240045
53300	0.061936313	53312	0.118151133
53300	0.047698006	53316	0.086551409
53300	0.022055923	53320	0.036970603
53300	0.053412659	53324	0.027442036
53300	0.05783583	53328	0.023663779
53300	0.034481898	53332	0.007464089
53300	0.037493021	53336	0.057333691
53300	0.091124632	53340	0.090782829
53300	0.08673803	53344	0.077674522
53300	0.052848314	53348	0.06514381
53400	0.024278892	53352	0.06510262
53400	0.033862903	53356	0.036820013
53400	0.045536843	53360	0.025154432

PowerSpectrumData

53400	0.024246696	53364	0.063199288
53400	0.048493599	53368	0.072269773
53400	0.030500873	53372	0.077660785
53400	0.045387398	53376	0.084928899
53400	0.086543398	53380	0.078906327
53400	0.096393123	53384	0.068786896
53400	0.052798849	53388	0.048601407
53400	0.042446296	53392	0.034890167
53400	0.076836768	53396	0.018860201
53400	0.055560711	53400	0.064357475
53400	0.019482504	53404	0.064718726
53400	0.01436383	53408	0.030059591
53400	0.032713779	53412	0.049384304
53400	0.041326588	53416	0.083079038
53400	0.029637982	53420	0.086051296
53400	0.04910972	53424	0.044569708
53400	0.062972569	53428	0.023754363
53400	0.054851153	53432	0.02994814
53400	0.04655126	53436	0.069977592
53400	0.038086419	53440	0.090583766
53400	0.010324856	53444	0.044110864
53400	0.037258749	53448	0.034974339
53500	0.045389592	53452	0.121674944
53500	0.034533441	53456	0.157413946
53500	0.061362684	53460	0.119038414
53500	0.035974026	53464	0.08782083
53500	0.030412659	53468	0.100278856
53500	0.049393915	53472	0.080077705
53500	0.040559458	53476	0.016925795
53500	0.029702243	53480	0.045375258
53500	0.0431925	53484	0.079880105
53500	0.066160021	53488	0.086136417
53500	0.056659817	53492	0.066222929
53500	0.078535792	53496	0.053163261

PowerSpectrumData

53500	0.071619143	53500	0.050382907
53500	0.043097847	53504	0.038906921
53500	0.014591007	53508	0.039556213
53500	0.057742767	53512	0.091385737
53500	0.068070083	53516	0.059243852
53500	0.071086877	53520	0.017765528
53500	0.079314428	53524	0.026365338
53500	0.054359742	53528	0.016375831
53500	0.006813208	53532	0.102281476
53500	0.058401347	53536	0.181759475
53500	0.078365265	53540	0.172092434
53500	0.065038177	53544	0.108304084
53500	0.050486731	53548	0.061708524
53600	0.035370369	53552	0.016092168
53600	0.005630341	53556	0.024393039
53600	0.048923426	53560	0.018154668
53600	0.057428479	53564	0.01897037
53600	0.042020867	53568	0.0413903
53600	0.027583121	53572	0.053557575
53600	0.038444236	53576	0.021381655
53600	0.034040386	53580	0.047732108
53600	0.041601015	53584	0.103783401
53600	0.035476856	53588	0.086664695
53600	0.070193732	53592	0.064579232
53600	0.109083681	53596	0.062041676
53600	0.082767452	53600	0.025529264
53600	0.0276016	53604	0.050257448
53600	0.015777492	53608	0.048857339
53600	0.032938169	53612	0.055579381
53600	0.006900518	53616	0.030941072
53600	0.040352661	53620	0.050143255
53600	0.039987335	53624	0.075023068
53600	0.026517257	53628	0.068926413
53600	0.057792026	53632	0.091749716

PowerSpectrumData

53600	0.0699558	53636	0.107564076
53600	0.056443772	53640	0.101176833
53600	0.025871472	53644	0.080015772
53600	0.02515988	53648	0.06495382
53700	0.061559869	53652	0.051546835
53700	0.074018928	53656	0.013145775
53700	0.03973196	53660	0.056571113
53700	0.020793428	53664	0.056861511
53700	0.017579609	53668	0.099850528
53700	0.01490132	53672	0.064082851
53700	0.041824573	53676	0.043834294
53700	0.07176707	53680	0.043938995
53700	0.06441927	53684	0.013010832
53700	0.054674722	53688	0.103406266
53700	0.065759698	53692	0.118540542
53700	0.03779235	53696	0.053089338
53700	0.009421154	53700	0.082579762
53700	0.01802573	53704	0.088177512
53700	0.025632129	53708	0.032036202
53700	0.071780683	53712	0.06234536
53700	0.095106378	53716	0.047813781
53700	0.066935376	53720	0.049306134
53700	0.041527012	53724	0.072528383
53700	0.029977064	53728	0.135926094
53700	0.029499495	53732	0.114372822
53700	0.038267917	53736	0.113147311
53700	0.035423273	53740	0.11827032
53700	0.025187952	53744	0.08097072
53700	0.024359091	53748	0.053889489
53800	0.027590633	53752	0.064975109
53800	0.053170228	53756	0.079407611
53800	0.07836047	53760	0.11912574
53800	0.059591996	53764	0.091401344
53800	0.019834579	53768	0.082576058

PowerSpectrumData

53800	0.023945062	53772	0.131948823
53800	0.043284741	53776	0.105482708
53800	0.042602304	53780	0.123633741
53800	0.022477219	53784	0.112796857
53800	0.034559471	53788	0.105237414
53800	0.038443079	53792	0.177335038
53800	0.010864855	53796	0.164269746
53800	0.031839081	53800	0.06473915
53800	0.05614827	53804	0.035242854
53800	0.061524166	53808	0.089940397
53800	0.067576279	53812	0.153904679
53800	0.059089016	53816	0.072194853
53800	0.059269467	53820	0.013339773
53800	0.083748106	53824	0.119515069
53800	0.090218804	53828	0.193213884
53800	0.06015494	53832	0.093068833
53800	0.018031571	53836	0.020205893
53800	0.01926692	53840	0.062797364
53800	0.053252421	53844	0.165197708
53800	0.072501956	53848	0.136809045
53900	0.059175462	53852	0.04971126
53900	0.039716077	53856	0.034002635
53900	0.048617847	53860	0.165117789
53900	0.043237807	53864	0.108315086
53900	0.011694598	53868	0.044372086
53900	0.07399296	53872	0.023660292
53900	0.083768769	53876	0.087307824
53900	0.040654239	53880	0.224302231
53900	0.025520933	53884	0.141630371
53900	0.034362318	53888	0.048099806
53900	0.020600894	53892	0.114464863
53900	0.031922926	53896	0.331661431
53900	0.061833591	53900	0.152303648
53900	0.06233008	53904	0.220961884

PowerSpectrumData

53900	0.05595047	53908	0.261638721
53900	0.048515485	53912	0.30395505
53900	0.042744832	53916	0.253886101
53900	0.032157121	53920	0.482862583
53900	0.012932776	53924	0.39259257
53900	0.018020233	53928	0.737021678
53900	0.03876994	53932	1.860443625
53900	0.051942443	53936	2.070107263
53900	0.024110988	53940	1.06788415
53900	0.051903353	53944	0.230183665
53900	0.086848195	53948	0.091344889
54000	0.070471004	53952	0.073620024
54000	0.053730579	53956	0.141710465
54000	0.03070843	53960	0.060135953
54000	0.007231041	53964	0.14878904
54000	0.050638933	53968	0.025432795
54000	0.067429857	53972	0.081179554
54000	0.033880388	53976	0.051972216
54000	0.034908062	53980	0.042755364
54000	0.064664731	53984	0.12570752
54000	0.045270484	53988	0.127896754
54000	0.042906562	53992	0.164267796
54000	0.03815287	53996	0.118406999
54000	0.018988452	54000	0.163245146
54000	0.046606805	54004	0.130024011
54000	0.061438004	54008	0.030730396
54000	0.032664142	54012	0.036783185
54000	0.022538387	54016	0.042280059
54000	0.070467766	54020	0.085725216
54000	0.08158834	54024	0.116803909
54000	0.061168532	54028	0.058030906
54000	0.040117888	54032	0.036267389
54000	0.039393221	54036	0.042776872
54000	0.038076927	54040	0.086810294

PowerSpectrumData

54000	0.026503201	54044	0.05193361
54000	0.033537854	54048	0.019050956
54100	0.055088691	54052	0.103605344
54100	0.035944973	54056	0.153304893
54100	0.011409019	54060	0.049493472
54100	0.020079602	54064	0.036679408
54100	0.024049028	54068	0.080566082
54100	0.035988982	54072	0.07071314
54100	0.043771266	54076	0.046240752
54100	0.040416227	54080	0.069956826
54100	0.027083188	54084	0.026667502
54100	0.043194381	54088	0.064538457
54100	0.050268962	54092	0.023091599
54100	0.034756373	54096	0.01010904
54100	0.070000329	54100	0.039855928
54100	0.061373226	54104	0.014235149
54100	0.055930188	54108	0.052131374
54100	0.047153106	54112	0.008582299
54100	0.019548497	54116	0.065942615
54100	0.067838751	54120	0.044995974
54100	0.067604669	54124	0.123474412
54100	0.0288263	54128	0.111865076
54100	0.022948498	54132	0.080041922
54100	0.050845694	54136	0.131850335
54100	0.051947642	54140	0.127129344
54100	0.021735725	54144	0.065782529
54100	0.022125692	54148	0.094329058
54200	0.015458392	54152	0.073875388
54200	0.040676969	54156	0.06217874
54200	0.065330278	54160	0.115527771
54200	0.032783752	54164	0.120855395
54200	0.00860726	54168	0.101970545
54200	0.0122871	54172	0.082467435
54200	0.044146833	54176	0.028019076

PowerSpectrumData

54200	0.078564284	54180	0.039146067
54200	0.084474625	54184	0.049915587
54200	0.061634833	54188	0.062123327
54200	0.038627331	54192	0.121814381
54200	0.031112344	54196	0.143819329
54200	0.006437373	54200	0.108294225
54200	0.061134422	54204	0.090417634
54200	0.099686899	54208	0.121165591
54200	0.091315167	54212	0.139312674
54200	0.076526332	54216	0.096688375
54200	0.06822688	54220	0.045470155
54200	0.069761496	54224	0.072927978
54200	0.072495459	54228	0.106728548
54200	0.04566681	54232	0.083855666
54200	0.061009061	54236	0.06455824
54200	0.064005952	54240	0.067491194
54200	0.065912383	54244	0.078066172
54200	0.077830031	54248	0.116067029
54300	0.059755224	54252	0.126140338
54300	0.024491923	54256	0.095802607
54300	0.024322137	54260	0.038501326
54300	0.031470641	54264	0.087890316
54300	0.035165675	54268	0.133828071
54300	0.026589023	54272	0.158324241
54300	0.03779378	54276	0.199839168
54300	0.05438564	54280	0.184988283
54300	0.02518146	54284	0.089628571
54300	0.014351068	54288	0.050909166
54300	0.02115676	54292	0.047608184
54300	0.034203509	54296	0.066072294
54300	0.032467568	54300	0.104119397
54300	0.038480204	54304	0.070259041
54300	0.044886623	54308	0.038464805
54300	0.02624254	54312	0.136610834

PowerSpectrumData

54300	0.03789628	54316	0.159846386
54300	0.046802088	54320	0.106704662
54300	0.110884656	54324	0.076403732
54300	0.16301767	54328	0.07073587
54300	0.112240959	54332	0.017384951
54300	0.040890813	54336	0.063539657
54300	0.02816551	54340	0.104748382
54300	0.01636436	54344	0.10120769
54300	0.022271888	54348	0.064784712
54400	0.03371455	54352	0.053267686
54400	0.034128814	54356	0.050229533
54400	0.025681398	54360	0.058921516
54400	0.015980952	54364	0.088595632
54400	0.017718599	54368	0.102758808
54400	0.026835563	54372	0.071438008
54400	0.074643096	54376	0.076272372
54400	0.072240713	54380	0.129938431
54400	0.03453327	54384	0.094973468
54400	0.028524224	54388	0.047595644
54400	0.040827687	54392	0.056896308
54400	0.029844718	54396	0.105332889
54400	0.04029561	54400	0.141902375
54400	0.076651028	54404	0.114584596
54400	0.086695611	54408	0.075885175
54400	0.067723202	54412	0.115184957
54400	0.053851414	54416	0.13230172
54400	0.030102805	54420	0.125830149
54400	0.008974447	54424	0.116018884
54400	0.019152887	54428	0.086788947
54400	0.032287044	54432	0.062530009
54400	0.02283024	54436	0.081940612
54400	0.049865877	54440	0.10967486
54400	0.065259737	54444	0.12245313
54400	0.057506146	54448	0.076265278

PowerSpectrumData

54500	0.042995649	54452	0.059996153
54500	0.052036692	54456	0.041758693
54500	0.081168029	54460	0.0225816
54500	0.097988122	54464	0.081579681
54500	0.080838756	54468	0.114166745
54500	0.052018251	54472	0.102012316
54500	0.051628216	54476	0.133383364
54500	0.057077828	54480	0.15479447
54500	0.056787361	54484	0.120037672
54500	0.031070584	54488	0.091852671
54500	0.002634161	54492	0.096139949
54500	0.05427784	54496	0.126589148
54500	0.070997645	54500	0.125517574
54500	0.030150655	54504	0.062305
54500	0.045958332	54508	0.046618177
54500	0.066623463	54512	0.087862718
54500	0.056855435	54516	0.104205072
54500	0.055148761	54520	0.067860463
54500	0.074080977	54524	0.085494408
54500	0.07471929	54528	0.093308467
54500	0.037348436	54532	0.04161194
54500	0.017444896	54536	0.101025391
54500	0.042825803	54540	0.143989251
54500	0.066782988	54544	0.124351544
54500	0.085966873	54548	0.095973846
54600	0.085977416	54552	0.130712971
54600	0.087358647	54556	0.1808776
54600	0.077320969	54560	0.147374026
54600	0.045768411	54564	0.100857447
54600	0.007983988	54568	0.099670011
54600	0.038965816	54572	0.085330205
54600	0.069758564	54576	0.082383951
54600	0.070846196	54580	0.0991282
54600	0.053729684	54584	0.073026298

PowerSpectrumData

54600	0.06867294	54588	0.02656478
54600	0.075302676	54592	0.045615798
54600	0.027655784	54596	0.067575449
54600	0.043447562	54600	0.056862009
54600	0.065471337	54604	0.03956489
54600	0.058513146	54608	0.035532095
54600	0.040077575	54612	0.02494408
54600	0.009132718	54616	0.021862898
54600	0.016002618	54620	0.078875208
54600	0.045526605	54624	0.112474008
54600	0.043216263	54628	0.093459203
54600	0.006977421	54632	0.07019315
54600	0.046783585	54636	0.053298398
54600	0.05336609	54640	0.02218639
54600	0.053266831	54644	0.012172957
54600	0.084304564	54648	0.043365497
54700	0.099123878	54652	0.10427714
54700	0.061107137	54656	0.119941688
54700	0.023231783	54660	0.081016544
54700	0.022571776	54664	0.023944493
54700	0.036602673	54668	0.0550595
54700	0.036323025	54672	0.072986426
54700	0.040053015	54676	0.076916069
54700	0.071755618	54680	0.083750914
54700	0.083699459	54684	0.05808055
54700	0.063730935	54688	0.034643028
54700	0.045834266	54692	0.061542771
54700	0.0333727	54696	0.069143294
54700	0.003337853	54700	0.099866455
54700	0.048593287	54704	0.099495206
54700	0.058319969	54708	0.069553513
54700	0.02278616	54712	0.05748406
54700	0.011145878	54716	0.047392729
54700	0.023982722	54720	0.021985235

PowerSpectrumData

54700	0.028200224	54724	0.031349257
54700	0.016495324	54728	0.047959708
54700	0.015142305	54732	0.046613546
54700	0.025230283	54736	0.082537001
54700	0.014764828	54740	0.095378106
54700	0.013896502	54744	0.070345501
54700	0.023426641	54748	0.070303671
54800	0.018430697	54752	0.070836744
54800	0.071210314	54756	0.050355775
54800	0.081802064	54760	0.059920028
54800	0.047914706	54764	0.063950218
54800	0.025966163	54768	0.021531956
54800	0.012689171	54772	0.042685115
54800	0.019664452	54776	0.055534536
54800	0.029643268	54780	0.047865462
54800	0.023109576	54784	0.042149753
54800	0.001495132	54788	0.04409203
54800	0.019639718	54792	0.073516632
54800	0.038833357	54796	0.099027959
54800	0.069737347	54800	0.113836723
54800	0.093989867	54804	0.076128832
54800	0.097761324	54808	0.019803758
54800	0.072527058	54812	0.022782897
54800	0.03093476	54816	0.040036102
54800	0.069732181	54820	0.057258818
54800	0.064735483	54824	0.054546323
54800	0.047626734	54828	0.007416605
54800	0.044442077	54832	0.055539324
54800	0.004635658	54836	0.044582244
54800	0.037371072	54840	0.016555685
54800	0.070233655	54844	0.030862735
54800	0.092814342	54848	0.044528744
54900	0.09099365	54852	0.041002524
54900	0.085525106	54856	0.042254796

PowerSpectrumData

54900	0.070001173	54860	0.021135425
54900	0.058468144	54864	0.033588614
54900	0.08055965	54868	0.029877852
54900	0.063985492	54872	0.010373104
54900	0.015354706	54876	0.018524479
54900	0.017105327	54880	0.035349171
54900	0.017078117	54884	0.055194003
54900	0.015558875	54888	0.031514341
54900	0.059377715	54892	0.030164483
54900	0.082371436	54896	0.031284872
54900	0.053328146	54900	0.037486047
54900	0.026258358	54904	0.051116582
54900	0.061775609	54908	0.044154771
54900	0.062523868	54912	0.055899065
54900	0.032058873	54916	0.041365078
54900	0.027377022	54920	0.024576017
54900	0.033668952	54924	0.058571517
54900	0.037717771	54928	0.082091196
54900	0.027281298	54932	0.050835843
54900	0.030612486	54936	0.014478486
54900	0.053998589	54940	0.014311752
54900	0.032010383	54944	0.009763558
54900	0.084809391	54948	0.013062053
55000	0.080969752	54952	0.02081801
55000	0.04373155	54956	0.026575573
55000	0.018292398	54960	0.021564452
55000	0.027488108	54964	0.055183202
55000	0.009740324	54968	0.069642374
55000	0.036726575	54972	0.029299017
55000	0.052166735	54976	0.046633468
55000	0.025131989	54980	0.04844525
55000	0.008328027	54984	0.04506671
55000	0.021777749	54988	0.072675
55000	0.037319936	54992	0.073937343

PowerSpectrumData

55000	0.040645591	54996	0.030631501
55000	0.046711855	55000	0.039236686
55000	0.034335935	55004	0.063068241
55000	0.014505637	55008	0.063446219
55000	0.029522589	55012	0.039034301
55000	0.032806831	55016	0.043763514
55000	0.034426321	55020	0.085544612
55000	0.099938035	55024	0.072102172
55000	0.090349211	55028	0.01647932
55000	0.028649229	55032	0.061210449
55000	0.030892996	55036	0.053883228
55000	0.065096538	55040	0.041795291
55000	0.107106309	55044	0.090350906
55000	0.145929676	55048	0.079086523
55100	0.146122883	55052	0.034138131
55100	0.111711466	55056	0.043889198
55100	0.074447067	55060	0.103349164
55100	0.032435932	55064	0.067248511
55100	0.048622806	55068	0.018398059
55100	0.079039681	55072	0.024988958
55100	0.06380507	55076	0.031463675
55100	0.038573398	55080	0.046021803
55100	0.026473355	55084	0.056871108
55100	0.023107496	55088	0.036336925
55100	0.024742178	55092	0.061112987
55100	0.017441822	55096	0.096366435
55100	0.017605031	55100	0.071337483
55100	0.045240126	55104	0.030867552
55100	0.042889973	55108	0.07106502
55100	0.005871934	55112	0.05373983
55100	0.03004861	55116	0.056717516
55100	0.087152468	55120	0.065128261
55100	0.093835894	55124	0.065663488
55100	0.047886588	55128	0.067758323

PowerSpectrumData

55100	0.023448494	55132	0.067218753
55100	0.04098261	55136	0.115583731
55100	0.038736926	55140	0.148732011
55100	0.035586734	55144	0.117311865
55100	0.055757497	55148	0.109852554
55200	0.065476837	55152	0.09645606
55200	0.043609685	55156	0.056545658
55200	0.030208004	55160	0.025259276
55200	0.016129279	55164	0.08214049
55200	0.021287728	55168	0.090791633
55200	0.030147319	55172	0.052955835
55200	0.052095384	55176	0.027243677
55200	0.073414056	55180	0.032837659
55200	0.090151072	55184	0.011301155
55200	0.111916823	55188	0.022959099
55200	0.078057572	55192	0.014741063
55200	0.017622693	55196	0.024628916
55200	0.061992978	55200	0.065524007
55200	0.030063628	55204	0.120641358
55200	0.067174202	55208	0.155636516
55200	0.081979866	55212	0.157156508
55200	0.0265299	55216	0.131943438
55200	0.021613054	55220	0.112598624
55200	0.038690585	55224	0.078518591
55200	0.045388209	55228	0.028076396
55200	0.041380605	55232	0.007672311
55200	0.051120715	55236	0.031122698
55200	0.064126893	55240	0.029094477
55200	0.041571679	55244	0.017848059
55200	0.005150174	55248	0.038290924
55300	0.043333472	55252	0.044792683
55300	0.045386005	55256	0.018249064
55300	0.045309989	55260	0.047990794
55300	0.07288839	55264	0.071017814

PowerSpectrumData

55300	0.06325842	55268	0.084244399
55300	0.029842286	55272	0.087078763
55300	0.042763324	55276	0.0617353
55300	0.056768957	55280	0.05809711
55300	0.07119029	55284	0.085557564
55300	0.057186997	55288	0.064876193
55300	0.032836597	55292	0.055774563
55300	0.07406652	55296	0.088957509
55300	0.075462835	55300	0.082124025
55300	0.041449388	55304	0.068572816
55300	0.008979697	55308	0.044062734
55300	0.021960517	55312	0.020005085
55300	0.065860884	55316	0.017595858
55300	0.090961083	55320	0.030165616
55300	0.083032348	55324	0.079816738
55300	0.068649584	55328	0.105237181
55300	0.043238884	55332	0.099202516
55300	0.01826655	55336	0.071516377
55300	0.01625804	55340	0.074093463
55300	0.016204293	55344	0.084531086
55300	0.035359935	55348	0.057580317
55400	0.046533856	55352	0.016598719
55400	0.027884611	55356	0.078713383
55400	0.059066184	55360	0.11899331
55400	0.075801458	55364	0.105937921
55400	0.043917276	55368	0.042813503
55400	0.013743435	55372	0.033886718
55400	0.03205943	55376	0.062720064
55400	0.024363031	55380	0.032876193
55400	0.041109641	55384	0.031092026
55400	0.050352399	55388	0.058382819
55400	0.058747206	55392	0.060457398
55400	0.055970453	55396	0.043063807
55400	0.053764605	55400	0.054479293

PowerSpectrumData

55400	0.061969186	55404	0.035950412
55400	0.078503836	55408	0.018728521
55400	0.045613946	55412	0.052942491
55400	0.013200967	55416	0.079514357
55400	0.019132625	55420	0.078236546
55400	0.004691549	55424	0.054639932
55400	0.019802503	55428	0.042766995
55400	0.031831045	55432	0.075141361
55400	0.037834965	55436	0.10190301
55400	0.055843611	55440	0.09990258
55400	0.053651394	55444	0.101321923
55400	0.01413729	55448	0.13479963
55500	0.017559223	55452	0.139492143
55500	0.02978766	55456	0.09849912
55500	0.032679749	55460	0.038806702
55500	0.062123654	55464	0.083888372
55500	0.056479581	55468	0.116361778
55500	0.03502248	55472	0.072698502
55500	0.026608521	55476	0.025969668
55500	0.031891086	55480	0.068867252
55500	0.034301305	55484	0.092473907
55500	0.063750107	55488	0.068943889
55500	0.075805925	55492	0.050035982
55500	0.057188594	55496	0.058211499
55500	0.056022283	55500	0.082124963
55500	0.046378282	55504	0.087484572
55500	0.004662291	55508	0.076141128
55500	0.038864146	55512	0.09240106
55500	0.026048616	55516	0.084293461
55500	0.020339181	55520	0.083653715
55500	0.040403054	55524	0.0879945
55500	0.021355922	55528	0.063640575
55500	0.027147971	55532	0.06555736
55500	0.045540925	55536	0.085100997

PowerSpectrumData

55500	0.026314649	55540	0.08057012
55500	0.0166462	55544	0.055936038
55500	0.016990691	55548	0.037318525
55600	0.044821878	55552	0.019613357
55600	0.044499109	55556	0.038467933
55600	0.02514086	55560	0.067930661
55600	0.034482946	55564	0.063170177
55600	0.053615226	55568	0.05950659
55600	0.03606618	55572	0.054060263
55600	0.040926003	55576	0.066056637
55600	0.074377997	55580	0.0985808
55600	0.076276527	55584	0.127459905
55600	0.059839043	55588	0.132465691
55600	0.054735618	55592	0.080667873
55600	0.071125891	55596	0.045933975
55600	0.134848684	55600	0.082751372
55600	0.174901449	55604	0.070721901
55600	0.145216953	55608	0.060448292
55600	0.075000076	55612	0.110213034
55600	0.018349263	55616	0.14631734
55600	0.012511852	55620	0.137438386
55600	0.025120058	55624	0.112080641
55600	0.060029219	55628	0.071512986
55600	0.064140571	55632	0.064751694
55600	0.095393523	55636	0.057922713
55600	0.121286917	55640	0.044305398
55600	0.113745235	55644	0.057918929
55600	0.092811824	55648	0.081276659
55700	0.070381015	55652	0.132525108
55700	0.039463233	55656	0.148050021
55700	0.086555432	55660	0.096388569
55700	0.18229436	55664	0.100160556
55700	0.182327334	55668	0.18170473
55700	0.105245243	55672	0.168812345

PowerSpectrumData

55700	0.065466695	55676	0.128406115
55700	0.083174477	55680	0.114217299
55700	0.084270447	55684	0.090593872
55700	0.067039073	55688	0.082475366
55700	0.132173024	55692	0.106231259
55700	0.158441835	55696	0.131218884
55700	0.077430421	55700	0.147643499
55700	0.071585964	55704	0.138081698
55700	0.12318432	55708	0.118712087
55700	0.067316083	55712	0.137408279
55700	0.053751268	55716	0.157669041
55700	0.145526458	55720	0.149384374
55700	0.21970534	55724	0.103598075
55700	0.233890649	55728	0.064686399
55700	0.122375772	55732	0.103325852
55700	0.116568561	55736	0.132779548
55700	0.129390551	55740	0.105372797
55700	0.056632493	55744	0.124194849
55700	0.108457585	55748	0.16877125
55800	0.287248433	55752	0.142039644
55800	0.332235359	55756	0.124410653
55800	0.234675099	55760	0.166490194
55800	0.125097723	55764	0.136284434
55800	0.11367842	55768	0.116009607
55800	0.024611081	55772	0.140367221
55800	0.269154698	55776	0.100910824
55800	0.685353996	55780	0.053053409
55800	1.127767144	55784	0.111094407
55800	1.055822708	55788	0.108522982
55800	0.463392644	55792	0.109918306
55800	0.649496971	55796	0.152454698
55800	0.46031797	55800	0.136998366
55800	0.976776704	55804	0.113124275
55800	3.195591738	55808	0.130199681

PowerSpectrumData

55800	4.8413258	55812	0.14287344
55800	4.355643413	55816	0.141419179
55800	2.228847238	55820	0.109358014
55800	0.532264763	55824	0.037244838
55800	0.584313414	55828	0.053081665
55800	0.507451361	55832	0.105462845
55800	0.612424978	55836	0.119604141
55800	1.282471238	55840	0.12971164
55800	1.76368805	55844	0.107434156
55800	1.050953754	55848	0.093504925
55900	0.822173548	55852	0.088089197
55900	0.954218034	55856	0.039039842
55900	1.027641934	55860	0.046981499
55900	3.787762025	55864	0.080564816
55900	9.024529713	55868	0.061242536
55900	10.90194191	55872	0.061444
55900	7.1823108	55876	0.099070931
55900	2.126034588	55880	0.109697969
55900	0.901449646	55884	0.096855016
55900	0.724813144	55888	0.085314263
55900	0.612838776	55892	0.083167302
55900	0.506972894	55896	0.088152236
55900	0.721942925	55900	0.068913629
55900	0.413205883	55904	0.098804871
55900	0.357494108	55908	0.115541137
55900	0.346400223	55912	0.116575298
55900	0.458269264	55916	0.133165856
55900	0.227608121	55920	0.125563674
55900	1.191688934	55924	0.08809712
55900	1.959894088	55928	0.052955875
55900	1.769405788	55932	0.076146498
55900	0.822666566	55936	0.063722204
55900	0.139088631	55940	0.015997111
55900	0.158982948	55944	0.041768853

PowerSpectrumData

55900	0.0893834	55948	0.067658184
56000	0.16413453	55952	0.104133542
56000	0.513114501	55956	0.114640956
56000	0.598675048	55960	0.074204698
56000	0.316629128	55964	0.033215001
56000	0.130031476	55968	0.051557941
56000	0.163683231	55972	0.040433457
56000	0.014244848	55976	0.032371034
56000	0.316060643	55980	0.059160735
56000	0.791504921	55984	0.023912668
56000	1.058805151	55988	0.053685744
56000	0.816681655	55992	0.056920784
56000	0.267399738	55996	0.033495562
56000	0.137240466	56000	0.050259154
56000	0.174532819	56004	0.07473689
56000	0.154647991	56008	0.069138463
56000	0.421137433	56012	0.041115112
56000	0.703980448	56016	0.035726003
56000	0.670451148	56020	0.08403742
56000	0.322880805	56024	0.08915837
56000	0.156921946	56028	0.084499807
56000	0.193894754	56032	0.056752168
56000	0.03969192	56036	0.02313949
56000	0.268939766	56040	0.08298843
56000	0.486116508	56044	0.102837992
56000	0.511201681	56048	0.073375799
56100	0.324838416	56052	0.026378984
56100	0.048083221	56056	0.030212799
56100	0.136258415	56060	0.023094277
56100	0.148153645	56064	0.031727297
56100	0.261450478	56068	0.0176914
56100	0.482320844	56072	0.018597089
56100	0.633019081	56076	0.026211761
56100	0.509008241	56080	0.047691599

PowerSpectrumData

56100	0.165771911	56084	0.058948484
56100	0.107827713	56088	0.079122125
56100	0.10315179	56092	0.075565113
56100	0.084404011	56096	0.070231596
56100	0.205597943	56100	0.074511605
56100	0.224913456	56104	0.068517664
56100	0.177726775	56108	0.03930485
56100	0.110686931	56112	0.044333658
56100	0.061944462	56116	0.051700106
56100	0.06286108	56120	0.030357736
56100	0.076377233	56124	0.019568852
56100	0.162258336	56128	0.079496254
56100	0.259876076	56132	0.132244371
56100	0.249112404	56136	0.112410264
56100	0.132569315	56140	0.055291144
56100	0.041526455	56144	0.057242854
56100	0.067169436	56148	0.063263484
56200	0.100584592	56152	0.059798556
56200	0.110843066	56156	0.06517311
56200	0.130655099	56160	0.051563708
56200	0.114747905	56164	0.0411687
56200	0.069854228	56168	0.024471485
56200	0.072160896	56172	0.026462625
56200	0.100285499	56176	0.030658324
56200	0.084295265	56180	0.00292984
56200	0.113720758	56184	0.028202278
56200	0.176067071	56188	0.045945126
56200	0.203981094	56192	0.070540024
56200	0.152798544	56196	0.075234624
56200	0.02783651	56200	0.059032642
56200	0.058033162	56204	0.033948316
56200	0.025428566	56208	0.030740644
56200	0.049015449	56212	0.035132816
56200	0.088747496	56216	0.018293038

PowerSpectrumData

56200	0.105333296	56220	0.057259877
56200	0.11365168	56224	0.042951553
56200	0.102206104	56228	0.017441618
56200	0.073711504	56232	0.050408838
56200	0.069589783	56236	0.047624872
56200	0.038344901	56240	0.017269054
56200	0.0415098	56244	0.047779464
56200	0.083123501	56248	0.022857803
56300	0.07779328	56252	0.017825314
56300	0.066401546	56256	0.018554945
56300	0.075643002	56260	0.061964332
56300	0.05691448	56264	0.077823803
56300	0.032785996	56268	0.081532468
56300	0.051650408	56272	0.050343682
56300	0.072204915	56276	0.005974247
56300	0.088573965	56280	0.035432859
56300	0.075728036	56284	0.046577166
56300	0.041199975	56288	0.033296219
56300	0.036909438	56292	0.054280896
56300	0.08293763	56296	0.091582064
56300	0.053492437	56300	0.096803073
56300	0.040500305	56304	0.053753738
56300	0.030481777	56308	0.019159954
56300	0.081993218	56312	0.040233696
56300	0.105469895	56316	0.047647245
56300	0.072652962	56320	0.026594709
56300	0.055669047	56324	0.019216879
56300	0.049248225	56328	0.022924187
56300	0.042002197	56332	0.038551243
56300	0.003528648	56336	0.085179876
56300	0.014268911	56340	0.106791566
56300	0.048590446	56344	0.104167368
56300	0.061585568	56348	0.090813228
56400	0.057303125	56352	0.064343076

PowerSpectrumData

56400	0.02772494	56356	0.081406979
56400	0.014629125	56360	0.089945344
56400	0.016047621	56364	0.052180698
56400	0.021378335	56368	0.038605769
56400	0.020952357	56372	0.066993947
56400	0.02577012	56376	0.092258757
56400	0.02545958	56380	0.095059426
56400	0.010422938	56384	0.045525354
56400	0.018647876	56388	0.045425444
56400	0.040074658	56392	0.084987376
56400	0.06374708	56396	0.099655576
56400	0.060220209	56400	0.076477925
56400	0.067196488	56404	0.062916734
56400	0.080627964	56408	0.073142168
56400	0.027708669	56412	0.082513019
56400	0.016751807	56416	0.078259902
56400	0.017097582	56420	0.045419092
56400	0.047235299	56424	0.061104969
56400	0.070345908	56428	0.050101597
56400	0.036572976	56432	0.082521605
56400	0.078636171	56436	0.143904719
56400	0.070426147	56440	0.160300639
56400	0.048692629	56444	0.152245891
56400	0.043455217	56448	0.129454988
56500	0.033221812	56452	0.076609736
56500	0.025576541	56456	0.061194864
56500	0.028088311	56460	0.076655779
56500	0.060427399	56464	0.078676145
56500	0.023223942	56468	0.072965369
56500	0.053123662	56472	0.040769075
56500	0.070426089	56476	0.034670677
56500	0.02233852	56480	0.083566934
56500	0.0772555	56484	0.077810983
56500	0.114149858	56488	0.053318188

PowerSpectrumData

56500	0.099288067	56492	0.056247245
56500	0.055713354	56496	0.080255195
56500	0.072954528	56500	0.070632152
56500	0.076876604	56504	0.04323565
56500	0.06770128	56508	0.083463056
56500	0.066068817	56512	0.115974573
56500	0.024884888	56516	0.103506063
56500	0.025957952	56520	0.070861934
56500	0.0376422	56524	0.016800448
56500	0.030585503	56528	0.049125159
56500	0.023039944	56532	0.085770378
56500	0.053908334	56536	0.133995694
56500	0.065233202	56540	0.193212516
56500	0.092217095	56544	0.187809361
56500	0.091645619	56548	0.102660866
56600	0.073717987	56552	0.091435759
56600	0.058659407	56556	0.105261381
56600	0.036678262	56560	0.070911694
56600	0.05823201	56564	0.041739702
56600	0.083100866	56568	0.059675305
56600	0.063134699	56572	0.099511519
56600	0.066365858	56576	0.110734909
56600	0.072450464	56580	0.094701063
56600	0.052504292	56584	0.09993618
56600	0.075123549	56588	0.1187007
56600	0.060910796	56592	0.117020209
56600	0.038394366	56596	0.087571163
56600	0.018428875	56600	0.057940368
56600	0.046995698	56604	0.062592582
56600	0.067865243	56608	0.08036619
56600	0.0319061	56612	0.060597977
56600	0.052344363	56616	0.049194736
56600	0.108065557	56620	0.075686694
56600	0.100648242	56624	0.084578715

PowerSpectrumData

56600	0.057277652	56628	0.079128971
56600	0.009721844	56632	0.075305419
56600	0.022797991	56636	0.040320338
56600	0.0441868	56640	0.036294303
56600	0.099987476	56644	0.090324356
56600	0.087589797	56648	0.095597141
56700	0.062740772	56652	0.089091547
56700	0.071677525	56656	0.098083197
56700	0.071509989	56660	0.103910308
56700	0.050068433	56664	0.090001617
56700	0.040959509	56668	0.039841536
56700	0.024117835	56672	0.021620695
56700	0.025487221	56676	0.049395199
56700	0.032275511	56680	0.050166877
56700	0.03564154	56684	0.063618179
56700	0.032315009	56688	0.069506343
56700	0.034311543	56692	0.077469231
56700	0.019871668	56696	0.094089686
56700	0.051080479	56700	0.110844325
56700	0.080326732	56704	0.092704366
56700	0.074779739	56708	0.045695502
56700	0.071170121	56712	0.096654694
56700	0.040848001	56716	0.091138092
56700	0.016935839	56720	0.048566664
56700	0.014149535	56724	0.041603875
56700	0.031712079	56728	0.050639606
56700	0.052081519	56732	0.013603211
56700	0.048780472	56736	0.056131179
56700	0.042130192	56740	0.074254116
56700	0.049530252	56744	0.025388836
56700	0.03950347	56748	0.028078326
56800	0.014836475	56752	0.038498998
56800	0.017480554	56756	0.026218351
56800	0.035611083	56760	0.044318847

PowerSpectrumData

56800	0.047059493	56764	0.041280386
56800	0.036530219	56768	0.037099653
56800	0.062273153	56772	0.045411849
56800	0.050888371	56776	0.058115587
56800	0.031693729	56780	0.053412732
56800	0.052929994	56784	0.067910391
56800	0.07296307	56788	0.097036609
56800	0.063187457	56792	0.065143686
56800	0.017789374	56796	0.014469228
56800	0.047089339	56800	0.039574392
56800	0.065560351	56804	0.065383509
56800	0.03976607	56808	0.076171404
56800	0.02065465	56812	0.040363564
56800	0.020370031	56816	0.039132243
56800	0.056131201	56820	0.053515869
56800	0.088017659	56824	0.043731161
56800	0.091686823	56828	0.051723175
56800	0.072873569	56832	0.046691686
56800	0.033123608	56836	0.034327666
56800	0.014022158	56840	0.048404498
56800	0.047741185	56844	0.093524381
56800	0.057345053	56848	0.10526213
56900	0.019824589	56852	0.07785557
56900	0.001503559	56856	0.041533382
56900	0.032739183	56860	0.049278733
56900	0.055929308	56864	0.062667132
56900	0.05129175	56868	0.057552319
56900	0.041973122	56872	0.069750502
56900	0.019209736	56876	0.090660818
56900	0.008481359	56880	0.078461228
56900	0.032010146	56884	0.079196121
56900	0.084667321	56888	0.109893728
56900	0.060244911	56892	0.101790021
56900	0.041518208	56896	0.046306825

PowerSpectrumData

56900	0.020949919	56900	0.031804746
56900	0.016236694	56904	0.007142953
56900	0.009371982	56908	0.045176261
56900	0.024364457	56912	0.05481717
56900	0.061505598	56916	0.031809657
56900	0.045699846	56920	0.038089522
56900	0.054777087	56924	0.06894199
56900	0.069035712	56928	0.073572337
56900	0.023138115	56932	0.048468482
56900	0.015817432	56936	0.028321576
56900	0.021846932	56940	0.031689309
56900	0.055036569	56944	0.041572916
56900	0.074196905	56948	0.054270397
57000	0.024184234	56952	0.056322417
57000	0.059169499	56956	0.055629585
57000	0.087967528	56960	0.060559774
57000	0.059546816	56964	0.029895829
57000	0.038892031	56968	0.050757779
57000	0.073212294	56972	0.102896687
57000	0.067147237	56976	0.082337523
57000	0.028231512	56980	0.032457203
57000	0.054291082	56984	0.016286156
57000	0.054018477	56988	0.026688709
57000	0.020492442	56992	0.035286601
57000	0.021936518	56996	0.046778121
57000	0.066767556	57000	0.044327764
57000	0.090215799	57004	0.049255337
57000	0.066451707	57008	0.04568684
57000	0.039687835	57012	0.049173465
57000	0.057020578	57016	0.043632801
57000	0.062803832	57020	0.058339658
57000	0.086557346	57024	0.067178982
57000	0.084163199	57028	0.047381229
57000	0.086349719	57032	0.057211706

PowerSpectrumData

57000	0.106532971	57036	0.090781672
57000	0.09305081	57040	0.063266867
57000	0.067993948	57044	0.03092509
57000	0.048676659	57048	0.079044454
57100	0.027213382	57052	0.073469535
57100	0.026017233	57056	0.064029649
57100	0.025664685	57060	0.040827028
57100	0.043964523	57064	0.020931304
57100	0.035881225	57068	0.049111703
57100	0.033158784	57072	0.043166449
57100	0.028665607	57076	0.009757588
57100	0.043065709	57080	0.029793169
57100	0.086136708	57084	0.023195389
57100	0.100137237	57088	0.060641851
57100	0.071961331	57092	0.102675993
57100	0.039694318	57096	0.082755825
57100	0.039272418	57100	0.032937987
57100	0.040897747	57104	0.017637602
57100	0.011648186	57108	0.04637746
57100	0.026228032	57112	0.073368174
57100	0.044664696	57116	0.088261229
57100	0.023812621	57120	0.06827039
57100	0.076531811	57124	0.062130195
57100	0.112276634	57128	0.064539672
57100	0.087293993	57132	0.054608801
57100	0.046950085	57136	0.037994752
57100	0.02828472	57140	0.020457408
57100	0.031064014	57144	0.042842785
57100	0.043421034	57148	0.048439815
57200	0.059058377	57152	0.005815879
57200	0.080108381	57156	0.039273604
57200	0.073490046	57160	0.059053615
57200	0.048289865	57164	0.070942195
57200	0.017802049	57168	0.065982116

PowerSpectrumData

57200	0.026612446	57172	0.030883475
57200	0.042365129	57176	0.015941248
57200	0.014747289	57180	0.032583703
57200	0.031745232	57184	0.057207704
57200	0.059609971	57188	0.059499587
57200	0.073346964	57192	0.054713491
57200	0.034621451	57196	0.059975377
57200	0.037875547	57200	0.048226437
57200	0.041064515	57204	0.069296642
57200	0.021908696	57208	0.066078472
57200	0.024783309	57212	0.074831216
57200	0.068329362	57216	0.076570759
57200	0.086107641	57220	0.07899577
57200	0.054300097	57224	0.086853659
57200	0.056122273	57228	0.041324973
57200	0.073885225	57232	0.030530802
57200	0.052815416	57236	0.037628015
57200	0.04708079	57240	0.027331278
57200	0.057153284	57244	0.033918892
57200	0.05587655	57248	0.048458333
57300	0.063044819	57252	0.011356178
57300	0.07091085	57256	0.053679443
57300	0.060486313	57260	0.069829264
57300	0.042463307	57264	0.047011417
57300	0.054283803	57268	0.027552762
57300	0.055255609	57272	0.045968787
57300	0.035231074	57276	0.036735492
57300	0.018141605	57280	0.044525641
57300	0.04629224	57284	0.066964654
57300	0.063667467	57288	0.066607317
57300	0.090742324	57292	0.055231598
57300	0.095553711	57296	0.038450773
57300	0.055551569	57300	0.039985211
57300	0.03799779	57304	0.015632499

PowerSpectrumData

57300	0.023255072	57308	0.048371079
57300	0.025871268	57312	0.062251413
57300	0.019599456	57316	0.098398981
57300	0.029788831	57320	0.140212578
57300	0.029503948	57324	0.099076409
57300	0.008850287	57328	0.076101591
57300	0.046608398	57332	0.096456904
57300	0.088502537	57336	0.073304895
57300	0.07952621	57340	0.042070085
57300	0.048001461	57344	0.026555426
57300	0.040265946	57348	0.045698391
57400	0.050645038	57352	0.047871556
57400	0.062590421	57356	0.023398019
57400	0.045406825	57360	0.029705774
57400	0.031328516	57364	0.04005853
57400	0.050115363	57368	0.063387859
57400	0.032409495	57372	0.026708578
57400	0.060413277	57376	0.072899362
57400	0.079242585	57380	0.041834552
57400	0.040807892	57384	0.014010295
57400	0.0327747	57388	0.076219345
57400	0.070240545	57392	0.096593663
57400	0.064000269	57396	0.017934983
57400	0.037239533	57400	0.032566219
57400	0.046503235	57404	0.022639422
57400	0.029300352	57408	0.044325047
57400	0.115654228	57412	0.100537473
57400	0.147704108	57416	0.081039165
57400	0.114144052	57420	0.05963002
57400	0.073743526	57424	0.079006291
57400	0.049215032	57428	0.074238407
57400	0.038377999	57432	0.04308363
57400	0.021202881	57436	0.064756794
57400	0.036107878	57440	0.078445999

PowerSpectrumData

57400	0.037761387	57444	0.083058287
57400	0.019303132	57448	0.04388746
57500	0.040127867	57452	0.037940958
57500	0.077514378	57456	0.042377564
57500	0.082485582	57460	0.06527792
57500	0.046655259	57464	0.081715356
57500	0.058623857	57468	0.049582021
57500	0.063590036	57472	0.058013022
57500	0.061370985	57476	0.052865238
57500	0.063405256	57480	0.041990468
57500	0.030645824	57484	0.033582513
57500	0.015524796	57488	0.035126745
57500	0.0336987	57492	0.019668456
57500	0.043429354	57496	0.061076404
57500	0.030950876	57500	0.101068647
57500	0.031141834	57504	0.067151079
57500	0.011394039	57508	0.028842836
57500	0.017498516	57512	0.051140109
57500	0.035931378	57516	0.089808033
57500	0.066722249	57520	0.081313439
57500	0.084136627	57524	0.034621466
57500	0.076066594	57528	0.076076627
57500	0.078107543	57532	0.114955903
57500	0.070327158	57536	0.083980434
57500	0.056431727	57540	0.028075523
57500	0.041575484	57544	0.015511876
57500	0.062750689	57548	0.041201583
57600	0.061618819	57552	0.039757819
57600	0.017770639	57556	0.024844547
57600	0.031518131	57560	0.078453559
57600	0.005183899	57564	0.078033605
57600	0.020478336	57568	0.053987413
57600	0.018469798	57572	0.075511132
57600	0.008408537	57576	0.068892921

PowerSpectrumData

57600	0.024291494	57580	0.035785764
57600	0.061534069	57584	0.038972699
57600	0.038407248	57588	0.068546651
57600	0.044432767	57592	0.096420823
57600	0.084560779	57596	0.068437563
57600	0.053672753	57600	0.006168004
57600	0.005269288	57604	0.030884196
57600	0.014793261	57608	0.034755147
57600	0.024848503	57612	0.068870213
57600	0.032275177	57616	0.106515348
57600	0.020069971	57620	0.117085183
57600	0.018648501	57624	0.121038676
57600	0.035517296	57628	0.088603236
57600	0.030396561	57632	0.035124671
57600	0.030843326	57636	0.015647343
57600	0.034496799	57640	0.023795752
57600	0.036239231	57644	0.04450079
57600	0.027973949	57648	0.06596552
57700	0.009957623	57652	0.068942478
57700	0.037110262	57656	0.035822613
57700	0.024524734	57660	0.030940824
57700	0.024554693	57664	0.063245017
57700	0.043929606	57668	0.049704078
57700	0.051351821	57672	0.016688045
57700	0.023206194	57676	0.02915868
57700	0.015746169	57680	0.055639179
57700	0.009685582	57684	0.080225866
57700	0.008506073	57688	0.112526162
57700	0.015549556	57692	0.110985915
57700	0.045840414	57696	0.084419102
57700	0.070288028	57700	0.031436586
57700	0.049019385	57704	0.07464239
57700	0.056796998	57708	0.079334954
57700	0.067165602	57712	0.024788522

PowerSpectrumData

57700	0.067680376	57716	0.048562437
57700	0.071420058	57720	0.080889884
57700	0.070222719	57724	0.069717062
57700	0.053337528	57728	0.066568129
57700	0.055500026	57732	0.044119744
57700	0.086576503	57736	0.016708264
57700	0.082527498	57740	0.007498804
57700	0.048667542	57744	0.063859094
57700	0.022175136	57748	0.084765954
57800	0.012853036	57752	0.056482295
57800	0.02908211	57756	0.063410014
57800	0.062340638	57760	0.050611034
57800	0.057259607	57764	0.048052196
57800	0.049063459	57768	0.069460235
57800	0.052430634	57772	0.064740736
57800	0.045787791	57776	0.035146404
57800	0.025939087	57780	0.022711307
57800	0.021846849	57784	0.037418628
57800	0.03238926	57788	0.04014522
57800	0.041964584	57792	0.041845575
57800	0.057394293	57796	0.049061597
57800	0.039980212	57800	0.038839455
57800	0.025144791	57804	0.05041172
57800	0.026598311	57808	0.088210421
57800	0.037750524	57812	0.092411443
57800	0.040287414	57816	0.020881682
57800	0.046631882	57820	0.046113695
57800	0.060884213	57824	0.058284066
57800	0.051205047	57828	0.073953474
57800	0.021213429	57832	0.073417461
57800	0.013567236	57836	0.044625118
57800	0.037520666	57840	0.049223476
57800	0.070410351	57844	0.01263698
57800	0.062815139	57848	0.074227282

PowerSpectrumData

57900	0.056526831	57852	0.085123502
57900	0.086436936	57856	0.071664079
57900	0.119859964	57860	0.033631644
57900	0.098274089	57864	0.023460791
57900	0.047402904	57868	0.058463913
57900	0.01552962	57872	0.063650616
57900	0.00950094	57876	0.010977134
57900	0.035192545	57880	0.012851038
57900	0.047787835	57884	0.024697265
57900	0.036308913	57888	0.010972852
57900	0.015811487	57892	0.027444288
57900	0.031961499	57896	0.035594174
57900	0.04690266	57900	0.048104856
57900	0.033234195	57904	0.046876576
57900	0.034961398	57908	0.059840186
57900	0.023641327	57912	0.084406027
57900	0.024918811	57916	0.072512434
57900	0.033560249	57920	0.063620915
57900	0.030061952	57924	0.026193966
57900	0.027692848	57928	0.036246875
57900	0.042983898	57932	0.009309549
57900	0.051143863	57936	0.024882726
57900	0.030958167	57940	0.037076487
57900	0.021833797	57944	0.027672741
57900	0.05122539	57948	0.040899104
58000	0.052038522	57952	0.055927179
58000	0.00879196	57956	0.045203829
58000	0.059607814	57960	0.036646368
58000	0.082008155	57964	0.051886607
58000	0.056739715	57968	0.056504563
58000	0.030165697	57972	0.090834401
58000	0.042223815	57976	0.095475632
58000	0.047014451	57980	0.050005165
58000	0.013550673	57984	0.018538067

PowerSpectrumData

58000	0.02623659	57988	0.0312222
58000	0.050144299	57992	0.025296695
58000	0.076237971	57996	0.057963218
58000	0.062348569	58000	0.083623265
58000	0.026748494	58004	0.054017652
58000	0.0025212	58008	0.040458104
58000	0.027036627	58012	0.048143636
58000	0.028372244	58016	0.030772462
58000	0.033246168	58020	0.082291124
58000	0.031579784	58024	0.108562155
58000	0.013372964	58028	0.120040459
58000	0.008645126	58032	0.116332296
58000	0.030760893	58036	0.118281823
58000	0.066117616	58040	0.117645883
58000	0.068661961	58044	0.09095994
58000	0.046613797	58048	0.065515538
58100	0.056185036	58052	0.042131702
58100	0.033091408	58056	0.007152522
58100	0.033993201	58060	0.044705015
58100	0.044096167	58064	0.05795345
58100	0.00244267	58068	0.024331097
58100	0.023113427	58072	0.073415213
58100	0.021884598	58076	0.084104555
58100	0.040982293	58080	0.078249286
58100	0.060372957	58084	0.091983398
58100	0.067008885	58088	0.079099824
58100	0.072899864	58092	0.056442652
58100	0.077001343	58096	0.079244281
58100	0.08636104	58100	0.098803816
58100	0.054024204	58104	0.040651292
58100	0.016100788	58108	0.030903913
58100	0.042589596	58112	0.027954269
58100	0.032836604	58116	0.045784007
58100	0.024733112	58120	0.031455984

PowerSpectrumData

58100	0.043107979	58124	0.025850626
58100	0.077459474	58128	0.042821252
58100	0.05670805	58132	0.054719028
58100	0.030484496	58136	0.075732562
58100	0.020635702	58140	0.060319158
58100	0.022459486	58144	0.007477253
58100	0.030699041	58148	0.044078028
58200	0.049258091	58152	0.089485628
58200	0.040274339	58156	0.121257166
58200	0.022282235	58160	0.110096313
58200	0.060393464	58164	0.076722507
58200	0.065618951	58168	0.053677937
58200	0.054299453	58172	0.023601699
58200	0.072791831	58176	0.016809556
58200	0.078089652	58180	0.051227002
58200	0.054814525	58184	0.078117613
58200	0.03970262	58188	0.058245256
58200	0.019758678	58192	0.029435269
58200	0.026918046	58196	0.032125267
58200	0.057567857	58200	0.053632943
58200	0.045534482	58204	0.053500789
58200	0.026052587	58208	0.040655814
58200	0.044543824	58212	0.060826285
58200	0.069590969	58216	0.044174361
58200	0.073206516	58220	0.031084783
58200	0.050955725	58224	0.043470125
58200	0.021137506	58228	0.035014073
58200	0.00398193	58232	0.030810286
58200	0.012026982	58236	0.03698084
58200	0.020150308	58240	0.031226318
58200	0.010611237	58244	0.011591464
58200	0.031619322	58248	0.034081542
58300	0.04858222	58252	0.041004128
58300	0.040826792	58256	0.098166383

PowerSpectrumData

58300	0.031670799	58260	0.094077557
58300	0.05868611	58264	0.014251441
58300	0.08783106	58268	0.070073664
58300	0.084866093	58272	0.064464213
58300	0.067410074	58276	0.04887132
58300	0.063533451	58280	0.095292031
58300	0.057345267	58284	0.122136436
58300	0.024843283	58288	0.072722156
58300	0.026602498	58292	0.042485855
58300	0.045754532	58296	0.047259142
58300	0.034530789	58300	0.050337461
58300	0.046053283	58304	0.061280494
58300	0.050027451	58308	0.067763751
58300	0.014020084	58312	0.061088947
58300	0.064129628	58316	0.038856964
58300	0.093907976	58320	0.044453944
58300	0.090494061	58324	0.053104683
58300	0.051779047	58328	0.053363139
58300	0.006847275	58332	0.01914942
58300	0.020124722	58336	0.02153507
58300	0.050013488	58340	0.02991074
58300	0.042730786	58344	0.041378702
58300	0.018530414	58348	0.026506508
58400	0.032725326	58352	0.048963841
58400	0.048115966	58356	0.06949441
58400	0.059007958	58360	0.051699066
58400	0.057858259	58364	0.062784355
58400	0.037242204	58368	0.096436503
58400	0.068969435	58372	0.080911916
58400	0.086273751	58376	0.053266438
58400	0.034505265	58380	0.077282974
58400	0.040731426	58384	0.079456404
58400	0.091426708	58388	0.044921952
58400	0.094225841	58392	0.02622677

PowerSpectrumData

58400	0.054936736	58396	0.045685611
58400	0.035609046	58400	0.03769964
58400	0.030246984	58404	0.025159521
58400	0.004215204	58408	0.007957605
58400	0.055258221	58412	0.015872634
58400	0.102795573	58416	0.036201054
58400	0.081010003	58420	0.021896778
58400	0.014126427	58424	0.027535823
58400	0.083957079	58428	0.043579985
58400	0.11882621	58432	0.061288192
58400	0.102200269	58436	0.065855718
58400	0.064376669	58440	0.037518814
58400	0.038958104	58444	0.105493651
58400	0.005246595	58448	0.201547213
58500	0.028892446	58452	0.222516843
58500	0.035225923	58456	0.135974769
58500	0.014535249	58460	0.063823049
58500	0.029057288	58464	0.056788449
58500	0.01892784	58468	0.032439537
58500	0.041322594	58472	0.051214389
58500	0.011868253	58476	0.052574273
58500	0.049600232	58480	0.055560886
58500	0.055209093	58484	0.01918902
58500	0.008376596	58488	0.042656109
58500	0.007136586	58492	0.08035684
58500	0.062998224	58496	0.102197053
58500	0.08373546	58500	0.073162097
58500	0.007478679	58504	0.060012018
58500	0.080289101	58508	0.049333936
58500	0.089708323	58512	0.062382584
58500	0.043167936	58516	0.082139144
58500	0.043415257	58520	0.069407601
58500	0.074240874	58524	0.088733337
58500	0.077302786	58528	0.083899715

PowerSpectrumData

58500	0.047926485	58532	0.061296822
58500	0.017631945	58536	0.048014201
58500	0.023410001	58540	0.06321077
58500	0.039731167	58544	0.10487008
58500	0.003281013	58548	0.144017104
58600	0.060030714	58552	0.130467771
58600	0.050135128	58556	0.069907874
58600	0.055487253	58560	0.029123157
58600	0.077067358	58564	0.06102007
58600	0.043011387	58568	0.068163608
58600	0.037937654	58572	0.042216994
58600	0.072050141	58576	0.023985731
58600	0.085941007	58580	0.009875708
58600	0.068068002	58584	0.022038659
58600	0.031828542	58588	0.01831716
58600	0.004657176	58592	0.070222042
58600	0.026738342	58596	0.077294506
58600	0.043077205	58600	0.086263273
58600	0.021295438	58604	0.1011942
58600	0.006260487	58608	0.075018099
58600	0.018825082	58612	0.048685382
58600	0.023304105	58616	0.068262263
58600	0.041592226	58620	0.124285376
58600	0.078489575	58624	0.108243614
58600	0.066681845	58628	0.044942979
58600	0.029535959	58632	0.030062885
58600	0.042181582	58636	0.077233126
58600	0.049669179	58640	0.077228535
58600	0.048683014	58644	0.044297351
58600	0.046037876	58648	0.025381027
58700	0.044374865	58652	0.088397836
58700	0.049314876	58656	0.126627361
58700	0.037945585	58660	0.100326775
58700	0.044879856	58664	0.069381043

PowerSpectrumData

58700	0.053567299	58668	0.081317216
58700	0.061404513	58672	0.088496563
58700	0.062870502	58676	0.085322929
58700	0.040034178	58680	0.084810308
58700	0.021062308	58684	0.06769543
58700	0.024496845	58688	0.051277089
58700	0.063243606	58692	0.036765006
58700	0.088334222	58696	0.034540175
58700	0.088788875	58700	0.048846669
58700	0.068552508	58704	0.057611043
58700	0.049112099	58708	0.052056064
58700	0.042081741	58712	0.061656647
58700	0.019236284	58716	0.068616173
58700	0.024832931	58720	0.06048584
58700	0.006062775	58724	0.07021855
58700	0.028501649	58728	0.057997579
58700	0.033953606	58732	0.059975031
58700	0.066047782	58736	0.052510557
58700	0.065756802	58740	0.053376461
58700	0.014474312	58744	0.078432822
58700	0.051696385	58748	0.105997038
58800	0.059701673	58752	0.140349134
58800	0.031977877	58756	0.114095645
58800	0.002582397	58760	0.061068611
58800	0.026739488	58764	0.071888797
58800	0.047569629	58768	0.100642901
58800	0.060994538	58772	0.071585964
58800	0.048119768	58776	0.024862915
58800	0.008319475	58780	0.01730272
58800	0.046872472	58784	0.034667239
58800	0.061801271	58788	0.076093631
58800	0.056939618	58792	0.066744324
58800	0.041498588	58796	0.034884459
58800	0.015513209	58800	0.056604797

PowerSpectrumData

58800	0.042306423	58804	0.098263576
58800	0.083822983	58808	0.051085241
58800	0.078327605	58812	0.032682252
58800	0.018232764	58816	0.030566775
58800	0.031629832	58820	0.033151242
58800	0.034780114	58824	0.050827461
58800	0.020000271	58828	0.082850063
58800	0.038979564	58832	0.100741323
58800	0.021953236	58836	0.072901879
58800	0.089858382	58840	0.044825214
58800	0.084961677	58844	0.080060418
58800	0.033993201	58848	0.069732414
58900	0.016967153	58852	0.071645613
58900	0.028235903	58856	0.095223091
58900	0.03424997	58860	0.065339475
58900	0.077726989	58864	0.023820283
58900	0.088958906	58868	0.031191456
58900	0.050908981	58872	0.035560617
58900	0.017496574	58876	0.036339923
58900	0.00649842	58880	0.03377302
58900	0.035687976	58884	0.006783016
58900	0.052519798	58888	0.029042143
58900	0.017560142	58892	0.07751416
58900	0.021485181	58896	0.048036873
58900	0.010133444	58900	0.041274408
58900	0.044776036	58904	0.049024813
58900	0.063907304	58908	0.010187846
58900	0.069620917	58912	0.033869845
58900	0.070245435	58916	0.073473653
58900	0.07420042	58920	0.055091474
58900	0.058313151	58924	0.004966336
58900	0.018939001	58928	0.023315386
58900	0.006239892	58932	0.007962127
58900	0.021559918	58936	0.017203525

PowerSpectrumData

58900	0.016561795	58940	0.079313926
58900	0.038632592	58944	0.116551571
58900	0.030822543	58948	0.081790633
59000	0.030651252	58952	0.045634555
59000	0.056044013	58956	0.021403164
59000	0.059426864	58960	0.01562867
59000	0.033147746	58964	0.013296941
59000	0.028242732	58968	0.02161958
59000	0.041956791	58972	0.045209814
59000	0.034923902	58976	0.069536531
59000	0.028061286	58980	0.078443663
59000	0.025827838	58984	0.048606824
59000	0.012887305	58988	0.030627165
59000	0.017816272	58992	0.03899587
59000	0.03599202	58996	0.0404821
59000	0.057767102	59000	0.034725883
59000	0.054383807	59004	0.01960151
59000	0.053761782	59008	0.067217923
59000	0.049591887	59012	0.057074121
59000	0.026603822	59016	0.029244386
59000	0.027047785	59020	0.047478625
59000	0.031510259	59024	0.053436088
59000	0.033828172	59028	0.038768747
59000	0.076274177	59032	0.03638514
59000	0.082582737	59036	0.043564636
59000	0.086670676	59040	0.033584583
59000	0.061450024	59044	0.026390426
59000	0.000284058	59048	0.012714076
59100	0.053545155	59052	0.019306783
59100	0.056815537	59056	0.020810005
59100	0.052613857	59060	0.015309595
59100	0.032716027	59064	0.028099936
59100	0.016826076	59068	0.027930344
59100	0.013513836	59072	0.021553085

PowerSpectrumData

59100	0.009089086	59076	0.012321487
59100	0.057387613	59080	0.031699685
59100	0.080557999	59084	0.044901801
59100	0.041929005	59088	0.085188513
59100	0.009310778	59092	0.103372353
59100	0.012068482	59096	0.090859045
59100	0.023466117	59100	0.073352552
59100	0.028614953	59104	0.036151418
59100	0.035830366	59108	0.016329841
59100	0.058462098	59112	0.035593283
59100	0.042765838	59116	0.057235859
59100	0.023835621	59120	0.046835106
59100	0.074765339	59124	0.02676089
59100	0.080078011	59128	0.042076594
59100	0.04604182	59132	0.059747876
59100	0.005598294	59136	0.081464103
59100	0.054174619	59140	0.086547203
59100	0.071441187	59144	0.07749904
59100	0.031575259	59148	0.079251717
59200	0.002361882	59152	0.076999866
59200	0.02833956	59156	0.063209438
59200	0.041030824	59160	0.064607681
59200	0.02224008	59164	0.055229069
59200	0.022683284	59168	0.041158219
59200	0.02919838	59172	0.054659598
59200	0.0280819	59176	0.09676331
59200	0.051151321	59180	0.102817692
59200	0.056749039	59184	0.046398836
59200	0.025371075	59188	0.012379528
59200	0.035989346	59192	0.022484315
59200	0.073114781	59196	0.024763704
59200	0.07876804	59200	0.019589648
59200	0.063681771	59204	0.059739366
59200	0.053360451	59208	0.046612699

PowerSpectrumData

59200	0.044827244	59212	0.017881162
59200	0.026249636	59216	0.006702484
59200	0.005812264	59220	0.009396613
59200	0.014807945	59224	0.019573579
59200	0.029091305	59228	0.038815153
59200	0.031158495	59232	0.055665612
59200	0.041376807	59236	0.042086842
59200	0.071057039	59240	0.037209724
59200	0.075932017	59244	0.033041026
59200	0.045739434	59248	0.013936196
59300	0.017508588	59252	0.024715686
59300	0.037946051	59256	0.05757506
59300	0.088544715	59260	0.087648215
59300	0.071804097	59264	0.089504851
59300	0.035117773	59268	0.045098732
59300	0.044928049	59272	0.044221142
59300	0.070911883	59276	0.048845031
59300	0.071714763	59280	0.05523202
59300	0.068967842	59284	0.039813887
59300	0.085865737	59288	0.03030534
59300	0.09453611	59292	0.028767139
59300	0.086773238	59296	0.032132317
59300	0.057080801	59300	0.05923754
59300	0.01085042	59304	0.05944095
59300	0.035621637	59308	0.028053091
59300	0.040381379	59312	0.022632094
59300	0.044500059	59316	0.061189661
59300	0.066697336	59320	0.086455279
59300	0.058725625	59324	0.048960625
59300	0.018571584	59328	0.029987908
59300	0.066344568	59332	0.063005784
59300	0.074578173	59336	0.070221169
59300	0.041263178	59340	0.018511442
59300	0.013133707	59344	0.029325023

PowerSpectrumData

59300	0.022232875	59348	0.035811008
59400	0.048422717	59352	0.04840628
59400	0.06474206	59356	0.051072653
59400	0.051087893	59360	0.021914601
59400	0.0568996	59364	0.037065209
59400	0.079754616	59368	0.063474909
59400	0.058832775	59372	0.022393722
59400	0.023008282	59376	0.065502223
59400	0.060032009	59380	0.065368957
59400	0.038677474	59384	0.09304467
59400	0.019885571	59388	0.092162583
59400	0.049726597	59392	0.060213119
59400	0.085305343	59396	0.067671732
59400	0.079907171	59400	0.066562665
59400	0.121921621	59404	0.073487579
59400	0.112942529	59408	0.078621328
59400	0.06318419	59412	0.073191222
59400	0.035129942	59416	0.059011516
59400	0.018628471	59420	0.032238495
59400	0.02978271	59424	0.085384636
59400	0.023669827	59428	0.109086759
59400	0.013154458	59432	0.08087021
59400	0.035002449	59436	0.061097846
59400	0.016483684	59440	0.023599445
59400	0.039516974	59444	0.028424342
59400	0.066340435	59448	0.050819614
59500	0.059307175	59452	0.070277296
59500	0.057952144	59456	0.057003679
59500	0.065569067	59460	0.058870937
59500	0.061086823	59464	0.078657562
59500	0.042293807	59468	0.072289353
59500	0.014845267	59472	0.061902625
59500	0.035596546	59476	0.045315559
59500	0.064779677	59480	0.067451205

PowerSpectrumData

59500	0.075550379	59484	0.077235352
59500	0.076292192	59488	0.019382382
59500	0.05568409	59492	0.034749013
59500	0.029806797	59496	0.024651356
59500	0.031878994	59500	0.031887877
59500	0.048019207	59504	0.071031136
59500	0.054216613	59508	0.072893097
59500	0.046717014	59512	0.029944811
59500	0.048026257	59516	0.00484575
59500	0.041368454	59520	0.019169134
59500	0.031406155	59524	0.051472085
59500	0.063584368	59528	0.077041957
59500	0.055990928	59532	0.07429035
59500	0.025400297	59536	0.041451516
59500	0.03396062	59540	0.050473318
59500	0.011272057	59544	0.016181437
59500	0.042972675	59548	0.050024253
59600	0.070678107	59552	0.056567766
59600	0.066889304	59556	0.04549888
59600	0.042202108	59560	0.070272574
59600	0.054148251	59564	0.088186644
59600	0.031853237	59568	0.065824483
59600	0.051419607	59572	0.074271098
59600	0.084393811	59576	0.06736206
59600	0.063348038	59580	0.01364601
59600	0.056641202	59584	0.024982075
59600	0.056803619	59588	0.046777659
59600	0.011525334	59592	0.058802027
59600	0.026522866	59596	0.082888226
59600	0.03722265	59600	0.051727802
59600	0.041503368	59604	0.006488418
59600	0.008713818	59608	0.021384331
59600	0.021495274	59612	0.041171355
59600	0.001303166	59616	0.056912137

PowerSpectrumData

59600	0.059438618	59620	0.045343852
59600	0.095921736	59624	0.05113786
59600	0.039549486	59628	0.085833657
59600	0.068630521	59632	0.074080577
59600	0.100212383	59636	0.055995981
59600	0.054149077	59640	0.09195177
59600	0.040518586	59644	0.096961267
59600	0.022750332	59648	0.084306565
59700	0.003736561	59652	0.024026018
59700	0.006251607	59656	0.048494279
59700	0.024080306	59660	0.074288444
59700	0.049293038	59664	0.078305842
59700	0.083295825	59668	0.050709798
59700	0.088445777	59672	0.069926879
59700	0.056357229	59676	0.078405421
59700	0.089263529	59680	0.095059229
59700	0.082781844	59684	0.118762036
59700	0.033056349	59688	0.1182208
59700	0.042702839	59692	0.103063234
59700	0.03832384	59696	0.076560507
59700	0.059365539	59700	0.035660687
59700	0.072119707	59704	0.011530095
59700	0.051226987	59708	0.041286767
59700	0.060951366	59712	0.058007026
59700	0.054642198	59716	0.06679901
59700	0.114727976	59720	0.05859981
59700	0.107518405	59724	0.055462664
59700	0.047201495	59728	0.091892747
59700	0.030044146	59732	0.123330974
59700	0.040722091	59736	0.109010623
59700	0.025442379	59740	0.073477742
59700	0.085794251	59744	0.058172958
59700	0.134011381	59748	0.049058883
59800	0.11099915	59752	0.062744824

PowerSpectrumData

59800	0.072113071	59756	0.0758479
59800	0.083237792	59760	0.057570374
59800	0.092166592	59764	0.050561626
59800	0.080261452	59768	0.073540883
59800	0.061756749	59772	0.118238924
59800	0.054356824	59776	0.116157018
59800	0.048277751	59780	0.074991142
59800	0.015905253	59784	0.051616462
59800	0.076486715	59788	0.040561932
59800	0.094707481	59792	0.041502099
59800	0.0400147	59796	0.086267937
59800	0.055580458	59800	0.110389738
59800	0.044152032	59804	0.07589376
59800	0.008572503	59808	0.069116431
59800	0.040921335	59812	0.125226986
59800	0.054817159	59816	0.165562669
59800	0.02546717	59820	0.166898798
59800	0.0646267	59824	0.114138333
59800	0.081932449	59828	0.061797015
59800	0.047398709	59832	0.043658645
59800	0.020225276	59836	0.078423116
59800	0.039709827	59840	0.110912981
59800	0.030828731	59844	0.100728699
59800	0.013320856	59848	0.11118855
59900	0.037728736	59852	0.12246285
59900	0.036300418	59856	0.145483413
59900	0.018472456	59860	0.131086418
59900	0.006651849	59864	0.080165621
59900	0.002465147	59868	0.090818336
59900	0.00464586	59872	0.108580542
59900	0.019701205	59876	0.101956845
59900	0.039081173	59880	0.08770937
59900	0.032916702	59884	0.062870618
59900	0.011738645	59888	0.085659267

PowerSpectrumData

59900	0.048052574	59892	0.154030408
59900	0.074212199	59896	0.131718101
59900	0.04181264	59900	0.066822926
59900	0.029941122	59904	0.058561349
59900	0.06630658	59908	0.083635147
59900	0.074424512	59912	0.076823984
59900	0.041256873	59916	0.081136241
59900	0.045546269	59920	0.124552054
59900	0.026773225	59924	0.114662209
59900	0.052016891	59928	0.056060679
59900	0.075031378	59932	0.07485009
59900	0.067567744	59936	0.102072874
59900	0.048506485	59940	0.089955196
59900	0.046440429	59944	0.091850532
59900	0.051840332	59948	0.106233681
60000	0.071638373	59952	0.068774018
60000	0.100044468	59956	0.052032596
60000	0.079150515	59960	0.062959603
60000	0.055442572	59964	0.027063175
60000	0.065074521	59968	0.031137137
60000	0.087372551	59972	0.045285571
60000	0.077905963	59976	0.030674706
60000	0.01544823	59980	0.045173321
60000	0.079486774	59984	0.073610572
60000	0.107682296	59988	0.058210568
60000	0.11138383	59992	0.024635707
60000	0.121808276	59996	0.043404085
60000	0.109642628	60000	0.115774317
60000	0.087196553	60004	0.132140864
60000	0.033732056	60008	0.08905177
60000	0.052549622	60012	0.06892877
60000	0.09612232	60016	0.106530475
60000	0.078463039	60020	0.085166124
60000	0.056698511	60024	0.060048755

PowerSpectrumData

60000	0.069581227	60028	0.077138408
60000	0.073472562	60032	0.110876012
60000	0.036501922	60036	0.097545548
60000	0.05053584	60040	0.055595701
60000	0.065505439	60044	0.07228546
60000	0.04111803	60048	0.070417184
60100	0.039113318	60052	0.090966772
60100	0.137338313	60056	0.10979511
60100	0.173179098	60060	0.094156203
60100	0.097018463	60064	0.075199299
60100	0.043024709	60068	0.039030485
60100	0.051847048	60072	0.041258696
60100	0.027007545	60076	0.06624189
60100	0.004893188	60080	0.031748881
60100	0.032581073	60084	0.026654499
60100	0.06575969	60088	0.052229145
60100	0.060320526	60092	0.075949698
60100	0.028785711	60096	0.072631148
60100	0.057177404	60100	0.053184253
60100	0.064271713	60104	0.028831104
60100	0.078398611	60108	0.012272373
60100	0.16469619	60112	0.012140757
60100	0.203997989	60116	0.030692019
60100	0.13391352	60120	0.040650262
60100	0.060683873	60124	0.051291052
60100	0.037912767	60128	0.059166032
60100	0.069016904	60132	0.062232546
60100	0.034786805	60136	0.066199071
60100	0.080635422	60140	0.044744927
60100	0.130790344	60144	0.011478826
60100	0.105956809	60148	0.019779311
60200	0.073399162	60152	0.014399845
60200	0.036165868	60156	0.022478436
60200	0.031834865	60160	0.021751313

PowerSpectrumData

60200	0.055286015	60164	0.048123071
60200	0.035330657	60168	0.020446329
60200	0.141436468	60172	0.029209312
60200	0.20264792	60176	0.036026588
60200	0.135110793	60180	0.027439075
60200	0.022574399	60184	0.033233875
60200	0.06476116	60188	0.030581723
60200	0.0565097	60192	0.06293878
60200	0.077660683	60196	0.10505219
60200	0.147155938	60200	0.120305165
60200	0.139352924	60204	0.110022971
60200	0.085302956	60208	0.080902559
60200	0.022566557	60212	0.077391822
60200	0.038422735	60216	0.058996106
60200	0.035485355	60220	0.03200116
60200	0.019480134	60224	0.076851029
60200	0.03509982	60228	0.056152614
60200	0.070194146	60232	0.030059073
60200	0.058446833	60236	0.061754668
60200	0.028780782	60240	0.07338891
60200	0.020472557	60244	0.060318354
60200	0.044142398	60248	0.049970797
60300	0.047633228	60252	0.045248624
60300	0.043679305	60256	0.031983731
60300	0.052241547	60260	0.004846954
60300	0.077248362	60264	0.030818246
60300	0.068747184	60268	0.076858938
60300	0.057818059	60272	0.111259789
60300	0.0565806	60276	0.084767824
60300	0.020448993	60280	0.044652283
60300	0.012845725	60284	0.046386838
60300	0.008438931	60288	0.044299864
60300	0.030906595	60292	0.049378661
60300	0.057761954	60296	0.092985953

PowerSpectrumData

60300	0.05756992	60300	0.130424698
60300	0.016910597	60304	0.120840581
60300	0.025385381	60308	0.044253651
60300	0.062783693	60312	0.060395552
60300	0.098410368	60316	0.103362116
60300	0.103590646	60320	0.126621308
60300	0.0698402	60324	0.111512614
60300	0.018509243	60328	0.065478656
60300	0.059802002	60332	0.042922206
60300	0.061129598	60336	0.030440267
60300	0.041279338	60340	0.049587281
60300	0.024882753	60344	0.085034051
60300	0.063319792	60348	0.07642743
60400	0.1057298	60352	0.065632448
60400	0.071621798	60356	0.078854879
60400	0.027499824	60360	0.090067886
60400	0.025541149	60364	0.090329384
60400	0.026369489	60368	0.092696238
60400	0.07947805	60372	0.079733225
60400	0.13726679	60376	0.028036371
60400	0.17357542	60380	0.066418128
60400	0.13537945	60384	0.115083618
60400	0.050839401	60388	0.153815796
60400	0.057367954	60392	0.172822053
60400	0.062908264	60396	0.144647653
60400	0.069834838	60400	0.101523619
60400	0.129955631	60404	0.081145096
60400	0.153323258	60408	0.079649137
60400	0.067589965	60412	0.089531248
60400	0.027839624	60416	0.103685023
60400	0.024250016	60420	0.119037934
60400	0.044704517	60424	0.124886385
60400	0.064230902	60428	0.138392265
60400	0.105393257	60432	0.129297769

PowerSpectrumData

60400	0.159380653	60436	0.090941634
60400	0.15091656	60440	0.059863134
60400	0.092517337	60444	0.034110191
60400	0.045115503	60448	0.054211399
60500	0.078133897	60452	0.069569876
60500	0.049123573	60456	0.058229027
60500	0.021223283	60460	0.028224911
60500	0.02797645	60464	0.060567279
60500	0.022921964	60468	0.078601464
60500	0.024111176	60472	0.09055969
60500	0.077075252	60476	0.125782623
60500	0.093501134	60480	0.141503043
60500	0.065308181	60484	0.130248314
60500	0.054100328	60488	0.127096443
60500	0.068903239	60492	0.142486548
60500	0.054973458	60496	0.157102768
60500	0.015916668	60500	0.162175929
60500	0.045132892	60504	0.132675763
60500	0.039616167	60508	0.13697923
60500	0.042833461	60512	0.124183731
60500	0.044484164	60516	0.082630395
60500	0.016812117	60520	0.08916761
60500	0.026723164	60524	0.108324217
60500	0.038697974	60528	0.080599129
60500	0.033811237	60532	0.03923116
60500	0.031036187	60536	0.057382738
60500	0.028072058	60540	0.07331591
60500	0.020516171	60544	0.100398589
60500	0.0116352	60548	0.119295655
60600	0.005731241	60552	0.114560586
60600	0.03677604	60556	0.111694833
60600	0.0462098	60560	0.138636883
60600	0.006229448	60564	0.148728388
60600	0.054225231	60568	0.122558282

PowerSpectrumData

60600	0.075966171	60572	0.121143676
60600	0.050625193	60576	0.093128969
60600	0.02831456	60580	0.030439138
60600	0.050304367	60584	0.106689033
60600	0.001245398	60588	0.140735909
60600	0.044971013	60592	0.120223682
60600	0.032233285	60596	0.125506355
60600	0.008388709	60600	0.11802317
60600	0.023579734	60604	0.066144523
60600	0.051004467	60608	0.034544642
60600	0.037905978	60612	0.066234868
60600	0.015728569	60616	0.106246094
60600	0.048153837	60620	0.105846186
60600	0.0470218	60624	0.07614772
60600	0.039056497	60628	0.08757264
60600	0.022341337	60632	0.108672881
60600	0.016367738	60636	0.124698156
60600	0.013460126	60640	0.073144722
60600	0.034416185	60644	0.050464409
60600	0.051029081	60648	0.12443641
60700	0.050249822	60652	0.130029454
60700	0.039145438	60656	0.096145544
60700	0.055961624	60660	0.060305087
60700	0.091601811	60664	0.083752733
60700	0.116520932	60668	0.121644567
60700	0.104810359	60672	0.129949491
60700	0.040051291	60676	0.112090347
60700	0.053713331	60680	0.103957726
60700	0.098208788	60684	0.082577484
60700	0.076288772	60688	0.068406902
60700	0.060950173	60692	0.131699766
60700	0.065878543	60696	0.141996439
60700	0.057906967	60700	0.048794234
60700	0.007681037	60704	0.070614646

PowerSpectrumData

60700	0.047533722	60708	0.115551564
60700	0.040519488	60712	0.106316467
60700	0.014938279	60716	0.118547767
60700	0.045723529	60720	0.14874569
60700	0.047568043	60724	0.169761464
60700	0.024702102	60728	0.170798929
60700	0.016903081	60732	0.137935873
60700	0.015451278	60736	0.108791799
60700	0.022377091	60740	0.108092972
60700	0.016185373	60744	0.1368019
60700	0.042917352	60748	0.172359244
60800	0.044781486	60752	0.170130079
60800	0.043544584	60756	0.138699834
60800	0.073568052	60760	0.11157601
60800	0.077357428	60764	0.091557842
60800	0.06380726	60768	0.083698586
60800	0.057850262	60772	0.058182348
60800	0.046838813	60776	0.011260347
60800	0.056004581	60780	0.026222846
60800	0.030842472	60784	0.059731647
60800	0.018870851	60788	0.08721371
60800	0.070081347	60792	0.108066364
60800	0.104563493	60796	0.130147891
60800	0.090624999	60800	0.125542414
60800	0.054683009	60804	0.095123425
60800	0.053608532	60808	0.095338139
60800	0.039071085	60812	0.1070047
60800	0.046790377	60816	0.098324221
60800	0.049077651	60820	0.057925554
60800	0.045319812	60824	0.045265795
60800	0.044414435	60828	0.058193411
60800	0.019037369	60832	0.089047921
60800	0.011708479	60836	0.130498709
60800	0.030527892	60840	0.079098332

PowerSpectrumData

60800	0.035147583	60844	0.007726882
60800	0.029275039	60848	0.049048773
60900	0.032681437	60852	0.058378955
60900	0.021748258	60856	0.056999565
60900	0.049717455	60860	0.041239811
60900	0.047177586	60864	0.012893705
60900	0.015053895	60868	0.005982148
60900	0.078746256	60872	0.037794202
60900	0.106434462	60876	0.035389614
60900	0.083191641	60880	0.023744135
60900	0.026985621	60884	0.051342904
60900	0.014461341	60888	0.075982614
60900	0.049722137	60892	0.058594425
60900	0.061201252	60896	0.045302066
60900	0.038930295	60900	0.073027004
60900	0.032231848	60904	0.073132294
60900	0.047373698	60908	0.045004326
60900	0.060684146	60912	0.076637218
60900	0.075957461	60916	0.075720171
60900	0.072450013	60920	0.046759877
60900	0.038639049	60924	0.066971545
60900	0.013232943	60928	0.030377571
60900	0.005561016	60932	0.012648478
60900	0.032440614	60936	0.019933192
60900	0.042578537	60940	0.04984171
60900	0.034602326	60944	0.070472335
60900	0.038746715	60948	0.064251064
61000	0.026690037	60952	0.055077573
61000	0.029077737	60956	0.087496883
61000	0.067799963	60960	0.078663761
61000	0.051752992	60964	0.041628468
61000	0.02925913	60968	0.043825541
61000	0.018294646	60972	0.051343552
61000	0.021621956	60976	0.057032939

PowerSpectrumData

61000	0.040658273	60980	0.067191053
61000	0.038132803	60984	0.032918386
61000	0.013545962	60988	0.011168898
61000	0.052101896	60992	0.017704429
61000	0.061742794	60996	0.01264558
61000	0.051720435	61000	0.024266583
61000	0.034264918	61004	0.023798139
61000	0.020586356	61008	0.066125947
61000	0.014201428	61012	0.092832706
61000	0.022264978	61016	0.090215464
61000	0.018893104	61020	0.034035533
61000	0.035422701	61024	0.045094308
61000	0.042449545	61028	0.073157324
61000	0.028800172	61032	0.072863724
61000	0.007372938	61036	0.073687945
61000	0.023783816	61040	0.066155422
61000	0.054240394	61044	0.053092193
61000	0.063548789	61048	0.03823185
61100	0.055775341	61052	0.035754521
61100	0.054470813	61056	0.031809635
61100	0.068168192	61060	0.037172889
61100	0.071954855	61064	0.059572132
61100	0.064787426	61068	0.023962857
61100	0.049035734	61072	0.061158062
61100	0.008468654	61076	0.085757551
61100	0.051646817	61080	0.05501269
61100	0.057742622	61084	0.069684167
61100	0.032372693	61088	0.097555603
61100	0.019489091	61092	0.061916064
61100	0.046055753	61096	0.034469365
61100	0.049660204	61100	0.059623948
61100	0.008952927	61104	0.067005472
61100	0.03160728	61108	0.077725839
61100	0.048111084	61112	0.06471196

PowerSpectrumData

61100	0.091517926	61116	0.04100554
61100	0.09366023	61120	0.038396167
61100	0.082697334	61124	0.058544916
61100	0.046736332	61128	0.04908874
61100	0.035793524	61132	0.034660916
61100	0.067380242	61136	0.044650707
61100	0.061368592	61140	0.045100678
61100	0.054598469	61144	0.048185822
61100	0.045167803	61148	0.054315278
61200	0.055691671	61152	0.071089547
61200	0.063791966	61156	0.112326678
61200	0.051008115	61160	0.12531245
61200	0.045983037	61164	0.099691111
61200	0.0393298	61168	0.157768228
61200	0.035069254	61172	0.194454129
61200	0.058447011	61176	0.115490337
61200	0.065331544	61180	0.043797441
61200	0.047221172	61184	0.07752113
61200	0.029994917	61188	0.103036182
61200	0.040375413	61192	0.050016741
61200	0.030834057	61196	0.036135079
61200	0.011144209	61200	0.079329788
61200	0.00945041	61204	0.104003819
61200	0.016077496	61208	0.09300674
61200	0.042726319	61212	0.08705927
61200	0.051856558	61216	0.088027417
61200	0.030950076	61220	0.069853842
61200	0.03535231	61224	0.033863609
61200	0.049708779	61228	0.01773799
61200	0.027183893	61232	0.050000323
61200	0.040857351	61236	0.073726391
61200	0.065426466	61240	0.084918742
61200	0.065615961	61244	0.009641098
61200	0.067145425	61248	0.051265313

PowerSpectrumData

61300	0.065140492	61252	0.07855468
61300	0.010965685	61256	0.088711058
61300	0.034690846	61260	0.032854441
61300	0.03004499	61264	0.027240873
61300	0.027555212	61268	0.024911135
61300	0.046668218	61272	0.047593254
61300	0.019713418	61276	0.066467044
61300	0.026854363	61280	0.057633875
61300	0.056633449	61284	0.057742578
61300	0.066043882	61288	0.049357303
61300	0.053593471	61292	0.045366604
61300	0.035422545	61296	0.028132467
61300	0.016197735	61300	0.084122607
61300	0.043463992	61304	0.096084514
61300	0.052088777	61308	0.055437209
61300	0.102944949	61312	0.075697266
61300	0.102639853	61316	0.14378583
61300	0.037285321	61320	0.135635841
61300	0.054116365	61324	0.120768578
61300	0.089186353	61328	0.115906572
61300	0.077264136	61332	0.077831661
61300	0.040633757	61336	0.039775365
61300	0.010607087	61340	0.098140969
61300	0.006589846	61344	0.098575751
61300	0.016180296	61348	0.092655493
61400	0.05260676	61352	0.075230717
61400	0.073039475	61356	0.064614818
61400	0.035466317	61360	0.083360021
61400	0.03426609	61364	0.088876383
61400	0.024149533	61368	0.071093411
61400	0.00985752	61372	0.060241411
61400	0.01415662	61376	0.048168982
61400	0.03669941	61380	0.075201409
61400	0.035709792	61384	0.102218044

61400	0.011139667	61388	0.121488047
61400	0.040066509	61392	0.112503411
61400	0.029524581	61396	0.076582401
61400	0.03544637	61400	0.063949497
61400	0.01158442	61404	0.108943568
61400	0.032686898	61408	0.084954154
61400	0.035764653	61412	0.056467074
61400	0.008719423	61416	0.080007754
61400	0.017661669	61420	0.095573239
61400	0.011243777	61424	0.108601438
61400	0.031630563	61428	0.095315409
61400	0.024434809	61432	0.103551844
61400	0.029961955	61436	0.120626144
61400	0.056987828	61440	0.080273974
61400	0.039191971	61444	0.013117911
61400	0.030861473	61448	0.034905221
61500	0.031079046	61452	0.063344611
61500	0.029939094	61456	0.054374552
61500	0.081670696	61460	0.110996865
61500	0.082973718	61464	0.123437145
61500	0.066911322	61468	0.072080118
61500	0.070342423	61472	0.009121757
61500	0.058656886	61476	0.086910637
61500	0.042019332	61480	0.117900745
61500	0.030264573	61484	0.10712981
61500	0.012287267	61488	0.096021991
61500	0.012011203	61492	0.090860878
61500	0.030229854	61496	0.094707284
61500	0.048885839	61500	0.10107024
61500	0.062975916	61504	0.133910376
61500	0.068564099	61508	0.1323506
61500	0.063780513	61512	0.054255015
61500	0.053173648	61516	0.046187866
61500	0.03907103	61520	0.051280265

PowerSpectrumData

61500	0.047915997	61524	0.069537295
61500	0.055284621	61528	0.103101251
61500	0.024634244	61532	0.096722608
61500	0.014423322	61536	0.046249854
61500	0.049695369	61540	0.010030572
61500	0.078401543	61544	0.071721108
61500	0.071996947	61548	0.116607604
61600	0.071126277	61552	0.145061728
61600	0.054124164	61556	0.120125384
61600	0.019824234	61560	0.089883914
61600	0.009612324	61564	0.105836734
61600	0.038850885	61568	0.128674889
61600	0.026817828	61572	0.155787988
61600	0.026901615	61576	0.13895797
61600	0.04971783	61580	0.087856875
61600	0.0264669	61584	0.063045176
61600	0.011682624	61588	0.033419474
61600	0.042002441	61592	0.025794494
61600	0.051639334	61596	0.00418716
61600	0.037252004	61600	0.007146866
61600	0.034102643	61604	0.042508753
61600	0.05507785	61608	0.083290819
61600	0.030482614	61612	0.136979303
61600	0.016374526	61616	0.155829955
61600	0.022211798	61620	0.097767937
61600	0.039197985	61624	0.024004381
61600	0.021295518	61628	0.020213931
61600	0.062293388	61632	0.029263805
61600	0.089202178	61636	0.017435285
61600	0.066394976	61640	0.044577992
61600	0.026533891	61644	0.105173902
61600	0.007593271	61648	0.119184726
61700	0.009898374	61652	0.105272942
61700	0.019735106	61656	0.087658846

61700	0.033738823	61660	0.044301251
61700	0.026668919	61664	0.011633369
61700	0.034646564	61668	0.045275578
61700	0.056160483	61672	0.072794981
61700	0.051512874	61676	0.070797411
61700	0.016116241	61680	0.021110667
61700	0.039370949	61684	0.035758181
61700	0.066922396	61688	0.067931796
61700	0.068022222	61692	0.067080895
61700	0.054886721	61696	0.040854386
61700	0.065985347	61700	0.06230372
61700	0.04135659	61704	0.047311671
61700	0.007176075	61708	0.035235971
61700	0.009473807	61712	0.012922191
61700	0.019414303	61716	0.041712669
61700	0.027547183	61720	0.050124534
61700	0.038094826	61724	0.034042463
61700	0.059486418	61728	0.056463599
61700	0.061881532	61732	0.059845977
61700	0.036604393	61736	0.066374298
61700	0.035166679	61740	0.062147628
61700	0.013075519	61744	0.013194128
61700	0.009852034	61748	0.051687628
61800	0.02624091	61752	0.055660981
61800	0.052354888	61756	0.01055813
61800	0.04240924	61760	0.02637428
61800	0.030495223	61764	0.051182989
61800	0.026498126	61768	0.091978887
61800	0.010470778	61772	0.074617288
61800	0.032583379	61776	0.056434059
61800	0.050399078	61780	0.034353569
61800	0.029087079	61784	0.021392025
61800	0.023098959	61788	0.045774425
61800	0.048998092	61792	0.046428486

PowerSpectrumData

61800	0.045285375	61796	0.046203324
61800	0.022326807	61800	0.030966225
61800	0.046982299	61804	0.016968668
61800	0.040995936	61808	0.044451033
61800	0.043275773	61812	0.065075416
61800	0.026580918	61816	0.034860568
61800	0.035737041	61820	0.024773101
61800	0.053203065	61824	0.045079985
61800	0.051291681	61828	0.066190485
61800	0.063255786	61832	0.051424173
61800	0.068443042	61836	0.046024379
61800	0.028783419	61840	0.067669156
61800	0.011235661	61844	0.049012044
61800	0.018786832	61848	0.013370336
61900	0.033623255	61852	0.023385381
61900	0.047082398	61856	0.008156795
61900	0.028864342	61860	0.041359996
61900	0.002936509	61864	0.061776053
61900	0.005355793	61868	0.041005631
61900	0.041884774	61872	0.035970254
61900	0.078909165	61876	0.072546151
61900	0.08152645	61880	0.088853201
61900	0.07520106	61884	0.027428061
61900	0.076411954	61888	0.053120428
61900	0.056770528	61892	0.048164548
61900	0.019920308	61896	0.011118534
61900	0.005087159	61900	0.052105075
61900	0.010578496	61904	0.031546053
61900	0.016686239	61908	0.03023645
61900	0.029121577	61912	0.04973626
61900	0.052710482	61916	0.03662442
61900	0.060217622	61920	0.032613927
61900	0.043621745	61924	0.022350851
61900	0.006034817	61928	0.006815973

PowerSpectrumData

61900	0.025397611	61932	0.065985507
61900	0.029682846	61936	0.089573441
61900	0.051843395	61940	0.042673364
61900	0.057175927	61944	0.031450836
61900	0.100955178	61948	0.096783777
62000	0.090768197	61952	0.115703973
62000	0.040892588	61956	0.088109991
62000	0.044195138	61960	0.080660837
62000	0.054344895	61964	0.087038468
62000	0.012090904	61968	0.088727938
62000	0.051840772	61972	0.072150935
62000	0.091834794	61976	0.018316314
62000	0.108568114	61980	0.032839824
62000	0.102989514	61984	0.045689681
62000	0.062942185	61988	0.043527922
62000	0.019800515	61992	0.029894254
62000	0.022502001	61996	0.054467237
62000	0.022464596	62000	0.065216074
62000	0.021152426	62004	0.063507767
62000	0.01742182	62008	0.054333708
62000	0.04139663	62012	0.039644208
62000	0.016152806	62016	0.048940357
62000	0.029865774	62020	0.053970212
62000	0.045166587	62024	0.042289405
62000	0.029030531	62028	0.039520808
62000	0.035687051	62032	0.07275126
62000	0.034838718	62036	0.101989739
62000	0.053671625	62040	0.086103282
62000	0.043536111	62044	0.055539011
62000	0.035689507	62048	0.034997247
62100	0.031825737	62052	0.08688189
62100	0.033563221	62056	0.139664029
62100	0.062148625	62060	0.114813724
62100	0.096182215	62064	0.079735299

PowerSpectrumData

62100	0.119322169	62068	0.075060583
62100	0.113776783	62072	0.081942555
62100	0.10615433	62076	0.114947405
62100	0.064025473	62080	0.102618091
62100	0.007375839	62084	0.067630528
62100	0.022051831	62088	0.080433521
62100	0.021085098	62092	0.112232367
62100	0.048659847	62096	0.111437425
62100	0.067615016	62100	0.083809231
62100	0.065135413	62104	0.052802348
62100	0.044322555	62108	0.058824342
62100	0.013305678	62112	0.052965952
62100	0.022297647	62116	0.018240804
62100	0.054005766	62120	0.063523745
62100	0.084850071	62124	0.130393528
62100	0.06229146	62128	0.137657873
62100	0.037683203	62132	0.079013495
62100	0.035426983	62136	0.026643093
62100	0.021460503	62140	0.034942543
62100	0.038945935	62144	0.057809168
62100	0.090463691	62148	0.050841085
62200	0.101680518	62152	0.048220616
62200	0.045658715	62156	0.086072374
62200	0.052915239	62160	0.102991362
62200	0.055937861	62164	0.069067282
62200	0.039087678	62168	0.094367286
62200	0.04092895	62172	0.145226411
62200	0.052640371	62176	0.152614841
62200	0.097321732	62180	0.136353585
62200	0.063974505	62184	0.116972442
62200	0.039256913	62188	0.099503428
62200	0.030500083	62192	0.095746713
62200	0.013152007	62196	0.066815104
62200	0.015473304	62200	0.052499279

PowerSpectrumData

62200	0.057400626	62204	0.050485829
62200	0.071160699	62208	0.065526096
62200	0.073234878	62212	0.057746311
62200	0.045325964	62216	0.055526332
62200	0.022175384	62220	0.053678472
62200	0.028069357	62224	0.03134321
62200	0.050317001	62228	0.088310255
62200	0.072950024	62232	0.102885701
62200	0.040679639	62236	0.049645863
62200	0.017444239	62240	0.050533727
62200	0.03089348	62244	0.074805765
62200	0.045336998	62248	0.055915338
62300	0.037308382	62252	0.062447769
62300	0.026555321	62256	0.055542772
62300	0.055362583	62260	0.065640204
62300	0.072287679	62264	0.071220311
62300	0.054405515	62268	0.051971041
62300	0.042457439	62272	0.025810085
62300	0.029397248	62276	0.033396129
62300	0.009228088	62280	0.030357982
62300	0.045832403	62284	0.025386722
62300	0.059786187	62288	0.039137496
62300	0.023458564	62292	0.059137958
62300	0.037742462	62296	0.054905417
62300	0.042835723	62300	0.042461721
62300	0.020690995	62304	0.066529981
62300	0.045466208	62308	0.075056349
62300	0.039663701	62312	0.04851347
62300	0.050437793	62316	0.013755431
62300	0.069229507	62320	0.01810594
62300	0.055135148	62324	0.02049115
62300	0.023637127	62328	0.023890325
62300	0.014947751	62332	0.029282664
62300	0.022817891	62336	0.054530203

PowerSpectrumData

62300	0.0414362	62340	0.058176058
62300	0.056096742	62344	0.029892979
62300	0.040740568	62348	0.025006159
62400	0.04243568	62352	0.073880488
62400	0.043668217	62356	0.112317008
62400	0.023645549	62360	0.094240917
62400	0.039512121	62364	0.076057986
62400	0.078665893	62368	0.083323757
62400	0.059031147	62372	0.053387386
62400	0.053994845	62376	0.020728303
62400	0.046450768	62380	0.042482589
62400	0.023428405	62384	0.052838193
62400	0.021487582	62388	0.067619156
62400	0.074407137	62392	0.078730314
62400	0.07341864	62396	0.065708991
62400	0.038848277	62400	0.076237688
62400	0.051293046	62404	0.092457172
62400	0.052462005	62408	0.074677759
62400	0.03387106	62412	0.056470839
62400	0.013186993	62416	0.055565895
62400	0.060922099	62420	0.045976278
62400	0.055034878	62424	0.06686589
62400	0.012372031	62428	0.09489448
62400	0.008893498	62432	0.091335875
62400	0.018374532	62436	0.086387787
62400	0.018070914	62440	0.081519109
62400	0.05406198	62444	0.074625175
62400	0.032934822	62448	0.038306407
62500	0.010178183	62452	0.022429493
62500	0.038329356	62456	0.017848392
62500	0.044842996	62460	0.031593132
62500	0.034749304	62464	0.06874479
62500	0.048819937	62468	0.047632828
62500	0.035785131	62472	0.046248937

PowerSpectrumData

62500	0.024019524	62476	0.042377422
62500	0.01397212	62480	0.04941734
62500	0.0436388	62484	0.039726347
62500	0.055209715	62488	0.039655861
62500	0.073043913	62492	0.067482833
62500	0.04529217	62496	0.081824255
62500	0.050542058	62500	0.072184521
62500	0.060937007	62504	0.082100836
62500	0.028653354	62508	0.073912226
62500	0.013210058	62512	0.026832951
62500	0.019247849	62516	0.083157211
62500	0.03450648	62520	0.083908039
62500	0.032731346	62524	0.064392028
62500	0.019204017	62528	0.057719077
62500	0.004503719	62532	0.059226088
62500	0.040037186	62536	0.042463656
62500	0.094208663	62540	0.044958033
62500	0.119626122	62544	0.062334962
62500	0.100366604	62548	0.062901148
62600	0.064523032	62552	0.013722884
62600	0.029256877	62556	0.077088364
62600	0.030276102	62560	0.093592738
62600	0.039363211	62564	0.084467261
62600	0.015401502	62568	0.069627102
62600	0.06961394	62572	0.049632323
62600	0.075096905	62576	0.033962075
62600	0.047051381	62580	0.083145169
62600	0.011914004	62584	0.097167598
62600	0.021690847	62588	0.048324739
62600	0.038918915	62592	0.046936912
62600	0.052957799	62596	0.076343022
62600	0.053655705	62600	0.089772497
62600	0.05797769	62604	0.067331857
62600	0.039450788	62608	0.027634644

PowerSpectrumData

62600	0.010147583	62612	0.022139711
62600	0.017646924	62616	0.053291944
62600	0.038969516	62620	0.037782764
62600	0.088106746	62624	0.033280063
62600	0.100714577	62628	0.020196943
62600	0.069612732	62632	0.042033109
62600	0.028235152	62636	0.038601018
62600	0.024607898	62640	0.016107179
62600	0.019520276	62644	0.045576897
62600	0.015789969	62648	0.069643036
62700	0.047009242	62652	0.059609793
62700	0.082943356	62656	0.032309119
62700	0.066825014	62660	0.044794499
62700	0.042090545	62664	0.061779567
62700	0.047520953	62668	0.043828502
62700	0.058107296	62672	0.052652093
62700	0.058618658	62676	0.093485352
62700	0.025024156	62680	0.068108311
62700	0.054033433	62684	0.06378525
62700	0.056950572	62688	0.124835889
62700	0.044854518	62692	0.115303155
62700	0.012654782	62696	0.061318118
62700	0.051458275	62700	0.042262556
62700	0.049800379	62704	0.0249935
62700	0.049331677	62708	0.030379315
62700	0.037616184	62712	0.049233211
62700	0.036402518	62716	0.032416152
62700	0.069996	62720	0.028519758
62700	0.052878582	62724	0.057474299
62700	0.023604476	62728	0.066393841
62700	0.034452718	62732	0.069084388
62700	0.085195759	62736	0.090363392
62700	0.106979278	62740	0.090634807
62700	0.069952723	62744	0.06687671

PowerSpectrumData

62700	0.02899758	62748	0.030367513
62800	0.027406821	62752	0.007052509
62800	0.015641848	62756	0.059035883
62800	0.028880959	62760	0.092140261
62800	0.047726164	62764	0.086001572
62800	0.035928268	62768	0.087724395
62800	0.05138179	62772	0.107843487
62800	0.071464856	62776	0.08827768
62800	0.062647247	62780	0.080655431
62800	0.061967447	62784	0.091925875
62800	0.031949978	62788	0.079565805
62800	0.0453986	62792	0.072155977
62800	0.055558499	62796	0.059924696
62800	0.034004508	62800	0.040139104
62800	0.045753845	62804	0.039333816
62800	0.064578155	62808	0.048419122
62800	0.058580463	62812	0.053412041
62800	0.051059767	62816	0.071993956
62800	0.044410954	62820	0.088246503
62800	0.056352732	62824	0.092313254
62800	0.067809124	62828	0.04040089
62800	0.050386498	62832	0.059129579
62800	0.003832736	62836	0.107315129
62800	0.03098854	62840	0.112155089
62800	0.039970608	62844	0.11762308
62800	0.068301641	62848	0.109196713
62900	0.084987645	62852	0.083341911
62900	0.068935471	62856	0.046023648
62900	0.056701829	62860	0.042938555
62900	0.057664893	62864	0.097269731
62900	0.012335251	62868	0.123812468
62900	0.039055467	62872	0.118282143
62900	0.058820639	62876	0.087552442
62900	0.05867083	62880	0.049514551

PowerSpectrumData

62900	0.046079	62884	0.031269578
62900	0.048226902	62888	0.061747218
62900	0.037079317	62892	0.081194354
62900	0.039574858	62896	0.092042348
62900	0.029874143	62900	0.085205
62900	0.01633739	62904	0.05387728
62900	0.03857063	62908	0.058098849
62900	0.065547123	62912	0.047549725
62900	0.078500147	62916	0.034685036
62900	0.066394394	62920	0.007868594
62900	0.043701533	62924	0.028872326
62900	0.026435328	62928	0.05440403
62900	0.01783211	62932	0.071433075
62900	0.018479654	62936	0.044034758
62900	0.027812603	62940	0.023134095
62900	0.041516196	62944	0.041067502
62900	0.040898282	62948	0.065712877
63000	0.02153027	62952	0.059528491
63000	0.023208653	62956	0.021000629
63000	0.04718659	62960	0.016377528
63000	0.069226255	62964	0.012844316
63000	0.071799688	62968	0.007845739
63000	0.037762711	62972	0.02275129
63000	0.007462065	62976	0.042679309
63000	0.017318522	62980	0.03750356
63000	0.03816457	62984	0.038284292
63000	0.039225968	62988	0.05129987
63000	0.036067318	62992	0.041059415
63000	0.035420559	62996	0.042727952
63000	0.038273447	63000	0.034058732
63000	0.043549891	63004	0.029632212
63000	0.033930141	63008	0.054996279
63000	0.018589642	63012	0.040489893
63000	0.016794353	63016	0.012481704

PowerSpectrumData

63000	0.027178074	63020	0.039840325
63000	0.043299719	63024	0.039010229
63000	0.073573865	63028	0.072455085
63000	0.077123033	63032	0.080451559
63000	0.051094903	63036	0.054731459
63000	0.051006453	63040	0.066805522
63000	0.048653383	63044	0.104710452
63000	0.016534006	63048	0.088410416
63100	0.020080906	63052	0.031104089
63100	0.025679638	63056	0.078888246
63100	0.045085188	63060	0.092603208
63100	0.054600125	63064	0.06032157
63100	0.020217285	63068	0.066896333
63100	0.024350291	63072	0.057359815
63100	0.02518576	63076	0.031850053
63100	0.019778407	63080	0.086181179
63100	0.017594148	63084	0.109693428
63100	0.026179721	63088	0.102795202
63100	0.053851651	63092	0.082521445
63100	0.046670535	63096	0.035350655
63100	0.013408635	63100	0.005638384
63100	0.015411381	63104	0.027261651
63100	0.029035324	63108	0.066768524
63100	0.052577252	63112	0.047784615
63100	0.055040968	63116	0.024379076
63100	0.024906269	63120	0.054568707
63100	0.026450995	63124	0.043711632
63100	0.063030384	63128	0.015167284
63100	0.086186105	63132	0.036083966
63100	0.072494375	63136	0.044761546
63100	0.059162507	63140	0.033069806
63100	0.069494628	63144	0.034244509
63100	0.049855553	63148	0.024347826
63200	0.020561205	63152	0.051719533

PowerSpectrumData

63200	0.058098871	63156	0.077009274
63200	0.013978927	63160	0.078236175
63200	0.04984238	63164	0.076891527
63200	0.043167689	63168	0.077509561
63200	0.05046367	63172	0.081985425
63200	0.080193589	63176	0.060091483
63200	0.089501351	63180	0.052463023
63200	0.07707913	63184	0.092980889
63200	0.039956103	63188	0.107128435
63200	0.023639233	63192	0.061360261
63200	0.031067622	63196	0.021505141
63200	0.016321497	63200	0.04922555
63200	0.030286301	63204	0.053747557
63200	0.048161135	63208	0.041962539
63200	0.046134919	63212	0.071906725
63200	0.046395053	63216	0.070008762
63200	0.026950273	63220	0.040823481
63200	0.006574019	63224	0.045011595
63200	0.049224233	63228	0.075584685
63200	0.085260886	63232	0.08914512
63200	0.101459096	63236	0.058424535
63200	0.106960346	63240	0.010623326
63200	0.069350717	63244	0.006909143
63200	0.043611137	63248	0.024801195
63300	0.05072941	63252	0.02170683
63300	0.052381547	63256	0.005748394
63300	0.04545975	63260	0.011504475
63300	0.041910131	63264	0.05222172
63300	0.015768599	63268	0.05813727
63300	0.029705188	63272	0.005590666
63300	0.056797133	63276	0.045656034
63300	0.04892379	63280	0.064410531
63300	0.021830241	63284	0.045979454
63300	0.029817016	63288	0.025495092

PowerSpectrumData

63300	0.032586966	63292	0.044746663
63300	0.064923057	63296	0.068346795
63300	0.084246662	63300	0.091037269
63300	0.053061147	63304	0.111023765
63300	0.040609833	63308	0.097411641
63300	0.079174519	63312	0.050217801
63300	0.044062395	63316	0.03678698
63300	0.043728811	63320	0.023914125
63300	0.065389206	63324	0.050667131
63300	0.065403729	63328	0.073712698
63300	0.061394741	63332	0.039713119
63300	0.052238967	63336	0.026634512
63300	0.072524701	63340	0.06020016
63300	0.122649624	63344	0.055564909
63300	0.132483954	63348	0.051851963
63400	0.091166163	63352	0.033206117
63400	0.053386189	63356	0.035906302
63400	0.028769584	63360	0.06524707
63400	0.019326089	63364	0.034878951
63400	0.04587958	63368	0.05693944
63400	0.030343135	63372	0.066416425
63400	0.019887577	63376	0.082284692
63400	0.051796807	63380	0.105290535
63400	0.051600157	63384	0.0716758
63400	0.04928992	63388	0.023619137
63400	0.046099583	63392	0.034633969
63400	0.080976446	63396	0.054698659
63400	0.091634443	63400	0.068981572
63400	0.049618422	63404	0.051833857
63400	0.05003317	63408	0.041967724
63400	0.038970218	63412	0.059687278
63400	0.01450424	63416	0.081521073
63400	0.025484831	63420	0.062561179
63400	0.018544104	63424	0.009887895

PowerSpectrumData

63400	0.009724683	63428	0.04060442
63400	0.024039735	63432	0.059835347
63400	0.009564136	63436	0.047792579
63400	0.031318457	63440	0.026212158
63400	0.076680066	63444	0.027246197
63400	0.080556354	63448	0.010054123
63500	0.033199642	63452	0.041535805
63500	0.029292423	63456	0.051236708
63500	0.015943815	63460	0.038741728
63500	0.032491262	63464	0.020345106
63500	0.026221958	63468	0.021312955
63500	0.023720399	63472	0.039794588
63500	0.043417585	63476	0.051050902
63500	0.055992648	63480	0.042946915
63500	0.061527848	63484	0.047103411
63500	0.034832869	63488	0.027802706
63500	0.006540846	63492	0.023270988
63500	0.014976911	63496	0.05775609
63500	0.027574013	63500	0.042898373
63500	0.036830952	63504	0.02591964
63500	0.024540554	63508	0.057361136
63500	0.022312835	63512	0.061428917
63500	0.045974732	63516	0.06764273
63500	0.022515951	63520	0.015743277
63500	0.023149021	63524	0.027294966
63500	0.039067047	63528	0.016541635
63500	0.029172963	63532	0.017733511
63500	0.042090433	63536	0.020788604
63500	0.036918198	63540	0.056349341
63500	0.03741085	63544	0.054619835
63500	0.034917597	63548	0.052882762
63600	0.041863656	63552	0.026748156
63600	0.028320721	63556	0.013685282
63600	0.013941995	63560	0.049385708

PowerSpectrumData

63600	0.050077502	63564	0.052944623
63600	0.090794558	63568	0.041461215
63600	0.121327808	63572	0.076336437
63600	0.098313685	63576	0.061964856
63600	0.053804357	63580	0.038524187
63600	0.028724462	63584	0.033470067
63600	0.030229967	63588	0.058730402
63600	0.054510983	63592	0.042676235
63600	0.054187782	63596	0.044129502
63600	0.027014894	63600	0.086125925
63600	0.033831489	63604	0.139014679
63600	0.053248841	63608	0.106689608
63600	0.056561796	63612	0.037057049
63600	0.052976171	63616	0.021747359
63600	0.047215879	63620	0.017338465
63600	0.06093712	63624	0.034734127
63600	0.062354447	63628	0.047583741
63600	0.044520966	63632	0.047296966
63600	0.055213619	63636	0.041427051
63600	0.096111988	63640	0.039058781
63600	0.089901383	63644	0.040756695
63600	0.073561823	63648	0.013408402
63700	0.070812472	63652	0.041935033
63700	0.018239269	63656	0.024869909
63700	0.028123201	63660	0.02671274
63700	0.009879989	63664	0.058303453
63700	0.030247713	63668	0.081270293
63700	0.055632052	63672	0.098505851
63700	0.081849968	63676	0.088669614
63700	0.078758327	63680	0.068290363
63700	0.034574565	63684	0.062114479
63700	0.015990321	63688	0.034453933
63700	0.010610631	63692	0.0652378
63700	0.036968308	63696	0.094119678

PowerSpectrumData

63700	0.069510323	63700	0.074715725
63700	0.057217971	63704	0.051079205
63700	0.017437662	63708	0.061585983
63700	0.03509011	63712	0.048172184
63700	0.055032582	63716	0.003665795
63700	0.045626533	63720	0.030482932
63700	0.021911214	63724	0.047893271
63700	0.04114976	63728	0.084606087
63700	0.092228773	63732	0.110702458
63700	0.076619755	63736	0.078220299
63700	0.07583436	63740	0.046360434
63700	0.09540467	63744	0.059474729
63700	0.079568345	63748	0.050242863
63800	0.089057154	63752	0.041410509
63800	0.105805731	63756	0.047930927
63800	0.080670427	63760	0.050527171
63800	0.035056779	63764	0.084694206
63800	0.021240521	63768	0.142578035
63800	0.027319407	63772	0.095578827
63800	0.013245338	63776	0.048040896
63800	0.017930033	63780	0.064349879
63800	0.004732639	63784	0.054238008
63800	0.037708855	63788	0.066064757
63800	0.0472549	63792	0.085764827
63800	0.065864908	63796	0.078814432
63800	0.089233778	63800	0.086146938
63800	0.076901735	63804	0.098897064
63800	0.043840621	63808	0.081305116
63800	0.061916493	63812	0.083153165
63800	0.113374619	63816	0.119590244
63800	0.094076961	63820	0.092663409
63800	0.033210268	63824	0.060962346
63800	0.038357328	63828	0.090673464
63800	0.044228353	63832	0.084678868

PowerSpectrumData

63800	0.036951511	63836	0.080450496
63800	0.040080646	63840	0.052244428
63800	0.045767843	63844	0.007878267
63800	0.039711558	63848	0.029232424
63900	0.022948585	63852	0.049009861
63900	0.010467759	63856	0.038345377
63900	0.002989303	63860	0.028993898
63900	0.022885115	63864	0.033789522
63900	0.028846029	63868	0.093064911
63900	0.012288923	63872	0.126168423
63900	0.014582465	63876	0.099831537
63900	0.016006319	63880	0.066597117
63900	0.021933814	63884	0.081699938
63900	0.043017339	63888	0.105365005
63900	0.002119759	63892	0.11566333
63900	0.053765132	63896	0.131347959
63900	0.062139916	63900	0.116884505
63900	0.031436848	63904	0.126038708
63900	0.023325645	63908	0.140348391
63900	0.069672533	63912	0.10445268
63900	0.063808955	63916	0.103415114
63900	0.038462389	63920	0.140094009
63900	0.051012954	63924	0.11626671
63900	0.041883035	63928	0.077329918
63900	0.049644827	63932	0.117236814
63900	0.051273837	63936	0.140758334
63900	0.05657076	63940	0.13066172
63900	0.064547028	63944	0.061942985
63900	0.057666341	63948	0.061224499
64000	0.072265437	63952	0.113265371
64000	0.093664909	63956	0.105593324
64000	0.121027915	63960	0.052259249
64000	0.097035605	63964	0.028371122
64000	0.047750593	63968	0.058369857

PowerSpectrumData

64000	0.072201779	63972	0.069997048
64000	0.066475535	63976	0.075000549
64000	0.04430216	63980	0.044769462
64000	0.029813935	63984	0.01277582
64000	0.019601275	63988	0.06096558
64000	0.0549016	63992	0.082641433
64000	0.072015813	63996	0.076699253
64000	0.025602391	64000	0.066357388
64000	0.032224172	64004	0.055963472
64000	0.039116869	64008	0.054899498
64000	0.016241433	64012	0.03279551
64000	0.039975486	64016	0.021384787
64000	0.030790896	64020	0.073169089
64000	0.039507529	64024	0.108894521
64000	0.070087728	64028	0.103769249
64000	0.07946774	64032	0.115409923
64000	0.0345351	64036	0.114997565
64000	0.03886253	64040	0.070724149
64000	0.06158503	64044	0.052029202
64000	0.053395634	64048	0.071870731
64100	0.038753293	64052	0.076786339
64100	0.022811593	64056	0.071785857
64100	0.009464485	64060	0.050865343
64100	0.027480355	64064	0.02772695
64100	0.020049627	64068	0.013300836
64100	0.022885728	64072	0.011587618
64100	0.001620573	64076	0.033902325
64100	0.052390926	64080	0.029638628
64100	0.077162709	64084	0.007277824
64100	0.068242865	64088	0.025602971
64100	0.062594481	64092	0.033273169
64100	0.049368806	64096	0.051137409
64100	0.036138772	64100	0.059045371
64100	0.025071415	64104	0.044645203

PowerSpectrumData

64100	0.020781519	64108	0.041225881
64100	0.010386851	64112	0.082126986
64100	0.034897297	64116	0.091764676
64100	0.043392694	64120	0.043191045
64100	0.018372959	64124	0.061382925
64100	0.025616535	64128	0.053969965
64100	0.025626983	64132	0.043515654
64100	0.009870495	64136	0.039273164
64100	0.017412538	64140	0.032758617
64100	0.022422088	64144	0.040822786
64100	0.020580639	64148	0.033442328
64200	0.022130265	64152	0.04156931
64200	0.040235525	64156	0.053535641
64200	0.068317524	64160	0.029925366
64200	0.062048144	64164	0.018540262
64200	0.067166642	64168	0.045885754
64200	0.065101958	64172	0.024661036
64200	0.058442314	64176	0.013743153
64200	0.063986161	64180	0.041703384
64200	0.096687552	64184	0.044023385
64200	0.101921738	64188	0.032141848
64200	0.067906542	64192	0.03967424
64200	0.035233072	64196	0.066522269
64200	0.061555089	64200	0.080977363
64200	0.043199158	64204	0.041647341
64200	0.050184717	64208	0.015165559
64200	0.056197991	64212	0.014773619
64200	0.042049011	64216	0.026316844
64200	0.041403426	64220	0.06072452
64200	0.075644493	64224	0.101485392
64200	0.089553148	64228	0.122445505
64200	0.052841693	64232	0.143557466
64200	0.010097989	64236	0.129870969
64200	0.03590891	64240	0.092309987

PowerSpectrumData

64200	0.069955939	64244	0.087444641
64200	0.074177653	64248	0.052000039
64300	0.055816116	64252	0.067188274
64300	0.042073833	64256	0.109189903
64300	0.042323685	64260	0.1143673
64300	0.048824728	64264	0.085324507
64300	0.02974326	64268	0.079875637
64300	0.024954437	64272	0.074108735
64300	0.023254223	64276	0.024427483
64300	0.082627739	64280	0.050884759
64300	0.146274295	64284	0.076223201
64300	0.116319032	64288	0.068162655
64300	0.089974412	64292	0.080179947
64300	0.079999838	64296	0.100561192
64300	0.026642572	64300	0.064495078
64300	0.064645494	64304	0.037585665
64300	0.094270261	64308	0.110761852
64300	0.092005059	64312	0.111696558
64300	0.095496645	64316	0.069841321
64300	0.081863618	64320	0.040626008
64300	0.046669709	64324	0.018568851
64300	0.028838087	64328	0.064139174
64300	0.041904837	64332	0.099685771
64300	0.056786048	64336	0.076785211
64300	0.047589751	64340	0.05118184
64300	0.013340185	64344	0.031931057
64300	0.047320638	64348	0.063499792
64400	0.052638279	64352	0.09754327
64400	0.039581388	64356	0.103702099
64400	0.052934069	64360	0.083983701
64400	0.051395531	64364	0.051824613
64400	0.042225322	64368	0.084949563
64400	0.049178714	64372	0.103780651
64400	0.051795047	64376	0.089061272

PowerSpectrumData

64400	0.057016958	64380	0.074123658
64400	0.062167645	64384	0.06389842
64400	0.046853587	64388	0.037414211
64400	0.033718723	64392	0.039233069
64400	0.031249118	64396	0.032686501
64400	0.03949504	64400	0.027824486
64400	0.031207215	64404	0.06812573
64400	0.01949095	64408	0.082230348
64400	0.045118093	64412	0.071545299
64400	0.064681612	64416	0.084676016
64400	0.080973667	64420	0.057997604
64400	0.0432165	64424	0.073298237
64400	0.04213268	64428	0.122556347
64400	0.076562108	64432	0.106851818
64400	0.027687076	64436	0.056226953
64400	0.021251362	64440	0.001971436
64400	0.013588804	64444	0.069627728
64400	0.01951161	64448	0.106518441
64500	0.046030997	64452	0.103578714
64500	0.028561879	64456	0.101466561
64500	0.020912554	64460	0.085179425
64500	0.037008598	64464	0.062470725
64500	0.018739964	64468	0.054217144
64500	0.021937149	64472	0.072001043
64500	0.046311852	64476	0.113416834
64500	0.058651054	64480	0.111680827
64500	0.039626641	64484	0.073314026
64500	0.025474417	64488	0.065367764
64500	0.065092027	64492	0.069874615
64500	0.064007763	64496	0.064532025
64500	0.058590758	64500	0.029365501
64500	0.041482872	64504	0.026026624
64500	0.034336841	64508	0.067911606
64500	0.038968366	64512	0.099002115

PowerSpectrumData

64500	0.008449655	64516	0.073141091
64500	0.04470459	64520	0.028333923
64500	0.040563544	64524	0.027834525
64500	0.04353833	64528	0.026940754
64500	0.055920424	64532	0.041025858
64500	0.065727247	64536	0.06213184
64500	0.040214833	64540	0.053620315
64500	0.030022668	64544	0.024501665
64500	0.031331641	64548	0.069691232
64600	0.024162644	64552	0.085037267
64600	0.037090638	64556	0.046776218
64600	0.027821099	64560	0.036232934
64600	0.013180087	64564	0.027835626
64600	0.037277023	64568	0.010552694
64600	0.034286517	64572	0.022468767
64600	0.028200524	64576	0.055937308
64600	0.021254036	64580	0.107815242
64600	0.058295322	64584	0.124237413
64600	0.060130162	64588	0.079945916
64600	0.065873035	64592	0.034237157
64600	0.025162513	64596	0.015302567
64600	0.038854534	64600	0.031912212
64600	0.032201428	64604	0.049048769
64600	0.026323323	64608	0.050248167
64600	0.016345604	64612	0.052576539
64600	0.041749234	64616	0.034117773
64600	0.067377936	64620	0.043108503
64600	0.071101262	64624	0.09077572
64600	0.059502439	64628	0.103874816
64600	0.034707526	64632	0.056984689
64600	0.022541553	64636	0.036208166
64600	0.014457563	64640	0.064472304
64600	0.018543191	64644	0.072736606
64600	0.016214728	64648	0.059019858

PowerSpectrumData

64700	0.037224196	64652	0.015380692
64700	0.042564334	64656	0.042780401
64700	0.027921326	64660	0.055505829
64700	0.017291797	64664	0.043254364
64700	0.042248852	64668	0.04547101
64700	0.029551664	64672	0.04168271
64700	0.037814749	64676	0.026082404
64700	0.071112641	64680	0.038997576
64700	0.074451717	64684	0.018162804
64700	0.052373674	64688	0.021616972
64700	0.014107002	64692	0.068673413
64700	0.030716932	64696	0.114313916
64700	0.033795946	64700	0.110603891
64700	0.029870509	64704	0.074510877
64700	0.038591552	64708	0.036512374
64700	0.032961478	64712	0.011521253
64700	0.012994525	64716	0.037199301
64700	0.029907704	64720	0.052297157
64700	0.028677909	64724	0.033115211
64700	0.040363739	64728	0.045732297
64700	0.071725859	64732	0.080152553
64700	0.086653323	64736	0.06600302
64700	0.070476868	64740	0.077982077
64700	0.05643419	64744	0.062252213
64700	0.026270349	64748	0.040401963
64800	0.031896561	64752	0.006455537
64800	0.037033493	64756	0.036324876
64800	0.008764237	64760	0.048079033
64800	0.02709643	64764	0.042402422
64800	0.036487723	64768	0.04392003
64800	0.031460622	64772	0.045718753
64800	0.029260331	64776	0.022394841
64800	0.028587638	64780	0.015627882
64800	0.017170059	64784	0.023984305

PowerSpectrumData

64800	0.059748065	64788	0.061009792
64800	0.056313518	64792	0.041290157
64800	0.028591932	64796	0.002231513
64800	0.0754633	64800	0.01606038
64800	0.057049878	64804	0.020304135
64800	0.023983446	64808	0.027887896
64800	0.019218696	64812	0.060161267
64800	0.019575615	64816	0.066958623
64800	0.038520186	64820	0.027717191
64800	0.055457524	64824	0.026219263
64800	0.031074978	64828	0.04128088
64800	0.008007119	64832	0.025242049
64800	0.019045645	64836	0.029430052
64800	0.049616352	64840	0.034160665
64800	0.07789438	64844	0.023441162
64800	0.064416265	64848	0.0509854
64900	0.047422858	64852	0.065245098
64900	0.023725619	64856	0.042742166
64900	0.022731734	64860	0.04441143
64900	0.039823481	64864	0.020556843
64900	0.021756692	64868	0.050233382
64900	0.048032212	64872	0.070147238
64900	0.06036185	64876	0.041206298
64900	0.054574171	64880	0.045083329
64900	0.060983675	64884	0.081173675
64900	0.073713032	64888	0.058882066
64900	0.097399221	64892	0.044872486
64900	0.079271682	64896	0.031782456
64900	0.030072151	64900	0.024008137
64900	0.018098654	64904	0.033232172
64900	0.038557118	64908	0.033780871
64900	0.033549852	64912	0.050368333
64900	0.028558487	64916	0.053747539
64900	0.025754676	64920	0.03886742

PowerSpectrumData

64900	0.080175181	64924	0.040105806
64900	0.111916925	64928	0.054745837
64900	0.07907088	64932	0.055264292
64900	0.023756747	64936	0.076947967
64900	0.023001592	64940	0.120348821
64900	0.035474968	64944	0.132968649
64900	0.058050107	64948	0.091585498
65000	0.070968192	64952	0.070716022
65000	0.051757394	64956	0.083348517
65000	0.059570055	64960	0.071903138
65000	0.078031626	64964	0.058320729
65000	0.051051513	64968	0.016230393
65000	0.005882548	64972	0.023622866
65000	0.051018444	64976	0.028288379
65000	0.053359749	64980	0.019133009
65000	0.037611149	64984	0.019921488
65000	0.059207629	64988	0.031493528
65000	0.03666689	64992	0.035071935
65000	0.022650836	64996	0.124003855
65000	0.019127649	65000	0.134769784
65000	0.026427233	65004	0.089040201
65000	0.051593659	65008	0.055828852
65000	0.067187153	65012	0.069479567
65000	0.051543568	65016	0.045243312
65000	0.036197387	65020	0.06744048
65000	0.059546255	65024	0.099035264
65000	0.069000656	65028	0.099571596
65000	0.062783955	65032	0.090371534
65000	0.062777646	65036	0.058498983
65000	0.06303229	65040	0.031015035
65000	0.058709211	65044	0.016402784
65000	0.038524751	65048	0.043295364
65100	0.026527981	65052	0.038412301
65100	0.078420198	65056	0.044642678

PowerSpectrumData

65100	0.070203627	65060	0.007403616
65100	0.046025605	65064	0.025395104
65100	0.037896163	65068	0.005078929
65100	0.043001779	65072	0.026089341
65100	0.066553592	65076	0.085947046
65100	0.042101088	65080	0.05859834
65100	0.028336463	65084	0.075453885
65100	0.022572536	65088	0.114030707
65100	0.011922521	65092	0.112534879
65100	0.030582662	65096	0.038131784
65100	0.05931795	65100	0.081515951
65100	0.078783407	65104	0.091518727
65100	0.074124575	65108	0.08800027
65100	0.084482606	65112	0.084928564
65100	0.086720262	65116	0.095161326
65100	0.066532673	65120	0.11305439
65100	0.034469587	65124	0.128375526
65100	0.032381253	65128	0.10307083
65100	0.038520648	65132	0.061164159
65100	0.018726238	65136	0.095051037
65100	0.012894788	65140	0.085825443
65100	0.037170463	65144	0.080096565
65100	0.045858622	65148	0.073925789
65200	0.051323088	65152	0.031774627
65200	0.050989416	65156	0.076736142
65200	0.037018661	65160	0.129282445
65200	0.053701766	65164	0.216210071
65200	0.053458945	65168	0.153650631
65200	0.027960889	65172	0.014222657
65200	0.037538808	65176	0.060996656
65200	0.048322963	65180	0.00609988
65200	0.03743223	65184	0.072796895
65200	0.04145857	65188	0.041258049
65200	0.054164415	65192	0.062735308

PowerSpectrumData

65200	0.057314865	65196	0.147131374
65200	0.044976074	65200	0.192695269
65200	0.029046136	65204	0.094813491
65200	0.024008929	65208	0.121140096
65200	0.02407841	65212	0.031171712
65200	0.016363365	65216	0.065106666
65200	0.033511024	65220	0.05745425
65200	0.066187204	65224	0.080194717
65200	0.041112697	65228	0.067004454
65200	0.0152486	65232	0.05335969
65200	0.028697785	65236	0.057998601
65200	0.035584166	65240	0.087090302
65200	0.024705922	65244	0.090308953
65200	0.027368937	65248	0.104048209
65300	0.038838196	65252	0.161988515
65300	0.023843966	65256	0.088771587
65300	0.007345168	65260	0.008582842
65300	0.050086066	65264	0.121813166
65300	0.064718617	65268	0.160514435
65300	0.029329609	65272	0.067413537
65300	0.009776263	65276	0.014826461
65300	0.035546469	65280	0.073458163
65300	0.065494889	65284	0.094498952
65300	0.065070373	65288	0.073029485
65300	0.053017444	65292	0.069313297
65300	0.032265281	65296	0.069326394
65300	0.014351355	65300	0.083166204
65300	0.044261618	65304	0.091350034
65300	0.045317607	65308	0.102896542
65300	0.043033004	65312	0.086363325
65300	0.059352155	65316	0.077431549
65300	0.061478757	65320	0.105841697
65300	0.043903758	65324	0.083001229
65300	0.044451048	65328	0.09228904

PowerSpectrumData

65300	0.033246219	65332	0.031942542
65300	0.016745034	65336	0.102541431
65300	0.051450679	65340	0.150832726
65300	0.046355894	65344	0.13377158
65300	0.018976047	65348	0.151942819
65400	0.050401763	65352	0.172865738
65400	0.015384698	65356	0.10204866
65400	0.031640404	65360	0.039385544
65400	0.03520562	65364	0.037664191
65400	0.025365938	65368	0.044642791
65400	0.046046771	65372	0.114529154
65400	0.046166118	65376	0.139318086
65400	0.024916586	65380	0.061714847
65400	0.031283304	65384	0.071424052
65400	0.048650589	65388	0.205408054
65400	0.042259559	65392	0.226667631
65400	0.021627115	65396	0.150379303
65400	0.030011021	65400	0.156262163
65400	0.00512199	65404	0.166069164
65400	0.031107378	65408	0.088908964
65400	0.043887907	65412	0.097446144
65400	0.038132817	65416	0.126506653
65400	0.036962134	65420	0.129732041
65400	0.04693868	65424	0.117039563
65400	0.036103167	65428	0.124230806
65400	0.024501569	65432	0.146106148
65400	0.04246163	65436	0.157018833
65400	0.053514497	65440	0.160090553
65400	0.034309829	65444	0.15558851
65400	0.016841415	65448	0.140480086
65500	0.012839589	65452	0.12866396
65500	0.030742267	65456	0.111880479
65500	0.057440699	65460	0.079566395
65500	0.072356634	65464	0.107012958

PowerSpectrumData

65500	0.03225957	65468	0.110799498
65500	0.004328565	65472	0.075799006
65500	0.021245598	65476	0.046210334
65500	0.043418328	65480	0.055647233
65500	0.028648969	65484	0.075930613
65500	0.018685418	65488	0.084288906
65500	0.027571823	65492	0.076233861
65500	0.027958007	65496	0.062748601
65500	0.022034143	65500	0.087956098
65500	0.034384091	65504	0.082407212
65500	0.041754225	65508	0.067490961
65500	0.0571815	65512	0.039054863
65500	0.066927096	65516	0.078222387
65500	0.070373018	65520	0.124994302
65500	0.100548088	65524	0.113618036
65500	0.088659952	65528	0.090599933
65500	0.06944385	65532	0.063852946
65500	0.05231674	65536	0.056693476
65500	0.042443233	65540	0.067193992
65500	0.094010626	65544	0.079890167
65500	0.106641921	65548	0.122004065
65600	0.073459785	65552	0.099696219
65600	0.042500655	65556	0.040282757
65600	0.033149543	65560	0.038918861
65600	0.025723381	65564	0.03750213
65600	0.01932797	65568	0.048253591
65600	0.016980031	65572	0.134162154
65600	0.032209271	65576	0.140094736
65600	0.052966883	65580	0.110236964
65600	0.055436143	65584	0.112793641
65600	0.04502268	65588	0.078854413
65600	0.02998293	65592	0.012100717
65600	0.020565163	65596	0.055382006
65600	0.016144833	65600	0.058932488

PowerSpectrumData

65600	0.032226493	65604	0.08909779
65600	0.039238992	65608	0.105392079
65600	0.033762131	65612	0.055064229
65600	0.035724846	65616	0.029725148
65600	0.002107313	65620	0.030765827
65600	0.06927806	65624	0.079194892
65600	0.102554979	65628	0.080615741
65600	0.073080242	65632	0.073274881
65600	0.043583346	65636	0.048240472
65600	0.053821557	65640	0.033720648
65600	0.055218909	65644	0.077954937
65600	0.048281407	65648	0.071368908
65700	0.034681623	65652	0.06601186
65700	0.011553105	65656	0.085410749
65700	0.013316364	65660	0.076938246
65700	0.059086306	65664	0.045527893
65700	0.0848524	65668	0.043882421
65700	0.040268435	65672	0.080985315
65700	0.034683515	65676	0.117949567
65700	0.054492597	65680	0.148428983
65700	0.055871275	65684	0.134212896
65700	0.053381868	65688	0.09088339
65700	0.028736842	65692	0.05809428
65700	0.015201889	65696	0.067395944
65700	0.022783574	65700	0.087777044
65700	0.025182739	65704	0.10449766
65700	0.026748194	65708	0.112421905
65700	0.029333332	65712	0.075601762
65700	0.040362458	65716	0.021116883
65700	0.030399799	65720	0.030551528
65700	0.017362403	65724	0.055660934
65700	0.030319692	65728	0.080365251
65700	0.034198856	65732	0.090868838
65700	0.027539152	65736	0.12197964

PowerSpectrumData

65700	0.039205654	65740	0.158782626
65700	0.039577313	65744	0.140365738
65700	0.004057331	65748	0.104314269
65800	0.009900538	65752	0.083458275
65800	0.017347815	65756	0.056632329
65800	0.045753524	65760	0.035988745
65800	0.084497959	65764	0.019432206
65800	0.077131874	65768	0.035818335
65800	0.00720238	65772	0.109057852
65800	0.04956 65776	0.121546087	
65800	0.026572587	65780	0.072539799
65800	0.016963686	65784	0.026328491
65800	0.039511702	65788	0.082254432
65800	0.056630215	65792	0.06826254
65800	0.040989722	65796	0.037231625
65800	0.023451417	65800	0.034228902
65800	0.06946236	65804	0.051239229
65800	0.073455172	65808	0.035695779
65800	0.045772929	65812	0.049448852
65800	0.036703095	65816	0.098319601
65800	0.026695434	65820	0.089353765
65800	0.012608964	65824	0.095238604
65800	0.017980801	65828	0.112117014
65800	0.025236976	65832	0.103564875
65800	0.019394511	65836	0.090521302
65800	0.011742639	65840	0.065176399
65800	0.029478637	65844	0.064670348
65800	0.056774548	65848	0.081406448
65900	0.061472616	65852	0.094564843
65900	0.039579751	65856	0.062171348
65900	0.015286316	65860	0.015467354
65900	0.031025025	65864	0.051260649
65900	0.064258391	65868	0.090906491
65900	0.071451388	65872	0.099082216

PowerSpectrumData

65900	0.054872529	65876	0.038372429
65900	0.051105086	65880	0.045406821
65900	0.025299027	65884	0.06756877
65900	0.014701393	65888	0.067005232
65900	0.016899274	65892	0.016879034
65900	0.029033936	65896	0.073433403
65900	0.074916723	65900	0.088822977
65900	0.103115708	65904	0.092430397
65900	0.067933717	65908	0.053588927
65900	0.010919675	65912	0.091121765
65900	0.032887005	65916	0.09143175
65900	0.01406853	65920	0.067297158
65900	0.007804973	65924	0.06509653
65900	0.02138426	65928	0.074175732
65900	0.019397709	65932	0.086722561
65900	0.018147428	65936	0.063437576
65900	0.045804267	65940	0.066960769
65900	0.039628943	65944	0.058205504
65900	0.045527315	65948	0.034313976
66000	0.068651214	65952	0.042868451
66000	0.056703291	65956	0.0456749
66000	0.028690001	65960	0.024100224
66000	0.003361456	65964	0.02729327
66000	0.038839189	65968	0.064762193
66000	0.053798824	65972	0.102840306
66000	0.038036036	65976	0.133527778
66000	0.054664684	65980	0.111825488
66000	0.079754485	65984	0.08264459
66000	0.078359401	65988	0.049907878
66000	0.060607581	65992	0.033006007
66000	0.034947771	65996	0.05569492
66000	0.00450982	66000	0.064690044
66000	0.025631722	66004	0.015731777
66000	0.013176262	66008	0.030867272

PowerSpectrumData

66000	0.032517794	66012	0.057034537
66000	0.040191113	66016	0.068724134
66000	0.033583765	66020	0.046804024
66000	0.012252778	66024	0.019691635
66000	0.025030407	66028	0.074736512
66000	0.036594189	66032	0.107555527
66000	0.043905577	66036	0.09460919
66000	0.06438006	66040	0.057503152
66000	0.070949398	66044	0.056558518
66000	0.053001902	66048	0.099662037
66100	0.035564255	66052	0.115046736
66100	0.032948039	66056	0.085754982
66100	0.053377527	66060	0.055898327
66100	0.03837483	66064	0.029553119
66100	0.03980663	66068	0.117401185
66100	0.043626271	66072	0.155281713
66100	0.008013139	66076	0.114943876
66100	0.0470171	66080	0.09229852
66100	0.057152036	66084	0.076342942
66100	0.023897981	66088	0.084149433
66100	0.008304677	66092	0.130395929
66100	0.003097444	66096	0.132923626
66100	0.016533528	66100	0.096590295
66100	0.0204628	66104	0.082721286
66100	0.015247657	66108	0.129342123
66100	0.014965411	66112	0.127700536
66100	0.009055821	66116	0.080060519
66100	0.019827459	66120	0.097853415
66100	0.060561004	66124	0.134472139
66100	0.078692567	66128	0.137950949
66100	0.059448372	66132	0.103254475
66100	0.044191686	66136	0.043503289
66100	0.048831964	66140	0.072455034
66100	0.059599213	66144	0.097664735

PowerSpectrumData

66100	0.033376215	66148	0.062909006
66200	0.004103097	66152	0.069314352
66200	0.020788064	66156	0.101451224
66200	0.063630345	66160	0.060261908
66200	0.081491387	66164	0.021307169
66200	0.075332733	66168	0.032834363
66200	0.061317325	66172	0.020680625
66200	0.043312888	66176	0.033002569
66200	0.043432388	66180	0.045312758
66200	0.033549637	66184	0.034436129
66200	0.067838802	66188	0.042479955
66200	0.106036321	66192	0.056753361
66200	0.109641092	66196	0.05616714
66200	0.06691779	66200	0.079764031
66200	0.015088732	66204	0.065984175
66200	0.05041881	66208	0.040033057
66200	0.059555798	66212	0.05781596
66200	0.082869759	66216	0.053818785
66200	0.085389627	66220	0.047546957
66200	0.031928503	66224	0.045507542
66200	0.022732809	66228	0.050394931
66200	0.020668622	66232	0.072139825
66200	0.052885032	66236	0.073140531
66200	0.023808545	66240	0.032162719
66200	0.02014746	66244	0.028428251
66200	0.031650307	66248	0.011848179
66300	0.046852638	66252	0.039745195
66300	0.076259399	66256	0.046537611
66300	0.05422462	66260	0.034649638
66300	0.026168169	66264	0.026098318
66300	0.036129262	66268	0.021513608
66300	0.015246019	66272	0.021545378
66300	0.035186189	66276	0.067038673
66300	0.044939458	66280	0.048515165

PowerSpectrumData

66300	0.044982695	66284	0.004662149
66300	0.045775214	66288	0.018421702
66300	0.016711494	66292	0.022651333
66300	0.054043798	66296	0.038609836
66300	0.036497397	66300	0.050423947
66300	0.022542525	66304	0.032903085
66300	0.037430618	66308	0.012283074
66300	0.021437574	66312	0.048441179
66300	0.019449386	66316	0.033890479
66300	0.040532021	66320	0.011445942
66300	0.039589002	66324	0.065135027
66300	0.034556579	66328	0.10255845
66300	0.001394491	66332	0.082476508
66300	0.032600387	66336	0.047707359
66300	0.053611409	66340	0.040512347
66300	0.057764191	66344	0.033025572
66300	0.05523342	66348	0.048001592
66400	0.053109605	66352	0.056495675
66400	0.050158447	66356	0.037665675
66400	0.040025348	66360	0.024973833
66400	0.014976165	66364	0.029288422
66400	0.021620237	66368	0.023716033
66400	0.027727921	66372	0.040835901
66400	0.005443886	66376	0.107034655
66400	0.053385589	66380	0.071595336
66400	0.081915998	66384	0.040918443
66400	0.066832683	66388	0.079938844
66400	0.042895157	66392	0.087300359
66400	0.045305522	66396	0.071069109
66400	0.05602356	66400	0.066059904
66400	0.055004151	66404	0.042802611
66400	0.028656299	66408	0.051887961
66400	0.095341988	66412	0.04933375
66400	0.084913125	66416	0.016168702

PowerSpectrumData

66400	0.022354325	66420	0.022709688
66400	0.035735044	66424	0.040055464
66400	0.074417876	66428	0.029087425
66400	0.078652112	66432	0.066904169
66400	0.052650834	66436	0.092281574
66400	0.031745847	66440	0.063683976
66400	0.017461496	66444	0.021152589
66400	0.027581926	66448	0.018372037
66500	0.03526298	66452	0.042589767
66500	0.041971445	66456	0.065493601
66500	0.063549065	66460	0.088155743
66500	0.082887331	66464	0.094854702
66500	0.076865246	66468	0.062389772
66500	0.036848749	66472	0.05277797
66500	0.021422828	66476	0.075421827
66500	0.061252984	66480	0.093730174
66500	0.067609202	66484	0.097540411
66500	0.047199603	66488	0.061645194
66500	0.014473311	66492	0.032068285
66500	0.043344571	66496	0.098712364
66500	0.024622807	66500	0.085612373
66500	0.047607864	66504	0.084740255
66500	0.072186384	66508	0.108641019
66500	0.04898079	66512	0.076999284
66500	0.019969222	66516	0.035286332
66500	0.022953071	66520	0.004253027
66500	0.040601455	66524	0.033156332
66500	0.060882929	66528	0.055590183
66500	0.086697895	66532	0.045182129
66500	0.080324462	66536	0.03084942
66500	0.038195944	66540	0.017117705
66500	0.043368043	66544	0.068218564
66500	0.024971629	66548	0.127235093
66600	0.006227552	66552	0.137018229

PowerSpectrumData

66600	0.008888371	66556	0.101263941
66600	0.027010543	66560	0.053172404
66600	0.067079993	66564	0.037907696
66600	0.057416724	66568	0.04593248
66600	0.021349877	66572	0.059822334
66600	0.02691189	66576	0.064241023
66600	0.050373215	66580	0.060331349
66600	0.040582763	66584	0.083354418
66600	0.01783922	66588	0.040755233
66600	0.006699838	66592	0.024187664
66600	0.065613131	66596	0.014497965
66600	0.066894136	66600	0.03964841
66600	0.033946326	66604	0.092756425
66600	0.066655754	66608	0.085723237
66600	0.073327072	66612	0.040035582
66600	0.077907935	66616	0.054236727
66600	0.054031079	66620	0.081429651
66600	0.017218865	66624	0.090283713
66600	0.027165876	66628	0.06505051
66600	0.03930218	66632	0.035857906
66600	0.025478959	66636	0.049409253
66600	0.035715872	66640	0.056423167
66600	0.065120607	66644	0.025557465
66600	0.093542374	66648	0.053231608
66700	0.092065071	66652	0.079048208
66700	0.06764297	66656	0.056229594
66700	0.062891697	66660	0.009848957
66700	0.064254447	66664	0.054458829
66700	0.045375808	66668	0.098123463
66700	0.048892031	66672	0.108649721
66700	0.057775804	66676	0.054598721
66700	0.039396553	66680	0.035118439
66700	0.021141914	66684	0.062548636
66700	0.020071631	66688	0.080694386

PowerSpectrumData

66700	0.045256955	66692	0.074183728
66700	0.043051623	66696	0.076096418
66700	0.024751502	66700	0.069068992
66700	0.0406162	66704	0.0586071
66700	0.077056808	66708	0.072786243
66700	0.092504575	66712	0.05475145
66700	0.098646102	66716	0.034863275
66700	0.117889686	66720	0.03977499
66700	0.102384685	66724	0.031311123
66700	0.046543664	66728	0.004436381
66700	0.021857057	66732	0.037024074
66700	0.04678515	66736	0.055088021
66700	0.067759414	66740	0.035220925
66700	0.033189099	66744	0.05497153
66700	0.026257938	66748	0.085986663
66800	0.029226578	66752	0.036762474
66800	0.030644456	66756	0.021197839
66800	0.051550574	66760	0.016614804
66800	0.049453709	66764	0.008118601
66800	0.030715371	66768	0.011338502
66800	0.00903006	66772	0.005746692
66800	0.024493695	66776	0.021268166
66800	0.024929925	66780	0.030150912
66800	0.073848227	66784	0.029326613
66800	0.084799533	66788	0.009786941
66800	0.045507914	66792	0.038092676
66800	0.043589793	66796	0.063456297
66800	0.037739323	66800	0.063617481
66800	0.025905552	66804	0.048959733
66800	0.057787038	66808	0.040530485
66800	0.062825689	66812	0.026569131
66800	0.029380371	66816	0.046086778
66800	0.011502803	66820	0.128117245
66800	0.018184013	66824	0.116032068

PowerSpectrumData

66800	0.046918289	66828	0.049189883
66800	0.076284247	66832	0.059738803
66800	0.062466716	66836	0.081702106
66800	0.024844805	66840	0.062330037
66800	0.029725912	66844	0.018960995
66800	0.058349502	66848	0.014641163
66900	0.050022507	66852	0.027677659
66900	0.028344832	66856	0.030696599
66900	0.008256251	66860	0.030320443
66900	0.026380485	66864	0.07641111
66900	0.050494055	66868	0.113323273
66900	0.051713359	66872	0.131199763
66900	0.052747077	66876	0.106690124
66900	0.044358305	66880	0.084301704
66900	0.048398575	66884	0.049821181
66900	0.033262215	66888	0.003854213
66900	0.0305586	66892	0.030735853
66900	0.063560081	66896	0.052100855
66900	0.087099179	66900	0.032882872
66900	0.083346386	66904	0.005406397
66900	0.084622268	66908	0.012216987
66900	0.069996553	66912	0.034513192
66900	0.042443837	66916	0.065824504
66900	0.038287686	66920	0.058056794
66900	0.120074496	66924	0.03980504
66900	0.150715773	66928	0.05638057
66900	0.074323747	66932	0.10117369
66900	0.015354492	66936	0.100573765
66900	0.049485047	66940	0.039040817
66900	0.098439035	66944	0.039049679
66900	0.103713297	66948	0.060885748
67000	0.065177432	66952	0.052437245
67000	0.023063965	66956	0.020523718
67000	0.054858203	66960	0.024437086

PowerSpectrumData

67000	0.06210099	66964	0.068696645
67000	0.053227166	66968	0.093499024
67000	0.044233668	66972	0.089152367
67000	0.043229695	66976	0.049440663
67000	0.036747606	66980	0.018290864
67000	0.053788721	66984	0.067532012
67000	0.071545801	66988	0.083256884
67000	0.059610306	66992	0.09349981
67000	0.031378437	66996	0.099008496
67000	0.009915282	67000	0.090261616
67000	0.010990034	67004	0.114015005
67000	0.035820202	67008	0.141594661
67000	0.073148345	67012	0.134938906
67000	0.097487035	67016	0.11012092
67000	0.079193393	67020	0.057158282
67000	0.055029821	67024	0.033392487
67000	0.025254354	67028	0.072315503
67000	0.036632089	67032	0.046366113
67000	0.076247008	67036	0.054434706
67000	0.043642383	67040	0.074784926
67000	0.01611367	67044	0.059851573
67000	0.01283107	67048	0.035433841
67100	0.007759614	67052	0.079349782
67100	0.043289405	67056	0.106261687
67100	0.059507645	67060	0.078997815
67100	0.05017902	67064	0.028487344
67100	0.067768786	67068	0.026950531
67100	0.048876646	67072	0.06894453
67100	0.00386674	67076	0.072808078
67100	0.052142474	67080	0.024348286
67100	0.083697989	67084	0.049419079
67100	0.089533853	67088	0.103562255
67100	0.064778767	67092	0.1038384
67100	0.036395897	67096	0.068059271

PowerSpectrumData

67100	0.030203393	67100	0.059243408
67100	0.024145449	67104	0.068151421
67100	0.01926111	67108	0.06096128
67100	0.020090673	67112	0.095163116
67100	0.030669238	67116	0.105884596
67100	0.012998493	67120	0.080515216
67100	0.039859064	67124	0.109855791
67100	0.070283	67128	0.13839404
67100	0.090322159	67132	0.115389812
67100	0.061511149	67136	0.085649954
67100	0.058666214	67140	0.056895984
67100	0.086524844	67144	0.009225394
67100	0.053792355	67148	0.061138286
67200	0.025746902	67152	0.092946779
67200	0.04909738	67156	0.090377325
67200	0.05232972	67160	0.030452608
67200	0.019909929	67164	0.053018128
67200	0.017773367	67168	0.100183701
67200	0.024017465	67172	0.09219459
67200	0.019952551	67176	0.028939876
67200	0.017009277	67180	0.082127961
67200	0.019142173	67184	0.111813693
67200	0.033138869	67188	0.081681392
67200	0.051668387	67192	0.039232218
67200	0.050512881	67196	0.036047954
67200	0.036261507	67200	0.08457563
67200	0.034985125	67204	0.076472563
67200	0.071895607	67208	0.070499576
67200	0.044270648	67212	0.059289079
67200	0.046042555	67216	0.049098428
67200	0.057136011	67220	0.122722311
67200	0.039660521	67224	0.149991436
67200	0.04274286	67228	0.11960639
67200	0.0152872	67232	0.084284817

PowerSpectrumData

67200	0.030148834	67236	0.088530862
67200	0.026751655	67240	0.067811328
67200	0.026791475	67244	0.05997522
67200	0.041972835	67248	0.044838896
67300	0.008527588	67252	0.031693984
67300	0.061652514	67256	0.095898431
67300	0.082525759	67260	0.124104583
67300	0.040660816	67264	0.130053915
67300	0.037052316	67268	0.12028186
67300	0.050413728	67272	0.153704314
67300	0.044219883	67276	0.172608009
67300	0.034838362	67280	0.14148996
67300	0.010792419	67284	0.074822165
67300	0.033979955	67288	0.076255878
67300	0.059020444	67292	0.08705174
67300	0.066815737	67296	0.087916407
67300	0.049066301	67300	0.084182204
67300	0.025609355	67304	0.065392051
67300	0.038796192	67308	0.057354453
67300	0.055292025	67312	0.069930829
67300	0.051810686	67316	0.071791837
67300	0.048034352	67320	0.051057632
67300	0.078604746	67324	0.042971398
67300	0.084609281	67328	0.015727914
67300	0.094297982	67332	0.037405142
67300	0.089960966	67336	0.063132618
67300	0.045893332	67340	0.035229543
67300	0.015217799	67344	0.05155983
67300	0.04159275	67348	0.120929472
67400	0.042786222	67352	0.17548431
67400	0.037962407	67356	0.166369603
67400	0.038543007	67360	0.086555992
67400	0.05240495	67364	0.049790215
67400	0.042322557	67368	0.080908183

PowerSpectrumData

67400	0.032767843	67372	0.058453228
67400	0.028229641	67376	0.061908613
67400	0.026848646	67380	0.086076398
67400	0.062401559	67384	0.0945801
67400	0.091271271	67388	0.08569131
67400	0.071011833	67392	0.055969038
67400	0.023060742	67396	0.079290876
67400	0.038978222	67400	0.095589057
67400	0.054121345	67404	0.105142106
67400	0.041770349	67408	0.089348636
67400	0.054338023	67412	0.013585765
67400	0.045405817	67416	0.049887156
67400	0.029375922	67420	0.051752511
67400	0.031851625	67424	0.065777618
67400	0.029190422	67428	0.037484158
67400	0.048562284	67432	0.027026339
67400	0.075532953	67436	0.056734738
67400	0.059685044	67440	0.067086039
67400	0.058727972	67444	0.075434808
67400	0.089627756	67448	0.049278569
67500	0.079302205	67452	0.038610942
67500	0.01817659	67456	0.061182051
67500	0.032908603	67460	0.028930121
67500	0.039686966	67464	0.052404117
67500	0.053230375	67468	0.075309814
67500	0.051237217	67472	0.043918717
67500	0.030231966	67476	0.039078539
67500	0.017876984	67480	0.103318474
67500	0.017671213	67484	0.12359205
67500	0.021376744	67488	0.085581494
67500	0.019157318	67492	0.03956796
67500	0.057295376	67496	0.024928613
67500	0.070711743	67500	0.027551008
67500	0.080716665	67504	0.045094072

PowerSpectrumData

67500	0.093325769	67508	0.071223825
67500	0.098900026	67512	0.081259852
67500	0.07139743	67516	0.060985283
67500	0.025356168	67520	0.028882927
67500	0.015599439	67524	0.068859648
67500	0.015727908	67528	0.101817612
67500	0.017527327	67532	0.098293509
67500	0.054740689	67536	0.075320946
67500	0.071960771	67540	0.119224314
67500	0.072412804	67544	0.12854625
67500	0.040220679	67548	0.060175167
67600	0.028933846	67552	0.054944339
67600	0.068369402	67556	0.135367169
67600	0.068484776	67560	0.138411648
67600	0.0640207	67564	0.138195654
67600	0.074315401	67568	0.119118457
67600	0.05512112	67572	0.059081587
67600	0.030623942	67576	0.03775283
67600	0.012813318	67580	0.062812302
67600	0.051387888	67584	0.086827538
67600	0.051961702	67588	0.083366889
67600	0.049963859	67592	0.024544957
67600	0.039744289	67596	0.09692664
67600	0.017794071	67600	0.071973183
67600	0.031683841	67604	0.029899575
67600	0.022343758	67608	0.068518297
67600	0.048788999	67612	0.093347087
67600	0.054437769	67616	0.116110838
67600	0.048810176	67620	0.114410337
67600	0.02128244	67624	0.069639718
67600	0.029412722	67628	0.074331401
67600	0.045488916	67632	0.14490707
67600	0.022305683	67636	0.145657555
67600	0.014499387	67640	0.102341517

PowerSpectrumData

67600	0.025966685	67644	0.106824955
67600	0.052774645	67648	0.114520299
67700	0.032492648	67652	0.105788953
67700	0.014582504	67656	0.147009094
67700	0.042895863	67660	0.183371681
67700	0.041537642	67664	0.133962633
67700	0.029017125	67668	0.039475359
67700	0.016673157	67672	0.044433069
67700	0.012803852	67676	0.086171531
67700	0.018088162	67680	0.102327998
67700	0.018992503	67684	0.099734461
67700	0.047746431	67688	0.063270745
67700	0.069661357	67692	0.072370291
67700	0.065692926	67696	0.106087326
67700	0.063256412	67700	0.104924009
67700	0.054076863	67704	0.094927556
67700	0.043744236	67708	0.086467189
67700	0.058396829	67712	0.107927917
67700	0.060475955	67716	0.094312396
67700	0.051160074	67720	0.025406218
67700	0.037259259	67724	0.042914609
67700	0.030306976	67728	0.081643419
67700	0.035659781	67732	0.11472882
67700	0.035486148	67736	0.122451515
67700	0.022392798	67740	0.088628069
67700	0.054249704	67744	0.071443152
67700	0.07707964	67748	0.122932936
67800	0.076885219	67752	0.110046552
67800	0.044803383	67756	0.056681241
67800	0.023466444	67760	0.075325639
67800	0.076566568	67764	0.089720183
67800	0.080143866	67768	0.085399195
67800	0.042797474	67772	0.098704964
67800	0.05528381	67776	0.09740192

PowerSpectrumData

67800	0.113614995	67780	0.092554255
67800	0.106547261	67784	0.067996654
67800	0.039164614	67788	0.043916305
67800	0.026443126	67792	0.046164572
67800	0.057952362	67796	0.042858745
67800	0.068156413	67800	0.032951972
67800	0.054368236	67804	0.078809222
67800	0.038040435	67808	0.095536394
67800	0.027590912	67812	0.086140783
67800	0.014138191	67816	0.066873348
67800	0.027559252	67820	0.065084278
67800	0.033125718	67824	0.092155024
67800	0.010141213	67828	0.059372746
67800	0.01460062	67832	0.03171181
67800	0.037493621	67836	0.113323717
67800	0.077100493	67840	0.141603726
67800	0.070799011	67844	0.098888122
67800	0.04021309	67848	0.069325251
67900	0.047406084	67852	0.037552763
67900	0.052742824	67856	0.033377135
67900	0.035338617	67860	0.07114694
67900	0.006853656	67864	0.074301934
67900	0.028090619	67868	0.117633106
67900	0.041830223	67872	0.15207543
67900	0.036527596	67876	0.108334767
67900	0.032007465	67880	0.060955274
67900	0.045213041	67884	0.062070059
67900	0.048402777	67888	0.052112835
67900	0.046871752	67892	0.025678091
67900	0.054873803	67896	0.031946493
67900	0.051912906	67900	0.008873712
67900	0.041756099	67904	0.021006341
67900	0.031100644	67908	0.020218246
67900	0.027013499	67912	0.001455072

PowerSpectrumData

67900	0.042258755	67916	0.017237953
67900	0.041679603	67920	0.045997458
67900	0.039912469	67924	0.090916263
67900	0.028791146	67928	0.115481402
67900	0.011547759	67932	0.1009548
67900	0.022258266	67936	0.077490331
67900	0.028899043	67940	0.048075093
67900	0.054156226	67944	0.02576921
67900	0.065274711	67948	0.024915464
68000	0.084894149	67952	0.009362635
68000	0.087643741	67956	0.01466629
68000	0.072560135	67960	0.033900531
68000	0.065959168	67964	0.042315682
68000	0.045584031	67968	0.027346101
68000	0.01605388	67972	0.025627645
68000	0.034040255	67976	0.027538083
68000	0.025361793	67980	0.016589575
68000	0.019689454	67984	0.051982395
68000	0.042563843	67988	0.090683694
68000	0.051630796	67992	0.08036942
68000	0.039815808	67996	0.037138794
68000	0.015890342	68000	0.057203084
68000	0.063262363	68004	0.073645206
68000	0.059325328	68008	0.045298253
68000	0.052735995	68012	0.028203014
68000	0.06363228	68016	0.034397755
68000	0.043881435	68020	0.048301397
68000	0.019673782	68024	0.068143061
68000	0.010674608	68028	0.069653739
68000	0.040363459	68032	0.060042228
68000	0.054738437	68036	0.030028074
68000	0.067693123	68040	0.029515681
68000	0.069017515	68044	0.054148826
68000	0.040644056	68048	0.032018103

PowerSpectrumData

68100	0.003572988	68052	0.033508241
68100	0.034060784	68056	0.022232367
68100	0.054891305	68060	0.009536082
68100	0.078560603	68064	0.024203524
68100	0.046768571	68068	0.027757676
68100	0.030706415	68072	0.029209119
68100	0.06920367	68076	0.007967163
68100	0.060052676	68080	0.032709198
68100	0.048264679	68084	0.038851755
68100	0.043740387	68088	0.00556043
68100	0.008290825	68092	0.045722285
68100	0.046475434	68096	0.079654601
68100	0.054230299	68100	0.102890874
68100	0.043141299	68104	0.076764438
68100	0.022933838	68108	0.022233371
68100	0.040995514	68112	0.058310234
68100	0.030896466	68116	0.096319061
68100	0.00942407	68120	0.101274905
68100	0.006966212	68124	0.060320017
68100	0.033823111	68128	0.02022047
68100	0.077569261	68132	0.040549861
68100	0.110839836	68136	0.056574394
68100	0.118152151	68140	0.064641099
68100	0.086565997	68144	0.067301487
68100	0.057795915	68148	0.076746685
68200	0.055633173	68152	0.105195759
68200	0.046139609	68156	0.117523603
68200	0.029436455	68160	0.088952686
68200	0.041469553	68164	0.040464202
68200	0.053039476	68168	0.027219885
68200	0.087148561	68172	0.052696072
68200	0.08623438	68176	0.072724979
68200	0.06520118	68180	0.093480914
68200	0.051583444	68184	0.118521202

PowerSpectrumData

68200	0.061674597	68188	0.079564015
68200	0.096415293	68192	0.060662154
68200	0.107973552	68196	0.094988434
68200	0.06920498	68200	0.099390054
68200	0.019326972	68204	0.079685487
68200	0.022303751	68208	0.046375157
68200	0.075646036	68212	0.023903485
68200	0.084350126	68216	0.060806695
68200	0.021737222	68220	0.066067012
68200	0.027959966	68224	0.064625747
68200	0.015938091	68228	0.063393454
68200	0.014177006	68232	0.036547695
68200	0.012513973	68236	0.043803626
68200	0.020782676	68240	0.056997429
68200	0.026965287	68244	0.054546254
68200	0.024016621	68248	0.05861
68300	0.036676862	68252	0.046215369
68300	0.040039071	68256	0.009781537
68300	0.022424894	68260	0.077940822
68300	0.010566213	68264	0.133237205
68300	0.020590747	68268	0.124900907
68300	0.031621275	68272	0.050852028
68300	0.014068053	68276	0.021072772
68300	0.022368538	68280	0.035516998
68300	0.032751726	68284	0.060493712
68300	0.035700057	68288	0.06820108
68300	0.042978307	68292	0.025845431
68300	0.042141091	68296	0.057543792
68300	0.021012182	68300	0.109249784
68300	0.019125975	68304	0.073443705
68300	0.049045826	68308	0.022629556
68300	0.059134258	68312	0.023863486
68300	0.063613632	68316	0.071372735
68300	0.071538263	68320	0.084037885

PowerSpectrumData

68300	0.06638276	68324	0.076407661
68300	0.017926908	68328	0.135169655
68300	0.053705942	68332	0.150148436
68300	0.077907076	68336	0.100414611
68300	0.042242049	68340	0.062085826
68300	0.037546026	68344	0.07972469
68300	0.062619591	68348	0.059979164
68400	0.061080653	68352	0.091368944
68400	0.052706975	68356	0.143814351
68400	0.063935549	68360	0.137840558
68400	0.071199873	68364	0.117661519
68400	0.074053416	68368	0.095957934
68400	0.038932634	68372	0.078271114
68400	0.037680198	68376	0.103478204
68400	0.062105894	68380	0.113782866
68400	0.061180894	68384	0.118354365
68400	0.050681072	68388	0.13277214
68400	0.071648487	68392	0.114437353
68400	0.09102968	68396	0.066716035
68400	0.053954627	68400	0.012649818
68400	0.004962119	68404	0.078309306
68400	0.028015847	68408	0.103805549
68400	0.048274775	68412	0.113752918
68400	0.066758126	68416	0.138184944
68400	0.066789035	68420	0.154141788
68400	0.061400264	68424	0.127613515
68400	0.038778035	68428	0.075131895
68400	0.041124069	68432	0.082774153
68400	0.041782361	68436	0.094514202
68400	0.039330447	68440	0.062307394
68400	0.022414413	68444	0.05310182
68400	0.023046829	68448	0.107444015
68500	0.018996485	68452	0.135548399
68500	0.010646627	68456	0.13834663

PowerSpectrumData

68500	0.041570165	68460	0.134982853
68500	0.055715951	68464	0.136978386
68500	0.081025231	68468	0.146546853
68500	0.108300461	68472	0.170623578
68500	0.070987604	68476	0.181320298
68500	0.028725082	68480	0.149475484
68500	0.074764488	68484	0.123355305
68500	0.037304162	68488	0.130235916
68500	0.045452911	68492	0.100812875
68500	0.084292791	68496	0.08153329
68500	0.070861715	68500	0.095649863
68500	0.063387306	68504	0.104635539
68500	0.074297466	68508	0.093366252
68500	0.071252885	68512	0.075562304
68500	0.049618742	68516	0.08991408
68500	0.005344891	68520	0.082895058
68500	0.054000004	68524	0.110886242
68500	0.080253522	68528	0.133847658
68500	0.079775615	68532	0.129182838
68500	0.068684836	68536	0.194960129
68500	0.037478458	68540	0.255150663
68500	0.019048015	68544	0.237277593
68500	0.01774955	68548	0.16128282
68600	0.019653429	68552	0.096170508
68600	0.053559936	68556	0.113771646
68600	0.065793109	68560	0.123072168
68600	0.056228379	68564	0.092711794
68600	0.046771609	68568	0.052373827
68600	0.054466455	68572	0.048462847
68600	0.047286441	68576	0.017391734
68600	0.038778977	68580	0.033300967
68600	0.030239686	68584	0.066672728
68600	0.036454778	68588	0.082276099
68600	0.064313943	68592	0.10925746

PowerSpectrumData

68600	0.09083077	68596	0.104675077
68600	0.084903346	68600	0.081861304
68600	0.097460339	68604	0.103643964
68600	0.096202035	68608	0.104578714
68600	0.053111431	68612	0.092098046
68600	0.01949398	68616	0.111753514
68600	0.026803287	68620	0.122046666
68600	0.038163049	68624	0.083355648
68600	0.034329583	68628	0.041090036
68600	0.005036011	68632	0.075498669
68600	0.028659562	68636	0.083399886
68600	0.04698705	68640	0.06615329
68600	0.045669345	68644	0.060380291
68600	0.041786734	68648	0.07081432
68700	0.031974076	68652	0.082787468
68700	0.019662057	68656	0.095742842
68700	0.022522465	68660	0.09806882
68700	0.072185554	68664	0.089888374
68700	0.120260535	68668	0.076510187
68700	0.097457029	68672	0.080598802
68700	0.039227034	68676	0.089070949
68700	0.02145418	68680	0.083842751
68700	0.037495534	68684	0.063369582
68700	0.032722255	68688	0.029902045
68700	0.007384445	68692	0.068078305
68700	0.024725874	68696	0.079051344
68700	0.025356707	68700	0.060373586
68700	0.05414445	68704	0.066713008
68700	0.029902543	68708	0.079444879
68700	0.018809184	68712	0.044865006
68700	0.016138858	68716	0.043771397
68700	0.020830423	68720	0.100091944
68700	0.037621074	68724	0.151108078
68700	0.068686517	68728	0.162495416

PowerSpectrumData

68700	0.082245926	68732	0.138944204
68700	0.053504813	68736	0.121745667
68700	0.05006211	68740	0.104377599
68700	0.086865191	68744	0.088387613
68700	0.120004414	68748	0.106350068
68800	0.107199405	68752	0.099482233
68800	0.053192751	68756	0.055027489
68800	0.01693724	68760	0.042068372
68800	0.019716163	68764	0.035929697
68800	0.048279344	68768	0.06283923
68800	0.055063974	68772	0.131734633
68800	0.032232008	68776	0.142531878
68800	0.010178447	68780	0.089953181
68800	0.02874659	68784	0.052355732
68800	0.036445297	68788	0.075819502
68800	0.024648445	68792	0.107803848
68800	0.034246499	68796	0.105618805
68800	0.054565484	68800	0.063082196
68800	0.024052321	68804	0.030098978
68800	0.023102764	68808	0.071733855
68800	0.011738215	68812	0.084657739
68800	0.010891135	68816	0.054046101
68800	0.03862799	68820	0.027259399
68800	0.054966618	68824	0.080584221
68800	0.054584722	68828	0.125820996
68800	0.039656374	68832	0.124137936
68800	0.019857442	68836	0.167528866
68800	0.047793736	68840	0.215796623
68800	0.059311813	68844	0.164358033
68800	0.023024775	68848	0.076886267
68900	0.03308629	68852	0.043986671
68900	0.028572433	68856	0.056045086
68900	0.019752621	68860	0.107812564
68900	0.003517602	68864	0.135710215

PowerSpectrumData

68900	0.024646315	68868	0.137950439
68900	0.04196987	68872	0.126467218
68900	0.05136602	68876	0.111877111
68900	0.032646411	68880	0.108310538
68900	0.037813967	68884	0.094247182
68900	0.031811494	68888	0.097377022
68900	0.026621859	68892	0.130705114
68900	0.040752631	68896	0.123241916
68900	0.045047407	68900	0.062412379
68900	0.052830492	68904	0.100010009
68900	0.044783534	68908	0.118860553
68900	0.018472976	68912	0.073253665
68900	0.007525513	68916	0.067809284
68900	0.030167061	68920	0.052778356
68900	0.04914563	68924	0.043261305
68900	0.037097205	68928	0.066838169
68900	0.027106551	68932	0.076489931
68900	0.055934335	68936	0.067806868
68900	0.044378176	68940	0.054802837
68900	0.016319487	68944	0.051469597
68900	0.018690631	68948	0.118878197
69000	0.030076441	68952	0.122701211
69000	0.048038652	68956	0.078838748
69000	0.057367888	68960	0.056717567
69000	0.022598326	68964	0.034324901
69000	0.027350154	68968	0.062905114
69000	0.061008577	68972	0.091400812
69000	0.074896365	68976	0.105344312
69000	0.03171666	68980	0.081198676
69000	0.030626085	68984	0.035181311
69000	0.047193127	68988	0.042415089
69000	0.034227822	68992	0.048850252
69000	0.05653609	68996	0.077878474
69000	0.095448915	69000	0.112246664

PowerSpectrumData

69000	0.093065384	69004	0.082445054
69000	0.080761049	69008	0.032299711
69000	0.076731441	69012	0.030098829
69000	0.047110108	69016	0.055874076
69000	0.00480644	69020	0.062336243
69000	0.008383229	69024	0.055495599
69000	0.021424337	69028	0.045838307
69000	0.019973544	69032	0.066190536
69000	0.030721782	69036	0.061651364
69000	0.043104581	69040	0.04062126
69000	0.018654435	69044	0.060350005
69000	0.048276332	69048	0.080266516
69100	0.023262193	69052	0.063393622
69100	0.075182339	69056	0.045380799
69100	0.070484071	69060	0.020710819
69100	0.0354285	69064	0.075922828
69100	0.008748914	69068	0.095840231
69100	0.049750153	69072	0.053422504
69100	0.07628835	69076	0.029598192
69100	0.062223684	69080	0.05971628
69100	0.035645015	69084	0.066158413
69100	0.008072573	69088	0.065443732
69100	0.006923702	69092	0.072516115
69100	0.021410877	69096	0.056411634
69100	0.021668127	69100	0.039752817
69100	0.014321171	69104	0.018089579
69100	0.050866689	69108	0.035913548
69100	0.0708472	69112	0.013323574
69100	0.072058596	69116	0.04384647
69100	0.047350197	69120	0.076297176
69100	0.025342341	69124	0.05020784
69100	0.024205123	69128	0.05790326
69100	0.038606941	69132	0.109030945
69100	0.066251545	69136	0.107729393

PowerSpectrumData

69100	0.062183535	69140	0.04685044
69100	0.039339382	69144	0.010308785
69100	0.033147018	69148	0.027546223
69200	0.027277814	69152	0.030486035
69200	0.015613647	69156	0.04072897
69200	0.022513401	69160	0.093838622
69200	0.029584524	69164	0.103029553
69200	0.045337303	69168	0.10190528
69200	0.045238801	69172	0.100210789
69200	0.023849045	69176	0.087236331
69200	0.033317716	69180	0.090790207
69200	0.056984136	69184	0.081494931
69200	0.089182766	69188	0.024138932
69200	0.09631084	69192	0.03049159
69200	0.07782469	69196	0.035299985
69200	0.047734873	69200	0.027363536
69200	0.00393634	69204	0.040194038
69200	0.052780972	69208	0.076952747
69200	0.07943809	69212	0.083299332
69200	0.091069291	69216	0.067139947
69200	0.086758424	69220	0.043388871
69200	0.071617265	69224	0.0152182
69200	0.064886146	69228	0.040574272
69200	0.041467534	69232	0.078809484
69200	0.045572968	69236	0.086512897
69200	0.052607749	69240	0.098683442
69200	0.04501615	69244	0.097263212
69200	0.048315727	69248	0.053558662
69300	0.016546184	69252	0.026075446
69300	0.034736953	69256	0.048687893
69300	0.049270395	69260	0.05064861
69300	0.008068861	69264	0.029642026
69300	0.030326492	69268	0.041464038
69300	0.022407663	69272	0.071720482

PowerSpectrumData

69300	0.024953099	69276	0.073193718
69300	0.031977426	69280	0.041866162
69300	0.110350986	69284	0.013285174
69300	0.119390199	69288	0.025667576
69300	0.052651034	69292	0.038446226
69300	0.021228576	69296	0.034571807
69300	0.043216416	69300	0.02943381
69300	0.031232921	69304	0.030862899
69300	0.022690394	69308	0.035107187
69300	0.038559261	69312	0.006580555
69300	0.050650004	69316	0.072471579
69300	0.076115415	69320	0.123534148
69300	0.111062313	69324	0.081790429
69300	0.103335289	69328	0.017394881
69300	0.052726377	69332	0.024828934
69300	0.013947338	69336	0.05129445
69300	0.06426956	69340	0.087587396
69300	0.093306117	69344	0.043380813
69300	0.091604044	69348	0.043761884
69400	0.05941408	69352	0.056512119
69400	0.039764593	69356	0.048695787
69400	0.021211272	69360	0.079959078
69400	0.025757619	69364	0.071490256
69400	0.00927871	69368	0.043680087
69400	0.039281375	69372	0.043776381
69400	0.045546265	69376	0.017433225
69400	0.019896703	69380	0.041567859
69400	0.055593457	69384	0.030498599
69400	0.080492828	69388	0.01514062
69400	0.037526777	69392	0.032727636
69400	0.024162122	69396	0.028678715
69400	0.042770571	69400	0.016920223
69400	0.02916013	69404	0.035156216
69400	0.010543905	69408	0.028909839

PowerSpectrumData

69400	0.051430838	69412	0.025298476
69400	0.076298638	69416	0.04541962
69400	0.074650401	69420	0.063347143
69400	0.083785395	69424	0.047587735
69400	0.091550122	69428	0.035793142
69400	0.045871166	69432	0.043392913
69400	0.058109734	69436	0.037197337
69400	0.074592426	69440	0.049693976
69400	0.032761531	69444	0.082068102
69400	0.026626758	69448	0.068145542
69500	0.012022501	69452	0.071145667
69500	0.049714392	69456	0.042552852
69500	0.088543093	69460	0.014128929
69500	0.100394151	69464	0.023250974
69500	0.069864305	69468	0.057939989
69500	0.017280338	69472	0.100071622
69500	0.055453616	69476	0.089917965
69500	0.080197657	69480	0.042937107
69500	0.085363543	69484	0.011608089
69500	0.072693474	69488	0.058941168
69500	0.032684206	69492	0.077147859
69500	0.016076509	69496	0.04004527
69500	0.0471432	69500	0.052959025
69500	0.070068949	69504	0.122594051
69500	0.062252322	69508	0.095206866
69500	0.064388078	69512	0.013790866
69500	0.066659821	69516	0.070143913
69500	0.050181643	69520	0.090346061
69500	0.038895207	69524	0.069600275
69500	0.063469444	69528	0.041061667
69500	0.088008113	69532	0.052773652
69500	0.069024049	69536	0.017935827
69500	0.029637839	69540	0.033507859
69500	0.010137585	69544	0.070446717

PowerSpectrumData

69500	0.023350993	69548	0.059576458
69600	0.053585161	69552	0.025544907
69600	0.085224427	69556	0.068825866
69600	0.084832122	69560	0.080545775
69600	0.069834139	69564	0.070669499
69600	0.022851511	69568	0.044101125
69600	0.012737518	69572	0.014562857
69600	0.052131378	69576	0.036746191
69600	0.081941253	69580	0.052114054
69600	0.018712099	69584	0.066915214
69600	0.05196581	69588	0.071763876
69600	0.050360748	69592	0.035624275
69600	0.01922602	69596	0.023228664
69600	0.016904829	69600	0.038376536
69600	0.044800465	69604	0.005725925
69600	0.098624289	69608	0.080988866
69600	0.091112343	69612	0.108323511
69600	0.060172872	69616	0.071903553
69600	0.071814626	69620	0.037466791
69600	0.057307771	69624	0.060124385
69600	0.0591631	69628	0.076851422
69600	0.046126304	69632	0.043982345
69600	0.022443195	69636	0.023342242
69600	0.059414106	69640	0.003132207
69600	0.141280253	69644	0.0555189
69600	0.196399196	69648	0.091947914
69700	0.161810953	69652	0.07307886
69700	0.0633237	69656	0.022999511
69700	0.020085186	69660	0.019454486
69700	0.038936578	69664	0.034765428
69700	0.053244694	69668	0.068666057
69700	0.079151701	69672	0.163655495
69700	0.108706205	69676	0.193081898
69700	0.108914319	69680	0.125316146

PowerSpectrumData

69700	0.089566027	69684	0.040262577
69700	0.064153232	69688	0.083375089
69700	0.209033475	69692	0.120638768
69700	0.210144499	69696	0.141526428
69700	0.262218499	69700	0.142493096
69700	0.715698523	69704	0.078624369
69700	0.942133251	69708	0.0522345
69700	0.737457129	69712	0.046986468
69700	0.301270629	69716	0.039634611
69700	0.072483883	69720	0.113012982
69700	0.062221741	69724	0.134776449
69700	0.231591578	69728	0.094347801
69700	0.44433659	69732	0.078396872
69700	0.550841448	69736	0.097993216
69700	0.554608646	69740	0.099449157
69700	0.336311263	69744	0.077995566
69700	0.190130858	69748	0.060095219
69800	0.51125983	69752	0.057963298
69800	0.203095435	69756	0.07215167
69800	1.375822813	69760	0.09827489
69800	2.66991905	69764	0.044083477
69800	2.742738463	69768	0.035854922
69800	1.553775275	69772	0.080910213
69800	0.419645221	69776	0.079290534
69800	0.245125964	69780	0.06843848
69800	0.150462104	69784	0.112547568
69800	0.652257819	69788	0.13508783
69800	1.688436488	69792	0.096595672
69800	2.50033965	69796	0.066857436
69800	2.153599175	69800	0.070982314
69800	1.009492669	69804	0.095075156
69800	0.81863493	69808	0.134864953
69800	0.958086224	69812	0.14270944
69800	1.452567288	69816	0.089214205

PowerSpectrumData

69800	3.919332288	69820	0.043067572
69800	5.216826225	69824	0.108949229
69800	3.943019075	69828	0.099546211
69800	1.465124888	69832	0.028984603
69800	0.35358197	69836	0.052711486
69800	0.649862108	69840	0.092294999
69800	0.490975566	69844	0.114875322
69800	3.075448563	69848	0.090356436
69900	5.636699963	69852	0.071989183
69900	5.5363942	69856	0.063880114
69900	2.949194988	69860	0.057573667
69900	0.837736821	69864	0.05296653
69900	0.87766134	69868	0.048139893
69900	0.382815954	69872	0.038299491
69900	1.552358388	69876	0.148126826
69900	3.25903855	69880	0.167273581
69900	3.582697825	69884	0.093514762
69900	2.136625588	69888	0.034038145
69900	0.414715934	69892	0.111862195
69900	0.291399513	69896	0.164032813
69900	0.241959104	69900	0.104048864
69900	1.046713558	69904	0.064859116
69900	2.925420413	69908	0.121673889
69900	4.0844595	69912	0.125950304
69900	3.286870663	69916	0.074604708
69900	1.243509701	69920	0.098999757
69900	0.671543938	69924	0.112851158
69900	0.708610053	69928	0.111150315
69900	0.291605451	69932	0.079308848
69900	0.885963789	69936	0.036312751
69900	1.042897464	69940	0.040132058
69900	0.759704388	69944	0.091593625
69900	0.295487931	69948	0.123400474
70000	0.217330511	69952	0.129871871

PowerSpectrumData

70000	0.130148401	69956	0.126853033
70000	0.104990919	69960	0.130496279
70000	0.698157411	69964	0.116069365
70000	1.606719688	69968	0.083377432
70000	1.878359475	69972	0.072299357
70000	1.204370055	69976	0.098321412
70000	0.316995808	69980	0.130087399
70000	0.422700018	69984	0.099462952
70000	0.263745548	69988	0.084171115
70000	0.243687523	69992	0.121743899
70000	0.308711635	69996	0.116960859
70000	0.205413555	70000	0.059010628
70000	0.236213004	70004	0.097748096
70000	0.230887323	70008	0.121653415
70000	0.202489508	70012	0.123644379
70000	0.158638256	70016	0.137189738
70000	0.106485481	70020	0.13271335
70000	0.572204998	70024	0.116999181
70000	0.879675383	70028	0.09474744
70000	0.721724936	70032	0.041519998
70000	0.251978025	70036	0.072368079
70000	0.155938571	70040	0.090576104
70000	0.174994363	70044	0.078281744
70000	0.027175476	70048	0.109223431
70100	0.090395755	70052	0.152717185
70100	0.14407777	70056	0.150920343
70100	0.217726614	70060	0.107104956
70100	0.166599974	70064	0.072045958
70100	0.119166594	70068	0.068851688
70100	0.145555518	70072	0.062972897
70100	0.078716243	70076	0.059188697
70100	0.189566839	70080	0.061110877
70100	0.332835596	70084	0.063587955
70100	0.323261745	70088	0.055422333

PowerSpectrumData

70100	0.197411049	70092	0.065026434
70100	0.074967764	70096	0.065901004
70100	0.034445344	70100	0.084971696
70100	0.052514199	70104	0.075601594
70100	0.048845577	70108	0.010641527
70100	0.040136551	70112	0.039255814
70100	0.149842599	70116	0.039843195
70100	0.166680009	70120	0.040468138
70100	0.128491301	70124	0.083743624
70100	0.101135272	70128	0.107132313
70100	0.053132415	70132	0.073535106
70100	0.013725427	70136	0.043204207
70100	0.092687325	70140	0.060819373
70100	0.114671726	70144	0.050079223
70100	0.070218368	70148	0.018991943
70200	0.066824629	70152	0.067697751
70200	0.057495574	70156	0.105528932
70200	0.018271379	70160	0.126276209
70200	0.068501809	70164	0.11127301
70200	0.118403848	70168	0.054804561
70200	0.086287997	70172	0.053511409
70200	0.061605075	70176	0.133750058
70200	0.070045724	70180	0.114531133
70200	0.090022782	70184	0.031377782
70200	0.102622798	70188	0.058557744
70200	0.074310796	70192	0.078507081
70200	0.045550292	70196	0.080819307
70200	0.029722376	70200	0.054669119
70200	0.037858958	70204	0.034442761
70200	0.055274548	70208	0.078922582
70200	0.0824749	70212	0.070246242
70200	0.094105475	70216	0.015951428
70200	0.053287309	70220	0.011308528
70200	0.033141707	70224	0.052589869

PowerSpectrumData

70200	0.021795391	70228	0.074778756
70200	0.014948359	70232	0.079817488
70200	0.013770567	70236	0.086796521
70200	0.057797799	70240	0.070983457
70200	0.087589324	70244	0.052409869
70200	0.07057083	70248	0.038708102
70300	0.032593867	70252	0.049700997
70300	0.019208166	70256	0.072217277
70300	0.025073439	70260	0.050188053
70300	0.02380411	70264	0.03952043
70300	0.001051012	70268	0.047701014
70300	0.024555839	70272	0.05821474
70300	0.018972572	70276	0.071713403
70300	0.006739614	70280	0.025407486
70300	0.052778822	70284	0.037606602
70300	0.086663196	70288	0.020631647
70300	0.053633019	70292	0.020849264
70300	0.026871938	70296	0.011883999
70300	0.041004474	70300	0.010651404
70300	0.059073238	70304	0.022545135
70300	0.100293488	70308	0.051348252
70300	0.099035708	70312	0.10678135
70300	0.050091509	70316	0.109848515
70300	0.020582147	70320	0.05132001
70300	0.03242031	70324	0.031868527
70300	0.049091425	70328	0.03902143
70300	0.058697631	70332	0.036167832
70300	0.070342489	70336	0.018928098
70300	0.061155552	70340	0.055200741
70300	0.027423188	70344	0.071763112
70300	0.019437477	70348	0.037660502
70400	0.049344639	70352	0.044646433
70400	0.072427727	70356	0.04409831
70400	0.06501739	70360	0.081635306

PowerSpectrumData

70400	0.032249733	70364	0.114124465
70400	0.017424925	70368	0.071487215
70400	0.009479438	70372	0.026402786
70400	0.009283553	70376	0.025066844
70400	0.03738144	70380	0.032314645
70400	0.062014347	70384	0.070106813
70400	0.05395542	70388	0.056426681
70400	0.039171908	70392	0.020074518
70400	0.031338335	70396	0.045190394
70400	0.019377751	70400	0.103378836
70400	0.030560572	70404	0.109767207
70400	0.082616534	70408	0.090429589
70400	0.097701442	70412	0.059036214
70400	0.057695866	70416	0.110199944
70400	0.01183929	70420	0.116960902
70400	0.02637129	70424	0.090383015
70400	0.022937322	70428	0.111635105
70400	0.063030479	70432	0.110383029
70400	0.05434324	70436	0.059859842
70400	0.020150701	70440	0.091903705
70400	0.048766447	70444	0.131512919
70400	0.073357798	70448	0.119531658
70500	0.097055483	70452	0.102021026
70500	0.061403742	70456	0.082834806
70500	0.035469209	70460	0.068366986
70500	0.038679726	70464	0.070613627
70500	0.063927007	70468	0.072021321
70500	0.075090582	70472	0.046435136
70500	0.058771719	70476	0.014709992
70500	0.043497756	70480	0.071646275
70500	0.044521446	70484	0.126001221
70500	0.064466847	70488	0.122857964
70500	0.064270418	70492	0.06536828
70500	0.03002284	70496	0.019603536

PowerSpectrumData

70500	0.025527363	70500	0.057967878
70500	0.077461365	70504	0.081849161
70500	0.086804124	70508	0.039440518
70500	0.047113888	70512	0.034466324
70500	0.020902158	70516	0.03296879
70500	0.002493496	70520	0.061387138
70500	0.015123669	70524	0.077678618
70500	0.015888441	70528	0.12302761
70500	0.020079597	70532	0.129395091
70500	0.049801118	70536	0.073382085
70500	0.046488829	70540	0.019287383
70500	0.013365586	70544	0.006033011
70500	0.017916671	70548	0.065188709
70600	0.039606955	70552	0.084035375
70600	0.032948516	70556	0.057960191
70600	0.01543464	70560	0.046313493
70600	0.039022074	70564	0.045924713
70600	0.064791086	70568	0.053975811
70600	0.070816357	70572	0.073452517
70600	0.074544405	70576	0.07393516
70600	0.076456323	70580	0.076570948
70600	0.024214396	70584	0.080240687
70600	0.03970513	70588	0.064903317
70600	0.057301004	70592	0.033870001
70600	0.048936585	70596	0.018085864
70600	0.046923524	70600	0.045915578
70600	0.037762591	70604	0.058084836
70600	0.043820557	70608	0.075432537
70600	0.062787454	70612	0.076005068
70600	0.061131294	70616	0.060776882
70600	0.044080629	70620	0.028193514
70600	0.053076095	70624	0.007338171
70600	0.06861896	70628	0.026795042
70600	0.061219733	70632	0.032206648

PowerSpectrumData

70600	0.030835519	70636	0.029525218
70600	0.022422702	70640	0.031946591
70600	0.058654208	70644	0.025811829
70600	0.074574695	70648	0.001575423
70700	0.032708944	70652	0.043574317
70700	0.041947424	70656	0.068571302
70700	0.070016307	70660	0.080686368
70700	0.052131629	70664	0.079531754
70700	0.06449924	70668	0.047989266
70700	0.052199819	70672	0.007090118
70700	0.016839855	70676	0.03843032
70700	0.029282619	70680	0.04709393
70700	0.058029389	70684	0.034570181
70700	0.069416099	70688	0.033446588
70700	0.059485079	70692	0.060860311
70700	0.038259288	70696	0.065713284
70700	0.043598106	70700	0.059738639
70700	0.032075543	70704	0.049831971
70700	0.055696517	70708	0.013306616
70700	0.031082622	70712	0.070994836
70700	0.018152203	70716	0.106396707
70700	0.033042907	70720	0.085367006
70700	0.031740172	70724	0.014532841
70700	0.018500281	70728	0.010517185
70700	0.012919433	70732	0.032830754
70700	0.016847835	70736	0.044369113
70700	0.030503996	70740	0.034608755
70700	0.006755501	70744	0.018805156
70700	0.037449572	70748	0.030727162
70800	0.063276057	70752	0.027222568
70800	0.093868301	70756	0.0145548
70800	0.058376165	70760	0.010497557
70800	0.049103383	70764	0.020443527
70800	0.053683711	70768	0.050260802

PowerSpectrumData

70800	0.034352961	70772	0.031756626
70800	0.020411413	70776	0.070886963
70800	0.044454446	70780	0.080644713
70800	0.090019064	70784	0.053063584
70800	0.070873808	70788	0.043668282
70800	0.020840224	70792	0.022457691
70800	0.026851709	70796	0.008321438
70800	0.079259902	70800	0.010593498
70800	0.066965309	70804	0.041773132
70800	0.028567507	70808	0.053352393
70800	0.023788	70812	0.014689378
70800	0.002581155	70816	0.050822069
70800	0.023382305	70820	0.060694034
70800	0.025523388	70824	0.063540785
70800	0.018018098	70828	0.062743551
70800	0.035329751	70832	0.025537551
70800	0.057790181	70836	0.047379639
70800	0.034483666	70840	0.053872598
70800	0.049051316	70844	0.038811431
70800	0.065351764	70848	0.020510577
70900	0.049653605	70852	0.079723905
70900	0.01744053	70856	0.090956572
70900	0.003806297	70860	0.060503389
70900	0.025050274	70864	0.022086502
70900	0.043529548	70868	0.031516403
70900	0.055997876	70872	0.06263399
70900	0.064250009	70876	0.074502787
70900	0.059465332	70880	0.079594633
70900	0.047250964	70884	0.066220295
70900	0.056082081	70888	0.030931813
70900	0.077377204	70892	0.002689113
70900	0.087935849	70896	0.024684856
70900	0.102109814	70900	0.06803633
70900	0.093977025	70904	0.0730471

PowerSpectrumData

70900	0.06049054	70908	0.072538125
70900	0.062387619	70912	0.087016633
70900	0.057438218	70916	0.076857235
70900	0.046854577	70920	0.056213139
70900	0.022695718	70924	0.050092596
70900	0.014491118	70928	0.071453949
70900	0.04936352	70932	0.094222123
70900	0.08236873	70936	0.058141814
70900	0.105651583	70940	0.068696631
70900	0.111829453	70944	0.063144144
70900	0.076118806	70948	0.056725556
71000	0.014761319	70952	0.081560531
71000	0.034314187	70956	0.086729233
71000	0.049706989	70960	0.087307613
71000	0.019459143	70964	0.085613603
71000	0.022926877	70968	0.049349048
71000	0.033971253	70972	0.045139695
71000	0.030106821	70976	0.090668684
71000	0.043856744	70980	0.071851267
71000	0.055972858	70984	0.066810993
71000	0.037252085	70988	0.04274735
71000	0.045331723	70992	0.030030644
71000	0.046171535	70996	0.075867138
71000	0.037866095	71000	0.092326714
71000	0.041785723	71004	0.08827947
71000	0.014424081	71008	0.07657057
71000	0.028906557	71012	0.053517673
71000	0.036444948	71016	0.012350746
71000	0.056381407	71020	0.077026518
71000	0.071621267	71024	0.113062466
71000	0.055607794	71028	0.063276792
71000	0.030084506	71032	0.037986658
71000	0.035203284	71036	0.063181367
71000	0.041044212	71040	0.030450792

PowerSpectrumData

71000	0.03840237	71044	0.043717559
71000	0.039476126	71048	0.063175925
71100	0.03128187	71052	0.054965803
71100	0.050553248	71056	0.050028451
71100	0.058368933	71060	0.045255525
71100	0.054809345	71064	0.062076761
71100	0.069195929	71068	0.082970822
71100	0.04758421	71072	0.05007612
71100	0.011668577	71076	0.067425746
71100	0.033396529	71080	0.0617185
71100	0.033165077	71084	0.024037727
71100	0.023686225	71088	0.045824014
71100	0.023294682	71092	0.095673138
71100	0.07185759	71096	0.082700171
71100	0.078348705	71100	0.051467909
71100	0.036682792	71104	0.03564281
71100	0.009441548	71108	0.003662601
71100	0.018065181	71112	0.049321345
71100	0.04217276	71116	0.061414008
71100	0.047143451	71120	0.066792527
71100	0.003745137	71124	0.048144
71100	0.046096255	71128	0.049778606
71100	0.050493021	71132	0.067441688
71100	0.0233868	71136	0.090089015
71100	0.021423613	71140	0.116578776
71100	0.044023425	71144	0.100029174
71100	0.063942855	71148	0.058745984
71200	0.064753564	71152	0.026470463
71200	0.053038501	71156	0.075669275
71200	0.032221502	71160	0.144537305
71200	0.041707732	71164	0.160496405
71200	0.062630112	71168	0.09644041
71200	0.060060862	71172	0.027819779
71200	0.050945902	71176	0.039866685

PowerSpectrumData

71200	0.065956505	71180	0.071536393
71200	0.072042232	71184	0.073895266
71200	0.034213084	71188	0.052412597
71200	0.075071337	71192	0.048753922
71200	0.12256304	71196	0.067688503
71200	0.123712409	71200	0.06115812
71200	0.105876708	71204	0.011374221
71200	0.091279355	71208	0.02937452
71200	0.08281682	71212	0.02157469
71200	0.056638175	71216	0.031037409
71200	0.010998896	71220	0.028749055
71200	0.024023428	71224	0.012004527
71200	0.032113341	71228	0.03796985
71200	0.028725912	71232	0.067478562
71200	0.040202329	71236	0.040396481
71200	0.097794648	71240	0.030621439
71200	0.092161885	71244	0.063370084
71200	0.054169865	71248	0.048097812
71300	0.041094452	71252	0.01416856
71300	0.048323545	71256	0.045777648
71300	0.041325176	71260	0.066116212
71300	0.012025917	71264	0.083454965
71300	0.038259826	71268	0.057102552
71300	0.024401921	71272	0.064591739
71300	0.016596303	71276	0.090596208
71300	0.042547112	71280	0.082410552
71300	0.080783393	71284	0.072146031
71300	0.094513394	71288	0.070187874
71300	0.072643205	71292	0.076257849
71300	0.046937257	71296	0.064026637
71300	0.035031069	71300	0.037715574
71300	0.039133105	71304	0.013045565
71300	0.034133915	71308	0.017615275
71300	0.044901815	71312	0.011774138

PowerSpectrumData

71300	0.053920627	71316	0.030584284
71300	0.046018067	71320	0.073944029
71300	0.057360412	71324	0.071117807
71300	0.062559382	71328	0.054373541
71300	0.035147983	71332	0.040400817
71300	0.036661335	71336	0.012171173
71300	0.023515504	71340	0.057176665
71300	0.06103731	71344	0.068400172
71300	0.031325704	71348	0.042037438
71400	0.040529521	71352	0.076004573
71400	0.048088801	71356	0.088690897
71400	0.06148246	71360	0.055825807
71400	0.042795335	71364	0.019270867
71400	0.022259426	71368	0.022139671
71400	0.033571796	71372	0.058099067
71400	0.040939234	71376	0.112271337
71400	0.044162058	71380	0.133975118
71400	0.035797242	71384	0.092592498
71400	0.071596871	71388	0.007970448
71400	0.058790571	71392	0.079357778
71400	0.015776072	71396	0.102779253
71400	0.011154563	71400	0.077124947
71400	0.026131049	71404	0.053308439
71400	0.046441768	71408	0.058454938
71400	0.053071282	71412	0.062258783
71400	0.04893485	71416	0.050256218
71400	0.057010526	71420	0.088861329
71400	0.0640785	71424	0.116555486
71400	0.049554692	71428	0.094840114
71400	0.028817165	71432	0.067048823
71400	0.006990954	71436	0.05681338
71400	0.047038433	71440	0.041647832
71400	0.069217873	71444	0.041133575
71400	0.070886461	71448	0.024595292

PowerSpectrumData

71500	0.090353766	71452	0.072680035
71500	0.104101295	71456	0.071199669
71500	0.060359002	71460	0.050701678
71500	0.068866277	71464	0.068588459
71500	0.103841405	71468	0.061879007
71500	0.078931684	71472	0.048014528
71500	0.032968128	71476	0.069884431
71500	0.026256212	71480	0.102029335
71500	0.056526958	71484	0.101877406
71500	0.068039422	71488	0.050688836
71500	0.070000308	71492	0.063062202
71500	0.053173942	71496	0.083997555
71500	0.044097127	71500	0.075388292
71500	0.022409584	71504	0.093124843
71500	0.009358855	71508	0.098301607
71500	0.038651939	71512	0.047456422
71500	0.059204216	71516	0.009241932
71500	0.019996774	71520	0.007920461
71500	0.04464461	71524	0.024374242
71500	0.044475444	71528	0.056257912
71500	0.006195378	71532	0.062973748
71500	0.027225664	71536	0.062480322
71500	0.038910665	71540	0.074804215
71500	0.036567399	71544	0.119875076
71500	0.041751908	71548	0.135787835
71600	0.070808885	71552	0.148921011
71600	0.077845769	71556	0.154813839
71600	0.049214905	71560	0.101497135
71600	0.019465426	71564	0.114714247
71600	0.025783271	71568	0.127694366
71600	0.03907561	71572	0.109056993
71600	0.026790274	71576	0.13418289
71600	0.022757118	71580	0.14828131
71600	0.010996984	71584	0.096431118

PowerSpectrumData

71600	0.019261868	71588	0.037565802
71600	0.016113181	71592	0.047166137
71600	0.009497007	71596	0.087412525
71600	0.045387511	71600	0.083300991
71600	0.054645927	71604	0.038176171
71600	0.051487754	71608	0.049745559
71600	0.063279702	71612	0.084230072
71600	0.056224279	71616	0.088310691
71600	0.046356734	71620	0.057483772
71600	0.029442186	71624	0.035685225
71600	0.033622713	71628	0.062674226
71600	0.052720403	71632	0.102796548
71600	0.055593973	71636	0.127589913
71600	0.063121945	71640	0.122201935
71600	0.046347177	71644	0.089432258
71600	0.051966857	71648	0.039013346
71700	0.053692394	71652	0.04281319
71700	0.070453003	71656	0.057823305
71700	0.045694305	71660	0.050979663
71700	0.046564597	71664	0.060738636
71700	0.039082584	71668	0.087028617
71700	0.056737037	71672	0.085382155
71700	0.065978646	71676	0.104053841
71700	0.036996113	71680	0.094625641
71700	0.033752909	71684	0.046544548
71700	0.027839524	71688	0.056209887
71700	0.020958556	71692	0.054543758
71700	0.020826488	71696	0.044804019
71700	0.044077515	71700	0.088963316
71700	0.03232967	71704	0.185712765
71700	0.025434507	71708	0.197840185
71700	0.055388595	71712	0.121625039
71700	0.033074997	71716	0.091927781
71700	0.022134895	71720	0.092603077

PowerSpectrumData

71700	0.01716877	71724	0.080196376
71700	0.048342114	71728	0.082765728
71700	0.041527736	71732	0.073744603
71700	0.036170717	71736	0.045196961
71700	0.055399476	71740	0.027672326
71700	0.03409059	71744	0.046715151
71700	0.031314405	71748	0.066865032
71800	0.016388902	71752	0.075390126
71800	0.052939537	71756	0.053170024
71800	0.039985556	71760	0.007518898
71800	0.068502413	71764	0.036607638
71800	0.093688032	71768	0.01995258
71800	0.066409419	71772	0.058915081
71800	0.028739889	71776	0.049736686
71800	0.048432012	71780	0.05398332
71800	0.072443661	71784	0.060565988
71800	0.028743481	71788	0.02618054
71800	0.036412403	71792	0.028197643
71800	0.063174855	71796	0.061007449
71800	0.035519828	71800	0.067852096
71800	0.031511259	71804	0.025482901
71800	0.037440801	71808	0.017099525
71800	0.049234503	71812	0.01232768
71800	0.051736708	71816	0.040330051
71800	0.028406306	71820	0.084628322
71800	0.036376096	71824	0.064179985
71800	0.034641238	71828	0.024491625
71800	0.022273367	71832	0.064689368
71800	0.032926237	71836	0.068761678
71800	0.031030431	71840	0.067727291
71800	0.013369912	71844	0.070754948
71800	0.036712452	71848	0.067557863
71900	0.055152974	71852	0.063942178
71900	0.087740453	71856	0.035973972

PowerSpectrumData

71900	0.085617583	71860	0.028607517
71900	0.024433481	71864	0.025198528
71900	0.060242757	71868	0.073822957
71900	0.046181711	71872	0.09056658
71900	0.022796201	71876	0.056415716
71900	0.039214232	71880	0.013093333
71900	0.061318977	71884	0.025477724
71900	0.061156199	71888	0.051541934
71900	0.022417367	71892	0.071583265
71900	0.034459459	71896	0.051648367
71900	0.071730225	71900	0.032900989
71900	0.052694719	71904	0.038048514
71900	0.017820841	71908	0.053732696
71900	0.046666282	71912	0.035366629
71900	0.04636803	71916	0.025216792
71900	0.020222591	71920	0.039241975
71900	0.034393906	71924	0.030348041
71900	0.063529587	71928	0.078611731
71900	0.063425796	71932	0.092153881
71900	0.040975992	71936	0.059661976
71900	0.005226159	71940	0.013437083
71900	0.03160736	71944	0.065470733
71900	0.016969434	71948	0.021804819
72000	0.027779894	71952	0.063755913
72000	0.021335211	71956	0.066200977
72000	0.042560016	71960	0.073173906
72000	0.041812629	71964	0.041248575
72000	0.029305784	71968	0.042809708
72000	0.016613714	71972	0.058870301
72000	0.027437032	71976	0.031971285
72000	0.034015411	71980	0.003084754
72000	0.028644394	71984	0.0445113
72000	0.050613809	71988	0.061361592
72000	0.036825964	71992	0.064021609

PowerSpectrumData

72000	0.027543547	71996	0.047912366
72000	0.033607095	72000	0.073155403
72000	0.052180913	72004	0.135156108
72000	0.037726215	72008	0.105976928
72000	0.026201786	72012	0.07187076
72000	0.02096408	72016	0.064372296
72000	0.019855623	72020	0.054467175
72000	0.060135135	72024	0.081529885
72000	0.052934436	72028	0.088137873
72000	0.028713394	72032	0.035069676
72000	0.035246409	72036	0.013910852
72000	0.039505099	72040	0.007619455
72000	0.03075289	72044	0.043849705
72000	0.035820136	72048	0.076822274
72100	0.036873622	72052	0.043708358
72100	0.034228444	72056	0.031570384
72100	0.054960467	72060	0.059100054
72100	0.05429544	72064	0.098968398
72100	0.026184129	72068	0.117681615
72100	0.00366161	72072	0.14314994
72100	0.022279788	72076	0.168474856
72100	0.017162856	72080	0.146805513
72100	0.033327593	72084	0.085708489
72100	0.030490288	72088	0.066478366
72100	0.054212436	72092	0.079248915
72100	0.080569909	72096	0.066533976
72100	0.063523694	72100	0.040365379
72100	0.038057602	72104	0.025882015
72100	0.006424142	72108	0.052619555
72100	0.011193647	72112	0.069003785
72100	0.021593052	72116	0.071735929
72100	0.021965305	72120	0.035179724
72100	0.031482279	72124	0.046984405
72100	0.085031017	72128	0.071061892

PowerSpectrumData

72100	0.070730865	72132	0.095937117
72100	0.029921839	72136	0.114160393
72100	0.007788061	72140	0.095414514
72100	0.024043415	72144	0.073503012
72100	0.045571102	72148	0.073805495
72200	0.105266285	72152	0.032783326
72200	0.131583336	72156	0.059729224
72200	0.099031931	72160	0.11935721
72200	0.044557914	72164	0.107652661
72200	0.010658015	72168	0.049381251
72200	0.038370392	72172	0.051274652
72200	0.044015134	72176	0.037136582
72200	0.032878739	72180	0.059740043
72200	0.03291473	72184	0.113982482
72200	0.028366298	72188	0.109083863
72200	0.012086138	72192	0.080441263
72200	0.016155611	72196	0.077446428
72200	0.01957958	72200	0.088706707
72200	0.030578518	72204	0.075256357
72200	0.061962943	72208	0.036078251
72200	0.049731538	72212	0.014074179
72200	0.006346319	72216	0.043316901
72200	0.027050317	72220	0.055080207
72200	0.027841263	72224	0.07246263
72200	0.04633722	72228	0.080956452
72200	0.053803258	72232	0.081663042
72200	0.055496235	72236	0.08269793
72200	0.061657309	72240	0.06503539
72200	0.070950249	72244	0.027543383
72200	0.070259193	72248	0.028981669
72300	0.041561387	72252	0.057561592
72300	0.014887829	72256	0.053898268
72300	0.039233313	72260	0.034903973
72300	0.074963777	72264	0.019126554

PowerSpectrumData

72300	0.070672206	72268	0.052492676
72300	0.045389916	72272	0.075293443
72300	0.05186717	72276	0.058222242
72300	0.0586887	72280	0.049479171
72300	0.039259812	72284	0.070311144
72300	0.039017403	72288	0.07122643
72300	0.02052972	72292	0.076964359
72300	0.018035426	72296	0.102851707
72300	0.029379271	72300	0.091902708
72300	0.019385489	72304	0.034898534
72300	0.043860149	72308	0.029500974
72300	0.038735492	72312	0.033390948
72300	0.052918131	72316	0.053695349
72300	0.059054466	72320	0.071679249
72300	0.05840517	72324	0.053878008
72300	0.070600589	72328	0.056805526
72300	0.055630353	72332	0.078914636
72300	0.076356635	72336	0.091910537
72300	0.088072193	72340	0.098409815
72300	0.048538477	72344	0.083502411
72300	0.025374484	72348	0.040119365
72400	0.034980985	72352	0.041879226
72400	0.051145493	72356	0.048037317
72400	0.048328249	72360	0.058630212
72400	0.051887397	72364	0.051516821
72400	0.050260696	72368	0.031270451
72400	0.033229757	72372	0.047304508
72400	0.022942069	72376	0.075594151
72400	0.025970752	72380	0.06526857
72400	0.031290143	72384	0.047656657
72400	0.031050447	72388	0.082427301
72400	0.022298586	72392	0.103269296
72400	0.014773332	72396	0.067247253
72400	0.041556232	72400	0.044190681

PowerSpectrumData

72400	0.073559539	72404	0.018137916
72400	0.056973262	72408	0.052989974
72400	0.017165874	72412	0.079468271
72400	0.008401247	72416	0.097434189
72400	0.012584959	72420	0.079614045
72400	0.049901344	72424	0.071048373
72400	0.095066214	72428	0.050011098
72400	0.094875832	72432	0.024815643
72400	0.042717526	72436	0.096275944
72400	0.081723862	72440	0.114216695
72400	0.110533343	72444	0.091674687
72400	0.053373096	72448	0.065989705
72500	0.043520879	72452	0.043217056
72500	0.072794916	72456	0.033703836
72500	0.069677386	72460	0.07274979
72500	0.06414453	72464	0.105942374
72500	0.049563147	72468	0.101723483
72500	0.008297364	72472	0.067961548
72500	0.044584307	72476	0.031396725
72500	0.073487136	72480	0.01344349
72500	0.09042953	72484	0.031994488
72500	0.106789135	72488	0.040109851
72500	0.105176703	72492	0.060770748
72500	0.071530165	72496	0.059612925
72500	0.043415941	72500	0.06696982
72500	0.085055479	72504	0.075721677
72500	0.073089897	72508	0.053597967
72500	0.036475776	72512	0.03321212
72500	0.0319188	72516	0.084571191
72500	0.034988567	72520	0.089234265
72500	0.067666486	72524	0.060787657
72500	0.037320049	72528	0.07606337
72500	0.023073921	72532	0.085155836
72500	0.034451601	72536	0.075540367

PowerSpectrumData

72500	0.017026845	72540	0.070026792
72500	0.036768346	72544	0.039067982
72500	0.021974893	72548	0.032308621
72600	0.00746332	72552	0.064105159
72600	0.01566541	72556	0.06530383
72600	0.007609836	72560	0.06854711
72600	0.035578643	72564	0.046134457
72600	0.020557794	72568	0.018723
72600	0.021093749	72572	0.020525697
72600	0.03677245	72576	0.050767605
72600	0.035962275	72580	0.06655066
72600	0.034184053	72584	0.036182311
72600	0.018825407	72588	0.017513341
72600	0.061585	72592	0.009388412
72600	0.03329488	72596	0.013572617
72600	0.055110133	72600	0.051814146
72600	0.077703087	72604	0.103381717
72600	0.065371241	72608	0.112735979
72600	0.06816406	72612	0.090380396
72600	0.05291342	72616	0.068514732
72600	0.013307097	72620	0.035930763
72600	0.023879788	72624	0.040667037
72600	0.013780042	72628	0.083048042
72600	0.050821876	72632	0.098289733
72600	0.091411355	72636	0.065670618
72600	0.051504187	72640	0.043786957
72600	0.027411876	72644	0.062794075
72600	0.064879176	72648	0.049252412
72700	0.054798147	72652	0.04198854
72700	0.032603908	72656	0.04365038
72700	0.027240514	72660	0.013466211
72700	0.043113181	72664	0.069127309
72700	0.058447291	72668	0.099969344
72700	0.042727446	72672	0.077977456

PowerSpectrumData

72700	0.003252135	72676	0.062332052
72700	0.029263854	72680	0.07847236
72700	0.027601944	72684	0.089709189
72700	0.031133768	72688	0.085128777
72700	0.030625961	72692	0.05131053
72700	0.012977737	72696	0.013475013
72700	0.052808027	72700	0.068495639
72700	0.075919073	72704	0.072451447
72700	0.07533934	72708	0.051767522
72700	0.060740142	72712	0.059397356
72700	0.020917649	72716	0.034734472
72700	0.042693166	72720	0.023330511
72700	0.044540524	72724	0.046896545
72700	0.059059952	72728	0.065619832
72700	0.068946305	72732	0.067557026
72700	0.052232885	72736	0.092828734
72700	0.04106151	72740	0.103382583
72700	0.011635578	72744	0.09336001
72700	0.027052136	72748	0.063994412
72800	0.03475869	72752	0.047904672
72800	0.041076444	72756	0.05541799
72800	0.052146639	72760	0.063145839
72800	0.019694446	72764	0.084011845
72800	0.047625654	72768	0.075309727
72800	0.056957269	72772	0.047109952
72800	0.029168592	72776	0.068584501
72800	0.020281037	72780	0.058797858
72800	0.061078776	72784	0.065156994
72800	0.057655532	72788	0.080992475
72800	0.026292426	72792	0.075171432
72800	0.053704731	72796	0.095776755
72800	0.078850266	72800	0.105800064
72800	0.048423673	72804	0.087490393
72800	0.027362181	72808	0.079840575

PowerSpectrumData

72800	0.024086021	72812	0.091996975
72800	0.032536576	72816	0.07208655
72800	0.083192084	72820	0.042580574
72800	0.120935998	72824	0.080120844
72800	0.109405002	72828	0.081661652
72800	0.083217048	72832	0.06706539
72800	0.061769912	72836	0.058312824
72800	0.037800033	72840	0.06945998
72800	0.036741447	72844	0.099219637
72800	0.027229615	72848	0.11457019
72900	0.035048386	72852	0.059080041
72900	0.067085181	72856	0.090493079
72900	0.075814191	72860	0.11524243
72900	0.04954094	72864	0.061255043
72900	0.015288892	72868	0.022392485
72900	0.041934611	72872	0.063249143
72900	0.055994864	72876	0.087458808
72900	0.030608189	72880	0.064837943
72900	0.048714934	72884	0.055754848
72900	0.067549707	72888	0.078422032
72900	0.063923493	72892	0.10319658
72900	0.054694527	72896	0.107573767
72900	0.013091918	72900	0.091502749
72900	0.054739761	72904	0.060264451
72900	0.063713269	72908	0.027424801
72900	0.034231296	72912	0.10358802
72900	0.012109072	72916	0.100898396
72900	0.022583812	72920	0.047943184
72900	0.007860657	72924	0.086318818
72900	0.050590275	72928	0.099156743
72900	0.092642818	72932	0.04218796
72900	0.071851842	72936	0.03512462
72900	0.047298436	72940	0.067887588
72900	0.046568071	72944	0.076054566

PowerSpectrumData

72900	0.032861932	72948	0.082691709
73000	0.039972631	72952	0.11723824
73000	0.053265765	72956	0.109212007
73000	0.059983468	72960	0.076606149
73000	0.054099917	72964	0.055419085
73000	0.071368093	72968	0.091112968
73000	0.085290754	72972	0.105328625
73000	0.043329063	72976	0.09546987
73000	0.014401508	72980	0.070725924
73000	0.011296405	72984	0.074045929
73000	0.041080937	72988	0.126198633
73000	0.061213992	72992	0.138605843
73000	0.018921282	72996	0.121802819
73000	0.032408196	73000	0.10237228
73000	0.022356597	73004	0.076423166
73000	0.048283873	73008	0.032269778
73000	0.048407535	73012	0.023209432
73000	0.050783539	73016	0.037028342
73000	0.07313503	73020	0.039934173
73000	0.073747797	73024	0.047707996
73000	0.087588305	73028	0.065298867
73000	0.082496197	73032	0.095383679
73000	0.048138125	73036	0.112149333
73000	0.007197647	73040	0.108310997
73000	0.036666792	73044	0.092132948
73000	0.047426631	73048	0.04633654
73100	0.028261116	73052	0.022181046
73100	0.01247415	73056	0.070119539
73100	0.025326728	73060	0.085961161
73100	0.06978387	73064	0.102474762
73100	0.089773639	73068	0.109841232
73100	0.059509912	73072	0.072099749
73100	0.024171515	73076	0.036620822
73100	0.028327648	73080	0.054427146

PowerSpectrumData

73100	0.03421586	73084	0.035413534
73100	0.044567678	73088	0.008962958
73100	0.011979353	73092	0.036995192
73100	0.053154694	73096	0.054858869
73100	0.044293855	73100	0.068669026
73100	0.027971364	73104	0.080687045
73100	0.073132847	73108	0.064471729
73100	0.062353596	73112	0.058348614
73100	0.041651118	73116	0.062928761
73100	0.046003479	73120	0.034893434
73100	0.044584867	73124	0.022443199
73100	0.014203247	73128	0.028283557
73100	0.018945244	73132	0.017771334
73100	0.032271706	73136	0.060112929
73100	0.037709866	73140	0.093080525
73100	0.040146049	73144	0.085675085
73100	0.032524058	73148	0.057484194
73200	0.06467072	73152	0.038581689
73200	0.078131168	73156	0.037078695
73200	0.057587502	73160	0.017967552
73200	0.034560311	73164	0.014558067
73200	0.032176085	73168	0.042718577
73200	0.056450659	73172	0.066480869
73200	0.047047375	73176	0.075247721
73200	0.009336001	73180	0.063721469
73200	0.026001095	73184	0.026358874
73200	0.050327195	73188	0.033853848
73200	0.035521753	73192	0.085717038
73200	0.033578734	73196	0.084271691
73200	0.033074874	73200	0.025817515
73200	0.02885418	73204	0.039550807
73200	0.006931938	73208	0.052883752
73200	0.0313692	73212	0.069199676
73200	0.047697249	73216	0.069976239

PowerSpectrumData

73200	0.036108242	73220	0.044512264
73200	0.044531156	73224	0.015775882
73200	0.034128061	73228	0.02129773
73200	0.014450526	73232	0.042769272
73200	0.020324025	73236	0.038949234
73200	0.066212771	73240	0.016473317
73200	0.092562041	73244	0.00566285
73200	0.094563635	73248	0.028301685
73300	0.094258765	73252	0.055683759
73300	0.081075043	73256	0.055324803
73300	0.051589064	73260	0.042810822
73300	0.068696943	73264	0.050319082
73300	0.079271144	73268	0.038410864
73300	0.064871936	73272	0.064394466
73300	0.057517809	73276	0.051672578
73300	0.046290505	73280	0.03960729
73300	0.038885701	73284	0.06229095
73300	0.033971639	73288	0.025554187
73300	0.044406603	73292	0.034607994
73300	0.076524513	73296	0.043366134
73300	0.043508713	73300	0.085233049
73300	0.015964906	73304	0.140205143
73300	0.048856247	73308	0.142934805
73300	0.085837673	73312	0.110023604
73300	0.078402431	73316	0.067463152
73300	0.042881722	73320	0.044330198
73300	0.022306609	73324	0.041545154
73300	0.057950532	73328	0.015493688
73300	0.057826423	73332	0.032014908
73300	0.035532816	73336	0.054367461
73300	0.032828481	73340	0.049738817
73300	0.013860369	73344	0.043190728
73300	0.046719706	73348	0.027585253
73400	0.084414925	73352	0.043940829

PowerSpectrumData

73400	0.085626933	73356	0.091320755
73400	0.066730805	73360	0.081088219
73400	0.042438875	73364	0.043109754
73400	0.025773737	73368	0.044254484
73400	0.033148037	73372	0.07401406
73400	0.027666431	73376	0.066539367
73400	0.032841515	73380	0.003369272
73400	0.071568218	73384	0.076756987
73400	0.109535111	73388	0.116374074
73400	0.131211665	73392	0.091692738
73400	0.127389023	73396	0.076282195
73400	0.087841479	73400	0.124458777
73400	0.080872014	73404	0.128421293
73400	0.064801534	73408	0.078122605
73400	0.030375084	73412	0.064515014
73400	0.105830121	73416	0.064697117
73400	0.11217744	73420	0.081319704
73400	0.069592148	73424	0.098853991
73400	0.012314114	73428	0.069469665
73400	0.035386165	73432	0.089215551
73400	0.063926309	73436	0.120476529
73400	0.123769496	73440	0.090940281
73400	0.126346131	73444	0.042304389
73400	0.051031642	73448	0.061827814
73500	0.022889715	73452	0.08108178
73500	0.022468534	73456	0.093976225
73500	0.042539334	73460	0.096399352
73500	0.097934157	73464	0.071856535
73500	0.107167245	73468	0.101967569
73500	0.071412556	73472	0.135719558
73500	0.051667754	73476	0.098083314
73500	0.045481556	73480	0.041209452
73500	0.036747806	73484	0.074439966
73500	0.049517825	73488	0.099461511

PowerSpectrumData

73500	0.086660759	73492	0.095641779
73500	0.114275485	73496	0.075964788
73500	0.094883173	73500	0.039396247
73500	0.047894573	73504	0.022598786
73500	0.042908403	73508	0.023777946
73500	0.037931357	73512	0.026652129
73500	0.024111439	73516	0.043670858
73500	0.072711882	73520	0.044078708
73500	0.063878768	73524	0.02651399
73500	0.033515302	73528	0.040854156
73500	0.014422531	73532	0.060748705
73500	0.011402026	73536	0.071369585
73500	0.029290031	73540	0.079157057
73500	0.047271082	73544	0.062796644
73500	0.052769767	73548	0.092160168
73600	0.034826833	73552	0.155915084
73600	0.030306423	73556	0.155914153
73600	0.0213407	73560	0.098463905
73600	0.023517599	73564	0.041228639
73600	0.016324229	73568	0.052631356
73600	0.032719148	73572	0.081862039
73600	0.055903067	73576	0.101791935
73600	0.043886521	73580	0.113525573
73600	0.022406501	73584	0.082623767
73600	0.019687012	73588	0.019092451
73600	0.042748121	73592	0.049033919
73600	0.071384027	73596	0.048762253
73600	0.065579945	73600	0.037510887
73600	0.066234461	73604	0.058606842
73600	0.080870363	73608	0.079707344
73600	0.074247815	73612	0.044458157
73600	0.041718242	73616	0.023998429
73600	0.026936192	73620	0.052074909
73600	0.022177803	73624	0.057183937

PowerSpectrumData

73600	0.025023985	73628	0.044703542
73600	0.024464542	73632	0.043506294
73600	0.037922986	73636	0.055160705
73600	0.052823834	73640	0.049667429
73600	0.058900801	73644	0.050469251
73600	0.046441368	73648	0.079675127
73700	0.046648122	73652	0.031716692
73700	0.016924802	73656	0.072690054
73700	0.045898782	73660	0.094854855
73700	0.061187398	73664	0.099661171
73700	0.060957653	73668	0.078015808
73700	0.06588603	73672	0.043273081
73700	0.061695799	73676	0.079796148
73700	0.04888112	73680	0.078285331
73700	0.038983522	73684	0.051930841
73700	0.03821075	73688	0.039189814
73700	0.048883157	73692	0.053844902
73700	0.056692625	73696	0.059364582
73700	0.047333506	73700	0.047869966
73700	0.032249947	73704	0.054813885
73700	0.031579799	73708	0.05623194
73700	0.043032913	73712	0.050509709
73700	0.072092058	73716	0.061258106
73700	0.066952431	73720	0.069053662
73700	0.02616557	73724	0.068604437
73700	0.02278073	73728	0.044791894
73700	0.056543184	73732	0.01923219
73700	0.068910718	73736	0.039634699
73700	0.061058381	73740	0.049127793
73700	0.050102448	73744	0.019698931
73700	0.053905424	73748	0.037676149
73800	0.027929604	73752	0.051019477
73800	0.034987414	73756	0.022156508
73800	0.076926648	73760	0.061426363

PowerSpectrumData

73800	0.086578017	73764	0.053439497
73800	0.102864215	73768	0.043131695
73800	0.101911399	73772	0.024864254
73800	0.062250692	73776	0.06048059
73800	0.025370135	73780	0.076886739
73800	0.02499404	73784	0.041207859
73800	0.033138407	73788	0.061325598
73800	0.021183916	73792	0.058601458
73800	0.017437049	73796	0.075613236
73800	0.040171417	73800	0.081019694
73800	0.038463881	73804	0.025182837
73800	0.038912163	73808	0.055683082
73800	0.056461984	73812	0.099551915
73800	0.060310085	73816	0.099122684
73800	0.038859009	73820	0.064847569
73800	0.037641636	73824	0.017762193
73800	0.032768425	73828	0.042073185
73800	0.042014068	73832	0.056425452
73800	0.071505296	73836	0.036485999
73800	0.07439089	73840	0.040068295
73800	0.04539486	73844	0.047080328
73800	0.041797015	73848	0.037825867
73900	0.081885155	73852	0.058372054
73900	0.12643743	73856	0.083083345
73900	0.136328235	73860	0.083858366
73900	0.092802744	73864	0.068394351
73900	0.038784514	73868	0.021109358
73900	0.017155135	73872	0.0333752
73900	0.050614526	73876	0.03504684
73900	0.018335282	73880	0.046183646
73900	0.038682592	73884	0.072780735
73900	0.026139333	73888	0.06377892
73900	0.062927487	73892	0.036859201
73900	0.088746339	73896	0.04661642

73900	0.08549817	73900	0.071184019
73900	0.091398419	73904	0.078903657
73900	0.065280408	73908	0.053181004
73900	0.029606628	73912	0.074246716
73900	0.064709711	73916	0.092091192
73900	0.044085278	73920	0.123145685
73900	0.053319825	73924	0.132734741
73900	0.056600864	73928	0.086926746
73900	0.050032199	73932	0.07333354
73900	0.039667011	73936	0.120817836
73900	0.035536694	73940	0.132935399
73900	0.075713739	73944	0.093699506
73900	0.118485834	73948	0.060057369
74000	0.142753168	73952	0.058040638
74000	0.150743741	73956	0.065093118
74000	0.121992038	73960	0.082345992
74000	0.06102429	73964	0.107834094
74000	0.015964384	73968	0.081270759
74000	0.002715673	73972	0.044832152
74000	0.01978018	73976	0.058608959
74000	0.074431504	73980	0.031492087
74000	0.121309626	73984	0.028622968
74000	0.099564742	73988	0.030555926
74000	0.017327751	73992	0.044630229
74000	0.047381713	73996	0.059717069
74000	0.035949168	74000	0.077505501
74000	0.053032982	74004	0.058930134
74000	0.154072318	74008	0.049075272
74000	0.200609356	74012	0.077212746
74000	0.171871914	74016	0.091460628
74000	0.123777616	74020	0.087337998
74000	0.088780231	74024	0.085320782
74000	0.11713292	74028	0.08906045
74000	0.047146186	74032	0.047513709

PowerSpectrumData

74000	0.142883131	74036	0.029197223
74000	0.269298355	74040	0.067763896
74000	0.234208331	74044	0.036683519
74000	0.114169394	74048	0.018199984
74100	0.036550748	74052	0.012505074
74100	0.112925234	74056	0.027900189
74100	0.10203429	74060	0.05856311
74100	0.057503687	74064	0.078201534
74100	0.129027729	74068	0.088084977
74100	0.127113148	74072	0.089839872
74100	0.085851832	74076	0.05847455
74100	0.045137764	74080	0.014876907
74100	0.112121292	74084	0.01236294
74100	0.12518304	74088	0.041034622
74100	0.100403093	74092	0.038859849
74100	0.37807686	74096	0.027382645
74100	0.50443405	74100	0.044146051
74100	0.362343039	74104	0.067010398
74100	0.121627621	74108	0.068271766
74100	0.027960314	74112	0.040683219
74100	0.075366428	74116	0.025735164
74100	0.06997231	74120	0.02763456
74100	0.051234551	74124	0.027821638
74100	0.08324095	74128	0.044689707
74100	0.090946	74132	0.050770592
74100	0.060358569	74136	0.036438036
74100	0.035448476	74140	0.01963381
74100	0.10345217	74144	0.015150544
74100	0.077476194	74148	0.017667289
74200	0.406556326	74152	0.035396013
74200	0.724308833	74156	0.024487174
74200	0.709146436	74160	0.005816249
74200	0.404143968	74164	0.029822468
74200	0.108222463	74168	0.034317145

PowerSpectrumData

74200	0.061482213	74172	0.040670555
74200	0.010903925	74176	0.023225541
74200	0.082255574	74180	0.01608757
74200	0.221102644	74184	0.032194992
74200	0.315304759	74188	0.079556186
74200	0.229296435	74192	0.100424877
74200	0.058410537	74196	0.071631934
74200	0.134812755	74200	0.028752158
74200	0.222778938	74204	0.024878345
74200	0.155275528	74208	0.029131763
74200	0.409829285	74212	0.001896468
74200	0.560625805	74216	0.020804779
74200	0.390742265	74220	0.041875901
74200	0.119471137	74224	0.022583534
74200	0.041460855	74228	0.050210449
74200	0.077648947	74232	0.042667791
74200	0.047934573	74236	0.057568508
74200	0.210857543	74240	0.048975908
74200	0.393228198	74244	0.066798457
74200	0.40408317	74248	0.048064689
74300	0.225467739	74252	0.045978253
74300	0.106994368	74256	0.019832902
74300	0.170478103	74260	0.020396528
74300	0.067881512	74264	0.038415157
74300	0.192849868	74268	0.063961285
74300	0.356634293	74272	0.071483519
74300	0.373438001	74276	0.106399035
74300	0.246571348	74280	0.098655859
74300	0.075281401	74284	0.060437775
74300	0.046004683	74288	0.04032813
74300	0.071536284	74292	0.014024412
74300	0.082862171	74296	0.030310586
74300	0.166948885	74300	0.055767156
74300	0.192599633	74304	0.04793036

PowerSpectrumData

74300	0.166160128	74308	0.054640608
74300	0.097691634	74312	0.059951526
74300	0.035978614	74316	0.071801391
74300	0.0402431	74320	0.082703482
74300	0.038526952	74324	0.099710996
74300	0.092166461	74328	0.078607212
74300	0.218600704	74332	0.012789368
74300	0.214679501	74336	0.036866321
74300	0.074841613	74340	0.041318202
74300	0.077842815	74344	0.034118421
74300	0.087114058	74348	0.012660134
74400	0.046500547	74352	0.048010701
74400	0.11885303	74356	0.093956071
74400	0.328641356	74360	0.0934852
74400	0.36415967	74364	0.033340162
74400	0.203697724	74368	0.049171034
74400	0.036640719	74372	0.073385367
74400	0.100267018	74376	0.066506866
74400	0.086822503	74380	0.017424847
74400	0.028669974	74384	0.044632565
74400	0.110652742	74388	0.062925385
74400	0.156050883	74392	0.074012038
74400	0.096381889	74396	0.114477436
74400	0.021148546	74400	0.123605089
74400	0.064446744	74404	0.082540275
74400	0.079907324	74408	0.062229214
74400	0.10326966	74412	0.053935055
74400	0.231068785	74416	0.032369506
74400	0.330766546	74420	0.013277298
74400	0.269151321	74424	0.024655792
74400	0.133492213	74428	0.059110822
74400	0.121740406	74432	0.074979274
74400	0.15004637	74436	0.04587989
74400	0.11322309	74440	0.044060133

PowerSpectrumData

74400	0.101868849	74444	0.074476215
74400	0.138791904	74448	0.057104226
74500	0.126027109	74452	0.017112565
74500	0.058373476	74456	0.016240374
74500	0.075324322	74460	0.022430255
74500	0.109054512	74464	0.03712413
74500	0.062288062	74468	0.061228777
74500	0.063679516	74472	0.064118707
74500	0.175328605	74476	0.031407661
74500	0.165316276	74480	0.031413056
74500	0.065983906	74484	0.013013706
74500	0.062516338	74488	0.018364601
74500	0.091952657	74492	0.016707074
74500	0.106534804	74496	0.014252645
74500	0.115642324	74500	0.046859892
74500	0.133039023	74504	0.061796367
74500	0.110799178	74508	0.058119753
74500	0.06857558	74512	0.044307119
74500	0.041420783	74516	0.036110239
74500	0.05988486	74520	0.046126548
74500	0.047243048	74524	0.065211163
74500	0.016721002	74528	0.071993061
74500	0.053507785	74532	0.087940061
74500	0.05176873	74536	0.084638763
74500	0.025277403	74540	0.06324437
74500	0.013682926	74544	0.028723245
74500	0.041170999	74548	0.038298516
74600	0.053270196	74552	0.053893513
74600	0.035498757	74556	0.067880625
74600	0.039295661	74560	0.089206951
74600	0.044133056	74564	0.073705873
74600	0.044597287	74568	0.051431132
74600	0.073821015	74572	0.057047171
74600	0.057400481	74576	0.054073524

PowerSpectrumData

74600	0.045412322	74580	0.046210371
74600	0.067877685	74584	0.056799087
74600	0.0559855	74588	0.065697132
74600	0.060432845	74592	0.065563792
74600	0.062872779	74596	0.042108706
74600	0.037227535	74600	0.03126794
74600	0.045176032	74604	0.042891723
74600	0.076543613	74608	0.060949831
74600	0.051678253	74612	0.064949833
74600	0.029819605	74616	0.027664388
74600	0.004295908	74620	0.012102717
74600	0.057945119	74624	0.025093588
74600	0.057208294	74628	0.027866638
74600	0.040579671	74632	0.005441676
74600	0.052076528	74636	0.046295838
74600	0.050813571	74640	0.036609275
74600	0.026566913	74644	0.037324982
74600	0.04622724	74648	0.021243537
74700	0.042708554	74652	0.049152903
74700	0.037372509	74656	0.067881978
74700	0.042638651	74660	0.080512589
74700	0.05015625	74664	0.071455455
74700	0.063524654	74668	0.038077254
74700	0.075902564	74672	0.033123502
74700	0.083966865	74676	0.025088641
74700	0.084442996	74680	0.02568986
74700	0.059754228	74684	0.031399912
74700	0.048795289	74688	0.044401153
74700	0.080856007	74692	0.054988534
74700	0.067072549	74696	0.033888471
74700	0.035403882	74700	0.00381541
74700	0.011718923	74704	0.006973884
74700	0.028971099	74708	0.013469887
74700	0.009181023	74712	0.016570548

PowerSpectrumData

74700	0.038356844	74716	0.01107635
74700	0.036697944	74720	0.032638087
74700	0.020255746	74724	0.066840621
74700	0.009546193	74728	0.093828778
74700	0.010152733	74732	0.095998948
74700	0.034574186	74736	0.081992796
74700	0.02407065	74740	0.09369351
74700	0.017268165	74744	0.084900406
74700	0.036403209	74748	0.013087744
74800	0.021843744	74752	0.067124369
74800	0.026917413	74756	0.09245293
74800	0.040475781	74760	0.087294102
74800	0.032915315	74764	0.065692148
74800	0.049670271	74768	0.025798889
74800	0.049328402	74772	0.033156775
74800	0.042337262	74776	0.050659521
74800	0.027092236	74780	0.068419773
74800	0.009673387	74784	0.045621779
74800	0.036926755	74788	0.029826819
74800	0.029725439	74792	0.043272521
74800	0.008723474	74796	0.042282074
74800	0.03324345	74800	0.068775786
74800	0.021059419	74804	0.080099744
74800	0.008518982	74808	0.048110484
74800	0.01506571	74812	0.028019515
74800	0.038288818	74816	0.079853606
74800	0.085820662	74820	0.091300331
74800	0.082377941	74824	0.058359077
74800	0.03697757	74828	0.039592436
74800	0.002843805	74832	0.045784051
74800	0.011411814	74836	0.019305777
74800	0.01765699	74840	0.030625397
74800	0.052227861	74844	0.051072515
74800	0.083860417	74848	0.082353239

PowerSpectrumData

74900	0.066065804	74852	0.087407832
74900	0.02366033	74856	0.06736166
74900	0.039893661	74860	0.039018782
74900	0.057996527	74864	0.027375258
74900	0.078512901	74868	0.040844283
74900	0.08074315	74872	0.058631133
74900	0.054914239	74876	0.021247795
74900	0.014656416	74880	0.043485175
74900	0.020235351	74884	0.035182682
74900	0.042187166	74888	0.04289674
74900	0.050710019	74892	0.040827777
74900	0.018057552	74896	0.040206363
74900	0.03818376	74900	0.050930536
74900	0.044595545	74904	0.063256463
74900	0.011100551	74908	0.052372889
74900	0.054336339	74912	0.038223552
74900	0.051158735	74916	0.074164484
74900	0.023253755	74920	0.094039322
74900	0.013700187	74924	0.066592242
74900	0.004470774	74928	0.043379787
74900	0.023276371	74932	0.062088344
74900	0.019459747	74936	0.08424485
74900	0.022840282	74940	0.114022449
74900	0.05823492	74944	0.133910391
74900	0.066625631	74948	0.107121326
75000	0.079481157	74952	0.068739333
75000	0.061347084	74956	0.060450613
75000	0.005576435	74960	0.058159749
75000	0.00738642	74964	0.089150155
75000	0.025599915	74968	0.105725841
75000	0.030774943	74972	0.072201838
75000	0.010192459	74976	0.049830589
75000	0.005592631	74980	0.034582707
75000	0.01412011	74984	0.025462578

PowerSpectrumData

75000	0.051467931	74988	0.057804693
75000	0.086811197	74992	0.085083659
75000	0.09402437	74996	0.086853077
75000	0.058049791	75000	0.079894082
75000	0.011207801	75004	0.060546743
75000	0.007407671	75008	0.040369294
75000	0.02982438	75012	0.010117225
75000	0.040571969	75016	0.049045928
75000	0.035253474	75020	0.03924303
75000	0.01231435	75024	0.012911448
75000	0.021088612	75028	0.076689496
75000	0.024241814	75032	0.083458028
75000	0.075808668	75036	0.03062834
75000	0.125799139	75040	0.064394721
75000	0.096756477	75044	0.07118682
75000	0.049237926	75048	0.047648082
75100	0.059618436	75052	0.024876787
75100	0.079437865	75056	0.027505741
75100	0.089702808	75060	0.034734334
75100	0.065740402	75064	0.059882907
75100	0.037992031	75068	0.066705434
75100	0.036059799	75072	0.0679285
75100	0.052852698	75076	0.079636462
75100	0.06617148	75080	0.047127363
75100	0.05994098	75084	0.043881333
75100	0.033317479	75088	0.087484215
75100	0.011673424	75092	0.100429876
75100	0.04339762	75096	0.081917002
75100	0.051882063	75100	0.050563427
75100	0.050149563	75104	0.106319843
75100	0.035979952	75108	0.100769619
75100	0.027516242	75112	0.071534931
75100	0.012791154	75116	0.070351824
75100	0.01369923	75120	0.069372589

PowerSpectrumData

75100	0.042354124	75124	0.049141672
75100	0.075552045	75128	0.04664774
75100	0.073381991	75132	0.065587054
75100	0.044686032	75136	0.06495772
75100	0.043233918	75140	0.063175052
75100	0.052803236	75144	0.098678953
75100	0.009784306	75148	0.107399974
75200	0.044344157	75152	0.079542835
75200	0.050360992	75156	0.058304748
75200	0.031152013	75160	0.038240007
75200	0.015234397	75164	0.011782893
75200	0.069743815	75168	0.049547711
75200	0.085754982	75172	0.055189921
75200	0.031100557	75176	0.041408766
75200	0.042292184	75180	0.023230427
75200	0.054185344	75184	0.047327831
75200	0.033133838	75188	0.089404181
75200	0.030042103	75192	0.063282409
75200	0.029831857	75196	0.020695799
75200	0.024859663	75200	0.035316592
75200	0.039675695	75204	0.043100099
75200	0.014585345	75208	0.017086963
75200	0.076602009	75212	0.014213227
75200	0.092332652	75216	0.023714567
75200	0.083549094	75220	0.068085996
75200	0.059130845	75224	0.089086985
75200	0.012102034	75228	0.053799922
75200	0.050714611	75232	0.043773554
75200	0.059976388	75236	0.069002294
75200	0.03455269	75240	0.058929858
75200	0.01890445	75244	0.016357037
75200	0.007025159	75248	0.043855511
75300	0.036346992	75252	0.024553725
75300	0.044770051	75256	0.047901307

PowerSpectrumData

75300	0.062333224	75260	0.042611988
75300	0.070927912	75264	0.028732135
75300	0.022391239	75268	0.065943976
75300	0.003150112	75272	0.074872703
75300	0.031741573	75276	0.053398053
75300	0.060001647	75280	0.037814181
75300	0.057576406	75284	0.02589922
75300	0.060978087	75288	0.004826167
75300	0.051362924	75292	0.022945074
75300	0.016934046	75296	0.062083229
75300	0.041506552	75300	0.07372407
75300	0.061090788	75304	0.058010657
75300	0.050870127	75308	0.034326837
75300	0.002294313	75312	0.030268988
75300	0.047934573	75316	0.046941979
75300	0.04380762	75320	0.055924862
75300	0.037671238	75324	0.077364377
75300	0.075112243	75328	0.091738053
75300	0.096404394	75332	0.082973376
75300	0.090711517	75336	0.047520323
75300	0.033079341	75340	0.016374494
75300	0.031563148	75344	0.029094672
75300	0.028530723	75348	0.031432239
75400	0.04051673	75352	0.049279628
75400	0.06067986	75356	0.07166966
75400	0.054980785	75360	0.094607836
75400	0.039611401	75364	0.085197957
75400	0.020544871	75368	0.069418995
75400	0.035764901	75372	0.066225606
75400	0.024614679	75376	0.059350045
75400	0.013850565	75380	0.056531226
75400	0.03391214	75384	0.058026235
75400	0.053613127	75388	0.043301818
75400	0.040176954	75392	0.013722971

PowerSpectrumData

75400	0.04828571	75396	0.074065451
75400	0.058837533	75400	0.107936692
75400	0.032410866	75404	0.077645804
75400	0.015865016	75408	0.038586699
75400	0.073072137	75412	0.039140003
75400	0.102412792	75416	0.01086493
75400	0.062557359	75420	0.011016692
75400	0.022550754	75424	0.020658557
75400	0.022345495	75428	0.042470951
75400	0.047670026	75432	0.040135816
75400	0.038636099	75436	0.027052724
75400	0.006080511	75440	0.112159083
75400	0.022762179	75444	0.138456991
75400	0.012142628	75448	0.089117006
75500	0.01152198	75452	0.043472272
75500	0.003253117	75456	0.023018058
75500	0.023949022	75460	0.027575412
75500	0.053890344	75464	0.052918
75500	0.069869973	75468	0.038344682
75500	0.055071971	75472	0.012678438
75500	0.070578579	75476	0.049260743
75500	0.06314621	75480	0.024262423
75500	0.03546054	75484	0.021318014
75500	0.015344542	75488	0.013963482
75500	0.02660493	75492	0.042088566
75500	0.047710695	75496	0.091637339
75500	0.048979084	75500	0.046246332
75500	0.061388935	75504	0.041474548
75500	0.075276388	75508	0.079906153
75500	0.071251372	75512	0.07555919
75500	0.051452076	75516	0.052433421
75500	0.023675862	75520	0.051415413
75500	0.007052612	75524	0.047010843
75500	0.033201312	75528	0.041908181

PowerSpectrumData

75500	0.097531134	75532	0.026740809
75500	0.125764899	75536	0.023240478
75500	0.092190989	75540	0.014672081
75500	0.047930931	75544	0.018464607
75500	0.026776439	75548	0.060251998
75600	0.046932029	75552	0.056825327
75600	0.077128534	75556	0.067086963
75600	0.095203468	75560	0.096346281
75600	0.069916699	75564	0.096738149
75600	0.043312612	75568	0.049960181
75600	0.047303696	75572	0.062906314
75600	0.047996236	75576	0.072453251
75600	0.047510348	75580	0.076154087
75600	0.054563334	75584	0.055371409
75600	0.047652768	75588	0.012317712
75600	0.021098556	75592	0.03762979
75600	0.032956341	75596	0.065655811
75600	0.063794243	75600	0.080467908
75600	0.076811688	75604	0.07281491
75600	0.05465739	75608	0.063521024
75600	0.030098419	75612	0.074090895
75600	0.070145252	75616	0.092401402
75600	0.03818257	75620	0.076826167
75600	0.018399111	75624	0.055317123
75600	0.027126531	75628	0.045664474
75600	0.030208092	75632	0.03857314
75600	0.028307124	75636	0.059666811
75600	0.036438578	75640	0.069281756
75600	0.067008885	75644	0.04596396
75600	0.076064229	75648	0.028465454
75700	0.037832386	75652	0.040454423
75700	0.01577783	75656	0.057133286
75700	0.055882334	75660	0.04433233
75700	0.06783627	75664	0.022277453

PowerSpectrumData

75700	0.052963602	75668	0.063487918
75700	0.051677733	75672	0.10771997
75700	0.055300447	75676	0.090637579
75700	0.049028469	75680	0.049535945
75700	0.031787477	75684	0.110693502
75700	0.020146932	75688	0.110002598
75700	0.033966793	75692	0.074588053
75700	0.038765164	75696	0.06061469
75700	0.026405329	75700	0.047865291
75700	0.038162434	75704	0.036258301
75700	0.057568126	75708	0.044442186
75700	0.027166274	75712	0.029948224
75700	0.040036586	75716	0.052440544
75700	0.057696481	75720	0.043854267
75700	0.016428627	75724	0.027539958
75700	0.038713286	75728	0.081904917
75700	0.058099267	75732	0.11327878
75700	0.045342807	75736	0.080547579
75700	0.007249746	75740	0.048229784
75700	0.036366	75744	0.088541798
75700	0.047193313	75748	0.097077747
75800	0.051935524	75752	0.015946285
75800	0.102934908	75756	0.079448648
75800	0.102150378	75760	0.076414319
75800	0.065051791	75764	0.041241354
75800	0.046486304	75768	0.039523453
75800	0.022711312	75772	0.031960866
75800	0.019008316	75776	0.039504892
75800	0.034693014	75780	0.052651238
75800	0.022885946	75784	0.054624848
75800	0.028375069	75788	0.060179558
75800	0.033696964	75792	0.029654018
75800	0.064855813	75796	0.053465603
75800	0.037027825	75800	0.12675162

PowerSpectrumData

75800	0.01271041	75804	0.115743264
75800	0.017286846	75808	0.063353364
75800	0.025608384	75812	0.068415669
75800	0.022143722	75816	0.10754267
75800	0.012185349	75820	0.114238203
75800	0.035740344	75824	0.069488124
75800	0.030031502	75828	0.068339854
75800	0.012782028	75832	0.086640714
75800	0.051539053	75836	0.034063109
75800	0.062913772	75840	0.085345542
75800	0.058916896	75844	0.084205487
75800	0.054437125	75848	0.032213608
75900	0.061112631	75852	0.050545601
75900	0.077707984	75856	0.081067505
75900	0.083242114	75860	0.087949251
75900	0.059116916	75864	0.081399521
75900	0.045396871	75868	0.077786521
75900	0.076813522	75872	0.064520689
75900	0.098977638	75876	0.0313196
75900	0.069201182	75880	0.052570118
75900	0.03769594	75884	0.073258831
75900	0.061918079	75888	0.066829976
75900	0.067464476	75892	0.061679675
75900	0.048441401	75896	0.059708047
75900	0.025447549	75900	0.07611492
75900	0.040114512	75904	0.09798375
75900	0.031552841	75908	0.08497938
75900	0.016814269	75912	0.046702975
75900	0.045610264	75916	0.081817656
75900	0.033856712	75920	0.130138811
75900	0.018510089	75924	0.158127776
75900	0.01479576	75928	0.147833024
75900	0.044768967	75932	0.112593312
75900	0.042077962	75936	0.117615047

PowerSpectrumData

75900	0.016879036	75940	0.1406144
75900	0.015094382	75944	0.157068396
75900	0.045050536	75948	0.147546744
76000	0.076776174	75952	0.125478735
76000	0.093960072	75956	0.177125636
76000	0.058127807	75960	0.125680119
76000	0.048976021	75964	0.071983086
76000	0.071443079	75968	0.123532373
76000	0.052240535	75972	0.139327341
76000	0.021497717	75976	0.133403388
76000	0.045109133	75980	0.129839813
76000	0.089831628	75984	0.140275443
76000	0.08558845	75988	0.139577955
76000	0.044031134	75992	0.101601261
76000	0.016969792	75996	0.069332535
76000	0.033452765	76000	0.135938171
76000	0.07345112	76004	0.172866873
76000	0.110714515	76008	0.112176735
76000	0.082670915	76012	0.045383185
76000	0.030095938	76016	0.045977264
76000	0.010062988	76020	0.09046006
76000	0.034054585	76024	0.145814498
76000	0.036256013	76028	0.139327764
76000	0.037127058	76032	0.113542046
76000	0.059733076	76036	0.126481944
76000	0.050959752	76040	0.144969585
76000	0.031887692	76044	0.160530574
76000	0.031472118	76048	0.165072023
76100	0.034018642	76052	0.130055079
76100	0.003873979	76056	0.095553151
76100	0.059387112	76060	0.12529848
76100	0.072830873	76064	0.139205236
76100	0.04080659	76068	0.130602406
76100	0.037643687	76072	0.13761138

PowerSpectrumData

76100	0.029761373	76076	0.161662581
76100	0.022289883	76080	0.151477186
76100	0.046629324	76084	0.148147271
76100	0.062627587	76088	0.169109103
76100	0.056966776	76092	0.152420078
76100	0.069849084	76096	0.131875335
76100	0.053057735	76100	0.122168989
76100	0.009363526	76104	0.087398883
76100	0.029296309	76108	0.103644758
76100	0.025147367	76112	0.124930579
76100	0.059567057	76116	0.080581885
76100	0.07337155	76120	0.06671888
76100	0.087446184	76124	0.070310678
76100	0.078923527	76128	0.080282451
76100	0.048043261	76132	0.079102902
76100	0.036821461	76136	0.10543059
76100	0.069326794	76140	0.128359433
76100	0.08880633	76144	0.11880105
76100	0.064064989	76148	0.121511934
76200	0.029897436	76152	0.158098585
76200	0.042531938	76156	0.14593311
76200	0.038010938	76160	0.078119439
76200	0.033749453	76164	0.098422643
76200	0.040532199	76168	0.124740196
76200	0.04222164	76172	0.097323027
76200	0.054891465	76176	0.081354541
76200	0.065288514	76180	0.118825017
76200	0.060449853	76184	0.111071153
76200	0.052714462	76188	0.09753638
76200	0.036239606	76192	0.129595661
76200	0.006302396	76196	0.12880929
76200	0.024389712	76200	0.118144562
76200	0.038195623	76204	0.110208319
76200	0.057250825	76208	0.063655796

PowerSpectrumData

76200	0.02575574	76212	0.066491419
76200	0.063245694	76216	0.108407061
76200	0.09881 76220	0.108889304	
76200	0.072700306	76224	0.10476738
76200	0.050490842	76228	0.102599857
76200	0.049091501	76232	0.0959311
76200	0.035175752	76236	0.076834571
76200	0.013526581	76240	0.077345976
76200	0.009068983	76244	0.059755806
76200	0.027061647	76248	0.019386673
76300	0.051043095	76252	0.046036683
76300	0.035599911	76256	0.065726737
76300	0.029315772	76260	0.023584242
76300	0.033327302	76264	0.09776048
76300	0.0281949	76268	0.134423623
76300	0.051641437	76272	0.108630491
76300	0.051749667	76276	0.094568335
76300	0.035771798	76280	0.098430603
76300	0.025691241	76284	0.108459361
76300	0.011412555	76288	0.109052453
76300	0.015446572	76292	0.069696951
76300	0.051852327	76296	0.079517355
76300	0.073954216	76300	0.114363444
76300	0.072759671	76304	0.107880427
76300	0.063705622	76308	0.073846386
76300	0.046838624	76312	0.029178709
76300	0.036539328	76316	0.064061169
76300	0.009432841	76320	0.060172351
76300	0.022992464	76324	0.077974626
76300	0.056499317	76328	0.101347781
76300	0.06605321	76332	0.088512861
76300	0.034228575	76336	0.063200263
76300	0.052359563	76340	0.05235527
76300	0.074150928	76344	0.041077095

PowerSpectrumData

76300	0.04903911	76348	0.04100797
76400	0.003100375	76352	0.060934919
76400	0.035194789	76356	0.069408896
76400	0.059741942	76360	0.067937617
76400	0.086812877	76364	0.006883399
76400	0.066981658	76368	0.079577229
76400	0.029536001	76372	0.096990334
76400	0.069281552	76376	0.077663783
76400	0.072619732	76380	0.052085248
76400	0.056526074	76384	0.013794628
76400	0.04928252	76388	0.034409455
76400	0.066935441	76392	0.022910581
76400	0.05417956	76396	0.06493448
76400	0.028464943	76400	0.087348555
76400	0.04665901	76404	0.115322655
76400	0.013758114	76408	0.105328574
76400	0.04314899	76412	0.049611394
76400	0.048296144	76416	0.022417384
76400	0.068320318	76420	0.005391728
76400	0.078482561	76424	0.044646055
76400	0.051681429	76428	0.05646464
76400	0.028507939	76432	0.025068508
76400	0.073960313	76436	0.041921696
76400	0.083291001	76440	0.06053062
76400	0.061591592	76444	0.036305457
76400	0.042469288	76448	0.022307482
76500	0.014517988	76452	0.030038716
76500	0.045129284	76456	0.02078786
76500	0.049423557	76460	0.059713479
76500	0.030950207	76464	0.069432004
76500	0.0182032	76468	0.051350413
76500	0.030396712	76472	0.053299631
76500	0.077718083	76476	0.056180812
76500	0.088492474	76480	0.064958535

PowerSpectrumData

76500	0.064043328	76484	0.076642318
76500	0.036746078	76488	0.06686712
76500	0.039524068	76492	0.042901025
76500	0.034233872	76496	0.049536277
76500	0.038429178	76500	0.062263411
76500	0.030159477	76504	0.034540874
76500	0.011106162	76508	0.025319479
76500	0.014506349	76512	0.0326111
76500	0.012703205	76516	0.042920146
76500	0.0122925	76520	0.089139117
76500	0.027601458	76524	0.119409495
76500	0.051402451	76528	0.114317358
76500	0.037227848	76532	0.097378827
76500	0.014301726	76536	0.084960877
76500	0.035029963	76540	0.03762931
76500	0.080710772	76544	0.030704618
76500	0.099887628	76548	0.00116498
76600	0.061998617	76552	0.046013276
76600	0.027253254	76556	0.072317154
76600	0.01064251	76560	0.092776929
76600	0.001620869	76564	0.046511763
76600	0.006397384	76568	0.012780014
76600	0.020499534	76572	0.035428475
76600	0.021733082	76576	0.034752084
76600	0.022637867	76580	0.027972315
76600	0.077586083	76584	0.037379126
76600	0.072345101	76588	0.046665264
76600	0.013728076	76592	0.041681375
76600	0.022966447	76596	0.049940751
76600	0.008583428	76600	0.044596483
76600	0.036691567	76604	0.023472638
76600	0.077555596	76608	0.019090101
76600	0.089935929	76612	0.020569027
76600	0.092253948	76616	0.027410597

PowerSpectrumData

76600	0.063372048	76620	0.025748324
76600	0.025382426	76624	0.048572892
76600	0.035366393	76628	0.024053763
76600	0.026349968	76632	0.017627957
76600	0.014633471	76636	0.034046327
76600	0.013671579	76640	0.049659495
76600	0.056224879	76644	0.066837412
76600	0.064519692	76648	0.057368612
76700	0.028378909	76652	0.0300804
76700	0.013928579	76656	0.0247961
76700	0.032147851	76660	0.025185929
76700	0.036204525	76664	0.05287712
76700	0.017729208	76668	0.097421136
76700	0.058011669	76672	0.107978485
76700	0.040671919	76676	0.083362662
76700	0.011794015	76680	0.060106424
76700	0.026997828	76684	0.040984593
76700	0.026661573	76688	0.023696322
76700	0.056075685	76692	0.034887951
76700	0.040479728	76696	0.054208405
76700	0.061788916	76700	0.061758707
76700	0.065315006	76704	0.024303823
76700	0.074438991	76708	0.038427512
76700	0.071029048	76712	0.013119502
76700	0.024563651	76716	0.005153116
76700	0.029905461	76720	0.026648442
76700	0.067019028	76724	0.057030648
76700	0.080708203	76728	0.044950699
76700	0.056309429	76732	0.026361206
76700	0.055954199	76736	0.052829455
76700	0.063041167	76740	0.061547798
76700	0.088901426	76744	0.082193808
76700	0.075043805	76748	0.071530434
76800	0.009259422	76752	0.026999465

PowerSpectrumData

76800	0.071580274	76756	0.030199597
76800	0.06940958	76760	0.03519149
76800	0.023018143	76764	0.043639429
76800	0.040754763	76768	0.087263179
76800	0.003385138	76772	0.06606504
76800	0.049410723	76776	0.016043106
76800	0.04400314	76780	0.033285782
76800	0.036942118	76784	0.082081991
76800	0.096015043	76788	0.092730523
76800	0.103320417	76792	0.098341479
76800	0.028591641	76796	0.089020461
76800	0.059893337	76800	0.057852823
76800	0.089204252	76804	0.049976192
76800	0.075125921	76808	0.070744463
76800	0.055906028	76812	0.046867404
76800	0.033049495	76816	0.015961905
76800	0.046665809	76820	0.056510748
76800	0.063359745	76824	0.067987196
76800	0.026311713	76828	0.028725945
76800	0.010188272	76832	0.033154149
76800	0.035869696	76836	0.083236468
76800	0.035316967	76840	0.08613112
76800	0.033564484	76844	0.006458457
76800	0.066657543	76848	0.073003299
76900	0.102046048	76852	0.067951507
76900	0.078314588	76856	0.022534256
76900	0.018843852	76860	0.048513612
76900	0.040191742	76864	0.055763059
76900	0.035617384	76868	0.053472377
76900	0.018858884	76872	0.020429701
76900	0.004770838	76876	0.035887271
76900	0.012894955	76880	0.052477659
76900	0.030373383	76884	0.03845925
76900	0.037396865	76888	0.045967805

PowerSpectrumData

76900	0.032271477	76892	0.055874203
76900	0.021032285	76896	0.05178951
76900	0.031059328	76900	0.048134618
76900	0.068265363	76904	0.056816363
76900	0.082916718	76908	0.032042179
76900	0.067575049	76912	0.036639158
76900	0.051195842	76916	0.048051647
76900	0.045471206	76920	0.045042714
76900	0.058065947	76924	0.055197699
76900	0.088471388	76928	0.067889901
76900	0.079520112	76932	0.069038128
76900	0.09175254	76936	0.071699549
76900	0.147408413	76940	0.06186025
76900	0.147631145	76944	0.051096013
76900	0.087787215	76948	0.062665378
77000	0.119217279	76952	0.043875949
77000	0.116501964	76956	0.085010914
77000	0.03242206	76960	0.117163501
77000	0.047730366	76964	0.09664549
77000	0.068489077	76968	0.056431709
77000	0.083735598	76972	0.047156296
77000	0.057166704	76976	0.051542069
77000	0.037906124	76980	0.058151989
77000	0.051591589	76984	0.036894548
77000	0.060874419	76988	0.011792855
77000	0.050752919	76992	0.034890189
77000	0.053713542	76996	0.076847362
77000	0.048560745	77000	0.114871284
77000	0.027121689	77004	0.107403881
77000	0.030836309	77008	0.046743891
77000	0.037198704	77012	0.042473195
77000	0.018597015	77016	0.014412329
77000	0.021533808	77020	0.032956374
77000	0.028065	77024	0.037960326

PowerSpectrumData

77000	0.027805685	77028	0.030551211
77000	0.035204244	77032	0.006039442
77000	0.043430751	77036	0.036925845
77000	0.050703045	77040	0.041476618
77000	0.065203945	77044	0.045746907
77000	0.036145459	77048	0.032777018
77100	0.033045137	77052	0.013201088
77100	0.056082787	77056	0.026096923
77100	0.040892446	77060	0.024472764
77100	0.046876696	77064	0.029853931
77100	0.06390913	77068	0.01768205
77100	0.04556033	77072	0.02338786
77100	0.00105187	77076	0.034842844
77100	0.034713608	77080	0.028820514
77100	0.028461727	77084	0.020756717
77100	0.033759152	77088	0.003604789
77100	0.061367136	77092	0.040960538
77100	0.040723637	77096	0.031932788
77100	0.046144109	77100	0.008202058
77100	0.047008532	77104	0.025050304
77100	0.02069087	77108	0.05422972
77100	0.062622741	77112	0.043416436
77100	0.05537922	77116	0.033169152
77100	0.018297491	77120	0.011083866
77100	0.041281339	77124	0.023664663
77100	0.023314029	77128	0.013362113
77100	0.022174267	77132	0.036469795
77100	0.030281866	77136	0.017666518
77100	0.003719251	77140	0.048729959
77100	0.036232399	77144	0.033670021
77100	0.066801971	77148	0.016488843
77200	0.073318675	77152	0.047253507
77200	0.062009123	77156	0.038869726
77200	0.039955576	77160	0.033293269

PowerSpectrumData

77200	0.042854827	77164	0.032525048
77200	0.044835801	77168	0.04934585
77200	0.039975686	77172	0.067428606
77200	0.02377387	77176	0.064759559
77200	0.015720971	77180	0.072187271
77200	0.03187625	77184	0.071239738
77200	0.060199716	77188	0.043366657
77200	0.059455615	77192	0.040336767
77200	0.035057001	77196	0.032146221
77200	0.036269943	77200	0.024784315
77200	0.055136996	77204	0.057389665
77200	0.04321702	77208	0.121373392
77200	0.058687692	77212	0.092142167
77200	0.076429969	77216	0.071724571
77200	0.042726908	77220	0.111738562
77200	0.024359364	77224	0.140717646
77200	0.052348234	77228	0.135249589
77200	0.061209881	77232	0.123643244
77200	0.04655317	77236	0.09585097
77200	0.02254301	77240	0.035021843
77200	0.01309544	77244	0.104951738
77200	0.036627298	77248	0.150001026
77300	0.025575746	77252	0.129937965
77300	0.013073917	77256	0.106706924
77300	0.042009553	77260	0.14387806
77300	0.042439919	77264	0.15415471
77300	0.037622322	77268	0.091814967
77300	0.052018611	77272	0.003532806
77300	0.061968465	77276	0.07254399
77300	0.034397293	77280	0.087774926
77300	0.043401473	77284	0.072738112
77300	0.070634436	77288	0.072391362
77300	0.076360841	77292	0.068608031
77300	0.071878341	77296	0.049501483

PowerSpectrumData

77300	0.055942463	77300	0.066114444
77300	0.051229807	77304	0.094257535
77300	0.05496461	77308	0.071520015
77300	0.070824681	77312	0.07580495
77300	0.091052927	77316	0.119238262
77300	0.110354791	77320	0.115762719
77300	0.106211395	77324	0.061597675
77300	0.054573255	77328	0.063867563
77300	0.019178197	77332	0.121099933
77300	0.012053336	77336	0.122763551
77300	0.014744795	77340	0.08217946
77300	0.038596681	77344	0.067475201
77300	0.056645768	77348	0.127394
77400	0.067671324	77352	0.137089926
77400	0.046806985	77356	0.104494997
77400	0.023213064	77360	0.097619886
77400	0.013721567	77364	0.130777655
77400	0.023431652	77368	0.189903949
77400	0.019787776	77372	0.199888935
77400	0.029768899	77376	0.156837181
77400	0.044648597	77380	0.13332619
77400	0.061774241	77384	0.133780661
77400	0.064555454	77388	0.113731192
77400	0.060245602	77392	0.115153351
77400	0.048984435	77396	0.148291496
77400	0.042444681	77400	0.14502494
77400	0.04718373	77404	0.121950398
77400	0.065797169	77408	0.109974608
77400	0.05906331	77412	0.122189827
77400	0.036035242	77416	0.112449707
77400	0.061559244	77420	0.085719599
77400	0.075095151	77424	0.107079351
77400	0.069835143	77428	0.101260936
77400	0.053113818	77432	0.082762301

PowerSpectrumData

77400	0.037577018	77436	0.12071176
77400	0.074500247	77440	0.142309276
77400	0.076190423	77444	0.136721835
77400	0.028337834	77448	0.144163103
77500	0.040565592	77452	0.149666201
77500	0.052923675	77456	0.125331164
77500	0.020353224	77460	0.147544678
77500	0.036307989	77464	0.160647309
77500	0.045508994	77468	0.100183708
77500	0.018001027	77472	0.077999888
77500	0.010660601	77476	0.082042272
77500	0.01158891	77480	0.080867452
77500	0.023051802	77484	0.094229705
77500	0.02502743	77488	0.120730074
77500	0.043165273	77492	0.13630146
77500	0.051210027	77496	0.12031274
77500	0.042102343	77500	0.091401453
77500	0.012810962	77504	0.130540633
77500	0.074514712	77508	0.181831944
77500	0.071113209	77512	0.187537124
77500	0.027697899	77516	0.14845944
77500	0.015777961	77520	0.121650111
77500	0.00677708	77524	0.125498336
77500	0.009138966	77528	0.13399568
77500	0.013217713	77532	0.131681576
77500	0.026694679	77536	0.118478165
77500	0.040975694	77540	0.127437685
77500	0.028226943	77544	0.127595806
77500	0.031437754	77548	0.144146383
77600	0.050862553	77552	0.138336356
77600	0.040563442	77556	0.086990309
77600	0.021614531	77560	0.104813247
77600	0.020341049	77564	0.148775536
77600	0.028428718	77568	0.156708528

PowerSpectrumData

77600	0.061160201	77572	0.158886076
77600	0.081466649	77576	0.14907126
77600	0.075089141	77580	0.109852765
77600	0.045067442	77584	0.092154682
77600	0.035575551	77588	0.099041266
77600	0.01287766	77592	0.109292101
77600	0.019553751	77596	0.085459687
77600	0.029335697	77600	0.055558947
77600	0.050976541	77604	0.05549422
77600	0.043109554	77608	0.097011682
77600	0.023171455	77612	0.114151335
77600	0.026598687	77616	0.111615853
77600	0.033480723	77620	0.143790749
77600	0.028209408	77624	0.124237733
77600	0.031435287	77628	0.029981527
77600	0.021666163	77632	0.088355897
77600	0.012721072	77636	0.159396878
77600	0.036091322	77640	0.197056186
77600	0.054830012	77644	0.142754143
77600	0.089498288	77648	0.105560888
77700	0.125782055	77652	0.09851389
77700	0.113637652	77656	0.072258059
77700	0.053379259	77660	0.11385676
77700	0.010913825	77664	0.146023361
77700	0.05089436	77668	0.136013201
77700	0.07535339	77672	0.101398786
77700	0.05452066	77676	0.078137746
77700	0.013508418	77680	0.111760768
77700	0.014981626	77684	0.096501201
77700	0.012482035	77688	0.068142916
77700	0.016780996	77692	0.080314429
77700	0.033262651	77696	0.116664116
77700	0.028407958	77700	0.126100014
77700	0.031616182	77704	0.115073912

PowerSpectrumData

77700	0.029038847	77708	0.094532967
77700	0.008443201	77712	0.039082323
77700	0.059971815	77716	0.024992804
77700	0.083504783	77720	0.041365263
77700	0.071579751	77724	0.048227306
77700	0.044611446	77728	0.101296653
77700	0.042833402	77732	0.111237299
77700	0.043411386	77736	0.068735171
77700	0.0188008	77740	0.038305192
77700	0.018913333	77744	0.052457523
77700	0.04847307	77748	0.052186941
77800	0.071569928	77752	0.034600227
77800	0.063677675	77756	0.0062606
77800	0.056292782	77760	0.041899224
77800	0.067141235	77764	0.080591868
77800	0.063728192	77768	0.070769616
77800	0.049716913	77772	0.067831992
77800	0.058280682	77776	0.077506571
77800	0.054409862	77780	0.069058631
77800	0.051004932	77784	0.073166171
77800	0.033815719	77788	0.059979338
77800	0.004655479	77792	0.026631322
77800	0.028997758	77796	0.013698131
77800	0.05730064	77800	0.052737363
77800	0.062596759	77804	0.077592558
77800	0.038335729	77808	0.058358197
77800	0.028883558	77812	0.030588952
77800	0.055502795	77816	0.036941663
77800	0.082723811	77820	0.045446235
77800	0.059789167	77824	0.082019091
77800	0.00499843	77828	0.086523571
77800	0.044928096	77832	0.039183524
77800	0.06401509	77836	0.017835973
77800	0.03385391	77840	0.043383054

77800	0.018253	77844	0.026655718
77800	0.021863067	77848	0.025282376
77900	0.045734527	77852	0.041965901
77900	0.079900703	77856	0.065299886
77900	0.08500462	77860	0.073697949
77900	0.056128913	77864	0.0487258
77900	0.021371548	77868	0.044477114
77900	0.01531118	77872	0.091314854
77900	0.015168194	77876	0.110271692
77900	0.028282595	77880	0.066199369
77900	0.038359489	77884	0.020745609
77900	0.018280343	77888	0.022131708
77900	0.033876491	77892	0.044875564
77900	0.061582403	77896	0.023610377
77900	0.064775733	77900	0.018767745
77900	0.044001783	77904	0.079101214
77900	0.035798526	77908	0.114023955
77900	0.046739438	77912	0.089975089
77900	0.009745533	77916	0.043486136
77900	0.047543035	77920	0.010833113
77900	0.061682455	77924	0.049606177
77900	0.045678135	77928	0.074434902
77900	0.044061249	77932	0.062837695
77900	0.07519668	77936	0.04101879
77900	0.12527802	77940	0.074050404
77900	0.111168425	77944	0.087791632
77900	0.056093541	77948	0.077112782
78000	0.012278591	77952	0.069329573
78000	0.01629414	77956	0.058367441
78000	0.02253377	77960	0.018764065
78000	0.023482475	77964	0.063520965
78000	0.004192753	77968	0.081564846
78000	0.041139891	77972	0.055548266
78000	0.055388122	77976	0.03225555

PowerSpectrumData

78000	0.019739897	77980	0.086348562
78000	0.026785785	77984	0.104077357
78000	0.051477913	77988	0.063697982
78000	0.052348736	77992	0.032716544
78000	0.035651727	77996	0.039901403
78000	0.041473591	78000	0.055955727
78000	0.050753162	78004	0.023426212
78000	0.050686904	78008	0.023539316
78000	0.03360954	78012	0.012897132
78000	0.061778483	78016	0.033063236
78000	0.06448957	78020	0.059348302
78000	0.041429281	78024	0.089742272
78000	0.003740069	78028	0.046989429
78000	0.044028318	78032	0.023984978
78000	0.045919256	78036	0.036925059
78000	0.04194539	78040	0.022163606
78000	0.0280513	78044	0.021473343
78000	0.035481469	78048	0.042183514
78100	0.05936372	78052	0.061343497
78100	0.060842638	78056	0.033602461
78100	0.026782427	78060	0.01892004
78100	0.016595415	78064	0.075020173
78100	0.022652328	78068	0.085463784
78100	0.036136851	78072	0.052247411
78100	0.043930046	78076	0.049916063
78100	0.052515043	78080	0.032270171
78100	0.062368643	78084	0.035539481
78100	0.048307233	78088	0.041452397
78100	0.043832475	78092	0.03028991
78100	0.028333374	78096	0.042108059
78100	0.009927017	78100	0.042025182
78100	0.020565081	78104	0.049136193
78100	0.027731507	78108	0.069037968
78100	0.035537756	78112	0.043401633

PowerSpectrumData

78100	0.042304244	78116	0.032144286
78100	0.030404306	78120	0.032771313
78100	0.006979451	78124	0.023460732
78100	0.0177042	78128	0.011205752
78100	0.021389353	78132	0.045930858
78100	0.035262532	78136	0.0590606
78100	0.047886602	78140	0.053285348
78100	0.032436408	78144	0.036370908
78100	0.01063072	78148	0.029483384
78200	0.03288333	78152	0.036534078
78200	0.056237652	78156	0.053397227
78200	0.046795183	78160	0.059487353
78200	0.028810302	78164	0.057566267
78200	0.055557721	78168	0.084260857
78200	0.078213532	78172	0.095648167
78200	0.090081776	78176	0.042433243
78200	0.057194899	78180	0.019681402
78200	0.022355604	78184	0.032519602
78200	0.015313281	78188	0.0380688
78200	0.023023702	78192	0.002022043
78200	0.063209314	78196	0.054345313
78200	0.08172918	78200	0.060490609
78200	0.07483684	78204	0.081388884
78200	0.08387796	78208	0.112591726
78200	0.082948871	78212	0.124980972
78200	0.043845463	78216	0.102637263
78200	0.037395777	78220	0.065261644
78200	0.008282988	78224	0.050840761
78200	0.02114545	78228	0.032438595
78200	0.033260531	78232	0.051949868
78200	0.069768546	78236	0.008426069
78200	0.06758517	78240	0.042945387
78200	0.028205996	78244	0.052123109
78200	0.044256438	78248	0.040580839

PowerSpectrumData

78300	0.035597066	78252	0.026735765
78300	0.069194895	78256	0.055258752
78300	0.107369982	78260	0.108963694
78300	0.074293195	78264	0.1422692
78300	0.055928977	78268	0.112826296
78300	0.068440699	78272	0.07531295
78300	0.041877531	78276	0.099461227
78300	0.048239246	78280	0.094955445
78300	0.058182079	78284	0.059679725
78300	0.05610368	78288	0.048246813
78300	0.040779094	78292	0.060628183
78300	0.051954259	78296	0.063668347
78300	0.094261464	78300	0.079356076
78300	0.098457276	78304	0.083129184
78300	0.087230648	78308	0.036532514
78300	0.106424872	78312	0.048218804
78300	0.123870923	78316	0.054461434
78300	0.121131088	78320	0.008023917
78300	0.087147215	78324	0.03441795
78300	0.067127869	78328	0.08711165
78300	0.067296271	78332	0.093954623
78300	0.077679586	78336	0.035515161
78300	0.071386668	78340	0.045953635
78300	0.045614721	78344	0.069347625
78300	0.04073988	78348	0.108528751
78400	0.046400917	78352	0.13459487
78400	0.048471331	78356	0.100961086
78400	0.05279544	78360	0.038016005
78400	0.060962389	78364	0.041738575
78400	0.054755117	78368	0.098435994
78400	0.042423188	78372	0.067117093
78400	0.036988084	78376	0.060710318
78400	0.037098929	78380	0.105143394
78400	0.045783399	78384	0.103711391

PowerSpectrumData

78400	0.058047848	78388	0.127381398
78400	0.06983283	78392	0.142659148
78400	0.069000766	78396	0.121925143
78400	0.041455485	78400	0.084369691
78400	0.019564481	78404	0.064924083
78400	0.064209795	78408	0.091298294
78400	0.056429937	78412	0.07329628
78400	0.04413633	78416	0.093486779
78400	0.04657183	78420	0.167078994
78400	0.042360342	78424	0.18535195
78400	0.023428163	78428	0.128564105
78400	0.020980948	78432	0.081329286
78400	0.01571951	78436	0.093649112
78400	0.043506509	78440	0.10399583
78400	0.087100983	78444	0.111469773
78400	0.087339628	78448	0.110739391
78500	0.042724918	78452	0.080315294
78500	0.002983096	78456	0.049015423
78500	0.013370621	78460	0.082558239
78500	0.045829653	78464	0.132688234
78500	0.061189021	78468	0.100065401
78500	0.063550499	78472	0.022743569
78500	0.075428834	78476	0.109109082
78500	0.074449083	78480	0.122043901
78500	0.058344464	78484	0.104135608
78500	0.066964931	78488	0.144697159
78500	0.056689823	78492	0.169020554
78500	0.050991032	78496	0.113935821
78500	0.069745962	78500	0.04213669
78500	0.051897423	78504	0.04606085
78500	0.026015046	78508	0.068879344
78500	0.06867927	78512	0.078679565
78500	0.10208691	78516	0.05146203
78500	0.085424763	78520	0.020637799

PowerSpectrumData

78500	0.019223682	78524	0.04552646
78500	0.053956825	78528	0.04824697
78500	0.067553672	78532	0.038010123
78500	0.059088819	78536	0.031255102
78500	0.036611258	78540	0.017240485
78500	0.038967839	78544	0.032606135
78500	0.078123951	78548	0.073361785
78600	0.073248106	78552	0.07613343
78600	0.038210153	78556	0.051156683
78600	0.053174277	78560	0.045937846
78600	0.10694057	78564	0.05560918
78600	0.038166509	78568	0.055047836
78600	0.038973431	78572	0.049014645
78600	0.023480314	78576	0.062390194
78600	0.048241254	78580	0.10861786
78600	0.033340988	78584	0.111000562
78600	0.028839362	78588	0.056955516
78600	0.072510884	78592	0.072272371
78600	0.089208275	78596	0.086699081
78600	0.07411289	78600	0.067109453
78600	0.071394308	78604	0.069001697
78600	0.072287345	78608	0.048186095
78600	0.044980523	78612	0.020594858
78600	0.009618161	78616	0.010947771
78600	0.040883639	78620	0.030148663
78600	0.04398005	78624	0.032940112
78600	0.092319686	78628	0.032114789
78600	0.091045287	78632	0.031603795
78600	0.030719108	78636	0.058980761
78600	0.042604475	78640	0.091250106
78600	0.074839503	78644	0.071631373
78600	0.045529443	78648	0.027503482
78700	0.009047584	78652	0.024882153
78700	0.041281848	78656	0.022234042

PowerSpectrumData

78700	0.060827973	78660	0.029027588
78700	0.046853664	78664	0.046367652
78700	0.007234552	78668	0.030154317
78700	0.065220309	78672	0.068149901
78700	0.09673796	78676	0.081847997
78700	0.096527256	78680	0.061314851
78700	0.056293124	78684	0.055287379
78700	0.01554594	78688	0.088412831
78700	0.014175936	78692	0.064059714
78700	0.015178027	78696	0.010667783
78700	0.026523008	78700	0.038933267
78700	0.029045739	78704	0.068260255
78700	0.046126319	78708	0.042270593
78700	0.049171918	78712	0.018190272
78700	0.05305887	78716	0.016888693
78700	0.059548624	78720	0.035898724
78700	0.033489348	78724	0.036035712
78700	0.026521873	78728	0.022501308
78700	0.031570933	78732	0.028645842
78700	0.067379056	78736	0.058591184
78700	0.113593902	78740	0.073527059
78700	0.103434832	78744	0.072597431
78700	0.070667949	78748	0.111698391
78800	0.041985273	78752	0.081689825
78800	0.012656077	78756	0.032547599
78800	0.016020877	78760	0.017269824
78800	0.012879361	78764	0.017893066
78800	0.010574598	78768	0.065600296
78800	0.027061977	78772	0.096592543
78800	0.024417173	78776	0.099947669
78800	0.021611337	78780	0.055809342
78800	0.022544144	78784	0.036793193
78800	0.022640901	78788	0.035884877
78800	0.040133447	78792	0.025766383

PowerSpectrumData

78800	0.041991829	78796	0.021517946
78800	0.065621978	78800	0.028140177
78800	0.051659332	78804	0.019554034
78800	0.02581106	78808	0.039953589
78800	0.032707794	78812	0.030999607
78800	0.043753065	78816	0.016802287
78800	0.028713444	78820	0.0270234
78800	0.039811541	78824	0.025488618
78800	0.076478857	78828	0.010706921
78800	0.094082163	78832	0.02242932
78800	0.075014286	78836	0.033230943
78800	0.04038394	78840	0.073465177
78800	0.036214489	78844	0.049685721
78800	0.046344543	78848	0.039588827
78900	0.052611438	78852	0.037711187
78900	0.055019344	78856	0.038187616
78900	0.056810895	78860	0.012105447
78900	0.050982686	78864	0.058228266
78900	0.050368206	78868	0.067594243
78900	0.053760843	78872	0.048274756
78900	0.050145674	78876	0.045199595
78900	0.030681051	78880	0.062738014
78900	0.025905396	78884	0.087258843
78900	0.044743403	78888	0.086875676
78900	0.052815743	78892	0.060614595
78900	0.023324667	78896	0.061237843
78900	0.016870716	78900	0.050883125
78900	0.028042761	78904	0.009199679
78900	0.056881443	78908	0.052780019
78900	0.045349661	78912	0.093804141
78900	0.022707216	78916	0.097065262
78900	0.057835398	78920	0.075914075
78900	0.071568022	78924	0.080725156
78900	0.050723851	78928	0.042295444

PowerSpectrumData

78900	0.041067833	78932	0.048162321
78900	0.069907721	78936	0.090038222
78900	0.110677291	78940	0.085891123
78900	0.089773181	78944	0.083446321
78900	0.024181545	78948	0.083145191
79000	0.028951801	78952	0.055831199
79000	0.043376233	78956	0.062786334
79000	0.020107376	78960	0.049434952
79000	0.038046866	78964	0.048678565
79000	0.061980878	78968	0.02636144
79000	0.03467967	78972	0.034290704
79000	0.022003931	78976	0.04140746
79000	0.021854319	78980	0.087020781
79000	0.080718681	78984	0.096677832
79000	0.115783827	78988	0.078949772
79000	0.097080614	78992	0.068374618
79000	0.034561104	78996	0.057886835
79000	0.025697131	79000	0.027006096
79000	0.0410055	79004	0.038018061
79000	0.02980482	79008	0.091206261
79000	0.038915485	79012	0.108283173
79000	0.052890984	79016	0.117157491
79000	0.035120524	79020	0.12268094
79000	0.042194773	79024	0.099227706
79000	0.048526679	79028	0.066265435
79000	0.060488623	79032	0.037881913
79000	0.041056828	79036	0.023404668
79000	0.006142759	79040	0.047110047
79000	0.02118352	79044	0.063413048
79000	0.01264592	79048	0.038169212
79100	0.024423425	79052	0.033562978
79100	0.044823733	79056	0.094772608
79100	0.020843936	79060	0.108015804
79100	0.022047912	79064	0.077768134

PowerSpectrumData

79100	0.047531812	79068	0.020618301
79100	0.062081897	79072	0.030067111
79100	0.063344938	79076	0.057820274
79100	0.028350823	79080	0.056397035
79100	0.027972743	79084	0.070251968
79100	0.064318825	79088	0.098943077
79100	0.061534585	79092	0.078571895
79100	0.080799866	79096	0.06254598
79100	0.077859382	79100	0.034828634
79100	0.045358189	79104	0.013146397
79100	0.050725059	79108	0.026044671
79100	0.052939449	79112	0.041626896
79100	0.052794832	79116	0.048114951
79100	0.043681983	79120	0.050329178
79100	0.046064011	79124	0.045465877
79100	0.060128965	79128	0.021000677
79100	0.064953216	79132	0.050362371
79100	0.063306499	79136	0.06249654
79100	0.047987607	79140	0.022849332
79100	0.034107576	79144	0.074807373
79100	0.040957893	79148	0.122973739
79200	0.059994138	79152	0.085801163
79200	0.072516625	79156	0.04247134
79200	0.065971901	79160	0.091390219
79200	0.051920208	79164	0.121923404
79200	0.046398145	79168	0.107069143
79200	0.062602558	79172	0.059147849
79200	0.061766557	79176	0.015212724
79200	0.050068618	79180	0.025637351
79200	0.03475188	79184	0.025105801
79200	0.030818803	79188	0.024081022
79200	0.027785398	79192	0.027818893
79200	0.038619543	79196	0.039016315
79200	0.052576419	79200	0.054147404

PowerSpectrumData

79200	0.045387496	79204	0.060980878
79200	0.029893938	79208	0.04894309
79200	0.060391809	79212	0.05135412
79200	0.074930395	79216	0.045308188
79200	0.05379946	79220	0.022976183
79200	0.017351147	79224	0.061277402
79200	0.02816061	79228	0.07440606
79200	0.066833847	79232	0.04927941
79200	0.070757094	79236	0.027048678
79200	0.056023582	79240	0.019197572
79200	0.044613553	79244	0.02622472
79200	0.006710056	79248	0.062236679
79300	0.037266098	79252	0.082090279
79300	0.048224767	79256	0.069543472
79300	0.046443951	79260	0.046778852
79300	0.059369453	79264	0.044688535
79300	0.059846767	79268	0.023324959
79300	0.031319134	79272	0.026372503
79300	0.041901345	79276	0.039731858
79300	0.04852876	79280	0.020447831
79300	0.034713361	79284	0.03180858
79300	0.018288189	79288	0.0606697
79300	0.008646313	79292	0.056777855
79300	0.031447758	79296	0.024000125
79300	0.0888941	79300	0.03703342
79300	0.11893257	79304	0.038599581
79300	0.114868853	79308	0.015209302
79300	0.096217467	79312	0.03770761
79300	0.069165428	79316	0.047592668
79300	0.054716616	79320	0.049621736
79300	0.061649545	79324	0.007430977
79300	0.071390445	79328	0.037980877
79300	0.062595871	79332	0.019046647
79300	0.046135865	79336	0.029413846

PowerSpectrumData

79300	0.052249037	79340	0.020246998
79300	0.033232027	79344	0.036271475
79300	0.029274283	79348	0.081071681
79400	0.046084628	79352	0.081979553
79400	0.028811664	79356	0.058699054
79400	0.032305568	79360	0.034924517
79400	0.055904602	79364	0.025671785
79400	0.016625227	79368	0.048064645
79400	0.04453757	79372	0.025276489
79400	0.039392784	79376	0.043112239
79400	0.018300285	79380	0.077175864
79400	0.043727268	79384	0.050267801
79400	0.077687924	79388	0.001995642
79400	0.079268721	79392	0.020676547
79400	0.086376589	79396	0.007500894
79400	0.115270392	79400	0.022858114
79400	0.123063946	79404	0.032654309
79400	0.073726027	79408	0.024426126
79400	0.031393964	79412	0.014423818
79400	0.025003967	79416	0.020530246
79400	0.022095595	79420	0.054200096
79400	0.031323867	79424	0.070105976
79400	0.038690949	79428	0.067290952
79400	0.007674808	79432	0.076584329
79400	0.026227463	79436	0.066793175
79400	0.025220668	79440	0.016687372
79400	0.017654787	79444	0.046835601
79400	0.025334737	79448	0.057378195
79500	0.022139519	79452	0.027886077
79500	0.016806351	79456	0.033487177
79500	0.006252206	79460	0.060784336
79500	0.029267434	79464	0.072573239
79500	0.043969631	79468	0.083914994
79500	0.044608252	79472	0.106262487

PowerSpectrumData

79500	0.071323135	79476	0.085169653
79500	0.075614975	79480	0.022958311
79500	0.067290363	79484	0.042524473
79500	0.066484623	79488	0.041398103
79500	0.0146198	79492	0.028502995
79500	0.039637238	79496	0.030427787
79500	0.040807299	79500	0.026938929
79500	0.037100443	79504	0.019236313
79500	0.061868625	79508	0.026924241
79500	0.04624189	79512	0.045710909
79500	0.035073666	79516	0.062812003
79500	0.013315447	79520	0.044096374
79500	0.026030453	79524	0.014498898
79500	0.024978042	79528	0.015107697
79500	0.012517277	79532	0.010325814
79500	0.047623234	79536	0.004155657
79500	0.081200386	79540	0.01478234
79500	0.073082127	79544	0.01315186
79500	0.058488968	79548	0.015384449
79600	0.065095643	79552	0.009537897
79600	0.068954956	79556	0.006629017
79600	0.034857749	79560	0.015901189
79600	0.04642969	79564	0.027297796
79600	0.020619631	79568	0.034394085
79600	0.043512482	79572	0.028774362
79600	0.066490262	79576	0.050795636
79600	0.028229984	79580	0.036929319
79600	0.022685417	79584	0.037535043
79600	0.056538516	79588	0.076806595
79600	0.042151154	79592	0.102613143
79600	0.024760393	79596	0.081993174
79600	0.047314028	79600	0.012534176
79600	0.08251812	79604	0.086521279
79600	0.092095244	79608	0.093128343

PowerSpectrumData

79600	0.080548365	79612	0.068051653
79600	0.069282483	79616	0.049175065
79600	0.080269791	79620	0.033927507
79600	0.102737293	79624	0.017148936
79600	0.11170825	79628	0.061711915
79600	0.090267786	79632	0.096347154
79600	0.050085331	79636	0.087256129
79600	0.033330918	79640	0.060559112
79600	0.028937568	79644	0.027225175
79600	0.061548723	79648	0.037686823
79700	0.085772226	79652	0.054180702
79700	0.080808371	79656	0.05162635
79700	0.057045363	79660	0.042689491
79700	0.031980482	79664	0.047208174
79700	0.008275756	79668	0.03533499
79700	0.016819333	79672	0.026996573
79700	0.028257242	79676	0.033856482
79700	0.09375401	79680	0.038131402
79700	0.092842733	79684	0.028357443
79700	0.042448879	79688	0.033856864
79700	0.041778498	79692	0.024819388
79700	0.067560613	79696	0.009055408
79700	0.059963517	79700	0.032354041
79700	0.032392574	79704	0.044549597
79700	0.070642942	79708	0.027243119
79700	0.060202496	79712	0.041070216
79700	0.022769131	79716	0.05596074
79700	0.05635956	79720	0.062365609
79700	0.056638735	79724	0.033234712
79700	0.04305338	79728	0.030037631
79700	0.066674686	79732	0.028124779
79700	0.05739422	79736	0.031626827
79700	0.02354113	79740	0.044892327
79700	0.000805481	79744	0.025057772

PowerSpectrumData

79700	0.018361583	79748	0.026102007
79800	0.029432376	79752	0.02154365
79800	0.051661362	79756	0.05584367
79800	0.056080098	79760	0.015626732
79800	0.045116358	79764	0.026973539
79800	0.020877496	79768	0.016599579
79800	0.024345416	79772	0.00836294
79800	0.031122985	79776	0.040157513
79800	0.050973224	79780	0.076939788
79800	0.083282015	79784	0.053715299
79800	0.051051316	79788	0.048704154
79800	0.011601021	79792	0.078793288
79800	0.044372144	79796	0.07983714
79800	0.050204613	79800	0.024298417
79800	0.016098175	79804	0.068333844
79800	0.044268221	79808	0.058861915
79800	0.058708421	79812	0.052713633
79800	0.036219564	79816	0.087208297
79800	0.023285615	79820	0.037518013
79800	0.042002943	79824	0.024550449
79800	0.055500426	79828	0.041422696
79800	0.048491391	79832	0.049297123
79800	0.064295491	79836	0.047781719
79800	0.085647458	79840	0.046836689
79800	0.102770449	79844	0.038885868
79800	0.081611281	79848	0.063383697
79900	0.009509651	79852	0.078908779
79900	0.045111534	79856	0.041813342
79900	0.02586429	79860	0.041865984
79900	0.023244496	79864	0.042305896
79900	0.009798554	79868	0.044908156
79900	0.065181739	79872	0.07691951
79900	0.078591882	79876	0.099077857
79900	0.034933673	79880	0.123798076

PowerSpectrumData

79900	0.051356972	79884	0.108839093
79900	0.065949484	79888	0.060949522
79900	0.09426631	79892	0.035304202
79900	0.102430829	79896	0.075562828
79900	0.068533438	79900	0.100189769
79900	0.050558079	79904	0.084590633
79900	0.045653942	79908	0.056068013
79900	0.041737785	79912	0.021998481
79900	0.043192231	79916	0.031513788
79900	0.065832901	79920	0.052306299
79900	0.067083885	79924	0.057025729
79900	0.033342632	79928	0.072423638
79900	0.035423389	79932	0.070036425
79900	0.009718612	79936	0.049025639
79900	0.028124705	79940	0.049588431
79900	0.030779782	79944	0.067282424
79900	0.014726032	79948	0.099987577
80000	0.003416978	79952	0.122245547
80000	0.063109721	79956	0.084122905
80000	0.111469941	79960	0.018853634
80000	0.089052577	79964	0.044367102
80000	0.044604607	79968	0.077690813
80000	0.030528441	79972	0.051436848
80000	0.055911383	79976	0.013336422
80000	0.089404413	79980	0.064827538
80000	0.107802312	79984	0.083932777
80000	0.103854349	79988	0.057000132
80000	0.079245045	79992	0.097425524
80000	0.048117003	79996	0.109603017
80000	0.027012195	80000	0.074315292
80000	0.024684767	80004	0.076642114
80000	0.032506541	80008	0.08174168
80000	0.036134363	80012	0.053475626
80000	0.015140189	80016	0.050840081

PowerSpectrumData

80000	0.008082002	80020	0.057390178
80000	0.001588658	80024	0.056882458
80000	0.030355268	80028	0.092639159
80000	0.052617292	80032	0.114379407
80000	0.061237508	80036	0.103368271
80000	0.071725757	80040	0.059029902
80000	0.086651548	80044	0.04960255
80000	0.072453091	80048	0.045387773
80100	0.028640088	80052	0.029130291
80100	0.021277634	80056	0.057526107
80100	0.038458631	80060	0.030200023
80100	0.064311847	80064	0.028028766
80100	0.064991509	80068	0.063000247
80100	0.067549496	80072	0.052333158
80100	0.063376407	80076	0.060300608
80100	0.035665984	80080	0.084095031
80100	0.014005292	80084	0.078267825
80100	0.014396913	80088	0.085254316
80100	0.01528008	80092	0.095596835
80100	0.006817979	80096	0.121258563
80100	0.032758122	80100	0.133119204
80100	0.030661231	80104	0.074163014
80100	0.039911181	80108	0.011173569
80100	0.016794909	80112	0.046650588
80100	0.050453589	80116	0.040132265
80100	0.096377429	80120	0.049884904
80100	0.078752652	80124	0.070212576
80100	0.039885639	80128	0.110176414
80100	0.044653873	80132	0.103822589
80100	0.078036152	80136	0.059974238
80100	0.109276407	80140	0.067468107
80100	0.095953197	80144	0.099718891
80100	0.080730562	80148	0.078227691
80200	0.117180243	80152	0.027206937

PowerSpectrumData

80200	0.145520535	80156	0.035746296
80200	0.109823122	80160	0.068669717
80200	0.033061515	80164	0.045425393
80200	0.033811113	80168	0.024753734
80200	0.05238441	80172	0.046808258
80200	0.046158904	80176	0.053012354
80200	0.072402101	80180	0.065467917
80200	0.085636842	80184	0.099161327
80200	0.04958552	80188	0.106994885
80200	0.032651511	80192	0.117554017
80200	0.04908413	80196	0.093986717
80200	0.033545086	80200	0.047606023
80200	0.034562025	80204	0.035294721
80200	0.062257997	80208	0.045275152
80200	0.039883922	80212	0.041761618
80200	0.017424471	80216	0.030369407
80200	0.022143515	80220	0.040092309
80200	0.008662124	80224	0.037289803
80200	0.027751039	80228	0.045825207
80200	0.039783536	80232	0.048410115
80200	0.034710214	80236	0.049082639
80200	0.022739194	80240	0.02982353
80200	0.024212417	80244	0.062816274
80200	0.043319276	80248	0.095783224
80300	0.047646823	80252	0.0879168
80300	0.031890937	80256	0.049271519
80300	0.024719679	80260	0.023365703
80300	0.04475272	80264	0.025020261
80300	0.042778233	80268	0.006633268
80300	0.060288538	80272	0.026992129
80300	0.074445488	80276	0.016091755
80300	0.051215222	80280	0.054309265
80300	0.072011702	80284	0.079876496
80300	0.069930909	80288	0.098712051

PowerSpectrumData

80300	0.015868056	80292	0.097739845
80300	0.038265938	80296	0.02811692
80300	0.051073956	80300	0.055525179
80300	0.033726334	80304	0.087403052
80300	0.038606588	80308	0.09239783
80300	0.050047616	80312	0.071912837
80300	0.027941422	80316	0.016247359
80300	0.04312312	80320	0.031139349
80300	0.054879703	80324	0.042725944
80300	0.007034148	80328	0.026958442
80300	0.059174847	80332	0.061454572
80300	0.067032845	80336	0.070198141
80300	0.034428187	80340	0.045869652
80300	0.052083586	80344	0.023692444
80300	0.045375869	80348	0.033485958
80400	0.054578493	80352	0.087779547
80400	0.050015773	80356	0.099111363
80400	0.033150143	80360	0.079072139
80400	0.071809547	80364	0.051780873
80400	0.046484805	80368	0.059299473
80400	0.021620992	80372	0.057388046
80400	0.050315375	80376	0.046275352
80400	0.033339453	80380	0.029489023
80400	0.011812526	80384	0.061188584
80400	0.026027454	80388	0.051856641
80400	0.055791486	80392	0.046999634
80400	0.054040469	80396	0.018201627
80400	0.032138865	80400	0.047461948
80400	0.035998521	80404	0.070112037
80400	0.03337492	80408	0.06175835
80400	0.020929927	80412	0.05850953
80400	0.027700642	80416	0.023694747
80400	0.027985741	80420	0.047511814
80400	0.014135948	80424	0.071534938

PowerSpectrumData

80400	0.010814754	80428	0.056211775
80400	0.027259521	80432	0.083061474
80400	0.016465518	80436	0.099037461
80400	0.011157523	80440	0.054036416
80400	0.013317609	80444	0.068588204
80400	0.006150035	80448	0.087427325
80500	0.011658825	80452	0.042784261
80500	0.027383969	80456	0.057238216
80500	0.030347628	80460	0.039782495
80500	0.033070821	80464	0.03091271
80500	0.042770385	80468	0.095757481
80500	0.052285719	80472	0.092971699
80500	0.044455068	80476	0.094112897
80500	0.03154295	80480	0.105096951
80500	0.007190894	80484	0.071959315
80500	0.063956992	80488	0.026614707
80500	0.089458408	80492	0.038963786
80500	0.066426081	80496	0.01860154
80500	0.035908244	80500	0.047723534
80500	0.026347572	80504	0.026503385
80500	0.045034856	80508	0.041606971
80500	0.041214589	80512	0.027608416
80500	0.02869596	80516	0.006240443
80500	0.057496098	80520	0.030539861
80500	0.061130442	80524	0.077463541
80500	0.047405152	80528	0.100709556
80500	0.071710078	80532	0.076170894
80500	0.101144251	80536	0.056682216
80500	0.080206359	80540	0.078413141
80500	0.03856088	80544	0.074803342
80500	0.029053654	80548	0.053034575
80600	0.04091968	80552	0.022027194
80600	0.043539261	80556	0.02805533
80600	0.042243537	80560	0.065140965

PowerSpectrumData

80600	0.034834597	80564	0.043959724
80600	0.050328526	80568	0.071779788
80600	0.066188637	80572	0.082618688
80600	0.04009638	80576	0.053791344
80600	0.041361847	80580	0.050250401
80600	0.067358174	80584	0.086832923
80600	0.047997335	80588	0.097161501
80600	0.010977026	80592	0.051768675
80600	0.021256879	80596	0.009977481
80600	0.041580945	80600	0.041729869
80600	0.045245401	80604	0.082305269
80600	0.026483131	80608	0.05607897
80600	0.033787757	80612	0.01510622
80600	0.020492425	80616	0.018374587
80600	0.056314231	80620	0.023827963
80600	0.080121259	80624	0.027729311
80600	0.027420239	80628	0.092099937
80600	0.039568706	80632	0.122426151
80600	0.055617718	80636	0.081062608
80600	0.06019654	80640	0.035341334
80600	0.051707983	80644	0.007910839
80600	0.029433948	80648	0.03225484
80700	0.036237208	80652	0.041042578
80700	0.01100139	80656	0.053813405
80700	0.039037343	80660	0.022499458
80700	0.053841119	80664	0.04555154
80700	0.046404821	80668	0.091843576
80700	0.029598667	80672	0.095917145
80700	0.006984971	80676	0.096897864
80700	0.024122521	80680	0.10130024
80700	0.045240773	80684	0.06252512
80700	0.070673043	80688	0.014024559
80700	0.094458374	80692	0.030425685
80700	0.0817783	80696	0.046011795

PowerSpectrumData

80700	0.023233817	80700	0.064616303
80700	0.019888101	80704	0.087147782
80700	0.04087995	80708	0.039169434
80700	0.077312281	80712	0.027677068
80700	0.071912684	80716	0.059153117
80700	0.050910458	80720	0.022935668
80700	0.017167527	80724	0.0698898
80700	0.014911432	80728	0.077035875
80700	0.044698707	80732	0.05255135
80700	0.07366814	80736	0.021860316
80700	0.068658228	80740	0.039489005
80700	0.043797121	80744	0.032447475
80700	0.025177085	80748	0.012305431
80800	0.043157041	80752	0.060734263
80800	0.046896323	80756	0.094399176
80800	0.031145915	80760	0.061037441
80800	0.032684096	80764	0.091753966
80800	0.049897619	80768	0.086443979
80800	0.030997031	80772	0.077714562
80800	0.074158284	80776	0.075830569
80800	0.107892389	80780	0.070347145
80800	0.090494956	80784	0.069183974
80800	0.062221501	80788	0.0696547
80800	0.03348196	80792	0.057339716
80800	0.050166884	80796	0.143017358
80800	0.043967775	80800	0.049503258
80800	0.020370029	80804	0.079980702
80800	0.025974367	80808	0.19569305
80800	0.064789339	80812	0.174825356
80800	0.10196212	80816	0.021899152
80800	0.086190798	80820	0.141252516
80800	0.042440108	80824	0.250049343
80800	0.063408385	80828	0.108600965
80800	0.051999785	80832	0.211304359

PowerSpectrumData

80800	0.00604697	80836	0.287194708
80800	0.070390612	80840	0.215870008
80800	0.104294086	80844	0.189360886
80800	0.083784733	80848	0.503241783
80900	0.056123186	80852	0.398218631
80900	0.042644751	80856	0.266948656
80900	0.024834573	80860	0.421068951
80900	0.011558938	80864	0.542861119
80900	0.002820563	80868	0.483448298
80900	0.048712973	80872	0.481440016
80900	0.101472535	80876	0.543662405
80900	0.090871406	80880	0.383431383
80900	0.030718973	80884	0.411351358
80900	0.023566981	80888	0.736462768
80900	0.058837955	80892	0.671870774
80900	0.099398727	80896	1.628835688
80900	0.087612636	80900	3.7104371
80900	0.023505176	80904	4.14858525
80900	0.016902357	80908	2.51310225
80900	0.051296098	80912	0.684886298
80900	0.064532753	80916	0.317946309
80900	0.059133559	80920	0.180910764
80900	0.072526644	80924	0.085758802
80900	0.084582629	80928	0.067966066
80900	0.074005991	80932	0.022433524
80900	0.032987646	80936	0.164610479
80900	0.035551675	80940	0.110424859
80900	0.047819875	80944	0.053294476
80900	0.027811459	80948	0.068169466
81000	0.058994308	80952	0.110580164
81000	0.038485468	80956	0.024606388
81000	0.015136354	80960	0.083341605
81000	0.026282183	80964	0.084952437
81000	0.021731068	80968	0.119055389

PowerSpectrumData

81000	0.031713491	80972	0.097710268
81000	0.052852603	80976	0.028749226
81000	0.021137665	80980	0.081371924
81000	0.040831736	80984	0.017907052
81000	0.03702136	80988	0.068509318
81000	0.047357902	80992	0.033549964
81000	0.068980058	80996	0.055331548
81000	0.057250283	81000	0.066464032
81000	0.038627604	81004	0.080114391
81000	0.051793693	81008	0.073348994
81000	0.024882562	81012	0.036125559
81000	0.032572898	81016	0.033996374
81000	0.060846418	81020	0.038435686
81000	0.059916831	81024	0.02405158
81000	0.040251351	81028	0.037430458
81000	0.040582632	81032	0.025027528
81000	0.064214903	81036	0.030984091
81000	0.052103384	81040	0.035116402
81000	0.026202015	81044	0.050836596
81000	0.062329091	81048	0.048375296
81100	0.070344373	81052	0.065311215
81100	0.008703642	81056	0.061129184
81100	0.056093253	81060	0.042416108
81100	0.048433081	81064	0.043504238
81100	0.056955752	81068	0.048191338
81100	0.089903784	81072	0.066383604
81100	0.107343549	81076	0.045976249
81100	0.102514197	81080	0.053733056
81100	0.084389467	81084	0.096250333
81100	0.067278284	81088	0.118922464
81100	0.060841216	81092	0.121812154
81100	0.07206032	81096	0.133850758
81100	0.082810431	81100	0.097835633
81100	0.088758796	81104	0.058481579

PowerSpectrumData

81100	0.096454591	81108	0.08698678
81100	0.109365647	81112	0.089803179
81100	0.087202767	81116	0.057653175
81100	0.040735733	81120	0.03089591
81100	0.038413939	81124	0.013676546
81100	0.034491888	81128	0.084524196
81100	0.047504003	81132	0.118246673
81100	0.043105789	81136	0.117432333
81100	0.022794677	81140	0.127125939
81100	0.033105771	81144	0.098777389
81100	0.036013491	81148	0.046763293
81200	0.006150909	81152	0.042457374
81200	0.020312633	81156	0.061689752
81200	0.020855228	81160	0.062424348
81200	0.026770853	81164	0.009015645
81200	0.03566681	81168	0.109769659
81200	0.045269495	81172	0.14541579
81200	0.049791073	81176	0.139770141
81200	0.028525206	81180	0.14372151
81200	0.035911933	81184	0.109309185
81200	0.050072427	81188	0.080318598
81200	0.066380882	81192	0.079717611
81200	0.041080173	81196	0.056310048
81200	0.026961508	81200	0.029644871
81200	0.069293063	81204	0.066922607
81200	0.082792503	81208	0.082110368
81200	0.079131598	81212	0.100245692
81200	0.063075218	81216	0.124828745
81200	0.037300364	81220	0.085680425
81200	0.057288926	81224	0.083302599
81200	0.064064007	81228	0.093533105
81200	0.056494337	81232	0.058622391
81200	0.042801399	81236	0.091980917
81200	0.033879143	81240	0.108077453

PowerSpectrumData

81200	0.058205769	81244	0.084919659
81200	0.044471257	81248	0.055656903
81300	0.006969724	81252	0.050513703
81300	0.035278328	81256	0.058594749
81300	0.047710732	81260	0.043204633
81300	0.032716544	81264	0.064010492
81300	0.010805864	81268	0.130814704
81300	0.007780389	81272	0.146700433
81300	0.010181485	81276	0.099739627
81300	0.021918186	81280	0.032811931
81300	0.036562935	81284	0.071916802
81300	0.057119531	81288	0.131163324
81300	0.038549035	81292	0.158770519
81300	0.046725825	81296	0.12856879
81300	0.083159917	81300	0.045383724
81300	0.082531245	81304	0.01097098
81300	0.056310368	81308	0.005116185
81300	0.030988904	81312	0.045130539
81300	0.064039741	81316	0.099972116
81300	0.074646829	81320	0.103847066
81300	0.034881301	81324	0.07271982
81300	0.031992899	81328	0.06280942
81300	0.049686969	81332	0.086818604
81300	0.043023589	81336	0.098437886
81300	0.035424797	81340	0.089693996
81300	0.062767562	81344	0.095441865
81300	0.040673862	81348	0.08556484
81400	0.017736089	81352	0.091093418
81400	0.024173955	81356	0.081769271
81400	0.018923873	81360	0.066355751
81400	0.017007658	81364	0.095028052
81400	0.017619885	81368	0.066124347
81400	0.025576957	81372	0.01996255
81400	0.032502438	81376	0.029239229

PowerSpectrumData

81400	0.068472349	81380	0.07330066
81400	0.093928771	81384	0.106626656
81400	0.07349738	81388	0.086550666
81400	0.018251874	81392	0.046555168
81400	0.061298932	81396	0.038634844
81400	0.064143969	81400	0.032371463
81400	0.031300875	81404	0.04979382
81400	0.065103777	81408	0.050058628
81400	0.086237276	81412	0.048211237
81400	0.063215637	81416	0.120205601
81400	0.012081375	81420	0.159149408
81400	0.049159549	81424	0.091121219
81400	0.037670063	81428	0.060880091
81400	0.03415014	81432	0.094056559
81400	0.045974164	81436	0.114156967
81400	0.049183367	81440	0.113498587
81400	0.023752444	81444	0.057607638
81400	0.035768007	81448	0.03984927
81500	0.07085636	81452	0.070268703
81500	0.058266291	81456	0.092827919
81500	0.034500517	81460	0.102476486
81500	0.033051274	81464	0.058568025
81500	0.038307619	81468	0.013612382
81500	0.066011424	81472	0.029254215
81500	0.071897506	81476	0.060013495
81500	0.045616031	81480	0.069858252
81500	0.063290354	81484	0.055338944
81500	0.064741282	81488	0.033040877
81500	0.045012068	81492	0.030762119
81500	0.045236462	81496	0.055990084
81500	0.026957783	81500	0.051233619
81500	0.05925283	81504	0.036156282
81500	0.052663065	81508	0.03426287
81500	0.033045915	81512	0.056621771

PowerSpectrumData

81500	0.029797993	81516	0.0782711
81500	0.04005574	81520	0.053576001
81500	0.033194265	81524	0.079156067
81500	0.022743187	81528	0.086817439
81500	0.021456895	81532	0.059710783
81500	0.042797907	81536	0.089648049
81500	0.060277835	81540	0.091644019
81500	0.039503426	81544	0.075313852
81500	0.002323238	81548	0.065661385
81600	0.032358035	81552	0.027577744
81600	0.047675167	81556	0.01687443
81600	0.04324524	81560	0.009178836
81600	0.045159377	81564	0.003551351
81600	0.046582318	81568	0.036857429
81600	0.062732666	81572	0.061226143
81600	0.082955194	81576	0.063735977
81600	0.045485333	81580	0.056419056
81600	0.024962183	81584	0.035069756
81600	0.036606441	81588	0.034299173
81600	0.031823125	81592	0.054324006
81600	0.016117272	81596	0.037072296
81600	0.037599588	81600	0.015432279
81600	0.038478454	81604	0.029250488
81600	0.032986754	81608	0.063674219
81600	0.035306028	81612	0.060220027
81600	0.04857422	81616	0.059328679
81600	0.032853535	81620	0.080645164
81600	0.03201899	81624	0.082388484
81600	0.018484079	81628	0.064872897
81600	0.015682597	81632	0.041857082
81600	0.031161799	81636	0.029047889
81600	0.018860286	81640	0.040695235
81600	0.04673142	81644	0.051940195
81600	0.031523166	81648	0.068773719

81700	0.038469847	81652	0.090207403
81700	0.073821677	81656	0.102581558
81700	0.049530812	81660	0.118315802
81700	0.021037979	81664	0.094779949
81700	0.025194564	81668	0.026858455
81700	0.00879135	81672	0.026527565
81700	0.008773533	81676	0.011214399
81700	0.033673412	81680	0.039582777
81700	0.05277741	81684	0.037218171
81700	0.045763205	81688	0.022204698
81700	0.0308046	81692	0.031486368
81700	0.025368852	81696	0.032310156
81700	0.040877196	81700	0.031706495
81700	0.025380639	81704	0.037668626
81700	0.021448395	81708	0.045369525
81700	0.044050234	81712	0.037486341
81700	0.038866972	81716	0.031200762
81700	0.017451648	81720	0.033623834
81700	0.002357859	81724	0.018601837
81700	0.014697817	81728	0.011392102
81700	0.023043969	81732	0.028001954
81700	0.042010033	81736	0.045608285
81700	0.093664356	81740	0.069906186
81700	0.113861133	81744	0.070045695
81700	0.105085019	81748	0.04718564
81800	0.074389158	81752	0.024738345
81800	0.036332811	81756	0.021751766
81800	0.004560191	81760	0.03878021
81800	0.017425517	81764	0.054894088
81800	0.033068285	81768	0.027748034
81800	0.057176691	81772	0.039368228
81800	0.051761559	81776	0.023201348
81800	0.054887856	81780	0.038714446
81800	0.076475502	81784	0.054461965

PowerSpectrumData

81800	0.059659349	81788	0.046426005
81800	0.050473209	81792	0.042362601
81800	0.042613334	81796	0.047310528
81800	0.025307851	81800	0.030201698
81800	0.02114863	81804	0.017664179
81800	0.037763621	81808	0.046939935
81800	0.049698931	81812	0.012535678
81800	0.068398636	81816	0.058601137
81800	0.068142072	81820	0.062346626
81800	0.036762649	81824	0.047393322
81800	0.012684775	81828	0.044385524
81800	0.001885843	81832	0.046975023
81800	0.015456813	81836	0.075307769
81800	0.051854051	81840	0.07765153
81800	0.067288303	81844	0.054002761
81800	0.032757896	81848	0.029477247
81900	0.036383557	81852	0.015274929
81900	0.057014382	81856	0.018630526
81900	0.054988675	81860	0.040871393
81900	0.033342301	81864	0.051646686
81900	0.033966182	81868	0.040185478
81900	0.043830292	81872	0.054541109
81900	0.019461493	81876	0.0861429
81900	0.031800002	81880	0.073889845
81900	0.02596454	81884	0.08631016
81900	0.023438795	81888	0.065783279
81900	0.034147059	81892	0.059431848
81900	0.05720093	81896	0.060600545
81900	0.057199606	81900	0.046116576
81900	0.061696432	81904	0.017172451
81900	0.04140977	81908	0.059918802
81900	0.028988025	81912	0.072350609
81900	0.042453801	81916	0.032706856
81900	0.009115964	81920	0.027456066

PowerSpectrumData

81900	0.003333913	81924	0.064721775
81900	0.0299733	81928	0.090532434
81900	0.041414431	81932	0.085948377
81900	0.040171795	81936	0.068114539
81900	0.055146138	81940	0.042957203
81900	0.051812476	81944	0.02600773
81900	0.051245275	81948	0.03202074
82000	0.074072654	81952	0.014501192
82000	0.043964188	81956	0.024835352
82000	0.021381457	81960	0.036096066
82000	0.050935432	81964	0.068886991
82000	0.06631844	81968	0.075253709
82000	0.048326427	81972	0.062611034
82000	0.02375809	81976	0.05494057
82000	0.038937582	81980	0.030530639
82000	0.040165753	81984	0.033613662
82000	0.02358008	81988	0.070536589
82000	0.036414946	81992	0.07575997
82000	0.025379832	81996	0.044389333
82000	0.019676432	82000	0.009215687
82000	0.012609132	82004	0.011408254
82000	0.021673555	82008	0.038117432
82000	0.025097888	82012	0.045164175
82000	0.023243369	82016	0.060881441
82000	0.039075625	82020	0.094297167
82000	0.035506921	82024	0.06217674
82000	0.025639556	82028	0.028037832
82000	0.069648602	82032	0.017513894
82000	0.081036786	82036	0.02942466
82000	0.04176439	82040	0.046330595
82000	0.020401345	82044	0.028558892
82000	0.025084881	82048	0.050614483
82100	0.041282136	82052	0.097050455
82100	0.034434874	82056	0.106426291

PowerSpectrumData

82100	0.034003286	82060	0.084941195
82100	0.081816368	82064	0.06253412
82100	0.096778102	82068	0.056748093
82100	0.08234971	82072	0.051674488
82100	0.104321844	82076	0.061478771
82100	0.139015581	82080	0.065033841
82100	0.131393201	82084	0.048245423
82100	0.086925822	82088	0.019550655
82100	0.048566162	82092	0.040932377
82100	0.065097396	82096	0.082487211
82100	0.064447428	82100	0.096236545
82100	0.056535118	82104	0.100578691
82100	0.049987357	82108	0.059853723
82100	0.069405025	82112	0.025829058
82100	0.092569964	82116	0.016629661
82100	0.05563446	82120	0.004866932
82100	0.025885045	82124	0.035074845
82100	0.042858941	82128	0.021936201
82100	0.036597168	82132	0.060245402
82100	0.04478098	82136	0.067887879
82100	0.052349653	82140	0.056108911
82100	0.016436405	82144	0.050822306
82100	0.002682283	82148	0.052033265
82200	0.027316095	82152	0.071197828
82200	0.037122667	82156	0.068495261
82200	0.03265671	82160	0.017413702
82200	0.046290665	82164	0.079633559
82200	0.029394329	82168	0.078505538
82200	0.01149589	82172	0.059623206
82200	0.047844675	82176	0.055297005
82200	0.045626573	82180	0.087296256
82200	0.027280124	82184	0.124795362
82200	0.038692433	82188	0.132524685
82200	0.044022756	82192	0.117347059

PowerSpectrumData

82200	0.012945202	82196	0.069802823
82200	0.04553876	82200	0.050861228
82200	0.046982437	82204	0.026131316
82200	0.039696672	82208	0.019327526
82200	0.037245751	82212	0.081970953
82200	0.00335095	82216	0.091817099
82200	0.037414775	82220	0.060446731
82200	0.036078596	82224	0.075279975
82200	0.056290224	82228	0.063935499
82200	0.06572072	82232	0.028796627
82200	0.053880423	82236	0.083894964
82200	0.046709269	82240	0.078693112
82200	0.060589693	82244	0.060856863
82200	0.042877378	82248	0.06947184
82300	0.040198887	82252	0.06285432
82300	0.022741342	82256	0.036687543
82300	0.037156879	82260	0.068939829
82300	0.020655838	82264	0.102667284
82300	0.006067165	82268	0.086872584
82300	0.02169632	82272	0.05262965
82300	0.046288562	82276	0.084875137
82300	0.071678718	82280	0.107982982
82300	0.067619709	82284	0.110301102
82300	0.026387421	82288	0.092614959
82300	0.021721642	82292	0.056204688
82300	0.067567497	82296	0.054279964
82300	0.076153745	82300	0.087061933
82300	0.052424588	82304	0.073439187
82300	0.056875128	82308	0.067592089
82300	0.075092947	82312	0.073102274
82300	0.054356955	82316	0.044093242
82300	0.050852657	82320	0.041605304
82300	0.052477746	82324	0.051578474
82300	0.008079071	82328	0.054840475

PowerSpectrumData

82300	0.051944113	82332	0.056544734
82300	0.053653152	82336	0.018907515
82300	0.042174677	82340	0.029808647
82300	0.029387142	82344	0.038813287
82300	0.050397448	82348	0.040879681
82400	0.045947461	82352	0.057968355
82400	0.015655262	82356	0.081501908
82400	0.034071029	82360	0.037044003
82400	0.031900963	82364	0.026983113
82400	0.039935439	82368	0.042610136
82400	0.087493012	82372	0.042439082
82400	0.06612431	82376	0.066304507
82400	0.031172018	82380	0.087839755
82400	0.039932762	82384	0.099859091
82400	0.028932083	82388	0.10240003
82400	0.029970448	82392	0.084947744
82400	0.039709379	82396	0.094222633
82400	0.042987525	82400	0.114442228
82400	0.041961765	82404	0.098021592
82400	0.052641703	82408	0.057547513
82400	0.051068262	82412	0.028409626
82400	0.030644915	82416	0.032962547
82400	0.039547511	82420	0.031598793
82400	0.041919786	82424	0.037877973
82400	0.036314028	82428	0.053541389
82400	0.031578322	82432	0.030299105
82400	0.040936513	82436	0.035286135
82400	0.047374721	82440	0.067883273
82400	0.023433939	82444	0.082675746
82400	0.013800646	82448	0.078320016
82500	0.008158529	82452	0.065517343
82500	0.034578421	82456	0.062435414
82500	0.04378787	82460	0.092383103
82500	0.052800737	82464	0.107776177

PowerSpectrumData

82500	0.057406523	82468	0.110083347
82500	0.018950841	82472	0.085660395
82500	0.035569305	82476	0.074156968
82500	0.039083949	82480	0.106009102
82500	0.00578041	82484	0.101606456
82500	0.030031753	82488	0.094792435
82500	0.031672003	82492	0.068593938
82500	0.065250257	82496	0.063045744
82500	0.082348204	82500	0.078033845
82500	0.046336791	82504	0.057225767
82500	0.007349453	82508	0.057301095
82500	0.024848108	82512	0.046794216
82500	0.035707508	82516	0.05851853
82500	0.040713971	82520	0.126418105
82500	0.021437658	82524	0.131594309
82500	0.032947595	82528	0.064689521
82500	0.047460082	82532	0.068063484
82500	0.056241435	82536	0.109037261
82500	0.101010577	82540	0.103464488
82500	0.092970891	82544	0.063109073
82500	0.030877902	82548	0.043040018
82600	0.008640447	82552	0.067593283
82600	0.037176997	82556	0.037111298
82600	0.075270473	82560	0.07382199
82600	0.070038514	82564	0.077504628
82600	0.043136388	82568	0.048171474
82600	0.037383958	82572	0.046095218
82600	0.055570843	82576	0.060367467
82600	0.070127637	82580	0.08245396
82600	0.077591787	82584	0.097592929
82600	0.072525138	82588	0.084122497
82600	0.061683728	82592	0.046293266
82600	0.040247483	82596	0.073567004
82600	0.004928179	82600	0.104919105

PowerSpectrumData

82600	0.042398948	82604	0.102104932
82600	0.038989187	82608	0.052959378
82600	0.006260495	82612	0.052352203
82600	0.02671678	82616	0.053772485
82600	0.060465463	82620	0.020171039
82600	0.114054317	82624	0.012667579
82600	0.132345841	82628	0.014261544
82600	0.078493533	82632	0.03730027
82600	0.055325061	82636	0.08253826
82600	0.063384418	82640	0.119207682
82600	0.057115587	82644	0.125409351
82600	0.028902034	82648	0.091907386
82700	0.038225368	82652	0.064712127
82700	0.038424827	82656	0.018760862
82700	0.010176782	82660	0.07392362
82700	0.022125218	82664	0.133472931
82700	0.038001341	82668	0.148102554
82700	0.075490178	82672	0.127759121
82700	0.098663179	82676	0.087956672
82700	0.088613451	82680	0.082247476
82700	0.075195429	82684	0.087373221
82700	0.100082165	82688	0.094347248
82700	0.101169178	82692	0.117319993
82700	0.063302767	82696	0.111007837
82700	0.029906934	82700	0.08981819
82700	0.066762055	82704	0.078338635
82700	0.067043446	82708	0.060960203
82700	0.022511727	82712	0.079303594
82700	0.058076541	82716	0.111949885
82700	0.037611961	82720	0.109962843
82700	0.013469343	82724	0.087647983
82700	0.044672546	82728	0.064342268
82700	0.075038719	82732	0.112781672
82700	0.08823994	82736	0.178866758

PowerSpectrumData

82700	0.06063842	82740	0.161674078
82700	0.03047471	82744	0.082201674
82700	0.021594236	82748	0.074453921
82800	0.034221641	82752	0.127094099
82800	0.104430255	82756	0.124948638
82800	0.112331618	82760	0.077878249
82800	0.027028564	82764	0.066971166
82800	0.045302451	82768	0.07043271
82800	0.0387365	82772	0.092239265
82800	0.012624736	82776	0.112562186
82800	0.011458975	82780	0.094062481
82800	0.034006738	82784	0.07774854
82800	0.080968785	82788	0.074606964
82800	0.100428944	82792	0.087346445
82800	0.050552793	82796	0.115190247
82800	0.020675503	82800	0.114712886
82800	0.035401448	82804	0.089440553
82800	0.007730113	82808	0.089639987
82800	0.052911684	82812	0.094302806
82800	0.045451114	82816	0.064608423
82800	0.02136482	82820	0.024553259
82800	0.069127891	82824	0.021682672
82800	0.102641141	82828	0.083379171
82800	0.09904203	82832	0.122275902
82800	0.047438116	82836	0.114479095
82800	0.017833971	82840	0.107741878
82800	0.011250599	82844	0.107031796
82800	0.036090525	82848	0.071735216
82900	0.065135348	82852	0.012814407
82900	0.065623462	82856	0.035107507
82900	0.045893328	82860	0.057488982
82900	0.039548209	82864	0.04201529
82900	0.067820824	82868	0.051075193
82900	0.080205347	82872	0.047730573

PowerSpectrumData

82900	0.027962928	82876	0.04767218
82900	0.054407214	82880	0.050703631
82900	0.061450803	82884	0.074062664
82900	0.053012398	82888	0.062084371
82900	0.051347313	82892	0.042477481
82900	0.020816397	82896	0.044184053
82900	0.015061033	82900	0.030564319
82900	0.017642358	82904	0.027369149
82900	0.029553637	82908	0.046550184
82900	0.027056789	82912	0.062096362
82900	0.052818195	82916	0.081345286
82900	0.040501433	82920	0.093792274
82900	0.016741873	82924	0.092043643
82900	0.021910961	82928	0.106781903
82900	0.034727807	82932	0.092558737
82900	0.016683605	82936	0.034937759
82900	0.058532198	82940	0.053768505
82900	0.056541663	82944	0.039801529
82900	0.059766051	82948	0.04541608
83000	0.026458822	82952	0.092918963
83000	0.027250075	82956	0.099059806
83000	0.070146787	82960	0.064387212
83000	0.068697991	82964	0.037123464
83000	0.051512208	82968	0.040363342
83000	0.049273931	82972	0.0336413
83000	0.074730095	82976	0.045871369
83000	0.086166649	82980	0.037476701
83000	0.073797732	82984	0.041858435
83000	0.061389204	82988	0.044786812
83000	0.051804109	82992	0.015788268
83000	0.032687458	82996	0.048144888
83000	0.042691219	83000	0.088812391
83000	0.042650103	83004	0.106995511
83000	0.059952032	83008	0.062067193

PowerSpectrumData

83000	0.059478425	83012	0.057142243
83000	0.018438128	83016	0.071685885
83000	0.039379214	83020	0.054727687
83000	0.037335387	83024	0.034499579
83000	0.066926579	83028	0.050442177
83000	0.082155384	83032	0.018927094
83000	0.057182708	83036	0.043410058
83000	0.03672771	83040	0.06962449
83000	0.042435386	83044	0.053684005
83000	0.059937665	83048	0.063981432
83100	0.057017467	83052	0.08727039
83100	0.046983299	83056	0.065914937
83100	0.023071298	83060	0.00922655
83100	0.005963467	83064	0.040080337
83100	0.019039515	83068	0.045632303
83100	0.045054942	83072	0.046048797
83100	0.053172975	83076	0.053307118
83100	0.050795748	83080	0.050149829
83100	0.049104248	83084	0.028637554
83100	0.013087509	83088	0.05132924
83100	0.023401695	83092	0.077566234
83100	0.010385083	83096	0.067705027
83100	0.014484304	83100	0.032136821
83100	0.024501387	83104	0.041441264
83100	0.030774965	83108	0.05536024
83100	0.025332787	83112	0.061244362
83100	0.060276885	83116	0.032607943
83100	0.044902205	83120	0.034960562
83100	0.042090185	83124	0.043747652
83100	0.034253873	83128	0.06309749
83100	0.052273648	83132	0.074844851
83100	0.03690827	83136	0.053365322
83100	0.054275963	83140	0.03861402
83100	0.108883774	83144	0.044927027

PowerSpectrumData

83100	0.088158893	83148	0.027285345
83200	0.044692442	83152	0.016690578
83200	0.025721653	83156	0.03258259
83200	0.043473025	83160	0.029296154
83200	0.037771853	83164	0.02087782
83200	0.037499434	83168	0.027359738
83200	0.086046603	83172	0.037556056
83200	0.05005355	83176	0.063488711
83200	0.024211793	83180	0.071874922
83200	0.029284969	83184	0.059514234
83200	0.016638558	83188	0.056005563
83200	0.017563625	83192	0.097043994
83200	0.009574847	83196	0.072025301
83200	0.020071067	83200	0.037316968
83200	0.014882096	83204	0.045301913
83200	0.056619574	83208	0.050383645
83200	0.082803548	83212	0.064902088
83200	0.062944171	83216	0.047171823
83200	0.013049664	83220	0.020351465
83200	0.039674411	83224	0.058051002
83200	0.051261446	83228	0.051458483
83200	0.023435763	83232	0.017594361
83200	0.036833706	83236	0.036822636
83200	0.051225248	83240	0.067836081
83200	0.021860702	83244	0.09149962
83200	0.021291275	83248	0.081929138
83300	0.038257211	83252	0.063286789
83300	0.029336125	83256	0.110527166
83300	0.02620215	83260	0.143762504
83300	0.063624335	83264	0.113817856
83300	0.100294688	83268	0.072035917
83300	0.103554528	83272	0.052616546
83300	0.090387744	83276	0.045003326
83300	0.048620426	83280	0.027246251

PowerSpectrumData

83300	0.051421663	83284	0.016918277
83300	0.061951796	83288	0.053470871
83300	0.055132681	83292	0.0753196
83300	0.0561936	83296	0.048978578
83300	0.036824818	83300	0.023889366
83300	0.023090237	83304	0.022774126
83300	0.072327035	83308	0.052182564
83300	0.061708401	83312	0.083221588
83300	0.042061365	83316	0.064020867
83300	0.082910745	83320	0.051800274
83300	0.111984235	83324	0.109382512
83300	0.084874911	83328	0.151021654
83300	0.035157238	83332	0.123576057
83300	0.02494882	83336	0.055297856
83300	0.021579319	83340	0.053951419
83300	0.006596841	83344	0.052166382
83300	0.022681337	83348	0.037059657
83400	0.05061807	83352	0.041945867
83400	0.047763373	83356	0.055260331
83400	0.019353724	83360	0.049826958
83400	0.011395473	83364	0.044514018
83400	0.040132214	83368	0.038441707
83400	0.051958432	83372	0.037458027
83400	0.060855571	83376	0.044133059
83400	0.064418135	83380	0.069614995
83400	0.040049839	83384	0.063219653
83400	0.051406802	83388	0.037925871
83400	0.071800088	83392	0.052847354
83400	0.053609005	83396	0.08845907
83400	0.03531218	83400	0.127868684
83400	0.037744598	83404	0.09921188
83400	0.041888339	83408	0.021570288
83400	0.040227995	83412	0.047728325
83400	0.032670552	83416	0.095884534

PowerSpectrumData

83400	0.090773414	83420	0.106234969
83400	0.078321318	83424	0.077185199
83400	0.022774055	83428	0.068069763
83400	0.024709589	83432	0.052284697
83400	0.074542259	83436	0.038163893
83400	0.06695545	83440	0.079437174
83400	0.043117096	83444	0.068634785
83400	0.042115626	83448	0.051961557
83500	0.051370116	83452	0.076303251
83500	0.026037125	83456	0.090336471
83500	0.008590281	83460	0.096137541
83500	0.019778654	83464	0.083909923
83500	0.015220834	83468	0.056023848
83500	0.037674134	83472	0.064893662
83500	0.040449537	83476	0.114868279
83500	0.052850719	83480	0.156741866
83500	0.072557676	83484	0.158434835
83500	0.068587731	83488	0.105816289
83500	0.062776693	83492	0.055396686
83500	0.083820683	83496	0.070130787
83500	0.050625509	83500	0.104335202
83500	0.035377419	83504	0.107382533
83500	0.112989037	83508	0.065351087
83500	0.127562845	83512	0.04494761
83500	0.07600226	83516	0.052173491
83500	0.084550258	83520	0.07103596
83500	0.071814888	83524	0.096972231
83500	0.024810308	83528	0.087139226
83500	0.043177097	83532	0.060634222
83500	0.022109349	83536	0.033545872
83500	0.055806588	83540	0.03829721
83500	0.082178332	83544	0.09519342
83500	0.058117101	83548	0.100183555
83600	0.053216285	83552	0.078124409

PowerSpectrumData

83600	0.055514593	83556	0.113617025
83600	0.053662901	83560	0.137486844
83600	0.05677019	83564	0.1042027
83600	0.04525614	83568	0.078699377
83600	0.047729813	83572	0.118115095
83600	0.065398475	83576	0.145585131
83600	0.039175291	83580	0.132561574
83600	0.055117358	83584	0.08851225
83600	0.06615786	83588	0.058227455
83600	0.062098698	83592	0.069875467
83600	0.053237498	83596	0.076661352
83600	0.01716346	83600	0.099456374
83600	0.048450653	83604	0.115761963
83600	0.007102354	83608	0.09679325
83600	0.036548121	83612	0.06093485
83600	0.052825089	83616	0.093982737
83600	0.075260279	83620	0.106510554
83600	0.045671884	83624	0.099385579
83600	0.079499077	83628	0.108872882
83600	0.130391768	83632	0.089456036
83600	0.12897914	83636	0.065322311
83600	0.115457762	83640	0.074228308
83600	0.108585824	83644	0.095149058
83600	0.059280734	83648	0.102064209
83700	0.049073122	83652	0.105349311
83700	0.173192515	83656	0.058947433
83700	0.30756669	83660	0.035506622
83700	0.362490449	83664	0.075504053
83700	0.222096569	83668	0.099175151
83700	0.11032894	83672	0.077694378
83700	0.091917034	83676	0.052201565
83700	0.099707788	83680	0.044853696
83700	0.393205934	83684	0.014960672
83700	0.61720045	83688	0.065202206

PowerSpectrumData

83700	0.59357495	83692	0.106512671
83700	0.40815954	83696	0.103700135
83700	0.228992765	83700	0.06392099
83700	0.07438429	83704	0.014520734
83700	0.026072128	83708	0.024026749
83700	0.121391975	83712	0.049810402
83700	0.389299938	83716	0.074118951
83700	0.825937605	83720	0.104873696
83700	0.84294827	83724	0.08944908
83700	0.366582274	83728	0.011013749
83700	0.5934785	83732	0.063555614
83700	0.419653515	83736	0.087596542
83700	1.076522749	83740	0.072478528
83700	3.071663438	83744	0.048015594
83700	4.291917663	83748	0.041099556
83800	3.606918738	83752	0.042074898
83800	1.716844738	83756	0.047985352
83800	0.367407978	83760	0.057429439
83800	0.467317179	83764	0.063735708
83800	0.259669178	83768	0.047894024
83800	0.468033658	83772	0.039409137
83800	1.116427593	83776	0.046502442
83800	1.471302938	83780	0.04786098
83800	0.79905265	83784	0.064968444
83800	0.59567421	83788	0.080424077
83800	0.743944431	83792	0.100543548
83800	0.74636942	83796	0.094475799
83800	2.95290235	83800	0.024916055
83800	6.573309188	83804	0.087608787
83800	7.612954825	83808	0.100007237
83800	4.753788938	83812	0.071171926
83800	1.305563138	83816	0.064226275
83800	0.717531948	83820	0.061362
83800	0.242394148	83824	0.047041329

PowerSpectrumData

83800	0.365569081	83828	0.088865119
83800	0.258742395	83832	0.092362549
83800	0.647458481	83836	0.041797266
83800	0.540797948	83840	0.053568288
83800	0.377565768	83844	0.100735262
83800	0.20489232	83848	0.120117453
83900	0.254034123	83852	0.080598504
83900	0.142910868	83856	0.031529951
83900	0.716503594	83860	0.052293828
83900	1.143869361	83864	0.05676597
83900	1.017672243	83868	0.053441003
83900	0.459928676	83872	0.063881533
83900	0.109268418	83876	0.045876066
83900	0.054619257	83880	0.015212749
83900	0.02893312	83884	0.032024731
83900	0.022578697	83888	0.030533753
83900	0.140612349	83892	0.00808988
83900	0.194344568	83896	0.021004753
83900	0.13144649	83900	0.035467336
83900	0.073396695	83904	0.029070492
83900	0.116231684	83908	0.023339122
83900	0.065082553	83912	0.01990399
83900	0.163141871	83916	0.02629841
83900	0.309726165	83920	0.038934519
83900	0.3711186	83924	0.035593155
83900	0.247336313	83928	0.054161876
83900	0.027838993	83932	0.048643713
83900	0.110723333	83936	0.055266504
83900	0.074315685	83940	0.101032951
83900	0.066838649	83944	0.100838108
83900	0.222355986	83948	0.058059675
84000	0.366900728	83952	0.052776759
84000	0.300206564	83956	0.05612653
84000	0.113087503	83960	0.079543272

PowerSpectrumData

84000	0.012052359	83964	0.06300422
84000	0.03937041	83968	0.028112985
84000	0.114898496	83972	0.080387472
84000	0.1865011	83976	0.084692751
84000	0.201754155	83980	0.048994345
84000	0.154715715	83984	0.025923187
84000	0.074111806	83988	0.006728063
84000	0.017826131	83992	0.03036996
84000	0.057175043	83996	0.050646992
84000	0.062701605	84000	0.044379147
84000	0.103267659	84004	0.019160512
84000	0.190199615	84008	0.008071374
84000	0.24637868	84012	0.052260799
84000	0.203103933	84016	0.089494635
84000	0.083612198	84020	0.068510824
84000	0.029528106	84024	0.069018541
84000	0.064368207	84028	0.088437868
84000	0.068166599	84032	0.056207729
84000	0.114366136	84036	0.019088342
84000	0.128883839	84040	0.016567765
84000	0.109510089	84044	0.057549627
84000	0.067652865	84048	0.092146926
84100	0.044786761	84052	0.073411422
84100	0.064122614	84056	0.080982594
84100	0.081799088	84060	0.07460179
84100	0.116401803	84064	0.051500192
84100	0.15441193	84068	0.038483653
84100	0.143246434	84072	0.017731682
84100	0.088059998	84076	0.026257905
84100	0.046564746	84080	0.023457033
84100	0.017752025	84084	0.025690457
84100	0.047378737	84088	0.054352844
84100	0.08470274	84092	0.079326164
84100	0.056450619	84096	0.047129157

PowerSpectrumData

84100	0.045630739	84100	0.044045319
84100	0.069097303	84104	0.098529112
84100	0.061542152	84108	0.113624745
84100	0.06667959	84112	0.086711254
84100	0.012995087	84116	0.043422799
84100	0.078040146	84120	0.033254175
84100	0.116786461	84124	0.015477226
84100	0.1352041	84128	0.055664721
84100	0.122861369	84132	0.070712755
84100	0.07303802	84136	0.065946515
84100	0.082247963	84140	0.042880634
84100	0.109665481	84144	0.017434682
84100	0.09492923	84148	0.010884914
84200	0.0675298	84152	0.032892745
84200	0.080327438	84156	0.035704001
84200	0.101967416	84160	0.017575563
84200	0.091263239	84164	0.027455522
84200	0.042445918	84168	0.022156377
84200	0.052914882	84172	0.013873484
84200	0.12110106	84176	0.025571071
84200	0.13554524	84180	0.02650486
84200	0.08732194	84184	0.01537129
84200	0.066120883	84188	0.029522376
84200	0.084486637	84192	0.06199917
84200	0.080233753	84196	0.067747882
84200	0.050741306	84200	0.079634898
84200	0.033867789	84204	0.074092954
84200	0.05162623	84208	0.04316702
84200	0.054048578	84212	0.024253113
84200	0.06646484	84216	0.048543789
84200	0.093782401	84220	0.062548948
84200	0.09299317	84224	0.098888319
84200	0.038390306	84228	0.106415777
84200	0.031564334	84232	0.049668801

PowerSpectrumData

84200	0.032053667	84236	0.042829968
84200	0.016042275	84240	0.069876973
84200	0.025605406	84244	0.039895356
84200	0.05021556	84248	0.021318469
84300	0.031739808	84252	0.050729541
84300	0.046348116	84256	0.059509275
84300	0.073748939	84260	0.06001861
84300	0.058551472	84264	0.035235593
84300	0.066258079	84268	0.04743605
84300	0.074744399	84272	0.070918708
84300	0.044410797	84276	0.049559007
84300	0.039625167	84280	0.050261366
84300	0.054290009	84284	0.108905231
84300	0.020020512	84288	0.113792215
84300	0.036009064	84292	0.067448025
84300	0.054448603	84296	0.061467865
84300	0.066619032	84300	0.074653872
84300	0.057053749	84304	0.071169015
84300	0.029848896	84308	0.037441139
84300	0.025423769	84312	0.022674996
84300	0.034937995	84316	0.075763594
84300	0.036222336	84320	0.095134994
84300	0.040009622	84324	0.062622123
84300	0.045431068	84328	0.045059023
84300	0.04454067	84332	0.051734649
84300	0.008379497	84336	0.075078016
84300	0.04987009	84340	0.075076052
84300	0.063312298	84344	0.067645538
84300	0.059967395	84348	0.03658505
84400	0.104931307	84352	0.020532869
84400	0.08232407	84356	0.061544153
84400	0.03292138	84360	0.102209808
84400	0.091147347	84364	0.1096708
84400	0.069201451	84368	0.117899763

PowerSpectrumData

84400	0.046731984	84372	0.090205074
84400	0.061376857	84376	0.038943388
84400	0.056915393	84380	0.016479107
84400	0.037559086	84384	0.029996976
84400	0.083777894	84388	0.055224737
84400	0.087648907	84392	0.044049142
84400	0.053146141	84396	0.045901132
84400	0.020331494	84400	0.048246984
84400	0.012606374	84404	0.031621861
84400	0.059650778	84408	0.044452183
84400	0.090714217	84412	0.063215506
84400	0.088119254	84416	0.073536372
84400	0.08332562	84420	0.067000859
84400	0.084415471	84424	0.061708939
84400	0.062628176	84428	0.049393711
84400	0.007120522	84432	0.033715012
84400	0.073400966	84436	0.041917916
84400	0.098599601	84440	0.037336944
84400	0.085379928	84444	0.013128268
84400	0.038152535	84448	0.014917062
84500	0.015848307	84452	0.03595902
84500	0.029877021	84456	0.03851116
84500	0.012600002	84460	0.030969837
84500	0.027116468	84464	0.019553907
84500	0.026511523	84468	0.012481745
84500	0.002368004	84472	0.041285828
84500	0.015965172	84476	0.036889836
84500	0.047064928	84480	0.014941328
84500	0.025694712	84484	0.018907638
84500	0.020785818	84488	0.046476507
84500	0.058723541	84492	0.059870574
84500	0.089225825	84496	0.063010928
84500	0.074908334	84500	0.061959399
84500	0.044778779	84504	0.048983198

PowerSpectrumData

84500	0.030230753	84508	0.03710053
84500	0.041689695	84512	0.044006218
84500	0.072877294	84516	0.050074781
84500	0.052877018	84520	0.054868418
84500	0.016335469	84524	0.051720883
84500	0.037549948	84528	0.028063851
84500	0.040382023	84532	0.035265777
84500	0.009024609	84536	0.052828873
84500	0.038660703	84540	0.069105896
84500	0.044245204	84544	0.04108527
84500	0.040328247	84548	0.040978026
84600	0.071073664	84552	0.093304567
84600	0.076585689	84556	0.085607324
84600	0.049855447	84560	0.062332372
84600	0.047903275	84564	0.018110841
84600	0.063905711	84568	0.018571132
84600	0.033678476	84572	0.019038462
84600	0.012045823	84576	0.007221982
84600	0.029488305	84580	0.010839856
84600	0.036864502	84584	0.042827316
84600	0.016380069	84588	0.085659893
84600	0.025396086	84592	0.095887845
84600	0.015127032	84596	0.104519291
84600	0.042220065	84600	0.122236656
84600	0.088973036	84604	0.109743814
84600	0.093584997	84608	0.095136464
84600	0.060219656	84612	0.067352179
84600	0.040988194	84616	0.020907408
84600	0.030809279	84620	0.033190758
84600	0.013703295	84624	0.049318325
84600	0.043235403	84628	0.074006348
84600	0.026584761	84632	0.094054842
84600	0.0561005	84636	0.09981091
84600	0.061037841	84640	0.092934548

PowerSpectrumData

84600	0.045455683	84644	0.065185479
84600	0.030272868	84648	0.013740546
84700	0.036663088	84652	0.046729427
84700	0.043798893	84656	0.065865585
84700	0.051469902	84660	0.053610944
84700	0.036809986	84664	0.087613866
84700	0.020663758	84668	0.096596843
84700	0.017011565	84672	0.078638106
84700	0.048182319	84676	0.084503161
84700	0.076294724	84680	0.067733949
84700	0.045750177	84684	0.028062721
84700	0.032358665	84688	0.056337682
84700	0.066473833	84692	0.065605666
84700	0.033801556	84696	0.042712829
84700	0.021510641	84700	0.02999999
84700	0.016954711	84704	0.052305095
84700	0.023052202	84708	0.058528545
84700	0.056696514	84712	0.040876319
84700	0.063418644	84716	0.04259478
84700	0.048316444	84720	0.033474164
84700	0.060102888	84724	0.028002494
84700	0.055198234	84728	0.065759428
84700	0.001574097	84732	0.088111905
84700	0.042050509	84736	0.052214607
84700	0.032283228	84740	0.012277917
84700	0.018708366	84744	0.021610773
84700	0.014852432	84748	0.059198159
84800	0.04650018	84752	0.056760477
84800	0.058857335	84756	0.03791937
84800	0.007616114	84760	0.006859079
84800	0.048357775	84764	0.044699766
84800	0.04140738	84768	0.045543609
84800	0.044153552	84772	0.022684886
84800	0.062434272	84776	0.035792527

PowerSpectrumData

84800	0.030670479	84780	0.056548397
84800	0.022345801	84784	0.043520646
84800	0.027786393	84788	0.019905852
84800	0.024614479	84792	0.039441813
84800	0.039746203	84796	0.029312765
84800	0.029817373	84800	0.064507178
84800	0.081012426	84804	0.074663665
84800	0.081036626	84808	0.019608182
84800	0.050926417	84812	0.028166693
84800	0.059331811	84816	0.041180396
84800	0.042455115	84820	0.051564588
84800	0.010209565	84824	0.047740825
84800	0.002145024	84828	0.02770846
84800	0.017743252	84832	0.007890346
84800	0.024306599	84836	0.032952947
84800	0.061859137	84840	0.059144404
84800	0.054619519	84844	0.063707776
84800	0.036837035	84848	0.101050886
84900	0.029125711	84852	0.104889638
84900	0.021453814	84856	0.078568395
84900	0.026504078	84860	0.044200981
84900	0.024059011	84864	0.037300168
84900	0.011150421	84868	0.051324601
84900	0.011528657	84872	0.041549305
84900	0.019108977	84876	0.018957549
84900	0.037915739	84880	0.05469258
84900	0.052012361	84884	0.048477832
84900	0.05128382	84888	0.02915681
84900	0.048186139	84892	0.033326083
84900	0.040715324	84896	0.057473579
84900	0.039320588	84900	0.065215107
84900	0.071208873	84904	0.084330961
84900	0.082378683	84908	0.08949935
84900	0.049535429	84912	0.060190883

PowerSpectrumData

84900	0.035707733	84916	0.02704201
84900	0.052502517	84920	0.002714122
84900	0.048485174	84924	0.012390797
84900	0.061499377	84928	0.039213635
84900	0.050207265	84932	0.016106204
84900	0.016858227	84936	0.032757966
84900	0.009966193	84940	0.043193762
84900	0.019745748	84944	0.026764925
84900	0.028538518	84948	0.030145542
85000	0.038811209	84952	0.03900774
85000	0.025746274	84956	0.01350128
85000	0.044487581	84960	0.044111101
85000	0.044950961	84964	0.039285013
85000	0.056714111	84968	0.058737238
85000	0.051141247	84972	0.071972958
85000	0.028283712	84976	0.087171633
85000	0.007344915	84980	0.0929897
85000	0.034520144	84984	0.066991815
85000	0.053664775	84988	0.049654969
85000	0.048129034	84992	0.01617093
85000	0.045658973	84996	0.062499144
85000	0.044346463	85000	0.049073547
85000	0.044393812	85004	0.042343148
85000	0.063089145	85008	0.103531696
85000	0.056283425	85012	0.109231929
85000	0.04294531	85016	0.091394963
85000	0.061800703	85020	0.056274959
85000	0.067181762	85024	0.027884929
85000	0.052399268	85028	0.066132023
85000	0.035486908	85032	0.058226236
85000	0.065495478	85036	0.055938512
85000	0.081526028	85040	0.083798688
85000	0.088311346	85044	0.072472329
85000	0.106068248	85048	0.033241144

PowerSpectrumData

85100	0.086557709	85052	0.039919363
85100	0.0477422	85056	0.039845734
85100	0.063090462	85060	0.019709307
85100	0.048195248	85064	0.017126584
85100	0.045481826	85068	0.062224834
85100	0.039754214	85072	0.111732989
85100	0.011633941	85076	0.115753115
85100	0.012474418	85080	0.073962459
85100	0.016666931	85084	0.067611349
85100	0.046567766	85088	0.075673379
85100	0.083761188	85092	0.062787469
85100	0.060619575	85096	0.046726571
85100	0.008354834	85100	0.102957347
85100	0.005414793	85104	0.126409869
85100	0.038961662	85108	0.080551006
85100	0.039261286	85112	0.074899115
85100	0.006123213	85116	0.103886028
85100	0.040266357	85120	0.085916523
85100	0.049829228	85124	0.031683256
85100	0.08896138	85128	0.047653168
85100	0.096055133	85132	0.06986245
85100	0.042785847	85136	0.044910565
85100	0.065347085	85140	0.029358012
85100	0.070756665	85144	0.050334711
85100	0.055275119	85148	0.048391707
85200	0.037114507	85152	0.027408896
85200	0.015053035	85156	0.029100673
85200	0.011325353	85160	0.024308447
85200	0.0255542	85164	0.035335317
85200	0.035378867	85168	0.06275608
85200	0.034886838	85172	0.067974594
85200	0.039998962	85176	0.035699144
85200	0.05011139	85180	0.013811688
85200	0.020002799	85184	0.027111177

PowerSpectrumData

85200	0.037649526	85188	0.018558523
85200	0.062581145	85192	0.011652416
85200	0.035210713	85196	0.034508277
85200	0.014706228	85200	0.056076529
85200	0.001905991	85204	0.061953069
85200	0.027952203	85208	0.070040966
85200	0.032006177	85212	0.050546645
85200	0.012761058	85216	0.004335744
85200	0.026145903	85220	0.054283868
85200	0.025103092	85224	0.066062879
85200	0.036564998	85228	0.042292319
85200	0.071063885	85232	0.050520888
85200	0.075923352	85236	0.048971815
85200	0.07965765	85240	0.017976965
85200	0.074604453	85244	0.032051838
85200	0.044916487	85248	0.029416404
85300	0.025792033	85252	0.04015927
85300	0.051677496	85256	0.028298538
85300	0.039185688	85260	0.080060279
85300	0.06633137	85264	0.075440716
85300	0.077612131	85268	0.045121054
85300	0.046702244	85272	0.007349915
85300	0.009855749	85276	0.044959536
85300	0.03320157	85280	0.054092485
85300	0.049689385	85284	0.021768477
85300	0.044386223	85288	0.077467463
85300	0.017836923	85292	0.110682682
85300	0.036079673	85296	0.079825164
85300	0.03655491	85300	0.035333524
85300	0.053203854	85304	0.004049141
85300	0.081467122	85308	0.057116129
85300	0.070197013	85312	0.096741736
85300	0.022768925	85316	0.112814356
85300	0.038043039	85320	0.110125824

PowerSpectrumData

85300	0.063202482	85324	0.060713363
85300	0.030889463	85328	0.032381566
85300	0.014975055	85332	0.033894772
85300	0.004972627	85336	0.029443081
85300	0.004940595	85340	0.041110317
85300	0.048404592	85344	0.089449641
85300	0.052859621	85348	0.112733244
85400	0.061783423	85352	0.078626443
85400	0.094536401	85356	0.051652933
85400	0.118788637	85360	0.040375238
85400	0.113302216	85364	0.033856253
85400	0.089249144	85368	0.058851951
85400	0.077667028	85372	0.061495135
85400	0.052275369	85376	0.004210973
85400	0.068555622	85380	0.029600869
85400	0.107056243	85384	0.016025235
85400	0.092253133	85388	0.01443733
85400	0.024628262	85392	0.039679475
85400	0.02887377	85396	0.044395612
85400	0.010166452	85400	0.045555677
85400	0.049248873	85404	0.054363442
85400	0.063862848	85408	0.080607242
85400	0.052597101	85412	0.079391801
85400	0.045363864	85416	0.065670043
85400	0.01853379	85420	0.103382015
85400	0.023787688	85424	0.138572111
85400	0.034948978	85428	0.116925105
85400	0.044355926	85432	0.074618038
85400	0.035874436	85436	0.046737259
85400	0.018449491	85440	0.045472501
85400	0.022503677	85444	0.062213068
85400	0.053966036	85448	0.048743365
85500	0.046701767	85452	0.011577625
85500	0.047434416	85456	0.076232129

PowerSpectrumData

85500	0.057851292	85460	0.077412857
85500	0.063294989	85464	0.079436431
85500	0.039832179	85468	0.085551612
85500	0.006544294	85472	0.081387574
85500	0.013905867	85476	0.045720863
85500	0.01606536	85480	0.024655714
85500	0.027555178	85484	0.059577909
85500	0.012909348	85488	0.081879349
85500	0.059707356	85492	0.074426927
85500	0.113945695	85496	0.063643944
85500	0.074312731	85500	0.104240491
85500	0.035026165	85504	0.136404735
85500	0.092176102	85508	0.118637981
85500	0.094708768	85512	0.056737725
85500	0.035458823	85516	0.021510234
85500	0.00202397	85520	0.045780198
85500	0.021356857	85524	0.088876514
85500	0.026250858	85528	0.142078425
85500	0.032911365	85532	0.158606141
85500	0.053023508	85536	0.116321469
85500	0.042309348	85540	0.032471489
85500	0.016902039	85544	0.110019428
85500	0.01000569	85548	0.16843139
85600	0.018239531	85552	0.147742306
85600	0.043506803	85556	0.112408678
85600	0.081688762	85560	0.123277947
85600	0.078806006	85564	0.114260918
85600	0.030282678	85568	0.09150117
85600	0.091120819	85572	0.115513787
85600	0.118187578	85576	0.143709389
85600	0.143887039	85580	0.124114959
85600	0.1197846	85584	0.076028679
85600	0.069485941	85588	0.045100594
85600	0.052738455	85592	0.067973735

PowerSpectrumData

85600	0.053767806	85596	0.111230358
85600	0.053785359	85600	0.089802823
85600	0.055833873	85604	0.076392942
85600	0.054676588	85608	0.087995046
85600	0.034911955	85612	0.093751638
85600	0.017273063	85616	0.155200294
85600	0.037897007	85620	0.161502423
85600	0.049891853	85624	0.107533058
85600	0.041484647	85628	0.08383907
85600	0.034945748	85632	0.09061303
85600	0.054557822	85636	0.062973209
85600	0.075603311	85640	0.047511261
85600	0.06523529	85644	0.086726068
85600	0.060193772	85648	0.130705361
85700	0.087762674	85652	0.146724939
85700	0.11532582	85656	0.134056754
85700	0.091218397	85660	0.130969333
85700	0.037887996	85664	0.124167418
85700	0.023938417	85668	0.106615094
85700	0.027417318	85672	0.142965568
85700	0.030728101	85676	0.150017339
85700	0.029174158	85680	0.095323267
85700	0.011496581	85684	0.079017365
85700	0.047146117	85688	0.106302119
85700	0.080481535	85692	0.104088118
85700	0.063869222	85696	0.105541272
85700	0.039916682	85700	0.131592736
85700	0.036551141	85704	0.15370459
85700	0.025054504	85708	0.119908807
85700	0.011084417	85712	0.099089448
85700	0.046996	85716	0.137841154
85700	0.089535541	85720	0.158553331
85700	0.11005647	85724	0.166456826
85700	0.087870452	85728	0.151562024

PowerSpectrumData

85700	0.038819704	85732	0.103531034
85700	0.021141368	85736	0.055458688
85700	0.024058427	85740	0.091814487
85700	0.021748176	85744	0.151634115
85700	0.051846539	85748	0.153880058
85800	0.095199677	85752	0.123638427
85800	0.095474272	85756	0.076952179
85800	0.053027667	85760	0.080303806
85800	0.033260705	85764	0.074115676
85800	0.0678098	85768	0.086087137
85800	0.049147082	85772	0.093796858
85800	0.004192342	85776	0.090675269
85800	0.034205063	85780	0.089334135
85800	0.068929592	85784	0.083923143
85800	0.058235521	85788	0.066728586
85800	0.004616571	85792	0.031066433
85800	0.031527568	85796	0.055511817
85800	0.011082884	85800	0.062279585
85800	0.02617825	85804	0.091684262
85800	0.061121878	85808	0.134598173
85800	0.088302688	85812	0.099466808
85800	0.083033825	85816	0.0466364
85800	0.03739087	85820	0.08122978
85800	0.030192054	85824	0.077815945
85800	0.037885602	85828	0.044138065
85800	0.016365561	85832	0.081254104
85800	0.028263277	85836	0.054828757
85800	0.026889942	85840	0.002933675
85800	0.021375541	85844	0.050935294
85800	0.058290036	85848	0.056137771
85900	0.049133647	85852	0.040111161
85900	0.007249977	85856	0.067988862
85900	0.034911831	85860	0.106316984
85900	0.033531454	85864	0.058413825

PowerSpectrumData

85900	0.052466512	85868	0.01967688
85900	0.072426534	85872	0.047466037
85900	0.082179147	85876	0.07689387
85900	0.068213543	85880	0.090598274
85900	0.062830673	85884	0.084178835
85900	0.063430212	85888	0.068555593
85900	0.07259319	85892	0.042072759
85900	0.050581126	85896	0.009261706
85900	0.012952874	85900	0.047086389
85900	0.06272184	85904	0.073497147
85900	0.087385153	85908	0.075379088
85900	0.053745062	85912	0.082176652
85900	0.009211515	85916	0.106585045
85900	0.016821912	85920	0.078900652
85900	0.00637482	85924	0.100536054
85900	0.009736299	85928	0.127203763
85900	0.027614724	85932	0.1146984
85900	0.015953223	85936	0.087877532
85900	0.014086311	85940	0.056519009
85900	0.010871159	85944	0.024323017
85900	0.015876341	85948	0.034428922
86000	0.033377637	85952	0.058406957
86000	0.044387103	85956	0.07369386
86000	0.041253366	85960	0.052823958
86000	0.021224143	85964	0.040964733
86000	0.008615316	85968	0.036864698
86000	0.007961696	85972	0.014794694
86000	0.012827663	85976	0.034642973
86000	0.019797506	85980	0.058064867
86000	0.043272325	85984	0.051847059
86000	0.03357933	85988	0.04242998
86000	0.025734666	85992	0.034964567
86000	0.051173673	85996	0.030672392
86000	0.046802186	86000	0.045743836

PowerSpectrumData

86000	0.029075869	86004	0.026473621
86000	0.020408956	86008	0.013196124
86000	0.025479374	86012	0.022377975
86000	0.034625176	86016	0.003876731
86000	0.036127378	86020	0.016396256
86000	0.0337778	86024	0.008327392
86000	0.00878427	86028	0.037168284
86000	0.033867375	86032	0.038875201
86000	0.048000471	86036	0.044311721
86000	0.031090403	86040	0.077485442
86000	0.029871369	86044	0.090440779
86000	0.087979897	86048	0.064394008
86100	0.113712267	86052	0.031647622
86100	0.090519476	86056	0.029195247
86100	0.060145456	86060	0.041701642
86100	0.032578224	86064	0.014489129
86100	0.008623858	86068	0.062308187
86100	0.030572141	86072	0.046390327
86100	0.073641924	86076	0.041356918
86100	0.088517976	86080	0.083106781
86100	0.055441968	86084	0.060420902
86100	0.047264846	86088	0.027016511
86100	0.057360507	86092	0.026882695
86100	0.062029641	86096	0.032498141
86100	0.030266678	86100	0.01086242
86100	0.07133848	86104	0.022512308
86100	0.068016088	86108	0.054568729
86100	0.073851574	86112	0.079490899
86100	0.066007939	86116	0.056365567
86100	0.049738635	86120	0.024089406
86100	0.051770418	86124	0.045119927
86100	0.049857834	86128	0.057032412
86100	0.021602677	86132	0.061069186
86100	0.008757995	86136	0.094946649

PowerSpectrumData

86100	0.041676278	86140	0.10376516
86100	0.064028798	86144	0.035862166
86100	0.030706124	86148	0.063237909
86200	0.024070798	86152	0.101764657
86200	0.050752151	86156	0.087243578
86200	0.053591812	86160	0.07397325
86200	0.059003829	86164	0.049303464
86200	0.066667293	86168	0.056269179
86200	0.058195776	86172	0.117000105
86200	0.041466425	86176	0.116411371
86200	0.039392067	86180	0.034683504
86200	0.041391668	86184	0.059697813
86200	0.059945567	86188	0.082175378
86200	0.071095783	86192	0.086050233
86200	0.045593501	86196	0.110181616
86200	0.053215088	86200	0.098986071
86200	0.07163149	86204	0.060963346
86200	0.050474002	86208	0.071934985
86200	0.027907425	86212	0.070498099
86200	0.023218217	86216	0.075318749
86200	0.030662017	86220	0.098685181
86200	0.043980592	86224	0.071553448
86200	0.045133766	86228	0.002237021
86200	0.029908397	86232	0.08772
86200	0.037985552	86236	0.13249903
86200	0.026754136	86240	0.129864289
86200	0.042604468	86244	0.140680495
86200	0.048118709	86248	0.131466266
86300	0.043957163	86252	0.069068519
86300	0.021881846	86256	0.042906555
86300	0.032965741	86260	0.051206473
86300	0.047190286	86264	0.065031556
86300	0.022137288	86268	0.099940786
86300	0.021594489	86272	0.099072

PowerSpectrumData

86300	0.009771917	86276	0.08998187
86300	0.048065573	86280	0.112072899
86300	0.057953275	86284	0.13837828
86300	0.040655083	86288	0.093228744
86300	0.029762792	86292	0.065494889
86300	0.07343779	86296	0.088083514
86300	0.072909512	86300	0.060608079
86300	0.030933072	86304	0.076121483
86300	0.035173165	86308	0.126318555
86300	0.079934245	86312	0.153117683
86300	0.089586363	86316	0.151342101
86300	0.055011722	86320	0.107989901
86300	0.052947737	86324	0.069365007
86300	0.064451408	86328	0.088746463
86300	0.052294854	86332	0.097136093
86300	0.018731724	86336	0.107545893
86300	0.011326331	86340	0.119226119
86300	0.007591304	86344	0.100250087
86300	0.007857491	86348	0.078383047
86400	0.022769558	86352	0.070088543
86400	0.045714522	86356	0.086209046
86400	0.032895689	86360	0.062888415
86400	0.038398022	86364	0.062741732
86400	0.049266026	86368	0.081561149
86400	0.043025208	86372	0.049970688
86400	0.045755412	86376	0.055776389
86400	0.035457175	86380	0.075481017
86400	0.017349859	86384	0.079644742
86400	0.037004305	86388	0.068450121
86400	0.047899379	86392	0.089003414
86400	0.039776962	86396	0.060800874
86400	0.01235626	86400	0.038831811
86400	0.022544626	86404	0.065485016
86400	0.025862086	86408	0.064225307

PowerSpectrumData

86400	0.026854199	86412	0.044843622
86400	0.073185976	86416	0.056830322
86400	0.077937468	86420	0.079282967
86400	0.043824195	86424	0.066936511
86400	0.049067545	86428	0.042948046
86400	0.072430266	86432	0.035191053
86400	0.05267272	86436	0.050436662
86400	0.025792557	86440	0.02587065
86400	0.01840605	86444	0.024232209
86400	0.029081903	86448	0.030002484
86500	0.030383582	86452	0.039300045
86500	0.026409021	86456	0.061227991
86500	0.005935178	86460	0.033369757
86500	0.005511733	86464	0.02478613
86500	0.037188755	86468	0.042230025
86500	0.080369086	86472	0.044021545
86500	0.071695147	86476	0.010642303
86500	0.04396274	86480	0.033505537
86500	0.032981323	86484	0.064086045
86500	0.022739605	86488	0.098729877
86500	0.04578229	86492	0.097378514
86500	0.051021696	86496	0.048448997
86500	0.040160492	86500	0.038271661
86500	0.049179736	86504	0.047621794
86500	0.009103622	86508	0.008678774
86500	0.061668201	86512	0.024805637
86500	0.062703497	86516	0.010253161
86500	0.031999938	86520	0.036754176
86500	0.04432568	86524	0.055195404
86500	0.068301095	86528	0.041986044
86500	0.0572233	86532	0.018230536
86500	0.019591273	86536	0.020089485
86500	0.03891049	86540	0.048533206
86500	0.074533746	86544	0.041627896

PowerSpectrumData

86500	0.068848211	86548	0.003305166
86600	0.075358614	86552	0.039263254
86600	0.101079662	86556	0.059438888
86600	0.081650222	86560	0.087333414
86600	0.039188621	86564	0.113296024
86600	0.036034919	86568	0.103383303
86600	0.03284472	86572	0.07396689
86600	0.04807701	86576	0.025758876
86600	0.032299584	86580	0.067520174
86600	0.066325272	86584	0.077789657
86600	0.063502586	86588	0.048000798
86600	0.08959401	86592	0.024574749
86600	0.102592901	86596	0.056577614
86600	0.093402086	86600	0.053962976
86600	0.075896314	86604	0.090212161
86600	0.039371553	86608	0.082743311
86600	0.035488749	86612	0.038353959
86600	0.037115613	86616	0.135594514
86600	0.018471445	86620	0.112272006
86600	0.007381854	86624	0.046479181
86600	0.01694891	86628	0.088556895
86600	0.019374804	86632	0.072342082
86600	0.023564984	86636	0.038855898
86600	0.051762836	86640	0.055236375
86600	0.017883731	86644	0.079839068
86600	0.041896983	86648	0.092525392
86700	0.048445185	86652	0.134346003
86700	0.025567066	86656	0.161609976
86700	0.035251364	86660	0.134205271
86700	0.028558086	86664	0.071354501
86700	0.030715346	86668	0.017325268
86700	0.039257608	86672	0.030003317
86700	0.055236444	86676	0.045584748
86700	0.062568317	86680	0.07751755

PowerSpectrumData

86700	0.05195538	86684	0.086238164
86700	0.026488664	86688	0.061957071
86700	0.013532754	86692	0.090579481
86700	0.029527711	86696	0.122803685
86700	0.036255118	86700	0.084874788
86700	0.042760552	86704	0.03667397
86700	0.061034916	86708	0.053431155
86700	0.05891726	86712	0.073147152
86700	0.025034859	86716	0.048786751
86700	0.024283281	86720	0.012908367
86700	0.020051952	86724	0.04353722
86700	0.023553395	86728	0.085423417
86700	0.047117846	86732	0.128631029
86700	0.068821602	86736	0.113196446
86700	0.081749917	86740	0.054465119
86700	0.080845734	86744	0.068384972
86700	0.066592009	86748	0.116674775
86800	0.03452873	86752	0.135055569
86800	0.010309528	86756	0.11676427
86800	0.030975993	86760	0.070206013
86800	0.031246382	86764	0.096127878
86800	0.04191163	86768	0.161615346
86800	0.052882293	86772	0.160984113
86800	0.042428703	86776	0.100089339
86800	0.035320831	86780	0.087072229
86800	0.041751748	86784	0.129403211
86800	0.031106582	86788	0.109450433
86800	0.047986101	86792	0.101216887
86800	0.059421796	86796	0.107901156
86800	0.070606431	86800	0.099204568
86800	0.061078397	86804	0.093528441
86800	0.054373806	86808	0.09975693
86800	0.060014289	86812	0.08355311
86800	0.037314356	86816	0.081033526

PowerSpectrumData

86800	0.002254314	86820	0.137106094
86800	0.021513308	86824	0.160618016
86800	0.015437283	86828	0.12312147
86800	0.016325821	86832	0.087970933
86800	0.029638804	86836	0.077085264
86800	0.012775045	86840	0.092659153
86800	0.017337918	86844	0.103859413
86800	0.051040115	86848	0.091223934
86900	0.066017899	86852	0.099774712
86900	0.067005982	86856	0.112760332
86900	0.064503605	86860	0.089559122
86900	0.046429017	86864	0.147402738
86900	0.064783504	86868	0.162519675
86900	0.074270916	86872	0.099414487
86900	0.057859172	86876	0.10302462
86900	0.0383355	86880	0.158538431
86900	0.038172264	86884	0.195078188
86900	0.029888863	86888	0.140277494
86900	0.041623705	86892	0.078511293
86900	0.039230767	86896	0.110230234
86900	0.03278696	86900	0.096650118
86900	0.049449882	86904	0.081591461
86900	0.070608963	86908	0.070733069
86900	0.066045039	86912	0.055861201
86900	0.086000022	86916	0.074435513
86900	0.096107149	86920	0.11152283
86900	0.069630492	86924	0.121896614
86900	0.051495648	86928	0.09779205
86900	0.036801506	86932	0.065503096
86900	0.005458237	86936	0.051480405
86900	0.051106668	86940	0.046029622
86900	0.077867844	86944	0.063683874
86900	0.069436494	86948	0.10059082
87000	0.066364308	86952	0.094347095

PowerSpectrumData

87000	0.051547762	86956	0.058766305
87000	0.041193187	86960	0.01977739
87000	0.0448598	86964	0.037659604
87000	0.036695823	86968	0.06548025
87000	0.063032654	86972	0.048573791
87000	0.10391965	86976	0.051652118
87000	0.066373752	86980	0.035075693
87000	0.013615138	86984	0.032754651
87000	0.039699415	86988	0.066084169
87000	0.038753751	86992	0.056965364
87000	0.026877857	86996	0.03223099
87000	0.075183903	87000	0.00555857
87000	0.095913005	87004	0.04312793
87000	0.065196735	87008	0.038293696
87000	0.030554307	87012	0.024509045
87000	0.025788753	87016	0.033559139
87000	0.053679338	87020	0.072468247
87000	0.05714954	87024	0.104907238
87000	0.041238291	87028	0.085966021
87000	0.056315705	87032	0.046885602
87000	0.092295406	87036	0.044008211
87000	0.096687261	87040	0.063944928
87000	0.044246779	87044	0.034297933
87000	0.041683925	87048	0.010342127
87100	0.049211627	87052	0.027154092
87100	0.022517501	87056	0.012670176
87100	0.010115823	87060	0.017233469
87100	0.014471906	87064	0.020817126
87100	0.036860391	87068	0.033144272
87100	0.06970996	87072	0.01672165
87100	0.059405487	87076	0.047718251
87100	0.032329604	87080	0.05902573
87100	0.065647306	87084	0.017848535
87100	0.059547387	87088	0.019661538

PowerSpectrumData

87100	0.035321627	87092	0.027879441
87100	0.016390803	87096	0.021579172
87100	0.075387565	87100	0.015687971
87100	0.138880045	87104	0.029128662
87100	0.116770178	87108	0.042781157
87100	0.058951679	87112	0.02576067
87100	0.041673575	87116	0.029285495
87100	0.04820052	87120	0.020776861
87100	0.02591122	87124	0.057320791
87100	0.051646177	87128	0.077626261
87100	0.092779992	87132	0.075810291
87100	0.096744014	87136	0.057242167
87100	0.080165126	87140	0.030738145
87100	0.06241534	87144	0.046180045
87100	0.041148549	87148	0.071027025
87200	0.026864316	87152	0.069595211
87200	0.030548614	87156	0.037857291
87200	0.013799004	87160	0.082483042
87200	0.031951418	87164	0.106333551
87200	0.049531875	87168	0.08997375
87200	0.025503499	87172	0.069533518
87200	0.025000643	87176	0.099045341
87200	0.0508757	87180	0.115955569
87200	0.048324298	87184	0.077045457
87200	0.029637004	87188	0.040495121
87200	0.004351301	87192	0.039178296
87200	0.043277603	87196	0.01419161
87200	0.064499443	87200	0.022068796
87200	0.057391681	87204	0.013544518
87200	0.0313036	87208	0.066552449
87200	0.036288438	87212	0.074687676
87200	0.051642062	87216	0.063763422
87200	0.040477869	87220	0.11074845
87200	0.009008113	87224	0.178876363

PowerSpectrumData

87200	0.017412156	87228	0.171823689
87200	0.034502937	87232	0.091835442
87200	0.040692143	87236	0.069144822
87200	0.023404511	87240	0.132772475
87200	0.027505086	87244	0.089323243
87200	0.060605224	87248	0.085394218
87300	0.048642942	87252	0.117722746
87300	0.041819156	87256	0.116539668
87300	0.044561122	87260	0.11434676
87300	0.055628218	87264	0.051829291
87300	0.047798265	87268	0.05313829
87300	0.009631073	87272	0.125866514
87300	0.044511351	87276	0.14920655
87300	0.0524067	87280	0.123664635
87300	0.040553361	87284	0.081478524
87300	0.009775688	87288	0.06479783
87300	0.043333974	87292	0.093927374
87300	0.065332693	87296	0.107386724
87300	0.053490425	87300	0.126955958
87300	0.01557539	87304	0.134731658
87300	0.017989079	87308	0.072235373
87300	0.041931307	87312	0.073322699
87300	0.05596851	87316	0.099304714
87300	0.063882479	87320	0.090381975
87300	0.059134731	87324	0.090605259
87300	0.017001032	87328	0.099290075
87300	0.041571067	87332	0.136382106
87300	0.034688619	87336	0.193182219
87300	0.013119543	87340	0.207135745
87300	0.051920564	87344	0.16084261
87300	0.051129449	87348	0.137179275
87400	0.030898238	87352	0.158404436
87400	0.036600734	87356	0.160402109
87400	0.017958875	87360	0.12761046

PowerSpectrumData

87400	0.02390477	87364	0.084549924
87400	0.028435736	87368	0.095677322
87400	0.018409659	87372	0.138419185
87400	0.006482895	87376	0.117542681
87400	0.035176639	87380	0.109063672
87400	0.044832908	87384	0.153061178
87400	0.027570022	87388	0.140963006
87400	0.011801731	87392	0.119924
87400	0.043205884	87396	0.099439043
87400	0.073603951	87400	0.110848341
87400	0.050161547	87404	0.138597578
87400	0.026830267	87408	0.13938974
87400	0.057795813	87412	0.1227709
87400	0.084734245	87416	0.127957086
87400	0.084427586	87420	0.146619131
87400	0.060510978	87424	0.14722193
87400	0.038515915	87428	0.152459805
87400	0.047888454	87432	0.175953726
87400	0.038426621	87436	0.188341918
87400	0.043491742	87440	0.163501304
87400	0.053780743	87444	0.109298177
87400	0.041777545	87448	0.10208552
87500	0.025206377	87452	0.140641205
87500	0.015323069	87456	0.127111649
87500	0.021911092	87460	0.082484236
87500	0.020243529	87464	0.085215579
87500	0.007344289	87468	0.148132181
87500	0.027870879	87472	0.184830089
87500	0.048869206	87476	0.167371749
87500	0.038077295	87480	0.119314325
87500	0.016700677	87484	0.060349739
87500	0.068944763	87488	0.084665175
87500	0.087366854	87492	0.120807104
87500	0.072101808	87496	0.115989482

PowerSpectrumData

87500	0.040369763	87500	0.103238039
87500	0.018019775	87504	0.087939727
87500	0.045000532	87508	0.074160409
87500	0.052018862	87512	0.078368787
87500	0.021209327	87516	0.106740459
87500	0.056925936	87520	0.116140618
87500	0.075709191	87524	0.124124548
87500	0.056697125	87528	0.134300499
87500	0.034303994	87532	0.131661465
87500	0.030372843	87536	0.151850501
87500	0.049928342	87540	0.114264003
87500	0.058767648	87544	0.068484209
87500	0.025071146	87548	0.118558099
87600	0.028448469	87552	0.119161923
87600	0.044228971	87556	0.085544714
87600	0.039732335	87560	0.057458728
87600	0.02388646	87564	0.039983712
87600	0.016903568	87568	0.066163178
87600	0.050537266	87572	0.098250719
87600	0.062896594	87576	0.104512576
87600	0.056716126	87580	0.096536365
87600	0.04830143	87584	0.074311603
87600	0.009936972	87588	0.067033936
87600	0.036706075	87592	0.062572392
87600	0.022427563	87596	0.031074749
87600	0.014247719	87600	0.0136745
87600	0.018822502	87604	0.069538619
87600	0.039034501	87608	0.109296198
87600	0.051797026	87612	0.081514016
87600	0.087504122	87616	0.039610866
87600	0.075293887	87620	0.077278499
87600	0.017482975	87624	0.122447716
87600	0.015990152	87628	0.105663188
87600	0.031430474	87632	0.085484753

PowerSpectrumData

87600	0.048113947	87636	0.048460333
87600	0.017738701	87640	0.069127309
87600	0.075268108	87644	0.076157157
87600	0.122046156	87648	0.069431015
87700	0.130376459	87652	0.119058364
87700	0.095826639	87656	0.149022963
87700	0.050980136	87660	0.119890297
87700	0.020549895	87664	0.113144706
87700	0.032473348	87668	0.1411845
87700	0.06529432	87672	0.108775021
87700	0.071070375	87676	0.057168498
87700	0.080771315	87680	0.093830982
87700	0.077370154	87684	0.075507574
87700	0.026879718	87688	0.022232593
87700	0.037537484	87692	0.074485572
87700	0.028601988	87696	0.084073792
87700	0.038834402	87700	0.077065633
87700	0.099006153	87704	0.075939606
87700	0.119357617	87708	0.092259252
87700	0.08159798	87712	0.131642083
87700	0.023650198	87716	0.118976372
87700	0.031762298	87720	0.094546325
87700	0.045724744	87724	0.011007802
87700	0.048055194	87728	0.071907722
87700	0.069878079	87732	0.102506616
87700	0.074684933	87736	0.109429821
87700	0.049981347	87740	0.130109896
87700	0.025148824	87744	0.097479591
87700	0.037161073	87748	0.083068509
87800	0.071300674	87752	0.116963267
87800	0.073428368	87756	0.120685043
87800	0.038221002	87760	0.08006132
87800	0.03398937	87764	0.038733346
87800	0.022112214	87768	0.010162134

PowerSpectrumData

87800	0.02316168	87772	0.031853895
87800	0.0325943	87776	0.016505012
87800	0.032228985	87780	0.06146713
87800	0.042580006	87784	0.093877643
87800	0.063944302	87788	0.114479146
87800	0.077566343	87792	0.110746805
87800	0.074842537	87796	0.063958403
87800	0.044984819	87800	0.038495247
87800	0.014280003	87804	0.039609411
87800	0.033652174	87808	0.021201042
87800	0.068789123	87812	0.050590152
87800	0.090381043	87816	0.01290764
87800	0.070764741	87820	0.042628024
87800	0.03877985	87824	0.090464579
87800	0.046954127	87828	0.126074185
87800	0.067012043	87832	0.121495781
87800	0.079778984	87836	0.075554832
87800	0.108768021	87840	0.01821037
87800	0.109725945	87844	0.044228007
87800	0.108981862	87848	0.10144394
87900	0.110135981	87852	0.104202809
87900	0.075654025	87856	0.06608713
87900	0.056537272	87860	0.042209471
87900	0.049061553	87864	0.049106719
87900	0.06151032	87868	0.063463696
87900	0.113780276	87872	0.076303848
87900	0.168157785	87876	0.050666193
87900	0.148192194	87880	0.040472947
87900	0.081687344	87884	0.076874421
87900	0.052193154	87888	0.03839692
87900	0.056502311	87892	0.042743995
87900	0.069806192	87896	0.092742819
87900	0.068364461	87900	0.098537632
87900	0.051689804	87904	0.080005339

PowerSpectrumData

87900	0.03641942	87908	0.073976124
87900	0.00845324	87912	0.060330513
87900	0.072251976	87916	0.021494523
87900	0.117297946	87920	0.087751243
87900	0.107268876	87924	0.088385292
87900	0.04658354	87928	0.062709638
87900	0.117397212	87932	0.076570301
87900	0.192945459	87936	0.079000449
87900	0.17377171	87940	0.064448192
87900	0.086212618	87944	0.054015029
87900	0.028208684	87948	0.038050388
88000	0.049759514	87952	0.037828962
88000	0.105105159	87956	0.020300091
88000	0.123559192	87960	0.012091476
88000	0.121009332	87964	0.023284083
88000	0.14623077	87968	0.04035325
88000	0.112086476	87972	0.052454354
88000	0.02558097	87976	0.073253868
88000	0.066711706	87980	0.057323636
88000	0.057556805	87984	0.008351097
88000	0.154057693	87988	0.036119596
88000	0.283192523	87992	0.018139492
88000	0.300805813	87996	0.013556973
88000	0.164926154	88000	0.039335879
88000	0.053430609	88004	0.058803544
88000	0.066889174	88008	0.07565735
88000	0.10194791	88012	0.065881388
88000	0.071132425	88016	0.035745881
88000	0.147080733	88020	0.04519197
88000	0.222137053	88024	0.053036791
88000	0.19021504	88028	0.034743265
88000	0.053798714	88032	0.010329623
88000	0.10271759	88036	0.042571359
88000	0.100437566	88040	0.056917757

PowerSpectrumData

88000	0.104441606	88044	0.053951822
88000	0.37794013	88048	0.091561102
88100	0.497209374	88052	0.104361541
88100	0.381005695	88056	0.091412658
88100	0.203099408	88060	0.046056732
88100	0.12826937	88064	0.094049501
88100	0.125818566	88068	0.11343418
88100	0.034901714	88072	0.06758541
88100	0.227560886	88076	0.020068306
88100	0.377343473	88080	0.020834954
88100	0.375503093	88084	0.013480401
88100	0.232607709	88088	0.077876051
88100	0.068460024	88092	0.105438485
88100	0.161795716	88096	0.096182077
88100	0.13748197	88100	0.073505937
88100	0.205503121	88104	0.05425053
88100	0.505389355	88108	0.042136606
88100	0.5749829	88112	0.031782525
88100	0.377887133	88116	0.070855822
88100	0.148750186	88120	0.094852228
88100	0.117350115	88124	0.086769804
88100	0.12827343	88128	0.101313111
88100	0.131761219	88132	0.103199767
88100	0.594952319	88136	0.069456153
88100	0.897616206	88140	0.040508421
88100	0.770469138	88144	0.042534412
88100	0.375692965	88148	0.041235257
88200	0.047124897	88152	0.032520333
88200	0.112379865	88156	0.061416933
88200	0.162645446	88160	0.067068519
88200	0.263824564	88164	0.047964462
88200	0.445937564	88168	0.050550563
88200	0.423697784	88172	0.021214573
88200	0.209852893	88176	0.065257256

PowerSpectrumData

88200	0.078492667	88180	0.101672857
88200	0.154529728	88184	0.096327742
88200	0.110086585	88188	0.061224739
88200	0.491694955	88192	0.06385758
88200	1.162501634	88196	0.134708505
88200	1.3339424	88200	0.113365626
88200	0.841211004	88204	0.082408638
88200	0.189352694	88208	0.083852749
88200	0.187408586	88212	0.03963133
88200	0.087808519	88216	0.066176188
88200	0.123353035	88220	0.068773341
88200	0.25167194	88224	0.012769507
88200	0.378694501	88228	0.021498288
88200	0.180012663	88232	0.086750544
88200	0.133942828	88236	0.089466077
88200	0.147238753	88240	0.066854096
88200	0.277399173	88244	0.063491934
88200	0.265860231	88248	0.075139098
88300	1.324932438	88252	0.068657362
88300	2.182648288	88256	0.066208428
88300	1.919132888	88260	0.089420595
88300	0.931143004	88264	0.087923327
88300	0.179057825	88268	0.075965108
88300	0.284922513	88272	0.051078812
88300	0.209864448	88276	0.047873546
88300	0.294931116	88280	0.071339855
88300	0.394927688	88284	0.072909141
88300	0.257590785	88288	0.063954918
88300	0.110417001	88292	0.073879652
88300	0.068092879	88296	0.080190337
88300	0.035047302	88300	0.066188804
88300	0.068998328	88304	0.046692956
88300	0.204770636	88308	0.042963347
88300	0.361369079	88312	0.044283959

PowerSpectrumData

88300	0.447609899	88316	0.03252543
88300	0.37987309	88320	0.078109442
88300	0.202390118	88324	0.100397992
88300	0.023629542	88328	0.093680719
88300	0.128007479	88332	0.066291555
88300	0.110387853	88336	0.078894402
88300	0.479011214	88340	0.094337906
88300	0.708788518	88344	0.069708913
88300	0.568949094	88348	0.053100361
88400	0.243234274	88352	0.077745397
88400	0.097891803	88356	0.101565747
88400	0.117291093	88360	0.087045206
88400	0.093293696	88364	0.073343334
88400	0.359109311	88368	0.087086191
88400	0.61734044	88372	0.105354666
88400	0.681398669	88376	0.102747406
88400	0.417021308	88380	0.075661708
88400	0.04669456	88384	0.067405323
88400	0.105364845	88388	0.105716201
88400	0.063242333	88392	0.135031093
88400	0.046449484	88396	0.133757203
88400	0.116185431	88400	0.113455404
88400	0.169526116	88404	0.066419401
88400	0.159567004	88408	0.071998242
88400	0.091793212	88412	0.093622199
88400	0.098814468	88416	0.064524575
88400	0.067853907	88420	0.060354429
88400	0.069890783	88424	0.100445868
88400	0.093426948	88428	0.111311478
88400	0.127511274	88432	0.086210981
88400	0.142029501	88436	0.062939827
88400	0.109648972	88440	0.025093681
88400	0.075803	88444	0.047725574
88400	0.044058787	88448	0.082275474

88500	0.028194219	88452	0.080753118
88500	0.130423796	88456	0.076338838
88500	0.157507573	88460	0.039015362
88500	0.094471056	88464	0.038078229
88500	0.062240077	88468	0.073726769
88500	0.06840608	88472	0.093612733
88500	0.05184815	88476	0.123237536
88500	0.020920465	88480	0.121771162
88500	0.007982057	88484	0.077193639
88500	0.008110593	88488	0.105597726
88500	0.033612883	88492	0.134805829
88500	0.059894293	88496	0.102100705
88500	0.058178557	88500	0.091268084
88500	0.043099033	88504	0.169287159
88500	0.057427733	88508	0.179802664
88500	0.066790388	88512	0.096177217
88500	0.059782746	88516	0.070554517
88500	0.06225441	88520	0.098839504
88500	0.058348189	88524	0.067369074
88500	0.025855907	88528	0.082836516
88500	0.013340391	88532	0.115635616
88500	0.014007385	88536	0.098124889
88500	0.019974959	88540	0.092980074
88500	0.020780082	88544	0.112276481
88500	0.018819404	88548	0.079027188
88600	0.021281046	88552	0.054053118
88600	0.044772605	88556	0.088413595
88600	0.057446741	88560	0.05241992
88600	0.053633645	88564	0.041697429
88600	0.023691167	88568	0.089435482
88600	0.026214042	88572	0.088810048
88600	0.044213593	88576	0.083262523
88600	0.033722026	88580	0.084091109
88600	0.020847374	88584	0.107677392

PowerSpectrumData

88600	0.013045682	88588	0.128039873
88600	0.043375971	88592	0.088550674
88600	0.047751695	88596	0.07992937
88600	0.016865359	88600	0.115228577
88600	0.027486471	88604	0.091110014
88600	0.062125851	88608	0.02625962
88600	0.059826278	88612	0.035729609
88600	0.029790614	88616	0.034245717
88600	0.046229681	88620	0.056537207
88600	0.036752586	88624	0.093292976
88600	0.010874401	88628	0.086930988
88600	0.008894397	88632	0.039750874
88600	0.00377947	88636	0.051764531
88600	0.01006253	88640	0.066743669
88600	0.034745743	88644	0.057112164
88600	0.064195105	88648	0.033385597
88700	0.062602667	88652	0.007134567
88700	0.037275575	88656	0.05053699
88700	0.046445595	88660	0.09049326
88700	0.064883345	88664	0.073531184
88700	0.070450136	88668	0.082730221
88700	0.060762457	88672	0.114254319
88700	0.034416094	88676	0.101406105
88700	0.029512876	88680	0.068305177
88700	0.071233175	88684	0.015659383
88700	0.111504829	88688	0.065273809
88700	0.106929248	88692	0.107345986
88700	0.071672606	88696	0.098188444
88700	0.062702027	88700	0.055122942
88700	0.045522884	88704	0.061587001
88700	0.014041852	88708	0.067175613
88700	0.015790629	88712	0.04729494
88700	0.048562182	88716	0.04717736
88700	0.056962577	88720	0.028208862

PowerSpectrumData

88700	0.037163158	88724	0.030210673
88700	0.032087923	88728	0.00712419
88700	0.036452897	88732	0.002317575
88700	0.061992687	88736	0.051686246
88700	0.060524115	88740	0.09027512
88700	0.045154578	88744	0.079923251
88700	0.028051083	88748	0.092907125
88800	0.019649708	88752	0.112880334
88800	0.045633409	88756	0.098371806
88800	0.045725152	88760	0.064383523
88800	0.037086607	88764	0.032525346
88800	0.028552728	88768	0.060180042
88800	0.031862935	88772	0.064736079
88800	0.064832188	88776	0.054295808
88800	0.078239013	88780	0.063865955
88800	0.051603238	88784	0.05972388
88800	0.059517952	88788	0.021085192
88800	0.05796941	88792	0.042552536
88800	0.042469503	88796	0.082061662
88800	0.033591048	88800	0.080617574
88800	0.022932405	88804	0.060704399
88800	0.024299197	88808	0.048017068
88800	0.033045773	88812	0.039265997
88800	0.02804318	88816	0.033191394
88800	0.033837325	88820	0.03491262
88800	0.049400522	88824	0.029775083
88800	0.051902815	88828	0.058676204
88800	0.052615575	88832	0.109116059
88800	0.020321639	88836	0.068966627
88800	0.051302981	88840	0.009555465
88800	0.069712522	88844	0.013506353
88800	0.038463182	88848	0.011959281
88900	0.030686686	88852	0.037400245
88900	0.021005646	88856	0.06116226

PowerSpectrumData

88900	0.044885208	88860	0.054032578
88900	0.079263315	88864	0.061750739
88900	0.067274043	88868	0.076131015
88900	0.046281813	88872	0.042847976
88900	0.036847021	88876	0.016789914
88900	0.054532586	88880	0.035727939
88900	0.055946348	88884	0.050083771
88900	0.053577955	88888	0.053618132
88900	0.026959235	88892	0.03952011
88900	0.016287411	88896	0.043552027
88900	0.042144242	88900	0.076976132
88900	0.071295312	88904	0.101111393
88900	0.067198023	88908	0.06909504
88900	0.037469858	88912	0.060567989
88900	0.039918359	88916	0.057438567
88900	0.013621208	88920	0.051482475
88900	0.071011949	88924	0.080709666
88900	0.106116808	88928	0.088434514
88900	0.07178793	88932	0.062570558
88900	0.040473209	88936	0.039469134
88900	0.044390163	88940	0.076503908
88900	0.041020681	88944	0.096265889
88900	0.040420182	88948	0.099476194
89000	0.019676225	88952	0.080629179
89000	0.01748809	88956	0.025704214
89000	0.045477536	88960	0.029699997
89000	0.071088187	88964	0.018820241
89000	0.066751643	88968	0.039131322
89000	0.035743844	88972	0.050142866
89000	0.060756225	88976	0.054399185
89000	0.050133007	88980	0.036707002
89000	0.015172871	88984	0.004027026
89000	0.078500074	88988	0.038252052
89000	0.109713175	88992	0.036074081

PowerSpectrumData

89000	0.087307519	88996	0.013238227
89000	0.059230933	89000	0.034843139
89000	0.045650504	89004	0.061405634
89000	0.066043533	89008	0.063544569
89000	0.087418703	89012	0.06654644
89000	0.064620486	89016	0.050021285
89000	0.023531924	89020	0.030368972
89000	0.027834711	89024	0.016600654
89000	0.02451591	89028	0.022193195
89000	0.015976408	89032	0.039375143
89000	0.050220122	89036	0.053640983
89000	0.054139619	89040	0.057107562
89000	0.013444039	89044	0.047971822
89000	0.06482513	89048	0.038037539
89100	0.065602966	89052	0.057661629
89100	0.005135764	89056	0.046856087
89100	0.040056653	89060	0.00903956
89100	0.030289639	89064	0.010576434
89100	0.012841359	89068	0.04155892
89100	0.02568488	89072	0.047468766
89100	0.073417388	89076	0.011794182
89100	0.066785717	89080	0.06198699
89100	0.038576698	89084	0.094832045
89100	0.036081368	89088	0.099183664
89100	0.040953713	89092	0.071419112
89100	0.03532452	89096	0.066154287
89100	0.053329106	89100	0.048951519
89100	0.041360148	89104	0.005323012
89100	0.045578308	89108	0.037479644
89100	0.067289235	89112	0.072209463
89100	0.056204153	89116	0.10074758
89100	0.048748621	89120	0.092413204
89100	0.042064658	89124	0.060944312
89100	0.049327791	89128	0.074968913

PowerSpectrumData

89100	0.032031676	89132	0.060453633
89100	0.005444052	89136	0.020308828
89100	0.0126375	89140	0.023182241
89100	0.048776805	89144	0.063276479
89100	0.044552617	89148	0.084710759
89200	0.009713282	89152	0.089167632
89200	0.022223345	89156	0.087433429
89200	0.030077501	89160	0.100217134
89200	0.057954589	89164	0.105967047
89200	0.075900985	89168	0.090176647
89200	0.074137308	89172	0.036415597
89200	0.03710348	89176	0.035591864
89200	0.054820186	89180	0.039114497
89200	0.065346736	89184	0.041549753
89200	0.045184712	89188	0.03655054
89200	0.043910462	89192	0.043457811
89200	0.068742913	89196	0.113359682
89200	0.081978818	89200	0.12435102
89200	0.075339143	89204	0.067571811
89200	0.057906182	89208	0.011790962
89200	0.028985036	89212	0.023262637
89200	0.033394437	89216	0.028608751
89200	0.022417557	89220	0.047136626
89200	0.025377831	89224	0.07499916
89200	0.024093621	89228	0.073311654
89200	0.019089695	89232	0.062983738
89200	0.006637943	89236	0.039410479
89200	0.021364751	89240	0.016730237
89200	0.019140991	89244	0.019081821
89200	0.031825228	89248	0.022327449
89300	0.031249107	89252	0.005928004
89300	0.034751898	89256	0.047526231
89300	0.023376366	89260	0.066573542
89300	0.012008466	89264	0.042486034

PowerSpectrumData

89300	0.034501376	89268	0.011735724
89300	0.069063564	89272	0.057414811
89300	0.09797531	89276	0.063095955
89300	0.10008234	89280	0.056315472
89300	0.082251652	89284	0.028015262
89300	0.072661213	89288	0.032192158
89300	0.058932521	89292	0.029444411
89300	0.036504614	89296	0.018261095
89300	0.003830174	89300	0.05021098
89300	0.057734505	89304	0.014793152
89300	0.085267187	89308	0.046065608
89300	0.066091379	89312	0.064816872
89300	0.028809644	89316	0.070318936
89300	0.043246633	89320	0.064107764
89300	0.071466166	89324	0.030639174
89300	0.06741783	89328	0.034913493
89300	0.038719962	89332	0.060746512
89300	0.017737833	89336	0.05930397
89300	0.058961909	89340	0.018256538
89300	0.08084358	89344	0.045321031
89300	0.082693383	89348	0.074902244
89400	0.062621475	89352	0.066581706
89400	0.030933868	89356	0.05429492
89400	0.012353861	89360	0.110760499
89400	0.022791697	89364	0.133547408
89400	0.040515253	89368	0.122520913
89400	0.075441865	89372	0.080315389
89400	0.095149364	89376	0.037108464
89400	0.065522829	89380	0.038597467
89400	0.037387039	89384	0.032865788
89400	0.043567856	89388	0.020261272
89400	0.033395467	89392	0.058326772
89400	0.037257298	89396	0.021870386
89400	0.059530736	89400	0.020570093

PowerSpectrumData

89400	0.049635924	89404	0.032262524
89400	0.075974007	89408	0.065023734
89400	0.124318001	89412	0.099043798
89400	0.076240758	89416	0.096733726
89400	0.010000917	89420	0.064602406
89400	0.026884178	89424	0.069261783
89400	0.025630539	89428	0.108022519
89400	0.06588504	89432	0.107333268
89400	0.057362446	89436	0.088292101
89400	0.052651172	89440	0.116101757
89400	0.063066669	89444	0.095745272
89400	0.055259043	89448	0.052744734
89500	0.049052	89452	0.038303999
89500	0.083568302	89456	0.009244599
89500	0.108364809	89460	0.074058735
89500	0.093668103	89464	0.101485472
89500	0.077959776	89468	0.118324613
89500	0.053181077	89472	0.144318969
89500	0.043380609	89476	0.148029401
89500	0.084199273	89480	0.114417802
89500	0.06640151	89484	0.054292792
89500	0.016927083	89488	0.028701254
89500	0.0102015	89492	0.093297967
89500	0.00537632	89496	0.115376541
89500	0.020691759	89500	0.101500758
89500	0.036616006	89504	0.1552506
89500	0.029045041	89508	0.185805664
89500	0.02923554	89512	0.155016445
89500	0.040284751	89516	0.107124302
89500	0.029370331	89520	0.041987067
89500	0.02653923	89524	0.025058957
89500	0.047939004	89528	0.061600265
89500	0.032371867	89532	0.065240398
89500	0.041712079	89536	0.023283761

PowerSpectrumData

89500	0.026462723	89540	0.062046172
89500	0.052814761	89544	0.120060147
89500	0.083802479	89548	0.093496747
89600	0.073382413	89552	0.071514631
89600	0.022058248	89556	0.099586665
89600	0.037850121	89560	0.078429883
89600	0.039461836	89564	0.052634121
89600	0.002902058	89568	0.037357877
89600	0.017059891	89572	0.016170014
89600	0.017710256	89576	0.028675568
89600	0.029805726	89580	0.063295469
89600	0.027328257	89584	0.059403788
89600	0.00317205	89588	0.063249558
89600	0.033515153	89592	0.071914241
89600	0.058324444	89596	0.063383624
89600	0.063251064	89600	0.038656057
89600	0.055582317	89604	0.028209273
89600	0.087334825	89608	0.060924282
89600	0.093173061	89612	0.071240676
89600	0.026023299	89616	0.038880939
89600	0.057619731	89620	0.043934644
89600	0.058016227	89624	0.052102441
89600	0.02245659	89628	0.0605446
89600	0.014705684	89632	0.07080465
89600	0.029194293	89636	0.048490398
89600	0.027144604	89640	0.015419184
89600	0.060542101	89644	0.039591003
89600	0.069607231	89648	0.039975072
89700	0.07824965	89652	0.051519241
89700	0.071960028	89656	0.048458122
89700	0.017787683	89660	0.04210589
89700	0.051744104	89664	0.06099317
89700	0.010646979	89668	0.080194594
89700	0.041872914	89672	0.052832427

PowerSpectrumData

89700	0.04126658	89676	0.069891052
89700	0.015184422	89680	0.114312374
89700	0.010768859	89684	0.077288743
89700	0.004674504	89688	0.079562291
89700	0.018881923	89692	0.080816477
89700	0.042954569	89696	0.045987221
89700	0.048574355	89700	0.047929243
89700	0.058692069	89704	0.080482023
89700	0.085720771	89708	0.060655184
89700	0.096178264	89712	0.041271091
89700	0.06840127	89716	0.038322341
89700	0.013221268	89720	0.050061546
89700	0.03317032	89724	0.097809803
89700	0.059697799	89728	0.10372894
89700	0.080961203	89732	0.116914023
89700	0.095983363	89736	0.13616735
89700	0.059385911	89740	0.108846965
89700	0.007856234	89744	0.048841579
89700	0.033335498	89748	0.043482749
89800	0.044468859	89752	0.04132999
89800	0.051673225	89756	0.036916339
89800	0.071498747	89760	0.022589082
89800	0.110772809	89764	0.033891738
89800	0.110618901	89768	0.039998831
89800	0.03867353	89772	0.04362996
89800	0.043350923	89776	0.053125583
89800	0.062795458	89780	0.014603355
89800	0.04070628	89784	0.100247271
89800	0.055616387	89788	0.166924394
89800	0.076638411	89792	0.164587895
89800	0.055211345	89796	0.126053259
89800	0.043107808	89800	0.099638513
89800	0.079255282	89804	0.08058524
89800	0.052970318	89808	0.055413169

PowerSpectrumData

89800	0.00943916	89812	0.064922715
89800	0.037093061	89816	0.070621987
89800	0.01003645	89820	0.053718079
89800	0.045215784	89824	0.070462367
89800	0.042234464	89828	0.070141985
89800	0.020480744	89832	0.038308481
89800	0.044999863	89836	0.046826215
89800	0.047576075	89840	0.08159945
89800	0.05271885	89844	0.099565361
89800	0.054915046	89848	0.092016941
89900	0.065328481	89852	0.080487138
89900	0.076747478	89856	0.047315691
89900	0.058937629	89860	0.019245046
89900	0.049890412	89864	0.057577378
89900	0.039821451	89868	0.057267433
89900	0.021629126	89872	0.043857923
89900	0.013109551	89876	0.045821114
89900	0.028100036	89880	0.058297046
89900	0.015958029	89884	0.048372192
89900	0.019152212	89888	0.071065464
89900	0.057104069	89892	0.086629458
89900	0.046759466	89896	0.074254145
89900	0.026512525	89900	0.037997656
89900	0.036124777	89904	0.049729606
89900	0.055552402	89908	0.030644857
89900	0.038173817	89912	0.023541694
89900	0.007568089	89916	0.007728841
89900	0.022818682	89920	0.049759405
89900	0.034264984	89924	0.065921151
89900	0.076396078	89928	0.042116932
89900	0.076621036	89932	0.046473837
89900	0.064619002	89936	0.086347041
89900	0.080204853	89940	0.07542668
89900	0.073154981	89944	0.043023065

PowerSpectrumData

89900	0.012764766	89948	0.057413508
90000	0.054235028	89952	0.072843883
90000	0.082612583	89956	0.051975261
90000	0.082285114	89960	0.054998425
90000	0.044054141	89964	0.068914473
90000	0.022333516	89968	0.075346092
90000	0.040695122	89972	0.064635933
90000	0.034786608	89976	0.008359292
90000	0.051048126	89980	0.090914386
90000	0.05343838	89984	0.126429164
90000	0.047829355	89988	0.077738318
90000	0.064416818	89992	0.042789739
90000	0.071797971	89996	0.017368719
90000	0.093164053	90000	0.056852387
90000	0.110173452	90004	0.093183677
90000	0.086798391	90008	0.100527999
90000	0.044691067	90012	0.075442287
90000	0.018846968	90016	0.042828731
90000	0.020482392	90020	0.034088032
90000	0.016216276	90024	0.035494784
90000	0.029777844	90028	0.04692909
90000	0.033003376	90032	0.02411716
90000	0.028906772	90036	0.019590163
90000	0.024327552	90040	0.02519456
90000	0.029746707	90044	0.011356826
90000	0.033226246	90048	0.076222001
90100	0.011185834	90052	0.077333018
90100	0.031819825	90056	0.035758785
90100	0.050419392	90060	0.035391895
90100	0.039511127	90064	0.054430719
90100	0.032689764	90068	0.056116322
90100	0.044216398	90072	0.057011363
90100	0.070504713	90076	0.06394642
90100	0.063581712	90080	0.047774538

PowerSpectrumData

90100	0.058268095	90084	0.012037198
90100	0.043042779	90088	0.048250447
90100	0.0260583	90092	0.053108055
90100	0.030173482	90096	0.029048137
90100	0.079038582	90100	0.012712961
90100	0.089849971	90104	0.019397356
90100	0.067234047	90108	0.03407582
90100	0.021213478	90112	0.080610364
90100	0.053857071	90116	0.117881056
90100	0.089305286	90120	0.116322051
90100	0.081181694	90124	0.098127035
90100	0.056634868	90128	0.078192068
90100	0.052105825	90132	0.047231642
90100	0.04558131	90136	0.017262995
90100	0.057246256	90140	0.051541683
90100	0.080937665	90144	0.062465682
90100	0.059719092	90148	0.04248487
90200	0.049410235	90152	0.039461338
90200	0.077708071	90156	0.03230539
90200	0.04372287	90160	0.049486178
90200	0.013166536	90164	0.105930041
90200	0.022206581	90168	0.104957791
90200	0.036537997	90172	0.08624592
90200	0.025837619	90176	0.070681752
90200	0.057404715	90180	0.067919755
90200	0.1075244	90184	0.09234243
90200	0.067490633	90188	0.093339942
90200	0.042289048	90192	0.066225803
90200	0.040178475	90196	0.021726066
90200	0.043090768	90200	0.062447805
90200	0.020393467	90204	0.098687764
90200	0.080529491	90208	0.089925132
90200	0.134903108	90212	0.061858977
90200	0.115161347	90216	0.0294481

PowerSpectrumData

90200	0.073334893	90220	0.03848107
90200	0.06054181	90224	0.105195315
90200	0.052883454	90228	0.138171439
90200	0.041828684	90232	0.102736383
90200	0.021740245	90236	0.034702483
90200	0.054213291	90240	0.019778045
90200	0.085066189	90244	0.058284433
90200	0.057376397	90248	0.075030112
90300	0.035892717	90252	0.06194762
90300	0.012340444	90256	0.04984788
90300	0.027258124	90260	0.018921126
90300	0.021109505	90264	0.019714606
90300	0.065012879	90268	0.039110168
90300	0.069233858	90272	0.055928245
90300	0.054057469	90276	0.082259678
90300	0.028231989	90280	0.091838934
90300	0.014135318	90284	0.056784695
90300	0.055569875	90288	0.031798187
90300	0.072626237	90292	0.076382945
90300	0.063465013	90296	0.081865816
90300	0.045471243	90300	0.036829144
90300	0.033714907	90304	0.03694258
90300	0.034043162	90308	0.032270098
90300	0.037064216	90312	0.02415071
90300	0.043584143	90316	0.013341251
90300	0.069022492	90320	0.007501714
90300	0.06683352	90324	0.001376425
90300	0.025998334	90328	0.007930423
90300	0.021217515	90332	0.028944476
90300	0.061913823	90336	0.072682808
90300	0.07010041	90340	0.078351783
90300	0.040793562	90344	0.060380411
90300	0.019679686	90348	0.028398539
90400	0.017790504	90352	0.031727348

PowerSpectrumData

90400	0.035669124	90356	0.048855422
90400	0.047490292	90360	0.057502017
90400	0.047342528	90364	0.058440779
90400	0.032289616	90368	0.023685938
90400	0.010294793	90372	0.016167771
90400	0.03538634	90376	0.021986378
90400	0.055225086	90380	0.026687052
90400	0.049361046	90384	0.072158546
90400	0.028777802	90388	0.057423902
90400	0.024917417	90392	0.02928994
90400	0.04086304	90396	0.008305908
90400	0.045903653	90400	0.020384485
90400	0.070442839	90404	0.006457448
90400	0.099263183	90408	0.040724313
90400	0.086239423	90412	0.045500292
90400	0.05193827	90416	0.075725548
90400	0.0046396	90420	0.082433893
90400	0.080597711	90424	0.048554284
90400	0.117936135	90428	0.023447981
90400	0.085584339	90432	0.04021448
90400	0.039778373	90436	0.057525096
90400	0.009009359	90440	0.046001816
90400	0.031684536	90444	0.034958877
90400	0.027564882	90448	0.043832817
90500	0.013986771	90452	0.049649694
90500	0.009451823	90456	0.057186742
90500	0.011393066	90460	0.059229071
90500	0.060480899	90464	0.052791296
90500	0.088572379	90468	0.05976747
90500	0.050590617	90472	0.072560535
90500	0.036097492	90476	0.056981968
90500	0.042106509	90480	0.047885922
90500	0.024825658	90484	0.065316512
90500	0.031274882	90488	0.089992187

PowerSpectrumData

90500	0.024243194	90492	0.067648587
90500	0.002926307	90496	0.019529121
90500	0.014520684	90500	0.054550172
90500	0.004114921	90504	0.053862317
90500	0.032468408	90508	0.045272886
90500	0.076345154	90512	0.091133581
90500	0.077413351	90516	0.103888291
90500	0.036299975	90520	0.033089273
90500	0.042728378	90524	0.066959881
90500	0.058362304	90528	0.097916869
90500	0.032841403	90532	0.091270362
90500	0.009942026	90536	0.065179163
90500	0.014732427	90540	0.02115783
90500	0.054106225	90544	0.027233016
90500	0.077068988	90548	0.02952431
90600	0.049873797	90552	0.039511502
90600	0.031713866	90556	0.080650563
90600	0.047457645	90560	0.109832814
90600	0.085200227	90564	0.09807483
90600	0.103956605	90568	0.048352165
90600	0.077790392	90572	0.041524731
90600	0.026853706	90576	0.057447218
90600	0.026557489	90580	0.030042891
90600	0.050694369	90584	0.008870566
90600	0.055066317	90588	0.00362499
90600	0.057627185	90592	0.009531174
90600	0.052401672	90596	0.017009108
90600	0.025854912	90600	0.027205335
90600	0.020201502	90604	0.038366914
90600	0.032486823	90608	0.056942914
90600	0.003663147	90612	0.04490334
90600	0.039852785	90616	0.014074468
90600	0.034214088	90620	0.04674997
90600	0.011562831	90624	0.057006404

PowerSpectrumData

90600	0.045536028	90628	0.061144063
90600	0.052571108	90632	0.075837386
90600	0.071610208	90636	0.067819448
90600	0.061831022	90640	0.039684135
90600	0.037219696	90644	0.033996708
90600	0.059944123	90648	0.072778224
90700	0.099772689	90652	0.105999236
90700	0.096321906	90656	0.069393107
90700	0.048797316	90660	0.018856106
90700	0.006944025	90664	0.047717706
90700	0.019754718	90668	0.069087917
90700	0.043403805	90672	0.107919674
90700	0.053220032	90676	0.137698516
90700	0.040916479	90680	0.117317883
90700	0.023665269	90684	0.096635682
90700	0.013204705	90688	0.089101675
90700	0.02508417	90692	0.065564163
90700	0.033783785	90696	0.030606487
90700	0.020855348	90700	0.054210348
90700	0.010078924	90704	0.063549836
90700	0.031512798	90708	0.024066187
90700	0.028703364	90712	0.042213222
90700	0.013478943	90716	0.08728345
90700	0.014430029	90720	0.104927749
90700	0.026614442	90724	0.087611719
90700	0.022198812	90728	0.035809968
90700	0.013886301	90732	0.040902938
90700	0.042851119	90736	0.055661687
90700	0.055784527	90740	0.068941125
90700	0.020442649	90744	0.083039748
90700	0.043024873	90748	0.101015998
90800	0.057759989	90752	0.109981578
90800	0.054856449	90756	0.107443259
90800	0.053272699	90760	0.076689154

PowerSpectrumData

90800	0.012079025	90764	0.050969706
90800	0.048385973	90768	0.074907446
90800	0.079968901	90772	0.090093883
90800	0.069327667	90776	0.067797831
90800	0.040982268	90780	0.020735099
90800	0.052899239	90784	0.030378466
90800	0.067109337	90788	0.055948753
90800	0.038478487	90792	0.068301044
90800	0.019426449	90796	0.074603973
90800	0.06220807	90800	0.075465752
90800	0.054523636	90804	0.050146624
90800	0.048276623	90808	0.039756484
90800	0.079426107	90812	0.063046027
90800	0.056159806	90816	0.091409631
90800	0.058494257	90820	0.094499206
90800	0.052560165	90824	0.051256804
90800	0.062822517	90828	0.020828036
90800	0.068902744	90832	0.030130446
90800	0.042885647	90836	0.017599332
90800	0.020764908	90840	0.03192373
90800	0.035296733	90844	0.029689687
90800	0.054053282	90848	0.079216123
90900	0.06450947	90852	0.106030821
90900	0.037299582	90856	0.056354489
90900	0.02343362	90860	0.017310242
90900	0.029687919	90864	0.058545029
90900	0.041360992	90868	0.071222268
90900	0.055453194	90872	0.059376067
90900	0.094769261	90876	0.045850458
90900	0.091051195	90880	0.034665245
90900	0.046567184	90884	0.089029076
90900	0.01587782	90888	0.136270188
90900	0.043224078	90892	0.087672001
90900	0.041982621	90896	0.014903794

PowerSpectrumData

90900	0.052541076	90900	0.092652641
90900	0.058721507	90904	0.110880479
90900	0.05791054	90908	0.055957502
90900	0.048139591	90912	0.000353662
90900	0.009557256	90916	0.032358872
90900	0.050227707	90920	0.09998888
90900	0.053056891	90924	0.121028119
90900	0.013734295	90928	0.090320085
90900	0.03667469	90932	0.071715076
90900	0.015272481	90936	0.081169099
90900	0.027174501	90940	0.115863069
90900	0.007924494	90944	0.138026356
90900	0.030798601	90948	0.1004652
91000	0.032192907	90952	0.088734036
91000	0.022486134	90956	0.086399377
91000	0.018790739	90960	0.037506816
91000	0.012320669	90964	0.039016501
91000	0.003937271	90968	0.046001325
91000	0.013218272	90972	0.010958086
91000	0.004663217	90976	0.025687215
91000	0.033580152	90980	0.074429168
91000	0.03938692	90984	0.116360374
91000	0.026894115	90988	0.134231581
91000	0.0310953	90992	0.1432399
91000	0.061167659	90996	0.097194665
91000	0.066633205	91000	0.037371152
91000	0.011109174	91004	0.097935888
91000	0.064218635	91008	0.12518253
91000	0.084584273	91012	0.082647704
91000	0.056994566	91016	0.080616766
91000	0.023216609	91020	0.039565599
91000	0.042179145	91024	0.047491201
91000	0.059460406	91028	0.040111976
91000	0.088082459	91032	0.061166713

PowerSpectrumData

91000	0.104014995	91036	0.100809542
91000	0.063771688	91040	0.069997666
91000	0.009367222	91044	0.032998934
91000	0.017415219	91048	0.016163698
91100	0.021728318	91052	0.022591843
91100	0.013870417	91056	0.05650954
91100	0.021924563	91060	0.074228941
91100	0.049835424	91064	0.071345297
91100	0.034423334	91068	0.085334519
91100	0.033933986	91072	0.046381487
91100	0.06299145	91076	0.022796452
91100	0.067253284	91080	0.055901219
91100	0.049887367	91084	0.077543351
91100	0.027012007	91088	0.055865978
91100	0.014043232	91092	0.040063591
91100	0.037917529	91096	0.032259319
91100	0.025272539	91100	0.032693937
91100	0.048255632	91104	0.104195387
91100	0.086915803	91108	0.10486917
91100	0.092157112	91112	0.061222665
91100	0.07936218	91116	0.042369644
91100	0.047998856	91120	0.065382832
91100	0.056452398	91124	0.036246205
91100	0.069877257	91128	0.019321577
91100	0.069340531	91132	0.037573318
91100	0.048955662	91136	0.060398113
91100	0.034175293	91140	0.069353875
91100	0.053478758	91144	0.053938657
91100	0.091515591	91148	0.055552559
91200	0.130362373	91152	0.088397494
91200	0.137298558	91156	0.076385979
91200	0.069639202	91160	0.046815807
91200	0.034717388	91164	0.044365745
91200	0.073280571	91168	0.051531319

PowerSpectrumData

91200	0.073715077	91172	0.049548846
91200	0.060421808	91176	0.004845223
91200	0.048085076	91180	0.067413748
91200	0.048965605	91184	0.085595537
91200	0.037505859	91188	0.065738837
91200	0.059839134	91192	0.0383222
91200	0.057647751	91196	0.056545996
91200	0.026178743	91200	0.051909632
91200	0.034021072	91204	0.039986815
91200	0.056482651	91208	0.043311324
91200	0.058715341	91212	0.083067287
91200	0.067269444	91216	0.097004522
91200	0.071283852	91220	0.06638185
91200	0.032932716	91224	0.035119239
91200	0.037110607	91228	0.08635706
91200	0.046911438	91232	0.082529186
91200	0.018212433	91236	0.068251538
91200	0.067880537	91240	0.076384713
91200	0.06869308	91244	0.079650512
91200	0.047470385	91248	0.064031883
91300	0.047655754	91252	0.050107716
91300	0.053554133	91256	0.024560401
91300	0.030832169	91260	0.035462475
91300	0.012317408	91264	0.06470758
91300	0.024806386	91268	0.056619621
91300	0.041235704	91272	0.053478336
91300	0.060929709	91276	0.046273224
91300	0.055419187	91280	0.089404421
91300	0.027459448	91284	0.10627164
91300	0.050044881	91288	0.087057459
91300	0.090502494	91292	0.07260128
91300	0.082424936	91296	0.056139241
91300	0.038912753	91300	0.008068141
91300	0.024710947	91304	0.083997424

91300	0.066809138	91308	0.141777811
91300	0.078372177	91312	0.132560498
91300	0.055363089	91316	0.074349431
91300	0.037162445	91320	0.029718018
91300	0.03700961	91324	0.040590134
91300	0.035167836	91328	0.061033752
91300	0.017705554	91332	0.068212074
91300	0.029825333	91336	0.058814225
91300	0.013895543	91340	0.028137123
91300	0.026292033	91344	0.03695397
91300	0.023255996	91348	0.092302878
91400	0.047669815	91352	0.11721014
91400	0.066165871	91356	0.121086872
91400	0.061912986	91360	0.105048137
91400	0.036490481	91364	0.107958753
91400	0.017772583	91368	0.108035478
91400	0.003953217	91372	0.082247454
91400	0.029033494	91376	0.09037456
91400	0.04181534	91380	0.113969771
91400	0.042336451	91384	0.07341968
91400	0.034086952	91388	0.039636339
91400	0.027503789	91392	0.084608735
91400	0.026730764	91396	0.082029685
91400	0.004576043	91400	0.031131964
91400	0.043727865	91404	0.011985959
91400	0.041790387	91408	0.026223001
91400	0.032251588	91412	0.037815495
91400	0.057314701	91416	0.069348535
91400	0.036942878	91420	0.104642277
91400	0.03994635	91424	0.085943306
91400	0.058203576	91428	0.038742168
91400	0.025981748	91432	0.067369656
91400	0.084005238	91436	0.052383191
91400	0.073861906	91440	0.039054143

PowerSpectrumData

91400	0.072045761	91444	0.044813158
91400	0.10323897	91448	0.084438761
91500	0.079247999	91452	0.052845091
91500	0.057925681	91456	0.043442495
91500	0.068897447	91460	0.082399849
91500	0.062027997	91464	0.084927022
91500	0.055684785	91468	0.035675941
91500	0.067901965	91472	0.047306949
91500	0.072977673	91476	0.06033924
91500	0.087170127	91480	0.059385999
91500	0.065843735	91484	0.056463945
91500	0.068110618	91488	0.077176403
91500	0.053098574	91492	0.077458775
91500	0.02986499	91496	0.037305803
91500	0.038542428	91500	0.015855476
91500	0.064570602	91504	0.047385565
91500	0.082788174	91508	0.093189323
91500	0.064220723	91512	0.058605456
91500	0.028790119	91516	0.013003111
91500	0.026378253	91520	0.058300622
91500	0.075213044	91524	0.067910383
91500	0.098308374	91528	0.03686209
91500	0.069851187	91532	0.040574265
91500	0.023318153	91536	0.026844886
91500	0.024098914	91540	0.084186802
91500	0.065107663	91544	0.092973103
91500	0.080765232	91548	0.057727488
91600	0.060030609	91552	0.030635551
91600	0.090071219	91556	0.004595486
91600	0.11694328	91560	0.016909593
91600	0.077742443	91564	0.025797943
91600	0.061729086	91568	0.049406812
91600	0.039408376	91572	0.032788983
91600	0.046906778	91576	0.016375154

PowerSpectrumData

91600	0.074065967	91580	0.025847972
91600	0.069773538	91584	0.011511475
91600	0.038530914	91588	0.027846536
91600	0.015364698	91592	0.017574776
91600	0.045364195	91596	0.017844128
91600	0.063348554	91600	0.030709252
91600	0.05413613	91604	0.03700277
91600	0.028524368	91608	0.024247014
91600	0.027842212	91612	0.015464953
91600	0.011517565	91616	0.014246966
91600	0.0027107	91620	0.040465831
91600	0.01793308	91624	0.023711014
91600	0.037655427	91628	0.039709001
91600	0.044423665	91632	0.021767166
91600	0.053647138	91636	0.024944497
91600	0.062328523	91640	0.028314873
91600	0.035918136	91644	0.017947099
91600	0.032852735	91648	0.037646591
91700	0.104584506	91652	0.047616482
91700	0.126885119	91656	0.063360014
91700	0.083712846	91660	0.070003582
91700	0.040943996	91664	0.045337205
91700	0.036751149	91668	0.011983132
91700	0.070420407	91672	0.044767869
91700	0.095681418	91676	0.060136754
91700	0.073534982	91680	0.053904056
91700	0.037321304	91684	0.043072774
91700	0.036873986	91688	0.018723285
91700	0.054894295	91692	0.040252897
91700	0.014468975	91696	0.0466436
91700	0.054113079	91700	0.024330098
91700	0.055317512	91704	0.021729562
91700	0.04053396	91708	0.043376065
91700	0.057116791	91712	0.082134699

PowerSpectrumData

91700	0.070946881	91716	0.088933419
91700	0.057548939	91720	0.052581039
91700	0.042707234	91724	0.059151687
91700	0.056842429	91728	0.074118638
91700	0.077389923	91732	0.055596254
91700	0.056171313	91736	0.057138263
91700	0.036992489	91740	0.089818168
91700	0.057622921	91744	0.104034851
91700	0.037840931	91748	0.06210724
91800	0.030388119	91752	0.060368027
91800	0.061292187	91756	0.049541221
91800	0.088172492	91760	0.025787325
91800	0.08490133	91764	0.059152324
91800	0.058215577	91768	0.066038243
91800	0.034404366	91772	0.069659072
91800	0.042296328	91776	0.05437815
91800	0.05889668	91780	0.047717516
91800	0.06252592	91784	0.071507231
91800	0.021008254	91788	0.06731099
91800	0.067130866	91792	0.05700492
91800	0.062048843	91796	0.055304885
91800	0.055429297	91800	0.058714064
91800	0.081237376	91804	0.055775508
91800	0.064357686	91808	0.03004413
91800	0.051344552	91812	0.034910045
91800	0.033748238	91816	0.032769014
91800	0.026054189	91820	0.065530039
91800	0.076967946	91824	0.099626501
91800	0.049615705	91828	0.110048037
91800	0.026084666	91832	0.081780367
91800	0.044649169	91836	0.053632022
91800	0.015067171	91840	0.05655739
91800	0.010081072	91844	0.078454912
91800	0.004994492	91848	0.067745234

PowerSpectrumData

91900	0.05661753	91852	0.037632348
91900	0.080183701	91856	0.071964547
91900	0.058281563	91860	0.088695095
91900	0.049133076	91864	0.091254718
91900	0.026233352	91868	0.072658171
91900	0.041354491	91872	0.024423751
91900	0.028941471	91876	0.006706931
91900	0.02840516	91880	0.029411642
91900	0.028443012	91884	0.03838928
91900	0.038843391	91888	0.035521927
91900	0.070457623	91892	0.055404016
91900	0.079557998	91896	0.060233917
91900	0.091857233	91900	0.034679491
91900	0.073657386	91904	0.03492702
91900	0.034688288	91908	0.06112027
91900	0.024292416	91912	0.093853691
91900	0.010546896	91916	0.120173267
91900	0.003371064	91920	0.140012563
91900	0.024997862	91924	0.105548934
91900	0.055510442	91928	0.056929424
91900	0.05315696	91932	0.040699211
91900	0.038185182	91936	0.037670718
91900	0.051760795	91940	0.036365709
91900	0.057891513	91944	0.044233457
91900	0.048790025	91948	0.026249396
92000	0.02919743	91952	0.0553094
92000	0.022763237	91956	0.059749258
92000	0.061474922	91960	0.044382905
92000	0.058634836	91964	0.061546598
92000	0.052595478	91968	0.096507916
92000	0.086653905	91972	0.060121474
92000	0.088005189	91976	0.023929888
92000	0.090770322	91980	0.089274232
92000	0.117690252	91984	0.110646288

PowerSpectrumData

92000	0.088410583	91988	0.066477114
92000	0.069387621	91992	0.034310844
92000	0.070094146	91996	0.056885565
92000	0.047236004	92000	0.10733735
92000	0.063977292	92004	0.062723877
92000	0.037172056	92008	0.037804151
92000	0.022841534	92012	0.055324978
92000	0.042013795	92016	0.091085654
92000	0.045495486	92020	0.090095484
92000	0.067906927	92024	0.055637935
92000	0.066755863	92028	0.032020136
92000	0.036723264	92032	0.106955908
92000	0.009678501	92036	0.110727793
92000	0.024110248	92040	0.04032912
92000	0.061741164	92044	0.028613675
92000	0.058286671	92048	0.063001084
92100	0.039457915	92052	0.032847398
92100	0.043669512	92056	0.054074753
92100	0.054662734	92060	0.040408006
92100	0.047491678	92064	0.052439202
92100	0.030595136	92068	0.065571934
92100	0.051658066	92072	0.039331706
92100	0.059997958	92076	0.068461894
92100	0.067811037	92080	0.058811598
92100	0.063270774	92084	0.0357588
92100	0.051005023	92088	0.057274607
92100	0.030927346	92092	0.158710158
92100	0.023217255	92096	0.117163916
92100	0.054144613	92100	0.074599186
92100	0.038822887	92104	0.133276931
92100	0.002496456	92108	0.175265974
92100	0.012870014	92112	0.106467422
92100	0.025962692	92116	0.099361023
92100	0.047631682	92120	0.287125556

PowerSpectrumData

92100	0.06059805	92124	0.667825225
92100	0.060324925	92128	1.0639088
92100	0.047471229	92132	1.065191464
92100	0.025715966	92136	0.57161384
92100	0.04038955	92140	0.152878624
92100	0.04843028	92144	0.134694419
92100	0.034602072	92148	0.206505574
92200	0.034456658	92152	0.176205576
92200	0.057181529	92156	0.167685678
92200	0.05941734	92160	0.051727762
92200	0.024700839	92164	0.212112413
92200	0.035511035	92168	0.220425194
92200	0.046265166	92172	0.171289779
92200	0.014114545	92176	0.064182219
92200	0.004983401	92180	0.104193801
92200	0.0203809	92184	0.062059888
92200	0.02102142	92188	0.001902316
92200	0.037091002	92192	0.080447419
92200	0.047237674	92196	0.051575298
92200	0.03946263	92200	0.07671745
92200	0.027977276	92204	0.103048398
92200	0.021403215	92208	0.094711839
92200	0.024278155	92212	0.075470314
92200	0.046606157	92216	0.086632987
92200	0.019022145	92220	0.124279875
92200	0.041708834	92224	0.037642341
92200	0.079015568	92228	0.05485657
92200	0.07731354	92232	0.150857144
92200	0.055837511	92236	0.121550416
92200	0.033627959	92240	0.065958018
92200	0.018533299	92244	0.04920418
92200	0.023084938	92248	0.081648257
92300	0.047173759	92252	0.05396652
92300	0.077558703	92256	0.022906332

PowerSpectrumData

92300	0.08647651	92260	0.047848811
92300	0.061328334	92264	0.104091952
92300	0.021473605	92268	0.061693907
92300	0.024986357	92272	0.081446764
92300	0.021591905	92276	0.103471219
92300	0.010554975	92280	0.129380234
92300	0.030166119	92284	0.142392019
92300	0.06259705	92288	0.127159918
92300	0.049363091	92292	0.062467996
92300	0.08605529	92296	0.075419448
92300	0.120737801	92300	0.121830701
92300	0.102032856	92304	0.090015084
92300	0.062973937	92308	0.048993657
92300	0.019710002	92312	0.095977972
92300	0.019400955	92316	0.127981039
92300	0.037363865	92320	0.062183361
92300	0.025718186	92324	0.071481496
92300	0.052099455	92328	0.054941989
92300	0.060012051	92332	0.073984236
92300	0.032990927	92336	0.128289335
92300	0.083310246	92340	0.11406486
92300	0.084495041	92344	0.093448645
92300	0.017908436	92348	0.112278038
92400	0.062935753	92352	0.066634093
92400	0.103810016	92356	0.048851489
92400	0.106664455	92360	0.085836131
92400	0.075023374	92364	0.098925106
92400	0.084691339	92368	0.081287559
92400	0.088399487	92372	0.09262687
92400	0.040243827	92376	0.089237867
92400	0.00741442	92380	0.055845543
92400	0.024000588	92384	0.03664006
92400	0.05216397	92388	0.082300132
92400	0.063499996	92392	0.12331894

PowerSpectrumData

92400	0.022881073	92396	0.099595389
92400	0.042074982	92400	0.053316104
92400	0.061233499	92404	0.05166655
92400	0.024796753	92408	0.095605166
92400	0.029752435	92412	0.083316612
92400	0.055525619	92416	0.073292176
92400	0.076210694	92420	0.095336232
92400	0.037594244	92424	0.096430835
92400	0.004433763	92428	0.041519081
92400	0.040017927	92432	0.07476029
92400	0.07001371	92436	0.094690753
92400	0.05634208	92440	0.051505351
92400	0.039068214	92444	0.014984568
92400	0.037264912	92448	0.041464657
92500	0.050882452	92452	0.032316842
92500	0.040106312	92456	0.038749546
92500	0.043691965	92460	0.051854477
92500	0.059092399	92464	0.040325464
92500	0.052656524	92468	0.068701294
92500	0.058772475	92472	0.118150958
92500	0.07744175	92476	0.123499864
92500	0.066607616	92480	0.072952302
92500	0.058553898	92484	0.002840068
92500	0.06031579	92488	0.037600443
92500	0.031252268	92492	0.045004905
92500	0.008587524	92496	0.058633224
92500	0.035807476	92500	0.065285676
92500	0.047558617	92504	0.020040543
92500	0.056803521	92508	0.047826881
92500	0.046259069	92512	0.089400724
92500	0.060209539	92516	0.106478481
92500	0.077200231	92520	0.103386701
92500	0.058197431	92524	0.10610863
92500	0.055039644	92528	0.084335617

PowerSpectrumData

92500	0.049543767	92532	0.030297475
92500	0.018130855	92536	0.050562088
92500	0.006803351	92540	0.055721237
92500	0.017323087	92544	0.018374232
92500	0.049600731	92548	0.016724578
92600	0.060844002	92552	0.060541555
92600	0.073001611	92556	0.02909533
92600	0.06858015	92560	0.023388444
92600	0.036874182	92564	0.019615791
92600	0.019611529	92568	0.009511588
92600	0.028190496	92572	0.016666396
92600	0.035326841	92576	0.038123737
92600	0.019044112	92580	0.039731531
92600	0.024343372	92584	0.067456465
92600	0.050862145	92588	0.109692046
92600	0.055523404	92592	0.125660415
92600	0.041427862	92596	0.108711574
92600	0.090169968	92600	0.080326536
92600	0.106289896	92604	0.054952252
92600	0.05934267	92608	0.043023258
92600	0.011692674	92612	0.036888243
92600	0.02179876	92616	0.037715789
92600	0.052156589	92620	0.027622111
92600	0.067014742	92624	0.067317065
92600	0.065659464	92628	0.092095077
92600	0.063059211	92632	0.082282779
92600	0.06202214	92636	0.085304739
92600	0.092570917	92640	0.090330272
92600	0.095774238	92644	0.059720067
92600	0.059008333	92648	0.040519986
92700	0.0141742	92652	0.057673839
92700	0.048849801	92656	0.044953893
92700	0.073915973	92660	0.047529156
92700	0.078151599	92664	0.051546595

PowerSpectrumData

92700	0.107296968	92668	0.06991611
92700	0.095139309	92672	0.100234262
92700	0.0570179	92676	0.090555193
92700	0.052766027	92680	0.051370873
92700	0.048600083	92684	0.013839976
92700	0.029749617	92688	0.027729402
92700	0.038606424	92692	0.045202967
92700	0.023442495	92696	0.059482187
92700	0.024691406	92700	0.080696016
92700	0.04317355	92704	0.097427968
92700	0.03166129	92708	0.096860786
92700	0.018256586	92712	0.086484317
92700	0.025051193	92716	0.052128795
92700	0.036729463	92720	0.034950266
92700	0.033067616	92724	0.025830845
92700	0.040726769	92728	0.013735442
92700	0.056729201	92732	0.024385638
92700	0.071430528	92736	0.050600876
92700	0.082125254	92740	0.043563301
92700	0.076700402	92744	0.017238988
92700	0.066253648	92748	0.019387322
92800	0.083587634	92752	0.019453721
92800	0.1009642	92756	0.091654241
92800	0.103100836	92760	0.113074071
92800	0.084387211	92764	0.064435211
92800	0.043233056	92768	0.029725845
92800	0.050201921	92772	0.047493464
92800	0.042472424	92776	0.048070746
92800	0.031258442	92780	0.066245091
92800	0.017457463	92784	0.093239461
92800	0.020029094	92788	0.097934317
92800	0.033236189	92792	0.083104525
92800	0.046339133	92796	0.043393055
92800	0.058251728	92800	0.069393289

PowerSpectrumData

92800	0.057562818	92804	0.100161684
92800	0.059185168	92808	0.065996799
92800	0.045553526	92812	0.008463085
92800	0.033188455	92816	0.053415464
92800	0.030114936	92820	0.087771681
92800	0.023864717	92824	0.0738159
92800	0.023266743	92828	0.01764308
92800	0.048938797	92832	0.048812814
92800	0.053591444	92836	0.097706783
92800	0.031521406	92840	0.116769945
92800	0.022336453	92844	0.077710829
92800	0.033814678	92848	0.003349243
92900	0.025663816	92852	0.050065428
92900	0.022207305	92856	0.051141222
92900	0.058780301	92860	0.072475952
92900	0.054843069	92864	0.131881185
92900	0.055515633	92868	0.108061664
92900	0.042884618	92872	0.051132403
92900	0.043206324	92876	0.050790251
92900	0.067504261	92880	0.076408091
92900	0.08928719	92884	0.076305412
92900	0.091627611	92888	0.043263906
92900	0.074302065	92892	0.04586369
92900	0.050093138	92896	0.101152866
92900	0.016888567	92900	0.102430291
92900	0.040309678	92904	0.078803787
92900	0.058722118	92908	0.061002917
92900	0.031966523	92912	0.059142582
92900	0.016548707	92916	0.031572519
92900	0.01754364	92920	0.046927245
92900	0.046442565	92924	0.043268636
92900	0.099213416	92928	0.023610721
92900	0.106773601	92932	0.025268195
92900	0.091251975	92936	0.043044198

PowerSpectrumData

92900	0.058708032	92940	0.043916181
92900	0.039804647	92944	0.045239143
92900	0.041350857	92948	0.029735254
93000	0.028737763	92952	0.026477428
93000	0.010756316	92956	0.03643144
93000	0.010656899	92960	0.062264058
93000	0.057103367	92964	0.085958585
93000	0.066082175	92968	0.059882739
93000	0.038237122	92972	0.065386877
93000	0.036004378	92976	0.060932118
93000	0.056084435	92980	0.045007149
93000	0.037273629	92984	0.050908166
93000	0.057365181	92988	0.037965823
93000	0.068736561	92992	0.039563365
93000	0.051575444	92996	0.115285242
93000	0.060700091	93000	0.125220948
93000	0.07794299	93004	0.114671108
93000	0.059976286	93008	0.113004506
93000	0.031635955	93012	0.066261826
93000	0.034246885	93016	0.060264636
93000	0.014767668	93020	0.064825872
93000	0.074216179	93024	0.038806415
93000	0.08850656	93028	0.084019601
93000	0.081826423	93032	0.122235462
93000	0.073600109	93036	0.116395066
93000	0.045538582	93040	0.11115527
93000	0.01302782	93044	0.120105928
93000	0.007859818	93048	0.086098648
93100	0.005380692	93052	0.042729233
93100	0.020652042	93056	0.086101842
93100	0.030239124	93060	0.129042179
93100	0.035881738	93064	0.142861653
93100	0.059152575	93068	0.102235761
93100	0.079535559	93072	0.063361207

PowerSpectrumData

93100	0.05673572	93076	0.063031192
93100	0.04610818	93080	0.076167497
93100	0.065002794	93084	0.071341703
93100	0.035042864	93088	0.072499213
93100	0.027601966	93092	0.131921028
93100	0.04905324	93096	0.1449134
93100	0.052048497	93100	0.074330346
93100	0.034331373	93104	0.012776656
93100	0.017839651	93108	0.021641343
93100	0.012586644	93112	0.053934458
93100	0.002702982	93116	0.062808118
93100	0.046696532	93120	0.05410238
93100	0.073537442	93124	0.069631526
93100	0.049480463	93128	0.082722363
93100	0.00732524	93132	0.081664628
93100	0.02959007	93136	0.087486245
93100	0.039500748	93140	0.070322763
93100	0.015527143	93144	0.059152415
93100	0.015726131	93148	0.037583264
93200	0.034303506	93152	0.017445069
93200	0.025273372	93156	0.052831147
93200	0.018801284	93160	0.079875419
93200	0.023665058	93164	0.087525899
93200	0.039773891	93168	0.056194644
93200	0.037410919	93172	0.060359122
93200	0.026308062	93176	0.066680863
93200	0.01135284	93180	0.021592436
93200	0.045223547	93184	0.060635575
93200	0.026690226	93188	0.075334901
93200	0.045411187	93192	0.0552612
93200	0.042383585	93196	0.039285678
93200	0.03326833	93200	0.013309233
93200	0.0675048	93204	0.044771507
93200	0.047051148	93208	0.051352185

PowerSpectrumData

93200	0.033139306	93212	0.033718421
93200	0.021249825	93216	0.065661792
93200	0.037651167	93220	0.071275768
93200	0.048655686	93224	0.030973024
93200	0.053263302	93228	0.016730337
93200	0.059718837	93232	0.018844434
93200	0.032364049	93236	0.031716372
93200	0.038037877	93240	0.037407095
93200	0.056881068	93244	0.05574209
93200	0.072018396	93248	0.077349607
93300	0.068034759	93252	0.04624845
93300	0.056465822	93256	0.022001677
93300	0.036771235	93260	0.012887828
93300	0.005889862	93264	0.038844799
93300	0.03930305	93268	0.03667995
93300	0.024599991	93272	0.050964503
93300	0.067653971	93276	0.053765456
93300	0.107144995	93280	0.038429273
93300	0.082031344	93284	0.038586084
93300	0.035756959	93288	0.012496288
93300	0.011109693	93292	0.020030058
93300	0.034230245	93296	0.058205973
93300	0.051214756	93300	0.068101457
93300	0.061566927	93304	0.051514438
93300	0.037665886	93308	0.040575418
93300	0.015033111	93312	0.047751466
93300	0.008045787	93316	0.055331504
93300	0.017538812	93320	0.060420342
93300	0.045798537	93324	0.064737746
93300	0.056138619	93328	0.051806554
93300	0.02678405	93332	0.026383139
93300	0.010098064	93336	0.021402766
93300	0.016712354	93340	0.007394964
93300	0.008927039	93344	0.019665533

PowerSpectrumData

93300	0.002199067	93348	0.011234813
93400	0.005137807	93352	0.026933374
93400	0.007148095	93356	0.055942521
93400	0.032802527	93360	0.079195743
93400	0.043946056	93364	0.049668342
93400	0.044655466	93368	0.018562687
93400	0.047017649	93372	0.028447088
93400	0.052408355	93376	0.041086332
93400	0.029340636	93380	0.038807841
93400	0.044922919	93384	0.015621739
93400	0.065336215	93388	0.089748828
93400	0.050209648	93392	0.126792336
93400	0.058093228	93396	0.093836097
93400	0.054278047	93400	0.02718864
93400	0.080063153	93404	0.058068596
93400	0.061233332	93408	0.049637794
93400	0.04111895	93412	0.023885292
93400	0.062176412	93416	0.026945796
93400	0.074855168	93420	0.043666183
93400	0.06416408	93424	0.057105597
93400	0.04849218	93428	0.083127765
93400	0.041999039	93432	0.091292495
93400	0.023428342	93436	0.041692132
93400	0.004057024	93440	0.023151413
93400	0.022329945	93444	0.048915325
93400	0.031179963	93448	0.036930876
93500	0.028657349	93452	0.041347848
93500	0.022975104	93456	0.047732388
93500	0.006769589	93460	0.033358803
93500	0.017054197	93464	0.048951541
93500	0.004362933	93468	0.065657499
93500	0.052479114	93472	0.079368729
93500	0.100723155	93476	0.065430839
93500	0.108456108	93480	0.060108418

PowerSpectrumData

93500	0.107465596	93484	0.113976705
93500	0.104921048	93488	0.085657717
93500	0.066376102	93492	0.029057679
93500	0.03460699	93496	0.028440987
93500	0.015292873	93500	0.083773761
93500	0.012088098	93504	0.112056179
93500	0.023534278	93508	0.099606528
93500	0.037853071	93512	0.053662381
93500	0.053349329	93516	0.017975772
93500	0.027188095	93520	0.026984002
93500	0.027895041	93524	0.008360287
93500	0.049665032	93528	0.039221945
93500	0.02398556	93532	0.022164617
93500	0.034658042	93536	0.028639252
93500	0.030669133	93540	0.038544855
93500	0.017658078	93544	0.056683013
93500	0.030355041	93548	0.073485717
93600	0.034262586	93552	0.072814626
93600	0.040860872	93556	0.055856639
93600	0.021493808	93560	0.05005307
93600	0.022916103	93564	0.073868592
93600	0.046980171	93568	0.044080869
93600	0.035536945	93572	0.040121428
93600	0.022375658	93576	0.072377225
93600	0.067342335	93580	0.077450102
93600	0.07334198	93584	0.059400139
93600	0.02575665	93588	0.070213187
93600	0.023278266	93592	0.075324802
93600	0.008088527	93596	0.041657953
93600	0.037559003	93600	0.0556612
93600	0.051714618	93604	0.097750984
93600	0.049662598	93608	0.073951065
93600	0.051481453	93612	0.027459688
93600	0.044436169	93616	0.011359043

PowerSpectrumData

93600	0.062973311	93620	0.011507856
93600	0.082034261	93624	0.027337532
93600	0.073761155	93628	0.037589121
93600	0.04250896	93632	0.039343398
93600	0.031742271	93636	0.041326326
93600	0.050262464	93640	0.051137817
93600	0.039182003	93644	0.034279194
93600	0.008981646	93648	0.007035661
93700	0.040055817	93652	0.020609352
93700	0.042873093	93656	0.039038776
93700	0.026820884	93660	0.032796597
93700	0.031572694	93664	0.055013032
93700	0.016409169	93668	0.087146604
93700	0.031978074	93672	0.060531409
93700	0.069627989	93676	0.038225102
93700	0.093839022	93680	0.043481388
93700	0.059896058	93684	0.043563559
93700	0.035984409	93688	0.037286183
93700	0.082785096	93692	0.045866109
93700	0.067892783	93696	0.03735545
93700	0.059023314	93700	0.035095331
93700	0.056742611	93704	0.035094374
93700	0.038153798	93708	0.041824449
93700	0.069573842	93712	0.023687737
93700	0.037204565	93716	0.035669964
93700	0.031536143	93720	0.074559248
93700	0.020348583	93724	0.075595424
93700	0.054628705	93728	0.071003095
93700	0.058285241	93732	0.067104193
93700	0.036985806	93736	0.040021736
93700	0.023771949	93740	0.016063248
93700	0.037906215	93744	0.049308979
93700	0.067659763	93748	0.055323468
93800	0.076859127	93752	0.059632504

PowerSpectrumData

93800	0.060975344	93756	0.088981171
93800	0.039646744	93760	0.088960704
93800	0.051549196	93764	0.034114662
93800	0.050936109	93768	0.016270429
93800	0.022252307	93772	0.025670253
93800	0.02941936	93776	0.053511081
93800	0.063140426	93780	0.05659974
93800	0.053132313	93784	0.068926776
93800	0.011606964	93788	0.082523751
93800	0.019268311	93792	0.081423357
93800	0.025540254	93796	0.066343884
93800	0.077333003	93800	0.042969714
93800	0.078258425	93804	0.023221826
93800	0.048408445	93808	0.021373509
93800	0.021653832	93812	0.046369256
93800	0.03172952	93816	0.070090115
93800	0.042221502	93820	0.068847083
93800	0.050736759	93824	0.055548309
93800	0.03536027	93828	0.064026397
93800	0.026174947	93832	0.062142608
93800	0.015589571	93836	0.022538186
93800	0.031468691	93840	0.047545258
93800	0.058068727	93844	0.06200694
93800	0.020312229	93848	0.056548859
93900	0.032891974	93852	0.054538399
93900	0.01281937	93856	0.039080042
93900	0.068688692	93860	0.013985882
93900	0.046381269	93864	0.029439629
93900	0.042669344	93868	0.024099571
93900	0.052215379	93872	0.024814794
93900	0.010996666	93876	0.066105233
93900	0.04453864	93880	0.074117124
93900	0.062669955	93884	0.045858382
93900	0.060083286	93888	0.040544055

PowerSpectrumData

93900	0.032468695	93892	0.040820833
93900	0.013583896	93896	0.054296266
93900	0.01540988	93900	0.071250899
93900	0.007557464	93904	0.057006688
93900	0.005184588	93908	0.040304269
93900	0.020544516	93912	0.035366622
93900	0.067947985	93916	0.068337191
93900	0.086607965	93920	0.042947875
93900	0.046729605	93924	0.026959486
93900	0.026871956	93928	0.01729499
93900	0.057002715	93932	0.021670508
93900	0.081943537	93936	0.041046398
93900	0.077608667	93940	0.046117406
93900	0.035827354	93944	0.036112237
93900	0.00229751	93948	0.010853994
94000	0.035033176	93952	0.052469535
94000	0.037128837	93956	0.05656436
94000	0.025910451	93960	0.051943636
94000	0.040721021	93964	0.10307278
94000	0.039957529	93968	0.085307787
94000	0.044250686	93972	0.051628802
94000	0.05686325	93976	0.057720154
94000	0.058746355	93980	0.095080002
94000	0.026591486	93984	0.109750581
94000	0.00333094	93988	0.070464106
94000	0.035491103	93992	0.038512691
94000	0.043159504	93996	0.057033616
94000	0.02038896	94000	0.058168374
94000	0.017478176	94004	0.041526746
94000	0.021662489	94008	0.036105208
94000	0.014794814	94012	0.046341324
94000	0.013299494	94016	0.032269425
94000	0.025251999	94020	0.017002618
94000	0.014000205	94024	0.039601342

PowerSpectrumData

94000	0.035079862	94028	0.042317435
94000	0.092824223	94032	0.030493096
94000	0.114377181	94036	0.090050467
94000	0.062824525	94040	0.096534968
94000	0.034578428	94044	0.044454329
94000	0.039916529	94048	0.008150023
94100	0.06086853	94052	0.027612945
94100	0.058067853	94056	0.069690083
94100	0.048368478	94060	0.115613671
94100	0.043428194	94064	0.100362908
94100	0.040545685	94068	0.047356261
94100	0.041805313	94072	0.016696666
94100	0.053719221	94076	0.015782845
94100	0.065298751	94080	0.030053101
94100	0.058036472	94084	0.035634585
94100	0.051202645	94088	0.031423257
94100	0.048709208	94092	0.026266636
94100	0.058222788	94096	0.026631244
94100	0.057947858	94100	0.033253782
94100	0.029581446	94104	0.065030654
94100	0.047913429	94108	0.084288564
94100	0.028531067	94112	0.088425753
94100	0.054204837	94116	0.064692547
94100	0.032959029	94120	0.064031541
94100	0.015572379	94124	0.094410956
94100	0.037994483	94128	0.076953009
94100	0.065303801	94132	0.049012171
94100	0.077872275	94136	0.043003802
94100	0.059625672	94140	0.049650385
94100	0.010069972	94144	0.035833531
94100	0.041177074	94148	0.024548266
94200	0.026742338	94152	0.038312188
94200	0.022751941	94156	0.010611518
94200	0.033804085	94160	0.01965401

PowerSpectrumData

94200	0.047742331	94164	0.032453587
94200	0.073956937	94168	0.034220022
94200	0.109362089	94172	0.034021919
94200	0.089227353	94176	0.024144385
94200	0.035936533	94180	0.065566776
94200	0.026146548	94184	0.069054091
94200	0.056891204	94188	0.058539732
94200	0.048366368	94192	0.078804413
94200	0.033503613	94196	0.072509014
94200	0.030041367	94200	0.060054059
94200	0.012360118	94204	0.053742908
94200	0.041485477	94208	0.028168175
94200	0.0232609	94212	0.052403357
94200	0.021936055	94216	0.082936116
94200	0.044666387	94220	0.090038499
94200	0.045944846	94224	0.133820911
94200	0.034386081	94228	0.123576931
94200	0.042344327	94232	0.027433191
94200	0.047084675	94236	0.052244657
94200	0.041869585	94240	0.06216651
94200	0.017218417	94244	0.073039781
94200	0.057487228	94248	0.080108286
94300	0.068801266	94252	0.029828914
94300	0.025470305	94256	0.0459443
94300	0.04913086	94260	0.074219657
94300	0.033688932	94264	0.066818939
94300	0.020790742	94268	0.063442101
94300	0.012573199	94272	0.049372055
94300	0.046596306	94276	0.051212915
94300	0.096087155	94280	0.043636643
94300	0.100177189	94284	0.04460011
94300	0.071991308	94288	0.071406852
94300	0.043981429	94292	0.10764342
94300	0.038820072	94296	0.111549307

PowerSpectrumData

94300	0.058186201	94300	0.116495372
94300	0.064860949	94304	0.095806288
94300	0.068883703	94308	0.045298333
94300	0.073888041	94312	0.023777524
94300	0.071329654	94316	0.020754906
94300	0.066741472	94320	0.052491712
94300	0.05466029	94324	0.115240255
94300	0.051262017	94328	0.1275947
94300	0.027945744	94332	0.086023625
94300	0.015330266	94336	0.010254271
94300	0.019302899	94340	0.032158525
94300	0.048616443	94344	0.004647859
94300	0.072203999	94348	0.02905476
94400	0.054507316	94352	0.013665566
94400	0.011213961	94356	0.028830082
94400	0.050374245	94360	0.039267587
94400	0.02494908	94364	0.036227921
94400	0.014431773	94368	0.069891823
94400	0.038556846	94372	0.099901525
94400	0.035744535	94376	0.088256194
94400	0.02936069	94380	0.070100978
94400	0.032250944	94384	0.063266372
94400	0.015615158	94388	0.060873404
94400	0.010837221	94392	0.028065189
94400	0.039912127	94396	0.026640666
94400	0.058545433	94400	0.04744023
94400	0.035763616	94404	0.039687042
94400	0.025723663	94408	0.022450386
94400	0.036568545	94412	0.010389688
94400	0.085329273	94416	0.009207824
94400	0.104586419	94420	0.008142188
94400	0.082023151	94424	0.031907101
94400	0.052596894	94428	0.064696345
94400	0.059983308	94432	0.048500733

PowerSpectrumData

94400	0.061565836	94436	0.015788884
94400	0.049211023	94440	0.030602158
94400	0.053335218	94444	0.04886098
94400	0.061704988	94448	0.025662001
94500	0.0535705	94452	0.039250277
94500	0.046769503	94456	0.073126277
94500	0.012584991	94460	0.084387604
94500	0.054113985	94464	0.10464689
94500	0.034766301	94468	0.10380413
94500	0.01066898	94472	0.025856331
94500	0.025902555	94476	0.031383061
94500	0.017600116	94480	0.042182128
94500	0.043125896	94484	0.076450582
94500	0.071803836	94488	0.102139777
94500	0.103977574	94492	0.070506067
94500	0.100873127	94496	0.083465006
94500	0.083083563	94500	0.081887731
94500	0.062185747	94504	0.036039975
94500	0.016474842	94508	0.080539125
94500	0.027542648	94512	0.104363026
94500	0.040013791	94516	0.11841744
94500	0.073317926	94520	0.100548183
94500	0.076230557	94524	0.061076295
94500	0.062662679	94528	0.061298444
94500	0.063645523	94532	0.089497939
94500	0.014742626	94536	0.118404409
94500	0.017400467	94540	0.104582716
94500	0.017399822	94544	0.091776965
94500	0.019959651	94548	0.109390341
94600	0.031725554	94552	0.102699269
94600	0.05955523	94556	0.058469905
94600	0.061111387	94560	0.054865064
94600	0.041288684	94564	0.115136456
94600	0.042212159	94568	0.143754529

PowerSpectrumData

94600	0.037115566	94572	0.118443415
94600	0.026988464	94576	0.073214935
94600	0.018165365	94580	0.041554624
94600	0.016302573	94584	0.014136975
94600	0.044554276	94588	0.032400647
94600	0.078134588	94592	0.066716209
94600	0.066116714	94596	0.080973987
94600	0.033662793	94600	0.088204848
94600	0.022652361	94604	0.098139382
94600	0.063722589	94608	0.114013885
94600	0.094558054	94612	0.087339606
94600	0.075701064	94616	0.029184168
94600	0.034763147	94620	0.094847892
94600	0.022099943	94624	0.181235475
94600	0.024852277	94628	0.211158171
94600	0.039193001	94632	0.148836058
94600	0.043199521	94636	0.057203997
94600	0.066945999	94640	0.064518506
94600	0.055203262	94644	0.081184015
94600	0.010222398	94648	0.073735224
94700	0.024995843	94652	0.049454549
94700	0.046869813	94656	0.055740704
94700	0.059698821	94660	0.103881037
94700	0.054791522	94664	0.101215323
94700	0.042734806	94668	0.09090508
94700	0.051048115	94672	0.085450789
94700	0.081661528	94676	0.030260682
94700	0.074525255	94680	0.058074962
94700	0.030698815	94684	0.086036234
94700	0.025902373	94688	0.067369088
94700	0.019898844	94692	0.055334946
94700	0.025497062	94696	0.076176257
94700	0.018374258	94700	0.072450748
94700	0.077379082	94704	0.011958903

PowerSpectrumData

94700	0.099391436	94708	0.056665609
94700	0.064510859	94712	0.109439039
94700	0.024468009	94716	0.124286657
94700	0.031432264	94720	0.070911985
94700	0.037167167	94724	0.06316146
94700	0.021222451	94728	0.091612441
94700	0.00754954	94732	0.099617209
94700	0.015880587	94736	0.061342857
94700	0.027830121	94740	0.056245946
94700	0.026266434	94744	0.079446974
94700	0.051328163	94748	0.080274142
94800	0.043308595	94752	0.064006592
94800	0.019543706	94756	0.051867297
94800	0.026966472	94760	0.049192975
94800	0.016803997	94764	0.048436512
94800	0.016396796	94768	0.044304052
94800	0.046056015	94772	0.05828921
94800	0.059943192	94776	0.058030804
94800	0.048295155	94780	0.059914662
94800	0.02486652	94784	0.056432185
94800	0.03402407	94788	0.06498702
94800	0.021301985	94792	0.104331593
94800	0.028200888	94796	0.106667045
94800	0.041121122	94800	0.11443413
94800	0.040182087	94804	0.123360165
94800	0.032590629	94808	0.120881021
94800	0.03695994	94812	0.100225392
94800	0.040719038	94816	0.070000402
94800	0.052114134	94820	0.028762637
94800	0.074118805	94824	0.011840538
94800	0.056103119	94828	0.04925067
94800	0.028111203	94832	0.067189474
94800	0.046129	94836	0.020740206
94800	0.087358378	94840	0.013779886

PowerSpectrumData

94800	0.101301368	94844	0.037662569
94800	0.094911753	94848	0.058567206
94900	0.058374	94852	0.043877641
94900	0.016277254	94856	0.044836746
94900	0.005171121	94860	0.044232955
94900	0.022168486	94864	0.063978987
94900	0.015466465	94868	0.094018265
94900	0.035262201	94872	0.091041926
94900	0.067366651	94876	0.081048558
94900	0.067068133	94880	0.105523097
94900	0.04826556	94884	0.089153102
94900	0.067766428	94888	0.013990682
94900	0.125944644	94892	0.066085384
94900	0.134750575	94896	0.049999657
94900	0.082218161	94900	0.013041566
94900	0.059391896	94904	0.021452803
94900	0.050415027	94908	0.019006107
94900	0.028935943	94912	0.0089473
94900	0.009245588	94916	0.019669589
94900	0.048682006	94920	0.053052678
94900	0.080412196	94924	0.057909969
94900	0.063856613	94928	0.01900791
94900	0.01998554	94932	0.014482785
94900	0.0820473	94936	0.037892765
94900	0.08329616	94940	0.082861006
94900	0.036792899	94944	0.108970191
94900	0.010155431	94948	0.110574249
95000	0.021802904	94952	0.099063167
95000	0.034381759	94956	0.071335482
95000	0.040793937	94960	0.069105903
95000	0.054212007	94964	0.075425734
95000	0.095113988	94968	0.061280443
95000	0.116748808	94972	0.004500788
95000	0.058233462	94976	0.039341925

PowerSpectrumData

95000	0.041067535	94980	0.025404464
95000	0.065582928	94984	0.019978639
95000	0.036446338	94988	0.016445609
95000	0.009714184	94992	0.008970215
95000	0.033120392	94996	0.007501188
95000	0.051740382	95000	0.035790308
95000	0.041199131	95004	0.083240717
95000	0.037277478	95008	0.077971228
95000	0.018321598	95012	0.04892638
95000	0.01752442	95016	0.059497572
95000	0.013369747	95020	0.047580998
95000	0.067514811	95024	0.054575601
95000	0.091166847	95028	0.091990245
95000	0.037044756	95032	0.087229913
95000	0.050891355	95036	0.057524565
95000	0.080487327	95040	0.039774179
95000	0.047770842	95044	0.009424843
95000	0.026415826	95048	0.030160298
95100	0.027290924	95052	0.068441885
95100	0.009787093	95056	0.089303285
95100	0.031040738	95060	0.047760488
95100	0.034582237	95064	0.019214704
95100	0.024758216	95068	0.070025948
95100	0.047922327	95072	0.113168513
95100	0.058311449	95076	0.108285283
95100	0.06301223	95080	0.08505374
95100	0.086957916	95084	0.081664897
95100	0.111491012	95088	0.081947386
95100	0.092291564	95092	0.082364277
95100	0.035108275	95096	0.064851382
95100	0.065135442	95100	0.025677426
95100	0.096703661	95104	0.055041415
95100	0.094233204	95108	0.073342206
95100	0.072961186	95112	0.057504429

PowerSpectrumData

95100	0.065047723	95116	0.052465675
95100	0.057227673	95120	0.054368848
95100	0.046121262	95124	0.080927079
95100	0.027503322	95128	0.100518315
95100	0.027765524	95132	0.094874391
95100	0.020901891	95136	0.081893762
95100	0.042456471	95140	0.099808014
95100	0.078071294	95144	0.103363105
95100	0.092311297	95148	0.051272611
95200	0.063168736	95152	0.033453722
95200	0.045201246	95156	0.111598856
95200	0.075357253	95160	0.120250144
95200	0.095951975	95164	0.079078527
95200	0.095071278	95168	0.112549205
95200	0.062603627	95172	0.126518876
95200	0.023098661	95176	0.111624133
95200	0.00289711	95180	0.086071043
95200	0.020714917	95184	0.036721063
95200	0.053004791	95188	0.060124799
95200	0.090853297	95192	0.13079535
95200	0.066372319	95196	0.143255151
95200	0.054433571	95200	0.121384022
95200	0.077753663	95204	0.085178079
95200	0.086299362	95208	0.091980161
95200	0.081015009	95212	0.125889914
95200	0.056627439	95216	0.127682316
95200	0.02677842	95220	0.100284502
95200	0.02866445	95224	0.095312949
95200	0.085859487	95228	0.112318034
95200	0.100172161	95232	0.109295113
95200	0.038077811	95236	0.1007397
95200	0.080186248	95240	0.095774703
95200	0.076420365	95244	0.110720634
95200	0.043528438	95248	0.12397916

PowerSpectrumData

95300	0.026252621	95252	0.090026202
95300	0.034820423	95256	0.03909344
95300	0.049384402	95260	0.028158705
95300	0.032461489	95264	0.091170601
95300	0.019091378	95268	0.107858032
95300	0.016070571	95272	0.068513713
95300	0.020115778	95276	0.08268698
95300	0.030370731	95280	0.063792861
95300	0.031703763	95284	0.058120557
95300	0.007219301	95288	0.081258833
95300	0.003974401	95292	0.082118299
95300	0.004473452	95296	0.064844826
95300	0.021347287	95300	0.061613784
95300	0.021222049	95304	0.056603501
95300	0.015891536	95308	0.109900531
95300	0.029075842	95312	0.121334815
95300	0.039509985	95316	0.117654054
95300	0.071345035	95320	0.122240133
95300	0.0633573	95324	0.08478541
95300	0.032355827	95328	0.019140893
95300	0.057194997	95332	0.068779373
95300	0.034866756	95336	0.10582598
95300	0.048166174	95340	0.099246398
95300	0.097051474	95344	0.084935578
95300	0.100536345	95348	0.09095166
95400	0.05760515	95352	0.074580785
95400	0.024754403	95356	0.083201077
95400	0.034771616	95360	0.147259693
95400	0.031113938	95364	0.178057395
95400	0.033449247	95368	0.138718111
95400	0.029077888	95372	0.088304063
95400	0.01329132	95376	0.076794808
95400	0.041608328	95380	0.074656084
95400	0.050416933	95384	0.08027575

PowerSpectrumData

95400	0.027979737	95388	0.079186779
95400	0.00573144	95392	0.134632094
95400	0.019480978	95396	0.176788031
95400	0.039309151	95400	0.1638861
95400	0.049616847	95404	0.113574992
95400	0.058296744	95408	0.112324655
95400	0.063113308	95412	0.109216962
95400	0.042060405	95416	0.093923874
95400	0.013623338	95420	0.124507264
95400	0.034146433	95424	0.137735173
95400	0.055991211	95428	0.078017169
95400	0.035174777	95432	0.082530918
95400	0.021713542	95436	0.149885484
95400	0.04572222	95440	0.142783014
95400	0.030866238	95444	0.122844576
95400	0.039167604	95448	0.090531939
95500	0.072816103	95452	0.057974052
95500	0.07868473	95456	0.091426293
95500	0.053008957	95460	0.085397798
95500	0.054297085	95464	0.090953472
95500	0.053431668	95468	0.126514235
95500	0.009320389	95472	0.114527713
95500	0.037147904	95476	0.100851197
95500	0.045168232	95480	0.103266226
95500	0.070740818	95484	0.092558323
95500	0.076738492	95488	0.080211728
95500	0.046402107	95492	0.059867281
95500	0.019077776	95496	0.09386443
95500	0.013828616	95500	0.128675325
95500	0.019028546	95504	0.141177778
95500	0.049238901	95508	0.146632069
95500	0.086516135	95512	0.086507149
95500	0.087735622	95516	0.060562004
95500	0.0490975	95520	0.138344753

PowerSpectrumData

95500	0.012415157	95524	0.150822889
95500	0.020678743	95528	0.118912962
95500	0.058930451	95532	0.062880492
95500	0.039819417	95536	0.092234877
95500	0.046852681	95540	0.082866187
95500	0.061186918	95544	0.109605862
95500	0.021456835	95548	0.169617546
95600	0.028213084	95552	0.145379585
95600	0.013969877	95556	0.115337731
95600	0.023048702	95560	0.147941698
95600	0.016763854	95564	0.160390249
95600	0.042077456	95568	0.152403388
95600	0.058162521	95572	0.120172088
95600	0.064077445	95576	0.076387216
95600	0.030128022	95580	0.104763305
95600	0.027823422	95584	0.138785399
95600	0.048226477	95588	0.113190399
95600	0.039094204	95592	0.086471424
95600	0.00947174	95596	0.087812681
95600	0.040419578	95600	0.065175686
95600	0.078454548	95604	0.051632786
95600	0.098935918	95608	0.093205134
95600	0.062745297	95612	0.080290323
95600	0.037617119	95616	0.058942878
95600	0.067041823	95620	0.132696921
95600	0.070801856	95624	0.122651152
95600	0.048630373	95628	0.084185369
95600	0.012752867	95632	0.120880417
95600	0.003727514	95636	0.120284334
95600	0.022322345	95640	0.064773019
95600	0.039091017	95644	0.076667187
95600	0.027423059	95648	0.113436116
95700	0.040403003	95652	0.093163304
95700	0.040858868	95656	0.076443896

PowerSpectrumData

95700	0.028174225	95660	0.064149026
95700	0.07270756	95664	0.028095554
95700	0.074064526	95668	0.056203717
95700	0.043874388	95672	0.092544273
95700	0.027275468	95676	0.091197951
95700	0.059008922	95680	0.049510807
95700	0.045868394	95684	0.030586343
95700	0.02537167	95688	0.050481231
95700	0.043832286	95692	0.048925514
95700	0.054588032	95696	0.052783562
95700	0.014129231	95700	0.052215295
95700	0.039806942	95704	0.057425812
95700	0.044136505	95708	0.07577808
95700	0.028201263	95712	0.063719228
95700	0.033692217	95716	0.078949415
95700	0.038761413	95720	0.099991921
95700	0.037273345	95724	0.091693408
95700	0.036529254	95728	0.074733391
95700	0.041111045	95732	0.010687946
95700	0.023358341	95736	0.065838874
95700	0.039277595	95740	0.105386047
95700	0.05639387	95744	0.121431833
95700	0.067837711	95748	0.12231851
95800	0.068157518	95752	0.1057595
95800	0.024062638	95756	0.06245097
95800	0.032125787	95760	0.024637908
95800	0.045549037	95764	0.059158403
95800	0.035547622	95768	0.057712477
95800	0.034041419	95772	0.032814685
95800	0.0289521	95776	0.026611209
95800	0.034457145	95780	0.01697542
95800	0.057752459	95784	0.081308186
95800	0.075523582	95788	0.100807942
95800	0.068711757	95792	0.081192658

PowerSpectrumData

95800	0.039517679	95796	0.061212617
95800	0.030314646	95800	0.05501098
95800	0.043134853	95804	0.049078779
95800	0.063756117	95808	0.018899236
95800	0.046525162	95812	0.021404852
95800	0.008971798	95816	0.025554233
95800	0.03665451	95820	0.055184817
95800	0.046716639	95824	0.067100053
95800	0.042771608	95828	0.043890723
95800	0.029211738	95832	0.057953592
95800	0.036501591	95836	0.064918342
95800	0.048542341	95840	0.028105296
95800	0.028259459	95844	0.046476111
95800	0.021794218	95848	0.104798528
95900	0.067411733	95852	0.08874973
95900	0.08760301	95856	0.064181673
95900	0.057354533	95860	0.087067827
95900	0.048713395	95864	0.063252715
95900	0.029769639	95868	0.017478933
95900	0.016447879	95872	0.039402494
95900	0.037478592	95876	0.075151387
95900	0.010045789	95880	0.075591277
95900	0.041995674	95884	0.021753827
95900	0.056697387	95888	0.031249827
95900	0.038707658	95892	0.024481225
95900	0.022506843	95896	0.031340642
95900	0.033138782	95900	0.03386338
95900	0.060701674	95904	0.00500994
95900	0.084310785	95908	0.020402726
95900	0.076897821	95912	0.043104181
95900	0.065738874	95916	0.058541009
95900	0.0586567	95920	0.062886858
95900	0.03209649	95924	0.068700916
95900	0.016569497	95928	0.039734579

PowerSpectrumData

95900	0.043819629	95932	0.046174162
95900	0.038226288	95936	0.05611376
95900	0.028041586	95940	0.067074412
95900	0.023273267	95944	0.070891794
95900	0.018469253	95948	0.056824414
96000	0.028027667	95952	0.065596199
96000	0.070078153	95956	0.06605449
96000	0.062728213	95960	0.083392981
96000	0.011522888	95964	0.033298838
96000	0.03999444	95968	0.053387517
96000	0.011101736	95972	0.061593892
96000	0.039931678	95976	0.030865966
96000	0.056610072	95980	0.014052142
96000	0.066775232	95984	0.008276917
96000	0.069862952	95988	0.014069638
96000	0.048162263	95992	0.023725786
96000	0.034780624	95996	0.078379744
96000	0.036311954	96000	0.141071476
96000	0.041944553	96004	0.130298024
96000	0.050116374	96008	0.074242307
96000	0.061126382	96012	0.066855238
96000	0.027110255	96016	0.080388825
96000	0.05415807	96020	0.028388262
96000	0.046880796	96024	0.031600652
96000	0.025411026	96028	0.060861563
96000	0.071444047	96032	0.082379498
96000	0.053242653	96036	0.080244216
96000	0.03731476	96040	0.040735449
96000	0.080624457	96044	0.01446109
96000	0.077379264	96048	0.069004054
96100	0.073997457	96052	0.076309196
96100	0.055588531	96056	0.0256678
96100	0.022384207	96060	0.018300676
96100	0.014593931	96064	0.045994966

PowerSpectrumData

96100	0.04077499	96068	0.043301399
96100	0.081507802	96072	0.021660066
96100	0.099825076	96076	0.027801296
96100	0.063597945	96080	0.028761926
96100	0.02589426	96084	0.045394478
96100	0.027428916	96088	0.087079927
96100	0.046924499	96092	0.064487831
96100	0.07015231	96096	0.012509061
96100	0.06876445	96100	0.024115672
96100	0.042323274	96104	0.062455736
96100	0.026667709	96108	0.09319721
96100	0.020099817	96112	0.117519783
96100	0.029701101	96116	0.095327072
96100	0.033547683	96120	0.06873344
96100	0.01971159	96124	0.084027677
96100	0.068473804	96128	0.097582757
96100	0.082831779	96132	0.10647202
96100	0.026377235	96136	0.05170027
96100	0.056818062	96140	0.039592891
96100	0.045893023	96144	0.078481426
96100	0.046231857	96148	0.065351633
96200	0.054521446	96152	0.019998808
96200	0.045208748	96156	0.049511727
96200	0.037719201	96160	0.073536015
96200	0.041074123	96164	0.05540597
96200	0.02583515	96168	0.039652743
96200	0.032985132	96172	0.087335757
96200	0.076371762	96176	0.083143837
96200	0.066538989	96180	0.053690725
96200	0.043166234	96184	0.073673902
96200	0.057187055	96188	0.055456378
96200	0.068606081	96192	0.033302971
96200	0.038249797	96196	0.089100562
96200	0.042875807	96200	0.093206989

PowerSpectrumData

96200	0.087745029	96204	0.067579895
96200	0.092068156	96208	0.055439065
96200	0.071934548	96212	0.03111377
96200	0.060501101	96216	0.105227307
96200	0.030620795	96220	0.133987531
96200	0.044650034	96224	0.072236449
96200	0.064696418	96228	0.038402573
96200	0.035870668	96232	0.076011522
96200	0.048707268	96236	0.050764171
96200	0.069178932	96240	0.046917783
96200	0.072050592	96244	0.066410852
96200	0.087475943	96248	0.051699226
96300	0.091799841	96252	0.017641205
96300	0.092884089	96256	0.027251657
96300	0.044108667	96260	0.03633407
96300	0.021921265	96264	0.044559452
96300	0.041477666	96268	0.0314434
96300	0.066813758	96272	0.026713906
96300	0.050013066	96276	0.042864827
96300	0.028838815	96280	0.028864664
96300	0.009457127	96284	0.021944456
96300	0.047775386	96288	0.026263964
96300	0.061377003	96292	0.012204376
96300	0.044927791	96296	0.039952949
96300	0.044616092	96300	0.092477632
96300	0.022793814	96304	0.074978394
96300	0.059116013	96308	0.009837495
96300	0.089296424	96312	0.102921506
96300	0.097092627	96316	0.119458484
96300	0.076051976	96320	0.077481338
96300	0.066389162	96324	0.079457139
96300	0.091375616	96328	0.092005255
96300	0.090110771	96332	0.07612873
96300	0.02611028	96336	0.052793192

PowerSpectrumData

96300	0.039550654	96340	0.026919128
96300	0.055297503	96344	0.016467988
96300	0.07301796	96348	0.053752126
96400	0.081745639	96352	0.063340056
96400	0.053667474	96356	0.055596556
96400	0.005007502	96360	0.060975657
96400	0.042940046	96364	0.05981842
96400	0.029152861	96368	0.017139731
96400	0.081341939	96372	0.017264762
96400	0.088869172	96376	0.019495465
96400	0.035103298	96380	0.009730831
96400	0.063586944	96384	0.083584084
96400	0.069365728	96388	0.111399888
96400	0.027503904	96392	0.046630463
96400	0.008775934	96396	0.019073434
96400	0.027594781	96400	0.032384836
96400	0.054479006	96404	0.04254789
96400	0.030271393	96408	0.053865664
96400	0.060787927	96412	0.062058214
96400	0.073842239	96416	0.05591883
96400	0.100577687	96420	0.047049845
96400	0.092867515	96424	0.054817785
96400	0.051156545	96428	0.053299289
96400	0.060039107	96432	0.020686959
96400	0.057903471	96436	0.034688492
96400	0.022646158	96440	0.055252272
96400	0.029593004	96444	0.033315027
96400	0.01844864	96448	0.042380398
96500	0.035731442	96452	0.067590736
96500	0.064057327	96456	0.056385477
96500	0.068247078	96460	0.019416746
96500	0.01342945	96464	0.011090735
96500	0.043552591	96468	0.007144135
96500	0.051775776	96472	0.010141436

PowerSpectrumData

96500	0.038500697	96476	0.030704825
96500	0.05264418	96480	0.076186603
96500	0.059361322	96484	0.066999513
96500	0.065152948	96488	0.029141307
96500	0.056594563	96492	0.02935806
96500	0.017305418	96496	0.033646586
96500	0.065297842	96500	0.047600308
96500	0.066513952	96504	0.084390536
96500	0.025511177	96508	0.06069016
96500	0.028457631	96512	0.019065816
96500	0.047479236	96516	0.079531339
96500	0.058041234	96520	0.080393424
96500	0.044614935	96524	0.029051571
96500	0.040977131	96528	0.050297404
96500	0.042551583	96532	0.048432736
96500	0.045535271	96536	0.036747606
96500	0.052265023	96540	0.040048068
96500	0.043336804	96544	0.045553301
96500	0.04490522	96548	0.039762141
96600	0.052487401	96552	0.019020392
96600	0.047829046	96556	0.022093656
96600	0.039001865	96560	0.015335596
96600	0.030440304	96564	0.027928196
96600	0.027318076	96568	0.052417334
96600	0.031581629	96572	0.035895377
96600	0.064653126	96576	0.059783655
96600	0.077428689	96580	0.036716236
96600	0.067067362	96584	0.01993433
96600	0.069355017	96588	0.048908936
96600	0.022760427	96592	0.068065376
96600	0.035265111	96596	0.073315256
96600	0.06075517	96600	0.043442749
96600	0.044607485	96604	0.04288301
96600	0.01702119	96608	0.028298517

PowerSpectrumData

96600	0.060553211	96612	0.035731595
96600	0.075904798	96616	0.083230421
96600	0.112903814	96620	0.092923569
96600	0.100072073	96624	0.04178625
96600	0.059244456	96628	0.082572697
96600	0.061775514	96632	0.087257067
96600	0.036938436	96636	0.063746324
96600	0.059603553	96640	0.08905906
96600	0.029013769	96644	0.116155476
96600	0.05778066	96648	0.079824487
96700	0.080765676	96652	0.053670046
96700	0.037066751	96656	0.092381895
96700	0.018499755	96660	0.072927665
96700	0.011051454	96664	0.050619503
96700	0.048355716	96668	0.059761329
96700	0.076622091	96672	0.0645896
96700	0.078802739	96676	0.058087266
96700	0.054111879	96680	0.08103178
96700	0.036416099	96684	0.097124233
96700	0.051668943	96688	0.035439287
96700	0.041877476	96692	0.079803831
96700	0.025099018	96696	0.081301492
96700	0.027543898	96700	0.060345225
96700	0.048058537	96704	0.138637188
96700	0.049649458	96708	0.200008915
96700	0.005799332	96712	0.162883618
96700	0.062649117	96716	0.082636943
96700	0.072340961	96720	0.068410453
96700	0.035468442	96724	0.065063316
96700	0.028435683	96728	0.06371041
96700	0.06268458	96732	0.076936667
96700	0.052178173	96736	0.120704273
96700	0.047769183	96740	0.172594388
96700	0.046256981	96744	0.15736073

PowerSpectrumData

96700	0.011446805	96748	0.080881102
96800	0.029433106	96752	0.030682808
96800	0.023302584	96756	0.141152268
96800	0.012721946	96760	0.155307338
96800	0.010395912	96764	0.112272886
96800	0.007549991	96768	0.101455829
96800	0.037016838	96772	0.083390973
96800	0.073393552	96776	0.038690781
96800	0.050521925	96780	0.079147125
96800	0.034424211	96784	0.109540604
96800	0.035528912	96788	0.12579457
96800	0.059516358	96792	0.126649903
96800	0.03581429	96796	0.069760507
96800	0.042929365	96800	0.029640778
96800	0.064528285	96804	0.104378443
96800	0.075182928	96808	0.136144009
96800	0.033404795	96812	0.099020326
96800	0.043536595	96816	0.059403941
96800	0.060766095	96820	0.067358742
96800	0.08074072	96824	0.092567854
96800	0.074512871	96828	0.0308048
96800	0.034417673	96832	0.089966088
96800	0.017442922	96836	0.12147981
96800	0.05733555	96840	0.085168918
96800	0.067080284	96844	0.0630223
96800	0.08109706	96848	0.081824495
96900	0.109527362	96852	0.092865696
96900	0.10289967	96856	0.086670676
96900	0.063237414	96860	0.071852279
96900	0.066399996	96864	0.058782924
96900	0.066065091	96868	0.071678551
96900	0.041733569	96872	0.084564344
96900	0.035796955	96876	0.076715754
96900	0.039446968	96880	0.069836067

PowerSpectrumData

96900	0.029625953	96884	0.087861881
96900	0.021089551	96888	0.080369311
96900	0.020338288	96892	0.119569864
96900	0.025912215	96896	0.131674416
96900	0.040564941	96900	0.110394874
96900	0.048564773	96904	0.10007136
96900	0.048813323	96908	0.045599343
96900	0.035065146	96912	0.055982564
96900	0.041745901	96916	0.122808357
96900	0.061505343	96920	0.142815101
96900	0.06991814	96924	0.146443359
96900	0.066546432	96928	0.154335721
96900	0.054313532	96932	0.144824488
96900	0.031106254	96936	0.104447237
96900	0.02416897	96940	0.056267247
96900	0.053802633	96944	0.091452006
96900	0.065128312	96948	0.11390623
97000	0.038167043	96952	0.081970844
97000	0.014084066	96956	0.08931732
97000	0.057537582	96960	0.107868393
97000	0.06224346	96964	0.10798053
97000	0.04063921	96968	0.091588867
97000	0.040858253	96972	0.0536847
97000	0.042371805	96976	0.057638055
97000	0.019667661	96980	0.09365884
97000	0.029902531	96984	0.089249254
97000	0.043659922	96988	0.09216959
97000	0.040123367	96992	0.107392851
97000	0.051795469	96996	0.075549811
97000	0.054125128	97000	0.032218886
97000	0.036278128	97004	0.032039952
97000	0.022428225	97008	0.095316304
97000	0.035479301	97012	0.124269267
97000	0.041741096	97016	0.105693478

PowerSpectrumData

97000	0.025782823	97020	0.113349153
97000	0.038155828	97024	0.108626889
97000	0.064987653	97028	0.058126378
97000	0.037503876	97032	0.119992503
97000	0.03138337	97036	0.143068523
97000	0.044856595	97040	0.134869348
97000	0.054635959	97044	0.121529018
97000	0.048708738	97048	0.090564259
97100	0.007850431	97052	0.078211851
97100	0.047106667	97056	0.101836646
97100	0.076173099	97060	0.096977165
97100	0.06369 97064	0.083090934	
97100	0.025670583	97068	0.052463798
97100	0.046261765	97072	0.017388218
97100	0.064624961	97076	0.044131008
97100	0.068774993	97080	0.097641438
97100	0.047554357	97084	0.135010166
97100	0.025574911	97088	0.153965375
97100	0.035398738	97092	0.127080551
97100	0.040413554	97096	0.065805085
97100	0.04841913	97100	0.071775889
97100	0.054413613	97104	0.071316921
97100	0.032193941	97108	0.053197073
97100	0.020517067	97112	0.041998152
97100	0.042148142	97116	0.081410843
97100	0.072024508	97120	0.082666877
97100	0.08402094	97124	0.095920361
97100	0.06363658	97128	0.126841696
97100	0.044172426	97132	0.137564319
97100	0.052311501	97136	0.099275341
97100	0.076958502	97140	0.061624021
97100	0.073039526	97144	0.110942398
97100	0.035688761	97148	0.137844996
97200	0.019634608	97152	0.115969531

97200	0.037775324	97156	0.074449548
97200	0.020234051	97160	0.051159506
97200	0.04125627	97164	0.057101093
97200	0.06376586	97168	0.051452924
97200	0.03634235	97172	0.06472104
97200	0.032287364	97176	0.058329948
97200	0.039262446	97180	0.064866297
97200	0.018514902	97184	0.093291848
97200	0.009130824	97188	0.09709219
97200	0.021040753	97192	0.087186127
97200	0.033610559	97196	0.070284848
97200	0.036612299	97200	0.039606861
97200	0.029196632	97204	0.041347998
97200	0.037218699	97208	0.066729743
97200	0.050525279	97212	0.086947148
97200	0.022891227	97216	0.095640557
97200	0.015152996	97220	0.10187531
97200	0.026661532	97224	0.067151763
97200	0.023246021	97228	0.026232619
97200	0.015193612	97232	0.042373835
97200	0.01634792	97236	0.022431219
97200	0.022316135	97240	0.020303876
97200	0.031995354	97244	0.021254518
97200	0.03501321	97248	0.045841687
97300	0.034990586	97252	0.041196774
97300	0.047942111	97256	0.052273219
97300	0.050937808	97260	0.051703784
97300	0.025999738	97264	0.028287641
97300	0.007309829	97268	0.037673166
97300	0.028172317	97272	0.038388946
97300	0.035864319	97276	0.043239379
97300	0.050519164	97280	0.052083295
97300	0.069045505	97284	0.026744745
97300	0.081075363	97288	0.052327516

PowerSpectrumData

97300	0.092677241	97292	0.09724233
97300	0.083360406	97296	0.080251906
97300	0.065157597	97300	0.047548794
97300	0.068406051	97304	0.046158435
97300	0.089652604	97308	0.052406442
97300	0.066247478	97312	0.046866197
97300	0.017186821	97316	0.033216584
97300	0.083234707	97320	0.012828771
97300	0.116445073	97324	0.061337807
97300	0.10890355	97328	0.078763856
97300	0.06284046	97332	0.042287175
97300	0.049272079	97336	0.013044479
97300	0.068193513	97340	0.049307175
97300	0.058721318	97344	0.040815972
97300	0.039660758	97348	0.041995212
97400	0.048719699	97352	0.070117421
97400	0.060238021	97356	0.035465011
97400	0.04318287	97360	0.057310368
97400	0.033607197	97364	0.057882844
97400	0.050681822	97368	0.034033106
97400	0.051403691	97372	0.033260902
97400	0.041248197	97376	0.065994034
97400	0.060089951	97380	0.052564912
97400	0.040801657	97384	0.012305701
97400	0.029866127	97388	0.020987522
97400	0.063350126	97392	0.022801962
97400	0.054742086	97396	0.047723028
97400	0.04458267	97400	0.08761861
97400	0.080968726	97404	0.084664884
97400	0.065750537	97408	0.060540682
97400	0.041171668	97412	0.04012269
97400	0.027755727	97416	0.01875331
97400	0.029041104	97420	0.014001747
97400	0.023665118	97424	0.038350547

PowerSpectrumData

97400	0.039601513	97428	0.050302024
97400	0.03618126	97432	0.029897676
97400	0.016281108	97436	0.067955574
97400	0.006546121	97440	0.065470951
97400	0.010165866	97444	0.023991897
97400	0.033384727	97448	0.011443142
97500	0.034837365	97452	0.020086895
97500	0.008179154	97456	0.019891466
97500	0.060720187	97460	0.025923153
97500	0.088690933	97464	0.043856875
97500	0.095909847	97468	0.044740053
97500	0.090287846	97472	0.037772654
97500	0.060547929	97476	0.049218874
97500	0.031429667	97480	0.041095787
97500	0.04527959	97484	0.048732447
97500	0.048644793	97488	0.033760451
97500	0.063965374	97492	0.0255373
97500	0.046768975	97496	0.050736035
97500	0.033708166	97500	0.064704866
97500	0.079421836	97504	0.047308393
97500	0.075784898	97508	0.051079966
97500	0.0334861	97512	0.048041238
97500	0.017364562	97516	0.03089822
97500	0.014107363	97520	0.045479319
97500	0.043548596	97524	0.006245255
97500	0.063375424	97528	0.057589292
97500	0.054306776	97532	0.080455619
97500	0.039498682	97536	0.085588581
97500	0.044859655	97540	0.051277901
97500	0.060832652	97544	0.012548375
97500	0.079761463	97548	0.033250442
97600	0.065714463	97552	0.032927139
97600	0.036092624	97556	0.024488845
97600	0.062385036	97560	0.056333258

PowerSpectrumData

97600	0.06002191	97564	0.03076933
97600	0.091212096	97568	0.0645986
97600	0.092139642	97572	0.090574278
97600	0.020092701	97576	0.03965326
97600	0.083672007	97580	0.005849533
97600	0.14406527	97584	0.035049532
97600	0.132422618	97588	0.051707932
97600	0.076471042	97592	0.020890469
97600	0.034321198	97596	0.033142576
97600	0.076852855	97600	0.068360867
97600	0.070375194	97604	0.049396353
97600	0.09292188	97608	0.016104948
97600	0.120463505	97612	0.039136099
97600	0.093804163	97616	0.05326224
97600	0.034283843	97620	0.047957976
97600	0.055426292	97624	0.021613361
97600	0.093119219	97628	0.032909549
97600	0.043255357	97632	0.037416186
97600	0.167423044	97636	0.032016957
97600	0.387595268	97640	0.027582973
97600	0.442468358	97644	0.023722245
97600	0.312100718	97648	0.048742848
97700	0.130892411	97652	0.050884308
97700	0.062703446	97656	0.04845842
97700	0.043252043	97660	0.077885401
97700	0.096957832	97664	0.063459513
97700	0.141923913	97668	0.023733153
97700	0.213249135	97672	0.047196227
97700	0.231161103	97676	0.070636903
97700	0.167605759	97680	0.042094736
97700	0.021432397	97684	0.01697524
97700	0.147679166	97688	0.025984786
97700	0.06952032	97692	0.078025762
97700	0.433881971	97696	0.088802684

PowerSpectrumData

97700	0.790000079	97700	0.035737172
97700	0.776099624	97704	0.020758671
97700	0.445778133	97708	0.024121264
97700	0.151813933	97712	0.01596503
97700	0.107395768	97716	0.06439913
97700	0.035290199	97720	0.092335555
97700	0.184751581	97724	0.078166115
97700	0.447026018	97728	0.039440125
97700	0.583669171	97732	0.038269813
97700	0.449957298	97736	0.067110261
97700	0.148321604	97740	0.069977265
97700	0.178581336	97744	0.022372364
97700	0.219597598	97748	0.024817533
97800	0.291207514	97752	0.031159816
97800	0.65502408	97756	0.035505174
97800	0.804865733	97760	0.019367028
97800	0.570455915	97764	0.034199296
97800	0.209615683	97768	0.055466368
97800	0.042986838	97772	0.013881296
97800	0.070639137	97776	0.081319544
97800	0.070423768	97780	0.064114713
97800	0.446328544	97784	0.001137445
97800	0.73779514	97788	0.028370552
97800	0.653696305	97792	0.04107663
97800	0.338512793	97796	0.034464367
97800	0.119577351	97800	0.031309042
97800	0.043186599	97804	0.072572017
97800	0.068460322	97808	0.082598563
97800	0.28052734	97812	0.074755859
97800	0.50125207	97816	0.048062811
97800	0.499700254	97820	0.023165521
97800	0.267286581	97824	0.076934281
97800	0.082643281	97828	0.13339
97800	0.063493339	97832	0.080872975

PowerSpectrumData

97800	0.044543634	97836	0.009856864
97800	0.158822731	97840	0.04421128
97800	0.474741711	97844	0.06545095
97800	0.658500765	97848	0.057824003
97900	0.480708928	97852	0.045754641
97900	0.130117521	97856	0.075241951
97900	0.098279299	97860	0.103691906
97900	0.052183415	97864	0.075707467
97900	0.065366578	97868	0.018783092
97900	0.112056623	97872	0.079092104
97900	0.165598409	97876	0.125524079
97900	0.148577251	97880	0.143392318
97900	0.057895813	97884	0.102274549
97900	0.015177301	97888	0.077988501
97900	0.007134388	97892	0.085588938
97900	0.086699554	97896	0.087266992
97900	0.3048199	97900	0.104077109
97900	0.526354531	97904	0.128256491
97900	0.542590045	97908	0.111452217
97900	0.316110848	97912	0.062746214
97900	0.111517053	97916	0.03086303
97900	0.170618994	97920	0.076683944
97900	0.059596627	97924	0.135644194
97900	0.097368989	97928	0.150980778
97900	0.103461956	97932	0.141197598
97900	0.053516709	97936	0.095894575
97900	0.057878617	97940	0.091432754
97900	0.090239133	97944	0.070450507
97900	0.079041645	97948	0.048709673
98000	0.039380997	97952	0.079713886
98000	0.028562483	97956	0.052426807
98000	0.161120231	97960	0.038952152
98000	0.282141235	97964	0.04448049
98000	0.276673585	97968	0.074536103

PowerSpectrumData

98000	0.13012084	97972	0.109969544
98000	0.043397944	97976	0.106478001
98000	0.075150085	97980	0.101995771
98000	0.07730064	97984	0.123277423
98000	0.124586994	97988	0.113567658
98000	0.105320323	97992	0.072221199
98000	0.079152618	97996	0.053930224
98000	0.092776732	98000	0.047875805
98000	0.081280807	98004	0.031590633
98000	0.047647111	98008	0.039209146
98000	0.002380292	98012	0.081766237
98000	0.047143767	98016	0.098505079
98000	0.071428265	98020	0.059701419
98000	0.077069228	98024	0.023483479
98000	0.078375153	98028	0.057569265
98000	0.070283903	98032	0.072993098
98000	0.061497252	98036	0.068459682
98000	0.047853318	98040	0.099614401
98000	0.109974659	98044	0.166542886
98000	0.137966054	98048	0.185469573
98100	0.079519188	98052	0.154462526
98100	0.023763119	98056	0.085874824
98100	0.005437926	98060	0.030674823
98100	0.029800487	98064	0.052563719
98100	0.055312816	98068	0.061983265
98100	0.072645969	98072	0.029263327
98100	0.031851552	98076	0.032946544
98100	0.02877873	98080	0.024926454
98100	0.03199702	98084	0.007800225
98100	0.019555009	98088	0.025923957
98100	0.017740127	98092	0.044973011
98100	0.033033604	98096	0.053171298
98100	0.032063465	98100	0.052251657
98100	0.031030191	98104	0.057049729

PowerSpectrumData

98100	0.067199922	98108	0.046301662
98100	0.09718533	98112	0.031600637
98100	0.088101246	98116	0.035853766
98100	0.044108223	98120	0.054358618
98100	0.054103581	98124	0.061021929
98100	0.075285301	98128	0.038877792
98100	0.059257927	98132	0.042737738
98100	0.01065883	98136	0.087962435
98100	0.026445876	98140	0.096295917
98100	0.033341716	98144	0.056694778
98100	0.019446952	98148	0.061312661
98200	0.05472072	98152	0.078206474
98200	0.050930044	98156	0.048489717
98200	0.061102801	98160	0.034813715
98200	0.048112357	98164	0.057821017
98200	0.029709958	98168	0.042402124
98200	0.026391637	98172	0.050539369
98200	0.034508757	98176	0.061418912
98200	0.093393406	98180	0.042230975
98200	0.104649618	98184	0.046348687
98200	0.041191346	98188	0.0536798
98200	0.024653269	98192	0.048498027
98200	0.045115805	98196	0.053485008
98200	0.041055551	98200	0.031502194
98200	0.033251356	98204	0.026854083
98200	0.052925541	98208	0.068283895
98200	0.069014146	98212	0.073643067
98200	0.058915251	98216	0.042861953
98200	0.023383498	98220	0.005951465
98200	0.016958491	98224	0.028408016
98200	0.055279168	98228	0.036187914
98200	0.088729452	98232	0.016292906
98200	0.093965915	98236	0.023686142
98200	0.089410554	98240	0.04118713

PowerSpectrumData

98200	0.080451893	98244	0.050039471
98200	0.095719632	98248	0.037066067
98300	0.079061254	98252	0.003727517
98300	0.03076229	98256	0.027306161
98300	0.032858421	98260	0.033822154
98300	0.015782531	98264	0.039788716
98300	0.06454956	98268	0.026657806
98300	0.080104379	98272	0.049415372
98300	0.04524261	98276	0.088482848
98300	0.037756479	98280	0.087143519
98300	0.0501904	98284	0.097237062
98300	0.069070811	98288	0.098279568
98300	0.090673399	98292	0.050203929
98300	0.03503159	98296	0.034231878
98300	0.07762602	98300	0.042732179
98300	0.102863327	98304	0.049508697
98300	0.065478052	98308	0.031827403
98300	0.039817955	98312	0.018187202
98300	0.041799656	98316	0.038996681
98300	0.023746003	98320	0.060209794
98300	0.022787946	98324	0.072667492
98300	0.033439061	98328	0.110427129
98300	0.032529937	98332	0.111198482
98300	0.04513641	98336	0.083930863
98300	0.081093269	98340	0.068181078
98300	0.09948502	98344	0.079239952
98300	0.073351563	98348	0.072154842
98400	0.009577566	98352	0.039986473
98400	0.063660264	98356	0.032989272
98400	0.055986206	98360	0.050244904
98400	0.030898969	98364	0.117416021
98400	0.023253884	98368	0.131030204
98400	0.02929718	98372	0.097387463
98400	0.044641802	98376	0.072879317

PowerSpectrumData

98400	0.073407653	98380	0.067966867
98400	0.071604503	98384	0.096525226
98400	0.045128501	98388	0.091730886
98400	0.035808964	98392	0.044777156
98400	0.015640564	98396	0.059193422
98400	0.044882258	98400	0.089350244
98400	0.032104959	98404	0.055199082
98400	0.069530273	98408	0.078609082
98400	0.059933907	98412	0.122901663
98400	0.034656114	98416	0.106540698
98400	0.015565071	98420	0.06578827
98400	0.019639141	98424	0.05866155
98400	0.012577962	98428	0.058465234
98400	0.016154727	98432	0.026648351
98400	0.002788591	98436	0.019170235
98400	0.020156358	98440	0.025952364
98400	0.036473455	98444	0.03721103
98400	0.041689651	98448	0.066947941
98500	0.026477881	98452	0.063329782
98500	0.047128913	98456	0.091646907
98500	0.036454992	98460	0.137231604
98500	0.043119984	98464	0.12240166
98500	0.023255187	98468	0.02175613
98500	0.013829232	98472	0.076464181
98500	0.028113598	98476	0.074534968
98500	0.036490106	98480	0.06676311
98500	0.068702393	98484	0.075316617
98500	0.081204213	98488	0.060255392
98500	0.044259581	98492	0.047535239
98500	0.03640194	98496	0.081347978
98500	0.065493085	98500	0.104798412
98500	0.081003862	98504	0.069102083
98500	0.081541752	98508	0.051190997
98500	0.062587664	98512	0.078854471

PowerSpectrumData

98500	0.049007293	98516	0.090174079
98500	0.05531303	98520	0.035971032
98500	0.084216415	98524	0.05733057
98500	0.09508025	98528	0.099002442
98500	0.06977078	98532	0.090477734
98500	0.050326529	98536	0.062663043
98500	0.050973984	98540	0.064328728
98500	0.055056509	98544	0.056045763
98500	0.042361131	98548	0.050706825
98600	0.015555235	98552	0.045981768
98600	0.030201596	98556	0.017618906
98600	0.052236326	98560	0.048614747
98600	0.058311558	98564	0.065844688
98600	0.03396688	98568	0.044984601
98600	0.025462203	98572	0.021021668
98600	0.029412977	98576	0.01151069
98600	0.032424981	98580	0.048669092
98600	0.033916462	98584	0.075992051
98600	0.031063959	98588	0.065726526
98600	0.014680185	98592	0.057255496
98600	0.04807509	98596	0.064722568
98600	0.083145991	98600	0.059159956
98600	0.084878055	98604	0.043660832
98600	0.095783624	98608	0.022340104
98600	0.089158246	98612	0.033090768
98600	0.039241539	98616	0.036256384
98600	0.017677565	98620	0.064604668
98600	0.031164316	98624	0.110975649
98600	0.023182942	98628	0.109093933
98600	0.054050208	98632	0.036431928
98600	0.091873488	98636	0.044209093
98600	0.087845059	98640	0.067415742
98600	0.056586774	98644	0.06111386
98600	0.030812571	98648	0.075645505

PowerSpectrumData

98700	0.025486048	98652	0.092759379
98700	0.025935147	98656	0.093377632
98700	0.011563779	98660	0.0698148
98700	0.010248586	98664	0.034386023
98700	0.027627815	98668	0.049970062
98700	0.035954097	98672	0.058544316
98700	0.064263448	98676	0.040795105
98700	0.107243221	98680	0.038681996
98700	0.115616451	98684	0.036382731
98700	0.106315471	98688	0.00071959
98700	0.081479993	98692	0.013740228
98700	0.02930245	98696	0.028322174
98700	0.058702306	98700	0.069029724
98700	0.087180422	98704	0.07241303
98700	0.10192232	98708	0.06674014
98700	0.084391679	98712	0.043172368
98700	0.050396637	98716	0.016163574
98700	0.063022148	98720	0.006891528
98700	0.083978754	98724	0.002367859
98700	0.065430817	98728	0.010936593
98700	0.061903782	98732	0.009291438
98700	0.039411847	98736	0.033111621
98700	0.000436611	98740	0.03884402
98700	0.024629926	98744	0.017217106
98700	0.026627218	98748	0.016450174
98800	0.00591104	98752	0.026969623
98800	0.028636399	98756	0.072559757
98800	0.056899498	98760	0.07036826
98800	0.059001166	98764	0.029047187
98800	0.052658023	98768	0.014880505
98800	0.072759453	98772	0.021270962
98800	0.099626101	98776	0.042814168
98800	0.098143457	98780	0.041359399
98800	0.075202726	98784	0.017159828

PowerSpectrumData

98800	0.061668754	98788	0.073324321
98800	0.07603463	98792	0.077039891
98800	0.078088546	98796	0.010022679
98800	0.051994626	98800	0.042053824
98800	0.049662427	98804	0.051318122
98800	0.054560544	98808	0.063545951
98800	0.049815564	98812	0.079307793
98800	0.037607984	98816	0.07810245
98800	0.017652235	98820	0.065341184
98800	0.048951817	98824	0.034523284
98800	0.077767785	98828	0.063574058
98800	0.061164275	98832	0.102648788
98800	0.049208415	98836	0.096408447
98800	0.054880438	98840	0.062620755
98800	0.048656882	98844	0.054263383
98800	0.043582066	98848	0.051348565
98900	0.030610783	98852	0.031753738
98900	0.019759928	98856	0.046408059
98900	0.019754863	98860	0.082296574
98900	0.017511136	98864	0.100623336
98900	0.006652919	98868	0.083657564
98900	0.004765421	98872	0.071726397
98900	0.020016269	98876	0.058962141
98900	0.075661228	98880	0.051707753
98900	0.120155361	98884	0.039972787
98900	0.113976996	98888	0.013358659
98900	0.071596245	98892	0.047030164
98900	0.032283067	98896	0.080149155
98900	0.022719718	98900	0.068466885
98900	0.017882678	98904	0.061512503
98900	0.033396136	98908	0.09082746
98900	0.070874288	98912	0.097877266
98900	0.088346533	98916	0.07205634
98900	0.058270867	98920	0.020017447

PowerSpectrumData

98900	0.02721338	98924	0.032317057
98900	0.032421409	98928	0.051123028
98900	0.033745022	98932	0.028831613
98900	0.027669952	98936	0.037152135
98900	0.027864029	98940	0.037479451
98900	0.052571413	98944	0.02473767
98900	0.059289898	98948	0.013194168
99000	0.08434023	98952	0.054441931
99000	0.058432557	98956	0.081629289
99000	0.033300625	98960	0.066995963
99000	0.008605824	98964	0.042401873
99000	0.044851571	98968	0.017048884
99000	0.076302364	98972	0.020482648
99000	0.047149337	98976	0.03344837
99000	0.013077864	98980	0.031619802
99000	0.033916243	98984	0.03224138
99000	0.030249459	98988	0.02501479
99000	0.009753164	98992	0.009976605
99000	0.03306727	98996	0.041270363
99000	0.046011504	99000	0.065599605
99000	0.058457001	99004	0.04105088
99000	0.059547641	99008	0.017296041
99000	0.042120133	99012	0.012125146
99000	0.030355463	99016	0.013304092
99000	0.019367852	99020	0.021234197
99000	0.055794051	99024	0.056540208
99000	0.045476503	99028	0.050944549
99000	0.050819079	99032	0.046564386
99000	0.058176109	99036	0.044658023
99000	0.055640805	99040	0.045568111
99000	0.045778117	99044	0.044267879
99000	0.040190986	99048	0.02634408
99100	0.041581112	99052	0.012394497
99100	0.021840853	99056	0.023260956

PowerSpectrumData

99100	0.033563312	99060	0.046270892
99100	0.037211994	99064	0.060855553
99100	0.034286295	99068	0.043698517
99100	0.05196327	99072	0.024964667
99100	0.064917585	99076	0.069956463
99100	0.059493628	99080	0.086196611
99100	0.050763341	99084	0.066503839
99100	0.028673909	99088	0.062807929
99100	0.044502962	99092	0.065825079
99100	0.067049106	99096	0.05964463
99100	0.042920572	99100	0.043440825
99100	0.010395404	99104	0.02524941
99100	0.028971423	99108	0.052611711
99100	0.054444281	99112	0.069828202
99100	0.039433147	99116	0.081229322
99100	0.016164304	99120	0.092571732
99100	0.033468667	99124	0.096337171
99100	0.045665394	99128	0.051518047
99100	0.049442206	99132	0.031989006
99100	0.025983272	99136	0.052114745
99100	0.029476465	99140	0.028357739
99100	0.048001737	99144	0.090693226
99100	0.044200173	99148	0.080351565
99200	0.020836571	99152	0.044815286
99200	0.014790169	99156	0.031989581
99200	0.027250102	99160	0.03937104
99200	0.018656565	99164	0.039893068
99200	0.007024827	99168	0.069299065
99200	0.009607436	99172	0.090290487
99200	0.037954011	99176	0.085647225
99200	0.045709458	99180	0.073672694
99200	0.01226332	99184	0.052601103
99200	0.040063813	99188	0.023250963
99200	0.026994161	99192	0.028621496

PowerSpectrumData

99200	0.035133518	99196	0.025256511
99200	0.040057388	99200	0.007348673
99200	0.028898228	99204	0.041440093
99200	0.039046929	99208	0.068530659
99200	0.035471701	99212	0.054911918
99200	0.0388523	99216	0.028169045
99200	0.015252097	99220	0.01464261
99200	0.065902736	99224	0.019298541
99200	0.064663407	99228	0.013909089
99200	0.04914129	99232	0.03833653
99200	0.061335915	99236	0.072829949
99200	0.064073349	99240	0.057847756
99200	0.049655904	99244	0.075658158
99200	0.048322138	99248	0.12052618
99300	0.020453046	99252	0.109756264
99300	0.040743409	99256	0.072246789
99300	0.074991949	99260	0.072609422
99300	0.05222188	99264	0.098016659
99300	0.047475471	99268	0.086603053
99300	0.06079394	99272	0.032711629
99300	0.056721387	99276	0.016878315
99300	0.051638999	99280	0.040447132
99300	0.072927505	99284	0.057076053
99300	0.095240393	99288	0.046660247
99300	0.057370766	99292	0.017075367
99300	0.012230709	99296	0.007465319
99300	0.03729506	99300	0.026329604
99300	0.053092725	99304	0.028643546
99300	0.043141154	99308	0.009928704
99300	0.012065114	99312	0.021900742
99300	0.031521293	99316	0.020631853
99300	0.058358426	99320	0.035190984
99300	0.070462294	99324	0.073640986
99300	0.070760565	99328	0.08834451

PowerSpectrumData

99300	0.058623955	99332	0.065007909
99300	0.04262826	99336	0.021851362
99300	0.018742574	99340	0.039075214
99300	0.029868374	99344	0.068440364
99300	0.054875138	99348	0.07144644
99400	0.085860251	99352	0.047041558
99400	0.085988781	99356	0.030554205
99400	0.05463136	99360	0.048335936
99400	0.024899697	99364	0.057662463
99400	0.005314896	99368	0.081583276
99400	0.041786945	99372	0.079113059
99400	0.062185434	99376	0.0577557
99400	0.051966814	99380	0.038547842
99400	0.065217071	99384	0.029713034
99400	0.081775885	99388	0.011079147
99400	0.069021829	99392	0.014224193
99400	0.066625726	99396	0.039671944
99400	0.090267262	99400	0.123047605
99400	0.102279111	99404	0.169686348
99400	0.074613723	99408	0.144753213
99400	0.027131444	99412	0.097432829
99400	0.013306684	99416	0.063251369
99400	0.008379007	99420	0.046381305
99400	0.041837793	99424	0.056451001
99400	0.061977145	99428	0.043779608
99400	0.01562752	99432	0.056002664
99400	0.028090941	99436	0.058453214
99400	0.024961859	99440	0.056543322
99400	0.047658195	99444	0.04082434
99400	0.072653922	99448	0.044889373
99500	0.087618806	99452	0.024978528
99500	0.070628143	99456	0.031815227
99500	0.043643064	99460	0.022635984
99500	0.023986255	99464	0.072889008

PowerSpectrumData

99500	0.024922247	99468	0.101062724
99500	0.019306626	99472	0.066742949
99500	0.01921161	99476	0.016390417
99500	0.021946391	99480	0.074128751
99500	0.001653424	99484	0.120230165
99500	0.04376665	99488	0.085039734
99500	0.074852746	99492	0.055532182
99500	0.078300356	99496	0.042265827
99500	0.085576205	99500	0.093555806
99500	0.081906008	99504	0.114042065
99500	0.067541871	99508	0.10079865
99500	0.049853436	99512	0.076382996
99500	0.048994385	99516	0.04538404
99500	0.038310787	99520	0.028347107
99500	0.013775368	99524	0.040604402
99500	0.001718911	99528	0.05549888
99500	0.02284457	99532	0.056828263
99500	0.040167819	99536	0.042904641
99500	0.025206522	99540	0.01889659
99500	0.036849975	99544	0.009081059
99500	0.028546505	99548	0.043946086
99600	0.019580702	99552	0.076323551
99600	0.040936779	99556	0.089722351
99600	0.033507487	99560	0.079771227
99600	0.030864925	99564	0.071123184
99600	0.034598117	99568	0.082717328
99600	0.03024917	99572	0.04157922
99600	0.028657092	99576	0.044750515
99600	0.046265046	99580	0.082621671
99600	0.046103258	99584	0.101986945
99600	0.051147694	99588	0.101667232
99600	0.035727237	99592	0.054276072
99600	0.045187346	99596	0.011966667
99600	0.043479737	99600	0.024543422

PowerSpectrumData

99600	0.029604073	99604	0.028598577
99600	0.002520972	99608	0.028025686
99600	0.037372447	99612	0.030108422
99600	0.07655903	99616	0.06989629
99600	0.082917803	99620	0.06656153
99600	0.051803876	99624	0.049599163
99600	0.040765008	99628	0.082065824
99600	0.032869102	99632	0.085378197
99600	0.018557488	99636	0.065978427
99600	0.069071597	99640	0.075801938
99600	0.105255182	99644	0.082267172
99600	0.081229882	99648	0.059707509
99700	0.04488054	99652	0.047591293
99700	0.05963341	99656	0.043316413
99700	0.029470934	99660	0.005510212
99700	0.008414482	99664	0.013504199
99700	0.034845947	99668	0.027683036
99700	0.060894803	99672	0.019862097
99700	0.051362422	99676	0.032831318
99700	0.015770957	99680	0.043500088
99700	0.014131633	99684	0.049075974
99700	0.024286854	99688	0.007559041
99700	0.039846425	99692	0.049454364
99700	0.046441441	99696	0.048682145
99700	0.009563015	99700	0.049878203
99700	0.038540224	99704	0.050823579
99700	0.045168752	99708	0.061293569
99700	0.03506384	99712	0.06568063
99700	0.029804816	99716	0.030610703
99700	0.037153586	99720	0.014848404
99700	0.026801106	99724	0.026308642
99700	0.05549127	99728	0.051634375
99700	0.094095354	99732	0.051261701
99700	0.065999549	99736	0.055064396

PowerSpectrumData

99700	0.022529359	99740	0.069201051
99700	0.037351372	99744	0.075550517
99700	0.032644264	99748	0.056075231
99800	0.028432562	99752	0.02599877
99800	0.025852753	99756	0.016881058
99800	0.031845597	99760	0.049348466
99800	0.030658237	99764	0.063684049
99800	0.035951725	99768	0.065229673
99800	0.068202935	99772	0.040693045
99800	0.054001677	99776	0.035709199
99800	0.05558729	99780	0.042600295
99800	0.053878644	99784	0.012515825
99800	0.058982427	99788	0.025398987
99800	0.050150695	99792	0.070216447
99800	0.042314259	99796	0.085388361
99800	0.030499727	99800	0.069241425
99800	0.019436136	99804	0.036361664
99800	0.056574758	99808	0.019326586
99800	0.053800937	99812	0.012446964
99800	0.010952908	99816	0.028728578
99800	0.024423409	99820	0.041191004
99800	0.04069756	99824	0.056299083
99800	0.031896791	99828	0.12832148
99800	0.020253454	99832	0.146998398
99800	0.028716195	99836	0.105352425
99800	0.030443935	99840	0.0464556
99800	0.031097781	99844	0.005724358
99800	0.015002151	99848	0.025335425
99900	0.036545196	99852	0.029346431
99900	0.042531119	99856	0.053289816
99900	0.028972372	99860	0.069505564
99900	0.059122089	99864	0.088181856
99900	0.084336331	99868	0.105457752
99900	0.055047738	99872	0.091698785

PowerSpectrumData

99900	0.034032924	99876	0.071614966
99900	0.053063111	99880	0.063396736
99900	0.043115208	99884	0.033478835
99900	0.021640262	99888	0.033336994
99900	0.019262305	99892	0.073144147
99900	0.023591738	99896	0.05805412
99900	0.032599262	99900	0.046904581
99900	0.0285111	99904	0.030035273
99900	0.027533024	99908	0.01980524
99900	0.035469551	99912	0.060221788
99900	0.03162718	99916	0.090894326
99900	0.054351414	99920	0.125142324
99900	0.083696679	99924	0.109399749
99900	0.051713141	99928	0.075802622
99900	0.029490655	99932	0.052590222
99900	0.076106873	99936	0.054565622
99900	0.105134423	99940	0.095537071
99900	0.115234347	99944	0.111919879
99900	0.091178714	99948	0.106886299
100000	0.075621414	99952	0.086600841
100000	0.082517254	99956	0.039526876
100000	0.068507041	99960	0.046666602
100000	0.034406487	99964	0.07053512
100000	0.003959171	99968	0.074660449
100000	0.010686541	99972	0.043631742
100000	0.04876246	99976	0.052773394
100000	0.05605612	99980	0.055550048
100000	0.046603596	99984	0.058020312
100000	0.029262325	99988	0.058217447
100000	0.048299673	99992	0.02920437
100000	0.070267452	99996	0.035836427
