# Supplementary material for: Understanding nonlinear vibration behaviours in high-power ultrasonic surgical devices
Source: Proc Math Phys Eng Sci. 2015 Apr 8;471(2176):20140906. doi: 10.1098/rspa.2014.0906 (PMC4991263; doi:10.1098/rspa.2014.0906)
Supplement: Bidirectional Sweep Data [file rspa20140906supp3.pdf]

Bi-Directional Sweeps

Response from Laser Doppler Vibrator (LDV) setting 125mm/s/V

I3 Piezoceramic stack temperatures are included for sweeps with a 1 sec time delay throughout and those with a 1 sec and 10 sec time delay.

I1
2V (LDV 125mm/s/V)   6V (LDV 125mm/s/V)   10V (LDV
125mm/s/V)   15V (LDV 125mm/s/V)   20V (LDV 125mm/s/V)
25V (LDV 125mm/s/V)   30V (LDV 125mm/s/V)   35V (LDV
125mm/s/V)   40V (LDV 125mm/s/V)   45V (LDV 125mm/s/V)
50V (LDV 125mm/s/V)
Frequency (Hz)   Response (V)   Frequency (Hz)   Response (V)   Frequency (Hz)
Response (V)   Frequency (Hz)   Response (V)   Frequency (Hz)   Response (V)
Frequency (Hz)   Response (V)   Frequency (Hz)   Response (V)   Frequency (Hz)
Response (V)   Frequency (Hz)   Response (V)   Frequency (Hz)   Response (V)
Frequency (Hz)   Response (V)
26200   2.04E-02   26200   6.03E-02   26200   1.08E-01   26190
1.88E-01   26190   3.33E-01   26130   2.88E-01   26130   3.70E-01
26130   4.76E-01   26120   5.52E-01   26120   6.70E-01   26120
8.15E-01
26201   2.05E-02   26201   6.09E-02   26201   1.10E-01   26192
1.88E-01   26192   3.40E-01   26132   2.91E-01   26132   3.88E-01
26132   5.19E-01   26122   6.05E-01   26122   7.54E-01   26122
9.32E-01
26202   2.07E-02   26202   6.14E-02   26202   1.11E-01   26194
1.91E-01   26194   3.48E-01   26134   2.94E-01   26134   3.98E-01
26134   5.38E-01   26124   6.22E-01   26124   7.68E-01   26124
9.67E-01
26203   2.08E-02   26203   6.21E-02   26203   1.11E-01   26196
1.95E-01   26196   3.58E-01   26136   2.97E-01   26136   4.07E-01
26136   5.46E-01   26126   6.35E-01   26126   7.83E-01   26126
9.95E-01
26204   2.09E-02   26204   6.24E-02   26204   7.03E-02   26198
1.99E-01   26198   3.74E-01   26138   3.02E-01   26138   4.15E-01
26138   5.58E-01   26128   6.50E-01   26128   8.00E-01   26128
1.02E+00
26205   2.11E-02   26205   6.29E-02   26205   8.09E-02   26200
2.03E-01   26200   3.86E-01   26140   3.06E-01   26140   4.23E-01
26140   5.70E-01   26130   6.61E-01   26130   8.16E-01   26130
1.04E+00
26206   2.14E-02   26206   6.36E-02   26206   1.12E-01   26202
2.06E-01   26202   4.02E-01   26142   3.12E-01   26142   4.32E-01
26142   5.83E-01   26132   6.73E-01   26132   8.33E-01   26132
1.07E+00
26207   2.15E-02   26207   6.38E-02   26207   7.83E-02   26204
2.12E-01   26204   4.29E-01   26144   3.17E-01   26144   4.43E-01
26144   5.95E-01   26134   6.88E-01   26134   8.52E-01   26134
1.10E+00
26208   2.17E-02   26208   6.47E-02   26208   1.18E-01   26206
2.18E-01   26206   4.51E-01   26146   3.22E-01   26146   4.51E-01
26146   6.09E-01   26136   6.99E-01   26136   8.72E-01   26136
1.14E+00
26209   2.18E-02   26209   6.49E-02   26209   1.19E-01   26208
2.23E-01   26208   4.82E-01   26148   3.28E-01   26148   4.62E-01
26148   6.23E-01   26138   7.17E-01   26138   8.92E-01   26138
1.17E+00
26210   2.20E-02   26210   6.58E-02   26210   1.19E-01   26210
2.28E-01   26210   5.11E-01   26150   3.34E-01   26150   4.72E-01
26150   6.39E-01   26140   7.33E-01   26140   9.13E-01   26140
1.21E+00
26211   2.22E-02   26211   6.62E-02   26211   5.76E-02   26212
2.35E-01   26212   5.41E-01   26152   3.41E-01   26152   4.83E-01
26152   6.53E-01   26142   7.49E-01   26142   9.38E-01   26142
1.26E+00
26212   2.24E-02   26212   6.68E-02   26212   1.20E-01   26214

```
2.42E-01          26214    5.80E-01          26154    3.48E-01          26154    4.96E-01
26154   6.69E-01          26144   7.68E-01          26144   9.65E-01          26144
1.30E+00
26213   2.27E-02          26213   6.71E-02          26213   1.22E-01          26216
2.50E-01          26216    6.24E-01          26156    3.55E-01          26156    5.08E-01
26156   6.89E-01          26146   7.91E-01          26146   9.90E-01          26146
1.36E+00
26214   2.28E-02          26214   6.78E-02          26214   1.25E-01          26218
2.60E-01          26218    6.76E-01          26158    3.64E-01          26158    5.21E-01
26158   7.08E-01          26148   8.10E-01          26148   1.02E+00          26148
1.42E+00
26215   2.30E-02          26215   6.85E-02          26215   1.25E-01          26220
2.68E-01          26220    7.41E-01          26160    3.72E-01          26160    5.34E-01
26160   7.25E-01          26150   8.32E-01          26150   1.06E+00          26150
1.49E+00
26216   2.32E-02          26216   6.91E-02          26216   1.26E-01          26222
2.79E-01          26222    8.22E-01          26162    3.82E-01          26162    5.51E-01
26162   7.46E-01          26152   8.59E-01          26152   1.10E+00          26152
1.58E+00
26217   2.33E-02          26217   6.97E-02          26217   1.27E-01          26224
2.91E-01          26224    9.83E-01          26164    3.91E-01          26164    5.66E-01
26164   7.70E-01          26154   8.86E-01          26154   1.15E+00          26154
1.71E+00
26218   2.35E-02          26218   7.06E-02          26218   1.29E-01          26226
3.03E-01          26226    1.17E+00          26166    4.04E-01          26166    5.83E-01
26166   8.00E-01          26156   9.13E-01          26156   1.19E+00          26156
1.96E+00
26219   2.38E-02          26219   7.10E-02          26219   1.30E-01          26228
3.16E-01          26228    1.55E+00          26168    4.15E-01          26168    6.00E-01
26168   8.24E-01          26158   9.40E-01          26158   1.25E+00          26158
4.48E+00
26220   2.40E-02          26220   7.19E-02          26220   1.31E-01          26230
3.35E-01          26230    1.79E+00          26170    4.27E-01          26170    6.18E-01
26170   8.55E-01          26160   9.74E-01          26160   1.32E+00          26160
4.42E+00
26221   2.43E-02          26221   7.24E-02          26221   1.33E-01          26232
3.55E-01          26232    1.76E+00          26172    4.39E-01          26172    6.39E-01
26172   8.89E-01          26162   1.01E+00          26162   1.39E+00          26162
4.36E+00
26222   2.43E-02          26222   7.32E-02          26222   1.33E-01          26234
3.78E-01          26234    1.69E+00          26174    4.52E-01          26174    6.59E-01
26174   9.29E-01          26164   1.05E+00          26164   1.48E+00          26164
4.29E+00
26223   2.47E-02          26223   7.36E-02          26223   1.34E-01          26236
4.05E-01          26236    1.63E+00          26176    4.67E-01          26176    6.85E-01
26176   9.67E-01          26166   1.10E+00          26166   1.62E+00          26166
4.23E+00
26224   2.52E-02          26224   7.44E-02          26224   1.37E-01          26238
4.52E-01          26238    1.58E+00          26178    4.83E-01          26178    7.12E-01
26178   1.01E+00          26168   1.15E+00          26168   2.43E+00          26168
4.18E+00
26225   2.51E-02          26225   7.57E-02          26225   1.38E-01          26240
4.89E-01          26240    1.53E+00          26180    5.02E-01          26180    7.38E-01
26180   1.06E+00          26170   1.23E+00          26170   4.22E+00          26170
4.12E+00
26226   2.52E-02          26226   7.60E-02          26226   1.40E-01          26242
5.59E-01          26242    1.48E+00          26182    5.19E-01          26182    7.68E-01
26182   1.13E+00          26172   1.32E+00          26172   4.15E+00          26172
4.06E+00
26227   2.57E-02          26227   7.69E-02          26227   1.41E-01          26244
6.26E-01          26244    1.43E+00          26184    5.38E-01          26184    8.04E-01
26184   1.21E+00          26174   1.44E+00          26174   4.08E+00          26174
4.00E+00
26228   2.56E-02          26228   7.78E-02          26228   1.43E-01          26246
6.97E-01          26246    1.38E+00          26186    5.62E-01          26186    8.43E-01
26186   1.35E+00          26176   1.66E+00          26176   4.01E+00          26176
3.94E+00
26229   2.62E-02          26229   7.82E-02          26229   1.44E-01          26248
```

7.80E-01 26248 1.33E+00 26188 5.85E-01 26188 8.84E-01
26188 1.58E+00 26178 3.95E+00 26178 3.94E+00 26178
3.88E+00
26230 2.64E-02 26230 7.90E-02 26230 1.46E-01 26250
8.30E-01 26250 1.29E+00 26190 6.12E-01 26190 9.36E-01
26190 3.56E+00 26180 3.86E+00 26180 3.87E+00 26180
3.82E+00
26231 2.66E-02 26231 7.99E-02 26231 1.48E-01 26252
8.55E-01 26252 1.24E+00 26192 6.39E-01 26192 1.00E+00
26192 3.47E+00 26182 3.78E+00 26182 3.80E+00 26182
3.76E+00
26232 2.71E-02 26232 8.08E-02 26232 1.49E-01 26254
8.71E-01 26254 1.20E+00 26194 6.69E-01 26194 1.07E+00
26194 3.40E+00 26184 3.72E+00 26184 3.73E+00 26184
3.70E+00
26233 2.71E-02 26233 8.19E-02 26233 1.51E-01 26256
8.64E-01 26256 1.16E+00 26196 7.03E-01 26196 1.17E+00
26196 3.34E+00 26186 3.64E+00 26186 3.67E+00 26186
3.64E+00
26234 2.76E-02 26234 8.26E-02 26234 1.54E-01 26258
8.37E-01 26258 1.12E+00 26198 7.43E-01 26198 1.32E+00
26198 3.26E+00 26188 3.57E+00 26188 3.60E+00 26188
3.57E+00
26235 2.78E-02 26235 8.42E-02 26235 1.55E-01 26260
8.15E-01 26260 1.08E+00 26200 7.86E-01 26200 3.15E+00
26200 3.20E+00 26190 3.50E+00 26190 3.54E+00 26190
3.52E+00
26236 2.80E-02 26236 8.41E-02 26236 1.57E-01 26262
7.88E-01 26262 1.04E+00 26202 8.38E-01 26202 3.05E+00
26202 3.12E+00 26192 3.42E+00 26192 3.47E+00 26192
3.45E+00
26237 2.83E-02 26237 8.59E-02 26237 1.58E-01 26264
7.60E-01 26264 1.00E+00 26204 9.08E-01 26204 2.98E+00
26204 3.05E+00 26194 3.36E+00 26194 3.41E+00 26194
3.40E+00
26238 2.86E-02 26238 8.61E-02 26238 1.61E-01 26266
7.35E-01 26266 9.66E-01 26206 1.01E+00 26206 2.90E+00
26206 2.98E+00 26196 3.29E+00 26196 3.34E+00 26196
3.34E+00
26239 2.92E-02 26239 8.88E-02 26239 1.64E-01 26268
7.09E-01 26268 9.30E-01 26208 1.13E+00 26208 2.83E+00
26208 2.91E+00 26198 3.22E+00 26198 3.28E+00 26198
3.28E+00
26240 2.91E-02 26240 8.98E-02 26240 1.66E-01 26270
6.82E-01 26270 8.98E-01 26210 1.33E+00 26210 2.75E+00
26210 2.85E+00 26200 3.16E+00 26200 3.22E+00 26200
3.22E+00
26241 2.97E-02 26241 8.83E-02 26241 1.68E-01 26272
6.59E-01 26272 8.69E-01 26212 2.59E+00 26212 2.68E+00
26212 2.79E+00 26202 3.09E+00 26202 3.16E+00 26202
3.17E+00
26242 2.97E-02 26242 9.15E-02 26242 1.71E-01 26274
6.37E-01 26274 8.43E-01 26214 2.50E+00 26214 2.61E+00
26214 2.73E+00 26204 3.02E+00 26204 3.10E+00 26204
3.12E+00
26243 2.99E-02 26243 9.21E-02 26243 1.73E-01 26276
6.15E-01 26276 8.17E-01 26216 2.42E+00 26216 2.55E+00
26216 2.66E+00 26206 2.96E+00 26206 3.04E+00 26206
3.07E+00
26244 3.05E-02 26244 9.27E-02 26244 1.76E-01 26278
5.95E-01 26278 7.92E-01 26218 2.35E+00 26218 2.48E+00
26218 2.59E+00 26208 2.90E+00 26208 2.98E+00 26208
3.01E+00
26245 3.13E-02 26245 9.49E-02 26245 1.78E-01 26280
5.77E-01 26280 7.66E-01 26220 2.28E+00 26220 2.42E+00
26220 2.53E+00 26210 2.84E+00 26210 2.92E+00 26210
2.96E+00
26246 3.08E-02 26246 9.47E-02 26246 1.81E-01 26282

5.57E-01 26282 7.43E-01 26222 2.22E+00 26222 2.36E+00
26222 2.47E+00 26212 2.78E+00 26212 2.87E+00 26212
2.91E+00
26247 3.17E-02 26247 9.63E-02 26247 1.85E-01 26284
5.39E-01 26284 7.22E-01 26224 2.15E+00 26224 2.29E+00
26224 2.41E+00 26214 2.72E+00 26214 2.81E+00 26214
2.85E+00
26248 3.14E-02 26248 9.79E-02 26248 1.88E-01 26286
5.23E-01 26286 7.01E-01 26226 2.09E+00 26226 2.24E+00
26226 2.35E+00 26216 2.66E+00 26216 2.76E+00 26216
2.80E+00
26249 3.31E-02 26249 9.87E-02 26249 1.91E-01 26288
5.07E-01 26288 6.81E-01 26228 2.02E+00 26228 2.18E+00
26228 2.29E+00 26218 2.61E+00 26218 2.71E+00 26218
2.75E+00
26250 3.37E-02 26250 1.00E-01 26250 1.95E-01 26290
4.92E-01 26290 6.63E-01 26230 1.96E+00 26230 2.12E+00
26230 2.24E+00 26220 2.55E+00 26220 2.65E+00 26220
2.70E+00
26251 3.31E-02 26251 1.01E-01 26251 1.98E-01 26292
4.78E-01 26292 6.45E-01 26232 1.90E+00 26232 2.06E+00
26232 2.19E+00 26222 2.50E+00 26222 2.60E+00 26222
2.65E+00
26252 3.41E-02 26252 1.03E-01 26252 2.02E-01 26294
4.64E-01 26294 6.27E-01 26234 1.84E+00 26234 2.01E+00
26234 2.13E+00 26224 2.45E+00 26224 2.55E+00 26224
2.60E+00
26253 3.47E-02 26253 1.04E-01 26253 2.06E-01 26296
4.52E-01 26296 6.10E-01 26236 1.78E+00 26236 1.95E+00
26236 2.08E+00 26226 2.40E+00 26226 2.50E+00 26226
2.56E+00
26254 3.44E-02 26254 1.06E-01 26254 2.10E-01 26298
4.40E-01 26298 5.93E-01 26238 1.73E+00 26238 1.89E+00
26238 2.03E+00 26228 2.34E+00 26228 2.45E+00 26228
2.51E+00
26255 3.45E-02 26255 1.08E-01 26255 2.13E-01 26300
4.29E-01 26300 5.77E-01 26240 1.68E+00 26240 1.84E+00
26240 1.98E+00 26230 2.29E+00 26230 2.40E+00 26230
2.46E+00
26256 3.50E-02 26256 1.09E-01 26256 2.20E-01 26302
4.19E-01 26302 5.63E-01 26242 1.62E+00 26242 1.79E+00
26242 1.93E+00 26232 2.24E+00 26232 2.35E+00 26232
2.41E+00
26257 3.63E-02 26257 1.10E-01 26257 2.25E-01 26304
4.08E-01 26304 5.49E-01 26244 1.57E+00 26244 1.74E+00
26244 1.88E+00 26234 2.19E+00 26234 2.30E+00 26234
2.36E+00
26258 3.59E-02 26258 1.13E-01 26258 2.30E-01 26306
3.99E-01 26306 5.34E-01 26246 1.53E+00 26246 1.70E+00
26246 1.84E+00 26236 2.14E+00 26236 2.25E+00 26236
2.31E+00
26259 3.68E-02 26259 1.14E-01 26259 2.36E-01 26308
3.90E-01 26308 5.27E-01 26248 1.48E+00 26248 1.65E+00
26248 1.80E+00 26238 2.09E+00 26238 2.20E+00 26238
2.27E+00
26260 3.69E-02 26260 1.16E-01 26260 2.42E-01 26310
3.81E-01 26310 5.08E-01 26250 1.44E+00 26250 1.61E+00
26250 1.75E+00 26240 2.05E+00 26240 2.16E+00 26240
2.23E+00
26261 3.75E-02 26261 1.17E-01 26261 2.48E-01 26312
3.72E-01 26312 4.96E-01 26252 1.39E+00 26252 1.57E+00
26252 1.71E+00 26242 2.00E+00 26242 2.11E+00 26242
2.18E+00
26262 3.80E-02 26262 1.19E-01 26262 2.53E-01 26314
3.64E-01 26314 4.84E-01 26254 1.35E+00 26254 1.53E+00
26254 1.66E+00 26244 1.95E+00 26244 2.07E+00 26244
2.14E+00
26263 3.88E-02 26263 1.21E-01 26263 2.61E-01 26314

3.64E-01 26314 4.84E-01 26254 1.35E+00 26254 1.53E+00
26254 1.67E+00 26244 1.96E+00 26244 2.07E+00 26244
2.14E+00
26264 3.93E-02 26264 1.23E-01 26264 2.69E-01 26312
3.73E-01 26312 4.95E-01 26252 1.40E+00 26252 1.58E+00
26252 1.71E+00 26242 2.01E+00 26242 2.12E+00 26242
2.20E+00
26265 3.96E-02 26265 1.25E-01 26265 2.76E-01 26310
3.81E-01 26310 5.07E-01 26250 1.44E+00 26250 1.62E+00
26250 1.76E+00 26240 2.06E+00 26240 2.17E+00 26240
2.25E+00
26266 4.08E-02 26266 1.27E-01 26266 2.85E-01 26308
3.90E-01 26308 5.21E-01 26248 1.49E+00 26248 1.67E+00
26248 1.81E+00 26238 2.11E+00 26238 2.22E+00 26238
2.30E+00
26267 4.11E-02 26267 1.29E-01 26267 2.92E-01 26306
4.00E-01 26306 5.33E-01 26246 1.53E+00 26246 1.72E+00
26246 1.85E+00 26236 2.17E+00 26236 2.27E+00 26236
2.35E+00
26268 4.14E-02 26268 1.31E-01 26268 3.02E-01 26304
4.10E-01 26304 5.47E-01 26244 1.58E+00 26244 1.77E+00
26244 1.90E+00 26234 2.22E+00 26234 2.33E+00 26234
2.40E+00
26269 4.18E-02 26269 1.33E-01 26269 3.11E-01 26302
4.22E-01 26302 5.61E-01 26242 1.63E+00 26242 1.82E+00
26242 1.94E+00 26232 2.28E+00 26232 2.38E+00 26232
2.46E+00
26270 4.25E-02 26270 1.35E-01 26270 3.18E-01 26300
4.33E-01 26300 5.76E-01 26240 1.67E+00 26240 1.88E+00
26240 2.00E+00 26230 2.34E+00 26230 2.43E+00 26230
2.52E+00
26271 4.29E-02 26271 1.37E-01 26271 3.25E-01 26298
4.46E-01 26298 5.94E-01 26238 1.73E+00 26238 1.93E+00
26238 2.05E+00 26228 2.39E+00 26228 2.48E+00 26228
2.57E+00
26272 4.39E-02 26272 1.40E-01 26272 3.32E-01 26296
4.58E-01 26296 6.08E-01 26236 1.78E+00 26236 1.98E+00
26236 2.10E+00 26226 2.45E+00 26226 2.54E+00 26226
2.62E+00
26273 4.46E-02 26273 1.42E-01 26273 3.41E-01 26294
4.70E-01 26294 6.27E-01 26234 1.83E+00 26234 2.04E+00
26234 2.15E+00 26224 2.51E+00 26224 2.59E+00 26224
2.67E+00
26274 4.56E-02 26274 1.45E-01 26274 3.44E-01 26292
4.85E-01 26292 6.46E-01 26232 1.89E+00 26232 2.10E+00
26232 2.21E+00 26222 2.57E+00 26222 2.64E+00 26222
2.72E+00
26275 4.57E-02 26275 1.47E-01 26275 3.48E-01 26290
5.00E-01 26290 6.63E-01 26230 1.94E+00 26230 2.15E+00
26230 2.25E+00 26220 2.62E+00 26220 2.70E+00 26220
2.77E+00
26276 4.70E-02 26276 1.50E-01 26276 3.52E-01 26288
5.15E-01 26288 6.85E-01 26228 2.00E+00 26228 2.21E+00
26228 2.31E+00 26218 2.68E+00 26218 2.75E+00 26218
2.82E+00
26277 4.76E-02 26277 1.52E-01 26277 3.56E-01 26286
5.32E-01 26286 7.04E-01 26226 2.05E+00 26226 2.27E+00
26226 2.36E+00 26216 2.74E+00 26216 2.81E+00 26216
2.88E+00
26278 4.84E-02 26278 1.55E-01 26278 3.51E-01 26284
5.48E-01 26284 7.28E-01 26224 2.11E+00 26224 2.33E+00
26224 2.41E+00 26214 2.79E+00 26214 2.86E+00 26214
2.94E+00
26279 4.89E-02 26279 1.58E-01 26279 3.54E-01 26282
5.66E-01 26282 7.50E-01 26222 2.17E+00 26222 2.38E+00
26222 2.48E+00 26212 2.85E+00 26212 2.92E+00 26212
2.99E+00
26280 4.98E-02 26280 1.60E-01 26280 3.54E-01 26280

```
5.85E-01        26280   7.75E-01        26220   2.23E+00        26220   2.44E+00
26220   2.53E+00        26210   2.91E+00        26210   2.98E+00        26210
3.05E+00
26281   5.04E-02        26281   1.64E-01        26281   3.51E-01        26278
6.05E-01        26278   8.01E-01        26218   2.29E+00        26218   2.51E+00
26218   2.59E+00        26208   2.97E+00        26208   3.04E+00        26208
3.10E+00
26282   5.16E-02        26282   1.67E-01        26282   3.53E-01        26276
6.28E-01        26276   8.29E-01        26216   2.34E+00        26216   2.58E+00
26216   2.65E+00        26206   3.03E+00        26206   3.09E+00        26206
3.16E+00
26283   5.20E-02        26283   1.70E-01        26283   3.51E-01        26274
6.48E-01        26274   8.56E-01        26214   2.40E+00        26214   2.63E+00
26214   2.71E+00        26204   3.09E+00        26204   3.14E+00        26204
3.22E+00
26284   5.33E-02        26284   1.73E-01        26284   3.50E-01        26272
6.72E-01        26272   8.84E-01        26212   2.46E+00        26212   2.69E+00
26212   2.76E+00        26202   3.14E+00        26202   3.20E+00        26202
3.27E+00
26285   5.39E-02        26285   1.76E-01        26285   3.46E-01        26270
6.97E-01        26270   9.15E-01        26210   2.51E+00        26210   2.76E+00
26210   2.82E+00        26200   3.21E+00        26200   3.25E+00        26200
3.32E+00
26286   5.47E-02        26286   1.79E-01        26286   3.47E-01        26268
7.23E-01        26268   9.47E-01        26208   2.57E+00        26208   2.81E+00
26208   2.88E+00        26198   3.26E+00        26198   3.31E+00        26198
3.38E+00
26287   5.59E-02        26287   1.82E-01        26287   3.43E-01        26266
7.48E-01        26266   9.80E-01        26206   2.65E+00        26206   2.87E+00
26206   2.93E+00        26196   3.32E+00        26196   3.36E+00        26196
3.43E+00
26288   5.69E-02        26288   1.85E-01        26288   3.40E-01        26264
7.79E-01        26264   1.02E+00        26204   2.56E+00        26204   2.93E+00
26204   2.99E+00        26194   3.38E+00        26194   3.42E+00        26194
3.49E+00
26289   5.75E-02        26289   1.88E-01        26289   3.37E-01        26262
8.03E-01        26262   1.05E+00        26202   1.73E+00        26202   2.99E+00
26202   3.05E+00        26192   3.44E+00        26192   3.47E+00        26192
3.54E+00
26290   5.91E-02        26290   1.91E-01        26290   3.35E-01        26260
8.26E-01        26260   1.09E+00        26200   1.24E+00        26200   3.06E+00
26200   3.11E+00        26190   3.50E+00        26190   3.52E+00        26190
3.60E+00
26291   5.96E-02        26291   1.94E-01        26291   3.33E-01        26258
8.42E-01        26258   1.14E+00        26198   1.05E+00        26198   3.11E+00
26198   3.17E+00        26188   3.56E+00        26188   3.58E+00        26188
3.65E+00
26292   6.08E-02        26292   1.96E-01        26292   3.31E-01        26256
8.56E-01        26256   1.18E+00        26196   8.97E-01        26196   3.17E+00
26196   3.21E+00        26186   3.61E+00        26186   3.63E+00        26186
3.70E+00
26293   6.17E-02        26293   1.99E-01        26293   3.27E-01        26254
8.62E-01        26254   1.22E+00        26194   8.26E-01        26194   3.24E+00
26194   3.27E+00        26184   3.67E+00        26184   3.68E+00        26184
3.75E+00
26294   6.28E-02        26294   2.01E-01        26294   3.24E-01        26252
8.46E-01        26252   1.26E+00        26192   7.67E-01        26192   2.72E+00
26192   3.32E+00        26182   3.72E+00        26182   3.74E+00        26182
3.80E+00
26295   6.39E-02        26295   2.03E-01        26295   3.21E-01        26250
8.14E-01        26250   1.31E+00        26190   7.18E-01        26190   1.51E+00
26190   3.38E+00        26180   3.77E+00        26180   3.80E+00        26180
3.85E+00
26296   6.49E-02        26296   2.05E-01        26296   3.18E-01        26248
7.71E-01        26248   1.35E+00        26188   6.78E-01        26188   1.23E+00
26188   3.44E+00        26178   3.82E+00        26178   3.85E+00        26178
3.90E+00
26297   6.62E-02        26297   2.06E-01        26297   3.16E-01        26246
```

7.17E-01 26246 1.39E+00 26186 6.43E-01 26186 1.10E+00
26186 3.49E+00 26176 3.88E+00 26176 3.89E+00 26176
3.96E+00
26298 6.69E-02 26298 2.08E-01 26298 3.12E-01 26244
6.59E-01 26244 1.43E+00 26184 6.12E-01 26184 1.00E+00
26184 3.55E+00 26174 3.93E+00 26174 3.95E+00 26174
4.01E+00
26299 6.78E-02 26299 2.09E-01 26299 3.10E-01 26242
5.80E-01 26242 1.47E+00 26182 5.82E-01 26182 9.09E-01
26182 3.61E+00 26172 3.98E+00 26172 4.00E+00 26172
4.06E+00
26300 6.90E-02 26300 2.11E-01 26300 3.08E-01 26240
5.22E-01 26240 1.52E+00 26180 5.57E-01 26180 8.54E-01
26180 3.68E+00 26170 4.04E+00 26170 4.05E+00 26170
4.10E+00
26301 6.99E-02 26301 2.12E-01 26301 3.07E-01 26238
4.69E-01 26238 1.56E+00 26178 5.36E-01 26178 8.09E-01
26178 3.69E+00 26168 3.91E+00 26168 4.10E+00 26168
4.16E+00
26302 7.11E-02 26302 2.12E-01 26302 3.03E-01 26236
4.29E-01 26236 1.60E+00 26176 5.13E-01 26176 7.74E-01
26176 1.78E+00 26166 1.79E+00 26166 4.14E+00 26166
4.20E+00
26303 7.17E-02 26303 2.12E-01 26303 3.01E-01 26234
3.99E-01 26234 1.65E+00 26174 4.94E-01 26174 7.40E-01
26174 1.39E+00 26164 1.47E+00 26164 4.18E+00 26164
4.25E+00
26304 7.27E-02 26304 2.12E-01 26304 2.98E-01 26232
3.69E-01 26232 1.70E+00 26172 4.75E-01 26172 7.09E-01
26172 1.24E+00 26162 1.33E+00 26162 4.22E+00 26162
4.29E+00
26305 7.34E-02 26305 2.12E-01 26305 2.95E-01 26230
3.49E-01 26230 1.76E+00 26170 4.57E-01 26170 6.85E-01
26170 1.14E+00 26160 1.24E+00 26160 4.27E+00 26160
4.32E+00
26306 7.37E-02 26306 2.11E-01 26306 2.91E-01 26228
3.31E-01 26228 1.80E+00 26168 4.42E-01 26168 6.60E-01
26168 1.06E+00 26158 1.16E+00 26158 4.31E+00 26158
4.36E+00
26307 7.48E-02 26307 2.10E-01 26307 2.88E-01 26226
3.16E-01 26226 1.82E+00 26166 4.27E-01 26166 6.35E-01
26166 9.99E-01 26156 1.10E+00 26156 4.36E+00 26156
4.41E+00
26308 7.55E-02 26308 2.10E-01 26308 2.85E-01 26224
3.01E-01 26224 1.76E+00 26164 4.12E-01 26164 6.15E-01
26164 9.47E-01 26154 1.05E+00 26154 2.21E+00 26154
4.45E+00
26309 7.59E-02 26309 2.09E-01 26309 2.83E-01 26222
2.90E-01 26222 1.58E+00 26162 4.00E-01 26162 5.96E-01
26162 8.98E-01 26152 1.00E+00 26152 1.66E+00 26152
4.49E+00
26310 7.64E-02 26310 2.08E-01 26310 2.85E-01 26220
2.79E-01 26220 1.30E+00 26160 3.88E-01 26160 5.79E-01
26160 8.41E-01 26150 9.64E-01 26150 1.48E+00 26150
4.54E+00
26311 7.66E-02 26311 2.07E-01 26311 2.82E-01 26218
2.70E-01 26218 1.06E+00 26158 3.77E-01 26158 5.59E-01
26158 8.04E-01 26148 9.23E-01 26148 1.36E+00 26148
4.59E+00
26312 7.68E-02 26312 2.05E-01 26312 2.79E-01 26216
2.59E-01 26216 8.92E-01 26156 3.67E-01 26156 5.44E-01
26156 7.75E-01 26146 8.91E-01 26146 1.28E+00 26146
2.23E+00
26313 7.73E-02 26313 2.04E-01 26313 2.76E-01 26214
2.51E-01 26214 7.86E-01 26154 3.58E-01 26154 5.30E-01
26154 7.47E-01 26144 8.62E-01 26144 1.21E+00 26144
1.74E+00
26314 7.74E-02 26314 2.02E-01 26314 2.73E-01 26212

2.42E-01 26212 6.94E-01 26152 3.48E-01 26152 5.14E-01
26152 7.21E-01 26142 8.34E-01 26142 1.15E+00 26142
1.50E+00
26315 7.77E-02 26315 2.01E-01 26315 2.70E-01 26210
2.36E-01 26210 6.38E-01 26150 3.42E-01 26150 5.03E-01
26150 6.96E-01 26140 8.10E-01 26140 1.10E+00 26140
1.41E+00
26316 7.80E-02 26316 2.00E-01 26316 2.66E-01 26208
2.29E-01 26208 5.86E-01 26148 3.33E-01 26148 4.91E-01
26148 6.79E-01 26138 7.89E-01 26138 1.06E+00 26138
1.34E+00
26317 7.74E-02 26317 1.98E-01 26317 2.64E-01 26206
2.23E-01 26206 5.35E-01 26146 3.27E-01 26146 4.79E-01
26146 6.60E-01 26136 7.66E-01 26136 1.02E+00 26136
1.28E+00
26318 7.81E-02 26318 1.97E-01 26318 2.60E-01 26204
2.17E-01 26204 5.00E-01 26144 3.19E-01 26144 4.67E-01
26144 6.42E-01 26134 7.39E-01 26134 9.82E-01 26134
1.22E+00
26319 7.75E-02 26319 1.95E-01 26319 2.58E-01 26202
2.13E-01 26202 4.67E-01 26142 3.13E-01 26142 4.56E-01
26142 6.25E-01 26132 7.21E-01 26132 9.49E-01 26132
1.18E+00
26320 7.74E-02 26320 1.94E-01 26320 2.55E-01 26200
2.08E-01 26200 4.42E-01 26140 3.06E-01 26140 4.46E-01
26140 6.10E-01 26130 7.04E-01 26130 9.22E-01 26130
1.14E+00
26321 7.75E-02 26321 1.92E-01 26321 2.57E-01 26198
2.03E-01 26198 4.19E-01 26138 3.01E-01 26138 4.36E-01
26138 5.95E-01 26128 6.89E-01 26128 8.95E-01 26128
1.09E+00
26322 7.68E-02 26322 1.90E-01 26322 2.56E-01 26196
1.99E-01 26196 3.95E-01 26136 2.95E-01 26136 4.26E-01
26136 5.80E-01 26126 6.73E-01 26126 8.71E-01 26126
1.06E+00
26323 7.60E-02 26323 1.89E-01 26323 2.08E-01 26194
1.95E-01 26194 3.77E-01 26134 2.90E-01 26134 4.16E-01
26134 5.67E-01 26124 6.59E-01 26124 8.45E-01 26124
1.02E+00
26324 7.57E-02 26324 1.88E-01 26324 1.06E-01 26192
1.90E-01 26192 3.62E-01 26132 2.85E-01 26132 4.07E-01
26132 5.55E-01 26122 6.44E-01 26122 8.24E-01 26122
9.96E-01
26325 7.51E-02 26325 1.86E-01 26325 1.25E-01 26190
1.87E-01 26190 3.50E-01 26130 2.80E-01 26130 3.98E-01
26130 5.43E-01 26120 6.30E-01 26120 8.03E-01 26120
9.67E-01
26326 7.43E-02 26326 1.85E-01 26326 2.17E-01

26327 7.40E-02 26327 1.84E-01 26327 1.64E-01

26328 7.32E-02 26328 1.82E-01 26328 4.90E-02

26329 7.27E-02 26329 1.80E-01 26329 7.02E-02

26330 7.18E-02 26330 1.79E-01 26330 2.35E-01

26331 7.16E-02 26331 1.77E-01 26331 2.43E-01

26332 7.01E-02 26332 1.76E-01 26332 1.56E-01

26333	6.92E-02	26333	1.73E-01	26333	1.95E-01
26334	6.91E-02	26334	1.72E-01	26334	2.04E-01
26335	6.80E-02	26335	1.70E-01	26335	2.33E-01
26336	6.72E-02	26336	1.68E-01	26336	2.25E-01
26337	6.64E-02	26337	1.66E-01	26337	2.28E-01
26338	6.53E-02	26338	1.65E-01	26338	2.26E-01
26339	6.46E-02	26339	1.63E-01	26339	2.23E-01
26340	6.33E-02	26340	1.61E-01	26340	2.20E-01
26341	6.27E-02	26341	1.59E-01	26341	2.18E-01
26342	6.14E-02	26342	1.58E-01	26342	2.16E-01
26343	6.10E-02	26343	1.56E-01	26343	2.15E-01
26344	5.97E-02	26344	1.54E-01	26344	2.11E-01
26345	5.87E-02	26345	1.53E-01	26345	2.09E-01
26346	5.80E-02	26346	1.51E-01	26346	2.07E-01
26347	5.75E-02	26347	1.49E-01	26347	2.06E-01
26348	5.65E-02	26348	1.48E-01	26348	2.03E-01
26349	5.58E-02	26349	1.45E-01	26349	2.02E-01
26350	5.46E-02	26350	1.44E-01	26350	1.98E-01
26351	5.42E-02	26351	1.42E-01	26351	1.97E-01
26352	5.34E-02	26352	1.40E-01	26352	1.94E-01
26353	5.28E-02	26353	1.39E-01	26353	1.94E-01
26354	5.19E-02	26354	1.38E-01	26354	1.93E-01
26355	5.12E-02	26355	1.35E-01	26355	1.90E-01

26356	5.04E-02	26356	1.34E-01	26356	1.89E-01
26357	4.95E-02	26357	1.33E-01	26357	1.87E-01
26358	4.84E-02	26358	1.31E-01	26358	1.85E-01
26359	4.82E-02	26359	1.30E-01	26359	1.83E-01
26360	4.76E-02	26360	1.28E-01	26360	1.81E-01
26361	4.57E-02	26361	1.26E-01	26361	1.80E-01
26362	4.59E-02	26362	1.25E-01	26362	1.79E-01
26363	4.59E-02	26363	1.23E-01	26363	1.77E-01
26364	4.40E-02	26364	1.22E-01	26364	1.75E-01
26365	4.46E-02	26365	1.21E-01	26365	1.75E-01
26366	4.30E-02	26366	1.19E-01	26366	1.73E-01
26367	4.43E-02	26367	1.18E-01	26367	1.72E-01
26368	4.19E-02	26368	1.16E-01	26368	1.71E-01
26369	4.06E-02	26369	1.15E-01	26369	1.68E-01
26370	4.02E-02	26370	1.14E-01	26370	1.68E-01
26371	4.05E-02	26371	1.12E-01	26371	1.65E-01
26372	4.13E-02	26372	1.11E-01	26372	1.65E-01
26373	3.87E-02	26373	1.09E-01	26373	1.63E-01
26374	3.84E-02	26374	1.08E-01	26374	1.61E-01
26375	3.78E-02	26375	1.07E-01	26375	1.60E-01
26376	3.73E-02	26376	1.06E-01	26376	1.58E-01
26377	3.66E-02	26377	1.04E-01	26377	1.58E-01
26378	3.69E-02	26378	1.03E-01	26378	1.56E-01

26379	3.57E-02	26379	1.02E-01	26379	1.54E-01
26380	3.56E-02	26380	1.01E-01	26380	1.54E-01
26381	3.48E-02	26381	9.96E-02	26381	1.54E-01
26382	3.44E-02	26382	9.85E-02	26382	1.52E-01
26383	3.40E-02	26383	9.74E-02	26383	1.51E-01
26384	3.37E-02	26384	9.66E-02	26384	1.49E-01
26385	3.34E-02	26385	9.53E-02	26385	1.47E-01
26386	3.27E-02	26386	9.43E-02	26386	1.47E-01
26387	3.23E-02	26387	9.33E-02	26387	1.46E-01
26388	3.20E-02	26388	9.22E-02	26388	1.45E-01
26389	3.18E-02	26389	9.13E-02	26389	1.43E-01
26390	3.09E-02	26390	9.04E-02	26390	1.41E-01
26391	3.06E-02	26391	8.94E-02	26391	1.39E-01
26392	3.04E-02	26392	8.79E-02	26392	1.38E-01
26393	3.03E-02	26393	8.76E-02	26393	1.39E-01
26394	2.98E-02	26394	8.67E-02	26394	1.36E-01
26395	2.94E-02	26395	8.58E-02	26395	1.35E-01
26396	2.91E-02	26396	8.47E-02	26396	1.34E-01
26397	2.89E-02	26397	8.38E-02	26397	1.34E-01
26398	2.85E-02	26398	8.34E-02	26398	1.33E-01
26399	2.83E-02	26399	8.26E-02	26399	1.31E-01
26400	2.77E-02	26400	8.14E-02	26400	1.29E-01

26400	2.78E-02	26400	8.17E-02	26400	1.30E-01
26399	2.82E-02	26399	8.27E-02	26399	1.31E-01
26398	2.86E-02	26398	8.32E-02	26398	1.33E-01
26397	2.87E-02	26397	8.40E-02	26397	1.33E-01
26396	2.93E-02	26396	8.51E-02	26396	1.35E-01
26395	2.95E-02	26395	8.61E-02	26395	1.36E-01
26394	3.00E-02	26394	8.71E-02	26394	1.37E-01
26393	3.02E-02	26393	8.84E-02	26393	1.39E-01
26392	3.06E-02	26392	8.94E-02	26392	1.39E-01
26391	3.09E-02	26391	8.99E-02	26391	1.41E-01
26390	3.13E-02	26390	9.02E-02	26390	1.43E-01
26389	3.18E-02	26389	9.19E-02	26389	1.43E-01
26388	3.21E-02	26388	9.25E-02	26388	1.45E-01
26387	3.25E-02	26387	9.34E-02	26387	1.46E-01
26386	3.30E-02	26386	9.53E-02	26386	1.47E-01
26385	3.35E-02	26385	9.64E-02	26385	1.49E-01
26384	3.38E-02	26384	9.72E-02	26384	1.50E-01
26383	3.42E-02	26383	9.84E-02	26383	1.51E-01
26382	3.48E-02	26382	9.89E-02	26382	1.53E-01
26381	3.55E-02	26381	1.00E-01	26381	1.54E-01
26380	3.58E-02	26380	1.03E-01	26380	1.56E-01
26379	3.64E-02	26379	1.04E-01	26379	1.57E-01
26378	3.68E-02	26378	1.05E-01	26378	1.59E-01

26377	3.73E-02	26377	1.03E-01	26377	1.60E-01
26376	3.78E-02	26376	1.04E-01	26376	1.62E-01
26375	3.83E-02	26375	1.06E-01	26375	1.62E-01
26374	3.88E-02	26374	1.10E-01	26374	1.64E-01
26373	3.95E-02	26373	1.11E-01	26373	1.66E-01
26372	4.01E-02	26372	1.13E-01	26372	1.67E-01
26371	4.06E-02	26371	1.14E-01	26371	1.68E-01
26370	4.13E-02	26370	1.16E-01	26370	1.69E-01
26369	4.18E-02	26369	1.16E-01	26369	1.71E-01
26368	4.25E-02	26368	1.19E-01	26368	1.73E-01
26367	4.32E-02	26367	1.20E-01	26367	1.74E-01
26366	4.39E-02	26366	1.22E-01	26366	1.76E-01
26365	4.47E-02	26365	1.22E-01	26365	1.78E-01
26364	4.53E-02	26364	1.24E-01	26364	1.79E-01
26363	4.60E-02	26363	1.26E-01	26363	1.80E-01
26362	4.68E-02	26362	1.27E-01	26362	1.82E-01
26361	4.76E-02	26361	1.29E-01	26361	1.83E-01
26360	4.82E-02	26360	1.30E-01	26360	1.85E-01
26359	4.89E-02	26359	1.32E-01	26359	1.86E-01
26358	4.99E-02	26358	1.34E-01	26358	1.88E-01
26357	5.03E-02	26357	1.36E-01	26357	1.90E-01
26356	5.12E-02	26356	1.37E-01	26356	1.92E-01
26355	5.21E-02	26355	1.39E-01	26355	1.93E-01

26354	5.30E-02	26354	1.41E-01	26354	1.95E-01
26353	5.40E-02	26353	1.43E-01	26353	1.97E-01
26352	5.48E-02	26352	1.44E-01	26352	1.99E-01
26351	5.57E-02	26351	1.45E-01	26351	2.01E-01
26350	5.65E-02	26350	1.48E-01	26350	2.03E-01
26349	5.75E-02	26349	1.50E-01	26349	2.05E-01
26348	5.83E-02	26348	1.51E-01	26348	2.07E-01
26347	5.92E-02	26347	1.53E-01	26347	2.09E-01
26346	5.96E-02	26346	1.55E-01	26346	2.12E-01
26345	6.10E-02	26345	1.56E-01	26345	2.13E-01
26344	6.17E-02	26344	1.58E-01	26344	2.15E-01
26343	6.28E-02	26343	1.60E-01	26343	2.17E-01
26342	6.36E-02	26342	1.62E-01	26342	2.19E-01
26341	6.45E-02	26341	1.63E-01	26341	2.21E-01
26340	6.53E-02	26340	1.66E-01	26340	2.23E-01
26339	6.62E-02	26339	1.67E-01	26339	2.25E-01
26338	6.70E-02	26338	1.68E-01	26338	2.27E-01
26337	6.80E-02	26337	1.70E-01	26337	2.29E-01
26336	6.88E-02	26336	1.72E-01	26336	2.32E-01
26335	6.98E-02	26335	1.74E-01	26335	2.34E-01
26334	7.11E-02	26334	1.75E-01	26334	2.36E-01
26333	7.16E-02	26333	1.78E-01	26333	2.39E-01

26332	7.24E-02	26332	1.79E-01	26332	2.41E-01
26331	7.32E-02	26331	1.79E-01	26331	2.42E-01
26330	7.40E-02	26330	1.81E-01	26330	2.44E-01
26329	7.50E-02	26329	1.84E-01	26329	2.46E-01
26328	7.57E-02	26328	1.83E-01	26328	2.47E-01
26327	7.59E-02	26327	1.86E-01	26327	2.50E-01
26326	7.63E-02	26326	1.87E-01	26326	2.53E-01
26325	7.64E-02	26325	1.88E-01	26325	2.55E-01
26324	7.68E-02	26324	1.90E-01	26324	2.56E-01
26323	7.78E-02	26323	1.91E-01	26323	2.59E-01
26322	7.83E-02	26322	1.92E-01	26322	2.60E-01
26321	7.87E-02	26321	1.93E-01	26321	2.63E-01
26320	7.88E-02	26320	1.95E-01	26320	2.66E-01
26319	7.91E-02	26319	1.96E-01	26319	2.68E-01
26318	7.85E-02	26318	1.98E-01	26318	2.70E-01
26317	7.78E-02	26317	1.99E-01	26317	2.72E-01
26316	7.71E-02	26316	2.00E-01	26316	2.76E-01
26315	7.81E-02	26315	2.02E-01	26315	2.78E-01
26314	7.91E-02	26314	2.03E-01	26314	2.80E-01
26313	7.50E-02	26313	2.04E-01	26313	2.82E-01
26312	7.44E-02	26312	2.05E-01	26312	2.85E-01
26311	7.87E-02	26311	2.07E-01	26311	2.88E-01
26310	7.60E-02	26310	2.08E-01	26310	2.91E-01

26309	7.52E-02	26309	2.08E-01	26309	2.93E-01
26308	7.46E-02	26308	2.09E-01	26308	2.97E-01
26307	7.46E-02	26307	2.09E-01	26307	2.99E-01
26306	7.33E-02	26306	2.10E-01	26306	3.02E-01
26305	7.26E-02	26305	2.10E-01	26305	3.03E-01
26304	7.12E-02	26304	2.10E-01	26304	3.05E-01
26303	7.07E-02	26303	2.11E-01	26303	3.07E-01
26302	7.03E-02	26302	2.11E-01	26302	3.09E-01
26301	6.82E-02	26301	2.10E-01	26301	3.12E-01
26300	6.74E-02	26300	2.10E-01	26300	3.14E-01
26299	6.65E-02	26299	2.09E-01	26299	3.18E-01
26298	6.57E-02	26298	2.08E-01	26298	3.19E-01
26297	6.50E-02	26297	2.07E-01	26297	3.21E-01
26296	6.32E-02	26296	2.05E-01	26296	3.25E-01
26295	6.30E-02	26295	2.04E-01	26295	3.26E-01
26294	6.17E-02	26294	2.02E-01	26294	3.28E-01
26293	6.00E-02	26293	2.00E-01	26293	3.30E-01
26292	5.94E-02	26292	1.98E-01	26292	3.33E-01
26291	5.83E-02	26291	1.96E-01	26291	3.35E-01
26290	5.75E-02	26290	1.93E-01	26290	3.37E-01
26289	5.71E-02	26289	1.91E-01	26289	3.39E-01
26288	5.58E-02	26288	1.88E-01	26288	3.41E-01
26287	5.44E-02	26287	1.85E-01	26287	3.43E-01

26286	5.41E-02	26286	1.82E-01	26286	3.45E-01
26285	5.24E-02	26285	1.80E-01	26285	3.48E-01
26284	5.15E-02	26284	1.77E-01	26284	3.51E-01
26283	5.12E-02	26283	1.73E-01	26283	3.52E-01
26282	5.02E-02	26282	1.70E-01	26282	3.54E-01
26281	4.93E-02	26281	1.68E-01	26281	3.55E-01
26280	4.86E-02	26280	1.65E-01	26280	3.56E-01
26279	4.80E-02	26279	1.62E-01	26279	3.56E-01
26278	4.70E-02	26278	1.59E-01	26278	3.59E-01
26277	4.64E-02	26277	1.56E-01	26277	3.58E-01
26276	4.58E-02	26276	1.53E-01	26276	3.57E-01
26275	4.50E-02	26275	1.51E-01	26275	3.55E-01
26274	4.44E-02	26274	1.48E-01	26274	3.55E-01
26273	4.36E-02	26273	1.46E-01	26273	3.55E-01
26272	4.32E-02	26272	1.43E-01	26272	3.51E-01
26271	4.24E-02	26271	1.41E-01	26271	3.48E-01
26270	4.17E-02	26270	1.39E-01	26270	3.44E-01
26269	4.10E-02	26269	1.36E-01	26269	3.40E-01
26268	4.06E-02	26268	1.34E-01	26268	3.36E-01
26267	3.99E-02	26267	1.32E-01	26267	3.32E-01
26266	3.92E-02	26266	1.30E-01	26266	3.27E-01
26265	3.87E-02	26265	1.28E-01	26265	3.21E-01

26264	3.84E-02	26264	1.26E-01	26264	3.13E-01
26263	3.78E-02	26263	1.24E-01	26263	3.09E-01
26262	3.71E-02	26262	1.22E-01	26262	3.04E-01
26261	3.68E-02	26261	1.20E-01	26261	2.94E-01
26260	3.62E-02	26260	1.18E-01	26260	2.89E-01
26259	3.58E-02	26259	1.16E-01	26259	2.83E-01
26258	3.54E-02	26258	1.14E-01	26258	2.75E-01
26257	3.49E-02	26257	1.13E-01	26257	2.67E-01
26256	3.45E-02	26256	1.12E-01	26256	2.61E-01
26255	3.41E-02	26255	1.10E-01	26255	2.55E-01
26254	3.35E-02	26254	1.08E-01	26254	2.49E-01
26253	3.31E-02	26253	1.07E-01	26253	2.43E-01
26252	3.28E-02	26252	1.05E-01	26252	2.37E-01
26251	3.21E-02	26251	1.04E-01	26251	2.32E-01
26250	3.20E-02	26250	1.02E-01	26250	2.26E-01
26249	3.16E-02	26249	1.01E-01	26249	2.20E-01
26248	3.13E-02	26248	9.96E-02	26248	2.17E-01
26247	3.09E-02	26247	9.85E-02	26247	2.10E-01
26246	3.05E-02	26246	9.71E-02	26246	2.04E-01
26245	3.01E-02	26245	9.58E-02	26245	2.00E-01
26244	2.98E-02	26244	9.46E-02	26244	1.95E-01
26243	2.97E-02	26243	9.35E-02	26243	1.91E-01
26242	2.86E-02	26242	9.24E-02	26242	1.88E-01

26241	2.84E-02	26241	9.11E-02	26241	1.84E-01
26240	2.84E-02	26240	9.01E-02	26240	1.81E-01
26239	2.83E-02	26239	8.91E-02	26239	1.78E-01
26238	2.80E-02	26238	8.80E-02	26238	1.74E-01
26237	2.76E-02	26237	8.69E-02	26237	1.69E-01
26236	2.72E-02	26236	8.61E-02	26236	1.68E-01
26235	2.70E-02	26235	8.50E-02	26235	1.66E-01
26234	2.64E-02	26234	8.39E-02	26234	1.64E-01
26233	2.65E-02	26233	8.29E-02	26233	1.61E-01
26232	2.62E-02	26232	8.21E-02	26232	1.59E-01
26231	2.59E-02	26231	8.11E-02	26231	1.56E-01
26230	2.54E-02	26230	8.03E-02	26230	1.53E-01
26229	2.56E-02	26229	7.96E-02	26229	1.52E-01
26228	2.54E-02	26228	7.87E-02	26228	1.50E-01
26227	2.49E-02	26227	7.77E-02	26227	1.48E-01
26226	2.47E-02	26226	7.70E-02	26226	1.47E-01
26225	2.45E-02	26225	7.62E-02	26225	1.43E-01
26224	2.42E-02	26224	7.54E-02	26224	1.41E-01
26223	2.40E-02	26223	7.46E-02	26223	1.40E-01
26222	2.38E-02	26222	7.42E-02	26222	1.38E-01
26221	2.36E-02	26221	7.32E-02	26221	1.37E-01
26220	2.33E-02	26220	7.27E-02	26220	1.35E-01
26219	2.33E-02	26219	7.19E-02	26219	1.34E-01

26218	2.32E-02	26218	7.12E-02	26218	1.32E-01
26217	2.28E-02	26217	7.04E-02	26217	1.31E-01
26216	2.28E-02	26216	7.01E-02	26216	1.29E-01
26215	2.23E-02	26215	6.94E-02	26215	1.28E-01
26214	2.22E-02	26214	6.87E-02	26214	1.27E-01
26213	2.20E-02	26213	6.77E-02	26213	1.25E-01
26212	2.19E-02	26212	6.73E-02	26212	1.24E-01
26211	2.14E-02	26211	6.65E-02	26211	1.23E-01
26210	2.16E-02	26210	6.62E-02	26210	1.21E-01
26209	2.11E-02	26209	6.53E-02	26209	1.21E-01
26208	2.10E-02	26208	6.49E-02	26208	1.20E-01
26207	2.08E-02	26207	6.46E-02	26207	1.19E-01
26206	2.10E-02	26206	6.36E-02	26206	1.17E-01
26205	2.06E-02	26205	6.31E-02	26205	1.16E-01
26204	2.09E-02	26204	6.29E-02	26204	1.15E-01
26203	2.01E-02	26203	6.22E-02	26203	1.13E-01
26202	2.02E-02	26202	6.16E-02	26202	1.13E-01
26201	2.00E-02	26201	6.13E-02	26201	1.11E-01
26200	1.96E-02	26200	6.06E-02	26200	1.11E-01

I2
2V (LDV 125mm/s/V) 6V (LDV 125mm/s/V) 10V (LDV 125mm/s/V) 15V (LDV 125mm/s/V) 20V (LDV 125mm/s/V)
25V (LDV 125mm/s/V) 30V (LDV 125mm/s/V) 35V (LDV 125mm/s/V) 40V (LDV 125mm/s/V) 45V (LDV 125mm/s/V)
50V (LDV 125mm/s/V)
Frequency (Hz) Response (V) Frequency (Hz) Response (V) Frequency (Hz)

BiDirectionalSweepData
Response (V)   Frequency (Hz) Response (V)   Frequency (Hz) Response (V)
Frequency (Hz) Response (V)   Frequency (Hz) Response (V)   Frequency (Hz)
Response (V)   Frequency (Hz) Response (V)   Frequency (Hz) Response (V)
Frequency (Hz) Response (V)
28500   4.37E-03   28500   1.26E-02   28500   2.24E-02   28475
3.28E-02   28475   5.55E-02   28475   8.58E-02   28420   6.13E-02
28420   7.51E-02   28420   9.43E-02   28400   1.16E-01   28400
1.42E-01
28501   4.61E-03   28501   1.23E-02   28501   2.27E-02   28477
3.28E-02   28477   5.65E-02   28477   8.87E-02   28422   7.15E-02
28422   9.14E-02   28422   1.25E-01   28402   1.28E-01   28402
1.63E-01
28502   4.32E-03   28502   1.25E-02   28502   2.29E-02   28479
3.31E-02   28479   5.78E-02   28479   9.14E-02   28424   7.49E-02
28424   9.70E-02   28424   1.28E-01   28404   1.32E-01   28404
1.68E-01
28503   4.55E-03   28503   1.30E-02   28503   2.32E-02   28481
3.36E-02   28481   5.90E-02   28481   9.43E-02   28426   7.65E-02
28426   9.93E-02   28426   1.31E-01   28406   1.36E-01   28406
1.71E-01
28504   4.62E-03   28504   1.25E-02   28504   2.34E-02   28483
3.41E-02   28483   6.04E-02   28483   9.76E-02   28428   7.78E-02
28428   1.01E-01   28428   1.34E-01   28408   1.39E-01   28408
1.71E-01
28505   4.44E-03   28505   1.29E-02   28505   2.36E-02   28485
3.47E-02   28485   6.19E-02   28485   1.01E-01   28430   7.90E-02
28430   1.03E-01   28430   1.36E-01   28410   1.41E-01   28410
1.76E-01
28506   4.73E-03   28506   1.34E-02   28506   2.37E-02   28487
3.52E-02   28487   6.37E-02   28487   1.04E-01   28432   8.04E-02
28432   1.05E-01   28432   1.39E-01   28412   1.43E-01   28412
1.75E-01
28507   4.58E-03   28507   1.28E-02   28507   2.41E-02   28489
3.59E-02   28489   6.53E-02   28489   1.07E-01   28434   8.19E-02
28434   1.07E-01   28434   1.43E-01   28414   1.46E-01   28414
1.80E-01
28508   4.58E-03   28508   1.32E-02   28508   2.42E-02   28491
3.65E-02   28491   6.69E-02   28491   1.11E-01   28436   8.31E-02
28436   1.09E-01   28436   1.46E-01   28416   1.49E-01   28416
1.85E-01
28509   4.79E-03   28509   1.32E-02   28509   2.45E-02   28493
3.70E-02   28493   6.87E-02   28493   1.15E-01   28438   8.48E-02
28438   1.11E-01   28438   1.50E-01   28418   1.52E-01   28418
1.89E-01
28510   5.08E-03   28510   1.33E-02   28510   2.45E-02   28495
3.79E-02   28495   7.09E-02   28495   1.20E-01   28440   8.62E-02
28440   1.14E-01   28440   1.54E-01   28420   1.55E-01   28420
1.94E-01
28511   4.89E-03   28511   1.34E-02   28511   2.49E-02   28497
3.83E-02   28497   7.38E-02   28497   1.26E-01   28442   8.77E-02
28442   1.16E-01   28442   1.58E-01   28422   1.58E-01   28422
2.01E-01
28512   4.96E-03   28512   1.36E-02   28512   2.51E-02   28499
3.90E-02   28499   7.64E-02   28499   1.32E-01   28444   8.92E-02
28444   1.19E-01   28444   1.63E-01   28424   1.62E-01   28424
2.08E-01
28513   4.70E-03   28513   1.37E-02   28513   2.54E-02   28501
3.99E-02   28501   7.90E-02   28501   1.40E-01   28446   9.09E-02
28446   1.21E-01   28446   1.68E-01   28426   1.66E-01   28426
2.15E-01
28514   5.28E-03   28514   1.39E-02   28514   2.54E-02   28503
4.07E-02   28503   8.21E-02   28503   1.48E-01   28448   9.29E-02
28448   1.25E-01   28448   1.72E-01   28428   1.70E-01   28428
2.23E-01
28515   4.96E-03   28515   1.39E-02   28515   2.56E-02   28505
4.19E-02   28505   8.53E-02   28505   1.61E-01   28450   9.46E-02
28450   1.27E-01   28450   1.78E-01   28430   1.74E-01   28430
2.32E-01

28516 5.12E-03 28516 1.40E-02 28516 2.61E-02 28507
4.31E-02 28507 8.85E-02 28507 1.74E-01 28452 9.64E-02
28452 1.31E-01 28452 1.84E-01 28432 1.79E-01 28432
2.41E-01
28517 5.25E-03 28517 1.45E-02 28517 2.64E-02 28509
4.42E-02 28509 9.23E-02 28509 1.97E-01 28454 9.88E-02
28454 1.34E-01 28454 1.91E-01 28434 1.84E-01 28434
2.53E-01
28518 5.23E-03 28518 1.46E-02 28518 2.65E-02 28511
4.55E-02 28511 9.63E-02 28511 2.47E-01 28456 1.01E-01
28456 1.39E-01 28456 1.98E-01 28436 1.90E-01 28436
2.68E-01
28519 5.20E-03 28519 1.47E-02 28519 2.67E-02 28513
4.67E-02 28513 1.01E-01 28513 4.42E-01 28458 1.03E-01
28458 1.45E-01 28458 2.06E-01 28438 1.96E-01 28438
2.89E-01
28520 5.23E-03 28520 1.50E-02 28520 2.72E-02 28515
4.82E-02 28515 1.08E-01 28515 4.32E-01 28460 1.06E-01
28460 1.50E-01 28460 2.16E-01 28440 2.03E-01 28440
3.38E-01
28521 5.19E-03 28521 1.50E-02 28521 2.73E-02 28517
4.97E-02 28517 1.16E-01 28517 4.23E-01 28462 1.08E-01
28462 1.56E-01 28462 2.29E-01 28442 2.11E-01 28442
6.83E-01
28522 5.43E-03 28522 1.52E-02 28522 2.77E-02 28519
5.15E-02 28519 1.24E-01 28519 4.14E-01 28464 1.11E-01
28464 1.62E-01 28464 2.46E-01 28444 2.19E-01 28444
6.20E-01
28523 5.24E-03 28523 1.53E-02 28523 2.80E-02 28521
5.34E-02 28521 1.35E-01 28521 4.05E-01 28466 1.14E-01
28466 1.68E-01 28466 2.72E-01 28446 2.29E-01 28446
6.52E-01
28524 5.56E-03 28524 1.54E-02 28524 2.83E-02 28523
5.52E-02 28523 1.50E-01 28523 3.96E-01 28468 1.18E-01
28468 1.75E-01 28468 6.26E-01 28448 2.41E-01 28448
6.54E-01
28525 5.71E-03 28525 1.56E-02 28525 2.85E-02 28525
5.74E-02 28525 1.95E-01 28525 3.87E-01 28470 1.21E-01
28470 1.84E-01 28470 6.17E-01 28450 2.55E-01 28450
6.50E-01
28526 5.55E-03 28526 1.58E-02 28526 2.90E-02 28527
6.03E-02 28527 2.52E-01 28527 3.79E-01 28472 1.25E-01
28472 1.94E-01 28472 6.08E-01 28452 2.77E-01 28452
6.45E-01
28527 5.46E-03 28527 1.57E-02 28527 2.93E-02 28529
6.31E-02 28529 3.47E-01 28529 3.71E-01 28474 1.29E-01
28474 2.05E-01 28474 6.00E-01 28454 6.64E-01 28454
6.39E-01
28528 5.49E-03 28528 1.59E-02 28528 2.96E-02 28531
6.63E-02 28531 3.40E-01 28531 3.62E-01 28476 1.33E-01
28476 2.19E-01 28476 5.92E-01 28456 6.56E-01 28456
6.33E-01
28529 5.67E-03 28529 1.61E-02 28529 3.00E-02 28533
7.06E-02 28533 3.30E-01 28533 3.55E-01 28478 1.38E-01
28478 2.39E-01 28478 5.84E-01 28458 6.47E-01 28458
6.27E-01
28530 5.63E-03 28530 1.63E-02 28530 3.04E-02 28535
7.58E-02 28535 3.22E-01 28535 3.47E-01 28480 1.44E-01
28480 2.81E-01 28480 5.77E-01 28460 6.40E-01 28460
6.20E-01
28531 5.71E-03 28531 1.64E-02 28531 3.08E-02 28537
8.22E-02 28537 3.14E-01 28537 3.39E-01 28482 1.51E-01
28482 5.76E-01 28482 5.69E-01 28462 6.32E-01 28462
6.14E-01
28532 5.98E-03 28532 1.67E-02 28532 3.11E-02 28539
8.99E-02 28539 3.06E-01 28539 3.31E-01 28484 1.62E-01
28484 5.68E-01 28484 5.62E-01 28464 6.25E-01 28464
6.07E-01

28533 6.18E-03 28533 1.69E-02 28533 3.15E-02 28541
9.87E-02 28541 2.98E-01 28541 3.23E-01 28486 1.73E-01
28486 5.59E-01 28486 5.56E-01 28466 6.18E-01 28466
6.01E-01
28534 5.89E-03 28534 1.71E-02 28534 3.19E-02 28543
1.09E-01 28543 2.90E-01 28543 3.16E-01 28488 1.87E-01
28488 5.51E-01 28488 5.49E-01 28468 6.10E-01 28468
5.94E-01
28535 5.97E-03 28535 1.73E-02 28535 3.24E-02 28545
1.28E-01 28545 2.83E-01 28545 3.08E-01 28490 2.01E-01
28490 5.43E-01 28490 5.41E-01 28470 6.03E-01 28470
5.88E-01
28536 6.25E-03 28536 1.75E-02 28536 3.28E-02 28547
1.47E-01 28547 2.76E-01 28547 3.01E-01 28492 2.24E-01
28492 5.35E-01 28492 5.34E-01 28472 5.96E-01 28472
5.82E-01
28537 6.15E-03 28537 1.76E-02 28537 3.32E-02 28549
1.77E-01 28549 2.69E-01 28549 2.94E-01 28494 2.81E-01
28494 5.26E-01 28494 5.26E-01 28474 5.87E-01 28474
5.75E-01
28538 6.60E-03 28538 1.78E-02 28538 3.36E-02 28551
1.92E-01 28551 2.62E-01 28551 2.87E-01 28496 5.17E-01
28496 5.18E-01 28496 5.18E-01 28476 5.80E-01 28476
5.69E-01
28539 6.31E-03 28539 1.81E-02 28539 3.41E-02 28553
1.99E-01 28553 2.54E-01 28553 2.79E-01 28498 5.08E-01
28498 5.10E-01 28498 5.11E-01 28478 5.73E-01 28478
5.42E-01
28540 6.41E-03 28540 1.81E-02 28540 3.46E-02 28555
1.98E-01 28555 2.47E-01 28555 2.72E-01 28500 4.99E-01
28500 5.02E-01 28500 5.03E-01 28480 5.65E-01 28480
5.55E-01
28541 6.35E-03 28541 1.87E-02 28541 3.51E-02 28557
1.94E-01 28557 2.40E-01 28557 2.65E-01 28502 4.90E-01
28502 4.94E-01 28502 4.96E-01 28482 5.58E-01 28482
5.49E-01
28542 6.40E-03 28542 1.86E-02 28542 3.55E-02 28559
1.89E-01 28559 2.33E-01 28559 2.58E-01 28504 4.82E-01
28504 4.87E-01 28504 4.88E-01 28484 5.51E-01 28484
5.42E-01
28543 6.70E-03 28543 1.90E-02 28543 3.60E-02 28561
1.83E-01 28561 2.27E-01 28561 2.51E-01 28506 4.73E-01
28506 4.80E-01 28506 4.81E-01 28486 5.44E-01 28486
5.35E-01
28544 6.72E-03 28544 1.92E-02 28544 3.67E-02 28563
1.77E-01 28563 2.20E-01 28563 2.45E-01 28508 4.64E-01
28508 4.72E-01 28508 4.73E-01 28488 5.38E-01 28488
5.29E-01
28545 7.04E-03 28545 1.95E-02 28545 3.71E-02 28565
1.72E-01 28565 2.14E-01 28565 2.38E-01 28510 4.56E-01
28510 4.65E-01 28510 4.66E-01 28490 5.31E-01 28490
5.22E-01
28546 6.99E-03 28546 1.97E-02 28546 3.79E-02 28567
1.67E-01 28567 2.07E-01 28567 2.32E-01 28512 4.48E-01
28512 4.58E-01 28512 4.60E-01 28492 5.25E-01 28492
5.16E-01
28547 7.17E-03 28547 1.98E-02 28547 3.85E-02 28569
1.61E-01 28569 2.01E-01 28569 2.26E-01 28514 4.40E-01
28514 4.50E-01 28514 4.53E-01 28494 5.19E-01 28494
5.09E-01
28548 7.31E-03 28548 2.02E-02 28548 3.92E-02 28571
1.56E-01 28571 1.95E-01 28571 2.20E-01 28516 4.33E-01
28516 4.43E-01 28516 4.45E-01 28496 5.13E-01 28496
5.03E-01
28549 7.19E-03 28549 2.07E-02 28549 3.98E-02 28573
1.51E-01 28573 1.90E-01 28573 2.14E-01 28518 4.26E-01
28518 4.35E-01 28518 4.38E-01 28498 5.06E-01 28498
4.97E-01

28550 7.23E-03 28550 2.08E-02 28550 4.07E-02 28575
1.45E-01 28575 1.85E-01 28575 2.08E-01 28520 4.18E-01
28520 4.27E-01 28520 4.30E-01 28500 4.99E-01 28500
4.90E-01
28551 7.52E-03 28551 2.13E-02 28551 4.14E-02 28577
1.41E-01 28577 1.79E-01 28577 2.02E-01 28522 4.10E-01
28522 4.19E-01 28522 4.23E-01 28502 4.94E-01 28502
4.83E-01
28552 7.60E-03 28552 2.14E-02 28552 4.22E-02 28579
1.36E-01 28579 1.74E-01 28579 1.97E-01 28524 4.03E-01
28524 4.12E-01 28524 4.16E-01 28504 4.87E-01 28504
4.77E-01
28553 7.60E-03 28553 2.16E-02 28553 4.30E-02 28581
1.32E-01 28581 1.69E-01 28581 1.92E-01 28526 3.95E-01
28526 4.05E-01 28526 4.09E-01 28506 4.80E-01 28506
4.71E-01
28554 7.68E-03 28554 2.22E-02 28554 4.41E-02 28583
1.28E-01 28583 1.64E-01 28583 1.87E-01 28528 3.87E-01
28528 3.98E-01 28528 4.02E-01 28508 4.72E-01 28508
4.65E-01
28555 7.75E-03 28555 2.24E-02 28555 4.51E-02 28585
1.25E-01 28585 1.60E-01 28585 1.82E-01 28530 3.80E-01
28530 3.91E-01 28530 3.95E-01 28510 4.65E-01 28510
4.59E-01
28556 7.80E-03 28556 2.30E-02 28556 4.61E-02 28587
1.21E-01 28587 1.56E-01 28587 1.77E-01 28532 3.73E-01
28532 3.84E-01 28532 3.89E-01 28512 4.58E-01 28512
4.53E-01
28557 7.85E-03 28557 2.30E-02 28557 4.73E-02 28589
1.18E-01 28589 1.52E-01 28589 1.73E-01 28534 3.66E-01
28534 3.76E-01 28534 3.82E-01 28514 4.52E-01 28514
4.46E-01
28558 8.25E-03 28558 2.32E-02 28558 4.86E-02 28591
1.14E-01 28591 1.48E-01 28591 1.69E-01 28536 3.59E-01
28536 3.69E-01 28536 3.76E-01 28516 4.46E-01 28516
4.40E-01
28559 8.42E-03 28559 2.39E-02 28559 4.99E-02 28593
1.11E-01 28593 1.44E-01 28593 1.65E-01 28538 3.52E-01
28538 3.62E-01 28538 3.69E-01 28518 4.38E-01 28518
4.34E-01
28560 8.43E-03 28560 2.40E-02 28560 5.12E-02 28595
1.08E-01 28595 1.40E-01 28595 1.61E-01 28540 3.45E-01
28540 3.55E-01 28540 3.62E-01 28520 4.31E-01 28520
4.28E-01
28561 8.59E-03 28561 2.49E-02 28561 5.27E-02 28597
1.05E-01 28597 1.36E-01 28597 1.57E-01 28542 3.38E-01
28542 3.48E-01 28542 3.56E-01 28522 4.25E-01 28522
4.22E-01
28562 8.54E-03 28562 2.51E-02 28562 5.42E-02 28599
1.02E-01 28599 1.33E-01 28599 1.53E-01 28544 3.30E-01
28544 3.42E-01 28544 3.50E-01 28524 4.18E-01 28524
4.16E-01
28563 8.95E-03 28563 2.53E-02 28563 5.61E-02 28599
1.02E-01 28599 1.33E-01 28599 1.54E-01 28544 3.30E-01
28544 3.42E-01 28544 3.50E-01 28526 4.12E-01 28526
4.10E-01
28564 8.77E-03 28564 2.56E-02 28564 5.81E-02 28597
1.06E-01 28597 1.37E-01 28597 1.58E-01 28542 3.38E-01
28542 3.49E-01 28542 3.57E-01 28528 4.06E-01 28528
4.04E-01
28565 9.22E-03 28565 2.64E-02 28565 6.02E-02 28595
1.09E-01 28595 1.41E-01 28595 1.62E-01 28540 3.45E-01
28540 3.56E-01 28540 3.64E-01 28530 3.99E-01 28530
3.97E-01
28566 9.35E-03 28566 2.64E-02 28566 6.24E-02 28593
1.13E-01 28593 1.44E-01 28593 1.67E-01 28538 3.52E-01
28538 3.64E-01 28538 3.71E-01 28530 3.99E-01 28530
3.98E-01

28567 9.15E-03 28567 2.73E-02 28567 6.51E-02 28591
1.16E-01 28591 1.48E-01 28591 1.71E-01 28536 3.59E-01
28536 3.71E-01 28536 3.78E-01 28528 4.07E-01 28528
4.05E-01
28568 9.64E-03 28568 2.77E-02 28568 6.78E-02 28589
1.20E-01 28589 1.52E-01 28589 1.76E-01 28534 3.67E-01
28534 3.78E-01 28534 3.86E-01 28526 4.14E-01 28526
4.11E-01
28569 9.48E-03 28569 2.82E-02 28569 7.11E-02 28587
1.24E-01 28587 1.57E-01 28587 1.80E-01 28532 3.74E-01
28532 3.85E-01 28532 3.93E-01 28524 4.21E-01 28524
4.18E-01
28570 1.00E-02 28570 2.86E-02 28570 7.44E-02 28585
1.28E-01 28585 1.61E-01 28585 1.85E-01 28530 3.81E-01
28530 3.92E-01 28530 4.00E-01 28522 4.28E-01 28522
4.25E-01
28571 1.01E-02 28571 2.90E-02 28571 7.75E-02 28583
1.32E-01 28583 1.66E-01 28583 1.90E-01 28528 3.88E-01
28528 3.99E-01 28528 4.06E-01 28520 4.34E-01 28520
4.31E-01
28572 1.00E-02 28572 2.98E-02 28572 8.03E-02 28581
1.37E-01 28581 1.71E-01 28581 1.95E-01 28526 3.95E-01
28526 4.07E-01 28526 4.13E-01 28518 4.41E-01 28518
4.38E-01
28573 1.01E-02 28573 3.03E-02 28573 8.30E-02 28579
1.41E-01 28579 1.76E-01 28579 2.00E-01 28524 4.02E-01
28524 4.14E-01 28524 4.20E-01 28516 4.48E-01 28516
4.45E-01
28574 1.05E-02 28574 3.08E-02 28574 8.47E-02 28577
1.43E-01 28577 1.81E-01 28577 2.05E-01 28522 4.09E-01
28522 4.21E-01 28522 4.27E-01 28514 4.54E-01 28514
4.51E-01
28575 1.06E-02 28575 3.14E-02 28575 8.60E-02 28575
1.47E-01 28575 1.87E-01 28575 2.11E-01 28520 4.16E-01
28520 4.28E-01 28520 4.34E-01 28512 4.61E-01 28512
4.58E-01
28576 1.10E-02 28576 3.21E-02 28576 8.68E-02 28573
1.51E-01 28573 1.93E-01 28573 2.17E-01 28518 4.23E-01
28518 4.35E-01 28518 4.41E-01 28510 4.67E-01 28510
4.65E-01
28577 1.09E-02 28577 3.27E-02 28577 8.75E-02 28571
1.56E-01 28571 1.98E-01 28571 2.23E-01 28516 4.30E-01
28516 4.42E-01 28516 4.47E-01 28508 4.74E-01 28508
4.71E-01
28578 1.14E-02 28578 3.33E-02 28578 8.78E-02 28569
1.61E-01 28569 2.03E-01 28569 2.29E-01 28514 4.37E-01
28514 4.49E-01 28514 4.54E-01 28506 4.81E-01 28506
4.77E-01
28579 1.15E-02 28579 3.41E-02 28579 8.76E-02 28567
1.66E-01 28567 2.00E-01 28567 2.35E-01 28512 4.44E-01
28512 4.56E-01 28512 4.61E-01 28504 4.87E-01 28504
4.62E-01
28580 1.14E-02 28580 3.48E-02 28580 8.74E-02 28565
1.71E-01 28565 2.01E-01 28565 2.41E-01 28510 4.51E-01
28510 4.63E-01 28510 4.68E-01 28502 4.94E-01 28502
4.91E-01
28581 1.17E-02 28581 3.56E-02 28581 8.69E-02 28563
1.76E-01 28563 2.17E-01 28563 2.48E-01 28508 4.58E-01
28508 4.70E-01 28508 4.75E-01 28500 5.00E-01 28500
4.98E-01
28582 1.19E-02 28582 3.64E-02 28582 8.64E-02 28561
1.82E-01 28561 2.23E-01 28561 2.54E-01 28506 4.65E-01
28506 4.76E-01 28506 4.81E-01 28498 5.07E-01 28498
5.04E-01
28583 1.22E-02 28583 3.73E-02 28583 8.56E-02 28559
1.87E-01 28559 2.30E-01 28559 2.62E-01 28504 4.71E-01
28504 4.83E-01 28504 4.87E-01 28496 5.14E-01 28496
5.10E-01

28584 1.25E-02 28584 3.78E-02 28584 8.49E-02 28557
1.92E-01 28557 2.36E-01 28557 2.68E-01 28502 4.78E-01
28502 4.90E-01 28502 4.94E-01 28494 5.21E-01 28494
5.17E-01
28585 1.26E-02 28585 3.85E-02 28585 8.41E-02 28555
1.97E-01 28555 2.44E-01 28555 2.75E-01 28500 4.85E-01
28500 4.97E-01 28500 5.00E-01 28492 5.28E-01 28492
5.23E-01
28586 1.28E-02 28586 3.96E-02 28586 8.32E-02 28553
2.01E-01 28553 2.52E-01 28553 2.82E-01 28498 4.91E-01
28498 5.04E-01 28498 5.07E-01 28490 5.33E-01 28490
5.29E-01
28587 1.31E-02 28587 4.01E-02 28587 8.21E-02 28551
2.02E-01 28551 2.59E-01 28551 2.89E-01 28496 4.98E-01
28496 5.11E-01 28496 5.13E-01 28488 5.39E-01 28488
5.35E-01
28588 1.32E-02 28588 4.12E-02 28588 8.13E-02 28549
1.95E-01 28549 2.66E-01 28549 2.96E-01 28494 5.05E-01
28494 5.18E-01 28494 5.20E-01 28486 5.45E-01 28486
5.41E-01
28589 1.35E-02 28589 4.22E-02 28589 8.03E-02 28547
1.84E-01 28547 2.73E-01 28547 3.03E-01 28492 5.12E-01
28492 5.23E-01 28492 5.26E-01 28484 5.51E-01 28484
5.48E-01
28590 1.38E-02 28590 4.28E-02 28590 7.93E-02 28545
1.68E-01 28545 2.80E-01 28545 3.10E-01 28490 5.19E-01
28490 5.29E-01 28490 5.33E-01 28482 5.58E-01 28482
5.54E-01
28591 1.39E-02 28591 4.37E-02 28591 7.83E-02 28543
1.46E-01 28543 2.87E-01 28543 3.18E-01 28488 5.26E-01
28488 5.36E-01 28488 5.39E-01 28480 5.64E-01 28480
5.60E-01
28592 1.43E-02 28592 4.45E-02 28592 7.73E-02 28541
1.26E-01 28541 2.94E-01 28541 3.25E-01 28486 5.33E-01
28486 5.42E-01 28486 5.46E-01 28478 5.70E-01 28478
5.65E-01
28593 1.45E-02 28593 4.52E-02 28593 7.63E-02 28539
1.11E-01 28539 3.02E-01 28539 3.32E-01 28484 4.42E-01
28484 5.48E-01 28484 5.52E-01 28476 5.76E-01 28476
5.71E-01
28594 1.47E-02 28594 4.58E-02 28594 7.52E-02 28537
1.01E-01 28537 3.09E-01 28537 3.40E-01 28482 2.44E-01
28482 5.54E-01 28482 5.58E-01 28474 5.82E-01 28474
5.77E-01
28595 1.50E-02 28595 4.63E-02 28595 7.43E-02 28535
9.11E-02 28535 3.16E-01 28535 3.47E-01 28480 2.09E-01
28480 5.60E-01 28480 5.64E-01 28472 5.87E-01 28472
5.83E-01
28596 1.52E-02 28596 4.67E-02 28596 7.33E-02 28533
8.24E-02 28533 3.23E-01 28533 3.54E-01 28478 1.83E-01
28478 5.66E-01 28478 5.62E-01 28470 5.94E-01 28470
5.88E-01
28597 1.55E-02 28597 4.71E-02 28597 7.23E-02 28531
7.70E-02 28531 3.30E-01 28531 3.61E-01 28476 1.71E-01
28476 5.71E-01 28476 5.65E-01 28468 5.99E-01 28468
5.94E-01
28598 1.56E-02 28598 4.73E-02 28598 7.13E-02 28529
7.19E-02 28529 3.37E-01 28529 3.68E-01 28474 1.61E-01
28474 5.77E-01 28474 5.67E-01 28466 6.05E-01 28466
5.99E-01
28599 1.58E-02 28599 4.75E-02 28599 7.04E-02 28527
6.72E-02 28527 3.45E-01 28527 3.75E-01 28472 1.53E-01
28472 5.83E-01 28472 5.77E-01 28464 6.11E-01 28464
6.05E-01
28600 1.60E-02 28600 4.75E-02 28600 6.95E-02 28525
6.38E-02 28525 3.53E-01 28525 3.82E-01 28470 1.45E-01
28470 5.91E-01 28470 5.85E-01 28462 6.16E-01 28462
6.10E-01

28601 1.63E-02 28601 4.77E-02 28601 6.85E-02 28523
6.11E-02 28523 3.51E-01 28523 3.90E-01 28468 1.39E-01
28468 4.42E-01 28468 5.93E-01 28460 6.21E-01 28460
6.15E-01
28602 1.65E-02 28602 4.76E-02 28602 6.76E-02 28521
5.81E-02 28521 3.00E-01 28521 3.97E-01 28466 1.33E-01
28466 2.55E-01 28466 6.00E-01 28458 6.26E-01 28458
6.20E-01
28603 1.66E-02 28603 4.76E-02 28603 6.66E-02 28519
5.59E-02 28519 2.24E-01 28519 4.04E-01 28464 1.28E-01
28464 2.10E-01 28464 6.06E-01 28456 6.31E-01 28456
6.26E-01
28604 1.69E-02 28604 4.72E-02 28604 6.58E-02 28517
5.36E-02 28517 1.78E-01 28517 4.11E-01 28462 1.24E-01
28462 1.95E-01 28462 6.06E-01 28454 6.35E-01 28454
6.31E-01
28605 1.69E-02 28605 4.71E-02 28605 6.50E-02 28515
5.18E-02 28515 1.48E-01 28515 4.17E-01 28460 1.19E-01
28460 1.84E-01 28460 6.14E-01 28452 6.42E-01 28452
6.36E-01
28606 1.72E-02 28606 4.67E-02 28606 6.41E-02 28513
5.02E-02 28513 1.35E-01 28513 4.24E-01 28458 1.15E-01
28458 1.75E-01 28458 6.14E-01 28450 6.47E-01 28450
6.41E-01
28607 1.72E-02 28607 4.64E-02 28607 6.33E-02 28511
4.85E-02 28511 1.23E-01 28511 4.31E-01 28456 1.11E-01
28456 1.67E-01 28456 6.22E-01 28448 6.51E-01 28448
6.46E-01
28608 1.73E-02 28608 4.61E-02 28608 6.25E-02 28509
4.73E-02 28509 1.14E-01 28509 4.38E-01 28454 1.07E-01
28454 1.58E-01 28454 6.24E-01 28446 6.55E-01 28446
6.51E-01
28609 1.74E-02 28609 4.59E-02 28609 6.17E-02 28507
4.60E-02 28507 1.06E-01 28507 4.46E-01 28452 1.04E-01
28452 1.53E-01 28452 6.25E-01 28444 6.59E-01 28444
6.56E-01
28610 1.74E-02 28610 4.54E-02 28610 6.10E-02 28505
4.48E-02 28505 9.98E-02 28505 4.53E-01 28450 1.01E-01
28450 1.48E-01 28450 4.30E-01 28442 6.64E-01 28442
6.60E-01
28611 1.74E-02 28611 4.53E-02 28611 6.02E-02 28503
4.38E-02 28503 9.46E-02 28503 4.63E-01 28448 9.89E-02
28448 1.43E-01 28448 2.47E-01 28440 6.68E-01 28440
6.65E-01
28612 1.75E-02 28612 4.48E-02 28612 5.95E-02 28501
4.27E-02 28501 9.00E-02 28501 3.62E-01 28446 9.67E-02
28446 1.38E-01 28446 2.27E-01 28438 6.74E-01 28438
6.69E-01
28613 1.74E-02 28613 4.44E-02 28613 5.88E-02 28499
4.17E-02 28499 8.51E-02 28499 2.32E-01 28444 9.45E-02
28444 1.34E-01 28444 2.11E-01 28436 4.56E-01 28436
6.74E-01
28614 1.75E-02 28614 4.38E-02 28614 5.80E-02 28497
4.06E-02 28497 8.15E-02 28497 1.91E-01 28442 9.22E-02
28442 1.30E-01 28442 1.99E-01 28434 2.85E-01 28434
6.78E-01
28615 1.74E-02 28615 4.34E-02 28615 5.74E-02 28495
4.00E-02 28495 7.83E-02 28495 1.70E-01 28440 8.97E-02
28440 1.27E-01 28440 1.87E-01 28432 2.42E-01 28432
6.83E-01
28616 1.73E-02 28616 4.31E-02 28616 5.67E-02 28493
3.92E-02 28493 7.55E-02 28493 1.51E-01 28438 8.75E-02
28438 1.23E-01 28438 1.80E-01 28430 2.25E-01 28430
6.87E-01
28617 1.72E-02 28617 4.27E-02 28617 5.61E-02 28491
3.80E-02 28491 7.26E-02 28491 1.41E-01 28436 8.54E-02
28436 1.20E-01 28436 1.69E-01 28428 2.14E-01 28428
6.92E-01

28618 1.71E-02 28618 4.24E-02 28618 5.54E-02 28489
3.76E-02 28489 6.99E-02 28489 1.33E-01 28434 8.37E-02
28434 1.17E-01 28434 1.64E-01 28426 2.03E-01 28426
6.96E-01
28619 1.70E-02 28619 4.20E-02 28619 5.47E-02 28487
3.66E-02 28487 1.84E-01 28487 1.25E-01 28432 8.20E-02
28432 1.14E-01 28432 1.59E-01 28424 1.95E-01 28424
7.01E-01
28620 1.70E-02 28620 4.15E-02 28620 5.43E-02 28485
3.61E-02 28485 6.96E-02 28485 1.19E-01 28430 8.06E-02
28430 1.11E-01 28430 1.55E-01 28422 1.88E-01 28422
7.05E-01
28621 1.67E-02 28621 4.14E-02 28621 5.36E-02 28483
3.55E-02 28483 5.94E-02 28483 1.13E-01 28428 7.91E-02
28428 1.09E-01 28428 1.50E-01 28420 1.82E-01 28420
7.10E-01
28622 1.66E-02 28622 4.07E-02 28622 5.29E-02 28481
3.50E-02 28481 5.79E-02 28481 1.08E-01 28426 7.76E-02
28426 1.06E-01 28426 1.46E-01 28418 1.76E-01 28418
2.90E-01
28623 1.65E-02 28623 4.06E-02 28623 5.25E-02 28479
3.43E-02 28479 5.65E-02 28479 1.03E-01 28424 7.61E-02
28424 1.04E-01 28424 1.42E-01 28416 1.71E-01 28416
2.64E-01
28624 1.63E-02 28624 4.03E-02 28624 5.20E-02 28477
3.37E-02 28477 5.53E-02 28477 9.98E-02 28422 7.49E-02
28422 1.02E-01 28422 1.38E-01 28414 1.66E-01 28414
2.46E-01
28625 1.60E-02 28625 3.97E-02 28625 5.14E-02 28475
3.31E-02 28475 5.40E-02 28475 9.60E-02 28420 7.36E-02
28420 1.00E-01 28420 1.35E-01 28412 1.61E-01 28412
2.33E-01
28626 1.59E-02 28626 3.94E-02 28626 5.09E-02

28410 1.57E-01 28410 2.22E-01
28627 1.57E-02 28627 3.89E-02 28627 5.05E-02

28408 1.53E-01 28408 2.13E-01
28628 1.54E-02 28628 3.85E-02 28628 4.99E-02

28406 1.50E-01 28406 2.05E-01
28629 1.53E-02 28629 3.79E-02 28629 4.95E-02

28404 1.46E-01 28404 1.98E-01
28630 1.50E-02 28630 3.77E-02 28630 4.89E-02

28402 1.43E-01 28402 1.91E-01
28631 1.49E-02 28631 3.74E-02 28631 4.85E-02

28400 1.39E-01 28400 1.85E-01
28632 1.47E-02 28632 3.68E-02 28632 4.80E-02

28633 1.45E-02 28633 3.64E-02 28633 4.76E-02

28634 1.42E-02 28634 3.63E-02 28634 4.71E-02

28635 1.40E-02 28635 3.58E-02 28635 4.67E-02

28636 1.38E-02 28636 3.55E-02 28636 4.62E-02

28637 1.36E-02 28637 3.53E-02 28637 4.58E-02

28638	1.34E-02	28638	3.48E-02	28638	4.55E-02
28639	1.32E-02	28639	3.44E-02	28639	4.50E-02
28640	1.30E-02	28640	3.40E-02	28640	4.45E-02
28641	1.26E-02	28641	3.34E-02	28641	4.41E-02
28642	1.25E-02	28642	3.32E-02	28642	4.37E-02
28643	1.24E-02	28643	3.27E-02	28643	4.34E-02
28644	1.22E-02	28644	3.24E-02	28644	4.30E-02
28645	1.18E-02	28645	3.20E-02	28645	4.26E-02
28646	1.18E-02	28646	3.15E-02	28646	4.22E-02
28647	1.16E-02	28647	3.11E-02	28647	4.18E-02
28648	1.13E-02	28648	3.07E-02	28648	4.16E-02
28649	1.13E-02	28649	3.04E-02	28649	4.11E-02
28650	1.11E-02	28650	3.01E-02	28650	4.08E-02
28651	1.09E-02	28651	2.97E-02	28651	4.04E-02
28652	1.06E-02	28652	2.93E-02	28652	4.02E-02
28653	1.03E-02	28653	2.89E-02	28653	3.97E-02
28654	1.02E-02	28654	2.85E-02	28654	3.94E-02
28655	9.99E-03	28655	2.79E-02	28655	3.90E-02
28656	9.76E-03	28656	2.78E-02	28656	3.88E-02
28657	9.85E-03	28657	2.75E-02	28657	3.84E-02
28658	9.71E-03	28658	2.70E-02	28658	3.82E-02
28659	9.33E-03	28659	2.69E-02	28659	3.79E-02
28660	9.47E-03	28660	2.65E-02	28660	3.75E-02

28661	9.23E-03	28661	2.62E-02	28661	3.73E-02
28662	9.20E-03	28662	2.58E-02	28662	3.70E-02
28663	8.94E-03	28663	2.52E-02	28663	3.66E-02
28664	8.78E-03	28664	2.52E-02	28664	3.64E-02
28665	8.79E-03	28665	2.51E-02	28665	3.59E-02
28666	8.33E-03	28666	2.47E-02	28666	3.57E-02
28667	8.37E-03	28667	2.43E-02	28667	3.54E-02
28668	8.12E-03	28668	2.41E-02	28668	3.51E-02
28669	8.33E-03	28669	2.38E-02	28669	3.49E-02
28670	8.20E-03	28670	2.34E-02	28670	3.45E-02
28671	8.13E-03	28671	2.33E-02	28671	3.43E-02
28672	7.78E-03	28672	2.30E-02	28672	3.41E-02
28673	7.67E-03	28673	2.25E-02	28673	3.36E-02
28674	7.80E-03	28674	2.25E-02	28674	3.35E-02
28675	7.44E-03	28675	2.23E-02	28675	3.31E-02
28676	7.29E-03	28676	2.14E-02	28676	3.29E-02
28677	7.51E-03	28677	2.16E-02	28677	3.27E-02
28678	7.16E-03	28678	2.10E-02	28678	3.24E-02
28679	7.33E-03	28679	2.11E-02	28679	3.22E-02
28680	7.14E-03	28680	2.07E-02	28680	3.19E-02
28681	7.13E-03	28681	2.07E-02	28681	3.16E-02
28682	7.05E-03	28682	2.03E-02	28682	3.14E-02
28683	6.68E-03	28683	2.02E-02	28683	3.11E-02

28684	6.86E-03	28684	1.99E-02	28684	3.08E-02
28685	6.48E-03	28685	1.98E-02	28685	3.05E-02
28686	6.39E-03	28686	1.95E-02	28686	3.03E-02
28687	6.56E-03	28687	1.92E-02	28687	3.00E-02
28688	6.26E-03	28688	1.92E-02	28688	2.98E-02
28689	6.26E-03	28689	1.88E-02	28689	2.95E-02
28690	6.33E-03	28690	1.87E-02	28690	2.92E-02
28691	6.06E-03	28691	1.85E-02	28691	2.91E-02
28692	6.00E-03	28692	1.83E-02	28692	2.88E-02
28693	5.91E-03	28693	1.82E-02	28693	2.86E-02
28694	6.01E-03	28694	1.79E-02	28694	2.83E-02
28695	5.86E-03	28695	1.77E-02	28695	2.81E-02
28696	5.71E-03	28696	1.75E-02	28696	2.79E-02
28697	5.89E-03	28697	1.73E-02	28697	2.77E-02
28698	5.82E-03	28698	1.72E-02	28698	2.74E-02
28699	5.75E-03	28699	1.70E-02	28699	2.70E-02
28700	5.36E-03	28700	1.68E-02	28700	2.69E-02
28700	5.39E-03	28700	1.69E-02	28700	2.68E-02
28699	5.55E-03	28699	1.70E-02	28699	2.73E-02
28698	5.53E-03	28698	1.72E-02	28698	2.75E-02
28697	5.71E-03	28697	1.74E-02	28697	2.77E-02
28696	5.98E-03	28696	1.75E-02	28696	2.79E-02

28695	5.74E-03	28695	1.77E-02	28695	2.83E-02
28694	5.86E-03	28694	1.79E-02	28694	2.85E-02
28693	6.10E-03	28693	1.82E-02	28693	2.88E-02
28692	6.23E-03	28692	1.83E-02	28692	2.90E-02
28691	6.04E-03	28691	1.85E-02	28691	2.93E-02
28690	6.16E-03	28690	1.87E-02	28690	2.96E-02
28689	6.51E-03	28689	1.89E-02	28689	2.98E-02
28688	6.35E-03	28688	1.91E-02	28688	3.02E-02
28687	6.75E-03	28687	1.94E-02	28687	3.04E-02
28686	6.84E-03	28686	1.95E-02	28686	3.07E-02
28685	6.57E-03	28685	2.01E-02	28685	3.10E-02
28684	6.88E-03	28684	2.02E-02	28684	3.12E-02
28683	7.00E-03	28683	2.02E-02	28683	3.16E-02
28682	7.14E-03	28682	2.10E-02	28682	3.18E-02
28681	7.28E-03	28681	2.10E-02	28681	3.22E-02
28680	7.19E-03	28680	2.14E-02	28680	3.24E-02
28679	7.35E-03	28679	2.12E-02	28679	3.27E-02
28678	7.52E-03	28678	2.16E-02	28678	3.30E-02
28677	7.34E-03	28677	2.18E-02	28677	3.34E-02
28676	7.43E-03	28676	2.19E-02	28676	3.36E-02
28675	7.87E-03	28675	2.26E-02	28675	3.40E-02
28674	7.89E-03	28674	2.25E-02	28674	3.42E-02
28673	7.84E-03	28673	2.30E-02	28673	3.46E-02

28672	7.86E-03	28672	2.35E-02	28672	3.49E-02
28671	8.31E-03	28671	2.35E-02	28671	3.51E-02
28670	8.39E-03	28670	2.41E-02	28670	3.54E-02
28669	8.32E-03	28669	2.45E-02	28669	3.59E-02
28668	8.26E-03	28668	2.45E-02	28668	3.62E-02
28667	8.45E-03	28667	2.52E-02	28667	3.64E-02
28666	8.75E-03	28666	2.53E-02	28666	3.68E-02
28665	9.05E-03	28665	2.56E-02	28665	3.70E-02
28664	8.92E-03	28664	2.58E-02	28664	3.73E-02
28663	9.23E-03	28663	2.64E-02	28663	3.77E-02
28662	9.22E-03	28662	2.68E-02	28662	3.80E-02
28661	9.39E-03	28661	2.68E-02	28661	3.85E-02
28660	9.55E-03	28660	2.71E-02	28660	3.88E-02
28659	9.84E-03	28659	2.75E-02	28659	3.91E-02
28658	1.01E-02	28658	2.81E-02	28658	3.95E-02
28657	1.00E-02	28657	2.83E-02	28657	3.98E-02
28656	1.01E-02	28656	2.86E-02	28656	4.02E-02
28655	1.06E-02	28655	2.90E-02	28655	4.06E-02
28654	1.07E-02	28654	2.96E-02	28654	4.09E-02
28653	1.06E-02	28653	2.98E-02	28653	4.12E-02
28652	1.11E-02	28652	3.03E-02	28652	4.15E-02
28651	1.14E-02	28651	3.07E-02	28651	4.20E-02
28650	1.15E-02	28650	3.10E-02	28650	4.23E-02

28649	1.16E-02	28649	3.14E-02	28649	4.27E-02
28648	1.17E-02	28648	3.19E-02	28648	4.30E-02
28647	1.20E-02	28647	3.24E-02	28647	4.34E-02
28646	1.23E-02	28646	3.27E-02	28646	4.38E-02
28645	1.23E-02	28645	3.32E-02	28645	4.42E-02
28644	1.26E-02	28644	3.36E-02	28644	4.44E-02
28643	1.29E-02	28643	3.40E-02	28643	4.48E-02
28642	1.32E-02	28642	3.44E-02	28642	4.52E-02
28641	1.33E-02	28641	3.50E-02	28641	4.56E-02
28640	1.36E-02	28640	3.52E-02	28640	4.60E-02
28639	1.39E-02	28639	3.56E-02	28639	4.64E-02
28638	1.40E-02	28638	3.61E-02	28638	4.69E-02
28637	1.44E-02	28637	3.65E-02	28637	4.72E-02
28636	1.46E-02	28636	3.70E-02	28636	4.76E-02
28635	1.49E-02	28635	3.69E-02	28635	4.80E-02
28634	1.51E-02	28634	3.72E-02	28634	4.84E-02
28633	1.53E-02	28633	3.79E-02	28633	4.89E-02
28632	1.55E-02	28632	3.82E-02	28632	4.93E-02
28631	1.57E-02	28631	3.86E-02	28631	4.97E-02
28630	1.60E-02	28630	3.87E-02	28630	5.03E-02
28629	1.62E-02	28629	3.92E-02	28629	5.06E-02
28628	1.64E-02	28628	3.97E-02	28628	5.12E-02

28627	1.66E-02	28627	4.02E-02	28627	5.16E-02
28626	1.68E-02	28626	4.07E-02	28626	5.21E-02
28625	1.70E-02	28625	4.09E-02	28625	5.27E-02
28624	1.70E-02	28624	4.14E-02	28624	5.30E-02
28623	1.73E-02	28623	4.17E-02	28623	5.36E-02
28622	1.74E-02	28622	4.22E-02	28622	5.42E-02
28621	1.74E-02	28621	4.24E-02	28621	5.46E-02
28620	1.76E-02	28620	4.27E-02	28620	5.51E-02
28619	1.77E-02	28619	4.32E-02	28619	5.57E-02
28618	1.77E-02	28618	4.37E-02	28618	5.61E-02
28617	1.78E-02	28617	4.39E-02	28617	5.67E-02
28616	1.78E-02	28616	4.42E-02	28616	5.73E-02
28615	1.78E-02	28615	4.46E-02	28615	5.79E-02
28614	1.78E-02	28614	4.49E-02	28614	5.86E-02
28613	1.77E-02	28613	4.53E-02	28613	5.93E-02
28612	1.76E-02	28612	4.57E-02	28612	5.99E-02
28611	1.75E-02	28611	4.60E-02	28611	6.06E-02
28610	1.74E-02	28610	4.62E-02	28610	6.13E-02
28609	1.74E-02	28609	4.64E-02	28609	6.21E-02
28608	1.71E-02	28608	4.67E-02	28608	6.28E-02
28607	1.70E-02	28607	4.69E-02	28607	6.35E-02
28606	1.68E-02	28606	4.70E-02	28606	6.43E-02
28605	1.66E-02	28605	4.71E-02	28605	6.52E-02

28604	1.64E-02	28604	4.73E-02	28604	6.59E-02
28603	1.62E-02	28603	4.73E-02	28603	6.66E-02
28602	1.59E-02	28602	4.72E-02	28602	6.75E-02
28601	1.57E-02	28601	4.76E-02	28601	6.83E-02
28600	1.54E-02	28600	4.75E-02	28600	6.91E-02
28599	1.52E-02	28599	4.74E-02	28599	7.00E-02
28598	1.49E-02	28598	4.73E-02	28598	7.09E-02
28597	1.47E-02	28597	4.72E-02	28597	7.17E-02
28596	1.44E-02	28596	4.69E-02	28596	7.27E-02
28595	1.41E-02	28595	4.65E-02	28595	7.36E-02
28594	1.39E-02	28594	4.61E-02	28594	7.45E-02
28593	1.35E-02	28593	4.56E-02	28593	7.54E-02
28592	1.33E-02	28592	4.51E-02	28592	7.63E-02
28591	1.30E-02	28591	4.44E-02	28591	7.75E-02
28590	1.29E-02	28590	4.40E-02	28590	7.83E-02
28589	1.26E-02	28589	4.31E-02	28589	7.93E-02
28588	1.22E-02	28588	4.25E-02	28588	8.03E-02
28587	1.20E-02	28587	4.17E-02	28587	8.13E-02
28586	1.18E-02	28586	4.10E-02	28586	8.24E-02
28585	1.18E-02	28585	4.02E-02	28585	8.32E-02
28584	1.15E-02	28584	3.93E-02	28584	8.42E-02
28583	1.11E-02	28583	3.84E-02	28583	8.51E-02
28582	1.10E-02	28582	3.76E-02	28582	8.60E-02

28581	1.10E-02	28581	3.71E-02	28581	8.69E-02
28580	1.08E-02	28580	3.63E-02	28580	8.77E-02
28579	1.05E-02	28579	3.57E-02	28579	8.85E-02
28578	1.04E-02	28578	3.49E-02	28578	8.90E-02
28577	1.01E-02	28577	3.40E-02	28577	8.97E-02
28576	1.01E-02	28576	3.33E-02	28576	9.00E-02
28575	9.55E-03	28575	3.27E-02	28575	9.03E-02
28574	9.67E-03	28574	3.20E-02	28574	9.05E-02
28573	9.58E-03	28573	3.14E-02	28573	9.05E-02
28572	9.44E-03	28572	3.08E-02	28572	9.03E-02
28571	9.35E-03	28571	3.00E-02	28571	9.01E-02
28570	9.09E-03	28570	2.96E-02	28570	8.93E-02
28569	8.75E-03	28569	2.90E-02	28569	8.84E-02
28568	8.61E-03	28568	2.84E-02	28568	8.71E-02
28567	8.78E-03	28567	2.78E-02	28567	8.60E-02
28566	8.67E-03	28566	2.75E-02	28566	8.43E-02
28565	8.17E-03	28565	2.69E-02	28565	8.27E-02
28564	8.43E-03	28564	2.67E-02	28564	8.08E-02
28563	8.05E-03	28563	2.62E-02	28563	7.86E-02
28562	8.20E-03	28562	2.55E-02	28562	7.64E-02
28561	7.66E-03	28561	2.52E-02	28561	7.40E-02
28560	7.94E-03	28560	2.46E-02	28560	7.15E-02

28559	7.95E-03	28559	2.44E-02	28559	6.92E-02
28558	7.39E-03	28558	2.38E-02	28558	6.68E-02
28557	7.40E-03	28557	2.33E-02	28557	6.44E-02
28556	7.47E-03	28556	2.33E-02	28556	6.21E-02
28555	7.26E-03	28555	2.28E-02	28555	6.00E-02
28554	7.12E-03	28554	2.25E-02	28554	5.78E-02
28553	6.87E-03	28553	2.20E-02	28553	5.56E-02
28552	6.88E-03	28552	2.19E-02	28552	5.40E-02
28551	6.75E-03	28551	2.15E-02	28551	5.21E-02
28550	7.09E-03	28550	2.12E-02	28550	5.07E-02
28549	6.59E-03	28549	2.09E-02	28549	4.92E-02
28548	6.46E-03	28548	2.05E-02	28548	4.79E-02
28547	6.46E-03	28547	2.04E-02	28547	4.65E-02
28546	6.47E-03	28546	2.00E-02	28546	4.52E-02
28545	6.17E-03	28545	1.97E-02	28545	4.41E-02
28544	6.52E-03	28544	1.95E-02	28544	4.31E-02
28543	6.44E-03	28543	1.92E-02	28543	4.22E-02
28542	6.32E-03	28542	1.90E-02	28542	4.11E-02
28541	6.31E-03	28541	1.88E-02	28541	4.01E-02
28540	6.01E-03	28540	1.85E-02	28540	3.93E-02
28539	5.75E-03	28539	1.83E-02	28539	3.84E-02
28538	6.01E-03	28538	1.81E-02	28538	3.76E-02
28537	5.67E-03	28537	1.79E-02	28537	3.68E-02

28536	6.01E-03	28536	1.77E-02	28536	3.61E-02
28535	5.62E-03	28535	1.75E-02	28535	3.54E-02
28534	5.99E-03	28534	1.73E-02	28534	3.45E-02
28533	5.80E-03	28533	1.71E-02	28533	3.41E-02
28532	5.51E-03	28532	1.69E-02	28532	3.35E-02
28531	5.30E-03	28531	1.67E-02	28531	3.28E-02
28530	5.27E-03	28530	1.66E-02	28530	3.23E-02
28529	5.22E-03	28529	1.64E-02	28529	3.19E-02
28528	5.59E-03	28528	1.62E-02	28528	3.14E-02
28527	5.45E-03	28527	1.59E-02	28527	3.09E-02
28526	5.02E-03	28526	1.59E-02	28526	3.05E-02
28525	5.15E-03	28525	1.58E-02	28525	3.00E-02
28524	5.08E-03	28524	1.55E-02	28524	2.96E-02
28523	5.02E-03	28523	1.54E-02	28523	2.93E-02
28522	4.88E-03	28522	1.51E-02	28522	2.89E-02
28521	4.98E-03	28521	1.50E-02	28521	2.84E-02
28520	5.05E-03	28520	1.48E-02	28520	2.83E-02
28519	4.84E-03	28519	1.48E-02	28519	2.79E-02
28518	4.86E-03	28518	1.47E-02	28518	2.75E-02
28517	4.72E-03	28517	1.44E-02	28517	2.72E-02
28516	4.82E-03	28516	1.44E-02	28516	2.69E-02
28515	4.54E-03	28515	1.44E-02	28515	2.67E-02
28514	4.54E-03	28514	1.41E-02	28514	2.64E-02

28513	4.52E-03	28513	1.41E-02	28513	2.59E-02
28512	4.54E-03	28512	1.40E-02	28512	2.57E-02
28511	4.50E-03	28511	1.39E-02	28511	2.54E-02
28510	4.53E-03	28510	1.35E-02	28510	2.53E-02
28509	4.61E-03	28509	1.34E-02	28509	2.49E-02
28508	4.16E-03	28508	1.32E-02	28508	2.49E-02
28507	4.27E-03	28507	1.32E-02	28507	2.46E-02
28506	4.19E-03	28506	1.31E-02	28506	2.43E-02
28505	4.00E-03	28505	1.32E-02	28505	2.40E-02
28504	4.10E-03	28504	1.31E-02	28504	2.37E-02
28503	4.25E-03	28503	1.28E-02	28503	2.36E-02
28502	4.22E-03	28502	1.27E-02	28502	2.35E-02
28501	3.94E-03	28501	1.24E-02	28501	2.31E-02
28500	4.29E-03	28500	1.26E-02	28500	2.29E-02

I3 (1 sec time delay below 10V and 10 sec time delay 10V and above)
2V (LDV 125mm/s/V)   6V (LDV 125mm/s/V)
10V (LDV 125mm/s/V)   15V (LDV 125mm/s/V)
20V (LDV 125mm/s/V)   25V (LDV 125mm/s/V)
30V (LDV 125mm/s/V)   35V (LDV 125mm/s/V)
40V (LDV 125mm/s/V)   45V (LDV 125mm/s/V)
50V (LDV 125mm/s/V)
Frequency (Hz) Response (V) Temperature Frequency (Hz) Response (V)
Temperature Frequency (Hz) Response (V) Temperature Frequency (Hz)
Response (V) Temperature Frequency (Hz) Response (V) Temperature
Frequency (Hz) Response (V) Temperature Frequency (Hz) Response (V)
Temperature Frequency (Hz) Response (V) Temperature Frequency (Hz)
Response (V) Temperature Frequency (Hz) Response (V) Temperature
Frequency (Hz) Response (V) Temperature

27925	2.06E-02	2.83E+01	27925	6.17E-02	2.81E+01
27925	1.07E-01	2.78E+01	27976	3.19E-01	2.83E+01
27976	5.11E-01	2.83E+01	27976	7.64E-01	2.84E+01
27950	8.30E-01	2.95E+01	27950	1.04E+00	2.89E+01
27910	6.64E-01	2.86E+01	27910	7.86E-01	2.89E+01
27900	8.42E-01	2.89E+01			
27926	2.06E-02	2.81E+01	27926	6.16E-02	2.81E+01
27926	1.08E-01	2.79E+01	27978	3.24E-01	2.82E+01

27978	5.27E-01	2.83E+01	27978	7.98E-01	2.84E+01
27952	8.55E-01	2.98E+01	27952	1.08E+00	2.89E+01
27912	7.30E-01	2.85E+01	27912	8.90E-01	2.89E+01
27902	9.57E-01	2.91E+01			
27927	2.04E-02	2.83E+01	27927	6.22E-02	2.82E+01
27927	1.09E-01	2.80E+01	27980	3.34E-01	2.83E+01
27980	5.45E-01	2.85E+01	27980	8.34E-01	2.84E+01
27954	8.85E-01	2.97E+01	27954	1.12E+00	2.89E+01
27914	7.56E-01	2.85E+01	27914	9.23E-01	2.89E+01
27904	9.90E-01	2.91E+01			
27928	2.04E-02	2.83E+01	27928	6.29E-02	2.82E+01
27928	1.10E-01	2.79E+01	27982	3.44E-01	2.82E+01
27982	5.65E-01	2.84E+01	27982	8.79E-01	2.83E+01
27956	9.19E-01	2.97E+01	27956	1.17E+00	2.87E+01
27916	7.73E-01	2.86E+01	27916	9.46E-01	2.89E+01
27906	1.01E+00	2.90E+01			
27929	2.11E-02	2.81E+01	27929	6.34E-02	2.82E+01
27929	1.11E-01	2.79E+01	27984	3.54E-01	2.83E+01
27984	5.88E-01	2.84E+01	27984	9.31E-01	2.83E+01
27958	9.57E-01	2.96E+01	27958	1.23E+00	2.87E+01
27918	7.89E-01	2.85E+01	27918	9.68E-01	2.88E+01
27908	1.04E+00	2.91E+01			
27930	2.13E-02	2.81E+01	27930	6.37E-02	2.81E+01
27930	1.12E-01	2.79E+01	27986	3.68E-01	2.83E+01
27986	6.15E-01	2.84E+01	27986	9.93E-01	2.84E+01
27960	1.00E+00	2.96E+01	27960	1.30E+00	2.88E+01
27920	8.07E-01	2.85E+01	27920	9.91E-01	2.87E+01
27910	1.06E+00	2.91E+01			
27931	2.15E-02	2.81E+01	27931	6.39E-02	2.83E+01
27931	1.13E-01	2.79E+01	27988	3.80E-01	2.83E+01
27988	6.43E-01	2.83E+01	27988	1.07E+00	2.85E+01
27962	1.05E+00	2.96E+01	27962	1.38E+00	2.88E+01
27922	8.26E-01	2.85E+01	27922	1.01E+00	2.89E+01
27912	1.08E+00	2.89E+01			
27932	2.16E-02	2.82E+01	27932	6.44E-02	2.81E+01
27932	1.14E-01	2.78E+01	27990	3.94E-01	2.83E+01
27990	6.75E-01	2.83E+01	27990	1.19E+00	2.83E+01
27964	1.10E+00	2.95E+01	27964	1.49E+00	2.89E+01
27924	8.43E-01	2.85E+01	27924	1.04E+00	2.87E+01
27914	1.11E+00	2.91E+01			
27933	2.18E-02	2.83E+01	27933	6.49E-02	2.80E+01
27933	1.15E-01	2.80E+01	27992	4.10E-01	2.82E+01
27992	7.11E-01	2.83E+01	27992	1.40E+00	2.83E+01
27966	1.17E+00	2.95E+01	27966	1.67E+00	2.87E+01
27926	8.65E-01	2.85E+01	27926	1.07E+00	2.87E+01
27916	1.13E+00	2.90E+01			
27934	2.23E-02	2.83E+01	27934	6.56E-02	2.81E+01
27934	1.15E-01	2.79E+01	27994	4.27E-01	2.83E+01
27994	7.53E-01	2.83E+01	27994	3.07E+00	2.85E+01
27968	1.26E+00	2.95E+01	27968	2.21E+00	2.88E+01
27928	8.86E-01	2.85E+01	27928	1.10E+00	2.87E+01
27918	1.16E+00	2.88E+01			
27935	2.19E-02	2.83E+01	27935	6.60E-02	2.82E+01
27935	1.16E-01	2.79E+01	27996	4.46E-01	2.83E+01
27996	8.00E-01	2.83E+01	27996	3.01E+00	2.84E+01
27970	1.38E+00	2.95E+01	27970	3.85E+00	2.88E+01
27930	9.09E-01	2.85E+01	27930	1.13E+00	2.89E+01
27920	1.20E+00	2.89E+01			
27936	2.21E-02	2.82E+01	27936	6.69E-02	2.79E+01
27936	1.17E-01	2.79E+01	27998	4.67E-01	2.83E+01
27998	8.57E-01	2.83E+01	27998	2.94E+00	2.84E+01
27972	1.56E+00	2.94E+01	27972	3.79E+00	2.88E+01
27932	9.34E-01	2.85E+01	27932	1.16E+00	2.87E+01
27922	1.23E+00	2.89E+01			
27937	2.25E-02	2.82E+01	27937	6.78E-02	2.80E+01
27937	1.18E-01	2.79E+01	28000	4.90E-01	2.82E+01
28000	9.30E-01	2.83E+01	28000	2.87E+00	2.85E+01
27974	2.30E+00	2.94E+01	27974	3.73E+00	2.90E+01

27934	9.60E-01	2.85E+01	27934	1.20E+00	2.87E+01
27924	1.27E+00	2.90E+01			
27938	2.22E-02	2.81E+01	27938	6.90E-02	2.81E+01
27938	1.19E-01	2.80E+01	28002	5.17E-01	2.83E+01
28002	1.03E+00	2.84E+01	28002	2.81E+00	2.85E+01
27976	3.44E+00	2.94E+01	27976	3.67E+00	2.88E+01
27936	9.86E-01	2.85E+01	27936	1.24E+00	2.88E+01
27926	1.31E+00	2.89E+01			
27939	2.29E-02	2.82E+01	27939	6.82E-02	2.81E+01
27939	1.21E-01	2.79E+01	28004	5.47E-01	2.82E+01
28004	1.20E+00	2.83E+01	28004	2.74E+00	2.85E+01
27978	3.38E+00	2.94E+01	27978	3.61E+00	2.89E+01
27938	1.02E+00	2.84E+01	27938	1.29E+00	2.87E+01
27928	1.35E+00	2.89E+01			
27940	2.29E-02	2.81E+01	27940	6.87E-02	2.81E+01
27940	1.22E-01	2.79E+01	28006	5.82E-01	2.83E+01
28006	2.53E+00	2.83E+01	28006	2.67E+00	2.85E+01
27980	3.32E+00	2.94E+01	27980	3.56E+00	2.89E+01
27940	1.05E+00	2.85E+01	27940	1.34E+00	2.87E+01
27930	1.40E+00	2.89E+01			
27941	2.32E-02	2.82E+01	27941	6.98E-02	2.81E+01
27941	1.23E-01	2.80E+01	28009	6.24E-01	2.83E+01
28009	2.45E+00	2.84E+01	28009	2.61E+00	2.85E+01
27982	3.26E+00	2.95E+01	27982	3.50E+00	2.89E+01
27942	1.08E+00	2.85E+01	27942	1.41E+00	2.89E+01
27932	1.45E+00	2.90E+01			
27942	2.32E-02	2.83E+01	27942	7.02E-02	2.80E+01
27942	1.24E-01	2.79E+01	28011	6.75E-01	2.85E+01
28011	2.38E+00	2.85E+01	28011	2.55E+00	2.85E+01
27984	3.20E+00	2.93E+01	27984	3.44E+00	2.89E+01
27944	1.12E+00	2.85E+01	27944	1.47E+00	2.89E+01
27934	1.52E+00	2.89E+01			
27943	2.37E-02	2.81E+01	27943	7.08E-02	2.81E+01
27943	1.25E-01	2.80E+01	28013	7.40E-01	2.83E+01
28013	2.32E+00	2.83E+01	28013	2.48E+00	2.85E+01
27986	3.14E+00	2.95E+01	27986	3.38E+00	2.89E+01
27946	1.16E+00	2.85E+01	27946	1.56E+00	2.89E+01
27936	1.59E+00	2.89E+01			
27944	2.35E-02	2.83E+01	27944	7.13E-02	2.81E+01
27944	1.26E-01	2.81E+01	28015	8.27E-01	2.83E+01
28015	2.26E+00	2.83E+01	28015	2.41E+00	2.83E+01
27988	3.08E+00	2.94E+01	27988	3.33E+00	2.88E+01
27948	1.21E+00	2.85E+01	27948	1.66E+00	2.89E+01
27938	1.67E+00	2.88E+01			
27945	2.41E-02	2.82E+01	27945	7.22E-02	2.82E+01
27945	1.27E-01	2.79E+01	28017	9.65E-01	2.83E+01
28017	2.19E+00	2.83E+01	28017	2.35E+00	2.87E+01
27990	3.02E+00	2.94E+01	27990	3.27E+00	2.89E+01
27950	1.26E+00	2.85E+01	27950	1.80E+00	2.89E+01
27940	1.78E+00	2.89E+01			
27946	2.42E-02	2.83E+01	27946	7.36E-02	2.81E+01
27946	1.28E-01	2.81E+01	28019	1.20E+00	2.84E+01
28019	2.13E+00	2.85E+01	28019	2.29E+00	2.85E+01
27992	2.96E+00	2.95E+01	27992	3.21E+00	2.90E+01
27952	1.33E+00	2.85E+01	27952	2.05E+00	2.88E+01
27942	1.93E+00	2.89E+01			
27947	2.45E-02	2.83E+01	27947	7.33E-02	2.81E+01
27947	1.30E-01	2.81E+01	28021	1.74E+00	2.83E+01
28021	2.06E+00	2.85E+01	28021	2.23E+00	2.85E+01
27994	2.90E+00	2.92E+01	27994	3.15E+00	2.89E+01
27954	1.40E+00	2.83E+01	27954	4.37E+00	2.87E+01
27944	2.25E+00	2.89E+01			
27948	2.47E-02	2.82E+01	27948	7.42E-02	2.81E+01
27948	1.31E-01	2.81E+01	28023	1.76E+00	2.84E+01
28023	2.00E+00	2.85E+01	28023	2.16E+00	2.85E+01
27996	2.85E+00	2.94E+01	27996	3.09E+00	2.91E+01
27956	1.50E+00	2.85E+01	27956	4.31E+00	2.89E+01
27946	4.50E+00	2.91E+01			

27949	2.46E-02	2.83E+01	27949	7.44E-02	2.79E+01
27949	1.32E-01	2.80E+01	28025	1.70E+00	2.84E+01
28025	1.93E+00	2.85E+01	28025	2.10E+00	2.85E+01
27998	2.79E+00	2.93E+01	27998	3.03E+00	2.90E+01
27958	1.61E+00	2.85E+01	27958	4.26E+00	2.89E+01
27948	4.46E+00	2.92E+01			
27950	2.50E-02	2.83E+01	27950	7.56E-02	2.81E+01
27950	1.33E-01	2.81E+01	28027	1.63E+00	2.83E+01
28027	1.87E+00	2.84E+01	28027	2.04E+00	2.85E+01
28000	2.74E+00	2.94E+01	28000	2.97E+00	2.89E+01
27960	1.80E+00	2.84E+01	27960	4.21E+00	2.89E+01
27950	4.41E+00	2.92E+01			
27951	2.50E-02	2.82E+01	27951	7.61E-02	2.81E+01
27951	1.35E-01	2.79E+01	28029	1.57E+00	2.83E+01
28029	1.81E+00	2.84E+01	28029	1.98E+00	2.87E+01
28002	2.68E+00	2.94E+01	28002	2.92E+00	2.91E+01
27962	2.38E+00	2.85E+01	27962	4.17E+00	2.89E+01
27952	4.37E+00	2.93E+01			
27952	2.53E-02	2.83E+01	27952	7.76E-02	2.80E+01
27952	1.36E-01	2.79E+01	28031	1.52E+00	2.83E+01
28031	1.75E+00	2.83E+01	28031	1.92E+00	2.85E+01
28004	2.62E+00	2.94E+01	28004	2.85E+00	2.89E+01
27964	4.13E+00	2.84E+01	27964	4.12E+00	2.89E+01
27954	4.33E+00	2.94E+01			
27953	2.57E-02	2.83E+01	27953	7.78E-02	2.81E+01
27953	1.38E-01	2.79E+01	28033	1.46E+00	2.85E+01
28033	1.69E+00	2.85E+01	28033	1.86E+00	2.84E+01
28006	2.56E+00	2.94E+01	28006	2.79E+00	2.89E+01
27966	4.07E+00	2.87E+01	27966	4.07E+00	2.89E+01
27956	4.29E+00	2.93E+01			
27954	2.59E-02	2.83E+01	27954	7.80E-02	2.81E+01
27954	1.39E-01	2.80E+01	28035	1.40E+00	2.85E+01
28035	1.63E+00	2.85E+01	28035	1.80E+00	2.85E+01
28008	2.50E+00	2.94E+01	28008	2.74E+00	2.91E+01
27968	4.01E+00	2.88E+01	27968	4.02E+00	2.91E+01
27958	4.25E+00	2.94E+01			
27955	2.60E-02	2.83E+01	27955	7.97E-02	2.81E+01
27955	1.40E-01	2.79E+01	28037	1.35E+00	2.83E+01
28037	1.58E+00	2.85E+01	28037	1.75E+00	2.85E+01
28010	2.45E+00	2.94E+01	28010	2.68E+00	2.89E+01
27970	3.96E+00	2.87E+01	27970	3.97E+00	2.91E+01
27960	4.20E+00	2.94E+01			
27956	2.60E-02	2.83E+01	27956	7.98E-02	2.81E+01
27956	1.42E-01	2.79E+01	28039	1.29E+00	2.83E+01
28039	1.52E+00	2.84E+01	28039	1.70E+00	2.85E+01
28012	2.39E+00	2.94E+01	28012	2.63E+00	2.91E+01
27972	3.90E+00	2.88E+01	27972	3.92E+00	2.91E+01
27962	4.16E+00	2.94E+01			
27957	2.64E-02	2.82E+01	27957	8.04E-02	2.81E+01
27957	1.43E-01	2.79E+01	28041	1.24E+00	2.83E+01
28041	1.47E+00	2.84E+01	28041	1.65E+00	2.85E+01
28014	2.33E+00	2.94E+01	28014	2.57E+00	2.89E+01
27974	3.85E+00	2.89E+01	27974	3.87E+00	2.92E+01
27964	4.12E+00	2.94E+01			
27958	2.74E-02	2.82E+01	27958	8.15E-02	2.79E+01
27958	1.45E-01	2.79E+01	28043	1.20E+00	2.83E+01
28043	1.42E+00	2.84E+01	28043	1.60E+00	2.85E+01
28016	2.28E+00	2.94E+01	28016	2.51E+00	2.89E+01
27976	3.79E+00	2.89E+01	27976	3.81E+00	2.93E+01
27966	4.07E+00	2.95E+01			
27959	2.70E-02	2.81E+01	27959	8.19E-02	2.79E+01
27959	1.46E-01	2.79E+01	28045	1.17E+00	2.83E+01
28045	1.37E+00	2.83E+01	28045	1.55E+00	2.85E+01
28018	2.22E+00	2.94E+01	28018	2.46E+00	2.91E+01
27978	3.74E+00	2.89E+01	27978	3.76E+00	2.93E+01
27968	4.03E+00	2.96E+01			
27960	2.76E-02	2.82E+01	27960	8.27E-02	2.81E+01
27960	1.48E-01	2.79E+01	28047	1.12E+00	2.82E+01

28047	1.32E+00	2.83E+01	28047	1.50E+00	2.85E+01
28020	2.17E+00	2.94E+01	28020	2.40E+00	2.91E+01
27980	3.69E+00	2.89E+01	27980	3.71E+00	2.92E+01
27970	3.98E+00	2.95E+01			
27961	2.73E-02	2.81E+01	27961	8.33E-02	2.82E+01
27961	1.50E-01	2.81E+01	28049	1.08E+00	2.83E+01
28049	1.28E+00	2.85E+01	28049	1.46E+00	2.85E+01
28022	2.11E+00	2.93E+01	28022	2.35E+00	2.90E+01
27982	3.63E+00	2.89E+01	27982	3.66E+00	2.91E+01
27972	3.94E+00	2.97E+01			
27962	2.79E-02	2.81E+01	27962	8.67E-02	2.81E+01
27962	1.51E-01	2.80E+01	28051	1.04E+00	2.84E+01
28051	1.23E+00	2.83E+01	28051	1.41E+00	2.86E+01
28024	2.06E+00	2.94E+01	28024	2.29E+00	2.89E+01
27984	3.58E+00	2.88E+01	27984	3.61E+00	2.91E+01
27974	3.89E+00	2.95E+01			
27963	2.82E-02	2.81E+01	27963	8.60E-02	2.81E+01
27963	1.53E-01	2.81E+01	28053	9.97E-01	2.83E+01
28053	1.19E+00	2.84E+01	28053	1.37E+00	2.85E+01
28026	2.01E+00	2.92E+01	28026	2.24E+00	2.90E+01
27986	3.52E+00	2.89E+01	27986	3.56E+00	2.92E+01
27976	3.84E+00	2.96E+01			
27964	2.84E-02	2.81E+01	27964	8.69E-02	2.81E+01
27964	1.54E-01	2.79E+01	28055	9.57E-01	2.84E+01
28055	1.16E+00	2.85E+01	28055	1.32E+00	2.85E+01
28028	1.96E+00	2.93E+01	28028	2.19E+00	2.90E+01
27988	3.46E+00	2.89E+01	27988	3.51E+00	2.93E+01
27978	3.80E+00	2.96E+01			
27965	2.90E-02	2.82E+01	27965	8.75E-02	2.81E+01
27965	1.57E-01	2.79E+01	28057	9.18E-01	2.83E+01
28057	1.12E+00	2.84E+01	28057	1.28E+00	2.85E+01
28030	1.91E+00	2.92E+01	28030	2.13E+00	2.89E+01
27990	3.41E+00	2.90E+01	27990	3.45E+00	2.91E+01
27980	3.75E+00	2.95E+01			
27966	2.92E-02	2.81E+01	27966	8.95E-02	2.80E+01
27966	1.59E-01	2.81E+01	28059	8.80E-01	2.82E+01
28059	1.09E+00	2.83E+01	28059	1.24E+00	2.85E+01
28032	1.86E+00	2.94E+01	28032	2.08E+00	2.91E+01
27992	3.36E+00	2.90E+01	27992	3.40E+00	2.91E+01
27982	3.70E+00	2.94E+01			
27967	2.95E-02	2.81E+01	27967	8.94E-02	2.81E+01
27967	1.60E-01	2.79E+01	28061	8.44E-01	2.84E+01
28061	1.05E+00	2.85E+01	28061	1.20E+00	2.85E+01
28034	1.81E+00	2.92E+01	28034	2.03E+00	2.90E+01
27994	3.30E+00	2.90E+01	27994	3.35E+00	2.91E+01
27984	3.65E+00	2.96E+01			
27968	2.99E-02	2.81E+01	27968	9.08E-02	2.81E+01
27968	1.62E-01	2.80E+01	28063	8.10E-01	2.83E+01
28063	1.02E+00	2.83E+01	28063	1.17E+00	2.85E+01
28036	1.77E+00	2.93E+01	28036	1.98E+00	2.88E+01
27996	3.24E+00	2.91E+01	27996	3.30E+00	2.93E+01
27986	3.60E+00	2.95E+01			
27969	3.05E-02	2.83E+01	27969	9.12E-02	2.81E+01
27969	1.64E-01	2.79E+01	28065	7.77E-01	2.83E+01
28065	9.83E-01	2.85E+01	28065	1.14E+00	2.85E+01
28038	1.72E+00	2.93E+01	28038	1.93E+00	2.88E+01
27998	3.19E+00	2.89E+01	27998	3.25E+00	2.91E+01
27988	3.55E+00	2.97E+01			
27970	3.03E-02	2.82E+01	27970	9.21E-02	2.81E+01
27970	1.67E-01	2.79E+01	28067	7.45E-01	2.83E+01
28067	9.54E-01	2.83E+01	28067	1.10E+00	2.85E+01
28040	1.68E+00	2.92E+01	28040	1.89E+00	2.89E+01
28000	3.13E+00	2.89E+01	28000	3.19E+00	2.91E+01
27990	3.50E+00	2.96E+01			
27971	3.09E-02	2.82E+01	27971	9.30E-02	2.79E+01
27971	1.68E-01	2.79E+01	28070	7.16E-01	2.84E+01
28070	9.22E-01	2.84E+01	28070	1.07E+00	2.85E+01
28042	1.63E+00	2.92E+01	28042	1.84E+00	2.88E+01

28002	3.07E+00	2.90E+01	28002	3.13E+00	2.93E+01
27992	3.45E+00	2.95E+01			
27972	3.08E-02	2.82E+01	27972	9.50E-02	2.81E+01
27972	1.70E-01	2.80E+01	28072	6.87E-01	2.83E+01
28072	8.92E-01	2.84E+01	28072	1.04E+00	2.85E+01
28044	1.59E+00	2.92E+01	28044	1.79E+00	2.89E+01
28004	3.02E+00	2.91E+01	28004	3.08E+00	2.93E+01
27994	3.40E+00	2.94E+01			
27973	3.14E-02	2.83E+01	27973	9.57E-02	2.80E+01
27973	1.72E-01	2.81E+01	28074	6.61E-01	2.84E+01
28074	8.63E-01	2.83E+01	28074	1.01E+00	2.85E+01
28046	1.55E+00	2.92E+01	28046	1.75E+00	2.89E+01
28006	2.96E+00	2.89E+01	28006	3.02E+00	2.93E+01
27996	3.35E+00	2.95E+01			
27974	3.19E-02	2.83E+01	27974	9.66E-02	2.79E+01
27974	1.75E-01	2.79E+01	28076	6.36E-01	2.84E+01
28076	8.36E-01	2.84E+01	28076	9.89E-01	2.85E+01
28048	1.51E+00	2.91E+01	28048	1.71E+00	2.89E+01
28008	2.90E+00	2.90E+01	28008	2.97E+00	2.94E+01
27998	3.30E+00	2.95E+01			
27975	3.23E-02	2.83E+01	27975	9.85E-02	2.81E+01
27975	1.77E-01	2.81E+01	28078	6.12E-01	2.83E+01
28078	8.09E-01	2.83E+01	28078	9.62E-01	2.85E+01
28050	1.47E+00	2.91E+01	28050	1.67E+00	2.89E+01
28010	2.85E+00	2.91E+01	28010	2.92E+00	2.92E+01
28000	3.25E+00	2.97E+01			
27976	3.27E-02	2.83E+01	27976	9.92E-02	2.81E+01
27976	1.79E-01	2.80E+01	28080	5.90E-01	2.85E+01
28080	7.84E-01	2.83E+01	28080	9.37E-01	2.85E+01
28050	1.47E+00	2.93E+01	28050	1.67E+00	2.87E+01
28010	2.85E+00	2.90E+01	28010	2.92E+00	2.94E+01
28000	3.25E+00	2.96E+01			
27977	3.30E-02	2.83E+01	27977	1.01E-01	2.79E+01
27977	1.82E-01	2.79E+01	28082	5.69E-01	2.83E+01
28082	7.59E-01	2.83E+01	28082	9.11E-01	2.85E+01
28048	1.52E+00	2.91E+01	28048	1.72E+00	2.89E+01
28008	2.91E+00	2.88E+01	28008	2.98E+00	2.93E+01
27998	3.31E+00	2.96E+01			
27978	3.32E-02	2.81E+01	27978	1.02E-01	2.79E+01
27978	1.84E-01	2.80E+01	28084	5.49E-01	2.83E+01
28084	7.35E-01	2.82E+01	28084	8.89E-01	2.85E+01
28046	1.57E+00	2.90E+01	28046	1.76E+00	2.88E+01
28006	2.96E+00	2.90E+01	28006	3.04E+00	2.91E+01
27996	3.36E+00	2.94E+01			
27979	3.39E-02	2.83E+01	27979	1.03E-01	2.79E+01
27979	1.87E-01	2.81E+01	28086	5.30E-01	2.85E+01
28086	7.11E-01	2.85E+01	28086	8.66E-01	2.84E+01
28044	1.62E+00	2.90E+01	28044	1.81E+00	2.88E+01
28004	3.02E+00	2.90E+01	28004	3.09E+00	2.92E+01
27994	3.41E+00	2.95E+01			
27980	3.41E-02	2.83E+01	27980	1.05E-01	2.81E+01
27980	1.90E-01	2.80E+01	28088	5.12E-01	2.85E+01
28088	6.90E-01	2.83E+01	28088	8.45E-01	2.85E+01
28042	1.66E+00	2.91E+01	28042	1.87E+00	2.88E+01
28002	3.08E+00	2.90E+01	28002	3.15E+00	2.93E+01
27992	3.47E+00	2.94E+01			
27981	3.46E-02	2.82E+01	27981	1.06E-01	2.82E+01
27981	1.92E-01	2.79E+01	28090	4.95E-01	2.84E+01
28090	6.70E-01	2.83E+01	28090	8.23E-01	2.85E+01
28040	1.71E+00	2.91E+01	28040	1.91E+00	2.88E+01
28000	3.14E+00	2.91E+01	28000	3.20E+00	2.94E+01
27990	3.52E+00	2.95E+01			
27982	3.50E-02	2.81E+01	27982	1.08E-01	2.81E+01
27982	1.96E-01	2.81E+01	28092	4.79E-01	2.83E+01
28092	6.50E-01	2.85E+01	28092	8.01E-01	2.85E+01
28038	1.76E+00	2.92E+01	28038	1.97E+00	2.89E+01
27998	3.19E+00	2.91E+01	27998	3.26E+00	2.93E+01
27988	3.57E+00	2.96E+01			

27983	3.56E-02	2.82E+01	27983	1.09E-01	2.81E+01
27983	1.98E-01	2.80E+01	28094	4.63E-01	2.83E+01
28094	6.32E-01	2.85E+01	28094	7.79E-01	2.85E+01
28036	1.81E+00	2.90E+01	28036	2.02E+00	2.87E+01
27996	3.24E+00	2.90E+01	27996	3.31E+00	2.93E+01
27986	3.61E+00	2.96E+01			
27984	3.59E-02	2.81E+01	27984	1.11E-01	2.81E+01
27984	2.01E-01	2.81E+01	28096	4.48E-01	2.83E+01
28096	6.13E-01	2.83E+01	28096	7.60E-01	2.85E+01
28034	1.86E+00	2.90E+01	28034	2.07E+00	2.87E+01
27994	3.30E+00	2.90E+01	27994	3.36E+00	2.94E+01
27984	3.66E+00	2.96E+01			
27985	3.66E-02	2.83E+01	27985	1.12E-01	2.80E+01
27985	2.04E-01	2.81E+01	28098	4.35E-01	2.83E+01
28098	5.96E-01	2.84E+01	28098	7.40E-01	2.85E+01
28032	1.92E+00	2.90E+01	28032	2.12E+00	2.88E+01
27992	3.35E+00	2.92E+01	27992	3.41E+00	2.92E+01
27982	3.71E+00	2.94E+01			
27986	3.73E-02	2.83E+01	27986	1.14E-01	2.80E+01
27986	2.07E-01	2.79E+01	28100	4.22E-01	2.83E+01
28100	5.79E-01	2.83E+01	28100	7.22E-01	2.85E+01
28030	1.97E+00	2.89E+01	28030	2.18E+00	2.88E+01
27990	3.40E+00	2.90E+01	27990	3.47E+00	2.92E+01
27980	3.76E+00	2.96E+01			
27987	3.79E-02	2.83E+01	27987	1.16E-01	2.79E+01
27987	2.10E-01	2.81E+01	28100	4.22E-01	2.81E+01
28100	5.79E-01	2.83E+01	28100	7.24E-01	2.83E+01
28028	2.03E+00	2.91E+01	28028	2.23E+00	2.88E+01
27988	3.45E+00	2.90E+01	27988	3.52E+00	2.93E+01
27978	3.80E+00	2.96E+01			
27988	3.79E-02	2.83E+01	27988	1.17E-01	2.81E+01
27988	2.14E-01	2.80E+01	28098	4.36E-01	2.83E+01
28098	5.97E-01	2.83E+01	28098	7.43E-01	2.83E+01
28026	2.09E+00	2.90E+01	28026	2.29E+00	2.88E+01
27986	3.50E+00	2.91E+01	27986	3.57E+00	2.92E+01
27976	3.85E+00	2.96E+01			
27989	3.83E-02	2.82E+01	27989	1.19E-01	2.81E+01
27989	2.18E-01	2.79E+01	28096	4.50E-01	2.83E+01
28096	6.15E-01	2.85E+01	28096	7.62E-01	2.84E+01
28024	2.14E+00	2.92E+01	28024	2.34E+00	2.87E+01
27984	3.55E+00	2.91E+01	27984	3.62E+00	2.93E+01
27974	3.89E+00	2.96E+01			
27990	3.87E-02	2.81E+01	27990	1.21E-01	2.81E+01
27990	2.21E-01	2.79E+01	28094	4.64E-01	2.81E+01
28094	6.33E-01	2.84E+01	28094	7.83E-01	2.85E+01
28022	2.21E+00	2.89E+01	28022	2.40E+00	2.88E+01
27982	3.60E+00	2.91E+01	27982	3.66E+00	2.91E+01
27972	3.93E+00	2.97E+01			
27991	3.95E-02	2.81E+01	27991	1.23E-01	2.81E+01
27991	2.25E-01	2.80E+01	28092	4.80E-01	2.83E+01
28092	6.53E-01	2.84E+01	28092	8.06E-01	2.85E+01
28020	2.26E+00	2.89E+01	28020	2.46E+00	2.87E+01
27980	3.64E+00	2.91E+01	27980	3.71E+00	2.92E+01
27970	3.97E+00	2.96E+01			
27992	3.98E-02	2.83E+01	27992	1.25E-01	2.81E+01
27992	2.29E-01	2.79E+01	28090	4.96E-01	2.82E+01
28090	6.73E-01	2.84E+01	28090	8.27E-01	2.85E+01
28018	2.32E+00	2.89E+01	28018	2.52E+00	2.88E+01
27978	3.69E+00	2.92E+01	27978	3.75E+00	2.94E+01
27968	4.01E+00	2.97E+01			
27993	4.05E-02	2.83E+01	27993	1.27E-01	2.81E+01
27993	2.34E-01	2.79E+01	28088	5.14E-01	2.83E+01
28088	6.95E-01	2.84E+01	28088	8.51E-01	2.83E+01
28016	2.38E+00	2.90E+01	28016	2.57E+00	2.88E+01
27976	3.74E+00	2.93E+01	27976	3.80E+00	2.94E+01
27966	4.05E+00	2.97E+01			
27994	4.17E-02	2.82E+01	27994	1.29E-01	2.81E+01
27994	2.37E-01	2.79E+01	28086	5.33E-01	2.83E+01

28086	7.17E-01	2.83E+01	28086	8.75E-01	2.85E+01
28014	2.44E+00	2.91E+01	28014	2.63E+00	2.88E+01
27974	3.78E+00	2.92E+01	27974	3.84E+00	2.94E+01
27964	4.09E+00	2.97E+01			
27995	4.20E-02	2.83E+01	27995	1.31E-01	2.81E+01
27995	2.42E-01	2.81E+01	28084	5.53E-01	2.83E+01
28084	7.41E-01	2.84E+01	28084	9.01E-01	2.85E+01
28012	2.50E+00	2.90E+01	28012	2.69E+00	2.89E+01
27972	3.82E+00	2.93E+01	27972	3.89E+00	2.96E+01
27962	4.12E+00	2.98E+01			
27996	4.29E-02	2.81E+01	27996	1.33E-01	2.80E+01
27996	2.47E-01	2.79E+01	28082	5.73E-01	2.83E+01
28082	7.65E-01	2.83E+01	28082	9.28E-01	2.85E+01
28010	2.56E+00	2.90E+01	28010	2.75E+00	2.88E+01
27970	3.87E+00	2.92E+01	27970	3.93E+00	2.95E+01
27960	4.16E+00	2.99E+01			
27997	4.32E-02	2.81E+01	27997	1.36E-01	2.79E+01
27997	2.52E-01	2.79E+01	28080	5.94E-01	2.82E+01
28080	7.91E-01	2.85E+01	28080	9.55E-01	2.84E+01
28008	2.62E+00	2.89E+01	28008	2.80E+00	2.87E+01
27968	3.91E+00	2.91E+01	27968	3.97E+00	2.94E+01
27958	4.19E+00	2.98E+01			
27998	4.42E-02	2.82E+01	27998	1.38E-01	2.80E+01
27998	2.56E-01	2.81E+01	28078	6.17E-01	2.83E+01
28078	8.18E-01	2.83E+01	28078	9.83E-01	2.83E+01
28006	2.68E+00	2.89E+01	28006	2.86E+00	2.89E+01
27966	3.95E+00	2.91E+01	27966	4.01E+00	2.96E+01
27956	4.23E+00	2.97E+01			
27999	4.46E-02	2.81E+01	27999	1.41E-01	2.80E+01
27999	2.62E-01	2.80E+01	28076	6.42E-01	2.83E+01
28076	8.47E-01	2.83E+01	28076	1.01E+00	2.83E+01
28004	2.73E+00	2.90E+01	28004	2.92E+00	2.88E+01
27964	3.99E+00	2.93E+01	27964	4.05E+00	2.95E+01
27954	4.26E+00	2.97E+01			
28000	4.55E-02	2.81E+01	28000	1.43E-01	2.81E+01
28000	2.67E-01	2.79E+01	28074	6.67E-01	2.81E+01
28074	8.76E-01	2.83E+01	28074	1.04E+00	2.83E+01
28002	2.79E+00	2.90E+01	28002	2.97E+00	2.90E+01
27962	4.03E+00	2.92E+01	27962	4.08E+00	2.96E+01
27952	4.29E+00	2.98E+01			
28001	4.63E-02	2.83E+01	28001	1.46E-01	2.81E+01
28001	2.73E-01	2.79E+01	28072	6.95E-01	2.82E+01
28072	9.07E-01	2.83E+01	28072	1.07E+00	2.84E+01
28000	2.85E+00	2.90E+01	28000	3.03E+00	2.87E+01
27960	4.07E+00	2.94E+01	27960	4.12E+00	2.95E+01
27950	4.33E+00	2.98E+01			
28002	4.67E-02	2.83E+01	28002	1.49E-01	2.81E+01
28002	2.80E-01	2.79E+01	28070	7.23E-01	2.83E+01
28070	9.38E-01	2.83E+01	28070	1.10E+00	2.85E+01
27998	2.91E+00	2.91E+01	27998	3.09E+00	2.88E+01
27958	4.10E+00	2.92E+01	27958	4.15E+00	2.94E+01
27948	4.36E+00	2.98E+01			
28003	4.79E-02	2.83E+01	28003	1.52E-01	2.80E+01
28003	2.85E-01	2.81E+01	28067	7.54E-01	2.83E+01
28067	9.70E-01	2.83E+01	28067	1.13E+00	2.85E+01
27996	2.97E+00	2.90E+01	27996	3.14E+00	2.89E+01
27956	4.14E+00	2.93E+01	27956	4.19E+00	2.95E+01
27946	4.38E+00	2.99E+01			
28004	4.84E-02	2.83E+01	28004	1.55E-01	2.81E+01
28004	2.92E-01	2.79E+01	28065	7.86E-01	2.82E+01
28065	1.00E+00	2.83E+01	28065	1.17E+00	2.84E+01
27994	3.03E+00	2.90E+01	27994	3.19E+00	2.88E+01
27954	4.19E+00	2.92E+01	27954	4.22E+00	2.96E+01
27944	4.41E+00	3.00E+01			
28005	4.97E-02	2.82E+01	28005	1.58E-01	2.81E+01
28005	2.99E-01	2.78E+01	28063	8.20E-01	2.83E+01
28063	1.04E+00	2.83E+01	28063	1.20E+00	2.83E+01
27992	3.08E+00	2.89E+01	27992	3.25E+00	2.89E+01

27952	1.91E+00	2.93E+01	27952	4.26E+00	2.96E+01
27942	4.44E+00	3.00E+01			
28006	5.04E-02	2.83E+01	28006	1.62E-01	2.81E+01
28006	3.07E-01	2.80E+01	28061	8.56E-01	2.84E+01
28061	1.08E+00	2.84E+01	28061	1.23E+00	2.83E+01
27990	3.13E+00	2.91E+01	27990	3.30E+00	2.89E+01
27950	1.62E+00	2.93E+01	27950	4.29E+00	2.97E+01
27940	4.47E+00	2.98E+01			
28007	5.11E-02	2.83E+01	28007	1.65E-01	2.82E+01
28007	3.15E-01	2.79E+01	28059	8.93E-01	2.83E+01
28059	1.11E+00	2.84E+01	28059	1.27E+00	2.83E+01
27988	3.18E+00	2.91E+01	27988	3.35E+00	2.89E+01
27948	1.48E+00	2.93E+01	27948	4.32E+00	2.97E+01
27938	4.50E+00	3.00E+01			
28008	5.23E-02	2.82E+01	28008	1.68E-01	2.82E+01
28008	3.23E-01	2.81E+01	28057	9.32E-01	2.83E+01
28057	1.15E+00	2.84E+01	28057	1.32E+00	2.83E+01
27986	3.24E+00	2.90E+01	27986	3.40E+00	2.91E+01
27946	1.38E+00	2.93E+01	27946	4.35E+00	2.98E+01
27936	4.52E+00	3.00E+01			
28009	5.34E-02	2.82E+01	28009	1.72E-01	2.81E+01
28009	3.33E-01	2.79E+01	28055	9.72E-01	2.82E+01
28055	1.18E+00	2.83E+01	28055	1.36E+00	2.83E+01
27984	3.29E+00	2.91E+01	27984	3.45E+00	2.89E+01
27944	1.30E+00	2.94E+01	27944	4.39E+00	2.98E+01
27934	4.55E+00	3.01E+01			
28010	5.45E-02	2.82E+01	28010	1.76E-01	2.81E+01
28010	3.42E-01	2.79E+01	28053	1.01E+00	2.83E+01
28053	1.22E+00	2.83E+01	28053	1.40E+00	2.83E+01
27982	3.34E+00	2.90E+01	27982	3.51E+00	2.89E+01
27942	1.23E+00	2.92E+01	27942	2.04E+00	2.98E+01
27932	2.11E+00	3.00E+01			
28011	5.53E-02	2.82E+01	28011	1.81E-01	2.80E+01
28011	3.52E-01	2.80E+01	28051	1.06E+00	2.83E+01
28051	1.26E+00	2.84E+01	28051	1.45E+00	2.84E+01
27980	3.39E+00	2.91E+01	27980	3.54E+00	2.89E+01
27940	1.18E+00	2.91E+01	27940	1.73E+00	2.96E+01
27930	1.82E+00	2.98E+01			
28012	5.66E-02	2.82E+01	28012	1.85E-01	2.81E+01
28012	3.63E-01	2.80E+01	28049	1.10E+00	2.83E+01
28049	1.30E+00	2.83E+01	28049	1.50E+00	2.84E+01
27978	3.45E+00	2.91E+01	27978	3.59E+00	2.89E+01
27938	1.13E+00	2.92E+01	27938	1.58E+00	2.96E+01
27928	1.68E+00	2.99E+01			
28013	5.78E-02	2.82E+01	28013	1.89E-01	2.79E+01
28013	3.75E-01	2.81E+01	28047	1.14E+00	2.82E+01
28047	1.35E+00	2.83E+01	28047	1.54E+00	2.83E+01
27976	3.50E+00	2.90E+01	27976	3.64E+00	2.91E+01
27936	1.08E+00	2.91E+01	27936	1.48E+00	2.94E+01
27926	1.57E+00	2.99E+01			
28014	5.89E-02	2.81E+01	28014	1.94E-01	2.81E+01
28014	3.88E-01	2.79E+01	28045	1.18E+00	2.83E+01
28045	1.40E+00	2.83E+01	28045	1.59E+00	2.85E+01
27974	3.55E+00	2.92E+01	27974	3.69E+00	2.89E+01
27934	1.05E+00	2.92E+01	27934	1.39E+00	2.96E+01
27924	1.49E+00	2.98E+01			
28015	6.04E-02	2.83E+01	28015	1.99E-01	2.81E+01
28015	4.01E-01	2.81E+01	28043	1.22E+00	2.83E+01
28043	1.45E+00	2.83E+01	28043	1.65E+00	2.84E+01
27972	2.38E+00	2.91E+01	27972	3.73E+00	2.89E+01
27932	1.01E+00	2.91E+01	27932	1.32E+00	2.96E+01
27922	1.42E+00	2.97E+01			
28016	6.13E-02	2.83E+01	28016	2.05E-01	2.81E+01
28016	4.15E-01	2.79E+01	28041	1.26E+00	2.82E+01
28041	1.51E+00	2.84E+01	28041	1.69E+00	2.84E+01
27970	1.53E+00	2.90E+01	27970	3.78E+00	2.91E+01
27930	9.79E-01	2.91E+01	27930	1.27E+00	2.95E+01
27920	1.34E+00	2.98E+01			

28017	6.33E-02	2.83E+01	28017	2.10E-01	2.81E+01
28017	4.33E-01	2.79E+01	28039	1.31E+00	2.82E+01
28039	1.55E+00	2.83E+01	28039	1.75E+00	2.83E+01
27968	1.32E+00	2.89E+01	27968	3.82E+00	2.90E+01
27928	9.47E-01	2.91E+01	27928	1.22E+00	2.94E+01
27918	1.29E+00	2.98E+01			
28018	6.47E-02	2.82E+01	28018	2.16E-01	2.81E+01
28018	4.51E-01	2.80E+01	28037	1.36E+00	2.83E+01
28037	1.61E+00	2.83E+01	28037	1.80E+00	2.85E+01
27966	1.20E+00	2.91E+01	27966	3.87E+00	2.92E+01
27926	9.20E-01	2.92E+01	27926	1.17E+00	2.94E+01
27916	1.24E+00	2.98E+01			
28019	6.62E-02	2.82E+01	28019	2.23E-01	2.81E+01
28019	4.70E-01	2.81E+01	28035	1.41E+00	2.84E+01
28035	1.67E+00	2.83E+01	28035	1.86E+00	2.84E+01
27964	1.11E+00	2.91E+01	27964	3.91E+00	2.91E+01
27924	8.94E-01	2.91E+01	27924	1.13E+00	2.94E+01
27914	1.20E+00	2.97E+01			
28020	6.79E-02	2.83E+01	28020	2.30E-01	2.81E+01
28020	4.92E-01	2.80E+01	28033	1.47E+00	2.83E+01
28033	1.72E+00	2.85E+01	28033	1.91E+00	2.86E+01
27962	1.04E+00	2.90E+01	27962	1.75E+00	2.91E+01
27922	8.70E-01	2.91E+01	27922	1.10E+00	2.94E+01
27912	1.17E+00	2.97E+01			
28021	6.95E-02	2.81E+01	28021	2.37E-01	2.81E+01
28021	5.16E-01	2.79E+01	28031	1.52E+00	2.83E+01
28031	1.78E+00	2.83E+01	28031	1.97E+00	2.85E+01
27960	9.87E-01	2.91E+01	27960	1.24E+00	2.90E+01
27920	8.47E-01	2.89E+01	27920	1.06E+00	2.94E+01
27910	1.13E+00	2.96E+01			
28022	7.13E-02	2.83E+01	28022	2.45E-01	2.80E+01
28022	5.44E-01	2.79E+01	28029	1.57E+00	2.83E+01
28029	1.84E+00	2.83E+01	28029	2.03E+00	2.86E+01
27958	9.38E-01	2.89E+01	27958	1.33E+00	2.91E+01
27918	8.25E-01	2.91E+01	27918	1.03E+00	2.96E+01
27908	1.10E+00	2.95E+01			
28023	7.33E-02	2.83E+01	28023	2.54E-01	2.81E+01
28023	5.77E-01	2.81E+01	28027	1.63E+00	2.82E+01
28027	1.89E+00	2.83E+01	28027	2.08E+00	2.85E+01
27956	8.95E-01	2.88E+01	27956	1.28E+00	2.91E+01
27916	8.06E-01	2.91E+01	27916	1.00E+00	2.94E+01
27906	1.07E+00	2.95E+01			
28025	7.53E-02	2.82E+01	28025	2.63E-01	2.81E+01
28025	6.15E-01	2.80E+01	28025	1.69E+00	2.83E+01
28025	1.95E+00	2.84E+01	28025	2.14E+00	2.85E+01
27954	8.57E-01	2.87E+01	27954	1.20E+00	2.90E+01
27914	7.87E-01	2.89E+01	27914	9.75E-01	2.92E+01
27904	1.05E+00	2.95E+01			
28026	7.71E-02	2.83E+01	28026	2.72E-01	2.81E+01
28026	6.61E-01	2.80E+01	28023	1.74E+00	2.83E+01
28023	2.01E+00	2.83E+01	28023	2.21E+00	2.85E+01
27952	8.22E-01	2.89E+01	27952	1.14E+00	2.91E+01
27912	7.70E-01	2.88E+01	27912	9.51E-01	2.92E+01
27902	1.02E+00	2.96E+01			
28027	7.96E-02	2.82E+01	28027	2.83E-01	2.79E+01
28027	7.19E-01	2.79E+01	28021	1.79E+00	2.85E+01
28021	2.07E+00	2.83E+01	28021	2.26E+00	2.85E+01
27950	7.92E-01	2.89E+01	27950	1.09E+00	2.91E+01
27910	7.52E-01	2.89E+01	27910	9.26E-01	2.92E+01
27900	9.98E-01	2.96E+01			
28028	8.19E-02	2.81E+01	28028	2.95E-01	2.80E+01
28028	7.87E-01	2.79E+01	28019	1.78E+00	2.83E+01
28019	2.13E+00	2.84E+01	28019	2.32E+00	2.84E+01

28029	8.43E-02	2.83E+01	28029	3.06E-01	2.80E+01
28029	8.60E-01	2.80E+01	28017	1.46E+00	2.84E+01
28017	2.19E+00	2.85E+01	28017	2.38E+00	2.84E+01

28030	8.67E-02	2.82E+01	28030	3.20E-01	2.80E+01
28030	9.59E-01	2.81E+01	28015	1.07E+00	2.84E+01
28015	2.25E+00	2.85E+01	28015	2.44E+00	2.85E+01
28031	8.93E-02	2.83E+01	28031	3.34E-01	2.79E+01
28031	1.00E+00	2.81E+01	28013	8.79E-01	2.83E+01
28013	2.31E+00	2.84E+01	28013	2.50E+00	2.85E+01
28032	9.22E-02	2.83E+01	28032	3.51E-01	2.80E+01
28032	1.02E+00	2.80E+01	28011	7.73E-01	2.84E+01
28011	2.36E+00	2.85E+01	28011	2.56E+00	2.85E+01
28033	9.49E-02	2.81E+01	28033	3.69E-01	2.79E+01
28033	1.03E+00	2.79E+01	28009	6.95E-01	2.85E+01
28009	2.42E+00	2.83E+01	28009	2.61E+00	2.84E+01
28034	9.85E-02	2.81E+01	28034	3.89E-01	2.80E+01
28034	1.02E+00	2.81E+01	28006	6.38E-01	2.85E+01
28006	2.48E+00	2.85E+01	28006	2.68E+00	2.85E+01
28035	1.02E-01	2.82E+01	28035	4.09E-01	2.79E+01
28035	1.01E+00	2.81E+01	28004	5.92E-01	2.84E+01
28004	2.55E+00	2.84E+01	28004	2.74E+00	2.85E+01
28036	1.05E-01	2.81E+01	28036	4.32E-01	2.81E+01
28036	9.93E-01	2.80E+01	28002	5.53E-01	2.83E+01
28002	1.84E+00	2.85E+01	28002	2.79E+00	2.85E+01
28037	1.09E-01	2.81E+01	28037	4.62E-01	2.79E+01
28037	9.76E-01	2.80E+01	28000	5.21E-01	2.84E+01
28000	1.20E+00	2.85E+01	28000	2.84E+00	2.85E+01
28038	1.13E-01	2.83E+01	28038	4.85E-01	2.80E+01
28038	9.55E-01	2.81E+01	27998	4.92E-01	2.83E+01
27998	9.95E-01	2.84E+01	27998	2.90E+00	2.85E+01
28039	1.17E-01	2.81E+01	28039	5.06E-01	2.81E+01
28039	9.35E-01	2.81E+01	27996	4.67E-01	2.85E+01
27996	8.94E-01	2.84E+01	27996	2.96E+00	2.85E+01
28040	1.22E-01	2.82E+01	28040	5.28E-01	2.79E+01
28040	9.16E-01	2.81E+01	27994	4.45E-01	2.84E+01
27994	8.21E-01	2.85E+01	27994	3.01E+00	2.85E+01
28041	1.27E-01	2.81E+01	28041	5.46E-01	2.81E+01
28041	8.95E-01	2.81E+01	27992	4.26E-01	2.83E+01
27992	7.65E-01	2.85E+01	27992	3.07E+00	2.85E+01
28042	1.32E-01	2.81E+01	28042	5.52E-01	2.81E+01
28042	8.75E-01	2.81E+01	27990	4.08E-01	2.83E+01
27990	7.12E-01	2.83E+01	27990	3.15E+00	2.86E+01
28043	1.37E-01	2.81E+01	28043	5.59E-01	2.79E+01

28043	8.55E-01	2.81E+01	27988	3.93E-01	2.83E+01
27988	6.75E-01	2.83E+01	27988	1.65E+00	2.86E+01
28044	1.42E-01	2.81E+01	28044	5.61E-01	2.79E+01
28044	8.33E-01	2.81E+01	27986	3.78E-01	2.83E+01
27986	6.41E-01	2.84E+01	27986	1.27E+00	2.85E+01
28045	1.46E-01	2.81E+01	28045	5.59E-01	2.81E+01
28045	8.13E-01	2.81E+01	27984	3.64E-01	2.83E+01
27984	6.13E-01	2.83E+01	27984	1.11E+00	2.87E+01
28046	1.51E-01	2.81E+01	28046	5.54E-01	2.81E+01
28046	7.94E-01	2.79E+01	27982	3.52E-01	2.83E+01
27982	5.86E-01	2.84E+01	27982	1.01E+00	2.86E+01
28047	1.56E-01	2.81E+01	28047	5.46E-01	2.81E+01
28047	7.75E-01	2.81E+01	27980	3.41E-01	2.83E+01
27980	5.62E-01	2.83E+01	27980	9.39E-01	2.86E+01
28048	1.59E-01	2.81E+01	28048	5.38E-01	2.82E+01
28048	7.56E-01	2.81E+01	27978	3.30E-01	2.83E+01
27978	5.40E-01	2.83E+01	27978	8.80E-01	2.86E+01
28049	1.62E-01	2.81E+01	28049	5.28E-01	2.81E+01
28049	7.37E-01	2.81E+01	27976	3.20E-01	2.83E+01
27976	5.21E-01	2.83E+01	27976	8.33E-01	2.86E+01
28050	1.65E-01	2.81E+01	28050	5.17E-01	2.81E+01
28050	7.18E-01	2.81E+01			
28051	1.68E-01	2.81E+01	28051	5.04E-01	2.80E+01
28051	7.01E-01	2.81E+01			
28052	1.69E-01	2.81E+01	28052	4.93E-01	2.81E+01
28052	6.84E-01	2.82E+01			
28053	1.69E-01	2.83E+01	28053	4.80E-01	2.79E+01
28053	6.66E-01	2.80E+01			
28054	1.69E-01	2.81E+01	28054	4.69E-01	2.81E+01
28054	6.50E-01	2.81E+01			
28055	1.68E-01	2.81E+01	28055	4.57E-01	2.81E+01
28055	6.35E-01	2.82E+01			
28056	1.67E-01	2.82E+01	28056	4.45E-01	2.81E+01
28056	6.21E-01	2.80E+01			
28057	1.64E-01	2.81E+01	28057	4.33E-01	2.81E+01
28057	6.06E-01	2.82E+01			
28058	1.61E-01	2.81E+01	28058	4.22E-01	2.81E+01
28058	5.92E-01	2.81E+01			

28059	1.59E-01	2.82E+01	28059	4.11E-01	2.79E+01
28059	5.79E-01	2.81E+01			
28060	1.56E-01	2.82E+01	28060	4.00E-01	2.81E+01
28060	5.66E-01	2.81E+01			
28061	1.52E-01	2.83E+01	28061	3.89E-01	2.80E+01
28061	5.54E-01	2.81E+01			
28062	1.49E-01	2.83E+01	28062	3.79E-01	2.81E+01
28062	5.40E-01	2.80E+01			
28063	1.45E-01	2.83E+01	28063	3.69E-01	2.81E+01
28063	5.29E-01	2.81E+01			
28064	1.41E-01	2.83E+01	28064	3.59E-01	2.81E+01
28064	5.18E-01	2.81E+01			
28065	1.38E-01	2.83E+01	28065	3.49E-01	2.79E+01
28065	5.07E-01	2.82E+01			
28066	1.34E-01	2.83E+01	28066	3.41E-01	2.81E+01
28066	4.96E-01	2.81E+01			
28067	1.31E-01	2.83E+01	28067	3.31E-01	2.82E+01
28067	4.84E-01	2.81E+01			
28068	1.27E-01	2.83E+01	28068	3.23E-01	2.81E+01
28068	4.74E-01	2.82E+01			
28069	1.24E-01	2.81E+01	28069	3.15E-01	2.81E+01
28069	4.64E-01	2.81E+01			
28070	1.21E-01	2.82E+01	28070	3.06E-01	2.81E+01
28070	4.54E-01	2.81E+01			
28071	1.17E-01	2.83E+01	28071	2.99E-01	2.81E+01
28071	4.43E-01	2.81E+01			
28072	1.14E-01	2.83E+01	28072	2.93E-01	2.81E+01
28072	4.34E-01	2.81E+01			
28073	1.10E-01	2.81E+01	28073	2.84E-01	2.81E+01
28073	4.25E-01	2.82E+01			
28074	1.08E-01	2.81E+01	28074	2.78E-01	2.81E+01
28074	4.16E-01	2.81E+01			
28075	1.04E-01	2.82E+01	28075	2.70E-01	2.81E+01
28075	4.07E-01	2.81E+01			

28076	1.01E-01	2.82E+01	28076	2.64E-01	2.81E+01
28076	3.99E-01	2.81E+01			
28077	9.89E-02	2.81E+01	28077	2.58E-01	2.79E+01
28077	3.90E-01	2.81E+01			
28078	9.61E-02	2.81E+01	28078	2.52E-01	2.80E+01
28078	3.82E-01	2.81E+01			
28079	9.38E-02	2.81E+01	28079	2.46E-01	2.80E+01
28079	3.74E-01	2.81E+01			
28080	9.16E-02	2.81E+01	28080	2.40E-01	2.81E+01
28080	3.67E-01	2.81E+01			
28081	8.93E-02	2.82E+01	28081	2.35E-01	2.81E+01
28081	3.60E-01	2.81E+01			
28082	8.68E-02	2.82E+01	28082	2.30E-01	2.79E+01
28082	3.52E-01	2.80E+01			
28083	8.47E-02	2.83E+01	28083	2.24E-01	2.79E+01
28083	3.46E-01	2.81E+01			
28084	8.24E-02	2.83E+01	28084	2.20E-01	2.81E+01
28084	3.39E-01	2.81E+01			
28085	8.06E-02	2.81E+01	28085	2.15E-01	2.79E+01
28085	3.33E-01	2.81E+01			
28086	7.84E-02	2.81E+01	28086	2.11E-01	2.80E+01
28086	3.26E-01	2.81E+01			
28087	7.66E-02	2.82E+01	28087	2.06E-01	2.81E+01
28087	3.21E-01	2.81E+01			
28088	7.49E-02	2.82E+01	28088	2.02E-01	2.81E+01
28088	3.15E-01	2.81E+01			
28089	7.30E-02	2.82E+01	28089	1.98E-01	2.81E+01
28089	3.09E-01	2.82E+01			
28090	7.14E-02	2.81E+01	28090	1.94E-01	2.81E+01
28090	3.04E-01	2.81E+01			
28091	6.98E-02	2.83E+01	28091	1.90E-01	2.81E+01
28091	2.98E-01	2.81E+01			
28092	6.81E-02	2.83E+01	28092	1.87E-01	2.80E+01
28092	2.93E-01	2.81E+01			

28093	6.67E-02	2.81E+01	28093	1.83E-01	2.81E+01
28093	2.88E-01	2.81E+01			
28094	6.54E-02	2.82E+01	28094	1.79E-01	2.80E+01
28094	2.83E-01	2.81E+01			
28095	6.38E-02	2.81E+01	28095	1.76E-01	2.79E+01
28095	2.79E-01	2.82E+01			
28096	6.26E-02	2.83E+01	28096	1.72E-01	2.81E+01
28096	2.74E-01	2.81E+01			
28097	6.14E-02	2.81E+01	28097	1.69E-01	2.81E+01
28097	2.69E-01	2.81E+01			
28098	6.01E-02	2.82E+01	28098	1.66E-01	2.79E+01
28098	2.65E-01	2.80E+01			
28099	5.90E-02	2.83E+01	28099	1.63E-01	2.81E+01
28099	2.61E-01	2.80E+01			
28100	5.80E-02	2.83E+01	28100	1.61E-01	2.82E+01
28100	2.57E-01	2.80E+01			
28101	5.68E-02	2.82E+01	28101	1.58E-01	2.79E+01
28101	2.52E-01	2.81E+01			
28102	5.57E-02	2.83E+01	28102	1.55E-01	2.81E+01
28102	2.48E-01	2.81E+01			
28103	5.46E-02	2.81E+01	28103	1.53E-01	2.80E+01
28103	2.45E-01	2.81E+01			
28104	5.36E-02	2.82E+01	28104	1.50E-01	2.79E+01
28104	2.41E-01	2.81E+01			
28105	5.23E-02	2.83E+01	28105	1.47E-01	2.81E+01
28105	2.37E-01	2.81E+01			
28106	5.15E-02	2.82E+01	28106	1.45E-01	2.80E+01
28106	2.34E-01	2.81E+01			
28107	5.07E-02	2.82E+01	28107	1.43E-01	2.81E+01
28107	2.30E-01	2.81E+01			
28108	4.99E-02	2.82E+01	28108	1.40E-01	2.81E+01
28108	2.27E-01	2.81E+01			
28109	4.89E-02	2.82E+01	28109	1.38E-01	2.81E+01
28109	2.23E-01	2.81E+01			

28110	4.81E-02	2.81E+01	28110	1.36E-01	2.81E+01
28110	2.21E-01	2.81E+01			
28111	4.70E-02	2.83E+01	28111	1.34E-01	2.81E+01
28111	2.18E-01	2.81E+01			
28112	4.67E-02	2.81E+01	28112	1.32E-01	2.80E+01
28112	2.14E-01	2.81E+01			
28113	4.58E-02	2.82E+01	28113	1.30E-01	2.80E+01
28113	2.11E-01	2.81E+01			
28114	4.50E-02	2.83E+01	28114	1.28E-01	2.79E+01
28114	2.09E-01	2.81E+01			
28115	4.41E-02	2.81E+01	28115	1.26E-01	2.81E+01
28115	2.06E-01	2.81E+01			
28116	4.40E-02	2.81E+01	28116	1.25E-01	2.81E+01
28116	2.03E-01	2.81E+01			
28117	4.29E-02	2.82E+01	28117	1.23E-01	2.79E+01
28117	2.00E-01	2.81E+01			
28118	4.26E-02	2.82E+01	28118	1.21E-01	2.79E+01
28118	1.98E-01	2.81E+01			
28119	4.17E-02	2.83E+01	28119	1.20E-01	2.79E+01
28119	1.95E-01	2.81E+01			
28120	4.10E-02	2.81E+01	28120	1.18E-01	2.79E+01
28120	1.93E-01	2.80E+01			
28121	4.03E-02	2.83E+01	28121	1.16E-01	2.79E+01
28121	1.90E-01	2.81E+01			
28122	3.98E-02	2.81E+01	28122	1.15E-01	2.81E+01
28122	1.88E-01	2.81E+01			
28123	3.90E-02	2.81E+01	28123	1.13E-01	2.81E+01
28123	1.86E-01	2.81E+01			
28124	3.87E-02	2.81E+01	28124	1.12E-01	2.80E+01
28124	1.84E-01	2.82E+01			
28125	3.82E-02	2.83E+01	28125	1.10E-01	2.79E+01
28125	1.82E-01	2.81E+01			
28125	3.82E-02	2.82E+01	28125	1.11E-01	2.81E+01
28125	1.81E-01	2.81E+01			

28124	3.88E-02	2.81E+01	28124	1.13E-01	2.79E+01
28124	1.84E-01	2.81E+01			
28123	3.93E-02	2.81E+01	28123	1.14E-01	2.79E+01
28123	1.86E-01	2.81E+01			
28122	4.00E-02	2.82E+01	28122	1.15E-01	2.79E+01
28122	1.88E-01	2.80E+01			
28121	4.06E-02	2.81E+01	28121	1.17E-01	2.81E+01
28121	1.91E-01	2.81E+01			
28120	4.11E-02	2.83E+01	28120	1.18E-01	2.80E+01
28120	1.94E-01	2.82E+01			
28119	4.21E-02	2.82E+01	28119	1.20E-01	2.79E+01
28119	1.96E-01	2.81E+01			
28118	4.22E-02	2.81E+01	28118	1.22E-01	2.80E+01
28118	1.98E-01	2.81E+01			
28117	4.33E-02	2.81E+01	28117	1.23E-01	2.81E+01
28117	2.01E-01	2.81E+01			
28116	4.39E-02	2.81E+01	28116	1.25E-01	2.81E+01
28116	2.04E-01	2.81E+01			
28115	4.46E-02	2.81E+01	28115	1.27E-01	2.81E+01
28115	2.07E-01	2.81E+01			
28114	4.53E-02	2.80E+01	28114	1.29E-01	2.81E+01
28114	2.10E-01	2.82E+01			
28113	4.58E-02	2.82E+01	28113	1.31E-01	2.79E+01
28113	2.13E-01	2.81E+01			
28112	4.68E-02	2.82E+01	28112	1.33E-01	2.80E+01
28112	2.16E-01	2.82E+01			
28111	4.76E-02	2.81E+01	28111	1.35E-01	2.80E+01
28111	2.19E-01	2.81E+01			
28110	4.83E-02	2.81E+01	28110	1.37E-01	2.81E+01
28110	2.22E-01	2.81E+01			
28109	4.91E-02	2.82E+01	28109	1.39E-01	2.79E+01
28109	2.25E-01	2.82E+01			
28108	5.00E-02	2.83E+01	28108	1.41E-01	2.81E+01
28108	2.29E-01	2.81E+01			

28107	5.09E-02	2.81E+01	28107	1.43E-01	2.81E+01
28107	2.33E-01	2.81E+01			
28106	5.20E-02	2.83E+01	28106	1.46E-01	2.79E+01
28106	2.36E-01	2.81E+01			
28105	5.30E-02	2.83E+01	28105	1.48E-01	2.81E+01
28105	2.40E-01	2.83E+01			
28104	5.39E-02	2.81E+01	28104	1.51E-01	2.81E+01
28104	2.44E-01	2.81E+01			
28103	5.50E-02	2.81E+01	28103	1.53E-01	2.81E+01
28103	2.48E-01	2.81E+01			
28102	5.60E-02	2.82E+01	28102	1.56E-01	2.80E+01
28102	2.51E-01	2.81E+01			
28101	5.70E-02	2.83E+01	28101	1.59E-01	2.81E+01
28101	2.56E-01	2.81E+01			
28100	5.82E-02	2.83E+01	28100	1.62E-01	2.79E+01
28100	2.59E-01	2.82E+01			
28099	5.93E-02	2.83E+01	28099	1.65E-01	2.81E+01
28099	2.64E-01	2.80E+01			
28098	6.06E-02	2.81E+01	28098	1.67E-01	2.81E+01
28098	2.69E-01	2.82E+01			
28097	6.17E-02	2.82E+01	28097	1.71E-01	2.79E+01
28097	2.73E-01	2.81E+01			
28096	6.32E-02	2.81E+01	28096	1.74E-01	2.80E+01
28096	2.78E-01	2.81E+01			
28095	6.45E-02	2.82E+01	28095	1.77E-01	2.80E+01
28095	2.82E-01	2.81E+01			
28094	6.58E-02	2.82E+01	28094	1.81E-01	2.81E+01
28094	2.88E-01	2.81E+01			
28093	6.71E-02	2.82E+01	28093	1.84E-01	2.81E+01
28093	2.93E-01	2.81E+01			
28092	6.88E-02	2.81E+01	28092	1.88E-01	2.81E+01
28092	2.99E-01	2.81E+01			
28091	7.05E-02	2.81E+01	28091	1.92E-01	2.81E+01
28091	3.03E-01	2.81E+01			

28090	7.20E-02	2.83E+01	28090	1.96E-01	2.81E+01
28090	3.11E-01	2.81E+01			
28089	7.38E-02	2.82E+01	28089	1.99E-01	2.81E+01
28089	3.16E-01	2.82E+01			
28088	7.52E-02	2.81E+01	28088	2.03E-01	2.80E+01
28088	3.21E-01	2.81E+01			
28087	7.72E-02	2.81E+01	28087	2.09E-01	2.80E+01
28087	3.27E-01	2.81E+01			
28086	7.93E-02	2.81E+01	28086	2.13E-01	2.79E+01
28086	3.33E-01	2.82E+01			
28085	8.13E-02	2.81E+01	28085	2.17E-01	2.81E+01
28085	3.39E-01	2.81E+01			
28084	8.31E-02	2.81E+01	28084	2.22E-01	2.81E+01
28084	3.47E-01	2.81E+01			
28083	8.55E-02	2.81E+01	28083	2.27E-01	2.81E+01
28083	3.54E-01	2.81E+01			
28082	8.79E-02	2.82E+01	28082	2.32E-01	2.79E+01
28082	3.61E-01	2.81E+01			
28081	9.04E-02	2.81E+01	28081	2.37E-01	2.80E+01
28081	3.68E-01	2.82E+01			
28080	9.28E-02	2.81E+01	28080	2.43E-01	2.81E+01
28080	3.76E-01	2.80E+01			
28079	9.50E-02	2.81E+01	28079	2.49E-01	2.81E+01
28079	3.84E-01	2.81E+01			
28078	9.81E-02	2.81E+01	28078	2.55E-01	2.81E+01
28078	3.92E-01	2.83E+01			
28077	1.01E-01	2.81E+01	28077	2.61E-01	2.79E+01
28077	4.00E-01	2.81E+01			
28076	1.04E-01	2.81E+01	28076	2.68E-01	2.80E+01
28076	4.08E-01	2.81E+01			
28075	1.07E-01	2.82E+01	28075	2.74E-01	2.81E+01
28075	4.18E-01	2.82E+01			
28074	1.09E-01	2.81E+01	28074	2.81E-01	2.80E+01
28074	4.27E-01	2.81E+01			

28073	1.13E-01	2.81E+01	28073	2.89E-01	2.81E+01
28073	4.36E-01	2.79E+01			
28072	1.16E-01	2.81E+01	28072	2.96E-01	2.79E+01
28072	4.46E-01	2.81E+01			
28071	1.19E-01	2.81E+01	28071	3.04E-01	2.81E+01
28071	4.56E-01	2.79E+01			
28070	1.22E-01	2.81E+01	28070	3.11E-01	2.80E+01
28070	4.66E-01	2.80E+01			
28069	1.26E-01	2.81E+01	28069	3.19E-01	2.81E+01
28069	4.76E-01	2.79E+01			
28068	1.29E-01	2.81E+01	28068	3.28E-01	2.81E+01
28068	4.88E-01	2.81E+01			
28067	1.33E-01	2.81E+01	28067	3.37E-01	2.81E+01
28067	4.99E-01	2.81E+01			
28066	1.37E-01	2.81E+01	28066	3.46E-01	2.81E+01
28066	5.10E-01	2.80E+01			
28065	1.40E-01	2.81E+01	28065	3.55E-01	2.81E+01
28065	5.23E-01	2.81E+01			
28064	1.44E-01	2.81E+01	28064	3.64E-01	2.81E+01
28064	5.35E-01	2.79E+01			
28063	1.47E-01	2.81E+01	28063	3.74E-01	2.81E+01
28063	5.47E-01	2.80E+01			
28062	1.51E-01	2.81E+01	28062	3.84E-01	2.81E+01
28062	5.60E-01	2.81E+01			
28061	1.54E-01	2.81E+01	28061	3.95E-01	2.80E+01
28061	5.74E-01	2.81E+01			
28060	1.57E-01	2.81E+01	28060	4.05E-01	2.81E+01
28060	5.87E-01	2.79E+01			
28059	1.61E-01	2.82E+01	28059	4.16E-01	2.81E+01
28059	6.01E-01	2.81E+01			
28058	1.63E-01	2.81E+01	28058	4.26E-01	2.81E+01
28058	6.14E-01	2.81E+01			
28057	1.66E-01	2.81E+01	28057	4.38E-01	2.80E+01
28057	6.31E-01	2.80E+01			

28056	1.68E-01	2.82E+01	28056	4.49E-01	2.81E+01
28056	6.44E-01	2.81E+01			
28055	1.69E-01	2.82E+01	28055	4.61E-01	2.79E+01
28055	6.60E-01	2.81E+01			
28054	1.70E-01	2.81E+01	28054	4.72E-01	2.81E+01
28054	6.75E-01	2.80E+01			
28053	1.70E-01	2.81E+01	28053	4.84E-01	2.81E+01
28053	6.91E-01	2.80E+01			
28052	1.69E-01	2.81E+01	28052	4.96E-01	2.81E+01
28052	7.07E-01	2.81E+01			
28051	1.68E-01	2.81E+01	28051	5.07E-01	2.81E+01
28051	7.24E-01	2.81E+01			
28050	1.66E-01	2.81E+01	28050	5.18E-01	2.79E+01
28050	7.41E-01	2.81E+01			
28049	1.63E-01	2.81E+01	28049	5.28E-01	2.81E+01
28049	7.58E-01	2.79E+01			
28048	1.60E-01	2.81E+01	28048	5.38E-01	2.81E+01
28048	7.74E-01	2.81E+01			
28047	1.56E-01	2.81E+01	28047	5.46E-01	2.81E+01
28047	7.92E-01	2.81E+01			
28046	1.52E-01	2.81E+01	28046	5.53E-01	2.79E+01
28046	8.09E-01	2.81E+01			
28045	1.47E-01	2.82E+01	28045	5.58E-01	2.81E+01
28045	8.26E-01	2.81E+01			
28044	1.41E-01	2.81E+01	28044	5.61E-01	2.81E+01
28044	8.47E-01	2.81E+01			
28043	1.37E-01	2.82E+01	28043	5.60E-01	2.81E+01
28043	8.65E-01	2.81E+01			
28042	1.33E-01	2.81E+01	28042	5.57E-01	2.81E+01
28042	8.82E-01	2.81E+01			
28041	1.28E-01	2.81E+01	28041	5.47E-01	2.81E+01
28041	8.97E-01	2.81E+01			
28040	1.23E-01	2.81E+01	28040	5.37E-01	2.81E+01
28040	9.16E-01	2.82E+01			

28039	1.19E-01	2.82E+01	28039	5.22E-01	2.81E+01
28039	9.36E-01	2.81E+01			
28038	1.15E-01	2.83E+01	28038	5.04E-01	2.81E+01
28038	9.54E-01	2.81E+01			
28037	1.11E-01	2.81E+01	28037	4.83E-01	2.82E+01
28037	9.72E-01	2.81E+01			
28036	1.07E-01	2.81E+01	28036	4.61E-01	2.81E+01
28036	9.89E-01	2.82E+01			
28035	1.03E-01	2.81E+01	28035	4.37E-01	2.81E+01
28035	1.00E+00	2.81E+01			
28034	9.97E-02	2.82E+01	28034	4.16E-01	2.82E+01
28034	1.02E+00	2.80E+01			
28033	9.64E-02	2.81E+01	28033	3.95E-01	2.81E+01
28033	1.03E+00	2.81E+01			
28032	9.29E-02	2.81E+01	28032	3.76E-01	2.81E+01
28032	1.03E+00	2.82E+01			
28031	9.00E-02	2.81E+01	28031	3.58E-01	2.81E+01
28031	1.02E+00	2.82E+01			
28030	8.74E-02	2.82E+01	28030	3.41E-01	2.81E+01
28030	1.01E+00	2.81E+01			
28029	8.49E-02	2.81E+01	28029	3.26E-01	2.82E+01
28029	9.82E-01	2.81E+01			
28028	8.24E-02	2.81E+01	28028	3.12E-01	2.81E+01
28028	9.42E-01	2.81E+01			
28027	7.99E-02	2.81E+01	28027	2.98E-01	2.81E+01
28027	8.88E-01	2.81E+01			
28026	7.78E-02	2.81E+01	28026	2.87E-01	2.81E+01
28026	8.29E-01	2.81E+01			
28025	7.56E-02	2.80E+01	28025	2.77E-01	2.81E+01
28025	7.66E-01	2.81E+01			
28023	7.37E-02	2.81E+01	28023	2.66E-01	2.81E+01
28023	7.07E-01	2.82E+01			
28022	7.17E-02	2.80E+01	28022	2.56E-01	2.81E+01
28022	6.53E-01	2.82E+01			

28021	6.97E-02	2.81E+01	28021	2.48E-01	2.81E+01
28021	6.06E-01	2.81E+01			
28020	6.82E-02	2.81E+01	28020	2.40E-01	2.81E+01
28020	5.65E-01	2.81E+01			
28019	6.66E-02	2.82E+01	28019	2.32E-01	2.82E+01
28019	5.31E-01	2.81E+01			
28018	6.49E-02	2.81E+01	28018	2.25E-01	2.81E+01
28018	5.03E-01	2.81E+01			
28017	6.34E-02	2.81E+01	28017	2.18E-01	2.81E+01
28017	4.79E-01	2.81E+01			
28016	6.18E-02	2.81E+01	28016	2.12E-01	2.81E+01
28016	4.59E-01	2.81E+01			
28015	6.07E-02	2.81E+01	28015	2.06E-01	2.81E+01
28015	4.39E-01	2.81E+01			
28014	5.94E-02	2.80E+01	28014	2.00E-01	2.81E+01
28014	4.21E-01	2.81E+01			
28013	5.80E-02	2.80E+01	28013	1.96E-01	2.81E+01
28013	4.06E-01	2.81E+01			
28012	5.69E-02	2.81E+01	28012	1.90E-01	2.81E+01
28012	3.91E-01	2.80E+01			
28011	5.56E-02	2.81E+01	28011	1.85E-01	2.81E+01
28011	3.78E-01	2.81E+01			
28010	5.46E-02	2.80E+01	28010	1.81E-01	2.81E+01
28010	3.66E-01	2.81E+01			
28009	5.34E-02	2.81E+01	28009	1.77E-01	2.81E+01
28009	3.54E-01	2.79E+01			
28008	5.25E-02	2.81E+01	28008	1.73E-01	2.81E+01
28008	3.43E-01	2.81E+01			
28007	5.15E-02	2.81E+01	28007	1.69E-01	2.81E+01
28007	3.33E-01	2.81E+01			
28006	5.06E-02	2.81E+01	28006	1.65E-01	2.81E+01
28006	3.24E-01	2.81E+01			
28005	4.96E-02	2.81E+01	28005	1.62E-01	2.81E+01
28005	3.16E-01	2.81E+01			

28004	4.87E-02	2.81E+01	28004	1.58E-01	2.81E+01
28004	3.07E-01	2.81E+01			
28003	4.76E-02	2.81E+01	28003	1.55E-01	2.81E+01
28003	2.99E-01	2.81E+01			
28002	4.70E-02	2.81E+01	28002	1.52E-01	2.80E+01
28002	2.92E-01	2.82E+01			
28001	4.64E-02	2.81E+01	28001	1.49E-01	2.81E+01
28001	2.84E-01	2.81E+01			
28000	4.54E-02	2.81E+01	28000	1.46E-01	2.81E+01
28000	2.79E-01	2.81E+01			
27999	4.49E-02	2.83E+01	27999	1.43E-01	2.81E+01
27999	2.72E-01	2.81E+01			
27998	4.39E-02	2.81E+01	27998	1.41E-01	2.81E+01
27998	2.66E-01	2.81E+01			
27997	4.33E-02	2.81E+01	27997	1.38E-01	2.79E+01
27997	2.61E-01	2.81E+01			
27996	4.27E-02	2.82E+01	27996	1.35E-01	2.81E+01
27996	2.56E-01	2.81E+01			
27995	4.23E-02	2.81E+01	27995	1.33E-01	2.81E+01
27995	2.51E-01	2.81E+01			
27994	4.16E-02	2.81E+01	27994	1.31E-01	2.82E+01
27994	2.46E-01	2.80E+01			
27993	4.07E-02	2.81E+01	27993	1.29E-01	2.81E+01
27993	2.41E-01	2.81E+01			
27992	4.01E-02	2.81E+01	27992	1.27E-01	2.81E+01
27992	2.36E-01	2.82E+01			
27991	3.96E-02	2.81E+01	27991	1.24E-01	2.80E+01
27991	2.32E-01	2.81E+01			
27990	3.92E-02	2.80E+01	27990	1.23E-01	2.81E+01
27990	2.28E-01	2.81E+01			
27989	3.85E-02	2.81E+01	27989	1.21E-01	2.81E+01
27989	2.24E-01	2.81E+01			
27988	3.79E-02	2.81E+01	27988	1.19E-01	2.80E+01
27988	2.20E-01	2.81E+01			

27987	3.77E-02	2.81E+01	27987	1.17E-01	2.82E+01
27987	2.16E-01	2.81E+01			
27986	3.70E-02	2.81E+01	27986	1.15E-01	2.80E+01
27986	2.13E-01	2.81E+01			
27985	3.65E-02	2.81E+01	27985	1.14E-01	2.81E+01
27985	2.09E-01	2.81E+01			
27984	3.59E-02	2.81E+01	27984	1.12E-01	2.81E+01
27984	2.06E-01	2.81E+01			
27983	3.56E-02	2.81E+01	27983	1.10E-01	2.79E+01
27983	2.02E-01	2.81E+01			
27982	3.53E-02	2.82E+01	27982	1.09E-01	2.81E+01
27982	2.00E-01	2.81E+01			
27981	3.46E-02	2.81E+01	27981	1.07E-01	2.81E+01
27981	1.97E-01	2.81E+01			
27980	3.41E-02	2.81E+01	27980	1.06E-01	2.81E+01
27980	1.94E-01	2.80E+01			
27979	3.39E-02	2.79E+01	27979	1.04E-01	2.80E+01
27979	1.91E-01	2.81E+01			
27978	3.33E-02	2.81E+01	27978	1.03E-01	2.81E+01
27978	1.88E-01	2.80E+01			
27977	3.30E-02	2.81E+01	27977	1.01E-01	2.81E+01
27977	1.85E-01	2.81E+01			
27976	3.25E-02	2.81E+01	27976	1.00E-01	2.81E+01
27976	1.83E-01	2.82E+01			
27975	3.22E-02	2.81E+01	27975	9.87E-02	2.81E+01
27975	1.81E-01	2.80E+01			
27974	3.17E-02	2.81E+01	27974	9.80E-02	2.81E+01
27974	1.77E-01	2.81E+01			
27973	3.17E-02	2.81E+01	27973	9.69E-02	2.81E+01
27973	1.76E-01	2.81E+01			
27972	3.10E-02	2.81E+01	27972	9.50E-02	2.80E+01
27972	1.73E-01	2.81E+01			
27971	3.07E-02	2.81E+01	27971	9.37E-02	2.80E+01
27971	1.71E-01	2.81E+01			

27970	3.05E-02	2.81E+01	27970	9.42E-02	2.82E+01
27970	1.69E-01	2.81E+01			
27969	3.01E-02	2.81E+01	27969	9.31E-02	2.81E+01
27969	1.67E-01	2.80E+01			
27968	2.97E-02	2.81E+01	27968	9.06E-02	2.81E+01
27968	1.65E-01	2.80E+01			
27967	2.93E-02	2.82E+01	27967	8.96E-02	2.81E+01
27967	1.63E-01	2.81E+01			
27966	2.91E-02	2.81E+01	27966	8.86E-02	2.81E+01
27966	1.61E-01	2.81E+01			
27965	2.88E-02	2.81E+01	27965	8.75E-02	2.80E+01
27965	1.59E-01	2.79E+01			
27964	2.83E-02	2.81E+01	27964	8.74E-02	2.81E+01
27964	1.57E-01	2.81E+01			
27963	2.80E-02	2.82E+01	27963	8.58E-02	2.78E+01
27963	1.55E-01	2.81E+01			
27962	2.79E-02	2.81E+01	27962	8.54E-02	2.81E+01
27962	1.54E-01	2.80E+01			
27961	2.76E-02	2.81E+01	27961	8.41E-02	2.79E+01
27961	1.52E-01	2.82E+01			
27960	2.74E-02	2.82E+01	27960	8.34E-02	2.80E+01
27960	1.50E-01	2.81E+01			
27959	2.71E-02	2.81E+01	27959	8.29E-02	2.80E+01
27959	1.48E-01	2.81E+01			
27958	2.66E-02	2.81E+01	27958	8.14E-02	2.80E+01
27958	1.46E-01	2.81E+01			
27957	2.65E-02	2.81E+01	27957	8.04E-02	2.80E+01
27957	1.45E-01	2.81E+01			
27956	2.64E-02	2.82E+01	27956	7.99E-02	2.81E+01
27956	1.44E-01	2.80E+01			
27955	2.60E-02	2.81E+01	27955	7.89E-02	2.80E+01
27955	1.42E-01	2.79E+01			
27954	2.58E-02	2.82E+01	27954	7.85E-02	2.80E+01
27954	1.40E-01	2.80E+01			

27953	2.56E-02	2.82E+01	27953	7.76E-02	2.80E+01
27953	1.39E-01	2.81E+01			
27952	2.52E-02	2.82E+01	27952	7.67E-02	2.80E+01
27952	1.37E-01	2.80E+01			
27951	2.51E-02	2.81E+01	27951	7.61E-02	2.79E+01
27951	1.37E-01	2.81E+01			
27950	2.49E-02	2.83E+01	27950	7.55E-02	2.80E+01
27950	1.35E-01	2.81E+01			
27949	2.47E-02	2.81E+01	27949	7.48E-02	2.79E+01
27949	1.34E-01	2.81E+01			
27948	2.44E-02	2.83E+01	27948	7.41E-02	2.79E+01
27948	1.33E-01	2.80E+01			
27947	2.42E-02	2.81E+01	27947	7.66E-02	2.81E+01
27947	1.30E-01	2.81E+01			
27946	2.39E-02	2.82E+01	27946	7.28E-02	2.80E+01
27946	1.30E-01	2.80E+01			
27945	2.38E-02	2.82E+01	27945	7.28E-02	2.80E+01
27945	1.29E-01	2.81E+01			
27944	2.36E-02	2.83E+01	27944	7.13E-02	2.81E+01
27944	1.28E-01	2.81E+01			
27943	2.34E-02	2.81E+01	27943	7.07E-02	2.79E+01
27943	1.26E-01	2.79E+01			
27942	2.32E-02	2.81E+01	27942	7.03E-02	2.81E+01
27942	1.25E-01	2.79E+01			
27941	2.32E-02	2.81E+01	27941	6.98E-02	2.81E+01
27941	1.23E-01	2.81E+01			
27940	2.30E-02	2.81E+01	27940	6.89E-02	2.80E+01
27940	1.23E-01	2.80E+01			
27939	2.27E-02	2.81E+01	27939	6.85E-02	2.81E+01
27939	1.22E-01	2.80E+01			
27938	2.23E-02	2.82E+01	27938	6.79E-02	2.81E+01
27938	1.21E-01	2.79E+01			
27937	2.22E-02	2.82E+01	27937	6.73E-02	2.81E+01
27937	1.20E-01	2.81E+01			

27936	2.20E-02	2.81E+01	27936	6.65E-02	2.80E+01
27936	1.19E-01	2.80E+01			
27935	2.16E-02	2.82E+01	27935	6.64E-02	2.81E+01
27935	1.17E-01	2.79E+01			
27934	2.17E-02	2.82E+01	27934	6.63E-02	2.81E+01
27934	1.17E-01	2.80E+01			
27933	2.17E-02	2.81E+01	27933	6.57E-02	2.79E+01
27933	1.16E-01	2.80E+01			
27932	2.13E-02	2.81E+01	27932	6.43E-02	2.81E+01
27932	1.14E-01	2.80E+01			
27931	2.13E-02	2.81E+01	27931	6.56E-02	2.81E+01
27931	1.14E-01	2.80E+01			
27930	2.10E-02	2.81E+01	27930	6.38E-02	2.80E+01
27930	1.12E-01	2.81E+01			
27929	2.09E-02	2.81E+01	27929	6.34E-02	2.80E+01
27929	1.11E-01	2.80E+01			
27928	2.08E-02	2.81E+01	27928	6.28E-02	2.81E+01
27928	1.10E-01	2.80E+01			
27927	2.06E-02	2.81E+01	27927	6.24E-02	2.80E+01
27927	1.10E-01	2.79E+01			
27926	2.03E-02	2.81E+01	27926	6.24E-02	2.81E+01
27926	1.09E-01	2.80E+01			
27925	2.04E-02	2.81E+01	27925	6.21E-02	2.79E+01
27925	1.08E-01	2.81E+01			

I3 (1 sec time delay throughout excitation range)
2V (LDV 125mm/s/V) 6V (LDV 125mm/s/V)
10V (LDV 125mm/s/V) 20V (LDV 125mm/s/V)
30V (LDV 125mm/s/V) 40V (LDV 125mm/s/V)
50V (LDV 125mm/s/V)
Frequency (Hz) Response (V) Temperature Frequency (Hz) Response (V)
Temperature Frequency (Hz) Response (V) Temperature Frequency (Hz)
Response (V) Temperature Frequency (Hz) Response (V) Temperature
Frequency (Hz) Response (V) Temperature Frequency (Hz) Response (V)
Temperature

27925	2.06E-02	2.83E+01	27925	6.17E-02	2.81E+01
27925	1.07E-01	2.78E+01	27925	3.02E-01	2.85E+01
27925	5.67E-01	2.87E+01	27900	6.74E-01	2.93E+01
27875	8.95E-01	3.33E+01			
27926	2.06E-02	2.81E+01	27926	6.16E-02	2.81E+01
27926	1.08E-01	2.79E+01	27927	3.08E-01	2.85E+01

27927	5.79E-01	2.87E+01	27902	7.14E-01	2.94E+01
27877	9.81E-01	3.31E+01			
27927	2.04E-02	2.83E+01	27927	6.22E-02	2.82E+01
27927	1.09E-01	2.80E+01	27929	3.12E-01	2.85E+01
27929	5.91E-01	2.88E+01	27904	7.32E-01	2.93E+01
27879	1.01E+00	3.31E+01			
27928	2.04E-02	2.83E+01	27928	6.29E-02	2.82E+01
27928	1.10E-01	2.79E+01	27931	3.18E-01	2.86E+01
27931	6.04E-01	2.86E+01	27906	7.46E-01	2.94E+01
27881	1.03E+00	3.31E+01			
27929	2.11E-02	2.81E+01	27929	6.34E-02	2.82E+01
27929	1.11E-01	2.79E+01	27933	3.25E-01	2.85E+01
27933	6.18E-01	2.88E+01	27908	7.61E-01	2.93E+01
27883	1.05E+00	3.32E+01			
27930	2.13E-02	2.81E+01	27930	6.37E-02	2.81E+01
27930	1.12E-01	2.79E+01	27935	3.31E-01	2.85E+01
27935	6.38E-01	2.86E+01	27910	7.77E-01	2.93E+01
27885	1.07E+00	3.31E+01			
27931	2.15E-02	2.81E+01	27931	6.39E-02	2.83E+01
27931	1.13E-01	2.79E+01	27937	3.37E-01	2.85E+01
27937	6.52E-01	2.88E+01	27912	7.93E-01	2.94E+01
27887	1.10E+00	3.31E+01			
27932	2.16E-02	2.82E+01	27932	6.44E-02	2.81E+01
27932	1.14E-01	2.78E+01	27939	3.45E-01	2.85E+01
27939	6.70E-01	2.89E+01	27914	8.11E-01	2.94E+01
27889	1.13E+00	3.30E+01			
27933	2.18E-02	2.83E+01	27933	6.49E-02	2.80E+01
27933	1.15E-01	2.80E+01	27941	3.52E-01	2.85E+01
27941	6.89E-01	2.87E+01	27916	8.30E-01	2.94E+01
27891	1.15E+00	3.31E+01			
27934	2.23E-02	2.83E+01	27934	6.56E-02	2.81E+01
27934	1.15E-01	2.79E+01	27943	3.60E-01	2.85E+01
27943	7.10E-01	2.87E+01	27918	8.48E-01	2.93E+01
27893	1.18E+00	3.31E+01			
27935	2.19E-02	2.83E+01	27935	6.60E-02	2.82E+01
27935	1.16E-01	2.79E+01	27945	3.68E-01	2.85E+01
27945	7.34E-01	2.88E+01	27920	8.70E-01	2.95E+01
27895	1.21E+00	3.31E+01			
27936	2.21E-02	2.82E+01	27936	6.69E-02	2.79E+01
27936	1.17E-01	2.79E+01	27947	3.77E-01	2.85E+01
27947	7.57E-01	2.87E+01	27922	8.92E-01	2.94E+01
27897	1.25E+00	3.31E+01			
27937	2.25E-02	2.82E+01	27937	6.78E-02	2.80E+01
27937	1.18E-01	2.79E+01	27949	3.87E-01	2.85E+01
27949	7.82E-01	2.89E+01	27924	9.14E-01	2.94E+01
27899	1.28E+00	3.31E+01			
27938	2.22E-02	2.81E+01	27938	6.90E-02	2.81E+01
27938	1.19E-01	2.80E+01	27951	3.95E-01	2.85E+01
27951	8.10E-01	2.89E+01	27926	9.39E-01	2.93E+01
27901	1.33E+00	3.30E+01			
27939	2.29E-02	2.82E+01	27939	6.82E-02	2.81E+01
27939	1.21E-01	2.79E+01	27953	4.06E-01	2.85E+01
27953	8.41E-01	2.87E+01	27928	9.66E-01	2.94E+01
27903	1.37E+00	3.31E+01			
27940	2.29E-02	2.81E+01	27940	6.87E-02	2.81E+01
27940	1.22E-01	2.79E+01	27955	4.17E-01	2.85E+01
27955	8.75E-01	2.89E+01	27930	9.96E-01	2.94E+01
27905	1.41E+00	3.29E+01			
27941	2.32E-02	2.82E+01	27941	6.98E-02	2.81E+01
27941	1.23E-01	2.80E+01	27957	4.28E-01	2.83E+01
27957	9.11E-01	2.87E+01	27932	1.03E+00	2.94E+01
27907	1.47E+00	3.30E+01			
27942	2.32E-02	2.83E+01	27942	7.02E-02	2.80E+01
27942	1.24E-01	2.79E+01	27959	4.41E-01	2.85E+01
27959	9.54E-01	2.86E+01	27934	1.06E+00	2.94E+01
27909	1.53E+00	3.29E+01			
27943	2.37E-02	2.81E+01	27943	7.08E-02	2.81E+01
27943	1.25E-01	2.80E+01	27961	4.54E-01	2.83E+01

27961	1.00E+00	2.85E+01	27936	1.10E+00	2.94E+01
27911	1.61E+00	3.29E+01			
27944	2.35E-02	2.83E+01	27944	7.13E-02	2.81E+01
27944	1.26E-01	2.81E+01	27963	4.67E-01	2.84E+01
27963	1.06E+00	2.88E+01	27938	1.14E+00	2.95E+01
27913	1.70E+00	3.29E+01			
27945	2.41E-02	2.82E+01	27945	7.22E-02	2.82E+01
27945	1.27E-01	2.79E+01	27965	4.82E-01	2.85E+01
27965	1.13E+00	2.87E+01	27940	1.18E+00	2.95E+01
27915	1.82E+00	3.30E+01			
27946	2.42E-02	2.83E+01	27946	7.36E-02	2.81E+01
27946	1.28E-01	2.81E+01	27967	4.99E-01	2.83E+01
27967	1.21E+00	2.87E+01	27942	1.24E+00	2.94E+01
27917	2.02E+00	3.30E+01			
27947	2.45E-02	2.83E+01	27947	7.33E-02	2.81E+01
27947	1.30E-01	2.81E+01	27969	5.16E-01	2.84E+01
27969	1.31E+00	2.87E+01	27944	1.30E+00	2.94E+01
27919	4.42E+00	3.33E+01			
27948	2.47E-02	2.82E+01	27948	7.42E-02	2.81E+01
27948	1.31E-01	2.81E+01	27971	5.35E-01	2.84E+01
27971	1.47E+00	2.87E+01	27946	1.36E+00	2.95E+01
27921	4.37E+00	3.35E+01			
27949	2.46E-02	2.83E+01	27949	7.44E-02	2.79E+01
27949	1.32E-01	2.80E+01	27973	5.55E-01	2.83E+01
27973	1.80E+00	2.87E+01	27948	1.47E+00	2.93E+01
27923	4.33E+00	3.36E+01			
27950	2.50E-02	2.83E+01	27950	7.56E-02	2.81E+01
27950	1.33E-01	2.81E+01	27975	5.79E-01	2.85E+01
27975	3.44E+00	2.90E+01	27950	1.57E+00	2.95E+01
27925	4.29E+00	3.38E+01			
27951	2.50E-02	2.82E+01	27951	7.61E-02	2.81E+01
27951	1.35E-01	2.79E+01	27977	6.05E-01	2.83E+01
27977	3.38E+00	2.90E+01	27952	1.72E+00	2.94E+01
27927	4.24E+00	3.39E+01			
27952	2.53E-02	2.83E+01	27952	7.76E-02	2.80E+01
27952	1.36E-01	2.79E+01	27979	6.33E-01	2.84E+01
27979	3.32E+00	2.91E+01	27954	2.03E+00	2.92E+01
27929	4.20E+00	3.40E+01			
27953	2.57E-02	2.83E+01	27953	7.78E-02	2.81E+01
27953	1.38E-01	2.79E+01	27981	6.67E-01	2.84E+01
27981	3.25E+00	2.90E+01	27956	4.10E+00	2.95E+01
27931	4.16E+00	3.42E+01			
27954	2.59E-02	2.83E+01	27954	7.80E-02	2.81E+01
27954	1.39E-01	2.80E+01	27983	7.04E-01	2.83E+01
27983	3.18E+00	2.91E+01	27958	4.04E+00	2.95E+01
27933	4.11E+00	3.44E+01			
27955	2.60E-02	2.83E+01	27955	7.97E-02	2.81E+01
27955	1.40E-01	2.79E+01	27985	7.51E-01	2.83E+01
27985	3.12E+00	2.92E+01	27960	3.99E+00	2.97E+01
27935	4.07E+00	3.42E+01			
27956	2.60E-02	2.83E+01	27956	7.98E-02	2.81E+01
27956	1.42E-01	2.79E+01	27988	8.27E-01	2.83E+01
27988	3.06E+00	2.92E+01	27962	3.93E+00	2.98E+01
27937	4.02E+00	3.42E+01			
27957	2.64E-02	2.82E+01	27957	8.04E-02	2.81E+01
27957	1.43E-01	2.79E+01	27990	8.96E-01	2.83E+01
27990	2.99E+00	2.92E+01	27963	3.88E+00	2.99E+01
27939	3.98E+00	3.44E+01			
27958	2.74E-02	2.82E+01	27958	8.15E-02	2.79E+01
27958	1.45E-01	2.79E+01	27992	9.88E-01	2.85E+01
27992	2.93E+00	2.91E+01	27965	3.82E+00	3.00E+01
27941	3.93E+00	3.42E+01			
27959	2.70E-02	2.81E+01	27959	8.19E-02	2.79E+01
27959	1.46E-01	2.79E+01	27994	1.14E+00	2.85E+01
27994	2.87E+00	2.93E+01	27967	3.76E+00	3.01E+01
27943	3.89E+00	3.43E+01			
27960	2.76E-02	2.82E+01	27960	8.27E-02	2.81E+01
27960	1.48E-01	2.79E+01	27996	1.50E+00	2.85E+01

27996	2.80E+00	2.92E+01	27969	3.71E+00	3.00E+01
27945	3.84E+00	3.44E+01			
27961	2.73E-02	2.81E+01	27961	8.33E-02	2.82E+01
27961	1.50E-01	2.81E+01	27998	2.44E+00	2.84E+01
27998	2.74E+00	2.92E+01	27971	3.65E+00	3.00E+01
27947	3.79E+00	3.42E+01			
27962	2.79E-02	2.81E+01	27962	8.67E-02	2.81E+01
27962	1.51E-01	2.80E+01	28000	2.37E+00	2.85E+01
28000	2.67E+00	2.92E+01	27973	3.59E+00	3.01E+01
27949	3.74E+00	3.41E+01			
27963	2.82E-02	2.81E+01	27963	8.60E-02	2.81E+01
27963	1.53E-01	2.81E+01	28002	2.31E+00	2.84E+01
28002	2.60E+00	2.92E+01	27975	3.53E+00	3.01E+01
27951	3.69E+00	3.44E+01			
27964	2.84E-02	2.81E+01	27964	8.69E-02	2.81E+01
27964	1.54E-01	2.79E+01	28004	2.25E+00	2.85E+01
28004	2.54E+00	2.92E+01	27977	3.47E+00	3.00E+01
27953	3.64E+00	3.43E+01			
27965	2.90E-02	2.82E+01	27965	8.75E-02	2.81E+01
27965	1.57E-01	2.79E+01	28006	2.18E+00	2.85E+01
28006	2.47E+00	2.92E+01	27979	3.41E+00	3.01E+01
27955	3.59E+00	3.42E+01			
27966	2.92E-02	2.81E+01	27966	8.95E-02	2.80E+01
27966	1.59E-01	2.81E+01	28008	2.12E+00	2.85E+01
28008	2.41E+00	2.92E+01	27981	3.35E+00	3.02E+01
27957	3.54E+00	3.42E+01			
27967	2.95E-02	2.81E+01	27967	8.94E-02	2.81E+01
27967	1.60E-01	2.79E+01	28010	2.06E+00	2.85E+01
28010	2.35E+00	2.92E+01	27983	3.29E+00	3.02E+01
27959	3.49E+00	3.43E+01			
27968	2.99E-02	2.81E+01	27968	9.08E-02	2.81E+01
27968	1.62E-01	2.80E+01	28012	1.99E+00	2.85E+01
28012	2.29E+00	2.92E+01	27985	3.23E+00	3.03E+01
27961	3.44E+00	3.42E+01			
27969	3.05E-02	2.83E+01	27969	9.12E-02	2.81E+01
27969	1.64E-01	2.79E+01	28014	1.93E+00	2.84E+01
28014	2.22E+00	2.92E+01	27987	3.17E+00	3.02E+01
27963	3.39E+00	3.43E+01			
27970	3.03E-02	2.82E+01	27970	9.21E-02	2.81E+01
27970	1.67E-01	2.79E+01	28016	1.87E+00	2.87E+01
28016	2.16E+00	2.92E+01	27989	3.11E+00	3.02E+01
27965	3.34E+00	3.41E+01			
27971	3.09E-02	2.82E+01	27971	9.30E-02	2.79E+01
27971	1.68E-01	2.79E+01	28018	1.81E+00	2.86E+01
28018	2.10E+00	2.92E+01	27991	3.05E+00	3.02E+01
27967	3.29E+00	3.42E+01			
27972	3.08E-02	2.82E+01	27972	9.50E-02	2.81E+01
27972	1.70E-01	2.80E+01	28020	1.74E+00	2.87E+01
28020	2.04E+00	2.92E+01	27993	2.98E+00	3.04E+01
27969	3.23E+00	3.42E+01			
27973	3.14E-02	2.83E+01	27973	9.57E-02	2.80E+01
27973	1.72E-01	2.81E+01	28022	1.69E+00	2.87E+01
28022	1.98E+00	2.92E+01	27995	2.92E+00	3.02E+01
27971	3.18E+00	3.42E+01			
27974	3.19E-02	2.83E+01	27974	9.66E-02	2.79E+01
27974	1.75E-01	2.79E+01	28024	1.62E+00	2.86E+01
28024	1.93E+00	2.93E+01	27997	2.86E+00	3.04E+01
27973	3.13E+00	3.40E+01			
27975	3.23E-02	2.83E+01	27975	9.85E-02	2.81E+01
27975	1.77E-01	2.81E+01	28026	1.57E+00	2.86E+01
28026	1.87E+00	2.92E+01	27999	2.80E+00	3.02E+01
27975	3.08E+00	3.42E+01			
27976	3.27E-02	2.83E+01	27976	9.92E-02	2.81E+01
27976	1.79E-01	2.80E+01	28028	1.51E+00	2.87E+01
28028	1.82E+00	2.92E+01	28001	2.74E+00	3.03E+01
27975	3.08E+00	3.42E+01			
27977	3.30E-02	2.83E+01	27977	1.01E-01	2.79E+01
27977	1.82E-01	2.79E+01	28030	1.46E+00	2.87E+01

28030	1.77E+00	2.92E+01	28003	2.68E+00	3.03E+01
27973	3.13E+00	3.42E+01			
27978	3.32E-02	2.81E+01	27978	1.02E-01	2.79E+01
27978	1.84E-01	2.80E+01	28032	1.40E+00	2.87E+01
28032	1.72E+00	2.93E+01	28005	2.62E+00	3.03E+01
27971	3.18E+00	3.41E+01			
27979	3.39E-02	2.83E+01	27979	1.03E-01	2.79E+01
27979	1.87E-01	2.81E+01	28034	1.35E+00	2.87E+01
28034	1.67E+00	2.94E+01	28007	2.56E+00	3.03E+01
27969	3.23E+00	3.42E+01			
27980	3.41E-02	2.83E+01	27980	1.05E-01	2.81E+01
27980	1.90E-01	2.80E+01	28036	1.30E+00	2.87E+01
28036	1.62E+00	2.92E+01	28009	2.50E+00	3.02E+01
27967	3.29E+00	3.41E+01			
27981	3.46E-02	2.82E+01	27981	1.06E-01	2.82E+01
27981	1.92E-01	2.79E+01	28038	1.25E+00	2.87E+01
28038	1.57E+00	2.92E+01	28011	2.45E+00	3.02E+01
27965	3.33E+00	3.40E+01			
27982	3.50E-02	2.81E+01	27982	1.08E-01	2.81E+01
27982	1.96E-01	2.81E+01	28040	1.21E+00	2.86E+01
28040	1.53E+00	2.92E+01	28013	2.39E+00	3.04E+01
27963	3.38E+00	3.41E+01			
27983	3.56E-02	2.82E+01	27983	1.09E-01	2.81E+01
27983	1.98E-01	2.80E+01	28042	1.16E+00	2.86E+01
28042	1.49E+00	2.92E+01	28015	2.34E+00	3.03E+01
27961	3.43E+00	3.42E+01			
27984	3.59E-02	2.81E+01	27984	1.11E-01	2.81E+01
27984	2.01E-01	2.81E+01	28044	1.12E+00	2.85E+01
28044	1.44E+00	2.92E+01	28017	2.28E+00	3.03E+01
27959	3.47E+00	3.41E+01			
27985	3.66E-02	2.83E+01	27985	1.12E-01	2.80E+01
27985	2.04E-01	2.81E+01	28046	1.08E+00	2.86E+01
28046	1.40E+00	2.93E+01	28019	2.23E+00	3.02E+01
27957	3.52E+00	3.42E+01			
27986	3.73E-02	2.83E+01	27986	1.14E-01	2.80E+01
27986	2.07E-01	2.79E+01	28048	1.04E+00	2.85E+01
28048	1.36E+00	2.92E+01	28021	2.18E+00	3.02E+01
27955	3.56E+00	3.42E+01			
27987	3.79E-02	2.83E+01	27987	1.16E-01	2.79E+01
27987	2.10E-01	2.81E+01	28050	1.01E+00	2.85E+01
28050	1.33E+00	2.92E+01	28023	2.13E+00	3.03E+01
27953	3.61E+00	3.42E+01			
27988	3.79E-02	2.83E+01	27988	1.17E-01	2.81E+01
27988	2.14E-01	2.80E+01	28050	1.01E+00	2.85E+01
28050	1.33E+00	2.92E+01	28025	2.08E+00	3.02E+01
27951	3.65E+00	3.44E+01			
27989	3.83E-02	2.82E+01	27989	1.19E-01	2.81E+01
27989	2.18E-01	2.79E+01	28048	1.05E+00	2.85E+01
28048	1.37E+00	2.93E+01	28025	2.08E+00	3.02E+01
27949	3.69E+00	3.43E+01			
27990	3.87E-02	2.81E+01	27990	1.21E-01	2.81E+01
27990	2.21E-01	2.79E+01	28046	1.09E+00	2.85E+01
28046	1.41E+00	2.92E+01	28023	2.13E+00	3.04E+01
27947	3.73E+00	3.44E+01			
27991	3.95E-02	2.81E+01	27991	1.23E-01	2.81E+01
27991	2.25E-01	2.80E+01	28044	1.13E+00	2.85E+01
28044	1.45E+00	2.93E+01	28021	2.18E+00	3.02E+01
27945	3.77E+00	3.43E+01			
27992	3.98E-02	2.83E+01	27992	1.25E-01	2.81E+01
27992	2.29E-01	2.79E+01	28042	1.18E+00	2.85E+01
28042	1.50E+00	2.93E+01	28019	2.24E+00	3.02E+01
27943	3.80E+00	3.44E+01			
27993	4.05E-02	2.83E+01	27993	1.27E-01	2.81E+01
27993	2.34E-01	2.79E+01	28040	1.22E+00	2.85E+01
28040	1.54E+00	2.92E+01	28017	2.29E+00	3.02E+01
27941	3.84E+00	3.45E+01			
27994	4.17E-02	2.82E+01	27994	1.29E-01	2.81E+01
27994	2.37E-01	2.79E+01	28038	1.27E+00	2.86E+01

28038	1.59E+00	2.92E+01	28015	2.35E+00	3.02E+01
27939	3.87E+00	3.45E+01			
27995	4.20E-02	2.83E+01	27995	1.31E-01	2.81E+01
27995	2.42E-01	2.81E+01	28036	1.32E+00	2.86E+01
28036	1.64E+00	2.92E+01	28013	2.40E+00	3.03E+01
27937	3.91E+00	3.46E+01			
27996	4.29E-02	2.81E+01	27996	1.33E-01	2.80E+01
27996	2.47E-01	2.79E+01	28034	1.37E+00	2.87E+01
28034	1.69E+00	2.93E+01	28011	2.46E+00	3.02E+01
27935	3.94E+00	3.46E+01			
27997	4.32E-02	2.81E+01	27997	1.36E-01	2.79E+01
27997	2.52E-01	2.79E+01	28032	1.42E+00	2.87E+01
28032	1.74E+00	2.92E+01	28009	2.51E+00	3.02E+01
27933	3.97E+00	3.47E+01			
27998	4.42E-02	2.82E+01	27998	1.38E-01	2.80E+01
27998	2.56E-01	2.81E+01	28030	1.47E+00	2.86E+01
28030	1.79E+00	2.92E+01	28007	2.57E+00	3.03E+01
27931	4.01E+00	3.47E+01			
27999	4.46E-02	2.81E+01	27999	1.41E-01	2.80E+01
27999	2.62E-01	2.80E+01	28028	1.53E+00	2.85E+01
28028	1.85E+00	2.92E+01	28005	2.62E+00	3.03E+01
27929	4.04E+00	3.48E+01			
28000	4.55E-02	2.81E+01	28000	1.43E-01	2.81E+01
28000	2.67E-01	2.79E+01	28026	1.58E+00	2.86E+01
28026	1.90E+00	2.92E+01	28003	2.68E+00	3.03E+01
27927	4.07E+00	3.49E+01			
28001	4.63E-02	2.83E+01	28001	1.46E-01	2.81E+01
28001	2.73E-01	2.79E+01	28024	1.64E+00	2.85E+01
28024	1.95E+00	2.92E+01	28001	2.73E+00	3.03E+01
27925	4.09E+00	3.50E+01			
28002	4.67E-02	2.83E+01	28002	1.49E-01	2.81E+01
28002	2.80E-01	2.79E+01	28022	1.69E+00	2.85E+01
28022	2.01E+00	2.93E+01	27999	2.79E+00	3.03E+01
27923	4.12E+00	3.49E+01			
28003	4.79E-02	2.83E+01	28003	1.52E-01	2.80E+01
28003	2.85E-01	2.81E+01	28020	1.75E+00	2.86E+01
28020	2.06E+00	2.93E+01	27997	2.84E+00	3.04E+01
27921	4.15E+00	3.50E+01			
28004	4.84E-02	2.83E+01	28004	1.55E-01	2.81E+01
28004	2.92E-01	2.79E+01	28018	1.80E+00	2.86E+01
28018	2.12E+00	2.92E+01	27995	2.90E+00	3.04E+01
27919	4.18E+00	3.52E+01			
28005	4.97E-02	2.82E+01	28005	1.58E-01	2.81E+01
28005	2.99E-01	2.78E+01	28016	1.86E+00	2.87E+01
28016	2.18E+00	2.93E+01	27993	2.95E+00	3.05E+01
27917	4.20E+00	3.51E+01			
28006	5.04E-02	2.83E+01	28006	1.62E-01	2.81E+01
28006	3.07E-01	2.80E+01	28014	1.92E+00	2.86E+01
28014	2.23E+00	2.92E+01	27991	3.00E+00	3.05E+01
27915	4.23E+00	3.53E+01			
28007	5.11E-02	2.83E+01	28007	1.65E-01	2.82E+01
28007	3.15E-01	2.79E+01	28012	1.98E+00	2.85E+01
28012	2.29E+00	2.94E+01	27989	3.06E+00	3.05E+01
27913	4.25E+00	3.52E+01			
28008	5.23E-02	2.82E+01	28008	1.68E-01	2.82E+01
28008	3.23E-01	2.81E+01	28010	2.04E+00	2.87E+01
28010	2.35E+00	2.94E+01	27987	3.11E+00	3.05E+01
27911	4.27E+00	3.52E+01			
28009	5.34E-02	2.82E+01	28009	1.72E-01	2.81E+01
28009	3.33E-01	2.79E+01	28008	2.10E+00	2.87E+01
28008	2.41E+00	2.93E+01	27985	3.16E+00	3.05E+01
27909	4.30E+00	3.53E+01			
28010	5.45E-02	2.82E+01	28010	1.76E-01	2.81E+01
28010	3.42E-01	2.79E+01	28006	2.16E+00	2.87E+01
28006	2.47E+00	2.94E+01	27983	3.21E+00	3.05E+01
27907	4.32E+00	3.54E+01			
28011	5.53E-02	2.82E+01	28011	1.81E-01	2.80E+01
28011	3.52E-01	2.80E+01	28004	2.21E+00	2.88E+01

28004	2.53E+00	2.94E+01	27981	3.26E+00	3.05E+01
27905	4.34E+00	3.55E+01			
28012	5.66E-02	2.82E+01	28012	1.85E-01	2.81E+01
28012	3.63E-01	2.80E+01	28002	2.27E+00	2.87E+01
28002	2.59E+00	2.94E+01	27979	3.31E+00	3.05E+01
27903	4.36E+00	3.56E+01			
28013	5.78E-02	2.82E+01	28013	1.89E-01	2.79E+01
28013	3.75E-01	2.81E+01	28000	2.32E+00	2.87E+01
28000	2.65E+00	2.94E+01	27977	3.36E+00	3.05E+01
27901	4.38E+00	3.57E+01			
28014	5.89E-02	2.81E+01	28014	1.94E-01	2.81E+01
28014	3.88E-01	2.79E+01	27998	2.37E+00	2.87E+01
27998	2.70E+00	2.96E+01	27975	3.40E+00	3.07E+01
27899	4.40E+00	3.56E+01			
28015	6.04E-02	2.83E+01	28015	1.99E-01	2.81E+01
28015	4.01E-01	2.81E+01	27996	2.42E+00	2.88E+01
27996	2.76E+00	2.96E+01	27973	3.45E+00	3.06E+01
27897	2.13E+00	3.56E+01			
28016	6.13E-02	2.83E+01	28016	2.05E-01	2.81E+01
28016	4.15E-01	2.79E+01	27994	2.48E+00	2.88E+01
27994	2.82E+00	2.96E+01	27971	3.50E+00	3.08E+01
27895	1.80E+00	3.56E+01			
28017	6.33E-02	2.83E+01	28017	2.10E-01	2.81E+01
28017	4.33E-01	2.79E+01	27992	2.45E+00	2.87E+01
27992	2.87E+00	2.97E+01	27969	3.54E+00	3.07E+01
27893	1.65E+00	3.54E+01			
28018	6.47E-02	2.82E+01	28018	2.16E-01	2.81E+01
28018	4.51E-01	2.80E+01	27990	1.67E+00	2.88E+01
27990	2.93E+00	2.96E+01	27967	3.58E+00	3.09E+01
27891	1.54E+00	3.52E+01			
28019	6.62E-02	2.82E+01	28019	2.23E-01	2.81E+01
28019	4.70E-01	2.81E+01	27988	1.19E+00	2.89E+01
27988	2.98E+00	2.97E+01	27965	3.63E+00	3.09E+01
27889	1.46E+00	3.51E+01			
28020	6.79E-02	2.83E+01	28020	2.30E-01	2.81E+01
28020	4.92E-01	2.80E+01	27985	1.01E+00	2.89E+01
27985	3.04E+00	2.98E+01	27963	3.67E+00	3.10E+01
27887	1.39E+00	3.51E+01			
28021	6.95E-02	2.81E+01	28021	2.37E-01	2.81E+01
28021	5.16E-01	2.79E+01	27983	9.01E-01	2.89E+01
27983	3.09E+00	2.96E+01	27962	3.71E+00	3.09E+01
27885	1.32E+00	3.49E+01			
28022	7.13E-02	2.83E+01	28022	2.45E-01	2.80E+01
28022	5.44E-01	2.79E+01	27981	8.25E-01	2.87E+01
27981	3.14E+00	2.98E+01	27960	3.75E+00	3.10E+01
27883	1.27E+00	3.49E+01			
28023	7.33E-02	2.83E+01	28023	2.54E-01	2.81E+01
28023	5.77E-01	2.81E+01	27979	7.57E-01	2.88E+01
27979	3.19E+00	2.98E+01	27958	3.79E+00	3.10E+01
27881	1.22E+00	3.47E+01			
28025	7.53E-02	2.82E+01	28025	2.63E-01	2.81E+01
28025	6.15E-01	2.80E+01	27977	7.10E-01	2.88E+01
27977	3.24E+00	2.98E+01	27956	3.82E+00	3.09E+01
27879	1.18E+00	3.48E+01			
28026	7.71E-02	2.83E+01	28026	2.72E-01	2.81E+01
28026	6.61E-01	2.80E+01	27975	6.70E-01	2.88E+01
27975	3.29E+00	2.98E+01	27954	3.86E+00	3.11E+01
27877	1.14E+00	3.47E+01			
28027	7.96E-02	2.82E+01	28027	2.83E-01	2.79E+01
28027	7.19E-01	2.79E+01	27973	6.35E-01	2.89E+01
27973	3.34E+00	2.99E+01	27952	3.90E+00	3.11E+01
27875	1.11E+00	3.47E+01			
28028	8.19E-02	2.81E+01	28028	2.95E-01	2.80E+01
28028	7.87E-01	2.79E+01	27971	6.05E-01	2.87E+01
27971	3.39E+00	2.98E+01	27950	3.93E+00	3.11E+01
28029	8.43E-02	2.83E+01	28029	3.06E-01	2.80E+01
28029	8.60E-01	2.80E+01	27969	5.78E-01	2.87E+01

27969	3.43E+00	2.99E+01	27948	3.97E+00	3.13E+01
28030	8.67E-02	2.82E+01	28030	3.20E-01	2.80E+01
28030	9.59E-01	2.81E+01	27967	5.53E-01	2.87E+01
27967	3.48E+00	2.99E+01	27946	4.00E+00	3.13E+01
28031	8.93E-02	2.83E+01	28031	3.34E-01	2.79E+01
28031	1.00E+00	2.81E+01	27965	5.31E-01	2.87E+01
27965	3.53E+00	3.00E+01	27944	4.03E+00	3.13E+01
28032	9.22E-02	2.83E+01	28032	3.51E-01	2.80E+01
28032	1.02E+00	2.80E+01	27963	5.11E-01	2.89E+01
27963	2.60E+00	2.99E+01	27942	4.06E+00	3.16E+01
28033	9.49E-02	2.81E+01	28033	3.69E-01	2.79E+01
28033	1.03E+00	2.79E+01	27961	4.92E-01	2.86E+01
27961	1.55E+00	3.00E+01	27940	4.10E+00	3.16E+01
28034	9.85E-02	2.81E+01	28034	3.89E-01	2.80E+01
28034	1.02E+00	2.81E+01	27959	4.74E-01	2.87E+01
27959	1.33E+00	2.98E+01	27938	4.13E+00	3.16E+01
28035	1.02E-01	2.82E+01	28035	4.09E-01	2.79E+01
28035	1.01E+00	2.81E+01	27957	4.59E-01	2.87E+01
27957	1.21E+00	2.98E+01	27936	2.11E+00	3.17E+01
28036	1.05E-01	2.81E+01	28036	4.32E-01	2.81E+01
28036	9.93E-01	2.80E+01	27955	4.45E-01	2.88E+01
27955	1.12E+00	2.99E+01	27934	1.67E+00	3.14E+01
28037	1.09E-01	2.81E+01	28037	4.62E-01	2.79E+01
28037	9.76E-01	2.80E+01	27953	4.31E-01	2.87E+01
27953	1.05E+00	2.97E+01	27932	1.51E+00	3.14E+01
28038	1.13E-01	2.83E+01	28038	4.85E-01	2.80E+01
28038	9.55E-01	2.81E+01	27951	4.19E-01	2.87E+01
27951	9.92E-01	2.98E+01	27930	1.40E+00	3.12E+01
28039	1.17E-01	2.81E+01	28039	5.06E-01	2.81E+01
28039	9.35E-01	2.81E+01	27949	4.07E-01	2.87E+01
27949	9.43E-01	2.98E+01	27928	1.32E+00	3.12E+01
28040	1.22E-01	2.82E+01	28040	5.28E-01	2.79E+01
28040	9.16E-01	2.81E+01	27947	3.97E-01	2.87E+01
27947	9.01E-01	2.97E+01	27926	1.25E+00	3.12E+01
28041	1.27E-01	2.81E+01	28041	5.46E-01	2.81E+01
28041	8.95E-01	2.81E+01	27945	3.87E-01	2.88E+01
27945	8.63E-01	2.96E+01	27924	1.19E+00	3.11E+01
28042	1.32E-01	2.81E+01	28042	5.52E-01	2.81E+01
28042	8.75E-01	2.81E+01	27943	3.77E-01	2.88E+01
27943	8.29E-01	2.96E+01	27922	1.14E+00	3.11E+01
28043	1.37E-01	2.81E+01	28043	5.59E-01	2.79E+01
28043	8.55E-01	2.81E+01	27941	3.68E-01	2.87E+01
27941	7.98E-01	2.97E+01	27920	1.10E+00	3.11E+01
28044	1.42E-01	2.81E+01	28044	5.61E-01	2.79E+01
28044	8.33E-01	2.81E+01	27939	3.59E-01	2.88E+01
27939	7.69E-01	2.96E+01	27918	1.06E+00	3.11E+01
28045	1.46E-01	2.81E+01	28045	5.59E-01	2.81E+01
28045	8.13E-01	2.81E+01	27937	3.51E-01	2.87E+01
27937	7.44E-01	2.96E+01	27916	1.02E+00	3.10E+01
28046	1.51E-01	2.81E+01	28046	5.54E-01	2.81E+01
28046	7.94E-01	2.79E+01	27935	3.44E-01	2.87E+01

27935	7.20E-01	2.96E+01	27914	9.85E-01	3.09E+01
28047	1.56E-01	2.81E+01	28047	5.46E-01	2.81E+01
28047	7.75E-01	2.81E+01	27933	3.37E-01	2.87E+01
27933	6.98E-01	2.97E+01	27912	9.53E-01	3.09E+01
28048	1.59E-01	2.81E+01	28048	5.38E-01	2.82E+01
28048	7.56E-01	2.81E+01	27931	3.29E-01	2.87E+01
27931	6.76E-01	2.96E+01	27910	9.23E-01	3.11E+01
28049	1.62E-01	2.81E+01	28049	5.28E-01	2.81E+01
28049	7.37E-01	2.81E+01	27929	3.23E-01	2.87E+01
27929	6.57E-01	2.96E+01	27908	8.99E-01	3.10E+01
28050	1.65E-01	2.81E+01	28050	5.17E-01	2.81E+01
28050	7.18E-01	2.81E+01	27927	3.16E-01	2.86E+01
27927	6.39E-01	2.96E+01	27906	8.74E-01	3.10E+01
28051	1.68E-01	2.81E+01	28051	5.04E-01	2.80E+01
28051	7.01E-01	2.81E+01	27925	3.10E-01	2.88E+01
27925	6.21E-01	2.96E+01	27904	8.50E-01	3.09E+01
28052	1.69E-01	2.81E+01	28052	4.93E-01	2.81E+01
28052	6.84E-01	2.82E+01			
27902	8.29E-01	3.09E+01			
28053	1.69E-01	2.83E+01	28053	4.80E-01	2.79E+01
28053	6.66E-01	2.80E+01			
27900	8.09E-01	3.10E+01			
28054	1.69E-01	2.81E+01	28054	4.69E-01	2.81E+01
28054	6.50E-01	2.81E+01			
28055	1.68E-01	2.81E+01	28055	4.57E-01	2.81E+01
28055	6.35E-01	2.82E+01			
28056	1.67E-01	2.82E+01	28056	4.45E-01	2.81E+01
28056	6.21E-01	2.80E+01			
28057	1.64E-01	2.81E+01	28057	4.33E-01	2.81E+01
28057	6.06E-01	2.82E+01			
28058	1.61E-01	2.81E+01	28058	4.22E-01	2.81E+01
28058	5.92E-01	2.81E+01			
28059	1.59E-01	2.82E+01	28059	4.11E-01	2.79E+01
28059	5.79E-01	2.81E+01			
28060	1.56E-01	2.82E+01	28060	4.00E-01	2.81E+01
28060	5.66E-01	2.81E+01			
28061	1.52E-01	2.83E+01	28061	3.89E-01	2.80E+01
28061	5.54E-01	2.81E+01			
28062	1.49E-01	2.83E+01	28062	3.79E-01	2.81E+01
28062	5.40E-01	2.80E+01			
28063	1.45E-01	2.83E+01	28063	3.69E-01	2.81E+01
28063	5.29E-01	2.81E+01			
28064	1.41E-01	2.83E+01	28064	3.59E-01	2.81E+01
28064	5.18E-01	2.81E+01			
28065	1.38E-01	2.83E+01	28065	3.49E-01	2.79E+01
28065	5.07E-01	2.82E+01			
28066	1.34E-01	2.83E+01	28066	3.41E-01	2.81E+01
28066	4.96E-01	2.81E+01			
28067	1.31E-01	2.83E+01	28067	3.31E-01	2.82E+01

28067	4.84E-01	2.81E+01			
28068	1.27E-01	2.83E+01	28068	3.23E-01	2.81E+01
28068	4.74E-01	2.82E+01			
28069	1.24E-01	2.81E+01	28069	3.15E-01	2.81E+01
28069	4.64E-01	2.81E+01			
28070	1.21E-01	2.82E+01	28070	3.06E-01	2.81E+01
28070	4.54E-01	2.81E+01			
28071	1.17E-01	2.83E+01	28071	2.99E-01	2.81E+01
28071	4.43E-01	2.81E+01			
28072	1.14E-01	2.83E+01	28072	2.93E-01	2.81E+01
28072	4.34E-01	2.81E+01			
28073	1.10E-01	2.81E+01	28073	2.84E-01	2.81E+01
28073	4.25E-01	2.82E+01			
28074	1.08E-01	2.81E+01	28074	2.78E-01	2.81E+01
28074	4.16E-01	2.81E+01			
28075	1.04E-01	2.82E+01	28075	2.70E-01	2.81E+01
28075	4.07E-01	2.81E+01			
28076	1.01E-01	2.82E+01	28076	2.64E-01	2.81E+01
28076	3.99E-01	2.81E+01			
28077	9.89E-02	2.81E+01	28077	2.58E-01	2.79E+01
28077	3.90E-01	2.81E+01			
28078	9.61E-02	2.81E+01	28078	2.52E-01	2.80E+01
28078	3.82E-01	2.81E+01			
28079	9.38E-02	2.81E+01	28079	2.46E-01	2.80E+01
28079	3.74E-01	2.81E+01			
28080	9.16E-02	2.81E+01	28080	2.40E-01	2.81E+01
28080	3.67E-01	2.81E+01			
28081	8.93E-02	2.82E+01	28081	2.35E-01	2.81E+01
28081	3.60E-01	2.81E+01			
28082	8.68E-02	2.82E+01	28082	2.30E-01	2.79E+01
28082	3.52E-01	2.80E+01			
28083	8.47E-02	2.83E+01	28083	2.24E-01	2.79E+01
28083	3.46E-01	2.81E+01			
28084	8.24E-02	2.83E+01	28084	2.20E-01	2.81E+01
28084	3.39E-01	2.81E+01			
28085	8.06E-02	2.81E+01	28085	2.15E-01	2.79E+01
28085	3.33E-01	2.81E+01			
28086	7.84E-02	2.81E+01	28086	2.11E-01	2.80E+01
28086	3.26E-01	2.81E+01			
28087	7.66E-02	2.82E+01	28087	2.06E-01	2.81E+01
28087	3.21E-01	2.81E+01			
28088	7.49E-02	2.82E+01	28088	2.02E-01	2.81E+01
28088	3.15E-01	2.81E+01			
28089	7.30E-02	2.82E+01	28089	1.98E-01	2.81E+01
28089	3.09E-01	2.82E+01			

28090	7.14E-02	2.81E+01	28090	1.94E-01	2.81E+01
28090	3.04E-01	2.81E+01			
28091	6.98E-02	2.83E+01	28091	1.90E-01	2.81E+01
28091	2.98E-01	2.81E+01			
28092	6.81E-02	2.83E+01	28092	1.87E-01	2.80E+01
28092	2.93E-01	2.81E+01			
28093	6.67E-02	2.81E+01	28093	1.83E-01	2.81E+01
28093	2.88E-01	2.81E+01			
28094	6.54E-02	2.82E+01	28094	1.79E-01	2.80E+01
28094	2.83E-01	2.81E+01			
28095	6.38E-02	2.81E+01	28095	1.76E-01	2.79E+01
28095	2.79E-01	2.82E+01			
28096	6.26E-02	2.83E+01	28096	1.72E-01	2.81E+01
28096	2.74E-01	2.81E+01			
28097	6.14E-02	2.81E+01	28097	1.69E-01	2.81E+01
28097	2.69E-01	2.81E+01			
28098	6.01E-02	2.82E+01	28098	1.66E-01	2.79E+01
28098	2.65E-01	2.80E+01			
28099	5.90E-02	2.83E+01	28099	1.63E-01	2.81E+01
28099	2.61E-01	2.80E+01			
28100	5.80E-02	2.83E+01	28100	1.61E-01	2.82E+01
28100	2.57E-01	2.80E+01			
28101	5.68E-02	2.82E+01	28101	1.58E-01	2.79E+01
28101	2.52E-01	2.81E+01			
28102	5.57E-02	2.83E+01	28102	1.55E-01	2.81E+01
28102	2.48E-01	2.81E+01			
28103	5.46E-02	2.81E+01	28103	1.53E-01	2.80E+01
28103	2.45E-01	2.81E+01			
28104	5.36E-02	2.82E+01	28104	1.50E-01	2.79E+01
28104	2.41E-01	2.81E+01			
28105	5.23E-02	2.83E+01	28105	1.47E-01	2.81E+01
28105	2.37E-01	2.81E+01			
28106	5.15E-02	2.82E+01	28106	1.45E-01	2.80E+01
28106	2.34E-01	2.81E+01			
28107	5.07E-02	2.82E+01	28107	1.43E-01	2.81E+01
28107	2.30E-01	2.81E+01			
28108	4.99E-02	2.82E+01	28108	1.40E-01	2.81E+01
28108	2.27E-01	2.81E+01			
28109	4.89E-02	2.82E+01	28109	1.38E-01	2.81E+01
28109	2.23E-01	2.81E+01			
28110	4.81E-02	2.81E+01	28110	1.36E-01	2.81E+01
28110	2.21E-01	2.81E+01			
28111	4.70E-02	2.83E+01	28111	1.34E-01	2.81E+01
28111	2.18E-01	2.81E+01			
28112	4.67E-02	2.81E+01	28112	1.32E-01	2.80E+01
28112	2.14E-01	2.81E+01			

28113	4.58E-02	2.82E+01	28113	1.30E-01	2.80E+01
28113	2.11E-01	2.81E+01			
28114	4.50E-02	2.83E+01	28114	1.28E-01	2.79E+01
28114	2.09E-01	2.81E+01			
28115	4.41E-02	2.81E+01	28115	1.26E-01	2.81E+01
28115	2.06E-01	2.81E+01			
28116	4.40E-02	2.81E+01	28116	1.25E-01	2.81E+01
28116	2.03E-01	2.81E+01			
28117	4.29E-02	2.82E+01	28117	1.23E-01	2.79E+01
28117	2.00E-01	2.81E+01			
28118	4.26E-02	2.82E+01	28118	1.21E-01	2.79E+01
28118	1.98E-01	2.81E+01			
28119	4.17E-02	2.83E+01	28119	1.20E-01	2.79E+01
28119	1.95E-01	2.81E+01			
28120	4.10E-02	2.81E+01	28120	1.18E-01	2.79E+01
28120	1.93E-01	2.80E+01			
28121	4.03E-02	2.83E+01	28121	1.16E-01	2.79E+01
28121	1.90E-01	2.81E+01			
28122	3.98E-02	2.81E+01	28122	1.15E-01	2.81E+01
28122	1.88E-01	2.81E+01			
28123	3.90E-02	2.81E+01	28123	1.13E-01	2.81E+01
28123	1.86E-01	2.81E+01			
28124	3.87E-02	2.81E+01	28124	1.12E-01	2.80E+01
28124	1.84E-01	2.82E+01			
28125	3.82E-02	2.83E+01	28125	1.10E-01	2.79E+01
28125	1.82E-01	2.81E+01			
28125	3.82E-02	2.82E+01	28125	1.11E-01	2.81E+01
28125	1.81E-01	2.81E+01			
28124	3.88E-02	2.81E+01	28124	1.13E-01	2.79E+01
28124	1.84E-01	2.81E+01			
28123	3.93E-02	2.81E+01	28123	1.14E-01	2.79E+01
28123	1.86E-01	2.81E+01			
28122	4.00E-02	2.82E+01	28122	1.15E-01	2.79E+01
28122	1.88E-01	2.80E+01			
28121	4.06E-02	2.81E+01	28121	1.17E-01	2.81E+01
28121	1.91E-01	2.81E+01			
28120	4.11E-02	2.83E+01	28120	1.18E-01	2.80E+01
28120	1.94E-01	2.82E+01			
28119	4.21E-02	2.82E+01	28119	1.20E-01	2.79E+01
28119	1.96E-01	2.81E+01			
28118	4.22E-02	2.81E+01	28118	1.22E-01	2.80E+01
28118	1.98E-01	2.81E+01			
28117	4.33E-02	2.81E+01	28117	1.23E-01	2.81E+01
28117	2.01E-01	2.81E+01			
28116	4.39E-02	2.81E+01	28116	1.25E-01	2.81E+01

28116	2.04E-01	2.81E+01			
28115	4.46E-02	2.81E+01	28115	1.27E-01	2.81E+01
28115	2.07E-01	2.81E+01			
28114	4.53E-02	2.80E+01	28114	1.29E-01	2.81E+01
28114	2.10E-01	2.82E+01			
28113	4.58E-02	2.82E+01	28113	1.31E-01	2.79E+01
28113	2.13E-01	2.81E+01			
28112	4.68E-02	2.82E+01	28112	1.33E-01	2.80E+01
28112	2.16E-01	2.82E+01			
28111	4.76E-02	2.81E+01	28111	1.35E-01	2.80E+01
28111	2.19E-01	2.81E+01			
28110	4.83E-02	2.81E+01	28110	1.37E-01	2.81E+01
28110	2.22E-01	2.81E+01			
28109	4.91E-02	2.82E+01	28109	1.39E-01	2.79E+01
28109	2.25E-01	2.82E+01			
28108	5.00E-02	2.83E+01	28108	1.41E-01	2.81E+01
28108	2.29E-01	2.81E+01			
28107	5.09E-02	2.81E+01	28107	1.43E-01	2.81E+01
28107	2.33E-01	2.81E+01			
28106	5.20E-02	2.83E+01	28106	1.46E-01	2.79E+01
28106	2.36E-01	2.81E+01			
28105	5.30E-02	2.83E+01	28105	1.48E-01	2.81E+01
28105	2.40E-01	2.83E+01			
28104	5.39E-02	2.81E+01	28104	1.51E-01	2.81E+01
28104	2.44E-01	2.81E+01			
28103	5.50E-02	2.81E+01	28103	1.53E-01	2.81E+01
28103	2.48E-01	2.81E+01			
28102	5.60E-02	2.82E+01	28102	1.56E-01	2.80E+01
28102	2.51E-01	2.81E+01			
28101	5.70E-02	2.83E+01	28101	1.59E-01	2.81E+01
28101	2.56E-01	2.81E+01			
28100	5.82E-02	2.83E+01	28100	1.62E-01	2.79E+01
28100	2.59E-01	2.82E+01			
28099	5.93E-02	2.83E+01	28099	1.65E-01	2.81E+01
28099	2.64E-01	2.80E+01			
28098	6.06E-02	2.81E+01	28098	1.67E-01	2.81E+01
28098	2.69E-01	2.82E+01			
28097	6.17E-02	2.82E+01	28097	1.71E-01	2.79E+01
28097	2.73E-01	2.81E+01			
28096	6.32E-02	2.81E+01	28096	1.74E-01	2.80E+01
28096	2.78E-01	2.81E+01			
28095	6.45E-02	2.82E+01	28095	1.77E-01	2.80E+01
28095	2.82E-01	2.81E+01			
28094	6.58E-02	2.82E+01	28094	1.81E-01	2.81E+01
28094	2.88E-01	2.81E+01			

28093	6.71E-02	2.82E+01	28093	1.84E-01	2.81E+01
28093	2.93E-01	2.81E+01			
28092	6.88E-02	2.81E+01	28092	1.88E-01	2.81E+01
28092	2.99E-01	2.81E+01			
28091	7.05E-02	2.81E+01	28091	1.92E-01	2.81E+01
28091	3.03E-01	2.81E+01			
28090	7.20E-02	2.83E+01	28090	1.96E-01	2.81E+01
28090	3.11E-01	2.81E+01			
28089	7.38E-02	2.82E+01	28089	1.99E-01	2.81E+01
28089	3.16E-01	2.82E+01			
28088	7.52E-02	2.81E+01	28088	2.03E-01	2.80E+01
28088	3.21E-01	2.81E+01			
28087	7.72E-02	2.81E+01	28087	2.09E-01	2.80E+01
28087	3.27E-01	2.81E+01			
28086	7.93E-02	2.81E+01	28086	2.13E-01	2.79E+01
28086	3.33E-01	2.82E+01			
28085	8.13E-02	2.81E+01	28085	2.17E-01	2.81E+01
28085	3.39E-01	2.81E+01			
28084	8.31E-02	2.81E+01	28084	2.22E-01	2.81E+01
28084	3.47E-01	2.81E+01			
28083	8.55E-02	2.81E+01	28083	2.27E-01	2.81E+01
28083	3.54E-01	2.81E+01			
28082	8.79E-02	2.82E+01	28082	2.32E-01	2.79E+01
28082	3.61E-01	2.81E+01			
28081	9.04E-02	2.81E+01	28081	2.37E-01	2.80E+01
28081	3.68E-01	2.82E+01			
28080	9.28E-02	2.81E+01	28080	2.43E-01	2.81E+01
28080	3.76E-01	2.80E+01			
28079	9.50E-02	2.81E+01	28079	2.49E-01	2.81E+01
28079	3.84E-01	2.81E+01			
28078	9.81E-02	2.81E+01	28078	2.55E-01	2.81E+01
28078	3.92E-01	2.83E+01			
28077	1.01E-01	2.81E+01	28077	2.61E-01	2.79E+01
28077	4.00E-01	2.81E+01			
28076	1.04E-01	2.81E+01	28076	2.68E-01	2.80E+01
28076	4.08E-01	2.81E+01			
28075	1.07E-01	2.82E+01	28075	2.74E-01	2.81E+01
28075	4.18E-01	2.82E+01			
28074	1.09E-01	2.81E+01	28074	2.81E-01	2.80E+01
28074	4.27E-01	2.81E+01			
28073	1.13E-01	2.81E+01	28073	2.89E-01	2.81E+01
28073	4.36E-01	2.79E+01			
28072	1.16E-01	2.81E+01	28072	2.96E-01	2.79E+01
28072	4.46E-01	2.81E+01			
28071	1.19E-01	2.81E+01	28071	3.04E-01	2.81E+01
28071	4.56E-01	2.79E+01			

28070	1.22E-01	2.81E+01	28070	3.11E-01	2.80E+01
28070	4.66E-01	2.80E+01			
28069	1.26E-01	2.81E+01	28069	3.19E-01	2.81E+01
28069	4.76E-01	2.79E+01			
28068	1.29E-01	2.81E+01	28068	3.28E-01	2.81E+01
28068	4.88E-01	2.81E+01			
28067	1.33E-01	2.81E+01	28067	3.37E-01	2.81E+01
28067	4.99E-01	2.81E+01			
28066	1.37E-01	2.81E+01	28066	3.46E-01	2.81E+01
28066	5.10E-01	2.80E+01			
28065	1.40E-01	2.81E+01	28065	3.55E-01	2.81E+01
28065	5.23E-01	2.81E+01			
28064	1.44E-01	2.81E+01	28064	3.64E-01	2.81E+01
28064	5.35E-01	2.79E+01			
28063	1.47E-01	2.81E+01	28063	3.74E-01	2.81E+01
28063	5.47E-01	2.80E+01			
28062	1.51E-01	2.81E+01	28062	3.84E-01	2.81E+01
28062	5.60E-01	2.81E+01			
28061	1.54E-01	2.81E+01	28061	3.95E-01	2.80E+01
28061	5.74E-01	2.81E+01			
28060	1.57E-01	2.81E+01	28060	4.05E-01	2.81E+01
28060	5.87E-01	2.79E+01			
28059	1.61E-01	2.82E+01	28059	4.16E-01	2.81E+01
28059	6.01E-01	2.81E+01			
28058	1.63E-01	2.81E+01	28058	4.26E-01	2.81E+01
28058	6.14E-01	2.81E+01			
28057	1.66E-01	2.81E+01	28057	4.38E-01	2.80E+01
28057	6.31E-01	2.80E+01			
28056	1.68E-01	2.82E+01	28056	4.49E-01	2.81E+01
28056	6.44E-01	2.81E+01			
28055	1.69E-01	2.82E+01	28055	4.61E-01	2.79E+01
28055	6.60E-01	2.81E+01			
28054	1.70E-01	2.81E+01	28054	4.72E-01	2.81E+01
28054	6.75E-01	2.80E+01			
28053	1.70E-01	2.81E+01	28053	4.84E-01	2.81E+01
28053	6.91E-01	2.80E+01			
28052	1.69E-01	2.81E+01	28052	4.96E-01	2.81E+01
28052	7.07E-01	2.81E+01			
28051	1.68E-01	2.81E+01	28051	5.07E-01	2.81E+01
28051	7.24E-01	2.81E+01			
28050	1.66E-01	2.81E+01	28050	5.18E-01	2.79E+01
28050	7.41E-01	2.81E+01			
28049	1.63E-01	2.81E+01	28049	5.28E-01	2.81E+01
28049	7.58E-01	2.79E+01			
28048	1.60E-01	2.81E+01	28048	5.38E-01	2.81E+01

28048	7.74E-01	2.81E+01			
28047	1.56E-01	2.81E+01	28047	5.46E-01	2.81E+01
28047	7.92E-01	2.81E+01			
28046	1.52E-01	2.81E+01	28046	5.53E-01	2.79E+01
28046	8.09E-01	2.81E+01			
28045	1.47E-01	2.82E+01	28045	5.58E-01	2.81E+01
28045	8.26E-01	2.81E+01			
28044	1.41E-01	2.81E+01	28044	5.61E-01	2.81E+01
28044	8.47E-01	2.81E+01			
28043	1.37E-01	2.82E+01	28043	5.60E-01	2.81E+01
28043	8.65E-01	2.81E+01			
28042	1.33E-01	2.81E+01	28042	5.57E-01	2.81E+01
28042	8.82E-01	2.81E+01			
28041	1.28E-01	2.81E+01	28041	5.47E-01	2.81E+01
28041	8.97E-01	2.81E+01			
28040	1.23E-01	2.81E+01	28040	5.37E-01	2.81E+01
28040	9.16E-01	2.82E+01			
28039	1.19E-01	2.82E+01	28039	5.22E-01	2.81E+01
28039	9.36E-01	2.81E+01			
28038	1.15E-01	2.83E+01	28038	5.04E-01	2.81E+01
28038	9.54E-01	2.81E+01			
28037	1.11E-01	2.81E+01	28037	4.83E-01	2.82E+01
28037	9.72E-01	2.81E+01			
28036	1.07E-01	2.81E+01	28036	4.61E-01	2.81E+01
28036	9.89E-01	2.82E+01			
28035	1.03E-01	2.81E+01	28035	4.37E-01	2.81E+01
28035	1.00E+00	2.81E+01			
28034	9.97E-02	2.82E+01	28034	4.16E-01	2.82E+01
28034	1.02E+00	2.80E+01			
28033	9.64E-02	2.81E+01	28033	3.95E-01	2.81E+01
28033	1.03E+00	2.81E+01			
28032	9.29E-02	2.81E+01	28032	3.76E-01	2.81E+01
28032	1.03E+00	2.82E+01			
28031	9.00E-02	2.81E+01	28031	3.58E-01	2.81E+01
28031	1.02E+00	2.82E+01			
28030	8.74E-02	2.82E+01	28030	3.41E-01	2.81E+01
28030	1.01E+00	2.81E+01			
28029	8.49E-02	2.81E+01	28029	3.26E-01	2.82E+01
28029	9.82E-01	2.81E+01			
28028	8.24E-02	2.81E+01	28028	3.12E-01	2.81E+01
28028	9.42E-01	2.81E+01			
28027	7.99E-02	2.81E+01	28027	2.98E-01	2.81E+01
28027	8.88E-01	2.81E+01			
28026	7.78E-02	2.81E+01	28026	2.87E-01	2.81E+01
28026	8.29E-01	2.81E+01			

28025	7.56E-02	2.80E+01	28025	2.77E-01	2.81E+01
28025	7.66E-01	2.81E+01			
28023	7.37E-02	2.81E+01	28023	2.66E-01	2.81E+01
28023	7.07E-01	2.82E+01			
28022	7.17E-02	2.80E+01	28022	2.56E-01	2.81E+01
28022	6.53E-01	2.82E+01			
28021	6.97E-02	2.81E+01	28021	2.48E-01	2.81E+01
28021	6.06E-01	2.81E+01			
28020	6.82E-02	2.81E+01	28020	2.40E-01	2.81E+01
28020	5.65E-01	2.81E+01			
28019	6.66E-02	2.82E+01	28019	2.32E-01	2.82E+01
28019	5.31E-01	2.81E+01			
28018	6.49E-02	2.81E+01	28018	2.25E-01	2.81E+01
28018	5.03E-01	2.81E+01			
28017	6.34E-02	2.81E+01	28017	2.18E-01	2.81E+01
28017	4.79E-01	2.81E+01			
28016	6.18E-02	2.81E+01	28016	2.12E-01	2.81E+01
28016	4.59E-01	2.81E+01			
28015	6.07E-02	2.81E+01	28015	2.06E-01	2.81E+01
28015	4.39E-01	2.81E+01			
28014	5.94E-02	2.80E+01	28014	2.00E-01	2.81E+01
28014	4.21E-01	2.81E+01			
28013	5.80E-02	2.80E+01	28013	1.96E-01	2.81E+01
28013	4.06E-01	2.81E+01			
28012	5.69E-02	2.81E+01	28012	1.90E-01	2.81E+01
28012	3.91E-01	2.80E+01			
28011	5.56E-02	2.81E+01	28011	1.85E-01	2.81E+01
28011	3.78E-01	2.81E+01			
28010	5.46E-02	2.80E+01	28010	1.81E-01	2.81E+01
28010	3.66E-01	2.81E+01			
28009	5.34E-02	2.81E+01	28009	1.77E-01	2.81E+01
28009	3.54E-01	2.79E+01			
28008	5.25E-02	2.81E+01	28008	1.73E-01	2.81E+01
28008	3.43E-01	2.81E+01			
28007	5.15E-02	2.81E+01	28007	1.69E-01	2.81E+01
28007	3.33E-01	2.81E+01			
28006	5.06E-02	2.81E+01	28006	1.65E-01	2.81E+01
28006	3.24E-01	2.81E+01			
28005	4.96E-02	2.81E+01	28005	1.62E-01	2.81E+01
28005	3.16E-01	2.81E+01			
28004	4.87E-02	2.81E+01	28004	1.58E-01	2.81E+01
28004	3.07E-01	2.81E+01			
28003	4.76E-02	2.81E+01	28003	1.55E-01	2.81E+01
28003	2.99E-01	2.81E+01			
28002	4.70E-02	2.81E+01	28002	1.52E-01	2.80E+01
28002	2.92E-01	2.82E+01			

28001	4.64E-02	2.81E+01	28001	1.49E-01	2.81E+01
28001	2.84E-01	2.81E+01			
28000	4.54E-02	2.81E+01	28000	1.46E-01	2.81E+01
28000	2.79E-01	2.81E+01			
27999	4.49E-02	2.83E+01	27999	1.43E-01	2.81E+01
27999	2.72E-01	2.81E+01			
27998	4.39E-02	2.81E+01	27998	1.41E-01	2.81E+01
27998	2.66E-01	2.81E+01			
27997	4.33E-02	2.81E+01	27997	1.38E-01	2.79E+01
27997	2.61E-01	2.81E+01			
27996	4.27E-02	2.82E+01	27996	1.35E-01	2.81E+01
27996	2.56E-01	2.81E+01			
27995	4.23E-02	2.81E+01	27995	1.33E-01	2.81E+01
27995	2.51E-01	2.81E+01			
27994	4.16E-02	2.81E+01	27994	1.31E-01	2.82E+01
27994	2.46E-01	2.80E+01			
27993	4.07E-02	2.81E+01	27993	1.29E-01	2.81E+01
27993	2.41E-01	2.81E+01			
27992	4.01E-02	2.81E+01	27992	1.27E-01	2.81E+01
27992	2.36E-01	2.82E+01			
27991	3.96E-02	2.81E+01	27991	1.24E-01	2.80E+01
27991	2.32E-01	2.81E+01			
27990	3.92E-02	2.80E+01	27990	1.23E-01	2.81E+01
27990	2.28E-01	2.81E+01			
27989	3.85E-02	2.81E+01	27989	1.21E-01	2.81E+01
27989	2.24E-01	2.81E+01			
27988	3.79E-02	2.81E+01	27988	1.19E-01	2.80E+01
27988	2.20E-01	2.81E+01			
27987	3.77E-02	2.81E+01	27987	1.17E-01	2.82E+01
27987	2.16E-01	2.81E+01			
27986	3.70E-02	2.81E+01	27986	1.15E-01	2.80E+01
27986	2.13E-01	2.81E+01			
27985	3.65E-02	2.81E+01	27985	1.14E-01	2.81E+01
27985	2.09E-01	2.81E+01			
27984	3.59E-02	2.81E+01	27984	1.12E-01	2.81E+01
27984	2.06E-01	2.81E+01			
27983	3.56E-02	2.81E+01	27983	1.10E-01	2.79E+01
27983	2.02E-01	2.81E+01			
27982	3.53E-02	2.82E+01	27982	1.09E-01	2.81E+01
27982	2.00E-01	2.81E+01			
27981	3.46E-02	2.81E+01	27981	1.07E-01	2.81E+01
27981	1.97E-01	2.81E+01			
27980	3.41E-02	2.81E+01	27980	1.06E-01	2.81E+01
27980	1.94E-01	2.80E+01			
27979	3.39E-02	2.79E+01	27979	1.04E-01	2.80E+01

27979	1.91E-01	2.81E+01			
27978	3.33E-02	2.81E+01	27978	1.03E-01	2.81E+01
27978	1.88E-01	2.80E+01			
27977	3.30E-02	2.81E+01	27977	1.01E-01	2.81E+01
27977	1.85E-01	2.81E+01			
27976	3.25E-02	2.81E+01	27976	1.00E-01	2.81E+01
27976	1.83E-01	2.82E+01			
27975	3.22E-02	2.81E+01	27975	9.87E-02	2.81E+01
27975	1.81E-01	2.80E+01			
27974	3.17E-02	2.81E+01	27974	9.80E-02	2.81E+01
27974	1.77E-01	2.81E+01			
27973	3.17E-02	2.81E+01	27973	9.69E-02	2.81E+01
27973	1.76E-01	2.81E+01			
27972	3.10E-02	2.81E+01	27972	9.50E-02	2.80E+01
27972	1.73E-01	2.81E+01			
27971	3.07E-02	2.81E+01	27971	9.37E-02	2.80E+01
27971	1.71E-01	2.81E+01			
27970	3.05E-02	2.81E+01	27970	9.42E-02	2.82E+01
27970	1.69E-01	2.81E+01			
27969	3.01E-02	2.81E+01	27969	9.31E-02	2.81E+01
27969	1.67E-01	2.80E+01			
27968	2.97E-02	2.81E+01	27968	9.06E-02	2.81E+01
27968	1.65E-01	2.80E+01			
27967	2.93E-02	2.82E+01	27967	8.96E-02	2.81E+01
27967	1.63E-01	2.81E+01			
27966	2.91E-02	2.81E+01	27966	8.86E-02	2.81E+01
27966	1.61E-01	2.81E+01			
27965	2.88E-02	2.81E+01	27965	8.75E-02	2.80E+01
27965	1.59E-01	2.79E+01			
27964	2.83E-02	2.81E+01	27964	8.74E-02	2.81E+01
27964	1.57E-01	2.81E+01			
27963	2.80E-02	2.82E+01	27963	8.58E-02	2.78E+01
27963	1.55E-01	2.81E+01			
27962	2.79E-02	2.81E+01	27962	8.54E-02	2.81E+01
27962	1.54E-01	2.80E+01			
27961	2.76E-02	2.81E+01	27961	8.41E-02	2.79E+01
27961	1.52E-01	2.82E+01			
27960	2.74E-02	2.82E+01	27960	8.34E-02	2.80E+01
27960	1.50E-01	2.81E+01			
27959	2.71E-02	2.81E+01	27959	8.29E-02	2.80E+01
27959	1.48E-01	2.81E+01			
27958	2.66E-02	2.81E+01	27958	8.14E-02	2.80E+01
27958	1.46E-01	2.81E+01			
27957	2.65E-02	2.81E+01	27957	8.04E-02	2.80E+01
27957	1.45E-01	2.81E+01			

27956	2.64E-02	2.82E+01	27956	7.99E-02	2.81E+01
27956	1.44E-01	2.80E+01			
27955	2.60E-02	2.81E+01	27955	7.89E-02	2.80E+01
27955	1.42E-01	2.79E+01			
27954	2.58E-02	2.82E+01	27954	7.85E-02	2.80E+01
27954	1.40E-01	2.80E+01			
27953	2.56E-02	2.82E+01	27953	7.76E-02	2.80E+01
27953	1.39E-01	2.81E+01			
27952	2.52E-02	2.82E+01	27952	7.67E-02	2.80E+01
27952	1.37E-01	2.80E+01			
27951	2.51E-02	2.81E+01	27951	7.61E-02	2.79E+01
27951	1.37E-01	2.81E+01			
27950	2.49E-02	2.83E+01	27950	7.55E-02	2.80E+01
27950	1.35E-01	2.81E+01			
27949	2.47E-02	2.81E+01	27949	7.48E-02	2.79E+01
27949	1.34E-01	2.81E+01			
27948	2.44E-02	2.83E+01	27948	7.41E-02	2.79E+01
27948	1.33E-01	2.80E+01			
27947	2.42E-02	2.81E+01	27947	7.66E-02	2.81E+01
27947	1.30E-01	2.81E+01			
27946	2.39E-02	2.82E+01	27946	7.28E-02	2.80E+01
27946	1.30E-01	2.80E+01			
27945	2.38E-02	2.82E+01	27945	7.28E-02	2.80E+01
27945	1.29E-01	2.81E+01			
27944	2.36E-02	2.83E+01	27944	7.13E-02	2.81E+01
27944	1.28E-01	2.81E+01			
27943	2.34E-02	2.81E+01	27943	7.07E-02	2.79E+01
27943	1.26E-01	2.79E+01			
27942	2.32E-02	2.81E+01	27942	7.03E-02	2.81E+01
27942	1.25E-01	2.79E+01			
27941	2.32E-02	2.81E+01	27941	6.98E-02	2.81E+01
27941	1.23E-01	2.81E+01			
27940	2.30E-02	2.81E+01	27940	6.89E-02	2.80E+01
27940	1.23E-01	2.80E+01			
27939	2.27E-02	2.81E+01	27939	6.85E-02	2.81E+01
27939	1.22E-01	2.80E+01			
27938	2.23E-02	2.82E+01	27938	6.79E-02	2.81E+01
27938	1.21E-01	2.79E+01			
27937	2.22E-02	2.82E+01	27937	6.73E-02	2.81E+01
27937	1.20E-01	2.81E+01			
27936	2.20E-02	2.81E+01	27936	6.65E-02	2.80E+01
27936	1.19E-01	2.80E+01			
27935	2.16E-02	2.82E+01	27935	6.64E-02	2.81E+01
27935	1.17E-01	2.79E+01			
27934	2.17E-02	2.82E+01	27934	6.63E-02	2.81E+01
27934	1.17E-01	2.80E+01			

27933	2.17E-02	2.81E+01	27933	6.57E-02	2.79E+01
27933	1.16E-01	2.80E+01			
27932	2.13E-02	2.81E+01	27932	6.43E-02	2.81E+01
27932	1.14E-01	2.80E+01			
27931	2.13E-02	2.81E+01	27931	6.56E-02	2.81E+01
27931	1.14E-01	2.80E+01			
27930	2.10E-02	2.81E+01	27930	6.38E-02	2.80E+01
27930	1.12E-01	2.81E+01			
27929	2.09E-02	2.81E+01	27929	6.34E-02	2.80E+01
27929	1.11E-01	2.80E+01			
27928	2.08E-02	2.81E+01	27928	6.28E-02	2.81E+01
27928	1.10E-01	2.80E+01			
27927	2.06E-02	2.81E+01	27927	6.24E-02	2.80E+01
27927	1.10E-01	2.79E+01			
27926	2.03E-02	2.81E+01	27926	6.24E-02	2.81E+01
27926	1.09E-01	2.80E+01			
27925	2.04E-02	2.81E+01	27925	6.21E-02	2.79E+01
27925	1.08E-01	2.81E+01			

I4
2V (LDV 125mm/s/V)   6V (LDV 125mm/s/V)   10V (LDV
125mm/s/V)   15V (LDV 125mm/s/V)   20V (LDV 125mm/s/V)
25V (LDV 125mm/s/V)   30V (LDV 125mm/s/V)   35V (LDV
125mm/s/V)   40V (LDV 125mm/s/V)   45V (LDV 125mm/s/V)
50V (LDV 125mm/s/V)
Frequency (Hz) Response (V) Frequency (Hz) Response (V) Frequency (Hz)
Response (V) Frequency (Hz) Response (V) Frequency (Hz) Response (V)
Frequency (Hz) Response (V) Frequency (Hz) Response (V) Frequency (Hz)
Response (V) Frequency (Hz) Response (V) Frequency (Hz) Response (V)
Frequency (Hz) Response (V)
27050 2.67E-02 27050 8.37E-02 27050 1.57E-01 26950
1.09E-01 27000 2.42E-01 27000 3.44E-01 27000 4.47E-01
26980 4.61E-01 26980 5.48E-01 26980 6.36E-01 26980
7.46E-01
27051 2.74E-02 27051 8.50E-02 27051 1.60E-01 26952
1.08E-01 27002 2.46E-01 27002 3.51E-01 27002 4.68E-01
26982 4.88E-01 26982 5.89E-01 26982 7.02E-01 26982
8.40E-01
27052 2.75E-02 27052 8.62E-02 27052 1.63E-01 26954
1.10E-01 27004 2.52E-01 27004 3.59E-01 27004 4.81E-01
26984 4.89E-01 26984 6.12E-01 26984 7.26E-01 26984
8.69E-01
27053 2.79E-02 27053 8.74E-02 27053 1.65E-01 26956
1.11E-01 27006 2.58E-01 27006 3.67E-01 27006 4.92E-01
26986 5.06E-01 26986 6.25E-01 26986 7.42E-01 26986
8.90E-01
27054 2.84E-02 27054 8.86E-02 27054 1.69E-01 26958
1.13E-01 27008 2.63E-01 27008 3.76E-01 27008 5.05E-01
26988 5.17E-01 26988 6.38E-01 26988 7.60E-01 26988
9.16E-01
27055 2.89E-02 27055 8.99E-02 27055 1.72E-01 26960
1.13E-01 27010 2.70E-01 27010 3.86E-01 27010 5.18E-01
26990 5.30E-01 26990 6.52E-01 26990 7.78E-01 26990
9.41E-01
27056 2.92E-02 27056 9.13E-02 27056 1.76E-01 26962
1.15E-01 27012 2.77E-01 27012 3.95E-01 27012 5.32E-01
26992 5.39E-01 26992 6.66E-01 26992 7.95E-01 26992

9.70E-01
27057 2.94E-02 27057 9.28E-02 27057 1.79E-01 26964
1.17E-01 27014 2.84E-01 27014 4.05E-01 27014 5.46E-01
26994 5.45E-01 26994 6.79E-01 26994 8.16E-01 26994
1.00E+00
27058 3.00E-02 27058 9.42E-02 27058 1.83E-01 26966
1.19E-01 27016 2.91E-01 27016 4.15E-01 27016 5.63E-01
26996 5.69E-01 26996 6.93E-01 26996 8.38E-01 26996
1.03E+00
27059 3.05E-02 27059 9.59E-02 27059 1.87E-01 26968
1.20E-01 27018 2.96E-01 27018 4.27E-01 27018 5.79E-01
26998 5.79E-01 26998 7.14E-01 26998 8.59E-01 26998
1.07E+00
27060 3.09E-02 27060 9.75E-02 27060 1.90E-01 26970
1.22E-01 27020 3.05E-01 27020 4.40E-01 27020 5.97E-01
27000 5.84E-01 27000 7.30E-01 27000 8.85E-01 27000
1.10E+00
27061 3.13E-02 27061 9.89E-02 27061 1.94E-01 26972
1.22E-01 27022 3.11E-01 27022 4.52E-01 27022 6.17E-01
27002 6.06E-01 27002 7.51E-01 27002 9.12E-01 27002
1.15E+00
27062 3.18E-02 27062 1.01E-01 27062 1.99E-01 26974
1.25E-01 27024 3.19E-01 27024 4.66E-01 27024 6.38E-01
27004 6.13E-01 27004 7.72E-01 27004 9.41E-01 27004
1.19E+00
27063 3.24E-02 27063 1.03E-01 27063 2.03E-01 26976
1.28E-01 27026 3.29E-01 27026 4.80E-01 27026 6.61E-01
27006 6.28E-01 27006 7.99E-01 27006 9.71E-01 27006
1.24E+00
27064 3.29E-02 27064 1.04E-01 27064 2.09E-01 26978
1.29E-01 27028 3.38E-01 27028 4.97E-01 27028 6.84E-01
27008 6.49E-01 27008 8.19E-01 27008 1.01E+00 27008
1.29E+00
27065 3.35E-02 27065 1.06E-01 27065 2.13E-01 26980
1.31E-01 27030 3.48E-01 27030 5.13E-01 27030 7.13E-01
27010 6.66E-01 27010 8.48E-01 27010 1.05E+00 27010
1.35E+00
27066 3.39E-02 27066 1.08E-01 27066 2.18E-01 26982
1.32E-01 27032 3.59E-01 27032 5.33E-01 27032 7.44E-01
27012 6.89E-01 27012 8.77E-01 27012 1.09E+00 27012
1.43E+00
27067 3.46E-02 27067 1.10E-01 27067 2.24E-01 26984
1.35E-01 27034 3.70E-01 27034 5.52E-01 27034 7.75E-01
27014 7.09E-01 27014 9.05E-01 27014 1.14E+00 27014
1.52E+00
27068 3.50E-02 27068 1.12E-01 27068 2.30E-01 26986
1.37E-01 27036 3.83E-01 27036 5.77E-01 27036 8.12E-01
27016 7.27E-01 27016 9.37E-01 27016 1.20E+00 27016
1.65E+00
27069 3.59E-02 27069 1.14E-01 27069 2.36E-01 26988
1.38E-01 27038 3.96E-01 27038 6.02E-01 27038 8.55E-01
27018 7.58E-01 27018 9.77E-01 27018 1.26E+00 27018
1.83E+00
27070 3.65E-02 27070 1.16E-01 27070 2.42E-01 26990
1.41E-01 27040 4.13E-01 27040 6.28E-01 27040 9.05E-01
27020 7.80E-01 27020 1.02E+00 27020 1.34E+00 27020
2.29E+00
27071 3.71E-02 27071 1.19E-01 27071 2.50E-01 26992
1.43E-01 27042 4.31E-01 27042 6.60E-01 27042 9.63E-01
27022 8.10E-01 27022 1.07E+00 27022 1.43E+00 27022
3.34E+00
27072 3.76E-02 27072 1.22E-01 27072 2.57E-01 26994
1.46E-01 27044 4.46E-01 27044 6.95E-01 27044 1.03E+00
27024 8.37E-01 27024 1.12E+00 27024 1.55E+00 27024
3.31E+00
27073 3.84E-02 27073 1.24E-01 27073 2.64E-01 26996
1.49E-01 27046 4.67E-01 27046 7.38E-01 27046 1.11E+00
27026 8.78E-01 27026 1.19E+00 27026 1.71E+00 27026

3.29E+00
27074 3.91E-02 27074 1.27E-01 27074 2.72E-01 26998
1.52E-01 27048 4.87E-01 27048 7.89E-01 27048 1.23E+00
27028 9.24E-01 27028 1.26E+00 27028 3.23E+00 27028
3.27E+00
27075 3.98E-02 27075 1.30E-01 27075 2.81E-01 27000
1.54E-01 27050 5.12E-01 27050 8.45E-01 27050 1.42E+00
27030 9.60E-01 27030 1.35E+00 27030 3.19E+00 27030
3.24E+00
27076 4.07E-02 27076 1.33E-01 27076 2.91E-01 27002
1.57E-01 27052 5.40E-01 27052 9.15E-01 27052 2.44E+00
27032 1.02E+00 27032 1.49E+00 27032 3.16E+00 27032
3.21E+00
27077 4.12E-02 27077 1.36E-01 27077 3.02E-01 27004
1.60E-01 27054 5.74E-01 27054 1.00E+00 27054 2.57E+00
27034 1.08E+00 27034 1.74E+00 27034 3.14E+00 27034
3.18E+00
27078 4.22E-02 27078 1.39E-01 27078 3.13E-01 27006
1.64E-01 27056 6.09E-01 27056 1.12E+00 27056 2.52E+00
27036 1.14E+00 27036 3.04E+00 27036 3.11E+00 27036
3.15E+00
27079 4.32E-02 27079 1.43E-01 27079 3.26E-01 27008
1.67E-01 27058 6.54E-01 27058 1.31E+00 27058 2.49E+00
27038 1.23E+00 27038 3.01E+00 27038 3.08E+00 27038
3.12E+00
27080 4.39E-02 27080 1.47E-01 27080 3.40E-01 27010
1.71E-01 27060 7.06E-01 27060 1.92E+00 27060 2.44E+00
27040 1.33E+00 27040 2.98E+00 27040 3.04E+00 27040
3.08E+00
27081 4.48E-02 27081 1.50E-01 27081 3.56E-01 27012
1.74E-01 27062 7.83E-01 27062 2.18E+00 27062 2.40E+00
27042 1.52E+00 27042 2.95E+00 27042 3.01E+00 27042
3.05E+00
27082 4.60E-02 27082 1.56E-01 27082 3.73E-01 27014
1.78E-01 27064 8.69E-01 27064 2.17E+00 27064 2.35E+00
27044 2.86E+00 27044 2.92E+00 27044 2.98E+00 27044
3.01E+00
27083 4.69E-02 27083 1.60E-01 27083 3.91E-01 27016
1.82E-01 27066 1.02E+00 27066 2.12E+00 27066 2.30E+00
27046 2.87E+00 27046 2.88E+00 27046 2.94E+00 27046
2.97E+00
27084 4.78E-02 27084 1.66E-01 27084 4.12E-01 27018
1.86E-01 27068 1.21E+00 27068 2.07E+00 27068 2.25E+00
27048 2.86E+00 27048 2.85E+00 27048 2.90E+00 27048
2.94E+00
27085 4.91E-02 27085 1.72E-01 27085 4.37E-01 27020
1.90E-01 27070 1.47E+00 27070 2.02E+00 27070 2.19E+00
27050 2.81E+00 27050 2.81E+00 27050 2.87E+00 27050
2.89E+00
27086 5.00E-02 27086 1.79E-01 27086 4.67E-01 27022
1.95E-01 27072 1.67E+00 27072 1.97E+00 27072 2.14E+00
27052 2.77E+00 27052 2.77E+00 27052 2.83E+00 27052
2.84E+00
27087 5.12E-02 27087 1.85E-01 27087 4.94E-01 27024
2.00E-01 27074 1.69E+00 27074 1.92E+00 27074 2.10E+00
27054 2.71E+00 27054 2.73E+00 27054 2.79E+00 27054
2.82E+00
27088 5.26E-02 27088 1.91E-01 27088 5.24E-01 27026
2.06E-01 27076 1.65E+00 27076 1.86E+00 27076 2.04E+00
27056 2.68E+00 27056 2.69E+00 27056 2.75E+00 27056
2.78E+00
27089 5.37E-02 27089 1.98E-01 27089 5.54E-01 27028
2.11E-01 27078 1.61E+00 27078 1.81E+00 27078 1.98E+00
27058 2.63E+00 27058 2.65E+00 27058 2.71E+00 27058
2.74E+00
27090 5.51E-02 27090 2.06E-01 27090 5.87E-01 27030
2.17E-01 27080 1.56E+00 27080 1.75E+00 27080 1.93E+00
27060 2.60E+00 27060 2.61E+00 27060 2.67E+00 27060

2.69E+00
27091 5.64E-02 27091 2.14E-01 27091 6.15E-01 27032
2.23E-01 27082 1.51E+00 27082 1.70E+00 27082 1.88E+00
27062 2.52E+00 27062 2.56E+00 27062 2.62E+00 27062
2.66E+00
27092 5.80E-02 27092 2.23E-01 27092 6.40E-01 27034
2.29E-01 27084 1.47E+00 27084 1.65E+00 27084 1.82E+00
27064 2.47E+00 27064 2.52E+00 27064 2.58E+00 27064
2.61E+00
27093 5.94E-02 27093 2.31E-01 27093 6.59E-01 27036
2.35E-01 27086 1.41E+00 27086 1.60E+00 27086 1.77E+00
27066 2.46E+00 27066 2.47E+00 27066 2.54E+00 27066
2.54E+00
27094 6.10E-02 27094 2.41E-01 27094 6.67E-01 27038
2.43E-01 27088 1.37E+00 27088 1.54E+00 27088 1.71E+00
27068 2.38E+00 27068 2.42E+00 27068 2.49E+00 27068
2.52E+00
27095 6.26E-02 27095 2.52E-01 27095 6.73E-01 27040
2.50E-01 27090 1.32E+00 27090 1.49E+00 27090 1.66E+00
27070 2.35E+00 27070 2.38E+00 27070 2.45E+00 27070
2.49E+00
27096 6.43E-02 27096 2.63E-01 27096 6.73E-01 27042
2.59E-01 27092 1.27E+00 27092 1.45E+00 27092 1.61E+00
27072 2.26E+00 27072 2.33E+00 27072 2.40E+00 27072
2.45E+00
27097 6.61E-02 27097 2.73E-01 27097 6.67E-01 27044
2.67E-01 27094 1.22E+00 27094 1.41E+00 27094 1.57E+00
27074 2.24E+00 27074 2.28E+00 27074 2.36E+00 27074
2.40E+00
27098 6.79E-02 27098 2.84E-01 27098 6.61E-01 27046
2.77E-01 27096 1.18E+00 27096 1.36E+00 27096 1.52E+00
27076 2.18E+00 27076 2.23E+00 27076 2.31E+00 27076
2.35E+00
27099 7.00E-02 27099 2.95E-01 27099 6.50E-01 27048
2.87E-01 27098 1.13E+00 27098 1.32E+00 27098 1.47E+00
27078 2.14E+00 27078 2.18E+00 27078 2.26E+00 27078
2.31E+00
27100 7.16E-02 27100 3.06E-01 27100 6.38E-01 27050
3.00E-01 27100 1.09E+00 27100 1.27E+00 27100 1.43E+00
27080 2.08E+00 27080 2.13E+00 27080 2.22E+00 27080
2.27E+00
27101 7.39E-02 27101 3.15E-01 27101 6.27E-01 27052
3.10E-01 27102 1.05E+00 27102 1.23E+00 27102 1.38E+00
27082 2.02E+00 27082 2.08E+00 27082 2.17E+00 27082
2.22E+00
27102 7.60E-02 27102 3.25E-01 27102 6.13E-01 27054
3.23E-01 27104 1.01E+00 27104 1.18E+00 27104 1.35E+00
27084 1.99E+00 27084 2.03E+00 27084 2.12E+00 27084
2.18E+00
27103 7.81E-02 27103 3.30E-01 27103 6.00E-01 27056
3.41E-01 27106 9.67E-01 27106 1.14E+00 27106 1.30E+00
27086 1.90E+00 27086 1.99E+00 27086 2.07E+00 27086
2.12E+00
27104 8.03E-02 27104 3.35E-01 27104 5.86E-01 27058
3.57E-01 27108 9.31E-01 27108 1.10E+00 27108 1.26E+00
27088 1.86E+00 27088 1.94E+00 27088 2.03E+00 27088
2.09E+00
27105 8.23E-02 27105 3.37E-01 27105 5.73E-01 27060
3.80E-01 27110 8.93E-01 27110 1.06E+00 27110 1.23E+00
27090 1.81E+00 27090 1.89E+00 27090 1.98E+00 27090
2.04E+00
27106 8.44E-02 27106 3.37E-01 27106 5.59E-01 27062
4.02E-01 27112 8.52E-01 27112 1.03E+00 27112 1.19E+00
27092 1.76E+00 27092 1.84E+00 27092 1.94E+00 27092
2.01E+00
27107 8.67E-02 27107 3.37E-01 27107 5.46E-01 27064
4.21E-01 27114 8.23E-01 27114 9.92E-01 27114 1.15E+00
27094 1.70E+00 27094 1.79E+00 27094 1.89E+00 27094

1.94E+00
27108 8.86E-02 27108 3.33E-01 27108 5.32E-01 27066
4.52E-01 27116 7.88E-01 27116 9.58E-01 27116 1.12E+00
27096 1.68E+00 27096 1.75E+00 27096 1.84E+00 27096
1.92E+00
27109 9.10E-02 27109 3.30E-01 27109 5.20E-01 27068
4.87E-01 27118 7.60E-01 27118 9.24E-01 27118 1.08E+00
27098 1.64E+00 27098 1.70E+00 27098 1.80E+00 27098
1.87E+00
27110 9.27E-02 27110 3.26E-01 27110 5.07E-01 27070
5.33E-01 27120 7.30E-01 27120 8.92E-01 27120 1.06E+00
27100 1.58E+00 27100 1.66E+00 27100 1.76E+00 27100
1.83E+00
27111 9.45E-02 27111 3.21E-01 27111 4.94E-01 27072
5.85E-01 27122 7.08E-01 27122 8.63E-01 27122 1.02E+00
27100 1.56E+00 27100 1.66E+00 27100 1.76E+00 27100
1.84E+00
27112 9.64E-02 27112 3.15E-01 27112 4.81E-01 27074
6.46E-01 27124 6.83E-01 27124 8.35E-01 27124 9.84E-01
27098 1.61E+00 27098 1.71E+00 27098 1.81E+00 27098
1.88E+00
27113 9.75E-02 27113 3.09E-01 27113 4.70E-01 27076
7.37E-01 27124 6.82E-01 27124 8.36E-01 27124 9.84E-01
27096 1.69E+00 27096 1.75E+00 27096 1.86E+00 27096
1.93E+00
27114 9.91E-02 27114 3.02E-01 27114 4.58E-01 27078
8.68E-01 27122 7.07E-01 27122 8.65E-01 27122 1.02E+00
27094 1.71E+00 27094 1.80E+00 27094 1.90E+00 27094
1.98E+00
27115 9.96E-02 27115 2.97E-01 27115 4.45E-01 27080
1.06E+00 27120 7.36E-01 27120 8.97E-01 27120 1.05E+00
27092 1.76E+00 27092 1.85E+00 27092 1.95E+00 27092
2.02E+00
27116 1.01E-01 27116 2.90E-01 27116 4.34E-01 27082
1.16E+00 27118 7.67E-01 27118 9.31E-01 27118 1.09E+00
27090 1.82E+00 27090 1.90E+00 27090 2.00E+00 27090
2.07E+00
27117 1.01E-01 27117 2.83E-01 27117 4.24E-01 27084
1.19E+00 27116 7.98E-01 27116 9.66E-01 27116 1.13E+00
27088 1.87E+00 27088 1.95E+00 27088 2.05E+00 27088
2.12E+00
27118 1.01E-01 27118 2.77E-01 27118 4.13E-01 27086
1.17E+00 27114 8.32E-01 27114 1.00E+00 27114 1.16E+00
27086 1.92E+00 27086 2.00E+00 27086 2.10E+00 27086
2.17E+00
27119 1.01E-01 27119 2.70E-01 27119 4.03E-01 27088
1.13E+00 27112 8.68E-01 27112 1.04E+00 27112 1.21E+00
27084 1.96E+00 27084 2.05E+00 27084 2.15E+00 27084
2.22E+00
27120 1.01E-01 27120 2.64E-01 27120 3.94E-01 27090
1.09E+00 27110 9.05E-01 27110 1.08E+00 27110 1.25E+00
27082 2.02E+00 27082 2.10E+00 27082 2.20E+00 27082
2.27E+00
27121 9.98E-02 27121 2.58E-01 27121 3.84E-01 27092
1.07E+00 27108 9.45E-01 27108 1.12E+00 27108 1.28E+00
27080 2.07E+00 27080 2.15E+00 27080 2.25E+00 27080
2.31E+00
27122 9.84E-02 27122 2.52E-01 27122 3.75E-01 27094
1.02E+00 27106 9.86E-01 27106 1.16E+00 27106 1.33E+00
27078 2.13E+00 27078 2.20E+00 27078 2.30E+00 27078
2.35E+00
27123 9.75E-02 27123 2.45E-01 27123 3.66E-01 27096
9.50E-01 27104 1.03E+00 27104 1.20E+00 27104 1.38E+00
27076 2.17E+00 27076 2.25E+00 27076 2.35E+00 27076
2.41E+00
27124 9.57E-02 27124 2.40E-01 27124 3.57E-01 27098
9.16E-01 27102 1.08E+00 27102 1.25E+00 27102 1.41E+00
27074 2.25E+00 27074 2.30E+00 27074 2.39E+00 27074

2.45E+00
27125 9.46E-02 27125 2.34E-01 27125 3.49E-01 27100
8.98E-01 27100 1.12E+00 27100 1.29E+00 27100 1.45E+00
27072 2.29E+00 27072 2.35E+00 27072 2.44E+00 27072
2.50E+00
27126 9.28E-02 27126 2.29E-01 27126 3.41E-01 27102
8.58E-01 27098 1.17E+00 27098 1.33E+00 27098 1.50E+00
27070 2.31E+00 27070 2.40E+00 27070 2.48E+00 27070
2.53E+00
27127 9.11E-02 27127 2.23E-01 27127 3.33E-01 27104
8.19E-01 27096 1.21E+00 27096 1.38E+00 27096 1.55E+00
27068 2.38E+00 27068 2.44E+00 27068 2.52E+00 27068
2.58E+00
27128 8.89E-02 27128 2.18E-01 27128 3.26E-01 27106
7.79E-01 27094 1.26E+00 27094 1.42E+00 27094 1.60E+00
27066 2.45E+00 27066 2.49E+00 27066 2.58E+00 27066
2.61E+00
27129 8.72E-02 27129 2.13E-01 27129 3.19E-01 27108
7.46E-01 27092 1.31E+00 27092 1.47E+00 27092 1.65E+00
27064 2.49E+00 27064 2.53E+00 27064 2.62E+00 27064
2.67E+00
27130 8.53E-02 27130 2.08E-01 27130 3.12E-01 27110
7.13E-01 27090 1.36E+00 27090 1.51E+00 27090 1.70E+00
27062 2.52E+00 27062 2.57E+00 27062 2.65E+00 27062
2.70E+00
27131 8.31E-02 27131 2.03E-01 27131 3.05E-01 27112
6.80E-01 27088 1.40E+00 27088 1.56E+00 27088 1.75E+00
27060 2.55E+00 27060 2.62E+00 27060 2.69E+00 27060
2.73E+00
27132 8.12E-02 27132 1.99E-01 27132 2.99E-01 27114
6.45E-01 27086 1.46E+00 27086 1.61E+00 27086 1.80E+00
27058 2.57E+00 27058 2.66E+00 27058 2.73E+00 27058
2.77E+00
27133 7.92E-02 27133 1.94E-01 27133 2.93E-01 27116
6.24E-01 27084 1.50E+00 27084 1.66E+00 27084 1.86E+00
27056 2.66E+00 27056 2.70E+00 27056 2.76E+00 27056
2.80E+00
27134 7.69E-02 27134 1.89E-01 27134 2.86E-01 27118
5.95E-01 27082 1.55E+00 27082 1.72E+00 27082 1.91E+00
27054 2.69E+00 27054 2.73E+00 27054 2.80E+00 27054
2.84E+00
27135 7.52E-02 27135 1.85E-01 27135 2.80E-01 27120
5.69E-01 27080 1.59E+00 27080 1.77E+00 27080 1.96E+00
27052 2.70E+00 27052 2.77E+00 27052 2.84E+00 27052
2.86E+00
27136 7.33E-02 27136 1.81E-01 27136 2.75E-01 27122
5.46E-01 27078 1.64E+00 27078 1.82E+00 27078 2.02E+00
27050 2.73E+00 27050 2.80E+00 27050 2.87E+00 27050
2.91E+00
27137 7.14E-02 27137 1.77E-01 27137 2.70E-01 27124
5.12E-01 27076 1.68E+00 27076 1.87E+00 27076 2.07E+00
27048 2.80E+00 27048 2.84E+00 27048 2.90E+00 27048
2.95E+00
27138 6.95E-02 27138 1.73E-01 27138 2.65E-01 27126
5.00E-01 27074 1.70E+00 27074 1.92E+00 27074 2.12E+00
27046 2.79E+00 27046 2.86E+00 27046 2.94E+00 27046
2.97E+00
27139 6.77E-02 27139 1.69E-01 27139 2.59E-01 27128
4.81E-01 27072 1.70E+00 27072 1.97E+00 27072 2.17E+00
27044 2.86E+00 27044 2.90E+00 27044 2.97E+00 27044
3.00E+00
27140 6.62E-02 27140 1.66E-01 27140 2.53E-01 27130
4.61E-01 27070 1.63E+00 27070 2.02E+00 27070 2.22E+00
27042 2.88E+00 27042 2.92E+00 27042 2.98E+00 27042
3.03E+00
27141 6.44E-02 27141 1.63E-01 27141 2.49E-01 27132
4.44E-01 27068 1.44E+00 27068 2.06E+00 27068 2.28E+00
27040 2.87E+00 27040 2.95E+00 27040 3.02E+00 27040

3.07E+00
27142 6.30E-02 27142 1.59E-01 27142 2.44E-01 27134
4.27E-01 27066 1.20E+00 27066 2.10E+00 27066 2.34E+00
27038 2.24E+00 27038 2.97E+00 27038 3.04E+00 27038
3.09E+00
27143 6.13E-02 27143 1.56E-01 27143 2.40E-01 27136
4.11E-01 27064 1.02E+00 27064 2.14E+00 27064 2.36E+00
27036 1.58E+00 27036 3.00E+00 27036 3.07E+00 27036
3.12E+00
27144 5.97E-02 27144 1.53E-01 27144 2.36E-01 27138
3.95E-01 27062 8.83E-01 27062 2.18E+00 27062 2.40E+00
27034 1.35E+00 27034 3.02E+00 27034 3.09E+00 27034
3.15E+00
27145 5.84E-02 27145 1.51E-01 27145 2.31E-01 27140
3.82E-01 27060 7.86E-01 27060 2.20E+00 27060 2.46E+00
27032 1.21E+00 27032 3.04E+00 27032 3.12E+00 27032
3.16E+00
27146 5.68E-02 27146 1.48E-01 27146 2.26E-01 27142
3.69E-01 27058 7.13E-01 27058 2.14E+00 27058 2.49E+00
27030 1.12E+00 27030 3.03E+00 27030 3.14E+00 27030
3.19E+00
27147 5.55E-02 27147 1.46E-01 27147 2.22E-01 27144
3.57E-01 27056 6.59E-01 27056 1.80E+00 27056 2.54E+00
27028 1.05E+00 27028 1.92E+00 27028 3.15E+00 27028
3.16E+00
27148 5.43E-02 27148 1.44E-01 27148 2.19E-01 27146
3.45E-01 27054 6.12E-01 27054 1.38E+00 27054 2.58E+00
27026 9.79E-01 27026 1.56E+00 27026 3.17E+00 27026
3.24E+00
27149 5.32E-02 27149 1.41E-01 27149 2.15E-01 27148
3.33E-01 27052 5.74E-01 27052 1.15E+00 27052 2.58E+00
27024 9.25E-01 27024 1.38E+00 27024 3.16E+00 27024
3.23E+00
27150 5.19E-02 27150 1.39E-01 27150 2.11E-01 27150
3.22E-01 27050 5.40E-01 27050 1.01E+00 27050 2.58E+00
27022 8.92E-01 27022 1.27E+00 27022 2.99E+00 27022
3.26E+00
27151 5.07E-02 27151 1.37E-01 27151 2.08E-01 27150
3.23E-01 27048 5.10E-01 27048 9.21E-01 27048 2.19E+00
27020 8.50E-01 27020 1.19E+00 27020 1.82E+00 27020
3.28E+00
27152 4.96E-02 27152 1.35E-01 27152 2.04E-01 27148
3.34E-01 27046 4.86E-01 27046 8.42E-01 27046 1.55E+00
27018 8.12E-01 27018 1.11E+00 27018 1.57E+00 27018
3.30E+00
27153 4.85E-02 27153 1.33E-01 27153 2.00E-01 27146
3.46E-01 27044 4.63E-01 27044 7.82E-01 27044 1.26E+00
27016 7.82E-01 27016 1.05E+00 27016 1.42E+00 27016
3.31E+00
27154 4.75E-02 27154 1.30E-01 27154 1.97E-01 27144
3.57E-01 27042 4.45E-01 27042 7.36E-01 27042 1.12E+00
27014 7.51E-01 27014 1.00E+00 27014 1.32E+00 27014
3.34E+00
27155 4.64E-02 27155 1.28E-01 27155 1.95E-01 27142
3.63E-01 27040 4.25E-01 27040 6.94E-01 27040 1.05E+00
27012 7.30E-01 27012 9.57E-01 27012 1.25E+00 27012
2.08E+00
27156 4.55E-02 27156 1.26E-01 27156 1.91E-01 27140
3.84E-01 27038 4.10E-01 27038 6.58E-01 27038 9.59E-01
27010 7.02E-01 27010 9.16E-01 27010 1.19E+00 27010
1.73E+00
27157 4.45E-02 27157 1.24E-01 27157 1.88E-01 27138
3.98E-01 27036 3.95E-01 27036 6.26E-01 27036 9.06E-01
27008 6.83E-01 27008 8.81E-01 27008 1.13E+00 27008
1.51E+00
27158 4.36E-02 27158 1.22E-01 27158 1.85E-01 27136
4.09E-01 27034 3.81E-01 27034 5.98E-01 27034 8.52E-01
27006 6.72E-01 27006 8.48E-01 27006 1.08E+00 27006

1.42E+00
27159 4.26E-02 27159 1.20E-01 27159 1.82E-01 27134
4.29E-01 27032 3.68E-01 27032 5.72E-01 27032 8.10E-01
27004 6.43E-01 27004 8.18E-01 27004 1.04E+00 27004
1.34E+00
27160 4.18E-02 27160 1.18E-01 27160 1.79E-01 27132
4.47E-01 27030 3.57E-01 27030 5.49E-01 27030 7.70E-01
27002 6.21E-01 27002 7.90E-01 27002 1.00E+00 27002
1.27E+00
27161 4.11E-02 27161 1.16E-01 27161 1.77E-01 27130
4.48E-01 27028 3.46E-01 27028 5.29E-01 27028 7.26E-01
27000 6.09E-01 27000 7.65E-01 27000 9.62E-01 27000
1.20E+00
27162 4.01E-02 27162 1.14E-01 27162 1.75E-01 27128
4.72E-01 27026 3.36E-01 27026 5.09E-01 27026 7.15E-01
26998 5.93E-01 26998 7.42E-01 26998 9.27E-01 26998
1.16E+00
27163 3.93E-02 27163 1.12E-01 27163 1.71E-01 27126
4.93E-01 27024 3.27E-01 27024 4.91E-01 27024 6.72E-01
26996 5.75E-01 26996 7.22E-01 26996 8.99E-01 26996
1.10E+00
27164 3.88E-02 27164 1.10E-01 27164 1.69E-01 27124
5.24E-01 27022 3.17E-01 27022 4.75E-01 27022 6.46E-01
26994 5.62E-01 26994 7.01E-01 26994 8.67E-01 26994
1.07E+00
27165 3.81E-02 27165 1.08E-01 27165 1.67E-01 27122
5.54E-01 27020 3.08E-01 27020 4.60E-01 27020 6.29E-01
26992 5.48E-01 26992 6.84E-01 26992 8.42E-01 26992
1.03E+00
27166 3.73E-02 27166 1.06E-01 27166 1.64E-01 27120
5.73E-01 27018 3.01E-01 27018 4.45E-01 27018 6.09E-01
26990 5.34E-01 26990 6.66E-01 26990 8.17E-01 26990
9.99E-01
27167 3.66E-02 27167 1.05E-01 27167 1.62E-01 27118
5.90E-01 27016 2.93E-01 27016 4.32E-01 27016 5.89E-01
26988 5.22E-01 26988 6.50E-01 26988 7.93E-01 26988
9.65E-01
27168 3.60E-02 27168 1.03E-01 27168 1.60E-01 27116
6.29E-01 27014 2.86E-01 27014 4.20E-01 27014 5.73E-01
26986 5.12E-01 26986 6.34E-01 26986 7.73E-01 26986
9.40E-01
27169 3.53E-02 27169 1.01E-01 27169 1.58E-01 27114
6.61E-01 27012 2.79E-01 27012 4.08E-01 27012 5.50E-01
26984 5.08E-01 26984 6.20E-01 26984 7.54E-01 26984
9.12E-01
27170 3.48E-02 27170 9.97E-02 27170 1.56E-01 27112
6.85E-01 27010 2.73E-01 27010 3.98E-01 27010 5.34E-01
26982 4.93E-01 26982 6.06E-01 26982 7.34E-01 26982
8.82E-01
27171 3.43E-02 27171 9.81E-02 27171 1.54E-01 27110
7.17E-01 27008 2.67E-01 27008 3.88E-01 27008 5.22E-01
26980 4.83E-01 26980 5.93E-01 26980 7.17E-01 26980
8.62E-01
27172 3.36E-02 27172 9.66E-02 27172 1.52E-01 27108
7.54E-01 27006 2.61E-01 27006 3.78E-01 27006 5.04E-01

27173 3.30E-02 27173 9.51E-02 27173 1.50E-01 27106
7.88E-01 27004 2.55E-01 27004 3.68E-01 27004 4.98E-01

27174 3.25E-02 27174 9.36E-02 27174 1.48E-01 27104
8.14E-01 27002 2.50E-01 27002 3.60E-01 27002 4.87E-01

27175 3.22E-02 27175 9.22E-02 27175 1.46E-01 27102
8.60E-01 27000 2.44E-01 27000 3.52E-01 27000 4.70E-01

27176 3.15E-02 27176 9.10E-02 27176 1.44E-01 27100
9.03E-01

27177	3.11E-02	27177	8.95E-02	27177	1.43E-01	27098	9.40E-01
27178	3.04E-02	27178	8.84E-02	27178	1.41E-01	27096	9.83E-01
27179	3.00E-02	27179	8.70E-02	27179	1.39E-01	27094	1.03E+00
27180	2.96E-02	27180	8.58E-02	27180	1.38E-01	27092	1.07E+00
27181	2.91E-02	27181	8.47E-02	27181	1.36E-01	27090	1.11E+00
27182	2.87E-02	27182	8.35E-02	27182	1.35E-01	27088	1.15E+00
27183	2.83E-02	27183	8.23E-02	27183	1.33E-01	27086	1.18E+00
27184	2.78E-02	27184	8.12E-02	27184	1.33E-01	27084	1.17E+00
27185	2.75E-02	27185	8.01E-02	27185	1.30E-01	27082	1.17E+00
27186	2.72E-02	27186	7.90E-02	27186	1.29E-01	27080	1.11E+00
27187	2.68E-02	27187	7.80E-02	27187	1.27E-01	27078	1.01E+00
27188	2.63E-02	27188	7.70E-02	27188	1.27E-01	27076	8.77E-01
27189	2.61E-02	27189	7.60E-02	27189	1.25E-01	27074	7.49E-01
27190	2.57E-02	27190	7.51E-02	27190	1.23E-01	27072	6.57E-01
27191	2.54E-02	27191	7.41E-02	27191	1.22E-01	27070	5.88E-01
27192	2.49E-02	27192	7.33E-02	27192	1.21E-01	27068	5.52E-01
27193	2.47E-02	27193	7.22E-02	27193	1.19E-01	27066	4.93E-01
27194	2.44E-02	27194	7.14E-02	27194	1.18E-01	27064	4.62E-01
27195	2.40E-02	27195	7.05E-02	27195	1.17E-01	27062	4.31E-01
27196	2.37E-02	27196	6.98E-02	27196	1.16E-01	27060	4.02E-01
27197	2.35E-02	27197	6.90E-02	27197	1.14E-01	27058	3.80E-01
27198	2.33E-02	27198	6.82E-02	27198	1.13E-01	27056	3.61E-01
27199	2.29E-02	27199	6.74E-02	27199	1.12E-01	27054	3.42E-01

27200	2.26E-02	27200	6.66E-02	27200	1.11E-01	27052	3.27E-01
27201	2.23E-02	27201	6.59E-02	27201	1.10E-01	27050	3.13E-01
27202	2.20E-02	27202	6.49E-02	27202	1.09E-01	27048	2.99E-01
27203	2.19E-02	27203	6.44E-02	27203	1.07E-01	27046	2.88E-01
27204	2.17E-02	27204	6.37E-02	27204	1.06E-01	27044	2.78E-01
27205	2.15E-02	27205	6.29E-02	27205	1.05E-01	27042	2.68E-01
27206	2.11E-02	27206	6.23E-02	27206	1.04E-01	27040	2.59E-01
27207	2.09E-02	27207	6.17E-02	27207	1.03E-01	27038	2.52E-01
27208	2.06E-02	27208	6.09E-02	27208	1.02E-01	27036	2.43E-01
27209	2.05E-02	27209	6.04E-02	27209	1.01E-01	27034	2.36E-01
27210	2.02E-02	27210	5.99E-02	27210	1.00E-01	27032	2.28E-01
27211	2.00E-02	27211	5.93E-02	27211	9.93E-02	27030	2.22E-01
27212	1.98E-02	27212	5.85E-02	27212	9.82E-02	27028	2.16E-01
27213	1.95E-02	27213	5.81E-02	27213	9.74E-02	27026	2.10E-01
27214	1.93E-02	27214	5.72E-02	27214	9.65E-02	27024	2.04E-01
27215	1.92E-02	27215	5.68E-02	27215	9.55E-02	27022	1.99E-01
27216	1.90E-02	27216	5.62E-02	27216	9.45E-02	27020	1.94E-01
27217	1.88E-02	27217	5.57E-02	27217	9.38E-02	27018	1.89E-01
27218	1.86E-02	27218	5.51E-02	27218	9.31E-02	27016	1.85E-01
27219	1.84E-02	27219	5.46E-02	27219	9.20E-02	27014	1.80E-01
27220	1.82E-02	27220	5.41E-02	27220	9.13E-02	27012	1.77E-01
27221	1.81E-02	27221	5.35E-02	27221	9.03E-02	27010	1.72E-01
27222	1.79E-02	27222	5.29E-02	27222	8.99E-02	27008	

1.69E-01

27223	1.77E-02	27223	5.26E-02	27223	8.85E-02	27006	1.66E-01
27224	1.75E-02	27224	5.21E-02	27224	8.78E-02	27004	1.62E-01
27225	1.73E-02	27225	5.17E-02	27225	8.74E-02	27002	1.58E-01
27226	1.72E-02	27226	5.11E-02	27226	8.67E-02	27000	1.55E-01
27227	1.70E-02	27227	5.07E-02	27227	8.56E-02	26998	1.53E-01
27228	1.69E-02	27228	5.01E-02	27228	8.49E-02	26996	1.49E-01
27229	1.67E-02	27229	4.97E-02	27229	8.42E-02	26994	1.47E-01
27230	1.67E-02	27230	4.93E-02	27230	8.35E-02	26992	1.43E-01
27231	1.65E-02	27231	4.90E-02	27231	8.29E-02	26990	1.42E-01
27232	1.63E-02	27232	4.85E-02	27232	8.20E-02	26988	1.39E-01
27233	1.61E-02	27233	4.81E-02	27233	8.13E-02	26986	1.37E-01
27234	1.60E-02	27234	4.76E-02	27234	8.08E-02	26984	1.35E-01
27235	1.58E-02	27235	4.72E-02	27235	8.00E-02	26982	1.33E-01
27236	1.57E-02	27236	4.70E-02	27236	7.97E-02	26980	1.31E-01
27237	1.56E-02	27237	4.64E-02	27237	7.88E-02	26978	1.30E-01
27238	1.55E-02	27238	4.61E-02	27238	7.82E-02	26976	1.27E-01
27239	1.53E-02	27239	4.57E-02	27239	7.76E-02	26974	1.25E-01
27240	1.52E-02	27240	4.53E-02	27240	7.70E-02	26972	1.24E-01
27241	1.50E-02	27241	4.50E-02	27241	7.62E-02	26970	1.22E-01
27242	1.50E-02	27242	4.46E-02	27242	7.56E-02	26968	1.20E-01
27243	1.48E-02	27243	4.44E-02	27243	7.52E-02	26966	1.17E-01
27244	1.47E-02	27244	4.40E-02	27244	7.46E-02	26964	1.16E-01

27245	1.46E-02	27245	4.36E-02	27245	7.40E-02	26962	1.14E-01
27246	1.45E-02	27246	4.33E-02	27246	7.34E-02	26960	1.13E-01
27247	1.44E-02	27247	4.29E-02	27247	7.30E-02		
27248	1.42E-02	27248	4.27E-02	27248	7.23E-02		
27249	1.41E-02	27249	4.22E-02	27249	7.21E-02		
27250	1.41E-02	27250	4.20E-02	27250	7.12E-02		
27250	1.40E-02	27250	4.19E-02	27250	7.10E-02		
27249	1.41E-02	27249	4.22E-02	27249	7.17E-02		
27248	1.42E-02	27248	4.25E-02	27248	7.22E-02		
27247	1.44E-02	27247	4.29E-02	27247	7.25E-02		
27246	1.44E-02	27246	4.32E-02	27246	7.32E-02		
27245	1.46E-02	27245	4.36E-02	27245	7.40E-02		
27244	1.48E-02	27244	4.40E-02	27244	7.48E-02		
27243	1.48E-02	27243	4.44E-02	27243	7.55E-02		
27242	1.49E-02	27242	4.47E-02	27242	7.58E-02		
27241	1.50E-02	27241	4.50E-02	27241	7.64E-02		
27240	1.51E-02	27240	4.54E-02	27240	7.69E-02		
27239	1.53E-02	27239	4.58E-02	27239	7.73E-02		
27238	1.55E-02	27238	4.62E-02	27238	7.82E-02		
27237	1.56E-02	27237	4.66E-02	27237	7.88E-02		
27236	1.58E-02	27236	4.69E-02	27236	7.92E-02		
27235	1.58E-02	27235	4.74E-02	27235	8.01E-02		
27234	1.61E-02	27234	4.77E-02	27234	8.09E-02		

27233	1.61E-02	27233	4.81E-02	27233	8.16E-02
27232	1.63E-02	27232	4.86E-02	27232	8.23E-02
27231	1.64E-02	27231	4.90E-02	27231	8.31E-02
27230	1.66E-02	27230	4.95E-02	27230	8.38E-02
27229	1.67E-02	27229	4.99E-02	27229	8.44E-02
27228	1.70E-02	27228	5.04E-02	27228	8.52E-02
27227	1.70E-02	27227	5.09E-02	27227	8.61E-02
27226	1.71E-02	27226	5.13E-02	27226	8.67E-02
27225	1.74E-02	27225	5.17E-02	27225	8.75E-02
27224	1.77E-02	27224	5.22E-02	27224	8.82E-02
27223	1.77E-02	27223	5.28E-02	27223	8.88E-02
27222	1.79E-02	27222	5.32E-02	27222	8.98E-02
27221	1.81E-02	27221	5.37E-02	27221	9.07E-02
27220	1.82E-02	27220	5.43E-02	27220	9.16E-02
27219	1.84E-02	27219	5.48E-02	27219	9.24E-02
27218	1.86E-02	27218	5.53E-02	27218	9.34E-02
27217	1.88E-02	27217	5.59E-02	27217	9.45E-02
27216	1.90E-02	27216	5.65E-02	27216	9.48E-02
27215	1.93E-02	27215	5.70E-02	27215	9.60E-02
27214	1.94E-02	27214	5.76E-02	27214	9.68E-02
27213	1.96E-02	27213	5.81E-02	27213	9.80E-02
27212	1.97E-02	27212	5.87E-02	27212	9.86E-02
27211	2.01E-02	27211	5.94E-02	27211	9.99E-02

27210	2.03E-02	27210	6.01E-02	27210	1.01E-01
27209	2.05E-02	27209	6.07E-02	27209	1.02E-01
27208	2.07E-02	27208	6.14E-02	27208	1.03E-01
27207	2.09E-02	27207	6.21E-02	27207	1.04E-01
27206	2.12E-02	27206	6.28E-02	27206	1.05E-01
27205	2.15E-02	27205	6.34E-02	27205	1.06E-01
27204	2.17E-02	27204	6.43E-02	27204	1.07E-01
27203	2.19E-02	27203	6.50E-02	27203	1.08E-01
27202	2.22E-02	27202	6.56E-02	27202	1.10E-01
27201	2.25E-02	27201	6.64E-02	27201	1.11E-01
27200	2.27E-02	27200	6.71E-02	27200	1.12E-01
27199	2.30E-02	27199	6.78E-02	27199	1.13E-01
27198	2.32E-02	27198	6.88E-02	27198	1.14E-01
27197	2.36E-02	27197	6.97E-02	27197	1.16E-01
27196	2.38E-02	27196	7.06E-02	27196	1.17E-01
27195	2.40E-02	27195	7.13E-02	27195	1.18E-01
27194	2.45E-02	27194	7.22E-02	27194	1.19E-01
27193	2.48E-02	27193	7.31E-02	27193	1.20E-01
27192	2.51E-02	27192	7.40E-02	27192	1.22E-01
27191	2.54E-02	27191	7.50E-02	27191	1.23E-01
27190	2.58E-02	27190	7.58E-02	27190	1.24E-01
27189	2.60E-02	27189	7.68E-02	27189	1.26E-01

27188	2.65E-02	27188	7.79E-02	27188	1.27E-01
27187	2.68E-02	27187	7.90E-02	27187	1.29E-01
27186	2.72E-02	27186	8.01E-02	27186	1.30E-01
27185	2.76E-02	27185	8.11E-02	27185	1.32E-01
27184	2.79E-02	27184	8.21E-02	27184	1.33E-01
27183	2.85E-02	27183	8.34E-02	27183	1.34E-01
27182	2.88E-02	27182	8.45E-02	27182	1.35E-01
27181	2.93E-02	27181	8.57E-02	27181	1.37E-01
27180	2.98E-02	27180	8.69E-02	27180	1.39E-01
27179	3.01E-02	27179	8.82E-02	27179	1.40E-01
27178	3.06E-02	27178	8.95E-02	27178	1.42E-01
27177	3.12E-02	27177	9.09E-02	27177	1.44E-01
27176	3.17E-02	27176	9.23E-02	27176	1.46E-01
27175	3.21E-02	27175	9.36E-02	27175	1.48E-01
27174	3.26E-02	27174	9.50E-02	27174	1.49E-01
27173	3.30E-02	27173	9.64E-02	27173	1.52E-01
27172	3.39E-02	27172	9.78E-02	27172	1.53E-01
27171	3.43E-02	27171	9.96E-02	27171	1.55E-01
27170	3.50E-02	27170	1.01E-01	27170	1.58E-01
27169	3.55E-02	27169	1.03E-01	27169	1.60E-01
27168	3.60E-02	27168	1.04E-01	27168	1.62E-01
27167	3.69E-02	27167	1.06E-01	27167	1.64E-01
27166	3.76E-02	27166	1.08E-01	27166	1.66E-01

27165	3.83E-02	27165	1.10E-01	27165	1.69E-01
27164	3.91E-02	27164	1.11E-01	27164	1.72E-01
27163	3.98E-02	27163	1.13E-01	27163	1.74E-01
27162	4.04E-02	27162	1.15E-01	27162	1.77E-01
27161	4.13E-02	27161	1.17E-01	27161	1.79E-01
27160	4.20E-02	27160	1.19E-01	27160	1.82E-01
27159	4.31E-02	27159	1.21E-01	27159	1.85E-01
27158	4.40E-02	27158	1.23E-01	27158	1.87E-01
27157	4.50E-02	27157	1.26E-01	27157	1.91E-01
27156	4.58E-02	27156	1.28E-01	27156	1.95E-01
27155	4.68E-02	27155	1.30E-01	27155	1.97E-01
27154	4.79E-02	27154	1.32E-01	27154	2.00E-01
27153	4.89E-02	27153	1.34E-01	27153	2.04E-01
27152	5.04E-02	27152	1.36E-01	27152	2.08E-01
27151	5.14E-02	27151	1.39E-01	27151	2.11E-01
27150	5.28E-02	27150	1.41E-01	27150	2.15E-01
27149	5.36E-02	27149	1.44E-01	27149	2.18E-01
27148	5.50E-02	27148	1.46E-01	27148	2.22E-01
27147	5.63E-02	27147	1.48E-01	27147	2.26E-01
27146	5.76E-02	27146	1.50E-01	27146	2.31E-01
27145	5.94E-02	27145	1.53E-01	27145	2.35E-01
27144	6.10E-02	27144	1.55E-01	27144	2.39E-01
27143	6.23E-02	27143	1.58E-01	27143	2.44E-01

27142	6.38E-02	27142	1.61E-01	27142	2.48E-01
27141	6.57E-02	27141	1.64E-01	27141	2.53E-01
27140	6.75E-02	27140	1.67E-01	27140	2.59E-01
27139	6.92E-02	27139	1.71E-01	27139	2.64E-01
27138	7.08E-02	27138	1.75E-01	27138	2.70E-01
27137	7.27E-02	27137	1.78E-01	27137	2.75E-01
27136	7.46E-02	27136	1.82E-01	27136	2.80E-01
27135	7.63E-02	27135	1.86E-01	27135	2.86E-01
27134	7.80E-02	27134	1.90E-01	27134	2.92E-01
27133	8.04E-02	27133	1.95E-01	27133	2.99E-01
27132	8.23E-02	27132	1.99E-01	27132	3.06E-01
27131	8.45E-02	27131	2.03E-01	27131	3.11E-01
27130	8.63E-02	27130	2.09E-01	27130	3.18E-01
27129	8.81E-02	27129	2.13E-01	27129	3.25E-01
27128	9.02E-02	27128	2.18E-01	27128	3.32E-01
27127	9.20E-02	27127	2.24E-01	27127	3.40E-01
27126	9.42E-02	27126	2.29E-01	27126	3.47E-01
27125	9.53E-02	27125	2.33E-01	27125	3.57E-01
27124	9.69E-02	27124	2.39E-01	27124	3.65E-01
27123	9.82E-02	27123	2.45E-01	27123	3.74E-01
27122	1.01E-01	27122	2.51E-01	27122	3.82E-01
27121	1.01E-01	27121	2.56E-01	27121	3.91E-01

27120	1.01E-01	27120	2.62E-01	27120	4.01E-01
27119	1.02E-01	27119	2.68E-01	27119	4.11E-01
27118	1.01E-01	27118	2.74E-01	27118	4.21E-01
27117	1.02E-01	27117	2.80E-01	27117	4.31E-01
27116	1.01E-01	27116	2.87E-01	27116	4.42E-01
27115	1.00E-01	27115	2.94E-01	27115	4.54E-01
27114	9.95E-02	27114	3.00E-01	27114	4.65E-01
27113	9.81E-02	27113	3.06E-01	27113	4.78E-01
27112	9.70E-02	27112	3.13E-01	27112	4.91E-01
27111	9.50E-02	27111	3.17E-01	27111	5.04E-01
27110	9.33E-02	27110	3.22E-01	27110	5.18E-01
27109	9.13E-02	27109	3.27E-01	27109	5.30E-01
27108	8.97E-02	27108	3.31E-01	27108	5.42E-01
27107	8.79E-02	27107	3.32E-01	27107	5.55E-01
27106	8.60E-02	27106	3.35E-01	27106	5.68E-01
27105	8.36E-02	27105	3.35E-01	27105	5.83E-01
27104	8.09E-02	27104	3.34E-01	27104	5.95E-01
27103	7.91E-02	27103	3.32E-01	27103	6.10E-01
27102	7.69E-02	27102	3.29E-01	27102	6.21E-01
27101	7.49E-02	27101	3.23E-01	27101	6.32E-01
27100	7.28E-02	27100	3.17E-01	27100	6.44E-01
27099	7.15E-02	27099	3.09E-01	27099	6.54E-01
27098	6.95E-02	27098	3.01E-01	27098	6.63E-01

27097	6.75E-02	27097	2.91E-01	27097	6.73E-01
27096	6.53E-02	27096	2.81E-01	27096	6.76E-01
27095	6.37E-02	27095	2.70E-01	27095	6.75E-01
27094	6.23E-02	27094	2.60E-01	27094	6.70E-01
27093	6.04E-02	27093	2.50E-01	27093	6.64E-01
27092	5.87E-02	27092	2.40E-01	27092	6.52E-01
27091	5.73E-02	27091	2.31E-01	27091	6.37E-01
27090	5.58E-02	27090	2.22E-01	27090	6.17E-01
27089	5.45E-02	27089	2.14E-01	27089	5.94E-01
27088	5.33E-02	27088	2.06E-01	27088	5.68E-01
27087	5.21E-02	27087	1.98E-01	27087	5.39E-01
27086	5.08E-02	27086	1.92E-01	27086	5.12E-01
27085	4.95E-02	27085	1.85E-01	27085	4.85E-01
27084	4.83E-02	27084	1.79E-01	27084	4.58E-01
27083	4.70E-02	27083	1.73E-01	27083	4.33E-01
27082	4.62E-02	27082	1.67E-01	27082	4.12E-01
27081	4.51E-02	27081	1.61E-01	27081	3.91E-01
27080	4.44E-02	27080	1.56E-01	27080	3.73E-01
27079	4.34E-02	27079	1.51E-01	27079	3.57E-01
27078	4.24E-02	27078	1.47E-01	27078	3.42E-01
27077	4.19E-02	27077	1.43E-01	27077	3.28E-01
27076	4.09E-02	27076	1.39E-01	27076	3.15E-01
27075	3.99E-02	27075	1.35E-01	27075	3.04E-01

27074	3.91E-02	27074	1.32E-01	27074	2.93E-01
27073	3.87E-02	27073	1.29E-01	27073	2.83E-01
27072	3.77E-02	27072	1.27E-01	27072	2.76E-01
27071	3.72E-02	27071	1.24E-01	27071	2.66E-01
27070	3.69E-02	27070	1.21E-01	27070	2.57E-01
27069	3.58E-02	27069	1.18E-01	27069	2.49E-01
27068	3.54E-02	27068	1.16E-01	27068	2.42E-01
27067	3.44E-02	27067	1.13E-01	27067	2.36E-01
27066	3.40E-02	27066	1.12E-01	27066	2.30E-01
27065	3.38E-02	27065	1.09E-01	27065	2.24E-01
27064	3.30E-02	27064	1.07E-01	27064	2.18E-01
27063	3.25E-02	27063	1.05E-01	27063	2.13E-01
27062	3.19E-02	27062	1.03E-01	27062	2.08E-01
27061	3.16E-02	27061	1.02E-01	27061	2.02E-01
27060	3.11E-02	27060	9.99E-02	27060	1.98E-01
27059	3.06E-02	27059	9.81E-02	27059	1.94E-01
27058	3.02E-02	27058	9.64E-02	27058	1.89E-01
27057	2.98E-02	27057	9.50E-02	27057	1.86E-01
27056	2.92E-02	27056	9.33E-02	27056	1.83E-01
27055	2.89E-02	27055	9.20E-02	27055	1.78E-01
27054	2.85E-02	27054	9.06E-02	27054	1.74E-01
27053	2.79E-02	27053	8.91E-02	27053	1.72E-01

27052 2.76E-02 27052 8.80E-02 27052 1.67E-01

27051 2.73E-02 27051 8.65E-02 27051 1.65E-01

27050 2.72E-02 27050 8.53E-02 27050 1.62E-01

OT7
2V (LDV 125mm/s/V) 6V (LDV 125mm/s/V) 10V (LDV
125mm/s/V) 15V (LDV 125mm/s/V) 20V (LDV 125mm/s/V)
25V (LDV 125mm/s/V) 30V (LDV 125mm/s/V) 45V (LDV
125mm/s/V) 50V (LDV 125mm/s/V)
Frequency (Hz) Response (V) Frequency (Hz) Response (V) Frequency (Hz)
Response (V) Frequency (Hz) Response (V) Frequency (Hz) Response (V)
Frequency (Hz) Response (V) Frequency (Hz) Response (V) Frequency (Hz)
Response (V) Frequency (Hz) Response (V)
26990 1.00E-01 26990 3.52E-01 26940 4.15E-01 26900
4.80E-01 26900 7.75E-01 26820 6.16E-01 26850 9.79E-01
26750 8.72E-01 26740 9.78E-01
26991 1.01E-01 26991 3.60E-01 26941 4.21E-01 26901
5.00E-01 26901 7.86E-01 26822 6.28E-01 26852 1.08E+00
26752 1.05E+00 26742 1.20E+00
26992 1.02E-01 26992 3.66E-01 26942 4.28E-01 26902
5.08E-01 26902 7.96E-01 26824 6.35E-01 26854 1.13E+00
26754 1.10E+00 26744 1.26E+00
26993 1.03E-01 26993 3.76E-01 26943 4.35E-01 26903
5.13E-01 26903 8.07E-01 26826 6.43E-01 26856 1.16E+00
26756 1.12E+00 26745 1.28E+00
26994 1.04E-01 26994 3.83E-01 26944 4.43E-01 26904
5.18E-01 26904 8.18E-01 26828 6.51E-01 26858 1.19E+00
26758 1.13E+00 26747 1.29E+00
26995 1.05E-01 26995 3.92E-01 26945 4.51E-01 26905
5.22E-01 26905 8.28E-01 26830 6.59E-01 26860 1.22E+00
26760 1.15E+00 26749 1.31E+00
26996 1.07E-01 26996 4.02E-01 26946 4.59E-01 26906
5.27E-01 26906 8.40E-01 26832 6.67E-01 26862 1.25E+00
26762 1.16E+00 26751 1.32E+00
26997 1.08E-01 26997 4.13E-01 26947 4.67E-01 26907
5.31E-01 26907 8.51E-01 26834 6.76E-01 26864 1.29E+00
26764 1.17E+00 26752 1.33E+00
26998 1.11E-01 26998 4.23E-01 26948 4.76E-01 26908
5.36E-01 26908 8.63E-01 26836 6.85E-01 26866 1.32E+00
26766 1.19E+00 26754 1.35E+00
26999 1.12E-01 26999 4.35E-01 26949 4.85E-01 26909
5.41E-01 26909 8.75E-01 26838 6.94E-01 26868 1.36E+00
26768 1.20E+00 26756 1.36E+00
27000 1.13E-01 27000 4.47E-01 26950 4.95E-01 26910
5.47E-01 26910 8.88E-01 26840 7.03E-01 26870 1.41E+00
26770 1.22E+00 26758 1.38E+00
27001 1.15E-01 27001 4.61E-01 26951 5.06E-01 26911
5.51E-01 26911 9.02E-01 26842 7.13E-01 26872 1.45E+00
26772 1.23E+00 26759 1.39E+00
27002 1.17E-01 27002 4.76E-01 26952 5.16E-01 26912
5.57E-01 26912 9.16E-01 26844 7.23E-01 26874 1.51E+00
26774 1.25E+00 26761 1.40E+00
27003 1.19E-01 27003 4.92E-01 26953 5.28E-01 26913
5.63E-01 26913 9.31E-01 26846 7.34E-01 26876 1.58E+00
26776 1.26E+00 26763 1.42E+00
27004 1.20E-01 27004 5.08E-01 26954 5.40E-01 26914
5.69E-01 26914 9.46E-01 26848 7.45E-01 26878 1.65E+00
26778 1.28E+00 26765 1.44E+00
27005 1.23E-01 27005 5.26E-01 26955 5.54E-01 26915
5.75E-01 26915 9.62E-01 26850 7.56E-01 26880 1.74E+00
26780 1.30E+00 26766 1.45E+00

27006 1.24E-01 27006 5.48E-01 26956 5.67E-01 26916
5.82E-01 26916 9.79E-01 26852 7.68E-01 26882 1.86E+00
26783 1.32E+00 26768 1.47E+00
27007 1.27E-01 27007 5.70E-01 26957 5.83E-01 26917
5.88E-01 26917 9.98E-01 26854 7.80E-01 26884 2.06E+00
26785 1.33E+00 26770 1.49E+00
27008 1.29E-01 27008 5.91E-01 26958 5.99E-01 26918
5.95E-01 26918 1.02E+00 26856 7.93E-01 26886 3.69E+00
26787 1.35E+00 26772 1.51E+00
27009 1.31E-01 27009 6.15E-01 26959 6.16E-01 26919
6.02E-01 26919 1.04E+00 26858 8.06E-01 26888 3.23E+00
26789 1.37E+00 26774 1.53E+00
27010 1.33E-01 27010 6.38E-01 26960 6.35E-01 26920
6.09E-01 26920 1.06E+00 26860 8.20E-01 26890 3.10E+00
26791 1.40E+00 26775 1.55E+00
27011 1.36E-01 27011 6.59E-01 26961 6.55E-01 26921
6.17E-01 26921 1.08E+00 26862 8.35E-01 26892 3.32E+00
26793 1.42E+00 26777 1.57E+00
27012 1.38E-01 27012 6.80E-01 26962 6.78E-01 26922
6.24E-01 26922 1.11E+00 26864 8.51E-01 26894 3.72E+00
26795 1.44E+00 26779 1.59E+00
27013 1.42E-01 27013 6.96E-01 26963 7.05E-01 26923
6.33E-01 26923 1.13E+00 26866 8.67E-01 26896 3.69E+00
26797 1.46E+00 26781 1.61E+00
27014 1.44E-01 27014 7.14E-01 26964 7.34E-01 26924
6.41E-01 26924 1.16E+00 26868 8.85E-01 26898 3.93E+00
26799 1.49E+00 26782 1.64E+00
27015 1.46E-01 27015 7.24E-01 26965 7.66E-01 26925
6.50E-01 26925 1.20E+00 26870 9.05E-01 26900 4.13E+00
26801 1.52E+00 26784 1.66E+00
27016 1.48E-01 27016 7.32E-01 26966 8.01E-01 26926
6.60E-01 26926 1.23E+00 26872 9.27E-01 26902 4.10E+00
26803 1.54E+00 26786 1.69E+00
27017 1.51E-01 27017 7.38E-01 26967 8.46E-01 26927
6.69E-01 26927 1.28E+00 26874 9.53E-01 26904 4.06E+00
26805 1.57E+00 26788 1.72E+00
27018 1.54E-01 27018 7.41E-01 26968 8.96E-01 26928
6.79E-01 26928 1.33E+00 26876 9.81E-01 26906 4.02E+00
26807 1.61E+00 26789 1.74E+00
27019 1.57E-01 27019 7.43E-01 26969 9.71E-01 26929
6.89E-01 26929 1.40E+00 26878 1.01E+00 26908 3.98E+00
26809 1.64E+00 26791 1.78E+00
27020 1.60E-01 27020 7.42E-01 26970 1.03E+00 26930
7.00E-01 26930 1.52E+00 26880 1.04E+00 26910 3.94E+00
26811 1.67E+00 26793 1.81E+00
27021 1.63E-01 27021 7.39E-01 26971 1.11E+00 26931
7.11E-01 26931 3.31E+00 26882 1.07E+00 26912 3.90E+00
26813 1.71E+00 26795 1.84E+00
27022 1.66E-01 27022 7.34E-01 26972 1.20E+00 26932
7.24E-01 26932 3.27E+00 26884 1.10E+00 26914 3.86E+00
26815 1.75E+00 26796 1.88E+00
27023 1.69E-01 27023 7.31E-01 26973 1.28E+00 26933
7.36E-01 26933 3.24E+00 26886 1.13E+00 26916 3.82E+00
26817 1.80E+00 26798 1.92E+00
27024 1.72E-01 27024 7.24E-01 26974 1.33E+00 26934
7.50E-01 26934 3.21E+00 26888 1.17E+00 26918 3.78E+00
26819 1.85E+00 26800 1.96E+00
27025 1.74E-01 27025 7.20E-01 26975 1.36E+00 26935
7.63E-01 26935 3.18E+00 26890 1.21E+00 26920 3.74E+00
26821 1.90E+00 26802 2.01E+00
27026 1.78E-01 27026 7.12E-01 26976 1.36E+00 26936
7.79E-01 26936 3.15E+00 26892 1.26E+00 26920 3.74E+00
26823 1.96E+00 26804 2.06E+00
27027 1.80E-01 27027 7.06E-01 26977 1.36E+00 26937
7.94E-01 26937 3.12E+00 26894 1.31E+00 26918 3.78E+00
26825 2.03E+00 26805 2.13E+00
27028 1.83E-01 27028 6.98E-01 26978 1.35E+00 26938
8.11E-01 26938 3.10E+00 26896 1.37E+00 26916 3.82E+00

26827 2.12E+00 26807 2.19E+00
27029 1.85E-01 27029 6.91E-01 26979 1.34E+00 26939
8.29E-01 26939 3.07E+00 26898 1.44E+00 26914 3.85E+00
26829 2.22E+00 26809 2.28E+00
27030 1.88E-01 27030 6.83E-01 26980 1.33E+00 26940
8.48E-01 26940 3.05E+00 26900 1.53E+00 26912 3.89E+00
26831 2.37E+00 26811 2.38E+00
27031 1.91E-01 27031 6.75E-01 26981 1.32E+00 26941
8.68E-01 26941 3.02E+00 26902 1.64E+00 26910 3.93E+00
26833 2.65E+00 26812 2.52E+00
27032 1.93E-01 27032 6.67E-01 26982 1.31E+00 26942
8.90E-01 26942 3.00E+00 26904 1.81E+00 26908 3.97E+00
26835 5.13E+00 26814 2.81E+00
27033 1.94E-01 27033 6.59E-01 26983 1.30E+00 26943
9.14E-01 26943 2.98E+00 26906 3.99E+00 26906 4.00E+00
26837 5.07E+00 26816 5.54E+00
27034 1.97E-01 27034 6.50E-01 26984 1.28E+00 26944
9.40E-01 26944 2.95E+00 26908 3.93E+00 26904 4.03E+00
26839 5.02E+00 26818 5.50E+00
27035 1.98E-01 27035 6.42E-01 26985 1.27E+00 26945
9.71E-01 26945 2.93E+00 26910 3.88E+00 26902 4.06E+00
26841 4.96E+00 26819 5.46E+00
27036 2.00E-01 27036 6.34E-01 26986 1.25E+00 26946
1.00E+00 26946 2.91E+00 26912 3.84E+00 26900 4.09E+00
26843 4.91E+00 26821 5.42E+00
27037 2.01E-01 27037 6.26E-01 26987 1.24E+00 26947
1.04E+00 26947 2.88E+00 26914 3.80E+00 26898 4.13E+00
26845 4.85E+00 26823 5.38E+00
27038 2.01E-01 27038 6.17E-01 26988 1.22E+00 26948
1.09E+00 26948 2.86E+00 26916 3.75E+00 26896 4.16E+00
26848 4.81E+00 26825 5.35E+00
27039 2.02E-01 27039 6.09E-01 26989 1.21E+00 26949
1.15E+00 26949 2.84E+00 26918 3.71E+00 26894 4.20E+00
26850 4.74E+00 26826 5.31E+00
27040 2.03E-01 27040 6.02E-01 26990 1.20E+00 26950
1.25E+00 26950 2.82E+00 26920 3.66E+00 26892 4.23E+00
26852 4.68E+00 26828 5.27E+00
27041 2.03E-01 27041 5.94E-01 26991 1.18E+00 26951
2.45E+00 26951 2.79E+00 26920 3.66E+00 26890 3.87E+00
26854 4.63E+00 26830 5.23E+00
27042 2.03E-01 27042 5.85E-01 26992 1.17E+00 26952
2.45E+00 26952 2.77E+00 26918 3.70E+00 26888 3.85E+00
26856 4.55E+00 26832 5.20E+00
27043 2.03E-01 27043 5.77E-01 26993 1.16E+00 26953
2.42E+00 26953 2.75E+00 26916 3.74E+00 26886 3.06E+00
26858 4.52E+00 26834 5.16E+00
27044 2.03E-01 27044 5.70E-01 26994 1.14E+00 26954
2.39E+00 26954 2.73E+00 26914 3.77E+00 26884 3.05E+00
26860 4.44E+00 26835 5.13E+00
27045 2.02E-01 27045 5.62E-01 26995 1.13E+00 26955
2.36E+00 26955 2.71E+00 26912 3.81E+00 26882 3.09E+00
26862 4.39E+00 26837 5.08E+00
27046 2.00E-01 27046 5.54E-01 26996 1.12E+00 26956
2.34E+00 26956 2.68E+00 26910 3.85E+00 26880 3.04E+00
26864 4.32E+00 26839 5.05E+00
27047 2.00E-01 27047 5.46E-01 26997 1.10E+00 26957
2.32E+00 26957 2.66E+00 26908 3.88E+00 26878 3.13E+00
26866 4.26E+00 26841 5.01E+00
27048 1.99E-01 27048 5.39E-01 26998 1.09E+00 26958
2.30E+00 26958 2.64E+00 26906 3.92E+00 26876 3.52E+00
26868 4.20E+00 26842 4.98E+00
27049 1.98E-01 27049 5.32E-01 26999 1.08E+00 26959
2.28E+00 26959 2.62E+00 26904 3.96E+00 26874 4.09E+00
26870 4.17E+00 26844 4.94E+00
27050 1.96E-01 27050 5.25E-01 27000 1.07E+00 26960
2.26E+00 26960 2.60E+00 26902 3.99E+00 26872 2.22E+00
26872 4.09E+00 26846 4.90E+00
27051 1.94E-01 27051 5.18E-01 27001 1.05E+00 26961

2.24E+00 26961 2.58E+00 26900 4.04E+00 26870 1.87E+00
26874 4.04E+00 26848 4.86E+00
27052 1.93E-01 27052 5.11E-01 27002 1.04E+00 26962
2.22E+00 26962 2.56E+00 26898 3.57E+00 26868 1.65E+00
26876 3.99E+00 26849 4.83E+00
27053 1.92E-01 27053 5.04E-01 27003 1.03E+00 26963
2.20E+00 26963 2.54E+00 26896 1.96E+00 26866 1.57E+00
26878 3.93E+00 26851 4.80E+00
27054 1.90E-01 27054 4.97E-01 27004 1.01E+00 26964
2.18E+00 26964 2.52E+00 26894 1.64E+00 26864 1.49E+00
26880 3.88E+00 26853 4.76E+00
27055 1.89E-01 27055 4.90E-01 27005 1.00E+00 26965
2.16E+00 26965 2.50E+00 26892 1.50E+00 26862 1.43E+00
26880 3.88E+00 26855 4.72E+00
27056 1.86E-01 27056 4.83E-01 27006 9.91E-01 26966
2.14E+00 26966 2.48E+00 26890 1.40E+00 26860 1.36E+00
26878 3.93E+00 26856 4.68E+00
27057 1.84E-01 27057 4.77E-01 27007 9.80E-01 26967
2.13E+00 26967 2.46E+00 26888 1.33E+00 26858 1.32E+00
26876 3.91E+00 26858 4.64E+00
27058 1.82E-01 27058 4.70E-01 27008 9.68E-01 26968
2.11E+00 26968 2.44E+00 26886 1.26E+00 26856 1.27E+00
26874 4.02E+00 26860 4.61E+00
27059 1.80E-01 27059 4.64E-01 27009 9.56E-01 26969
2.09E+00 26969 2.42E+00 26884 1.21E+00 26854 1.24E+00
26872 4.08E+00 26862 4.57E+00
27060 1.78E-01 27060 4.57E-01 27010 9.44E-01 26970
2.07E+00 26970 2.40E+00 26882 1.16E+00 26852 1.20E+00
26870 4.13E+00 26864 4.53E+00
27061 1.76E-01 27061 4.52E-01 27011 9.31E-01 26971
2.05E+00 26971 2.38E+00 26880 1.12E+00 26850 1.17E+00
26868 4.17E+00 26865 4.49E+00
27062 1.74E-01 27062 4.45E-01 27012 9.20E-01 26972
2.03E+00 26972 2.36E+00 26878 1.08E+00
26866 4.21E+00 26867 4.45E+00
27063 1.72E-01 27063 4.40E-01 27013 9.09E-01 26973
2.01E+00 26973 2.34E+00 26876 1.05E+00
26864 4.26E+00 26869 4.42E+00
27064 1.70E-01 27064 4.33E-01 27014 8.98E-01 26974
1.99E+00 26974 2.32E+00 26874 1.02E+00
26862 4.32E+00 26871 4.37E+00
27065 1.67E-01 27065 4.28E-01 27015 8.87E-01 26975
1.98E+00 26975 2.30E+00 26872 9.88E-01
26860 4.35E+00 26872 4.34E+00
27066 1.65E-01 27066 4.23E-01 27016 8.76E-01 26976
1.96E+00 26976 2.28E+00 26870 9.61E-01
26858 4.42E+00 26874 4.29E+00
27067 1.63E-01 27067 4.17E-01 27017 8.66E-01 26977
1.94E+00 26977 2.26E+00 26868 9.35E-01
26856 4.45E+00 26876 4.25E+00
27068 1.61E-01 27068 4.12E-01 27018 8.56E-01 26978
1.92E+00 26978 2.24E+00 26866 9.09E-01
26854 4.50E+00 26878 4.21E+00
27069 1.59E-01 27069 4.07E-01 27019 8.46E-01 26979
1.90E+00 26979 2.22E+00 26864 8.85E-01
26852 4.55E+00 26879 4.17E+00
27070 1.57E-01 27070 4.01E-01 27020 8.36E-01 26980
1.88E+00 26980 2.20E+00 26862 8.62E-01
26850 4.60E+00 26881 4.13E+00
27070 1.57E-01 27070 4.01E-01 27021 8.24E-01 26981
1.86E+00 26981 2.18E+00 26860 8.41E-01
26848 4.64E+00 26883 4.08E+00
27069 1.59E-01 27069 4.08E-01 27022 8.16E-01 26982
1.84E+00 26982 2.17E+00 26858 8.22E-01
26845 4.68E+00 26885 4.04E+00
27068 1.61E-01 27068 4.13E-01 27023 8.06E-01 26983
1.83E+00 26983 2.15E+00 26856 8.06E-01
26843 4.73E+00 26886 4.01E+00

27067 1.64E-01 27067 4.19E-01 27024 7.96E-01 26984
1.81E+00 26984 2.13E+00 26854 7.90E-01
26841 4.77E+00 26888 3.97E+00
27066 1.66E-01 27066 4.25E-01 27025 7.87E-01 26985
1.79E+00 26985 2.11E+00 26852 7.76E-01
26839 4.81E+00 26890 3.93E+00
27065 1.68E-01 27065 4.31E-01 27026 7.77E-01 26986
1.77E+00 26986 2.09E+00 26850 7.63E-01
26837 4.85E+00 26890 3.92E+00
27064 1.71E-01 27064 4.37E-01 27027 7.68E-01 26987
1.76E+00 26987 2.07E+00 26848 7.50E-01
26835 4.89E+00 26888 3.97E+00
27063 1.72E-01 27063 4.43E-01 27028 7.58E-01 26988
1.74E+00 26988 2.05E+00 26846 7.37E-01
26833 4.92E+00 26886 4.00E+00
27062 1.75E-01 27062 4.49E-01 27029 7.49E-01 26989
1.72E+00 26989 2.04E+00 26844 7.26E-01
26831 4.96E+00 26885 4.04E+00
27061 1.77E-01 27061 4.56E-01 27030 7.40E-01 26990
1.70E+00 26990 2.02E+00 26842 7.14E-01
26829 5.00E+00 26883 4.07E+00
27060 1.80E-01 27060 4.62E-01 27031 7.30E-01 26991
1.69E+00 26991 2.00E+00 26840 7.03E-01
26827 5.03E+00 26881 4.11E+00
27059 1.81E-01 27059 4.68E-01 27032 7.22E-01 26992
1.67E+00 26992 1.98E+00 26838 6.92E-01
26825 5.06E+00 26879 4.15E+00
27058 1.84E-01 27058 4.75E-01 27033 7.13E-01 26993
1.65E+00 26993 1.97E+00 26836 6.82E-01
26823 5.10E+00 26878 4.19E+00
27057 1.86E-01 27057 4.81E-01 27034 7.04E-01 26994
1.64E+00 26994 1.95E+00 26834 6.72E-01
26821 5.13E+00 26876 4.22E+00
27056 1.88E-01 27056 4.89E-01 27035 6.96E-01 26995
1.62E+00 26995 1.93E+00 26832 6.63E-01
26819 5.16E+00 26874 4.27E+00
27055 1.90E-01 27055 4.95E-01 27036 6.87E-01 26996
1.60E+00 26996 1.91E+00 26830 6.52E-01
26817 5.19E+00 26872 4.30E+00
27054 1.92E-01 27054 5.02E-01 27037 6.79E-01 26997
1.59E+00 26997 1.90E+00 26828 6.44E-01
26815 5.22E+00 26871 4.33E+00
27053 1.94E-01 27053 5.09E-01 27038 6.71E-01 26998
1.57E+00 26998 1.88E+00 26826 6.35E-01
26813 5.25E+00 26869 4.37E+00
27052 1.96E-01 27052 5.16E-01 27039 6.63E-01 26999
1.56E+00 26999 1.86E+00 26824 6.27E-01
26811 5.25E+00 26867 4.41E+00
27051 1.97E-01 27051 5.23E-01 27040 6.55E-01 27000
1.54E+00 27000 1.85E+00 26822 6.19E-01
26809 2.72E+00 26865 4.44E+00
27050 1.98E-01 27050 5.31E-01 27040 6.56E-01 27000
1.54E+00 27000 1.85E+00 26820 6.11E-01
26807 2.30E+00 26864 4.48E+00
27049 2.00E-01 27049 5.38E-01 27039 6.65E-01 26999
1.56E+00 26999 1.87E+00 26805
2.12E+00 26862 4.51E+00
27048 2.02E-01 27048 5.46E-01 27038 6.74E-01 26998
1.58E+00 26998 1.89E+00 26803
2.00E+00 26860 4.55E+00
27047 2.02E-01 27047 5.53E-01 27037 6.83E-01 26997
1.60E+00 26997 1.91E+00 26801
1.91E+00 26858 4.58E+00
27046 2.03E-01 27046 5.60E-01 27036 6.93E-01 26996
1.62E+00 26996 1.93E+00 26799
1.84E+00 26856 4.61E+00
27045 2.03E-01 27045 5.68E-01 27035 7.02E-01 26995
1.64E+00 26995 1.94E+00 26797

1.78E+00		26855	4.65E+00				
27044	2.04E-01		27044	5.76E-01	27034	7.12E-01	26994
1.66E+00		26994	1.96E+00				26795
1.72E+00		26853	4.68E+00				
27043	2.04E-01		27043	5.84E-01	27033	7.22E-01	26993
1.67E+00		26993	1.98E+00				26793
1.67E+00		26851	4.71E+00				
27042	2.04E-01		27042	5.92E-01	27032	7.32E-01	26992
1.69E+00		26992	2.00E+00				26791
1.63E+00		26849	4.73E+00				
27041	2.04E-01		27041	6.00E-01	27031	7.41E-01	26991
1.71E+00		26991	2.02E+00				26789
1.59E+00		26848	4.77E+00				
27040	2.04E-01		27040	6.07E-01	27030	7.51E-01	26990
1.73E+00		26990	2.04E+00				26787
1.55E+00		26846	4.80E+00				
27039	2.03E-01		27039	6.16E-01	27029	7.61E-01	26989
1.75E+00		26989	2.06E+00				26785
1.52E+00		26844	4.83E+00				
27038	2.02E-01		27038	6.24E-01	27028	7.71E-01	26988
1.77E+00		26988	2.08E+00				26783
1.48E+00		26842	4.85E+00				
27037	2.00E-01		27037	6.32E-01	27027	7.82E-01	26987
1.79E+00		26987	2.10E+00				26780
1.45E+00		26841	4.89E+00				
27036	2.00E-01		27036	6.39E-01	27026	7.92E-01	26986
1.81E+00		26986	2.12E+00				26778
1.42E+00		26839	4.93E+00				
27035	1.97E-01		27035	6.47E-01	27025	8.02E-01	26985
1.83E+00		26985	2.14E+00				26776
1.40E+00		26837	4.96E+00				
27034	1.95E-01		27034	6.56E-01	27024	8.13E-01	26984
1.85E+00		26984	2.16E+00				26774
1.37E+00		26835	4.99E+00				
27033	1.94E-01		27033	6.63E-01	27023	8.24E-01	26983
1.87E+00		26983	2.18E+00				26772
1.35E+00		26834	5.01E+00				
27032	1.89E-01		27032	6.71E-01	27022	8.34E-01	26982
1.89E+00		26982	2.20E+00				26770
1.32E+00		26832	5.04E+00				
27031	1.88E-01		27031	6.79E-01	27021	8.45E-01	26981
1.91E+00		26981	2.21E+00				26768
1.30E+00		26830	5.07E+00				
27030	1.85E-01		27030	6.87E-01	27020	8.56E-01	26980
1.93E+00		26980	2.23E+00				26766
1.28E+00		26828	5.09E+00				
27029	1.82E-01		27029	6.95E-01	27019	8.67E-01	26979
1.95E+00		26979	2.25E+00				26764
1.26E+00		26826	5.12E+00				
27028	1.80E-01		27028	7.02E-01	27018	8.78E-01	26978
1.96E+00		26978	2.27E+00				26762
1.24E+00		26825	5.15E+00				
27027	1.76E-01		27027	7.08E-01	27017	8.89E-01	26977
1.98E+00		26977	2.29E+00				26760
1.22E+00		26823	5.17E+00				
27026	1.74E-01		27026	7.16E-01	27016	9.00E-01	26976
2.00E+00		26976	2.31E+00				26758
1.20E+00		26821	5.20E+00				
27025	1.70E-01		27025	7.20E-01	27015	9.11E-01	26975
2.02E+00		26975	2.33E+00				26756
1.18E+00		26819	5.22E+00				
27024	1.67E-01		27024	7.28E-01	27014	9.22E-01	26974
2.05E+00		26974	2.35E+00				26754
1.17E+00		26818	5.25E+00				
27023	1.64E-01		27023	7.32E-01	27013	9.33E-01	26973
2.07E+00		26973	2.37E+00				26752
1.15E+00		26816	5.27E+00				
27022	1.60E-01		27022	7.37E-01	27012	9.45E-01	26972

2.08E+00 26972 2.39E+00 26750
1.14E+00 26814 5.30E+00
27021 1.57E-01 27021 7.41E-01 27011 9.57E-01 26971
2.11E+00 26971 2.41E+00
26812 5.32E+00
27020 1.55E-01 27020 7.44E-01 27010 9.69E-01 26970
2.13E+00 26970 2.43E+00
26811 5.34E+00
27019 1.52E-01 27019 7.44E-01 27009 9.82E-01 26969
2.15E+00 26969 2.45E+00
26809 5.37E+00
27018 1.49E-01 27018 7.43E-01 27008 9.96E-01 26968
2.16E+00 26968 2.47E+00
26807 5.39E+00
27017 1.47E-01 27017 7.44E-01 27007 1.01E+00 26967
2.18E+00 26967 2.49E+00
26805 5.41E+00
27016 1.43E-01 27016 7.41E-01 27006 1.02E+00 26966
2.20E+00 26966 2.51E+00
26804 5.43E+00
27015 1.41E-01 27015 7.34E-01 27005 1.04E+00 26965
2.22E+00 26965 2.53E+00
26802 5.45E+00
27014 1.38E-01 27014 7.28E-01 27004 1.05E+00 26964
2.24E+00 26964 2.55E+00
26800 5.48E+00
27013 1.35E-01 27013 7.18E-01 27003 1.06E+00 26963
2.26E+00 26963 2.57E+00
26798 5.50E+00
27012 1.33E-01 27012 7.05E-01 27002 1.07E+00 26962
2.28E+00 26962 2.59E+00
26796 5.52E+00
27011 1.31E-01 27011 6.90E-01 27001 1.08E+00 26961
2.30E+00 26961 2.61E+00
26795 5.54E+00
27010 1.28E-01 27010 6.73E-01 27000 1.10E+00 26960
2.32E+00 26960 2.63E+00
26793 5.56E+00
27009 1.25E-01 27009 6.56E-01 26999 1.11E+00 26959
2.33E+00 26959 2.65E+00
26791 5.58E+00
27008 1.23E-01 27008 6.35E-01 26998 1.12E+00 26958
2.35E+00 26958 2.67E+00
26789 5.61E+00
27007 1.21E-01 27007 6.15E-01 26997 1.13E+00 26957
2.37E+00 26957 2.69E+00
26788 5.63E+00
27006 1.19E-01 27006 5.97E-01 26996 1.14E+00 26956
2.40E+00 26956 2.71E+00
26786 4.68E+00
27005 1.17E-01 27005 5.74E-01 26995 1.15E+00 26955
2.42E+00 26955 2.73E+00
26784 2.62E+00
27004 1.15E-01 27004 5.55E-01 26994 1.16E+00 26954
2.44E+00 26954 2.75E+00
26782 2.34E+00
27003 1.13E-01 27003 5.35E-01 26993 1.17E+00 26953
2.47E+00 26953 2.77E+00
26781 2.20E+00
27002 1.11E-01 27002 5.17E-01 26992 1.19E+00 26952
2.48E+00 26952 2.79E+00
26779 2.09E+00
27001 1.09E-01 27001 4.99E-01 26991 1.20E+00 26951
2.46E+00 26951 2.81E+00
26777 2.01E+00
27000 1.08E-01 27000 4.83E-01 26990 1.22E+00 26950
2.34E+00 26950 2.83E+00
26775 1.94E+00

26999 1.06E-01 26999 4.66E-01 26989 1.23E+00 26949
2.05E+00 26949 2.84E+00
26774 1.88E+00
26998 1.04E-01 26998 4.52E-01 26988 1.24E+00 26948
1.70E+00 26948 2.87E+00
26772 1.83E+00
26997 1.03E-01 26997 4.38E-01 26987 1.26E+00 26947
1.40E+00 26947 2.88E+00
26770 1.79E+00
26996 1.01E-01 26996 4.26E-01 26986 1.27E+00 26946
1.23E+00 26946 2.90E+00
26768 1.74E+00
26995 9.95E-02 26995 4.13E-01 26985 1.28E+00 26945
1.13E+00 26945 2.92E+00
26766 1.71E+00
26994 9.81E-02 26994 4.02E-01 26984 1.29E+00 26944
1.06E+00 26944 2.94E+00
26765 1.67E+00
26993 9.63E-02 26993 3.92E-01 26983 1.31E+00 26943
1.01E+00 26943 2.96E+00
26763 1.64E+00
26992 9.52E-02 26992 3.82E-01 26982 1.32E+00 26942
9.69E-01 26942 2.98E+00
26761 1.61E+00
26991 9.42E-02 26991 3.72E-01 26981 1.33E+00 26941
9.32E-01 26941 3.00E+00
26759 1.58E+00
26990 9.26E-02 26990 3.64E-01 26980 1.34E+00 26940
8.86E-01 26940 3.02E+00
26758 1.55E+00
26979 1.35E+00 26939 8.60E-01
26939 3.04E+00 26756
1.52E+00
26978 1.36E+00 26938 8.35E-01
26938 3.06E+00 26754
1.50E+00
26977 1.37E+00 26937 8.13E-01
26937 3.08E+00 26752
1.47E+00
26976 1.37E+00 26936 7.93E-01
26936 3.10E+00 26751
1.45E+00
26975 1.38E+00 26935 7.75E-01
26935 3.12E+00 26749
1.43E+00
26974 1.37E+00 26934 7.57E-01
26934 3.14E+00 26747
1.41E+00
26973 1.37E+00 26933 7.41E-01
26933 3.16E+00 26745
1.39E+00
26972 1.35E+00 26932 7.25E-01
26932 3.18E+00 26744
1.37E+00
26971 1.32E+00 26931 7.10E-01
26931 3.20E+00 26742
1.35E+00
26970 1.29E+00 26930 6.97E-01
26930 3.21E+00 26740
1.34E+00
26969 1.24E+00 26929 6.83E-01
26929 3.23E+00
26968 1.19E+00 26928 6.70E-01
26928 3.25E+00
26967 1.13E+00 26927 6.59E-01
26927 3.27E+00
26966 1.06E+00 26926 6.47E-01
26926 3.29E+00

26925	3.31E+00	26965	1.00E+00	26925	6.36E-01
26924	3.33E+00	26964	9.46E-01	26924	6.26E-01
26923	3.35E+00	26963	8.93E-01	26923	6.16E-01
26922	3.38E+00	26962	8.49E-01	26922	6.06E-01
26921	3.41E+00	26961	8.06E-01	26921	5.98E-01
26920	3.37E+00	26960	7.69E-01	26920	5.89E-01
26919	3.18E+00	26959	7.35E-01	26919	5.80E-01
26918	2.34E+00	26958	7.07E-01	26918	5.72E-01
26917	1.81E+00	26957	6.80E-01	26917	5.64E-01
26916	1.54E+00	26956	6.56E-01	26916	5.57E-01
26915	1.41E+00	26955	6.33E-01	26915	5.50E-01
26914	1.32E+00	26954	6.14E-01	26914	5.42E-01
26913	1.26E+00	26953	5.95E-01	26913	5.35E-01
26912	1.20E+00	26952	5.79E-01	26912	5.29E-01
26911	1.16E+00	26951	5.63E-01	26911	5.22E-01
26910	1.12E+00	26950	5.48E-01	26910	5.16E-01
26909	1.08E+00	26949	5.34E-01	26909	5.10E-01
26908	1.05E+00	26948	5.22E-01	26908	5.04E-01
26907	1.02E+00	26947	5.09E-01	26907	4.98E-01
26906	9.96E-01	26946	4.98E-01	26906	4.93E-01
26905	9.69E-01	26945	4.88E-01	26905	4.87E-01
26904	9.44E-01	26944	4.78E-01	26904	4.82E-01
26903	9.18E-01	26943	4.68E-01	26903	4.77E-01
26902	8.96E-01	26942	4.58E-01	26902	4.72E-01
26901	8.75E-01	26941	4.50E-01	26901	4.67E-01
26900	8.57E-01	26940	4.41E-01	26900	4.62E-01

BI
2V (LDV 125mm/s/V)		6V (LDV 125mm/s/V)		10V (LDV 125mm/s/V)
15V (LDV 125mm/s/V)		20V (LDV 125mm/s/V)
25V (LDV 125mm/s/V)		30V (LDV 125mm/s/V)		35V (LDV 125mm/s/V)
40V (LDV 125mm/s/V)		45V (LDV 125mm/s/V)
50V (LDV 125mm/s/V)
Frequency (Hz)	Response (V)	Frequency (Hz)	Response (V)	Frequency (Hz)
Response (V)	Frequency (Hz)	Response (V)	Frequency (Hz)	Response (V)
Frequency (Hz)	Response (V)	Frequency (Hz)	Response (V)	Frequency (Hz)
Response (V)	Frequency (Hz)	Response (V)	Frequency (Hz)	Response (V)
Frequency (Hz)	Response (V)
28670	1.53E-01		28610	2.85E-01		28610	4.89E-01		28600
1.91E+00		28560	1.03E+00		28530	1.15E+00		28495	1.11E+00
28480	1.25E+00		28450	1.24E+00		28430	1.29E+00		28410

1.33E+00
28671 1.55E-01 28611 2.87E-01 28611 4.89E-01 28602
2.61E+00 28562 1.05E+00 28532 1.18E+00 28497 1.22E+00
28482 1.49E+00 28452 1.53E+00 28432 1.57E+00 28412
1.66E+00
28672 1.57E-01 28612 2.89E-01 28612 4.94E-01 28604
2.58E+00 28564 1.08E+00 28534 1.21E+00 28499 1.27E+00
28484 1.52E+00 28454 1.55E+00 28434 1.66E+00 28414
1.76E+00
28673 1.59E-01 28613 2.91E-01 28613 5.00E-01 28606
2.55E+00 28566 1.11E+00 28536 1.25E+00 28501 1.30E+00
28486 1.55E+00 28456 1.58E+00 28436 1.70E+00 28416
1.79E+00
28674 1.62E-01 28614 2.94E-01 28614 5.07E-01 28608
2.52E+00 28568 1.14E+00 28538 1.28E+00 28503 1.33E+00
28488 1.58E+00 28458 1.60E+00 28438 1.73E+00 28418
1.82E+00
28675 1.63E-01 28615 2.96E-01 28615 5.13E-01 28610
2.50E+00 28570 1.18E+00 28540 1.32E+00 28505 1.35E+00
28490 1.61E+00 28460 1.62E+00 28440 1.75E+00 28420
1.85E+00
28676 1.66E-01 28616 2.99E-01 28616 5.20E-01 28612
2.47E+00 28572 1.23E+00 28542 1.36E+00 28507 1.39E+00
28492 1.65E+00 28462 1.65E+00 28442 1.78E+00 28422
1.88E+00
28677 1.67E-01 28617 3.02E-01 28617 5.28E-01 28614
2.44E+00 28574 1.27E+00 28544 1.40E+00 28509 1.42E+00
28494 1.69E+00 28464 1.68E+00 28444 1.81E+00 28424
1.91E+00
28678 1.70E-01 28618 3.05E-01 28618 5.35E-01 28616
2.41E+00 28576 1.34E+00 28546 1.45E+00 28511 1.45E+00
28496 1.74E+00 28466 1.71E+00 28446 1.84E+00 28426
1.94E+00
28679 1.72E-01 28619 3.08E-01 28619 5.43E-01 28618
2.39E+00 28578 1.45E+00 28548 1.51E+00 28513 1.49E+00
28498 1.78E+00 28468 1.74E+00 28448 1.87E+00 28428
1.97E+00
28680 1.75E-01 28620 3.11E-01 28620 5.50E-01 28620
2.36E+00 28580 1.58E+00 28550 1.57E+00 28515 1.53E+00
28500 1.84E+00 28470 1.77E+00 28450 1.91E+00 28430
2.01E+00
28681 1.78E-01 28621 3.14E-01 28621 5.59E-01 28622
2.34E+00 28582 1.77E+00 28552 1.65E+00 28517 1.58E+00
28502 1.89E+00 28472 1.81E+00 28452 1.94E+00 28432
2.04E+00
28682 1.82E-01 28622 3.18E-01 28622 5.67E-01 28624
2.31E+00 28584 2.30E+00 28554 1.74E+00 28519 1.62E+00
28504 1.96E+00 28474 1.86E+00 28454 1.98E+00 28434
2.08E+00
28683 1.84E-01 28623 3.21E-01 28623 5.77E-01 28626
2.28E+00 28586 3.53E+00 28556 1.89E+00 28521 1.68E+00
28506 2.04E+00 28476 1.90E+00 28456 2.02E+00 28436
2.13E+00
28684 1.87E-01 28624 3.25E-01 28624 5.87E-01 28628
2.25E+00 28588 3.49E+00 28558 2.14E+00 28523 1.74E+00
28508 2.13E+00 28478 1.95E+00 28458 2.07E+00 28438
2.17E+00
28685 1.90E-01 28625 3.28E-01 28625 5.96E-01 28630
2.22E+00 28590 3.44E+00 28560 4.02E+00 28525 1.81E+00
28510 2.28E+00 28480 2.00E+00 28460 2.13E+00 28440
2.22E+00
28686 1.94E-01 28626 3.33E-01 28626 6.08E-01 28632
2.19E+00 28592 3.40E+00 28562 4.17E+00 28527 1.91E+00
28512 2.54E+00 28482 2.06E+00 28462 2.19E+00 28442
2.28E+00
28687 1.96E-01 28627 3.36E-01 28627 6.18E-01 28634
2.18E+00 28594 3.36E+00 28564 4.14E+00 28529 2.03E+00
28514 5.17E+00 28484 2.13E+00 28464 2.26E+00 28444

2.35E+00
28688 2.00E-01 28628 3.41E-01 28628 6.30E-01 28636
2.14E+00 28596 3.33E+00 28566 4.10E+00 28531 2.22E+00
28516 5.13E+00 28486 2.21E+00 28466 2.34E+00 28446
2.42E+00
28689 2.04E-01 28629 3.45E-01 28629 6.42E-01 28638
2.10E+00 28598 3.29E+00 28568 4.06E+00 28533 4.74E+00
28518 5.10E+00 28488 2.32E+00 28468 2.45E+00 28448
2.51E+00
28690 2.07E-01 28630 3.50E-01 28630 6.55E-01 28640
2.07E+00 28600 3.25E+00 28570 4.02E+00 28535 4.70E+00
28520 5.06E+00 28490 2.47E+00 28470 2.61E+00 28450
2.64E+00
28691 2.10E-01 28631 3.54E-01 28631 6.70E-01 28642
2.04E+00 28602 3.22E+00 28572 3.99E+00 28537 4.66E+00
28522 5.03E+00 28492 5.59E+00 28472 5.93E+00 28452
2.90E+00
28692 2.14E-01 28632 3.59E-01 28632 6.83E-01 28644
2.01E+00 28604 3.18E+00 28574 3.95E+00 28539 4.62E+00
28524 5.00E+00 28494 5.55E+00 28474 5.90E+00 28454
6.21E+00
28693 2.17E-01 28633 3.64E-01 28633 6.99E-01 28646
1.97E+00 28606 3.15E+00 28576 3.92E+00 28541 4.59E+00
28526 4.96E+00 28496 5.52E+00 28476 5.86E+00 28456
6.17E+00
28694 2.21E-01 28634 3.69E-01 28634 7.14E-01 28648
1.94E+00 28608 3.11E+00 28578 3.88E+00 28543 4.55E+00
28528 4.93E+00 28498 5.48E+00 28478 5.83E+00 28458
6.14E+00
28695 2.24E-01 28635 3.74E-01 28635 7.33E-01 28650
5.71E-01 28610 3.08E+00 28580 3.85E+00 28545 4.52E+00
28530 4.90E+00 28500 5.45E+00 28480 5.80E+00 28460
6.11E+00
28696 2.27E-01 28636 3.80E-01 28636 7.51E-01 28652
1.89E+00 28612 3.04E+00 28582 3.81E+00 28547 4.48E+00
28532 4.86E+00 28502 5.42E+00 28482 5.77E+00 28462
6.08E+00
28697 2.30E-01 28637 3.85E-01 28637 7.71E-01 28654
1.86E+00 28614 3.00E+00 28584 3.78E+00 28549 4.45E+00
28534 4.83E+00 28504 5.38E+00 28484 5.74E+00 28464
6.05E+00
28698 2.32E-01 28638 3.91E-01 28638 7.95E-01 28656
1.48E+00 28616 2.97E+00 28586 3.75E+00 28551 4.42E+00
28536 4.80E+00 28506 5.35E+00 28486 5.71E+00 28466
6.02E+00
28699 2.35E-01 28639 3.97E-01 28639 8.18E-01 28658
1.82E+00 28618 2.93E+00 28588 3.71E+00 28553 4.38E+00
28538 4.77E+00 28508 5.32E+00 28488 5.68E+00 28468
5.99E+00
28700 2.38E-01 28640 4.03E-01 28640 8.46E-01 28660
1.78E+00 28620 2.90E+00 28590 3.70E+00 28555 4.35E+00
28540 4.74E+00 28510 5.29E+00 28490 5.65E+00 28470
5.96E+00
28701 2.40E-01 28641 4.09E-01 28641 8.74E-01 28662
1.74E+00 28622 2.86E+00 28592 3.58E+00 28557 4.32E+00
28542 4.71E+00 28512 5.26E+00 28492 5.62E+00 28472
5.94E+00
28702 2.43E-01 28642 4.16E-01 28642 9.09E-01 28664
1.71E+00 28624 2.83E+00 28594 3.64E+00 28559 4.29E+00
28544 4.68E+00 28514 5.23E+00 28494 5.60E+00 28474
5.91E+00
28703 2.45E-01 28643 4.23E-01 28643 9.55E-01 28666
1.68E+00 28626 2.79E+00 28596 3.61E+00 28561 4.26E+00
28546 4.64E+00 28516 5.20E+00 28496 5.57E+00 28476
5.88E+00
28704 2.46E-01 28644 4.30E-01 28644 9.96E-01 28668
1.66E+00 28628 2.76E+00 28598 3.57E+00 28563 4.23E+00
28548 4.61E+00 28518 5.17E+00 28498 5.54E+00 28478

5.85E+00
28705 2.48E-01 28645 4.38E-01 28645 1.05E+00 28670
1.62E+00 28630 2.73E+00 28600 3.53E+00 28565 4.19E+00
28550 4.58E+00 28520 5.14E+00 28500 5.51E+00 28480
5.82E+00
28706 2.49E-01 28646 4.45E-01 28646 1.11E+00 28670
1.63E+00 28630 2.73E+00 28600 2.69E+00 28565 4.19E+00
28550 4.58E+00 28520 5.14E+00 28500 5.50E+00 28480
5.82E+00
28707 2.50E-01 28647 4.53E-01 28647 1.18E+00 28668
1.66E+00 28628 2.77E+00 28598 3.57E+00 28563 4.22E+00
28548 4.61E+00 28518 5.17E+00 28498 5.53E+00 28478
5.84E+00
28708 2.50E-01 28648 4.62E-01 28648 1.30E+00 28666
1.69E+00 28626 2.81E+00 28596 3.35E+00 28561 4.26E+00
28546 4.64E+00 28516 5.19E+00 28496 5.55E+00 28476
5.86E+00
28709 2.52E-01 28649 4.70E-01 28649 1.42E+00 28664
1.73E+00 28624 2.85E+00 28594 3.64E+00 28559 4.29E+00
28544 4.67E+00 28514 5.22E+00 28494 5.57E+00 28474
5.88E+00
28710 2.52E-01 28650 4.81E-01 28650 1.50E+00 28662
1.76E+00 28622 2.88E+00 28592 3.65E+00 28557 4.32E+00
28542 4.70E+00 28512 5.24E+00 28492 5.59E+00 28472
5.90E+00
28711 2.51E-01 28651 4.90E-01 28651 1.54E+00 28660
1.79E+00 28620 2.92E+00 28590 3.70E+00 28555 4.35E+00
28540 4.73E+00 28510 5.27E+00 28490 5.61E+00 28470
5.92E+00
28712 2.52E-01 28652 5.02E-01 28652 1.56E+00 28658
1.82E+00 28618 2.96E+00 28588 3.74E+00 28553 4.37E+00
28538 4.75E+00 28508 5.29E+00 28488 5.64E+00 28468
5.94E+00
28713 2.51E-01 28653 5.12E-01 28653 1.57E+00 28656
1.86E+00 28616 2.99E+00 28586 3.77E+00 28551 4.40E+00
28536 4.78E+00 28506 5.31E+00 28486 5.66E+00 28466
5.96E+00
28714 2.51E-01 28654 5.24E-01 28654 1.57E+00 28654
1.89E+00 28614 3.02E+00 28584 3.80E+00 28549 4.43E+00
28534 4.81E+00 28504 5.33E+00 28484 5.68E+00 28464
5.97E+00
28715 2.50E-01 28655 5.35E-01 28655 1.56E+00 28652
1.92E+00 28612 3.06E+00 28582 3.83E+00 28547 4.46E+00
28532 4.83E+00 28502 5.35E+00 28482 5.70E+00 28462
5.99E+00
28716 2.49E-01 28656 5.49E-01 28656 1.56E+00 28650
1.95E+00 28610 3.09E+00 28580 3.85E+00 28545 4.49E+00
28530 4.86E+00 28500 5.37E+00 28480 5.72E+00 28460
6.01E+00
28717 2.48E-01 28657 5.62E-01 28657 1.55E+00 28648
1.99E+00 28608 3.13E+00 28578 3.87E+00 28543 4.52E+00
28528 4.89E+00 28498 5.40E+00 28478 5.75E+00 28458
6.03E+00
28718 2.46E-01 28658 5.77E-01 28658 1.53E+00 28646
2.02E+00 28606 3.16E+00 28576 3.90E+00 28541 4.55E+00
28526 4.91E+00 28496 5.42E+00 28476 5.77E+00 28456
6.05E+00
28719 2.44E-01 28659 5.91E-01 28659 1.52E+00 28644
2.06E+00 28604 3.20E+00 28574 3.93E+00 28539 4.57E+00
28524 4.94E+00 28494 5.44E+00 28474 5.79E+00 28454
6.07E+00
28720 2.43E-01 28660 6.08E-01 28660 1.51E+00 28642
2.09E+00 28602 3.23E+00 28572 3.95E+00 28537 4.60E+00
28522 4.97E+00 28492 5.46E+00 28472 5.82E+00 28452
6.09E+00
28721 2.41E-01 28661 6.24E-01 28661 1.49E+00 28640
2.13E+00 28600 3.26E+00 28570 3.98E+00 28535 4.63E+00
28520 5.00E+00 28490 5.48E+00 28470 5.84E+00 28450

```
6.11E+00
28722   2.39E-01          28662   6.43E-01          28662   1.48E+00          28638
2.17E+00          28598   3.30E+00          28568   4.01E+00          28533   4.66E+00
28518   5.02E+00          28488   5.50E+00          28468   5.86E+00          28448
6.13E+00
28723   2.37E-01          28663   6.60E-01          28663   1.47E+00          28636
2.21E+00          28596   3.33E+00          28566   4.04E+00          28531   4.69E+00
28516   5.05E+00          28486   5.53E+00          28466   5.88E+00          28446
6.15E+00
28724   2.35E-01          28664   6.80E-01          28664   1.45E+00          28634
2.24E+00          28594   3.37E+00          28564   4.06E+00          28529   4.72E+00
28514   5.07E+00          28484   5.55E+00          28464   5.90E+00          28444
6.17E+00
28725   2.33E-01          28665   6.97E-01          28665   1.44E+00          28632
2.28E+00          28592   3.40E+00          28562   4.09E+00          28527   4.75E+00
28512   5.10E+00          28482   5.57E+00          28462   5.92E+00          28442
6.18E+00
28726   2.31E-01          28666   7.16E-01          28666   1.42E+00          28630
2.31E+00          28590   3.44E+00          28560   4.12E+00          28525   4.78E+00
28510   5.12E+00          28480   5.59E+00          28460   5.94E+00          28440
6.20E+00
28727   2.28E-01          28667   7.37E-01          28667   1.41E+00          28628
2.35E+00          28588   3.47E+00          28558   4.15E+00          28523   4.39E+00
28508   5.15E+00          28478   5.62E+00          28458   5.96E+00          28438
6.22E+00
28728   2.27E-01          28668   7.54E-01          28668   1.40E+00          28626
2.39E+00          28586   3.51E+00          28556   4.18E+00          28521   2.35E+00
28506   5.17E+00          28476   3.23E+00          28456   2.95E+00          28436
6.24E+00
28729   2.24E-01          28669   7.74E-01          28669   1.38E+00          28624
2.43E+00          28584   3.54E+00          28554   4.21E+00          28519   2.01E+00
28504   5.20E+00          28474   2.44E+00          28454   2.48E+00          28434
6.26E+00
28730   2.22E-01          28670   7.89E-01          28670   1.37E+00          28622
2.46E+00          28582   3.58E+00          28552   4.24E+00          28517   1.85E+00
28502   5.23E+00          28472   2.20E+00          28452   2.30E+00          28432
2.79E+00
28731   2.19E-01          28671   8.05E-01          28671   1.36E+00          28620
2.49E+00          28580   3.62E+00          28550   4.23E+00          28515   1.74E+00
28500   4.67E+00          28470   2.08E+00          28450   2.19E+00          28430
2.50E+00
28732   2.17E-01          28672   8.18E-01          28672   1.34E+00          28618
2.53E+00          28578   3.37E+00          28548   3.47E+00          28513   1.67E+00
28498   2.42E+00          28468   1.99E+00          28448   2.10E+00          28428
2.36E+00
28733   2.14E-01          28673   8.28E-01          28673   1.33E+00          28616
2.56E+00          28576   2.12E+00          28546   2.09E+00          28511   1.60E+00
28496   2.12E+00          28466   1.92E+00          28446   2.03E+00          28426
2.26E+00
28734   2.12E-01          28674   8.39E-01          28674   1.32E+00          28614
2.61E+00          28574   1.67E+00          28544   1.72E+00          28509   1.54E+00
28494   2.00E+00          28464   1.85E+00          28444   1.97E+00          28424
2.18E+00
28735   2.10E-01          28675   8.45E-01          28675   1.30E+00          28612
2.65E+00          28572   1.48E+00          28542   1.62E+00          28507   1.48E+00
28492   1.90E+00          28462   1.80E+00          28442   1.92E+00          28422
2.11E+00
28736   2.08E-01          28676   8.52E-01          28676   1.29E+00          28610
2.64E+00          28570   1.37E+00          28540   1.53E+00          28505   1.43E+00
28490   1.83E+00          28460   1.75E+00          28440   1.87E+00          28420
2.06E+00
28737   2.05E-01          28677   8.55E-01          28677   1.28E+00          28608
2.22E+00          28568   1.28E+00          28538   1.47E+00          28503   1.40E+00
28488   1.76E+00          28458   1.70E+00          28438   1.83E+00          28418
2.01E+00
28738   2.03E-01          28678   8.58E-01          28678   1.27E+00          28606
1.63E+00          28566   1.21E+00          28536   1.41E+00          28501   1.36E+00
28486   1.70E+00          28456   1.67E+00          28436   1.79E+00          28416
```

1.96E+00
28739 2.01E-01 28679 8.58E-01 28679 1.25E+00 28604
1.27E+00 28564 1.14E+00 28534 1.36E+00 28499 1.33E+00
28484 1.65E+00 28454 1.63E+00 28434 1.75E+00 28414
1.92E+00
28740 1.98E-01 28680 8.59E-01 28680 1.25E+00 28602
1.16E+00 28562 1.09E+00 28532 1.31E+00 28497 1.30E+00
28482 1.60E+00 28452 1.60E+00 28432 1.72E+00 28412
1.89E+00
28741 1.96E-01 28681 8.57E-01 28681 1.23E+00 28600
1.08E+00 28560 1.04E+00 28530 1.27E+00 28495 1.27E+00
28480 1.56E+00 28450 1.57E+00 28430 1.69E+00 28410
1.85E+00
28742 1.94E-01 28682 8.55E-01 28682 1.22E+00

28743 1.92E-01 28683 8.52E-01 28683 1.21E+00

28744 1.89E-01 28684 8.47E-01 28684 1.20E+00

28745 1.87E-01 28685 8.43E-01 28685 1.19E+00

28746 1.85E-01 28686 8.38E-01 28686 1.18E+00

28747 1.83E-01 28687 8.33E-01 28687 1.17E+00

28748 1.81E-01 28688 8.26E-01 28688 1.16E+00

28749 1.78E-01 28689 8.19E-01 28689 1.14E+00

28750 1.76E-01 28690 8.13E-01 28690 1.13E+00

28750 1.76E-01 28691 8.07E-01 28691 1.12E+00

28749 1.78E-01 28692 8.00E-01 28692 1.11E+00

28748 1.81E-01 28693 7.92E-01 28693 1.10E+00

28747 1.84E-01 28694 7.86E-01 28694 1.09E+00

28746 1.86E-01 28695 7.78E-01 28695 1.08E+00

28745 1.88E-01 28696 7.71E-01 28696 1.07E+00

28744 1.90E-01 28697 7.63E-01 28697 1.06E+00

28743 1.93E-01 28698 7.57E-01 28698 1.05E+00

28742 1.95E-01 28699 7.50E-01 28699 1.04E+00

28741 1.97E-01 28700 7.41E-01 28700 1.03E+00

28740	2.00E-01	28701	7.34E-01	28701	1.03E+00
28739	2.02E-01	28702	7.27E-01	28702	1.02E+00
28738	2.05E-01	28703	7.20E-01	28703	1.01E+00
28737	2.08E-01	28704	7.12E-01	28704	1.00E+00
28736	2.09E-01	28705	7.04E-01	28705	9.92E-01
28735	2.12E-01	28706	6.97E-01	28706	9.84E-01
28734	2.15E-01	28707	6.90E-01	28707	9.76E-01
28733	2.17E-01	28708	6.83E-01	28708	9.68E-01
28732	2.20E-01	28709	6.75E-01	28709	9.60E-01
28731	2.22E-01	28710	6.68E-01	28710	9.53E-01
28730	2.24E-01	28710	6.68E-01	28710	9.54E-01
28729	2.26E-01	28709	6.77E-01	28709	9.66E-01
28728	2.29E-01	28708	6.86E-01	28708	9.76E-01
28727	2.31E-01	28707	6.94E-01	28707	9.87E-01
28726	2.33E-01	28706	7.03E-01	28706	9.97E-01
28725	2.36E-01	28705	7.11E-01	28705	1.01E+00
28724	2.38E-01	28704	7.20E-01	28704	1.02E+00
28723	2.41E-01	28703	7.28E-01	28703	1.03E+00
28722	2.42E-01	28702	7.36E-01	28702	1.04E+00
28721	2.43E-01	28701	7.44E-01	28701	1.05E+00
28720	2.46E-01	28700	7.53E-01	28700	1.05E+00
28719	2.48E-01	28699	7.61E-01	28699	1.07E+00

28718	2.48E-01	28698	7.69E-01	28698	1.08E+00
28717	2.49E-01	28697	7.77E-01	28697	1.09E+00
28716	2.51E-01	28696	7.84E-01	28696	1.10E+00
28715	2.52E-01	28695	7.93E-01	28695	1.11E+00
28714	2.53E-01	28694	8.00E-01	28694	1.12E+00
28713	2.54E-01	28693	8.08E-01	28693	1.13E+00
28712	2.54E-01	28692	8.16E-01	28692	1.14E+00
28711	2.54E-01	28691	8.21E-01	28691	1.16E+00
28710	2.52E-01	28690	8.28E-01	28690	1.17E+00
28709	2.52E-01	28689	8.34E-01	28689	1.18E+00
28708	2.52E-01	28688	8.41E-01	28688	1.20E+00
28707	2.51E-01	28687	8.45E-01	28687	1.21E+00
28706	2.50E-01	28686	8.50E-01	28686	1.22E+00
28705	2.48E-01	28685	8.54E-01	28685	1.24E+00
28704	2.45E-01	28684	8.59E-01	28684	1.25E+00
28703	2.45E-01	28683	8.60E-01	28683	1.26E+00
28702	2.42E-01	28682	8.61E-01	28682	1.27E+00
28701	2.39E-01	28681	8.62E-01	28681	1.28E+00
28700	2.36E-01	28680	8.61E-01	28680	1.30E+00
28699	2.34E-01	28679	8.59E-01	28679	1.31E+00
28698	2.30E-01	28678	8.54E-01	28678	1.32E+00
28697	2.28E-01	28677	8.49E-01	28677	1.33E+00
28696	2.24E-01	28676	8.40E-01	28676	1.34E+00

28695	2.19E-01	28675	8.31E-01	28675	1.35E+00
28694	2.17E-01	28674	8.20E-01	28674	1.36E+00
28693	2.12E-01	28673	8.06E-01	28673	1.37E+00
28692	2.10E-01	28672	7.90E-01	28672	1.39E+00
28691	2.06E-01	28671	7.73E-01	28671	1.40E+00
28690	2.03E-01	28670	7.53E-01	28670	1.41E+00
28689	1.98E-01	28669	7.34E-01	28669	1.42E+00
28688	1.96E-01	28668	7.14E-01	28668	1.43E+00
28687	1.91E-01	28667	6.92E-01	28667	1.44E+00
28686	1.88E-01	28666	6.71E-01	28666	1.45E+00
28685	1.84E-01	28665	6.51E-01	28665	1.47E+00
28684	1.82E-01	28664	6.33E-01	28664	1.48E+00
28683	1.77E-01	28663	6.12E-01	28663	1.49E+00
28682	1.74E-01	28662	5.92E-01	28662	1.50E+00
28681	1.70E-01	28661	5.75E-01	28661	1.51E+00
28680	1.68E-01	28660	5.59E-01	28660	1.52E+00
28679	1.65E-01	28659	5.44E-01	28659	1.53E+00
28678	1.61E-01	28658	5.30E-01	28658	1.54E+00
28677	1.58E-01	28657	5.16E-01	28657	1.55E+00
28676	1.56E-01	28656	5.03E-01	28656	1.56E+00
28675	1.52E-01	28655	4.91E-01	28655	1.57E+00
28674	1.51E-01	28654	4.78E-01	28654	1.57E+00
28673	1.47E-01	28653	4.67E-01	28653	1.57E+00

28672	1.45E-01	28652	4.57E-01	28652	1.57E+00
28671	1.42E-01	28651	4.46E-01	28651	1.56E+00
28670	1.40E-01	28650	4.38E-01	28650	1.54E+00
		28649	4.28E-01	28649	1.51E+00
		28648	4.19E-01	28648	1.47E+00
		28647	4.11E-01	28647	1.42E+00
		28646	4.03E-01	28646	1.36E+00
		28645	3.95E-01	28645	1.30E+00
		28644	3.89E-01	28644	1.23E+00
		28643	3.82E-01	28643	1.16E+00
		28642	3.75E-01	28642	1.10E+00
		28641	3.68E-01	28641	1.05E+00
		28640	3.62E-01	28640	1.00E+00
		28639	3.56E-01	28639	9.53E-01
		28638	3.50E-01	28638	9.13E-01
		28637	3.44E-01	28637	8.77E-01
		28636	3.40E-01	28636	8.44E-01
		28635	3.34E-01	28635	8.16E-01
		28634	3.29E-01	28634	7.90E-01
		28633	3.24E-01	28633	7.67E-01
		28632	3.20E-01	28632	7.44E-01
		28631	3.15E-01	28631	7.23E-01

28630	3.11E-01	28630	7.06E-01
28629	3.07E-01	28629	6.88E-01
28628	3.03E-01	28628	6.71E-01
28627	2.99E-01	28627	6.57E-01
28626	2.95E-01	28626	6.43E-01
28625	2.91E-01	28625	6.28E-01
28624	2.88E-01	28624	6.16E-01
28623	2.84E-01	28623	6.04E-01
28622	2.81E-01	28622	5.92E-01
28621	2.77E-01	28621	5.81E-01
28620	2.74E-01	28620	5.70E-01
28619	2.71E-01	28619	5.60E-01
28618	2.67E-01	28618	5.50E-01
28617	2.64E-01	28617	5.41E-01
28616	2.61E-01	28616	5.32E-01
28615	2.58E-01	28615	5.24E-01
28614	2.55E-01	28614	5.16E-01
28613	2.53E-01	28613	5.07E-01
28612	2.50E-01	28612	5.01E-01
28611	2.47E-01	28611	4.94E-01
28610	2.45E-01	28610	4.87E-01
